# DNA barcoding and comparative RNA-Seq analysis provide new insights into leaf formation using a novel resource of high-yielding *Epimedium koreanum*

**DOI:** 10.3389/fpls.2023.1290836

**Published:** 2023-12-18

**Authors:** Jiaxin Yang, Siqing Fan, Min Guo, Zhaoqi Xie, Qiqing Cheng, Puxin Gao, Chunsong Cheng

**Affiliations:** ^1^ Lushan Botanical Garden, Chinese Academic of Sciences, Jiujiang, China; ^2^ School of Pharmacy, Hubei University of Science and Technology, Xianning, China; ^3^ National Resource Center for Chinese Materia Medica, Chinese Academy of Chinese Medical Sciences, Beijing, China

**Keywords:** *Epimedium*, DEGs, compound leaf, *EkTCP*, plant selection, leaf yield

## Abstract

*Epimedium koreanum* Nakai, a well-known traditional Chinese medicinal herb, has been widely used to treat osteoporosis and sexual dysfunction for thousands of years. However, due to the decreasing population of East Asian natural resources, yearly output of Epimedium crude herb has been in low supply year by year. In this study, an unusual variety of *E. koreanum* was discovered in Dunhua, Jilin Province, the northernmost area where this variety was found containing 6 individuals, with three branches that had 27 leaflets, which is much more than the typical leaflet number of 9. Firstly, the novel *E. koreanum* varety was identified using DNA barcodes. Then, 1171 differentially expressed genes (DEGs) were discovered through parallel RNA-seq analysis between the newly discovered variety and wild type (WT) *E. koreanum* plant. Furthermore, the results of bioinformatics investigation revealed that 914 positively and 619 negatively correlated genes associated with the number of leaflets. Additionally, based on RNA-Seq and qRT-PCR analysis, two homologous hub *TCP* genes, which were commonly implicated in plant leaf development, and shown to be up regulated and down regulated in the discovered newly variety, respectively. Thus, our study discovered a novel wild resource for leaf yield rewarding medicinal *Epimedium* plant breeding, provided insights into the relationship between plant compound leaf formation and gene expression of *TCPs* transcription factors and other gene candidates, providing bases for creating high yield cultivated *Epimedium* variety by using further molecular selection and breeding techniques in the future.

## 1 Introduction

*Epimedium* is the largest herbaceous genus in berberidaceae family (Zhang et al., 2007; Zhang et al., 2022). Herbal *Epimedium*, first described in the *Shen Nong Herbal Classics*, is a prominent traditional Chinese medicinal plant, that has been widely used to treat osteoporosis and sexual dysfunction for thousands of years. There are currently 62 *Epimedium* species, 52 species are indigenous to China (Zhang et al., 2022). The published papers showed that Epimedium leaves contain a large number of flavonoid chemical components, which provides beneficial properties including anti-cancer effects and the treatment of cardiovascular diseases, rheumatoid arthritis, osteoporosis, and immune enhancement (Li et al., 2001; Ma et al., 2011; Jiang et al., 2015; Indran et al., 2016; Jiao et al., 2021; Liu et al., 2021; Zhu et al., 2021). In the *Pharmacopoeia of the People’s Republic of China* (*Ch. P*), total flavonoids and flavonoid glycoside quantitation serve as indicators of the herbal Epimedium quality. Additionally, polysaccharides, another medicinal component of Epimedium, have antiviral, anti-aging, and immune-regulating activities.

The majority of Epimedium research focused on the therapeutic benefit and chemical potential of its metabolites. However, there have been few studies that dived into botany and plant physiology, specifically the molecular processes that regulate the growth and development of herbal Epimedium. Among the statutory 5 medicinal *Epimedium* species recorded in the *Ch. P*, *Epimedium koreanum* is one of the species with a large amount of wild resources across in the two northeastern provinces of China, Jilin and Liaoning provinces. It primarily spreads in Eastern Asia, with notable distribution in China, Korea, and Japan (Lee et al., 2016; Qian et al., 2023). While the published papers on *E. koreanum* have mainly focused on recourse protection and sustainable utilization, including resource collection, cultivation, geographical distribution characteristics, and medical and pharmaceutical applications of its metabolites (Zhong et al., 2017; Zhang et al., 2020), and there have been few investigations on its plant physiology and molecular characteristics. (Lee et al., 2016). *E. koreanum* has been in low supply in the Chinese herbal medicine market in recent years due to the depletion of natural resources across East Asia, and its price has been rising year by year. With the foreseeable rising market demands, the crude herb of wild *E. koreanum* supplies are depleting, and cultivated *E. koreanum* will become the most important raw materials in the primary market. So, it is critical to use molecular techniques to create and select new *E. koreanum* varieties.

The principal photosynthetic organs of flowering plants, with great diversity in number, shape, and structure. Leaves are scientifical ciencclassified into two categories based on the number of leaflets and the structure of the leaf: simple leaves and complex leaves with many leaflets. A compound leaf, distinguished by its potential to take on many forms such as pinnate and palmate compound leaves, is made up of numerous discontinuous leaf units attached to the rachis and petiole, as opposed to a simple leaf, which is a single unit (Kim et al., 2003). Each leaflet in a complex leaf provides the same photosynthetic function as a simple leaf. From a functional aspect, each leaflet fulfills the same job as a simple leaf; hence, the development of complex leaves may boost plants’ capacity for photosynthetic energy generation and plant survival rate (Laura et al., 2010).

At present, the mechanism of compound leaf development and formation is not well understood. Leaf shape develops from the apical meristem (SAM) of the plant, while the leaf primordium cells develop from the flanks of SAM. Leaf development must arises in three distinct and overlapping stages: The first stage is leaf initiation, in which the leaf primordium differentiates from the flank of SAM; this is followed by primary morphogenesis (PM), in which leaf margin structures such as blade, serrate, and lobes begin to form; and finally, secondary morphogenesis (SM), which determines the final size and shape of the leaf (Dengler and Tsukaya, 2001; Bar and Ori, 2015). Leaf development is regulated by transcription factors and phytohormone networks. One of the essential genes involved in regulating leaf growth is the plant specific transcription factors *TEOSINTE BRANCHED1 CYCLOIDEA PROLIFERATING CELL FACTOR* (*TCP*). Various *TCP* transcription factors have been identified as critical modulators of leaf architecture. For instance, in tomato (*Solanum lycopersicum*), the *TCP* gene family member *LANCEOLATE* (*LA*) was showed exhibiting the premature leaf differentiation in the gain-of-function mutant *La-2*, resulting in a single-leaf pattern. Conversely, the loss of function mutant *la-6* displayed highly fragmented leaf margin shape, indicating the involvement of *LA* in leaf development (Ori et al., 2007). In *Arabidopsis*, the microRNA *miR319* was found to down-regulate the expression of *TCP* gene family members, thereby influencing leaf morphogenesis (Koyama et al., 2017). Similarly, *TCP13* was showed to regulate leaf and root growth in response to drought conditions in *Arabidopsis* (Urano et al., 2022). Furthermore, in lettuce (*Lactuca sativa L.*), the *LsAP2* gene promoted the leaf division by inhibiting *TCP* transcription factor activity, emphasizing the significance of *TCPs* in leaf development across different plant species (Luo et al., 2021). In addition, aside from the compound leaf plant tomato, there have few number of studies investigating compound leaf development in other plant species such as medicago (*Medicago truncatula*) and pea (*Pisum sativum*). In medicago, the *PINNATE LIKE PENTAFOLIATA1* (*PINNA1*) gene was identified as a key regulator of terminal leaflet morphogenesis. It works by inhibiting the expression of the *FLORICAULA*/*LFY* homologous gene, *SINGLE LEAFLET1* (*SGL1*), thereby suppressing the formation of lateral leaflets (He et al., 2020). Similarly, in peas, research focused on the *afila* (*af*) mutant, which exhibits increased leaf complexity. This increase in leaf complexity is accompanied by elevated expression of the *UNIFOLIATA* (*UNI*) gene. Further investigations involving the double mutant *af tendril* (*tl*) also revealed a synergistic effect, with heightened *UNI* expression suggesting that *AF* and *TL* jointly inhibit leaflet formation (Mishra et al., 2009; Demason et al., 2013). While there has been considerable research on compound leaf development in tomato, pea, and medicago, studies investigating the mechanism of compound leaf development in medicinal plants are currently lacking for scientific community. This knowledge gap is especially concerning in light of the scientific and practical consequences for medicinal or commercial plants that relys on complex leaves as harvesting and therapeutic components. Understanding the development and regulation of leaflets in medicinal plant species holds immense potential for enhancing their cultivation, yield, and medicinal properties. Therefore, imperious demands are expected to investigate compound leaf development in medicinal plants and uncover the underlying molecular mechanisms.

During extensive field investigations and resource collection encompassing various species of *Epimedium*, our research team made an intriguing discovery that the presence of a distinct *Epimedium* plant displayed remarkable characteristics in the primary growth region of *E. koreanum* in northeast China. This unique wild resource exhibited an unprecedented number of leaflets, ranging from 11 to 27, surpassing the typical 9 leaflets observed in *E. koreanum*. To unravel the taxonomic implications associated with this finding, we rigorously employed DNA barcode analysis and constructed an evolutionary tree, aiming to ascertain whether this variant represented a new species of *E. koreanum*. Subsequently, we performed comprehensive investigations on this “super *Epimedium*” plant. Fresh leaf tissues were carefully sampled and subjected to global RNA sequencing (RNA-Seq) and qRT-PCR analysis, allowing us to delve into the intricate molecular mechanisms underlying its exceptional leaf development. Through rigorous analysis of differential gene expression (DEGs) and comparison of gene expression patterns, we gained valuable insights into the critical signaling pathways involved in leaf development. By integrating these findings with important molecular pathways, we aimed to gain a holistic understanding of compound leaf morphogenesis in *E. koreanum*. Ultimately, our study endeavored to improve our understanding of compound leaf morphogenesis in *E. koreanum* while also opening up novel prospects for future selection and breeding.

## 2 Materials and methods

### 2.1 Sample collection

The material used in this study were *E.koreanum* and the discovered newly variation. The newly variation named *E. koreanum* var. *polyphylla* CS Cheng (EKP) in this study collected from in Dunhua City Jilin Province (E: 128.0369, N: 43.1156, A: 670) and ex-situ cultivated in the E115°59′, N29°51′ at Lushan Botanical Garden.

### 2.2 Sequence mining and primer design

The nucleic acid sequences of each species within the genus *Epimedium* were downloaded from the NCBI website (https://www.ncbi.nlm.nih.gov) by searching with “ ‘*Epimedium*’ [organism] and ‘*matK*’ [All fileds] “. The *ITS*, *rbcL*, and *trnL-trnF* sequences were also searched and downloaded in a similar manner. Save the downloaded sequences with Geneious primer 2021 software (Kearse et al., 2012) for the subsequent steps of nucleic acid sequence SNP analysis. The *matK* sequence (GenBank: AB069837.1) of *E. koreanum* species was used as the reference sequence and Primer premier 5.0 was used for primer design (Li et al., 2009). The primer sequences were designed as:

*matK* forward primer (FmatK): 5’-TATGACAATAAATCCAGTTC-3’

*matK* reverse primer (RmatK): 5’-ATGCCCCGATACGTTACAAA-3’

### 2.3 DNA extraction and DNA sequencing

Genomic DNA from leaves was isolated using the standard Cetyltrimethyl ammonium bromid (CTAB) extraction protocol (Helliwell et al., 2016). The targeted sequences were amplified with specific primers. The standard 50 μL PCR reaction mixture contained 25 μL of 2 × PrimeSTAR^®^ Max DNA Polymerase (Takara, Code NO. R045A) and 10 ng of template DNA, 0.2 μM of each primer. The samples were amplified using a Verit 96- Well Fast Thermal Cycler (Applied Biosystems, Foster City, CA, USA) under the following conditions: initial denaturation at 94 °C for 5 min, followed by 30 cycles of denaturation at 98 °C for 10 s, annealing at 54 °C for 10 s, extension at 72 °C for 30 s, and a final elongation step at 72 °C for 7 min. The PCR products were confirmed by 1.0% agarose gel electrophoresis in 1 × TAE buffer to detect whether the target sequences were cloned successfully. The amplicons were purified with an TaKaRaMinBEST Agarose Gel DNA Extraction Kit Ver.4.0 (Takara, Code No.9762) and quantified with a NaoDrop 2000 spectrophptometer (Thermo Fisher Scientific) (Lin et al., 2021). And then the target fragments were sent to BGI (Wuhan, China) for bidirectional sequencing.

### 2.4 Sequence analysis

To discover the SNP of sequences, sequenced sequences and *matK* sequences downloaded from NCBI were subjected to multiple sequence alignments through Geneious primer 2021 software with default settings. Polymorphic locis were counted after multiple alignment for different species. In the *Geneious* software, the head and tail of the aligned *matK* sequences were cut off while removing the gaps in different species, respectively. Then, intraspecific genetic distances were calculated as SNP%. Six species in the *Epimedium* genus were randomly selected, and all *matK* sequences of each species were randomly sampled with put-back six times for sequence alignments, and then the calculated SNP% was used as the interspecific genetic distance. The *ITS*, *rbcL*, and t*rnL-trnF* sequences were also handled as described above.

### 2.5 Phylogenetic analysis

Test sequences from the collected samples and *matK* sequences downloaded from NCBI for all species of the *Epimedium* genus were used for multiple sequence alignments through MEGA-X (Kumar et al., 2018) with the default setting. Then, they were performed cutting the head and tail, as well as aligning the gaps in the sequence. To compare the evolutionary relationships, the results of the above alignments and processing were used to construct the phylogenetic tree using MEGA-X with Maximum Likelihood (ML) method. The phylogenetic tree was then visualized by EVOLVIEW. (https://www.evolgenius.info/evolview/#login).

To build phylogenetic tree of *TCP* proteins, the *TCP* protein sequences were first aligned with MAFFT (Version 7.037b) (model: “BLOSUM62”, strategy: “L-INS-i”) (Katoh and Standley, 2013), then refined conserved sequences from the alignments by Gblocks (Version 0.91b) (Maximum number of contiguous noncom served positions: 32000, Minimum length of a block: 2, Allowed gap positions: all) (Castresana, 2000). The neighbor-joining phylogenetic tree was finally generated with Mega 6 (Tamura et al., 2013).

### 2.6 Geographic analysis

According to the origin information of different species of *Epimedium* genus (http://www.iplant.cn/, http://www.plantsoftheworldonline.org/), the latitude and longitude of origins were also found and recorded by Google Earth. The locations were projected to the provincial boundary map of China depended on the latitude and longitude data by ArcGIS10 software to observe and analysis the distribution of different groups.

### 2.7 mRNA sequencing

The newly developed leaves of *E.koreanum* in the rainy season of August in Jilin Province were collected for molecular sequencing analyses. The two groups of *E. koreanum* with significant differences in leaf shape and number of leaflets of compound leaves were named variety and normal. And, each sample was blended with several leaflets from the same biological source, and at least three biological duplicate samples were chosen for each group in this investigation. All samples were powdered by liquid nitrogen quick-freezing and then RNA was extracted. Nanodrop 2000 (ThermoFisher) was used for purity and concentration detection of the extracted RNA, RNA integrity was detected by agarose gel electrophoresis, and Agilent 2100 was used to determine the RIN value. Four samples were send to BGI Genomics Co., Ltd. (East Lake Development Area, Wuhan, China) for library preparation and RNA sequencing. Using the BGISEQ-500 sequencing platform, sequencing data quality control included sequencing data statistics, original data statistics and quality control data statistics.

### 2.8 Transcriptome data analysis

Data filtering: The raw data obtained from sequencing was filtered using the filtering software fastp (Chen et al., 2018) to remove reads containing adapters (adapter contamination), reads with unknown base N content greater than 5%, and low-quality reads (reads with a quality value below 15 that account for more than 20% of the total bases in the read). *De novo* assembly and quality assessment: Clean reads were assembled *de novo* using Trinity (Grabherr et al., 2011), and their assembly quality was evaluated using BUSCO. Reference gene alignment: Clean data was aligned to reference gene sequences using Bowtie 2 (v2.2.5) (Langmead and Salzberg, 2012)software, and gene and transcript expression levels were calculated using RSEM software (Li and Dewey, 2011). CDS prediction: Candidate coding regions within transcripts were identified using Transdecode software (Kim et al., 2015), and BLASTed against SwissProt and searched for Pfam protein homologous sequences were using the Hmmscan to predict coding regions. Gene annotation: Transcripts were annotated with seven major functional databases (KEGG, GO, NR, NT, SwissProt, Pfam, and KOG). WGCNA analysis: Gene co-expression networks were analyzed using WGCNA (v1.48). differentially expressed genes: Group difference gene analysis was performed using DESeq 2 (Love et al., 2014), with the condition that Fold Change ≥ 1 and padj value (after multiple correction) was less than 0.05. Based on GO annotation results and official classification, differentially expressed genes were classified functionally, and GO enrichment analysis was performed using the clusterProfile package (Wu et al., 2021). A threshold of qvalue ≤ 0.05 was used in where a definition of significant enrichment in candidate genes was met.

### 2.9 Validation of differential gene expression by qRT-PCR analysis

RNA extraction and cDNA synthesis: Total RNA was extracted from the frozen leaf samples with the MiniBEST Plant RNA Extration Kit (TaKaRa, China). The NanoDrop ND1000 spectrophotometer (Thermo, USA) was used to calculate the RNA concentration and assess purity. The RNA samples with a 260/280 nm absorbance ratio of 1.8–2.0 were retained for further analyses. The RNA integrity was evaluated by 1% agarose gel electrophoresis. The HiScript@III RT SuperMix for qPCR (Vazyme) was used to synthesize cDNA.The qRT-PCR assay was completed with SYBR qPCR Master Mix (Vazyme) and The LightCycle 480 Instrument II(Roche). The reaction solution consisted of 5 μL SYBR qPCR Master Mix (Vazyme), 4 μL cDNA (100 ng), 0.5 μL 10 µM forward primer, 0.5 μL 10 µM reverse primer for a final volume of 10 μL (**Table 1**). The amplification conditions were as follows: 95°C for 3min s; 40 cycles of 95°C for 10 s, 58°C for 10 s, and 72°C for 25 s, followed by a melting curve analysis from 60 to 95°C. The gene expression levels for each sample were determined based on three replicates.

**Table 1:** Primer sequences used in this study.

Primer name	Primer sequence
*EkTCP14*-QF *EkTCP14*-QR *EkTCP9-*QF *EkTCP9*-QR *EkACT2-*QF *EkACT2*-QR *EkSERK1*-QF *EkSERK1*-QR *EkACC2*-QF *EkACC2*-QR *EkSUS4*-QF *EkSUS4*-QR *EkSPL1*-QF *EkSPL1*-QR *EkARP8*-QF *EkARP8*-QR *EkCDKE-1*-QF *EkCDKE-1*-QR	5’-ATGGGAGATACCAAACCAAGTGAAA-3’ 5’-TCCACCTTTGTGTGTCTGTCCT-3’ 5’-CCGATGTGGGCGATGGT-3’ 5’-GAACTTGAATAGCGTTTGCCAT-3’ 5'-GCCATTCAGGCTGTTCTTTC-3' 5'-GGTAAGATCGCGACCTGCTA-3' 5'-CACTTTTCCTATTTGCTGTCGC -3' 5'-TTTACCAAACCCTCCTCTACCC -3' 5’-GAGACGAATCATACCGCTCACC-3’ 5’-GAGCGAAGAGCCGAACCTAC-3’ 5’-GCTTTCATTCCCTTTCCCGT-3’ 5’-AGAAGAGAGACTAATTCGTTGCG-3’ 5’-TGGATTTCTGGGGGCATAG-3’ 5’-CCAATAAACCAAACCAAGCCTT-3’ 5’-TTGGGATACTATTGTTTTCGCC-3’ 5’-CGAGGCATATTTACTCCAACCA-3’ 5’-ATCTTCTACCCGCCCTACTTTT-3’ 5’-GCCTGTTGGGATTGTTGTTAGT-3’

### 2.10 Statistical analysis

Graphpad Prism 7 software was used to analyze the data. All numerical values were presented as mean ± SEM and the sequence type was indicated in the legends. Statistically significant differences between inter- and intraspecific distance were determined by pair using t-test, with *p* values < 0.05. The three-dimensional structure of TCP proteins were builtin SWISS-MODEL (https://swissmodel.expasy.org) (Waterhouse et al., 2018).

## 3 Results

### 3.1 Distinctive resources discovered in the wild population of *Epimedium koreanum*

**Figure 1:**
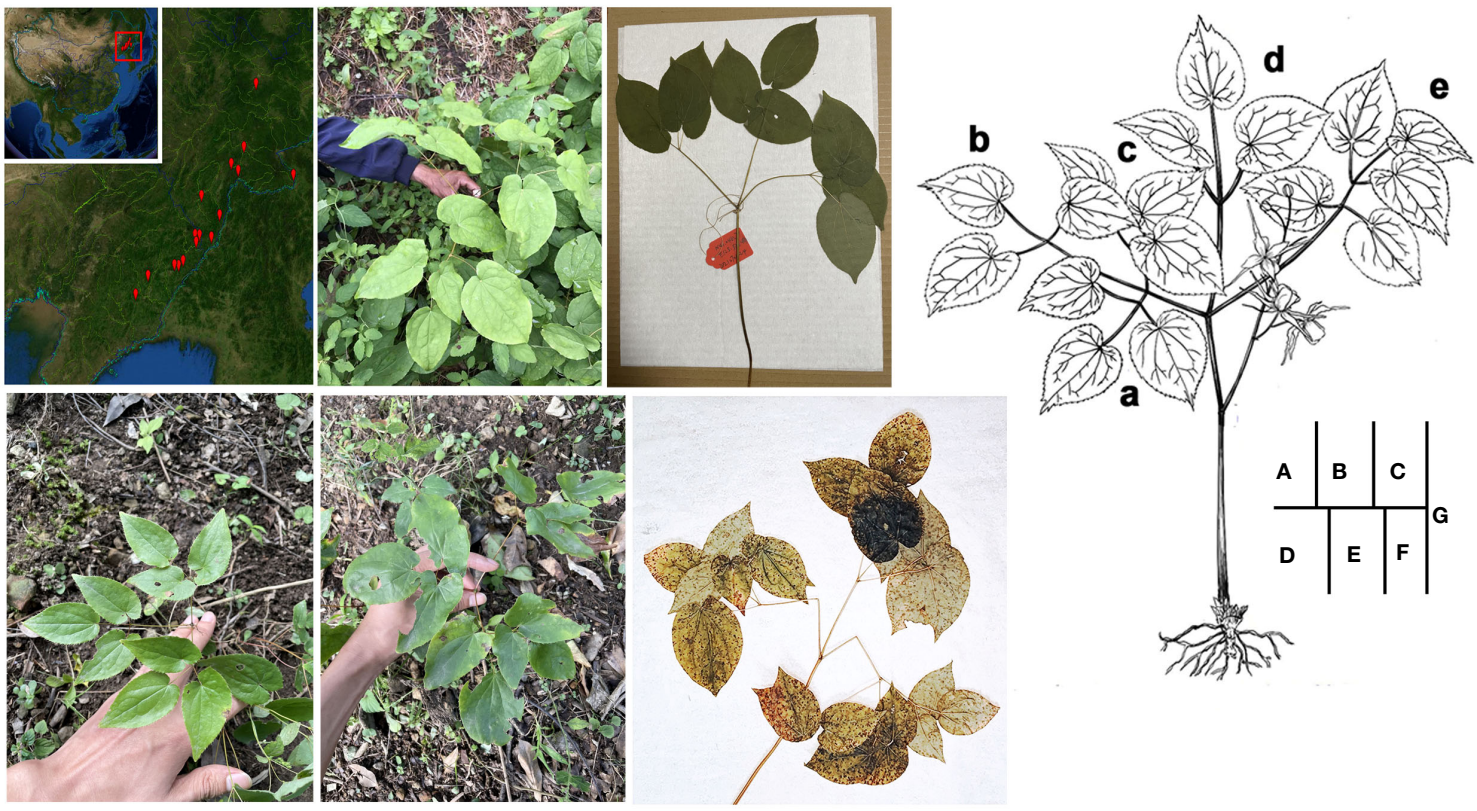
Discovery of a novel variety of *E koreanum*. **(A)** Red marks represent the areas where the *Epimedium* resources are collected; **(B)** The picture of wild *E koreanum* var. *polyphylla* CS Cheng with 11 blades; **(C)** Specimen of normal *E koreanum*; **(D)** Ex-situ cultivated normal *E koreanum* in Lushan botanical garden; **(E)** Ex-situ cultivated *E koreanum* var. *polyphylla* CS Cheng in Lushan botanical garden; **(F)** Specimen of *E koreanum* var.*polyphylla* CS Cheng with 24 blades; **(G)** Hand drawing of *E koreanum* var. *polyphylla* CS Cheng, the labels (a-e) represent different petiole areas.

Referring to the records of plant specimens collected in China mainland. The sample collection of resources of *E. koreanum* was implemented in August 2021, covering the whole areas along the Yalu River in Jilin and Liaoning provinces (**Figure 1A**). A distinctive wild population of *Epimedium* was discovered in Dunhua City Jilin Province (E: 128.0369, N: 43.1156, A: 670). The decovered newly resource contains 6 individuals which grow mixed with ordinary *E. koreanum* under the same masson pine forest. It was hard to tell whether a newly discovered exceptional resource belongs to a budding mutation or a new species on the spot. Its reproductive organs were not appreciably different from those of the typical *E. koreanum* (**Figures 1C, D**). The number of their leaves generally exceeds 9, and reaching a maximum of 27, its flower stem often has more than 1 biternate leaf, which is a significant feature of this population (**Figures 1B, E–G**). In details, this wild *Epimedium* is also a perennial herb, with 20-45 cm tall, rhizome creeping, triternate leaves, 11-27 foliolate, leaflets ovate, abaxially pallid but adaxially dark green, 6-16 × 4-12 cm, papery, glabrous, base deeply cordate with usually round lobes, margin minutely serrate, apex acute or acuminate. Flowering stem with 2-3 biternate leaf. Simple raceme inflorescence 12-17 cm with 6-16 flowered, glabrous. Pedicel 1-2 cm, flowers yellowish-white, 2-5 cm in diam. Outer sepals reddish or pale yellow, 5-8 mm, inner sepals narrowly ovate to lanceolate and apically acute. Petals nearly twice longer than the inner sepals, spurs slender, elongate and tapering subulate, 1-2 cm. The stamens ca 6.5 mm, anther ca 5 mm, filaments ca 1.5 mm,pistil ca 8 mm, ovary ca 5 mm, capsules 6-12 mm long, and 2-4 mm broad. Seeds usually 5-7. Fl. May, fr. May. Based on the phenotype of the newly resource with strong biomass advantage and the identification of medicinal plant taxonomy by expert Dr. Cheng Chung, we suspected that this is a variety of *E. koreanum*. So, we tentatively named the distinctive resource with *E. koreanum* var. *polyphylla* CS Cheng (EKP) in this study. This exceptional germplasm resource was grown ex-situ at the Lushan Botanical Garden. Moreover, after one year of phenological records performed, most of the phenotypes of complex leaf leaflets may still be maintained (**Figure 1E**), however the number of leaflets may fluctuate due to soil nutrition or climatic environment.

### 3.2 Molecular identification and phylogenetic tree analysis

**Figure 2:**
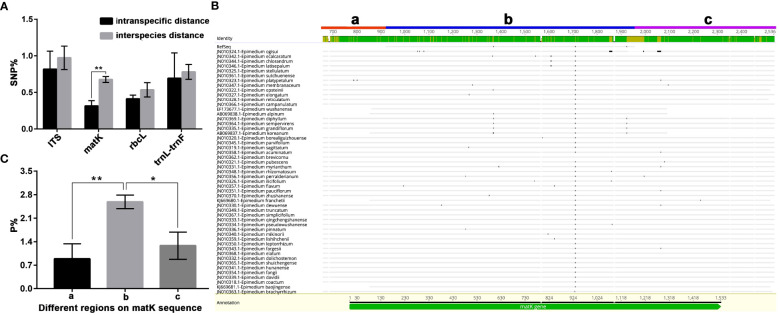
Scientific screening of commonly used DNA barcodes for assisted identification of Epimedium species. **(A)** The SNP statistics of *Epimedium* genus DNA barcodes within and between species; **(B)** Homologous alignment analysis of matK sequence in the chloroplast genome fingerprint region of Epimedium species, a and c represent the 3 ‘and 5’ ends of the matK sequence, respectively (prone to sequencing errors or deletions due to primers), and b represents the region with high degree of sequence homology in *Epimedium* genus; **(C)** The distribution and statistics of SNPs in the *matK* sequence of the genus *Epimedium*. Data presented as means ± SEM (*n* > 6). T-test, **p < 0.01 *p < 0.05; the dots represent single nucleotide mutation sites, with the first sequence as a reference.

The morphological identification of *Epimedium* species and the molecular classification based on DNA barcoding are both challenging (Zhang et al., 2016; Ren et al., 2018; Zhang et al., 2022). However, the application of DNA sequencing and barcoding is undoubtedly crucial for evaluating a new plant resource. In this study, the applied DNA sequences (DNA barcodes) (Vijayan and Tsou, 2010; Kim et al., 2016), including *ITS*, *matK*, *rabL* and *trnL-trnF* were evaluated for usability by using the degree of differences in DNA sequences within and between species, also known as intraspecific distance and interspecific distance. According to the DNA sequences statistics of *Epimedium* plants published by NCBI, only *matK* sequence was considered to be suitable for next step of genetic analysis and species identification of the new plant resource, because its interspecific difference was significantly higher than intraspecific difference (P<0.01, N>6) (**Figure 2A**). The other investigated sequences included *ITS*, *rabL*, and*trnL-trnF*, although all showed an average interspecific distance greater than the average intraspecific distance, but the *t-test* result showed no significant differences (P > 0.05, N > 6). Herein, specific primers were designed at the beginning and end of the referenced sequence to maximize the amplification of the *matK* sequence. In order to balance the credibility of public sequences with the longest possible sequence length, the referenced matK sequences were artificially divided into three parts (a, b, and c), differential DNA base count results showed that part b covers the most SNPs in *Epimedium* plants (N > 50, P < 0.001) (**Figures 2B, C**).

**Figure 3:**
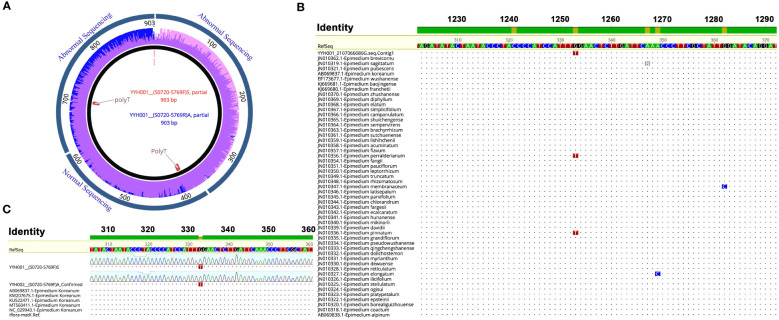
Discovery of mutation sites on the*matK* sequence of *E koreanum* var. *polyphylla* CS Cheng. **(A)** The sequencing electropherogram of the *matK* sequence of *E koreanum* var. *polyphylla* CS Cheng. **(B)** Alignment of *matK* sequences among species of the *Epimedium* genus. **(C)** Alignment of *matK* sequences from *E koreanum*.

**Figure 4:**
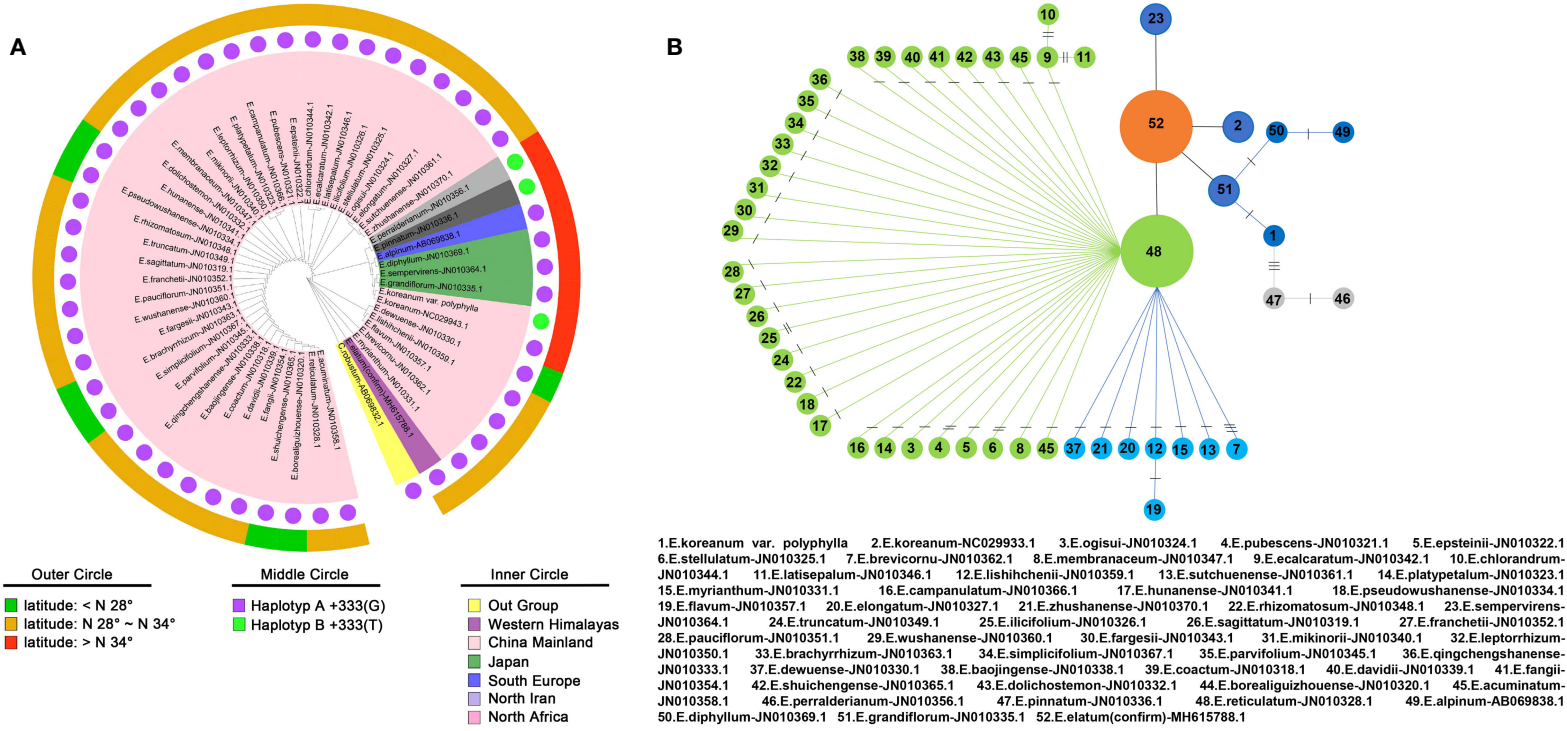
Research on the monophyletic origin of the Epimedium genus. **(A)** Phylogenetic tree based on the maximum likelihood method. **(B)** Haplotype parsimony network analysis of the diversity of 52 matK sequences in the Epimedium genus.

The PCR products were sequenced by Sanger sequencing and verified by positive and negative sequencing (**Figure 3A**). A specific homozygous mutation (T) was generated at site 333, compared to the typical *E. koreanum* population (G) (**Figures 3B, C**). Mutations specific to this site appeared to be rare, since all public *matK* sequences showed that only *E. perralderianum* and *E. pinnatum* from the highest latitudes or altitudes (N > 34°) have genotype T/T at this site (**Figures 3B**, **4A**, **S1**). This homozygous SNP site was obviously not created by sequencing errors (**Figure 3A**), and the function of the *matK* gene corresponding to the SNP at this site may be relevant to the evolution of high-latitude plant species. Overall, the confirmation of this discovered SNP verified our idea that this novel resource discovered in wild *E. koreanum* population can be identified as a variety. Although the study of the novel SNP and its kinase function corresponding to the matK gene belongs to an interest scientific issue, as one of the rapidly evolving genes in plant chloroplast genome, matK sequence is often used to assist in the identification of species below plant genera. Therefore, in this study, only the phylogenetic tree of matK sequence was discussed.

As shown in **Figure 4A**, the maximum likelihood method based phylogenetic tree was conducted and revealed that the monophyletic origin in *Epimedium* genus, which was consistent with other reports (Zhang et al., 2007; Guo et al., 2022). An important group including the *E. koreanum*, which is entirely located at high latitudes and is of particular concern to this study. There were 8 *Epimedium* species in this group, including *E. pinnatum, EKP*, *E. grandiflorum, E. sempervirens, E. diphyllum, E. alpinum, E. pinnatum* and *E. perralderianum.* Furthermore, haplotype parsimony network analysis of matK sequences diversity obtained from sampling 52 sequences (**Figure 4B**). The network analysis revealed a more distinct genealogical connect among the eight Epimedium species indicated above, which are found in high latitudes all over the world.

### 3.3 DEGs analysis for exploring molecular mechanism of leaflets increasing

**Figure 5:**
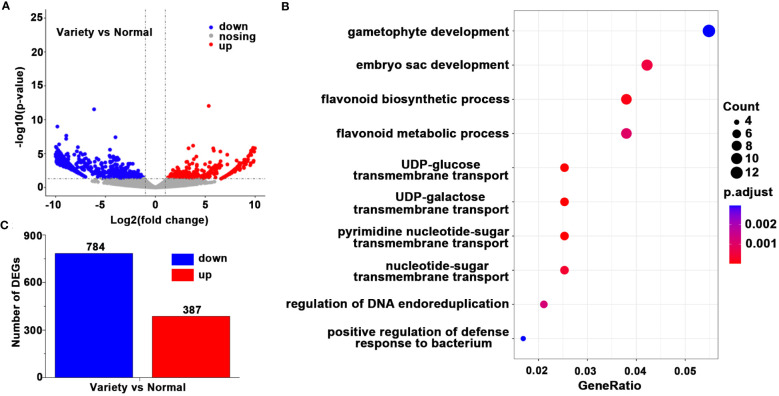
The differences in transcription expression levels between *E koreanum* var. *polyphylla* CS Cheng and *E koreanum*. **(A)** The volcano plots. **(B)** The number of DEGs. **(C)** GO enrichment analysis of DEGs.

We investigated the differences in transcription expression levels between *EKP* and *E. koreanum* by second-generation transcriptome sequencing. We used Trimmed Mean of M-values (TMM) to analyze gene transcription levels. The volcano map showed the number of differenced genes between *EKP* and normal *E. koreanum*, and showed significantly up-regulated, down-regulated, and non-significant genes in different colors (**Figure 5A**). The genes that were considerably up-regulated and down-regulated in *EKP* were statistically analyzed in a bar chart, and it was found that 387 genes were significantly up-regulated and 784 genes were significantly down-regulated when compared to the typical *E. koreanum* (**Figure 5C**). Table was used to show DEGs in this study (**Table S1**). With these transcripts, we then performed GO enrichment analysis, and we chose the top 10 modules to present (**Figure 5B**). It was discovered that DEGs were primarily enriched in the development of gametophytes, embryo sacs, flavonoid biosynthetic pathways, and flavonoid metabolic pathways. The majority of the genes involved in the differentiation and proliferation of leaf primordium cells were associated with gametophyte and embryo sac development. The involvement of plant hormones such as auxin and ethylene is widely regarded as important roles in the synergistic regulation of gametophyte and embryo sac development. So these findings provided new insights into *E. koreanum* leaf growth and flavonoid metabolism.

### 3.4 Weighted gene co-expression network analysis for discovery of leaf development related genes

**Figure 6:**
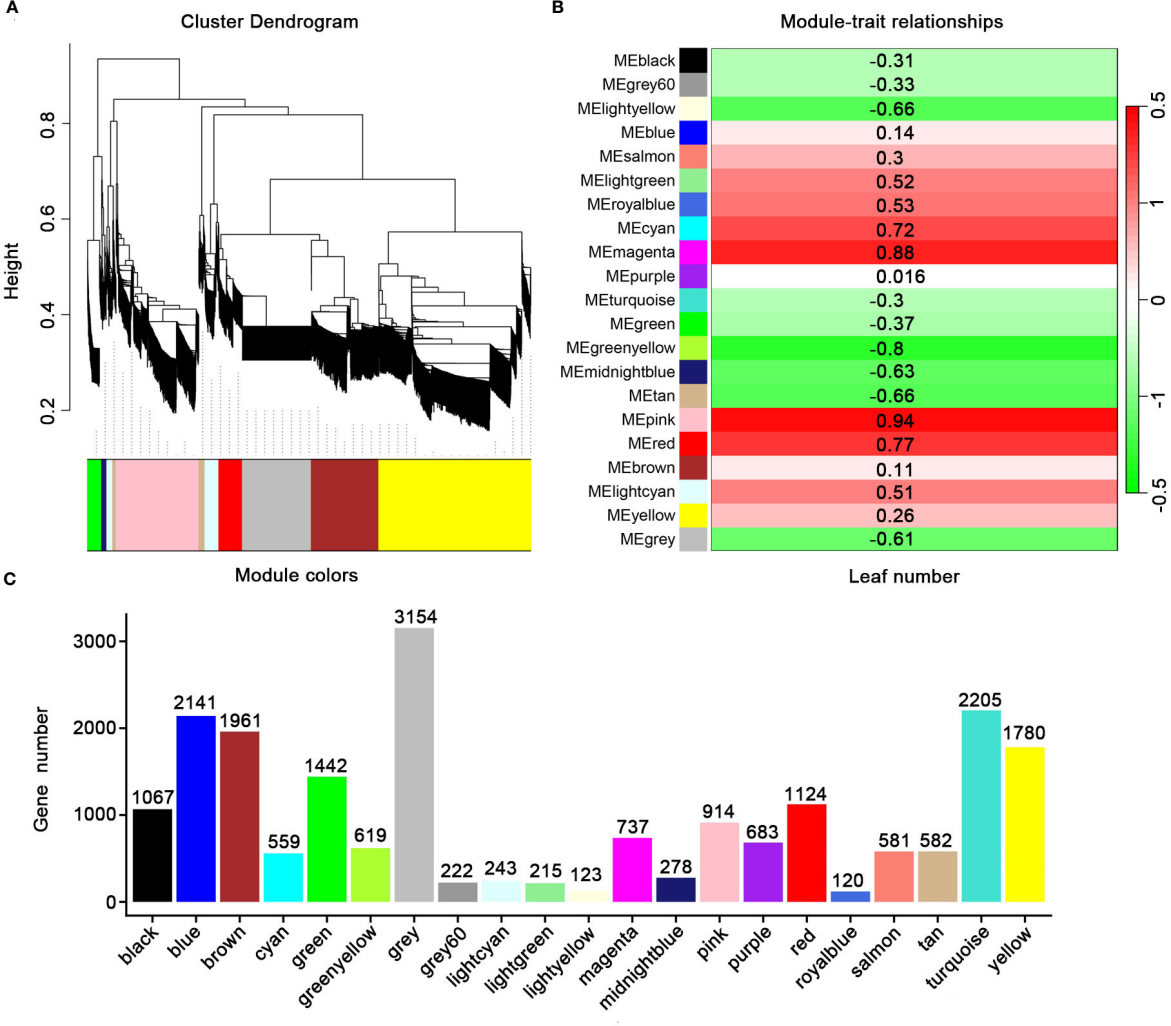
WGCNA analysis of genes related to leaf biomass in *E koreanum*. **(A)** Clustering dendrogram of genes. Similarity is based on topological overlap, with module colors assigned. The 21 co-expression modules are displayed in different colors. **(B)** Correlation between the 21 modules. **(C)** Member count in the 21 modules.

In order to identify the DEGs related to leaf development in *E. koreanum*, we further analyzed leaf development as a trait by WGCNA (Langfelder and Horvath, 2008). Twenty-one modules were discovered and colored differently. The twenty-one modules’ gene counted ranged from 120 to 3154 (**Figures 6A, C**). In this research, we focused on the two main modules. The pink module involved 914transcripts, was positively correlated with leaf development. The green yellow module involved 619 transcripts were negatively correlated with leaf development (**Figure 6B**). Thus, these results provided a further understanding of leaf development of *E. koreanum*.

### 3.5 Identification of hub genes regulate to the leaf development in *E. koreanum*

**Figure 7:**
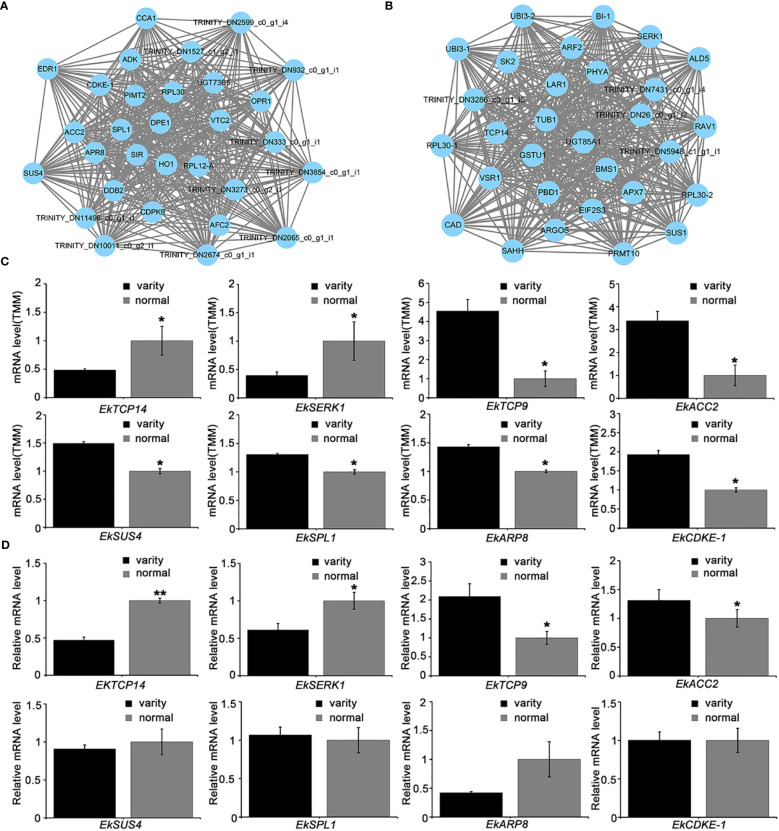
Visual analysis of modules and expression of hub gene transcripts. **(A)** Hub genes in the pink module. **(B)** Hub genes in the greenyellow module; **(C)** Transcript levels of part hub genes determined by RNA-seq. TMM, Trimmed Mean of M-values; **(D)** Validation of differential gene expressions by qRT-PCR analysis. T-test, **p < 0.01 *p < 0.05.

We performed a visual network analysis of these two modules, identified the top 30 most reliable nodes for visualization, and discovered that these hub genes were involved in leaf development (**Figures 7A, B**). Because no whole genome data for *E. koreanum* is now publicly available, we used homologous alignment to annotate these genes and discovered *TRINITY_DN4142_c0_g1_i1*, a *TCP14* transcription factor homologous gene in green yellow module, which has previously been reported to inhibit cell proliferation in leaf tissues (Kieffer et al., 2011). Besides, we also found other genes may involve in regulating leaf development, such as *SERK1* in pink module, *SPL1*, *ARP8* and *SUS4* in green yellow module. Although the homologous genes of these genes in other species have rarely been reported to regulate leaf development in other species, they may be involved in leaf development in *Epimedium*. The expression levels of these genes were shown in **Figure 7C**.

Based on the visual network’s cues, we used the *TCP* conserved domain homologous comparison method to identify potential *TCP* transcription factors from our transcript data and demonstrated their expression levels (**Figure 7C**). The transcription of *TRINITY_DN4142_c0_g1_i1* was found to be lower in *E. koreanum* var. *polyphylla* CS Cheng than in the typical *E. koreanum*, the decrease of *EkTCP14* expression may promote the proliferation of cells and thus increase the number of leaves (Kieffer et al., 2011; Challa et al., 2021) (**Figure 7C**). Additionally, we discovered another *TCP9* similar gene, *TRINITY_DN3273_c2_g1_i2*, which is a member of the class I *TCP* family and whose transcripts were up-regulated in *E. koreanum* var. *polyphylla* CS Cheng. Class I *TCP* usually promotes cell proliferation, so its increased expression in *EKP* is also consistent with the phenotype (Li, 2015). So, these results first suggested that different TCP transcription factors have antagonistic effects on compound leaf development.

In this study, the *EkTCP* genes and other hub genes mentioned in **Figure 7D** were then validated by qRT-PCR analysis. The results showed that the gene expression of *EkTCP14, EkTCP9, EkSERK1 and EkACC2* was consistent with that in RNA-Seq analysis (t-test: N=3, **P < 0.01; *P < 0.05), but the expression of other 4 genes showed no significant difference compared to the discovered newly variety and the normal *E. koreanum*. We also investigated the sequence differences of *EkTCP* between the variety and normal *E.koreanum*, as well as the probable changes in spatial structure and evolutionary relationships. The results showed that *EkTCP9* and *EkTCP14* had multiple SNP sites, however, SNP sites were showed not in the *EkTCP* domain, and only the spatial structure of *EkTCP9* was slightly changed, so we speculated that there was no significant difference in the function of *EkTCP9* and *EkTCP14* (**Figures S2A, B**). In addition, we also carried out evolutionary analysis on the *EkTCP* of berberaceae, and found that the variation of *EkTCP* did not change much in evolution, were closest relatives to *Papaver somniferum* and *Coptis chinensis* (**Figure S2C**). Overall, the findings in this work implied that *EkTCP* transcription factors may play an essential role in controlling *E. koreanum* leaf development.

## 4 Discussion

### 4.1 Discovery and identification of new variety of *Epimedium koreanum*

*E. koreanum* is a commonly used Chinese herb, and the *Ch. P* records five species of *Epimedium*, which include *E. koreanum*, *E. wushanense*, *E. sagittatum*, *E. pubescens*, and *E. brevicornu*. Our research team has been committed to comprehensively collecting wild *Epimedium* resources on a large scale and across latitudes. During our investigations in the growing area of *E. koreanum* in Dunhua, Jilin Province, we discovered a striking variety of *Epimedium* exhibiting more than 9 and up to 27 leaflets. Through meticulous DNA barcoding analysis, this variety was conclusively identified as a distinctive variety of *E. koreanum* with a remarkable leaf yield. The distribution of this variety within its growing area appears to occur in concentrated patches. Notably, the *Ch. P* has recognized the aboveground parts of *E. koreanum* as a clinical use of Chinese medicine, with leaves gradually replacing other therapeutic portions since 2010 (Qian et al., 2023). The increased number of leaflets in this newly discovered variety significantly enhances its application value. Furthermore, and the utilization of this resource on breeding and cultivation may contribute to alleviating the existing shortage of *E. koreanumin* the herb market. It is conceivable that the yield of this variety will be much higher than that of other Epimedium species. So, we used comparative RNA-Seq analysis to investigate the process of leaf development after discovering the novel *E. koreanum* varieties.

Currently, the descovered newly variety of *E. koreanum* has been cultivated in the Lushan Botanical Garden for one year, completing a phenological observation of the reproductive cycle. Although the newly identified germplasm still has an advantage in terms of the number of leaflets, the number of leaflets in ex situ grown plants has reduced in comparison to the original plants. The instability in the number of small leaf blades was observed, pointing to an innovative field of investigation into the molecular mechanism of complex leaf development.

### 4.2 Identification of hub genes regulating leaf biomass in *E. koreanum* var. *polyphylla* CS Cheng

The regulation of leaf development has been extensively studied in various plant species, including *Arabidopsis* (Koyama et al., 2017), tomato (Ori et al., 2007), andmedicago (He et al., 2020). However, the complete genome sequence of *E. koreanum* is not yet available, making the functional study of its genes particularly challenging. *Epimedium* species, including *E. koreanum*, possess relatively large genomes and undergo numerous interspecific hybridizations, further complicating gene function analysis. To overcome these challenges, we employed RNA-seq, a powerful technique that allows for the analysis of gene expression and phenotypic characteristics. In this study, we performed comparative RNA-seq analysis on different varieties of *E. koreanum* and a control sample of normal *E. koreanum*. Our analysis identified 387 up-regulated genes and 784 down-regulated genes (**Figure 1A**). Actually, we intended to uncover essential genes with significant alterations in the hot reported genes associated with plant compound leaf production, and then confirm their function using RT-PCR with a larger sample size (**Table S2**), However, we found that none of the 10 hot genes mentioned in the study had significant differences between the newly resources and the typical *E. koreanum*. To gain insights into the functional implications of these differentially expressed genes, we conducted Gene Ontology (GO) enrichment analysis, which revealed the top ten most enriched gene categories (**Figure 1B**). These findings significantly contributed to our understanding of *E. koreanum*’s flavonoid metabolism and leaf growth processes.

Furthermore, we employed weighted gene co-expression network analysis (WGCNA) to uncover the modular structure and regulatory relationships among the identified genes. By performing network analysis, we successfully identified several hub genes that play a pivotal role in the regulation of leaf development in *Epimedium* (**Figure 7A**). Notably, our investigation highlighted the relevance of *TCP* family genes in leaf development, a finding supported by studies in other plant species. *TCP* genes have been demonstrated to affect various aspects of leaf development, such as leaf shape (Palatnik et al., 2003; Qin et al., 2005; Schommer et al., 2008), size (Efroni et al., 2008; Tao et al., 2013), senescence (Danisman et al., 2013) and complexity (Koyama et al., 2010; Rubio-Somoza et al., 2014; Viola et al., 2023). Based on the integration of RNA-seq data and bioinformatics analysis, we made a significant discovery by identifying hub genes that are crucial for the regulation of leaf growth and development in *Epimedium*. These findings enhance our understanding of the molecular mechanisms underlying leaf growth and hold promise for potential applications in *Epimedium* breeding and leaf biomass improvement.

Currently, Transcriptome sequencing is the most effective approach for identifying differentially expressed genes in wild samples. It is required to investigate the spatiotemporal expression of genes involved to compound leaf development using real-time fluorescence quantification under an environmental control conditions. Specifically, absolute quantification of gene expression levels in samples with a gradient in leaflet number can assist discover the critical genes and regulatory mechanisms determining compound leaf biomass. In our validation study of differential gene expressions by using qRT-PCR analysis under an equal environmental control conditions, the results showed that the gene expression of EkTCP14, EkTCP9, EkSERK1 and EkACC2 was obvious consistent with that in RNA-Seq analysis, but the other 4 gene expressions showed no significant difference compared to the discovered newly variety and the normal *E. koreanum.* We speculate that the reason for this predicament is that direct transcriptome sequencing utilizing wild plant samples might provide some false positive findings. Therefore, the results of the qRT-PCR investigation revealed the regulatory involvement of the *EkTCP* transcription factors in the development of compound leaves.

### 4.3 Phytohormone regulates leaf development

Leaf development in plants is regulated by various phytohormones, including auxin and cytokinin (Navarro-Cartagena and Micol, 2023). The morphology of leaves varies across different plant species, primarily due to the distinct arrangement of leaf lobes or teeth in single leaves and leaflets in compound leaves (Hay and Tsiantis, 2006; Runions et al., 2017; Kierzkowski et al., 2019). Cytokinin plays a significant role in regulating leaf complexity in compound leaf species such as tomato and cardamine (*Cardamine hirsuta*). Additionally, in simple leaf species like *Arabidopsis thaliana*, both cytokinin and auxin co-regulate the morphogenesis of leaf margins. Transcription factors belonging to the *TCP* family are essential for maintaining the balance between cell proliferation and differentiation during leaf development. Class I *TCP* family members promote cell proliferation, while class II family members repress cell proliferation (Li, 2015). In *Arabidopsis*, Class I TCPs have been found to promote cytokinin responses (Steiner et al., 2012; Steiner et al., 2016).Notably, the up-regulation of Class II *TCPs* and *KNOX2* genes has been shown to repress the expression levels of *KNOX1* and *CUP-SHAPED COTYLEDON*(*CUC2*), thereby enhancing cytokinin response in Nicotiana tabacum *BY-2* protoplasts and increasing the levels of active cytokinin (Cucinotta et al., 2018; Cucinotta et al., 2020; Challa et al., 2021).

Our findings indicate that the expression of *TCPs* in *EKP* significantly differed from that of normal *E. koreanum*. This suggested that abnormal leaf development in *EKP* might be regulated through phytohormone mediated abnormal expression of *TCPs*. However, it is important to note that we were unable to identify any genes directly involved in cytokinin regulation in this study. Although these genes may potentially exist, their specific functions remain unknown due to the lack of comprehensive genomic information. In conclusion, our study highlights the crucial roles of auxin and cytokinin in regulating leaf development. The complex interplay between phytohormones and key transcription factors, such as *TCPs*, is fundamental for determining leaf morphology and complexity across plant species. Further research is needed to unravel the precise mechanisms underlying cytokine in regulation and its direct genetic targets, which will contribute to a comprehensive understanding of leaf development in plants.

## 5 Conclusion

In this study, we have discovered a new class of *Epimedium* resources. Through DNA barcoding and phylogenetic tree analysis, it is believed that it belongs to the *E. koreanum* variety. This discovery not only identified a high leaf biomass of *Epimedium* and expanded the number of varieties of *Epimedium*, but also alleviated the current situation of resource shortage of *Epimedium*. Then, we analyzed the hub genes regulating the leaf biomass of *E. koreanum* through RNA-seq data. Based on the literature reports of related homologous genes and validation of differential gene expressions by qRT-PCR analysis, we suggested that *EkTCP9* and *EkTCP14* transcription factors play an important role in the leaf development of *E. koreanum*. The level of their expression was in line with expectations. We also investigated the SNP sites and possible spatial structure of these two *EkTCP*, and found that the *EkTCP* of these two *E. koreanum* has multiple SNP sites, but there was no significant effect on their spatial structure. In conclusion, our study not only found a new variety of *E. koreanum*, but also preliminarily analyzed the hub genes that regulate leaf development, providing new insights for future breeding of *Epimedium* with high leaf biomass.

## Data availability statement

The datasets presented in this study can be found in online repositories. The names of the repository/repositories and accession number(s) can be found below: Bioproject accession number PRJNA1008345.

## Author contributions

JY: Investigation, Writing – original draft, Formal analysis, Methodology, Software. SF: Investigation, Methodology, Software, Data curation. ZX & MG: Methodology, Validation. QC: Methodology, Validation, Investigation, Software. PG: Writing – review & editing. CC: Investigation, Writing – review & editing, Conceptualization, Funding acquisition, Project administration, Resources, Supervision, Writing – original draft.

## Supplementary material

Supplementary Figure 1 Distribution of *E. perralderianum, E. pinnatum*and *E. koreanum.* The distribution of three species of *Epimedium* on the map. The red triangles represent different sample aera.

Supplementary Figure 2 Evolutionary relationships of TCP-containing proteins of *E. koreanum.* **(A)** SNP sites of *EkTCP9* and *EkTCP14. EkTCP14-1* and *EkTCP9-1* extracted from *E. kroeanum*, *EkTCP14-2* and *EkTCP9-2* extracted from *E. koreanum*var.*polyphylla* CS Cheng. **(B)**The spatial structure of TCP9 and TCP14.The red arrows represent the distinct areas. **(C)** The optimal tree with the sum of branch length = 14.79 is shown. The tree is drawn to scale, with branch lengths in the same units as those of the evolutionary distances used to infer the phylogenetic tree. The evolutionary distances are in the units of the number of amino acid substitutions per site. The analysis involved 298 amino acid sequences with *TCP* domains, of which 294 were from the order Ranunculales. All positions containing gaps and missing data were eliminated.

Supplementary Table 1 Differentially expressed transcripts in variety Compared with normal *E. koreanum.*
