# Supplementary figures and images for: DNA barcoding and comparative RNA-Seq analysis provide new insights into leaf formation using a novel resource of high-yielding Epimedium koreanum
Source: Front Plant Sci. 2023 Dec 18;14:1290836. doi: 10.3389/fpls.2023.1290836 (PMC10760978; doi:10.3389/fpls.2023.1290836)

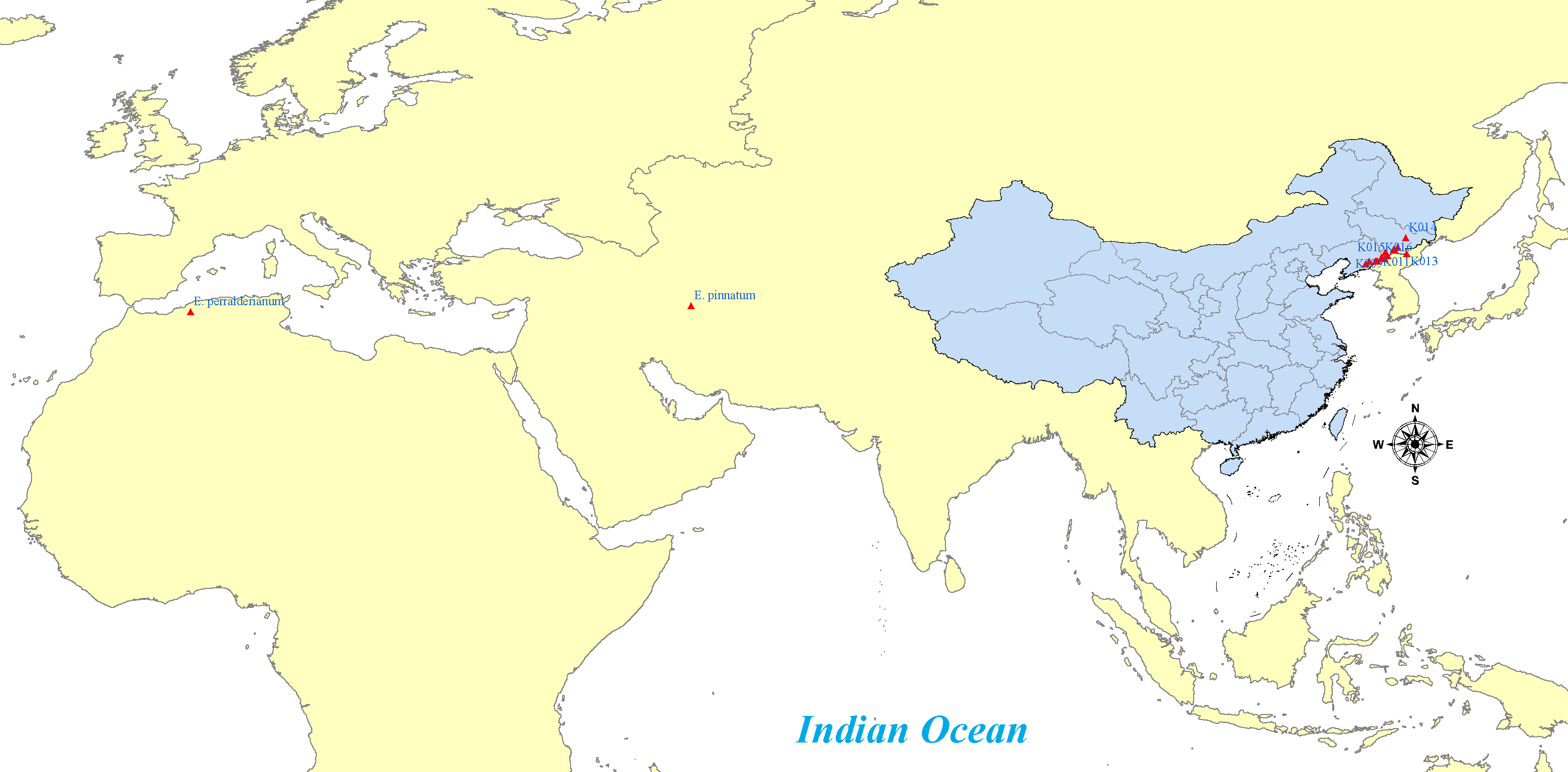

Supplement: Supplementary Figure 1 — Distribution of E. perralderianum, E. pinnatumand E. koreanum. The distribution of three species of Epimedium on the map. The red triangles represent different sample aera. [file Image_1.jpeg]

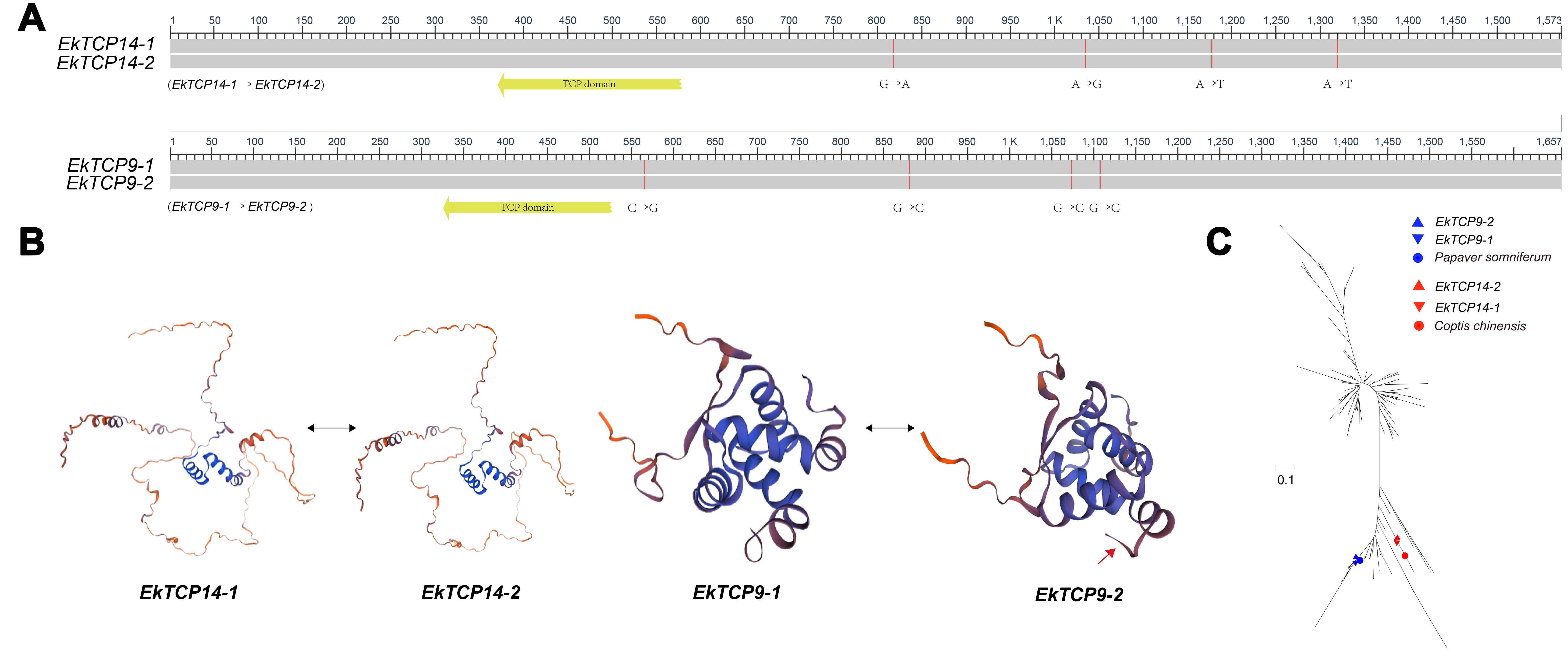

Supplement: Supplementary Figure 2 — Evolutionary relationships of TCP-containing proteins of E. koreanum. (A) SNP sites of EkTCP9 and EkTCP14. EkTCP14-1 and EkTCP9-1 extracted from E. kroeanum, EkTCP14-2 and EkTCP9-2 extracted from E. koreanumvar.polyphylla CS Cheng. (B)The spatial structure of TCP9 and TCP14.The red arrows represent the distinct areas. (C) The optimal tree with the sum of branch length = 14.79 is shown. The tree is drawn to scale, with branch lengths in the same units as those of the evolutionary distances used to infer the phylogenetic tree. The evolutionary distances are in the units of the number of amino acid substitutions per site. The analysis involved 298 amino acid sequences with TCP domains, of which 294 were from the order Ranunculales. All positions containing gaps and missing data were eliminated. [file Image_2.jpeg]
