# Supplementary material for: DNA barcoding and comparative RNA-Seq analysis provide new insights into leaf formation using a novel resource of high-yielding Epimedium koreanum
Source: Front Plant Sci. 2023 Dec 18;14:1290836. doi: 10.3389/fpls.2023.1290836 (PMC10760978; doi:10.3389/fpls.2023.1290836)
Supplement: Supplementary file 4 [file Table_2.docx]

**Table S2.** Blastp and difference analysis of reported hot genes related to plant compound leaf formation in RNA-Seq results of this study

| Reported hot genes related to plant compound leaf formation (NCBI ID) | | The homologous sequence in RNA-Seq data | Gene expression difference |
| --- | --- | --- | --- |
| KANT2 | at1g70510 | TRINITY_DN1571_c0_g1_i5 | N |
|  |  | TRINITY_DN1571_c0_g1_i7 | N |
|  |  | TRINITY_DN1571_c0_g1_i4 | N |
|  |  | TRINITY_DN1571_c0_g1_i3 | N |
|  |  | TRINITY_DN1571_c0_g1_i2 | N |
| BP | at4g08150 | TRINITY_DN1571_c0_g1_i5 | N |
|  |  | TRINITY_DN1571_c0_g1_i3 | N |
|  |  | TRINITY_DN1571_c0_g1_i7 | N |
|  |  | TRINITY_DN1571_c0_g1_i4 | N |
|  |  | TRINITY_DN1571_c0_g1_i2 | N |
| KNAT6 | at1g23380 | TRINITY_DN1571_c0_g1_i5 | N |
|  |  | TRINITY_DN1571_c0_g1_i3 | N |
|  |  | TRINITY_DN1571_c0_g1_i7 | N |
|  |  | TRINITY_DN1571_c0_g1_i4 | N |
|  |  | TRINITY_DN1571_c0_g1_i2 | N |
| STM | at1g62360 | TRINITY_DN1571_c0_g1_i5 | N |
|  |  | TRINITY_DN1571_c0_g1_i3 | N |
|  |  | TRINITY_DN1571_c0_g1_i7 | N |
|  |  | TRINITY_DN1571_c0_g1_i4 | N |
|  |  | TRINITY_DN1571_c0_g1_i2 | N |
| PALM1 | MtrunA17_Chr5g040057 | None | NA |
| SGL1 | Newentry by Eukaryota | None | NA |
| AS1 | at2g37630 | TRINITY_DN30659_c0_g1_i3 | N |
|  |  | TRINITY_DN3413_c0_g1_i4 | N |
|  |  | TRINITY_DN5357_c0_g1_i3 | N |
|  |  | TRINITY_DN6550_c0_g1_i6 | N |
|  |  | TRINITY_DN6550_c0_g1_i5 | N |
|  |  | TRINITY_DN3413_c0_g1_i5 | N |
|  |  | TRINITY_DN18224_c0_g1_i1 | N |
|  |  | TRINITY_DN256_c0_g1_i6 | N |
|  |  | TRINITY_DN256_c0_g1_i4 | N |
|  |  | TRINITY_DN256_c0_g1_i2 | N |
|  |  | TRINITY_DN13942_c0_g1_i3 | N |
|  |  | TRINITY_DN6521_c0_g1_i1 | N |
|  |  | TRINITY_DN101244_c0_g1_i1 | N |
|  |  | TRINITY_DN1713_c0_g1_i2 | N |
|  |  | TRINITY_DN30659_c0_g1_i6 | N |
|  |  | TRINITY_DN22311_c0_g2_i1 | N |
|  |  | TRINITY_DN8091_c0_g1_i2 | N |
|  |  | TRINITY_DN4002_c0_g1_i7 | N |
|  |  | TRINITY_DN4002_c0_g1_i6 | N |
|  |  | TRINITY_DN4002_c0_g1_i1 | N |
|  |  | TRINITY_DN967_c1_g1_i1 | N |
|  |  | TRINITY_DN7228_c0_g1_i4 | N |
|  |  | TRINITY_DN7228_c0_g1_i2 | N |
|  |  | TRINITY_DN7228_c0_g1_i1 | N |
|  |  | TRINITY_DN7228_c0_g1_i3 | N |
|  |  | TRINITY_DN4002_c0_g1_i9 | N |
|  |  | TRINITY_DN4002_c0_g1_i2 | N |
|  |  | TRINITY_DN5654_c0_g1_i2 | N |
|  |  | TRINITY_DN222592_c0_g1_i1 | N |
|  |  | TRINITY_DN967_c1_g1_i4 | N |
|  |  | TRINITY_DN967_c1_g1_i6 | N |
|  |  | TRINITY_DN4016_c0_g1_i3 | N |
|  |  | TRINITY_DN967_c1_g1_i3 | N |
|  |  | TRINITY_DN967_c1_g1_i2 | N |
|  |  | TRINITY_DN4016_c0_g1_i2 | N |
|  |  | TRINITY_DN3413_c0_g1_i9 | N |
|  |  | TRINITY_DN967_c1_g1_i7 | N |
|  |  | TRINITY_DN967_c1_g1_i8 | N |
|  |  | TRINITY_DN8893_c1_g1_i1 | N |
|  |  | TRINITY_DN9454_c0_g1_i1 | N |
|  |  | TRINITY_DN4205_c1_g1_i1 | N |
|  |  | TRINITY_DN6521_c0_g1_i2 | N |
|  |  | TRINITY_DN1271_c0_g1_i1 | N |
|  |  | TRINITY_DN1884_c0_g1_i3 | N |
|  |  | TRINITY_DN31366_c0_g1_i1 | N |
|  |  | TRINITY_DN1884_c0_g1_i1 | N |
|  |  | TRINITY_DN31366_c0_g1_i2 | N |
|  |  | TRINITY_DN140511_c0_g1_i1 | N |
|  |  | TRINITY_DN8937_c0_g1_i2 | N |
|  |  | TRINITY_DN13942_c0_g1_i2 | N |
|  |  | TRINITY_DN107631_c0_g1_i1 | N |
|  |  | TRINITY_DN6521_c0_g1_i5 | N |
|  |  | TRINITY_DN228690_c0_g1_i1 | N |
|  |  | TRINITY_DN21528_c0_g1_i2 | N |
|  |  | TRINITY_DN195118_c0_g1_i1 | N |
|  |  | TRINITY_DN29644_c0_g1_i3 | N |
|  |  | TRINITY_DN29644_c0_g1_i3 | N |
|  |  | TRINITY_DN29644_c0_g1_i4 | N |
|  |  | TRINITY_DN29644_c0_g1_i4 | N |
|  |  | TRINITY_DN29644_c0_g1_i1 | N |
|  |  | TRINITY_DN29644_c0_g1_i1 | N |
|  |  | TRINITY_DN4016_c0_g1_i4 | N |
|  |  | TRINITY_DN256_c0_g1_i7 | N |
|  |  | TRINITY_DN256_c0_g1_i5 | N |
|  |  | TRINITY_DN256_c1_g2_i1 | N |
| LFY | at5g61850 | None | NA |
| YUCCA1 | at4g32540 | TRINITY_DN222417_c0_g1_i1 | N |
|  |  | TRINITY_DN20595_c0_g1_i1 | N |
|  |  | TRINITY_DN1813_c5_g2_i1 | N |
|  |  | TRINITY_DN1813_c5_g2_i2 | N |
|  |  | TRINITY_DN202158_c0_g1_i1 | N |
|  |  | TRINITY_DN94242_c0_g1_i1 | N |
|  |  | TRINITY_DN81817_c0_g1_i1 | N |
|  |  | TRINITY_DN81817_c0_g1_i2 | N |
|  |  | TRINITY_DN20595_c0_g1_i2 | N |
|  |  | TRINITY_DN27429_c0_g1_i1 | N |
|  |  | TRINITY_DN107865_c0_g1_i1 | N |
|  |  | TRINITY_DN81817_c0_g1_i8 | N |
|  |  | TRINITY_DN81817_c0_g1_i4 | N |
|  |  | TRINITY_DN81817_c0_g1_i7 | N |
|  |  | TRINITY_DN44477_c0_g1_i1 | N |
|  |  | TRINITY_DN42388_c0_g3_i1 | N |
|  |  | TRINITY_DN28259_c0_g1_i1 | N |
|  |  | TRINITY_DN41091_c0_g1_i1 | N |
|  |  | TRINITY_DN54689_c0_g1_i1 | N |
|  |  | TRINITY_DN137664_c0_g1_i1 | N |
|  |  | TRINITY_DN24032_c0_g1_i2 | N |
|  |  | TRINITY_DN24032_c0_g1_i1 | N |
|  |  | TRINITY_DN51022_c0_g1_i1 | N |
|  |  | TRINITY_DN13449_c0_g1_i1 | N |
|  |  | TRINITY_DN13449_c0_g1_i14 | N |
|  |  | TRINITY_DN13449_c0_g1_i7 | N |
|  |  | TRINITY_DN13449_c0_g1_i5 | N |
|  |  | TRINITY_DN13449_c0_g1_i13 | N |
|  |  | TRINITY_DN13449_c0_g1_i11 | N |
|  |  | TRINITY_DN13449_c0_g1_i4 | N |
|  |  | TRINITY_DN13449_c0_g1_i8 | N |
|  |  | TRINITY_DN263912_c0_g1_i1 | N |
| PINNA1 | MtrunA17_Chr3g0141931 | TRINITY_DN196348_c0_g1_i1 | N |
|  |  | TRINITY_DN34431_c0_g2_i1 | N |
