# Supplementary material for: DNA barcoding and comparative RNA-Seq analysis provide new insights into leaf formation using a novel resource of high-yielding Epimedium koreanum
Source: Front Plant Sci. 2023 Dec 18;14:1290836. doi: 10.3389/fpls.2023.1290836 (PMC10760978; doi:10.3389/fpls.2023.1290836)
Supplement: Supplementary Table 1 — Differentially expressed transcripts in variety Compared with normal E. koreanum. [file Table_1.docx]

| **transcript_id** | **logFC** | **pvalue** | **padj** | **Var_1** | **Var_2** | **Nor_1** | **Nor_2** | **Gene_Name** |
| --- | --- | --- | --- | --- | --- | --- | --- | --- |
| TRINITY_DN6164_c0_g1_i1 | -15.6421736 | 6.38E-10 | 8.02E-07 | 0 | 0 | 7.011 | 125.641 | - |
| TRINITY_DN14910_c0_g1_i1 | -14.8224767 | 5.46E-11 | 1.19E-07 | 0 | 0 | 76.906 | 15.412 | Glutaredoxin |
| TRINITY_DN542_c0_g1_i10 | -14.7637518 | 1.12E-15 | 3.66E-11 | 0 | 0 | 605.719 | 1182.496 | NA |
| TRINITY_DN615_c0_g1_i11 | -14.5159924 | 1.04E-10 | 1.80E-07 | 0 | 0 | 17.588 | 123.781 | NA |
| TRINITY_DN2478_c0_g1_i2 | -14.4054858 | 4.94E-15 | 8.07E-11 | 0 | 0 | 23.865 | 17.865 | NA |
| TRINITY_DN1329_c1_g1_i1 | -13.8525117 | 8.57E-06 | 0.001608537 | 0 | 0 | 117.304 | 0.935 | NA |
| TRINITY_DN1074_c0_g1_i3 | -13.2339193 | 7.13E-08 | 3.19E-05 | 0 | 0 | 54.797 | 6.304 | NA |
| TRINITY_DN2610_c0_g1_i2 | -13.1024999 | 5.31E-12 | 1.93E-08 | 0 | 0 | 14.566 | 33.569 | EGF_CA |
| TRINITY_DN3917_c0_g1_i4 | -13.0693268 | 4.95E-08 | 2.42E-05 | 0 | 0 | 33.698 | 4.755 | Glyco_trans_1_4 |
| TRINITY_DN2679_c0_g1_i3 | -13.0421575 | 3.95E-12 | 1.88E-08 | 0 | 0 | 43.831 | 30.282 | UDPGT |
| TRINITY_DN3286_c0_g1_i8 | -12.9228609 | 2.21E-11 | 5.56E-08 | 0 | 0 | 35.209 | 20.489 | p450 |
| TRINITY_DN3286_c0_g1_i4 | -12.8722604 | 9.28E-09 | 6.89E-06 | 0 | 0 | 68.474 | 15.714 | p450 |
| TRINITY_DN2336_c0_g1_i2 | -12.8686183 | 2.01E-11 | 5.46E-08 | 0 | 0 | 47.086 | 28.643 | NA |
| TRINITY_DN8249_c0_g1_i4 | -12.8641942 | 3.28E-12 | 1.88E-08 | 0 | 0 | 10.299 | 18.8 | UDPGT |
| TRINITY_DN19209_c0_g1_i8 | -12.8237433 | 5.09E-12 | 1.93E-08 | 0 | 0 | 56.241 | 43.362 | - |
| TRINITY_DN2860_c0_g1_i4 | -12.8224529 | 8.02E-10 | 9.70E-07 | 0 | 0 | 54.641 | 19.846 | NA |
| TRINITY_DN5275_c0_g2_i2 | -12.7661975 | 7.30E-08 | 3.22E-05 | 0 | 0 | 67.429 | 10.516 | - |
| TRINITY_DN30338_c0_g3_i3 | -12.6974699 | 1.05E-10 | 1.80E-07 | 0 | 0 | 38.464 | 20.369 | NA |
| TRINITY_DN4191_c0_g1_i1 | -12.5898981 | 8.37E-12 | 2.74E-08 | 0 | 0 | 17.199 | 14.779 | NIR_SIR |
| TRINITY_DN10976_c0_g1_i3 | -12.5776892 | 4.04E-12 | 1.88E-08 | 0 | 0 | 11.288 | 15.714 | APH |
| TRINITY_DN6458_c0_g1_i2 | -12.5412979 | 2.58E-09 | 2.29E-06 | 0 | 0 | 29.087 | 10.255 | - |
| TRINITY_DN2564_c1_g1_i1 | -12.519462 | 1.15E-09 | 1.25E-06 | 0 | 0 | 591.664 | 229.787 | Glycolytic |
| TRINITY_DN3270_c0_g2_i4 | -12.4341496 | 1.09E-06 | 0.000299796 | 0 | 0 | 15.632 | 1.418 | NA |
| TRINITY_DN6368_c1_g1_i5 | -12.4262442 | 6.37E-11 | 1.30E-07 | 0 | 0 | 76.818 | 163.222 | NA |
| TRINITY_DN1681_c0_g1_i6 | -12.4228728 | 1.30E-11 | 3.85E-08 | 0 | 0 | 13.41 | 12.416 | NAM |
| TRINITY_DN679_c0_g1_i4 | -12.3867549 | 3.02E-09 | 2.60E-06 | 0 | 0 | 13.144 | 51.344 | NA |
| TRINITY_DN732_c0_g1_i2 | -12.3530914 | 1.55E-07 | 5.97E-05 | 0 | 0 | 6.444 | 55.336 | NAM |
| TRINITY_DN3959_c0_g1_i1 | -12.3008445 | 2.10E-09 | 1.97E-06 | 0 | 0 | 11.077 | 38.727 | NA |
| TRINITY_DN1547_c0_g1_i3 | -12.298113 | 5.66E-07 | 0.000187248 | 0 | 0 | 6.177 | 73.03 | NA |
| TRINITY_DN2073_c0_g1_i5 | -12.1481167 | 9.24E-07 | 0.000270631 | 0 | 0 | 33.687 | 4.052 | NA |
| TRINITY_DN33_c0_g1_i1 | -12.0910349 | 7.66E-07 | 0.00023179 | 0 | 0 | 21.676 | 2.895 | NA |
| TRINITY_DN2001_c0_g1_i1 | -12.0510837 | 1.48E-07 | 5.77E-05 | 0 | 0 | 32.365 | 6.917 | NA |
| TRINITY_DN2670_c0_g1_i2 | -12.0329669 | 4.58E-10 | 6.51E-07 | 0 | 0 | 6.711 | 15.332 | NA |
| TRINITY_DN2764_c0_g1_i7 | -11.9910497 | 2.00E-10 | 2.96E-07 | 0 | 0 | 15.566 | 12.165 | UAA |
| TRINITY_DN131_c0_g1_i5 | -11.9761676 | 2.85E-06 | 0.000662954 | 0 | 0 | 14.644 | 1.377 | NA |
| TRINITY_DN3256_c0_g1_i1 | -11.9503325 | 1.28E-06 | 0.000340214 | 0 | 0 | 1.944 | 22.661 | NA |
| TRINITY_DN62269_c0_g2_i3 | -11.8901097 | 3.10E-08 | 1.64E-05 | 0 | 0 | 3.944 | 17.353 | NA |
| TRINITY_DN1444_c1_g1_i8 | -11.8872766 | 2.94E-08 | 1.57E-05 | 0 | 0 | 11.044 | 47.624 | NA |
| TRINITY_DN614_c0_g1_i8 | -11.8847864 | 9.65E-11 | 1.80E-07 | 0 | 0 | 6.933 | 8.656 | NA |
| TRINITY_DN1660_c0_g1_i4 | -11.8634915 | 5.38E-06 | 0.001112428 | 0 | 0 | 19.243 | 1.558 | Glyco_transf_5 |
| TRINITY_DN3885_c0_g1_i2 | -11.8586555 | 1.21E-10 | 1.92E-07 | 0 | 0 | 9.388 | 12.909 | NA |
| TRINITY_DN4030_c0_g1_i5 | -11.8343888 | 1.23E-10 | 1.92E-07 | 0 | 0 | 8.744 | 10.305 | Ribonuclease_T2 |
| TRINITY_DN12_c0_g1_i1 | -11.7878969 | 2.11E-09 | 1.97E-06 | 0 | 0 | 10.777 | 6.374 | NA |
| TRINITY_DN2971_c0_g1_i6 | -11.652769 | 2.59E-09 | 2.29E-06 | 0 | 0 | 11.766 | 7.42 | NA |
| TRINITY_DN252_c0_g1_i6 | -11.6432173 | 1.20E-09 | 1.26E-06 | 0 | 0 | 10.533 | 7.822 | NA |
| TRINITY_DN859_c0_g1_i1 | -11.6249977 | 1.52E-08 | 9.84E-06 | 0 | 0.01 | 22.121 | 10.194 | NA |
| TRINITY_DN14520_c0_g1_i1 | -11.623701 | 1.55E-08 | 9.84E-06 | 0 | 0 | 15.655 | 7.219 | p450 |
| TRINITY_DN4152_c0_g1_i3 | -11.6005128 | 7.08E-09 | 5.38E-06 | 0 | 0 | 7.966 | 21.555 | TPR_8 |
| TRINITY_DN1660_c0_g1_i1 | 11.59974632 | 3.74E-05 | 0.005203975 | 0.727 | 23.14 | 0 | 0 | Glyco_transf_5 |
| TRINITY_DN970_c0_g1_i2 | -11.5912281 | 1.00E-07 | 4.14E-05 | 0 | 0 | 31.454 | 10.124 | NA |
| TRINITY_DN6820_c0_g1_i2 | -11.5871892 | 1.47E-08 | 9.79E-06 | 0 | 0 | 3.722 | 11.511 | DnaJ |
| TRINITY_DN614_c0_g1_i4 | -11.5791539 | 5.05E-10 | 6.87E-07 | 0 | 0 | 6.433 | 6.575 | NA |
| TRINITY_DN2554_c0_g2_i3 | -11.5734817 | 3.54E-09 | 2.89E-06 | 0 | 0 | 46.775 | 106.247 | NA |
| TRINITY_DN11496_c0_g1_i1 | -11.5409982 | 5.64E-10 | 7.37E-07 | 0 | 0 | 35.198 | 48.378 | zf-C2H2_jaz |
| TRINITY_DN15284_c0_g1_i2 | -11.4973299 | 1.49E-09 | 1.52E-06 | 0 | 0 | 18.343 | 15.02 | GLTP |
| TRINITY_DN2848_c0_g1_i5 | -11.4894068 | 4.54E-09 | 3.53E-06 | 0 | 0 | 23.754 | 15.272 | NA |
| TRINITY_DN26287_c0_g2_i3 | -11.4219067 | 3.50E-09 | 2.89E-06 | 0 | 0 | 10.033 | 20.409 | NA |
| TRINITY_DN2015_c0_g1_i1 | -11.3775075 | 1.11E-09 | 1.25E-06 | 0 | 0 | 5.822 | 6.756 | NA |
| TRINITY_DN6636_c0_g1_i5 | -11.317961 | 3.81E-09 | 3.04E-06 | 0 | 0 | 6.311 | 5.017 | - |
| TRINITY_DN4747_c1_g1_i6 | -11.300191 | 1.05E-08 | 7.47E-06 | 0 | 0 | 11.333 | 7.249 | NA |
| TRINITY_DN230_c0_g1_i6 | -11.2963072 | 7.57E-08 | 3.30E-05 | 0 | 0 | 26.998 | 11.471 | NA |
| TRINITY_DN3521_c0_g1_i5 | -11.2903194 | 3.64E-08 | 1.89E-05 | 0 | 0 | 5.911 | 17.393 | NA |
| TRINITY_DN1532_c0_g2_i3 | -11.2845032 | 1.81E-09 | 1.79E-06 | 0 | 0 | 11.677 | 15.533 | Aldo_ket_red |
| TRINITY_DN473_c0_g1_i4 | 11.26266729 | 1.07E-07 | 4.29E-05 | 5.087 | 1.695 | 0 | 0 | AAA_11 |
| TRINITY_DN485_c0_g1_i2 | -11.2444323 | 1.59E-06 | 0.000402496 | 0 | 0 | 5.711 | 39.531 | NA |
| TRINITY_DN1685_c0_g1_i3 | -11.2421174 | 1.32E-08 | 9.18E-06 | 0 | 0 | 18.899 | 42.738 | Ribosomal_L7Ae |
| TRINITY_DN1878_c0_g1_i1 | 11.23293641 | 8.22E-05 | 0.009719175 | 11.137 | 0.345 | 0 | 0 | NA |
| TRINITY_DN3109_c0_g1_i2 | -11.2190038 | 6.01E-08 | 2.84E-05 | 0 | 0 | 56.708 | 26.582 | NA |
| TRINITY_DN5488_c0_g1_i1 | -11.1873575 | 9.60E-09 | 6.97E-06 | 0 | 0 | 259.118 | 179.298 | NA |
| TRINITY_DN6221_c1_g1_i1 | -11.1656545 | 1.05E-07 | 4.29E-05 | 0 | 0 | 21.199 | 9.32 | NA |
| TRINITY_DN7684_c0_g1_i2 | -11.1411628 | 5.29E-07 | 0.000179943 | 0 | 0 | 5.4 | 1.679 | K_trans |
| TRINITY_DN33_c0_g1_i3 | -11.1390902 | 3.63E-11 | 8.47E-08 | 0.018 | 0.039 | 99.027 | 12.989 | NA |
| TRINITY_DN438_c0_g1_i7 | -11.1360439 | 3.46E-05 | 0.00487874 | 0.064 | 0 | 0.567 | 137.022 | NA |

| TRINITY_DN2152_c0_g1_i5 | -11.1093602 | 1.34E-07 | 5.28E-05 | 0 | 0 | 4.055 | 13.643 | DUF247 |
| --- | --- | --- | --- | --- | --- | --- | --- | --- |
| TRINITY_DN3988_c0_g1_i2 | -11.0898562 | 4.80E-06 | 0.00103167 | 0 | 0 | 6.133 | 1.046 | Phosphorylase |
| TRINITY_DN89924_c0_g1_i2 | -11.0859774 | 9.28E-07 | 0.000270631 | 0 | 0 | 9.966 | 2.795 | NA |
| TRINITY_DN14_c0_g1_i5 | -11.0796263 | 2.22E-08 | 1.25E-05 | 0 | 0 | 2.633 | 5.811 | Gelsolin |
| TRINITY_DN1750_c0_g1_i2 | -11.0766496 | 1.67E-06 | 0.000410173 | 0 | 0 | 9.311 | 2.252 | SRP54 |
| TRINITY_DN1458_c0_g1_i3 | -11.0481924 | 2.00E-08 | 1.19E-05 | 0 | 0 | 15.31 | 31.699 | NA |
| TRINITY_DN3269_c1_g1_i2 | -11.0389797 | 1.62E-07 | 6.16E-05 | 0 | 0 | 7.633 | 3.398 | Med26 |
| TRINITY_DN460_c0_g1_i5 | -11.0327916 | 1.98E-07 | 7.29E-05 | 0 | 0 | 4.4 | 15.191 | NA |
| TRINITY_DN905_c0_g1_i2 | -11.0245504 | 1.57E-08 | 9.84E-06 | 0 | 0 | 5.022 | 9.551 | NA |
| TRINITY_DN1681_c0_g1_i3 | 11.02296951 | 5.03E-06 | 0.001067015 | 10.964 | 1.597 | 0 | 0 | NAM |
| TRINITY_DN2015_c0_g1_i4 | 10.99069365 | 1.07E-06 | 0.000298647 | 2.135 | 9.51 | 0 | 0 | NA |
| TRINITY_DN587_c0_g1_i7 | 10.95460988 | 7.90E-07 | 0.000236866 | 8.63 | 2.286 | 0 | 0 | NA |
| TRINITY_DN1505_c0_g1_i4 | -10.9530965 | 4.23E-08 | 2.10E-05 | 0 | 0 | 8.288 | 5.409 | NA |
| TRINITY_DN1917_c0_g1_i1 | -10.9284444 | 1.95E-08 | 1.18E-05 | 0 | 0 | 11.533 | 20.449 | NA |
| TRINITY_DN3967_c0_g1_i2 | -10.9117826 | 1.70E-08 | 1.05E-05 | 0 | 0 | 10.599 | 9.35 | DUF2470 |
| TRINITY_DN3794_c0_g2_i2 | -10.887992 | 4.63E-06 | 0.001001099 | 0 | 0 | 6.944 | 1.448 | GAF |
| TRINITY_DN327_c0_g1_i2 | -10.8874893 | 6.82E-08 | 3.09E-05 | 0 | 0 | 12.599 | 7.802 | NA |
| TRINITY_DN1123_c0_g1_i3 | -10.8776836 | 1.46E-08 | 9.79E-06 | 0 | 0 | 6.722 | 6.776 | NA |
| TRINITY_DN3662_c0_g1_i1 | -10.867751 | 0.000154697 | 0.016199551 | 0 | 0 | 10.777 | 0.432 | NA |
| TRINITY_DN67766_c0_g1_i6 | -10.8322629 | 5.84E-07 | 0.000190682 | 0 | 0 | 292.516 | 108.168 | NA |
| TRINITY_DN17232_c0_g2_i2 | -10.832248 | 3.70E-08 | 1.89E-05 | 0 | 0 | 4.078 | 7.721 | NAD_binding_1 |
| TRINITY_DN14484_c0_g1_i3 | -10.8249607 | 2.16E-07 | 7.85E-05 | 0 | 0 | 3.322 | 1.669 | DCB |
| TRINITY_DN3646_c0_g1_i5 | -10.8228585 | 1.23E-06 | 0.000328855 | 0 | 0 | 1.989 | 8.918 | Dimerisation |
| TRINITY_DN7192_c0_g1_i2 | -10.8192889 | 4.57E-06 | 0.000999219 | 0 | 0 | 9.611 | 2.141 | NA |
| TRINITY_DN682_c0_g2_i1 | -10.8102196 | 5.57E-06 | 0.001144568 | 0 | 0 | 2.133 | 14.93 | DUF247 |
| TRINITY_DN934_c0_g1_i3 | -10.8085138 | 1.73E-07 | 6.44E-05 | 0 | 0 | 8 | 4.313 | NA |
| TRINITY_DN3627_c0_g1_i1 | -10.8040169 | 2.46E-08 | 1.36E-05 | 0 | 0 | 2.044 | 3.257 | Pro_CA |
| TRINITY_DN1471_c0_g1_i1 | -10.7949519 | 2.11E-08 | 1.23E-05 | 0 | 0 | 5.566 | 8.043 | LRRNT_2 |
| TRINITY_DN156_c0_g1_i13 | -10.7750098 | 5.61E-06 | 0.001144568 | 0 | 0 | 4.1 | 0.895 | DNA_pol_phi |
| TRINITY_DN3646_c0_g1_i1 | -10.7635377 | 9.89E-05 | 0.011255548 | 0 | 0 | 0.978 | 23.646 | NA |
| TRINITY_DN1704_c0_g1_i2 | -10.741038 | 1.99E-05 | 0.003127782 | 0 | 0 | 7.655 | 1.076 | Kri1 |
| TRINITY_DN1537_c0_g1_i1 | -10.7270768 | 6.82E-05 | 0.008479853 | 0 | 0 | 13.588 | 1.106 | NA |
| TRINITY_DN12648_c0_g1_i1 | -10.7245593 | 7.99E-08 | 3.41E-05 | 0 | 0 | 13.055 | 9.249 | RmlD_sub_bind |
| TRINITY_DN105_c0_g1_i4 | -10.7181816 | 2.67E-08 | 1.46E-05 | 0 | 0 | 3.755 | 4.765 | NA |
| TRINITY_DN476_c0_g3_i1 | -10.7179534 | 7.41E-05 | 0.009028509 | 0 | 0 | 1.411 | 27.065 | NA |
| TRINITY_DN476_c0_g2_i1 | -10.7023995 | 3.30E-05 | 0.004746199 | 0 | 0 | 7.244 | 86.985 | NA |
| TRINITY_DN2444_c0_g1_i3 | -10.6942468 | 9.25E-08 | 3.87E-05 | 0 | 0 | 4.122 | 8.596 | Pyr_redox_2 |
| TRINITY_DN4486_c1_g1_i2 | -10.6829418 | 6.71E-05 | 0.008437216 | 0 | 0 | 246.618 | 19.936 | NA |
| TRINITY_DN1032_c0_g1_i7 | -10.6525879 | 1.91E-05 | 0.003042917 | 0 | 0 | 0.811 | 7.621 | Lipoxygenase |
| TRINITY_DN3054_c0_g2_i2 | -10.6369926 | 1.08E-07 | 4.29E-05 | 0 | 0 | 6.411 | 4.675 | Pkinase |
| TRINITY_DN5000_c0_g2_i5 | -10.6243244 | 4.64E-05 | 0.006160063 | 0 | 0 | 0.911 | 12.165 | NA |
| TRINITY_DN1321_c1_g1_i4 | -10.6225692 | 6.80E-07 | 0.000211452 | 0 | 0 | 9.766 | 4.464 | CpeT |
| TRINITY_DN699_c0_g1_i2 | -10.6052513 | 1.83E-05 | 0.002936866 | 0 | 0 | 13.299 | 2.232 | NA |
| TRINITY_DN4969_c0_g1_i2 | -10.5940487 | 6.87E-05 | 0.008499525 | 0 | 0 | 6.111 | 0.563 | NA |
| TRINITY_DN664_c0_g1_i3 | -10.5705123 | 8.41E-06 | 0.001592938 | 0 | 0 | 4.244 | 0.985 | NA |
| TRINITY_DN5400_c0_g2_i1 | -10.5671522 | 6.01E-05 | 0.00776591 | 0 | 0 | 8.555 | 0.885 | NA |
| TRINITY_DN7687_c0_g1_i4 | -10.5527544 | 1.05E-06 | 0.000295961 | 0 | 0 | 4.833 | 2.111 | NA |
| TRINITY_DN156_c0_g1_i1 | 10.55113923 | 0.000135102 | 0.014467093 | 0.3 | 5.933 | 0 | 0 | DNA_pol_phi |
| TRINITY_DN9882_c0_g1_i4 | -10.5499724 | 9.71E-06 | 0.001792816 | 2.471 | 26.44 | 32859.36 | 104.79 | NA |
| TRINITY_DN1023_c0_g1_i8 | -10.5366566 | 6.46E-08 | 3.02E-05 | 0 | 0 | 6.722 | 7.681 | NA |
| TRINITY_DN12239_c0_g1_i2 | -10.5173014 | 1.73E-07 | 6.44E-05 | 0 | 0 | 2.722 | 2.031 | ABC2_membrane |
| TRINITY_DN1099_c0_g1_i3 | -10.5091574 | 8.03E-08 | 3.41E-05 | 0 | 0 | 1.578 | 1.649 | RNA_pol_Rpb1_1 |
| TRINITY_DN6032_c0_g1_i2 | 10.50325465 | 5.36E-08 | 2.57E-05 | 2.489 | 2.385 | 0 | 0 | NA |
| TRINITY_DN2844_c0_g1_i8 | -10.4794065 | 2.17E-06 | 0.000524465 | 0 | 0 | 2.211 | 8.515 | - |
| TRINITY_DN9832_c0_g1_i3 | -10.4733011 | 2.86E-06 | 0.000662954 | 0 | 0 | 3.889 | 15.945 | NA |
| TRINITY_DN325_c1_g1_i5 | -10.4696676 | 1.75E-05 | 0.002834678 | 0 | 0 | 5.255 | 1.036 | Sugar_tr |
| TRINITY_DN392_c2_g1_i3 | -10.458816 | 1.67E-06 | 0.000410173 | 0 | 0 | 12.944 | 5.439 | NUDIX_2 |
| TRINITY_DN357_c0_g1_i4 | -10.4582322 | 2.21E-07 | 7.94E-05 | 0 | 0 | 11.477 | 22.44 | NA |
| TRINITY_DN5242_c0_g1_i2 | -10.4280735 | 0.000210491 | 0.020652118 | 0 | 0 | 0.5 | 12.366 | LysM |
| TRINITY_DN5283_c0_g2_i1 | -10.4180813 | 1.33E-05 | 0.002280938 | 0 | 0 | 1.822 | 11.562 | NA |
| TRINITY_DN4362_c0_g1_i2 | -10.4143184 | 8.37E-06 | 0.001592938 | 0 | 0 | 4.133 | 1.116 | NA |
| TRINITY_DN14_c0_g1_i6 | -10.3951217 | 5.39E-07 | 0.000181694 | 0 | 0 | 3.589 | 2.212 | Gelsolin |
| TRINITY_DN14069_c0_g1_i1 | -10.3892273 | 4.74E-07 | 0.000164738 | 0 | 0 | 2.811 | 1.82 | NA |
| TRINITY_DN1985_c0_g1_i14 | -10.386719 | 9.76E-07 | 0.000282068 | 0 | 0 | 9.399 | 4.936 | zf-C3HC4_3 |
| TRINITY_DN934_c0_g1_i15 | -10.3779141 | 7.12E-07 | 0.000219558 | 0 | 0 | 5.522 | 3.217 | NA |
| TRINITY_DN7054_c0_g1_i2 | -10.3778798 | 2.95E-06 | 0.000677853 | 0 | 0 | 11.322 | 4.393 | NA |
| TRINITY_DN325_c0_g2_i1 | -10.341078 | 1.10E-06 | 0.000299796 | 0 | 0 | 9.711 | 26.331 | NA |
| TRINITY_DN23187_c0_g1_i1 | -10.3061684 | 1.40E-06 | 0.000360759 | 0 | 0 | 14.277 | 7.379 | - |
| TRINITY_DN1643_c0_g1_i3 | -10.2916976 | 6.37E-07 | 0.000203894 | 0 | 0 | 3.422 | 7.56 | NA |
| TRINITY_DN3486_c0_g1_i6 | -10.2827113 | 4.63E-05 | 0.006160063 | 0 | 0 | 6.1 | 1.005 | MatE |
| TRINITY_DN108_c1_g1_i3 | 10.28044276 | 7.50E-06 | 0.001466817 | 1.272 | 5.263 | 0 | 0 | NA |
| TRINITY_DN7589_c0_g1_i2 | -10.2775885 | 7.79E-06 | 0.001515667 | 0 | 0 | 1.444 | 6.686 | MFS_1 |
| TRINITY_DN3686_c0_g1_i2 | 10.26621378 | 1.70E-05 | 0.002769911 | 1.072 | 5.795 | 0 | 0 | NA |
| TRINITY_DN4090_c0_g1_i1 | -10.2397719 | 2.05E-05 | 0.003168904 | 0 | 0 | 7.188 | 1.719 | TOM20_plant |

| TRINITY_DN387_c0_g1_i1 | 10.22503029 | 2.69E-06 | 0.000637019 | 5.369 | 15.35 | 0 | 0 | NA |
| --- | --- | --- | --- | --- | --- | --- | --- | --- |
| TRINITY_DN1243_c1_g1_i7 | -10.1966371 | 2.71E-06 | 0.0006374 | 0 | 0 | 3.622 | 1.759 | RRM_1 |
| TRINITY_DN1251_c0_g1_i2 | -10.1800047 | 1.35E-06 | 0.000352602 | 0 | 0 | 4.066 | 2.493 | bZIP_1 |
| TRINITY_DN1643_c0_g2_i3 | -10.174848 | 9.27E-05 | 0.010665042 | 0 | 0 | 0.444 | 4.926 | NA |
| TRINITY_DN6750_c0_g1_i9 | -10.1705554 | 6.09E-07 | 0.000197048 | 0 | 0 | 3.822 | 3.147 | NA |
| TRINITY_DN57_c0_g1_i1 | -10.1610047 | 6.60E-07 | 0.000209213 | 0 | 0 | 0.9 | 1.649 | NA |
| TRINITY_DN5882_c0_g1_i1 | -10.1595127 | 3.47E-05 | 0.00487874 | 0 | 0 | 6.422 | 1.357 | DUF1777 |
| TRINITY_DN7314_c0_g1_i1 | -10.1594292 | 5.67E-07 | 0.000187248 | 0 | 0 | 2.378 | 2.061 | NA |
| TRINITY_DN8248_c0_g1_i3 | -10.1523469 | 4.13E-06 | 0.000918443 | 0 | 0 | 1.878 | 6.153 | TB2_DP1_HVA22 |
| TRINITY_DN2798_c0_g1_i9 | -10.1503171 | 8.67E-05 | 0.010120852 | 0 | 0 | 3.333 | 0.483 | - |
| TRINITY_DN1322_c1_g1_i1 | 10.14587826 | 4.87E-05 | 0.006436713 | 0.572 | 4.11 | 0 | 0 | NA |
| TRINITY_DN548_c0_g2_i2 | -10.1290039 | 1.08E-05 | 0.001951502 | 0 | 0 | 4.211 | 1.428 | NA |
| TRINITY_DN20437_c0_g1_i2 | -10.1285497 | 2.61E-05 | 0.003896779 | 0 | 0 | 1.233 | 7.319 | NA |
| TRINITY_DN916_c0_g1_i2 | 10.11638719 | 4.35E-07 | 0.000152674 | 2.416 | 3.331 | 0 | 0 | PWWP |
| TRINITY_DN2143_c0_g1_i3 | -10.1038562 | 0.000120719 | 0.01319102 | 0 | 0 | 0.667 | 7.822 | AAA |
| TRINITY_DN1163_c0_g1_i1 | -10.1007218 | 1.10E-06 | 0.000299796 | 0 | 0 | 5.077 | 3.72 | DUF3110 |
| TRINITY_DN587_c0_g1_i4 | -10.0983161 | 5.15E-07 | 0.00017726 | 0 | 0 | 2.189 | 2.684 | NA |
| TRINITY_DN21232_c0_g1_i4 | -10.0272622 | 7.27E-07 | 0.000222024 | 0 | 0 | 1.867 | 2.151 | NA |
| TRINITY_DN907_c0_g1_i1 | -10.018409 | 8.36E-05 | 0.00982373 | 0 | 0 | 4.744 | 0.804 | RibD_C |
| TRINITY_DN587_c0_g1_i9 | -10.0112454 | 1.53E-05 | 0.002544579 | 0 | 0 | 3.255 | 1.116 | NA |
| TRINITY_DN3928_c0_g1_i2 | -10.0062194 | 9.10E-07 | 0.000270163 | 0 | 0 | 3.1 | 3.066 | NA |
| TRINITY_DN24859_c0_g1_i1 | -9.99779247 | 1.27E-05 | 0.002209196 | 0 | 0 | 1.811 | 7.158 | Per1 |
| TRINITY_DN6273_c0_g1_i1 | -9.99102104 | 2.58E-05 | 0.00387062 | 0 | 0 | 8.155 | 2.363 | NA |
| TRINITY_DN3744_c0_g1_i10 | 9.979838587 | 1.61E-06 | 0.000405472 | 2.389 | 4.425 | 0 | 0 | NA |
| TRINITY_DN2807_c1_g1_i3 | -9.97048598 | 9.90E-07 | 0.000283725 | 0 | 0 | 3.522 | 3.78 | RINGv |
| TRINITY_DN15168_c0_g1_i3 | -9.95750085 | 1.03E-06 | 0.000292418 | 0 | 0 | 2.7 | 3.579 | NA |
| TRINITY_DN13100_c3_g1_i2 | -9.92044474 | 0.000120617 | 0.01319102 | 0 | 0 | 13.099 | 2.101 | NA |
| TRINITY_DN468_c0_g1_i1 | -9.91935229 | 0.000178305 | 0.017980144 | 0 | 0 | 2.811 | 30.644 | NA |
| TRINITY_DN4022_c0_g1_i2 | -9.91483929 | 1.35E-06 | 0.000352602 | 0 | 0 | 3.489 | 3.549 | DnaJ |
| TRINITY_DN2672_c0_g1_i2 | -9.91069119 | 6.08E-06 | 0.001210468 | 0 | 0 | 12.577 | 6.575 | NA |
| TRINITY_DN673_c0_g1_i2 | 9.90801377 | 5.16E-06 | 0.001079049 | 5.087 | 12.59 | 0 | 0 | NA |
| TRINITY_DN10034_c0_g1_i2 | -9.90692747 | 1.99E-05 | 0.003127782 | 0 | 0 | 9.099 | 3.187 | Acetyltransf_1 |
| TRINITY_DN2020_c0_g1_i6 | -9.89844234 | 1.44E-06 | 0.000366676 | 0 | 0 | 1.955 | 2.805 | NA |
| TRINITY_DN3764_c0_g1_i5 | -9.89515782 | 1.83E-05 | 0.002936866 | 0 | 0 | 5.466 | 2.011 | NA |
| TRINITY_DN477_c0_g1_i5 | -9.89085236 | 1.93E-05 | 0.003057468 | 0 | 0 | 5.033 | 1.82 | NA |
| TRINITY_DN1065_c0_g1_i9 | -9.89003219 | 4.58E-05 | 0.006136415 | 0 | 0 | 5.222 | 1.347 | CRS1_YhbY |
| TRINITY_DN466_c0_g1_i8 | -9.88110647 | 6.95E-05 | 0.008563111 | 0 | 0 | 2.444 | 0.543 | NA |
| TRINITY_DN9447_c0_g1_i1 | -9.86444661 | 8.50E-05 | 0.009953852 | 0 | 0 | 1 | 7.249 | NA |
| TRINITY_DN9882_c0_g1_i3 | -9.86241325 | 0.000152559 | 0.016027047 | 1.072 | 0 | 746.022 | 7.611 | Copper-bind |
| TRINITY_DN1178_c0_g1_i1 | 9.855623179 | 0.000112683 | 0.012608153 | 0.699 | 5.322 | 0 | 0 | Methyltr_RsmB-F |
| TRINITY_DN799_c0_g1_i5 | -9.84351347 | 5.94E-06 | 0.001194488 | 0 | 0.158 | 103.316 | 15.181 | FA_desaturase |
| TRINITY_DN72571_c0_g1_i1 | -9.82835004 | 0.000307626 | 0.028153385 | 0 | 0 | 8.955 | 0.955 | NA |
| TRINITY_DN545_c0_g1_i6 | -9.82740888 | 1.02E-09 | 1.19E-06 | 0 | 0.099 | 19.465 | 62.775 | ABC_membrane |
| TRINITY_DN6980_c0_g1_i2 | -9.82468192 | 4.19E-05 | 0.00573233 | 0 | 0 | 4.411 | 1.277 | Pkinase |
| TRINITY_DN105_c0_g1_i5 | 9.824190809 | 0.000168929 | 0.017430668 | 0.509 | 4.583 | 0 | 0 | NA |
| TRINITY_DN2833_c0_g1_i13 | -9.82330268 | 1.19E-05 | 0.002099432 | 0 | 0 | 6.788 | 3.177 | NA |
| TRINITY_DN664_c0_g1_i1 | -9.8218417 | 4.02E-05 | 0.00552107 | 0 | 0 | 0.589 | 2.926 | NA |
| TRINITY_DN2764_c0_g1_i8 | -9.82015738 | 0.000119424 | 0.013137482 | 0 | 0 | 4.877 | 0.895 | UAA |
| TRINITY_DN4543_c2_g1_i1 | -9.81363522 | 5.72E-05 | 0.007450849 | 0 | 0 | 5.3 | 1.377 | NA |
| TRINITY_DN64_c0_g1_i2 | -9.81221164 | 0.000102159 | 0.011549269 | 0 | 0 | 6.077 | 1.227 | NA |
| TRINITY_DN155_c0_g1_i2 | 9.800675022 | 1.40E-06 | 0.000360759 | 5.487 | 5.243 | 0 | 0 | Ank |
| TRINITY_DN209_c0_g1_i1 | -9.77996799 | 5.98E-05 | 0.007754288 | 0 | 0 | 1.767 | 9.722 | NA |
| TRINITY_DN519_c1_g1_i2 | -9.77954485 | 3.85E-06 | 0.000860605 | 0 | 0 | 4.155 | 3.237 | BPL_LplA_LipB |
| TRINITY_DN11204_c0_g1_i2 | -9.77902019 | 5.19E-06 | 0.001079049 | 0 | 0 | 1.078 | 2.312 | NA |
| TRINITY_DN5183_c0_g1_i2 | -9.77446782 | 2.24E-05 | 0.003446134 | 0 | 0 | 2.811 | 1.116 | HisG |
| TRINITY_DN958_c0_g1_i5 | -9.76948549 | 3.14E-05 | 0.004589895 | 0 | 0 | 2.789 | 0.985 | CBS |
| TRINITY_DN765_c0_g1_i6 | -9.76688224 | 1.15E-05 | 0.002062027 | 0 | 0 | 3.744 | 1.91 | UBX |
| TRINITY_DN5246_c0_g1_i3 | -9.74962767 | 2.97E-06 | 0.000679524 | 0 | 0 | 2.689 | 2.644 | COPIIcoated_ERV |
| TRINITY_DN185_c1_g2_i1 | -9.74368612 | 0.00033531 | 0.030014382 | 0 | 0 | 1.167 | 14.829 | NA |
| TRINITY_DN750_c0_g1_i4 | 9.738385788 | 2.39E-06 | 0.00057336 | 4.46 | 3.42 | 0 | 0 | NA |
| TRINITY_DN3916_c0_g1_i9 | -9.70457912 | 5.14E-06 | 0.001079049 | 0 | 0 | 1.9 | 1.498 | BET |
| TRINITY_DN1347_c0_g3_i3 | -9.68031566 | 0.000317512 | 0.028736141 | 0 | 0 | 2.189 | 0.292 | AARP2CN |
| TRINITY_DN131_c0_g1_i6 | -9.66843018 | 4.00E-05 | 0.005520475 | 0 | 0 | 1.655 | 0.603 | - |
| TRINITY_DN5909_c0_g1_i6 | -9.6658646 | 8.07E-05 | 0.009622084 | 0 | 0 | 1.867 | 10.174 | zf-RING_2 |
| TRINITY_DN27869_c0_g1_i1 | -9.65596925 | 4.34E-07 | 0.000152674 | 0.69 | 0 | 386.965 | 74.136 | NA |
| TRINITY_DN450_c0_g1_i7 | -9.64887276 | 1.22E-05 | 0.002134408 | 0 | 0 | 2.555 | 1.518 | RCC1 |
| TRINITY_DN7165_c0_g1_i2 | -9.64529782 | 4.31E-05 | 0.005814943 | 0 | 0 | 5.555 | 2.011 | NA |
| TRINITY_DN466_c0_g1_i3 | 9.64184632 | 4.44E-06 | 0.000980902 | 1.817 | 1.242 | 0 | 0 | NA |
| TRINITY_DN542_c0_g1_i12 | -9.62901562 | 9.45E-06 | 0.001764561 | 0 | 0 | 33.409 | 22.128 | NA |
| TRINITY_DN9443_c0_g2_i1 | -9.6159429 | 3.48E-05 | 0.00487874 | 0 | 0 | 3.833 | 1.578 | NA |
| TRINITY_DN4597_c0_g1_i10 | 9.60901291 | 1.62E-05 | 0.002658839 | 3.043 | 7.589 | 0 | 0 | F-box |
| TRINITY_DN690_c0_g1_i8 | -9.60729212 | 6.30E-06 | 0.001247882 | 0 | 0 | 8.244 | 7.349 | SHS2_Rpb7-N |
| TRINITY_DN5242_c0_g1_i1 | -9.60421762 | 5.78E-06 | 0.001172591 | 0 | 0 | 2.6 | 3.931 | LysM |
| TRINITY_DN18248_c0_g1_i10 | 9.597885434 | 3.64E-06 | 0.000820253 | 1.226 | 1.439 | 0 | 0 | ABC_membrane |

| TRINITY_DN6229_c0_g1_i1 | -9.59675224 | 6.76E-06 | 0.00133137 | 0 | 0 | 7.366 | 6.465 | NA |
| --- | --- | --- | --- | --- | --- | --- | --- | --- |
| TRINITY_DN2004_c0_g1_i1 | -9.59141646 | 8.43E-06 | 0.001592938 | 0 | 0 | 1.822 | 3.418 | F-box |
| TRINITY_DN5781_c0_g1_i1 | -9.58500254 | 5.96E-06 | 0.001194488 | 0 | 0 | 38.431 | 51.988 | GTP_EFTU |
| TRINITY_DN71880_c0_g1_i1 | -9.58427432 | 0.000129692 | 0.014030766 | 0 | 0 | 12.699 | 3.036 | NA |
| TRINITY_DN2373_c0_g1_i5 | -9.58422873 | 0.0001134 | 0.01264508 | 0 | 0 | 2.022 | 11.421 | NA |
| TRINITY_DN156_c0_g2_i2 | 9.57639716 | 0.000257535 | 0.024318444 | 1.054 | 8.554 | 0 | 0 | FHA |
| TRINITY_DN2404_c0_g1_i11 | -9.57033274 | 1.20E-05 | 0.002106832 | 0 | 0 | 2.522 | 1.749 | - |
| TRINITY_DN5769_c1_g1_i1 | -9.56751638 | 0.000317365 | 0.028736141 | 0 | 0 | 8.744 | 1.337 | NA |
| TRINITY_DN1617_c0_g1_i16 | 9.56115892 | 7.98E-06 | 0.001534444 | 2.008 | 1.222 | 0 | 0 | NA |
| TRINITY_DN5052_c0_g1_i2 | -9.55533415 | 0.000510179 | 0.041591705 | 0 | 0 | 6.622 | 0.774 | NA |
| TRINITY_DN6022_c0_g1_i7 | -9.54063311 | 0.00030744 | 0.028153385 | 0 | 0 | 1.689 | 0.271 | PNRC |
| TRINITY_DN3744_c0_g1_i6 | -9.53403862 | 9.31E-05 | 0.01067015 | 0 | 0 | 0.733 | 3.589 | NA |
| TRINITY_DN4845_c0_g1_i5 | -9.53293128 | 1.69E-05 | 0.002753811 | 0 | 0 | 1.611 | 3.79 | NA |
| TRINITY_DN4517_c0_g1_i4 | 9.518923433 | 0.000507594 | 0.041564159 | 0.209 | 2.395 | 0 | 0 | NA |
| TRINITY_DN255_c1_g1_i11 | -9.51533932 | 0.00033686 | 0.030070775 | 0 | 0 | 7.788 | 1.237 | NA |
| TRINITY_DN1474_c0_g1_i2 | -9.50203316 | 4.36E-05 | 0.005864285 | 0 | 0 | 3.3 | 1.458 | NA |
| TRINITY_DN387_c0_g1_i3 | -9.49043721 | 1.13E-05 | 0.002047559 | 0 | 0 | 3.344 | 5.801 | NA |
| TRINITY_DN6065_c0_g1_i2 | -9.48646714 | 9.70E-06 | 0.001792816 | 0 | 0 | 3.689 | 5.55 | - |
| TRINITY_DN614_c0_g1_i9 | -9.48046619 | 1.32E-05 | 0.002274514 | 0 | 0 | 1.089 | 2.021 | NA |
| TRINITY_DN13793_c1_g1_i1 | -9.46034727 | 1.16E-05 | 0.002062027 | 0 | 0 | 2.189 | 2.001 | DUF630 |
| TRINITY_DN33981_c0_g1_i2 | -9.45966806 | 1.04E-05 | 0.001901554 | 0 | 0 | 3.966 | 4.082 | Frigida |
| TRINITY_DN2764_c0_g1_i5 | -9.45356239 | 0.000236604 | 0.02266957 | 0 | 0 | 1.467 | 0.322 | UAA |
| TRINITY_DN2212_c0_g2_i1 | 9.453201851 | 1.59E-05 | 0.002644295 | 0.654 | 1.301 | 0 | 0 | RRM_1 |
| TRINITY_DN6286_c0_g1_i1 | -9.4286589 | 2.37E-05 | 0.003628945 | 0 | 0 | 32.965 | 20.57 | NA |
| TRINITY_DN978_c0_g1_i4 | -9.41418819 | 1.61E-05 | 0.002655235 | 0 | 0 | 2.278 | 1.9 | NA |
| TRINITY_DN5383_c0_g1_i6 | -9.405579 | 3.48E-05 | 0.00487874 | 0 | 0 | 2.211 | 5.731 | PP2C |
| TRINITY_DN18854_c0_g1_i3 | -9.40495919 | 0.000383181 | 0.032859019 | 0 | 0 | 11.444 | 1.971 | NA |
| TRINITY_DN8726_c0_g1_i5 | -9.3960416 | 2.03E-05 | 0.003151448 | 0 | 0 | 2.866 | 5.519 | Myosin_TH1 |
| TRINITY_DN45551_c0_g1_i1 | -9.38060679 | 0.000510476 | 0.041591705 | 0 | 0 | 6.944 | 1.046 | NA |
| TRINITY_DN658_c0_g1_i3 | -9.37161452 | 4.26E-05 | 0.005781192 | 0 | 0 | 2.011 | 5.409 | SKI |
| TRINITY_DN2603_c0_g1_i3 | -9.36236943 | 0.000178136 | 0.017980144 | 0 | 0 | 1.367 | 7.078 | NA |
| TRINITY_DN4408_c0_g1_i2 | -9.34826545 | 5.44E-05 | 0.007111587 | 0 | 0 | 1.044 | 3.016 | NA |
| TRINITY_DN1232_c0_g1_i1 | -9.33301019 | 0.00030749 | 0.028153385 | 0 | 0 | 7.211 | 1.588 | NA |
| TRINITY_DN583_c1_g1_i3 | 9.323745831 | 2.52E-05 | 0.003788281 | 1.944 | 1.074 | 0 | 0 | Pkinase |
| TRINITY_DN3269_c0_g5_i2 | 9.317430722 | 2.46E-05 | 0.003730894 | 1.408 | 0.798 | 0 | 0 | NA |
| TRINITY_DN4845_c0_g1_i3 | 9.292520009 | 7.26E-05 | 0.008884086 | 1.59 | 4.74 | 0 | 0 | NA |
| TRINITY_DN9254_c0_g1_i2 | -9.26924761 | 0.000344833 | 0.030532237 | 0 | 0 | 0.644 | 4.092 | NA |
| TRINITY_DN5632_c0_g2_i1 | -9.26580645 | 3.38E-05 | 0.004842769 | 0 | 0 | 3.089 | 2.363 | Retrotran_gag_2 |
| TRINITY_DN10005_c0_g1_i2 | -9.26396454 | 0.000236441 | 0.02266957 | 0 | 0 | 3.322 | 0.925 | DHHC |
| TRINITY_DN7969_c0_g1_i2 | -9.26317194 | 2.47E-05 | 0.003739549 | 0 | 0 | 1.511 | 2.212 | NA |
| TRINITY_DN3228_c0_g1_i3 | -9.26176136 | 0.000177293 | 0.017980144 | 0 | 0 | 0.6 | 2.694 | Serpin |
| TRINITY_DN3297_c0_g1_i4 | -9.26176136 | 0.000177293 | 0.017980144 | 0 | 0 | 0.856 | 3.881 | Ist1 |
| TRINITY_DN6545_c0_g1_i4 | -9.25088398 | 0.00047214 | 0.038953959 | 0 | 0 | 1 | 7.57 | NA |
| TRINITY_DN10522_c0_g1_i2 | 9.248633661 | 0.000131685 | 0.014176372 | 0.945 | 3.439 | 0 | 0 | NA |
| TRINITY_DN61_c0_g1_i2 | -9.21572815 | 0.000818935 | 0.060809637 | 0 | 0 | 8.266 | 1.146 | DUF4220 |
| TRINITY_DN4180_c0_g2_i2 | -9.21529872 | 0.000355605 | 0.031232035 | 0 | 0 | 6.988 | 1.689 | NA |
| TRINITY_DN903_c0_g1_i14 | -9.21494159 | 3.22E-05 | 0.004659799 | 0 | 0 | 1.122 | 1.759 | ANF_receptor |
| TRINITY_DN11932_c0_g1_i1 | -9.21124257 | 3.41E-05 | 0.004866198 | 0 | 0 | 5.311 | 4.725 | NA |
| TRINITY_DN223_c0_g1_i6 | -9.20669072 | 0.000173431 | 0.017762816 | 0 | 0 | 2.311 | 0.845 | CUE |
| TRINITY_DN2603_c0_g1_i7 | -9.1987242 | 0.000434963 | 0.036438759 | 0 | 0 | 1.278 | 8.395 | NA |
| TRINITY_DN9719_c0_g1_i2 | -9.19492754 | 3.15E-05 | 0.004589895 | 0 | 0 | 1.455 | 1.93 | NA |
| TRINITY_DN1147_c1_g1_i3 | -9.18009689 | 9.07E-05 | 0.010509596 | 0 | 0 | 1.611 | 0.855 | NA |
| TRINITY_DN11894_c0_g1_i6 | -9.17191859 | 0.000418616 | 0.035432727 | 0 | 0 | 0.9 | 5.58 | NA |
| TRINITY_DN2656_c0_g1_i4 | -9.165843 | 0.000268198 | 0.025179823 | 0 | 0 | 3.511 | 1.076 | Amidohydro_1 |
| TRINITY_DN1948_c0_g2_i1 | -9.16106165 | 6.79E-05 | 0.008479853 | 0 | 0 | 5.689 | 3.639 | NA |
| TRINITY_DN7300_c0_g1_i3 | -9.13794899 | 0.000532222 | 0.043030224 | 0 | 0 | 2.211 | 0.483 | NA |
| TRINITY_DN13038_c0_g1_i4 | 9.122863384 | 0.000142276 | 0.015141474 | 1.099 | 0.375 | 0 | 0 | BHD_1 |
| TRINITY_DN8456_c0_g1_i2 | 9.10433429 | 0.000135496 | 0.014467093 | 0.563 | 1.685 | 0 | 0 | NA |
| TRINITY_DN48408_c0_g1_i9 | 9.083283542 | 0.000367354 | 0.031920714 | 1.263 | 0.276 | 0 | 0 | NA |
| TRINITY_DN28519_c0_g3_i4 | -9.0704013 | 0.000445548 | 0.037071259 | 0 | 0 | 0.333 | 1.8 | CRM1_C |
| TRINITY_DN1945_c0_g1_i1 | -9.06363931 | 0.000117877 | 0.013074949 | 0 | 0 | 1.389 | 3.559 | EF-hand_1 |
| TRINITY_DN555_c1_g1_i12 | -9.03270088 | 0.000124553 | 0.013564616 | 0 | 0 | 3.622 | 8.988 | NA |
| TRINITY_DN1528_c1_g1_i11 | -9.03054146 | 6.48E-05 | 0.008238689 | 0 | 0 | 1.233 | 1.769 | zf-C2H2 |
| TRINITY_DN11190_c0_g1_i3 | -9.02742551 | 6.65E-05 | 0.008401951 | 0 | 0 | 0.989 | 0.985 | Myb_DNA-bind_ |
| TRINITY_DN19274_c0_g1_i2 | -9.02651944 | 6.29E-05 | 0.008028933 | 0 | 0 | 2.078 | 2.413 | HLH |
| TRINITY_DN438_c0_g1_i14 | -9.01747254 | 0.000235023 | 0.022650991 | 1.354 | 0 | 33.942 | 672.773 | NA |
| TRINITY_DN1295_c0_g1_i6 | -9.01626933 | 0.000753712 | 0.057002983 | 0 | 0 | 2.722 | 0.573 | NA |
| TRINITY_DN4239_c1_g1_i2 | -9.01606118 | 7.50E-05 | 0.009110861 | 0 | 0 | 1.711 | 1.548 | NA |
| TRINITY_DN4511_c0_g1_i5 | 9.012164668 | 7.93E-05 | 0.009528408 | 1.244 | 2.355 | 0 | 0 | NA |
| TRINITY_DN3067_c0_g1_i2 | -9.0060918 | 8.91E-05 | 0.010358699 | 0 | 0 | 2.9 | 2.312 | AAA |
| TRINITY_DN12650_c0_g1_i2 | -9.00218825 | 0.000650005 | 0.05008718 | 0 | 0 | 1.5 | 9.35 | NA |
| TRINITY_DN1428_c0_g2_i17 | -8.9801149 | 7.80E-05 | 0.00940908 | 0 | 0 | 3.111 | 3.308 | NA |
| TRINITY_DN4583_c0_g1_i2 | -8.97009029 | 0.000854493 | 0.062737102 | 0 | 0 | 4.855 | 1.005 | Usp |
| TRINITY_DN9230_c0_g1_i4 | -8.96497909 | 0.001049499 | 0.073111355 | 0 | 0 | 1.011 | 0.191 | PMD |

| TRINITY_DN8880_c0_g1_i1 | 8.954733632 | 0.000243611 | 0.02307033 | 1.272 | 0.424 | 0 | 0 | Helicase_C |
| --- | --- | --- | --- | --- | --- | --- | --- | --- |
| TRINITY_DN2282_c0_g1_i2 | -8.94703032 | 6.56E-08 | 3.02E-05 | 0 | 0.089 | 32.276 | 13.844 | Heme_oxygenase |
| TRINITY_DN11548_c0_g1_i2 | -8.9413336 | 0.000221976 | 0.021648979 | 0 | 0 | 9.666 | 4.826 | NA |
| TRINITY_DN6416_c0_g1_i1 | -8.93780956 | 0.000125259 | 0.013596209 | 0 | 0 | 2.389 | 1.79 | NA |
| TRINITY_DN2906_c0_g1_i5 | -8.93497475 | 2.22E-08 | 1.25E-05 | 0 | 0.118 | 28.154 | 19.866 | - |
| TRINITY_DN4368_c0_g1_i6 | -8.92716379 | 0.000374238 | 0.032261519 | 0 | 0 | 8.155 | 3.026 | NA |
| TRINITY_DN71880_c0_g1_i2 | -8.92265175 | 1.36E-05 | 0.002333086 | 0.191 | 0 | 76.051 | 22.189 | NA |
| TRINITY_DN1437_c2_g1_i14 | 8.912726268 | 8.07E-05 | 0.009622084 | 4.079 | 2.957 | 0 | 0.01 | NA |
| TRINITY_DN655_c0_g1_i2 | -8.90443825 | 0.000259609 | 0.024443613 | 0 | 0 | 2.633 | 1.307 | Semialdhyde_dh |
| TRINITY_DN8509_c0_g1_i2 | 8.899639561 | 0.000200195 | 0.019941385 | 1.026 | 2.651 | 0 | 0 | NA |
| TRINITY_DN218_c0_g1_i4 | 8.886184617 | 0.001303898 | 0.087297046 | 0.727 | 5.874 | 0 | 0 | KIX_2 |
| TRINITY_DN3228_c0_g1_i7 | -8.88260861 | 0.000886474 | 0.064361969 | 0 | 0 | 0.344 | 2.091 | Serpin |
| TRINITY_DN1428_c0_g2_i11 | -8.87409772 | 0.000118056 | 0.013074949 | 0 | 0 | 2.433 | 3.267 | NA |
| TRINITY_DN559_c0_g1_i1 | -8.8628632 | 0.000169121 | 0.017430668 | 0 | 0 | 1.255 | 0.935 | NA |
| TRINITY_DN72323_c0_g1_i1 | -8.86155888 | 0.000725938 | 0.055157803 | 0 | 0 | 44.664 | 12.054 | NA |
| TRINITY_DN425_c0_g1_i1 | 8.850742198 | 0.000833483 | 0.061749525 | 5.014 | 0.966 | 0 | 0 | NA |
| TRINITY_DN3020_c0_g1_i12 | -8.82327635 | 0.000239929 | 0.02279843 | 0 | 0 | 1.167 | 2.704 | Autophagy_C |
| TRINITY_DN1090_c0_g1_i6 | -8.82312474 | 0.000608358 | 0.04755091 | 0 | 0 | 1.833 | 0.623 | NA |
| TRINITY_DN10390_c0_g1_i1 | -8.81399679 | 0.000151516 | 0.015968782 | 0 | 0 | 5.511 | 7.611 | NA |
| TRINITY_DN599_c0_g1_i1 | -8.80721473 | 0.000165369 | 0.01715222 | 0 | 0 | 2.167 | 3.388 | NA |
| TRINITY_DN4368_c0_g1_i2 | -8.79860899 | 1.01E-05 | 0.001846725 | 0 | 0.138 | 12.155 | 45.282 | NA |
| TRINITY_DN7340_c0_g1_i6 | -8.79747126 | 0.000795422 | 0.059198268 | 0 | 0 | 0.967 | 0.292 | NA |
| TRINITY_DN15490_c0_g1_i5 | -8.79687989 | 0.001151809 | 0.078802863 | 0 | 0 | 2.544 | 0.583 | NA |
| TRINITY_DN4053_c0_g1_i1 | -8.78772903 | 0.000422271 | 0.035649711 | 0 | 0 | 3.766 | 1.709 | Linker_histone |
| TRINITY_DN5545_c0_g1_i2 | 8.784365718 | 0.000227876 | 0.022158206 | 1.163 | 0.562 | 0 | 0 | NA |
| TRINITY_DN3037_c0_g1_i4 | -8.7838088 | 0.000230759 | 0.022372008 | 0 | 0 | 1.889 | 1.357 | Rho_N |
| TRINITY_DN9946_c0_g1_i7 | -8.77895155 | 0.000920863 | 0.066184243 | 0 | 0 | 2.755 | 0.774 | NA |
| TRINITY_DN11327_c0_g4_i1 | -8.77781514 | 0.000615641 | 0.047890989 | 0 | 0 | 8.788 | 3.187 | NA |
| TRINITY_DN3221_c0_g1_i2 | -8.74549483 | 0.000361668 | 0.031679369 | 0 | 0 | 1.555 | 0.885 | NA |
| TRINITY_DN12988_c0_g1_i4 | -8.74421367 | 0.000648109 | 0.050059126 | 0 | 0 | 6.033 | 2.272 | PetM |
| TRINITY_DN434_c0_g1_i8 | -8.73480879 | 6.83E-05 | 0.008479853 | 10.001 | 0 | 2017.667 | 1725.667 | NA |
| TRINITY_DN6679_c0_g1_i3 | -8.73077553 | 0.000209449 | 0.020611765 | 0 | 0 | 4.333 | 4.454 | NA |
| TRINITY_DN10299_c0_g1_i8 | -8.71559816 | 2.65E-05 | 0.003923429 | 0 | 0.02 | 2.167 | 10.888 | NA |
| TRINITY_DN2091_c0_g1_i8 | -8.7149889 | 0.00023241 | 0.022465429 | 0 | 0 | 0.933 | 0.895 | LRR_6 |
| TRINITY_DN12118_c0_g1_i10 | -8.69262803 | 0.000603211 | 0.047261631 | 0 | 0 | 0.611 | 0.271 | NA |
| TRINITY_DN57_c0_g1_i2 | -8.68707851 | 0.000526477 | 0.042682499 | 0 | 0 | 0.589 | 0.292 | NA |
| TRINITY_DN4314_c0_g1_i1 | -8.66827187 | 0.000297403 | 0.027604424 | 0 | 0 | 2.411 | 4.142 | DUF842 |
| TRINITY_DN6293_c0_g1_i2 | -8.66348619 | 0.000981382 | 0.069252047 | 0 | 0 | 0.944 | 4.102 | DUF1640 |
| TRINITY_DN54_c0_g1_i1 | -8.65400587 | 0.001420863 | 0.093782692 | 0 | 0 | 0.911 | 5.158 | NA |
| TRINITY_DN2323_c0_g1_i6 | -8.65150247 | 0.000300758 | 0.02783677 | 0 | 0 | 0.978 | 0.895 | SAP |
| TRINITY_DN13105_c0_g1_i1 | -8.6436614 | 0.00048378 | 0.039713683 | 0 | 0 | 25.454 | 14.427 | NA |
| TRINITY_DN31821_c0_g1_i1 | -8.6410515 | 0.000598758 | 0.047025552 | 0 | 0 | 1.244 | 0.623 | - |
| TRINITY_DN2199_c0_g1_i1 | -8.63583534 | 0.000333438 | 0.029928823 | 0 | 0 | 1.289 | 2.222 | NA |
| TRINITY_DN4173_c0_g1_i2 | -8.63482014 | 0.000342856 | 0.030439656 | 0 | 0 | 1.733 | 1.448 | NA |
| TRINITY_DN2692_c0_g1_i1 | -8.62496795 | 0.000563976 | 0.044832625 | 0 | 0 | 2.344 | 1.287 | NA |
| TRINITY_DN565_c0_g1_i4 | -8.62357255 | 0.000354056 | 0.031179809 | 0 | 0 | 2.033 | 3.549 | zf-DNL |
| TRINITY_DN16022_c0_g1_i1 | -8.61531038 | 0.000685924 | 0.052483596 | 0 | 0 | 0.322 | 0.985 | NA |
| TRINITY_DN4001_c0_g1_i3 | -8.60411726 | 0.000544311 | 0.043481036 | 0 | 0 | 2.5 | 6.062 | NA |
| TRINITY_DN8092_c0_g1_i2 | -8.59695672 | 0.000896016 | 0.064910508 | 0 | 0 | 0.8 | 2.835 | - |
| TRINITY_DN2052_c0_g1_i9 | -8.59676357 | 0.000415309 | 0.035244115 | 0 | 0 | 0.989 | 1.86 | NA |
| TRINITY_DN19455_c0_g1_i8 | -8.56637922 | 0.000393329 | 0.03364092 | 0 | 0 | 0.722 | 0.714 | PMI_typeI |
| TRINITY_DN14623_c0_g1_i3 | -8.56542722 | 0.000539047 | 0.043272065 | 0 | 0 | 0.878 | 1.93 | NA |
| TRINITY_DN3595_c0_g1_i1 | -8.55678346 | 0.000475641 | 0.039143942 | 0 | 0 | 2.9 | 2.252 | NA |
| TRINITY_DN4079_c0_g1_i2 | 8.54599724 | 0.000275955 | 0.025716989 | 2.798 | 3.104 | 0 | 0 | NA |
| TRINITY_DN4387_c0_g2_i2 | -8.53052314 | 0.001073768 | 0.074326565 | 0 | 0 | 1.6 | 0.653 | NA |
| TRINITY_DN2939_c0_g1_i2 | -8.51835118 | 0.000467345 | 0.038723635 | 0 | 0 | 1.289 | 1.267 | NA |
| TRINITY_DN2091_c0_g1_i4 | -8.51468938 | 0.000611845 | 0.04770932 | 0 | 0 | 0.9 | 0.643 | LRR_6 |
| TRINITY_DN1637_c0_g1_i5 | -8.502143 | 0.000534203 | 0.043030224 | 0 | 0 | 1.4 | 1.206 | NA |
| TRINITY_DN4894_c0_g1_i11 | 8.499337621 | 0.001761581 | 0.111756054 | 0.854 | 0.177 | 0 | 0 | Peptidase_C48 |
| TRINITY_DN1388_c0_g1_i2 | 8.490299515 | 0.000468163 | 0.038723635 | 0.572 | 1.025 | 0 | 0 | NA |
| TRINITY_DN21318_c0_g1_i17 | 8.486004544 | 0.00192714 | 0.120389164 | 0.391 | 2.079 | 0 | 0 | PA |
| TRINITY_DN1890_c0_g1_i8 | 8.485956054 | 0.001525495 | 0.099482968 | 2.08 | 0.493 | 0 | 0 | NA |
| TRINITY_DN1980_c0_g1_i5 | -8.48478573 | 0.002044363 | 0.126434167 | 0 | 0 | 4.989 | 1.357 | NA |
| TRINITY_DN12807_c0_g1_i1 | 8.467755394 | 0.000524578 | 0.042634325 | 3.325 | 1.902 | 0 | 0 | NA |
| TRINITY_DN7593_c0_g1_i5 | 8.463285446 | 0.002583178 | 0.152617691 | 0.273 | 1.813 | 0 | 0 | NA |
| TRINITY_DN2349_c0_g2_i1 | -8.4487002 | 0.000757229 | 0.057124317 | 0 | 0 | 1.255 | 2.634 | NA |
| TRINITY_DN4012_c0_g2_i2 | -8.43829275 | 0.000646372 | 0.050043265 | 0 | 0 | 1.567 | 2.554 | Cu_bind_like |
| TRINITY_DN25703_c0_g1_i1 | 8.434012595 | 0.000412189 | 0.035070423 | 0.954 | 1.005 | 0 | 0 | NA |
| TRINITY_DN13349_c0_g1_i15 | 8.431170508 | 0.000934772 | 0.066537861 | 1.154 | 0.453 | 0 | 0 | NA |
| TRINITY_DN3744_c0_g1_i4 | -8.42727786 | 0.000767594 | 0.05758255 | 0 | 0 | 0.756 | 1.478 | NA |
| TRINITY_DN23_c1_g1_i17 | 8.427256172 | 0.000238944 | 0.02279843 | 10.12 | 0.493 | 0 | 0.03 | NA |
| TRINITY_DN686_c0_g1_i1 | 8.425432921 | 0.000540964 | 0.043319565 | 1.254 | 2.109 | 0 | 0 | NA |
| TRINITY_DN5653_c0_g1_i1 | -8.41636052 | 0.000749427 | 0.056810383 | 0 | 0 | 0.678 | 0.553 | NA |
| TRINITY_DN80282_c0_g1_i2 | -8.41533074 | 0.000789782 | 0.059047477 | 0 | 0 | 2.344 | 4.464 | NA |

| TRINITY_DN5025_c0_g1_i3 | -8.41020868 | 0.001064381 | 0.073833218 | 0 | 0 | 1.222 | 0.694 | Inhibitor_I9 |
| --- | --- | --- | --- | --- | --- | --- | --- | --- |
| TRINITY_DN2052_c0_g1_i13 | -8.4012184 | 0.000930457 | 0.06652056 | 0 | 0 | 1.233 | 0.824 | NA |
| TRINITY_DN10302_c0_g1_i2 | -8.39113523 | 0.00083736 | 0.061890383 | 0 | 0 | 0.878 | 1.619 | NA |
| TRINITY_DN29373_c0_g1_i1 | -8.38656506 | 0.002367912 | 0.142587073 | 0 | 0 | 3.089 | 0.905 | NA |
| TRINITY_DN10709_c0_g2_i5 | -8.38449999 | 0.000934013 | 0.066537861 | 0 | 0 | 0.989 | 0.684 | NA |
| TRINITY_DN1838_c0_g2_i2 | -8.37394843 | 0.000758813 | 0.057124317 | 0 | 0 | 0.689 | 0.724 | NA |
| TRINITY_DN9457_c0_g1_i6 | 8.373283243 | 0.000551 | 0.043907955 | 1.826 | 2.434 | 0 | 0 | NA |
| TRINITY_DN35644_c0_g1_i4 | 8.366034479 | 0.000588712 | 0.046459897 | 0.772 | 0.572 | 0 | 0 | NA |
| TRINITY_DN18676_c0_g1_i2 | -8.36445094 | 0.002048846 | 0.126434167 | 0 | 0 | 1.833 | 7.631 | NA |
| TRINITY_DN39444_c0_g1_i4 | -8.36440717 | 0.001122763 | 0.077064945 | 0 | 0 | 1.511 | 0.925 | Iso_dh |
| TRINITY_DN105_c0_g2_i3 | -8.36362251 | 0.001508831 | 0.098593039 | 0 | 0 | 1.355 | 4.343 | NA |
| TRINITY_DN533_c0_g1_i18 | -8.35870708 | 0.000858543 | 0.062752378 | 0.009 | 0 | 1.511 | 2.463 | NA |
| TRINITY_DN1671_c0_g1_i2 | -8.35722897 | 0.002475619 | 0.1475975 | 0 | 0 | 2.667 | 0.814 | zf-C3HC4_3 |
| TRINITY_DN25036_c0_g1_i3 | -8.3569158 | 0.000793913 | 0.059198268 | 0 | 0 | 0.678 | 0.734 | PPR |
| TRINITY_DN6668_c1_g1_i19 | -8.34872208 | 0.001475623 | 0.096810336 | 0 | 0 | 1.744 | 0.865 | NA |
| TRINITY_DN512_c0_g1_i1 | -8.34410126 | 0.001862131 | 0.116999102 | 0 | 0 | 0.389 | 1.428 | CCT |
| TRINITY_DN589_c0_g1_i17 | -8.34002882 | 0.001177487 | 0.080147587 | 0 | 0 | 1.222 | 2.825 | NA |
| TRINITY_DN6668_c1_g1_i10 | 8.323548826 | 0.000682972 | 0.05238043 | 1.381 | 1.015 | 0 | 0 | NA |
| TRINITY_DN10529_c0_g1_i5 | -8.31893572 | 0.000913741 | 0.065902326 | 0 | 0 | 0.656 | 0.684 | CBM_20 |
| TRINITY_DN1512_c0_g2_i2 | -8.31860382 | 0.001562033 | 0.101460739 | 0 | 0 | 6.077 | 3.046 | IGR |
| TRINITY_DN50126_c0_g1_i9 | -8.31710729 | 0.001195897 | 0.081062948 | 0 | 0 | 0.667 | 0.452 | AMP_N |
| TRINITY_DN15006_c0_g1_i1 | -8.30973121 | 0.00136889 | 0.090718829 | 0 | 0 | 41.175 | 94.384 | NA |
| TRINITY_DN1363_c0_g1_i2 | -8.30217233 | 0.000960324 | 0.068060068 | 0 | 0 | 1.111 | 1.498 | Dimerisation |
| TRINITY_DN17351_c0_g2_i8 | -8.28182469 | 0.001059666 | 0.073662554 | 0 | 0 | 0.989 | 1.488 | NA |
| TRINITY_DN563_c0_g1_i1 | 8.264416047 | 0.001027622 | 0.0720482 | 0.345 | 0.66 | 0 | 0 | PPR |
| TRINITY_DN10249_c0_g1_i7 | 8.256607417 | 0.000768425 | 0.05758255 | 1.381 | 1.35 | 0 | 0 | Motile_Sperm |
| TRINITY_DN1890_c0_g1_i16 | -8.25332507 | 0.001173719 | 0.080057912 | 0 | 0 | 0.611 | 0.583 | NA |
| TRINITY_DN6288_c0_g1_i4 | -8.24295811 | 0.001878895 | 0.117825859 | 0 | 0 | 13.899 | 6.907 | NA |
| TRINITY_DN2515_c0_g1_i2 | -8.2417889 | 0.001187788 | 0.080680652 | 0 | 0 | 1.355 | 1.981 | NA |
| TRINITY_DN785_c0_g1_i9 | 8.228168225 | 0.000874568 | 0.063781026 | 2.325 | 1.981 | 0 | 0 | GLTSCR1 |
| TRINITY_DN12775_c0_g1_i4 | 8.217954406 | 0.001746486 | 0.111013971 | 1.045 | 2.907 | 0 | 0 | HSP20 |
| TRINITY_DN3254_c0_g1_i4 | -8.19577048 | 0.002547729 | 0.151069719 | 0 | 0 | 0.756 | 2.554 | - |
| TRINITY_DN2156_c0_g1_i11 | 8.182168211 | 0.001232983 | 0.083403752 | 1.944 | 1.183 | 0 | 0 | NA |
| TRINITY_DN2294_c0_g1_i4 | -8.17471122 | 0.00207012 | 0.127133391 | 0 | 0 | 2.866 | 1.679 | NA |
| TRINITY_DN222482_c0_g1_i2 | -8.15718154 | 0.001554741 | 0.101188251 | 0 | 0 | 1.089 | 1.116 | Glyco_transf_92 |
| TRINITY_DN12215_c0_g1_i3 | -8.15614956 | 0.001730073 | 0.110185103 | 0 | 0 | 2.044 | 1.669 | NA |
| TRINITY_DN3586_c0_g1_i3 | 8.15546834 | 0.001278534 | 0.085951154 | 2.035 | 1.33 | 0 | 0 | NA |
| TRINITY_DN5158_c0_g1_i5 | -8.15046948 | 0.00341839 | 0.192230005 | 0 | 0 | 1.189 | 0.442 | NA |
| TRINITY_DN455_c0_g1_i12 | -8.14977868 | 0.001964125 | 0.122232188 | 0 | 0 | 1.889 | 1.307 | NA |
| TRINITY_DN279_c0_g2_i2 | 8.145495088 | 0.000104948 | 0.011783022 | 1.408 | 5.943 | 0.011 | 0 | LSM |
| TRINITY_DN97_c0_g1_i1 | -8.14275909 | 0.001636559 | 0.105255223 | 0 | 0 | 8.066 | 7.852 | NA |
| TRINITY_DN12828_c0_g1_i2 | -8.13420671 | 1.40E-05 | 0.002375227 | 0 | 0.01 | 3.666 | 3.247 | NA |
| TRINITY_DN39125_c0_g1_i1 | -8.12715543 | 1.40E-05 | 0.002375227 | 0 | 0.049 | 3.778 | 6.082 | PsbP |
| TRINITY_DN2108_c0_g1_i3 | -8.1248669 | 0.001856067 | 0.11684283 | 0 | 0 | 2.255 | 3.891 | NA |
| TRINITY_DN4989_c0_g1_i2 | -8.12441574 | 0.001911174 | 0.11962048 | 0 | 0 | 2.478 | 1.94 | NA |
| TRINITY_DN4946_c0_g1_i1 | 8.121630227 | 0.002054865 | 0.126434167 | 1.008 | 0.434 | 0 | 0 | Anticodon_1 |
| TRINITY_DN7495_c0_g1_i4 | -8.12145528 | 0.003070032 | 0.175663883 | 0 | 0 | 0.6 | 2.021 | Elong_Iki1 |
| TRINITY_DN6158_c0_g1_i9 | 8.108472851 | 0.002103752 | 0.128956431 | 1.944 | 0.838 | 0.011 | 0 | NA |
| TRINITY_DN15379_c0_g1_i11 | -8.10137691 | 0.00194091 | 0.121017967 | 0 | 0 | 0.567 | 0.503 | NA |
| TRINITY_DN67652_c0_g1_i1 | 8.100541722 | 0.001400376 | 0.092617578 | 1.072 | 0.798 | 0 | 0 | Pkinase |
| TRINITY_DN20302_c0_g1_i1 | 8.089400929 | 0.002397895 | 0.143750522 | 0.245 | 0.66 | 0 | 0 | NA |
| TRINITY_DN4380_c0_g1_i7 | 8.079729272 | 0.001685789 | 0.107784932 | 0.236 | 0.404 | 0 | 0 | NA |
| TRINITY_DN6187_c0_g1_i4 | -8.06034032 | 0.002707377 | 0.157956267 | 0 | 0 | 0.567 | 1.327 | NA |
| TRINITY_DN488_c0_g1_i2 | 8.047545558 | 0.001683365 | 0.107784932 | 0.572 | 0.857 | 0 | 0 | UCH |
| TRINITY_DN5415_c0_g1_i1 | -8.04044231 | 0.00244224 | 0.146140776 | 0 | 0 | 2.644 | 2.111 | NA |
| TRINITY_DN20222_c0_g1_i4 | 8.039256261 | 0.002739007 | 0.159516647 | 0.79 | 2.079 | 0 | 0 | F-box |
| TRINITY_DN4894_c0_g1_i14 | 8.03369364 | 0.001782423 | 0.112640835 | 0.645 | 0.986 | 0 | 0 | ABC_membrane |
| TRINITY_DN464_c0_g1_i3 | -8.02749252 | 0.003830915 | 0.212863332 | 0 | 0 | 1.2 | 3.891 | RRM_1 |
| TRINITY_DN10302_c0_g1_i6 | -8.02162945 | 0.004339383 | 0.236293846 | 0 | 0 | 1.055 | 3.891 | NA |
| TRINITY_DN3420_c1_g1_i2 | -8.01650209 | 0.002857139 | 0.165805414 | 0 | 0 | 0.811 | 1.689 | NA |
| TRINITY_DN11491_c0_g1_i2 | -8.01422341 | 0.002369759 | 0.142587073 | 0 | 0 | 1.044 | 1.287 | NA |
| TRINITY_DN4655_c0_g1_i10 | -8.01171212 | 0.003937247 | 0.21803005 | 0 | 0 | 0.544 | 1.78 | - |
| TRINITY_DN2837_c0_g2_i2 | 7.988364875 | 0.004353768 | 0.236682701 | 2.017 | 0.552 | 0 | 0 | NA |
| TRINITY_DN452_c0_g1_i3 | -7.9784916 | 0.003331187 | 0.188952313 | 0 | 0 | 2 | 4.514 | NA |
| TRINITY_DN1868_c0_g1_i13 | 7.978347709 | 0.00270738 | 0.157956267 | 111.1 | 2.237 | 0 | 0.452 | NA |
| TRINITY_DN3015_c0_g1_i1 | -7.97546933 | 0.003488616 | 0.195506113 | 0 | 0 | 1.633 | 3.951 | NA |
| TRINITY_DN19116_c0_g1_i3 | 7.972948809 | 0.00292371 | 0.16817509 | 0.69 | 0.306 | 0 | 0 | PPR |
| TRINITY_DN8974_c0_g1_i2 | 7.967031338 | 0.002183009 | 0.13331455 | 0.545 | 0.818 | 0 | 0 | ERCC4 |
| TRINITY_DN9262_c0_g3_i1 | -7.96635897 | 0.004052502 | 0.223277162 | 0 | 0 | 1.022 | 2.936 | NA |
| TRINITY_DN25036_c0_g1_i2 | 7.96269174 | 0.002038391 | 0.126372475 | 0.718 | 0.611 | 0 | 0 | PPR |
| TRINITY_DN8259_c1_g1_i1 | -7.96165016 | 0.002902713 | 0.167298656 | 0 | 0 | 3 | 2.745 | NA |
| TRINITY_DN15762_c0_g1_i4 | 7.960908403 | 0.00477344 | 0.256932198 | 0.636 | 0.168 | 0 | 0 | NA |
| TRINITY_DN2717_c0_g1_i6 | -7.94632877 | 0.002899654 | 0.167298656 | 0 | 0 | 1.011 | 1.176 | NA |
| TRINITY_DN1629_c0_g1_i2 | 7.941068936 | 0.002311608 | 0.139602307 | 1.917 | 1.399 | 0 | 0 | NA |

| TRINITY_DN4195_c0_g1_i5 | -7.93721797 | 0.000377998 | 0.032499843 | 0 | 0.02 | 0.333 | 2.332 | NA |
| --- | --- | --- | --- | --- | --- | --- | --- | --- |
| TRINITY_DN3186_c0_g1_i1 | -7.92176861 | 0.000325675 | 0.029312579 | 0 | 0.108 | 19.799 | 1.629 | NA |
| TRINITY_DN4316_c0_g1_i5 | -7.92024451 | 0.003158024 | 0.180382781 | 0 | 0 | 0.144 | 0.201 | Helicase_C |
| TRINITY_DN3228_c0_g1_i2 | 7.912358425 | 0.002537909 | 0.150760994 | 0.581 | 0.857 | 0 | 0 | Serpin |
| TRINITY_DN5831_c0_g1_i13 | -7.90832591 | 0.003416264 | 0.192230005 | 0 | 0 | 18.743 | 16.066 | NA |
| TRINITY_DN5025_c0_g1_i4 | -7.90515915 | 0.003392039 | 0.191407068 | 0 | 0 | 0.722 | 0.674 | Inhibitor_I9 |
| TRINITY_DN5093_c0_g1_i4 | 7.895843048 | 0.002582074 | 0.152617691 | 1.026 | 0.788 | 0 | 0 | NA |
| TRINITY_DN5307_c0_g1_i3 | -7.87774989 | 4.59E-06 | 0.000999219 | 0 | 0.02 | 3.089 | 1.578 | LRRNT_2 |
| TRINITY_DN6518_c0_g1_i1 | -7.87458358 | 0.005969277 | 0.313830058 | 0 | 0 | 123.581 | 48.067 | NA |
| TRINITY_DN5952_c0_g1_i3 | 7.863506808 | 0.004252506 | 0.232726737 | 0.609 | 1.567 | 0 | 0 | DUF296 |
| TRINITY_DN7342_c0_g2_i1 | -7.86278595 | 0.004219291 | 1 | 0 | 0 | 0.389 | 0.292 | NA |
| TRINITY_DN6717_c2_g1_i13 | 7.845784719 | 0.002865288 | 0.165983482 | 0.718 | 0.631 | 0 | 0 | NA |
| TRINITY_DN16468_c0_g1_i5 | -7.84565311 | 0.004273257 | 1 | 0 | 0 | 0.411 | 0.734 | TPR_16 |
| TRINITY_DN7982_c0_g1_i1 | -7.84565311 | 0.004273257 | 1 | 0 | 0 | 1.222 | 2.262 | NA |
| TRINITY_DN8005_c0_g1_i5 | -7.82901911 | 0.005267729 | 1 | 0 | 0 | 0.522 | 1.327 | NA |
| TRINITY_DN51673_c0_g1_i4 | -7.82175402 | 0.004192686 | 1 | 0 | 0 | 0.567 | 0.603 | Glyco_transf_20 |
| TRINITY_DN67766_c0_g1_i4 | -7.81672797 | 0.001049315 | 0.073111355 | 0 | 24.19 | 3732.452 | 536.918 | NA |
| TRINITY_DN2120_c0_g1_i11 | -7.8108937 | 0.005453501 | 1 | 0 | 0 | 0.733 | 1.86 | NA |
| TRINITY_DN4872_c1_g1_i1 | -7.80560333 | 0.005018999 | 1 | 0 | 0 | 1.222 | 0.865 | NA |
| TRINITY_DN86257_c0_g2_i3 | -7.80353145 | 0.004428972 | 1 | 0 | 0 | 0.878 | 0.895 | NA |
| TRINITY_DN1818_c0_g1_i3 | -7.79121439 | 0.00482814 | 1 | 0 | 0 | 1.622 | 2.825 | BolA |
| TRINITY_DN3232_c0_g1_i2 | -7.78971792 | 0.000997355 | 0.070227519 | 0 | 0.069 | 13.966 | 0.593 | NA |
| TRINITY_DN1343_c0_g1_i7 | -7.78909945 | 0.005925033 | 1 | 0 | 0 | 0.433 | 1.146 | NA |
| TRINITY_DN1899_c0_g1_i4 | 7.780227447 | 0.003828378 | 0.212863332 | 1.435 | 0.946 | 0 | 0 | NA |
| TRINITY_DN16130_c0_g1_i1 | -7.77817206 | 0.00469551 | 1 | 0 | 0 | 0.9 | 0.995 | AAR2 |
| TRINITY_DN2280_c0_g1_i1 | -7.77422604 | 1.13E-06 | 0.000304516 | 0 | 0.108 | 9.599 | 11.18 | AAA |
| TRINITY_DN179_c0_g1_i5 | -7.76912159 | 0.005192967 | 1 | 0 | 0 | 0.567 | 1.036 | CK_II_beta |
| TRINITY_DN673_c0_g1_i16 | 7.767108363 | 0.004125456 | 0.226913946 | 2.153 | 1.291 | 0 | 0 | NA |
| TRINITY_DN2755_c0_g1_i2 | 7.756012741 | 0.004363132 | 0.236797748 | 0.954 | 1.705 | 0 | 0 | NA |
| TRINITY_DN21318_c0_g1_i12 | -7.74906487 | 0.000186771 | 0.018775917 | 0 | 0.02 | 1.022 | 3.639 | PA |
| TRINITY_DN3898_c1_g1_i4 | -7.74449608 | 0.007039174 | 1 | 0 | 0 | 0.178 | 0.493 | MatE |
| TRINITY_DN1131_c1_g1_i4 | -7.72187754 | 0.007723063 | 1 | 0 | 0 | 1.7 | 0.804 | NA |
| TRINITY_DN16178_c0_g1_i4 | 7.705613818 | 0.00432252 | 0.235768593 | 0.282 | 0.355 | 0 | 0 | NA |
| TRINITY_DN2738_c0_g1_i1 | 7.69815815 | 0.00468791 | 0.25274486 | 1.172 | 1.774 | 0 | 0 | NA |
| TRINITY_DN1412_c0_g2_i13 | -7.68169926 | 0.006207814 | 1 | 0 | 0 | 1.944 | 1.86 | NA |
| TRINITY_DN5260_c0_g1_i5 | -7.67963796 | 2.45E-05 | 0.003730894 | 0 | 0.256 | 28.865 | 18.981 | NA |
| TRINITY_DN5705_c0_g1_i14 | 7.678226319 | 0.006172728 | 1 | 1.453 | 0.67 | 0 | 0 | DnaJ |
| TRINITY_DN3297_c0_g1_i6 | -7.67281847 | 0.006786477 | 1 | 0 | 0 | 0.811 | 0.613 | Ist1 |
| TRINITY_DN6675_c0_g1_i7 | -7.66908973 | 0.006701251 | 1 | 0 | 0 | 0.433 | 0.352 | NA |
| TRINITY_DN477_c0_g1_i13 | -7.66830147 | 0.006643037 | 1 | 0 | 0 | 0.6 | 1.015 | NA |
| TRINITY_DN3814_c0_g1_i2 | -7.66142094 | 6.27E-05 | 0.008028933 | 0 | 0.059 | 3.689 | 4.263 | NA |
| TRINITY_DN11955_c0_g1_i11 | 7.653375108 | 0.004935111 | 1 | 0.536 | 0.473 | 0 | 0 | AAA_11 |
| TRINITY_DN4288_c0_g1_i6 | -7.64949169 | 0.008010182 | 1 | 0 | 0 | 0.456 | 1.106 | NA |
| TRINITY_DN6923_c0_g1_i3 | 7.647987384 | 0.00720016 | 1 | 0.336 | 0.138 | 0 | 0 | DUF2428 |
| TRINITY_DN13094_c0_g1_i2 | -7.64652571 | 0.006641754 | 1 | 0 | 0 | 0.856 | 1.005 | NA |
| TRINITY_DN18151_c0_g2_i1 | 7.63593069 | 0.005304066 | 1 | 0.681 | 0.917 | 0 | 0 | NA |
| TRINITY_DN10289_c0_g3_i3 | -7.63592218 | 0.007880012 | 1 | 0 | 0 | 2.344 | 1.568 | NA |
| TRINITY_DN7260_c0_g1_i2 | -7.63278373 | 0.007966235 | 1 | 0 | 0 | 0.889 | 1.971 | NA |
| TRINITY_DN14461_c0_g1_i2 | -7.62360419 | 0.007474968 | 1 | 0 | 0 | 1.311 | 2.292 | NA |
| TRINITY_DN2895_c0_g1_i13 | 7.606767282 | 0.005569505 | 1 | 0.618 | 0.739 | 0 | 0 | PP2C |
| TRINITY_DN14920_c0_g1_i1 | -7.6050409 | 0.007403044 | 1 | 0 | 0 | 1.1 | 1.257 | NA |
| TRINITY_DN15517_c0_g2_i8 | 7.593969292 | 0.005711178 | 1 | 0.454 | 0.483 | 0 | 0 | NA |
| TRINITY_DN5743_c0_g1_i2 | -7.59090423 | 0.008642446 | 1 | 0 | 0 | 0.544 | 1.146 | F-box |
| TRINITY_DN12781_c0_g1_i2 | 7.585917284 | 0.005962943 | 1 | 0.572 | 0.729 | 0 | 0 | Metallophos |
| TRINITY_DN17769_c0_g1_i1 | -7.5838422 | 0.007827968 | 1 | 0 | 0 | 1.733 | 1.88 | NA |
| TRINITY_DN8077_c1_g2_i1 | 7.582666958 | 0.00761594 | 1 | 0.327 | 0.739 | 0 | 0 | AP2 |
| TRINITY_DN15404_c0_g1_i3 | -7.57832225 | 0.000178179 | 0.017980144 | 0 | 0.414 | 59.241 | 10.345 | NA |
| TRINITY_DN9778_c0_g1_i11 | -7.57432923 | 0.008362225 | 1 | 0 | 0 | 0.256 | 0.221 | ABC_membrane |
| TRINITY_DN9011_c0_g1_i3 | 7.567616686 | 0.007567779 | 1 | 0.291 | 0.611 | 0 | 0 | NA |
| TRINITY_DN2024_c0_g1_i5 | -7.56549108 | 0.0093849 | 1 | 0 | 0 | 3.678 | 7.952 | NA |
| TRINITY_DN8477_c0_g1_i2 | 7.556538371 | 0.007086042 | 1 | 0.391 | 0.68 | 0 | 0 | PPR |
| TRINITY_DN22400_c0_g1_i5 | -7.53230829 | 0.008862687 | 1 | 0 | 0 | 0.867 | 0.995 | NA |
| TRINITY_DN4908_c0_g2_i1 | -7.52571086 | 0.009824002 | 1 | 0 | 0 | 0.5 | 0.975 | B3 |
| TRINITY_DN8259_c0_g1_i3 | -7.51242484 | 7.94E-06 | 0.001534082 | 0 | 0.187 | 19.354 | 10.406 | NA |
| TRINITY_DN3706_c0_g1_i9 | -7.49998741 | 0.009946602 | 1 | 0 | 0 | 0.667 | 0.603 | NA |
| TRINITY_DN6717_c2_g1_i11 | -7.49157482 | 0.009904418 | 1 | 0 | 0 | 0.411 | 0.422 | NA |
| TRINITY_DN5487_c0_g2_i1 | 7.47032303 | 0.010347331 | 1 | 1.154 | 2.769 | 0 | 0 | Transcrip_act |
| TRINITY_DN8246_c0_g1_i6 | -7.46263362 | 0.011579368 | 1 | 0 | 0 | 0.222 | 0.161 | - |
| TRINITY_DN0_c0_g2_i3 | -7.45241191 | 0.000208431 | 0.0205736 | 0.018 | 0 | 0.667 | 1.448 | NA |
| TRINITY_DN7340_c0_g1_i5 | -7.45058364 | 0.001152907 | 0.078802863 | 0.109 | 0 | 13.91 | 1.196 | NA |
| TRINITY_DN44810_c0_g1_i6 | -7.44946048 | 0.000104823 | 0.011783022 | 0 | 1.114 | 145.925 | 34.766 | NA |
| TRINITY_DN8704_c0_g1_i5 | -7.44094658 | 0.011168817 | 1 | 0 | 0 | 0.378 | 0.382 | PPR |
| TRINITY_DN5541_c1_g2_i9 | 7.429049043 | 0.008750057 | 1 | 0.472 | 0.434 | 0 | 0 | Actin |
| TRINITY_DN16849_c0_g1_i3 | 7.420930598 | 0.010218644 | 1 | 0.3 | 0.552 | 0 | 0 | NA |

| TRINITY_DN4202_c0_g1_i13 | -7.41894055 | 0.011795914 | 1 | 0 | 0 | 0.422 | 0.633 | Nramp |
| --- | --- | --- | --- | --- | --- | --- | --- | --- |
| TRINITY_DN25443_c0_g1_i1 | -7.41095888 | 0.013088556 | 1 | 0 | 0 | 30.92 | 20.439 | NA |
| TRINITY_DN250089_c0_g1_i1 | -7.39930153 | 0.012218807 | 1 | 0 | 0 | 1.744 | 2.272 | NA |
| TRINITY_DN11565_c0_g1_i1 | -7.3975087 | 0.012655798 | 1 | 0 | 0 | 2.378 | 2.071 | NA |
| TRINITY_DN7651_c0_g1_i2 | -7.3975087 | 0.012655798 | 1 | 0 | 0 | 0.833 | 0.724 | DNA_pol3_delta2 |
| TRINITY_DN1935_c0_g1_i19 | -7.34526801 | 0.013829771 | 1 | 0 | 0 | 1.989 | 2.644 | NA |
| TRINITY_DN486_c0_g1_i13 | -7.32459055 | 0.014441906 | 1 | 0 | 0 | 3.178 | 3.72 | NA |
| TRINITY_DN104_c0_g1_i3 | 7.322563157 | 0.012285833 | 1 | 0.808 | 1.35 | 0 | 0 | GUB_WAK_bind |
| TRINITY_DN9837_c0_g1_i3 | 7.322563157 | 0.012285833 | 1 | 0.454 | 0.779 | 0 | 0 | NA |
| TRINITY_DN16084_c0_g2_i6 | 7.316725983 | 0.011445441 | 1 | 0.2 | 0.227 | 0 | 0 | CBM_4_9 |
| TRINITY_DN5302_c0_g1_i6 | -7.31031936 | 0.015138169 | 1 | 0 | 0 | 0.389 | 0.583 | NA |
| TRINITY_DN5056_c0_g1_i11 | -7.30955086 | 0.002054662 | 0.126434167 | 0.073 | 0 | 9.366 | 0.483 | DHquinase_I |
| TRINITY_DN3407_c0_g1_i2 | -7.3055297 | 0.015496681 | 1 | 0 | 0 | 0.456 | 0.744 | TAXi_C |
| TRINITY_DN3930_c0_g1_i1 | -7.30089006 | 1.42E-05 | 0.002384242 | 0 | 0.207 | 15.843 | 16.568 | NA |
| TRINITY_DN15889_c0_g1_i3 | -7.30072312 | 0.015925002 | 1 | 0 | 0 | 0.444 | 0.784 | NA |
| TRINITY_DN5286_c0_g1_i1 | -7.2995821 | 0.001023536 | 0.071916058 | 0 | 0.68 | 11.899 | 89.106 | NA |
| TRINITY_DN3488_c0_g1_i3 | -7.27450222 | 0.016738471 | 1 | 0 | 0 | 1.667 | 2.845 | NA |
| TRINITY_DN36097_c0_g2_i1 | -7.26071696 | 0.017300789 | 1 | 0 | 0 | 2.378 | 1.971 | NA |
| TRINITY_DN2384_c0_g2_i11 | -7.25774634 | 0.01684792 | 1 | 0 | 0 | 0.489 | 0.704 | NA |
| TRINITY_DN23565_c0_g1_i2 | -7.25078595 | 0.017196702 | 1 | 0 | 0 | 0.889 | 0.855 | NA |
| TRINITY_DN12300_c0_g2_i1 | 7.219979935 | 0.000395636 | 0.033749894 | 4.36 | 9.432 | 0 | 0.08 | NA |
| TRINITY_DN4773_c0_g1_i2 | -7.21838415 | 1.00E-04 | 0.011340045 | 0.463 | 0 | 39.709 | 24.762 | NA |
| TRINITY_DN269_c0_g1_i8 | 7.207984376 | 1.44E-05 | 0.002415913 | 6.64 | 3.42 | 0 | 0.07 | ArgoL1 |
| TRINITY_DN31185_c0_g1_i2 | 7.201751187 | 0.015651986 | 1 | 0.409 | 0.286 | 0 | 0 | NA |
| TRINITY_DN37130_c0_g1_i1 | -7.20111785 | 0.019636256 | 1 | 0 | 0 | 1.8 | 1.478 | NA |
| TRINITY_DN12788_c0_g2_i3 | -7.18556616 | 0.019586698 | 1 | 0 | 0 | 0.389 | 0.412 | NA |
| TRINITY_DN4958_c0_g1_i4 | 7.180535351 | 0.01563415 | 1 | 0.409 | 0.483 | 0 | 0 | Trypsin_2 |
| TRINITY_DN8130_c0_g1_i3 | 7.175182011 | 0.016068139 | 1 | 0.227 | 0.296 | 0 | 0 | AFG1_ATPase |
| TRINITY_DN46570_c0_g1_i1 | -7.16984926 | 0.020250156 | 1 | 0 | 0 | 0.333 | 0.442 | NA |
| TRINITY_DN21359_c0_g1_i2 | -7.16984926 | 0.020250156 | 1 | 0 | 0 | 0.889 | 1.216 | AAA |
| TRINITY_DN12007_c0_g3_i1 | -7.16773238 | 0.02087349 | 1 | 0 | 0 | 1.467 | 1.277 | NA |
| TRINITY_DN247_c1_g4_i2 | -7.16760081 | 0.001032698 | 0.072249079 | 0 | 0.079 | 0.778 | 11.109 | Peptidase_S10 |
| TRINITY_DN11586_c0_g1_i2 | 7.164409873 | 0.017569902 | 1 | 0.509 | 0.917 | 0 | 0 | NA |
| TRINITY_DN10932_c0_g1_i16 | -7.15181703 | 0.020953518 | 1 | 0 | 0 | 0.644 | 0.714 | PPR |
| TRINITY_DN8768_c0_g3_i1 | -7.15181703 | 0.020953518 | 1 | 0 | 0 | 3.933 | 4.253 | NA |
| TRINITY_DN9054_c0_g1_i19 | 7.141509783 | 0.018295425 | 1 | 0.563 | 0.966 | 0 | 0 | NA |
| TRINITY_DN56973_c0_g1_i3 | -7.13396926 | 0.000214872 | 0.021018873 | 1.108 | 21.1 | 2185.234 | 210.826 | NA |
| TRINITY_DN799_c0_g1_i6 | -7.13279651 | 0.002623217 | 0.154424748 | 0 | 1.084 | 60.685 | 77.896 | FA_desaturase |
| TRINITY_DN8887_c0_g1_i2 | -7.1062937 | 0.023074383 | 1 | 0 | 0 | 0.744 | 1.025 | - |
| TRINITY_DN542_c0_g1_i5 | -7.0985116 | 6.14E-05 | 0.007900562 | 0.191 | 0 | 12.144 | 13.985 | NA |
| TRINITY_DN10597_c0_g2_i3 | 7.096258019 | 0.018918996 | 1 | 0.263 | 0.217 | 0 | 0 | NA |
| TRINITY_DN4753_c0_g1_i2 | 7.087738527 | 0.01900432 | 1 | 0.527 | 0.552 | 0 | 0 | F-box |
| TRINITY_DN2707_c0_g1_i7 | -7.08198699 | 0.000566637 | 0.044934874 | 0 | 0.02 | 1.278 | 0.925 | NA |
| TRINITY_DN16275_c0_g1_i1 | -7.07624684 | 0.024438466 | 1 | 0 | 0 | 0.978 | 1.307 | NA |
| TRINITY_DN885_c0_g1_i1 | -7.06684353 | 1.16E-05 | 0.002062027 | 0.018 | 0.03 | 0.711 | 4.313 | CWC25 |
| TRINITY_DN42143_c0_g1_i3 | -7.02333776 | 0.000269233 | 0.025204547 | 0 | 0.739 | 37.398 | 53.023 | - |
| TRINITY_DN2963_c0_g1_i8 | 7.015232423 | 0.02373516 | 1 | 0.327 | 0.572 | 0 | 0 | NA |
| TRINITY_DN24873_c0_g1_i1 | -7.01420657 | 0.027513023 | 1 | 0 | 0 | 0.322 | 0.392 | NA |
| TRINITY_DN13629_c0_g1_i2 | -7.01420657 | 0.027513023 | 1 | 0 | 0 | 1.2 | 1.498 | GUB_WAK_bind |
| TRINITY_DN1428_c0_g2_i2 | -6.96065139 | 4.90E-05 | 0.006456086 | 0 | 0.049 | 2.789 | 2.433 | NA |
| TRINITY_DN9256_c0_g1_i5 | 6.947047746 | 0.025559946 | 1 | 0.445 | 0.562 | 0 | 0 | PP2C |
| TRINITY_DN4654_c0_g1_i8 | 6.929947582 | 0.026204561 | 1 | 0.645 | 0.601 | 0 | 0 | LRR_1 |
| TRINITY_DN9141_c1_g1_i4 | 6.906197411 | 0.027642352 | 1 | 0.327 | 0.276 | 0 | 0 | NA |
| TRINITY_DN10282_c0_g2_i3 | 6.872165648 | 0.029283346 | 1 | 0.827 | 0.798 | 0 | 0 | NA |
| TRINITY_DN2164_c0_g1_i5 | -6.85192118 | 0.000954841 | 0.067818627 | 1.19 | 1.222 | 228.564 | 1.408 | NA |
| TRINITY_DN485_c0_g1_i4 | -6.84710952 | 0.000640147 | 0.049679073 | 0 | 0.138 | 2.255 | 12.859 | NA |
| TRINITY_DN26616_c0_g1_i6 | 6.833922018 | 0.031569177 | 1 | 0.418 | 0.503 | 0 | 0 | PPR |
| TRINITY_DN27178_c0_g1_i5 | 6.822275142 | 0.032881789 | 1 | 14.879 | 11.05 | 0 | 0 | NA |
| TRINITY_DN8730_c0_g1_i3 | 6.798120681 | 0.034163182 | 1 | 0.518 | 0.7 | 0 | 0 | NA |
| TRINITY_DN8201_c0_g1_i6 | 6.782666761 | 0.034625047 | 1 | 0.491 | 0.463 | 0 | 0 | NA |
| TRINITY_DN1032_c0_g1_i5 | -6.76719536 | 0.001355044 | 0.08998374 | 0.554 | 0 | 23.999 | 31.287 | Lipoxygenase |
| TRINITY_DN4618_c0_g1_i4 | -6.75642292 | 0.000340754 | 0.030335426 | 0.045 | 0 | 3.233 | 1.649 | NA |
| TRINITY_DN1428_c0_g2_i1 | 6.75275305 | 0.036667934 | 1 | 0.445 | 0.404 | 0 | 0 | NA |
| TRINITY_DN223_c0_g1_i9 | -6.69961127 | 0.000119294 | 0.013137482 | 0 | 0.108 | 4.122 | 6.454 | CUE |
| TRINITY_DN279_c0_g2_i3 | -6.69338742 | 9.49E-05 | 0.010838457 | 0.082 | 0 | 2.933 | 3.609 | LSM |
| TRINITY_DN17019_c0_g1_i7 | -6.67387651 | 0.002123657 | 0.129932808 | 0 | 0.138 | 8.966 | 3.006 | Cyclin |
| TRINITY_DN3459_c0_g1_i7 | -6.64326386 | 0.002189064 | 0.133434873 | 0.118 | 0 | 4.133 | 2.564 | NA |
| TRINITY_DN284_c0_g1_i8 | 6.610583285 | 0.000582917 | 0.04611397 | 8.394 | 2.759 | 0 | 0.111 | NA |
| TRINITY_DN6895_c0_g1_i2 | 6.5959396 | 0.049517268 | 0.999970678 | 0 | 645 | 0.356 | 6.113 | NA |
| TRINITY_DN1180_c0_g1_i5 | 6.542032316 | 0.000131906 | 0.014176372 | 7.458 | 8.742 | 0 | 0.181 | NA |
| TRINITY_DN8359_c0_g1_i3 | -6.53784642 | 0.000195302 | 0.01951351 | 0.073 | 0 | 3.689 | 3.358 | NA |
| TRINITY_DN1517_c0_g1_i12 | 6.522029362 | 4.96E-06 | 0.001058297 | 9.911 | 3.755 | 0.1 | 0.02 | NA |
| TRINITY_DN750_c0_g1_i7 | -6.48457267 | 0.000921701 | 0.066184243 | 0.073 | 0 | 1.178 | 4.755 | NA |
| TRINITY_DN251_c0_g1_i2 | 6.438723697 | 0.001254257 | 0.084667515 | 18.032 | 3.755 | 0 | 0.251 | NA |

| TRINITY_DN1363_c0_g1_i1 | -6.43488347 | 0.004013117 | 0.22148069 | 0.963 | 0 | 40.264 | 36.123 | Dimerisation |
| --- | --- | --- | --- | --- | --- | --- | --- | --- |
| TRINITY_DN1589_c0_g1_i8 | 6.424529545 | 0.00034989 | 0.030896259 | 3.688 | 4.248 | 0.089 | 0 | Beta-Casp |
| TRINITY_DN4492_c0_g1_i1 | -6.41187697 | 0.001346756 | 0.08961549 | 0 | 0.315 | 13.832 | 5.258 | NA |
| TRINITY_DN168831_c0_g1_i1 | -6.31071836 | 0.114278603 | 0.999970678 | 1.145 | 0 | 76.118 | 0 | NA |
| TRINITY_DN4652_c0_g1_i7 | -6.2930605 | 0.011161162 | 0.531570661 | 0.672 | 0 | 40.553 | 4.192 | Sigma70_r2 |
| TRINITY_DN5846_c1_g2_i3 | -6.2391166 | 0.005550413 | 1 | 0.036 | 0 | 1.4 | 0.915 | NA |
| TRINITY_DN61617_c0_g1_i1 | -6.23309646 | 0.116346032 | 0.999970678 | 0 | 0.572 | 0 | 42.346 | NA |
| TRINITY_DN1412_c0_g2_i5 | 6.207767917 | 0.000596572 | 0.046966759 | 2.589 | 5.706 | 0 | 0.121 | NA |
| TRINITY_DN2514_c1_g1_i11 | 6.160858365 | 0.008306288 | 1 | 0.454 | 1.449 | 0.011 | 0 | NA |
| TRINITY_DN4452_c0_g1_i8 | 6.155729051 | 0.00162718 | 0.105099561 | 1.526 | 0.69 | 0.022 | 0 | NA |
| TRINITY_DN31565_c0_g1_i4 | -6.14522877 | 2.90E-12 | 1.88E-08 | 0.218 | 0.207 | 14.355 | 11.602 | CUE |
| TRINITY_DN7138_c0_g1_i1 | 6.12676633 | 0.004884546 | 1 | 3.161 | 2.622 | 0 | 0.08 | NA |
| TRINITY_DN11664_c0_g1_i2 | -6.09652117 | 0.004976653 | 0.266116559 | 0.073 | 0 | 0.955 | 5.127 | NA |
| TRINITY_DN2555_c0_g1_i2 | -6.07942177 | 0.004858818 | 0.261097534 | 0 | 0.217 | 9.111 | 3.388 | Anticodon_1 |
| TRINITY_DN131_c0_g1_i7 | -6.07920686 | 0.00015617 | 0.016301557 | 0.009 | 0.375 | 12.244 | 11.542 | NA |
| TRINITY_DN12055_c0_g1_i1 | 6.075064787 | 0.0056722 | 1 | 0.336 | 0.276 | 0.011 | 0 | NA |
| TRINITY_DN3261_c0_g1_i2 | -6.06390662 | 0.130664067 | 0.999970678 | 0.345 | 0 | 19.477 | 0 | DUF2358 |
| TRINITY_DN2637_c0_g1_i1 | -6.04992224 | 0.000699182 | 0.053248624 | 0 | 0.128 | 3.2 | 4.323 | Translin |
| TRINITY_DN1645_c0_g1_i7 | -6.03995832 | 0.008419938 | 0.419353962 | 0 | 0.256 | 2.989 | 13.12 | NA |
| TRINITY_DN5717_c0_g1_i2 | 6.032564766 | 0.032889426 | 0.999970678 | 203.24 | 8.131 | 0 | 3.288 | RPE65 |
| TRINITY_DN155_c5_g1_i4 | 6.008406599 | 0.000534717 | 0.043030224 | 1.944 | 1.675 | 0 | 0.06 | NA |
| TRINITY_DN12807_c0_g2_i1 | 5.973899982 | 0.002609295 | 0.153882483 | 3.479 | 1.212 | 0.067 | 0 | NA |
| TRINITY_DN2656_c0_g1_i9 | 5.933923109 | 0.009654987 | 1 | 1.181 | 0.611 | 0.033 | 0 | Amidohydro_1 |
| TRINITY_DN1867_c0_g1_i8 | -5.93091464 | 0.062122723 | 0.999970678 | 0.182 | 2.592 | 0 | 158.949 | B_lectin |
| TRINITY_DN1437_c2_g1_i13 | -5.92772611 | 0.002448607 | 0.146253911 | 0.345 | 0 | 7.233 | 13.572 | NA |
| TRINITY_DN7697_c0_g2_i3 | 5.925081692 | 0.138270869 | 0.999970678 | 0 | 406.5 | 0 | 6.183 | NA |
| TRINITY_DN1656_c0_g1_i1 | 5.903033869 | 0.010824634 | 0.521362441 | 2.498 | 25.51 | 0.367 | 0 | Aldo_ket_red |
| TRINITY_DN4401_c0_g1_i3 | -5.89231091 | 0.003239736 | 0.184405313 | 0.082 | 0 | 3.566 | 1.488 | zf-C3HC4 |
| TRINITY_DN1789_c0_g2_i1 | -5.8901977 | 4.94E-05 | 0.006488287 | 0.382 | 0.463 | 3.822 | 45.774 | NA |
| TRINITY_DN1829_c0_g1_i5 | -5.87701279 | 0.001627705 | 0.105099561 | 0.2 | 0 | 4.344 | 5.489 | - |
| TRINITY_DN8615_c0_g1_i10 | 5.864525967 | 3.62E-06 | 0.000820253 | 3.743 | 1.666 | 0.056 | 0.03 | NA |
| TRINITY_DN13234_c0_g1_i7 | 5.837830224 | 0.00097426 | 0.068898298 | 0.763 | 0.995 | 0 | 0.03 | NA |
| TRINITY_DN1519_c0_g1_i4 | -5.837136 | 0.007826563 | 0.391591832 | 0.727 | 0 | 29.232 | 6.515 | NA |
| TRINITY_DN3155_c0_g1_i1 | -5.81735788 | 0.001729214 | 0.110185103 | 0 | 0.246 | 4.411 | 7.631 | NA |
| TRINITY_DN1711_c1_g2_i1 | -5.80568395 | 0.006747609 | 0.346087749 | 0.4 | 0 | 7.677 | 13.009 | RRM_1 |
| TRINITY_DN1428_c0_g2_i10 | 5.804883487 | 1.69E-06 | 0.000411737 | 4.478 | 8.16 | 0.144 | 0.05 | NA |
| TRINITY_DN1416_c1_g1_i14 | -5.77665528 | 0.026246295 | 0.999970678 | 0 | 2.809 | 86.017 | 47.624 | GDPD |
| TRINITY_DN458_c0_g1_i17 | -5.76260906 | 0.007295532 | 0.370123637 | 0 | 0.256 | 1.989 | 12.768 | NA |
| TRINITY_DN1993_c2_g1_i1 | -5.75935426 | 0.021041508 | 0.880240892 | 0 | 1.34 | 53.286 | 7.178 | TENA_THI-4 |
| TRINITY_DN15666_c0_g1_i6 | 5.757954965 | 0.007238559 | 0.367804374 | 1.653 | 10.82 | 0 | 0.211 | GTP_EFTU |
| TRINITY_DN537_c0_g1_i7 | -5.75515759 | 0.151579803 | 0.999970678 | 0.936 | 0 | 0 | 50.309 | NA |
| TRINITY_DN2377_c0_g1_i1 | -5.70156219 | 7.80E-05 | 0.00940908 | 0.127 | 0.237 | 1.722 | 17.222 | NA |
| TRINITY_DN750_c0_g1_i5 | -5.68895195 | 0.002794918 | 0.162483228 | 0.082 | 0 | 1.989 | 1.86 | NA |
| TRINITY_DN2469_c2_g1_i1 | -5.68822687 | 0.001772134 | 0.112207653 | 0.254 | 0.089 | 13.921 | 1.005 | NA |
| TRINITY_DN5206_c0_g1_i3 | -5.68395232 | 0.003572942 | 0.199547275 | 0 | 0.197 | 5 | 2.895 | NA |
| TRINITY_DN7381_c0_g1_i1 | -5.68390811 | 0.006329535 | 0.328775799 | 0.055 | 0 | 1.7 | 0.935 | NA |
| TRINITY_DN2689_c0_g1_i2 | -5.65867852 | 0.007172035 | 0.364991812 | 0 | 0.089 | 2.811 | 0.895 | Lipase_GDSL |
| TRINITY_DN3898_c1_g1_i8 | 5.653905716 | 0.174902127 | 0.999970678 | 0 | 48.07 | 0.767 | 0 | MatE |
| TRINITY_DN12120_c0_g1_i4 | 5.645337169 | 0.00227743 | 0.137840931 | 3.307 | 1.941 | 0 | 0.09 | NA |
| TRINITY_DN2730_c0_g1_i1 | 5.634733228 | 0.001469421 | 0.09659745 | 5.959 | 0.65 | 0.022 | 0.111 | DUF4413 |
| TRINITY_DN3138_c0_g1_i18 | -5.61818132 | 0.009494703 | 0.465083883 | 0 | 0.049 | 1.867 | 0.714 | NA |
| TRINITY_DN23547_c0_g2_i1 | -5.60908674 | 0.018250994 | 1 | 0.082 | 0 | 1.6 | 2.121 | NA |
| TRINITY_DN11335_c0_g1_i9 | -5.55704166 | 0.014236712 | 0.645134337 | 0.145 | 0 | 4.789 | 0.814 | - |
| TRINITY_DN14495_c0_g1_i1 | -5.55259342 | 0.013236855 | 0.610839734 | 0.2 | 0 | 6.955 | 1.337 | NA |
| TRINITY_DN12453_c0_g2_i6 | 5.498707921 | 0.003605909 | 0.201044824 | 1.354 | 2.641 | 0 | 0.08 | RPAP3_C |
| TRINITY_DN1424_c0_g1_i1 | -5.49368203 | 0.014355213 | 0.649603204 | 0.654 | 0 | 3.589 | 24.109 | NA |
| TRINITY_DN9320_c0_g1_i16 | 5.475777148 | 0.000194658 | 0.019508828 | 1.344 | 0.434 | 0.022 | 0.02 | LRR_8 |
| TRINITY_DN3103_c1_g1_i1 | 5.457226777 | 0.022187329 | 1 | 0.263 | 0.424 | 0.011 | 0 | NA |
| TRINITY_DN2307_c1_g1_i4 | 5.453429252 | 0.009878285 | 0.479559196 | 1.299 | 0.562 | 0.033 | 0 | NA |
| TRINITY_DN3775_c0_g1_i3 | 5.451741838 | 0.183520151 | 0.999970678 | 61.753 | 0 | 0 | 1.438 | - |
| TRINITY_DN1354_c1_g1_i8 | -5.44147282 | 0.000363208 | 0.031729244 | 1.217 | 0.365 | 50.686 | 8.284 | NA |
| TRINITY_DN15379_c1_g1_i1 | 5.438362859 | 0.016060032 | 0.707160878 | 1.917 | 0.325 | 0.044 | 0 | NA |
| TRINITY_DN693_c0_g1_i3 | -5.40136127 | 0.000240042 | 0.02279843 | 0.572 | 0.168 | 21.888 | 4.102 | - |
| TRINITY_DN1374_c0_g1_i6 | -5.39856817 | 0.022346787 | 0.916078067 | 0 | 0.769 | 2.233 | 28.894 | NA |
| TRINITY_DN5416_c0_g1_i4 | -5.3829864 | 0.000316897 | 0.028736141 | 0.545 | 0.099 | 17.754 | 5.027 | NA |
| TRINITY_DN1078_c0_g1_i4 | 5.372042447 | 0.014903222 | 0.668843522 | 0.045 | 9.067 | 0.044 | 0.171 | LRRNT_2 |
| TRINITY_DN1354_c0_g1_i5 | -5.37104393 | 0.013405393 | 0.615732479 | 0 | 1.163 | 6.466 | 37.44 | NA |
| TRINITY_DN12166_c0_g1_i7 | 5.369260859 | 0.000886016 | 0.064361969 | 5.596 | 17.83 | 0.244 | 0.342 | NA |
| TRINITY_DN15294_c0_g1_i1 | 5.365162639 | 0.031743846 | 0.999970678 | 6.55 | 0.345 | 0.144 | 0 | NA |
| TRINITY_DN6354_c0_g1_i1 | -5.36269242 | 0.013979297 | 0.635467193 | 0 | 1.281 | 15.766 | 33.449 | NA |
| TRINITY_DN1589_c0_g1_i4 | -5.3626908 | 0.009158017 | 0.452663717 | 0.164 | 0 | 2.289 | 3.961 | Beta-Casp |
| TRINITY_DN3294_c0_g1_i3 | -5.35773593 | 0.008474581 | 0.421433028 | 0.227 | 0 | 2.3 | 5.952 | ADH_zinc_N |
| TRINITY_DN1216_c0_g1_i4 | -5.35657288 | 0.020563135 | 0.862437427 | 0.318 | 0 | 9.066 | 1.408 | NA |
| TRINITY_DN8786_c0_g1_i7 | -5.35458731 | 0.01344482 | 0.616085779 | 0 | 0.591 | 4.311 | 18.981 | NA |

| TRINITY_DN1037_c0_g1_i3 | 5.354367326 | 9.28E-13 | 1.01E-08 | 7.594 | 6.849 | 0.111 | 0.221 | Cpn60_TCP1 |
| --- | --- | --- | --- | --- | --- | --- | --- | --- |
| TRINITY_DN1202_c0_g1_i2 | -5.34207542 | 0.018738279 | 0.804979513 | 0 | 0.887 | 9.188 | 24.109 | NA |
| TRINITY_DN1918_c0_g1_i1 | -5.33873649 | 0.00564915 | 0.298655391 | 0.699 | 0.069 | 1.933 | 28.07 | NA |
| TRINITY_DN10102_c0_g1_i7 | -5.33470743 | 0.007321777 | 0.370879236 | 0.391 | 0 | 7.811 | 8.747 | NA |
| TRINITY_DN4397_c0_g2_i3 | -5.32767011 | 0.050210379 | 0.999970678 | 0.463 | 0 | 15.388 | 0.442 | AA_kinase |
| TRINITY_DN836_c0_g1_i7 | 5.318169738 | 0.018749676 | 0.804979513 | 0.409 | 3.371 | 0 | 0.09 | RRM_1 |
| TRINITY_DN1304_c0_g1_i25 | -5.31268607 | 0.00092665 | 0.066393675 | 0.173 | 0.039 | 6.622 | 0.824 | NA |
| TRINITY_DN7009_c0_g1_i1 | -5.31003302 | 0.00163224 | 0.105184519 | 0.554 | 1.853 | 5.866 | 84.411 | NA |
| TRINITY_DN2517_c0_g1_i1 | 5.300139582 | 0.013629131 | 0.622784572 | 1.535 | 0.611 | 0 | 0.06 | NA |
| TRINITY_DN9067_c0_g1_i2 | 5.29906474 | 0.007153621 | 0.364622606 | 0.945 | 0.67 | 0.033 | 0 | NA |
| TRINITY_DN4421_c0_g1_i1 | 5.291830378 | 0.010505561 | 0.507748052 | 0.427 | 0.621 | 0.022 | 0 | NA |
| TRINITY_DN15995_c0_g1_i11 | 5.238652755 | 0.009051189 | 0.448061299 | 0.808 | 1.33 | 0.044 | 0 | NA |
| TRINITY_DN3805_c0_g1_i2 | 5.214127623 | 0.198764612 | 0.999970678 | 25.381 | 0 | 0.578 | 0 | AP2 |
| TRINITY_DN6514_c0_g1_i1 | -5.21315738 | 0.024126209 | 0.974352908 | 0 | 0.404 | 10.388 | 1.599 | NA |
| TRINITY_DN1874_c0_g1_i6 | 5.199722469 | 0.012475486 | 0.580678249 | 3.843 | 1.114 | 0 | 0.141 | NA |
| TRINITY_DN9911_c0_g1_i3 | -5.19705813 | 0.012477171 | 0.580678249 | 0 | 0.049 | 1.133 | 0.503 | FAM178 |
| TRINITY_DN566_c0_g1_i4 | -5.19268044 | 0.020353969 | 0.854762061 | 0 | 0.522 | 11.333 | 4.977 | NA |
| TRINITY_DN7391_c0_g1_i2 | -5.19021445 | 0.01439028 | 0.650289405 | 0 | 0.069 | 0.389 | 1.709 | NA |
| TRINITY_DN8622_c0_g1_i2 | -5.17312577 | 0.019409533 | 0.824640132 | 0.036 | 0 | 1 | 0.352 | NB-ARC |
| TRINITY_DN20818_c0_g1_i3 | -5.15400852 | 0.047190825 | 0.999970678 | 0.754 | 0 | 21.188 | 1.136 | NA |
| TRINITY_DN13_c0_g1_i3 | -5.15026812 | 0.205727505 | 0.999970678 | 1.29 | 0 | 0 | 46.488 | NA |
| TRINITY_DN44145_c0_g1_i1 | -5.09198948 | 0.004314755 | 0.235738607 | 9.084 | 9.964 | 505.069 | 16.347 | Catalase |
| TRINITY_DN2714_c0_g1_i3 | 5.09175443 | 0.215851142 | 0.999970678 | 81.238 | 0 | 0 | 2.403 | PB1 |
| TRINITY_DN393_c0_g1_i4 | 5.080147072 | 0.209115894 | 0.999970678 | 9.356 | 0 | 0.233 | 0 | NA |
| TRINITY_DN9775_c0_g1_i2 | 5.0609324 | 0.206629828 | 0.999970678 | 0 | 11.41 | 0 | 0.332 | Adaptin_N |
| TRINITY_DN275_c0_g1_i5 | 5.060368992 | 0.206860152 | 0.999970678 | 0 | 7.155 | 0.167 | 0 | ABC_membrane |
| TRINITY_DN12_c0_g1_i2 | 5.053657689 | 0.02983123 | 0.999970678 | 16.269 | 9.234 | 0.633 | 0 | NA |
| TRINITY_DN539_c0_g1_i9 | -5.04872515 | 0.064706334 | 0.999970678 | 0.009 | 2.681 | 1.755 | 85.306 | NA |
| TRINITY_DN3160_c0_g1_i3 | 5.02872301 | 0.016166973 | 0.709003157 | 0.954 | 3.233 | 0 | 0.121 | Myb_DNA-bind_ |
| TRINITY_DN2729_c0_g1_i2 | 5.023255517 | 0.210714006 | 0.999970678 | 21.384 | 0 | 0 | 0.674 | NA |
| TRINITY_DN1978_c0_g1_i4 | -5.02223163 | 0.000205057 | 0.020363556 | 0.064 | 0.128 | 1.278 | 4.162 | NA |
| TRINITY_DN10842_c0_g1_i2 | 5.022053372 | 0.129499697 | 0.999970678 | 0.173 | 207.7 | 0 | 6.304 | NA |
| TRINITY_DN18248_c0_g1_i8 | 5.019845079 | 0.025568065 | 0.999970678 | 6.786 | 5.44 | 0 | 0.382 | ABC_membrane |
| TRINITY_DN153_c0_g1_i4 | -4.99684601 | 0.000856752 | 0.062752378 | 0.045 | 0.049 | 1.967 | 0.533 | bZIP_2 |
| TRINITY_DN509_c0_g1_i13 | 4.99472654 | 0.110441905 | 0.999970678 | 0 | 153.6 | 3.2 | 0.875 | NA |
| TRINITY_DN98_c0_g1_i7 | -4.98865831 | 0.033068552 | 0.999970678 | 0 | 0.907 | 18.288 | 6.223 | Sigma70_r2 |
| TRINITY_DN1771_c0_g1_i5 | 4.985046303 | 0.013138221 | 0.607145616 | 5.568 | 2.759 | 0 | 0.241 | NA |
| TRINITY_DN6501_c1_g1_i3 | 4.984834789 | 0.000372448 | 0.032192113 | 2.253 | 0.877 | 0.056 | 0.04 | NA |
| TRINITY_DN717_c0_g1_i8 | 4.949443147 | 0.016113493 | 0.708478394 | 0.427 | 64.11 | 1.111 | 0.714 | Band_7 |
| TRINITY_DN751_c0_g1_i2 | -4.94192554 | 0.006456992 | 0.333801986 | 0.427 | 7.667 | 27.554 | 192.237 | NA |
| TRINITY_DN5718_c1_g1_i3 | -4.94092872 | 0.215164296 | 0.999970678 | 0.89 | 0 | 22.91 | 0 | Ferritin_2 |
| TRINITY_DN1149_c0_g1_i12 | 4.940875162 | 0.22732901 | 0.999970678 | 19.839 | 0 | 0 | 0.653 | NA |
| TRINITY_DN4093_c0_g1_i9 | 4.929295357 | 0.021316672 | 0.889474213 | 2.871 | 0.769 | 0 | 0.121 | - |
| TRINITY_DN2915_c0_g1_i3 | -4.91507815 | 0.030486375 | 0.999970678 | 0 | 0.818 | 16.888 | 4.122 | NA |
| TRINITY_DN717_c0_g1_i3 | 4.877373114 | 0.004185537 | 0.229446085 | 1.181 | 31.71 | 0.422 | 0.593 | Band_7 |
| TRINITY_DN3915_c0_g1_i8 | 4.870596274 | 0.012956352 | 0.599589134 | 2.861 | 2.513 | 0 | 0.181 | TRAPP |
| TRINITY_DN1009_c0_g1_i9 | 4.8540661 | 0.017367981 | 0.754583333 | 0.718 | 1.055 | 0 | 0.06 | NA |
| TRINITY_DN1326_c0_g1_i9 | -4.84538425 | 0.031268222 | 0.999970678 | 0.236 | 0 | 4.555 | 1.327 | - |
| TRINITY_DN8138_c0_g1_i5 | -4.81912094 | 0.228513014 | 0.999970678 | 0.59 | 0 | 14.077 | 0 | NA |
| TRINITY_DN8435_c0_g1_i2 | 4.817361061 | 0.039985269 | 0.999970678 | 0.763 | 6.455 | 0 | 0.261 | NA |
| TRINITY_DN1090_c0_g1_i1 | 4.811772825 | 0.035117697 | 0.999970678 | 0.618 | 4.208 | 0 | 0.171 | NA |
| TRINITY_DN9995_c0_g1_i2 | -4.78981814 | 0.026636037 | 0.999970678 | 0.154 | 0 | 2.5 | 1.367 | NA |
| TRINITY_DN1783_c0_g1_i10 | -4.78970367 | 0.24081438 | 0.999970678 | 0.772 | 0 | 0 | 21.877 | Orn_Arg_deC_N |
| TRINITY_DN751_c0_g1_i1 | -4.77299938 | 0.005974605 | 0.313830058 | 1.508 | 17.91 | 44.264 | 451.723 | NA |
| TRINITY_DN5717_c0_g1_i1 | -4.76614612 | 0.090030874 | 0.999970678 | 0 | 0.591 | 12.755 | 0.241 | RPE65 |
| TRINITY_DN20832_c0_g1_i1 | -4.7641798 | 0.027047672 | 0.999970678 | 0.282 | 0 | 3.666 | 3.147 | NA |
| TRINITY_DN4747_c1_g1_i5 | -4.76080683 | 0.057404894 | 0.999970678 | 0 | 0.512 | 0.789 | 12.467 | NA |
| TRINITY_DN2132_c1_g2_i6 | 4.74426877 | 0.027741158 | 0.999970678 | 0.454 | 1.124 | 0.056 | 0 | TAXi_C |
| TRINITY_DN509_c0_g1_i8 | 4.740301573 | 0.11272941 | 0.999970678 | 0 | 34.94 | 0.867 | 0.251 | NA |
| TRINITY_DN539_c0_g1_i7 | 4.706786388 | 0.017643223 | 0.765523747 | 2.289 | 236.7 | 5 | 2.936 | - |
| TRINITY_DN12055_c0_g1_i2 | 4.700058609 | 0.023105495 | 0.938933761 | 0.245 | 0.296 | 0 | 0.02 | NA |
| TRINITY_DN459_c0_g1_i8 | -4.69105063 | 0.003543943 | 0.198266619 | 0.263 | 1.212 | 2.933 | 33.569 | - |
| TRINITY_DN1689_c0_g1_i1 | -4.67384299 | 0.037097127 | 0.999970678 | 0.191 | 0 | 3.222 | 1.116 | NA |
| TRINITY_DN8030_c0_g1_i4 | 4.671521583 | 0.048413369 | 0.999970678 | 4.451 | 0.769 | 0.167 | 0 | NA |
| TRINITY_DN2786_c0_g1_i3 | -4.6680985 | 0.029652736 | 0.999970678 | 0 | 0.079 | 0.467 | 1.428 | NA |
| TRINITY_DN5438_c0_g1_i2 | 4.668049329 | 0.002903353 | 0.167298656 | 17.269 | 2.769 | 0.311 | 0.442 | NA |
| TRINITY_DN535_c0_g1_i4 | -4.66734008 | 0.020322511 | 0.854762061 | 0.173 | 0.995 | 0.5 | 28.733 | Pkinase |
| TRINITY_DN2796_c0_g1_i6 | 4.667006402 | 0.025797857 | 0.999970678 | 23.927 | 10.93 | 0 | 1.327 | NA |
| TRINITY_DN217_c0_g1_i6 | -4.65896916 | 0.046524373 | 0.999970678 | 0 | 0.621 | 10.433 | 3.127 | NA |
| TRINITY_DN42769_c0_g1_i1 | -4.65891138 | 0.12696224 | 0.999970678 | 0 | 0.621 | 12.755 | 0.06 | DPBB_1 |
| TRINITY_DN2046_c0_g1_i1 | -4.65061805 | 0.053652992 | 0.999970678 | 0.799 | 31.47 | 3.722 | 787.345 | NA |
| TRINITY_DN551_c0_g1_i7 | 4.649725149 | 0.031316146 | 0.999970678 | 1.517 | 2.868 | 0.144 | 0 | Cellulose_synt |
| TRINITY_DN8993_c0_g1_i16 | 4.64452008 | 0.036350625 | 1 | 0.945 | 1.005 | 0 | 0.09 | NA |
| TRINITY_DN721_c0_g2_i2 | -4.63426097 | 0.074222295 | 0.999970678 | 0 | 1.124 | 21.065 | 1.578 | UDPGT |

| TRINITY_DN2018_c0_g1_i3 | -4.63122736 | 0.078157453 | 0.999970678 | 0 | 1.715 | 33.22 | 2.021 | NA |
| --- | --- | --- | --- | --- | --- | --- | --- | --- |
| TRINITY_DN3297_c0_g1_i3 | -4.62942363 | 0.031516275 | 1 | 0 | 0.069 | 0.611 | 0.794 | Ist1 |
| TRINITY_DN2764_c0_g1_i10 | 4.627788478 | 0.025921843 | 0.999970678 | 1.544 | 0.956 | 0 | 0.101 | UAA |
| TRINITY_DN3389_c0_g1_i9 | -4.62225744 | 0.042649889 | 0.999970678 | 0 | 1.626 | 25.843 | 8.686 | NA |
| TRINITY_DN2514_c0_g1_i1 | 4.618959404 | 0.021957574 | 0.907518796 | 0.699 | 36.86 | 0.156 | 1.297 | NA |
| TRINITY_DN7217_c0_g1_i3 | 4.610350314 | 0.023045671 | 0.938838124 | 6.359 | 9.51 | 0 | 0.613 | NA |
| TRINITY_DN2536_c8_g3_i1 | 4.603581932 | 0.249964025 | 0.999970678 | 22.501 | 0 | 0 | 0.955 | NA |
| TRINITY_DN3286_c0_g1_i6 | -4.59599745 | 2.01E-05 | 0.003147466 | 0.818 | 1.429 | 10.144 | 42.075 | p450 |
| TRINITY_DN4897_c0_g1_i2 | -4.58053208 | 0.043989878 | 0.999970678 | 0 | 0.67 | 9.599 | 4.021 | NA |
| TRINITY_DN3898_c1_g1_i2 | -4.57953496 | 0.051110403 | 0.999970678 | 0 | 0.306 | 4.9 | 1.166 | MatE |
| TRINITY_DN751_c0_g1_i5 | -4.56777759 | 0.002976186 | 0.1708561 | 0.754 | 7.963 | 48.753 | 134.066 | Hydrophob_seed |
| TRINITY_DN4545_c2_g1_i1 | -4.55463145 | 0.035110128 | 0.999970678 | 0 | 0.207 | 2.6 | 1.78 | ABC1 |
| TRINITY_DN1035_c0_g2_i6 | -4.55411077 | 2.64E-06 | 0.000629544 | 0.372 | 1.094 | 11.399 | 20.489 | Pyrophosphatase |
| TRINITY_DN4328_c0_g1_i2 | -4.54696248 | 0.097562785 | 0.999970678 | 0 | 0.601 | 11.266 | 0.322 | NA |
| TRINITY_DN3256_c0_g1_i3 | 4.544831221 | 0.066344738 | 0.999970678 | 10.619 | 33.23 | 0 | 1.87 | NA |
| TRINITY_DN3504_c1_g1_i4 | -4.54174362 | 0.007737094 | 0.388304686 | 0.808 | 1.537 | 1.589 | 52.712 | DEAD |
| TRINITY_DN1354_c1_g1_i6 | -4.53725484 | 0.000145959 | 0.015483006 | 1.381 | 1.508 | 37.931 | 20.218 | NA |
| TRINITY_DN5277_c1_g1_i1 | -4.53207829 | 3.16E-05 | 0.004589895 | 0.136 | 0.217 | 4.766 | 2.232 | - |
| TRINITY_DN14813_c0_g1_i9 | 4.526229092 | 0.256325277 | 0.999970678 | 0 | 10.96 | 0 | 0.462 | NA |
| TRINITY_DN7075_c0_g1_i1 | -4.5201077 | 0.003459652 | 0.194216094 | 0.282 | 1.025 | 21.043 | 4.212 | NA |
| TRINITY_DN8115_c0_g1_i1 | -4.50414011 | 0.000206853 | 0.020479675 | 0.045 | 0.03 | 0.756 | 0.885 | NA |
| TRINITY_DN1337_c0_g1_i4 | -4.49239638 | 0.017297196 | 0.753512004 | 0.209 | 1.38 | 1.022 | 34.112 | p450 |
| TRINITY_DN370_c0_g1_i3 | -4.48637047 | 0.11945563 | 0.999970678 | 3.806 | 0 | 66.718 | 6.002 | NA |
| TRINITY_DN5426_c0_g2_i4 | -4.47959186 | 0.265217586 | 0.999970678 | 8.13 | 0 | 147.98 | 0 | NA |
| TRINITY_DN6346_c0_g1_i4 | -4.47756313 | 0.000841471 | 0.061920118 | 0.445 | 0.552 | 15.943 | 2.664 | S6PP |
| TRINITY_DN1151_c0_g2_i1 | -4.47602891 | 0.037156981 | 0.999970678 | 0.182 | 0 | 1.922 | 2.312 | NA |
| TRINITY_DN438_c0_g1_i11 | -4.46837346 | 0.054434231 | 0.999970678 | 1.308 | 0 | 19.177 | 5.278 | NA |
| TRINITY_DN73_c1_g1_i8 | 4.46742608 | 0.040843695 | 0.999970678 | 0.836 | 1.863 | 0 | 0.121 | NA |
| TRINITY_DN478_c0_g2_i4 | 4.465183294 | 0.036499476 | 1 | 0.781 | 0.818 | 0 | 0.07 | P34-Arc |
| TRINITY_DN3108_c0_g1_i3 | 4.461424576 | 0.040821987 | 0.999970678 | 11.9 | 9.895 | 0 | 0.965 | Bystin |
| TRINITY_DN2714_c0_g1_i1 | 4.461076448 | 0.160275991 | 0.999970678 | 63.606 | 0.108 | 0 | 2.946 | PB1 |
| TRINITY_DN5381_c0_g1_i8 | 4.454667998 | 0.07102471 | 0.999970678 | 0.618 | 5.568 | 0.233 | 0 | ABC_membrane |
| TRINITY_DN7068_c0_g1_i2 | -4.45369813 | 0.039717972 | 0.999970678 | 0.354 | 0 | 3.433 | 2.966 | Ribosomal_L30 |
| TRINITY_DN482_c0_g3_i2 | 4.452706328 | 0.03474789 | 0.999970678 | 2.725 | 5.844 | 0 | 0.382 | EPF |
| TRINITY_DN921_c28_g1_i1 | 4.449862687 | 0.028387458 | 0.999970678 | 492.1 | 4.011 | 13.888 | 6.253 | PAPS_reduct |
| TRINITY_DN84_c0_g1_i4 | -4.4412066 | 0.045492565 | 0.999970678 | 0 | 0.207 | 0.967 | 3.318 | NA |
| TRINITY_DN19740_c0_g1_i5 | -4.43823222 | 0.001814704 | 0.114459456 | 0.036 | 0.039 | 0.878 | 0.905 | - |
| TRINITY_DN10806_c0_g1_i8 | -4.42387238 | 0.042552048 | 0.999970678 | 0 | 0.158 | 2.089 | 1.056 | UDPGT |
| TRINITY_DN6833_c1_g1_i8 | 4.411485948 | 0.058110316 | 1 | 6.722 | 5.046 | 0.444 | 0 | NA |
| TRINITY_DN1438_c0_g1_i4 | 4.397285258 | 0.041922332 | 0.999970678 | 1.081 | 0.532 | 0 | 0.08 | NA |
| TRINITY_DN284_c0_g1_i5 | -4.39105914 | 0.108450381 | 0.999970678 | 0.427 | 0 | 7.211 | 0.271 | NA |
| TRINITY_DN117_c0_g1_i1 | -4.38965857 | 0.055968487 | 0.999970678 | 0.336 | 8.939 | 1.455 | 188.326 | LOR |
| TRINITY_DN1868_c1_g1_i8 | -4.38584451 | 0.010836481 | 0.521362441 | 0.463 | 0.168 | 10.011 | 0.824 | NA |
| TRINITY_DN843_c1_g1_i3 | -4.37676025 | 0.038989898 | 0.999970678 | 0 | 0.168 | 1.678 | 1.639 | NAM |
| TRINITY_DN48762_c0_g1_i3 | 4.366037088 | 0.016562078 | 0.72438582 | 0.663 | 4.987 | 0.011 | 0.251 | NA |
| TRINITY_DN2141_c0_g1_i1 | -4.3653455 | 0.000150611 | 0.01592475 | 0.145 | 0.148 | 1.611 | 3.851 | NA |
| TRINITY_DN5765_c1_g1_i3 | -4.36429536 | 0.050211341 | 0.999970678 | 0 | 0.118 | 0.556 | 1.991 | NA |
| TRINITY_DN8953_c0_g1_i2 | -4.36378097 | 0.002698659 | 0.157956267 | 0.127 | 0.128 | 0.589 | 4.434 | NA |
| TRINITY_DN2100_c0_g1_i20 | 4.354913936 | 0.036816844 | 0.999970678 | 1.226 | 1.005 | 0 | 0.111 | NA |
| TRINITY_DN15778_c1_g1_i3 | 4.354235099 | 0.050023484 | 0.999970678 | 2.771 | 1.37 | 0.156 | 0 | Cation_ATPase |
| TRINITY_DN12795_c0_g1_i6 | 4.347882463 | 0.048489406 | 0.999970678 | 0.454 | 1.36 | 0 | 0.09 | ABC2_membrane |
| TRINITY_DN627_c0_g1_i1 | -4.33610903 | 0.047065319 | 0.999970678 | 0.109 | 0 | 0.9 | 1.247 | NA |
| TRINITY_DN751_c0_g1_i3 | -4.32769764 | 0.004876898 | 0.261638756 | 5.005 | 58.03 | 227.397 | 948.727 | Hydrophob_seed |
| TRINITY_DN29558_c0_g1_i1 | 4.312844945 | 0.061067522 | 1 | 1.026 | 0.453 | 0.067 | 0 | NA |
| TRINITY_DN7441_c0_g1_i9 | 4.312746543 | 0.056300668 | 1 | 0.354 | 0.808 | 0 | 0.05 | - |
| TRINITY_DN8688_c0_g1_i9 | 4.311700754 | 0.042968074 | 0.999970678 | 0.545 | 1.163 | 0 | 0.09 | NA |
| TRINITY_DN1100_c0_g1_i3 | -4.29961 | 0.285904767 | 0.999970678 | 0.972 | 0 | 15.821 | 0 | PUNUT |
| TRINITY_DN26629_c0_g1_i1 | -4.29837494 | 0.053332674 | 0.999970678 | 0.845 | 0 | 9.111 | 6.153 | NA |
| TRINITY_DN6954_c0_g2_i1 | -4.29189013 | 0.07832238 | 0.999970678 | 0 | 1.084 | 3.778 | 16.337 | NA |
| TRINITY_DN9427_c0_g1_i2 | 4.28913186 | 0.055644805 | 0.999970678 | 10.219 | 13.53 | 0 | 1.216 | NA |
| TRINITY_DN16776_c0_g1_i1 | -4.28305365 | 3.53E-05 | 0.004923514 | 0.327 | 0.148 | 2.933 | 5.68 | Glyco_hydro_16 |
| TRINITY_DN3343_c0_g2_i6 | -4.28219964 | 0.062452241 | 0.999970678 | 0.2 | 0 | 2.367 | 0.865 | CRAL_TRIO |
| TRINITY_DN3988_c0_g1_i18 | 4.282067585 | 0.089570838 | 0.999970678 | 0.372 | 4.297 | 0.2 | 0 | Phosphorylase |
| TRINITY_DN3151_c0_g1_i2 | -4.28170417 | 0.000699164 | 0.053248624 | 0.936 | 0.434 | 6.233 | 18.72 | NA |
| TRINITY_DN4383_c0_g1_i1 | -4.26722805 | 0.010878407 | 0.521907966 | 0.154 | 0.148 | 4.533 | 0.312 | CarD_CdnL_TRCF |
| TRINITY_DN1658_c0_g1_i1 | -4.26367618 | 0.082220866 | 0.999970678 | 0.436 | 0 | 6.144 | 1.005 | NA |
| TRINITY_DN9987_c0_g1_i6 | 4.257476777 | 0.187235556 | 0.999970678 | 0 | 235 | 0.422 | 10.989 | NA |
| TRINITY_DN14939_c0_g1_i5 | 4.252055798 | 0.002660887 | 0.156079885 | 3.161 | 3.4 | 0.167 | 0.181 | NA |
| TRINITY_DN8186_c0_g1_i3 | -4.25182058 | 0.066721881 | 0.999970678 | 0.154 | 0 | 1.967 | 0.684 | F-box |
| TRINITY_DN1645_c1_g1_i1 | -4.24114515 | 0.101034691 | 0.999970678 | 3.261 | 0 | 10.633 | 50.027 | NA |
| TRINITY_DN5393_c0_g1_i3 | 4.239525402 | 0.137902091 | 0.999970678 | 0.282 | 21.63 | 0 | 1.146 | MENTAL |
| TRINITY_DN1015_c0_g2_i8 | 4.232426294 | 0.009463156 | 1 | 0.627 | 0.532 | 0.033 | 0.01 | NA |
| TRINITY_DN3249_c1_g1_i3 | 4.227244906 | 0.05585608 | 1 | 0.654 | 0.631 | 0 | 0.08 | NA |
| TRINITY_DN2156_c0_g1_i6 | -4.22337081 | 0.070138339 | 0.999970678 | 0.518 | 6.485 | 0.444 | 127.008 | NA |

| TRINITY_DN1128_c0_g1_i3 | -4.22319858 | 0.039384333 | 0.999970678 | 0.345 | 2.286 | 0.556 | 47.624 | GDP_Man_Dehyd |
| --- | --- | --- | --- | --- | --- | --- | --- | --- |
| TRINITY_DN13473_c0_g1_i1 | 4.217850467 | 0.07493262 | 1 | 0.536 | 0.542 | 0 | 0.06 | NA |
| TRINITY_DN3541_c1_g1_i2 | -4.21754696 | 2.64E-05 | 0.00391424 | 1.699 | 2.75 | 51.241 | 20.59 | Glyco_hydro_32C |
| TRINITY_DN8314_c0_g1_i1 | -4.21537321 | 0.065848367 | 0.999970678 | 0 | 0.601 | 2.289 | 8.425 | NA |
| TRINITY_DN6867_c0_g1_i4 | 4.214853908 | 0.292530878 | 0.999970678 | 5.187 | 0 | 0 | 0.282 | TCP |
| TRINITY_DN2469_c0_g1_i9 | -4.20842982 | 0.002278223 | 0.137840931 | 0.082 | 0.256 | 3.378 | 1.91 | Glyco_hydro_14 |
| TRINITY_DN8904_c0_g1_i6 | -4.19591461 | 0.057373687 | 0.999970678 | 0.091 | 0 | 0.667 | 1.015 | - |
| TRINITY_DN1234_c0_g1_i10 | -4.19160692 | 0.108632294 | 0.999970678 | 0 | 1.36 | 18.977 | 1.518 | NA |
| TRINITY_DN12805_c0_g2_i1 | -4.1900481 | 0.060737449 | 0.999970678 | 0 | 0.601 | 6.355 | 3.046 | NA |
| TRINITY_DN102_c0_g1_i7 | 4.186611639 | 0.297795415 | 0.999970678 | 0 | 11.32 | 0.5 | 0 | NA |
| TRINITY_DN10999_c0_g1_i2 | -4.18528302 | 0.060005677 | 1 | 0 | 0.049 | 0.411 | 0.422 | NA |
| TRINITY_DN1023_c0_g1_i9 | 4.184839888 | 0.01357261 | 0.62107045 | 0.736 | 2.484 | 0.122 | 0.04 | NA |
| TRINITY_DN1371_c0_g1_i20 | -4.18410074 | 0.057690321 | 0.999970678 | 0.263 | 0 | 1.589 | 2.614 | Terpene_synth |
| TRINITY_DN8293_c0_g1_i10 | -4.18199906 | 0.002480895 | 0.147642598 | 0.481 | 0.187 | 2.033 | 10.365 | YIF1 |
| TRINITY_DN70956_c0_g1_i2 | 4.180488363 | 0.157153369 | 0.999970678 | 0 | 34.47 | 0.4 | 1.397 | NA |
| TRINITY_DN2012_c0_g1_i7 | 4.177977192 | 0.049017071 | 0.999970678 | 3.152 | 2.355 | 0 | 0.282 | NA |
| TRINITY_DN3413_c0_g1_i2 | 4.173561406 | 0.068835139 | 1 | 0.563 | 0.335 | 0.044 | 0 | Myb_DNA-bindin |
| TRINITY_DN2915_c0_g1_i5 | 4.16147196 | 0.000438485 | 0.036639885 | 2.961 | 10.74 | 0.278 | 0.392 | NA |
| TRINITY_DN2964_c0_g1_i7 | -4.15942498 | 0.081174414 | 0.999970678 | 0.263 | 0 | 0.656 | 3.841 | - |
| TRINITY_DN271_c0_g1_i7 | -4.15756647 | 0.153432472 | 0.999970678 | 0 | 3.518 | 0.644 | 60.302 | NA |
| TRINITY_DN3140_c0_g1_i6 | 4.156439005 | 0.300944516 | 0.999970678 | 0 | 14.34 | 0 | 0.784 | PGM_PMM_I |
| TRINITY_DN2040_c0_g1_i6 | -4.15551113 | 0.306442448 | 0.999970678 | 1.572 | 0 | 0 | 28.462 | Abhydrolase_1 |
| TRINITY_DN2183_c0_g2_i1 | 4.15024213 | 0.000679639 | 0.052247469 | 29.368 | 11.82 | 0.767 | 1.397 | NA |
| TRINITY_DN4990_c0_g1_i1 | 4.149325774 | 0.333041148 | 0.999970678 | 0 | 58.79 | 0 | 3.267 | NA |
| TRINITY_DN2507_c0_g1_i3 | 4.148084252 | 0.167758294 | 0.999970678 | 48.29 | 0 | 1.533 | 0.915 | - |
| TRINITY_DN3806_c0_g1_i1 | 4.148083872 | 0.065088634 | 0.999970678 | 0.772 | 2.553 | 0 | 0.181 | Glyco_transf_34 |
| TRINITY_DN20776_c0_g1_i1 | -4.14634888 | 8.11E-05 | 0.009632822 | 2.861 | 2.01 | 18.499 | 62.212 | NA |
| TRINITY_DN707_c0_g1_i16 | 4.145897797 | 0.300675022 | 0.999970678 | 2.362 | 0 | 0 | 0.141 | NA |
| TRINITY_DN2389_c0_g1_i2 | 4.139115451 | 0.091091906 | 0.999970678 | 16.878 | 8.653 | 1.211 | 0 | 3HCDH |
| TRINITY_DN37478_c0_g1_i2 | 4.13286454 | 0.023270173 | 0.943279256 | 427.55 | 275.9 | 0.644 | 37.199 | Pro_CA |
| TRINITY_DN251_c0_g1_i4 | 4.129643866 | 0.00554322 | 0.294006635 | 16.705 | 6.751 | 0.133 | 1.146 | NA |
| TRINITY_DN1413_c0_g1_i2 | -4.12948511 | 0.15069472 | 0.999970678 | 0 | 2.799 | 38.92 | 1.166 | NA |
| TRINITY_DN2787_c0_g1_i6 | -4.11683185 | 0.078253742 | 1 | 0.427 | 0 | 2.944 | 3.438 | CIA30 |
| TRINITY_DN1883_c0_g1_i1 | 4.115629844 | 0.178868704 | 0.999970678 | 74.506 | 0 | 1.244 | 2.865 | NA |
| TRINITY_DN3356_c0_g1_i8 | 4.115334835 | 0.317646445 | 0.999970678 | 0 | 13.46 | 0 | 0.764 | NA |
| TRINITY_DN3152_c0_g2_i3 | -4.11085264 | 0.315924365 | 0.999970678 | 1.199 | 0 | 17.21 | 0 | NA |
| TRINITY_DN30748_c0_g1_i2 | 4.10991658 | 2.74E-05 | 0.00402665 | 200.98 | 67.38 | 6.089 | 8.164 | NA |
| TRINITY_DN15372_c0_g1_i2 | 4.107655659 | 0.069328044 | 0.999970678 | 0.863 | 0.503 | 0.067 | 0 | RRM_1 |
| TRINITY_DN16963_c0_g1_i7 | -4.10734099 | 0.131388984 | 0.999970678 | 0.572 | 0 | 8 | 0.422 | NA |
| TRINITY_DN958_c0_g1_i7 | 4.105072144 | 0.001994183 | 0.12386685 | 0.6 | 0.976 | 0.033 | 0.06 | CBS |
| TRINITY_DN8126_c1_g1_i2 | 4.096724123 | 0.012188483 | 0.571635293 | 84.108 | 6.475 | 0.8 | 4.424 | AP2 |
| TRINITY_DN284_c0_g1_i6 | -4.09590885 | 0.099369101 | 0.999970678 | 0.754 | 0 | 2.011 | 10.737 | NA |
| TRINITY_DN721_c1_g1_i2 | -4.09558961 | 0.151466194 | 0.999970678 | 1.054 | 0.71 | 0 | 30.231 | NA |
| TRINITY_DN3155_c0_g1_i2 | -4.09415791 | 0.063485473 | 0.999970678 | 0 | 0.729 | 3.455 | 7.52 | NA |
| TRINITY_DN8087_c0_g2_i2 | -4.09374001 | 0.031494418 | 0.999970678 | 4.288 | 7.234 | 2.133 | 192.94 | Lipase_GDSL |
| TRINITY_DN45_c1_g1_i4 | -4.09044483 | 0.0812311 | 0.999970678 | 0.064 | 0 | 0.2 | 0.754 | GAF |
| TRINITY_DN1268_c0_g1_i5 | 4.086756287 | 0.021726236 | 0.901956259 | 0.745 | 8.022 | 0.067 | 0.422 | NA |
| TRINITY_DN2152_c0_g1_i1 | -4.08635526 | 0.068328473 | 0.999970678 | 0.727 | 0 | 6.733 | 4.776 | DUF247 |
| TRINITY_DN1683_c0_g1_i4 | -4.08206537 | 0.065420436 | 1 | 0 | 0.128 | 1.033 | 1.156 | NA |
| TRINITY_DN2154_c0_g1_i4 | -4.07982367 | 0.069657661 | 0.999970678 | 0 | 0.483 | 2.167 | 5.71 | NA |
| TRINITY_DN1827_c0_g1_i5 | -4.07878381 | 0.006377126 | 1 | 0.082 | 0.099 | 1.533 | 1.397 | AP2 |
| TRINITY_DN825_c0_g1_i4 | 4.062689184 | 0.007464839 | 0.376192409 | 5.35 | 1.833 | 0.044 | 0.382 | NA |
| TRINITY_DN5795_c0_g2_i8 | 4.060087623 | 0.074602252 | 1 | 0.627 | 0.473 | 0 | 0.07 | NA |
| TRINITY_DN14139_c0_g1_i1 | -4.04675588 | 0.002383163 | 0.143129943 | 1.244 | 2.306 | 24.876 | 25.305 | NA |
| TRINITY_DN1304_c0_g1_i10 | 4.042715688 | 0.005739269 | 0.302929583 | 15.061 | 2.809 | 0.756 | 0.181 | NA |
| TRINITY_DN16573_c0_g1_i1 | -4.03303524 | 0.039533198 | 0.999970678 | 0.391 | 3.518 | 1.467 | 61.539 | NA |
| TRINITY_DN1354_c1_g1_i2 | -4.02905781 | 0.000372154 | 0.032192113 | 1.326 | 1.557 | 9.155 | 33.861 | NA |
| TRINITY_DN1459_c0_g1_i5 | 4.024347947 | 0.092664927 | 0.999970678 | 0.445 | 2.937 | 0 | 0.211 | NA |
| TRINITY_DN5073_c0_g1_i2 | 4.023873244 | 0.06316538 | 0.999970678 | 1.78 | 3.035 | 0 | 0.292 | NA |
| TRINITY_DN2297_c0_g1_i1 | 4.02193139 | 0.315063713 | 0.999970678 | 0 | 6.83 | 0 | 0.412 | NA |
| TRINITY_DN4826_c0_g1_i4 | -4.01993713 | 0.06761625 | 0.999970678 | 0 | 0.138 | 0.844 | 1.196 | NA |
| TRINITY_DN5533_c0_g1_i1 | 4.019888047 | 0.316329806 | 0.999970678 | 0 | 17.56 | 0 | 1.056 | Per1 |
| TRINITY_DN5976_c0_g1_i3 | 4.016715312 | 0.174937705 | 0.999970678 | 0 | 19.47 | 0.767 | 0.251 | ARD |
| TRINITY_DN18122_c0_g1_i1 | -4.01192185 | 0.320821084 | 0.999970678 | 0.763 | 0 | 10.555 | 0 | NA |
| TRINITY_DN203_c0_g1_i12 | 4.010107834 | 0.037096555 | 0.999970678 | 0.681 | 9.806 | 0.033 | 0.583 | NA |
| TRINITY_DN3102_c0_g1_i5 | 4.006442404 | 0.324492451 | 0.999970678 | 109.12 | 0 | 5.7 | 0 | Ras |
| TRINITY_DN1899_c0_g1_i6 | -4.00561138 | 0.012116599 | 0.570422952 | 0.182 | 0.089 | 3.344 | 0.573 | NA |
| TRINITY_DN2724_c0_g1_i6 | -4.00308714 | 3.85E-08 | 1.94E-05 | 0.363 | 0.473 | 5.155 | 7.088 | EGF_CA |
| TRINITY_DN799_c0_g2_i7 | -3.9964549 | 0.032470779 | 0.999970678 | 0.345 | 0.01 | 3.622 | 1.347 | FA_desaturase |
| TRINITY_DN34_c1_g1_i4 | 3.996118548 | 0.078880207 | 0.999970678 | 1.499 | 3.745 | 0.267 | 0 | NA |
| TRINITY_DN13234_c0_g1_i3 | -3.99116264 | 0.079607182 | 0.999970678 | 0.036 | 0 | 0.189 | 0.432 | NA |
| TRINITY_DN9636_c0_g1_i17 | 3.990492999 | 0.067981653 | 0.999970678 | 4.188 | 5.815 | 0 | 0.603 | NA |
| TRINITY_DN3250_c0_g1_i11 | -3.9846691 | 0.072418754 | 0.999970678 | 46.265 | 0.177 | 119.237 | 590.986 | NA |
| TRINITY_DN86_c0_g1_i1 | -3.97693962 | 0.129917422 | 0.999970678 | 3.179 | 0 | 5.444 | 44.186 | NA |

| TRINITY_DN21888_c0_g1_i1 | -3.96543574 | 0.080801725 | 0.999970678 | 0.227 | 0 | 1.011 | 2.242 | NA |
| --- | --- | --- | --- | --- | --- | --- | --- | --- |
| TRINITY_DN754_c1_g1_i3 | 3.961529601 | 0.326454239 | 0.999970678 | 0 | 10.85 | 0.556 | 0 | LRR_6 |
| TRINITY_DN2_c0_g1_i1 | 3.960493504 | 0.32542247 | 0.999970678 | 3.824 | 0 | 0 | 0.251 | NA |
| TRINITY_DN1139_c0_g1_i5 | 3.959814162 | 0.081231669 | 0.999970678 | 0.1 | 38.27 | 1.211 | 0.925 | Band_7 |
| TRINITY_DN3481_c0_g1_i1 | 3.958255908 | 0.095429733 | 0.999970678 | 0.391 | 2.119 | 0 | 0.151 | Arm |
| TRINITY_DN5024_c0_g1_i9 | 3.956278054 | 0.012190912 | 0.571635293 | 0.209 | 1.271 | 0.033 | 0.06 | NA |
| TRINITY_DN3772_c0_g1_i7 | 3.952373467 | 0.082469086 | 0.999970678 | 3.824 | 4.908 | 0 | 0.553 | Cu_amine_oxid |
| TRINITY_DN5283_c0_g1_i6 | -3.9518737 | 9.23E-05 | 0.010660727 | 0.427 | 1.025 | 12.244 | 7.359 | CitMHS |
| TRINITY_DN3819_c0_g2_i7 | 3.951303586 | 0.104595257 | 0.999970678 | 0.518 | 3.922 | 0 | 0.282 | Cellulose_synt |
| TRINITY_DN5470_c0_g1_i2 | -3.95081965 | 0.007472725 | 0.376192409 | 0.082 | 0.246 | 3.089 | 1.337 | NA |
| TRINITY_DN453_c0_g2_i2 | -3.94919407 | 0.00133419 | 0.088960514 | 0.254 | 0.089 | 1.989 | 2.795 | Ribosomal_S17e |
| TRINITY_DN2326_c0_g2_i4 | 3.941828704 | 0.208930883 | 0.999970678 | 0.209 | 74 | 0 | 4.544 | NA |
| TRINITY_DN808_c3_g1_i2 | -3.93932679 | 4.24E-05 | 0.005775559 | 0.409 | 0.266 | 6.144 | 2.986 | RVT_1 |
| TRINITY_DN3476_c0_g1_i1 | -3.93421996 | 0.118776138 | 0.999970678 | 0 | 0.394 | 0.511 | 5.379 | B_lectin |
| TRINITY_DN3188_c0_g1_i5 | 3.933412524 | 0.079284379 | 0.999970678 | 1.78 | 1.064 | 0.156 | 0 | NA |
| TRINITY_DN1786_c0_g1_i1 | -3.9304193 | 0.176393097 | 0.999970678 | 0.291 | 1.045 | 0 | 20.138 | NA |
| TRINITY_DN6565_c0_g1_i1 | -3.92592574 | 0.079063871 | 0.999970678 | 0 | 0.276 | 1.567 | 2.101 | NA |
| TRINITY_DN576_c0_g1_i4 | -3.91904595 | 0.104492829 | 0.999970678 | 0 | 0.611 | 1.278 | 7.56 | NA |
| TRINITY_DN8942_c0_g1_i3 | 3.917849306 | 0.076498976 | 0.999970678 | 1.889 | 0.936 | 0 | 0.191 | Motile_Sperm |
| TRINITY_DN2740_c1_g1_i5 | 3.917649288 | 0.077791839 | 0.999970678 | 2.235 | 2.562 | 0.256 | 0 | Lectin_legB |
| TRINITY_DN13048_c0_g1_i4 | -3.90723098 | 0.335233249 | 0.999970678 | 0.799 | 0 | 10.177 | 0 | NA |
| TRINITY_DN9852_c0_g1_i22 | 3.905253508 | 0.09366551 | 1 | 2.816 | 1.429 | 0.256 | 0 | NA |
| TRINITY_DN23_c0_g1_i5 | 3.90344133 | 0.3359385 | 0.999970678 | 15.052 | 0 | 0 | 1.025 | NA |
| TRINITY_DN1878_c0_g1_i3 | 3.903286002 | 0.143454166 | 0.999970678 | 13.281 | 0.69 | 0 | 0.955 | NA |
| TRINITY_DN4586_c0_g1_i3 | 3.900362791 | 0.333225368 | 0.999970678 | 0 | 8.101 | 0.433 | 0 | Zip |
| TRINITY_DN10_c0_g2_i4 | -3.8926247 | 0.137842086 | 0.999970678 | 1.135 | 0 | 1.255 | 15.654 | NA |
| TRINITY_DN23035_c0_g1_i1 | -3.88917821 | 0.006635637 | 0.341955073 | 0.136 | 0.256 | 1.155 | 4.383 | Glyoxalase |
| TRINITY_DN2887_c0_g1_i3 | 3.888824843 | 0.08863763 | 1 | 2.525 | 1.429 | 0 | 0.231 | NA |
| TRINITY_DN6692_c1_g1_i1 | -3.87890211 | 0.089643895 | 1 | 0 | 0.345 | 2.544 | 2.151 | NA |
| TRINITY_DN3093_c0_g3_i2 | -3.87743441 | 0.00107981 | 0.074586759 | 0.118 | 0.315 | 1.533 | 4.464 | PTR2 |
| TRINITY_DN6929_c0_g1_i1 | -3.87458795 | 0.091363207 | 0.999970678 | 0.173 | 0 | 0.789 | 1.438 | NA |
| TRINITY_DN7280_c0_g1_i2 | 3.874328951 | 0.187618221 | 0.999970678 | 0 | 9.638 | 0.433 | 0.121 | NA |
| TRINITY_DN373_c0_g1_i5 | 3.872639205 | 0.142912422 | 0.999970678 | 14.271 | 3.617 | 1.022 | 0 | NA |
| TRINITY_DN3092_c0_g1_i3 | 3.869563898 | 0.339449278 | 0.999970678 | 11.637 | 0 | 0 | 0.804 | YTH |
| TRINITY_DN5145_c0_g1_i2 | -3.86624424 | 0.121214123 | 0.999970678 | 0 | 1.192 | 4.433 | 11.843 | ADH_N |
| TRINITY_DN21143_c0_g1_i4 | 3.865155991 | 0.098812732 | 1 | 0.6 | 0.818 | 0 | 0.101 | CAP |
| TRINITY_DN7065_c0_g1_i7 | 3.859142091 | 0.077228038 | 0.999970678 | 2.307 | 3.006 | 0 | 0.362 | NA |
| TRINITY_DN4349_c1_g1_i1 | 3.857724548 | 6.66E-05 | 0.008401951 | 1.008 | 1.281 | 0.056 | 0.09 | NA |
| TRINITY_DN2514_c0_g1_i2 | 3.852126325 | 0.035479785 | 0.999970678 | 1.217 | 28.74 | 0.367 | 1.619 | NA |
| TRINITY_DN997_c1_g1_i6 | 3.85150788 | 0.005116292 | 0.272690872 | 6.822 | 1.064 | 0.289 | 0.201 | NA |
| TRINITY_DN1341_c0_g1_i1 | -3.84971042 | 0.000423906 | 0.0356955 | 2.153 | 4.937 | 64.307 | 23.898 | Hydrolase_6 |
| TRINITY_DN9653_c0_g1_i2 | 3.848957363 | 0.107501702 | 1 | 0.754 | 0.473 | 0.078 | 0 | NA |
| TRINITY_DN12773_c0_g2_i2 | 3.845224794 | 0.095888493 | 1 | 2.952 | 1.606 | 0 | 0.332 | NA |
| TRINITY_DN20756_c0_g1_i5 | 3.83980512 | 0.035343105 | 0.999970678 | 19.349 | 1066 | 35.176 | 30.553 | NA |
| TRINITY_DN4572_c0_g1_i8 | 3.836508645 | 0.361420144 | 0.999970678 | 18.05 | 0 | 0 | 1.287 | ANF_receptor |
| TRINITY_DN3072_c0_g1_i1 | -3.83323814 | 0.341935456 | 0.999970678 | 0 | 3.824 | 0 | 52.862 | B_lectin |
| TRINITY_DN16034_c0_g1_i1 | -3.83001386 | 0.002250018 | 0.136640526 | 0.145 | 0.059 | 0.811 | 2.031 | NA |
| TRINITY_DN1564_c0_g1_i1 | -3.82987438 | 0.139542303 | 0.999970678 | 2.08 | 0 | 22.288 | 3.167 | CIAPIN1 |
| TRINITY_DN3201_c0_g1_i9 | -3.82752362 | 0.00150413 | 0.098482851 | 0.109 | 0.158 | 1.444 | 2.051 | NA |
| TRINITY_DN728_c0_g2_i2 | -3.82685792 | 0.183757225 | 0.999970678 | 0.273 | 0.158 | 5.033 | 0 | NA |
| TRINITY_DN1414_c0_g1_i1 | -3.82536356 | 0.14252618 | 0.999970678 | 1.372 | 0 | 15.021 | 1.478 | NA |
| TRINITY_DN2656_c0_g1_i10 | 3.822440803 | 0.007465164 | 0.376192409 | 1.054 | 2.198 | 0.156 | 0.06 | Amidohydro_1 |
| TRINITY_DN3190_c0_g1_i3 | -3.81902883 | 0.115128813 | 0.999970678 | 0.954 | 0 | 8.344 | 3.67 | NA |
| TRINITY_DN14898_c0_g1_i8 | -3.81722634 | 0.096870632 | 0.999970678 | 0 | 0.453 | 2.9 | 2.704 | UDPGT |
| TRINITY_DN751_c0_g1_i4 | -3.81462134 | 3.93E-05 | 0.005439185 | 1.826 | 4.12 | 36.431 | 37.882 | Hydrophob_seed |
| TRINITY_DN3278_c0_g1_i2 | -3.80798928 | 0.093574646 | 0.999970678 | 0 | 2.316 | 13.133 | 15.593 | PTPLA |
| TRINITY_DN2457_c0_g1_i4 | 3.804684738 | 0.187851239 | 0.999970678 | 0 | 2.868 | 0.056 | 0.151 | THOC2_N |
| TRINITY_DN7334_c0_g2_i1 | -3.8018865 | 0.009843603 | 0.478586582 | 0.382 | 0.384 | 1.4 | 9.29 | NA |
| TRINITY_DN2920_c0_g1_i1 | 3.800169467 | 0.082922437 | 0.999970678 | 1.599 | 1.616 | 0 | 0.231 | NA |
| TRINITY_DN18209_c0_g1_i2 | 3.799403923 | 0.084953399 | 0.999970678 | 1.499 | 2.178 | 0 | 0.271 | NA |
| TRINITY_DN16369_c0_g2_i2 | 3.799054026 | 0.097777935 | 0.999970678 | 2.126 | 0.986 | 0.2 | 0 | TAXi_C |
| TRINITY_DN5000_c0_g1_i3 | -3.79486976 | 0.148482521 | 0.999970678 | 2.117 | 0 | 22.221 | 2.966 | NA |
| TRINITY_DN406_c0_g1_i2 | -3.79129871 | 0.103606097 | 0.999970678 | 0.154 | 0 | 0.5 | 1.438 | Peptidase_S10 |
| TRINITY_DN393_c0_g1_i13 | 3.790247207 | 0.15526037 | 0.999970678 | 3.888 | 0.197 | 0 | 0.302 | NA |
| TRINITY_DN41609_c0_g1_i4 | 3.784755807 | 0.011177601 | 1 | 0.245 | 0.503 | 0.033 | 0.02 | NA |
| TRINITY_DN8611_c0_g2_i2 | 3.783404529 | 6.78E-07 | 0.000211452 | 8.421 | 8.032 | 0.4 | 0.714 | NA |
| TRINITY_DN1099_c0_g1_i7 | 3.780808368 | 0.130206247 | 0.999970678 | 3.852 | 8.564 | 0 | 0.905 | RNA_pol_Rpb1_1 |
| TRINITY_DN1242_c0_g2_i6 | 3.77771888 | 0.085302852 | 0.999970678 | 2.562 | 1.922 | 0 | 0.342 | NA |
| TRINITY_DN51007_c0_g1_i1 | -3.77637173 | 0.108372908 | 1 | 0 | 0.049 | 0.411 | 0.251 | Kinesin |
| TRINITY_DN7473_c0_g1_i1 | -3.77436584 | 0.023334487 | 1 | 0.636 | 0.168 | 4.255 | 4.474 | CSD |
| TRINITY_DN2595_c0_g1_i5 | -3.77026335 | 0.01058275 | 0.510723222 | 0.136 | 0.148 | 0.656 | 2.936 | Dimerisation |
| TRINITY_DN165701_c0_g1_i1 | -3.76874081 | 0.352810786 | 0.999970678 | 1.717 | 0 | 19.454 | 0 | NA |
| TRINITY_DN2882_c0_g1_i7 | -3.76434349 | 0.002980778 | 0.1708561 | 0.191 | 0.266 | 4.166 | 1.337 | NA |
| TRINITY_DN1293_c0_g1_i2 | 3.763585836 | 0.097875662 | 0.999970678 | 0.427 | 0.759 | 0.078 | 0 | NA |

| TRINITY_DN9778_c0_g1_i5 | 3.757830297 | 0.103448087 | 0.999970678 | 0.491 | 0.266 | 0.044 | 0 | ABC_membrane |
| --- | --- | --- | --- | --- | --- | --- | --- | --- |
| TRINITY_DN3386_c0_g1_i3 | -3.75675236 | 0.232835902 | 0.999970678 | 3.307 | 0.246 | 40.32 | 0 | NA |
| TRINITY_DN41918_c0_g1_i1 | -3.75485821 | 8.24E-05 | 0.009719175 | 1.008 | 0.424 | 8.033 | 9.662 | Dimerisation |
| TRINITY_DN7761_c0_g1_i1 | 3.752564175 | 0.118045546 | 1 | 0.936 | 0.749 | 0.1 | 0 | NAM |
| TRINITY_DN2486_c1_g1_i2 | -3.75020583 | 0.105590764 | 1 | 0 | 0.079 | 0.5 | 0.462 | NAM |
| TRINITY_DN9620_c0_g1_i4 | 3.748614883 | 0.038210185 | 0.999970678 | 20.957 | 4.77 | 1.455 | 0.241 | NA |
| TRINITY_DN10244_c0_g1_i5 | 3.745342635 | 0.223353928 | 0.999970678 | 6.286 | 0 | 0.033 | 0.442 | NAM |
| TRINITY_DN2887_c0_g1_i5 | -3.74004886 | 0.354536052 | 0.999970678 | 0 | 0.453 | 4.9 | 0 | 2OG-FeII_Oxy_2 |
| TRINITY_DN1955_c0_g1_i4 | -3.74003411 | 0.006152523 | 0.321897538 | 0.1 | 0.286 | 0.989 | 3.77 | Na_H_Exchanger |
| TRINITY_DN3589_c0_g1_i9 | 3.73384624 | 0.128192946 | 0.999970678 | 1.026 | 0.158 | 0 | 0.09 | NA |
| TRINITY_DN1877_c0_g1_i9 | 3.730583788 | 0.357492736 | 0.999970678 | 17.541 | 0 | 0 | 1.347 | NA |
| TRINITY_DN9515_c0_g1_i1 | 3.717121729 | 0.18687785 | 0.999970678 | 27.615 | 0.68 | 0 | 2.172 | PHM7_cyt |
| TRINITY_DN8246_c0_g1_i1 | 3.716870728 | 0.000276281 | 0.025716989 | 0.636 | 0.739 | 0.033 | 0.06 | - |
| TRINITY_DN352_c0_g1_i5 | 3.711502289 | 0.127190803 | 0.999970678 | 4.015 | 0.65 | 0 | 0.362 | NA |
| TRINITY_DN4486_c1_g2_i4 | 3.705525039 | 0.093961324 | 0.999970678 | 7.385 | 5.194 | 0 | 0.965 | NA |
| TRINITY_DN1467_c0_g1_i7 | -3.70201439 | 0.113558065 | 0.999970678 | 0.463 | 0 | 3.589 | 1.88 | NA |
| TRINITY_DN6042_c0_g1_i1 | -3.69032793 | 0.367493117 | 0.999970678 | 2.008 | 0 | 21.588 | 0 | NA |
| TRINITY_DN885_c0_g1_i3 | 3.686687929 | 0.364812652 | 0.999970678 | 6.014 | 0 | 0.389 | 0 | CWC25 |
| TRINITY_DN3949_c0_g1_i1 | -3.68526185 | 0.07345699 | 0.999970678 | 0.227 | 0.503 | 0.089 | 9.461 | NA |
| TRINITY_DN1337_c0_g1_i2 | -3.68309047 | 0.025998618 | 0.999970678 | 0.536 | 1.606 | 1.289 | 25.707 | p450 |
| TRINITY_DN9178_c0_g1_i2 | 3.682335366 | 0.113466914 | 0.999970678 | 0.772 | 0.414 | 0.078 | 0 | Inhibitor_I9 |
| TRINITY_DN3426_c0_g1_i18 | -3.67879377 | 6.98E-05 | 0.008574192 | 1.081 | 1.084 | 9.066 | 16.156 | NA |
| TRINITY_DN5158_c0_g1_i1 | -3.67781207 | 0.115900441 | 0.999970678 | 0.545 | 0 | 3.222 | 3.157 | NA |
| TRINITY_DN37122_c0_g1_i1 | -3.67741725 | 0.016040398 | 0.707160878 | 0.082 | 0.049 | 1.078 | 0.352 | NA |
| TRINITY_DN2175_c0_g1_i2 | 3.67664313 | 0.365082741 | 0.999970678 | 8.412 | 0 | 0 | 0.664 | NA |
| TRINITY_DN775_c1_g2_i3 | 3.676086387 | 0.360678813 | 0.999970678 | 0 | 7.352 | 0 | 0.563 | NA |
| TRINITY_DN7288_c0_g1_i5 | 3.673740651 | 0.114725724 | 0.999970678 | 0.972 | 0.434 | 0.089 | 0 | NA |
| TRINITY_DN45_c1_g1_i6 | -3.67183815 | 0.169433144 | 0.999970678 | 2.852 | 0 | 8.344 | 27.065 | GAF |
| TRINITY_DN2444_c0_g1_i1 | -3.67158825 | 0.361172264 | 0.999970678 | 0 | 0.503 | 0 | 6.414 | Pyr_redox_2 |
| TRINITY_DN527_c0_g2_i1 | 3.67113217 | 0.36647117 | 0.999970678 | 0 | 9.56 | 0 | 0.744 | NA |
| TRINITY_DN4195_c0_g1_i8 | 3.667907994 | 0.131344249 | 0.999970678 | 0.273 | 1.311 | 0.1 | 0 | NA |
| TRINITY_DN544_c0_g1_i3 | -3.66284714 | 0.133202154 | 0.999970678 | 0 | 11.27 | 62.818 | 62.313 | NA |
| TRINITY_DN2796_c0_g1_i14 | 3.660436426 | 0.102496721 | 0.999970678 | 6.958 | 3.942 | 0 | 0.845 | NA |
| TRINITY_DN7589_c0_g1_i1 | 3.66016499 | 0.124160687 | 0.999970678 | 3.879 | 7.125 | 0.722 | 0 | MFS_1 |
| TRINITY_DN6653_c0_g2_i1 | 3.65884412 | 0.018733169 | 0.804979513 | 25.517 | 1.705 | 0.833 | 1.166 | NA |
| TRINITY_DN0_c0_g2_i13 | 3.657156509 | 0.038496067 | 0.999970678 | 9.929 | 3.597 | 0.033 | 1.046 | NA |
| TRINITY_DN1316_c0_g1_i9 | 3.657013293 | 0.054127705 | 0.999970678 | 0.763 | 15.43 | 0.955 | 0.121 | LRRNT_2 |
| TRINITY_DN11120_c0_g1_i9 | -3.6455257 | 0.064251984 | 0.999970678 | 5.378 | 0.118 | 7.7 | 60.543 | NA |
| TRINITY_DN8023_c0_g1_i3 | 3.643624506 | 0.116839004 | 1 | 0.763 | 0.719 | 0 | 0.111 | NA |
| TRINITY_DN438_c0_g1_i1 | -3.63534202 | 0.118417373 | 0.999970678 | 1.354 | 0 | 7.244 | 8.254 | NA |
| TRINITY_DN544_c0_g1_i14 | -3.63496084 | 0.022205855 | 0.913225113 | 0.109 | 0.66 | 3.433 | 5.107 | NA |
| TRINITY_DN6998_c0_g1_i8 | 3.634398778 | 0.236977661 | 0.999970678 | 0 | 90.26 | 0.955 | 6.022 | NA |
| TRINITY_DN1184_c0_g1_i9 | 3.632698294 | 0.369376409 | 0.999970678 | 5.232 | 0 | 0 | 0.432 | NA |
| TRINITY_DN9657_c0_g1_i1 | -3.63229892 | 0.015104996 | 0.673274788 | 0.79 | 2.336 | 3.422 | 34.072 | GST_C |
| TRINITY_DN20185_c0_g2_i3 | -3.62830756 | 0.124734945 | 0.999970678 | 0 | 0.769 | 4.677 | 3.649 | GDI |
| TRINITY_DN5579_c0_g1_i3 | 3.625790478 | 0.149679256 | 0.999970678 | 1.917 | 9.136 | 0 | 0.885 | NA |
| TRINITY_DN6310_c0_g2_i2 | -3.62335286 | 0.114132454 | 0.999970678 | 0 | 0.365 | 1.722 | 2.373 | NA |
| TRINITY_DN265_c1_g2_i4 | -3.62310596 | 0.165838644 | 0.999970678 | 0 | 0.7 | 6.544 | 0.633 | NA |
| TRINITY_DN0_c0_g1_i4 | -3.61967155 | 0.181359014 | 0.999970678 | 14.207 | 0 | 101.538 | 54.159 | NA |
| TRINITY_DN1514_c0_g1_i2 | 3.61952969 | 0.047432697 | 0.999970678 | 0.327 | 14.64 | 0.478 | 0.633 | NA |
| TRINITY_DN1373_c0_g1_i4 | 3.618735355 | 0.125735059 | 1 | 0.418 | 1.222 | 0 | 0.131 | NA |
| TRINITY_DN1901_c0_g1_i8 | -3.61665752 | 0.200748367 | 0.999970678 | 0.618 | 0.621 | 0 | 15.272 | NA |
| TRINITY_DN8066_c0_g1_i3 | -3.60900171 | 0.012661078 | 0.587589113 | 0.309 | 0.099 | 1.589 | 3.278 | NA |
| TRINITY_DN2942_c0_g2_i9 | -3.60893057 | 0.118507512 | 0.999970678 | 0.009 | 1.863 | 9.211 | 11.139 | NA |
| TRINITY_DN6453_c0_g1_i1 | 3.605087755 | 0.373707953 | 0.999970678 | 0 | 9.5 | 0.644 | 0 | NA |
| TRINITY_DN12838_c0_g1_i2 | -3.60378748 | 0.14434566 | 0.999970678 | 0 | 3.853 | 9.833 | 33.891 | NA |
| TRINITY_DN20600_c0_g1_i5 | 3.60166686 | 0.145835916 | 0.999970678 | 1.517 | 7.993 | 0.644 | 0.01 | NA |
| TRINITY_DN8350_c0_g1_i4 | -3.60093699 | 0.160854373 | 0.999970678 | 0.845 | 0 | 0.9 | 9.159 | NA |
| TRINITY_DN6912_c0_g1_i1 | -3.59848471 | 0.174521413 | 0.999970678 | 0 | 2.217 | 1.467 | 24.571 | NA |
| TRINITY_DN754_c0_g1_i13 | -3.59809507 | 0.138198716 | 0.999970678 | 0.472 | 0 | 1.111 | 4.223 | NA |
| TRINITY_DN1894_c0_g1_i1 | -3.59575134 | 0.02168639 | 0.90144749 | 0.318 | 0.788 | 1.5 | 11.119 | NA |
| TRINITY_DN7503_c0_g1_i2 | 3.594433543 | 0.388274041 | 0.999970678 | 39.279 | 0 | 0 | 3.318 | NAM |
| TRINITY_DN7937_c0_g1_i1 | 3.594055392 | 0.15726853 | 0.999970678 | 11.173 | 1.459 | 0.867 | 0 | EamA |
| TRINITY_DN79_c0_g1_i29 | -3.58872665 | 0.140154641 | 0.999970678 | 0 | 0.099 | 0.8 | 0.211 | NA |
| TRINITY_DN1519_c0_g1_i7 | -3.58853562 | 0.378808437 | 0.999970678 | 0 | 4.001 | 39.32 | 0 | NA |
| TRINITY_DN9721_c0_g1_i4 | 3.58761924 | 0.114753223 | 1 | 0.899 | 1.321 | 0 | 0.181 | NA |
| TRINITY_DN1985_c0_g1_i8 | 3.587308839 | 0.107670243 | 0.999970678 | 2.771 | 3.873 | 0 | 0.533 | zf-C3HC4_3 |
| TRINITY_DN18337_c0_g1_i1 | -3.58338407 | 0.057037377 | 0.999970678 | 23.328 | 1.715 | 14.688 | 281.443 | NA |
| TRINITY_DN12055_c0_g1_i9 | 3.583013374 | 0.015161743 | 0.674883463 | 0.145 | 0.384 | 0.022 | 0.02 | NA |
| TRINITY_DN7042_c0_g2_i4 | 3.580333458 | 0.127731042 | 0.999970678 | 2.353 | 6.84 | 0 | 0.774 | AAA_18 |
| TRINITY_DN5981_c0_g1_i5 | 3.579527107 | 0.123058473 | 0.999970678 | 2.643 | 2.424 | 0 | 0.432 | adh_short |
| TRINITY_DN564_c0_g1_i9 | -3.5709486 | 0.011126615 | 0.531474809 | 23.7 | 16.38 | 35.12 | 428.488 | NA |
| TRINITY_DN155_c0_g1_i7 | 3.569988005 | 0.124732339 | 0.999970678 | 4.133 | 4.967 | 0 | 0.764 | Ank |
| TRINITY_DN70956_c0_g1_i1 | 3.569229184 | 0.378806461 | 0.999970678 | 0 | 18.37 | 1.278 | 0 | NA |

| TRINITY_DN40303_c0_g1_i1 | -3.56171073 | 0.128120101 | 0.999970678 | 0 | 0.276 | 1.744 | 1.066 | F-box |
| --- | --- | --- | --- | --- | --- | --- | --- | --- |
| TRINITY_DN948_c0_g2_i4 | 3.559808973 | 0.377102387 | 0.999970678 | 0 | 3.646 | 0 | 0.312 | TPR_MLP1_2 |
| TRINITY_DN1607_c0_g1_i12 | 3.554431978 | 0.383994759 | 0.999970678 | 10.265 | 0 | 0 | 0.895 | NA |
| TRINITY_DN75712_c0_g1_i1 | -3.55359287 | 0.038301105 | 0.999970678 | 20.421 | 1.143 | 101.738 | 109.927 | NA |
| TRINITY_DN22937_c0_g1_i11 | 3.548648297 | 0.149834221 | 1 | 1.081 | 1.261 | 0.167 | 0 | NA |
| TRINITY_DN3922_c0_g1_i2 | -3.54512389 | 0.142583194 | 1 | 0.082 | 0 | 0.244 | 0.643 | B_lectin |
| TRINITY_DN2146_c0_g1_i3 | 3.544960408 | 0.116849576 | 0.999970678 | 3.016 | 2.838 | 0 | 0.513 | NA |
| TRINITY_DN1815_c1_g1_i2 | 3.543617528 | 0.162041151 | 0.999970678 | 0.554 | 5.075 | 0 | 0.483 | SLH |
| TRINITY_DN256_c1_g2_i5 | 3.542067481 | 0.065847133 | 0.999970678 | 13.826 | 0.591 | 0.889 | 0.181 | NA |
| TRINITY_DN209_c0_g1_i6 | -3.53785582 | 0.143368734 | 0.999970678 | 1.09 | 0 | 2.755 | 9.42 | NA |
| TRINITY_DN3011_c0_g1_i1 | 3.536565582 | 0.085134503 | 0.999970678 | 48.09 | 1.252 | 3.322 | 0.302 | NA |
| TRINITY_DN2405_c0_g1_i6 | -3.53401028 | 0.132343093 | 0.999970678 | 0 | 0.897 | 4.611 | 4.695 | Sugar_tr |
| TRINITY_DN2330_c0_g1_i7 | 3.531228708 | 0.118808462 | 0.999970678 | 1.562 | 2.109 | 0 | 0.312 | LRR_8 |
| TRINITY_DN4991_c0_g1_i2 | -3.52291003 | 0.134204515 | 0.999970678 | 0 | 0.7 | 4.733 | 2.373 | ELO |
| TRINITY_DN532_c0_g2_i1 | -3.52269845 | 0.006453381 | 0.333801986 | 0.336 | 0.089 | 1.922 | 2.513 | NA |
| TRINITY_DN8626_c0_g1_i4 | 3.517943578 | 0.058338253 | 0.999970678 | 63.224 | 15.78 | 5.611 | 0.191 | MIP |
| TRINITY_DN3119_c0_g1_i1 | -3.51116067 | 0.235535897 | 0.999970678 | 0 | 2.454 | 0.267 | 26.874 | NA |
| TRINITY_DN7676_c0_g1_i17 | 3.510614142 | 0.133854159 | 0.999970678 | 0.59 | 0.887 | 0.1 | 0 | NA |
| TRINITY_DN461_c1_g1_i8 | 3.509479597 | 0.142294489 | 0.999970678 | 2.144 | 3.942 | 0.444 | 0 | LisH |
| TRINITY_DN5451_c0_g1_i1 | -3.50910534 | 0.043806951 | 0.999970678 | 1.326 | 3.213 | 1.4 | 49.746 | p450 |
| TRINITY_DN15_c0_g1_i25 | 3.508402848 | 0.021808453 | 0.904220523 | 1.953 | 18.68 | 0.711 | 0.935 | NA |
| TRINITY_DN7148_c0_g1_i8 | 3.508375741 | 0.016624116 | 1 | 0.454 | 0.581 | 0.044 | 0.03 | Exostosin |
| TRINITY_DN3826_c0_g1_i4 | -3.5081819 | 0.16023278 | 0.999970678 | 0.799 | 0 | 1.2 | 7.591 | NA |
| TRINITY_DN1147_c0_g1_i2 | -3.507784 | 0.389340383 | 0.999970678 | 0.781 | 0 | 7.477 | 0 | NA |
| TRINITY_DN697_c0_g1_i5 | -3.50560944 | 0.182150193 | 0.999970678 | 1.989 | 0 | 16.088 | 3.469 | NA |
| TRINITY_DN15744_c0_g1_i2 | -3.50079128 | 0.140733038 | 1 | 0.1 | 0 | 0.522 | 0.633 | ERCC4 |
| TRINITY_DN7213_c0_g1_i3 | 3.497959047 | 0.29995278 | 0.999970678 | 17.623 | 0.02 | 1.311 | 0 | NA |
| TRINITY_DN545_c0_g1_i4 | 3.497568154 | 0.150212672 | 1 | 2.18 | 5.46 | 0.589 | 0 | NA |
| TRINITY_DN3280_c0_g1_i4 | 3.494845864 | 0.143383483 | 0.999970678 | 0.699 | 2.828 | 0 | 0.302 | NA |
| TRINITY_DN24305_c0_g2_i1 | 3.492295717 | 0.022437204 | 0.917482269 | 15.951 | 1.252 | 0.6 | 0.814 | MatE |
| TRINITY_DN3281_c0_g1_i3 | 3.489485626 | 0.139005286 | 1 | 0.936 | 1.005 | 0.144 | 0 | NA |
| TRINITY_DN2221_c0_g1_i1 | -3.48895815 | 0.222893781 | 0.999970678 | 0.836 | 0.306 | 0 | 12.859 | LisH |
| TRINITY_DN1043_c0_g1_i8 | 3.486321971 | 0.388412674 | 0.999970678 | 0 | 44.07 | 0 | 3.82 | ADH_N |
| TRINITY_DN730_c0_g2_i5 | -3.48510804 | 0.396293181 | 0.999970678 | 0 | 0.532 | 0 | 5.891 | Frigida |
| TRINITY_DN3341_c0_g1_i16 | -3.48497019 | 0.000839172 | 0.061890383 | 0.245 | 0.128 | 1.555 | 2.232 | NA |
| TRINITY_DN11172_c0_g1_i2 | 3.482574987 | 0.010851079 | 0.521362441 | 0.627 | 2.099 | 0.078 | 0.141 | NA |
| TRINITY_DN319_c0_g1_i12 | -3.48253323 | 0.171754525 | 0.999970678 | 0.045 | 1.33 | 0.033 | 14.99 | MFS_1 |
| TRINITY_DN20600_c0_g1_i6 | -3.48132291 | 0.178175877 | 0.999970678 | 1.735 | 0 | 2.5 | 16.568 | NA |
| TRINITY_DN373_c1_g1_i9 | 3.479353893 | 0.160361294 | 0.999970678 | 21.002 | 4.179 | 1.844 | 0 | NA |
| TRINITY_DN5838_c0_g2_i15 | -3.47791793 | 0.145950375 | 0.999970678 | 0.282 | 0 | 1.944 | 0.945 | NA |
| TRINITY_DN9659_c0_g3_i3 | 3.476155118 | 0.145356854 | 0.999970678 | 0.79 | 0.246 | 0 | 0.101 | NA |
| TRINITY_DN237_c0_g1_i7 | -3.47543806 | 0.129301428 | 0.999970678 | 0 | 0.611 | 2.722 | 3.388 | NA |
| TRINITY_DN1760_c0_g1_i1 | 3.473373881 | 0.394534877 | 0.999970678 | 5.977 | 0 | 0.456 | 0 | NA |
| TRINITY_DN69251_c0_g1_i1 | -3.4706803 | 0.196564959 | 0.999970678 | 4.705 | 0 | 40.298 | 3.207 | NA |
| TRINITY_DN19937_c0_g1_i8 | -3.46728632 | 0.14099626 | 0.999970678 | 0 | 0.217 | 1.278 | 0.784 | NA |
| TRINITY_DN5819_c1_g2_i4 | -3.46685905 | 0.203764217 | 0.999970678 | 1.944 | 0 | 1.111 | 20.489 | NA |
| TRINITY_DN1970_c1_g1_i6 | 3.466621354 | 0.172634991 | 0.999970678 | 0.563 | 4.021 | 0.344 | 0 | NA |
| TRINITY_DN269_c0_g1_i6 | 3.465791052 | 0.048368244 | 0.999970678 | 4.578 | 0.335 | 0.3 | 0.08 | ArgoL1 |
| TRINITY_DN338_c0_g1_i1 | -3.46229001 | 0.169610894 | 0.999970678 | 1.662 | 0 | 2.433 | 15.362 | NA |
| TRINITY_DN799_c0_g2_i8 | 3.462257328 | 0.15910334 | 0.999970678 | 3.243 | 0.818 | 0.311 | 0 | FA_desaturase |
| TRINITY_DN5814_c0_g1_i2 | -3.46089153 | 0.002240581 | 0.136320811 | 0.245 | 0.956 | 4.911 | 7.198 | - |
| TRINITY_DN4259_c1_g1_i5 | -3.46057759 | 0.404821587 | 0.999970678 | 2.553 | 0 | 0 | 28.673 | PAE |
| TRINITY_DN3744_c0_g1_i1 | 3.458236546 | 0.157626551 | 0.999970678 | 8.512 | 4.543 | 0 | 1.196 | NA |
| TRINITY_DN1036_c1_g2_i5 | 3.456828118 | 0.393991459 | 0.999970678 | 4.115 | 0 | 0 | 0.382 | NA |
| TRINITY_DN2307_c1_g1_i3 | 3.452408501 | 0.125940996 | 0.999970678 | 1.063 | 0.808 | 0 | 0.181 | NA |
| TRINITY_DN4608_c0_g1_i7 | -3.44076478 | 0.014497978 | 0.653348889 | 0.1 | 0.266 | 1.733 | 1.488 | Ldh_1_C |
| TRINITY_DN5079_c0_g1_i1 | 3.440611583 | 0.164773919 | 0.999970678 | 2.871 | 10.97 | 1.055 | 0 | NA |
| TRINITY_DN16130_c0_g1_i4 | 3.438430953 | 0.029672065 | 0.999970678 | 1.381 | 1.153 | 0.011 | 0.201 | AAR2 |
| TRINITY_DN2809_c0_g4_i4 | 3.437603759 | 0.209944863 | 0.999970678 | 0.354 | 5.006 | 0.4 | 0 | COPI_C |
| TRINITY_DN11724_c0_g2_i4 | 3.437291358 | 0.13253726 | 0.999970678 | 2.316 | 1.991 | 0 | 0.392 | NA |
| TRINITY_DN16963_c0_g1_i5 | 3.435966542 | 0.128841962 | 0.999970678 | 1.435 | 1.143 | 0 | 0.241 | NA |
| TRINITY_DN5158_c0_g1_i2 | -3.43503106 | 0.162231175 | 0.999970678 | 0.681 | 0 | 4.633 | 1.769 | NA |
| TRINITY_DN7192_c0_g1_i3 | -3.43447851 | 0.042268484 | 0.999970678 | 0.109 | 2.198 | 14.599 | 6.746 | NA |
| TRINITY_DN1596_c0_g1_i1 | -3.43390683 | 0.398094206 | 0.999970678 | 0 | 0.848 | 7.355 | 0 | NA |
| TRINITY_DN4977_c0_g1_i1 | 3.432212256 | 0.397479865 | 0.999970678 | 3.942 | 0 | 0 | 0.372 | NA |
| TRINITY_DN11615_c0_g1_i14 | 3.430544693 | 0.133597614 | 0.999970678 | 1.29 | 1.528 | 0 | 0.251 | NA |
| TRINITY_DN10905_c1_g1_i1 | -3.42390869 | 0.082694668 | 0.999970678 | 0.799 | 7.5 | 1.933 | 85.547 | NA |
| TRINITY_DN6998_c0_g1_i1 | 3.423212833 | 0.400274569 | 0.999970678 | 0 | 2.641 | 0.2 | 0 | NA |
| TRINITY_DN47_c0_g1_i11 | 3.420680873 | 0.039864574 | 0.999970678 | 3.606 | 0.227 | 0.222 | 0.101 | PTR2 |
| TRINITY_DN11648_c0_g1_i9 | -3.42066686 | 0.029569223 | 0.999970678 | 0.763 | 0.187 | 1.1 | 9.008 | NA |
| TRINITY_DN406_c0_g1_i11 | -3.41967144 | 0.048848734 | 0.999970678 | 0.2 | 0.463 | 0.256 | 6.786 | Peptidase_S10 |
| TRINITY_DN4590_c0_g1_i2 | -3.41953551 | 0.148024347 | 0.999970678 | 1.145 | 0 | 4.744 | 6.404 | RALF |
| TRINITY_DN56_c1_g1_i3 | 3.419390666 | 0.401406877 | 0.999970678 | 0 | 30.87 | 0 | 2.845 | GATase |
| TRINITY_DN2330_c0_g1_i4 | -3.41873368 | 0.015568027 | 0.689212187 | 0.227 | 0.039 | 0.856 | 2.001 | LRR_8 |

| TRINITY_DN10004_c0_g1_i12 | -3.41659564 | 0.145669724 | 0.999970678 | 0.327 | 0 | 1.667 | 1.518 | NA |
| --- | --- | --- | --- | --- | --- | --- | --- | --- |
| TRINITY_DN6094_c0_g1_i4 | 3.411800738 | 0.398615974 | 0.999970678 | 0 | 5.598 | 0 | 0.523 | NA |
| TRINITY_DN1540_c1_g1_i1 | 3.407074834 | 0.170587285 | 0.999970678 | 3.452 | 17.83 | 1.633 | 0 | NA |
| TRINITY_DN2354_c0_g1_i6 | 3.406731517 | 0.225762671 | 0.999970678 | 51.697 | 1.981 | 0 | 5.127 | NA |
| TRINITY_DN14116_c0_g3_i1 | -3.40617951 | 0.404931523 | 0.999970678 | 0.672 | 0 | 6.077 | 0 | NA |
| TRINITY_DN2493_c0_g1_i4 | 3.404731115 | 0.000425922 | 0.035773105 | 5.587 | 5.696 | 0.344 | 0.684 | - |
| TRINITY_DN550_c0_g1_i4 | 3.403569901 | 0.073812498 | 0.999970678 | 12.418 | 1.045 | 0.989 | 0.09 | NA |
| TRINITY_DN3683_c0_g2_i5 | -3.40335853 | 0.165839333 | 0.999970678 | 0 | 1.124 | 7.977 | 2.252 | NA |
| TRINITY_DN6303_c0_g1_i7 | -3.40095302 | 0.15641045 | 0.999970678 | 0.363 | 0 | 2.167 | 1.307 | NA |
| TRINITY_DN7163_c0_g1_i5 | 3.398778711 | 0.157639782 | 0.999970678 | 1.853 | 0.71 | 0.2 | 0 | RNA_pol_Rpb1_1 |
| TRINITY_DN825_c0_g1_i8 | 3.397829277 | 0.152402301 | 0.999970678 | 4.378 | 1.409 | 0 | 0.553 | NA |
| TRINITY_DN4170_c0_g1_i1 | -3.39649147 | 0.001284099 | 0.086148032 | 0.291 | 0.414 | 1.878 | 5.067 | p450 |
| TRINITY_DN5735_c0_g1_i2 | 3.396265322 | 0.153260663 | 0.999970678 | 0.863 | 2.168 | 0.244 | 0 | NA |
| TRINITY_DN3019_c0_g1_i1 | 3.395802565 | 0.006176422 | 0.322357931 | 3.061 | 2.296 | 0.156 | 0.332 | NA |
| TRINITY_DN7862_c0_g1_i5 | 3.394693692 | 0.152234867 | 1 | 0.69 | 0.473 | 0 | 0.101 | NA |
| TRINITY_DN424_c0_g1_i14 | -3.39451798 | 0.16948091 | 0.999970678 | 0.745 | 0 | 4.866 | 1.769 | NA |
| TRINITY_DN859_c0_g1_i6 | -3.3925305 | 0.201500065 | 0.999970678 | 7.549 | 0 | 32.476 | 41.27 | NA |
| TRINITY_DN2809_c0_g4_i2 | 3.390779893 | 0.170060313 | 0.999970678 | 0.327 | 1.38 | 0.133 | 0 | COPI_C |
| TRINITY_DN103_c0_g1_i1 | -3.39018309 | 0.052028159 | 0.999970678 | 4.506 | 0.71 | 2.711 | 51.917 | NA |
| TRINITY_DN12364_c0_g1_i1 | 3.389972832 | 0.001461289 | 0.096256533 | 0.609 | 0.65 | 0.033 | 0.08 | - |
| TRINITY_DN1073_c0_g1_i2 | -3.38980666 | 0.126580339 | 0.999970678 | 0.872 | 7.51 | 0.611 | 84.652 | peroxidase |
| TRINITY_DN120_c2_g1_i5 | -3.38952505 | 0.041277916 | 0.999970678 | 0.845 | 0.197 | 0.889 | 10.184 | NA |
| TRINITY_DN7790_c0_g1_i1 | -3.38877246 | 0.156702298 | 0.999970678 | 0.345 | 0 | 2.067 | 1.086 | - |
| TRINITY_DN1357_c0_g1_i1 | 3.38696679 | 0.154735415 | 0.999970678 | 1.181 | 1.429 | 0.211 | 0 | Arm |
| TRINITY_DN10899_c0_g1_i7 | 3.383292601 | 0.415732091 | 0.999970678 | 0 | 22.53 | 1.744 | 0 | Peptidase_S28 |
| TRINITY_DN15705_c0_g1_i2 | -3.38259803 | 0.037250807 | 0.999970678 | 0.999 | 0.621 | 0.922 | 15.473 | - |
| TRINITY_DN2282_c0_g1_i4 | -3.38139226 | 0.180348693 | 0.999970678 | 0 | 5.351 | 21.365 | 28.874 | Heme_oxygenase |
| TRINITY_DN1907_c0_g1_i3 | 3.381259452 | 0.179941412 | 0.999970678 | 0.009 | 2.779 | 0.2 | 0.02 | DUF4283 |
| TRINITY_DN5393_c0_g1_i5 | -3.37728973 | 0.215113478 | 0.999970678 | 4.215 | 0 | 3.889 | 39.782 | MENTAL |
| TRINITY_DN2146_c1_g3_i2 | -3.37549338 | 0.15844472 | 0.999970678 | 0 | 0.443 | 1.1 | 3.298 | NA |
| TRINITY_DN9712_c0_g1_i1 | -3.371201 | 0.022783709 | 0.929325028 | 1.09 | 2.71 | 2.689 | 36.053 | Clathrin |
| TRINITY_DN13011_c0_g1_i1 | 3.370380247 | 0.186585032 | 0.999970678 | 1.463 | 0.207 | 0.133 | 0 | NA |
| TRINITY_DN16632_c0_g1_i2 | 3.367486551 | 0.172960448 | 0.999970678 | 0.845 | 4.977 | 0 | 0.563 | NA |
| TRINITY_DN14123_c0_g1_i1 | 3.365857576 | 0.16223581 | 1 | 0.609 | 0.926 | 0.122 | 0 | NA |
| TRINITY_DN6466_c0_g1_i4 | -3.3641183 | 0.025107466 | 0.999970678 | 0.173 | 0.7 | 6.344 | 1.79 | NA |
| TRINITY_DN211_c0_g1_i6 | 3.361552613 | 0.186445355 | 0.999970678 | 5.623 | 27.3 | 2.633 | 0 | NA |
| TRINITY_DN1867_c0_g1_i5 | -3.36110192 | 0.277566136 | 0.999970678 | 0 | 5.371 | 0.289 | 52.581 | B_lectin |
| TRINITY_DN4908_c0_g1_i6 | 3.360522585 | 0.171845919 | 0.999970678 | 0.736 | 4.139 | 0 | 0.473 | B3 |
| TRINITY_DN2140_c0_g1_i3 | 3.358017209 | 0.169688717 | 0.999970678 | 5.487 | 1.419 | 0 | 0.674 | Ank |
| TRINITY_DN14898_c0_g1_i1 | -3.35769709 | 0.17140164 | 0.999970678 | 0 | 1.084 | 5.866 | 3.911 | UDPGT |
| TRINITY_DN8803_c0_g1_i1 | 3.354006801 | 0.412172938 | 0.999970678 | 7.876 | 0 | 0.644 | 0 | Pkinase |
| TRINITY_DN3460_c0_g2_i2 | 3.353463588 | 0.238694382 | 0.999970678 | 0.254 | 10.72 | 0.856 | 0 | NA |
| TRINITY_DN1064_c0_g2_i2 | -3.35339144 | 0.004001965 | 0.221238942 | 0.118 | 0.207 | 2.122 | 0.905 | NA |
| TRINITY_DN108918_c0_g1_i1 | -3.34714761 | 0.01182507 | 0.557501736 | 1.562 | 4.878 | 8.911 | 53.888 | Polyketide_cyc2 |
| TRINITY_DN373_c1_g1_i3 | 3.343545055 | 0.173814923 | 0.999970678 | 13.045 | 3.173 | 1.355 | 0 | NA |
| TRINITY_DN9645_c0_g1_i2 | 3.339694012 | 0.175166813 | 0.999970678 | 1.317 | 0.335 | 0.133 | 0 | Lipoxygenase |
| TRINITY_DN414_c0_g1_i12 | -3.33934874 | 0.185489686 | 0.999970678 | 5.269 | 0 | 20.621 | 29.126 | Oxidored_FMN |
| TRINITY_DN104_c0_g1_i18 | -3.33169678 | 0.169847691 | 0.999970678 | 1.717 | 0 | 7.377 | 8.515 | GUB_WAK_bind |
| TRINITY_DN8342_c0_g1_i10 | 3.331145247 | 0.294151202 | 0.999970678 | 0 | 7.943 | 0.033 | 0.744 | NA |
| TRINITY_DN748_c0_g3_i1 | 3.328158422 | 1.65E-06 | 0.000410173 | 44.611 | 29.19 | 3.144 | 3.579 | NA |
| TRINITY_DN20199_c1_g1_i1 | -3.31936852 | 0.417184504 | 0.999970678 | 1.017 | 0 | 8.644 | 0 | NA |
| TRINITY_DN5507_c0_g1_i1 | -3.31898795 | 0.029825811 | 0.999970678 | 0.1 | 0.68 | 1.644 | 5.7 | UDPGT |
| TRINITY_DN6154_c0_g1_i6 | 3.318578826 | 0.255950718 | 0.999970678 | 3.706 | 0 | 0.078 | 0.292 | - |
| TRINITY_DN2573_c2_g1_i4 | -3.31578929 | 0.288904309 | 0.999970678 | 0 | 10.19 | 81.784 | 0.814 | Ribosomal_S8e |
| TRINITY_DN463_c0_g2_i7 | 3.31503748 | 0.027510446 | 0.999970678 | 1.254 | 7.598 | 0.489 | 0.332 | NA |
| TRINITY_DN1437_c2_g1_i12 | 3.314558148 | 0.165154046 | 0.999970678 | 0.136 | 23.15 | 1.8 | 0.141 | NA |
| TRINITY_DN2806_c0_g1_i3 | -3.31202047 | 0.102428833 | 0.999970678 | 15.506 | 0.296 | 12.621 | 144.25 | NA |
| TRINITY_DN5831_c0_g1_i6 | -3.31161258 | 0.005597419 | 0.296400114 | 1.889 | 1.035 | 7.8 | 18.76 | NA |
| TRINITY_DN550_c0_g1_i1 | 3.309674873 | 0.091311396 | 0.999970678 | 123 | 1.656 | 2.455 | 9.822 | NA |
| TRINITY_DN4770_c0_g1_i1 | 3.307780168 | 0.19742826 | 0.999970678 | 13.935 | 12.99 | 0 | 2.735 | NA |
| TRINITY_DN45147_c0_g1_i1 | -3.30761481 | 0.42031268 | 0.999970678 | 1.244 | 0 | 10.288 | 0 | NA |
| TRINITY_DN7720_c2_g1_i2 | -3.30611621 | 0.026068076 | 1 | 0.191 | 0.118 | 1.278 | 1.468 | NA |
| TRINITY_DN14315_c0_g1_i4 | 3.305601083 | 0.155435693 | 0.999970678 | 0.345 | 0.71 | 0 | 0.101 | BRCT |
| TRINITY_DN1868_c0_g1_i9 | 3.304173519 | 0.275238753 | 0.999970678 | 11.319 | 0.108 | 0.978 | 0 | NA |
| TRINITY_DN3447_c0_g1_i1 | -3.30320002 | 0.037005155 | 0.999970678 | 0.89 | 0.296 | 0.967 | 10.888 | NA |
| TRINITY_DN4776_c0_g1_i1 | -3.29817629 | 0.181542793 | 0.999970678 | 0.509 | 0 | 1.022 | 3.871 | NA |
| TRINITY_DN1478_c0_g1_i12 | -3.29813793 | 0.417282674 | 0.999970678 | 0.663 | 0 | 0 | 6.726 | F-box |
| TRINITY_DN4283_c0_g1_i7 | -3.29680521 | 0.42034951 | 0.999970678 | 1.426 | 0 | 11.855 | 0 | NA |
| TRINITY_DN2510_c0_g1_i2 | -3.29623417 | 0.42055037 | 0.999970678 | 1.017 | 0 | 8.499 | 0 | Mito_carr |
| TRINITY_DN29833_c0_g1_i1 | -3.29522246 | 0.416514568 | 0.999970678 | 0 | 1.281 | 0 | 12.416 | NA |
| TRINITY_DN6223_c0_g1_i2 | -3.29479628 | 0.220572873 | 0.999970678 | 0 | 1.34 | 9.944 | 0.794 | FKBP_C |
| TRINITY_DN14264_c0_g1_i2 | -3.29436094 | 0.223550202 | 0.999970678 | 3.897 | 0 | 4.711 | 33.167 | NA |
| TRINITY_DN8_c1_g2_i1 | 3.294113476 | 0.077217038 | 0.999970678 | 0.663 | 28.41 | 1.033 | 1.679 | NA |
| TRINITY_DN1744_c0_g1_i4 | -3.29403511 | 0.03144023 | 0.999970678 | 1.835 | 0.483 | 2.322 | 20.258 | Calmodulin_bind |

| TRINITY_DN7011_c0_g1_i1 | -3.29358946 | 0.425548878 | 0.999970678 | 2.235 | 0 | 18.343 | 0 | Abhydrolase_1 |
| --- | --- | --- | --- | --- | --- | --- | --- | --- |
| TRINITY_DN1568_c0_g1_i4 | 3.289168647 | 0.164388749 | 0.999970678 | 1.063 | 0.956 | 0.178 | 0 | Glyco_hydro_1 |
| TRINITY_DN564_c0_g1_i4 | -3.2887429 | 0.01380401 | 0.629894699 | 6.659 | 8.781 | 13.644 | 133.161 | NA |
| TRINITY_DN3676_c0_g1_i3 | -3.28625468 | 0.173685842 | 1 | 0.481 | 0 | 1.755 | 2.614 | NA |
| TRINITY_DN5231_c0_g1_i4 | 3.285280737 | 0.166186781 | 0.999970678 | 3.025 | 1.626 | 0.4 | 0 | NA |
| TRINITY_DN1424_c0_g1_i3 | -3.28359915 | 0.213371697 | 0.999970678 | 12.981 | 0 | 72.54 | 39.682 | NA |
| TRINITY_DN2279_c0_g1_i3 | -3.28312306 | 0.000365194 | 0.031817625 | 1.135 | 0.976 | 11.688 | 6.364 | Cyclase |
| TRINITY_DN31309_c0_g1_i1 | 3.282376222 | 0.184177864 | 1 | 0.709 | 0.907 | 0.133 | 0 | NA |
| TRINITY_DN3991_c0_g1_i1 | -3.28124154 | 0.030713508 | 0.999970678 | 0.999 | 0.453 | 1.922 | 11.883 | Mito_carr |
| TRINITY_DN2634_c3_g1_i1 | -3.27604272 | 0.114719646 | 0.999970678 | 0.164 | 2.612 | 0.8 | 25.456 | AP2 |
| TRINITY_DN17230_c1_g1_i1 | -3.2758336 | 0.044163186 | 0.999970678 | 1.281 | 0.296 | 10.355 | 2.604 | NA |
| TRINITY_DN18903_c0_g1_i1 | -3.27542758 | 0.185934516 | 1 | 0.154 | 0 | 0.6 | 0.764 | PTR2 |
| TRINITY_DN2985_c0_g1_i8 | 3.273602689 | 0.156202076 | 0.999970678 | 2.353 | 1.932 | 0 | 0.442 | NA |
| TRINITY_DN8882_c0_g1_i1 | 3.272724617 | 0.421819767 | 0.999970678 | 3.57 | 0 | 0 | 0.382 | NA |
| TRINITY_DN1202_c0_g1_i3 | 3.272230223 | 0.033981322 | 0.999970678 | 1.335 | 18.9 | 0.711 | 1.196 | NA |
| TRINITY_DN897_c2_g1_i5 | 3.272173096 | 0.030247299 | 1 | 4.833 | 14.28 | 0.978 | 0.724 | NA |
| TRINITY_DN3118_c0_g1_i7 | -3.2711008 | 0.190500926 | 0.999970678 | 0.609 | 0 | 1.033 | 4.816 | TPP_enzyme_C |
| TRINITY_DN10643_c0_g1_i6 | 3.270785357 | 0.005995087 | 0.314400475 | 1.326 | 2.04 | 0.067 | 0.261 | DEAD |
| TRINITY_DN1813_c0_g2_i2 | 3.270127407 | 0.006535958 | 0.337350423 | 22.592 | 4.809 | 1.233 | 1.377 | NA |
| TRINITY_DN1824_c0_g1_i14 | 3.270008653 | 0.049463269 | 1 | 0.881 | 0.453 | 0.011 | 0.121 | NA |
| TRINITY_DN1036_c1_g3_i2 | -3.26930991 | 0.178007164 | 0.999970678 | 0.209 | 0 | 1.011 | 0.684 | NA |
| TRINITY_DN356_c0_g1_i1 | -3.26746157 | 0.015887841 | 0.702418884 | 0.291 | 0.897 | 7.566 | 2.343 | NA |
| TRINITY_DN5867_c0_g1_i1 | -3.26476012 | 0.266215823 | 0.999970678 | 0 | 8.081 | 61.196 | 2.111 | NA |
| TRINITY_DN5018_c0_g1_i1 | -3.25924621 | 0.173188748 | 0.999970678 | 0.382 | 0 | 1.144 | 2.272 | NA |
| TRINITY_DN1815_c1_g1_i5 | -3.25919491 | 0.186846039 | 0.999970678 | 1.108 | 0 | 5.155 | 4.544 | SLH |
| TRINITY_DN3138_c0_g1_i11 | 3.258970676 | 0.286764534 | 0.999970678 | 0 | 4.622 | 0.033 | 0.432 | NA |
| TRINITY_DN4795_c0_g1_i1 | -3.25824659 | 0.423703301 | 0.999970678 | 0 | 0.66 | 0 | 6.213 | VARLMGL |
| TRINITY_DN1293_c0_g1_i14 | 3.257040392 | 0.164110746 | 0.999970678 | 0.6 | 0.512 | 0.089 | 0 | NA |
| TRINITY_DN104_c0_g1_i16 | 3.255617487 | 0.163275224 | 0.999970678 | 3.234 | 5.913 | 0 | 0.945 | GUB_WAK_bind |
| TRINITY_DN614_c0_g1_i1 | -3.2540981 | 0.012372251 | 0.577465958 | 3.207 | 0.512 | 10.755 | 22.973 | NA |
| TRINITY_DN8290_c0_g1_i2 | 3.253485162 | 0.425379247 | 0.999970678 | 0 | 3.508 | 0.311 | 0 | NA |
| TRINITY_DN1211_c0_g1_i8 | 3.252325367 | 0.018613836 | 0.803370226 | 1.799 | 0.966 | 0.2 | 0.04 | NA |
| TRINITY_DN7720_c0_g1_i8 | -3.25099078 | 0.190623327 | 1 | 1.154 | 0 | 4.889 | 5.389 | NA |
| TRINITY_DN13234_c0_g1_i10 | 3.246420797 | 0.16408658 | 0.999970678 | 1.054 | 0.66 | 0 | 0.181 | NA |
| TRINITY_DN3527_c0_g1_i5 | 3.24534799 | 0.164890785 | 0.999970678 | 1.181 | 0.798 | 0 | 0.201 | NA |
| TRINITY_DN801_c1_g1_i3 | 3.245300338 | 0.268743235 | 0.999970678 | 0.563 | 28.38 | 0 | 3.016 | Peptidase_S10 |
| TRINITY_DN1862_c0_g1_i4 | -3.2424816 | 0.00158254 | 0.102588754 | 0.263 | 0.345 | 1.667 | 3.71 | NA |
| TRINITY_DN735_c1_g1_i1 | 3.23922075 | 0.107518698 | 0.999970678 | 12.236 | 1404 | 30.643 | 111.093 | Chloroa_b-bind |
| TRINITY_DN1644_c0_g1_i1 | -3.23918211 | 0.44000085 | 0.999970678 | 0 | 3.725 | 28.632 | 0 | PAP_fibrillin |
| TRINITY_DN1898_c1_g1_i7 | 3.239071927 | 0.189751827 | 0.999970678 | 1.726 | 5.667 | 0.644 | 0 | NA |
| TRINITY_DN363_c2_g1_i1 | -3.2381324 | 0.252646669 | 0.999970678 | 1.372 | 0 | 0.356 | 12.708 | NA |
| TRINITY_DN3332_c0_g2_i3 | -3.23726896 | 0.429475547 | 0.999970678 | 0.59 | 0 | 4.611 | 0 | ABC1 |
| TRINITY_DN2844_c0_g1_i13 | -3.23676155 | 0.225648271 | 0.999970678 | 7.004 | 0 | 27.832 | 33.439 | - |
| TRINITY_DN4502_c0_g1_i2 | -3.23462178 | 0.045484573 | 0.999970678 | 1.998 | 0.926 | 1.855 | 25.305 | NA |
| TRINITY_DN2832_c0_g1_i6 | -3.23461081 | 0.020348149 | 0.854762061 | 0.69 | 0.197 | 1.189 | 6.967 | NA |
| TRINITY_DN843_c1_g1_i7 | -3.23456867 | 0.225597698 | 0.999970678 | 0 | 9.836 | 47.464 | 33.831 | NAM |
| TRINITY_DN581_c0_g1_i5 | -3.23168536 | 0.178680311 | 1 | 0 | 0.296 | 1.2 | 1.387 | NA |
| TRINITY_DN247_c0_g1_i17 | 3.228018867 | 0.174613443 | 1 | 0.445 | 0.67 | 0 | 0.111 | NA |
| TRINITY_DN5143_c1_g1_i3 | -3.22654039 | 0.208851649 | 0.999970678 | 1.281 | 0 | 8.599 | 1.88 | NA |
| TRINITY_DN6074_c0_g1_i2 | -3.22219017 | 0.431516693 | 0.999970678 | 1.172 | 0 | 9.288 | 0 | Grp1_Fun34_Yaa |
| TRINITY_DN492_c0_g1_i6 | -3.22158573 | 0.042382517 | 0.999970678 | 0.309 | 0.148 | 3.289 | 0.372 | NA |
| TRINITY_DN9659_c0_g3_i1 | 3.217208433 | 0.176540006 | 0.999970678 | 2.353 | 0.946 | 0 | 0.352 | NA |
| TRINITY_DN1652_c0_g1_i1 | 3.216029978 | 0.427889284 | 0.999970678 | 0 | 3.922 | 0 | 0.412 | NA |
| TRINITY_DN843_c3_g1_i1 | -3.21600486 | 0.434016654 | 0.999970678 | 1.081 | 0 | 0 | 10.205 | PHM7_cyt |
| TRINITY_DN1822_c0_g1_i5 | -3.21486689 | 0.279867295 | 0.999970678 | 0 | 16.29 | 1.755 | 141.506 | NA |
| TRINITY_DN50319_c0_g1_i2 | 3.211387897 | 0.019873709 | 0.842170964 | 29.823 | 5.48 | 1.933 | 1.458 | NA |
| TRINITY_DN13011_c0_g1_i3 | 3.210584228 | 0.224806089 | 0.999970678 | 2.507 | 0.237 | 0.244 | 0 | NA |
| TRINITY_DN19610_c0_g1_i2 | 3.210168597 | 0.431571059 | 0.999970678 | 0 | 9.284 | 0.811 | 0 | Xan_ur_permease |
| TRINITY_DN196_c0_g1_i7 | -3.20857966 | 0.242820735 | 0.999970678 | 0.963 | 0 | 0.433 | 8.626 | Lectin_legB |
| TRINITY_DN1233_c0_g1_i5 | 3.204042177 | 0.257818221 | 0.999970678 | 87.551 | 31.33 | 10.733 | 0 | NA |
| TRINITY_DN7687_c0_g1_i2 | 3.203070752 | 0.205199087 | 0.999970678 | 11.709 | 11.99 | 0 | 2.584 | NA |
| TRINITY_DN6954_c0_g2_i3 | 3.201718897 | 0.163840838 | 0.999970678 | 1.862 | 1.587 | 0 | 0.372 | NA |
| TRINITY_DN12343_c0_g1_i4 | -3.20169655 | 0.021627291 | 0.900136118 | 0.527 | 1.084 | 3.011 | 10.788 | NA |
| TRINITY_DN12118_c0_g1_i3 | 3.201594676 | 0.17294415 | 0.999970678 | 0.59 | 0.621 | 0.111 | 0 | NA |
| TRINITY_DN175_c0_g1_i6 | 3.197354376 | 0.000158457 | 0.016487613 | 10.092 | 9.895 | 1.078 | 0.865 | NA |
| TRINITY_DN4489_c0_g1_i3 | 3.197198994 | 0.308812648 | 0.999970678 | 20.375 | 0.089 | 0 | 2.272 | zf-Dof |
| TRINITY_DN6902_c0_g1_i9 | -3.19703388 | 0.032616984 | 0.999970678 | 0.055 | 0.158 | 0.4 | 1.528 | NA |
| TRINITY_DN8849_c0_g1_i1 | -3.19454659 | 0.188624096 | 1 | 0.282 | 0 | 1.344 | 1.116 | SBF |
| TRINITY_DN4806_c0_g1_i1 | -3.19048413 | 0.435745184 | 0.999970678 | 1.099 | 0 | 0 | 10.255 | NA |
| TRINITY_DN7340_c0_g1_i8 | 3.189461866 | 0.434920798 | 0.999970678 | 0 | 4.721 | 0.433 | 0 | Terpene_synth |
| TRINITY_DN7747_c0_g1_i4 | 3.186740252 | 0.061869889 | 1 | 1.59 | 2.533 | 0.311 | 0.07 | NA |
| TRINITY_DN5145_c0_g1_i4 | 3.186347673 | 0.206828398 | 0.999970678 | 1.435 | 9.855 | 0 | 1.196 | NA |
| TRINITY_DN3436_c0_g1_i3 | -3.18587336 | 0.195688153 | 0.999970678 | 1.862 | 0 | 4.322 | 11.944 | ACAS_N |
| TRINITY_DN117_c0_g1_i7 | -3.18194893 | 0.045983269 | 0.999970678 | 1.481 | 0.414 | 2.311 | 14.065 | LOR |

| TRINITY_DN681_c2_g1_i3 | 3.177994566 | 0.286636446 | 0.999970678 | 0.082 | 6.376 | 0.578 | 0 | NA |
| --- | --- | --- | --- | --- | --- | --- | --- | --- |
| TRINITY_DN14_c0_g1_i7 | 3.177738135 | 0.207733779 | 0.999970678 | 2.071 | 7.697 | 0 | 1.066 | Gelsolin |
| TRINITY_DN20185_c0_g2_i2 | 3.177100245 | 0.187738807 | 0.999970678 | 5.36 | 6.633 | 0 | 1.327 | GDI |
| TRINITY_DN3720_c0_g2_i6 | 3.175621752 | 0.207763455 | 0.999970678 | 1.226 | 6.376 | 0.7 | 0 | C1_2 |
| TRINITY_DN21660_c0_g1_i1 | -3.17359537 | 0.18045627 | 0.999970678 | 0 | 0.276 | 0.889 | 1.387 | WD40 |
| TRINITY_DN6364_c2_g1_i1 | 3.172577586 | 0.197776281 | 1 | 1.008 | 1.537 | 0.233 | 0 | NA |
| TRINITY_DN1868_c0_g1_i6 | 3.171871244 | 0.279310839 | 0.999970678 | 18.159 | 0.453 | 1.733 | 0 | NA |
| TRINITY_DN4226_c0_g1_i7 | 3.169659094 | 0.011387743 | 0.5407854 | 5.659 | 1.36 | 0.222 | 0.523 | NA |
| TRINITY_DN3465_c0_g1_i4 | 3.169245244 | 0.043495614 | 0.999970678 | 9.792 | 8.899 | 0.1 | 1.91 | NA |
| TRINITY_DN7582_c0_g1_i2 | 3.167963786 | 0.154654906 | 0.999970678 | 0.182 | 29.5 | 0.3 | 2.906 | MatE |
| TRINITY_DN18386_c0_g1_i2 | 3.165147969 | 0.182214255 | 1 | 0.527 | 0.818 | 0 | 0.151 | NA |
| TRINITY_DN743_c0_g1_i15 | 3.162812547 | 0.440327544 | 0.999970678 | 0 | 2.789 | 0.256 | 0 | NA |
| TRINITY_DN15056_c0_g1_i1 | 3.159342407 | 0.442099217 | 0.999970678 | 0 | 61.44 | 5.577 | 0 | NA |
| TRINITY_DN1186_c0_g1_i15 | -3.15931051 | 0.031859206 | 0.999970678 | 1.526 | 0.335 | 2.178 | 14.156 | NA |
| TRINITY_DN9798_c0_g1_i4 | 3.158811489 | 0.207932168 | 1 | 0.309 | 0.375 | 0.067 | 0 | SYS1 |
| TRINITY_DN1972_c0_g1_i1 | -3.15855694 | 0.152043 | 0.999970678 | 0.055 | 1.518 | 0.522 | 13.17 | LEA_2 |
| TRINITY_DN11894_c0_g1_i1 | 3.155155305 | 0.202443534 | 1 | 1.063 | 0.68 | 0.178 | 0 | NA |
| TRINITY_DN376_c0_g1_i5 | 3.154990059 | 0.172257003 | 0.999970678 | 0.645 | 0.798 | 0 | 0.161 | Pkinase |
| TRINITY_DN1808_c0_g1_i2 | -3.15404126 | 0.204516794 | 0.999970678 | 2.889 | 0 | 4.822 | 19.816 | NA |
| TRINITY_DN11178_c0_g1_i1 | -3.15397504 | 0.437392757 | 0.999970678 | 0 | 1.399 | 0 | 12.205 | polyprenyl_synt |
| TRINITY_DN10819_c0_g1_i1 | -3.15345897 | 0.038441076 | 0.999970678 | 2.189 | 4.957 | 3.711 | 58.884 | Glyco_hydro_17 |
| TRINITY_DN16122_c0_g1_i14 | -3.15179274 | 0.201825295 | 1 | 0.109 | 0 | 0.311 | 0.633 | NA |
| TRINITY_DN1695_c0_g1_i1 | 3.151131669 | 0.153814781 | 0.999970678 | 9.956 | 0.177 | 0.867 | 0.09 | NA |
| TRINITY_DN288_c0_g1_i1 | -3.14860227 | 0.132627257 | 0.999970678 | 0.472 | 10.7 | 2.422 | 94.575 | NA |
| TRINITY_DN2850_c0_g1_i1 | -3.14686654 | 0.072980919 | 0.999970678 | 10.528 | 11.33 | 3.833 | 189.17 | CAP160 |
| TRINITY_DN1354_c0_g1_i1 | 3.146654243 | 0.080222187 | 0.999970678 | 3.67 | 92.49 | 1.667 | 8.475 | NA |
| TRINITY_DN10838_c0_g1_i2 | -3.14382683 | 0.291713158 | 0.999970678 | 0.927 | 0 | 0.1 | 8.234 | NA |
| TRINITY_DN362_c0_g1_i1 | -3.14264622 | 0.126479995 | 0.999970678 | 0.036 | 0.532 | 0.211 | 4.705 | NA |
| TRINITY_DN6935_c0_g1_i3 | -3.14242038 | 0.205069764 | 0.999970678 | 0.672 | 0 | 1.122 | 4.534 | SNARE_assoc |
| TRINITY_DN1270_c1_g1_i3 | 3.140969429 | 0.26376182 | 0.999970678 | 20.92 | 0.798 | 2.078 | 0 | NA |
| TRINITY_DN13750_c0_g1_i2 | -3.13866254 | 0.290269709 | 0.999970678 | 4.851 | 0 | 34.965 | 0.613 | ABC_tran |
| TRINITY_DN13576_c0_g3_i1 | 3.138441772 | 0.212020748 | 1 | 0.527 | 0.463 | 0.111 | 0 | NA |
| TRINITY_DN680_c0_g2_i1 | 3.137969484 | 0.085936626 | 0.999970678 | 111.72 | 2.06 | 5.533 | 6.434 | Arm |
| TRINITY_DN639_c0_g1_i8 | 3.137883388 | 0.042094172 | 0.999970678 | 0.409 | 5.105 | 0.289 | 0.282 | NA |
| TRINITY_DN1262_c1_g1_i4 | -3.13617001 | 0.193157866 | 0.999970678 | 0 | 0.404 | 0.822 | 2.473 | - |
| TRINITY_DN373_c1_g1_i11 | 3.135637592 | 0.037850414 | 0.999970678 | 20.421 | 4.681 | 2.044 | 0.402 | NA |
| TRINITY_DN205_c0_g1_i4 | 3.134658366 | 0.202983716 | 0.999970678 | 3.915 | 7.372 | 0 | 1.287 | - |
| TRINITY_DN3875_c0_g2_i2 | 3.13123261 | 0.150298127 | 0.999970678 | 8.439 | 84.21 | 0.078 | 10.325 | NA |
| TRINITY_DN5280_c1_g1_i3 | -3.13009784 | 0.019978079 | 0.84440338 | 0.418 | 0.148 | 1.322 | 3.237 | NA |
| TRINITY_DN15745_c0_g1_i1 | -3.12936616 | 0.280022486 | 0.999970678 | 2.135 | 0 | 15.221 | 0.452 | NA |
| TRINITY_DN859_c0_g1_i9 | 3.128148714 | 0.001090145 | 0.075141781 | 43.776 | 74.05 | 8.066 | 3.68 | NA |
| TRINITY_DN3924_c0_g1_i6 | -3.12673176 | 0.452730062 | 0.999970678 | 3.079 | 0 | 22.532 | 0 | NA |
| TRINITY_DN1298_c0_g1_i10 | 3.126643349 | 0.443659805 | 0.999970678 | 4.042 | 0 | 0 | 0.473 | NB-ARC |
| TRINITY_DN4033_c0_g1_i2 | 3.125748177 | 0.287299923 | 0.999970678 | 14.271 | 0 | 1.089 | 0.332 | NA |
| TRINITY_DN10779_c0_g1_i2 | -3.1254042 | 0.20730178 | 1 | 0.282 | 0 | 1.389 | 0.804 | NA |
| TRINITY_DN17897_c0_g1_i5 | 3.125023318 | 0.033723238 | 1 | 0.382 | 0.286 | 0.022 | 0.05 | NA |
| TRINITY_DN10484_c0_g1_i11 | 3.123593402 | 0.189489801 | 0.999970678 | 1.553 | 0.591 | 0 | 0.251 | NA |
| TRINITY_DN649_c0_g1_i2 | 3.12324537 | 0.186186831 | 0.999970678 | 1.408 | 1.715 | 0.3 | 0 | NA |
| TRINITY_DN4024_c1_g1_i2 | 3.122642825 | 0.444918913 | 0.999970678 | 0 | 4.464 | 0.422 | 0 | Gal_Lectin |
| TRINITY_DN1384_c0_g1_i13 | 3.12065544 | 0.210628679 | 0.999970678 | 11.064 | 13 | 0 | 2.765 | NA |
| TRINITY_DN3527_c0_g1_i6 | 3.119780894 | 0.008380042 | 0.41800418 | 1.726 | 0.995 | 0.122 | 0.171 | NA |
| TRINITY_DN1268_c0_g1_i7 | -3.11834031 | 0.195380595 | 0.999970678 | 0.382 | 0 | 1.111 | 2.051 | NA |
| TRINITY_DN108638_c0_g1_i1 | -3.11745946 | 0.081580202 | 0.999970678 | 0.427 | 1.439 | 0.689 | 15.151 | NA |
| TRINITY_DN7897_c0_g1_i1 | 3.115895159 | 0.294804307 | 0.999970678 | 0 | 11.21 | 0.922 | 0.161 | OTU |
| TRINITY_DN9648_c0_g2_i1 | -3.11585961 | 0.195566601 | 0.999970678 | 0.627 | 0 | 2.478 | 2.544 | PPR |
| TRINITY_DN14599_c0_g1_i6 | 3.115265629 | 0.151462331 | 0.999970678 | 27.397 | 0.483 | 2.467 | 0.241 | NA |
| TRINITY_DN7228_c0_g1_i1 | 3.115168956 | 0.108855249 | 0.999970678 | 33.247 | 1.064 | 2.878 | 0.533 | Myb_DNA-bindin |
| TRINITY_DN5018_c0_g1_i4 | 3.113268252 | 0.00017013 | 0.017479553 | 1.998 | 2.198 | 0.2 | 0.261 | NA |
| TRINITY_DN166_c0_g1_i2 | -3.11299636 | 0.085432496 | 0.999970678 | 6.659 | 5.371 | 1.755 | 101.261 | NA |
| TRINITY_DN45_c1_g1_i5 | 3.111806627 | 0.269881772 | 0.999970678 | 6.313 | 42.75 | 0 | 5.65 | GAF |
| TRINITY_DN2207_c0_g1_i3 | -3.11137771 | 0.215706928 | 0.999970678 | 0 | 2.139 | 12.377 | 3.388 | NA |
| TRINITY_DN10594_c0_g1_i7 | 3.110254082 | 0.201220777 | 1 | 0.409 | 0.473 | 0.089 | 0 | NA |
| TRINITY_DN1183_c0_g1_i9 | 3.105636528 | 0.029282049 | 0.999970678 | 1.671 | 0.65 | 0.078 | 0.191 | LRR_8 |
| TRINITY_DN396_c0_g1_i6 | -3.10523132 | 0.198968196 | 0.999970678 | 0 | 1.567 | 6.289 | 5.64 | NA |
| TRINITY_DN11723_c0_g2_i5 | 3.101704569 | 0.449684451 | 0.999970678 | 3.252 | 0 | 0 | 0.382 | NA |
| TRINITY_DN11559_c0_g2_i5 | -3.10087341 | 0.021166752 | 0.884347995 | 0.382 | 0.306 | 3.733 | 1.237 | NA |
| TRINITY_DN260_c1_g1_i5 | 3.099981735 | 0.079845032 | 0.999970678 | 1.635 | 3.765 | 0.5 | 0.05 | NA |
| TRINITY_DN4733_c0_g1_i5 | -3.09821592 | 0.272688293 | 0.999970678 | 0.836 | 0 | 5.733 | 0.271 | NA |
| TRINITY_DN2123_c0_g1_i1 | 3.097643172 | 0.02725989 | 0.999970678 | 4.006 | 8.407 | 0.144 | 1.277 | AICARFT_IMPCH |
| TRINITY_DN5400_c1_g1_i1 | -3.09588265 | 0.009296945 | 0.457454478 | 0.818 | 0.345 | 2.278 | 7.229 | NA |
| TRINITY_DN197698_c0_g1_i1 | -3.09533083 | 0.303503265 | 0.999970678 | 0 | 1.794 | 0.178 | 14.668 | Tryp_alpha_amyl |
| TRINITY_DN438_c0_g1_i9 | -3.09218889 | 0.466680333 | 0.999970678 | 12.254 | 0 | 0 | 105.071 | NA |
| TRINITY_DN10456_c0_g5_i1 | -3.09186078 | 0.295223269 | 0.999970678 | 0 | 0.729 | 0.089 | 6.062 | Glyco_hydro_1 |
| TRINITY_DN4273_c2_g1_i1 | -3.08643697 | 0.345475684 | 0.999970678 | 2.253 | 0.039 | 16.321 | 0 | GTP_EFTU |

| TRINITY_DN8050_c0_g1_i3 | 3.085025652 | 0.205793831 | 0.999970678 | 0.372 | 1.005 | 0.133 | 0 | NA |
| --- | --- | --- | --- | --- | --- | --- | --- | --- |
| TRINITY_DN4656_c0_g1_i3 | 3.083771855 | 0.449647217 | 0.999970678 | 0 | 15.79 | 0 | 1.84 | Yip1 |
| TRINITY_DN6391_c0_g1_i2 | 3.082103345 | 0.200419143 | 0.999970678 | 1.017 | 0.384 | 0 | 0.171 | NA |
| TRINITY_DN39_c4_g1_i8 | 3.081053852 | 0.30913479 | 0.999970678 | 12.082 | 63.71 | 7.333 | 0 | GATase_2 |
| TRINITY_DN4517_c0_g1_i1 | 3.080708249 | 0.454779722 | 0.999970678 | 1.69 | 0 | 0.167 | 0 | NA |
| TRINITY_DN236_c0_g3_i2 | -3.08040921 | 0.243889412 | 0.999970678 | 0 | 5.371 | 22.332 | 17.695 | LTP_2 |
| TRINITY_DN45_c0_g2_i4 | 3.078434347 | 0.00330635 | 0.187869688 | 1.045 | 1.183 | 0.133 | 0.101 | GAF |
| TRINITY_DN9357_c0_g1_i1 | -3.07187007 | 0.22572661 | 0.999970678 | 0.627 | 0 | 0.789 | 4.323 | NA |
| TRINITY_DN6131_c0_g1_i1 | -3.07136604 | 0.01219484 | 0.571635293 | 0.109 | 0.286 | 1.467 | 1.468 | NA |
| TRINITY_DN5625_c0_g2_i4 | 3.067269366 | 0.211993529 | 1 | 1.099 | 0.581 | 0.167 | 0 | UbiA |
| TRINITY_DN18594_c0_g1_i1 | 3.06487164 | 0.026832791 | 1 | 1.281 | 1.794 | 0.189 | 0.141 | NA |
| TRINITY_DN5757_c0_g1_i1 | -3.06198618 | 0.028720113 | 0.999970678 | 0.908 | 0.197 | 2.267 | 6.615 | Gal-bind_lectin |
| TRINITY_DN406_c0_g1_i12 | -3.06176877 | 0.07133642 | 0.999970678 | 0.291 | 0.995 | 0.478 | 10.184 | Peptidase_S10 |
| TRINITY_DN6534_c0_g1_i4 | -3.06064017 | 0.319830599 | 0.999970678 | 0 | 0.917 | 0.044 | 7.591 | Solute_trans_a |
| TRINITY_DN352_c0_g1_i4 | 3.060581675 | 0.021900327 | 0.906878947 | 24.418 | 3.597 | 1.755 | 1.267 | NA |
| TRINITY_DN6469_c0_g1_i7 | 3.057380555 | 0.458431044 | 0.999970678 | 0 | 3.932 | 0.389 | 0 | NA |
| TRINITY_DN686_c0_g1_i4 | 3.05694508 | 0.304401753 | 0.999970678 | 40.133 | 0 | 0.978 | 3.73 | NA |
| TRINITY_DN4195_c0_g1_i14 | -3.05629857 | 0.456019152 | 0.999970678 | 0 | 0.246 | 1.7 | 0 | NA |
| TRINITY_DN373_c1_g1_i6 | 3.055051812 | 0.05538682 | 0.999970678 | 27.334 | 4.336 | 2.778 | 0.513 | NA |
| TRINITY_DN3551_c0_g1_i5 | -3.05473494 | 0.278968776 | 0.999970678 | 0 | 0.976 | 0.222 | 7.772 | Hyccin |
| TRINITY_DN8625_c0_g1_i5 | -3.05083876 | 0.113771653 | 0.999970678 | 0.055 | 0.306 | 2.233 | 0.171 | NA |
| TRINITY_DN7120_c0_g2_i5 | 3.04868238 | 0.013298548 | 0.611957988 | 2.335 | 6.14 | 0.644 | 0.231 | Glyco_transf_28 |
| TRINITY_DN9525_c1_g2_i2 | -3.04853565 | 0.207927173 | 0.999970678 | 0.372 | 0 | 0.978 | 1.89 | EF-hand_1 |
| TRINITY_DN4907_c0_g1_i1 | -3.04838365 | 0.07303096 | 0.999970678 | 36.599 | 4.888 | 23.732 | 306.849 | Sugar_tr |
| TRINITY_DN2307_c1_g1_i8 | 3.04620817 | 0.193172697 | 0.999970678 | 5.142 | 4.297 | 0 | 1.086 | NA |
| TRINITY_DN14756_c0_g1_i1 | -3.04525212 | 0.46086816 | 0.999970678 | 1.235 | 0 | 8.533 | 0 | NA |
| TRINITY_DN3572_c0_g1_i2 | -3.04442871 | 0.215310095 | 0.999970678 | 0.618 | 0 | 1.333 | 3.408 | NA |
| TRINITY_DN14436_c0_g1_i1 | 3.044268011 | 0.223521637 | 0.999970678 | 2.934 | 2.444 | 0.533 | 0 | NA |
| TRINITY_DN10619_c0_g1_i1 | -3.03977143 | 0.049899986 | 0.999970678 | 0.164 | 0.946 | 5.655 | 2.151 | NA |
| TRINITY_DN514_c0_g1_i4 | 3.03602852 | 0.46011504 | 0.999970678 | 0 | 4.12 | 0.411 | 0 | STAS |
| TRINITY_DN3051_c1_g3_i4 | 3.033764316 | 0.218281718 | 0.999970678 | 4.024 | 0.956 | 0 | 0.603 | NA |
| TRINITY_DN1304_c0_g1_i6 | 3.028832855 | 0.221920197 | 0.999970678 | 1.19 | 0.424 | 0.156 | 0 | NA |
| TRINITY_DN242_c1_g1_i6 | 3.028043393 | 0.026161603 | 0.999970678 | 7.294 | 2.05 | 0.533 | 0.452 | B_lectin |
| TRINITY_DN9480_c0_g1_i4 | 3.027707506 | 0.199738043 | 0.999970678 | 0.927 | 0.818 | 0 | 0.221 | zf-CCHC |
| TRINITY_DN9123_c0_g1_i9 | -3.02724294 | 0.003354425 | 0.189940662 | 0.472 | 0.335 | 2.667 | 3.308 | NA |
| TRINITY_DN3159_c0_g1_i2 | 3.026239418 | 0.306221156 | 0.999970678 | 0 | 4.583 | 0.078 | 0.473 | - |
| TRINITY_DN27718_c0_g1_i2 | 3.026134064 | 0.209166751 | 0.999970678 | 1.399 | 2.415 | 0 | 0.473 | - |
| TRINITY_DN11887_c0_g1_i7 | 3.022824389 | 0.214926545 | 1 | 0.409 | 0.187 | 0 | 0.07 | NA |
| TRINITY_DN2351_c0_g1_i4 | 3.019435695 | 0.465182427 | 0.999970678 | 0 | 8.614 | 0 | 1.056 | NA |
| TRINITY_DN3459_c0_g1_i14 | -3.01893445 | 0.317268108 | 0.999970678 | 0 | 1.892 | 0.133 | 14.92 | NA |
| TRINITY_DN920_c0_g2_i5 | 3.018778835 | 0.460517383 | 0.999970678 | 11.373 | 0 | 0 | 1.408 | NA |
| TRINITY_DN4589_c0_g1_i1 | -3.01810958 | 0.043615086 | 0.999970678 | 0.863 | 3.144 | 23.321 | 3.81 | NA |
| TRINITY_DN56221_c0_g1_i2 | 3.017519948 | 0.46450589 | 0.999970678 | 5.132 | 0 | 0.544 | 0 | NA |
| TRINITY_DN471_c0_g1_i12 | -3.01690177 | 0.098131416 | 0.999970678 | 2.571 | 0.296 | 1.033 | 22.46 | LRRNT_2 |
| TRINITY_DN4288_c0_g1_i3 | 3.015161591 | 0.204252448 | 0.999970678 | 1.145 | 0.641 | 0 | 0.211 | NA |
| TRINITY_DN1734_c0_g1_i1 | 3.014993492 | 0.506659787 | 0.999970678 | 0 | 141.7 | 14.255 | 0 | NA |
| TRINITY_DN521_c0_g1_i2 | -3.01317594 | 0.260394635 | 0.999970678 | 5.759 | 0 | 4.411 | 41.371 | NA |
| TRINITY_DN25703_c0_g1_i2 | -3.01308694 | 0.216847111 | 0.999970678 | 0.282 | 0 | 1.289 | 0.754 | NA |
| TRINITY_DN5143_c0_g3_i1 | 3.013047234 | 0.210860779 | 1 | 0.636 | 0.375 | 0 | 0.121 | NA |
| TRINITY_DN1783_c0_g1_i4 | -3.01278747 | 0.226468546 | 0.999970678 | 0.382 | 0 | 1.933 | 0.774 | Orn_Arg_deC_N |
| TRINITY_DN4972_c0_g1_i2 | 3.012513055 | 0.055731425 | 1 | 3.67 | 1.715 | 0.422 | 0.151 | DUF3537 |
| TRINITY_DN256_c0_g1_i4 | 3.011306859 | 0.040713114 | 0.999970678 | 2.871 | 1.882 | 0.078 | 0.513 | Myb_DNA-bindin |
| TRINITY_DN12359_c0_g1_i1 | -3.0086637 | 0.265568978 | 0.999970678 | 0.945 | 0 | 0.5 | 7.048 | NA |
| TRINITY_DN6322_c0_g1_i2 | 3.008128558 | 0.219787765 | 0.999970678 | 2.852 | 1.547 | 0.467 | 0 | B3 |
| TRINITY_DN11876_c0_g1_i2 | -3.00799467 | 0.464760577 | 0.999970678 | 1.208 | 0 | 8.122 | 0 | NA |
| TRINITY_DN1037_c0_g1_i6 | 3.004808071 | 0.212473845 | 0.999970678 | 0.336 | 0.237 | 0.067 | 0 | Cpn60_TCP1 |
| TRINITY_DN3664_c0_g2_i1 | -3.00454022 | 0.46522766 | 0.999970678 | 25.181 | 0 | 164.979 | 0 | NA |
| TRINITY_DN55430_c0_g2_i2 | -3.00323875 | 0.212331795 | 0.999970678 | 3.061 | 0 | 11.055 | 10.657 | adh_short |
| TRINITY_DN728_c0_g1_i16 | 3.003111817 | 0.225503319 | 0.999970678 | 0.354 | 1.084 | 0.156 | 0 | Glyco_hydro_79n |
| TRINITY_DN3431_c0_g1_i1 | -3.00272427 | 0.465138582 | 0.999970678 | 1.762 | 0 | 11.722 | 0 | NA |
| TRINITY_DN2266_c0_g1_i6 | 3.001929057 | 0.197480008 | 0.999970678 | 1.69 | 1.725 | 0 | 0.412 | NA |
| TRINITY_DN846_c0_g1_i11 | 3.001107527 | 0.009389415 | 0.461309735 | 3.479 | 1.35 | 0.178 | 0.392 | ATP-cone |
| TRINITY_DN31287_c0_g1_i5 | 3.001014825 | 0.465993104 | 0.999970678 | 2.071 | 0 | 0 | 0.261 | JmjC |
| TRINITY_DN2123_c0_g1_i9 | -2.99740071 | 0.050491304 | 0.999970678 | 0.491 | 2.257 | 2.078 | 19.444 | AICARFT_IMPCH |
| TRINITY_DN3794_c0_g3_i1 | 2.997385914 | 0.260273741 | 0.999970678 | 8.784 | 15.44 | 2.5 | 0 | NA |
| TRINITY_DN665_c1_g1_i11 | -2.99665585 | 0.135389758 | 0.999970678 | 14.08 | 2.937 | 110.571 | 2.111 | - |
| TRINITY_DN8270_c0_g1_i2 | -2.99646607 | 0.249156842 | 0.999970678 | 4.097 | 0 | 15.032 | 15.02 | Glyco_hydro_14 |
| TRINITY_DN6954_c0_g1_i1 | -2.99584023 | 0.054879313 | 0.999970678 | 3.188 | 1.725 | 2.267 | 36.073 | NA |
| TRINITY_DN8463_c0_g1_i1 | 2.994649744 | 0.20618052 | 0.999970678 | 1.526 | 3.242 | 0 | 0.593 | NA |
| TRINITY_DN3420_c1_g1_i7 | 2.992889614 | 0.206119733 | 0.999970678 | 1.953 | 1.104 | 0 | 0.382 | NA |
| TRINITY_DN3453_c1_g1_i1 | -2.99252381 | 0.18889316 | 0.999970678 | 5.423 | 0.256 | 36.998 | 0.714 | NA |
| TRINITY_DN4759_c0_g1_i2 | -2.99233455 | 0.058949776 | 0.999970678 | 0.291 | 0.493 | 0.444 | 5.6 | NA |
| TRINITY_DN4004_c0_g1_i6 | -2.99188397 | 0.046721304 | 0.999970678 | 0.118 | 0.434 | 2.622 | 1.076 | NA |
| TRINITY_DN3263_c1_g1_i1 | -2.99153809 | 0.003369759 | 0.190478806 | 2.516 | 2.247 | 7.4 | 28.945 | NA |

| TRINITY_DN2524_c0_g1_i10 | 2.990982442 | 0.050948651 | 0.999970678 | 0.627 | 5.726 | 0.311 | 0.412 | NA |
| --- | --- | --- | --- | --- | --- | --- | --- | --- |
| TRINITY_DN6095_c0_g1_i4 | 2.990499256 | 0.469269478 | 0.999970678 | 2.943 | 0 | 0.322 | 0 | NA |
| TRINITY_DN7446_c1_g1_i1 | -2.98879405 | 0.001257796 | 0.084731363 | 10.02 | 4.652 | 34.22 | 75.242 | Chlorophyllase |
| TRINITY_DN24332_c0_g1_i4 | -2.98808374 | 0.215547679 | 0.999970678 | 0.391 | 0 | 1.322 | 1.639 | PORR |
| TRINITY_DN4301_c0_g1_i9 | 2.987481008 | 0.465296337 | 0.999970678 | 5.414 | 0 | 0 | 0.694 | WRKY |
| TRINITY_DN1734_c0_g1_i4 | -2.98738327 | 0.38521935 | 0.999970678 | 4.533 | 0 | 30.165 | 0.03 | NA |
| TRINITY_DN23839_c0_g1_i5 | 2.986912699 | 0.222767324 | 1 | 0.554 | 0.788 | 0.144 | 0 | DNA_methylase |
| TRINITY_DN2640_c0_g2_i4 | 2.986723278 | 0.467402167 | 0.999970678 | 1.653 | 0 | 0 | 0.211 | RVT_1 |
| TRINITY_DN1962_c0_g1_i3 | -2.98588082 | 0.490867813 | 0.999970678 | 0 | 32.72 | 0 | 251.614 | 60KD_IMP |
| TRINITY_DN5208_c0_g1_i5 | 2.985742823 | 0.016133323 | 0.708478394 | 5.578 | 1.636 | 0.311 | 0.543 | EGF_CA |
| TRINITY_DN2175_c0_g1_i5 | 2.985235164 | 0.045707335 | 0.999970678 | 5.796 | 31.43 | 0.522 | 4.032 | NA |
| TRINITY_DN5361_c0_g1_i1 | -2.98479417 | 0.007103494 | 0.362633392 | 4.297 | 1.853 | 9.933 | 36.977 | p450 |
| TRINITY_DN994_c0_g1_i1 | 2.983570939 | 0.216545548 | 0.999970678 | 1.617 | 4.878 | 0 | 0.814 | NA |
| TRINITY_DN30724_c0_g1_i1 | 2.983550991 | 0.227471077 | 1 | 0.79 | 0.631 | 0.156 | 0 | NA |
| TRINITY_DN6601_c0_g3_i2 | -2.98349888 | 0.471931587 | 0.999970678 | 0.827 | 0 | 5.455 | 0 | Bac_rhodopsin |
| TRINITY_DN2862_c0_g1_i5 | -2.98318854 | 0.237146021 | 0.999970678 | 3.425 | 0 | 6.033 | 20.178 | NA |
| TRINITY_DN1868_c0_g1_i1 | 2.981397349 | 0.165039015 | 0.999970678 | 66.731 | 0.611 | 6.177 | 1.146 | NA |
| TRINITY_DN7233_c0_g1_i6 | -2.98108506 | 0.470830217 | 0.999970678 | 0.6 | 0 | 4.011 | 0 | Sugar_tr |
| TRINITY_DN13832_c2_g1_i1 | -2.98020809 | 0.467807481 | 0.999970678 | 0 | 143.7 | 870.458 | 0 | NA |
| TRINITY_DN13228_c0_g1_i7 | -2.97975198 | 0.029271628 | 1 | 0.109 | 0.158 | 0.833 | 0.955 | NA |
| TRINITY_DN1173_c0_g2_i5 | 2.979058727 | 0.470346231 | 0.999970678 | 2.871 | 0 | 0.3 | 0 | NA |
| TRINITY_DN1293_c0_g1_i5 | 2.977459728 | 0.214978696 | 0.999970678 | 0.472 | 0.788 | 0.133 | 0 | NA |
| TRINITY_DN124_c0_g1_i1 | 2.977446293 | 0.305112099 | 0.999970678 | 0 | 36.9 | 0.811 | 3.448 | NA |
| TRINITY_DN3627_c0_g1_i15 | -2.97710834 | 0.470030047 | 0.999970678 | 0.636 | 0 | 4.2 | 0 | Pro_CA |
| TRINITY_DN3395_c0_g1_i3 | 2.975749244 | 0.207361651 | 0.999970678 | 5.687 | 10.53 | 0 | 1.971 | NA |
| TRINITY_DN11022_c0_g1_i2 | -2.97421085 | 0.222497876 | 0.999970678 | 0 | 2.05 | 7.211 | 7.138 | OTU |
| TRINITY_DN26906_c0_g1_i2 | -2.97201364 | 0.226551615 | 0.999970678 | 0 | 1.271 | 5.689 | 3.227 | NA |
| TRINITY_DN7397_c0_g1_i4 | -2.97037873 | 0.232531433 | 0.999970678 | 0.781 | 0 | 1.467 | 4.393 | NA |
| TRINITY_DN3280_c0_g1_i9 | -2.96999748 | 0.246294965 | 0.999970678 | 1.281 | 0 | 6 | 2.956 | NA |
| TRINITY_DN2094_c0_g1_i4 | 2.967812488 | 0.129167024 | 0.999970678 | 0.254 | 1.478 | 0.167 | 0.02 | NA |
| TRINITY_DN334_c0_g1_i7 | -2.96774467 | 0.345014038 | 0.999970678 | 12.145 | 0 | 1.289 | 95.148 | NA |
| TRINITY_DN671_c0_g2_i4 | -2.96748627 | 0.177525374 | 0.999970678 | 2.353 | 0.118 | 15.599 | 0.503 | 2OG-FeII_Oxy |
| TRINITY_DN5056_c0_g1_i12 | -2.96727229 | 0.148841742 | 0.999970678 | 0.2 | 1.114 | 0.178 | 9.983 | DHquinase_I |
| TRINITY_DN255_c1_g1_i6 | 2.967151534 | 0.469870081 | 0.999970678 | 3.297 | 0 | 0.011 | 0.432 | NA |
| TRINITY_DN6318_c0_g3_i8 | -2.96706948 | 0.222353765 | 0.999970678 | 0 | 0.325 | 1.1 | 1.156 | Myb_DNA-bindin |
| TRINITY_DN16151_c0_g1_i7 | -2.967043 | 0.221520436 | 0.999970678 | 0.618 | 0 | 1.644 | 2.926 | NA |
| TRINITY_DN79_c0_g1_i36 | -2.96544192 | 0.226110516 | 0.999970678 | 0 | 0.581 | 2.744 | 1.196 | NA |
| TRINITY_DN8688_c0_g1_i5 | 2.964964677 | 0.218622327 | 1 | 0.927 | 0.601 | 0 | 0.201 | NA |
| TRINITY_DN525_c1_g2_i6 | 2.96470892 | 0.216454202 | 0.999970678 | 1.653 | 0.7 | 0 | 0.302 | FKBP_C |
| TRINITY_DN872_c0_g1_i9 | 2.964204578 | 0.322840815 | 0.999970678 | 8.475 | 0.099 | 0 | 1.126 | NA |
| TRINITY_DN108_c0_g1_i2 | -2.96402473 | 0.038525132 | 0.999970678 | 2.689 | 0.907 | 20.643 | 3.308 | - |
| TRINITY_DN748_c0_g1_i5 | -2.9638146 | 0.22257019 | 0.999970678 | 0 | 1.626 | 6.922 | 4.001 | NA |
| TRINITY_DN16151_c0_g2_i1 | 2.961988096 | 0.005242162 | 0.278944494 | 1.39 | 2.809 | 0.211 | 0.261 | NA |
| TRINITY_DN1894_c0_g1_i3 | 2.961858585 | 0.033390596 | 0.999970678 | 9.102 | 5.627 | 1.4 | 0.201 | NA |
| TRINITY_DN435_c0_g1_i1 | 2.961036668 | 0.087533173 | 0.999970678 | 0.836 | 12.9 | 0.367 | 1.307 | AA_permease_2 |
| TRINITY_DN5703_c0_g1_i4 | -2.96047338 | 0.040496797 | 0.999970678 | 14.38 | 1.311 | 38.431 | 76.991 | NA |
| TRINITY_DN1784_c1_g1_i2 | 2.959899078 | 0.153784977 | 0.999970678 | 0.372 | 48.11 | 2.033 | 3.619 | Redoxin |
| TRINITY_DN5265_c2_g1_i1 | -2.95949522 | 0.252959868 | 0.999970678 | 0.654 | 0 | 0.633 | 4.333 | NA |
| TRINITY_DN10008_c0_g1_i2 | 2.95910413 | 0.227365132 | 0.999970678 | 1.426 | 0.591 | 0.211 | 0 | NA |
| TRINITY_DN87730_c0_g1_i1 | -2.95849022 | 0.220291453 | 0.999970678 | 0.808 | 0 | 2.655 | 3.127 | NA |
| TRINITY_DN7656_c0_g1_i7 | -2.95847834 | 0.006711301 | 0.344945664 | 0.445 | 0.266 | 1.333 | 3.881 | NT-C2 |
| TRINITY_DN2054_c0_g1_i14 | 2.957818818 | 0.226731326 | 0.999970678 | 10.51 | 4.593 | 1.589 | 0 | NA |
| TRINITY_DN5527_c0_g1_i3 | 2.95479406 | 0.270484566 | 0.999970678 | 0.527 | 7.313 | 0 | 0.995 | LRR_6 |
| TRINITY_DN559_c0_g1_i6 | -2.95425699 | 0.283306588 | 0.999970678 | 0 | 4.642 | 24.687 | 5.58 | NA |
| TRINITY_DN7644_c0_g1_i1 | -2.95372119 | 0.251757527 | 0.999970678 | 0 | 0.522 | 2.733 | 0.633 | NA |
| TRINITY_DN4980_c0_g2_i3 | -2.95364793 | 0.473287724 | 0.999970678 | 0.863 | 0 | 5.633 | 0 | NA |
| TRINITY_DN1280_c0_g1_i5 | 2.953544258 | 0.474948812 | 0.999970678 | 0 | 75.11 | 0 | 9.4 | NA |
| TRINITY_DN3498_c0_g1_i1 | -2.95313358 | 0.014187842 | 0.643812744 | 0.109 | 0.089 | 0.489 | 0.975 | DEAD |
| TRINITY_DN8498_c0_g1_i2 | 2.952053061 | 0.228680924 | 0.999970678 | 1.572 | 2.681 | 0 | 0.553 | Big_2 |
| TRINITY_DN340_c1_g1_i11 | 2.952009081 | 0.217083011 | 0.999970678 | 0.736 | 0.404 | 0 | 0.141 | NA |
| TRINITY_DN3246_c0_g1_i2 | -2.95195865 | 0.21795712 | 0.999970678 | 0 | 0.306 | 1.067 | 1.005 | NA |
| TRINITY_DN226_c0_g1_i7 | -2.95117136 | 0.017965328 | 0.778465791 | 0.4 | 0.375 | 0.878 | 5.027 | ABC2_membrane |
| TRINITY_DN3541_c0_g1_i2 | 2.949968624 | 0.218133316 | 1 | 2.235 | 2.129 | 0 | 0.533 | NA |
| TRINITY_DN615_c0_g1_i3 | -2.94988364 | 0.112212175 | 0.999970678 | 10.637 | 24.51 | 3.9 | 266.232 | NA |
| TRINITY_DN4093_c0_g1_i11 | -2.94809712 | 0.012351786 | 0.577335532 | 0.263 | 0.256 | 2.589 | 0.905 | - |
| TRINITY_DN3237_c0_g1_i5 | 2.947163089 | 0.262573939 | 0.999970678 | 0.917 | 9.53 | 0 | 1.327 | Linker_histone |
| TRINITY_DN3103_c1_g1_i3 | 2.947108221 | 0.21663621 | 0.999970678 | 1.472 | 0.68 | 0 | 0.282 | NA |
| TRINITY_DN748_c0_g1_i2 | 2.947036827 | 0.328841816 | 0.999970678 | 11.736 | 0 | 0.2 | 1.297 | NA |
| TRINITY_DN4104_c0_g1_i13 | -2.94651871 | 0.23991715 | 0.999970678 | 0 | 1.754 | 8.366 | 2.835 | NA |
| TRINITY_DN10484_c0_g1_i16 | 2.946246609 | 0.223717709 | 0.999970678 | 0.89 | 1.084 | 0.222 | 0 | NA |
| TRINITY_DN39693_c0_g1_i1 | -2.94586779 | 0.027027043 | 1 | 0.236 | 0.266 | 1.255 | 2.322 | NA |
| TRINITY_DN12574_c0_g1_i1 | -2.94424505 | 0.3056466 | 0.999970678 | 0 | 1.902 | 0.322 | 13.995 | LRRNT_2 |
| TRINITY_DN3260_c0_g1_i5 | 2.942879009 | 0.23525097 | 1 | 0.5 | 0.591 | 0.122 | 0 | Guanylate_kin |
| TRINITY_DN13474_c0_g1_i9 | 2.940788733 | 0.219697139 | 0.999970678 | 0.772 | 1.35 | 0 | 0.282 | NA |

| TRINITY_DN561_c0_g1_i16 | 2.932784552 | 0.472082908 | 0.999970678 | 0 | 1.557 | 0 | 0.201 | NA |
| --- | --- | --- | --- | --- | --- | --- | --- | --- |
| TRINITY_DN1115_c0_g1_i2 | 2.932459942 | 0.473828964 | 0.999970678 | 20.185 | 0 | 0 | 2.654 | UQ_con |
| TRINITY_DN9275_c0_g1_i1 | -2.93157307 | 0.227483555 | 0.999970678 | 0 | 1.114 | 4.622 | 2.694 | NA |
| TRINITY_DN94_c0_g1_i2 | 2.931095103 | 0.228986647 | 0.999970678 | 5.923 | 5.962 | 0 | 1.558 | NA |
| TRINITY_DN12361_c0_g1_i1 | 2.928227765 | 0.236954233 | 0.999970678 | 0.5 | 1.636 | 0.233 | 0 | NA |
| TRINITY_DN2416_c0_g1_i15 | 2.927783816 | 0.223845863 | 0.999970678 | 2.634 | 2.237 | 0.522 | 0 | Pkinase |
| TRINITY_DN813_c0_g1_i4 | 2.926857751 | 0.230175547 | 0.999970678 | 3.061 | 1.597 | 0.511 | 0 | DUF659 |
| TRINITY_DN3785_c0_g2_i2 | 2.926375372 | 0.477517988 | 0.999970678 | 9.611 | 0 | 0 | 1.287 | NA |
| TRINITY_DN75_c0_g1_i11 | -2.9251444 | 0.261071505 | 0.999970678 | 2.235 | 0.099 | 14.277 | 0.141 | - |
| TRINITY_DN1642_c1_g1_i2 | -2.92506688 | 0.076626338 | 0.999970678 | 0.89 | 0.611 | 0.478 | 10.788 | PH_3 |
| TRINITY_DN1824_c0_g1_i9 | -2.92396653 | 0.22707056 | 0.999970678 | 0 | 0.296 | 0.633 | 1.408 | NA |
| TRINITY_DN2630_c0_g1_i6 | 2.922951232 | 0.240348767 | 0.999970678 | 0.418 | 1.705 | 0 | 0.282 | NA |
| TRINITY_DN5701_c0_g3_i1 | 2.922554012 | 0.234010305 | 1 | 0.4 | 0.404 | 0 | 0.101 | DYW_deaminase |
| TRINITY_DN615_c0_g2_i1 | -2.92155443 | 0.143963561 | 0.999970678 | 1.054 | 1.005 | 12.721 | 0.201 | NA |
| TRINITY_DN2334_c0_g1_i4 | 2.921551142 | 0.293443422 | 0.999970678 | 0.227 | 4.228 | 0.478 | 0 | NA |
| TRINITY_DN8338_c0_g1_i1 | -2.92120758 | 0.242431334 | 0.999970678 | 2.544 | 0 | 9.733 | 7.741 | RINGv |
| TRINITY_DN1207_c0_g1_i2 | -2.91931171 | 0.25386469 | 0.999970678 | 1.653 | 0 | 2.655 | 9.601 | Ank_2 |
| TRINITY_DN11258_c0_g1_i2 | -2.918649 | 0.478664494 | 0.999970678 | 0.863 | 0 | 5.433 | 0 | NA |
| TRINITY_DN2941_c1_g1_i10 | -2.91785859 | 0.226880132 | 0.999970678 | 0 | 0.197 | 0.589 | 0.734 | NA |
| TRINITY_DN2742_c0_g1_i3 | 2.9168328 | 0.47860895 | 0.999970678 | 0 | 24.32 | 0 | 3.177 | ADH_N |
| TRINITY_DN10719_c1_g1_i1 | 2.916149854 | 0.053927318 | 0.999970678 | 294.72 | 21.2 | 22.065 | 15.563 | NA |
| TRINITY_DN13094_c0_g1_i8 | -2.91581669 | 0.013984479 | 0.635467193 | 0.372 | 0.207 | 1.189 | 3.086 | NA |
| TRINITY_DN13890_c0_g1_i2 | 2.913188713 | 0.349514263 | 0.999970678 | 20.711 | 0 | 2.189 | 0.181 | LRRNT_2 |
| TRINITY_DN7562_c0_g1_i1 | 2.912718947 | 0.041396683 | 0.999970678 | 24.617 | 105.6 | 1.944 | 14.769 | NA |
| TRINITY_DN13011_c0_g1_i4 | 2.90989041 | 0.118080741 | 0.999970678 | 14.562 | 3.242 | 1.878 | 0.111 | NA |
| TRINITY_DN2846_c0_g1_i2 | -2.90885938 | 0.251560805 | 0.999970678 | 0.645 | 0 | 0.9 | 3.971 | NA |
| TRINITY_DN15817_c0_g1_i1 | -2.90829552 | 0.083186481 | 0.999970678 | 5.659 | 0.739 | 10.21 | 33.157 | NA |
| TRINITY_DN1258_c0_g1_i14 | 2.908149538 | 0.340299391 | 0.999970678 | 0 | 10.35 | 1.022 | 0.09 | - |
| TRINITY_DN6039_c0_g1_i2 | -2.90385132 | 0.236946728 | 1 | 0 | 8.988 | 26.876 | 26.622 | NA |
| TRINITY_DN18311_c0_g1_i5 | 2.902248011 | 0.113851934 | 0.999970678 | 1.299 | 0.355 | 0.011 | 0.201 | NA |
| TRINITY_DN10008_c0_g1_i1 | 2.901686008 | 0.216162891 | 0.999970678 | 1.217 | 1.192 | 0 | 0.322 | NA |
| TRINITY_DN1478_c0_g1_i4 | 2.900539355 | 0.48169236 | 0.999970678 | 0 | 7.924 | 0.867 | 0 | F-box |
| TRINITY_DN1354_c0_g1_i9 | 2.900168516 | 0.206272998 | 0.999970678 | 0.064 | 9.363 | 0.156 | 1.076 | NA |
| TRINITY_DN6668_c1_g1_i20 | -2.8987663 | 0.289630106 | 0.999970678 | 0.363 | 0 | 2.111 | 0.191 | NA |
| TRINITY_DN2910_c1_g1_i8 | -2.89868126 | 0.12777469 | 0.999970678 | 1.317 | 2.572 | 0.567 | 27.859 | NA |
| TRINITY_DN2691_c0_g1_i1 | -2.89856369 | 0.269854305 | 0.999970678 | 3.724 | 0 | 5.4 | 21.736 | NA |
| TRINITY_DN3898_c1_g1_i5 | -2.89621118 | 0.483374132 | 0.999970678 | 1.217 | 0 | 0 | 9.209 | MatE |
| TRINITY_DN393_c0_g1_i1 | 2.892055384 | 0.230567189 | 0.999970678 | 36.09 | 0.237 | 3.955 | 0.201 | NA |
| TRINITY_DN970_c1_g1_i6 | 2.890396607 | 0.482415907 | 0.999970678 | 2.916 | 0 | 0 | 0.402 | Myb_DNA-bindin |
| TRINITY_DN8475_c0_g1_i5 | -2.88938685 | 0.134159302 | 0.999970678 | 0.045 | 0.7 | 0.778 | 4.454 | NA |
| TRINITY_DN7011_c2_g1_i1 | -2.88904844 | 0.483320671 | 0.999970678 | 1.135 | 0 | 7.111 | 0 | NA |
| TRINITY_DN9679_c0_g1_i1 | 2.888286657 | 0.241142359 | 0.999970678 | 1.889 | 1.281 | 0.356 | 0 | ArgoL1 |
| TRINITY_DN4322_c0_g1_i1 | -2.88737218 | 0.319393938 | 0.999970678 | 0.718 | 3.292 | 0 | 29.126 | NA |
| TRINITY_DN8172_c0_g1_i1 | -2.88611839 | 0.347700828 | 0.999970678 | 1.962 | 0.138 | 12.933 | 0 | NA |
| TRINITY_DN12368_c0_g1_i3 | 2.886025381 | 0.23951932 | 0.999970678 | 0.999 | 2.503 | 0.378 | 0 | DHFR_1 |
| TRINITY_DN15795_c0_g1_i2 | -2.8856159 | 0.307416829 | 0.999970678 | 2.48 | 0 | 0.611 | 17.896 | NA |
| TRINITY_DN2633_c0_g1_i1 | 2.885070108 | 0.3706943 | 0.999970678 | 0 | 54.15 | 0.367 | 6.816 | LrgB |
| TRINITY_DN4581_c0_g1_i1 | -2.88439294 | 0.393152818 | 0.999970678 | 8.076 | 0.069 | 50.175 | 0 | GTP_cyclohydro2 |
| TRINITY_DN6764_c0_g1_i5 | -2.88327414 | 0.239570331 | 0.999970678 | 1.081 | 0 | 4.355 | 2.805 | NA |
| TRINITY_DN5265_c1_g1_i4 | -2.8817239 | 0.249637991 | 0.999970678 | 0.663 | 0 | 1.044 | 3.82 | NA |
| TRINITY_DN11335_c0_g1_i3 | -2.87994691 | 0.081515134 | 0.999970678 | 0.027 | 0.158 | 0.856 | 0.332 | - |
| TRINITY_DN749_c0_g2_i1 | 2.876850462 | 0.48286832 | 0.999970678 | 0 | 61.13 | 0 | 8.033 | NA |
| TRINITY_DN19944_c0_g1_i5 | -2.87543138 | 0.247986643 | 0.999970678 | 0 | 0.256 | 1.089 | 0.523 | NA |
| TRINITY_DN20699_c0_g1_i1 | -2.87023948 | 0.142362095 | 0.999970678 | 0.227 | 3.213 | 1.267 | 23.144 | Fasciclin |
| TRINITY_DN3570_c1_g1_i5 | 2.870174895 | 0.235744414 | 1 | 0.445 | 0.552 | 0 | 0.131 | NA |
| TRINITY_DN6198_c0_g1_i13 | 2.869502841 | 0.110108994 | 0.999970678 | 0.936 | 4.031 | 0.533 | 0.04 | NA |
| TRINITY_DN5904_c0_g1_i5 | -2.86915434 | 0.242214323 | 0.999970678 | 3.089 | 0 | 11.944 | 8.576 | NA |
| TRINITY_DN10516_c0_g1_i1 | -2.86770927 | 0.124837888 | 0.999970678 | 0.136 | 1.636 | 0.833 | 11.723 | NA |
| TRINITY_DN267_c0_g1_i5 | 2.867544193 | 0.232276551 | 1 | 0.899 | 0.848 | 0 | 0.241 | - |
| TRINITY_DN2724_c0_g1_i7 | -2.86598929 | 0.239501906 | 0.999970678 | 0.291 | 0 | 0.733 | 1.277 | EGF_CA |
| TRINITY_DN10033_c0_g1_i3 | -2.86513194 | 0.338299441 | 0.999970678 | 153.07 | 0 | 371.555 | 677.378 | PSI_PSAK |
| TRINITY_DN14910_c0_g1_i2 | 2.864385377 | 0.322645272 | 0.999970678 | 76.959 | 20.74 | 0 | 13.613 | Glutaredoxin |
| TRINITY_DN10976_c0_g1_i1 | 2.86312225 | 0.065595828 | 0.999970678 | 1.989 | 0.798 | 0.278 | 0.05 | APH |
| TRINITY_DN1107_c0_g1_i7 | -2.8624809 | 0.483292375 | 0.999970678 | 0 | 0.345 | 0 | 2.473 | Sugar_tr |
| TRINITY_DN18161_c0_g1_i3 | 2.86243031 | 0.30002007 | 0.999970678 | 0.772 | 16.37 | 0 | 2.343 | NA |
| TRINITY_DN6002_c0_g1_i1 | 2.860390414 | 0.247091503 | 0.999970678 | 1.526 | 1.252 | 0.322 | 0 | NA |
| TRINITY_DN46073_c0_g1_i1 | 2.859537238 | 0.254090688 | 1 | 1.399 | 1.853 | 0.367 | 0 | Sod_Cu |
| TRINITY_DN1304_c0_g1_i20 | 2.859051211 | 0.510477165 | 0.999970678 | 47.482 | 0 | 0 | 6.666 | NA |
| TRINITY_DN7884_c0_g1_i7 | 2.858685394 | 0.011537953 | 0.544751459 | 0.6 | 1.261 | 0.122 | 0.111 | NA |
| TRINITY_DN1138_c0_g1_i2 | -2.85821745 | 0.257524401 | 0.999970678 | 2.952 | 0 | 11.066 | 8.254 | NA |
| TRINITY_DN13083_c0_g1_i1 | 2.857513603 | 0.246718097 | 0.999970678 | 0.036 | 11.17 | 0.122 | 1.377 | ADH_N |
| TRINITY_DN9464_c0_g1_i3 | 2.856289107 | 0.246137174 | 0.999970678 | 0.481 | 0.956 | 0.167 | 0 | NA |
| TRINITY_DN6764_c0_g1_i8 | 2.854999185 | 0.164911023 | 0.999970678 | 0.391 | 3.814 | 0.022 | 0.553 | NA |
| TRINITY_DN6921_c0_g1_i2 | -2.85393138 | 0.317517818 | 0.999970678 | 0 | 0.788 | 0.144 | 5.369 | DUF588 |

| TRINITY_DN917_c0_g1_i3 | 2.853377157 | 0.257236848 | 1 | 0.445 | 0.7 | 0.133 | 0 | NA |
| --- | --- | --- | --- | --- | --- | --- | --- | --- |
| TRINITY_DN7121_c0_g2_i5 | -2.8527147 | 0.039586817 | 0.999970678 | 0.345 | 0.177 | 2.111 | 1.227 | NA |
| TRINITY_DN4722_c0_g2_i3 | 2.852075776 | 0.24238722 | 0.999970678 | 2.18 | 3.164 | 0.644 | 0 | NA |
| TRINITY_DN1786_c0_g1_i5 | -2.85122623 | 0.262878036 | 0.999970678 | 1.272 | 0 | 1.633 | 7.299 | NA |
| TRINITY_DN3683_c0_g2_i8 | 2.850369303 | 0.084584263 | 0.999970678 | 1.154 | 5.48 | 0.1 | 0.804 | NA |
| TRINITY_DN11190_c0_g1_i2 | 2.850006148 | 0.230046739 | 0.999970678 | 0.754 | 1.311 | 0 | 0.292 | Myb_DNA-bind_ |
| TRINITY_DN2813_c0_g1_i11 | -2.84841191 | 0.264373014 | 0.999970678 | 0 | 2.415 | 11.522 | 3.056 | NA |
| TRINITY_DN2175_c0_g1_i1 | -2.84755162 | 0.108794897 | 0.999970678 | 0.436 | 0.049 | 1.133 | 2.011 | NA |
| TRINITY_DN610_c0_g1_i2 | 2.846092824 | 0.252076321 | 0.999970678 | 3.307 | 7.204 | 1.211 | 0 | NA |
| TRINITY_DN919_c0_g1_i5 | 2.845200385 | 0.251345169 | 0.999970678 | 0.818 | 0.335 | 0.133 | 0 | NA |
| TRINITY_DN20564_c0_g1_i4 | 2.845020441 | 0.242810714 | 0.999970678 | 2.471 | 0.976 | 0 | 0.473 | NA |
| TRINITY_DN7593_c0_g1_i6 | 2.844363377 | 0.272099276 | 0.999970678 | 0.327 | 2.562 | 0 | 0.392 | NA |
| TRINITY_DN5151_c0_g2_i2 | -2.84311927 | 0.16864064 | 0.999970678 | 1.154 | 0.246 | 8.2 | 0.171 | NA |
| TRINITY_DN2844_c0_g1_i6 | -2.8430299 | 0.289805814 | 0.999970678 | 0.372 | 0 | 0.211 | 2.503 | - |
| TRINITY_DN5412_c0_g1_i19 | 2.842926723 | 0.490646501 | 0.999970678 | 6.322 | 0 | 0 | 0.895 | NA |
| TRINITY_DN9482_c0_g1_i1 | -2.8428733 | 0.070522348 | 0.999970678 | 4.006 | 4.849 | 2.7 | 60.302 | Transferase |
| TRINITY_DN4508_c0_g1_i5 | 2.84251879 | 0.059800173 | 0.999970678 | 1.017 | 8.505 | 0.822 | 0.312 | Nucleotid_trans |
| TRINITY_DN4209_c0_g1_i3 | -2.84207996 | 0.501057816 | 0.999970678 | 0 | 9.087 | 52.93 | 0 | NA |
| TRINITY_DN7663_c0_g1_i3 | 2.84176067 | 0.066391454 | 0.999970678 | 5.968 | 1.951 | 0.722 | 0.241 | NA |
| TRINITY_DN30841_c0_g1_i1 | 2.841376577 | 0.047593273 | 0.999970678 | 143.17 | 19.21 | 14.366 | 5.429 | GST_C |
| TRINITY_DN382_c4_g1_i2 | -2.84033022 | 0.281360387 | 0.999970678 | 0 | 1.173 | 0.9 | 7.158 | DUF1191 |
| TRINITY_DN5710_c0_g1_i4 | 2.840161872 | 0.24138442 | 0.999970678 | 4.642 | 3.351 | 0 | 1.126 | WRKY |
| TRINITY_DN23233_c0_g1_i9 | 2.83996147 | 0.244235242 | 1 | 0.999 | 1.518 | 0 | 0.332 | CCT |
| TRINITY_DN295_c0_g1_i5 | 2.839866619 | 0.50605936 | 0.999970678 | 0 | 12.45 | 0 | 1.719 | NA |
| TRINITY_DN52862_c0_g2_i1 | 2.839180011 | 0.080545255 | 0.999970678 | 0.572 | 8.998 | 0.689 | 0.493 | NA |
| TRINITY_DN7693_c0_g1_i3 | 2.838946518 | 0.235682783 | 0.999970678 | 2.998 | 1.547 | 0 | 0.633 | NA |
| TRINITY_DN527_c0_g1_i4 | 2.838873613 | 0.258994551 | 0.999970678 | 2.126 | 0.522 | 0 | 0.372 | A_deaminase |
| TRINITY_DN4480_c0_g1_i3 | -2.83728288 | 0.02616288 | 0.999970678 | 0.763 | 0.828 | 1.7 | 9.471 | NA |
| TRINITY_DN5667_c0_g1_i6 | 2.836882113 | 0.038467644 | 0.999970678 | 1.408 | 0.581 | 0.089 | 0.171 | NA |
| TRINITY_DN5334_c0_g1_i4 | -2.83630205 | 0.275217077 | 0.999970678 | 0.518 | 0 | 0.467 | 3.147 | NA |
| TRINITY_DN4026_c0_g1_i8 | -2.83629219 | 0.268414827 | 0.999970678 | 0 | 0.493 | 0.444 | 2.885 | NA |
| TRINITY_DN10637_c0_g1_i7 | -2.83593702 | 0.154715788 | 0.999970678 | 2.18 | 0.335 | 14.599 | 0.533 | NA |
| TRINITY_DN1433_c0_g3_i1 | -2.83203892 | 0.510971416 | 0.999970678 | 8.766 | 0 | 52.308 | 0 | NA |
| TRINITY_DN3071_c1_g1_i3 | 2.831915659 | 0.491943711 | 0.999970678 | 0 | 3.568 | 0.411 | 0 | NA |
| TRINITY_DN2120_c0_g1_i2 | 2.831590707 | 0.260401417 | 0.999970678 | 7.022 | 1.597 | 0 | 1.216 | NA |
| TRINITY_DN17874_c0_g1_i1 | -2.83141856 | 0.090045853 | 0.999970678 | 0.218 | 0.503 | 0.289 | 4.776 | NA |
| TRINITY_DN271_c0_g1_i6 | -2.83126027 | 0.054303505 | 0.999970678 | 12.817 | 11.48 | 13.355 | 142.551 | NA |
| TRINITY_DN2313_c0_g1_i2 | -2.82893758 | 0.357549561 | 0.999970678 | 4.151 | 0.306 | 0 | 32.081 | F-box |
| TRINITY_DN31494_c0_g1_i2 | -2.82889317 | 0.246166016 | 0.999970678 | 0 | 0.512 | 1.255 | 1.93 | NA |
| TRINITY_DN938_c0_g1_i14 | -2.82838243 | 0.271142209 | 0.999970678 | 1.108 | 0 | 5.5 | 1.418 | NA |
| TRINITY_DN7687_c1_g1_i1 | -2.82781415 | 0.269464256 | 0.999970678 | 0 | 2.493 | 2.855 | 14.045 | NA |
| TRINITY_DN48421_c0_g1_i1 | 2.827620653 | 0.318134766 | 0.999970678 | 0 | 10.07 | 0.544 | 0.724 | NA |
| TRINITY_DN542_c0_g1_i6 | -2.82726167 | 0.492299557 | 0.999970678 | 4.724 | 0 | 0 | 32.805 | NA |
| TRINITY_DN2984_c0_g1_i8 | -2.82226362 | 0.25053894 | 0.999970678 | 1.408 | 0 | 3.355 | 6.082 | GTP1_OBG |
| TRINITY_DN14112_c0_g1_i2 | -2.82168935 | 0.095812962 | 0.999970678 | 0.554 | 0.177 | 3.844 | 0.462 | NA |
| TRINITY_DN1423_c0_g1_i4 | 2.821417456 | 0.096951983 | 0.999970678 | 0.436 | 1.459 | 0.011 | 0.251 | NA |
| TRINITY_DN12422_c0_g2_i2 | 2.820658843 | 0.061218436 | 0.999970678 | 0.745 | 2.385 | 0.111 | 0.302 | NA |
| TRINITY_DN8792_c0_g1_i6 | 2.816594584 | 0.067568164 | 0.999970678 | 3.888 | 25.5 | 2.578 | 0.975 | NA |
| TRINITY_DN3931_c0_g1_i1 | -2.81646425 | 0.321638829 | 0.999970678 | 9.956 | 0 | 3.8 | 65.922 | NA |
| TRINITY_DN1043_c0_g1_i1 | 2.809193439 | 0.494486467 | 0.999970678 | 0 | 7.313 | 0 | 1.025 | ADH_N |
| TRINITY_DN1039_c1_g1_i11 | -2.80910522 | 0.497284614 | 0.999970678 | 0 | 0.394 | 2.222 | 0 | B_lectin |
| TRINITY_DN1583_c0_g1_i4 | -2.80755416 | 0.001306783 | 0.0873113 | 5.178 | 9.284 | 55.419 | 33.66 | - |
| TRINITY_DN3132_c0_g1_i2 | 2.806637266 | 0.49679252 | 0.999970678 | 6.05 | 0 | 0 | 0.885 | TPR_1 |
| TRINITY_DN5772_c0_g1_i4 | 2.80463177 | 0.238127476 | 0.999970678 | 0.854 | 1.202 | 0 | 0.282 | NA |
| TRINITY_DN14248_c0_g1_i2 | -2.80373595 | 0.257548215 | 0.999970678 | 0.454 | 0 | 1.211 | 1.699 | NA |
| TRINITY_DN964_c1_g2_i4 | 2.80365467 | 0.246143973 | 0.999970678 | 1.017 | 1.084 | 0.244 | 0 | NA |
| TRINITY_DN5416_c0_g1_i1 | 2.803077692 | 0.322686638 | 0.999970678 | 0 | 22.02 | 1.155 | 1.709 | NA |
| TRINITY_DN5045_c0_g1_i4 | -2.80289404 | 0.496865939 | 0.999970678 | 6.513 | 0 | 37.742 | 0 | NA |
| TRINITY_DN5804_c0_g1_i7 | 2.802472409 | 0.008129599 | 0.406131893 | 1.117 | 1.754 | 0.2 | 0.151 | NA |
| TRINITY_DN2934_c0_g1_i9 | -2.80221809 | 0.257102501 | 0.999970678 | 0 | 0.355 | 0.678 | 1.649 | - |
| TRINITY_DN13103_c0_g1_i1 | -2.8020145 | 0.50143656 | 0.999970678 | 2.816 | 0 | 16.021 | 0 | NA |
| TRINITY_DN4628_c0_g1_i1 | 2.801932167 | 0.314747776 | 0.999970678 | 0.055 | 1.252 | 0 | 0.191 | Helicase_C |
| TRINITY_DN11212_c0_g3_i1 | -2.80042591 | 0.296310505 | 0.999970678 | 4.433 | 0 | 22.699 | 3.79 | NA |
| TRINITY_DN5643_c0_g1_i1 | -2.79949357 | 0.140629404 | 0.999970678 | 0.245 | 1.242 | 0.511 | 9.44 | NA |
| TRINITY_DN2419_c0_g1_i2 | 2.799479814 | 0.004386421 | 0.237666899 | 5.723 | 2.631 | 0.389 | 0.734 | Myb_DNA-bindin |
| TRINITY_DN62269_c0_g2_i5 | -2.79827983 | 0.022249241 | 0.913225113 | 0.481 | 0.552 | 1.544 | 5.268 | NA |
| TRINITY_DN62269_c0_g3_i5 | -2.79827983 | 0.022249241 | 0.913225113 | 0.481 | 0.552 | 1.544 | 5.268 | NA |
| TRINITY_DN2700_c0_g1_i1 | 2.797937336 | 0.132591335 | 0.999970678 | 61.916 | 114.8 | 0.333 | 24.933 | Calreticulin |
| TRINITY_DN18802_c1_g2_i3 | 2.797929326 | 0.244665067 | 1 | 0.5 | 0.483 | 0 | 0.141 | NA |
| TRINITY_DN3938_c0_g1_i8 | 2.797874969 | 0.264402102 | 0.999970678 | 0.627 | 2.089 | 0.322 | 0 | NA |
| TRINITY_DN325_c1_g1_i2 | -2.7977892 | 0.097963265 | 0.999970678 | 0.073 | 0.877 | 4.166 | 1.468 | Sugar_tr |
| TRINITY_DN48408_c0_g1_i5 | 2.796966809 | 0.255266309 | 0.999970678 | 1.326 | 0.917 | 0.267 | 0 | NA |
| TRINITY_DN903_c0_g2_i1 | -2.79678902 | 0.267471662 | 1 | 0.245 | 0 | 0.667 | 0.925 | NA |
| TRINITY_DN75992_c0_g1_i1 | -2.79485745 | 0.08431538 | 0.999970678 | 0.036 | 0.453 | 1.4 | 1.659 | NA |

| TRINITY_DN18791_c0_g1_i4 | 2.794733538 | 0.01818864 | 0.787098346 | 1.544 | 5.371 | 0.344 | 0.583 | Mlo |
| --- | --- | --- | --- | --- | --- | --- | --- | --- |
| TRINITY_DN3645_c0_g1_i4 | 2.794477726 | 0.177384834 | 0.999970678 | 0.082 | 8.407 | 0.378 | 0.744 | NA |
| TRINITY_DN5030_c0_g1_i1 | 2.794068548 | 0.250493583 | 0.999970678 | 0.736 | 1.291 | 0.244 | 0 | NA |
| TRINITY_DN204_c0_g1_i3 | 2.794057609 | 0.137021855 | 0.999970678 | 2.353 | 0.946 | 0.011 | 0.473 | Gelsolin |
| TRINITY_DN23082_c0_g1_i5 | -2.79240658 | 0.502580673 | 0.999970678 | 19.921 | 0 | 114.604 | 0 | NA |
| TRINITY_DN2269_c0_g2_i3 | -2.79229121 | 0.026588089 | 0.999970678 | 1.181 | 0.907 | 3.622 | 9.501 | NA |
| TRINITY_DN1610_c0_g1_i5 | 2.79203836 | 0.498619118 | 0.999970678 | 1.862 | 0 | 0.222 | 0 | - |
| TRINITY_DN543_c0_g2_i4 | -2.79195052 | 0.496257045 | 0.999970678 | 0.963 | 0 | 0 | 6.726 | MFS_1 |
| TRINITY_DN4459_c0_g1_i18 | -2.79145454 | 0.502623333 | 0.999970678 | 0.3 | 0 | 1.711 | 0 | NA |
| TRINITY_DN2813_c0_g1_i9 | -2.78888774 | 0.034736493 | 0.999970678 | 0.454 | 0.424 | 3.478 | 1.89 | NA |
| TRINITY_DN2678_c1_g1_i9 | 2.788794036 | 0.265375401 | 0.999970678 | 0.681 | 3.026 | 0 | 0.523 | NA |
| TRINITY_DN4356_c0_g1_i8 | -2.78855544 | 0.277675612 | 0.999970678 | 0 | 2.553 | 3.078 | 13.532 | Pkinase |
| TRINITY_DN4065_c0_g1_i4 | -2.78640472 | 0.501377208 | 0.999970678 | 0.718 | 0 | 4.189 | 0 | NA |
| TRINITY_DN295_c0_g1_i1 | 2.785398867 | 0.223331971 | 0.999970678 | 0.009 | 2.079 | 0.133 | 0.141 | NA |
| TRINITY_DN6698_c0_g3_i2 | -2.78511904 | 0.499900632 | 0.999970678 | 2.871 | 0 | 16.543 | 0 | NA |
| TRINITY_DN1065_c0_g1_i3 | 2.784445648 | 0.500207636 | 0.999970678 | 0 | 3.932 | 0 | 0.563 | CRS1_YhbY |
| TRINITY_DN7848_c0_g1_i3 | -2.78417003 | 0.291423447 | 0.999970678 | 0.936 | 0 | 0.678 | 5.61 | Aldo_ket_red |
| TRINITY_DN2315_c0_g1_i2 | -2.78176946 | 0.15171503 | 0.999970678 | 2.662 | 0.325 | 0.678 | 19.836 | Rib_5-P_isom_A |
| TRINITY_DN247_c1_g4_i1 | 2.781443609 | 0.530233991 | 0.999970678 | 99.878 | 0 | 0 | 14.779 | Peptidase_S10 |
| TRINITY_DN6483_c0_g1_i3 | 2.781211073 | 0.29848159 | 0.999970678 | 0.627 | 7.253 | 0 | 1.126 | - |
| TRINITY_DN863_c0_g2_i6 | -2.78119374 | 0.068688161 | 0.999970678 | 8.693 | 1.656 | 7.566 | 62.152 | NA |
| TRINITY_DN1583_c0_g1_i6 | 2.780894985 | 0.498960276 | 0.999970678 | 0 | 53.65 | 0 | 7.55 | - |
| TRINITY_DN11124_c0_g1_i2 | 2.780869003 | 0.015000628 | 0.669536213 | 174.72 | 443.3 | 57.83 | 19.273 | NA |
| TRINITY_DN1979_c0_g1_i2 | -2.77962412 | 0.500985178 | 0.999970678 | 0.418 | 0 | 0 | 2.926 | NA |
| TRINITY_DN4194_c0_g1_i1 | 2.77916851 | 0.500511264 | 0.999970678 | 0 | 5.982 | 0 | 0.875 | NA |
| TRINITY_DN325_c0_g1_i1 | -2.77880714 | 0.109122187 | 0.999970678 | 34.9 | 3.459 | 19.066 | 243.541 | NA |
| TRINITY_DN2915_c1_g1_i15 | -2.77730094 | 0.030336477 | 0.999970678 | 0.318 | 0.207 | 2.322 | 0.845 | NA |
| TRINITY_DN3758_c0_g1_i2 | -2.77725552 | 0.269612493 | 0.999970678 | 0.59 | 0 | 0.833 | 3.036 | NA |
| TRINITY_DN5358_c0_g1_i1 | -2.77617738 | 0.097568312 | 0.999970678 | 0.282 | 5.312 | 7.711 | 28.432 | NA |
| TRINITY_DN8724_c0_g1_i2 | -2.7754488 | 0.000906444 | 0.065520645 | 1.526 | 1.951 | 12.588 | 8.566 | NA |
| TRINITY_DN435_c0_g1_i3 | -2.77516356 | 0.040325798 | 0.999970678 | 0.654 | 1.882 | 2.089 | 14.799 | AA_permease_2 |
| TRINITY_DN6017_c1_g2_i1 | -2.77441431 | 0.504068302 | 0.999970678 | 2.171 | 0 | 12.399 | 0 | NA |
| TRINITY_DN319_c0_g1_i13 | -2.773903 | 0.257533437 | 0.999970678 | 0.463 | 0 | 1.078 | 1.9 | MFS_1 |
| TRINITY_DN1692_c0_g2_i2 | 2.773068311 | 0.507725704 | 0.999970678 | 32.121 | 0 | 0 | 4.765 | NA |
| TRINITY_DN9141_c1_g1_i6 | -2.77198927 | 0.013831381 | 0.630263436 | 0.118 | 0.197 | 0.944 | 1.036 | NA |
| TRINITY_DN3653_c0_g1_i14 | 2.771632029 | 0.193639513 | 0.999970678 | 0.145 | 6.13 | 0.667 | 0.101 | NA |
| TRINITY_DN11087_c0_g2_i6 | 2.771502234 | 0.290864128 | 0.999970678 | 8.112 | 35.39 | 0 | 6.274 | NA |
| TRINITY_DN3938_c0_g1_i15 | -2.77068039 | 0.2582708 | 0.999970678 | 0.409 | 0 | 0.978 | 1.619 | NA |
| TRINITY_DN10623_c0_g1_i2 | 2.769522237 | 0.014745684 | 0.662683599 | 2.235 | 4.12 | 0.433 | 0.392 | - |
| TRINITY_DN1320_c0_g1_i2 | 2.769295246 | 0.081302673 | 0.999970678 | 78.067 | 5.509 | 7.711 | 3.086 | NA |
| TRINITY_DN1384_c0_g1_i14 | 2.768622855 | 0.267504594 | 0.999970678 | 5.841 | 1.823 | 0 | 1.136 | NA |
| TRINITY_DN5426_c0_g2_i8 | -2.76766042 | 0.400243936 | 0.999970678 | 14.825 | 0.227 | 84.45 | 0 | NA |
| TRINITY_DN4665_c0_g1_i6 | 2.765755679 | 0.026510026 | 0.999970678 | 5.232 | 2.217 | 0.744 | 0.211 | NA |
| TRINITY_DN12625_c1_g1_i3 | -2.76570507 | 0.368094667 | 0.999970678 | 0 | 1.833 | 0.089 | 12.215 | Grp1_Fun34_Yaa |
| TRINITY_DN1675_c0_g1_i6 | 2.763986216 | 0.009183134 | 0.453219561 | 4.633 | 3.016 | 0.478 | 0.523 | NA |
| TRINITY_DN2710_c0_g1_i2 | -2.76368821 | 0.268060165 | 1 | 0.173 | 0 | 0.522 | 0.583 | NA |
| TRINITY_DN1607_c0_g1_i7 | 2.761112135 | 0.502008146 | 0.999970678 | 0 | 2.848 | 0.344 | 0 | Sugar_tr |
| TRINITY_DN21009_c0_g1_i2 | -2.76045664 | 0.323202473 | 0.999970678 | 1.09 | 0 | 0.389 | 6.927 | DUF4228 |
| TRINITY_DN1677_c0_g1_i7 | 2.760399729 | 0.062959008 | 0.999970678 | 0.354 | 0.158 | 0.044 | 0.02 | ANAPC4_WD40 |
| TRINITY_DN9370_c0_g2_i3 | -2.75890864 | 0.340177913 | 0.999970678 | 0.754 | 0 | 0.133 | 5.037 | NA |
| TRINITY_DN983_c0_g1_i9 | 2.758878624 | 0.27346177 | 0.999970678 | 4.96 | 1.084 | 0 | 0.905 | CBM_4_9 |
| TRINITY_DN76874_c0_g1_i1 | -2.75725834 | 0.260592561 | 0.999970678 | 0.69 | 0 | 1.711 | 2.654 | NA |
| TRINITY_DN13_c0_g1_i6 | -2.75685967 | 0.280904175 | 1 | 0.227 | 0 | 0.633 | 0.794 | NA |
| TRINITY_DN6376_c0_g1_i4 | -2.75542147 | 0.504599462 | 0.999970678 | 4.388 | 0 | 24.854 | 0 | GMC_oxred_C |
| TRINITY_DN81_c0_g1_i3 | 2.752863605 | 0.167660294 | 0.999970678 | 4.206 | 0.897 | 0.022 | 0.754 | Diphthamide_syn |
| TRINITY_DN564_c0_g1_i5 | -2.75065464 | 0.028640993 | 0.999970678 | 41.586 | 9.471 | 211.209 | 87.457 | NA |
| TRINITY_DN2461_c0_g1_i3 | 2.750546938 | 0.201438799 | 0.999970678 | 4.506 | 0.069 | 0.089 | 0.583 | TAXi_N |
| TRINITY_DN2027_c0_g2_i4 | 2.750043017 | 0.006311867 | 0.328775799 | 1.054 | 2.631 | 0.167 | 0.352 | PGG |
| TRINITY_DN1000_c0_g2_i17 | 2.749950156 | 0.014948859 | 0.669536213 | 2.153 | 1.498 | 0.289 | 0.191 | DS |
| TRINITY_DN1872_c0_g1_i9 | 2.74749502 | 0.14943208 | 0.999970678 | 3.134 | 0.207 | 0.378 | 0.05 | NA |
| TRINITY_DN484_c0_g1_i8 | -2.74714917 | 0.323313168 | 0.999970678 | 0 | 10.44 | 5.677 | 61.911 | NA |
| TRINITY_DN8065_c0_g1_i1 | 2.747002086 | 0.279621503 | 1 | 0.391 | 0.552 | 0.122 | 0 | DUF1726 |
| TRINITY_DN543_c0_g2_i2 | -2.7463936 | 0.03753459 | 0.999970678 | 2.135 | 2.277 | 2.866 | 26.039 | MFS_1 |
| TRINITY_DN1261_c0_g1_i9 | -2.7461241 | 0.019246887 | 0.822005624 | 0.191 | 0.572 | 2.922 | 1.558 | NA |
| TRINITY_DN521_c0_g1_i1 | -2.74489065 | 0.505594592 | 0.999970678 | 0 | 4.75 | 25.832 | 0 | NA |
| TRINITY_DN556_c0_g1_i7 | -2.74477939 | 0.261822922 | 0.999970678 | 1.617 | 0 | 4.9 | 5.037 | NA |
| TRINITY_DN5528_c0_g1_i3 | 2.742423273 | 0.505044705 | 0.999970678 | 0 | 8.535 | 1.033 | 0 | Abhydrolase_1 |
| TRINITY_DN4133_c0_g1_i1 | 2.741469857 | 0.054205076 | 0.999970678 | 33.492 | 11.83 | 0.733 | 5.891 | 2_5_RNA_ligase2 |
| TRINITY_DN816_c0_g1_i2 | 2.741351469 | 0.249611208 | 0.999970678 | 1.417 | 2.079 | 0 | 0.513 | NA |
| TRINITY_DN1903_c0_g1_i6 | 2.74072278 | 0.131921764 | 0.999970678 | 22.428 | 0.562 | 1.033 | 2.242 | NA |
| TRINITY_DN15460_c0_g1_i5 | -2.74030205 | 0.509049281 | 0.999970678 | 0.663 | 0 | 3.744 | 0 | Catalase |
| TRINITY_DN1128_c0_g1_i4 | -2.73981587 | 0.077341762 | 0.999970678 | 2.634 | 5.075 | 2.722 | 48.057 | GDP_Man_Dehyd |
| TRINITY_DN1345_c1_g1_i2 | 2.739783565 | 0.236680779 | 0.999970678 | 25.626 | 0.227 | 2.833 | 0.442 | LRRNT_2 |
| TRINITY_DN74_c0_g1_i17 | -2.73941324 | 0.018929971 | 0.810589796 | 4.969 | 3.893 | 9.222 | 47.303 | NA |

| TRINITY_DN13880_c0_g2_i3 | 2.738282528 | 0.267198409 | 1 | 0.454 | 0.453 | 0.122 | 0 | NA |
| --- | --- | --- | --- | --- | --- | --- | --- | --- |
| TRINITY_DN19385_c0_g1_i1 | -2.73702087 | 0.393433004 | 0.999970678 | 0.999 | 0 | 5.555 | 0.04 | NA |
| TRINITY_DN2250_c0_g1_i3 | -2.73688543 | 0.505275352 | 0.999970678 | 1.008 | 0 | 0 | 6.806 | G-patch |
| TRINITY_DN23055_c0_g1_i5 | -2.73659524 | 0.263660795 | 0.999970678 | 0.554 | 0 | 1.611 | 1.83 | NA |
| TRINITY_DN1744_c0_g1_i1 | -2.73620618 | 0.041609829 | 0.999970678 | 2.489 | 1.212 | 2.955 | 21.002 | Calmodulin_bind |
| TRINITY_DN1028_c0_g1_i1 | -2.73412093 | 0.519766329 | 0.999970678 | 0 | 9.146 | 49.397 | 0 | RRM_1 |
| TRINITY_DN534_c0_g1_i6 | 2.732793466 | 0.252489115 | 0.999970678 | 0.363 | 0.453 | 0 | 0.121 | DPPIV_N |
| TRINITY_DN561_c0_g1_i7 | 2.732022586 | 0.506127359 | 0.999970678 | 2.098 | 0 | 0 | 0.322 | NA |
| TRINITY_DN5049_c0_g1_i2 | 2.731729365 | 0.504750962 | 0.999970678 | 0 | 1.784 | 0 | 0.261 | BRCT |
| TRINITY_DN9578_c0_g1_i2 | -2.73096712 | 0.279840244 | 1 | 0 | 0.227 | 0.656 | 0.623 | Myb_DNA-bindin |
| TRINITY_DN14663_c0_g1_i3 | -2.7301965 | 0.274037677 | 0.999970678 | 0.136 | 0 | 0.5 | 0.302 | MAP65_ASE1 |
| TRINITY_DN3720_c0_g1_i13 | -2.72879954 | 0.28739466 | 0.999970678 | 0.79 | 0 | 0.933 | 4.202 | - |
| TRINITY_DN114_c0_g1_i4 | 2.728415612 | 0.076203314 | 0.999970678 | 5.523 | 4.662 | 0.111 | 1.387 | NA |
| TRINITY_DN1063_c0_g1_i4 | 2.72828548 | 0.135968671 | 0.999970678 | 0.645 | 15.66 | 0.289 | 2.091 | NA |
| TRINITY_DN2473_c0_g1_i4 | -2.72699377 | 0.519559419 | 0.999970678 | 0 | 4.11 | 22.154 | 0 | Ank_2 |
| TRINITY_DN2112_c0_g2_i3 | 2.72642933 | 0.290952817 | 0.999970678 | 19.094 | 12.32 | 0 | 4.765 | ATP-synt_ab |
| TRINITY_DN1444_c1_g1_i11 | -2.72627244 | 0.017287347 | 0.753512004 | 0.3 | 0.168 | 1.1 | 1.85 | CSD |
| TRINITY_DN3250_c0_g1_i7 | -2.72612637 | 0.149767695 | 0.999970678 | 2.08 | 68.22 | 45.942 | 394.567 | NA |
| TRINITY_DN2130_c0_g1_i3 | -2.72467008 | 0.198921629 | 0.999970678 | 3.288 | 0.168 | 0.522 | 22.52 | p450 |
| TRINITY_DN546_c0_g1_i2 | -2.72435047 | 0.197030321 | 0.999970678 | 0.291 | 1.685 | 10.499 | 0.191 | ATP_bind_1 |
| TRINITY_DN1350_c0_g1_i12 | -2.72362412 | 0.161877211 | 0.999970678 | 0.854 | 48.26 | 212.998 | 56.974 | NA |
| TRINITY_DN3154_c0_g1_i12 | -2.72183973 | 0.08951745 | 0.999970678 | 1.054 | 0.069 | 3.255 | 3.569 | NA |
| TRINITY_DN10728_c0_g1_i1 | -2.72119657 | 0.134643047 | 0.999970678 | 0.136 | 1.577 | 8.133 | 1.377 | NA |
| TRINITY_DN3305_c0_g1_i12 | -2.72110237 | 0.048744064 | 0.999970678 | 0.145 | 0.631 | 3.011 | 1.458 | NA |
| TRINITY_DN2273_c0_g1_i3 | -2.7190505 | 0.037287399 | 0.999970678 | 1.026 | 0.453 | 6.788 | 1.599 | CBS |
| TRINITY_DN12599_c0_g1_i1 | 2.718836753 | 0.04739973 | 0.999970678 | 1.49 | 0.335 | 0.089 | 0.171 | DIL |
| TRINITY_DN1213_c0_g1_i11 | -2.71848338 | 0.279313902 | 1 | 0.291 | 0 | 0.644 | 1.036 | NA |
| TRINITY_DN13011_c0_g1_i5 | 2.717906839 | 0.096302335 | 0.999970678 | 7.976 | 0.838 | 0.955 | 0.201 | NA |
| TRINITY_DN724_c0_g1_i5 | -2.71720519 | 0.293768844 | 0.999970678 | 0 | 1.074 | 0.911 | 5.741 | NA |
| TRINITY_DN8667_c0_g1_i1 | -2.71705667 | 0.286739818 | 0.999970678 | 0.045 | 1.025 | 0.022 | 6.927 | NA |
| TRINITY_DN1326_c0_g1_i6 | 2.715878463 | 0.506914101 | 0.999970678 | 0 | 8.82 | 0 | 1.317 | - |
| TRINITY_DN863_c0_g1_i4 | 2.715329693 | 0.352270116 | 0.999970678 | 0 | 4.12 | 0.411 | 0.111 | BACK |
| TRINITY_DN2937_c0_g1_i10 | 2.715159978 | 0.343317032 | 0.999970678 | 0 | 11.3 | 0.422 | 1.176 | NA |
| TRINITY_DN15750_c1_g2_i3 | 2.714637834 | 0.288887611 | 0.999970678 | 0.481 | 1.715 | 0.278 | 0 | NA |
| TRINITY_DN28997_c0_g1_i1 | 2.714569001 | 0.070196851 | 0.999970678 | 1.026 | 10.87 | 0.633 | 1.025 | NA |
| TRINITY_DN10628_c0_g1_i2 | 2.713207954 | 0.132422326 | 0.999970678 | 0.69 | 0.355 | 0.122 | 0.01 | NA |
| TRINITY_DN2460_c0_g1_i1 | 2.711193818 | 0.511242818 | 0.999970678 | 7.712 | 0 | 0 | 1.186 | NA |
| TRINITY_DN367_c9_g1_i1 | 2.710806012 | 0.275944179 | 1 | 0.69 | 1.232 | 0 | 0.302 | NA |
| TRINITY_DN5265_c1_g2_i3 | -2.71043061 | 0.118734792 | 0.999970678 | 0.073 | 0.591 | 2.866 | 0.885 | NA |
| TRINITY_DN458_c0_g1_i4 | -2.71029426 | 0.000314413 | 0.028694152 | 0.845 | 0.67 | 3.578 | 5.62 | Epimerase |
| TRINITY_DN809_c0_g1_i4 | 2.709232737 | 0.51358711 | 0.999970678 | 0 | 4.957 | 0.611 | 0 | DUF3493 |
| TRINITY_DN391_c1_g2_i4 | -2.70902208 | 0.030219219 | 0.999970678 | 0.363 | 1.045 | 1.678 | 7.098 | NA |
| TRINITY_DN2419_c0_g1_i3 | 2.707648208 | 0.311729349 | 0.999970678 | 18.84 | 5.026 | 0 | 3.7 | Myb_DNA-bindin |
| TRINITY_DN4784_c0_g1_i6 | -2.70747747 | 0.266550232 | 0.999970678 | 0 | 0.355 | 0.711 | 1.408 | FAD-oxidase_C |
| TRINITY_DN2023_c0_g3_i6 | -2.70735631 | 0.353399541 | 0.999970678 | 20.239 | 0 | 69.018 | 50.992 | NA |
| TRINITY_DN6198_c0_g1_i3 | -2.70731406 | 0.269279618 | 0.999970678 | 0.609 | 0 | 1.578 | 2.101 | NA |
| TRINITY_DN6563_c0_g1_i1 | -2.70416631 | 0.048382169 | 0.999970678 | 17.35 | 7.283 | 46.042 | 92.052 | NA |
| TRINITY_DN5409_c0_g1_i3 | -2.70274673 | 0.280460207 | 0.999970678 | 0.754 | 0 | 1.178 | 3.509 | NA |
| TRINITY_DN16322_c0_g1_i7 | -2.70266383 | 0.052374914 | 1 | 0.164 | 0.158 | 0.856 | 1.025 | NA |
| TRINITY_DN3094_c0_g1_i1 | -2.70266295 | 0.121572196 | 0.999970678 | 1.308 | 13.13 | 6.411 | 84.743 | peroxidase |
| TRINITY_DN3656_c0_g1_i1 | 2.699243166 | 0.266941929 | 1 | 2.044 | 1.784 | 0 | 0.613 | NA |
| TRINITY_DN446_c0_g1_i7 | -2.69851488 | 0.055213426 | 0.999970678 | 2.48 | 2.306 | 2.5 | 28.01 | NA |
| TRINITY_DN1131_c0_g1_i4 | -2.69708887 | 0.271343163 | 0.999970678 | 1.971 | 0 | 5.411 | 6.404 | NA |
| TRINITY_DN6988_c0_g1_i2 | -2.69618202 | 0.274854823 | 0.999970678 | 1.272 | 0 | 4.044 | 3.338 | NA |
| TRINITY_DN242_c1_g1_i3 | -2.69516137 | 0.03358778 | 0.999970678 | 0.2 | 0.237 | 0.489 | 2.282 | B_lectin |
| TRINITY_DN3794_c0_g2_i1 | 2.693708141 | 0.304494726 | 0.999970678 | 12.745 | 7.253 | 2.567 | 0 | GAF |
| TRINITY_DN1442_c0_g1_i2 | -2.69287154 | 0.140671488 | 0.999970678 | 2.989 | 16.75 | 4.1 | 119.619 | NA |
| TRINITY_DN471_c0_g1_i3 | -2.69209465 | 0.0791589 | 0.999970678 | 1.317 | 0.926 | 11.233 | 0.945 | LRRNT_2 |
| TRINITY_DN265_c0_g1_i1 | -2.69148554 | 0.286170177 | 0.999970678 | 1.808 | 0 | 4.866 | 5.992 | NA |
| TRINITY_DN220_c0_g2_i8 | 2.690588146 | 0.345122482 | 0.999970678 | 4.842 | 0 | 0.333 | 0.372 | NA |
| TRINITY_DN2914_c0_g2_i1 | 2.689987413 | 0.202880319 | 0.999970678 | 0.073 | 8.653 | 0.378 | 0.885 | NA |
| TRINITY_DN16151_c0_g1_i6 | -2.68994184 | 0.162321279 | 0.999970678 | 0.963 | 0.039 | 3.689 | 2.061 | NA |
| TRINITY_DN19714_c0_g1_i1 | -2.68984275 | 0.000445917 | 0.037071259 | 4.206 | 4.445 | 17.754 | 33.78 | NA |
| TRINITY_DN1652_c0_g1_i6 | 2.689744749 | 0.275059411 | 0.999970678 | 3.252 | 3.568 | 0 | 1.066 | NA |
| TRINITY_DN5265_c1_g1_i3 | -2.68894712 | 0.306460319 | 0.999970678 | 1.535 | 0 | 6.944 | 1.578 | NA |
| TRINITY_DN3277_c2_g1_i7 | 2.68790919 | 0.134773434 | 0.999970678 | 1.835 | 2.454 | 0.522 | 0.03 | NA |
| TRINITY_DN17399_c0_g1_i1 | -2.68719989 | 0.273020266 | 0.999970678 | 0 | 1.202 | 3.322 | 3.629 | NA |
| TRINITY_DN2156_c0_g1_i4 | 2.686877249 | 0.006329578 | 0.328775799 | 12.39 | 6.554 | 0.789 | 2.001 | NA |
| TRINITY_DN13232_c0_g1_i2 | 2.686219425 | 0.27792759 | 1 | 0.945 | 2.03 | 0 | 0.483 | UPRTase |
| TRINITY_DN15231_c0_g1_i1 | -2.68493343 | 0.515919612 | 0.999970678 | 0.536 | 0 | 2.889 | 0 | NA |
| TRINITY_DN283_c0_g2_i7 | -2.68419465 | 0.296892124 | 0.999970678 | 6.777 | 0 | 10.355 | 31.518 | NA |
| TRINITY_DN1007_c0_g1_i7 | -2.6814147 | 0.518630982 | 0.999970678 | 0.99 | 0 | 0 | 6.364 | NA |
| TRINITY_DN4730_c0_g1_i5 | 2.680781449 | 0.066313015 | 0.999970678 | 3.007 | 0.729 | 0.122 | 0.462 | NA |
| TRINITY_DN1969_c1_g1_i6 | 2.679122305 | 0.294832071 | 0.999970678 | 2.135 | 5.913 | 1.033 | 0 | Anticodon_1 |

| TRINITY_DN1660_c0_g1_i5 | 2.678278917 | 0.310960874 | 0.999970678 | 20.194 | 6.435 | 0 | 4.212 | Glyco_transf_5 |
| --- | --- | --- | --- | --- | --- | --- | --- | --- |
| TRINITY_DN7537_c0_g1_i8 | -2.67824356 | 0.317833415 | 0.999970678 | 1.145 | 0 | 0.678 | 6.565 | NA |
| TRINITY_DN2991_c0_g2_i1 | -2.67818565 | 0.07104004 | 0.999970678 | 0.917 | 0.848 | 1.067 | 10.034 | NA |
| TRINITY_DN108_c3_g1_i1 | -2.67612275 | 0.289169174 | 0.999970678 | 0.681 | 0 | 0.955 | 3.267 | p450 |
| TRINITY_DN615_c0_g1_i7 | 2.673928137 | 0.333595232 | 0.999970678 | 11.637 | 0.818 | 0 | 1.981 | NA |
| TRINITY_DN3020_c0_g1_i10 | 2.672495565 | 0.323135853 | 0.999970678 | 0.418 | 5.686 | 0 | 0.955 | Autophagy_C |
| TRINITY_DN6668_c1_g1_i9 | 2.672236539 | 0.306931586 | 0.999970678 | 2.344 | 0.325 | 0 | 0.422 | NA |
| TRINITY_DN3140_c0_g1_i4 | 2.672012979 | 0.078181666 | 0.999970678 | 0.618 | 4.474 | 0.478 | 0.221 | PGM_PMM_I |
| TRINITY_DN5663_c0_g1_i1 | 2.671212504 | 0.059421805 | 0.999970678 | 1.154 | 0.591 | 0.067 | 0.201 | DEAD |
| TRINITY_DN946_c0_g1_i6 | 2.67092744 | 0.323596941 | 0.999970678 | 1.063 | 9.757 | 0 | 1.679 | TRAM_LAG1_CLN |
| TRINITY_DN13789_c0_g1_i4 | 2.670562471 | 0.121926296 | 1 | 0.863 | 0.67 | 0.044 | 0.181 | NA |
| TRINITY_DN373_c1_g1_i2 | 2.669881821 | 0.098180631 | 0.999970678 | 19.094 | 3.508 | 2.633 | 0.412 | NA |
| TRINITY_DN4608_c0_g1_i2 | -2.66859677 | 0.29137247 | 0.999970678 | 0.518 | 0 | 0.911 | 2.162 | Ldh_1_C |
| TRINITY_DN7421_c1_g1_i1 | -2.66828224 | 0.533149267 | 0.999970678 | 5.214 | 0 | 27.776 | 0 | NA |
| TRINITY_DN46871_c0_g1_i1 | -2.66791261 | 0.031035111 | 0.999970678 | 1.108 | 0.936 | 1.689 | 10.979 | Lectin_legB |
| TRINITY_DN8182_c0_g1_i7 | 2.667617091 | 0.281978248 | 0.999970678 | 0.472 | 1.173 | 0 | 0.251 | NA |
| TRINITY_DN38812_c0_g1_i2 | -2.66543339 | 0.051968512 | 0.999970678 | 0.2 | 0.177 | 0.333 | 1.981 | NA |
| TRINITY_DN3707_c0_g1_i1 | 2.66537548 | 0.127810913 | 0.999970678 | 0.6 | 14.28 | 0.578 | 1.629 | Mito_carr |
| TRINITY_DN4102_c0_g1_i1 | 2.665151339 | 0.277215009 | 0.999970678 | 3.97 | 7.096 | 0 | 1.729 | ACP_syn_III |
| TRINITY_DN2582_c0_g1_i6 | 2.663935496 | 0.387958244 | 0.999970678 | 2.107 | 0.01 | 0 | 0.342 | NA |
| TRINITY_DN6534_c0_g1_i5 | 2.663752652 | 0.173887481 | 0.999970678 | 4.96 | 0.108 | 0.444 | 0.261 | Solute_trans_a |
| TRINITY_DN1868_c0_g1_i16 | -2.66363404 | 0.370829184 | 0.999970678 | 3.361 | 0 | 17.343 | 0.633 | NA |
| TRINITY_DN10741_c0_g1_i2 | -2.66361655 | 0.289828415 | 1 | 0 | 1.498 | 2.978 | 5.58 | DUF247 |
| TRINITY_DN1811_c0_g3_i8 | -2.66338106 | 0.372603162 | 0.999970678 | 1.417 | 0.177 | 8.644 | 0 | Grp1_Fun34_Yaa |
| TRINITY_DN6203_c0_g1_i5 | -2.66310798 | 0.333763903 | 0.999970678 | 0.69 | 0 | 0.267 | 4.122 | LRRNT_2 |
| TRINITY_DN6897_c0_g1_i1 | -2.66210182 | 0.312269638 | 0.999970678 | 0.645 | 0 | 0.5 | 3.539 | RCC1 |
| TRINITY_DN25634_c0_g1_i1 | 2.661115418 | 0.519369206 | 0.999970678 | 0 | 23.55 | 0 | 3.66 | NA |
| TRINITY_DN3724_c0_g2_i12 | 2.660509313 | 0.2937377 | 1 | 0.618 | 0.857 | 0.2 | 0 | NA |
| TRINITY_DN2928_c0_g1_i11 | 2.660342431 | 0.046078999 | 0.999970678 | 4.569 | 5.549 | 0.389 | 1.086 | NB-ARC |
| TRINITY_DN7441_c0_g1_i11 | -2.6597428 | 0.291247159 | 0.999970678 | 0.273 | 0 | 0.9 | 0.623 | - |
| TRINITY_DN70618_c0_g1_i1 | 2.658690916 | 0.542072163 | 0.999970678 | 69.192 | 0 | 0 | 11.139 | NA |
| TRINITY_DN1153_c0_g1_i1 | -2.65658376 | 0.292226344 | 0.999970678 | 0.772 | 0 | 1.133 | 3.599 | NA |
| TRINITY_DN12476_c0_g1_i1 | -2.65578886 | 0.303104548 | 0.999970678 | 1.771 | 0.039 | 9.488 | 0.08 | NA |
| TRINITY_DN6563_c0_g1_i2 | -2.65570582 | 0.165053293 | 0.999970678 | 0.609 | 12.97 | 14.921 | 60.091 | NA |
| TRINITY_DN9380_c0_g1_i1 | 2.655385296 | 0.268197823 | 0.999970678 | 2.017 | 2.572 | 0 | 0.734 | Herpes_UL52 |
| TRINITY_DN10954_c0_g1_i1 | 2.654466316 | 0.296997906 | 1 | 0.663 | 1.074 | 0.233 | 0 | PIR |
| TRINITY_DN2052_c2_g1_i2 | 2.654343859 | 0.519753201 | 0.999970678 | 4.342 | 0 | 0 | 0.704 | NA |
| TRINITY_DN6564_c0_g3_i2 | -2.65289305 | 0.385955002 | 0.999970678 | 0 | 1.064 | 0.056 | 6.515 | NA |
| TRINITY_DN2340_c0_g2_i11 | 2.652434943 | 0.26811205 | 0.999970678 | 0.917 | 1.281 | 0 | 0.352 | NA |
| TRINITY_DN53324_c0_g1_i5 | -2.65237071 | 0.522920635 | 0.999970678 | 0.472 | 0 | 2.478 | 0 | NA |
| TRINITY_DN8260_c0_g1_i8 | -2.65232947 | 0.284338855 | 0.999970678 | 0.581 | 0 | 1.178 | 2.222 | NA |
| TRINITY_DN3615_c0_g1_i2 | 2.651432664 | 0.038848699 | 0.999970678 | 3.733 | 21 | 0.933 | 2.785 | ADH_N |
| TRINITY_DN2582_c0_g1_i3 | 2.651241335 | 0.385348353 | 0.999970678 | 11.119 | 0.138 | 0 | 1.83 | NA |
| TRINITY_DN5194_c0_g1_i2 | 2.650678116 | 0.098591466 | 0.999970678 | 1.79 | 36.3 | 2.144 | 3.398 | WRKY |
| TRINITY_DN15223_c0_g1_i1 | -2.65050489 | 0.107232073 | 0.999970678 | 0.245 | 1.271 | 0.989 | 8.174 | NA |
| TRINITY_DN3063_c0_g1_i5 | -2.64918733 | 0.053017414 | 0.999970678 | 3.279 | 1.823 | 22.332 | 4.896 | NA |
| TRINITY_DN868_c0_g1_i12 | -2.64884589 | 0.321149172 | 0.999970678 | 1.272 | 0 | 5.844 | 0.945 | NA |
| TRINITY_DN65171_c0_g1_i5 | 2.646454878 | 0.366916556 | 0.999970678 | 0 | 10.53 | 0.267 | 1.337 | NA |
| TRINITY_DN3615_c0_g1_i6 | -2.64579436 | 0.333367437 | 0.999970678 | 7.649 | 0 | 7.088 | 39.943 | ADH_N |
| TRINITY_DN7413_c0_g1_i1 | -2.64520736 | 0.287294566 | 1 | 0 | 0.276 | 0.778 | 0.694 | NA |
| TRINITY_DN8612_c9_g2_i2 | 2.644630285 | 0.524615349 | 0.999970678 | 3.207 | 0 | 0 | 0.523 | NA |
| TRINITY_DN4242_c0_g2_i5 | 2.64422727 | 0.142578864 | 0.999970678 | 7.249 | 5.558 | 1.589 | 0.141 | NA |
| TRINITY_DN9906_c0_g1_i3 | -2.64364918 | 0.07864684 | 0.999970678 | 0.527 | 0.946 | 0.756 | 8.345 | NA |
| TRINITY_DN8814_c1_g1_i3 | 2.643551755 | 0.066023001 | 1 | 0.254 | 0.621 | 0.078 | 0.05 | Pkinase |
| TRINITY_DN5173_c0_g2_i2 | 2.643203583 | 0.282696302 | 0.999970678 | 6.095 | 3.558 | 1.255 | 0 | LANC_like |
| TRINITY_DN9480_c0_g1_i3 | -2.64085114 | 0.05775449 | 0.999970678 | 0.091 | 0.345 | 1.478 | 0.915 | zf-CCHC |
| TRINITY_DN1384_c0_g1_i2 | 2.640694567 | 0.276138781 | 0.999970678 | 2.834 | 1.488 | 0 | 0.694 | NA |
| TRINITY_DN11157_c0_g1_i4 | 2.639319664 | 0.014657922 | 0.659646884 | 8.848 | 20.23 | 2.367 | 1.659 | NA |
| TRINITY_DN4572_c0_g1_i5 | 2.638591782 | 0.039895369 | 0.999970678 | 30.095 | 11.3 | 0.9 | 5.64 | ANF_receptor |
| TRINITY_DN5143_c1_g1_i2 | -2.63735762 | 0.318808297 | 0.999970678 | 0 | 1.39 | 6.189 | 1.015 | NA |
| TRINITY_DN196_c0_g1_i2 | -2.63700307 | 0.059511539 | 0.999970678 | 0.454 | 0.719 | 0.722 | 6.465 | Lectin_legB |
| TRINITY_DN13880_c0_g3_i1 | 2.636427912 | 0.283640921 | 0.999970678 | 1.799 | 1.202 | 0.411 | 0 | NA |
| TRINITY_DN20887_c0_g1_i3 | -2.63629001 | 0.357001257 | 0.999970678 | 1.799 | 0.581 | 0 | 14.839 | NA |
| TRINITY_DN1484_c0_g1_i3 | -2.63513975 | 0.284948099 | 0.999970678 | 0 | 0.503 | 0.989 | 1.9 | WRKY |
| TRINITY_DN1447_c0_g1_i1 | 2.635112164 | 0.323450373 | 0.999970678 | 2.698 | 23.01 | 0 | 4.092 | NA |
| TRINITY_DN4452_c0_g1_i2 | 2.634910303 | 0.292091497 | 0.999970678 | 1.753 | 0.719 | 0.333 | 0 | NA |
| TRINITY_DN314_c0_g2_i1 | 2.634670823 | 0.033879437 | 0.999970678 | 1.308 | 4.248 | 0.333 | 0.493 | NA |
| TRINITY_DN3408_c0_g1_i4 | -2.63456757 | 0.187975248 | 0.999970678 | 0.999 | 1.932 | 14.61 | 0.372 | NA |
| TRINITY_DN3032_c0_g1_i11 | 2.6344515 | 0.275555625 | 0.999970678 | 1.081 | 0.71 | 0 | 0.292 | NA |
| TRINITY_DN6809_c0_g1_i1 | 2.632990378 | 0.006157749 | 0.321897538 | 3.297 | 6.82 | 0.944 | 0.493 | Chorein_N |
| TRINITY_DN9189_c0_g1_i1 | 2.632761535 | 0.281523218 | 1 | 0.59 | 0.414 | 0 | 0.171 | zf-U11-48K |
| TRINITY_DN1329_c0_g3_i3 | 2.630850736 | 0.287996328 | 0.999970678 | 3.961 | 1.38 | 0 | 0.845 | NA |
| TRINITY_DN373_c0_g1_i2 | 2.630364216 | 0.051735759 | 0.999970678 | 20.948 | 9.895 | 3.644 | 0.603 | NA |
| TRINITY_DN7254_c0_g1_i2 | 2.630094439 | 0.525288852 | 0.999970678 | 1.19 | 0 | 0 | 0.201 | NA |

| TRINITY_DN14939_c0_g1_i8 | -2.62742021 | 0.039769122 | 0.999970678 | 0.681 | 0.512 | 4.289 | 2.232 | NA |
| --- | --- | --- | --- | --- | --- | --- | --- | --- |
| TRINITY_DN6736_c0_g2_i7 | 2.627260863 | 0.298692374 | 0.999970678 | 2.462 | 0.788 | 0.433 | 0 | NA |
| TRINITY_DN4571_c0_g1_i3 | 2.625999043 | 0.044034397 | 0.999970678 | 0.727 | 2.06 | 0.133 | 0.271 | Epimerase |
| TRINITY_DN5703_c0_g1_i1 | -2.62575317 | 0.05400322 | 0.999970678 | 0.672 | 0.187 | 2.355 | 2.302 | NA |
| TRINITY_DN668_c33_g1_i1 | 2.625238573 | 0.1594726 | 0.999970678 | 38.153 | 1.498 | 4.722 | 0.814 | NA |
| TRINITY_DN9036_c0_g1_i2 | -2.62512667 | 0.143870954 | 0.999970678 | 0.799 | 27.86 | 33.154 | 134.307 | AP2 |
| TRINITY_DN136646_c0_g1_i1 | -2.62489856 | 0.048742086 | 0.999970678 | 2.307 | 1.429 | 2.6 | 19.906 | NA |
| TRINITY_DN6364_c1_g1_i5 | 2.623423759 | 0.27277029 | 0.999970678 | 1.435 | 1.695 | 0 | 0.503 | Pkinase |
| TRINITY_DN1067_c0_g1_i8 | 2.623088131 | 0.345238416 | 0.999970678 | 28.015 | 5.962 | 0 | 5.6 | Peptidase_S9 |
| TRINITY_DN9149_c0_g1_i1 | -2.62224681 | 0.080805305 | 0.999970678 | 0.073 | 0.631 | 1.411 | 2.584 | NA |
| TRINITY_DN533_c0_g1_i26 | -2.62164884 | 0.314600258 | 0.999970678 | 1.263 | 0 | 5.455 | 1.297 | NA |
| TRINITY_DN4059_c0_g1_i1 | -2.62075999 | 0.023218872 | 0.942368945 | 2.28 | 0.7 | 8.055 | 8.596 | UBA |
| TRINITY_DN17181_c0_g1_i4 | 2.619815956 | 0.283660216 | 0.999970678 | 0.581 | 1.409 | 0 | 0.312 | MMR_HSR1 |
| TRINITY_DN450_c0_g1_i8 | 2.619553224 | 0.316202138 | 0.999970678 | 4.342 | 10.53 | 1.989 | 0 | RCC1 |
| TRINITY_DN10419_c0_g1_i1 | 2.618804453 | 0.298943422 | 0.999970678 | 0.745 | 0.256 | 0.133 | 0 | NA |
| TRINITY_DN2422_c0_g1_i3 | 2.61723654 | 0.525375342 | 0.999970678 | 5.532 | 0 | 0 | 0.915 | NA |
| TRINITY_DN960_c0_g1_i8 | 2.615919215 | 0.290924607 | 0.999970678 | 3.225 | 5.164 | 1.122 | 0 | Epimerase |
| TRINITY_DN6469_c0_g1_i4 | 2.615779703 | 0.526913272 | 0.999970678 | 0 | 1.192 | 0.156 | 0 | NA |
| TRINITY_DN17342_c0_g2_i2 | -2.61510416 | 0.528903347 | 0.999970678 | 0.699 | 0 | 0 | 4.404 | NA |
| TRINITY_DN164_c0_g2_i1 | -2.61465365 | 0.337298094 | 0.999970678 | 14.198 | 0 | 51.264 | 25.979 | NAC |
| TRINITY_DN4456_c1_g1_i5 | 2.614022522 | 0.288585972 | 0.999970678 | 2.907 | 1.656 | 0.622 | 0.01 | NA |
| TRINITY_DN4652_c1_g1_i2 | 2.613864912 | 0.29967852 | 1 | 0.972 | 0.788 | 0.244 | 0 | NA |
| TRINITY_DN35587_c0_g2_i1 | 2.613855643 | 0.076204879 | 0.999970678 | 0.436 | 1.143 | 0.056 | 0.191 | NA |
| TRINITY_DN19973_c0_g1_i2 | -2.61345169 | 0.292860504 | 0.999970678 | 0.972 | 0 | 2.189 | 3.388 | NA |
| TRINITY_DN1261_c1_g1_i7 | -2.61327128 | 0.032942147 | 0.999970678 | 15.143 | 13.31 | 119.204 | 26.341 | NA |
| TRINITY_DN2091_c0_g1_i13 | -2.61290361 | 0.531607774 | 0.999970678 | 0 | 0.266 | 1.333 | 0 | LRR_6 |
| TRINITY_DN3914_c0_g1_i1 | -2.61282246 | 0.104488137 | 0.999970678 | 2.18 | 3.035 | 1.555 | 29.679 | NA |
| TRINITY_DN10029_c0_g1_i3 | 2.612720161 | 0.31006838 | 1 | 0.59 | 0.512 | 0.144 | 0 | NA |
| TRINITY_DN18437_c0_g1_i2 | -2.60995537 | 0.34048621 | 0.999970678 | 0 | 0.621 | 0.244 | 3.539 | DUF1929 |
| TRINITY_DN3459_c0_g1_i4 | 2.609661381 | 0.532186054 | 0.999970678 | 4.415 | 0 | 0.6 | 0 | NA |
| TRINITY_DN1258_c0_g1_i7 | 2.609338192 | 0.367758271 | 0.999970678 | 2.916 | 0 | 0.3 | 0.141 | - |
| TRINITY_DN7190_c0_g1_i4 | -2.60841412 | 0.368629389 | 0.999970678 | 1.135 | 3.686 | 0 | 29.186 | NA |
| TRINITY_DN10583_c0_g1_i1 | 2.607038062 | 0.111804203 | 0.999970678 | 0.545 | 4.228 | 0.522 | 0.141 | NA |
| TRINITY_DN3775_c0_g1_i1 | -2.60519038 | 0.543326708 | 0.999970678 | 0 | 9.303 | 46.019 | 0 | - |
| TRINITY_DN7900_c0_g1_i1 | -2.60450406 | 0.103702056 | 0.999970678 | 2.371 | 1.695 | 1.355 | 22.882 | NA |
| TRINITY_DN6950_c0_g2_i1 | -2.60364079 | 0.285794947 | 0.999970678 | 0.055 | 5.627 | 0.6 | 33.137 | NA |
| TRINITY_DN4741_c0_g1_i3 | 2.603564085 | 0.290147843 | 0.999970678 | 0.727 | 2.04 | 0 | 0.452 | LRR_4 |
| TRINITY_DN5945_c0_g1_i2 | 2.602756415 | 0.114039461 | 0.999970678 | 2.271 | 8.407 | 1.355 | 0.111 | NA |
| TRINITY_DN867_c0_g1_i1 | 2.602708218 | 0.052714923 | 0.999970678 | 6.94 | 2.838 | 1.133 | 0.251 | ANF_receptor |
| TRINITY_DN43487_c0_g1_i7 | 2.601287531 | 0.07060237 | 1 | 4.933 | 5.016 | 0.678 | 0.855 | AAA |
| TRINITY_DN3311_c0_g1_i2 | -2.60075569 | 0.550292884 | 0.999970678 | 5.641 | 0 | 0 | 34.947 | AUX_IAA |
| TRINITY_DN2931_c0_g1_i9 | 2.60022917 | 0.084727757 | 0.999970678 | 3.016 | 38.7 | 1.867 | 4.544 | Glycos_trans_3N |
| TRINITY_DN191_c0_g1_i4 | -2.59926601 | 0.117028234 | 0.999970678 | 0.091 | 1.508 | 5.666 | 2.735 | Inos-1-P_synth |
| TRINITY_DN6827_c1_g2_i1 | -2.59625776 | 0.005785223 | 0.304862602 | 0.954 | 1.518 | 4.2 | 9.782 | Gb3_synth |
| TRINITY_DN3416_c0_g1_i8 | 2.59574658 | 0.307769638 | 1 | 0.218 | 0.197 | 0.056 | 0 | - |
| TRINITY_DN8420_c1_g1_i4 | 2.595219652 | 0.043200213 | 0.999970678 | 1.617 | 0.463 | 0.189 | 0.121 | BRCT_2 |
| TRINITY_DN46570_c0_g1_i2 | 2.593545004 | 0.295286492 | 0.999970678 | 0.609 | 0.621 | 0.167 | 0 | NA |
| TRINITY_DN10456_c0_g1_i11 | 2.593415558 | 0.532876152 | 0.999970678 | 2.325 | 0 | 0 | 0.392 | NA |
| TRINITY_DN1860_c0_g2_i2 | -2.59305321 | 0.078597863 | 0.999970678 | 1.063 | 2.07 | 2.233 | 16.096 | Phi_1 |
| TRINITY_DN2569_c0_g1_i1 | -2.59294613 | 0.042239086 | 0.999970678 | 2.107 | 11.4 | 19.732 | 56.431 | NA |
| TRINITY_DN4477_c0_g1_i2 | 2.592848529 | 0.289547836 | 0.999970678 | 14.453 | 13.9 | 0 | 4.645 | NA |
| TRINITY_DN7234_c0_g2_i1 | -2.59242278 | 0.03651851 | 0.999970678 | 0.409 | 0.503 | 1.111 | 4.062 | NA |
| TRINITY_DN11311_c0_g1_i6 | 2.591955558 | 0.342914963 | 0.999970678 | 1.217 | 9.658 | 1.478 | 0 | NA |
| TRINITY_DN169_c0_g3_i1 | 2.591202971 | 0.099462439 | 0.999970678 | 8.194 | 6.199 | 0.178 | 2.101 | LSM |
| TRINITY_DN5600_c0_g1_i1 | 2.590982302 | 0.076949518 | 0.999970678 | 1.363 | 5.647 | 0.633 | 0.392 | NA |
| TRINITY_DN6970_c0_g1_i5 | 2.590924429 | 0.031463014 | 0.999970678 | 0.845 | 1.577 | 0.233 | 0.121 | Acetyltransf_1 |
| TRINITY_DN7231_c0_g1_i2 | -2.58972001 | 0.038475437 | 0.999970678 | 2.898 | 0.887 | 5.333 | 16.237 | NA |
| TRINITY_DN6090_c1_g2_i2 | -2.58815204 | 0.30494754 | 0.999970678 | 0.59 | 0 | 2.178 | 1.046 | F-box |
| TRINITY_DN10358_c0_g1_i1 | -2.58800041 | 0.530267632 | 0.999970678 | 0 | 0.641 | 3.1 | 0 | RabGAP-TBC |
| TRINITY_DN4829_c0_g3_i1 | -2.58733165 | 0.294922449 | 0.999970678 | 2.026 | 0 | 4.689 | 6.605 | NA |
| TRINITY_DN4243_c0_g2_i6 | 2.586281707 | 0.311026632 | 0.999970678 | 2.089 | 0.434 | 0 | 0.422 | PPR |
| TRINITY_DN99_c1_g1_i2 | 2.585441889 | 0.174288137 | 0.999970678 | 0.236 | 8.328 | 0.789 | 0.462 | adh_short |
| TRINITY_DN11950_c0_g2_i3 | 2.58469131 | 0.302202659 | 1 | 1.335 | 1.882 | 0 | 0.553 | NA |
| TRINITY_DN8685_c0_g1_i3 | 2.58192316 | 0.297876103 | 0.999970678 | 0.245 | 0.532 | 0 | 0.131 | NA |
| TRINITY_DN1486_c2_g1_i1 | -2.58190987 | 0.15333252 | 1 | 0.345 | 0.03 | 1.011 | 0.965 | adh_short |
| TRINITY_DN13287_c0_g1_i1 | 2.579513758 | 0.04191585 | 0.999970678 | 31.894 | 9.954 | 4.733 | 1.347 | Raffinose_syn |
| TRINITY_DN23552_c0_g1_i3 | 2.578838465 | 0.301299531 | 0.999970678 | 9.638 | 4.484 | 1.955 | 0 | peroxidase |
| TRINITY_DN44113_c0_g1_i1 | 2.578604931 | 0.07869006 | 1 | 1.217 | 0.69 | 0.189 | 0.08 | NA |
| TRINITY_DN12479_c0_g1_i5 | 2.576710199 | 0.021424215 | 0.892821374 | 18.068 | 12.34 | 2.5 | 2.001 | NA |
| TRINITY_DN7192_c0_g1_i8 | -2.5748396 | 0.539520063 | 0.999970678 | 0 | 0.355 | 1.733 | 0 | NA |
| TRINITY_DN2150_c1_g1_i1 | -2.57425153 | 0.262920913 | 0.999970678 | 0.136 | 1.725 | 8.866 | 0.201 | DUF866 |
| TRINITY_DN2626_c0_g3_i1 | 2.573859407 | 0.53455624 | 0.999970678 | 0 | 1.981 | 0 | 0.332 | NUMOD3 |
| TRINITY_DN3531_c0_g1_i7 | -2.57307304 | 0.003841455 | 0.213086597 | 0.354 | 0.365 | 1.389 | 2.674 | UDPGT |
| TRINITY_DN1543_c0_g1_i2 | 2.570428954 | 0.303059258 | 0.999970678 | 2.934 | 3.302 | 0 | 1.056 | Pept_tRNA_hydro |

| TRINITY_DN1322_c0_g1_i7 | 2.569835299 | 0.536348033 | 0.999970678 | 0 | 15.78 | 2.122 | 0 | NA |
| --- | --- | --- | --- | --- | --- | --- | --- | --- |
| TRINITY_DN51165_c1_g1_i1 | -2.56895229 | 0.299964721 | 0.999970678 | 0.681 | 0 | 1.8 | 1.9 | NA |
| TRINITY_DN16963_c0_g1_i3 | 2.5686439 | 0.095812932 | 0.999970678 | 3.198 | 0.394 | 0.389 | 0.141 | NA |
| TRINITY_DN24243_c0_g1_i2 | -2.568283 | 0.058705866 | 0.999970678 | 0.472 | 0.177 | 1.333 | 2.312 | NA |
| TRINITY_DN2707_c0_g2_i3 | -2.56568887 | 0.14555767 | 0.999970678 | 1.517 | 11.27 | 3.855 | 69.813 | NA |
| TRINITY_DN4620_c0_g1_i1 | 2.564175492 | 0.144691342 | 0.999970678 | 3.043 | 1.794 | 0.056 | 0.744 | NA |
| TRINITY_DN18724_c0_g1_i5 | 2.562985879 | 0.301069147 | 0.999970678 | 5.587 | 5.43 | 0 | 1.87 | DUF707 |
| TRINITY_DN14265_c0_g1_i1 | -2.56287241 | 0.540949823 | 0.999970678 | 1.09 | 0 | 5.377 | 0 | - |
| TRINITY_DN1775_c0_g1_i2 | 2.562248806 | 0.533939388 | 0.999970678 | 9.375 | 0 | 0 | 1.609 | - |
| TRINITY_DN8326_c0_g1_i5 | 2.562241986 | 0.075718441 | 0.999970678 | 0.927 | 2.217 | 0.356 | 0.111 | RVT_1 |
| TRINITY_DN108_c0_g1_i13 | -2.56203616 | 0.081257341 | 0.999970678 | 1.363 | 0.719 | 9.311 | 1.146 | NA |
| TRINITY_DN10399_c1_g1_i2 | -2.56168224 | 0.318030506 | 1 | 0.454 | 0 | 1.289 | 1.297 | NA |
| TRINITY_DN287_c0_g1_i3 | 2.560114571 | 0.312604892 | 0.999970678 | 1.662 | 7.194 | 0 | 1.488 | NA |
| TRINITY_DN635_c0_g1_i8 | -2.55991254 | 0.295172418 | 0.999970678 | 0 | 1.508 | 4 | 3.921 | - |
| TRINITY_DN3666_c0_g1_i6 | 2.559725 | 0.316322369 | 1 | 0.663 | 0.739 | 0.178 | 0 | NA |
| TRINITY_DN28584_c0_g1_i4 | 2.559015892 | 0.533723349 | 0.999970678 | 0 | 5.618 | 0 | 0.945 | NA |
| TRINITY_DN3132_c0_g1_i1 | 2.558394509 | 0.537553751 | 0.999970678 | 1.899 | 0 | 0 | 0.332 | TPR_1 |
| TRINITY_DN6733_c0_g1_i3 | 2.557856558 | 0.372797388 | 0.999970678 | 0 | 7.52 | 0.322 | 0.855 | Bax1-I |
| TRINITY_DN31769_c0_g1_i1 | -2.55760682 | 0.285814841 | 0.999970678 | 0.173 | 1.33 | 0.044 | 8.676 | Catalase |
| TRINITY_DN247_c1_g5_i3 | 2.557362955 | 0.066061864 | 1 | 1.19 | 0.67 | 0.156 | 0.111 | Peptidase_S10 |
| TRINITY_DN872_c0_g1_i11 | 2.557144295 | 0.026547755 | 0.999970678 | 1.072 | 0.838 | 0.078 | 0.231 | NA |
| TRINITY_DN29585_c0_g2_i1 | -2.55696729 | 0.312697932 | 1 | 0.245 | 0 | 0.644 | 0.704 | NA |
| TRINITY_DN5906_c0_g1_i4 | 2.555604673 | 0.287724728 | 0.999970678 | 2.008 | 2.464 | 0 | 0.754 | NA |
| TRINITY_DN98_c0_g1_i15 | 2.555177287 | 0.225350723 | 0.999970678 | 46.274 | 0.434 | 0.9 | 7.017 | Sigma70_r2 |
| TRINITY_DN17351_c0_g2_i4 | -2.55509284 | 0.308509641 | 0.999970678 | 1.444 | 0 | 4.578 | 3.016 | NA |
| TRINITY_DN9078_c0_g1_i1 | 2.554553231 | 0.076575298 | 0.999970678 | 1.635 | 2.444 | 0.5 | 0.09 | NA |
| TRINITY_DN2973_c0_g1_i2 | -2.55392785 | 0.358340715 | 0.999970678 | 0.908 | 0 | 4.089 | 0.372 | NA |
| TRINITY_DN4260_c0_g1_i2 | 2.553678462 | 0.404866879 | 0.999970678 | 66.703 | 0 | 0.978 | 10.275 | - |
| TRINITY_DN15481_c1_g2_i1 | 2.5533469 | 0.32448512 | 0.999970678 | 0.463 | 2.966 | 0 | 0.573 | NA |
| TRINITY_DN12154_c0_g1_i1 | -2.55322485 | 0.340194876 | 0.999970678 | 2.307 | 0.049 | 11.51 | 0.06 | NA |
| TRINITY_DN1441_c0_g1_i4 | 2.551925091 | 0.324356129 | 0.999970678 | 4.006 | 20.72 | 0 | 4.162 | NA |
| TRINITY_DN3856_c0_g1_i1 | -2.55141785 | 0.544143373 | 0.999970678 | 0 | 4.405 | 20.954 | 0 | Ribosomal_L18p |
| TRINITY_DN16151_c0_g1_i2 | 2.550920357 | 0.295373025 | 0.999970678 | 1.19 | 2.503 | 0 | 0.633 | NA |
| TRINITY_DN1621_c0_g1_i9 | -2.55046103 | 0.30765057 | 0.999970678 | 0 | 1.025 | 2.9 | 2.373 | ERCC3_RAD25_C |
| TRINITY_DN799_c0_g2_i1 | -2.54919946 | 0.192942961 | 0.999970678 | 215.19 | 2.02 | 595.931 | 573.292 | FA_desaturase |
| TRINITY_DN434_c0_g1_i1 | -2.54724166 | 0.355420033 | 0.999970678 | 20.693 | 0 | 10.277 | 106.418 | NA |
| TRINITY_DN5025_c0_g1_i8 | -2.54706537 | 0.0391903 | 0.999970678 | 2.471 | 4.188 | 5.6 | 31.8 | Inhibitor_I9 |
| TRINITY_DN3998_c0_g1_i3 | 2.546630788 | 0.300705495 | 0.999970678 | 1.408 | 2.188 | 0.522 | 0 | NA |
| TRINITY_DN11602_c0_g1_i5 | 2.545225437 | 0.542222723 | 0.999970678 | 0 | 1.311 | 0.189 | 0 | Lgl_C |
| TRINITY_DN17280_c1_g1_i1 | -2.544726 | 0.325409701 | 0.999970678 | 2.226 | 0 | 2.167 | 10.345 | NA |
| TRINITY_DN4532_c0_g1_i2 | -2.54359427 | 0.416899139 | 0.999970678 | 1.826 | 0.089 | 9.344 | 0 | NA |
| TRINITY_DN7396_c0_g1_i13 | 2.543151306 | 0.105659141 | 1 | 0.818 | 0.857 | 0.189 | 0.06 | NA |
| TRINITY_DN2273_c0_g1_i5 | -2.5430738 | 0.055180806 | 0.999970678 | 1.817 | 4.218 | 3.855 | 30.453 | CBS |
| TRINITY_DN2444_c0_g1_i4 | -2.54223812 | 0.077616688 | 0.999970678 | 3.025 | 1.124 | 2.311 | 21.485 | Pyr_redox_2 |
| TRINITY_DN14175_c0_g1_i4 | -2.54055464 | 0.307212573 | 0.999970678 | 0.418 | 0 | 0.911 | 1.387 | NA |
| TRINITY_DN11776_c0_g1_i1 | -2.54049977 | 0.316849247 | 1 | 0.309 | 0 | 0.644 | 0.925 | NA |
| TRINITY_DN250035_c0_g1_i1 | -2.53915047 | 0.539884849 | 0.999970678 | 5.723 | 0 | 27.709 | 0 | GFA |
| TRINITY_DN51058_c0_g1_i2 | 2.53822089 | 0.032362062 | 0.999970678 | 1.753 | 2.06 | 0.311 | 0.271 | NA |
| TRINITY_DN3022_c0_g1_i7 | -2.53820337 | 0.393549486 | 0.999970678 | 2.053 | 0.237 | 0 | 13.482 | NA |
| TRINITY_DN9839_c0_g2_i2 | -2.53818618 | 0.327337276 | 0.999970678 | 0 | 2.759 | 3.622 | 11.431 | NA |
| TRINITY_DN12529_c1_g1_i2 | -2.5379996 | 0.390314431 | 0.999970678 | 0.173 | 1.419 | 0 | 9.179 | Fasciclin |
| TRINITY_DN13038_c0_g1_i9 | -2.53799059 | 0.303083751 | 0.999970678 | 0 | 0.345 | 0.811 | 0.965 | BHD_1 |
| TRINITY_DN22201_c0_g1_i3 | -2.53778516 | 0.064289389 | 0.999970678 | 0.309 | 0.857 | 3.666 | 2.292 | NA |
| TRINITY_DN2837_c0_g2_i3 | -2.53699665 | 0.3457573 | 0.999970678 | 0 | 0.552 | 2.344 | 0.352 | NA |
| TRINITY_DN1564_c0_g1_i2 | -2.53639491 | 0.034227754 | 0.999970678 | 29.932 | 5.529 | 96.105 | 90.855 | CIAPIN1 |
| TRINITY_DN13720_c0_g1_i3 | 2.535699677 | 0.536794726 | 0.999970678 | 0 | 5.056 | 0 | 0.865 | NA |
| TRINITY_DN182_c0_g1_i2 | -2.53537519 | 0.139497812 | 0.999970678 | 0.082 | 0.848 | 0.689 | 4.454 | NA |
| TRINITY_DN11182_c0_g1_i7 | 2.534904693 | 0.167008688 | 0.999970678 | 173.63 | 3.696 | 16.721 | 10.446 | Ank_2 |
| TRINITY_DN3472_c0_g1_i4 | 2.534614406 | 0.311217213 | 0.999970678 | 4.279 | 9.412 | 0 | 2.353 | - |
| TRINITY_DN186_c2_g2_i3 | 2.534503717 | 0.566132421 | 0.999970678 | 19.322 | 0 | 0 | 3.408 | Ras |
| TRINITY_DN3775_c0_g2_i1 | -2.53438492 | 0.066688012 | 1 | 0.136 | 0.296 | 0.711 | 1.649 | NA |
| TRINITY_DN9526_c0_g1_i2 | -2.53407021 | 0.541671902 | 0.999970678 | 1.408 | 0 | 6.822 | 0 | NA |
| TRINITY_DN10365_c0_g1_i4 | -2.53398151 | 0.3145801 | 1 | 0 | 0.296 | 0.767 | 0.824 | Lipase_GDSL |
| TRINITY_DN45294_c1_g1_i1 | -2.53268239 | 0.345830213 | 0.999970678 | 0 | 0.71 | 3.011 | 0.412 | NA |
| TRINITY_DN8461_c0_g2_i3 | -2.53241505 | 0.365683598 | 0.999970678 | 0 | 2.513 | 11.177 | 0.794 | NA |
| TRINITY_DN2144_c0_g1_i2 | -2.53211701 | 0.077161832 | 0.999970678 | 4.133 | 6.514 | 46.264 | 5.308 | DUF3474 |
| TRINITY_DN5541_c1_g1_i9 | -2.53157361 | 0.54118871 | 0.999970678 | 0.59 | 0 | 2.878 | 0 | NA |
| TRINITY_DN142_c0_g2_i4 | -2.53152632 | 0.335703825 | 0.999970678 | 4.542 | 0 | 13.221 | 10.587 | NA |
| TRINITY_DN7160_c1_g1_i2 | 2.530417081 | 0.312227518 | 0.999970678 | 0.818 | 0.384 | 0.178 | 0 | NA |
| TRINITY_DN21_c0_g1_i9 | -2.5302388 | 0.315583894 | 1 | 0 | 0.424 | 0.778 | 1.428 | NA |
| TRINITY_DN474_c0_g2_i4 | -2.53012328 | 0.000303748 | 0.028034072 | 2.098 | 1.616 | 8.977 | 10.516 | BSP |
| TRINITY_DN2253_c0_g1_i6 | 2.530001047 | 0.107961131 | 0.999970678 | 1.635 | 1.528 | 0.4 | 0.07 | TPX2 |
| TRINITY_DN4776_c0_g1_i3 | 2.528498856 | 0.390174529 | 0.999970678 | 4.315 | 0 | 0.111 | 0.623 | NA |
| TRINITY_DN2653_c0_g1_i1 | 2.528039464 | 0.058086679 | 0.999970678 | 1.226 | 5.253 | 0.378 | 0.643 | NA |

| TRINITY_DN9229_c1_g1_i2 | -2.52721013 | 0.542554396 | 0.999970678 | 2.162 | 0 | 10.41 | 0 | NA |
| --- | --- | --- | --- | --- | --- | --- | --- | --- |
| TRINITY_DN43487_c0_g1_i1 | 2.527132277 | 0.036154897 | 0.999970678 | 155.55 | 117.9 | 32.42 | 7.4 | AAA |
| TRINITY_DN2847_c0_g1_i3 | 2.52548782 | 0.343819358 | 0.999970678 | 3.007 | 0.335 | 0 | 0.593 | NA |
| TRINITY_DN1637_c0_g1_i12 | 2.525396845 | 0.34821077 | 0.999970678 | 0.6 | 6.761 | 0 | 1.267 | NA |
| TRINITY_DN1602_c0_g1_i2 | -2.52518146 | 0.071135013 | 0.999970678 | 0.082 | 0.217 | 0.367 | 1.267 | Acyl-CoA_dh_1 |
| TRINITY_DN38674_c0_g1_i1 | -2.52353363 | 0.543567796 | 0.999970678 | 3.061 | 0 | 14.732 | 0 | - |
| TRINITY_DN2701_c0_g1_i2 | -2.52282673 | 0.305019631 | 0.999970678 | 0 | 0.65 | 1.455 | 1.95 | NA |
| TRINITY_DN4380_c0_g1_i1 | 2.522357092 | 0.029209412 | 0.999970678 | 2.689 | 3.016 | 0.678 | 0.171 | NA |
| TRINITY_DN2729_c0_g1_i3 | -2.52175348 | 0.332104212 | 0.999970678 | 0 | 1.922 | 7.188 | 2.151 | NA |
| TRINITY_DN1708_c0_g1_i1 | 2.520460218 | 0.54153891 | 0.999970678 | 0 | 24.1 | 3.422 | 0 | NA |
| TRINITY_DN13713_c0_g1_i2 | -2.52027729 | 0.349630962 | 0.999970678 | 1.572 | 0 | 6.611 | 1.146 | NA |
| TRINITY_DN13011_c0_g1_i6 | 2.520113892 | 0.31262858 | 0.999970678 | 0.808 | 0.375 | 0.178 | 0 | NA |
| TRINITY_DN498_c0_g1_i3 | 2.518288908 | 0.146399583 | 0.999970678 | 0.345 | 5.44 | 0.578 | 0.302 | - |
| TRINITY_DN251_c0_g1_i13 | 2.517188051 | 0.031181399 | 0.999970678 | 3.615 | 1.439 | 0.378 | 0.432 | NA |
| TRINITY_DN2940_c0_g1_i2 | -2.51716011 | 0.100403174 | 0.999970678 | 0.327 | 3.213 | 13.155 | 4.212 | Jacalin |
| TRINITY_DN11258_c0_g1_i3 | -2.51663182 | 0.547400181 | 0.999970678 | 0.672 | 0 | 3.233 | 0 | NA |
| TRINITY_DN406_c0_g1_i5 | -2.51659088 | 0.108017664 | 0.999970678 | 0.291 | 0.069 | 0.289 | 1.739 | Peptidase_S10 |
| TRINITY_DN20144_c0_g1_i6 | 2.515496191 | 0.328583925 | 0.999970678 | 2.153 | 3.637 | 0.833 | 0 | Sec16 |
| TRINITY_DN4283_c0_g1_i4 | -2.51481288 | 0.545495206 | 0.999970678 | 1.272 | 0 | 6.022 | 0 | NA |
| TRINITY_DN2371_c1_g1_i8 | 2.514665156 | 0.118611942 | 1 | 4.887 | 2.671 | 0.911 | 0.231 | NA |
| TRINITY_DN6051_c0_g1_i2 | -2.51278858 | 0.326122554 | 0.999970678 | 0.354 | 0 | 1.278 | 0.573 | NA |
| TRINITY_DN7683_c0_g1_i3 | -2.51247421 | 0.316176323 | 0.999970678 | 0 | 0.71 | 1.255 | 2.413 | NA |
| TRINITY_DN1913_c0_g1_i5 | -2.51138641 | 0.030757482 | 0.999970678 | 0.472 | 0.483 | 3.633 | 1.096 | NA |
| TRINITY_DN10925_c0_g1_i1 | 2.510479623 | 0.22258477 | 0.999970678 | 0.454 | 15.29 | 0.356 | 2.252 | NA |
| TRINITY_DN22393_c0_g1_i1 | 2.509965813 | 0.35483396 | 0.999970678 | 0.263 | 3.173 | 0 | 0.603 | NA |
| TRINITY_DN4848_c3_g1_i14 | 2.509910888 | 0.063567128 | 0.999970678 | 0.808 | 1.34 | 0.056 | 0.302 | NA |
| TRINITY_DN23107_c0_g1_i2 | -2.50983657 | 0.335379449 | 0.999970678 | 1.944 | 0 | 3.389 | 7.219 | NA |
| TRINITY_DN17238_c0_g2_i3 | 2.50924186 | 0.310809587 | 0.999970678 | 0.681 | 0.818 | 0.222 | 0 | NA |
| TRINITY_DN24_c0_g3_i1 | -2.50903138 | 0.18163737 | 0.999970678 | 1.944 | 19.22 | 5.2 | 109.062 | NA |
| TRINITY_DN11120_c0_g1_i3 | -2.50900694 | 0.337411342 | 0.999970678 | 4.079 | 0 | 14.61 | 5.962 | NA |
| TRINITY_DN4572_c0_g1_i3 | 2.507168082 | 0.056796791 | 0.999970678 | 3.225 | 1.025 | 0.144 | 0.593 | ANF_receptor |
| TRINITY_DN5963_c0_g1_i2 | 2.506776129 | 0.067371598 | 0.999970678 | 1.235 | 0.453 | 0.167 | 0.111 | NA |
| TRINITY_DN75010_c0_g1_i5 | 2.506480828 | 0.304567683 | 0.999970678 | 3.47 | 2.838 | 0 | 1.086 | Metallophos |
| TRINITY_DN8126_c0_g2_i6 | 2.505066375 | 0.308978804 | 0.999970678 | 1.226 | 0.581 | 0 | 0.332 | NA |
| TRINITY_DN6761_c0_g1_i1 | 2.504827747 | 0.109354773 | 0.999970678 | 121.78 | 17.4 | 17.754 | 3.267 | Cu_bind_like |
| TRINITY_DN1909_c0_g1_i1 | 2.504340861 | 0.033278522 | 0.999970678 | 2.534 | 2.69 | 0.289 | 0.593 | - |
| TRINITY_DN6778_c0_g1_i6 | -2.50389478 | 0.211053004 | 0.999970678 | 0.055 | 0.611 | 0.267 | 3.539 | zf-RING_2 |
| TRINITY_DN2825_c1_g1_i1 | 2.503645237 | 0.19839524 | 0.999970678 | 11.818 | 1.183 | 1.8 | 0.141 | LRRNT_2 |
| TRINITY_DN2246_c0_g1_i5 | 2.503554717 | 0.396814099 | 0.999970678 | 0 | 9.944 | 0.244 | 1.428 | Tom22 |
| TRINITY_DN1847_c0_g1_i1 | -2.50336424 | 0.119207563 | 0.999970678 | 6.577 | 1.764 | 2.922 | 44.156 | Pkinase |
| TRINITY_DN8410_c0_g1_i4 | -2.50273355 | 0.356843717 | 0.999970678 | 0 | 0.956 | 4.011 | 0.483 | FAM86 |
| TRINITY_DN712_c0_g2_i3 | 2.502570638 | 0.304914162 | 0.999970678 | 0.699 | 0.631 | 0 | 0.241 | NA |
| TRINITY_DN2847_c0_g1_i1 | 2.502566296 | 0.029902635 | 0.999970678 | 0.963 | 3.193 | 0.322 | 0.332 | NA |
| TRINITY_DN5501_c0_g1_i3 | 2.50252168 | 0.044650006 | 1 | 0.581 | 0.69 | 0.089 | 0.111 | NA |
| TRINITY_DN885_c0_g1_i10 | 2.502468342 | 0.40355016 | 0.999970678 | 24.663 | 0 | 0.667 | 3.619 | CWC25 |
| TRINITY_DN29453_c0_g3_i1 | 2.502450563 | 0.153830372 | 0.999970678 | 2.671 | 0.424 | 0.411 | 0.06 | NA |
| TRINITY_DN18727_c0_g1_i2 | 2.501703119 | 0.301903844 | 0.999970678 | 2.634 | 2.651 | 0 | 0.925 | - |
| TRINITY_DN12881_c1_g1_i2 | -2.5011139 | 0.083218779 | 0.999970678 | 0.663 | 0.197 | 0.756 | 4.011 | PPO1_DWL |
| TRINITY_DN17038_c0_g1_i16 | -2.50055835 | 0.322710096 | 0.999970678 | 0.581 | 0 | 1.978 | 0.975 | mTERF |
| TRINITY_DN920_c0_g1_i6 | 2.500026835 | 0.049593263 | 0.999970678 | 13.263 | 2.809 | 1.622 | 0.905 | FBPase |
| TRINITY_DN8417_c0_g1_i2 | 2.498996014 | 0.319456686 | 0.999970678 | 0.536 | 1.173 | 0.256 | 0 | NA |
| TRINITY_DN1720_c2_g3_i3 | 2.498025494 | 0.546358633 | 0.999970678 | 0 | 24.94 | 3.6 | 0 | WRKY |
| TRINITY_DN6939_c0_g1_i3 | -2.49695219 | 0.552996417 | 0.999970678 | 3.87 | 0 | 18.199 | 0 | NA |
| TRINITY_DN6357_c0_g1_i2 | 2.494955352 | 0.545057915 | 0.999970678 | 7.549 | 0 | 0 | 1.357 | NA |
| TRINITY_DN25606_c0_g1_i1 | 2.494141604 | 0.086076516 | 0.999970678 | 4.242 | 4.524 | 1.078 | 0.251 | NA |
| TRINITY_DN6011_c0_g1_i2 | -2.49411444 | 0.549945543 | 0.999970678 | 4.669 | 0 | 0 | 26.552 | NA |
| TRINITY_DN4553_c0_g1_i3 | -2.4938513 | 0.138457159 | 0.999970678 | 0.191 | 2.198 | 1.933 | 10.828 | NA |
| TRINITY_DN2656_c0_g1_i11 | 2.493229983 | 0.032832443 | 0.999970678 | 1.235 | 3.568 | 0.367 | 0.412 | Amidohydro_1 |
| TRINITY_DN2755_c0_g1_i3 | -2.49273244 | 0.087872257 | 0.999970678 | 0.917 | 1.34 | 0.889 | 11.652 | Glyco_hydro_16 |
| TRINITY_DN14822_c0_g1_i6 | 2.491356471 | 0.102850776 | 1 | 1.09 | 0.788 | 0.067 | 0.251 | Chromo |
| TRINITY_DN14794_c0_g1_i9 | -2.49130177 | 0.549101264 | 0.999970678 | 3.179 | 0 | 14.955 | 0 | NA |
| TRINITY_DN3586_c0_g1_i1 | 2.491279282 | 0.033269159 | 0.999970678 | 5.932 | 2.01 | 0.411 | 0.905 | NA |
| TRINITY_DN4608_c0_g1_i3 | -2.49125359 | 0.106911245 | 0.999970678 | 2.107 | 0.394 | 3.033 | 10.285 | Ldh_1_C |
| TRINITY_DN4052_c0_g1_i8 | -2.49016122 | 0.164193159 | 0.999970678 | 2.162 | 2.838 | 0.567 | 27.336 | NA |
| TRINITY_DN15231_c0_g1_i2 | -2.48986352 | 0.548311044 | 0.999970678 | 0.481 | 0 | 2.3 | 0 | NA |
| TRINITY_DN4819_c0_g1_i7 | 2.489359015 | 0.152420478 | 0.999970678 | 11.155 | 0.621 | 1.444 | 0.392 | NAF |
| TRINITY_DN558_c1_g2_i1 | -2.48898392 | 0.552066572 | 0.999970678 | 18.113 | 0 | 84.284 | 0 | NA |
| TRINITY_DN2099_c1_g1_i3 | -2.48888414 | 0.408672822 | 0.999970678 | 0.372 | 4.06 | 0 | 23.807 | NA |
| TRINITY_DN600_c0_g1_i7 | 2.48870471 | 0.070966664 | 0.999970678 | 0.4 | 0.719 | 0.122 | 0.05 | ENOD93 |
| TRINITY_DN10958_c0_g1_i1 | -2.48815442 | 0.552669858 | 0.999970678 | 0 | 0.562 | 2.544 | 0 | DUF674 |
| TRINITY_DN186_c2_g2_i6 | -2.48794811 | 0.565346117 | 0.999970678 | 0 | 1.981 | 9.066 | 0 | Ras |
| TRINITY_DN1467_c0_g1_i10 | -2.48749572 | 0.126217878 | 0.999970678 | 1.644 | 2.247 | 0.978 | 20.58 | NA |
| TRINITY_DN7023_c0_g1_i2 | 2.487495569 | 0.412821894 | 0.999970678 | 3.279 | 0.049 | 0.5 | 0 | NA |
| TRINITY_DN10009_c0_g1_i1 | 2.487314427 | 0.55987252 | 0.999970678 | 0 | 65.28 | 0 | 11.441 | PLAC8 |

| TRINITY_DN1001_c0_g1_i4 | 2.486566125 | 0.392778139 | 0.999970678 | 0 | 4.681 | 0.556 | 0.161 | MAP70 |
| --- | --- | --- | --- | --- | --- | --- | --- | --- |
| TRINITY_DN4079_c0_g1_i5 | 2.486092875 | 0.117761442 | 0.999970678 | 54.476 | 3.144 | 3.378 | 6.314 | NA |
| TRINITY_DN1245_c0_g1_i11 | 2.485842107 | 0.069937813 | 0.999970678 | 0.509 | 3.055 | 0.211 | 0.382 | F-box |
| TRINITY_DN2251_c0_g1_i4 | 2.485458157 | 0.149865873 | 0.999970678 | 2.616 | 0.552 | 0.044 | 0.523 | NA |
| TRINITY_DN31_c0_g1_i1 | 2.485036049 | 0.550241618 | 0.999970678 | 14.925 | 0 | 0 | 2.704 | AAA |
| TRINITY_DN6122_c0_g1_i3 | 2.484954464 | 0.009810708 | 0.478411122 | 1.572 | 3.321 | 0.389 | 0.402 | Ion_trans |
| TRINITY_DN4474_c0_g1_i3 | 2.484625877 | 0.085271132 | 0.999970678 | 0.59 | 3.518 | 0.444 | 0.181 | Jas |
| TRINITY_DN4301_c0_g1_i4 | -2.48451365 | 0.049988534 | 0.999970678 | 0.082 | 0.246 | 0.889 | 0.744 | WRKY |
| TRINITY_DN9288_c0_g3_i1 | 2.483232443 | 0.55144056 | 0.999970678 | 0.654 | 0 | 0 | 0.121 | NA |
| TRINITY_DN4534_c0_g1_i3 | 2.482969518 | 0.549620314 | 0.999970678 | 49.417 | 0 | 0 | 8.787 | NA |
| TRINITY_DN10834_c0_g1_i2 | -2.48261602 | 0.084126561 | 0.999970678 | 0.082 | 0.138 | 0.156 | 1.025 | NA |
| TRINITY_DN10702_c0_g3_i1 | -2.4815641 | 0.339942874 | 0.999970678 | 0.863 | 0 | 0.778 | 3.921 | Metallophos |
| TRINITY_DN5751_c0_g1_i2 | -2.48057305 | 0.223941368 | 0.999970678 | 1.79 | 0.108 | 0.478 | 10.104 | NA |
| TRINITY_DN837_c0_g1_i1 | -2.47917488 | 0.328371771 | 0.999970678 | 0 | 3.913 | 12.255 | 6.666 | Ribosomal_L6e |
| TRINITY_DN1268_c2_g1_i21 | -2.47889067 | 0.14982797 | 0.999970678 | 0.1 | 0.552 | 2.589 | 0.422 | NA |
| TRINITY_DN1037_c0_g1_i5 | 2.47886739 | 0.31479453 | 0.999970678 | 0.654 | 0.788 | 0.211 | 0 | Cpn60_TCP1 |
| TRINITY_DN6754_c0_g2_i2 | -2.47812006 | 0.448589851 | 0.999970678 | 0 | 15.67 | 0.222 | 84.139 | NA |
| TRINITY_DN1179_c0_g1_i3 | -2.47805191 | 0.435190248 | 0.999970678 | 7.431 | 0 | 0.322 | 41.683 | NA |
| TRINITY_DN8761_c0_g1_i4 | -2.47776988 | 0.206779834 | 0.999970678 | 5.641 | 0.049 | 15.321 | 13.663 | NA |
| TRINITY_DN1320_c0_g1_i20 | -2.4764246 | 0.549389024 | 0.999970678 | 1.244 | 0 | 0 | 7.048 | NA |
| TRINITY_DN72_c0_g1_i11 | 2.47628979 | 0.553595766 | 0.999970678 | 12.845 | 0 | 0 | 2.363 | NA |
| TRINITY_DN5283_c0_g4_i1 | -2.47532029 | 0.335180875 | 0.999970678 | 3.652 | 0 | 12.155 | 5.851 | NA |
| TRINITY_DN28836_c0_g1_i1 | 2.474922045 | 0.05802079 | 1 | 0.536 | 0.424 | 0.078 | 0.08 | NA |
| TRINITY_DN1412_c0_g2_i3 | -2.47478107 | 0.325144172 | 0.999970678 | 0.5 | 0 | 1.567 | 0.965 | Ras |
| TRINITY_DN292_c2_g1_i1 | 2.474353804 | 0.327234267 | 0.999970678 | 1.717 | 0.926 | 0.4 | 0 | NA |
| TRINITY_DN14543_c0_g1_i1 | -2.47378408 | 0.553009638 | 0.999970678 | 0.872 | 0 | 4.033 | 0 | CAP |
| TRINITY_DN6591_c2_g2_i2 | -2.47291087 | 0.549745232 | 0.999970678 | 2.525 | 0 | 0 | 14.286 | NA |
| TRINITY_DN11225_c0_g1_i2 | -2.47246249 | 0.559660692 | 0.999970678 | 1.381 | 0 | 6.411 | 0 | Pyrid_ox_like |
| TRINITY_DN9673_c0_g1_i3 | 2.471699112 | 0.316771381 | 0.999970678 | 1.744 | 0.857 | 0 | 0.473 | p450 |
| TRINITY_DN461_c2_g1_i2 | 2.471576866 | 0.548929821 | 0.999970678 | 14.353 | 0 | 0 | 2.584 | NA |
| TRINITY_DN51007_c0_g1_i2 | 2.469062961 | 0.326967012 | 1 | 0.236 | 0.365 | 0 | 0.101 | Kinesin |
| TRINITY_DN1867_c0_g1_i10 | -2.46888639 | 0.298490829 | 0.999970678 | 0.254 | 1.981 | 0.111 | 12.004 | B_lectin |
| TRINITY_DN3589_c0_g1_i5 | 2.468382878 | 0.311777121 | 0.999970678 | 3.316 | 2.06 | 0 | 0.985 | NA |
| TRINITY_DN3938_c0_g1_i1 | -2.46832291 | 0.320796737 | 0.999970678 | 0.481 | 0 | 1.022 | 1.438 | NA |
| TRINITY_DN24957_c0_g1_i4 | 2.468234817 | 0.317665758 | 0.999970678 | 18.695 | 0.128 | 0.078 | 3.368 | NA |
| TRINITY_DN13775_c0_g1_i3 | -2.46729799 | 0.338725269 | 1 | 0.418 | 0 | 1.344 | 0.804 | NA |
| TRINITY_DN2671_c0_g1_i3 | 2.466392673 | 0.327968767 | 0.999970678 | 6.904 | 8.653 | 0 | 2.815 | NA |
| TRINITY_DN2536_c8_g2_i3 | 2.464656503 | 0.406298675 | 0.999970678 | 5.441 | 0 | 0.711 | 0.151 | NAD_binding_10 |
| TRINITY_DN22_c0_g2_i7 | 2.464192034 | 0.548808593 | 0.999970678 | 0 | 3.962 | 0 | 0.714 | AA_kinase |
| TRINITY_DN12775_c0_g1_i5 | -2.46363007 | 0.319968324 | 0.999970678 | 0 | 1.36 | 2.333 | 4.584 | HSP20 |
| TRINITY_DN2323_c0_g1_i1 | 2.463067844 | 0.550467772 | 0.999970678 | 0 | 6.948 | 0 | 1.247 | SAP |
| TRINITY_DN220_c0_g1_i7 | 2.463013939 | 0.386862095 | 0.999970678 | 4.033 | 0 | 0.3 | 0.382 | EGF_CA |
| TRINITY_DN4022_c0_g1_i3 | 2.461219324 | 0.170081499 | 0.999970678 | 2.471 | 13.18 | 2.211 | 0.141 | DnaJ |
| TRINITY_DN1397_c0_g1_i19 | 2.46058994 | 0.551046286 | 0.999970678 | 3.098 | 0 | 0 | 0.573 | NA |
| TRINITY_DN6189_c0_g1_i7 | 2.460256124 | 0.083224644 | 0.999970678 | 2.852 | 1.646 | 0.156 | 0.623 | NA |
| TRINITY_DN1773_c0_g1_i7 | 2.459401398 | 0.341777266 | 0.999970678 | 5.532 | 1.074 | 0 | 1.216 | NA |
| TRINITY_DN72_c1_g1_i2 | 2.456373413 | 0.338698743 | 0.999970678 | 14.725 | 4.829 | 0 | 3.589 | NA |
| TRINITY_DN7897_c0_g1_i3 | -2.45607212 | 0.372593569 | 0.999970678 | 5.505 | 0 | 3.189 | 26.813 | OTU |
| TRINITY_DN960_c0_g1_i3 | 2.456042293 | 0.00445295 | 0.240872165 | 128.15 | 190.5 | 14.632 | 40.175 | Epimerase |
| TRINITY_DN2950_c0_g1_i2 | 2.454759048 | 0.393896242 | 0.999970678 | 0 | 12.2 | 0.944 | 1.056 | AIG1 |
| TRINITY_DN1148_c0_g1_i6 | 2.454399167 | 0.429209762 | 0.999970678 | 0 | 24.4 | 0.256 | 4.092 | Glyco_hydro_1 |
| TRINITY_DN4235_c1_g1_i3 | 2.453486768 | 0.55403364 | 0.999970678 | 1.172 | 0 | 0 | 0.221 | HEAT |
| TRINITY_DN28440_c0_g1_i6 | 2.45342352 | 0.03776306 | 0.999970678 | 0.518 | 0.572 | 0.078 | 0.101 | NA |
| TRINITY_DN4836_c0_g1_i1 | -2.45288086 | 0.022396516 | 0.916966136 | 3.833 | 2.355 | 7.044 | 25.285 | Pollen_Ole_e_I |
| TRINITY_DN40966_c2_g1_i1 | 2.45011104 | 0.13154135 | 0.999970678 | 0.563 | 1.084 | 0.211 | 0.04 | NA |
| TRINITY_DN17795_c0_g1_i2 | -2.44944725 | 0.330321348 | 0.999970678 | 0.718 | 0 | 1.211 | 2.423 | NA |
| TRINITY_DN2723_c0_g1_i5 | 2.448132481 | 0.336997345 | 1 | 1.008 | 0.71 | 0.267 | 0 | NA |
| TRINITY_DN16882_c0_g2_i3 | -2.44712368 | 0.559848119 | 0.999970678 | 0 | 0.532 | 2.367 | 0 | EAP30 |
| TRINITY_DN12550_c0_g1_i1 | -2.44452417 | 0.1369093 | 0.999970678 | 0.282 | 5.243 | 14.366 | 12.135 | NA |
| TRINITY_DN395_c1_g1_i3 | 2.444347611 | 0.555091593 | 0.999970678 | 0 | 7.184 | 1.078 | 0 | Glyco_transf_8 |
| TRINITY_DN775_c2_g1_i1 | -2.44426964 | 0.358635461 | 0.999970678 | 0.936 | 0 | 0.578 | 4.404 | NA |
| TRINITY_DN22661_c0_g5_i1 | -2.44405527 | 0.381231892 | 0.999970678 | 1.681 | 0 | 7.077 | 0.694 | NA |
| TRINITY_DN1442_c0_g1_i4 | -2.44397906 | 0.2351356 | 0.999970678 | 3.007 | 50.98 | 6.488 | 276.255 | DUF4228 |
| TRINITY_DN23_c1_g1_i15 | 2.443352886 | 0.12699491 | 0.999970678 | 27.652 | 1.577 | 1.911 | 3.127 | NA |
| TRINITY_DN2023_c0_g4_i1 | 2.443165307 | 0.043832539 | 0.999970678 | 2.898 | 16.47 | 1.567 | 1.629 | Cation_ATPase_N |
| TRINITY_DN10588_c0_g2_i4 | 2.443031583 | 0.335728592 | 1 | 0.699 | 0.857 | 0 | 0.292 | NA |
| TRINITY_DN165_c0_g1_i3 | 2.442866635 | 0.415926759 | 0.999970678 | 0 | 24.46 | 3.244 | 0.503 | ADH_N |
| TRINITY_DN6014_c0_g1_i5 | 2.442851412 | 0.184870842 | 0.999970678 | 2.662 | 4.041 | 0.955 | 0.08 | NA |
| TRINITY_DN4899_c0_g1_i3 | 2.442343841 | 0.070849411 | 0.999970678 | 0.454 | 1.685 | 0.189 | 0.151 | RRM_1 |
| TRINITY_DN74309_c0_g1_i1 | 2.441318345 | 0.141680637 | 0.999970678 | 0.418 | 2.208 | 0.333 | 0.08 | NA |
| TRINITY_DN5412_c0_g1_i18 | 2.440508831 | 0.186501093 | 0.999970678 | 14.552 | 0.907 | 0.267 | 2.574 | NA |
| TRINITY_DN2160_c0_g1_i2 | -2.44020197 | 0.077304856 | 0.999970678 | 0.282 | 0.384 | 0.556 | 2.865 | NA |
| TRINITY_DN3228_c0_g1_i13 | 2.439982551 | 0.558489948 | 0.999970678 | 0 | 1.744 | 0 | 0.322 | Serpin |
| TRINITY_DN9357_c0_g1_i3 | -2.43897582 | 0.340696575 | 0.999970678 | 0.345 | 0 | 1.111 | 0.583 | NA |

| TRINITY_DN20691_c0_g1_i3 | -2.43890954 | 0.347854229 | 1 | 0.273 | 0 | 0.678 | 0.643 | Kelch_1 |
| --- | --- | --- | --- | --- | --- | --- | --- | --- |
| TRINITY_DN251_c0_g1_i8 | -2.43809039 | 0.335770491 | 0.999970678 | 0.672 | 0 | 1.678 | 1.599 | NA |
| TRINITY_DN13513_c0_g1_i2 | -2.43722164 | 0.121184025 | 0.999970678 | 0.236 | 0.039 | 0.389 | 1.066 | NA |
| TRINITY_DN424_c0_g1_i7 | -2.43511252 | 0.349744842 | 0.999970678 | 2.28 | 0 | 8.311 | 2.383 | NA |
| TRINITY_DN3627_c0_g1_i11 | -2.43482417 | 0.561567313 | 0.999970678 | 0.309 | 0 | 1.389 | 0 | Pro_CA |
| TRINITY_DN420_c0_g1_i7 | 2.433282571 | 0.120185496 | 0.999970678 | 0.672 | 9.333 | 0.544 | 1.166 | NA |
| TRINITY_DN1467_c0_g1_i11 | -2.43310355 | 0.004897677 | 0.262322807 | 3.561 | 2.474 | 17.477 | 11.411 | NA |
| TRINITY_DN8656_c0_g1_i2 | -2.43176134 | 0.351161279 | 0.999970678 | 0 | 1.311 | 4.666 | 1.377 | - |
| TRINITY_DN9839_c0_g1_i2 | 2.431602598 | 0.217153631 | 0.999970678 | 3.824 | 0.138 | 0.122 | 0.583 | Exostosin |
| TRINITY_DN504_c0_g1_i9 | 2.431313478 | 0.327275278 | 0.999970678 | 2.017 | 1.695 | 0.567 | 0 | DUF3522 |
| TRINITY_DN1867_c0_g1_i6 | -2.43088729 | 0.431671392 | 0.999970678 | 0 | 20.01 | 1.022 | 103.161 | B_lectin |
| TRINITY_DN478_c0_g1_i3 | 2.430403176 | 0.224334465 | 0.999970678 | 9.574 | 0.266 | 1.367 | 0.211 | Seryl_tRNA_N |
| TRINITY_DN10463_c0_g1_i1 | -2.4301325 | 0.566205854 | 0.999970678 | 0.245 | 0 | 1.144 | 0 | NA |
| TRINITY_DN1183_c0_g1_i7 | 2.429855184 | 0.395075042 | 0.999970678 | 5.741 | 0 | 0.567 | 0.402 | LRR_8 |
| TRINITY_DN7449_c0_g1_i2 | -2.42901618 | 0.043234752 | 0.999970678 | 0.273 | 0.857 | 3.189 | 2.141 | NA |
| TRINITY_DN5749_c0_g2_i3 | -2.42897412 | 0.188448345 | 0.999970678 | 5.723 | 0.443 | 2.244 | 30.805 | NA |
| TRINITY_DN4357_c0_g2_i8 | 2.428946907 | 0.318430299 | 0.999970678 | 3.597 | 4.632 | 0 | 1.488 | NA |
| TRINITY_DN1293_c0_g1_i1 | 2.428626347 | 0.325300219 | 0.999970678 | 0.627 | 0.542 | 0.178 | 0 | NA |
| TRINITY_DN2609_c3_g1_i1 | -2.4274632 | 0.011155041 | 0.531570661 | 252.32 | 100.2 | 562.499 | 1231.377 | NA |
| TRINITY_DN1344_c1_g1_i9 | 2.42739035 | 0.562746313 | 0.999970678 | 0 | 2.907 | 0.433 | 0 | NA |
| TRINITY_DN10337_c0_g3_i2 | -2.42681833 | 0.353438966 | 0.999970678 | 3.561 | 0 | 5.4 | 12.979 | NA |
| TRINITY_DN825_c0_g1_i7 | 2.425758831 | 0.092302921 | 0.999970678 | 2.698 | 0.69 | 0.178 | 0.422 | NA |
| TRINITY_DN5653_c0_g1_i2 | 2.42553699 | 0.316924666 | 0.999970678 | 0.727 | 0.867 | 0 | 0.302 | NA |
| TRINITY_DN36_c1_g1_i5 | -2.42541764 | 0.07201573 | 0.999970678 | 0.318 | 0.118 | 1.411 | 0.653 | RVT_2 |
| TRINITY_DN9382_c0_g1_i2 | 2.425356539 | 0.00995236 | 0.48243843 | 2.571 | 3.863 | 0.311 | 0.824 | NA |
| TRINITY_DN1424_c0_g2_i3 | -2.42435904 | 0.050492845 | 0.999970678 | 5.178 | 21.15 | 27.698 | 106.258 | NA |
| TRINITY_DN520_c1_g2_i1 | -2.42415278 | 0.230387667 | 0.999970678 | 0.145 | 1.932 | 0.744 | 9.923 | LRR_1 |
| TRINITY_DN2051_c0_g1_i4 | -2.42389647 | 0.362674217 | 0.999970678 | 3.343 | 0 | 10.866 | 5.127 | RabGAP-TBC |
| TRINITY_DN11654_c0_g1_i1 | -2.42373172 | 0.365396232 | 0.999970678 | 0.727 | 0.02 | 3.322 | 0.02 | ACAS_N |
| TRINITY_DN1607_c0_g1_i1 | 2.423038886 | 0.44998866 | 0.999970678 | 1.735 | 0.01 | 0 | 0.332 | NA |
| TRINITY_DN2740_c1_g1_i6 | -2.42243956 | 0.339280879 | 0.999970678 | 1.226 | 0 | 1.978 | 4.303 | Lectin_legB |
| TRINITY_DN95804_c0_g1_i5 | 2.422327567 | 0.43279187 | 0.999970678 | 7.912 | 0 | 1.167 | 0.09 | NA |
| TRINITY_DN2359_c0_g1_i2 | 2.42132929 | 0.340007774 | 0.999970678 | 0.263 | 1.074 | 0 | 0.251 | UCH |
| TRINITY_DN5279_c0_g1_i1 | 2.421049112 | 0.558947796 | 0.999970678 | 5.823 | 0 | 0 | 1.086 | NA |
| TRINITY_DN11267_c0_g1_i1 | -2.41989264 | 0.048125912 | 0.999970678 | 0.309 | 0.227 | 0.744 | 2.031 | - |
| TRINITY_DN49_c0_g2_i2 | 2.419286023 | 0.087033657 | 0.999970678 | 12.172 | 1.597 | 1.522 | 0.784 | G-alpha |
| TRINITY_DN4532_c0_g2_i1 | -2.41886165 | 0.560192747 | 0.999970678 | 2.843 | 0 | 12.644 | 0 | NA |
| TRINITY_DN20963_c0_g1_i8 | 2.418824333 | 0.321233918 | 0.999970678 | 9.048 | 13.57 | 0 | 4.142 | NA |
| TRINITY_DN393_c0_g1_i14 | 2.418199755 | 0.57658988 | 0.999970678 | 21.565 | 0 | 3.389 | 0 | NA |
| TRINITY_DN4987_c0_g1_i5 | 2.417247709 | 0.096461108 | 0.999970678 | 36.754 | 38 | 0.911 | 12.768 | NA |
| TRINITY_DN3590_c0_g1_i2 | -2.41710143 | 0.348259247 | 0.999970678 | 1.508 | 0 | 1.555 | 6.314 | NA |
| TRINITY_DN3248_c0_g1_i3 | -2.41689795 | 0.380812823 | 0.999970678 | 8.984 | 0 | 19.51 | 25.174 | NA |
| TRINITY_DN8010_c0_g1_i2 | -2.41539819 | 0.0783904 | 0.999970678 | 0.209 | 0.246 | 0.422 | 1.94 | Glyco_hydro_17 |
| TRINITY_DN15556_c0_g1_i5 | -2.41534035 | 0.56342252 | 0.999970678 | 0 | 0.414 | 1.8 | 0 | NA |
| TRINITY_DN4333_c0_g1_i10 | -2.41531505 | 0.39259997 | 0.999970678 | 0.709 | 0 | 3.011 | 0.211 | NA |
| TRINITY_DN3300_c1_g1_i2 | 2.415296902 | 0.075813256 | 1 | 1.363 | 2.109 | 0.367 | 0.211 | NA |
| TRINITY_DN14280_c0_g1_i2 | 2.415279435 | 0.091735131 | 0.999970678 | 5.196 | 9.047 | 1.911 | 0.322 | NA |
| TRINITY_DN5847_c0_g1_i3 | -2.41522368 | 0.334463058 | 0.999970678 | 0 | 0.453 | 0.867 | 1.307 | Melibiase_2 |
| TRINITY_DN2841_c0_g1_i6 | 2.415119802 | 0.351010046 | 0.999970678 | 0.609 | 2.661 | 0.511 | 0 | NA |
| TRINITY_DN4349_c1_g1_i4 | 2.415064446 | 0.117265849 | 0.999970678 | 1.135 | 1.774 | 0.078 | 0.432 | NA |
| TRINITY_DN698_c2_g1_i13 | 2.414509018 | 0.339603016 | 0.999970678 | 7.204 | 3.055 | 1.611 | 0 | DUF659 |
| TRINITY_DN5093_c0_g1_i1 | -2.41439524 | 0.334143035 | 0.999970678 | 0.509 | 0 | 0.978 | 1.599 | NA |
| TRINITY_DN9148_c0_g1_i2 | 2.414179725 | 0.352258841 | 0.999970678 | 0.318 | 1.222 | 0.233 | 0 | Coilin_N |
| TRINITY_DN4952_c0_g1_i16 | 2.413989701 | 0.34976268 | 1 | 0.4 | 0.276 | 0.111 | 0 | DUF659 |
| TRINITY_DN4934_c0_g1_i6 | 2.413851756 | 0.014978888 | 0.669536213 | 2.253 | 1.35 | 0.2 | 0.442 | NA |
| TRINITY_DN7623_c0_g2_i9 | -2.4136548 | 0.563376665 | 0.999970678 | 1.708 | 0 | 7.666 | 0 | adh_short |
| TRINITY_DN1158_c2_g1_i1 | 2.4121671 | 0.327177283 | 0.999970678 | 3.988 | 0.02 | 0.567 | 0.08 | NA |
| TRINITY_DN9403_c0_g1_i1 | 2.409246122 | 0.095772923 | 0.999970678 | 1.726 | 2.296 | 0.489 | 0.191 | RHD3 |
| TRINITY_DN435_c0_g1_i6 | 2.408925143 | 0.560451467 | 0.999970678 | 0 | 2.602 | 0 | 0.493 | AA_permease_2 |
| TRINITY_DN6303_c0_g1_i5 | 2.408853274 | 0.330093094 | 0.999970678 | 1.472 | 1.399 | 0 | 0.543 | NA |
| TRINITY_DN20697_c0_g1_i3 | 2.40778613 | 0.333976963 | 1 | 0.754 | 0.621 | 0 | 0.261 | NA |
| TRINITY_DN2984_c0_g1_i12 | 2.407051376 | 0.342817111 | 0.999970678 | 1.244 | 5.085 | 0 | 1.186 | GTP1_OBG |
| TRINITY_DN186_c2_g2_i2 | 2.406363387 | 0.521817818 | 0.999970678 | 34.128 | 0 | 5.4 | 0.01 | Ras |
| TRINITY_DN6318_c0_g3_i5 | 2.406288749 | 0.338795906 | 0.999970678 | 1.272 | 2.06 | 0 | 0.633 | Myb_DNA-bindin |
| TRINITY_DN236_c0_g1_i6 | 2.405602414 | 0.414531444 | 0.999970678 | 1.026 | 0.02 | 0 | 0.201 | HAT_KAT11 |
| TRINITY_DN10324_c0_g1_i4 | -2.40539198 | 0.354969207 | 0.999970678 | 9.874 | 0 | 25.71 | 21.646 | Ribosomal_L7Ae |
| TRINITY_DN47571_c0_g1_i2 | -2.40509298 | 0.179876629 | 0.999970678 | 0.136 | 0.71 | 0.322 | 4.042 | p450 |
| TRINITY_DN7024_c0_g1_i1 | -2.40371275 | 0.089013513 | 0.999970678 | 0.772 | 0.365 | 4.289 | 0.895 | NA |
| TRINITY_DN8542_c0_g1_i3 | 2.403267389 | 0.102586199 | 0.999970678 | 0.727 | 2.907 | 0.389 | 0.181 | NA |
| TRINITY_DN3616_c0_g1_i1 | -2.40316933 | 0.562178093 | 0.999970678 | 0 | 2.04 | 8.766 | 0 | PPR |
| TRINITY_DN17457_c0_g1_i1 | 2.402933286 | 0.324050202 | 0.999970678 | 6.077 | 5.677 | 0 | 2.212 | NA |
| TRINITY_DN4301_c0_g1_i7 | -2.40284463 | 0.398960933 | 0.999970678 | 0.754 | 0 | 0.167 | 3.81 | WRKY |
| TRINITY_DN18_c0_g2_i1 | 2.40239382 | 0.33752343 | 0.999970678 | 1.344 | 1.873 | 0 | 0.613 | NA |
| TRINITY_DN692_c0_g3_i2 | 2.401949583 | 0.117985817 | 0.999970678 | 0.672 | 2.208 | 0.356 | 0.111 | NA |

| TRINITY_DN4958_c0_g1_i1 | 2.401646606 | 0.334660786 | 0.999970678 | 0.781 | 1.173 | 0.3 | 0 | Trypsin_2 |
| --- | --- | --- | --- | --- | --- | --- | --- | --- |
| TRINITY_DN14123_c0_g3_i1 | -2.40102428 | 0.346563258 | 0.999970678 | 1.626 | 0 | 4.444 | 3.398 | NA |
| TRINITY_DN2927_c0_g1_i4 | -2.40069572 | 0.335888369 | 0.999970678 | 0 | 0.325 | 0.589 | 1.015 | DND1_DSRM |
| TRINITY_DN24305_c0_g1_i1 | -2.39963554 | 0.043358668 | 0.999970678 | 0.182 | 0.424 | 1.011 | 1.9 | NA |
| TRINITY_DN12937_c0_g1_i3 | -2.39952896 | 0.354361081 | 0.999970678 | 1.163 | 0 | 1.367 | 4.494 | NA |
| TRINITY_DN9580_c0_g1_i1 | -2.39952046 | 0.101395151 | 0.999970678 | 0.681 | 3.548 | 10.088 | 9.32 | NA |
| TRINITY_DN3894_c0_g2_i1 | -2.3995001 | 0.290596925 | 0.999970678 | 0.327 | 3.587 | 16.521 | 0.322 | NA |
| TRINITY_DN2146_c0_g1_i2 | 2.399349532 | 0.326503923 | 0.999970678 | 2.97 | 2.148 | 0 | 0.975 | NA |
| TRINITY_DN1041_c1_g1_i10 | -2.39930967 | 0.226144894 | 0.999970678 | 5.85 | 0.227 | 23.532 | 3.67 | Aldedh |
| TRINITY_DN1292_c3_g1_i1 | -2.39922748 | 0.076925742 | 1 | 0.118 | 0.099 | 0.411 | 0.633 | UDPGT |
| TRINITY_DN455_c0_g1_i11 | -2.39837683 | 0.34418533 | 0.999970678 | 0 | 1.804 | 2.433 | 6.414 | NA |
| TRINITY_DN406_c0_g1_i6 | -2.39798657 | 0.077155514 | 0.999970678 | 0.409 | 0.148 | 0.522 | 2.373 | Peptidase_S10 |
| TRINITY_DN3453_c1_g1_i5 | -2.39787828 | 0.439572308 | 0.999970678 | 4.115 | 0.217 | 18.977 | 0 | NA |
| TRINITY_DN15788_c0_g1_i4 | -2.39781116 | 0.211681826 | 0.999970678 | 1.226 | 0.237 | 6.255 | 0.282 | NA |
| TRINITY_DN983_c0_g1_i12 | 2.397491398 | 0.11594508 | 0.999970678 | 5.65 | 1.754 | 0.156 | 1.206 | NA |
| TRINITY_DN2369_c0_g1_i1 | 2.396496011 | 0.346944625 | 0.999970678 | 0.318 | 1.34 | 0 | 0.312 | LRR_1 |
| TRINITY_DN7408_c0_g1_i2 | 2.396074377 | 0.157255329 | 0.999970678 | 0.836 | 8.702 | 0.278 | 1.458 | - |
| TRINITY_DN9796_c0_g1_i10 | 2.395898181 | 0.188837776 | 1 | 1.163 | 0.976 | 0.278 | 0.05 | NA |
| TRINITY_DN761_c0_g1_i5 | 2.394732428 | 0.027653916 | 0.999970678 | 114.01 | 42.37 | 6.911 | 21.404 | NA |
| TRINITY_DN1486_c1_g1_i7 | -2.39283779 | 0.168637731 | 0.999970678 | 0.309 | 1.478 | 0.678 | 8.455 | Reticulon |
| TRINITY_DN223_c0_g1_i7 | -2.39281068 | 0.342498808 | 0.999970678 | 0 | 2.237 | 6.766 | 3.237 | NA |
| TRINITY_DN4219_c0_g1_i7 | 2.392782444 | 0.354308466 | 0.999970678 | 2.28 | 1.498 | 0.6 | 0 | NA |
| TRINITY_DN810_c0_g1_i2 | 2.392047641 | 0.250909271 | 0.999970678 | 1.099 | 23.03 | 3.633 | 0.161 | NA |
| TRINITY_DN3938_c0_g1_i2 | -2.39173485 | 0.354296206 | 0.999970678 | 0.699 | 0 | 2.2 | 1.046 | NA |
| TRINITY_DN33838_c0_g1_i4 | -2.39140396 | 0.419040057 | 0.999970678 | 1.535 | 0 | 0.222 | 7.993 | NA |
| TRINITY_DN4429_c0_g1_i4 | 2.390316858 | 0.338281786 | 0.999970678 | 0.718 | 1.38 | 0.333 | 0 | DUF5110 |
| TRINITY_DN14267_c0_g1_i1 | 2.389673178 | 0.32719525 | 0.999970678 | 1.172 | 95.94 | 0.156 | 18.107 | p450 |
| TRINITY_DN3104_c0_g2_i3 | 2.389604788 | 0.16840548 | 0.999970678 | 4.46 | 0.67 | 0.067 | 0.905 | 2OG-FeII_Oxy |
| TRINITY_DN12199_c0_g1_i5 | 2.389464667 | 0.563466461 | 0.999970678 | 7.903 | 0 | 0 | 1.538 | NA |
| TRINITY_DN1808_c0_g2_i4 | -2.38910737 | 0.344551498 | 0.999970678 | 1.317 | 0 | 2.322 | 4.142 | NA |
| TRINITY_DN11087_c0_g2_i1 | -2.38899698 | 0.567984306 | 0.999970678 | 0 | 5.184 | 22.054 | 0 | NA |
| TRINITY_DN2903_c0_g1_i1 | 2.388853865 | 0.571243287 | 0.999970678 | 0 | 32.36 | 0 | 6.082 | RRM_1 |
| TRINITY_DN1699_c1_g1_i3 | 2.388844908 | 0.120748143 | 1 | 0.536 | 0.818 | 0.056 | 0.211 | SH3_9 |
| TRINITY_DN1442_c0_g1_i5 | -2.38877604 | 0.260534836 | 0.999970678 | 1.099 | 26.07 | 3.144 | 134.157 | NA |
| TRINITY_DN3947_c0_g1_i5 | 2.388197251 | 0.418888783 | 0.999970678 | 3.624 | 0 | 0.478 | 0.121 | NA |
| TRINITY_DN2915_c0_g1_i8 | 2.388040652 | 0.030695242 | 0.999970678 | 1.49 | 1.636 | 0.322 | 0.201 | NA |
| TRINITY_DN2928_c0_g1_i7 | 2.387835169 | 0.205103448 | 0.999970678 | 40.206 | 2.907 | 1.233 | 6.565 | NB-ARC |
| TRINITY_DN1877_c0_g1_i4 | -2.3873618 | 0.271637519 | 0.999970678 | 0.917 | 4.937 | 24.643 | 0.241 | NA |
| TRINITY_DN3809_c0_g1_i4 | -2.38721903 | 0.097341078 | 0.999970678 | 11.882 | 10.88 | 90.695 | 9.28 | Calreticulin |
| TRINITY_DN12159_c0_g2_i1 | -2.38701224 | 0.360271995 | 1 | 0.173 | 0 | 0.367 | 0.473 | NA |
| TRINITY_DN3878_c1_g1_i3 | 2.386007977 | 0.355241184 | 1 | 0.309 | 0.434 | 0.122 | 0 | NA |
| TRINITY_DN1354_c0_g1_i2 | 2.385527025 | 0.162099696 | 0.999970678 | 5.151 | 93.21 | 2.667 | 15.362 | NA |
| TRINITY_DN207_c0_g2_i1 | -2.3853439 | 0.37041301 | 0.999970678 | 5.36 | 0 | 10.422 | 15.845 | NA |
| TRINITY_DN2377_c0_g1_i7 | 2.38530128 | 0.394201971 | 0.999970678 | 17.541 | 1.528 | 0 | 3.71 | NA |
| TRINITY_DN2369_c0_g1_i6 | -2.38504064 | 0.023668057 | 0.957033127 | 0.109 | 0.148 | 0.511 | 0.684 | LRR_1 |
| TRINITY_DN434_c0_g1_i10 | -2.38419341 | 0.008638667 | 0.428940015 | 24.39 | 20.9 | 123.248 | 76.579 | NA |
| TRINITY_DN10982_c0_g1_i3 | 2.383512212 | 0.562768165 | 0.999970678 | 0 | 3.676 | 0 | 0.704 | Pkinase |
| TRINITY_DN6002_c0_g1_i3 | 2.382246818 | 0.352586223 | 0.999970678 | 1.69 | 2.01 | 0.589 | 0 | NA |
| TRINITY_DN634_c0_g1_i4 | 2.381351881 | 0.564251237 | 0.999970678 | 11.409 | 0 | 0 | 2.232 | Caleosin |
| TRINITY_DN5710_c0_g1_i3 | 2.379489085 | 0.354924211 | 0.999970678 | 9.892 | 5.43 | 0 | 2.966 | WRKY |
| TRINITY_DN11797_c0_g1_i1 | -2.37935639 | 0.567146401 | 0.999970678 | 1.69 | 0 | 7.344 | 0 | MIP |
| TRINITY_DN3844_c0_g1_i8 | -2.37885696 | 0.111858056 | 0.999970678 | 0.336 | 0.069 | 0.744 | 1.287 | NA |
| TRINITY_DN537_c0_g1_i6 | -2.37878905 | 0.415354039 | 0.999970678 | 0 | 5.943 | 1.211 | 29.025 | NA |
| TRINITY_DN1952_c0_g2_i2 | -2.37845811 | 0.407324577 | 0.999970678 | 0.491 | 1.36 | 0 | 9.561 | NA |
| TRINITY_DN45_c1_g1_i2 | -2.37736187 | 0.348152753 | 0.999970678 | 0.182 | 0 | 0.489 | 0.352 | GAF |
| TRINITY_DN3125_c0_g2_i3 | 2.377012001 | 0.112568252 | 0.999970678 | 2.071 | 0.838 | 0.378 | 0.101 | NA |
| TRINITY_DN231_c0_g1_i1 | 2.374896511 | 0.586008012 | 0.999970678 | 0 | 110.2 | 0 | 20.892 | Arf |
| TRINITY_DN13208_c0_g1_i5 | -2.37469526 | 0.374983464 | 0.999970678 | 0 | 0.276 | 1.033 | 0.171 | - |
| TRINITY_DN4451_c1_g1_i1 | -2.37434119 | 0.103518628 | 0.999970678 | 0.681 | 0.177 | 1.244 | 2.845 | Aa_trans |
| TRINITY_DN1719_c0_g1_i2 | 2.373453404 | 0.013418264 | 0.615732479 | 41.532 | 82.76 | 5.711 | 17.021 | NA |
| TRINITY_DN6514_c0_g1_i3 | 2.372903586 | 0.430138073 | 0.999970678 | 0 | 10.78 | 1.544 | 0.201 | NA |
| TRINITY_DN191_c0_g1_i3 | -2.37186374 | 0.473012832 | 0.999970678 | 0 | 16 | 66.996 | 0.382 | Inos-1-P_synth |
| TRINITY_DN19973_c0_g1_i1 | 2.371356274 | 0.019852332 | 0.842170964 | 6.468 | 4.74 | 1.322 | 0.573 | NA |
| TRINITY_DN1043_c0_g1_i7 | 2.370681975 | 0.333493588 | 0.999970678 | 0.173 | 11.69 | 0.056 | 2.202 | ADH_N |
| TRINITY_DN1023_c0_g1_i10 | 2.370528062 | 0.009796426 | 0.478411122 | 5.523 | 11.06 | 1.6 | 1.247 | NA |
| TRINITY_DN0_c1_g1_i3 | -2.36954615 | 0.254166174 | 0.999970678 | 2.135 | 0.168 | 0.389 | 11.582 | - |
| TRINITY_DN26213_c0_g7_i2 | 2.36927669 | 0.340608027 | 1 | 1.517 | 1.439 | 0 | 0.563 | NA |
| TRINITY_DN4052_c0_g1_i9 | -2.36919613 | 0.234134021 | 0.999970678 | 1.39 | 0.296 | 0.189 | 8.475 | NA |
| TRINITY_DN2650_c0_g2_i1 | -2.36840515 | 0.271746475 | 0.999970678 | 6.222 | 1.301 | 32.031 | 0.322 | NA |
| TRINITY_DN1158_c0_g4_i1 | 2.368051083 | 0.210283139 | 0.999970678 | 9.111 | 17.7 | 4.211 | 0.07 | NA |
| TRINITY_DN221_c0_g1_i2 | -2.36694106 | 0.375590959 | 0.999970678 | 0 | 0.857 | 3.178 | 0.533 | NA |
| TRINITY_DN3307_c0_g1_i4 | 2.366439846 | 0.342117578 | 0.999970678 | 3.361 | 2.296 | 0 | 1.106 | NA |
| TRINITY_DN30748_c0_g1_i5 | -2.36620188 | 0.135934395 | 0.999970678 | 40.124 | 16.07 | 225.953 | 17.111 | NA |
| TRINITY_DN10515_c0_g1_i2 | -2.36558912 | 0.568571792 | 0.999970678 | 0 | 17.96 | 72.351 | 0 | NA |

| TRINITY_DN3334_c0_g1_i8 | -2.36442624 | 0.359534972 | 0.999970678 | 2.371 | 0 | 6.311 | 4.786 | BCDHK_Adom3 |
| --- | --- | --- | --- | --- | --- | --- | --- | --- |
| TRINITY_DN19205_c0_g1_i4 | 2.364072095 | 0.089482914 | 0.999970678 | 0.709 | 2.7 | 0.233 | 0.392 | Motile_Sperm |
| TRINITY_DN1318_c0_g2_i2 | 2.363807771 | 0.352692981 | 0.999970678 | 4.805 | 3.893 | 0 | 1.709 | NA |
| TRINITY_DN5438_c0_g1_i7 | 2.363661368 | 0.151040896 | 0.999970678 | 19.857 | 4.287 | 3.522 | 0.513 | NA |
| TRINITY_DN4301_c0_g1_i5 | 2.363304312 | 0.415536781 | 0.999970678 | 18.231 | 0 | 1.9 | 1.317 | WRKY |
| TRINITY_DN12159_c0_g2_i4 | -2.36147718 | 0.362966546 | 1 | 0.2 | 0 | 0.389 | 0.513 | NA |
| TRINITY_DN16153_c0_g1_i1 | 2.361308467 | 0.344743266 | 0.999970678 | 0.491 | 0.443 | 0.156 | 0 | Condensin2nSMC |
| TRINITY_DN3844_c0_g1_i9 | -2.36106068 | 0.358456698 | 1 | 0.872 | 0 | 2.278 | 1.699 | NA |
| TRINITY_DN3214_c1_g1_i6 | 2.360779013 | 0.568087729 | 0.999970678 | 6.268 | 0 | 0 | 1.247 | NA |
| TRINITY_DN3389_c0_g1_i8 | -2.35936847 | 0.388968748 | 0.999970678 | 24.754 | 0 | 89.761 | 18.539 | NA |
| TRINITY_DN10364_c0_g1_i3 | -2.35935729 | 0.36394196 | 0.999970678 | 1.026 | 0 | 3.289 | 1.387 | NA |
| TRINITY_DN1100_c0_g1_i2 | -2.3592147 | 0.352000526 | 0.999970678 | 2.507 | 0 | 4.255 | 8.003 | PUNUT |
| TRINITY_DN4811_c0_g1_i6 | 2.359071583 | 0.568120966 | 0.999970678 | 3.979 | 0 | 0 | 0.794 | NA |
| TRINITY_DN3154_c0_g1_i17 | -2.35858223 | 0.188424956 | 0.999970678 | 65.123 | 1.409 | 150.424 | 162.81 | NA |
| TRINITY_DN139184_c0_g1_i1 | 2.358193251 | 0.577248206 | 0.999970678 | 2.425 | 0 | 0.4 | 0 | NA |
| TRINITY_DN2308_c2_g1_i4 | 2.35817644 | 0.348103406 | 0.999970678 | 1.045 | 1.961 | 0 | 0.583 | - |
| TRINITY_DN7298_c0_g5_i2 | -2.3575522 | 0.465457063 | 0.999970678 | 0.754 | 0.039 | 3.4 | 0 | NA |
| TRINITY_DN414_c0_g1_i3 | -2.35617208 | 0.044399194 | 0.999970678 | 0.981 | 2.434 | 10.866 | 4.102 | Oxidored_FMN |
| TRINITY_DN12881_c1_g1_i3 | 2.356141827 | 0.371539287 | 0.999970678 | 0.427 | 2.178 | 0.411 | 0 | PPO1_DWL |
| TRINITY_DN13_c2_g1_i6 | 2.356018126 | 0.052932826 | 0.999970678 | 3.597 | 4.139 | 0.378 | 1.086 | NA |
| TRINITY_DN2754_c0_g1_i1 | 2.35525936 | 0.571320045 | 0.999970678 | 0 | 28.16 | 0 | 5.419 | NA |
| TRINITY_DN1907_c0_g1_i5 | 2.355162338 | 0.480378534 | 0.999970678 | 0.136 | 84.51 | 0 | 16.096 | NA |
| TRINITY_DN18557_c0_g1_i2 | -2.35396495 | 0.578065714 | 0.999970678 | 3.779 | 0 | 16.01 | 0 | NA |
| TRINITY_DN6816_c0_g1_i3 | 2.353952081 | 0.034755913 | 0.999970678 | 2.171 | 3.045 | 0.667 | 0.231 | DUF3452 |
| TRINITY_DN193_c0_g1_i23 | 2.353642135 | 0.423697634 | 0.999970678 | 189.3 | 64.25 | 0 | 50.088 | NA |
| TRINITY_DN555_c1_g1_i4 | -2.3523513 | 0.368809309 | 0.999970678 | 0 | 3.085 | 2.222 | 12.426 | NA |
| TRINITY_DN13744_c0_g1_i4 | -2.35171177 | 0.575198427 | 0.999970678 | 0.491 | 0 | 2.089 | 0 | WD40 |
| TRINITY_DN5546_c0_g1_i3 | 2.351653304 | 0.297248223 | 0.999970678 | 0.073 | 8.16 | 1.111 | 0.231 | Aldo_ket_red |
| TRINITY_DN18847_c2_g1_i3 | 2.351243059 | 0.412591081 | 0.999970678 | 0 | 3.252 | 0.156 | 0.442 | HSP70 |
| TRINITY_DN5374_c0_g1_i1 | -2.35090364 | 0.441825127 | 0.999970678 | 0.109 | 1.104 | 0 | 6.062 | AUX_IAA |
| TRINITY_DN3537_c0_g1_i7 | 2.350695644 | 0.375434543 | 0.999970678 | 8.176 | 7.874 | 2.611 | 0 | NABP |
| TRINITY_DN3459_c0_g1_i8 | 2.350617275 | 0.096025188 | 0.999970678 | 6.232 | 0.936 | 0.644 | 0.643 | NA |
| TRINITY_DN32709_c0_g1_i1 | 2.350544722 | 0.345334184 | 0.999970678 | 0.609 | 0.512 | 0.178 | 0 | NA |
| TRINITY_DN12808_c0_g1_i1 | -2.35043127 | 0.266311812 | 0.999970678 | 0.763 | 0.049 | 3.255 | 0.241 | NA |
| TRINITY_DN304_c0_g1_i2 | -2.34935516 | 0.38338398 | 0.999970678 | 1.072 | 0 | 0.556 | 4.866 | NA |
| TRINITY_DN6750_c0_g1_i5 | 2.349293409 | 0.348975201 | 0.999970678 | 5.578 | 3.824 | 0 | 1.86 | NA |
| TRINITY_DN9034_c0_g1_i8 | 2.349164072 | 0.148409954 | 0.999970678 | 2.298 | 3.627 | 0.811 | 0.141 | - |
| TRINITY_DN26793_c0_g1_i10 | -2.34806308 | 0.061165118 | 0.999970678 | 0.572 | 0.118 | 1.755 | 1.438 | - |
| TRINITY_DN1922_c3_g1_i12 | 2.347329626 | 0.18294546 | 0.999970678 | 0.536 | 1.813 | 0.344 | 0.04 | NA |
| TRINITY_DN8810_c0_g1_i3 | -2.34712759 | 0.080375393 | 0.999970678 | 2.725 | 1.764 | 15.666 | 3.77 | - |
| TRINITY_DN2598_c0_g1_i4 | 2.345982133 | 0.030618958 | 0.999970678 | 8.43 | 31.19 | 4.078 | 2.815 | NA |
| TRINITY_DN5212_c0_g1_i1 | 2.345975525 | 0.190420131 | 0.999970678 | 0.627 | 13.08 | 0.567 | 1.96 | NA |
| TRINITY_DN7254_c0_g1_i1 | 2.345329383 | 0.115403188 | 0.999970678 | 0.509 | 0.345 | 0.122 | 0.03 | NA |
| TRINITY_DN22498_c0_g1_i2 | -2.34393092 | 0.580592351 | 0.999970678 | 0.981 | 0 | 4.178 | 0 | MAPEG |
| TRINITY_DN4451_c1_g1_i3 | 2.343156986 | 0.357654087 | 0.999970678 | 1.081 | 4.386 | 0 | 1.076 | Aa_trans |
| TRINITY_DN58009_c0_g1_i4 | -2.34295826 | 0.101850183 | 0.999970678 | 3.67 | 9.461 | 5.655 | 59.407 | DUF569 |
| TRINITY_DN29463_c0_g1_i1 | -2.34193537 | 0.153347467 | 0.999970678 | 0.836 | 0.867 | 0.456 | 8.224 | NA |
| TRINITY_DN469_c0_g1_i1 | -2.34151385 | 0.31459923 | 0.999970678 | 0.009 | 5.716 | 2.133 | 26.21 | p450 |
| TRINITY_DN2881_c0_g1_i6 | 2.341239047 | 0.144662838 | 0.999970678 | 22.637 | 1.587 | 1.778 | 2.634 | NA |
| TRINITY_DN11134_c2_g1_i1 | 2.34090565 | 0.065617519 | 1 | 0.699 | 0.65 | 0.144 | 0.101 | Pkinase |
| TRINITY_DN5000_c0_g2_i2 | 2.340621367 | 0.585535554 | 0.999970678 | 0 | 37.37 | 6 | 0 | NA |
| TRINITY_DN29_c1_g2_i2 | -2.34010471 | 0.378791463 | 0.999970678 | 0.899 | 0 | 0.6 | 3.951 | NA |
| TRINITY_DN641_c0_g1_i8 | 2.339960044 | 0.587994993 | 0.999970678 | 0 | 52.84 | 0 | 10.285 | NA |
| TRINITY_DN12000_c0_g1_i1 | -2.33983332 | 0.087314275 | 0.999970678 | 0.663 | 0.237 | 1.1 | 3.036 | NA |
| TRINITY_DN11073_c0_g2_i1 | -2.33974765 | 0.055712403 | 1 | 0.409 | 0.375 | 1.867 | 1.749 | NA |
| TRINITY_DN5223_c0_g1_i3 | -2.33870784 | 0.405946514 | 0.999970678 | 0.418 | 0 | 1.622 | 0.151 | R3H |
| TRINITY_DN19200_c0_g2_i5 | -2.3381281 | 0.583383381 | 0.999970678 | 0.509 | 0 | 2.111 | 0 | NA |
| TRINITY_DN18112_c0_g1_i2 | 2.337775209 | 0.169800092 | 0.999970678 | 1.699 | 2.562 | 0.656 | 0.05 | NA |
| TRINITY_DN1005_c0_g1_i8 | -2.33679399 | 0.189952101 | 0.999970678 | 0.736 | 0.177 | 0.378 | 4.112 | NA |
| TRINITY_DN2156_c0_g1_i2 | 2.335820519 | 0.43197677 | 0.999970678 | 10.437 | 0 | 0.244 | 1.78 | NA |
| TRINITY_DN2136_c0_g1_i3 | -2.33508949 | 0.38211929 | 0.999970678 | 0 | 2.868 | 9.855 | 1.89 | NA |
| TRINITY_DN9228_c0_g1_i8 | -2.33409733 | 0.324845256 | 0.999970678 | 9.329 | 0.276 | 38.531 | 1.468 | NA |
| TRINITY_DN1467_c0_g1_i1 | 2.333953556 | 0.071657522 | 0.999970678 | 1.508 | 4.701 | 0.711 | 0.372 | NA |
| TRINITY_DN16_c0_g1_i6 | -2.33208586 | 0.422823772 | 0.999970678 | 10.156 | 0 | 10.288 | 39.732 | UDPGT |
| TRINITY_DN6262_c0_g1_i1 | -2.33140184 | 0.009280142 | 0.457316457 | 0.854 | 0.67 | 3.2 | 3.82 | NA |
| TRINITY_DN2801_c0_g1_i5 | 2.331354578 | 0.469530804 | 0.999970678 | 11.419 | 0.069 | 0 | 2.332 | Gelsolin |
| TRINITY_DN11226_c0_g1_i4 | -2.32903834 | 0.352968615 | 0.999970678 | 0 | 0.512 | 0.689 | 1.709 | WEMBL |
| TRINITY_DN3532_c0_g1_i5 | 2.328586601 | 0.349505869 | 0.999970678 | 8.275 | 4.731 | 0 | 2.614 | - |
| TRINITY_DN6320_c3_g1_i2 | -2.32855513 | 0.357422893 | 0.999970678 | 0 | 0.641 | 1.578 | 1.267 | TPR_1 |
| TRINITY_DN973_c0_g1_i4 | 2.328523664 | 0.231874248 | 0.999970678 | 0.718 | 0.246 | 0.156 | 0.01 | Cyclin_N |
| TRINITY_DN1492_c0_g2_i2 | -2.32821443 | 0.076654636 | 0.999970678 | 0.836 | 0.65 | 0.789 | 6.565 | NA |
| TRINITY_DN722_c0_g1_i14 | 2.327951949 | 0.022663312 | 0.92556968 | 3.624 | 4.947 | 0.878 | 0.623 | NA |
| TRINITY_DN2207_c0_g1_i5 | 2.327010918 | 0.219377062 | 0.999970678 | 59.027 | 6.958 | 0.422 | 12.778 | NA |
| TRINITY_DN7657_c0_g1_i6 | -2.32694106 | 0.016781174 | 0.732987328 | 0.318 | 0.463 | 1.311 | 2.373 | NA |

| TRINITY_DN2590_c0_g3_i1 | 2.326468926 | 0.354194552 | 0.999970678 | 1.608 | 1.951 | 0.589 | 0 | NA |
| --- | --- | --- | --- | --- | --- | --- | --- | --- |
| TRINITY_DN550_c0_g1_i11 | -2.32462199 | 0.026656665 | 0.999970678 | 1.717 | 3.508 | 5.722 | 19.172 | NA |
| TRINITY_DN7943_c0_g1_i1 | 2.324053406 | 0.015554791 | 0.689212187 | 4.242 | 3.469 | 0.911 | 0.432 | NA |
| TRINITY_DN2845_c0_g2_i10 | 2.323977939 | 0.574707216 | 0.999970678 | 6.086 | 0 | 0 | 1.227 | NA |
| TRINITY_DN2613_c0_g2_i2 | 2.323905518 | 0.042284196 | 0.999970678 | 8.975 | 30.61 | 1.811 | 5.66 | EamA |
| TRINITY_DN4187_c0_g1_i1 | 2.32378664 | 0.345425524 | 0.999970678 | 4.651 | 5.322 | 0 | 1.971 | zf-RING_2 |
| TRINITY_DN75_c0_g1_i1 | -2.32378324 | 0.330128022 | 0.999970678 | 22.446 | 1.252 | 97.005 | 0.674 | - |
| TRINITY_DN528_c1_g1_i3 | -2.32327853 | 0.039298877 | 0.999970678 | 2.816 | 2.474 | 5.455 | 19.323 | EGF_CA |
| TRINITY_DN5098_c0_g2_i1 | 2.323042952 | 0.203612443 | 1 | 0.518 | 0.443 | 0.144 | 0.03 | NA |
| TRINITY_DN8736_c0_g3_i2 | 2.322597918 | 0.57444461 | 0.999970678 | 4.769 | 0 | 0 | 0.965 | IBR |
| TRINITY_DN25617_c0_g1_i1 | -2.32238323 | 0.030225022 | 0.999970678 | 0.164 | 0.315 | 0.944 | 1.267 | ABC_membrane |
| TRINITY_DN961_c5_g1_i8 | -2.32228767 | 0.574920276 | 0.999970678 | 0 | 1.124 | 4.544 | 0 | NA |
| TRINITY_DN46349_c0_g1_i2 | -2.32124994 | 0.417540285 | 0.999970678 | 2.444 | 0 | 0.489 | 11.743 | NA |
| TRINITY_DN3359_c0_g1_i4 | 2.320621297 | 0.104029824 | 0.999970678 | 1.853 | 1.202 | 0.422 | 0.101 | NA |
| TRINITY_DN4457_c0_g1_i6 | 2.320603964 | 0.093938312 | 1 | 0.382 | 0.926 | 0.111 | 0.131 | NA |
| TRINITY_DN1732_c0_g1_i9 | 2.320151671 | 0.020306991 | 0.854762061 | 1.562 | 0.857 | 0.222 | 0.211 | PWI |
| TRINITY_DN4540_c0_g1_i3 | -2.31945161 | 0.576659276 | 0.999970678 | 0.481 | 0 | 0 | 2.413 | Methyltransf_29 |
| TRINITY_DN5064_c0_g1_i8 | 2.318240751 | 0.067179703 | 0.999970678 | 2.598 | 2.907 | 0.789 | 0.151 | NA |
| TRINITY_DN310_c0_g1_i2 | 2.318188861 | 0.124101667 | 0.999970678 | 1.944 | 12.51 | 1.555 | 0.985 | NA |
| TRINITY_DN5821_c0_g1_i7 | -2.31810252 | 0.36655118 | 0.999970678 | 0 | 0.404 | 0.433 | 1.448 | HSP70 |
| TRINITY_DN3277_c2_g1_i4 | 2.317373281 | 0.150552349 | 0.999970678 | 6.995 | 0.69 | 0.3 | 1.196 | UPF0016 |
| TRINITY_DN5056_c0_g1_i3 | 2.317301095 | 0.594627022 | 0.999970678 | 42.967 | 0 | 0 | 8.787 | DHquinase_I |
| TRINITY_DN8110_c0_g1_i2 | 2.31703232 | 0.039191791 | 0.999970678 | 6.795 | 5.085 | 0.433 | 1.84 | NA |
| TRINITY_DN867_c0_g1_i8 | 2.316836684 | 0.153461886 | 0.999970678 | 4.033 | 1.606 | 0.889 | 0.08 | ANF_receptor |
| TRINITY_DN12840_c0_g1_i4 | 2.316485281 | 0.355333083 | 0.999970678 | 3.652 | 1.932 | 0 | 1.136 | NA |
| TRINITY_DN21232_c0_g1_i3 | 2.315192397 | 0.018659117 | 0.804262105 | 1.172 | 2.602 | 0.356 | 0.332 | NA |
| TRINITY_DN2698_c0_g1_i2 | -2.31456233 | 0.388964595 | 0.999970678 | 6.995 | 0 | 4.355 | 29.839 | NA |
| TRINITY_DN2419_c0_g1_i1 | -2.31451713 | 0.381132671 | 0.999970678 | 0 | 3.627 | 8.788 | 7.158 | Myb_DNA-bindin |
| TRINITY_DN11126_c0_g1_i4 | 2.313102326 | 0.074366534 | 0.999970678 | 2.48 | 6.465 | 0.267 | 1.468 | NA |
| TRINITY_DN7824_c0_g1_i4 | -2.31293151 | 0.380070368 | 0.999970678 | 1.953 | 0 | 1.533 | 7.973 | NA |
| TRINITY_DN2536_c0_g1_i7 | -2.31278534 | 0.389836949 | 0.999970678 | 7.294 | 0 | 8.111 | 26.884 | NA |
| TRINITY_DN15062_c0_g1_i2 | 2.31136375 | 0.352813387 | 0.999970678 | 0.718 | 0.759 | 0.244 | 0 | DNA_pol_B |
| TRINITY_DN1284_c0_g1_i2 | -2.31098454 | 0.066061119 | 0.999970678 | 0.227 | 0.404 | 1.278 | 1.699 | NA |
| TRINITY_DN2045_c1_g1_i4 | 2.310952933 | 0.577203262 | 0.999970678 | 0 | 5.361 | 0.878 | 0 | NA |
| TRINITY_DN5326_c0_g1_i7 | -2.31081116 | 0.414457052 | 0.999970678 | 0 | 0.581 | 0.133 | 2.704 | Glyco_hydro_16 |
| TRINITY_DN31120_c0_g1_i2 | 2.310712434 | 0.364628288 | 1 | 0.382 | 0.404 | 0 | 0.151 | NA |
| TRINITY_DN2436_c0_g1_i4 | 2.309972344 | 0.025261145 | 0.999970678 | 9.22 | 4.957 | 1.711 | 0.794 | NA |
| TRINITY_DN674_c0_g1_i6 | 2.309878148 | 0.440475192 | 0.999970678 | 13.59 | 0 | 0.311 | 2.403 | NA |
| TRINITY_DN8605_c0_g1_i2 | -2.30982248 | 0.585446293 | 0.999970678 | 0 | 0.168 | 0.667 | 0 | NA |
| TRINITY_DN583_c0_g2_i3 | -2.30913865 | 0.419444627 | 0.999970678 | 0.917 | 0 | 3.644 | 0.211 | NA |
| TRINITY_DN251_c0_g1_i15 | 2.308520461 | 0.048263877 | 0.999970678 | 19.63 | 27.38 | 1.4 | 7.741 | NA |
| TRINITY_DN5871_c0_g1_i2 | -2.30848671 | 0.353297343 | 0.999970678 | 0 | 1.941 | 4.311 | 4.253 | NA |
| TRINITY_DN1288_c0_g1_i1 | -2.30836919 | 0.58625909 | 0.999970678 | 0 | 12.67 | 51.008 | 0 | Fe-S_biosyn |
| TRINITY_DN2054_c0_g1_i1 | 2.30805639 | 0.105123153 | 0.999970678 | 9.075 | 1.902 | 1.222 | 0.754 | NA |
| TRINITY_DN1813_c1_g1_i4 | -2.30750815 | 0.371603738 | 0.999970678 | 4.46 | 0 | 11.522 | 7.369 | NA |
| TRINITY_DN2796_c0_g1_i1 | 2.306653422 | 0.586356992 | 0.999970678 | 24.763 | 0 | 4.166 | 0 | NA |
| TRINITY_DN2784_c1_g1_i2 | -2.30645912 | 0.577008999 | 0.999970678 | 2.162 | 0 | 0 | 10.878 | NA |
| TRINITY_DN1136_c0_g1_i2 | 2.306355379 | 0.396198118 | 0.999970678 | 29.605 | 28.6 | 0 | 11.813 | MFS_MOT1 |
| TRINITY_DN196_c0_g1_i6 | -2.30576769 | 0.073104842 | 0.999970678 | 2.117 | 2.099 | 2.2 | 18.197 | Lectin_legB |
| TRINITY_DN2110_c0_g1_i6 | -2.30568762 | 0.411618054 | 0.999970678 | 1.008 | 1.626 | 0 | 12.879 | NA |
| TRINITY_DN2798_c0_g1_i10 | 2.304752919 | 0.077695006 | 0.999970678 | 1.744 | 3.371 | 0.511 | 0.432 | NA |
| TRINITY_DN1474_c1_g1_i1 | 2.303405204 | 0.0199242 | 0.843216931 | 1.208 | 1.744 | 0.333 | 0.191 | NA |
| TRINITY_DN203_c0_g1_i4 | -2.30292225 | 0.30382999 | 0.999970678 | 26.952 | 0.108 | 97.383 | 17.936 | NA |
| TRINITY_DN1956_c0_g1_i20 | 2.3025991 | 0.577608525 | 0.999970678 | 2.071 | 0 | 0 | 0.432 | Filamin |
| TRINITY_DN843_c0_g1_i6 | -2.30214091 | 0.062575205 | 1 | 0.382 | 0.434 | 1.755 | 1.709 | NA |
| TRINITY_DN1218_c0_g2_i1 | -2.30172989 | 0.074595631 | 0.999970678 | 4.669 | 4.179 | 4.989 | 37.43 | - |
| TRINITY_DN20222_c0_g1_i15 | -2.30078958 | 0.047288318 | 0.999970678 | 0.245 | 0.227 | 0.689 | 1.468 | F-box |
| TRINITY_DN17313_c0_g1_i4 | 2.300785907 | 0.088958673 | 0.999970678 | 1.444 | 1.074 | 0.333 | 0.09 | NA |
| TRINITY_DN809_c0_g1_i2 | 2.300323381 | 0.42119233 | 0.999970678 | 8.348 | 0 | 0.467 | 1.146 | DUF3493 |
| TRINITY_DN8783_c0_g1_i1 | -2.30013834 | 0.416165992 | 0.999970678 | 0.491 | 1.33 | 0 | 8.847 | DUF3741 |
| TRINITY_DN11804_c0_g1_i1 | 2.299549207 | 0.36608936 | 0.999970678 | 0.681 | 0.414 | 0.189 | 0 | NA |
| TRINITY_DN164_c0_g2_i4 | -2.29930433 | 0.466178759 | 0.999970678 | 0 | 6.426 | 25.543 | 0.221 | NAC |
| TRINITY_DN1535_c0_g1_i5 | 2.299150002 | 0.35687645 | 0.999970678 | 2.162 | 2.937 | 0.856 | 0 | NA |
| TRINITY_DN6051_c0_g1_i1 | 2.299106026 | 0.352190185 | 0.999970678 | 0.745 | 0.887 | 0 | 0.322 | NA |
| TRINITY_DN8456_c0_g1_i7 | -2.29904039 | 0.362574361 | 0.999970678 | 0.59 | 0 | 1.067 | 1.639 | NA |
| TRINITY_DN1962_c0_g1_i2 | 2.298817199 | 0.582403687 | 0.999970678 | 2.371 | 0.01 | 0 | 0.483 | 60KD_IMP |
| TRINITY_DN8182_c0_g1_i8 | 2.298783352 | 0.352171071 | 0.999970678 | 0.709 | 1.449 | 0 | 0.442 | NA |
| TRINITY_DN5000_c0_g2_i3 | -2.29837802 | 0.054707113 | 0.999970678 | 2.471 | 1.163 | 3.011 | 14.437 | NA |
| TRINITY_DN649_c1_g1_i1 | 2.298159365 | 0.001652529 | 0.106073537 | 5.805 | 5.361 | 1.189 | 0.845 | NA |
| TRINITY_DN6703_c0_g1_i2 | 2.298043415 | 0.15127816 | 0.999970678 | 1.281 | 4.07 | 0.122 | 0.925 | NA |
| TRINITY_DN12517_c0_g1_i1 | 2.297547406 | 0.147917155 | 0.999970678 | 3.833 | 11.63 | 2.389 | 0.231 | NA |
| TRINITY_DN4355_c0_g1_i1 | 2.296231728 | 0.364078426 | 0.999970678 | 6.204 | 5.834 | 0 | 2.453 | NA |
| TRINITY_DN12789_c0_g1_i4 | -2.29555666 | 0.589169843 | 0.999970678 | 0.236 | 0 | 0.978 | 0 | NA |
| TRINITY_DN288_c0_g1_i4 | -2.29476126 | 0.05946777 | 0.999970678 | 53.395 | 9.313 | 110.638 | 176.07 | NA |

| TRINITY_DN10204_c0_g1_i1 | -2.29463039 | 0.581062551 | 0.999970678 | 1.517 | 0 | 6.244 | 0 | MFS_1 |
| --- | --- | --- | --- | --- | --- | --- | --- | --- |
| TRINITY_DN67516_c1_g1_i1 | 2.293037898 | 0.083176951 | 1 | 0.572 | 0.463 | 0.067 | 0.131 | NA |
| TRINITY_DN4944_c0_g1_i14 | -2.29295349 | 0.367163013 | 0.999970678 | 0.363 | 0 | 0.811 | 0.794 | NA |
| TRINITY_DN1665_c0_g1_i1 | 2.29274069 | 0.580314307 | 0.999970678 | 0 | 7.539 | 1.267 | 0 | MFS_1 |
| TRINITY_DN951_c2_g1_i2 | -2.29255028 | 0.588935898 | 0.999970678 | 0.972 | 0 | 3.978 | 0 | NA |
| TRINITY_DN5746_c0_g1_i2 | -2.29248259 | 0.364770991 | 0.999970678 | 0.636 | 0 | 1.555 | 1.337 | Lung_7-TM_R |
| TRINITY_DN17399_c0_g1_i2 | 2.292356912 | 0.34795197 | 0.999970678 | 1.917 | 2.04 | 0 | 0.814 | NA |
| TRINITY_DN6889_c0_g1_i4 | -2.29234962 | 0.366890035 | 0.999970678 | 0 | 0.631 | 1.844 | 0.824 | DEAD |
| TRINITY_DN62269_c0_g2_i2 | 2.291288277 | 0.118301228 | 0.999970678 | 16.033 | 48.29 | 9.744 | 1.257 | NA |
| TRINITY_DN8673_c0_g1_i5 | 2.291250483 | 0.355628407 | 0.999970678 | 1.008 | 1.478 | 0 | 0.503 | LpxK |
| TRINITY_DN18917_c0_g1_i3 | 2.291103312 | 0.372793305 | 0.999970678 | 0.681 | 2.365 | 0.511 | 0 | NA |
| TRINITY_DN227_c0_g1_i1 | -2.29107442 | 0.399854422 | 0.999970678 | 9.293 | 0 | 23.243 | 17.996 | Pollen_Ole_e_I |
| TRINITY_DN220_c0_g2_i5 | 2.290995391 | 0.028951851 | 0.999970678 | 6.186 | 4.514 | 1.311 | 0.613 | NA |
| TRINITY_DN2194_c0_g1_i1 | -2.28994945 | 0.584317818 | 0.999970678 | 1.435 | 0 | 5.911 | 0 | NA |
| TRINITY_DN1713_c0_g1_i2 | -2.28987736 | 0.102976635 | 0.999970678 | 2.098 | 2.109 | 1.9 | 18.268 | Myb_DNA-bindin |
| TRINITY_DN5575_c0_g1_i1 | 2.289785269 | 0.403778748 | 0.999970678 | 1.135 | 11.99 | 2.178 | 0 | NA |
| TRINITY_DN2181_c0_g1_i2 | 2.289225201 | 0.457374333 | 0.999970678 | 14.952 | 0 | 0.144 | 2.895 | DUF707 |
| TRINITY_DN1342_c0_g1_i1 | 2.288455649 | 0.286108781 | 0.999970678 | 5.06 | 0.069 | 0.333 | 0.653 | - |
| TRINITY_DN1105_c0_g1_i3 | -2.28843206 | 0.372685595 | 0.999970678 | 0 | 0.995 | 2.322 | 1.991 | Peptidase_S9 |
| TRINITY_DN72474_c0_g1_i4 | 2.288406195 | 0.371068452 | 1 | 1.072 | 0.897 | 0 | 0.412 | NA |
| TRINITY_DN8201_c0_g2_i2 | -2.28837615 | 0.047093452 | 0.999970678 | 0.318 | 0.345 | 2.011 | 0.804 | NA |
| TRINITY_DN48_c0_g1_i5 | -2.28796018 | 0.402512566 | 0.999970678 | 11.755 | 0 | 23.999 | 29.256 | E1_dh |
| TRINITY_DN20506_c0_g1_i4 | -2.28742754 | 0.365818437 | 0.999970678 | 0 | 0.512 | 1.433 | 0.744 | NA |
| TRINITY_DN4486_c1_g2_i2 | -2.28734567 | 0.221242726 | 0.999970678 | 2.425 | 0.177 | 5.811 | 5.389 | NA |
| TRINITY_DN477_c0_g1_i10 | -2.285676 | 0.174443483 | 0.999970678 | 0.736 | 0.325 | 4.011 | 0.372 | NA |
| TRINITY_DN3537_c0_g1_i6 | 2.285233741 | 0.379648207 | 0.999970678 | 0.927 | 0.266 | 0.2 | 0 | NABP |
| TRINITY_DN4426_c0_g1_i6 | -2.28398339 | 0.108285327 | 0.999970678 | 0.409 | 0.217 | 0.467 | 2.493 | DUF247 |
| TRINITY_DN257_c1_g1_i3 | -2.28354926 | 0.378563141 | 0.999970678 | 5.45 | 0 | 11.455 | 12.929 | Abhydrolase_1 |
| TRINITY_DN1724_c0_g1_i5 | 2.283531163 | 0.376493483 | 0.999970678 | 1.98 | 7.441 | 1.578 | 0 | NA |
| TRINITY_DN777_c0_g1_i1 | 2.283475083 | 0.587440342 | 0.999970678 | 0 | 8.338 | 1.389 | 0 | NA |
| TRINITY_DN5907_c0_g2_i2 | -2.28323787 | 0.170084459 | 0.999970678 | 0.818 | 1.912 | 1.044 | 11.391 | NA |
| TRINITY_DN1056_c1_g1_i6 | -2.28296633 | 0.397052028 | 0.999970678 | 0 | 1.025 | 3.589 | 0.553 | DUF4283 |
| TRINITY_DN9153_c0_g1_i1 | 2.282852955 | 0.379111378 | 0.999970678 | 0.681 | 0.246 | 0.156 | 0 | NA |
| TRINITY_DN19576_c1_g1_i1 | 2.280815047 | 0.213142466 | 1 | 0.736 | 0.838 | 0.244 | 0.04 | NA |
| TRINITY_DN5915_c0_g1_i5 | -2.28041625 | 0.375590574 | 1 | 7.712 | 0 | 14.677 | 17.845 | NA |
| TRINITY_DN1354_c0_g1_i6 | 2.280067296 | 0.315197312 | 0.999970678 | 0.1 | 28.87 | 3.966 | 1.086 | NA |
| TRINITY_DN1280_c0_g1_i6 | -2.27916163 | 0.384450824 | 0.999970678 | 12.609 | 0 | 36.476 | 17.443 | NA |
| TRINITY_DN1555_c0_g1_i10 | 2.278830189 | 0.132242799 | 0.999970678 | 0.799 | 0.286 | 0.144 | 0.06 | NA |
| TRINITY_DN11777_c0_g1_i1 | 2.277704195 | 0.358853607 | 0.999970678 | 1.762 | 1.508 | 0 | 0.674 | NA |
| TRINITY_DN23436_c0_g1_i1 | -2.27681571 | 0.584192534 | 0.999970678 | 3.979 | 0 | 16.11 | 0 | NA |
| TRINITY_DN10928_c0_g1_i1 | -2.27529773 | 0.370852349 | 0.999970678 | 0 | 0.828 | 2.111 | 1.408 | NA |
| TRINITY_DN1408_c0_g1_i19 | -2.27509339 | 0.377539975 | 0.999970678 | 0.182 | 0 | 0.433 | 0.352 | NA |
| TRINITY_DN3305_c0_g1_i10 | -2.27506959 | 0.030223434 | 0.999970678 | 0.736 | 0.325 | 2.522 | 2.081 | NA |
| TRINITY_DN8509_c0_g1_i1 | -2.27419879 | 0.055809472 | 0.999970678 | 0.645 | 0.197 | 1.544 | 2.302 | NA |
| TRINITY_DN7720_c2_g1_i1 | 2.273726935 | 0.397002695 | 0.999970678 | 0.636 | 4.721 | 0 | 1.086 | NA |
| TRINITY_DN37105_c0_g1_i2 | -2.27372277 | 0.585881977 | 0.999970678 | 0.954 | 0 | 3.844 | 0 | Glyco_hydro_114 |
| TRINITY_DN3968_c0_g1_i2 | 2.273697022 | 0.059819098 | 0.999970678 | 2.28 | 8.485 | 1.178 | 0.794 | NA |
| TRINITY_DN50227_c0_g1_i1 | 2.273434696 | 0.3595719 | 0.999970678 | 1.281 | 1.252 | 0 | 0.523 | NA |
| TRINITY_DN75_c0_g1_i14 | -2.273072 | 0.278958392 | 0.999970678 | 7.131 | 0.867 | 31.665 | 0.613 | NA |
| TRINITY_DN1280_c0_g1_i2 | -2.27291764 | 0.393268502 | 0.999970678 | 17.005 | 0 | 48.808 | 23.717 | NA |
| TRINITY_DN13629_c0_g1_i1 | -2.27273701 | 0.187013456 | 0.999970678 | 1.817 | 1.291 | 0.667 | 14.186 | GUB_WAK_bind |
| TRINITY_DN1588_c0_g1_i3 | -2.27243765 | 0.000318616 | 0.028756426 | 0.999 | 1.153 | 4.511 | 4.916 | CBS |
| TRINITY_DN27552_c2_g2_i6 | 2.272388069 | 0.587218768 | 0.999970678 | 1.235 | 0 | 0.211 | 0 | RVT_1 |
| TRINITY_DN5158_c0_g1_i4 | -2.27184753 | 0.366656828 | 0.999970678 | 0.654 | 0 | 1.5 | 1.397 | NA |
| TRINITY_DN31337_c0_g1_i2 | 2.271449223 | 0.417471932 | 0.999970678 | 11.291 | 17.53 | 4.922 | 0 | ABC_membrane |
| TRINITY_DN958_c0_g1_i6 | 2.271257438 | 0.583422634 | 0.999970678 | 5.396 | 0 | 0 | 1.136 | CBS |
| TRINITY_DN416_c4_g1_i1 | 2.269849504 | 0.486647629 | 0.999970678 | 0.036 | 12.14 | 0 | 2.483 | Lipase_GDSL |
| TRINITY_DN45105_c0_g1_i1 | -2.26974712 | 0.106277256 | 0.999970678 | 0.236 | 0.552 | 0.622 | 3.056 | Glyco_hydro_79n |
| TRINITY_DN603_c0_g1_i3 | -2.26966287 | 0.201891536 | 0.999970678 | 309.27 | 7.766 | 427.863 | 1024.864 | NA |
| TRINITY_DN7717_c0_g3_i1 | -2.26961585 | 0.381493039 | 0.999970678 | 1.344 | 0 | 1.622 | 4.524 | NA |
| TRINITY_DN7555_c0_g1_i2 | 2.269449463 | 0.367124163 | 0.999970678 | 49.017 | 22.23 | 0 | 13.804 | NA |
| TRINITY_DN18758_c0_g1_i1 | -2.26839236 | 0.582787024 | 0.999970678 | 0 | 25.08 | 0 | 112.873 | NA |
| TRINITY_DN5899_c1_g1_i1 | 2.268361123 | 0.363332184 | 0.999970678 | 2.062 | 1.39 | 0 | 0.714 | NA |
| TRINITY_DN1028_c0_g1_i11 | -2.26829587 | 0.334715965 | 0.999970678 | 0.282 | 3.627 | 15.332 | 0.08 | RRM_1 |
| TRINITY_DN32239_c0_g1_i1 | 2.267456345 | 0.489577804 | 0.999970678 | 0 | 117.3 | 0.278 | 22.239 | PsbX |
| TRINITY_DN9659_c0_g3_i2 | 2.266732018 | 0.178803889 | 0.999970678 | 3.752 | 1.232 | 0.067 | 0.965 | NA |
| TRINITY_DN19269_c0_g1_i1 | 2.265964494 | 0.32937856 | 0.999970678 | 0.055 | 2.681 | 0.444 | 0.02 | Beta_helix |
| TRINITY_DN5676_c0_g1_i4 | -2.26595932 | 0.596235265 | 0.999970678 | 0.291 | 0 | 1.2 | 0 | PPR_long |
| TRINITY_DN1049_c0_g1_i2 | 2.265741865 | 0.022023278 | 0.907518796 | 3.388 | 7.983 | 1.267 | 0.824 | - |
| TRINITY_DN8015_c0_g1_i4 | 2.265320537 | 0.222587371 | 0.999970678 | 1.826 | 10.73 | 2.044 | 0.131 | NA |
| TRINITY_DN9334_c0_g1_i5 | 2.265059302 | 0.585142873 | 0.999970678 | 5.705 | 0 | 0 | 1.216 | Peptidase_C48 |
| TRINITY_DN2113_c0_g1_i5 | -2.26495191 | 0.586042795 | 0.999970678 | 1.117 | 0 | 4.511 | 0 | GMC_oxred_C |
| TRINITY_DN4030_c0_g1_i4 | -2.26480371 | 0.595134647 | 0.999970678 | 0 | 0.857 | 3.322 | 0 | NA |
| TRINITY_DN939_c0_g1_i6 | 2.26459128 | 0.457704403 | 0.999970678 | 0 | 6.8 | 1.067 | 0.08 | NA |

| TRINITY_DN1226_c0_g2_i4 | -2.26444788 | 0.244856101 | 0.999970678 | 0.536 | 6.229 | 1.178 | 30.493 | NA |
| --- | --- | --- | --- | --- | --- | --- | --- | --- |
| TRINITY_DN1956_c0_g1_i9 | 2.264080508 | 0.08278009 | 0.999970678 | 0.963 | 1.764 | 0.389 | 0.09 | Filamin |
| TRINITY_DN3042_c0_g1_i22 | 2.263994143 | 0.383595021 | 1 | 0.273 | 0.424 | 0.111 | 0 | NA |
| TRINITY_DN3998_c0_g1_i5 | 2.263811407 | 0.584687536 | 0.999970678 | 3.216 | 0 | 0 | 0.694 | NA |
| TRINITY_DN3280_c0_g1_i14 | 2.263491622 | 0.283424089 | 0.999970678 | 0.109 | 5.696 | 0.1 | 1.076 | NA |
| TRINITY_DN2619_c0_g1_i5 | 2.26308733 | 0.381285588 | 0.999970678 | 8.793 | 4.671 | 2.322 | 0 | - |
| TRINITY_DN1507_c0_g1_i5 | 2.26307854 | 0.591443542 | 0.999970678 | 1.117 | 0 | 0 | 0.241 | BRCT_2 |
| TRINITY_DN6857_c0_g1_i1 | -2.26295389 | 0.005285436 | 0.280789874 | 0.818 | 1.34 | 4.189 | 5.278 | NA |
| TRINITY_DN43_c0_g2_i1 | -2.26270984 | 0.420802925 | 0.999970678 | 10.746 | 0 | 14.055 | 35.51 | NA |
| TRINITY_DN9014_c0_g1_i1 | -2.26230578 | 0.315604645 | 0.999970678 | 7.04 | 0.059 | 2.133 | 31.92 | Transferase |
| TRINITY_DN19610_c0_g1_i3 | -2.26192969 | 0.025331362 | 0.999970678 | 0.527 | 1.133 | 4.344 | 2.674 | Xan_ur_permease |
| TRINITY_DN188_c0_g1_i12 | -2.26158897 | 0.015342292 | 0.681064358 | 3.325 | 6.179 | 14.499 | 26.823 | NA |
| TRINITY_DN4833_c0_g1_i10 | -2.26153027 | 0.385892686 | 1 | 0 | 0.207 | 0.422 | 0.483 | NA |
| TRINITY_DN10578_c0_g1_i2 | 2.261208888 | 0.222523831 | 0.999970678 | 1.235 | 1.045 | 0.378 | 0.02 | NA |
| TRINITY_DN658_c0_g1_i2 | 2.261208213 | 0.091583691 | 0.999970678 | 16.942 | 20.34 | 0.7 | 6.897 | SKI |
| TRINITY_DN10713_c0_g1_i1 | 2.260522214 | 0.381121958 | 0.999970678 | 3.443 | 3.026 | 1.122 | 0 | NA |
| TRINITY_DN14670_c0_g1_i1 | 2.259994397 | 0.164039672 | 1 | 3.443 | 1.577 | 0.156 | 0.855 | NA |
| TRINITY_DN10736_c2_g1_i1 | 2.259982814 | 0.162859758 | 1 | 0.372 | 0.306 | 0.089 | 0.03 | NA |
| TRINITY_DN5007_c0_g2_i1 | 2.25922158 | 0.158192223 | 0.999970678 | 1.072 | 3.666 | 0.089 | 0.875 | NA |
| TRINITY_DN621_c0_g1_i2 | 2.258984523 | 0.381433232 | 0.999970678 | 0.645 | 2.937 | 0 | 0.744 | NA |
| TRINITY_DN12721_c0_g1_i2 | -2.25867151 | 0.182760924 | 0.999970678 | 0.808 | 0.128 | 0.667 | 3.639 | Peptidase_S10 |
| TRINITY_DN3311_c0_g1_i7 | 2.25791741 | 0.064761546 | 1 | 2.026 | 2.562 | 0.378 | 0.493 | NA |
| TRINITY_DN1845_c0_g1_i7 | 2.257552831 | 0.391557336 | 0.999970678 | 3.606 | 6.317 | 1.711 | 0 | Cation_ATPase |
| TRINITY_DN17019_c0_g1_i1 | 2.257104821 | 0.19062347 | 0.999970678 | 44.121 | 1.36 | 3.011 | 6.052 | Cyclin |
| TRINITY_DN478_c0_g2_i1 | 2.257079453 | 0.369151823 | 0.999970678 | 3.843 | 6.889 | 0 | 2.242 | P34-Arc |
| TRINITY_DN6108_c0_g1_i2 | -2.25493835 | 0.455108144 | 0.999970678 | 5.078 | 0 | 0.456 | 23.325 | NA |
| TRINITY_DN7258_c0_g1_i8 | -2.25440395 | 0.394255421 | 0.999970678 | 1.399 | 0 | 4.533 | 1.176 | NA |
| TRINITY_DN5996_c0_g1_i1 | -2.25315502 | 0.231007747 | 0.999970678 | 14.235 | 1.094 | 54.33 | 6.786 | NA |
| TRINITY_DN12372_c0_g1_i2 | -2.25281614 | 0.100742787 | 0.999970678 | 0.064 | 0.089 | 0.467 | 0.141 | DDT |
| TRINITY_DN4179_c0_g1_i1 | -2.25281518 | 0.022026886 | 0.907518796 | 24.999 | 60.94 | 228.431 | 126.506 | NA |
| TRINITY_DN7211_c0_g1_i1 | 2.252695879 | 0.063005182 | 0.999970678 | 0.772 | 0.453 | 0.1 | 0.141 | BOP1NT |
| TRINITY_DN6196_c0_g1_i22 | 2.252463573 | 0.59106323 | 0.999970678 | 3.715 | 0 | 0 | 0.794 | NA |
| TRINITY_DN8394_c0_g1_i2 | 2.252341984 | 0.119884065 | 0.999970678 | 1.045 | 0.798 | 0.289 | 0.04 | AAA |
| TRINITY_DN4996_c0_g2_i2 | -2.25223886 | 0.588442018 | 0.999970678 | 1.662 | 0 | 6.633 | 0 | NA |
| TRINITY_DN1779_c0_g1_i2 | -2.25216197 | 0.321990135 | 0.999970678 | 0.509 | 14.26 | 56.574 | 0.875 | NA |
| TRINITY_DN2319_c1_g3_i2 | 2.251559088 | 0.165193733 | 0.999970678 | 8.239 | 1.33 | 1.433 | 0.282 | NA |
| TRINITY_DN4592_c0_g1_i6 | 2.251450129 | 0.589995084 | 0.999970678 | 2.943 | 0 | 0 | 0.623 | NA |
| TRINITY_DN1014_c0_g1_i1 | 2.251434445 | 0.067455208 | 0.999970678 | 6.259 | 5.243 | 1.7 | 0.362 | NA |
| TRINITY_DN610_c0_g1_i1 | -2.25096993 | 0.195227475 | 0.999970678 | 1.354 | 6.376 | 1.522 | 34.665 | NA |
| TRINITY_DN8293_c0_g1_i6 | 2.249841586 | 0.007454285 | 0.376192409 | 6.395 | 6.081 | 0.878 | 1.528 | YIF1 |
| TRINITY_DN19247_c0_g1_i4 | -2.24978021 | 0.380442211 | 1 | 0 | 0.187 | 0.333 | 0.462 | NA |
| TRINITY_DN3788_c0_g1_i1 | -2.24923437 | 0.14659101 | 0.999970678 | 1.735 | 2.681 | 1.1 | 19.564 | TAXi_C |
| TRINITY_DN7182_c0_g1_i11 | 2.248966966 | 0.172166612 | 0.999970678 | 1.099 | 0.759 | 0.3 | 0.05 | NA |
| TRINITY_DN16579_c0_g1_i3 | -2.24822371 | 0.35487324 | 0.999970678 | 0.064 | 0.71 | 0.011 | 3.629 | NA |
| TRINITY_DN2679_c0_g1_i2 | -2.24756321 | 0.006428655 | 0.333392088 | 2.471 | 2.168 | 12.277 | 7.208 | UDPGT |
| TRINITY_DN1032_c0_g1_i2 | -2.2474008 | 0.390854479 | 0.999970678 | 0 | 2.769 | 1.889 | 10.587 | Lipoxygenase |
| TRINITY_DN3916_c0_g1_i4 | 2.245128826 | 0.593363037 | 0.999970678 | 10.401 | 0 | 0 | 2.232 | BET |
| TRINITY_DN4591_c0_g1_i2 | -2.24433784 | 0.107306356 | 0.999970678 | 0.291 | 0.719 | 0.589 | 4.072 | NA |
| TRINITY_DN81534_c0_g1_i8 | 2.243262684 | 0.296349178 | 0.999970678 | 33.611 | 1.823 | 5.9 | 0.462 | NA |
| TRINITY_DN5703_c0_g2_i1 | 2.242861415 | 0.298751321 | 0.999970678 | 1.399 | 104.9 | 1.178 | 20.65 | adh_short |
| TRINITY_DN1771_c0_g1_i3 | 2.242633983 | 0.039290928 | 0.999970678 | 7.758 | 17.63 | 3.189 | 1.468 | NA |
| TRINITY_DN1549_c0_g1_i5 | 2.241463247 | 0.291215395 | 0.999970678 | 9.302 | 0.079 | 0.489 | 1.408 | LRRNT_2 |
| TRINITY_DN3126_c0_g1_i2 | -2.2412514 | 0.586774302 | 0.999970678 | 0 | 4.859 | 0 | 22.158 | NA |
| TRINITY_DN1107_c0_g1_i3 | -2.24116069 | 0.598332391 | 0.999970678 | 1.581 | 0 | 0 | 7.661 | Sugar_tr |
| TRINITY_DN10499_c0_g1_i6 | -2.2407964 | 0.096693552 | 0.999970678 | 3.37 | 0.473 | 5.377 | 11.924 | NA |
| TRINITY_DN2451_c1_g1_i4 | -2.2407484 | 0.02582309 | 0.999970678 | 0.064 | 0.089 | 0.411 | 0.211 | NADHdh |
| TRINITY_DN11637_c0_g1_i1 | 2.24059801 | 0.184188128 | 0.999970678 | 2.344 | 0.946 | 0.044 | 0.633 | NA |
| TRINITY_DN1437_c2_g1_i5 | -2.24054293 | 0.013275277 | 0.611748743 | 0.754 | 1.045 | 3.889 | 3.639 | NA |
| TRINITY_DN4413_c0_g1_i3 | -2.23998416 | 0.10597564 | 0.999970678 | 3.179 | 8.407 | 5.533 | 47.655 | NA |
| TRINITY_DN847_c0_g1_i6 | -2.2394199 | 0.128832962 | 0.999970678 | 1.008 | 0.522 | 0.867 | 6.072 | NA |
| TRINITY_DN3332_c0_g2_i4 | -2.23841315 | 0.369477475 | 0.999970678 | 0 | 0.611 | 1.222 | 1.377 | ABC1 |
| TRINITY_DN13268_c0_g1_i5 | -2.23751485 | 0.217719701 | 0.999970678 | 0.082 | 0.483 | 1.911 | 0.271 | NA |
| TRINITY_DN3117_c0_g1_i6 | -2.23713439 | 0.382538855 | 0.999970678 | 1.835 | 0 | 2.222 | 6.103 | DUF538 |
| TRINITY_DN1213_c0_g1_i9 | 2.236970608 | 0.070088766 | 0.999970678 | 4.179 | 1.725 | 0.789 | 0.312 | NA |
| TRINITY_DN4532_c0_g1_i3 | -2.23648854 | 0.489687725 | 0.999970678 | 0.899 | 0.049 | 3.789 | 0 | NA |
| TRINITY_DN9540_c0_g1_i6 | 2.234803318 | 0.362530816 | 0.999970678 | 1.662 | 1.557 | 0 | 0.694 | NA |
| TRINITY_DN848_c1_g1_i1 | 2.234695306 | 0.490128438 | 0.999970678 | 11.128 | 0 | 0.044 | 2.363 | NA |
| TRINITY_DN1195_c0_g2_i4 | -2.23445701 | 0.432265293 | 0.999970678 | 36.554 | 0 | 68.085 | 91.961 | PfkB |
| TRINITY_DN2251_c1_g2_i1 | 2.234001127 | 0.372757554 | 0.999970678 | 0.763 | 0.532 | 0.233 | 0 | zf-C3HC4_3 |
| TRINITY_DN2877_c0_g1_i1 | 2.23259976 | 0.089203002 | 0.999970678 | 2.843 | 4.011 | 0.222 | 1.176 | GST_C |
| TRINITY_DN1610_c0_g1_i3 | 2.232599723 | 0.590265058 | 0.999970678 | 0 | 1.764 | 0.311 | 0 | - |
| TRINITY_DN522_c0_g1_i7 | -2.23257149 | 0.454331925 | 0.999970678 | 4.506 | 0 | 16.466 | 1.619 | SPX |
| TRINITY_DN967_c0_g1_i2 | -2.23230999 | 0.153962331 | 0.999970678 | 1.744 | 3.962 | 2.011 | 23.455 | NA |
| TRINITY_DN70702_c0_g2_i1 | -2.23206722 | 0.383685685 | 1 | 0 | 1.025 | 1.522 | 2.865 | NA |

| TRINITY_DN1955_c0_g1_i1 | -2.23186651 | 0.595520204 | 0.999970678 | 0.4 | 0 | 0 | 1.88 | Na_H_Exchanger |
| --- | --- | --- | --- | --- | --- | --- | --- | --- |
| TRINITY_DN1563_c0_g1_i5 | 2.231441002 | 0.591073121 | 0.999970678 | 13.462 | 0 | 0 | 2.906 | HhH-GPD |
| TRINITY_DN1272_c0_g2_i4 | -2.23138227 | 0.069512924 | 0.999970678 | 0.854 | 2.316 | 2.844 | 11.26 | NA |
| TRINITY_DN5712_c0_g1_i11 | -2.2295994 | 0.234788036 | 0.999970678 | 1.935 | 21.71 | 4.322 | 104.287 | NA |
| TRINITY_DN1915_c0_g1_i8 | -2.22941062 | 0.443924415 | 0.999970678 | 0.191 | 0.857 | 4.033 | 0 | - |
| TRINITY_DN14535_c0_g6_i1 | -2.22866725 | 0.385598226 | 1 | 0 | 0.779 | 1.444 | 1.84 | NA |
| TRINITY_DN2105_c0_g1_i1 | -2.22849234 | 0.079591568 | 1 | 0.872 | 0.601 | 2.389 | 3.72 | NA |
| TRINITY_DN672_c0_g1_i8 | 2.227882967 | 0.590552448 | 0.999970678 | 3.443 | 0 | 0 | 0.754 | NA |
| TRINITY_DN49722_c0_g1_i1 | -2.22771889 | 0.594245494 | 0.999970678 | 0 | 1.025 | 0 | 4.745 | MIP |
| TRINITY_DN14915_c2_g1_i1 | 2.22753591 | 0.08703092 | 0.999970678 | 1.326 | 0.995 | 0.322 | 0.101 | NA |
| TRINITY_DN1028_c0_g1_i13 | -2.22750487 | 0.593817596 | 0.999970678 | 0 | 3.745 | 14.232 | 0 | RRM_1 |
| TRINITY_DN4755_c0_g2_i2 | -2.22649203 | 0.043913812 | 0.999970678 | 2.389 | 6.091 | 8.311 | 29.447 | NA |
| TRINITY_DN2637_c0_g1_i10 | 2.22575725 | 0.365733873 | 0.999970678 | 2.353 | 2.484 | 0 | 1.036 | Translin |
| TRINITY_DN4_c0_g1_i12 | -2.22521713 | 0.08636455 | 0.999970678 | 0.681 | 2.454 | 2.622 | 11.451 | - |
| TRINITY_DN1529_c0_g1_i1 | -2.22509107 | 0.204399156 | 0.999970678 | 185.45 | 4.662 | 341.013 | 484.91 | NA |
| TRINITY_DN13828_c0_g1_i4 | -2.22460285 | 0.084172417 | 0.999970678 | 1.017 | 0.256 | 3.422 | 1.85 | NA |
| TRINITY_DN5656_c0_g1_i1 | -2.22450962 | 0.051691021 | 0.999970678 | 0.327 | 0.306 | 0.733 | 2.071 | - |
| TRINITY_DN4834_c0_g1_i1 | 2.224134649 | 0.13963657 | 0.999970678 | 1.163 | 1.567 | 0.411 | 0.08 | NA |
| TRINITY_DN4736_c1_g1_i3 | -2.22373979 | 0.399332418 | 0.999970678 | 0.536 | 0 | 0.378 | 2.061 | NA |
| TRINITY_DN2117_c0_g2_i10 | -2.22360928 | 0.59472758 | 0.999970678 | 1.699 | 0 | 0 | 8.003 | NA |
| TRINITY_DN1245_c0_g1_i2 | 2.223378888 | 0.045649226 | 0.999970678 | 1.226 | 4.257 | 0.411 | 0.664 | F-box |
| TRINITY_DN5557_c0_g1_i1 | -2.22328896 | 0.392505471 | 0.999970678 | 1.054 | 0 | 1.667 | 3.006 | DUF4205 |
| TRINITY_DN5583_c0_g1_i1 | -2.22310042 | 0.400376041 | 0.999970678 | 0 | 3.016 | 8.633 | 3.418 | Exostosin |
| TRINITY_DN5871_c0_g1_i7 | 2.222582286 | 0.595920149 | 0.999970678 | 7.485 | 0 | 0 | 1.619 | NA |
| TRINITY_DN256_c1_g1_i1 | 2.222492357 | 0.38602198 | 0.999970678 | 4.796 | 1.242 | 0 | 1.297 | Pantoate_transf |
| TRINITY_DN2009_c0_g1_i10 | -2.22242947 | 0.171673665 | 0.999970678 | 2.098 | 0.335 | 2.611 | 7.641 | - |
| TRINITY_DN83_c0_g1_i13 | -2.22232117 | 0.264504164 | 0.999970678 | 0.145 | 11.59 | 19.832 | 29.236 | NA |
| TRINITY_DN10444_c0_g2_i5 | -2.2218959 | 0.103114554 | 0.999970678 | 0.3 | 0.384 | 0.444 | 2.704 | Rab5ip |
| TRINITY_DN3650_c0_g1_i2 | -2.22172862 | 0.151873207 | 0.999970678 | 3.406 | 10.29 | 4.111 | 58.191 | NA |
| TRINITY_DN8942_c0_g1_i7 | 2.221065834 | 0.381524563 | 0.999970678 | 2.852 | 0.897 | 0 | 0.804 | Motile_Sperm |
| TRINITY_DN5246_c0_g1_i1 | 2.220584717 | 0.593951289 | 0.999970678 | 3.098 | 0 | 0 | 0.684 | COPIIcoated_ERV |
| TRINITY_DN8769_c0_g2_i1 | -2.21967087 | 0.391325255 | 1 | 0.136 | 0 | 0.289 | 0.292 | NA |
| TRINITY_DN274_c0_g1_i12 | 2.219131213 | 0.014986914 | 0.669536213 | 2.734 | 2.346 | 0.567 | 0.412 | RRM_1 |
| TRINITY_DN1171_c0_g1_i3 | 2.218551145 | 0.084531052 | 0.999970678 | 1.599 | 1.192 | 0.1 | 0.503 | Myb_Cef |
| TRINITY_DN7281_c0_g1_i5 | 2.215883631 | 0.380773051 | 0.999970678 | 0.345 | 1.015 | 0 | 0.292 | NA |
| TRINITY_DN2251_c0_g1_i17 | -2.21582374 | 0.384035067 | 0.999970678 | 0 | 0.394 | 0.467 | 1.237 | NA |
| TRINITY_DN1464_c0_g1_i7 | 2.215371849 | 0.039243992 | 0.999970678 | 13.571 | 10.81 | 3.366 | 1.186 | NA |
| TRINITY_DN920_c0_g2_i1 | -2.21496333 | 0.475864082 | 0.999970678 | 0 | 1.744 | 6.522 | 0.08 | NA |
| TRINITY_DN1455_c0_g1_i4 | 2.214938734 | 0.183374881 | 0.999970678 | 9.52 | 0.926 | 1.367 | 0.593 | NA |
| TRINITY_DN550_c0_g1_i13 | 2.21485435 | 0.203988576 | 0.999970678 | 27.979 | 0.867 | 2.766 | 2.976 | NA |
| TRINITY_DN7503_c0_g1_i3 | -2.21419441 | 0.594505266 | 0.999970678 | 0 | 3.045 | 11.477 | 0 | NAM |
| TRINITY_DN1812_c0_g1_i3 | 2.214018783 | 0.367951736 | 0.999970678 | 1.453 | 2.05 | 0 | 0.744 | Solute_trans_a |
| TRINITY_DN20600_c0_g1_i2 | -2.21360663 | 0.406746904 | 0.999970678 | 5.087 | 0 | 6 | 16.739 | NA |
| TRINITY_DN6198_c0_g1_i2 | -2.21357798 | 0.385488713 | 0.999970678 | 6.504 | 0 | 9.477 | 18.519 | NA |
| TRINITY_DN1082_c0_g1_i5 | 2.212936965 | 0.594654296 | 0.999970678 | 0 | 1.468 | 0 | 0.312 | PRP21_like_P |
| TRINITY_DN8822_c0_g1_i1 | 2.212810452 | 0.216770401 | 0.999970678 | 30.032 | 1.744 | 4.711 | 1.176 | DUF4228 |
| TRINITY_DN6121_c0_g1_i1 | -2.21263412 | 0.593839721 | 0.999970678 | 0 | 1.567 | 5.933 | 0 | Str_synth |
| TRINITY_DN4489_c0_g1_i2 | -2.21248363 | 0.17902228 | 0.999970678 | 4.269 | 0.828 | 17.832 | 2.141 | zf-Dof |
| TRINITY_DN937_c0_g1_i6 | -2.21190754 | 0.026533424 | 0.999970678 | 0.254 | 0.187 | 0.578 | 1.367 | NA |
| TRINITY_DN3912_c0_g1_i2 | 2.211011253 | 0.05549877 | 0.999970678 | 0.827 | 2.562 | 0.256 | 0.412 | NA |
| TRINITY_DN2648_c0_g1_i3 | 2.210816629 | 0.47237796 | 0.999970678 | 0 | 12.85 | 2.1 | 0.191 | NUP50 |
| TRINITY_DN24647_c0_g1_i2 | 2.21079029 | 0.112249352 | 1 | 0.518 | 1.035 | 0.1 | 0.211 | NA |
| TRINITY_DN28015_c0_g2_i1 | -2.21031466 | 0.392056676 | 0.999970678 | 0.418 | 0 | 1.155 | 0.543 | NA |
| TRINITY_DN2603_c0_g1_i2 | 2.210165595 | 0.350434298 | 0.999970678 | 100.86 | 90.81 | 0.044 | 41.461 | NA |
| TRINITY_DN1802_c0_g1_i3 | 2.21011476 | 0.404294598 | 0.999970678 | 4.424 | 15.72 | 0 | 4.313 | NA |
| TRINITY_DN4692_c0_g1_i2 | 2.21003834 | 0.00109903 | 0.075594775 | 7.412 | 5.223 | 1.1 | 1.418 | NA |
| TRINITY_DN5976_c0_g1_i4 | 2.210001653 | 0.615747083 | 0.999970678 | 56.702 | 0 | 0 | 12.477 | ARD |
| TRINITY_DN759_c0_g1_i1 | 2.209268215 | 0.4644479 | 0.999970678 | 7.322 | 0.108 | 0 | 1.639 | NA |
| TRINITY_DN4362_c0_g1_i1 | 2.209264177 | 0.065174054 | 0.999970678 | 13.608 | 4.415 | 0.778 | 3.006 | NA |
| TRINITY_DN3593_c0_g1_i1 | -2.20791816 | 0.395969408 | 0.999970678 | 0 | 2.336 | 1.789 | 8.515 | zf-RING_2 |
| TRINITY_DN2341_c0_g1_i1 | 2.207724644 | 0.16210128 | 0.999970678 | 4.369 | 0.384 | 0.344 | 0.633 | NA |
| TRINITY_DN728_c0_g2_i4 | 2.207691116 | 0.011448472 | 0.54288022 | 10.701 | 11.13 | 2.766 | 1.397 | NA |
| TRINITY_DN8260_c0_g1_i4 | 2.207289413 | 0.267116914 | 0.999970678 | 0.4 | 27.55 | 3.933 | 1.196 | NA |
| TRINITY_DN16970_c0_g3_i2 | 2.206811836 | 0.105074784 | 1 | 1.135 | 0.65 | 0.211 | 0.131 | NA |
| TRINITY_DN10592_c0_g1_i5 | 2.206762899 | 0.389923317 | 1 | 1.29 | 1.892 | 0 | 0.674 | NA |
| TRINITY_DN4912_c0_g1_i6 | 2.206395152 | 0.23315836 | 0.999970678 | 1.145 | 35.16 | 1.5 | 5.811 | NA |
| TRINITY_DN1197_c1_g2_i2 | 2.205652621 | 0.440908674 | 0.999970678 | 26.498 | 26.55 | 0 | 11.582 | CLU_N |
| TRINITY_DN478_c0_g1_i13 | -2.20557895 | 0.595064037 | 0.999970678 | 0 | 0.917 | 3.422 | 0 | Seryl_tRNA_N |
| TRINITY_DN3201_c0_g1_i5 | 2.205566355 | 0.370098228 | 0.999970678 | 3.724 | 3.302 | 0 | 1.508 | NA |
| TRINITY_DN1875_c0_g1_i17 | 2.205198523 | 0.420765226 | 0.999970678 | 3.107 | 0.266 | 0 | 0.744 | PHD |
| TRINITY_DN11473_c0_g1_i1 | -2.20513358 | 0.184438639 | 0.999970678 | 0.4 | 1.478 | 0.622 | 7.842 | NA |
| TRINITY_DN6421_c0_g1_i2 | -2.20487722 | 0.022020307 | 0.907518796 | 32.53 | 14.08 | 59.408 | 143.336 | NA |
| TRINITY_DN947_c0_g1_i8 | -2.20479784 | 0.110871152 | 0.999970678 | 2.189 | 5.588 | 3.611 | 31.257 | NA |
| TRINITY_DN22_c0_g2_i1 | 2.204772647 | 0.38668951 | 0.999970678 | 3.933 | 2.05 | 1.078 | 0 | AA_kinase |

| TRINITY_DN16449_c0_g1_i1 | 2.204670884 | 0.033374044 | 0.999970678 | 2.734 | 1.409 | 0.444 | 0.352 | ABC2_membrane |
| --- | --- | --- | --- | --- | --- | --- | --- | --- |
| TRINITY_DN2113_c0_g2_i1 | -2.20444266 | 0.597055208 | 0.999970678 | 5.014 | 0 | 19.188 | 0 | GMC_oxred_C |
| TRINITY_DN5272_c0_g4_i1 | 2.204408258 | 0.149480202 | 0.999970678 | 4.824 | 2.612 | 1.067 | 0.322 | NA |
| TRINITY_DN1679_c0_g1_i3 | 2.204120579 | 0.601985142 | 0.999970678 | 0 | 2.562 | 0 | 0.543 | SPC22 |
| TRINITY_DN1676_c0_g1_i4 | -2.20367909 | 0.434759282 | 0.999970678 | 2.008 | 2.01 | 0 | 18.438 | NA |
| TRINITY_DN14520_c0_g1_i2 | -2.20360048 | 0.100164836 | 0.999970678 | 0.636 | 5.174 | 10.199 | 14.115 | p450 |
| TRINITY_DN1298_c0_g1_i4 | 2.203256428 | 0.594639482 | 0.999970678 | 2.925 | 0 | 0 | 0.643 | NB-ARC |
| TRINITY_DN17303_c1_g1_i2 | 2.203244683 | 0.384722702 | 1 | 0.518 | 0.325 | 0 | 0.181 | NA |
| TRINITY_DN1335_c0_g1_i1 | -2.20323824 | 0.432586396 | 0.999970678 | 18.077 | 0 | 33.476 | 44.126 | ERp29 |
| TRINITY_DN478_c0_g2_i2 | -2.20313344 | 0.394606202 | 0.999970678 | 3.034 | 0 | 6.266 | 6.555 | P34-Arc |
| TRINITY_DN9717_c0_g1_i1 | -2.20294978 | 0.176746185 | 0.999970678 | 5.178 | 2.375 | 2.355 | 30.955 | NA |
| TRINITY_DN4975_c0_g1_i1 | 2.202543095 | 0.24240959 | 0.999970678 | 0.2 | 6.13 | 0.3 | 0.995 | Glyco_hydro_1 |
| TRINITY_DN8814_c1_g1_i4 | 2.202531633 | 0.058854884 | 0.999970678 | 0.872 | 1.35 | 0.278 | 0.141 | Pkinase |
| TRINITY_DN4138_c0_g1_i11 | -2.20232575 | 0.595911625 | 0.999970678 | 0 | 1.104 | 4.133 | 0 | NA |
| TRINITY_DN4209_c0_g1_i4 | 2.201506336 | 0.172847307 | 0.999970678 | 35.918 | 9.974 | 0.622 | 9.259 | NA |
| TRINITY_DN6904_c0_g2_i3 | -2.20124978 | 0.111424852 | 0.999970678 | 0.654 | 0.739 | 0.889 | 5.268 | NA |
| TRINITY_DN1711_c0_g1_i1 | 2.200972595 | 0.107920262 | 0.999970678 | 2.117 | 0.581 | 0.378 | 0.131 | NA |
| TRINITY_DN604_c1_g2_i5 | -2.20080022 | 0.112734784 | 0.999970678 | 42.44 | 122.4 | 73.673 | 648.624 | NA |
| TRINITY_DN1738_c0_g1_i4 | -2.20077808 | 0.410385492 | 0.999970678 | 3.143 | 0 | 6.577 | 6.756 | NA |
| TRINITY_DN68_c0_g1_i1 | -2.20067452 | 0.440279634 | 0.999970678 | 28.705 | 0 | 86.384 | 29.075 | Ribonuc_L-PSP |
| TRINITY_DN843_c3_g1_i6 | -2.20059363 | 0.041882096 | 0.999970678 | 0.118 | 0.177 | 0.533 | 0.754 | PHM7_cyt |
| TRINITY_DN443_c0_g1_i6 | -2.20033834 | 0.400153712 | 0.999970678 | 1.989 | 0 | 2.833 | 5.861 | Amidohydro_1 |
| TRINITY_DN11746_c0_g1_i4 | 2.199748481 | 0.378870824 | 0.999970678 | 2.244 | 4.1 | 0 | 1.367 | NA |
| TRINITY_DN6576_c0_g3_i1 | 2.19972075 | 0.104171205 | 0.999970678 | 1.235 | 0.453 | 0.144 | 0.191 | LRRNT_2 |
| TRINITY_DN1028_c0_g1_i2 | 2.198979998 | 0.191746992 | 0.999970678 | 19.966 | 1.114 | 0.989 | 3.458 | RRM_1 |
| TRINITY_DN1482_c1_g1_i1 | -2.19841499 | 0.244518878 | 0.999970678 | 1.744 | 2.592 | 15.955 | 0.543 | NA |
| TRINITY_DN6310_c0_g2_i5 | -2.1980443 | 0.601371375 | 0.999970678 | 0 | 0.424 | 1.544 | 0 | NA |
| TRINITY_DN12542_c0_g1_i4 | -2.19798809 | 0.604405181 | 0.999970678 | 0.563 | 0 | 2.2 | 0 | NA |
| TRINITY_DN708_c0_g1_i1 | 2.195256099 | 0.493515193 | 0.999970678 | 0 | 6.495 | 0.044 | 1.347 | NA |
| TRINITY_DN6027_c0_g1_i1 | 2.194415631 | 0.271361026 | 0.999970678 | 27.424 | 0.463 | 3.766 | 1.588 | NA |
| TRINITY_DN2054_c0_g1_i9 | 2.194130807 | 0.410170701 | 0.999970678 | 29.65 | 21.06 | 0 | 11.109 | LRRNT_2 |
| TRINITY_DN1845_c0_g1_i5 | 2.19410426 | 0.405443395 | 0.999970678 | 0.808 | 0.148 | 0 | 0.211 | Cation_ATPase |
| TRINITY_DN1011_c0_g1_i2 | -2.19402063 | 0.409216855 | 0.999970678 | 7.921 | 0 | 14.288 | 19.454 | NA |
| TRINITY_DN545_c0_g2_i2 | 2.19374192 | 0.059124864 | 0.999970678 | 18.413 | 11.74 | 4.5 | 1.176 | NA |
| TRINITY_DN692_c0_g2_i1 | -2.19372906 | 0.397721291 | 0.999970678 | 0.927 | 0 | 2.667 | 1.046 | UDPGT |
| TRINITY_DN804_c3_g1_i3 | 2.193666112 | 0.599251187 | 0.999970678 | 2.634 | 0 | 0 | 0.583 | NA |
| TRINITY_DN3532_c0_g1_i6 | -2.19335702 | 0.146775669 | 0.999970678 | 8.92 | 0.867 | 9.277 | 34.233 | - |
| TRINITY_DN72_c0_g1_i10 | 2.193288653 | 0.455357963 | 0.999970678 | 4.878 | 0 | 0.156 | 0.905 | NA |
| TRINITY_DN4218_c0_g1_i20 | 2.193254826 | 0.598321844 | 0.999970678 | 1.926 | 0 | 0 | 0.432 | NA |
| TRINITY_DN3091_c0_g1_i7 | 2.193228044 | 0.069428184 | 0.999970678 | 4.542 | 2.06 | 0.389 | 0.985 | NA |
| TRINITY_DN250977_c0_g1_i1 | -2.19278241 | 0.234474714 | 0.999970678 | 2.807 | 0.118 | 3.111 | 9.662 | NA |
| TRINITY_DN9963_c0_g1_i7 | -2.19245285 | 0.39559177 | 0.999970678 | 0 | 2.168 | 6.244 | 2.212 | NA |
| TRINITY_DN20071_c0_g1_i1 | 2.192331049 | 0.329540985 | 0.999970678 | 0.145 | 7.894 | 0.211 | 1.448 | NA |
| TRINITY_DN1719_c0_g1_i3 | 2.191508192 | 0.596861181 | 0.999970678 | 5.85 | 0 | 0 | 1.297 | NA |
| TRINITY_DN3838_c0_g1_i2 | 2.190560922 | 0.391078847 | 0.999970678 | 2.298 | 0.966 | 0.6 | 0 | Frigida |
| TRINITY_DN1041_c7_g1_i1 | -2.19049816 | 0.31324077 | 0.999970678 | 21.411 | 2.868 | 88.495 | 1.649 | Aldedh |
| TRINITY_DN3897_c0_g1_i3 | 2.189090566 | 0.177242896 | 1 | 1.299 | 1.005 | 0.333 | 0.08 | NA |
| TRINITY_DN2507_c0_g1_i5 | 2.187215807 | 0.466232081 | 0.999970678 | 13.035 | 0 | 0.278 | 2.584 | - |
| TRINITY_DN3378_c0_g1_i5 | -2.1869448 | 0.246988054 | 0.999970678 | 5.85 | 7.391 | 0.722 | 59.226 | Chal_sti_synt_C |
| TRINITY_DN10702_c0_g2_i1 | 2.186376095 | 0.383630821 | 0.999970678 | 0.672 | 0.621 | 0.244 | 0 | Metallophos |
| TRINITY_DN1649_c0_g1_i4 | 2.186115555 | 0.045513425 | 0.999970678 | 1.553 | 0.66 | 0.189 | 0.261 | NA |
| TRINITY_DN1437_c1_g1_i7 | 2.185841337 | 0.192142781 | 0.999970678 | 9.792 | 0.572 | 1.067 | 1.046 | NA |
| TRINITY_DN1245_c0_g1_i6 | -2.18560038 | 0.007055114 | 0.360727208 | 0.59 | 0.887 | 3.189 | 2.885 | F-box |
| TRINITY_DN10412_c2_g1_i2 | 2.184869698 | 0.398336117 | 1 | 0.636 | 0.572 | 0.222 | 0 | NA |
| TRINITY_DN3315_c0_g1_i4 | 2.184860848 | 0.599411904 | 0.999970678 | 21.774 | 0 | 0 | 4.725 | NA |
| TRINITY_DN7687_c1_g1_i2 | -2.18467324 | 0.074398946 | 0.999970678 | 5.023 | 24.13 | 31.909 | 92.393 | NA |
| TRINITY_DN2093_c0_g1_i4 | -2.18451802 | 0.598058114 | 0.999970678 | 0.663 | 0 | 0 | 3.056 | NA |
| TRINITY_DN3260_c0_g1_i6 | -2.18404302 | 0.401688069 | 1 | 0.282 | 0 | 0.678 | 0.483 | Guanylate_kin |
| TRINITY_DN2844_c0_g1_i1 | -2.18399773 | 0.408683043 | 1 | 0.109 | 0 | 0.211 | 0.221 | - |
| TRINITY_DN9930_c0_g1_i6 | -2.18387788 | 0.205686661 | 0.999970678 | 0.045 | 0.493 | 1.422 | 0.744 | NA |
| TRINITY_DN468_c1_g1_i9 | 2.183570392 | 0.601685613 | 0.999970678 | 3.652 | 0 | 0 | 0.804 | NA |
| TRINITY_DN1304_c0_g1_i19 | 2.183569275 | 0.01447196 | 0.653077164 | 43.548 | 17.46 | 5.633 | 6.736 | NA |
| TRINITY_DN2382_c1_g1_i4 | 2.183147244 | 0.019341436 | 0.823889711 | 0.545 | 0.926 | 0.133 | 0.161 | NA |
| TRINITY_DN427_c3_g1_i1 | 2.182909474 | 0.601145199 | 0.999970678 | 8.612 | 0 | 1.589 | 0 | NA |
| TRINITY_DN5767_c0_g1_i9 | 2.182655358 | 0.285652297 | 0.999970678 | 2.253 | 0.03 | 0.167 | 0.302 | NA |
| TRINITY_DN3071_c1_g1_i1 | -2.18231496 | 0.427339323 | 0.999970678 | 0.427 | 0 | 0.211 | 1.759 | NA |
| TRINITY_DN1431_c0_g1_i3 | -2.18207263 | 0.403378735 | 0.999970678 | 5.487 | 0 | 9.711 | 13.552 | PsbP |
| TRINITY_DN14636_c0_g1_i2 | 2.181068898 | 0.38753699 | 0.999970678 | 1.998 | 1.33 | 0.6 | 0 | NA |
| TRINITY_DN8707_c0_g1_i1 | -2.18095932 | 0.604140354 | 0.999970678 | 1.544 | 0 | 5.966 | 0 | NA |
| TRINITY_DN1582_c0_g3_i3 | 2.180661439 | 0.258483775 | 0.999970678 | 5.341 | 24.9 | 0.111 | 6.515 | CCT |
| TRINITY_DN2953_c0_g2_i1 | 2.180529902 | 0.252191909 | 0.999970678 | 5.142 | 0.197 | 0.322 | 0.804 | NA |
| TRINITY_DN14444_c0_g1_i3 | 2.180469074 | 0.135672423 | 0.999970678 | 1.526 | 3.213 | 0.767 | 0.121 | NA |
| TRINITY_DN1148_c0_g1_i7 | -2.17995085 | 0.163366947 | 0.999970678 | 14.08 | 0.986 | 17.732 | 47.574 | Glyco_hydro_1 |
| TRINITY_DN9382_c0_g1_i1 | -2.17948059 | 0.410267208 | 0.999970678 | 1.181 | 0 | 3.6 | 1.086 | NA |

| TRINITY_DN7238_c0_g1_i5 | 2.178275904 | 0.120115121 | 1 | 0.327 | 0.719 | 0.111 | 0.09 | NA |
| --- | --- | --- | --- | --- | --- | --- | --- | --- |
| TRINITY_DN3154_c0_g1_i5 | -2.1779026 | 0.023376712 | 0.946423711 | 14.643 | 8.939 | 63.985 | 29.608 | NA |
| TRINITY_DN108_c0_g1_i5 | -2.17784264 | 0.179941381 | 0.999970678 | 3.479 | 0.759 | 14.688 | 1.578 | - |
| TRINITY_DN45361_c0_g1_i1 | 2.177819358 | 0.125155923 | 0.999970678 | 0.6 | 1.39 | 0.078 | 0.322 | NA |
| TRINITY_DN1186_c0_g1_i8 | -2.17771233 | 0.602342094 | 0.999970678 | 0 | 0.503 | 0 | 2.232 | NA |
| TRINITY_DN8206_c0_g2_i1 | 2.17736723 | 0.600400191 | 0.999970678 | 0 | 9.993 | 0 | 2.162 | NA |
| TRINITY_DN1767_c0_g2_i3 | 2.17691877 | 0.1713101 | 0.999970678 | 5.868 | 0.503 | 0.8 | 0.483 | NA |
| TRINITY_DN5395_c0_g1_i2 | -2.17631763 | 0.599540029 | 0.999970678 | 0.945 | 0 | 0 | 4.373 | Aldedh |
| TRINITY_DN30_c3_g2_i2 | 2.176258254 | 0.434895844 | 0.999970678 | 44.766 | 32.38 | 0 | 17.172 | NA |
| TRINITY_DN1711_c1_g1_i4 | 2.175208402 | 0.387763867 | 0.999970678 | 1.917 | 1.163 | 0.567 | 0 | NAF |
| TRINITY_DN2013_c0_g1_i1 | 2.175178861 | 0.039462532 | 0.999970678 | 4.07 | 6.633 | 0.478 | 1.79 | NA |
| TRINITY_DN26_c0_g2_i2 | 2.174371712 | 0.228572403 | 0.999970678 | 24.018 | 0.917 | 1.322 | 3.931 | - |
| TRINITY_DN16246_c0_g1_i3 | 2.173634775 | 0.05907891 | 0.999970678 | 0.336 | 0.404 | 0.067 | 0.08 | STAS |
| TRINITY_DN24863_c0_g1_i1 | -2.17329362 | 0.611866317 | 0.999970678 | 0.418 | 0 | 1.589 | 0 | Pkinase |
| TRINITY_DN193_c0_g1_i11 | 2.172875376 | 0.190084393 | 0.999970678 | 63.006 | 3.617 | 2.866 | 11.511 | NA |
| TRINITY_DN4762_c0_g1_i6 | 2.172356424 | 0.405894859 | 0.999970678 | 0.3 | 0.907 | 0.211 | 0 | NA |
| TRINITY_DN1486_c0_g1_i25 | 2.17207398 | 0.410586119 | 0.999970678 | 1.045 | 6.583 | 0 | 1.679 | NA |
| TRINITY_DN557_c0_g1_i6 | 2.171770458 | 0.042955228 | 0.999970678 | 27.915 | 9.56 | 4.7 | 2.714 | Cpn60_TCP1 |
| TRINITY_DN6915_c0_g1_i2 | 2.170259678 | 0.099733325 | 0.999970678 | 1.299 | 0.414 | 0.2 | 0.161 | CUE |
| TRINITY_DN1633_c0_g1_i5 | 2.170226282 | 0.395150125 | 1 | 0.863 | 0.562 | 0 | 0.312 | F-box |
| TRINITY_DN3138_c0_g1_i6 | 2.170169801 | 0.601521524 | 0.999970678 | 7.049 | 0 | 0 | 1.609 | NA |
| TRINITY_DN473_c0_g1_i5 | 2.170037139 | 0.241079136 | 1 | 3.597 | 4.997 | 1.455 | 0.211 | NA |
| TRINITY_DN4274_c0_g1_i10 | -2.16959005 | 0.414284925 | 0.999970678 | 0 | 1.735 | 5.377 | 1.106 | Ank |
| TRINITY_DN22150_c0_g1_i2 | -2.16944638 | 0.608896185 | 0.999970678 | 0.808 | 0 | 3.044 | 0 | DUF4243 |
| TRINITY_DN1163_c0_g1_i3 | 2.169444063 | 0.441247734 | 0.999970678 | 0 | 8.298 | 0.711 | 0.955 | DUF3110 |
| TRINITY_DN12461_c0_g1_i3 | -2.16850215 | 0.218550346 | 0.999970678 | 5.287 | 0.434 | 19.277 | 2.725 | AMP-binding |
| TRINITY_DN9648_c0_g1_i4 | -2.16824906 | 0.414305083 | 0.999970678 | 4.515 | 0 | 9.333 | 9.28 | NA |
| TRINITY_DN4773_c0_g1_i5 | 2.166034632 | 0.600029973 | 0.999970678 | 0 | 15.16 | 0 | 3.278 | NA |
| TRINITY_DN174_c0_g1_i1 | -2.16542363 | 0.477924207 | 0.999970678 | 3.143 | 0.217 | 0 | 15.06 | NA |
| TRINITY_DN5004_c0_g1_i6 | -2.16438676 | 0.176051087 | 0.999970678 | 0.454 | 0.611 | 0.256 | 4.494 | Lipase_3 |
| TRINITY_DN5222_c0_g1_i6 | -2.16428406 | 0.102689979 | 0.999970678 | 0.709 | 0.207 | 1.5 | 2.242 | COMPASS-Shg1 |
| TRINITY_DN1218_c6_g1_i1 | 2.163464403 | 0.383299051 | 0.999970678 | 1.653 | 3.016 | 0 | 1.036 | NA |
| TRINITY_DN18085_c1_g1_i3 | 2.163425672 | 0.064998979 | 0.999970678 | 3.397 | 1.784 | 0.7 | 0.312 | NA |
| TRINITY_DN1903_c0_g1_i9 | -2.16161959 | 0.485994416 | 0.999970678 | 0.854 | 0 | 3.155 | 0.05 | NA |
| TRINITY_DN5715_c0_g1_i1 | -2.16159206 | 0.635643643 | 0.999970678 | 69.919 | 0 | 261.851 | 0 | MFS_1 |
| TRINITY_DN7626_c0_g2_i5 | 2.16107916 | 0.183271711 | 1 | 0.454 | 0.532 | 0.144 | 0.04 | NA |
| TRINITY_DN3237_c0_g1_i2 | 2.160598821 | 0.400309236 | 0.999970678 | 1.517 | 6.091 | 0 | 1.669 | Linker_histone |
| TRINITY_DN2542_c0_g1_i3 | -2.16035432 | 0.427110313 | 0.999970678 | 0.018 | 2.523 | 0.933 | 9.943 | NA |
| TRINITY_DN23552_c0_g1_i1 | 2.159494791 | 0.03005807 | 0.999970678 | 28.006 | 21.49 | 5.555 | 3.901 | peroxidase |
| TRINITY_DN16764_c0_g1_i4 | -2.15921915 | 0.05872267 | 0.999970678 | 0.4 | 0.532 | 2.678 | 0.915 | DNA_gyraseB |
| TRINITY_DN26687_c0_g1_i1 | 2.158998635 | 0.476235844 | 0.999970678 | 1.671 | 0.049 | 0.322 | 0 | NA |
| TRINITY_DN113_c0_g2_i1 | 2.15858565 | 0.391358378 | 0.999970678 | 0.699 | 0.601 | 0.244 | 0 | NA |
| TRINITY_DN6241_c0_g1_i13 | -2.15814578 | 0.603372397 | 0.999970678 | 0 | 1.064 | 3.889 | 0 | NA |
| TRINITY_DN8615_c0_g1_i2 | 2.157711128 | 0.40991247 | 1 | 0.354 | 0.512 | 0.167 | 0 | NA |
| TRINITY_DN9839_c0_g2_i1 | -2.15748774 | 0.402610174 | 1 | 0 | 0.325 | 0.444 | 0.925 | NA |
| TRINITY_DN4692_c0_g1_i4 | 2.157479389 | 0.401708814 | 0.999970678 | 0.881 | 0.306 | 0.222 | 0 | NA |
| TRINITY_DN5660_c0_g1_i11 | 2.157114112 | 0.110257037 | 0.999970678 | 0.609 | 1.971 | 0.278 | 0.241 | Mlo |
| TRINITY_DN1625_c0_g1_i1 | -2.15614508 | 0.433708621 | 0.999970678 | 0 | 32.93 | 80.14 | 46.78 | Ribosomal_S17 |
| TRINITY_DN8325_c1_g1_i6 | 2.15606709 | 0.604877197 | 0.999970678 | 7.803 | 0 | 1.478 | 0 | NA |
| TRINITY_DN707_c0_g1_i7 | 2.156031847 | 0.603686557 | 0.999970678 | 0 | 1.035 | 0.189 | 0 | NA |
| TRINITY_DN808_c3_g1_i6 | 2.155567396 | 0.606362085 | 0.999970678 | 3.216 | 0 | 0.6 | 0 | RVT_1 |
| TRINITY_DN26_c0_g2_i1 | 2.154892772 | 0.178679202 | 0.999970678 | 27.697 | 2.503 | 2.9 | 3.247 | - |
| TRINITY_DN7125_c0_g1_i22 | -2.15442962 | 0.615253194 | 0.999970678 | 0.59 | 0 | 2.233 | 0 | NA |
| TRINITY_DN2060_c0_g1_i10 | -2.15319969 | 0.415517217 | 0.999970678 | 3.661 | 0 | 2.522 | 12.989 | NA |
| TRINITY_DN15211_c0_g1_i6 | 2.152797745 | 0.395423948 | 0.999970678 | 0.545 | 0.552 | 0.2 | 0 | NA |
| TRINITY_DN8050_c0_g1_i9 | 2.152741943 | 0.40818686 | 0.999970678 | 1.135 | 0.286 | 0 | 0.332 | NA |
| TRINITY_DN527_c0_g1_i9 | 2.151525911 | 0.392486922 | 0.999970678 | 1.735 | 1.202 | 0 | 0.664 | A_deaminase |
| TRINITY_DN7906_c0_g1_i3 | -2.1508029 | 0.425824001 | 0.999970678 | 0 | 1.311 | 0.567 | 5.027 | Branch |
| TRINITY_DN7833_c0_g1_i2 | -2.15080094 | 0.26598605 | 0.999970678 | 0.454 | 0.059 | 1.7 | 0.221 | NA |
| TRINITY_DN12260_c0_g1_i3 | -2.15052496 | 0.082952609 | 0.999970678 | 1.09 | 0.266 | 2.3 | 3.298 | NA |
| TRINITY_DN2310_c0_g1_i2 | -2.15045838 | 0.252602558 | 0.999970678 | 0.4 | 0.049 | 1.589 | 0.151 | UDPGT |
| TRINITY_DN831_c2_g1_i1 | -2.14969832 | 0.189056812 | 0.999970678 | 1.771 | 2.119 | 0.8 | 16.207 | NA |
| TRINITY_DN1032_c0_g1_i12 | 2.149558813 | 0.184225753 | 0.999970678 | 0.491 | 7.855 | 0.922 | 0.754 | Lipoxygenase |
| TRINITY_DN1397_c0_g1_i16 | 2.149066632 | 0.605563615 | 0.999970678 | 0 | 2.868 | 0.522 | 0 | NA |
| TRINITY_DN549_c0_g1_i4 | -2.1485263 | 0.428783845 | 0.999970678 | 2.671 | 0 | 1.489 | 10.194 | NA |
| TRINITY_DN1258_c0_g1_i12 | -2.14785502 | 0.181232357 | 0.999970678 | 2.607 | 0.512 | 1.144 | 12.678 | - |
| TRINITY_DN8858_c0_g1_i1 | -2.14770925 | 0.405889221 | 0.999970678 | 1.054 | 0 | 2.011 | 2.443 | NA |
| TRINITY_DN51506_c0_g1_i1 | 2.147157981 | 0.411292629 | 1 | 1.281 | 0.808 | 0.367 | 0 | MOR2-PAG1_C |
| TRINITY_DN76812_c0_g1_i1 | -2.14714649 | 0.094605788 | 0.999970678 | 0.4 | 0.434 | 0.811 | 2.684 | NA |
| TRINITY_DN4126_c0_g1_i1 | 2.146362754 | 0.313645188 | 0.999970678 | 1.735 | 14.02 | 0.056 | 3.438 | NA |
| TRINITY_DN606_c0_g1_i2 | 2.146350237 | 0.44934373 | 0.999970678 | 0 | 28.44 | 2.067 | 3.82 | NA |
| TRINITY_DN983_c0_g1_i8 | 2.146140148 | 0.606450961 | 0.999970678 | 2.289 | 0 | 0 | 0.523 | Glyco_hydro_10 |
| TRINITY_DN3340_c0_g1_i1 | 2.146077792 | 0.385561012 | 0.999970678 | 2.089 | 2.385 | 0 | 0.995 | NA |
| TRINITY_DN2589_c0_g1_i10 | 2.145821704 | 0.614371402 | 0.999970678 | 0 | 4.405 | 0.811 | 0 | DBINO |

| TRINITY_DN271_c0_g1_i5 | -2.14548996 | 0.245688488 | 0.999970678 | 9.702 | 8.466 | 1.233 | 78.198 | NA |
| --- | --- | --- | --- | --- | --- | --- | --- | --- |
| TRINITY_DN938_c0_g1_i10 | 2.144645739 | 0.131812494 | 0.999970678 | 27.661 | 2.986 | 3.511 | 2.765 | NA |
| TRINITY_DN10349_c0_g1_i6 | 2.144359478 | 0.406513835 | 0.999970678 | 1.626 | 0.512 | 0 | 0.503 | NA |
| TRINITY_DN12008_c0_g1_i3 | 2.144332447 | 0.295868063 | 0.999970678 | 0.118 | 9.776 | 0.544 | 1.558 | NA |
| TRINITY_DN3471_c0_g1_i1 | -2.14396209 | 0.213049188 | 0.999970678 | 0.082 | 0.936 | 2.378 | 1.558 | HLH |
| TRINITY_DN3015_c0_g1_i2 | 2.143913804 | 0.042696289 | 0.999970678 | 3.17 | 5.342 | 0.867 | 0.845 | NA |
| TRINITY_DN4593_c0_g1_i2 | 2.143322359 | 0.450748299 | 0.999970678 | 18.086 | 16.3 | 6.444 | 0 | Myb_DNA-bindin |
| TRINITY_DN2603_c0_g1_i1 | 2.143008822 | 0.414935306 | 0.999970678 | 7.767 | 1.498 | 0 | 2.121 | NA |
| TRINITY_DN3124_c0_g1_i5 | -2.14259306 | 0.284747838 | 0.999970678 | 0.854 | 0.365 | 0.067 | 5.268 | DEAD |
| TRINITY_DN475_c0_g1_i2 | -2.14258924 | 0.613136776 | 0.999970678 | 5.85 | 0 | 21.065 | 0 | NA |
| TRINITY_DN10522_c0_g1_i1 | -2.14226049 | 0.40405486 | 0.999970678 | 1.681 | 0 | 3.344 | 3.519 | NA |
| TRINITY_DN10848_c0_g1_i1 | 2.142245795 | 0.288862165 | 0.999970678 | 4.569 | 226.8 | 38.853 | 4.645 | NA |
| TRINITY_DN10424_c0_g1_i7 | 2.141259528 | 0.411134057 | 1 | 3.87 | 2.809 | 1.211 | 0 | NA |
| TRINITY_DN2661_c1_g1_i3 | 2.140437345 | 0.423074391 | 0.999970678 | 98.77 | 49.47 | 27.654 | 0 | NA |
| TRINITY_DN105506_c0_g1_i1 | -2.14017569 | 0.247907434 | 0.999970678 | 0.391 | 0.68 | 0.178 | 4.494 | NA |
| TRINITY_DN1845_c0_g1_i6 | 2.140022074 | 0.409154259 | 0.999970678 | 1.281 | 0.384 | 0.311 | 0 | Cation_ATPase |
| TRINITY_DN5142_c1_g1_i1 | 2.13992391 | 0.609861763 | 0.999970678 | 0 | 6.692 | 1.222 | 0 | NA |
| TRINITY_DN2138_c0_g1_i7 | -2.1398679 | 0.616989689 | 0.999970678 | 1.19 | 0 | 4.378 | 0 | Peroxidase_2 |
| TRINITY_DN27581_c0_g1_i6 | 2.13961162 | 0.25126471 | 0.999970678 | 0.736 | 1.478 | 0.022 | 0.473 | NA |
| TRINITY_DN2163_c0_g1_i1 | -2.13950354 | 0.137550597 | 0.999970678 | 2.507 | 21.71 | 21.588 | 79.464 | NA |
| TRINITY_DN8734_c1_g1_i2 | 2.139459855 | 0.097947362 | 0.999970678 | 1.163 | 1.261 | 0.311 | 0.171 | NA |
| TRINITY_DN4113_c0_g1_i4 | -2.13786001 | 0.447439303 | 0.999970678 | 0 | 1.399 | 4.7 | 0.372 | GAGA_bind |
| TRINITY_DN1158_c0_g5_i3 | -2.13746454 | 0.240903882 | 0.999970678 | 2.017 | 0.128 | 5.777 | 2.483 | Ribosomal_L28e |
| TRINITY_DN10587_c0_g1_i3 | 2.137201742 | 0.401776832 | 0.999970678 | 0.436 | 1.025 | 0.267 | 0 | NA |
| TRINITY_DN3637_c0_g1_i6 | 2.13626978 | 0.241849357 | 0.999970678 | 8.021 | 0.404 | 0.378 | 1.468 | LRRNT_2 |
| TRINITY_DN3330_c0_g1_i1 | 2.1360814 | 0.193793586 | 0.999970678 | 199.1 | 9.215 | 26.332 | 16.046 | NA |
| TRINITY_DN1269_c1_g1_i1 | -2.13588434 | 0.458936828 | 0.999970678 | 3.915 | 0 | 13.744 | 0.774 | NA |
| TRINITY_DN15186_c0_g1_i2 | -2.13578625 | 0.190953036 | 0.999970678 | 24.954 | 70.73 | 17.988 | 384.494 | NA |
| TRINITY_DN8991_c0_g2_i6 | -2.13574406 | 0.056481836 | 0.999970678 | 0.854 | 0.68 | 1.555 | 4.977 | - |
| TRINITY_DN28234_c0_g2_i2 | -2.13563418 | 0.432068521 | 0.999970678 | 0 | 18.1 | 12.099 | 62.926 | Barwin |
| TRINITY_DN29909_c0_g1_i1 | -2.13555192 | 0.399832219 | 0.999970678 | 0.572 | 0 | 1.133 | 1.166 | AMP-binding |
| TRINITY_DN21085_c0_g1_i5 | -2.13531572 | 0.618688859 | 0.999970678 | 0.563 | 0 | 2.089 | 0 | Fasciclin |
| TRINITY_DN11437_c0_g1_i2 | -2.13531572 | 0.618688859 | 0.999970678 | 1.244 | 0 | 4.655 | 0 | NA |
| TRINITY_DN198_c0_g1_i10 | 2.135101592 | 0.078711091 | 0.999970678 | 6.032 | 1.38 | 0.7 | 0.845 | NA |
| TRINITY_DN3406_c0_g1_i2 | -2.13500316 | 0.120710099 | 0.999970678 | 2.462 | 0.394 | 4.422 | 7.178 | Response_reg |
| TRINITY_DN13876_c1_g1_i1 | -2.13491681 | 0.371094364 | 0.999970678 | 0.491 | 0.02 | 1.833 | 0.08 | Beta_helix |
| TRINITY_DN10890_c0_g1_i1 | -2.13491174 | 0.341866731 | 0.999970678 | 0.654 | 4.809 | 0.256 | 22.812 | NA |
| TRINITY_DN6870_c0_g1_i2 | 2.134906667 | 0.398279161 | 0.999970678 | 0.981 | 0.424 | 0 | 0.332 | NA |
| TRINITY_DN20600_c0_g1_i1 | -2.13387818 | 0.419859763 | 0.999970678 | 1.971 | 0 | 1.533 | 7.007 | NA |
| TRINITY_DN1031_c2_g1_i2 | 2.1336441 | 0.288498672 | 0.999970678 | 0.136 | 7.51 | 0.256 | 1.418 | NA |
| TRINITY_DN542_c0_g1_i2 | -2.13280953 | 0.174430236 | 0.999970678 | 2.253 | 0.207 | 2.978 | 7.329 | NA |
| TRINITY_DN3724_c0_g2_i14 | 2.13212088 | 0.256079159 | 0.999970678 | 1.426 | 2.503 | 0.678 | 0.08 | NA |
| TRINITY_DN3531_c0_g1_i5 | -2.13187067 | 0.140935535 | 0.999970678 | 7.104 | 12.1 | 5.633 | 77.162 | UDPGT |
| TRINITY_DN3746_c0_g1_i1 | -2.13120207 | 0.400992367 | 0.999970678 | 0.963 | 0 | 1.489 | 2.473 | DUF1685 |
| TRINITY_DN34233_c0_g1_i2 | 2.129868442 | 0.416984996 | 1 | 0.336 | 0.453 | 0.144 | 0 | NA |
| TRINITY_DN9270_c0_g1_i2 | 2.129654912 | 0.398805391 | 0.999970678 | 2.307 | 2.553 | 0.911 | 0 | NA |
| TRINITY_DN61768_c0_g1_i3 | 2.129377745 | 0.111221771 | 0.999970678 | 0.509 | 1.045 | 0.167 | 0.131 | NA |
| TRINITY_DN1197_c1_g1_i3 | 2.129066926 | 0.614084471 | 0.999970678 | 2.198 | 0 | 0.422 | 0 | CLU |
| TRINITY_DN16992_c0_g1_i2 | -2.12813636 | 0.058010178 | 0.999970678 | 8.975 | 10.86 | 19.51 | 58.452 | NA |
| TRINITY_DN6183_c0_g1_i2 | 2.12761215 | 0.6092671 | 0.999970678 | 0 | 7.667 | 1.422 | 0 | NA |
| TRINITY_DN3334_c0_g1_i1 | -2.12673422 | 0.414097715 | 0.999970678 | 2.943 | 0 | 5.689 | 6.123 | BCDHK_Adom3 |
| TRINITY_DN13067_c0_g1_i1 | 2.126653906 | 0.075612609 | 0.999970678 | 2.725 | 3.223 | 0.244 | 1.066 | PPR |
| TRINITY_DN5355_c0_g1_i1 | 2.126631729 | 0.20800583 | 0.999970678 | 5.078 | 0.306 | 0.356 | 0.845 | OPT |
| TRINITY_DN9231_c0_g1_i1 | -2.12635151 | 0.401153196 | 0.999970678 | 0.645 | 0 | 1.2 | 1.347 | NA |
| TRINITY_DN2561_c0_g1_i2 | -2.12610716 | 0.170337701 | 0.999970678 | 4.869 | 0.463 | 14.788 | 5.66 | Rubis-subs-bind |
| TRINITY_DN6221_c0_g1_i8 | 2.126057208 | 0.004151607 | 0.227968593 | 5.75 | 5.144 | 1.133 | 1.116 | ubiquitin |
| TRINITY_DN5548_c0_g1_i5 | -2.12576991 | 0.161446526 | 0.999970678 | 2.498 | 6.692 | 3.7 | 34.283 | NA |
| TRINITY_DN817_c0_g1_i11 | -2.12445896 | 0.313932608 | 0.999970678 | 0.6 | 0.01 | 0.333 | 2.272 | DUF4413 |
| TRINITY_DN1610_c0_g1_i7 | 2.123945402 | 0.612332071 | 0.999970678 | 0 | 0.926 | 0.167 | 0 | NA |
| TRINITY_DN1202_c0_g1_i7 | 2.122157681 | 0.296025003 | 0.999970678 | 1.335 | 13.77 | 2.711 | 0.161 | NA |
| TRINITY_DN1027_c0_g1_i2 | -2.12196585 | 0.065186181 | 0.999970678 | 2.098 | 2.75 | 3.189 | 17.282 | NA |
| TRINITY_DN3654_c0_g1_i2 | 2.121920331 | 0.128075609 | 0.999970678 | 1.29 | 7.204 | 1.033 | 0.674 | zf-RING_2 |
| TRINITY_DN4960_c0_g1_i2 | 2.121855412 | 0.163357265 | 0.999970678 | 0.218 | 1.478 | 0.233 | 0.111 | adh_short_C2 |
| TRINITY_DN566_c0_g1_i11 | -2.12121289 | 0.435334107 | 0.999970678 | 1.354 | 0 | 0.6 | 5.248 | NA |
| TRINITY_DN14445_c0_g1_i2 | 2.120416151 | 0.24850487 | 0.999970678 | 0.3 | 4.06 | 0.7 | 0.141 | Abhydrolase_3 |
| TRINITY_DN1031_c2_g1_i1 | -2.12031619 | 0.41729603 | 0.999970678 | 1.671 | 0 | 1.667 | 5.369 | NA |
| TRINITY_DN6731_c0_g1_i2 | 2.119181726 | 0.206979805 | 0.999970678 | 0.409 | 2.001 | 0.389 | 0.08 | - |
| TRINITY_DN186_c0_g1_i6 | -2.11880456 | 0.139403533 | 0.999970678 | 0.218 | 0.443 | 0.356 | 2.413 | Pkinase |
| TRINITY_DN21768_c0_g2_i7 | -2.11864664 | 0.61949654 | 0.999970678 | 0.5 | 0 | 0 | 2.222 | NA |
| TRINITY_DN19677_c0_g1_i1 | -2.11843716 | 0.111297101 | 0.999970678 | 0.345 | 0.375 | 0.567 | 2.413 | BBE |
| TRINITY_DN4543_c4_g1_i3 | 2.118213407 | 0.614680721 | 0.999970678 | 0 | 4.287 | 0 | 0.975 | NA |
| TRINITY_DN6886_c0_g1_i3 | -2.1172692 | 0.617147944 | 0.999970678 | 0.609 | 0 | 2.2 | 0 | NA |
| TRINITY_DN189_c1_g1_i2 | -2.11700854 | 0.070533734 | 0.999970678 | 0.436 | 0.207 | 1.067 | 1.508 | NA |
| TRINITY_DN5056_c0_g1_i2 | 2.116312581 | 0.137707681 | 0.999970678 | 2.371 | 23.07 | 3.111 | 2.041 | DHquinase_I |

| TRINITY_DN1183_c1_g1_i4 | 2.116171123 | 0.467057512 | 0.999970678 | 6.368 | 0 | 0.322 | 1.086 | NA |
| --- | --- | --- | --- | --- | --- | --- | --- | --- |
| TRINITY_DN1326_c0_g1_i5 | -2.11612999 | 0.421106284 | 0.999970678 | 0.936 | 0 | 0.8 | 3.177 | - |
| TRINITY_DN12971_c0_g1_i1 | 2.116030366 | 0.20684562 | 0.999970678 | 10.846 | 0.926 | 1.844 | 0.513 | NA |
| TRINITY_DN4411_c0_g1_i4 | -2.11585872 | 0.073338667 | 0.999970678 | 1.154 | 1.557 | 2.233 | 8.807 | NA |
| TRINITY_DN13479_c0_g2_i1 | -2.11578998 | 0.616092824 | 0.999970678 | 1.272 | 0 | 4.633 | 0 | NA |
| TRINITY_DN1646_c0_g1_i7 | 2.115012723 | 0.067118357 | 0.999970678 | 0.808 | 2.681 | 0.267 | 0.483 | NA |
| TRINITY_DN4086_c0_g1_i4 | -2.11496121 | 0.426343607 | 0.999970678 | 1.299 | 0 | 3.8 | 1.156 | NA |
| TRINITY_DN33476_c0_g1_i2 | -2.11489151 | 0.405274794 | 0.999970678 | 0 | 0.946 | 1.855 | 1.649 | NA |
| TRINITY_DN41841_c0_g1_i2 | 2.114273957 | 0.407479708 | 0.999970678 | 1.154 | 0.69 | 0.344 | 0 | NA |
| TRINITY_DN3928_c0_g1_i1 | 2.114224414 | 0.409025009 | 0.999970678 | 6.867 | 3.775 | 0 | 2.473 | NA |
| TRINITY_DN273_c0_g1_i6 | 2.113791804 | 0.057802604 | 0.999970678 | 0.345 | 0.315 | 0.056 | 0.08 | Aconitase |
| TRINITY_DN15762_c0_g1_i7 | -2.11378219 | 0.408486478 | 0.999970678 | 0.245 | 0 | 0.333 | 0.714 | NA |
| TRINITY_DN10371_c0_g1_i1 | -2.11376905 | 0.004990451 | 0.266418328 | 2.671 | 4.297 | 10.333 | 17.594 | NA |
| TRINITY_DN1268_c0_g1_i1 | -2.1132017 | 0.491929568 | 0.999970678 | 0.836 | 0 | 0.067 | 3.539 | NA |
| TRINITY_DN325_c0_g2_i2 | -2.1126713 | 0.217337894 | 0.999970678 | 34.119 | 4.859 | 10.488 | 155.229 | NA |
| TRINITY_DN96322_c0_g1_i2 | 2.11119636 | 0.22647488 | 0.999970678 | 4.26 | 0.867 | 0.1 | 1.056 | NA |
| TRINITY_DN3168_c0_g1_i1 | -2.11093514 | 0.247835254 | 0.999970678 | 1.363 | 12.37 | 3.111 | 54.019 | - |
| TRINITY_DN12147_c0_g1_i2 | -2.1108245 | 0.296083836 | 0.999970678 | 0.073 | 1.508 | 1.067 | 5.449 | Lipase_3 |
| TRINITY_DN7206_c1_g2_i1 | 2.110570853 | 0.179578991 | 1 | 1.126 | 0.995 | 0.089 | 0.372 | NA |
| TRINITY_DN1868_c0_g1_i11 | 2.110275858 | 0.481104902 | 0.999970678 | 4.842 | 0.108 | 0.967 | 0 | NA |
| TRINITY_DN492_c0_g1_i8 | -2.10960073 | 0.133359902 | 0.999970678 | 0.2 | 0.335 | 1.611 | 0.352 | NA |
| TRINITY_DN4906_c0_g1_i2 | -2.10954994 | 0.406180462 | 0.999970678 | 1.172 | 0 | 2.022 | 2.584 | - |
| TRINITY_DN1037_c0_g1_i10 | 2.109422156 | 0.456489952 | 0.999970678 | 17.023 | 17.26 | 0 | 8.003 | Cpn60_TCP1 |
| TRINITY_DN255_c1_g1_i10 | -2.10923425 | 0.611535651 | 0.999970678 | 0 | 1.084 | 3.8 | 0.01 | NA |
| TRINITY_DN4411_c0_g1_i7 | -2.1091167 | 0.445146003 | 0.999970678 | 4.578 | 0 | 3.166 | 16.257 | NA |
| TRINITY_DN1051_c2_g1_i3 | 2.108920786 | 0.151114838 | 0.999970678 | 5.005 | 0.838 | 0.822 | 0.362 | NA |
| TRINITY_DN4525_c0_g1_i2 | -2.10839535 | 0.420851724 | 0.999970678 | 6.995 | 0 | 12.233 | 15.704 | NA |
| TRINITY_DN7884_c0_g1_i4 | 2.107764478 | 0.176878299 | 0.999970678 | 2.534 | 0.414 | 0.444 | 0.141 | NA |
| TRINITY_DN934_c0_g1_i1 | -2.10764368 | 0.418669911 | 0.999970678 | 3.561 | 0 | 5.389 | 9.089 | NA |
| TRINITY_DN3690_c0_g1_i1 | -2.10673152 | 0.058459852 | 0.999970678 | 1.644 | 0.552 | 4.066 | 4.514 | NA |
| TRINITY_DN193_c0_g1_i3 | 2.105711652 | 0.266076289 | 0.999970678 | 180.29 | 2.602 | 13.533 | 26.602 | NA |
| TRINITY_DN712_c0_g2_i2 | 2.105389957 | 0.184996948 | 0.999970678 | 0.881 | 6.268 | 0.211 | 1.397 | NA |
| TRINITY_DN6741_c0_g1_i2 | -2.10526487 | 0.161721682 | 0.999970678 | 0.745 | 1.025 | 5.677 | 0.744 | NA |
| TRINITY_DN1308_c0_g1_i1 | 2.104780124 | 0.405416441 | 1 | 1.626 | 1.143 | 0 | 0.623 | NA |
| TRINITY_DN4227_c0_g1_i3 | 2.104379514 | 0.416788178 | 0.999970678 | 16.397 | 3.863 | 0 | 4.635 | NA |
| TRINITY_DN820_c0_g1_i2 | -2.10414614 | 0.628806324 | 0.999970678 | 0 | 20.24 | 70.762 | 0 | NA |
| TRINITY_DN220_c0_g1_i16 | -2.10368937 | 0.102419625 | 0.999970678 | 1.544 | 0.286 | 2.689 | 4.705 | EGF_CA |
| TRINITY_DN6928_c0_g2_i2 | 2.102375696 | 0.078490691 | 0.999970678 | 1.535 | 1.666 | 0.378 | 0.322 | NA |
| TRINITY_DN1572_c1_g1_i1 | -2.10192345 | 0.360457305 | 0.999970678 | 0.073 | 2.444 | 0.411 | 10.034 | NA |
| TRINITY_DN1296_c0_g1_i5 | -2.10155996 | 0.120155142 | 0.999970678 | 1.399 | 0.808 | 1.089 | 8.204 | - |
| TRINITY_DN4470_c0_g1_i1 | -2.10019413 | 0.417945466 | 0.999970678 | 1.072 | 0 | 2.655 | 1.418 | Dev_Cell_Death |
| TRINITY_DN1601_c0_g1_i4 | 2.099686687 | 0.231001953 | 0.999970678 | 0.5 | 2.158 | 0.456 | 0.06 | NA |
| TRINITY_DN4739_c0_g1_i3 | 2.099566272 | 0.062763637 | 0.999970678 | 1.562 | 1.557 | 0.156 | 0.553 | Glyco_trans_1_4 |
| TRINITY_DN11256_c0_g1_i2 | 2.099517262 | 0.243536191 | 1 | 1.408 | 1.409 | 0.067 | 0.573 | NA |
| TRINITY_DN8422_c0_g1_i4 | 2.097304824 | 0.613237049 | 0.999970678 | 6.768 | 0 | 0 | 1.619 | ETF |
| TRINITY_DN2179_c0_g1_i6 | 2.096978695 | 0.326314685 | 0.999970678 | 2.807 | 0.049 | 0.111 | 0.533 | NA |
| TRINITY_DN6188_c0_g1_i1 | 2.096943375 | 0.10228237 | 0.999970678 | 1.871 | 8.436 | 0.633 | 1.629 | Aa_trans |
| TRINITY_DN1991_c0_g1_i2 | -2.09616052 | 0.109979322 | 0.999970678 | 0.273 | 0.532 | 0.967 | 2.252 | Serpin |
| TRINITY_DN24910_c0_g1_i1 | -2.09598519 | 0.107756787 | 0.999970678 | 0.436 | 0.197 | 1.6 | 0.774 | Malate_synthase |
| TRINITY_DN757_c1_g1_i1 | -2.09591954 | 0.614863133 | 0.999970678 | 0 | 0.66 | 2.3 | 0 | AUX_IAA |
| TRINITY_DN98406_c0_g1_i6 | -2.09577477 | 0.412322276 | 0.999970678 | 0.618 | 0 | 1.411 | 0.955 | NA |
| TRINITY_DN16322_c0_g1_i8 | -2.09574799 | 0.418271392 | 1 | 0 | 0.463 | 0.833 | 0.995 | NA |
| TRINITY_DN538_c0_g1_i1 | 2.095648401 | 0.504953521 | 0.999970678 | 0 | 17.48 | 3.211 | 0.131 | NA |
| TRINITY_DN5103_c0_g1_i1 | -2.09564406 | 0.619011106 | 0.999970678 | 0 | 8.446 | 0 | 35.5 | NA |
| TRINITY_DN226_c0_g1_i8 | -2.09466125 | 0.244331595 | 0.999970678 | 2.017 | 2.651 | 0.422 | 19.414 | ABC2_membrane |
| TRINITY_DN6198_c0_g1_i5 | -2.09459272 | 0.421377219 | 0.999970678 | 0.409 | 0 | 0.922 | 0.613 | NA |
| TRINITY_DN16107_c0_g1_i3 | 2.094506135 | 0.218526378 | 0.999970678 | 1.344 | 0.286 | 0.278 | 0.06 | NA |
| TRINITY_DN63_c0_g1_i3 | -2.0940427 | 0.034728932 | 0.999970678 | 2.516 | 0.897 | 6.9 | 6.334 | FBPase |
| TRINITY_DN2212_c0_g1_i5 | 2.092965963 | 0.520400818 | 0.999970678 | 0 | 1.35 | 0.256 | 0 | NA |
| TRINITY_DN1153_c0_g1_i5 | -2.09284716 | 0.473223176 | 0.999970678 | 0 | 1.991 | 0.322 | 7.912 | NA |
| TRINITY_DN2483_c0_g1_i4 | -2.09236832 | 0.089446517 | 0.999970678 | 0.709 | 0.325 | 2.689 | 1.206 | NA |
| TRINITY_DN1773_c0_g1_i4 | -2.09182908 | 0.457278713 | 0.999970678 | 0 | 2.02 | 6.533 | 0.553 | NA |
| TRINITY_DN3932_c0_g1_i9 | -2.09024017 | 0.026862697 | 0.999970678 | 0.327 | 0.611 | 1.433 | 2.262 | Pkinase |
| TRINITY_DN4730_c0_g1_i1 | -2.09022325 | 0.129596369 | 0.999970678 | 0.427 | 0.355 | 2.333 | 0.503 | NA |
| TRINITY_DN1219_c2_g1_i4 | 2.08896014 | 0.621414011 | 0.999970678 | 1.635 | 0 | 0 | 0.392 | NA |
| TRINITY_DN309_c0_g1_i1 | -2.08884865 | 0.421939892 | 1 | 0 | 0.207 | 0.444 | 0.362 | PPR |
| TRINITY_DN2968_c0_g1_i6 | 2.08845428 | 0.61323259 | 0.999970678 | 0 | 12.42 | 0 | 2.885 | GST_C |
| TRINITY_DN527_c0_g1_i8 | 2.088317391 | 0.235037982 | 0.999970678 | 1.154 | 0.118 | 0.044 | 0.251 | A_deaminase |
| TRINITY_DN5326_c0_g1_i8 | -2.0883103 | 0.18458465 | 0.999970678 | 0.709 | 2.129 | 0.756 | 11.109 | Glyco_hydro_16 |
| TRINITY_DN849_c0_g1_i8 | -2.08817217 | 0.489390119 | 0.999970678 | 4.442 | 0 | 15.41 | 0.513 | NA |
| TRINITY_DN10682_c0_g1_i11 | 2.088103804 | 0.462601442 | 0.999970678 | 1.417 | 0.079 | 0 | 0.362 | Exo_endo_phos |
| TRINITY_DN28350_c0_g1_i1 | 2.087581304 | 0.131019323 | 1 | 0.627 | 0.355 | 0.122 | 0.09 | NA |
| TRINITY_DN2654_c0_g1_i2 | 2.08684906 | 0.011523316 | 0.544751459 | 2.471 | 4.494 | 0.756 | 0.714 | DUF247 |
| TRINITY_DN3154_c0_g1_i4 | -2.08676285 | 0.420126528 | 0.999970678 | 1.19 | 0 | 1.189 | 3.7 | NA |

| TRINITY_DN17105_c0_g1_i3 | -2.08545931 | 0.627421056 | 0.999970678 | 0 | 0.424 | 1.467 | 0 | NA |
| --- | --- | --- | --- | --- | --- | --- | --- | --- |
| TRINITY_DN10302_c0_g1_i3 | 2.085235113 | 0.143110845 | 0.999970678 | 0.518 | 0.966 | 0.233 | 0.07 | NA |
| TRINITY_DN1612_c0_g2_i1 | -2.08475417 | 0.291010259 | 0.999970678 | 5.441 | 62.31 | 6.522 | 277.1 | NA |
| TRINITY_DN9654_c1_g1_i3 | -2.08450874 | 0.106064573 | 0.999970678 | 0.418 | 0.355 | 1.889 | 0.905 | NA |
| TRINITY_DN2573_c1_g1_i5 | -2.08332586 | 0.082359297 | 0.999970678 | 4.587 | 13.43 | 13.199 | 59.93 | NA |
| TRINITY_DN9977_c0_g1_i7 | 2.083036323 | 0.269068383 | 0.999970678 | 4.378 | 0.443 | 0.856 | 0.131 | NA |
| TRINITY_DN6150_c0_g1_i10 | 2.082825151 | 0.4122926 | 0.999970678 | 0.808 | 0.473 | 0 | 0.302 | GRAS |
| TRINITY_DN3697_c0_g1_i5 | -2.08276442 | 0.619022608 | 0.999970678 | 0 | 1.951 | 6.8 | 0 | NA |
| TRINITY_DN10216_c0_g1_i1 | 2.082133042 | 0.140036737 | 0.999970678 | 1.372 | 0.522 | 0.111 | 0.312 | - |
| TRINITY_DN1945_c0_g1_i2 | -2.08193679 | 0.508754022 | 0.999970678 | 0.118 | 4.573 | 0 | 19.554 | EF-hand_1 |
| TRINITY_DN11463_c0_g1_i1 | 2.081928798 | 0.408339017 | 0.999970678 | 0.727 | 0.729 | 0.278 | 0 | NA |
| TRINITY_DN4113_c0_g1_i1 | 2.081910659 | 0.139379804 | 0.999970678 | 4.778 | 4.336 | 0.189 | 1.91 | GAGA_bind |
| TRINITY_DN854_c0_g1_i1 | -2.08117672 | 0.469719106 | 0.999970678 | 1.535 | 0 | 0.3 | 6.163 | CS |
| TRINITY_DN14894_c0_g2_i2 | 2.080809772 | 0.103227664 | 0.999970678 | 10.456 | 3.075 | 0.9 | 2.101 | NA |
| TRINITY_DN10774_c0_g1_i6 | -2.08014635 | 0.416580071 | 0.999970678 | 1.326 | 0 | 1.9 | 3.227 | NA |
| TRINITY_DN3420_c1_g1_i5 | 2.079995384 | 0.41798243 | 0.999970678 | 3.152 | 4.819 | 0 | 1.89 | NA |
| TRINITY_DN6032_c0_g1_i4 | -2.07985171 | 0.415901098 | 0.999970678 | 0 | 0.897 | 1.411 | 2.031 | NA |
| TRINITY_DN2941_c1_g1_i8 | 2.079590342 | 0.082991463 | 0.999970678 | 0.509 | 1.35 | 0.222 | 0.161 | NA |
| TRINITY_DN6090_c1_g2_i5 | 2.079417648 | 0.097351538 | 0.999970678 | 1.226 | 1.764 | 0.444 | 0.171 | F-box |
| TRINITY_DN16744_c0_g1_i1 | -2.07919425 | 0.10856336 | 0.999970678 | 0.3 | 0.828 | 1.055 | 3.438 | UQ_con |
| TRINITY_DN31095_c0_g1_i1 | 2.078945406 | 0.137256664 | 1 | 4.487 | 8.663 | 0.867 | 1.93 | NA |
| TRINITY_DN95804_c0_g1_i2 | 2.078188826 | 0.474653473 | 0.999970678 | 0 | 5.282 | 0.844 | 0.231 | NA |
| TRINITY_DN73651_c0_g1_i4 | 2.077879699 | 0.0693352 | 0.999970678 | 1.135 | 0.532 | 0.189 | 0.171 | NA |
| TRINITY_DN4231_c0_g1_i6 | -2.07760392 | 0.031737771 | 0.999970678 | 0.799 | 0.443 | 2.411 | 2.282 | PDZ |
| TRINITY_DN917_c1_g1_i3 | -2.07749711 | 0.058268246 | 0.999970678 | 3.47 | 2.079 | 4.366 | 18.247 | NA |
| TRINITY_DN954_c0_g1_i17 | 2.077411043 | 0.6241243 | 0.999970678 | 1.681 | 0 | 0 | 0.402 | NA |
| TRINITY_DN2530_c0_g1_i6 | 2.076685229 | 0.617912077 | 0.999970678 | 2.625 | 0 | 0 | 0.633 | NA |
| TRINITY_DN9293_c0_g1_i1 | -2.07644655 | 0.465765166 | 0.999970678 | 0.763 | 0 | 2.522 | 0.221 | 1-cysPrx_C |
| TRINITY_DN4454_c1_g1_i1 | -2.07641217 | 0.268055192 | 0.999970678 | 0.182 | 0.986 | 3.655 | 0.432 | F-box |
| TRINITY_DN1643_c0_g2_i4 | 2.076145974 | 0.452754827 | 0.999970678 | 19.558 | 11.56 | 6.133 | 0 | NA |
| TRINITY_DN18667_c1_g1_i2 | 2.075932189 | 0.204820511 | 1 | 1.09 | 1.055 | 0.344 | 0.101 | NA |
| TRINITY_DN3102_c0_g1_i2 | -2.07577451 | 0.496160729 | 0.999970678 | 1.762 | 0.158 | 6.666 | 0 | Ras |
| TRINITY_DN243_c1_g1_i1 | 2.07560505 | 0.260182895 | 0.999970678 | 15.643 | 0.907 | 0.533 | 3.257 | Myb_DNA-bindin |
| TRINITY_DN537_c0_g1_i4 | -2.07521342 | 0.477697439 | 0.999970678 | 4.224 | 0 | 0.711 | 17.282 | NA |
| TRINITY_DN1455_c1_g1_i2 | 2.075086273 | 0.135501424 | 1 | 0.382 | 0.424 | 0.111 | 0.06 | NA |
| TRINITY_DN168441_c0_g1_i1 | 2.074992413 | 0.317152245 | 0.999970678 | 20.43 | 0.227 | 3.255 | 1.036 | Pkinase |
| TRINITY_DN11839_c0_g1_i1 | -2.07461709 | 0.267663635 | 0.999970678 | 0.164 | 0.631 | 2.655 | 0.171 | NA |
| TRINITY_DN461_c1_g2_i1 | 2.07455622 | 0.431930798 | 0.999970678 | 1.49 | 5.391 | 1.344 | 0 | LisH |
| TRINITY_DN901_c0_g1_i2 | -2.07427994 | 0.398541387 | 0.999970678 | 1.635 | 0.02 | 5.655 | 0.211 | TIM21 |
| TRINITY_DN776_c0_g1_i2 | 2.074193316 | 0.464895095 | 0.999970678 | 0 | 4.829 | 0.378 | 0.674 | NA |
| TRINITY_DN3143_c1_g1_i4 | 2.073669242 | 0.480596203 | 0.999970678 | 0.69 | 19.26 | 3.866 | 0 | LRRNT_2 |
| TRINITY_DN367_c0_g1_i2 | -2.07357865 | 0.277557851 | 0.999970678 | 0.754 | 0.286 | 0.122 | 4.192 | Band_7 |
| TRINITY_DN11439_c0_g1_i1 | -2.07327972 | 0.023091806 | 0.938933761 | 0.936 | 0.759 | 3.4 | 2.976 | NA |
| TRINITY_DN3250_c0_g1_i6 | -2.07275597 | 0.064160513 | 0.999970678 | 66.885 | 65.84 | 82.017 | 446.374 | NA |
| TRINITY_DN14239_c0_g1_i5 | -2.0727173 | 0.628264661 | 0.999970678 | 1.008 | 0 | 3.622 | 0 | NA |
| TRINITY_DN1268_c2_g1_i17 | -2.07255812 | 0.20681165 | 0.999970678 | 0.409 | 0.079 | 1.311 | 0.442 | NA |
| TRINITY_DN5711_c0_g1_i3 | 2.070650606 | 0.475912368 | 0.999970678 | 4.224 | 0.158 | 0 | 1.066 | NA |
| TRINITY_DN739_c0_g1_i1 | 2.070302737 | 0.03041622 | 0.999970678 | 13.299 | 5.154 | 2.067 | 1.92 | NA |
| TRINITY_DN30035_c0_g2_i3 | -2.07004936 | 0.069410634 | 0.999970678 | 0.345 | 0.315 | 0.778 | 1.82 | Cation_efflux |
| TRINITY_DN0_c6_g1_i1 | 2.070023328 | 0.617732793 | 0.999970678 | 4.942 | 0 | 0 | 1.196 | NA |
| TRINITY_DN857_c0_g1_i3 | 2.069100235 | 0.472010488 | 0.999970678 | 0 | 10.84 | 0.544 | 1.83 | Ribosomal_L21e |
| TRINITY_DN12998_c0_g1_i1 | -2.06904692 | 0.416594535 | 0.999970678 | 0.936 | 0 | 1.944 | 1.629 | GDA1_CD39 |
| TRINITY_DN10854_c0_g1_i1 | -2.06882313 | 0.262230267 | 0.999970678 | 0.291 | 1.981 | 0.767 | 8.375 | C2 |
| TRINITY_DN6056_c0_g1_i1 | 2.068003334 | 0.620582362 | 0.999970678 | 0 | 18.05 | 3.5 | 0 | Myb_DNA-bindin |
| TRINITY_DN5426_c0_g2_i3 | -2.06796504 | 0.631778006 | 0.999970678 | 7.558 | 0 | 26.254 | 0 | NA |
| TRINITY_DN18834_c0_g1_i3 | 2.067868426 | 0.436764008 | 0.999970678 | 2.998 | 0.601 | 0.722 | 0 | PTR2 |
| TRINITY_DN3222_c0_g1_i4 | 2.067735581 | 0.502117244 | 0.999970678 | 0 | 20.8 | 3.711 | 0.412 | Glycos_transf_1 |
| TRINITY_DN4891_c0_g1_i3 | 2.067656799 | 0.639259955 | 0.999970678 | 62.497 | 0 | 0 | 15.171 | NA |
| TRINITY_DN1794_c0_g1_i4 | -2.06740372 | 0.421009554 | 0.999970678 | 0.736 | 0 | 1.744 | 1.025 | NA |
| TRINITY_DN2290_c0_g1_i2 | 2.067081651 | 0.431253895 | 0.999970678 | 3.479 | 1.823 | 1.055 | 0 | COPI_C |
| TRINITY_DN6933_c0_g1_i4 | -2.0670229 | 0.626676715 | 0.999970678 | 1.054 | 0 | 3.689 | 0 | Grp1_Fun34_Yaa |
| TRINITY_DN88158_c0_g1_i1 | 2.066454962 | 0.157024302 | 0.999970678 | 3.061 | 2.247 | 0.2 | 1.005 | HLH |
| TRINITY_DN19836_c0_g1_i8 | -2.06633433 | 0.058818546 | 0.999970678 | 0.327 | 0.522 | 1.511 | 1.719 | XH |
| TRINITY_DN1939_c0_g2_i2 | -2.06613925 | 0.119176371 | 0.999970678 | 6.341 | 1.35 | 6.033 | 25.365 | NA |
| TRINITY_DN6060_c0_g1_i1 | -2.06596635 | 0.169105386 | 0.999970678 | 6.604 | 9.638 | 51.775 | 4.906 | HSP20 |
| TRINITY_DN2175_c0_g1_i4 | -2.0653101 | 0.477384876 | 0.999970678 | 25.853 | 0 | 48.786 | 51.033 | NA |
| TRINITY_DN5916_c0_g2_i1 | 2.065260817 | 0.109877268 | 0.999970678 | 42.168 | 9.471 | 2.411 | 9.591 | Lyase_aromatic |
| TRINITY_DN1252_c0_g1_i2 | 2.065122205 | 0.479823504 | 0.999970678 | 10.546 | 0 | 1.722 | 0.473 | - |
| TRINITY_DN20252_c0_g1_i1 | 2.065071771 | 0.140039075 | 1 | 1.19 | 0.601 | 0.233 | 0.151 | NA |
| TRINITY_DN7342_c0_g2_i5 | -2.06500062 | 0.427986499 | 1 | 0.2 | 0 | 0.311 | 0.473 | NA |
| TRINITY_DN5846_c0_g1_i2 | 2.064667762 | 0.091564445 | 0.999970678 | 41.341 | 14.08 | 8.555 | 2.956 | NA |
| TRINITY_DN14483_c0_g2_i3 | -2.06401806 | 0.406424795 | 0.999970678 | 0.027 | 0.917 | 0.067 | 3.841 | ABC_membrane |
| TRINITY_DN2980_c0_g1_i1 | -2.06312583 | 0.329817749 | 0.999970678 | 329.11 | 1.311 | 666.849 | 589.931 | NifU_N |
| TRINITY_DN44479_c0_g3_i1 | 2.062652491 | 0.409588415 | 0.999970678 | 3.861 | 3.144 | 0 | 1.689 | NA |

| TRINITY_DN4101_c1_g1_i1 | 2.062383001 | 0.421818794 | 0.999970678 | 1.099 | 0.956 | 0.411 | 0 | NA |
| --- | --- | --- | --- | --- | --- | --- | --- | --- |
| TRINITY_DN7181_c0_g1_i3 | 2.06225186 | 0.198799185 | 0.999970678 | 2.525 | 2.148 | 0.111 | 0.985 | HLH |
| TRINITY_DN175_c0_g1_i17 | 2.062128004 | 0.146625397 | 0.999970678 | 4.315 | 35.34 | 3.689 | 4.715 | NA |
| TRINITY_DN1594_c0_g1_i8 | -2.06177776 | 0.492527548 | 0.999970678 | 5.45 | 0.601 | 20.732 | 0 | NA |
| TRINITY_DN2575_c0_g1_i10 | 2.061724149 | 0.431237361 | 0.999970678 | 1.081 | 2.671 | 0.767 | 0 | Methyltransf_11 |
| TRINITY_DN4737_c1_g2_i2 | 2.061658546 | 0.189834336 | 0.999970678 | 6.867 | 3.459 | 1.833 | 0.261 | NA |
| TRINITY_DN16723_c0_g1_i5 | 2.061368315 | 0.290013361 | 0.999970678 | 1.208 | 0.148 | 0.022 | 0.302 | NA |
| TRINITY_DN10091_c0_g1_i16 | 2.060853503 | 0.423627525 | 0.999970678 | 0.927 | 2.562 | 0 | 0.834 | NA |
| TRINITY_DN7314_c0_g1_i2 | 2.059966751 | 0.623080009 | 0.999970678 | 0 | 1.833 | 0.356 | 0 | NA |
| TRINITY_DN11588_c0_g2_i1 | 2.059787313 | 0.086805529 | 0.999970678 | 3.334 | 11.74 | 1.789 | 1.397 | FBPase |
| TRINITY_DN823_c0_g1_i4 | 2.059274831 | 0.624150344 | 0.999970678 | 0 | 8.82 | 1.722 | 0 | FAD_binding_8 |
| TRINITY_DN10420_c0_g1_i7 | 2.059158611 | 0.113231426 | 1 | 1.363 | 2.533 | 0.367 | 0.493 | NA |
| TRINITY_DN1207_c0_g1_i6 | -2.05875166 | 0.462848226 | 0.999970678 | 1.463 | 0 | 0.4 | 5.751 | Ank_2 |
| TRINITY_DN511_c0_g3_i1 | -2.0585801 | 0.61839225 | 0.999970678 | 0 | 0.917 | 0 | 3.8 | NA |
| TRINITY_DN1043_c1_g1_i1 | 2.058296121 | 0.514277762 | 0.999970678 | 0.273 | 42.25 | 0 | 9.863 | ADH_N |
| TRINITY_DN1327_c0_g1_i6 | -2.05827716 | 0.62446094 | 0.999970678 | 1.417 | 0 | 4.911 | 0 | NA |
| TRINITY_DN6998_c0_g1_i3 | 2.057598279 | 0.528886359 | 0.999970678 | 1.699 | 0.01 | 0.344 | 0 | NA |
| TRINITY_DN545_c0_g2_i5 | 2.056134489 | 0.053181573 | 0.999970678 | 8.612 | 2.631 | 1.333 | 1.126 | NA |
| TRINITY_DN3890_c0_g1_i8 | -2.05543165 | 0.431795807 | 1 | 0.291 | 0 | 0.511 | 0.573 | DUF1771 |
| TRINITY_DN4934_c0_g1_i4 | 2.055337059 | 0.126223444 | 0.999970678 | 1.263 | 0.562 | 0.3 | 0.08 | NA |
| TRINITY_DN7194_c0_g1_i1 | 2.054940391 | 0.286231081 | 0.999970678 | 1.208 | 0.591 | 0.344 | 0.02 | NA |
| TRINITY_DN7986_c0_g1_i2 | 2.054546859 | 0.410919478 | 0.999970678 | 0.663 | 0.503 | 0 | 0.271 | Glyco_hydro_47 |
| TRINITY_DN13132_c0_g2_i1 | -2.05433418 | 0.631578803 | 0.999970678 | 0.772 | 0 | 2.711 | 0 | ADH_N |
| TRINITY_DN4656_c0_g1_i1 | 2.053893279 | 0.486198179 | 0.999970678 | 9.738 | 0.256 | 0 | 2.453 | Yip1 |
| TRINITY_DN1612_c0_g2_i2 | -2.05388824 | 0.272788457 | 0.999970678 | 0.164 | 1.192 | 0.222 | 5.328 | NA |
| TRINITY_DN6411_c0_g1_i3 | 2.053753856 | 0.230449041 | 0.999970678 | 2.589 | 15.19 | 0.267 | 3.951 | NA |
| TRINITY_DN635_c0_g1_i3 | -2.0525713 | 0.437121679 | 0.999970678 | 0 | 2.838 | 7.688 | 2.302 | - |
| TRINITY_DN2359_c0_g1_i8 | 2.052187961 | 0.623896668 | 0.999970678 | 0 | 1.124 | 0.222 | 0 | UCH |
| TRINITY_DN10448_c0_g2_i2 | 2.052063668 | 0.416435989 | 0.999970678 | 0.554 | 0.67 | 0.244 | 0 | WD40 |
| TRINITY_DN3521_c0_g1_i1 | -2.05154296 | 0.631657051 | 0.999970678 | 0.672 | 0 | 0 | 2.906 | NA |
| TRINITY_DN65193_c0_g1_i1 | -2.05082197 | 0.428861492 | 0.999970678 | 0.236 | 0 | 0.3 | 0.623 | NA |
| TRINITY_DN156_c0_g1_i4 | 2.050474011 | 0.621112977 | 0.999970678 | 2.553 | 0 | 0 | 0.633 | DNA_pol_phi |
| TRINITY_DN4546_c0_g1_i1 | 2.050312826 | 0.102041595 | 1 | 0.681 | 0.611 | 0.1 | 0.191 | NA |
| TRINITY_DN1337_c0_g1_i3 | -2.04998391 | 0.070175065 | 0.999970678 | 1.681 | 3.242 | 3.778 | 15.774 | p450 |
| TRINITY_DN2212_c1_g1_i1 | -2.04985193 | 0.505947454 | 0.999970678 | 0 | 130.8 | 280.261 | 194.81 | DnaJ |
| TRINITY_DN4553_c0_g1_i6 | -2.04964685 | 0.484517953 | 0.999970678 | 0 | 0.335 | 0.056 | 1.357 | NA |
| TRINITY_DN779_c2_g1_i2 | -2.04906854 | 0.431191487 | 0.999970678 | 0.854 | 0 | 0.878 | 2.574 | STAS |
| TRINITY_DN190_c2_g1_i4 | 2.049030983 | 0.071416719 | 0.999970678 | 0.763 | 1.261 | 0.278 | 0.151 | NA |
| TRINITY_DN63572_c0_g1_i1 | -2.04880856 | 0.511291985 | 0.999970678 | 1.426 | 0.118 | 5.322 | 0 | NA |
| TRINITY_DN8853_c0_g1_i2 | -2.04835869 | 0.453706742 | 0.999970678 | 2.244 | 0 | 0.9 | 8.294 | Arm |
| TRINITY_DN847_c0_g1_i7 | -2.04759846 | 0.625856137 | 0.999970678 | 4.742 | 0 | 0 | 19.926 | NA |
| TRINITY_DN3443_c0_g1_i13 | 2.045389411 | 0.412165021 | 0.999970678 | 0.609 | 0.473 | 0 | 0.261 | NA |
| TRINITY_DN1803_c0_g1_i4 | -2.04531323 | 0.046608531 | 0.999970678 | 3.288 | 3.952 | 5.611 | 23.093 | Glyco_hydro_1 |
| TRINITY_DN4195_c0_g1_i12 | 2.045247021 | 0.187514322 | 0.999970678 | 1.735 | 1.715 | 0.056 | 0.774 | NA |
| TRINITY_DN2040_c0_g1_i8 | 2.04523756 | 0.624688059 | 0.999970678 | 0 | 4.248 | 0 | 1.005 | Abhydrolase_1 |
| TRINITY_DN11111_c0_g1_i2 | -2.0451248 | 0.429111189 | 0.999970678 | 0 | 1.242 | 2.733 | 1.639 | NA |
| TRINITY_DN7323_c0_g1_i3 | 2.044927196 | 0.474523332 | 0.999970678 | 2.153 | 0 | 0.289 | 0.181 | LRRNT_2 |
| TRINITY_DN1785_c0_g1_i3 | 2.044607937 | 0.418959516 | 0.999970678 | 2.244 | 1.971 | 0.844 | 0 | DUF659 |
| TRINITY_DN5676_c0_g1_i3 | 2.044439435 | 0.437259568 | 0.999970678 | 0.263 | 1.222 | 0 | 0.352 | PPR_long |
| TRINITY_DN6025_c0_g1_i5 | -2.04384251 | 0.386298666 | 0.999970678 | 6.25 | 0.532 | 22.888 | 0.503 | NA |
| TRINITY_DN1314_c0_g1_i1 | 2.043311808 | 0.425581892 | 0.999970678 | 0.69 | 1.725 | 0.478 | 0 | NA |
| TRINITY_DN57289_c0_g1_i4 | -2.04269527 | 0.043819708 | 0.999970678 | 1.226 | 0.729 | 4.611 | 2.503 | NA |
| TRINITY_DN335_c0_g1_i7 | 2.0419248 | 0.546452731 | 0.999970678 | 28.315 | 0 | 0.089 | 6.927 | LRRNT_2 |
| TRINITY_DN10564_c0_g1_i1 | -2.04151435 | 0.29801208 | 0.999970678 | 0.164 | 1.163 | 0.278 | 5.027 | NA |
| TRINITY_DN1255_c0_g1_i5 | 2.04118524 | 0.092851674 | 0.999970678 | 0.245 | 0.463 | 0.078 | 0.08 | Mur_ligase_M |
| TRINITY_DN15364_c0_g1_i1 | -2.04096513 | 0.424815061 | 0.999970678 | 0.391 | 0 | 0.556 | 0.945 | NA |
| TRINITY_DN1617_c0_g1_i8 | 2.03989725 | 0.412058839 | 0.999970678 | 0.636 | 0.966 | 0 | 0.392 | FAR1 |
| TRINITY_DN6025_c0_g1_i15 | -2.03988551 | 0.313210776 | 0.999970678 | 6.758 | 0.562 | 22.065 | 2.906 | NA |
| TRINITY_DN37401_c0_g1_i6 | -2.03895678 | 0.144340224 | 0.999970678 | 1.181 | 0.729 | 0.789 | 6.927 | NA |
| TRINITY_DN8201_c0_g1_i2 | -2.03877288 | 0.454650804 | 0.999970678 | 0 | 0.581 | 1.722 | 0.292 | NA |
| TRINITY_DN3268_c0_g1_i2 | 2.037803297 | 0.054474704 | 0.999970678 | 0.654 | 0.808 | 0.156 | 0.191 | NA |
| TRINITY_DN7449_c0_g1_i10 | 2.037414699 | 0.475520874 | 0.999970678 | 4.533 | 0 | 0.311 | 0.744 | NA |
| TRINITY_DN11735_c0_g1_i1 | 2.036951329 | 0.444144965 | 0.999970678 | 0.218 | 1.202 | 0.278 | 0 | DUF3730 |
| TRINITY_DN1061_c0_g1_i6 | -2.03670516 | 0.00769773 | 0.386923436 | 2.734 | 5.144 | 12.821 | 16.719 | NA |
| TRINITY_DN649_c0_g1_i3 | -2.03657251 | 0.436316433 | 0.999970678 | 1.917 | 0 | 4.944 | 2.011 | NA |
| TRINITY_DN82558_c0_g1_i1 | 2.036109415 | 0.432272914 | 0.999970678 | 1.799 | 3.784 | 1.122 | 0 | DUF3527 |
| TRINITY_DN1682_c0_g1_i5 | 2.035847677 | 0.631350304 | 0.999970678 | 0 | 7.086 | 1.4 | 0 | NA |
| TRINITY_DN2915_c0_g1_i1 | 2.035054453 | 0.655547244 | 0.999970678 | 0 | 308.8 | 0 | 74.287 | NA |
| TRINITY_DN37312_c0_g1_i1 | -2.03455029 | 0.62814093 | 0.999970678 | 0 | 0.67 | 0 | 2.704 | GMC_oxred_C |
| TRINITY_DN6158_c0_g1_i23 | 2.034306927 | 0.625475926 | 0.999970678 | 0 | 3.893 | 0 | 0.925 | NA |
| TRINITY_DN934_c0_g1_i12 | -2.0339948 | 0.280725246 | 0.999970678 | 1.135 | 0.443 | 0.144 | 6.374 | NA |
| TRINITY_DN4153_c0_g1_i1 | 2.033939247 | 0.417105175 | 0.999970678 | 1.481 | 2.779 | 0 | 1.036 | Pollen_Ole_e_I |
| TRINITY_DN855_c0_g1_i13 | -2.03346708 | 0.117449157 | 0.999970678 | 0.936 | 0.739 | 0.889 | 5.831 | - |
| TRINITY_DN3509_c0_g1_i3 | -2.03340627 | 0.544601823 | 0.999970678 | 67.93 | 1.932 | 239.23 | 0 | NA |

| TRINITY_DN31733_c0_g1_i1 | -2.03332769 | 0.146257359 | 0.999970678 | 2.071 | 0.296 | 3.266 | 5.831 | U-box |
| --- | --- | --- | --- | --- | --- | --- | --- | --- |
| TRINITY_DN477_c0_g1_i14 | -2.03310866 | 0.626471682 | 0.999970678 | 0 | 4.129 | 13.677 | 0 | NA |
| TRINITY_DN11830_c0_g1_i4 | 2.032926524 | 0.631540097 | 0.999970678 | 2.171 | 0 | 0 | 0.543 | NA |
| TRINITY_DN11211_c0_g1_i10 | 2.032846752 | 0.62570567 | 0.999970678 | 0 | 2.365 | 0.467 | 0 | - |
| TRINITY_DN394_c1_g1_i6 | 2.032561871 | 0.093904032 | 0.999970678 | 1.78 | 0.887 | 0.2 | 0.392 | NA |
| TRINITY_DN17599_c0_g1_i1 | -2.03227453 | 0.154740838 | 0.999970678 | 3.325 | 7.372 | 27.609 | 9.159 | NA |
| TRINITY_DN3772_c0_g1_i1 | -2.03178176 | 0.440482817 | 0.999970678 | 1.453 | 0 | 3.722 | 1.548 | Cu_amine_oxid |
| TRINITY_DN4321_c0_g1_i2 | 2.031275645 | 0.142391089 | 1 | 0.654 | 0.65 | 0.1 | 0.171 | SAC3_GANP |
| TRINITY_DN3915_c0_g1_i11 | -2.03091673 | 0.370525083 | 0.999970678 | 1.108 | 0.03 | 0.233 | 4.363 | TRAPP |
| TRINITY_DN10244_c0_g1_i1 | 2.030378803 | 0.235981017 | 0.999970678 | 0.273 | 3.065 | 0.556 | 0.141 | DUF4283 |
| TRINITY_DN2081_c0_g1_i6 | -2.03025349 | 0.648119821 | 0.999970678 | 0 | 15.99 | 0 | 64.615 | NA |
| TRINITY_DN76961_c0_g1_i1 | -2.02966025 | 0.629831814 | 0.999970678 | 0.736 | 0 | 2.522 | 0 | NA |
| TRINITY_DN10211_c0_g1_i1 | 2.028657386 | 0.155533642 | 0.999970678 | 42.458 | 4.169 | 5.622 | 4.765 | C1_2 |
| TRINITY_DN12234_c0_g1_i2 | -2.02849028 | 0.632132867 | 0.999970678 | 1.69 | 0 | 5.8 | 0 | adh_short_C2 |
| TRINITY_DN3651_c0_g1_i4 | -2.02798429 | 0.633167106 | 0.999970678 | 1.145 | 0 | 3.989 | 0 | Glyoxalase |
| TRINITY_DN136648_c0_g1_i1 | -2.02783819 | 0.439041243 | 1 | 0 | 0.286 | 0.522 | 0.533 | NA |
| TRINITY_DN2917_c0_g1_i1 | 2.026628347 | 0.291878087 | 0.999970678 | 16.914 | 0.424 | 2.8 | 0.925 | Auxin_inducible |
| TRINITY_DN3452_c0_g1_i3 | -2.02631003 | 0.304100594 | 0.999970678 | 0.009 | 0.7 | 1.389 | 1.166 | NA |
| TRINITY_DN21283_c1_g1_i1 | 2.02611862 | 0.430946526 | 0.999970678 | 1.163 | 0.522 | 0 | 0.432 | NA |
| TRINITY_DN9496_c0_g1_i6 | -2.02610586 | 0.628654473 | 0.999970678 | 1.899 | 0 | 6.466 | 0 | NA |
| TRINITY_DN13011_c0_g1_i7 | 2.025473934 | 0.190468435 | 0.999970678 | 12.191 | 5.184 | 3.066 | 0.583 | NA |
| TRINITY_DN4851_c0_g1_i2 | 2.0240592 | 0.627151074 | 0.999970678 | 0 | 3.095 | 0.611 | 0 | - |
| TRINITY_DN37401_c0_g1_i5 | 2.023683568 | 0.634856124 | 0.999970678 | 16.705 | 0 | 0 | 4.202 | NA |
| TRINITY_DN10612_c2_g1_i3 | -2.02302561 | 0.463344969 | 0.999970678 | 0.854 | 0 | 0.289 | 3.117 | NA |
| TRINITY_DN11723_c0_g2_i2 | 2.022910084 | 0.474138182 | 0.999970678 | 0.182 | 3.361 | 0 | 0.865 | NA |
| TRINITY_DN8893_c1_g1_i1 | 2.022837534 | 0.269070897 | 0.999970678 | 13.599 | 1.212 | 2.778 | 0.322 | Myb_DNA-bindin |
| TRINITY_DN26985_c0_g3_i1 | -2.02225496 | 0.110058265 | 0.999970678 | 1.045 | 1.074 | 2.267 | 5.7 | NA |
| TRINITY_DN6337_c0_g2_i1 | 2.022110766 | 0.438570203 | 1 | 1.008 | 0.897 | 0.389 | 0 | NA |
| TRINITY_DN28875_c0_g3_i2 | -2.0220723 | 0.434767793 | 0.999970678 | 0 | 0.296 | 0.667 | 0.402 | NA |
| TRINITY_DN6171_c0_g1_i12 | 2.021627396 | 0.199633164 | 1 | 0.263 | 0.591 | 0.144 | 0.05 | EF_assoc_1 |
| TRINITY_DN5850_c0_g1_i3 | 2.021433469 | 0.047832453 | 0.999970678 | 1.017 | 2.395 | 0.344 | 0.412 | NA |
| TRINITY_DN205_c0_g1_i6 | -2.02131609 | 0.447088454 | 0.999970678 | 0.981 | 0 | 2.722 | 0.764 | - |
| TRINITY_DN4918_c0_g1_i1 | -2.02129635 | 0.165225437 | 0.999970678 | 0.636 | 0.335 | 0.344 | 3.609 | MFS_1 |
| TRINITY_DN346_c0_g1_i2 | -2.02098648 | 0.438291243 | 0.999970678 | 10.892 | 0 | 21.788 | 18.177 | NA |
| TRINITY_DN928_c0_g1_i4 | -2.02066012 | 0.628525345 | 0.999970678 | 2.126 | 0 | 7.277 | 0 | NA |
| TRINITY_DN1315_c1_g1_i3 | 2.020560104 | 0.63526515 | 0.999970678 | 0 | 22.58 | 4.522 | 0 | AUX_IAA |
| TRINITY_DN7787_c0_g1_i1 | -2.01916878 | 0.056476299 | 0.999970678 | 1.681 | 2.365 | 4.689 | 10.416 | NA |
| TRINITY_DN732_c0_g1_i1 | 2.018609205 | 0.072422289 | 0.999970678 | 111.49 | 30.93 | 9.444 | 24.008 | NAM |
| TRINITY_DN2343_c0_g1_i4 | -2.01814716 | 0.440784033 | 0.999970678 | 0.609 | 0 | 0.6 | 1.8 | NA |
| TRINITY_DN556_c0_g1_i2 | 2.017358749 | 0.625691217 | 0.999970678 | 0 | 22.44 | 0 | 5.379 | NA |
| TRINITY_DN1213_c0_g1_i10 | 2.016805096 | 0.090782795 | 0.999970678 | 0.554 | 0.631 | 0.089 | 0.201 | NA |
| TRINITY_DN24_c0_g2_i8 | -2.01556486 | 0.166683521 | 0.999970678 | 20.757 | 104.3 | 56.141 | 431.002 | - |
| TRINITY_DN24907_c0_g1_i3 | -2.01397938 | 0.158277431 | 1 | 1.026 | 0.424 | 2.544 | 2.714 | NA |
| TRINITY_DN5022_c0_g3_i1 | 2.013743412 | 0.628782023 | 0.999970678 | 0 | 4.612 | 0.933 | 0 | NA |
| TRINITY_DN2508_c0_g1_i4 | 2.013513904 | 0.267378906 | 0.999970678 | 0.472 | 10.34 | 0.489 | 2.061 | TPR_19 |
| TRINITY_DN8763_c0_g1_i1 | -2.01348071 | 0.636863359 | 0.999970678 | 0.472 | 0 | 0 | 1.91 | PDZ_2 |
| TRINITY_DN6104_c0_g1_i1 | 2.01303044 | 0.631281976 | 0.999970678 | 24.835 | 0 | 0 | 6.253 | NA |
| TRINITY_DN5597_c0_g1_i7 | -2.01297104 | 0.181575427 | 0.999970678 | 0.136 | 0.641 | 1.389 | 1.458 | - |
| TRINITY_DN8164_c0_g1_i2 | 2.012288667 | 0.437137796 | 0.999970678 | 1.045 | 3.203 | 0.856 | 0 | NA |
| TRINITY_DN1344_c1_g2_i2 | -2.01148555 | 0.631544903 | 0.999970678 | 0 | 1.064 | 3.522 | 0 | NA |
| TRINITY_DN5857_c0_g2_i3 | -2.01148292 | 0.148461996 | 0.999970678 | 0.509 | 1.114 | 0.822 | 5.54 | NA |
| TRINITY_DN3032_c0_g1_i7 | 2.011305828 | 0.109987392 | 0.999970678 | 1.072 | 1.311 | 0.189 | 0.392 | NA |
| TRINITY_DN1053_c0_g1_i7 | -2.01076002 | 0.344142409 | 0.999970678 | 0.191 | 0.877 | 3.366 | 0.131 | Abhydrolase_6 |
| TRINITY_DN1345_c0_g1_i4 | 2.010468627 | 0.004513016 | 0.243717803 | 22.919 | 18.12 | 4.211 | 5.107 | LRRNT_2 |
| TRINITY_DN3110_c1_g1_i2 | -2.01041194 | 0.37986533 | 0.999970678 | 0.027 | 0.641 | 0.111 | 2.513 | DEAD |
| TRINITY_DN2152_c0_g1_i4 | -2.01008591 | 0.128124393 | 0.999970678 | 0.173 | 0.848 | 1.433 | 2.373 | DUF247 |
| TRINITY_DN1143_c0_g1_i1 | 2.009844571 | 0.46491259 | 0.999970678 | 4.669 | 0.522 | 1.078 | 0 | NA |
| TRINITY_DN2558_c0_g1_i21 | -2.00855387 | 0.639097342 | 0.999970678 | 1.299 | 0 | 4.333 | 0 | Abhydrolase_1 |
| TRINITY_DN3824_c0_g1_i3 | -2.00829475 | 0.434459801 | 0.999970678 | 0.245 | 0 | 0.5 | 0.412 | DHHC |
| TRINITY_DN29066_c0_g2_i4 | 2.007951913 | 0.339246894 | 0.999970678 | 1.426 | 78.75 | 1.6 | 17.202 | NA |
| TRINITY_DN37126_c0_g1_i1 | -2.00695373 | 0.437929316 | 0.999970678 | 0.645 | 0 | 1.433 | 0.875 | NA |
| TRINITY_DN4180_c0_g3_i2 | -2.00692283 | 0.63196271 | 0.999970678 | 0 | 5.874 | 19.121 | 0 | NA |
| TRINITY_DN863_c0_g2_i16 | -2.00675797 | 0.431014542 | 0.999970678 | 0.672 | 0 | 1.144 | 1.357 | NA |
| TRINITY_DN1433_c1_g1_i4 | 2.00664005 | 0.635082415 | 0.999970678 | 4.669 | 0 | 0.967 | 0 | PIR |
| TRINITY_DN3135_c0_g1_i7 | -2.00662671 | 0.438174758 | 0.999970678 | 1.108 | 0 | 2.567 | 1.468 | NA |
| TRINITY_DN1868_c0_g1_i8 | 2.006324721 | 0.512225811 | 0.999970678 | 5.296 | 0 | 0.978 | 0.141 | NA |
| TRINITY_DN6101_c0_g1_i1 | 2.006260302 | 0.63428914 | 0.999970678 | 2.616 | 0 | 0 | 0.664 | NA |
| TRINITY_DN6469_c0_g1_i2 | 2.005545982 | 0.631743335 | 0.999970678 | 0 | 1.252 | 0.256 | 0 | NA |
| TRINITY_DN35325_c0_g1_i1 | 2.005545982 | 0.631743335 | 0.999970678 | 0 | 2.7 | 0.556 | 0 | NA |
| TRINITY_DN9001_c0_g2_i6 | -2.00415341 | 0.631175853 | 0.999970678 | 1.79 | 0 | 6.044 | 0 | NA |
| TRINITY_DN1269_c0_g1_i8 | 2.004141538 | 0.49513617 | 0.999970678 | 0.164 | 5.361 | 1.122 | 0 | NA |
| TRINITY_DN3920_c0_g1_i4 | 2.003053433 | 0.145307915 | 0.999970678 | 3.952 | 20.46 | 3.322 | 1.991 | NA |
| TRINITY_DN4791_c0_g1_i2 | -2.00274552 | 0.47356949 | 0.999970678 | 13.59 | 0 | 27.398 | 22.209 | Cpn60_TCP1 |
| TRINITY_DN255_c1_g1_i14 | 2.002609394 | 0.489921331 | 0.999970678 | 6.059 | 0 | 0.4 | 1.046 | NA |

| TRINITY_DN13736_c0_g1_i3 | -2.00210294 | 0.455418508 | 0.999970678 | 0 | 0.788 | 2.111 | 0.543 | Chal_sti_synt_C |
| --- | --- | --- | --- | --- | --- | --- | --- | --- |
| TRINITY_DN1665_c0_g1_i8 | 2.002093151 | 0.638682998 | 0.999970678 | 0 | 2.04 | 0.422 | 0 | MFS_1 |
| TRINITY_DN9251_c0_g2_i3 | -2.00167741 | 0.630402284 | 0.999970678 | 1.363 | 0 | 0 | 5.6 | NA |
| TRINITY_DN1471_c0_g1_i2 | -2.00127742 | 0.213523463 | 0.999970678 | 0.827 | 0.079 | 1.022 | 2.453 | LRRNT_2 |
| TRINITY_DN1471_c0_g1_i14 | -2.00127742 | 0.213523463 | 0.999970678 | 0.827 | 0.079 | 1.022 | 2.453 | LRRNT_2 |
| TRINITY_DN6364_c1_g1_i8 | 2.000698114 | 0.420928613 | 0.999970678 | 1.072 | 1.409 | 0 | 0.623 | Pkinase |
| TRINITY_DN658_c0_g1_i5 | -2.00019953 | 0.469788136 | 0.999970678 | 7.467 | 0 | 21.054 | 4.856 | SKI |
| TRINITY_DN7056_c0_g2_i1 | -2.0001595 | 0.446851103 | 0.999970678 | 0.736 | 0 | 1.8 | 0.784 | NA |
| TRINITY_DN4335_c0_g1_i5 | 2.000017424 | 0.143353918 | 0.999970678 | 5.496 | 51.69 | 6.366 | 6.444 | NA |
| TRINITY_DN7126_c0_g1_i6 | 1.999826479 | 0.429992794 | 0.999970678 | 0.763 | 0.907 | 0.344 | 0 | FKBP_C |
| TRINITY_DN3305_c0_g1_i7 | 1.999737766 | 0.037590934 | 0.999970678 | 3.661 | 6.091 | 0.644 | 1.649 | NA |
| TRINITY_DN12171_c1_g1_i1 | -1.99933042 | 0.418614725 | 0.999970678 | 0.055 | 4.228 | 0.167 | 16.709 | Aldedh |
| TRINITY_DN7235_c0_g1_i1 | 1.998959823 | 0.518967469 | 0.999970678 | 0 | 6.11 | 0.078 | 1.408 | NA |
| TRINITY_DN3531_c0_g1_i1 | -1.99855287 | 0.10803366 | 0.999970678 | 0.99 | 0.621 | 1.255 | 5.027 | UDPGT |
| TRINITY_DN85038_c0_g1_i2 | 1.998118815 | 0.20432452 | 0.999970678 | 4.942 | 89.21 | 10.599 | 10.325 | NA |
| TRINITY_DN29240_c0_g1_i6 | 1.997539244 | 0.430515676 | 0.999970678 | 0.818 | 0.798 | 0.333 | 0 | zf-C3HC4_3 |
| TRINITY_DN3138_c0_g1_i1 | -1.9971487 | 0.440382573 | 0.999970678 | 0 | 1.035 | 2.522 | 1.015 | NA |
| TRINITY_DN2421_c0_g1_i5 | 1.996571257 | 0.637843625 | 0.999970678 | 0 | 65.4 | 0 | 16.005 | - |
| TRINITY_DN9800_c1_g1_i2 | -1.99575643 | 0.250314863 | 0.999970678 | 13.526 | 2.089 | 3.178 | 59.246 | ABC_tran |
| TRINITY_DN211_c0_g1_i3 | -1.99575428 | 0.631692358 | 0.999970678 | 5.505 | 0 | 0 | 21.636 | NA |
| TRINITY_DN30599_c0_g1_i1 | 1.99571055 | 0.638367696 | 0.999970678 | 0 | 1.675 | 0.344 | 0 | DUF4005 |
| TRINITY_DN12712_c0_g1_i3 | -1.9954813 | 0.478075318 | 0.999970678 | 0 | 0.375 | 0.1 | 1.307 | NA |
| TRINITY_DN15824_c0_g1_i3 | -1.99513022 | 0.199232651 | 0.999970678 | 3.198 | 0.483 | 2.567 | 11.612 | NAR2 |
| TRINITY_DN2175_c0_g1_i6 | 1.994788477 | 0.64824734 | 0.999970678 | 38.034 | 0 | 0 | 9.732 | NA |
| TRINITY_DN99_c0_g2_i5 | 1.994452981 | 0.655252076 | 0.999970678 | 222.12 | 0 | 0 | 56.18 | NA |
| TRINITY_DN4662_c0_g1_i2 | 1.994291808 | 0.25986116 | 0.999970678 | 0.454 | 4.208 | 0.856 | 0.121 | NA |
| TRINITY_DN11677_c0_g1_i1 | -1.99391708 | 0.13795912 | 0.999970678 | 0.627 | 1.153 | 0.978 | 5.841 | U-box |
| TRINITY_DN2213_c0_g1_i2 | 1.993745232 | 0.445890289 | 1 | 0.881 | 0.503 | 0.289 | 0 | NA |
| TRINITY_DN48422_c0_g1_i12 | -1.99368002 | 0.437175275 | 0.999970678 | 0 | 1.232 | 2.811 | 1.448 | NA |
| TRINITY_DN2494_c0_g1_i1 | 1.993433294 | 0.445370837 | 0.999970678 | 5.677 | 8.495 | 0 | 3.569 | NA |
| TRINITY_DN7687_c0_g1_i3 | 1.993351038 | 0.03189721 | 0.999970678 | 1.172 | 2.02 | 0.344 | 0.392 | NA |
| TRINITY_DN1461_c0_g1_i1 | -1.99279821 | 0.443791634 | 0.999970678 | 1.699 | 0 | 1.589 | 4.956 | DUF966 |
| TRINITY_DN5263_c0_g2_i2 | -1.99256507 | 0.467482475 | 0.999970678 | 11.346 | 0 | 16.188 | 26.311 | DAP_epimerase |
| TRINITY_DN41_c0_g1_i4 | 1.992503061 | 0.516694851 | 0.999970678 | 0.173 | 13.19 | 0 | 3.308 | NA |
| TRINITY_DN1949_c0_g1_i1 | 1.992310473 | 0.460195376 | 0.999970678 | 2.189 | 0.286 | 0 | 0.633 | Glyco_transf_8 |
| TRINITY_DN2152_c0_g1_i6 | -1.99180377 | 0.205893156 | 0.999970678 | 2.889 | 9.717 | 2.989 | 46.157 | DUF247 |
| TRINITY_DN563_c2_g2_i1 | 1.991707399 | 0.425716244 | 0.999970678 | 1.971 | 3.075 | 0 | 1.267 | Kelch_1 |
| TRINITY_DN475_c0_g1_i10 | -1.99147864 | 0.63591028 | 0.999970678 | 6.431 | 0 | 21.343 | 0 | NA |
| TRINITY_DN1860_c0_g1_i1 | -1.99139118 | 0.23791137 | 0.999970678 | 0.718 | 2.71 | 1.344 | 11.511 | Phi_1 |
| TRINITY_DN273_c0_g1_i4 | -1.99133527 | 0.048820601 | 0.999970678 | 0.845 | 1.587 | 5.489 | 3.036 | Aconitase |
| TRINITY_DN5394_c1_g1_i5 | 1.990936816 | 0.152305514 | 0.999970678 | 2.589 | 1.567 | 0.689 | 0.241 | NA |
| TRINITY_DN2513_c0_g2_i2 | 1.990822397 | 0.533126402 | 0.999970678 | 71.491 | 0.7 | 0 | 18.438 | TatC |
| TRINITY_DN25110_c0_g1_i1 | 1.990084109 | 0.482318577 | 0.999970678 | 0 | 6.593 | 0.844 | 0.613 | NA |
| TRINITY_DN29696_c0_g1_i1 | 1.989945161 | 0.165536698 | 0.999970678 | 0.59 | 3.4 | 0.356 | 0.573 | NA |
| TRINITY_DN2877_c0_g1_i6 | 1.989911075 | 0.0898984 | 0.999970678 | 2.416 | 4.386 | 0.478 | 1.066 | GST_C |
| TRINITY_DN6891_c4_g2_i2 | 1.989782576 | 0.096685181 | 0.999970678 | 0.372 | 0.394 | 0.1 | 0.06 | NA |
| TRINITY_DN758_c0_g1_i4 | -1.98959955 | 0.523749306 | 0.999970678 | 0.491 | 0 | 1.589 | 0.04 | Sugar_tr |
| TRINITY_DN13175_c0_g1_i2 | -1.98944921 | 0.633026768 | 0.999970678 | 0 | 1.616 | 0 | 6.304 | HsbA |
| TRINITY_DN21318_c0_g1_i22 | 1.989425645 | 0.639425543 | 0.999970678 | 1.88 | 0 | 0 | 0.493 | Rhodanese |
| TRINITY_DN2934_c0_g1_i17 | -1.98942492 | 0.452884451 | 0.999970678 | 0.681 | 0 | 0.6 | 2.051 | DWNN |
| TRINITY_DN1732_c0_g1_i8 | 1.989127279 | 0.009023168 | 0.447351945 | 4.324 | 3.834 | 1.044 | 0.794 | PWI |
| TRINITY_DN3925_c0_g1_i2 | -1.98828395 | 0.444061361 | 0.999970678 | 3.87 | 0 | 3.8 | 10.878 | NA |
| TRINITY_DN255_c4_g1_i1 | -1.98811883 | 0.635984704 | 0.999970678 | 0 | 0.473 | 1.511 | 0 | NA |
| TRINITY_DN10314_c0_g1_i2 | 1.988063149 | 0.505692293 | 0.999970678 | 3.897 | 0.089 | 0.856 | 0 | LRRNT_2 |
| TRINITY_DN2013_c0_g1_i2 | -1.98798905 | 0.455698037 | 0.999970678 | 3.27 | 0 | 5.422 | 6.666 | NA |
| TRINITY_DN8979_c0_g1_i4 | 1.987953196 | 0.492123415 | 0.999970678 | 0 | 3.341 | 0.544 | 0.161 | NA |
| TRINITY_DN670_c0_g1_i10 | 1.987698609 | 0.635503576 | 0.999970678 | 5.014 | 0 | 0 | 1.267 | - |
| TRINITY_DN1918_c0_g1_i3 | -1.987523 | 0.125087788 | 0.999970678 | 7.958 | 20.11 | 13.71 | 93.68 | Pollen_Ole_e_I |
| TRINITY_DN2586_c0_g1_i4 | -1.98638239 | 0.105247745 | 0.999970678 | 1.172 | 0.828 | 2.3 | 5.007 | Histone |
| TRINITY_DN19348_c0_g1_i1 | -1.98622849 | 0.444533939 | 0.999970678 | 0.936 | 0 | 1.678 | 1.709 | NA |
| TRINITY_DN1258_c0_g1_i11 | 1.986190035 | 0.636480226 | 0.999970678 | 0 | 13.64 | 2.8 | 0 | - |
| TRINITY_DN934_c0_g1_i10 | 1.986086396 | 0.221345966 | 0.999970678 | 3.07 | 17.13 | 0.456 | 4.514 | NA |
| TRINITY_DN3395_c0_g1_i4 | 1.986055418 | 0.044598825 | 0.999970678 | 8.257 | 9.53 | 1.178 | 3.056 | NA |
| TRINITY_DN33395_c0_g1_i8 | -1.98585712 | 0.396958776 | 0.999970678 | 5.968 | 0.522 | 0.456 | 23.837 | NA |
| TRINITY_DN1498_c0_g1_i10 | 1.985811061 | 0.438164346 | 0.999970678 | 0.936 | 0.315 | 0 | 0.322 | - |
| TRINITY_DN9067_c0_g1_i3 | 1.985668902 | 0.069764906 | 0.999970678 | 1.326 | 2.227 | 0.167 | 0.684 | NA |
| TRINITY_DN1_c0_g1_i3 | -1.98535348 | 0.463929417 | 0.999970678 | 7.567 | 0 | 9.044 | 19.534 | KH_1 |
| TRINITY_DN1625_c0_g1_i3 | -1.98515834 | 0.484940206 | 0.999970678 | 0 | 85.81 | 160.501 | 137.917 | Ribosomal_S17 |
| TRINITY_DN162_c0_g1_i8 | -1.98489704 | 0.443676456 | 0.999970678 | 2.207 | 0 | 4.455 | 3.539 | NA |
| TRINITY_DN760_c4_g1_i2 | -1.98458286 | 0.137953679 | 0.999970678 | 2.707 | 3.242 | 3.111 | 19.444 | NA |
| TRINITY_DN2813_c0_g1_i10 | 1.984105348 | 0.072427336 | 0.999970678 | 8.348 | 9.944 | 0.789 | 3.629 | NA |
| TRINITY_DN12348_c0_g1_i2 | -1.98384394 | 0.29856347 | 0.999970678 | 0.073 | 0.315 | 1.178 | 0.08 | NA |
| TRINITY_DN606_c0_g1_i9 | -1.98369814 | 0.466766195 | 0.999970678 | 13.562 | 0 | 10.299 | 41.652 | NA |
| TRINITY_DN1985_c0_g1_i1 | 1.983455079 | 0.633207591 | 0.999970678 | 12.409 | 0 | 0 | 3.187 | zf-C3HC4_3 |

| TRINITY_DN4665_c0_g1_i4 | 1.983215876 | 0.117695797 | 0.999970678 | 1.562 | 0.68 | 0.2 | 0.322 | NA |
| --- | --- | --- | --- | --- | --- | --- | --- | --- |
| TRINITY_DN4451_c0_g1_i4 | -1.98320105 | 0.642991959 | 0.999970678 | 12.218 | 0 | 40.153 | 0 | NA |
| TRINITY_DN13409_c0_g1_i1 | 1.983157707 | 0.445908785 | 1 | 0.236 | 0.306 | 0 | 0.131 | NA |
| TRINITY_DN14919_c0_g1_i1 | -1.98301956 | 0.149469316 | 0.999970678 | 0.509 | 0.187 | 0.767 | 1.83 | Branch |
| TRINITY_DN1698_c0_g2_i1 | 1.982737284 | 0.403184597 | 0.999970678 | 18.241 | 0.207 | 3.733 | 0.231 | NA |
| TRINITY_DN1549_c0_g1_i2 | -1.9820996 | 0.541970329 | 0.999970678 | 0.091 | 3.755 | 12.399 | 0 | LRRNT_2 |
| TRINITY_DN1891_c0_g1_i1 | 1.981952584 | 0.634043477 | 0.999970678 | 5.042 | 0 | 0 | 1.297 | TFIIB |
| TRINITY_DN937_c0_g1_i3 | -1.98143221 | 0.351976858 | 0.999970678 | 0.999 | 0.237 | 0.033 | 4.886 | NA |
| TRINITY_DN37206_c0_g1_i1 | 1.981429441 | 0.085171949 | 0.999970678 | 8.512 | 9.934 | 0.8 | 3.7 | NA |
| TRINITY_DN504_c0_g1_i5 | -1.98107566 | 0.043422736 | 0.999970678 | 0.227 | 0.434 | 0.922 | 1.508 | DUF3522 |
| TRINITY_DN4647_c0_g1_i6 | 1.980907505 | 0.442020855 | 0.999970678 | 5.841 | 3.134 | 1.911 | 0 | NA |
| TRINITY_DN15922_c0_g1_i1 | -1.98088496 | 0.113484857 | 0.999970678 | 0.245 | 0.522 | 0.578 | 2.312 | HLH |
| TRINITY_DN23233_c0_g1_i1 | -1.98047803 | 0.634309551 | 0.999970678 | 1.099 | 0 | 0 | 4.414 | NA |
| TRINITY_DN5799_c0_g2_i2 | -1.97951243 | 0.202935445 | 0.999970678 | 0.454 | 0.099 | 0.867 | 1.116 | NA |
| TRINITY_DN3273_c2_g1_i2 | 1.979279272 | 0.075120352 | 0.999970678 | 2.698 | 3.528 | 0.967 | 0.402 | TCP |
| TRINITY_DN4030_c0_g1_i1 | 1.979160771 | 0.555414898 | 0.999970678 | 0 | 7.411 | 1.511 | 0.02 | Ribonuclease_T2 |
| TRINITY_DN2113_c0_g1_i12 | -1.97907532 | 0.213892527 | 0.999970678 | 1.653 | 0.256 | 5.455 | 1.005 | GMC_oxred_C |
| TRINITY_DN69570_c0_g5_i1 | -1.97850904 | 0.542963189 | 0.999970678 | 1.381 | 0.059 | 4.744 | 0 | Ribosomal_S5 |
| TRINITY_DN2899_c0_g1_i21 | 1.978442436 | 0.443580249 | 1 | 0.863 | 0.503 | 0 | 0.352 | NA |
| TRINITY_DN12758_c0_g1_i12 | -1.97836594 | 0.64530859 | 0.999970678 | 0.636 | 0 | 2.144 | 0 | Ish1 |
| TRINITY_DN4579_c0_g1_i2 | 1.97834634 | 0.465410293 | 0.999970678 | 1.617 | 0.246 | 0 | 0.493 | NA |
| TRINITY_DN6361_c2_g1_i3 | -1.97830457 | 0.495907669 | 0.999970678 | 0.218 | 0.986 | 0 | 4.735 | CPSase_L_D2 |
| TRINITY_DN514_c0_g1_i3 | 1.978126188 | 0.241102446 | 0.999970678 | 1.09 | 3.982 | 0.122 | 1.136 | STAS |
| TRINITY_DN1424_c0_g2_i1 | -1.97780605 | 0.133746664 | 0.999970678 | 1.762 | 7.322 | 6.077 | 28.04 | NA |
| TRINITY_DN808_c3_g1_i1 | 1.977665224 | 0.634381274 | 0.999970678 | 2.498 | 0 | 0 | 0.643 | RVT_1 |
| TRINITY_DN162_c0_g1_i6 | 1.977646623 | 0.155713171 | 0.999970678 | 1.472 | 6.445 | 1.333 | 0.372 | NA |
| TRINITY_DN2755_c0_g1_i4 | -1.97763676 | 0.14309111 | 0.999970678 | 6.777 | 15.81 | 9.977 | 74.377 | Glyco_hydro_16 |
| TRINITY_DN8369_c0_g3_i1 | -1.97744448 | 0.110019878 | 0.999970678 | 0.3 | 0.424 | 0.511 | 2.222 | NA |
| TRINITY_DN9672_c0_g2_i2 | -1.97718149 | 0.468475888 | 0.999970678 | 0 | 0.828 | 2.378 | 0.352 | NA |
| TRINITY_DN9659_c0_g4_i2 | 1.977011051 | 0.157495798 | 1 | 1.181 | 0.69 | 0.144 | 0.292 | NA |
| TRINITY_DN8887_c0_g1_i4 | 1.976799384 | 0.442701143 | 0.999970678 | 1.408 | 1.429 | 0.611 | 0 | - |
| TRINITY_DN17437_c0_g1_i9 | -1.97675972 | 0.64352263 | 0.999970678 | 0.436 | 0 | 1.411 | 0 | NA |
| TRINITY_DN9799_c0_g1_i4 | -1.97656196 | 0.441180824 | 0.999970678 | 0.699 | 0 | 1.244 | 1.337 | NA |
| TRINITY_DN5543_c0_g2_i2 | -1.97640707 | 0.30209159 | 1 | 2.298 | 0.227 | 5.011 | 3.78 | NA |
| TRINITY_DN5761_c0_g1_i1 | 1.976165193 | 0.087915387 | 0.999970678 | 2.062 | 9.441 | 1.089 | 1.588 | MACPF |
| TRINITY_DN9253_c0_g1_i2 | -1.97612468 | 0.468755832 | 0.999970678 | 8.53 | 0 | 9.688 | 22.41 | EF-hand_1 |
| TRINITY_DN6577_c0_g1_i1 | 1.975195822 | 0.484920246 | 0.999970678 | 0 | 13.81 | 1.811 | 1.247 | Solute_trans_a |
| TRINITY_DN748_c0_g1_i8 | 1.975138926 | 0.108331973 | 0.999970678 | 2.871 | 4.287 | 0.233 | 1.538 | NA |
| TRINITY_DN1808_c0_g2_i1 | 1.975029669 | 0.487639414 | 0.999970678 | 0 | 8.811 | 0.589 | 1.508 | NA |
| TRINITY_DN4019_c0_g1_i8 | 1.974699444 | 0.240452507 | 0.999970678 | 4.26 | 1.035 | 1.033 | 0.111 | Amino_oxidase |
| TRINITY_DN11564_c0_g3_i7 | 1.974540796 | 0.449076243 | 0.999970678 | 0.89 | 4.001 | 0 | 1.237 | AMP-binding |
| TRINITY_DN2292_c0_g1_i1 | 1.974366949 | 0.65932184 | 0.999970678 | 0 | 95.15 | 19.699 | 0 | NA |
| TRINITY_DN18727_c0_g1_i1 | -1.97413971 | 0.439280571 | 0.999970678 | 2.107 | 0 | 3.289 | 4.454 | - |
| TRINITY_DN23_c1_g1_i7 | 1.973759407 | 0.487074066 | 0.999970678 | 6.677 | 0 | 0.789 | 0.764 | NA |
| TRINITY_DN8790_c0_g1_i2 | 1.973250218 | 0.120422141 | 1 | 0.563 | 0.71 | 0.1 | 0.201 | NA |
| TRINITY_DN13541_c0_g1_i8 | 1.972197839 | 0.450724847 | 0.999970678 | 0.581 | 0.256 | 0.167 | 0 | NA |
| TRINITY_DN10982_c0_g1_i1 | -1.97047381 | 0.47257983 | 0.999970678 | 0.799 | 0 | 0.3 | 2.785 | Pkinase |
| TRINITY_DN2033_c0_g1_i2 | 1.970037542 | 0.522781596 | 0.999970678 | 0 | 14.4 | 0.156 | 3.428 | NA |
| TRINITY_DN7455_c0_g1_i1 | -1.96893962 | 0.098260113 | 0.999970678 | 0.427 | 0.641 | 1.355 | 2.473 | NA |
| TRINITY_DN537_c0_g1_i1 | -1.96871822 | 0.32576016 | 0.999970678 | 0.79 | 0.089 | 0.222 | 3.187 | NA |
| TRINITY_DN1211_c0_g1_i25 | -1.96846921 | 0.636465129 | 0.999970678 | 0 | 0.818 | 2.622 | 0 | NA |
| TRINITY_DN23601_c0_g1_i2 | -1.96783815 | 0.456527903 | 0.999970678 | 0 | 0.325 | 0.256 | 0.945 | FAR1 |
| TRINITY_DN1261_c1_g1_i1 | 1.967362773 | 0.336078258 | 0.999970678 | 0.091 | 0.897 | 0.2 | 0.01 | Asp_protease_2 |
| TRINITY_DN5609_c0_g1_i2 | 1.967327234 | 0.346802296 | 0.999970678 | 0.109 | 12.39 | 0.578 | 2.443 | 2OG-FeII_Oxy |
| TRINITY_DN3107_c0_g1_i2 | -1.96726945 | 0.055936634 | 0.999970678 | 8.866 | 3.893 | 12.155 | 35.49 | NA |
| TRINITY_DN27199_c0_g3_i1 | -1.96723502 | 0.469201293 | 0.999970678 | 1.09 | 0 | 0.489 | 3.76 | NA |
| TRINITY_DN10336_c0_g2_i4 | 1.967210195 | 0.494672566 | 0.999970678 | 4.26 | 0 | 0.689 | 0.282 | DBD_Tnp_Mut |
| TRINITY_DN897_c0_g1_i6 | 1.966795141 | 0.236671718 | 0.999970678 | 3.543 | 0.246 | 0.544 | 0.332 | NA |
| TRINITY_DN10726_c0_g1_i1 | -1.96663113 | 0.141230736 | 0.999970678 | 3.361 | 1.971 | 11.466 | 6.344 | PPO1_DWL |
| TRINITY_DN287_c0_g2_i1 | 1.966374913 | 0.518622408 | 0.999970678 | 25.998 | 0 | 5 | 0.694 | BAG |
| TRINITY_DN56532_c0_g1_i2 | 1.966149949 | 0.071835126 | 0.999970678 | 1.88 | 3.085 | 0.389 | 0.784 | Abhydrolase_1 |
| TRINITY_DN3110_c1_g1_i8 | 1.965975999 | 0.162847382 | 0.999970678 | 6.332 | 1.37 | 1.311 | 0.412 | DEAD |
| TRINITY_DN1850_c0_g1_i13 | -1.96591713 | 0.12984894 | 1 | 0.218 | 0.177 | 0.811 | 0.613 | PALP |
| TRINITY_DN225_c0_g1_i7 | -1.96517651 | 0.641999707 | 0.999970678 | 0.563 | 0 | 1.833 | 0 | NA |
| TRINITY_DN14878_c1_g1_i1 | -1.96421142 | 0.431515258 | 0.999970678 | 1.081 | 15.95 | 53.063 | 0.261 | NA |
| TRINITY_DN2700_c0_g1_i2 | -1.96371244 | 0.516845239 | 0.999970678 | 95.845 | 0 | 143.247 | 206.634 | Calreticulin |
| TRINITY_DN179_c0_g1_i7 | -1.96317024 | 0.479573122 | 0.999970678 | 3.87 | 0 | 1.411 | 13.613 | CK_II_beta |
| TRINITY_DN8228_c0_g1_i2 | 1.962954517 | 0.090239697 | 0.999970678 | 1.517 | 1.853 | 0.222 | 0.593 | NA |
| TRINITY_DN393_c0_g1_i11 | 1.962304842 | 0.638850604 | 0.999970678 | 2.126 | 0 | 0.456 | 0 | NA |
| TRINITY_DN1497_c0_g1_i6 | -1.96228766 | 0.44507545 | 0.999970678 | 0 | 6.051 | 6.722 | 15.03 | NA |
| TRINITY_DN1838_c0_g1_i4 | 1.962265549 | 0.636896643 | 0.999970678 | 2.707 | 0 | 0 | 0.704 | Myb_DNA-bindin |
| TRINITY_DN10459_c0_g1_i2 | 1.962037554 | 0.643809362 | 0.999970678 | 1.254 | 0 | 0 | 0.332 | KH_1 |
| TRINITY_DN4463_c0_g1_i7 | -1.96198491 | 0.638994569 | 0.999970678 | 0.536 | 0 | 0 | 2.111 | NA |
| TRINITY_DN185_c0_g1_i5 | 1.961695453 | 0.498610844 | 0.999970678 | 12.381 | 0.532 | 0 | 3.368 | TPR_1 |

| TRINITY_DN8944_c0_g1_i1 | -1.96144303 | 0.137202214 | 0.999970678 | 2.244 | 3.489 | 2.811 | 18.64 | CCT_2 |
| --- | --- | --- | --- | --- | --- | --- | --- | --- |
| TRINITY_DN1105_c0_g1_i6 | 1.961308852 | 0.640481533 | 0.999970678 | 5.014 | 0 | 0 | 1.317 | Peptidase_S9 |
| TRINITY_DN11648_c0_g1_i3 | -1.96130537 | 0.103167836 | 0.999970678 | 1.444 | 0.897 | 1.544 | 7.269 | NA |
| TRINITY_DN9367_c0_g1_i7 | -1.96061355 | 0.101000824 | 0.999970678 | 0.772 | 0.217 | 1.722 | 1.769 | NA |
| TRINITY_DN2652_c0_g1_i4 | -1.96025475 | 0.648075264 | 0.999970678 | 0 | 2.06 | 0 | 7.711 | NA |
| TRINITY_DN10289_c0_g1_i2 | -1.96023189 | 0.190559598 | 0.999970678 | 0.5 | 0.128 | 0.767 | 1.488 | NA |
| TRINITY_DN4546_c0_g4_i1 | 1.959864031 | 0.197931691 | 1 | 0.818 | 1.064 | 0.3 | 0.131 | ABC2_membrane |
| TRINITY_DN3094_c0_g1_i2 | -1.95967176 | 0.347369473 | 0.999970678 | 0.136 | 4.208 | 0.8 | 15.764 | peroxidase |
| TRINITY_DN30_c3_g1_i1 | 1.959152301 | 0.099542296 | 0.999970678 | 4.705 | 9.372 | 1.755 | 1.448 | NA |
| TRINITY_DN3048_c0_g1_i11 | 1.959057599 | 0.446322394 | 0.999970678 | 1.735 | 1.005 | 0.589 | 0 | NA |
| TRINITY_DN6615_c0_g1_i1 | -1.95833637 | 0.459913355 | 1 | 0.173 | 0 | 0.367 | 0.251 | HECT |
| TRINITY_DN2400_c0_g2_i1 | -1.95832892 | 0.456114591 | 0.999970678 | 0.527 | 0 | 1.311 | 0.493 | PC-Esterase |
| TRINITY_DN1173_c5_g1_i2 | 1.957817612 | 0.638462001 | 0.999970678 | 6.023 | 0 | 0 | 1.568 | NA |
| TRINITY_DN73_c1_g1_i5 | 1.957116567 | 0.641967669 | 0.999970678 | 0.899 | 0 | 0.2 | 0 | NA |
| TRINITY_DN344_c1_g1_i1 | -1.95698965 | 0.462513686 | 0.999970678 | 13.535 | 0 | 33.198 | 12.627 | Pyrid_oxidase_2 |
| TRINITY_DN5625_c0_g3_i2 | 1.956982033 | 0.638109711 | 0.999970678 | 9.747 | 0 | 0 | 2.544 | SGTA_dimer |
| TRINITY_DN3326_c0_g1_i1 | 1.95650871 | 0.258708381 | 0.999970678 | 12.718 | 0.779 | 1.889 | 1.237 | zf-C2H2_6 |
| TRINITY_DN14367_c0_g1_i3 | -1.95645645 | 0.107716695 | 0.999970678 | 2.571 | 2.907 | 11.488 | 6.585 | NA |
| TRINITY_DN9150_c0_g1_i7 | -1.95645489 | 0.450535096 | 0.999970678 | 0.463 | 0 | 0.589 | 1.076 | NA |
| TRINITY_DN1329_c0_g1_i1 | 1.955874945 | 0.204430858 | 0.999970678 | 13.163 | 1.37 | 0.822 | 2.765 | NA |
| TRINITY_DN11681_c0_g2_i3 | -1.95584851 | 0.454863246 | 0.999970678 | 0 | 1.222 | 0.833 | 3.68 | NA |
| TRINITY_DN6934_c2_g1_i2 | -1.95576232 | 0.275728248 | 0.999970678 | 9.611 | 0.867 | 31.331 | 3.207 | NA |
| TRINITY_DN1680_c0_g1_i3 | 1.955506644 | 0.447868485 | 0.999970678 | 7.858 | 9.658 | 3.711 | 0 | NA |
| TRINITY_DN5395_c0_g1_i4 | 1.955482822 | 0.484805726 | 0.999970678 | 0.945 | 8.407 | 1.967 | 0 | Aldedh |
| TRINITY_DN15540_c0_g1_i1 | -1.95459712 | 0.119385424 | 0.999970678 | 0.79 | 0.296 | 1.733 | 2.141 | NA |
| TRINITY_DN1480_c0_g1_i1 | -1.95442585 | 0.391780763 | 0.999970678 | 7.594 | 0.355 | 24.665 | 0.935 | NA |
| TRINITY_DN31387_c2_g1_i4 | 1.954217899 | 0.169701959 | 0.999970678 | 1.808 | 0.719 | 0.144 | 0.493 | AAA_18 |
| TRINITY_DN9087_c0_g1_i1 | 1.953778321 | 0.13461466 | 0.999970678 | 1.335 | 4.78 | 0.289 | 1.227 | Homeobox |
| TRINITY_DN1515_c0_g1_i7 | 1.953627871 | 0.166820165 | 0.999970678 | 0.5 | 1.587 | 0.167 | 0.332 | NA |
| TRINITY_DN460_c2_g1_i3 | 1.953402775 | 0.568544082 | 0.999970678 | 97.434 | 0 | 0.3 | 25.285 | TPR_17 |
| TRINITY_DN1333_c1_g1_i5 | 1.953324935 | 0.457535636 | 1 | 0.554 | 0.7 | 0.278 | 0 | NA |
| TRINITY_DN843_c2_g1_i2 | 1.952800757 | 0.164601625 | 0.999970678 | 1.063 | 1.38 | 0.456 | 0.101 | NA |
| TRINITY_DN2725_c1_g1_i6 | -1.95243652 | 0.474148037 | 0.999970678 | 7.858 | 0 | 10.255 | 18.469 | NA |
| TRINITY_DN4169_c0_g1_i1 | -1.95237557 | 0.12757232 | 0.999970678 | 0.491 | 1.35 | 3.366 | 2.996 | Kunitz_legume |
| TRINITY_DN2832_c0_g3_i1 | 1.952126884 | 0.218142344 | 0.999970678 | 1.562 | 3.991 | 0.178 | 1.196 | NA |
| TRINITY_DN1582_c0_g1_i3 | 1.951988875 | 0.357844123 | 0.999970678 | 1.417 | 0.089 | 0.311 | 0.03 | ORC3_N |
| TRINITY_DN75_c0_g1_i13 | -1.95178548 | 0.437323217 | 0.999970678 | 4.088 | 0.128 | 13.188 | 0.221 | - |
| TRINITY_DN4457_c0_g1_i7 | 1.951277253 | 0.073764385 | 0.999970678 | 3.997 | 1.547 | 0.511 | 0.814 | NA |
| TRINITY_DN4195_c0_g1_i1 | 1.951129933 | 0.074461801 | 0.999970678 | 2.689 | 0.857 | 0.422 | 0.422 | NA |
| TRINITY_DN1288_c0_g1_i2 | -1.95074991 | 0.491923055 | 0.999970678 | 48.881 | 0 | 70.007 | 106.328 | Fe-S_biosyn |
| TRINITY_DN2768_c1_g1_i2 | 1.950492104 | 0.64177314 | 0.999970678 | 13.98 | 0 | 2.989 | 0 | - |
| TRINITY_DN10244_c0_g1_i6 | 1.950472767 | 0.100223339 | 0.999970678 | 47.482 | 10.75 | 4.389 | 9.843 | NAM |
| TRINITY_DN5855_c1_g2_i1 | 1.950326641 | 0.495592862 | 0.999970678 | 0 | 7.47 | 1.144 | 0.513 | NA |
| TRINITY_DN1704_c0_g1_i3 | 1.950189353 | 0.437445051 | 0.999970678 | 1.971 | 1.114 | 0 | 0.804 | Kri1 |
| TRINITY_DN2764_c0_g1_i2 | 1.949568488 | 0.009573204 | 0.468227105 | 4.16 | 3.577 | 0.889 | 0.945 | UAA |
| TRINITY_DN2905_c0_g2_i2 | 1.948429252 | 0.640260277 | 0.999970678 | 4.578 | 0 | 0 | 1.196 | NA |
| TRINITY_DN4488_c0_g2_i1 | 1.94826824 | 0.646095284 | 0.999970678 | 16.188 | 0 | 0 | 4.273 | NA |
| TRINITY_DN155_c0_g1_i4 | -1.94826601 | 0.048069989 | 0.999970678 | 3.107 | 6.741 | 21.965 | 11.27 | Ank |
| TRINITY_DN3614_c0_g1_i2 | -1.9479277 | 0.21938281 | 0.999970678 | 0.863 | 2.444 | 9.644 | 1.036 | NA |
| TRINITY_DN4490_c0_g1_i1 | 1.947867172 | 0.184280817 | 0.999970678 | 0.554 | 0.798 | 0.244 | 0.06 | NA |
| TRINITY_DN944_c0_g1_i9 | -1.94763486 | 0.081463187 | 0.999970678 | 1.472 | 0.995 | 2.766 | 5.841 | PPO1_DWL |
| TRINITY_DN1005_c0_g1_i1 | -1.94759019 | 0.216541492 | 0.999970678 | 1.036 | 3.439 | 1.178 | 15.724 | NA |
| TRINITY_DN2132_c0_g1_i7 | -1.94701264 | 0.507665643 | 0.999970678 | 0 | 33.12 | 97.627 | 3.71 | NA |
| TRINITY_DN355_c0_g2_i3 | -1.94686458 | 0.461475844 | 0.999970678 | 3.261 | 0 | 7.877 | 3.227 | NA |
| TRINITY_DN182_c0_g1_i9 | -1.94681297 | 0.105501812 | 0.999970678 | 7.485 | 5.696 | 6.588 | 42.959 | NA |
| TRINITY_DN13648_c0_g1_i1 | 1.94670129 | 0.11866899 | 0.999970678 | 0.718 | 1.597 | 0.244 | 0.282 | NA |
| TRINITY_DN29137_c0_g1_i1 | 1.946279989 | 0.45071251 | 0.999970678 | 4.724 | 1.449 | 0 | 1.629 | NA |
| TRINITY_DN2299_c0_g1_i3 | -1.94595985 | 0.481705893 | 0.999970678 | 0 | 54.11 | 73.429 | 114.391 | NA |
| TRINITY_DN591_c0_g1_i15 | -1.94583595 | 0.490246856 | 0.999970678 | 1.372 | 0 | 0.322 | 4.946 | NA |
| TRINITY_DN11128_c0_g1_i9 | -1.94537566 | 0.504299633 | 0.999970678 | 1.626 | 0 | 0.233 | 6.022 | Pkinase |
| TRINITY_DN12129_c0_g1_i2 | 1.94522374 | 0.463349824 | 1 | 0.382 | 0.522 | 0.2 | 0 | MCM_bind |
| TRINITY_DN3850_c0_g1_i6 | 1.944948491 | 0.643218156 | 0.999970678 | 3.016 | 0 | 0 | 0.804 | Pkinase |
| TRINITY_DN8737_c0_g1_i1 | 1.944909912 | 0.271476183 | 0.999970678 | 46.61 | 783.1 | 155.813 | 23.717 | CAP |
| TRINITY_DN1582_c0_g1_i7 | -1.9447302 | 0.475978086 | 0.999970678 | 0.391 | 0 | 0.2 | 1.307 | ORC3_N |
| TRINITY_DN1165_c0_g1_i4 | 1.944659604 | 0.449401104 | 0.999970678 | 7.567 | 4.809 | 0 | 3.217 | NA |
| TRINITY_DN1195_c0_g2_i1 | 1.94449409 | 0.486079928 | 0.999970678 | 63.379 | 56.58 | 0 | 31.177 | PfkB |
| TRINITY_DN7737_c0_g1_i9 | -1.94446126 | 0.647735261 | 0.999970678 | 0.772 | 0 | 2.478 | 0 | Pro-kuma_activ |
| TRINITY_DN1221_c0_g1_i4 | -1.94443014 | 0.46935863 | 0.999970678 | 9.811 | 0 | 18.143 | 16.357 | NA |
| TRINITY_DN9231_c0_g1_i7 | 1.944421965 | 0.096599724 | 0.999970678 | 0.89 | 1.035 | 0.278 | 0.151 | NA |
| TRINITY_DN1226_c0_g2_i3 | -1.94440288 | 0.215087918 | 0.999970678 | 0.354 | 4.12 | 6.166 | 9.36 | NA |
| TRINITY_DN9587_c0_g1_i1 | 1.943841585 | 0.485432037 | 0.999970678 | 0.972 | 13.2 | 0 | 3.609 | - |
| TRINITY_DN4105_c0_g1_i1 | -1.9437072 | 0.505021913 | 0.999970678 | 27.879 | 0 | 10.022 | 96.304 | LRRNT_2 |
| TRINITY_DN11607_c0_g1_i2 | -1.94349465 | 0.298411335 | 0.999970678 | 0.718 | 0.059 | 1.989 | 0.623 | NA |
| TRINITY_DN2053_c0_g1_i2 | 1.943432489 | 0.298536304 | 0.999970678 | 5.741 | 0.404 | 1.211 | 0.161 | NA |

| TRINITY_DN453_c0_g3_i1 | -1.9432638 | 0.480169811 | 0.999970678 | 14.271 | 0 | 8.211 | 45.222 | Ribosomal_S17e |
| --- | --- | --- | --- | --- | --- | --- | --- | --- |
| TRINITY_DN632_c0_g1_i4 | -1.94320586 | 0.057385722 | 0.999970678 | 1.072 | 0.907 | 3.333 | 3.378 | NADHdh |
| TRINITY_DN9525_c1_g2_i1 | 1.9428519 | 0.436803072 | 0.999970678 | 1.681 | 1.37 | 0 | 0.804 | EF-hand_1 |
| TRINITY_DN2240_c0_g1_i1 | -1.94281269 | 0.033712423 | 0.999970678 | 19.24 | 32.2 | 47.73 | 138.922 | NA |
| TRINITY_DN6820_c0_g1_i3 | 1.942447285 | 0.030374454 | 0.999970678 | 9.783 | 7.598 | 2.633 | 1.357 | DnaJ |
| TRINITY_DN21657_c0_g1_i6 | -1.94237648 | 0.152160793 | 0.999970678 | 0.908 | 1.932 | 7.2 | 2.373 | NA |
| TRINITY_DN10177_c0_g1_i3 | 1.942130426 | 0.644505182 | 0.999970678 | 0 | 23.8 | 5.022 | 0 | NA |
| TRINITY_DN1880_c1_g1_i9 | 1.942015029 | 0.507839748 | 0.999970678 | 5.587 | 0.256 | 1.255 | 0 | NA |
| TRINITY_DN20222_c0_g1_i10 | -1.94123052 | 0.462128548 | 0.999970678 | 0.681 | 0 | 1.655 | 0.653 | F-box |
| TRINITY_DN12158_c0_g3_i3 | 1.940820326 | 0.100653304 | 0.999970678 | 1.008 | 0.798 | 0.289 | 0.131 | DUF4371 |
| TRINITY_DN16824_c0_g1_i2 | 1.940322954 | 0.464775696 | 1 | 0.427 | 0.325 | 0.167 | 0 | NA |
| TRINITY_DN49821_c1_g2_i1 | -1.94029709 | 0.459611426 | 0.999970678 | 1.054 | 0 | 2.111 | 1.508 | NA |
| TRINITY_DN1898_c0_g1_i3 | 1.940202903 | 0.199665627 | 0.999970678 | 3.843 | 3.893 | 0.222 | 1.759 | NA |
| TRINITY_DN5970_c0_g1_i3 | 1.939498168 | 0.457452721 | 0.999970678 | 0.936 | 3.893 | 0 | 1.257 | MORN |
| TRINITY_DN358_c0_g1_i4 | -1.93947645 | 0.106420498 | 0.999970678 | 0.263 | 0.601 | 1.955 | 0.995 | PPR |
| TRINITY_DN1522_c0_g1_i6 | 1.939359185 | 0.255221884 | 0.999970678 | 2.725 | 20.35 | 0.411 | 5.449 | NA |
| TRINITY_DN19247_c0_g1_i8 | -1.93892223 | 0.066408877 | 0.999970678 | 0.318 | 0.562 | 1.033 | 2.111 | NA |
| TRINITY_DN9491_c0_g1_i12 | -1.93882766 | 0.652474116 | 0.999970678 | 1.036 | 0 | 3.311 | 0 | NA |
| TRINITY_DN5697_c0_g1_i2 | 1.938660203 | 0.176025863 | 0.999970678 | 3.179 | 4.208 | 1.233 | 0.392 | CHDCT2 |
| TRINITY_DN3737_c0_g1_i5 | -1.93843819 | 0.183378663 | 0.999970678 | 3.207 | 2.237 | 1.933 | 18.308 | NA |
| TRINITY_DN3199_c0_g1_i8 | 1.937662992 | 0.502344866 | 0.999970678 | 0 | 5.539 | 0.933 | 0.282 | NA |
| TRINITY_DN26146_c0_g1_i3 | -1.93722707 | 0.650088023 | 0.999970678 | 0.6 | 0 | 1.878 | 0 | NA |
| TRINITY_DN835_c0_g1_i10 | -1.93709134 | 0.454796326 | 0.999970678 | 0.69 | 0 | 0.711 | 1.81 | LRR_8 |
| TRINITY_DN438_c0_g1_i13 | -1.93668401 | 0.44619526 | 0.999970678 | 0 | 1.666 | 2.966 | 2.574 | NA |
| TRINITY_DN1873_c3_g1_i11 | 1.936333132 | 0.244289402 | 0.999970678 | 5.732 | 0.384 | 0.744 | 0.714 | NA |
| TRINITY_DN5592_c0_g1_i9 | -1.93524652 | 0.520729079 | 0.999970678 | 64.85 | 0 | 81.328 | 153.902 | Ribosomal_S5 |
| TRINITY_DN2146_c0_g1_i1 | -1.93518573 | 0.083424619 | 0.999970678 | 3.152 | 1.449 | 11.066 | 4.353 | NA |
| TRINITY_DN1126_c0_g1_i8 | 1.935070608 | 0.467277746 | 0.999970678 | 12.472 | 19.69 | 0 | 8.375 | NA |
| TRINITY_DN14081_c0_g1_i4 | 1.934702313 | 0.144808621 | 0.999970678 | 0.654 | 1.114 | 0.289 | 0.131 | NA |
| TRINITY_DN1904_c0_g1_i6 | 1.934415019 | 0.537471024 | 0.999970678 | 21.638 | 0 | 4.5 | 0.292 | PMD |
| TRINITY_DN4946_c0_g1_i5 | 1.934201684 | 0.08143477 | 0.999970678 | 0.709 | 0.572 | 0.178 | 0.121 | Anticodon_1 |
| TRINITY_DN814_c0_g1_i2 | -1.93412503 | 0.464143913 | 1 | 0.254 | 0 | 0.378 | 0.493 | - |
| TRINITY_DN12472_c0_g1_i1 | -1.9333866 | 0.644895365 | 0.999970678 | 3.843 | 0 | 12.233 | 0 | NA |
| TRINITY_DN5262_c0_g1_i8 | -1.93335002 | 0.078426831 | 0.999970678 | 0.999 | 0.394 | 1.755 | 3.177 | NA |
| TRINITY_DN4016_c0_g1_i3 | -1.933068 | 0.497324801 | 0.999970678 | 0 | 0.65 | 1.855 | 0.191 | Myb_DNA-bindin |
| TRINITY_DN8442_c0_g1_i1 | -1.93295916 | 0.009831993 | 0.478586582 | 1.017 | 1.468 | 3.811 | 4.856 | NA |
| TRINITY_DN411_c0_g1_i6 | 1.932886175 | 0.282793587 | 0.999970678 | 1.744 | 24.25 | 0.7 | 5.841 | NA |
| TRINITY_DN10407_c0_g1_i1 | 1.932866689 | 0.08529679 | 0.999970678 | 0.518 | 0.611 | 0.167 | 0.09 | NA |
| TRINITY_DN859_c0_g1_i7 | 1.932618543 | 0.494407113 | 0.999970678 | 103.04 | 25.82 | 0 | 33.931 | NA |
| TRINITY_DN4627_c0_g2_i1 | -1.93257879 | 0.485502837 | 0.999970678 | 0 | 7.116 | 19.565 | 3.036 | NA |
| TRINITY_DN2590_c0_g1_i5 | 1.932021922 | 0.08567033 | 0.999970678 | 2.18 | 3.39 | 0.811 | 0.483 | NA |
| TRINITY_DN45929_c0_g1_i3 | 1.931891636 | 0.151773803 | 0.999970678 | 105.22 | 34.02 | 25.987 | 5.339 | - |
| TRINITY_DN3766_c0_g1_i4 | 1.931864204 | 0.514977314 | 0.999970678 | 48.817 | 24.14 | 15.888 | 0 | EFG_C |
| TRINITY_DN8621_c0_g1_i2 | 1.931273527 | 0.646552758 | 0.999970678 | 0 | 15.46 | 3.311 | 0 | NA |
| TRINITY_DN516_c0_g1_i2 | 1.93109954 | 0.644872819 | 0.999970678 | 0 | 6.702 | 1.444 | 0 | NA |
| TRINITY_DN4555_c0_g1_i6 | -1.93089626 | 0.463330546 | 1 | 0.5 | 0 | 0.822 | 0.855 | NA |
| TRINITY_DN2269_c0_g1_i1 | -1.9307387 | 0.027599255 | 0.999970678 | 3.897 | 3.311 | 10.11 | 14.719 | NA |
| TRINITY_DN4461_c0_g1_i2 | -1.93011206 | 0.462474589 | 0.999970678 | 2.525 | 0 | 2.322 | 6.907 | NA |
| TRINITY_DN34437_c0_g1_i1 | 1.929804752 | 0.071601163 | 0.999970678 | 2.026 | 1.133 | 0.444 | 0.312 | NA |
| TRINITY_DN8359_c0_g1_i1 | 1.929800514 | 0.520130657 | 0.999970678 | 11.9 | 0 | 0.256 | 2.865 | NA |
| TRINITY_DN2514_c1_g1_i6 | 1.929654117 | 0.215669658 | 0.999970678 | 1.135 | 1.124 | 0.444 | 0.05 | NA |
| TRINITY_DN1126_c2_g1_i2 | 1.929575378 | 0.317183607 | 0.999970678 | 66.476 | 1.232 | 10.222 | 5.359 | NA |
| TRINITY_DN4928_c0_g1_i1 | 1.928949001 | 0.237585445 | 0.999970678 | 5.323 | 1.991 | 0.189 | 1.699 | NA |
| TRINITY_DN2043_c0_g1_i6 | 1.928935912 | 0.510698129 | 0.999970678 | 2.453 | 0 | 0.122 | 0.513 | NA |
| TRINITY_DN6485_c0_g1_i6 | -1.92880711 | 0.655997727 | 0.999970678 | 1.453 | 0 | 4.644 | 0 | Asp |
| TRINITY_DN1355_c0_g2_i1 | -1.92874993 | 0.643691664 | 0.999970678 | 0 | 7.401 | 22.999 | 0 | NA |
| TRINITY_DN2343_c0_g1_i3 | -1.92839042 | 0.345772256 | 0.999970678 | 1.662 | 0.059 | 0.578 | 6.002 | NA |
| TRINITY_DN1915_c0_g1_i17 | 1.928298899 | 0.514032327 | 0.999970678 | 5.659 | 0 | 0.178 | 1.307 | - |
| TRINITY_DN8612_c3_g1_i1 | 1.9282031 | 0.4617681 | 0.999970678 | 0.418 | 0.887 | 0.278 | 0 | NA |
| TRINITY_DN518_c0_g1_i3 | -1.92812046 | 0.344459566 | 0.999970678 | 0.164 | 2.07 | 0.289 | 8.003 | Tubulin |
| TRINITY_DN8131_c0_g1_i5 | 1.927855202 | 0.441907685 | 0.999970678 | 0.754 | 0.503 | 0 | 0.332 | NA |
| TRINITY_DN7279_c0_g1_i1 | 1.927756049 | 0.148952911 | 1 | 0.836 | 0.414 | 0.122 | 0.181 | NA |
| TRINITY_DN1509_c0_g1_i1 | 1.927611708 | 0.03115527 | 0.999970678 | 9.783 | 4.228 | 1.567 | 1.82 | NA |
| TRINITY_DN2332_c0_g1_i5 | -1.92750485 | 0.649622636 | 0.999970678 | 0 | 1.666 | 0 | 6.042 | NA |
| TRINITY_DN3570_c0_g2_i3 | -1.92675658 | 0.145135348 | 0.999970678 | 0.372 | 0.71 | 0.878 | 2.966 | NA |
| TRINITY_DN1417_c0_g1_i5 | -1.92670374 | 0.532239051 | 0.999970678 | 76.986 | 0 | 116.393 | 157.28 | H_PPase |
| TRINITY_DN1460_c0_g2_i3 | 1.926217044 | 0.025069089 | 0.999970678 | 10.365 | 21.5 | 3.166 | 4.444 | NA |
| TRINITY_DN4545_c2_g1_i2 | 1.925930284 | 0.06060846 | 0.999970678 | 3.243 | 2.503 | 0.9 | 0.432 | ABC1 |
| TRINITY_DN1949_c0_g1_i3 | -1.92428486 | 0.647761733 | 0.999970678 | 0 | 0.434 | 1.355 | 0 | Glyco_transf_8 |
| TRINITY_DN16979_c0_g1_i3 | -1.92392265 | 0.21647115 | 0.999970678 | 0.064 | 0.355 | 0.389 | 1.116 | NA |
| TRINITY_DN4133_c0_g1_i4 | -1.92389392 | 0.474808955 | 0.999970678 | 0 | 23.8 | 36.009 | 44.789 | 2_5_RNA_ligase2 |
| TRINITY_DN12800_c0_g2_i1 | 1.923636783 | 0.137073882 | 1 | 0.418 | 0.759 | 0.111 | 0.171 | NA |
| TRINITY_DN227_c0_g1_i2 | 1.923632627 | 0.477298347 | 0.999970678 | 9.293 | 24.81 | 0 | 8.948 | Pollen_Ole_e_I |
| TRINITY_DN745_c0_g1_i1 | 1.92335727 | 0.24721619 | 0.999970678 | 2.044 | 2.937 | 0.078 | 1.206 | NA |

| TRINITY_DN7281_c0_g1_i13 | -1.92308962 | 0.469328197 | 0.999970678 | 0.173 | 0 | 0.4 | 0.181 | NA |
| --- | --- | --- | --- | --- | --- | --- | --- | --- |
| TRINITY_DN58_c0_g1_i7 | 1.92291655 | 0.535852694 | 0.999970678 | 2.017 | 0 | 0.033 | 0.493 | NA |
| TRINITY_DN4912_c0_g1_i4 | 1.922711538 | 0.105432468 | 0.999970678 | 1.98 | 3.548 | 0.422 | 0.985 | NA |
| TRINITY_DN1027_c0_g1_i6 | 1.922659483 | 0.329559807 | 0.999970678 | 19.022 | 0.266 | 2.3 | 2.363 | NA |
| TRINITY_DN6989_c0_g1_i2 | 1.9226231 | 0.093758792 | 0.999970678 | 0.872 | 0.591 | 0.156 | 0.191 | DUF547 |
| TRINITY_DN1363_c0_g1_i8 | 1.922619639 | 0.256077781 | 0.999970678 | 17.977 | 10.01 | 0.278 | 6.806 | Dimerisation |
| TRINITY_DN9637_c0_g2_i8 | 1.92211082 | 0.472174378 | 1 | 0.681 | 0.621 | 0.289 | 0 | RVT_2 |
| TRINITY_DN1320_c0_g1_i16 | 1.922077043 | 0.662884655 | 0.999970678 | 52.514 | 0 | 0 | 14.125 | NA |
| TRINITY_DN20464_c0_g1_i1 | -1.92186479 | 0.282804771 | 0.999970678 | 0.781 | 0.187 | 0.3 | 3.338 | NA |
| TRINITY_DN18843_c0_g1_i1 | 1.921524072 | 0.647103073 | 0.999970678 | 0 | 2.631 | 0 | 0.674 | Vps62 |
| TRINITY_DN8689_c0_g1_i1 | 1.921226753 | 0.026296937 | 0.999970678 | 4.578 | 5.893 | 1.089 | 1.448 | NA |
| TRINITY_DN17703_c0_g1_i3 | -1.92084217 | 0.375430437 | 0.999970678 | 0.173 | 0.798 | 0.078 | 3.499 | - |
| TRINITY_DN2573_c1_g1_i4 | -1.92070485 | 0.469389308 | 0.999970678 | 0 | 1.035 | 0.556 | 3.197 | NA |
| TRINITY_DN5265_c1_g3_i1 | 1.920468622 | 0.471560602 | 0.999970678 | 3.098 | 11.32 | 0 | 3.79 | NA |
| TRINITY_DN13288_c1_g1_i3 | 1.920095353 | 0.647885794 | 0.999970678 | 0 | 49.77 | 10.677 | 0 | NA |
| TRINITY_DN2135_c0_g1_i9 | 1.919808764 | 0.060118696 | 0.999970678 | 0.6 | 0.877 | 0.133 | 0.221 | NA |
| TRINITY_DN7627_c0_g1_i3 | -1.91907682 | 0.52271807 | 0.999970678 | 1.045 | 0 | 0.156 | 3.79 | NA |
| TRINITY_DN3875_c0_g2_i3 | -1.91853073 | 0.644913489 | 0.999970678 | 0 | 3.193 | 9.822 | 0 | NA |
| TRINITY_DN686_c0_g1_i11 | 1.918149265 | 0.180026837 | 0.999970678 | 2.198 | 1.597 | 0.122 | 0.855 | NA |
| TRINITY_DN748_c1_g1_i4 | -1.91770482 | 0.048024677 | 0.999970678 | 209.63 | 250.7 | 343.535 | 1323.257 | Thi4 |
| TRINITY_DN1453_c0_g1_i1 | 1.917377823 | 0.036106163 | 0.999970678 | 8.312 | 13.82 | 1.467 | 4.082 | C2 |
| TRINITY_DN1536_c0_g1_i1 | -1.91731908 | 0.214407524 | 0.999970678 | 2.998 | 5.312 | 1.678 | 29.317 | Lipase_GDSL |
| TRINITY_DN34701_c0_g1_i1 | 1.917204283 | 0.461877898 | 0.999970678 | 1.69 | 0.877 | 0.556 | 0 | NA |
| TRINITY_DN4454_c0_g1_i9 | 1.917044115 | 0.180737767 | 1 | 1.026 | 0.808 | 0.278 | 0.161 | NA |
| TRINITY_DN5146_c0_g1_i5 | -1.91676081 | 0.240310008 | 0.999970678 | 1.008 | 0.207 | 3.078 | 0.885 | mTERF |
| TRINITY_DN3214_c1_g1_i8 | -1.91665732 | 0.456661709 | 0.999970678 | 0 | 1.951 | 3.111 | 3.529 | NA |
| TRINITY_DN980_c0_g2_i4 | 1.916220726 | 0.157663737 | 0.999970678 | 4.206 | 3.302 | 1.433 | 0.271 | Cellulose_synt |
| TRINITY_DN10212_c1_g1_i1 | 1.916100372 | 0.229222432 | 0.999970678 | 17.714 | 1.36 | 1.689 | 2.996 | GUB_WAK_bind |
| TRINITY_DN2140_c1_g1_i2 | 1.916029386 | 0.444618396 | 0.999970678 | 1.299 | 1.419 | 0 | 0.724 | NA |
| TRINITY_DN24953_c0_g1_i1 | -1.91573025 | 0.188776269 | 0.999970678 | 2.434 | 5.036 | 2.778 | 24.219 | NA |
| TRINITY_DN11362_c0_g1_i2 | 1.914914186 | 0.448511379 | 0.999970678 | 0.518 | 0.522 | 0 | 0.271 | C1_1 |
| TRINITY_DN2939_c0_g1_i6 | 1.914080206 | 0.652428332 | 0.999970678 | 0 | 2.119 | 0 | 0.553 | NA |
| TRINITY_DN8689_c0_g1_i2 | -1.9140424 | 0.338566602 | 0.999970678 | 3.134 | 0.039 | 5.155 | 5.781 | NA |
| TRINITY_DN2407_c0_g1_i3 | 1.913558011 | 0.648513214 | 0.999970678 | 0 | 4.179 | 0.9 | 0 | EamA |
| TRINITY_DN6228_c0_g1_i2 | 1.9134566 | 0.649209306 | 0.999970678 | 9.347 | 0 | 0 | 2.534 | NA |
| TRINITY_DN20162_c0_g1_i2 | -1.91343001 | 0.46395954 | 1 | 0 | 0.641 | 1.089 | 1.116 | NA |
| TRINITY_DN2358_c2_g1_i5 | -1.91285984 | 0.235928279 | 0.999970678 | 0.972 | 0.118 | 1.922 | 1.769 | - |
| TRINITY_DN1607_c0_g1_i17 | -1.91239843 | 0.483912078 | 0.999970678 | 0.363 | 0 | 0.167 | 1.186 | NA |
| TRINITY_DN12887_c0_g1_i1 | 1.911268825 | 0.467362069 | 0.999970678 | 9.238 | 13.13 | 0 | 5.942 | FAD-oxidase_C |
| TRINITY_DN5438_c0_g1_i6 | 1.910105979 | 0.345158762 | 0.999970678 | 26.434 | 2.424 | 6.144 | 0.332 | NA |
| TRINITY_DN5149_c0_g1_i1 | -1.91005882 | 0.47944828 | 0.999970678 | 1.735 | 0 | 4.478 | 1.277 | NA |
| TRINITY_DN1334_c0_g1_i8 | -1.90986708 | 0.474212528 | 0.999970678 | 0 | 1.252 | 3.178 | 0.754 | NA |
| TRINITY_DN9037_c0_g1_i4 | 1.909740447 | 0.038902358 | 0.999970678 | 5.759 | 3.38 | 0.844 | 1.428 | NA |
| TRINITY_DN29656_c0_g1_i5 | -1.90971585 | 0.557770717 | 0.999970678 | 4.478 | 0.207 | 14.144 | 0 | Actin |
| TRINITY_DN11401_c0_g1_i2 | -1.90881091 | 0.651468225 | 0.999970678 | 0 | 2.868 | 0 | 10.526 | HsbA |
| TRINITY_DN5973_c0_g5_i1 | 1.908576705 | 0.169558512 | 0.999970678 | 1.363 | 7.086 | 0.667 | 1.408 | NA |
| TRINITY_DN272_c0_g2_i2 | -1.90844267 | 0.135496821 | 0.999970678 | 4.378 | 6.307 | 4.344 | 34.806 | BPS1 |
| TRINITY_DN1258_c0_g1_i3 | 1.908345397 | 0.46810769 | 0.999970678 | 0.545 | 1.991 | 0.556 | 0 | - |
| TRINITY_DN3707_c0_g1_i5 | 1.90812646 | 0.131140161 | 0.999970678 | 1.562 | 6.603 | 0.644 | 1.377 | Mito_carr |
| TRINITY_DN1699_c0_g2_i8 | -1.90806017 | 0.453787312 | 0.999970678 | 0 | 0.976 | 1.822 | 1.367 | NA |
| TRINITY_DN3444_c0_g1_i4 | 1.907810601 | 0.648038938 | 0.999970678 | 6.023 | 0 | 0 | 1.639 | NA |
| TRINITY_DN2796_c0_g1_i10 | 1.90747071 | 0.424279476 | 0.999970678 | 137.59 | 2.513 | 29.776 | 0.845 | NA |
| TRINITY_DN8758_c0_g1_i4 | 1.906978446 | 0.646378536 | 0.999970678 | 5.36 | 0 | 0 | 1.468 | Trigger_N |
| TRINITY_DN3159_c0_g1_i1 | -1.90679499 | 0.651680228 | 0.999970678 | 0.636 | 0 | 0 | 2.413 | - |
| TRINITY_DN1388_c0_g1_i3 | 1.9066718 | 0.125675503 | 0.999970678 | 0.481 | 0.483 | 0.156 | 0.07 | NA |
| TRINITY_DN1425_c0_g2_i4 | -1.90648238 | 0.649384652 | 0.999970678 | 0 | 3.351 | 10.244 | 0.01 | Aminotran_5 |
| TRINITY_DN8972_c1_g1_i2 | 1.90634864 | 0.541699221 | 0.999970678 | 13.135 | 0.128 | 2.978 | 0 | NA |
| TRINITY_DN1126_c2_g1_i7 | 1.906046484 | 0.177576632 | 0.999970678 | 6.668 | 7.52 | 2.822 | 0.372 | NA |
| TRINITY_DN461_c1_g1_i3 | 1.905527412 | 0.035228554 | 0.999970678 | 10.955 | 19.91 | 4.644 | 2.594 | LisH |
| TRINITY_DN4_c0_g1_i2 | -1.90510626 | 0.230560038 | 0.999970678 | 1.117 | 0.138 | 0.978 | 3.529 | - |
| TRINITY_DN1384_c0_g1_i20 | -1.90501758 | 0.656448727 | 0.999970678 | 0 | 0.641 | 0 | 2.393 | NA |
| TRINITY_DN1009_c0_g1_i4 | -1.90490976 | 0.171165748 | 0.999970678 | 0.309 | 2.277 | 5.255 | 3.288 | NA |
| TRINITY_DN2352_c0_g1_i3 | 1.904837234 | 0.504036064 | 0.999970678 | 4.951 | 0 | 0.489 | 0.754 | Abhydrolase_1 |
| TRINITY_DN13076_c0_g1_i5 | -1.90446242 | 0.39105601 | 0.999970678 | 0.036 | 0.867 | 0.211 | 3.056 | Pkinase |
| TRINITY_DN825_c0_g1_i9 | 1.904408004 | 0.226561651 | 0.999970678 | 6.032 | 2.631 | 0.311 | 1.87 | NA |
| TRINITY_DN645_c0_g1_i4 | 1.90431572 | 0.645789522 | 0.999970678 | 0 | 6.593 | 0 | 1.739 | NA |
| TRINITY_DN9632_c0_g1_i4 | 1.904117795 | 0.531827483 | 0.999970678 | 0 | 58.81 | 8.588 | 5.117 | NA |
| TRINITY_DN5805_c0_g1_i4 | -1.90402764 | 0.485688737 | 0.999970678 | 0.845 | 0 | 2.189 | 0.543 | NA |
| TRINITY_DN20144_c0_g1_i7 | 1.904025267 | 0.457382461 | 0.999970678 | 0.845 | 1.39 | 0.489 | 0 | Sec16 |
| TRINITY_DN874_c0_g1_i3 | -1.90398931 | 0.500902342 | 0.999970678 | 0.509 | 0.217 | 0 | 2.765 | PEPCK_ATP |
| TRINITY_DN606_c0_g1_i5 | -1.9039418 | 0.536804306 | 0.999970678 | 69.447 | 0 | 148.358 | 84.903 | TLC |
| TRINITY_DN11040_c0_g1_i1 | 1.903898024 | 0.655739286 | 0.999970678 | 0 | 98.64 | 20.388 | 0 | NA |
| TRINITY_DN20607_c0_g1_i3 | -1.90338651 | 0.46840364 | 0.999970678 | 0.818 | 0 | 1.722 | 0.915 | NA |
| TRINITY_DN862_c0_g1_i18 | 1.903013366 | 0.650988666 | 0.999970678 | 0 | 3.035 | 0.667 | 0 | NA |

| TRINITY_DN960_c0_g1_i2 | 1.902485036 | 0.095512206 | 0.999970678 | 7.185 | 8.298 | 0.767 | 3.167 | Epimerase |
| --- | --- | --- | --- | --- | --- | --- | --- | --- |
| TRINITY_DN1107_c0_g1_i4 | -1.90246322 | 0.128072074 | 0.999970678 | 6.004 | 3.154 | 4.289 | 29.377 | Sugar_tr |
| TRINITY_DN1396_c0_g3_i2 | -1.90239781 | 0.123639884 | 0.999970678 | 0.3 | 0.512 | 0.822 | 2.111 | NA |
| TRINITY_DN534_c0_g1_i10 | 1.902159125 | 0.648884099 | 0.999970678 | 1.662 | 0 | 0 | 0.452 | PDCD7 |
| TRINITY_DN17019_c0_g1_i4 | 1.901368998 | 0.018431661 | 0.796559811 | 6.141 | 5.736 | 1.755 | 1.066 | ParBc |
| TRINITY_DN335_c0_g1_i3 | 1.900587392 | 0.361896693 | 0.999970678 | 17.169 | 0.089 | 1.589 | 2.795 | LRRNT_2 |
| TRINITY_DN28435_c0_g2_i1 | -1.90054314 | 0.650516941 | 0.999970678 | 1.272 | 0 | 3.989 | 0 | zf-C2H2 |
| TRINITY_DN967_c0_g1_i4 | -1.90046499 | 0.015288729 | 0.679610015 | 10.319 | 7.342 | 19.699 | 42.386 | AAA |
| TRINITY_DN5266_c0_g1_i12 | -1.90011435 | 0.171421708 | 0.999970678 | 0.136 | 0.365 | 0.544 | 1.156 | NA |
| TRINITY_DN6368_c1_g1_i4 | -1.90007488 | 0.655895769 | 0.999970678 | 0 | 24.89 | 75.118 | 0 | NA |
| TRINITY_DN3280_c0_g1_i2 | 1.899987216 | 0.650080879 | 0.999970678 | 5.232 | 0 | 1.167 | 0 | NA |
| TRINITY_DN1031_c1_g1_i5 | -1.89965189 | 0.166265851 | 0.999970678 | 0.59 | 0.217 | 1.667 | 1.096 | NA |
| TRINITY_DN3071_c1_g1_i4 | 1.899529969 | 0.303983454 | 0.999970678 | 0.409 | 8.259 | 1.633 | 0.322 | NA |
| TRINITY_DN4545_c2_g1_i6 | -1.89931198 | 0.139646931 | 1 | 0.145 | 0.108 | 0.467 | 0.432 | ABC1 |
| TRINITY_DN4397_c0_g2_i2 | -1.89899096 | 0.06541626 | 0.999970678 | 2.171 | 1.459 | 7.955 | 3.871 | AA_kinase |
| TRINITY_DN835_c0_g1_i9 | -1.8989387 | 0.455319798 | 0.999970678 | 0 | 0.512 | 0.922 | 0.744 | LRR_8 |
| TRINITY_DN102689_c0_g1_i5 | 1.898820682 | 0.247785825 | 0.999970678 | 0.509 | 1.33 | 0.356 | 0.08 | NA |
| TRINITY_DN6689_c0_g1_i3 | 1.898536835 | 0.153394527 | 1 | 2.434 | 1.616 | 0.5 | 0.462 | NA |
| TRINITY_DN30413_c0_g1_i4 | -1.89791054 | 0.180680023 | 1 | 0.327 | 0.976 | 1.722 | 2.805 | NA |
| TRINITY_DN750_c1_g1_i2 | 1.897476024 | 0.50891385 | 0.999970678 | 0 | 1.606 | 0.267 | 0.101 | zf-CCCH |
| TRINITY_DN750_c1_g1_i11 | 1.897476024 | 0.50891385 | 0.999970678 | 0 | 1.606 | 0.267 | 0.101 | zf-CCCH |
| TRINITY_DN10578_c0_g1_i16 | 1.896937982 | 0.470181231 | 0.999970678 | 4.787 | 1.971 | 1.533 | 0 | NA |
| TRINITY_DN701_c1_g2_i3 | 1.89667897 | 0.502495857 | 0.999970678 | 0 | 8.436 | 0.767 | 1.297 | NA |
| TRINITY_DN1922_c1_g1_i13 | 1.895955632 | 0.392623452 | 0.999970678 | 2.344 | 0.059 | 0.522 | 0.03 | HSF_DNA-bind |
| TRINITY_DN3316_c1_g1_i2 | 1.895741786 | 0.187386961 | 0.999970678 | 1.744 | 11.68 | 2.022 | 1.106 | Aldo_ket_red |
| TRINITY_DN38680_c0_g1_i1 | -1.89551433 | 0.178641142 | 0.999970678 | 2.162 | 0.601 | 5.955 | 3.046 | GASA |
| TRINITY_DN11615_c0_g1_i10 | 1.895407383 | 0.1205482 | 0.999970678 | 2.153 | 1.34 | 0.4 | 0.432 | NA |
| TRINITY_DN825_c0_g1_i2 | 1.895187086 | 0.450151299 | 0.999970678 | 5.469 | 3.637 | 0 | 2.433 | NA |
| TRINITY_DN4667_c0_g1_i6 | -1.89489437 | 0.410573666 | 0.999970678 | 2.825 | 45.07 | 0.833 | 173.125 | NA |
| TRINITY_DN4773_c0_g1_i4 | 1.894293518 | 0.473555596 | 0.999970678 | 13.126 | 4.09 | 3.844 | 0 | NA |
| TRINITY_DN2066_c0_g2_i1 | -1.89426895 | 0.089002712 | 0.999970678 | 3.497 | 11.39 | 13.344 | 38.797 | NA |
| TRINITY_DN20691_c1_g1_i1 | 1.893890926 | 0.452881226 | 0.999970678 | 2.298 | 2.72 | 0 | 1.347 | NA |
| TRINITY_DN22602_c0_g2_i3 | 1.893766025 | 0.525651769 | 0.999970678 | 0.191 | 4.819 | 0 | 1.317 | - |
| TRINITY_DN3793_c1_g2_i6 | -1.89293788 | 0.659939159 | 0.999970678 | 0 | 1.754 | 5.289 | 0 | NA |
| TRINITY_DN7104_c0_g1_i2 | 1.891796388 | 0.188393981 | 1 | 0.327 | 0.345 | 0.089 | 0.06 | NA |
| TRINITY_DN11784_c0_g1_i4 | 1.89156626 | 0.508928606 | 0.999970678 | 2.453 | 0.187 | 0.6 | 0 | GMC_oxred_C |
| TRINITY_DN14060_c0_g1_i6 | 1.891559627 | 0.069482683 | 0.999970678 | 0.336 | 0.512 | 0.111 | 0.09 | NA |
| TRINITY_DN5730_c0_g2_i1 | -1.89145746 | 0.508821751 | 0.999970678 | 0 | 7.756 | 1.867 | 26.099 | NA |
| TRINITY_DN21201_c0_g2_i1 | -1.89125625 | 0.659654797 | 0.999970678 | 1.453 | 0 | 4.511 | 0 | DLH |
| TRINITY_DN3966_c0_g1_i12 | -1.89125625 | 0.659654797 | 0.999970678 | 1.217 | 0 | 3.733 | 0 | NA |
| TRINITY_DN12376_c0_g2_i3 | 1.891220104 | 0.65040848 | 0.999970678 | 0 | 13.42 | 0 | 3.519 | NA |
| TRINITY_DN1890_c0_g1_i2 | 1.890842233 | 0.16510624 | 0.999970678 | 7.412 | 3.035 | 2.033 | 0.382 | NA |
| TRINITY_DN1467_c0_g1_i14 | -1.89079096 | 0.060436487 | 0.999970678 | 8.412 | 7.943 | 12.255 | 45.222 | NA |
| TRINITY_DN15743_c0_g1_i1 | 1.890610763 | 0.560141868 | 0.999970678 | 0 | 4.997 | 1.055 | 0.04 | NA |
| TRINITY_DN3921_c0_g1_i3 | 1.890227327 | 0.4517673 | 0.999970678 | 2.571 | 3.558 | 0 | 1.649 | NA |
| TRINITY_DN2595_c1_g1_i1 | -1.89000842 | 0.489814158 | 0.999970678 | 0 | 1.005 | 2.578 | 0.563 | ACC_central |
| TRINITY_DN11421_c0_g1_i7 | 1.889873642 | 0.271462436 | 0.999970678 | 4.787 | 2.671 | 0.067 | 1.96 | HLH |
| TRINITY_DN11421_c0_g1_i19 | 1.889873642 | 0.271462436 | 0.999970678 | 4.787 | 2.671 | 0.067 | 1.96 | HLH |
| TRINITY_DN8175_c0_g1_i3 | 1.889741952 | 0.246450001 | 0.999970678 | 3.715 | 0.522 | 0.722 | 0.292 | - |
| TRINITY_DN8463_c0_g2_i2 | 1.888913253 | 0.044472039 | 0.999970678 | 4.124 | 4.829 | 0.678 | 1.568 | NA |
| TRINITY_DN7046_c0_g1_i6 | 1.888863843 | 0.65305216 | 0.999970678 | 0 | 3.055 | 0 | 0.804 | DUF647 |
| TRINITY_DN6486_c0_g1_i1 | -1.88868151 | 0.198829322 | 0.999970678 | 5.623 | 4.395 | 2.322 | 34.444 | NA |
| TRINITY_DN2931_c0_g1_i5 | -1.88842866 | 0.508016865 | 0.999970678 | 23.2 | 0 | 42.242 | 36.173 | Glycos_trans_3N |
| TRINITY_DN10838_c0_g2_i1 | 1.887956741 | 0.655193799 | 0.999970678 | 0 | 1.922 | 0.411 | 0 | NA |
| TRINITY_DN4126_c0_g1_i3 | 1.887354064 | 0.329417303 | 0.999970678 | 1.453 | 8.998 | 0.111 | 2.644 | NA |
| TRINITY_DN9733_c0_g1_i12 | -1.88733823 | 0.664530044 | 0.999970678 | 1.381 | 0 | 4.311 | 0 | NA |
| TRINITY_DN1760_c0_g1_i4 | -1.88724906 | 0.302938188 | 0.999970678 | 2.507 | 3.577 | 0.367 | 22.048 | NA |
| TRINITY_DN2092_c0_g2_i2 | -1.88713393 | 0.481291998 | 0.999970678 | 0 | 1.281 | 3.244 | 0.724 | NA |
| TRINITY_DN6814_c0_g1_i4 | -1.88656498 | 0.298286615 | 0.999970678 | 3.216 | 0.552 | 10.61 | 1.056 | NA |
| TRINITY_DN6865_c0_g1_i4 | -1.88653819 | 0.49579001 | 0.999970678 | 15.043 | 0 | 31.265 | 18.589 | NA |
| TRINITY_DN8451_c2_g1_i1 | 1.886178778 | 0.658237329 | 0.999970678 | 0 | 1.735 | 0 | 0.462 | ZF-HD_dimer |
| TRINITY_DN914_c0_g1_i5 | 1.885968283 | 0.223107748 | 1 | 10.774 | 7.303 | 3.111 | 0.935 | NA |
| TRINITY_DN1824_c0_g1_i15 | 1.885822831 | 0.465927925 | 1 | 0.463 | 0.256 | 0 | 0.201 | NA |
| TRINITY_DN69257_c0_g1_i1 | -1.88516914 | 0.151847924 | 1 | 1.889 | 1.222 | 4.855 | 5.62 | DUF247 |
| TRINITY_DN7095_c0_g1_i2 | 1.885013228 | 0.285932134 | 0.999970678 | 17.45 | 11.15 | 6 | 0.392 | NA |
| TRINITY_DN18067_c0_g1_i5 | -1.88495803 | 0.095173545 | 0.999970678 | 0.445 | 0.453 | 1.1 | 1.94 | Glyco_hydro_16 |
| TRINITY_DN52424_c0_g1_i1 | -1.88472906 | 0.12506592 | 0.999970678 | 2.126 | 10.51 | 14.855 | 27.688 | NA |
| TRINITY_DN9728_c0_g1_i2 | -1.88440616 | 0.20112436 | 0.999970678 | 0.273 | 1.202 | 0.778 | 4.494 | Lectin_legB |
| TRINITY_DN6857_c0_g1_i2 | 1.884339919 | 0.652421978 | 0.999970678 | 0 | 13.2 | 2.9 | 0 | NA |
| TRINITY_DN6820_c0_g2_i1 | -1.88430562 | 0.006991696 | 0.358044988 | 1.935 | 2.7 | 7.933 | 7.51 | PPR |
| TRINITY_DN11816_c0_g1_i2 | 1.883730958 | 0.651387314 | 0.999970678 | 4.878 | 0 | 0 | 1.347 | NA |
| TRINITY_DN2499_c0_g1_i3 | -1.88308816 | 0.216567662 | 0.999970678 | 2.453 | 0.246 | 4.989 | 4.032 | NA |
| TRINITY_DN3254_c0_g1_i1 | 1.883040023 | 0.457179079 | 0.999970678 | 1.235 | 2.326 | 0 | 0.965 | - |
| TRINITY_DN2159_c0_g1_i4 | 1.882970799 | 0.352023506 | 0.999970678 | 1.281 | 0.355 | 0.356 | 0.02 | NA |

| TRINITY_DN4644_c1_g1_i5 | -1.88285483 | 0.127656877 | 0.999970678 | 0.209 | 0.848 | 1.933 | 1.528 | GTP-bdg_M |
| --- | --- | --- | --- | --- | --- | --- | --- | --- |
| TRINITY_DN71738_c0_g1_i3 | -1.88253831 | 0.194947198 | 0.999970678 | 2.48 | 0.71 | 2.478 | 8.485 | NA |
| TRINITY_DN395_c1_g1_i2 | -1.8823599 | 0.473558834 | 1 | 0.309 | 0 | 0.533 | 0.543 | Glyco_transf_8 |
| TRINITY_DN3886_c0_g1_i1 | -1.88087843 | 0.346562573 | 0.999970678 | 0.481 | 55.25 | 118.526 | 58.643 | Cupin_1 |
| TRINITY_DN15168_c0_g1_i2 | -1.88074999 | 0.50601557 | 0.999970678 | 2.371 | 0 | 0.544 | 8.154 | NA |
| TRINITY_DN12054_c0_g1_i11 | -1.88025846 | 0.656686702 | 0.999970678 | 1.935 | 0 | 6.011 | 0 | NA |
| TRINITY_DN4686_c0_g2_i1 | 1.880041973 | 0.548381619 | 0.999970678 | 3.706 | 0 | 0.789 | 0.06 | NA |
| TRINITY_DN9941_c0_g1_i3 | -1.87992106 | 0.491402302 | 0.999970678 | 0.736 | 0 | 1.878 | 0.523 | Pro_isomerase |
| TRINITY_DN1877_c0_g1_i7 | -1.87984044 | 0.167970471 | 0.999970678 | 0.772 | 4.287 | 11.31 | 4.786 | NA |
| TRINITY_DN3758_c0_g1_i4 | -1.87983052 | 0.051564079 | 0.999970678 | 0.917 | 0.493 | 1.9 | 2.966 | NA |
| TRINITY_DN9039_c0_g1_i1 | 1.879750845 | 0.179927474 | 1 | 0.818 | 0.828 | 0.2 | 0.201 | NA |
| TRINITY_DN5772_c0_g1_i1 | 1.879694567 | 0.508769466 | 0.999970678 | 0 | 3.183 | 0.211 | 0.573 | NA |
| TRINITY_DN282_c1_g1_i1 | -1.87946342 | 0.103804835 | 0.999970678 | 1.081 | 0.719 | 1.167 | 5.228 | NA |
| TRINITY_DN954_c0_g1_i8 | 1.879425876 | 0.513255135 | 0.999970678 | 2.008 | 0 | 0.311 | 0.181 | NA |
| TRINITY_DN22_c2_g1_i1 | 1.879113893 | 0.351971418 | 0.999970678 | 1.899 | 5.982 | 1.711 | 0.05 | NA |
| TRINITY_DN9369_c0_g1_i1 | -1.87906456 | 0.075168995 | 0.999970678 | 2.398 | 5.263 | 6.333 | 20.349 | NA |
| TRINITY_DN9578_c0_g1_i4 | -1.87836186 | 0.166331474 | 0.999970678 | 0.436 | 1.853 | 1.733 | 6.243 | Myb_DNA-bindin |
| TRINITY_DN23107_c0_g1_i1 | 1.878291296 | 0.099876819 | 0.999970678 | 3.134 | 6.997 | 1.755 | 0.633 | NA |
| TRINITY_DN20190_c0_g1_i1 | -1.87804403 | 0.659291456 | 0.999970678 | 2.144 | 0 | 6.588 | 0 | NA |
| TRINITY_DN1966_c0_g1_i2 | -1.87754577 | 0.476602455 | 0.999970678 | 0 | 57.07 | 95.883 | 83.456 | NA |
| TRINITY_DN6687_c0_g1_i4 | 1.876739645 | 0.124505936 | 0.999970678 | 0.4 | 0.404 | 0.056 | 0.161 | NA |
| TRINITY_DN9259_c0_g1_i2 | 1.876217579 | 0.193150351 | 0.999970678 | 10.628 | 4.07 | 0.589 | 3.217 | NA |
| TRINITY_DN11477_c0_g1_i3 | 1.875943041 | 0.516541208 | 0.999970678 | 0.908 | 13.13 | 3.155 | 0 | RRM_1 |
| TRINITY_DN170_c1_g1_i2 | -1.87505221 | 0.656827604 | 0.999970678 | 2.062 | 0 | 6.433 | 0 | NA |
| TRINITY_DN2297_c0_g1_i3 | 1.874892554 | 0.287282639 | 0.999970678 | 0.291 | 2.69 | 0.556 | 0.131 | NA |
| TRINITY_DN4457_c0_g1_i5 | -1.87443025 | 0.259690774 | 0.999970678 | 0.372 | 0.621 | 2.744 | 0.271 | NA |
| TRINITY_DN1183_c0_g1_i11 | 1.874166412 | 0.06158599 | 0.999970678 | 3.852 | 1.853 | 0.589 | 0.834 | LRR_8 |
| TRINITY_DN6816_c0_g1_i1 | -1.87386035 | 0.067323065 | 0.999970678 | 0.699 | 0.286 | 1.4 | 1.92 | DUF3452 |
| TRINITY_DN7060_c0_g1_i1 | -1.87374179 | 0.166541864 | 0.999970678 | 4.079 | 7.086 | 4.044 | 35.66 | NA |
| TRINITY_DN3297_c0_g1_i1 | -1.87361502 | 0.483005351 | 0.999970678 | 1.008 | 0 | 0.589 | 3.006 | Ist1 |
| TRINITY_DN60242_c0_g1_i2 | 1.87327645 | 0.470355231 | 1 | 2.489 | 1.192 | 0 | 0.995 | NA |
| TRINITY_DN3449_c0_g1_i2 | -1.87316604 | 0.495515307 | 0.999970678 | 11.446 | 0 | 27.309 | 9.43 | NA |
| TRINITY_DN4533_c1_g1_i1 | 1.872795153 | 0.468256283 | 0.999970678 | 10.837 | 10.84 | 4.844 | 0 | NA |
| TRINITY_DN5112_c0_g1_i4 | 1.87247097 | 0.487490486 | 0.999970678 | 18.213 | 18.44 | 0 | 10.024 | NA |
| TRINITY_DN4784_c0_g1_i3 | 1.872369657 | 0.059981147 | 0.999970678 | 2.353 | 1.488 | 0.611 | 0.322 | FAD-oxidase_C |
| TRINITY_DN6801_c0_g1_i1 | 1.872253919 | 0.153186518 | 0.999970678 | 0.79 | 0.443 | 0.211 | 0.08 | - |
| TRINITY_DN7676_c0_g2_i2 | 1.872241543 | 0.207445154 | 0.999970678 | 4.351 | 10.51 | 3.055 | 0.342 | NA |
| TRINITY_DN8529_c0_g1_i2 | -1.87215535 | 0.187562968 | 0.999970678 | 23.318 | 23.59 | 11.588 | 157.2 | Methyltransf_11 |
| TRINITY_DN687_c0_g1_i5 | -1.87205886 | 0.65915468 | 0.999970678 | 1.308 | 0 | 4.011 | 0 | NA |
| TRINITY_DN5675_c0_g1_i1 | 1.872048017 | 0.457338086 | 0.999970678 | 2.653 | 2.178 | 0 | 1.327 | NA |
| TRINITY_DN2062_c0_g1_i3 | -1.87171032 | 0.041845017 | 0.999970678 | 0.463 | 0.266 | 1.1 | 1.357 | LRRNT_2 |
| TRINITY_DN3815_c0_g1_i6 | -1.87133675 | 0.653339094 | 0.999970678 | 0 | 3.045 | 0 | 10.898 | NA |
| TRINITY_DN1808_c0_g2_i3 | 1.8712521 | 0.508649068 | 0.999970678 | 0 | 5.45 | 0.822 | 0.473 | NA |
| TRINITY_DN5836_c0_g1_i1 | -1.87084006 | 0.657442941 | 0.999970678 | 0 | 29.51 | 83.062 | 0 | DUF2854 |
| TRINITY_DN18115_c0_g1_i2 | 1.870825242 | 0.458601068 | 0.999970678 | 0.772 | 1.488 | 0 | 0.623 | NA |
| TRINITY_DN1197_c1_g1_i2 | 1.87023924 | 0.32375997 | 0.999970678 | 13.117 | 2.247 | 3.411 | 0.121 | CLU_N |
| TRINITY_DN11203_c0_g1_i1 | 1.869977125 | 0.291195005 | 0.999970678 | 2.38 | 1.35 | 0.822 | 0.03 | NA |
| TRINITY_DN2159_c0_g1_i7 | 1.869485367 | 0.654670261 | 0.999970678 | 6.332 | 0 | 0 | 1.759 | NA |
| TRINITY_DN13208_c0_g1_i1 | 1.869470289 | 0.103991805 | 0.999970678 | 1.771 | 0.503 | 0.189 | 0.402 | AAA |
| TRINITY_DN3022_c0_g1_i4 | -1.86946974 | 0.146767036 | 0.999970678 | 8.584 | 16.17 | 9.855 | 78.228 | - |
| TRINITY_DN7473_c0_g1_i2 | -1.86942422 | 0.481970297 | 1 | 1.145 | 0 | 1.544 | 2.222 | NA |
| TRINITY_DN8314_c0_g1_i3 | 1.86940586 | 0.520047743 | 0.999970678 | 0 | 23 | 2.144 | 3.619 | NA |
| TRINITY_DN14311_c0_g1_i1 | 1.868991104 | 0.655146069 | 0.999970678 | 9.856 | 0 | 0 | 2.725 | NA |
| TRINITY_DN728_c0_g2_i3 | 1.868984692 | 0.343846472 | 0.999970678 | 3.297 | 3.085 | 1.422 | 0.03 | NA |
| TRINITY_DN6543_c0_g1_i1 | -1.86848027 | 0.468114362 | 0.999970678 | 2.516 | 0 | 4.311 | 4.072 | NA |
| TRINITY_DN2508_c0_g1_i3 | -1.86845303 | 0.472888647 | 0.999970678 | 0 | 0.591 | 0.611 | 1.418 | TPR_19 |
| TRINITY_DN9768_c0_g1_i1 | -1.86830395 | 0.170945529 | 0.999970678 | 3.906 | 9.52 | 5.155 | 42.306 | NA |
| TRINITY_DN11955_c0_g1_i8 | -1.86805742 | 0.4826983 | 0.999970678 | 0 | 0.818 | 0.533 | 2.302 | AAA_11 |
| TRINITY_DN581_c0_g1_i12 | 1.867731218 | 0.128762441 | 0.999970678 | 3.343 | 7.391 | 1.267 | 1.408 | NA |
| TRINITY_DN7597_c0_g1_i7 | -1.86708164 | 0.659068387 | 0.999970678 | 1.572 | 0 | 4.811 | 0 | HsbA |
| TRINITY_DN9043_c0_g1_i2 | 1.866910596 | 0.080170378 | 0.999970678 | 1.889 | 1.34 | 0.333 | 0.473 | NA |
| TRINITY_DN2026_c0_g1_i2 | 1.866433322 | 0.510890901 | 0.999970678 | 0 | 2.986 | 0.278 | 0.473 | NA |
| TRINITY_DN9427_c0_g1_i1 | 1.866384903 | 0.379616831 | 0.999970678 | 11.137 | 2.365 | 0.044 | 3.69 | NA |
| TRINITY_DN594_c0_g1_i1 | 1.865872045 | 0.076810201 | 0.999970678 | 2.643 | 3.942 | 1.122 | 0.452 | NA |
| TRINITY_DN8036_c0_g1_i10 | -1.86382594 | 0.662342212 | 0.999970678 | 2.098 | 0 | 6.488 | 0 | - |
| TRINITY_DN1418_c1_g1_i1 | -1.86377027 | 0.551371324 | 0.999970678 | 2.144 | 0.168 | 7.011 | 0 | Ribosomal_S7 |
| TRINITY_DN4730_c0_g1_i13 | 1.863680908 | 0.065321413 | 0.999970678 | 1.699 | 0.986 | 0.244 | 0.452 | - |
| TRINITY_DN548_c0_g1_i5 | 1.863619844 | 0.129630082 | 0.999970678 | 1.363 | 0.749 | 0.378 | 0.141 | NA |
| TRINITY_DN9118_c0_g1_i1 | -1.86303094 | 0.184636216 | 0.999970678 | 0.118 | 0.365 | 0.922 | 0.613 | NA |
| TRINITY_DN5310_c0_g1_i1 | 1.86302954 | 0.134013137 | 0.999970678 | 2.952 | 2.828 | 0.278 | 1.247 | XPG_I |
| TRINITY_DN12514_c0_g1_i1 | -1.86291312 | 0.195009137 | 0.999970678 | 9.266 | 4.218 | 4.022 | 44.407 | NA |
| TRINITY_DN2764_c0_g1_i4 | 1.862722752 | 0.010360986 | 0.501502424 | 35.963 | 22.76 | 5.866 | 9.139 | UAA |
| TRINITY_DN5499_c0_g1_i3 | -1.86242495 | 0.537036192 | 0.999970678 | 1.544 | 0.217 | 5.233 | 0 | NA |
| TRINITY_DN15776_c0_g2_i1 | 1.862344605 | 0.101476901 | 0.999970678 | 3.843 | 4.612 | 0.611 | 1.548 | NA |

| TRINITY_DN4352_c0_g1_i2 | 1.861974075 | 0.470398811 | 0.999970678 | 8.711 | 5.312 | 0 | 3.871 | NA |
| --- | --- | --- | --- | --- | --- | --- | --- | --- |
| TRINITY_DN181_c0_g1_i1 | 1.861902009 | 0.270671304 | 0.999970678 | 10.001 | 0.542 | 1.855 | 0.694 | CBM-like |
| TRINITY_DN735_c1_g1_i5 | -1.86177354 | 0.111969527 | 0.999970678 | 584.55 | 145.8 | 1553.406 | 802.516 | Chloroa_b-bind |
| TRINITY_DN8394_c0_g1_i4 | -1.86128033 | 0.180775328 | 0.999970678 | 0.309 | 0.187 | 0.233 | 1.538 | AAA |
| TRINITY_DN25067_c0_g1_i2 | -1.86115117 | 0.477166171 | 0.999970678 | 0.663 | 0 | 1.3 | 0.865 | PP2C |
| TRINITY_DN39_c4_g1_i4 | 1.86077905 | 0.191556265 | 0.999970678 | 1.717 | 0.325 | 0.344 | 0.151 | GATase_2 |
| TRINITY_DN4706_c0_g1_i2 | 1.86063567 | 0.317181417 | 0.999970678 | 1.244 | 0.463 | 0.378 | 0.01 | NA |
| TRINITY_DN7460_c2_g1_i5 | 1.860543487 | 0.036718369 | 0.999970678 | 0.627 | 0.621 | 0.156 | 0.151 | NA |
| TRINITY_DN3341_c0_g1_i1 | 1.859820738 | 0.663678257 | 0.999970678 | 3.652 | 0 | 0 | 1.025 | NA |
| TRINITY_DN6248_c0_g2_i1 | -1.8597699 | 0.262702609 | 0.999970678 | 1.254 | 11 | 27.076 | 9.883 | NA |
| TRINITY_DN7968_c0_g1_i1 | -1.8595847 | 0.114950923 | 0.999970678 | 1.444 | 3.558 | 3.589 | 13.643 | NA |
| TRINITY_DN10446_c0_g1_i3 | 1.859264475 | 0.1214975 | 0.999970678 | 2.017 | 0.818 | 0.211 | 0.523 | PLAC8 |
| TRINITY_DN29484_c0_g1_i1 | 1.859159411 | 0.176982412 | 1 | 0.5 | 0.394 | 0.067 | 0.161 | AAA |
| TRINITY_DN252257_c0_g1_i2 | 1.859073069 | 0.331833919 | 0.999970678 | 0.827 | 1.439 | 0.489 | 0.03 | NA |
| TRINITY_DN9567_c2_g1_i3 | -1.85887866 | 0.305578419 | 0.999970678 | 0.845 | 0.266 | 3.1 | 0.261 | DUF4283 |
| TRINITY_DN6616_c0_g1_i2 | -1.85880196 | 0.485695379 | 0.999970678 | 0 | 1.074 | 0.589 | 3.137 | LysM |
| TRINITY_DN936_c0_g1_i1 | -1.85754033 | 0.132496766 | 0.999970678 | 8.993 | 1.843 | 20.532 | 14.739 | PP-binding |
| TRINITY_DN120_c2_g1_i7 | -1.85695787 | 0.507448094 | 0.999970678 | 1.244 | 0 | 0.467 | 4.062 | NA |
| TRINITY_DN722_c0_g1_i1 | 1.856914914 | 0.145134083 | 0.999970678 | 2.117 | 5.48 | 1.067 | 0.774 | NA |
| TRINITY_DN937_c0_g1_i1 | -1.8559014 | 0.05407662 | 0.999970678 | 0.227 | 0.434 | 1.222 | 0.935 | NA |
| TRINITY_DN33476_c0_g1_i4 | 1.855892987 | 0.145514064 | 0.999970678 | 2.08 | 3.489 | 0.256 | 1.206 | NA |
| TRINITY_DN6689_c0_g2_i6 | 1.855772481 | 0.318799799 | 0.999970678 | 1.499 | 7.884 | 1.989 | 0.151 | NA |
| TRINITY_DN518_c0_g1_i8 | -1.8549104 | 0.557559826 | 0.999970678 | 52.296 | 0 | 5.4 | 185.159 | Tubulin |
| TRINITY_DN327_c0_g1_i1 | -1.85478358 | 0.011120323 | 0.531474809 | 3.316 | 5.154 | 13.832 | 13.743 | NA |
| TRINITY_DN7337_c0_g1_i9 | 1.85460423 | 0.020046805 | 0.846213449 | 1.671 | 1.557 | 0.4 | 0.402 | - |
| TRINITY_DN11591_c0_g1_i3 | 1.854545817 | 0.315985582 | 0.999970678 | 63.824 | 11.19 | 15.277 | 1.588 | LRRNT_2 |
| TRINITY_DN504_c0_g1_i4 | 1.854299703 | 0.165976731 | 1 | 0.372 | 0.384 | 0.122 | 0.06 | DUF3522 |
| TRINITY_DN10782_c1_g1_i1 | -1.85377739 | 0.477148496 | 0.999970678 | 1.408 | 0 | 1.278 | 3.599 | NA |
| TRINITY_DN9142_c0_g1_i1 | -1.85370321 | 0.390310676 | 0.999970678 | 0.445 | 7.145 | 0.6 | 25.959 | NA |
| TRINITY_DN6189_c0_g1_i1 | -1.85345479 | 0.533615261 | 0.999970678 | 0.109 | 1.153 | 0 | 4.504 | NA |
| TRINITY_DN12111_c0_g3_i5 | -1.85290335 | 0.208371035 | 0.999970678 | 0.3 | 0.079 | 0.589 | 0.593 | PORR |
| TRINITY_DN5889_c0_g1_i1 | 1.852701251 | 0.195488352 | 0.999970678 | 0.245 | 1.084 | 0.2 | 0.131 | NA |
| TRINITY_DN8246_c0_g1_i4 | 1.852619641 | 0.520432346 | 0.999970678 | 0.645 | 0.039 | 0 | 0.191 | - |
| TRINITY_DN261_c0_g1_i1 | -1.85170994 | 0.027308567 | 0.999970678 | 2.08 | 1.932 | 4.089 | 9.631 | Metallophos |
| TRINITY_DN13687_c0_g1_i1 | 1.851662498 | 0.359486769 | 0.999970678 | 6.386 | 909.5 | 105.138 | 109.585 | NA |
| TRINITY_DN1154_c0_g1_i5 | 1.8512599 | 0.104854984 | 0.999970678 | 1.126 | 3.962 | 0.622 | 0.643 | NA |
| TRINITY_DN4048_c0_g1_i4 | -1.85125827 | 0.053061662 | 0.999970678 | 3.879 | 1.715 | 7.522 | 11.16 | Arf |
| TRINITY_DN2471_c0_g2_i3 | -1.85064282 | 0.65694734 | 0.999970678 | 0 | 0.69 | 0 | 2.463 | MORN |
| TRINITY_DN10616_c0_g1_i1 | -1.85034185 | 0.663918284 | 0.999970678 | 0.463 | 0 | 0 | 1.709 | vATP-synt_E |
| TRINITY_DN5111_c0_g1_i1 | 1.850156937 | 0.100856832 | 0.999970678 | 1.135 | 1.321 | 0.422 | 0.161 | NA |
| TRINITY_DN13584_c0_g1_i1 | 1.84991077 | 0.48399984 | 1 | 1.09 | 1.587 | 0.6 | 0 | NA |
| TRINITY_DN5465_c0_g1_i4 | 1.849888649 | 0.662163294 | 0.999970678 | 45.819 | 0 | 0 | 12.808 | NA |
| TRINITY_DN13195_c0_g1_i3 | -1.84974441 | 0.66595997 | 0.999970678 | 8.094 | 0 | 24.087 | 0 | Peroxidase_2 |
| TRINITY_DN15999_c0_g1_i1 | 1.84915245 | 0.340699293 | 0.999970678 | 0.963 | 8.958 | 2.089 | 0.241 | NA |
| TRINITY_DN12903_c0_g1_i4 | -1.84790261 | 0.440665933 | 0.999970678 | 151.1 | 0.108 | 256.184 | 239.982 | CAAD |
| TRINITY_DN7537_c0_g1_i2 | 1.847478906 | 0.518924819 | 0.999970678 | 0 | 35.11 | 4.211 | 4.504 | NA |
| TRINITY_DN7276_c0_g2_i4 | -1.84696301 | 0.553818894 | 0.999970678 | 1.072 | 0 | 3.144 | 0.09 | 2-Hacid_dh |
| TRINITY_DN10359_c0_g1_i1 | 1.846578738 | 0.482539188 | 0.999970678 | 0.727 | 2.582 | 0.756 | 0 | NA |
| TRINITY_DN2416_c0_g1_i17 | 1.8465742 | 0.479812917 | 0.999970678 | 0.481 | 1.133 | 0.367 | 0 | Pkinase |
| TRINITY_DN252638_c0_g1_i1 | -1.84650511 | 0.114566501 | 0.999970678 | 7.376 | 17.95 | 15.588 | 71.2 | NA |
| TRINITY_DN1039_c0_g1_i3 | 1.846455926 | 0.488485612 | 0.999970678 | 6.749 | 1.173 | 0 | 2.232 | B_lectin |
| TRINITY_DN6308_c0_g2_i2 | 1.845934407 | 0.07928322 | 0.999970678 | 0.999 | 0.976 | 0.2 | 0.312 | NA |
| TRINITY_DN4150_c0_g1_i2 | -1.84584452 | 0.47387782 | 0.999970678 | 0 | 1.291 | 2.555 | 1.488 | Methyltransf_4 |
| TRINITY_DN14340_c0_g1_i1 | 1.845537073 | 0.486437568 | 1 | 0.927 | 1.035 | 0.456 | 0 | NA |
| TRINITY_DN9637_c0_g2_i12 | 1.845421415 | 0.468398748 | 0.999970678 | 1.026 | 0.926 | 0.444 | 0 | Retrotran_gag_2 |
| TRINITY_DN107_c1_g1_i4 | 1.845120184 | 0.659295611 | 0.999970678 | 8.675 | 0 | 0 | 2.433 | - |
| TRINITY_DN6825_c0_g1_i1 | -1.84489284 | 0.325030911 | 0.999970678 | 0.354 | 4.179 | 0.867 | 15.05 | Orn_Arg_deC_N |
| TRINITY_DN6122_c0_g1_i1 | -1.84480902 | 0.29177698 | 0.999970678 | 3.624 | 0.453 | 1.522 | 12.778 | Ion_trans |
| TRINITY_DN867_c0_g1_i9 | 1.844230159 | 0.493916318 | 0.999970678 | 2.226 | 0.335 | 0 | 0.724 | ANF_receptor |
| TRINITY_DN461_c1_g1_i4 | -1.84390925 | 0.531203298 | 0.999970678 | 0.6 | 0.099 | 2.1 | 0 | LisH |
| TRINITY_DN325_c1_g1_i1 | 1.843908338 | 0.40688798 | 0.999970678 | 3.861 | 0.039 | 0.789 | 0.141 | Sugar_tr |
| TRINITY_DN3277_c0_g1_i2 | -1.84352087 | 0.48772385 | 0.999970678 | 0 | 7.51 | 12.499 | 11.401 | NA |
| TRINITY_DN2377_c0_g1_i3 | -1.84344914 | 0.179810416 | 0.999970678 | 6.431 | 22.34 | 11.744 | 88.452 | NA |
| TRINITY_DN297_c1_g1_i3 | -1.84344542 | 0.296117086 | 0.999970678 | 0.127 | 3.804 | 4.366 | 8.606 | NA |
| TRINITY_DN1194_c0_g1_i3 | -1.84342171 | 0.322931698 | 0.999970678 | 27.806 | 5.223 | 2.866 | 116.281 | NA |
| TRINITY_DN13290_c0_g1_i4 | -1.8431609 | 0.493990824 | 0.999970678 | 10.31 | 0 | 15.177 | 19.233 | - |
| TRINITY_DN3138_c0_g1_i5 | 1.84296999 | 0.661150983 | 0.999970678 | 2.18 | 0 | 0 | 0.623 | NA |
| TRINITY_DN394_c0_g1_i7 | -1.84221132 | 0.061803699 | 0.999970678 | 1.908 | 2.277 | 3.322 | 10.948 | Polysacc_synt_4 |
| TRINITY_DN57_c0_g4_i1 | -1.84209762 | 0.345411489 | 0.999970678 | 1.172 | 34.58 | 8.155 | 116.894 | PTR2 |
| TRINITY_DN3152_c0_g2_i1 | -1.84188082 | 0.659786307 | 0.999970678 | 1.499 | 0 | 4.533 | 0 | NA |
| TRINITY_DN514_c0_g1_i1 | 1.84177995 | 0.05859374 | 0.999970678 | 2.407 | 1.981 | 0.678 | 0.442 | STAS |
| TRINITY_DN2310_c0_g1_i1 | 1.841679805 | 0.499692709 | 0.999970678 | 0.908 | 0.118 | 0 | 0.292 | UDPGT |
| TRINITY_DN5412_c0_g1_i16 | 1.84157239 | 0.528313785 | 0.999970678 | 3.534 | 0 | 0.2 | 0.784 | NA |
| TRINITY_DN13458_c1_g1_i1 | -1.8413112 | 0.665775731 | 0.999970678 | 0 | 17.05 | 47.819 | 0 | Ribosomal_60s |

| TRINITY_DN366_c0_g1_i7 | -1.84119752 | 0.138228086 | 0.999970678 | 33.52 | 8.012 | 37.42 | 98.255 | NA |
| --- | --- | --- | --- | --- | --- | --- | --- | --- |
| TRINITY_DN3288_c0_g2_i3 | 1.841188178 | 0.469631162 | 0.999970678 | 7.467 | 3.292 | 0 | 3.006 | NA |
| TRINITY_DN5818_c0_g1_i12 | 1.841185951 | 0.470966978 | 0.999970678 | 0.59 | 0.522 | 0.256 | 0 | - |
| TRINITY_DN2477_c0_g1_i1 | -1.84113353 | 0.661144842 | 0.999970678 | 0 | 2.533 | 7.377 | 0 | NA |
| TRINITY_DN2968_c0_g1_i2 | -1.84098392 | 0.48111638 | 0.999970678 | 1.935 | 0 | 4.144 | 2.001 | GST_C |
| TRINITY_DN6580_c0_g1_i1 | -1.84038387 | 0.252369043 | 0.999970678 | 0.672 | 0.384 | 2.844 | 0.322 | NA |
| TRINITY_DN4754_c0_g1_i1 | -1.8388651 | 0.293475197 | 0.999970678 | 0.481 | 5.273 | 2.078 | 17.705 | Lysine_decarbox |
| TRINITY_DN1497_c0_g1_i1 | -1.83882993 | 0.066132506 | 0.999970678 | 6.313 | 6.248 | 9.755 | 32.584 | NA |
| TRINITY_DN9987_c0_g1_i4 | 1.838510902 | 0.515336195 | 0.999970678 | 0 | 112.1 | 8.855 | 18.72 | NA |
| TRINITY_DN7658_c0_g1_i4 | -1.83835554 | 0.054766356 | 0.999970678 | 9.138 | 3.735 | 15.388 | 27.447 | GDPD |
| TRINITY_DN3374_c0_g2_i2 | 1.838293368 | 0.508885641 | 0.999970678 | 11.028 | 14.34 | 5.855 | 0 | Nucleoporin2 |
| TRINITY_DN33956_c0_g1_i1 | 1.838245835 | 0.083741813 | 0.999970678 | 10.556 | 6.475 | 2.555 | 1.659 | NA |
| TRINITY_DN1536_c0_g1_i3 | -1.83819552 | 0.277992026 | 0.999970678 | 0.572 | 1.459 | 0.378 | 6.676 | Lipase_GDSL |
| TRINITY_DN1617_c0_g1_i11 | -1.83763778 | 0.530652249 | 0.999970678 | 0.663 | 0.108 | 2.333 | 0 | NA |
| TRINITY_DN5412_c0_g1_i10 | 1.837626403 | 0.387244521 | 0.999970678 | 6.431 | 0.069 | 0.767 | 0.915 | NA |
| TRINITY_DN88_c0_g1_i2 | 1.83762501 | 0.321623861 | 0.999970678 | 4.097 | 0.227 | 0.789 | 0.261 | NA |
| TRINITY_DN8426_c0_g2_i3 | 1.83759819 | 0.537849741 | 0.999970678 | 35.091 | 0.956 | 8.377 | 0 | NA |
| TRINITY_DN18871_c0_g1_i1 | 1.837555902 | 0.230492107 | 1 | 0.545 | 0.483 | 0.189 | 0.05 | NA |
| TRINITY_DN2340_c0_g2_i15 | 1.837444637 | 0.571151914 | 0.999970678 | 0 | 1.991 | 0.444 | 0.02 | NA |
| TRINITY_DN2253_c1_g1_i3 | 1.837028972 | 0.295832537 | 0.999970678 | 3.679 | 0.286 | 0.789 | 0.171 | CMAS |
| TRINITY_DN2317_c0_g1_i4 | 1.837023311 | 0.079028234 | 0.999970678 | 5.096 | 18.64 | 2.444 | 3.649 | NA |
| TRINITY_DN3183_c0_g1_i2 | -1.83681044 | 0.280939237 | 0.999970678 | 19.167 | 1.784 | 7.133 | 66.917 | Ammonium_trans |
| TRINITY_DN1712_c0_g1_i10 | 1.836693505 | 0.661577174 | 0.999970678 | 3.733 | 0 | 0 | 1.076 | NA |
| TRINITY_DN1293_c0_g1_i11 | -1.83665583 | 0.481857231 | 0.999970678 | 1.081 | 0 | 1.889 | 1.659 | NA |
| TRINITY_DN1085_c0_g1_i1 | -1.83627025 | 0.568041215 | 0.999970678 | 0.836 | 15.66 | 0 | 53.154 | UbiA |
| TRINITY_DN214_c0_g1_i2 | 1.835879715 | 0.540760648 | 0.999970678 | 2.571 | 0 | 0.067 | 0.623 | NA |
| TRINITY_DN120_c0_g1_i3 | 1.835869763 | 0.057959924 | 0.999970678 | 3.897 | 8.19 | 1.778 | 1.196 | NA |
| TRINITY_DN9250_c0_g1_i2 | -1.8354859 | 0.49322527 | 1 | 0.218 | 0 | 0.344 | 0.372 | NA |
| TRINITY_DN1883_c0_g1_i5 | 1.835455112 | 0.53002919 | 0.999970678 | 1.708 | 0.099 | 0.422 | 0 | NA |
| TRINITY_DN7287_c0_g3_i3 | 1.834978009 | 0.186655605 | 0.999970678 | 1.608 | 6.711 | 0.989 | 1.076 | NA |
| TRINITY_DN13646_c1_g2_i2 | 1.834749503 | 0.668968745 | 0.999970678 | 4.206 | 0 | 0 | 1.186 | NA |
| TRINITY_DN799_c0_g2_i4 | 1.834366091 | 0.472087334 | 0.999970678 | 0.699 | 1.666 | 0 | 0.664 | Glyco_hydro_20 |
| TRINITY_DN28244_c0_g1_i5 | -1.83401173 | 0.670763329 | 0.999970678 | 0.927 | 0 | 2.755 | 0 | NA |
| TRINITY_DN424_c0_g2_i1 | -1.83353394 | 0.066900131 | 0.999970678 | 3.098 | 3.124 | 6.444 | 13.683 | NA |
| TRINITY_DN6422_c0_g1_i2 | -1.83334588 | 0.154785479 | 0.999970678 | 0.5 | 0.335 | 0.467 | 2.403 | Glyco_hydro_2 |
| TRINITY_DN238_c1_g1_i3 | 1.833277514 | 0.277180391 | 0.999970678 | 1.145 | 0.365 | 0.322 | 0.04 | NA |
| TRINITY_DN1711_c1_g1_i5 | -1.83313971 | 0.670221733 | 0.999970678 | 0.59 | 0 | 1.744 | 0 | NAF |
| TRINITY_DN3810_c0_g1_i1 | 1.833123038 | 0.015969087 | 0.705056757 | 6.84 | 7.569 | 1.222 | 2.574 | - |
| TRINITY_DN2252_c0_g1_i7 | 1.833029681 | 0.478386707 | 0.999970678 | 3.052 | 6.504 | 2.211 | 0 | DUF561 |
| TRINITY_DN9492_c0_g1_i1 | -1.83254166 | 0.662314415 | 0.999970678 | 6.459 | 0 | 19.243 | 0 | CHRD |
| TRINITY_DN4682_c0_g1_i2 | -1.83246448 | 0.495445975 | 0.999970678 | 0 | 15.27 | 20.632 | 28.432 | Ras |
| TRINITY_DN8133_c0_g1_i9 | 1.832229638 | 0.4891093 | 0.999970678 | 1.926 | 0.483 | 0.556 | 0 | Cyclin_N |
| TRINITY_DN8126_c1_g1_i1 | 1.831761872 | 0.561604293 | 0.999970678 | 260.67 | 0 | 46.031 | 18.549 | AP2 |
| TRINITY_DN12434_c0_g1_i2 | 1.831440884 | 0.163857267 | 0.999970678 | 1.272 | 0.611 | 0.256 | 0.231 | Guanylate_cyc_2 |
| TRINITY_DN1312_c0_g1_i2 | 1.831262871 | 0.090251864 | 0.999970678 | 0.963 | 2.237 | 0.489 | 0.302 | Dabb |
| TRINITY_DN4335_c0_g1_i2 | 1.831122375 | 0.359896804 | 0.999970678 | 0.936 | 27.31 | 5.989 | 0.573 | NA |
| TRINITY_DN40800_c0_g1_i2 | -1.83110125 | 0.061286005 | 0.999970678 | 0.291 | 0.365 | 1.133 | 0.965 | Ion_trans |
| TRINITY_DN3658_c0_g1_i13 | -1.83105916 | 0.34747807 | 0.999970678 | 6.195 | 0.424 | 3.033 | 18.901 | NA |
| TRINITY_DN711_c0_g1_i13 | -1.83054557 | 0.324975905 | 0.999970678 | 0.99 | 0.059 | 2.6 | 0.653 | peroxidase |
| TRINITY_DN13474_c0_g1_i5 | 1.830290023 | 0.274705272 | 0.999970678 | 1.626 | 5.608 | 0.144 | 1.84 | NA |
| TRINITY_DN29526_c0_g1_i2 | 1.830224145 | 0.373552358 | 0.999970678 | 1.508 | 290.1 | 35.731 | 37.661 | Aldedh |
| TRINITY_DN107700_c0_g1_i1 | 1.829354412 | 0.213209398 | 0.999970678 | 21.511 | 2.927 | 2.3 | 4.042 | NA |
| TRINITY_DN7701_c0_g1_i2 | -1.82884763 | 0.213123852 | 0.999970678 | 1.017 | 1.528 | 0.722 | 8.214 | EF-hand_1 |
| TRINITY_DN7701_c0_g1_i1 | -1.82884763 | 0.213123852 | 0.999970678 | 1.017 | 1.528 | 0.722 | 8.214 | EF-hand_1 |
| TRINITY_DN13007_c1_g3_i3 | -1.82868311 | 0.199785119 | 0.999970678 | 0.118 | 0.463 | 0.822 | 1.086 | NA |
| TRINITY_DN12357_c0_g1_i6 | -1.82859531 | 0.667416678 | 0.999970678 | 1.263 | 0 | 3.755 | 0 | FAD_binding_2 |
| TRINITY_DN9623_c0_g1_i6 | -1.82846163 | 0.232847243 | 0.999970678 | 0.536 | 3.104 | 6.844 | 4.353 | NA |
| TRINITY_DN4489_c0_g1_i7 | 1.828290236 | 0.49294304 | 0.999970678 | 29.695 | 15.02 | 0 | 12.617 | zf-Dof |
| TRINITY_DN1753_c1_g2_i4 | -1.82828281 | 0.158682159 | 1 | 0.045 | 0.069 | 0.2 | 0.181 | NA |
| TRINITY_DN415_c0_g1_i11 | -1.82819656 | 0.092319801 | 0.999970678 | 4.978 | 9.875 | 33.398 | 12.044 | NA |
| TRINITY_DN2939_c0_g1_i1 | -1.82815513 | 0.481391454 | 0.999970678 | 1.98 | 0 | 3.578 | 2.825 | NA |
| TRINITY_DN778_c1_g1_i11 | 1.82735384 | 0.163329162 | 0.999970678 | 0.845 | 0.463 | 0.233 | 0.08 | NA |
| TRINITY_DN1548_c0_g1_i1 | -1.8269886 | 0.196008056 | 0.999970678 | 1.472 | 0.345 | 4.422 | 1.206 | NA |
| TRINITY_DN672_c0_g1_i6 | 1.826653563 | 0.661430256 | 0.999970678 | 1.899 | 0 | 0 | 0.543 | NA |
| TRINITY_DN5613_c0_g2_i2 | 1.826262777 | 0.520353013 | 0.999970678 | 35.01 | 59.49 | 0 | 26.612 | WD40 |
| TRINITY_DN1246_c0_g4_i1 | -1.82584377 | 0.511016763 | 0.999970678 | 3.325 | 0 | 1.044 | 10.587 | NA |
| TRINITY_DN8952_c0_g1_i2 | 1.825766756 | 0.552915021 | 0.999970678 | 8.884 | 0 | 1.967 | 0.141 | NA |
| TRINITY_DN9357_c0_g1_i5 | -1.82557089 | 0.490594073 | 0.999970678 | 1.063 | 0 | 0.711 | 2.966 | NA |
| TRINITY_DN5871_c0_g1_i4 | -1.82542834 | 0.531430141 | 0.999970678 | 0 | 2.119 | 5.811 | 0.332 | NA |
| TRINITY_DN3205_c2_g1_i2 | 1.825038329 | 0.062464381 | 0.999970678 | 2.198 | 4.415 | 0.756 | 0.925 | EF_TS |
| TRINITY_DN2432_c0_g1_i5 | 1.824999274 | 0.512970307 | 0.999970678 | 6.468 | 0.512 | 0 | 2.001 | NA |
| TRINITY_DN17640_c0_g2_i1 | 1.824952809 | 0.204641333 | 1 | 0.781 | 0.375 | 0.189 | 0.101 | NA |
| TRINITY_DN478_c2_g1_i2 | 1.82458992 | 0.031092289 | 0.999970678 | 4.26 | 2.661 | 0.622 | 1.206 | NA |
| TRINITY_DN12529_c1_g1_i5 | -1.82443462 | 0.359807308 | 0.999970678 | 0.145 | 1.606 | 0.389 | 5.55 | Fasciclin |

| TRINITY_DN5528_c0_g1_i2 | -1.82426805 | 0.494619827 | 0.999970678 | 6.295 | 0 | 9.344 | 11.32 | Abhydrolase_1 |
| --- | --- | --- | --- | --- | --- | --- | --- | --- |
| TRINITY_DN9266_c0_g1_i1 | 1.823836305 | 0.285413101 | 0.999970678 | 5.323 | 0.286 | 0.856 | 0.563 | AP2 |
| TRINITY_DN4436_c0_g1_i4 | -1.82383157 | 0.495064781 | 0.999970678 | 0.872 | 0 | 1.955 | 0.744 | PTH2 |
| TRINITY_DN1877_c0_g1_i6 | 1.823714276 | 0.317190533 | 0.999970678 | 7.849 | 0.384 | 1.622 | 0.402 | NA |
| TRINITY_DN5468_c0_g1_i3 | -1.8235795 | 0.498295334 | 0.999970678 | 0.672 | 0 | 0.444 | 1.91 | Cyt-b5 |
| TRINITY_DN5133_c0_g1_i1 | -1.82313863 | 0.51383335 | 0.999970678 | 0 | 9.589 | 22.654 | 5.952 | - |
| TRINITY_DN2197_c0_g1_i2 | 1.822985388 | 0.470058266 | 0.999970678 | 4.279 | 3.144 | 0 | 2.111 | IspD |
| TRINITY_DN591_c0_g1_i11 | -1.82275041 | 0.019283482 | 0.822493375 | 0.581 | 0.69 | 1.911 | 2.212 | NA |
| TRINITY_DN3240_c0_g1_i4 | 1.822655991 | 0.183782003 | 0.999970678 | 0.945 | 1.518 | 0.489 | 0.101 | NA |
| TRINITY_DN413_c0_g1_i1 | -1.82263617 | 0.067418947 | 0.999970678 | 5.687 | 3.922 | 20.877 | 8.817 | NA |
| TRINITY_DN5272_c0_g3_i3 | 1.822436369 | 0.575406143 | 0.999970678 | 0 | 12.42 | 0.056 | 3.358 | NA |
| TRINITY_DN2395_c0_g1_i5 | 1.822335802 | 0.520093137 | 0.999970678 | 0 | 3.233 | 0.278 | 0.563 | Methyltr_RsmB-F |
| TRINITY_DN8893_c0_g2_i2 | 1.821468321 | 0.286560608 | 0.999970678 | 6.777 | 5.696 | 2.778 | 0.191 | NA |
| TRINITY_DN3366_c0_g1_i2 | -1.82143016 | 0.578053571 | 0.999970678 | 2.171 | 0 | 6.377 | 0.04 | NA |
| TRINITY_DN5272_c0_g2_i7 | -1.82058406 | 0.139167495 | 0.999970678 | 12.309 | 18.13 | 75.773 | 15.473 | DUF3007 |
| TRINITY_DN146_c0_g1_i2 | 1.820284713 | 0.296519435 | 0.999970678 | 0.354 | 11.54 | 1.433 | 1.599 | KH_dom-like |
| TRINITY_DN2284_c0_g1_i1 | 1.82007074 | 0.531135604 | 0.999970678 | 0.654 | 14.96 | 0 | 4.363 | Metallophos |
| TRINITY_DN5787_c0_g1_i1 | -1.81963008 | 0.564466097 | 0.999970678 | 6.459 | 0.256 | 19.654 | 0 | NA |
| TRINITY_DN208_c0_g1_i5 | -1.81911995 | 0.033798709 | 0.999970678 | 59.609 | 25.96 | 131.848 | 141.104 | Ribosomal_L36e |
| TRINITY_DN50306_c0_g1_i7 | 1.819079858 | 0.48218422 | 0.999970678 | 0.554 | 1.311 | 0.433 | 0 | JmjC |
| TRINITY_DN152_c1_g1_i16 | -1.81906593 | 0.490424686 | 0.999970678 | 0.763 | 0 | 0.667 | 1.88 | NA |
| TRINITY_DN68692_c1_g1_i1 | -1.81868605 | 0.234650183 | 0.999970678 | 0.145 | 0.641 | 1.589 | 0.845 | NA |
| TRINITY_DN1232_c0_g1_i6 | 1.818159742 | 0.098709032 | 0.999970678 | 3.579 | 1.991 | 0.5 | 0.935 | NA |
| TRINITY_DN8652_c0_g1_i4 | -1.81807637 | 0.517475928 | 0.999970678 | 1.126 | 0 | 2.933 | 0.432 | NA |
| TRINITY_DN5025_c0_g1_i5 | -1.81804453 | 0.082115632 | 0.999970678 | 1.453 | 0.926 | 2.755 | 4.997 | Inhibitor_I9 |
| TRINITY_DN12823_c0_g1_i1 | 1.817847327 | 0.079221053 | 0.999970678 | 6.213 | 8.367 | 0.9 | 3.016 | NA |
| TRINITY_DN947_c0_g1_i4 | -1.81782662 | 0.513067452 | 0.999970678 | 0.382 | 0.335 | 0 | 2.534 | NA |
| TRINITY_DN9223_c0_g1_i9 | 1.81762851 | 0.162250285 | 1 | 0.418 | 0.66 | 0.133 | 0.141 | NA |
| TRINITY_DN1354_c1_g1_i3 | -1.81746633 | 0.318244404 | 0.999970678 | 5.895 | 0.641 | 2.689 | 19.233 | NA |
| TRINITY_DN1877_c0_g1_i16 | -1.81731614 | 0.66675627 | 0.999970678 | 0 | 4.11 | 11.777 | 0 | NA |
| TRINITY_DN788_c0_g1_i8 | -1.81703553 | 0.257896182 | 0.999970678 | 1.045 | 0.099 | 1.211 | 2.574 | NA |
| TRINITY_DN3378_c0_g1_i11 | -1.81677026 | 0.178590524 | 0.999970678 | 6.232 | 57.59 | 78.84 | 127.38 | Chal_sti_synt_C |
| TRINITY_DN7555_c1_g1_i1 | -1.81674158 | 0.13856174 | 0.999970678 | 13.362 | 30.04 | 82.695 | 42.658 | NA |
| TRINITY_DN255_c1_g1_i4 | -1.81655683 | 0.400255676 | 0.999970678 | 0.164 | 2.415 | 7.311 | 0.131 | NA |
| TRINITY_DN175_c0_g1_i13 | 1.816341483 | 0.212624189 | 0.999970678 | 3.752 | 19.33 | 4.222 | 1.287 | NA |
| TRINITY_DN2758_c0_g1_i7 | -1.81597666 | 0.519015534 | 0.999970678 | 19.331 | 0 | 39.62 | 21.133 | MIP |
| TRINITY_DN18650_c0_g1_i1 | -1.81422554 | 0.387417633 | 0.999970678 | 0.327 | 0.788 | 0.044 | 3.891 | NA |
| TRINITY_DN18129_c1_g1_i4 | 1.814113341 | 0.2851558 | 0.999970678 | 0.318 | 0.256 | 0.122 | 0.01 | NA |
| TRINITY_DN8249_c0_g1_i1 | -1.81400919 | 0.662443569 | 0.999970678 | 0 | 1.449 | 0 | 4.987 | NA |
| TRINITY_DN1003_c0_g1_i6 | 1.813767689 | 0.526689239 | 0.999970678 | 0.245 | 4.317 | 1.055 | 0 | RVT_1 |
| TRINITY_DN14088_c0_g1_i1 | 1.813732814 | 0.479920558 | 1 | 0.336 | 0.483 | 0 | 0.231 | NA |
| TRINITY_DN10904_c0_g1_i2 | -1.81357103 | 0.242924202 | 0.999970678 | 0.681 | 0.443 | 0.367 | 3.509 | NA |
| TRINITY_DN1350_c0_g1_i14 | -1.81338488 | 0.542887136 | 0.999970678 | 0.799 | 6.288 | 0 | 23.666 | NA |
| TRINITY_DN9237_c0_g1_i3 | -1.81329519 | 0.677013958 | 0.999970678 | 2.725 | 0 | 8.066 | 0 | NA |
| TRINITY_DN14543_c0_g1_i4 | -1.8132779 | 0.676108818 | 0.999970678 | 0.572 | 0 | 1.722 | 0 | CAP |
| TRINITY_DN24006_c0_g1_i2 | -1.81326214 | 0.675279705 | 0.999970678 | 0.527 | 0 | 1.522 | 0 | NA |
| TRINITY_DN85817_c0_g1_i1 | -1.81326214 | 0.675279705 | 0.999970678 | 1.662 | 0 | 4.889 | 0 | NA |
| TRINITY_DN54201_c0_g1_i6 | -1.81320994 | 0.672606913 | 0.999970678 | 2.226 | 0 | 6.377 | 0 | NA |
| TRINITY_DN7729_c0_g1_i1 | -1.8131705 | 0.670630502 | 0.999970678 | 0.418 | 0 | 1.244 | 0 | PHM7_cyt |
| TRINITY_DN5189_c0_g1_i4 | -1.81316217 | 0.670220412 | 0.999970678 | 0.618 | 0 | 1.822 | 0 | NA |
| TRINITY_DN46917_c0_g1_i1 | -1.81306662 | 0.666100685 | 0.999970678 | 1.59 | 0 | 4.689 | 0 | Inhibitor_I9 |
| TRINITY_DN7990_c0_g2_i3 | -1.81237819 | 0.501140553 | 0.999970678 | 0 | 0.995 | 0.5 | 2.875 | Serpin |
| TRINITY_DN3222_c0_g1_i7 | -1.81226206 | 0.163907361 | 0.999970678 | 0.79 | 5.558 | 6.988 | 13.663 | Glycos_transf_1 |
| TRINITY_DN2469_c0_g1_i6 | 1.812109138 | 0.210769898 | 0.999970678 | 6.886 | 1.616 | 1.722 | 0.372 | Glyco_hydro_14 |
| TRINITY_DN239_c0_g2_i1 | 1.81209164 | 0.011529238 | 0.544751459 | 64.223 | 78.59 | 12.688 | 25.044 | NA |
| TRINITY_DN917_c1_g1_i2 | 1.811280415 | 0.208300586 | 0.999970678 | 0.745 | 1.005 | 0.344 | 0.08 | NA |
| TRINITY_DN13801_c0_g1_i1 | -1.81124834 | 0.126321048 | 0.999970678 | 3.561 | 3.538 | 3.822 | 20.107 | DPBB_1 |
| TRINITY_DN239_c0_g2_i4 | -1.81093765 | 0.525290223 | 0.999970678 | 39.379 | 0 | 90.161 | 30.473 | NA |
| TRINITY_DN3488_c0_g1_i1 | 1.810409789 | 0.546810642 | 0.999970678 | 0 | 15.4 | 0.356 | 3.881 | NA |
| TRINITY_DN3395_c0_g1_i1 | 1.810198161 | 0.29703283 | 0.999970678 | 9.447 | 35 | 0.578 | 11.361 | NA |
| TRINITY_DN6832_c0_g1_i1 | 1.809763307 | 0.257703932 | 1 | 3.143 | 6.357 | 1.833 | 0.452 | PCI |
| TRINITY_DN81461_c0_g3_i1 | 1.809736734 | 0.360878616 | 0.999970678 | 0.273 | 16.98 | 1.744 | 2.714 | NA |
| TRINITY_DN3240_c0_g1_i8 | -1.8091206 | 0.024488616 | 0.985335046 | 0.6 | 1.015 | 2.389 | 2.735 | NA |
| TRINITY_DN5265_c2_g2_i1 | -1.80901571 | 0.675884758 | 0.999970678 | 2.017 | 0 | 0 | 7.048 | NA |
| TRINITY_DN19623_c0_g1_i2 | -1.80886591 | 0.489858078 | 0.999970678 | 7.667 | 0 | 6.266 | 18.951 | AAA |
| TRINITY_DN113375_c0_g1_i1 | -1.8079243 | 0.49396303 | 1 | 2.398 | 0 | 3.833 | 3.7 | NA |
| TRINITY_DN4223_c0_g1_i1 | -1.80782546 | 0.277021532 | 0.999970678 | 2.089 | 0.818 | 0.489 | 9.631 | PP2C |
| TRINITY_DN916_c1_g2_i1 | 1.807815589 | 0.557808928 | 0.999970678 | 0 | 143 | 1.944 | 36.606 | GASA |
| TRINITY_DN2486_c1_g1_i4 | 1.807557842 | 0.220186738 | 0.999970678 | 20.466 | 2.188 | 3.9 | 1.84 | NAM |
| TRINITY_DN4370_c0_g1_i1 | 1.807242141 | 0.044476048 | 0.999970678 | 9.257 | 15.37 | 3.922 | 2.272 | p450 |
| TRINITY_DN1245_c0_g1_i13 | -1.80712689 | 0.135280797 | 0.999970678 | 0.372 | 0.917 | 2.744 | 1.136 | F-box |
| TRINITY_DN1564_c0_g1_i4 | 1.80643025 | 0.45751211 | 0.999970678 | 0.118 | 28.96 | 6.4 | 0.452 | CIAPIN1 |
| TRINITY_DN1867_c0_g1_i4 | -1.8062683 | 0.670251296 | 0.999970678 | 0 | 2.631 | 0 | 9.028 | B_lectin |
| TRINITY_DN15795_c0_g1_i3 | -1.80537249 | 0.173418537 | 0.999970678 | 5.078 | 3.834 | 3.244 | 27.034 | NA |

| TRINITY_DN296_c1_g1_i2 | 1.805359226 | 0.074964005 | 0.999970678 | 25.916 | 13.84 | 2.6 | 8.224 | zf-RING_2 |
| --- | --- | --- | --- | --- | --- | --- | --- | --- |
| TRINITY_DN6503_c0_g1_i2 | -1.80516955 | 0.224145362 | 0.999970678 | 0.082 | 0.434 | 0.922 | 0.694 | Branch |
| TRINITY_DN2282_c0_g1_i1 | 1.805046898 | 0.199227145 | 0.999970678 | 48.717 | 73.79 | 2.567 | 31.82 | Heme_oxygenase |
| TRINITY_DN7497_c0_g1_i3 | -1.80492701 | 0.665877925 | 0.999970678 | 0.563 | 0 | 0 | 1.981 | Ist1 |
| TRINITY_DN13377_c0_g2_i10 | -1.80492185 | 0.491224672 | 0.999970678 | 0.972 | 0 | 0.778 | 2.473 | NA |
| TRINITY_DN91711_c0_g1_i1 | -1.80427708 | 0.667088008 | 0.999970678 | 0.972 | 0 | 2.822 | 0 | zf-C2H2 |
| TRINITY_DN9253_c0_g1_i1 | 1.804090126 | 0.099943923 | 0.999970678 | 16.496 | 19.71 | 6.855 | 2.051 | EF-hand_1 |
| TRINITY_DN4739_c0_g1_i5 | 1.803974101 | 0.485658005 | 1 | 0.318 | 0.601 | 0 | 0.261 | Glyco_trans_1_4 |
| TRINITY_DN85038_c0_g1_i1 | -1.80391286 | 0.204578567 | 0.999970678 | 26.007 | 3.853 | 19.899 | 80.47 | NA |
| TRINITY_DN2195_c1_g1_i5 | 1.803851255 | 0.482729324 | 0.999970678 | 0.999 | 0.867 | 0.444 | 0 | NA |
| TRINITY_DN649_c0_g1_i1 | -1.80376646 | 0.338261357 | 0.999970678 | 0.418 | 0.237 | 1.811 | 0.101 | NA |
| TRINITY_DN863_c0_g2_i10 | -1.80294054 | 0.506341358 | 0.999970678 | 10.846 | 0 | 16.321 | 18.69 | NA |
| TRINITY_DN7564_c0_g1_i3 | -1.80234905 | 0.507250763 | 0.999970678 | 0 | 1.084 | 0.411 | 3.217 | NA |
| TRINITY_DN867_c0_g1_i2 | 1.80231819 | 0.103788584 | 0.999970678 | 10.592 | 5.056 | 2.855 | 1.076 | ANF_receptor |
| TRINITY_DN7840_c0_g1_i2 | 1.80146196 | 0.061841607 | 0.999970678 | 0.681 | 0.611 | 0.189 | 0.131 | Mon1 |
| TRINITY_DN1706_c0_g1_i1 | 1.800780924 | 0.53060655 | 0.999970678 | 0.699 | 7.037 | 1.811 | 0 | NA |
| TRINITY_DN6564_c1_g1_i2 | 1.800765625 | 0.511516337 | 0.999970678 | 4.569 | 0.7 | 1.255 | 0 | NA |
| TRINITY_DN4138_c0_g1_i5 | 1.8002165 | 0.670516629 | 0.999970678 | 5.341 | 0 | 0 | 1.538 | NA |
| TRINITY_DN1901_c0_g1_i1 | -1.79999091 | 0.547698279 | 0.999970678 | 0.145 | 1.666 | 0 | 6.324 | NA |
| TRINITY_DN1513_c0_g1_i1 | 1.79985152 | 0.478318674 | 0.999970678 | 0.972 | 1.991 | 0 | 0.855 | NA |
| TRINITY_DN504_c0_g1_i12 | 1.799676317 | 0.214399173 | 0.999970678 | 0.345 | 1.173 | 0.278 | 0.101 | DUF3522 |
| TRINITY_DN2911_c0_g1_i3 | 1.799631055 | 0.092493726 | 0.999970678 | 4.351 | 1.508 | 0.656 | 0.915 | NA |
| TRINITY_DN4714_c0_g1_i3 | -1.79863696 | 0.200660951 | 0.999970678 | 3.007 | 3.134 | 14.599 | 3.187 | NA |
| TRINITY_DN5266_c0_g1_i7 | -1.79859634 | 0.216780871 | 0.999970678 | 1.771 | 5.194 | 16.099 | 4.122 | NA |
| TRINITY_DN9931_c1_g1_i4 | 1.797838155 | 0.61095367 | 0.999970678 | 384.84 | 0.867 | 0 | 112.621 | 2OG-FeII_Oxy |
| TRINITY_DN3906_c0_g1_i2 | -1.79779623 | 0.506456759 | 0.999970678 | 0.2 | 0 | 0.456 | 0.141 | Na_H_Exchanger |
| TRINITY_DN8631_c0_g3_i5 | -1.79775522 | 0.492966177 | 0.999970678 | 1.644 | 0 | 1.344 | 4.192 | GFO_IDH_MocA |
| TRINITY_DN8428_c0_g1_i4 | 1.797710251 | 0.361844833 | 0.999970678 | 0.5 | 17.24 | 1.244 | 3.388 | NA |
| TRINITY_DN5001_c0_g1_i2 | -1.79747024 | 0.203607508 | 0.999970678 | 0.382 | 1.961 | 1.633 | 6.062 | NA |
| TRINITY_DN1228_c0_g1_i1 | -1.79710602 | 0.085963 | 0.999970678 | 5.478 | 5.844 | 7 | 30.865 | NA |
| TRINITY_DN1168_c0_g1_i2 | -1.79687559 | 0.209176871 | 0.999970678 | 2.798 | 17.71 | 11.844 | 55.708 | Myb_DNA-bindin |
| TRINITY_DN559_c0_g1_i5 | 1.796688569 | 0.513337404 | 0.999970678 | 19.93 | 13.89 | 0 | 9.802 | NA |
| TRINITY_DN12118_c0_g1_i2 | 1.796628584 | 0.66548777 | 0.999970678 | 0 | 1.35 | 0 | 0.382 | NA |
| TRINITY_DN477_c0_g1_i15 | 1.796296909 | 0.606318149 | 0.999970678 | 23.473 | 0 | 0.022 | 6.857 | NA |
| TRINITY_DN1665_c0_g1_i3 | 1.796037881 | 0.149099423 | 0.999970678 | 0.772 | 2.089 | 0.511 | 0.221 | MFS_1 |
| TRINITY_DN19484_c0_g2_i2 | -1.79601942 | 0.260325615 | 0.999970678 | 0.145 | 0.631 | 0.289 | 2.332 | NA |
| TRINITY_DN901_c0_g1_i1 | -1.79538638 | 0.091898905 | 0.999970678 | 1.017 | 2.907 | 3.511 | 9.32 | TIM21 |
| TRINITY_DN18693_c0_g1_i1 | 1.794922713 | 0.230866036 | 0.999970678 | 2.97 | 37.33 | 4.911 | 5.469 | Glyco_hydro_14 |
| TRINITY_DN5038_c0_g2_i4 | -1.79434634 | 0.482494239 | 0.999970678 | 0 | 1.685 | 2.722 | 2.443 | SNARE |
| TRINITY_DN18465_c0_g2_i2 | -1.79426027 | 0.667377053 | 0.999970678 | 2.353 | 0 | 0 | 8.304 | UBA_4 |
| TRINITY_DN800_c0_g1_i2 | -1.79400489 | 0.107701475 | 0.999970678 | 5.214 | 11.8 | 20.754 | 30.131 | NA |
| TRINITY_DN536_c0_g1_i1 | 1.793279267 | 0.531420229 | 0.999970678 | 2.871 | 0 | 0.211 | 0.593 | NA |
| TRINITY_DN11315_c0_g1_i2 | 1.793186302 | 0.667163323 | 0.999970678 | 0 | 5.371 | 1.267 | 0 | NA |
| TRINITY_DN1354_c0_g1_i4 | 1.792501414 | 0.421647768 | 0.999970678 | 1.026 | 157 | 2.933 | 41.401 | NA |
| TRINITY_DN34324_c0_g1_i4 | 1.792490675 | 0.15096355 | 0.999970678 | 3.089 | 0.769 | 0.556 | 0.462 | NA |
| TRINITY_DN8315_c0_g1_i1 | -1.79239635 | 0.123176636 | 0.999970678 | 1.354 | 2.277 | 2.033 | 10.094 | Glutaredoxin |
| TRINITY_DN2707_c0_g1_i9 | -1.79229127 | 0.677987658 | 0.999970678 | 0 | 0.404 | 0 | 1.357 | NA |
| TRINITY_DN10182_c0_g1_i8 | -1.79218024 | 0.678384256 | 0.999970678 | 0.654 | 0 | 1.967 | 0 | p450 |
| TRINITY_DN3916_c0_g1_i7 | -1.79139193 | 0.491662421 | 0.999970678 | 0.427 | 0 | 0.822 | 0.523 | BET |
| TRINITY_DN13_c1_g1_i4 | 1.7912512 | 0.309982327 | 0.999970678 | 3.806 | 1.892 | 1.278 | 0.161 | NA |
| TRINITY_DN69214_c0_g1_i2 | 1.791187349 | 0.115251082 | 0.999970678 | 1.553 | 4.583 | 0.7 | 0.915 | ADH_N |
| TRINITY_DN2787_c0_g1_i4 | 1.790975171 | 0.116476837 | 0.999970678 | 1.054 | 0.532 | 0.189 | 0.231 | CIA30 |
| TRINITY_DN3901_c0_g1_i1 | -1.7909717 | 0.072095924 | 0.999970678 | 2.852 | 5.992 | 7.544 | 21.374 | NA |
| TRINITY_DN2072_c0_g1_i2 | 1.790880655 | 0.499471804 | 0.999970678 | 0.781 | 0.227 | 0.244 | 0 | FAT |
| TRINITY_DN34908_c0_g1_i2 | -1.79064614 | 0.486542565 | 0.999970678 | 1.117 | 0 | 1.633 | 1.95 | PPR |
| TRINITY_DN1156_c0_g1_i7 | -1.79061507 | 0.313302389 | 0.999970678 | 0.154 | 2.966 | 4.466 | 5.228 | NA |
| TRINITY_DN7502_c0_g1_i1 | -1.79058747 | 0.271982069 | 0.999970678 | 1.054 | 4.514 | 2.555 | 15.402 | - |
| TRINITY_DN90914_c0_g1_i1 | -1.78944481 | 0.671636978 | 0.999970678 | 1.226 | 0 | 3.555 | 0 | NA |
| TRINITY_DN53877_c0_g2_i1 | -1.78904002 | 0.681434857 | 0.999970678 | 0.718 | 0 | 2.089 | 0 | CDH-cyt |
| TRINITY_DN2688_c1_g1_i2 | -1.78903461 | 0.672954193 | 0.999970678 | 0 | 1.291 | 0 | 4.404 | NA |
| TRINITY_DN564_c0_g1_i3 | -1.78870429 | 0.076901738 | 0.999970678 | 14.852 | 17.36 | 22.776 | 80.198 | NA |
| TRINITY_DN2746_c1_g1_i1 | -1.78864228 | 0.190011083 | 0.999970678 | 2.044 | 0.71 | 6.577 | 1.599 | NA |
| TRINITY_DN12513_c0_g1_i8 | -1.78855562 | 0.679633891 | 0.999970678 | 0.336 | 0 | 0 | 1.206 | NA |
| TRINITY_DN1665_c0_g1_i4 | 1.788428077 | 0.509525536 | 0.999970678 | 0.927 | 6.022 | 1.644 | 0 | MFS_1 |
| TRINITY_DN8713_c0_g1_i2 | -1.78836642 | 0.676496574 | 0.999970678 | 0.427 | 0 | 0 | 1.478 | NA |
| TRINITY_DN2678_c1_g1_i12 | 1.78801946 | 0.676997682 | 0.999970678 | 0 | 1.863 | 0 | 0.523 | NA |
| TRINITY_DN5200_c0_g1_i1 | -1.78777032 | 0.682659494 | 0.999970678 | 6.895 | 0 | 19.754 | 0 | Cupin_7 |
| TRINITY_DN6154_c0_g1_i3 | -1.78754419 | 0.488168269 | 0.999970678 | 0 | 0.818 | 1.255 | 1.267 | - |
| TRINITY_DN1266_c0_g1_i14 | -1.78730383 | 0.374312403 | 0.999970678 | 9.547 | 0.719 | 2.067 | 32.122 | NA |
| TRINITY_DN16142_c1_g1_i1 | -1.78694285 | 0.400753845 | 0.999970678 | 1.317 | 0.148 | 0.156 | 4.916 | NA |
| TRINITY_DN12129_c0_g1_i3 | 1.78629185 | 0.260792405 | 0.999970678 | 0.318 | 2.562 | 0.489 | 0.231 | MCM_bind |
| TRINITY_DN13608_c1_g1_i3 | -1.78608575 | 0.498097216 | 0.999970678 | 0.336 | 0 | 0.367 | 0.764 | NA |
| TRINITY_DN2812_c0_g4_i3 | 1.78597668 | 0.08485189 | 0.999970678 | 0.627 | 1.882 | 0.289 | 0.382 | FKS1_dom1 |
| TRINITY_DN13900_c1_g1_i1 | 1.785741817 | 0.672270565 | 0.999970678 | 20.339 | 0 | 0 | 5.992 | FAD_binding_6 |

| TRINITY_DN270_c1_g2_i2 | 1.78573286 | 0.116007227 | 0.999970678 | 4.624 | 18.18 | 2.067 | 4.052 | p450 |
| --- | --- | --- | --- | --- | --- | --- | --- | --- |
| TRINITY_DN2509_c0_g1_i4 | 1.78547173 | 0.327379663 | 0.999970678 | 0.445 | 4.573 | 0.1 | 1.337 | RRM_1 |
| TRINITY_DN11443_c0_g1_i1 | -1.78516171 | 0.217046945 | 0.999970678 | 1.726 | 0.572 | 1.055 | 6.666 | peroxidase |
| TRINITY_DN28074_c0_g1_i3 | 1.785007485 | 0.219883436 | 0.999970678 | 0.89 | 0.424 | 0.244 | 0.09 | NA |
| TRINITY_DN7396_c0_g1_i14 | 1.78489981 | 0.488852367 | 0.999970678 | 0.681 | 0.365 | 0.256 | 0 | NA |
| TRINITY_DN477_c0_g1_i8 | -1.78483529 | 0.065886164 | 0.999970678 | 0.636 | 0.71 | 2.544 | 1.609 | NA |
| TRINITY_DN1873_c3_g1_i5 | 1.784621913 | 0.463722299 | 0.999970678 | 15.197 | 0.059 | 0.211 | 4.233 | NA |
| TRINITY_DN854_c0_g1_i5 | 1.784186755 | 0.362007163 | 0.999970678 | 14.689 | 0.306 | 0.944 | 3.257 | CS |
| TRINITY_DN2844_c0_g1_i9 | -1.78403465 | 0.668954059 | 0.999970678 | 0.563 | 0 | 0 | 1.971 | - |
| TRINITY_DN2507_c0_g1_i2 | -1.7839908 | 0.516876723 | 0.999970678 | 0 | 7.943 | 5.311 | 20.55 | - |
| TRINITY_DN2458_c0_g1_i4 | 1.783636036 | 0.486454882 | 0.999970678 | 0.863 | 0.838 | 0.4 | 0 | GLE1 |
| TRINITY_DN2633_c0_g1_i4 | 1.783420497 | 0.234709287 | 0.999970678 | 7.458 | 1.863 | 0.356 | 2.322 | LrgB |
| TRINITY_DN6398_c0_g1_i4 | 1.783109839 | 0.341373167 | 0.999970678 | 0.154 | 4.543 | 0.811 | 0.362 | NA |
| TRINITY_DN11189_c0_g1_i1 | -1.78285472 | 0.18890678 | 0.999970678 | 0.454 | 1.242 | 1.144 | 4.454 | NA |
| TRINITY_DN54103_c0_g1_i1 | -1.78273089 | 0.2649309 | 0.999970678 | 0.2 | 1.715 | 1.678 | 4.474 | NA |
| TRINITY_DN2106_c2_g1_i1 | -1.78272113 | 0.278787011 | 0.999970678 | 0.245 | 0.788 | 2.711 | 0.241 | ABC_tran |
| TRINITY_DN1474_c1_g1_i7 | -1.78253192 | 0.36107528 | 0.999970678 | 1.49 | 0.079 | 0.756 | 4.474 | NA |
| TRINITY_DN3407_c0_g1_i1 | 1.781939644 | 0.347584657 | 0.999970678 | 0.372 | 12.15 | 0.511 | 2.996 | TAXi_C |
| TRINITY_DN242_c0_g3_i2 | 1.781676993 | 0.235856523 | 0.999970678 | 64.605 | 4.662 | 6.855 | 12.165 | B_lectin |
| TRINITY_DN4176_c0_g1_i10 | -1.78143348 | 0.131223852 | 0.999970678 | 0.799 | 0.227 | 1.111 | 2.242 | NA |
| TRINITY_DN4299_c0_g1_i3 | 1.7812711 | 0.67135689 | 0.999970678 | 8.384 | 0 | 0 | 2.493 | WD40 |
| TRINITY_DN14181_c0_g1_i2 | 1.781209752 | 0.675727243 | 0.999970678 | 1.59 | 0 | 0 | 0.462 | NA |
| TRINITY_DN50841_c0_g1_i1 | 1.781097383 | 0.671874296 | 0.999970678 | 4.587 | 0 | 1.122 | 0 | Aldedh |
| TRINITY_DN2941_c1_g2_i2 | 1.78080511 | 0.327717128 | 0.999970678 | 2.053 | 6.573 | 0.111 | 2.332 | NA |
| TRINITY_DN3313_c0_g1_i1 | -1.7805808 | 0.250324313 | 0.999970678 | 0.282 | 2.188 | 1.355 | 6.736 | Sugar_tr |
| TRINITY_DN10536_c0_g1_i1 | 1.780218932 | 0.118782732 | 0.999970678 | 12.054 | 20.26 | 4.433 | 3.549 | NA |
| TRINITY_DN3019_c0_g2_i4 | 1.780091143 | 0.466439879 | 0.999970678 | 3.079 | 0.02 | 0.044 | 0.885 | NA |
| TRINITY_DN2387_c2_g1_i1 | 1.779886684 | 0.190288564 | 1 | 1.535 | 1.784 | 0.522 | 0.332 | NA |
| TRINITY_DN11993_c0_g1_i2 | -1.7788682 | 0.497049494 | 1 | 0 | 0.611 | 0.744 | 1.156 | NA |
| TRINITY_DN9850_c0_g1_i1 | 1.778460929 | 0.187077041 | 0.999970678 | 1.817 | 3.883 | 1.011 | 0.402 | Aldedh |
| TRINITY_DN3196_c0_g1_i2 | 1.778148796 | 0.102553999 | 0.999970678 | 0.709 | 1.429 | 0.256 | 0.312 | NA |
| TRINITY_DN2690_c0_g1_i17 | -1.77790461 | 0.504738705 | 0.999970678 | 1.09 | 0 | 2.489 | 0.824 | DUF247 |
| TRINITY_DN5691_c0_g1_i2 | 1.777827631 | 0.146362722 | 0.999970678 | 5.36 | 22.13 | 1.944 | 5.59 | NA |
| TRINITY_DN14535_c0_g7_i1 | 1.7775887 | 0.104106314 | 0.999970678 | 2.044 | 3.43 | 0.8 | 0.623 | NA |
| TRINITY_DN397_c1_g1_i9 | 1.777408353 | 0.15800074 | 0.999970678 | 10.156 | 1.863 | 1.655 | 1.548 | PUNUT |
| TRINITY_DN228_c0_g1_i3 | -1.7766286 | 0.336790257 | 0.999970678 | 0.2 | 0.266 | 1.289 | 0.06 | UDPGT |
| TRINITY_DN2844_c0_g1_i11 | 1.776580714 | 0.368236209 | 0.999970678 | 0.055 | 1.941 | 0.122 | 0.432 | - |
| TRINITY_DN136684_c0_g1_i1 | 1.776437387 | 0.489436874 | 0.999970678 | 7.821 | 6.435 | 0 | 4.172 | NA |
| TRINITY_DN5334_c0_g1_i5 | -1.77633156 | 0.541494981 | 0.999970678 | 1.372 | 0.237 | 0 | 5.59 | NA |
| TRINITY_DN1212_c0_g1_i4 | 1.776024987 | 0.533870164 | 0.999970678 | 7.849 | 17.52 | 6.089 | 0 | Glutaredoxin |
| TRINITY_DN4874_c0_g1_i2 | -1.77590868 | 0.191115539 | 0.999970678 | 0.718 | 0.443 | 2.667 | 0.784 | NA |
| TRINITY_DN7101_c0_g1_i6 | -1.7754547 | 0.538357693 | 0.999970678 | 0.917 | 0 | 0.222 | 2.986 | NA |
| TRINITY_DN5270_c0_g2_i3 | -1.77542167 | 0.193640137 | 0.999970678 | 0.481 | 2.326 | 5.844 | 2.483 | NA |
| TRINITY_DN3459_c0_g1_i1 | 1.775163431 | 0.236004532 | 0.999970678 | 87.278 | 25.42 | 24.71 | 3.227 | NA |
| TRINITY_DN9343_c0_g1_i4 | -1.77506983 | 0.493017371 | 0.999970678 | 5.868 | 0 | 6.855 | 11.773 | Ribosomal_L11 |
| TRINITY_DN7572_c0_g2_i4 | 1.774916671 | 0.350604455 | 0.999970678 | 0.454 | 1.005 | 0.022 | 0.402 | NA |
| TRINITY_DN1023_c0_g1_i5 | -1.77487805 | 0.562204057 | 0.999970678 | 7.558 | 0.424 | 0 | 27.326 | DUF1685 |
| TRINITY_DN6425_c0_g1_i4 | 1.77478314 | 0.219909479 | 0.999970678 | 1.299 | 0.562 | 0.1 | 0.432 | NA |
| TRINITY_DN10448_c0_g2_i1 | -1.77450265 | 0.224133559 | 0.999970678 | 0.291 | 0.493 | 0.244 | 2.393 | WD40 |
| TRINITY_DN5321_c0_g1_i3 | 1.774336619 | 0.489702463 | 0.999970678 | 1.208 | 1.143 | 0.567 | 0 | NA |
| TRINITY_DN5162_c0_g1_i2 | -1.77424865 | 0.669505351 | 0.999970678 | 0 | 0.552 | 0 | 1.88 | MafB19-deam |
| TRINITY_DN16950_c0_g1_i3 | -1.77421872 | 0.680310236 | 0.999970678 | 0.763 | 0 | 2.178 | 0 | NA |
| TRINITY_DN3627_c0_g1_i10 | 1.774166623 | 0.391690068 | 0.999970678 | 5.777 | 0.394 | 1.467 | 0.05 | Pro_CA |
| TRINITY_DN7843_c0_g1_i1 | -1.77344888 | 0.521088221 | 0.999970678 | 0.854 | 0 | 0.378 | 2.513 | NA |
| TRINITY_DN3844_c0_g1_i1 | 1.773254223 | 0.220790771 | 0.999970678 | 3.806 | 4.021 | 0.256 | 1.93 | NA |
| TRINITY_DN2077_c0_g1_i12 | -1.77320051 | 0.085516622 | 0.999970678 | 1.217 | 0.414 | 2.555 | 2.564 | NA |
| TRINITY_DN2183_c0_g2_i3 | 1.773109719 | 0.682474509 | 0.999970678 | 11.555 | 0 | 2.866 | 0 | NA |
| TRINITY_DN1117_c0_g1_i1 | -1.77284665 | 0.533686754 | 0.999970678 | 16.242 | 0 | 16.066 | 37.078 | Ammonium_trans |
| TRINITY_DN2857_c2_g2_i2 | 1.772805179 | 0.516259075 | 0.999970678 | 0.518 | 3.765 | 1.022 | 0 | NA |
| TRINITY_DN808_c1_g1_i3 | 1.772641217 | 0.225414443 | 0.999970678 | 0.845 | 3.38 | 0.3 | 0.834 | NA |
| TRINITY_DN155_c0_g1_i18 | 1.772467574 | 0.50057027 | 0.999970678 | 8.285 | 4.79 | 0 | 3.861 | Ank |
| TRINITY_DN7463_c0_g1_i5 | 1.771645177 | 0.379512157 | 0.999970678 | 208.94 | 2.553 | 42.675 | 11.19 | NA |
| TRINITY_DN2049_c0_g1_i6 | -1.77124136 | 0.007752761 | 0.388494197 | 0.899 | 0.897 | 2.567 | 3.026 | DYW_deaminase |
| TRINITY_DN804_c1_g1_i4 | 1.770768318 | 0.508845045 | 0.999970678 | 7.476 | 1.537 | 0 | 2.684 | NA |
| TRINITY_DN6641_c0_g1_i4 | -1.77072022 | 0.166246595 | 0.999970678 | 0.273 | 0.197 | 0.922 | 0.543 | NA |
| TRINITY_DN6809_c0_g1_i2 | 1.769889208 | 0.127624398 | 0.999970678 | 0.291 | 0.512 | 0.144 | 0.06 | Chorein_N |
| TRINITY_DN45200_c0_g1_i2 | -1.76869101 | 0.470960842 | 0.999970678 | 2.235 | 0.108 | 0.156 | 7.611 | NA |
| TRINITY_DN1202_c0_g2_i6 | -1.76853923 | 0.107138068 | 0.999970678 | 2.598 | 0.917 | 6.855 | 3.77 | Cellulose_synt |
| TRINITY_DN18614_c0_g1_i2 | 1.768362012 | 0.294104737 | 1 | 0.409 | 0.463 | 0.167 | 0.05 | NA |
| TRINITY_DN3007_c1_g1_i2 | -1.76826566 | 0.151389793 | 0.999970678 | 3.406 | 1.399 | 8.655 | 5.881 | NA |
| TRINITY_DN3438_c0_g1_i1 | 1.768191119 | 0.511992058 | 0.999970678 | 14.371 | 19.05 | 0 | 9.812 | GTP1_OBG |
| TRINITY_DN7758_c0_g1_i2 | 1.768130569 | 0.49389973 | 0.999970678 | 3.134 | 1.547 | 1.144 | 0 | EloA-BP1 |
| TRINITY_DN6877_c0_g2_i3 | 1.768118783 | 0.165543399 | 0.999970678 | 1.226 | 3.942 | 0.822 | 0.503 | Acetyltransf_11 |
| TRINITY_DN356_c0_g1_i3 | -1.76809147 | 0.535752178 | 0.999970678 | 3.034 | 1.468 | 12.71 | 0 | NA |

| TRINITY_DN392_c1_g1_i5 | 1.767531276 | 0.328543025 | 0.999970678 | 0.763 | 4.514 | 1.155 | 0.121 | NA |
| --- | --- | --- | --- | --- | --- | --- | --- | --- |
| TRINITY_DN1877_c0_g1_i1 | 1.767513214 | 0.486232655 | 0.999970678 | 2.216 | 1.183 | 0 | 1.005 | NA |
| TRINITY_DN4739_c0_g1_i8 | 1.765998317 | 0.280101012 | 1 | 0.363 | 0.483 | 0.022 | 0.211 | Glyco_trans_1_4 |
| TRINITY_DN7431_c0_g1_i4 | -1.76598731 | 0.179896119 | 0.999970678 | 0.754 | 5.342 | 6.688 | 12.487 | F-box |
| TRINITY_DN1776_c1_g1_i11 | 1.765942748 | 0.678523412 | 0.999970678 | 1.871 | 0 | 0 | 0.563 | NA |
| TRINITY_DN3664_c0_g1_i1 | -1.76584698 | 0.678829337 | 0.999970678 | 14.979 | 0 | 41.631 | 0 | NA |
| TRINITY_DN5421_c0_g1_i3 | -1.76519174 | 0.494568693 | 0.999970678 | 1.063 | 0 | 1.244 | 2.192 | DUF2828 |
| TRINITY_DN21318_c0_g2_i1 | 1.765174306 | 0.492055699 | 0.999970678 | 2.534 | 1.567 | 1 | 0 | NA |
| TRINITY_DN748_c0_g1_i6 | 1.764625762 | 0.002629347 | 0.154507232 | 13.926 | 15.91 | 3.878 | 4.102 | NA |
| TRINITY_DN312_c0_g1_i4 | 1.764323474 | 0.086893694 | 0.999970678 | 2.825 | 2.641 | 1 | 0.412 | NA |
| TRINITY_DN485_c0_g1_i1 | -1.76419968 | 0.352172972 | 0.999970678 | 2.598 | 12.63 | 0.967 | 50.208 | NA |
| TRINITY_DN3106_c0_g1_i3 | 1.763784334 | 0.492842635 | 0.999970678 | 3.261 | 1.804 | 0 | 1.508 | UDPGT |
| TRINITY_DN4371_c0_g1_i1 | -1.76254438 | 0.006714785 | 0.344945664 | 16.36 | 14.62 | 41.686 | 52.722 | NA |
| TRINITY_DN13451_c0_g1_i6 | -1.76254327 | 0.680291682 | 0.999970678 | 1.308 | 0 | 3.678 | 0 | NA |
| TRINITY_DN514_c0_g1_i9 | 1.762337643 | 0.285358453 | 0.999970678 | 2.253 | 8.15 | 2.344 | 0.201 | STAS |
| TRINITY_DN13935_c1_g1_i1 | -1.76213806 | 0.35094748 | 0.999970678 | 4.46 | 3.242 | 0.733 | 24.37 | PUNUT |
| TRINITY_DN1726_c0_g1_i9 | 1.762008842 | 0.67215201 | 0.999970678 | 3.988 | 0 | 0 | 1.196 | CTD_bind |
| TRINITY_DN23979_c0_g1_i2 | -1.76176648 | 0.687399942 | 0.999970678 | 0.672 | 0 | 1.944 | 0 | NA |
| TRINITY_DN5474_c0_g1_i1 | -1.76162681 | 0.139036785 | 0.999970678 | 0.627 | 2.06 | 2.766 | 5.67 | HLH |
| TRINITY_DN2251_c1_g1_i5 | 1.760595353 | 0.302978899 | 0.999970678 | 2.008 | 2.415 | 1.044 | 0.04 | NA |
| TRINITY_DN4191_c0_g1_i3 | 1.760183568 | 0.092692254 | 0.999970678 | 1.572 | 2.631 | 0.722 | 0.372 | NIR_SIR |
| TRINITY_DN1140_c0_g1_i2 | -1.75991962 | 0.233833321 | 0.999970678 | 0.854 | 2.661 | 8.655 | 1.337 | - |
| TRINITY_DN4811_c0_g1_i4 | -1.75981533 | 0.496688682 | 0.999970678 | 0.999 | 0 | 1.733 | 1.347 | NA |
| TRINITY_DN4486_c1_g1_i4 | 1.759445435 | 0.505642099 | 0.999970678 | 2.88 | 11.38 | 0 | 4.182 | NA |
| TRINITY_DN632_c0_g1_i5 | -1.7591677 | 0.114083698 | 0.999970678 | 2.68 | 1.242 | 8.444 | 3.177 | NADHdh |
| TRINITY_DN15038_c0_g1_i2 | -1.75877427 | 0.512081357 | 1 | 0.908 | 0 | 1.4 | 1.508 | LRRNT_2 |
| TRINITY_DN5595_c0_g2_i5 | -1.75873874 | 0.68197948 | 0.999970678 | 2.362 | 0 | 6.7 | 0 | NA |
| TRINITY_DN2845_c0_g2_i8 | -1.75821959 | 0.515191009 | 0.999970678 | 0 | 0.552 | 1.3 | 0.292 | NA |
| TRINITY_DN17978_c0_g1_i5 | -1.75820852 | 0.151102433 | 0.999970678 | 0.881 | 0.404 | 1.789 | 2.212 | CorA |
| TRINITY_DN2733_c0_g1_i12 | -1.75812412 | 0.250266147 | 0.999970678 | 0.654 | 3.972 | 10.666 | 2.503 | NA |
| TRINITY_DN4987_c0_g1_i2 | 1.757875606 | 0.673536905 | 0.999970678 | 26.834 | 0 | 0 | 7.942 | NA |
| TRINITY_DN16849_c0_g1_i8 | 1.757422692 | 0.486173785 | 0.999970678 | 0.609 | 0.68 | 0 | 0.392 | NA |
| TRINITY_DN2135_c0_g1_i4 | 1.757373793 | 0.551133094 | 0.999970678 | 0 | 6.16 | 1.289 | 0.241 | NA |
| TRINITY_DN2015_c0_g1_i3 | 1.757085366 | 0.031900136 | 0.999970678 | 1.453 | 1.271 | 0.322 | 0.422 | NA |
| TRINITY_DN14605_c0_g2_i2 | -1.75661281 | 0.682922592 | 0.999970678 | 1.154 | 0 | 3.233 | 0 | - |
| TRINITY_DN23786_c0_g1_i4 | 1.756380373 | 0.525081435 | 0.999970678 | 5.014 | 0.641 | 0 | 1.689 | NA |
| TRINITY_DN8846_c0_g1_i3 | -1.75620656 | 0.249869845 | 0.999970678 | 0.972 | 0.611 | 3.955 | 0.563 | MMR_HSR1 |
| TRINITY_DN8846_c0_g1_i2 | -1.75620656 | 0.249869845 | 0.999970678 | 0.972 | 0.611 | 3.955 | 0.563 | NA |
| TRINITY_DN3758_c0_g1_i5 | 1.756178848 | 0.678952379 | 0.999970678 | 0 | 19.64 | 4.733 | 0 | NA |
| TRINITY_DN7660_c0_g1_i10 | 1.755963168 | 0.303388608 | 0.999970678 | 3.434 | 3.183 | 0.078 | 1.88 | NA |
| TRINITY_DN22575_c0_g1_i1 | 1.755470727 | 0.333920137 | 0.999970678 | 1.381 | 4.1 | 1.233 | 0.121 | NA |
| TRINITY_DN1233_c0_g1_i11 | 1.755465534 | 0.452122107 | 0.999970678 | 88.741 | 0.108 | 15.344 | 8.184 | NA |
| TRINITY_DN3237_c0_g1_i1 | -1.75531131 | 0.50117816 | 0.999970678 | 3.57 | 0 | 5.433 | 5.6 | Linker_histone |
| TRINITY_DN1212_c0_g1_i1 | -1.75497516 | 0.546766301 | 0.999970678 | 7.24 | 0 | 4.133 | 19.926 | Glutaredoxin |
| TRINITY_DN16131_c0_g1_i4 | 1.754928015 | 0.17009027 | 0.999970678 | 0.536 | 0.581 | 0.211 | 0.08 | RRM_1 |
| TRINITY_DN3826_c0_g1_i1 | 1.754884021 | 0.254808935 | 0.999970678 | 6.095 | 29.89 | 7.688 | 1.227 | NA |
| TRINITY_DN2453_c0_g1_i7 | -1.75451755 | 0.679063136 | 0.999970678 | 0.481 | 0 | 0 | 1.649 | CBS |
| TRINITY_DN1121_c0_g1_i3 | -1.75446163 | 0.018825487 | 0.807173619 | 36.581 | 23.53 | 100.294 | 81.606 | NA |
| TRINITY_DN3858_c0_g1_i1 | -1.75390818 | 0.559936696 | 0.999970678 | 0 | 62.94 | 162.612 | 11.049 | - |
| TRINITY_DN8259_c0_g1_i1 | -1.75373824 | 0.563398847 | 0.999970678 | 2.135 | 0 | 0.2 | 7.017 | NA |
| TRINITY_DN7290_c0_g1_i3 | -1.75363493 | 0.680304777 | 0.999970678 | 0.999 | 0 | 2.866 | 0 | NA |
| TRINITY_DN12205_c0_g1_i1 | -1.75356952 | 0.499112372 | 0.999970678 | 0.073 | 1.774 | 5.011 | 0.07 | NA |
| TRINITY_DN1698_c0_g2_i5 | 1.753239612 | 0.53278269 | 0.999970678 | 78.367 | 6.751 | 20.788 | 0 | NA |
| TRINITY_DN730_c0_g2_i6 | 1.75298247 | 0.216276512 | 0.999970678 | 0.981 | 3.706 | 0.889 | 0.302 | NA |
| TRINITY_DN5803_c0_g1_i1 | -1.75288244 | 0.373406055 | 0.999970678 | 2.089 | 0.049 | 3.033 | 3.589 | NA |
| TRINITY_DN4180_c1_g1_i1 | -1.75287637 | 0.678610434 | 0.999970678 | 3.824 | 0 | 10.799 | 0 | NA |
| TRINITY_DN5255_c0_g1_i5 | -1.75281444 | 0.09360148 | 0.999970678 | 0.282 | 0.197 | 0.9 | 0.533 | p450 |
| TRINITY_DN1541_c0_g1_i2 | -1.75278885 | 0.598898357 | 0.999970678 | 0.245 | 14.86 | 0 | 50.389 | Glyco_transf_5 |
| TRINITY_DN4195_c0_g1_i15 | 1.752755757 | 0.534745765 | 0.999970678 | 2.68 | 0.177 | 0 | 0.865 | NA |
| TRINITY_DN2758_c0_g1_i14 | -1.75257431 | 0.565793605 | 0.999970678 | 0.663 | 0 | 1.778 | 0.08 | MIP |
| TRINITY_DN6687_c0_g1_i3 | -1.75248559 | 0.5334111 | 0.999970678 | 0.282 | 0 | 0.7 | 0.111 | NA |
| TRINITY_DN4262_c2_g1_i1 | 1.752466002 | 0.587964048 | 0.999970678 | 5.487 | 0.059 | 0 | 1.649 | NA |
| TRINITY_DN9229_c0_g2_i6 | -1.75181752 | 0.685044865 | 0.999970678 | 4.551 | 0 | 12.721 | 0 | NA |
| TRINITY_DN3396_c0_g1_i1 | -1.75167532 | 0.205568775 | 0.999970678 | 2.198 | 0.877 | 1.733 | 8.003 | NA |
| TRINITY_DN5759_c0_g1_i7 | -1.75109826 | 0.512769394 | 0.999970678 | 0.99 | 0 | 0.756 | 2.453 | NA |
| TRINITY_DN4349_c1_g1_i5 | 1.750682768 | 0.185137031 | 0.999970678 | 4.787 | 5.992 | 0.4 | 2.694 | NA |
| TRINITY_DN1015_c0_g2_i7 | 1.75062007 | 0.135159004 | 0.999970678 | 0.727 | 1.183 | 0.311 | 0.191 | NA |
| TRINITY_DN29_c1_g1_i1 | 1.750598976 | 0.362107902 | 0.999970678 | 1.008 | 78.99 | 6.277 | 15.865 | NA |
| TRINITY_DN354_c0_g1_i3 | 1.750481128 | 0.217145191 | 1 | 0.472 | 0.808 | 0.089 | 0.261 | NA |
| TRINITY_DN6787_c0_g2_i1 | -1.75025343 | 0.506465336 | 0.999970678 | 0.273 | 0 | 0.544 | 0.282 | IKI3 |
| TRINITY_DN3987_c0_g1_i6 | 1.749785425 | 0.246183803 | 0.999970678 | 0.917 | 1.212 | 0.467 | 0.08 | NA |
| TRINITY_DN975_c0_g1_i7 | -1.74957718 | 0.564160029 | 0.999970678 | 0.681 | 6.327 | 0 | 23.345 | RRM_1 |
| TRINITY_DN10671_c1_g2_i1 | 1.748429973 | 0.498685139 | 0.999970678 | 0.445 | 0.69 | 0.289 | 0 | NA |
| TRINITY_DN2829_c0_g1_i2 | 1.748329016 | 0.501308196 | 0.999970678 | 4.887 | 2.129 | 1.733 | 0 | Glyco_hydro_17 |

| TRINITY_DN29210_c0_g1_i4 | 1.748282292 | 0.487335268 | 0.999970678 | 3.379 | 3.242 | 0 | 1.971 | NA |
| --- | --- | --- | --- | --- | --- | --- | --- | --- |
| TRINITY_DN1769_c0_g1_i5 | -1.74817681 | 0.170053127 | 0.999970678 | 0.672 | 2.178 | 5.244 | 3.137 | - |
| TRINITY_DN1977_c0_g1_i7 | -1.74791887 | 0.676512816 | 0.999970678 | 0.936 | 0 | 2.644 | 0 | Aldedh |
| TRINITY_DN1309_c0_g1_i1 | 1.747852271 | 0.269397967 | 0.999970678 | 1.935 | 23.23 | 2.978 | 3.77 | Transferase |
| TRINITY_DN12590_c0_g2_i2 | 1.747797932 | 0.470955817 | 0.999970678 | 11.101 | 0.365 | 2.733 | 0.121 | NA |
| TRINITY_DN15995_c0_g1_i2 | 1.747601145 | 0.161014662 | 0.999970678 | 1.299 | 0.818 | 0.233 | 0.352 | NA |
| TRINITY_DN8940_c0_g1_i1 | -1.74754137 | 0.505326292 | 0.999970678 | 0.518 | 0 | 1.033 | 0.503 | - |
| TRINITY_DN7391_c0_g1_i4 | 1.747461265 | 0.256735587 | 0.999970678 | 1.29 | 1.183 | 0.567 | 0.05 | - |
| TRINITY_DN7015_c0_g1_i4 | -1.7465463 | 0.558477589 | 0.999970678 | 0.636 | 0.099 | 2.022 | 0 | PEPCK_ATP |
| TRINITY_DN12329_c1_g2_i1 | 1.745845915 | 0.501641079 | 1 | 0.409 | 0.256 | 0 | 0.201 | NA |
| TRINITY_DN1727_c0_g1_i9 | -1.74578619 | 0.236534027 | 0.999970678 | 0.917 | 0.187 | 1.9 | 1.387 | NA |
| TRINITY_DN771_c0_g3_i1 | 1.74569645 | 0.210900299 | 0.999970678 | 134.55 | 280.2 | 11.544 | 98.657 | NA |
| TRINITY_DN1011_c0_g1_i5 | 1.745354266 | 0.502342136 | 1 | 10.41 | 12.5 | 0 | 6.384 | NA |
| TRINITY_DN388_c0_g1_i5 | -1.74514135 | 0.141057977 | 0.999970678 | 1.572 | 1.577 | 3.389 | 5.741 | NA |
| TRINITY_DN27196_c0_g1_i1 | -1.74492788 | 0.546589486 | 0.999970678 | 0 | 0.65 | 0.133 | 2.001 | p450 |
| TRINITY_DN22603_c0_g1_i1 | 1.744734632 | 0.193775951 | 1 | 0.872 | 0.976 | 0.244 | 0.241 | NA |
| TRINITY_DN13391_c0_g1_i1 | -1.74468725 | 0.113023371 | 0.999970678 | 6.277 | 4.356 | 18.466 | 12.095 | NA |
| TRINITY_DN1555_c0_g1_i8 | 1.74449346 | 0.50336338 | 0.999970678 | 3.634 | 1.252 | 0 | 1.478 | NA |
| TRINITY_DN403_c0_g2_i2 | -1.74431865 | 0.199791676 | 0.999970678 | 1.235 | 0.522 | 3.733 | 1.277 | NA |
| TRINITY_DN1568_c0_g1_i18 | 1.744153048 | 0.51319585 | 0.999970678 | 0.354 | 1.055 | 0.356 | 0 | Glyco_hydro_1 |
| TRINITY_DN3086_c0_g1_i5 | 1.743954249 | 0.296580431 | 0.999970678 | 2.843 | 4.129 | 1.655 | 0.09 | BLM10_mid |
| TRINITY_DN4984_c0_g1_i1 | -1.74340353 | 0.058759991 | 0.999970678 | 2.017 | 1.094 | 5.311 | 4.042 | NA |
| TRINITY_DN8400_c0_g1_i2 | -1.74322968 | 0.512502762 | 1 | 0 | 0.237 | 0.311 | 0.392 | NA |
| TRINITY_DN1995_c0_g1_i9 | -1.74315814 | 0.343754763 | 0.999970678 | 28.614 | 20.78 | 2.389 | 160.95 | NA |
| TRINITY_DN12706_c0_g1_i2 | -1.74236349 | 0.50735567 | 0.999970678 | 0 | 3.173 | 2.233 | 7.792 | ANTH |
| TRINITY_DN8259_c0_g1_i8 | 1.74215601 | 0.566003316 | 0.999970678 | 0.263 | 11.54 | 2.855 | 0 | NA |
| TRINITY_DN1484_c0_g1_i2 | 1.742039665 | 0.495637695 | 0.999970678 | 1.735 | 1.695 | 0.844 | 0 | WRKY |
| TRINITY_DN4811_c0_g1_i5 | -1.7419698 | 0.519007878 | 0.999970678 | 1.244 | 0 | 0.622 | 3.398 | NA |
| TRINITY_DN15379_c0_g1_i7 | -1.74166422 | 0.504744059 | 0.999970678 | 0.69 | 0 | 0.611 | 1.619 | NA |
| TRINITY_DN4274_c0_g1_i8 | 1.741577567 | 0.680440209 | 0.999970678 | 14.87 | 0 | 0 | 4.524 | Ank |
| TRINITY_DN9642_c0_g1_i2 | -1.74146315 | 0.515104792 | 1 | 0.972 | 0 | 1.278 | 1.759 | NA |
| TRINITY_DN10869_c0_g1_i11 | 1.740282349 | 0.679872487 | 0.999970678 | 1.481 | 0 | 0 | 0.452 | Aa_trans |
| TRINITY_DN5226_c0_g1_i7 | -1.7401236 | 0.169419777 | 0.999970678 | 0.545 | 0.158 | 1.211 | 0.925 | - |
| TRINITY_DN10387_c0_g1_i1 | -1.74010339 | 0.534865195 | 0.999970678 | 1.19 | 0 | 0.378 | 3.569 | Transferase |
| TRINITY_DN1206_c0_g1_i1 | 1.739992617 | 0.195791296 | 1 | 1.998 | 4.346 | 0.933 | 0.704 | NA |
| TRINITY_DN1940_c1_g3_i1 | -1.73939255 | 0.35766299 | 0.999970678 | 0.427 | 0.857 | 0.156 | 4.112 | UBA |
| TRINITY_DN2928_c0_g1_i9 | 1.739375505 | 0.143717494 | 0.999970678 | 3.997 | 6.219 | 1.644 | 1.116 | LRR_8 |
| TRINITY_DN79_c0_g1_i26 | 1.739206894 | 0.677389209 | 0.999970678 | 2.789 | 0 | 0 | 0.855 | NA |
| TRINITY_DN2205_c0_g1_i7 | 1.738254604 | 0.317614212 | 0.999970678 | 1.117 | 1.153 | 0.533 | 0.05 | NA |
| TRINITY_DN1884_c0_g1_i3 | -1.73782 | 0.531532651 | 0.999970678 | 0 | 4.819 | 4.633 | 10.285 | Myb_DNA-bindin |
| TRINITY_DN5596_c0_g1_i1 | -1.73779921 | 0.291191783 | 0.999970678 | 0.391 | 0.049 | 0.344 | 1.086 | PHD |
| TRINITY_DN7019_c0_g1_i3 | 1.737785927 | 0.285898707 | 1 | 0.518 | 0.542 | 0.044 | 0.261 | NA |
| TRINITY_DN2152_c0_g1_i7 | -1.73728941 | 0.551012765 | 0.999970678 | 0.191 | 0.572 | 2.078 | 0 | DUF247 |
| TRINITY_DN1870_c0_g1_i1 | -1.73710173 | 0.135028034 | 0.999970678 | 0.772 | 0.335 | 1.355 | 2.031 | GST_C_6 |
| TRINITY_DN917_c1_g1_i1 | 1.737056424 | 0.311070229 | 0.999970678 | 1.063 | 1.124 | 0.511 | 0.03 | NA |
| TRINITY_DN30_c0_g1_i2 | 1.736727903 | 0.174332381 | 0.999970678 | 2.58 | 2.02 | 0.944 | 0.251 | Amino_oxidase |
| TRINITY_DN166684_c0_g1_i1 | -1.73630609 | 0.150476557 | 0.999970678 | 0.954 | 1.715 | 1.455 | 7.118 | NA |
| TRINITY_DN2983_c0_g2_i2 | -1.73624727 | 0.524582478 | 0.999970678 | 3.07 | 0 | 1.344 | 8.727 | NA |
| TRINITY_DN1676_c0_g1_i5 | 1.73615125 | 0.564714588 | 0.999970678 | 0 | 10.03 | 0.211 | 2.714 | NA |
| TRINITY_DN16469_c0_g1_i3 | 1.736134956 | 0.025298602 | 0.999970678 | 22.61 | 26.12 | 5.4 | 7.852 | NA |
| TRINITY_DN15942_c0_g1_i2 | -1.73548769 | 0.370628229 | 0.999970678 | 0.009 | 0.355 | 0.622 | 0.442 | MutS_III |
| TRINITY_DN6035_c0_g1_i1 | -1.73542137 | 0.50302287 | 0.999970678 | 3.116 | 0 | 4.855 | 4.625 | NA |
| TRINITY_DN166_c0_g1_i3 | -1.73525912 | 0.318563263 | 0.999970678 | 48.245 | 25.18 | 6.044 | 232.612 | NA |
| TRINITY_DN15373_c0_g1_i1 | 1.7352497 | 0.456499141 | 0.999970678 | 12.59 | 0.315 | 3.033 | 0.221 | NA |
| TRINITY_DN897_c2_g1_i4 | -1.7350892 | 0.173978425 | 0.999970678 | 0.263 | 0.306 | 1.133 | 0.573 | NA |
| TRINITY_DN16590_c1_g2_i1 | 1.734901657 | 0.502367165 | 1 | 3.161 | 2.681 | 0 | 1.759 | NA |
| TRINITY_DN2384_c0_g2_i8 | 1.734885959 | 0.575592829 | 0.999970678 | 4.006 | 0.089 | 0 | 1.216 | PABP |
| TRINITY_DN1877_c0_g1_i10 | -1.73474411 | 0.52279874 | 0.999970678 | 0 | 0.887 | 0.411 | 2.423 | NA |
| TRINITY_DN6148_c0_g2_i1 | -1.73455976 | 0.507898353 | 0.999970678 | 1.599 | 0 | 1.522 | 3.599 | NA |
| TRINITY_DN362_c0_g1_i3 | -1.73444555 | 0.067650838 | 0.999970678 | 3.416 | 1.419 | 8.055 | 6.485 | Bet_v_1 |
| TRINITY_DN2378_c0_g1_i3 | 1.734125209 | 0.12147767 | 0.999970678 | 0.881 | 2.464 | 0.478 | 0.432 | zf-C3HC4_3 |
| TRINITY_DN2009_c0_g1_i2 | -1.73393909 | 0.15399608 | 0.999970678 | 2.089 | 1.597 | 2.766 | 8.475 | NA |
| TRINITY_DN2597_c0_g1_i6 | -1.73319925 | 0.489048014 | 0.999970678 | 0.4 | 0.039 | 1.2 | 0.01 | NA |
| TRINITY_DN1099_c0_g1_i5 | 1.733101191 | 0.58756116 | 0.999970678 | 2.107 | 0 | 0.011 | 0.623 | RNA_pol_Rpb1_1 |
| TRINITY_DN1922_c2_g1_i2 | -1.73303956 | 0.107978383 | 0.999970678 | 1.417 | 0.483 | 3.422 | 2.212 | MFS_1 |
| TRINITY_DN10505_c0_g1_i4 | 1.732108292 | 0.136855889 | 0.999970678 | 0.536 | 1.636 | 0.344 | 0.241 | RVP_2 |
| TRINITY_DN12618_c0_g1_i1 | -1.73121936 | 0.036311643 | 0.999970678 | 8.821 | 7.628 | 18.677 | 29.92 | NA |
| TRINITY_DN2_c0_g1_i6 | 1.731199312 | 0.681330896 | 0.999970678 | 0 | 1.025 | 0.256 | 0 | NA |
| TRINITY_DN4499_c1_g1_i1 | 1.73071407 | 0.508409967 | 0.999970678 | 2.398 | 1.311 | 0.933 | 0 | NA |
| TRINITY_DN659_c0_g2_i2 | -1.73066553 | 0.25611749 | 0.999970678 | 0.99 | 0.217 | 0.644 | 3.207 | NA |
| TRINITY_DN1854_c0_g1_i3 | 1.730454254 | 0.259237181 | 0.999970678 | 3.797 | 1.301 | 1.1 | 0.261 | Pyr_redox_2 |
| TRINITY_DN6159_c0_g1_i6 | 1.730439763 | 0.084620907 | 0.999970678 | 4.451 | 3.597 | 1.489 | 0.633 | NA |
| TRINITY_DN11288_c0_g2_i1 | 1.730429313 | 0.215349794 | 0.999970678 | 0.808 | 1.252 | 0.1 | 0.503 | NA |
| TRINITY_DN3017_c0_g1_i8 | 1.729739903 | 0.2667347 | 0.999970678 | 7.794 | 0.601 | 0.867 | 1.508 | TFIID_20kDa |

| TRINITY_DN3217_c0_g1_i2 | 1.729490677 | 0.505938242 | 0.999970678 | 0.79 | 0.443 | 0.322 | 0 | Myb_CC_LHEQLE |
| --- | --- | --- | --- | --- | --- | --- | --- | --- |
| TRINITY_DN9799_c0_g1_i1 | 1.729424226 | 0.25101516 | 0.999970678 | 0.6 | 1.449 | 0.1 | 0.483 | NA |
| TRINITY_DN49937_c0_g1_i4 | -1.72931509 | 0.575645472 | 0.999970678 | 4.288 | 0 | 11.633 | 0.271 | NA |
| TRINITY_DN5461_c0_g1_i1 | 1.729312116 | 0.104476666 | 0.999970678 | 9.865 | 5.115 | 2.611 | 1.357 | NA |
| TRINITY_DN372_c0_g1_i12 | -1.72921419 | 0.523266104 | 0.999970678 | 1.463 | 0 | 0.8 | 3.861 | NA |
| TRINITY_DN5700_c0_g1_i4 | -1.72907774 | 0.176344147 | 0.999970678 | 0.981 | 0.808 | 1.411 | 4.313 | NA |
| TRINITY_DN1195_c0_g2_i3 | -1.72896852 | 0.003223191 | 0.183783767 | 8.194 | 7.47 | 21.332 | 26.14 | PfkB |
| TRINITY_DN10_c1_g1_i2 | -1.72886035 | 0.163273288 | 0.999970678 | 3.089 | 0.759 | 5.689 | 5.811 | NA |
| TRINITY_DN3721_c0_g1_i7 | 1.728694936 | 0.153738804 | 0.999970678 | 0.727 | 1.981 | 0.444 | 0.302 | NA |
| TRINITY_DN2332_c0_g1_i7 | -1.7285148 | 0.188807964 | 0.999970678 | 0.382 | 0.207 | 1.2 | 0.483 | NA |
| TRINITY_DN14880_c0_g1_i1 | -1.72827724 | 0.685091492 | 0.999970678 | 0 | 0.562 | 0 | 1.83 | Methyltransf_4 |
| TRINITY_DN2132_c2_g1_i3 | 1.72820443 | 0.171338539 | 1 | 0.772 | 1.409 | 0.289 | 0.322 | NA |
| TRINITY_DN4508_c0_g1_i4 | -1.72772601 | 0.530549535 | 0.999970678 | 2.316 | 0 | 5.489 | 1.176 | Nucleotid_trans |
| TRINITY_DN3912_c0_g2_i1 | 1.727493135 | 0.214233959 | 0.999970678 | 3.733 | 35.57 | 5.555 | 5.007 | Sugar_tr |
| TRINITY_DN16118_c0_g1_i6 | 1.726918326 | 0.564674749 | 0.999970678 | 0 | 1.143 | 0.233 | 0.05 | NA |
| TRINITY_DN80970_c0_g1_i2 | -1.72641754 | 0.172404512 | 0.999970678 | 48.172 | 18.88 | 32.687 | 174.874 | NA |
| TRINITY_DN647_c0_g4_i1 | 1.726186687 | 0.096204133 | 0.999970678 | 17.55 | 60.15 | 7.533 | 14.166 | NA |
| TRINITY_DN1862_c0_g1_i3 | -1.72595098 | 0.235081445 | 0.999970678 | 0.336 | 1.478 | 2.244 | 3.338 | NA |
| TRINITY_DN799_c0_g2_i9 | -1.72580499 | 0.076543465 | 0.999970678 | 1027.5 | 424 | 2669.35 | 1563.691 | FA_desaturase |
| TRINITY_DN373_c0_g1_i1 | 1.725784304 | 0.054415213 | 0.999970678 | 8.439 | 3.676 | 1.778 | 1.548 | NA |
| TRINITY_DN2632_c0_g1_i6 | -1.72572679 | 0.549112464 | 1 | 0 | 3.193 | 7.711 | 0.955 | NA |
| TRINITY_DN10963_c0_g1_i1 | 1.725286548 | 0.506810484 | 0.999970678 | 0.4 | 0.443 | 0.211 | 0 | NA |
| TRINITY_DN16632_c0_g1_i3 | -1.72526001 | 0.500743054 | 0.999970678 | 0 | 0.887 | 1.067 | 1.629 | NA |
| TRINITY_DN5694_c0_g1_i4 | -1.72478424 | 0.691434371 | 0.999970678 | 0 | 2.06 | 5.622 | 0 | NA |
| TRINITY_DN5168_c0_g1_i1 | 1.724573166 | 0.681437786 | 0.999970678 | 0 | 1.636 | 0.411 | 0 | Bromo_TP |
| TRINITY_DN6418_c0_g2_i14 | 1.724245922 | 0.513580618 | 1 | 1.753 | 1.567 | 0.856 | 0 | NA |
| TRINITY_DN8146_c2_g1_i7 | 1.724041418 | 0.193161901 | 0.999970678 | 16.079 | 2.809 | 2.089 | 3.237 | NA |
| TRINITY_DN18909_c0_g1_i2 | -1.72399167 | 0.163914762 | 0.999970678 | 1.244 | 2.72 | 5.033 | 6.826 | NA |
| TRINITY_DN25724_c0_g1_i1 | -1.72374021 | 0.313402056 | 0.999970678 | 0.118 | 0.818 | 0.478 | 2.483 | p450 |
| TRINITY_DN2597_c0_g1_i11 | 1.723587166 | 0.681871126 | 0.999970678 | 2.544 | 0 | 0.644 | 0 | NA |
| TRINITY_DN135_c0_g3_i2 | -1.72349302 | 0.135640694 | 0.999970678 | 1.835 | 1.961 | 8.266 | 2.503 | LRRNT_2 |
| TRINITY_DN254_c2_g1_i5 | -1.72334898 | 0.206976369 | 0.999970678 | 0.164 | 0.907 | 1.411 | 1.79 | NA |
| TRINITY_DN3272_c0_g1_i5 | -1.72324958 | 0.241724881 | 0.999970678 | 11.755 | 27.68 | 8.966 | 118.523 | NA |
| TRINITY_DN2253_c1_g1_i1 | -1.7230721 | 0.353101016 | 0.999970678 | 0.263 | 0.404 | 0.056 | 2.151 | CMAS |
| TRINITY_DN18468_c0_g1_i1 | -1.7229299 | 0.157704051 | 0.999970678 | 0.763 | 0.522 | 2.567 | 1.176 | NA |
| TRINITY_DN8570_c0_g2_i1 | -1.72239938 | 0.526108339 | 0.999970678 | 0.481 | 0 | 1 | 0.412 | NA |
| TRINITY_DN1907_c0_g1_i8 | -1.72235748 | 0.215430333 | 0.999970678 | 0.59 | 2.484 | 1.944 | 7.772 | NA |
| TRINITY_DN21359_c0_g1_i4 | 1.722242158 | 0.040719317 | 0.999970678 | 1.744 | 2.03 | 0.444 | 0.603 | AAA |
| TRINITY_DN7666_c1_g1_i5 | -1.72215669 | 0.527245561 | 0.999970678 | 0.963 | 0 | 0.611 | 2.413 | NA |
| TRINITY_DN4356_c0_g1_i9 | 1.722010538 | 0.137794549 | 0.999970678 | 0.618 | 0.857 | 0.2 | 0.211 | AAA |
| TRINITY_DN8001_c0_g1_i1 | 1.721728506 | 0.303578988 | 0.999970678 | 1.354 | 2.217 | 0.1 | 0.945 | Myb_DNA-bindin |
| TRINITY_DN834_c0_g1_i11 | 1.721609744 | 0.162735887 | 0.999970678 | 2.071 | 1.774 | 0.756 | 0.261 | NA |
| TRINITY_DN5681_c0_g1_i1 | -1.7214309 | 0.402020847 | 0.999970678 | 2.425 | 5.578 | 0.256 | 25.546 | NA |
| TRINITY_DN8115_c0_g1_i7 | 1.721379447 | 0.554132824 | 0.999970678 | 0.836 | 0.059 | 0 | 0.282 | NA |
| TRINITY_DN34025_c0_g1_i1 | -1.72090273 | 0.406963831 | 0.999970678 | 0.917 | 2.316 | 0.222 | 10.084 | NA |
| TRINITY_DN3755_c0_g1_i5 | 1.720577919 | 0.228716307 | 0.999970678 | 1.117 | 0.631 | 0.344 | 0.131 | NA |
| TRINITY_DN8761_c0_g1_i2 | 1.720281707 | 0.084314013 | 0.999970678 | 62.924 | 132.1 | 13.433 | 42.467 | NA |
| TRINITY_DN3912_c0_g2_i2 | -1.72015942 | 0.203343099 | 0.999970678 | 6.976 | 5.194 | 3.5 | 36.123 | Sugar_tr |
| TRINITY_DN12599_c0_g1_i3 | 1.720063226 | 0.682910241 | 0.999970678 | 1.363 | 0 | 0.356 | 0 | DIL |
| TRINITY_DN45_c1_g1_i1 | -1.72003659 | 0.539767147 | 0.999970678 | 7.73 | 0 | 11.744 | 11.723 | GAF |
| TRINITY_DN3459_c0_g1_i9 | -1.71963398 | 0.077122108 | 0.999970678 | 8.176 | 5.696 | 22.01 | 17.142 | NA |
| TRINITY_DN5718_c0_g4_i3 | 1.719597489 | 0.420234595 | 0.999970678 | 0.145 | 5.046 | 1.122 | 0.171 | Ferritin_2 |
| TRINITY_DN2453_c0_g1_i1 | 1.71904557 | 0.554366569 | 0.999970678 | 11.673 | 0 | 2.155 | 0.895 | RNA_pol_A_bac |
| TRINITY_DN1870_c0_g1_i2 | 1.718757579 | 0.380875204 | 0.999970678 | 1.808 | 2.178 | 0.978 | 0.02 | GST_C_6 |
| TRINITY_DN11149_c0_g1_i3 | 1.71871821 | 0.150468978 | 0.999970678 | 5.559 | 7.411 | 2.5 | 0.905 | Cupin_1 |
| TRINITY_DN798_c0_g2_i9 | 1.718562962 | 0.558720936 | 0.999970678 | 0 | 0.986 | 0.056 | 0.231 | NA |
| TRINITY_DN2023_c0_g2_i3 | -1.71845199 | 0.136142507 | 0.999970678 | 2.416 | 1.035 | 2.6 | 8.335 | NA |
| TRINITY_DN1720_c1_g1_i5 | 1.718099098 | 0.497037001 | 0.999970678 | 0.881 | 0.966 | 0 | 0.563 | NA |
| TRINITY_DN3298_c0_g1_i1 | -1.71799246 | 0.046001969 | 0.999970678 | 2.843 | 3.568 | 11.544 | 7.028 | - |
| TRINITY_DN5051_c0_g1_i3 | -1.71797391 | 0.252273108 | 0.999970678 | 2.78 | 0.572 | 2.467 | 7.963 | NA |
| TRINITY_DN2582_c0_g1_i1 | -1.7178414 | 0.510947089 | 0.999970678 | 0 | 1.133 | 2.078 | 1.186 | NA |
| TRINITY_DN5686_c0_g4_i1 | 1.717758849 | 0.563492804 | 0.999970678 | 0.336 | 10.74 | 0 | 3.278 | NA |
| TRINITY_DN9138_c0_g1_i1 | 1.717723144 | 0.297179837 | 0.999970678 | 0.418 | 5.489 | 0.467 | 1.216 | LRRNT_2 |
| TRINITY_DN5139_c0_g3_i1 | -1.71766312 | 0.11480686 | 0.999970678 | 7.403 | 12 | 10.977 | 50.409 | NA |
| TRINITY_DN5889_c0_g1_i11 | 1.717584974 | 0.567068918 | 0.999970678 | 0 | 1.173 | 0.244 | 0.05 | NA |
| TRINITY_DN7811_c0_g1_i6 | 1.717540498 | 0.180325221 | 0.999970678 | 6.004 | 4.08 | 1.789 | 0.845 | NA |
| TRINITY_DN19040_c0_g1_i2 | -1.71753282 | 0.17400255 | 0.999970678 | 1.98 | 1.666 | 7.944 | 2.534 | NA |
| TRINITY_DN799_c0_g1_i1 | 1.717020292 | 0.010998466 | 0.52689423 | 226.73 | 175.5 | 41.286 | 73.03 | FA_desaturase |
| TRINITY_DN703_c0_g1_i9 | 1.716652714 | 0.549852805 | 0.999970678 | 19.467 | 1.853 | 5.433 | 0 | NA |
| TRINITY_DN961_c0_g2_i3 | 1.716641551 | 0.515922749 | 0.999970678 | 9.247 | 3.676 | 0 | 3.971 | NA |
| TRINITY_DN2291_c0_g1_i4 | -1.71659483 | 0.682941619 | 0.999970678 | 0 | 0.986 | 0 | 3.197 | Hexapep |
| TRINITY_DN46665_c2_g1_i2 | 1.71637023 | 0.513954518 | 0.999970678 | 0.581 | 1.232 | 0.467 | 0 | NA |
| TRINITY_DN8326_c0_g1_i9 | 1.716305728 | 0.135323681 | 0.999970678 | 0.418 | 0.621 | 0.156 | 0.121 | ABC_tran |
| TRINITY_DN9824_c0_g1_i1 | 1.716186038 | 0.174185064 | 0.999970678 | 0.89 | 2.582 | 0.544 | 0.402 | NA |

| TRINITY_DN665_c0_g1_i12 | 1.716011897 | 0.265780712 | 0.999970678 | 5.305 | 3.794 | 2.078 | 0.292 | LRR_8 |
| --- | --- | --- | --- | --- | --- | --- | --- | --- |
| TRINITY_DN9719_c0_g1_i1 | 1.715998411 | 0.331379966 | 0.999970678 | 4.515 | 5.627 | 2.478 | 0.09 | NA |
| TRINITY_DN441_c1_g1_i1 | 1.715933009 | 0.164137342 | 0.999970678 | 3.543 | 0.946 | 0.356 | 0.955 | Ion_trans_2 |
| TRINITY_DN188_c0_g2_i1 | -1.71551468 | 0.118300207 | 0.999970678 | 33.338 | 62.26 | 53.53 | 247.763 | NA |
| TRINITY_DN1621_c0_g1_i3 | 1.71506948 | 0.565577677 | 0.999970678 | 7.249 | 0.187 | 0 | 2.302 | ERCC3_RAD25_C |
| TRINITY_DN1151_c1_g1_i3 | -1.71495621 | 0.147326825 | 0.999970678 | 1.154 | 0.611 | 1.155 | 4.424 | NA |
| TRINITY_DN19836_c0_g1_i6 | -1.71493511 | 0.508148348 | 0.999970678 | 0 | 0.779 | 1.078 | 1.196 | XH |
| TRINITY_DN1207_c0_g1_i7 | 1.714480409 | 0.684616029 | 0.999970678 | 3.252 | 0 | 0.844 | 0 | Ank_2 |
| TRINITY_DN2008_c0_g1_i3 | -1.71421061 | 0.310743236 | 0.999970678 | 0.781 | 8.426 | 3.255 | 25.737 | NA |
| TRINITY_DN24_c0_g2_i5 | -1.71416657 | 0.459700269 | 0.999970678 | 0.5 | 27.38 | 1.278 | 88.151 | - |
| TRINITY_DN6635_c1_g1_i1 | 1.714002502 | 0.377669199 | 0.999970678 | 1.889 | 2.71 | 1.122 | 0.04 | NA |
| TRINITY_DN3717_c0_g1_i1 | -1.71365293 | 0.691838494 | 0.999970678 | 20.094 | 0 | 55.23 | 0 | Peroxidase_2 |
| TRINITY_DN14_c0_g1_i1 | 1.713324589 | 0.043260037 | 0.999970678 | 9.992 | 8.751 | 3.255 | 1.8 | Gelsolin |
| TRINITY_DN698_c1_g1_i16 | -1.71243436 | 0.52274816 | 0.999970678 | 1.744 | 0 | 1 | 4.595 | NA |
| TRINITY_DN1227_c0_g1_i1 | -1.71238091 | 0.541022099 | 0.999970678 | 36.499 | 0 | 15.588 | 99.873 | NA |
| TRINITY_DN9204_c1_g1_i4 | 1.712300514 | 0.148876231 | 0.999970678 | 0.454 | 0.375 | 0.111 | 0.121 | BTB |
| TRINITY_DN3076_c0_g1_i9 | 1.711832586 | 0.681476639 | 0.999970678 | 11.909 | 0 | 0 | 3.68 | GrpE |
| TRINITY_DN1422_c4_g1_i3 | -1.71180179 | 0.017341276 | 0.754426342 | 1.889 | 2.326 | 5.789 | 6.796 | CDI |
| TRINITY_DN1767_c0_g1_i4 | 1.711685592 | 0.172833724 | 0.999970678 | 0.709 | 2.809 | 0.444 | 0.523 | NA |
| TRINITY_DN13654_c0_g1_i6 | 1.711491034 | 0.456382253 | 0.999970678 | 9.093 | 0.296 | 2.222 | 0.201 | NA |
| TRINITY_DN16151_c0_g3_i2 | 1.711420123 | 0.106084766 | 0.999970678 | 0.327 | 0.828 | 0.144 | 0.191 | DUF4283 |
| TRINITY_DN1250_c0_g1_i1 | -1.71133791 | 0.266368414 | 0.999970678 | 1.853 | 0.542 | 1.189 | 6.545 | NA |
| TRINITY_DN81297_c0_g1_i5 | -1.71126521 | 0.584099145 | 0.999970678 | 0.999 | 0 | 2.6 | 0.09 | NA |
| TRINITY_DN5272_c0_g2_i3 | 1.711249393 | 0.5099173 | 1 | 0.79 | 0.512 | 0 | 0.392 | DUF3007 |
| TRINITY_DN6802_c0_g2_i8 | 1.711130462 | 0.684529214 | 0.999970678 | 18.231 | 0 | 4.633 | 0 | - |
| TRINITY_DN6013_c0_g1_i1 | 1.710751564 | 0.314122265 | 0.999970678 | 13.671 | 11.43 | 6.022 | 0.382 | ABC2_membrane |
| TRINITY_DN4217_c0_g1_i2 | 1.710569567 | 0.685015163 | 0.999970678 | 0 | 2.434 | 0 | 0.734 | Cation_ATPase |
| TRINITY_DN5887_c0_g2_i1 | 1.710472913 | 0.296973721 | 0.999970678 | 39.388 | 12.06 | 0.789 | 14.769 | NA |
| TRINITY_DN5741_c0_g1_i3 | -1.71022808 | 0.233543118 | 1 | 0.127 | 0.375 | 0.633 | 0.834 | NA |
| TRINITY_DN3471_c0_g1_i2 | 1.71017774 | 0.092885376 | 0.999970678 | 8.984 | 5.46 | 1.055 | 3.127 | HLH |
| TRINITY_DN5061_c0_g1_i2 | -1.7101736 | 0.034385112 | 0.999970678 | 60.118 | 39.63 | 176.056 | 114.019 | PEX11 |
| TRINITY_DN14057_c0_g1_i1 | 1.710027193 | 0.520928553 | 0.999970678 | 0.663 | 0.246 | 0.222 | 0 | BSD |
| TRINITY_DN7687_c0_g1_i1 | -1.70998852 | 0.159820027 | 0.999970678 | 0.273 | 0.345 | 1.255 | 0.493 | NA |
| TRINITY_DN3237_c0_g1_i3 | -1.70988869 | 0.524583437 | 0.999970678 | 8.03 | 0 | 11.255 | 13.03 | Linker_histone |
| TRINITY_DN7916_c0_g1_i2 | 1.70983149 | 0.568916617 | 0.999970678 | 0 | 13.4 | 0.278 | 3.649 | NA |
| TRINITY_DN269_c0_g1_i13 | 1.709421304 | 0.294192839 | 0.999970678 | 4.615 | 0.808 | 1.122 | 0.322 | ArgoL1 |
| TRINITY_DN1255_c0_g1_i7 | -1.70937141 | 0.047472006 | 0.999970678 | 1.299 | 1.015 | 2.055 | 5.117 | Mur_ligase_M |
| TRINITY_DN40507_c0_g1_i12 | -1.70937084 | 0.692796084 | 0.999970678 | 0.481 | 0 | 1.322 | 0 | TRP |
| TRINITY_DN114_c0_g1_i1 | 1.709148108 | 0.05180599 | 0.999970678 | 11.555 | 7.648 | 2.733 | 2.473 | NA |
| TRINITY_DN2563_c0_g3_i9 | 1.709145407 | 0.526942248 | 0.999970678 | 13.898 | 12.66 | 0 | 8.154 | RRM_1 |
| TRINITY_DN1860_c0_g2_i1 | -1.70912871 | 0.353147028 | 0.999970678 | 1.363 | 2.7 | 0.5 | 12.175 | Phi_1 |
| TRINITY_DN1955_c0_g3_i3 | 1.709023598 | 0.296585318 | 0.999970678 | 0.772 | 4.149 | 0.189 | 1.257 | NA |
| TRINITY_DN2252_c0_g1_i4 | -1.70887914 | 0.282595221 | 0.999970678 | 0.254 | 1.853 | 1.767 | 4.514 | DUF561 |
| TRINITY_DN35456_c1_g2_i2 | 1.708624644 | 0.315294951 | 1 | 0.681 | 0.611 | 0.3 | 0.05 | PTR2 |
| TRINITY_DN12144_c0_g1_i3 | 1.708489918 | 0.194598995 | 0.999970678 | 0.999 | 0.65 | 0.089 | 0.382 | NA |
| TRINITY_DN38936_c0_g1_i1 | -1.70830916 | 0.697150093 | 1 | 0.936 | 0 | 2.555 | 0 | NA |
| TRINITY_DN9892_c0_g2_i3 | 1.70821894 | 0.510460657 | 0.999970678 | 1.145 | 1.685 | 0.722 | 0 | NA |
| TRINITY_DN1118_c1_g1_i1 | -1.70821663 | 0.468126878 | 0.999970678 | 0.064 | 24.71 | 8.133 | 69.33 | NA |
| TRINITY_DN8099_c0_g1_i2 | 1.708189736 | 0.514638694 | 1 | 0.654 | 0.384 | 0 | 0.322 | - |
| TRINITY_DN546_c0_g1_i4 | -1.70815865 | 0.211579277 | 0.999970678 | 0.509 | 1.261 | 4.011 | 0.895 | ATP_bind_1 |
| TRINITY_DN4093_c0_g1_i7 | 1.707263618 | 0.178611307 | 0.999970678 | 1.844 | 0.739 | 0.511 | 0.181 | - |
| TRINITY_DN2809_c0_g4_i3 | -1.70723486 | 0.540100239 | 0.999970678 | 4.469 | 0 | 4.977 | 8.867 | COPI_C |
| TRINITY_DN7463_c0_g1_i6 | 1.707113461 | 0.427404266 | 0.999970678 | 140.44 | 1.212 | 32.387 | 4.816 | NA |
| TRINITY_DN26085_c0_g1_i5 | 1.707007615 | 0.516915184 | 0.999970678 | 0.254 | 0.473 | 0.178 | 0 | DEAD |
| TRINITY_DN9141_c2_g1_i8 | 1.706229232 | 0.688265743 | 0.999970678 | 0 | 0.956 | 0 | 0.282 | NA |
| TRINITY_DN3313_c0_g1_i2 | -1.70579525 | 0.158603715 | 0.999970678 | 0.291 | 1.212 | 2.467 | 1.83 | Sugar_tr |
| TRINITY_DN18724_c0_g1_i1 | -1.70569963 | 0.524251168 | 0.999970678 | 2.226 | 0 | 1.378 | 5.7 | DUF707 |
| TRINITY_DN6638_c0_g1_i2 | 1.705574324 | 0.687615624 | 0.999970678 | 0 | 1.981 | 0.489 | 0 | GRAS |
| TRINITY_DN2424_c0_g1_i3 | -1.70527462 | 0.510846808 | 0.999970678 | 5.16 | 0 | 7.855 | 7.47 | NA |
| TRINITY_DN1626_c0_g1_i8 | -1.7050563 | 0.168633749 | 0.999970678 | 0.327 | 0.848 | 1.9 | 1.558 | NA |
| TRINITY_DN15379_c0_g1_i3 | 1.704442284 | 0.142725094 | 0.999970678 | 0.709 | 1.616 | 0.4 | 0.241 | NA |
| TRINITY_DN10592_c0_g1_i14 | -1.70413343 | 0.583600548 | 0.999970678 | 0.445 | 0.02 | 0 | 1.578 | NA |
| TRINITY_DN918_c0_g2_i5 | 1.703581433 | 0.532516561 | 0.999970678 | 0.273 | 1.557 | 0 | 0.553 | EF-hand_1 |
| TRINITY_DN33964_c0_g1_i3 | -1.70349371 | 0.535017111 | 0.999970678 | 0 | 1.271 | 2.911 | 0.513 | NA |
| TRINITY_DN1390_c0_g1_i2 | -1.70331748 | 0.551773787 | 0.999970678 | 0 | 35.15 | 37.331 | 67.591 | ECH_1 |
| TRINITY_DN1607_c0_g1_i2 | -1.70329661 | 0.533767615 | 0.999970678 | 0 | 4.149 | 3.644 | 8.918 | Sugar_tr |
| TRINITY_DN11421_c0_g1_i12 | 1.703135695 | 0.091385534 | 0.999970678 | 3.788 | 2.336 | 0.989 | 0.684 | HLH |
| TRINITY_DN3894_c0_g1_i5 | 1.702997424 | 0.348009561 | 0.999970678 | 2.607 | 0.079 | 0.456 | 0.292 | - |
| TRINITY_DN5355_c0_g1_i3 | 1.70286543 | 0.683477892 | 0.999970678 | 6.295 | 0 | 0 | 1.971 | OPT |
| TRINITY_DN3007_c0_g2_i4 | 1.702678885 | 0.097038258 | 0.999970678 | 4.66 | 13.25 | 3.022 | 1.82 | DBC1 |
| TRINITY_DN17586_c0_g1_i6 | -1.70254366 | 0.691659468 | 0.999970678 | 0 | 0.404 | 1.055 | 0 | RabGAP-TBC |
| TRINITY_DN10899_c0_g1_i2 | 1.702294625 | 0.683461061 | 0.999970678 | 8.103 | 0 | 0 | 2.534 | Peptidase_S28 |
| TRINITY_DN1377_c0_g1_i3 | -1.70227816 | 0.344366586 | 0.999970678 | 0.836 | 0.099 | 0.389 | 2.634 | SpoU_methylase |
| TRINITY_DN2813_c0_g1_i8 | -1.70212331 | 0.683639109 | 0.999970678 | 0.89 | 0 | 0 | 2.956 | NA |

| TRINITY_DN3124_c0_g1_i8 | 1.701857882 | 0.012341198 | 0.577335532 | 6.332 | 6.14 | 1.855 | 1.588 | DEAD |
| --- | --- | --- | --- | --- | --- | --- | --- | --- |
| TRINITY_DN5331_c0_g3_i1 | 1.701781008 | 0.694308586 | 0.999970678 | 0 | 4.602 | 1.144 | 0 | NA |
| TRINITY_DN587_c0_g1_i8 | 1.701717447 | 0.551114389 | 0.999970678 | 1.917 | 0 | 0.178 | 0.382 | NA |
| TRINITY_DN6651_c0_g2_i3 | 1.701636844 | 0.508555277 | 0.999970678 | 1.817 | 1.429 | 0.833 | 0 | DUF4283 |
| TRINITY_DN11705_c0_g1_i3 | -1.70116778 | 0.215672241 | 0.999970678 | 1.045 | 0.69 | 3.922 | 0.865 | - |
| TRINITY_DN1474_c1_g1_i2 | 1.701034449 | 0.684277757 | 0.999970678 | 0 | 4.248 | 1.067 | 0 | NA |
| TRINITY_DN8089_c0_g1_i2 | -1.70100517 | 0.237016687 | 0.999970678 | 0.472 | 0.138 | 0.656 | 1.227 | AA_permease_2 |
| TRINITY_DN18831_c0_g1_i4 | -1.70039714 | 0.186974965 | 0.999970678 | 0.2 | 0.473 | 0.933 | 1.116 | NA |
| TRINITY_DN4353_c1_g1_i1 | 1.700257281 | 0.199485259 | 0.999970678 | 0.672 | 3.518 | 0.578 | 0.573 | XPG_I |
| TRINITY_DN536_c0_g1_i7 | 1.699851801 | 0.393566157 | 0.999970678 | 2.126 | 6.958 | 2.267 | 0.04 | NA |
| TRINITY_DN344_c1_g1_i5 | -1.69981892 | 0.09150921 | 0.999970678 | 5.487 | 6.465 | 23.743 | 9.802 | NA |
| TRINITY_DN14867_c0_g1_i4 | -1.69977485 | 0.148978396 | 0.999970678 | 0.254 | 0.503 | 0.833 | 1.418 | NA |
| TRINITY_DN2118_c0_g1_i1 | -1.699658 | 0.685003044 | 0.999970678 | 11.528 | 0 | 31.054 | 0 | NA |
| TRINITY_DN6750_c0_g1_i7 | -1.69946013 | 0.112776922 | 0.999970678 | 1.163 | 3.745 | 5.144 | 9.601 | NA |
| TRINITY_DN25279_c0_g1_i4 | 1.699427528 | 0.532593897 | 0.999970678 | 2.934 | 0.66 | 0.922 | 0 | Bromodomain |
| TRINITY_DN41416_c0_g1_i1 | -1.69900661 | 0.285127252 | 0.999970678 | 1.581 | 3.814 | 2.111 | 14.226 | - |
| TRINITY_DN29298_c0_g1_i1 | -1.69890627 | 0.28240627 | 0.999970678 | 0.064 | 0.335 | 0.411 | 0.754 | NA |
| TRINITY_DN2985_c0_g1_i3 | -1.69854724 | 0.51002792 | 0.999970678 | 0 | 0.788 | 0.744 | 1.649 | NA |
| TRINITY_DN10181_c0_g1_i10 | 1.6984663 | 0.112850675 | 0.999970678 | 1.172 | 1.38 | 0.456 | 0.231 | DUF4283 |
| TRINITY_DN17351_c0_g4_i2 | -1.69837856 | 0.698008526 | 1 | 0 | 1.34 | 3.511 | 0 | NA |
| TRINITY_DN761_c0_g1_i4 | -1.69818069 | 0.20133341 | 0.999970678 | 78.149 | 11.11 | 160.89 | 96.887 | NA |
| TRINITY_DN10970_c0_g1_i1 | -1.69805892 | 0.204282165 | 0.999970678 | 2.017 | 0.907 | 1.5 | 7.711 | - |
| TRINITY_DN2818_c0_g1_i2 | 1.697532138 | 0.019404775 | 0.824640132 | 19.367 | 22.3 | 3.944 | 8.093 | NA |
| TRINITY_DN1282_c0_g1_i3 | -1.69747483 | 0.28772857 | 0.999970678 | 0.127 | 0.828 | 1.422 | 1.327 | Ribosomal_L4 |
| TRINITY_DN1384_c0_g1_i11 | 1.697179322 | 0.539969936 | 0.999970678 | 2.961 | 0.325 | 0 | 1.025 | NA |
| TRINITY_DN7260_c0_g1_i15 | 1.697025313 | 0.691557853 | 0.999970678 | 0 | 5.213 | 1.311 | 0 | NA |
| TRINITY_DN10986_c0_g1_i1 | 1.69701436 | 0.54718934 | 0.999970678 | 0 | 1.419 | 0.222 | 0.161 | LRRNT_2 |
| TRINITY_DN19740_c0_g1_i3 | 1.696897775 | 0.505721541 | 0.999970678 | 2.126 | 2.878 | 0 | 1.518 | - |
| TRINITY_DN7131_c0_g1_i1 | 1.696367533 | 0.575846011 | 0.999970678 | 0.091 | 2.661 | 0.711 | 0 | NA |
| TRINITY_DN5837_c0_g1_i2 | 1.696351969 | 0.082742071 | 0.999970678 | 2.907 | 4.967 | 1.422 | 0.704 | NA |
| TRINITY_DN800_c0_g1_i1 | -1.69603557 | 0.164896922 | 0.999970678 | 5.169 | 3.972 | 19.177 | 6.203 | NA |
| TRINITY_DN9604_c0_g1_i1 | -1.69584005 | 0.684565417 | 0.999970678 | 0 | 108 | 269.75 | 0 | NA |
| TRINITY_DN2622_c0_g1_i2 | -1.69581097 | 0.529190455 | 0.999970678 | 3.252 | 0 | 2.233 | 8.023 | Hydrolase_4 |
| TRINITY_DN3042_c0_g1_i7 | 1.695720277 | 0.692970426 | 0.999970678 | 0.89 | 0 | 0.233 | 0 | NA |
| TRINITY_DN13703_c0_g1_i2 | 1.695673772 | 0.688856646 | 0.999970678 | 0 | 29.09 | 0 | 8.365 | ADH_N |
| TRINITY_DN13377_c0_g2_i3 | 1.695551033 | 0.131575527 | 0.999970678 | 2.752 | 2.139 | 1 | 0.312 | NA |
| TRINITY_DN376_c0_g1_i2 | -1.69500986 | 0.277179395 | 0.999970678 | 0.227 | 1.439 | 0.689 | 4.544 | Pkinase |
| TRINITY_DN1248_c2_g1_i5 | 1.694866578 | 0.501662021 | 0.999970678 | 851.1 | 3.341 | 3.678 | 254.258 | Inhibitor_I29 |
| TRINITY_DN5658_c0_g1_i1 | 1.69405773 | 0.620538987 | 0.999970678 | 0 | 8.426 | 2.111 | 0.01 | DSPc |
| TRINITY_DN2554_c0_g2_i4 | -1.69371422 | 0.522656055 | 0.999970678 | 0 | 6.455 | 7.655 | 11.351 | NA |
| TRINITY_DN1698_c0_g2_i3 | 1.693526042 | 0.557520838 | 0.999970678 | 49.244 | 3.193 | 13.066 | 0 | NA |
| TRINITY_DN7749_c0_g1_i5 | 1.69301356 | 0.23115464 | 1 | 0.436 | 0.296 | 0.056 | 0.171 | NA |
| TRINITY_DN8314_c1_g1_i1 | -1.69289679 | 0.695159005 | 0.999970678 | 2.434 | 0 | 6.466 | 0 | NA |
| TRINITY_DN8879_c0_g1_i4 | 1.692766608 | 0.296282015 | 0.999970678 | 3.434 | 1.419 | 0.089 | 1.408 | NA |
| TRINITY_DN8680_c0_g2_i2 | -1.69241599 | 0.141391755 | 0.999970678 | 1.562 | 1.192 | 4.466 | 3.237 | Histone |
| TRINITY_DN7880_c0_g1_i1 | 1.692308681 | 0.231479856 | 0.999970678 | 1.281 | 9.914 | 1.567 | 1.528 | NA |
| TRINITY_DN12764_c0_g1_i3 | 1.692232077 | 0.194498388 | 1 | 2.616 | 2.375 | 0.789 | 0.523 | NA |
| TRINITY_DN2962_c0_g1_i2 | 1.692086142 | 0.081889307 | 0.999970678 | 2.38 | 3.794 | 0.889 | 0.845 | R3H |
| TRINITY_DN3041_c0_g1_i1 | -1.69195919 | 0.564243165 | 0.999970678 | 0 | 19.17 | 44.342 | 7.329 | La |
| TRINITY_DN9857_c0_g1_i1 | -1.69175629 | 0.266190927 | 0.999970678 | 2.507 | 1.616 | 9.699 | 1.558 | NA |
| TRINITY_DN10578_c0_g1_i18 | 1.691267669 | 0.264841398 | 0.999970678 | 3.752 | 2.503 | 1.433 | 0.241 | NA |
| TRINITY_DN22148_c0_g1_i1 | -1.69125149 | 0.106939895 | 0.999970678 | 1.054 | 1.202 | 4.089 | 2.373 | NA |
| TRINITY_DN548_c0_g3_i1 | 1.690701947 | 0.182963421 | 0.999970678 | 0.654 | 0.266 | 0.144 | 0.121 | HMA |
| TRINITY_DN99_c1_g1_i3 | 1.690618012 | 0.173076169 | 0.999970678 | 24.863 | 165.5 | 23.432 | 29.88 | adh_short |
| TRINITY_DN2212_c0_g2_i16 | 1.690393951 | 0.691705851 | 0.999970678 | 0 | 0.641 | 0.167 | 0 | RRM_1 |
| TRINITY_DN3165_c0_g1_i4 | 1.690297197 | 0.087347864 | 0.999970678 | 9.711 | 11.27 | 1.678 | 4.313 | NA |
| TRINITY_DN2924_c0_g1_i8 | 1.690111958 | 0.294101342 | 0.999970678 | 0.254 | 3.321 | 0.344 | 0.674 | B_lectin |
| TRINITY_DN9131_c0_g1_i2 | -1.68949663 | 0.604687279 | 0.999970678 | 0.6 | 0 | 1.578 | 0.02 | NA |
| TRINITY_DN387_c0_g1_i9 | 1.689362349 | 0.472426546 | 0.999970678 | 0.045 | 10.77 | 0.467 | 2.745 | NA |
| TRINITY_DN4839_c0_g1_i1 | -1.68801717 | 0.300723861 | 0.999970678 | 0.236 | 0.828 | 0.333 | 3.006 | NA |
| TRINITY_DN7007_c1_g1_i1 | -1.68776334 | 0.214000994 | 1 | 0.309 | 0.808 | 1.411 | 1.86 | NA |
| TRINITY_DN1740_c0_g1_i8 | 1.687744344 | 0.265383047 | 0.999970678 | 1.535 | 2.622 | 0.978 | 0.101 | RVT_1 |
| TRINITY_DN947_c0_g1_i5 | -1.68774379 | 0.298416119 | 0.999970678 | 0.745 | 0.286 | 0.356 | 2.936 | NA |
| TRINITY_DN2558_c0_g1_i24 | -1.68759864 | 0.692715124 | 0.999970678 | 3.161 | 0 | 8.566 | 0 | Abhydrolase_1 |
| TRINITY_DN7020_c0_g1_i2 | 1.68731351 | 0.15903705 | 0.999970678 | 1.862 | 3.903 | 1.155 | 0.382 | RRM_1 |
| TRINITY_DN3846_c0_g1_i3 | 1.686119421 | 0.565128436 | 0.999970678 | 0 | 38.07 | 6.166 | 4.182 | TPR_12 |
| TRINITY_DN961_c0_g2_i2 | -1.68592226 | 0.519665638 | 0.999970678 | 0 | 3.558 | 4.622 | 5.7 | NA |
| TRINITY_DN12_c2_g1_i5 | 1.685736586 | 0.511838881 | 0.999970678 | 1.072 | 0.788 | 0.478 | 0 | NA |
| TRINITY_DN5562_c0_g1_i6 | -1.68554548 | 0.555921586 | 0.999970678 | 0 | 8.702 | 5.489 | 21.042 | 60KD_IMP |
| TRINITY_DN5251_c0_g1_i1 | -1.68553759 | 0.040795332 | 0.999970678 | 1.226 | 1.409 | 4.344 | 3.177 | GRAS |
| TRINITY_DN27278_c0_g1_i2 | 1.68504258 | 0.258417523 | 0.999970678 | 1.572 | 1.183 | 0.633 | 0.121 | Pro_isomerase |
| TRINITY_DN7865_c0_g3_i1 | 1.684797116 | 0.370360799 | 0.999970678 | 7.031 | 1.222 | 1.989 | 0.191 | LRRNT_2 |
| TRINITY_DN8521_c5_g1_i1 | 1.684460956 | 0.697018745 | 0.999970678 | 6.777 | 0 | 1.755 | 0 | Pro_isomerase |
| TRINITY_DN8972_c1_g1_i1 | -1.68392512 | 0.387484558 | 0.999970678 | 3.806 | 0.759 | 11.877 | 0.382 | NA |

| TRINITY_DN362_c0_g2_i4 | -1.68340513 | 0.395040378 | 0.999970678 | 4.606 | 0.059 | 7.655 | 5.942 | NA |
| --- | --- | --- | --- | --- | --- | --- | --- | --- |
| TRINITY_DN4562_c1_g2_i8 | 1.683322362 | 0.573012854 | 0.999970678 | 2.098 | 0 | 0.444 | 0.121 | NA |
| TRINITY_DN9134_c0_g1_i2 | -1.68310804 | 0.224273294 | 0.999970678 | 1.535 | 0.325 | 2.1 | 3.579 | - |
| TRINITY_DN808_c3_g1_i4 | -1.68292484 | 0.264412958 | 0.999970678 | 0.554 | 0.069 | 0.489 | 1.448 | RVT_1 |
| TRINITY_DN485_c0_g1_i3 | 1.682787238 | 0.547612862 | 0.999970678 | 33.756 | 26.93 | 15.643 | 0 | NA |
| TRINITY_DN2784_c1_g1_i1 | -1.6824275 | 0.538861192 | 0.999970678 | 0 | 4.514 | 1.7 | 12.205 | NA |
| TRINITY_DN5651_c2_g1_i2 | -1.68226509 | 0.570864803 | 0.999970678 | 0 | 1.606 | 3.944 | 0.312 | GDP_Man_Dehyd |
| TRINITY_DN3062_c0_g1_i7 | -1.6819829 | 0.225619889 | 0.999970678 | 0.627 | 2.217 | 1.489 | 7.259 | RraA-like |
| TRINITY_DN4208_c0_g2_i1 | 1.681936381 | 0.690841336 | 0.999970678 | 9.375 | 0 | 0 | 2.926 | NA |
| TRINITY_DN4596_c0_g5_i1 | -1.68187048 | 0.534495589 | 0.999970678 | 0 | 0.591 | 1.278 | 0.322 | Choline_transpo |
| TRINITY_DN9020_c0_g1_i5 | -1.68178886 | 0.011258716 | 0.535436368 | 545.63 | 647.4 | 1874.642 | 1447.591 | Chloroa_b-bind |
| TRINITY_DN3717_c0_g1_i2 | -1.68128544 | 0.691082427 | 0.999970678 | 2.089 | 0 | 5.644 | 0 | Peroxidase_2 |
| TRINITY_DN6098_c0_g1_i6 | -1.68128544 | 0.691082427 | 0.999970678 | 0.554 | 0 | 1.522 | 0 | DUF4449 |
| TRINITY_DN1641_c0_g1_i15 | -1.68112674 | 0.521832385 | 0.999970678 | 0.99 | 0 | 0.878 | 2.172 | NA |
| TRINITY_DN79_c0_g1_i21 | 1.681095685 | 0.227178235 | 0.999970678 | 0.382 | 1.646 | 0.333 | 0.231 | NA |
| TRINITY_DN10309_c0_g1_i5 | 1.680070099 | 0.132963766 | 0.999970678 | 0.718 | 0.887 | 0.111 | 0.372 | JmjC |
| TRINITY_DN2269_c0_g2_i2 | -1.67964973 | 0.348722119 | 0.999970678 | 1.799 | 2.917 | 11.755 | 0.724 | NA |
| TRINITY_DN13061_c0_g1_i4 | 1.679562447 | 0.578311307 | 0.999970678 | 0 | 1.439 | 0.056 | 0.372 | NA |
| TRINITY_DN1212_c0_g1_i11 | -1.67945463 | 0.700628964 | 0.999970678 | 0 | 6.465 | 0 | 20.51 | NA |
| TRINITY_DN10062_c0_g1_i2 | -1.67931964 | 0.520565604 | 0.999970678 | 6.45 | 0 | 7.366 | 11.924 | NA |
| TRINITY_DN6932_c0_g1_i7 | -1.67842655 | 0.530747652 | 1 | 0 | 1.192 | 1.667 | 1.82 | NA |
| TRINITY_DN519_c1_g1_i1 | 1.67838119 | 0.223637658 | 0.999970678 | 2.58 | 3.824 | 0.233 | 1.729 | BPL_LplA_LipB |
| TRINITY_DN9533_c0_g1_i5 | -1.67803922 | 0.329793074 | 0.999970678 | 231.89 | 8.909 | 480.238 | 197.123 | Ribosomal_L12 |
| TRINITY_DN591_c0_g1_i4 | 1.677987122 | 0.522560713 | 0.999970678 | 0.818 | 0.473 | 0.333 | 0 | NA |
| TRINITY_DN1153_c0_g1_i2 | 1.677833126 | 0.35466115 | 0.999970678 | 0.59 | 27.94 | 3.311 | 4.776 | NA |
| TRINITY_DN343_c1_g1_i4 | 1.677803412 | 0.516176012 | 0.999970678 | 9.52 | 5.47 | 3.866 | 0.03 | Glutaredoxin |
| TRINITY_DN13755_c0_g1_i6 | 1.677658615 | 0.513648542 | 0.999970678 | 22.719 | 9.826 | 0 | 9.802 | NA |
| TRINITY_DN583_c0_g2_i1 | -1.67757055 | 0.540827273 | 0.999970678 | 0 | 0.581 | 1.267 | 0.312 | NA |
| TRINITY_DN3472_c0_g1_i1 | -1.67748649 | 0.530818361 | 0.999970678 | 6.268 | 0 | 9.244 | 9.169 | - |
| TRINITY_DN42420_c0_g3_i5 | -1.67697259 | 0.51755386 | 0.999970678 | 0 | 1.961 | 1.855 | 3.901 | DSPc |
| TRINITY_DN13121_c0_g1_i1 | -1.6767772 | 0.688502683 | 0.999970678 | 1.635 | 0 | 0 | 5.298 | NA |
| TRINITY_DN8444_c0_g1_i3 | 1.676649016 | 0.292758453 | 0.999970678 | 0.372 | 3.706 | 0.589 | 0.563 | A_thal_3526 |
| TRINITY_DN7662_c0_g1_i7 | 1.676487648 | 0.224882711 | 1 | 3.906 | 3.302 | 1.244 | 0.784 | Aldedh |
| TRINITY_DN725_c1_g1_i8 | 1.676051144 | 0.514742287 | 0.999970678 | 0.845 | 1.232 | 0.533 | 0 | EloA-BP1 |
| TRINITY_DN6307_c0_g1_i2 | -1.67587508 | 0.196043636 | 0.999970678 | 7.04 | 1.537 | 7.688 | 18.026 | - |
| TRINITY_DN6050_c1_g1_i6 | 1.67579031 | 0.563616221 | 0.999970678 | 4.606 | 0.256 | 1.278 | 0 | LRRNT_2 |
| TRINITY_DN461_c1_g1_i7 | -1.67573894 | 0.323041939 | 0.999970678 | 0.999 | 8.614 | 2.367 | 27.567 | LisH |
| TRINITY_DN19130_c0_g1_i3 | -1.67557976 | 0.529193912 | 0.999970678 | 1.181 | 0 | 2.044 | 1.347 | NA |
| TRINITY_DN410_c0_g1_i9 | -1.67537783 | 0.536718359 | 0.999970678 | 5.223 | 0 | 2.3 | 13.904 | NA |
| TRINITY_DN4087_c0_g1_i1 | -1.6753159 | 0.310565702 | 0.999970678 | 0.827 | 18.84 | 33.387 | 21.414 | Abhydrolase_1 |
| TRINITY_DN2405_c0_g1_i2 | -1.67516336 | 0.689493089 | 0.999970678 | 0 | 1.035 | 0 | 3.227 | Sugar_tr |
| TRINITY_DN600_c0_g1_i6 | 1.674861726 | 0.18567631 | 0.999970678 | 2.262 | 11.37 | 1.911 | 1.93 | NA |
| TRINITY_DN535_c2_g1_i1 | 1.674352384 | 0.255280192 | 0.999970678 | 4.033 | 4.07 | 1.855 | 0.292 | NA |
| TRINITY_DN2755_c0_g1_i6 | -1.67403508 | 0.314621542 | 0.999970678 | 0.536 | 1.685 | 0.367 | 6.635 | Glyco_hydro_16 |
| TRINITY_DN34629_c0_g1_i2 | -1.67398623 | 0.696461928 | 0.999970678 | 0.899 | 0 | 2.433 | 0 | NA |
| TRINITY_DN3618_c0_g1_i1 | -1.67398623 | 0.696461928 | 0.999970678 | 1.181 | 0 | 3.1 | 0 | NA |
| TRINITY_DN317_c0_g1_i3 | -1.67365428 | 0.049369425 | 0.999970678 | 0.718 | 0.729 | 1.322 | 3.046 | TIP120 |
| TRINITY_DN186_c0_g2_i1 | 1.67352875 | 0.533417016 | 0.999970678 | 6.55 | 15.72 | 0 | 6.957 | NA |
| TRINITY_DN20111_c0_g1_i1 | 1.673489038 | 0.47712163 | 0.999970678 | 7.231 | 0.158 | 1.844 | 0.121 | NA |
| TRINITY_DN2030_c0_g1_i4 | 1.672313926 | 0.130529238 | 0.999970678 | 2.989 | 3.755 | 0.444 | 1.568 | NA |
| TRINITY_DN10529_c0_g1_i3 | -1.67219941 | 0.519676201 | 0.999970678 | 1.126 | 0 | 1.511 | 1.79 | CBM_20 |
| TRINITY_DN2473_c0_g2_i1 | 1.672070396 | 0.264191966 | 0.999970678 | 18.177 | 1.606 | 2.678 | 2.996 | NA |
| TRINITY_DN2832_c0_g1_i1 | -1.67159309 | 0.351806724 | 0.999970678 | 6.931 | 0.187 | 6.544 | 15.111 | NA |
| TRINITY_DN7828_c0_g1_i2 | -1.67154923 | 0.35007353 | 0.999970678 | 0.436 | 19.14 | 26.465 | 29.206 | Pro_isomerase |
| TRINITY_DN37_c0_g2_i1 | -1.67132474 | 0.706588109 | 0.999970678 | 23.655 | 0 | 63.163 | 0 | NA |
| TRINITY_DN13814_c0_g1_i2 | 1.671062674 | 0.530628759 | 0.999970678 | 7.858 | 1.705 | 0 | 2.966 | Glutaredoxin |
| TRINITY_DN1432_c0_g2_i1 | 1.670621088 | 0.523078613 | 0.999970678 | 1.172 | 0.493 | 0.433 | 0 | - |
| TRINITY_DN4276_c0_g1_i3 | 1.670609798 | 0.688726883 | 0.999970678 | 5.741 | 0 | 0 | 1.83 | NA |
| TRINITY_DN701_c1_g1_i1 | 1.670508838 | 0.277207291 | 0.999970678 | 0.863 | 6.8 | 0.7 | 1.528 | NA |
| TRINITY_DN1274_c0_g2_i3 | 1.670508534 | 0.552659246 | 0.999970678 | 1.535 | 0.168 | 0.456 | 0 | MatE |
| TRINITY_DN3515_c0_g1_i1 | 1.670490985 | 0.400072258 | 0.999970678 | 1.681 | 1.104 | 0.711 | 0.02 | NA |
| TRINITY_DN7309_c0_g1_i3 | -1.66995443 | 0.526960174 | 0.999970678 | 0.536 | 0 | 0.622 | 0.955 | Myb_DNA-bind_ |
| TRINITY_DN155_c0_g1_i17 | 1.669715414 | 0.366791855 | 0.999970678 | 12.69 | 23.25 | 0.211 | 10.989 | Ank |
| TRINITY_DN13453_c0_g1_i1 | -1.66963535 | 0.604107525 | 0.999970678 | 0 | 1.301 | 0.044 | 4.032 | NA |
| TRINITY_DN12844_c0_g1_i2 | -1.66874823 | 0.300042929 | 0.999970678 | 0.263 | 0.65 | 0.3 | 2.554 | Glyco_transf_34 |
| TRINITY_DN2008_c0_g1_i4 | 1.668202859 | 0.38587535 | 0.999970678 | 0.118 | 3.834 | 0.411 | 0.744 | NA |
| TRINITY_DN1052_c0_g1_i1 | -1.66811768 | 0.362000614 | 0.999970678 | 2.498 | 22.07 | 56.308 | 7.138 | NA |
| TRINITY_DN827_c0_g1_i2 | -1.66776004 | 0.125460567 | 0.999970678 | 25.172 | 25.05 | 25.143 | 129.1 | peroxidase |
| TRINITY_DN606_c0_g1_i7 | -1.66774371 | 0.702802568 | 0.999970678 | 38.779 | 0 | 0 | 124.103 | NA |
| TRINITY_DN1738_c0_g1_i3 | -1.66759744 | 0.185474774 | 0.999970678 | 1.017 | 2.158 | 1.367 | 8.425 | NA |
| TRINITY_DN9514_c0_g1_i1 | -1.66751328 | 0.289876993 | 0.999970678 | 1.699 | 4.612 | 1.233 | 18.428 | 2OG-FeII_Oxy |
| TRINITY_DN3143_c1_g1_i2 | 1.667409649 | 0.115985192 | 0.999970678 | 1.508 | 3.627 | 0.756 | 0.684 | LRRNT_2 |
| TRINITY_DN9433_c1_g1_i2 | -1.66739689 | 0.266364185 | 0.999970678 | 2.335 | 1.005 | 7.644 | 1.357 | LRRNT_2 |
| TRINITY_DN1710_c0_g1_i2 | 1.667343548 | 0.535391793 | 0.999970678 | 63.887 | 10.73 | 0 | 21.917 | NA |

| TRINITY_DN4784_c0_g1_i1 | -1.66727609 | 0.563611451 | 0.999970678 | 0.509 | 0 | 1.267 | 0.101 | FAD-oxidase_C |
| --- | --- | --- | --- | --- | --- | --- | --- | --- |
| TRINITY_DN967_c1_g1_i3 | 1.666894748 | 0.689804094 | 0.999970678 | 12.672 | 0 | 0 | 4.042 | Myb_DNA-bindin |
| TRINITY_DN2362_c0_g1_i3 | 1.66658363 | 0.550749151 | 0.999970678 | 8.657 | 17.77 | 6.844 | 0 | NA |
| TRINITY_DN13125_c0_g1_i3 | 1.666318888 | 0.167179839 | 0.999970678 | 1.117 | 4.179 | 0.511 | 1.036 | Malectin_like |
| TRINITY_DN944_c0_g1_i2 | -1.66626128 | 0.158813951 | 0.999970678 | 59.727 | 28.4 | 42.564 | 230.441 | PPO1_DWL |
| TRINITY_DN1533_c0_g1_i10 | 1.666228097 | 0.222639585 | 1 | 6.486 | 12.12 | 3.155 | 1.85 | NA |
| TRINITY_DN2152_c0_g1_i3 | -1.66584258 | 0.517376215 | 0.999970678 | 0 | 2.247 | 2.755 | 3.589 | DUF247 |
| TRINITY_DN8972_c0_g1_i20 | -1.66563754 | 0.700768053 | 0.999970678 | 1.108 | 0 | 2.889 | 0 | NA |
| TRINITY_DN7153_c0_g1_i2 | 1.66556012 | 0.137297046 | 0.999970678 | 18.595 | 4.593 | 2.766 | 4.032 | PMEI |
| TRINITY_DN730_c0_g1_i2 | 1.665437538 | 0.513664934 | 0.999970678 | 2.498 | 4.859 | 0 | 2.292 | ACC_central |
| TRINITY_DN13255_c0_g1_i7 | 1.665242391 | 0.542657236 | 0.999970678 | 1.226 | 0.168 | 0 | 0.442 | NA |
| TRINITY_DN4668_c0_g2_i5 | 1.665142353 | 0.247490538 | 0.999970678 | 3.079 | 1.488 | 1.033 | 0.201 | Myb_CC_LHEQLE |
| TRINITY_DN3040_c0_g1_i3 | 1.665086327 | 0.34051657 | 0.999970678 | 11.409 | 0.473 | 1.233 | 2.322 | NA |
| TRINITY_DN2149_c0_g1_i3 | 1.665035651 | 0.202754931 | 0.999970678 | 1.335 | 0.749 | 0.433 | 0.131 | NA |
| TRINITY_DN1489_c0_g2_i3 | -1.66489746 | 0.520076571 | 0.999970678 | 0 | 2.572 | 3.7 | 3.569 | MORN |
| TRINITY_DN6078_c0_g1_i2 | 1.664823532 | 0.700111071 | 0.999970678 | 3.025 | 0 | 0 | 0.965 | RNase_T |
| TRINITY_DN13474_c0_g1_i15 | 1.664293328 | 0.538706921 | 0.999970678 | 0.354 | 2.02 | 0.611 | 0 | NA |
| TRINITY_DN536_c0_g1_i6 | -1.66399434 | 0.542563429 | 0.999970678 | 0 | 1.074 | 0.467 | 2.735 | NA |
| TRINITY_DN19857_c0_g1_i1 | -1.66396839 | 0.019014438 | 0.813141001 | 11.945 | 12.27 | 33.087 | 34.715 | NA |
| TRINITY_DN33476_c0_g1_i1 | 1.663917414 | 0.514706699 | 0.999970678 | 4.842 | 5.893 | 0 | 3.378 | NA |
| TRINITY_DN35858_c0_g5_i3 | 1.663664016 | 0.516926698 | 0.999970678 | 0.509 | 0.503 | 0.267 | 0 | GYF |
| TRINITY_DN8643_c0_g1_i2 | -1.66350923 | 0.358245979 | 0.999970678 | 0.372 | 0.552 | 0.111 | 2.785 | Kinesin |
| TRINITY_DN411_c0_g1_i7 | -1.66314499 | 0.234106407 | 0.999970678 | 30.649 | 18.37 | 12.321 | 140.229 | NA |
| TRINITY_DN721_c0_g2_i3 | -1.66298004 | 0.440803349 | 0.999970678 | 0.064 | 2.178 | 5.4 | 0.462 | UDPGT |
| TRINITY_DN22225_c0_g1_i1 | -1.66264544 | 0.279187998 | 0.999970678 | 0.581 | 1.074 | 3.955 | 0.452 | DUF3475 |
| TRINITY_DN18759_c0_g1_i1 | -1.66254777 | 0.300746624 | 0.999970678 | 3.116 | 1.912 | 1.233 | 14.125 | NA |
| TRINITY_DN3750_c0_g1_i3 | -1.66253446 | 0.52084394 | 0.999970678 | 1.217 | 0 | 1.633 | 1.94 | Cpn60_TCP1 |
| TRINITY_DN7924_c0_g1_i1 | -1.66249196 | 0.333716742 | 0.999970678 | 4.106 | 3.814 | 0.844 | 23.777 | NA |
| TRINITY_DN73_c1_g1_i6 | 1.662459418 | 0.695021947 | 0.999970678 | 0.754 | 0 | 0.2 | 0 | NA |
| TRINITY_DN964_c4_g1_i5 | 1.662447796 | 0.204059834 | 0.999970678 | 2.562 | 1.429 | 0.778 | 0.312 | NA |
| TRINITY_DN48469_c0_g1_i1 | -1.66220548 | 0.57280089 | 0.999970678 | 0 | 329.7 | 759.754 | 60.242 | NA |
| TRINITY_DN3495_c0_g1_i4 | -1.6621784 | 0.183899403 | 0.999970678 | 0.318 | 1.35 | 1.911 | 2.936 | NA |
| TRINITY_DN1331_c0_g2_i2 | -1.66195069 | 0.153467339 | 0.999970678 | 12.027 | 10.36 | 48.708 | 11.873 | NA |
| TRINITY_DN3042_c0_g1_i6 | -1.66189517 | 0.699117956 | 0.999970678 | 0 | 0.256 | 0.644 | 0 | NA |
| TRINITY_DN1801_c0_g1_i1 | 1.661798106 | 0.524529459 | 0.999970678 | 2.08 | 5.243 | 1.911 | 0 | - |
| TRINITY_DN4333_c0_g1_i8 | -1.66162974 | 0.562066238 | 0.999970678 | 0.745 | 0 | 1.822 | 0.181 | NA |
| TRINITY_DN1549_c0_g1_i12 | 1.661628171 | 0.301206604 | 0.999970678 | 12.118 | 1.666 | 3.089 | 0.674 | LRRNT_2 |
| TRINITY_DN17383_c0_g1_i4 | -1.66126503 | 0.229261484 | 0.999970678 | 0.391 | 0.197 | 1.189 | 0.442 | RVP_2 |
| TRINITY_DN4918_c0_g1_i4 | 1.661251519 | 0.211184922 | 0.999970678 | 0.927 | 0.217 | 0.189 | 0.131 | MFS_1 |
| TRINITY_DN74605_c0_g1_i1 | -1.66117306 | 0.529368935 | 1 | 0 | 0.424 | 0.567 | 0.633 | NA |
| TRINITY_DN5919_c0_g3_i3 | -1.66079511 | 0.237499411 | 0.999970678 | 0.536 | 0.148 | 0.689 | 1.297 | Tfb4 |
| TRINITY_DN5191_c0_g1_i1 | -1.660515 | 0.54973486 | 0.999970678 | 3.77 | 0 | 6.744 | 3.991 | ABC2_membrane |
| TRINITY_DN8404_c0_g1_i10 | 1.660353169 | 0.700270198 | 0.999970678 | 0 | 13.67 | 3.444 | 0 | LRRNT_2 |
| TRINITY_DN235_c0_g1_i7 | 1.660089388 | 0.537748473 | 0.999970678 | 10.238 | 21.18 | 0 | 9.893 | NA |
| TRINITY_DN564_c0_g1_i6 | -1.65988501 | 0.040852201 | 0.999970678 | 15.125 | 8.495 | 36.831 | 30.191 | NA |
| TRINITY_DN1987_c0_g1_i5 | 1.659719138 | 0.26978152 | 0.999970678 | 4.279 | 0.877 | 1.067 | 0.362 | Peptidase_M48 |
| TRINITY_DN2957_c0_g1_i1 | -1.6594941 | 0.697486003 | 0.999970678 | 6.659 | 0 | 17.143 | 0 | EthD |
| TRINITY_DN20529_c0_g1_i1 | 1.65938695 | 0.366825209 | 0.999970678 | 3.125 | 2.907 | 1.544 | 0.04 | FH2 |
| TRINITY_DN85546_c0_g1_i4 | -1.65929946 | 0.703350051 | 0.999970678 | 1.135 | 0 | 0 | 3.569 | - |
| TRINITY_DN17105_c0_g1_i2 | -1.65922283 | 0.542302229 | 0.999970678 | 0.654 | 0 | 1.389 | 0.392 | NA |
| TRINITY_DN18127_c0_g1_i5 | 1.658959442 | 0.612160124 | 0.999970678 | 4.642 | 0 | 1.167 | 0.07 | NA |
| TRINITY_DN29457_c0_g1_i1 | -1.65887707 | 0.702706101 | 0.999970678 | 0.818 | 0 | 2.167 | 0 | NA |
| TRINITY_DN26563_c0_g3_i2 | -1.65887707 | 0.702706101 | 0.999970678 | 0.536 | 0 | 1.422 | 0 | Sugar_tr |
| TRINITY_DN12264_c0_g1_i5 | -1.65851877 | 0.691738729 | 0.999970678 | 2.589 | 0 | 0 | 8.314 | NA |
| TRINITY_DN8963_c0_g1_i2 | 1.658186537 | 0.524145109 | 0.999970678 | 0.382 | 1.242 | 0 | 0.513 | NA |
| TRINITY_DN1061_c0_g1_i13 | -1.65786175 | 0.142222858 | 0.999970678 | 22.328 | 7.983 | 20.165 | 72.055 | NA |
| TRINITY_DN7340_c0_g1_i3 | 1.65784516 | 0.695567644 | 0.999970678 | 2.053 | 0 | 0 | 0.664 | Terpene_synth |
| TRINITY_DN665_c0_g1_i20 | 1.657677114 | 0.693329876 | 0.999970678 | 0 | 3.173 | 0 | 1.005 | LRR_8 |
| TRINITY_DN33985_c0_g1_i3 | -1.65763334 | 0.039442273 | 0.999970678 | 1.381 | 1.675 | 3.844 | 4.956 | adh_short_C2 |
| TRINITY_DN26656_c0_g1_i3 | -1.65752004 | 0.44099862 | 0.999970678 | 0.009 | 0.503 | 1.178 | 0.191 | Ketoacyl-synt_C |
| TRINITY_DN1617_c0_g1_i15 | 1.657441385 | 0.514815865 | 0.999970678 | 0.718 | 1.271 | 0 | 0.633 | NA |
| TRINITY_DN535_c2_g1_i4 | -1.65738218 | 0.381464435 | 0.999970678 | 1.581 | 0.256 | 4.455 | 0.452 | NA |
| TRINITY_DN33568_c0_g1_i2 | -1.65732799 | 0.522217937 | 0.999970678 | 0.554 | 0 | 0.789 | 0.834 | NA |
| TRINITY_DN1038_c0_g1_i2 | -1.65730905 | 0.550993296 | 0.999970678 | 5.487 | 0 | 12.488 | 2.463 | RuvX |
| TRINITY_DN3280_c0_g1_i12 | -1.65699491 | 0.292570872 | 0.999970678 | 3.343 | 12.86 | 3.3 | 46.699 | NA |
| TRINITY_DN1899_c0_g2_i1 | -1.65664848 | 0.544060945 | 0.999970678 | 12.936 | 0 | 8.566 | 30.815 | NA |
| TRINITY_DN1262_c0_g1_i2 | 1.656570349 | 0.302726862 | 0.999970678 | 10.755 | 2.651 | 3.122 | 0.503 | bZIP_1 |
| TRINITY_DN11050_c0_g1_i1 | 1.656561611 | 0.518406016 | 0.999970678 | 1.381 | 3.154 | 0 | 1.438 | GUB_WAK_bind |
| TRINITY_DN7693_c0_g2_i2 | -1.65646227 | 0.537617062 | 0.999970678 | 2.153 | 0 | 4.333 | 1.699 | NA |
| TRINITY_DN691_c1_g1_i1 | 1.65617443 | 0.016428273 | 0.719496707 | 6.513 | 5.884 | 1.344 | 2.322 | EF-hand_7 |
| TRINITY_DN18437_c0_g1_i1 | 1.655642364 | 0.697109189 | 0.999970678 | 0 | 2.71 | 0 | 0.834 | DUF1929 |
| TRINITY_DN2060_c0_g1_i4 | 1.655444224 | 0.327502339 | 0.999970678 | 0.718 | 5.962 | 1.333 | 0.503 | NA |
| TRINITY_DN296_c0_g1_i10 | 1.65538753 | 0.045931481 | 0.999970678 | 4.587 | 2.828 | 0.989 | 1.176 | NA |
| TRINITY_DN32400_c3_g1_i1 | 1.655069601 | 0.57041349 | 0.999970678 | 0 | 2649 | 398.043 | 278.256 | NA |

| TRINITY_DN846_c0_g2_i13 | 1.654474865 | 0.692241602 | 0.999970678 | 0 | 3.252 | 0 | 1.015 | NA |
| --- | --- | --- | --- | --- | --- | --- | --- | --- |
| TRINITY_DN50325_c0_g1_i3 | -1.65442497 | 0.698779847 | 0.999970678 | 0.863 | 0 | 2.278 | 0 | NA |
| TRINITY_DN28390_c0_g1_i1 | 1.654417993 | 0.570311161 | 0.999970678 | 1.935 | 0.138 | 0 | 0.664 | NA |
| TRINITY_DN11100_c0_g1_i1 | 1.654259592 | 0.393571293 | 0.999970678 | 164.69 | 3.035 | 10.71 | 38.898 | NA |
| TRINITY_DN19199_c0_g1_i1 | 1.654087642 | 0.694016971 | 0.999970678 | 12.854 | 0 | 0 | 4.152 | UQ_con |
| TRINITY_DN8761_c0_g1_i1 | 1.65378101 | 0.259279628 | 0.999970678 | 11.818 | 69.2 | 3.311 | 21.434 | NA |
| TRINITY_DN11813_c0_g1_i6 | -1.65358732 | 0.157032537 | 0.999970678 | 5.069 | 2.937 | 3.933 | 20.61 | NA |
| TRINITY_DN2946_c0_g2_i5 | 1.653573377 | 0.162554366 | 0.999970678 | 4.279 | 1.064 | 0.844 | 0.694 | Ank_2 |
| TRINITY_DN21_c0_g1_i4 | -1.65321474 | 0.312948197 | 0.999970678 | 0.89 | 6.347 | 2.522 | 19.193 | NA |
| TRINITY_DN12262_c0_g2_i1 | -1.65303054 | 0.691691566 | 0.999970678 | 1.989 | 0 | 0 | 6.374 | NA |
| TRINITY_DN606_c0_g1_i4 | 1.652901927 | 0.218226032 | 0.999970678 | 69.883 | 286.9 | 15.721 | 93.841 | TLC |
| TRINITY_DN1517_c0_g1_i13 | -1.65251259 | 0.543956302 | 0.999970678 | 0 | 7.874 | 13.533 | 8.043 | RAP |
| TRINITY_DN3936_c0_g1_i1 | 1.652511743 | 0.246865796 | 0.999970678 | 0.945 | 5.854 | 1.3 | 0.553 | Ldh_1_C |
| TRINITY_DN10081_c3_g1_i10 | 1.652335236 | 0.57005084 | 0.999970678 | 0 | 3.962 | 0.822 | 0.241 | NA |
| TRINITY_DN1767_c0_g1_i8 | 1.651918557 | 0.16197148 | 0.999970678 | 8.993 | 19.79 | 6.077 | 1.759 | NA |
| TRINITY_DN613_c0_g1_i9 | -1.65166682 | 0.349143959 | 0.999970678 | 0.445 | 4.415 | 1.2 | 13.673 | p450 |
| TRINITY_DN20251_c0_g4_i1 | -1.6514638 | 0.70482134 | 0.999970678 | 1.208 | 0 | 3.178 | 0 | Peroxidase_2 |
| TRINITY_DN11563_c0_g1_i2 | -1.6514638 | 0.70482134 | 0.999970678 | 0.627 | 0 | 1.678 | 0 | NA |
| TRINITY_DN155_c0_g1_i11 | 1.651369089 | 0.528810156 | 0.999970678 | 4.188 | 9.441 | 0 | 4.313 | Ank |
| TRINITY_DN9966_c0_g1_i6 | -1.65096856 | 0.18525848 | 0.999970678 | 3.198 | 16.65 | 34.887 | 19.343 | NA |
| TRINITY_DN2037_c0_g1_i8 | -1.65089348 | 0.488434604 | 0.999970678 | 9.247 | 0.128 | 24.065 | 0.633 | ADH_N |
| TRINITY_DN5415_c0_g1_i2 | 1.650546768 | 0.542160641 | 0.999970678 | 2.389 | 0.66 | 0.789 | 0 | - |
| TRINITY_DN978_c0_g1_i5 | 1.650391408 | 0.563528778 | 0.999970678 | 0 | 9.964 | 0.744 | 2.242 | NA |
| TRINITY_DN237_c0_g1_i6 | 1.650363714 | 0.551514714 | 0.999970678 | 4.887 | 0.473 | 0 | 1.739 | NA |
| TRINITY_DN3839_c0_g1_i1 | -1.65003223 | 0.562150652 | 0.999970678 | 5.968 | 6.82 | 0 | 39.994 | Ctr |
| TRINITY_DN550_c0_g1_i3 | 1.649885296 | 0.477808629 | 0.999970678 | 6.322 | 0.02 | 0.489 | 1.448 | NA |
| TRINITY_DN29240_c0_g1_i4 | 1.649672459 | 0.531448115 | 0.999970678 | 2.316 | 2.661 | 1.311 | 0 | Helicase_C |
| TRINITY_DN15694_c0_g1_i8 | -1.64965282 | 0.701395473 | 0.999970678 | 1.154 | 0 | 3 | 0 | - |
| TRINITY_DN12357_c0_g1_i10 | -1.64965282 | 0.701395473 | 0.999970678 | 0.99 | 0 | 2.578 | 0 | FAD_binding_2 |
| TRINITY_DN60452_c0_g1_i5 | 1.649489974 | 0.214184381 | 0.999970678 | 1.208 | 1.311 | 0.156 | 0.603 | NA |
| TRINITY_DN1246_c0_g1_i4 | -1.64927451 | 0.699723715 | 0.999970678 | 2.244 | 0 | 0 | 7.158 | NA |
| TRINITY_DN760_c0_g2_i1 | -1.64918353 | 0.368397711 | 0.999970678 | 4.814 | 0.315 | 2.755 | 12.879 | Cation_ATPase |
| TRINITY_DN694_c0_g1_i5 | 1.648961539 | 0.445180609 | 0.999970678 | 23.418 | 1.409 | 0.289 | 7.42 | NA |
| TRINITY_DN5111_c0_g1_i11 | -1.64857548 | 0.545234823 | 0.999970678 | 5.241 | 0 | 9.611 | 5.057 | NA |
| TRINITY_DN12074_c0_g1_i6 | -1.64847833 | 0.699157029 | 0.999970678 | 2.952 | 0 | 7.655 | 0 | Glyco_hydro_25 |
| TRINITY_DN3250_c0_g1_i8 | -1.64827801 | 0.360476485 | 0.999970678 | 55.012 | 3.755 | 14.288 | 167.404 | NA |
| TRINITY_DN10859_c0_g1_i1 | 1.64826879 | 0.241027593 | 0.999970678 | 0.518 | 0.71 | 0.256 | 0.09 | DNA_methylase |
| TRINITY_DN7402_c0_g1_i1 | -1.6480885 | 0.243061995 | 0.999970678 | 20.239 | 14.98 | 8.111 | 100.185 | DPBB_1 |
| TRINITY_DN38194_c0_g1_i1 | -1.64802955 | 0.546245493 | 0.999970678 | 1.199 | 0 | 0.778 | 2.895 | NA |
| TRINITY_DN1544_c0_g1_i8 | -1.6477826 | 0.163485825 | 0.999970678 | 7.476 | 3.223 | 6.544 | 25.295 | Motile_Sperm |
| TRINITY_DN22930_c0_g1_i7 | -1.64773634 | 0.613763124 | 0.999970678 | 0 | 3.164 | 8.011 | 0.08 | LRRNT_2 |
| TRINITY_DN13476_c2_g2_i1 | 1.647458941 | 0.703784553 | 0.999970678 | 2.662 | 0 | 0.7 | 0 | NA |
| TRINITY_DN4359_c0_g1_i1 | -1.64734389 | 0.703066559 | 0.999970678 | 0.527 | 0 | 0 | 1.659 | CDT1 |
| TRINITY_DN7337_c0_g1_i7 | 1.647334989 | 0.11349743 | 0.999970678 | 2.044 | 3.203 | 1.011 | 0.442 | - |
| TRINITY_DN843_c4_g1_i2 | 1.647109611 | 0.691868851 | 0.999970678 | 0 | 6.83 | 0 | 2.151 | - |
| TRINITY_DN2772_c0_g1_i2 | -1.64708459 | 0.411239828 | 0.999970678 | 0.745 | 6.455 | 0.6 | 21.344 | NA |
| TRINITY_DN4243_c0_g2_i10 | -1.64693928 | 0.522772376 | 0.999970678 | 0 | 0.355 | 0.5 | 0.513 | PPR |
| TRINITY_DN6623_c0_g1_i2 | 1.646904108 | 0.69515949 | 0.999970678 | 3.261 | 0 | 0.878 | 0 | LRRNT_2 |
| TRINITY_DN6064_c0_g1_i5 | 1.646665276 | 0.094621288 | 0.999970678 | 2.643 | 2.119 | 0.955 | 0.372 | C2 |
| TRINITY_DN3325_c0_g1_i2 | -1.64629775 | 0.209300989 | 0.999970678 | 1.372 | 7.086 | 15.188 | 7.691 | - |
| TRINITY_DN836_c0_g1_i8 | -1.64610634 | 0.566517677 | 0.999970678 | 6.486 | 0 | 15.732 | 1.558 | NA |
| TRINITY_DN14555_c0_g1_i1 | -1.64589007 | 0.077250318 | 0.999970678 | 52.196 | 35.8 | 80.073 | 158.909 | NA |
| TRINITY_DN3305_c0_g1_i2 | 1.645662175 | 0.247419302 | 0.999970678 | 3.879 | 2.592 | 0.2 | 1.83 | NA |
| TRINITY_DN1607_c0_g1_i13 | 1.645623914 | 0.697569181 | 0.999970678 | 13.299 | 0 | 0 | 4.333 | NA |
| TRINITY_DN1732_c0_g1_i10 | 1.645517207 | 0.172904346 | 0.999970678 | 2.38 | 1.252 | 0.756 | 0.251 | PWI |
| TRINITY_DN103_c0_g1_i3 | -1.64548614 | 0.278662914 | 0.999970678 | 0.154 | 0.936 | 1.389 | 1.639 | NA |
| TRINITY_DN4137_c0_g1_i4 | -1.64525177 | 0.534844984 | 0.999970678 | 2.734 | 0 | 3.033 | 4.806 | NA |
| TRINITY_DN18318_c0_g1_i1 | 1.644821989 | 0.362948259 | 0.999970678 | 19.703 | 5.608 | 6.255 | 0.493 | NA |
| TRINITY_DN1261_c0_g1_i3 | -1.64477723 | 0.070662348 | 0.999970678 | 1.408 | 2.178 | 6.333 | 3.539 | NA |
| TRINITY_DN27690_c0_g1_i5 | 1.644395196 | 0.588446597 | 0.999970678 | 0.091 | 3.055 | 0.822 | 0 | NA |
| TRINITY_DN125_c0_g2_i1 | 1.643987518 | 0.02662697 | 0.999970678 | 9.093 | 6.357 | 2.255 | 2.232 | UDPGT |
| TRINITY_DN314_c0_g2_i4 | -1.64377285 | 0.532025054 | 1 | 0 | 0.641 | 0.789 | 1.066 | NA |
| TRINITY_DN1028_c0_g1_i8 | 1.643702853 | 0.695349309 | 0.999970678 | 2.871 | 0 | 0 | 0.935 | RRM_1 |
| TRINITY_DN3057_c0_g1_i2 | -1.64365587 | 0.137564948 | 0.999970678 | 8.403 | 10.33 | 39.042 | 11.18 | FAF |
| TRINITY_DN57901_c0_g1_i1 | -1.64330072 | 0.707137688 | 0.999970678 | 0.527 | 0 | 1.378 | 0 | GMC_oxred_C |
| TRINITY_DN5012_c1_g1_i4 | -1.64330072 | 0.707137688 | 0.999970678 | 0.463 | 0 | 1.2 | 0 | NA |
| TRINITY_DN14732_c0_g1_i4 | 1.643292772 | 0.263157243 | 1 | 0.609 | 0.611 | 0.233 | 0.131 | NA |
| TRINITY_DN716_c0_g1_i6 | 1.642973948 | 0.167145392 | 0.999970678 | 7.34 | 2.878 | 0.611 | 2.554 | NA |
| TRINITY_DN3738_c0_g1_i2 | -1.6424334 | 0.559481693 | 0.999970678 | 0.5 | 0 | 1.133 | 0.181 | NA |
| TRINITY_DN997_c1_g1_i5 | 1.642233129 | 0.701338105 | 0.999970678 | 0 | 18.22 | 0 | 5.781 | NA |
| TRINITY_DN1042_c0_g1_i5 | 1.642215711 | 0.318300063 | 0.999970678 | 3.334 | 0.296 | 0.533 | 0.543 | NA |
| TRINITY_DN256_c0_g1_i6 | 1.642116331 | 0.560358058 | 0.999970678 | 8.321 | 0 | 1.167 | 1.307 | Myb_DNA-bindin |
| TRINITY_DN511_c0_g3_i3 | -1.64207543 | 0.704820481 | 0.999970678 | 0 | 0.296 | 0 | 0.905 | NA |
| TRINITY_DN3389_c0_g1_i10 | -1.64207543 | 0.704820481 | 0.999970678 | 0 | 2.208 | 0 | 6.615 | NA |

| TRINITY_DN1669_c0_g1_i6 | 1.641924095 | 0.56110223 | 0.999970678 | 0.636 | 7.342 | 0 | 2.534 | ANF_receptor |
| --- | --- | --- | --- | --- | --- | --- | --- | --- |
| TRINITY_DN2882_c0_g1_i3 | -1.64159232 | 0.697797636 | 0.999970678 | 1.272 | 0 | 0 | 3.971 | NA |
| TRINITY_DN6131_c0_g1_i5 | -1.64158197 | 0.213282783 | 0.999970678 | 0.263 | 1.429 | 2.444 | 2.232 | NA |
| TRINITY_DN15799_c0_g1_i1 | -1.64145197 | 0.144225975 | 0.999970678 | 8.73 | 4.11 | 19.299 | 15.02 | NA |
| TRINITY_DN8848_c0_g1_i9 | 1.641126358 | 0.567000445 | 0.999970678 | 0 | 90.59 | 17.31 | 7.138 | NA |
| TRINITY_DN18962_c0_g1_i1 | 1.641076525 | 0.530090562 | 0.999970678 | 0.581 | 0.867 | 0.378 | 0 | ABC_membrane |
| TRINITY_DN4643_c0_g1_i3 | 1.640874238 | 0.386175961 | 0.999970678 | 0.827 | 20.36 | 4.955 | 0.714 | NA |
| TRINITY_DN12118_c0_g1_i8 | 1.640266696 | 0.142579653 | 0.999970678 | 0.409 | 0.325 | 0.133 | 0.07 | NA |
| TRINITY_DN1287_c0_g1_i5 | -1.63988301 | 0.17802019 | 0.999970678 | 6.986 | 2.257 | 5.544 | 22.359 | NA |
| TRINITY_DN5948_c1_g1_i1 | -1.63983175 | 0.127417478 | 0.999970678 | 1.335 | 2.484 | 3.122 | 8.063 | Auxin_inducible |
| TRINITY_DN95804_c0_g1_i3 | 1.639446641 | 0.695405875 | 0.999970678 | 3.479 | 0 | 0 | 1.136 | NA |
| TRINITY_DN13720_c1_g1_i9 | -1.63904244 | 0.331887423 | 0.999970678 | 0.436 | 0.158 | 1.378 | 0.171 | RabGAP-TBC |
| TRINITY_DN566_c0_g1_i9 | -1.63892826 | 0.171210979 | 0.999970678 | 3.643 | 0.749 | 5.111 | 7.671 | NA |
| TRINITY_DN2086_c0_g1_i2 | 1.638852381 | 0.232859303 | 0.999970678 | 0.945 | 2.237 | 0.689 | 0.171 | Thioredoxin |
| TRINITY_DN10824_c0_g1_i1 | -1.63881021 | 0.536478948 | 0.999970678 | 1.444 | 0 | 0.978 | 3.318 | NA |
| TRINITY_DN19800_c0_g2_i5 | 1.638789909 | 0.525612896 | 0.999970678 | 1.817 | 1.399 | 0 | 1.025 | NA |
| TRINITY_DN6320_c1_g1_i4 | 1.638777499 | 0.320192308 | 0.999970678 | 1.09 | 0.187 | 0.3 | 0.06 | zf-CCHC |
| TRINITY_DN7302_c0_g3_i1 | -1.6386704 | 0.262686859 | 0.999970678 | 0.536 | 0.976 | 3.344 | 0.633 | NA |
| TRINITY_DN20910_c0_g1_i7 | 1.638494171 | 0.203864643 | 0.999970678 | 0.345 | 1.045 | 0.156 | 0.261 | GYF |
| TRINITY_DN9038_c0_g1_i13 | -1.63840735 | 0.548993344 | 1 | 0 | 0.739 | 0.389 | 1.81 | NA |
| TRINITY_DN863_c0_g2_i21 | -1.63829844 | 0.693519674 | 0.999970678 | 0 | 5.962 | 0 | 18.066 | NA |
| TRINITY_DN1890_c0_g1_i5 | 1.638289144 | 0.453278398 | 0.999970678 | 3.525 | 0.572 | 1.089 | 0.02 | NA |
| TRINITY_DN2077_c0_g1_i13 | -1.63811088 | 0.535628667 | 0.999970678 | 1.372 | 0 | 1.689 | 2.312 | Glycos_transf_1 |
| TRINITY_DN2687_c0_g2_i1 | -1.63784308 | 0.566044083 | 0.999970678 | 2.734 | 0.926 | 9.444 | 0 | Bac_rhodopsin |
| TRINITY_DN51_c0_g1_i1 | -1.63763444 | 0.399562911 | 0.999970678 | 2.144 | 5.677 | 0.267 | 23.948 | NA |
| TRINITY_DN2864_c0_g1_i6 | 1.637616203 | 0.702087588 | 0.999970678 | 25.217 | 0 | 0 | 7.812 | NA |
| TRINITY_DN18702_c0_g1_i2 | -1.63749534 | 0.592857378 | 0.999970678 | 3.615 | 0.237 | 0 | 11.954 | NA |
| TRINITY_DN63030_c0_g1_i1 | -1.63732061 | 0.119050864 | 0.999970678 | 1629.6 | 1365 | 5823.114 | 1752.068 | NA |
| TRINITY_DN14444_c0_g1_i2 | 1.63664695 | 0.258190412 | 0.999970678 | 0.581 | 0.315 | 0.178 | 0.07 | NA |
| TRINITY_DN5337_c0_g1_i7 | 1.636498904 | 0.701806904 | 0.999970678 | 0.945 | 0 | 0 | 0.312 | NA |
| TRINITY_DN22166_c0_g1_i1 | -1.63637678 | 0.537789301 | 1 | 0 | 2.277 | 2.689 | 3.619 | NA |
| TRINITY_DN8586_c0_g2_i1 | -1.6357636 | 0.614204311 | 0.999970678 | 3.706 | 0.099 | 0 | 11.723 | NA |
| TRINITY_DN7043_c0_g1_i3 | 1.635624475 | 0.196403483 | 0.999970678 | 10.628 | 10.1 | 1.411 | 4.514 | NA |
| TRINITY_DN327_c0_g1_i3 | 1.635367853 | 0.552745089 | 0.999970678 | 40.342 | 20.25 | 0 | 19.605 | NA |
| TRINITY_DN2154_c0_g1_i6 | 1.635345747 | 0.566552674 | 0.999970678 | 6.259 | 0 | 0.511 | 1.438 | NA |
| TRINITY_DN4988_c0_g1_i1 | -1.63487829 | 0.530084376 | 0.999970678 | 1.917 | 0 | 2.989 | 2.453 | Hexapep |
| TRINITY_DN3459_c0_g1_i5 | 1.63484481 | 0.215848709 | 0.999970678 | 70.083 | 11.93 | 14.732 | 8.596 | NA |
| TRINITY_DN673_c0_g1_i3 | -1.63441064 | 0.524441889 | 0.999970678 | 0 | 1.754 | 2.3 | 2.544 | NA |
| TRINITY_DN1164_c0_g2_i1 | -1.63428494 | 0.536006893 | 0.999970678 | 0.627 | 0 | 0.589 | 1.247 | GUB_WAK_bind |
| TRINITY_DN13164_c0_g1_i6 | -1.63399746 | 0.617815235 | 0.999970678 | 9.148 | 0.384 | 23.576 | 0 | Actin |
| TRINITY_DN10497_c0_g1_i2 | -1.63387265 | 0.152151728 | 0.999970678 | 0.273 | 0.227 | 0.644 | 0.734 | ELMO_CED12 |
| TRINITY_DN16881_c1_g1_i2 | 1.633679041 | 0.562827344 | 0.999970678 | 0 | 2.513 | 0.367 | 0.352 | NA |
| TRINITY_DN4474_c0_g1_i1 | -1.63362151 | 0.53574438 | 0.999970678 | 1.026 | 0 | 0.767 | 2.282 | Jas |
| TRINITY_DN3161_c0_g1_i6 | -1.63325605 | 0.707237401 | 0.999970678 | 0.263 | 0 | 0 | 0.845 | RRM_1 |
| TRINITY_DN2698_c0_g1_i3 | -1.63303635 | 0.537956433 | 0.999970678 | 0 | 1.498 | 0.833 | 3.589 | NA |
| TRINITY_DN1447_c0_g1_i3 | 1.632875059 | 0.561072592 | 0.999970678 | 11.01 | 56.47 | 0 | 21.465 | NA |
| TRINITY_DN4405_c0_g1_i9 | 1.632431545 | 0.19971077 | 0.999970678 | 1.517 | 1.399 | 0.189 | 0.684 | NA |
| TRINITY_DN6477_c0_g1_i5 | -1.63207864 | 0.219407402 | 0.999970678 | 0.445 | 0.769 | 2.189 | 1.036 | NA |
| TRINITY_DN1274_c0_g4_i1 | 1.631919946 | 0.267279129 | 0.999970678 | 4.442 | 3.814 | 1.978 | 0.271 | MatE |
| TRINITY_DN5064_c0_g1_i6 | -1.63143463 | 0.538092368 | 0.999970678 | 1.063 | 0 | 1.033 | 2.091 | NA |
| TRINITY_DN4803_c0_g1_i2 | 1.631127068 | 0.70047827 | 0.999970678 | 1.299 | 0 | 0 | 0.432 | F-box |
| TRINITY_DN262_c1_g1_i2 | 1.630779044 | 0.302940128 | 0.999970678 | 0.336 | 2.03 | 0.456 | 0.221 | NA |
| TRINITY_DN2656_c0_g2_i8 | -1.63070023 | 0.267881164 | 0.999970678 | 0.627 | 0.424 | 0.378 | 2.765 | Telomere_reg-2 |
| TRINITY_DN102689_c0_g2_i4 | -1.63069119 | 0.570976378 | 0.999970678 | 0.481 | 0.177 | 0 | 2.081 | NA |
| TRINITY_DN31112_c0_g1_i1 | 1.630656345 | 0.70141616 | 0.999970678 | 2.998 | 0 | 0.811 | 0 | AMP-binding |
| TRINITY_DN3969_c1_g1_i4 | -1.63012112 | 0.560192419 | 0.999970678 | 0 | 1.981 | 0.711 | 5.057 | NA |
| TRINITY_DN5548_c0_g1_i6 | -1.62990683 | 0.280736625 | 0.999970678 | 6.359 | 7.135 | 2.678 | 38.033 | NA |
| TRINITY_DN1043_c2_g1_i3 | -1.62988696 | 0.487883914 | 0.999970678 | 0.209 | 3.104 | 0.189 | 9.672 | ADH_N |
| TRINITY_DN4333_c0_g1_i14 | 1.629701536 | 0.245263151 | 0.999970678 | 1.208 | 0.542 | 0.367 | 0.131 | NA |
| TRINITY_DN338_c0_g1_i5 | -1.62935567 | 0.433079047 | 0.999970678 | 8.557 | 0.138 | 5.766 | 19.745 | NA |
| TRINITY_DN3386_c0_g1_i2 | 1.629257329 | 0.3854103 | 0.999970678 | 11.573 | 0.522 | 0.411 | 3.469 | NA |
| TRINITY_DN23130_c0_g1_i4 | 1.629018391 | 0.567012892 | 0.999970678 | 0 | 11.14 | 2.1 | 0.995 | NA |
| TRINITY_DN1812_c1_g1_i6 | 1.628780536 | 0.264976291 | 0.999970678 | 2.38 | 13.44 | 2.511 | 1.92 | NA |
| TRINITY_DN10395_c0_g1_i5 | 1.628699355 | 0.550731202 | 0.999970678 | 0.536 | 0.089 | 0.167 | 0 | Chromo |
| TRINITY_DN2586_c0_g1_i2 | -1.62854191 | 0.512575739 | 0.999970678 | 3.025 | 0.118 | 7.844 | 0.241 | Histone |
| TRINITY_DN2265_c0_g1_i1 | -1.62853116 | 0.547030442 | 0.999970678 | 1.408 | 0 | 0.678 | 3.589 | NA |
| TRINITY_DN3135_c0_g1_i3 | 1.628178905 | 0.192663181 | 0.999970678 | 2.716 | 1.961 | 0.289 | 1.146 | NA |
| TRINITY_DN694_c0_g1_i7 | 1.628173548 | 0.034116034 | 0.999970678 | 1.435 | 1.459 | 0.444 | 0.412 | NA |
| TRINITY_DN41633_c0_g1_i3 | 1.628010131 | 0.57727161 | 0.999970678 | 6.313 | 0 | 0.344 | 1.659 | NA |
| TRINITY_DN3138_c0_g1_i34 | 1.627715111 | 0.707027694 | 0.999970678 | 0 | 1.143 | 0.311 | 0 | NA |
| TRINITY_DN11542_c0_g1_i3 | 1.627694887 | 0.143561995 | 0.999970678 | 1.108 | 0.483 | 0.3 | 0.161 | PHD |
| TRINITY_DN2846_c0_g1_i1 | -1.6269183 | 0.538408414 | 0.999970678 | 0 | 1.025 | 0.656 | 2.322 | NA |
| TRINITY_DN13552_c1_g1_i1 | -1.62679417 | 0.709332794 | 0.999970678 | 0.981 | 0 | 2.522 | 0 | NA |
| TRINITY_DN8468_c0_g1_i1 | 1.626498358 | 0.59794798 | 0.999970678 | 11.973 | 0.158 | 0 | 3.981 | NA |

| TRINITY_DN804_c0_g1_i3 | 1.626229284 | 0.430972088 | 0.999970678 | 11.827 | 0.217 | 2.278 | 1.126 | NA |
| --- | --- | --- | --- | --- | --- | --- | --- | --- |
| TRINITY_DN14473_c0_g1_i2 | 1.626223872 | 0.169686078 | 0.999970678 | 6.777 | 3.558 | 0.567 | 2.684 | PPR |
| TRINITY_DN20688_c0_g1_i1 | 1.625731972 | 0.227772991 | 0.999970678 | 2.78 | 1.902 | 0.167 | 1.317 | NA |
| TRINITY_DN33636_c0_g1_i2 | 1.625665931 | 0.294620284 | 0.999970678 | 100.83 | 7.007 | 20.221 | 10.969 | zf-RING_2 |
| TRINITY_DN5894_c0_g1_i1 | -1.62558615 | 0.541031582 | 0.999970678 | 0.254 | 0 | 0.411 | 0.312 | NA |
| TRINITY_DN420_c0_g1_i6 | -1.62543167 | 0.278987694 | 0.999970678 | 6.786 | 0.542 | 10.499 | 10.194 | NA |
| TRINITY_DN40458_c0_g1_i2 | 1.625267721 | 0.133505071 | 0.999970678 | 0.999 | 0.877 | 0.167 | 0.412 | NA |
| TRINITY_DN20716_c1_g1_i4 | 1.625265986 | 0.124395278 | 0.999970678 | 2.925 | 2.04 | 0.4 | 1.136 | DUF599 |
| TRINITY_DN7072_c0_g2_i3 | 1.625249251 | 0.23034776 | 0.999970678 | 2.598 | 14.8 | 2.622 | 2.312 | B_lectin |
| TRINITY_DN4507_c0_g1_i2 | 1.625118788 | 0.309539811 | 0.999970678 | 7.594 | 3.035 | 2.655 | 0.251 | NA |
| TRINITY_DN25110_c0_g1_i4 | -1.62505262 | 0.172457967 | 0.999970678 | 1.699 | 0.542 | 2.255 | 4.162 | NA |
| TRINITY_DN381_c0_g1_i21 | 1.6250476 | 0.701344296 | 0.999970678 | 5.142 | 0 | 1.411 | 0 | NB-ARC |
| TRINITY_DN834_c0_g1_i21 | 1.624918657 | 0.190055761 | 0.999970678 | 2.671 | 6.928 | 0.922 | 1.89 | NA |
| TRINITY_DN581_c0_g1_i16 | -1.62489945 | 0.706496783 | 0.999970678 | 0.545 | 0 | 0 | 1.719 | PTS_2-RNA |
| TRINITY_DN6694_c0_g1_i1 | -1.62481923 | 0.564064011 | 0.999970678 | 16.66 | 0 | 15.755 | 33.177 | NA |
| TRINITY_DN7822_c0_g1_i1 | 1.624780501 | 0.590357643 | 0.999970678 | 1.145 | 40.85 | 0 | 12.507 | NA |
| TRINITY_DN4235_c1_g1_i5 | -1.6243874 | 0.211893696 | 0.999970678 | 1.344 | 0.641 | 0.778 | 5.238 | HEAT |
| TRINITY_DN27340_c0_g1_i1 | -1.62421571 | 0.712495933 | 1 | 0.509 | 0 | 1.278 | 0 | NA |
| TRINITY_DN37261_c0_g2_i1 | -1.62421571 | 0.712495933 | 1 | 2.48 | 0 | 6.344 | 0 | NA |
| TRINITY_DN1518_c0_g1_i4 | 1.624036658 | 0.703949413 | 0.999970678 | 0 | 1.863 | 0 | 0.593 | Ion_trans_2 |
| TRINITY_DN1883_c0_g1_i2 | 1.623852087 | 0.561540783 | 0.999970678 | 3.843 | 0.404 | 1.155 | 0 | NA |
| TRINITY_DN1391_c0_g1_i1 | -1.62383204 | 0.31700269 | 0.999970678 | 10.855 | 3.203 | 33.854 | 2.735 | NA |
| TRINITY_DN1261_c1_g1_i8 | -1.6235068 | 0.351523094 | 0.999970678 | 0.572 | 0.237 | 2 | 0.101 | Asp_protease_2 |
| TRINITY_DN3951_c0_g1_i1 | -1.62330479 | 0.309738994 | 0.999970678 | 6.922 | 2.553 | 22.465 | 2.182 | NA |
| TRINITY_DN2044_c0_g1_i1 | -1.62311545 | 0.253038003 | 0.999970678 | 0.618 | 1.873 | 1.367 | 5.891 | FAD_binding_3 |
| TRINITY_DN17824_c0_g1_i1 | -1.6230871 | 0.603298333 | 0.999970678 | 0.427 | 4.376 | 11.744 | 0 | NA |
| TRINITY_DN76958_c0_g1_i1 | 1.62294819 | 0.599314489 | 0.999970678 | 0 | 4.346 | 0.056 | 1.327 | - |
| TRINITY_DN1196_c0_g2_i2 | -1.6225429 | 0.240291641 | 0.999970678 | 494.31 | 173.2 | 1428.436 | 248.517 | NA |
| TRINITY_DN6246_c0_g1_i3 | 1.622395377 | 0.240615231 | 0.999970678 | 0.5 | 2.454 | 0.3 | 0.583 | NA |
| TRINITY_DN1478_c0_g1_i3 | -1.62227591 | 0.532006597 | 0.999970678 | 0 | 0.759 | 1.233 | 0.784 | F-box |
| TRINITY_DN1999_c0_g1_i6 | 1.621926644 | 0.698340415 | 0.999970678 | 0 | 1.202 | 0.322 | 0 | NA |
| TRINITY_DN2786_c0_g1_i4 | -1.62184524 | 0.568789604 | 0.999970678 | 0.545 | 0 | 0.133 | 1.508 | NA |
| TRINITY_DN17132_c0_g1_i2 | -1.62153754 | 0.69979039 | 0.999970678 | 0.4 | 0 | 1.044 | 0 | NA |
| TRINITY_DN375_c0_g2_i1 | 1.621280514 | 0.281704348 | 0.999970678 | 14.825 | 94.24 | 4.133 | 30.04 | CLP_protease |
| TRINITY_DN973_c0_g1_i3 | 1.620672362 | 0.266740505 | 0.999970678 | 2.107 | 3.006 | 1.255 | 0.141 | Cyclin_N |
| TRINITY_DN160_c0_g1_i4 | -1.62053702 | 0.709021449 | 0.999970678 | 2.807 | 0 | 7.3 | 0 | NA |
| TRINITY_DN2573_c1_g1_i8 | 1.620391689 | 0.180225078 | 0.999970678 | 0.609 | 1.055 | 0.178 | 0.322 | NA |
| TRINITY_DN8999_c0_g1_i5 | 1.620339343 | 0.326066884 | 0.999970678 | 2.062 | 11.76 | 3.178 | 0.553 | NA |
| TRINITY_DN4154_c0_g1_i5 | 1.620252567 | 0.525352189 | 0.999970678 | 1.054 | 0.808 | 0 | 0.603 | BRCT |
| TRINITY_DN1038_c0_g1_i1 | 1.62022401 | 0.529045076 | 0.999970678 | 5.096 | 7.352 | 0 | 4.011 | RuvX |
| TRINITY_DN1195_c0_g2_i2 | 1.62000268 | 0.125723658 | 0.999970678 | 17.805 | 18.56 | 2.067 | 9.36 | PfkB |
| TRINITY_DN4836_c0_g1_i2 | -1.61998737 | 0.22273921 | 0.999970678 | 12.272 | 5.608 | 6.588 | 46.659 | Pollen_Ole_e_I |
| TRINITY_DN1711_c1_g1_i8 | 1.61997177 | 0.058985886 | 0.999970678 | 2.362 | 2.247 | 0.667 | 0.674 | NAF |
| TRINITY_DN2239_c0_g2_i1 | 1.619935547 | 0.152216289 | 0.999970678 | 5.187 | 2.424 | 0.756 | 1.619 | NA |
| TRINITY_DN26494_c0_g1_i6 | 1.619727169 | 0.567638004 | 0.999970678 | 0.173 | 2.079 | 0 | 0.714 | NA |
| TRINITY_DN25172_c0_g2_i2 | -1.61942494 | 0.16846314 | 0.999970678 | 1.135 | 0.532 | 3.022 | 1.498 | NA |
| TRINITY_DN5524_c0_g2_i14 | -1.61902094 | 0.704419459 | 0.999970678 | 0 | 0.788 | 1.955 | 0 | NA |
| TRINITY_DN10846_c0_g1_i9 | 1.618324923 | 0.428418974 | 0.999970678 | 16.942 | 2.346 | 5.044 | 0.221 | NA |
| TRINITY_DN4406_c0_g1_i8 | 1.618183584 | 0.257059181 | 1 | 0.445 | 0.306 | 0.056 | 0.191 | NA |
| TRINITY_DN17835_c3_g1_i2 | 1.618132275 | 0.265292868 | 0.999970678 | 0.854 | 1.242 | 0.111 | 0.553 | NA |
| TRINITY_DN1437_c0_g1_i1 | 1.618095196 | 0.024741616 | 0.994290387 | 29.595 | 17.46 | 6.611 | 7.45 | UQ_con |
| TRINITY_DN7101_c0_g1_i2 | -1.618054 | 0.542333537 | 0.999970678 | 4.233 | 0 | 7.422 | 4.182 | NA |
| TRINITY_DN252561_c0_g1_i1 | -1.61794498 | 0.699934639 | 0.999970678 | 5.005 | 0 | 12.877 | 0 | NA |
| TRINITY_DN10718_c0_g1_i3 | -1.61775809 | 0.247419781 | 0.999970678 | 1.317 | 2.346 | 1.255 | 9.742 | NA |
| TRINITY_DN5140_c0_g1_i4 | 1.617748674 | 0.291690398 | 0.999970678 | 4.842 | 1.005 | 1.367 | 0.271 | - |
| TRINITY_DN2225_c0_g1_i1 | 1.617748673 | 0.296773685 | 0.999970678 | 23.882 | 40.2 | 16.232 | 1.176 | Pyr_redox_2 |
| TRINITY_DN4745_c0_g1_i1 | 1.617715098 | 0.234365093 | 0.999970678 | 64.378 | 7.687 | 7.866 | 14.246 | NA |
| TRINITY_DN2650_c0_g1_i2 | -1.617636 | 0.488572082 | 0.999970678 | 11.028 | 1.705 | 32.298 | 0.161 | NA |
| TRINITY_DN16_c1_g1_i1 | 1.617347404 | 0.445299113 | 0.999970678 | 12.063 | 0.503 | 3.155 | 0.312 | NA |
| TRINITY_DN2812_c0_g3_i2 | 1.617282324 | 0.442078856 | 0.999970678 | 6.613 | 0.128 | 0.711 | 1.317 | NA |
| TRINITY_DN13755_c0_g1_i2 | 1.616923981 | 0.060829768 | 0.999970678 | 19.994 | 19.09 | 5.133 | 6.163 | NA |
| TRINITY_DN2775_c0_g1_i6 | -1.61672166 | 0.70236968 | 0.999970678 | 2.453 | 0 | 0 | 7.651 | NA |
| TRINITY_DN203_c0_g1_i7 | 1.616695346 | 0.192643235 | 0.999970678 | 10.483 | 69.63 | 9.622 | 14.286 | NA |
| TRINITY_DN686_c0_g1_i16 | 1.615924407 | 0.544454181 | 0.999970678 | 3.37 | 0.719 | 0 | 1.347 | NA |
| TRINITY_DN2575_c0_g1_i4 | -1.61559948 | 0.145829127 | 0.999970678 | 9.702 | 4.139 | 25.81 | 11.129 | Methyltransf_11 |
| TRINITY_DN10797_c0_g4_i1 | -1.61557957 | 0.54128322 | 0.999970678 | 1.472 | 0 | 2.155 | 2.001 | NA |
| TRINITY_DN4407_c0_g1_i2 | 1.615189027 | 0.584429339 | 0.999970678 | 0.127 | 4.356 | 0 | 1.438 | - |
| TRINITY_DN397_c0_g1_i1 | 1.614852223 | 0.174653827 | 0.999970678 | 0.627 | 0.808 | 0.3 | 0.111 | PUNUT |
| TRINITY_DN9357_c0_g4_i1 | -1.61476694 | 0.555174055 | 1 | 0.881 | 0 | 1.678 | 0.714 | NA |
| TRINITY_DN13183_c4_g1_i1 | -1.61439122 | 0.207376928 | 0.999970678 | 2.716 | 0.719 | 2.533 | 7.52 | NA |
| TRINITY_DN627_c0_g1_i11 | -1.61433643 | 0.363063722 | 0.999970678 | 0.064 | 1.025 | 1.211 | 1.82 | NA |
| TRINITY_DN6893_c0_g1_i1 | -1.61397206 | 0.212298724 | 0.999970678 | 2.407 | 6.928 | 18.854 | 5.469 | NA |
| TRINITY_DN14483_c0_g2_i4 | 1.613884421 | 0.476768193 | 0.999970678 | 0.136 | 4.07 | 0.022 | 1.317 | ABC_membrane |
| TRINITY_DN2172_c0_g1_i1 | -1.61380634 | 0.19858743 | 0.999970678 | 5.296 | 2.089 | 15.466 | 4.052 | Transferase |

| TRINITY_DN3892_c0_g2_i4 | 1.613724032 | 0.528418062 | 0.999970678 | 1.145 | 2.178 | 0 | 1.076 | NA |
| --- | --- | --- | --- | --- | --- | --- | --- | --- |
| TRINITY_DN3143_c0_g1_i1 | -1.61357166 | 0.264376912 | 0.999970678 | 1.799 | 2.286 | 1.089 | 11.099 | EF-hand_1 |
| TRINITY_DN21793_c0_g1_i5 | -1.6134069 | 0.541555395 | 0.999970678 | 0.6 | 0 | 0.511 | 1.267 | NA |
| TRINITY_DN2702_c0_g1_i6 | -1.61327423 | 0.541429994 | 0.999970678 | 0.2 | 0 | 0.244 | 0.332 | NA |
| TRINITY_DN1784_c0_g1_i5 | -1.61310241 | 0.202616468 | 0.999970678 | 0.336 | 0.158 | 0.722 | 0.653 | FPN1 |
| TRINITY_DN868_c0_g1_i5 | -1.612675 | 0.60151282 | 0.999970678 | 0.082 | 1.833 | 4.755 | 0 | NA |
| TRINITY_DN7419_c0_g1_i1 | -1.61267054 | 0.326003891 | 0.999970678 | 1.799 | 1.212 | 0.4 | 8.767 | Lipase_3 |
| TRINITY_DN17442_c0_g1_i1 | 1.612607135 | 0.711794027 | 0.999970678 | 2.616 | 0 | 0.722 | 0 | NA |
| TRINITY_DN2349_c0_g1_i6 | 1.612381357 | 0.57301929 | 0.999970678 | 7.694 | 0 | 1.3 | 0.985 | NA |
| TRINITY_DN9001_c0_g1_i2 | 1.612071575 | 0.701597573 | 0.999970678 | 9.638 | 0 | 2.644 | 0 | NA |
| TRINITY_DN16867_c0_g1_i1 | 1.611935389 | 0.210253393 | 1 | 1.036 | 1.902 | 0.4 | 0.462 | NA |
| TRINITY_DN729_c0_g2_i2 | 1.611814454 | 0.526852878 | 0.999970678 | 2.48 | 1.606 | 0 | 1.347 | BAG |
| TRINITY_DN1586_c0_g1_i3 | 1.611550548 | 0.386810642 | 0.999970678 | 0.545 | 19.21 | 1.644 | 4.283 | NA |
| TRINITY_DN3658_c0_g1_i14 | -1.61120606 | 0.132792734 | 0.999970678 | 28.887 | 10.23 | 64.174 | 39.712 | NA |
| TRINITY_DN2382_c0_g1_i1 | -1.61098909 | 0.210218038 | 0.999970678 | 5.323 | 18.07 | 10.51 | 58.412 | K_trans |
| TRINITY_DN826_c0_g3_i3 | -1.61077903 | 0.451559071 | 0.999970678 | 7.24 | 0.237 | 16.232 | 3.328 | NA |
| TRINITY_DN11258_c0_g1_i4 | -1.6106503 | 0.708537602 | 0.999970678 | 0.718 | 0 | 1.867 | 0 | NA |
| TRINITY_DN2906_c0_g1_i8 | 1.610351969 | 0.31200583 | 0.999970678 | 11.818 | 7.184 | 0.3 | 5.891 | - |
| TRINITY_DN6597_c0_g1_i4 | 1.610349585 | 0.057651292 | 0.999970678 | 2.961 | 5.608 | 1.155 | 1.397 | zf-RING_2 |
| TRINITY_DN2561_c0_g1_i1 | 1.610080621 | 0.150097795 | 0.999970678 | 3.416 | 13.08 | 1.755 | 3.237 | Rubis-subs-bind |
| TRINITY_DN1437_c0_g1_i3 | -1.60982527 | 0.184602261 | 0.999970678 | 2.053 | 0.581 | 3.011 | 4.474 | NA |
| TRINITY_DN22344_c0_g1_i2 | 1.609688127 | 0.009479707 | 0.46504651 | 18.858 | 24.62 | 5.933 | 7.068 | Cation_efflux |
| TRINITY_DN1410_c0_g2_i1 | 1.60937717 | 0.580814654 | 0.999970678 | 6.613 | 0.276 | 0 | 2.302 | NA |
| TRINITY_DN1039_c1_g1_i3 | 1.608368391 | 0.570954196 | 0.999970678 | 5.496 | 0 | 0.589 | 1.126 | B_lectin |
| TRINITY_DN9731_c0_g1_i3 | 1.608288849 | 0.226622298 | 1 | 1.662 | 1.281 | 0.533 | 0.282 | NA |
| TRINITY_DN2143_c0_g1_i5 | 1.608172629 | 0.181434345 | 0.999970678 | 1.581 | 0.818 | 0.456 | 0.251 | AAA |
| TRINITY_DN5146_c0_g1_i1 | 1.608162492 | 0.529207479 | 0.999970678 | 2.344 | 1.675 | 0 | 1.297 | mTERF |
| TRINITY_DN3805_c0_g1_i1 | 1.607882868 | 0.47220723 | 0.999970678 | 28.26 | 0.443 | 7.6 | 0.362 | AP2 |
| TRINITY_DN7324_c0_g1_i15 | -1.60751134 | 0.538662448 | 0.999970678 | 0.409 | 0 | 0.533 | 0.623 | LRR_8 |
| TRINITY_DN10427_c0_g1_i14 | -1.60750453 | 0.543983509 | 0.999970678 | 0.69 | 0 | 0.8 | 1.166 | NA |
| TRINITY_DN1632_c0_g1_i6 | 1.607457781 | 0.27598867 | 0.999970678 | 2.643 | 0.355 | 0.567 | 0.292 | NA |
| TRINITY_DN671_c0_g2_i2 | -1.60721999 | 0.568547452 | 0.999970678 | 3.352 | 0 | 7.766 | 0.935 | 2OG-FeII_Oxy |
| TRINITY_DN2146_c0_g1_i4 | 1.606982626 | 0.530115439 | 0.999970678 | 3.443 | 2.188 | 0 | 1.86 | NA |
| TRINITY_DN1315_c1_g1_i4 | -1.60694526 | 0.418085464 | 0.999970678 | 7.185 | 0.118 | 3.6 | 18.278 | AUX_IAA |
| TRINITY_DN6218_c0_g1_i10 | -1.60678711 | 0.553365161 | 0.999970678 | 0.908 | 0 | 1.667 | 0.724 | NA |
| TRINITY_DN16529_c1_g1_i1 | 1.606544215 | 0.550802154 | 1 | 2.98 | 2.671 | 1.522 | 0 | NA |
| TRINITY_DN2707_c0_g2_i1 | -1.60640247 | 0.237605291 | 0.999970678 | 13.253 | 18.2 | 8.111 | 85.406 | NA |
| TRINITY_DN3341_c0_g1_i2 | -1.60637499 | 0.708349639 | 0.999970678 | 0 | 1.212 | 3 | 0 | NA |
| TRINITY_DN7252_c0_g1_i8 | 1.605406489 | 0.17968896 | 0.999970678 | 0.6 | 0.857 | 0.256 | 0.171 | RRM_1 |
| TRINITY_DN4519_c0_g1_i1 | 1.605365532 | 0.286226264 | 0.999970678 | 196.4 | 19.85 | 45.642 | 16.649 | Staygreen |
| TRINITY_DN836_c0_g1_i3 | 1.604536902 | 0.319774549 | 0.999970678 | 0.736 | 5.095 | 1.367 | 0.241 | RRM_1 |
| TRINITY_DN8775_c0_g1_i3 | -1.60410176 | 0.209216503 | 0.999970678 | 0.69 | 1.124 | 1.267 | 4.001 | NA |
| TRINITY_DN8069_c0_g2_i2 | -1.60373561 | 0.192932078 | 1 | 0.118 | 0.138 | 0.3 | 0.432 | NA |
| TRINITY_DN4544_c0_g1_i1 | 1.603507331 | 0.575522619 | 0.999970678 | 10.147 | 0 | 1.055 | 2.131 | - |
| TRINITY_DN2120_c0_g1_i3 | -1.60348903 | 0.606398636 | 0.999970678 | 0 | 0.424 | 1.033 | 0.03 | NA |
| TRINITY_DN22020_c0_g2_i5 | 1.603378254 | 0.273183514 | 1 | 1.962 | 1.804 | 0.778 | 0.282 | NA |
| TRINITY_DN2661_c1_g1_i1 | -1.60317968 | 0.342611553 | 0.999970678 | 25.653 | 33.75 | 144.147 | 5.69 | ubiquitin |
| TRINITY_DN2922_c0_g3_i2 | 1.602893806 | 0.540028029 | 0.999970678 | 2.026 | 4.937 | 0 | 2.292 | NA |
| TRINITY_DN2318_c0_g1_i6 | 1.602493644 | 0.555422829 | 0.999970678 | 0.254 | 1.291 | 0.411 | 0 | NA |
| TRINITY_DN12451_c0_g1_i3 | 1.602023376 | 0.266952594 | 0.999970678 | 0.463 | 0.739 | 0.078 | 0.302 | NA |
| TRINITY_DN10732_c0_g1_i1 | 1.601771031 | 0.297560489 | 0.999970678 | 109.02 | 8.702 | 23.776 | 10.556 | PNRC |
| TRINITY_DN7929_c0_g1_i2 | 1.601204659 | 0.147333048 | 0.999970678 | 2.489 | 0.986 | 0.411 | 0.633 | Lactamase_B |
| TRINITY_DN1703_c0_g1_i3 | 1.600594024 | 0.052103891 | 0.999970678 | 7.149 | 6.642 | 2.322 | 1.759 | Glyco_transf_20 |
| TRINITY_DN7885_c0_g1_i5 | -1.60048773 | 0.624166317 | 0.999970678 | 0 | 4.326 | 10.555 | 0.08 | NA |
| TRINITY_DN140_c0_g2_i3 | -1.60044887 | 0.146079555 | 0.999970678 | 9.475 | 6.702 | 8.288 | 39.159 | ADH_N |
| TRINITY_DN466_c1_g1_i6 | 1.60041641 | 0.293130638 | 0.999970678 | 0.945 | 0.306 | 0.267 | 0.101 | Glyco_transf_5 |
| TRINITY_DN3318_c0_g1_i8 | -1.60014538 | 0.427479497 | 0.999970678 | 0.109 | 2.986 | 6.555 | 1.287 | NA |
| TRINITY_DN7434_c0_g1_i4 | 1.600115276 | 0.261734604 | 0.999970678 | 3.288 | 2.661 | 0.333 | 1.508 | NA |
| TRINITY_DN7292_c0_g1_i1 | -1.59980692 | 0.148504151 | 0.999970678 | 2.117 | 1.537 | 2.278 | 8.355 | NA |
| TRINITY_DN7656_c0_g1_i8 | 1.599585086 | 0.558074814 | 0.999970678 | 0.263 | 2.109 | 0 | 0.774 | NT-C2 |
| TRINITY_DN4631_c0_g1_i4 | 1.599360776 | 0.552579946 | 1 | 2.816 | 2.907 | 1.622 | 0 | NA |
| TRINITY_DN14595_c0_g1_i5 | -1.59933142 | 0.149847365 | 0.999970678 | 88.559 | 31.31 | 195.988 | 109.947 | NA |
| TRINITY_DN10906_c0_g1_i1 | -1.59923623 | 0.172512395 | 0.999970678 | 2.371 | 2.848 | 2.467 | 12.798 | NA |
| TRINITY_DN940_c0_g1_i1 | -1.59891093 | 0.034381648 | 0.999970678 | 5.042 | 6.504 | 17.866 | 13.251 | 40S_SA_C |
| TRINITY_DN1209_c0_g1_i10 | 1.598830966 | 0.440807659 | 0.999970678 | 0.518 | 6.16 | 0.167 | 1.93 | NA |
| TRINITY_DN798_c0_g2_i12 | -1.59874337 | 0.54166969 | 0.999970678 | 1.254 | 0 | 1.455 | 2.121 | NA |
| TRINITY_DN7337_c0_g1_i1 | 1.598676971 | 0.594874452 | 0.999970678 | 1.063 | 0.049 | 0.311 | 0 | - |
| TRINITY_DN6506_c0_g1_i1 | -1.59862139 | 0.172892543 | 0.999970678 | 1.917 | 1.764 | 6 | 3.73 | Ldh_1_C |
| TRINITY_DN1583_c0_g1_i1 | 1.598418298 | 0.557301176 | 0.999970678 | 35.418 | 4.888 | 0 | 12.768 | - |
| TRINITY_DN12747_c0_g2_i3 | 1.598070334 | 0.533565836 | 0.999970678 | 7.494 | 13.46 | 0 | 6.475 | NA |
| TRINITY_DN2864_c0_g1_i5 | 1.597822747 | 0.180431758 | 0.999970678 | 12.39 | 8.387 | 2.122 | 4.001 | NA |
| TRINITY_DN2813_c0_g1_i4 | -1.59765045 | 0.701689035 | 0.999970678 | 3.379 | 0 | 0 | 10.305 | NA |
| TRINITY_DN2665_c0_g1_i1 | 1.597542251 | 0.583556769 | 0.999970678 | 132.66 | 115.1 | 0 | 81.817 | GST_C_2 |
| TRINITY_DN1381_c0_g2_i4 | -1.59702286 | 0.47552055 | 0.999970678 | 2.335 | 0.099 | 0.2 | 7.239 | NA |

| TRINITY_DN2113_c0_g1_i7 | -1.59698369 | 0.27851831 | 0.999970678 | 1.435 | 0.256 | 3.455 | 1.005 | GMC_oxred_C |
| --- | --- | --- | --- | --- | --- | --- | --- | --- |
| TRINITY_DN8672_c0_g1_i4 | 1.59647103 | 0.549996594 | 1 | 0.518 | 0.414 | 0.244 | 0 | NA |
| TRINITY_DN6416_c0_g1_i3 | 1.596462679 | 0.70803835 | 0.999970678 | 0 | 3.459 | 0.911 | 0 | NA |
| TRINITY_DN28317_c0_g1_i1 | -1.59597834 | 0.036963673 | 0.999970678 | 0.827 | 0.641 | 2 | 2.021 | Arm |
| TRINITY_DN29753_c0_g1_i1 | 1.59582979 | 0.703100273 | 0.999970678 | 3.761 | 0 | 0 | 1.267 | RINGv |
| TRINITY_DN8600_c0_g1_i3 | -1.59581865 | 0.360360439 | 0.999970678 | 0.808 | 0.187 | 0.378 | 2.664 | NA |
| TRINITY_DN15182_c0_g1_i1 | -1.59574957 | 0.056698968 | 0.999970678 | 86.724 | 54.3 | 119.348 | 282.288 | NA |
| TRINITY_DN1924_c0_g1_i3 | -1.59538851 | 0.040119177 | 0.999970678 | 0.981 | 1.202 | 2.244 | 3.83 | HSP70 |
| TRINITY_DN1177_c0_g1_i1 | -1.59520538 | 0.314362588 | 0.999970678 | 1.535 | 2.316 | 0.889 | 10.597 | NA |
| TRINITY_DN803_c0_g1_i1 | -1.59506667 | 0.551201946 | 0.999970678 | 3.561 | 0 | 6.933 | 2.433 | NA |
| TRINITY_DN13992_c0_g1_i1 | -1.59491385 | 0.576735179 | 0.999970678 | 0 | 2.217 | 5.089 | 0.442 | NA |
| TRINITY_DN21069_c0_g1_i5 | 1.594793535 | 0.45091358 | 0.999970678 | 30.84 | 3.095 | 8.966 | 0.322 | NA |
| TRINITY_DN3404_c0_g1_i6 | -1.59442283 | 0.547832846 | 0.999970678 | 1.372 | 0 | 1.244 | 2.714 | NA |
| TRINITY_DN4662_c0_g1_i3 | -1.59422548 | 0.256931764 | 0.999970678 | 0.064 | 0.256 | 0.367 | 0.503 | NA |
| TRINITY_DN12236_c0_g1_i6 | -1.59414549 | 0.550103354 | 1 | 0.3 | 0 | 0.333 | 0.533 | TPR_16 |
| TRINITY_DN10637_c0_g1_i9 | 1.593753259 | 0.568575597 | 0.999970678 | 3.743 | 0.542 | 1.178 | 0 | NA |
| TRINITY_DN20302_c0_g1_i7 | 1.5936834 | 0.544571966 | 0.999970678 | 0.718 | 0.296 | 0 | 0.342 | NA |
| TRINITY_DN9986_c0_g1_i2 | -1.59347341 | 0.41747534 | 0.999970678 | 7.458 | 12.35 | 0.556 | 58.211 | DUF1685 |
| TRINITY_DN2208_c0_g1_i3 | 1.593255199 | 0.23266211 | 1 | 0.418 | 0.562 | 0.1 | 0.201 | NA |
| TRINITY_DN23055_c0_g1_i3 | -1.59284674 | 0.093130947 | 0.999970678 | 2.544 | 1.173 | 5.7 | 4.373 | NA |
| TRINITY_DN1219_c1_g1_i3 | -1.59250089 | 0.544987265 | 0.999970678 | 1.599 | 0 | 2.5 | 1.91 | HGTP_anticodon |
| TRINITY_DN15509_c1_g1_i1 | -1.59244523 | 0.199856436 | 0.999970678 | 0.536 | 0.365 | 0.811 | 1.729 | NA |
| TRINITY_DN12431_c0_g1_i2 | 1.592270848 | 0.706039541 | 0.999970678 | 4.451 | 0 | 0 | 1.498 | NA |
| TRINITY_DN1838_c0_g2_i3 | 1.592123149 | 0.555068756 | 1 | 0.254 | 0.315 | 0.167 | 0 | NA |
| TRINITY_DN8493_c0_g1_i7 | 1.592123115 | 0.242154369 | 0.999970678 | 0.572 | 1.892 | 0.133 | 0.664 | Acetyltransf_1 |
| TRINITY_DN6802_c0_g2_i5 | -1.59193264 | 0.705028899 | 0.999970678 | 5.505 | 0 | 13.855 | 0 | - |
| TRINITY_DN157_c0_g1_i2 | -1.59114991 | 0.152240841 | 0.999970678 | 0.69 | 0.788 | 1.122 | 3.117 | HMA |
| TRINITY_DN5098_c0_g1_i7 | 1.590994844 | 0.547492828 | 0.999970678 | 0.763 | 0.355 | 0.3 | 0 | NA |
| TRINITY_DN6551_c5_g1_i3 | -1.59078211 | 0.219630661 | 0.999970678 | 0.972 | 2.602 | 7.366 | 1.84 | - |
| TRINITY_DN10755_c0_g1_i4 | -1.59074531 | 0.712185243 | 0.999970678 | 1.263 | 0 | 3.178 | 0 | NA |
| TRINITY_DN2393_c0_g1_i1 | -1.59073348 | 0.615484501 | 0.999970678 | 3.107 | 0 | 0.078 | 9.42 | Abhydrolase_3 |
| TRINITY_DN8168_c0_g1_i1 | -1.59063464 | 0.222316378 | 0.999970678 | 15.425 | 4.77 | 9.099 | 50.268 | NA |
| TRINITY_DN14187_c0_g1_i2 | -1.59050939 | 0.191749154 | 0.999970678 | 0.354 | 0.384 | 1.255 | 0.794 | NA |
| TRINITY_DN2724_c0_g1_i3 | -1.59041538 | 0.543950499 | 0.999970678 | 0 | 0.365 | 0.356 | 0.653 | EGF_CA |
| TRINITY_DN466_c0_g1_i6 | 1.590117461 | 0.152758564 | 0.999970678 | 9.393 | 3.351 | 0.989 | 3.086 | NA |
| TRINITY_DN98406_c0_g1_i3 | -1.59005233 | 0.716068606 | 1 | 0.282 | 0 | 0 | 0.855 | NA |
| TRINITY_DN970_c0_g1_i4 | 1.589929161 | 0.639634746 | 0.999970678 | 21.738 | 0.049 | 0 | 7.329 | NA |
| TRINITY_DN6014_c0_g1_i4 | 1.589869997 | 0.077394998 | 0.999970678 | 4.351 | 4.464 | 1.422 | 1.196 | NA |
| TRINITY_DN1941_c0_g1_i5 | -1.5896242 | 0.244401803 | 0.999970678 | 8.748 | 6.918 | 4.455 | 41.341 | NA |
| TRINITY_DN142_c0_g2_i3 | -1.58960403 | 0.57162017 | 0.999970678 | 17.514 | 0 | 20.454 | 28.864 | NA |
| TRINITY_DN6148_c0_g1_i3 | -1.58937185 | 0.537367737 | 0.999970678 | 0 | 1.873 | 2.633 | 2.373 | NA |
| TRINITY_DN11692_c0_g1_i2 | 1.589309681 | 0.540897082 | 0.999970678 | 2.498 | 1.991 | 1.233 | 0 | DEAD |
| TRINITY_DN1610_c0_g1_i6 | 1.589207055 | 0.704650025 | 0.999970678 | 2.943 | 0 | 0 | 0.995 | - |
| TRINITY_DN1832_c0_g1_i7 | 1.58916514 | 0.25783563 | 0.999970678 | 0.881 | 2.217 | 0.6 | 0.312 | NHL |
| TRINITY_DN8758_c0_g1_i6 | -1.58912019 | 0.537524756 | 0.999970678 | 0 | 0.838 | 1.078 | 1.196 | Trigger_N |
| TRINITY_DN4071_c0_g1_i1 | -1.58904686 | 0.578459835 | 0.999970678 | 0 | 7.451 | 16.91 | 1.578 | NA |
| TRINITY_DN10293_c0_g1_i12 | 1.589023597 | 0.229548611 | 0.999970678 | 3.388 | 9.619 | 1.811 | 2.182 | NB-ARC |
| TRINITY_DN10482_c0_g1_i17 | 1.589010334 | 0.177684794 | 0.999970678 | 1.717 | 2.119 | 0.678 | 0.452 | NA |
| TRINITY_DN4252_c0_g1_i5 | 1.588939051 | 0.139001126 | 0.999970678 | 3.761 | 1.468 | 0.967 | 0.583 | Sugar_tr |
| TRINITY_DN44013_c0_g1_i5 | 1.588918637 | 0.39749746 | 0.999970678 | 3.752 | 121.4 | 14.188 | 20.801 | NA |
| TRINITY_DN8758_c0_g1_i3 | -1.58887174 | 0.715395857 | 0.999970678 | 0.427 | 0 | 0 | 1.287 | Trigger_N |
| TRINITY_DN6980_c0_g1_i3 | 1.588808297 | 0.556962149 | 0.999970678 | 5.632 | 0.956 | 0 | 2.222 | Pkinase |
| TRINITY_DN2661_c1_g1_i2 | -1.58829751 | 0.567797128 | 0.999970678 | 0 | 0.65 | 0.222 | 1.669 | - |
| TRINITY_DN103_c0_g1_i6 | -1.58814973 | 0.580638677 | 0.999970678 | 0 | 10.71 | 23.143 | 3.76 | NA |
| TRINITY_DN858_c0_g1_i4 | 1.58793259 | 0.411945227 | 0.999970678 | 7.449 | 2.878 | 2.778 | 0.08 | ABC2_membrane |
| TRINITY_DN55_c1_g1_i4 | 1.587902798 | 0.227340332 | 1 | 0.79 | 0.838 | 0.289 | 0.191 | NA |
| TRINITY_DN2049_c0_g1_i5 | 1.587489103 | 0.703808973 | 0.999970678 | 3.452 | 0 | 0 | 1.176 | DYW_deaminase |
| TRINITY_DN3661_c0_g1_i2 | -1.58736164 | 0.576636718 | 0.999970678 | 18.422 | 0 | 18.699 | 33.69 | NA |
| TRINITY_DN59308_c0_g1_i1 | 1.58710075 | 0.345759567 | 0.999970678 | 26.625 | 9.905 | 9.077 | 1.025 | NA |
| TRINITY_DN4299_c0_g1_i2 | -1.58708407 | 0.541349412 | 0.999970678 | 0 | 1.143 | 1.844 | 1.156 | WD40 |
| TRINITY_DN1978_c0_g1_i6 | -1.58707331 | 0.56446613 | 0.999970678 | 0 | 2.799 | 0.989 | 7.128 | NA |
| TRINITY_DN2965_c1_g1_i2 | 1.586896871 | 0.58383117 | 0.999970678 | 17.041 | 0 | 3.666 | 1.297 | - |
| TRINITY_DN1894_c0_g1_i4 | -1.58658882 | 0.565145809 | 0.999970678 | 6.84 | 0 | 11.044 | 7.52 | NA |
| TRINITY_DN2239_c0_g1_i1 | 1.586523434 | 0.13208438 | 0.999970678 | 23.936 | 9.343 | 4.278 | 5.751 | NA |
| TRINITY_DN3240_c0_g1_i17 | 1.586157287 | 0.544074291 | 0.999970678 | 1.026 | 0.355 | 0 | 0.473 | NA |
| TRINITY_DN14141_c2_g3_i2 | 1.586145931 | 0.549873616 | 1 | 0.463 | 0.365 | 0.233 | 0 | NA |
| TRINITY_DN10349_c0_g1_i5 | -1.58614532 | 0.550058493 | 0.999970678 | 1.008 | 0 | 1.778 | 0.915 | NA |
| TRINITY_DN3722_c0_g1_i2 | -1.5861265 | 0.157932286 | 0.999970678 | 2.28 | 4.987 | 4.111 | 16.729 | BTB |
| TRINITY_DN5058_c0_g1_i5 | 1.585989676 | 0.190652138 | 0.999970678 | 1.017 | 2.001 | 0.678 | 0.181 | Cnd1 |
| TRINITY_DN5841_c0_g1_i2 | -1.58595071 | 0.542129028 | 0.999970678 | 1.835 | 0 | 2.644 | 2.403 | Nup54 |
| TRINITY_DN2764_c0_g1_i9 | 1.585948325 | 0.167214431 | 0.999970678 | 1.117 | 0.394 | 0.244 | 0.211 | RVT_1 |
| TRINITY_DN6429_c0_g2_i1 | 1.585837895 | 0.424333895 | 0.999970678 | 7.83 | 0.414 | 1.844 | 0.533 | NA |
| TRINITY_DN30147_c0_g1_i1 | -1.58578512 | 0.552350187 | 0.999970678 | 0.736 | 0 | 0.633 | 1.488 | NA |
| TRINITY_DN3404_c0_g1_i3 | 1.585769415 | 0.550940469 | 1 | 0.345 | 0.424 | 0.2 | 0 | NA |

| TRINITY_DN1193_c0_g1_i3 | 1.585465277 | 0.704846475 | 0.999970678 | 0 | 14.02 | 0 | 4.615 | NA |
| --- | --- | --- | --- | --- | --- | --- | --- | --- |
| TRINITY_DN6062_c0_g1_i6 | 1.585448444 | 0.172678568 | 0.999970678 | 9.82 | 5.864 | 3.455 | 1.076 | Retrotran_gag_2 |
| TRINITY_DN2135_c0_g1_i2 | 1.58491957 | 0.094815593 | 0.999970678 | 0.654 | 0.877 | 0.211 | 0.261 | NA |
| TRINITY_DN1637_c0_g1_i1 | 1.584899418 | 0.577930298 | 0.999970678 | 0 | 12.43 | 1.067 | 2.795 | NA |
| TRINITY_DN1320_c0_g1_i3 | -1.58463451 | 0.562466356 | 0.999970678 | 0 | 11.43 | 20.254 | 9.249 | NA |
| TRINITY_DN1771_c0_g1_i2 | 1.584484842 | 0.077199632 | 0.999970678 | 9.366 | 7.125 | 1.644 | 3.489 | NA |
| TRINITY_DN23_c1_g1_i16 | -1.58443054 | 0.253678827 | 0.999970678 | 3.606 | 1.143 | 10.155 | 2.121 | NA |
| TRINITY_DN1394_c0_g1_i2 | -1.58426877 | 0.307285055 | 0.999970678 | 2.825 | 0.207 | 4.389 | 3.941 | Ribosomal_S4 |
| TRINITY_DN13928_c0_g1_i2 | 1.583934361 | 0.219528398 | 0.999970678 | 0.863 | 0.729 | 0.122 | 0.402 | NA |
| TRINITY_DN1035_c0_g2_i10 | 1.583903427 | 0.70613448 | 0.999970678 | 0 | 11.15 | 3.022 | 0 | Pyrophosphatase |
| TRINITY_DN2533_c0_g2_i2 | -1.5837958 | 0.095118483 | 0.999970678 | 126.13 | 94.39 | 405.164 | 170.078 | NA |
| TRINITY_DN2180_c0_g1_i8 | -1.58375769 | 0.706074183 | 0.999970678 | 3.843 | 0 | 9.633 | 0 | NA |
| TRINITY_DN7740_c0_g1_i1 | 1.583755135 | 0.589997188 | 0.999970678 | 0.118 | 3.351 | 0 | 1.146 | Jas |
| TRINITY_DN5749_c0_g1_i4 | 1.583043501 | 0.233253406 | 0.999970678 | 2.771 | 1.567 | 1.033 | 0.211 | DEAD |
| TRINITY_DN127_c0_g1_i4 | 1.583010441 | 0.584646552 | 0.999970678 | 0 | 229 | 36.709 | 29.246 | NA |
| TRINITY_DN7513_c0_g1_i1 | -1.58289406 | 0.545033325 | 0.999970678 | 3.743 | 0 | 5.255 | 4.856 | NA |
| TRINITY_DN5543_c0_g2_i3 | -1.58186548 | 0.19763132 | 0.999970678 | 4.533 | 1.242 | 8.722 | 6.726 | NA |
| TRINITY_DN91711_c0_g1_i2 | -1.58181766 | 0.7143378 | 0.999970678 | 0.454 | 0 | 1.167 | 0 | zf-C2H2 |
| TRINITY_DN16220_c0_g1_i1 | -1.58169831 | 0.164925243 | 0.999970678 | 0.254 | 0.542 | 0.978 | 1.176 | NA |
| TRINITY_DN11539_c0_g1_i1 | -1.58164335 | 0.706149269 | 0.999970678 | 4.378 | 0 | 10.966 | 0 | NA |
| TRINITY_DN4024_c0_g1_i3 | -1.58141996 | 0.157381139 | 0.999970678 | 36.654 | 51.56 | 39.986 | 213.581 | NA |
| TRINITY_DN2214_c0_g1_i1 | -1.58130271 | 0.376833714 | 0.999970678 | 0.981 | 0.512 | 0.211 | 4.223 | NA |
| TRINITY_DN252_c0_g1_i5 | -1.58121215 | 0.609166117 | 0.999970678 | 1.117 | 12.32 | 0 | 39.853 | NA |
| TRINITY_DN16886_c0_g1_i2 | 1.581195005 | 0.070845641 | 0.999970678 | 1.726 | 2.957 | 0.6 | 0.834 | zf-C3HC4_3 |
| TRINITY_DN39397_c0_g1_i1 | 1.581072375 | 0.264816875 | 1 | 1.526 | 1.34 | 0.267 | 0.613 | NA |
| TRINITY_DN7165_c0_g1_i1 | 1.580908843 | 0.096884333 | 0.999970678 | 9.665 | 4.464 | 1.578 | 2.825 | NA |
| TRINITY_DN26971_c0_g1_i1 | -1.58077469 | 0.223670588 | 0.999970678 | 0.263 | 0.483 | 0.489 | 1.609 | NA |
| TRINITY_DN4203_c1_g1_i6 | -1.58062671 | 0.076103246 | 0.999970678 | 2.135 | 1.399 | 3.211 | 6.706 | NA |
| TRINITY_DN2436_c0_g1_i11 | 1.580443308 | 0.108983948 | 0.999970678 | 2.553 | 4.11 | 1.289 | 0.674 | NA |
| TRINITY_DN7792_c0_g1_i2 | -1.58042856 | 0.546833877 | 0.999970678 | 0.681 | 0 | 1.111 | 0.744 | NA |
| TRINITY_DN8790_c0_g1_i4 | -1.58031196 | 0.36077311 | 0.999970678 | 0.127 | 1.212 | 1.833 | 1.739 | NA |
| TRINITY_DN5702_c0_g2_i2 | -1.58013265 | 0.557449837 | 0.999970678 | 0 | 0.66 | 1.3 | 0.332 | DUF4283 |
| TRINITY_DN1555_c0_g1_i12 | 1.580110981 | 0.712139643 | 0.999970678 | 1.335 | 0 | 0.378 | 0 | NA |
| TRINITY_DN1408_c0_g1_i8 | 1.580053554 | 0.580211167 | 0.999970678 | 2.298 | 0 | 0.211 | 0.533 | NA |
| TRINITY_DN6590_c0_g1_i4 | -1.57985501 | 0.587224671 | 0.999970678 | 0.227 | 1.212 | 3.511 | 0 | BRX |
| TRINITY_DN11277_c0_g1_i3 | 1.579841822 | 0.705038963 | 0.999970678 | 0 | 3.548 | 0 | 1.176 | TMEM135_C_rich |
| TRINITY_DN37206_c0_g1_i3 | -1.57974249 | 0.546717804 | 0.999970678 | 6.113 | 0 | 8.233 | 8.455 | NA |
| TRINITY_DN9949_c0_g1_i8 | 1.57964798 | 0.538426999 | 0.999970678 | 1.163 | 0.986 | 0 | 0.714 | Mem_trans |
| TRINITY_DN6131_c0_g1_i2 | 1.579553713 | 0.137058087 | 0.999970678 | 1.426 | 1.478 | 0.3 | 0.623 | NA |
| TRINITY_DN87714_c0_g1_i3 | 1.579503057 | 0.240721818 | 0.999970678 | 0.718 | 1.429 | 0.178 | 0.503 | NA |
| TRINITY_DN960_c0_g1_i7 | 1.579425523 | 0.242661145 | 0.999970678 | 35.954 | 17.62 | 1.878 | 15.593 | Epimerase |
| TRINITY_DN2268_c0_g1_i5 | -1.57941201 | 0.073214021 | 0.999970678 | 0.445 | 0.552 | 0.933 | 1.85 | Chloroa_b-bind |
| TRINITY_DN134_c0_g1_i1 | 1.5792056 | 0.024419517 | 0.983766272 | 6.877 | 10.03 | 2.589 | 2.523 | FAD_binding_8 |
| TRINITY_DN4276_c0_g1_i9 | 1.579129604 | 0.708925581 | 0.999970678 | 2.961 | 0 | 0 | 0.995 | NA |
| TRINITY_DN103_c0_g1_i9 | -1.57908455 | 0.595934894 | 0.999970678 | 13.362 | 0 | 30.12 | 4.142 | NA |
| TRINITY_DN112_c0_g1_i9 | -1.57883091 | 0.399927004 | 0.999970678 | 1.299 | 0.148 | 3.333 | 0.372 | NA |
| TRINITY_DN6470_c0_g1_i3 | 1.578720444 | 0.088131873 | 0.999970678 | 0.718 | 1.143 | 0.211 | 0.372 | MutS_I |
| TRINITY_DN8645_c0_g1_i1 | -1.57839646 | 0.293611482 | 0.999970678 | 17.041 | 23.54 | 6.422 | 113.255 | PP2C |
| TRINITY_DN250458_c0_g1_i1 | -1.57831034 | 0.367395836 | 0.999970678 | 3.706 | 0.128 | 5.455 | 5.007 | DUF4283 |
| TRINITY_DN6561_c0_g1_i1 | 1.577960545 | 0.176947675 | 0.999970678 | 0.336 | 0.779 | 0.178 | 0.161 | DHHC |
| TRINITY_DN3591_c0_g1_i1 | 1.577790132 | 0.059874093 | 0.999970678 | 53.096 | 85.24 | 25.576 | 15.292 | NA |
| TRINITY_DN36770_c0_g1_i3 | -1.5776875 | 0.564022293 | 0.999970678 | 0.345 | 0 | 0.678 | 0.211 | NA |
| TRINITY_DN42724_c0_g1_i1 | 1.57713918 | 0.148128671 | 0.999970678 | 1.735 | 2.178 | 0.667 | 0.503 | NA |
| TRINITY_DN5061_c2_g1_i1 | -1.57685304 | 0.551078484 | 0.999970678 | 16.006 | 0 | 18.988 | 24.853 | NA |
| TRINITY_DN393_c0_g1_i6 | 1.576734242 | 0.360483646 | 0.999970678 | 117.74 | 3.863 | 22.332 | 14.246 | NA |
| TRINITY_DN563_c1_g1_i4 | -1.57628078 | 0.217022059 | 0.999970678 | 0.672 | 0.315 | 0.844 | 1.83 | NA |
| TRINITY_DN335_c1_g1_i1 | -1.57599873 | 0.563462967 | 0.999970678 | 4.397 | 0 | 1.889 | 10.948 | LRRNT_2 |
| TRINITY_DN414_c0_g1_i9 | -1.57586879 | 0.176642391 | 0.999970678 | 7.712 | 12.63 | 8.722 | 49.917 | Oxidored_FMN |
| TRINITY_DN7235_c0_g3_i2 | -1.57499114 | 0.603815942 | 0.999970678 | 0.845 | 0 | 0.067 | 2.473 | NA |
| TRINITY_DN9946_c0_g1_i9 | 1.574967307 | 0.715930888 | 0.999970678 | 0 | 1.991 | 0.533 | 0 | NA |
| TRINITY_DN3818_c0_g1_i5 | -1.57484947 | 0.073264421 | 0.999970678 | 21.538 | 33.54 | 89.672 | 51.787 | NA |
| TRINITY_DN162_c0_g2_i4 | -1.57464407 | 0.552153309 | 0.999970678 | 4.061 | 0 | 2.933 | 8.696 | PCI |
| TRINITY_DN278_c0_g1_i4 | 1.5746226 | 0.15287376 | 0.999970678 | 10.81 | 49 | 7.933 | 10.345 | NA |
| TRINITY_DN6592_c0_g2_i3 | 1.574201736 | 0.553382048 | 0.999970678 | 1.79 | 0.532 | 0.656 | 0 | PH |
| TRINITY_DN30467_c1_g1_i1 | -1.57398141 | 0.717547104 | 0.999970678 | 0.527 | 0 | 1.322 | 0 | NA |
| TRINITY_DN3894_c0_g1_i7 | 1.57396553 | 0.598020038 | 0.999970678 | 1.59 | 0 | 0.056 | 0.473 | - |
| TRINITY_DN4451_c0_g1_i1 | -1.57385192 | 0.709659715 | 0.999970678 | 5.823 | 0 | 14.31 | 0 | NA |
| TRINITY_DN5922_c0_g1_i1 | -1.57380812 | 0.577861061 | 0.999970678 | 3.597 | 0 | 8.088 | 1.076 | FGGY_C |
| TRINITY_DN660_c0_g1_i1 | -1.57365474 | 0.712093518 | 0.999970678 | 0 | 0.325 | 0.8 | 0 | NA |
| TRINITY_DN2273_c0_g1_i4 | -1.57351298 | 0.583196628 | 0.999970678 | 0 | 0.719 | 1.622 | 0.141 | CBS |
| TRINITY_DN1699_c1_g1_i2 | 1.573133346 | 0.197120365 | 1 | 0.89 | 0.828 | 0.2 | 0.322 | SH3_9 |
| TRINITY_DN1397_c0_g1_i7 | 1.573002367 | 0.555472857 | 0.999970678 | 2.198 | 0.601 | 0.789 | 0 | NA |
| TRINITY_DN5583_c0_g1_i2 | 1.572825024 | 0.597930641 | 0.999970678 | 0 | 6.633 | 0.178 | 2.011 | Exostosin |
| TRINITY_DN520_c0_g1_i6 | 1.572316933 | 0.224502054 | 0.999970678 | 17.668 | 2.543 | 2.989 | 3.278 | LRR_1 |

| TRINITY_DN1924_c0_g1_i1 | -1.5722689 | 0.573145824 | 0.999970678 | 2.117 | 0 | 0.611 | 5.66 | HSP70 |
| --- | --- | --- | --- | --- | --- | --- | --- | --- |
| TRINITY_DN956_c0_g2_i2 | 1.572039107 | 0.187119197 | 0.999970678 | 1.145 | 2.927 | 0.289 | 1.005 | NA |
| TRINITY_DN7323_c0_g1_i1 | 1.571794562 | 0.345507742 | 0.999970678 | 1.917 | 0.187 | 0.433 | 0.191 | LRRNT_2 |
| TRINITY_DN15014_c0_g2_i1 | -1.57178572 | 0.362617883 | 0.999970678 | 10.001 | 1.882 | 2.611 | 31.538 | NA |
| TRINITY_DN1162_c0_g1_i7 | -1.57163184 | 0.074237866 | 0.999970678 | 2.634 | 1.439 | 6.488 | 4.333 | mTERF |
| TRINITY_DN1004_c0_g1_i8 | -1.57156484 | 0.542502303 | 0.999970678 | 0 | 1.222 | 1.444 | 1.84 | Metallophos |
| TRINITY_DN706_c0_g1_i1 | -1.57149785 | 0.154304552 | 0.999970678 | 2.516 | 9.195 | 10.288 | 22.058 | NA |
| TRINITY_DN9463_c0_g1_i1 | -1.57126038 | 0.02420638 | 0.976383772 | 2.353 | 1.961 | 5.244 | 6.525 | Y_phosphatase2 |
| TRINITY_DN8656_c0_g1_i1 | 1.571130525 | 0.543933438 | 0.999970678 | 3.815 | 2.74 | 0 | 2.222 | - |
| TRINITY_DN1540_c1_g1_i9 | 1.571073627 | 0.541348089 | 0.999970678 | 2.58 | 1.36 | 0 | 1.317 | NA |
| TRINITY_DN3938_c0_g1_i6 | 1.571035991 | 0.550125371 | 0.999970678 | 0.672 | 0.266 | 0.267 | 0 | NA |
| TRINITY_DN255_c1_g1_i1 | 1.570693595 | 0.730632669 | 0.999970678 | 62.198 | 0 | 0 | 21.404 | NA |
| TRINITY_DN77394_c0_g1_i1 | 1.570489757 | 0.189182687 | 0.999970678 | 2.316 | 1.675 | 0.356 | 0.905 | NA |
| TRINITY_DN6203_c0_g1_i3 | 1.570324605 | 0.540340591 | 0.999970678 | 0.4 | 0.562 | 0 | 0.322 | LRRNT_2 |
| TRINITY_DN193700_c0_g1_i1 | 1.570242278 | 0.346221549 | 0.999970678 | 2.444 | 5.361 | 0.189 | 2.363 | NA |
| TRINITY_DN5759_c0_g1_i6 | -1.57017352 | 0.586390953 | 0.999970678 | 1.363 | 0 | 0.367 | 3.67 | NA |
| TRINITY_DN577_c0_g1_i8 | 1.569771754 | 0.584868596 | 0.999970678 | 15.07 | 1.626 | 4.711 | 0 | NA |
| TRINITY_DN843_c0_g1_i12 | 1.569748544 | 0.71170981 | 0.999970678 | 0 | 6.711 | 0 | 2.222 | NA |
| TRINITY_DN6721_c0_g1_i4 | 1.569483801 | 0.160653672 | 0.999970678 | 0.718 | 0.394 | 0.189 | 0.151 | DRY_EERY |
| TRINITY_DN2354_c0_g1_i2 | 1.569430674 | 0.239723724 | 0.999970678 | 45.293 | 5.578 | 6.833 | 9.119 | BURP |
| TRINITY_DN2964_c0_g1_i2 | 1.569351977 | 0.252357805 | 0.999970678 | 2.943 | 4.198 | 1.767 | 0.271 | - |
| TRINITY_DN391_c1_g2_i3 | -1.56921339 | 0.251225594 | 0.999970678 | 0.282 | 2.07 | 2.978 | 3.237 | NA |
| TRINITY_DN8999_c0_g1_i4 | 1.568854449 | 0.300233757 | 0.999970678 | 0.454 | 3.844 | 0.756 | 0.503 | HSP70 |
| TRINITY_DN8728_c0_g1_i2 | -1.56835406 | 0.556817289 | 0.999970678 | 5.968 | 0 | 8.299 | 7.942 | NA |
| TRINITY_DN9902_c0_g4_i1 | 1.568082536 | 0.26412592 | 0.999970678 | 9.611 | 1.34 | 1.022 | 2.503 | NA |
| TRINITY_DN15117_c0_g1_i2 | -1.56792594 | 0.552437519 | 0.999970678 | 0 | 1.715 | 1.055 | 3.7 | F-box |
| TRINITY_DN3589_c0_g1_i4 | 1.567663519 | 0.081788329 | 0.999970678 | 0.917 | 1.025 | 0.356 | 0.221 | NA |
| TRINITY_DN1611_c0_g1_i9 | -1.56757924 | 0.547727531 | 0.999970678 | 1.644 | 0 | 2.422 | 2.001 | NA |
| TRINITY_DN1632_c0_g1_i12 | -1.56736921 | 0.546562045 | 0.999970678 | 0.173 | 0.01 | 0.433 | 0.01 | NA |
| TRINITY_DN4349_c1_g1_i7 | 1.567338431 | 0.239027099 | 0.999970678 | 1.208 | 1.744 | 0.156 | 0.804 | NA |
| TRINITY_DN11183_c0_g1_i4 | -1.56721535 | 0.720326379 | 0.999970678 | 0 | 1.38 | 3.278 | 0 | TIC20 |
| TRINITY_DN1347_c0_g3_i2 | 1.567041826 | 0.567901683 | 0.999970678 | 7.74 | 3.38 | 3.122 | 0 | AARP2CN |
| TRINITY_DN5857_c0_g1_i1 | 1.567018452 | 0.365498557 | 0.999970678 | 1.226 | 0.296 | 0.033 | 0.462 | NA |
| TRINITY_DN6717_c2_g1_i12 | 1.566942522 | 0.359614799 | 0.999970678 | 2.771 | 0.168 | 0.478 | 0.422 | NA |
| TRINITY_DN10073_c0_g1_i8 | -1.56661538 | 0.632285503 | 0.999970678 | 3.316 | 0.118 | 8.355 | 0 | eIF-5a |
| TRINITY_DN46130_c0_g1_i7 | -1.56625649 | 0.485017818 | 0.999970678 | 0.745 | 0.069 | 1.933 | 0.121 | Tyrosinase |
| TRINITY_DN3192_c2_g1_i2 | -1.56621685 | 0.205278232 | 0.999970678 | 0.372 | 0.591 | 0.7 | 2.011 | NA |
| TRINITY_DN56820_c0_g1_i2 | 1.565965377 | 0.124657805 | 0.999970678 | 4.615 | 5.391 | 0.856 | 2.322 | NA |
| TRINITY_DN562_c0_g1_i3 | -1.56580901 | 0.273986592 | 0.999970678 | 0.954 | 0.276 | 0.656 | 2.895 | Dynamin_N |
| TRINITY_DN4906_c0_g1_i1 | 1.565748587 | 0.24425365 | 0.999970678 | 1.608 | 0.867 | 0.478 | 0.271 | - |
| TRINITY_DN36_c1_g1_i7 | 1.565636654 | 0.368740434 | 1 | 0.654 | 0.434 | 0.267 | 0.05 | NA |
| TRINITY_DN304_c0_g1_i11 | -1.5655111 | 0.550266218 | 0.999970678 | 0.69 | 0 | 0.733 | 1.176 | NA |
| TRINITY_DN1268_c1_g1_i6 | 1.565438288 | 0.38520309 | 0.999970678 | 1.69 | 13.81 | 3.778 | 0.603 | NA |
| TRINITY_DN7692_c0_g1_i4 | -1.56541697 | 0.352039225 | 0.999970678 | 0.064 | 0.641 | 0.556 | 1.387 | NA |
| TRINITY_DN252_c0_g2_i1 | 1.565405018 | 0.315668143 | 0.999970678 | 3.606 | 0.65 | 0.955 | 0.292 | NA |
| TRINITY_DN9067_c0_g1_i4 | -1.56518075 | 0.574273025 | 0.999970678 | 0.182 | 0.365 | 0 | 1.629 | NA |
| TRINITY_DN9170_c0_g1_i5 | 1.564962015 | 0.103517461 | 0.999970678 | 1.599 | 3.055 | 0.744 | 0.674 | NA |
| TRINITY_DN1338_c0_g1_i3 | -1.56432101 | 0.545461771 | 0.999970678 | 0 | 2.158 | 2.467 | 3.278 | NA |
| TRINITY_DN1056_c1_g1_i9 | -1.56409739 | 0.578000503 | 0.999970678 | 0 | 0.887 | 1.9 | 0.271 | DUF4283 |
| TRINITY_DN4571_c0_g1_i1 | 1.564062952 | 0.259360806 | 0.999970678 | 28.424 | 55.47 | 2.378 | 25.365 | Epimerase |
| TRINITY_DN1898_c0_g1_i15 | 1.563909072 | 0.266376527 | 0.999970678 | 13.317 | 9.274 | 1.167 | 5.912 | NA |
| TRINITY_DN2436_c0_g2_i6 | -1.56351273 | 0.321611628 | 0.999970678 | 0.118 | 1.409 | 1.611 | 2.513 | NA |
| TRINITY_DN1675_c0_g1_i2 | -1.56340088 | 0.231780789 | 0.999970678 | 0.445 | 0.187 | 0.722 | 1.086 | NA |
| TRINITY_DN13194_c0_g2_i1 | 1.563397137 | 0.528401561 | 0.999970678 | 3.098 | 0.049 | 0.056 | 0.965 | NA |
| TRINITY_DN9367_c0_g1_i1 | 1.563277259 | 0.713724421 | 0.999970678 | 0 | 1.409 | 0 | 0.462 | NA |
| TRINITY_DN698_c1_g1_i15 | 1.563250465 | 0.234940327 | 1 | 0.372 | 0.473 | 0.111 | 0.161 | NA |
| TRINITY_DN3108_c0_g1_i9 | 1.563225407 | 0.209411615 | 0.999970678 | 5.841 | 3.765 | 2.189 | 0.603 | Bystin |
| TRINITY_DN5705_c0_g1_i2 | -1.56315378 | 0.556215235 | 0.999970678 | 0 | 2.957 | 5.277 | 2.252 | DnaJ |
| TRINITY_DN7100_c0_g1_i2 | 1.562922732 | 0.209347878 | 0.999970678 | 3.207 | 5.292 | 0.389 | 2.403 | NA |
| TRINITY_DN649_c1_g1_i5 | 1.56287677 | 0.36563553 | 0.999970678 | 0.899 | 0.759 | 0.444 | 0.03 | - |
| TRINITY_DN15781_c0_g1_i1 | -1.56274803 | 0.403886041 | 0.999970678 | 19.049 | 6.248 | 60.796 | 1.568 | NA |
| TRINITY_DN12212_c0_g1_i5 | 1.562610376 | 0.06585902 | 0.999970678 | 2.098 | 2.217 | 0.733 | 0.593 | NA |
| TRINITY_DN82_c0_g2_i1 | -1.56208329 | 0.145844741 | 0.999970678 | 5.877 | 6.238 | 23.476 | 7.359 | NA |
| TRINITY_DN564_c0_g1_i8 | -1.56180178 | 0.036227098 | 0.999970678 | 109.96 | 61.59 | 221.487 | 235.086 | NA |
| TRINITY_DN17333_c0_g1_i1 | -1.56173639 | 0.183227677 | 0.999970678 | 9.338 | 12.84 | 9.399 | 53.395 | - |
| TRINITY_DN750_c1_g1_i6 | 1.561643565 | 0.025343511 | 0.999970678 | 2.471 | 2.966 | 0.811 | 0.865 | zf-CCCH |
| TRINITY_DN104973_c0_g1_i6 | -1.56137869 | 0.156150648 | 0.999970678 | 1.281 | 1.311 | 1.322 | 6.123 | Ank_2 |
| TRINITY_DN491_c3_g1_i4 | 1.561191433 | 0.251716304 | 0.999970678 | 1.154 | 7.628 | 1.122 | 1.599 | NA |
| TRINITY_DN4384_c0_g1_i2 | 1.561172416 | 0.145045974 | 0.999970678 | 2.834 | 2.03 | 0.711 | 0.804 | Methyltransf_7 |
| TRINITY_DN601_c0_g1_i3 | -1.56074543 | 0.146856535 | 0.999970678 | 0.545 | 0.463 | 1.033 | 1.739 | NA |
| TRINITY_DN1596_c0_g1_i3 | 1.5604861 | 0.544273161 | 0.999970678 | 4.406 | 5.686 | 0 | 3.408 | NA |
| TRINITY_DN2595_c1_g2_i1 | 1.560336257 | 0.111896712 | 0.999970678 | 51.088 | 39.97 | 19.454 | 7.47 | ACC_central |
| TRINITY_DN3776_c0_g1_i1 | 1.560285679 | 0.709729899 | 0.999970678 | 12.899 | 0 | 0 | 4.454 | NA |
| TRINITY_DN17879_c0_g2_i1 | 1.560049067 | 0.439293809 | 0.999970678 | 0.772 | 0.276 | 0.289 | 0.01 | NA |

| TRINITY_DN6594_c0_g1_i1 | -1.56000434 | 0.717341182 | 0.999970678 | 3.161 | 0 | 7.711 | 0 | NA |
| --- | --- | --- | --- | --- | --- | --- | --- | --- |
| TRINITY_DN526_c0_g1_i2 | 1.559937626 | 0.26418008 | 0.999970678 | 1.026 | 0.404 | 0.289 | 0.131 | EF-hand_5 |
| TRINITY_DN7633_c0_g1_i2 | -1.55966125 | 0.147200346 | 0.999970678 | 7.294 | 3.508 | 19.266 | 8.656 | NA |
| TRINITY_DN10930_c0_g2_i1 | 1.559529804 | 0.585166231 | 0.999970678 | 8.012 | 0.562 | 2.444 | 0 | ABC_tran |
| TRINITY_DN2180_c0_g1_i6 | -1.55950819 | 0.710448825 | 0.999970678 | 3.906 | 0 | 9.622 | 0 | NA |
| TRINITY_DN8585_c1_g2_i2 | 1.559495871 | 0.252025019 | 0.999970678 | 1.699 | 0.907 | 0.544 | 0.231 | NA |
| TRINITY_DN9672_c0_g1_i5 | 1.559397015 | 0.098575028 | 0.999970678 | 1.008 | 1.528 | 0.367 | 0.412 | NA |
| TRINITY_DN6954_c0_g2_i2 | 1.558965756 | 0.720339591 | 0.999970678 | 1.199 | 0 | 0 | 0.422 | NA |
| TRINITY_DN96322_c0_g1_i6 | 1.558964432 | 0.159171395 | 0.999970678 | 7.839 | 4.09 | 0.922 | 2.926 | NA |
| TRINITY_DN105_c0_g2_i2 | 1.558902204 | 0.195262215 | 0.999970678 | 4.833 | 5.164 | 0.589 | 2.674 | NA |
| TRINITY_DN2119_c0_g1_i5 | 1.5587464 | 0.078011002 | 0.999970678 | 20.475 | 22.26 | 3.422 | 10.406 | NA |
| TRINITY_DN11588_c0_g1_i2 | -1.55825409 | 0.39726424 | 0.999970678 | 34.092 | 0.65 | 55.708 | 36.404 | NA |
| TRINITY_DN60718_c0_g1_i1 | 1.558217316 | 0.188202206 | 0.999970678 | 0.454 | 0.532 | 0.167 | 0.141 | Helicase_C |
| TRINITY_DN2114_c1_g1_i3 | 1.557973099 | 0.382761545 | 0.999970678 | 1.408 | 9.806 | 0.567 | 3.006 | NA |
| TRINITY_DN56_c5_g1_i1 | 1.557801333 | 0.304383007 | 0.999970678 | 2.643 | 1.715 | 0.167 | 1.277 | NA |
| TRINITY_DN11955_c0_g1_i1 | 1.557614691 | 0.249298283 | 0.999970678 | 0.463 | 0.315 | 0.167 | 0.07 | AAA_11 |
| TRINITY_DN2277_c0_g1_i4 | -1.55701305 | 0.260983481 | 0.999970678 | 1.481 | 2.897 | 2.233 | 9.943 | NA |
| TRINITY_DN3760_c0_g1_i1 | 1.556999527 | 0.259221726 | 0.999970678 | 1.944 | 2.474 | 0.244 | 1.196 | NA |
| TRINITY_DN29495_c0_g1_i1 | -1.55674177 | 0.253342454 | 0.999970678 | 17.786 | 11.95 | 7.588 | 78.348 | - |
| TRINITY_DN27283_c0_g1_i1 | -1.55659955 | 0.353611373 | 0.999970678 | 7.331 | 1.892 | 19.266 | 3.649 | NA |
| TRINITY_DN11585_c0_g1_i2 | 1.556292538 | 0.252508268 | 0.999970678 | 0.917 | 0.325 | 0.089 | 0.322 | DUF4219 |
| TRINITY_DN114351_c0_g1_i1 | 1.55609024 | 0.194687797 | 1 | 1.108 | 1.163 | 0.311 | 0.392 | ANTH |
| TRINITY_DN4168_c0_g1_i5 | 1.555893054 | 0.146014561 | 0.999970678 | 17.95 | 8.436 | 1.855 | 6.806 | DBINO |
| TRINITY_DN3724_c0_g2_i2 | 1.555891124 | 0.354575328 | 0.999970678 | 1.363 | 0.168 | 0.089 | 0.422 | Ank |
| TRINITY_DN1295_c0_g1_i3 | -1.55573582 | 0.578325765 | 0.999970678 | 7.948 | 0 | 4.066 | 18.821 | NA |
| TRINITY_DN250008_c0_g1_i1 | -1.55564081 | 0.712584224 | 0.999970678 | 2.816 | 0 | 6.922 | 0 | NA |
| TRINITY_DN1904_c0_g1_i3 | 1.555480365 | 0.075789865 | 0.999970678 | 2.898 | 4.031 | 1.211 | 0.925 | NA |
| TRINITY_DN1675_c0_g1_i7 | 1.555191518 | 0.16105657 | 0.999970678 | 1.544 | 2.247 | 0.711 | 0.442 | NA |
| TRINITY_DN23130_c0_g1_i1 | 1.555093232 | 0.58030095 | 0.999970678 | 0 | 4.159 | 0.7 | 0.533 | NA |
| TRINITY_DN5096_c3_g1_i1 | -1.55473602 | 0.301952621 | 0.999970678 | 1.526 | 1.991 | 0.933 | 9.068 | NA |
| TRINITY_DN1909_c0_g1_i5 | 1.554664726 | 0.590402376 | 0.999970678 | 2.262 | 0.128 | 0 | 0.834 | DUF3475 |
| TRINITY_DN1956_c0_g1_i4 | 1.554655832 | 0.720447962 | 0.999970678 | 0 | 0.611 | 0.167 | 0 | Filamin |
| TRINITY_DN5273_c0_g1_i1 | -1.55441716 | 0.574583812 | 0.999970678 | 1.263 | 0 | 0.511 | 3.167 | NA |
| TRINITY_DN321_c0_g1_i1 | -1.55437293 | 0.311009208 | 0.999970678 | 2.798 | 0.217 | 4.989 | 2.956 | NA |
| TRINITY_DN16764_c0_g1_i6 | 1.553964499 | 0.555539354 | 0.999970678 | 0.209 | 0.483 | 0 | 0.231 | DNA_gyraseB |
| TRINITY_DN49_c0_g2_i5 | -1.55394877 | 0.189211245 | 0.999970678 | 1.599 | 1.399 | 6.033 | 1.548 | G-alpha |
| TRINITY_DN872_c0_g1_i1 | 1.553915227 | 0.148118218 | 0.999970678 | 0.527 | 0.424 | 0.111 | 0.191 | NA |
| TRINITY_DN1344_c1_g1_i2 | 1.553722183 | 0.420310538 | 0.999970678 | 0.182 | 1.735 | 0.078 | 0.543 | NA |
| TRINITY_DN25279_c0_g1_i5 | 1.553614985 | 0.710683331 | 0.999970678 | 0 | 2.474 | 0.689 | 0 | Bromodomain |
| TRINITY_DN254003_c0_g1_i1 | 1.553374316 | 0.403670222 | 0.999970678 | 6.095 | 59.62 | 1.022 | 20.52 | NA |
| TRINITY_DN26105_c0_g3_i1 | 1.552668547 | 0.20863624 | 0.999970678 | 1.235 | 1.064 | 0.144 | 0.613 | Raffinose_syn |
| TRINITY_DN30323_c0_g1_i1 | 1.55246011 | 0.17880362 | 0.999970678 | 1.135 | 1.281 | 0.533 | 0.181 | NA |
| TRINITY_DN1798_c0_g1_i2 | 1.552450021 | 0.714097405 | 0.999970678 | 3.852 | 0 | 0 | 1.327 | NA |
| TRINITY_DN3295_c0_g1_i3 | -1.55244889 | 0.710146047 | 0.999970678 | 5.142 | 0 | 0 | 15.201 | KH_2 |
| TRINITY_DN1404_c0_g1_i4 | 1.552448313 | 0.277453446 | 0.999970678 | 1.79 | 5.223 | 0.322 | 1.991 | Ion_trans_2 |
| TRINITY_DN355_c0_g1_i3 | -1.55144391 | 0.550841581 | 0.999970678 | 1.835 | 0 | 2.211 | 2.735 | NA |
| TRINITY_DN5061_c1_g1_i5 | -1.55137818 | 0.560888707 | 0.999970678 | 1.026 | 0 | 1.089 | 1.669 | NA |
| TRINITY_DN20825_c0_g1_i5 | 1.551339768 | 0.620349766 | 0.999970678 | 4.742 | 0.049 | 1.378 | 0 | B_lectin |
| TRINITY_DN655_c0_g1_i7 | -1.55104623 | 0.554235715 | 0.999970678 | 2.062 | 0 | 3.089 | 2.393 | Semialdhyde_dh |
| TRINITY_DN10022_c0_g1_i1 | -1.55094968 | 0.280964414 | 0.999970678 | 1.608 | 9.678 | 5.233 | 26.331 | NA |
| TRINITY_DN6071_c0_g1_i2 | 1.550141576 | 0.248696434 | 0.999970678 | 1.472 | 0.483 | 0.322 | 0.271 | NA |
| TRINITY_DN2195_c0_g2_i1 | -1.55008344 | 0.257287071 | 0.999970678 | 0.472 | 0.089 | 0.878 | 0.613 | NA |
| TRINITY_DN6523_c0_g1_i1 | -1.55006611 | 0.717903087 | 0.999970678 | 1.508 | 0 | 3.733 | 0 | NA |
| TRINITY_DN6594_c0_g1_i13 | -1.55005489 | 0.71719088 | 0.999970678 | 28.097 | 0 | 66.84 | 0 | NA |
| TRINITY_DN12259_c0_g4_i1 | 1.550050442 | 0.493598126 | 0.999970678 | 0.018 | 1.468 | 0.111 | 0.362 | NA |
| TRINITY_DN3715_c0_g1_i4 | -1.55000708 | 0.714334436 | 0.999970678 | 3.216 | 0 | 7.888 | 0 | Peroxidase_2 |
| TRINITY_DN2486_c1_g1_i7 | -1.54960504 | 0.711943506 | 0.999970678 | 0 | 4.208 | 9.988 | 0 | NAM |
| TRINITY_DN120_c0_g1_i6 | 1.549449659 | 0.320919197 | 0.999970678 | 1.344 | 2.04 | 0.867 | 0.121 | NA |
| TRINITY_DN16590_c0_g1_i1 | -1.54939567 | 0.564583548 | 0.999970678 | 1.535 | 0 | 0.833 | 3.519 | NA |
| TRINITY_DN2404_c0_g1_i12 | 1.549257781 | 0.55127598 | 0.999970678 | 1.244 | 2.296 | 0.989 | 0 | - |
| TRINITY_DN1838_c0_g1_i1 | 1.549155639 | 0.485189163 | 0.999970678 | 1.581 | 0.03 | 0.411 | 0.05 | Myb_DNA-bindin |
| TRINITY_DN9752_c0_g1_i7 | 1.548595308 | 0.610427183 | 0.999970678 | 0.154 | 160.8 | 2.789 | 47.383 | NA |
| TRINITY_DN7516_c1_g1_i2 | -1.54858891 | 0.2456617 | 0.999970678 | 0.536 | 0.207 | 0.644 | 1.418 | NA |
| TRINITY_DN18109_c0_g1_i1 | -1.54844088 | 0.503549866 | 0.999970678 | 3.279 | 0.276 | 0.333 | 9.953 | NA |
| TRINITY_DN2573_c1_g1_i3 | 1.548370039 | 0.089438423 | 0.999970678 | 4.833 | 2.326 | 0.911 | 1.357 | NA |
| TRINITY_DN3561_c0_g1_i9 | -1.54822441 | 0.579629409 | 0.999970678 | 9.102 | 0 | 14.877 | 9.099 | Pyr_redox_2 |
| TRINITY_DN2164_c0_g1_i6 | -1.54788678 | 0.299587982 | 0.999970678 | 0.818 | 6.031 | 4.677 | 13.864 | NA |
| TRINITY_DN355_c0_g1_i1 | 1.547731766 | 0.170196574 | 0.999970678 | 4.397 | 18.38 | 2.8 | 4.333 | NA |
| TRINITY_DN4940_c0_g1_i1 | -1.54770747 | 0.066843743 | 0.999970678 | 2.48 | 3.568 | 9.477 | 6.183 | NA |
| TRINITY_DN8066_c0_g1_i7 | -1.54763604 | 0.576759625 | 0.999970678 | 1.235 | 0 | 0.589 | 2.895 | NA |
| TRINITY_DN13234_c0_g1_i1 | -1.54756279 | 0.582334201 | 0.999970678 | 0 | 0.128 | 0.267 | 0.05 | NA |
| TRINITY_DN9304_c0_g2_i1 | 1.547335186 | 0.57763587 | 0.999970678 | 10.537 | 1.873 | 3.511 | 0 | Aldedh |
| TRINITY_DN4180_c0_g2_i4 | 1.547213927 | 0.552969209 | 0.999970678 | 1.208 | 2.493 | 0 | 1.227 | NA |
| TRINITY_DN1193_c1_g1_i1 | 1.54699466 | 0.719857593 | 0.999970678 | 3.07 | 0 | 0 | 1.066 | NA |

| TRINITY_DN3237_c3_g1_i1 | -1.54674212 | 0.264626415 | 0.999970678 | 1.444 | 5.884 | 13.31 | 4.967 | NA |
| --- | --- | --- | --- | --- | --- | --- | --- | --- |
| TRINITY_DN4183_c0_g1_i3 | -1.54674053 | 0.377937718 | 0.999970678 | 8.366 | 0.315 | 13.844 | 8.847 | NA |
| TRINITY_DN2305_c0_g1_i2 | 1.54661966 | 0.318205182 | 0.999970678 | 0.345 | 2.296 | 0.244 | 0.583 | NA |
| TRINITY_DN7980_c0_g1_i1 | 1.546308706 | 0.335266767 | 0.999970678 | 0.79 | 1.508 | 0.089 | 0.684 | - |
| TRINITY_DN534_c0_g1_i1 | -1.54622696 | 0.559777077 | 0.999970678 | 0 | 1.823 | 2.855 | 1.79 | DPPIV_N |
| TRINITY_DN29066_c0_g2_i1 | -1.54622394 | 0.562610549 | 0.999970678 | 0 | 0.749 | 1.4 | 0.483 | NA |
| TRINITY_DN2330_c1_g1_i2 | -1.54622026 | 0.550914057 | 0.999970678 | 0 | 1.281 | 1.789 | 1.498 | LRR_8 |
| TRINITY_DN7947_c1_g1_i1 | -1.54594226 | 0.576719101 | 0.999970678 | 0.636 | 0 | 0.322 | 1.498 | NA |
| TRINITY_DN461_c1_g1_i5 | 1.54557214 | 0.115100289 | 0.999970678 | 5.687 | 5.322 | 2.333 | 0.955 | LisH |
| TRINITY_DN5224_c0_g1_i2 | 1.545338023 | 0.405060084 | 0.999970678 | 1.835 | 0.079 | 0.167 | 0.473 | NA |
| TRINITY_DN1165_c0_g1_i3 | 1.545273184 | 0.243179178 | 0.999970678 | 0.681 | 1.606 | 0.167 | 0.583 | NA |
| TRINITY_DN3204_c0_g2_i7 | 1.545232471 | 0.351943913 | 1 | 2.253 | 2.07 | 0.2 | 1.206 | NA |
| TRINITY_DN3268_c0_g2_i4 | 1.545035402 | 0.202837167 | 0.999970678 | 0.309 | 0.493 | 0.111 | 0.131 | NA |
| TRINITY_DN11072_c0_g4_i2 | 1.544908226 | 0.102249362 | 0.999970678 | 1.372 | 1.35 | 0.4 | 0.452 | CRAL_TRIO |
| TRINITY_DN5850_c0_g1_i7 | -1.54422312 | 0.195924556 | 0.999970678 | 0.581 | 0.483 | 2.022 | 0.674 | NA |
| TRINITY_DN26906_c0_g1_i1 | 1.54419424 | 0.284871407 | 0.999970678 | 9.874 | 3.853 | 0.756 | 3.79 | NA |
| TRINITY_DN17045_c0_g1_i4 | -1.54395702 | 0.425463488 | 0.999970678 | 1.517 | 0.315 | 4.1 | 0.332 | Lectin_legB |
| TRINITY_DN3370_c0_g1_i1 | -1.54385772 | 0.301640842 | 0.999970678 | 28.042 | 32.07 | 9.366 | 163.262 | NA |
| TRINITY_DN5096_c1_g1_i2 | 1.543753894 | 0.390747473 | 0.999970678 | 1.217 | 11.2 | 0.333 | 3.81 | NA |
| TRINITY_DN2027_c0_g2_i3 | 1.543641203 | 0.066067757 | 0.999970678 | 0.636 | 0.621 | 0.189 | 0.201 | DUF4283 |
| TRINITY_DN4642_c0_g1_i2 | 1.543393339 | 0.551940784 | 0.999970678 | 0.454 | 0.572 | 0.289 | 0 | NA |
| TRINITY_DN580_c0_g1_i2 | 1.543383164 | 0.341077665 | 0.999970678 | 1.326 | 12.27 | 3.055 | 0.905 | NA |
| TRINITY_DN6045_c0_g1_i1 | 1.543321172 | 0.323407042 | 0.999970678 | 3.161 | 43.21 | 4.289 | 10.355 | Cu_bind_like |
| TRINITY_DN21318_c0_g1_i1 | -1.54267431 | 0.153292416 | 0.999970678 | 0.545 | 1.705 | 2.644 | 3.288 | PA |
| TRINITY_DN6071_c0_g1_i4 | 1.542472511 | 0.578863631 | 0.999970678 | 2.816 | 0.335 | 0.011 | 1.106 | NA |
| TRINITY_DN5183_c0_g1_i1 | -1.54241114 | 0.570049046 | 0.999970678 | 5.269 | 0 | 6.344 | 7.912 | HisG |
| TRINITY_DN57447_c0_g1_i1 | 1.542315701 | 0.722319721 | 0.999970678 | 1.708 | 0 | 0.489 | 0 | NAD_binding_8 |
| TRINITY_DN1676_c0_g1_i2 | -1.54227702 | 0.712389716 | 0.999970678 | 1.09 | 0 | 0 | 3.237 | NA |
| TRINITY_DN4572_c0_g1_i1 | 1.542200028 | 0.306775608 | 0.999970678 | 4.787 | 0.818 | 1.233 | 0.452 | ANF_receptor |
| TRINITY_DN459_c0_g1_i4 | -1.5419991 | 0.599514349 | 0.999970678 | 2.08 | 13.34 | 0 | 43.945 | - |
| TRINITY_DN1618_c0_g1_i4 | 1.541660646 | 0.383927227 | 0.999970678 | 3.997 | 9.392 | 0.122 | 4.434 | NA |
| TRINITY_DN38_c5_g1_i1 | 1.54165316 | 0.456558984 | 0.999970678 | 6.686 | 4.77 | 3.233 | 0.03 | - |
| TRINITY_DN1391_c1_g1_i1 | 1.541548164 | 0.266990737 | 0.999970678 | 4.215 | 2.73 | 1.678 | 0.352 | CRAL_TRIO |
| TRINITY_DN5242_c0_g1_i4 | -1.54144154 | 0.565078915 | 0.999970678 | 0 | 2.562 | 1.222 | 5.891 | LysM |
| TRINITY_DN7396_c0_g1_i10 | 1.541406827 | 0.253101842 | 0.999970678 | 0.509 | 0.493 | 0.233 | 0.06 | NA |
| TRINITY_DN15762_c0_g1_i1 | 1.541065675 | 0.554569278 | 0.999970678 | 0.472 | 0.966 | 0.411 | 0 | NA |
| TRINITY_DN4522_c0_g2_i2 | 1.541001364 | 0.275606561 | 0.999970678 | 0.454 | 0.158 | 0.056 | 0.151 | NA |
| TRINITY_DN8272_c0_g1_i1 | 1.540869823 | 0.328639498 | 1 | 3.643 | 3.824 | 1.678 | 0.503 | LRRNT_2 |
| TRINITY_DN3071_c0_g2_i3 | 1.540595414 | 0.36332571 | 0.999970678 | 11.855 | 7.155 | 0.211 | 6.304 | NA |
| TRINITY_DN2136_c0_g1_i7 | 1.540529021 | 0.713527588 | 0.999970678 | 6.668 | 0 | 0 | 2.332 | NA |
| TRINITY_DN3517_c1_g1_i6 | -1.54021013 | 0.365112142 | 0.999970678 | 0.963 | 0.079 | 1.211 | 1.599 | NA |
| TRINITY_DN2575_c0_g1_i6 | 1.540119956 | 0.623388176 | 0.999970678 | 5.123 | 0 | 0.067 | 1.709 | NA |
| TRINITY_DN1783_c0_g1_i2 | -1.53980461 | 0.17632454 | 0.999970678 | 4.96 | 5.568 | 4.455 | 25.315 | Orn_Arg_deC_N |
| TRINITY_DN125_c2_g1_i8 | 1.53973987 | 0.565995228 | 0.999970678 | 0.672 | 1.675 | 0.667 | 0 | NA |
| TRINITY_DN15372_c0_g1_i7 | -1.53967143 | 0.565516142 | 0.999970678 | 0.4 | 0 | 0.356 | 0.764 | RRM_1 |
| TRINITY_DN4171_c0_g1_i3 | 1.539592899 | 0.618285812 | 0.999970678 | 0.036 | 1.587 | 0 | 0.543 | zf-CCCH |
| TRINITY_DN760_c0_g2_i6 | -1.53958721 | 0.356822029 | 0.999970678 | 3.098 | 0.759 | 0.833 | 10.174 | Cation_ATPase |
| TRINITY_DN3104_c1_g1_i2 | 1.539336153 | 0.3892423 | 0.999970678 | 1.844 | 0.227 | 0.111 | 0.583 | NA |
| TRINITY_DN6096_c0_g1_i2 | 1.538767546 | 0.71332193 | 0.999970678 | 0 | 6.475 | 1.8 | 0 | NA |
| TRINITY_DN30952_c0_g1_i1 | 1.538417199 | 0.520722763 | 0.999970678 | 2.653 | 0.108 | 0.767 | 0.03 | NA |
| TRINITY_DN15875_c0_g1_i1 | 1.538085395 | 0.42760731 | 1 | 0.636 | 0.641 | 0.344 | 0.03 | NA |
| TRINITY_DN9000_c0_g1_i11 | 1.538020908 | 0.560743217 | 0.999970678 | 0.999 | 3.065 | 1.133 | 0 | NA |
| TRINITY_DN638_c0_g1_i8 | -1.53750446 | 0.558725345 | 0.999970678 | 0 | 0.719 | 1.211 | 0.583 | NA |
| TRINITY_DN4177_c0_g1_i1 | -1.53701825 | 0.039915464 | 0.999970678 | 18.531 | 22.62 | 35.253 | 76.237 | GST_C |
| TRINITY_DN5710_c0_g1_i1 | -1.53683975 | 0.101531846 | 0.999970678 | 2.171 | 3.233 | 9.199 | 4.544 | WRKY |
| TRINITY_DN12235_c0_g1_i7 | 1.536787562 | 0.495561025 | 0.999970678 | 0.091 | 3.775 | 1 | 0.101 | NA |
| TRINITY_DN10229_c0_g1_i2 | 1.536624578 | 0.336314426 | 0.999970678 | 1.808 | 25.71 | 2.311 | 6.525 | MtN3_slv |
| TRINITY_DN13828_c0_g2_i4 | 1.536587739 | 0.428830698 | 0.999970678 | 5.986 | 8.722 | 4.122 | 0.06 | NA |
| TRINITY_DN17012_c0_g3_i1 | 1.536491405 | 0.315534438 | 1 | 49.498 | 65.7 | 5.1 | 27.949 | NA |
| TRINITY_DN3379_c1_g2_i2 | 1.536399955 | 0.713363905 | 0.999970678 | 0 | 14.78 | 4.144 | 0 | - |
| TRINITY_DN2195_c2_g1_i6 | -1.53625277 | 0.200092525 | 0.999970678 | 2.017 | 1.39 | 1.755 | 7.681 | NA |
| TRINITY_DN14048_c0_g1_i2 | 1.536219604 | 0.591060541 | 0.999970678 | 0 | 3.735 | 0.3 | 0.905 | EF-hand_7 |
| TRINITY_DN2213_c0_g1_i8 | 1.536014909 | 0.276569462 | 0.999970678 | 2.28 | 0.956 | 0.789 | 0.181 | NA |
| TRINITY_DN1219_c0_g1_i4 | -1.53595707 | 0.386804713 | 0.999970678 | 83.272 | 839.6 | 145.013 | 2315.587 | NA |
| TRINITY_DN10764_c0_g1_i2 | -1.5358085 | 0.174214381 | 0.999970678 | 0.409 | 0.355 | 1.1 | 0.824 | NA |
| TRINITY_DN10287_c0_g1_i1 | 1.535802251 | 0.720359616 | 0.999970678 | 8.203 | 0 | 0 | 2.755 | NA |
| TRINITY_DN10512_c0_g1_i7 | -1.53571664 | 0.090984372 | 0.999970678 | 1.045 | 0.867 | 2.722 | 2.282 | NA |
| TRINITY_DN1645_c2_g1_i1 | -1.53567247 | 0.145137299 | 0.999970678 | 3.052 | 2.927 | 3.122 | 13.653 | B_lectin |
| TRINITY_DN4917_c0_g1_i1 | -1.5348618 | 0.247067852 | 0.999970678 | 5.305 | 10.85 | 4.866 | 40.768 | NA |
| TRINITY_DN2034_c0_g1_i5 | 1.534472992 | 0.272267212 | 0.999970678 | 3.434 | 1.39 | 1.144 | 0.302 | NA |
| TRINITY_DN8339_c0_g1_i1 | 1.534395615 | 0.652163062 | 0.999970678 | 74.924 | 0 | 0.144 | 25.496 | Glycolytic |
| TRINITY_DN936_c0_g1_i2 | 1.534360399 | 0.354954412 | 0.999970678 | 9.384 | 38.7 | 0.922 | 15.231 | PP-binding |
| TRINITY_DN3341_c0_g1_i5 | 1.53420316 | 0.182589068 | 0.999970678 | 3.597 | 1.045 | 0.611 | 0.895 | NA |
| TRINITY_DN2105_c0_g1_i16 | 1.534046602 | 0.213358077 | 0.999970678 | 3.606 | 6.968 | 0.7 | 2.765 | NA |

| TRINITY_DN1619_c0_g1_i4 | 1.533692851 | 0.611947461 | 0.999970678 | 0 | 7.943 | 2.011 | 0.251 | NA |
| --- | --- | --- | --- | --- | --- | --- | --- | --- |
| TRINITY_DN15853_c1_g2_i3 | 1.533150317 | 0.373745859 | 1 | 0.863 | 0.966 | 0.456 | 0.08 | NA |
| TRINITY_DN4184_c0_g1_i2 | 1.533121591 | 0.35444018 | 0.999970678 | 0.645 | 0.335 | 0.233 | 0.04 | - |
| TRINITY_DN6914_c0_g1_i7 | -1.53302752 | 0.588973671 | 0.999970678 | 11.273 | 0 | 12.71 | 17.835 | - |
| TRINITY_DN26432_c0_g1_i1 | -1.53282359 | 0.565060566 | 0.999970678 | 21.656 | 0 | 19.732 | 35.54 | NA |
| TRINITY_DN537_c0_g1_i5 | 1.532591102 | 0.562560238 | 0.999970678 | 6.886 | 5.026 | 3.4 | 0 | NA |
| TRINITY_DN6313_c0_g1_i1 | -1.53257315 | 0.425833712 | 0.999970678 | 0.454 | 2.237 | 0.3 | 7.309 | EF-hand_8 |
| TRINITY_DN711_c0_g1_i3 | -1.53242384 | 0.025987531 | 0.999970678 | 51.515 | 45.49 | 143.147 | 108.097 | peroxidase |
| TRINITY_DN7089_c0_g1_i1 | 1.532129957 | 0.228165315 | 0.999970678 | 7.958 | 16.82 | 5.689 | 1.599 | NA |
| TRINITY_DN5492_c0_g1_i10 | 1.532072413 | 0.565093458 | 0.999970678 | 12.436 | 0.099 | 3.555 | 0.121 | NA |
| TRINITY_DN34_c0_g3_i1 | -1.53204571 | 0.185382169 | 0.999970678 | 0.872 | 2.158 | 2.744 | 5.298 | zf-C2H2_6 |
| TRINITY_DN2341_c0_g1_i2 | 1.531934697 | 0.178708507 | 0.999970678 | 15.025 | 3.38 | 2.411 | 3.529 | NA |
| TRINITY_DN2558_c0_g1_i5 | -1.53156885 | 0.715264946 | 0.999970678 | 3.652 | 0 | 8.877 | 0 | Abhydrolase_1 |
| TRINITY_DN2477_c0_g1_i2 | 1.53119734 | 0.570785503 | 0.999970678 | 10.31 | 8.909 | 0 | 6.686 | NA |
| TRINITY_DN5595_c0_g2_i3 | -1.53064184 | 0.718053554 | 0.999970678 | 2.98 | 0 | 7.166 | 0 | NA |
| TRINITY_DN864_c0_g1_i7 | 1.530468136 | 0.575920201 | 0.999970678 | 0.109 | 0.641 | 0.211 | 0 | PDCD2_C |
| TRINITY_DN9864_c0_g1_i3 | -1.53033943 | 0.556183217 | 0.999970678 | 0.009 | 1.084 | 1.555 | 1.227 | PCRF |
| TRINITY_DN2251_c0_g2_i1 | -1.52995092 | 0.253381838 | 0.999970678 | 0.327 | 0.838 | 1.155 | 1.93 | NA |
| TRINITY_DN7528_c0_g1_i1 | -1.52986719 | 0.299455426 | 1 | 0.182 | 0.719 | 1.011 | 1.277 | NA |
| TRINITY_DN5158_c0_g1_i3 | 1.529853531 | 0.606256368 | 0.999970678 | 0 | 6.968 | 1.767 | 0.241 | NA |
| TRINITY_DN20713_c0_g1_i1 | -1.52984087 | 0.061596851 | 0.999970678 | 8.448 | 9.422 | 20.01 | 25.546 | NA |
| TRINITY_DN10558_c0_g1_i1 | 1.529765885 | 0.294697972 | 0.999970678 | 1.426 | 0.424 | 0.156 | 0.462 | NA |
| TRINITY_DN3911_c1_g1_i1 | -1.52953031 | 0.214241351 | 1 | 0.273 | 0.286 | 0.733 | 0.754 | NA |
| TRINITY_DN9659_c0_g2_i1 | -1.52937192 | 0.56353292 | 0.999970678 | 1.154 | 0 | 1.7 | 1.317 | NA |
| TRINITY_DN53_c1_g1_i2 | 1.529203395 | 0.041087976 | 0.999970678 | 96.19 | 59.79 | 18.766 | 31.237 | NA |
| TRINITY_DN3185_c0_g1_i3 | 1.528984516 | 0.266337441 | 0.999970678 | 2.716 | 0.67 | 0.711 | 0.332 | HALZ |
| TRINITY_DN3362_c0_g1_i2 | -1.52891563 | 0.020797997 | 0.871169434 | 21.52 | 22.13 | 41.942 | 74.598 | NA |
| TRINITY_DN30792_c0_g2_i1 | 1.528836053 | 0.230544907 | 0.999970678 | 0.463 | 0.621 | 0.244 | 0.08 | NA |
| TRINITY_DN583_c1_g1_i13 | 1.528796158 | 0.717650989 | 0.999970678 | 0 | 0.581 | 0 | 0.201 | Pkinase |
| TRINITY_DN2636_c0_g1_i2 | -1.52868626 | 0.715714412 | 0.999970678 | 2.498 | 0 | 0 | 7.208 | NA |
| TRINITY_DN9966_c0_g1_i8 | 1.528399761 | 0.612493142 | 0.999970678 | 54.931 | 0.01 | 2.678 | 16.026 | NA |
| TRINITY_DN28477_c0_g1_i1 | -1.52817536 | 0.418805821 | 0.999970678 | 0.064 | 0.68 | 0.189 | 1.9 | Pkinase_Tyr |
| TRINITY_DN478_c2_g1_i7 | 1.528159909 | 0.353324373 | 0.999970678 | 4.796 | 1.399 | 1.678 | 0.141 | NA |
| TRINITY_DN7760_c0_g1_i2 | 1.527947999 | 0.557229842 | 1 | 1.163 | 0.907 | 0 | 0.734 | NA |
| TRINITY_DN6193_c0_g1_i15 | -1.52785456 | 0.570741711 | 1 | 0.509 | 0 | 0.433 | 0.925 | NA |
| TRINITY_DN6902_c0_g1_i6 | -1.52784558 | 0.143196527 | 0.999970678 | 1.39 | 0.877 | 1.878 | 4.323 | NA |
| TRINITY_DN7564_c0_g1_i1 | 1.527817927 | 0.554513621 | 0.999970678 | 1.081 | 1.36 | 0.711 | 0 | NA |
| TRINITY_DN46_c0_g1_i8 | 1.52771594 | 0.639970955 | 0.999970678 | 2.589 | 0.02 | 0 | 0.915 | RRM_1 |
| TRINITY_DN2915_c0_g1_i4 | -1.52742921 | 0.169013212 | 0.999970678 | 173.66 | 184.8 | 700.402 | 185.038 | NA |
| TRINITY_DN33764_c0_g1_i3 | -1.52727515 | 0.082146776 | 0.999970678 | 0.917 | 0.838 | 1.633 | 3.086 | Gpi16 |
| TRINITY_DN1391_c1_g1_i4 | 1.52718434 | 0.073807635 | 0.999970678 | 6.077 | 4.819 | 1.255 | 2.262 | CRAL_TRIO |
| TRINITY_DN31120_c0_g1_i3 | 1.526990338 | 0.111921419 | 0.999970678 | 1.853 | 2.553 | 0.844 | 0.493 | NA |
| TRINITY_DN2528_c0_g1_i1 | 1.526954117 | 0.22706504 | 0.999970678 | 0.527 | 0.946 | 0.322 | 0.131 | BAH |
| TRINITY_DN468_c0_g2_i1 | 1.526489012 | 0.059923831 | 0.999970678 | 10.628 | 11.54 | 2.311 | 4.876 | NA |
| TRINITY_DN414_c0_g1_i11 | -1.52641554 | 0.239667226 | 0.999970678 | 0.663 | 3.972 | 6.322 | 5.519 | Oxidored_FMN |
| TRINITY_DN7515_c0_g2_i2 | -1.52641139 | 0.575463028 | 0.999970678 | 1.135 | 0 | 0.5 | 2.704 | NA |
| TRINITY_DN1056_c0_g5_i1 | 1.526376494 | 0.550935826 | 0.999970678 | 4.26 | 6.692 | 0 | 3.69 | NA |
| TRINITY_DN1410_c0_g1_i1 | -1.52631658 | 0.599094947 | 0.999970678 | 36.599 | 0 | 69.74 | 22.44 | NA |
| TRINITY_DN2164_c3_g1_i1 | 1.526133244 | 0.121521709 | 0.999970678 | 7.285 | 2.976 | 1.178 | 2.172 | NA |
| TRINITY_DN1322_c0_g1_i3 | -1.52593638 | 0.717778525 | 0.999970678 | 0 | 1.794 | 4.2 | 0 | HMA |
| TRINITY_DN1762_c0_g3_i1 | -1.52556099 | 0.565491849 | 0.999970678 | 6.032 | 0 | 9.311 | 6.284 | NA |
| TRINITY_DN890_c0_g1_i4 | 1.525257147 | 0.346097023 | 0.999970678 | 2.253 | 2.01 | 1.144 | 0.101 | NA |
| TRINITY_DN17985_c0_g1_i1 | 1.525126112 | 0.551551917 | 0.999970678 | 1.017 | 1.291 | 0 | 0.814 | NA |
| TRINITY_DN7256_c0_g1_i1 | -1.52511858 | 0.330508762 | 0.999970678 | 1.935 | 1.114 | 0.567 | 8.123 | NA |
| TRINITY_DN2348_c0_g1_i5 | 1.524756021 | 0.716585366 | 0.999970678 | 2.562 | 0 | 0.744 | 0 | NA |
| TRINITY_DN2210_c0_g1_i1 | -1.52465279 | 0.715593784 | 0.999970678 | 0 | 2.375 | 5.566 | 0 | Cpn60_TCP1 |
| TRINITY_DN4495_c0_g1_i1 | 1.524320661 | 0.063868136 | 0.999970678 | 50.425 | 79.73 | 12.566 | 30.01 | Lyase_aromatic |
| TRINITY_DN2796_c0_g1_i19 | 1.524149481 | 0.45179247 | 0.999970678 | 535.65 | 39.18 | 157.868 | 5.831 | NA |
| TRINITY_DN1539_c0_g1_i2 | -1.52413922 | 0.58735691 | 0.999970678 | 0 | 1.764 | 3.766 | 0.442 | Amidase |
| TRINITY_DN12937_c1_g1_i1 | -1.52376248 | 0.579945976 | 0.999970678 | 5.814 | 0 | 1.989 | 14.407 | NA |
| TRINITY_DN60452_c0_g1_i3 | -1.52346952 | 0.22362466 | 0.999970678 | 0.672 | 0.345 | 1.667 | 0.925 | NA |
| TRINITY_DN59_c0_g1_i1 | 1.523443432 | 0.560106611 | 0.999970678 | 0.854 | 1.705 | 0.733 | 0 | NA |
| TRINITY_DN28234_c0_g2_i3 | -1.52342352 | 0.180256962 | 0.999970678 | 40.623 | 14.5 | 33.398 | 117.296 | Barwin |
| TRINITY_DN28269_c0_g1_i3 | -1.52337111 | 0.569028941 | 0.999970678 | 1.844 | 0 | 1.633 | 3.328 | NA |
| TRINITY_DN743_c0_g1_i6 | 1.523140957 | 0.289791697 | 0.999970678 | 7.367 | 1.133 | 1.255 | 1.488 | NA |
| TRINITY_DN406_c0_g1_i7 | -1.52274226 | 0.562625349 | 0.999970678 | 4.633 | 0 | 4.189 | 8.415 | NA |
| TRINITY_DN1020_c0_g1_i5 | 1.52253101 | 0.259598704 | 0.999970678 | 0.781 | 5.164 | 0.667 | 1.257 | PUB |
| TRINITY_DN2131_c0_g1_i3 | 1.522422738 | 0.324876936 | 0.999970678 | 2.453 | 1.941 | 1.133 | 0.211 | NA |
| TRINITY_DN589_c0_g1_i1 | -1.52221665 | 0.563150018 | 0.999970678 | 0 | 1.419 | 1.055 | 2.775 | NA |
| TRINITY_DN1461_c0_g1_i5 | -1.52203877 | 0.715411839 | 0.999970678 | 0 | 1.675 | 0 | 4.735 | DUF966 |
| TRINITY_DN3916_c0_g1_i8 | 1.521969994 | 0.503177943 | 0.999970678 | 2.026 | 0.168 | 0.633 | 0.01 | BET |
| TRINITY_DN11734_c1_g1_i1 | -1.52172284 | 0.726933359 | 0.999970678 | 0.4 | 0 | 0 | 1.126 | NA |
| TRINITY_DN1413_c0_g1_i4 | -1.5215238 | 0.585735318 | 0.999970678 | 0 | 8.259 | 16.343 | 3.579 | NA |
| TRINITY_DN703_c0_g1_i2 | 1.521483511 | 0.633504185 | 0.999970678 | 10.41 | 0.079 | 3.066 | 0 | NA |

| TRINITY_DN4987_c0_g1_i6 | 1.520961428 | 0.094259275 | 0.999970678 | 40.087 | 63.97 | 20.777 | 10.768 | NA |
| --- | --- | --- | --- | --- | --- | --- | --- | --- |
| TRINITY_DN2595_c0_g1_i2 | -1.52089571 | 0.19610084 | 0.999970678 | 35.673 | 67.16 | 42.053 | 242.746 | Dimerisation |
| TRINITY_DN5410_c0_g1_i1 | 1.520880975 | 0.012765045 | 0.591573834 | 38.916 | 32.19 | 10.766 | 11.793 | NA |
| TRINITY_DN1562_c0_g2_i3 | 1.520477823 | 0.307678841 | 0.999970678 | 8.294 | 0.71 | 1.389 | 1.508 | NA |
| TRINITY_DN2107_c0_g1_i2 | 1.520370255 | 0.727448492 | 0.999970678 | 24.3 | 0 | 7.111 | 0 | NA |
| TRINITY_DN962_c0_g1_i8 | 1.520261193 | 0.723489828 | 0.999970678 | 0 | 2.671 | 0.756 | 0 | CCB2_CCB4 |
| TRINITY_DN6411_c0_g1_i1 | -1.51975022 | 0.572987272 | 0.999970678 | 5.096 | 0 | 8.422 | 4.675 | NA |
| TRINITY_DN5795_c0_g3_i2 | 1.519703768 | 0.564363574 | 0.999970678 | 1.326 | 0.562 | 0.544 | 0 | NA |
| TRINITY_DN18579_c0_g1_i2 | -1.51969211 | 0.424415711 | 0.999970678 | 1.508 | 0.177 | 3.522 | 0.694 | NA |
| TRINITY_DN4655_c0_g1_i2 | 1.518843283 | 0.561109071 | 0.999970678 | 1.054 | 0.986 | 0.6 | 0 | - |
| TRINITY_DN1985_c0_g1_i10 | 1.518573817 | 0.034452986 | 0.999970678 | 10.474 | 15.24 | 4.055 | 4.042 | zf-C3HC4_3 |
| TRINITY_DN4806_c0_g1_i4 | 1.518563081 | 0.37001646 | 0.999970678 | 0.999 | 4.78 | 1.389 | 0.292 | NA |
| TRINITY_DN15052_c0_g2_i4 | 1.518393025 | 0.201997548 | 0.999970678 | 1.272 | 0.986 | 0.467 | 0.221 | - |
| TRINITY_DN2733_c0_g1_i11 | -1.51783638 | 0.383664061 | 0.999970678 | 0.263 | 6.386 | 9.044 | 7.761 | NA |
| TRINITY_DN1498_c0_g1_i1 | -1.51773222 | 0.129028032 | 0.999970678 | 3.034 | 2.543 | 9.899 | 4.021 | - |
| TRINITY_DN872_c0_g1_i6 | 1.517619002 | 0.640390079 | 0.999970678 | 0.799 | 0.01 | 0 | 0.292 | NA |
| TRINITY_DN41_c0_g1_i3 | 1.517493489 | 0.286356098 | 0.999970678 | 24.617 | 4.08 | 6.722 | 2.011 | NA |
| TRINITY_DN472_c0_g2_i1 | -1.51738639 | 0.088861427 | 0.999970678 | 9.574 | 4.898 | 13.088 | 25.597 | Dirigent |
| TRINITY_DN1499_c0_g1_i5 | -1.51735436 | 0.189695744 | 0.999970678 | 0.2 | 0.256 | 0.6 | 0.573 | NA |
| TRINITY_DN6400_c0_g2_i2 | 1.517321256 | 0.72083487 | 0.999970678 | 12.799 | 0 | 3.755 | 0 | - |
| TRINITY_DN4848_c3_g1_i6 | 1.517245118 | 0.592849618 | 0.999970678 | 1.835 | 0 | 0.278 | 0.312 | NA |
| TRINITY_DN106_c1_g1_i2 | 1.517099075 | 0.265998018 | 0.999970678 | 1.072 | 0.631 | 0.1 | 0.473 | NA |
| TRINITY_DN4762_c0_g1_i1 | 1.516881471 | 0.559196455 | 0.999970678 | 0.799 | 1.34 | 0.611 | 0 | NA |
| TRINITY_DN1017_c0_g1_i9 | -1.51661516 | 0.588317637 | 1 | 1.753 | 0 | 3.444 | 0.905 | NA |
| TRINITY_DN3660_c0_g1_i2 | 1.516013678 | 0.625011695 | 0.999970678 | 11.518 | 0.128 | 0 | 4.122 | NA |
| TRINITY_DN5772_c0_g1_i2 | -1.51597941 | 0.199294236 | 0.999970678 | 0.345 | 1.36 | 2.055 | 2.343 | NA |
| TRINITY_DN3647_c0_g1_i4 | -1.51585251 | 0.40831615 | 0.999970678 | 0.209 | 2.789 | 0.856 | 7.44 | NA |
| TRINITY_DN3197_c0_g1_i7 | -1.51577706 | 0.334169639 | 0.999970678 | 0.336 | 1.646 | 0.744 | 4.765 | LANC_like |
| TRINITY_DN30542_c0_g1_i3 | 1.515379892 | 0.395248408 | 0.999970678 | 1.217 | 0.315 | 0.422 | 0.03 | RSB_motif |
| TRINITY_DN4258_c0_g1_i1 | 1.515286066 | 0.547861411 | 0.999970678 | 68.293 | 0.128 | 0.744 | 23.485 | NA |
| TRINITY_DN12595_c0_g1_i6 | 1.515283937 | 0.204399637 | 0.999970678 | 1.426 | 1.192 | 0.6 | 0.191 | PHD |
| TRINITY_DN4642_c0_g1_i5 | 1.51525699 | 0.276502609 | 0.999970678 | 1.072 | 0.256 | 0.133 | 0.312 | NA |
| TRINITY_DN12624_c0_g1_i6 | 1.515118744 | 0.425862387 | 0.999970678 | 3.27 | 0.197 | 0.9 | 0.141 | - |
| TRINITY_DN3642_c0_g1_i3 | 1.5148705 | 0.250958125 | 0.999970678 | 55.312 | 7.51 | 9.999 | 10.104 | F-box |
| TRINITY_DN2461_c0_g1_i1 | 1.514632797 | 0.623140903 | 0.999970678 | 6.522 | 0.099 | 0 | 2.363 | TAXi_N |
| TRINITY_DN1922_c3_g1_i5 | 1.51444431 | 0.230343166 | 0.999970678 | 3.107 | 2.227 | 1.267 | 0.332 | NA |
| TRINITY_DN2169_c0_g1_i3 | -1.51371772 | 0.392972716 | 0.999970678 | 0.899 | 4.731 | 0.811 | 14.799 | NA |
| TRINITY_DN304_c0_g1_i10 | 1.513651381 | 0.603325289 | 0.999970678 | 14.516 | 0 | 3.333 | 1.116 | NA |
| TRINITY_DN1422_c2_g2_i1 | 1.513521624 | 0.127233729 | 0.999970678 | 1.671 | 3.597 | 1 | 0.633 | NTF2 |
| TRINITY_DN21537_c0_g1_i14 | 1.513161039 | 0.728764216 | 0.999970678 | 13.263 | 0 | 0 | 4.534 | NA |
| TRINITY_DN4507_c0_g2_i6 | 1.513161039 | 0.728764216 | 0.999970678 | 1.154 | 0 | 0 | 0.412 | NA |
| TRINITY_DN11109_c0_g2_i2 | 1.513111394 | 0.305554522 | 0.999970678 | 4.433 | 0.818 | 1.089 | 0.533 | XAP5 |
| TRINITY_DN49_c0_g2_i1 | -1.51288134 | 0.185018552 | 0.999970678 | 0.854 | 3.715 | 4.278 | 7.761 | G-alpha |
| TRINITY_DN19937_c0_g1_i5 | -1.51286728 | 0.389919919 | 0.999970678 | 0.182 | 0.542 | 0.167 | 1.91 | NA |
| TRINITY_DN18371_c0_g1_i6 | -1.51256495 | 0.727959599 | 0.999970678 | 0 | 0.621 | 0 | 1.749 | FMO-like |
| TRINITY_DN4160_c0_g2_i4 | 1.512173547 | 0.231086498 | 0.999970678 | 0.3 | 0.897 | 0.167 | 0.211 | NA |
| TRINITY_DN6764_c0_g1_i4 | 1.512025228 | 0.452183154 | 0.999970678 | 0.899 | 2.582 | 0.022 | 1.176 | NA |
| TRINITY_DN12881_c2_g1_i6 | 1.511888834 | 0.558869317 | 0.999970678 | 1.408 | 1.626 | 0.878 | 0 | Myb_DNA-bind_ |
| TRINITY_DN1970_c0_g1_i10 | -1.5117705 | 0.577197747 | 0.999970678 | 1.871 | 0 | 3.566 | 1.106 | NA |
| TRINITY_DN1133_c0_g1_i13 | 1.511232121 | 0.71806196 | 0.999970678 | 0 | 3.932 | 1.133 | 0 | NA |
| TRINITY_DN589_c0_g1_i6 | 1.510936234 | 0.512939084 | 0.999970678 | 0.027 | 4.869 | 0.267 | 1.357 | NA |
| TRINITY_DN9367_c0_g1_i3 | -1.51051343 | 0.616036359 | 0.999970678 | 0 | 1.094 | 0.1 | 2.916 | NA |
| TRINITY_DN850_c0_g1_i4 | 1.510143783 | 0.725212883 | 0.999970678 | 0 | 30.33 | 0 | 10.506 | NA |
| TRINITY_DN16720_c0_g1_i2 | 1.509925691 | 0.728951064 | 0.999970678 | 1.735 | 0 | 0.511 | 0 | peroxidase |
| TRINITY_DN13191_c0_g1_i2 | -1.50969703 | 0.418516269 | 0.999970678 | 4.933 | 0.542 | 1.7 | 13.452 | NA |
| TRINITY_DN9015_c0_g1_i1 | -1.50939235 | 0.470134443 | 0.999970678 | 0.055 | 4.563 | 1.755 | 10.818 | NA |
| TRINITY_DN22930_c0_g1_i6 | 1.509064135 | 0.316049609 | 0.999970678 | 13.154 | 1.567 | 2.811 | 1.79 | LRRNT_2 |
| TRINITY_DN758_c0_g1_i7 | 1.509025583 | 0.299924007 | 0.999970678 | 2.716 | 0.749 | 0.744 | 0.302 | Sugar_tr |
| TRINITY_DN6651_c0_g3_i1 | 1.508782769 | 0.501254959 | 0.999970678 | 7.64 | 0.118 | 2.067 | 0.261 | zf-Dof |
| TRINITY_DN226_c0_g1_i5 | -1.50858994 | 0.468212415 | 0.999970678 | 4.696 | 0.177 | 0.833 | 13.02 | NA |
| TRINITY_DN43487_c0_g1_i10 | 1.508301298 | 0.185546604 | 0.999970678 | 561.41 | 2032 | 223.542 | 539.924 | NA |
| TRINITY_DN24798_c0_g1_i1 | -1.50823163 | 0.580581898 | 0.999970678 | 0.554 | 0 | 1.078 | 0.302 | NA |
| TRINITY_DN114_c0_g1_i8 | -1.50822701 | 0.588836328 | 0.999970678 | 2.889 | 0 | 6.011 | 1.076 | NA |
| TRINITY_DN4888_c0_g1_i10 | 1.507902471 | 0.25134158 | 0.999970678 | 0.908 | 4.563 | 0.556 | 1.237 | NA |
| TRINITY_DN2966_c0_g1_i1 | -1.50772715 | 0.444110442 | 0.999970678 | 1.826 | 8.85 | 24.254 | 0.623 | NA |
| TRINITY_DN2318_c0_g1_i5 | -1.50771724 | 0.210695026 | 0.999970678 | 0.327 | 0.788 | 0.778 | 2.172 | NA |
| TRINITY_DN133_c0_g2_i2 | 1.507710849 | 0.569453982 | 0.999970678 | 9.429 | 7.953 | 0 | 6.133 | zinc_ribbon_10 |
| TRINITY_DN4894_c0_g1_i9 | -1.50770273 | 0.168774229 | 0.999970678 | 0.2 | 0.227 | 0.744 | 0.342 | DUF4216 |
| TRINITY_DN3632_c0_g1_i1 | 1.507533189 | 0.056700025 | 0.999970678 | 4.76 | 7.017 | 1.489 | 2.322 | EF1G |
| TRINITY_DN2750_c1_g1_i9 | 1.507103681 | 0.568944979 | 0.999970678 | 0.182 | 0.315 | 0.144 | 0 | Jas |
| TRINITY_DN26360_c0_g1_i3 | -1.50689271 | 0.567527068 | 0.999970678 | 0 | 0.798 | 1.089 | 0.905 | NA |
| TRINITY_DN3231_c0_g1_i1 | 1.506836473 | 0.180374745 | 0.999970678 | 0.845 | 1.399 | 0.356 | 0.362 | NA |
| TRINITY_DN3982_c0_g1_i3 | 1.506815283 | 0.268257414 | 0.999970678 | 1.953 | 1.449 | 0.833 | 0.201 | NA |
| TRINITY_DN13925_c0_g1_i2 | 1.506691131 | 0.600294722 | 0.999970678 | 0.263 | 2.72 | 0.867 | 0 | C2 |

| TRINITY_DN1268_c1_g1_i4 | 1.506499971 | 0.596712826 | 0.999970678 | 0 | 19.2 | 3.644 | 2.202 | NA |
| --- | --- | --- | --- | --- | --- | --- | --- | --- |
| TRINITY_DN5632_c0_g1_i9 | 1.5064058 | 0.58895215 | 0.999970678 | 5.505 | 0.71 | 0 | 2.172 | NA |
| TRINITY_DN776_c0_g1_i4 | -1.5059749 | 0.288994766 | 0.999970678 | 0.754 | 1.321 | 4.189 | 0.824 | NA |
| TRINITY_DN897_c0_g1_i9 | -1.50585037 | 0.730834495 | 0.999970678 | 0 | 0.315 | 0.722 | 0 | NA |
| TRINITY_DN2238_c0_g1_i3 | -1.5052711 | 0.479078983 | 0.999970678 | 114.44 | 0.404 | 135.57 | 166.208 | NA |
| TRINITY_DN48285_c1_g1_i1 | -1.50511039 | 0.108911845 | 0.999970678 | 280.71 | 746.6 | 1456.045 | 1022.692 | NA |
| TRINITY_DN4153_c0_g1_i3 | 1.505074957 | 0.123264532 | 0.999970678 | 37.489 | 80.65 | 24.01 | 12.296 | AMPK1_CBM |
| TRINITY_DN1154_c0_g1_i2 | -1.50501784 | 0.565745601 | 0.999970678 | 1.535 | 0 | 1.389 | 2.704 | NA |
| TRINITY_DN41_c0_g1_i1 | 1.504768024 | 0.234012516 | 0.999970678 | 2.671 | 0.68 | 0.656 | 0.402 | NA |
| TRINITY_DN37_c0_g1_i3 | -1.50471646 | 0.073455794 | 0.999970678 | 6.995 | 5.775 | 20.532 | 11.461 | - |
| TRINITY_DN1904_c0_g1_i5 | 1.504582906 | 0.383797013 | 0.999970678 | 21.511 | 2.277 | 6.311 | 0.834 | PMD |
| TRINITY_DN19869_c0_g1_i2 | 1.5043101 | 0.729308635 | 0.999970678 | 0 | 2.523 | 0 | 0.885 | NA |
| TRINITY_DN439_c0_g1_i4 | 1.504290507 | 0.162528423 | 0.999970678 | 1.163 | 1.064 | 0.344 | 0.352 | NA |
| TRINITY_DN1697_c0_g1_i1 | 1.504035291 | 0.348509487 | 0.999970678 | 2.08 | 15.95 | 0.7 | 5.439 | Amidohydro_2 |
| TRINITY_DN25999_c0_g1_i1 | 1.503881958 | 0.426825256 | 1 | 1.59 | 1.35 | 0.067 | 0.945 | NA |
| TRINITY_DN29674_c0_g1_i1 | -1.50387742 | 0.734454432 | 1 | 5.877 | 0 | 13.71 | 0 | NA |
| TRINITY_DN14510_c0_g1_i6 | -1.50379792 | 0.198049761 | 0.999970678 | 1.426 | 6.298 | 6.855 | 13.351 | NA |
| TRINITY_DN2562_c0_g1_i1 | -1.5036275 | 0.486291807 | 0.999970678 | 3.552 | 0.049 | 6.966 | 1.88 | NA |
| TRINITY_DN2266_c0_g1_i4 | -1.50354985 | 0.589213828 | 0.999970678 | 0 | 4.494 | 8.899 | 1.759 | NA |
| TRINITY_DN9374_c0_g1_i5 | 1.503542426 | 0.577350481 | 0.999970678 | 2.144 | 10.02 | 0 | 4.253 | Glyco_hydro_1 |
| TRINITY_DN12724_c0_g1_i2 | 1.503483315 | 0.721177241 | 0.999970678 | 11.927 | 0 | 3.522 | 0 | NA |
| TRINITY_DN20425_c0_g1_i1 | 1.503298867 | 0.561527067 | 0.999970678 | 3.77 | 5.016 | 0 | 3.097 | NA |
| TRINITY_DN91503_c0_g1_i1 | -1.50290035 | 0.196286189 | 0.999970678 | 16.769 | 16.38 | 52.197 | 25.727 | NA |
| TRINITY_DN3858_c0_g1_i9 | 1.502848239 | 0.125521363 | 0.999970678 | 118.08 | 236.3 | 29.632 | 83.013 | NA |
| TRINITY_DN93_c0_g1_i2 | -1.50279037 | 0.215582243 | 0.999970678 | 0.509 | 0.315 | 1.533 | 0.503 | DUF538 |
| TRINITY_DN568_c0_g2_i2 | -1.50277213 | 0.444826632 | 0.999970678 | 0.127 | 2.917 | 0.622 | 7.751 | Glyco_hydro_17 |
| TRINITY_DN186_c1_g1_i1 | 1.502550217 | 0.597568155 | 0.999970678 | 0.709 | 11.25 | 0 | 4.122 | NA |
| TRINITY_DN406_c0_g1_i4 | -1.50251394 | 0.051337987 | 0.999970678 | 0.727 | 1.163 | 2.1 | 2.845 | Peptidase_S10 |
| TRINITY_DN4522_c0_g2_i4 | 1.502335086 | 0.233885634 | 0.999970678 | 0.382 | 0.394 | 0.056 | 0.211 | NA |
| TRINITY_DN1813_c4_g1_i1 | -1.50223686 | 0.368179216 | 0.999970678 | 5.696 | 2.208 | 0.9 | 21.505 | GH3 |
| TRINITY_DN7480_c0_g2_i1 | -1.50164475 | 0.348482065 | 0.999970678 | 0.391 | 3.952 | 1.933 | 9.802 | LRRNT_2 |
| TRINITY_DN6877_c0_g2_i1 | -1.50151435 | 0.042860663 | 0.999970678 | 3.161 | 2.74 | 8.022 | 6.977 | Acetyltransf_11 |
| TRINITY_DN6706_c0_g1_i4 | 1.501352601 | 0.282662348 | 0.999970678 | 12.808 | 2.641 | 0.9 | 4.424 | - |
| TRINITY_DN1837_c0_g2_i2 | -1.50117765 | 0.268729961 | 0.999970678 | 27.007 | 4.08 | 20.621 | 63.962 | Longin |
| TRINITY_DN51_c0_g1_i4 | -1.50078658 | 0.376270332 | 0.999970678 | 1.69 | 2.01 | 0.322 | 10.114 | NA |
| TRINITY_DN5702_c0_g2_i3 | 1.50071347 | 0.416619671 | 0.999970678 | 2.516 | 0.434 | 0.822 | 0.06 | DUF4283 |
| TRINITY_DN2813_c0_g1_i5 | -1.50058367 | 0.719483717 | 0.999970678 | 3.125 | 0 | 0 | 8.968 | NA |
| TRINITY_DN6484_c0_g2_i3 | -1.49988165 | 0.562280581 | 0.999970678 | 0 | 1.932 | 2.022 | 2.966 | NA |
| TRINITY_DN1821_c0_g1_i2 | -1.49982177 | 0.597665142 | 0.999970678 | 0 | 16.1 | 32.631 | 5.268 | DUF1990 |
| TRINITY_DN3250_c0_g1_i4 | 1.499614772 | 0.390997137 | 0.999970678 | 126.23 | 3.558 | 20.354 | 21.505 | NA |
| TRINITY_DN3898_c1_g1_i10 | 1.499310455 | 0.72023744 | 0.999970678 | 0 | 3.124 | 0.9 | 0 | MatE |
| TRINITY_DN37735_c0_g1_i1 | 1.499223974 | 0.456444168 | 0.999970678 | 4.951 | 0.089 | 0.767 | 0.885 | NA |
| TRINITY_DN21037_c0_g1_i3 | 1.499213213 | 0.28912573 | 0.999970678 | 0.318 | 1.36 | 0.133 | 0.432 | Na_sulph_symp |
| TRINITY_DN46256_c0_g1_i5 | 1.499148652 | 0.562902177 | 0.999970678 | 1.426 | 2.365 | 0 | 1.317 | NA |
| TRINITY_DN1579_c0_g1_i2 | 1.498878078 | 0.579605221 | 0.999970678 | 14.943 | 7.638 | 0 | 8.063 | Hydrolase_4 |
| TRINITY_DN10163_c0_g1_i2 | -1.49879961 | 0.221496682 | 0.999970678 | 0.663 | 0.375 | 0.589 | 2.212 | Ank_2 |
| TRINITY_DN12568_c1_g2_i2 | -1.49844879 | 0.566154747 | 0.999970678 | 0 | 0.384 | 0.5 | 0.473 | NA |
| TRINITY_DN1171_c0_g1_i8 | 1.498342728 | 0.101166056 | 0.999970678 | 4.26 | 3.489 | 0.667 | 1.94 | Myb_Cef |
| TRINITY_DN3106_c0_g1_i1 | -1.4983418 | 0.72113283 | 0.999970678 | 0 | 2.602 | 0 | 7.208 | UDPGT |
| TRINITY_DN188_c0_g1_i1 | -1.49790362 | 0.338075232 | 0.999970678 | 16.469 | 9.195 | 6.055 | 60.322 | NA |
| TRINITY_DN6473_c0_g1_i2 | -1.49770576 | 0.579034311 | 0.999970678 | 0 | 1.271 | 0.522 | 2.906 | PNRC |
| TRINITY_DN2384_c0_g1_i1 | 1.49764298 | 0.572675505 | 0.999970678 | 0.936 | 2.454 | 0.978 | 0 | NA |
| TRINITY_DN41839_c0_g2_i1 | 1.497249711 | 0.729930494 | 0.999970678 | 2.316 | 0 | 0.689 | 0 | NA |
| TRINITY_DN7446_c0_g1_i1 | -1.49679097 | 0.617455026 | 0.999970678 | 0 | 26.82 | 57.319 | 5.067 | Abhydrolase_3 |
| TRINITY_DN1363_c0_g1_i4 | 1.496770717 | 0.60487269 | 0.999970678 | 78.558 | 57.22 | 0 | 48.308 | Dimerisation |
| TRINITY_DN3724_c0_g2_i4 | -1.49673212 | 0.724016819 | 0.999970678 | 0 | 0.335 | 0.778 | 0 | Ank |
| TRINITY_DN2486_c1_g1_i5 | 1.496502434 | 0.337747635 | 0.999970678 | 90.503 | 5.105 | 15.843 | 15.251 | NAM |
| TRINITY_DN3104_c1_g1_i1 | 1.496200867 | 0.246329105 | 0.999970678 | 0.527 | 1.715 | 0.233 | 0.493 | NA |
| TRINITY_DN1499_c0_g1_i16 | 1.496061466 | 0.348079308 | 0.999970678 | 4.715 | 2.05 | 1.633 | 0.382 | NA |
| TRINITY_DN6749_c0_g1_i2 | -1.49605458 | 0.589795332 | 0.999970678 | 7.849 | 0 | 10.222 | 10.164 | Mur_ligase |
| TRINITY_DN7878_c0_g1_i1 | -1.49597587 | 0.218675313 | 0.999970678 | 4.769 | 19.11 | 14.81 | 48.469 | Cu_bind_like |
| TRINITY_DN4657_c2_g1_i2 | -1.49592834 | 0.387895031 | 0.999970678 | 4.215 | 0.749 | 0.8 | 13.17 | STAS |
| TRINITY_DN14248_c0_g1_i8 | -1.49586438 | 0.240276004 | 0.999970678 | 3.515 | 0.956 | 7.8 | 3.318 | NA |
| TRINITY_DN4822_c0_g1_i2 | 1.49526279 | 0.57720915 | 0.999970678 | 1.045 | 1.646 | 0.789 | 0 | NA |
| TRINITY_DN5142_c1_g1_i2 | -1.49498956 | 0.416438469 | 0.999970678 | 4.715 | 0.128 | 5.811 | 6.776 | NA |
| TRINITY_DN12798_c0_g1_i1 | 1.494912634 | 0.330415172 | 0.999970678 | 4.369 | 0.532 | 0.8 | 0.784 | zf-Dof |
| TRINITY_DN4384_c0_g1_i1 | -1.49484134 | 0.244819794 | 0.999970678 | 0.191 | 0.719 | 1.167 | 1.166 | Methyltransf_7 |
| TRINITY_DN214_c0_g2_i3 | -1.49425657 | 0.271550433 | 0.999970678 | 0.218 | 0.355 | 0.3 | 1.287 | NA |
| TRINITY_DN41_c0_g1_i5 | 1.494233555 | 0.264589063 | 0.999970678 | 1.281 | 0.384 | 0.2 | 0.342 | NA |
| TRINITY_DN1811_c0_g1_i2 | -1.49392921 | 0.63490583 | 0.999970678 | 0.59 | 0.049 | 1.5 | 0 | Grp1_Fun34_Yaa |
| TRINITY_DN710_c0_g1_i6 | 1.493825453 | 0.393031914 | 0.999970678 | 3.198 | 45.48 | 1.955 | 14.528 | NA |
| TRINITY_DN340_c1_g1_i4 | -1.49372369 | 0.601770794 | 1 | 0 | 0.394 | 0.122 | 0.955 | NA |
| TRINITY_DN44482_c2_g1_i2 | 1.49361261 | 0.728456928 | 0.999970678 | 2.925 | 0 | 0.867 | 0 | NA |
| TRINITY_DN4609_c0_g1_i7 | -1.4935953 | 0.428363752 | 0.999970678 | 0.082 | 0.424 | 0.1 | 1.307 | NA |

| TRINITY_DN5931_c0_g2_i5 | -1.49346489 | 0.586103617 | 0.999970678 | 0 | 2.641 | 5.222 | 0.985 | NA |
| --- | --- | --- | --- | --- | --- | --- | --- | --- |
| TRINITY_DN7653_c0_g1_i4 | -1.49304527 | 0.575704823 | 0.999970678 | 1.308 | 0 | 2.289 | 0.985 | NA |
| TRINITY_DN1283_c0_g2_i2 | 1.493038739 | 0.612102147 | 0.999970678 | 5.142 | 0 | 0.211 | 1.599 | NA |
| TRINITY_DN1146_c0_g1_i2 | 1.492907099 | 0.288729416 | 0.999970678 | 0.291 | 0.611 | 0.211 | 0.06 | NA |
| TRINITY_DN23710_c0_g1_i6 | 1.492800698 | 0.333520877 | 0.999970678 | 0.191 | 0.581 | 0.189 | 0.05 | Bromodomain |
| TRINITY_DN5015_c0_g1_i1 | 1.492673237 | 0.291239048 | 1 | 0.745 | 0.562 | 0.267 | 0.141 | NA |
| TRINITY_DN16401_c0_g2_i5 | 1.492451081 | 0.575739883 | 0.999970678 | 1.453 | 2.976 | 1.278 | 0 | NA |
| TRINITY_DN11970_c0_g1_i2 | 1.491838998 | 0.722367732 | 0.999970678 | 11.31 | 0 | 3.378 | 0 | Fer2_3 |
| TRINITY_DN4931_c0_g1_i1 | -1.49171523 | 0.578668754 | 1 | 1.544 | 0 | 1.589 | 2.463 | NA |
| TRINITY_DN23601_c0_g1_i4 | 1.491694452 | 0.56478259 | 0.999970678 | 1.463 | 1.232 | 0.789 | 0 | FAR1 |
| TRINITY_DN461_c1_g1_i1 | -1.49158302 | 0.604585588 | 0.999970678 | 1.335 | 0.365 | 4 | 0 | LisH |
| TRINITY_DN17835_c2_g3_i1 | 1.491549074 | 0.724834738 | 0.999970678 | 1.799 | 0 | 0 | 0.653 | NA |
| TRINITY_DN4349_c2_g1_i2 | -1.4914758 | 0.095525817 | 0.999970678 | 42.231 | 38.47 | 51.786 | 164.559 | Ribosomal_S13 |
| TRINITY_DN16906_c0_g1_i12 | 1.491268908 | 0.72381647 | 0.999970678 | 1.662 | 0 | 0.5 | 0 | NA |
| TRINITY_DN6997_c0_g4_i1 | 1.491267669 | 0.163525117 | 0.999970678 | 2.126 | 1.094 | 0.678 | 0.342 | NA |
| TRINITY_DN12074_c0_g1_i4 | -1.49120743 | 0.732079061 | 0.999970678 | 1.835 | 0 | 4.189 | 0 | Glyco_hydro_25 |
| TRINITY_DN1857_c0_g1_i4 | 1.490905716 | 0.727369763 | 0.999970678 | 1.853 | 0 | 0.544 | 0 | Homeobox |
| TRINITY_DN10172_c0_g1_i1 | -1.49076721 | 0.12017302 | 0.999970678 | 6.722 | 2.74 | 11.477 | 12.718 | Dirigent |
| TRINITY_DN6017_c0_g1_i2 | 1.490742518 | 0.132285226 | 0.999970678 | 1.117 | 1.863 | 0.256 | 0.764 | DUF4283 |
| TRINITY_DN57999_c0_g1_i7 | -1.49060017 | 0.375479066 | 0.999970678 | 21.266 | 6.357 | 54.208 | 9.36 | NA |
| TRINITY_DN10369_c0_g1_i14 | 1.490456325 | 0.155469386 | 0.999970678 | 0.427 | 0.641 | 0.222 | 0.111 | DUF2934 |
| TRINITY_DN9189_c0_g1_i6 | -1.49006611 | 0.73122467 | 0.999970678 | 0 | 0.365 | 0.844 | 0 | zf-U11-48K |
| TRINITY_DN8045_c0_g2_i2 | -1.48995727 | 0.393372947 | 0.999970678 | 0.536 | 4.198 | 1.7 | 10.697 | NA |
| TRINITY_DN12973_c0_g1_i2 | -1.48985931 | 0.587598639 | 0.999970678 | 0 | 9.146 | 14.099 | 8.264 | Biotin_carb_C |
| TRINITY_DN3093_c0_g1_i1 | 1.489819354 | 0.259524012 | 0.999970678 | 6.041 | 43.89 | 5.011 | 11.542 | PTR2 |
| TRINITY_DN2027_c0_g2_i5 | 1.489588418 | 0.56528637 | 0.999970678 | 0.454 | 0.434 | 0.267 | 0 | DUF4283 |
| TRINITY_DN16408_c0_g1_i1 | -1.48957351 | 0.475910276 | 0.999970678 | 4.869 | 1.468 | 0.178 | 17.493 | NA |
| TRINITY_DN2710_c0_g1_i4 | -1.48954427 | 0.231826774 | 0.999970678 | 0.327 | 0.138 | 0.5 | 0.744 | NA |
| TRINITY_DN18962_c0_g1_i5 | 1.489280734 | 0.385722871 | 0.999970678 | 1.49 | 2.661 | 1.167 | 0.07 | ABC_membrane |
| TRINITY_DN970_c0_g1_i5 | 1.488922656 | 0.577296991 | 0.999970678 | 22.038 | 17.01 | 0 | 13.924 | NA |
| TRINITY_DN452_c1_g2_i3 | -1.48864438 | 0.301110591 | 0.999970678 | 0.273 | 1.636 | 1.289 | 3.841 | FA_desaturase |
| TRINITY_DN4649_c0_g1_i1 | 1.488595523 | 0.163340223 | 0.999970678 | 0.518 | 1.449 | 0.333 | 0.292 | NA |
| TRINITY_DN34900_c0_g1_i3 | -1.48828705 | 0.735710981 | 1 | 0.681 | 0 | 0 | 1.95 | NA |
| TRINITY_DN2612_c0_g1_i1 | 1.488241745 | 0.739533959 | 0.999970678 | 129.79 | 0 | 38.742 | 0 | DUF3129 |
| TRINITY_DN10795_c0_g1_i1 | 1.488152441 | 0.560938159 | 0.999970678 | 2.444 | 2.651 | 0 | 1.82 | NA |
| TRINITY_DN1860_c0_g1_i6 | -1.4880722 | 0.50590951 | 0.999970678 | 0.1 | 2.917 | 0.578 | 7.57 | Phi_1 |
| TRINITY_DN1039_c1_g1_i4 | 1.488032627 | 0.5869433 | 0.999970678 | 7.34 | 1.301 | 0 | 3.117 | B_lectin |
| TRINITY_DN15095_c0_g2_i3 | -1.48735755 | 0.571851176 | 0.999970678 | 1.971 | 0 | 2.011 | 3.187 | NA |
| TRINITY_DN7676_c0_g2_i1 | -1.48733686 | 0.164507302 | 0.999970678 | 2.416 | 1.202 | 2.1 | 7.701 | NA |
| TRINITY_DN1184_c0_g1_i2 | 1.486830058 | 0.124090283 | 0.999970678 | 0.636 | 0.848 | 0.244 | 0.221 | NA |
| TRINITY_DN1719_c0_g1_i6 | -1.48676201 | 0.623624014 | 0.999970678 | 32.539 | 0 | 64.707 | 14.377 | NA |
| TRINITY_DN14380_c0_g1_i10 | 1.486551681 | 0.285323799 | 1 | 0.291 | 0.542 | 0.178 | 0.09 | NA |
| TRINITY_DN1970_c0_g1_i13 | 1.486514757 | 0.28206329 | 0.999970678 | 2.653 | 0.532 | 0.278 | 0.814 | NA |
| TRINITY_DN86_c0_g1_i5 | 1.48637736 | 0.723846159 | 0.999970678 | 8.466 | 0 | 0 | 3.066 | NA |
| TRINITY_DN3265_c0_g1_i5 | -1.48629148 | 0.217201017 | 0.999970678 | 2.371 | 2.07 | 8.499 | 2.202 | NA |
| TRINITY_DN449_c1_g1_i7 | 1.486212136 | 0.433981699 | 0.999970678 | 146.34 | 2.227 | 28.643 | 18.75 | Dimerisation |
| TRINITY_DN20742_c0_g2_i1 | -1.48564586 | 0.573515816 | 0.999970678 | 4.369 | 0 | 3.389 | 8.244 | NA |
| TRINITY_DN731_c0_g2_i1 | 1.485254594 | 0.561166638 | 0.999970678 | 7.812 | 9.244 | 0 | 6.012 | TraB |
| TRINITY_DN30542_c0_g1_i4 | -1.48525157 | 0.108629921 | 0.999970678 | 1.808 | 0.966 | 2.522 | 4.776 | RSB_motif |
| TRINITY_DN16178_c0_g1_i3 | -1.48513684 | 0.155460828 | 0.999970678 | 0.491 | 0.325 | 0.533 | 1.659 | NA |
| TRINITY_DN2598_c0_g1_i6 | 1.48488447 | 0.297311205 | 0.999970678 | 5.986 | 34.49 | 9.455 | 2.805 | NA |
| TRINITY_DN1806_c0_g1_i3 | 1.484807091 | 0.199039341 | 0.999970678 | 7.294 | 35.12 | 5.389 | 8.455 | NA |
| TRINITY_DN4954_c0_g1_i7 | -1.48433875 | 0.574539636 | 0.999970678 | 0 | 5.618 | 6.4 | 7.198 | LRRNT_2 |
| TRINITY_DN512_c0_g2_i6 | 1.484208242 | 0.732622584 | 0.999970678 | 0 | 1.725 | 0.489 | 0 | EXS |
| TRINITY_DN7160_c1_g1_i1 | 1.483993512 | 0.734938615 | 1 | 0.681 | 0 | 0 | 0.251 | NA |
| TRINITY_DN8800_c0_g1_i3 | 1.483993512 | 0.734938615 | 1 | 0.908 | 0 | 0 | 0.342 | HUN |
| TRINITY_DN7720_c1_g1_i2 | 1.483993512 | 0.734938615 | 1 | 2.071 | 0 | 0 | 0.744 | NA |
| TRINITY_DN2702_c0_g1_i8 | -1.48376096 | 0.573639132 | 0.999970678 | 0.472 | 0 | 0.7 | 0.503 | NA |
| TRINITY_DN1924_c0_g2_i1 | -1.48284335 | 0.578430023 | 0.999970678 | 3.361 | 0 | 4.389 | 4.243 | HSP70 |
| TRINITY_DN19836_c1_g3_i3 | -1.48276822 | 0.146655021 | 0.999970678 | 2.098 | 1.794 | 2.711 | 7.55 | NA |
| TRINITY_DN214_c0_g2_i1 | -1.48257125 | 0.085539671 | 0.999970678 | 7.104 | 9.225 | 11.533 | 31.659 | NA |
| TRINITY_DN6543_c0_g1_i3 | 1.482472135 | 0.611663501 | 0.999970678 | 0 | 6.268 | 0.289 | 1.86 | NA |
| TRINITY_DN5846_c1_g2_i13 | 1.482110708 | 0.604025235 | 0.999970678 | 0 | 3.508 | 0.322 | 0.875 | NA |
| TRINITY_DN3982_c0_g1_i1 | 1.481927758 | 0.061765162 | 0.999970678 | 37.353 | 34.33 | 7.011 | 17.212 | NA |
| TRINITY_DN4345_c1_g2_i1 | 1.481505156 | 0.296414743 | 1 | 0.5 | 0.365 | 0.189 | 0.09 | NA |
| TRINITY_DN1121_c0_g2_i1 | -1.48121304 | 0.570677251 | 0.999970678 | 4.914 | 0 | 6.066 | 6.404 | NA |
| TRINITY_DN16131_c0_g1_i3 | 1.481118488 | 0.287980465 | 0.999970678 | 0.681 | 0.473 | 0.078 | 0.322 | RRM_1 |
| TRINITY_DN6895_c0_g1_i1 | 1.481085085 | 0.318968885 | 0.999970678 | 385.6 | 1473 | 54.563 | 593.992 | NA |
| TRINITY_DN338_c0_g1_i2 | -1.4809306 | 0.601215226 | 0.999970678 | 46.664 | 0.069 | 0.389 | 131.814 | NA |
| TRINITY_DN34324_c0_g1_i6 | 1.480837456 | 0.256326647 | 0.999970678 | 4.415 | 0.848 | 0.611 | 1.166 | NA |
| TRINITY_DN5757_c0_g1_i3 | 1.480645728 | 0.619875405 | 0.999970678 | 4.17 | 0 | 0.122 | 1.367 | Gal-bind_lectin |
| TRINITY_DN2595_c1_g2_i2 | -1.48063849 | 0.359633001 | 0.999970678 | 1.608 | 7.047 | 1.411 | 22.329 | ACC_central |
| TRINITY_DN5111_c0_g1_i14 | -1.48063223 | 0.355594677 | 0.999970678 | 2.734 | 0.473 | 1.589 | 7.028 | NA |
| TRINITY_DN1284_c0_g1_i1 | -1.48051869 | 0.10911588 | 0.999970678 | 1.899 | 4.208 | 6.6 | 8.807 | NA |

| TRINITY_DN1163_c0_g1_i2 | 1.47978953 | 0.40737995 | 0.999970678 | 1.008 | 1.892 | 0.811 | 0.07 | DUF3110 |
| --- | --- | --- | --- | --- | --- | --- | --- | --- |
| TRINITY_DN16174_c0_g1_i4 | -1.47974775 | 0.73703951 | 1 | 0.472 | 0 | 1.078 | 0 | - |
| TRINITY_DN1158_c2_g1_i2 | 1.479579777 | 0.605682341 | 0.999970678 | 10.583 | 0 | 2.289 | 1.086 | NA |
| TRINITY_DN569_c0_g1_i10 | 1.479250318 | 0.726348736 | 0.999970678 | 10.937 | 0 | 0 | 4.001 | NA |
| TRINITY_DN8414_c0_g1_i3 | -1.47919743 | 0.615936629 | 0.999970678 | 1.572 | 0 | 3.489 | 0.251 | WD40 |
| TRINITY_DN5312_c0_g1_i7 | 1.479180155 | 0.239324239 | 0.999970678 | 2.171 | 0.857 | 0.489 | 0.503 | Pro_isomerase |
| TRINITY_DN7216_c0_g1_i4 | -1.47899814 | 0.086931435 | 0.999970678 | 18.586 | 15.14 | 25.343 | 60.956 | p450 |
| TRINITY_DN2023_c0_g3_i2 | -1.47893748 | 0.602759783 | 0.999970678 | 1.017 | 0 | 0.2 | 2.654 | NA |
| TRINITY_DN467_c0_g1_i3 | -1.47888784 | 0.581887132 | 0.999970678 | 10.674 | 0 | 18.954 | 6.676 | NA |
| TRINITY_DN67_c0_g1_i1 | -1.47877708 | 0.440946228 | 0.999970678 | 2.798 | 0.118 | 1.544 | 6.284 | NA |
| TRINITY_DN35154_c0_g1_i1 | -1.47868322 | 0.732270537 | 0.999970678 | 0.572 | 0 | 1.344 | 0 | NA |
| TRINITY_DN23703_c0_g1_i1 | 1.478068177 | 0.583298151 | 1 | 1.063 | 0.917 | 0.611 | 0.01 | NA |
| TRINITY_DN5379_c0_g1_i2 | 1.478022445 | 0.539266566 | 0.999970678 | 0.164 | 12.54 | 3.255 | 0.543 | NA |
| TRINITY_DN3237_c0_g1_i4 | 1.477864517 | 0.574042532 | 0.999970678 | 7.449 | 12.38 | 0 | 7.068 | Linker_histone |
| TRINITY_DN5268_c0_g1_i1 | -1.47774473 | 0.732185583 | 0.999970678 | 6.704 | 0 | 15.655 | 0 | Glyco_hydro_47 |
| TRINITY_DN3460_c0_g2_i1 | -1.47760317 | 0.144310058 | 0.999970678 | 1.917 | 3.016 | 3.378 | 9.601 | NA |
| TRINITY_DN9335_c2_g1_i1 | -1.47753811 | 0.531646595 | 0.999970678 | 0.064 | 3.834 | 0.222 | 10.416 | NA |
| TRINITY_DN5870_c0_g1_i2 | -1.47729926 | 0.573937514 | 0.999970678 | 3.288 | 0 | 4.344 | 4.052 | NA |
| TRINITY_DN5357_c0_g1_i3 | 1.477225391 | 0.161857331 | 0.999970678 | 4.751 | 4.445 | 0.767 | 2.393 | Myb_DNA-bindin |
| TRINITY_DN4793_c0_g1_i6 | -1.47722267 | 0.332861123 | 0.999970678 | 20.666 | 7.293 | 59.396 | 6.374 | NA |
| TRINITY_DN3201_c0_g1_i4 | 1.477011084 | 0.284060503 | 1 | 0.718 | 1.212 | 0.167 | 0.503 | NA |
| TRINITY_DN4537_c0_g2_i2 | 1.47686209 | 0.74364126 | 0.999970678 | 70.391 | 0 | 0 | 25.798 | NA |
| TRINITY_DN1732_c0_g1_i6 | -1.47669605 | 0.102104068 | 0.999970678 | 0.881 | 0.512 | 1.311 | 2.332 | PWI |
| TRINITY_DN3836_c1_g1_i3 | -1.47667113 | 0.576732784 | 0.999970678 | 2.207 | 0 | 2.167 | 3.619 | Myb_DNA-bindin |
| TRINITY_DN3138_c0_g1_i33 | 1.476599549 | 0.65101694 | 0.999970678 | 7.113 | 0 | 0.033 | 2.564 | NA |
| TRINITY_DN4796_c0_g1_i1 | 1.476186686 | 0.382783017 | 0.999970678 | 7.44 | 113.4 | 12.244 | 24.943 | NA |
| TRINITY_DN11776_c0_g3_i1 | -1.47611594 | 0.189476436 | 0.999970678 | 1.953 | 1.429 | 3.666 | 4.816 | NA |
| TRINITY_DN474_c0_g2_i1 | -1.47607176 | 0.122731211 | 0.999970678 | 9.32 | 7.539 | 10.51 | 34.122 | BSP |
| TRINITY_DN13664_c0_g1_i3 | -1.47592992 | 0.735360178 | 0.999970678 | 1.481 | 0 | 0 | 4.132 | NA |
| TRINITY_DN5766_c0_g3_i6 | -1.47574777 | 0.586014102 | 1 | 0.681 | 0 | 0.922 | 0.784 | Carb_kinase |
| TRINITY_DN2173_c0_g1_i2 | -1.47570653 | 0.594507492 | 0.999970678 | 0 | 11.71 | 20.443 | 7.269 | NA |
| TRINITY_DN13358_c0_g1_i1 | 1.475639649 | 0.286869027 | 0.999970678 | 1.944 | 1.153 | 0.811 | 0.141 | NA |
| TRINITY_DN2809_c0_g2_i3 | -1.47553769 | 0.205110355 | 0.999970678 | 1.971 | 2.267 | 1.655 | 9.812 | COPI_C |
| TRINITY_DN971_c0_g1_i2 | -1.47539331 | 0.132232265 | 0.999970678 | 0.472 | 0.463 | 1.044 | 1.357 | NA |
| TRINITY_DN4067_c0_g1_i19 | 1.475300884 | 0.406797471 | 0.999970678 | 8.966 | 144.8 | 6.277 | 44.196 | NA |
| TRINITY_DN19687_c0_g1_i1 | 1.475240946 | 0.578778576 | 0.999970678 | 0.5 | 1.153 | 0.489 | 0 | DUF620 |
| TRINITY_DN14939_c0_g1_i4 | 1.474943697 | 0.247372229 | 0.999970678 | 3.597 | 1.961 | 1.1 | 0.734 | NA |
| TRINITY_DN25089_c0_g1_i1 | 1.474937493 | 0.240835634 | 0.999970678 | 4.16 | 2.001 | 0.678 | 1.327 | Cytochrom_C |
| TRINITY_DN5625_c0_g2_i3 | 1.474701618 | 0.261212421 | 0.999970678 | 1.808 | 1.271 | 0.756 | 0.201 | UbiA |
| TRINITY_DN2005_c0_g1_i2 | -1.47461401 | 0.731009067 | 0.999970678 | 0 | 0.946 | 0 | 2.584 | Aldo_ket_red |
| TRINITY_DN1772_c0_g1_i1 | -1.4743165 | 0.304290531 | 0.999970678 | 0.372 | 1.104 | 2.7 | 0.814 | NA |
| TRINITY_DN9588_c0_g1_i1 | 1.474083607 | 0.724256207 | 0.999970678 | 17.259 | 0 | 0 | 6.233 | NA |
| TRINITY_DN4277_c0_g1_i3 | -1.47398731 | 0.056648568 | 0.999970678 | 4.424 | 6.83 | 15.677 | 12.255 | NA |
| TRINITY_DN3651_c1_g1_i12 | -1.47386286 | 0.730864035 | 0.999970678 | 3.643 | 0 | 8.455 | 0 | Glyoxalase |
| TRINITY_DN13831_c0_g1_i1 | -1.47358283 | 0.7296128 | 0.999970678 | 0 | 1.577 | 0 | 4.243 | HsbA |
| TRINITY_DN7120_c0_g2_i1 | -1.47351186 | 0.208067279 | 0.999970678 | 1.199 | 0.995 | 1.178 | 4.635 | Glyco_transf_28 |
| TRINITY_DN332_c0_g1_i2 | 1.473334576 | 0.323827136 | 0.999970678 | 5.986 | 63.09 | 5.244 | 18.217 | NA |
| TRINITY_DN5621_c0_g2_i1 | 1.473145286 | 0.327956667 | 0.999970678 | 0.245 | 1.636 | 0.167 | 0.473 | NA |
| TRINITY_DN1722_c0_g1_i4 | 1.472812637 | 0.580688122 | 0.999970678 | 8.957 | 6.938 | 4.744 | 0 | ACT |
| TRINITY_DN13598_c0_g2_i1 | 1.472781647 | 0.412696417 | 0.999970678 | 0.563 | 0.463 | 0.289 | 0.02 | NA |
| TRINITY_DN21172_c0_g1_i6 | 1.47275638 | 0.576400692 | 1 | 1.045 | 0.818 | 0 | 0.653 | Fe_bilin_red |
| TRINITY_DN18246_c0_g1_i3 | 1.472679745 | 0.201628479 | 0.999970678 | 1.344 | 0.631 | 0.256 | 0.402 | Anticodon_1 |
| TRINITY_DN4002_c0_g1_i9 | 1.472516223 | 0.567865043 | 0.999970678 | 1.671 | 2.474 | 0 | 1.478 | Myb_DNA-bindin |
| TRINITY_DN484_c0_g1_i7 | -1.4723886 | 0.263228424 | 0.999970678 | 3.434 | 3.223 | 2.522 | 15.161 | NA |
| TRINITY_DN50983_c0_g1_i1 | -1.47215664 | 0.740208202 | 1 | 0.164 | 0 | 0.389 | 0 | RasGEF |
| TRINITY_DN1099_c0_g1_i6 | 1.472117188 | 0.117589819 | 0.999970678 | 6.45 | 9.964 | 3.533 | 1.629 | RNA_pol_Rpb1_1 |
| TRINITY_DN6049_c0_g1_i1 | 1.472069713 | 0.270230293 | 0.999970678 | 3.334 | 0.857 | 0.456 | 0.965 | NA |
| TRINITY_DN1966_c0_g1_i7 | -1.47194491 | 0.089963743 | 0.999970678 | 3.615 | 2.74 | 9.244 | 6.384 | NA |
| TRINITY_DN4242_c0_g2_i2 | -1.47189944 | 0.241278088 | 0.999970678 | 2.416 | 0.739 | 2.622 | 5.68 | NA |
| TRINITY_DN12144_c0_g1_i1 | -1.47186157 | 0.466372583 | 0.999970678 | 2.834 | 0.315 | 0.356 | 8.435 | NA |
| TRINITY_DN24015_c0_g1_i1 | 1.471705899 | 0.726764686 | 0.999970678 | 14.344 | 0 | 4.333 | 0 | NA |
| TRINITY_DN169_c0_g2_i1 | -1.4715963 | 0.570522367 | 0.999970678 | 0 | 4.464 | 4.211 | 7.017 | NA |
| TRINITY_DN7449_c0_g1_i7 | 1.471521816 | 0.726956667 | 0.999970678 | 0 | 2.937 | 0.867 | 0 | NA |
| TRINITY_DN2697_c0_g1_i4 | -1.47135739 | 0.57447248 | 0.999970678 | 0 | 0.798 | 0.689 | 1.357 | NA |
| TRINITY_DN5125_c0_g1_i3 | -1.47110918 | 0.257145915 | 0.999970678 | 1.254 | 0.512 | 2.378 | 2.001 | NA |
| TRINITY_DN339_c0_g1_i1 | 1.470876122 | 0.292530222 | 0.999970678 | 5.105 | 29.85 | 2.2 | 9.802 | NA |
| TRINITY_DN19116_c0_g1_i14 | 1.470469242 | 0.736766263 | 0.999970678 | 0 | 0.641 | 0.189 | 0 | PPR |
| TRINITY_DN2612_c0_g1_i2 | 1.470379664 | 0.731112695 | 0.999970678 | 40.224 | 0 | 12.144 | 0 | DUF3129 |
| TRINITY_DN4043_c0_g4_i3 | 1.47034896 | 0.603833161 | 0.999970678 | 10.401 | 0 | 2.089 | 1.277 | NA |
| TRINITY_DN4658_c0_g1_i1 | 1.470287978 | 0.574647094 | 0.999970678 | 0.999 | 0.493 | 0 | 0.553 | NA |
| TRINITY_DN10236_c0_g1_i2 | -1.47026173 | 0.318662986 | 0.999970678 | 0.127 | 0.493 | 0.911 | 0.593 | NA |
| TRINITY_DN491_c3_g1_i2 | -1.47008494 | 0.351741385 | 0.999970678 | 6.159 | 0.384 | 6.766 | 10.144 | NA |
| TRINITY_DN1064_c0_g1_i7 | 1.469988 | 0.616143432 | 0.999970678 | 7.758 | 0 | 1.6 | 0.915 | LRRNT_2 |
| TRINITY_DN843_c1_g1_i4 | -1.46994855 | 0.133099673 | 0.999970678 | 3.316 | 6.79 | 14.832 | 9.621 | NA |

| TRINITY_DN6957_c0_g1_i1 | -1.46976276 | 0.125462419 | 0.999970678 | 1.281 | 1.449 | 2.578 | 4.414 | NA |
| --- | --- | --- | --- | --- | --- | --- | --- | --- |
| TRINITY_DN5578_c0_g1_i7 | 1.469627728 | 0.253761479 | 0.999970678 | 2.126 | 0.562 | 0.544 | 0.322 | NA |
| TRINITY_DN3017_c0_g1_i12 | 1.469588065 | 0.293174481 | 0.999970678 | 6.568 | 0.867 | 0.944 | 1.588 | TFIID_20kDa |
| TRINITY_DN8762_c0_g1_i2 | 1.469550427 | 0.337116941 | 0.999970678 | 0.654 | 2.799 | 0.256 | 0.945 | DUF868 |
| TRINITY_DN439_c0_g1_i15 | 1.469290915 | 0.601967553 | 0.999970678 | 0 | 16.28 | 1.722 | 3.67 | NA |
| TRINITY_DN5931_c2_g1_i1 | 1.469196701 | 0.422840865 | 0.999970678 | 3.143 | 4.474 | 2.211 | 0.07 | FH2 |
| TRINITY_DN1615_c0_g1_i2 | -1.46902497 | 0.251350167 | 0.999970678 | 11.655 | 24.03 | 11.955 | 82.822 | zf-FLZ |
| TRINITY_DN1699_c1_g1_i7 | 1.468878473 | 0.581572138 | 1 | 0.854 | 0.601 | 0.433 | 0 | SH3_9 |
| TRINITY_DN11026_c0_g1_i3 | 1.468764439 | 0.726074162 | 0.999970678 | 0 | 4.602 | 0 | 1.629 | - |
| TRINITY_DN2303_c0_g1_i2 | -1.46864544 | 0.074170133 | 0.999970678 | 6.032 | 3.233 | 10.566 | 12.929 | NA |
| TRINITY_DN19373_c0_g1_i1 | 1.468096031 | 0.736618885 | 0.999970678 | 2.816 | 0 | 0.856 | 0 | NA |
| TRINITY_DN3420_c0_g1_i2 | 1.467943989 | 0.177187703 | 0.999970678 | 0.699 | 1.163 | 0.267 | 0.352 | - |
| TRINITY_DN14060_c0_g1_i10 | 1.467920585 | 0.734416468 | 0.999970678 | 0.427 | 0 | 0 | 0.161 | NA |
| TRINITY_DN10834_c0_g1_i1 | 1.467842767 | 0.102615722 | 0.999970678 | 1.217 | 1.804 | 0.589 | 0.382 | NA |
| TRINITY_DN1915_c0_g1_i3 | -1.46782237 | 0.487994162 | 0.999970678 | 0.382 | 1.104 | 3.3 | 0.08 | - |
| TRINITY_DN32292_c0_g1_i3 | 1.467791609 | 0.477007558 | 0.999970678 | 10.31 | 0.769 | 3.011 | 0.352 | NA |
| TRINITY_DN9230_c0_g1_i20 | 1.467719816 | 0.349017388 | 0.999970678 | 1.562 | 1.587 | 0.111 | 0.965 | NA |
| TRINITY_DN3737_c0_g1_i7 | 1.46761537 | 0.600276661 | 0.999970678 | 4.733 | 4.1 | 2.644 | 0 | Beach |
| TRINITY_DN471_c0_g1_i2 | -1.46754198 | 0.185766585 | 0.999970678 | 4.769 | 3.203 | 3.589 | 17.886 | LRRNT_2 |
| TRINITY_DN255_c1_g1_i15 | -1.46745003 | 0.486897782 | 0.999970678 | 1.626 | 49.39 | 111.382 | 4.383 | NA |
| TRINITY_DN1245_c0_g1_i8 | 1.467246663 | 0.326945165 | 0.999970678 | 0.273 | 2.365 | 0.344 | 0.533 | F-box |
| TRINITY_DN2484_c0_g3_i2 | 1.467133604 | 0.58724519 | 0.999970678 | 7.967 | 14.37 | 0 | 8.073 | NA |
| TRINITY_DN472_c0_g1_i3 | -1.4669924 | 0.49788573 | 0.999970678 | 7.921 | 2.543 | 23.854 | 0.201 | Dirigent |
| TRINITY_DN17596_c0_g1_i4 | -1.46688786 | 0.486300464 | 0.999970678 | 0.018 | 1.015 | 0.489 | 2.202 | Methyltransf_29 |
| TRINITY_DN6660_c0_g1_i7 | 1.466761047 | 0.726998838 | 0.999970678 | 3.334 | 0 | 0 | 1.227 | NA |
| TRINITY_DN13124_c0_g1_i1 | -1.46614415 | 0.522550341 | 0.999970678 | 2.861 | 0.197 | 0.089 | 8.425 | NA |
| TRINITY_DN119_c0_g1_i6 | -1.46606647 | 0.143511357 | 0.999970678 | 25.39 | 66.1 | 64.807 | 172.381 | Ferritin |
| TRINITY_DN5857_c0_g1_i2 | 1.465792692 | 0.729292467 | 0.999970678 | 0 | 1.222 | 0.356 | 0 | NA |
| TRINITY_DN4379_c0_g1_i1 | -1.46550457 | 0.724778614 | 0.999970678 | 0 | 6.652 | 0 | 17.966 | NA |
| TRINITY_DN5625_c0_g2_i5 | -1.46469834 | 0.169300477 | 0.999970678 | 0.663 | 0.512 | 1.011 | 1.991 | UbiA |
| TRINITY_DN1862_c0_g1_i5 | -1.46448807 | 0.414497044 | 0.999970678 | 1.281 | 0.355 | 3.389 | 0.513 | NA |
| TRINITY_DN15166_c0_g1_i2 | 1.464229215 | 0.410298192 | 0.999970678 | 0.863 | 0.424 | 0.367 | 0.03 | NA |
| TRINITY_DN5058_c0_g1_i6 | 1.464107888 | 0.295325081 | 0.999970678 | 1.372 | 2.493 | 0.878 | 0.352 | NA |
| TRINITY_DN30154_c0_g1_i2 | 1.463782307 | 0.321011107 | 0.999970678 | 2.144 | 7.835 | 2.422 | 0.694 | DUF588 |
| TRINITY_DN2756_c0_g1_i2 | -1.46371507 | 0.190460937 | 0.999970678 | 2.525 | 3.252 | 2.889 | 12.376 | - |
| TRINITY_DN4775_c0_g1_i1 | -1.46350269 | 0.158828647 | 0.999970678 | 0.808 | 0.917 | 1.722 | 2.574 | NA |
| TRINITY_DN2458_c0_g1_i5 | 1.463474201 | 0.592076535 | 0.999970678 | 2.562 | 0.463 | 0.911 | 0 | GLE1 |
| TRINITY_DN7154_c0_g1_i1 | 1.462890423 | 0.738336144 | 0.999970678 | 0 | 47.89 | 14.121 | 0 | TPR_8 |
| TRINITY_DN4756_c0_g1_i1 | 1.462793287 | 0.297660612 | 0.999970678 | 0.681 | 1.094 | 0.422 | 0.141 | Peptidase_M24 |
| TRINITY_DN6534_c1_g1_i6 | 1.462501388 | 0.514275354 | 0.999970678 | 0.055 | 6.554 | 1.278 | 0.814 | NA |
| TRINITY_DN1304_c0_g1_i1 | 1.462411951 | 0.617953153 | 0.999970678 | 0.572 | 0.039 | 0.189 | 0 | NA |
| TRINITY_DN10264_c0_g1_i1 | 1.462393117 | 0.145871892 | 0.999970678 | 0.863 | 1.055 | 0.256 | 0.392 | Dynamin_M |
| TRINITY_DN1287_c0_g1_i1 | -1.46200314 | 0.606291012 | 0.999970678 | 11.746 | 0 | 5 | 26.813 | NA |
| TRINITY_DN3619_c0_g1_i3 | 1.461947395 | 0.588750342 | 0.999970678 | 5.523 | 9.767 | 4.566 | 0 | WD40 |
| TRINITY_DN16151_c0_g1_i3 | -1.46178237 | 0.310288709 | 0.999970678 | 2.662 | 0.572 | 5.877 | 1.85 | NA |
| TRINITY_DN28528_c0_g1_i2 | -1.46172854 | 0.127362231 | 0.999970678 | 0.963 | 1.597 | 1.944 | 4.695 | NA |
| TRINITY_DN1871_c0_g1_i1 | 1.461215311 | 0.21511938 | 0.999970678 | 29.405 | 6.8 | 6.855 | 4.946 | NA |
| TRINITY_DN14767_c0_g1_i3 | 1.461043563 | 0.573853572 | 0.999970678 | 2.38 | 2.641 | 1.511 | 0 | Spt5-NGN |
| TRINITY_DN1291_c0_g1_i4 | 1.460966078 | 0.187525103 | 0.999970678 | 0.917 | 0.808 | 0.222 | 0.372 | NA |
| TRINITY_DN382_c0_g1_i1 | 1.46085956 | 0.229702917 | 0.999970678 | 11.082 | 2.966 | 3.011 | 1.528 | NA |
| TRINITY_DN2850_c0_g1_i9 | -1.46069479 | 0.27924915 | 0.999970678 | 10.801 | 10.87 | 5.277 | 53.063 | NA |
| TRINITY_DN1845_c0_g1_i4 | 1.460629163 | 0.605160921 | 0.999970678 | 2.616 | 0.256 | 0.867 | 0 | Cation_ATPase |
| TRINITY_DN7227_c0_g1_i5 | 1.460569461 | 0.234510148 | 0.999970678 | 0.382 | 0.759 | 0.233 | 0.141 | ArgoL1 |
| TRINITY_DN3773_c0_g1_i1 | -1.46052298 | 0.580069634 | 0.999970678 | 0 | 2.217 | 3.466 | 1.83 | NA |
| TRINITY_DN11879_c0_g1_i5 | 1.460472274 | 0.728718731 | 0.999970678 | 0 | 11.04 | 3.222 | 0 | HALZ |
| TRINITY_DN2032_c0_g1_i4 | 1.460415534 | 0.430743509 | 0.999970678 | 29.459 | 2.395 | 8.411 | 1.448 | NA |
| TRINITY_DN6305_c0_g1_i6 | 1.460136928 | 0.60829821 | 0.999970678 | 1.989 | 0 | 0.244 | 0.442 | NA |
| TRINITY_DN9734_c0_g1_i1 | 1.45988457 | 0.275837325 | 0.999970678 | 3.089 | 1.064 | 0.944 | 0.412 | NA |
| TRINITY_DN2212_c0_g2_i12 | -1.45975922 | 0.600027865 | 0.999970678 | 0 | 2.395 | 1.055 | 5.238 | RRM_1 |
| TRINITY_DN7705_c0_g3_i2 | -1.45945963 | 0.327381281 | 0.999970678 | 1.635 | 4.533 | 1.522 | 14.92 | DPBB_1 |
| TRINITY_DN10082_c0_g1_i2 | 1.45941275 | 0.735527239 | 0.999970678 | 0 | 1.508 | 0 | 0.543 | Terpene_synth |
| TRINITY_DN4616_c0_g1_i3 | 1.458945752 | 0.178063396 | 0.999970678 | 1.989 | 2.582 | 0.967 | 0.483 | Calmodulin_bind |
| TRINITY_DN8342_c1_g1_i8 | 1.458742639 | 0.729731524 | 0.999970678 | 0 | 8.14 | 2.411 | 0 | GH3 |
| TRINITY_DN817_c0_g1_i6 | 1.458676549 | 0.586661308 | 1 | 0.273 | 0.404 | 0.211 | 0 | DUF4413 |
| TRINITY_DN1043_c0_g1_i17 | 1.458619588 | 0.640381131 | 0.999970678 | 0 | 14.45 | 0.256 | 4.796 | ADH_N |
| TRINITY_DN259_c0_g1_i4 | -1.45855682 | 0.417775585 | 0.999970678 | 0.781 | 0.059 | 0.811 | 1.297 | NA |
| TRINITY_DN274_c0_g1_i1 | -1.4582626 | 0.619859474 | 0.999970678 | 0.654 | 0 | 1.389 | 0.131 | RRM_1 |
| TRINITY_DN42694_c0_g1_i4 | -1.45808936 | 0.657512571 | 1 | 0.309 | 0.01 | 0.756 | 0 | F-box-like |
| TRINITY_DN901_c0_g1_i7 | -1.45808219 | 0.05045635 | 0.999970678 | 3.143 | 2.257 | 7.111 | 6.294 | NA |
| TRINITY_DN414_c0_g1_i2 | -1.45801287 | 0.178118518 | 0.999970678 | 6.313 | 2.858 | 15.377 | 6.716 | Oxidored_FMN |
| TRINITY_DN2725_c1_g1_i9 | -1.45801236 | 0.356679741 | 0.999970678 | 0.509 | 0.128 | 0.311 | 1.418 | NA |
| TRINITY_DN6444_c0_g1_i5 | -1.45797757 | 0.238670851 | 1 | 0.5 | 0.512 | 1.155 | 1.377 | Castor_Poll_mid |
| TRINITY_DN10362_c3_g1_i1 | 1.457945518 | 0.607693073 | 0.999970678 | 0 | 1.784 | 0.378 | 0.181 | NA |
| TRINITY_DN3448_c0_g1_i2 | -1.45764288 | 0.242714724 | 0.999970678 | 1.045 | 3.39 | 2.9 | 8.556 | NA |

| TRINITY_DN457_c0_g1_i2 | 1.457615653 | 0.383093471 | 0.999970678 | 10.946 | 16.56 | 0.311 | 9.621 | NA |
| --- | --- | --- | --- | --- | --- | --- | --- | --- |
| TRINITY_DN17797_c0_g1_i1 | -1.45759399 | 0.486278144 | 0.999970678 | 0.445 | 0.049 | 0.978 | 0.121 | NA |
| TRINITY_DN3209_c0_g1_i4 | -1.45750831 | 0.615415115 | 0.999970678 | 0 | 33.95 | 17.466 | 70.718 | ADH_N |
| TRINITY_DN2200_c0_g1_i2 | -1.45728597 | 0.146509077 | 0.999970678 | 1.662 | 0.828 | 2.255 | 4.122 | F-box |
| TRINITY_DN4739_c0_g2_i4 | -1.45728139 | 0.584122658 | 0.999970678 | 0 | 2.139 | 3.544 | 1.508 | Glyco_trans_1_4 |
| TRINITY_DN5503_c0_g2_i1 | 1.457223855 | 0.16303636 | 0.999970678 | 1.544 | 0.917 | 0.389 | 0.432 | NA |
| TRINITY_DN706_c0_g1_i4 | 1.456884212 | 0.07528489 | 0.999970678 | 9.002 | 8.397 | 1.844 | 4.082 | NA |
| TRINITY_DN3646_c0_g1_i3 | 1.456804511 | 0.159550424 | 0.999970678 | 5.614 | 20.2 | 4.133 | 4.353 | Dimerisation |
| TRINITY_DN1318_c0_g2_i3 | -1.45604871 | 0.59056104 | 0.999970678 | 2.825 | 0 | 4.266 | 2.735 | NA |
| TRINITY_DN6606_c0_g1_i1 | -1.45597082 | 0.651009702 | 0.999970678 | 14.126 | 0.286 | 33.065 | 0 | PIR |
| TRINITY_DN3443_c0_g1_i11 | 1.455916939 | 0.737862195 | 0.999970678 | 0.681 | 0 | 0 | 0.251 | NA |
| TRINITY_DN13543_c1_g1_i1 | -1.45589592 | 0.733303934 | 0.999970678 | 1.508 | 0 | 3.489 | 0 | Aldedh |
| TRINITY_DN57883_c0_g1_i1 | -1.45587841 | 0.524623653 | 0.999970678 | 2567.7 | 3.302 | 3045.238 | 3108.251 | NA |
| TRINITY_DN4268_c0_g1_i4 | 1.455870167 | 0.42393768 | 0.999970678 | 4.342 | 0.65 | 0.167 | 1.629 | NA |
| TRINITY_DN2813_c0_g1_i6 | -1.45580547 | 0.55025365 | 0.999970678 | 3.652 | 0.306 | 0.056 | 10.808 | NA |
| TRINITY_DN2796_c0_g1_i18 | -1.45578972 | 0.621863875 | 0.999970678 | 7.222 | 0.956 | 18.61 | 0 | NA |
| TRINITY_DN362_c0_g1_i2 | -1.45575935 | 0.735591175 | 0.999970678 | 7.758 | 0 | 0 | 21.686 | Bet_v_1 |
| TRINITY_DN381_c0_g1_i1 | -1.45575576 | 0.590701492 | 0.999970678 | 0 | 2.631 | 4.855 | 1.247 | NB-ARC |
| TRINITY_DN2515_c0_g1_i5 | 1.455015396 | 0.27441125 | 0.999970678 | 8.939 | 4.267 | 3.366 | 0.734 | NA |
| TRINITY_DN5748_c0_g1_i1 | -1.45500426 | 0.314468053 | 0.999970678 | 4.796 | 0.719 | 7.033 | 6.565 | LRRNT_2 |
| TRINITY_DN10982_c0_g1_i2 | -1.45447567 | 0.579332057 | 0.999970678 | 0.636 | 0 | 0.678 | 0.965 | Pkinase |
| TRINITY_DN4104_c0_g1_i1 | -1.45432832 | 0.206396145 | 0.999970678 | 12.645 | 33.11 | 30.365 | 80.037 | NA |
| TRINITY_DN3714_c0_g1_i15 | 1.45418821 | 0.612144207 | 0.999970678 | 2.026 | 0 | 0.167 | 0.553 | Peptidase_M16 |
| TRINITY_DN5850_c0_g1_i4 | 1.454173744 | 0.313502537 | 0.999970678 | 0.609 | 4.208 | 0.644 | 0.965 | NA |
| TRINITY_DN1811_c0_g3_i6 | -1.45411048 | 0.730627801 | 0.999970678 | 2.498 | 0 | 5.7 | 0 | Grp1_Fun34_Yaa |
| TRINITY_DN10661_c0_g1_i1 | 1.454015119 | 0.248522073 | 0.999970678 | 213.5 | 68.01 | 69.562 | 19.494 | NA |
| TRINITY_DN3293_c0_g1_i2 | -1.45384197 | 0.729678076 | 0.999970678 | 0 | 5.144 | 0 | 13.583 | NA |
| TRINITY_DN7074_c0_g1_i2 | 1.453588535 | 0.615864786 | 0.999970678 | 6.104 | 0.306 | 0 | 2.363 | 2OG-FeII_Oxy |
| TRINITY_DN471_c0_g1_i8 | -1.45348471 | 0.310859452 | 0.999970678 | 0.491 | 1.035 | 2.866 | 0.643 | LRR_8 |
| TRINITY_DN9979_c0_g1_i3 | 1.453375438 | 0.730299366 | 0.999970678 | 0 | 2.237 | 0.656 | 0 | Pkinase |
| TRINITY_DN9656_c0_g1_i1 | 1.453356518 | 0.373459834 | 0.999970678 | 0.872 | 1.646 | 0.078 | 0.824 | NA |
| TRINITY_DN3087_c0_g1_i3 | -1.45333792 | 0.039133945 | 0.999970678 | 9.393 | 10.51 | 27.365 | 21.243 | NA |
| TRINITY_DN2588_c0_g1_i3 | -1.4531907 | 0.601933157 | 0.999970678 | 6.277 | 0 | 4.011 | 12.668 | NA |
| TRINITY_DN558_c0_g1_i6 | 1.452925264 | 0.731287322 | 0.999970678 | 38.634 | 0 | 11.788 | 0 | NA |
| TRINITY_DN1797_c0_g2_i1 | 1.452809195 | 0.728492316 | 0.999970678 | 0 | 7.155 | 2.133 | 0 | NA |
| TRINITY_DN934_c0_g1_i5 | -1.45279621 | 0.580340628 | 0.999970678 | 1.844 | 0 | 2.3 | 2.353 | NA |
| TRINITY_DN6991_c0_g1_i6 | -1.45266822 | 0.591330307 | 1 | 0.663 | 0 | 0.922 | 0.684 | NA |
| TRINITY_DN4222_c0_g1_i4 | 1.452583402 | 0.642399048 | 0.999970678 | 3.343 | 0 | 0.078 | 1.166 | NA |
| TRINITY_DN2389_c1_g1_i4 | -1.45233295 | 0.732207859 | 0.999970678 | 0 | 0.473 | 1.055 | 0 | NA |
| TRINITY_DN1467_c0_g1_i13 | 1.45223301 | 0.330538265 | 0.999970678 | 1.799 | 4.898 | 0.256 | 2.101 | NA |
| TRINITY_DN6815_c0_g1_i2 | -1.45218377 | 0.580348393 | 0.999970678 | 0.927 | 0 | 1.322 | 0.965 | LRRNT_2 |
| TRINITY_DN8722_c0_g1_i5 | 1.452142774 | 0.31206642 | 0.999970678 | 2.162 | 0.562 | 0.167 | 0.804 | BRCT |
| TRINITY_DN693_c0_g1_i8 | 1.451651403 | 0.308733924 | 0.999970678 | 1.98 | 0.542 | 0.244 | 0.643 | - |
| TRINITY_DN8283_c0_g1_i1 | -1.45129871 | 0.589320839 | 1 | 0 | 6.288 | 4.578 | 10.667 | NA |
| TRINITY_DN1582_c0_g3_i1 | 1.45116607 | 0.215762736 | 0.999970678 | 2.444 | 6.189 | 2 | 0.724 | Response_reg |
| TRINITY_DN9712_c0_g1_i3 | 1.450748869 | 0.601587999 | 0.999970678 | 1.681 | 0.227 | 0.578 | 0 | Clathrin |
| TRINITY_DN10182_c0_g1_i9 | -1.4505912 | 0.74236905 | 1 | 0.618 | 0 | 1.411 | 0 | p450 |
| TRINITY_DN4808_c0_g1_i1 | 1.450281431 | 0.175320471 | 0.999970678 | 3.579 | 11.87 | 2 | 3.187 | NA |
| TRINITY_DN3103_c0_g1_i4 | 1.449796838 | 0.586253265 | 0.999970678 | 11.509 | 16.04 | 8.288 | 0 | NA |
| TRINITY_DN6592_c0_g2_i1 | 1.449762433 | 0.371024444 | 1 | 0.545 | 0.414 | 0.267 | 0.05 | PH |
| TRINITY_DN2010_c0_g1_i3 | 1.449583478 | 0.167074028 | 0.999970678 | 12.036 | 11.33 | 1.644 | 6.535 | XS |
| TRINITY_DN251607_c0_g1_i1 | 1.449530088 | 0.604529303 | 0.999970678 | 0 | 11.32 | 1.367 | 2.423 | NA |
| TRINITY_DN1211_c0_g2_i4 | 1.449486817 | 0.228426675 | 0.999970678 | 2.425 | 2.296 | 0.267 | 1.387 | CRS1_YhbY |
| TRINITY_DN4616_c0_g1_i9 | 1.449393258 | 0.378103648 | 0.999970678 | 1.254 | 1.666 | 0.833 | 0.06 | Calmodulin_bind |
| TRINITY_DN2044_c1_g3_i2 | -1.44934424 | 0.612035224 | 0.999970678 | 22.265 | 0 | 12.455 | 46.609 | NA |
| TRINITY_DN2123_c1_g1_i1 | -1.44933198 | 0.601244418 | 0.999970678 | 12.318 | 0 | 16.71 | 13.944 | PCI |
| TRINITY_DN3452_c0_g2_i3 | -1.44920325 | 0.38363689 | 0.999970678 | 0.055 | 0.375 | 0.611 | 0.392 | NA |
| TRINITY_DN266_c0_g1_i5 | -1.44920124 | 0.587834698 | 0.999970678 | 0 | 1.784 | 3.089 | 1.025 | NA |
| TRINITY_DN3517_c1_g1_i9 | -1.44916638 | 0.73187798 | 0.999970678 | 0.727 | 0 | 0 | 2.031 | NA |
| TRINITY_DN4274_c0_g1_i19 | 1.448952132 | 0.733775146 | 0.999970678 | 1.926 | 0 | 0 | 0.724 | Ank |
| TRINITY_DN9023_c0_g1_i2 | -1.44894773 | 0.584669681 | 0.999970678 | 0 | 0.542 | 0.9 | 0.362 | AAA_25 |
| TRINITY_DN2309_c0_g1_i1 | -1.44892809 | 0.583748823 | 0.999970678 | 1.226 | 0 | 1.911 | 1.076 | NA |
| TRINITY_DN8683_c0_g1_i1 | -1.44889324 | 0.334501091 | 0.999970678 | 0.282 | 0.562 | 0.3 | 1.89 | NA |
| TRINITY_DN411_c0_g1_i2 | -1.4488799 | 0.38019847 | 0.999970678 | 11.001 | 13.49 | 2.211 | 63.761 | NA |
| TRINITY_DN2750_c1_g1_i3 | -1.44842757 | 0.577547733 | 0.999970678 | 0 | 0.394 | 0.456 | 0.503 | Jas |
| TRINITY_DN30257_c0_g1_i2 | -1.44837541 | 0.180893396 | 0.999970678 | 11.537 | 30.13 | 65.385 | 31.89 | NA |
| TRINITY_DN1285_c0_g1_i3 | 1.448295303 | 0.130921311 | 0.999970678 | 19.558 | 12.66 | 2.689 | 8.626 | CDC50 |
| TRINITY_DN4739_c0_g3_i1 | -1.44817994 | 0.268434077 | 0.999970678 | 0.645 | 1.261 | 1.044 | 3.82 | Glyco_trans_1_4 |
| TRINITY_DN5058_c0_g1_i3 | -1.44793069 | 0.098186889 | 0.999970678 | 0.409 | 0.71 | 1.167 | 1.619 | Cnd1 |
| TRINITY_DN846_c0_g2_i10 | -1.44790253 | 0.597139672 | 0.999970678 | 4.56 | 0 | 7.8 | 3.177 | NA |
| TRINITY_DN7964_c0_g1_i1 | -1.44773075 | 0.378194621 | 0.999970678 | 4.533 | 12.96 | 2 | 45.071 | EF-hand_5 |
| TRINITY_DN1278_c0_g1_i1 | 1.447670024 | 0.584567457 | 0.999970678 | 5.45 | 2.641 | 2.467 | 0 | NA |
| TRINITY_DN1455_c1_g1_i4 | 1.447496311 | 0.592561259 | 0.999970678 | 9.211 | 6.652 | 4.811 | 0 | NA |
| TRINITY_DN10344_c0_g1_i2 | 1.447349847 | 0.314848894 | 1 | 1.163 | 1.577 | 0.2 | 0.754 | NA |

| TRINITY_DN18839_c0_g1_i2 | -1.44694038 | 0.600581222 | 0.999970678 | 1.363 | 1.212 | 0 | 7.048 | NA |
| --- | --- | --- | --- | --- | --- | --- | --- | --- |
| TRINITY_DN4536_c0_g1_i5 | -1.44692898 | 0.080284227 | 0.999970678 | 2.634 | 1.557 | 5.255 | 5.117 | Cu-oxidase |
| TRINITY_DN2186_c0_g1_i2 | 1.4467175 | 0.740538258 | 0.999970678 | 5.078 | 0 | 1.522 | 0 | SnoaL_2 |
| TRINITY_DN697_c0_g1_i3 | -1.44661167 | 0.615666938 | 0.999970678 | 2.834 | 0 | 6.033 | 0.553 | NA |
| TRINITY_DN1903_c0_g1_i3 | 1.446604589 | 0.472225891 | 0.999970678 | 15.942 | 0.808 | 4.933 | 0.271 | NA |
| TRINITY_DN4339_c0_g1_i1 | 1.446573784 | 0.729169642 | 0.999970678 | 4.279 | 0 | 0 | 1.599 | NA |
| TRINITY_DN7390_c0_g1_i2 | 1.44634671 | 0.121389296 | 0.999970678 | 6.05 | 2.799 | 1.322 | 1.649 | NA |
| TRINITY_DN10671_c0_g1_i12 | -1.44632384 | 0.465483339 | 0.999970678 | 0.182 | 5.923 | 2.555 | 13.09 | NA |
| TRINITY_DN1428_c0_g2_i7 | 1.446315309 | 0.333094652 | 0.999970678 | 3.016 | 2.474 | 1.511 | 0.191 | NA |
| TRINITY_DN137529_c0_g1_i1 | -1.44622545 | 0.059970175 | 0.999970678 | 5.042 | 3.134 | 8.233 | 12.386 | - |
| TRINITY_DN5138_c0_g1_i1 | 1.445913363 | 0.264664877 | 1 | 1.971 | 2.227 | 0.522 | 0.885 | NA |
| TRINITY_DN5579_c0_g1_i2 | -1.44581897 | 0.588670542 | 0.999970678 | 1.926 | 0 | 2.822 | 1.92 | NA |
| TRINITY_DN12142_c0_g1_i5 | -1.44569118 | 0.593387496 | 0.999970678 | 6.241 | 0 | 3.289 | 13.15 | NA |
| TRINITY_DN2573_c0_g1_i2 | 1.445288838 | 0.201676215 | 0.999970678 | 24.127 | 104.8 | 24.821 | 16.92 | AP2 |
| TRINITY_DN720_c0_g1_i5 | -1.44512719 | 0.587843953 | 0.999970678 | 2.762 | 0 | 4.1 | 2.684 | NA |
| TRINITY_DN4475_c0_g1_i3 | -1.44505127 | 0.596989067 | 0.999970678 | 0 | 8.968 | 14.344 | 6.645 | NA |
| TRINITY_DN6712_c0_g1_i1 | -1.44504546 | 0.012494393 | 0.580678249 | 19.785 | 19.07 | 47.219 | 48.881 | NA |
| TRINITY_DN10510_c0_g1_i2 | 1.444675928 | 0.585827122 | 1 | 0.245 | 0.384 | 0 | 0.231 | CRT-like |
| TRINITY_DN25952_c0_g1_i2 | 1.44467402 | 0.458732411 | 0.999970678 | 0.091 | 2.976 | 0.644 | 0.332 | G6PD_C |
| TRINITY_DN1724_c0_g1_i3 | 1.444351629 | 0.221631064 | 0.999970678 | 3.37 | 12.46 | 3.155 | 1.96 | NA |
| TRINITY_DN4059_c1_g1_i1 | -1.44434186 | 0.385843135 | 0.999970678 | 0.727 | 0.138 | 1.655 | 0.402 | NA |
| TRINITY_DN1607_c0_g1_i6 | 1.444336905 | 0.026677473 | 0.999970678 | 5.251 | 5.677 | 1.711 | 1.94 | Sugar_tr |
| TRINITY_DN2989_c0_g1_i1 | 1.444315041 | 0.608761028 | 0.999970678 | 0 | 14.28 | 1.778 | 3.016 | - |
| TRINITY_DN8342_c1_g1_i5 | -1.44426312 | 0.347221752 | 0.999970678 | 11.264 | 0.759 | 17.077 | 12.547 | GH3 |
| TRINITY_DN636_c0_g1_i2 | -1.44414359 | 0.051158595 | 0.999970678 | 7.194 | 4.849 | 13.01 | 16.92 | - |
| TRINITY_DN3585_c0_g1_i1 | -1.44403167 | 0.591427182 | 0.999970678 | 0 | 0.68 | 0.311 | 1.448 | B_lectin |
| TRINITY_DN1261_c0_g1_i4 | -1.44393872 | 0.179759474 | 0.999970678 | 2.707 | 2.868 | 2.633 | 12.064 | NA |
| TRINITY_DN8139_c0_g1_i4 | 1.443920238 | 0.332680964 | 0.999970678 | 5.469 | 2.484 | 0.344 | 2.493 | NA |
| TRINITY_DN9344_c0_g1_i3 | -1.44389562 | 0.272062236 | 0.999970678 | 2.78 | 1.606 | 2.211 | 9.109 | NA |
| TRINITY_DN2567_c0_g1_i6 | 1.443878973 | 0.11737246 | 0.999970678 | 13.072 | 36.2 | 8.477 | 7.782 | Hemerythrin |
| TRINITY_DN6615_c0_g1_i5 | -1.44384842 | 0.73781155 | 0.999970678 | 0.345 | 0 | 0.8 | 0 | HECT |
| TRINITY_DN5381_c0_g1_i7 | -1.44359154 | 0.468977147 | 0.999970678 | 0.027 | 0.217 | 0.044 | 0.603 | ABC_membrane |
| TRINITY_DN11078_c0_g1_i2 | -1.44349983 | 0.408564428 | 0.999970678 | 3.543 | 0.187 | 5.644 | 3.438 | NA |
| TRINITY_DN5024_c0_g2_i3 | 1.443298982 | 0.584764439 | 1 | 0.709 | 0.581 | 0 | 0.462 | NA |
| TRINITY_DN3250_c0_g1_i10 | -1.44317432 | 0.462336452 | 0.999970678 | 1.481 | 78.26 | 26.198 | 178.835 | NA |
| TRINITY_DN18161_c0_g1_i4 | -1.44286861 | 0.278446518 | 0.999970678 | 2.017 | 7.007 | 16.299 | 4.524 | NA |
| TRINITY_DN8950_c0_g1_i2 | 1.442759081 | 0.74055991 | 0.999970678 | 1.753 | 0 | 0.544 | 0 | NA |
| TRINITY_DN82644_c0_g1_i1 | -1.44266028 | 0.303960241 | 0.999970678 | 3.034 | 1.133 | 8.144 | 1.578 | DNA_photolyase |
| TRINITY_DN11926_c0_g1_i2 | 1.442646935 | 0.580311423 | 0.999970678 | 0.654 | 1.409 | 0 | 0.754 | AAA |
| TRINITY_DN5392_c0_g1_i6 | 1.442506478 | 0.585540951 | 0.999970678 | 0.908 | 1.104 | 0.611 | 0 | DCB |
| TRINITY_DN2658_c0_g4_i1 | -1.44249097 | 0.223640727 | 0.999970678 | 0.59 | 0.542 | 0.878 | 2.121 | NA |
| TRINITY_DN101375_c0_g1_i1 | -1.44247223 | 0.333582148 | 0.999970678 | 0.1 | 0.434 | 0.411 | 0.955 | NA |
| TRINITY_DN24924_c0_g1_i3 | 1.44245483 | 0.164303971 | 0.999970678 | 13.263 | 17.95 | 5.755 | 4.333 | NB-ARC |
| TRINITY_DN22462_c0_g1_i6 | 1.442329036 | 0.303972851 | 0.999970678 | 1.608 | 0.552 | 0.478 | 0.221 | DA1-like |
| TRINITY_DN7460_c2_g1_i3 | -1.4422745 | 0.108575151 | 0.999970678 | 0.336 | 0.256 | 0.511 | 1.005 | NA |
| TRINITY_DN545_c2_g1_i10 | 1.44223044 | 0.250737428 | 1 | 9.983 | 15.2 | 3.633 | 4.182 | NA |
| TRINITY_DN8879_c0_g1_i11 | 1.442148707 | 0.461221167 | 0.999970678 | 1.117 | 0.591 | 0.022 | 0.613 | NA |
| TRINITY_DN3452_c1_g1_i1 | 1.442087517 | 0.573674573 | 0.999970678 | 0.845 | 1.291 | 0 | 0.784 | NA |
| TRINITY_DN7386_c0_g1_i3 | 1.441936471 | 0.589233142 | 0.999970678 | 0.645 | 0.384 | 0.311 | 0 | NA |
| TRINITY_DN1266_c0_g1_i3 | -1.44184346 | 0.29086917 | 0.999970678 | 24.518 | 25.86 | 11.033 | 122.695 | NA |
| TRINITY_DN1900_c0_g1_i15 | -1.44174297 | 0.228219193 | 0.999970678 | 1.208 | 1.932 | 2.5 | 5.339 | NA |
| TRINITY_DN1076_c0_g1_i7 | 1.441733648 | 0.232305127 | 0.999970678 | 3.234 | 2.188 | 1.244 | 0.513 | Cellulose_synt |
| TRINITY_DN10275_c0_g1_i2 | 1.441533847 | 0.365962077 | 0.999970678 | 1.172 | 3.637 | 1.333 | 0.151 | NA |
| TRINITY_DN1180_c0_g1_i10 | -1.44119313 | 0.629700369 | 0.999970678 | 3.761 | 0 | 0.278 | 10.003 | NA |
| TRINITY_DN7737_c0_g1_i10 | -1.44106135 | 0.734691808 | 0.999970678 | 0.945 | 0 | 2.178 | 0.01 | Pro-kuma_activ |
| TRINITY_DN82220_c0_g1_i11 | -1.44106135 | 0.734691808 | 0.999970678 | 1.935 | 0 | 4.389 | 0 | NA |
| TRINITY_DN203_c0_g1_i3 | -1.44081758 | 0.313976865 | 0.999970678 | 3.661 | 0.424 | 4.644 | 5.59 | NA |
| TRINITY_DN1493_c0_g1_i3 | 1.440747448 | 0.330400291 | 0.999970678 | 1.217 | 11.14 | 1.389 | 2.795 | Rad60-SLD_2 |
| TRINITY_DN5070_c0_g1_i1 | -1.44043897 | 0.578034704 | 0.999970678 | 7.621 | 0.237 | 17.599 | 0.131 | NA |
| TRINITY_DN502_c0_g3_i2 | -1.44020787 | 0.04757225 | 0.999970678 | 11.954 | 21.38 | 39.709 | 42.065 | Tubulin |
| TRINITY_DN1091_c0_g1_i6 | 1.440187751 | 0.263795164 | 0.999970678 | 5.132 | 7.391 | 0.733 | 3.639 | NA |
| TRINITY_DN50853_c0_g1_i1 | 1.439988962 | 0.460625853 | 0.999970678 | 2.244 | 146.1 | 16.855 | 30.513 | Chloroa_b-bind |
| TRINITY_DN10175_c0_g1_i3 | 1.439952995 | 0.249980046 | 0.999970678 | 0.218 | 0.818 | 0.156 | 0.201 | NA |
| TRINITY_DN40079_c0_g1_i1 | -1.4397227 | 0.746123802 | 1 | 0.3 | 0 | 0.689 | 0 | NA |
| TRINITY_DN10873_c0_g1_i1 | -1.4397227 | 0.746123802 | 1 | 1.453 | 0 | 3.278 | 0 | NA |
| TRINITY_DN1783_c0_g1_i8 | -1.43954365 | 0.396914868 | 0.999970678 | 1.463 | 10.8 | 2.055 | 30.603 | Orn_Arg_deC_N |
| TRINITY_DN5958_c3_g1_i1 | 1.439536219 | 0.29993303 | 1 | 0.463 | 0.828 | 0.289 | 0.141 | NA |
| TRINITY_DN81_c0_g1_i1 | -1.4393492 | 0.62597127 | 0.999970678 | 0 | 24.32 | 4.5 | 59.407 | NA |
| TRINITY_DN3879_c0_g1_i4 | -1.4393131 | 0.633192869 | 0.999970678 | 176.47 | 0 | 158.035 | 292.341 | TPM_phosphatas |
| TRINITY_DN93_c0_g1_i4 | -1.43928595 | 0.247713962 | 0.999970678 | 0.409 | 0.463 | 1.589 | 0.422 | DUF538 |
| TRINITY_DN6310_c0_g2_i12 | -1.43919065 | 0.128216356 | 0.999970678 | 0.872 | 1.695 | 2.267 | 4.102 | NA |
| TRINITY_DN7396_c0_g1_i1 | 1.439116426 | 0.162087838 | 0.999970678 | 1.29 | 0.68 | 0.411 | 0.241 | NA |
| TRINITY_DN22454_c0_g1_i1 | -1.4388139 | 0.404954943 | 0.999970678 | 0.963 | 0.118 | 0.744 | 2.041 | DUF1635 |
| TRINITY_DN6238_c0_g1_i7 | -1.43865033 | 0.606133156 | 0.999970678 | 0 | 1.675 | 3.222 | 0.553 | NA |

| TRINITY_DN4376_c0_g1_i2 | 1.438505776 | 0.732586597 | 0.999970678 | 10.501 | 0 | 0 | 3.951 | NA |
| --- | --- | --- | --- | --- | --- | --- | --- | --- |
| TRINITY_DN60363_c1_g1_i8 | -1.43842068 | 0.339783355 | 0.999970678 | 1.799 | 0.473 | 0.867 | 5.228 | Abhydrolase_1 |
| TRINITY_DN1958_c0_g1_i2 | 1.438390259 | 0.093270536 | 0.999970678 | 15.661 | 29.03 | 4.722 | 10.747 | NA |
| TRINITY_DN614_c0_g1_i7 | 1.438193811 | 0.150235622 | 0.999970678 | 128.33 | 53.83 | 38.798 | 20.831 | NA |
| TRINITY_DN1955_c0_g1_i2 | 1.438125615 | 0.19959693 | 0.999970678 | 2.771 | 6.031 | 2.022 | 0.794 | Na_H_Exchanger |
| TRINITY_DN2143_c0_g1_i2 | 1.43795115 | 0.095008033 | 0.999970678 | 4.533 | 2.651 | 1.144 | 1.277 | AAA |
| TRINITY_DN577_c0_g1_i2 | 1.437935523 | 0.391886866 | 0.999970678 | 23.328 | 3.41 | 2 | 7.048 | NA |
| TRINITY_DN20127_c0_g1_i5 | -1.43789369 | 0.739815663 | 0.999970678 | 0 | 0.926 | 2.022 | 0 | NA |
| TRINITY_DN20691_c0_g1_i2 | 1.437827001 | 0.593305797 | 0.999970678 | 0.472 | 2.02 | 0 | 0.925 | Kelch_1 |
| TRINITY_DN125_c3_g1_i3 | -1.4377614 | 0.164116305 | 0.999970678 | 3.624 | 1.429 | 7.088 | 5.127 | NA |
| TRINITY_DN34087_c0_g1_i1 | -1.43748131 | 0.733033223 | 0.999970678 | 2.607 | 0 | 5.911 | 0 | NA |
| TRINITY_DN1054_c0_g1_i1 | 1.437236942 | 0.386025442 | 0.999970678 | 9.384 | 5.972 | 0.2 | 5.469 | NA |
| TRINITY_DN40419_c0_g1_i2 | -1.4362371 | 0.740070479 | 0.999970678 | 0.545 | 0 | 1.244 | 0 | DUF2722 |
| TRINITY_DN19338_c0_g1_i3 | -1.4362371 | 0.740070479 | 0.999970678 | 1.154 | 0 | 2.578 | 0 | NA |
| TRINITY_DN966_c0_g1_i2 | 1.436209688 | 0.617738269 | 0.999970678 | 0 | 3.272 | 0.733 | 0.292 | PB1 |
| TRINITY_DN2654_c0_g2_i2 | 1.43599048 | 0.314160998 | 0.999970678 | 1.208 | 3.164 | 0.378 | 1.136 | DUF247 |
| TRINITY_DN11514_c0_g2_i1 | 1.435804865 | 0.740613439 | 0.999970678 | 1.989 | 0 | 0.622 | 0 | FMO-like |
| TRINITY_DN14021_c0_g1_i1 | -1.43571651 | 0.605166235 | 0.999970678 | 0.4 | 0.394 | 0 | 2.121 | NA |
| TRINITY_DN29393_c0_g1_i3 | 1.435669023 | 0.157593157 | 0.999970678 | 0.917 | 0.65 | 0.322 | 0.191 | Lipase_3 |
| TRINITY_DN4153_c0_g1_i4 | 1.435665314 | 0.224218428 | 0.999970678 | 0.69 | 2.237 | 0.378 | 0.603 | AMPK1_CBM |
| TRINITY_DN79_c0_g1_i10 | -1.43544814 | 0.731789194 | 0.999970678 | 0 | 0.66 | 1.455 | 0 | NA |
| TRINITY_DN3197_c0_g1_i4 | -1.4354224 | 0.182149323 | 0.999970678 | 14.425 | 12.53 | 11.922 | 58.824 | LANC_like |
| TRINITY_DN4488_c0_g2_i3 | -1.43539532 | 0.379827563 | 0.999970678 | 0.872 | 0.345 | 2.433 | 0.392 | NA |
| TRINITY_DN612_c0_g1_i5 | 1.435373676 | 0.613249617 | 0.999970678 | 0.127 | 1.055 | 0.367 | 0 | - |
| TRINITY_DN10478_c0_g1_i1 | -1.4353568 | 0.435909052 | 0.999970678 | 5.914 | 1.399 | 0.556 | 19.333 | NA |
| TRINITY_DN6906_c0_g1_i1 | 1.435285883 | 0.123891071 | 0.999970678 | 3.098 | 4.248 | 1.589 | 0.794 | NA |
| TRINITY_DN6317_c0_g1_i1 | -1.4344531 | 0.337414143 | 0.999970678 | 1.036 | 11.56 | 8.066 | 23.918 | PMEI |
| TRINITY_DN4628_c0_g1_i8 | -1.43423499 | 0.354715481 | 0.999970678 | 0.1 | 0.315 | 0.789 | 0.161 | Helicase_C |
| TRINITY_DN8808_c0_g1_i3 | 1.434208906 | 0.731913559 | 0.999970678 | 0 | 14.06 | 4.233 | 0 | Peptidase_S28 |
| TRINITY_DN1663_c0_g1_i8 | -1.43415173 | 0.58388123 | 0.999970678 | 8.166 | 0 | 8.933 | 11.331 | FolB |
| TRINITY_DN5682_c0_g1_i1 | -1.43408216 | 0.22035188 | 0.999970678 | 3.443 | 0.897 | 5.989 | 4.605 | NA |
| TRINITY_DN2236_c0_g1_i1 | 1.434053018 | 0.222313708 | 0.999970678 | 11.191 | 7.707 | 4.455 | 1.639 | NA |
| TRINITY_DN2024_c0_g1_i2 | -1.43381914 | 0.367153488 | 0.999970678 | 2.734 | 3.775 | 1.289 | 15.563 | NA |
| TRINITY_DN4841_c0_g1_i1 | 1.433365971 | 0.744239313 | 1 | 3.543 | 0 | 0 | 1.347 | NA |
| TRINITY_DN23390_c0_g1_i1 | 1.433334294 | 0.371529113 | 0.999970678 | 2.743 | 0.335 | 0.789 | 0.191 | NA |
| TRINITY_DN236_c0_g1_i7 | -1.43304431 | 0.604957086 | 0.999970678 | 0 | 2.434 | 4.733 | 0.784 | NA |
| TRINITY_DN289_c0_g1_i5 | -1.43244603 | 0.226791019 | 0.999970678 | 0.618 | 0.848 | 0.611 | 3.237 | DUF659 |
| TRINITY_DN9307_c0_g1_i1 | 1.432296784 | 0.056454266 | 0.999970678 | 2.553 | 2.74 | 0.878 | 0.905 | NA |
| TRINITY_DN2005_c0_g1_i8 | 1.432283625 | 0.614765384 | 0.999970678 | 0 | 5.164 | 1.133 | 0.503 | Aldo_ket_red |
| TRINITY_DN8010_c0_g1_i1 | 1.432194299 | 0.617452251 | 0.999970678 | 0.191 | 2.02 | 0.678 | 0 | Glyco_hydro_17 |
| TRINITY_DN1410_c0_g1_i3 | 1.432133841 | 0.619355577 | 0.999970678 | 102.66 | 80.64 | 0 | 67.872 | NA |
| TRINITY_DN4776_c0_g1_i2 | 1.431898869 | 0.305894235 | 0.999970678 | 2.58 | 8.801 | 2.966 | 0.603 | NA |
| TRINITY_DN14982_c0_g1_i2 | 1.431897631 | 0.497712893 | 0.999970678 | 0.363 | 6.091 | 1.711 | 0.271 | - |
| TRINITY_DN1913_c0_g1_i7 | -1.43176854 | 0.148071949 | 0.999970678 | 0.917 | 0.503 | 1.444 | 2.111 | NA |
| TRINITY_DN4002_c0_g1_i6 | -1.43154121 | 0.738964714 | 0.999970678 | 1.381 | 0 | 0 | 3.8 | Myb_DNA-bindin |
| TRINITY_DN86262_c0_g1_i3 | 1.431400692 | 0.6623101 | 0.999970678 | 2.562 | 0.03 | 0.811 | 0 | NA |
| TRINITY_DN5107_c0_g1_i8 | -1.43137208 | 0.596807674 | 1 | 0 | 0.207 | 0.3 | 0.171 | NUFIP1 |
| TRINITY_DN3637_c0_g1_i5 | -1.43135847 | 0.065148406 | 0.999970678 | 5.45 | 3.913 | 7.844 | 15.875 | LRRNT_2 |
| TRINITY_DN7197_c0_g1_i6 | 1.431257786 | 0.73197364 | 0.999970678 | 16.333 | 0 | 0 | 6.103 | NA |
| TRINITY_DN39677_c0_g1_i1 | 1.431154349 | 0.521623868 | 0.999970678 | 2.88 | 0.306 | 0.944 | 0.04 | NA |
| TRINITY_DN112_c1_g1_i4 | -1.43055151 | 0.102504235 | 0.999970678 | 2.307 | 2.079 | 3.144 | 8.053 | NA |
| TRINITY_DN6628_c0_g1_i1 | -1.43037771 | 0.232953425 | 0.999970678 | 8.012 | 27.59 | 18.588 | 72.547 | NA |
| TRINITY_DN1408_c0_g1_i14 | -1.43023553 | 0.597658842 | 0.999970678 | 0.472 | 0 | 0.789 | 0.342 | NA |
| TRINITY_DN19202_c2_g2_i2 | -1.43015021 | 0.661181144 | 0.999970678 | 0.018 | 0.512 | 1.155 | 0 | NA |
| TRINITY_DN1248_c2_g1_i4 | -1.43005336 | 0.497021313 | 0.999970678 | 147.94 | 32.03 | 2.855 | 485.071 | NA |
| TRINITY_DN36320_c0_g1_i1 | -1.42980603 | 0.238090865 | 0.999970678 | 3.724 | 2.681 | 5.455 | 10.164 | NA |
| TRINITY_DN1003_c0_g1_i4 | -1.42966659 | 0.734756459 | 0.999970678 | 0.972 | 0 | 2.167 | 0 | NA |
| TRINITY_DN14872_c0_g1_i4 | 1.429663905 | 0.370345961 | 1 | 0.554 | 0.581 | 0.289 | 0.08 | NA |
| TRINITY_DN668_c2_g1_i9 | 1.429663905 | 0.370345961 | 1 | 3.743 | 4.001 | 1.922 | 0.523 | DUF659 |
| TRINITY_DN14895_c0_g1_i3 | -1.42963428 | 0.601343307 | 0.999970678 | 0.999 | 0 | 1.833 | 0.503 | NA |
| TRINITY_DN72_c0_g1_i12 | -1.42927253 | 0.442476732 | 0.999970678 | 2.716 | 3.982 | 0.4 | 17.383 | NA |
| TRINITY_DN8726_c0_g1_i4 | 1.429189609 | 0.325051045 | 0.999970678 | 13.181 | 10.41 | 0.633 | 7.993 | Myosin_TH1 |
| TRINITY_DN18298_c0_g1_i1 | 1.429097374 | 0.449704602 | 0.999970678 | 0.427 | 2.986 | 0.089 | 1.156 | NA |
| TRINITY_DN4795_c0_g1_i2 | -1.42897536 | 0.597672004 | 0.999970678 | 2.235 | 0 | 1.011 | 4.876 | VARLMGL |
| TRINITY_DN4436_c0_g1_i1 | 1.428913059 | 0.576594363 | 0.999970678 | 3.079 | 2.296 | 0 | 2.011 | PTH2 |
| TRINITY_DN469_c0_g1_i5 | 1.428733298 | 0.033624271 | 0.999970678 | 64.768 | 42.81 | 16.388 | 20.308 | p450 |
| TRINITY_DN30694_c0_g1_i1 | -1.42866788 | 0.532692975 | 0.999970678 | 0.418 | 2.001 | 0.056 | 6.344 | DUF674 |
| TRINITY_DN1070_c0_g1_i7 | 1.428554485 | 0.204093154 | 0.999970678 | 0.681 | 0.542 | 0.156 | 0.251 | Pkinase |
| TRINITY_DN1489_c0_g1_i1 | 1.42850343 | 0.358003122 | 0.999970678 | 2.144 | 2.109 | 1.167 | 0.221 | NA |
| TRINITY_DN6629_c0_g1_i15 | 1.428409335 | 0.584159197 | 0.999970678 | 0.545 | 0.808 | 0.411 | 0 | NA |
| TRINITY_DN21821_c0_g1_i4 | -1.42762934 | 0.440857747 | 0.999970678 | 0.536 | 1.744 | 4.433 | 0.462 | NA |
| TRINITY_DN67652_c0_g1_i5 | -1.42748536 | 0.37635615 | 0.999970678 | 1.39 | 0.177 | 2.844 | 0.845 | Pkinase |
| TRINITY_DN1444_c1_g1_i6 | -1.4270549 | 0.22004456 | 0.999970678 | 0.917 | 0.453 | 1.289 | 2.121 | NA |
| TRINITY_DN748_c0_g3_i3 | 1.426380883 | 0.159527684 | 0.999970678 | 20.72 | 10.33 | 2.778 | 8.274 | NA |

| TRINITY_DN2755_c0_g1_i12 | -1.42631943 | 0.374511643 | 0.999970678 | 0.345 | 2.444 | 0.811 | 6.475 | Glyco_hydro_16 |
| --- | --- | --- | --- | --- | --- | --- | --- | --- |
| TRINITY_DN7446_c1_g1_i2 | -1.42590622 | 0.59604735 | 1 | 0.972 | 0 | 1 | 1.408 | Chlorophyllase |
| TRINITY_DN4052_c0_g1_i6 | -1.42581804 | 0.273863997 | 0.999970678 | 0.491 | 1.478 | 2.244 | 2.473 | NA |
| TRINITY_DN7620_c1_g1_i2 | 1.425538731 | 0.409407732 | 0.999970678 | 2.743 | 1.094 | 1.133 | 0.07 | NA |
| TRINITY_DN382_c4_g1_i3 | 1.425319916 | 0.60527951 | 0.999970678 | 2.162 | 7.214 | 2.866 | 0 | DUF1191 |
| TRINITY_DN11446_c0_g1_i1 | 1.425236857 | 0.276925135 | 1 | 0.836 | 1.794 | 0.489 | 0.402 | NA |
| TRINITY_DN3772_c0_g1_i2 | 1.424996615 | 0.635795809 | 1 | 0.055 | 0.986 | 0 | 0.382 | Cu_amine_oxid |
| TRINITY_DN3385_c0_g1_i3 | -1.42490199 | 0.303276992 | 0.999970678 | 0.936 | 0.453 | 2.633 | 0.573 | 3HCDH |
| TRINITY_DN698_c6_g1_i3 | -1.42480908 | 0.598816057 | 0.999970678 | 1.572 | 0 | 2.755 | 0.965 | CBF |
| TRINITY_DN5777_c0_g1_i1 | 1.424666485 | 0.367830267 | 0.999970678 | 9.465 | 25.5 | 1.055 | 11.109 | NA |
| TRINITY_DN6945_c0_g1_i1 | -1.42457323 | 0.586200859 | 0.999970678 | 1.026 | 0 | 1.278 | 1.267 | Ribosomal_L12 |
| TRINITY_DN4466_c0_g1_i2 | -1.42439639 | 0.734565795 | 0.999970678 | 3.443 | 0 | 7.744 | 0 | Peroxidase_2 |
| TRINITY_DN5_c3_g2_i3 | 1.423843186 | 0.229141594 | 0.999970678 | 5.087 | 1.38 | 1.178 | 0.995 | WRKY |
| TRINITY_DN9966_c0_g1_i4 | 1.423358596 | 0.423554431 | 0.999970678 | 4.578 | 1.094 | 0.1 | 1.981 | NA |
| TRINITY_DN6198_c0_g1_i1 | 1.423200758 | 0.468709219 | 0.999970678 | 4.996 | 17.46 | 0.167 | 8.023 | NA |
| TRINITY_DN1722_c0_g1_i6 | 1.423179498 | 0.301563273 | 0.999970678 | 19.83 | 4.967 | 1.278 | 7.812 | ACT |
| TRINITY_DN800_c1_g1_i3 | -1.42307683 | 0.182499209 | 0.999970678 | 8.93 | 4.77 | 13.855 | 19.283 | NA |
| TRINITY_DN1898_c1_g1_i1 | -1.42289339 | 0.593266375 | 0.999970678 | 0 | 0.611 | 0.522 | 0.975 | NA |
| TRINITY_DN4433_c1_g2_i1 | 1.422784156 | 0.286682779 | 0.999970678 | 0.899 | 1.528 | 0.578 | 0.201 | NA |
| TRINITY_DN10796_c0_g1_i4 | 1.42278019 | 0.277572589 | 1 | 0.345 | 0.443 | 0.133 | 0.141 | CRT-like |
| TRINITY_DN29259_c0_g3_i2 | -1.42238964 | 0.098801141 | 0.999970678 | 1.572 | 2.671 | 4.366 | 5.972 | MAM33 |
| TRINITY_DN8339_c0_g1_i2 | 1.422168384 | 0.740474171 | 0.999970678 | 89.232 | 0 | 0 | 30.543 | NA |
| TRINITY_DN4452_c2_g1_i2 | -1.42208451 | 0.607767364 | 0.999970678 | 7.049 | 0 | 7.3 | 10.406 | LRRNT_2 |
| TRINITY_DN1729_c0_g1_i2 | -1.42206163 | 0.610317585 | 0.999970678 | 0 | 0.798 | 0.322 | 1.769 | - |
| TRINITY_DN84_c0_g2_i3 | -1.42190729 | 0.596016776 | 1 | 1.045 | 0 | 1.155 | 1.448 | TPT |
| TRINITY_DN5922_c0_g1_i5 | -1.42182756 | 0.202901894 | 0.999970678 | 1.49 | 1.015 | 4.222 | 1.629 | FGGY_C |
| TRINITY_DN2099_c0_g1_i4 | 1.421740039 | 0.649188368 | 0.999970678 | 0.154 | 6.061 | 1.855 | 0 | NA |
| TRINITY_DN1411_c0_g1_i1 | 1.421539206 | 0.733096234 | 0.999970678 | 0 | 16.51 | 0 | 6.012 | NA |
| TRINITY_DN2844_c0_g1_i4 | 1.4214605 | 0.336705837 | 0.999970678 | 16.569 | 37.21 | 1.355 | 18.368 | - |
| TRINITY_DN5079_c0_g1_i3 | 1.421424035 | 0.59982809 | 0.999970678 | 5.314 | 15.78 | 0 | 7.832 | NA |
| TRINITY_DN58272_c0_g1_i5 | 1.421055303 | 0.508173941 | 0.999970678 | 0.182 | 1.321 | 0.011 | 0.533 | LRR_8 |
| TRINITY_DN5562_c0_g1_i1 | 1.420949278 | 0.742645171 | 0.999970678 | 0 | 0.641 | 0 | 0.231 | 60KD_IMP |
| TRINITY_DN2011_c1_g2_i1 | 1.420867342 | 0.270901072 | 0.999970678 | 93.883 | 20.94 | 14.044 | 23.878 | NA |
| TRINITY_DN6980_c0_g1_i1 | -1.42084924 | 0.612393028 | 0.999970678 | 0 | 1.547 | 0.322 | 3.71 | Pkinase |
| TRINITY_DN34324_c0_g1_i5 | 1.420833858 | 0.23233239 | 0.999970678 | 2.18 | 0.641 | 0.378 | 0.603 | NA |
| TRINITY_DN2957_c0_g1_i6 | -1.42083333 | 0.747807135 | 1 | 5.541 | 0 | 12.199 | 0 | EthD |
| TRINITY_DN2138_c0_g1_i1 | -1.42073996 | 0.74063987 | 0.999970678 | 2.271 | 0 | 5.089 | 0 | Peroxidase_2 |
| TRINITY_DN14935_c0_g1_i5 | -1.42061561 | 0.737828552 | 0.999970678 | 5.568 | 0 | 12.477 | 0 | NA |
| TRINITY_DN3207_c0_g2_i4 | -1.42057641 | 0.402226703 | 0.999970678 | 0.772 | 4.839 | 0.922 | 13.814 | - |
| TRINITY_DN8634_c0_g1_i1 | -1.42051343 | 0.443017643 | 0.999970678 | 0.227 | 1.833 | 0.244 | 5.198 | DUF674 |
| TRINITY_DN3707_c0_g1_i4 | -1.42036326 | 0.077672516 | 0.999970678 | 6.622 | 4.75 | 9.211 | 19.313 | Mito_carr |
| TRINITY_DN860_c0_g1_i6 | 1.420227258 | 0.435302869 | 0.999970678 | 7.04 | 8.584 | 0.156 | 5.519 | DUF2052 |
| TRINITY_DN2865_c1_g1_i2 | 1.42010334 | 0.743434597 | 0.999970678 | 0 | 0.71 | 0 | 0.261 | NRDE-2 |
| TRINITY_DN743_c0_g1_i3 | -1.42003878 | 0.320179183 | 0.999970678 | 5.95 | 1.36 | 2.533 | 16.76 | NA |
| TRINITY_DN18209_c0_g1_i3 | -1.42001052 | 0.185283527 | 0.999970678 | 0.545 | 0.503 | 1.155 | 1.438 | NA |
| TRINITY_DN93_c1_g2_i1 | 1.419891072 | 0.43876069 | 0.999970678 | 0.309 | 7.776 | 0.556 | 2.332 | NA |
| TRINITY_DN5400_c1_g1_i2 | -1.41972864 | 0.635373195 | 0.999970678 | 0.127 | 1.192 | 0 | 3.489 | NA |
| TRINITY_DN20809_c0_g1_i9 | -1.41961393 | 0.248124983 | 0.999970678 | 1.998 | 1.055 | 3.355 | 4.072 | NA |
| TRINITY_DN1390_c0_g1_i4 | 1.419512723 | 0.63054291 | 0.999970678 | 44.275 | 0 | 4.689 | 11.139 | ECH_1 |
| TRINITY_DN2380_c0_g1_i1 | -1.4194787 | 0.605737096 | 1 | 0 | 0.71 | 1.189 | 0.452 | NA |
| TRINITY_DN22333_c0_g2_i1 | 1.41937098 | 0.609655796 | 0.999970678 | 16.733 | 21.6 | 11.822 | 0 | AMP-binding |
| TRINITY_DN1391_c1_g2_i2 | 1.419281754 | 0.578704524 | 0.999970678 | 9.82 | 11.24 | 0 | 7.53 | CRAL_TRIO |
| TRINITY_DN759_c0_g1_i2 | 1.419265607 | 0.734945224 | 0.999970678 | 9.375 | 0 | 0 | 3.569 | NA |
| TRINITY_DN26175_c0_g1_i2 | 1.419231815 | 0.744232444 | 0.999970678 | 1.88 | 0 | 0.589 | 0 | NA |
| TRINITY_DN6449_c0_g1_i2 | -1.41891563 | 0.236664425 | 0.999970678 | 0.79 | 1.882 | 3.389 | 3.016 | NA |
| TRINITY_DN1862_c0_g1_i9 | 1.418824679 | 0.141335533 | 0.999970678 | 1.644 | 3.765 | 0.689 | 1.166 | NA |
| TRINITY_DN2966_c0_g2_i1 | -1.41881373 | 0.249215562 | 0.999970678 | 0.727 | 0.562 | 0.833 | 2.433 | NA |
| TRINITY_DN11687_c0_g1_i2 | -1.41854399 | 0.171605031 | 0.999970678 | 10.901 | 7.579 | 31.054 | 11.793 | TPK_B1_binding |
| TRINITY_DN6178_c0_g1_i5 | -1.41806091 | 0.467441452 | 0.999970678 | 18.477 | 2.484 | 41.486 | 3.981 | NA |
| TRINITY_DN11111_c0_g1_i15 | 1.417979698 | 0.288559979 | 0.999970678 | 2.598 | 1.232 | 0.422 | 0.895 | NA |
| TRINITY_DN2245_c0_g1_i2 | -1.41773444 | 0.223312717 | 0.999970678 | 0.727 | 2.129 | 3.233 | 3.599 | NA |
| TRINITY_DN37254_c0_g1_i1 | -1.41760572 | 0.466003501 | 0.999970678 | 0.191 | 0.788 | 0.156 | 2.403 | Thioredoxin |
| TRINITY_DN1118_c1_g1_i5 | -1.41697265 | 0.591720679 | 0.999970678 | 0 | 36.33 | 23.687 | 62.695 | NA |
| TRINITY_DN539_c0_g1_i3 | -1.41664218 | 0.671968939 | 0.999970678 | 47.872 | 0.108 | 0.356 | 121.227 | NA |
| TRINITY_DN5408_c0_g1_i4 | -1.41663233 | 0.604317738 | 0.999970678 | 6.822 | 0 | 11.766 | 4.212 | NA |
| TRINITY_DN2863_c0_g1_i7 | 1.416591772 | 0.495492736 | 0.999970678 | 0.145 | 11.39 | 2 | 1.79 | Aa_trans |
| TRINITY_DN3915_c0_g1_i4 | -1.41649781 | 0.599444382 | 1 | 0.827 | 0 | 0.778 | 1.317 | TRAPP |
| TRINITY_DN4473_c0_g1_i6 | 1.416293075 | 0.734976714 | 0.999970678 | 7.667 | 0 | 0 | 2.926 | NA |
| TRINITY_DN9966_c0_g1_i5 | 1.416150139 | 0.061734253 | 0.999970678 | 8.303 | 6.12 | 2.367 | 2.544 | NA |
| TRINITY_DN4813_c0_g1_i1 | 1.41611909 | 0.253280462 | 0.999970678 | 1.172 | 1.547 | 0.156 | 0.834 | NA |
| TRINITY_DN3926_c0_g1_i5 | -1.41609557 | 0.257486142 | 0.999970678 | 17.886 | 7.825 | 9.311 | 57.909 | NA |
| TRINITY_DN665_c0_g1_i19 | 1.416079915 | 0.736161059 | 0.999970678 | 6.513 | 0.01 | 2.044 | 0 | LRR_8 |
| TRINITY_DN4090_c0_g1_i2 | 1.415732372 | 0.205037439 | 0.999970678 | 12.599 | 10.17 | 1.378 | 6.887 | TOM20_plant |
| TRINITY_DN7693_c0_g1_i2 | -1.4155465 | 0.094099616 | 0.999970678 | 1.681 | 1.084 | 3.522 | 3.157 | NA |

| TRINITY_DN3191_c0_g1_i8 | -1.41530372 | 0.612260309 | 0.999970678 | 0.554 | 0 | 0.178 | 1.277 | NA |
| --- | --- | --- | --- | --- | --- | --- | --- | --- |
| TRINITY_DN7609_c0_g1_i1 | 1.415302607 | 0.10890148 | 0.999970678 | 8.094 | 4.021 | 2.167 | 1.96 | LRR_1 |
| TRINITY_DN3503_c0_g1_i2 | -1.4147441 | 0.601463241 | 0.999970678 | 5.959 | 0 | 3.178 | 12.266 | Rtf2 |
| TRINITY_DN3495_c1_g1_i2 | -1.41473584 | 0.231391409 | 0.999970678 | 0.173 | 0.394 | 0.5 | 0.925 | NA |
| TRINITY_DN1954_c0_g1_i4 | -1.41457876 | 0.184894567 | 0.999970678 | 17.169 | 5.332 | 28.876 | 24.712 | Ribosom_S12_S2 |
| TRINITY_DN6694_c0_g3_i1 | 1.414572105 | 0.639575259 | 0.999970678 | 23.636 | 0 | 6.455 | 1.176 | NA |
| TRINITY_DN8746_c0_g1_i2 | -1.41452394 | 0.594893532 | 0.999970678 | 0.345 | 0 | 0.456 | 0.382 | - |
| TRINITY_DN3136_c0_g1_i1 | -1.41447639 | 0.281291779 | 0.999970678 | 9.311 | 26.97 | 67.296 | 14.528 | NA |
| TRINITY_DN10233_c0_g1_i1 | -1.41414726 | 0.641353907 | 0.999970678 | 2.08 | 0 | 0.222 | 5.288 | NA |
| TRINITY_DN846_c0_g2_i3 | 1.41391789 | 0.589136867 | 0.999970678 | 4.642 | 4.869 | 0 | 3.579 | NA |
| TRINITY_DN1550_c0_g1_i3 | 1.413847131 | 0.740419951 | 0.999970678 | 13.935 | 0 | 0 | 5.328 | HATPase_c |
| TRINITY_DN10046_c0_g1_i1 | 1.413551216 | 0.609069271 | 0.999970678 | 1.88 | 9.905 | 3.589 | 0 | NA |
| TRINITY_DN1280_c0_g1_i1 | -1.41322826 | 0.594025346 | 0.999970678 | 2.534 | 0 | 2.433 | 3.891 | NA |
| TRINITY_DN2319_c1_g2_i3 | 1.413048935 | 0.675127624 | 0.999970678 | 0 | 18.63 | 0.044 | 6.867 | p450 |
| TRINITY_DN4917_c0_g1_i2 | -1.41282393 | 0.618981226 | 0.999970678 | 0.227 | 0.473 | 0 | 1.84 | NA |
| TRINITY_DN1786_c0_g1_i2 | -1.41282045 | 0.252652629 | 0.999970678 | 1.126 | 0.522 | 1.044 | 3.187 | NA |
| TRINITY_DN10233_c1_g1_i1 | -1.41281023 | 0.5915898 | 0.999970678 | 3.016 | 0 | 2.455 | 5.147 | NA |
| TRINITY_DN40733_c0_g1_i1 | -1.41274146 | 0.309963301 | 0.999970678 | 0.245 | 0.729 | 1.344 | 0.955 | Mito_carr |
| TRINITY_DN804_c2_g1_i9 | 1.412717546 | 0.646811883 | 0.999970678 | 0.972 | 0 | 0.278 | 0.03 | NA |
| TRINITY_DN16934_c0_g1_i2 | -1.4126 | 0.748524703 | 1 | 0.69 | 0 | 1.511 | 0 | NA |
| TRINITY_DN16970_c0_g3_i3 | 1.412545068 | 0.357776294 | 0.999970678 | 5.877 | 2.592 | 2.378 | 0.312 | NA |
| TRINITY_DN7737_c0_g1_i3 | -1.41244735 | 0.737500684 | 0.999970678 | 1.426 | 0 | 3.189 | 0.01 | Pro-kuma_activ |
| TRINITY_DN9704_c0_g1_i3 | -1.41244332 | 0.58576645 | 0.999970678 | 0 | 1.557 | 1.678 | 2.031 | NA |
| TRINITY_DN2317_c0_g1_i3 | -1.41237276 | 0.614896902 | 0.999970678 | 7.249 | 0 | 11.944 | 5.188 | NA |
| TRINITY_DN2212_c1_g1_i9 | -1.4123371 | 0.400334644 | 0.999970678 | 0.954 | 0.187 | 0.333 | 2.674 | NA |
| TRINITY_DN34324_c0_g1_i1 | 1.412007178 | 0.359534762 | 0.999970678 | 1.126 | 0.187 | 0.267 | 0.171 | NA |
| TRINITY_DN7340_c0_g1_i2 | 1.411985897 | 0.735641522 | 0.999970678 | 5.578 | 0 | 0 | 2.141 | Terpene_synth |
| TRINITY_DN1527_c0_g1_i1 | -1.41191229 | 0.596310168 | 0.999970678 | 2.38 | 0 | 2.444 | 3.438 | NA |
| TRINITY_DN688_c0_g1_i3 | -1.41172579 | 0.616208401 | 0.999970678 | 0.681 | 0.404 | 0 | 2.875 | Pkinase |
| TRINITY_DN2239_c0_g2_i2 | 1.411489889 | 0.26957218 | 0.999970678 | 108.99 | 16.19 | 17.754 | 25.788 | NA |
| TRINITY_DN9149_c0_g1_i3 | -1.41144088 | 0.737712263 | 0.999970678 | 0 | 0.759 | 0 | 1.991 | NA |
| TRINITY_DN9067_c0_g1_i1 | 1.411342939 | 0.598209886 | 1 | 0.154 | 0.246 | 0.122 | 0 | NA |
| TRINITY_DN133_c0_g1_i2 | 1.411284193 | 0.737020354 | 0.999970678 | 0 | 13.82 | 4.211 | 0 | NA |
| TRINITY_DN1043_c0_g1_i11 | -1.41118874 | 0.516135863 | 0.999970678 | 0.636 | 6.702 | 0.2 | 19.042 | ADH_N |
| TRINITY_DN3092_c0_g1_i2 | 1.411155051 | 0.735629377 | 0.999970678 | 3.288 | 0 | 0 | 1.267 | YTH |
| TRINITY_DN12451_c0_g1_i6 | 1.411122888 | 0.629787283 | 0.999970678 | 1.108 | 0.089 | 0 | 0.452 | NA |
| TRINITY_DN28876_c0_g3_i2 | 1.41095838 | 0.209446984 | 0.999970678 | 0.391 | 0.739 | 0.178 | 0.221 | ABC2_membrane |
| TRINITY_DN3916_c0_g1_i2 | 1.410747453 | 0.261236751 | 0.999970678 | 0.263 | 0.572 | 0.133 | 0.151 | BET |
| TRINITY_DN3584_c0_g2_i2 | -1.41023145 | 0.614614211 | 0.999970678 | 3.388 | 1.991 | 0 | 14.387 | NA |
| TRINITY_DN1622_c0_g1_i6 | -1.41022381 | 0.301089038 | 0.999970678 | 2.362 | 4.711 | 2.133 | 16.036 | NA |
| TRINITY_DN2573_c1_g1_i7 | 1.410176082 | 0.258518027 | 0.999970678 | 5.123 | 3.43 | 2.244 | 0.523 | NA |
| TRINITY_DN336_c0_g1_i1 | 1.409804136 | 0.182036819 | 0.999970678 | 0.709 | 0.779 | 0.211 | 0.302 | - |
| TRINITY_DN3903_c0_g1_i1 | 1.409793397 | 0.110188454 | 0.999970678 | 3.198 | 1.843 | 0.622 | 1.156 | NA |
| TRINITY_DN185_c0_g1_i6 | 1.40976994 | 0.121651057 | 0.999970678 | 4.442 | 10.81 | 2.111 | 3.157 | TPR_1 |
| TRINITY_DN1870_c0_g2_i1 | 1.409564054 | 0.68781399 | 0.999970678 | 136.84 | 0 | 0.422 | 52.038 | IlvC |
| TRINITY_DN4421_c0_g1_i5 | 1.40949054 | 0.627545771 | 0.999970678 | 0.881 | 0.069 | 0 | 0.362 | NA |
| TRINITY_DN20058_c0_g1_i2 | 1.409024851 | 0.283618897 | 0.999970678 | 4.115 | 2.612 | 0.422 | 1.971 | NA |
| TRINITY_DN7708_c0_g1_i6 | 1.408702652 | 0.315640241 | 0.999970678 | 1.208 | 1.015 | 0.6 | 0.111 | NA |
| TRINITY_DN1843_c0_g1_i10 | -1.40859339 | 0.60587756 | 0.999970678 | 0.672 | 0 | 0.422 | 1.277 | zf-C3HC4_3 |
| TRINITY_DN15081_c0_g3_i2 | -1.40859133 | 0.296323705 | 0.999970678 | 0.69 | 0.276 | 1.544 | 0.714 | NA |
| TRINITY_DN12172_c0_g1_i2 | -1.4082405 | 0.601848508 | 0.999970678 | 0.781 | 0 | 0.544 | 1.448 | NA |
| TRINITY_DN4517_c0_g1_i2 | 1.408215335 | 0.604835744 | 0.999970678 | 1.699 | 5.923 | 0 | 2.865 | NA |
| TRINITY_DN1088_c0_g2_i1 | -1.40791858 | 0.03687872 | 0.999970678 | 20.611 | 16.84 | 33.998 | 58.12 | CCT_2 |
| TRINITY_DN561_c0_g1_i6 | 1.407619554 | 0.736374033 | 0.999970678 | 0 | 2.582 | 0 | 0.965 | NA |
| TRINITY_DN2893_c0_g1_i1 | -1.40748654 | 0.060218365 | 0.999970678 | 441.28 | 439.2 | 1210.171 | 751.524 | HSP70 |
| TRINITY_DN3988_c0_g1_i12 | 1.407010004 | 0.589273379 | 0.999970678 | 1.163 | 0.779 | 0.6 | 0 | Phosphorylase |
| TRINITY_DN7869_c0_g1_i1 | -1.40674275 | 0.166684577 | 0.999970678 | 2.425 | 1.764 | 6.677 | 3.026 | NA |
| TRINITY_DN44574_c0_g1_i1 | -1.40670923 | 0.259702725 | 1 | 0.899 | 1.34 | 2.189 | 3.187 | EF-hand_1 |
| TRINITY_DN664_c0_g1_i2 | 1.406621255 | 0.0715405 | 0.999970678 | 9.883 | 15.64 | 2.966 | 6.042 | NA |
| TRINITY_DN2369_c0_g1_i5 | 1.40657784 | 0.743617563 | 0.999970678 | 1.054 | 0 | 0.344 | 0 | LRR_1 |
| TRINITY_DN11856_c0_g1_i1 | -1.40654359 | 0.752171869 | 1 | 2.989 | 0 | 6.566 | 0 | NA |
| TRINITY_DN850_c0_g1_i1 | -1.40631281 | 0.739058115 | 0.999970678 | 2.689 | 0 | 6 | 0 | NA |
| TRINITY_DN2362_c0_g2_i1 | 1.406290733 | 0.366766937 | 0.999970678 | 5.578 | 7.953 | 3.866 | 0.402 | NA |
| TRINITY_DN19516_c0_g1_i3 | 1.406058733 | 0.74089077 | 0.999970678 | 1.644 | 0 | 0 | 0.633 | NA |
| TRINITY_DN14484_c0_g1_i6 | -1.40573477 | 0.453309452 | 0.999970678 | 0.109 | 2.415 | 0.578 | 5.952 | DCB |
| TRINITY_DN334_c0_g1_i8 | 1.405483723 | 0.646985818 | 0.999970678 | 0 | 19.56 | 5.677 | 0.362 | NA |
| TRINITY_DN1141_c0_g2_i10 | 1.405378492 | 0.282318617 | 0.999970678 | 3.079 | 1.39 | 1.2 | 0.251 | NA |
| TRINITY_DN2827_c0_g1_i1 | -1.40480975 | 0.508798097 | 0.999970678 | 1.072 | 25.49 | 1.5 | 67.309 | PUNUT |
| TRINITY_DN458_c1_g1_i1 | -1.40451886 | 0.27668055 | 1 | 0.89 | 1.744 | 3.211 | 2.966 | NA |
| TRINITY_DN4819_c1_g1_i2 | -1.40430901 | 0.258648884 | 0.999970678 | 0.173 | 0.414 | 0.389 | 1.066 | NA |
| TRINITY_DN3048_c0_g1_i8 | 1.404305559 | 0.586597119 | 0.999970678 | 0.545 | 1.084 | 0 | 0.613 | NA |
| TRINITY_DN121_c0_g1_i3 | -1.40428459 | 0.738433219 | 0.999970678 | 0 | 1.242 | 0 | 3.267 | NA |
| TRINITY_DN3302_c0_g2_i7 | 1.404247963 | 0.548490751 | 0.999970678 | 0.045 | 8.022 | 2.089 | 0.473 | NA |
| TRINITY_DN7892_c0_g3_i1 | 1.403988286 | 0.403855685 | 1 | 0.481 | 0.493 | 0.044 | 0.302 | SMC_N |

| TRINITY_DN82_c0_g2_i2 | -1.40352975 | 0.083913876 | 0.999970678 | 1.254 | 1.419 | 2.322 | 4.243 | NA |
| --- | --- | --- | --- | --- | --- | --- | --- | --- |
| TRINITY_DN474_c0_g1_i8 | 1.403414968 | 0.602448813 | 0.999970678 | 1.181 | 0.345 | 0.478 | 0 | NA |
| TRINITY_DN148_c1_g1_i3 | 1.403370071 | 0.372202254 | 0.999970678 | 25.226 | 3.371 | 7.011 | 2.433 | NA |
| TRINITY_DN5200_c0_g1_i9 | -1.4031962 | 0.745290008 | 0.999970678 | 6.304 | 0 | 13.688 | 0 | Cupin_7 |
| TRINITY_DN104_c0_g1_i20 | 1.402919676 | 0.61109007 | 0.999970678 | 0.554 | 4.79 | 0 | 2.001 | GUB_WAK_bind |
| TRINITY_DN1489_c0_g2_i1 | -1.4028166 | 0.592245728 | 0.999970678 | 0 | 3.006 | 3.689 | 3.388 | MORN |
| TRINITY_DN1008_c0_g1_i14 | -1.40259573 | 0.121890441 | 0.999970678 | 0.781 | 0.66 | 1.411 | 2.131 | NA |
| TRINITY_DN11234_c0_g1_i4 | 1.402435233 | 0.220484356 | 0.999970678 | 0.518 | 0.542 | 0.167 | 0.201 | NA |
| TRINITY_DN813_c1_g1_i7 | 1.402159578 | 0.48850078 | 0.999970678 | 0.627 | 8.367 | 0.256 | 2.986 | NA |
| TRINITY_DN7768_c0_g1_i3 | -1.4019729 | 0.427592778 | 0.999970678 | 0.309 | 1.941 | 4.178 | 0.814 | NA |
| TRINITY_DN12453_c0_g2_i4 | 1.401762611 | 0.590147068 | 0.999970678 | 1.499 | 0.68 | 0 | 0.814 | RPAP3_C |
| TRINITY_DN4471_c1_g1_i3 | -1.40157474 | 0.738847338 | 0.999970678 | 0 | 2.69 | 5.789 | 0 | NA |
| TRINITY_DN605_c0_g1_i3 | -1.4013476 | 0.593784323 | 0.999970678 | 3.388 | 0 | 3.033 | 5.409 | NA |
| TRINITY_DN12863_c0_g1_i3 | 1.401290431 | 0.257505482 | 0.999970678 | 15.116 | 73.85 | 16.288 | 12.708 | Glyco_transf_5 |
| TRINITY_DN2044_c0_g1_i4 | -1.40113678 | 0.6031295 | 0.999970678 | 0 | 0.966 | 0.6 | 1.81 | FAD_binding_3 |
| TRINITY_DN6998_c0_g1_i4 | 1.401090906 | 0.632629089 | 0.999970678 | 9.102 | 0 | 2.055 | 1.025 | NA |
| TRINITY_DN862_c0_g1_i26 | 1.400908391 | 0.744990018 | 0.999970678 | 0 | 1.735 | 0.533 | 0 | NA |
| TRINITY_DN4173_c0_g1_i1 | 1.40090394 | 0.378177246 | 0.999970678 | 1.708 | 0.788 | 0.1 | 0.834 | NA |
| TRINITY_DN3180_c0_g1_i2 | -1.40071451 | 0.738126023 | 0.999970678 | 1.899 | 0 | 0 | 5.117 | NA |
| TRINITY_DN406_c0_g1_i1 | -1.40033824 | 0.627868972 | 0.999970678 | 2.634 | 0 | 5.411 | 0.553 | Peptidase_S10 |
| TRINITY_DN1687_c0_g2_i1 | -1.4002894 | 0.61360277 | 0.999970678 | 0.009 | 39.92 | 33.22 | 62.866 | Ribosomal_S13_N |
| TRINITY_DN4475_c0_g1_i2 | 1.400148836 | 0.609937197 | 0.999970678 | 8.23 | 24.24 | 0 | 12.235 | NA |
| TRINITY_DN16075_c0_g1_i14 | 1.400095843 | 0.602463883 | 1 | 0.799 | 0.69 | 0.478 | 0 | NA |
| TRINITY_DN2850_c0_g1_i7 | -1.39989511 | 0.59846612 | 0.999970678 | 0.718 | 0 | 0.967 | 0.774 | CAP160 |
| TRINITY_DN29793_c0_g1_i3 | 1.399294382 | 0.275498442 | 0.999970678 | 0.754 | 3.272 | 0.411 | 1.025 | NA |
| TRINITY_DN3921_c0_g1_i2 | -1.39897876 | 0.601917132 | 0.999970678 | 3.07 | 0 | 4.877 | 2.292 | NA |
| TRINITY_DN164759_c0_g1_i1 | -1.39896375 | 0.373828186 | 0.999970678 | 0.309 | 1.124 | 0.322 | 3.408 | p450 |
| TRINITY_DN164759_c0_g1_i2 | -1.39896375 | 0.373828186 | 0.999970678 | 0.309 | 1.124 | 0.322 | 3.408 | p450 |
| TRINITY_DN288_c0_g1_i3 | -1.39881749 | 0.377951874 | 0.999970678 | 0.763 | 0.099 | 0.944 | 1.106 | NA |
| TRINITY_DN6123_c0_g1_i6 | 1.398815382 | 0.3150405 | 0.999970678 | 18.368 | 24.34 | 1.2 | 14.789 | Kinase-PPPase |
| TRINITY_DN1265_c0_g1_i3 | -1.39879647 | 0.351450823 | 0.999970678 | 13.308 | 1.399 | 9.233 | 27.758 | Sedlin_N |
| TRINITY_DN50306_c0_g1_i6 | -1.39862239 | 0.332837469 | 0.999970678 | 0.273 | 0.306 | 0.189 | 1.307 | JmjC |
| TRINITY_DN5854_c0_g1_i1 | 1.398612089 | 0.235330981 | 0.999970678 | 5.232 | 7.667 | 0.767 | 3.961 | NA |
| TRINITY_DN4624_c0_g1_i1 | 1.398444005 | 0.737604837 | 0.999970678 | 0 | 9.776 | 0 | 3.609 | NA |
| TRINITY_DN5160_c0_g1_i1 | -1.39822579 | 0.458067316 | 0.999970678 | 5.114 | 0.177 | 7.477 | 4.926 | NA |
| TRINITY_DN7324_c0_g1_i11 | 1.398191263 | 0.378007062 | 0.999970678 | 0.899 | 2.286 | 0.889 | 0.121 | LRR_8 |
| TRINITY_DN1592_c0_g1_i6 | -1.39811572 | 0.752807125 | 1 | 1.962 | 0 | 4.278 | 0 | Pro_isomerase |
| TRINITY_DN23078_c0_g1_i9 | -1.39807797 | 0.74978555 | 0.999970678 | 1.008 | 0 | 2.211 | 0 | Asp |
| TRINITY_DN6812_c0_g1_i7 | -1.39807797 | 0.74978555 | 0.999970678 | 3.543 | 0 | 7.766 | 0 | NA |
| TRINITY_DN12070_c0_g1_i3 | -1.39804987 | 0.747561435 | 0.999970678 | 0.972 | 0 | 2.122 | 0 | NA |
| TRINITY_DN2456_c0_g1_i1 | -1.39802892 | 0.595727789 | 0.999970678 | 3.434 | 0 | 2.866 | 5.69 | NA |
| TRINITY_DN23413_c0_g1_i2 | -1.39802864 | 0.745853055 | 0.999970678 | 0.59 | 0 | 1.3 | 0 | Carn_acyltransf |
| TRINITY_DN3136_c0_g1_i2 | 1.397936036 | 0.741634915 | 0.999970678 | 19.14 | 0 | 0 | 7.379 | NA |
| TRINITY_DN7660_c0_g1_i9 | 1.397730144 | 0.464386318 | 0.999970678 | 2.616 | 0.375 | 0.078 | 1.066 | NA |
| TRINITY_DN134_c0_g1_i2 | 1.397590048 | 0.307578831 | 1 | 0.481 | 0.355 | 0.189 | 0.101 | FAD_binding_8 |
| TRINITY_DN155_c0_g1_i19 | 1.397374949 | 0.315886323 | 0.999970678 | 7.303 | 2.553 | 0.444 | 3.247 | Ank |
| TRINITY_DN1851_c0_g1_i2 | 1.397306634 | 0.618613488 | 0.999970678 | 0 | 5.667 | 0.622 | 1.377 | NA |
| TRINITY_DN1875_c0_g1_i14 | 1.397296173 | 0.628589425 | 0.999970678 | 9.629 | 0.966 | 3.355 | 0 | PHD |
| TRINITY_DN2498_c0_g1_i8 | 1.397259134 | 0.59547604 | 0.999970678 | 1.444 | 4.583 | 0 | 2.252 | NA |
| TRINITY_DN7940_c1_g2_i1 | 1.397145273 | 0.325661814 | 0.999970678 | 4.033 | 6.455 | 2.878 | 0.483 | NA |
| TRINITY_DN341_c0_g1_i1 | -1.39712739 | 0.266657035 | 0.999970678 | 2.716 | 1.528 | 1.467 | 9.481 | NA |
| TRINITY_DN18123_c0_g1_i1 | -1.39688357 | 0.474955323 | 0.999970678 | 0.064 | 2.296 | 4.211 | 1.036 | Amino_oxidase |
| TRINITY_DN651_c0_g1_i10 | -1.39677973 | 0.312106626 | 0.999970678 | 25.163 | 56.57 | 157.868 | 22.882 | NAD-GH |
| TRINITY_DN14842_c0_g1_i1 | -1.39668353 | 0.285573762 | 0.999970678 | 2.216 | 2.099 | 7.188 | 2.513 | NA |
| TRINITY_DN11540_c0_g1_i3 | 1.39647115 | 0.617770456 | 0.999970678 | 0.363 | 2.503 | 0.878 | 0 | HLH |
| TRINITY_DN2910_c1_g1_i2 | 1.396140597 | 0.245974358 | 0.999970678 | 0.854 | 1.459 | 0.256 | 0.563 | NA |
| TRINITY_DN3192_c2_g1_i1 | -1.39613173 | 0.748646105 | 0.999970678 | 0.754 | 0 | 0 | 2.071 | NA |
| TRINITY_DN41_c0_g1_i12 | 1.395761068 | 0.028206726 | 0.999970678 | 19.794 | 17.72 | 6.633 | 6.253 | NA |
| TRINITY_DN4480_c0_g1_i1 | 1.39569735 | 0.335277928 | 0.999970678 | 13.889 | 14.89 | 0.733 | 10.003 | NA |
| TRINITY_DN12099_c0_g1_i1 | 1.39568007 | 0.439189457 | 0.999970678 | 0.254 | 4.898 | 1.222 | 0.442 | NA |
| TRINITY_DN4259_c1_g1_i4 | 1.395655101 | 0.632966205 | 0.999970678 | 0 | 34.11 | 3.278 | 8.797 | PAE |
| TRINITY_DN7042_c0_g2_i5 | -1.39519319 | 0.603000728 | 0.999970678 | 5.241 | 0 | 6.377 | 6.274 | AAA_18 |
| TRINITY_DN15545_c0_g2_i1 | -1.39464967 | 0.26718621 | 0.999970678 | 0.263 | 0.818 | 1.022 | 1.518 | NA |
| TRINITY_DN832_c0_g1_i7 | 1.394447966 | 0.308579332 | 0.999970678 | 28.696 | 3.745 | 3.333 | 8.505 | NA |
| TRINITY_DN14398_c0_g3_i2 | 1.394304582 | 0.738722287 | 0.999970678 | 0 | 6.16 | 0 | 2.302 | NA |
| TRINITY_DN665_c1_g1_i5 | -1.3939602 | 0.244002609 | 0.999970678 | 37.807 | 41.58 | 26.61 | 171.054 | - |
| TRINITY_DN2833_c0_g1_i7 | 1.393857456 | 0.739531093 | 0.999970678 | 14.244 | 0 | 0 | 5.489 | NA |
| TRINITY_DN520_c0_g1_i4 | 1.393643433 | 0.366765187 | 0.999970678 | 5.023 | 0.463 | 0.533 | 1.468 | LRR_1 |
| TRINITY_DN32868_c0_g2_i1 | 1.39342143 | 0.447812377 | 0.999970678 | 0.2 | 2.306 | 0.2 | 0.704 | NA |
| TRINITY_DN7583_c0_g3_i1 | -1.39340026 | 0.738855637 | 0.999970678 | 1.717 | 0 | 0 | 4.574 | LysM |
| TRINITY_DN5880_c0_g1_i1 | -1.39332775 | 0.245346874 | 0.999970678 | 31.103 | 9.422 | 30.487 | 64.756 | NA |
| TRINITY_DN3593_c0_g1_i3 | -1.39321144 | 0.603545648 | 0.999970678 | 1.644 | 0 | 2.589 | 1.206 | zf-RING_2 |
| TRINITY_DN10918_c0_g2_i1 | 1.392584721 | 0.306062623 | 0.999970678 | 2.425 | 1.567 | 1.078 | 0.221 | NA |
| TRINITY_DN2132_c0_g1_i4 | -1.39257495 | 0.129063796 | 0.999970678 | 3.252 | 1.528 | 6.677 | 4.584 | SHMT |

| TRINITY_DN6221_c0_g1_i6 | -1.39256446 | 0.630675612 | 0.999970678 | 0.518 | 0 | 0.144 | 1.206 | ubiquitin |
| --- | --- | --- | --- | --- | --- | --- | --- | --- |
| TRINITY_DN12069_c0_g1_i1 | 1.392463633 | 0.598828779 | 0.999970678 | 0.836 | 1.074 | 0.6 | 0 | NA |
| TRINITY_DN1489_c0_g1_i3 | 1.392330341 | 0.277440753 | 0.999970678 | 1.59 | 3.104 | 0.933 | 0.664 | NA |
| TRINITY_DN70585_c0_g1_i1 | -1.39225158 | 0.318230748 | 0.999970678 | 5064.3 | 2305 | 13848.25 | 1841.103 | NA |
| TRINITY_DN3517_c2_g2_i1 | 1.392213398 | 0.596781318 | 0.999970678 | 0.89 | 0.798 | 0.533 | 0 | NA |
| TRINITY_DN976_c1_g1_i3 | 1.392136299 | 0.743653423 | 0.999970678 | 0 | 2.247 | 0.7 | 0 | ubiquitin |
| TRINITY_DN2841_c0_g1_i2 | -1.39207554 | 0.620497522 | 0.999970678 | 0.718 | 0 | 1.4 | 0.201 | NA |
| TRINITY_DN8942_c0_g1_i2 | -1.3917899 | 0.161019649 | 0.999970678 | 1.862 | 1.666 | 5.633 | 2.453 | Motile_Sperm |
| TRINITY_DN13147_c1_g1_i8 | -1.39164198 | 0.75034292 | 0.999970678 | 1.526 | 0 | 3.311 | 0 | adh_short_C2 |
| TRINITY_DN1201_c0_g1_i4 | 1.391588276 | 0.379059667 | 0.999970678 | 1.036 | 4.326 | 0.278 | 1.639 | NA |
| TRINITY_DN1006_c0_g2_i6 | -1.39147977 | 0.596511994 | 0.999970678 | 11.682 | 0 | 11.199 | 16.83 | Ribosomal_L40e |
| TRINITY_DN7634_c0_g1_i1 | 1.391470286 | 0.233149546 | 0.999970678 | 4.742 | 15.96 | 1.789 | 5.67 | NA |
| TRINITY_DN7329_c0_g1_i1 | -1.39134141 | 0.219752975 | 0.999970678 | 2.934 | 4.74 | 12.899 | 4.424 | LOB |
| TRINITY_DN3898_c0_g1_i2 | 1.391099137 | 0.596461332 | 1 | 1.263 | 1.547 | 0 | 1.056 | NA |
| TRINITY_DN9011_c0_g2_i6 | -1.39097809 | 0.742832125 | 0.999970678 | 0 | 0.443 | 0.944 | 0 | NA |
| TRINITY_DN17619_c0_g1_i1 | -1.39095249 | 0.743176556 | 0.999970678 | 0 | 1.192 | 2.533 | 0 | NA |
| TRINITY_DN1177_c0_g1_i2 | -1.39092584 | 0.459850862 | 0.999970678 | 0.609 | 5.332 | 0.878 | 14.065 | NA |
| TRINITY_DN616_c1_g2_i1 | 1.390902114 | 0.522292574 | 0.999970678 | 3.089 | 0.197 | 0.056 | 1.206 | NA |
| TRINITY_DN3284_c0_g2_i2 | -1.39083457 | 0.19985065 | 0.999970678 | 47.718 | 60.45 | 187.589 | 54.752 | NA |
| TRINITY_DN590_c1_g1_i1 | -1.39079199 | 0.532674264 | 0.999970678 | 0.908 | 169.7 | 319.614 | 41.612 | NA |
| TRINITY_DN6427_c0_g1_i6 | -1.39061377 | 0.600146909 | 0.999970678 | 4.596 | 0 | 6.622 | 4.162 | - |
| TRINITY_DN9112_c0_g1_i12 | -1.39044879 | 0.753357893 | 1 | 2.916 | 0 | 6.322 | 0 | NA |
| TRINITY_DN8934_c0_g1_i15 | -1.39044879 | 0.753357893 | 1 | 2.698 | 0 | 5.811 | 0 | NA |
| TRINITY_DN2341_c0_g2_i2 | 1.390270731 | 0.744121021 | 0.999970678 | 10.592 | 0 | 0 | 4.122 | NA |
| TRINITY_DN1676_c1_g1_i1 | 1.390086445 | 0.607146908 | 1 | 0.263 | 0.601 | 0.267 | 0 | NA |
| TRINITY_DN51233_c0_g1_i1 | 1.389998782 | 0.131088678 | 0.999970678 | 1.871 | 2.582 | 0.811 | 0.714 | NA |
| TRINITY_DN618_c0_g1_i4 | -1.38972156 | 0.078839689 | 0.999970678 | 5.35 | 3.302 | 11.144 | 9.3 | NA |
| TRINITY_DN8912_c0_g4_i3 | 1.389444412 | 0.738684321 | 0.999970678 | 0 | 8.347 | 0 | 3.127 | TPT |
| TRINITY_DN8594_c0_g1_i5 | 1.389397321 | 0.593842926 | 0.999970678 | 1.372 | 1.025 | 0.756 | 0 | Nexin_C |
| TRINITY_DN1173_c5_g1_i6 | -1.38910227 | 0.748741617 | 0.999970678 | 1.063 | 0 | 0 | 2.795 | NA |
| TRINITY_DN536_c0_g1_i3 | 1.388935127 | 0.191802344 | 0.999970678 | 2.171 | 0.808 | 0.344 | 0.724 | NA |
| TRINITY_DN7203_c0_g1_i2 | -1.38892303 | 0.60645989 | 1 | 0.263 | 0 | 0.322 | 0.342 | Suf |
| TRINITY_DN15132_c0_g2_i4 | -1.38856817 | 0.551474484 | 1 | 7.703 | 0.7 | 17.188 | 0.714 | Actin |
| TRINITY_DN61_c0_g1_i6 | 1.388377426 | 0.596385255 | 0.999970678 | 3.579 | 8.761 | 0 | 4.665 | DUF4220 |
| TRINITY_DN8894_c0_g1_i1 | 1.388321499 | 0.746248802 | 0.999970678 | 4.987 | 0 | 1.6 | 0 | NA |
| TRINITY_DN7047_c0_g1_i1 | -1.38810944 | 0.423527006 | 0.999970678 | 0.427 | 2.385 | 5.3 | 0.885 | NA |
| TRINITY_DN1219_c2_g1_i1 | 1.387944098 | 0.747137137 | 0.999970678 | 0 | 1.143 | 0 | 0.442 | NA |
| TRINITY_DN2054_c0_g1_i2 | 1.387885118 | 0.355428457 | 0.999970678 | 61.108 | 5.657 | 15.521 | 7.058 | LRRNT_2 |
| TRINITY_DN8743_c1_g1_i1 | -1.38764996 | 0.263592402 | 1 | 0.164 | 0.138 | 0.378 | 0.332 | NA |
| TRINITY_DN671_c0_g1_i1 | 1.387610474 | 0.645150391 | 0.999970678 | 5.768 | 0.158 | 0 | 2.302 | NA |
| TRINITY_DN2792_c0_g1_i1 | 1.38753252 | 0.088725166 | 0.999970678 | 3.924 | 2.395 | 0.989 | 1.247 | Myb_DNA-bindin |
| TRINITY_DN5322_c0_g1_i1 | -1.38744242 | 0.595664612 | 0.999970678 | 18.704 | 0.197 | 40.253 | 0.533 | Aldedh |
| TRINITY_DN62_c0_g1_i2 | 1.387405636 | 0.519013088 | 0.999970678 | 0.064 | 0.907 | 0.033 | 0.332 | NA |
| TRINITY_DN2965_c1_g2_i1 | 1.387353742 | 0.746097636 | 0.999970678 | 5.051 | 0 | 1.589 | 0 | NA |
| TRINITY_DN24894_c0_g1_i1 | -1.38713685 | 0.291879788 | 0.999970678 | 1.281 | 0.887 | 1.133 | 4.263 | NA |
| TRINITY_DN7470_c0_g1_i2 | 1.387129673 | 0.146907812 | 0.999970678 | 7.522 | 21.64 | 4.155 | 6.042 | - |
| TRINITY_DN11807_c0_g1_i2 | -1.3869927 | 0.631645856 | 0.999970678 | 0.282 | 1.794 | 0 | 5.389 | NA |
| TRINITY_DN1901_c0_g1_i9 | -1.38692044 | 0.740118041 | 0.999970678 | 3.97 | 0 | 0 | 10.496 | NA |
| TRINITY_DN9690_c0_g2_i2 | -1.38668339 | 0.739650078 | 0.999970678 | 0 | 1.961 | 0 | 5.087 | F-box-like |
| TRINITY_DN8100_c0_g1_i9 | -1.38646295 | 0.743061784 | 0.999970678 | 0.754 | 0 | 1.644 | 0 | CFEM |
| TRINITY_DN2965_c1_g1_i1 | 1.386200763 | 0.547589145 | 0.999970678 | 10.328 | 0.049 | 2.433 | 1.066 | NA |
| TRINITY_DN5129_c0_g1_i1 | 1.386179484 | 0.146686843 | 0.999970678 | 0.899 | 0.788 | 0.344 | 0.221 | Pkinase |
| TRINITY_DN7428_c0_g1_i1 | -1.38613187 | 0.385165289 | 0.999970678 | 1.181 | 1.961 | 0.544 | 7.54 | NA |
| TRINITY_DN5177_c0_g1_i1 | 1.386095547 | 0.605916347 | 0.999970678 | 0.69 | 3.065 | 0 | 1.418 | NA |
| TRINITY_DN4884_c0_g1_i1 | -1.38579983 | 0.258629835 | 0.999970678 | 279.07 | 307.5 | 172.367 | 1307.895 | LEA_2 |
| TRINITY_DN4937_c0_g1_i6 | -1.38524211 | 0.360368026 | 0.999970678 | 0.245 | 0.375 | 1.122 | 0.241 | NA |
| TRINITY_DN12361_c0_g1_i4 | -1.38516027 | 0.596917192 | 0.999970678 | 0.736 | 0 | 0.811 | 0.975 | NA |
| TRINITY_DN6017_c0_g1_i5 | 1.385005716 | 0.11428422 | 0.999970678 | 1.099 | 0.69 | 0.344 | 0.271 | DUF4283 |
| TRINITY_DN4608_c0_g1_i11 | -1.38469197 | 0.306312374 | 0.999970678 | 1.508 | 0.404 | 2.8 | 1.639 | Ldh_1_C |
| TRINITY_DN1032_c0_g1_i13 | 1.384601796 | 0.340125021 | 0.999970678 | 2.289 | 20.76 | 5.277 | 2.332 | Lipoxygenase |
| TRINITY_DN1649_c0_g1_i6 | 1.384493778 | 0.24354425 | 0.999970678 | 4.033 | 0.956 | 0.878 | 0.885 | NA |
| TRINITY_DN9307_c0_g1_i5 | 1.38425926 | 0.749852129 | 0.999970678 | 1.145 | 0 | 0 | 0.442 | NA |
| TRINITY_DN966_c1_g1_i3 | 1.384216416 | 0.202237702 | 0.999970678 | 10.156 | 6.071 | 4 | 1.397 | NA |
| TRINITY_DN687_c0_g1_i1 | 1.383788498 | 0.258064376 | 0.999970678 | 2.053 | 1.488 | 0.844 | 0.352 | NA |
| TRINITY_DN13543_c1_g1_i4 | -1.38365764 | 0.742234276 | 0.999970678 | 2.098 | 0 | 4.622 | 0 | Aldedh |
| TRINITY_DN2243_c0_g1_i1 | 1.383610188 | 0.631298397 | 0.999970678 | 0 | 25.08 | 6.244 | 1.93 | IF4E |
| TRINITY_DN273_c0_g1_i11 | 1.383559152 | 0.304484259 | 1 | 0.736 | 0.739 | 0.322 | 0.171 | Aconitase |
| TRINITY_DN4948_c0_g2_i1 | -1.38345907 | 0.249270713 | 0.999970678 | 23.073 | 13.99 | 14.188 | 77.574 | NA |
| TRINITY_DN1202_c0_g1_i5 | 1.383301561 | 0.48934538 | 0.999970678 | 0.99 | 1.902 | 0.9 | 0.05 | NA |
| TRINITY_DN565_c0_g1_i2 | 1.383091922 | 0.124201822 | 0.999970678 | 3.225 | 3.223 | 0.878 | 1.418 | zf-DNL |
| TRINITY_DN33696_c0_g2_i1 | 1.38306735 | 0.753788561 | 1 | 8.848 | 0 | 2.811 | 0 | NA |
| TRINITY_DN438_c0_g1_i2 | -1.38303626 | 0.417091207 | 0.999970678 | 0.391 | 8.574 | 8.111 | 13.12 | NA |
| TRINITY_DN5300_c0_g1_i3 | 1.382897278 | 0.300543115 | 0.999970678 | 1.099 | 1.015 | 0.578 | 0.111 | NA |
| TRINITY_DN10370_c0_g1_i3 | 1.382770591 | 0.630553083 | 1 | 0.109 | 0.798 | 0.278 | 0 | Pkinase |

| TRINITY_DN73_c1_g1_i1 | 1.382714087 | 0.601418232 | 0.999970678 | 1.299 | 1.577 | 0.911 | 0 | NA |
| --- | --- | --- | --- | --- | --- | --- | --- | --- |
| TRINITY_DN13704_c0_g1_i2 | -1.38268515 | 0.597935408 | 0.999970678 | 6.986 | 0 | 7.988 | 8.727 | Ribosomal_L14 |
| TRINITY_DN2729_c0_g1_i1 | 1.382666018 | 0.499449592 | 0.999970678 | 9.011 | 0.66 | 2.978 | 0.141 | NA |
| TRINITY_DN8561_c0_g1_i12 | 1.382664657 | 0.391232383 | 0.999970678 | 15.088 | 2.355 | 1.355 | 4.997 | - |
| TRINITY_DN520_c1_g1_i1 | 1.382570252 | 0.510762211 | 0.999970678 | 3.952 | 0.069 | 0.844 | 0.563 | NA |
| TRINITY_DN7687_c0_g1_i6 | -1.38226116 | 0.292302184 | 0.999970678 | 1.935 | 0.552 | 4.111 | 1.538 | NA |
| TRINITY_DN6216_c0_g1_i2 | 1.382185271 | 0.745721051 | 0.999970678 | 10.546 | 0 | 0 | 4.122 | NA |
| TRINITY_DN3844_c0_g1_i3 | -1.38212068 | 0.292461657 | 0.999970678 | 0.808 | 0.217 | 1.044 | 1.468 | NA |
| TRINITY_DN7836_c0_g1_i5 | -1.38201593 | 0.452833708 | 0.999970678 | 1.026 | 0.049 | 1.233 | 1.347 | NA |
| TRINITY_DN9590_c0_g1_i1 | 1.381994448 | 0.424913788 | 0.999970678 | 0.291 | 2.72 | 0.178 | 0.935 | NA |
| TRINITY_DN220_c0_g2_i7 | -1.38172559 | 0.456070287 | 0.999970678 | 2.889 | 0.424 | 6.744 | 0.543 | NA |
| TRINITY_DN1632_c0_g1_i9 | 1.38157516 | 0.446031954 | 0.999970678 | 1.299 | 0.079 | 0.344 | 0.121 | NA |
| TRINITY_DN2724_c0_g1_i8 | -1.38143587 | 0.194814286 | 0.999970678 | 2.344 | 0.867 | 2.422 | 5.509 | EGF_CA |
| TRINITY_DN4235_c1_g1_i6 | 1.381268174 | 0.749352147 | 0.999970678 | 0.481 | 0 | 0 | 0.191 | HEAT |
| TRINITY_DN698_c1_g1_i12 | 1.381254887 | 0.6255532 | 0.999970678 | 0 | 9.343 | 1.022 | 2.302 | NA |
| TRINITY_DN25487_c0_g1_i5 | -1.38109743 | 0.743198054 | 0.999970678 | 3.933 | 0 | 8.533 | 0 | NA |
| TRINITY_DN6418_c0_g1_i2 | 1.380821186 | 0.476696808 | 0.999970678 | 2.044 | 1.281 | 0.033 | 1.237 | NA |
| TRINITY_DN131_c0_g3_i2 | 1.380617904 | 0.476788678 | 0.999970678 | 11.627 | 7.451 | 6.011 | 0.08 | NA |
| TRINITY_DN6804_c0_g1_i1 | -1.37998251 | 0.538871322 | 0.999970678 | 1.145 | 20.12 | 3.733 | 43.693 | NA |
| TRINITY_DN47849_c0_g1_i1 | -1.37992104 | 0.224597437 | 0.999970678 | 0.372 | 0.887 | 0.889 | 2.192 | F-box |
| TRINITY_DN5952_c0_g1_i2 | -1.37991355 | 0.59874728 | 0.999970678 | 1.463 | 0 | 1.767 | 1.719 | DUF296 |
| TRINITY_DN2212_c0_g2_i14 | -1.37958768 | 0.744721099 | 0.999970678 | 1.626 | 0 | 0 | 4.343 | RRM_1 |
| TRINITY_DN15481_c0_g1_i1 | -1.37945693 | 0.632296583 | 0.999970678 | 1.163 | 0 | 0.256 | 2.714 | NA |
| TRINITY_DN1143_c0_g1_i3 | 1.379086998 | 0.127367292 | 0.999970678 | 76.668 | 32.08 | 15.277 | 23.194 | NA |
| TRINITY_DN2552_c0_g1_i1 | 1.378973238 | 0.332932648 | 0.999970678 | 0.509 | 2.415 | 0.3 | 0.744 | Trehalose_PPase |
| TRINITY_DN7903_c0_g1_i1 | 1.37889273 | 0.751054739 | 0.999970678 | 0 | 1.705 | 0 | 0.653 | NA |
| TRINITY_DN6047_c0_g1_i6 | 1.37889273 | 0.751054739 | 0.999970678 | 0 | 2.947 | 0 | 1.136 | NA |
| TRINITY_DN15440_c0_g1_i1 | -1.37849243 | 0.479494924 | 0.999970678 | 0.727 | 0.108 | 0.167 | 1.981 | NA |
| TRINITY_DN9063_c0_g2_i2 | -1.37802997 | 0.148882266 | 0.999970678 | 18.758 | 7.943 | 22.599 | 41.602 | NA |
| TRINITY_DN8247_c0_g1_i1 | -1.37791475 | 0.608035289 | 0.999970678 | 0 | 3.016 | 1.389 | 6.032 | NA |
| TRINITY_DN179_c0_g1_i9 | -1.37787249 | 0.620313419 | 0.999970678 | 17.45 | 0 | 21.999 | 19.414 | CK_II_beta |
| TRINITY_DN10338_c0_g1_i2 | -1.37724901 | 0.101799345 | 0.999970678 | 1.072 | 1.301 | 2.111 | 3.599 | NA |
| TRINITY_DN892_c0_g1_i2 | 1.377054322 | 0.25035816 | 0.999970678 | 26.334 | 5.972 | 5.966 | 5.298 | NA |
| TRINITY_DN2926_c0_g1_i2 | -1.37701799 | 0.754312508 | 0.999970678 | 3.606 | 0 | 7.744 | 0 | Glutaredoxin |
| TRINITY_DN705_c0_g1_i4 | 1.376755432 | 0.744534572 | 0.999970678 | 2.425 | 0 | 0 | 0.955 | NA |
| TRINITY_DN292_c1_g1_i3 | 1.376556432 | 0.61282632 | 0.999970678 | 1.072 | 5.361 | 2.022 | 0 | Peptidase_M14 |
| TRINITY_DN7829_c0_g1_i2 | -1.37639696 | 0.612375528 | 0.999970678 | 5.923 | 0 | 5.589 | 8.877 | NA |
| TRINITY_DN5534_c0_g1_i1 | -1.37619777 | 0.272722967 | 0.999970678 | 1.135 | 0.562 | 0.667 | 3.66 | RRM_1 |
| TRINITY_DN3236_c0_g1_i1 | -1.37617309 | 0.305266257 | 0.999970678 | 1.49 | 11.02 | 13.188 | 15.945 | NA |
| TRINITY_DN22881_c0_g1_i6 | 1.37616846 | 0.193140585 | 0.999970678 | 2.771 | 1.35 | 0.367 | 1.166 | B-block_TFIIIC |
| TRINITY_DN9443_c0_g2_i2 | 1.376116048 | 0.744640421 | 0.999970678 | 3.47 | 0 | 1.122 | 0 | NA |
| TRINITY_DN4386_c1_g1_i4 | 1.376008355 | 0.599739313 | 0.999970678 | 1.145 | 2.158 | 1.055 | 0 | Ketoacyl-synt_C |
| TRINITY_DN93_c1_g2_i2 | -1.375707 | 0.442950855 | 0.999970678 | 8.303 | 0.197 | 9.166 | 11.341 | NA |
| TRINITY_DN148_c0_g1_i7 | 1.375701695 | 0.60529699 | 0.999970678 | 5.941 | 4.238 | 0 | 3.951 | NA |
| TRINITY_DN7653_c0_g1_i5 | -1.37552425 | 0.122123875 | 0.999970678 | 5.687 | 2.72 | 7.722 | 12.607 | NA |
| TRINITY_DN2396_c0_g1_i11 | 1.375411225 | 0.750196067 | 0.999970678 | 1.39 | 0 | 0.444 | 0 | NA |
| TRINITY_DN1207_c0_g1_i5 | 1.375411225 | 0.750196067 | 0.999970678 | 1.844 | 0 | 0.589 | 0 | Ank_2 |
| TRINITY_DN1628_c0_g1_i3 | 1.375329057 | 0.743081914 | 0.999970678 | 16.805 | 0 | 0 | 6.575 | NA |
| TRINITY_DN5911_c0_g1_i1 | 1.375242535 | 0.742482384 | 0.999970678 | 0 | 4.829 | 0 | 1.83 | CcmH |
| TRINITY_DN5595_c0_g2_i4 | -1.37490315 | 0.747279385 | 0.999970678 | 2.707 | 0 | 5.933 | 0 | NA |
| TRINITY_DN633_c0_g1_i4 | -1.37487919 | 0.2110637 | 0.999970678 | 0.69 | 2.71 | 3.989 | 3.941 | NA |
| TRINITY_DN2169_c0_g1_i2 | -1.37485744 | 0.541208717 | 0.999970678 | 0.954 | 3.371 | 0.089 | 10.898 | NA |
| TRINITY_DN3821_c0_g2_i1 | 1.374823392 | 0.338370555 | 0.999970678 | 2.671 | 0.315 | 0.456 | 0.623 | NA |
| TRINITY_DN5215_c0_g1_i4 | -1.37467492 | 0.309079722 | 0.999970678 | 0.182 | 0.552 | 0.511 | 1.257 | NA |
| TRINITY_DN2573_c1_g1_i11 | -1.37466861 | 0.304682812 | 0.999970678 | 6.976 | 8.042 | 3.466 | 34.796 | NA |
| TRINITY_DN6178_c1_g1_i9 | -1.3742848 | 0.748989798 | 0.999970678 | 49.517 | 0 | 106.46 | 0 | NA |
| TRINITY_DN5041_c0_g1_i1 | -1.37423539 | 0.656203423 | 0.999970678 | 0 | 2.809 | 5.8 | 0.141 | DUF383 |
| TRINITY_DN186_c1_g1_i4 | 1.373976743 | 0.418372582 | 0.999970678 | 0.3 | 1.912 | 0.122 | 0.694 | NA |
| TRINITY_DN26832_c0_g1_i1 | -1.37393169 | 0.743675525 | 0.999970678 | 3.043 | 0 | 0 | 7.912 | AP2 |
| TRINITY_DN10236_c0_g1_i5 | 1.373705651 | 0.295409181 | 0.999970678 | 0.509 | 1.537 | 0.344 | 0.352 | NA |
| TRINITY_DN13044_c0_g1_i4 | 1.373541421 | 0.608121044 | 0.999970678 | 1.453 | 3.282 | 1.5 | 0 | HGTP_anticodon |
| TRINITY_DN46199_c0_g1_i1 | -1.37350437 | 0.261798772 | 0.999970678 | 0.491 | 0.7 | 0.889 | 2.051 | NA |
| TRINITY_DN9516_c0_g1_i2 | -1.37306119 | 0.341608009 | 0.999970678 | 10.737 | 4.859 | 4.989 | 32.634 | NA |
| TRINITY_DN1016_c0_g1_i1 | 1.373023958 | 0.614336501 | 0.999970678 | 1.79 | 0.483 | 0.733 | 0 | NA |
| TRINITY_DN12461_c0_g1_i2 | -1.37300295 | 0.249540539 | 0.999970678 | 1.081 | 0.788 | 0.878 | 3.8 | AMP-binding |
| TRINITY_DN1118_c1_g1_i4 | -1.37291511 | 0.46800734 | 0.999970678 | 30.032 | 286.1 | 26.243 | 711.671 | NA |
| TRINITY_DN4921_c0_g1_i3 | 1.372802502 | 0.630843827 | 0.999970678 | 4.824 | 0 | 0.955 | 0.714 | NA |
| TRINITY_DN6692_c1_g1_i2 | -1.37265935 | 0.360183154 | 0.999970678 | 11.291 | 0.966 | 11.222 | 18.398 | NA |
| TRINITY_DN28172_c0_g1_i2 | -1.37258239 | 0.75835832 | 1 | 0.227 | 0 | 0.511 | 0.01 | RVT_2 |
| TRINITY_DN27930_c0_g1_i1 | -1.37258239 | 0.75835832 | 1 | 1.154 | 0 | 2.555 | 0 | NA |
| TRINITY_DN35453_c0_g1_i1 | -1.37258239 | 0.75835832 | 1 | 0.418 | 0 | 0.922 | 0 | NA |
| TRINITY_DN9805_c0_g2_i3 | -1.37258239 | 0.75835832 | 1 | 0.427 | 0 | 0.9 | 0 | NA |
| TRINITY_DN9391_c0_g1_i1 | -1.3724432 | 0.600771199 | 0.999970678 | 1.426 | 0 | 1.489 | 1.94 | Med17 |
| TRINITY_DN2212_c0_g1_i6 | 1.372284907 | 0.652986248 | 0.999970678 | 8.839 | 0.306 | 0 | 3.609 | NA |

| TRINITY_DN8213_c0_g1_i3 | 1.372283884 | 0.661616911 | 0.999970678 | 0 | 21.99 | 0.189 | 8.033 | NA |
| --- | --- | --- | --- | --- | --- | --- | --- | --- |
| TRINITY_DN3002_c0_g1_i1 | -1.37224003 | 0.288575234 | 0.999970678 | 7.812 | 5.893 | 4.255 | 29.96 | NA |
| TRINITY_DN5316_c0_g1_i10 | 1.371971398 | 0.438546667 | 0.999970678 | 0.154 | 1.863 | 0.133 | 0.603 | NA |
| TRINITY_DN22690_c0_g3_i1 | 1.370916043 | 0.312092993 | 1 | 0.736 | 0.798 | 0.367 | 0.161 | NA |
| TRINITY_DN835_c1_g1_i7 | 1.370562649 | 0.208253076 | 0.999970678 | 1.508 | 1.34 | 0.644 | 0.322 | NA |
| TRINITY_DN7330_c0_g1_i3 | -1.3705137 | 0.61688773 | 1 | 0.372 | 0 | 0.544 | 0.302 | WD40 |
| TRINITY_DN518_c0_g1_i7 | -1.37040309 | 0.38468065 | 0.999970678 | 5.305 | 34.41 | 9.266 | 90.443 | Tubulin |
| TRINITY_DN4730_c0_g1_i11 | -1.37021004 | 0.334493765 | 0.999970678 | 1.39 | 0.296 | 2.822 | 1.036 | - |
| TRINITY_DN9347_c0_g1_i3 | -1.37013054 | 0.752224848 | 0.999970678 | 1.29 | 0 | 2.811 | 0 | NA |
| TRINITY_DN875_c0_g1_i4 | -1.36977432 | 0.744782274 | 0.999970678 | 0 | 4.031 | 8.477 | 0 | NA |
| TRINITY_DN10367_c0_g1_i1 | -1.36972204 | 0.165909153 | 0.999970678 | 1.354 | 1.015 | 1.6 | 4.202 | NA |
| TRINITY_DN336_c0_g1_i8 | 1.369523596 | 0.615643022 | 0.999970678 | 1.399 | 0.325 | 0.556 | 0 | - |
| TRINITY_DN13257_c0_g1_i3 | -1.36939041 | 0.35030474 | 0.999970678 | 1.072 | 0.158 | 1.222 | 1.729 | NA |
| TRINITY_DN1363_c0_g1_i6 | 1.369250321 | 0.598225642 | 0.999970678 | 1.172 | 2.759 | 0 | 1.518 | Dimerisation |
| TRINITY_DN4587_c1_g1_i4 | 1.369220697 | 0.603222463 | 0.999970678 | 1.417 | 1.459 | 0.933 | 0 | CNH |
| TRINITY_DN19534_c0_g1_i4 | -1.36886877 | 0.744973409 | 0.999970678 | 1.653 | 0 | 3.589 | 0.01 | NA |
| TRINITY_DN19860_c0_g1_i2 | -1.36883595 | 0.32924078 | 0.999970678 | 0.327 | 1.981 | 2.744 | 2.544 | NA |
| TRINITY_DN12201_c1_g2_i2 | 1.36882577 | 0.293939502 | 0.999970678 | 0.382 | 0.611 | 0.233 | 0.101 | ERAP1_C |
| TRINITY_DN3434_c0_g1_i1 | -1.36870673 | 0.389543434 | 0.999970678 | 1.635 | 4.307 | 1.378 | 13.241 | NA |
| TRINITY_DN800_c1_g1_i5 | -1.36842588 | 0.337995828 | 0.999970678 | 1.59 | 3.814 | 2.011 | 11.15 | NA |
| TRINITY_DN22768_c0_g1_i1 | 1.368394748 | 0.750420136 | 0.999970678 | 1.453 | 0 | 0 | 0.573 | NA |
| TRINITY_DN7352_c0_g1_i2 | 1.368343729 | 0.388823841 | 0.999970678 | 1.653 | 0.848 | 0.722 | 0.101 | AA_kinase |
| TRINITY_DN349_c0_g1_i1 | 1.368191193 | 0.745929191 | 0.999970678 | 13.653 | 0 | 0 | 5.379 | DUF3531 |
| TRINITY_DN3802_c0_g1_i1 | 1.368132457 | 0.473281293 | 0.999970678 | 0.091 | 2.671 | 0.467 | 0.493 | NA |
| TRINITY_DN6474_c0_g1_i13 | 1.367960249 | 0.204382361 | 0.999970678 | 0.336 | 0.66 | 0.1 | 0.261 | Asparaginase_2 |
| TRINITY_DN660_c0_g1_i3 | 1.3679523 | 0.747360845 | 0.999970678 | 6.976 | 0 | 0 | 2.755 | NA |
| TRINITY_DN3269_c0_g1_i6 | -1.36779214 | 0.241781881 | 0.999970678 | 0.291 | 0.128 | 0.667 | 0.302 | NA |
| TRINITY_DN27908_c0_g1_i7 | 1.367788682 | 0.497651993 | 0.999970678 | 25.944 | 15.35 | 0.2 | 15.101 | LRRNT_2 |
| TRINITY_DN2474_c0_g1_i1 | 1.367553977 | 0.630010955 | 0.999970678 | 3.733 | 0 | 0.378 | 1.015 | GCFC |
| TRINITY_DN6418_c0_g2_i11 | 1.367517289 | 0.374217911 | 0.999970678 | 1.472 | 5.243 | 1.822 | 0.402 | NA |
| TRINITY_DN12762_c0_g1_i6 | -1.36661508 | 0.235380545 | 0.999970678 | 1.599 | 2.217 | 2.7 | 6.485 | NA |
| TRINITY_DN3810_c0_g1_i3 | -1.36641427 | 0.098309237 | 0.999970678 | 3.643 | 5.805 | 13.099 | 8.495 | - |
| TRINITY_DN2427_c0_g1_i1 | -1.3662873 | 0.0440997 | 0.999970678 | 13.326 | 10.47 | 21.276 | 35.781 | NA |
| TRINITY_DN3808_c0_g1_i10 | 1.366254136 | 0.374205575 | 0.999970678 | 1.453 | 0.532 | 0.556 | 0.101 | - |
| TRINITY_DN1461_c0_g2_i11 | 1.366165487 | 0.295695388 | 0.999970678 | 0.418 | 0.788 | 0.133 | 0.302 | DUF966 |
| TRINITY_DN6966_c0_g1_i3 | 1.3660689 | 0.36903215 | 0.999970678 | 1.699 | 3.932 | 1.633 | 0.201 | NA |
| TRINITY_DN5660_c0_g1_i10 | -1.36592278 | 0.745428572 | 0.999970678 | 0 | 1.055 | 0 | 2.684 | Mlo |
| TRINITY_DN3931_c0_g1_i3 | 1.365883543 | 0.683684074 | 0.999970678 | 46.837 | 0.177 | 0 | 18.559 | NA |
| TRINITY_DN7759_c0_g2_i12 | -1.36563877 | 0.755112731 | 0.999970678 | 1.308 | 0 | 2.866 | 0 | NA |
| TRINITY_DN18956_c0_g1_i3 | 1.365585439 | 0.279390063 | 0.999970678 | 0.727 | 1.941 | 0.489 | 0.432 | NA |
| TRINITY_DN10151_c0_g3_i4 | 1.365569151 | 0.418905041 | 0.999970678 | 0.917 | 0.512 | 0.411 | 0.05 | NA |
| TRINITY_DN6074_c0_g1_i9 | -1.36498567 | 0.227955301 | 0.999970678 | 0.563 | 0.512 | 1.189 | 1.317 | Grp1_Fun34_Yaa |
| TRINITY_DN391_c0_g1_i4 | -1.36476384 | 0.337227115 | 0.999970678 | 2.789 | 5.746 | 2.078 | 19.233 | Ribosomal_L18 |
| TRINITY_DN4887_c0_g1_i3 | 1.36467746 | 0.60470832 | 0.999970678 | 1.126 | 0.522 | 0.533 | 0 | NA |
| TRINITY_DN1897_c0_g1_i3 | -1.36422081 | 0.260861446 | 0.999970678 | 3.079 | 4.228 | 4.066 | 13.301 | NA |
| TRINITY_DN20288_c0_g1_i1 | 1.363890122 | 0.746456952 | 0.999970678 | 0 | 2.562 | 0 | 0.985 | - |
| TRINITY_DN4390_c0_g1_i6 | -1.36317968 | 0.60759237 | 0.999970678 | 3.661 | 0 | 3.033 | 5.65 | NA |
| TRINITY_DN10914_c0_g1_i6 | 1.362751016 | 0.216105958 | 0.999970678 | 0.79 | 1.084 | 0.189 | 0.513 | DEAD |
| TRINITY_DN864_c0_g1_i11 | -1.36274502 | 0.144315396 | 0.999970678 | 1.181 | 0.601 | 1.322 | 3.016 | PDCD2_C |
| TRINITY_DN8670_c0_g2_i1 | -1.36269894 | 0.74877092 | 0.999970678 | 3.27 | 0 | 7.022 | 0 | NA |
| TRINITY_DN559_c0_g1_i4 | 1.362685478 | 0.598995414 | 0.999970678 | 2.516 | 1.459 | 0 | 1.558 | NA |
| TRINITY_DN5578_c0_g1_i11 | 1.361990131 | 0.608530248 | 0.999970678 | 0.372 | 0.887 | 0.4 | 0 | NA |
| TRINITY_DN8213_c0_g1_i6 | -1.36184424 | 0.606842964 | 0.999970678 | 7.676 | 0 | 5.633 | 13.03 | NA |
| TRINITY_DN6670_c0_g1_i5 | 1.361702813 | 0.746308507 | 0.999970678 | 2.198 | 0 | 0 | 0.875 | NA |
| TRINITY_DN9041_c0_g1_i5 | -1.36166682 | 0.12372477 | 0.999970678 | 1.69 | 1.833 | 4.6 | 3.519 | NA |
| TRINITY_DN9937_c0_g1_i7 | 1.36166342 | 0.355848524 | 0.999970678 | 11.237 | 1.459 | 2.189 | 2.322 | NA |
| TRINITY_DN15978_c3_g1_i1 | -1.36154293 | 0.458244324 | 0.999970678 | 4.388 | 72.95 | 120.604 | 37.169 | NA |
| TRINITY_DN25587_c0_g1_i1 | -1.36136556 | 0.752988979 | 0.999970678 | 1.562 | 0 | 3.378 | 0 | NA |
| TRINITY_DN19234_c0_g1_i3 | -1.3612409 | 0.75670138 | 1 | 0 | 0.542 | 1.122 | 0 | NA |
| TRINITY_DN46638_c0_g1_i11 | 1.360948793 | 0.608271648 | 0.999970678 | 0.282 | 0.325 | 0.2 | 0 | Asn_synthase |
| TRINITY_DN8029_c0_g1_i5 | 1.360879994 | 0.360940104 | 0.999970678 | 1.426 | 1.902 | 0.144 | 1.136 | PPTA |
| TRINITY_DN489_c0_g1_i4 | 1.360775397 | 0.286182891 | 0.999970678 | 0.263 | 0.621 | 0.133 | 0.191 | NA |
| TRINITY_DN16410_c0_g1_i1 | -1.3607612 | 0.287028593 | 0.999970678 | 0.327 | 0.453 | 1.178 | 0.573 | NA |
| TRINITY_DN813_c0_g1_i2 | -1.36073178 | 0.630005657 | 0.999970678 | 0.581 | 1.37 | 4.1 | 0 | DUF659 |
| TRINITY_DN591_c0_g1_i9 | -1.36068969 | 0.315285194 | 0.999970678 | 5.777 | 1.744 | 2.644 | 16.357 | NA |
| TRINITY_DN34788_c0_g1_i9 | -1.36057718 | 0.755466768 | 0.999970678 | 0.572 | 0 | 1.255 | 0 | Peptidase_S8 |
| TRINITY_DN28625_c0_g1_i1 | -1.36057718 | 0.755466768 | 0.999970678 | 1.036 | 0 | 2.222 | 0 | NA |
| TRINITY_DN367_c0_g1_i11 | -1.3603567 | 0.352772046 | 0.999970678 | 15.57 | 10.06 | 4.189 | 61.056 | Band_7 |
| TRINITY_DN883_c0_g1_i1 | -1.36027661 | 0.60812805 | 0.999970678 | 2.925 | 0 | 2.355 | 4.765 | NA |
| TRINITY_DN3420_c0_g1_i6 | -1.36016768 | 0.271776202 | 0.999970678 | 4.415 | 0.828 | 4.7 | 7.922 | - |
| TRINITY_DN2965_c1_g1_i3 | 1.360033913 | 0.572082143 | 0.999970678 | 9.302 | 0.039 | 2.855 | 0.241 | - |
| TRINITY_DN1336_c0_g1_i2 | 1.359749016 | 0.747748795 | 0.999970678 | 13.617 | 0 | 0 | 5.419 | NA |
| TRINITY_DN155_c2_g1_i2 | -1.35967097 | 0.614246002 | 0.999970678 | 9.974 | 0 | 16.443 | 5.751 | PsbP |
| TRINITY_DN9135_c0_g1_i2 | -1.35965193 | 0.171896054 | 0.999970678 | 2.153 | 3.361 | 7.944 | 4.434 | NA |

| TRINITY_DN9536_c0_g1_i6 | 1.359646168 | 0.409960725 | 0.999970678 | 5.623 | 0.355 | 0.844 | 1.357 | NT-C2 |
| --- | --- | --- | --- | --- | --- | --- | --- | --- |
| TRINITY_DN57289_c0_g1_i3 | -1.35941061 | 0.759025694 | 1 | 0.518 | 0 | 1.111 | 0 | NA |
| TRINITY_DN1471_c3_g1_i3 | 1.359285471 | 0.750017333 | 0.999970678 | 0 | 4.208 | 1.344 | 0 | NA |
| TRINITY_DN3108_c0_g1_i8 | -1.35879982 | 0.122521124 | 0.999970678 | 5.959 | 2.858 | 9.544 | 11.18 | Bystin |
| TRINITY_DN90803_c0_g1_i1 | -1.35840529 | 0.747032032 | 0.999970678 | 3.343 | 0 | 7.166 | 0 | NA |
| TRINITY_DN28119_c1_g1_i2 | 1.357969724 | 0.758377508 | 1 | 3.316 | 0 | 1.067 | 0 | Peroxidase_2 |
| TRINITY_DN167746_c0_g1_i1 | 1.357927127 | 0.757566252 | 0.999970678 | 5.614 | 0 | 1.822 | 0 | NA |
| TRINITY_DN960_c0_g1_i4 | 1.357837602 | 0.754947429 | 0.999970678 | 0 | 2.415 | 0 | 0.915 | Epimerase |
| TRINITY_DN29814_c0_g1_i1 | 1.357818003 | 0.755511228 | 0.999970678 | 2.353 | 0 | 0.767 | 0 | But2 |
| TRINITY_DN16941_c0_g1_i3 | 1.357730587 | 0.45301433 | 0.999970678 | 3.997 | 1.045 | 0.122 | 1.87 | NA |
| TRINITY_DN2268_c0_g1_i3 | -1.35758141 | 0.640597599 | 0.999970678 | 0.436 | 0.079 | 0 | 1.317 | Chloroa_b-bind |
| TRINITY_DN7061_c0_g1_i8 | -1.35737649 | 0.753338182 | 0.999970678 | 4.587 | 0 | 9.555 | 0 | NA |
| TRINITY_DN4904_c0_g1_i2 | -1.35723839 | 0.186737182 | 0.999970678 | 0.663 | 1.104 | 1.744 | 2.403 | - |
| TRINITY_DN2785_c0_g1_i2 | -1.35711331 | 0.315322067 | 0.999970678 | 1.154 | 0.286 | 2.278 | 0.955 | NA |
| TRINITY_DN3582_c0_g1_i1 | 1.356736072 | 0.446890377 | 0.999970678 | 2.062 | 32.74 | 2.155 | 10.426 | NA |
| TRINITY_DN2330_c1_g1_i1 | 1.356729326 | 0.598529792 | 0.999970678 | 2.008 | 1.833 | 0 | 1.498 | LRR_8 |
| TRINITY_DN6346_c0_g1_i3 | 1.356712144 | 0.295749653 | 0.999970678 | 52.124 | 7.204 | 10.377 | 10.908 | S6PP |
| TRINITY_DN11687_c0_g2_i2 | 1.356496721 | 0.319493896 | 0.999970678 | 23.664 | 4.494 | 3.866 | 6.223 | NA |
| TRINITY_DN1064_c0_g1_i1 | 1.356281049 | 0.526651915 | 0.999970678 | 10.946 | 0.039 | 1.489 | 2.584 | LRRNT_2 |
| TRINITY_DN6361_c0_g1_i1 | -1.35612345 | 0.652723104 | 0.999970678 | 0 | 2.789 | 0.167 | 6.857 | CPSase_L_D2 |
| TRINITY_DN31404_c0_g1_i2 | 1.356100039 | 0.603828618 | 0.999970678 | 1.008 | 0.798 | 0.589 | 0 | ANAPC1 |
| TRINITY_DN3627_c0_g1_i17 | 1.355968945 | 0.748897443 | 0.999970678 | 0 | 5.677 | 1.811 | 0 | Pro_CA |
| TRINITY_DN31654_c0_g1_i2 | 1.355828639 | 0.179348313 | 0.999970678 | 3.752 | 2.119 | 1.3 | 0.724 | Aldedh |
| TRINITY_DN950_c0_g1_i4 | -1.35542264 | 0.155885596 | 0.999970678 | 4.124 | 2.267 | 9.155 | 5.349 | Mito_carr |
| TRINITY_DN16815_c0_g1_i4 | -1.35541804 | 0.760745846 | 1 | 0 | 0.601 | 1.267 | 0 | NA |
| TRINITY_DN1061_c0_g2_i1 | 1.354723547 | 0.291908046 | 0.999970678 | 3.252 | 2.04 | 0.356 | 1.659 | NA |
| TRINITY_DN528_c0_g1_i9 | -1.35462566 | 0.316784095 | 0.999970678 | 0.118 | 0.365 | 0.511 | 0.623 | LRRNT_2 |
| TRINITY_DN13468_c0_g1_i4 | -1.35433554 | 0.348915233 | 0.999970678 | 0.636 | 1.202 | 0.478 | 4.112 | Lipase_GDSL |
| TRINITY_DN813_c1_g1_i3 | 1.354208687 | 0.495292026 | 0.999970678 | 0.318 | 7.559 | 0.522 | 2.413 | NA |
| TRINITY_DN7168_c0_g1_i2 | -1.35418697 | 0.244897874 | 0.999970678 | 5.705 | 3.528 | 15.21 | 5.248 | PAR1 |
| TRINITY_DN623_c0_g1_i11 | 1.354025163 | 0.645780267 | 0.999970678 | 2.698 | 0 | 0.122 | 0.925 | - |
| TRINITY_DN458_c0_g1_i5 | -1.35394496 | 0.52751326 | 0.999970678 | 6.477 | 138 | 7.266 | 325.006 | NA |
| TRINITY_DN435_c0_g1_i7 | 1.353846403 | 0.404721546 | 0.999970678 | 1.635 | 1.084 | 0.822 | 0.07 | AA_permease_2 |
| TRINITY_DN11783_c1_g1_i4 | -1.35372618 | 0.354736445 | 1 | 4.297 | 1.192 | 6.111 | 6.183 | NA |
| TRINITY_DN14935_c0_g1_i4 | -1.35361945 | 0.753668182 | 0.999970678 | 2.489 | 0 | 5.355 | 0 | NA |
| TRINITY_DN1080_c0_g1_i6 | -1.35361724 | 0.242734497 | 0.999970678 | 10.601 | 16.68 | 10.166 | 57.135 | LRRNT_2 |
| TRINITY_DN3091_c0_g1_i16 | 1.353584624 | 0.282429944 | 1 | 2.162 | 1.715 | 0.511 | 0.885 | NA |
| TRINITY_DN11546_c0_g1_i1 | 1.353377949 | 0.184435889 | 0.999970678 | 0.854 | 1.449 | 0.5 | 0.292 | FH2 |
| TRINITY_DN5273_c0_g2_i1 | -1.35323274 | 0.462925622 | 0.999970678 | 4.36 | 0.118 | 3.078 | 7.912 | NA |
| TRINITY_DN4974_c0_g1_i1 | 1.353171115 | 0.6131875 | 0.999970678 | 0.972 | 0.325 | 0.422 | 0 | Myb_DNA-bind_ |
| TRINITY_DN14858_c0_g1_i5 | -1.35314901 | 0.669938247 | 0.999970678 | 1.681 | 0 | 3.555 | 0.04 | NA |
| TRINITY_DN519_c4_g1_i2 | 1.353077273 | 0.337511002 | 0.999970678 | 4.079 | 1.38 | 1.5 | 0.332 | NA |
| TRINITY_DN6689_c0_g1_i1 | 1.352964058 | 0.607696223 | 0.999970678 | 6.74 | 4.563 | 0 | 4.444 | NA |
| TRINITY_DN845_c0_g1_i5 | 1.352790477 | 0.207695247 | 0.999970678 | 3.87 | 10.7 | 1.844 | 3.428 | NA |
| TRINITY_DN641_c0_g1_i6 | 1.352693692 | 0.363460855 | 0.999970678 | 4.333 | 1.261 | 1.567 | 0.312 | NA |
| TRINITY_DN11043_c0_g1_i5 | -1.35248891 | 0.629297744 | 0.999970678 | 2.643 | 1.114 | 0 | 9.501 | NA |
| TRINITY_DN30127_c0_g4_i1 | 1.351844946 | 0.31471629 | 0.999970678 | 0.936 | 3.745 | 0.689 | 0.985 | NA |
| TRINITY_DN174_c0_g1_i3 | -1.3515391 | 0.411816084 | 0.999970678 | 17.832 | 2.148 | 6.311 | 42.728 | NA |
| TRINITY_DN14718_c0_g1_i6 | -1.35149478 | 0.75610039 | 0.999970678 | 0.472 | 0 | 1 | 0 | NA |
| TRINITY_DN45465_c0_g1_i1 | -1.35149275 | 0.669884994 | 0.999970678 | 1.299 | 0.049 | 2.878 | 0 | NA |
| TRINITY_DN1043_c0_g2_i1 | -1.35090577 | 0.754229111 | 0.999970678 | 12.318 | 0 | 26.343 | 0 | ADH_N |
| TRINITY_DN3561_c0_g1_i6 | -1.35090009 | 0.614211623 | 0.999970678 | 2.098 | 0 | 1.167 | 4.042 | NA |
| TRINITY_DN3250_c0_g1_i15 | -1.35086985 | 0.034943945 | 0.999970678 | 50.143 | 37.97 | 90.95 | 111.405 | NA |
| TRINITY_DN4634_c0_g1_i7 | 1.350737783 | 0.447255098 | 0.999970678 | 9.42 | 2.917 | 3.6 | 0.503 | NA |
| TRINITY_DN7771_c0_g1_i11 | 1.350619745 | 0.451579132 | 0.999970678 | 4.079 | 5.903 | 3.011 | 0.241 | EGF_CA |
| TRINITY_DN165_c0_g1_i1 | -1.35043945 | 0.185640961 | 0.999970678 | 20.539 | 6.13 | 26.143 | 36.887 | ADH_N |
| TRINITY_DN1877_c0_g1_i5 | -1.35022136 | 0.643959101 | 0.999970678 | 0 | 0.68 | 1.322 | 0.131 | NA |
| TRINITY_DN6641_c0_g1_i1 | -1.34969603 | 0.139122249 | 0.999970678 | 5.396 | 2.257 | 8.577 | 9.219 | NA |
| TRINITY_DN3360_c0_g1_i5 | 1.34952925 | 0.748734557 | 0.999970678 | 2.934 | 0 | 0.955 | 0 | NA |
| TRINITY_DN479_c0_g1_i6 | 1.349515556 | 0.263561858 | 0.999970678 | 8.648 | 5.893 | 2.955 | 2.051 | DUF4228 |
| TRINITY_DN1117_c0_g1_i2 | 1.34944115 | 0.654175202 | 0.999970678 | 0 | 28.87 | 1.367 | 9.531 | Ammonium_trans |
| TRINITY_DN294_c0_g1_i2 | -1.34943478 | 0.451267904 | 0.999970678 | 2.38 | 0.375 | 4.9 | 1.036 | NA |
| TRINITY_DN209_c0_g1_i3 | -1.34937267 | 0.613997289 | 0.999970678 | 7.104 | 0 | 5.366 | 11.863 | NA |
| TRINITY_DN4033_c0_g1_i3 | 1.349320486 | 0.197195706 | 0.999970678 | 82.364 | 29.18 | 11.51 | 30.262 | NA |
| TRINITY_DN1214_c0_g1_i2 | 1.349289944 | 0.215361084 | 0.999970678 | 15.67 | 24.99 | 10.466 | 3.267 | NA |
| TRINITY_DN3248_c0_g1_i4 | 1.34893745 | 0.315542657 | 0.999970678 | 4.769 | 22.97 | 1.833 | 8.606 | NA |
| TRINITY_DN2990_c0_g1_i1 | 1.348856803 | 0.348083464 | 0.999970678 | 2.053 | 0.572 | 0.278 | 0.714 | NA |
| TRINITY_DN4870_c0_g1_i1 | -1.34885383 | 0.608909087 | 0.999970678 | 84.762 | 0 | 92.006 | 93.298 | NA |
| TRINITY_DN2152_c0_g1_i8 | -1.34881356 | 0.355592079 | 0.999970678 | 0.273 | 2.375 | 2 | 4.273 | DUF247 |
| TRINITY_DN2009_c0_g1_i15 | -1.34870452 | 0.493746953 | 0.999970678 | 7.24 | 0.65 | 15.444 | 1.558 | NA |
| TRINITY_DN1347_c0_g3_i6 | -1.34866482 | 0.273088855 | 0.999970678 | 4.333 | 3.41 | 2.533 | 16.76 | AARP2CN |
| TRINITY_DN2390_c0_g1_i4 | 1.348451787 | 0.63059619 | 0.999970678 | 0 | 4.07 | 0.833 | 0.563 | NA |
| TRINITY_DN253291_c0_g1_i1 | -1.3484391 | 0.75954377 | 0.999970678 | 0.754 | 0 | 1.611 | 0 | Fungal_trans |
| TRINITY_DN8044_c0_g2_i3 | -1.34836481 | 0.607889258 | 0.999970678 | 3.806 | 0 | 3.655 | 5.339 | NA |

| TRINITY_DN902_c0_g1_i1 | -1.34819649 | 0.27203275 | 0.999970678 | 3.47 | 7.835 | 4.122 | 23.697 | NA |
| --- | --- | --- | --- | --- | --- | --- | --- | --- |
| TRINITY_DN6762_c0_g1_i3 | -1.34785762 | 0.341844523 | 0.999970678 | 8.094 | 3.853 | 21.888 | 3.871 | NA |
| TRINITY_DN4583_c0_g1_i3 | 1.347462484 | 0.74652274 | 0.999970678 | 0 | 8.722 | 0 | 3.388 | Usp |
| TRINITY_DN3247_c0_g1_i1 | 1.347383244 | 0.351627637 | 0.999970678 | 4.715 | 32.82 | 9.622 | 2.956 | - |
| TRINITY_DN519_c0_g1_i4 | 1.34728876 | 0.104341571 | 0.999970678 | 5.714 | 3.4 | 1.378 | 1.92 | NA |
| TRINITY_DN2786_c0_g1_i2 | 1.346940512 | 0.273894446 | 0.999970678 | 0.491 | 0.542 | 0.256 | 0.09 | NA |
| TRINITY_DN235_c0_g1_i1 | -1.34686389 | 0.633340972 | 0.999970678 | 8.448 | 0 | 15.244 | 3.368 | NA |
| TRINITY_DN3827_c0_g1_i4 | -1.34665636 | 0.749368511 | 0.999970678 | 2.398 | 0 | 5.1 | 0 | NA |
| TRINITY_DN897_c2_g1_i6 | 1.346471605 | 0.429324556 | 0.999970678 | 0.609 | 1.794 | 0.089 | 0.824 | NA |
| TRINITY_DN7848_c0_g1_i6 | 1.346289577 | 0.627478314 | 0.999970678 | 0.354 | 1.794 | 0.7 | 0 | Aldo_ket_red |
| TRINITY_DN6651_c0_g1_i10 | 1.346019594 | 0.453432882 | 0.999970678 | 0.1 | 1.922 | 0.156 | 0.603 | Glyco_hydro_1 |
| TRINITY_DN1957_c0_g1_i12 | -1.3459618 | 0.631359359 | 0.999970678 | 0.6 | 0.355 | 2.011 | 0 | NA |
| TRINITY_DN3788_c0_g1_i3 | 1.34586886 | 0.612708005 | 0.999970678 | 1.835 | 5.361 | 2.3 | 0 | TAXi_C |
| TRINITY_DN7527_c2_g1_i3 | 1.345794685 | 0.18770944 | 0.999970678 | 0.781 | 1.36 | 0.489 | 0.251 | NA |
| TRINITY_DN12095_c1_g1_i2 | 1.34576118 | 0.748516061 | 0.999970678 | 1.862 | 0 | 0 | 0.754 | NA |
| TRINITY_DN1555_c0_g1_i14 | 1.345551925 | 0.759386849 | 1 | 0 | 0.71 | 0 | 0.282 | NA |
| TRINITY_DN4390_c1_g1_i2 | 1.345240473 | 0.641118103 | 0.999970678 | 0.218 | 2.079 | 0.733 | 0 | NA |
| TRINITY_DN3389_c0_g1_i7 | -1.34516475 | 0.613586738 | 0.999970678 | 10.41 | 0 | 10.366 | 13.18 | NA |
| TRINITY_DN1189_c0_g1_i1 | 1.345136958 | 0.653182816 | 0.999970678 | 0 | 41.94 | 2.789 | 12.909 | NA |
| TRINITY_DN15166_c0_g1_i3 | 1.344987495 | 0.429192699 | 0.999970678 | 1.154 | 2.001 | 0.978 | 0.05 | NA |
| TRINITY_DN9852_c0_g1_i7 | 1.344927543 | 0.468307803 | 0.999970678 | 1.581 | 0.177 | 0.056 | 0.623 | NA |
| TRINITY_DN383_c0_g1_i2 | -1.34466399 | 0.27724038 | 0.999970678 | 1.217 | 4.494 | 9.044 | 3.448 | NA |
| TRINITY_DN6324_c0_g1_i2 | -1.34460099 | 0.632835099 | 0.999970678 | 4.124 | 0 | 1 | 9.45 | NA |
| TRINITY_DN3886_c0_g1_i2 | -1.34441005 | 0.474632115 | 0.999970678 | 0.718 | 50.75 | 63.429 | 51.917 | Cupin_1 |
| TRINITY_DN2882_c0_g1_i5 | -1.34418123 | 0.086088709 | 0.999970678 | 63.006 | 133.6 | 199.455 | 256.027 | NA |
| TRINITY_DN278_c0_g1_i8 | 1.34413337 | 0.492999725 | 0.999970678 | 10.274 | 0.355 | 0.333 | 3.871 | NA |
| TRINITY_DN22930_c0_g1_i2 | 1.343975943 | 0.371821545 | 1 | 1.181 | 1.321 | 0.589 | 0.271 | NA |
| TRINITY_DN8606_c2_g1_i3 | 1.343975943 | 0.371821545 | 1 | 0.6 | 0.66 | 0.3 | 0.121 | NA |
| TRINITY_DN397_c0_g1_i2 | 1.343745581 | 0.359546403 | 0.999970678 | 2.743 | 3.814 | 1.955 | 0.211 | PUNUT |
| TRINITY_DN8942_c0_g1_i12 | -1.3433923 | 0.361783677 | 0.999970678 | 0.818 | 0.512 | 2.467 | 0.402 | Glyoxalase |
| TRINITY_DN5080_c0_g1_i2 | -1.34318559 | 0.279758034 | 0.999970678 | 19.939 | 4.1 | 24.821 | 29.769 | p450 |
| TRINITY_DN85_c0_g1_i7 | 1.343080803 | 0.324205539 | 0.999970678 | 0.681 | 1.675 | 0.589 | 0.221 | 2-oxogl_dehyd_N |
| TRINITY_DN728_c0_g1_i13 | 1.343058613 | 0.258122262 | 0.999970678 | 4.197 | 1.498 | 0.844 | 1.227 | NA |
| TRINITY_DN833_c2_g1_i2 | 1.342871565 | 0.651409075 | 0.999970678 | 0 | 4.908 | 0.189 | 1.679 | NA |
| TRINITY_DN1874_c0_g5_i1 | 1.342802169 | 0.615719745 | 0.999970678 | 5.841 | 5.194 | 3.611 | 0 | NA |
| TRINITY_DN220_c0_g2_i11 | 1.342591528 | 0.400145036 | 0.999970678 | 8.239 | 2.7 | 0.3 | 3.951 | NA |
| TRINITY_DN34114_c0_g1_i1 | -1.34258634 | 0.293699041 | 0.999970678 | 45.002 | 55.7 | 50.353 | 169.938 | NA |
| TRINITY_DN3147_c0_g1_i4 | -1.34248723 | 0.133327988 | 0.999970678 | 1.744 | 1.39 | 2.811 | 4.504 | NA |
| TRINITY_DN9230_c0_g1_i14 | -1.34239737 | 0.431333358 | 0.999970678 | 0.1 | 0.365 | 0.878 | 0.111 | PMD |
| TRINITY_DN9230_c0_g1_i17 | -1.34239737 | 0.431333358 | 0.999970678 | 0.1 | 0.365 | 0.878 | 0.111 | PMD |
| TRINITY_DN7757_c0_g3_i1 | 1.342339102 | 0.2940397 | 1 | 1.39 | 1.951 | 0.644 | 0.523 | NA |
| TRINITY_DN46_c0_g1_i10 | 1.341996034 | 0.2413776 | 0.999970678 | 1.799 | 0.818 | 0.344 | 0.613 | RRM_1 |
| TRINITY_DN4480_c0_g2_i2 | 1.341866232 | 0.322066318 | 0.999970678 | 0.745 | 1.025 | 0.444 | 0.151 | NA |
| TRINITY_DN5324_c0_g2_i1 | 1.34140868 | 0.299797393 | 0.999970678 | 8.039 | 12.29 | 5.166 | 1.669 | LRRNT_2 |
| TRINITY_DN6094_c0_g1_i1 | -1.34113862 | 0.205198947 | 0.999970678 | 2.834 | 0.857 | 3.9 | 4.725 | NA |
| TRINITY_DN11120_c0_g1_i10 | -1.34100598 | 0.64297325 | 0.999970678 | 25.635 | 0 | 10.777 | 52.852 | NA |
| TRINITY_DN2485_c0_g1_i2 | 1.340682493 | 0.641171871 | 0.999970678 | 0 | 9.974 | 2.489 | 0.824 | NA |
| TRINITY_DN798_c0_g2_i2 | 1.340554939 | 0.211038411 | 0.999970678 | 3.797 | 12.31 | 1.967 | 3.921 | NA |
| TRINITY_DN2626_c0_g2_i1 | 1.340361069 | 0.353247861 | 0.999970678 | 5.16 | 0.779 | 0.978 | 1.206 | Ribosomal_S4 |
| TRINITY_DN11727_c0_g1_i1 | 1.340339042 | 0.163013304 | 0.999970678 | 2.344 | 1.547 | 0.789 | 0.593 | NA |
| TRINITY_DN19619_c0_g1_i1 | -1.33999806 | 0.624198759 | 0.999970678 | 0.972 | 0 | 1.511 | 0.694 | Metallophos |
| TRINITY_DN6948_c0_g1_i2 | -1.33958777 | 0.213477175 | 0.999970678 | 1.78 | 0.621 | 2.244 | 3.448 | NA |
| TRINITY_DN1439_c0_g1_i2 | 1.339315911 | 0.511846855 | 0.999970678 | 2.562 | 56.75 | 16.866 | 2.061 | NA |
| TRINITY_DN5192_c0_g1_i5 | -1.33911845 | 0.74988032 | 0.999970678 | 1.381 | 0 | 0 | 3.549 | NA |
| TRINITY_DN6164_c1_g1_i2 | -1.33905891 | 0.608601529 | 0.999970678 | 0 | 3.272 | 4.211 | 3.046 | NA |
| TRINITY_DN48766_c0_g1_i1 | 1.338890458 | 0.221714406 | 0.999970678 | 4.842 | 7.204 | 1.533 | 2.805 | NA |
| TRINITY_DN3194_c0_g1_i11 | 1.338858635 | 0.22614428 | 0.999970678 | 0.981 | 1.774 | 0.5 | 0.473 | NLE |
| TRINITY_DN7380_c0_g1_i2 | 1.338633683 | 0.188833994 | 0.999970678 | 16.551 | 4.987 | 3.544 | 4.333 | Mem_trans |
| TRINITY_DN192_c0_g1_i3 | -1.33859145 | 0.615635752 | 0.999970678 | 5.441 | 0 | 5.155 | 7.711 | Ribosomal_L3 |
| TRINITY_DN8288_c0_g1_i2 | 1.338232904 | 0.461221593 | 0.999970678 | 0.663 | 10.63 | 0.678 | 3.529 | DUF212 |
| TRINITY_DN23187_c0_g1_i2 | 1.338073287 | 0.216807747 | 0.999970678 | 6.849 | 22.73 | 3.689 | 7.068 | - |
| TRINITY_DN1684_c1_g1_i1 | 1.338039168 | 0.507625898 | 0.999970678 | 2.171 | 4.543 | 2.122 | 0.06 | NA |
| TRINITY_DN7666_c0_g1_i3 | 1.33798503 | 0.753990326 | 0.999970678 | 0 | 3.154 | 0 | 1.227 | NA |
| TRINITY_DN1932_c0_g2_i2 | -1.33774548 | 0.612228985 | 0.999970678 | 4.533 | 0 | 4.933 | 5.449 | NA |
| TRINITY_DN60363_c1_g1_i3 | -1.3375938 | 0.588046362 | 1 | 0.663 | 0.069 | 1.5 | 0.03 | NA |
| TRINITY_DN7872_c0_g1_i1 | 1.337584042 | 0.561799161 | 0.999970678 | 0.091 | 14.75 | 0.478 | 5.188 | Glyco_hydro_16 |
| TRINITY_DN3338_c0_g1_i5 | -1.33740251 | 0.082754872 | 0.999970678 | 1.326 | 1.488 | 3.166 | 3.298 | G6PD_C |
| TRINITY_DN2135_c0_g1_i5 | -1.33734988 | 0.340545351 | 0.999970678 | 0.091 | 0.384 | 0.367 | 0.764 | NA |
| TRINITY_DN6950_c0_g1_i1 | 1.337342212 | 0.646360426 | 0.999970678 | 2.598 | 0.168 | 0 | 1.126 | - |
| TRINITY_DN2690_c0_g1_i12 | -1.33730551 | 0.340810838 | 0.999970678 | 0.436 | 0.128 | 0.589 | 0.744 | - |
| TRINITY_DN284_c0_g1_i11 | 1.337295386 | 0.22927295 | 0.999970678 | 132.6 | 32.35 | 33.142 | 25.848 | NA |
| TRINITY_DN50272_c1_g1_i1 | -1.33721867 | 0.638714498 | 0.999970678 | 0.427 | 0 | 0.1 | 0.985 | Methyltransf_10 |
| TRINITY_DN3240_c0_g1_i13 | -1.33719344 | 0.610665789 | 0.999970678 | 1.272 | 0 | 1.2 | 1.8 | NA |
| TRINITY_DN14858_c0_g1_i4 | -1.33717965 | 0.674539922 | 0.999970678 | 1.944 | 0 | 4.078 | 0.04 | NA |

| TRINITY_DN38797_c0_g1_i1 | -1.33704827 | 0.139305388 | 0.999970678 | 0.699 | 0.798 | 1.455 | 2.021 | Band_7 |
| --- | --- | --- | --- | --- | --- | --- | --- | --- |
| TRINITY_DN12364_c0_g1_i5 | -1.33691746 | 0.46359019 | 0.999970678 | 0.436 | 0.02 | 0.433 | 0.664 | - |
| TRINITY_DN4404_c0_g1_i3 | -1.33668614 | 0.649164921 | 0.999970678 | 0 | 34.46 | 63.318 | 8.928 | NA |
| TRINITY_DN192472_c0_g1_i1 | 1.336667616 | 0.513975253 | 0.999970678 | 1.553 | 39.45 | 0.967 | 14.427 | NA |
| TRINITY_DN3653_c0_g1_i11 | 1.336621492 | 0.419946288 | 0.999970678 | 0.863 | 1.34 | 0.656 | 0.111 | - |
| TRINITY_DN14811_c0_g1_i11 | -1.33661278 | 0.757162542 | 0.999970678 | 0.736 | 0 | 1.589 | 0 | Aldedh |
| TRINITY_DN72_c0_g1_i14 | -1.33638592 | 0.62899668 | 0.999970678 | 1.263 | 1.34 | 0 | 6.625 | NA |
| TRINITY_DN4656_c1_g1_i5 | 1.336369217 | 0.75561347 | 0.999970678 | 0 | 1.439 | 0.467 | 0 | NA |
| TRINITY_DN84_c0_g2_i1 | -1.3363359 | 0.235635216 | 0.999970678 | 2.28 | 3.095 | 2.2 | 10.918 | TPT |
| TRINITY_DN1639_c1_g1_i1 | -1.33630283 | 0.173607018 | 0.999970678 | 17.85 | 6.16 | 20.91 | 35.791 | Pkinase |
| TRINITY_DN10762_c0_g1_i1 | -1.33628007 | 0.328032159 | 0.999970678 | 2.362 | 4.908 | 1.967 | 15.865 | TRAM_LAG1_CLN |
| TRINITY_DN2065_c0_g1_i1 | -1.33615912 | 0.027981208 | 0.999970678 | 12.073 | 10.73 | 23.743 | 28.744 | HAD_2 |
| TRINITY_DN1962_c0_g1_i1 | 1.336146903 | 0.159984051 | 0.999970678 | 48.208 | 21.15 | 7.766 | 18.328 | 60KD_IMP |
| TRINITY_DN934_c0_g1_i13 | -1.33567294 | 0.678319456 | 0.999970678 | 4.051 | 0.069 | 0 | 10.627 | NA |
| TRINITY_DN6502_c0_g1_i2 | -1.33566579 | 0.624537772 | 0.999970678 | 0.681 | 0 | 0.4 | 1.257 | NA |
| TRINITY_DN13928_c0_g1_i3 | -1.33525884 | 0.281938948 | 1 | 0.254 | 0.315 | 0.656 | 0.613 | NA |
| TRINITY_DN5107_c0_g1_i5 | -1.33523963 | 0.61435865 | 0.999970678 | 0 | 0.621 | 0.356 | 1.136 | NUFIP1 |
| TRINITY_DN1265_c0_g1_i1 | -1.33498394 | 0.625961196 | 0.999970678 | 2.48 | 0 | 1 | 5.047 | Sedlin_N |
| TRINITY_DN249_c0_g1_i5 | 1.334852546 | 0.545694279 | 0.999970678 | 0.254 | 17.63 | 0.867 | 5.821 | NA |
| TRINITY_DN1119_c0_g1_i3 | 1.334781242 | 0.378329972 | 0.999970678 | 9.502 | 8.111 | 5.433 | 0.432 | Agenet |
| TRINITY_DN52706_c0_g1_i1 | -1.33420803 | 0.304129114 | 0.999970678 | 0.99 | 2.464 | 1.289 | 7.108 | NA |
| TRINITY_DN17854_c0_g1_i1 | -1.33420156 | 0.511890643 | 0.999970678 | 2.371 | 0.03 | 1.689 | 4.102 | F-box |
| TRINITY_DN13805_c0_g1_i2 | -1.33391242 | 0.248640642 | 0.999970678 | 0.845 | 0.394 | 1.144 | 1.769 | NA |
| TRINITY_DN760_c0_g2_i5 | -1.33369524 | 0.384319357 | 0.999970678 | 1.69 | 0.256 | 0.722 | 4.122 | Cation_ATPase_C |
| TRINITY_DN2847_c0_g3_i1 | -1.33361574 | 0.612436177 | 0.999970678 | 0 | 1.774 | 2 | 1.94 | NA |
| TRINITY_DN4712_c0_g1_i3 | 1.333165097 | 0.18405656 | 0.999970678 | 0.609 | 1.005 | 0.344 | 0.221 | FYRC |
| TRINITY_DN14701_c0_g1_i4 | 1.333103937 | 0.52274477 | 0.999970678 | 0.136 | 13.01 | 0.856 | 4.092 | NA |
| TRINITY_DN8846_c1_g1_i5 | 1.333084679 | 0.6395113 | 0.999970678 | 1.626 | 0 | 0.344 | 0.241 | NA |
| TRINITY_DN9093_c0_g1_i5 | 1.332930443 | 0.619550597 | 0.999970678 | 0.164 | 0.463 | 0 | 0.251 | PPR |
| TRINITY_DN3118_c0_g1_i2 | 1.332881597 | 0.355739503 | 0.999970678 | 2.117 | 7.253 | 2.655 | 0.473 | TPP_enzyme_C |
| TRINITY_DN3449_c0_g1_i1 | 1.332783186 | 0.636908312 | 0.999970678 | 0 | 22.83 | 3.289 | 4.916 | NA |
| TRINITY_DN2906_c1_g1_i1 | 1.332744795 | 0.300885607 | 1 | 0.799 | 0.808 | 0.3 | 0.292 | MAGE |
| TRINITY_DN15692_c0_g1_i2 | -1.3326661 | 0.31719794 | 0.999970678 | 308.17 | 884.4 | 338.557 | 2550.24 | NA |
| TRINITY_DN153_c0_g1_i6 | 1.332588301 | 0.070706151 | 0.999970678 | 10.883 | 7.845 | 2.522 | 4.414 | bZIP_2 |
| TRINITY_DN6747_c0_g1_i2 | 1.332295578 | 0.371771963 | 0.999970678 | 1.635 | 2.346 | 0.244 | 1.287 | NA |
| TRINITY_DN5700_c0_g2_i2 | 1.331913928 | 0.749362464 | 0.999970678 | 0 | 3.558 | 0 | 1.397 | NA |
| TRINITY_DN1254_c0_g1_i17 | -1.33171032 | 0.133761317 | 0.999970678 | 1.408 | 1.646 | 3.422 | 3.479 | NA |
| TRINITY_DN3787_c0_g1_i2 | -1.3316988 | 0.34183523 | 0.999970678 | 3.316 | 3.637 | 1.433 | 15.754 | Cation_efflux |
| TRINITY_DN13092_c0_g1_i8 | -1.33166467 | 0.754331758 | 0.999970678 | 1.581 | 0 | 3.355 | 0 | NA |
| TRINITY_DN4026_c0_g1_i1 | 1.331236768 | 0.162355825 | 0.999970678 | 4.36 | 1.922 | 1.311 | 0.945 | DUF3741 |
| TRINITY_DN490_c0_g1_i17 | 1.331224483 | 0.612355734 | 0.999970678 | 1.417 | 0.779 | 0.722 | 0 | Asp_protease_2 |
| TRINITY_DN373_c0_g1_i3 | 1.331156296 | 0.093618557 | 0.999970678 | 41.259 | 24.02 | 8.388 | 15.985 | NA |
| TRINITY_DN396_c0_g1_i1 | -1.33106669 | 0.130651457 | 0.999970678 | 0.908 | 1.311 | 1.711 | 3.479 | NA |
| TRINITY_DN4949_c0_g1_i3 | 1.330932791 | 0.323492489 | 0.999970678 | 2.898 | 0.601 | 0.667 | 0.593 | NA |
| TRINITY_DN6689_c0_g2_i9 | 1.330907964 | 0.391606662 | 0.999970678 | 4.033 | 4.159 | 2.467 | 0.271 | NA |
| TRINITY_DN2813_c0_g3_i1 | -1.33082431 | 0.750565611 | 0.999970678 | 2.107 | 0 | 0 | 5.379 | NA |
| TRINITY_DN5050_c0_g2_i9 | -1.3306261 | 0.275654357 | 0.999970678 | 0.2 | 0.099 | 0.289 | 0.422 | NA |
| TRINITY_DN15404_c0_g1_i1 | -1.33049815 | 0.277811142 | 0.999970678 | 6.513 | 34.73 | 29.065 | 67.038 | NA |
| TRINITY_DN2084_c0_g1_i7 | 1.330156579 | 0.170656895 | 0.999970678 | 3.906 | 4.888 | 0.8 | 2.534 | Methyltransf_29 |
| TRINITY_DN1771_c1_g1_i2 | -1.33011526 | 0.146750347 | 0.999970678 | 29.241 | 22.6 | 77.74 | 36.183 | - |
| TRINITY_DN90888_c0_g1_i1 | -1.33009522 | 0.302190277 | 0.999970678 | 26.907 | 44.27 | 18.532 | 155.611 | Phi_1 |
| TRINITY_DN8531_c0_g1_i2 | -1.32979794 | 0.662370941 | 0.999970678 | 5.014 | 0 | 0.433 | 11.843 | NA |
| TRINITY_DN2674_c0_g1_i6 | -1.32967779 | 0.752880737 | 0.999970678 | 0 | 1.252 | 2.544 | 0 | zf-FLZ |
| TRINITY_DN2213_c0_g2_i1 | 1.329669409 | 0.620188842 | 0.999970678 | 2.416 | 0.759 | 1.055 | 0 | NA |
| TRINITY_DN1875_c1_g1_i1 | 1.329631558 | 0.295424961 | 0.999970678 | 0.627 | 1.281 | 0.467 | 0.201 | Sugar_tr |
| TRINITY_DN1216_c0_g1_i2 | -1.32961129 | 0.215861098 | 0.999970678 | 5.895 | 23.54 | 32.876 | 33.368 | NA |
| TRINITY_DN16036_c0_g1_i3 | 1.329484922 | 0.494821586 | 0.999970678 | 0.091 | 4.218 | 0.955 | 0.543 | NA |
| TRINITY_DN5231_c0_g1_i3 | 1.329158936 | 0.290640715 | 0.999970678 | 1.435 | 0.611 | 0.389 | 0.332 | NA |
| TRINITY_DN805_c1_g2_i1 | 1.329132079 | 0.755313152 | 0.999970678 | 0 | 2.424 | 0.778 | 0 | NA |
| TRINITY_DN1284_c1_g1_i11 | -1.32912559 | 0.762361422 | 1 | 0.563 | 0 | 0 | 1.448 | NA |
| TRINITY_DN10591_c0_g1_i1 | 1.328994728 | 0.756264417 | 0.999970678 | 3.27 | 0 | 1.1 | 0 | Peroxidase_2 |
| TRINITY_DN27590_c1_g1_i2 | 1.328994728 | 0.756264417 | 0.999970678 | 1.272 | 0 | 0.422 | 0 | SPOC |
| TRINITY_DN8371_c0_g1_i4 | -1.32892496 | 0.372597387 | 0.999970678 | 0.282 | 0.838 | 1.744 | 0.674 | NA |
| TRINITY_DN3185_c0_g1_i1 | 1.328833518 | 0.294265389 | 0.999970678 | 47.509 | 7.815 | 11.544 | 8.345 | HALZ |
| TRINITY_DN2164_c0_g2_i5 | 1.328673519 | 0.219544621 | 0.999970678 | 1.226 | 3.587 | 0.922 | 0.794 | NA |
| TRINITY_DN2240_c0_g2_i3 | -1.32861635 | 0.130755634 | 0.999970678 | 21.91 | 45.7 | 52.108 | 102.638 | NA |
| TRINITY_DN1424_c0_g1_i8 | 1.328586957 | 0.441485758 | 0.999970678 | 8.067 | 146.9 | 34.353 | 16.066 | NA |
| TRINITY_DN4632_c0_g3_i1 | 1.32841082 | 0.549244272 | 0.999970678 | 0.055 | 5.45 | 0.667 | 1.347 | NA |
| TRINITY_DN3259_c0_g1_i1 | 1.32793085 | 0.618950807 | 0.999970678 | 2.889 | 4.336 | 2.344 | 0 | NA |
| TRINITY_DN2195_c2_g1_i4 | -1.32788345 | 0.632135328 | 0.999970678 | 1.154 | 0 | 2.055 | 0.462 | NA |
| TRINITY_DN2350_c0_g1_i3 | -1.32771846 | 0.310203181 | 0.999970678 | 0.936 | 2.237 | 1.533 | 6.123 | DUF1365 |
| TRINITY_DN10547_c0_g1_i1 | 1.327641845 | 0.460853059 | 0.999970678 | 13.862 | 0.591 | 3.644 | 1.418 | NA |
| TRINITY_DN5541_c1_g2_i4 | -1.32764116 | 0.760485645 | 0.999970678 | 0.681 | 0 | 1.467 | 0 | Actin |
| TRINITY_DN3715_c0_g1_i8 | -1.32764116 | 0.760485645 | 0.999970678 | 0.89 | 0 | 1.867 | 0 | Peroxidase_2 |

| TRINITY_DN2957_c0_g1_i7 | -1.32760994 | 0.757801949 | 0.999970678 | 9.738 | 0 | 20.065 | 0 | EthD |
| --- | --- | --- | --- | --- | --- | --- | --- | --- |
| TRINITY_DN12504_c0_g1_i2 | -1.32747686 | 0.349218371 | 1 | 12.908 | 37.75 | 52.841 | 52.641 | NA |
| TRINITY_DN2177_c0_g1_i3 | 1.327292008 | 0.61641054 | 0.999970678 | 13.235 | 14.95 | 0 | 11.19 | NA |
| TRINITY_DN9987_c0_g1_i8 | 1.327223094 | 0.372026654 | 0.999970678 | 96.19 | 1344 | 214.254 | 265.106 | NA |
| TRINITY_DN2909_c0_g1_i1 | 1.32714842 | 0.58427777 | 0.999970678 | 0.118 | 212.7 | 45.964 | 28.02 | NA |
| TRINITY_DN18674_c0_g1_i1 | -1.32688744 | 0.639196895 | 0.999970678 | 18.649 | 0 | 18.51 | 25.185 | NA |
| TRINITY_DN7897_c0_g1_i4 | -1.3267269 | 0.315908962 | 0.999970678 | 1.017 | 0.434 | 0.8 | 2.714 | OTU |
| TRINITY_DN5894_c0_g1_i2 | 1.32647921 | 0.610681334 | 0.999970678 | 0.391 | 0.798 | 0 | 0.483 | NA |
| TRINITY_DN1059_c0_g1_i1 | 1.326017137 | 0.262130494 | 0.999970678 | 0.972 | 3.784 | 0.611 | 1.146 | NA |
| TRINITY_DN670_c0_g1_i4 | 1.326008357 | 0.640822857 | 0.999970678 | 0 | 8.84 | 0.7 | 2.634 | - |
| TRINITY_DN2631_c0_g1_i1 | -1.32599066 | 0.184452848 | 0.999970678 | 0.818 | 0.769 | 1.7 | 1.93 | NA |
| TRINITY_DN7916_c2_g1_i1 | -1.32584339 | 0.293466358 | 0.999970678 | 454.99 | 786.3 | 2116.55 | 386.816 | NA |
| TRINITY_DN2535_c0_g1_i7 | 1.325631037 | 0.757999562 | 0.999970678 | 0 | 3.164 | 1.033 | 0 | NA |
| TRINITY_DN94_c0_g1_i1 | -1.32558512 | 0.758890752 | 0.999970678 | 0 | 0.621 | 1.278 | 0 | NA |
| TRINITY_DN8790_c0_g1_i7 | 1.325545466 | 0.237761546 | 0.999970678 | 0.999 | 2.217 | 0.367 | 0.834 | NA |
| TRINITY_DN8243_c0_g1_i5 | 1.325437622 | 0.620172972 | 1 | 0.491 | 1.094 | 0 | 0.623 | NA |
| TRINITY_DN731_c0_g1_i9 | 1.325338669 | 0.413063067 | 0.999970678 | 2.78 | 4.79 | 2.333 | 0.221 | TraB |
| TRINITY_DN6594_c0_g1_i5 | -1.3251001 | 0.752976808 | 0.999970678 | 8.593 | 0 | 17.877 | 0 | NA |
| TRINITY_DN24611_c0_g1_i8 | 1.324935985 | 0.376006709 | 0.999970678 | 1.771 | 1.114 | 0.8 | 0.201 | Glycolytic |
| TRINITY_DN4903_c0_g1_i14 | -1.32458709 | 0.753718341 | 0.999970678 | 1.399 | 0 | 2.933 | 0 | NA |
| TRINITY_DN66463_c1_g1_i4 | 1.324559765 | 0.263677793 | 0.999970678 | 0.318 | 0.493 | 0.156 | 0.131 | NA |
| TRINITY_DN22333_c1_g1_i4 | -1.32439471 | 0.644888482 | 1 | 0.463 | 0 | 0.8 | 0.181 | AMP-binding |
| TRINITY_DN3701_c0_g1_i3 | -1.32436247 | 0.652375465 | 0.999970678 | 0.954 | 0.335 | 2.744 | 0 | NA |
| TRINITY_DN3750_c0_g1_i2 | 1.323958477 | 0.619396728 | 0.999970678 | 1.381 | 0.572 | 0.644 | 0 | Cpn60_TCP1 |
| TRINITY_DN38665_c0_g3_i1 | -1.32389164 | 0.307557407 | 0.999970678 | 4.124 | 7.244 | 3.089 | 24.682 | NA |
| TRINITY_DN12361_c0_g2_i1 | -1.32363235 | 0.634041187 | 0.999970678 | 0.672 | 0 | 1.167 | 0.312 | NA |
| TRINITY_DN8445_c0_g1_i2 | 1.323590996 | 0.615349515 | 0.999970678 | 0.727 | 0.463 | 0.4 | 0 | KH_1 |
| TRINITY_DN50235_c0_g2_i1 | 1.323540368 | 0.75693935 | 0.999970678 | 0 | 2.523 | 0.822 | 0 | NA |
| TRINITY_DN6392_c0_g1_i3 | -1.3232151 | 0.262482882 | 0.999970678 | 1.163 | 0.463 | 0.944 | 2.946 | FAR1 |
| TRINITY_DN23680_c0_g1_i4 | -1.32317818 | 0.187203462 | 0.999970678 | 0.527 | 0.384 | 1.133 | 0.905 | DUF4283 |
| TRINITY_DN2156_c0_g1_i10 | 1.322963397 | 0.167293217 | 0.999970678 | 2.244 | 2.02 | 0.844 | 0.694 | NA |
| TRINITY_DN4338_c0_g1_i2 | 1.322444763 | 0.647594725 | 0.999970678 | 6.123 | 0 | 0.378 | 2.021 | Abhydrolase_3 |
| TRINITY_DN13_c0_g2_i1 | -1.32210823 | 0.366360808 | 0.999970678 | 1.844 | 6.485 | 2.278 | 17.745 | NA |
| TRINITY_DN9516_c2_g1_i1 | 1.322023422 | 0.305134589 | 0.999970678 | 3.216 | 1.34 | 0.856 | 0.794 | NA |
| TRINITY_DN2757_c0_g1_i9 | 1.321886129 | 0.490093746 | 1 | 0.699 | 0.71 | 0.022 | 0.513 | NA |
| TRINITY_DN12376_c0_g2_i2 | -1.32165391 | 0.616181165 | 0.999970678 | 8.203 | 0 | 6.933 | 12.165 | NA |
| TRINITY_DN1844_c0_g1_i2 | 1.321579502 | 0.352413469 | 0.999970678 | 1.072 | 5.016 | 1.567 | 0.513 | NA |
| TRINITY_DN131_c0_g1_i10 | 1.321491511 | 0.148601474 | 0.999970678 | 12.082 | 5.627 | 3.778 | 2.584 | NA |
| TRINITY_DN10508_c0_g1_i2 | 1.321236602 | 0.321665889 | 0.999970678 | 0.209 | 0.424 | 0.167 | 0.05 | TamB |
| TRINITY_DN8809_c0_g1_i1 | -1.32091896 | 0.379464794 | 0.999970678 | 1.045 | 2.05 | 0.678 | 6.816 | C2 |
| TRINITY_DN133_c0_g1_i6 | -1.32080979 | 0.630619501 | 0.999970678 | 15.524 | 0 | 11.533 | 25.305 | NA |
| TRINITY_DN1813_c0_g2_i1 | 1.320473895 | 0.653156375 | 0.999970678 | 47.327 | 0 | 10.522 | 6.475 | NA |
| TRINITY_DN2104_c0_g2_i1 | 1.320329445 | 0.15789868 | 0.999970678 | 3.198 | 3.203 | 0.633 | 1.8 | LRRNT_2 |
| TRINITY_DN23411_c0_g2_i2 | -1.32028771 | 0.755593374 | 0.999970678 | 1.644 | 0 | 3.444 | 0 | NA |
| TRINITY_DN64931_c0_g1_i1 | -1.32019524 | 0.479579099 | 0.999970678 | 0.273 | 1.725 | 0.267 | 4.625 | NA |
| TRINITY_DN3337_c0_g1_i1 | 1.319969476 | 0.117198947 | 0.999970678 | 3.788 | 5.115 | 1.278 | 2.031 | Fis1_TPR_C |
| TRINITY_DN4583_c0_g1_i1 | 1.319896499 | 0.201766055 | 0.999970678 | 37.217 | 11.66 | 6.766 | 11.461 | Usp |
| TRINITY_DN2959_c0_g1_i2 | 1.319751602 | 0.650714705 | 0.999970678 | 9.747 | 0 | 0.611 | 3.237 | NA |
| TRINITY_DN17677_c0_g2_i1 | -1.31928297 | 0.617227819 | 0.999970678 | 4.351 | 0 | 4.6 | 5.429 | NA |
| TRINITY_DN17591_c0_g1_i5 | -1.31928276 | 0.764879006 | 1 | 0.427 | 0 | 0.9 | 0 | DUF3425 |
| TRINITY_DN9278_c0_g1_i6 | -1.31918992 | 0.756801687 | 0.999970678 | 1.199 | 0 | 2.522 | 0 | FMO-like |
| TRINITY_DN2301_c1_g1_i1 | 1.319122309 | 0.640761939 | 0.999970678 | 16.996 | 0 | 3.778 | 2.363 | NA |
| TRINITY_DN92_c3_g1_i2 | 1.319070804 | 0.615719702 | 0.999970678 | 2.316 | 1.705 | 1.333 | 0 | NA |
| TRINITY_DN4815_c0_g1_i1 | -1.31883745 | 0.333524456 | 0.999970678 | 3.198 | 1.951 | 1.211 | 11.481 | NA |
| TRINITY_DN20100_c0_g1_i2 | 1.318761988 | 0.35540715 | 0.999970678 | 0.69 | 1.912 | 0.2 | 0.794 | Aa_trans |
| TRINITY_DN10352_c0_g1_i1 | 1.318757249 | 0.761585501 | 0.999970678 | 3.07 | 0 | 1.022 | 0 | NA |
| TRINITY_DN1952_c0_g1_i1 | 1.318606982 | 0.362060476 | 0.999970678 | 3.624 | 3.972 | 2.311 | 0.251 | DnaJ |
| TRINITY_DN2060_c0_g1_i3 | 1.318539423 | 0.627779851 | 0.999970678 | 0.527 | 2.484 | 0 | 1.186 | NA |
| TRINITY_DN3252_c0_g1_i6 | 1.31852699 | 0.752755064 | 0.999970678 | 10.374 | 0 | 0 | 4.233 | NA |
| TRINITY_DN9220_c0_g1_i9 | -1.3184085 | 0.766805639 | 1 | 1.998 | 0 | 0 | 4.997 | NA |
| TRINITY_DN5383_c0_g1_i5 | 1.318395471 | 0.451838975 | 0.999970678 | 1.671 | 9.816 | 0.278 | 4.233 | PP2C |
| TRINITY_DN10446_c0_g1_i2 | -1.31832973 | 0.754881941 | 0.999970678 | 0 | 1.133 | 2.311 | 0 | PLAC8 |
| TRINITY_DN4546_c0_g1_i4 | 1.318301431 | 0.320868735 | 0.999970678 | 3.561 | 0.986 | 0.422 | 1.327 | NA |
| TRINITY_DN731_c0_g1_i1 | 1.318226299 | 0.753752726 | 0.999970678 | 3.824 | 0 | 0 | 1.568 | TraB |
| TRINITY_DN2735_c0_g1_i2 | -1.31821212 | 0.083223261 | 0.999970678 | 32.075 | 38.17 | 50.175 | 113.546 | Chalcone_3 |
| TRINITY_DN3636_c0_g1_i2 | -1.31817276 | 0.229385738 | 0.999970678 | 0.872 | 1.035 | 0.978 | 3.579 | NA |
| TRINITY_DN65409_c0_g1_i2 | 1.317722799 | 0.754160678 | 0.999970678 | 2.943 | 0 | 0.989 | 0 | DNA_photolyase |
| TRINITY_DN4891_c0_g1_i2 | -1.31768304 | 0.645579968 | 0.999970678 | 0 | 7.736 | 13.932 | 2.101 | NA |
| TRINITY_DN4801_c0_g1_i1 | -1.31742278 | 0.298036709 | 0.999970678 | 2.08 | 1.449 | 1.6 | 6.746 | NA |
| TRINITY_DN7448_c0_g1_i6 | 1.317402641 | 0.290123592 | 0.999970678 | 2.816 | 0.995 | 0.444 | 0.995 | NA |
| TRINITY_DN108_c3_g1_i2 | 1.317248187 | 0.655798876 | 0.999970678 | 0.309 | 8.397 | 0 | 3.458 | p450 |
| TRINITY_DN8377_c0_g2_i1 | 1.317136793 | 0.762402406 | 0.999970678 | 1.535 | 0 | 0.522 | 0 | NA |
| TRINITY_DN14595_c0_g1_i2 | -1.31688404 | 0.152702213 | 0.999970678 | 517.31 | 428.9 | 1325.364 | 598.476 | NA |
| TRINITY_DN1422_c1_g1_i6 | 1.316871187 | 0.160189988 | 0.999970678 | 2.861 | 3.499 | 0.744 | 1.619 | NA |

| TRINITY_DN5362_c0_g1_i2 | -1.31681148 | 0.1766654 | 0.999970678 | 1.835 | 2.139 | 2.2 | 7.279 | PB1 |
| --- | --- | --- | --- | --- | --- | --- | --- | --- |
| TRINITY_DN7463_c0_g1_i3 | 1.31678465 | 0.495905294 | 0.999970678 | 20.875 | 0.473 | 2.8 | 5.147 | NA |
| TRINITY_DN1637_c0_g1_i9 | 1.316635404 | 0.638015883 | 0.999970678 | 0 | 6.386 | 0.811 | 1.548 | NA |
| TRINITY_DN17181_c0_g1_i3 | -1.31662071 | 0.623418234 | 0.999970678 | 0.672 | 0 | 1.011 | 0.493 | MMR_HSR1 |
| TRINITY_DN1297_c0_g1_i4 | 1.316441605 | 0.047539429 | 0.999970678 | 21.783 | 21.48 | 7.811 | 7.812 | NA |
| TRINITY_DN49_c0_g1_i2 | 1.316441213 | 0.163834187 | 0.999970678 | 1.081 | 2.119 | 0.544 | 0.623 | G-alpha |
| TRINITY_DN35858_c0_g5_i1 | 1.316212118 | 0.303031628 | 0.999970678 | 3.179 | 3.706 | 1.978 | 0.372 | GYF |
| TRINITY_DN1555_c0_g1_i1 | 1.316051604 | 0.614203895 | 0.999970678 | 0.854 | 1.202 | 0.678 | 0 | NA |
| TRINITY_DN6221_c0_g1_i5 | -1.31580502 | 0.633471708 | 1 | 1.354 | 0 | 0.8 | 2.433 | ubiquitin |
| TRINITY_DN4242_c0_g2_i1 | 1.315650086 | 0.182433333 | 0.999970678 | 3.198 | 4.632 | 0.911 | 1.991 | NA |
| TRINITY_DN1090_c0_g1_i2 | -1.31564308 | 0.129330526 | 0.999970678 | 1.135 | 1.941 | 3.889 | 2.916 | NA |
| TRINITY_DN481_c0_g2_i2 | -1.31558941 | 0.764900103 | 0.999970678 | 1.399 | 0 | 2.944 | 0 | NA |
| TRINITY_DN4482_c0_g1_i4 | -1.31558941 | 0.764900103 | 0.999970678 | 0.454 | 0 | 0.933 | 0 | NA |
| TRINITY_DN10835_c0_g1_i8 | -1.31558941 | 0.764900103 | 0.999970678 | 0.609 | 0 | 1.267 | 0 | NA |
| TRINITY_DN4532_c0_g1_i5 | -1.31558941 | 0.764900103 | 0.999970678 | 1.363 | 0 | 2.789 | 0 | NA |
| TRINITY_DN73_c1_g2_i2 | 1.315474336 | 0.627876707 | 0.999970678 | 0.336 | 1.38 | 0.567 | 0 | DSPc |
| TRINITY_DN2392_c0_g1_i2 | 1.315280553 | 0.754473686 | 0.999970678 | 0 | 16.78 | 5.455 | 0 | NA |
| TRINITY_DN9435_c0_g1_i5 | -1.31520551 | 0.638684352 | 0.999970678 | 0.845 | 0 | 0.244 | 1.86 | NA |
| TRINITY_DN1290_c0_g1_i2 | -1.31520111 | 0.364335837 | 0.999970678 | 0.227 | 1.055 | 1.9 | 0.814 | DUF1712 |
| TRINITY_DN6171_c0_g1_i9 | 1.315127073 | 0.466684412 | 0.999970678 | 1.617 | 0.168 | 0.1 | 0.603 | EF_assoc_1 |
| TRINITY_DN7363_c0_g1_i2 | 1.314936493 | 0.125620486 | 0.999970678 | 1.254 | 1.104 | 0.411 | 0.462 | FA_desaturase_2 |
| TRINITY_DN1293_c0_g1_i4 | 1.314550045 | 0.617725156 | 0.999970678 | 0.709 | 1.38 | 0.7 | 0 | NA |
| TRINITY_DN7266_c0_g1_i4 | 1.314523883 | 0.45536795 | 0.999970678 | 1.281 | 0.729 | 0.044 | 0.764 | dCMP_cyt_deam_ |
| TRINITY_DN519_c0_g1_i2 | -1.31451182 | 0.233878601 | 0.999970678 | 0.418 | 1.045 | 1.855 | 1.377 | NA |
| TRINITY_DN105_c0_g1_i2 | 1.314395868 | 0.175577125 | 0.999970678 | 6.177 | 11.29 | 1.7 | 4.936 | NA |
| TRINITY_DN7101_c0_g1_i4 | -1.31431816 | 0.631230616 | 0.999970678 | 2.553 | 0 | 0.944 | 5.318 | NA |
| TRINITY_DN8313_c0_g1_i4 | 1.31428596 | 0.610858152 | 0.999970678 | 1.354 | 2.365 | 0 | 1.488 | PPR |
| TRINITY_DN668_c0_g1_i20 | 1.314135165 | 0.614679918 | 0.999970678 | 1.535 | 1.173 | 0.911 | 0 | NA |
| TRINITY_DN1875_c1_g1_i7 | 1.313852026 | 0.625387429 | 0.999970678 | 1.108 | 0.404 | 0.5 | 0 | Sugar_tr |
| TRINITY_DN428_c0_g1_i2 | -1.31384982 | 0.042123354 | 0.999970678 | 33.129 | 49.79 | 85.461 | 101.572 | GTP_EFTU |
| TRINITY_DN8942_c0_g1_i1 | 1.313781001 | 0.308399459 | 0.999970678 | 1.998 | 1.744 | 1.022 | 0.282 | Glyoxalase |
| TRINITY_DN2478_c0_g1_i1 | 1.313688441 | 0.309401055 | 0.999970678 | 47.545 | 36.61 | 24.743 | 4.042 | NA |
| TRINITY_DN3629_c0_g1_i3 | 1.313534774 | 0.216012925 | 0.999970678 | 2.571 | 1.153 | 0.456 | 0.945 | NA |
| TRINITY_DN22491_c1_g2_i2 | -1.31344429 | 0.75883782 | 0.999970678 | 1.099 | 0 | 2.311 | 0 | NA |
| TRINITY_DN5267_c0_g1_i1 | 1.313245584 | 0.514903882 | 0.999970678 | 0.627 | 26.49 | 0.811 | 9.782 | Pyridoxal_deC |
| TRINITY_DN688_c0_g1_i1 | -1.31323201 | 0.654610472 | 0.999970678 | 0.89 | 0.168 | 0 | 2.664 | Pkinase |
| TRINITY_DN4855_c0_g1_i1 | -1.31291349 | 0.32080083 | 0.999970678 | 1.081 | 1.301 | 0.7 | 5.067 | Lectin_legB |
| TRINITY_DN17050_c0_g1_i5 | 1.312771933 | 0.575738661 | 0.999970678 | 0.091 | 1.537 | 0.5 | 0.03 | NA |
| TRINITY_DN869_c0_g1_i2 | -1.31268003 | 0.622073243 | 0.999970678 | 3.615 | 0 | 4.877 | 3.227 | NA |
| TRINITY_DN11692_c0_g1_i5 | 1.312640342 | 0.169602977 | 0.999970678 | 3.779 | 3.095 | 1.655 | 0.784 | DEAD |
| TRINITY_DN1141_c0_g2_i1 | 1.312592805 | 0.754476466 | 0.999970678 | 0 | 0.798 | 0.267 | 0 | NA |
| TRINITY_DN4609_c0_g1_i5 | 1.312450504 | 0.321633881 | 0.999970678 | 0.627 | 0.769 | 0.367 | 0.131 | NA |
| TRINITY_DN651_c0_g1_i8 | -1.31229635 | 0.369267935 | 0.999970678 | 19.449 | 6.061 | 47.075 | 6.826 | NAD-GH |
| TRINITY_DN15328_c0_g1_i1 | 1.3120031 | 0.757482568 | 0.999970678 | 3.079 | 0 | 1.044 | 0 | NA |
| TRINITY_DN2370_c0_g1_i3 | -1.31192005 | 0.24682127 | 0.999970678 | 2.516 | 0.877 | 3.489 | 4.192 | NA |
| TRINITY_DN80_c0_g1_i4 | -1.3118752 | 0.370426278 | 0.999970678 | 12.49 | 50.1 | 14.532 | 136.348 | Epimerase |
| TRINITY_DN2057_c0_g1_i8 | -1.31172809 | 0.630497788 | 1 | 0.463 | 0.01 | 0.478 | 0.603 | NA |
| TRINITY_DN26184_c0_g1_i3 | -1.31165476 | 0.525476725 | 0.999970678 | 1.744 | 0.099 | 3.389 | 0.553 | NA |
| TRINITY_DN2443_c0_g1_i7 | 1.311544257 | 0.660691331 | 0.999970678 | 4.442 | 0 | 0.2 | 1.578 | NA |
| TRINITY_DN11213_c0_g2_i3 | -1.31148326 | 0.474895776 | 0.999970678 | 2.098 | 0.779 | 5.5 | 0.473 | Glyco_hydro_1 |
| TRINITY_DN28025_c0_g1_i3 | -1.31146587 | 0.758987184 | 0.999970678 | 2.316 | 0 | 4.777 | 0 | Abhydrolase_1 |
| TRINITY_DN4598_c1_g1_i3 | 1.311457106 | 0.411130927 | 0.999970678 | 0.681 | 5.568 | 1.3 | 0.935 | NA |
| TRINITY_DN7144_c0_g1_i9 | 1.311362804 | 0.669291171 | 0.999970678 | 3.506 | 0.158 | 1.255 | 0 | NA |
| TRINITY_DN20_c1_g1_i12 | 1.311334504 | 0.765354583 | 0.999970678 | 1.272 | 0 | 0.433 | 0 | NA |
| TRINITY_DN1719_c0_g1_i10 | 1.31121884 | 0.195898008 | 0.999970678 | 1.099 | 2.72 | 0.722 | 0.653 | NA |
| TRINITY_DN43_c0_g2_i4 | 1.311111948 | 0.052759446 | 0.999970678 | 11.2 | 17.48 | 5.166 | 5.318 | OPT |
| TRINITY_DN863_c0_g1_i13 | -1.3110407 | 0.621688516 | 0.999970678 | 1.681 | 0 | 1.433 | 2.523 | BACK |
| TRINITY_DN3022_c0_g1_i5 | -1.3110214 | 0.396689814 | 0.999970678 | 2.789 | 0.483 | 1.333 | 6.535 | NA |
| TRINITY_DN8005_c0_g1_i2 | -1.31090008 | 0.277386024 | 0.999970678 | 0.309 | 0.729 | 0.722 | 1.699 | NA |
| TRINITY_DN2192_c0_g2_i2 | -1.31081408 | 0.360831588 | 0.999970678 | 1.635 | 1.124 | 4.833 | 0.895 | - |
| TRINITY_DN1215_c0_g1_i2 | -1.31052538 | 0.267806052 | 0.999970678 | 0.627 | 0.306 | 1.1 | 0.995 | WD40 |
| TRINITY_DN23_c1_g1_i8 | 1.310276901 | 0.450722634 | 0.999970678 | 1.562 | 0.128 | 0.2 | 0.432 | NA |
| TRINITY_DN439_c0_g1_i8 | -1.31021853 | 0.620591905 | 0.999970678 | 1.88 | 0 | 1.367 | 3.066 | NA |
| TRINITY_DN11648_c0_g1_i6 | 1.310129206 | 0.36716402 | 0.999970678 | 3.188 | 32.57 | 5.855 | 7.058 | NA |
| TRINITY_DN6268_c0_g1_i6 | 1.3100559 | 0.300513373 | 1 | 2.907 | 4.12 | 1.144 | 1.438 | NA |
| TRINITY_DN1054_c1_g1_i4 | 1.31001765 | 0.619249774 | 0.999970678 | 1.79 | 1.084 | 0.967 | 0 | NA |
| TRINITY_DN693_c0_g1_i13 | 1.309856852 | 0.109680597 | 0.999970678 | 11.346 | 12.16 | 4.911 | 3.509 | - |
| TRINITY_DN1492_c0_g2_i1 | 1.30969932 | 0.209059718 | 0.999970678 | 15.752 | 13.08 | 7.566 | 2.513 | NA |
| TRINITY_DN3021_c0_g1_i3 | 1.309648909 | 0.1276433 | 0.999970678 | 2.58 | 4.041 | 0.889 | 1.578 | NA |
| TRINITY_DN2685_c0_g1_i1 | -1.30954088 | 0.300939466 | 0.999970678 | 2.889 | 9.688 | 5.055 | 24.873 | Methyltransf_11 |
| TRINITY_DN2469_c4_g1_i1 | -1.30947046 | 0.494916344 | 0.999970678 | 2.371 | 1.991 | 0.289 | 10.225 | RVT_1 |
| TRINITY_DN3278_c0_g2_i2 | -1.30946334 | 0.406695664 | 0.999970678 | 2.18 | 0.719 | 4.977 | 1.287 | PTPLA |
| TRINITY_DN2626_c0_g5_i1 | -1.30930113 | 0.521588526 | 0.999970678 | 1.081 | 12.64 | 0.844 | 32.041 | NA |
| TRINITY_DN4644_c2_g1_i4 | -1.30903126 | 0.764928328 | 0.999970678 | 0.309 | 0 | 0.656 | 0 | NA |

| TRINITY_DN12049_c0_g1_i2 | 1.309025308 | 0.285818295 | 0.999970678 | 0.99 | 0.345 | 0.2 | 0.312 | NA |
| --- | --- | --- | --- | --- | --- | --- | --- | --- |
| TRINITY_DN224998_c0_g1_i1 | 1.309004986 | 0.766520255 | 0.999970678 | 11.927 | 0 | 3.978 | 0 | NA |
| TRINITY_DN731_c0_g1_i5 | -1.30897063 | 0.075711127 | 0.999970678 | 2.053 | 1.685 | 3.578 | 4.987 | TraB |
| TRINITY_DN5310_c0_g1_i4 | -1.30877308 | 0.092015072 | 0.999970678 | 3.361 | 2.07 | 5.677 | 6.666 | XPG_I |
| TRINITY_DN2378_c0_g1_i4 | 1.308762327 | 0.643994662 | 0.999970678 | 0.89 | 0.158 | 0.367 | 0 | zf-C3HC4_3 |
| TRINITY_DN207_c0_g2_i5 | -1.30874327 | 0.25005317 | 0.999970678 | 2.807 | 12.19 | 18.51 | 14.387 | NA |
| TRINITY_DN7769_c0_g1_i13 | -1.30845318 | 0.627672165 | 1 | 0.363 | 0 | 0.389 | 0.452 | NA |
| TRINITY_DN100300_c0_g1_i2 | -1.3082663 | 0.640110504 | 0.999970678 | 183.84 | 0 | 183.389 | 222.9 | NA |
| TRINITY_DN3651_c1_g1_i1 | -1.3082402 | 0.76137434 | 0.999970678 | 3.679 | 0 | 7.488 | 0 | Glyoxalase |
| TRINITY_DN2117_c1_g1_i2 | 1.308124204 | 0.334773509 | 0.999970678 | 20.521 | 9.432 | 8.833 | 1.478 | NA |
| TRINITY_DN1194_c0_g1_i2 | -1.30769917 | 0.291022541 | 0.999970678 | 263.24 | 125.5 | 125.981 | 806.849 | NA |
| TRINITY_DN136456_c0_g1_i1 | 1.307657926 | 0.628845482 | 0.999970678 | 13.481 | 7.618 | 0 | 8.576 | NA |
| TRINITY_DN4376_c0_g1_i4 | 1.307593393 | 0.209497763 | 0.999970678 | 0.845 | 1.449 | 0.433 | 0.392 | NA |
| TRINITY_DN3277_c0_g1_i1 | -1.30724126 | 0.049946463 | 0.999970678 | 2.989 | 3.173 | 5.944 | 8.093 | NA |
| TRINITY_DN468_c0_g2_i2 | -1.30722806 | 0.632164993 | 0.999970678 | 8.739 | 0 | 13.921 | 5.037 | NA |
| TRINITY_DN16034_c0_g1_i13 | -1.30709513 | 0.445985694 | 0.999970678 | 1.608 | 0.256 | 0.578 | 3.871 | NA |
| TRINITY_DN397_c0_g1_i10 | -1.3070815 | 0.509855242 | 0.999970678 | 8.648 | 121 | 8.299 | 306.859 | PUNUT |
| TRINITY_DN3911_c1_g1_i2 | -1.30663772 | 0.631387348 | 0.999970678 | 0 | 6.041 | 9.699 | 2.986 | NA |
| TRINITY_DN1626_c0_g1_i1 | -1.30656445 | 0.236128351 | 0.999970678 | 1.799 | 2.651 | 2.3 | 8.133 | NA |
| TRINITY_DN13541_c0_g1_i2 | -1.30647832 | 0.372979915 | 0.999970678 | 33.483 | 9.983 | 9.377 | 97.38 | NA |
| TRINITY_DN6665_c0_g1_i23 | 1.306353946 | 0.630628091 | 1 | 0.79 | 0.887 | 0.578 | 0 | NA |
| TRINITY_DN749_c0_g2_i2 | -1.30635091 | 0.082467111 | 0.999970678 | 35.664 | 21.86 | 51.63 | 77.997 | NA |
| TRINITY_DN2449_c0_g1_i10 | -1.3063394 | 0.241039879 | 0.999970678 | 0.5 | 0.443 | 0.978 | 1.106 | NA |
| TRINITY_DN6724_c0_g1_i3 | -1.30602806 | 0.758109406 | 0.999970678 | 0.936 | 0 | 1.944 | 0 | Aft1_HRA |
| TRINITY_DN9067_c0_g1_i7 | 1.305891582 | 0.618147377 | 0.999970678 | 0.881 | 0.572 | 0.489 | 0 | NA |
| TRINITY_DN5066_c1_g1_i14 | -1.30586139 | 0.638453046 | 0.999970678 | 0.318 | 0.434 | 0 | 1.86 | NA |
| TRINITY_DN175_c0_g1_i10 | 1.30584592 | 0.097290192 | 0.999970678 | 8.684 | 9.215 | 3.055 | 3.398 | NA |
| TRINITY_DN1597_c0_g1_i6 | -1.30571689 | 0.755127149 | 0.999970678 | 3.879 | 0 | 0 | 9.631 | NA |
| TRINITY_DN4515_c7_g1_i2 | 1.305689001 | 0.210586114 | 0.999970678 | 1.163 | 2.759 | 0.689 | 0.744 | NA |
| TRINITY_DN10627_c0_g1_i1 | 1.30543929 | 0.764965236 | 0.999970678 | 1.626 | 0 | 0 | 0.674 | DUF707 |
| TRINITY_DN2329_c0_g1_i1 | -1.30543894 | 0.312577044 | 0.999970678 | 1.163 | 2.996 | 1.522 | 8.375 | NA |
| TRINITY_DN2338_c0_g1_i5 | -1.30529054 | 0.090078219 | 0.999970678 | 15.742 | 28.65 | 50.541 | 47.162 | NA |
| TRINITY_DN1626_c0_g1_i3 | -1.30476197 | 0.265672152 | 0.999970678 | 4.288 | 1.646 | 8.744 | 4.202 | NA |
| TRINITY_DN2027_c0_g2_i7 | 1.304751276 | 0.238849741 | 0.999970678 | 0.4 | 1.242 | 0.311 | 0.271 | DUF4283 |
| TRINITY_DN533_c0_g1_i7 | -1.30474322 | 0.219933778 | 0.999970678 | 2.48 | 4.001 | 8.811 | 5.007 | NA |
| TRINITY_DN70841_c0_g1_i1 | -1.30469508 | 0.303166352 | 1 | 0.336 | 0.463 | 0.867 | 0.925 | NA |
| TRINITY_DN3738_c0_g1_i1 | -1.30443541 | 0.419363812 | 0.999970678 | 0.827 | 2.937 | 7.022 | 0.774 | NA |
| TRINITY_DN1489_c0_g2_i4 | -1.30440016 | 0.639758674 | 0.999970678 | 0 | 4.208 | 1.111 | 8.938 | MORN |
| TRINITY_DN3378_c0_g1_i1 | -1.30438518 | 0.402123431 | 0.999970678 | 26.552 | 71.04 | 12.888 | 223.343 | NA |
| TRINITY_DN5973_c0_g2_i1 | 1.304322491 | 0.653604995 | 0.999970678 | 0 | 4.514 | 0.233 | 1.508 | NA |
| TRINITY_DN12908_c0_g1_i1 | 1.304155721 | 0.351644156 | 0.999970678 | 2.434 | 1.133 | 0.933 | 0.332 | NA |
| TRINITY_DN12_c4_g1_i1 | -1.30410075 | 0.662610884 | 0.999970678 | 4.669 | 0 | 9.088 | 0.583 | NA |
| TRINITY_DN9639_c0_g1_i3 | 1.304033752 | 0.461759403 | 0.999970678 | 4.914 | 4.622 | 2.922 | 0.312 | NA |
| TRINITY_DN8257_c1_g1_i7 | 1.304025791 | 0.757108395 | 0.999970678 | 5.35 | 0 | 0 | 2.182 | OTU |
| TRINITY_DN17047_c0_g1_i3 | 1.303862503 | 0.633920483 | 0.999970678 | 0.6 | 0.168 | 0.256 | 0 | ABC_membrane |
| TRINITY_DN6330_c0_g1_i2 | -1.3038391 | 0.250107765 | 0.999970678 | 0.336 | 0.453 | 0.889 | 0.804 | - |
| TRINITY_DN7400_c0_g1_i2 | -1.30378272 | 0.326415971 | 0.999970678 | 2.634 | 3.735 | 10.955 | 2.383 | NA |
| TRINITY_DN13894_c0_g1_i1 | -1.30365198 | 0.27549921 | 0.999970678 | 1.381 | 3.233 | 2.011 | 8.908 | Na_Ca_ex |
| TRINITY_DN6608_c0_g1_i4 | 1.30353465 | 0.756084063 | 0.999970678 | 3.924 | 0 | 0 | 1.629 | TRAPPC-Trs85 |
| TRINITY_DN1587_c0_g2_i1 | -1.30342826 | 0.479248939 | 0.999970678 | 0.136 | 0.266 | 0.789 | 0.04 | - |
| TRINITY_DN1015_c0_g2_i2 | -1.30338441 | 0.624893842 | 0.999970678 | 0.899 | 0 | 1.255 | 0.734 | NA |
| TRINITY_DN16120_c0_g2_i1 | -1.30323665 | 0.622534846 | 0.999970678 | 3.788 | 0 | 5.044 | 3.378 | NA |
| TRINITY_DN1049_c0_g1_i1 | 1.303140918 | 0.649851927 | 0.999970678 | 1.517 | 0 | 0.167 | 0.432 | - |
| TRINITY_DN6522_c0_g1_i1 | -1.30305776 | 0.211180183 | 0.999970678 | 2.961 | 1.291 | 2.467 | 7.641 | ABC2_membrane |
| TRINITY_DN11640_c0_g1_i7 | -1.3030408 | 0.060745582 | 0.999970678 | 38.616 | 41.12 | 95.283 | 76.016 | NA |
| TRINITY_DN4176_c0_g1_i4 | 1.302801737 | 0.407414398 | 0.999970678 | 2.943 | 6.169 | 2.855 | 0.231 | KH_1 |
| TRINITY_DN3393_c0_g3_i1 | -1.30269373 | 0.505291708 | 0.999970678 | 46.473 | 2.927 | 97.272 | 5.64 | NA |
| TRINITY_DN2384_c0_g1_i4 | 1.302593238 | 0.411471273 | 0.999970678 | 6.849 | 11.2 | 5.7 | 0.392 | RRM_1 |
| TRINITY_DN2692_c0_g1_i2 | 1.30241851 | 0.14986583 | 0.999970678 | 7.376 | 15.95 | 4.789 | 3.629 | NA |
| TRINITY_DN17577_c0_g1_i1 | -1.30240285 | 0.212844687 | 0.999970678 | 0.917 | 0.581 | 1.033 | 2.453 | NTP_transferase |
| TRINITY_DN2935_c0_g1_i2 | -1.30237851 | 0.632165664 | 0.999970678 | 17.06 | 0 | 25.154 | 11.914 | NA |
| TRINITY_DN6098_c0_g1_i4 | -1.30216424 | 0.771170028 | 1 | 0.354 | 0 | 0.733 | 0 | DUF4449 |
| TRINITY_DN166288_c0_g1_i1 | -1.30216424 | 0.771170028 | 1 | 0.354 | 0 | 0.722 | 0 | NA |
| TRINITY_DN27686_c0_g1_i1 | -1.30171086 | 0.247374585 | 0.999970678 | 21.029 | 60.48 | 45.864 | 134.428 | PsbQ |
| TRINITY_DN2353_c0_g1_i5 | -1.30157686 | 0.497262019 | 0.999970678 | 44.139 | 0.532 | 55.019 | 45.362 | FAF |
| TRINITY_DN8104_c0_g1_i5 | 1.301540729 | 0.481252048 | 0.999970678 | 5.587 | 0.375 | 0.578 | 1.719 | NA |
| TRINITY_DN4706_c0_g1_i4 | 1.301374706 | 0.757099901 | 0.999970678 | 0 | 1.508 | 0.5 | 0 | NA |
| TRINITY_DN8613_c0_g2_i1 | 1.301335117 | 0.324003547 | 1 | 0.418 | 0.424 | 0.144 | 0.171 | NA |
| TRINITY_DN3931_c0_g1_i2 | -1.30131227 | 0.631894505 | 0.999970678 | 1.599 | 0 | 0.989 | 2.795 | NA |
| TRINITY_DN5266_c0_g1_i13 | -1.30128941 | 0.213559862 | 0.999970678 | 0.663 | 1.183 | 2.355 | 1.659 | NA |
| TRINITY_DN1143_c0_g1_i2 | 1.30124247 | 0.438217809 | 0.999970678 | 2.825 | 0.384 | 0.2 | 1.066 | NA |
| TRINITY_DN10826_c0_g1_i1 | 1.301195869 | 0.269504578 | 0.999970678 | 26.752 | 9.806 | 9.611 | 3.318 | NA |
| TRINITY_DN24607_c0_g1_i2 | -1.30115039 | 0.179330519 | 0.999970678 | 8.375 | 5.253 | 18.321 | 10.989 | NA |
| TRINITY_DN2877_c0_g1_i10 | -1.30113038 | 0.434157097 | 0.999970678 | 6.041 | 1.478 | 1.255 | 17.111 | GST_C |

| TRINITY_DN10011_c0_g2_i2 | -1.30098613 | 0.757040222 | 0.999970678 | 0 | 6.209 | 0 | 15.091 | C2 |
| --- | --- | --- | --- | --- | --- | --- | --- | --- |
| TRINITY_DN1281_c0_g1_i4 | 1.300830335 | 0.283208428 | 0.999970678 | 3.198 | 1.735 | 0.467 | 1.468 | HMG_box |
| TRINITY_DN35196_c0_g1_i7 | -1.30079586 | 0.364751759 | 0.999970678 | 1.045 | 0.355 | 2.344 | 0.643 | NA |
| TRINITY_DN1328_c0_g1_i2 | -1.30045511 | 0.155016035 | 0.999970678 | 1.553 | 1.754 | 4.389 | 2.845 | zf-RING_2 |
| TRINITY_DN79209_c1_g1_i1 | -1.30041961 | 0.25587536 | 0.999970678 | 3.988 | 6.711 | 11.455 | 11.24 | UDPGT |
| TRINITY_DN3244_c0_g3_i6 | 1.300357431 | 0.756997201 | 0.999970678 | 11.827 | 0 | 0 | 4.896 | - |
| TRINITY_DN17879_c0_g1_i6 | -1.30021951 | 0.438100785 | 0.999970678 | 0.118 | 1.045 | 1.733 | 0.784 | NA |
| TRINITY_DN11640_c0_g1_i5 | -1.2999966 | 0.210999372 | 0.999970678 | 24.69 | 17.56 | 63.452 | 25.154 | NA |
| TRINITY_DN10742_c0_g1_i3 | -1.29983561 | 0.211862901 | 0.999970678 | 1.272 | 2.434 | 2.244 | 6.414 | NA |
| TRINITY_DN7926_c0_g1_i9 | 1.299768067 | 0.661568995 | 0.999970678 | 5.096 | 0.424 | 1.911 | 0 | NA |
| TRINITY_DN7516_c1_g1_i8 | -1.29959329 | 0.489743105 | 0.999970678 | 0.227 | 1.567 | 0.244 | 4.072 | NA |
| TRINITY_DN3393_c0_g2_i1 | -1.29957613 | 0.382836871 | 0.999970678 | 58.873 | 23.02 | 153.146 | 16.981 | NA |
| TRINITY_DN5842_c0_g1_i1 | -1.29924748 | 0.62152239 | 0.999970678 | 1.49 | 0 | 1.733 | 1.629 | Spermine_synth |
| TRINITY_DN468_c0_g2_i5 | -1.29916052 | 0.273042759 | 0.999970678 | 57.656 | 60.54 | 37.998 | 233.728 | NA |
| TRINITY_DN8135_c0_g1_i1 | 1.298973209 | 0.441638403 | 0.999970678 | 0.354 | 3.085 | 0.333 | 0.955 | NA |
| TRINITY_DN226_c0_g1_i3 | 1.298662931 | 0.306560064 | 0.999970678 | 2.28 | 0.542 | 0.611 | 0.412 | ABC2_membrane |
| TRINITY_DN18782_c0_g1_i4 | -1.29854202 | 0.770930927 | 1 | 0.79 | 0 | 1.578 | 0 | Hpt |
| TRINITY_DN12070_c0_g1_i4 | -1.2977892 | 0.695567471 | 0.999970678 | 2.861 | 0.03 | 5.955 | 0 | NA |
| TRINITY_DN3732_c1_g1_i2 | 1.297777199 | 0.488416308 | 0.999970678 | 3.025 | 0.601 | 1.078 | 0.201 | HLH |
| TRINITY_DN19357_c0_g5_i2 | 1.29770796 | 0.186125888 | 0.999970678 | 1.417 | 0.887 | 0.344 | 0.523 | NA |
| TRINITY_DN6346_c0_g1_i1 | -1.29769888 | 0.762184296 | 0.999970678 | 0 | 0.936 | 1.878 | 0 | S6PP |
| TRINITY_DN13289_c0_g1_i1 | 1.297564217 | 0.510857969 | 1 | 0.563 | 0.562 | 0.356 | 0.03 | NA |
| TRINITY_DN1674_c0_g1_i1 | 1.297005625 | 0.478892805 | 0.999970678 | 0.781 | 9.471 | 0.433 | 3.589 | rRNA_processing |
| TRINITY_DN5337_c0_g1_i6 | 1.296926802 | 0.268658423 | 0.999970678 | 1.844 | 3.42 | 1.4 | 0.442 | NA |
| TRINITY_DN188_c0_g1_i2 | -1.29687115 | 0.294980804 | 0.999970678 | 9.238 | 3.449 | 5.355 | 24.823 | NA |
| TRINITY_DN10094_c0_g1_i4 | 1.296791811 | 0.263875231 | 0.999970678 | 6.867 | 20.31 | 2.167 | 8.385 | NA |
| TRINITY_DN11674_c0_g1_i10 | -1.29651919 | 0.631082701 | 0.999970678 | 1.853 | 0 | 0.878 | 3.549 | Pkinase |
| TRINITY_DN7042_c0_g1_i7 | 1.296503888 | 0.291530525 | 1 | 1.145 | 0.729 | 0.333 | 0.382 | EMP70 |
| TRINITY_DN1429_c1_g1_i2 | -1.2960369 | 0.62822724 | 0.999970678 | 0 | 2.07 | 3.111 | 1.247 | NA |
| TRINITY_DN11507_c0_g1_i4 | 1.295996525 | 0.651214812 | 0.999970678 | 1.626 | 0 | 0.411 | 0.171 | SHR-BD |
| TRINITY_DN11288_c0_g2_i7 | 1.295893219 | 0.651127282 | 0.999970678 | 1.553 | 0.187 | 0 | 0.704 | NA |
| TRINITY_DN8296_c0_g1_i3 | 1.295874862 | 0.544167856 | 0.999970678 | 10.328 | 0.296 | 3.389 | 0.282 | peroxidase |
| TRINITY_DN948_c0_g2_i6 | 1.295864051 | 0.292636261 | 0.999970678 | 3.543 | 2.336 | 1.678 | 0.382 | TPR_MLP1_2 |
| TRINITY_DN11559_c0_g4_i1 | -1.295723 | 0.390601709 | 0.999970678 | 3.543 | 0.946 | 7.288 | 2.202 | NA |
| TRINITY_DN967_c3_g1_i1 | 1.295704325 | 0.315083456 | 0.999970678 | 2.762 | 2.602 | 1.355 | 0.543 | - |
| TRINITY_DN17752_c0_g1_i2 | 1.295578569 | 0.175312101 | 0.999970678 | 2.671 | 6.169 | 1.322 | 2.001 | NA |
| TRINITY_DN3600_c0_g1_i4 | 1.295459742 | 0.239234304 | 0.999970678 | 1.844 | 3.696 | 0.656 | 1.428 | NA |
| TRINITY_DN1215_c0_g1_i4 | -1.29539228 | 0.420923689 | 0.999970678 | 0.5 | 0.069 | 0.433 | 0.885 | WD40 |
| TRINITY_DN1888_c0_g2_i2 | -1.29514301 | 0.637673027 | 0.999970678 | 3.161 | 0 | 1.733 | 5.459 | NA |
| TRINITY_DN10002_c0_g1_i1 | -1.2950315 | 0.636966953 | 0.999970678 | 0.999 | 0 | 0.389 | 2.011 | Pkinase |
| TRINITY_DN2273_c0_g1_i1 | 1.294937091 | 0.169261536 | 0.999970678 | 13.508 | 29.95 | 9.811 | 5.771 | CBS |
| TRINITY_DN14994_c0_g1_i4 | -1.29472801 | 0.48928265 | 0.999970678 | 2.017 | 0.049 | 2.333 | 2.312 | SAND |
| TRINITY_DN9860_c0_g1_i4 | 1.294627698 | 0.299477578 | 0.999970678 | 3.915 | 2.572 | 0.433 | 2.111 | NA |
| TRINITY_DN2270_c0_g1_i2 | 1.29400476 | 0.386723312 | 0.999970678 | 0.754 | 1.459 | 0.622 | 0.131 | TPT |
| TRINITY_DN4747_c0_g3_i1 | 1.293891258 | 0.368918661 | 0.999970678 | 23.473 | 4.001 | 6.855 | 2.916 | NA |
| TRINITY_DN2712_c0_g1_i3 | 1.293833966 | 0.179888658 | 0.999970678 | 4.215 | 2.257 | 0.778 | 1.719 | DSPc |
| TRINITY_DN6750_c0_g1_i4 | -1.29377028 | 0.449752991 | 0.999970678 | 0.182 | 1.804 | 3.122 | 1.005 | NA |
| TRINITY_DN1334_c0_g1_i7 | 1.293748 | 0.390777358 | 0.999970678 | 1.117 | 9.974 | 2.667 | 1.227 | NA |
| TRINITY_DN1441_c0_g1_i5 | -1.29362419 | 0.635289746 | 0.999970678 | 13.008 | 0 | 7.8 | 22.721 | NA |
| TRINITY_DN87_c0_g1_i4 | -1.29347899 | 0.630559255 | 0.999970678 | 0.954 | 0 | 1 | 1.136 | Ras |
| TRINITY_DN1861_c0_g2_i2 | 1.293233251 | 0.268576792 | 0.999970678 | 0.627 | 0.798 | 0.278 | 0.261 | DDOST_48kD |
| TRINITY_DN2253_c1_g2_i1 | 1.293114952 | 0.448069035 | 1 | 0.772 | 0.946 | 0.089 | 0.583 | 4HBT |
| TRINITY_DN34795_c0_g1_i4 | 1.292995743 | 0.588172731 | 0.999970678 | 0.018 | 1.646 | 0.067 | 0.593 | NA |
| TRINITY_DN1315_c0_g1_i2 | -1.29299414 | 0.485487107 | 0.999970678 | 3.597 | 0.394 | 0.5 | 9.31 | DUF789 |
| TRINITY_DN1363_c0_g1_i5 | 1.292943233 | 0.681793566 | 0.999970678 | 29.15 | 0.256 | 0 | 11.944 | Dimerisation |
| TRINITY_DN63052_c0_g1_i6 | 1.292696948 | 0.769392394 | 1 | 0 | 1.547 | 0.511 | 0 | NA |
| TRINITY_DN716_c0_g1_i4 | 1.292676166 | 0.409510301 | 0.999970678 | 2.462 | 0.256 | 0.578 | 0.422 | NA |
| TRINITY_DN920_c0_g1_i3 | 1.292406282 | 0.381944289 | 0.999970678 | 1.562 | 0.227 | 0.367 | 0.282 | FBPase |
| TRINITY_DN9170_c0_g1_i6 | -1.29231271 | 0.640408549 | 0.999970678 | 0.736 | 0 | 0.333 | 1.438 | NA |
| TRINITY_DN7283_c0_g1_i2 | -1.29226724 | 0.630474794 | 0.999970678 | 0.881 | 0 | 1.255 | 0.674 | NA |
| TRINITY_DN13966_c0_g1_i6 | 1.292233002 | 0.276210792 | 0.999970678 | 0.854 | 0.335 | 0.233 | 0.211 | PP2C |
| TRINITY_DN9802_c0_g1_i5 | -1.29220368 | 0.764986721 | 0.999970678 | 4.587 | 0 | 9.188 | 0 | NA |
| TRINITY_DN4494_c0_g1_i7 | -1.29213005 | 0.759106721 | 0.999970678 | 2.78 | 0 | 5.744 | 0 | Asp |
| TRINITY_DN6437_c0_g2_i1 | 1.291874629 | 0.398472171 | 0.999970678 | 1.099 | 0.365 | 0.4 | 0.121 | NA |
| TRINITY_DN384_c0_g2_i1 | 1.291816162 | 0.377886066 | 0.999970678 | 0.781 | 1.252 | 0.111 | 0.694 | NA |
| TRINITY_DN2160_c0_g1_i7 | -1.29163142 | 0.438112392 | 0.999970678 | 0.073 | 1.222 | 1 | 1.93 | NA |
| TRINITY_DN10015_c0_g1_i3 | 1.291610447 | 0.635158195 | 0.999970678 | 0.69 | 0.256 | 0.311 | 0 | NA |
| TRINITY_DN12487_c0_g1_i1 | 1.291552507 | 0.620642622 | 0.999970678 | 0.491 | 0.611 | 0.367 | 0 | NA |
| TRINITY_DN22_c0_g1_i1 | 1.291520698 | 0.111029269 | 0.999970678 | 1.771 | 1.606 | 0.622 | 0.623 | BET |
| TRINITY_DN184_c0_g4_i6 | -1.29145666 | 0.647504403 | 0.999970678 | 0 | 21.59 | 33.376 | 11.592 | Ribosomal_L31e |
| TRINITY_DN3819_c0_g2_i6 | -1.29141956 | 0.767334743 | 0.999970678 | 0.709 | 0 | 0 | 1.759 | Cellulose_synt |
| TRINITY_DN23_c0_g2_i1 | -1.29115334 | 0.096693086 | 0.999970678 | 9.911 | 5.391 | 13.433 | 21.525 | NA |
| TRINITY_DN5853_c0_g1_i1 | -1.29109794 | 0.350255974 | 0.999970678 | 3.843 | 3.853 | 1.7 | 16.739 | LRR_8 |
| TRINITY_DN4957_c0_g1_i2 | -1.2910098 | 0.646450683 | 0.999970678 | 6.64 | 0 | 1.644 | 14.105 | NA |

| TRINITY_DN2997_c0_g1_i1 | -1.29098325 | 0.2321026 | 0.999970678 | 10.674 | 15.17 | 10.71 | 50.067 | Annexin |
| --- | --- | --- | --- | --- | --- | --- | --- | --- |
| TRINITY_DN25671_c0_g1_i2 | -1.29079279 | 0.63345624 | 1 | 0 | 0.66 | 0.489 | 1.005 | zf-RING_2 |
| TRINITY_DN1898_c0_g1_i4 | 1.29054427 | 0.326746621 | 0.999970678 | 3.443 | 7.983 | 0.955 | 3.418 | NA |
| TRINITY_DN798_c0_g2_i5 | 1.290488237 | 0.643835473 | 0.999970678 | 0 | 1.833 | 0.322 | 0.342 | NA |
| TRINITY_DN8917_c0_g1_i3 | -1.29048637 | 0.633146361 | 0.999970678 | 2.398 | 0 | 1.211 | 4.554 | NA |
| TRINITY_DN653_c2_g1_i3 | 1.290409105 | 0.388863151 | 0.999970678 | 1.808 | 1.37 | 0.111 | 1.156 | NA |
| TRINITY_DN3199_c0_g1_i6 | -1.29029094 | 0.766591612 | 0.999970678 | 0 | 0.345 | 0 | 0.834 | NA |
| TRINITY_DN2410_c0_g1_i1 | -1.2899744 | 0.321223608 | 0.999970678 | 4.342 | 4.179 | 2.555 | 17.544 | EF-hand_1 |
| TRINITY_DN3192_c1_g1_i1 | 1.289624507 | 0.758091177 | 0.999970678 | 2.861 | 0 | 0 | 1.196 | NA |
| TRINITY_DN2887_c0_g1_i6 | -1.28954596 | 0.757953065 | 0.999970678 | 2.107 | 0 | 0 | 5.218 | 2OG-FeII_Oxy_2 |
| TRINITY_DN74442_c0_g1_i1 | 1.289370007 | 0.328657958 | 1 | 0.372 | 0.355 | 0.144 | 0.121 | NA |
| TRINITY_DN1922_c2_g1_i1 | -1.28926841 | 0.65534893 | 0.999970678 | 0 | 8.229 | 13.11 | 3.971 | MFS_1 |
| TRINITY_DN3527_c0_g1_i21 | -1.28919038 | 0.629099431 | 0.999970678 | 0.899 | 0 | 1.044 | 1.005 | NA |
| TRINITY_DN9014_c0_g1_i3 | -1.28915765 | 0.499865224 | 0.999970678 | 0.082 | 3.952 | 3.066 | 5.992 | Transferase |
| TRINITY_DN25212_c0_g1_i1 | -1.28894776 | 0.223874868 | 0.999970678 | 1.417 | 2.562 | 2.544 | 6.575 | NA |
| TRINITY_DN6713_c0_g1_i1 | -1.28894631 | 0.228591925 | 0.999970678 | 0.409 | 0.197 | 0.744 | 0.583 | PPR |
| TRINITY_DN6713_c0_g1_i3 | -1.28894631 | 0.228591925 | 0.999970678 | 0.409 | 0.197 | 0.744 | 0.583 | PPR |
| TRINITY_DN5988_c0_g1_i3 | -1.28894318 | 0.415900091 | 0.999970678 | 0.981 | 0.71 | 0.356 | 3.68 | NA |
| TRINITY_DN1023_c0_g1_i2 | 1.288908833 | 0.62753987 | 0.999970678 | 9.593 | 3.597 | 0 | 5.449 | NA |
| TRINITY_DN289_c1_g1_i2 | 1.288892925 | 0.757871496 | 0.999970678 | 0 | 3.095 | 0 | 1.247 | UDPGT |
| TRINITY_DN2359_c0_g1_i3 | -1.28858211 | 0.630348754 | 0.999970678 | 1.39 | 0 | 1.344 | 1.85 | UCH |
| TRINITY_DN7721_c0_g1_i3 | 1.288382074 | 0.75951545 | 0.999970678 | 10.792 | 0 | 0 | 4.494 | NA |
| TRINITY_DN7065_c0_g1_i8 | 1.28837389 | 0.065561461 | 0.999970678 | 8.321 | 6.11 | 2.567 | 2.825 | - |
| TRINITY_DN3581_c0_g1_i3 | 1.288331824 | 0.625256069 | 0.999970678 | 1.217 | 1.38 | 0.878 | 0 | NA |
| TRINITY_DN13163_c0_g1_i1 | -1.28833101 | 0.210518071 | 0.999970678 | 35.146 | 87.47 | 171.934 | 84.944 | 2OG-FeII_Oxy |
| TRINITY_DN2240_c0_g1_i2 | -1.28818172 | 0.276816108 | 0.999970678 | 0.999 | 0.788 | 1.011 | 3.137 | NA |
| TRINITY_DN14909_c2_g1_i1 | -1.28780802 | 0.385588579 | 0.999970678 | 1.017 | 5.342 | 3.089 | 11.421 | - |
| TRINITY_DN3473_c0_g1_i4 | 1.287805351 | 0.481583901 | 0.999970678 | 0.863 | 1.252 | 0.689 | 0.04 | FGGY_C |
| TRINITY_DN2083_c0_g1_i1 | 1.287783622 | 0.348630785 | 0.999970678 | 95.736 | 11.8 | 12.455 | 29.477 | NA |
| TRINITY_DN23213_c0_g1_i10 | -1.28770345 | 0.626873954 | 0.999970678 | 0.563 | 0 | 0.5 | 0.774 | Helicase_C |
| TRINITY_DN26374_c0_g1_i7 | 1.287439605 | 0.121754969 | 0.999970678 | 6.268 | 10.94 | 2.522 | 3.951 | DnaJ |
| TRINITY_DN1429_c0_g1_i2 | 1.287431002 | 0.764296716 | 0.999970678 | 0 | 5.292 | 1.778 | 0 | NA |
| TRINITY_DN80116_c0_g1_i3 | 1.287407222 | 0.325321753 | 0.999970678 | 1.962 | 0.946 | 0.256 | 0.875 | Agenet |
| TRINITY_DN193609_c0_g1_i1 | 1.287300025 | 0.286113351 | 0.999970678 | 3.288 | 10.89 | 1.722 | 3.629 | NA |
| TRINITY_DN5000_c3_g1_i4 | 1.287259196 | 0.334496013 | 1 | 0.545 | 0.542 | 0.2 | 0.201 | NA |
| TRINITY_DN1898_c1_g1_i4 | 1.287148137 | 0.761008878 | 0.999970678 | 2.38 | 0 | 0 | 0.975 | NA |
| TRINITY_DN522_c0_g1_i3 | 1.287082486 | 0.281467101 | 0.999970678 | 56.13 | 10.13 | 12.01 | 13.009 | SPX |
| TRINITY_DN2495_c0_g1_i1 | -1.28692629 | 0.762438395 | 0.999970678 | 0.173 | 0 | 0.356 | 0 | ABC_membrane |
| TRINITY_DN29238_c0_g2_i3 | 1.28687686 | 0.770355235 | 1 | 1.426 | 0 | 0 | 0.593 | NA |
| TRINITY_DN1961_c0_g1_i5 | 1.286726164 | 0.113152931 | 0.999970678 | 2.298 | 3.499 | 1.067 | 1.086 | NA |
| TRINITY_DN1226_c0_g2_i1 | -1.28666642 | 0.373353711 | 0.999970678 | 0.627 | 5.647 | 4.322 | 9.903 | NA |
| TRINITY_DN2396_c0_g1_i5 | 1.286344253 | 0.662325149 | 0.999970678 | 3.552 | 0 | 1.044 | 0.211 | NA |
| TRINITY_DN5371_c0_g1_i1 | 1.286223588 | 0.758814075 | 0.999970678 | 3.234 | 0 | 0 | 1.347 | Cation_ATPase |
| TRINITY_DN10756_c0_g1_i3 | 1.286101561 | 0.104751268 | 0.999970678 | 2.271 | 2.277 | 0.767 | 0.955 | NA |
| TRINITY_DN31120_c0_g1_i6 | -1.28600904 | 0.092170573 | 0.999970678 | 1.172 | 1.34 | 2.933 | 2.574 | NA |
| TRINITY_DN3911_c0_g1_i3 | -1.28597117 | 0.650497925 | 0.999970678 | 0 | 73.92 | 86.617 | 71.612 | - |
| TRINITY_DN16272_c0_g1_i2 | -1.28592653 | 0.239014874 | 0.999970678 | 2.734 | 2.247 | 2.433 | 9.109 | NA |
| TRINITY_DN7132_c0_g1_i4 | 1.285706732 | 0.633767329 | 0.999970678 | 0.681 | 2.474 | 0 | 1.297 | NA |
| TRINITY_DN2772_c0_g1_i6 | -1.28564457 | 0.473540823 | 0.999970678 | 0.327 | 2.7 | 0.5 | 6.686 | NA |
| TRINITY_DN11406_c0_g1_i4 | 1.285477634 | 0.341173836 | 0.999970678 | 1.971 | 2.119 | 1.189 | 0.241 | MT-A70 |
| TRINITY_DN18791_c0_g1_i1 | 1.285404868 | 0.370557477 | 0.999970678 | 3.643 | 0.828 | 1.155 | 0.473 | Mlo |
| TRINITY_DN4652_c0_g1_i2 | 1.285329249 | 0.365626485 | 0.999970678 | 6.25 | 0.877 | 1.722 | 0.885 | Sigma70_r2 |
| TRINITY_DN21610_c0_g1_i1 | -1.2851453 | 0.19129172 | 0.999970678 | 0.917 | 1.153 | 1.433 | 3.308 | NA |
| TRINITY_DN2199_c0_g1_i5 | 1.284797005 | 0.461630846 | 0.999970678 | 3.506 | 2.631 | 2.022 | 0.09 | NA |
| TRINITY_DN4235_c1_g1_i1 | 1.28450514 | 0.646301199 | 0.999970678 | 3.179 | 7.037 | 3.455 | 0 | HEAT |
| TRINITY_DN1956_c0_g1_i17 | -1.28448994 | 0.641667122 | 1 | 0.164 | 0 | 0.222 | 0.141 | Filamin |
| TRINITY_DN192_c0_g1_i9 | -1.28447555 | 0.658338207 | 0.999970678 | 0.481 | 0 | 0.889 | 0.141 | Ribosomal_L3 |
| TRINITY_DN2309_c0_g1_i2 | -1.28441914 | 0.635636044 | 0.999970678 | 1.381 | 0 | 2.233 | 0.754 | NA |
| TRINITY_DN125_c3_g1_i2 | 1.284403599 | 0.359584941 | 0.999970678 | 1.317 | 5.204 | 0.456 | 2.101 | NA |
| TRINITY_DN1238_c0_g2_i1 | -1.28424842 | 0.380412733 | 0.999970678 | 0.254 | 2.07 | 2.933 | 2.041 | NA |
| TRINITY_DN1111_c0_g1_i1 | -1.28401978 | 0.448260533 | 0.999970678 | 1.017 | 3.42 | 0.511 | 10.114 | MFS_1 |
| TRINITY_DN3541_c1_g2_i3 | 1.283735072 | 0.474151246 | 0.999970678 | 9.82 | 2.641 | 4.055 | 0.211 | NA |
| TRINITY_DN31565_c0_g1_i2 | 1.282874938 | 0.292449497 | 0.999970678 | 31.449 | 8.86 | 3.433 | 12.547 | CUE |
| TRINITY_DN6309_c0_g1_i2 | -1.28284811 | 0.644529536 | 0.999970678 | 0.954 | 1.606 | 0 | 6.233 | NA |
| TRINITY_DN9738_c0_g1_i4 | -1.28274949 | 0.299129994 | 0.999970678 | 1.226 | 1.261 | 1.089 | 4.685 | TatD_DNase |
| TRINITY_DN14210_c0_g1_i1 | -1.28260178 | 0.634046415 | 0.999970678 | 1.599 | 0 | 2.267 | 1.196 | NA |
| TRINITY_DN5676_c3_g1_i6 | -1.28255086 | 0.769787817 | 0.999970678 | 0.463 | 0 | 0.967 | 0 | NA |
| TRINITY_DN16243_c0_g1_i3 | -1.28253109 | 0.696032448 | 0.999970678 | 0.917 | 0 | 1.855 | 0.02 | NA |
| TRINITY_DN6933_c0_g1_i7 | -1.28249976 | 0.765020632 | 0.999970678 | 1.59 | 0 | 3.233 | 0 | Grp1_Fun34_Yaa |
| TRINITY_DN12838_c0_g1_i1 | -1.28248533 | 0.085780229 | 0.999970678 | 21.12 | 21.46 | 55.508 | 36.425 | NA |
| TRINITY_DN3607_c0_g1_i5 | -1.28246609 | 0.175707138 | 0.999970678 | 0.854 | 0.867 | 2.233 | 1.498 | Fer4 |
| TRINITY_DN4589_c0_g1_i3 | 1.282177182 | 0.759751446 | 0.999970678 | 0 | 14.41 | 4.833 | 0 | NA |
| TRINITY_DN9078_c0_g1_i2 | -1.28207157 | 0.205261375 | 0.999970678 | 0.345 | 0.631 | 1.133 | 0.995 | NA |
| TRINITY_DN8046_c0_g1_i1 | 1.2819278 | 0.309756625 | 0.999970678 | 2.889 | 0.798 | 0.889 | 0.462 | NA |

| TRINITY_DN1303_c0_g1_i9 | 1.281925275 | 0.525589986 | 0.999970678 | 1.026 | 33.26 | 8.588 | 3.338 | GUB_WAK_bind |
| --- | --- | --- | --- | --- | --- | --- | --- | --- |
| TRINITY_DN7438_c0_g2_i1 | -1.28187789 | 0.505358572 | 0.999970678 | 0.3 | 0.601 | 0.078 | 2.091 | NA |
| TRINITY_DN2339_c0_g1_i2 | 1.281836897 | 0.376727556 | 0.999970678 | 1.081 | 3.4 | 1.311 | 0.251 | NA |
| TRINITY_DN4946_c0_g1_i3 | -1.28183302 | 0.293556369 | 0.999970678 | 2.534 | 2.631 | 1.667 | 10.546 | Anticodon_1 |
| TRINITY_DN4908_c3_g1_i1 | 1.281811611 | 0.436408056 | 0.999970678 | 1.072 | 1.311 | 0.711 | 0.111 | NA |
| TRINITY_DN15062_c0_g2_i1 | -1.28161966 | 0.136593448 | 0.999970678 | 0.191 | 0.256 | 0.467 | 0.533 | NA |
| TRINITY_DN81817_c0_g1_i1 | 1.281527769 | 0.657613748 | 0.999970678 | 0.154 | 1.794 | 0 | 0.784 | FMO-like |
| TRINITY_DN832_c0_g1_i6 | -1.28144298 | 0.529987654 | 0.999970678 | 12.808 | 22.17 | 0.456 | 84.441 | NA |
| TRINITY_DN4474_c0_g1_i2 | -1.28124086 | 0.627443715 | 0.999970678 | 0.6 | 0 | 0.689 | 0.653 | Jas |
| TRINITY_DN2558_c0_g1_i19 | -1.28108402 | 0.761363358 | 0.999970678 | 28.405 | 0 | 56.952 | 0 | Abhydrolase_1 |
| TRINITY_DN1362_c0_g1_i1 | 1.281027869 | 0.512197113 | 0.999970678 | 6.986 | 8.604 | 5.244 | 0.07 | ARID |
| TRINITY_DN2725_c0_g2_i8 | 1.280972735 | 0.778879076 | 0.999970678 | 200.46 | 0 | 69.151 | 0 | NA |
| TRINITY_DN14880_c0_g1_i7 | 1.280899268 | 0.354293691 | 0.999970678 | 0.409 | 1.695 | 0.456 | 0.302 | Methyltransf_4 |
| TRINITY_DN7015_c0_g1_i7 | -1.28061372 | 0.696819229 | 0.999970678 | 0.609 | 0.02 | 1.289 | 0 | PEPCK_ATP |
| TRINITY_DN17504_c0_g1_i4 | -1.28043601 | 0.292546762 | 0.999970678 | 6.068 | 9.727 | 4.822 | 32.564 | NA |
| TRINITY_DN1347_c0_g2_i3 | 1.280053005 | 0.218399715 | 0.999970678 | 2.444 | 2.592 | 1.289 | 0.533 | NA |
| TRINITY_DN12201_c0_g1_i1 | -1.27980201 | 0.497057135 | 0.999970678 | 1.008 | 35.73 | 60.541 | 14.166 | - |
| TRINITY_DN370_c0_g1_i2 | 1.279661757 | 0.177428423 | 0.999970678 | 23.791 | 59.97 | 9.877 | 22.45 | NA |
| TRINITY_DN1487_c0_g1_i8 | 1.279550177 | 0.769599912 | 0.999970678 | 0 | 11.23 | 3.733 | 0 | NA |
| TRINITY_DN5557_c0_g1_i2 | 1.279514417 | 0.183581306 | 0.999970678 | 2.044 | 5.992 | 1.311 | 1.709 | DUF4205 |
| TRINITY_DN4647_c0_g1_i2 | 1.279481514 | 0.380428861 | 0.999970678 | 1.917 | 2.395 | 1.167 | 0.322 | NA |
| TRINITY_DN1503_c0_g1_i6 | -1.27946572 | 0.362157977 | 0.999970678 | 5.16 | 7.865 | 2.489 | 28.542 | NA |
| TRINITY_DN4242_c0_g2_i10 | -1.27929845 | 0.630587592 | 0.999970678 | 7.249 | 0 | 9.833 | 5.831 | NA |
| TRINITY_DN369_c0_g1_i1 | -1.27927156 | 0.115730324 | 0.999970678 | 667.31 | 425.9 | 762.909 | 1719.463 | NA |
| TRINITY_DN3850_c0_g1_i4 | -1.2792099 | 0.762230914 | 0.999970678 | 0.709 | 0 | 0 | 1.759 | Pkinase |
| TRINITY_DN4657_c2_g1_i4 | 1.27915681 | 0.361647665 | 0.999970678 | 2.97 | 13.94 | 0.989 | 5.721 | STAS |
| TRINITY_DN1678_c0_g1_i2 | -1.27873141 | 0.663471609 | 0.999970678 | 0 | 35.78 | 36.242 | 41.803 | Aminotran_1_2 |
| TRINITY_DN5548_c0_g1_i1 | -1.27866583 | 0.377158396 | 0.999970678 | 47.827 | 7.066 | 23.454 | 103.623 | NA |
| TRINITY_DN8146_c2_g1_i6 | 1.278657178 | 0.348134359 | 0.999970678 | 312.54 | 45.82 | 33.498 | 108.479 | PNRC |
| TRINITY_DN3143_c1_g3_i1 | -1.27853098 | 0.325612146 | 1 | 1.09 | 0.729 | 1.844 | 2.141 | Zeta_toxin |
| TRINITY_DN19353_c0_g1_i7 | 1.278452884 | 0.400845825 | 1 | 0.282 | 0.276 | 0.156 | 0.05 | NA |
| TRINITY_DN5676_c1_g1_i2 | 1.278391825 | 0.254780885 | 0.999970678 | 5.06 | 2.02 | 1.733 | 0.845 | NA |
| TRINITY_DN2207_c0_g1_i1 | -1.27833032 | 0.765771248 | 0.999970678 | 0 | 1.439 | 2.811 | 0 | NA |
| TRINITY_DN3020_c0_g1_i3 | 1.278120825 | 0.410298565 | 0.999970678 | 1.399 | 8.909 | 2.9 | 0.694 | Autophagy_C |
| TRINITY_DN4364_c0_g1_i3 | 1.278100808 | 0.508667086 | 0.999970678 | 4.615 | 5.361 | 3.366 | 0.05 | Med23 |
| TRINITY_DN2180_c0_g1_i4 | -1.27792963 | 0.715539213 | 0.999970678 | 17.368 | 0 | 35.22 | 0.04 | NA |
| TRINITY_DN532_c0_g1_i1 | 1.277902314 | 0.16545294 | 0.999970678 | 7.458 | 16.04 | 2.966 | 6.052 | NA |
| TRINITY_DN4960_c0_g1_i10 | 1.277878193 | 0.628329131 | 0.999970678 | 0.79 | 1.902 | 0 | 1.106 | AP2 |
| TRINITY_DN938_c0_g1_i17 | 1.277838152 | 0.342523328 | 0.999970678 | 3.534 | 1.705 | 0.356 | 1.719 | NA |
| TRINITY_DN6332_c0_g1_i2 | 1.277626119 | 0.769163368 | 0.999970678 | 0 | 3.873 | 0 | 1.538 | NA |
| TRINITY_DN3434_c0_g1_i2 | -1.27750333 | 0.329530882 | 0.999970678 | 0.59 | 2.385 | 1.378 | 5.54 | Methyltransf_29 |
| TRINITY_DN4180_c0_g2_i1 | 1.27735661 | 0.622404983 | 0.999970678 | 4.179 | 7.51 | 0 | 4.776 | NA |
| TRINITY_DN4853_c0_g1_i11 | -1.27730934 | 0.761429527 | 0.999970678 | 6.822 | 0 | 13.844 | 0 | NA |
| TRINITY_DN534_c0_g1_i13 | 1.2772226 | 0.261296865 | 0.999970678 | 0.391 | 0.779 | 0.133 | 0.322 | NA |
| TRINITY_DN37276_c0_g1_i1 | 1.277172303 | 0.419098382 | 1 | 0.427 | 0.818 | 0.356 | 0.09 | NA |
| TRINITY_DN8509_c0_g1_i3 | -1.27715392 | 0.760652629 | 0.999970678 | 0 | 1.764 | 0 | 4.223 | NA |
| TRINITY_DN30816_c0_g1_i1 | -1.27699578 | 0.769338059 | 0.999970678 | 3.225 | 0 | 6.511 | 0 | NA |
| TRINITY_DN249_c0_g1_i2 | 1.276504626 | 0.669893868 | 0.999970678 | 0 | 81.14 | 4.366 | 27.507 | Ribosomal_L5 |
| TRINITY_DN3269_c1_g1_i1 | 1.276360077 | 0.37684145 | 0.999970678 | 13.299 | 8.85 | 0.644 | 8.435 | Med26 |
| TRINITY_DN2819_c0_g1_i1 | -1.27634809 | 0.649574012 | 0.999970678 | 7.776 | 0 | 13.377 | 2.895 | Pectinesterase |
| TRINITY_DN1003_c0_g1_i2 | -1.27625967 | 0.515197279 | 0.999970678 | 4.851 | 0.069 | 6.033 | 4.796 | NA |
| TRINITY_DN2396_c0_g1_i18 | 1.276245662 | 0.771603351 | 1 | 2.098 | 0 | 0 | 0.885 | NA |
| TRINITY_DN7981_c0_g1_i1 | -1.27608474 | 0.763777464 | 0.999970678 | 0.609 | 0 | 0 | 1.478 | UVR |
| TRINITY_DN5346_c0_g1_i2 | 1.276071548 | 0.624074701 | 0.999970678 | 1.381 | 1.656 | 0 | 1.257 | - |
| TRINITY_DN5574_c1_g2_i1 | -1.27602376 | 0.640002025 | 0.999970678 | 6.086 | 0 | 2.433 | 11.763 | NA |
| TRINITY_DN44627_c0_g1_i1 | -1.27599707 | 0.347545508 | 1 | 2.28 | 5.992 | 8.044 | 9.31 | NA |
| TRINITY_DN2015_c0_g1_i7 | -1.27577359 | 0.130907129 | 0.999970678 | 2.498 | 1.33 | 3.4 | 5.188 | NA |
| TRINITY_DN2786_c0_g1_i1 | 1.275711939 | 0.165446846 | 0.999970678 | 0.936 | 1.971 | 0.544 | 0.543 | NA |
| TRINITY_DN4311_c0_g1_i1 | -1.27566925 | 0.629842755 | 0.999970678 | 1.108 | 0 | 0.856 | 1.709 | NA |
| TRINITY_DN191_c0_g1_i2 | 1.275503399 | 0.383831743 | 0.999970678 | 297.78 | 39.39 | 92.972 | 28.422 | Inos-1-P_synth |
| TRINITY_DN10314_c0_g1_i5 | -1.27531038 | 0.285087808 | 0.999970678 | 0.236 | 0.897 | 0.9 | 1.669 | LRRNT_2 |
| TRINITY_DN1549_c0_g1_i8 | -1.27524738 | 0.651695256 | 0.999970678 | 0.681 | 0.995 | 3.3 | 0 | LRRNT_2 |
| TRINITY_DN16036_c0_g1_i2 | 1.275075126 | 0.779869062 | 0.999970678 | 0 | 205.8 | 69.207 | 0 | NA |
| TRINITY_DN3476_c0_g1_i3 | -1.27496628 | 0.270684425 | 0.999970678 | 2.071 | 1.163 | 1.378 | 6.223 | - |
| TRINITY_DN5602_c0_g1_i5 | 1.274888169 | 0.763025072 | 0.999970678 | 1.399 | 0 | 0.489 | 0 | NA |
| TRINITY_DN9987_c0_g1_i1 | 1.274845123 | 0.319395474 | 0.999970678 | 7.912 | 51.61 | 7.822 | 14.789 | Chloroa_b-bind |
| TRINITY_DN2280_c0_g1_i2 | 1.274690514 | 0.276999911 | 0.999970678 | 32.193 | 8.988 | 4.033 | 12.326 | AAA |
| TRINITY_DN11190_c0_g1_i4 | 1.274639246 | 0.197290651 | 0.999970678 | 3.897 | 1.478 | 0.978 | 1.056 | Myb_DNA-bind_ |
| TRINITY_DN4902_c0_g1_i1 | -1.27459056 | 0.509497405 | 0.999970678 | 0.481 | 0.099 | 1.111 | 0.08 | NA |
| TRINITY_DN2496_c0_g1_i6 | -1.27447225 | 0.382783902 | 0.999970678 | 2.017 | 1.744 | 0.633 | 8.385 | NA |
| TRINITY_DN299_c0_g1_i4 | -1.27436566 | 0.649252715 | 0.999970678 | 0.763 | 1.055 | 3.678 | 0 | Aldo_ket_red |
| TRINITY_DN10529_c0_g1_i2 | 1.274322233 | 0.629642793 | 0.999970678 | 2.289 | 2.513 | 1.644 | 0 | CBM_20 |
| TRINITY_DN1571_c0_g1_i1 | -1.27411753 | 0.351018235 | 0.999970678 | 6.132 | 2.888 | 2.367 | 19.001 | ELK |
| TRINITY_DN45_c0_g2_i5 | 1.273960961 | 0.281377704 | 0.999970678 | 4.733 | 9.579 | 3.922 | 1.146 | GAF |

| TRINITY_DN5265_c0_g2_i1 | -1.27393194 | 0.760900426 | 0.999970678 | 7.185 | 0 | 0 | 17.514 | NA |
| --- | --- | --- | --- | --- | --- | --- | --- | --- |
| TRINITY_DN64_c0_g1_i5 | 1.273816227 | 0.159971351 | 0.999970678 | 10.519 | 20.02 | 3.478 | 8.355 | NA |
| TRINITY_DN7737_c0_g1_i5 | -1.27381456 | 0.763301296 | 0.999970678 | 1.935 | 0 | 3.955 | 0 | Pro-kuma_activ |
| TRINITY_DN2069_c0_g1_i4 | -1.27341438 | 0.307662936 | 0.999970678 | 149.33 | 74.36 | 70.662 | 426.377 | NA |
| TRINITY_DN5962_c0_g1_i2 | 1.273265861 | 0.252436594 | 0.999970678 | 0.618 | 0.532 | 0.189 | 0.241 | NA |
| TRINITY_DN321_c0_g1_i8 | -1.2730594 | 0.314532457 | 0.999970678 | 0.336 | 0.68 | 1.6 | 0.513 | NA |
| TRINITY_DN11048_c0_g1_i1 | -1.27276574 | 0.461489963 | 0.999970678 | 2.68 | 0.227 | 2.755 | 3.639 | NA |
| TRINITY_DN1777_c0_g1_i2 | 1.272361001 | 0.770495349 | 0.999970678 | 14.225 | 0 | 0 | 5.66 | NA |
| TRINITY_DN1922_c0_g1_i1 | 1.272119291 | 0.273628681 | 0.999970678 | 2.762 | 10.16 | 1.6 | 3.358 | NA |
| TRINITY_DN18076_c0_g1_i1 | -1.27201786 | 0.4769087 | 0.999970678 | 0.045 | 0.729 | 0.778 | 0.915 | NA |
| TRINITY_DN3459_c3_g3_i1 | 1.272004556 | 0.270051915 | 0.999970678 | 1.453 | 1.301 | 0.489 | 0.543 | RVT_1 |
| TRINITY_DN7428_c0_g2_i1 | 1.271872776 | 0.668837816 | 0.999970678 | 1.244 | 0 | 0.089 | 0.412 | NA |
| TRINITY_DN4503_c0_g1_i5 | -1.27151723 | 0.421730207 | 0.999970678 | 0.472 | 0.059 | 0.444 | 0.774 | PRP1_N |
| TRINITY_DN10064_c0_g1_i12 | 1.271360653 | 0.362768975 | 0.999970678 | 0.327 | 1.084 | 0.1 | 0.462 | NA |
| TRINITY_DN829_c0_g2_i6 | 1.271358282 | 0.763175321 | 0.999970678 | 5.26 | 0 | 0 | 2.192 | Med21 |
| TRINITY_DN6576_c0_g4_i1 | -1.27130503 | 0.762931045 | 0.999970678 | 0 | 1.252 | 2.455 | 0 | NA |
| TRINITY_DN11277_c0_g3_i2 | -1.27125092 | 0.465930566 | 0.999970678 | 0.5 | 2.415 | 5.144 | 0.784 | TMEM135_C_rich |
| TRINITY_DN2195_c2_g1_i1 | -1.27120141 | 0.65583334 | 0.999970678 | 33.038 | 0 | 43.642 | 28.02 | NA |
| TRINITY_DN12285_c0_g1_i1 | -1.27116161 | 0.29928706 | 0.999970678 | 13.299 | 5.026 | 17.454 | 21.103 | NA |
| TRINITY_DN13043_c0_g1_i2 | 1.270816529 | 0.643050159 | 0.999970678 | 1.281 | 0.276 | 0.533 | 0 | NA |
| TRINITY_DN43276_c0_g1_i1 | 1.270763234 | 0.201743859 | 0.999970678 | 3.016 | 3.991 | 0.789 | 1.93 | NA |
| TRINITY_DN1110_c0_g1_i5 | -1.27074137 | 0.28158554 | 0.999970678 | 25.281 | 7.293 | 22.676 | 48.851 | CENP-T_C |
| TRINITY_DN2190_c0_g1_i4 | -1.27055576 | 0.214135801 | 0.999970678 | 2.253 | 1.528 | 2.044 | 6.686 | NA |
| TRINITY_DN13563_c0_g1_i3 | 1.270497932 | 0.659401188 | 0.999970678 | 0.164 | 1.656 | 0 | 0.754 | NA |
| TRINITY_DN86257_c0_g2_i4 | -1.27039393 | 0.643355164 | 0.999970678 | 10.692 | 0 | 11.388 | 12.436 | NA |
| TRINITY_DN2937_c0_g1_i11 | -1.27024792 | 0.64447884 | 0.999970678 | 11.718 | 0 | 17.421 | 7.54 | NA |
| TRINITY_DN1619_c0_g1_i1 | -1.27020424 | 0.683383546 | 0.999970678 | 7.885 | 0.256 | 0 | 19.876 | NA |
| TRINITY_DN1821_c0_g1_i1 | 1.270170294 | 0.636303028 | 0.999970678 | 16.733 | 26.53 | 0 | 17.785 | DUF1990 |
| TRINITY_DN12795_c0_g1_i8 | -1.27007231 | 0.773428426 | 1 | 0 | 0.099 | 0.2 | 0 | ABC2_membrane |
| TRINITY_DN3110_c1_g1_i3 | 1.270062457 | 0.245613712 | 0.999970678 | 2.189 | 1.449 | 0.344 | 1.086 | DEAD |
| TRINITY_DN1362_c0_g1_i2 | -1.27003497 | 0.077875061 | 0.999970678 | 4.351 | 4.06 | 6.422 | 12.567 | ARID |
| TRINITY_DN2942_c0_g2_i2 | 1.269552433 | 0.286299199 | 0.999970678 | 4.533 | 15.6 | 4.666 | 2.574 | NA |
| TRINITY_DN84015_c0_g1_i8 | -1.26938512 | 0.63425389 | 0.999970678 | 2.126 | 0 | 2.989 | 1.599 | NA |
| TRINITY_DN2448_c0_g2_i2 | 1.269187897 | 0.277403194 | 0.999970678 | 1.444 | 1.252 | 0.733 | 0.231 | NA |
| TRINITY_DN11950_c0_g1_i3 | 1.268955423 | 0.633176385 | 0.999970678 | 1.599 | 0.532 | 0 | 0.905 | NA |
| TRINITY_DN11475_c0_g1_i2 | 1.268947681 | 0.12476371 | 0.999970678 | 3.588 | 2.72 | 1.233 | 1.136 | NA |
| TRINITY_DN2591_c0_g1_i1 | 1.26873201 | 0.197172239 | 0.999970678 | 4.842 | 2.001 | 1.511 | 1.046 | DUF3453 |
| TRINITY_DN3609_c0_g1_i2 | 1.268552573 | 0.299500922 | 0.999970678 | 0.836 | 1.311 | 0.567 | 0.201 | NA |
| TRINITY_DN35492_c0_g1_i2 | 1.268286437 | 0.649723733 | 0.999970678 | 2.371 | 0.512 | 1 | 0 | NA |
| TRINITY_DN2981_c0_g1_i1 | -1.26823166 | 0.539437416 | 0.999970678 | 3.343 | 22.54 | 50.275 | 0.734 | NA |
| TRINITY_DN804_c1_g1_i5 | -1.2681693 | 0.643754187 | 1 | 0 | 0.325 | 0.433 | 0.251 | NA |
| TRINITY_DN15004_c0_g1_i1 | -1.26810662 | 0.51047562 | 0.999970678 | 0.073 | 2.316 | 0.933 | 4.544 | p450 |
| TRINITY_DN858_c0_g1_i6 | 1.268084737 | 0.523961396 | 0.999970678 | 54.44 | 4.267 | 18.777 | 1.518 | NA |
| TRINITY_DN322_c0_g2_i1 | 1.267813257 | 0.640197509 | 0.999970678 | 1.426 | 8.012 | 0 | 3.851 | NA |
| TRINITY_DN6454_c0_g1_i2 | -1.26779562 | 0.645108038 | 1 | 1.063 | 0 | 0.678 | 1.78 | Myb_DNA-bindin |
| TRINITY_DN10299_c0_g1_i11 | -1.26779562 | 0.645108038 | 1 | 0.899 | 0 | 0.544 | 1.488 | NA |
| TRINITY_DN755_c0_g1_i2 | 1.267709754 | 0.500718071 | 0.999970678 | 3.079 | 0.197 | 0.233 | 1.086 | F-box-like |
| TRINITY_DN2184_c0_g3_i1 | 1.267582804 | 0.223402135 | 0.999970678 | 0.745 | 1.38 | 0.422 | 0.372 | NA |
| TRINITY_DN4593_c0_g1_i1 | -1.26747389 | 0.297787405 | 0.999970678 | 8.63 | 1.547 | 8.488 | 14.598 | Myb_DNA-bindin |
| TRINITY_DN34900_c0_g1_i2 | 1.267448711 | 0.426293405 | 0.999970678 | 0.609 | 4.405 | 0.411 | 1.558 | NA |
| TRINITY_DN766_c0_g2_i4 | -1.26741654 | 0.406395573 | 0.999970678 | 0.917 | 0.177 | 1 | 1.397 | NA |
| TRINITY_DN843_c3_g1_i3 | -1.26661421 | 0.246280099 | 0.999970678 | 0.427 | 0.246 | 0.533 | 0.995 | PHM7_cyt |
| TRINITY_DN834_c0_g1_i16 | -1.26660322 | 0.07627551 | 0.999970678 | 4.206 | 2.976 | 7.777 | 7.892 | NA |
| TRINITY_DN3450_c0_g1_i21 | 1.266582992 | 0.531778608 | 0.999970678 | 4.015 | 0.108 | 1.178 | 0.322 | NA |
| TRINITY_DN197_c0_g1_i3 | -1.26655041 | 0.34762712 | 0.999970678 | 35.173 | 49.16 | 17.11 | 181.891 | NA |
| TRINITY_DN209_c0_g1_i4 | 1.266303479 | 0.098297793 | 0.999970678 | 14.416 | 26.61 | 6.788 | 8.767 | NA |
| TRINITY_DN154_c0_g1_i1 | -1.26609204 | 0.554717644 | 0.999970678 | 0.064 | 12.48 | 19.354 | 6.324 | zf-Dof |
| TRINITY_DN610_c0_g1_i4 | -1.26592598 | 0.230269641 | 0.999970678 | 4.306 | 4.563 | 3.855 | 16.659 | NA |
| TRINITY_DN2757_c0_g1_i18 | 1.265807407 | 0.207183207 | 0.999970678 | 0.581 | 0.66 | 0.189 | 0.282 | NA |
| TRINITY_DN36772_c0_g1_i4 | 1.265785574 | 0.400755202 | 0.999970678 | 12.109 | 1.133 | 2.733 | 2.262 | NA |
| TRINITY_DN236_c0_g1_i2 | -1.26573637 | 0.646747962 | 0.999970678 | 0 | 2.947 | 4.966 | 0.965 | NA |
| TRINITY_DN16685_c0_g2_i11 | -1.26562312 | 0.777808554 | 1 | 0.309 | 0 | 0.633 | 0 | FAD_binding_3 |
| TRINITY_DN32110_c0_g1_i1 | -1.26562312 | 0.777808554 | 1 | 0.572 | 0 | 1.155 | 0 | NA |
| TRINITY_DN78699_c0_g2_i1 | -1.26562312 | 0.777808554 | 1 | 0.627 | 0 | 1.255 | 0 | NA |
| TRINITY_DN9961_c0_g1_i4 | -1.26557443 | 0.146245563 | 0.999970678 | 3.325 | 3.371 | 4.4 | 10.747 | Acetyltransf_3 |
| TRINITY_DN2244_c0_g1_i3 | 1.265362358 | 0.770711402 | 0.999970678 | 0 | 44.53 | 15.055 | 0 | NA |
| TRINITY_DN836_c0_g1_i9 | 1.265333975 | 0.285350426 | 0.999970678 | 2.716 | 4.573 | 2.022 | 0.573 | RRM_1 |
| TRINITY_DN3888_c0_g1_i2 | -1.26525145 | 0.087743519 | 0.999970678 | 11.055 | 9.905 | 16.521 | 29.9 | Ribosomal_L11 |
| TRINITY_DN542_c0_g1_i4 | -1.26511598 | 0.656844442 | 0.999970678 | 13.735 | 0 | 2.711 | 28.502 | NA |
| TRINITY_DN2693_c0_g1_i4 | 1.264881335 | 0.6537204 | 0.999970678 | 1.126 | 0.227 | 0.478 | 0 | NA |
| TRINITY_DN10765_c0_g1_i1 | -1.26486311 | 0.763881363 | 0.999970678 | 0 | 0.779 | 0 | 1.83 | NA |
| TRINITY_DN1422_c3_g1_i5 | 1.264685501 | 0.690915536 | 0.999970678 | 0.118 | 16.12 | 0 | 6.656 | NA |
| TRINITY_DN11608_c0_g1_i1 | 1.264663635 | 0.238775889 | 0.999970678 | 2.889 | 2.365 | 0.844 | 1.096 | NA |
| TRINITY_DN12487_c0_g1_i2 | 1.264371188 | 0.646799123 | 0.999970678 | 4.769 | 3.242 | 2.766 | 0 | NA |

| TRINITY_DN1507_c0_g1_i6 | 1.264319042 | 0.337991285 | 0.999970678 | 1.108 | 0.897 | 0.556 | 0.171 | PPR |
| --- | --- | --- | --- | --- | --- | --- | --- | --- |
| TRINITY_DN7168_c0_g1_i1 | 1.26390711 | 0.156928594 | 0.999970678 | 70.246 | 42.41 | 26.387 | 14.95 | PAR1 |
| TRINITY_DN21404_c0_g1_i1 | -1.26382825 | 0.355444074 | 0.999970678 | 1.036 | 0.355 | 1.233 | 1.85 | NA |
| TRINITY_DN24820_c0_g1_i6 | -1.2637916 | 0.596104661 | 0.999970678 | 0.845 | 0.03 | 1.678 | 0.111 | NA |
| TRINITY_DN15_c0_g1_i16 | -1.26378755 | 0.649761824 | 0.999970678 | 11.973 | 0 | 20.299 | 3.961 | NA |
| TRINITY_DN27402_c0_g1_i8 | -1.26376259 | 0.777262182 | 1 | 0.772 | 0 | 1.555 | 0 | NA |
| TRINITY_DN10076_c0_g3_i2 | 1.263680868 | 0.335182279 | 1 | 0.481 | 0.443 | 0.122 | 0.241 | NA |
| TRINITY_DN608_c0_g1_i2 | 1.263659334 | 0.278501723 | 0.999970678 | 1.126 | 2.503 | 0.533 | 0.865 | AAA |
| TRINITY_DN432_c0_g1_i4 | -1.26338548 | 0.559856223 | 0.999970678 | 7.667 | 1.183 | 0.167 | 21.264 | NA |
| TRINITY_DN10639_c0_g1_i10 | 1.263302061 | 0.442318023 | 0.999970678 | 5.514 | 0.572 | 0.878 | 1.498 | NA |
| TRINITY_DN3840_c0_g1_i1 | -1.26325723 | 0.29289944 | 0.999970678 | 0.718 | 2.444 | 1.767 | 5.399 | Alliinase_C |
| TRINITY_DN28458_c0_g1_i1 | 1.263228055 | 0.774166627 | 0.999970678 | 2.816 | 0 | 0.978 | 0 | - |
| TRINITY_DN16075_c0_g1_i24 | -1.26319051 | 0.395328618 | 0.999970678 | 0.227 | 0.611 | 1.278 | 0.462 | NA |
| TRINITY_DN2400_c0_g1_i1 | 1.263171895 | 0.633902456 | 0.999970678 | 0.781 | 1.863 | 0.9 | 0 | PC-Esterase |
| TRINITY_DN16862_c0_g1_i1 | -1.26296575 | 0.699498246 | 0.999970678 | 0.018 | 0.473 | 0.967 | 0 | NA |
| TRINITY_DN8912_c0_g4_i2 | -1.26284936 | 0.63736173 | 0.999970678 | 5.332 | 0 | 6.9 | 4.605 | TPT |
| TRINITY_DN3343_c0_g2_i3 | 1.262529198 | 0.339987244 | 0.999970678 | 3.506 | 2.129 | 0.278 | 2.001 | CRAL_TRIO |
| TRINITY_DN846_c0_g2_i7 | 1.262506904 | 0.77321247 | 1 | 0 | 1.045 | 0 | 0.432 | NA |
| TRINITY_DN12664_c0_g1_i3 | -1.26235493 | 0.58199819 | 0.999970678 | 0.064 | 1.439 | 2.689 | 0.271 | NA |
| TRINITY_DN11547_c0_g1_i5 | -1.2623186 | 0.644379425 | 1 | 0 | 0.276 | 0.367 | 0.231 | PPR |
| TRINITY_DN18215_c0_g1_i1 | 1.262245966 | 0.766102216 | 0.999970678 | 11.064 | 0 | 3.844 | 0 | NA |
| TRINITY_DN31397_c0_g1_i3 | 1.262204937 | 0.482135692 | 0.999970678 | 2.089 | 48.21 | 14.377 | 3.237 | ADH_N |
| TRINITY_DN1927_c0_g1_i2 | -1.26215774 | 0.552272421 | 0.999970678 | 0.218 | 1.715 | 0.167 | 4.373 | NA |
| TRINITY_DN6341_c0_g1_i5 | 1.262136529 | 0.771510087 | 0.999970678 | 7.449 | 0 | 2.578 | 0 | NA |
| TRINITY_DN10633_c0_g2_i3 | -1.26181048 | 0.313471551 | 0.999970678 | 3.143 | 4.908 | 4.9 | 12.678 | NA |
| TRINITY_DN901_c0_g2_i1 | -1.26167885 | 0.234251434 | 0.999970678 | 21.247 | 16.42 | 15.743 | 71.351 | BTB |
| TRINITY_DN26639_c0_g1_i4 | 1.261664244 | 0.635686494 | 0.999970678 | 0.491 | 0.424 | 0.311 | 0 | NA |
| TRINITY_DN5063_c0_g1_i7 | 1.261655374 | 0.776741003 | 0.999970678 | 95.926 | 0 | 0 | 40.587 | CCT_2 |
| TRINITY_DN1435_c0_g2_i8 | -1.26164185 | 0.560706781 | 0.999970678 | 3.843 | 0.138 | 7.644 | 0.523 | - |
| TRINITY_DN170_c0_g1_i1 | -1.26150675 | 0.378746065 | 0.999970678 | 33.82 | 14.29 | 86.506 | 11.3 | AP2 |
| TRINITY_DN1063_c0_g1_i1 | -1.261456 | 0.178300562 | 0.999970678 | 1.317 | 2.868 | 2.911 | 6.515 | NA |
| TRINITY_DN3471_c0_g1_i4 | -1.26141025 | 0.770398087 | 0.999970678 | 0 | 1.468 | 2.878 | 0 | HLH |
| TRINITY_DN2860_c0_g1_i6 | 1.261293599 | 0.0330397 | 0.999970678 | 34.791 | 37.78 | 11.522 | 16.287 | NA |
| TRINITY_DN3311_c1_g2_i1 | 1.261281294 | 0.657042264 | 0.999970678 | 3.37 | 0 | 0.833 | 0.412 | OTU |
| TRINITY_DN23416_c0_g2_i6 | -1.26123607 | 0.645666162 | 1 | 1.017 | 0 | 0.667 | 1.659 | NA |
| TRINITY_DN4463_c0_g1_i4 | 1.261212023 | 0.276154979 | 0.999970678 | 3.97 | 9.974 | 3.689 | 1.317 | NA |
| TRINITY_DN6693_c0_g1_i9 | -1.26118506 | 0.689429666 | 0.999970678 | 0.772 | 0.049 | 1.633 | 0 | NA |
| TRINITY_DN1757_c0_g1_i8 | -1.26114585 | 0.359777998 | 0.999970678 | 4.133 | 1.626 | 8.488 | 3.438 | PCI |
| TRINITY_DN3305_c0_g1_i1 | -1.26095204 | 0.654270271 | 0.999970678 | 1.263 | 0 | 0.344 | 2.654 | NA |
| TRINITY_DN608_c0_g2_i1 | -1.26093144 | 0.481581141 | 0.999970678 | 11.782 | 0.404 | 5.8 | 22.581 | NA |
| TRINITY_DN7443_c0_g1_i4 | -1.26064245 | 0.652267928 | 0.999970678 | 0.254 | 0.345 | 1.178 | 0 | His_Phos_1 |
| TRINITY_DN1186_c0_g1_i7 | -1.26060614 | 0.036963267 | 0.999970678 | 24.835 | 20.71 | 41.953 | 58.573 | NA |
| TRINITY_DN196758_c0_g1_i1 | -1.26054287 | 0.776312226 | 1 | 0.627 | 0 | 1.267 | 0 | NA |
| TRINITY_DN12803_c0_g1_i1 | 1.260233456 | 0.352287441 | 0.999970678 | 0.518 | 0.838 | 0.122 | 0.422 | NA |
| TRINITY_DN44465_c0_g1_i7 | -1.26008306 | 0.653800111 | 1 | 0 | 0.532 | 0.811 | 0.271 | - |
| TRINITY_DN16963_c0_g1_i6 | 1.260049848 | 0.254032852 | 0.999970678 | 0.672 | 0.483 | 0.278 | 0.141 | NA |
| TRINITY_DN1014_c0_g1_i4 | -1.25985486 | 0.289012997 | 0.999970678 | 6.332 | 2.543 | 4.044 | 16.448 | NA |
| TRINITY_DN2475_c0_g1_i1 | 1.259841048 | 0.406863184 | 0.999970678 | 2.97 | 0.848 | 1.167 | 0.201 | NA |
| TRINITY_DN4202_c0_g1_i3 | 1.259787586 | 0.561679219 | 0.999970678 | 0.509 | 3.292 | 0.022 | 1.538 | Nramp |
| TRINITY_DN55_c1_g1_i3 | 1.259557873 | 0.493098584 | 0.999970678 | 0.872 | 2.208 | 0.067 | 1.216 | NA |
| TRINITY_DN18360_c0_g1_i5 | -1.25914078 | 0.775877189 | 1 | 0.79 | 0 | 1.555 | 0 | NA |
| TRINITY_DN7945_c0_g1_i3 | 1.258919888 | 0.764212216 | 0.999970678 | 2.444 | 0 | 0 | 1.046 | NA |
| TRINITY_DN11057_c0_g1_i4 | -1.25862147 | 0.229170469 | 0.999970678 | 7.676 | 8.958 | 8.966 | 27.426 | NA |
| TRINITY_DN1336_c0_g1_i4 | 1.258548949 | 0.764981962 | 0.999970678 | 3.098 | 0 | 0 | 1.307 | NA |
| TRINITY_DN1877_c0_g1_i3 | 1.258462478 | 0.580838726 | 0.999970678 | 10.247 | 0.079 | 0.322 | 4.001 | NA |
| TRINITY_DN1192_c0_g1_i1 | 1.258271333 | 0.777500872 | 0.999970678 | 65.277 | 0 | 0 | 27.768 | NA |
| TRINITY_DN3596_c0_g2_i3 | 1.258262167 | 0.764201641 | 0.999970678 | 3.043 | 0 | 0 | 1.307 | Methyltransf_11 |
| TRINITY_DN1004_c0_g1_i9 | 1.258093784 | 0.374141188 | 0.999970678 | 3.325 | 0.591 | 0.944 | 0.513 | Metallophos |
| TRINITY_DN4647_c0_g1_i5 | 1.257916464 | 0.334941966 | 0.999970678 | 31.231 | 19.68 | 15.266 | 2.835 | NA |
| TRINITY_DN19940_c0_g1_i10 | -1.25785435 | 0.775471258 | 0.999970678 | 0.454 | 0 | 0.911 | 0 | NA |
| TRINITY_DN87511_c0_g1_i3 | -1.25777794 | 0.238986161 | 0.999970678 | 6.486 | 2.582 | 7.811 | 11.813 | NA |
| TRINITY_DN11218_c2_g4_i2 | -1.25774717 | 0.650585709 | 1 | 0.799 | 0 | 0.444 | 1.418 | NA |
| TRINITY_DN6745_c0_g1_i2 | 1.25768073 | 0.637682737 | 0.999970678 | 1.926 | 1.183 | 1.067 | 0 | PPR |
| TRINITY_DN3730_c0_g1_i7 | -1.25742766 | 0.429408291 | 0.999970678 | 4.596 | 1.537 | 1.222 | 13.06 | NA |
| TRINITY_DN7010_c0_g1_i3 | 1.257417963 | 0.510223064 | 0.999970678 | 24.318 | 4.041 | 9.577 | 0.372 | NA |
| TRINITY_DN564_c0_g1_i1 | -1.25741073 | 0.107335078 | 0.999970678 | 181.68 | 92.67 | 284.472 | 308.648 | NA |
| TRINITY_DN22930_c0_g1_i4 | -1.25724272 | 0.488862927 | 1 | 0.182 | 1.715 | 2.489 | 1.428 | LRRNT_2 |
| TRINITY_DN5632_c1_g1_i2 | 1.257125499 | 0.402696959 | 0.999970678 | 0.454 | 2.631 | 0.344 | 0.875 | DWNN |
| TRINITY_DN1278_c0_g1_i4 | -1.25670908 | 0.318086738 | 0.999970678 | 19.14 | 3.045 | 21.41 | 27.607 | NA |
| TRINITY_DN4093_c0_g1_i3 | -1.25655378 | 0.765225693 | 0.999970678 | 1.054 | 0 | 2.1 | 0 | - |
| TRINITY_DN6778_c0_g1_i4 | 1.256312165 | 0.186983653 | 0.999970678 | 1.962 | 2.139 | 0.856 | 0.694 | zf-RING_2 |
| TRINITY_DN10188_c0_g1_i1 | 1.256240593 | 0.646082471 | 0.999970678 | 0.2 | 0.611 | 0.278 | 0 | RINT1_TIP1 |
| TRINITY_DN20481_c0_g2_i2 | -1.25622139 | 0.485834572 | 0.999970678 | 0.645 | 10.26 | 18.721 | 2.946 | NA |
| TRINITY_DN230569_c0_g1_i1 | 1.25602229 | 0.46521093 | 0.999970678 | 0.609 | 12.62 | 2.311 | 2.644 | DOG1 |

| TRINITY_DN10849_c0_g1_i7 | 1.256010602 | 0.773317268 | 0.999970678 | 0 | 1.409 | 0.478 | 0 | - |
| --- | --- | --- | --- | --- | --- | --- | --- | --- |
| TRINITY_DN18248_c0_g1_i1 | 1.255727039 | 0.50581128 | 0.999970678 | 0.182 | 0.591 | 0.244 | 0.02 | ABC_membrane |
| TRINITY_DN28871_c0_g1_i4 | 1.255671433 | 0.421469495 | 0.999970678 | 20.911 | 43.47 | 3.189 | 20.389 | NA |
| TRINITY_DN2489_c0_g1_i4 | -1.25567102 | 0.644837116 | 0.999970678 | 3.288 | 0 | 1.622 | 5.992 | NA |
| TRINITY_DN555_c1_g1_i3 | -1.25549986 | 0.132037023 | 0.999970678 | 40.269 | 25.13 | 44.731 | 102.226 | Mito_carr |
| TRINITY_DN12595_c0_g1_i4 | -1.2551575 | 0.411539558 | 0.999970678 | 0.273 | 0.463 | 0.167 | 1.568 | PHD |
| TRINITY_DN18707_c0_g1_i1 | 1.255123839 | 0.478950143 | 1 | 0.454 | 0.434 | 0.278 | 0.03 | NA |
| TRINITY_DN6972_c0_g1_i3 | -1.25504062 | 0.416292531 | 0.999970678 | 1.099 | 2.641 | 0.856 | 7.711 | NA |
| TRINITY_DN1640_c0_g4_i2 | -1.25502998 | 0.651357583 | 1 | 0 | 0.7 | 1.033 | 0.412 | NA |
| TRINITY_DN15792_c0_g2_i1 | 1.254842974 | 0.283218795 | 0.999970678 | 0.69 | 0.828 | 0.2 | 0.382 | NA |
| TRINITY_DN18650_c0_g1_i4 | -1.25481371 | 0.638877496 | 0.999970678 | 2.425 | 0 | 2.189 | 3.247 | NA |
| TRINITY_DN750_c5_g1_i4 | -1.2547684 | 0.250442012 | 0.999970678 | 3.225 | 1.587 | 2.522 | 8.455 | NA |
| TRINITY_DN221444_c0_g1_i1 | -1.25456132 | 0.77440445 | 0.999970678 | 0.836 | 0 | 1.667 | 0 | NA |
| TRINITY_DN8547_c0_g1_i4 | 1.25454039 | 0.222323843 | 0.999970678 | 0.881 | 1.183 | 0.522 | 0.231 | - |
| TRINITY_DN2396_c0_g1_i6 | 1.254323086 | 0.663937071 | 0.999970678 | 6.368 | 0 | 1.778 | 0.523 | NA |
| TRINITY_DN3596_c0_g2_i4 | 1.254170688 | 0.684135631 | 0.999970678 | 0.109 | 6.327 | 2.189 | 0 | Methyltransf_11 |
| TRINITY_DN3202_c0_g1_i2 | -1.25406953 | 0.648361336 | 0.999970678 | 7.821 | 0 | 5.577 | 12.215 | NA |
| TRINITY_DN5266_c0_g1_i11 | -1.25401677 | 0.389454609 | 0.999970678 | 0.427 | 1.784 | 2.089 | 2.503 | NA |
| TRINITY_DN802_c0_g1_i1 | 1.253950333 | 0.642952363 | 0.999970678 | 4.923 | 6.8 | 4.055 | 0 | - |
| TRINITY_DN62805_c1_g1_i1 | 1.253558082 | 0.767584082 | 0.999970678 | 3.161 | 0 | 1.111 | 0 | NA |
| TRINITY_DN16785_c0_g1_i1 | -1.25322014 | 0.664625051 | 0.999970678 | 0.881 | 0.355 | 2.433 | 0 | GMC_oxred_C |
| TRINITY_DN2845_c0_g2_i5 | -1.2531995 | 0.646763046 | 0.999970678 | 0 | 1.143 | 1.744 | 0.563 | NA |
| TRINITY_DN2269_c0_g2_i1 | -1.25289375 | 0.636219193 | 0.999970678 | 12.699 | 0 | 14.855 | 12.396 | NA |
| TRINITY_DN5699_c0_g1_i1 | 1.252774064 | 0.765266149 | 0.999970678 | 0 | 3.371 | 0 | 1.387 | CTD_bind |
| TRINITY_DN4466_c0_g1_i8 | -1.25274129 | 0.773799631 | 0.999970678 | 1.453 | 0 | 2.911 | 0 | Peroxidase_2 |
| TRINITY_DN337_c0_g1_i1 | -1.25270459 | 0.657281926 | 0.999970678 | 6.368 | 0 | 8.488 | 5.178 | ADK |
| TRINITY_DN834_c0_g1_i13 | 1.252149546 | 0.447620911 | 0.999970678 | 17.196 | 1.528 | 5.489 | 1.317 | NA |
| TRINITY_DN1430_c0_g2_i1 | -1.25208573 | 0.680634869 | 0.999970678 | 73.062 | 0 | 43.164 | 124.817 | NA |
| TRINITY_DN1267_c0_g1_i7 | -1.25195086 | 0.489345719 | 0.999970678 | 25.39 | 6.465 | 51.83 | 9.31 | NA |
| TRINITY_DN2254_c0_g1_i4 | -1.25185921 | 0.649079844 | 0.999970678 | 0 | 0.394 | 0.189 | 0.694 | Cellulose_synt |
| TRINITY_DN22_c0_g2_i3 | -1.25162941 | 0.339380363 | 0.999970678 | 0.327 | 0.099 | 0.344 | 0.613 | AA_kinase |
| TRINITY_DN9236_c0_g1_i6 | -1.25159847 | 0.643389338 | 1 | 1.481 | 0 | 1.578 | 1.659 | RNase_P_Rpp14 |
| TRINITY_DN24810_c0_g1_i1 | 1.251476138 | 0.325644131 | 1 | 1.017 | 1.005 | 0.367 | 0.412 | Longin |
| TRINITY_DN1002_c0_g2_i1 | -1.25125919 | 0.768613558 | 0.999970678 | 0 | 8.82 | 0 | 20.721 | - |
| TRINITY_DN5126_c1_g1_i3 | 1.251165654 | 0.410426796 | 0.999970678 | 9.729 | 1.626 | 3.366 | 0.764 | OPT |
| TRINITY_DN2836_c1_g1_i7 | 1.251135636 | 0.639213247 | 0.999970678 | 3.634 | 4.721 | 0 | 3.519 | DEAD |
| TRINITY_DN10713_c0_g1_i2 | -1.25063521 | 0.640770768 | 0.999970678 | 0 | 2.395 | 2.155 | 3.036 | NA |
| TRINITY_DN3541_c1_g1_i3 | -1.25046625 | 0.225117834 | 0.999970678 | 0.6 | 0.641 | 1.289 | 1.357 | Glyco_hydro_32C |
| TRINITY_DN3609_c0_g1_i8 | -1.25035077 | 0.649879424 | 0.999970678 | 0.981 | 0 | 0.511 | 1.739 | NA |
| TRINITY_DN6469_c0_g1_i9 | 1.250300141 | 0.142197436 | 0.999970678 | 3.025 | 1.468 | 0.778 | 0.965 | NA |
| TRINITY_DN10094_c0_g1_i8 | -1.25024492 | 0.668009083 | 0.999970678 | 11.437 | 0 | 19.332 | 4.233 | NA |
| TRINITY_DN12560_c0_g1_i5 | 1.250161098 | 0.238917525 | 0.999970678 | 1.335 | 0.917 | 0.4 | 0.462 | Abhydrolase_6 |
| TRINITY_DN2484_c0_g3_i3 | -1.24997465 | 0.125713195 | 0.999970678 | 5.505 | 10.49 | 12.41 | 22.902 | NA |
| TRINITY_DN6042_c0_g2_i1 | 1.249951732 | 0.092107538 | 0.999970678 | 59.527 | 33.26 | 16.61 | 19.182 | PA |
| TRINITY_DN3566_c0_g2_i4 | -1.2499194 | 0.692952145 | 0.999970678 | 6.132 | 0.246 | 12.399 | 0 | NA |
| TRINITY_DN4336_c0_g1_i4 | -1.24983434 | 0.647698651 | 0.999970678 | 0 | 36.44 | 50.719 | 23.093 | NA |
| TRINITY_DN20270_c0_g1_i10 | -1.24978835 | 0.541875043 | 0.999970678 | 1.753 | 0.453 | 4.066 | 0.271 | NA |
| TRINITY_DN12215_c0_g1_i1 | -1.24971159 | 0.646975173 | 0.999970678 | 1.045 | 0 | 1.5 | 0.724 | NA |
| TRINITY_DN156_c0_g2_i1 | -1.24962839 | 0.514908984 | 0.999970678 | 7.331 | 0.108 | 6.133 | 10.536 | FHA |
| TRINITY_DN6018_c0_g1_i1 | 1.2495549 | 0.336053048 | 0.999970678 | 0.718 | 1.833 | 0.289 | 0.714 | WD40 |
| TRINITY_DN1168_c0_g1_i4 | 1.249316935 | 0.682240901 | 0.999970678 | 0.082 | 1.715 | 0.633 | 0 | NA |
| TRINITY_DN935_c0_g1_i1 | -1.24921173 | 0.224823551 | 0.999970678 | 29.069 | 15.05 | 22.088 | 78.982 | N_BRCA1_IG |
| TRINITY_DN3351_c0_g1_i3 | 1.249193796 | 0.369926541 | 0.999970678 | 82.673 | 9.619 | 21.443 | 13.311 | NA |
| TRINITY_DN1397_c0_g1_i9 | 1.24914308 | 0.534079712 | 0.999970678 | 0.173 | 3.479 | 1.167 | 0.111 | NA |
| TRINITY_DN168699_c0_g1_i1 | -1.2491209 | 0.772596356 | 0.999970678 | 1.644 | 0 | 3.266 | 0 | NA |
| TRINITY_DN7335_c0_g1_i2 | 1.248987842 | 0.76687364 | 0.999970678 | 3.997 | 0 | 1.422 | 0 | NA |
| TRINITY_DN1489_c0_g1_i2 | 1.248918027 | 0.361991504 | 0.999970678 | 9.565 | 5.371 | 4.533 | 0.824 | NA |
| TRINITY_DN2422_c0_g1_i6 | -1.24874853 | 0.518835256 | 0.999970678 | 4.833 | 0.434 | 9.899 | 0.653 | NA |
| TRINITY_DN380_c1_g1_i5 | -1.24864008 | 0.223677264 | 0.999970678 | 3.888 | 6.731 | 12.055 | 9.903 | NA |
| TRINITY_DN2200_c0_g1_i4 | 1.248600066 | 0.264119797 | 0.999970678 | 4.097 | 2.375 | 0.622 | 1.96 | F-box |
| TRINITY_DN3420_c0_g1_i3 | 1.248505455 | 0.191013811 | 0.999970678 | 4.26 | 5.627 | 2.444 | 1.206 | - |
| TRINITY_DN14191_c0_g1_i5 | 1.248504597 | 0.36706516 | 0.999970678 | 8.103 | 20.47 | 7.1 | 2.885 | NA |
| TRINITY_DN478_c0_g1_i11 | 1.248449019 | 0.462144577 | 0.999970678 | 0.173 | 3.439 | 0.633 | 0.744 | - |
| TRINITY_DN8150_c0_g1_i1 | 1.248405182 | 0.429065479 | 0.999970678 | 0.327 | 1.843 | 0.544 | 0.251 | NA |
| TRINITY_DN946_c0_g1_i1 | 1.248404588 | 0.765541277 | 0.999970678 | 2.925 | 0 | 0 | 1.257 | TRAM_LAG1_CLN |
| TRINITY_DN14291_c0_g1_i1 | 1.248258456 | 0.321355967 | 0.999970678 | 1.953 | 0.591 | 0.411 | 0.583 | NA |
| TRINITY_DN5721_c0_g2_i1 | 1.248204764 | 0.292621311 | 0.999970678 | 0.472 | 0.473 | 0.2 | 0.161 | NA |
| TRINITY_DN7345_c0_g1_i2 | -1.24805381 | 0.352567349 | 0.999970678 | 5.278 | 14.75 | 13.21 | 28 | NA |
| TRINITY_DN5408_c1_g2_i12 | 1.247786922 | 0.137155789 | 0.999970678 | 6.25 | 5.509 | 2.533 | 1.9 | NA |
| TRINITY_DN1620_c0_g1_i1 | -1.24741951 | 0.646525549 | 0.999970678 | 5.023 | 0 | 3.611 | 7.731 | EMP70 |
| TRINITY_DN3737_c0_g1_i6 | -1.2472741 | 0.766626377 | 0.999970678 | 0 | 0.936 | 0 | 2.212 | Beach |
| TRINITY_DN15112_c0_g1_i1 | 1.247043996 | 0.549363265 | 0.999970678 | 8.048 | 0.148 | 0.689 | 2.674 | EF-hand_1 |
| TRINITY_DN2291_c0_g1_i10 | -1.24701436 | 0.765821312 | 0.999970678 | 0 | 1.735 | 0 | 4.052 | Hexapep |
| TRINITY_DN1783_c0_g1_i6 | -1.24664915 | 0.447971094 | 0.999970678 | 1.653 | 18.65 | 5.5 | 41.13 | Orn_Arg_deC_N |

| TRINITY_DN112_c1_g1_i5 | -1.24651153 | 0.765841757 | 0.999970678 | 2.325 | 0 | 0 | 5.65 | NA |
| --- | --- | --- | --- | --- | --- | --- | --- | --- |
| TRINITY_DN7657_c0_g1_i2 | 1.246064917 | 0.223915375 | 0.999970678 | 3.924 | 3.371 | 1.878 | 0.794 | NA |
| TRINITY_DN2316_c0_g1_i2 | 1.246058647 | 0.231570132 | 0.999970678 | 21.156 | 7.904 | 3.444 | 8.143 | S1-P1_nuclease |
| TRINITY_DN6267_c0_g1_i1 | -1.24602807 | 0.295268959 | 0.999970678 | 6.813 | 9.757 | 5.6 | 32.212 | DUF4228 |
| TRINITY_DN17887_c0_g1_i1 | -1.24594633 | 0.637020141 | 0.999970678 | 1.054 | 0 | 0.933 | 1.438 | p450 |
| TRINITY_DN3994_c0_g1_i1 | -1.24570522 | 0.217914756 | 0.999970678 | 18.822 | 13.74 | 15.044 | 59.256 | Hs1pro-1_C |
| TRINITY_DN913_c5_g1_i3 | 1.245660913 | 0.307720274 | 0.999970678 | 5.478 | 2.188 | 2.111 | 0.724 | Aldedh |
| TRINITY_DN7902_c0_g1_i2 | 1.24549135 | 0.341158524 | 0.999970678 | 1.017 | 1.114 | 0.167 | 0.694 | - |
| TRINITY_DN4301_c0_g1_i1 | 1.245324078 | 0.666097052 | 0.999970678 | 6.468 | 0 | 0.489 | 2.192 | WRKY |
| TRINITY_DN9073_c0_g1_i1 | 1.245078844 | 0.706050343 | 0.999970678 | 0.073 | 16.62 | 5.733 | 0 | NA |
| TRINITY_DN659_c0_g2_i6 | -1.2446853 | 0.417887789 | 0.999970678 | 6.25 | 0.503 | 4.722 | 10.486 | NA |
| TRINITY_DN5579_c0_g1_i6 | 1.244406241 | 0.647555916 | 0.999970678 | 0.772 | 3.863 | 0 | 1.95 | NA |
| TRINITY_DN2421_c0_g1_i2 | -1.24427101 | 0.572207284 | 0.999970678 | 36.372 | 0.099 | 32.742 | 47.665 | - |
| TRINITY_DN4510_c0_g1_i2 | -1.24426306 | 0.287574578 | 0.999970678 | 1.644 | 0.71 | 1.367 | 3.891 | PRA1 |
| TRINITY_DN1660_c0_g1_i2 | -1.24412144 | 0.46887937 | 0.999970678 | 3.007 | 12.54 | 28.698 | 1.729 | Glyco_transf_5 |
| TRINITY_DN9367_c0_g1_i4 | 1.244021025 | 0.282716167 | 0.999970678 | 5.332 | 1.735 | 1.722 | 0.935 | NA |
| TRINITY_DN13966_c0_g1_i3 | 1.243934787 | 0.537516997 | 1 | 0.363 | 0.177 | 0.178 | 0.01 | PP2C |
| TRINITY_DN3087_c0_g1_i4 | -1.24381039 | 0.330247343 | 1 | 0.363 | 0.71 | 0.911 | 1.498 | NA |
| TRINITY_DN4964_c0_g1_i1 | 1.243765151 | 0.208478013 | 0.999970678 | 6.259 | 7.973 | 1.722 | 3.911 | NA |
| TRINITY_DN5745_c0_g1_i4 | 1.243673751 | 0.277332067 | 0.999970678 | 1.145 | 3.065 | 0.656 | 0.975 | NA |
| TRINITY_DN1704_c0_g1_i5 | 1.243494074 | 0.649151135 | 0.999970678 | 11.727 | 5.391 | 0 | 7.299 | Kri1 |
| TRINITY_DN713_c0_g1_i7 | 1.24346464 | 0.162922809 | 0.999970678 | 3.316 | 6.603 | 1.378 | 2.513 | NA |
| TRINITY_DN31160_c0_g2_i1 | -1.2431983 | 0.272050105 | 0.999970678 | 0.808 | 1.242 | 1.367 | 3.127 | NA |
| TRINITY_DN5975_c0_g1_i2 | -1.24319484 | 0.766903927 | 0.999970678 | 14.371 | 0 | 0 | 33.78 | NA |
| TRINITY_DN41955_c0_g1_i8 | 1.243167959 | 0.311819724 | 0.999970678 | 22.383 | 15.14 | 3.822 | 10.627 | LRRNT_2 |
| TRINITY_DN4658_c0_g1_i4 | -1.24300946 | 0.644035356 | 0.999970678 | 1.09 | 0 | 1.578 | 0.744 | NA |
| TRINITY_DN17247_c0_g1_i1 | 1.242623303 | 0.469075913 | 0.999970678 | 0.409 | 0.798 | 0.378 | 0.05 | NA |
| TRINITY_DN37800_c0_g1_i1 | 1.242536373 | 0.569237825 | 0.999970678 | 1.417 | 0.227 | 0.567 | 0.02 | NA |
| TRINITY_DN691_c1_g1_i4 | 1.242004779 | 0.264593997 | 0.999970678 | 1.026 | 3.548 | 0.667 | 1.126 | EF-hand_7 |
| TRINITY_DN4067_c0_g1_i8 | 1.241592514 | 0.328922687 | 0.999970678 | 4.787 | 14.11 | 1.633 | 5.902 | NA |
| TRINITY_DN758_c0_g1_i1 | 1.24135691 | 0.243689075 | 0.999970678 | 3.852 | 1.37 | 1 | 1.005 | Sugar_tr |
| TRINITY_DN46655_c0_g1_i2 | 1.240807746 | 0.773802657 | 0.999970678 | 1.263 | 0 | 0.444 | 0 | NDT80_PhoG |
| TRINITY_DN2927_c0_g1_i7 | 1.240733836 | 0.658031163 | 0.999970678 | 0 | 2.602 | 0.567 | 0.412 | DND1_DSRM |
| TRINITY_DN4618_c0_g1_i2 | 1.240712523 | 0.779548296 | 1 | 1.244 | 0 | 0 | 0.543 | NA |
| TRINITY_DN885_c0_g1_i5 | 1.240712523 | 0.779548296 | 1 | 0.69 | 0 | 0 | 0.292 | CWC25 |
| TRINITY_DN6564_c0_g2_i2 | 1.240415664 | 0.261803231 | 0.999970678 | 0.836 | 1.005 | 0.211 | 0.533 | NA |
| TRINITY_DN5464_c0_g2_i1 | 1.240401468 | 0.169743918 | 0.999970678 | 3.752 | 9.727 | 2.333 | 2.865 | NA |
| TRINITY_DN272_c0_g2_i1 | -1.24034637 | 0.249030206 | 0.999970678 | 13.735 | 11.83 | 10.055 | 48.368 | BPS1 |
| TRINITY_DN2596_c0_g1_i1 | -1.24030082 | 0.662526592 | 0.999970678 | 1.299 | 0 | 2.167 | 0.513 | NA |
| TRINITY_DN27504_c0_g2_i2 | -1.24002564 | 0.694590716 | 0.999970678 | 0.872 | 0.069 | 1.855 | 0 | PIR |
| TRINITY_DN2319_c1_g2_i4 | 1.240002388 | 0.429888188 | 0.999970678 | 1.708 | 0.67 | 0.767 | 0.09 | NB-ARC |
| TRINITY_DN953_c0_g1_i2 | 1.239950221 | 0.267520764 | 0.999970678 | 2.852 | 2.927 | 1.578 | 0.543 | TFIID_20kDa |
| TRINITY_DN6599_c3_g1_i1 | -1.23986577 | 0.358102795 | 0.999970678 | 2.653 | 5.578 | 13.644 | 2.795 | NA |
| TRINITY_DN525_c1_g2_i2 | -1.23973794 | 0.446343359 | 0.999970678 | 1.735 | 0.138 | 2.578 | 1.387 | FKBP_C |
| TRINITY_DN976_c0_g1_i5 | -1.23968951 | 0.775459436 | 0.999970678 | 0 | 0.286 | 0 | 0.674 | DNase-RNase |
| TRINITY_DN8982_c0_g1_i2 | 1.239507278 | 0.190932702 | 0.999970678 | 1.989 | 2.424 | 0.678 | 1.036 | NA |
| TRINITY_DN583_c1_g1_i12 | 1.239417243 | 0.341549803 | 0.999970678 | 1.308 | 2.03 | 0.178 | 1.186 | Pkinase |
| TRINITY_DN7152_c0_g1_i4 | -1.23927408 | 0.766364728 | 0.999970678 | 0 | 3.913 | 0 | 9.109 | NA |
| TRINITY_DN2788_c0_g1_i1 | 1.239248527 | 0.234913271 | 0.999970678 | 73.262 | 21.57 | 14.688 | 22.42 | NA |
| TRINITY_DN624_c0_g1_i2 | -1.2392076 | 0.644649465 | 1 | 0 | 1.321 | 1.322 | 1.397 | Sgf11 |
| TRINITY_DN3021_c0_g1_i4 | -1.23916122 | 0.780924988 | 0.999970678 | 30.504 | 0 | 0 | 73.221 | NA |
| TRINITY_DN152_c1_g1_i5 | 1.238925868 | 0.652919562 | 1 | 0.209 | 0.591 | 0.267 | 0 | NA |
| TRINITY_DN1761_c0_g1_i3 | 1.238795347 | 0.699161577 | 0.999970678 | 190.58 | 0 | 18.932 | 59.367 | NA |
| TRINITY_DN4444_c0_g1_i10 | -1.23871423 | 0.355433176 | 0.999970678 | 3.234 | 12.02 | 11.888 | 19.715 | NA |
| TRINITY_DN23706_c0_g1_i1 | -1.23870151 | 0.500141688 | 1 | 4.987 | 0.719 | 2.278 | 10.315 | NA |
| TRINITY_DN1353_c0_g1_i4 | 1.238667796 | 0.777608242 | 1 | 0 | 2.819 | 0 | 1.196 | RNase_PH |
| TRINITY_DN2349_c0_g1_i9 | 1.238512299 | 0.704139067 | 0.999970678 | 0 | 5.056 | 1.711 | 0.03 | NA |
| TRINITY_DN2604_c0_g1_i2 | -1.23841033 | 0.422976832 | 0.999970678 | 3.861 | 0.434 | 6.266 | 2.664 | 2_5_RNA_ligase2 |
| TRINITY_DN5919_c1_g1_i2 | -1.23835589 | 0.779241452 | 1 | 0 | 0.828 | 1.6 | 0 | DUF2040 |
| TRINITY_DN14059_c0_g1_i1 | -1.23829474 | 0.678190503 | 1 | 2.434 | 0 | 0.444 | 5.097 | ABC2_membrane |
| TRINITY_DN258_c0_g2_i5 | 1.238182595 | 0.677157985 | 0.999970678 | 5.932 | 0.463 | 2.222 | 0 | NA |
| TRINITY_DN6026_c0_g1_i1 | -1.2379196 | 0.367842417 | 0.999970678 | 2.58 | 0.384 | 1.989 | 4.705 | F-box-like |
| TRINITY_DN1621_c0_g1_i8 | 1.237866264 | 0.131379691 | 0.999970678 | 2.135 | 2.089 | 0.589 | 1.086 | ERCC3_RAD25_C |
| TRINITY_DN7018_c0_g1_i1 | 1.237850811 | 0.312510818 | 0.999970678 | 0.745 | 1.981 | 0.722 | 0.271 | NA |
| TRINITY_DN761_c0_g2_i2 | -1.23769938 | 0.345876455 | 0.999970678 | 22.664 | 3.321 | 33.442 | 21.605 | NA |
| TRINITY_DN1032_c0_g1_i4 | -1.23768324 | 0.460210426 | 0.999970678 | 10.737 | 220.6 | 97.772 | 421.491 | Lipoxygenase |
| TRINITY_DN3146_c0_g1_i1 | -1.23765243 | 0.168850141 | 0.999970678 | 12.182 | 10.46 | 12.444 | 38.455 | Glyco_transf_34 |
| TRINITY_DN8314_c1_g1_i3 | -1.23742706 | 0.770602984 | 0.999970678 | 18.286 | 0 | 35.953 | 0 | NA |
| TRINITY_DN8124_c0_g3_i1 | 1.237283914 | 0.308971999 | 0.999970678 | 0.818 | 2.484 | 0.522 | 0.774 | NA |
| TRINITY_DN315_c0_g1_i2 | 1.23697119 | 0.431926219 | 0.999970678 | 1.063 | 20.03 | 4.333 | 3.619 | MA3 |
| TRINITY_DN29017_c0_g1_i1 | 1.236936812 | 0.686710863 | 0.999970678 | 0 | 2.237 | 0.722 | 0.101 | Pro_CA |
| TRINITY_DN18248_c0_g1_i9 | -1.23692523 | 0.659811793 | 0.999970678 | 0.808 | 0.473 | 2.5 | 0 | ABC_membrane |
| TRINITY_DN5012_c0_g1_i2 | 1.236864473 | 0.575279917 | 0.999970678 | 17.814 | 0.089 | 5.222 | 1.397 | NA |
| TRINITY_DN1735_c0_g1_i7 | -1.2368355 | 0.257776801 | 0.999970678 | 11.219 | 6.298 | 7.477 | 32.463 | NA |

| TRINITY_DN753_c0_g1_i1 | 1.236547549 | 0.175960192 | 0.999970678 | 16.079 | 19.68 | 3.478 | 10.959 | NA |
| --- | --- | --- | --- | --- | --- | --- | --- | --- |
| TRINITY_DN8626_c0_g1_i8 | 1.236545692 | 0.390080073 | 0.999970678 | 11.31 | 1.508 | 3.322 | 1.478 | MIP |
| TRINITY_DN1087_c0_g1_i4 | 1.236442168 | 0.242484968 | 0.999970678 | 4.061 | 3.706 | 2.111 | 0.764 | B3_4 |
| TRINITY_DN8133_c0_g1_i8 | -1.23627707 | 0.381078088 | 0.999970678 | 0.572 | 0.365 | 0.244 | 1.95 | Cyclin_N |
| TRINITY_DN15245_c0_g1_i3 | 1.23617134 | 0.406027926 | 0.999970678 | 3.334 | 12.41 | 3.966 | 1.689 | NA |
| TRINITY_DN10746_c0_g4_i1 | 1.235914317 | 0.665027282 | 0.999970678 | 8.13 | 0 | 0.756 | 2.554 | NA |
| TRINITY_DN37091_c0_g1_i6 | 1.235834772 | 0.777034422 | 0.999970678 | 0 | 0.995 | 0.344 | 0 | EF_assoc_1 |
| TRINITY_DN3356_c0_g1_i2 | 1.235825883 | 0.67032699 | 0.999970678 | 9.647 | 0 | 2.233 | 1.468 | NA |
| TRINITY_DN266_c0_g1_i2 | -1.23571053 | 0.305628121 | 0.999970678 | 22.374 | 54.88 | 122.048 | 32.966 | NA |
| TRINITY_DN2565_c0_g1_i1 | -1.23569725 | 0.644864479 | 1 | 0 | 1.419 | 1.422 | 1.619 | - |
| TRINITY_DN673_c0_g1_i1 | -1.2353929 | 0.645535811 | 0.999970678 | 0 | 11.13 | 8.577 | 15.422 | NA |
| TRINITY_DN5523_c0_g1_i2 | -1.2352888 | 0.30009714 | 0.999970678 | 0.6 | 0.424 | 1.389 | 0.724 | NA |
| TRINITY_DN3990_c0_g1_i4 | -1.23472418 | 0.665239845 | 0.999970678 | 21.593 | 0 | 18.343 | 29.477 | NA |
| TRINITY_DN5541_c1_g2_i11 | -1.23469886 | 0.465842169 | 0.999970678 | 0.545 | 0.059 | 0.533 | 0.804 | Actin |
| TRINITY_DN2469_c0_g1_i8 | 1.234521734 | 0.451428728 | 0.999970678 | 1.036 | 19.89 | 2.2 | 6.113 | Glyco_hydro_14 |
| TRINITY_DN5707_c0_g1_i1 | -1.23440082 | 0.409292059 | 0.999970678 | 3.843 | 2.592 | 11.51 | 1.277 | NA |
| TRINITY_DN5_c1_g4_i4 | 1.234104598 | 0.644120751 | 0.999970678 | 5.369 | 2.533 | 2.8 | 0 | NA |
| TRINITY_DN1746_c0_g2_i2 | -1.23405421 | 0.47762678 | 1 | 0.254 | 0.039 | 0.389 | 0.211 | - |
| TRINITY_DN2138_c0_g2_i1 | -1.23392626 | 0.769403156 | 0.999970678 | 10.683 | 0 | 20.943 | 0 | Peroxidase_2 |
| TRINITY_DN2586_c0_g1_i1 | -1.23387292 | 0.707814451 | 0.999970678 | 11.409 | 0.404 | 22.71 | 0 | Histone |
| TRINITY_DN6861_c0_g1_i13 | -1.23387292 | 0.707814451 | 0.999970678 | 3.525 | 0.128 | 7.1 | 0 | NA |
| TRINITY_DN502_c0_g2_i4 | -1.23365743 | 0.091785921 | 0.999970678 | 4.197 | 3.518 | 6.033 | 10.848 | Tubulin |
| TRINITY_DN15988_c0_g5_i1 | -1.23363753 | 0.637686177 | 0.999970678 | 0 | 1.577 | 1.389 | 1.971 | zf-RRN7 |
| TRINITY_DN485_c0_g1_i10 | -1.23363558 | 0.313452667 | 0.999970678 | 13.78 | 17.24 | 8.777 | 62.001 | NA |
| TRINITY_DN10695_c0_g1_i1 | 1.233603858 | 0.276455212 | 0.999970678 | 88.114 | 313.8 | 39.786 | 120.996 | Epimerase |
| TRINITY_DN236_c2_g1_i1 | -1.23354223 | 0.54877865 | 0.999970678 | 8.911 | 0.089 | 5.033 | 15.302 | NA |
| TRINITY_DN304_c0_g1_i1 | -1.23353467 | 0.654403898 | 0.999970678 | 1.762 | 0 | 0.567 | 3.519 | NA |
| TRINITY_DN1306_c0_g1_i11 | -1.23351389 | 0.678862978 | 0.999970678 | 0 | 87.01 | 53.086 | 137.112 | NA |
| TRINITY_DN1361_c0_g1_i5 | 1.233476604 | 0.279554337 | 0.999970678 | 0.99 | 0.779 | 0.444 | 0.221 | NA |
| TRINITY_DN10484_c0_g1_i8 | -1.23334811 | 0.661471217 | 0.999970678 | 0.227 | 0.256 | 0.955 | 0 | NA |
| TRINITY_DN1126_c1_g1_i11 | 1.233221942 | 0.769524543 | 0.999970678 | 2.534 | 0 | 0.911 | 0 | NA |
| TRINITY_DN21267_c0_g1_i3 | 1.233214857 | 0.440826717 | 0.999970678 | 6.577 | 1.419 | 2.1 | 0.824 | NA |
| TRINITY_DN22071_c0_g1_i1 | -1.2331679 | 0.461939149 | 0.999970678 | 0.291 | 1.025 | 0.211 | 2.845 | NA |
| TRINITY_DN13320_c0_g1_i2 | 1.233030546 | 0.386030982 | 0.999970678 | 0.309 | 1.646 | 0.411 | 0.322 | NA |
| TRINITY_DN3570_c0_g2_i6 | 1.232906482 | 0.675790801 | 0.999970678 | 3.261 | 0 | 0.922 | 0.292 | NA |
| TRINITY_DN6222_c0_g2_i4 | 1.232599727 | 0.52534665 | 0.999970678 | 0.863 | 0.769 | 0.033 | 0.674 | NA |
| TRINITY_DN12815_c0_g1_i4 | 1.232529185 | 0.6734458 | 0.999970678 | 1.935 | 0 | 0.2 | 0.583 | NA |
| TRINITY_DN6266_c0_g1_i1 | -1.23247615 | 0.214595281 | 0.999970678 | 1.417 | 1.971 | 1.844 | 5.731 | NA |
| TRINITY_DN10299_c0_g1_i13 | -1.23234902 | 0.647566513 | 0.999970678 | 1.917 | 0 | 1.044 | 3.288 | NA |
| TRINITY_DN5933_c0_g1_i1 | -1.23233614 | 0.448663655 | 0.999970678 | 0.254 | 0.532 | 0.133 | 1.659 | DUF3675 |
| TRINITY_DN16034_c0_g1_i7 | -1.23220605 | 0.772327863 | 0.999970678 | 0.781 | 0 | 0 | 1.89 | NA |
| TRINITY_DN796_c0_g1_i4 | -1.2320901 | 0.21760327 | 0.999970678 | 83.163 | 43.96 | 66.385 | 217.19 | NA |
| TRINITY_DN17464_c0_g2_i3 | -1.23190615 | 0.506562531 | 0.999970678 | 1.026 | 0.177 | 0.289 | 2.473 | NA |
| TRINITY_DN20346_c0_g1_i5 | 1.2317388 | 0.640974981 | 0.999970678 | 0.609 | 0.562 | 0.411 | 0 | HLH |
| TRINITY_DN915_c0_g1_i1 | 1.231708572 | 0.769932787 | 0.999970678 | 53.532 | 0 | 18.877 | 0 | NA |
| TRINITY_DN13234_c0_g1_i8 | -1.23165031 | 0.645915014 | 0.999970678 | 0.191 | 0 | 0.144 | 0.292 | NA |
| TRINITY_DN11035_c0_g1_i5 | 1.231322401 | 0.414367879 | 0.999970678 | 2.235 | 0.394 | 0.411 | 0.653 | NA |
| TRINITY_DN1029_c0_g1_i2 | 1.231264877 | 0.242490423 | 0.999970678 | 43.785 | 55.72 | 7.388 | 33.238 | - |
| TRINITY_DN439_c0_g1_i16 | -1.23118499 | 0.641934253 | 0.999970678 | 10.828 | 0 | 9.988 | 13.502 | NA |
| TRINITY_DN56963_c0_g1_i1 | 1.231065588 | 0.454035196 | 0.999970678 | 56.002 | 761.7 | 54.43 | 244.395 | NA |
| TRINITY_DN934_c0_g1_i4 | 1.230881527 | 0.780842585 | 0.999970678 | 0 | 28.42 | 9.855 | 0 | NA |
| TRINITY_DN1584_c0_g1_i9 | -1.23069892 | 0.333024744 | 0.999970678 | 1.444 | 0.828 | 0.822 | 4.393 | B_lectin |
| TRINITY_DN1544_c0_g1_i4 | 1.230586037 | 0.69560206 | 0.999970678 | 4.369 | 0 | 0.067 | 1.81 | Motile_Sperm |
| TRINITY_DN449_c1_g1_i3 | 1.230585227 | 0.406817932 | 0.999970678 | 30.976 | 3.676 | 8.466 | 4.554 | Dimerisation |
| TRINITY_DN2393_c0_g1_i3 | -1.23053868 | 0.243590393 | 0.999970678 | 5.151 | 8.505 | 6.133 | 24.521 | Abhydrolase_3 |
| TRINITY_DN3662_c0_g1_i2 | 1.230429945 | 0.301825491 | 0.999970678 | 15.125 | 3.804 | 2.122 | 5.59 | NA |
| TRINITY_DN1869_c0_g1_i1 | -1.230298 | 0.523652136 | 0.999970678 | 6.677 | 0.177 | 9.766 | 4.363 | NA |
| TRINITY_DN382_c2_g1_i4 | 1.23027074 | 0.205040916 | 0.999970678 | 7.621 | 3.311 | 2.022 | 2.262 | G-patch |
| TRINITY_DN974_c0_g1_i3 | 1.23009022 | 0.768547554 | 0.999970678 | 0 | 4.514 | 0 | 1.89 | Branch |
| TRINITY_DN955_c0_g1_i4 | 1.229730137 | 0.770625413 | 0.999970678 | 0 | 4.721 | 1.633 | 0 | NA |
| TRINITY_DN3091_c0_g1_i4 | 1.229702156 | 0.406266293 | 0.999970678 | 0.908 | 0.365 | 0.367 | 0.121 | NA |
| TRINITY_DN9042_c0_g1_i1 | 1.229608001 | 0.379180023 | 0.999970678 | 2.643 | 7.865 | 3.178 | 0.593 | - |
| TRINITY_DN8433_c0_g1_i1 | 1.229581559 | 0.342616516 | 1 | 0.718 | 1.153 | 0.244 | 0.513 | NA |
| TRINITY_DN9340_c0_g1_i1 | -1.22939838 | 0.151920384 | 0.999970678 | 2.062 | 1.33 | 3.644 | 3.589 | NA |
| TRINITY_DN6240_c0_g1_i5 | 1.229316079 | 0.644861987 | 0.999970678 | 0.345 | 0.838 | 0.411 | 0 | TMEM135_C_rich |
| TRINITY_DN5700_c1_g1_i2 | 1.229147625 | 0.644979166 | 1 | 0.663 | 1.311 | 0 | 0.834 | NA |
| TRINITY_DN1089_c0_g1_i16 | -1.22907948 | 0.706934786 | 0.999970678 | 0 | 2.217 | 4.2 | 0.05 | NA |
| TRINITY_DN7630_c0_g1_i4 | -1.22906402 | 0.680757705 | 0.999970678 | 6.986 | 0 | 0.544 | 15.975 | BAG |
| TRINITY_DN2869_c0_g1_i1 | -1.22895285 | 0.769228836 | 0.999970678 | 0 | 8.278 | 0 | 19.052 | CCT_2 |
| TRINITY_DN1433_c1_g1_i1 | -1.22892776 | 0.684426595 | 0.999970678 | 1.88 | 0.177 | 4.022 | 0 | PIR |
| TRINITY_DN3276_c0_g1_i6 | -1.22884043 | 0.645030133 | 0.999970678 | 0.845 | 0 | 0.533 | 1.367 | GRAS |
| TRINITY_DN10439_c0_g1_i1 | 1.228793121 | 0.237453417 | 0.999970678 | 12.481 | 43.87 | 12.077 | 9.28 | Alpha-amylase |
| TRINITY_DN4222_c0_g3_i1 | -1.22860257 | 0.657328954 | 1 | 1.226 | 0 | 0.778 | 1.95 | NA |
| TRINITY_DN3927_c0_g2_i2 | 1.228590242 | 0.19183255 | 0.999970678 | 1.072 | 1.34 | 0.422 | 0.503 | NA |

| TRINITY_DN6689_c0_g1_i2 | 1.228397161 | 0.237771768 | 0.999970678 | 0.736 | 1.094 | 0.267 | 0.462 | NA |
| --- | --- | --- | --- | --- | --- | --- | --- | --- |
| TRINITY_DN15824_c0_g1_i1 | -1.22834233 | 0.500611529 | 0.999970678 | 1.154 | 0.089 | 1.222 | 1.448 | NA |
| TRINITY_DN18386_c0_g1_i5 | 1.228285329 | 0.293878557 | 0.999970678 | 0.4 | 0.404 | 0.122 | 0.201 | NA |
| TRINITY_DN34574_c0_g1_i10 | -1.22812419 | 0.783743465 | 1 | 0.709 | 0 | 1.4 | 0 | NA |
| TRINITY_DN4853_c0_g1_i5 | -1.22812419 | 0.783743465 | 1 | 3.788 | 0 | 7.188 | 0 | NA |
| TRINITY_DN2646_c0_g1_i10 | -1.22810293 | 0.314805579 | 0.999970678 | 0.418 | 0.335 | 0.478 | 1.166 | HMG_box |
| TRINITY_DN12019_c0_g1_i1 | -1.22808098 | 0.779266327 | 0.999970678 | 0.69 | 0 | 1.333 | 0 | NA |
| TRINITY_DN72954_c0_g1_i2 | -1.22808098 | 0.779266327 | 0.999970678 | 0.718 | 0 | 1.433 | 0 | - |
| TRINITY_DN5476_c0_g1_i1 | -1.22808098 | 0.779266327 | 0.999970678 | 1.544 | 0 | 3 | 0 | - |
| TRINITY_DN7737_c0_g1_i8 | -1.22803804 | 0.774846458 | 0.999970678 | 1.09 | 0 | 2.144 | 0 | Pro-kuma_activ |
| TRINITY_DN11326_c0_g1_i2 | 1.228036816 | 0.652403159 | 0.999970678 | 0.209 | 0.818 | 0.356 | 0 | FAR1 |
| TRINITY_DN1675_c0_g1_i11 | -1.22795059 | 0.241603931 | 0.999970678 | 1.29 | 0.69 | 2.155 | 1.981 | NA |
| TRINITY_DN1078_c0_g1_i8 | -1.22775949 | 0.326321584 | 0.999970678 | 2.48 | 0.611 | 1.589 | 5.419 | LRRNT_2 |
| TRINITY_DN6721_c0_g1_i5 | 1.227342674 | 0.39713061 | 0.999970678 | 0.863 | 0.296 | 0.311 | 0.121 | DRY_EERY |
| TRINITY_DN1844_c0_g2_i2 | -1.2273304 | 0.438404478 | 0.999970678 | 2.752 | 0.542 | 2.022 | 5.158 | NA |
| TRINITY_DN897_c1_g1_i12 | -1.22720543 | 0.772615519 | 0.999970678 | 8.148 | 0 | 0 | 19.394 | NA |
| TRINITY_DN208_c0_g1_i1 | 1.227129174 | 0.652460716 | 0.999970678 | 24.608 | 58.47 | 0 | 34.675 | Ribosomal_L36e |
| TRINITY_DN3952_c0_g1_i1 | -1.22708097 | 0.339839449 | 0.999970678 | 6.195 | 23.41 | 14.632 | 48.378 | NA |
| TRINITY_DN867_c0_g1_i5 | -1.22688658 | 0.322830331 | 0.999970678 | 1.19 | 0.266 | 1.5 | 1.639 | ANF_receptor |
| TRINITY_DN6863_c0_g2_i3 | 1.226482085 | 0.423865922 | 1 | 0.745 | 0.71 | 0.422 | 0.111 | NA |
| TRINITY_DN4453_c0_g1_i6 | -1.22639124 | 0.652057141 | 1 | 0.79 | 0 | 0.778 | 0.935 | NA |
| TRINITY_DN828_c0_g1_i2 | 1.226381659 | 0.63796497 | 0.999970678 | 0.136 | 29.44 | 0.256 | 11.733 | NA |
| TRINITY_DN5421_c0_g1_i1 | 1.226356407 | 0.769046561 | 0.999970678 | 0 | 3.844 | 0 | 1.619 | DUF2828 |
| TRINITY_DN33092_c0_g2_i1 | 1.226181487 | 0.249735062 | 0.999970678 | 1.508 | 1.794 | 0.889 | 0.332 | DEAD |
| TRINITY_DN29767_c1_g2_i1 | 1.226045281 | 0.273233056 | 0.999970678 | 0.89 | 0.65 | 0.356 | 0.231 | BRX |
| TRINITY_DN8846_c0_g1_i5 | 1.225922559 | 0.664764345 | 0.999970678 | 0 | 3.233 | 0.833 | 0.372 | MMR_HSR1 |
| TRINITY_DN9976_c0_g1_i3 | -1.22583629 | 0.522768982 | 0.999970678 | 0.581 | 0.079 | 0.144 | 1.387 | NA |
| TRINITY_DN9976_c0_g1_i2 | -1.22583629 | 0.522768982 | 0.999970678 | 0.581 | 0.079 | 0.144 | 1.387 | NA |
| TRINITY_DN7475_c0_g1_i4 | 1.225690019 | 0.395437224 | 0.999970678 | 14.516 | 14.02 | 1.778 | 9.39 | p450 |
| TRINITY_DN2080_c0_g1_i3 | -1.22567246 | 0.360649065 | 0.999970678 | 1.862 | 0.877 | 3.755 | 1.86 | Lectin_legB |
| TRINITY_DN45811_c0_g1_i2 | 1.225657764 | 0.773613911 | 0.999970678 | 7.958 | 0 | 2.844 | 0 | 2014/3/3 |
| TRINITY_DN55655_c0_g1_i1 | 1.225483511 | 0.782572728 | 1 | 1.472 | 0 | 0.533 | 0 | AMP-binding |
| TRINITY_DN5541_c1_g1_i7 | 1.225340205 | 0.636773781 | 0.999970678 | 1.699 | 1.045 | 0 | 1.176 | NA |
| TRINITY_DN9011_c0_g2_i1 | -1.22523259 | 0.673328587 | 1 | 0.227 | 0 | 0.389 | 0.08 | NA |
| TRINITY_DN8338_c0_g1_i2 | 1.224471128 | 0.343699809 | 0.999970678 | 3.043 | 20.57 | 4.322 | 4.765 | RINGv |
| TRINITY_DN4016_c0_g1_i2 | 1.224278957 | 0.672380711 | 0.999970678 | 7.267 | 0 | 2.133 | 0.563 | Myb_DNA-bindin |
| TRINITY_DN2056_c0_g1_i6 | 1.224146711 | 0.258188273 | 0.999970678 | 1.526 | 0.769 | 0.522 | 0.372 | NA |
| TRINITY_DN5882_c0_g3_i1 | -1.22411928 | 0.659277994 | 0.999970678 | 3.188 | 0 | 5.3 | 1.156 | NA |
| TRINITY_DN2928_c0_g1_i13 | 1.224105862 | 0.393974441 | 0.999970678 | 2.18 | 7.825 | 1.144 | 2.815 | NB-ARC |
| TRINITY_DN1632_c0_g1_i4 | -1.22384773 | 0.327724722 | 0.999970678 | 0.881 | 0.414 | 2.022 | 0.603 | NA |
| TRINITY_DN2138_c0_g1_i5 | -1.22369496 | 0.771933877 | 0.999970678 | 11.01 | 0 | 21.454 | 0 | Peroxidase_2 |
| TRINITY_DN1219_c1_g1_i2 | 1.223684588 | 0.665380039 | 0.999970678 | 0 | 7.47 | 1.622 | 1.206 | HGTP_anticodon |
| TRINITY_DN2615_c0_g1_i3 | 1.223656462 | 0.521208571 | 0.999970678 | 6.495 | 2.02 | 2.844 | 0.221 | NA |
| TRINITY_DN2366_c0_g1_i1 | -1.22365231 | 0.777852116 | 0.999970678 | 12.49 | 0 | 24.454 | 0 | Grp1_Fun34_Yaa |
| TRINITY_DN9151_c0_g1_i1 | 1.223628783 | 0.430171762 | 0.999970678 | 0.981 | 9.648 | 1.133 | 3.066 | Lipase_GDSL |
| TRINITY_DN4112_c0_g1_i2 | -1.22357215 | 0.656417737 | 0.999970678 | 6.631 | 0 | 2.866 | 12.245 | PCO_ADO |
| TRINITY_DN1846_c0_g3_i1 | 1.223111417 | 0.240710835 | 0.999970678 | 6.195 | 5.056 | 2.6 | 1.689 | NA |
| TRINITY_DN25527_c1_g1_i11 | -1.22299515 | 0.457058187 | 0.999970678 | 2.289 | 0.473 | 1.322 | 4.796 | NA |
| TRINITY_DN13097_c0_g1_i1 | -1.222817 | 0.221885685 | 0.999970678 | 0.836 | 0.739 | 1.678 | 1.629 | NA |
| TRINITY_DN9209_c0_g3_i2 | -1.22278911 | 0.644037997 | 0.999970678 | 1.181 | 0 | 1.022 | 1.568 | NA |
| TRINITY_DN2804_c0_g1_i1 | 1.222674763 | 0.661892324 | 0.999970678 | 0 | 6.751 | 1.022 | 1.619 | DUF4588 |
| TRINITY_DN4652_c0_g1_i1 | 1.222590637 | 0.097841718 | 0.999970678 | 65.659 | 49.21 | 15.277 | 30.905 | Sigma70_r2 |
| TRINITY_DN7912_c0_g1_i1 | 1.222447618 | 0.412471626 | 0.999970678 | 12.309 | 96.56 | 7.366 | 36.726 | Pollen_Ole_e_I |
| TRINITY_DN5322_c0_g1_i3 | -1.22241202 | 0.645027741 | 0.999970678 | 14.516 | 0.227 | 28.587 | 0.191 | Aldedh |
| TRINITY_DN2337_c0_g2_i1 | -1.22238097 | 0.278442148 | 0.999970678 | 5.605 | 2.346 | 3.822 | 14.055 | NA |
| TRINITY_DN4535_c0_g2_i1 | -1.22233003 | 0.29274816 | 0.999970678 | 2.198 | 3.696 | 2.067 | 11.27 | NA |
| TRINITY_DN12140_c0_g1_i3 | -1.22215499 | 0.664394916 | 0.999970678 | 0.836 | 0.414 | 0 | 2.966 | NA |
| TRINITY_DN2722_c1_g1_i8 | 1.222040034 | 0.566843029 | 0.999970678 | 0.209 | 5.696 | 1.755 | 0.322 | NA |
| TRINITY_DN2746_c2_g1_i6 | -1.22192372 | 0.308073798 | 0.999970678 | 0.2 | 0.453 | 0.578 | 0.824 | NA |
| TRINITY_DN1812_c1_g1_i5 | 1.221897434 | 0.649629196 | 0.999970678 | 3.861 | 13.03 | 5.944 | 0 | NA |
| TRINITY_DN2524_c0_g1_i4 | -1.22167895 | 0.645132205 | 0.999970678 | 1.244 | 0 | 1.3 | 1.397 | NA |
| TRINITY_DN1563_c0_g1_i1 | 1.221675929 | 0.455191366 | 0.999970678 | 12.636 | 0.779 | 2.055 | 3.338 | HhH-GPD |
| TRINITY_DN15089_c0_g1_i3 | -1.22154295 | 0.773821972 | 0.999970678 | 0 | 1.222 | 0 | 2.785 | NA |
| TRINITY_DN14473_c0_g1_i1 | -1.22137231 | 0.249008521 | 0.999970678 | 6.322 | 3.302 | 13.944 | 5.68 | PPR |
| TRINITY_DN6797_c0_g1_i3 | 1.221283863 | 0.63812264 | 0.999970678 | 2.771 | 4.139 | 0 | 2.966 | NA |
| TRINITY_DN5597_c0_g1_i1 | -1.22097333 | 0.20872424 | 0.999970678 | 1.135 | 1.675 | 2.6 | 3.318 | Cauli_VI |
| TRINITY_DN15634_c0_g4_i2 | -1.2208899 | 0.029595494 | 0.999970678 | 24.381 | 24.92 | 47.386 | 57.517 | HLH |
| TRINITY_DN710_c0_g1_i4 | -1.22078115 | 0.517768525 | 0.999970678 | 4.133 | 0.217 | 1.711 | 8.083 | NA |
| TRINITY_DN7055_c0_g2_i6 | -1.22073137 | 0.396853816 | 0.999970678 | 0.509 | 0.443 | 1.533 | 0.372 | NA |
| TRINITY_DN15379_c0_g1_i1 | 1.220634129 | 0.108312611 | 0.999970678 | 2.189 | 2.01 | 0.833 | 0.804 | NA |
| TRINITY_DN9885_c0_g2_i6 | 1.220588198 | 0.649845785 | 1 | 1.363 | 1.636 | 1.055 | 0 | NA |
| TRINITY_DN13692_c0_g1_i2 | -1.22035335 | 0.272360286 | 0.999970678 | 3.888 | 4.731 | 8.533 | 8.596 | NA |
| TRINITY_DN4352_c0_g1_i3 | -1.21979416 | 0.646195416 | 0.999970678 | 3.861 | 0 | 3.266 | 4.906 | NA |
| TRINITY_DN3893_c0_g1_i2 | 1.219583507 | 0.378355168 | 0.999970678 | 5.823 | 9.776 | 4.955 | 0.674 | NA |

| TRINITY_DN1397_c0_g1_i13 | 1.21957659 | 0.182622921 | 0.999970678 | 3.007 | 2.158 | 1.233 | 0.744 | NA |
| --- | --- | --- | --- | --- | --- | --- | --- | --- |
| TRINITY_DN18532_c1_g2_i6 | 1.219505259 | 0.306948532 | 0.999970678 | 3.815 | 10.21 | 2.789 | 2.503 | NA |
| TRINITY_DN332_c0_g1_i1 | -1.21950087 | 0.265968311 | 0.999970678 | 5.741 | 1.626 | 6.244 | 9.682 | NA |
| TRINITY_DN7044_c0_g1_i8 | 1.219231356 | 0.436467874 | 1 | 1.054 | 1.114 | 0.156 | 0.734 | NA |
| TRINITY_DN43284_c0_g1_i1 | -1.21916567 | 0.772398397 | 0.999970678 | 6.931 | 0 | 13.455 | 0 | NA |
| TRINITY_DN3460_c0_g2_i3 | -1.21905936 | 0.651729258 | 0.999970678 | 7.294 | 0 | 4.6 | 11.642 | NA |
| TRINITY_DN817_c0_g1_i10 | -1.21876438 | 0.779823479 | 0.999970678 | 0 | 0.384 | 0 | 0.865 | DUF4413 |
| TRINITY_DN16554_c0_g1_i6 | -1.21867304 | 0.629282662 | 0.999970678 | 0.182 | 4.07 | 0.033 | 9.752 | 2OG-FeII_Oxy |
| TRINITY_DN24611_c0_g1_i4 | 1.218392862 | 0.51858741 | 0.999970678 | 0.236 | 2.178 | 0.733 | 0.151 | Glycolytic |
| TRINITY_DN2693_c0_g3_i1 | 1.217744735 | 0.466388697 | 0.999970678 | 1.135 | 0.877 | 0.644 | 0.09 | NA |
| TRINITY_DN1219_c3_g1_i1 | 1.217719165 | 0.453649872 | 0.999970678 | 97.652 | 5.026 | 12.266 | 29.739 | - |
| TRINITY_DN6867_c0_g1_i2 | -1.21766635 | 0.653369622 | 0.999970678 | 0 | 2.405 | 3.444 | 1.327 | TCP |
| TRINITY_DN1062_c0_g1_i3 | -1.21766079 | 0.65302308 | 0.999970678 | 0 | 27.76 | 29.687 | 27.105 | NA |
| TRINITY_DN4665_c0_g1_i7 | 1.217614664 | 0.415232749 | 0.999970678 | 1.508 | 0.306 | 0.511 | 0.171 | NA |
| TRINITY_DN8958_c0_g1_i3 | -1.21750107 | 0.660390596 | 1 | 1.163 | 0 | 1.644 | 0.774 | NA |
| TRINITY_DN265_c1_g2_i3 | -1.21747115 | 0.648242443 | 0.999970678 | 9.774 | 0 | 7.322 | 14.015 | NA |
| TRINITY_DN611_c0_g1_i6 | -1.21743806 | 0.556504202 | 0.999970678 | 2.344 | 0.03 | 2.689 | 2.292 | NA |
| TRINITY_DN11572_c0_g1_i5 | -1.2171219 | 0.620359567 | 1 | 0.427 | 4.386 | 8.944 | 0.201 | NA |
| TRINITY_DN1720_c2_g3_i1 | -1.2171171 | 0.658907941 | 0.999970678 | 20.33 | 0 | 12.677 | 32.373 | WRKY |
| TRINITY_DN5207_c1_g2_i2 | -1.21705237 | 0.693290423 | 1 | 2.298 | 0.306 | 4.989 | 0 | adh_short_C2 |
| TRINITY_DN8314_c1_g1_i11 | -1.2170288 | 0.773514734 | 0.999970678 | 8.366 | 0 | 16.266 | 0 | NA |
| TRINITY_DN4242_c0_g2_i11 | 1.217025437 | 0.414839723 | 0.999970678 | 0.463 | 2.493 | 0.711 | 0.382 | NA |
| TRINITY_DN3772_c0_g1_i3 | -1.21702452 | 0.22133686 | 0.999970678 | 1.481 | 2.444 | 2.144 | 6.545 | Cu_amine_oxid |
| TRINITY_DN5834_c0_g1_i1 | -1.21696029 | 0.542121292 | 0.999970678 | 2.562 | 0.069 | 3.989 | 1.327 | NA |
| TRINITY_DN1216_c0_g1_i1 | -1.21670641 | 0.120096758 | 0.999970678 | 2.843 | 3.085 | 4.633 | 8.123 | NA |
| TRINITY_DN13121_c0_g1_i4 | -1.21669298 | 0.77464006 | 0.999970678 | 2.453 | 0 | 0 | 5.7 | NA |
| TRINITY_DN2970_c0_g1_i2 | -1.21664267 | 0.771929306 | 0.999970678 | 6.704 | 0 | 0 | 15.804 | CSN8_PSD8_EIF3 |
| TRINITY_DN634_c0_g1_i1 | 1.216530311 | 0.301781875 | 0.999970678 | 7.512 | 27.71 | 3.555 | 10.667 | Caleosin |
| TRINITY_DN3225_c0_g1_i5 | -1.21651279 | 0.669408556 | 1 | 0 | 0.769 | 0.256 | 1.448 | NA |
| TRINITY_DN15412_c0_g1_i1 | 1.216505718 | 0.264638051 | 0.999970678 | 0.772 | 0.463 | 0.222 | 0.271 | PPR |
| TRINITY_DN4111_c0_g1_i10 | 1.216395435 | 0.6319387 | 0.999970678 | 46.128 | 0.848 | 16.499 | 0.221 | NA |
| TRINITY_DN77180_c0_g1_i1 | -1.2159325 | 0.774170905 | 0.999970678 | 2.389 | 0 | 4.655 | 0 | NA |
| TRINITY_DN14189_c0_g1_i3 | 1.215887395 | 0.676608088 | 0.999970678 | 0.082 | 1.488 | 0.556 | 0 | DUF863 |
| TRINITY_DN17403_c0_g3_i1 | 1.215690232 | 0.555805592 | 0.999970678 | 0.073 | 4.021 | 0.889 | 0.653 | NA |
| TRINITY_DN15328_c0_g1_i2 | -1.21551636 | 0.774429882 | 0.999970678 | 1.426 | 0 | 2.778 | 0 | NA |
| TRINITY_DN8615_c0_g1_i5 | 1.215452314 | 0.257488456 | 0.999970678 | 0.581 | 1.104 | 0.256 | 0.422 | NA |
| TRINITY_DN1699_c0_g1_i1 | -1.21542067 | 0.559494615 | 0.999970678 | 0.145 | 27 | 17.799 | 40.607 | AP2 |
| TRINITY_DN9156_c0_g2_i1 | 1.215352129 | 0.298561136 | 0.999970678 | 0.645 | 2.079 | 0.467 | 0.603 | Cyclin_C |
| TRINITY_DN3775_c2_g2_i3 | 1.215249935 | 0.647877278 | 1 | 3.47 | 3.331 | 0 | 2.785 | Histone |
| TRINITY_DN2791_c0_g1_i3 | -1.21522939 | 0.468751339 | 0.999970678 | 0.581 | 0.217 | 1.344 | 0.241 | NA |
| TRINITY_DN3586_c0_g1_i2 | 1.215104318 | 0.474799526 | 0.999970678 | 2.716 | 0.926 | 0.111 | 1.448 | NA |
| TRINITY_DN3799_c0_g1_i1 | 1.21502799 | 0.135504381 | 0.999970678 | 84.217 | 42.37 | 25.876 | 23.506 | C1_2 |
| TRINITY_DN11102_c0_g1_i3 | -1.21490349 | 0.308159522 | 0.999970678 | 2.335 | 0.601 | 3.211 | 3.006 | PALP |
| TRINITY_DN5036_c0_g1_i5 | 1.214897699 | 0.782234241 | 1 | 0.563 | 0 | 0 | 0.241 | HIRAN |
| TRINITY_DN2319_c1_g2_i2 | 1.214804627 | 0.479774297 | 0.999970678 | 0.363 | 0.848 | 0.4 | 0.04 | NB-ARC |
| TRINITY_DN355_c0_g2_i1 | 1.214767989 | 0.651263573 | 0.999970678 | 14.562 | 8.564 | 0 | 10.003 | NA |
| TRINITY_DN23286_c0_g1_i1 | 1.214656789 | 0.698956253 | 0.999970678 | 0 | 1.37 | 0.444 | 0.05 | NA |
| TRINITY_DN1890_c0_g1_i7 | 1.214610197 | 0.537122308 | 0.999970678 | 10.892 | 0.237 | 3.455 | 0.684 | NA |
| TRINITY_DN1081_c0_g1_i2 | 1.214448447 | 0.368126977 | 0.999970678 | 51.152 | 6.613 | 9.644 | 13.321 | SelK_SelG |
| TRINITY_DN6327_c0_g1_i5 | 1.214336636 | 0.447992829 | 1 | 0.972 | 1.419 | 0.144 | 0.885 | BolA |
| TRINITY_DN3546_c0_g1_i13 | 1.214076952 | 0.423586266 | 0.999970678 | 4.933 | 1.537 | 2.033 | 0.372 | NA |
| TRINITY_DN7908_c0_g1_i4 | 1.213905849 | 0.65020049 | 0.999970678 | 1.744 | 4.612 | 2.267 | 0 | NA |
| TRINITY_DN361_c0_g1_i4 | -1.21373973 | 0.350863015 | 0.999970678 | 0.372 | 0.838 | 0.6 | 2.031 | Ins134_P3_kin |
| TRINITY_DN3435_c0_g1_i2 | -1.21369064 | 0.773600485 | 0.999970678 | 4.805 | 0 | 0 | 11.18 | NA |
| TRINITY_DN3692_c0_g1_i2 | -1.21354129 | 0.351221482 | 0.999970678 | 0.254 | 1.222 | 1.533 | 1.588 | HLH |
| TRINITY_DN31574_c0_g1_i1 | 1.213462642 | 0.783276481 | 0.999970678 | 2.362 | 0 | 0.856 | 0 | Sdh5 |
| TRINITY_DN5156_c0_g2_i1 | 1.21333363 | 0.271884557 | 0.999970678 | 0.99 | 1.991 | 0.644 | 0.513 | MatE |
| TRINITY_DN14231_c1_g1_i1 | 1.213133993 | 0.777903053 | 0.999970678 | 0 | 458.5 | 0 | 167.585 | NA |
| TRINITY_DN8002_c0_g1_i4 | 1.213049517 | 0.500825768 | 0.999970678 | 51.724 | 2730 | 467.694 | 594.797 | NA |
| TRINITY_DN4021_c0_g1_i1 | 1.212547738 | 0.673799657 | 0.999970678 | 0 | 3.932 | 1.133 | 0.302 | NA |
| TRINITY_DN4105_c0_g1_i2 | -1.21224757 | 0.24102647 | 0.999970678 | 31.358 | 11.04 | 27.543 | 65.279 | LRRNT_2 |
| TRINITY_DN8192_c0_g1_i1 | -1.2119948 | 0.480696709 | 0.999970678 | 22.41 | 145.6 | 19.21 | 360.083 | - |
| TRINITY_DN1392_c1_g1_i5 | 1.211952293 | 0.343269428 | 0.999970678 | 0.182 | 0.158 | 0.089 | 0.05 | Alpha-mann_mid |
| TRINITY_DN939_c0_g1_i7 | 1.211934115 | 0.681145445 | 0.999970678 | 0 | 1.587 | 0.433 | 0.141 | NA |
| TRINITY_DN914_c0_g1_i8 | 1.211876165 | 0.172598401 | 0.999970678 | 2.362 | 3.824 | 1.189 | 1.216 | NA |
| TRINITY_DN9946_c0_g1_i10 | -1.21160127 | 0.773242814 | 0.999970678 | 7.712 | 0 | 0 | 18.026 | NA |
| TRINITY_DN12731_c1_g3_i1 | -1.21157191 | 0.781531701 | 0.999970678 | 0 | 2.622 | 4.844 | 0 | NA |
| TRINITY_DN4085_c0_g1_i5 | 1.211550361 | 0.306376163 | 0.999970678 | 10.383 | 3.666 | 2.9 | 2.534 | NA |
| TRINITY_DN11395_c0_g1_i4 | 1.211503412 | 0.523647475 | 0.999970678 | 0.3 | 10.75 | 3.089 | 0.955 | NA |
| TRINITY_DN1610_c0_g1_i13 | 1.211471258 | 0.779114993 | 0.999970678 | 0 | 0.483 | 0.167 | 0 | - |
| TRINITY_DN3932_c0_g1_i6 | 1.211469365 | 0.693101978 | 0.999970678 | 0 | 2.543 | 0.044 | 1.015 | Pkinase |
| TRINITY_DN2220_c0_g1_i5 | 1.211450646 | 0.772308537 | 0.999970678 | 3.27 | 0 | 0 | 1.438 | OKR_DC_1 |
| TRINITY_DN2322_c0_g1_i1 | 1.211443737 | 0.268742539 | 0.999970678 | 1.072 | 0.966 | 0.333 | 0.473 | NA |
| TRINITY_DN16209_c1_g1_i4 | -1.21115443 | 0.777328901 | 0.999970678 | 2.117 | 0 | 4.078 | 0 | - |

| TRINITY_DN1942_c0_g1_i3 | -1.21102787 | 0.368885239 | 0.999970678 | 2.634 | 2.543 | 1.5 | 10.003 | NA |
| --- | --- | --- | --- | --- | --- | --- | --- | --- |
| TRINITY_DN3022_c0_g1_i12 | -1.21101453 | 0.368947142 | 0.999970678 | 3.852 | 3.538 | 1.789 | 14.779 | - |
| TRINITY_DN2373_c0_g1_i6 | 1.210953081 | 0.200416405 | 0.999970678 | 1.244 | 2.957 | 0.756 | 0.885 | NA |
| TRINITY_DN2132_c1_g1_i1 | 1.210649104 | 0.289640813 | 0.999970678 | 5.005 | 9.52 | 2.778 | 2.755 | NA |
| TRINITY_DN2567_c0_g1_i5 | 1.210578912 | 0.153895703 | 0.999970678 | 0.963 | 1.311 | 0.478 | 0.412 | Hemerythrin |
| TRINITY_DN3658_c0_g1_i9 | -1.21057423 | 0.372078798 | 0.999970678 | 55.348 | 8.574 | 42.586 | 90.936 | NA |
| TRINITY_DN967_c2_g1_i2 | 1.210538646 | 0.63943108 | 0.999970678 | 4.115 | 3.085 | 0 | 3.127 | DUF177 |
| TRINITY_DN4813_c0_g1_i2 | -1.21038605 | 0.055685977 | 0.999970678 | 1.671 | 1.754 | 3.389 | 3.83 | NA |
| TRINITY_DN11440_c0_g1_i1 | -1.20999192 | 0.502532449 | 0.999970678 | 0.654 | 0.256 | 0.167 | 1.92 | NA |
| TRINITY_DN53245_c0_g1_i3 | -1.20988684 | 0.778202688 | 0.999970678 | 0.609 | 0 | 1.167 | 0 | NA |
| TRINITY_DN2348_c0_g1_i14 | 1.209847158 | 0.775860789 | 0.999970678 | 3.161 | 0 | 0 | 1.377 | NA |
| TRINITY_DN9334_c0_g1_i4 | 1.209816142 | 0.652672134 | 0.999970678 | 0.3 | 0.749 | 0.367 | 0 | Peptidase_C48 |
| TRINITY_DN30569_c0_g1_i1 | 1.209475618 | 0.348020629 | 0.999970678 | 0.209 | 0.65 | 0.144 | 0.191 | NA |
| TRINITY_DN3537_c0_g1_i2 | -1.2094467 | 0.388948183 | 0.999970678 | 3.615 | 1.833 | 1.211 | 11.23 | NABP |
| TRINITY_DN478_c0_g2_i3 | -1.20931385 | 0.66082974 | 0.999970678 | 1.026 | 0 | 1.589 | 0.473 | P34-Arc |
| TRINITY_DN8893_c0_g1_i3 | 1.209125175 | 0.362015592 | 0.999970678 | 4.515 | 1.202 | 1.378 | 0.824 | NA |
| TRINITY_DN5847_c0_g1_i5 | -1.20901771 | 0.121882519 | 0.999970678 | 9.138 | 11.2 | 13.477 | 30.624 | Melibiase_2 |
| TRINITY_DN32195_c0_g1_i1 | -1.20892975 | 0.778865195 | 0.999970678 | 0.99 | 0 | 1.922 | 0 | NA |
| TRINITY_DN3237_c4_g1_i1 | 1.20879356 | 0.774601046 | 0.999970678 | 4.388 | 0 | 0 | 1.92 | PP2C |
| TRINITY_DN3773_c1_g1_i3 | 1.208768414 | 0.300395577 | 0.999970678 | 15.997 | 9.274 | 7.422 | 2.011 | NA |
| TRINITY_DN6881_c0_g1_i20 | 1.208749386 | 0.495342025 | 1 | 1.662 | 1.912 | 1.1 | 0.211 | NA |
| TRINITY_DN4307_c0_g2_i1 | 1.208613049 | 0.672332498 | 0.999970678 | 0 | 4.287 | 1.2 | 0.392 | NA |
| TRINITY_DN803_c0_g1_i3 | 1.208559989 | 0.344998095 | 0.999970678 | 40.16 | 10.81 | 13.91 | 5.228 | NA |
| TRINITY_DN4459_c0_g1_i7 | 1.208539077 | 0.669038831 | 0.999970678 | 1.508 | 0 | 0.256 | 0.362 | NA |
| TRINITY_DN4668_c0_g1_i1 | -1.20851701 | 0.475127132 | 0.999970678 | 0.736 | 0.089 | 1.289 | 0.372 | DUF659 |
| TRINITY_DN11661_c0_g1_i5 | 1.208479415 | 0.772879064 | 0.999970678 | 8.593 | 0 | 0 | 3.77 | NA |
| TRINITY_DN2215_c4_g1_i3 | -1.20844779 | 0.660512705 | 0.999970678 | 0 | 22.83 | 33.02 | 11.572 | NA |
| TRINITY_DN778_c0_g1_i2 | 1.20842787 | 0.347868523 | 0.999970678 | 1.108 | 0.601 | 0.456 | 0.191 | NA |
| TRINITY_DN5676_c4_g1_i2 | -1.20760204 | 0.665675014 | 0.999970678 | 0.345 | 0.572 | 1.767 | 0 | PPR |
| TRINITY_DN8625_c0_g1_i19 | 1.207555243 | 0.776840294 | 0.999970678 | 1.544 | 0 | 0 | 0.674 | NA |
| TRINITY_DN5854_c0_g1_i2 | 1.207420547 | 0.097807672 | 0.999970678 | 8.821 | 9.845 | 3.166 | 4.233 | NA |
| TRINITY_DN5183_c0_g1_i3 | 1.207416346 | 0.44217032 | 0.999970678 | 3.833 | 12.7 | 0.444 | 6.595 | HisG |
| TRINITY_DN1505_c0_g1_i1 | 1.207360965 | 0.400757922 | 0.999970678 | 1.862 | 1.715 | 0.167 | 1.327 | NA |
| TRINITY_DN1566_c0_g2_i5 | 1.207081425 | 0.777195929 | 0.999970678 | 0 | 2.03 | 0.711 | 0 | NA |
| TRINITY_DN11867_c1_g2_i1 | 1.207007791 | 0.647767923 | 0.999970678 | 1.998 | 0.02 | 0.711 | 0.03 | NA |
| TRINITY_DN3179_c0_g1_i4 | 1.206936927 | 0.07965238 | 0.999970678 | 3.515 | 2.976 | 1.233 | 1.317 | Glyco_transf_41 |
| TRINITY_DN15038_c0_g1_i1 | -1.20674238 | 0.523089379 | 0.999970678 | 3.434 | 5.273 | 15.488 | 0.664 | LRRNT_2 |
| TRINITY_DN24875_c0_g1_i2 | 1.206710501 | 0.573249567 | 0.999970678 | 0.345 | 4.888 | 0.1 | 2.111 | NA |
| TRINITY_DN3737_c0_g1_i9 | 1.206648698 | 0.773805343 | 0.999970678 | 0 | 0.877 | 0.311 | 0 | Beach |
| TRINITY_DN3696_c0_g2_i2 | -1.20650232 | 0.67321268 | 0.999970678 | 9.529 | 0 | 10.066 | 10.225 | NA |
| TRINITY_DN1176_c1_g1_i1 | -1.20649675 | 0.50901595 | 1 | 1.099 | 0.099 | 1.378 | 1.076 | NA |
| TRINITY_DN5444_c0_g1_i3 | 1.206489327 | 0.545544059 | 0.999970678 | 3.706 | 3.784 | 2.6 | 0.101 | NA |
| TRINITY_DN8893_c1_g1_i2 | 1.206168275 | 0.591548971 | 0.999970678 | 4.951 | 0.108 | 1.7 | 0.181 | Myb_DNA-bindin |
| TRINITY_DN13054_c0_g1_i1 | 1.206058508 | 0.712976535 | 0.999970678 | 0 | 13.16 | 4.478 | 0.211 | NA |
| TRINITY_DN6771_c0_g1_i1 | -1.20602906 | 0.302213259 | 0.999970678 | 2.625 | 0.719 | 3.122 | 4.001 | UDPGT |
| TRINITY_DN15191_c0_g1_i1 | -1.20597223 | 0.775560971 | 0.999970678 | 2.144 | 0 | 4.155 | 0 | But2 |
| TRINITY_DN4533_c0_g2_i2 | -1.20594742 | 0.132705171 | 0.999970678 | 126.74 | 73.71 | 146.224 | 278.477 | NA |
| TRINITY_DN387_c0_g1_i12 | -1.20591532 | 0.282065216 | 0.999970678 | 8.012 | 9.668 | 6.3 | 33.127 | NA |
| TRINITY_DN6762_c0_g1_i2 | 1.205708893 | 0.773982047 | 0.999970678 | 0 | 16.92 | 5.955 | 0 | NA |
| TRINITY_DN2422_c0_g1_i9 | 1.20569104 | 0.636324961 | 0.999970678 | 0.045 | 10.28 | 3.511 | 0.151 | NA |
| TRINITY_DN24422_c1_g1_i2 | 1.205646616 | 0.348467675 | 0.999970678 | 0.872 | 1.636 | 0.3 | 0.734 | NA |
| TRINITY_DN4628_c0_g1_i2 | -1.20542356 | 0.77388005 | 0.999970678 | 0.645 | 0 | 0 | 1.518 | Helicase_C |
| TRINITY_DN406_c1_g1_i2 | 1.205088284 | 0.162190384 | 0.999970678 | 11.21 | 7.717 | 2.311 | 5.449 | - |
| TRINITY_DN7267_c0_g2_i6 | -1.20464332 | 0.27495591 | 0.999970678 | 0.209 | 0.719 | 1 | 0.935 | NA |
| TRINITY_DN439_c0_g1_i12 | 1.204548894 | 0.679511075 | 0.999970678 | 0.2 | 3.213 | 1.211 | 0 | NA |
| TRINITY_DN11017_c0_g1_i1 | -1.20441141 | 0.354242509 | 0.999970678 | 1.017 | 0.434 | 2.033 | 0.935 | Pkinase |
| TRINITY_DN6830_c0_g1_i5 | 1.204329763 | 0.458878694 | 0.999970678 | 2.153 | 0.611 | 0.9 | 0.121 | NA |
| TRINITY_DN3565_c0_g1_i4 | -1.20429546 | 0.657547783 | 0.999970678 | 8.757 | 0 | 8.411 | 10.295 | Pex2_Pex12 |
| TRINITY_DN4102_c0_g1_i2 | -1.20412211 | 0.25756398 | 0.999970678 | 2.961 | 10.31 | 9.555 | 18.78 | ACP_syn_III |
| TRINITY_DN1095_c0_g2_i3 | 1.203995132 | 0.58305131 | 0.999970678 | 0.109 | 6.524 | 1.967 | 0.462 | NA |
| TRINITY_DN2756_c0_g1_i1 | 1.203603921 | 0.230079139 | 0.999970678 | 9.52 | 18.05 | 7.266 | 3.147 | - |
| TRINITY_DN7024_c0_g2_i1 | 1.203600354 | 0.405054858 | 1 | 0.527 | 0.503 | 0.278 | 0.111 | NA |
| TRINITY_DN14653_c0_g1_i7 | -1.20351766 | 0.66254375 | 0.999970678 | 1.962 | 0 | 1.078 | 3.318 | NA |
| TRINITY_DN650_c0_g1_i2 | -1.20350698 | 0.454907661 | 0.999970678 | 0.182 | 1.222 | 1.911 | 0.865 | Ssu72 |
| TRINITY_DN3135_c0_g1_i8 | 1.203264074 | 0.34774533 | 1 | 0.536 | 0.956 | 0.211 | 0.372 | NA |
| TRINITY_DN12802_c0_g1_i1 | 1.20323777 | 0.784792187 | 1 | 0 | 0.828 | 0 | 0.352 | ABC2_membrane |
| TRINITY_DN4196_c0_g1_i2 | 1.203208766 | 0.648290038 | 0.999970678 | 4.951 | 3.499 | 0 | 3.68 | NA |
| TRINITY_DN8268_c0_g1_i8 | 1.203056028 | 0.417231069 | 0.999970678 | 4.433 | 15.74 | 5.3 | 2.292 | NA |
| TRINITY_DN2957_c0_g1_i5 | -1.20299982 | 0.776347454 | 0.999970678 | 76.469 | 0 | 144.425 | 0 | EthD |
| TRINITY_DN1010_c0_g2_i1 | -1.20286044 | 0.232670152 | 0.999970678 | 14.861 | 21.23 | 16.21 | 63.147 | NA |
| TRINITY_DN11743_c0_g2_i14 | -1.20269283 | 0.657731546 | 1 | 1.154 | 0 | 0.978 | 1.468 | NA |
| TRINITY_DN8417_c0_g1_i1 | -1.20254133 | 0.774541586 | 0.999970678 | 1.335 | 0 | 0 | 3.137 | DUF1336 |
| TRINITY_DN4357_c0_g1_i8 | -1.20228577 | 0.409901239 | 0.999970678 | 2.089 | 1.971 | 6.511 | 1.347 | LRRNT_2 |
| TRINITY_DN34621_c0_g1_i1 | -1.20204654 | 0.373839846 | 0.999970678 | 31.385 | 10.66 | 12.755 | 79.253 | EF-hand_1 |

| TRINITY_DN1367_c0_g1_i2 | 1.201853048 | 0.299907538 | 0.999970678 | 12.263 | 41.36 | 5.177 | 16.8 | HLH |
| --- | --- | --- | --- | --- | --- | --- | --- | --- |
| TRINITY_DN6476_c0_g1_i9 | 1.201582561 | 0.782639186 | 0.999970678 | 0.372 | 0 | 0.133 | 0 | RVT_1 |
| TRINITY_DN992_c0_g1_i1 | 1.201546128 | 0.150261967 | 0.999970678 | 5.759 | 6.554 | 1.655 | 3.328 | Motile_Sperm |
| TRINITY_DN10083_c0_g1_i2 | 1.201446513 | 0.65062963 | 0.999970678 | 0.273 | 0.453 | 0 | 0.322 | EGF_2 |
| TRINITY_DN13829_c0_g1_i7 | 1.201364663 | 0.711335229 | 0.999970678 | 0.036 | 1.37 | 0 | 0.603 | GMC_oxred_C |
| TRINITY_DN7285_c0_g1_i7 | 1.20134502 | 0.395433314 | 0.999970678 | 2.762 | 0.857 | 0.278 | 1.247 | DUF4477 |
| TRINITY_DN7639_c0_g1_i1 | -1.20134221 | 0.371312196 | 0.999970678 | 7.322 | 2.424 | 15.221 | 4.192 | - |
| TRINITY_DN5013_c0_g1_i1 | 1.201295914 | 0.458878613 | 0.999970678 | 3.761 | 48.91 | 3.5 | 18.368 | PC-Esterase |
| TRINITY_DN3326_c0_g1_i9 | -1.20113746 | 0.655951127 | 0.999970678 | 2.716 | 0 | 2.567 | 3.147 | zf-C2H2_6 |
| TRINITY_DN40246_c0_g1_i2 | 1.201110626 | 0.321442341 | 0.999970678 | 0.445 | 0.493 | 0.111 | 0.261 | PPR |
| TRINITY_DN13255_c0_g1_i5 | 1.201079205 | 0.129973413 | 0.999970678 | 2.852 | 2.7 | 1.278 | 0.875 | NA |
| TRINITY_DN15504_c0_g1_i6 | -1.20095544 | 0.244736703 | 0.999970678 | 2.889 | 1.833 | 6.533 | 2.986 | NA |
| TRINITY_DN794_c0_g1_i1 | -1.20068082 | 0.259189638 | 0.999970678 | 297.96 | 198.9 | 199.477 | 839.926 | Dimerisation |
| TRINITY_DN7431_c0_g1_i3 | 1.200602878 | 0.651065472 | 0.999970678 | 1.499 | 4.829 | 0 | 2.725 | F-box |
| TRINITY_DN4100_c0_g1_i2 | 1.200433756 | 0.653530701 | 0.999970678 | 0.491 | 1.281 | 0 | 0.754 | NA |
| TRINITY_DN372_c0_g1_i8 | -1.20039975 | 0.66258821 | 0.999970678 | 1.59 | 0 | 0.8 | 2.755 | NA |
| TRINITY_DN1699_c0_g1_i2 | -1.20019096 | 0.342218262 | 0.999970678 | 23.909 | 34.71 | 14.499 | 116.824 | AP2 |
| TRINITY_DN9010_c0_g1_i5 | 1.199997624 | 0.644851653 | 0.999970678 | 2.053 | 1.39 | 0 | 1.508 | NA |
| TRINITY_DN15637_c0_g1_i4 | -1.19999422 | 0.672604615 | 0.999970678 | 0.245 | 0.315 | 1.055 | 0 | Laminin_G_3 |
| TRINITY_DN1740_c0_g1_i13 | -1.19998965 | 0.360467116 | 1 | 0.554 | 0.276 | 0.889 | 0.865 | NA |
| TRINITY_DN756_c0_g1_i4 | 1.199988889 | 0.690496505 | 0.999970678 | 0 | 94.16 | 16.977 | 19.826 | Thioredoxin |
| TRINITY_DN1074_c0_g1_i2 | 1.199965699 | 0.677661729 | 0.999970678 | 37.871 | 47.04 | 0 | 37.008 | NA |
| TRINITY_DN1144_c0_g1_i5 | 1.199824695 | 0.134949786 | 0.999970678 | 7.204 | 9.836 | 3.189 | 3.448 | - |
| TRINITY_DN12_c2_g1_i3 | -1.19980138 | 0.503207124 | 0.999970678 | 0.418 | 4.967 | 0.867 | 11.21 | NA |
| TRINITY_DN988_c0_g1_i1 | -1.19946843 | 0.672176476 | 0.999970678 | 11.6 | 0 | 4.444 | 21.706 | NA |
| TRINITY_DN745_c0_g1_i6 | 1.199452446 | 0.123390821 | 0.999970678 | 16.033 | 20.14 | 4.855 | 9.762 | NA |
| TRINITY_DN3447_c0_g1_i4 | -1.19934482 | 0.651999905 | 0.999970678 | 1.998 | 0 | 1.433 | 2.895 | NA |
| TRINITY_DN11124_c0_g1_i1 | -1.19923509 | 0.095611529 | 0.999970678 | 345.15 | 238.2 | 667.526 | 534.444 | NA |
| TRINITY_DN2981_c0_g1_i7 | 1.199203356 | 0.784727439 | 0.999970678 | 46.201 | 0 | 0 | 20.469 | NA |
| TRINITY_DN11008_c0_g1_i2 | -1.19906624 | 0.123295348 | 0.999970678 | 8.457 | 11.77 | 22.754 | 18.258 | NA |
| TRINITY_DN1180_c0_g2_i2 | 1.19898731 | 0.32819696 | 1 | 0.518 | 0.384 | 0.144 | 0.201 | DSPc |
| TRINITY_DN2111_c1_g1_i2 | -1.1988444 | 0.676023989 | 0.999970678 | 22.537 | 0 | 18.177 | 30.634 | Metallophos |
| TRINITY_DN766_c0_g2_i3 | 1.198835813 | 0.378098629 | 0.999970678 | 0.209 | 0.473 | 0.178 | 0.08 | NA |
| TRINITY_DN11934_c0_g1_i4 | -1.19880321 | 0.384856445 | 0.999970678 | 2.553 | 8.87 | 8.144 | 14.869 | Aldedh |
| TRINITY_DN838_c1_g1_i1 | -1.19865205 | 0.167489431 | 0.999970678 | 2.444 | 3.371 | 3.922 | 8.556 | Exostosin |
| TRINITY_DN4021_c2_g1_i1 | -1.19845798 | 0.361694197 | 0.999970678 | 0.89 | 0.187 | 1.133 | 1.096 | NA |
| TRINITY_DN138_c0_g3_i4 | 1.198263972 | 0.488670509 | 0.999970678 | 0.572 | 6.514 | 0.544 | 2.413 | NA |
| TRINITY_DN12439_c0_g1_i10 | 1.198153513 | 0.463277979 | 0.999970678 | 30.94 | 2.139 | 5.711 | 7.5 | NA |
| TRINITY_DN468_c1_g1_i10 | 1.198110705 | 0.652329798 | 0.999970678 | 2.253 | 1.025 | 1.178 | 0 | NA |
| TRINITY_DN9312_c0_g1_i7 | 1.198109069 | 0.644256839 | 0.999970678 | 0.99 | 0.729 | 0 | 0.754 | AAA |
| TRINITY_DN1008_c0_g1_i18 | -1.19792378 | 0.344848272 | 1 | 0.2 | 0.315 | 0.489 | 0.583 | NA |
| TRINITY_DN1836_c0_g1_i17 | 1.19789829 | 0.310593358 | 0.999970678 | 2.017 | 0.769 | 0.389 | 0.764 | Maf1 |
| TRINITY_DN19994_c0_g1_i1 | -1.19786413 | 0.65939831 | 0.999970678 | 8.339 | 0 | 8.588 | 9.018 | NA |
| TRINITY_DN19994_c0_g1_i3 | -1.19786413 | 0.65939831 | 0.999970678 | 8.339 | 0 | 8.588 | 9.018 | NA |
| TRINITY_DN5149_c0_g1_i2 | 1.197789496 | 0.455666925 | 0.999970678 | 2.562 | 8.052 | 0.433 | 3.991 | NA |
| TRINITY_DN11764_c0_g1_i5 | -1.19773634 | 0.381431944 | 0.999970678 | 0.118 | 0.483 | 0.6 | 0.613 | NA |
| TRINITY_DN45086_c0_g1_i2 | -1.19771254 | 0.786472931 | 1 | 1.054 | 0 | 2.033 | 0 | NA |
| TRINITY_DN7478_c0_g1_i3 | 1.19767729 | 0.67309536 | 0.999970678 | 4.306 | 0 | 0.456 | 1.347 | HAUS6_N |
| TRINITY_DN12887_c0_g1_i2 | -1.19760285 | 0.213694522 | 0.999970678 | 2.961 | 4.06 | 9.633 | 4.434 | FAD-oxidase_C |
| TRINITY_DN10010_c0_g1_i3 | 1.197462372 | 0.787681874 | 1 | 1.49 | 0 | 0.544 | 0 | - |
| TRINITY_DN3197_c0_g1_i8 | 1.197224227 | 0.457814349 | 0.999970678 | 1.126 | 0.276 | 0.422 | 0.101 | Retrotran_gag_2 |
| TRINITY_DN3132_c0_g1_i4 | 1.196923528 | 0.777544768 | 0.999970678 | 6.395 | 0 | 0 | 2.855 | TPR_1 |
| TRINITY_DN78495_c0_g1_i2 | 1.196690555 | 0.537834257 | 0.999970678 | 38.407 | 0.848 | 12.155 | 2.664 | Phosphoesterase |
| TRINITY_DN138438_c0_g1_i1 | -1.19638247 | 0.787340993 | 1 | 1.862 | 0 | 3.555 | 0 | NA |
| TRINITY_DN2023_c0_g3_i7 | 1.196241216 | 0.43218339 | 0.999970678 | 25.153 | 215.4 | 14.499 | 86.643 | NA |
| TRINITY_DN2384_c0_g1_i5 | 1.196202481 | 0.44995597 | 0.999970678 | 0.472 | 0.601 | 0.344 | 0.05 | RRM_1 |
| TRINITY_DN5111_c0_g1_i3 | 1.196199625 | 0.470292569 | 0.999970678 | 6.104 | 0.857 | 2.167 | 0.442 | NA |
| TRINITY_DN19484_c0_g2_i1 | -1.19613596 | 0.212503751 | 0.999970678 | 9.193 | 13.53 | 11.422 | 38.033 | NA |
| TRINITY_DN412_c0_g1_i1 | 1.196121357 | 0.242398994 | 0.999970678 | 2.098 | 1.202 | 0.8 | 0.473 | RRM_1 |
| TRINITY_DN872_c0_g1_i7 | 1.196054427 | 0.789552596 | 0.999970678 | 41.713 | 0 | 0 | 18.579 | NA |
| TRINITY_DN8999_c0_g2_i5 | -1.19605223 | 0.420542561 | 0.999970678 | 1.481 | 0.237 | 2.155 | 1.347 | NA |
| TRINITY_DN11025_c0_g1_i5 | 1.19589303 | 0.662710412 | 0.999970678 | 0.263 | 1.183 | 0 | 0.623 | NA |
| TRINITY_DN3419_c0_g1_i3 | 1.195884687 | 0.138692283 | 0.999970678 | 2.144 | 1.882 | 0.8 | 0.794 | NA |
| TRINITY_DN29137_c0_g1_i8 | 1.195831839 | 0.661325853 | 0.999970678 | 2.689 | 0.759 | 0 | 1.498 | NA |
| TRINITY_DN7254_c0_g1_i3 | -1.19578122 | 0.77657408 | 0.999970678 | 0 | 0.503 | 0.933 | 0 | NA |
| TRINITY_DN13541_c0_g1_i3 | 1.195608812 | 0.373751275 | 0.999970678 | 2.044 | 0.552 | 0.233 | 0.865 | NA |
| TRINITY_DN841_c0_g1_i2 | 1.195538935 | 0.320048607 | 0.999970678 | 5.877 | 1.685 | 1.578 | 1.377 | ATP_bind_3 |
| TRINITY_DN3996_c0_g1_i10 | 1.195469538 | 0.480260448 | 0.999970678 | 0.318 | 1.035 | 0.422 | 0.09 | NA |
| TRINITY_DN1720_c0_g2_i1 | 1.195438666 | 0.262614025 | 0.999970678 | 114.26 | 31.71 | 31.976 | 25.486 | WRKY |
| TRINITY_DN1677_c0_g1_i3 | -1.19543416 | 0.307139892 | 0.999970678 | 0.881 | 1.025 | 1.522 | 2.473 | ANAPC4_WD40 |
| TRINITY_DN7065_c0_g1_i12 | 1.195321065 | 0.786463443 | 1 | 2.135 | 0 | 0 | 0.935 | NA |
| TRINITY_DN1758_c0_g1_i10 | -1.19528493 | 0.430986457 | 0.999970678 | 1.635 | 2.267 | 0.567 | 8.224 | O-FucT |
| TRINITY_DN4315_c0_g1_i1 | -1.19523371 | 0.163331721 | 0.999970678 | 7.803 | 3.814 | 11.755 | 12.446 | NA |
| TRINITY_DN12202_c0_g1_i4 | -1.19516143 | 0.595899678 | 0.999970678 | 0.055 | 0.621 | 0.044 | 1.488 | PTR2 |

| TRINITY_DN1022_c0_g1_i1 | -1.19504467 | 0.294239611 | 0.999970678 | 1.826 | 6.889 | 10.055 | 7.611 | NA |
| --- | --- | --- | --- | --- | --- | --- | --- | --- |
| TRINITY_DN3008_c0_g1_i1 | 1.195007757 | 0.580458598 | 0.999970678 | 0.254 | 30.22 | 8.988 | 2.192 | NA |
| TRINITY_DN1680_c0_g2_i1 | -1.19493071 | 0.788252698 | 1 | 1.499 | 0 | 2.9 | 0 | - |
| TRINITY_DN1416_c1_g1_i16 | -1.19489221 | 0.625081255 | 0.999970678 | 3.661 | 0.01 | 6.644 | 0.533 | GDPD |
| TRINITY_DN6381_c0_g1_i2 | 1.194736714 | 0.77552528 | 0.999970678 | 0 | 4.307 | 0 | 1.85 | NA |
| TRINITY_DN2881_c0_g1_i11 | 1.194730227 | 0.515308683 | 0.999970678 | 46.137 | 4.829 | 15.466 | 3.086 | NA |
| TRINITY_DN3931_c0_g1_i7 | 1.194634375 | 0.505272065 | 0.999970678 | 0.945 | 24.99 | 1.778 | 9.048 | NA |
| TRINITY_DN5062_c0_g1_i2 | -1.19462489 | 0.680323924 | 0.999970678 | 3.243 | 0 | 0.467 | 6.987 | Calmodulin_bind |
| TRINITY_DN14148_c0_g1_i4 | 1.194566799 | 0.784079085 | 0.999970678 | 0 | 2.957 | 1.055 | 0 | NA |
| TRINITY_DN220_c0_g1_i8 | 1.194520634 | 0.783572002 | 0.999970678 | 0 | 1.774 | 0.644 | 0 | EGF_CA |
| TRINITY_DN12373_c0_g1_i1 | -1.19443219 | 0.776838006 | 0.999970678 | 27.642 | 0 | 52.164 | 0 | NA |
| TRINITY_DN1422_c2_g3_i3 | 1.194366254 | 0.52600123 | 0.999970678 | 0.154 | 2.198 | 0.622 | 0.251 | DYW_deaminase |
| TRINITY_DN701_c0_g1_i6 | 1.194339339 | 0.339177635 | 1 | 0.318 | 0.404 | 0.156 | 0.131 | NB-ARC |
| TRINITY_DN2656_c0_g1_i8 | 1.194317838 | 0.660790999 | 0.999970678 | 1.135 | 0.493 | 0.6 | 0 | Amidohydro_1 |
| TRINITY_DN4164_c0_g1_i7 | 1.194213475 | 0.31529139 | 0.999970678 | 1.126 | 1.37 | 0.722 | 0.211 | - |
| TRINITY_DN11406_c0_g2_i1 | 1.193956107 | 0.382613762 | 0.999970678 | 4.378 | 4.11 | 2.689 | 0.452 | MT-A70 |
| TRINITY_DN1823_c0_g1_i6 | -1.19393882 | 0.216404803 | 0.999970678 | 0.945 | 1.508 | 1.544 | 3.74 | F-box |
| TRINITY_DN5554_c0_g1_i1 | -1.19384141 | 0.657964306 | 0.999970678 | 1.826 | 0 | 2.444 | 1.277 | - |
| TRINITY_DN628_c0_g1_i2 | -1.1938207 | 0.241017846 | 0.999970678 | 0.2 | 0.375 | 0.567 | 0.633 | NA |
| TRINITY_DN905_c0_g1_i4 | -1.19328611 | 0.547079162 | 0.999970678 | 0.681 | 3.558 | 7.544 | 0.422 | NA |
| TRINITY_DN419_c0_g1_i8 | 1.193275653 | 0.658839703 | 0.999970678 | 3.515 | 4.8 | 3 | 0 | Cation_ATPase |
| TRINITY_DN772_c1_g1_i2 | -1.19322387 | 0.651541117 | 0.999970678 | 6.668 | 0 | 6.222 | 7.751 | NA |
| TRINITY_DN3511_c0_g3_i1 | 1.193138481 | 0.283182479 | 0.999970678 | 10.91 | 2.957 | 2.744 | 2.805 | HALZ |
| TRINITY_DN586_c0_g1_i3 | 1.193054033 | 0.511785957 | 0.999970678 | 4.669 | 2.828 | 2.489 | 0.231 | NA |
| TRINITY_DN2653_c0_g1_i2 | -1.19300792 | 0.660001268 | 0.999970678 | 1.944 | 0 | 0.944 | 3.348 | NA |
| TRINITY_DN2180_c0_g1_i3 | -1.19266738 | 0.778313646 | 0.999970678 | 2.952 | 0 | 5.644 | 0 | NA |
| TRINITY_DN16079_c0_g1_i1 | -1.19262794 | 0.365598024 | 0.999970678 | 27.361 | 12.12 | 10.199 | 78.338 | - |
| TRINITY_DN2936_c0_g1_i4 | -1.1926102 | 0.655116229 | 0.999970678 | 1.099 | 0 | 1.178 | 1.076 | C2 |
| TRINITY_DN3517_c0_g1_i4 | 1.192569228 | 0.214579526 | 0.999970678 | 1.808 | 1.685 | 0.678 | 0.734 | Thioredoxin |
| TRINITY_DN2174_c0_g1_i6 | 1.192452351 | 0.477650802 | 0.999970678 | 0.727 | 1.064 | 0.578 | 0.07 | NA |
| TRINITY_DN2713_c1_g1_i2 | -1.19239374 | 0.229489327 | 0.999970678 | 2.135 | 4.731 | 5.377 | 8.928 | NA |
| TRINITY_DN7790_c0_g1_i4 | 1.192354184 | 0.421878068 | 0.999970678 | 0.481 | 3.765 | 0.955 | 0.684 | - |
| TRINITY_DN5963_c0_g1_i3 | -1.19219896 | 0.357127376 | 0.999970678 | 0.645 | 0.355 | 0.489 | 1.649 | NA |
| TRINITY_DN14645_c0_g1_i3 | 1.191952171 | 0.270900672 | 0.999970678 | 0.663 | 0.907 | 0.244 | 0.382 | - |
| TRINITY_DN1824_c0_g2_i2 | 1.191873295 | 0.491622285 | 0.999970678 | 3.252 | 1.419 | 1.522 | 0.221 | NA |
| TRINITY_DN15512_c0_g1_i6 | -1.19158323 | 0.790381621 | 1 | 0.772 | 0 | 1.467 | 0 | EHN |
| TRINITY_DN72697_c0_g1_i1 | -1.19158323 | 0.790381621 | 1 | 2.389 | 0 | 4.533 | 0 | EthD |
| TRINITY_DN175_c0_g1_i25 | 1.19155968 | 0.257135622 | 0.999970678 | 9.048 | 27.44 | 7.777 | 6.374 | NA |
| TRINITY_DN169486_c0_g1_i1 | 1.191483842 | 0.334759733 | 1 | 0.981 | 0.67 | 0.311 | 0.352 | NA |
| TRINITY_DN955_c0_g1_i1 | 1.191451817 | 0.776452104 | 0.999970678 | 5.478 | 0 | 0 | 2.423 | NA |
| TRINITY_DN5670_c0_g1_i3 | 1.191448984 | 0.671669223 | 0.999970678 | 6.922 | 0 | 1.522 | 1.237 | NA |
| TRINITY_DN798_c0_g2_i10 | -1.19143549 | 0.664173705 | 0.999970678 | 0.763 | 0 | 0.267 | 1.438 | NA |
| TRINITY_DN21226_c0_g2_i4 | 1.191303846 | 0.439102659 | 1 | 5.005 | 5.893 | 3.155 | 0.834 | NB-ARC |
| TRINITY_DN1740_c0_g1_i10 | 1.191171872 | 0.671300257 | 0.999970678 | 1.699 | 0 | 0.267 | 0.432 | RVT_1 |
| TRINITY_DN152_c1_g1_i9 | 1.191099627 | 0.682027686 | 0.999970678 | 0 | 2.592 | 0.678 | 0.302 | NA |
| TRINITY_DN2444_c0_g1_i2 | -1.19087349 | 0.672899035 | 0.999970678 | 3.697 | 4.642 | 0 | 19.042 | Pyr_redox_2 |
| TRINITY_DN14481_c0_g1_i3 | -1.1906289 | 0.284047495 | 0.999970678 | 0.372 | 0.887 | 1.089 | 1.538 | NA |
| TRINITY_DN1071_c0_g1_i3 | -1.1905208 | 0.361196738 | 0.999970678 | 3.779 | 10.8 | 4.922 | 26.853 | NA |
| TRINITY_DN944_c1_g1_i1 | -1.19044102 | 0.371221024 | 0.999970678 | 4.097 | 3.725 | 12.999 | 2.162 | DHQ_synthase |
| TRINITY_DN843_c0_g1_i15 | -1.19008212 | 0.243548532 | 0.999970678 | 6.241 | 9.027 | 11.444 | 18.831 | NA |
| TRINITY_DN24448_c0_g1_i5 | -1.19002161 | 0.777951333 | 0.999970678 | 2.689 | 0 | 5.144 | 0 | NA |
| TRINITY_DN17504_c0_g1_i3 | -1.18993472 | 0.211489112 | 0.999970678 | 0.427 | 0.443 | 0.756 | 1.056 | NA |
| TRINITY_DN1442_c0_g1_i3 | -1.18965853 | 0.389708566 | 0.999970678 | 5.523 | 11.19 | 3.566 | 33.418 | DUF4228 |
| TRINITY_DN6485_c0_g1_i7 | -1.18964181 | 0.791557216 | 1 | 0.654 | 0 | 1.244 | 0 | Asp |
| TRINITY_DN17407_c0_g1_i1 | -1.18950805 | 0.777609196 | 0.999970678 | 4.215 | 0 | 8.044 | 0 | DJ-1_PfpI |
| TRINITY_DN8992_c0_g1_i3 | -1.1894422 | 0.683636093 | 0.999970678 | 0 | 85.93 | 94.028 | 78.66 | NA |
| TRINITY_DN1018_c0_g1_i1 | -1.1892121 | 0.189098441 | 0.999970678 | 30.086 | 23.23 | 28.254 | 87.598 | NA |
| TRINITY_DN999_c0_g1_i6 | -1.18915664 | 0.666751294 | 0.999970678 | 14.098 | 0 | 19.299 | 9.179 | NA |
| TRINITY_DN4882_c0_g1_i1 | -1.18910537 | 0.117749571 | 0.999970678 | 11.446 | 8.554 | 23.843 | 16.91 | NA |
| TRINITY_DN1082_c0_g1_i7 | 1.189095113 | 0.684898965 | 0.999970678 | 1.19 | 0 | 0.367 | 0.09 | PRP21_like_P |
| TRINITY_DN4438_c0_g2_i2 | -1.18899914 | 0.659549469 | 0.999970678 | 0.99 | 0 | 0.522 | 1.659 | NA |
| TRINITY_DN603_c0_g1_i1 | 1.18891731 | 0.708509618 | 0.999970678 | 0 | 809.8 | 155.902 | 159.723 | NA |
| TRINITY_DN70_c0_g1_i3 | -1.18886681 | 0.282034918 | 0.999970678 | 11.346 | 7.539 | 28.465 | 8.747 | UDPGT |
| TRINITY_DN11128_c0_g1_i8 | -1.18872678 | 0.679940637 | 0.999970678 | 0.936 | 0 | 0.244 | 1.82 | Pkinase |
| TRINITY_DN8062_c0_g1_i4 | 1.188620201 | 0.47073186 | 1 | 2.998 | 3.104 | 1.855 | 0.332 | DUF659 |
| TRINITY_DN94_c2_g1_i7 | 1.188427124 | 0.777878292 | 0.999970678 | 0 | 3.913 | 0 | 1.699 | Ribosomal_L18p |
| TRINITY_DN636_c1_g2_i5 | 1.188274137 | 0.780067677 | 0.999970678 | 14.498 | 0 | 0 | 6.485 | Snf7 |
| TRINITY_DN1952_c1_g1_i1 | -1.18812934 | 0.706835122 | 0.999970678 | 3.77 | 0.276 | 7.688 | 0 | NA |
| TRINITY_DN32622_c0_g1_i1 | 1.187921142 | 0.787949061 | 1 | 1.708 | 0 | 0.633 | 0 | AdoHcyase |
| TRINITY_DN2126_c0_g1_i1 | -1.18785988 | 0.120768106 | 0.999970678 | 18.886 | 34.48 | 56.385 | 52.138 | Copper-bind |
| TRINITY_DN6410_c0_g1_i1 | -1.18778986 | 0.350636279 | 0.999970678 | 2.335 | 12.6 | 8.644 | 23.184 | DUF1645 |
| TRINITY_DN7796_c1_g1_i5 | -1.18748676 | 0.663287416 | 0.999970678 | 0 | 3.784 | 5.444 | 1.83 | NA |
| TRINITY_DN2775_c0_g1_i1 | -1.18748606 | 0.434332318 | 0.999970678 | 0.672 | 5.46 | 3.355 | 9.611 | NA |
| TRINITY_DN5812_c0_g1_i2 | 1.187420564 | 0.230112352 | 0.999970678 | 3.252 | 1.744 | 1.133 | 0.845 | WRKY |

| TRINITY_DN9356_c0_g1_i1 | 1.18738951 | 0.31969855 | 0.999970678 | 8.157 | 24.37 | 3.333 | 9.822 | NA |
| --- | --- | --- | --- | --- | --- | --- | --- | --- |
| TRINITY_DN14795_c0_g1_i2 | 1.187301356 | 0.569464017 | 0.999970678 | 0.045 | 4.297 | 0.733 | 0.975 | Terpene_synth |
| TRINITY_DN22524_c0_g1_i1 | 1.187290186 | 0.658768558 | 0.999970678 | 0.309 | 0.838 | 0 | 0.513 | FAR1 |
| TRINITY_DN10461_c0_g2_i1 | 1.187289761 | 0.65865473 | 1 | 1.998 | 1.725 | 1.378 | 0.01 | NA |
| TRINITY_DN3017_c0_g1_i10 | -1.1872387 | 0.592270188 | 0.999970678 | 0.309 | 0.552 | 1.611 | 0.02 | TFIID_20kDa |
| TRINITY_DN1753_c1_g1_i7 | -1.18722187 | 0.146482969 | 0.999970678 | 0.481 | 0.394 | 0.978 | 0.794 | CcmF_C |
| TRINITY_DN1544_c0_g1_i7 | -1.18721055 | 0.406710724 | 0.999970678 | 6.486 | 1.202 | 3.1 | 13.884 | Motile_Sperm |
| TRINITY_DN7947_c1_g1_i8 | -1.18716934 | 0.671354963 | 1 | 0 | 0.601 | 0.833 | 0.312 | NA |
| TRINITY_DN2308_c0_g1_i1 | 1.187118593 | 0.062335136 | 0.999970678 | 12.318 | 13.3 | 5.311 | 4.846 | - |
| TRINITY_DN2903_c0_g1_i2 | -1.18688834 | 0.677768081 | 0.999970678 | 24.79 | 0 | 31.431 | 19.253 | RRM_1 |
| TRINITY_DN13584_c0_g2_i6 | 1.186612807 | 0.209586933 | 0.999970678 | 4.715 | 11.92 | 3.455 | 3.097 | NA |
| TRINITY_DN5672_c0_g1_i3 | 1.186519344 | 0.782637059 | 0.999970678 | 28.751 | 0 | 0 | 12.829 | C2 |
| TRINITY_DN888_c0_g2_i1 | -1.18638772 | 0.224847178 | 0.999970678 | 17.405 | 27.02 | 22.665 | 71.251 | NA |
| TRINITY_DN4515_c0_g1_i2 | 1.186317293 | 0.340383237 | 0.999970678 | 3.361 | 2.543 | 0.456 | 2.001 | NA |
| TRINITY_DN6531_c1_g1_i5 | 1.186162984 | 0.287263966 | 0.999970678 | 0.654 | 1.074 | 0.311 | 0.372 | RVP_2 |
| TRINITY_DN11318_c0_g1_i6 | -1.18598521 | 0.378155716 | 0.999970678 | 0.999 | 2.838 | 4.166 | 3.579 | NA |
| TRINITY_DN4616_c0_g1_i5 | 1.185669793 | 0.393214089 | 0.999970678 | 2.144 | 1.626 | 0.167 | 1.468 | Calmodulin_bind |
| TRINITY_DN9567_c2_g1_i6 | -1.18499378 | 0.318515779 | 0.999970678 | 0.382 | 0.227 | 0.778 | 0.473 | DUF4283 |
| TRINITY_DN6337_c0_g1_i4 | 1.184795785 | 0.296776017 | 0.999970678 | 2.407 | 2.582 | 1.478 | 0.412 | NA |
| TRINITY_DN21678_c0_g1_i1 | -1.18473481 | 0.345030206 | 0.999970678 | 0.754 | 2.582 | 2.067 | 4.997 | PMEI |
| TRINITY_DN2855_c0_g1_i3 | 1.184531608 | 0.323637413 | 0.999970678 | 31.013 | 6.485 | 8.644 | 6.183 | EF-hand_5 |
| TRINITY_DN8281_c0_g1_i1 | -1.18436074 | 0.496445908 | 0.999970678 | 1.29 | 0.069 | 1.689 | 1.066 | MS_channel |
| TRINITY_DN48408_c0_g1_i4 | -1.18428917 | 0.663945911 | 0.999970678 | 0 | 0.995 | 1.478 | 0.442 | NA |
| TRINITY_DN330_c0_g1_i2 | 1.184267418 | 0.654624314 | 0.999970678 | 2.371 | 1.675 | 1.478 | 0 | EF-hand_1 |
| TRINITY_DN5084_c0_g1_i7 | 1.183924865 | 0.783842319 | 0.999970678 | 156.66 | 0 | 0 | 63.037 | NA |
| TRINITY_DN1148_c0_g1_i2 | -1.18385497 | 0.381470805 | 0.999970678 | 0.563 | 0.424 | 0.411 | 1.739 | Glyco_hydro_1 |
| TRINITY_DN3490_c0_g1_i2 | -1.18381612 | 0.780362495 | 0.999970678 | 0 | 6.623 | 12.244 | 0 | NA |
| TRINITY_DN9573_c0_g2_i7 | -1.18373221 | 0.685944511 | 0.999970678 | 0 | 6.386 | 0.989 | 12.547 | ADH_N |
| TRINITY_DN5672_c0_g1_i5 | -1.18369532 | 0.107576528 | 0.999970678 | 60.844 | 71.59 | 88.317 | 193.765 | C2 |
| TRINITY_DN4490_c0_g1_i4 | 1.183591165 | 0.654233925 | 0.999970678 | 1.599 | 1.222 | 1.033 | 0 | NA |
| TRINITY_DN12100_c0_g1_i1 | 1.183309541 | 0.623801886 | 0.999970678 | 3.824 | 0.079 | 1.4 | 0.05 | NA |
| TRINITY_DN7039_c3_g1_i1 | -1.18313345 | 0.291744445 | 0.999970678 | 2.562 | 2.07 | 6.611 | 2.513 | NmrA |
| TRINITY_DN166_c0_g1_i4 | -1.18310576 | 0.698498659 | 0.999970678 | 2.898 | 0.138 | 0 | 7.038 | NA |
| TRINITY_DN6189_c0_g1_i12 | -1.18309593 | 0.431435407 | 0.999970678 | 0.145 | 1.33 | 1.2 | 1.82 | NA |
| TRINITY_DN4778_c0_g1_i1 | 1.183001611 | 0.347842152 | 0.999970678 | 1.199 | 5.529 | 0.978 | 1.78 | RNA_pol_Rpb4 |
| TRINITY_DN11951_c0_g1_i1 | 1.182944562 | 0.351535182 | 0.999970678 | 10.301 | 16.05 | 1.655 | 9.35 | NA |
| TRINITY_DN3898_c1_g1_i12 | -1.18292276 | 0.442534441 | 0.999970678 | 20.294 | 4.662 | 4.6 | 51.897 | MatE |
| TRINITY_DN19513_c0_g1_i6 | 1.182752658 | 0.450909618 | 1 | 0.227 | 0.375 | 0.189 | 0.05 | NA |
| TRINITY_DN5382_c0_g1_i3 | 1.182724021 | 0.780290773 | 0.999970678 | 3.016 | 0 | 0 | 1.347 | Tic22 |
| TRINITY_DN220_c1_g1_i1 | 1.182634145 | 0.134411955 | 0.999970678 | 6.631 | 6.504 | 3.033 | 2.121 | NA |
| TRINITY_DN38577_c0_g1_i1 | -1.18251594 | 0.462776597 | 0.999970678 | 0.672 | 3.114 | 0.8 | 7.55 | NA |
| TRINITY_DN108_c1_g1_i18 | -1.1822825 | 0.436997528 | 0.999970678 | 1.344 | 0.286 | 0.589 | 3.026 | NA |
| TRINITY_DN1148_c0_g1_i4 | -1.18213065 | 0.339565578 | 0.999970678 | 41.486 | 90.39 | 38.698 | 250.619 | Glyco_hydro_1 |
| TRINITY_DN8459_c0_g1_i12 | -1.1820642 | 0.658237031 | 0.999970678 | 0 | 1.242 | 1.111 | 1.428 | EamA |
| TRINITY_DN5381_c1_g1_i1 | 1.182000193 | 0.472538784 | 1 | 0.572 | 0.611 | 0.378 | 0.07 | NA |
| TRINITY_DN234_c2_g1_i9 | 1.181901239 | 0.487380943 | 0.999970678 | 0.5 | 7.618 | 1.589 | 1.609 | NA |
| TRINITY_DN9898_c0_g1_i2 | -1.18173879 | 0.670159927 | 0.999970678 | 0.99 | 0 | 0.344 | 1.87 | Creatinase_N |
| TRINITY_DN23710_c0_g1_i3 | 1.181699361 | 0.193681647 | 0.999970678 | 0.563 | 0.621 | 0.189 | 0.302 | Bromodomain |
| TRINITY_DN1499_c0_g1_i7 | 1.181615618 | 0.68113815 | 0.999970678 | 3.406 | 0 | 0.989 | 0.332 | NA |
| TRINITY_DN13778_c2_g1_i3 | -1.1815324 | 0.423342368 | 0.999970678 | 0.772 | 0.197 | 1.255 | 0.704 | Pyrophosphatase |
| TRINITY_DN56_c4_g1_i2 | -1.18148837 | 0.271496374 | 0.999970678 | 2.852 | 1.784 | 2.544 | 7.43 | NA |
| TRINITY_DN2382_c1_g1_i3 | 1.181089453 | 0.782023438 | 0.999970678 | 0 | 0.719 | 0.256 | 0 | NA |
| TRINITY_DN3589_c0_g1_i7 | 1.181066544 | 0.552472122 | 1 | 1.535 | 1.34 | 1.033 | 0.08 | NA |
| TRINITY_DN4095_c0_g1_i11 | 1.180959895 | 0.387247815 | 0.999970678 | 0.754 | 0.345 | 0.289 | 0.151 | NA |
| TRINITY_DN7473_c1_g1_i2 | -1.18090289 | 0.65544452 | 0.999970678 | 2.362 | 0 | 1.9 | 3.137 | NA |
| TRINITY_DN284_c0_g1_i2 | 1.180865535 | 0.718605337 | 0.999970678 | 13.172 | 0.059 | 0 | 5.962 | NA |
| TRINITY_DN1700_c0_g2_i2 | -1.18054572 | 0.213643376 | 0.999970678 | 0.472 | 0.473 | 1.122 | 0.814 | B_lectin |
| TRINITY_DN23_c0_g3_i1 | 1.180541628 | 0.368634636 | 0.999970678 | 77.377 | 43.93 | 38.553 | 6.786 | RVT_1 |
| TRINITY_DN5745_c0_g1_i6 | -1.18052455 | 0.69576219 | 0.999970678 | 0.636 | 0 | 1.133 | 0.101 | NA |
| TRINITY_DN17867_c0_g1_i4 | 1.18036197 | 0.155516639 | 0.999970678 | 1.553 | 1.912 | 0.756 | 0.613 | NA |
| TRINITY_DN26749_c0_g1_i5 | -1.18018857 | 0.782514654 | 0.999970678 | 0.836 | 0 | 1.611 | 0 | NA |
| TRINITY_DN2654_c0_g1_i5 | 1.180112866 | 0.318463958 | 0.999970678 | 1.526 | 4.1 | 1.567 | 0.583 | DUF247 |
| TRINITY_DN489_c0_g1_i2 | 1.180070281 | 0.353324725 | 0.999970678 | 0.327 | 1.163 | 0.289 | 0.312 | NA |
| TRINITY_DN4003_c1_g1_i1 | 1.180054099 | 0.455292642 | 0.999970678 | 0.999 | 2.385 | 0.211 | 1.206 | GATase |
| TRINITY_DN18274_c0_g1_i6 | -1.17992767 | 0.688946909 | 0.999970678 | 1.117 | 0.217 | 2.478 | 0 | Mannitol_dh |
| TRINITY_DN6973_c0_g2_i1 | 1.179872082 | 0.483691789 | 0.999970678 | 56.747 | 1363 | 143.591 | 392.225 | NA |
| TRINITY_DN4153_c0_g1_i7 | 1.179778289 | 0.262468514 | 0.999970678 | 0.818 | 1.005 | 0.378 | 0.352 | AMPK1_CBM |
| TRINITY_DN4555_c0_g1_i8 | -1.17970075 | 0.661259966 | 0.999970678 | 1.817 | 0 | 2.378 | 1.347 | NA |
| TRINITY_DN9427_c0_g1_i3 | -1.17969859 | 0.288427644 | 0.999970678 | 100.84 | 32.61 | 70.94 | 219.181 | NA |
| TRINITY_DN10738_c0_g1_i1 | 1.179679725 | 0.179967643 | 0.999970678 | 11.3 | 5.637 | 3.733 | 3.016 | WEMBL |
| TRINITY_DN1041_c1_g1_i1 | -1.17959649 | 0.599999328 | 0.999970678 | 30.994 | 1.074 | 59.952 | 1.066 | Aldedh |
| TRINITY_DN8839_c0_g1_i1 | 1.179544965 | 0.235372583 | 0.999970678 | 2.689 | 7.717 | 2.011 | 2.121 | DA1-like |
| TRINITY_DN1802_c0_g1_i2 | -1.1794485 | 0.140590203 | 0.999970678 | 7.458 | 12.65 | 14.055 | 28.482 | NA |
| TRINITY_DN10983_c1_g1_i2 | -1.1793873 | 0.242779841 | 0.999970678 | 3.152 | 1.478 | 3.078 | 6.756 | NA |

| TRINITY_DN2092_c0_g1_i1 | -1.17918551 | 0.473081186 | 0.999970678 | 1.844 | 2.74 | 0.456 | 9.802 | NA |
| --- | --- | --- | --- | --- | --- | --- | --- | --- |
| TRINITY_DN7555_c0_g1_i1 | -1.17882496 | 0.46440553 | 0.999970678 | 119.32 | 11.25 | 185.578 | 61.609 | NA |
| TRINITY_DN9985_c0_g1_i12 | 1.178748728 | 0.383712736 | 0.999970678 | 2.598 | 0.68 | 0.744 | 0.543 | NA |
| TRINITY_DN616_c1_g1_i2 | 1.178714111 | 0.677983756 | 0.999970678 | 1.744 | 0.217 | 0 | 0.875 | EF_assoc_1 |
| TRINITY_DN5380_c0_g1_i2 | 1.178591127 | 0.357295154 | 0.999970678 | 4.76 | 7.037 | 3.089 | 1.337 | NA |
| TRINITY_DN13530_c0_g1_i1 | -1.17850081 | 0.393756358 | 0.999970678 | 1.154 | 0.621 | 0.544 | 3.388 | MatE |
| TRINITY_DN4674_c1_g1_i9 | -1.17849474 | 0.203168333 | 0.999970678 | 0.4 | 0.237 | 0.678 | 0.623 | HLH |
| TRINITY_DN2456_c0_g1_i8 | -1.17826028 | 0.49668591 | 0.999970678 | 2.571 | 0.138 | 2.189 | 3.549 | NA |
| TRINITY_DN10983_c1_g1_i6 | -1.17819516 | 0.317769089 | 0.999970678 | 1.699 | 2.769 | 1.989 | 7.661 | NA |
| TRINITY_DN538_c0_g2_i1 | 1.178097538 | 0.492580229 | 0.999970678 | 2.916 | 59.41 | 5.389 | 20.228 | NA |
| TRINITY_DN3682_c0_g1_i12 | -1.17792949 | 0.565498298 | 0.999970678 | 2.444 | 0.168 | 4.233 | 0.804 | 2014/3/3 |
| TRINITY_DN492_c0_g1_i2 | 1.177878385 | 0.312782086 | 0.999970678 | 2.571 | 1.961 | 1.333 | 0.392 | NA |
| TRINITY_DN14946_c0_g1_i1 | 1.177727065 | 0.658121761 | 0.999970678 | 5.087 | 2.178 | 2.667 | 0 | ABC_membrane |
| TRINITY_DN5858_c0_g1_i2 | 1.177663511 | 0.778917543 | 0.999970678 | 7.14 | 0 | 0 | 3.187 | NA |
| TRINITY_DN3091_c0_g1_i8 | 1.17755505 | 0.783840885 | 0.999970678 | 0 | 1.133 | 0 | 0.493 | NA |
| TRINITY_DN8279_c3_g3_i1 | 1.17740441 | 0.564943787 | 1 | 0.209 | 2.74 | 0.156 | 1.096 | NA |
| TRINITY_DN24684_c0_g1_i1 | -1.17732809 | 0.666923153 | 1 | 0.273 | 0 | 0.222 | 0.362 | LRRNT_2 |
| TRINITY_DN2199_c0_g1_i6 | 1.177251265 | 0.789572237 | 1 | 0 | 0.65 | 0 | 0.292 | NA |
| TRINITY_DN20736_c0_g1_i1 | 1.177156512 | 0.2020781 | 0.999970678 | 32.884 | 47.82 | 17.799 | 12.829 | PD40 |
| TRINITY_DN950_c0_g1_i1 | 1.177139899 | 0.451898208 | 0.999970678 | 0.509 | 3.558 | 1.122 | 0.422 | Mito_carr |
| TRINITY_DN3017_c0_g1_i5 | 1.177038624 | 0.260220048 | 0.999970678 | 2.58 | 1.212 | 0.889 | 0.623 | TFIID_20kDa |
| TRINITY_DN12417_c0_g1_i4 | 1.176972478 | 0.655139326 | 0.999970678 | 0.563 | 0.956 | 0.544 | 0 | DEAD |
| TRINITY_DN9256_c0_g1_i4 | 1.176876326 | 0.272908869 | 0.999970678 | 3.089 | 3.755 | 0.589 | 2.292 | PP2C |
| TRINITY_DN11059_c0_g1_i1 | -1.17681881 | 0.600653223 | 0.999970678 | 0.018 | 0.187 | 0.011 | 0.452 | - |
| TRINITY_DN267_c0_g1_i2 | -1.17668372 | 0.664428687 | 0.999970678 | 20.221 | 0 | 16.355 | 26.21 | - |
| TRINITY_DN2077_c0_g1_i4 | 1.176656436 | 0.65946789 | 0.999970678 | 4.469 | 4.721 | 3.355 | 0 | Hist_deacetyl |
| TRINITY_DN4067_c0_g1_i11 | 1.176630279 | 0.276225816 | 0.999970678 | 235.88 | 68.36 | 45.197 | 74.367 | NA |
| TRINITY_DN9638_c0_g1_i3 | 1.176591928 | 0.424976055 | 0.999970678 | 0.736 | 4.494 | 0.978 | 1.116 | NA |
| TRINITY_DN4607_c0_g1_i2 | -1.17624609 | 0.308891145 | 0.999970678 | 13.199 | 8.387 | 32.209 | 9.702 | Pirin |
| TRINITY_DN9846_c0_g1_i1 | -1.17614791 | 0.32802894 | 0.999970678 | 7.403 | 11.94 | 5.822 | 36.565 | NA |
| TRINITY_DN3051_c1_g3_i3 | 1.175742833 | 0.673678642 | 0.999970678 | 3.806 | 0.453 | 0 | 1.9 | NA |
| TRINITY_DN13946_c0_g1_i1 | -1.17570751 | 0.27411131 | 0.999970678 | 1.372 | 0.542 | 1.967 | 2.011 | NA |
| TRINITY_DN4702_c0_g1_i3 | -1.17565678 | 0.554352154 | 0.999970678 | 0.382 | 7.707 | 0.955 | 16.86 | NA |
| TRINITY_DN7323_c0_g1_i4 | 1.175436489 | 0.557414614 | 0.999970678 | 1.263 | 0.03 | 0.178 | 0.362 | LRRNT_2 |
| TRINITY_DN3683_c0_g1_i3 | 1.175384603 | 0.513989012 | 0.999970678 | 2.68 | 0.335 | 0.933 | 0.221 | NA |
| TRINITY_DN1315_c1_g3_i1 | 1.175271394 | 0.438106933 | 0.999970678 | 4.033 | 9.954 | 4.666 | 0.513 | NA |
| TRINITY_DN1385_c0_g3_i2 | 1.175092532 | 0.096657621 | 0.999970678 | 20.929 | 30.47 | 10.799 | 9.591 | WRKY |
| TRINITY_DN6210_c0_g1_i4 | -1.17502748 | 0.264463968 | 0.999970678 | 0.745 | 0.857 | 1.622 | 1.699 | NUDIX |
| TRINITY_DN1041_c1_g1_i4 | 1.174896408 | 0.442826289 | 0.999970678 | 2.008 | 7.244 | 2.855 | 0.623 | Aldedh |
| TRINITY_DN15387_c0_g1_i9 | 1.174786583 | 0.786732104 | 0.999970678 | 4.606 | 0 | 1.711 | 0 | Ribosomal_L2 |
| TRINITY_DN1425_c0_g2_i1 | 1.174783015 | 0.439874516 | 0.999970678 | 32.239 | 4.051 | 2.333 | 13.542 | Aminotran_5 |
| TRINITY_DN4737_c0_g1_i4 | -1.17476535 | 0.413882299 | 0.999970678 | 4.469 | 1.291 | 9.399 | 1.749 | NA |
| TRINITY_DN2_c0_g1_i4 | 1.174680795 | 0.110502506 | 0.999970678 | 10.283 | 12.02 | 2.944 | 6.354 | NA |
| TRINITY_DN4628_c0_g1_i3 | -1.17447748 | 0.682870049 | 0.999970678 | 0.236 | 0 | 0.4 | 0.06 | Helicase_C |
| TRINITY_DN2713_c1_g1_i1 | 1.174445998 | 0.656179977 | 0.999970678 | 2.725 | 7.874 | 0 | 4.645 | NA |
| TRINITY_DN3246_c0_g1_i4 | -1.17405461 | 0.654845352 | 0.999970678 | 0 | 1.656 | 1.222 | 2.202 | NA |
| TRINITY_DN1386_c0_g1_i1 | -1.17404145 | 0.493754952 | 0.999970678 | 9.647 | 0.986 | 17.021 | 3.519 | HSP70 |
| TRINITY_DN944_c0_g1_i5 | -1.17386074 | 0.176415253 | 0.999970678 | 0.672 | 1.025 | 1.478 | 2.021 | PPO1_DWL |
| TRINITY_DN2324_c0_g1_i4 | -1.17376309 | 0.480384407 | 0.999970678 | 0.972 | 13.48 | 21.465 | 6.133 | NA |
| TRINITY_DN112622_c0_g1_i1 | -1.17361677 | 0.789261544 | 0.999970678 | 1.553 | 0 | 2.922 | 0 | GST_C |
| TRINITY_DN19300_c0_g1_i1 | 1.173361145 | 0.421035029 | 0.999970678 | 6.94 | 0.995 | 2.089 | 1.025 | NA |
| TRINITY_DN2962_c0_g1_i1 | -1.17324917 | 0.657644477 | 0.999970678 | 2.68 | 0 | 2.966 | 2.554 | R3H |
| TRINITY_DN54871_c0_g1_i1 | -1.17315266 | 0.781251405 | 0.999970678 | 2.734 | 0 | 0 | 6.233 | NA |
| TRINITY_DN10191_c0_g1_i11 | -1.17295971 | 0.685029156 | 0.999970678 | 0.354 | 0 | 0.589 | 0.101 | NA |
| TRINITY_DN7742_c0_g1_i1 | 1.172929094 | 0.150530692 | 0.999970678 | 13.444 | 14.75 | 6.788 | 4.293 | NA |
| TRINITY_DN94_c1_g1_i1 | 1.172372403 | 0.390439663 | 0.999970678 | 15.861 | 2.69 | 4.055 | 3.378 | NA |
| TRINITY_DN11561_c0_g1_i4 | 1.1723334 | 0.394993087 | 0.999970678 | 15.425 | 2.148 | 4.155 | 2.855 | NA |
| TRINITY_DN11226_c0_g1_i1 | 1.172208179 | 0.664494584 | 0.999970678 | 0.654 | 2.424 | 1.122 | 0 | WEMBL |
| TRINITY_DN9629_c0_g1_i2 | -1.17219798 | 0.314110762 | 0.999970678 | 2.235 | 2.208 | 1.611 | 8.063 | DUF247 |
| TRINITY_DN4885_c0_g1_i2 | 1.172057191 | 0.572976755 | 0.999970678 | 2.126 | 0.089 | 0.667 | 0.191 | NA |
| TRINITY_DN5967_c0_g1_i1 | -1.1720134 | 0.395105631 | 0.999970678 | 1.717 | 0.503 | 1.189 | 3.559 | NA |
| TRINITY_DN8154_c0_g1_i1 | 1.172011964 | 0.306183718 | 0.999970678 | 8.166 | 3.824 | 1.4 | 3.589 | NA |
| TRINITY_DN1934_c0_g1_i5 | -1.17181957 | 0.427145939 | 0.999970678 | 0.273 | 0.266 | 0.156 | 1.046 | Exo_endo_phos |
| TRINITY_DN6785_c1_g1_i6 | -1.17154638 | 0.345365232 | 0.999970678 | 0.336 | 0.187 | 0.622 | 0.422 | NA |
| TRINITY_DN17236_c0_g1_i1 | -1.17148407 | 0.790154661 | 0.999970678 | 0.799 | 0 | 1.5 | 0 | NA |
| TRINITY_DN385_c0_g1_i6 | 1.171467447 | 0.156143846 | 0.999970678 | 2.026 | 2.257 | 1.033 | 0.653 | RPAP1_C |
| TRINITY_DN10572_c0_g1_i7 | 1.171395064 | 0.790326955 | 0.999970678 | 3.07 | 0 | 1.155 | 0 | NA |
| TRINITY_DN564_c0_g1_i7 | -1.1713084 | 0.063433815 | 0.999970678 | 9.193 | 9.569 | 15.555 | 23.405 | NA |
| TRINITY_DN10865_c0_g1_i1 | -1.17128597 | 0.45006984 | 0.999970678 | 0.309 | 0.148 | 0.144 | 0.865 | NA |
| TRINITY_DN2319_c0_g1_i1 | 1.170874969 | 0.499584405 | 0.999970678 | 0.536 | 1.537 | 0.078 | 0.845 | Carb_anhydrase |
| TRINITY_DN12102_c0_g1_i1 | -1.17065407 | 0.781534999 | 0.999970678 | 3.988 | 0 | 7.5 | 0 | NA |
| TRINITY_DN3349_c0_g1_i1 | -1.17062847 | 0.317977953 | 0.999970678 | 43.676 | 44.16 | 26.087 | 163.996 | PRA1 |
| TRINITY_DN6188_c0_g1_i3 | 1.170598905 | 0.41982229 | 0.999970678 | 10.056 | 67.56 | 5.255 | 27.728 | Aa_trans |
| TRINITY_DN3538_c0_g2_i1 | -1.17041351 | 0.200882007 | 0.999970678 | 1.862 | 4.317 | 6.189 | 6.344 | Beta_elim_lyase |

| TRINITY_DN8112_c1_g1_i1 | 1.170407102 | 0.204277446 | 0.999970678 | 1.036 | 1.439 | 0.333 | 0.684 | NA |
| --- | --- | --- | --- | --- | --- | --- | --- | --- |
| TRINITY_DN5915_c0_g1_i1 | -1.17039134 | 0.392366358 | 0.999970678 | 0.845 | 4.928 | 2.878 | 9.4 | NA |
| TRINITY_DN3623_c0_g1_i2 | -1.17038627 | 0.667723789 | 0.999970678 | 4.606 | 0 | 2.3 | 7.772 | NA |
| TRINITY_DN256_c1_g1_i2 | -1.17038564 | 0.416078755 | 0.999970678 | 4.487 | 0.552 | 6.355 | 3.82 | Pantoate_transf |
| TRINITY_DN20679_c0_g1_i1 | -1.17037048 | 0.525509351 | 0.999970678 | 0.3 | 1.084 | 0.144 | 2.926 | NA |
| TRINITY_DN147_c0_g1_i4 | -1.17034999 | 0.572277779 | 0.999970678 | 2.235 | 0.03 | 2.378 | 2.292 | NA |
| TRINITY_DN3269_c0_g3_i1 | 1.170143892 | 0.524851375 | 0.999970678 | 28.178 | 1.567 | 10.055 | 1.257 | Ribosomal_L2 |
| TRINITY_DN6313_c1_g1_i1 | -1.17008602 | 0.342969905 | 0.999970678 | 10.083 | 5.006 | 4.7 | 28.532 | NA |
| TRINITY_DN144_c0_g1_i4 | 1.169696679 | 0.661229371 | 0.999970678 | 1.453 | 5.273 | 0 | 2.966 | EF-hand_5 |
| TRINITY_DN6803_c0_g1_i1 | 1.169527319 | 0.480407606 | 0.999970678 | 3.534 | 42.64 | 13.61 | 3.66 | Tryp_alpha_amyl |
| TRINITY_DN27199_c0_g1_i4 | 1.169425507 | 0.40620984 | 0.999970678 | 0.754 | 0.256 | 0.267 | 0.141 | NA |
| TRINITY_DN79906_c0_g1_i1 | 1.169416981 | 0.370579456 | 0.999970678 | 10.437 | 5.489 | 1.922 | 4.494 | NA |
| TRINITY_DN1093_c0_g1_i1 | 1.16929645 | 0.51765894 | 0.999970678 | 2.534 | 2.759 | 0.089 | 2.272 | NA |
| TRINITY_DN469_c0_g1_i4 | 1.169270655 | 0.189537321 | 0.999970678 | 40.896 | 18.88 | 13.666 | 10.225 | p450 |
| TRINITY_DN17345_c0_g1_i3 | 1.169226286 | 0.438395703 | 0.999970678 | 1.344 | 0.926 | 0.078 | 0.915 | LRR_8 |
| TRINITY_DN8857_c0_g1_i5 | 1.169223916 | 0.236164922 | 0.999970678 | 2.162 | 2.858 | 1.355 | 0.603 | Myb_DNA-bind_ |
| TRINITY_DN1252_c0_g1_i1 | 1.169191333 | 0.242034447 | 0.999970678 | 5.559 | 5.805 | 1.333 | 3.418 | - |
| TRINITY_DN1624_c0_g1_i2 | -1.16917758 | 0.791113586 | 0.999970678 | 0.581 | 0 | 1.122 | 0 | NA |
| TRINITY_DN1009_c0_g1_i13 | 1.16904736 | 0.655536518 | 0.999970678 | 1.081 | 2.592 | 0 | 1.619 | NA |
| TRINITY_DN2010_c0_g1_i2 | -1.16889677 | 0.670627107 | 0.999970678 | 0 | 9.205 | 11.399 | 6.585 | XS |
| TRINITY_DN4811_c0_g1_i10 | 1.168884807 | 0.792864114 | 1 | 0.863 | 0 | 0.322 | 0 | NA |
| TRINITY_DN5682_c0_g1_i8 | 1.168750737 | 0.268696704 | 0.999970678 | 1.971 | 3.794 | 0.7 | 1.729 | NA |
| TRINITY_DN4232_c0_g1_i5 | 1.16858943 | 0.784127751 | 0.999970678 | 0 | 2.148 | 0.767 | 0 | NA |
| TRINITY_DN15726_c0_g1_i4 | 1.168580287 | 0.787033642 | 0.999970678 | 1.989 | 0 | 0 | 0.905 | NA |
| TRINITY_DN4592_c0_g1_i17 | 1.168580287 | 0.787033642 | 0.999970678 | 1.562 | 0 | 0 | 0.714 | NA |
| TRINITY_DN5821_c0_g1_i1 | 1.168580287 | 0.787033642 | 0.999970678 | 1.735 | 0 | 0 | 0.794 | HSP70 |
| TRINITY_DN2056_c0_g2_i2 | -1.16852404 | 0.434897667 | 0.999970678 | 0.173 | 0.621 | 1.1 | 0.473 | NA |
| TRINITY_DN15455_c0_g1_i1 | -1.16844489 | 0.264139701 | 0.999970678 | 0.754 | 0.71 | 0.933 | 2.192 | AAA |
| TRINITY_DN2408_c0_g1_i3 | -1.16843137 | 0.785648889 | 0.999970678 | 0 | 0.828 | 1.511 | 0 | NA |
| TRINITY_DN6414_c0_g1_i6 | 1.167961072 | 0.341910342 | 0.999970678 | 446.56 | 114.8 | 52.597 | 176.101 | NA |
| TRINITY_DN6711_c0_g1_i3 | -1.16788489 | 0.681342868 | 1 | 1.699 | 0 | 2.444 | 0.885 | NA |
| TRINITY_DN6591_c0_g2_i1 | 1.167738275 | 0.781965672 | 0.999970678 | 3.579 | 0 | 0 | 1.619 | NA |
| TRINITY_DN7012_c0_g1_i2 | -1.16763247 | 0.418493427 | 0.999970678 | 61.553 | 34.7 | 164.168 | 18.097 | YGGT |
| TRINITY_DN2542_c0_g1_i6 | -1.16762844 | 0.460653856 | 0.999970678 | 1.771 | 12.04 | 3.244 | 26.652 | NA |
| TRINITY_DN12983_c0_g1_i3 | 1.167627623 | 0.384334941 | 0.999970678 | 4.742 | 0.808 | 1.078 | 1.206 | Myb_DNA-bindin |
| TRINITY_DN808_c0_g4_i4 | -1.16756676 | 0.127369931 | 0.999970678 | 3.425 | 5.223 | 6.7 | 11.29 | ubiquitin |
| TRINITY_DN3188_c0_g1_i4 | 1.167539644 | 0.361259992 | 0.999970678 | 0.472 | 1.429 | 0.444 | 0.302 | NA |
| TRINITY_DN4406_c0_g1_i10 | -1.16741201 | 0.190281807 | 0.999970678 | 0.763 | 0.749 | 1.6 | 1.438 | NA |
| TRINITY_DN4416_c0_g1_i2 | 1.167405404 | 0.679820039 | 0.999970678 | 2.544 | 0.227 | 0 | 1.257 | NA |
| TRINITY_DN9558_c1_g1_i1 | 1.167401284 | 0.588447264 | 0.999970678 | 0.654 | 18.64 | 0.289 | 8.063 | NA |
| TRINITY_DN18045_c0_g1_i1 | -1.16731212 | 0.360094517 | 0.999970678 | 6.095 | 0.986 | 6.488 | 8.224 | NA |
| TRINITY_DN2109_c0_g1_i6 | -1.16712865 | 0.546498875 | 0.999970678 | 1.453 | 0.246 | 0.222 | 3.529 | Pro_CA |
| TRINITY_DN641_c0_g1_i7 | 1.167079355 | 0.272762232 | 0.999970678 | 1.09 | 2.484 | 0.6 | 0.834 | NA |
| TRINITY_DN4521_c0_g1_i2 | 1.167070559 | 0.267845821 | 0.999970678 | 2.716 | 2.759 | 0.6 | 1.689 | Cullin |
| TRINITY_DN2732_c0_g1_i1 | 1.167025756 | 0.143459832 | 0.999970678 | 2.389 | 3.459 | 1.255 | 1.096 | Sugar_tr |
| TRINITY_DN2804_c0_g1_i2 | -1.16700437 | 0.49948567 | 0.999970678 | 3.897 | 0.227 | 2.5 | 6.304 | DUF4588 |
| TRINITY_DN5522_c0_g1_i2 | 1.166954201 | 0.339449911 | 0.999970678 | 0.445 | 1.143 | 0.289 | 0.352 | NA |
| TRINITY_DN14753_c0_g1_i1 | -1.16688236 | 0.722608459 | 0.999970678 | 1.054 | 0 | 1.978 | 0.03 | Ras |
| TRINITY_DN8685_c0_g1_i2 | -1.16667467 | 0.792145647 | 1 | 0.254 | 0 | 0.478 | 0 | NA |
| TRINITY_DN3086_c0_g1_i3 | 1.166665774 | 0.388543218 | 0.999970678 | 2.598 | 3.449 | 1.978 | 0.302 | BLM10_mid |
| TRINITY_DN23565_c0_g1_i1 | 1.166284553 | 0.232586143 | 0.999970678 | 2.08 | 2.247 | 0.678 | 1.106 | NA |
| TRINITY_DN639_c0_g1_i1 | -1.16624586 | 0.614200365 | 0.999970678 | 1.835 | 0.148 | 0.056 | 4.393 | NA |
| TRINITY_DN8151_c0_g1_i5 | 1.166186838 | 0.267609707 | 0.999970678 | 0.754 | 1.666 | 0.556 | 0.392 | NA |
| TRINITY_DN835_c1_g1_i2 | 1.166047603 | 0.379673079 | 1 | 0.936 | 1.183 | 0.244 | 0.623 | NA |
| TRINITY_DN18236_c0_g5_i1 | -1.16600008 | 0.720514621 | 1 | 0.036 | 0.729 | 0 | 1.679 | NA |
| TRINITY_DN3422_c0_g1_i2 | 1.165929213 | 0.78478722 | 0.999970678 | 45.274 | 0 | 0 | 19.042 | NA |
| TRINITY_DN3984_c1_g1_i6 | -1.16589117 | 0.675846625 | 1 | 0 | 3.075 | 1.167 | 5.258 | NA |
| TRINITY_DN8575_c0_g1_i1 | -1.16581709 | 0.150799808 | 0.999970678 | 2.771 | 3.331 | 7.277 | 4.876 | NA |
| TRINITY_DN46_c0_g1_i7 | -1.1658152 | 0.242614053 | 0.999970678 | 0.899 | 1.321 | 1.522 | 3.137 | RRM_1 |
| TRINITY_DN12022_c0_g1_i3 | 1.16570159 | 0.368302831 | 0.999970678 | 0.518 | 1.587 | 0.3 | 0.583 | NA |
| TRINITY_DN1041_c1_g1_i6 | -1.16568724 | 0.380960511 | 0.999970678 | 9.711 | 8.998 | 30.843 | 4.625 | Aldedh |
| TRINITY_DN3298_c0_g1_i2 | 1.165517476 | 0.462928214 | 0.999970678 | 6.568 | 3.765 | 3.555 | 0.332 | - |
| TRINITY_DN2318_c0_g1_i4 | 1.165494041 | 0.697786539 | 0.999970678 | 0 | 41.15 | 9.444 | 6.666 | NA |
| TRINITY_DN4774_c0_g1_i2 | 1.165413063 | 0.659492819 | 0.999970678 | 1.889 | 1.015 | 1.078 | 0 | zf-RING_2 |
| TRINITY_DN162_c0_g1_i2 | -1.16540714 | 0.288810866 | 0.999970678 | 4.578 | 1.183 | 5.8 | 6.042 | NA |
| TRINITY_DN8670_c0_g1_i2 | -1.16527707 | 0.785112845 | 0.999970678 | 4.833 | 0 | 8.999 | 0 | NA |
| TRINITY_DN74778_c0_g1_i1 | 1.165151909 | 0.250821148 | 0.999970678 | 5.223 | 6.248 | 2.6 | 1.87 | NA |
| TRINITY_DN335_c0_g1_i8 | -1.16508572 | 0.155594728 | 0.999970678 | 1.217 | 0.946 | 1.622 | 2.885 | LRRNT_2 |
| TRINITY_DN10515_c0_g1_i1 | 1.16507825 | 0.540744256 | 0.999970678 | 1294.3 | 27.61 | 388.177 | 107.514 | NA |
| TRINITY_DN3627_c0_g1_i3 | -1.16506499 | 0.505232259 | 0.999970678 | 1.735 | 1.321 | 5.2 | 0.483 | NA |
| TRINITY_DN10121_c0_g1_i2 | 1.16497995 | 0.404695822 | 0.999970678 | 4.242 | 3.558 | 2.333 | 0.633 | NA |
| TRINITY_DN27620_c0_g2_i3 | 1.164928315 | 0.787155608 | 0.999970678 | 1.299 | 0 | 0.489 | 0 | NA |
| TRINITY_DN59987_c0_g1_i1 | -1.16475361 | 0.669796026 | 0.999970678 | 1.099 | 0 | 1.567 | 0.603 | NA |
| TRINITY_DN2841_c0_g1_i5 | 1.16453132 | 0.352407913 | 0.999970678 | 0.336 | 1.409 | 0.378 | 0.322 | NA |

| TRINITY_DN1883_c0_g1_i3 | 1.164466925 | 0.387811767 | 0.999970678 | 24.563 | 40.55 | 21.332 | 3.117 | NA |
| --- | --- | --- | --- | --- | --- | --- | --- | --- |
| TRINITY_DN584_c0_g1_i6 | 1.164225743 | 0.256796591 | 0.999970678 | 8.067 | 24.97 | 7.122 | 5.952 | NA |
| TRINITY_DN5002_c0_g2_i4 | 1.16421456 | 0.279957293 | 0.999970678 | 4.569 | 3.351 | 0.8 | 2.523 | NA |
| TRINITY_DN758_c0_g1_i12 | 1.164062214 | 0.656475391 | 0.999970678 | 0.936 | 2.139 | 0 | 1.377 | Sugar_tr |
| TRINITY_DN8972_c0_g1_i15 | -1.16394905 | 0.79325926 | 1 | 0.354 | 0 | 0.656 | 0 | BTB |
| TRINITY_DN22789_c0_g1_i5 | -1.16394905 | 0.79325926 | 1 | 0.872 | 0 | 1.622 | 0 | UDPGP |
| TRINITY_DN699_c0_g1_i3 | -1.16394028 | 0.667463947 | 0.999970678 | 6.25 | 0 | 8.866 | 3.398 | NA |
| TRINITY_DN10639_c0_g1_i11 | -1.16370447 | 0.469026462 | 0.999970678 | 1.054 | 0.375 | 0.411 | 2.735 | NA |
| TRINITY_DN35926_c0_g1_i4 | -1.16365378 | 0.670872098 | 1 | 0 | 0.611 | 0.767 | 0.392 | NA |
| TRINITY_DN10845_c0_g1_i1 | -1.16350898 | 0.317660814 | 0.999970678 | 0.609 | 0.227 | 0.711 | 0.985 | NA |
| TRINITY_DN459_c0_g1_i7 | -1.16350825 | 0.501557596 | 0.999970678 | 4.478 | 17.52 | 1.822 | 46.518 | - |
| TRINITY_DN1474_c1_g1_i9 | -1.16346873 | 0.66512231 | 0.999970678 | 2.216 | 0 | 1.978 | 2.664 | NA |
| TRINITY_DN2850_c0_g1_i5 | -1.16330756 | 0.307824267 | 0.999970678 | 3.416 | 6.307 | 3.633 | 17.333 | CAP160 |
| TRINITY_DN473_c0_g1_i9 | 1.163200575 | 0.673543133 | 1 | 0.363 | 0.946 | 0.467 | 0 | NA |
| TRINITY_DN793_c0_g1_i2 | 1.163128896 | 0.126579717 | 0.999970678 | 16.115 | 11.81 | 4.1 | 7.53 | adh_short |
| TRINITY_DN9632_c0_g1_i7 | -1.16309477 | 0.782966531 | 0.999970678 | 0 | 3.863 | 0 | 8.566 | NA |
| TRINITY_DN5819_c1_g1_i6 | 1.162867933 | 0.678859607 | 0.999970678 | 2.816 | 0 | 0.611 | 0.543 | Pkinase |
| TRINITY_DN1389_c0_g1_i3 | -1.16282857 | 0.389704188 | 0.999970678 | 0.2 | 1.055 | 1.467 | 1.005 | UPF0052 |
| TRINITY_DN6428_c0_g1_i4 | 1.162754049 | 0.260691202 | 0.999970678 | 0.618 | 0.838 | 0.267 | 0.332 | DDT |
| TRINITY_DN21985_c0_g1_i1 | -1.16242692 | 0.785977472 | 0.999970678 | 0.881 | 0 | 1.633 | 0 | NA |
| TRINITY_DN6721_c0_g1_i6 | 1.162282397 | 0.272130648 | 0.999970678 | 0.818 | 0.828 | 0.211 | 0.493 | DRY_EERY |
| TRINITY_DN2282_c0_g1_i5 | -1.16224104 | 0.335879228 | 0.999970678 | 10.437 | 2.267 | 8.666 | 18.177 | Heme_oxygenase |
| TRINITY_DN238_c0_g1_i9 | 1.161871038 | 0.388327486 | 0.999970678 | 5.777 | 2.415 | 2.6 | 0.543 | NA |
| TRINITY_DN3217_c0_g1_i1 | -1.16168094 | 0.468360661 | 1 | 0.245 | 0.069 | 0.144 | 0.553 | Myb_CC_LHEQLE |
| TRINITY_DN0_c0_g1_i3 | -1.16167727 | 0.673916695 | 1 | 1.817 | 0 | 1.011 | 2.956 | NA |
| TRINITY_DN4722_c0_g1_i4 | 1.161657188 | 0.37054369 | 0.999970678 | 0.872 | 2.858 | 0.644 | 0.885 | NA |
| TRINITY_DN8108_c0_g1_i7 | -1.16161311 | 0.411656665 | 0.999970678 | 0.209 | 0.808 | 1.278 | 0.704 | p450 |
| TRINITY_DN7972_c0_g1_i4 | 1.16158467 | 0.374641756 | 0.999970678 | 1.599 | 2.819 | 0.3 | 1.609 | NA |
| TRINITY_DN2318_c1_g1_i5 | -1.16137417 | 0.580811535 | 0.999970678 | 0.418 | 19.07 | 7.611 | 31.488 | NA |
| TRINITY_DN2401_c0_g1_i1 | -1.16135819 | 0.680057401 | 0.999970678 | 0.736 | 0 | 0.289 | 1.367 | - |
| TRINITY_DN5605_c0_g1_i1 | 1.161352025 | 0.611419022 | 0.999970678 | 80.502 | 0.158 | 22.554 | 9.28 | GDPD |
| TRINITY_DN387_c0_g1_i15 | -1.16116817 | 0.4108149 | 0.999970678 | 10.328 | 20.13 | 5.533 | 60.775 | NA |
| TRINITY_DN10866_c0_g1_i1 | -1.16112392 | 0.447879539 | 0.999970678 | 3.779 | 2.553 | 1.133 | 12.668 | - |
| TRINITY_DN37220_c0_g1_i1 | -1.16096992 | 0.794441561 | 1 | 0.545 | 0 | 1.022 | 0 | NA |
| TRINITY_DN60575_c0_g1_i1 | -1.16096992 | 0.794441561 | 1 | 1.399 | 0 | 2.611 | 0 | NA |
| TRINITY_DN15238_c0_g1_i4 | 1.160848669 | 0.787336115 | 0.999970678 | 1.962 | 0 | 0.722 | 0 | NA |
| TRINITY_DN430_c0_g1_i3 | -1.16082661 | 0.674769181 | 0.999970678 | 0 | 6.288 | 9.644 | 2.131 | Proteasome |
| TRINITY_DN1474_c2_g1_i2 | -1.16081556 | 0.360147688 | 0.999970678 | 1.644 | 0.512 | 2.555 | 1.769 | NA |
| TRINITY_DN302_c0_g1_i6 | 1.160815169 | 0.788103838 | 0.999970678 | 0 | 12.13 | 4.366 | 0 | NA |
| TRINITY_DN2209_c0_g1_i3 | 1.160732656 | 0.301539443 | 0.999970678 | 0.818 | 0.424 | 0.256 | 0.241 | DnaJ |
| TRINITY_DN1840_c0_g1_i3 | -1.16070448 | 0.780920679 | 0.999970678 | 0 | 1.222 | 0 | 2.714 | NA |
| TRINITY_DN2818_c0_g1_i1 | -1.16065817 | 0.689520984 | 0.999970678 | 21.529 | 0 | 29.765 | 12.859 | NA |
| TRINITY_DN10053_c0_g1_i7 | -1.16063093 | 0.782553856 | 0.999970678 | 0 | 10.85 | 19.621 | 0 | NA |
| TRINITY_DN5772_c0_g1_i5 | 1.160250028 | 0.787902786 | 0.999970678 | 1.408 | 0 | 0 | 0.653 | NA |
| TRINITY_DN10597_c0_g2_i1 | 1.160212924 | 0.34789452 | 0.999970678 | 1.099 | 1.173 | 0.689 | 0.181 | NA |
| TRINITY_DN3250_c0_g1_i9 | -1.16016601 | 0.734181204 | 0.999970678 | 0 | 25.42 | 0.1 | 55.004 | NA |
| TRINITY_DN8845_c0_g2_i3 | 1.159949729 | 0.412630933 | 1 | 0.572 | 0.857 | 0.389 | 0.151 | NA |
| TRINITY_DN13110_c0_g1_i1 | -1.15985669 | 0.481993011 | 0.999970678 | 0.4 | 3.302 | 1.233 | 6.666 | LRRNT_2 |
| TRINITY_DN11746_c0_g1_i5 | -1.15970355 | 0.675603285 | 1 | 0 | 2.957 | 3.922 | 1.759 | NA |
| TRINITY_DN27723_c0_g1_i1 | 1.159612708 | 0.70894891 | 0.999970678 | 0.073 | 1.991 | 0 | 0.905 | NA |
| TRINITY_DN3147_c0_g1_i2 | -1.15934611 | 0.47779667 | 0.999970678 | 1.135 | 2.444 | 0.667 | 7.058 | NA |
| TRINITY_DN13902_c0_g1_i2 | 1.159281704 | 0.133690684 | 0.999970678 | 3.615 | 4.602 | 1.822 | 1.488 | NA |
| TRINITY_DN2558_c0_g1_i4 | -1.15916683 | 0.784281224 | 0.999970678 | 6.659 | 0 | 12.455 | 0 | Abhydrolase_1 |
| TRINITY_DN1008_c0_g1_i13 | -1.15916483 | 0.671515601 | 1 | 1.29 | 0 | 1.111 | 1.478 | NA |
| TRINITY_DN549_c0_g1_i2 | 1.159099606 | 0.49017511 | 0.999970678 | 1.272 | 1.153 | 0.8 | 0.101 | NA |
| TRINITY_DN6546_c0_g1_i1 | 1.158875542 | 0.325026522 | 0.999970678 | 1.626 | 0.749 | 0.311 | 0.684 | NA |
| TRINITY_DN1097_c0_g1_i4 | -1.15879856 | 0.673726021 | 0.999970678 | 13.017 | 0 | 10.466 | 16.79 | NA |
| TRINITY_DN4341_c0_g1_i11 | -1.15877978 | 0.789929516 | 0.999970678 | 3.161 | 0 | 5.833 | 0 | NA |
| TRINITY_DN11551_c1_g2_i2 | 1.158677266 | 0.462078457 | 0.999970678 | 2.089 | 1.882 | 0.156 | 1.578 | NA |
| TRINITY_DN4685_c0_g1_i3 | -1.1586745 | 0.225280486 | 0.999970678 | 1.272 | 0.769 | 2.3 | 1.78 | NA |
| TRINITY_DN19247_c0_g1_i6 | -1.15856335 | 0.671320769 | 1 | 0 | 0.355 | 0.433 | 0.251 | NA |
| TRINITY_DN3034_c0_g1_i2 | 1.15853924 | 0.183381367 | 0.999970678 | 112.22 | 60.51 | 22.199 | 50.972 | NA |
| TRINITY_DN3569_c0_g1_i4 | 1.158492566 | 0.432416164 | 0.999970678 | 4.887 | 1.261 | 0.333 | 2.373 | Biotin_carb_C |
| TRINITY_DN1328_c0_g1_i3 | -1.15825184 | 0.685783298 | 0.999970678 | 11.637 | 0 | 3.078 | 22.621 | zf-RING_2 |
| TRINITY_DN7786_c0_g1_i3 | 1.158181318 | 0.42599368 | 0.999970678 | 1.708 | 0.306 | 0.356 | 0.503 | NA |
| TRINITY_DN6745_c0_g1_i14 | 1.158169469 | 0.67082574 | 1 | 0.6 | 0.631 | 0.456 | 0 | NA |
| TRINITY_DN760_c0_g1_i6 | -1.15806124 | 0.214039044 | 0.999970678 | 12.509 | 14.35 | 12.921 | 44.427 | NA |
| TRINITY_DN37234_c0_g1_i6 | -1.15793239 | 0.675285416 | 1 | 0 | 2.966 | 3.589 | 2.081 | NA |
| TRINITY_DN4996_c0_g1_i5 | -1.15785749 | 0.593488559 | 1 | 2.107 | 0.089 | 3.366 | 0.845 | NA |
| TRINITY_DN6545_c0_g1_i2 | 1.157835125 | 0.286006741 | 0.999970678 | 3.525 | 2.079 | 0.611 | 1.8 | NA |
| TRINITY_DN2044_c1_g3_i3 | -1.15780115 | 0.723752686 | 0.999970678 | 1.817 | 0 | 0.022 | 4.112 | NA |
| TRINITY_DN8166_c0_g1_i4 | 1.157768477 | 0.785538246 | 0.999970678 | 15.515 | 0 | 0 | 7.088 | DUF1644 |
| TRINITY_DN12357_c0_g1_i12 | -1.15769919 | 0.795754982 | 1 | 0.899 | 0 | 1.644 | 0 | FAD_binding_2 |
| TRINITY_DN2558_c0_g1_i13 | -1.15762995 | 0.787436919 | 0.999970678 | 4.197 | 0 | 7.711 | 0 | Abhydrolase_1 |

| TRINITY_DN3878_c0_g1_i4 | -1.15762995 | 0.787436919 | 0.999970678 | 1.626 | 0 | 3.055 | 0 | NA |
| --- | --- | --- | --- | --- | --- | --- | --- | --- |
| TRINITY_DN397_c1_g1_i4 | -1.15761791 | 0.364615453 | 0.999970678 | 0.291 | 1.449 | 1.744 | 1.759 | PUNUT |
| TRINITY_DN928_c0_g1_i1 | -1.15759322 | 0.783721241 | 0.999970678 | 3.134 | 0 | 5.877 | 0 | NA |
| TRINITY_DN16710_c0_g1_i1 | -1.15753105 | 0.21027396 | 0.999970678 | 0.527 | 0.562 | 0.889 | 1.357 | LRRNT_2 |
| TRINITY_DN862_c0_g1_i10 | 1.157519125 | 0.683641859 | 0.999970678 | 0 | 3.587 | 0.311 | 1.196 | NA |
| TRINITY_DN160_c0_g1_i1 | -1.15720577 | 0.783585425 | 0.999970678 | 4.624 | 0 | 8.611 | 0 | NA |
| TRINITY_DN8143_c0_g1_i2 | 1.157109578 | 0.67348804 | 0.999970678 | 0.409 | 1.37 | 0.656 | 0 | Transferase |
| TRINITY_DN633_c0_g1_i2 | 1.157091347 | 0.781648544 | 0.999970678 | 0 | 4.376 | 0 | 1.94 | NA |
| TRINITY_DN8253_c0_g1_i2 | -1.15706391 | 0.17069797 | 0.999970678 | 5.469 | 4.78 | 5.922 | 15.764 | NA |
| TRINITY_DN16725_c0_g1_i2 | 1.156909147 | 0.672006396 | 0.999970678 | 0.581 | 0.237 | 0.3 | 0 | NA |
| TRINITY_DN2276_c0_g1_i1 | 1.15679971 | 0.731875043 | 0.999970678 | 0 | 87.43 | 31.609 | 0.312 | CS |
| TRINITY_DN9434_c0_g1_i5 | -1.15674264 | 0.098183824 | 0.999970678 | 13.144 | 10.21 | 19.743 | 27.688 | NA |
| TRINITY_DN16944_c0_g1_i1 | -1.15660586 | 0.199426571 | 0.999970678 | 249.43 | 489.4 | 721.223 | 620.273 | NA |
| TRINITY_DN8445_c0_g1_i3 | 1.15657309 | 0.257466601 | 0.999970678 | 23.491 | 10.7 | 9.155 | 4.373 | KH_1 |
| TRINITY_DN16259_c0_g1_i1 | 1.156528848 | 0.138060124 | 0.999970678 | 9.475 | 8.623 | 4.355 | 2.875 | NA |
| TRINITY_DN1478_c0_g1_i1 | -1.15649775 | 0.783226054 | 0.999970678 | 0 | 2.819 | 5.122 | 0 | F-box |
| TRINITY_DN1218_c1_g1_i3 | 1.15640392 | 0.510749413 | 0.999970678 | 4.451 | 0.296 | 1.311 | 0.603 | NA |
| TRINITY_DN17970_c0_g1_i1 | -1.15631401 | 0.710266619 | 0.999970678 | 0.618 | 9.678 | 0 | 21.726 | NA |
| TRINITY_DN4142_c0_g1_i1 | -1.15630542 | 0.092758242 | 0.999970678 | 5.777 | 5.263 | 8.533 | 14.306 | TCP |
| TRINITY_DN7015_c0_g1_i10 | 1.156172748 | 0.701946649 | 0.999970678 | 1.326 | 0.079 | 0.511 | 0 | PEPCK_ATP |
| TRINITY_DN2991_c0_g1_i1 | -1.15611704 | 0.269257489 | 0.999970678 | 0.463 | 0.493 | 0.667 | 1.337 | - |
| TRINITY_DN11049_c0_g1_i5 | -1.15607768 | 0.72526172 | 0.999970678 | 1.79 | 0.059 | 3.389 | 0 | NA |
| TRINITY_DN708_c0_g1_i6 | 1.156052364 | 0.792467772 | 1 | 2.589 | 0 | 0 | 1.146 | NA |
| TRINITY_DN13390_c0_g1_i1 | 1.15600916 | 0.673335844 | 0.999970678 | 4.533 | 5.164 | 3.6 | 0 | NA |
| TRINITY_DN3287_c0_g1_i8 | -1.15536226 | 0.783911875 | 0.999970678 | 0 | 1.705 | 0 | 3.74 | PPR |
| TRINITY_DN2615_c0_g1_i2 | 1.155238411 | 0.346079616 | 1 | 2.943 | 1.902 | 1.033 | 0.965 | NA |
| TRINITY_DN4405_c0_g1_i7 | -1.15518235 | 0.29261533 | 0.999970678 | 3.034 | 3.844 | 2.922 | 11.733 | NA |
| TRINITY_DN2196_c0_g1_i3 | -1.15488305 | 0.186541482 | 0.999970678 | 1.463 | 2.415 | 4.133 | 3.599 | PPR |
| TRINITY_DN372_c0_g1_i3 | -1.15484098 | 0.670726392 | 0.999970678 | 7.185 | 0 | 3.111 | 12.346 | NA |
| TRINITY_DN2159_c0_g1_i3 | 1.154741695 | 0.463588249 | 0.999970678 | 1.063 | 0.69 | 0.578 | 0.09 | NA |
| TRINITY_DN2360_c0_g1_i1 | -1.15466184 | 0.443857165 | 0.999970678 | 0.799 | 0.197 | 0.489 | 1.629 | NA |
| TRINITY_DN91_c0_g1_i2 | 1.154536594 | 0.486450373 | 0.999970678 | 0.282 | 2.582 | 0.456 | 0.704 | NA |
| TRINITY_DN1487_c0_g1_i12 | -1.15439881 | 0.669455052 | 0.999970678 | 6.768 | 0 | 9.466 | 3.77 | NA |
| TRINITY_DN16280_c0_g1_i3 | -1.15415122 | 0.681628639 | 0.999970678 | 1.217 | 0 | 1.933 | 0.412 | NA |
| TRINITY_DN49681_c0_g1_i4 | -1.15409285 | 0.797145489 | 1 | 0.709 | 0 | 1.311 | 0 | NA |
| TRINITY_DN35152_c0_g1_i1 | -1.15409285 | 0.797145489 | 1 | 0.654 | 0 | 1.211 | 0 | DLH |
| TRINITY_DN14845_c0_g1_i1 | -1.15409285 | 0.797145489 | 1 | 0.454 | 0 | 0.867 | 0 | FAD_binding_2 |
| TRINITY_DN6693_c0_g1_i1 | -1.15399597 | 0.785648633 | 0.999970678 | 2.725 | 0 | 5.077 | 0 | NA |
| TRINITY_DN955_c0_g1_i2 | 1.153889329 | 0.678168762 | 0.999970678 | 26.289 | 7.894 | 0 | 15.503 | NA |
| TRINITY_DN7883_c1_g1_i8 | 1.153819463 | 0.686533316 | 0.999970678 | 3.207 | 0 | 0.5 | 0.855 | NA |
| TRINITY_DN5676_c3_g1_i11 | -1.15377287 | 0.5004506 | 0.999970678 | 0.609 | 0.059 | 0.578 | 0.804 | NA |
| TRINITY_DN3953_c0_g1_i20 | 1.15359856 | 0.354167059 | 1 | 1.544 | 1.941 | 0.778 | 0.553 | NA |
| TRINITY_DN564_c0_g1_i11 | -1.1534962 | 0.322235914 | 0.999970678 | 19.14 | 4.277 | 21.61 | 25.818 | NA |
| TRINITY_DN5693_c0_g1_i3 | 1.153439452 | 0.686613692 | 0.999970678 | 10.21 | 0 | 0.933 | 3.479 | - |
| TRINITY_DN1243_c1_g1_i6 | -1.15330407 | 0.352003899 | 0.999970678 | 1.217 | 2.72 | 5.777 | 1.729 | RRM_1 |
| TRINITY_DN3458_c0_g1_i5 | 1.153280172 | 0.213711725 | 0.999970678 | 1.808 | 2.769 | 0.678 | 1.206 | p450 |
| TRINITY_DN743_c0_g1_i9 | -1.15312873 | 0.125838792 | 0.999970678 | 3.107 | 5.608 | 8.333 | 9.259 | NA |
| TRINITY_DN8666_c1_g1_i7 | -1.15275564 | 0.421167273 | 0.999970678 | 0.318 | 2.434 | 1.489 | 4.243 | DUF1475 |
| TRINITY_DN3673_c0_g1_i4 | 1.15262496 | 0.310818591 | 0.999970678 | 0.4 | 0.434 | 0.156 | 0.201 | EF-hand_5 |
| TRINITY_DN16151_c0_g3_i1 | 1.152552998 | 0.328258397 | 0.999970678 | 0.182 | 0.532 | 0.144 | 0.151 | DUF4283 |
| TRINITY_DN809_c0_g1_i5 | -1.15254818 | 0.688718106 | 0.999970678 | 18.15 | 0 | 27.965 | 7.088 | DUF3493 |
| TRINITY_DN2690_c0_g1_i4 | 1.152395125 | 0.438435879 | 0.999970678 | 1.126 | 1.537 | 0.844 | 0.171 | - |
| TRINITY_DN1756_c0_g1_i3 | -1.15235161 | 0.258584135 | 0.999970678 | 0.454 | 0.365 | 0.844 | 0.824 | PGI |
| TRINITY_DN99805_c0_g1_i2 | 1.152247759 | 0.278264844 | 0.999970678 | 1.117 | 3.134 | 0.789 | 0.975 | CH |
| TRINITY_DN11683_c0_g1_i2 | -1.15222777 | 0.229033364 | 0.999970678 | 67.103 | 26.23 | 66.718 | 123.218 | NA |
| TRINITY_DN3_c0_g1_i2 | -1.15208163 | 0.299180147 | 0.999970678 | 26.452 | 15.18 | 15.977 | 73.08 | HSP70 |
| TRINITY_DN8118_c0_g2_i2 | 1.152064792 | 0.790442795 | 0.999970678 | 3.497 | 0 | 0 | 1.619 | NA |
| TRINITY_DN999_c0_g2_i1 | 1.152025743 | 0.135382785 | 0.999970678 | 66.186 | 51.74 | 28.165 | 18.881 | NA |
| TRINITY_DN2392_c0_g1_i1 | 1.15186738 | 0.228052437 | 0.999970678 | 20.248 | 22.85 | 4.133 | 14.306 | NA |
| TRINITY_DN1502_c0_g1_i4 | 1.151782936 | 0.505643188 | 0.999970678 | 0.372 | 5.657 | 1.267 | 1.176 | NA |
| TRINITY_DN17761_c0_g1_i2 | 1.151777629 | 0.367778694 | 0.999970678 | 1.562 | 3.903 | 0.667 | 1.609 | NA |
| TRINITY_DN11248_c0_g2_i2 | -1.15167248 | 0.508490366 | 0.999970678 | 0.999 | 0.177 | 1.811 | 0.412 | NUDIX |
| TRINITY_DN16107_c0_g2_i3 | 1.151290036 | 0.508155656 | 0.999970678 | 8.257 | 0.345 | 1.078 | 2.634 | Myb_DNA-bindin |
| TRINITY_DN7078_c0_g1_i1 | -1.15128806 | 0.79645687 | 1 | 0.363 | 0 | 0 | 0.814 | GAF |
| TRINITY_DN1811_c0_g3_i4 | 1.151265804 | 0.792370078 | 0.999970678 | 2.88 | 0 | 1.1 | 0 | Grp1_Fun34_Yaa |
| TRINITY_DN14514_c2_g1_i3 | 1.151252954 | 0.535483231 | 0.999970678 | 0.372 | 0.66 | 0.367 | 0.02 | NA |
| TRINITY_DN2820_c0_g1_i3 | -1.15124137 | 0.632206246 | 0.999970678 | 0.082 | 33.9 | 55.563 | 6.877 | NA |
| TRINITY_DN2951_c1_g2_i2 | 1.1510474 | 0.792850515 | 0.999970678 | 4.088 | 0 | 0 | 1.85 | NA |
| TRINITY_DN2268_c0_g1_i2 | -1.15098707 | 0.514184526 | 0.999970678 | 0.1 | 3.252 | 2.255 | 4.625 | Chloroa_b-bind |
| TRINITY_DN2404_c0_g1_i1 | 1.150805728 | 0.170617157 | 0.999970678 | 3.479 | 3.302 | 0.9 | 1.971 | - |
| TRINITY_DN13234_c0_g1_i4 | -1.15076847 | 0.69258184 | 1 | 0.145 | 0 | 0.233 | 0.05 | NA |
| TRINITY_DN1939_c0_g2_i1 | 1.150412639 | 0.115955555 | 0.999970678 | 29.823 | 26.93 | 13.51 | 9.29 | NA |
| TRINITY_DN9945_c0_g1_i4 | 1.150345846 | 0.470299439 | 0.999970678 | 0.2 | 2.158 | 0.478 | 0.473 | NA |
| TRINITY_DN6322_c0_g1_i1 | 1.150280059 | 0.26335184 | 0.999970678 | 4.033 | 1.754 | 1.233 | 1.146 | B3 |

| TRINITY_DN20_c0_g1_i5 | 1.150263063 | 0.337251893 | 0.999970678 | 1.472 | 6.682 | 1.844 | 1.408 | NA |
| --- | --- | --- | --- | --- | --- | --- | --- | --- |
| TRINITY_DN3762_c0_g1_i2 | 1.150245552 | 0.561961214 | 0.999970678 | 13.017 | 2.602 | 5.377 | 0.503 | NA |
| TRINITY_DN9734_c1_g1_i1 | 1.150168454 | 0.425897505 | 0.999970678 | 1.762 | 0.68 | 0.722 | 0.231 | NA |
| TRINITY_DN5912_c0_g1_i9 | -1.15009604 | 0.79872004 | 1 | 1.453 | 0 | 2.7 | 0 | GST_C |
| TRINITY_DN7852_c0_g1_i19 | -1.15009604 | 0.79872004 | 1 | 0.945 | 0 | 1.722 | 0 | Fungal_trans_2 |
| TRINITY_DN2541_c0_g1_i1 | -1.15000276 | 0.250859942 | 0.999970678 | 6.622 | 3.844 | 5.255 | 16.951 | NA |
| TRINITY_DN7913_c0_g1_i9 | -1.14998685 | 0.78695739 | 0.999970678 | 3.706 | 0 | 0 | 8.395 | CBM_20 |
| TRINITY_DN12_c0_g1_i4 | -1.14997783 | 0.33544634 | 0.999970678 | 1.726 | 0.453 | 1.633 | 2.885 | NA |
| TRINITY_DN1245_c0_g1_i14 | 1.149851809 | 0.394798796 | 0.999970678 | 0.491 | 2.957 | 0.778 | 0.613 | F-box |
| TRINITY_DN2338_c0_g1_i6 | -1.14967699 | 0.330819373 | 0.999970678 | 18.059 | 52.72 | 28.165 | 120.896 | NA |
| TRINITY_DN250466_c0_g1_i1 | -1.1495233 | 0.787281756 | 0.999970678 | 9.356 | 0 | 17.388 | 0 | ADH_N |
| TRINITY_DN145_c0_g1_i6 | 1.149463663 | 0.190751761 | 0.999970678 | 3.761 | 3.311 | 1.633 | 1.216 | NA |
| TRINITY_DN3037_c0_g1_i1 | 1.149162731 | 0.384253133 | 0.999970678 | 1.081 | 2.01 | 0.244 | 1.106 | Rho_N |
| TRINITY_DN2436_c0_g1_i8 | 1.148785691 | 0.269984607 | 0.999970678 | 4.188 | 11.68 | 3.7 | 2.664 | NA |
| TRINITY_DN3244_c0_g3_i1 | -1.14870842 | 0.528495355 | 0.999970678 | 0.209 | 8.111 | 5.111 | 12.004 | - |
| TRINITY_DN733_c0_g1_i2 | 1.148532807 | 0.789192368 | 0.999970678 | 40.178 | 0 | 0 | 18.328 | Rhodanese |
| TRINITY_DN555_c0_g1_i1 | -1.14816937 | 0.33146848 | 0.999970678 | 3.198 | 8.722 | 5.955 | 18.64 | NA |
| TRINITY_DN0_c1_g1_i1 | 1.148132498 | 0.423723701 | 0.999970678 | 31.667 | 13.78 | 15.244 | 2.121 | - |
| TRINITY_DN2769_c1_g1_i3 | -1.14813026 | 0.685797742 | 0.999970678 | 16.251 | 0 | 11.599 | 22.581 | Cation_efflux |
| TRINITY_DN213_c0_g1_i1 | -1.1480285 | 0.135419736 | 0.999970678 | 54.903 | 105.4 | 164.457 | 152.726 | Ribosomal_L22 |
| TRINITY_DN5729_c0_g2_i1 | 1.147952764 | 0.506492878 | 0.999970678 | 0.245 | 3.607 | 0.611 | 0.975 | zf-FLZ |
| TRINITY_DN3578_c0_g1_i2 | -1.14779073 | 0.506997932 | 0.999970678 | 29.396 | 2.405 | 51.73 | 8.505 | LTP_2 |
| TRINITY_DN38492_c0_g1_i1 | 1.147738398 | 0.398466647 | 0.999970678 | 6.559 | 5.273 | 3.611 | 0.935 | NA |
| TRINITY_DN1426_c1_g1_i3 | 1.14745201 | 0.67898991 | 0.999970678 | 1.908 | 9.343 | 4.155 | 0 | NA |
| TRINITY_DN3768_c0_g1_i1 | 1.147357961 | 0.392241804 | 0.999970678 | 116.12 | 14.13 | 25.076 | 28.754 | NA |
| TRINITY_DN6969_c0_g1_i1 | -1.14726746 | 0.282896391 | 0.999970678 | 2.889 | 2.129 | 2.244 | 8.435 | AP2 |
| TRINITY_DN9328_c0_g1_i8 | -1.147079 | 0.314841505 | 0.999970678 | 0.391 | 0.818 | 1.067 | 1.357 | NA |
| TRINITY_DN32687_c0_g1_i1 | -1.14703727 | 0.718742718 | 0.999970678 | 0.327 | 0 | 0.6 | 0.01 | NA |
| TRINITY_DN1121_c0_g1_i2 | -1.14695399 | 0.58816842 | 0.999970678 | 11.346 | 0.384 | 0.889 | 25.285 | NA |
| TRINITY_DN13100_c0_g1_i3 | 1.146727628 | 0.565831405 | 0.999970678 | 2.516 | 0.059 | 0.811 | 0.201 | NA |
| TRINITY_DN2547_c0_g1_i13 | -1.1466432 | 0.455992045 | 0.999970678 | 2.226 | 0.256 | 2.222 | 2.845 | NA |
| TRINITY_DN10068_c0_g1_i1 | -1.14664014 | 0.790827311 | 0.999970678 | 8.484 | 0 | 15.766 | 0 | NA |
| TRINITY_DN1549_c0_g2_i3 | 1.146387253 | 0.681668752 | 0.999970678 | 0 | 13.08 | 2.389 | 2.926 | NA |
| TRINITY_DN8331_c0_g1_i2 | -1.14632752 | 0.154608656 | 0.999970678 | 3.77 | 4.977 | 5.589 | 12.617 | IQ |
| TRINITY_DN757_c0_g1_i13 | 1.146307561 | 0.706278486 | 0.999970678 | 0 | 0.956 | 0.044 | 0.362 | NA |
| TRINITY_DN10724_c0_g1_i2 | -1.14623542 | 0.668039945 | 0.999970678 | 2.68 | 0 | 3.111 | 2.232 | p450 |
| TRINITY_DN6492_c0_g1_i7 | 1.146224111 | 0.664054072 | 0.999970678 | 0.745 | 2.089 | 0 | 1.277 | APG9 |
| TRINITY_DN3416_c0_g1_i5 | 1.145946873 | 0.32280891 | 0.999970678 | 0.881 | 1.163 | 0.6 | 0.191 | - |
| TRINITY_DN20798_c0_g1_i1 | -1.14578917 | 0.293366291 | 0.999970678 | 1.481 | 2.553 | 4.933 | 2.936 | NA |
| TRINITY_DN10163_c0_g1_i3 | 1.145447614 | 0.11565455 | 0.999970678 | 2.907 | 2.533 | 1.1 | 1.156 | Ank_2 |
| TRINITY_DN14971_c1_g2_i1 | -1.14533022 | 0.666446999 | 0.999970678 | 3.316 | 0 | 2.422 | 4.474 | NA |
| TRINITY_DN64441_c0_g1_i2 | 1.145302684 | 0.340570189 | 0.999970678 | 1.063 | 2.148 | 0.4 | 0.975 | NA |
| TRINITY_DN6189_c0_g1_i4 | 1.145143466 | 0.667676559 | 1 | 0.427 | 0.453 | 0 | 0.402 | NA |
| TRINITY_DN29444_c0_g1_i3 | 1.14514231 | 0.668251852 | 0.999970678 | 7.095 | 6.396 | 5.044 | 0 | NA |
| TRINITY_DN2986_c0_g1_i2 | 1.144994317 | 0.792630392 | 0.999970678 | 0 | 7.076 | 0 | 3.086 | NA |
| TRINITY_DN2123_c0_g1_i7 | 1.144945566 | 0.340542325 | 0.999970678 | 24.018 | 10.48 | 10.522 | 2.986 | AICARFT_IMPCH |
| TRINITY_DN772_c0_g4_i1 | -1.14488379 | 0.683039157 | 0.999970678 | 74.007 | 0 | 56.341 | 95.038 | - |
| TRINITY_DN9587_c0_g1_i2 | -1.1447317 | 0.237918886 | 0.999970678 | 7.403 | 3.834 | 7.555 | 15.503 | - |
| TRINITY_DN75_c0_g1_i6 | 1.144701881 | 0.786077032 | 0.999970678 | 10.092 | 0 | 3.811 | 0 | NA |
| TRINITY_DN7543_c0_g1_i1 | -1.14449403 | 0.158337858 | 0.999970678 | 3.588 | 3.351 | 4.522 | 9.873 | NA |
| TRINITY_DN269_c0_g1_i1 | 1.144243604 | 0.380705253 | 0.999970678 | 4.896 | 0.897 | 1.411 | 0.955 | ArgoL1 |
| TRINITY_DN18650_c0_g1_i2 | 1.144218928 | 0.297407876 | 0.999970678 | 0.981 | 1.764 | 0.744 | 0.342 | NA |
| TRINITY_DN899_c0_g1_i1 | 1.144026434 | 0.683171282 | 0.999970678 | 0 | 14.03 | 1.955 | 3.881 | NA |
| TRINITY_DN1912_c0_g1_i2 | -1.14394804 | 0.310445761 | 0.999970678 | 5.205 | 2.878 | 3.311 | 13.965 | NA |
| TRINITY_DN7197_c0_g1_i4 | 1.14391023 | 0.796531026 | 1 | 0.781 | 0 | 0 | 0.362 | NA |
| TRINITY_DN4471_c1_g1_i5 | 1.143741604 | 0.786862226 | 0.999970678 | 0 | 21.77 | 8.022 | 0 | NA |
| TRINITY_DN520_c0_g1_i8 | 1.14372473 | 0.381606345 | 0.999970678 | 1.899 | 9.471 | 1.089 | 3.79 | LRR_1 |
| TRINITY_DN3326_c0_g1_i5 | -1.14370617 | 0.365542557 | 0.999970678 | 2.88 | 1.074 | 2.911 | 5.168 | zf-C2H2_6 |
| TRINITY_DN2291_c0_g1_i3 | -1.14360727 | 0.78483072 | 0.999970678 | 0 | 2.129 | 0 | 4.625 | Hexapep |
| TRINITY_DN1873_c3_g1_i14 | -1.14349524 | 0.794776509 | 1 | 0 | 0.759 | 0 | 1.629 | NA |
| TRINITY_DN17464_c0_g1_i1 | -1.14334011 | 0.451981625 | 0.999970678 | 3.597 | 1.025 | 7.444 | 1.297 | NA |
| TRINITY_DN4153_c0_g1_i10 | 1.143074514 | 0.380335053 | 0.999970678 | 1.017 | 3.508 | 0.444 | 1.488 | AMPK1_CBM |
| TRINITY_DN6444_c0_g1_i2 | -1.14300557 | 0.682457244 | 1 | 0.291 | 0 | 0.367 | 0.211 | Castor_Poll_mid |
| TRINITY_DN92_c2_g1_i2 | 1.142885596 | 0.385170977 | 0.999970678 | 3.515 | 5.962 | 3.089 | 0.553 | Vps52 |
| TRINITY_DN1941_c1_g1_i2 | -1.14285143 | 0.357442232 | 0.999970678 | 3.961 | 1.419 | 7.688 | 2.674 | p450 |
| TRINITY_DN8246_c0_g1_i18 | -1.14259632 | 0.347637278 | 0.999970678 | 0.672 | 1.045 | 0.656 | 3.026 | - |
| TRINITY_DN443_c0_g1_i7 | -1.1423927 | 0.680353434 | 0.999970678 | 1.081 | 0 | 1.689 | 0.382 | Amidohydro_1 |
| TRINITY_DN98406_c0_g1_i2 | 1.142292735 | 0.256453122 | 0.999970678 | 1.254 | 3.518 | 0.844 | 1.136 | NA |
| TRINITY_DN197967_c0_g1_i1 | 1.14201273 | 0.794575351 | 0.999970678 | 1.581 | 0 | 0.6 | 0 | NA |
| TRINITY_DN4288_c0_g1_i2 | -1.14188595 | 0.680955576 | 0.999970678 | 0 | 4.819 | 1.333 | 8.898 | NA |
| TRINITY_DN4423_c0_g1_i1 | -1.14175243 | 0.096550935 | 0.999970678 | 6.431 | 7.579 | 10.733 | 17.835 | NA |
| TRINITY_DN761_c0_g1_i9 | -1.1417231 | 0.500162144 | 0.999970678 | 2.18 | 45.77 | 19.51 | 80.188 | NA |
| TRINITY_DN46239_c0_g2_i1 | 1.141718406 | 0.670108985 | 0.999970678 | 13.362 | 8.682 | 0 | 9.993 | NA |
| TRINITY_DN17126_c0_g1_i1 | -1.14128308 | 0.788006987 | 0.999970678 | 0.69 | 0 | 1.278 | 0 | NA |

| TRINITY_DN12345_c0_g1_i1 | 1.141018846 | 0.247194243 | 0.999970678 | 22.937 | 15.75 | 4.644 | 11.471 | NA |
| --- | --- | --- | --- | --- | --- | --- | --- | --- |
| TRINITY_DN779_c2_g1_i6 | 1.141001618 | 0.172404972 | 0.999970678 | 4.905 | 4.435 | 1.122 | 2.895 | STAS |
| TRINITY_DN4588_c1_g1_i1 | 1.14060595 | 0.344053747 | 0.999970678 | 36.1 | 8.82 | 12.088 | 5.962 | NA |
| TRINITY_DN9682_c0_g1_i1 | -1.14048543 | 0.390202177 | 0.999970678 | 20.076 | 68.04 | 25.198 | 161.482 | NA |
| TRINITY_DN2659_c4_g1_i3 | -1.14039278 | 0.719546007 | 0.999970678 | 0 | 73.12 | 121.048 | 12.245 | PRK |
| TRINITY_DN3861_c0_g1_i1 | 1.140302515 | 0.250206348 | 0.999970678 | 0.881 | 1.764 | 0.633 | 0.432 | IBN_N |
| TRINITY_DN4023_c0_g1_i1 | 1.140286665 | 0.509598788 | 0.999970678 | 75.46 | 3.292 | 23.41 | 7.892 | NA |
| TRINITY_DN479_c0_g1_i3 | 1.140112094 | 0.201598107 | 0.999970678 | 30.177 | 21.42 | 8.422 | 12.507 | NA |
| TRINITY_DN2285_c0_g2_i2 | -1.13972246 | 0.397534936 | 0.999970678 | 10.864 | 1.557 | 7.433 | 18.78 | NA |
| TRINITY_DN4645_c0_g1_i1 | 1.13967141 | 0.24847162 | 0.999970678 | 3.915 | 2.346 | 1.322 | 1.227 | NA |
| TRINITY_DN468_c1_g1_i6 | -1.13959254 | 0.680480848 | 0.999970678 | 10.065 | 0 | 5.677 | 15.664 | NA |
| TRINITY_DN545_c0_g1_i10 | 1.139500421 | 0.414587831 | 0.999970678 | 9.411 | 2.385 | 1.022 | 4.122 | - |
| TRINITY_DN3120_c0_g1_i1 | -1.13924167 | 0.686049341 | 0.999970678 | 17.705 | 0 | 17.277 | 18.74 | NA |
| TRINITY_DN2535_c0_g1_i5 | 1.13914955 | 0.183326433 | 0.999970678 | 11.927 | 7.391 | 2.766 | 5.449 | NA |
| TRINITY_DN11288_c0_g2_i2 | 1.139137291 | 0.343172497 | 0.999970678 | 2.062 | 1.281 | 1 | 0.322 | NA |
| TRINITY_DN8420_c1_g1_i1 | -1.13913346 | 0.675140746 | 0.999970678 | 0 | 0.907 | 1.311 | 0.382 | BRCT_2 |
| TRINITY_DN2262_c0_g1_i2 | -1.13895294 | 0.543981463 | 0.999970678 | 21.384 | 3.292 | 1.933 | 50.972 | NA |
| TRINITY_DN10483_c0_g1_i2 | -1.13881412 | 0.688040622 | 0.999970678 | 10.328 | 0 | 15.432 | 3.469 | NA |
| TRINITY_DN419_c0_g1_i5 | -1.13879509 | 0.797437561 | 1 | 0 | 0.286 | 0.522 | 0 | Cation_ATPase |
| TRINITY_DN12758_c0_g1_i10 | -1.13879342 | 0.79667158 | 1 | 0.772 | 0 | 1.455 | 0 | Ish1 |
| TRINITY_DN2384_c0_g2_i7 | -1.1387927 | 0.789171802 | 0.999970678 | 0 | 0.966 | 1.733 | 0 | NA |
| TRINITY_DN16585_c0_g1_i2 | -1.13877111 | 0.578697471 | 0.999970678 | 2.589 | 0.039 | 2.633 | 2.674 | NA |
| TRINITY_DN4541_c0_g1_i8 | -1.13853562 | 0.309217168 | 0.999970678 | 1.226 | 0.68 | 0.867 | 3.177 | AAA_11 |
| TRINITY_DN13905_c0_g1_i2 | -1.13849774 | 0.683186854 | 1 | 0 | 0.463 | 0.222 | 0.744 | NA |
| TRINITY_DN1985_c0_g1_i5 | 1.138332202 | 0.709145834 | 0.999970678 | 0.191 | 6.603 | 2.511 | 0.01 | zf-C3HC4_3 |
| TRINITY_DN14939_c1_g1_i1 | -1.13822763 | 0.702190716 | 1 | 0.509 | 0 | 0.856 | 0.111 | NA |
| TRINITY_DN1114_c0_g1_i1 | 1.138140988 | 0.668639989 | 0.999970678 | 4.751 | 7.096 | 0 | 5.389 | Pentapeptide |
| TRINITY_DN3774_c0_g1_i1 | -1.13805872 | 0.190915363 | 0.999970678 | 10.91 | 16.09 | 15.332 | 40.607 | NA |
| TRINITY_DN6827_c1_g1_i5 | -1.13790929 | 0.670184752 | 0.999970678 | 0 | 0.325 | 0.333 | 0.322 | NA |
| TRINITY_DN516_c0_g1_i8 | -1.13787345 | 0.209462877 | 0.999970678 | 37.326 | 37.73 | 36.953 | 119.961 | NA |
| TRINITY_DN30866_c0_g1_i4 | -1.13779199 | 0.788195948 | 0.999970678 | 3.634 | 0 | 6.633 | 0 | NA |
| TRINITY_DN3711_c0_g1_i1 | 1.137480944 | 0.28365135 | 0.999970678 | 73.389 | 19.34 | 18.243 | 20.459 | NA |
| TRINITY_DN380_c1_g1_i3 | 1.137359131 | 0.37135188 | 1 | 3.915 | 5.834 | 1.689 | 2.363 | NA |
| TRINITY_DN382_c0_g1_i2 | 1.137275739 | 0.609703104 | 0.999970678 | 63.142 | 3.952 | 24.91 | 0.452 | NA |
| TRINITY_DN3916_c0_g1_i3 | 1.137267849 | 0.489035952 | 0.999970678 | 1.753 | 0.197 | 0.178 | 0.674 | BET |
| TRINITY_DN10736_c0_g1_i2 | 1.137180919 | 0.412440401 | 1 | 2.044 | 1.961 | 1.122 | 0.503 | NA |
| TRINITY_DN9483_c0_g1_i1 | -1.1370976 | 0.139733968 | 0.999970678 | 16.079 | 14.82 | 25.076 | 35.288 | NA |
| TRINITY_DN25178_c0_g1_i6 | -1.13690648 | 0.364438839 | 0.999970678 | 21.038 | 4.987 | 35.765 | 14.558 | NAD_binding_10 |
| TRINITY_DN799_c0_g1_i3 | -1.13671899 | 0.397116894 | 0.999970678 | 24.427 | 18.88 | 8.255 | 85.617 | NA |
| TRINITY_DN5084_c0_g1_i1 | 1.136496976 | 0.515814228 | 0.999970678 | 21.747 | 9.284 | 0.578 | 13.04 | NA |
| TRINITY_DN5041_c0_g2_i1 | -1.13646202 | 0.678976757 | 0.999970678 | 12.908 | 0.02 | 13.466 | 12.467 | NA |
| TRINITY_DN2341_c1_g1_i5 | 1.136325549 | 0.402292008 | 0.999970678 | 1.444 | 2.966 | 1.233 | 0.513 | NA |
| TRINITY_DN468_c1_g1_i7 | 1.136253378 | 0.085975018 | 0.999970678 | 11.827 | 15.98 | 5.589 | 5.861 | NA |
| TRINITY_DN1250_c0_g4_i1 | -1.13609435 | 0.42565815 | 0.999970678 | 2.898 | 1.38 | 1.189 | 7.942 | NA |
| TRINITY_DN2645_c0_g4_i1 | -1.13606821 | 0.579870226 | 1 | 0.036 | 0.739 | 0.389 | 1.196 | NA |
| TRINITY_DN477_c0_g1_i6 | 1.136018063 | 0.175611925 | 0.999970678 | 39.533 | 23.82 | 15.432 | 10.245 | NA |
| TRINITY_DN3546_c0_g1_i10 | -1.13600873 | 0.440704689 | 0.999970678 | 1.426 | 0.276 | 2.044 | 1.277 | NA |
| TRINITY_DN23206_c0_g1_i1 | -1.1360002 | 0.679270003 | 0.999970678 | 0 | 65.01 | 77.718 | 44.075 | NA |
| TRINITY_DN8378_c0_g1_i3 | 1.13562138 | 0.787444885 | 0.999970678 | 30.776 | 0 | 11.622 | 0 | NA |
| TRINITY_DN9281_c0_g1_i8 | 1.135604909 | 0.363275242 | 0.999970678 | 4.569 | 1.597 | 1.389 | 1.106 | NA |
| TRINITY_DN3443_c0_g1_i14 | 1.135397519 | 0.305470527 | 0.999970678 | 1.108 | 0.818 | 0.511 | 0.251 | NA |
| TRINITY_DN415_c0_g1_i14 | -1.13530631 | 0.317731922 | 0.999970678 | 1.026 | 2.464 | 4.278 | 2.473 | NA |
| TRINITY_DN2827_c0_g1_i4 | -1.13521788 | 0.55436922 | 0.999970678 | 2.752 | 17.97 | 1 | 43.814 | PUNUT |
| TRINITY_DN6341_c0_g1_i3 | 1.134983142 | 0.789432844 | 0.999970678 | 11.2 | 0 | 4.222 | 0 | NA |
| TRINITY_DN11721_c0_g1_i7 | -1.13490849 | 0.792072426 | 0.999970678 | 1.163 | 0 | 2.133 | 0 | NA |
| TRINITY_DN25016_c0_g1_i1 | -1.13490544 | 0.427427308 | 0.999970678 | 5.178 | 14.26 | 3.933 | 37.47 | Usp |
| TRINITY_DN2671_c0_g1_i6 | -1.13489828 | 0.681487757 | 0.999970678 | 0 | 5.381 | 7.755 | 2.292 | NA |
| TRINITY_DN9497_c0_g1_i1 | -1.13486619 | 0.301643536 | 0.999970678 | 0.79 | 1.37 | 1.055 | 3.489 | B_lectin |
| TRINITY_DN27787_c0_g1_i1 | 1.134798806 | 0.787612815 | 0.999970678 | 14.225 | 0 | 5.411 | 0 | NA |
| TRINITY_DN541_c0_g1_i1 | 1.134796487 | 0.66398976 | 0.999970678 | 7.894 | 14.08 | 0 | 9.591 | NA |
| TRINITY_DN8594_c0_g1_i2 | -1.13469105 | 0.668270984 | 0.999970678 | 0 | 0.434 | 0.4 | 0.462 | Nexin_C |
| TRINITY_DN4276_c0_g1_i5 | 1.134518679 | 0.692867697 | 0.999970678 | 5.941 | 0 | 1.411 | 0.995 | NA |
| TRINITY_DN1_c0_g1_i5 | 1.134297071 | 0.484292499 | 0.999970678 | 2.571 | 1.074 | 1.267 | 0.141 | KH_1 |
| TRINITY_DN13720_c0_g1_i2 | -1.13428899 | 0.239627982 | 0.999970678 | 2.144 | 0.917 | 3.066 | 3.056 | NA |
| TRINITY_DN9124_c0_g1_i3 | -1.13425085 | 0.67196727 | 0.999970678 | 1.989 | 0 | 1.355 | 2.775 | NA |
| TRINITY_DN24455_c0_g1_i4 | 1.134225642 | 0.246891528 | 0.999970678 | 1.671 | 3.942 | 1.178 | 1.116 | Sec20 |
| TRINITY_DN3808_c0_g1_i2 | 1.134088422 | 0.388984787 | 0.999970678 | 1.036 | 0.788 | 0.567 | 0.141 | - |
| TRINITY_DN13768_c0_g2_i5 | -1.13399936 | 0.68075002 | 0.999970678 | 1.499 | 0 | 0.522 | 2.725 | BRAP2 |
| TRINITY_DN1866_c0_g1_i5 | -1.13399687 | 0.473848906 | 0.999970678 | 0.3 | 0.69 | 1.533 | 0.312 | CK2S |
| TRINITY_DN10806_c0_g1_i2 | -1.13393881 | 0.52286936 | 0.999970678 | 0.382 | 3.173 | 0.544 | 7.068 | UDPGT |
| TRINITY_DN3517_c1_g1_i1 | 1.133776106 | 0.698462755 | 0.999970678 | 2.026 | 0 | 0.189 | 0.694 | NA |
| TRINITY_DN3034_c0_g1_i4 | 1.13355014 | 0.35027661 | 0.999970678 | 3.733 | 1.222 | 0.589 | 1.588 | NA |
| TRINITY_DN255_c1_g1_i16 | -1.13344179 | 0.45367844 | 0.999970678 | 3.397 | 1.311 | 7.788 | 0.965 | NA |
| TRINITY_DN2139_c0_g1_i2 | -1.13343426 | 0.69305905 | 0.999970678 | 30.44 | 0 | 31.109 | 30.151 | Epimerase |

| TRINITY_DN7509_c0_g3_i1 | -1.13329493 | 0.389173167 | 0.999970678 | 10.646 | 1.666 | 14.288 | 9.963 | NA |
| --- | --- | --- | --- | --- | --- | --- | --- | --- |
| TRINITY_DN1334_c0_g1_i9 | 1.133273317 | 0.495285966 | 0.999970678 | 0.736 | 9.914 | 3.078 | 1.066 | NA |
| TRINITY_DN8141_c0_g1_i2 | -1.13319688 | 0.464369607 | 0.999970678 | 0.5 | 0.099 | 0.856 | 0.302 | Xpo1 |
| TRINITY_DN2158_c0_g1_i1 | 1.133120059 | 0.526118686 | 0.999970678 | 5.096 | 1.685 | 2.311 | 0.282 | NA |
| TRINITY_DN12_c2_g1_i6 | 1.133023542 | 0.531337406 | 0.999970678 | 6.132 | 0.325 | 0.367 | 2.554 | NA |
| TRINITY_DN2064_c0_g1_i5 | 1.132884937 | 0.684589684 | 0.999970678 | 0 | 3.765 | 0.744 | 0.794 | Pkinase |
| TRINITY_DN2191_c0_g1_i3 | 1.132747513 | 0.663663063 | 0.999970678 | 2.425 | 1.616 | 0 | 1.86 | NA |
| TRINITY_DN986_c0_g1_i2 | 1.132736146 | 0.277229541 | 0.999970678 | 3.879 | 4.08 | 2.311 | 0.855 | NA |
| TRINITY_DN8435_c0_g1_i1 | -1.13254434 | 0.348196626 | 0.999970678 | 2.889 | 0.986 | 1.789 | 6.394 | NA |
| TRINITY_DN1202_c0_g2_i2 | -1.13250082 | 0.324795622 | 0.999970678 | 0.536 | 1.38 | 2.433 | 1.257 | Cellulose_synt |
| TRINITY_DN1508_c0_g1_i7 | -1.13232529 | 0.526438946 | 0.999970678 | 4.251 | 7.037 | 0.711 | 23.204 | NA |
| TRINITY_DN12547_c1_g1_i3 | 1.132167586 | 0.688852173 | 0.999970678 | 4.351 | 0 | 0.567 | 1.347 | NA |
| TRINITY_DN612_c0_g1_i9 | 1.132044091 | 0.422439854 | 0.999970678 | 3.17 | 2.474 | 1.922 | 0.271 | - |
| TRINITY_DN1921_c0_g1_i6 | 1.131673777 | 0.270910725 | 0.999970678 | 7.758 | 10.81 | 5.377 | 1.95 | NA |
| TRINITY_DN12795_c0_g1_i7 | -1.13151581 | 0.669218019 | 0.999970678 | 0 | 0.798 | 0.944 | 0.573 | ABC2_membrane |
| TRINITY_DN3478_c0_g1_i1 | -1.13150487 | 0.385231564 | 0.999970678 | 55.621 | 8.002 | 73.029 | 52.681 | NA |
| TRINITY_DN3425_c0_g1_i2 | -1.13143192 | 0.609523861 | 0.999970678 | 4.724 | 2.72 | 13.333 | 0.141 | NA |
| TRINITY_DN2937_c0_g1_i7 | -1.13136098 | 0.713603204 | 1 | 3.942 | 0 | 6.633 | 0.593 | NA |
| TRINITY_DN4328_c0_g1_i1 | 1.131187089 | 0.671024307 | 0.999970678 | 8.057 | 4.021 | 0 | 5.54 | NA |
| TRINITY_DN100129_c0_g1_i1 | -1.13113682 | 0.555004239 | 0.999970678 | 3.97 | 0.089 | 5.077 | 2.865 | NA |
| TRINITY_DN11079_c0_g1_i2 | -1.13112418 | 0.481365786 | 0.999970678 | 2.88 | 0.207 | 2.555 | 3.77 | NA |
| TRINITY_DN3715_c0_g1_i7 | -1.13077047 | 0.799308523 | 1 | 2.852 | 0 | 5.244 | 0 | Peroxidase_2 |
| TRINITY_DN9573_c2_g1_i1 | 1.130534001 | 0.787941123 | 0.999970678 | 0 | 9.579 | 0 | 4.263 | ADH_N |
| TRINITY_DN199_c0_g1_i2 | 1.130447663 | 0.385629258 | 0.999970678 | 2.489 | 0.591 | 0.711 | 0.583 | MFAP1 |
| TRINITY_DN3288_c0_g2_i2 | 1.130358486 | 0.357386693 | 0.999970678 | 12.354 | 3.026 | 3.344 | 2.986 | NA |
| TRINITY_DN1659_c0_g1_i4 | -1.13007417 | 0.798218639 | 1 | 1.399 | 0 | 0 | 3.086 | NA |
| TRINITY_DN1737_c1_g2_i2 | 1.129927665 | 0.425770731 | 0.999970678 | 11.864 | 18.02 | 1.133 | 12.155 | NA |
| TRINITY_DN1870_c0_g2_i3 | -1.12982703 | 0.594474908 | 0.999970678 | 0.827 | 159.8 | 224.142 | 75.342 | IlvC |
| TRINITY_DN6459_c0_g1_i2 | 1.129824549 | 0.697596677 | 0.999970678 | 1.744 | 0 | 0.533 | 0.181 | NA |
| TRINITY_DN9959_c0_g1_i2 | -1.12967242 | 0.668847612 | 0.999970678 | 0 | 1.813 | 1.244 | 2.433 | NA |
| TRINITY_DN6948_c0_g1_i6 | -1.12958416 | 0.284637346 | 0.999970678 | 0.563 | 0.995 | 1.711 | 1.297 | NA |
| TRINITY_DN296_c0_g1_i2 | 1.129398543 | 0.684874391 | 0.999970678 | 0 | 4.553 | 0.767 | 1.136 | Glyco_transf_4 |
| TRINITY_DN8751_c0_g1_i9 | 1.12922789 | 0.513428076 | 1 | 4.733 | 2.208 | 2.278 | 0.372 | NA |
| TRINITY_DN7658_c0_g2_i2 | -1.12919878 | 0.279761723 | 0.999970678 | 9.874 | 7.746 | 25.043 | 8.395 | Aldose_epim |
| TRINITY_DN18871_c0_g2_i3 | 1.12911923 | 0.206443533 | 0.999970678 | 3.788 | 2.582 | 1.633 | 0.945 | ALMT |
| TRINITY_DN7684_c0_g1_i1 | 1.129107911 | 0.073102108 | 0.999970678 | 7.485 | 6.258 | 2.422 | 3.378 | K_trans |
| TRINITY_DN184_c0_g4_i3 | -1.12881674 | 0.419525643 | 0.999970678 | 0.5 | 1.252 | 2.489 | 0.774 | Ribosomal_L31e |
| TRINITY_DN1668_c0_g1_i1 | -1.12867082 | 0.354882735 | 0.999970678 | 14.825 | 41.51 | 19.121 | 99.431 | MFS_1 |
| TRINITY_DN11241_c0_g1_i1 | -1.1285038 | 0.796234513 | 0.999970678 | 1.899 | 0 | 3.466 | 0 | TB2_DP1_HVA22 |
| TRINITY_DN14590_c0_g1_i6 | -1.12834791 | 0.485772795 | 0.999970678 | 0.681 | 0.108 | 0.289 | 1.397 | NA |
| TRINITY_DN3277_c2_g1_i6 | -1.12823372 | 0.683871413 | 0.999970678 | 0 | 11.15 | 7.777 | 14.628 | UPF0016 |
| TRINITY_DN11351_c0_g1_i2 | 1.128224282 | 0.338624065 | 0.999970678 | 3.134 | 1.025 | 0.633 | 1.156 | NA |
| TRINITY_DN3846_c0_g1_i2 | -1.12811943 | 0.138619361 | 0.999970678 | 10.982 | 6.761 | 13.777 | 22.229 | TPR_12 |
| TRINITY_DN2089_c0_g1_i3 | -1.12798019 | 0.668950866 | 0.999970678 | 0 | 1.35 | 1.022 | 1.669 | NA |
| TRINITY_DN3032_c0_g1_i5 | -1.12728746 | 0.671828402 | 0.999970678 | 2.153 | 0 | 1.489 | 2.966 | NA |
| TRINITY_DN590_c1_g2_i1 | -1.12721667 | 0.616530808 | 0.999970678 | 13.653 | 1577 | 207.554 | 2714.407 | NA |
| TRINITY_DN5062_c0_g1_i4 | 1.127119185 | 0.26139699 | 0.999970678 | 7.54 | 23.59 | 7.1 | 5.58 | Calmodulin_bind |
| TRINITY_DN415_c6_g1_i2 | -1.12709657 | 0.590003111 | 0.999970678 | 5.251 | 0.089 | 6.811 | 3.448 | - |
| TRINITY_DN22605_c0_g1_i1 | -1.12709294 | 0.794249439 | 0.999970678 | 2.189 | 0 | 3.989 | 0 | NA |
| TRINITY_DN9155_c0_g1_i1 | -1.12708828 | 0.802282813 | 1 | 0 | 0.867 | 1.533 | 0 | Acetyltransf_1 |
| TRINITY_DN2297_c0_g1_i2 | -1.12696928 | 0.503354554 | 0.999970678 | 4.424 | 0.177 | 4.466 | 4.816 | NA |
| TRINITY_DN114_c0_g1_i7 | -1.12673099 | 0.484454048 | 0.999970678 | 17.587 | 2.405 | 27.198 | 9.822 | NA |
| TRINITY_DN3540_c0_g1_i1 | 1.126551542 | 0.675888318 | 0.999970678 | 3.079 | 5.125 | 3.089 | 0 | NA |
| TRINITY_DN645_c0_g1_i6 | -1.12653028 | 0.074604556 | 0.999970678 | 4.351 | 5.775 | 8.388 | 12.004 | NA |
| TRINITY_DN15786_c1_g1_i2 | -1.12619075 | 0.800773196 | 1 | 0.699 | 0 | 1.3 | 0 | AMP-binding |
| TRINITY_DN178_c0_g1_i3 | 1.126125014 | 0.362828831 | 0.999970678 | 7.703 | 44.58 | 9.699 | 11.803 | 2OG-FeII_Oxy |
| TRINITY_DN314_c0_g2_i2 | -1.12593647 | 0.677148779 | 0.999970678 | 0.463 | 0 | 0.356 | 0.603 | NA |
| TRINITY_DN722_c0_g1_i9 | -1.12566913 | 0.402886986 | 0.999970678 | 7.049 | 1.873 | 3.022 | 16.016 | Cu-oxidase |
| TRINITY_DN6891_c2_g2_i6 | 1.125651046 | 0.618897003 | 0.999970678 | 2.553 | 0.276 | 1.055 | 0.04 | NA |
| TRINITY_DN2322_c0_g1_i8 | 1.125531297 | 0.69447539 | 0.999970678 | 0.363 | 4.297 | 0 | 2.111 | NA |
| TRINITY_DN1906_c1_g1_i2 | 1.125504507 | 0.570172452 | 0.999970678 | 2.398 | 0.503 | 1.033 | 0.101 | NA |
| TRINITY_DN13225_c2_g1_i1 | -1.12529786 | 0.704760882 | 0.999970678 | 1.054 | 0.197 | 2.233 | 0 | NA |
| TRINITY_DN11910_c0_g1_i2 | -1.12528391 | 0.46468705 | 0.999970678 | 0.481 | 1.232 | 2.755 | 0.392 | CBM49 |
| TRINITY_DN695_c1_g1_i3 | 1.125202569 | 0.448528651 | 0.999970678 | 585.68 | 48.25 | 162.446 | 97.843 | 2OG-FeII_Oxy |
| TRINITY_DN5676_c0_g1_i2 | 1.125081873 | 0.515806979 | 0.999970678 | 0.663 | 0.138 | 0.256 | 0.06 | PPR_long |
| TRINITY_DN7145_c0_g1_i4 | 1.12499503 | 0.675038183 | 0.999970678 | 1.744 | 0.631 | 0.911 | 0 | FAR1 |
| TRINITY_DN75_c0_g1_i4 | 1.124964109 | 0.722147443 | 0.999970678 | 4.179 | 0 | 1.467 | 0.141 | NA |
| TRINITY_DN2379_c0_g1_i3 | -1.12495773 | 0.464630035 | 0.999970678 | 1.354 | 0.256 | 1.755 | 1.458 | NA |
| TRINITY_DN10058_c0_g1_i2 | 1.124957572 | 0.67828864 | 1 | 0.554 | 0.355 | 0.356 | 0 | NA |
| TRINITY_DN10833_c0_g1_i6 | 1.12480312 | 0.692187068 | 1 | 0.491 | 2.415 | 1.089 | 0 | NA |
| TRINITY_DN883_c0_g1_i3 | -1.12452234 | 0.311635867 | 0.999970678 | 2.97 | 9.037 | 6.177 | 18.589 | NA |
| TRINITY_DN11593_c0_g2_i1 | -1.12439612 | 0.144211882 | 0.999970678 | 198.2 | 201.6 | 239.985 | 555.416 | NA |
| TRINITY_DN6437_c0_g2_i4 | -1.12436692 | 0.683561591 | 0.999970678 | 0 | 0.828 | 1.211 | 0.312 | NA |
| TRINITY_DN2616_c0_g2_i3 | -1.12435425 | 0.444322523 | 0.999970678 | 2.207 | 0.246 | 2.178 | 2.795 | NOP5NT |

| TRINITY_DN3082_c0_g1_i6 | -1.12428131 | 0.435078907 | 0.999970678 | 0.309 | 0.059 | 0.222 | 0.563 | NA |
| --- | --- | --- | --- | --- | --- | --- | --- | --- |
| TRINITY_DN3233_c0_g1_i2 | 1.124267054 | 0.7885932 | 0.999970678 | 5.995 | 0 | 0 | 2.785 | NA |
| TRINITY_DN13906_c0_g1_i4 | -1.1241372 | 0.78956691 | 0.999970678 | 5.587 | 0 | 10.222 | 0 | Ferritin_2 |
| TRINITY_DN15678_c1_g3_i1 | -1.12412499 | 0.798704265 | 1 | 1.281 | 0 | 0 | 2.815 | NA |
| TRINITY_DN897_c2_g1_i3 | 1.123967726 | 0.535927421 | 0.999970678 | 3.016 | 9.638 | 0.167 | 5.57 | NA |
| TRINITY_DN1404_c0_g1_i3 | -1.12393206 | 0.383915197 | 0.999970678 | 11.373 | 9.5 | 4.677 | 39.893 | Ion_trans_2 |
| TRINITY_DN8831_c0_g1_i1 | -1.12375122 | 0.678841656 | 0.999970678 | 74.506 | 0 | 77.095 | 60.121 | NA |
| TRINITY_DN138_c0_g3_i3 | -1.12368504 | 0.672412074 | 0.999970678 | 3.397 | 0 | 2.4 | 4.574 | NA |
| TRINITY_DN5201_c0_g1_i4 | 1.123625878 | 0.790423144 | 0.999970678 | 13.381 | 0 | 0 | 6.243 | QRPTase_C |
| TRINITY_DN2482_c0_g1_i1 | 1.123464927 | 0.29063618 | 0.999970678 | 5.605 | 13.73 | 5.177 | 2.554 | NA |
| TRINITY_DN6042_c0_g1_i2 | -1.12323402 | 0.805073066 | 0.999970678 | 74.506 | 0 | 135.903 | 0 | NA |
| TRINITY_DN3793_c1_g1_i1 | 1.123191848 | 0.690397428 | 0.999970678 | 4.397 | 0 | 0.6 | 1.327 | NA |
| TRINITY_DN7144_c0_g1_i2 | -1.12303594 | 0.632972828 | 1 | 0.89 | 0.168 | 1.855 | 0.06 | NA |
| TRINITY_DN8246_c0_g1_i5 | 1.122601352 | 0.34848284 | 0.999970678 | 0.518 | 1.124 | 0.211 | 0.483 | - |
| TRINITY_DN635_c0_g1_i1 | 1.122601293 | 0.692550948 | 0.999970678 | 12.699 | 0 | 1.389 | 4.253 | - |
| TRINITY_DN4338_c0_g1_i1 | -1.12251305 | 0.165230676 | 0.999970678 | 2.653 | 5.164 | 6.433 | 9.169 | Abhydrolase_3 |
| TRINITY_DN135_c0_g1_i6 | -1.12223881 | 0.205157823 | 0.999970678 | 1.653 | 1.892 | 3.955 | 2.946 | NA |
| TRINITY_DN10225_c0_g1_i1 | -1.12219927 | 0.222386014 | 0.999970678 | 3.325 | 5.549 | 4.955 | 13.281 | NA |
| TRINITY_DN2733_c1_g1_i1 | 1.122125283 | 0.459029065 | 0.999970678 | 1.562 | 0.749 | 0.744 | 0.211 | NA |
| TRINITY_DN4297_c0_g1_i3 | 1.121909435 | 0.300659754 | 0.999970678 | 0.427 | 0.473 | 0.233 | 0.131 | AA_permease_2 |
| TRINITY_DN5255_c0_g1_i4 | -1.12190902 | 0.292239607 | 0.999970678 | 10.737 | 3.095 | 9.388 | 19.132 | p450 |
| TRINITY_DN1948_c0_g1_i4 | 1.121792688 | 0.34647062 | 0.999970678 | 4.351 | 1.941 | 1.389 | 1.216 | EF_assoc_1 |
| TRINITY_DN2291_c0_g1_i6 | -1.12167302 | 0.465474463 | 0.999970678 | 0.518 | 6.298 | 3.778 | 10.084 | Hexapep |
| TRINITY_DN18967_c0_g1_i1 | -1.1215044 | 0.718783045 | 1 | 0 | 1.104 | 0.111 | 2.212 | NA |
| TRINITY_DN14510_c0_g1_i2 | 1.121501965 | 0.501281122 | 0.999970678 | 0.236 | 5.253 | 1.211 | 1.025 | NA |
| TRINITY_DN39437_c0_g1_i1 | 1.121400416 | 0.471943312 | 0.999970678 | 31.231 | 175.2 | 23.876 | 57.336 | NA |
| TRINITY_DN2842_c0_g1_i2 | 1.121321274 | 0.216513716 | 0.999970678 | 55.903 | 21.45 | 15.344 | 17.292 | NA |
| TRINITY_DN1897_c0_g1_i5 | -1.12131009 | 0.325763095 | 0.999970678 | 6.849 | 3.607 | 12.41 | 7.229 | NA |
| TRINITY_DN4189_c0_g1_i6 | -1.12117843 | 0.672129003 | 0.999970678 | 3.861 | 0 | 3.178 | 4.564 | NA |
| TRINITY_DN3233_c0_g1_i1 | -1.12116639 | 0.672419462 | 0.999970678 | 0 | 4.228 | 4.589 | 3.499 | NA |
| TRINITY_DN22918_c0_g1_i12 | -1.12115837 | 0.802392543 | 1 | 0.527 | 0 | 0.967 | 0 | COesterase |
| TRINITY_DN2506_c0_g1_i5 | -1.12095517 | 0.597625187 | 0.999970678 | 0.363 | 6.504 | 0.622 | 13.824 | NA |
| TRINITY_DN13775_c1_g1_i1 | -1.12093293 | 0.322877241 | 0.999970678 | 31.803 | 7.116 | 29.387 | 50.168 | RRM_1 |
| TRINITY_DN29173_c0_g1_i1 | 1.12090677 | 0.450044984 | 0.999970678 | 2.698 | 1.577 | 1.344 | 0.342 | NA |
| TRINITY_DN4294_c0_g1_i1 | -1.12089426 | 0.114678475 | 0.999970678 | 33.529 | 24.96 | 42.364 | 75.574 | NA |
| TRINITY_DN187_c0_g1_i1 | -1.12085806 | 0.152497359 | 0.999970678 | 34.355 | 25.06 | 37.92 | 83.094 | - |
| TRINITY_DN354_c0_g1_i2 | 1.120794815 | 0.396264442 | 0.999970678 | 0.636 | 0.798 | 0.411 | 0.161 | NA |
| TRINITY_DN17809_c0_g1_i2 | -1.12074803 | 0.681951321 | 0.999970678 | 12.727 | 0 | 11.51 | 14.135 | NA |
| TRINITY_DN2368_c0_g1_i6 | -1.12059065 | 0.674318961 | 0.999970678 | 0 | 1.409 | 1.678 | 0.985 | DUF565 |
| TRINITY_DN45304_c0_g1_i3 | 1.120587644 | 0.580281908 | 0.999970678 | 14.534 | 13.77 | 0.122 | 12.457 | NA |
| TRINITY_DN4674_c1_g1_i11 | -1.1205792 | 0.722707863 | 0.999970678 | 1.099 | 0 | 1.944 | 0.05 | HLH |
| TRINITY_DN1139_c0_g1_i1 | -1.12014022 | 0.740459339 | 0.999970678 | 32.203 | 0 | 0.211 | 70.527 | Band_7 |
| TRINITY_DN2205_c0_g1_i9 | 1.119986705 | 0.532072962 | 1 | 0.636 | 0.7 | 0.478 | 0.05 | NA |
| TRINITY_DN3517_c1_g1_i7 | -1.11989267 | 0.464883943 | 0.999970678 | 1.771 | 0.207 | 2.011 | 1.94 | NA |
| TRINITY_DN15995_c0_g1_i9 | 1.119859469 | 0.685308436 | 1 | 0.409 | 1.429 | 0 | 0.845 | NA |
| TRINITY_DN411_c0_g1_i5 | -1.11982414 | 0.523818633 | 0.999970678 | 1.808 | 7.106 | 1.1 | 17.644 | NA |
| TRINITY_DN7199_c0_g1_i1 | 1.119823999 | 0.286057047 | 0.999970678 | 1.126 | 1.35 | 0.278 | 0.804 | NA |
| TRINITY_DN32204_c0_g1_i2 | 1.119812715 | 0.333530037 | 0.999970678 | 0.718 | 1.114 | 0.244 | 0.533 | SCAMP |
| TRINITY_DN1284_c1_g1_i9 | 1.119747241 | 0.791180572 | 0.999970678 | 10.819 | 0 | 0 | 5.077 | NA |
| TRINITY_DN175_c0_g1_i20 | -1.11970867 | 0.594036904 | 0.999970678 | 0.273 | 2.966 | 0.122 | 6.877 | NA |
| TRINITY_DN6346_c0_g1_i10 | 1.119537816 | 0.220447964 | 0.999970678 | 20.384 | 8.278 | 6.089 | 5.932 | S6PP |
| TRINITY_DN3252_c0_g1_i4 | -1.11949841 | 0.674547909 | 0.999970678 | 4.769 | 0 | 3.811 | 5.902 | NA |
| TRINITY_DN8002_c0_g1_i1 | 1.119436957 | 0.533484839 | 0.999970678 | 1.372 | 41.18 | 8.799 | 8.254 | NA |
| TRINITY_DN4259_c0_g1_i5 | -1.11936797 | 0.581647717 | 0.999970678 | 0.118 | 0.897 | 0.1 | 2.101 | NA |
| TRINITY_DN1649_c0_g1_i3 | -1.11926008 | 0.545026431 | 0.999970678 | 1.299 | 1.439 | 0.111 | 5.801 | NA |
| TRINITY_DN11177_c0_g1_i8 | 1.119234618 | 0.587305752 | 0.999970678 | 2.325 | 0.069 | 0.733 | 0.241 | NA |
| TRINITY_DN7824_c0_g1_i2 | 1.119177956 | 0.372583427 | 0.999970678 | 2.643 | 5.302 | 2.522 | 0.593 | NA |
| TRINITY_DN576_c0_g1_i1 | -1.11914941 | 0.405711893 | 0.999970678 | 1.208 | 0.384 | 1.411 | 1.699 | NA |
| TRINITY_DN5113_c0_g2_i3 | -1.11913936 | 0.675724437 | 0.999970678 | 2.244 | 0 | 1.333 | 3.348 | HSP20 |
| TRINITY_DN186_c7_g1_i2 | -1.11911753 | 0.62780652 | 0.999970678 | 4.124 | 523.3 | 40.775 | 966.019 | NA |
| TRINITY_DN21_c0_g1_i7 | -1.11887047 | 0.444391049 | 0.999970678 | 2.498 | 14.86 | 6.022 | 29.548 | NA |
| TRINITY_DN2844_c0_g1_i2 | 1.118855028 | 0.710943005 | 0.999970678 | 0.073 | 2.385 | 0.922 | 0 | - |
| TRINITY_DN11501_c0_g1_i2 | -1.11874853 | 0.199923446 | 0.999970678 | 4.869 | 8.16 | 8.011 | 18.499 | NA |
| TRINITY_DN193_c0_g1_i24 | 1.118722993 | 0.792046114 | 0.999970678 | 18.168 | 0 | 0 | 8.485 | NA |
| TRINITY_DN8569_c0_g1_i2 | 1.118659191 | 0.322716689 | 0.999970678 | 0.345 | 0.453 | 0.178 | 0.151 | NA |
| TRINITY_DN208_c0_g1_i4 | 1.11865265 | 0.789795728 | 0.999970678 | 30.295 | 0 | 0 | 13.904 | Ribosomal_L36e |
| TRINITY_DN13049_c0_g1_i5 | -1.11862993 | 0.79915128 | 1 | 0.572 | 0 | 0 | 1.297 | DUF812 |
| TRINITY_DN2902_c0_g1_i1 | -1.11842891 | 0.507473627 | 0.999970678 | 3.252 | 0.276 | 4.989 | 1.719 | NA |
| TRINITY_DN4218_c0_g1_i14 | 1.118412069 | 0.3886098 | 0.999970678 | 6.867 | 4.09 | 3.555 | 0.784 | NA |
| TRINITY_DN3175_c0_g1_i2 | 1.118321416 | 0.613354733 | 0.999970678 | 3.379 | 0.099 | 1.289 | 0.08 | NA |
| TRINITY_DN5411_c1_g1_i1 | -1.11826104 | 0.363524068 | 0.999970678 | 0.972 | 0.611 | 0.578 | 2.755 | NA |
| TRINITY_DN6364_c1_g1_i6 | -1.11817266 | 0.311002431 | 0.999970678 | 1.517 | 0.581 | 1.178 | 3.177 | Pkinase |
| TRINITY_DN1211_c0_g2_i20 | -1.11812913 | 0.675688927 | 0.999970678 | 1.154 | 0 | 1.389 | 0.865 | CRS1_YhbY |
| TRINITY_DN223_c0_g1_i1 | -1.1181285 | 0.303424799 | 0.999970678 | 5.923 | 14.58 | 10.177 | 31.438 | Str_synth |

| TRINITY_DN11126_c0_g1_i3 | -1.11806649 | 0.354962993 | 0.999970678 | 1.744 | 4.376 | 2.267 | 10.496 | NA |
| --- | --- | --- | --- | --- | --- | --- | --- | --- |
| TRINITY_DN14623_c0_g1_i4 | 1.118025832 | 0.334595206 | 0.999970678 | 1.88 | 0.867 | 0.689 | 0.442 | NA |
| TRINITY_DN2112_c1_g1_i6 | 1.117940129 | 0.57272372 | 0.999970678 | 83.3 | 4.139 | 30.187 | 3.7 | NA |
| TRINITY_DN7065_c1_g1_i4 | 1.117847989 | 0.802007972 | 1 | 2.389 | 0 | 0 | 1.096 | PfkB |
| TRINITY_DN3897_c0_g1_i8 | 1.117842898 | 0.076048578 | 0.999970678 | 2.544 | 3.016 | 1.067 | 1.277 | NA |
| TRINITY_DN4439_c0_g2_i2 | 1.117730438 | 0.348418261 | 0.999970678 | 3.597 | 1.143 | 0.744 | 1.287 | NA |
| TRINITY_DN800_c1_g1_i6 | -1.1176654 | 0.326555663 | 0.999970678 | 15.742 | 21.79 | 12.055 | 66.495 | NA |
| TRINITY_DN8942_c0_g1_i11 | 1.117631233 | 0.675663901 | 0.999970678 | 6.014 | 3.371 | 0 | 4.373 | Motile_Sperm |
| TRINITY_DN3512_c0_g1_i2 | 1.117576598 | 0.373587063 | 0.999970678 | 17.714 | 3.479 | 4.444 | 4.454 | NA |
| TRINITY_DN17105_c0_g1_i1 | -1.11757022 | 0.444650018 | 0.999970678 | 0.354 | 3.331 | 4.166 | 2.865 | NA |
| TRINITY_DN16485_c0_g1_i3 | -1.11748363 | 0.673809295 | 0.999970678 | 0 | 1.025 | 0.822 | 1.206 | NA |
| TRINITY_DN20836_c0_g1_i2 | -1.11746831 | 0.79929513 | 0.999970678 | 0 | 0.493 | 0.856 | 0 | Nodulin-like |
| TRINITY_DN1993_c1_g1_i6 | 1.117258296 | 0.53902332 | 0.999970678 | 0.3 | 4.169 | 0.578 | 1.317 | NA |
| TRINITY_DN4515_c5_g1_i4 | -1.1172286 | 0.696605327 | 1 | 1.444 | 0 | 2.133 | 0.543 | NA |
| TRINITY_DN8245_c1_g1_i1 | 1.117212394 | 0.422558494 | 1 | 2.789 | 2.602 | 1.455 | 0.643 | NA |
| TRINITY_DN2133_c0_g1_i3 | -1.11719722 | 0.685860298 | 0.999970678 | 10.074 | 0 | 11.022 | 8.847 | NA |
| TRINITY_DN662_c0_g1_i1 | -1.11703934 | 0.681304495 | 0.999970678 | 3.515 | 0 | 3.044 | 4.072 | - |
| TRINITY_DN99805_c0_g1_i1 | -1.11699438 | 0.69416948 | 0.999970678 | 1.163 | 0 | 0.333 | 2.101 | CH |
| TRINITY_DN10082_c0_g2_i1 | -1.11692893 | 0.284004917 | 0.999970678 | 5.441 | 7.047 | 4.889 | 21.233 | Cu_amine_oxid |
| TRINITY_DN2927_c0_g1_i2 | 1.116891995 | 0.696782695 | 0.999970678 | 2.589 | 0 | 0.233 | 0.925 | DND1_DSRM |
| TRINITY_DN3063_c0_g1_i4 | -1.11665112 | 0.129272277 | 0.999970678 | 54.204 | 47.5 | 68.007 | 131.563 | NA |
| TRINITY_DN57_c0_g1_i3 | 1.116299777 | 0.151091376 | 0.999970678 | 1.526 | 1.035 | 0.5 | 0.583 | NA |
| TRINITY_DN349_c0_g1_i2 | -1.11617652 | 0.686171438 | 0.999970678 | 0 | 6.406 | 8.877 | 2.966 | DUF3531 |
| TRINITY_DN21572_c0_g1_i2 | -1.11609965 | 0.692234886 | 1 | 2.716 | 0 | 1.344 | 4.313 | NA |
| TRINITY_DN17541_c0_g1_i6 | 1.116047455 | 0.337401133 | 0.999970678 | 5.278 | 5.135 | 2.733 | 1.478 | DUF659 |
| TRINITY_DN4046_c0_g1_i2 | -1.11595725 | 0.387445487 | 0.999970678 | 1.335 | 0.739 | 3 | 0.925 | NA |
| TRINITY_DN5076_c0_g1_i6 | 1.115808475 | 0.795551962 | 0.999970678 | 1.581 | 0 | 0 | 0.734 | BAH |
| TRINITY_DN5940_c0_g1_i1 | 1.11572074 | 0.106473228 | 0.999970678 | 12.808 | 9.047 | 4.733 | 4.424 | SMC_N |
| TRINITY_DN14584_c1_g2_i1 | 1.115646159 | 0.577686363 | 1 | 3.079 | 2.217 | 1.922 | 0.111 | DUF1761 |
| TRINITY_DN37105_c0_g1_i4 | -1.11560224 | 0.804126538 | 1 | 0.781 | 0 | 1.422 | 0 | Glyco_hydro_114 |
| TRINITY_DN15387_c0_g1_i1 | -1.11560224 | 0.804126538 | 1 | 1.581 | 0 | 2.822 | 0 | Ribosomal_L2 |
| TRINITY_DN9316_c0_g1_i4 | -1.11560224 | 0.804126538 | 1 | 0.618 | 0 | 1.133 | 0 | - |
| TRINITY_DN33675_c0_g1_i3 | -1.11560224 | 0.804126538 | 1 | 0.963 | 0 | 1.767 | 0 | NA |
| TRINITY_DN18323_c0_g1_i9 | -1.11560224 | 0.804126538 | 1 | 2.961 | 0 | 5.255 | 0 | NA |
| TRINITY_DN13191_c0_g2_i3 | -1.11556329 | 0.363003874 | 0.999970678 | 0.999 | 0.581 | 2.055 | 0.935 | NA |
| TRINITY_DN6222_c0_g1_i1 | 1.115532829 | 0.288571839 | 0.999970678 | 6.441 | 5.125 | 3.422 | 1.216 | Lzipper-MIP1 |
| TRINITY_DN633_c0_g3_i1 | 1.115290919 | 0.693139627 | 0.999970678 | 0 | 10.37 | 1.844 | 2.503 | C2 |
| TRINITY_DN2690_c0_g1_i3 | -1.11514431 | 0.503311844 | 0.999970678 | 0.927 | 0.936 | 3.122 | 0.292 | - |
| TRINITY_DN8798_c0_g1_i1 | -1.11494835 | 0.346613589 | 0.999970678 | 0.581 | 0.453 | 1.111 | 0.935 | - |
| TRINITY_DN1799_c0_g1_i8 | 1.1149138 | 0.677607708 | 0.999970678 | 1.535 | 0.719 | 0.867 | 0 | PPR |
| TRINITY_DN1920_c0_g1_i3 | -1.11479953 | 0.383565068 | 0.999970678 | 10.038 | 9.717 | 28.421 | 7.711 | - |
| TRINITY_DN78495_c0_g1_i1 | -1.11465443 | 0.718264485 | 0.999970678 | 7.24 | 0 | 12.844 | 0.372 | Phosphoesterase |
| TRINITY_DN2613_c0_g2_i1 | -1.11457898 | 0.051955613 | 0.999970678 | 9.538 | 10.08 | 17.01 | 21.987 | EamA |
| TRINITY_DN2723_c0_g1_i8 | -1.11455572 | 0.689848215 | 0.999970678 | 0 | 0.779 | 0.278 | 1.307 | NA |
| TRINITY_DN3362_c0_g1_i4 | 1.114464801 | 0.681619455 | 0.999970678 | 20.112 | 27.88 | 0 | 21.957 | NA |
| TRINITY_DN2757_c0_g1_i6 | 1.114403052 | 0.682698985 | 1 | 0.309 | 0.266 | 0.222 | 0 | NA |
| TRINITY_DN38784_c0_g1_i1 | -1.11402153 | 0.391881416 | 0.999970678 | 0.554 | 0.207 | 0.778 | 0.744 | PseudoU_synth_2 |
| TRINITY_DN3640_c0_g1_i2 | 1.113917677 | 0.722387609 | 0.999970678 | 63.251 | 0 | 22.132 | 2.895 | NA |
| TRINITY_DN4797_c0_g1_i4 | -1.11388259 | 0.410606039 | 0.999970678 | 5.696 | 8.466 | 2.733 | 27.306 | NA |
| TRINITY_DN15379_c0_g2_i1 | 1.11388138 | 0.675755907 | 0.999970678 | 1.326 | 0.7 | 0.789 | 0 | NA |
| TRINITY_DN1175_c0_g1_i6 | 1.113611713 | 0.692211027 | 0.999970678 | 2.244 | 20.02 | 0 | 10.144 | NA |
| TRINITY_DN5689_c0_g1_i7 | 1.113461184 | 0.305889057 | 0.999970678 | 3.37 | 1.409 | 1.322 | 0.643 | NA |
| TRINITY_DN3530_c0_g1_i6 | 1.113449559 | 0.376799727 | 0.999970678 | 0.382 | 1.705 | 0.478 | 0.382 | NA |
| TRINITY_DN4879_c0_g1_i3 | -1.11338686 | 0.697590173 | 0.999970678 | 23.255 | 0 | 5.8 | 43.663 | NA |
| TRINITY_DN1788_c0_g1_i7 | 1.113296667 | 0.354345203 | 0.999970678 | 0.554 | 0.867 | 0.367 | 0.211 | DapB_C |
| TRINITY_DN351_c0_g1_i7 | -1.11328444 | 0.67831075 | 0.999970678 | 0.699 | 0 | 0.456 | 0.995 | NA |
| TRINITY_DN1416_c1_g1_i4 | 1.113152196 | 0.565200685 | 0.999970678 | 34.628 | 0.601 | 10.822 | 3.448 | GDPD |
| TRINITY_DN2010_c0_g1_i9 | 1.113014402 | 0.298875619 | 0.999970678 | 55.04 | 28.41 | 23.776 | 9.571 | NA |
| TRINITY_DN1031_c5_g1_i1 | -1.11233845 | 0.195803245 | 0.999970678 | 4.197 | 5.824 | 5.9 | 14.467 | NA |
| TRINITY_DN1979_c0_g1_i1 | 1.112314687 | 0.340088984 | 0.999970678 | 3.615 | 7.628 | 3.378 | 1.096 | NA |
| TRINITY_DN10671_c0_g1_i11 | 1.112252345 | 0.398252869 | 0.999970678 | 10.401 | 2.878 | 4.111 | 1.227 | NA |
| TRINITY_DN11731_c0_g1_i14 | -1.11223899 | 0.377603908 | 0.999970678 | 0.291 | 0.473 | 0.433 | 1.156 | F-box |
| TRINITY_DN5143_c1_g1_i5 | 1.112209849 | 0.313089376 | 0.999970678 | 10.764 | 9.146 | 1.522 | 7.379 | NA |
| TRINITY_DN533_c0_g1_i3 | -1.11214034 | 0.299074928 | 0.999970678 | 1.998 | 4.218 | 5.989 | 5.932 | NA |
| TRINITY_DN7579_c0_g1_i2 | 1.112122608 | 0.694941022 | 0.999970678 | 0 | 3.035 | 0.5 | 0.794 | NA |
| TRINITY_DN4022_c0_g1_i4 | 1.111931264 | 0.580185548 | 0.999970678 | 0.727 | 7.253 | 2.911 | 0.131 | DnaJ |
| TRINITY_DN4316_c0_g1_i4 | 1.11192609 | 0.194184339 | 0.999970678 | 8.693 | 8.19 | 4.555 | 2.332 | Helicase_C |
| TRINITY_DN482_c0_g3_i1 | -1.11185904 | 0.228937862 | 0.999970678 | 6.104 | 4.474 | 13.155 | 6.937 | EPF |
| TRINITY_DN1506_c0_g1_i2 | 1.111678504 | 0.790971307 | 0.999970678 | 8.521 | 0 | 0 | 4.001 | NA |
| TRINITY_DN804_c0_g1_i7 | 1.111632598 | 0.676597168 | 0.999970678 | 5.105 | 1.961 | 0 | 3.308 | NA |
| TRINITY_DN2911_c0_g1_i8 | 1.111512651 | 0.299486181 | 0.999970678 | 5.223 | 3.016 | 2.355 | 0.975 | NA |
| TRINITY_DN6529_c0_g1_i4 | -1.11149723 | 0.536780415 | 1 | 0.154 | 1.064 | 0.478 | 2.021 | NA |
| TRINITY_DN3492_c0_g1_i2 | -1.11126289 | 0.456317635 | 0.999970678 | 3.243 | 0.917 | 6.444 | 1.307 | NA |
| TRINITY_DN4903_c0_g1_i4 | -1.1111625 | 0.792173475 | 0.999970678 | 4.36 | 0 | 7.844 | 0 | NA |

| TRINITY_DN1631_c0_g2_i1 | 1.11097065 | 0.204523171 | 0.999970678 | 7.921 | 6.367 | 3.633 | 2.242 | NA |
| --- | --- | --- | --- | --- | --- | --- | --- | --- |
| TRINITY_DN348_c0_g1_i2 | 1.11091949 | 0.795930351 | 0.999970678 | 3.652 | 0 | 0 | 1.729 | NA |
| TRINITY_DN2037_c0_g1_i4 | -1.11076164 | 0.702004355 | 0.999970678 | 21.674 | 0.03 | 39.153 | 0.111 | ADH_N |
| TRINITY_DN1254_c0_g1_i12 | -1.11066258 | 0.500330182 | 0.999970678 | 4.951 | 0.848 | 1.222 | 11.089 | NA |
| TRINITY_DN4314_c0_g1_i3 | 1.110306665 | 0.794398497 | 0.999970678 | 4.842 | 0 | 0 | 2.272 | DUF842 |
| TRINITY_DN14231_c0_g1_i2 | -1.11025326 | 0.08766073 | 0.999970678 | 45.456 | 42.31 | 65.618 | 107.142 | NA |
| TRINITY_DN3334_c0_g1_i4 | -1.1102484 | 0.689495139 | 0.999970678 | 7.149 | 0 | 3.344 | 11.672 | BCDHK_Adom3 |
| TRINITY_DN645_c0_g1_i1 | -1.11018372 | 0.695223104 | 0.999970678 | 12.272 | 0 | 16.555 | 6.877 | NA |
| TRINITY_DN791_c1_g1_i8 | -1.11014763 | 0.442423659 | 0.999970678 | 4.46 | 6.751 | 15.943 | 4.112 | NA |
| TRINITY_DN618_c0_g1_i2 | -1.1101424 | 0.79324862 | 0.999970678 | 6.259 | 0 | 0 | 13.764 | NA |
| TRINITY_DN28431_c0_g1_i1 | 1.109973895 | 0.803589899 | 1 | 1.463 | 0 | 0.567 | 0 | NA |
| TRINITY_DN863_c0_g1_i7 | 1.109684735 | 0.477190831 | 0.999970678 | 1.453 | 0.306 | 0.133 | 0.674 | BACK |
| TRINITY_DN4299_c0_g1_i4 | 1.109585209 | 0.802152275 | 1 | 0.763 | 0 | 0 | 0.352 | WD40 |
| TRINITY_DN6391_c0_g1_i9 | -1.10955516 | 0.696865206 | 0.999970678 | 0.182 | 0.394 | 1.011 | 0 | NA |
| TRINITY_DN2339_c0_g1_i3 | 1.10955474 | 0.509945423 | 0.999970678 | 0.2 | 3.045 | 0.389 | 1.005 | NA |
| TRINITY_DN13386_c0_g1_i1 | -1.10943677 | 0.806072326 | 1 | 0.282 | 0 | 0.522 | 0 | PPR |
| TRINITY_DN1413_c0_g1_i5 | -1.10934752 | 0.676302345 | 0.999970678 | 0 | 4.997 | 3.766 | 6.082 | NA |
| TRINITY_DN2924_c0_g1_i1 | -1.10925632 | 0.544366937 | 0.999970678 | 0.845 | 0.118 | 0.167 | 1.93 | B_lectin |
| TRINITY_DN2798_c0_g1_i14 | 1.109046905 | 0.198079745 | 0.999970678 | 1.435 | 2.286 | 0.744 | 0.834 | NA |
| TRINITY_DN3775_c0_g3_i1 | -1.1089407 | 0.615462797 | 1 | 0.273 | 2.119 | 0.178 | 4.816 | NA |
| TRINITY_DN9835_c0_g1_i2 | -1.10880835 | 0.799947228 | 0.999970678 | 1.417 | 0 | 0 | 3.117 | NA |
| TRINITY_DN2517_c0_g2_i1 | 1.108693066 | 0.499472172 | 0.999970678 | 18.64 | 1.429 | 6.233 | 1.9 | zf-RING_2 |
| TRINITY_DN423_c0_g1_i1 | -1.10856657 | 0.522897908 | 0.999970678 | 19.412 | 11.19 | 1.633 | 64.052 | NA |
| TRINITY_DN10771_c0_g2_i5 | -1.10855794 | 0.29778015 | 0.999970678 | 0.536 | 0.611 | 0.856 | 1.488 | Rft-1 |
| TRINITY_DN7626_c0_g4_i3 | 1.108502532 | 0.298755636 | 0.999970678 | 0.754 | 0.473 | 0.211 | 0.332 | LRR_4 |
| TRINITY_DN1514_c0_g1_i1 | 1.108372986 | 0.460062801 | 0.999970678 | 1.79 | 13.85 | 1.178 | 5.761 | NA |
| TRINITY_DN16670_c0_g1_i1 | -1.10836585 | 0.678493044 | 0.999970678 | 233.85 | 0 | 189.422 | 246.537 | NA |
| TRINITY_DN284_c0_g1_i1 | -1.10832224 | 0.794640219 | 0.999970678 | 0 | 4.79 | 8.411 | 0 | NA |
| TRINITY_DN11575_c0_g1_i2 | -1.10826998 | 0.690644777 | 0.999970678 | 0.554 | 0.749 | 0 | 2.825 | NA |
| TRINITY_DN4336_c0_g1_i5 | -1.10820518 | 0.677649918 | 0.999970678 | 0 | 3.213 | 2.533 | 3.78 | NA |
| TRINITY_DN458_c0_g1_i1 | -1.10803853 | 0.448533537 | 0.999970678 | 0.836 | 4.731 | 2.255 | 9.099 | Epimerase |
| TRINITY_DN6515_c0_g1_i7 | 1.108030483 | 0.796780615 | 0.999970678 | 0 | 0.779 | 0 | 0.352 | NA |
| TRINITY_DN42827_c0_g1_i1 | -1.10800834 | 0.474171104 | 0.999970678 | 0.563 | 1.685 | 0.744 | 3.881 | NA |
| TRINITY_DN10709_c0_g2_i2 | 1.107915447 | 0.18786279 | 0.999970678 | 2.825 | 1.735 | 0.911 | 1.025 | NA |
| TRINITY_DN33763_c0_g1_i1 | -1.10788209 | 0.327813227 | 0.999970678 | 0.827 | 0.897 | 1.922 | 1.387 | NA |
| TRINITY_DN1893_c0_g1_i5 | 1.107868934 | 0.39840876 | 0.999970678 | 1.908 | 2.769 | 1.555 | 0.292 | APG9 |
| TRINITY_DN1712_c0_g1_i12 | -1.10783857 | 0.337005856 | 0.999970678 | 0.954 | 0.581 | 0.9 | 2.202 | NA |
| TRINITY_DN51673_c0_g1_i1 | 1.107721001 | 0.691737025 | 0.999970678 | 0 | 2.02 | 0.467 | 0.362 | Glyco_transf_20 |
| TRINITY_DN959_c0_g1_i1 | -1.10770181 | 0.525185684 | 0.999970678 | 0.927 | 0.246 | 0.167 | 2.312 | NA |
| TRINITY_DN6868_c0_g1_i1 | -1.10762437 | 0.24274839 | 0.999970678 | 2.898 | 2.947 | 2.978 | 8.978 | - |
| TRINITY_DN367_c0_g1_i5 | -1.10760999 | 0.180414519 | 0.999970678 | 72.008 | 82.88 | 84.639 | 231.506 | Band_7 |
| TRINITY_DN3296_c0_g1_i1 | 1.10758673 | 0.301043566 | 0.999970678 | 97.97 | 30.43 | 18.043 | 36.967 | Ribosomal_L22e |
| TRINITY_DN43615_c0_g1_i1 | -1.10742317 | 0.656440575 | 0.999970678 | 78.858 | 0.246 | 4.755 | 162.438 | CP12 |
| TRINITY_DN29244_c0_g1_i1 | -1.10739727 | 0.381699005 | 0.999970678 | 15.506 | 47.82 | 19.877 | 111.395 | Pkinase |
| TRINITY_DN2865_c0_g1_i2 | -1.10727446 | 0.579370896 | 0.999970678 | 0.109 | 5.302 | 1.633 | 9.501 | NA |
| TRINITY_DN10869_c0_g1_i8 | 1.107252277 | 0.717442017 | 0.999970678 | 0.064 | 1.557 | 0.611 | 0 | Aa_trans |
| TRINITY_DN9229_c0_g2_i4 | 1.107218592 | 0.801495043 | 0.999970678 | 5.523 | 0 | 2.144 | 0 | NA |
| TRINITY_DN713_c0_g1_i1 | 1.1072085 | 0.471266187 | 0.999970678 | 0.318 | 2.05 | 0.411 | 0.593 | NA |
| TRINITY_DN808_c0_g2_i2 | -1.10716922 | 0.141901119 | 0.999970678 | 2.752 | 3.095 | 4.366 | 7.269 | - |
| TRINITY_DN1282_c0_g1_i4 | -1.10714191 | 0.520957716 | 0.999970678 | 13.281 | 2.198 | 25.854 | 2.373 | Ribosomal_L4 |
| TRINITY_DN265_c1_g3_i1 | -1.10684076 | 0.679963192 | 0.999970678 | 9.093 | 0 | 8.722 | 9.259 | NA |
| TRINITY_DN14484_c0_g1_i5 | 1.106829459 | 0.287209046 | 0.999970678 | 13.471 | 17.38 | 9.288 | 3.086 | DCB |
| TRINITY_DN1754_c0_g2_i2 | -1.106786 | 0.682583997 | 1 | 0 | 0.217 | 0.178 | 0.241 | Jas |
| TRINITY_DN23186_c0_g2_i2 | 1.106478263 | 0.454964564 | 0.999970678 | 1.69 | 1.567 | 1.044 | 0.312 | NA |
| TRINITY_DN1632_c0_g1_i11 | 1.106322978 | 0.399200591 | 0.999970678 | 6.649 | 0.986 | 1.444 | 1.85 | NA |
| TRINITY_DN1159_c0_g2_i2 | 1.106145308 | 0.430403406 | 0.999970678 | 1.299 | 9.077 | 1.178 | 3.358 | NA |
| TRINITY_DN1515_c0_g2_i1 | 1.106122913 | 0.406891993 | 0.999970678 | 7.631 | 1.252 | 1.467 | 2.393 | NA |
| TRINITY_DN7252_c0_g1_i3 | 1.106080686 | 0.323273949 | 0.999970678 | 1.308 | 0.788 | 0.522 | 0.352 | RRM_1 |
| TRINITY_DN10646_c0_g1_i1 | -1.10602373 | 0.44794933 | 0.999970678 | 0.709 | 0.276 | 1.389 | 0.452 | p450 |
| TRINITY_DN12731_c1_g2_i1 | -1.10596386 | 0.487372625 | 0.999970678 | 1.154 | 4.642 | 8.622 | 1.86 | NA |
| TRINITY_DN249_c0_g1_i6 | -1.10596133 | 0.360242621 | 0.999970678 | 74.216 | 13.2 | 85.262 | 86.009 | Ribosomal_L5 |
| TRINITY_DN9213_c0_g1_i1 | 1.105952717 | 0.558552887 | 0.999970678 | 39.742 | 16.32 | 19.71 | 1.247 | ABC2_membrane |
| TRINITY_DN284_c0_g3_i1 | -1.10580792 | 0.205030219 | 0.999970678 | 201.37 | 147.3 | 184.7 | 520.279 | NA |
| TRINITY_DN11727_c0_g1_i3 | -1.1054988 | 0.286681374 | 0.999970678 | 2.725 | 1.025 | 2.778 | 4.765 | NA |
| TRINITY_DN928_c0_g1_i2 | -1.10549264 | 0.793316731 | 0.999970678 | 3.216 | 0 | 5.8 | 0 | NA |
| TRINITY_DN21218_c0_g1_i1 | 1.105474407 | 0.676701818 | 0.999970678 | 1.108 | 0.631 | 0.678 | 0 | RTT107_BRCT_5 |
| TRINITY_DN155_c0_g1_i6 | 1.105131972 | 0.680276093 | 0.999970678 | 4.705 | 11.8 | 0 | 7.601 | Ank |
| TRINITY_DN718_c0_g1_i1 | 1.105039986 | 0.120713358 | 0.999970678 | 5.696 | 4.238 | 1.978 | 2.242 | UBA |
| TRINITY_DN50368_c0_g1_i2 | -1.10501978 | 0.793498749 | 0.999970678 | 1.045 | 0 | 0 | 2.302 | 2-Hacid_dh_C |
| TRINITY_DN3712_c0_g1_i1 | 1.105011569 | 0.291294063 | 0.999970678 | 2.089 | 5.401 | 2.044 | 0.995 | zf-UBR |
| TRINITY_DN16529_c1_g2_i3 | 1.104690969 | 0.294561519 | 0.999970678 | 1.935 | 1.853 | 0.867 | 0.724 | NA |
| TRINITY_DN5307_c0_g1_i1 | -1.10466556 | 0.286965677 | 0.999970678 | 0.645 | 1.38 | 2.489 | 1.357 | LRRNT_2 |
| TRINITY_DN8461_c0_g3_i2 | -1.10460339 | 0.545583373 | 0.999970678 | 4.433 | 0.404 | 1.622 | 8.355 | NA |
| TRINITY_DN215_c0_g1_i1 | 1.104499181 | 0.370415124 | 0.999970678 | 16.814 | 4.662 | 2.778 | 6.575 | NA |

| TRINITY_DN85038_c0_g1_i4 | -1.10449489 | 0.702615496 | 0.999970678 | 68.62 | 0 | 26.743 | 115.547 | NA |
| --- | --- | --- | --- | --- | --- | --- | --- | --- |
| TRINITY_DN131_c0_g1_i2 | 1.104254762 | 0.216119945 | 0.999970678 | 15.079 | 8.249 | 6.011 | 3.649 | NA |
| TRINITY_DN39023_c0_g1_i1 | -1.10416858 | 0.3589534 | 0.999970678 | 0.327 | 0.286 | 0.833 | 0.312 | QLQ |
| TRINITY_DN4226_c0_g1_i5 | -1.10413572 | 0.411691958 | 0.999970678 | 4.624 | 0.71 | 4.266 | 6.394 | NA |
| TRINITY_DN4283_c3_g1_i3 | -1.10403543 | 0.689538681 | 0.999970678 | 4.188 | 0.039 | 7.622 | 0.04 | NA |
| TRINITY_DN649_c1_g1_i4 | -1.10357827 | 0.392814901 | 0.999970678 | 3.043 | 0.788 | 2.567 | 5.268 | NA |
| TRINITY_DN14109_c0_g1_i3 | 1.103576115 | 0.477611316 | 0.999970678 | 0.318 | 0.522 | 0.044 | 0.332 | NA |
| TRINITY_DN2684_c0_g1_i2 | -1.10349985 | 0.680730372 | 0.999970678 | 4.469 | 0 | 2.667 | 6.454 | NA |
| TRINITY_DN2443_c0_g1_i6 | 1.103497531 | 0.598340233 | 0.999970678 | 0.409 | 3.499 | 1.389 | 0.151 | NA |
| TRINITY_DN7646_c0_g1_i2 | -1.10349271 | 0.727409955 | 1 | 3.043 | 0 | 5.166 | 0.342 | NA |
| TRINITY_DN372_c0_g1_i15 | -1.10333298 | 0.690351037 | 0.999970678 | 1.045 | 0 | 0.456 | 1.709 | NA |
| TRINITY_DN6477_c0_g1_i3 | -1.10329678 | 0.486101426 | 0.999970678 | 0.427 | 1.37 | 2.478 | 0.774 | NA |
| TRINITY_DN441_c0_g1_i1 | -1.10323374 | 0.726065018 | 0.999970678 | 0.154 | 6.8 | 0 | 14.709 | NA |
| TRINITY_DN11731_c0_g1_i21 | -1.10316449 | 0.271360092 | 0.999970678 | 0.472 | 0.719 | 0.989 | 1.357 | NA |
| TRINITY_DN331_c0_g1_i4 | -1.10308886 | 0.486030251 | 0.999970678 | 11.982 | 0.779 | 6.922 | 19.504 | NA |
| TRINITY_DN15576_c0_g1_i17 | -1.10305448 | 0.726771227 | 0.999970678 | 2.907 | 0 | 5.089 | 0.181 | Ribosomal_L5 |
| TRINITY_DN37342_c0_g1_i1 | 1.10290976 | 0.706169782 | 0.999970678 | 0.118 | 1.971 | 0 | 0.955 | zf-MYND |
| TRINITY_DN7776_c0_g1_i4 | -1.10271305 | 0.389787345 | 0.999970678 | 31.567 | 10.96 | 62.407 | 16.126 | NA |
| TRINITY_DN5126_c1_g1_i4 | 1.10266223 | 0.696065233 | 0.999970678 | 3.216 | 0 | 0.4 | 1.025 | OPT |
| TRINITY_DN37802_c0_g1_i1 | 1.102580173 | 0.462453797 | 1 | 0.645 | 0.414 | 0.333 | 0.111 | NA |
| TRINITY_DN3827_c0_g1_i1 | -1.10252108 | 0.803857979 | 1 | 1.026 | 0 | 1.833 | 0 | NA |
| TRINITY_DN31862_c0_g1_i1 | -1.10252108 | 0.803857979 | 1 | 1.354 | 0 | 2.411 | 0 | NA |
| TRINITY_DN6693_c0_g1_i8 | -1.10252108 | 0.803857979 | 1 | 2.026 | 0 | 3.655 | 0 | NA |
| TRINITY_DN1239_c0_g1_i1 | -1.10247667 | 0.797649317 | 0.999970678 | 3.534 | 0 | 6.333 | 0 | NA |
| TRINITY_DN2052_c0_g1_i11 | 1.102457154 | 0.699529219 | 0.999970678 | 3.697 | 0 | 0.344 | 1.337 | NA |
| TRINITY_DN5510_c0_g1_i1 | 1.102454817 | 0.79285083 | 0.999970678 | 0 | 3.144 | 1.189 | 0 | HEAT |
| TRINITY_DN2558_c0_g1_i6 | -1.10245449 | 0.794755072 | 0.999970678 | 3.661 | 0 | 6.622 | 0 | Abhydrolase_1 |
| TRINITY_DN9231_c0_g1_i2 | 1.102395553 | 0.709268876 | 0.999970678 | 0 | 1.597 | 0.522 | 0.111 | NA |
| TRINITY_DN10112_c0_g1_i2 | 1.102258194 | 0.67118262 | 0.999970678 | 2.925 | 2.277 | 0 | 2.443 | NA |
| TRINITY_DN97_c0_g1_i5 | -1.10223614 | 0.454589439 | 0.999970678 | 41.159 | 34.69 | 123.248 | 11.984 | NA |
| TRINITY_DN13470_c0_g2_i1 | 1.102216095 | 0.675037618 | 0.999970678 | 2.017 | 1.853 | 1.489 | 0 | zf-C3HC4_3 |
| TRINITY_DN4730_c1_g1_i1 | -1.10218422 | 0.408578774 | 0.999970678 | 13.853 | 2.927 | 23.454 | 8.033 | Glyco_hydro_1 |
| TRINITY_DN9247_c0_g1_i5 | 1.102113437 | 0.606139396 | 0.999970678 | 1.272 | 20.25 | 7.622 | 0.593 | NA |
| TRINITY_DN612_c0_g1_i11 | 1.102093791 | 0.578465921 | 0.999970678 | 1.235 | 0.296 | 0.567 | 0.03 | - |
| TRINITY_DN5651_c0_g1_i4 | 1.101206049 | 0.471942832 | 0.999970678 | 4.515 | 0.631 | 0.5 | 1.84 | GDP_Man_Dehyd |
| TRINITY_DN13_c2_g1_i2 | -1.10112059 | 0.707943678 | 0.999970678 | 2.071 | 0.394 | 0 | 5.278 | NA |
| TRINITY_DN378_c0_g1_i4 | 1.101082356 | 0.796208735 | 0.999970678 | 140.6 | 0 | 54.041 | 0 | NA |
| TRINITY_DN2913_c0_g1_i1 | -1.10106911 | 0.68519712 | 0.999970678 | 2.434 | 0 | 3.078 | 1.478 | NA |
| TRINITY_DN1258_c0_g1_i13 | -1.10090028 | 0.503662178 | 0.999970678 | 5.95 | 0.315 | 3.722 | 9.129 | - |
| TRINITY_DN4466_c0_g1_i1 | -1.10072824 | 0.795320578 | 0.999970678 | 7.612 | 0 | 13.666 | 0 | Peroxidase_2 |
| TRINITY_DN9323_c0_g2_i7 | -1.10066547 | 0.806319213 | 1 | 0 | 3.686 | 6.377 | 0 | LRRNT_2 |
| TRINITY_DN2687_c0_g1_i1 | 1.100648574 | 0.70638102 | 0.999970678 | 4.969 | 0.493 | 2.155 | 0 | Bac_rhodopsin |
| TRINITY_DN2928_c0_g1_i1 | -1.10057434 | 0.541096115 | 0.999970678 | 6.232 | 0.384 | 4.166 | 9.089 | NB-ARC |
| TRINITY_DN6266_c0_g1_i2 | 1.10056207 | 0.100442964 | 0.999970678 | 8.548 | 11.95 | 3.666 | 5.117 | NA |
| TRINITY_DN668_c1_g1_i1 | -1.10042094 | 0.23022389 | 0.999970678 | 4.66 | 3.144 | 4.911 | 10.757 | CYTH |
| TRINITY_DN1680_c0_g2_i3 | 1.100319346 | 0.457673705 | 0.999970678 | 1.354 | 4.484 | 1.8 | 0.553 | - |
| TRINITY_DN5643_c0_g1_i2 | -1.10026368 | 0.330892322 | 0.999970678 | 1.562 | 2.957 | 1.733 | 7.58 | NA |
| TRINITY_DN2820_c1_g2_i4 | 1.10015488 | 0.703384011 | 0.999970678 | 25.753 | 0 | 1.967 | 9.712 | NA |
| TRINITY_DN7977_c0_g1_i4 | 1.099968772 | 0.671018373 | 0.999970678 | 1.626 | 1.616 | 0 | 1.518 | Glycos_transf_1 |
| TRINITY_DN24395_c0_g1_i1 | -1.09994635 | 0.398358554 | 0.999970678 | 0.727 | 0.404 | 0.411 | 1.93 | NA |
| TRINITY_DN13722_c0_g2_i2 | 1.099745654 | 0.499142922 | 0.999970678 | 2.235 | 2.74 | 1.833 | 0.101 | NA |
| TRINITY_DN1836_c0_g1_i15 | -1.09947862 | 0.29370461 | 0.999970678 | 3.961 | 1.537 | 3.811 | 7.219 | Maf1 |
| TRINITY_DN1341_c0_g1_i3 | -1.09947257 | 0.798720564 | 0.999970678 | 0.527 | 0 | 0 | 1.136 | Hydrolase_6 |
| TRINITY_DN17835_c0_g1_i1 | 1.098902691 | 0.436055324 | 0.999970678 | 3.116 | 1.399 | 0.311 | 1.739 | NA |
| TRINITY_DN234_c2_g1_i5 | 1.098871961 | 0.447424532 | 0.999970678 | 0.609 | 4.464 | 1.022 | 1.096 | NA |
| TRINITY_DN4098_c0_g1_i4 | -1.09874771 | 0.695962267 | 1 | 0 | 0.404 | 0.178 | 0.633 | NA |
| TRINITY_DN15245_c0_g1_i9 | 1.098602369 | 0.295563365 | 0.999970678 | 8.866 | 11.86 | 2.989 | 5.741 | NA |
| TRINITY_DN4713_c0_g1_i2 | -1.09849861 | 0.156014236 | 0.999970678 | 23.873 | 19.68 | 50.075 | 32.554 | NA |
| TRINITY_DN4317_c0_g1_i1 | -1.09845386 | 0.426267519 | 0.999970678 | 7.004 | 1.291 | 9.366 | 6.384 | NA |
| TRINITY_DN9986_c0_g1_i3 | -1.09841511 | 0.714114185 | 0.999970678 | 31.085 | 0 | 3.566 | 62.745 | DUF1685 |
| TRINITY_DN4663_c1_g3_i3 | -1.09825099 | 0.657727474 | 0.999970678 | 4.206 | 0.286 | 7.955 | 0.101 | NA |
| TRINITY_DN5924_c0_g1_i2 | 1.098094583 | 0.799037539 | 0.999970678 | 0 | 1.882 | 0.711 | 0 | NA |
| TRINITY_DN216_c0_g1_i3 | 1.09809438 | 0.499891003 | 0.999970678 | 1.526 | 24.91 | 2.778 | 8.807 | NA |
| TRINITY_DN2637_c0_g1_i9 | 1.097909654 | 0.299557341 | 0.999970678 | 7.912 | 9.195 | 1.544 | 6.113 | Translin |
| TRINITY_DN1643_c0_g2_i5 | 1.097818447 | 0.350668508 | 0.999970678 | 2.153 | 1.537 | 1.144 | 0.352 | NA |
| TRINITY_DN7376_c0_g1_i3 | -1.09781836 | 0.535144524 | 0.999970678 | 0.545 | 3.164 | 0.422 | 7.389 | Tetraspannin |
| TRINITY_DN3260_c0_g1_i8 | -1.09770278 | 0.280496186 | 0.999970678 | 2.362 | 3.449 | 2.622 | 9.27 | Guanylate_kin |
| TRINITY_DN7766_c0_g1_i5 | 1.097610382 | 0.694608552 | 0.999970678 | 0 | 6.642 | 1.644 | 1.076 | NA |
| TRINITY_DN993_c0_g2_i11 | -1.09758929 | 0.74367846 | 0.999970678 | 5.36 | 0 | 9.555 | 0.04 | 2-Hacid_dh |
| TRINITY_DN4928_c0_g1_i4 | 1.097484005 | 0.398100003 | 0.999970678 | 3.952 | 2.05 | 0.689 | 2.031 | NA |
| TRINITY_DN7432_c0_g1_i2 | -1.09743105 | 0.469970977 | 0.999970678 | 0.327 | 0.68 | 0.256 | 1.82 | NA |
| TRINITY_DN10671_c1_g2_i2 | 1.097428744 | 0.524859811 | 0.999970678 | 10.519 | 5.411 | 5.977 | 0.251 | NA |
| TRINITY_DN12930_c0_g1_i3 | 1.097230482 | 0.363419717 | 0.999970678 | 147.82 | 37.83 | 51.975 | 23.707 | NA |
| TRINITY_DN11854_c0_g1_i1 | -1.09705257 | 0.182775498 | 0.999970678 | 1.853 | 2.897 | 4 | 5.278 | NA |

| TRINITY_DN10502_c0_g1_i2 | -1.09691137 | 0.116093304 | 0.999970678 | 23.228 | 22.28 | 30.198 | 61.167 | NA |
| --- | --- | --- | --- | --- | --- | --- | --- | --- |
| TRINITY_DN5095_c0_g1_i4 | 1.096876756 | 0.695155993 | 0.999970678 | 0 | 3.686 | 0.911 | 0.603 | Utp12 |
| TRINITY_DN5734_c0_g1_i2 | -1.09685547 | 0.68926573 | 0.999970678 | 2.998 | 0 | 4.311 | 1.257 | PMEI |
| TRINITY_DN73279_c0_g2_i1 | -1.09680982 | 0.805471811 | 1 | 0.745 | 0 | 1.333 | 0 | NA |
| TRINITY_DN40366_c0_g1_i5 | -1.09680982 | 0.805471811 | 1 | 2.207 | 0 | 3.922 | 0 | NA |
| TRINITY_DN5637_c2_g1_i1 | 1.096767915 | 0.299721807 | 0.999970678 | 1.544 | 3.41 | 1.2 | 0.885 | NA |
| TRINITY_DN34041_c0_g1_i5 | -1.09619621 | 0.79613197 | 0.999970678 | 1.78 | 0 | 3.189 | 0 | - |
| TRINITY_DN22683_c0_g1_i3 | 1.096080435 | 0.14634718 | 0.999970678 | 8.412 | 6.357 | 3.111 | 3.167 | NA |
| TRINITY_DN8404_c0_g1_i5 | -1.09599211 | 0.727910917 | 0.999970678 | 9.456 | 0.315 | 0 | 20.298 | LRRNT_2 |
| TRINITY_DN1254_c0_g1_i7 | -1.09585531 | 0.447087295 | 0.999970678 | 1.835 | 12.55 | 8.188 | 19.796 | NA |
| TRINITY_DN7100_c0_g1_i1 | -1.09572263 | 0.688283642 | 0.999970678 | 0 | 5.351 | 6.133 | 3.841 | NA |
| TRINITY_DN5769_c0_g3_i1 | 1.095672437 | 0.68698038 | 0.999970678 | 2.316 | 0.621 | 1.144 | 0 | NA |
| TRINITY_DN234_c0_g1_i2 | 1.095578933 | 0.369671627 | 0.999970678 | 2.916 | 13.89 | 3.889 | 3.046 | NA |
| TRINITY_DN2915_c2_g1_i3 | -1.09557407 | 0.690895718 | 0.999970678 | 0 | 1.774 | 2.533 | 0.684 | NA |
| TRINITY_DN4243_c0_g2_i3 | 1.095564138 | 0.713784302 | 0.999970678 | 0 | 0.926 | 0.311 | 0.06 | PPR |
| TRINITY_DN2296_c0_g1_i1 | -1.09550944 | 0.409949868 | 0.999970678 | 18.867 | 75.12 | 28.687 | 164.257 | - |
| TRINITY_DN8171_c0_g2_i15 | 1.095464046 | 0.707096164 | 0.999970678 | 0.563 | 0 | 0.156 | 0.08 | NA |
| TRINITY_DN2656_c0_g1_i7 | 1.095166991 | 0.306752507 | 0.999970678 | 1.653 | 1.498 | 0.856 | 0.462 | Amidohydro_1 |
| TRINITY_DN1277_c1_g1_i1 | -1.09469029 | 0.280977342 | 0.999970678 | 1.871 | 3.4 | 2.933 | 7.641 | NA |
| TRINITY_DN15378_c1_g3_i3 | -1.09467843 | 0.504609812 | 0.999970678 | 6.822 | 0.335 | 6.811 | 7.269 | NA |
| TRINITY_DN2769_c1_g1_i2 | -1.09450896 | 0.686862028 | 0.999970678 | 2.107 | 0 | 2.644 | 1.387 | Cation_efflux |
| TRINITY_DN23667_c0_g1_i1 | -1.09430623 | 0.413668176 | 0.999970678 | 78.603 | 15.98 | 77.595 | 94.575 | NA |
| TRINITY_DN3153_c0_g1_i3 | 1.09426271 | 0.709555387 | 0.999970678 | 46.419 | 0 | 7.244 | 13.301 | NA |
| TRINITY_DN691_c1_g1_i3 | -1.09397445 | 0.128665676 | 0.999970678 | 17.932 | 32.3 | 42.242 | 55.868 | EF-hand_7 |
| TRINITY_DN2340_c0_g2_i14 | 1.093928637 | 0.684803759 | 0.999970678 | 1.072 | 0.463 | 0 | 0.734 | NA |
| TRINITY_DN6590_c0_g1_i10 | 1.093813253 | 0.34888729 | 0.999970678 | 1.917 | 0.818 | 0.7 | 0.452 | BRX |
| TRINITY_DN19051_c0_g3_i3 | -1.09371314 | 0.680270805 | 0.999970678 | 0 | 0.581 | 0.667 | 0.422 | NA |
| TRINITY_DN8434_c0_g1_i6 | -1.09369543 | 0.415524922 | 0.999970678 | 1.126 | 1.242 | 3.266 | 1.025 | NA |
| TRINITY_DN14264_c0_g1_i4 | 1.093543073 | 0.709976228 | 0.999970678 | 17.378 | 0 | 5.511 | 1.588 | NA |
| TRINITY_DN3336_c0_g1_i2 | -1.09326553 | 0.462645412 | 0.999970678 | 0.327 | 0.956 | 0.4 | 2.272 | NA |
| TRINITY_DN1008_c0_g1_i11 | -1.09320344 | 0.19877706 | 0.999970678 | 4.451 | 5.972 | 10.877 | 8.837 | NA |
| TRINITY_DN2276_c0_g1_i2 | -1.09307321 | 0.383082853 | 0.999970678 | 77.159 | 19.62 | 39.698 | 159.602 | CS |
| TRINITY_DN20825_c0_g1_i8 | 1.092994794 | 0.707214955 | 0.999970678 | 1.926 | 0.128 | 0.811 | 0 | B_lectin |
| TRINITY_DN1465_c0_g1_i5 | 1.092936489 | 0.380565495 | 1 | 0.881 | 0.759 | 0.278 | 0.432 | NA |
| TRINITY_DN11608_c0_g2_i2 | -1.0929019 | 0.703241711 | 1 | 2.616 | 0 | 3.566 | 1.196 | NA |
| TRINITY_DN3229_c0_g1_i4 | 1.092893877 | 0.689099987 | 0.999970678 | 2.098 | 0.798 | 1.144 | 0 | NA |
| TRINITY_DN9308_c0_g1_i1 | -1.09272721 | 0.242543785 | 0.999970678 | 1.962 | 1.843 | 1.9 | 5.851 | NA |
| TRINITY_DN11034_c1_g1_i7 | 1.09272204 | 0.795116953 | 0.999970678 | 0 | 2.907 | 1.1 | 0 | NA |
| TRINITY_DN3114_c0_g1_i1 | 1.092644101 | 0.795356042 | 0.999970678 | 0 | 3.656 | 1.4 | 0 | NA |
| TRINITY_DN4500_c0_g1_i2 | -1.09259302 | 0.684961883 | 0.999970678 | 1.126 | 0 | 0.678 | 1.619 | PSS |
| TRINITY_DN25960_c0_g1_i3 | -1.09235502 | 0.800174976 | 0.999970678 | 0.917 | 0 | 1.622 | 0 | NA |
| TRINITY_DN155_c0_g1_i16 | 1.092220606 | 0.333715357 | 0.999970678 | 11.855 | 14.48 | 1.933 | 9.993 | Ank |
| TRINITY_DN4594_c0_g1_i3 | 1.09210944 | 0.431619393 | 0.999970678 | 8.457 | 2.858 | 3.755 | 0.784 | NA |
| TRINITY_DN234_c0_g1_i3 | -1.09207391 | 0.610842959 | 0.999970678 | 1.054 | 0.039 | 0.4 | 1.86 | NA |
| TRINITY_DN1322_c0_g1_i2 | 1.092045868 | 0.317706862 | 0.999970678 | 10.02 | 3.233 | 2.144 | 3.629 | HMA |
| TRINITY_DN4067_c0_g1_i20 | 1.092043145 | 0.105585046 | 0.999970678 | 40.787 | 50.43 | 15.277 | 23.536 | NA |
| TRINITY_DN2537_c0_g2_i1 | 1.092012207 | 0.355576795 | 0.999970678 | 2.389 | 3.035 | 1.6 | 0.603 | OSR1_C |
| TRINITY_DN796_c0_g1_i6 | -1.09188273 | 0.243044728 | 0.999970678 | 19.421 | 19.36 | 18.088 | 60.332 | NA |
| TRINITY_DN2416_c0_g1_i12 | -1.09110459 | 0.68149318 | 0.999970678 | 0.754 | 0 | 0.789 | 0.684 | Pkinase |
| TRINITY_DN46401_c0_g1_i1 | -1.09078048 | 0.322973542 | 0.999970678 | 2.625 | 1.192 | 3.166 | 4.353 | Steroid_dh |
| TRINITY_DN16124_c0_g1_i2 | -1.09076931 | 0.7001602 | 0.999970678 | 0.736 | 1.488 | 0 | 4.755 | NA |
| TRINITY_DN1904_c0_g1_i4 | 1.090765927 | 0.551424506 | 0.999970678 | 0.354 | 10.83 | 0.878 | 4.122 | PMD |
| TRINITY_DN411_c0_g1_i4 | 1.0907214 | 0.243769889 | 0.999970678 | 8.194 | 17.83 | 4.022 | 7.168 | NA |
| TRINITY_DN18568_c0_g1_i2 | -1.0905526 | 0.807254006 | 1 | 0.354 | 0 | 0.633 | 0 | NA |
| TRINITY_DN8422_c0_g1_i1 | -1.09049232 | 0.300050263 | 0.999970678 | 1.998 | 1.409 | 1.667 | 5.208 | ETF |
| TRINITY_DN711_c0_g1_i9 | 1.090347295 | 0.328828401 | 0.999970678 | 14.198 | 65.83 | 16.032 | 17.825 | peroxidase |
| TRINITY_DN2327_c0_g1_i2 | -1.09016038 | 0.257641748 | 0.999970678 | 2.916 | 2.001 | 5.489 | 3.8 | NA |
| TRINITY_DN7966_c0_g1_i1 | -1.09000725 | 0.333144935 | 0.999970678 | 9.184 | 9.727 | 6.289 | 32.483 | NA |
| TRINITY_DN9084_c0_g1_i1 | 1.089988382 | 0.689074615 | 1 | 0.636 | 0.355 | 0.378 | 0 | BT1 |
| TRINITY_DN199_c1_g1_i3 | -1.08994379 | 0.464578618 | 0.999970678 | 3.27 | 2.947 | 1.067 | 11.853 | CGI-121 |
| TRINITY_DN6052_c0_g1_i2 | 1.089939696 | 0.365822741 | 0.999970678 | 0.799 | 1.311 | 0.556 | 0.322 | WAK_assoc |
| TRINITY_DN2666_c0_g1_i6 | 1.089924726 | 0.380016598 | 0.999970678 | 6.377 | 2.976 | 2.955 | 0.845 | NA |
| TRINITY_DN302_c0_g1_i3 | -1.08991237 | 0.498840927 | 0.999970678 | 1.662 | 0.759 | 3.578 | 0.774 | NA |
| TRINITY_DN11955_c0_g1_i9 | 1.089542803 | 0.73035594 | 1 | 0.609 | 0.02 | 0.244 | 0 | AAA_11 |
| TRINITY_DN176_c0_g1_i2 | 1.089529206 | 0.083740167 | 0.999970678 | 30.05 | 25.59 | 12.121 | 11.542 | NA |
| TRINITY_DN703_c0_g1_i11 | 1.089435564 | 0.591566709 | 0.999970678 | 14.87 | 1.124 | 6.055 | 0.261 | NA |
| TRINITY_DN5485_c3_g2_i1 | -1.08937415 | 0.517873079 | 0.999970678 | 1.354 | 27.9 | 38.809 | 14.387 | Cullin |
| TRINITY_DN13659_c0_g1_i3 | -1.08928694 | 0.739544254 | 0.999970678 | 717.85 | 0 | 899.968 | 461.012 | NA |
| TRINITY_DN1655_c0_g1_i11 | -1.08927521 | 0.380032139 | 0.999970678 | 4.978 | 11.01 | 19.777 | 9.008 | NA |
| TRINITY_DN11570_c0_g1_i8 | -1.08902894 | 0.28406649 | 0.999970678 | 1.626 | 0.611 | 2.155 | 2.202 | Kelch_4 |
| TRINITY_DN2850_c0_g1_i8 | -1.088957 | 0.37346746 | 0.999970678 | 1.626 | 0.867 | 0.922 | 4.212 | CAP160 |
| TRINITY_DN8465_c0_g1_i1 | -1.08872352 | 0.681459058 | 0.999970678 | 1.254 | 0 | 1.155 | 1.317 | PPR |
| TRINITY_DN9447_c0_g1_i2 | 1.088591503 | 0.231163436 | 0.999970678 | 3.706 | 2.641 | 1.578 | 1.086 | NA |
| TRINITY_DN19202_c0_g1_i1 | -1.08851772 | 0.683627899 | 0.999970678 | 0.79 | 0 | 0.667 | 0.895 | NA |

| TRINITY_DN7780_c0_g1_i2 | -1.0884713 | 0.366305123 | 0.999970678 | 1.726 | 2.917 | 1.589 | 7.912 | Asp_Glu_race |
| --- | --- | --- | --- | --- | --- | --- | --- | --- |
| TRINITY_DN7645_c0_g1_i1 | -1.0883632 | 0.604259198 | 1 | 0.154 | 1.468 | 0.322 | 2.996 | NA |
| TRINITY_DN20723_c0_g1_i1 | -1.08834548 | 0.682423082 | 0.999970678 | 0 | 56.61 | 46.942 | 57.346 | NA |
| TRINITY_DN3284_c0_g1_i1 | -1.08823936 | 0.485085246 | 0.999970678 | 15.234 | 1.429 | 21.765 | 9.29 | NA |
| TRINITY_DN4825_c0_g1_i5 | -1.08799326 | 0.231825546 | 0.999970678 | 5.469 | 4.021 | 4.9 | 14.337 | NA |
| TRINITY_DN1039_c0_g1_i7 | -1.08791195 | 0.485531653 | 0.999970678 | 0.827 | 3.39 | 6.311 | 1.216 | B_lectin |
| TRINITY_DN6466_c0_g1_i2 | 1.087809188 | 0.400713985 | 0.999970678 | 7.167 | 3.39 | 0.811 | 4.032 | NA |
| TRINITY_DN6338_c0_g1_i2 | 1.087800751 | 0.580330003 | 0.999970678 | 1.326 | 1.528 | 0.033 | 1.327 | NA |
| TRINITY_DN11215_c0_g1_i3 | -1.0877959 | 0.638131599 | 0.999970678 | 0.109 | 0.69 | 0.022 | 1.659 | NA |
| TRINITY_DN4386_c1_g1_i3 | -1.0876902 | 0.298058204 | 0.999970678 | 0.481 | 0.848 | 0.9 | 1.759 | Ketoacyl-synt_C |
| TRINITY_DN3042_c0_g1_i4 | 1.08768679 | 0.450708973 | 0.999970678 | 0.699 | 0.453 | 0.367 | 0.101 | NA |
| TRINITY_DN11427_c0_g1_i9 | -1.08759154 | 0.350785488 | 0.999970678 | 3.043 | 1.528 | 4.933 | 3.569 | - |
| TRINITY_DN1426_c1_g1_i4 | 1.087519482 | 0.510434396 | 0.999970678 | 3.079 | 0.897 | 1.344 | 0.261 | NA |
| TRINITY_DN12039_c0_g1_i2 | -1.0874492 | 0.394386303 | 0.999970678 | 1.272 | 1.735 | 0.856 | 5.318 | p450 |
| TRINITY_DN2347_c0_g1_i4 | 1.087411611 | 0.28247941 | 0.999970678 | 16.869 | 39.1 | 6.144 | 18.841 | NmrA |
| TRINITY_DN4845_c0_g1_i4 | -1.08722681 | 0.30622035 | 0.999970678 | 1.081 | 0.986 | 0.989 | 3.207 | NA |
| TRINITY_DN5855_c1_g1_i9 | 1.087207128 | 0.302403122 | 0.999970678 | 0.645 | 1.183 | 0.267 | 0.543 | NA |
| TRINITY_DN3286_c0_g1_i1 | 1.08712392 | 0.459307925 | 0.999970678 | 9.048 | 18.76 | 0.911 | 11.954 | p450 |
| TRINITY_DN14813_c0_g1_i11 | -1.08711204 | 0.540259044 | 0.999970678 | 8.784 | 0.256 | 10.544 | 6.756 | NA |
| TRINITY_DN4231_c0_g1_i3 | 1.086966492 | 0.456268068 | 0.999970678 | 0.945 | 3.233 | 0.289 | 1.619 | PDZ |
| TRINITY_DN44466_c0_g1_i3 | 1.086965301 | 0.802486697 | 0.999970678 | 1.09 | 0 | 0 | 0.533 | NA |
| TRINITY_DN9182_c1_g1_i1 | -1.08693307 | 0.661200637 | 0.999970678 | 1.871 | 0.059 | 3.378 | 0.06 | NA |
| TRINITY_DN9736_c0_g1_i1 | 1.086878391 | 0.417243876 | 0.999970678 | 1.535 | 0.404 | 0.422 | 0.402 | NA |
| TRINITY_DN1382_c0_g1_i1 | -1.08682001 | 0.702997538 | 0.999970678 | 36.209 | 0 | 36.776 | 33.378 | NA |
| TRINITY_DN99087_c0_g1_i4 | -1.08665373 | 0.561988101 | 0.999970678 | 34.383 | 3.607 | 64.152 | 3.77 | Ribosomal_L30 |
| TRINITY_DN3092_c0_g1_i4 | 1.086528706 | 0.474062263 | 0.999970678 | 0.881 | 0.67 | 0.533 | 0.09 | YTH |
| TRINITY_DN1612_c0_g1_i6 | 1.086212472 | 0.376814239 | 0.999970678 | 1.354 | 0.581 | 0.244 | 0.613 | NA |
| TRINITY_DN5334_c0_g1_i10 | -1.08599693 | 0.709129041 | 0.999970678 | 1.971 | 0.325 | 0 | 4.866 | NA |
| TRINITY_DN1012_c0_g1_i2 | -1.08579353 | 0.356718988 | 0.999970678 | 1.235 | 1.498 | 2.989 | 2.031 | NA |
| TRINITY_DN3286_c0_g1_i5 | -1.08579247 | 0.086032004 | 0.999970678 | 6.068 | 6.554 | 9.611 | 15.181 | p450 |
| TRINITY_DN8584_c0_g1_i1 | 1.085545281 | 0.333229223 | 0.999970678 | 175.93 | 223 | 42.042 | 119.076 | NA |
| TRINITY_DN8438_c0_g1_i1 | 1.085511907 | 0.41153181 | 0.999970678 | 1.199 | 5.618 | 1.955 | 0.824 | NA |
| TRINITY_DN22428_c0_g1_i1 | 1.085490331 | 0.274376181 | 0.999970678 | 15.597 | 6.84 | 3.444 | 6.384 | NA |
| TRINITY_DN10548_c0_g1_i1 | 1.085470909 | 0.79699116 | 0.999970678 | 1.935 | 0 | 0 | 0.935 | NA |
| TRINITY_DN1969_c1_g1_i2 | 1.085415778 | 0.796114211 | 0.999970678 | 0 | 2.681 | 1.033 | 0 | Anticodon_1 |
| TRINITY_DN5676_c0_g1_i8 | 1.085375914 | 0.796746466 | 0.999970678 | 0 | 1.981 | 0 | 0.925 | PPR_long |
| TRINITY_DN39732_c0_g1_i1 | -1.08525499 | 0.809864562 | 1 | 0 | 1.222 | 2.111 | 0 | PsbR |
| TRINITY_DN367_c0_g1_i8 | -1.08520171 | 0.171530005 | 0.999970678 | 15.37 | 10.04 | 26.176 | 21.806 | NA |
| TRINITY_DN10091_c0_g1_i5 | -1.08508975 | 0.728999555 | 0.999970678 | 0.917 | 0 | 1.6 | 0.03 | NA |
| TRINITY_DN21_c0_g1_i14 | -1.08508433 | 0.432617538 | 0.999970678 | 3.179 | 7.007 | 3.644 | 16.237 | NA |
| TRINITY_DN4994_c0_g1_i2 | -1.08505674 | 0.692994172 | 0.999970678 | 0.963 | 0 | 0.544 | 1.377 | ADH_N |
| TRINITY_DN38801_c0_g1_i1 | 1.084595773 | 0.802980649 | 0.999970678 | 2.607 | 0 | 1.033 | 0 | Asp |
| TRINITY_DN2510_c0_g1_i3 | -1.08423201 | 0.374941028 | 0.999970678 | 7.703 | 10.06 | 26.421 | 5.6 | Mito_carr |
| TRINITY_DN1444_c1_g1_i4 | -1.08409833 | 0.536626003 | 0.999970678 | 11.064 | 0.345 | 11.855 | 10.154 | NA |
| TRINITY_DN10094_c0_g1_i1 | -1.08408513 | 0.280789942 | 0.999970678 | 4.769 | 5.608 | 13.888 | 5.188 | NA |
| TRINITY_DN733_c0_g1_i5 | 1.084038896 | 0.285075511 | 0.999970678 | 22.574 | 10.45 | 8.2 | 5.61 | Rhodanese |
| TRINITY_DN2076_c0_g2_i2 | 1.084020064 | 0.451069438 | 0.999970678 | 0.845 | 0.897 | 0.111 | 0.694 | DNA_pol_A_exo1 |
| TRINITY_DN931_c0_g1_i1 | 1.083900777 | 0.52084145 | 0.999970678 | 0.936 | 10.13 | 1.944 | 2.714 | NA |
| TRINITY_DN3307_c0_g1_i9 | -1.08376041 | 0.686526483 | 0.999970678 | 0 | 1.892 | 2.344 | 1.106 | NA |
| TRINITY_DN23793_c0_g1_i1 | 1.083459961 | 0.564534638 | 0.999970678 | 3.352 | 0.384 | 0.089 | 1.679 | Mcl1_mid |
| TRINITY_DN3852_c0_g1_i2 | -1.08344478 | 0.799035442 | 0.999970678 | 0 | 8.298 | 14.299 | 0 | ABC_tran |
| TRINITY_DN6785_c0_g1_i2 | -1.08337552 | 0.610905246 | 1 | 1.208 | 0.108 | 2.089 | 0.282 | NA |
| TRINITY_DN12534_c0_g1_i1 | 1.083304447 | 0.545114968 | 0.999970678 | 11.546 | 1.045 | 1.567 | 3.8 | NA |
| TRINITY_DN155_c2_g1_i1 | 1.082996033 | 0.135478936 | 0.999970678 | 8.821 | 13.62 | 3.844 | 5.891 | PsbP |
| TRINITY_DN2837_c0_g1_i1 | 1.082989362 | 0.802544466 | 0.999970678 | 3.924 | 0 | 0 | 1.86 | zf-RING_2 |
| TRINITY_DN6564_c0_g3_i1 | -1.08265601 | 0.497226906 | 0.999970678 | 0.209 | 2.434 | 1.589 | 3.619 | NA |
| TRINITY_DN11224_c1_g1_i9 | 1.082626371 | 0.352635073 | 1 | 0.618 | 0.611 | 0.278 | 0.282 | SRF-TF |
| TRINITY_DN2509_c0_g1_i3 | -1.08260049 | 0.704333238 | 0.999970678 | 0.3 | 0 | 0.456 | 0.101 | RRM_1 |
| TRINITY_DN2509_c0_g1_i8 | -1.08260049 | 0.704333238 | 0.999970678 | 0.3 | 0 | 0.456 | 0.101 | RRM_1 |
| TRINITY_DN7679_c0_g1_i3 | -1.08259391 | 0.800377082 | 0.999970678 | 0.981 | 0 | 0 | 2.121 | NA |
| TRINITY_DN14626_c0_g1_i2 | 1.082466329 | 0.225836412 | 0.999970678 | 0.954 | 1.005 | 0.322 | 0.543 | NA |
| TRINITY_DN2177_c0_g1_i1 | -1.08238897 | 0.16024892 | 0.999970678 | 4.778 | 5.332 | 10.899 | 8.123 | NA |
| TRINITY_DN12933_c0_g1_i6 | -1.08236499 | 0.690233533 | 0.999970678 | 1.244 | 0 | 1.455 | 0.834 | NA |
| TRINITY_DN1688_c0_g2_i1 | -1.08231446 | 0.710402837 | 0.999970678 | 58.764 | 0 | 37.853 | 80.239 | NA |
| TRINITY_DN11227_c0_g1_i1 | -1.08224015 | 0.807210048 | 1 | 0 | 2.089 | 3.589 | 0 | NA |
| TRINITY_DN1317_c0_g1_i2 | 1.082215672 | 0.253971077 | 0.999970678 | 4.651 | 2.464 | 1.611 | 1.428 | Chal_sti_synt_C |
| TRINITY_DN7554_c0_g1_i5 | 1.082137689 | 0.347949371 | 0.999970678 | 0.899 | 1.577 | 0.333 | 0.744 | Exostosin |
| TRINITY_DN4691_c0_g1_i4 | 1.082038315 | 0.326335206 | 0.999970678 | 0.454 | 0.808 | 0.278 | 0.261 | FAD_binding_2 |
| TRINITY_DN665_c0_g1_i18 | 1.081992753 | 0.703271357 | 0.999970678 | 0 | 7.303 | 1.8 | 1.216 | LRR_8 |
| TRINITY_DN6202_c0_g1_i2 | -1.08192477 | 0.282190308 | 0.999970678 | 1.344 | 0.779 | 2.622 | 1.367 | NA |
| TRINITY_DN20407_c0_g2_i2 | -1.08187874 | 0.498040705 | 0.999970678 | 2.843 | 0.936 | 1.089 | 6.575 | NA |
| TRINITY_DN2334_c0_g1_i1 | -1.08184985 | 0.278482783 | 0.999970678 | 4.805 | 3.321 | 10.744 | 4.293 | NA |
| TRINITY_DN9528_c0_g1_i1 | -1.08173633 | 0.240107792 | 0.999970678 | 4.669 | 8.653 | 8.277 | 17.855 | NA |
| TRINITY_DN4352_c0_g1_i5 | -1.0817004 | 0.699304888 | 0.999970678 | 0 | 6.642 | 9.711 | 2.101 | NA |

| TRINITY_DN2179_c0_g1_i2 | -1.08165534 | 0.357324421 | 0.999970678 | 0.763 | 0.355 | 1.3 | 0.774 | NA |
| --- | --- | --- | --- | --- | --- | --- | --- | --- |
| TRINITY_DN14720_c0_g1_i1 | -1.08142597 | 0.170981555 | 0.999970678 | 2.97 | 2.277 | 4.677 | 5.429 | NA |
| TRINITY_DN686_c0_g1_i9 | 1.08141773 | 0.802128638 | 0.999970678 | 0 | 2.454 | 0.955 | 0 | NA |
| TRINITY_DN4672_c0_g1_i1 | 1.08135381 | 0.39437248 | 0.999970678 | 4.015 | 6.702 | 3.233 | 1.116 | LRR_8 |
| TRINITY_DN2069_c0_g1_i3 | -1.08118138 | 0.293525966 | 0.999970678 | 39.815 | 27.07 | 27.054 | 108.499 | NA |
| TRINITY_DN1153_c0_g1_i8 | -1.08107917 | 0.564700631 | 0.999970678 | 0.182 | 2.286 | 3.7 | 0.744 | NA |
| TRINITY_DN7143_c0_g1_i2 | -1.08103859 | 0.796837482 | 0.999970678 | 0.781 | 0 | 0 | 1.699 | NA |
| TRINITY_DN2963_c0_g1_i6 | 1.08102006 | 0.42438104 | 0.999970678 | 1.244 | 1.873 | 1.022 | 0.231 | NA |
| TRINITY_DN22462_c0_g1_i3 | 1.080995171 | 0.693913903 | 1 | 0.382 | 0.926 | 0.5 | 0 | DA1-like |
| TRINITY_DN51475_c0_g1_i2 | 1.080978477 | 0.807485907 | 1 | 5.959 | 0 | 2.333 | 0 | NA |
| TRINITY_DN1665_c0_g1_i2 | 1.080747847 | 0.24268477 | 0.999970678 | 13.362 | 10.14 | 2.589 | 8.043 | MFS_1 |
| TRINITY_DN1441_c0_g1_i2 | 1.080741616 | 0.505617639 | 0.999970678 | 0.327 | 2.188 | 0.322 | 0.784 | NA |
| TRINITY_DN8881_c0_g1_i1 | -1.08067369 | 0.461801775 | 0.999970678 | 1.163 | 3.38 | 0.955 | 8.345 | NA |
| TRINITY_DN289_c0_g1_i6 | -1.08032018 | 0.681148343 | 0.999970678 | 0 | 1.537 | 1.355 | 1.588 | Meth_synt_1 |
| TRINITY_DN1135_c0_g1_i6 | -1.08027392 | 0.578474258 | 0.999970678 | 3.397 | 0.227 | 5.422 | 1.196 | NA |
| TRINITY_DN16_c0_g1_i4 | 1.080256841 | 0.558306292 | 0.999970678 | 0.572 | 1.104 | 0.622 | 0.05 | NA |
| TRINITY_DN193_c0_g1_i4 | 1.080242905 | 0.585781917 | 0.999970678 | 14.126 | 1.991 | 0.211 | 7.42 | NA |
| TRINITY_DN9743_c0_g1_i2 | 1.080235341 | 0.298004332 | 0.999970678 | 1.008 | 0.788 | 0.467 | 0.292 | Myb_DNA-bindin |
| TRINITY_DN9605_c1_g1_i2 | -1.08013045 | 0.333497398 | 0.999970678 | 3.979 | 14.66 | 10.61 | 26.26 | PTR2 |
| TRINITY_DN1316_c0_g1_i10 | -1.07995511 | 0.412696084 | 0.999970678 | 14.062 | 2.355 | 9.577 | 23.274 | LRRNT_2 |
| TRINITY_DN16459_c0_g1_i1 | 1.079907151 | 0.798501572 | 0.999970678 | 9.529 | 0 | 3.778 | 0 | NA |
| TRINITY_DN8281_c0_g2_i1 | -1.07982687 | 0.457100884 | 0.999970678 | 0.99 | 0.631 | 0.4 | 2.946 | NA |
| TRINITY_DN5117_c0_g1_i3 | -1.07978992 | 0.709779598 | 0.999970678 | 0 | 2.148 | 3.444 | 0.312 | DEAD |
| TRINITY_DN7118_c0_g1_i1 | -1.07974229 | 0.34873879 | 0.999970678 | 5.096 | 2.671 | 3.311 | 12.406 | NA |
| TRINITY_DN100316_c0_g1_i1 | 1.079591367 | 0.809113884 | 1 | 1.226 | 0 | 0.489 | 0 | NA |
| TRINITY_DN5613_c0_g2_i1 | -1.0794716 | 0.160183664 | 0.999970678 | 35.591 | 39.38 | 86.684 | 53.546 | WD40 |
| TRINITY_DN15744_c0_g1_i4 | 1.079297644 | 0.686833577 | 0.999970678 | 0.554 | 0.788 | 0.522 | 0 | ERCC4 |
| TRINITY_DN2537_c1_g1_i1 | -1.07927974 | 0.352398027 | 0.999970678 | 0.509 | 1.291 | 1.1 | 2.443 | NA |
| TRINITY_DN8819_c1_g1_i4 | 1.079203702 | 0.699575056 | 0.999970678 | 0.082 | 0.434 | 0.2 | 0 | NA |
| TRINITY_DN2260_c0_g1_i1 | 1.079105409 | 0.436654689 | 0.999970678 | 4.297 | 12.79 | 2.2 | 5.047 | NA |
| TRINITY_DN78564_c0_g1_i6 | -1.07882038 | 0.698389417 | 1 | 2.525 | 0 | 1.822 | 3.026 | NA |
| TRINITY_DN644_c0_g1_i1 | -1.07875359 | 0.362685566 | 0.999970678 | 42.74 | 143.2 | 76.062 | 291.758 | SQAPI |
| TRINITY_DN11692_c0_g1_i6 | 1.078617004 | 0.421966504 | 0.999970678 | 4.042 | 1.882 | 2.011 | 0.392 | DEAD |
| TRINITY_DN4891_c0_g1_i1 | 1.078564903 | 0.728225655 | 0.999970678 | 0.2 | 11.42 | 4.489 | 0 | NA |
| TRINITY_DN1943_c0_g1_i6 | 1.078529877 | 0.341317291 | 0.999970678 | 11.182 | 5.834 | 5.066 | 1.991 | NA |
| TRINITY_DN4033_c1_g1_i1 | 1.078512801 | 0.44930037 | 0.999970678 | 64.823 | 7.076 | 19.465 | 10.908 | NAF |
| TRINITY_DN3193_c0_g1_i2 | -1.07842264 | 0.697127294 | 0.999970678 | 13.399 | 0 | 6.877 | 20.379 | Abi |
| TRINITY_DN6976_c0_g1_i2 | 1.078175786 | 0.37288946 | 0.999970678 | 1.235 | 2.267 | 0.444 | 1.096 | NA |
| TRINITY_DN1443_c0_g1_i1 | -1.07812186 | 0.742284371 | 0.999970678 | 0 | 2.937 | 0.022 | 6.113 | Tcp11 |
| TRINITY_DN935_c0_g1_i2 | 1.078050128 | 0.198024866 | 0.999970678 | 28.233 | 65.85 | 19.988 | 20.208 | N_BRCA1_IG |
| TRINITY_DN6574_c0_g1_i1 | 1.078047678 | 0.691112688 | 0.999970678 | 7.712 | 2.198 | 0 | 4.745 | zf-C3HC4_2 |
| TRINITY_DN21072_c0_g1_i3 | -1.07783927 | 0.330263096 | 0.999970678 | 0.99 | 1.005 | 2.389 | 1.267 | Ribosomal_L14 |
| TRINITY_DN14248_c0_g1_i9 | -1.07770995 | 0.685557641 | 0.999970678 | 2.807 | 0 | 3.022 | 2.353 | NA |
| TRINITY_DN10303_c0_g1_i1 | -1.07759068 | 0.566836228 | 1 | 0.609 | 0.079 | 0.233 | 1.196 | EGF |
| TRINITY_DN9174_c0_g2_i1 | -1.07732721 | 0.685380269 | 0.999970678 | 8.93 | 0 | 7.677 | 9.732 | NA |
| TRINITY_DN450_c0_g1_i2 | 1.077204013 | 0.246215738 | 0.999970678 | 5.414 | 3.085 | 2.167 | 1.438 | RCC1 |
| TRINITY_DN20302_c0_g1_i6 | -1.07705965 | 0.68480006 | 0.999970678 | 0 | 0.621 | 0.467 | 0.714 | NA |
| TRINITY_DN7806_c0_g1_i1 | -1.07670163 | 0.513738409 | 0.999970678 | 2.044 | 1.025 | 4.844 | 0.613 | NA |
| TRINITY_DN3721_c0_g1_i6 | 1.076617603 | 0.455154897 | 0.999970678 | 5.659 | 2.464 | 2.844 | 0.452 | NA |
| TRINITY_DN20558_c0_g1_i1 | 1.076616847 | 0.472042372 | 0.999970678 | 0.391 | 1.281 | 0.078 | 0.694 | DUF3883 |
| TRINITY_DN3710_c0_g1_i2 | 1.076465331 | 0.339598183 | 0.999970678 | 0.481 | 0.848 | 0.189 | 0.402 | CBS |
| TRINITY_DN56812_c0_g2_i1 | 1.076364336 | 0.434362004 | 0.999970678 | 1.135 | 0.818 | 0.178 | 0.704 | NA |
| TRINITY_DN2634_c1_g1_i2 | -1.07633689 | 0.199661144 | 0.999970678 | 15.088 | 22.47 | 21.454 | 52.822 | DUF179 |
| TRINITY_DN1920_c0_g1_i2 | 1.076336841 | 0.392134289 | 0.999970678 | 20.875 | 7.303 | 4.2 | 7.782 | - |
| TRINITY_DN16485_c0_g1_i2 | 1.07623757 | 0.473081983 | 1 | 0.672 | 0.798 | 0.1 | 0.553 | NA |
| TRINITY_DN2895_c0_g1_i14 | -1.07621153 | 0.48059382 | 0.999970678 | 0.082 | 0.562 | 0.511 | 0.744 | PP2C |
| TRINITY_DN800_c0_g1_i15 | -1.07605953 | 0.561813927 | 0.999970678 | 0.245 | 5.263 | 3.489 | 6.997 | NA |
| TRINITY_DN14212_c0_g2_i1 | -1.07605663 | 0.811287488 | 1 | 1.889 | 0 | 3.311 | 0 | NA |
| TRINITY_DN1436_c0_g1_i11 | -1.07603544 | 0.808139496 | 1 | 28.742 | 0 | 49.097 | 0 | NA |
| TRINITY_DN19490_c0_g1_i1 | -1.07603544 | 0.808139496 | 1 | 0.972 | 0 | 1.711 | 0 | NA |
| TRINITY_DN18731_c0_g1_i1 | -1.07598975 | 0.801228897 | 0.999970678 | 1.335 | 0 | 2.355 | 0 | - |
| TRINITY_DN453_c0_g2_i1 | 1.075989585 | 0.685255424 | 0.999970678 | 0.781 | 1.005 | 0 | 0.834 | Ribosomal_S17e |
| TRINITY_DN2824_c0_g1_i6 | -1.07590005 | 0.696183419 | 0.999970678 | 1.326 | 0 | 0.556 | 2.172 | NA |
| TRINITY_DN4853_c0_g1_i4 | -1.07580601 | 0.744902708 | 1 | 7.631 | 0.315 | 13.844 | 0 | NA |
| TRINITY_DN6967_c0_g2_i4 | 1.075801087 | 0.699278887 | 0.999970678 | 1.599 | 0.286 | 0.756 | 0 | NA |
| TRINITY_DN6906_c0_g1_i7 | -1.0757768 | 0.225372444 | 0.999970678 | 1.98 | 0.907 | 2.422 | 3.197 | NA |
| TRINITY_DN3915_c0_g1_i9 | -1.07556373 | 0.688514416 | 0.999970678 | 4.424 | 0 | 5.277 | 3.006 | TRAPP |
| TRINITY_DN7515_c1_g1_i2 | -1.07544298 | 0.687446969 | 0.999970678 | 0.654 | 0 | 0.689 | 0.573 | PPR |
| TRINITY_DN934_c0_g1_i9 | -1.07536324 | 0.454916782 | 0.999970678 | 2.625 | 1.685 | 0.756 | 8.214 | NA |
| TRINITY_DN6451_c0_g1_i2 | 1.075298923 | 0.629169115 | 0.999970678 | 0.027 | 3.853 | 0.911 | 0.684 | NA |
| TRINITY_DN4839_c0_g1_i2 | -1.07520729 | 0.43748736 | 0.999970678 | 1.644 | 11.02 | 5.544 | 19.715 | NA |
| TRINITY_DN315_c0_g1_i4 | -1.07520191 | 0.364027931 | 0.999970678 | 14.616 | 2.976 | 11.822 | 23.304 | MA3 |
| TRINITY_DN6204_c0_g1_i6 | -1.0751513 | 0.475470807 | 0.999970678 | 0.227 | 0.808 | 0.389 | 1.719 | WLM |
| TRINITY_DN301_c0_g1_i2 | 1.075108744 | 0.402917799 | 0.999970678 | 0.99 | 3.124 | 1.133 | 0.563 | NA |

| TRINITY_DN1126_c2_g1_i5 | 1.075003135 | 0.801079926 | 0.999970678 | 7.04 | 0 | 2.8 | 0 | NA |
| --- | --- | --- | --- | --- | --- | --- | --- | --- |
| TRINITY_DN4354_c0_g2_i2 | 1.074946503 | 0.685371168 | 0.999970678 | 2.643 | 1.429 | 1.6 | 0 | NA |
| TRINITY_DN79_c0_g1_i16 | -1.07493828 | 0.807563357 | 1 | 0 | 0.138 | 0.244 | 0 | NA |
| TRINITY_DN16688_c0_g1_i1 | -1.07489883 | 0.709455034 | 1 | 2.017 | 0 | 0.556 | 3.589 | NA |
| TRINITY_DN8672_c0_g1_i2 | 1.074436404 | 0.429359732 | 0.999970678 | 1.217 | 0.453 | 0.189 | 0.583 | NA |
| TRINITY_DN30807_c0_g1_i2 | 1.074352641 | 0.432463382 | 0.999970678 | 11.155 | 28.18 | 2.011 | 16.046 | NA |
| TRINITY_DN1701_c2_g1_i1 | -1.07430979 | 0.697831988 | 1 | 0.709 | 0 | 0.822 | 0.523 | NA |
| TRINITY_DN3250_c0_g1_i3 | -1.07426298 | 0.80427548 | 0.999970678 | 0 | 73.9 | 0 | 150.142 | NA |
| TRINITY_DN1721_c0_g1_i4 | 1.07414618 | 0.708392801 | 0.999970678 | 0.699 | 0.108 | 0.322 | 0 | Fasciclin |
| TRINITY_DN79583_c0_g1_i1 | 1.074114208 | 0.801543684 | 0.999970678 | 1.971 | 0 | 0.789 | 0 | Glyco_hydro_71 |
| TRINITY_DN2390_c0_g1_i5 | 1.073970502 | 0.518250384 | 0.999970678 | 0.509 | 6.229 | 0.489 | 2.574 | NA |
| TRINITY_DN6185_c0_g1_i2 | -1.07396593 | 0.699354607 | 0.999970678 | 0.509 | 0.631 | 1.978 | 0 | PCI |
| TRINITY_DN9487_c0_g1_i3 | 1.073882063 | 0.084161711 | 0.999970678 | 13.272 | 11.33 | 5.166 | 5.479 | MOSC |
| TRINITY_DN9487_c0_g1_i2 | 1.073882063 | 0.084161711 | 0.999970678 | 13.272 | 11.33 | 5.166 | 5.479 | MOSC |
| TRINITY_DN4347_c0_g1_i3 | 1.073879964 | 0.338845406 | 0.999970678 | 0.563 | 0.71 | 0.233 | 0.322 | - |
| TRINITY_DN5370_c0_g1_i3 | -1.07375826 | 0.264555749 | 0.999970678 | 2.062 | 1.813 | 2 | 5.751 | Translin |
| TRINITY_DN6275_c0_g1_i1 | -1.0737465 | 0.223467075 | 0.999970678 | 40.869 | 77.97 | 68.996 | 162.82 | NA |
| TRINITY_DN2014_c0_g1_i7 | 1.073697974 | 0.129630193 | 0.999970678 | 2.789 | 4.425 | 1.478 | 1.649 | - |
| TRINITY_DN2014_c0_g1_i6 | 1.073697974 | 0.129630193 | 0.999970678 | 2.789 | 4.425 | 1.478 | 1.649 | - |
| TRINITY_DN4048_c0_g1_i2 | 1.073657624 | 0.681884463 | 0.999970678 | 4.887 | 8.781 | 0 | 6.394 | Arf |
| TRINITY_DN1031_c2_g2_i2 | -1.0734667 | 0.368253871 | 0.999970678 | 1.808 | 0.986 | 1.211 | 4.454 | NA |
| TRINITY_DN783_c0_g1_i1 | -1.0733559 | 0.13653143 | 0.999970678 | 4.833 | 7.184 | 11.999 | 10.707 | DUF4922 |
| TRINITY_DN1609_c0_g1_i8 | -1.07329545 | 0.698370987 | 0.999970678 | 8.257 | 0 | 11.866 | 3.237 | - |
| TRINITY_DN1655_c0_g1_i9 | -1.07328088 | 0.420025237 | 0.999970678 | 36.79 | 16.78 | 77.54 | 16.88 | NA |
| TRINITY_DN5291_c0_g1_i1 | -1.07325561 | 0.465914319 | 0.999970678 | 1.408 | 0.611 | 0.611 | 3.519 | NA |
| TRINITY_DN12850_c0_g1_i2 | 1.073215816 | 0.543143732 | 0.999970678 | 0.173 | 1.163 | 0.089 | 0.533 | LRR_8 |
| TRINITY_DN2848_c0_g1_i6 | -1.0731896 | 0.515584696 | 0.999970678 | 0.481 | 0.552 | 1.633 | 0.211 | NA |
| TRINITY_DN11942_c0_g1_i1 | -1.07314404 | 0.375365993 | 0.999970678 | 21.665 | 40.33 | 18.532 | 103.633 | NA |
| TRINITY_DN1755_c0_g1_i4 | 1.072976888 | 0.40318669 | 0.999970678 | 0.581 | 1.173 | 0.456 | 0.282 | NA |
| TRINITY_DN2733_c0_g1_i3 | 1.072959589 | 0.519565654 | 1 | 0.145 | 0.177 | 0.111 | 0.02 | NA |
| TRINITY_DN3921_c0_g1_i1 | 1.072655555 | 0.323703961 | 0.999970678 | 1.59 | 3.656 | 1.389 | 0.824 | NA |
| TRINITY_DN2543_c0_g1_i1 | 1.072612268 | 0.243706833 | 0.999970678 | 9.238 | 23.66 | 5.733 | 8.616 | K-box |
| TRINITY_DN2195_c1_g1_i13 | -1.07254125 | 0.687472966 | 0.999970678 | 1.008 | 0 | 0.7 | 1.307 | NA |
| TRINITY_DN492_c0_g1_i4 | 1.072355631 | 0.141905628 | 0.999970678 | 5.114 | 3.991 | 1.9 | 2.031 | NA |
| TRINITY_DN11109_c0_g2_i5 | -1.0723162 | 0.47328592 | 0.999970678 | 0.382 | 4.248 | 4.078 | 4.735 | XAP5 |
| TRINITY_DN5578_c0_g1_i5 | 1.072260031 | 0.701551409 | 0.999970678 | 2.362 | 0 | 0.467 | 0.583 | NA |
| TRINITY_DN587_c0_g1_i3 | -1.07197128 | 0.526429025 | 0.999970678 | 8.003 | 0.542 | 3.278 | 14.105 | NA |
| TRINITY_DN8685_c0_g2_i4 | -1.07185025 | 0.573565795 | 0.999970678 | 3.997 | 0.089 | 3.9 | 3.981 | NA |
| TRINITY_DN16084_c0_g2_i8 | -1.07172215 | 0.308519987 | 0.999970678 | 0.609 | 0.463 | 0.644 | 1.458 | CBM_4_9 |
| TRINITY_DN4545_c4_g1_i2 | -1.07166954 | 0.442175169 | 0.999970678 | 2.198 | 0.414 | 3.544 | 1.277 | NA |
| TRINITY_DN8999_c0_g2_i2 | 1.071591029 | 0.481233247 | 0.999970678 | 0.618 | 8.219 | 1.633 | 2.182 | NA |
| TRINITY_DN616_c2_g1_i4 | -1.0715437 | 0.7109825 | 0.999970678 | 0 | 29.41 | 33.32 | 20.59 | NA |
| TRINITY_DN2173_c0_g1_i1 | 1.071465096 | 0.70054373 | 0.999970678 | 28.796 | 13.47 | 0 | 20.238 | NA |
| TRINITY_DN4100_c0_g1_i1 | 1.071381408 | 0.272448536 | 0.999970678 | 10.283 | 13.31 | 2.389 | 8.324 | NA |
| TRINITY_DN13248_c0_g1_i3 | 1.0712859 | 0.710591387 | 1 | 1.744 | 0.325 | 0.822 | 0 | NA |
| TRINITY_DN7993_c0_g1_i1 | -1.07097588 | 0.352805412 | 0.999970678 | 0.4 | 0.286 | 0.6 | 0.714 | NA |
| TRINITY_DN2094_c0_g1_i3 | 1.070915301 | 0.496521605 | 0.999970678 | 3.207 | 4.869 | 2.989 | 0.241 | NA |
| TRINITY_DN10696_c0_g1_i5 | 1.070908277 | 0.400621165 | 1 | 4.36 | 3.38 | 1.711 | 1.458 | NA |
| TRINITY_DN1791_c0_g2_i2 | 1.070893166 | 0.502722176 | 0.999970678 | 0.436 | 0.67 | 0.378 | 0.08 | HATPase_c_3 |
| TRINITY_DN4191_c0_g1_i4 | 1.070645506 | 0.072113159 | 0.999970678 | 51.052 | 48.99 | 17.399 | 26.713 | NIR_SIR |
| TRINITY_DN509_c0_g1_i6 | -1.07049589 | 0.204445086 | 0.999970678 | 20.684 | 10.42 | 23.087 | 37.128 | NA |
| TRINITY_DN15929_c1_g1_i1 | -1.07033123 | 0.366068947 | 0.999970678 | 1.172 | 0.65 | 2.267 | 1.086 | NA |
| TRINITY_DN616_c0_g2_i4 | 1.070309292 | 0.565403227 | 0.999970678 | 9.247 | 7.086 | 6.311 | 0.151 | NA |
| TRINITY_DN1437_c0_g1_i5 | 1.070250362 | 0.422731712 | 0.999970678 | 16.86 | 3.42 | 6.289 | 2.182 | UQ_con |
| TRINITY_DN5235_c0_g1_i1 | 1.070246737 | 0.172229949 | 0.999970678 | 6.25 | 11.96 | 3.933 | 3.891 | NA |
| TRINITY_DN8182_c0_g1_i5 | -1.0701072 | 0.710287313 | 0.999970678 | 1.172 | 0 | 0.178 | 2.292 | NA |
| TRINITY_DN1455_c0_g1_i1 | -1.06980542 | 0.280595776 | 0.999970678 | 0.854 | 1.045 | 2.311 | 1.196 | Myb_DNA-bindin |
| TRINITY_DN3329_c0_g1_i2 | -1.06977669 | 0.358607855 | 0.999970678 | 10.129 | 13.65 | 7.222 | 40.898 | NUDIX |
| TRINITY_DN191_c0_g1_i1 | 1.069627721 | 0.716821742 | 0.999970678 | 10.692 | 0.542 | 4.478 | 0 | Inos-1-P_synth |
| TRINITY_DN29741_c0_g1_i1 | 1.069489514 | 0.681919158 | 0.999970678 | 0.718 | 1.084 | 0 | 0.865 | NA |
| TRINITY_DN13692_c0_g1_i1 | -1.06942013 | 0.514373164 | 0.999970678 | 11.264 | 1.163 | 16.843 | 5.65 | NA |
| TRINITY_DN35585_c0_g1_i4 | -1.06934258 | 0.809907622 | 1 | 0.89 | 0 | 1.589 | 0 | MmgE_PrpD |
| TRINITY_DN61078_c0_g1_i3 | -1.06934192 | 0.089028025 | 0.999970678 | 840.72 | 859.1 | 1189.361 | 1952.438 | NA |
| TRINITY_DN8969_c0_g1_i2 | 1.06924348 | 0.50614552 | 0.999970678 | 3.379 | 16.63 | 6.144 | 1.78 | Str_synth |
| TRINITY_DN2408_c0_g1_i5 | -1.06920046 | 0.800449577 | 0.999970678 | 0 | 1.37 | 2.344 | 0 | NA |
| TRINITY_DN2384_c0_g1_i9 | 1.069174211 | 0.68237435 | 0.999970678 | 3.134 | 1.804 | 0 | 2.363 | NA |
| TRINITY_DN2320_c0_g1_i5 | -1.06916383 | 0.127913817 | 0.999970678 | 10.592 | 9.116 | 13.844 | 24.611 | NA |
| TRINITY_DN16992_c0_g1_i4 | -1.06913099 | 0.471214776 | 0.999970678 | 19.694 | 4.455 | 8.477 | 38.345 | NA |
| TRINITY_DN5408_c0_g3_i1 | 1.069027092 | 0.257272747 | 0.999970678 | 54.649 | 19.69 | 13.821 | 18.931 | NA |
| TRINITY_DN1557_c0_g1_i2 | 1.06887394 | 0.450240996 | 0.999970678 | 1.217 | 1.804 | 0.978 | 0.271 | DUF2232 |
| TRINITY_DN1637_c0_g1_i6 | -1.06884303 | 0.417938112 | 0.999970678 | 7.303 | 1.301 | 4.589 | 12.698 | NA |
| TRINITY_DN1163_c0_g1_i8 | 1.068747298 | 0.295816231 | 0.999970678 | 10.183 | 3.637 | 3.311 | 2.634 | TspO_MBR |
| TRINITY_DN9547_c0_g1_i4 | 1.068440293 | 0.358936476 | 0.999970678 | 0.963 | 1.074 | 0.267 | 0.643 | NA |
| TRINITY_DN4418_c0_g1_i2 | 1.068317463 | 0.725678244 | 0.999970678 | 0 | 106 | 14.61 | 32.152 | NA |

| TRINITY_DN7508_c0_g1_i1 | -1.06829766 | 0.698678089 | 0.999970678 | 0 | 10.38 | 3.511 | 17.111 | NA |
| --- | --- | --- | --- | --- | --- | --- | --- | --- |
| TRINITY_DN7241_c0_g1_i6 | 1.068276235 | 0.809191524 | 1 | 3.234 | 0 | 1.267 | 0 | NA |
| TRINITY_DN3141_c0_g1_i7 | -1.06808066 | 0.746935048 | 0.999970678 | 3.479 | 0.02 | 6.077 | 0.05 | NA |
| TRINITY_DN4536_c0_g1_i3 | 1.067949936 | 0.711151663 | 1 | 0.245 | 1.666 | 0 | 0.895 | Cu-oxidase |
| TRINITY_DN686_c0_g1_i7 | 1.067764881 | 0.539593189 | 0.999970678 | 26.225 | 1.38 | 1.889 | 11.109 | NA |
| TRINITY_DN9392_c0_g1_i1 | -1.06765406 | 0.275932858 | 0.999970678 | 5.069 | 5.322 | 13.521 | 5.429 | NA |
| TRINITY_DN14075_c0_g1_i1 | -1.0676488 | 0.69448782 | 0.999970678 | 0 | 1.991 | 2.722 | 0.845 | NA |
| TRINITY_DN10650_c0_g1_i1 | -1.06764578 | 0.341602256 | 0.999970678 | 7.213 | 1.912 | 7.722 | 9.893 | NA |
| TRINITY_DN22101_c0_g2_i6 | -1.06759685 | 0.813624431 | 1 | 0.881 | 0 | 1.589 | 0 | UPF0121 |
| TRINITY_DN14935_c0_g1_i3 | -1.06759685 | 0.813624431 | 1 | 0.945 | 0 | 1.667 | 0 | NA |
| TRINITY_DN79810_c0_g1_i1 | -1.06754095 | 0.805002887 | 0.999970678 | 2.307 | 0 | 4.044 | 0 | NA |
| TRINITY_DN2037_c0_g1_i5 | -1.06754095 | 0.805002887 | 0.999970678 | 11.518 | 0 | 19.788 | 0 | ADH_N |
| TRINITY_DN11512_c0_g1_i5 | 1.067255579 | 0.392095677 | 0.999970678 | 1.081 | 0.611 | 0.3 | 0.442 | NA |
| TRINITY_DN1991_c0_g1_i1 | -1.06725197 | 0.448643845 | 0.999970678 | 2.253 | 0.355 | 2.211 | 2.815 | Serpin |
| TRINITY_DN9714_c0_g1_i1 | -1.06724401 | 0.415449515 | 0.999970678 | 7.803 | 1.902 | 4.566 | 14.819 | - |
| TRINITY_DN1922_c1_g1_i11 | -1.06721524 | 0.492582677 | 0.999970678 | 2.634 | 0.848 | 5.589 | 0.583 | HSF_DNA-bind |
| TRINITY_DN15371_c0_g1_i1 | 1.066998723 | 0.336229069 | 0.999970678 | 1.562 | 1.912 | 0.367 | 1.216 | Helicase_C |
| TRINITY_DN40034_c0_g1_i1 | -1.06682826 | 0.64735273 | 0.999970678 | 1.09 | 0.049 | 1.955 | 0.06 | DUF1644 |
| TRINITY_DN3380_c0_g2_i1 | 1.06678297 | 0.506685773 | 0.999970678 | 3.543 | 29.2 | 2.144 | 12.617 | BTB |
| TRINITY_DN561_c0_g1_i13 | 1.066780986 | 0.800339154 | 0.999970678 | 2.026 | 0 | 0.811 | 0 | NA |
| TRINITY_DN31515_c0_g1_i3 | -1.06669622 | 0.359215205 | 0.999970678 | 0.536 | 0.306 | 0.667 | 0.935 | NA |
| TRINITY_DN2670_c3_g1_i3 | 1.066625357 | 0.765361013 | 0.999970678 | 150.9 | 0 | 0.167 | 72.93 | S1 |
| TRINITY_DN86_c1_g1_i1 | -1.06656226 | 0.359875827 | 0.999970678 | 20.33 | 44.28 | 21.121 | 109.384 | 2-Hacid_dh |
| TRINITY_DN9679_c0_g1_i4 | -1.06654589 | 0.367714465 | 0.999970678 | 0.609 | 2.868 | 3.378 | 3.157 | NA |
| TRINITY_DN11531_c0_g1_i2 | 1.06650957 | 0.423710371 | 0.999970678 | 3.416 | 1.261 | 1.533 | 0.402 | K_trans |
| TRINITY_DN53884_c0_g1_i1 | -1.06626314 | 0.806638765 | 0.999970678 | 0.572 | 0 | 1.011 | 0 | NA |
| TRINITY_DN1123_c0_g1_i2 | -1.06619903 | 0.697137866 | 0.999970678 | 6.613 | 0 | 4.155 | 9.078 | NA |
| TRINITY_DN10577_c0_g1_i1 | -1.06616221 | 0.257592813 | 0.999970678 | 1.98 | 1.39 | 2 | 4.655 | PP2C |
| TRINITY_DN1727_c0_g1_i5 | -1.06615001 | 0.686747414 | 0.999970678 | 0 | 1.941 | 1.8 | 1.84 | NA |
| TRINITY_DN155_c0_g1_i20 | 1.065959832 | 0.800926501 | 0.999970678 | 0 | 5.499 | 2.122 | 0 | Ank |
| TRINITY_DN15466_c0_g1_i2 | -1.06575019 | 0.491050325 | 0.999970678 | 0.536 | 4.504 | 1.989 | 8.053 | NA |
| TRINITY_DN2688_c0_g1_i5 | -1.06571475 | 0.73783876 | 0.999970678 | 2.616 | 0.079 | 4.722 | 0 | NA |
| TRINITY_DN2189_c0_g1_i1 | 1.065669532 | 0.810917832 | 1 | 0.954 | 0 | 0.389 | 0 | AAA |
| TRINITY_DN7718_c0_g1_i3 | -1.0654752 | 0.425033529 | 0.999970678 | 0.89 | 6.731 | 7.044 | 7.259 | IPPT |
| TRINITY_DN808_c3_g1_i3 | 1.065338317 | 0.58452617 | 0.999970678 | 14.098 | 0.177 | 3.911 | 2.212 | RVT_1 |
| TRINITY_DN7113_c1_g1_i2 | -1.06527914 | 0.696534124 | 0.999970678 | 2.253 | 0 | 0.955 | 3.609 | NA |
| TRINITY_DN29626_c0_g1_i1 | -1.06491516 | 0.7158204 | 0.999970678 | 0.69 | 0.187 | 1.544 | 0 | Na_Ca_ex |
| TRINITY_DN31_c0_g1_i3 | -1.06473906 | 0.210799377 | 0.999970678 | 12.727 | 5.765 | 16.61 | 18.861 | AAA |
| TRINITY_DN7109_c0_g1_i4 | -1.06473244 | 0.690117059 | 0.999970678 | 0 | 3.568 | 2.067 | 4.866 | NA |
| TRINITY_DN229_c0_g1_i5 | -1.06469278 | 0.132229478 | 0.999970678 | 87.288 | 128.5 | 148.047 | 271.57 | AdoHcyase |
| TRINITY_DN11874_c0_g1_i1 | -1.06466616 | 0.607267843 | 0.999970678 | 0.581 | 0.01 | 0.678 | 0.432 | NA |
| TRINITY_DN3046_c0_g1_i1 | -1.06463114 | 0.393278577 | 0.999970678 | 24.454 | 13.83 | 11.299 | 64.042 | NA |
| TRINITY_DN20528_c0_g1_i5 | 1.064627109 | 0.42840443 | 1 | 0.654 | 0.631 | 0.378 | 0.181 | NA |
| TRINITY_DN11726_c0_g1_i1 | -1.06450053 | 0.804682995 | 0.999970678 | 1.699 | 0 | 2.978 | 0 | - |
| TRINITY_DN1100_c0_g1_i4 | 1.064494884 | 0.171467933 | 0.999970678 | 10.265 | 13.05 | 5.755 | 4.132 | PUNUT |
| TRINITY_DN1095_c1_g2_i1 | 1.06445295 | 0.599205322 | 0.999970678 | 0.318 | 6.455 | 2.378 | 0.312 | - |
| TRINITY_DN4597_c0_g1_i4 | -1.06440009 | 0.716836968 | 0.999970678 | 0.236 | 1.971 | 0 | 4.595 | F-box |
| TRINITY_DN14283_c0_g1_i1 | 1.064293151 | 0.662812645 | 0.999970678 | 2.825 | 0.03 | 1.044 | 0.121 | NA |
| TRINITY_DN1601_c0_g2_i1 | -1.06418813 | 0.474618884 | 0.999970678 | 0.409 | 0.217 | 0.211 | 1.046 | LRRNT_2 |
| TRINITY_DN2847_c0_g5_i1 | -1.06418326 | 0.369710207 | 0.999970678 | 0.663 | 1.222 | 0.7 | 3.086 | NA |
| TRINITY_DN5273_c0_g2_i3 | 1.064107814 | 0.356834749 | 0.999970678 | 5.405 | 1.636 | 1.389 | 1.679 | NA |
| TRINITY_DN224_c0_g1_i3 | 1.064012701 | 0.594937 | 0.999970678 | 6.74 | 0.138 | 0.522 | 2.725 | TRM |
| TRINITY_DN994_c0_g1_i12 | -1.06386469 | 0.477767902 | 0.999970678 | 4.024 | 2.365 | 10.11 | 1.206 | NA |
| TRINITY_DN14443_c0_g1_i1 | 1.063723238 | 0.567853877 | 0.999970678 | 0.527 | 12.46 | 0.689 | 5.288 | NA |
| TRINITY_DN28964_c0_g1_i1 | 1.06371291 | 0.558290128 | 0.999970678 | 2.289 | 1.863 | 1.533 | 0.131 | NA |
| TRINITY_DN6196_c0_g1_i5 | -1.06361503 | 0.491173512 | 0.999970678 | 0.772 | 0.138 | 0.844 | 0.895 | NA |
| TRINITY_DN1642_c1_g1_i3 | 1.063432796 | 0.689719295 | 0.999970678 | 0.681 | 1.005 | 0.656 | 0 | NA |
| TRINITY_DN31120_c0_g1_i4 | -1.06313096 | 0.69241234 | 0.999970678 | 0.818 | 0 | 0.622 | 0.995 | NA |
| TRINITY_DN446_c0_g1_i1 | -1.0631069 | 0.36325326 | 0.999970678 | 6.722 | 35.57 | 33.642 | 46.539 | NA |
| TRINITY_DN5919_c0_g1_i4 | -1.06304516 | 0.698318981 | 0.999970678 | 4.769 | 0 | 1.867 | 7.852 | CPP1-like |
| TRINITY_DN21498_c0_g1_i1 | -1.06279852 | 0.479500507 | 0.999970678 | 0.681 | 1.015 | 2.467 | 0.553 | - |
| TRINITY_DN2558_c0_g1_i12 | -1.06274347 | 0.800947176 | 0.999970678 | 7.467 | 0 | 13.055 | 0 | Abhydrolase_1 |
| TRINITY_DN8539_c0_g1_i2 | 1.062385605 | 0.398599997 | 0.999970678 | 0.418 | 2.02 | 0.467 | 0.603 | NA |
| TRINITY_DN175110_c0_g1_i1 | -1.06231828 | 0.431889296 | 0.999970678 | 33.002 | 107.7 | 35.442 | 241.349 | NA |
| TRINITY_DN43020_c0_g1_i5 | 1.062263524 | 0.700143902 | 1 | 1.372 | 0.394 | 0 | 0.855 | NA |
| TRINITY_DN2758_c0_g1_i5 | 1.062167006 | 0.501682789 | 0.999970678 | 4.206 | 35.95 | 2.311 | 16.186 | MIP |
| TRINITY_DN452_c0_g1_i1 | 1.062104914 | 0.424007366 | 0.999970678 | 6.032 | 8.416 | 0.922 | 5.731 | NA |
| TRINITY_DN19353_c0_g1_i3 | 1.062063983 | 0.704049642 | 0.999970678 | 0.899 | 0.158 | 0.422 | 0 | NA |
| TRINITY_DN28328_c0_g1_i10 | -1.06203075 | 0.811803328 | 1 | 0.3 | 0 | 0.544 | 0 | NA |
| TRINITY_DN1880_c1_g1_i17 | 1.062009874 | 0.530716337 | 0.999970678 | 8.003 | 0.631 | 0.678 | 3.378 | NA |
| TRINITY_DN585_c0_g1_i2 | -1.0619922 | 0.411723459 | 0.999970678 | 6.232 | 1.074 | 7.277 | 6.555 | adh_short |
| TRINITY_DN6950_c0_g1_i6 | -1.06198426 | 0.476847359 | 0.999970678 | 2.544 | 1.468 | 0.889 | 7.329 | NA |
| TRINITY_DN33955_c0_g1_i1 | 1.061976106 | 0.457273213 | 0.999970678 | 2.189 | 0.838 | 0.867 | 0.382 | NA |
| TRINITY_DN4238_c0_g2_i9 | -1.06197049 | 0.43363213 | 0.999970678 | 0.445 | 3.538 | 3.9 | 3.519 | FAR1 |

| TRINITY_DN10121_c0_g2_i1 | -1.06184663 | 0.261012229 | 0.999970678 | 4.678 | 1.981 | 5.077 | 7.832 | NA |
| --- | --- | --- | --- | --- | --- | --- | --- | --- |
| TRINITY_DN3878_c0_g1_i1 | -1.06163279 | 0.345417499 | 0.999970678 | 0.318 | 0.611 | 1.055 | 0.674 | NA |
| TRINITY_DN1403_c0_g1_i4 | -1.06158676 | 0.475693857 | 0.999970678 | 67.821 | 5.194 | 78.684 | 59.206 | NA |
| TRINITY_DN880_c0_g1_i3 | -1.06154862 | 0.243825269 | 0.999970678 | 4.733 | 9.5 | 15.632 | 10.627 | - |
| TRINITY_DN2770_c0_g1_i1 | -1.06154255 | 0.172133656 | 0.999970678 | 26.071 | 46.31 | 48.741 | 91.841 | NA |
| TRINITY_DN913_c5_g1_i1 | 1.061538779 | 0.362314444 | 0.999970678 | 8.248 | 3.095 | 3.433 | 1.327 | Aldedh |
| TRINITY_DN2221_c0_g1_i4 | 1.061399605 | 0.262134729 | 0.999970678 | 5.605 | 4.198 | 1.167 | 3.288 | LisH |
| TRINITY_DN16178_c0_g1_i2 | 1.061257782 | 0.692628545 | 0.999970678 | 1.145 | 2.089 | 1.278 | 0 | NA |
| TRINITY_DN698_c6_g1_i1 | 1.061234012 | 0.689776478 | 0.999970678 | 2.589 | 3.627 | 0 | 2.986 | CBF |
| TRINITY_DN7296_c0_g1_i9 | 1.061232562 | 0.452336048 | 0.999970678 | 1.108 | 0.857 | 0.656 | 0.151 | NA |
| TRINITY_DN3103_c0_g1_i2 | -1.06116265 | 0.168185059 | 0.999970678 | 8.23 | 14.57 | 16.977 | 26.662 | NA |
| TRINITY_DN6542_c0_g1_i2 | -1.06114657 | 0.279203014 | 0.999970678 | 5.441 | 1.833 | 6.488 | 7.48 | Pkinase |
| TRINITY_DN1495_c0_g1_i1 | -1.06105023 | 0.132114907 | 0.999970678 | 10.51 | 15.41 | 19.399 | 30.221 | - |
| TRINITY_DN5224_c0_g1_i4 | -1.06093837 | 0.469563496 | 0.999970678 | 0.136 | 0.719 | 0.667 | 0.965 | NA |
| TRINITY_DN5882_c0_g1_i4 | 1.060617505 | 0.230810061 | 0.999970678 | 3.852 | 4.1 | 1.033 | 2.564 | DUF1777 |
| TRINITY_DN17507_c0_g1_i1 | -1.06060444 | 0.215659459 | 0.999970678 | 5.977 | 4.918 | 8.011 | 12.778 | NA |
| TRINITY_DN5429_c0_g1_i1 | -1.06052807 | 0.323989071 | 0.999970678 | 6.913 | 13.32 | 9.199 | 30.161 | NA |
| TRINITY_DN729_c0_g2_i4 | -1.06043276 | 0.237676895 | 0.999970678 | 0.636 | 0.936 | 1.289 | 1.689 | BAG |
| TRINITY_DN1695_c0_g1_i4 | -1.06036752 | 0.549715152 | 0.999970678 | 10.065 | 1.084 | 17.966 | 1.82 | NA |
| TRINITY_DN800_c0_g1_i13 | -1.05991072 | 0.318120451 | 0.999970678 | 2.998 | 2.375 | 6.511 | 3.328 | NA |
| TRINITY_DN11143_c0_g1_i1 | -1.05982521 | 0.509385264 | 0.999970678 | 1.017 | 0.503 | 2.278 | 0.422 | NA |
| TRINITY_DN3275_c0_g1_i4 | -1.05977086 | 0.25734101 | 0.999970678 | 2.253 | 4.464 | 7.777 | 4.554 | - |
| TRINITY_DN4_c0_g1_i4 | 1.059582404 | 0.532549268 | 0.999970678 | 1.49 | 14.16 | 0.711 | 6.575 | - |
| TRINITY_DN9876_c0_g1_i7 | 1.059582259 | 0.414972887 | 0.999970678 | 2.008 | 1.429 | 1.011 | 0.462 | NA |
| TRINITY_DN2181_c1_g1_i1 | -1.05953907 | 0.806566125 | 0.999970678 | 0 | 1.035 | 1.767 | 0 | NA |
| TRINITY_DN17098_c0_g1_i2 | -1.0594467 | 0.689921625 | 0.999970678 | 0 | 0.936 | 0.767 | 0.995 | NA |
| TRINITY_DN8550_c0_g1_i3 | 1.059311603 | 0.697748265 | 1 | 1.135 | 2.001 | 1.267 | 0 | - |
| TRINITY_DN19267_c0_g1_i1 | 1.059119735 | 0.432675441 | 0.999970678 | 2.28 | 17.76 | 4.644 | 3.891 | NA |
| TRINITY_DN5544_c0_g1_i5 | 1.05906894 | 0.661041955 | 0.999970678 | 29.695 | 1.291 | 12.077 | 0.191 | NA |
| TRINITY_DN2351_c1_g1_i2 | 1.059065103 | 0.490475618 | 0.999970678 | 1.045 | 0.857 | 0.667 | 0.121 | - |
| TRINITY_DN23_c0_g2_i5 | 1.058907036 | 0.708897285 | 0.999970678 | 0 | 2.424 | 0.233 | 0.885 | NA |
| TRINITY_DN15611_c0_g1_i4 | -1.05888082 | 0.560121125 | 0.999970678 | 2.462 | 3.558 | 0.633 | 11.501 | Lectin_legB |
| TRINITY_DN43293_c0_g1_i4 | 1.058865981 | 0.629808948 | 0.999970678 | 13.481 | 2.7 | 6.344 | 0.08 | NA |
| TRINITY_DN4852_c0_g1_i1 | 1.058822529 | 0.435178601 | 0.999970678 | 12.045 | 2.119 | 1.989 | 4.404 | NA |
| TRINITY_DN13234_c0_g1_i2 | -1.05880537 | 0.305917036 | 0.999970678 | 0.518 | 0.335 | 0.844 | 0.734 | NA |
| TRINITY_DN23620_c0_g1_i2 | 1.058805274 | 0.458302132 | 0.999970678 | 2.807 | 0.67 | 0.556 | 1.036 | NA |
| TRINITY_DN28_c0_g1_i1 | -1.05876244 | 0.708953115 | 0.999970678 | 0.781 | 2.247 | 5.166 | 0 | PP2C |
| TRINITY_DN18726_c0_g1_i3 | 1.058619042 | 0.545458642 | 0.999970678 | 0.145 | 1.804 | 0.467 | 0.332 | NA |
| TRINITY_DN3730_c0_g2_i4 | -1.05861828 | 0.719803965 | 0.999970678 | 1.417 | 0 | 0.267 | 2.654 | NA |
| TRINITY_DN6062_c0_g1_i9 | 1.058587564 | 0.486862787 | 0.999970678 | 1.081 | 0.69 | 0.567 | 0.171 | Retrotran_gag_2 |
| TRINITY_DN12189_c0_g1_i2 | 1.058546296 | 0.480376335 | 0.999970678 | 0.527 | 2.237 | 0.289 | 0.985 | NA |
| TRINITY_DN11879_c0_g1_i4 | 1.058475462 | 0.490572018 | 0.999970678 | 0.5 | 3.085 | 0.478 | 1.156 | HALZ |
| TRINITY_DN325_c0_g1_i3 | 1.058218258 | 0.803708877 | 0.999970678 | 34.991 | 0 | 0 | 16.991 | NA |
| TRINITY_DN8769_c0_g2_i5 | 1.058104979 | 0.216662297 | 0.999970678 | 0.981 | 1.261 | 0.367 | 0.643 | NA |
| TRINITY_DN9229_c0_g1_i1 | -1.05806942 | 0.805320211 | 0.999970678 | 4.233 | 0 | 7.3 | 0 | NA |
| TRINITY_DN3827_c0_g1_i2 | -1.05804585 | 0.802022322 | 0.999970678 | 3.079 | 0 | 5.366 | 0 | NA |
| TRINITY_DN24947_c0_g1_i1 | 1.057995743 | 0.806916424 | 0.999970678 | 3.325 | 0 | 1.344 | 0 | NA |
| TRINITY_DN756_c0_g1_i6 | 1.057846352 | 0.424174975 | 0.999970678 | 1.626 | 5.509 | 0.644 | 2.604 | Thioredoxin |
| TRINITY_DN672_c0_g1_i5 | -1.05783105 | 0.339678317 | 0.999970678 | 0.89 | 0.365 | 0.733 | 1.749 | NA |
| TRINITY_DN9357_c0_g1_i11 | -1.05776368 | 0.706677928 | 0.999970678 | 0.963 | 3.203 | 0 | 8.586 | NA |
| TRINITY_DN6076_c5_g1_i5 | 1.057643211 | 0.360103275 | 0.999970678 | 1.344 | 1.794 | 0.422 | 1.015 | NA |
| TRINITY_DN1605_c0_g1_i4 | -1.05758939 | 0.705859311 | 0.999970678 | 0 | 5.509 | 7.777 | 1.9 | NA |
| TRINITY_DN2264_c0_g1_i1 | -1.05751461 | 0.717263963 | 0.999970678 | 2.135 | 0 | 3.378 | 0.352 | Patatin |
| TRINITY_DN1292_c0_g1_i9 | -1.05748705 | 0.546575859 | 0.999970678 | 0.99 | 0.473 | 2.4 | 0.161 | NA |
| TRINITY_DN8694_c0_g1_i1 | 1.057322761 | 0.413740878 | 0.999970678 | 58.646 | 167.3 | 22.021 | 71.974 | NA |
| TRINITY_DN1107_c0_g1_i6 | 1.057256418 | 0.681064271 | 0.999970678 | 1.026 | 16.04 | 6.677 | 0.01 | Sugar_tr |
| TRINITY_DN10203_c0_g1_i3 | 1.057220975 | 0.369890178 | 0.999970678 | 0.818 | 2.966 | 0.889 | 0.734 | K_trans |
| TRINITY_DN13513_c0_g3_i1 | 1.057119294 | 0.599989292 | 0.999970678 | 0.4 | 0.286 | 0.267 | 0.01 | NA |
| TRINITY_DN2752_c0_g1_i2 | 1.057116334 | 0.433332337 | 0.999970678 | 1.671 | 0.818 | 0.233 | 0.925 | NA |
| TRINITY_DN5076_c0_g1_i2 | 1.057027522 | 0.708025848 | 0.999970678 | 2.816 | 0.306 | 0 | 1.528 | BAH |
| TRINITY_DN3794_c0_g3_i2 | -1.05677897 | 0.696999138 | 0.999970678 | 0 | 2.671 | 1.444 | 3.74 | NA |
| TRINITY_DN1922_c1_g1_i10 | 1.056740749 | 0.582215392 | 0.999970678 | 0.836 | 0.118 | 0.356 | 0.04 | HSF_DNA-bind |
| TRINITY_DN4264_c0_g1_i4 | -1.05670704 | 0.803190237 | 0.999970678 | 3.098 | 0 | 5.422 | 0 | NA |
| TRINITY_DN1215_c0_g1_i10 | -1.05659515 | 0.699124474 | 0.999970678 | 3.397 | 0 | 2.5 | 4.172 | DUF167 |
| TRINITY_DN2713_c1_g2_i1 | -1.05656795 | 0.725158333 | 1 | 0 | 0.867 | 1.333 | 0.191 | NA |
| TRINITY_DN6485_c0_g1_i2 | -1.05650283 | 0.8039348 | 0.999970678 | 1.971 | 0 | 3.455 | 0 | Asp |
| TRINITY_DN1713_c0_g2_i3 | 1.056098049 | 0.25897829 | 0.999970678 | 6.895 | 4.277 | 3.122 | 1.629 | Clathrin |
| TRINITY_DN8313_c0_g1_i1 | -1.0558713 | 0.690944953 | 0.999970678 | 2.335 | 0 | 2.022 | 2.473 | PPR |
| TRINITY_DN10058_c0_g1_i4 | 1.055787738 | 0.172599916 | 0.999970678 | 4.442 | 5.194 | 1.433 | 2.906 | NA |
| TRINITY_DN2038_c0_g2_i1 | 1.055779079 | 0.811659686 | 0.999970678 | 32.375 | 0 | 0 | 15.895 | DBP10CT |
| TRINITY_DN2481_c0_g1_i3 | -1.055658 | 0.694233981 | 0.999970678 | 4.487 | 0 | 3.766 | 4.896 | NA |
| TRINITY_DN9712_c0_g1_i2 | 1.055484673 | 0.447004634 | 0.999970678 | 36.49 | 40.7 | 27.976 | 3.308 | Clathrin |
| TRINITY_DN7940_c0_g1_i6 | -1.05517856 | 0.809343869 | 0.999970678 | 3.025 | 0 | 5.244 | 0 | Ferritin_2 |
| TRINITY_DN1156_c0_g1_i6 | 1.055151683 | 0.801578035 | 0.999970678 | 13.989 | 0 | 0 | 6.837 | NA |

| TRINITY_DN7940_c0_g1_i1 | -1.05514412 | 0.803839054 | 0.999970678 | 6.441 | 0 | 11.222 | 0 | Ferritin_2 |
| --- | --- | --- | --- | --- | --- | --- | --- | --- |
| TRINITY_DN8096_c0_g1_i1 | 1.055132686 | 0.727969164 | 0.999970678 | 0 | 44.85 | 15.255 | 2.785 | NA |
| TRINITY_DN4615_c1_g2_i5 | -1.05494709 | 0.701498299 | 0.999970678 | 3.733 | 0 | 1.255 | 6.374 | NA |
| TRINITY_DN13888_c0_g1_i5 | 1.054895664 | 0.286973137 | 0.999970678 | 0.799 | 0.917 | 0.233 | 0.543 | NA |
| TRINITY_DN14495_c0_g1_i3 | 1.054848787 | 0.49950637 | 0.999970678 | 3.47 | 0.621 | 1.278 | 0.432 | NA |
| TRINITY_DN946_c0_g1_i2 | -1.05484747 | 0.705229831 | 0.999970678 | 7.176 | 0 | 5.955 | 7.973 | TRAM_LAG1_CLN |
| TRINITY_DN999_c0_g1_i5 | -1.05474364 | 0.80267945 | 0.999970678 | 9.883 | 0 | 0 | 20.821 | NA |
| TRINITY_DN4513_c0_g1_i3 | 1.05469683 | 0.523988087 | 0.999970678 | 1.036 | 8.899 | 2.789 | 1.297 | PsaN |
| TRINITY_DN1632_c0_g1_i3 | 1.054679058 | 0.511594668 | 0.999970678 | 1.644 | 0.148 | 0.233 | 0.593 | NA |
| TRINITY_DN2135_c0_g1_i10 | 1.054660105 | 0.449454426 | 0.999970678 | 1.481 | 6.337 | 1.311 | 2.131 | NA |
| TRINITY_DN412_c0_g1_i6 | 1.054569841 | 0.569865997 | 0.999970678 | 0.064 | 0.68 | 0.067 | 0.261 | RRM_1 |
| TRINITY_DN3387_c0_g1_i8 | -1.05446409 | 0.65908043 | 0.999970678 | 10.774 | 1.281 | 20.832 | 0.141 | NA |
| TRINITY_DN788_c0_g1_i1 | 1.054306191 | 0.432570948 | 0.999970678 | 0.382 | 1.626 | 0.456 | 0.412 | NA |
| TRINITY_DN9558_c0_g3_i1 | 1.054106397 | 0.226687395 | 0.999970678 | 9.929 | 11.98 | 3.866 | 5.65 | SBP |
| TRINITY_DN3110_c1_g1_i9 | -1.05410355 | 0.436816129 | 0.999970678 | 0.3 | 0.739 | 1.322 | 0.533 | DEAD |
| TRINITY_DN19761_c0_g1_i1 | 1.054046375 | 0.624933696 | 0.999970678 | 16.215 | 3.449 | 7.5 | 0.292 | Actin |
| TRINITY_DN40303_c0_g1_i2 | 1.054039091 | 0.688254322 | 0.999970678 | 1.308 | 2.73 | 0 | 1.95 | F-box |
| TRINITY_DN8760_c0_g2_i7 | -1.05401189 | 0.813890831 | 1 | 3.797 | 0 | 6.655 | 0 | NA |
| TRINITY_DN6886_c0_g1_i7 | -1.05401189 | 0.813890831 | 1 | 0.5 | 0 | 0.867 | 0 | NA |
| TRINITY_DN3213_c0_g1_i2 | -1.05395973 | 0.717681555 | 1 | 4.76 | 0 | 1.222 | 7.952 | NA |
| TRINITY_DN2195_c3_g1_i2 | 1.053624768 | 0.445887639 | 0.999970678 | 0.808 | 1.114 | 0.6 | 0.231 | NA |
| TRINITY_DN12060_c0_g1_i4 | -1.05344798 | 0.749478703 | 0.999970678 | 2.216 | 0 | 3.789 | 0.08 | CRIM |
| TRINITY_DN2054_c0_g1_i3 | 1.053327105 | 0.243711986 | 0.999970678 | 2.298 | 1.853 | 0.767 | 1.066 | LRRNT_2 |
| TRINITY_DN6588_c0_g1_i3 | -1.05330489 | 0.80462623 | 0.999970678 | 2.562 | 0 | 4.433 | 0 | - |
| TRINITY_DN10381_c0_g1_i4 | -1.05323071 | 0.753792897 | 0.999970678 | 1.19 | 0.01 | 0 | 2.544 | NA |
| TRINITY_DN2968_c0_g1_i1 | 1.0531768 | 0.740747442 | 0.999970678 | 9.792 | 0 | 0.111 | 4.625 | GST_C |
| TRINITY_DN50210_c0_g1_i3 | -1.05298231 | 0.704850638 | 0.999970678 | 0 | 1.991 | 0.456 | 3.509 | NA |
| TRINITY_DN2457_c0_g1_i3 | 1.05255186 | 0.284110338 | 0.999970678 | 6.132 | 2.227 | 1.333 | 2.463 | THOC2_N |
| TRINITY_DN1721_c0_g1_i16 | -1.05249539 | 0.802233567 | 0.999970678 | 0 | 1.951 | 3.289 | 0 | Fasciclin |
| TRINITY_DN1533_c0_g1_i5 | -1.05242548 | 0.579442778 | 0.999970678 | 1.481 | 6.307 | 12.688 | 0.623 | NA |
| TRINITY_DN17546_c0_g1_i11 | -1.05235572 | 0.425705776 | 0.999970678 | 0.227 | 1.074 | 1.111 | 1.337 | - |
| TRINITY_DN4065_c0_g1_i1 | 1.05233216 | 0.350528245 | 0.999970678 | 4.923 | 2.868 | 0.767 | 2.845 | NA |
| TRINITY_DN1989_c0_g1_i6 | -1.05232483 | 0.378798483 | 0.999970678 | 5.868 | 1.449 | 5.889 | 8.113 | NA |
| TRINITY_DN8165_c0_g1_i2 | 1.052278805 | 0.607539789 | 0.999970678 | 27.27 | 0.581 | 8.933 | 2.604 | NA |
| TRINITY_DN337_c0_g1_i3 | 1.05217626 | 0.40601555 | 0.999970678 | 1.099 | 5.736 | 1.833 | 1.056 | ADK |
| TRINITY_DN6244_c0_g1_i1 | 1.052119157 | 0.384133251 | 0.999970678 | 1.535 | 0.798 | 0.678 | 0.322 | FAD_binding_4 |
| TRINITY_DN7595_c0_g1_i1 | 1.052071719 | 0.189514381 | 0.999970678 | 16.569 | 18.9 | 4.8 | 11.24 | NA |
| TRINITY_DN2241_c0_g1_i2 | 1.051859701 | 0.376703006 | 0.999970678 | 3.906 | 1.853 | 1.733 | 0.664 | NA |
| TRINITY_DN10629_c0_g1_i12 | -1.05169663 | 0.534206435 | 0.999970678 | 4.596 | 0.867 | 7.444 | 2.121 | Actin |
| TRINITY_DN44199_c0_g1_i1 | -1.05162024 | 0.349740981 | 0.999970678 | 1.181 | 1.311 | 2.833 | 1.709 | Inhibitor_I9 |
| TRINITY_DN1507_c1_g1_i4 | -1.05133844 | 0.450978042 | 0.999970678 | 0.436 | 1.212 | 0.6 | 2.664 | CbiQ |
| TRINITY_DN2179_c0_g1_i4 | 1.051206073 | 0.456824122 | 0.999970678 | 9.329 | 1.971 | 0.856 | 4.474 | NA |
| TRINITY_DN1830_c0_g1_i4 | -1.0511339 | 0.569969525 | 0.999970678 | 5.432 | 0.118 | 6.189 | 4.192 | NA |
| TRINITY_DN3001_c0_g1_i2 | -1.05095505 | 0.290894134 | 0.999970678 | 7.494 | 8.83 | 21.021 | 8.314 | Thioredoxin |
| TRINITY_DN68803_c0_g1_i1 | 1.050828285 | 0.423176512 | 0.999970678 | 0.318 | 0.69 | 0.289 | 0.141 | RVP_2 |
| TRINITY_DN16268_c0_g1_i3 | 1.050781164 | 0.456113469 | 1 | 1.036 | 1.064 | 0.222 | 0.754 | GDPD |
| TRINITY_DN2840_c0_g1_i4 | 1.050767809 | 0.71883972 | 0.999970678 | 0 | 21.19 | 6.733 | 1.94 | PRP38 |
| TRINITY_DN1686_c0_g1_i6 | 1.050656344 | 0.343531584 | 0.999970678 | 4.306 | 6.504 | 3.411 | 1.086 | Vps53_N |
| TRINITY_DN4021_c2_g1_i2 | -1.05062929 | 0.272500275 | 0.999970678 | 1.408 | 1.301 | 3.333 | 1.588 | NADHdh |
| TRINITY_DN1822_c0_g1_i10 | 1.050596701 | 0.223242417 | 0.999970678 | 9.366 | 5.312 | 2.411 | 4.212 | DUF4228 |
| TRINITY_DN6966_c0_g1_i4 | -1.05049932 | 0.694887964 | 0.999970678 | 1.617 | 0 | 0.944 | 2.262 | NA |
| TRINITY_DN8706_c0_g1_i2 | 1.050371906 | 0.385907935 | 0.999970678 | 7.767 | 25.9 | 9.966 | 4.001 | Myb_DNA-bindin |
| TRINITY_DN8290_c0_g1_i3 | -1.05029241 | 0.434991071 | 0.999970678 | 0.727 | 0.177 | 0.522 | 1.257 | NA |
| TRINITY_DN1106_c0_g1_i3 | -1.0501774 | 0.436705288 | 0.999970678 | 0.291 | 1.015 | 1.5 | 0.895 | NA |
| TRINITY_DN7620_c0_g1_i4 | 1.049968097 | 0.497283984 | 0.999970678 | 0.609 | 3.922 | 0.422 | 1.649 | TAXi_C |
| TRINITY_DN6808_c0_g1_i3 | -1.04996685 | 0.69391697 | 0.999970678 | 0.89 | 0 | 0.933 | 0.744 | Methyltransf_15 |
| TRINITY_DN6392_c0_g2_i1 | 1.049925138 | 0.353263455 | 0.999970678 | 0.254 | 0.7 | 0.178 | 0.241 | NA |
| TRINITY_DN1158_c1_g1_i6 | 1.049872968 | 0.710836546 | 0.999970678 | 7.558 | 0 | 1.955 | 1.267 | MIF |
| TRINITY_DN1013_c0_g1_i4 | 1.049870903 | 0.57238189 | 0.999970678 | 1.108 | 0.729 | 0.711 | 0.04 | NA |
| TRINITY_DN1753_c1_g1_i4 | -1.0498396 | 0.486331624 | 0.999970678 | 0.109 | 0.177 | 0.422 | 0.09 | CcmF_C |
| TRINITY_DN25018_c0_g1_i1 | -1.04973052 | 0.716443826 | 1 | 0 | 0.572 | 0.156 | 0.965 | ACP_syn_III_C |
| TRINITY_DN3628_c1_g1_i1 | 1.049607395 | 0.62534759 | 1 | 1.635 | 0.158 | 0.667 | 0.07 | NA |
| TRINITY_DN8318_c0_g1_i1 | -1.0495995 | 0.446207884 | 0.999970678 | 1.399 | 4.287 | 1.567 | 9.782 | NA |
| TRINITY_DN477_c0_g1_i12 | -1.04955007 | 0.718188138 | 0.999970678 | 6.441 | 0 | 1.044 | 12.306 | NA |
| TRINITY_DN23947_c0_g1_i2 | 1.049548574 | 0.810603048 | 0.999970678 | 2.88 | 0 | 1.167 | 0 | NA |
| TRINITY_DN6132_c0_g1_i5 | -1.0494986 | 0.495801164 | 0.999970678 | 1.835 | 0.197 | 1.722 | 2.121 | EGF_CA |
| TRINITY_DN7287_c0_g1_i2 | -1.04947079 | 0.471620128 | 0.999970678 | 0.127 | 0.739 | 0.867 | 0.724 | NA |
| TRINITY_DN534_c0_g1_i8 | -1.04941226 | 0.128025413 | 0.999970678 | 8.548 | 13.97 | 19.788 | 22.55 | NA |
| TRINITY_DN3452_c0_g1_i2 | -1.04910022 | 0.368837443 | 0.999970678 | 4.042 | 2.996 | 9.966 | 2.564 | NA |
| TRINITY_DN4060_c0_g1_i1 | -1.04907785 | 0.172304722 | 0.999970678 | 8.612 | 11.1 | 21.488 | 14.709 | NA |
| TRINITY_DN6255_c0_g1_i1 | 1.048863152 | 0.36582597 | 0.999970678 | 1.263 | 1.951 | 0.4 | 1.025 | LysM |
| TRINITY_DN56871_c0_g2_i1 | 1.048787424 | 0.65102285 | 0.999970678 | 0.045 | 3.351 | 1.111 | 0.261 | NA |
| TRINITY_DN2426_c0_g1_i3 | -1.0487781 | 0.692871014 | 0.999970678 | 1.681 | 0 | 1.333 | 1.92 | NA |
| TRINITY_DN4995_c0_g2_i1 | 1.048742193 | 0.710306854 | 0.999970678 | 4.933 | 0 | 0.722 | 1.568 | NA |

| TRINITY_DN6389_c0_g1_i1 | -1.04867953 | 0.197159616 | 0.999970678 | 10.601 | 20.35 | 29.265 | 27.748 | Ribosomal_60s |
| --- | --- | --- | --- | --- | --- | --- | --- | --- |
| TRINITY_DN655_c0_g1_i5 | 1.048588561 | 0.524956741 | 0.999970678 | 1.072 | 2.986 | 0.122 | 1.78 | Semialdhyde_dh |
| TRINITY_DN188_c0_g1_i7 | -1.04848412 | 0.225761709 | 0.999970678 | 7.503 | 3.784 | 9.377 | 11.954 | NA |
| TRINITY_DN11944_c0_g1_i2 | 1.048471269 | 0.689343452 | 0.999970678 | 0.618 | 1.242 | 0 | 0.895 | PPR |
| TRINITY_DN1061_c0_g1_i4 | -1.04841162 | 0.163851921 | 0.999970678 | 1.562 | 2.336 | 3.166 | 4.243 | NA |
| TRINITY_DN15326_c0_g1_i1 | -1.04827895 | 0.694292914 | 0.999970678 | 0 | 0.591 | 0.333 | 0.814 | NA |
| TRINITY_DN636_c1_g2_i10 | 1.047950469 | 0.803504486 | 0.999970678 | 8.566 | 0 | 0 | 4.212 | Snf7 |
| TRINITY_DN5173_c0_g1_i2 | -1.04788429 | 0.695129068 | 0.999970678 | 4.378 | 0 | 4.655 | 3.469 | NA |
| TRINITY_DN960_c0_g1_i6 | 1.047870034 | 0.727951295 | 0.999970678 | 7.958 | 0 | 0.256 | 3.599 | Epimerase |
| TRINITY_DN1753_c0_g1_i1 | 1.047796829 | 0.751825825 | 0.999970678 | 0 | 28.47 | 11.088 | 0.151 | Ribosomal_60s |
| TRINITY_DN8249_c0_g1_i7 | 1.047747686 | 0.806599825 | 0.999970678 | 0 | 1.124 | 0.444 | 0 | UDPGT |
| TRINITY_DN325_c0_g3_i3 | 1.047704794 | 0.74073265 | 0.999970678 | 4.197 | 0.079 | 1.744 | 0 | NA |
| TRINITY_DN162_c0_g1_i3 | 1.047671394 | 0.510780553 | 0.999970678 | 3.715 | 8.545 | 4.589 | 0.352 | NA |
| TRINITY_DN1604_c0_g1_i1 | -1.04767062 | 0.711082432 | 0.999970678 | 1.535 | 0.572 | 3.633 | 0 | LTP_2 |
| TRINITY_DN4137_c0_g1_i1 | 1.047484447 | 0.395107375 | 0.999970678 | 3.234 | 8.574 | 1.733 | 3.458 | NA |
| TRINITY_DN4032_c0_g1_i4 | -1.04746514 | 0.813565504 | 1 | 0 | 2.257 | 3.744 | 0 | NA |
| TRINITY_DN973_c5_g1_i1 | 1.047425083 | 0.568399373 | 0.999970678 | 0.327 | 2.838 | 1.044 | 0.241 | NA |
| TRINITY_DN11004_c0_g1_i2 | -1.04737895 | 0.266986457 | 0.999970678 | 0.818 | 0.532 | 1.511 | 0.985 | NA |
| TRINITY_DN856_c0_g1_i2 | -1.04733921 | 0.810191015 | 0.999970678 | 0 | 3.006 | 0 | 6.002 | NA |
| TRINITY_DN9538_c0_g1_i1 | 1.047072165 | 0.804000725 | 0.999970678 | 7.476 | 0 | 3.033 | 0 | ADH_N |
| TRINITY_DN33703_c0_g1_i2 | -1.04684185 | 0.406542957 | 0.999970678 | 12.681 | 27.85 | 15.321 | 59.347 | Glyco_transf_8 |
| TRINITY_DN12449_c0_g2_i1 | -1.04681743 | 0.459660716 | 0.999970678 | 8.121 | 46.05 | 27.243 | 69.24 | NA |
| TRINITY_DN13828_c0_g1_i2 | 1.046762161 | 0.511740929 | 0.999970678 | 1.263 | 0.365 | 0.556 | 0.131 | NA |
| TRINITY_DN16060_c0_g1_i2 | -1.04671959 | 0.695589358 | 0.999970678 | 3.47 | 0 | 3.733 | 2.745 | NA |
| TRINITY_DN4390_c1_g1_i1 | -1.04670848 | 0.693581874 | 0.999970678 | 3.016 | 0 | 2.555 | 3.217 | NA |
| TRINITY_DN1812_c1_g1_i4 | 1.046668359 | 0.119450161 | 0.999970678 | 6.014 | 7.953 | 3.2 | 2.906 | RNA_pol_Rpb2_1 |
| TRINITY_DN2075_c0_g1_i1 | -1.04640557 | 0.363347744 | 0.999970678 | 7.004 | 12.06 | 6.011 | 32.051 | NA |
| TRINITY_DN13080_c0_g1_i1 | -1.0462913 | 0.806358731 | 0.999970678 | 0 | 1.488 | 0 | 3.056 | NA |
| TRINITY_DN40398_c0_g1_i3 | -1.04616205 | 0.808955171 | 0.999970678 | 1.544 | 0 | 2.667 | 0 | NA |
| TRINITY_DN4987_c0_g1_i3 | 1.046091854 | 0.434273524 | 0.999970678 | 1.481 | 3.035 | 1.344 | 0.523 | NA |
| TRINITY_DN3244_c0_g1_i4 | 1.046070086 | 0.81215589 | 1 | 1.517 | 0 | 0 | 0.744 | NA |
| TRINITY_DN6392_c0_g1_i6 | 1.045996018 | 0.490191447 | 0.999970678 | 2.553 | 1.301 | 1.4 | 0.181 | FAR1 |
| TRINITY_DN555_c1_g1_i6 | 1.045896579 | 0.718738833 | 0.999970678 | 19.912 | 1.33 | 0 | 9.973 | NA |
| TRINITY_DN13835_c0_g2_i1 | -1.04585192 | 0.373930909 | 0.999970678 | 1.272 | 1.39 | 0.767 | 4.605 | LRRNT_2 |
| TRINITY_DN1838_c0_g1_i3 | -1.04573277 | 0.805466315 | 0.999970678 | 0 | 3.568 | 6 | 0 | Myb_DNA-bindin |
| TRINITY_DN3169_c0_g1_i5 | 1.045730474 | 0.285635987 | 0.999970678 | 1.135 | 1.311 | 0.511 | 0.583 | NA |
| TRINITY_DN639_c0_g1_i6 | -1.04571683 | 0.803133708 | 0.999970678 | 1.263 | 0 | 0 | 2.654 | NA |
| TRINITY_DN5293_c0_g1_i5 | 1.045457119 | 0.400690782 | 0.999970678 | 1.335 | 4.317 | 0.822 | 1.749 | NA |
| TRINITY_DN8616_c0_g1_i1 | -1.04541706 | 0.814323909 | 1 | 0.754 | 0 | 0 | 1.588 | - |
| TRINITY_DN8281_c0_g2_i2 | 1.045416247 | 0.692506757 | 0.999970678 | 1.735 | 2.05 | 1.511 | 0 | NA |
| TRINITY_DN184_c0_g1_i3 | 1.045191318 | 0.258163929 | 0.999970678 | 1.263 | 1.183 | 0.444 | 0.643 | NA |
| TRINITY_DN22522_c0_g1_i3 | -1.04517764 | 0.816133524 | 1 | 0.927 | 0 | 1.633 | 0 | NA |
| TRINITY_DN5538_c0_g1_i7 | -1.04517609 | 0.708054324 | 0.999970678 | 0.59 | 1.646 | 0 | 4.605 | NA |
| TRINITY_DN9961_c0_g1_i7 | -1.04509982 | 0.705413506 | 0.999970678 | 10.328 | 0 | 4.044 | 16.689 | Acetyltransf_3 |
| TRINITY_DN4231_c0_g1_i4 | 1.045082694 | 0.393909564 | 0.999970678 | 4.315 | 17.3 | 6.3 | 2.755 | PDZ |
| TRINITY_DN1961_c0_g1_i3 | 1.045076016 | 0.234190041 | 0.999970678 | 146.84 | 79.88 | 60.541 | 37.309 | NA |
| TRINITY_DN3560_c0_g1_i6 | 1.04495382 | 0.711003193 | 0.999970678 | 0 | 4.829 | 0.544 | 1.649 | - |
| TRINITY_DN1195_c0_g2_i5 | 1.04492974 | 0.548824928 | 0.999970678 | 2.798 | 43.27 | 15.71 | 2.966 | PfkB |
| TRINITY_DN37379_c0_g1_i4 | -1.04478348 | 0.513021274 | 0.999970678 | 0.663 | 0.838 | 0.233 | 2.815 | NA |
| TRINITY_DN167_c0_g1_i3 | 1.044689499 | 0.360445434 | 0.999970678 | 0.636 | 1.478 | 0.3 | 0.664 | EMP70 |
| TRINITY_DN7747_c0_g1_i8 | 1.044642547 | 0.711916872 | 1 | 2.689 | 0.759 | 1.378 | 0 | NA |
| TRINITY_DN3615_c0_g1_i3 | -1.044511 | 0.702872187 | 0.999970678 | 3.561 | 0 | 2.6 | 4.323 | ADH_N |
| TRINITY_DN8479_c0_g1_i2 | 1.044415781 | 0.315329522 | 0.999970678 | 1.644 | 3.932 | 0.811 | 1.729 | NA |
| TRINITY_DN1583_c0_g1_i3 | 1.044270438 | 0.61422357 | 0.999970678 | 35.836 | 40.06 | 0.178 | 36.575 | - |
| TRINITY_DN5808_c0_g1_i1 | -1.04425817 | 0.478997279 | 0.999970678 | 6.341 | 5.696 | 1.567 | 22.973 | NA |
| TRINITY_DN1433_c0_g4_i1 | -1.04423678 | 0.606636253 | 0.999970678 | 7.258 | 0.473 | 11.022 | 2.463 | NA |
| TRINITY_DN4478_c0_g1_i4 | 1.044223959 | 0.446013557 | 0.999970678 | 4.887 | 3.666 | 2.955 | 0.573 | Pro_isomerase |
| TRINITY_DN2405_c0_g1_i8 | -1.04397324 | 0.751166581 | 0.999970678 | 0.045 | 5.765 | 0 | 11.863 | Sugar_tr |
| TRINITY_DN4311_c0_g1_i2 | 1.043898229 | 0.344808652 | 0.999970678 | 1.054 | 3.292 | 1.111 | 0.764 | NA |
| TRINITY_DN70_c0_g1_i1 | -1.04382982 | 0.152966613 | 0.999970678 | 5.886 | 10.24 | 12.91 | 17.554 | UDPGT |
| TRINITY_DN13208_c0_g1_i2 | 1.043709846 | 0.22809328 | 0.999970678 | 2.053 | 3.439 | 1.455 | 0.895 | AAA |
| TRINITY_DN6163_c0_g1_i2 | -1.04364188 | 0.433814977 | 0.999970678 | 4.015 | 1.399 | 1.822 | 9.048 | NA |
| TRINITY_DN2858_c0_g1_i7 | -1.04360218 | 0.814058953 | 1 | 2.053 | 0 | 3.533 | 0 | F_bP_aldolase |
| TRINITY_DN2523_c1_g1_i2 | -1.04360218 | 0.814058953 | 1 | 1.744 | 0 | 3.078 | 0 | Aldo_ket_red |
| TRINITY_DN1621_c0_g1_i5 | 1.043375545 | 0.3846959 | 0.999970678 | 6.668 | 2.336 | 0.878 | 3.358 | ERCC3_RAD25_C |
| TRINITY_DN5958_c0_g2_i2 | -1.04320603 | 0.486560811 | 0.999970678 | 1.135 | 0.591 | 2.511 | 0.543 | peroxidase |
| TRINITY_DN7702_c0_g2_i3 | 1.043179667 | 0.340207396 | 0.999970678 | 1.844 | 4.888 | 0.767 | 2.332 | Exo70 |
| TRINITY_DN7689_c0_g1_i1 | -1.04312988 | 0.311242772 | 0.999970678 | 0.854 | 2.907 | 2.844 | 4.283 | NA |
| TRINITY_DN1332_c0_g4_i2 | 1.042991586 | 0.391800695 | 0.999970678 | 1.372 | 2.809 | 1.111 | 0.653 | NA |
| TRINITY_DN80226_c0_g2_i4 | 1.042963346 | 0.346506501 | 0.999970678 | 3.652 | 1.882 | 1.622 | 0.754 | SRP40_C |
| TRINITY_DN15682_c0_g1_i2 | -1.04292997 | 0.805619986 | 0.999970678 | 5.36 | 0 | 9.288 | 0 | NA |
| TRINITY_DN2264_c1_g1_i5 | -1.04288464 | 0.612175749 | 0.999970678 | 0.881 | 0.03 | 1.022 | 0.674 | NA |
| TRINITY_DN4379_c0_g1_i2 | -1.04269848 | 0.671267018 | 0.999970678 | 0.173 | 6.475 | 0.167 | 13.03 | NA |
| TRINITY_DN503_c0_g1_i5 | -1.04264533 | 0.274791092 | 0.999970678 | 1.072 | 1.981 | 2.689 | 2.946 | S10_plectin |

| TRINITY_DN2588_c0_g1_i1 | 1.042622269 | 0.728631862 | 0.999970678 | 0 | 22.13 | 7.4 | 1.629 | NA |
| --- | --- | --- | --- | --- | --- | --- | --- | --- |
| TRINITY_DN8375_c0_g1_i4 | -1.0425246 | 0.478023094 | 0.999970678 | 0.218 | 0.966 | 1.278 | 0.885 | LIM |
| TRINITY_DN234047_c0_g1_i1 | -1.04246033 | 0.812439182 | 0.999970678 | 1.054 | 0 | 1.822 | 0 | Lipase_3 |
| TRINITY_DN9496_c0_g1_i11 | -1.04246033 | 0.812439182 | 0.999970678 | 1.254 | 0 | 2.2 | 0 | NA |
| TRINITY_DN46762_c0_g1_i1 | -1.04242539 | 0.806551771 | 0.999970678 | 1.244 | 0 | 2.144 | 0 | NA |
| TRINITY_DN12070_c0_g1_i6 | -1.04240963 | 0.804652095 | 0.999970678 | 4.006 | 0 | 6.911 | 0 | NA |
| TRINITY_DN32709_c0_g1_i7 | 1.042148266 | 0.58207034 | 0.999970678 | 2.725 | 0.207 | 0.211 | 1.186 | NA |
| TRINITY_DN2482_c0_g1_i9 | -1.04210679 | 0.716533463 | 0.999970678 | 0 | 0.986 | 1.433 | 0.292 | NA |
| TRINITY_DN3988_c0_g1_i4 | 1.042090675 | 0.502951336 | 0.999970678 | 0.645 | 0.995 | 0.578 | 0.101 | Phosphorylase |
| TRINITY_DN6318_c0_g3_i6 | -1.04201446 | 0.706469299 | 0.999970678 | 1.417 | 0 | 0.556 | 2.292 | Myb_DNA-bindin |
| TRINITY_DN750_c0_g1_i9 | -1.04196912 | 0.439142815 | 0.999970678 | 3.043 | 0.453 | 3.289 | 3.298 | NA |
| TRINITY_DN28394_c0_g1_i1 | -1.04178877 | 0.435659569 | 1 | 0.572 | 0.246 | 0.756 | 0.784 | - |
| TRINITY_DN28394_c0_g1_i4 | -1.04178877 | 0.435659569 | 1 | 0.572 | 0.246 | 0.756 | 0.784 | - |
| TRINITY_DN7517_c0_g1_i2 | -1.04159413 | 0.811169588 | 0.999970678 | 6.649 | 0 | 11.233 | 0 | NA |
| TRINITY_DN8753_c0_g1_i1 | -1.04159413 | 0.811169588 | 0.999970678 | 0.545 | 0 | 0.944 | 0 | NA |
| TRINITY_DN26287_c0_g2_i2 | -1.04145911 | 0.355883129 | 0.999970678 | 2.244 | 0.976 | 1.655 | 4.655 | NA |
| TRINITY_DN5860_c0_g1_i1 | 1.041436708 | 0.444191228 | 0.999970678 | 2.716 | 2.326 | 1.5 | 0.623 | NA |
| TRINITY_DN7491_c0_g1_i4 | -1.04139877 | 0.498183312 | 0.999970678 | 11.945 | 2.651 | 7.922 | 19.052 | NA |
| TRINITY_DN7676_c0_g1_i1 | -1.04134726 | 0.695272572 | 0.999970678 | 0 | 1.163 | 1.311 | 0.784 | NA |
| TRINITY_DN9264_c0_g1_i2 | 1.041311645 | 0.703729368 | 0.999970678 | 3.152 | 2.089 | 2.111 | 0 | NA |
| TRINITY_DN11735_c0_g1_i3 | -1.04130221 | 0.703835088 | 0.999970678 | 0.291 | 0 | 0.133 | 0.452 | DUF3730 |
| TRINITY_DN754_c1_g1_i8 | -1.04110837 | 0.695561779 | 0.999970678 | 1.608 | 0 | 1.622 | 1.408 | LRR_6 |
| TRINITY_DN2717_c0_g1_i4 | 1.041083419 | 0.740209229 | 0.999970678 | 2.198 | 0 | 0.067 | 1.015 | NA |
| TRINITY_DN961_c5_g1_i5 | -1.04101312 | 0.816930903 | 0.999970678 | 0 | 33.42 | 55.941 | 0 | NA |
| TRINITY_DN846_c0_g1_i2 | -1.04092331 | 0.264383178 | 0.999970678 | 2.498 | 3.045 | 6.766 | 3.207 | ATP-cone |
| TRINITY_DN11351_c1_g1_i1 | -1.04084253 | 0.70861611 | 0.999970678 | 0 | 2.996 | 4.266 | 0.885 | NA |
| TRINITY_DN336_c1_g1_i2 | -1.04078925 | 0.694894544 | 0.999970678 | 0 | 5.312 | 4.689 | 5.087 | WRKY |
| TRINITY_DN3807_c0_g1_i2 | -1.04045673 | 0.145402904 | 0.999970678 | 35.382 | 38.28 | 46.453 | 94.183 | NA |
| TRINITY_DN9883_c0_g1_i5 | 1.040379777 | 0.439186516 | 0.999970678 | 2.135 | 1.498 | 1.289 | 0.201 | - |
| TRINITY_DN9636_c0_g1_i3 | 1.040306321 | 0.813004317 | 0.999970678 | 0 | 15.38 | 5.933 | 0 | NA |
| TRINITY_DN59040_c0_g1_i1 | 1.040164326 | 0.81191061 | 0.999970678 | 1.435 | 0 | 0.589 | 0 | NA |
| TRINITY_DN17555_c0_g1_i8 | 1.040099617 | 0.625094803 | 1 | 0.055 | 0.641 | 0.022 | 0.302 | Cation_ATPase_N |
| TRINITY_DN542_c0_g3_i1 | 1.039758827 | 0.092856224 | 0.999970678 | 7.948 | 8.15 | 3.689 | 3.398 | NA |
| TRINITY_DN1519_c0_g1_i1 | 1.039650148 | 0.581283359 | 0.999970678 | 52.841 | 43.33 | 0.611 | 45.493 | NA |
| TRINITY_DN1020_c0_g1_i3 | 1.039584315 | 0.494749123 | 0.999970678 | 57.574 | 6.761 | 4.833 | 25.949 | PUB |
| TRINITY_DN1067_c0_g1_i2 | 1.039558832 | 0.302571262 | 0.999970678 | 17.241 | 24.17 | 12.755 | 4.695 | Peptidase_S9 |
| TRINITY_DN1969_c0_g2_i3 | -1.03955588 | 0.457590141 | 0.999970678 | 2.616 | 1.163 | 1.322 | 6.153 | WD40 |
| TRINITY_DN1641_c0_g1_i12 | 1.039437522 | 0.707347175 | 1 | 0.309 | 1.055 | 0 | 0.684 | NA |
| TRINITY_DN7199_c0_g1_i2 | 1.039426659 | 0.555403143 | 1 | 0.609 | 0.158 | 0.267 | 0.06 | NA |
| TRINITY_DN11211_c0_g2_i1 | -1.03937599 | 0.58034478 | 1 | 0.091 | 0.68 | 1.078 | 0.271 | NA |
| TRINITY_DN74442_c0_g1_i2 | -1.03928425 | 0.488167669 | 0.999970678 | 0.409 | 0.128 | 0.256 | 0.824 | NA |
| TRINITY_DN106_c0_g1_i1 | -1.03923677 | 0.386428573 | 0.999970678 | 0.345 | 0.641 | 1.144 | 0.603 | TPR_10 |
| TRINITY_DN10595_c0_g1_i2 | -1.03923393 | 0.351657054 | 0.999970678 | 1.59 | 3.696 | 5.911 | 3.66 | NA |
| TRINITY_DN1878_c0_g1_i2 | 1.039156028 | 0.452164956 | 0.999970678 | 9.138 | 1.252 | 2.355 | 2.282 | NA |
| TRINITY_DN2288_c0_g1_i2 | 1.039022626 | 0.709338436 | 0.999970678 | 0 | 2.828 | 0.6 | 0.623 | DUF5311 |
| TRINITY_DN5099_c0_g1_i1 | 1.038980869 | 0.429316615 | 0.999970678 | 17.577 | 11.39 | 10.11 | 1.86 | NA |
| TRINITY_DN641_c0_g1_i4 | 1.038847252 | 0.519072668 | 0.999970678 | 5.732 | 1.173 | 2.522 | 0.352 | NA |
| TRINITY_DN9700_c0_g1_i2 | 1.038738509 | 0.329976495 | 0.999970678 | 0.745 | 1.064 | 0.233 | 0.593 | NA |
| TRINITY_DN4730_c1_g1_i3 | -1.03873054 | 0.559853052 | 0.999970678 | 1.036 | 0.227 | 1.967 | 0.221 | Glyco_hydro_1 |
| TRINITY_DN8099_c0_g1_i4 | -1.03860905 | 0.449219758 | 0.999970678 | 0.609 | 0.148 | 0.678 | 0.744 | - |
| TRINITY_DN9424_c0_g1_i4 | 1.038182366 | 0.417832189 | 0.999970678 | 1.463 | 0.483 | 0.333 | 0.563 | NA |
| TRINITY_DN8619_c0_g1_i4 | -1.03817037 | 0.507294398 | 0.999970678 | 3.225 | 1.932 | 7.611 | 1.327 | NA |
| TRINITY_DN7258_c0_g1_i11 | 1.038042706 | 0.239958486 | 0.999970678 | 1.399 | 2.07 | 0.933 | 0.563 | NA |
| TRINITY_DN11369_c0_g1_i1 | 1.038033935 | 0.616044627 | 0.999970678 | 6.159 | 2.464 | 3.378 | 0.111 | NA |
| TRINITY_DN9937_c0_g1_i1 | 1.038000049 | 0.197238785 | 0.999970678 | 11.791 | 10.38 | 3.566 | 6.314 | NA |
| TRINITY_DN14694_c0_g1_i2 | -1.03798911 | 0.333211103 | 0.999970678 | 0.245 | 0.315 | 0.567 | 0.452 | NA |
| TRINITY_DN2081_c0_g1_i5 | -1.03795355 | 0.495553131 | 0.999970678 | 0.354 | 0.926 | 0.244 | 2.322 | NA |
| TRINITY_DN4738_c0_g1_i1 | -1.03794446 | 0.811975144 | 0.999970678 | 19.312 | 0 | 33.242 | 0 | NA |
| TRINITY_DN9229_c0_g2_i1 | -1.03785935 | 0.805785386 | 0.999970678 | 31.194 | 0 | 53.141 | 0 | NA |
| TRINITY_DN4242_c0_g2_i8 | 1.037813848 | 0.708243925 | 1 | 0.491 | 0.138 | 0 | 0.312 | NA |
| TRINITY_DN1568_c0_g1_i8 | 1.037797934 | 0.450161239 | 0.999970678 | 0.827 | 0.976 | 0.556 | 0.201 | Glyco_hydro_1 |
| TRINITY_DN583_c1_g1_i16 | 1.037704471 | 0.378305305 | 0.999970678 | 3.47 | 1.192 | 1.3 | 0.724 | Pkinase |
| TRINITY_DN1158_c0_g4_i3 | 1.037688736 | 0.495607532 | 0.999970678 | 6.922 | 1.991 | 3.278 | 0.422 | NA |
| TRINITY_DN9958_c0_g1_i8 | -1.03764369 | 0.713188075 | 1 | 2.162 | 0 | 0.889 | 3.267 | NA |
| TRINITY_DN3291_c0_g1_i1 | 1.037616473 | 0.733900722 | 0.999970678 | 0 | 14.24 | 5.322 | 0.382 | BES1_N |
| TRINITY_DN3436_c0_g1_i2 | 1.037611129 | 0.126945274 | 0.999970678 | 4.787 | 5.558 | 2.467 | 2.061 | ACAS_N |
| TRINITY_DN458_c0_g1_i16 | -1.03723688 | 0.244602444 | 0.999970678 | 5.587 | 6.692 | 14.055 | 8.164 | Epimerase |
| TRINITY_DN8732_c0_g1_i1 | -1.03707068 | 0.594726513 | 0.999970678 | 4.269 | 0.099 | 2.555 | 5.932 | NA |
| TRINITY_DN10381_c0_g1_i10 | -1.03701299 | 0.754487252 | 0.999970678 | 0.009 | 0.779 | 1.322 | 0 | NA |
| TRINITY_DN4948_c1_g1_i3 | -1.03697069 | 0.406579471 | 0.999970678 | 3.67 | 6.307 | 2.722 | 17.091 | NA |
| TRINITY_DN12239_c0_g1_i1 | 1.036817804 | 0.304646737 | 0.999970678 | 9.647 | 8.328 | 1.678 | 6.776 | ABC2_membrane |
| TRINITY_DN8905_c0_g1_i1 | -1.03679039 | 0.613004163 | 1 | 0.609 | 0.049 | 0.978 | 0.201 | NA |
| TRINITY_DN14595_c0_g1_i4 | -1.0367151 | 0.291175413 | 0.999970678 | 400.45 | 292.8 | 857.348 | 328.243 | NA |
| TRINITY_DN7342_c0_g1_i7 | -1.03657034 | 0.817271848 | 1 | 0 | 0.266 | 0.456 | 0 | NA |

| TRINITY_DN40348_c0_g1_i1 | -1.03651176 | 0.691364196 | 1 | 1.463 | 0.059 | 2.544 | 0.06 | NA |
| --- | --- | --- | --- | --- | --- | --- | --- | --- |
| TRINITY_DN277_c0_g1_i5 | -1.03642585 | 0.197959715 | 0.999970678 | 24.427 | 22.33 | 25.798 | 64.595 | NA |
| TRINITY_DN794_c0_g1_i3 | -1.03636823 | 0.249134131 | 0.999970678 | 156.12 | 107.8 | 129.503 | 385.62 | Dimerisation |
| TRINITY_DN3244_c0_g2_i1 | -1.03629128 | 0.367469163 | 0.999970678 | 0.409 | 1.192 | 1.367 | 1.639 | - |
| TRINITY_DN10468_c0_g1_i2 | -1.03612074 | 0.391880634 | 0.999970678 | 0.309 | 0.848 | 1.022 | 1.156 | - |
| TRINITY_DN4404_c0_g1_i1 | -1.03605273 | 0.620065906 | 1 | 1.871 | 0.394 | 3.555 | 0.332 | NA |
| TRINITY_DN3256_c0_g1_i4 | 1.035949962 | 0.807628478 | 0.999970678 | 0 | 12.18 | 4.844 | 0 | NA |
| TRINITY_DN5155_c0_g1_i1 | -1.03594124 | 0.72036301 | 1 | 0 | 0.473 | 0.656 | 0.161 | Abhydrolase_3 |
| TRINITY_DN13044_c0_g1_i2 | -1.0359366 | 0.714208572 | 0.999970678 | 0.718 | 0 | 0.156 | 1.327 | HGTP_anticodon |
| TRINITY_DN9773_c0_g1_i11 | 1.035934208 | 0.348256794 | 0.999970678 | 0.881 | 1.153 | 0.378 | 0.523 | NA |
| TRINITY_DN25046_c0_g2_i3 | 1.03591376 | 0.816332987 | 1 | 2.407 | 0 | 0.978 | 0 | Glyco_hydro_114 |
| TRINITY_DN8246_c0_g1_i3 | 1.03591376 | 0.816332987 | 1 | 0.391 | 0 | 0.167 | 0 | - |
| TRINITY_DN16594_c1_g1_i1 | 1.035859518 | 0.45943366 | 0.999970678 | 0.227 | 0.7 | 0.256 | 0.141 | NA |
| TRINITY_DN832_c1_g2_i2 | 1.035774781 | 0.460601748 | 0.999970678 | 9.311 | 7.096 | 5.744 | 1.066 | NA |
| TRINITY_DN297_c0_g1_i5 | 1.035765535 | 0.288751466 | 0.999970678 | 11.509 | 5.194 | 2.4 | 5.268 | NAM |
| TRINITY_DN2160_c0_g1_i3 | -1.03569941 | 0.404274669 | 0.999970678 | 2.117 | 2.858 | 1.611 | 8.194 | NA |
| TRINITY_DN8570_c0_g2_i2 | -1.03560444 | 0.705922284 | 0.999970678 | 7.885 | 0 | 8.777 | 5.771 | NA |
| TRINITY_DN7752_c1_g1_i2 | -1.0354156 | 0.258762692 | 0.999970678 | 1.399 | 0.897 | 2.244 | 1.991 | Epimerase |
| TRINITY_DN40424_c0_g1_i1 | -1.03527373 | 0.709654019 | 0.999970678 | 0.954 | 0 | 1.267 | 0.452 | NA |
| TRINITY_DN1946_c0_g1_i5 | 1.035205849 | 0.695051274 | 0.999970678 | 1.989 | 2.336 | 1.755 | 0 | NA |
| TRINITY_DN6615_c0_g1_i2 | 1.035192896 | 0.700695771 | 0.999970678 | 0.273 | 1.045 | 0 | 0.643 | HECT |
| TRINITY_DN13317_c1_g1_i1 | 1.035104129 | 0.378872838 | 0.999970678 | 0.79 | 1.34 | 0.2 | 0.794 | DEAD |
| TRINITY_DN3570_c0_g2_i7 | 1.03504005 | 0.702438326 | 0.999970678 | 3.207 | 1.192 | 1.767 | 0 | NA |
| TRINITY_DN1324_c0_g2_i2 | 1.035012014 | 0.735777273 | 0.999970678 | 5.514 | 0 | 0.178 | 2.463 | NA |
| TRINITY_DN8288_c0_g1_i3 | -1.03500884 | 0.253654192 | 0.999970678 | 7.512 | 8.485 | 8.122 | 22.762 | DUF212 |
| TRINITY_DN2542_c0_g1_i7 | -1.03496973 | 0.751345546 | 0.999970678 | 1.59 | 0 | 0.033 | 3.278 | NA |
| TRINITY_DN5774_c0_g2_i1 | -1.03487333 | 0.450898352 | 0.999970678 | 0.5 | 0.345 | 1.111 | 0.412 | NA |
| TRINITY_DN6222_c0_g1_i2 | -1.03485354 | 0.247474218 | 0.999970678 | 0.772 | 0.552 | 1.311 | 1.126 | Lzipper-MIP1 |
| TRINITY_DN1508_c0_g1_i2 | 1.034841153 | 0.574614252 | 0.999970678 | 13.217 | 42.64 | 0.856 | 24.32 | NA |
| TRINITY_DN416_c0_g1_i7 | 1.034785432 | 0.3312543 | 0.999970678 | 20.039 | 69.52 | 19.099 | 19.293 | NA |
| TRINITY_DN8953_c0_g1_i4 | -1.03467124 | 0.711442441 | 0.999970678 | 0 | 1.133 | 0.267 | 1.971 | NA |
| TRINITY_DN72474_c0_g1_i3 | 1.034668623 | 0.712994792 | 0.999970678 | 11.528 | 0 | 1.678 | 3.66 | NA |
| TRINITY_DN9939_c0_g1_i1 | -1.03448319 | 0.274797167 | 0.999970678 | 0.663 | 1.261 | 1.4 | 2.222 | NA |
| TRINITY_DN5000_c1_g1_i1 | -1.03442741 | 0.202170562 | 0.999970678 | 2.843 | 2.927 | 3.511 | 7.58 | DUF1977 |
| TRINITY_DN5919_c0_g1_i1 | -1.03420284 | 0.719546767 | 0.999970678 | 0 | 6.721 | 10.255 | 1.156 | CPP1-like |
| TRINITY_DN13234_c0_g1_i6 | -1.03417735 | 0.301970816 | 0.999970678 | 0.436 | 0.394 | 0.833 | 0.694 | NA |
| TRINITY_DN1483_c0_g1_i2 | 1.034102117 | 0.265934161 | 0.999970678 | 4.106 | 2.602 | 1.333 | 1.649 | NA |
| TRINITY_DN19354_c0_g2_i11 | 1.034074664 | 0.256441854 | 0.999970678 | 1.135 | 0.877 | 0.378 | 0.523 | NA |
| TRINITY_DN12042_c0_g1_i1 | -1.03390804 | 0.565452102 | 1 | 0.055 | 0.641 | 0.567 | 0.724 | NA |
| TRINITY_DN6644_c0_g1_i3 | 1.033636438 | 0.696001442 | 0.999970678 | 0.273 | 0.168 | 0 | 0.211 | zf-CW |
| TRINITY_DN5966_c0_g1_i4 | -1.03345541 | 0.424251382 | 0.999970678 | 0.563 | 2.237 | 1.3 | 4.102 | LRRNT_2 |
| TRINITY_DN1039_c1_g1_i10 | 1.032964167 | 0.566683125 | 0.999970678 | 0.4 | 18.08 | 4.389 | 3.609 | B_lectin |
| TRINITY_DN16151_c0_g1_i1 | 1.032919957 | 0.302934088 | 0.999970678 | 14.198 | 10.94 | 7.655 | 3.056 | NA |
| TRINITY_DN462_c0_g1_i1 | -1.03269219 | 0.698685801 | 0.999970678 | 3.652 | 0 | 2.567 | 4.484 | NA |
| TRINITY_DN4847_c2_g1_i2 | -1.03258449 | 0.389418902 | 0.999970678 | 2.707 | 0.828 | 1.955 | 4.866 | NA |
| TRINITY_DN7258_c0_g1_i7 | -1.03240569 | 0.227519908 | 0.999970678 | 1.717 | 1.537 | 2.2 | 4.021 | NA |
| TRINITY_DN2401_c0_g1_i3 | -1.03220481 | 0.543111152 | 0.999970678 | 7.149 | 0.532 | 3.455 | 11.431 | - |
| TRINITY_DN1454_c0_g1_i7 | -1.03220323 | 0.572559584 | 0.999970678 | 0.145 | 6.041 | 6.277 | 4.896 | NA |
| TRINITY_DN8110_c0_g1_i1 | -1.03218988 | 0.706803246 | 0.999970678 | 10.846 | 0 | 11.233 | 8.948 | NA |
| TRINITY_DN4059_c0_g1_i5 | 1.032176248 | 0.360471264 | 0.999970678 | 2.38 | 8.574 | 2.378 | 2.443 | UBA |
| TRINITY_DN13674_c0_g1_i5 | -1.03217587 | 0.509981635 | 0.999970678 | 5.005 | 0.779 | 3.3 | 7.55 | NA |
| TRINITY_DN1613_c0_g1_i1 | 1.032091311 | 0.751173248 | 0.999970678 | 17.169 | 0 | 0.1 | 8.425 | NA |
| TRINITY_DN17536_c0_g1_i6 | -1.03194158 | 0.411741057 | 0.999970678 | 5.932 | 2.631 | 7.511 | 7.952 | NA |
| TRINITY_DN1507_c0_g1_i1 | 1.031906651 | 0.394111634 | 0.999970678 | 2.226 | 0.818 | 0.856 | 0.462 | BRCT_2 |
| TRINITY_DN1584_c0_g1_i7 | -1.03179283 | 0.246187419 | 0.999970678 | 2.298 | 4.228 | 3.8 | 8.727 | B_lectin |
| TRINITY_DN4167_c0_g1_i1 | 1.031602275 | 0.412849232 | 0.999970678 | 2.861 | 2.139 | 0.467 | 1.87 | NA |
| TRINITY_DN16191_c0_g1_i3 | 1.031599886 | 0.623818186 | 0.999970678 | 0.291 | 2.513 | 1.067 | 0.06 | NA |
| TRINITY_DN11262_c0_g2_i1 | -1.03143557 | 0.468968163 | 1 | 0.227 | 0.749 | 0.678 | 1.156 | NA |
| TRINITY_DN8501_c0_g1_i6 | 1.031395606 | 0.813135923 | 0.999970678 | 0.917 | 0 | 0.367 | 0 | NA |
| TRINITY_DN6722_c0_g1_i13 | 1.031395606 | 0.813135923 | 0.999970678 | 1.39 | 0 | 0.556 | 0 | NA |
| TRINITY_DN4067_c0_g1_i15 | 1.031263877 | 0.284379843 | 0.999970678 | 91.439 | 221.8 | 80.806 | 50.178 | NA |
| TRINITY_DN5667_c0_g1_i5 | -1.03118263 | 0.255079651 | 0.999970678 | 2.725 | 2.434 | 5.889 | 3.418 | NA |
| TRINITY_DN13748_c0_g1_i6 | 1.031009848 | 0.516149424 | 0.999970678 | 1.626 | 1.212 | 1.055 | 0.111 | NA |
| TRINITY_DN1200_c0_g1_i4 | 1.030891027 | 0.810280698 | 0.999970678 | 0 | 43.08 | 17.11 | 0 | NA |
| TRINITY_DN13131_c0_g1_i1 | 1.030830487 | 0.343720367 | 0.999970678 | 1.145 | 1.124 | 0.322 | 0.724 | NA |
| TRINITY_DN1721_c0_g1_i8 | 1.030798985 | 0.276716867 | 0.999970678 | 1.081 | 0.729 | 0.422 | 0.382 | Fasciclin |
| TRINITY_DN2142_c0_g1_i4 | -1.03078821 | 0.514022273 | 0.999970678 | 1.326 | 7.667 | 1.844 | 15.935 | A_deaminase |
| TRINITY_DN11_c0_g1_i3 | 1.030768238 | 0.469062529 | 0.999970678 | 21.02 | 13.35 | 12.544 | 1.629 | NA |
| TRINITY_DN8913_c0_g1_i2 | 1.030759138 | 0.700420272 | 0.999970678 | 0.182 | 0.522 | 0 | 0.342 | PB1 |
| TRINITY_DN2156_c1_g1_i1 | -1.03070016 | 0.172732992 | 0.999970678 | 21.047 | 12.52 | 25.232 | 38.053 | Ribonuclease_T2 |
| TRINITY_DN10233_c1_g2_i1 | 1.030694239 | 0.806394544 | 0.999970678 | 0 | 3.341 | 1.333 | 0 | NA |
| TRINITY_DN7709_c0_g1_i10 | 1.030660166 | 0.431333269 | 0.999970678 | 0.763 | 1.291 | 0.567 | 0.302 | MaoC_dehydrata |
| TRINITY_DN869_c0_g1_i1 | 1.030637218 | 0.277876269 | 0.999970678 | 5.532 | 9.816 | 1.922 | 5.137 | NA |
| TRINITY_DN28225_c0_g3_i1 | -1.03052751 | 0.745453468 | 1 | 0.5 | 0.049 | 0.933 | 0 | NA |

| TRINITY_DN12295_c0_g1_i1 | 1.030511703 | 0.430893442 | 0.999970678 | 7.267 | 5.785 | 4.133 | 1.337 | NA |
| --- | --- | --- | --- | --- | --- | --- | --- | --- |
| TRINITY_DN6863_c0_g2_i8 | -1.03040508 | 0.726514056 | 0.999970678 | 0.754 | 0.168 | 1.6 | 0 | NA |
| TRINITY_DN526_c0_g2_i1 | 1.030316677 | 0.458606222 | 0.999970678 | 0.718 | 5.154 | 1.511 | 1.015 | EF-hand_5 |
| TRINITY_DN1285_c0_g1_i1 | -1.03031449 | 0.202858537 | 0.999970678 | 14.471 | 10.94 | 14.421 | 34.725 | CDC50 |
| TRINITY_DN382_c2_g1_i2 | 1.030298165 | 0.521584386 | 0.999970678 | 3.098 | 0.769 | 1.344 | 0.261 | G-patch |
| TRINITY_DN983_c0_g1_i1 | 1.03029011 | 0.742823808 | 0.999970678 | 3.125 | 0.069 | 0 | 1.588 | NA |
| TRINITY_DN23240_c0_g1_i7 | 1.030208919 | 0.55805205 | 0.999970678 | 6.268 | 0.453 | 2.267 | 0.573 | NA |
| TRINITY_DN15051_c0_g1_i2 | 1.03020473 | 0.34646586 | 0.999970678 | 0.917 | 0.522 | 0.256 | 0.402 | - |
| TRINITY_DN11288_c0_g2_i4 | 1.030143961 | 0.411503355 | 1 | 0.409 | 0.552 | 0.133 | 0.292 | NA |
| TRINITY_DN8088_c0_g1_i6 | -1.03013178 | 0.717573679 | 0.999970678 | 0.291 | 0.187 | 0.822 | 0 | NA |
| TRINITY_DN13947_c0_g1_i5 | 1.030079973 | 0.264800392 | 0.999970678 | 6.268 | 5.292 | 1.444 | 3.911 | NA |
| TRINITY_DN23669_c0_g1_i1 | -1.02991007 | 0.812683264 | 0.999970678 | 0 | 0.621 | 0 | 1.237 | PPR |
| TRINITY_DN9699_c0_g2_i2 | -1.02977272 | 0.5652551 | 0.999970678 | 0.218 | 0.443 | 1.033 | 0.08 | NMD3 |
| TRINITY_DN12937_c0_g1_i2 | 1.029702257 | 0.735289529 | 0.999970678 | 0 | 4.514 | 0.156 | 1.9 | NA |
| TRINITY_DN2598_c0_g1_i2 | -1.02968189 | 0.806020728 | 0.999970678 | 2.053 | 0 | 0 | 4.283 | NA |
| TRINITY_DN5714_c0_g1_i5 | -1.02967661 | 0.267147567 | 0.999970678 | 8.23 | 5.302 | 7.011 | 19.182 | NA |
| TRINITY_DN778_c1_g1_i1 | -1.02963977 | 0.361193138 | 0.999970678 | 0.245 | 0.335 | 0.322 | 0.794 | NA |
| TRINITY_DN19994_c0_g2_i1 | 1.029510098 | 0.141979186 | 0.999970678 | 18.023 | 27.56 | 10.266 | 9.812 | CS |
| TRINITY_DN11050_c0_g1_i2 | -1.02948947 | 0.158727421 | 0.999970678 | 2.353 | 2.888 | 3.7 | 6.243 | GUB_WAK_bind |
| TRINITY_DN2373_c0_g1_i4 | 1.029445873 | 0.336078579 | 0.999970678 | 1.163 | 1.242 | 0.722 | 0.302 | NA |
| TRINITY_DN3917_c0_g1_i3 | 1.029444737 | 0.202247617 | 0.999970678 | 45.265 | 33.74 | 10.722 | 25.949 | Glyco_trans_1_4 |
| TRINITY_DN854_c0_g1_i4 | -1.02923432 | 0.262044077 | 0.999970678 | 2.653 | 1.794 | 3.078 | 5.268 | CS |
| TRINITY_DN1303_c0_g1_i4 | 1.028952253 | 0.2529242 | 0.999970678 | 14.934 | 26.43 | 9.122 | 8.737 | GUB_WAK_bind |
| TRINITY_DN53760_c0_g1_i1 | 1.028946477 | 0.714671266 | 1 | 0.636 | 0.187 | 0.333 | 0 | Retrotran_gag_2 |
| TRINITY_DN4299_c0_g1_i1 | -1.02894291 | 0.711186812 | 0.999970678 | 0 | 8.801 | 8.311 | 7.671 | WD40 |
| TRINITY_DN7012_c0_g1_i1 | 1.028905622 | 0.110679125 | 0.999970678 | 52.95 | 39.13 | 18.188 | 23.023 | YGGT |
| TRINITY_DN5155_c0_g1_i2 | 1.028837667 | 0.281665337 | 0.999970678 | 1.19 | 1.488 | 0.589 | 0.593 | Abhydrolase_3 |
| TRINITY_DN8781_c0_g1_i1 | -1.0287548 | 0.385334143 | 0.999970678 | 3.942 | 2.316 | 3.266 | 8.807 | NA |
| TRINITY_DN22211_c0_g1_i2 | 1.028727559 | 0.628932939 | 0.999970678 | 1.608 | 0.059 | 0.578 | 0.121 | NA |
| TRINITY_DN13784_c0_g1_i7 | -1.02810403 | 0.811258656 | 0.999970678 | 0 | 1.301 | 0 | 2.634 | - |
| TRINITY_DN12536_c0_g1_i2 | 1.027976443 | 0.703737163 | 0.999970678 | 1.581 | 0.7 | 0.933 | 0 | NA |
| TRINITY_DN850_c0_g1_i5 | 1.027973822 | 0.177243284 | 0.999970678 | 8.539 | 9.599 | 4.722 | 3.187 | NA |
| TRINITY_DN218_c0_g3_i1 | 1.027965211 | 0.459588283 | 0.999970678 | 6.123 | 4.721 | 3.578 | 0.955 | KIX_2 |
| TRINITY_DN18_c0_g1_i6 | -1.02782927 | 0.174792234 | 0.999970678 | 2.353 | 2.523 | 4.633 | 4.353 | NA |
| TRINITY_DN1986_c0_g1_i2 | 1.027802108 | 0.132278198 | 0.999970678 | 98.979 | 113.8 | 33.731 | 62.182 | NA |
| TRINITY_DN184_c0_g3_i1 | -1.02776973 | 0.388524135 | 0.999970678 | 11.437 | 11.68 | 5.966 | 40.024 | Glyco_transf_17 |
| TRINITY_DN6036_c0_g1_i3 | 1.02774358 | 0.331121591 | 0.999970678 | 0.972 | 2.02 | 0.633 | 0.704 | HLH |
| TRINITY_DN18455_c0_g1_i1 | -1.02771985 | 0.70130431 | 0.999970678 | 15.515 | 0 | 16.877 | 10.737 | NA |
| TRINITY_DN45959_c0_g1_i3 | -1.02771698 | 0.815991853 | 0.999970678 | 0.6 | 0 | 1.033 | 0 | NA |
| TRINITY_DN2077_c0_g1_i3 | -1.02769146 | 0.279071769 | 0.999970678 | 0.718 | 0.503 | 1.389 | 0.814 | Glycos_transf_1 |
| TRINITY_DN1267_c2_g1_i4 | 1.02768197 | 0.361956634 | 0.999970678 | 1.354 | 1.163 | 0.789 | 0.282 | NA |
| TRINITY_DN2778_c0_g1_i5 | -1.02756385 | 0.334989178 | 0.999970678 | 16.796 | 9.008 | 32.187 | 13.613 | NA |
| TRINITY_DN3510_c0_g1_i2 | 1.027434368 | 0.545985781 | 0.999970678 | 1.254 | 1.823 | 1.089 | 0.171 | Rer1 |
| TRINITY_DN5333_c0_g1_i1 | -1.02734821 | 0.300015682 | 0.999970678 | 2.98 | 6.337 | 4.8 | 13.09 | p450 |
| TRINITY_DN2850_c0_g1_i6 | -1.02733659 | 0.408847273 | 0.999970678 | 24.854 | 17.73 | 10.488 | 73.482 | NA |
| TRINITY_DN1437_c2_g1_i8 | 1.027275809 | 0.711263483 | 0.999970678 | 0 | 9.027 | 1.767 | 2.182 | NA |
| TRINITY_DN1848_c1_g1_i2 | 1.027243997 | 0.808669083 | 0.999970678 | 10.347 | 0 | 0 | 5.178 | NA |
| TRINITY_DN4290_c0_g1_i4 | -1.02717855 | 0.444215478 | 0.999970678 | 0.609 | 0.217 | 0.889 | 0.674 | NA |
| TRINITY_DN284_c0_g2_i4 | -1.02708991 | 0.384176549 | 0.999970678 | 198.33 | 44.68 | 136.725 | 325.81 | NA |
| TRINITY_DN12229_c0_g1_i1 | 1.02696394 | 0.486674845 | 0.999970678 | 0.745 | 0.276 | 0.344 | 0.09 | NA |
| TRINITY_DN1219_c0_g1_i5 | -1.02696076 | 0.310019133 | 0.999970678 | 9.175 | 5.982 | 6.322 | 23.405 | RPE65 |
| TRINITY_DN13718_c1_g1_i1 | -1.02690748 | 0.361168681 | 0.999970678 | 50.325 | 67.82 | 160.357 | 43.281 | HSP70 |
| TRINITY_DN12850_c0_g1_i1 | 1.026859083 | 0.538294411 | 0.999970678 | 0.409 | 2.227 | 0.222 | 1.046 | NA |
| TRINITY_DN1196_c0_g1_i4 | -1.02677328 | 0.196813663 | 0.999970678 | 348.31 | 667.4 | 673.081 | 1227.818 | NA |
| TRINITY_DN1361_c0_g1_i7 | 1.026759787 | 0.443651816 | 0.999970678 | 2.189 | 1.301 | 1.044 | 0.442 | ARGLU |
| TRINITY_DN509_c0_g1_i2 | -1.0266821 | 0.200424192 | 0.999970678 | 41.659 | 20.43 | 54.874 | 60.785 | NA |
| TRINITY_DN1361_c0_g1_i4 | 1.026655527 | 0.329726501 | 0.999970678 | 7.776 | 5.598 | 1.678 | 4.444 | ARGLU |
| TRINITY_DN10958_c0_g1_i2 | -1.02657724 | 0.710466821 | 0.999970678 | 1.799 | 0.966 | 0 | 5.69 | DUF674 |
| TRINITY_DN85836_c0_g1_i2 | -1.0265186 | 0.471820028 | 0.999970678 | 0.472 | 0.177 | 0.8 | 0.362 | NA |
| TRINITY_DN3235_c1_g1_i5 | -1.02608865 | 0.443933786 | 0.999970678 | 1.181 | 2.444 | 0.911 | 6.193 | NA |
| TRINITY_DN583_c1_g1_i19 | -1.0260602 | 0.330072472 | 0.999970678 | 0.182 | 0.256 | 0.433 | 0.372 | Pkinase |
| TRINITY_DN5062_c0_g1_i1 | -1.02590686 | 0.551022123 | 0.999970678 | 1.145 | 1.192 | 0.167 | 4.574 | Calmodulin_bind |
| TRINITY_DN4374_c0_g1_i1 | -1.0258839 | 0.063113615 | 0.999970678 | 95.518 | 102.3 | 169.245 | 196.66 | DUF1118 |
| TRINITY_DN5739_c0_g1_i3 | -1.02575571 | 0.699976977 | 0.999970678 | 4.687 | 0 | 4.355 | 4.323 | NA |
| TRINITY_DN5159_c0_g1_i1 | -1.02572403 | 0.420509595 | 0.999970678 | 0.681 | 0.749 | 1.922 | 0.583 | BT1 |
| TRINITY_DN164_c0_g2_i3 | -1.02571217 | 0.579258806 | 0.999970678 | 26.607 | 0.483 | 18.688 | 33.268 | NAC |
| TRINITY_DN6310_c0_g2_i13 | 1.025665117 | 0.698343555 | 0.999970678 | 1.599 | 1.025 | 1.078 | 0 | NA |
| TRINITY_DN12668_c0_g1_i2 | -1.0256484 | 0.525852213 | 0.999970678 | 0.463 | 0.69 | 1.744 | 0.241 | NA |
| TRINITY_DN2195_c1_g1_i1 | -1.02561453 | 0.189753415 | 0.999970678 | 232.97 | 129.4 | 250.74 | 437.487 | Cpn10 |
| TRINITY_DN7104_c1_g1_i1 | 1.025517905 | 0.242419647 | 0.999970678 | 0.672 | 0.887 | 0.322 | 0.382 | NA |
| TRINITY_DN31779_c0_g1_i1 | -1.02534528 | 0.292335829 | 0.999970678 | 6.949 | 14.68 | 17.721 | 20.691 | NA |
| TRINITY_DN3017_c0_g1_i4 | -1.02526474 | 0.584305201 | 0.999970678 | 0.672 | 0.877 | 2.522 | 0.08 | TFIID_20kDa |
| TRINITY_DN11257_c0_g1_i1 | -1.02518561 | 0.615151083 | 0.999970678 | 7.194 | 38.04 | 0.889 | 89.599 | NA |
| TRINITY_DN8631_c1_g1_i5 | 1.025169264 | 0.570242208 | 1 | 0.563 | 0.552 | 0.411 | 0.05 | NA |

| TRINITY_DN6164_c0_g1_i7 | -1.02487317 | 0.607684733 | 0.999970678 | 0.145 | 0.138 | 0.011 | 0.563 | - |
| --- | --- | --- | --- | --- | --- | --- | --- | --- |
| TRINITY_DN760_c0_g2_i3 | 1.024803664 | 0.339770472 | 0.999970678 | 23.664 | 10.59 | 10.51 | 4.263 | Cation_ATPase_C |
| TRINITY_DN9958_c0_g1_i3 | 1.024753446 | 0.495008645 | 0.999970678 | 0.854 | 0.158 | 0.178 | 0.271 | NA |
| TRINITY_DN11681_c0_g2_i6 | 1.024737474 | 0.318258206 | 0.999970678 | 2.271 | 3.568 | 1.778 | 0.714 | NA |
| TRINITY_DN389_c0_g3_i4 | -1.02469043 | 0.418676666 | 0.999970678 | 0.772 | 0.66 | 1.933 | 0.563 | NA |
| TRINITY_DN13218_c0_g2_i2 | 1.024662113 | 0.297265987 | 0.999970678 | 62.943 | 21.32 | 14.944 | 23.485 | NA |
| TRINITY_DN24974_c0_g1_i3 | 1.024652258 | 0.388769085 | 0.999970678 | 0.536 | 0.976 | 0.4 | 0.251 | NA |
| TRINITY_DN6394_c0_g1_i5 | -1.02452545 | 0.638646947 | 0.999970678 | 0.218 | 3.469 | 0.444 | 6.656 | NA |
| TRINITY_DN31334_c0_g1_i1 | 1.024520608 | 0.755989907 | 0.999970678 | 1.163 | 0.02 | 0.489 | 0 | ABC_tran |
| TRINITY_DN17623_c0_g1_i1 | -1.02450771 | 0.82144183 | 1 | 0.763 | 0 | 1.3 | 0 | MFS_1 |
| TRINITY_DN22702_c0_g1_i1 | -1.02450771 | 0.82144183 | 1 | 0.318 | 0 | 0.544 | 0 | NA |
| TRINITY_DN13509_c0_g1_i7 | -1.02450771 | 0.82144183 | 1 | 0.99 | 0 | 1.744 | 0 | Ras |
| TRINITY_DN25_c0_g1_i3 | 1.024356292 | 0.414650335 | 0.999970678 | 0.391 | 1.035 | 0.289 | 0.352 | NA |
| TRINITY_DN19325_c0_g2_i1 | 1.024352428 | 0.248744748 | 0.999970678 | 1.735 | 1.508 | 0.867 | 0.563 | OPT |
| TRINITY_DN867_c0_g1_i6 | 1.024209856 | 0.710702886 | 0.999970678 | 4.933 | 0.966 | 0 | 2.946 | ANF_receptor |
| TRINITY_DN9672_c0_g1_i2 | -1.02417214 | 0.410320147 | 0.999970678 | 0.563 | 0.207 | 0.656 | 0.784 | NA |
| TRINITY_DN1942_c0_g1_i5 | -1.0237937 | 0.483204973 | 0.999970678 | 0.309 | 1.705 | 1.389 | 2.363 | NA |
| TRINITY_DN1721_c0_g1_i5 | -1.02377964 | 0.807904386 | 0.999970678 | 0 | 0.818 | 0 | 1.649 | Fasciclin |
| TRINITY_DN18270_c0_g4_i2 | 1.02365228 | 0.569716723 | 1 | 0.5 | 0.552 | 0.378 | 0.05 | NA |
| TRINITY_DN773_c0_g1_i1 | 1.023585087 | 0.377382523 | 0.999970678 | 2.198 | 10.77 | 2.911 | 2.805 | SH3_1 |
| TRINITY_DN3988_c0_g1_i1 | 1.023344586 | 0.579857032 | 0.999970678 | 3.261 | 1.104 | 1.744 | 0.07 | Phosphorylase |
| TRINITY_DN585_c1_g1_i7 | 1.023188991 | 0.727136525 | 0.999970678 | 1.853 | 0 | 0.567 | 0.221 | NA |
| TRINITY_DN2950_c0_g1_i3 | -1.02318245 | 0.706298211 | 0.999970678 | 2.716 | 0 | 1.6 | 3.71 | AIG1 |
| TRINITY_DN8084_c0_g1_i2 | -1.02318063 | 0.8142697 | 0.999970678 | 0.863 | 0 | 1.455 | 0 | NA |
| TRINITY_DN39125_c0_g1_i2 | -1.02312791 | 0.186315126 | 0.999970678 | 32.284 | 41.3 | 80.717 | 51.364 | PsbP |
| TRINITY_DN2806_c0_g1_i2 | -1.02275574 | 0.509474773 | 0.999970678 | 15.125 | 69.63 | 12.91 | 155.179 | NA |
| TRINITY_DN1004_c0_g1_i6 | -1.02258226 | 0.160036451 | 0.999970678 | 2.798 | 3.942 | 4.655 | 8.093 | Metallophos |
| TRINITY_DN4803_c0_g1_i3 | 1.022542169 | 0.695522103 | 0.999970678 | 0.863 | 1.409 | 0 | 1.126 | F-box |
| TRINITY_DN92_c2_g1_i3 | -1.02250652 | 0.55669981 | 0.999970678 | 1.989 | 0.069 | 1.233 | 2.795 | Vps52 |
| TRINITY_DN18_c0_g1_i2 | -1.02249723 | 0.274009272 | 0.999970678 | 6.332 | 5.47 | 5.622 | 17.192 | NA |
| TRINITY_DN289_c0_g1_i17 | 1.022466538 | 0.583025632 | 0.999970678 | 2.471 | 0.404 | 1.122 | 0.07 | DUF659 |
| TRINITY_DN1873_c3_g1_i6 | 1.02242581 | 0.722641255 | 0.999970678 | 5.532 | 0 | 1.778 | 0.623 | NA |
| TRINITY_DN30846_c0_g2_i1 | -1.02235494 | 0.544253828 | 0.999970678 | 0.509 | 0.177 | 1 | 0.191 | NA |
| TRINITY_DN8973_c1_g2_i3 | -1.02228129 | 0.711965463 | 1 | 9.302 | 0 | 7.211 | 9.511 | NA |
| TRINITY_DN757_c1_g1_i5 | -1.02225382 | 0.174600473 | 0.999970678 | 1.88 | 1.577 | 3.4 | 2.926 | AUX_IAA |
| TRINITY_DN10541_c0_g2_i2 | -1.02215957 | 0.371840585 | 0.999970678 | 2.58 | 2.966 | 2.333 | 8.284 | - |
| TRINITY_DN57_c0_g3_i5 | 1.022072049 | 0.597416864 | 0.999970678 | 0.854 | 0.148 | 0.389 | 0.04 | PTR2 |
| TRINITY_DN3802_c0_g1_i3 | 1.022068205 | 0.446309098 | 0.999970678 | 1.49 | 3.686 | 1.733 | 0.442 | NA |
| TRINITY_DN38796_c0_g1_i4 | -1.02195195 | 0.40302602 | 0.999970678 | 0.418 | 0.907 | 0.9 | 1.609 | NA |
| TRINITY_DN44480_c0_g1_i3 | 1.021900272 | 0.642463647 | 0.999970678 | 1.508 | 12.56 | 0.311 | 6.374 | NA |
| TRINITY_DN4674_c1_g1_i3 | 1.021736728 | 0.361220182 | 0.999970678 | 0.536 | 0.641 | 0.356 | 0.141 | HLH |
| TRINITY_DN22666_c0_g1_i1 | 1.021683938 | 0.75511465 | 0.999970678 | 0.091 | 7.756 | 3.133 | 0 | NA |
| TRINITY_DN18323_c0_g1_i6 | -1.02155673 | 0.815888427 | 0.999970678 | 4.497 | 0 | 7.522 | 0 | NA |
| TRINITY_DN53871_c0_g1_i1 | -1.02153588 | 0.812095237 | 0.999970678 | 0.999 | 0 | 1.7 | 0 | NA |
| TRINITY_DN11134_c0_g1_i1 | -1.02145318 | 0.609058342 | 0.999970678 | 0.136 | 10.44 | 13.044 | 5.339 | NA |
| TRINITY_DN7426_c0_g1_i1 | -1.0213285 | 0.470398604 | 0.999970678 | 0.854 | 0.67 | 0.511 | 2.493 | PPR |
| TRINITY_DN10175_c0_g1_i9 | 1.021310942 | 0.326113816 | 0.999970678 | 3.388 | 1.577 | 0.633 | 1.699 | NA |
| TRINITY_DN1667_c0_g1_i4 | 1.02129481 | 0.812090281 | 0.999970678 | 0 | 52.94 | 0 | 23.063 | NA |
| TRINITY_DN296_c0_g1_i4 | -1.02120787 | 0.146459664 | 0.999970678 | 1.108 | 1.064 | 2.055 | 1.94 | PPR |
| TRINITY_DN47360_c0_g1_i3 | 1.02120733 | 0.297867887 | 0.999970678 | 0.727 | 0.966 | 0.267 | 0.513 | TRM13 |
| TRINITY_DN1133_c0_g1_i4 | -1.02109332 | 0.351523461 | 0.999970678 | 4.651 | 2.001 | 3.233 | 9.611 | NA |
| TRINITY_DN2213_c0_g1_i1 | 1.021056154 | 0.728467405 | 0.999970678 | 4.987 | 0.355 | 2.2 | 0 | NA |
| TRINITY_DN10518_c0_g1_i1 | 1.020696138 | 0.718044536 | 0.999970678 | 0 | 21.54 | 5.611 | 3.519 | NA |
| TRINITY_DN10933_c0_g1_i5 | 1.020503676 | 0.385206474 | 0.999970678 | 1.699 | 4.445 | 1.655 | 0.985 | Transcrip_reg |
| TRINITY_DN3769_c0_g1_i2 | -1.02031356 | 0.289605126 | 0.999970678 | 20.521 | 13.44 | 41.62 | 18.609 | NA |
| TRINITY_DN10578_c0_g1_i22 | 1.020280984 | 0.554241512 | 1 | 3.316 | 1.685 | 1.855 | 0.271 | NA |
| TRINITY_DN12975_c0_g1_i3 | -1.02026971 | 0.212788157 | 0.999970678 | 63.07 | 61.06 | 64.974 | 172.129 | NA |
| TRINITY_DN37_c0_g4_i1 | -1.02026606 | 0.232593534 | 0.999970678 | 9.792 | 9.294 | 22.554 | 11.522 | NA |
| TRINITY_DN4703_c0_g1_i5 | -1.02026456 | 0.309105476 | 0.999970678 | 1.908 | 2.572 | 2.355 | 6.183 | V-SNARE_C |
| TRINITY_DN7806_c0_g3_i2 | 1.020257524 | 0.621670163 | 0.999970678 | 0.572 | 10.91 | 4.2 | 0.473 | NA |
| TRINITY_DN784_c0_g1_i4 | 1.02021934 | 0.727577946 | 0.999970678 | 30.722 | 0 | 5.277 | 9.018 | CBM_20 |
| TRINITY_DN2533_c0_g2_i1 | 1.020189209 | 0.770603347 | 0.999970678 | 0 | 109.7 | 43.775 | 0.151 | NA |
| TRINITY_DN14311_c2_g1_i1 | -1.01987215 | 0.409958429 | 0.999970678 | 1.217 | 1.399 | 3.322 | 1.257 | NA |
| TRINITY_DN863_c0_g1_i9 | 1.019822136 | 0.421262566 | 0.999970678 | 0.572 | 2.819 | 0.778 | 0.724 | BACK |
| TRINITY_DN22420_c0_g1_i1 | 1.019743891 | 0.275162418 | 0.999970678 | 2.444 | 1.932 | 1.178 | 0.754 | NA |
| TRINITY_DN2770_c0_g1_i3 | 1.019739436 | 0.708586639 | 0.999970678 | 2.444 | 8.18 | 0 | 5.228 | NA |
| TRINITY_DN2605_c0_g1_i1 | -1.01972253 | 0.569874407 | 0.999970678 | 2.162 | 22.66 | 2.8 | 46.378 | - |
| TRINITY_DN8583_c0_g1_i2 | 1.01964019 | 0.574984342 | 0.999970678 | 12.609 | 0.65 | 4.378 | 1.297 | NA |
| TRINITY_DN872_c0_g1_i10 | 1.019638271 | 0.80788455 | 0.999970678 | 2.353 | 0 | 0 | 1.176 | NA |
| TRINITY_DN3532_c0_g1_i3 | -1.01955172 | 0.384734301 | 0.999970678 | 0.963 | 3.863 | 2.555 | 6.625 | - |
| TRINITY_DN3756_c0_g1_i2 | -1.01954494 | 0.698892137 | 0.999970678 | 0 | 4.376 | 4 | 3.861 | NA |
| TRINITY_DN101860_c0_g1_i1 | -1.01942859 | 0.81797186 | 1 | 2.444 | 0 | 4.122 | 0 | NA |
| TRINITY_DN13442_c0_g1_i2 | -1.01942859 | 0.81797186 | 1 | 0.4 | 0 | 0.689 | 0 | Aconitase |
| TRINITY_DN4618_c0_g1_i1 | 1.019339288 | 0.809670829 | 0.999970678 | 3.116 | 0 | 0 | 1.548 | NA |

| TRINITY_DN42807_c0_g2_i1 | 1.019287339 | 0.654211218 | 0.999970678 | 0.045 | 3.932 | 1.267 | 0.402 | NA |
| --- | --- | --- | --- | --- | --- | --- | --- | --- |
| TRINITY_DN6463_c0_g1_i1 | 1.019257373 | 0.810398875 | 0.999970678 | 0.917 | 0 | 0 | 0.462 | NA |
| TRINITY_DN10362_c2_g1_i1 | -1.01920947 | 0.551664279 | 0.999970678 | 0.481 | 0.788 | 1.978 | 0.201 | NA |
| TRINITY_DN8198_c0_g1_i1 | 1.019182798 | 0.703479817 | 0.999970678 | 1.181 | 0.66 | 0.756 | 0 | Glyco_hydro_17 |
| TRINITY_DN8326_c0_g1_i7 | 1.019112608 | 0.70422904 | 0.999970678 | 6.958 | 3.006 | 4.089 | 0 | RVT_1 |
| TRINITY_DN735_c1_g1_i4 | -1.01899793 | 0.653390931 | 0.999970678 | 548.43 | 0.936 | 290.427 | 782.027 | Chloroa_b-bind |
| TRINITY_DN13170_c0_g3_i2 | -1.01894526 | 0.349371461 | 0.999970678 | 6.649 | 6.899 | 10.355 | 13.633 | NA |
| TRINITY_DN1422_c3_g1_i1 | 1.018801335 | 0.703928525 | 0.999970678 | 6.404 | 14.05 | 8.266 | 0 | NA |
| TRINITY_DN270_c2_g1_i2 | -1.01876848 | 0.450378189 | 0.999970678 | 9.202 | 1.34 | 8.022 | 11.723 | PB1 |
| TRINITY_DN28814_c0_g1_i1 | 1.018753341 | 0.815461737 | 0.999970678 | 8.884 | 0 | 0 | 4.313 | NA |
| TRINITY_DN24504_c1_g1_i2 | 1.018753341 | 0.815461737 | 0.999970678 | 5.532 | 0 | 0 | 2.745 | NA |
| TRINITY_DN8184_c0_g3_i1 | -1.0186597 | 0.54013261 | 0.999970678 | 1.045 | 0.108 | 1.111 | 1.015 | NA |
| TRINITY_DN4135_c0_g1_i1 | -1.01864097 | 0.711561972 | 0.999970678 | 18.695 | 0 | 15.044 | 20.138 | NA |
| TRINITY_DN5554_c0_g1_i2 | 1.018568745 | 0.373790752 | 0.999970678 | 1.235 | 4.257 | 1.5 | 0.885 | - |
| TRINITY_DN3280_c0_g1_i15 | -1.01851392 | 0.705065753 | 0.999970678 | 2.135 | 0 | 1.389 | 2.725 | NA |
| TRINITY_DN7913_c0_g1_i7 | 1.018287312 | 0.807866266 | 0.999970678 | 0 | 2.01 | 0 | 0.985 | CBM_20 |
| TRINITY_DN5739_c0_g1_i6 | 1.0182738 | 0.736160645 | 0.999970678 | 0 | 1.843 | 0.622 | 0.141 | Pkinase |
| TRINITY_DN1031_c1_g1_i9 | -1.01815602 | 0.45726698 | 0.999970678 | 0.899 | 0.542 | 0.6 | 2.252 | NA |
| TRINITY_DN1302_c2_g1_i3 | -1.01802858 | 0.716299869 | 0.999970678 | 0 | 4.681 | 6.322 | 1.749 | NA |
| TRINITY_DN8506_c0_g1_i3 | 1.017953746 | 0.563217687 | 0.999970678 | 6.104 | 0.453 | 0.955 | 2.041 | NA |
| TRINITY_DN167143_c0_g1_i2 | -1.01791116 | 0.724976492 | 0.999970678 | 0 | 17.18 | 25.976 | 2.674 | SOUL |
| TRINITY_DN10959_c0_g1_i1 | -1.01772252 | 0.312206466 | 0.999970678 | 2.017 | 4.898 | 3.933 | 9.159 | PC-Esterase |
| TRINITY_DN1915_c0_g1_i6 | -1.01766729 | 0.359073469 | 0.999970678 | 1.544 | 3.696 | 5.289 | 4.072 | NA |
| TRINITY_DN1540_c1_g1_i2 | 1.017552176 | 0.580605627 | 0.999970678 | 5.405 | 0.325 | 1.789 | 0.694 | NA |
| TRINITY_DN2530_c0_g1_i1 | 1.017468905 | 0.404291386 | 0.999970678 | 0.536 | 0.434 | 0.122 | 0.322 | NA |
| TRINITY_DN11087_c0_g2_i7 | -1.01738867 | 0.718461624 | 0.999970678 | 0 | 33.19 | 37.487 | 20.701 | NA |
| TRINITY_DN2557_c0_g1_i3 | -1.0172821 | 0.36947711 | 0.999970678 | 14.098 | 60.11 | 80.228 | 51.777 | ATP-synt_DE_N |
| TRINITY_DN20680_c1_g1_i1 | -1.01724011 | 0.711900482 | 1 | 31.876 | 0 | 21.41 | 34.524 | NA |
| TRINITY_DN53736_c0_g1_i5 | -1.01718547 | 0.699427706 | 0.999970678 | 0 | 3.183 | 2.9 | 2.855 | Prenyltrans |
| TRINITY_DN7186_c0_g1_i3 | 1.01709459 | 0.596020849 | 0.999970678 | 0.518 | 6.288 | 0.3 | 2.916 | - |
| TRINITY_DN3138_c0_g1_i27 | -1.01708381 | 0.462788076 | 0.999970678 | 0.218 | 0.986 | 1.211 | 0.955 | NA |
| TRINITY_DN2857_c3_g1_i1 | 1.017011741 | 0.608472478 | 0.999970678 | 0.2 | 3.666 | 1.3 | 0.352 | NA |
| TRINITY_DN6418_c0_g2_i2 | -1.01688193 | 0.716638779 | 0.999970678 | 1.844 | 0 | 0.5 | 3.207 | NA |
| TRINITY_DN19873_c0_g1_i1 | 1.016837029 | 0.820505263 | 1 | 6.186 | 0 | 2.544 | 0 | 2-Hacid_dh |
| TRINITY_DN703_c0_g3_i5 | 1.016808134 | 0.525866216 | 0.999970678 | 5.714 | 10.89 | 6.422 | 0.402 | NA |
| TRINITY_DN10682_c0_g1_i3 | -1.01661419 | 0.719226018 | 0.999970678 | 1.653 | 0 | 2.467 | 0.402 | Exo_endo_phos |
| TRINITY_DN9198_c0_g1_i4 | -1.01649598 | 0.81579245 | 0.999970678 | 1.017 | 0 | 1.722 | 0 | NA |
| TRINITY_DN1199_c0_g1_i1 | -1.01632505 | 0.712205289 | 0.999970678 | 8.721 | 0 | 3.544 | 13.572 | NA |
| TRINITY_DN23741_c0_g1_i2 | -1.01628675 | 0.289443498 | 0.999970678 | 2.289 | 5.105 | 4.244 | 9.742 | NA |
| TRINITY_DN858_c0_g1_i3 | 1.016265055 | 0.370080315 | 0.999970678 | 132.55 | 67.86 | 65.94 | 19.916 | ABC2_membrane |
| TRINITY_DN2231_c0_g1_i2 | -1.01594211 | 0.306505552 | 0.999970678 | 0.663 | 0.66 | 0.833 | 1.689 | NA |
| TRINITY_DN12413_c0_g1_i6 | 1.01567272 | 0.811597563 | 0.999970678 | 4.933 | 0 | 2.022 | 0 | NA |
| TRINITY_DN905_c0_g1_i5 | 1.015667212 | 0.225448084 | 0.999970678 | 10.919 | 9.234 | 2.678 | 6.746 | NA |
| TRINITY_DN22761_c0_g1_i1 | -1.01537303 | 0.352115029 | 0.999970678 | 1.072 | 1.606 | 2.633 | 2.202 | DUF688 |
| TRINITY_DN3173_c0_g1_i2 | 1.015288953 | 0.145154309 | 0.999970678 | 9.611 | 7.707 | 3.066 | 4.896 | NA |
| TRINITY_DN14188_c0_g1_i1 | -1.0151139 | 0.191460234 | 0.999970678 | 265.62 | 321.9 | 651.805 | 395.412 | NA |
| TRINITY_DN13129_c0_g1_i6 | -1.01489173 | 0.708173982 | 0.999970678 | 1.553 | 0 | 1.133 | 1.769 | NA |
| TRINITY_DN13145_c0_g1_i1 | 1.014797251 | 0.392437104 | 0.999970678 | 0.491 | 0.749 | 0.156 | 0.422 | NA |
| TRINITY_DN1995_c0_g1_i7 | -1.01453271 | 0.461207475 | 0.999970678 | 87.297 | 25.35 | 29.698 | 181.851 | NA |
| TRINITY_DN7776_c0_g1_i2 | -1.01447067 | 0.670187931 | 0.999970678 | 5.868 | 0.099 | 9.388 | 0.664 | NA |
| TRINITY_DN15475_c0_g1_i2 | 1.014052234 | 0.73501671 | 0.999970678 | 0 | 4.494 | 1.655 | 0.181 | NA |
| TRINITY_DN8590_c0_g1_i7 | 1.013960154 | 0.385339211 | 0.999970678 | 0.745 | 0.838 | 0.378 | 0.352 | NA |
| TRINITY_DN40928_c0_g1_i2 | -1.01395242 | 0.276771967 | 0.999970678 | 1.717 | 1.488 | 1.889 | 4.182 | NA |
| TRINITY_DN7906_c0_g1_i2 | -1.01347144 | 0.74621601 | 0.999970678 | 1.199 | 0 | 0.089 | 2.353 | Branch |
| TRINITY_DN1334_c0_g1_i2 | -1.01333414 | 0.80847384 | 0.999970678 | 0 | 1.843 | 0 | 3.68 | NA |
| TRINITY_DN1056_c0_g2_i2 | 1.013202679 | 0.810129151 | 0.999970678 | 8.194 | 0 | 0 | 4.132 | DEAD |
| TRINITY_DN12618_c0_g1_i3 | -1.0131307 | 0.326608817 | 0.999970678 | 24.799 | 18.28 | 49.364 | 24.41 | NA |
| TRINITY_DN681_c0_g1_i2 | -1.0129283 | 0.315043805 | 0.999970678 | 35.809 | 21.93 | 72.34 | 29.126 | CRS1_YhbY |
| TRINITY_DN1251_c0_g1_i1 | 1.012849825 | 0.238308051 | 0.999970678 | 9.184 | 6.002 | 2.144 | 4.967 | bZIP_1 |
| TRINITY_DN7460_c2_g1_i4 | 1.012754321 | 0.746552382 | 0.999970678 | 5.814 | 0.118 | 2.467 | 0 | NA |
| TRINITY_DN10688_c0_g1_i2 | 1.012559186 | 0.415874637 | 0.999970678 | 0.6 | 1.222 | 0.567 | 0.221 | NA |
| TRINITY_DN2044_c0_g1_i2 | -1.01255314 | 0.390579008 | 0.999970678 | 3.134 | 6.465 | 3.322 | 15.171 | FAD_binding_3 |
| TRINITY_DN6474_c0_g1_i8 | 1.012286578 | 0.726634273 | 0.999970678 | 0.481 | 0 | 0.044 | 0.181 | Asparaginase_2 |
| TRINITY_DN6363_c0_g1_i1 | -1.01182197 | 0.397194704 | 0.999970678 | 10.91 | 22.25 | 13.033 | 47.323 | Mito_carr |
| TRINITY_DN8279_c2_g1_i1 | 1.01181902 | 0.435259857 | 0.999970678 | 2.425 | 1.222 | 0.267 | 1.508 | NA |
| TRINITY_DN19526_c0_g1_i6 | -1.01168572 | 0.749250635 | 0.999970678 | 1.971 | 0.128 | 3.511 | 0 | Ribosomal_L19e |
| TRINITY_DN3459_c0_g1_i11 | -1.01148285 | 0.503037385 | 0.999970678 | 2.271 | 9.185 | 2.289 | 20.047 | NA |
| TRINITY_DN24_c0_g2_i6 | -1.01143495 | 0.181369628 | 0.999970678 | 37.78 | 73.68 | 87.428 | 117.146 | - |
| TRINITY_DN10656_c0_g2_i1 | 1.01136275 | 0.631455335 | 1 | 0.999 | 0.306 | 0.522 | 0.03 | NA |
| TRINITY_DN14012_c0_g1_i4 | 1.01132197 | 0.535061927 | 0.999970678 | 0.254 | 0.729 | 0.367 | 0.05 | NA |
| TRINITY_DN4884_c0_g1_i2 | -1.01125397 | 0.483097658 | 0.999970678 | 5.75 | 12.53 | 3.078 | 32.604 | NA |
| TRINITY_DN9424_c0_g1_i12 | 1.011205098 | 0.385304821 | 0.999970678 | 1.581 | 1.961 | 0.878 | 0.724 | NA |
| TRINITY_DN7522_c1_g1_i5 | 1.011204427 | 0.481448663 | 0.999970678 | 1.326 | 2.799 | 1.433 | 0.322 | NA |
| TRINITY_DN2525_c0_g1_i2 | 1.011153488 | 0.561863068 | 0.999970678 | 0.354 | 4.701 | 1.133 | 1.086 | NA |

| TRINITY_DN1863_c0_g1_i6 | 1.010881936 | 0.160772644 | 0.999970678 | 9.938 | 13.17 | 5.555 | 4.705 | Exostosin |
| --- | --- | --- | --- | --- | --- | --- | --- | --- |
| TRINITY_DN11149_c0_g1_i1 | 1.010385589 | 0.556464842 | 0.999970678 | 0.5 | 5.549 | 1.722 | 0.875 | Cupin_1 |
| TRINITY_DN18202_c0_g1_i2 | -1.0102682 | 0.3649541 | 0.999970678 | 0.699 | 0.542 | 1.033 | 1.247 | NA |
| TRINITY_DN381_c0_g1_i3 | 1.010240118 | 0.414697831 | 0.999970678 | 0.863 | 0.848 | 0.233 | 0.543 | NB-ARC |
| TRINITY_DN779_c2_g1_i1 | -1.01016142 | 0.715145221 | 0.999970678 | 0.372 | 0.798 | 0 | 2.363 | STAS |
| TRINITY_DN1607_c0_g1_i10 | -1.01001598 | 0.214770487 | 0.999970678 | 1.017 | 1.518 | 1.6 | 3.187 | Peptidase_A22B |
| TRINITY_DN5475_c0_g1_i3 | -1.00999583 | 0.726127109 | 0.999970678 | 0.436 | 2.72 | 0 | 6.314 | NA |
| TRINITY_DN21360_c0_g1_i1 | -1.00998336 | 0.347533237 | 0.999970678 | 2.28 | 3.528 | 7.355 | 2.775 | PTR2 |
| TRINITY_DN15699_c0_g2_i2 | -1.00986003 | 0.716193909 | 1 | 1.008 | 0 | 0.689 | 1.176 | EF_assoc_1 |
| TRINITY_DN701_c1_g2_i1 | 1.009846038 | 0.666421617 | 0.999970678 | 0.154 | 15.95 | 0.267 | 7.54 | NA |
| TRINITY_DN4235_c2_g1_i4 | 1.009799246 | 0.450579791 | 0.999970678 | 1.099 | 5.884 | 1.467 | 1.649 | NA |
| TRINITY_DN999_c0_g1_i4 | 1.009728847 | 0.508927309 | 0.999970678 | 2.434 | 15.22 | 5.944 | 1.438 | NA |
| TRINITY_DN10986_c0_g1_i2 | -1.00972848 | 0.81914489 | 1 | 0.182 | 0 | 0 | 0.382 | LRRNT_2 |
| TRINITY_DN12058_c0_g2_i5 | -1.00972701 | 0.696166827 | 0.999970678 | 3.406 | 0.049 | 5.722 | 0.111 | NA |
| TRINITY_DN3228_c0_g1_i5 | -1.00968756 | 0.543819597 | 0.999970678 | 0.445 | 2.562 | 0.578 | 5.268 | Serpin |
| TRINITY_DN17809_c0_g1_i1 | 1.009641684 | 0.810612495 | 0.999970678 | 0 | 13.53 | 5.444 | 0 | NA |
| TRINITY_DN16228_c0_g1_i4 | 1.009635133 | 0.490001452 | 0.999970678 | 0.709 | 0.168 | 0.233 | 0.151 | NA |
| TRINITY_DN7047_c0_g1_i2 | 1.009513139 | 0.505016303 | 0.999970678 | 20.33 | 6.79 | 10.044 | 1.408 | NA |
| TRINITY_DN29456_c0_g2_i1 | -1.00938269 | 0.449105853 | 0.999970678 | 0.881 | 0.266 | 0.711 | 1.448 | NB-ARC |
| TRINITY_DN4747_c0_g1_i4 | 1.009322497 | 0.710453895 | 0.999970678 | 0.372 | 1.813 | 0 | 1.086 | - |
| TRINITY_DN179_c0_g1_i4 | 1.009293604 | 0.173847847 | 0.999970678 | 7.912 | 11.57 | 4.622 | 4.052 | CK_II_beta |
| TRINITY_DN5505_c0_g1_i1 | -1.00913303 | 0.378642303 | 0.999970678 | 5.06 | 7.943 | 6.122 | 17.755 | NA |
| TRINITY_DN3195_c0_g1_i3 | -1.00907396 | 0.563052606 | 0.999970678 | 3.325 | 0.266 | 4.566 | 1.769 | NA |
| TRINITY_DN185_c1_g1_i4 | -1.00886347 | 0.393951186 | 0.999970678 | 2.307 | 8.682 | 4.866 | 16.116 | - |
| TRINITY_DN19353_c0_g1_i6 | -1.00886232 | 0.561402917 | 1 | 0.073 | 0.276 | 0.5 | 0.111 | NA |
| TRINITY_DN3384_c0_g1_i1 | -1.00866561 | 0.296998067 | 0.999970678 | 7.276 | 3.39 | 7.255 | 12.627 | SRP9-21 |
| TRINITY_DN23_c1_g1_i5 | 1.008624507 | 0.722430039 | 0.999970678 | 13.935 | 0 | 3.933 | 2.252 | NA |
| TRINITY_DN7999_c0_g1_i2 | -1.00856533 | 0.713972875 | 1 | 0 | 0.404 | 0.444 | 0.261 | NA |
| TRINITY_DN725_c0_g1_i5 | -1.00852274 | 0.342070929 | 0.999970678 | 2.525 | 2.267 | 4.411 | 4.223 | Fer2 |
| TRINITY_DN13468_c0_g1_i3 | -1.0084862 | 0.174945704 | 0.999970678 | 11.882 | 14.93 | 16.199 | 34.323 | Lipase_GDSL |
| TRINITY_DN3617_c0_g1_i1 | -1.00846265 | 0.436084731 | 0.999970678 | 16.397 | 30.38 | 9.999 | 81.676 | NA |
| TRINITY_DN31589_c0_g1_i1 | -1.00842502 | 0.433271708 | 0.999970678 | 1.844 | 0.867 | 1.022 | 4.202 | DUF778 |
| TRINITY_DN369_c0_g2_i1 | -1.00836871 | 0.724751725 | 0.999970678 | 611.14 | 0 | 453.15 | 632.156 | NA |
| TRINITY_DN17179_c0_g1_i1 | 1.008248373 | 0.813138818 | 0.999970678 | 24.654 | 0 | 10.266 | 0 | Ferritin_2 |
| TRINITY_DN37301_c0_g1_i1 | 1.008210065 | 0.415689175 | 0.999970678 | 1.908 | 2.651 | 0.544 | 1.588 | DUF588 |
| TRINITY_DN8756_c0_g1_i1 | -1.00798695 | 0.19422672 | 0.999970678 | 9.12 | 7.529 | 10.055 | 21.324 | NA |
| TRINITY_DN2054_c0_g1_i8 | 1.007897159 | 0.309971591 | 0.999970678 | 50.461 | 18.73 | 10.144 | 22.46 | LRRNT_2 |
| TRINITY_DN27278_c0_g1_i6 | -1.00785735 | 0.495963298 | 0.999970678 | 0.436 | 0.335 | 1.1 | 0.231 | Pro_isomerase |
| TRINITY_DN2369_c0_g1_i2 | 1.007772228 | 0.429860203 | 0.999970678 | 1.099 | 0.335 | 0.289 | 0.382 | LRR_1 |
| TRINITY_DN117_c1_g1_i1 | -1.0075364 | 0.445827827 | 0.999970678 | 0.845 | 0.256 | 0.689 | 1.397 | NA |
| TRINITY_DN7881_c0_g1_i4 | 1.007531028 | 0.818030257 | 1 | 0 | 0.936 | 0 | 0.462 | NA |
| TRINITY_DN5066_c1_g1_i13 | -1.00753043 | 0.454247254 | 0.999970678 | 0.1 | 0.256 | 0.411 | 0.241 | NA |
| TRINITY_DN1791_c0_g2_i4 | 1.007251278 | 0.700188196 | 0.999970678 | 0.745 | 1.055 | 0 | 0.905 | HATPase_c_3 |
| TRINITY_DN907_c1_g2_i4 | -1.00721171 | 0.338839417 | 0.999970678 | 248.66 | 560 | 332.124 | 1220.891 | 2OG-FeII_Oxy |
| TRINITY_DN2038_c0_g1_i13 | -1.0071737 | 0.465411575 | 0.999970678 | 0.182 | 0.66 | 0.633 | 0.935 | NA |
| TRINITY_DN24948_c0_g1_i1 | -1.00699481 | 0.224713642 | 0.999970678 | 4.051 | 3.548 | 8.277 | 5.278 | NA |
| TRINITY_DN4574_c0_g3_i1 | 1.006959916 | 0.516296503 | 0.999970678 | 34.664 | 3.321 | 12.199 | 4.373 | NA |
| TRINITY_DN1480_c0_g1_i3 | -1.00675144 | 0.589821767 | 0.999970678 | 2.235 | 3.134 | 8.566 | 0.402 | NA |
| TRINITY_DN633_c0_g1_i1 | -1.00662495 | 0.728126126 | 0.999970678 | 9.92 | 13.75 | 0 | 47.484 | NA |
| TRINITY_DN8073_c0_g1_i1 | -1.00658218 | 0.818335769 | 0.999970678 | 7.531 | 0 | 0 | 14.548 | NA |
| TRINITY_DN11043_c0_g1_i6 | -1.00651588 | 0.571106067 | 0.999970678 | 0.354 | 3.666 | 1.089 | 6.656 | NA |
| TRINITY_DN4848_c1_g1_i4 | -1.00645347 | 0.247827657 | 0.999970678 | 3.843 | 7.066 | 7.122 | 13.1 | NA |
| TRINITY_DN544_c0_g1_i1 | -1.00641482 | 0.374327052 | 0.999970678 | 4.76 | 1.715 | 6.633 | 4.956 | NA |
| TRINITY_DN1065_c0_g1_i1 | -1.00634688 | 0.333590481 | 0.999970678 | 2.852 | 1.153 | 2.489 | 5.077 | CRS1_YhbY |
| TRINITY_DN10456_c1_g2_i1 | 1.006315429 | 0.500189486 | 0.999970678 | 1.808 | 5.391 | 0.622 | 2.755 | Tim17 |
| TRINITY_DN4076_c1_g2_i1 | 1.0061445 | 0.394149054 | 0.999970678 | 0.382 | 0.532 | 0.222 | 0.181 | FancD2 |
| TRINITY_DN18748_c0_g1_i4 | 1.00609311 | 0.498996564 | 0.999970678 | 3.797 | 0.443 | 1.144 | 0.754 | Pkinase |
| TRINITY_DN5334_c0_g1_i2 | 1.005969858 | 0.748891532 | 0.999970678 | 3.824 | 0 | 0.089 | 1.82 | NA |
| TRINITY_DN1843_c0_g1_i3 | 1.005848774 | 0.821783763 | 1 | 0 | 2.129 | 0.856 | 0 | zf-C3HC4_3 |
| TRINITY_DN4603_c0_g1_i2 | 1.00566206 | 0.40823002 | 0.999970678 | 1.036 | 1.478 | 0.811 | 0.271 | NA |
| TRINITY_DN32220_c0_g1_i1 | -1.00563216 | 0.823292587 | 1 | 0.818 | 0 | 1.378 | 0 | Beta-lactamase |
| TRINITY_DN37407_c0_g1_i1 | -1.00561343 | 0.819602379 | 0.999970678 | 1.263 | 0 | 2.133 | 0 | NA |
| TRINITY_DN7125_c0_g1_i19 | -1.00558864 | 0.814439062 | 0.999970678 | 2.026 | 0 | 3.378 | 0 | NA |
| TRINITY_DN135_c0_g1_i3 | 1.005531472 | 0.82185659 | 1 | 1.826 | 0 | 0.756 | 0 | NA |
| TRINITY_DN9087_c0_g1_i4 | -1.00538454 | 0.724179441 | 0.999970678 | 4.378 | 0 | 6.289 | 1.317 | Homeobox |
| TRINITY_DN3473_c0_g1_i3 | 1.005352423 | 0.415360642 | 0.999970678 | 0.881 | 0.365 | 0.289 | 0.271 | FGGY_C |
| TRINITY_DN10682_c0_g1_i5 | -1.00526622 | 0.81870555 | 0.999970678 | 0 | 0.375 | 0.611 | 0 | Exo_endo_phos |
| TRINITY_DN4444_c0_g1_i2 | -1.00507772 | 0.579594974 | 0.999970678 | 0.818 | 10.91 | 3.822 | 17.926 | NA |
| TRINITY_DN8390_c0_g1_i1 | 1.005067733 | 0.354392448 | 0.999970678 | 0.827 | 1.222 | 0.411 | 0.523 | NA |
| TRINITY_DN6794_c0_g1_i1 | 1.005005687 | 0.575988775 | 0.999970678 | 4.569 | 2.622 | 0.222 | 3.237 | NA |
| TRINITY_DN441_c0_g1_i2 | 1.004950508 | 0.38951507 | 0.999970678 | 59.427 | 11.93 | 14.666 | 18.147 | NA |
| TRINITY_DN9319_c0_g2_i1 | 1.004786829 | 0.446894987 | 0.999970678 | 0.254 | 0.769 | 0.167 | 0.302 | NA |
| TRINITY_DN108484_c0_g1_i1 | -1.00472768 | 0.811709281 | 0.999970678 | 6.213 | 0 | 10.444 | 0 | zf-LITAF-like |
| TRINITY_DN4691_c0_g1_i3 | 1.004685369 | 0.283473121 | 0.999970678 | 1.671 | 3.085 | 0.744 | 1.468 | FAD_binding_2 |

| TRINITY_DN4488_c0_g2_i10 | 1.004679528 | 0.615847278 | 0.999970678 | 0.145 | 2.385 | 0.767 | 0.282 | NA |
| --- | --- | --- | --- | --- | --- | --- | --- | --- |
| TRINITY_DN5024_c0_g1_i1 | -1.00467566 | 0.751131893 | 1 | 0.382 | 0 | 0.622 | 0.04 | NA |
| TRINITY_DN3928_c0_g1_i5 | 1.004583066 | 0.7138125 | 0.999970678 | 1.608 | 0.404 | 0.833 | 0 | NA |
| TRINITY_DN7644_c0_g1_i4 | 1.004411321 | 0.23938342 | 0.999970678 | 8.13 | 4.09 | 3.033 | 2.473 | NA |
| TRINITY_DN3240_c0_g1_i1 | 1.004188998 | 0.291295074 | 0.999970678 | 2.544 | 2.759 | 0.667 | 1.83 | NA |
| TRINITY_DN835_c31_g1_i3 | 1.004167711 | 0.617803614 | 1 | 1.944 | 1.646 | 1.411 | 0.08 | Chromo |
| TRINITY_DN13680_c0_g1_i5 | 1.004102622 | 0.393626843 | 0.999970678 | 3.334 | 7.332 | 2.489 | 2.151 | NA |
| TRINITY_DN577_c0_g1_i10 | -1.00397796 | 0.485461363 | 0.999970678 | 13.072 | 9.116 | 3.422 | 39.984 | NA |
| TRINITY_DN26213_c0_g2_i4 | 1.00373916 | 0.75965569 | 0.999970678 | 4.578 | 0 | 0.033 | 2.292 | NA |
| TRINITY_DN192531_c0_g1_i1 | -1.0037174 | 0.393756456 | 0.999970678 | 39.724 | 76.29 | 33.465 | 190.678 | GRAM |
| TRINITY_DN12517_c0_g1_i2 | -1.00356751 | 0.548346999 | 0.999970678 | 1.517 | 1.192 | 0.222 | 5.147 | NA |
| TRINITY_DN7796_c0_g1_i1 | 1.003488468 | 0.274248424 | 0.999970678 | 108.21 | 45.64 | 39.342 | 29.548 | NA |
| TRINITY_DN52442_c0_g1_i2 | -1.00347725 | 0.400107493 | 0.999970678 | 19.694 | 4.691 | 13.51 | 32.725 | DUF1218 |
| TRINITY_DN5625_c0_g2_i7 | 1.003423082 | 0.398122043 | 0.999970678 | 0.609 | 0.749 | 0.178 | 0.473 | UbiA |
| TRINITY_DN12909_c0_g1_i1 | -1.0033673 | 0.658936357 | 0.999970678 | 1.49 | 46.35 | 7.066 | 75.795 | NA |
| TRINITY_DN381_c0_g1_i8 | 1.003242023 | 0.448242056 | 0.999970678 | 1.871 | 12.56 | 3.444 | 2.976 | NB-ARC |
| TRINITY_DN6756_c0_g1_i4 | -1.00321629 | 0.721671092 | 0.999970678 | 8.176 | 0 | 9.077 | 5.69 | ACCA |
| TRINITY_DN12297_c1_g1_i6 | -1.00307653 | 0.728970693 | 1 | 0 | 0.877 | 0.267 | 1.448 | Grp1_Fun34_Yaa |
| TRINITY_DN13268_c0_g1_i3 | 1.003059806 | 0.527675233 | 0.999970678 | 1.29 | 0.404 | 0.111 | 0.724 | NA |
| TRINITY_DN2669_c0_g1_i1 | -1.00290202 | 0.162052067 | 0.999970678 | 410.99 | 730.1 | 989.94 | 1000.865 | PsaN |
| TRINITY_DN9925_c0_g1_i1 | 1.002844557 | 0.420897992 | 1 | 1.217 | 2.099 | 0.633 | 0.895 | NA |
| TRINITY_DN9533_c0_g1_i4 | -1.0027061 | 0.824104734 | 0.999970678 | 0 | 100.3 | 163.079 | 0 | Ribosomal_L12 |
| TRINITY_DN4508_c0_g1_i2 | -1.00268434 | 0.720023924 | 0.999970678 | 3.615 | 0 | 1.089 | 6.082 | Nucleotid_trans |
| TRINITY_DN44008_c0_g1_i1 | -1.00267651 | 0.548072659 | 1 | 0.154 | 1.114 | 1.089 | 1.206 | NA |
| TRINITY_DN1151_c1_g1_i2 | 1.00253735 | 0.572010077 | 0.999970678 | 126.41 | 5.48 | 45.942 | 11.17 | NA |
| TRINITY_DN14122_c0_g1_i10 | 1.002404652 | 0.428330517 | 0.999970678 | 1.98 | 2.188 | 1.3 | 0.513 | NA |
| TRINITY_DN13905_c0_g1_i1 | 1.002124103 | 0.625171778 | 0.999970678 | 0.718 | 35.42 | 1.222 | 16.337 | NA |
| TRINITY_DN289_c0_g2_i1 | 1.002024896 | 0.204489709 | 0.999970678 | 6.477 | 5.115 | 2.944 | 2.232 | NA |
| TRINITY_DN11773_c0_g1_i1 | 1.002003949 | 0.41604081 | 0.999970678 | 1.889 | 0.729 | 0.433 | 0.784 | NA |
| TRINITY_DN14611_c0_g1_i1 | -1.00187752 | 0.199403254 | 0.999970678 | 9.901 | 5.992 | 15.155 | 13.572 | NA |
| TRINITY_DN8077_c1_g2_i2 | -1.00176126 | 0.311018227 | 0.999970678 | 0.945 | 1.833 | 1.478 | 3.78 | AP2 |
| TRINITY_DN7125_c0_g1_i21 | -1.00172312 | 0.819170684 | 0.999970678 | 1.326 | 0 | 2.222 | 0 | NA |
| TRINITY_DN1720_c1_g1_i2 | 1.001650144 | 0.489936172 | 0.999970678 | 0.681 | 2.947 | 1.189 | 0.352 | NA |
| TRINITY_DN8928_c0_g1_i1 | 1.001453649 | 0.740999541 | 1 | 0.5 | 7.943 | 0 | 4.011 | NA |
| TRINITY_DN5527_c0_g1_i1 | 1.001387343 | 0.705275438 | 0.999970678 | 1.163 | 1.784 | 1.211 | 0 | LRR_6 |
| TRINITY_DN4712_c0_g1_i2 | 1.001130914 | 0.4893992 | 0.999970678 | 3.325 | 4.997 | 3.144 | 0.352 | FYRC |
| TRINITY_DN5279_c0_g1_i2 | -1.0011001 | 0.517771898 | 0.999970678 | 37.271 | 3.164 | 17.032 | 60.925 | NA |
| TRINITY_DN5226_c0_g1_i5 | -1.00095064 | 0.250126649 | 0.999970678 | 3.561 | 2.72 | 7.111 | 4.011 | - |
| TRINITY_DN1849_c0_g1_i1 | 1.000937921 | 0.296042774 | 0.999970678 | 10.21 | 3.873 | 3.478 | 2.906 | - |
| TRINITY_DN3840_c0_g1_i5 | -1.00090856 | 0.708940936 | 0.999970678 | 1.326 | 0 | 1.378 | 1.025 | NA |
| TRINITY_DN6310_c0_g2_i10 | -1.00087087 | 0.210234846 | 0.999970678 | 1.49 | 1.311 | 2.089 | 3.026 | NA |
| TRINITY_DN472_c0_g1_i2 | -1.00082733 | 0.56255404 | 0.999970678 | 0.245 | 1.488 | 0.667 | 2.604 | Dirigent |
| TRINITY_DN251_c0_g1_i12 | -1.00071277 | 0.724231957 | 0.999970678 | 0.545 | 1.597 | 3.522 | 0 | NA |
| TRINITY_DN2238_c0_g1_i5 | 1.000645963 | 0.728298931 | 0.999970678 | 52.387 | 72.77 | 0 | 62.283 | NA |
| TRINITY_DN1889_c0_g1_i1 | -1.00053981 | 0.212836294 | 0.999970678 | 26.007 | 26.4 | 27.654 | 71.713 | NA |
| TRINITY_DN9922_c0_g2_i1 | -1.00048478 | 0.40577558 | 0.999970678 | 46.646 | 243.2 | 282.372 | 202.823 | NA |
| TRINITY_DN17296_c0_g1_i2 | -1.0004432 | 0.186305883 | 0.999970678 | 3.089 | 2.296 | 5.155 | 4.564 | SPG48 |
| TRINITY_DN75225_c0_g1_i1 | -1.00019852 | 0.278818425 | 0.999970678 | 1.172 | 0.848 | 2.033 | 1.588 | NA |
| TRINITY_DN19209_c0_g1_i6 | 1.000030776 | 0.199738556 | 0.999970678 | 224.76 | 114.7 | 67.785 | 88.101 | - |
| TRINITY_DN22643_c0_g1_i10 | -0.99997506 | 0.618933449 | 0.999970678 | 18.985 | 1.518 | 27.287 | 6.957 | NA |
| TRINITY_DN545_c0_g2_i4 | 0.999893851 | 0.735345548 | 0.999970678 | 0 | 7.825 | 0.378 | 3.408 | NA |
| TRINITY_DN15740_c0_g1_i2 | 0.999842624 | 0.462668764 | 0.999970678 | 0.736 | 1.212 | 0.622 | 0.251 | B_lectin |
| TRINITY_DN858_c0_g1_i5 | 0.999820784 | 0.497756747 | 0.999970678 | 37.835 | 12.77 | 19.032 | 2.473 | ABC2_membrane |
| TRINITY_DN2363_c0_g1_i3 | -0.999815 | 0.483771489 | 0.999970678 | 2.28 | 1.045 | 4.5 | 1.287 | NA |
| TRINITY_DN1148_c0_g1_i5 | 0.999621111 | 0.578622591 | 0.999970678 | 0.263 | 4.386 | 0.833 | 1.267 | Glyco_hydro_1 |
| TRINITY_DN15900_c0_g1_i1 | 0.999574958 | 0.496797048 | 0.999970678 | 2.362 | 0.306 | 0.5 | 0.744 | Pkinase |
| TRINITY_DN2633_c0_g1_i3 | -0.99954176 | 0.361286672 | 0.999970678 | 33.756 | 51.83 | 113.449 | 33.529 | LrgB |
| TRINITY_DN3300_c1_g1_i5 | 0.999485491 | 0.53997873 | 0.999970678 | 0.545 | 6.81 | 0.9 | 2.544 | NA |
| TRINITY_DN46566_c0_g1_i1 | 0.999126982 | 0.398678669 | 0.999970678 | 15.515 | 80.76 | 23.587 | 19.042 | Glyco_transf_9 |
| TRINITY_DN3007_c0_g2_i2 | -0.99911697 | 0.712914911 | 0.999970678 | 2.489 | 0 | 2.678 | 1.82 | DBC1 |
| TRINITY_DN131_c1_g1_i3 | 0.999084245 | 0.472461136 | 0.999970678 | 51.697 | 6.022 | 15.521 | 10.426 | NA |
| TRINITY_DN1664_c0_g2_i2 | 0.999069522 | 0.336877943 | 0.999970678 | 20.384 | 8.436 | 3.7 | 10.003 | - |
| TRINITY_DN620_c0_g1_i12 | -0.99898873 | 0.825090382 | 0.999970678 | 0 | 10.79 | 0 | 21.394 | DUF3458 |
| TRINITY_DN241_c0_g1_i4 | -0.99888641 | 0.115804468 | 0.999970678 | 17.15 | 23.7 | 30.865 | 44.096 | NA |
| TRINITY_DN1163_c0_g1_i5 | 0.998797685 | 0.811806227 | 0.999970678 | 6.459 | 0 | 0 | 3.288 | DUF3110 |
| TRINITY_DN6639_c0_g1_i2 | -0.99852571 | 0.71257487 | 0.999970678 | 8.275 | 0 | 5.244 | 10.416 | DUF1751 |
| TRINITY_DN35236_c0_g1_i3 | 0.998383423 | 0.722979674 | 1 | 0.99 | 0.296 | 0.533 | 0 | NCA2 |
| TRINITY_DN31577_c0_g1_i5 | 0.998302811 | 0.733679964 | 0.999970678 | 0 | 3.331 | 0.244 | 1.357 | NA |
| TRINITY_DN4245_c0_g1_i1 | -0.99827664 | 0.364017697 | 0.999970678 | 16.651 | 7.736 | 30.931 | 11.542 | Aldedh |
| TRINITY_DN40271_c0_g1_i5 | -0.99813855 | 0.703769857 | 0.999970678 | 3332.8 | 0.443 | 3828.235 | 1947.21 | RbcS |
| TRINITY_DN300_c0_g1_i3 | 0.998084938 | 0.71161357 | 0.999970678 | 1.072 | 1.557 | 1.089 | 0 | NA |
| TRINITY_DN8114_c0_g1_i9 | 0.998076303 | 0.327148822 | 0.999970678 | 1.217 | 0.946 | 0.367 | 0.623 | NA |
| TRINITY_DN4972_c0_g1_i7 | 0.997913905 | 0.237662355 | 0.999970678 | 9.247 | 6.573 | 4.2 | 2.885 | DUF3537 |
| TRINITY_DN2771_c0_g1_i1 | 0.997861593 | 0.812225619 | 0.999970678 | 9.883 | 0 | 0 | 5.027 | NA |

| TRINITY_DN13573_c0_g1_i3 | 0.997853794 | 0.702388965 | 0.999970678 | 2.589 | 4.149 | 0 | 3.338 | NA |
| --- | --- | --- | --- | --- | --- | --- | --- | --- |
| TRINITY_DN638_c0_g1_i4 | -0.99784823 | 0.263180171 | 0.999970678 | 4.197 | 9.619 | 13.521 | 11.059 | NA |
| TRINITY_DN8126_c2_g1_i1 | 0.997843337 | 0.224888463 | 0.999970678 | 12.272 | 6.79 | 3.433 | 5.439 | - |
| TRINITY_DN32197_c0_g2_i2 | 0.997756439 | 0.295606897 | 0.999970678 | 3.824 | 3.39 | 2.044 | 1.146 | NA |
| TRINITY_DN23233_c0_g1_i2 | 0.997731499 | 0.661318627 | 0.999970678 | 0.027 | 5.765 | 0.633 | 2.091 | NA |
| TRINITY_DN7460_c2_g1_i1 | 0.997641719 | 0.484073198 | 0.999970678 | 0.382 | 0.769 | 0.411 | 0.07 | NA |
| TRINITY_DN10682_c0_g1_i1 | 0.99750893 | 0.818052388 | 0.999970678 | 0.881 | 0 | 0 | 0.442 | Exo_endo_phos |
| TRINITY_DN5919_c0_g3_i1 | 0.99727949 | 0.339403161 | 0.999970678 | 2.97 | 1.912 | 1.411 | 0.764 | Tfb4 |
| TRINITY_DN4722_c0_g1_i5 | -0.99723276 | 0.250768786 | 0.999970678 | 2.871 | 2.277 | 5.444 | 3.75 | NA |
| TRINITY_DN3747_c0_g1_i1 | 0.997047229 | 0.740179883 | 0.999970678 | 1.208 | 35.73 | 14.955 | 0 | NA |
| TRINITY_DN895_c0_g1_i3 | -0.99700364 | 0.383252173 | 0.999970678 | 39.506 | 102.9 | 51.697 | 217.602 | NA |
| TRINITY_DN13523_c0_g1_i2 | -0.99698704 | 0.522860882 | 0.999970678 | 1.29 | 3.085 | 0.656 | 7.902 | PC-Esterase |
| TRINITY_DN11326_c0_g1_i1 | -0.99683276 | 0.577892898 | 0.999970678 | 0.563 | 0.03 | 0.422 | 0.684 | FAR1 |
| TRINITY_DN12439_c0_g1_i9 | -0.99679706 | 0.713814615 | 0.999970678 | 3.025 | 0 | 3.144 | 2.222 | NA |
| TRINITY_DN4530_c0_g1_i3 | -0.9967752 | 0.817198092 | 0.999970678 | 0 | 9.826 | 15.955 | 0 | WRKY |
| TRINITY_DN690_c2_g1_i1 | 0.996771627 | 0.476291231 | 0.999970678 | 3.279 | 9.156 | 3.855 | 1.458 | NA |
| TRINITY_DN44977_c1_g1_i4 | -0.9967112 | 0.490611175 | 0.999970678 | 0.836 | 0.187 | 0.967 | 0.885 | NA |
| TRINITY_DN12656_c0_g1_i2 | -0.9966793 | 0.821667155 | 1 | 0.672 | 0 | 1.122 | 0 | NA |
| TRINITY_DN13048_c0_g1_i1 | -0.99665006 | 0.815475003 | 0.999970678 | 1.399 | 0 | 2.367 | 0 | NA |
| TRINITY_DN10835_c0_g1_i9 | -0.99665006 | 0.815475003 | 0.999970678 | 1.908 | 0 | 3.222 | 0 | NA |
| TRINITY_DN1329_c0_g2_i3 | 0.99664038 | 0.712109872 | 0.999970678 | 5.505 | 1.409 | 0 | 3.489 | NA |
| TRINITY_DN2723_c0_g1_i6 | -0.99663733 | 0.715088793 | 0.999970678 | 0 | 11.88 | 8.677 | 12.869 | NA |
| TRINITY_DN0_c0_g1_i1 | 0.996634829 | 0.598254052 | 0.999970678 | 60.054 | 0.976 | 8.677 | 20.469 | NA |
| TRINITY_DN3517_c1_g1_i3 | 0.996536511 | 0.753699689 | 0.999970678 | 6.686 | 0.059 | 0 | 3.448 | NA |
| TRINITY_DN8450_c1_g2_i2 | 0.996487312 | 0.485889553 | 0.999970678 | 0.672 | 0.946 | 0.567 | 0.121 | Arm |
| TRINITY_DN6100_c0_g2_i4 | 0.996486674 | 0.734560875 | 0.999970678 | 1.635 | 0 | 0.6 | 0.111 | - |
| TRINITY_DN28172_c0_g1_i1 | 0.996453044 | 0.308739855 | 0.999970678 | 0.536 | 0.66 | 0.222 | 0.322 | RVT_2 |
| TRINITY_DN1001_c0_g1_i6 | -0.99640322 | 0.718572376 | 0.999970678 | 2.716 | 0 | 0.967 | 4.363 | MAP70 |
| TRINITY_DN10349_c0_g1_i1 | -0.99625886 | 0.210522029 | 0.999970678 | 1.254 | 1.163 | 1.855 | 2.564 | NA |
| TRINITY_DN13067_c0_g1_i3 | -0.99618462 | 0.215022635 | 0.999970678 | 1.09 | 1.005 | 1.544 | 2.322 | PPR |
| TRINITY_DN5786_c0_g1_i1 | -0.99587211 | 0.174404969 | 0.999970678 | 13.481 | 20.21 | 31.965 | 27.859 | NA |
| TRINITY_DN2678_c0_g1_i1 | 0.995801726 | 0.590167285 | 0.999970678 | 3.852 | 5.411 | 0.144 | 4.424 | NA |
| TRINITY_DN237_c0_g1_i8 | 0.995712322 | 0.329359043 | 0.999970678 | 1.771 | 2.01 | 0.456 | 1.347 | NA |
| TRINITY_DN439_c0_g1_i1 | -0.99567983 | 0.266471962 | 0.999970678 | 2.126 | 3.528 | 3.466 | 7.038 | SAWADEE |
| TRINITY_DN1689_c0_g1_i4 | 0.995618538 | 0.812340081 | 0.999970678 | 6.431 | 0 | 0 | 3.278 | NA |
| TRINITY_DN1007_c0_g1_i3 | -0.99558324 | 0.345811595 | 0.999970678 | 7.067 | 11.03 | 7.155 | 26.994 | NA |
| TRINITY_DN21605_c0_g1_i1 | -0.99552041 | 0.424813408 | 0.999970678 | 15.97 | 9.323 | 6.211 | 43.191 | NA |
| TRINITY_DN6009_c0_g1_i1 | -0.99543687 | 0.598736955 | 1 | 1.717 | 0.355 | 3.044 | 0.462 | NA |
| TRINITY_DN468_c0_g1_i2 | 0.995308897 | 0.199945706 | 0.999970678 | 18.132 | 13.14 | 6.222 | 7.912 | NA |
| TRINITY_DN7584_c0_g3_i1 | 0.995258429 | 0.63159078 | 0.999970678 | 0.709 | 1.202 | 0.778 | 0.01 | NA |
| TRINITY_DN10358_c0_g1_i2 | 0.995154569 | 0.262804648 | 0.999970678 | 11.31 | 5.874 | 2.589 | 5.57 | RabGAP-TBC |
| TRINITY_DN14896_c0_g1_i3 | -0.99512592 | 0.48976744 | 0.999970678 | 33.91 | 8.259 | 9.222 | 73.925 | NA |
| TRINITY_DN2045_c0_g1_i10 | 0.995064023 | 0.410617265 | 0.999970678 | 2.48 | 4.326 | 1.522 | 1.528 | NA |
| TRINITY_DN10084_c0_g1_i3 | -0.99503944 | 0.815205169 | 0.999970678 | 7.067 | 0 | 11.822 | 0 | Malate_synthase |
| TRINITY_DN415_c1_g1_i2 | 0.995013595 | 0.818139711 | 0.999970678 | 0 | 5.864 | 2.389 | 0 | NA |
| TRINITY_DN6079_c0_g1_i4 | 0.995010247 | 0.712587294 | 0.999970678 | 0.927 | 2.631 | 0 | 1.729 | NA |
| TRINITY_DN748_c0_g1_i3 | 0.994983976 | 0.531850008 | 0.999970678 | 22.574 | 28.96 | 20.299 | 1.237 | NA |
| TRINITY_DN37_c0_g1_i9 | -0.99466418 | 0.263661546 | 0.999970678 | 36.472 | 55.92 | 107.005 | 54.23 | NA |
| TRINITY_DN668_c0_g1_i21 | 0.99459826 | 0.436972762 | 0.999970678 | 1.971 | 1.675 | 1.233 | 0.322 | NA |
| TRINITY_DN416_c0_g1_i4 | 0.994575523 | 0.298574395 | 0.999970678 | 43.812 | 89.3 | 28.72 | 27.638 | NA |
| TRINITY_DN9992_c0_g3_i1 | 0.99450367 | 0.819935885 | 0.999970678 | 3.879 | 0 | 0 | 1.96 | NA |
| TRINITY_DN2733_c1_g1_i6 | -0.994474 | 0.514516973 | 0.999970678 | 0.382 | 0.68 | 1.422 | 0.372 | NA |
| TRINITY_DN2069_c0_g1_i1 | -0.99434564 | 0.446813882 | 0.999970678 | 17.305 | 42.24 | 17.399 | 89.096 | NA |
| TRINITY_DN261_c0_g1_i3 | -0.99434508 | 0.603158484 | 0.999970678 | 51.397 | 8.811 | 97.516 | 2.996 | Metallophos |
| TRINITY_DN1640_c0_g2_i2 | -0.99425231 | 0.323258233 | 0.999970678 | 4.596 | 2.237 | 3.611 | 9.32 | NA |
| TRINITY_DN5096_c0_g1_i3 | 0.994209794 | 0.634798661 | 0.999970678 | 0.064 | 3.292 | 0.367 | 1.196 | NA |
| TRINITY_DN1794_c0_g1_i3 | 0.994028613 | 0.45049918 | 0.999970678 | 4.914 | 3.016 | 0.633 | 3.207 | NA |
| TRINITY_DN967_c0_g1_i5 | -0.99395244 | 0.40723395 | 0.999970678 | 5.414 | 7.096 | 3.3 | 20.932 | AAA |
| TRINITY_DN1510_c0_g1_i1 | 0.993733226 | 0.520755616 | 0.999970678 | 13.253 | 1.626 | 5 | 1.508 | LRR_1 |
| TRINITY_DN3470_c3_g1_i2 | 0.99371029 | 0.623251639 | 0.999970678 | 0.89 | 0.611 | 0.6 | 0.02 | NA |
| TRINITY_DN665_c0_g1_i1 | 0.99367666 | 0.407829775 | 0.999970678 | 2.171 | 2.493 | 0.7 | 1.478 | LRR_8 |
| TRINITY_DN805_c0_g1_i7 | -0.99367287 | 0.305359119 | 0.999970678 | 4.006 | 1.567 | 5.311 | 4.776 | p450 |
| TRINITY_DN3820_c0_g1_i2 | 0.993632089 | 0.495581775 | 0.999970678 | 0.736 | 4.711 | 0.889 | 1.599 | zf-RING_2 |
| TRINITY_DN5512_c0_g1_i2 | 0.99361063 | 0.414694565 | 0.999970678 | 0.927 | 0.759 | 0.167 | 0.643 | FimP |
| TRINITY_DN27245_c0_g1_i1 | -0.99355776 | 0.826186519 | 1 | 2.525 | 0 | 4.178 | 0 | Ribosomal_L29 |
| TRINITY_DN524_c0_g1_i2 | -0.99351574 | 0.197774512 | 0.999970678 | 59.954 | 88.81 | 156.079 | 105.785 | Ribosomal_L13 |
| TRINITY_DN55159_c0_g1_i2 | 0.993311919 | 0.716216793 | 1 | 0.191 | 0.463 | 0 | 0.342 | PWWP |
| TRINITY_DN880_c0_g1_i1 | 0.993296868 | 0.710232645 | 0.999970678 | 5.478 | 12.91 | 0 | 9.139 | - |
| TRINITY_DN12453_c0_g2_i3 | -0.99309309 | 0.712466661 | 0.999970678 | 3.57 | 0 | 3.455 | 3.026 | RPAP3_C |
| TRINITY_DN5102_c0_g1_i2 | 0.993011239 | 0.389266502 | 0.999970678 | 12.272 | 3.026 | 3.666 | 3.318 | NA |
| TRINITY_DN2730_c0_g1_i13 | -0.99289456 | 0.73498632 | 0.999970678 | 0 | 5.411 | 0.589 | 9.933 | OTCace |
| TRINITY_DN16778_c0_g1_i2 | -0.99282668 | 0.45441616 | 0.999970678 | 0.409 | 0.384 | 1.044 | 0.352 | DLD |
| TRINITY_DN2725_c1_g1_i2 | 0.992677889 | 0.821061777 | 0.999970678 | 1.145 | 0 | 0 | 0.573 | NA |
| TRINITY_DN7734_c0_g1_i9 | -0.99263385 | 0.579203677 | 0.999970678 | 0.127 | 0.483 | 0.122 | 1.076 | WD40 |

| TRINITY_DN6135_c0_g2_i3 | -0.99242776 | 0.292967875 | 0.999970678 | 0.863 | 0.759 | 1.322 | 1.679 | NA |
| --- | --- | --- | --- | --- | --- | --- | --- | --- |
| TRINITY_DN4609_c0_g1_i1 | 0.992321289 | 0.484216379 | 0.999970678 | 1.635 | 0.7 | 0.722 | 0.282 | NA |
| TRINITY_DN4486_c1_g1_i5 | -0.99231563 | 0.196800205 | 0.999970678 | 7.058 | 6.258 | 13.844 | 9.752 | NA |
| TRINITY_DN211_c0_g1_i7 | -0.99225664 | 0.732201167 | 0.999970678 | 15.933 | 15.05 | 50.852 | 0 | NA |
| TRINITY_DN247_c1_g2_i4 | 0.992132651 | 0.503740032 | 0.999970678 | 1.88 | 0.572 | 0.878 | 0.191 | Peptidase_S10 |
| TRINITY_DN13_c0_g1_i9 | 0.992116413 | 0.324695846 | 0.999970678 | 75.097 | 105.3 | 57.508 | 20.902 | NA |
| TRINITY_DN3561_c0_g1_i11 | 0.992077306 | 0.093827634 | 0.999970678 | 15.452 | 17.87 | 6.522 | 8.877 | Pyr_redox_2 |
| TRINITY_DN4656_c0_g1_i4 | -0.99204392 | 0.725153062 | 0.999970678 | 0 | 22.22 | 26.065 | 11.944 | Yip1 |
| TRINITY_DN9344_c0_g1_i2 | -0.99198491 | 0.421406541 | 0.999970678 | 27.615 | 29.67 | 13.321 | 96.556 | NA |
| TRINITY_DN14708_c0_g1_i1 | 0.991972939 | 0.598954974 | 0.999970678 | 34.546 | 1.971 | 14.077 | 1.538 | NA |
| TRINITY_DN10718_c0_g1_i4 | -0.99194105 | 0.723913358 | 0.999970678 | 1.671 | 3.321 | 0 | 9.903 | NA |
| TRINITY_DN7363_c0_g1_i4 | -0.99191454 | 0.412529309 | 0.999970678 | 0.908 | 1.192 | 0.756 | 3.227 | NA |
| TRINITY_DN9152_c0_g1_i1 | 0.991902315 | 0.103905427 | 0.999970678 | 6.913 | 7.559 | 2.711 | 4.021 | NA |
| TRINITY_DN1283_c0_g1_i4 | 0.991829754 | 0.3938963 | 0.999970678 | 3.279 | 1.015 | 0.856 | 1.136 | AKAP7_NLS |
| TRINITY_DN15_c0_g1_i28 | 0.991719443 | 0.336838651 | 0.999970678 | 22.328 | 18.92 | 4.933 | 14.085 | NA |
| TRINITY_DN8369_c0_g1_i5 | -0.9916776 | 0.714444403 | 0.999970678 | 0.709 | 0 | 0.478 | 0.865 | Exo70 |
| TRINITY_DN1262_c0_g1_i1 | 0.991652889 | 0.711617965 | 0.999970678 | 3.37 | 1.557 | 2.067 | 0 | bZIP_1 |
| TRINITY_DN1984_c0_g1_i6 | 0.99164746 | 0.269571934 | 0.999970678 | 1.054 | 1.202 | 0.567 | 0.452 | NA |
| TRINITY_DN16003_c1_g1_i1 | -0.99161473 | 0.331978019 | 0.999970678 | 2.653 | 1.892 | 5.444 | 2.433 | NYN |
| TRINITY_DN6554_c0_g1_i3 | -0.99155056 | 0.492487933 | 0.999970678 | 0.645 | 0.838 | 2.044 | 0.483 | NA |
| TRINITY_DN668_c2_g1_i1 | 0.991385782 | 0.500976935 | 0.999970678 | 1.962 | 2.208 | 1.467 | 0.352 | DUF659 |
| TRINITY_DN4164_c0_g1_i1 | 0.991225746 | 0.325451241 | 0.999970678 | 1.463 | 1.005 | 0.722 | 0.372 | - |
| TRINITY_DN5857_c0_g2_i1 | 0.991153289 | 0.39726365 | 0.999970678 | 3.952 | 5.026 | 3.078 | 0.794 | NA |
| TRINITY_DN2725_c0_g2_i9 | 0.991095517 | 0.710409406 | 0.999970678 | 2.244 | 2.503 | 0 | 2.403 | NA |
| TRINITY_DN1299_c0_g1_i1 | 0.991071213 | 0.459932994 | 0.999970678 | 1.617 | 9.974 | 2.933 | 2.202 | NA |
| TRINITY_DN2991_c0_g1_i8 | 0.991006435 | 0.342553666 | 0.999970678 | 0.763 | 1.242 | 0.389 | 0.523 | - |
| TRINITY_DN2031_c0_g2_i1 | 0.990991828 | 0.24231701 | 0.999970678 | 5.36 | 3.242 | 2.311 | 1.558 | - |
| TRINITY_DN12583_c0_g1_i1 | -0.99095349 | 0.820495496 | 0.999970678 | 6.422 | 0 | 10.633 | 0 | NA |
| TRINITY_DN9711_c0_g1_i1 | -0.99095349 | 0.820495496 | 0.999970678 | 0.681 | 0 | 1.144 | 0 | NA |
| TRINITY_DN19202_c2_g2_i1 | -0.99093579 | 0.563712749 | 0.999970678 | 1.272 | 0.079 | 1.167 | 1.317 | Amidase |
| TRINITY_DN10316_c0_g1_i2 | 0.990873917 | 0.726399284 | 0.999970678 | 86.079 | 0 | 21.91 | 16.267 | NA |
| TRINITY_DN3360_c2_g1_i4 | 0.990679691 | 0.514747814 | 0.999970678 | 6.25 | 0.641 | 1.533 | 1.639 | Aminotran_4 |
| TRINITY_DN11591_c0_g1_i8 | 0.99062516 | 0.527947561 | 0.999970678 | 56.52 | 7.805 | 18.232 | 9.099 | LRRNT_2 |
| TRINITY_DN8132_c0_g1_i1 | -0.99040621 | 0.663839327 | 0.999970678 | 5.759 | 1.882 | 12.51 | 0.07 | LTP_2 |
| TRINITY_DN1031_c1_g2_i2 | -0.99038471 | 0.106091856 | 0.999970678 | 4.624 | 5.953 | 8.022 | 11.351 | Peptidase_M16 |
| TRINITY_DN12529_c1_g1_i4 | -0.99032338 | 0.549530079 | 0.999970678 | 0.445 | 5.726 | 1.833 | 9.913 | Fasciclin |
| TRINITY_DN589_c0_g1_i12 | -0.99014065 | 0.329325176 | 0.999970678 | 3.17 | 1.252 | 3.233 | 4.896 | NA |
| TRINITY_DN9694_c0_g2_i3 | 0.990133148 | 0.747543681 | 1 | 0 | 1.873 | 0.678 | 0.111 | PsaD |
| TRINITY_DN7836_c0_g1_i4 | -0.9900357 | 0.70753863 | 0.999970678 | 0 | 1.35 | 1.122 | 1.267 | NA |
| TRINITY_DN175_c0_g1_i12 | 0.990014949 | 0.367520257 | 0.999970678 | 2.489 | 1.882 | 1.155 | 0.834 | NA |
| TRINITY_DN6024_c0_g1_i9 | -0.98998962 | 0.5629898 | 1 | 61.834 | 9.057 | 52.697 | 66.334 | NA |
| TRINITY_DN2067_c0_g1_i1 | 0.98992591 | 0.243720655 | 0.999970678 | 14.861 | 14.25 | 3.778 | 10.104 | NA |
| TRINITY_DN16474_c1_g1_i3 | 0.989904268 | 0.493281532 | 0.999970678 | 0.2 | 0.976 | 0.133 | 0.422 | PMEI |
| TRINITY_DN8439_c0_g1_i1 | -0.98985209 | 0.824919679 | 1 | 1.244 | 0 | 2.067 | 0 | NA |
| TRINITY_DN37_c0_g1_i6 | -0.98960814 | 0.311067824 | 0.999970678 | 1.908 | 2.06 | 4.733 | 2.162 | Proton_antipo_M |
| TRINITY_DN56_c2_g1_i2 | 0.989544475 | 0.322107089 | 0.999970678 | 0.845 | 0.936 | 0.478 | 0.322 | DNA_mis_repair |
| TRINITY_DN3420_c1_g1_i4 | -0.98946806 | 0.725916805 | 0.999970678 | 0 | 3.173 | 4.433 | 0.845 | NA |
| TRINITY_DN2341_c1_g2_i1 | 0.989389759 | 0.823050204 | 1 | 4.96 | 0 | 0 | 2.453 | LRRNT_2 |
| TRINITY_DN3636_c0_g1_i6 | 0.989339777 | 0.436960078 | 0.999970678 | 1.826 | 0.808 | 0.256 | 1.005 | NA |
| TRINITY_DN17848_c0_g1_i8 | 0.989051729 | 0.50706747 | 1 | 1.599 | 1.567 | 1.067 | 0.302 | NA |
| TRINITY_DN6827_c1_g1_i4 | 0.989045983 | 0.71742791 | 0.999970678 | 0.209 | 0.621 | 0.344 | 0 | NA |
| TRINITY_DN8270_c0_g1_i6 | -0.98899313 | 0.554064124 | 0.999970678 | 1.662 | 8.2 | 14.932 | 1.337 | Glyco_hydro_14 |
| TRINITY_DN3060_c0_g1_i8 | -0.98895014 | 0.5517108 | 0.999970678 | 1.835 | 0.848 | 4.178 | 0.302 | NA |
| TRINITY_DN3842_c0_g1_i3 | 0.988885737 | 0.819890259 | 0.999970678 | 0 | 0.887 | 0 | 0.442 | NA |
| TRINITY_DN1967_c0_g1_i3 | -0.98886324 | 0.415763668 | 0.999970678 | 1.472 | 1.301 | 1.089 | 4.122 | NA |
| TRINITY_DN10076_c0_g3_i3 | 0.988861358 | 0.562309525 | 1 | 0.3 | 0.483 | 0.289 | 0.04 | NA |
| TRINITY_DN15439_c0_g1_i3 | 0.988776975 | 0.538477662 | 1 | 2.144 | 2.454 | 0.278 | 1.84 | NA |
| TRINITY_DN1489_c0_g2_i2 | 0.98866761 | 0.480755469 | 0.999970678 | 12.463 | 4.06 | 0.944 | 7.259 | MORN |
| TRINITY_DN3240_c0_g1_i6 | 0.988627603 | 0.813912851 | 0.999970678 | 5.014 | 0 | 0 | 2.584 | NA |
| TRINITY_DN2103_c0_g1_i7 | 0.988546076 | 0.735251779 | 0.999970678 | 0 | 1477 | 190.177 | 440.221 | NA |
| TRINITY_DN6497_c0_g1_i7 | 0.988394879 | 0.529050752 | 0.999970678 | 0.972 | 6.031 | 0.889 | 2.393 | NA |
| TRINITY_DN3755_c0_g1_i1 | 0.988391658 | 0.353681504 | 0.999970678 | 2.162 | 1.823 | 0.578 | 1.317 | NA |
| TRINITY_DN415_c0_g1_i16 | -0.98833087 | 0.239961024 | 0.999970678 | 24.663 | 43.09 | 45.297 | 73.131 | NA |
| TRINITY_DN4696_c0_g1_i5 | -0.98829793 | 0.370052875 | 0.999970678 | 0.382 | 0.375 | 0.589 | 0.804 | NA |
| TRINITY_DN14813_c0_g1_i4 | 0.988098522 | 0.55101396 | 0.999970678 | 2.525 | 59.86 | 8.2 | 21.173 | NA |
| TRINITY_DN8542_c0_g1_i2 | -0.9880247 | 0.342651977 | 0.999970678 | 0.618 | 0.375 | 0.933 | 0.885 | NA |
| TRINITY_DN192825_c0_g1_i1 | -0.98800649 | 0.353035645 | 0.999970678 | 0.363 | 0.355 | 0.511 | 0.804 | PPR |
| TRINITY_DN7685_c0_g1_i1 | 0.988000133 | 0.194348952 | 0.999970678 | 25.789 | 29.84 | 8 | 18.418 | NA |
| TRINITY_DN372_c0_g1_i5 | -0.98799892 | 0.719180093 | 0.999970678 | 1.935 | 0 | 0.8 | 2.855 | NA |
| TRINITY_DN13740_c0_g1_i5 | 0.987955877 | 0.330882609 | 0.999970678 | 1.036 | 0.897 | 0.556 | 0.312 | PPR |
| TRINITY_DN8848_c0_g1_i4 | 0.987662403 | 0.601361143 | 0.999970678 | 7.222 | 413.9 | 59.663 | 121.77 | NA |
| TRINITY_DN8850_c0_g1_i6 | -0.98765149 | 0.723064072 | 0.999970678 | 21.956 | 0 | 20.632 | 19.112 | NA |
| TRINITY_DN189_c5_g1_i1 | 0.987590682 | 0.466257936 | 1 | 0.808 | 0.69 | 0.178 | 0.543 | NA |
| TRINITY_DN3199_c0_g1_i9 | 0.987350663 | 0.564262702 | 0.999970678 | 1.944 | 0.808 | 0.144 | 1.216 | NA |

| TRINITY_DN24480_c0_g1_i1 | -0.98730517 | 0.292164824 | 0.999970678 | 4.279 | 5.815 | 8.633 | 9.089 | NA |
| --- | --- | --- | --- | --- | --- | --- | --- | --- |
| TRINITY_DN2189_c0_g1_i2 | 0.987147466 | 0.43608623 | 0.999970678 | 0.663 | 0.867 | 0.189 | 0.553 | AAA |
| TRINITY_DN17549_c0_g1_i3 | 0.987046167 | 0.724327346 | 0.999970678 | 0.2 | 1.084 | 0.533 | 0 | NA |
| TRINITY_DN1270_c1_g1_i1 | 0.986825768 | 0.308916105 | 0.999970678 | 81.092 | 28.3 | 26.087 | 23.807 | NA |
| TRINITY_DN3497_c0_g1_i3 | 0.986807106 | 0.343788948 | 0.999970678 | 32.13 | 8.968 | 9.311 | 9.702 | RabGAP-TBC |
| TRINITY_DN28370_c0_g1_i1 | -0.98673617 | 0.427120012 | 0.999970678 | 2.716 | 5.45 | 2.478 | 13.04 | NA |
| TRINITY_DN70422_c0_g1_i1 | -0.98667419 | 0.301772065 | 0.999970678 | 77.341 | 202 | 164.301 | 326.182 | NA |
| TRINITY_DN1601_c0_g1_i6 | 0.986673224 | 0.466990329 | 0.999970678 | 3.806 | 3.873 | 2.833 | 0.462 | NA |
| TRINITY_DN1332_c0_g4_i1 | 0.986454546 | 0.330128758 | 0.999970678 | 7.431 | 21.03 | 4.644 | 8.606 | SelR |
| TRINITY_DN203_c0_g1_i14 | 0.986426611 | 0.481832945 | 0.999970678 | 1.299 | 0.335 | 0.489 | 0.241 | NA |
| TRINITY_DN8606_c2_g1_i6 | -0.98632167 | 0.603001385 | 0.999970678 | 1.108 | 0.296 | 2.2 | 0.141 | NA |
| TRINITY_DN16490_c0_g1_i1 | -0.98630204 | 0.519307531 | 0.999970678 | 0.654 | 3.784 | 1.533 | 6.786 | adh_short_C2 |
| TRINITY_DN3856_c0_g1_i2 | 0.986176138 | 0.725409887 | 0.999970678 | 22.147 | 7.382 | 0 | 15.06 | Ribosomal_L18p |
| TRINITY_DN2488_c0_g1_i2 | -0.98615409 | 0.353821574 | 0.999970678 | 2.098 | 0.917 | 3.033 | 2.312 | NA |
| TRINITY_DN1678_c0_g1_i1 | 0.986033273 | 0.825456468 | 0.999970678 | 44.866 | 0 | 0 | 23.093 | Aminotran_1_2 |
| TRINITY_DN1686_c0_g1_i2 | 0.985904798 | 0.343439282 | 0.999970678 | 4.342 | 3.755 | 2.589 | 0.965 | Vps53_N |
| TRINITY_DN1334_c1_g1_i9 | 0.985879498 | 0.490111214 | 0.999970678 | 2.88 | 6.573 | 3.411 | 0.633 | NA |
| TRINITY_DN6860_c0_g1_i1 | -0.9858038 | 0.71323666 | 0.999970678 | 69.011 | 0 | 42.931 | 79.304 | NA |
| TRINITY_DN1505_c0_g1_i2 | 0.985584103 | 0.565044944 | 0.999970678 | 0.327 | 2.957 | 0.933 | 0.533 | NA |
| TRINITY_DN103_c0_g1_i4 | -0.98550095 | 0.725126067 | 0.999970678 | 3.506 | 0 | 5.033 | 0.995 | NA |
| TRINITY_DN189_c0_g1_i3 | 0.985379018 | 0.468954955 | 0.999970678 | 3.543 | 0.828 | 1.378 | 0.573 | NA |
| TRINITY_DN8019_c0_g2_i2 | -0.9852975 | 0.711487571 | 0.999970678 | 2.017 | 0 | 1.9 | 1.769 | NA |
| TRINITY_DN5838_c0_g1_i1 | 0.985296272 | 0.174390503 | 0.999970678 | 3.77 | 4.652 | 2.155 | 1.659 | NA |
| TRINITY_DN11206_c0_g1_i2 | 0.985199912 | 0.825494847 | 1 | 0.945 | 0 | 0 | 0.493 | NA |
| TRINITY_DN32674_c0_g1_i5 | 0.985178384 | 0.748642665 | 0.999970678 | 0 | 4.08 | 1.533 | 0.231 | NA |
| TRINITY_DN16912_c0_g1_i6 | -0.98478499 | 0.469590413 | 0.999970678 | 0.109 | 0.256 | 0.389 | 0.261 | Chloroa_b-bind |
| TRINITY_DN3628_c0_g1_i1 | -0.98472587 | 0.823025226 | 0.999970678 | 5.033 | 0 | 8.188 | 0 | NA |
| TRINITY_DN8991_c0_g2_i1 | -0.9846655 | 0.751903264 | 1 | 0 | 1.439 | 0.111 | 2.574 | - |
| TRINITY_DN13049_c0_g1_i4 | 0.984663917 | 0.472020839 | 0.999970678 | 1.844 | 1.34 | 1.111 | 0.251 | DUF812 |
| TRINITY_DN5898_c0_g1_i4 | 0.984660672 | 0.576184567 | 0.999970678 | 1.081 | 1.114 | 0.889 | 0.05 | NA |
| TRINITY_DN883_c0_g1_i2 | 0.984497995 | 0.815598859 | 0.999970678 | 0 | 10.06 | 4.144 | 0 | NA |
| TRINITY_DN511_c0_g1_i4 | -0.98448495 | 0.722541364 | 1 | 0 | 0.857 | 0.989 | 0.483 | NA |
| TRINITY_DN2810_c0_g2_i3 | -0.98434349 | 0.736394423 | 0.999970678 | 2.662 | 0.739 | 5.577 | 0 | NA |
| TRINITY_DN16677_c0_g1_i2 | -0.98432159 | 0.246587586 | 0.999970678 | 1.099 | 1.183 | 1.822 | 2.282 | NA |
| TRINITY_DN695_c1_g1_i4 | -0.9842884 | 0.093731893 | 0.999970678 | 65.577 | 60.01 | 112.693 | 112.38 | 2OG-FeII_Oxy |
| TRINITY_DN1877_c0_g1_i12 | 0.984162945 | 0.573787597 | 0.999970678 | 4.106 | 0.207 | 1 | 1.025 | NA |
| TRINITY_DN26494_c0_g1_i1 | 0.984156231 | 0.562733799 | 0.999970678 | 1.681 | 20.85 | 1.2 | 9.802 | NA |
| TRINITY_DN1498_c0_g1_i7 | -0.98412336 | 0.818548257 | 0.999970678 | 0 | 0.384 | 0 | 0.764 | - |
| TRINITY_DN9516_c1_g1_i1 | 0.984024603 | 0.816110307 | 0.999970678 | 15.161 | 0 | 0 | 7.651 | NA |
| TRINITY_DN8752_c0_g1_i3 | -0.98392102 | 0.72630203 | 1 | 0.854 | 0 | 0.356 | 1.287 | p450 |
| TRINITY_DN133_c0_g1_i5 | 0.983919787 | 0.817081988 | 0.999970678 | 0 | 27.51 | 11.299 | 0 | NA |
| TRINITY_DN71700_c0_g2_i1 | 0.983861417 | 0.311957911 | 0.999970678 | 1.481 | 1.597 | 0.955 | 0.402 | FATC |
| TRINITY_DN10381_c0_g4_i1 | -0.98379273 | 0.340076613 | 0.999970678 | 3.897 | 10.31 | 15.066 | 9.591 | NA |
| TRINITY_DN786_c0_g1_i3 | -0.98378957 | 0.480433496 | 0.999970678 | 7.485 | 1.183 | 3.366 | 13.331 | DEAD |
| TRINITY_DN15565_c0_g1_i2 | 0.983682127 | 0.596197795 | 0.999970678 | 0.254 | 3.035 | 1.078 | 0.342 | NA |
| TRINITY_DN1963_c0_g1_i2 | -0.98358012 | 0.720248941 | 0.999970678 | 0 | 15.07 | 7.277 | 20.52 | OST3_OST6 |
| TRINITY_DN1720_c1_g1_i7 | 0.983536311 | 0.826422911 | 1 | 0.736 | 0 | 0 | 0.372 | NA |
| TRINITY_DN6024_c0_g1_i5 | -0.98345994 | 0.140221851 | 0.999970678 | 39.897 | 51.45 | 67.774 | 91.73 | NA |
| TRINITY_DN534_c0_g1_i2 | -0.98343579 | 0.513741155 | 0.999970678 | 0.363 | 0.453 | 1.233 | 0.121 | DPPIV_N |
| TRINITY_DN1031_c1_g1_i6 | -0.98325128 | 0.268274042 | 0.999970678 | 1.381 | 1.045 | 2.455 | 1.85 | NA |
| TRINITY_DN3843_c0_g1_i1 | -0.98315285 | 0.550140891 | 0.999970678 | 14.925 | 2.484 | 26.398 | 2.906 | GatB_N |
| TRINITY_DN2360_c0_g1_i9 | -0.98302615 | 0.637568327 | 1 | 0.073 | 0.552 | 0.9 | 0.101 | NA |
| TRINITY_DN8796_c0_g1_i2 | 0.982832236 | 0.301325028 | 0.999970678 | 1.544 | 1.409 | 0.378 | 1.036 | NUDIX |
| TRINITY_DN1054_c0_g1_i3 | -0.98238063 | 0.599785207 | 0.999970678 | 0.899 | 0.69 | 0.167 | 3.036 | NA |
| TRINITY_DN1587_c0_g2_i4 | 0.982342748 | 0.713180255 | 0.999970678 | 1.344 | 1.064 | 1.011 | 0 | - |
| TRINITY_DN3492_c1_g1_i1 | 0.982312143 | 0.522650278 | 0.999970678 | 3.188 | 2.888 | 2.367 | 0.211 | NA |
| TRINITY_DN2721_c0_g1_i1 | -0.98225624 | 0.124291695 | 0.999970678 | 20.957 | 24.89 | 40.298 | 40.667 | Stress-antifung |
| TRINITY_DN1028_c0_g1_i7 | 0.982251453 | 0.74008997 | 0.999970678 | 21.211 | 1.399 | 0 | 11.612 | RRM_1 |
| TRINITY_DN2061_c0_g2_i1 | 0.98202792 | 0.39904903 | 0.999970678 | 0.672 | 0.7 | 0.333 | 0.302 | NA |
| TRINITY_DN592_c0_g2_i3 | -0.9820215 | 0.747501682 | 0.999970678 | 0 | 4.336 | 0.256 | 8.033 | Ribosomal_L37 |
| TRINITY_DN4847_c3_g1_i2 | -0.98195282 | 0.234393059 | 0.999970678 | 4.551 | 3.449 | 4.789 | 10.013 | NA |
| TRINITY_DN9963_c0_g1_i3 | -0.98182997 | 0.359071508 | 0.999970678 | 1.199 | 2.947 | 4.078 | 3.167 | NA |
| TRINITY_DN6145_c0_g1_i8 | -0.98182813 | 0.550786674 | 0.999970678 | 0.127 | 0.266 | 0.089 | 0.674 | B_lectin |
| TRINITY_DN9679_c1_g1_i2 | 0.98177269 | 0.721063933 | 1 | 0.436 | 0.453 | 0.367 | 0 | NA |
| TRINITY_DN763_c0_g1_i2 | -0.9817444 | 0.205378289 | 0.999970678 | 3.752 | 3.715 | 4.611 | 9.078 | NA |
| TRINITY_DN837_c0_g1_i2 | -0.98170728 | 0.161262536 | 0.999970678 | 11.337 | 8.643 | 18.454 | 17.142 | Ribosomal_L6e |
| TRINITY_DN32144_c0_g1_i4 | -0.98161864 | 0.651824647 | 0.999970678 | 0.863 | 0.059 | 1.4 | 0.151 | PIR |
| TRINITY_DN1513_c0_g1_i3 | 0.981561141 | 0.710787163 | 0.999970678 | 0.536 | 0.976 | 0 | 0.764 | NA |
| TRINITY_DN7516_c1_g1_i4 | 0.981506121 | 0.423544175 | 0.999970678 | 1.036 | 0.453 | 0.289 | 0.412 | NA |
| TRINITY_DN3596_c0_g2_i2 | -0.98148568 | 0.712325245 | 0.999970678 | 1.998 | 0 | 1.522 | 2.182 | Methyltransf_11 |
| TRINITY_DN2043_c0_g1_i3 | 0.981424929 | 0.276004179 | 0.999970678 | 7.34 | 5.125 | 3.633 | 1.95 | NA |
| TRINITY_DN16060_c0_g1_i1 | -0.98142133 | 0.82731585 | 1 | 1.263 | 0 | 0 | 2.534 | NA |
| TRINITY_DN2026_c0_g2_i3 | -0.98142133 | 0.82731585 | 1 | 1.79 | 0 | 0 | 3.499 | NA |
| TRINITY_DN2553_c0_g1_i5 | 0.98111927 | 0.285196517 | 0.999970678 | 1.226 | 0.65 | 0.367 | 0.523 | NA |

| TRINITY_DN372_c0_g1_i16 | -0.98109004 | 0.823454341 | 1 | 2.08 | 0 | 0 | 4.152 | NA |
| --- | --- | --- | --- | --- | --- | --- | --- | --- |
| TRINITY_DN958_c0_g1_i1 | 0.981028928 | 0.460271257 | 0.999970678 | 17.795 | 2.986 | 5.944 | 3.418 | CBS |
| TRINITY_DN92177_c0_g1_i1 | 0.980926232 | 0.601273317 | 0.999970678 | 3.279 | 0.355 | 0.267 | 1.518 | PG_binding_1 |
| TRINITY_DN8626_c0_g1_i5 | 0.980900363 | 0.487066746 | 0.999970678 | 5.741 | 1.242 | 2.322 | 0.754 | MIP |
| TRINITY_DN2353_c0_g1_i6 | -0.9808962 | 0.097876087 | 0.999970678 | 62.234 | 82.3 | 120.026 | 139.797 | FAF |
| TRINITY_DN5915_c0_g1_i2 | -0.98068817 | 0.81878913 | 0.999970678 | 1.39 | 0 | 0 | 2.765 | NA |
| TRINITY_DN740_c0_g1_i2 | -0.98054238 | 0.591472613 | 0.999970678 | 0.663 | 27.76 | 32.809 | 15.412 | NA |
| TRINITY_DN1317_c0_g1_i4 | -0.98040843 | 0.220662726 | 0.999970678 | 191.3 | 234.9 | 223.542 | 569.934 | Chal_sti_synt_C |
| TRINITY_DN6422_c0_g1_i4 | 0.98028468 | 0.411160365 | 0.999970678 | 6.195 | 6.081 | 4.333 | 0.995 | Glyco_hydro_2 |
| TRINITY_DN4457_c0_g1_i1 | 0.980271702 | 0.49545366 | 0.999970678 | 0.472 | 0.591 | 0.367 | 0.111 | NA |
| TRINITY_DN8_c1_g2_i2 | -0.98021337 | 0.284940907 | 0.999970678 | 56.239 | 53.56 | 47.508 | 158.627 | NA |
| TRINITY_DN7442_c0_g1_i3 | -0.98009233 | 0.829409449 | 1 | 1.953 | 0 | 3.2 | 0 | NA |
| TRINITY_DN7625_c0_g1_i2 | -0.98009233 | 0.829409449 | 1 | 0.2 | 0 | 0.344 | 0 | FYRC |
| TRINITY_DN7045_c0_g1_i10 | -0.98009233 | 0.829409449 | 1 | 2.489 | 0 | 4.144 | 0 | NA |
| TRINITY_DN29126_c0_g2_i1 | -0.98009233 | 0.829409449 | 1 | 3.043 | 0 | 4.911 | 0 | Cyclase |
| TRINITY_DN12245_c0_g1_i6 | -0.98007393 | 0.823851799 | 1 | 0 | 0.917 | 0 | 1.79 | NA |
| TRINITY_DN46_c0_g1_i3 | -0.98005319 | 0.460456208 | 0.999970678 | 2.489 | 0.493 | 1.755 | 3.81 | RRM_1 |
| TRINITY_DN1873_c3_g1_i1 | 0.980041712 | 0.717304395 | 0.999970678 | 1.317 | 0.69 | 0.856 | 0 | NA |
| TRINITY_DN636_c0_g1_i5 | 0.979967031 | 0.28426968 | 0.999970678 | 9.883 | 19.52 | 8.044 | 5.087 | - |
| TRINITY_DN12800_c0_g1_i1 | 0.979940026 | 0.447500279 | 0.999970678 | 3.134 | 0.936 | 1.222 | 0.583 | NA |
| TRINITY_DN10176_c0_g1_i5 | -0.97973103 | 0.40666499 | 0.999970678 | 1.681 | 1.478 | 1.044 | 4.956 | NA |
| TRINITY_DN1074_c1_g1_i7 | -0.97962764 | 0.562703641 | 0.999970678 | 0.454 | 0.306 | 1.155 | 0.101 | CH |
| TRINITY_DN3195_c0_g1_i1 | -0.97946597 | 0.714932879 | 0.999970678 | 0 | 1.321 | 0.6 | 1.86 | Hydrolase_6 |
| TRINITY_DN7425_c0_g1_i3 | 0.97938978 | 0.540598281 | 0.999970678 | 2.107 | 1.212 | 0.122 | 1.528 | BTB_2 |
| TRINITY_DN490_c1_g3_i1 | 0.979328985 | 0.579400057 | 0.999970678 | 21.093 | 1.202 | 7.844 | 1.93 | Usp |
| TRINITY_DN1149_c0_g1_i9 | -0.97927065 | 0.713946906 | 0.999970678 | 0.972 | 0 | 0.7 | 1.116 | NA |
| TRINITY_DN15809_c0_g1_i2 | -0.97912865 | 0.228456608 | 0.999970678 | 18.577 | 41.13 | 45.908 | 61.398 | RRM_1 |
| TRINITY_DN88_c0_g3_i2 | -0.97912844 | 0.181022303 | 0.999970678 | 23.4 | 39.15 | 58.163 | 52.058 | NA |
| TRINITY_DN369_c0_g2_i3 | -0.97909709 | 0.750448144 | 0.999970678 | 1314.6 | 0 | 944.576 | 1397.423 | NA |
| TRINITY_DN7593_c0_g1_i1 | -0.9790425 | 0.713550121 | 0.999970678 | 4.869 | 0 | 3.266 | 5.66 | NA |
| TRINITY_DN51018_c0_g1_i1 | -0.97895323 | 0.820725502 | 0.999970678 | 2.407 | 0 | 3.966 | 0 | NA |
| TRINITY_DN4668_c0_g1_i2 | -0.97881713 | 0.729175274 | 1 | 0.372 | 0 | 0.133 | 0.583 | DUF659 |
| TRINITY_DN13268_c0_g1_i6 | -0.97879375 | 0.362147893 | 0.999970678 | 3.116 | 7.687 | 12.71 | 5.771 | NA |
| TRINITY_DN1165_c1_g1_i3 | 0.978734099 | 0.362160781 | 0.999970678 | 0.627 | 0.473 | 0.278 | 0.221 | NA |
| TRINITY_DN7890_c1_g2_i1 | -0.97859523 | 0.729226978 | 1 | 1.417 | 0 | 0.578 | 2.121 | NA |
| TRINITY_DN188_c0_g1_i13 | -0.97858798 | 0.297846955 | 0.999970678 | 28.015 | 12.72 | 42.064 | 28.975 | NA |
| TRINITY_DN1404_c0_g1_i2 | -0.97858704 | 0.411458051 | 0.999970678 | 0.445 | 0.867 | 1.011 | 1.357 | Ion_trans_2 |
| TRINITY_DN7845_c0_g1_i3 | 0.978557886 | 0.741159298 | 0.999970678 | 0 | 4.681 | 1.744 | 0.251 | NA |
| TRINITY_DN708_c0_g1_i10 | -0.9784188 | 0.719005423 | 0.999970678 | 2.589 | 0 | 2.833 | 1.769 | NA |
| TRINITY_DN16832_c0_g1_i1 | 0.978380164 | 0.423684637 | 0.999970678 | 0.6 | 0.798 | 0.444 | 0.181 | DNA_pol3_delta2 |
| TRINITY_DN13490_c0_g1_i1 | -0.97834812 | 0.717457383 | 0.999970678 | 2.943 | 0 | 1.389 | 4.212 | Ank |
| TRINITY_DN4668_c0_g2_i3 | 0.978301586 | 0.509364982 | 0.999970678 | 44.275 | 17.58 | 23.887 | 2.725 | Myb_CC_LHEQLE |
| TRINITY_DN55430_c0_g2_i3 | 0.978264645 | 0.086583675 | 0.999970678 | 183.79 | 178.9 | 74.029 | 90.966 | adh_short |
| TRINITY_DN28273_c0_g2_i7 | -0.97820537 | 0.723632165 | 0.999970678 | 0 | 2.789 | 0.867 | 4.313 | NA |
| TRINITY_DN6889_c0_g1_i2 | 0.978173586 | 0.724276431 | 1 | 0.2 | 0.67 | 0 | 0.432 | DEAD |
| TRINITY_DN24127_c0_g1_i1 | -0.97811197 | 0.661178771 | 1 | 0.345 | 1.153 | 0.056 | 2.845 | AP2 |
| TRINITY_DN45099_c0_g1_i3 | -0.97803135 | 0.827662412 | 1 | 3.297 | 0 | 5.411 | 0 | NA |
| TRINITY_DN23744_c0_g1_i1 | -0.97803135 | 0.827662412 | 1 | 0.436 | 0 | 0.722 | 0 | DUF1996 |
| TRINITY_DN6104_c0_g1_i2 | -0.97787623 | 0.43305647 | 0.999970678 | 6.041 | 36.29 | 42.764 | 30.533 | NA |
| TRINITY_DN14123_c0_g2_i2 | 0.977831079 | 0.542663636 | 0.999970678 | 0.273 | 1.537 | 0.189 | 0.664 | NA |
| TRINITY_DN1388_c0_g1_i6 | 0.977821099 | 0.721539188 | 0.999970678 | 2.398 | 0.601 | 1.267 | 0 | NA |
| TRINITY_DN12199_c0_g1_i2 | -0.97769225 | 0.48970538 | 0.999970678 | 0.236 | 1.094 | 1.289 | 1.025 | NA |
| TRINITY_DN1072_c0_g1_i3 | -0.97762518 | 0.418852284 | 0.999970678 | 6.858 | 1.301 | 7.255 | 7.5 | DUF2828 |
| TRINITY_DN18370_c0_g1_i4 | 0.977615066 | 0.526965233 | 0.999970678 | 0.845 | 0.631 | 0.544 | 0.121 | - |
| TRINITY_DN1272_c0_g2_i2 | -0.97760834 | 0.260896794 | 0.999970678 | 1.871 | 3.991 | 4.489 | 6.093 | NA |
| TRINITY_DN79_c0_g1_i37 | -0.97749611 | 0.445825785 | 0.999970678 | 1.508 | 8.071 | 4.444 | 13.351 | NA |
| TRINITY_DN72302_c0_g1_i1 | -0.97742329 | 0.817821884 | 0.999970678 | 0.972 | 0 | 1.611 | 0 | - |
| TRINITY_DN8290_c0_g1_i4 | -0.97700913 | 0.146130186 | 0.999970678 | 2.398 | 1.932 | 3.655 | 4.142 | NA |
| TRINITY_DN781_c0_g1_i1 | -0.97677699 | 0.477905677 | 0.999970678 | 3.57 | 2.828 | 8.977 | 1.709 | NA |
| TRINITY_DN2023_c0_g3_i5 | -0.97674415 | 0.279092644 | 0.999970678 | 2.017 | 4.001 | 3.411 | 7.691 | NA |
| TRINITY_DN9394_c0_g1_i2 | -0.97674155 | 0.32585228 | 0.999970678 | 4.206 | 1.823 | 3.478 | 7.731 | NA |
| TRINITY_DN37126_c0_g1_i3 | 0.976668009 | 0.5627675 | 0.999970678 | 0.972 | 0.522 | 0.067 | 0.674 | NA |
| TRINITY_DN24734_c0_g1_i18 | -0.97646324 | 0.826295008 | 1 | 0.69 | 0 | 1.144 | 0 | NA |
| TRINITY_DN8527_c0_g1_i7 | -0.97646324 | 0.826295008 | 1 | 2.725 | 0 | 4.5 | 0 | NA |
| TRINITY_DN12662_c0_g1_i4 | -0.97646324 | 0.826295008 | 1 | 0.972 | 0 | 1.633 | 0 | - |
| TRINITY_DN10592_c0_g1_i2 | -0.97643474 | 0.724552401 | 1 | 0.391 | 0 | 0.233 | 0.503 | NA |
| TRINITY_DN3910_c0_g1_i4 | 0.97636035 | 0.456118284 | 0.999970678 | 0.236 | 0.473 | 0.189 | 0.121 | NA |
| TRINITY_DN3350_c0_g2_i3 | -0.97630127 | 0.256518891 | 0.999970678 | 3.37 | 2.208 | 3.733 | 6.465 | His_biosynth |
| TRINITY_DN13720_c1_g1_i22 | 0.976296978 | 0.708310492 | 0.999970678 | 1.263 | 1.242 | 0 | 1.277 | RabGAP-TBC |
| TRINITY_DN49762_c0_g1_i1 | 0.976242634 | 0.207569889 | 0.999970678 | 4.487 | 3.469 | 2.1 | 1.528 | AAA_23 |
| TRINITY_DN602_c0_g1_i4 | 0.97620434 | 0.387226202 | 0.999970678 | 2.38 | 1.39 | 0.411 | 1.428 | NA |
| TRINITY_DN225_c0_g1_i2 | 0.976135965 | 0.45497992 | 0.999970678 | 231.59 | 189.9 | 156.391 | 24.189 | NA |
| TRINITY_DN2567_c0_g1_i2 | -0.97603197 | 0.478868069 | 0.999970678 | 13.689 | 1.853 | 18.143 | 9.038 | Hemerythrin |
| TRINITY_DN41777_c0_g1_i1 | 0.97588611 | 0.497958287 | 0.999970678 | 0.627 | 5.302 | 1.078 | 1.679 | NA |

| TRINITY_DN449_c1_g1_i5 | -0.97583221 | 0.459671226 | 0.999970678 | 30.667 | 5.903 | 15.777 | 52.782 | Dimerisation |
| --- | --- | --- | --- | --- | --- | --- | --- | --- |
| TRINITY_DN6634_c1_g1_i2 | -0.97578528 | 0.819468174 | 0.999970678 | 2.934 | 0 | 4.789 | 0 | NA |
| TRINITY_DN602_c0_g1_i3 | 0.97578126 | 0.275090741 | 0.999970678 | 2.989 | 6.889 | 2.2 | 2.332 | NA |
| TRINITY_DN1527_c0_g1_i5 | 0.97570203 | 0.674663641 | 0.999970678 | 0.036 | 2.484 | 0.889 | 0.201 | NA |
| TRINITY_DN2968_c0_g1_i4 | -0.97567456 | 0.568568056 | 0.999970678 | 0.963 | 4.77 | 0.733 | 10.245 | GST_C |
| TRINITY_DN2909_c0_g1_i2 | 0.975543693 | 0.149293846 | 0.999970678 | 21.711 | 15.89 | 6.944 | 10.808 | NA |
| TRINITY_DN1593_c0_g1_i4 | -0.9754476 | 0.82493592 | 1 | 0 | 0.424 | 0.678 | 0 | NA |
| TRINITY_DN1815_c1_g1_i3 | 0.975399375 | 0.731313053 | 0.999970678 | 3.979 | 0 | 0.456 | 1.518 | SLH |
| TRINITY_DN3808_c0_g1_i1 | -0.97538898 | 0.738640775 | 0.999970678 | 1.899 | 0 | 0.222 | 3.529 | - |
| TRINITY_DN12881_c1_g1_i1 | 0.975190915 | 0.755723734 | 0.999970678 | 0 | 17.4 | 0.244 | 8.445 | PPO1_DWL |
| TRINITY_DN9497_c0_g1_i2 | 0.975184612 | 0.746770791 | 1 | 0.999 | 0 | 0.078 | 0.412 | B_lectin |
| TRINITY_DN701_c0_g1_i9 | -0.97500564 | 0.721887208 | 1 | 0.263 | 0 | 0.2 | 0.302 | NB-ARC |
| TRINITY_DN11612_c0_g1_i14 | 0.974902866 | 0.752136014 | 1 | 9.911 | 0.68 | 4.455 | 0 | NA |
| TRINITY_DN6438_c0_g1_i1 | 0.974687174 | 0.539095425 | 0.999970678 | 0.545 | 3.824 | 0.767 | 1.257 | NA |
| TRINITY_DN1191_c0_g1_i4 | 0.974629964 | 0.711712489 | 0.999970678 | 0.709 | 1.636 | 0 | 1.196 | NA |
| TRINITY_DN22377_c0_g1_i1 | -0.97462312 | 0.536221902 | 0.999970678 | 0.836 | 1.774 | 3.733 | 0.573 | ER_lumen_recept |
| TRINITY_DN5526_c0_g1_i2 | -0.97460719 | 0.506415009 | 0.999970678 | 4.487 | 0.562 | 2.455 | 7.088 | PP2C |
| TRINITY_DN224942_c0_g1_i1 | 0.974603105 | 0.383309309 | 0.999970678 | 0.718 | 1.36 | 0.422 | 0.543 | NA |
| TRINITY_DN7833_c0_g1_i5 | 0.974588799 | 0.583691087 | 0.999970678 | 0.627 | 9.165 | 3.466 | 0.744 | NA |
| TRINITY_DN85493_c0_g1_i1 | 0.974495676 | 0.827492881 | 1 | 1.453 | 0 | 0.622 | 0 | NA |
| TRINITY_DN7986_c1_g1_i1 | 0.97425321 | 0.448661612 | 0.999970678 | 1.971 | 3.449 | 1.678 | 0.714 | NA |
| TRINITY_DN7907_c0_g1_i1 | -0.97411192 | 0.397648225 | 0.999970678 | 0.572 | 1.35 | 1.755 | 1.578 | NA |
| TRINITY_DN2094_c0_g1_i1 | 0.973962502 | 0.198361275 | 0.999970678 | 5.051 | 4.376 | 2.4 | 1.89 | NA |
| TRINITY_DN745_c0_g1_i15 | 0.973945959 | 0.412365934 | 0.999970678 | 0.917 | 3.193 | 0.6 | 1.367 | NA |
| TRINITY_DN3875_c0_g1_i1 | -0.97381932 | 0.399912758 | 0.999970678 | 46.337 | 10.65 | 33.931 | 71.693 | - |
| TRINITY_DN5611_c0_g1_i2 | 0.973720256 | 0.187054204 | 0.999970678 | 4.206 | 3.242 | 1.355 | 2.172 | B_lectin |
| TRINITY_DN15168_c0_g1_i4 | -0.97370726 | 0.455834894 | 0.999970678 | 1.635 | 1.991 | 0.844 | 6.093 | NA |
| TRINITY_DN1504_c0_g2_i4 | 0.973682828 | 0.440669649 | 1 | 0.908 | 0.926 | 0.5 | 0.352 | LRR_1 |
| TRINITY_DN2557_c0_g1_i1 | 0.973578215 | 0.74513563 | 0.999970678 | 49.38 | 0 | 14.521 | 7.993 | ATP-synt_DE_N |
| TRINITY_DN11064_c0_g2_i1 | 0.973455513 | 0.467666118 | 0.999970678 | 0.545 | 1.202 | 0.511 | 0.271 | NA |
| TRINITY_DN241_c0_g1_i3 | -0.9734518 | 0.723699501 | 0.999970678 | 22.437 | 0 | 13.099 | 28.552 | NA |
| TRINITY_DN28835_c0_g2_i1 | 0.973440504 | 0.521220564 | 0.999970678 | 1.472 | 1.064 | 0.955 | 0.141 | DYW_deaminase |
| TRINITY_DN4859_c0_g1_i1 | -0.97341351 | 0.823426682 | 0.999970678 | 5.95 | 0 | 9.666 | 0 | NA |
| TRINITY_DN8853_c0_g1_i1 | -0.97340478 | 0.405006427 | 0.999970678 | 1.599 | 0.867 | 0.878 | 3.82 | Arm |
| TRINITY_DN7947_c1_g1_i9 | 0.973221634 | 0.737365107 | 0.999970678 | 0.164 | 1.399 | 0.644 | 0 | NA |
| TRINITY_DN13_c2_g1_i4 | 0.972977947 | 0.366400778 | 0.999970678 | 4.188 | 4.77 | 0.889 | 3.458 | NA |
| TRINITY_DN8000_c0_g1_i3 | -0.97291647 | 0.567125391 | 0.999970678 | 0.718 | 0.069 | 0.833 | 0.583 | NA |
| TRINITY_DN1055_c0_g1_i7 | -0.97285315 | 0.413913836 | 0.999970678 | 9.465 | 3.311 | 16.21 | 5.69 | NA |
| TRINITY_DN56275_c0_g1_i1 | 0.972826978 | 0.395451333 | 0.999970678 | 0.318 | 0.719 | 0.267 | 0.211 | NA |
| TRINITY_DN811_c0_g1_i8 | -0.97279984 | 0.602781453 | 0.999970678 | 0.491 | 18.35 | 5.533 | 29.719 | NA |
| TRINITY_DN12444_c0_g1_i5 | -0.97272655 | 0.822767186 | 0.999970678 | 1.326 | 0 | 2.211 | 0 | NA |
| TRINITY_DN20421_c0_g1_i4 | -0.97272655 | 0.822767186 | 0.999970678 | 1.835 | 0 | 3.044 | 0 | NA |
| TRINITY_DN2287_c2_g1_i1 | 0.972625658 | 0.721673096 | 0.999970678 | 0.354 | 1.281 | 0 | 0.834 | p450 |
| TRINITY_DN9695_c0_g2_i1 | -0.97257743 | 0.330858832 | 0.999970678 | 0.363 | 0.67 | 0.967 | 0.845 | NA |
| TRINITY_DN3636_c0_g1_i3 | 0.972527565 | 0.512477283 | 0.999970678 | 1.599 | 2.148 | 1.422 | 0.191 | NA |
| TRINITY_DN10416_c0_g1_i2 | 0.972282246 | 0.32071996 | 0.999970678 | 3.606 | 2.937 | 0.933 | 2.202 | NA |
| TRINITY_DN6384_c0_g1_i1 | -0.97214417 | 0.335805004 | 0.999970678 | 0.463 | 0.838 | 1.255 | 1.015 | NA |
| TRINITY_DN58035_c0_g1_i1 | -0.97214104 | 0.8221923 | 0.999970678 | 2.598 | 0 | 4.255 | 0 | NA |
| TRINITY_DN75934_c0_g1_i1 | -0.97212042 | 0.632464766 | 0.999970678 | 0.6 | 0.237 | 0.056 | 1.588 | NA |
| TRINITY_DN1849_c0_g1_i5 | 0.972107875 | 0.71637829 | 0.999970678 | 1.744 | 3.144 | 0 | 2.493 | - |
| TRINITY_DN8108_c0_g1_i3 | 0.972100157 | 0.709689023 | 0.999970678 | 2.335 | 3.371 | 0 | 2.895 | p450 |
| TRINITY_DN2678_c1_g1_i6 | 0.97208794 | 0.732726055 | 0.999970678 | 1.572 | 0 | 0.222 | 0.543 | NA |
| TRINITY_DN2062_c0_g1_i2 | 0.972085661 | 0.346392167 | 0.999970678 | 2.553 | 5.736 | 1.033 | 2.966 | LRRNT_2 |
| TRINITY_DN152_c0_g1_i4 | -0.97208237 | 0.321296612 | 0.999970678 | 4.942 | 7.303 | 5.511 | 17.212 | Ribosomal_L9_C |
| TRINITY_DN14069_c0_g1_i2 | 0.972039276 | 0.127022567 | 0.999970678 | 8.057 | 8.751 | 3.033 | 4.926 | NA |
| TRINITY_DN3455_c0_g1_i8 | -0.97188964 | 0.676875998 | 0.999970678 | 1.899 | 0.039 | 0.244 | 3.539 | NA |
| TRINITY_DN6808_c0_g1_i12 | -0.97182685 | 0.719972825 | 0.999970678 | 0.6 | 0 | 0.367 | 0.734 | Methyltransf_15 |
| TRINITY_DN115569_c0_g1_i1 | -0.97174249 | 0.492067833 | 0.999970678 | 9.711 | 1.419 | 10.988 | 8.515 | NA |
| TRINITY_DN3210_c1_g1_i1 | 0.971735204 | 0.554276447 | 0.999970678 | 1.099 | 1.892 | 1.178 | 0.09 | Act-Frag_cataly |
| TRINITY_DN7208_c0_g1_i1 | -0.97173463 | 0.500536609 | 0.999970678 | 2.898 | 18.22 | 6.1 | 33.71 | NA |
| TRINITY_DN9986_c0_g1_i1 | 0.971613607 | 0.81924888 | 0.999970678 | 0 | 35.93 | 14.877 | 0 | DUF1685 |
| TRINITY_DN19325_c1_g1_i1 | 0.971606501 | 0.59212687 | 1 | 0.418 | 0.384 | 0.311 | 0.03 | NA |
| TRINITY_DN4192_c0_g1_i5 | 0.97160614 | 0.395143269 | 0.999970678 | 1.172 | 0.453 | 0.356 | 0.412 | NA |
| TRINITY_DN843_c0_g1_i11 | 0.971527014 | 0.35749515 | 0.999970678 | 7.694 | 10.1 | 5.422 | 2.463 | NA |
| TRINITY_DN10368_c0_g1_i1 | -0.97145538 | 0.155087085 | 0.999970678 | 24.845 | 19.91 | 42.864 | 35.892 | 2014/3/3 |
| TRINITY_DN834_c0_g1_i17 | 0.971378617 | 0.352025661 | 0.999970678 | 1.853 | 4.701 | 1.3 | 1.759 | NA |
| TRINITY_DN45051_c1_g1_i1 | 0.971334918 | 0.541088493 | 0.999970678 | 13.535 | 96.71 | 15.399 | 31.981 | NA |
| TRINITY_DN1035_c0_g2_i2 | 0.971142658 | 0.831431528 | 0.999970678 | 85.716 | 0 | 0 | 44.578 | Pyrophosphatase |
| TRINITY_DN324_c0_g1_i3 | -0.97110092 | 0.150221737 | 0.999970678 | 49.071 | 78.8 | 106.127 | 119.951 | Ribosomal_L23 |
| TRINITY_DN983_c0_g1_i3 | 0.97096084 | 0.552130729 | 0.999970678 | 5.169 | 0.296 | 1.222 | 1.357 | CBM_4_9 |
| TRINITY_DN25620_c0_g1_i1 | 0.970930578 | 0.3136222 | 0.999970678 | 5.687 | 3.282 | 2.489 | 1.578 | NA |
| TRINITY_DN8530_c0_g1_i4 | -0.97070111 | 0.745135532 | 0.999970678 | 0 | 49.11 | 46.686 | 38.455 | HD_4 |
| TRINITY_DN2351_c0_g1_i6 | -0.97064208 | 0.726291673 | 0.999970678 | 3.788 | 0 | 4.333 | 2.302 | NA |
| TRINITY_DN606_c0_g1_i6 | -0.97061487 | 0.743487976 | 0.999970678 | 3.606 | 0 | 0.367 | 6.696 | NA |

| TRINITY_DN7410_c0_g1_i1 | -0.97051251 | 0.463135448 | 0.999970678 | 0.609 | 3.755 | 3.011 | 4.846 | Sec20 |
| --- | --- | --- | --- | --- | --- | --- | --- | --- |
| TRINITY_DN28307_c0_g1_i1 | -0.97047558 | 0.820573496 | 0.999970678 | 1.317 | 0 | 2.167 | 0 | NA |
| TRINITY_DN40044_c0_g1_i1 | -0.97042208 | 0.392525907 | 0.999970678 | 0.554 | 1.626 | 2.344 | 1.408 | NA |
| TRINITY_DN2141_c0_g1_i4 | -0.9704131 | 0.721460559 | 0.999970678 | 0 | 2.957 | 3.722 | 1.186 | NA |
| TRINITY_DN172_c0_g1_i6 | 0.970336271 | 0.478887294 | 0.999970678 | 6.577 | 8.968 | 5.8 | 0.895 | NA |
| TRINITY_DN1727_c0_g5_i1 | -0.97012685 | 0.717319093 | 0.999970678 | 4.787 | 0 | 3.011 | 5.761 | NA |
| TRINITY_DN3853_c0_g1_i3 | -0.9700487 | 0.535827563 | 0.999970678 | 11.773 | 0.808 | 12.955 | 9.3 | - |
| TRINITY_DN642_c0_g1_i6 | -0.9699593 | 0.732204596 | 1 | 0 | 1.764 | 2.055 | 0.885 | NA |
| TRINITY_DN4853_c0_g1_i12 | -0.96989083 | 0.818430644 | 0.999970678 | 10.319 | 0 | 16.943 | 0 | NA |
| TRINITY_DN1235_c0_g1_i2 | 0.969797345 | 0.453507174 | 0.999970678 | 3.252 | 1.754 | 0.644 | 1.759 | NA |
| TRINITY_DN18650_c0_g1_i7 | 0.969797321 | 0.498881528 | 0.999970678 | 0.627 | 0.631 | 0.456 | 0.08 | NA |
| TRINITY_DN7401_c0_g1_i2 | -0.96979558 | 0.609186271 | 0.999970678 | 0.745 | 7.135 | 11.599 | 1.156 | NA |
| TRINITY_DN1188_c0_g1_i5 | 0.969740633 | 0.5254481 | 0.999970678 | 0.79 | 4.612 | 0.433 | 2.202 | Pyridoxal_deC |
| TRINITY_DN1120_c1_g1_i3 | 0.969633556 | 0.81751088 | 0.999970678 | 0 | 10.55 | 4.366 | 0 | NA |
| TRINITY_DN2077_c0_g1_i10 | -0.96960171 | 0.468012278 | 0.999970678 | 30.686 | 4.129 | 20.065 | 44.91 | NA |
| TRINITY_DN10562_c0_g1_i1 | -0.9695304 | 0.496686482 | 0.999970678 | 5.95 | 8.545 | 2.455 | 24.963 | NA |
| TRINITY_DN1876_c1_g1_i12 | -0.96952808 | 0.829012525 | 1 | 0 | 4.79 | 0 | 8.998 | HsbA |
| TRINITY_DN5272_c0_g2_i2 | -0.9694706 | 0.135112051 | 0.999970678 | 10.02 | 11.24 | 19.699 | 17.715 | DUF3007 |
| TRINITY_DN3067_c0_g1_i4 | 0.969336256 | 0.546203152 | 0.999970678 | 0.4 | 4.484 | 0.955 | 1.307 | AAA |
| TRINITY_DN197798_c0_g1_i1 | 0.969261623 | 0.630698567 | 1 | 0.327 | 0.266 | 0.244 | 0.01 | NA |
| TRINITY_DN4703_c1_g1_i2 | 0.9691709 | 0.182219009 | 0.999970678 | 1.572 | 2.168 | 0.878 | 0.855 | NA |
| TRINITY_DN6656_c0_g1_i3 | -0.9691607 | 0.742257951 | 0.999970678 | 1.862 | 0 | 2.878 | 0.211 | NA |
| TRINITY_DN8630_c0_g1_i4 | 0.969157674 | 0.540442324 | 0.999970678 | 27.588 | 1.685 | 8.366 | 5.067 | - |
| TRINITY_DN21007_c0_g1_i1 | 0.969136637 | 0.8262565 | 0.999970678 | 3.179 | 0 | 1.355 | 0 | NA |
| TRINITY_DN3009_c0_g1_i1 | 0.969053029 | 0.418578631 | 0.999970678 | 4.878 | 3.765 | 3.033 | 0.764 | WD40 |
| TRINITY_DN18607_c0_g1_i1 | -0.96886527 | 0.561236469 | 0.999970678 | 3.942 | 1.301 | 7.655 | 1.015 | NA |
| TRINITY_DN10661_c0_g1_i2 | 0.968833188 | 0.316948447 | 0.999970678 | 1.372 | 2.562 | 0.822 | 0.985 | NA |
| TRINITY_DN24332_c0_g1_i3 | 0.968813613 | 0.715729558 | 0.999970678 | 1.426 | 1.064 | 1.067 | 0 | PORR |
| TRINITY_DN2560_c0_g1_i4 | 0.968762472 | 0.826129554 | 1 | 0 | 17.45 | 7.222 | 0 | NA |
| TRINITY_DN10461_c0_g1_i3 | -0.96875794 | 0.751222226 | 1 | 1.472 | 0 | 0.178 | 2.694 | - |
| TRINITY_DN7222_c0_g1_i2 | 0.968752589 | 0.125838116 | 0.999970678 | 37.571 | 32.13 | 12.666 | 20.389 | Sigma70_r1_2 |
| TRINITY_DN22277_c0_g1_i8 | -0.96862723 | 0.768833447 | 0.999970678 | 2.825 | 0.059 | 4.744 | 0 | HSP70 |
| TRINITY_DN18214_c0_g1_i1 | 0.968591482 | 0.71432591 | 0.999970678 | 10.746 | 8.623 | 8.122 | 0 | NA |
| TRINITY_DN1806_c0_g1_i2 | 0.968466379 | 0.535958718 | 0.999970678 | 0.463 | 1.557 | 0.678 | 0.211 | NA |
| TRINITY_DN8362_c1_g1_i3 | 0.968458819 | 0.717172058 | 1 | 1.254 | 1.035 | 0 | 1.176 | ARID |
| TRINITY_DN7508_c0_g1_i3 | -0.96840782 | 0.383757925 | 0.999970678 | 17.786 | 10.92 | 9.599 | 44.608 | NA |
| TRINITY_DN3067_c1_g1_i2 | 0.968283974 | 0.544578115 | 0.999970678 | 7.34 | 3.262 | 3.944 | 0.603 | NA |
| TRINITY_DN10418_c0_g2_i6 | -0.9682626 | 0.520473729 | 0.999970678 | 0.481 | 2.375 | 2.433 | 2.554 | B_lectin |
| TRINITY_DN4597_c0_g1_i7 | -0.96821642 | 0.331068489 | 0.999970678 | 1.299 | 0.976 | 1.022 | 3.237 | F-box |
| TRINITY_DN5049_c0_g1_i1 | -0.96815778 | 0.721535376 | 0.999970678 | 1.871 | 0 | 1.844 | 1.518 | BRCT |
| TRINITY_DN5538_c0_g1_i3 | -0.96814892 | 0.494463059 | 0.999970678 | 0.263 | 0.926 | 0.533 | 1.659 | NA |
| TRINITY_DN30808_c0_g1_i9 | -0.96813761 | 0.610786384 | 1 | 3.089 | 1.084 | 6.177 | 0.543 | NA |
| TRINITY_DN794_c0_g1_i4 | -0.96812017 | 0.338840109 | 0.999970678 | 180.94 | 72.41 | 126.592 | 345.003 | Dimerisation |
| TRINITY_DN9852_c0_g1_i20 | 0.968085027 | 0.825184421 | 0.999970678 | 0.954 | 0 | 0 | 0.493 | NA |
| TRINITY_DN4902_c0_g1_i15 | 0.968060551 | 0.581937076 | 0.999970678 | 0.908 | 2.73 | 1.467 | 0.08 | NA |
| TRINITY_DN6758_c0_g1_i1 | 0.967952947 | 0.555608433 | 0.999970678 | 2.298 | 1.005 | 0.111 | 1.568 | NA |
| TRINITY_DN8685_c0_g2_i6 | 0.967912941 | 0.721449456 | 0.999970678 | 0.291 | 0.798 | 0 | 0.563 | NA |
| TRINITY_DN9045_c0_g1_i2 | -0.96788156 | 0.425678702 | 0.999970678 | 4.342 | 10.09 | 4.311 | 22.832 | ADK |
| TRINITY_DN7258_c0_g1_i4 | 0.967853934 | 0.466376379 | 0.999970678 | 0.164 | 0.128 | 0.089 | 0.04 | NA |
| TRINITY_DN703_c0_g3_i4 | 0.967656715 | 0.334070511 | 0.999970678 | 5.723 | 3.42 | 2.089 | 2.172 | - |
| TRINITY_DN2467_c0_g1_i1 | -0.96760939 | 0.257410936 | 0.999970678 | 30.231 | 45.07 | 38.431 | 99.401 | NA |
| TRINITY_DN1856_c0_g1_i4 | 0.967585955 | 0.381981605 | 0.999970678 | 0.863 | 2.129 | 0.6 | 0.794 | NOB1_Zn_bind |
| TRINITY_DN654_c0_g1_i1 | -0.96742524 | 0.361869691 | 0.999970678 | 76.305 | 20.44 | 71.551 | 102.598 | NA |
| TRINITY_DN3515_c0_g2_i5 | 0.96733758 | 0.39396481 | 0.999970678 | 0.418 | 0.503 | 0.267 | 0.161 | RRM_1 |
| TRINITY_DN46886_c0_g1_i1 | 0.967312755 | 0.314986815 | 0.999970678 | 1.181 | 2.661 | 0.889 | 0.885 | NA |
| TRINITY_DN5587_c0_g1_i1 | -0.96729277 | 0.503862347 | 0.999970678 | 0.291 | 1.321 | 1.978 | 0.764 | mTERF |
| TRINITY_DN9097_c0_g1_i1 | 0.967210952 | 0.563723698 | 0.999970678 | 0.336 | 3.469 | 0.433 | 1.428 | NA |
| TRINITY_DN12223_c0_g1_i4 | -0.96714028 | 0.610478085 | 1 | 0.091 | 0.69 | 0.211 | 1.267 | NA |
| TRINITY_DN135_c0_g1_i10 | -0.96708877 | 0.113305767 | 0.999970678 | 45.211 | 37.29 | 68.229 | 77.343 | NA |
| TRINITY_DN1432_c0_g1_i5 | 0.967086222 | 0.521154402 | 0.999970678 | 0.427 | 0.71 | 0.411 | 0.09 | DUF1995 |
| TRINITY_DN1033_c2_g1_i1 | -0.96708175 | 0.668171069 | 0.999970678 | 71.763 | 0.355 | 11.477 | 129.301 | TPT |
| TRINITY_DN1880_c1_g1_i27 | 0.967071579 | 0.745946006 | 0.999970678 | 0 | 2.533 | 0.856 | 0.231 | NA |
| TRINITY_DN2347_c0_g1_i5 | 0.966925789 | 0.345089541 | 0.999970678 | 27.225 | 19.65 | 15.288 | 5.57 | NmrA |
| TRINITY_DN1188_c0_g1_i6 | -0.96692308 | 0.607246568 | 0.999970678 | 0.509 | 0.355 | 0.067 | 1.599 | Pyridoxal_deC |
| TRINITY_DN5981_c0_g1_i4 | 0.966761832 | 0.212930598 | 0.999970678 | 253.96 | 153.5 | 105.882 | 81.425 | adh_short |
| TRINITY_DN1371_c0_g1_i3 | -0.96671131 | 0.719824806 | 0.999970678 | 2.852 | 0 | 1.678 | 3.629 | NA |
| TRINITY_DN3517_c2_g1_i1 | 0.966552373 | 0.474051551 | 0.999970678 | 1.226 | 0.976 | 0.711 | 0.261 | NA |
| TRINITY_DN3334_c0_g1_i3 | 0.966539621 | 0.421803956 | 0.999970678 | 8.884 | 25.27 | 2.978 | 13.774 | BCDHK_Adom3 |
| TRINITY_DN18893_c0_g4_i1 | -0.9663989 | 0.270793781 | 0.999970678 | 95.49 | 77.63 | 80.717 | 240.183 | REF |
| TRINITY_DN5218_c0_g1_i2 | -0.96627316 | 0.237319179 | 0.999970678 | 10.528 | 7.638 | 10.11 | 23.355 | NA |
| TRINITY_DN8156_c0_g1_i2 | 0.966203375 | 0.825930664 | 0.999970678 | 4.578 | 0 | 0 | 2.363 | Aminotran_3 |
| TRINITY_DN2909_c0_g1_i5 | -0.96600098 | 0.440485985 | 0.999970678 | 2.625 | 8.86 | 3.944 | 17.544 | NA |
| TRINITY_DN504_c0_g1_i14 | -0.96592825 | 0.379553492 | 0.999970678 | 0.218 | 0.197 | 0.322 | 0.442 | DUF3522 |
| TRINITY_DN2515_c0_g1_i1 | 0.965884793 | 0.247177381 | 0.999970678 | 6.204 | 3.913 | 2.355 | 2.363 | NA |

| TRINITY_DN10838_c1_g1_i4 | -0.96576143 | 0.465119073 | 0.999970678 | 0.227 | 0.621 | 0.867 | 0.623 | NA |
| --- | --- | --- | --- | --- | --- | --- | --- | --- |
| TRINITY_DN461_c0_g2_i1 | -0.9655374 | 0.335796043 | 0.999970678 | 11.746 | 13.82 | 9.933 | 37.591 | NA |
| TRINITY_DN45106_c0_g1_i1 | 0.965470612 | 0.82850448 | 1 | 1.572 | 0 | 0.689 | 0 | NA |
| TRINITY_DN1248_c2_g1_i2 | 0.965367727 | 0.225336556 | 0.999970678 | 24.563 | 17.93 | 6.466 | 13.924 | Inhibitor_I29 |
| TRINITY_DN8728_c0_g1_i1 | 0.965219047 | 0.306955028 | 0.999970678 | 4.224 | 9.156 | 2.078 | 4.283 | NA |
| TRINITY_DN17969_c0_g1_i1 | -0.96515804 | 0.637233007 | 1 | 1.272 | 0.049 | 0.922 | 1.478 | NA |
| TRINITY_DN4848_c2_g1_i4 | 0.96507594 | 0.724772936 | 1 | 1.363 | 0.581 | 0 | 0.985 | NA |
| TRINITY_DN17281_c0_g1_i1 | -0.96497319 | 0.830640114 | 1 | 0.672 | 0 | 1.1 | 0 | NA |
| TRINITY_DN16745_c0_g1_i1 | -0.96497319 | 0.830640114 | 1 | 1.953 | 0 | 3.178 | 0 | NA |
| TRINITY_DN255177_c0_g1_i1 | -0.96497319 | 0.830640114 | 1 | 0.981 | 0 | 1.6 | 0 | Mpv17_PMP22 |
| TRINITY_DN4264_c0_g1_i1 | -0.96495466 | 0.82627515 | 0.999970678 | 1.39 | 0 | 2.289 | 0 | NA |
| TRINITY_DN10432_c0_g1_i5 | -0.96495065 | 0.825281383 | 0.999970678 | 1.326 | 0 | 2.167 | 0 | NA |
| TRINITY_DN4264_c0_g1_i3 | -0.96492515 | 0.819025999 | 0.999970678 | 3.588 | 0 | 5.9 | 0 | NA |
| TRINITY_DN872_c0_g1_i3 | 0.96491417 | 0.470108147 | 0.999970678 | 0.391 | 1.094 | 0.156 | 0.583 | NA |
| TRINITY_DN2683_c0_g1_i6 | 0.964878567 | 0.147959496 | 0.999970678 | 9.756 | 12 | 5.389 | 4.655 | NAD_binding_10 |
| TRINITY_DN8478_c0_g1_i1 | -0.96470013 | 0.734997684 | 0.999970678 | 2.816 | 0 | 0.511 | 4.946 | Pur_DNA_glyco |
| TRINITY_DN9973_c0_g1_i2 | -0.96467945 | 0.740351839 | 0.999970678 | 0.3 | 1.084 | 2.189 | 0 | NA |
| TRINITY_DN1486_c2_g1_i3 | -0.9645693 | 0.73069142 | 0.999970678 | 1.217 | 0 | 1.578 | 0.462 | adh_short |
| TRINITY_DN1399_c0_g1_i9 | 0.964564458 | 0.425080206 | 0.999970678 | 1.354 | 1.577 | 0.422 | 0.985 | TAFII28 |
| TRINITY_DN1987_c0_g1_i6 | 0.96452447 | 0.410851824 | 0.999970678 | 3.325 | 1.133 | 1.211 | 0.824 | Peptidase_M48 |
| TRINITY_DN92_c1_g2_i1 | 0.96449238 | 0.160290554 | 0.999970678 | 42.622 | 27.92 | 16.11 | 16.84 | NA |
| TRINITY_DN5156_c0_g2_i3 | 0.964475957 | 0.437004697 | 0.999970678 | 2.425 | 2.326 | 0.544 | 1.769 | MatE |
| TRINITY_DN6519_c0_g1_i1 | 0.964457851 | 0.338673679 | 0.999970678 | 2.97 | 2.631 | 0.633 | 2.121 | CBF |
| TRINITY_DN4113_c0_g1_i9 | 0.964288562 | 0.819119278 | 0.999970678 | 0 | 1.754 | 0.733 | 0 | GAGA_bind |
| TRINITY_DN4999_c0_g1_i1 | -0.96421228 | 0.719389002 | 0.999970678 | 0 | 2.71 | 1.311 | 3.629 | NA |
| TRINITY_DN74309_c0_g1_i5 | -0.96406441 | 0.71837365 | 0.999970678 | 1.399 | 0 | 0.911 | 1.679 | NA |
| TRINITY_DN28955_c0_g2_i7 | 0.964033426 | 0.388587825 | 0.999970678 | 0.382 | 0.995 | 0.267 | 0.372 | NA |
| TRINITY_DN34113_c0_g1_i6 | -0.96395089 | 0.321121393 | 0.999970678 | 7.894 | 24.78 | 25.932 | 31.75 | DLH |
| TRINITY_DN2866_c0_g1_i1 | -0.96393073 | 0.517266674 | 0.999970678 | 5.469 | 19.53 | 4.344 | 42.799 | Dirigent |
| TRINITY_DN71775_c0_g1_i1 | -0.96374884 | 0.443342048 | 0.999970678 | 0.273 | 0.769 | 0.789 | 1.046 | Glyco_hydro_17 |
| TRINITY_DN725_c1_g1_i3 | 0.96374037 | 0.747298329 | 0.999970678 | 0.854 | 0.059 | 0.389 | 0 | EloA-BP1 |
| TRINITY_DN3448_c0_g1_i1 | -0.96360877 | 0.144676821 | 0.999970678 | 6.577 | 6.642 | 10.544 | 12.899 | NA |
| TRINITY_DN1401_c0_g3_i1 | 0.96353711 | 0.303047851 | 0.999970678 | 2.126 | 2.119 | 0.955 | 1.046 | NA |
| TRINITY_DN936_c0_g1_i3 | -0.96352528 | 0.7277084 | 0.999970678 | 21.375 | 0 | 23.876 | 13.241 | PP-binding |
| TRINITY_DN835_c0_g1_i6 | 0.963467943 | 0.734739148 | 0.999970678 | 4.469 | 0 | 0.589 | 1.639 | LRR_8 |
| TRINITY_DN616_c0_g1_i1 | 0.963452028 | 0.283918775 | 0.999970678 | 6.132 | 3.558 | 2.455 | 1.991 | NA |
| TRINITY_DN3241_c0_g2_i3 | 0.963431793 | 0.377804831 | 0.999970678 | 0.363 | 0.779 | 0.189 | 0.362 | NA |
| TRINITY_DN16376_c1_g2_i6 | 0.96338127 | 0.651238148 | 0.999970678 | 1.181 | 0.315 | 0.622 | 0.03 | NA |
| TRINITY_DN7376_c0_g1_i2 | -0.96327357 | 0.460278529 | 0.999970678 | 98.197 | 187.2 | 56.797 | 485.533 | Tetraspannin |
| TRINITY_DN6111_c0_g1_i6 | -0.9631097 | 0.594380036 | 0.999970678 | 1.026 | 0.138 | 0.322 | 1.94 | - |
| TRINITY_DN6111_c0_g1_i4 | -0.9631097 | 0.594380036 | 0.999970678 | 1.026 | 0.138 | 0.322 | 1.94 | - |
| TRINITY_DN5289_c0_g1_i5 | 0.962801743 | 0.281280272 | 0.999970678 | 1.608 | 0.956 | 0.511 | 0.704 | NA |
| TRINITY_DN1740_c0_g1_i5 | 0.962753448 | 0.379100556 | 0.999970678 | 3.161 | 4.031 | 1.167 | 2.212 | NA |
| TRINITY_DN1105_c0_g1_i1 | 0.96252628 | 0.236490048 | 0.999970678 | 4.406 | 3.193 | 1.144 | 2.544 | Peptidase_S9 |
| TRINITY_DN1502_c0_g1_i7 | 0.962524069 | 0.583025213 | 0.999970678 | 0.954 | 32.33 | 7.233 | 8.003 | NA |
| TRINITY_DN7894_c0_g1_i1 | -0.96249241 | 0.509025869 | 0.999970678 | 0.064 | 0.315 | 0.311 | 0.372 | Adenine_glyco |
| TRINITY_DN50272_c1_g1_i3 | 0.962353775 | 0.517204904 | 0.999970678 | 0.545 | 3.223 | 1.155 | 0.513 | NA |
| TRINITY_DN91089_c0_g1_i1 | 0.962293667 | 0.326103786 | 0.999970678 | 0.6 | 0.611 | 0.322 | 0.241 | NA |
| TRINITY_DN4655_c0_g3_i1 | -0.96226826 | 0.378264373 | 0.999970678 | 0.727 | 1.124 | 1.233 | 2.051 | Med11 |
| TRINITY_DN26227_c0_g1_i1 | -0.96209002 | 0.686739272 | 0.999970678 | 0.854 | 0.02 | 1.344 | 0.101 | NA |
| TRINITY_DN4283_c3_g1_i1 | -0.96206685 | 0.819668931 | 0.999970678 | 12.854 | 0 | 20.677 | 0 | NA |
| TRINITY_DN9697_c0_g1_i1 | -0.96204149 | 0.328999933 | 0.999970678 | 1.181 | 2.976 | 2.844 | 4.605 | DELLA |
| TRINITY_DN19455_c0_g1_i13 | 0.961935399 | 0.27791653 | 0.999970678 | 2.425 | 1.321 | 0.889 | 0.855 | PMI_typeI |
| TRINITY_DN2352_c0_g1_i1 | -0.96192484 | 0.17881781 | 0.999970678 | 21.375 | 29.09 | 31.498 | 59.819 | Abhydrolase_1 |
| TRINITY_DN4974_c0_g1_i2 | 0.961713803 | 0.539425053 | 0.999970678 | 4.36 | 2.7 | 2.778 | 0.292 | PolyA_pol |
| TRINITY_DN15200_c0_g1_i1 | 0.961470618 | 0.480486637 | 0.999970678 | 0.409 | 0.621 | 0.1 | 0.412 | NA |
| TRINITY_DN11681_c0_g1_i2 | 0.961466371 | 0.729171106 | 0.999970678 | 0.736 | 2.897 | 0 | 1.86 | NA |
| TRINITY_DN24077_c0_g1_i4 | -0.96145909 | 0.373520164 | 0.999970678 | 1.944 | 1.922 | 1.422 | 5.851 | NA |
| TRINITY_DN361_c0_g1_i1 | 0.961057342 | 0.482448915 | 0.999970678 | 3.525 | 2.395 | 0.356 | 2.654 | Ins134_P3_kin |
| TRINITY_DN23839_c0_g1_i1 | -0.96102279 | 0.405357543 | 0.999970678 | 0.391 | 0.217 | 0.544 | 0.563 | DNA_methylase |
| TRINITY_DN2910_c1_g1_i4 | -0.96097868 | 0.749169847 | 0.999970678 | 0.127 | 1.38 | 0 | 2.875 | NA |
| TRINITY_DN9906_c0_g1_i1 | 0.960976967 | 0.759631217 | 0.999970678 | 1.172 | 0 | 0.033 | 0.573 | NA |
| TRINITY_DN23401_c0_g1_i2 | -0.96070139 | 0.735785542 | 0.999970678 | 17.768 | 0 | 24.621 | 4.544 | NA |
| TRINITY_DN3329_c0_g1_i4 | -0.96059818 | 0.320052161 | 0.999970678 | 0.808 | 0.976 | 1.689 | 1.458 | NUDIX |
| TRINITY_DN4026_c0_g1_i5 | 0.960466971 | 0.457152582 | 0.999970678 | 2.044 | 0.493 | 0.778 | 0.372 | DUF3741 |
| TRINITY_DN17154_c0_g2_i5 | -0.96045628 | 0.771018136 | 0.999970678 | 0.827 | 0 | 1.322 | 0.03 | Transket_pyr |
| TRINITY_DN37401_c0_g1_i3 | -0.96034864 | 0.746481687 | 0.999970678 | 1.744 | 0.177 | 0 | 3.75 | NA |
| TRINITY_DN7411_c0_g1_i1 | 0.960130448 | 0.471951685 | 0.999970678 | 19.331 | 4.622 | 2.966 | 8.596 | Glutaredoxin |
| TRINITY_DN15354_c0_g1_i4 | 0.960126979 | 0.627761175 | 0.999970678 | 4.115 | 0.237 | 1.567 | 0.392 | NA |
| TRINITY_DN756_c0_g1_i5 | 0.960094669 | 0.314307557 | 0.999970678 | 55.939 | 32.51 | 10.666 | 32.805 | Thioredoxin |
| TRINITY_DN2972_c1_g1_i1 | -0.9599706 | 0.362390395 | 0.999970678 | 3.07 | 1.242 | 2.3 | 5.68 | Dimerisation |
| TRINITY_DN2972_c1_g1_i2 | -0.9599706 | 0.362390395 | 0.999970678 | 3.07 | 1.242 | 2.3 | 5.68 | Dimerisation |
| TRINITY_DN30599_c0_g1_i2 | -0.95993496 | 0.719336408 | 0.999970678 | 0.963 | 0 | 0.678 | 1.076 | DUF4005 |
| TRINITY_DN16260_c0_g1_i2 | 0.959861061 | 0.761803337 | 1 | 1.163 | 0.039 | 0.533 | 0 | NA |

| TRINITY_DN267_c0_g1_i1 | -0.95967194 | 0.569877186 | 0.999970678 | 1.508 | 0.562 | 0.267 | 3.73 | - |
| --- | --- | --- | --- | --- | --- | --- | --- | --- |
| TRINITY_DN1412_c0_g4_i1 | -0.95962844 | 0.729641439 | 0.999970678 | 0 | 14.13 | 4.422 | 21.545 | Ras |
| TRINITY_DN7425_c0_g1_i4 | -0.95954649 | 0.549763143 | 0.999970678 | 0.382 | 0.601 | 1.355 | 0.231 | BTB_2 |
| TRINITY_DN11648_c0_g1_i10 | 0.959478687 | 0.607405688 | 0.999970678 | 12.336 | 0.443 | 2.267 | 3.871 | NA |
| TRINITY_DN14436_c0_g1_i6 | 0.95924989 | 0.140393984 | 0.999970678 | 2.207 | 2.73 | 1.122 | 1.186 | NA |
| TRINITY_DN372_c0_g1_i2 | -0.95924482 | 0.73319107 | 0.999970678 | 1.453 | 0 | 0.533 | 2.212 | NA |
| TRINITY_DN1549_c0_g1_i10 | -0.95917491 | 0.664987981 | 1 | 0.073 | 0.877 | 1.422 | 0.141 | LRRNT_2 |
| TRINITY_DN156_c0_g1_i14 | -0.95916451 | 0.462454797 | 0.999970678 | 6.014 | 1.005 | 3.644 | 9.42 | DNA_pol_phi |
| TRINITY_DN7485_c0_g1_i1 | -0.95916264 | 0.727806256 | 1 | 7.194 | 0 | 4.411 | 7.942 | AAA |
| TRINITY_DN874_c0_g2_i1 | -0.95897075 | 0.356126119 | 0.999970678 | 30.686 | 20.75 | 18.621 | 77.846 | DUF4283 |
| TRINITY_DN5813_c0_g1_i1 | 0.95892851 | 0.546419676 | 1 | 1.953 | 1.794 | 1.367 | 0.271 | ABC_tran |
| TRINITY_DN17433_c0_g4_i1 | 0.958916732 | 0.351903841 | 0.999970678 | 6.531 | 16.45 | 3.244 | 7.772 | NA |
| TRINITY_DN1165_c0_g1_i1 | 0.958904664 | 0.553322807 | 1 | 0.681 | 0.414 | 0.411 | 0.08 | NA |
| TRINITY_DN5252_c0_g1_i4 | -0.95888595 | 0.825358592 | 0.999970678 | 4.542 | 0 | 0 | 8.686 | NA |
| TRINITY_DN33527_c0_g1_i1 | -0.95886953 | 0.526000292 | 0.999970678 | 1440.7 | 293.5 | 2410.465 | 368.317 | NA |
| TRINITY_DN9838_c0_g1_i4 | -0.95885256 | 0.618062075 | 0.999970678 | 1.299 | 1.567 | 4.433 | 0.211 | NA |
| TRINITY_DN2404_c0_g1_i10 | -0.95873028 | 0.828402365 | 1 | 0 | 0.443 | 0.711 | 0 | - |
| TRINITY_DN4624_c0_g1_i3 | -0.95871968 | 0.457564297 | 0.999970678 | 1.49 | 0.365 | 1.189 | 2.182 | MBD |
| TRINITY_DN9403_c0_g1_i2 | 0.958672754 | 0.464071698 | 1 | 1.644 | 1.626 | 0.933 | 0.613 | RHD3 |
| TRINITY_DN1870_c0_g1_i3 | -0.95850586 | 0.640427123 | 1 | 0.027 | 0.591 | 0.333 | 0.824 | GST_C_6 |
| TRINITY_DN1031_c1_g1_i1 | 0.958492486 | 0.460207475 | 1 | 1.208 | 0.828 | 0.556 | 0.412 | NA |
| TRINITY_DN49143_c1_g1_i1 | -0.95820074 | 0.258927047 | 0.999970678 | 592 | 1378 | 1314.576 | 2084.845 | PsbQ |
| TRINITY_DN2626_c0_g2_i2 | -0.95819917 | 0.135032635 | 0.999970678 | 22.41 | 27.86 | 44.886 | 42.567 | Ribosomal_S4 |
| TRINITY_DN1469_c0_g2_i1 | -0.95819259 | 0.445321378 | 0.999970678 | 9.138 | 3.568 | 9.799 | 11.894 | NA |
| TRINITY_DN3792_c0_g1_i1 | -0.95808975 | 0.443070931 | 0.999970678 | 4.642 | 11.2 | 10.677 | 16.016 | NA |
| TRINITY_DN1091_c0_g1_i2 | 0.958051506 | 0.205643464 | 0.999970678 | 4.896 | 4.997 | 2.333 | 2.262 | NA |
| TRINITY_DN3664_c0_g3_i1 | -0.95803782 | 0.820282899 | 0.999970678 | 62.743 | 0 | 99.605 | 0 | NA |
| TRINITY_DN2036_c0_g1_i2 | 0.957913476 | 0.607376893 | 0.999970678 | 31.748 | 52.38 | 1.144 | 38.274 | NA |
| TRINITY_DN28071_c0_g1_i2 | -0.95788654 | 0.824625208 | 0.999970678 | 1.272 | 0 | 2.067 | 0 | ADH_N |
| TRINITY_DN7579_c0_g1_i1 | 0.957877178 | 0.414059448 | 0.999970678 | 13.998 | 75.02 | 19.01 | 22.229 | NA |
| TRINITY_DN2228_c0_g1_i2 | 0.957862182 | 0.254464633 | 0.999970678 | 3.043 | 2.405 | 1.378 | 1.146 | NA |
| TRINITY_DN12960_c0_g2_i1 | 0.957693637 | 0.74292661 | 1 | 2.398 | 0.335 | 1.178 | 0 | NA |
| TRINITY_DN5766_c0_g3_i3 | 0.957416562 | 0.720103654 | 0.999970678 | 0.772 | 1.764 | 0 | 1.297 | Carb_kinase |
| TRINITY_DN4230_c1_g1_i1 | 0.957294333 | 0.379295228 | 0.999970678 | 0.563 | 0.838 | 0.344 | 0.312 | NA |
| TRINITY_DN9484_c0_g1_i1 | 0.95721695 | 0.829454897 | 1 | 2.171 | 0 | 0.922 | 0 | NA |
| TRINITY_DN38457_c0_g1_i1 | 0.957139854 | 0.49694516 | 0.999970678 | 1.081 | 0.956 | 0.133 | 0.885 | NA |
| TRINITY_DN2382_c0_g1_i4 | -0.95706788 | 0.62870278 | 0.999970678 | 0.109 | 1.015 | 0.111 | 2.011 | K_trans |
| TRINITY_DN11514_c0_g1_i1 | -0.9566785 | 0.826243422 | 0.999970678 | 3.307 | 0 | 5.266 | 0 | FMO-like |
| TRINITY_DN9974_c0_g1_i2 | -0.9566785 | 0.826243422 | 0.999970678 | 2.089 | 0 | 3.422 | 0 | NA |
| TRINITY_DN3193_c1_g1_i1 | -0.95650782 | 0.360633868 | 0.999970678 | 1.917 | 0.779 | 1.544 | 3.378 | HLH |
| TRINITY_DN1127_c0_g3_i1 | -0.95650674 | 0.376951325 | 0.999970678 | 1.526 | 5.233 | 4.4 | 7.701 | PC-Esterase |
| TRINITY_DN1183_c0_g2_i2 | 0.956504677 | 0.617174926 | 0.999970678 | 17.514 | 1.508 | 7.688 | 0.593 | LRR_8 |
| TRINITY_DN3714_c0_g1_i20 | -0.95633973 | 0.718018451 | 0.999970678 | 0 | 0.936 | 0.878 | 0.724 | Peptidase_M16 |
| TRINITY_DN4959_c0_g1_i1 | 0.956336635 | 0.308178955 | 0.999970678 | 4.687 | 3.863 | 2.355 | 1.558 | NA |
| TRINITY_DN6743_c0_g1_i3 | -0.95631128 | 0.718155646 | 0.999970678 | 0 | 2.533 | 1.867 | 2.574 | NA |
| TRINITY_DN9229_c0_g2_i2 | -0.95629091 | 0.82188119 | 0.999970678 | 12.872 | 0 | 20.732 | 0 | NA |
| TRINITY_DN15710_c0_g1_i4 | 0.956152701 | 0.613223663 | 0.999970678 | 1.39 | 0.187 | 0.611 | 0.07 | NA |
| TRINITY_DN1709_c0_g1_i1 | -0.95615018 | 0.596697526 | 0.999970678 | 0.055 | 0.907 | 0.411 | 1.357 | NA |
| TRINITY_DN37755_c0_g1_i11 | -0.95608665 | 0.728243157 | 1 | 0.436 | 0 | 0.378 | 0.402 | B3 |
| TRINITY_DN29066_c0_g2_i2 | -0.95608357 | 0.820007436 | 0.999970678 | 1.59 | 0 | 0 | 3.137 | NA |
| TRINITY_DN2930_c0_g1_i1 | -0.95606547 | 0.723638468 | 0.999970678 | 3.052 | 0 | 1.678 | 3.991 | - |
| TRINITY_DN13632_c0_g1_i7 | 0.956053191 | 0.820042049 | 0.999970678 | 0 | 9.816 | 4.122 | 0 | NA |
| TRINITY_DN1898_c1_g1_i8 | 0.955980524 | 0.39105348 | 0.999970678 | 1.054 | 1.281 | 0.367 | 0.754 | NA |
| TRINITY_DN9719_c0_g1_i4 | 0.955959472 | 0.716783417 | 0.999970678 | 3.661 | 1.744 | 0 | 2.805 | NA |
| TRINITY_DN8805_c0_g1_i1 | -0.95590397 | 0.82725696 | 0.999970678 | 3.488 | 0 | 5.577 | 0 | GFA |
| TRINITY_DN13063_c0_g1_i4 | -0.95590397 | 0.82725696 | 0.999970678 | 1.426 | 0 | 2.311 | 0 | NA |
| TRINITY_DN1568_c0_g1_i14 | 0.955881136 | 0.536454507 | 0.999970678 | 3.961 | 2.73 | 2.589 | 0.362 | Glyco_hydro_1 |
| TRINITY_DN23182_c1_g1_i1 | 0.955806471 | 0.819762745 | 0.999970678 | 5.768 | 0 | 0 | 3.026 | NA |
| TRINITY_DN12381_c0_g1_i1 | -0.95571248 | 0.345121901 | 0.999970678 | 6.531 | 5.933 | 4.933 | 18.157 | NA |
| TRINITY_DN1196_c0_g2_i1 | -0.95565522 | 0.311095642 | 0.999970678 | 3600.3 | 3668 | 8610.59 | 3359.412 | PsbQ |
| TRINITY_DN1605_c0_g1_i1 | 0.95565426 | 0.561306174 | 0.999970678 | 1.235 | 0.848 | 0.078 | 1.005 | NA |
| TRINITY_DN7645_c2_g1_i4 | 0.955393815 | 0.724463893 | 0.999970678 | 0.6 | 2.523 | 0 | 1.588 | ZF-HD_dimer |
| TRINITY_DN2205_c0_g1_i1 | 0.955338699 | 0.733335326 | 0.999970678 | 0 | 8.278 | 1.544 | 2.343 | NA |
| TRINITY_DN1942_c0_g1_i2 | -0.95533838 | 0.464555378 | 0.999970678 | 0.745 | 0.463 | 0.411 | 1.85 | NA |
| TRINITY_DN99804_c0_g1_i1 | -0.95527596 | 0.821131685 | 0.999970678 | 1.926 | 0 | 3.133 | 0 | LMBR1 |
| TRINITY_DN13543_c1_g1_i2 | -0.95527418 | 0.821191336 | 0.999970678 | 4.796 | 0 | 7.8 | 0 | Aldedh |
| TRINITY_DN7158_c0_g1_i1 | 0.955266219 | 0.733740002 | 0.999970678 | 7.022 | 1.567 | 3.7 | 0 | NYN |
| TRINITY_DN4117_c0_g1_i1 | 0.955237508 | 0.528449534 | 0.999970678 | 14.525 | 79.9 | 4.8 | 42.507 | NA |
| TRINITY_DN805_c0_g1_i3 | 0.955236627 | 0.47270493 | 0.999970678 | 19.885 | 4.652 | 7.922 | 3.147 | p450 |
| TRINITY_DN9434_c0_g1_i2 | -0.95506953 | 0.201497867 | 0.999970678 | 103.04 | 79.26 | 184.333 | 127.903 | NA |
| TRINITY_DN9487_c0_g1_i1 | -0.95499271 | 0.72896668 | 0.999970678 | 9.093 | 0 | 11.044 | 4.454 | MOSC |
| TRINITY_DN7667_c0_g3_i3 | -0.9549686 | 0.828476035 | 0.999970678 | 2.353 | 0 | 3.811 | 0 | NA |
| TRINITY_DN29454_c0_g1_i3 | -0.9549686 | 0.828476035 | 0.999970678 | 1.417 | 0 | 2.278 | 0 | NA |
| TRINITY_DN32885_c0_g1_i4 | 0.954891586 | 0.82798165 | 0.999970678 | 7.767 | 0 | 3.311 | 0 | Ribosomal_S10 |

| TRINITY_DN1890_c0_g1_i12 | 0.954808522 | 0.561818254 | 0.999970678 | 5.142 | 0.926 | 2.367 | 0.302 | NA |
| --- | --- | --- | --- | --- | --- | --- | --- | --- |
| TRINITY_DN3842_c0_g1_i5 | 0.954621968 | 0.36059408 | 0.999970678 | 0.718 | 1.813 | 0.489 | 0.704 | NA |
| TRINITY_DN1221_c0_g1_i2 | 0.954426876 | 0.424485722 | 0.999970678 | 14.707 | 35.47 | 4.133 | 20.691 | NA |
| TRINITY_DN6492_c0_g1_i5 | 0.9542939 | 0.830520818 | 1 | 0.581 | 0 | 0 | 0.302 | APG9 |
| TRINITY_DN1539_c0_g1_i1 | -0.95404876 | 0.335138913 | 0.999970678 | 4.288 | 1.646 | 3.944 | 6.806 | Amidase |
| TRINITY_DN3420_c0_g1_i5 | -0.95401936 | 0.4046282 | 0.999970678 | 0.972 | 0.335 | 1.044 | 1.287 | - |
| TRINITY_DN12757_c0_g1_i2 | -0.95399527 | 0.763649328 | 1 | 0.518 | 0.039 | 0.9 | 0 | NA |
| TRINITY_DN18421_c0_g1_i1 | -0.95378422 | 0.6450159 | 0.999970678 | 4.251 | 0.187 | 0.978 | 7.299 | NA |
| TRINITY_DN1609_c0_g1_i2 | -0.95351821 | 0.753082762 | 1 | 1.19 | 0 | 1.733 | 0.231 | - |
| TRINITY_DN11019_c0_g1_i1 | -0.95332194 | 0.532382759 | 0.999970678 | 22.065 | 10.3 | 48.375 | 4.424 | Cyclin_C |
| TRINITY_DN5534_c0_g1_i6 | 0.953199807 | 0.346421041 | 0.999970678 | 0.427 | 0.591 | 0.167 | 0.322 | RRM_1 |
| TRINITY_DN8336_c0_g1_i3 | -0.95315241 | 0.479226487 | 0.999970678 | 0.245 | 0.995 | 1.055 | 1.076 | WD40 |
| TRINITY_DN3609_c0_g1_i4 | 0.952990841 | 0.294103119 | 0.999970678 | 1.835 | 3.164 | 1.055 | 1.297 | NA |
| TRINITY_DN39868_c0_g1_i2 | -0.95287342 | 0.753767655 | 1 | 0 | 3.676 | 0.533 | 6.143 | NA |
| TRINITY_DN2251_c0_g1_i7 | 0.95283922 | 0.821646429 | 0.999970678 | 1.799 | 0 | 0.778 | 0 | NA |
| TRINITY_DN2332_c0_g1_i8 | -0.95278287 | 0.225842868 | 0.999970678 | 32.221 | 18.72 | 49.408 | 39.36 | NA |
| TRINITY_DN15637_c0_g1_i2 | 0.95274768 | 0.529314813 | 0.999970678 | 0.618 | 0.986 | 0.589 | 0.121 | Laminin_G_3 |
| TRINITY_DN8076_c0_g1_i2 | -0.95271729 | 0.55335349 | 0.999970678 | 1.39 | 0.394 | 2.478 | 0.473 | UDPGT |
| TRINITY_DN19798_c0_g2_i1 | 0.952579466 | 0.420603996 | 0.999970678 | 1.299 | 1.813 | 1 | 0.402 | mTERF |
| TRINITY_DN15372_c0_g1_i6 | 0.952577695 | 0.405874167 | 0.999970678 | 0.672 | 1.064 | 0.5 | 0.282 | RRM_1 |
| TRINITY_DN10643_c0_g1_i5 | 0.952473207 | 0.271989263 | 0.999970678 | 8.421 | 4.405 | 3.422 | 2.534 | DEAD |
| TRINITY_DN5447_c0_g1_i1 | -0.95240437 | 0.428288489 | 0.999970678 | 2.743 | 0.739 | 2.411 | 3.861 | - |
| TRINITY_DN11719_c0_g1_i6 | -0.95236199 | 0.831718693 | 1 | 21.474 | 0 | 33.231 | 0 | NA |
| TRINITY_DN27842_c0_g2_i2 | -0.95236199 | 0.831718693 | 1 | 0.218 | 0 | 0.344 | 0 | WD40 |
| TRINITY_DN11500_c0_g1_i12 | -0.95236199 | 0.831718693 | 1 | 1.499 | 0 | 2.389 | 0 | NA |
| TRINITY_DN18822_c0_g1_i8 | -0.95236199 | 0.831718693 | 1 | 0.699 | 0 | 1.133 | 0 | Iso_dh |
| TRINITY_DN53386_c0_g1_i1 | -0.9520785 | 0.568376591 | 0.999970678 | 0.273 | 1.912 | 0.411 | 3.68 | FH2 |
| TRINITY_DN175_c0_g1_i8 | 0.952039794 | 0.323443668 | 0.999970678 | 18.813 | 9.707 | 7.966 | 5.097 | NA |
| TRINITY_DN8736_c0_g3_i1 | -0.9519459 | 0.833965629 | 1 | 0 | 0.375 | 0.567 | 0 | IBR |
| TRINITY_DN4010_c1_g2_i2 | -0.95189355 | 0.552212265 | 0.999970678 | 0.79 | 4.75 | 1.2 | 9.169 | NA |
| TRINITY_DN11251_c0_g1_i1 | -0.95174187 | 0.514293938 | 0.999970678 | 1.908 | 0.877 | 3.344 | 1.317 | NA |
| TRINITY_DN2400_c0_g2_i5 | -0.95146621 | 0.823341746 | 0.999970678 | 0.491 | 0 | 0 | 0.965 | PC-Esterase |
| TRINITY_DN7657_c0_g1_i8 | -0.95134652 | 0.396163592 | 0.999970678 | 0.554 | 0.296 | 0.611 | 0.895 | NA |
| TRINITY_DN43029_c0_g1_i1 | 0.951318309 | 0.557254161 | 0.999970678 | 1.263 | 0.601 | 0.1 | 0.845 | NA |
| TRINITY_DN1306_c0_g1_i14 | 0.951307878 | 0.822255897 | 0.999970678 | 0 | 3.794 | 1.611 | 0 | NA |
| TRINITY_DN6564_c0_g2_i4 | -0.95126984 | 0.59463682 | 0.999970678 | 0.618 | 1.853 | 0.189 | 4.514 | NA |
| TRINITY_DN1962_c0_g2_i5 | -0.95121623 | 0.422397768 | 0.999970678 | 2.88 | 4.504 | 9.588 | 2.574 | Complex1_LYR |
| TRINITY_DN50949_c0_g1_i1 | -0.95112428 | 0.823120754 | 0.999970678 | 3.479 | 0 | 5.633 | 0 | NA |
| TRINITY_DN4237_c1_g1_i1 | -0.95099762 | 0.540141088 | 0.999970678 | 1.045 | 4.011 | 1.144 | 8.234 | NA |
| TRINITY_DN8246_c0_g1_i8 | 0.950893786 | 0.82150338 | 0.999970678 | 0 | 1.143 | 0.478 | 0 | - |
| TRINITY_DN1329_c1_g1_i3 | 0.950755409 | 0.403689241 | 0.999970678 | 26.053 | 114.2 | 21.099 | 46.368 | NA |
| TRINITY_DN8056_c0_g1_i1 | 0.950651913 | 0.360865954 | 0.999970678 | 2.462 | 7.529 | 1.989 | 2.725 | NA |
| TRINITY_DN186_c1_g1_i2 | 0.950649877 | 0.47170214 | 0.999970678 | 4.142 | 29.05 | 6 | 9.591 | NA |
| TRINITY_DN250071_c0_g1_i1 | 0.950573205 | 0.639206821 | 0.999970678 | 0.064 | 2.198 | 0.389 | 0.684 | NA |
| TRINITY_DN186_c0_g1_i3 | 0.950526419 | 0.32433635 | 0.999970678 | 3.307 | 7.736 | 3.033 | 2.021 | Pkinase |
| TRINITY_DN25147_c0_g1_i1 | -0.95046879 | 0.834004936 | 1 | 0.527 | 0 | 0.844 | 0 | NA |
| TRINITY_DN60432_c0_g1_i1 | -0.95046879 | 0.834004936 | 1 | 0.681 | 0 | 1.111 | 0 | NA |
| TRINITY_DN6346_c0_g1_i8 | -0.95043576 | 0.557345701 | 0.999970678 | 0.4 | 0.414 | 1.133 | 0.171 | S6PP |
| TRINITY_DN937_c0_g2_i1 | 0.950193644 | 0.160445634 | 0.999970678 | 10.174 | 15.31 | 4.911 | 7.249 | Motile_Sperm |
| TRINITY_DN6665_c0_g1_i2 | 0.950147155 | 0.730474183 | 1 | 1.399 | 2.168 | 1.555 | 0 | DUF659 |
| TRINITY_DN782_c1_g1_i8 | -0.9500676 | 0.422189274 | 0.999970678 | 31.258 | 39.53 | 18.366 | 111.747 | NA |
| TRINITY_DN3185_c0_g1_i4 | 0.950061248 | 0.49913279 | 0.999970678 | 6.958 | 0.956 | 1.189 | 2.714 | HALZ |
| TRINITY_DN41069_c0_g1_i2 | 0.949890876 | 0.506301736 | 0.999970678 | 0.872 | 1.252 | 0.167 | 0.895 | FoP_duplication |
| TRINITY_DN25202_c0_g3_i1 | 0.949639108 | 0.830309624 | 1 | 1.581 | 0 | 0.689 | 0 | NA |
| TRINITY_DN13444_c0_g2_i1 | 0.94962648 | 0.331595823 | 0.999970678 | 5.405 | 3.114 | 1.078 | 3.147 | Glyco_transf_90 |
| TRINITY_DN13444_c0_g2_i2 | 0.94962648 | 0.331595823 | 0.999970678 | 5.405 | 3.114 | 1.078 | 3.147 | Glyco_transf_90 |
| TRINITY_DN3117_c0_g1_i1 | 0.949517966 | 0.344863006 | 0.999970678 | 38.316 | 131.4 | 32.054 | 48.006 | DUF538 |
| TRINITY_DN1030_c0_g2_i4 | 0.949479437 | 0.43932947 | 0.999970678 | 5.587 | 4.188 | 0.867 | 4.011 | NA |
| TRINITY_DN23950_c0_g1_i1 | -0.94945488 | 0.594879895 | 0.999970678 | 1.453 | 3.637 | 0.644 | 8.747 | NA |
| TRINITY_DN12284_c0_g2_i2 | -0.94943148 | 0.694567401 | 0.999970678 | 4.124 | 0.069 | 0.3 | 7.782 | NA |
| TRINITY_DN2405_c0_g1_i3 | -0.94900865 | 0.73143392 | 0.999970678 | 0 | 1.587 | 1.8 | 0.774 | Sugar_tr |
| TRINITY_DN3988_c0_g1_i3 | 0.948948014 | 0.290511623 | 0.999970678 | 1.862 | 3.173 | 1.4 | 0.905 | Phosphorylase |
| TRINITY_DN1173_c5_g1_i3 | 0.948795401 | 0.821319915 | 0.999970678 | 7.44 | 0 | 0 | 3.901 | NA |
| TRINITY_DN938_c0_g1_i5 | 0.948732774 | 0.50276807 | 0.999970678 | 3.334 | 1.301 | 0.378 | 1.94 | NA |
| TRINITY_DN5881_c0_g1_i1 | -0.94872238 | 0.105569314 | 0.999970678 | 7.658 | 7.756 | 12.788 | 14.316 | NA |
| TRINITY_DN2284_c0_g1_i4 | -0.94864764 | 0.349025636 | 0.999970678 | 13.026 | 4.001 | 12.833 | 17.654 | Metallophos |
| TRINITY_DN9950_c0_g1_i1 | 0.948547278 | 0.323031863 | 0.999970678 | 7.585 | 11.08 | 5.677 | 2.765 | NA |
| TRINITY_DN8588_c0_g1_i25 | 0.948277723 | 0.620610815 | 1 | 0.854 | 0.306 | 0.456 | 0.06 | NA |
| TRINITY_DN2384_c0_g1_i12 | 0.948135857 | 0.82164074 | 0.999970678 | 0 | 9.816 | 4.122 | 0 | NA |
| TRINITY_DN5646_c0_g1_i1 | -0.94804709 | 0.719929146 | 0.999970678 | 0 | 1.35 | 1.144 | 1.206 | zf-Dof |
| TRINITY_DN486_c0_g1_i5 | 0.947923295 | 0.821733345 | 0.999970678 | 0 | 21.01 | 8.866 | 0 | NA |
| TRINITY_DN4520_c0_g1_i1 | -0.94787105 | 0.828131036 | 0.999970678 | 2.798 | 0 | 4.5 | 0 | NA |
| TRINITY_DN8934_c0_g1_i5 | -0.94787105 | 0.828131036 | 0.999970678 | 4.369 | 0 | 6.988 | 0 | NA |
| TRINITY_DN2128_c0_g1_i3 | -0.9477662 | 0.501673829 | 0.999970678 | 7.076 | 12.87 | 4.033 | 32.343 | Zip |

| TRINITY_DN10754_c0_g1_i1 | 0.947725548 | 0.47229019 | 0.999970678 | 27.933 | 6.642 | 11.922 | 3.7 | NA |
| --- | --- | --- | --- | --- | --- | --- | --- | --- |
| TRINITY_DN920_c0_g1_i8 | 0.947715985 | 0.618336979 | 0.999970678 | 14.126 | 0.67 | 5.166 | 1.438 | NA |
| TRINITY_DN6601_c0_g2_i5 | -0.94767706 | 0.635511817 | 0.999970678 | 2.189 | 0.306 | 3.822 | 0.251 | Bac_rhodopsin |
| TRINITY_DN3078_c1_g1_i2 | 0.947461508 | 0.632043721 | 0.999970678 | 2.607 | 0.69 | 1.367 | 0.07 | NA |
| TRINITY_DN201_c0_g2_i1 | -0.94746063 | 0.112172441 | 0.999970678 | 10.374 | 8.584 | 14.999 | 18.579 | UDPGT |
| TRINITY_DN2525_c0_g1_i6 | -0.9473909 | 0.27974105 | 0.999970678 | 4.878 | 3.045 | 5.011 | 9.189 | NA |
| TRINITY_DN7556_c0_g1_i2 | 0.947321893 | 0.425084515 | 0.999970678 | 38.852 | 21.98 | 21.71 | 5.439 | NA |
| TRINITY_DN1716_c0_g1_i2 | -0.94715756 | 0.461233872 | 0.999970678 | 2.38 | 0.532 | 1.822 | 3.428 | NA |
| TRINITY_DN1716_c0_g1_i1 | -0.94715756 | 0.461233872 | 0.999970678 | 2.38 | 0.532 | 1.822 | 3.428 | NA |
| TRINITY_DN1347_c1_g1_i7 | 0.947125201 | 0.377126895 | 0.999970678 | 3.997 | 8.416 | 3.989 | 1.588 | Gelsolin |
| TRINITY_DN2965_c1_g1_i4 | 0.946972001 | 0.576599985 | 0.999970678 | 256.59 | 9.274 | 76.451 | 47.262 | - |
| TRINITY_DN16695_c0_g1_i1 | -0.94691184 | 0.720261289 | 0.999970678 | 0 | 2.927 | 1.978 | 3.187 | NA |
| TRINITY_DN1000_c0_g2_i3 | -0.94685783 | 0.476581215 | 0.999970678 | 0.154 | 0.828 | 0.756 | 0.965 | DS |
| TRINITY_DN18767_c0_g2_i2 | 0.946768413 | 0.744149293 | 0.999970678 | 1.017 | 0.138 | 0.5 | 0 | NA |
| TRINITY_DN4442_c0_g1_i7 | 0.946764973 | 0.436497727 | 0.999970678 | 1.826 | 3.725 | 1.433 | 1.176 | NA |
| TRINITY_DN13940_c0_g1_i2 | -0.94660901 | 0.459233777 | 0.999970678 | 1.472 | 1.291 | 1.089 | 4.011 | - |
| TRINITY_DN125_c2_g1_i3 | 0.946562708 | 0.732052713 | 1 | 0.827 | 1.399 | 0.933 | 0 | NA |
| TRINITY_DN139558_c0_g1_i4 | -0.94647909 | 0.532979982 | 0.999970678 | 1.562 | 0.246 | 0.878 | 2.483 | - |
| TRINITY_DN9944_c0_g1_i5 | -0.94644084 | 0.734701793 | 0.999970678 | 0.463 | 0 | 0.567 | 0.211 | NA |
| TRINITY_DN8289_c0_g1_i5 | 0.946385845 | 0.499408099 | 0.999970678 | 0.772 | 1.015 | 0.644 | 0.131 | GDA1_CD39 |
| TRINITY_DN3515_c0_g2_i3 | -0.94623386 | 0.200044191 | 0.999970678 | 9.366 | 10.32 | 19.066 | 14.668 | RRM_1 |
| TRINITY_DN5307_c0_g1_i2 | -0.94609712 | 0.464195276 | 0.999970678 | 2.026 | 2.227 | 5.466 | 1.548 | LRRNT_2 |
| TRINITY_DN3294_c0_g1_i2 | 0.94607639 | 0.416816417 | 0.999970678 | 0.354 | 0.572 | 0.244 | 0.191 | Syja_N |
| TRINITY_DN8293_c0_g1_i1 | -0.9459967 | 0.347487354 | 0.999970678 | 0.917 | 1.794 | 2.389 | 2.302 | YIF1 |
| TRINITY_DN115_c0_g3_i1 | -0.94585793 | 0.269257353 | 0.999970678 | 12.863 | 18.14 | 15.643 | 40.466 | NA |
| TRINITY_DN52316_c0_g1_i4 | 0.945589535 | 0.336334416 | 0.999970678 | 2.235 | 1.616 | 0.9 | 0.915 | NA |
| TRINITY_DN2423_c2_g1_i1 | 0.945581318 | 0.437300166 | 0.999970678 | 0.463 | 0.838 | 0.189 | 0.452 | NA |
| TRINITY_DN5073_c0_g1_i4 | -0.9455782 | 0.354891854 | 0.999970678 | 6.422 | 10.31 | 19.91 | 7.963 | NA |
| TRINITY_DN1178_c0_g1_i7 | -0.94543049 | 0.744462098 | 1 | 0 | 0.335 | 0.433 | 0.131 | Methyltr_RsmB-F |
| TRINITY_DN3205_c0_g1_i9 | 0.945400311 | 0.741975055 | 1 | 0.663 | 0.138 | 0.356 | 0 | NA |
| TRINITY_DN7046_c0_g1_i3 | -0.945289 | 0.727203641 | 0.999970678 | 2.825 | 0 | 3.289 | 1.548 | DUF647 |
| TRINITY_DN3570_c0_g2_i1 | -0.94528616 | 0.464429772 | 0.999970678 | 0.736 | 2.139 | 1.389 | 3.82 | NA |
| TRINITY_DN1182_c0_g1_i1 | 0.94526007 | 0.615153686 | 0.999970678 | 36.336 | 2.346 | 15.532 | 1.508 | NA |
| TRINITY_DN2270_c0_g1_i3 | -0.94518278 | 0.414680323 | 0.999970678 | 0.336 | 0.453 | 0.789 | 0.563 | TPT |
| TRINITY_DN636_c1_g2_i1 | -0.94515998 | 0.616039418 | 0.999970678 | 0.082 | 4.445 | 4.278 | 3.428 | Snf7 |
| TRINITY_DN4640_c0_g1_i1 | 0.945026615 | 0.174553229 | 0.999970678 | 4.742 | 6.485 | 2.778 | 2.473 | ANAPC4_WD40 |
| TRINITY_DN4518_c0_g1_i13 | -0.94480125 | 0.474328212 | 0.999970678 | 0.318 | 1.202 | 1.044 | 1.639 | NA |
| TRINITY_DN5811_c0_g1_i1 | 0.94478828 | 0.391630565 | 0.999970678 | 46.019 | 11.89 | 10.11 | 18.036 | NA |
| TRINITY_DN8077_c0_g2_i3 | -0.94475821 | 0.508054712 | 1 | 1.444 | 0.532 | 1.067 | 2.483 | NA |
| TRINITY_DN2576_c0_g1_i1 | -0.9446726 | 0.319283952 | 0.999970678 | 8.176 | 7.125 | 9.133 | 17.473 | NA |
| TRINITY_DN91157_c0_g1_i1 | 0.944346959 | 0.425222367 | 1 | 0.518 | 0.384 | 0.178 | 0.251 | NA |
| TRINITY_DN7206_c1_g1_i4 | 0.944340818 | 0.30175127 | 0.999970678 | 41.15 | 21.24 | 17.966 | 10.838 | NA |
| TRINITY_DN3356_c0_g1_i4 | -0.9443047 | 0.55815115 | 0.999970678 | 5.578 | 0.296 | 3.544 | 7.219 | NA |
| TRINITY_DN668_c0_g1_i13 | 0.944194848 | 0.55418286 | 0.999970678 | 0.609 | 0.956 | 0.611 | 0.06 | NA |
| TRINITY_DN979_c0_g1_i1 | 0.944094661 | 0.601138673 | 0.999970678 | 0.436 | 7.658 | 0.533 | 3.519 | NA |
| TRINITY_DN1322_c2_g1_i1 | 0.944089134 | 0.525530297 | 0.999970678 | 0.418 | 2.533 | 0.444 | 0.985 | - |
| TRINITY_DN55478_c0_g1_i1 | -0.94408805 | 0.648360287 | 0.999970678 | 0.209 | 1.36 | 0.156 | 2.795 | NA |
| TRINITY_DN10249_c0_g1_i3 | 0.944034464 | 0.822140607 | 0.999970678 | 3.988 | 0 | 0 | 2.101 | Motile_Sperm |
| TRINITY_DN23_c1_g1_i3 | 0.94375594 | 0.627857365 | 0.999970678 | 16.842 | 0.217 | 3.989 | 4.202 | NA |
| TRINITY_DN2740_c0_g1_i9 | -0.94366828 | 0.776477991 | 1 | 0.391 | 0 | 0.611 | 0.02 | NA |
| TRINITY_DN1180_c0_g1_i3 | 0.94362804 | 0.704906397 | 0.999970678 | 2.689 | 0.069 | 0.056 | 1.397 | NA |
| TRINITY_DN2778_c1_g1_i1 | -0.94354526 | 0.439974662 | 0.999970678 | 2.58 | 5.371 | 2.533 | 12.105 | NA |
| TRINITY_DN7309_c0_g1_i6 | 0.943538921 | 0.728778626 | 0.999970678 | 0.491 | 1.015 | 0.644 | 0 | Myb_DNA-bind_ |
| TRINITY_DN4311_c0_g2_i1 | -0.94349707 | 0.256055212 | 0.999970678 | 1.226 | 1.892 | 2.789 | 2.624 | NA |
| TRINITY_DN179_c0_g1_i6 | 0.943418344 | 0.150550079 | 0.999970678 | 21.302 | 29.37 | 11.91 | 11.853 | CK_II_beta |
| TRINITY_DN8363_c0_g1_i1 | 0.943416169 | 0.455447589 | 0.999970678 | 1.608 | 0.66 | 0.467 | 0.613 | Bromodomain |
| TRINITY_DN10094_c0_g1_i6 | -0.94326594 | 0.724724829 | 0.999970678 | 2.08 | 0 | 1.422 | 2.353 | NA |
| TRINITY_DN9752_c0_g1_i5 | 0.942986949 | 0.739113133 | 0.999970678 | 0 | 71.87 | 6.922 | 26.361 | NA |
| TRINITY_DN75_c0_g1_i5 | -0.94297958 | 0.62851158 | 0.999970678 | 6.368 | 0.828 | 11.033 | 0.603 | NA |
| TRINITY_DN1504_c0_g3_i2 | 0.942968212 | 0.732949089 | 0.999970678 | 7.431 | 8.949 | 7.044 | 0 | LRRNT_2 |
| TRINITY_DN331_c0_g1_i1 | -0.94294156 | 0.558016699 | 0.999970678 | 2.852 | 0.227 | 1.322 | 4.414 | NA |
| TRINITY_DN4545_c2_g1_i3 | 0.942938486 | 0.46158223 | 1 | 0.609 | 0.946 | 0.244 | 0.533 | NA |
| TRINITY_DN5478_c0_g1_i2 | 0.942866846 | 0.822602141 | 0.999970678 | 7.303 | 0 | 0 | 3.871 | NA |
| TRINITY_DN1369_c0_g2_i2 | 0.942595984 | 0.256782449 | 0.999970678 | 1.472 | 2.158 | 1 | 0.684 | Glyco_hydro_17 |
| TRINITY_DN3831_c1_g1_i2 | -0.94249513 | 0.172787717 | 0.999970678 | 5.151 | 3.952 | 6.211 | 10.074 | NA |
| TRINITY_DN10434_c0_g1_i7 | -0.94246063 | 0.363149563 | 0.999970678 | 154.05 | 81.33 | 155.857 | 227.606 | NA |
| TRINITY_DN8862_c0_g1_i1 | 0.942388188 | 0.466984265 | 1 | 0.481 | 0.512 | 0.144 | 0.342 | NA |
| TRINITY_DN6464_c0_g1_i2 | -0.9422974 | 0.375286362 | 0.999970678 | 16.188 | 8.269 | 29.309 | 11.703 | NA |
| TRINITY_DN11022_c0_g1_i4 | 0.942082606 | 0.487143248 | 0.999970678 | 6.695 | 1.035 | 1.278 | 2.503 | OTU |
| TRINITY_DN5915_c0_g1_i6 | -0.94193082 | 0.728946981 | 0.999970678 | 0 | 2.592 | 0.955 | 3.78 | NA |
| TRINITY_DN4313_c0_g1_i4 | 0.94180681 | 0.519682548 | 0.999970678 | 1.126 | 10.29 | 2.8 | 2.463 | NA |
| TRINITY_DN4914_c0_g1_i5 | 0.941805303 | 0.39655494 | 0.999970678 | 10.937 | 44.25 | 9.177 | 17.312 | NA |
| TRINITY_DN16072_c0_g1_i5 | 0.941735763 | 0.336260865 | 0.999970678 | 0.936 | 0.611 | 0.3 | 0.452 | F-box |
| TRINITY_DN10260_c0_g1_i6 | -0.94168434 | 0.832602728 | 1 | 1.762 | 0 | 2.844 | 0 | NA |

| TRINITY_DN2831_c0_g1_i2 | -0.94159545 | 0.479690719 | 0.999970678 | 2.026 | 0.414 | 2.289 | 1.971 | NA |
| --- | --- | --- | --- | --- | --- | --- | --- | --- |
| TRINITY_DN8130_c0_g1_i2 | 0.941433982 | 0.358794174 | 0.999970678 | 1.099 | 3.41 | 1.055 | 1.056 | AFG1_ATPase |
| TRINITY_DN2026_c0_g2_i1 | -0.94137783 | 0.82454618 | 0.999970678 | 6.604 | 0 | 0 | 12.467 | NA |
| TRINITY_DN1991_c0_g1_i5 | -0.94134607 | 0.523303281 | 0.999970678 | 1.344 | 0.246 | 1.611 | 1.116 | Serpin |
| TRINITY_DN1967_c0_g1_i1 | -0.9412683 | 0.413717126 | 0.999970678 | 155.71 | 371.7 | 169.401 | 784.399 | NA |
| TRINITY_DN7397_c0_g1_i1 | 0.941267007 | 0.74314248 | 0.999970678 | 0.609 | 6.376 | 2.978 | 0 | NA |
| TRINITY_DN97_c0_g1_i3 | -0.94120777 | 0.529407718 | 0.999970678 | 6.531 | 1.597 | 11.444 | 1.92 | NA |
| TRINITY_DN3017_c0_g1_i1 | 0.941174722 | 0.369655101 | 0.999970678 | 5.45 | 1.784 | 1.444 | 2.051 | TFIID_20kDa |
| TRINITY_DN1120_c0_g2_i3 | -0.94086994 | 0.36946717 | 0.999970678 | 18.404 | 30.96 | 17.777 | 72.668 | NA |
| TRINITY_DN6574_c0_g1_i2 | -0.94079542 | 0.738107011 | 0.999970678 | 0 | 5.174 | 6.944 | 1.367 | zf-C3HC4_2 |
| TRINITY_DN15052_c0_g1_i2 | 0.940743271 | 0.423343211 | 0.999970678 | 6.695 | 2.168 | 2.689 | 1.408 | - |
| TRINITY_DN70956_c0_g1_i3 | -0.94072853 | 0.167104519 | 0.999970678 | 9.02 | 12.1 | 14.044 | 23.536 | NA |
| TRINITY_DN11888_c0_g1_i1 | -0.94069633 | 0.338051132 | 0.999970678 | 9.048 | 16 | 10.777 | 34.766 | NA |
| TRINITY_DN9571_c0_g1_i1 | -0.94069324 | 0.828928382 | 0.999970678 | 0.999 | 0 | 1.589 | 0 | MFS_1 |
| TRINITY_DN2637_c0_g1_i11 | -0.94061051 | 0.434236084 | 0.999970678 | 0.527 | 0.483 | 1.022 | 0.684 | Translin |
| TRINITY_DN5012_c0_g1_i3 | 0.940524174 | 0.542169702 | 0.999970678 | 1.272 | 0.325 | 0.522 | 0.221 | NA |
| TRINITY_DN1794_c0_g2_i1 | 0.940372656 | 0.382516571 | 0.999970678 | 1.699 | 2.139 | 1.3 | 0.432 | NA |
| TRINITY_DN10448_c0_g2_i3 | 0.940367996 | 0.728333294 | 0.999970678 | 2.68 | 3.035 | 2.455 | 0 | WD40 |
| TRINITY_DN8015_c0_g1_i5 | -0.94027397 | 0.124935061 | 0.999970678 | 19.131 | 19.64 | 35.309 | 31.87 | Aminotran_1_2 |
| TRINITY_DN443_c0_g1_i5 | 0.940218797 | 0.426388839 | 0.999970678 | 5.505 | 4.435 | 2.378 | 2.041 | NA |
| TRINITY_DN273_c0_g1_i8 | -0.94014647 | 0.578840777 | 0.999970678 | 0.036 | 0.434 | 0.544 | 0.241 | Aconitase |
| TRINITY_DN100876_c0_g1_i1 | -0.93993566 | 0.114407683 | 0.999970678 | 12.39 | 11.13 | 17.777 | 23.687 | ADH_N_2 |
| TRINITY_DN6597_c0_g1_i2 | 0.939914427 | 0.502370806 | 0.999970678 | 0.164 | 0.503 | 0.189 | 0.111 | zf-RING_2 |
| TRINITY_DN3744_c0_g1_i8 | -0.93987431 | 0.310014807 | 0.999970678 | 3.297 | 6.317 | 4.955 | 12.356 | NA |
| TRINITY_DN39076_c0_g1_i1 | -0.93980858 | 0.641496745 | 0.999970678 | 0.118 | 1.084 | 0.189 | 2.041 | Myb_DNA-bindin |
| TRINITY_DN4849_c0_g1_i2 | 0.939675871 | 0.37654415 | 0.999970678 | 2.044 | 3.223 | 1.678 | 0.714 | NA |
| TRINITY_DN1096_c0_g1_i8 | 0.939381355 | 0.383077725 | 0.999970678 | 1.79 | 1.784 | 0.489 | 1.257 | - |
| TRINITY_DN18012_c0_g1_i1 | 0.939291085 | 0.477498563 | 0.999970678 | 1.117 | 1.321 | 0.811 | 0.292 | NA |
| TRINITY_DN5195_c0_g1_i3 | -0.93928847 | 0.518279835 | 0.999970678 | 0.69 | 0.986 | 0.367 | 2.775 | NA |
| TRINITY_DN12827_c0_g1_i3 | -0.93928464 | 0.421871651 | 0.999970678 | 0.354 | 0.729 | 1.078 | 0.774 | NA |
| TRINITY_DN1189_c0_g1_i4 | -0.93926642 | 0.533270734 | 0.999970678 | 5.205 | 49.85 | 68.207 | 21.746 | NA |
| TRINITY_DN19878_c0_g1_i4 | 0.939264244 | 0.825411084 | 0.999970678 | 11.473 | 0 | 4.989 | 0 | NA |
| TRINITY_DN2850_c0_g1_i10 | 0.939261265 | 0.29999503 | 0.999970678 | 1.853 | 2.858 | 1.233 | 0.955 | CAP160 |
| TRINITY_DN13739_c0_g1_i5 | -0.93923578 | 0.646745807 | 0.999970678 | 2.643 | 0.079 | 2.411 | 2.272 | NA |
| TRINITY_DN2910_c1_g1_i9 | 0.939201926 | 0.509834972 | 0.999970678 | 10.247 | 117.6 | 24.043 | 36.565 | NA |
| TRINITY_DN1144_c0_g2_i1 | 0.938933286 | 0.288876559 | 0.999970678 | 11.936 | 6.169 | 4.622 | 3.891 | 2OG-FeII_Oxy_2 |
| TRINITY_DN7095_c0_g2_i1 | 0.93887698 | 0.674699168 | 0.999970678 | 16.978 | 0.276 | 2.555 | 5.821 | NA |
| TRINITY_DN1503_c0_g1_i7 | 0.938868381 | 0.722885132 | 0.999970678 | 1.735 | 1.597 | 1.444 | 0 | NA |
| TRINITY_DN274_c0_g1_i8 | -0.93883751 | 0.58542976 | 0.999970678 | 0.073 | 1.025 | 0.756 | 1.206 | RRM_1 |
| TRINITY_DN2156_c2_g1_i2 | 0.938766342 | 0.750772151 | 0.999970678 | 0 | 7.352 | 0.322 | 3.378 | NA |
| TRINITY_DN4169_c0_g3_i1 | 0.938698757 | 0.522881047 | 0.999970678 | 3.77 | 2.02 | 2.155 | 0.432 | Kunitz_legume |
| TRINITY_DN20094_c0_g1_i2 | -0.93847055 | 0.830152906 | 0.999970678 | 1.063 | 0 | 1.733 | 0 | NA |
| TRINITY_DN9278_c0_g1_i7 | -0.93846309 | 0.827669666 | 0.999970678 | 1.163 | 0 | 1.833 | 0 | FMO-like |
| TRINITY_DN30640_c0_g1_i1 | -0.93845337 | 0.824684631 | 0.999970678 | 1.344 | 0 | 2.155 | 0 | BBE |
| TRINITY_DN5849_c0_g1_i4 | -0.93844699 | 0.74591016 | 1 | 0 | 1.153 | 0.278 | 1.86 | NA |
| TRINITY_DN26149_c0_g1_i1 | -0.93841529 | 0.725169994 | 0.999970678 | 1.381 | 0 | 1.022 | 1.488 | NA |
| TRINITY_DN647_c0_g4_i2 | -0.93839568 | 0.389125382 | 0.999970678 | 82.146 | 19.76 | 81.762 | 97.993 | NA |
| TRINITY_DN50_c0_g1_i1 | -0.93779856 | 0.302064886 | 0.999970678 | 109.36 | 60.9 | 184.778 | 102.186 | EF-hand_8 |
| TRINITY_DN9352_c0_g1_i3 | 0.937762709 | 0.303774779 | 0.999970678 | 9.538 | 22.01 | 6.977 | 7.761 | NA |
| TRINITY_DN1643_c0_g1_i1 | 0.937747131 | 0.40313905 | 0.999970678 | 7.803 | 3.035 | 3.344 | 1.669 | NA |
| TRINITY_DN1712_c0_g1_i8 | -0.93766114 | 0.320087033 | 0.999970678 | 1.117 | 1.449 | 1.455 | 3.187 | NA |
| TRINITY_DN967_c3_g5_i1 | 0.937618471 | 0.159673792 | 0.999970678 | 7.303 | 5.529 | 2.811 | 3.338 | - |
| TRINITY_DN3541_c2_g1_i2 | 0.937592105 | 0.58420233 | 0.999970678 | 1.653 | 0.443 | 0.778 | 0.161 | NA |
| TRINITY_DN1035_c0_g2_i11 | 0.937387274 | 0.745043601 | 0.999970678 | 6.386 | 0 | 2.189 | 0.724 | NA |
| TRINITY_DN2981_c2_g1_i2 | 0.937328713 | 0.405651299 | 0.999970678 | 2.035 | 4.356 | 1.133 | 1.95 | Lipocalin_2 |
| TRINITY_DN14656_c0_g1_i6 | 0.937323456 | 0.644518938 | 0.999970678 | 1.562 | 0.099 | 0.611 | 0.151 | NYN_YacP |
| TRINITY_DN13608_c1_g1_i2 | 0.937054758 | 0.729814245 | 0.999970678 | 0.354 | 0.828 | 0.5 | 0 | NA |
| TRINITY_DN9223_c0_g1_i11 | 0.936976798 | 0.727867031 | 0.999970678 | 0.273 | 0.69 | 0.411 | 0 | NA |
| TRINITY_DN617_c0_g1_i4 | -0.93695702 | 0.73289328 | 0.999970678 | 11.064 | 0 | 10.122 | 9.27 | - |
| TRINITY_DN4908_c0_g1_i13 | -0.93684298 | 0.728800554 | 0.999970678 | 2.544 | 0 | 2.855 | 1.468 | B3 |
| TRINITY_DN12650_c0_g1_i1 | 0.936616441 | 0.171642098 | 0.999970678 | 15.588 | 20.68 | 7.244 | 10.054 | NA |
| TRINITY_DN5684_c0_g1_i2 | 0.936491434 | 0.490683708 | 0.999970678 | 0.563 | 0.857 | 0.456 | 0.181 | NA |
| TRINITY_DN23081_c0_g2_i2 | -0.93644745 | 0.36359988 | 0.999970678 | 14.325 | 23.79 | 13.944 | 56.029 | Pkinase |
| TRINITY_DN4656_c3_g1_i1 | 0.936425659 | 0.62724388 | 0.999970678 | 1.208 | 0.237 | 0.589 | 0.05 | NA |
| TRINITY_DN623_c0_g1_i10 | 0.936361103 | 0.824516798 | 0.999970678 | 2.353 | 0 | 1.033 | 0 | - |
| TRINITY_DN56_c0_g1_i4 | 0.936337209 | 0.427597044 | 0.999970678 | 20.366 | 18.78 | 14.277 | 3.217 | NA |
| TRINITY_DN7459_c1_g1_i2 | -0.93629404 | 0.748694217 | 1 | 0.164 | 0.522 | 0 | 1.297 | NA |
| TRINITY_DN3975_c1_g1_i4 | 0.936278092 | 0.209515213 | 0.999970678 | 6.304 | 7.51 | 2.489 | 4.233 | NA |
| TRINITY_DN65171_c0_g1_i6 | -0.93626367 | 0.53855089 | 0.999970678 | 0.363 | 3.292 | 2.455 | 3.921 | NA |
| TRINITY_DN635_c0_g1_i7 | -0.93622478 | 0.727277054 | 0.999970678 | 0 | 4.543 | 5.033 | 2.473 | - |
| TRINITY_DN20807_c0_g1_i12 | 0.936125271 | 0.682792948 | 0.999970678 | 0.772 | 8.397 | 3.766 | 0.131 | NA |
| TRINITY_DN3081_c0_g5_i1 | 0.936117951 | 0.678236126 | 0.999970678 | 31.158 | 0.059 | 5.589 | 9.822 | Transferase |
| TRINITY_DN6196_c0_g1_i14 | -0.93610668 | 0.829902385 | 0.999970678 | 0 | 1.025 | 1.6 | 0 | NA |
| TRINITY_DN13358_c0_g1_i2 | -0.93601677 | 0.565020206 | 0.999970678 | 0.109 | 0.384 | 0.111 | 0.814 | NA |

| TRINITY_DN8904_c0_g1_i5 | 0.935989325 | 0.736901625 | 0.999970678 | 0.236 | 0.946 | 0.511 | 0 | - |
| --- | --- | --- | --- | --- | --- | --- | --- | --- |
| TRINITY_DN6906_c0_g1_i4 | -0.93594365 | 0.731858511 | 0.999970678 | 1.753 | 0 | 2.067 | 0.915 | NA |
| TRINITY_DN10796_c0_g1_i3 | 0.935918713 | 0.423451437 | 0.999970678 | 1.617 | 2.01 | 1.233 | 0.422 | CRT-like |
| TRINITY_DN24734_c0_g1_i9 | -0.93579874 | 0.831620342 | 0.999970678 | 0.745 | 0 | 1.2 | 0 | NA |
| TRINITY_DN11455_c0_g3_i1 | 0.935745757 | 0.437788583 | 0.999970678 | 3.243 | 2.947 | 1.978 | 0.834 | zf-RING_2 |
| TRINITY_DN104973_c0_g1_i5 | 0.935616378 | 0.269144384 | 0.999970678 | 13.154 | 7.451 | 3.266 | 6.897 | Ank_2 |
| TRINITY_DN1665_c0_g2_i1 | 0.93544811 | 0.507268406 | 0.999970678 | 1.681 | 2.917 | 1.578 | 0.473 | NA |
| TRINITY_DN10361_c0_g1_i1 | -0.9354397 | 0.533972212 | 0.999970678 | 0.154 | 0.739 | 0.889 | 0.603 | Myb_DNA-bindin |
| TRINITY_DN10633_c0_g1_i9 | 0.935432821 | 0.324876785 | 0.999970678 | 4.097 | 11.44 | 3.189 | 4.233 | NA |
| TRINITY_DN1958_c0_g1_i3 | -0.93537446 | 0.743388614 | 0.999970678 | 13.544 | 0 | 11.644 | 12.266 | NA |
| TRINITY_DN1048_c0_g1_i5 | -0.93534216 | 0.266581175 | 0.999970678 | 2.834 | 5.578 | 7.5 | 6.927 | UDPGT |
| TRINITY_DN32171_c0_g1_i1 | -0.93524814 | 0.451498249 | 0.999970678 | 0.173 | 0.207 | 0.422 | 0.211 | NA |
| TRINITY_DN20825_c0_g1_i3 | 0.935156061 | 0.57114982 | 0.999970678 | 1.063 | 0.266 | 0.489 | 0.111 | B_lectin |
| TRINITY_DN9728_c0_g1_i1 | -0.93505342 | 0.733687401 | 0.999970678 | 0.672 | 1.045 | 0 | 3.288 | Lectin_legB |
| TRINITY_DN88188_c0_g2_i2 | -0.93502962 | 0.486664466 | 0.999970678 | 4.015 | 4.317 | 1.889 | 13.341 | NA |
| TRINITY_DN2504_c0_g1_i4 | -0.93502642 | 0.564598804 | 1 | 0.463 | 2.651 | 1.8 | 3.901 | NA |
| TRINITY_DN13858_c0_g1_i3 | -0.93500083 | 0.833040105 | 1 | 0 | 0.67 | 0 | 1.247 | NA |
| TRINITY_DN1276_c0_g1_i10 | 0.93498259 | 0.823740696 | 0.999970678 | 2.262 | 0 | 0 | 1.206 | BPS1 |
| TRINITY_DN3657_c0_g1_i3 | -0.93496391 | 0.548945102 | 0.999970678 | 6.213 | 2.03 | 1.8 | 13.432 | NA |
| TRINITY_DN2832_c0_g1_i4 | -0.9348967 | 0.479424602 | 0.999970678 | 1.19 | 3.203 | 1.133 | 6.987 | NA |
| TRINITY_DN8715_c0_g1_i1 | -0.93483875 | 0.501284455 | 0.999970678 | 4.524 | 2.828 | 2.589 | 10.466 | Glyco_hydro_16 |
| TRINITY_DN4616_c0_g1_i4 | 0.934838745 | 0.44469104 | 0.999970678 | 3.552 | 0.946 | 1.178 | 0.935 | Calmodulin_bind |
| TRINITY_DN24109_c0_g1_i1 | 0.934821247 | 0.572511766 | 1 | 0.69 | 1.143 | 0.1 | 0.834 | NA |
| TRINITY_DN39799_c0_g1_i2 | -0.93476192 | 0.400493384 | 0.999970678 | 0.754 | 0.512 | 0.844 | 1.408 | NA |
| TRINITY_DN11267_c0_g1_i2 | -0.93466468 | 0.466964658 | 0.999970678 | 0.572 | 1.163 | 0.7 | 2.433 | - |
| TRINITY_DN1032_c0_g1_i9 | 0.934625529 | 0.660517982 | 0.999970678 | 0.045 | 1.971 | 0.167 | 0.824 | Lipoxygenase |
| TRINITY_DN9977_c0_g1_i1 | -0.93457077 | 0.375843765 | 0.999970678 | 32.566 | 12.33 | 20.965 | 61.167 | NA |
| TRINITY_DN2391_c0_g1_i3 | 0.934477155 | 0.486405961 | 0.999970678 | 1.744 | 1.005 | 0.989 | 0.241 | Paf1 |
| TRINITY_DN23081_c0_g2_i3 | -0.93442387 | 0.745203486 | 0.999970678 | 14.507 | 0 | 15.555 | 9.41 | Pkinase |
| TRINITY_DN23892_c1_g1_i1 | -0.93431345 | 0.625277483 | 0.999970678 | 0.69 | 0.286 | 1.489 | 0.08 | NA |
| TRINITY_DN509_c0_g1_i15 | 0.934298567 | 0.546585232 | 0.999970678 | 2.634 | 4.514 | 0.233 | 3.458 | NA |
| TRINITY_DN2445_c2_g1_i2 | 0.934267604 | 0.613941474 | 0.999970678 | 66.313 | 7.589 | 30.365 | 2.111 | NA |
| TRINITY_DN40445_c0_g1_i1 | -0.93426626 | 0.837614072 | 1 | 0.382 | 0 | 0.622 | 0 | HEAT |
| TRINITY_DN82480_c0_g2_i3 | -0.93426626 | 0.837614072 | 1 | 0.836 | 0 | 1.344 | 0 | NA |
| TRINITY_DN4157_c0_g1_i2 | -0.93415812 | 0.651873047 | 1 | 1.117 | 0.177 | 1.811 | 0.261 | NA |
| TRINITY_DN4785_c0_g1_i2 | -0.93415812 | 0.651873047 | 1 | 1.299 | 0.187 | 2.111 | 0.271 | NA |
| TRINITY_DN1808_c0_g2_i5 | -0.93411044 | 0.726341623 | 0.999970678 | 2.316 | 0 | 2.044 | 2.021 | NA |
| TRINITY_DN571_c0_g2_i2 | 0.934097881 | 0.458262646 | 0.999970678 | 0.709 | 1.804 | 0.356 | 0.855 | RRF |
| TRINITY_DN10856_c0_g1_i6 | -0.93403396 | 0.674745661 | 0.999970678 | 0.318 | 5.618 | 0.378 | 10.607 | NA |
| TRINITY_DN251_c0_g1_i5 | -0.93402814 | 0.422509962 | 0.999970678 | 8.821 | 20.33 | 10.466 | 41.482 | NA |
| TRINITY_DN7449_c0_g1_i12 | -0.9340063 | 0.385234553 | 0.999970678 | 2.344 | 8.456 | 9.833 | 8.596 | NA |
| TRINITY_DN2112_c1_g1_i7 | 0.933977748 | 0.4907651 | 0.999970678 | 23.073 | 8.18 | 8.333 | 5.59 | NA |
| TRINITY_DN5184_c0_g1_i4 | -0.93396884 | 0.46683281 | 0.999970678 | 0.363 | 1.242 | 0.767 | 2.091 | p450 |
| TRINITY_DN4828_c0_g1_i1 | 0.933628755 | 0.729623101 | 1 | 0.572 | 0.581 | 0 | 0.583 | NA |
| TRINITY_DN12310_c0_g1_i1 | -0.93350505 | 0.424138505 | 0.999970678 | 0.6 | 0.986 | 0.667 | 2.252 | PP2C |
| TRINITY_DN1219_c1_g1_i5 | -0.93342017 | 0.580074531 | 0.999970678 | 0.818 | 0.739 | 2.333 | 0.161 | HGTP_anticodon |
| TRINITY_DN2355_c0_g1_i1 | 0.933295982 | 0.73992413 | 0.999970678 | 0 | 17.82 | 4.933 | 3.227 | NA |
| TRINITY_DN4674_c0_g1_i2 | -0.93323918 | 0.757198107 | 0.999970678 | 0 | 137.4 | 105.183 | 130.778 | CLP_protease |
| TRINITY_DN2170_c0_g2_i3 | -0.93318507 | 0.824025955 | 0.999970678 | 1.544 | 0 | 0 | 3.016 | F-box |
| TRINITY_DN1763_c1_g1_i8 | -0.93315936 | 0.511874972 | 0.999970678 | 0.1 | 0.207 | 0.378 | 0.131 | FAR1 |
| TRINITY_DN1266_c0_g1_i12 | -0.93315843 | 0.684000866 | 0.999970678 | 19.521 | 0.138 | 3.511 | 32.433 | NA |
| TRINITY_DN1175_c0_g1_i7 | 0.933110035 | 0.606948391 | 0.999970678 | 3.034 | 109.2 | 11.255 | 43.744 | NA |
| TRINITY_DN3455_c0_g1_i4 | -0.93304245 | 0.733450054 | 0.999970678 | 0 | 1.666 | 1.989 | 0.714 | NA |
| TRINITY_DN4908_c0_g1_i12 | -0.93300049 | 0.740289224 | 0.999970678 | 1.317 | 0 | 0.489 | 1.971 | B3 |
| TRINITY_DN2399_c0_g1_i7 | 0.932946018 | 0.721016811 | 0.999970678 | 2.189 | 2.04 | 0 | 2.222 | Fer4_14 |
| TRINITY_DN1412_c0_g2_i12 | 0.932786315 | 0.563105824 | 0.999970678 | 26.271 | 5.45 | 1.389 | 14.95 | Ras |
| TRINITY_DN19720_c0_g1_i4 | -0.9325261 | 0.833382462 | 1 | 1.817 | 0 | 2.889 | 0 | Ribosomal_L13e |
| TRINITY_DN1161_c0_g1_i2 | 0.932476868 | 0.247100153 | 0.999970678 | 15.97 | 17.98 | 9.433 | 6.253 | Glyoxalase |
| TRINITY_DN12759_c0_g2_i1 | 0.932383844 | 0.742209933 | 0.999970678 | 0 | 5.48 | 1.8 | 0.653 | Exo_endo_phos |
| TRINITY_DN6510_c0_g1_i1 | 0.932370905 | 0.390575304 | 0.999970678 | 9.747 | 8.407 | 6.311 | 1.9 | NA |
| TRINITY_DN2335_c0_g1_i3 | 0.932082281 | 0.825416409 | 0.999970678 | 0 | 13.86 | 0 | 7.148 | UbiA |
| TRINITY_DN3831_c1_g2_i1 | -0.93205059 | 0.384236408 | 0.999970678 | 3.843 | 3.745 | 3.011 | 10.747 | CaATP_NAI |
| TRINITY_DN42399_c0_g2_i2 | 0.931885698 | 0.401389692 | 0.999970678 | 1.317 | 1.212 | 0.822 | 0.342 | E1-E2_ATPase |
| TRINITY_DN1267_c2_g1_i3 | 0.931863405 | 0.333925337 | 0.999970678 | 0.999 | 1.38 | 0.7 | 0.402 | NA |
| TRINITY_DN4903_c0_g1_i3 | -0.93175846 | 0.826583034 | 0.999970678 | 5.905 | 0 | 9.444 | 0 | NA |
| TRINITY_DN12271_c0_g1_i2 | -0.9315804 | 0.830855979 | 0.999970678 | 0.954 | 0 | 1.533 | 0 | NA |
| TRINITY_DN527_c0_g1_i6 | -0.93148863 | 0.508086752 | 0.999970678 | 0.781 | 0.631 | 2 | 0.292 | A_deaminase |
| TRINITY_DN13091_c0_g1_i7 | -0.93147896 | 0.750558584 | 1 | 0 | 0.414 | 0.544 | 0.121 | NA |
| TRINITY_DN5630_c0_g1_i1 | -0.93146713 | 0.382943479 | 0.999970678 | 5.687 | 5.223 | 10.499 | 7.52 | NA |
| TRINITY_DN6024_c0_g1_i7 | -0.93142235 | 0.489506749 | 0.999970678 | 9.883 | 48.11 | 21.354 | 79.505 | NA |
| TRINITY_DN1513_c0_g1_i4 | 0.93129967 | 0.215650742 | 0.999970678 | 9.084 | 5.913 | 3.678 | 3.458 | NA |
| TRINITY_DN2688_c0_g1_i3 | -0.93129661 | 0.783327139 | 0.999970678 | 15.724 | 0.079 | 25.221 | 0 | NA |
| TRINITY_DN4597_c0_g1_i6 | -0.93127633 | 0.387305562 | 0.999970678 | 0.754 | 1.646 | 2.7 | 1.297 | F-box |
| TRINITY_DN907_c1_g1_i2 | 0.931265851 | 0.829662097 | 0.999970678 | 0 | 2.07 | 0.889 | 0 | NA |

| TRINITY_DN14333_c0_g1_i1 | 0.930915406 | 0.728853745 | 0.999970678 | 0.563 | 0.434 | 0.433 | 0 | - |
| --- | --- | --- | --- | --- | --- | --- | --- | --- |
| TRINITY_DN1020_c0_g1_i2 | -0.93081259 | 0.74562706 | 0.999970678 | 0 | 5.253 | 7.255 | 1.076 | PUB |
| TRINITY_DN2881_c0_g1_i4 | 0.930718671 | 0.6834142 | 0.999970678 | 33.72 | 0.552 | 13.932 | 1.206 | NA |
| TRINITY_DN2328_c0_g1_i2 | 0.93070448 | 0.257122061 | 0.999970678 | 11.146 | 9.225 | 5.966 | 3.499 | NA |
| TRINITY_DN3754_c1_g2_i1 | -0.93067042 | 0.45171424 | 0.999970678 | 4.206 | 6.041 | 12.044 | 4.574 | NA |
| TRINITY_DN17115_c0_g1_i1 | -0.93053748 | 0.31747908 | 0.999970678 | 3.297 | 1.754 | 3.789 | 5.047 | NA |
| TRINITY_DN79719_c0_g1_i1 | -0.93049741 | 0.154904054 | 0.999970678 | 62.352 | 88.2 | 127.759 | 126.445 | NA |
| TRINITY_DN6114_c0_g1_i1 | -0.93043588 | 0.411193182 | 0.999970678 | 0.808 | 2.02 | 1.667 | 3.328 | NA |
| TRINITY_DN2781_c0_g2_i6 | 0.930407904 | 0.495593825 | 0.999970678 | 3.715 | 0.65 | 1.278 | 0.784 | MutS_III |
| TRINITY_DN5943_c0_g1_i2 | -0.93033664 | 0.728304774 | 0.999970678 | 0 | 3.725 | 4.033 | 2.061 | NA |
| TRINITY_DN573_c0_g1_i3 | -0.93026332 | 0.392514261 | 0.999970678 | 1.935 | 1.39 | 1.967 | 3.991 | NA |
| TRINITY_DN2731_c0_g1_i1 | 0.930224796 | 0.827626685 | 0.999970678 | 15.306 | 0 | 0 | 8.174 | NA |
| TRINITY_DN894_c0_g1_i7 | -0.93020075 | 0.275846261 | 0.999970678 | 39.034 | 49.94 | 97.939 | 50.862 | p450 |
| TRINITY_DN13673_c0_g1_i1 | -0.93016908 | 0.421397914 | 0.999970678 | 0.636 | 2.02 | 1.867 | 2.765 | DUF868 |
| TRINITY_DN4127_c0_g1_i2 | 0.930090713 | 0.518097516 | 1 | 0.672 | 0.917 | 0.522 | 0.211 | NA |
| TRINITY_DN6762_c0_g1_i4 | 0.929963763 | 0.434628284 | 0.999970678 | 9.493 | 3.489 | 1.611 | 4.886 | NA |
| TRINITY_DN2996_c0_g1_i1 | -0.92986484 | 0.573358269 | 0.999970678 | 20.321 | 0.946 | 11.355 | 27.447 | NA |
| TRINITY_DN9424_c0_g1_i3 | -0.92985548 | 0.45495205 | 0.999970678 | 0.545 | 1.685 | 2.511 | 1.196 | NA |
| TRINITY_DN1261_c10_g1_i6 | 0.929789903 | 0.40012274 | 0.999970678 | 1.172 | 0.867 | 0.544 | 0.402 | NA |
| TRINITY_DN2023_c0_g4_i2 | 0.92973753 | 0.530506178 | 0.999970678 | 0.854 | 1.36 | 0.856 | 0.121 | Cation_ATPase_N |
| TRINITY_DN10251_c0_g1_i1 | 0.929698203 | 0.759330307 | 1 | 0.1 | 1.192 | 0 | 0.653 | Dimerisation |
| TRINITY_DN1238_c0_g1_i3 | 0.929548619 | 0.276244771 | 0.999970678 | 10.256 | 5.144 | 2.955 | 4.574 | NA |
| TRINITY_DN2984_c0_g1_i9 | -0.92935342 | 0.582442784 | 0.999970678 | 0.082 | 1.557 | 1.333 | 1.458 | GTP1_OBG |
| TRINITY_DN15771_c0_g1_i2 | -0.92934457 | 0.490224271 | 0.999970678 | 6.749 | 22.03 | 7.244 | 45.242 | NA |
| TRINITY_DN2872_c0_g1_i2 | 0.92933702 | 0.192317184 | 0.999970678 | 7.376 | 12.61 | 4.733 | 4.765 | NA |
| TRINITY_DN8428_c0_g1_i5 | 0.929200076 | 0.565260581 | 0.999970678 | 1.026 | 10.38 | 2.367 | 2.956 | NA |
| TRINITY_DN315_c0_g1_i3 | 0.928796967 | 0.538605689 | 0.999970678 | 0.327 | 3.43 | 0.5 | 1.337 | PPR |
| TRINITY_DN5112_c0_g1_i1 | -0.92877527 | 0.743849405 | 0.999970678 | 18.213 | 0 | 17.61 | 13.955 | NA |
| TRINITY_DN3862_c0_g1_i1 | -0.92873231 | 0.233804818 | 0.999970678 | 2.943 | 2.336 | 5.133 | 3.861 | ComA |
| TRINITY_DN9742_c0_g1_i1 | -0.92866727 | 0.751244522 | 1 | 0 | 3.361 | 4.611 | 0.694 | NA |
| TRINITY_DN11869_c0_g1_i1 | 0.928648199 | 0.830053449 | 0.999970678 | 7.258 | 0 | 3.178 | 0 | NA |
| TRINITY_DN1412_c0_g2_i18 | 0.928631238 | 0.234612047 | 0.999970678 | 9.738 | 15.66 | 4.655 | 7.661 | NA |
| TRINITY_DN2925_c0_g1_i5 | 0.928576622 | 0.361505485 | 0.999970678 | 8.92 | 8.239 | 1.8 | 6.826 | CDP-OH_P_trans |
| TRINITY_DN11759_c1_g1_i1 | -0.92852305 | 0.521077292 | 0.999970678 | 0.845 | 0.601 | 1.978 | 0.362 | NA |
| TRINITY_DN20602_c0_g1_i6 | -0.92842453 | 0.835517001 | 1 | 1.617 | 0 | 2.544 | 0.02 | NA |
| TRINITY_DN21232_c0_g1_i2 | 0.92841005 | 0.12510203 | 0.999970678 | 5.877 | 6.337 | 2.811 | 3.036 | NA |
| TRINITY_DN6543_c0_g1_i5 | 0.928358192 | 0.753170191 | 0.999970678 | 2.525 | 0 | 0.222 | 1.086 | NA |
| TRINITY_DN12551_c0_g1_i2 | 0.928351467 | 0.489815435 | 0.999970678 | 4.397 | 9.757 | 4.889 | 1.377 | NA |
| TRINITY_DN1412_c0_g2_i8 | 0.928197088 | 0.468788558 | 0.999970678 | 6.368 | 1.281 | 1.878 | 1.79 | Ras |
| TRINITY_DN7160_c0_g4_i2 | -0.92819637 | 0.743002698 | 0.999970678 | 0.727 | 0 | 0.256 | 1.116 | NA |
| TRINITY_DN3930_c0_g1_i4 | 0.928132503 | 0.764199361 | 0.999970678 | 17.968 | 0 | 0.467 | 8.777 | NA |
| TRINITY_DN4354_c0_g2_i4 | 0.928002876 | 0.731719737 | 0.999970678 | 3.352 | 1.133 | 1.967 | 0 | NA |
| TRINITY_DN28699_c0_g1_i3 | 0.927885691 | 0.52806777 | 0.999970678 | 19.485 | 3.213 | 1.778 | 9.903 | - |
| TRINITY_DN1151_c1_g1_i4 | 0.927819139 | 0.597021948 | 0.999970678 | 10.483 | 0.335 | 2.444 | 2.825 | NA |
| TRINITY_DN997_c1_g1_i4 | 0.927634718 | 0.653701728 | 0.999970678 | 4.279 | 0.059 | 0.478 | 1.739 | NA |
| TRINITY_DN13504_c0_g1_i1 | 0.927591661 | 0.77811387 | 0.999970678 | 0.045 | 5.815 | 2.5 | 0 | NA |
| TRINITY_DN2493_c0_g1_i1 | -0.9275253 | 0.59635809 | 0.999970678 | 0.245 | 2.296 | 3.3 | 0.804 | - |
| TRINITY_DN2324_c0_g1_i7 | -0.92737056 | 0.74716694 | 1 | 1.626 | 0 | 2.044 | 0.583 | NA |
| TRINITY_DN7686_c0_g1_i4 | -0.92735965 | 0.452139541 | 0.999970678 | 3.416 | 0.956 | 4.355 | 3.086 | CBFD_NFYB_HMF |
| TRINITY_DN7002_c0_g1_i1 | -0.92734989 | 0.519826215 | 0.999970678 | 3.57 | 8.574 | 2.133 | 20.268 | NA |
| TRINITY_DN54563_c0_g1_i1 | -0.92732625 | 0.586292942 | 0.999970678 | 85.498 | 2258 | 2534.891 | 1047.665 | NA |
| TRINITY_DN2630_c0_g1_i7 | 0.927197378 | 0.636323983 | 1 | 0.1 | 0.887 | 0.356 | 0.08 | NA |
| TRINITY_DN6243_c0_g1_i1 | 0.927144556 | 0.386509173 | 0.999970678 | 0.899 | 0.503 | 0.322 | 0.352 | Amino_oxidase |
| TRINITY_DN0_c0_g1_i5 | -0.92709506 | 0.599727269 | 0.999970678 | 4.088 | 0.286 | 4.8 | 2.493 | NA |
| TRINITY_DN26189_c0_g1_i9 | 0.927036543 | 0.435125777 | 0.999970678 | 0.491 | 0.848 | 0.267 | 0.382 | RNA_pol_Rpb2_1 |
| TRINITY_DN2880_c0_g2_i4 | 0.926915359 | 0.831409287 | 0.999970678 | 1.299 | 0 | 0.578 | 0 | NA |
| TRINITY_DN6956_c0_g1_i4 | -0.92690806 | 0.32598678 | 0.999970678 | 3.797 | 3.637 | 3.222 | 10.225 | NA |
| TRINITY_DN6739_c0_g1_i1 | -0.92686393 | 0.343620987 | 0.999970678 | 1.172 | 0.749 | 1.722 | 1.578 | PPR |
| TRINITY_DN26092_c0_g1_i2 | 0.926780664 | 0.826257004 | 0.999970678 | 7.948 | 0 | 3.5 | 0 | Pyrid_ox_like |
| TRINITY_DN57_c0_g2_i1 | -0.92673889 | 0.638451766 | 0.999970678 | 0.045 | 1.744 | 2.278 | 0.613 | PTR2 |
| TRINITY_DN775_c2_g1_i2 | 0.92666685 | 0.400741746 | 0.999970678 | 0.908 | 1.636 | 0.7 | 0.483 | NA |
| TRINITY_DN5658_c0_g1_i5 | -0.92660113 | 0.571306781 | 0.999970678 | 0.363 | 0.079 | 0.133 | 0.704 | DSPc |
| TRINITY_DN13722_c0_g2_i1 | 0.92658637 | 0.339400955 | 0.999970678 | 0.618 | 1.37 | 0.311 | 0.664 | NA |
| TRINITY_DN1403_c0_g1_i6 | 0.926492215 | 0.296282853 | 0.999970678 | 43.258 | 20.25 | 10.51 | 20.952 | NA |
| TRINITY_DN55430_c0_g2_i5 | 0.926453648 | 0.740173642 | 0.999970678 | 10.31 | 2.277 | 5.433 | 0 | adh_short |
| TRINITY_DN10642_c0_g1_i1 | 0.926427319 | 0.769617148 | 0.999970678 | 0 | 6.268 | 2.611 | 0.08 | NA |
| TRINITY_DN3887_c0_g1_i2 | 0.926368139 | 0.252858579 | 0.999970678 | 3.161 | 5.371 | 1.455 | 2.725 | NABP |
| TRINITY_DN77124_c0_g1_i1 | 0.926275644 | 0.829489403 | 0.999970678 | 3.706 | 0 | 1.633 | 0 | NA |
| TRINITY_DN2318_c0_g1_i8 | -0.9262374 | 0.583058542 | 0.999970678 | 0.136 | 0.69 | 0.233 | 1.257 | NA |
| TRINITY_DN30679_c0_g1_i2 | 0.926157332 | 0.829144144 | 0.999970678 | 0 | 1.33 | 0 | 0.694 | Sec15 |
| TRINITY_DN3281_c0_g1_i2 | -0.92599379 | 0.728400216 | 0.999970678 | 3.134 | 0 | 2.444 | 3.097 | NA |
| TRINITY_DN24957_c0_g1_i1 | 0.92580974 | 0.393339945 | 0.999970678 | 24.418 | 9.836 | 10.844 | 4.997 | NA |
| TRINITY_DN15444_c1_g1_i1 | 0.925784148 | 0.51949779 | 1 | 0.745 | 0.946 | 0.178 | 0.674 | NA |
| TRINITY_DN26213_c0_g2_i3 | 0.925705376 | 0.831945292 | 0.999970678 | 1.908 | 0 | 0 | 1.025 | NA |

| TRINITY_DN16970_c0_g4_i3 | 0.925666002 | 0.736051003 | 1 | 1.045 | 0.621 | 0.733 | 0 | NA |
| --- | --- | --- | --- | --- | --- | --- | --- | --- |
| TRINITY_DN199_c0_g1_i5 | 0.925585604 | 0.539849904 | 0.999970678 | 6.504 | 8.682 | 5.922 | 0.814 | NA |
| TRINITY_DN1890_c0_g1_i3 | -0.92555648 | 0.739095833 | 0.999970678 | 0.327 | 0.483 | 1.278 | 0 | NA |
| TRINITY_DN20620_c0_g1_i1 | -0.92549985 | 0.758548122 | 1 | 0 | 16.13 | 21.399 | 2.765 | NA |
| TRINITY_DN3153_c0_g2_i2 | 0.925359191 | 0.515724056 | 0.999970678 | 1.272 | 3.43 | 0.356 | 2.041 | peroxidase |
| TRINITY_DN9902_c0_g2_i3 | -0.92521653 | 0.616319935 | 0.999970678 | 1.354 | 0.237 | 2.378 | 0.211 | Ion_trans |
| TRINITY_DN1444_c1_g1_i9 | -0.92519607 | 0.500351501 | 0.999970678 | 17.859 | 15.36 | 5.133 | 56.673 | NA |
| TRINITY_DN23221_c0_g2_i1 | 0.925184984 | 0.299839231 | 0.999970678 | 1.145 | 1.232 | 0.456 | 0.704 | NA |
| TRINITY_DN11315_c0_g1_i1 | -0.92516755 | 0.732556335 | 0.999970678 | 4.388 | 0 | 3.8 | 3.871 | NA |
| TRINITY_DN1636_c0_g1_i1 | 0.924539828 | 0.352482165 | 0.999970678 | 1.135 | 2.415 | 0.489 | 1.267 | PWWP |
| TRINITY_DN1993_c0_g2_i1 | -0.92446569 | 0.44724249 | 0.999970678 | 4.415 | 1.409 | 6.811 | 2.895 | NA |
| TRINITY_DN2014_c1_g1_i2 | 0.924454837 | 0.682296861 | 0.999970678 | 0.118 | 2.395 | 0.089 | 1.227 | BTB |
| TRINITY_DN44910_c0_g1_i1 | 0.92442656 | 0.82906482 | 0.999970678 | 6.168 | 0 | 2.722 | 0 | NA |
| TRINITY_DN35971_c0_g1_i1 | -0.9243075 | 0.778129643 | 1 | 0.027 | 0.522 | 0 | 1.036 | NA |
| TRINITY_DN2162_c0_g1_i4 | 0.924239195 | 0.625868146 | 0.999970678 | 3.788 | 0.089 | 0.578 | 1.377 | NA |
| TRINITY_DN2137_c0_g1_i1 | -0.92416329 | 0.703886081 | 0.999970678 | 0.082 | 6.091 | 0.2 | 11.32 | - |
| TRINITY_DN5143_c0_g3_i5 | 0.924042992 | 0.293743543 | 0.999970678 | 1.235 | 1.321 | 0.656 | 0.553 | NA |
| TRINITY_DN1543_c0_g1_i4 | -0.92398921 | 0.743538299 | 0.999970678 | 0 | 3.252 | 4.278 | 0.905 | Pept_tRNA_hydro |
| TRINITY_DN325_c0_g3_i1 | 0.92348406 | 0.756150214 | 0.999970678 | 44.52 | 0 | 16.555 | 3.71 | NA |
| TRINITY_DN7962_c0_g2_i1 | -0.92338793 | 0.565805186 | 0.999970678 | 4.56 | 2.907 | 10.599 | 1.257 | NA |
| TRINITY_DN4668_c0_g3_i2 | 0.923379462 | 0.365083439 | 0.999970678 | 0.954 | 1.222 | 0.644 | 0.362 | DUF659 |
| TRINITY_DN4707_c0_g2_i1 | -0.92331739 | 0.387738952 | 0.999970678 | 30.504 | 7.796 | 31.509 | 35.329 | NA |
| TRINITY_DN31377_c0_g1_i4 | -0.92313355 | 0.838186479 | 1 | 0.3 | 0 | 0.467 | 0 | zf-C2H2 |
| TRINITY_DN20346_c0_g1_i6 | -0.92312772 | 0.742084711 | 0.999970678 | 0.454 | 0 | 0.567 | 0.191 | HLH |
| TRINITY_DN188_c0_g1_i10 | -0.92306565 | 0.495202661 | 0.999970678 | 50.516 | 9.254 | 37.764 | 61.358 | NA |
| TRINITY_DN94_c0_g2_i1 | 0.923057264 | 0.734566028 | 0.999970678 | 3.025 | 1.961 | 2.167 | 0 | PPR |
| TRINITY_DN463_c0_g2_i8 | 0.923001661 | 0.418732665 | 0.999970678 | 27.679 | 12.81 | 13.61 | 4.906 | NA |
| TRINITY_DN19202_c0_g1_i3 | 0.922996415 | 0.204766667 | 0.999970678 | 3.016 | 3.765 | 1.833 | 1.367 | NA |
| TRINITY_DN5827_c0_g1_i2 | 0.922916177 | 0.344442008 | 0.999970678 | 1.017 | 1.025 | 0.544 | 0.422 | HD |
| TRINITY_DN1711_c1_g1_i3 | 0.922899686 | 0.378741048 | 0.999970678 | 1.254 | 2.7 | 0.644 | 1.287 | NAF |
| TRINITY_DN4869_c0_g1_i1 | -0.92269509 | 0.165756374 | 0.999970678 | 24.127 | 34.07 | 38.709 | 63.409 | Bax1-I |
| TRINITY_DN5567_c0_g1_i5 | -0.92267331 | 0.393981985 | 0.999970678 | 2.389 | 1.055 | 2.655 | 3.318 | NA |
| TRINITY_DN12142_c0_g1_i4 | -0.92263299 | 0.493302823 | 0.999970678 | 2.262 | 0.591 | 1.244 | 3.941 | NA |
| TRINITY_DN12363_c0_g1_i2 | 0.922553887 | 0.506918079 | 0.999970678 | 0.191 | 0.808 | 0.2 | 0.271 | NA |
| TRINITY_DN284_c0_g1_i13 | 0.922203125 | 0.155252328 | 0.999970678 | 55.53 | 44.84 | 25.198 | 22.631 | NA |
| TRINITY_DN13447_c1_g1_i1 | 0.922013949 | 0.607890015 | 0.999970678 | 0.581 | 3.4 | 1.422 | 0.362 | NA |
| TRINITY_DN7482_c0_g1_i1 | 0.922009232 | 0.518064283 | 0.999970678 | 1.335 | 5.066 | 0.9 | 2.222 | NA |
| TRINITY_DN1397_c1_g1_i2 | 0.922001851 | 0.731299689 | 0.999970678 | 2.244 | 1.528 | 1.678 | 0 | LRRNT_2 |
| TRINITY_DN7883_c1_g3_i1 | 0.921928238 | 0.771639437 | 0.999970678 | 5.105 | 0.049 | 0 | 2.775 | NA |
| TRINITY_DN30504_c0_g1_i1 | 0.921909803 | 0.322179511 | 0.999970678 | 9.347 | 17.36 | 8.133 | 4.223 | NA |
| TRINITY_DN698_c2_g1_i10 | -0.92188697 | 0.743986625 | 0.999970678 | 1.017 | 0.404 | 2.233 | 0 | DUF659 |
| TRINITY_DN16921_c0_g2_i2 | 0.92188429 | 0.690353758 | 1 | 0.027 | 0.601 | 0.244 | 0.02 | NA |
| TRINITY_DN2671_c0_g1_i5 | -0.92186824 | 0.83291812 | 0.999970678 | 0.836 | 0 | 1.322 | 0 | NA |
| TRINITY_DN5592_c0_g1_i5 | 0.921825195 | 0.605440696 | 0.999970678 | 1.726 | 78.54 | 13.677 | 25.275 | Ribosomal_S5 |
| TRINITY_DN314_c0_g2_i10 | -0.92179188 | 0.620553942 | 0.999970678 | 3.243 | 0.099 | 2.555 | 3.328 | NA |
| TRINITY_DN2402_c0_g1_i1 | -0.9217457 | 0.375977062 | 0.999970678 | 1.381 | 3.824 | 5.289 | 3.438 | NA |
| TRINITY_DN14311_c1_g4_i1 | 0.921563361 | 0.740054979 | 1 | 1.054 | 0.522 | 0.689 | 0 | NA |
| TRINITY_DN14502_c0_g1_i5 | -0.92151302 | 0.409121443 | 0.999970678 | 3.47 | 10.4 | 14.488 | 8.445 | - |
| TRINITY_DN87639_c0_g1_i2 | -0.92136746 | 0.348678715 | 0.999970678 | 1.09 | 0.857 | 1.733 | 1.588 | Ribosomal_L11 |
| TRINITY_DN13671_c0_g2_i1 | -0.92135379 | 0.677956842 | 0.999970678 | 2.089 | 1.045 | 4.877 | 0.05 | NA |
| TRINITY_DN5262_c0_g1_i2 | 0.921296668 | 0.431104907 | 0.999970678 | 0.572 | 1.094 | 0.367 | 0.432 | NA |
| TRINITY_DN2384_c0_g1_i3 | -0.92128508 | 0.28723581 | 0.999970678 | 78.013 | 50.35 | 63.563 | 167.826 | RRM_1 |
| TRINITY_DN10206_c0_g1_i3 | 0.921278581 | 0.45372935 | 0.999970678 | 15.906 | 3.252 | 3.389 | 6.093 | NA |
| TRINITY_DN1062_c0_g1_i4 | 0.921194037 | 0.828048434 | 0.999970678 | 41.886 | 0 | 0 | 22.168 | NA |
| TRINITY_DN1456_c0_g1_i4 | 0.921123585 | 0.399302056 | 0.999970678 | 1.771 | 0.966 | 0.622 | 0.694 | PPR |
| TRINITY_DN1649_c0_g1_i7 | -0.92111125 | 0.750808113 | 0.999970678 | 0 | 2.493 | 3.566 | 0.332 | NA |
| TRINITY_DN32336_c0_g1_i7 | -0.92109445 | 0.301880875 | 0.999970678 | 1.626 | 1.37 | 1.722 | 3.579 | BAH |
| TRINITY_DN13745_c0_g1_i13 | -0.92100038 | 0.660202254 | 0.999970678 | 1.126 | 0.118 | 1.8 | 0.181 | - |
| TRINITY_DN61794_c0_g1_i1 | 0.920883785 | 0.397516851 | 0.999970678 | 1.19 | 2.07 | 0.856 | 0.674 | NA |
| TRINITY_DN14535_c0_g5_i4 | 0.920802965 | 0.831248446 | 0.999970678 | 3.188 | 0 | 0 | 1.699 | NA |
| TRINITY_DN5411_c0_g1_i4 | 0.920779237 | 0.156710664 | 0.999970678 | 12.817 | 16.68 | 7.477 | 6.555 | WRKY |
| TRINITY_DN8090_c0_g2_i5 | -0.92076062 | 0.335608653 | 0.999970678 | 0.709 | 0.946 | 0.844 | 2.091 | DEAD |
| TRINITY_DN1734_c0_g1_i7 | -0.920726 | 0.502884106 | 0.999970678 | 74.843 | 7.825 | 65.607 | 79.394 | NA |
| TRINITY_DN9209_c0_g1_i1 | 0.920405488 | 0.314437799 | 0.999970678 | 0.763 | 1.015 | 0.389 | 0.462 | NA |
| TRINITY_DN5618_c0_g1_i5 | 0.920309059 | 0.74532423 | 1 | 0.808 | 0.187 | 0 | 0.533 | NA |
| TRINITY_DN13720_c1_g1_i5 | 0.92024146 | 0.599730418 | 0.999970678 | 0.391 | 2.661 | 0.156 | 1.397 | RabGAP-TBC |
| TRINITY_DN189_c0_g1_i2 | 0.920166649 | 0.462113711 | 0.999970678 | 11.228 | 2.306 | 3.922 | 2.483 | NA |
| TRINITY_DN10305_c0_g1_i6 | 0.919880763 | 0.54938922 | 0.999970678 | 29.55 | 2.907 | 6.322 | 9.27 | NA |
| TRINITY_DN2986_c0_g1_i1 | -0.91977698 | 0.73491027 | 0.999970678 | 3.806 | 0 | 3.8 | 2.614 | NA |
| TRINITY_DN9068_c0_g1_i2 | 0.919772787 | 0.745692425 | 0.999970678 | 0.89 | 0 | 0.167 | 0.282 | DYW_deaminase |
| TRINITY_DN8411_c0_g1_i2 | -0.91975297 | 0.192626492 | 0.999970678 | 2.525 | 2.602 | 4.355 | 4.434 | Zip |
| TRINITY_DN14636_c0_g1_i3 | 0.919546105 | 0.836756519 | 1 | 0 | 1.419 | 0 | 0.724 | NA |
| TRINITY_DN10324_c0_g1_i1 | -0.91948161 | 0.165353037 | 0.999970678 | 33.638 | 32.23 | 60.93 | 50.148 | Ribosomal_L7Ae |
| TRINITY_DN2712_c0_g1_i6 | -0.91946515 | 0.732645702 | 0.999970678 | 0 | 5.529 | 3.911 | 5.6 | DSPc |

| TRINITY_DN9352_c0_g1_i2 | 0.919453114 | 0.633827926 | 0.999970678 | 7.676 | 1.429 | 3.6 | 0.362 | NA |
| --- | --- | --- | --- | --- | --- | --- | --- | --- |
| TRINITY_DN13591_c0_g2_i4 | -0.91938357 | 0.386053976 | 0.999970678 | 0.418 | 0.936 | 1.011 | 1.317 | LRRNT_2 |
| TRINITY_DN10864_c0_g1_i5 | 0.919293079 | 0.49964189 | 0.999970678 | 59.118 | 169.2 | 86.606 | 15.211 | NA |
| TRINITY_DN56257_c0_g1_i1 | 0.919203577 | 0.781041791 | 1 | 2.008 | 0.039 | 0.911 | 0 | NA |
| TRINITY_DN2364_c0_g1_i2 | 0.919147309 | 0.756661381 | 0.999970678 | 7.231 | 0 | 0.344 | 3.448 | NA |
| TRINITY_DN17974_c0_g1_i1 | 0.918969686 | 0.246672552 | 0.999970678 | 8.811 | 5.204 | 2.944 | 3.861 | NA |
| TRINITY_DN5128_c0_g1_i1 | -0.91890332 | 0.386365962 | 0.999970678 | 5.932 | 10.13 | 5.7 | 23.435 | PARP |
| TRINITY_DN913_c0_g1_i2 | 0.91885806 | 0.446856316 | 0.999970678 | 3.743 | 5.509 | 0.833 | 3.851 | NA |
| TRINITY_DN3391_c0_g2_i1 | 0.918817423 | 0.424790434 | 0.999970678 | 1.135 | 2.001 | 0.378 | 1.176 | TPT |
| TRINITY_DN1202_c0_g2_i4 | 0.918599312 | 0.836779139 | 0.999970678 | 0 | 41.1 | 17.699 | 0 | Cellulose_synt |
| TRINITY_DN6131_c0_g1_i4 | 0.918418497 | 0.323965989 | 0.999970678 | 3.552 | 4.662 | 1.122 | 2.996 | NA |
| TRINITY_DN252442_c0_g1_i1 | 0.918399346 | 0.828695017 | 0.999970678 | 6.286 | 0 | 2.789 | 0 | Uricase |
| TRINITY_DN944_c1_g1_i3 | -0.91838742 | 0.218191563 | 0.999970678 | 168.02 | 128.3 | 293.649 | 203.467 | DHQ_synthase |
| TRINITY_DN11879_c0_g1_i3 | 0.918251413 | 0.535619193 | 0.999970678 | 2.462 | 1.409 | 0.256 | 1.729 | HALZ |
| TRINITY_DN7267_c0_g2_i7 | 0.918218102 | 0.439237448 | 0.999970678 | 0.518 | 2.158 | 0.733 | 0.513 | NA |
| TRINITY_DN847_c0_g1_i2 | -0.91819382 | 0.496720967 | 0.999970678 | 2.117 | 2.523 | 1.078 | 7.44 | NA |
| TRINITY_DN9652_c1_g1_i5 | -0.91811977 | 0.743219853 | 0.999970678 | 0.363 | 0.325 | 1.067 | 0 | NA |
| TRINITY_DN6317_c0_g1_i2 | -0.91803871 | 0.734556438 | 0.999970678 | 0 | 0.69 | 0.367 | 0.834 | PMEI |
| TRINITY_DN59649_c0_g1_i1 | -0.91797366 | 0.781348111 | 1 | 0.609 | 0.02 | 1 | 0 | NA |
| TRINITY_DN15379_c0_g2_i2 | 0.917970271 | 0.457336165 | 0.999970678 | 0.99 | 0.867 | 0.633 | 0.221 | NA |
| TRINITY_DN13415_c0_g1_i1 | 0.917965494 | 0.572141011 | 0.999970678 | 14.489 | 6.455 | 7.944 | 1.337 | NA |
| TRINITY_DN516_c0_g1_i6 | -0.9179596 | 0.257092506 | 0.999970678 | 2.107 | 2.681 | 3.489 | 4.786 | NA |
| TRINITY_DN7658_c0_g1_i5 | 0.917937129 | 0.568172644 | 0.999970678 | 0.164 | 1.271 | 0.189 | 0.523 | GDPD |
| TRINITY_DN1027_c0_g1_i4 | -0.91774787 | 0.450453931 | 0.999970678 | 2.325 | 4.77 | 8.611 | 2.885 | NA |
| TRINITY_DN2912_c0_g1_i3 | -0.91772868 | 0.227646136 | 0.999970678 | 8.902 | 7.845 | 16.966 | 11.18 | NA |
| TRINITY_DN4466_c0_g1_i4 | -0.91761634 | 0.82808255 | 0.999970678 | 3.179 | 0 | 5.044 | 0 | Peroxidase_2 |
| TRINITY_DN7388_c0_g1_i3 | -0.91759645 | 0.730725681 | 0.999970678 | 3.416 | 0 | 2.6 | 3.358 | NA |
| TRINITY_DN1541_c0_g1_i5 | 0.917586618 | 0.261672056 | 0.999970678 | 23.164 | 50.07 | 17.799 | 17.051 | Glyco_transf_5 |
| TRINITY_DN79_c0_g1_i2 | -0.91753421 | 0.754178228 | 0.999970678 | 0.091 | 0.749 | 1.311 | 0 | NA |
| TRINITY_DN1617_c0_g1_i17 | 0.91724222 | 0.571202502 | 0.999970678 | 2.907 | 3.006 | 2.444 | 0.171 | NA |
| TRINITY_DN22739_c0_g1_i3 | -0.9171499 | 0.837105089 | 1 | 1.217 | 0 | 0 | 2.312 | NA |
| TRINITY_DN940_c0_g1_i4 | -0.91705867 | 0.42687662 | 0.999970678 | 6.668 | 2.779 | 11.033 | 4.574 | 40S_SA_C |
| TRINITY_DN1343_c0_g1_i17 | 0.917039266 | 0.526472792 | 0.999970678 | 2.135 | 0.483 | 0.289 | 1.056 | NA |
| TRINITY_DN868_c0_g1_i7 | -0.9169941 | 0.756627005 | 0.999970678 | 6.831 | 0 | 10.122 | 0.865 | NA |
| TRINITY_DN4706_c0_g1_i6 | 0.916971491 | 0.524605782 | 0.999970678 | 1.29 | 0.256 | 0.489 | 0.231 | NA |
| TRINITY_DN11087_c0_g3_i1 | -0.91691651 | 0.50481723 | 0.999970678 | 46.183 | 6.849 | 52.752 | 34.253 | NA |
| TRINITY_DN583_c2_g1_i3 | 0.916915071 | 0.735775987 | 0.999970678 | 17.904 | 0.118 | 7.8 | 0.131 | NA |
| TRINITY_DN6823_c0_g1_i1 | 0.916900066 | 0.73155622 | 0.999970678 | 0.572 | 0.907 | 0 | 0.784 | NA |
| TRINITY_DN490_c1_g2_i1 | 0.916860099 | 0.527134645 | 0.999970678 | 10.074 | 1.744 | 4.266 | 1.186 | NA |
| TRINITY_DN31638_c0_g2_i1 | 0.916841194 | 0.664922174 | 0.999970678 | 1.108 | 30.73 | 0.556 | 15.825 | zf-FLZ |
| TRINITY_DN1312_c0_g2_i1 | -0.91680414 | 0.434888205 | 0.999970678 | 1.444 | 5.292 | 3.2 | 8.767 | Peptidase_S10 |
| TRINITY_DN10882_c0_g1_i3 | -0.91672206 | 0.775398034 | 0.999970678 | 6.113 | 0 | 0.1 | 11.501 | NA |
| TRINITY_DN3988_c0_g1_i13 | 0.916696941 | 0.133204665 | 0.999970678 | 89.232 | 72.9 | 32.109 | 47.463 | Phosphorylase |
| TRINITY_DN61617_c0_g1_i2 | -0.91665229 | 0.298767764 | 0.999970678 | 4.279 | 7.953 | 12.221 | 8.164 | NA |
| TRINITY_DN1403_c0_g1_i7 | 0.916593189 | 0.734228233 | 0.999970678 | 0.709 | 1.232 | 0.856 | 0 | NA |
| TRINITY_DN6483_c0_g1_i1 | -0.91656564 | 0.373538794 | 0.999970678 | 7.73 | 2.336 | 8.2 | 9.27 | - |
| TRINITY_DN358_c0_g1_i3 | 0.91655535 | 0.594111964 | 0.999970678 | 4.415 | 0.207 | 1.244 | 0.975 | PPR |
| TRINITY_DN58618_c0_g2_i2 | 0.916518485 | 0.345465437 | 0.999970678 | 1.699 | 2.858 | 0.744 | 1.508 | NA |
| TRINITY_DN49_c0_g2_i4 | 0.916383495 | 0.420541691 | 0.999970678 | 4.796 | 5.292 | 0.822 | 4.373 | G-alpha |
| TRINITY_DN583_c1_g1_i18 | -0.91633848 | 0.837961436 | 1 | 0 | 0.266 | 0.4 | 0 | Pkinase |
| TRINITY_DN22881_c0_g1_i7 | 0.916258594 | 0.590055669 | 0.999970678 | 0.917 | 0.769 | 0.711 | 0.04 | B-block_TFIIIC |
| TRINITY_DN5861_c0_g1_i4 | 0.916219616 | 0.739454574 | 1 | 2.062 | 0.729 | 0 | 1.478 | Cornichon |
| TRINITY_DN1391_c1_g1_i3 | 0.916208291 | 0.418297999 | 0.999970678 | 14.525 | 7.165 | 6.933 | 3.036 | NA |
| TRINITY_DN13803_c0_g1_i1 | -0.91610944 | 0.542275622 | 0.999970678 | 6.304 | 3.597 | 2.311 | 15.181 | NA |
| TRINITY_DN11192_c0_g1_i1 | -0.91603109 | 0.129053125 | 0.999970678 | 14.598 | 13.42 | 21.71 | 26.461 | NA |
| TRINITY_DN20214_c0_g1_i1 | 0.916030825 | 0.830908587 | 0.999970678 | 16.687 | 0 | 7.322 | 0 | NA |
| TRINITY_DN5102_c0_g1_i1 | 0.916005681 | 0.74232307 | 0.999970678 | 0 | 3.321 | 0.689 | 0.895 | NA |
| TRINITY_DN1071_c0_g1_i7 | -0.91596383 | 0.46105319 | 0.999970678 | 3.879 | 10.54 | 4.155 | 22.008 | NA |
| TRINITY_DN826_c0_g3_i2 | -0.91585868 | 0.383517945 | 0.999970678 | 1.526 | 3.765 | 4.822 | 4.112 | NA |
| TRINITY_DN1234_c0_g2_i1 | -0.91585172 | 0.144776322 | 0.999970678 | 4.942 | 5.824 | 7.611 | 11.089 | NA |
| TRINITY_DN5541_c1_g1_i5 | 0.915770188 | 0.219755462 | 0.999970678 | 2.825 | 2.789 | 1.333 | 1.367 | NA |
| TRINITY_DN7725_c0_g1_i2 | -0.91576312 | 0.321684676 | 0.999970678 | 2.371 | 3.469 | 6.122 | 3.579 | UDPGT |
| TRINITY_DN25148_c0_g1_i1 | -0.91571294 | 0.429636092 | 0.999970678 | 0.781 | 1.882 | 1.389 | 3.318 | His_Phos_1 |
| TRINITY_DN3086_c0_g1_i1 | 0.915711206 | 0.227751492 | 0.999970678 | 1.499 | 2.306 | 0.733 | 1.126 | BLM10_mid |
| TRINITY_DN35585_c0_g2_i1 | 0.915684575 | 0.836026222 | 1 | 1.426 | 0 | 0.633 | 0 | MmgE_PrpD |
| TRINITY_DN30373_c0_g1_i2 | -0.91564886 | 0.582891704 | 0.999970678 | 4.642 | 1.705 | 9.288 | 0.814 | NA |
| TRINITY_DN1505_c0_g1_i3 | 0.915529816 | 0.472767683 | 0.999970678 | 43.031 | 11.47 | 5.1 | 23.033 | NA |
| TRINITY_DN102868_c0_g2_i1 | 0.915461462 | 0.459230439 | 0.999970678 | 0.827 | 0.572 | 0.411 | 0.261 | AAA |
| TRINITY_DN93753_c0_g1_i1 | 0.915353869 | 0.469591595 | 0.999970678 | 1.472 | 0.621 | 0.544 | 0.462 | Exo_endo_phos |
| TRINITY_DN3726_c0_g1_i1 | -0.91528163 | 0.58843985 | 0.999970678 | 0.282 | 1.242 | 0.344 | 2.433 | NA |
| TRINITY_DN3637_c0_g1_i3 | 0.915266205 | 0.564501954 | 0.999970678 | 0.273 | 1.153 | 0.167 | 0.543 | LRRNT_2 |
| TRINITY_DN601_c0_g1_i2 | 0.915216988 | 0.827778517 | 0.999970678 | 0 | 6.11 | 2.633 | 0 | NA |
| TRINITY_DN1474_c0_g1_i1 | 0.915051196 | 0.731819724 | 0.999970678 | 4.869 | 2.148 | 0 | 3.75 | NA |
| TRINITY_DN6107_c0_g1_i1 | -0.91489488 | 0.461800157 | 0.999970678 | 2.816 | 6.386 | 3.133 | 13.281 | NA |

| TRINITY_DN6785_c0_g4_i1 | -0.91487626 | 0.227956408 | 0.999970678 | 0.936 | 1.084 | 1.644 | 1.83 | NA |
| --- | --- | --- | --- | --- | --- | --- | --- | --- |
| TRINITY_DN16551_c0_g1_i2 | 0.914844844 | 0.332230159 | 0.999970678 | 7.049 | 3.183 | 2.7 | 2.212 | NA |
| TRINITY_DN3425_c0_g1_i3 | -0.91484193 | 0.772730806 | 0.999970678 | 1.481 | 0.069 | 2.478 | 0 | NA |
| TRINITY_DN9730_c0_g1_i2 | 0.914819167 | 0.469420659 | 1 | 0.917 | 0.897 | 0.444 | 0.422 | NA |
| TRINITY_DN5856_c0_g1_i1 | 0.914783415 | 0.757716786 | 0.999970678 | 7.758 | 0 | 0.467 | 3.559 | NA |
| TRINITY_DN34583_c0_g1_i1 | 0.914696361 | 0.570771118 | 0.999970678 | 0.663 | 1.39 | 0.122 | 0.935 | DUF4005 |
| TRINITY_DN37502_c0_g1_i3 | 0.914638651 | 0.579935114 | 0.999970678 | 0.59 | 2.602 | 1.233 | 0.201 | Trp_Tyr_perm |
| TRINITY_DN12159_c0_g2_i8 | 0.914483848 | 0.403796141 | 0.999970678 | 0.609 | 1.045 | 0.289 | 0.513 | NA |
| TRINITY_DN2463_c0_g1_i2 | -0.91445192 | 0.746087426 | 0.999970678 | 8.512 | 0 | 9.155 | 5.248 | NA |
| TRINITY_DN5964_c0_g1_i2 | -0.9144378 | 0.528757777 | 0.999970678 | 0.699 | 0.71 | 1.855 | 0.432 | NA |
| TRINITY_DN68625_c0_g1_i3 | 0.914372456 | 0.836582775 | 1 | 0 | 12.62 | 0 | 6.404 | NA |
| TRINITY_DN26213_c0_g2_i7 | 0.914330777 | 0.829592092 | 0.999970678 | 3.288 | 0 | 0 | 1.78 | NA |
| TRINITY_DN4285_c0_g1_i2 | 0.91423639 | 0.664676351 | 1 | 0.736 | 0.246 | 0.411 | 0.02 | NA |
| TRINITY_DN9127_c0_g1_i4 | 0.914149192 | 0.584601873 | 0.999970678 | 0.5 | 0.769 | 0.044 | 0.603 | Peptidase_M50 |
| TRINITY_DN4111_c0_g1_i9 | 0.914142701 | 0.516279486 | 0.999970678 | 7.313 | 6.051 | 4.655 | 1.397 | NA |
| TRINITY_DN1292_c0_g1_i8 | -0.91412985 | 0.833798871 | 0.999970678 | 0 | 0.759 | 0 | 1.397 | NA |
| TRINITY_DN4559_c0_g1_i4 | 0.914070246 | 0.588274501 | 1 | 0.645 | 0.769 | 0.556 | 0.08 | NA |
| TRINITY_DN3029_c0_g1_i1 | -0.91393612 | 0.303590439 | 0.999970678 | 32.193 | 44.54 | 34.098 | 103.603 | UDPG_MGDP_dh |
| TRINITY_DN10444_c0_g3_i2 | -0.91391417 | 0.742075493 | 0.999970678 | 8.748 | 0 | 5.044 | 10.627 | DEAD |
| TRINITY_DN1490_c0_g1_i6 | 0.913888479 | 0.74648353 | 0.999970678 | 2.78 | 0 | 0.878 | 0.442 | AP2 |
| TRINITY_DN10091_c0_g1_i8 | -0.91387438 | 0.309116 | 0.999970678 | 2.78 | 3.42 | 3.011 | 8.013 | NA |
| TRINITY_DN46826_c0_g1_i1 | -0.91387173 | 0.838688599 | 1 | 0.291 | 0 | 0.456 | 0 | Fungal_trans |
| TRINITY_DN12320_c0_g1_i9 | -0.91387173 | 0.838688599 | 1 | 0.627 | 0 | 1 | 0 | p450 |
| TRINITY_DN10518_c0_g2_i1 | -0.91383754 | 0.565176419 | 0.999970678 | 1.535 | 0.808 | 3.155 | 0.583 | NA |
| TRINITY_DN5662_c0_g1_i1 | 0.91353039 | 0.204147906 | 0.999970678 | 7.54 | 11.3 | 3.889 | 5.258 | NA |
| TRINITY_DN7753_c1_g1_i6 | -0.91352554 | 0.828093821 | 0.999970678 | 0 | 0.936 | 0 | 1.739 | Utp12 |
| TRINITY_DN3287_c1_g1_i5 | -0.91350898 | 0.529447085 | 0.999970678 | 0.282 | 0.779 | 1.267 | 0.412 | NA |
| TRINITY_DN2807_c1_g1_i6 | 0.913411066 | 0.228646805 | 0.999970678 | 21.883 | 17.41 | 6.289 | 13.311 | RINGv |
| TRINITY_DN4_c0_g1_i3 | 0.913361913 | 0.417781776 | 0.999970678 | 5.369 | 10.97 | 1.622 | 6.686 | - |
| TRINITY_DN1582_c0_g3_i4 | -0.9133586 | 0.748389051 | 0.999970678 | 11.655 | 0 | 7.933 | 12.748 | CCT |
| TRINITY_DN107257_c0_g1_i1 | -0.91334266 | 0.427767098 | 0.999970678 | 4.397 | 21.25 | 23.165 | 19.816 | Phi_1 |
| TRINITY_DN367_c1_g1_i1 | -0.91315254 | 0.417508129 | 0.999970678 | 36.763 | 82.9 | 40.486 | 170.189 | NA |
| TRINITY_DN1366_c0_g1_i2 | -0.91298581 | 0.741170524 | 0.999970678 | 2.852 | 0 | 0.867 | 4.434 | FH2 |
| TRINITY_DN1358_c0_g1_i7 | 0.912882836 | 0.761774995 | 0.999970678 | 0 | 2.612 | 1.055 | 0.09 | HECT |
| TRINITY_DN2257_c0_g1_i1 | 0.912795866 | 0.465967727 | 0.999970678 | 7.612 | 3.489 | 3.778 | 1.307 | NA |
| TRINITY_DN78_c0_g2_i3 | 0.912772625 | 0.513056537 | 0.999970678 | 3.543 | 2.631 | 2.255 | 0.563 | NA |
| TRINITY_DN2554_c0_g1_i2 | -0.91258513 | 0.48220515 | 0.999970678 | 0.972 | 2.178 | 0.856 | 4.896 | NTP_transf_2 |
| TRINITY_DN57157_c0_g1_i2 | -0.91256444 | 0.530091705 | 0.999970678 | 0.109 | 0.325 | 0.489 | 0.231 | NA |
| TRINITY_DN93_c1_g1_i4 | -0.91233575 | 0.482844179 | 0.999970678 | 1.662 | 5.549 | 2.244 | 10.757 | NA |
| TRINITY_DN1727_c0_g1_i1 | -0.91225786 | 0.730378939 | 0.999970678 | 0 | 2.119 | 1.855 | 1.689 | NA |
| TRINITY_DN2630_c0_g1_i1 | -0.91177968 | 0.352159144 | 0.999970678 | 10.101 | 5.499 | 7.255 | 20.68 | PLAC8 |
| TRINITY_DN519_c1_g1_i6 | 0.911743447 | 0.613254321 | 0.999970678 | 1.617 | 4.908 | 0.111 | 3.278 | BPL_LplA_LipB |
| TRINITY_DN13647_c0_g2_i6 | 0.911600039 | 0.832292054 | 0.999970678 | 0 | 4.524 | 0 | 2.332 | NA |
| TRINITY_DN2416_c0_g1_i16 | 0.911555035 | 0.318393849 | 0.999970678 | 4.07 | 2.129 | 1.6 | 1.387 | Pkinase |
| TRINITY_DN1880_c0_g1_i6 | -0.91151874 | 0.318067974 | 0.999970678 | 1.553 | 3.449 | 3.933 | 4.595 | YEATS |
| TRINITY_DN1949_c0_g1_i5 | -0.91135533 | 0.772545065 | 0.999970678 | 0.209 | 6.248 | 9.899 | 0 | Glyco_transf_8 |
| TRINITY_DN35637_c0_g2_i2 | -0.91130647 | 0.449542087 | 0.999970678 | 0.236 | 0.463 | 0.6 | 0.593 | GST_C_2 |
| TRINITY_DN2931_c0_g2_i4 | -0.91125846 | 0.249231267 | 0.999970678 | 45.71 | 22.8 | 54.986 | 63.087 | - |
| TRINITY_DN9659_c1_g1_i8 | 0.911198081 | 0.741174156 | 1 | 0.409 | 0.266 | 0.3 | 0 | NA |
| TRINITY_DN32027_c0_g2_i1 | -0.91099795 | 0.768035247 | 1 | 3.806 | 0 | 5.544 | 0.462 | Ribosomal_L44 |
| TRINITY_DN967_c0_g1_i6 | -0.91098452 | 0.57627985 | 0.999970678 | 9.465 | 1.429 | 1.844 | 18.499 | AAA |
| TRINITY_DN11932_c0_g1_i4 | 0.910797811 | 0.473441488 | 0.999970678 | 57.41 | 8.82 | 14.232 | 18.278 | NA |
| TRINITY_DN8410_c0_g1_i2 | 0.910794391 | 0.325309138 | 0.999970678 | 3.661 | 6.938 | 1.778 | 3.458 | FAM86 |
| TRINITY_DN10869_c0_g1_i13 | 0.91078761 | 0.450036168 | 1 | 0.318 | 0.237 | 0.133 | 0.141 | Aa_trans |
| TRINITY_DN3777_c0_g2_i1 | -0.91066161 | 0.319014062 | 0.999970678 | 1.572 | 2.612 | 2.489 | 4.816 | NA |
| TRINITY_DN1353_c1_g1_i3 | -0.91062435 | 0.523942884 | 0.999970678 | 1.562 | 7.46 | 2.478 | 13.764 | NA |
| TRINITY_DN5638_c0_g1_i2 | 0.9105883 | 0.49923732 | 0.999970678 | 8.821 | 15.86 | 9.588 | 1.498 | NA |
| TRINITY_DN653_c1_g1_i6 | 0.910578314 | 0.511355173 | 0.999970678 | 0.917 | 2.178 | 0.311 | 1.247 | RBM39linker |
| TRINITY_DN1514_c0_g2_i2 | -0.91050324 | 0.401694603 | 0.999970678 | 8.839 | 5.627 | 4.944 | 21.344 | NA |
| TRINITY_DN108_c0_g1_i1 | -0.91045884 | 0.446750482 | 0.999970678 | 4.142 | 2.927 | 9.166 | 2.242 | - |
| TRINITY_DN15778_c0_g1_i3 | 0.910407007 | 0.445901326 | 0.999970678 | 3.288 | 15.69 | 5 | 3.931 | MOZ_SAS |
| TRINITY_DN81297_c0_g1_i2 | 0.91034664 | 0.839121815 | 1 | 6.477 | 0 | 2.844 | 0 | - |
| TRINITY_DN11964_c0_g1_i14 | -0.91022292 | 0.833076651 | 0.999970678 | 0.981 | 0 | 1.533 | 0 | NA |
| TRINITY_DN4365_c0_g2_i1 | 0.910196122 | 0.258227648 | 0.999970678 | 4.051 | 4.267 | 2.3 | 1.649 | ANAPC8 |
| TRINITY_DN2895_c0_g1_i7 | -0.91019374 | 0.735451048 | 0.999970678 | 0 | 0.65 | 0.322 | 0.814 | PP2C |
| TRINITY_DN1343_c0_g1_i16 | 0.910104357 | 0.834793815 | 0.999970678 | 1.308 | 0 | 0.578 | 0 | NA |
| TRINITY_DN6924_c0_g2_i1 | -0.90997684 | 0.506970855 | 0.999970678 | 4.079 | 2.208 | 7.688 | 2.403 | NA |
| TRINITY_DN604_c0_g1_i8 | -0.90985538 | 0.737821856 | 0.999970678 | 27.824 | 0 | 18.199 | 30.211 | NA |
| TRINITY_DN13028_c0_g1_i4 | -0.90982639 | 0.784069234 | 0.999970678 | 6.949 | 0.069 | 10.977 | 0 | NA |
| TRINITY_DN6433_c0_g1_i1 | -0.90979162 | 0.521312733 | 0.999970678 | 0.091 | 0.394 | 0.389 | 0.442 | LRRNT_2 |
| TRINITY_DN24786_c0_g1_i4 | 0.909777021 | 0.743533842 | 0.999970678 | 4.905 | 36.65 | 0 | 21.032 | NA |
| TRINITY_DN8326_c0_g1_i4 | 0.909716736 | 0.448339284 | 0.999970678 | 3.715 | 2.523 | 2.178 | 0.694 | RVT_1 |
| TRINITY_DN3548_c0_g1_i1 | -0.90967316 | 0.31647582 | 0.999970678 | 17.514 | 16.46 | 14.499 | 46.297 | NERD |
| TRINITY_DN4509_c0_g1_i1 | 0.909671309 | 0.570162681 | 0.999970678 | 5.905 | 0.581 | 0.733 | 2.574 | NA |

| TRINITY_DN8190_c0_g1_i1 | 0.909425537 | 0.832727889 | 0.999970678 | 2.18 | 0 | 0 | 1.176 | NA |
| --- | --- | --- | --- | --- | --- | --- | --- | --- |
| TRINITY_DN2820_c1_g2_i1 | 0.909425537 | 0.832727889 | 0.999970678 | 5.587 | 0 | 0 | 2.966 | NA |
| TRINITY_DN14341_c1_g1_i4 | -0.90930165 | 0.558120275 | 1 | 2.062 | 0.562 | 2.744 | 1.508 | Sugar_tr |
| TRINITY_DN19375_c0_g1_i1 | 0.909290221 | 0.75468434 | 0.999970678 | 0 | 6.623 | 0.411 | 2.966 | NA |
| TRINITY_DN37541_c0_g1_i1 | -0.90926266 | 0.299933264 | 0.999970678 | 342.47 | 150.6 | 310.481 | 553.064 | EF-hand_1 |
| TRINITY_DN5066_c1_g1_i4 | -0.90924175 | 0.439077328 | 0.999970678 | 3.961 | 7.48 | 13.921 | 4.524 | NA |
| TRINITY_DN519_c0_g1_i3 | 0.909181154 | 0.389183282 | 0.999970678 | 9.484 | 3.006 | 3.044 | 3.016 | NA |
| TRINITY_DN12842_c0_g1_i1 | 0.909149719 | 0.736513318 | 0.999970678 | 0.581 | 0.296 | 0.389 | 0 | NA |
| TRINITY_DN432_c0_g1_i5 | -0.90912205 | 0.304926406 | 0.999970678 | 11.864 | 21.2 | 17.355 | 40.335 | NA |
| TRINITY_DN7337_c0_g1_i5 | -0.90906418 | 0.245205788 | 0.999970678 | 3.661 | 3.223 | 4.222 | 7.761 | AAA_11 |
| TRINITY_DN2348_c0_g1_i1 | -0.90897911 | 0.258664934 | 0.999970678 | 1.617 | 1.055 | 2.278 | 2.292 | NA |
| TRINITY_DN18117_c0_g1_i1 | -0.90870822 | 0.573163231 | 0.999970678 | 1.317 | 0.227 | 1.733 | 0.824 | NA |
| TRINITY_DN21_c0_g1_i1 | -0.90865188 | 0.607915948 | 0.999970678 | 1.263 | 8.692 | 1.244 | 16.84 | NA |
| TRINITY_DN6171_c0_g1_i4 | 0.908634267 | 0.591677887 | 0.999970678 | 0.118 | 1.38 | 0.389 | 0.312 | EF_assoc_1 |
| TRINITY_DN6425_c0_g1_i2 | 0.908458845 | 0.493468137 | 0.999970678 | 2.607 | 2.405 | 0.356 | 2.232 | NA |
| TRINITY_DN11724_c0_g2_i7 | -0.90823724 | 0.421204157 | 0.999970678 | 0.454 | 0.355 | 0.844 | 0.483 | NA |
| TRINITY_DN20818_c0_g1_i1 | -0.90818928 | 0.323427155 | 0.999970678 | 9.883 | 12.57 | 9.411 | 30.764 | Ribosomal_L18_c |
| TRINITY_DN20818_c0_g1_i5 | -0.90818928 | 0.323427155 | 0.999970678 | 9.883 | 12.57 | 9.411 | 30.764 | Ribosomal_L18_c |
| TRINITY_DN3721_c0_g1_i11 | -0.90815457 | 0.755994309 | 0.999970678 | 1.444 | 0.217 | 2.6 | 0 | NA |
| TRINITY_DN1548_c0_g1_i4 | -0.90812223 | 0.274974828 | 0.999970678 | 29.714 | 32.77 | 29.809 | 81.033 | NA |
| TRINITY_DN1147_c1_g1_i2 | 0.908107534 | 0.238246813 | 0.999970678 | 4.206 | 6.307 | 1.811 | 3.398 | NA |
| TRINITY_DN5584_c0_g1_i1 | -0.90795282 | 0.295008199 | 0.999970678 | 0.881 | 1.399 | 1.455 | 2.503 | PK |
| TRINITY_DN858_c1_g2_i1 | 0.907835567 | 0.410857179 | 0.999970678 | 39.052 | 33.78 | 26.165 | 7.249 | NA |
| TRINITY_DN30615_c0_g1_i3 | 0.907629495 | 0.484509571 | 0.999970678 | 1.817 | 2.355 | 1.522 | 0.392 | NA |
| TRINITY_DN6052_c0_g1_i13 | 0.907548574 | 0.481842416 | 0.999970678 | 3.143 | 6.258 | 3.166 | 1.126 | NA |
| TRINITY_DN3051_c1_g2_i2 | 0.907534805 | 0.291623306 | 0.999970678 | 2.798 | 5.361 | 1.555 | 2.463 | MFS_1 |
| TRINITY_DN2643_c0_g1_i2 | -0.90749622 | 0.742894093 | 0.999970678 | 16.378 | 0 | 8.922 | 20.288 | NA |
| TRINITY_DN5316_c0_g1_i6 | -0.90743909 | 0.684869095 | 0.999970678 | 0.572 | 147.3 | 195.433 | 36.455 | ThylakoidFormat |
| TRINITY_DN12_c2_g1_i4 | -0.90732975 | 0.469266767 | 0.999970678 | 0.391 | 0.65 | 0.344 | 1.548 | NA |
| TRINITY_DN7933_c0_g4_i2 | -0.90724984 | 0.184582006 | 0.999970678 | 16.297 | 23.08 | 26.21 | 41.683 | NA |
| TRINITY_DN1373_c0_g1_i5 | -0.90689111 | 0.579429723 | 0.999970678 | 0.972 | 0.108 | 1.378 | 0.392 | NA |
| TRINITY_DN6496_c1_g1_i1 | -0.90688031 | 0.425468028 | 0.999970678 | 0.827 | 1.301 | 2.155 | 1.367 | zf-met |
| TRINITY_DN10990_c0_g2_i2 | -0.90682398 | 0.46115845 | 0.999970678 | 0.618 | 0.404 | 1.155 | 0.553 | NA |
| TRINITY_DN11120_c0_g1_i6 | -0.90678912 | 0.601384087 | 0.999970678 | 2.371 | 53.92 | 16.543 | 83.999 | NA |
| TRINITY_DN16547_c0_g1_i1 | 0.906725213 | 0.739754608 | 1 | 0.463 | 0.946 | 0 | 0.744 | NA |
| TRINITY_DN9409_c0_g2_i2 | 0.906623177 | 0.30899251 | 0.999970678 | 25.935 | 43.16 | 10.266 | 23.596 | NA |
| TRINITY_DN2915_c1_g1_i5 | 0.90658587 | 0.740235903 | 0.999970678 | 4.878 | 1.025 | 0 | 3.187 | NA |
| TRINITY_DN4424_c0_g1_i2 | 0.906335587 | 0.241667886 | 0.999970678 | 3.543 | 2.69 | 1.344 | 1.719 | - |
| TRINITY_DN3926_c0_g1_i4 | 0.906330095 | 0.687171192 | 0.999970678 | 3.207 | 0.414 | 1.589 | 0.04 | NA |
| TRINITY_DN17931_c0_g2_i1 | -0.90629354 | 0.429228387 | 0.999970678 | 0.291 | 0.848 | 0.744 | 1.216 | PPR_2 |
| TRINITY_DN467_c0_g1_i6 | 0.906283504 | 0.745903978 | 0.999970678 | 71.454 | 0 | 16.321 | 17.182 | NA |
| TRINITY_DN697_c1_g1_i1 | 0.906245616 | 0.428996104 | 0.999970678 | 0.972 | 3.262 | 0.656 | 1.458 | DAGK_cat |
| TRINITY_DN5919_c0_g5_i2 | -0.90620546 | 0.507439173 | 0.999970678 | 5.668 | 0.897 | 3.511 | 8.184 | RNA_pol_Rbc25 |
| TRINITY_DN3313_c0_g1_i4 | -0.90620318 | 0.408846751 | 0.999970678 | 55.312 | 121.6 | 60.652 | 257.043 | Sugar_tr |
| TRINITY_DN2042_c0_g1_i1 | 0.906159868 | 0.422124517 | 0.999970678 | 1.081 | 1.784 | 0.733 | 0.664 | NA |
| TRINITY_DN11204_c0_g1_i1 | 0.906068571 | 0.153281812 | 0.999970678 | 6.613 | 5.726 | 2.944 | 3.036 | NA |
| TRINITY_DN5006_c0_g1_i4 | 0.906058491 | 0.574232558 | 0.999970678 | 0.863 | 11.39 | 3.9 | 1.719 | - |
| TRINITY_DN6858_c0_g1_i5 | -0.90605355 | 0.841968526 | 1 | 1.117 | 0 | 1.789 | 0 | DUF1993 |
| TRINITY_DN17158_c0_g1_i7 | -0.90605355 | 0.841968526 | 1 | 0.472 | 0 | 0.722 | 0 | NA |
| TRINITY_DN5912_c0_g1_i16 | -0.90604645 | 0.839081937 | 1 | 2.598 | 0 | 4.022 | 0 | GST_C |
| TRINITY_DN36_c0_g1_i2 | -0.90603544 | 0.27460992 | 0.999970678 | 37.162 | 27.51 | 33.576 | 79.052 | NA |
| TRINITY_DN20270_c0_g1_i2 | -0.90595555 | 0.768465853 | 1 | 0.164 | 1.498 | 2.622 | 0 | HLH |
| TRINITY_DN72_c0_g1_i17 | -0.90591609 | 0.396497454 | 0.999970678 | 3.397 | 1.744 | 5.877 | 2.564 | NA |
| TRINITY_DN3376_c0_g1_i5 | 0.905912601 | 0.623969919 | 0.999970678 | 10.192 | 1.202 | 0.289 | 5.831 | Mlo |
| TRINITY_DN2663_c0_g1_i2 | -0.90572938 | 0.474660124 | 0.999970678 | 1.281 | 6.633 | 5.111 | 8.475 | NA |
| TRINITY_DN783_c0_g1_i3 | 0.905688703 | 0.83124835 | 0.999970678 | 8.684 | 0 | 0 | 4.735 | DUF4922 |
| TRINITY_DN220_c0_g1_i17 | 0.905581075 | 0.572465808 | 1 | 0.772 | 1.153 | 0.722 | 0.181 | EGF_CA |
| TRINITY_DN7786_c0_g2_i1 | 0.905548348 | 0.436543905 | 0.999970678 | 5.568 | 4.671 | 0.889 | 4.383 | NA |
| TRINITY_DN11005_c0_g1_i5 | -0.90553304 | 0.739324024 | 0.999970678 | 0.745 | 0 | 0.456 | 0.845 | NA |
| TRINITY_DN15298_c0_g1_i2 | 0.905496791 | 0.418640233 | 0.999970678 | 1.889 | 1.311 | 1.033 | 0.462 | NA |
| TRINITY_DN6960_c0_g1_i4 | 0.905475703 | 0.589142075 | 0.999970678 | 1.444 | 1.961 | 1.422 | 0.121 | NA |
| TRINITY_DN1241_c0_g1_i1 | 0.905432364 | 0.268983368 | 0.999970678 | 7.767 | 7.727 | 3.566 | 3.871 | EXS |
| TRINITY_DN1422_c1_g1_i3 | -0.90537151 | 0.354426519 | 0.999970678 | 1.026 | 1.784 | 2.744 | 1.93 | NA |
| TRINITY_DN2028_c0_g1_i3 | 0.905368001 | 0.537311779 | 0.999970678 | 2.226 | 0.286 | 0.567 | 0.674 | NA |
| TRINITY_DN21250_c0_g2_i1 | 0.905357189 | 0.580658563 | 0.999970678 | 0.917 | 7.086 | 2.211 | 1.538 | Aldedh |
| TRINITY_DN2701_c0_g1_i1 | 0.905215417 | 0.214801473 | 0.999970678 | 7.458 | 5.154 | 3.166 | 2.936 | NA |
| TRINITY_DN26306_c1_g1_i2 | -0.90518739 | 0.570978344 | 0.999970678 | 1.79 | 1.271 | 4.2 | 0.664 | NA |
| TRINITY_DN1873_c3_g1_i4 | -0.90518003 | 0.829631124 | 0.999970678 | 0 | 4.277 | 6.488 | 0 | NA |
| TRINITY_DN28836_c2_g1_i1 | 0.904951315 | 0.593326446 | 1 | 1.126 | 1.035 | 0.856 | 0.131 | NA |
| TRINITY_DN4825_c0_g1_i2 | 0.904923885 | 0.50635892 | 0.999970678 | 1.726 | 1.735 | 1.322 | 0.241 | NA |
| TRINITY_DN6902_c0_g1_i7 | -0.9048343 | 0.534667607 | 0.999970678 | 0.745 | 0.306 | 0.411 | 1.438 | NA |
| TRINITY_DN12546_c0_g1_i9 | -0.90480959 | 0.785378827 | 1 | 2.335 | 0.099 | 3.744 | 0 | Ribosomal_L1 |
| TRINITY_DN478_c1_g1_i1 | 0.904708549 | 0.740360199 | 1 | 3.688 | 2.493 | 2.733 | 0 | NA |
| TRINITY_DN5272_c0_g2_i8 | 0.904660119 | 0.731122468 | 0.999970678 | 0.936 | 0.877 | 0 | 0.985 | DUF3007 |

| TRINITY_DN3807_c0_g1_i1 | 0.904568684 | 0.436819006 | 0.999970678 | 7.83 | 5.854 | 1.222 | 5.811 | NA |
| --- | --- | --- | --- | --- | --- | --- | --- | --- |
| TRINITY_DN41259_c0_g1_i1 | 0.904474334 | 0.770467699 | 0.999970678 | 0 | 2476 | 711.124 | 390.335 | NA |
| TRINITY_DN3920_c0_g1_i3 | -0.9044339 | 0.542551579 | 0.999970678 | 6.995 | 1.557 | 10.522 | 3.318 | NA |
| TRINITY_DN1438_c0_g1_i2 | -0.90442271 | 0.433469573 | 0.999970678 | 37.807 | 7.766 | 32.542 | 46.951 | NA |
| TRINITY_DN14712_c0_g1_i3 | -0.90439639 | 0.651103573 | 0.999970678 | 1.263 | 0.276 | 2.278 | 0.141 | NA |
| TRINITY_DN18906_c0_g1_i5 | 0.904383028 | 0.759309509 | 1 | 1.799 | 0.227 | 0.889 | 0 | Glyco_hydro_6 |
| TRINITY_DN26074_c0_g1_i1 | 0.904339909 | 0.428193577 | 0.999970678 | 0.69 | 0.897 | 0.178 | 0.633 | Kinesin |
| TRINITY_DN29686_c0_g1_i2 | 0.904326529 | 0.556642023 | 0.999970678 | 0.445 | 2.848 | 0.644 | 0.945 | Usp |
| TRINITY_DN2542_c0_g2_i2 | 0.904318804 | 0.750667149 | 0.999970678 | 15.316 | 0 | 3.911 | 3.569 | NA |
| TRINITY_DN20528_c0_g1_i1 | -0.90430309 | 0.834754684 | 0.999970678 | 18.613 | 0 | 0 | 35.389 | NA |
| TRINITY_DN8067_c0_g1_i2 | 0.904113077 | 0.379755199 | 0.999970678 | 3.47 | 4.376 | 1.233 | 2.644 | NA |
| TRINITY_DN1991_c0_g1_i7 | 0.904029177 | 0.764480005 | 0.999970678 | 4.805 | 0.138 | 0 | 2.694 | Serpin |
| TRINITY_DN698_c0_g1_i4 | -0.90395373 | 0.31814526 | 0.999970678 | 13.39 | 36.17 | 40.309 | 43.171 | NA |
| TRINITY_DN2770_c0_g1_i2 | -0.90387067 | 0.26041144 | 0.999970678 | 30.504 | 50.07 | 44.031 | 97.29 | NA |
| TRINITY_DN91777_c0_g1_i1 | 0.903785674 | 0.476692055 | 0.999970678 | 1.463 | 1.725 | 0.411 | 1.196 | NA |
| TRINITY_DN22664_c0_g1_i4 | -0.90373242 | 0.776035863 | 1 | 0.89 | 0.059 | 1.5 | 0 | NA |
| TRINITY_DN11950_c0_g2_i6 | 0.903673551 | 0.73424809 | 0.999970678 | 0.917 | 1.124 | 0 | 1.116 | NA |
| TRINITY_DN5331_c0_g4_i1 | -0.90362364 | 0.55441297 | 0.999970678 | 3.997 | 0.434 | 4.2 | 3.288 | NA |
| TRINITY_DN16265_c0_g2_i4 | -0.90355729 | 0.501919312 | 0.999970678 | 3.207 | 0.552 | 3.455 | 2.976 | NA |
| TRINITY_DN146_c0_g1_i4 | 0.903323726 | 0.830092207 | 0.999970678 | 2.407 | 0 | 0 | 1.307 | KH_dom-like |
| TRINITY_DN51673_c0_g1_i5 | 0.903297824 | 0.738072864 | 0.999970678 | 0.908 | 2.395 | 1.455 | 0 | Glyco_transf_20 |
| TRINITY_DN1347_c2_g1_i2 | 0.903287499 | 0.732024307 | 0.999970678 | 0.527 | 0.788 | 0 | 0.694 | Frigida |
| TRINITY_DN1035_c0_g2_i9 | 0.903231278 | 0.632447011 | 0.999970678 | 34.083 | 0.917 | 12.988 | 3.267 | Pyrophosphatase |
| TRINITY_DN1092_c0_g1_i5 | 0.903093062 | 0.830966986 | 0.999970678 | 0 | 11.11 | 4.833 | 0 | NA |
| TRINITY_DN53331_c0_g1_i2 | 0.903011766 | 0.303704847 | 0.999970678 | 3.406 | 2.582 | 1.167 | 1.8 | NA |
| TRINITY_DN801_c1_g1_i4 | -0.90296381 | 0.267126364 | 0.999970678 | 20.984 | 10.35 | 22.199 | 32.061 | Peptidase_S10 |
| TRINITY_DN3063_c0_g1_i6 | -0.90291616 | 0.541764078 | 0.999970678 | 5.986 | 1.646 | 4.111 | 8.847 | NA |
| TRINITY_DN64889_c0_g1_i1 | 0.902890529 | 0.504705272 | 0.999970678 | 0.872 | 4.06 | 1.2 | 1.176 | - |
| TRINITY_DN18671_c0_g1_i1 | -0.90259456 | 0.838966916 | 1 | 0 | 2.385 | 0 | 4.313 | NA |
| TRINITY_DN194920_c0_g1_i1 | 0.90233762 | 0.631009532 | 0.999970678 | 1.617 | 0.177 | 0.156 | 0.774 | NA |
| TRINITY_DN2789_c0_g1_i1 | -0.9022729 | 0.188968564 | 0.999970678 | 3.679 | 3.883 | 6.733 | 6.002 | NA |
| TRINITY_DN4038_c0_g1_i1 | 0.90217969 | 0.544508501 | 1 | 2.144 | 1.242 | 0.356 | 1.397 | Sybindin |
| TRINITY_DN8736_c0_g3_i4 | -0.90216339 | 0.501811989 | 0.999970678 | 0.454 | 2.641 | 3.166 | 1.95 | IBR |
| TRINITY_DN10914_c0_g1_i4 | -0.90210561 | 0.454967452 | 0.999970678 | 0.745 | 0.503 | 0.744 | 1.468 | DEAD |
| TRINITY_DN750_c5_g1_i3 | -0.90204303 | 0.478266813 | 0.999970678 | 6.713 | 1.202 | 4.722 | 9.219 | NA |
| TRINITY_DN2259_c0_g1_i1 | -0.90187249 | 0.738906475 | 0.999970678 | 0 | 0.808 | 0.5 | 0.895 | NA |
| TRINITY_DN9749_c0_g2_i2 | 0.901835573 | 0.460155841 | 0.999970678 | 0.745 | 0.769 | 0.211 | 0.563 | NA |
| TRINITY_DN3311_c0_g1_i3 | 0.901697631 | 0.370600644 | 0.999970678 | 55.53 | 38.01 | 31.698 | 11.944 | AUX_IAA |
| TRINITY_DN24298_c0_g2_i1 | -0.90169296 | 0.835542059 | 0.999970678 | 0 | 6.258 | 9.433 | 0 | NA |
| TRINITY_DN107_c0_g1_i1 | -0.90169195 | 0.610330774 | 0.999970678 | 7.14 | 1.991 | 13.633 | 0.714 | DOG1 |
| TRINITY_DN6773_c0_g1_i3 | -0.90167649 | 0.764449757 | 1 | 0 | 1.281 | 1.767 | 0.211 | NA |
| TRINITY_DN2052_c2_g1_i1 | 0.901574379 | 0.546066706 | 0.999970678 | 0.245 | 1.015 | 0.189 | 0.452 | NA |
| TRINITY_DN15564_c1_g1_i2 | 0.901461628 | 0.374337453 | 0.999970678 | 7.085 | 2.415 | 2.267 | 2.393 | TruD |
| TRINITY_DN395_c1_g1_i5 | 0.901333604 | 0.478921633 | 0.999970678 | 0.772 | 2.641 | 1.078 | 0.523 | Glyco_transf_8 |
| TRINITY_DN13649_c0_g1_i4 | -0.90132671 | 0.449549389 | 0.999970678 | 0.89 | 0.473 | 1.344 | 0.925 | NA |
| TRINITY_DN12372_c0_g1_i1 | 0.901275264 | 0.239740968 | 0.999970678 | 2.952 | 2.562 | 1.555 | 1.086 | DDT |
| TRINITY_DN2140_c0_g2_i4 | 0.901270481 | 0.332173662 | 0.999970678 | 2.562 | 2.079 | 1.378 | 0.845 | CTD_bind |
| TRINITY_DN6689_c0_g2_i3 | 0.90124032 | 0.657951375 | 0.999970678 | 3.043 | 4.395 | 3.166 | 0.101 | NA |
| TRINITY_DN2456_c0_g1_i6 | -0.90122178 | 0.746457651 | 0.999970678 | 27.979 | 0 | 22.91 | 25.114 | NA |
| TRINITY_DN4336_c0_g1_i2 | -0.90116249 | 0.752241519 | 0.999970678 | 0 | 17.81 | 13.588 | 16.377 | NA |
| TRINITY_DN633_c0_g1_i6 | 0.901159651 | 0.441931069 | 0.999970678 | 2.525 | 3.814 | 0.522 | 2.765 | NA |
| TRINITY_DN15682_c0_g1_i13 | -0.90115332 | 0.835976992 | 0.999970678 | 1.399 | 0 | 2.178 | 0 | NA |
| TRINITY_DN765_c0_g1_i4 | 0.900987587 | 0.357817391 | 0.999970678 | 10.456 | 7.135 | 2.089 | 6.937 | UBX |
| TRINITY_DN19983_c0_g1_i1 | -0.90092116 | 0.752229025 | 1 | 0.581 | 0 | 0.244 | 0.804 | - |
| TRINITY_DN2124_c0_g1_i1 | -0.90091573 | 0.294355827 | 0.999970678 | 19.676 | 18.84 | 18.254 | 49.132 | NA |
| TRINITY_DN10392_c0_g1_i1 | 0.900691839 | 0.754002989 | 1 | 1.653 | 0.355 | 0.889 | 0 | NA |
| TRINITY_DN1571_c0_g1_i2 | 0.90065341 | 0.46947218 | 0.999970678 | 31.53 | 5.263 | 8.355 | 9.802 | ELK |
| TRINITY_DN23313_c0_g1_i4 | 0.900557018 | 0.602987679 | 0.999970678 | 0.136 | 1.439 | 0.211 | 0.583 | PPR |
| TRINITY_DN5819_c1_g3_i1 | 0.900522987 | 0.628308218 | 0.999970678 | 2.026 | 0.237 | 0.833 | 0.221 | NA |
| TRINITY_DN3541_c0_g2_i4 | -0.90051247 | 0.456227273 | 0.999970678 | 3.034 | 3.666 | 7.233 | 3.639 | A_thal_3526 |
| TRINITY_DN2287_c2_g1_i2 | 0.900504079 | 0.631963725 | 0.999970678 | 1.135 | 29.26 | 1.522 | 14.226 | p450 |
| TRINITY_DN3024_c0_g1_i1 | -0.90018433 | 0.553333 | 0.999970678 | 0.236 | 1.794 | 0.978 | 2.564 | NA |
| TRINITY_DN1594_c0_g1_i3 | 0.900157527 | 0.336478471 | 0.999970678 | 8.33 | 21.94 | 6.311 | 8.465 | NA |
| TRINITY_DN388_c0_g1_i7 | -0.90014096 | 0.761546223 | 1 | 0 | 0.877 | 1.167 | 0.211 | NA |
| TRINITY_DN4644_c2_g1_i3 | 0.899918658 | 0.496677278 | 0.999970678 | 1.172 | 3.518 | 0.367 | 2.061 | NA |
| TRINITY_DN361_c0_g1_i5 | -0.8998345 | 0.733653083 | 0.999970678 | 0 | 1.932 | 1.489 | 1.719 | Ins134_P3_kin |
| TRINITY_DN10976_c0_g1_i2 | 0.899756609 | 0.149239345 | 0.999970678 | 22.065 | 16.93 | 8.399 | 10.848 | APH |
| TRINITY_DN469_c0_g1_i2 | 0.899576925 | 0.394002052 | 0.999970678 | 49.389 | 16.57 | 9.677 | 23.958 | p450 |
| TRINITY_DN17504_c0_g1_i1 | -0.89950577 | 0.511868722 | 0.999970678 | 5.305 | 19.9 | 5.844 | 39.682 | NA |
| TRINITY_DN679_c0_g1_i7 | -0.89950003 | 0.442476423 | 0.999970678 | 0.818 | 2.079 | 3.144 | 1.599 | NA |
| TRINITY_DN4612_c0_g1_i8 | 0.899315242 | 0.590553689 | 0.999970678 | 0.164 | 2.759 | 0.833 | 0.533 | NA |
| TRINITY_DN18933_c2_g1_i1 | -0.89926687 | 0.24891724 | 0.999970678 | 104.47 | 132 | 240.652 | 147.548 | UPF0047 |
| TRINITY_DN8422_c0_g1_i6 | -0.89924888 | 0.739718538 | 0.999970678 | 4.16 | 0 | 4.522 | 2.363 | ETF |
| TRINITY_DN11418_c0_g1_i2 | -0.89919798 | 0.516924839 | 0.999970678 | 16.278 | 2.385 | 20.932 | 9.812 | NA |

| TRINITY_DN19247_c0_g1_i1 | 0.899171407 | 0.533480043 | 0.999970678 | 0.254 | 0.769 | 0.344 | 0.131 | NA |
| --- | --- | --- | --- | --- | --- | --- | --- | --- |
| TRINITY_DN29240_c0_g1_i2 | 0.899138832 | 0.710560194 | 0.999970678 | 0.418 | 0.02 | 0.189 | 0.01 | zf-C3HC4_3 |
| TRINITY_DN1279_c0_g1_i1 | 0.899128933 | 0.282952964 | 0.999970678 | 1.935 | 1.754 | 0.833 | 0.965 | DUF1639 |
| TRINITY_DN835_c3_g1_i2 | 0.899120683 | 0.583143064 | 0.999970678 | 1.508 | 1.981 | 1.467 | 0.09 | NA |
| TRINITY_DN298_c0_g2_i4 | 0.899038557 | 0.589337656 | 0.999970678 | 1.499 | 1.094 | 0.089 | 1.297 | NA |
| TRINITY_DN11477_c0_g1_i1 | 0.898922861 | 0.5515398 | 1 | 0.736 | 0.66 | 0.511 | 0.131 | RRM_1 |
| TRINITY_DN2819_c0_g1_i2 | 0.898922557 | 0.741131549 | 0.999970678 | 10.655 | 15.73 | 0 | 14.115 | Pectinesterase |
| TRINITY_DN3155_c0_g1_i3 | -0.89873284 | 0.357743883 | 0.999970678 | 3.661 | 4.494 | 3.811 | 10.365 | NA |
| TRINITY_DN7212_c0_g1_i2 | 0.898307326 | 0.840175401 | 1 | 5.523 | 0 | 2.5 | 0 | NA |
| TRINITY_DN4318_c0_g1_i2 | 0.898267074 | 0.280669426 | 0.999970678 | 1.753 | 2.365 | 0.856 | 1.166 | Ank |
| TRINITY_DN3996_c0_g1_i2 | 0.898232722 | 0.839694685 | 1 | 0.945 | 0 | 0 | 0.523 | NA |
| TRINITY_DN41955_c0_g1_i6 | -0.89818944 | 0.547031656 | 0.999970678 | 17.559 | 3.085 | 23.81 | 9.068 | LRRNT_2 |
| TRINITY_DN6285_c0_g1_i2 | 0.898166143 | 0.602264827 | 0.999970678 | 1.88 | 0.286 | 0.833 | 0.171 | NA |
| TRINITY_DN45501_c0_g1_i1 | 0.898082791 | 0.836187084 | 0.999970678 | 3.016 | 0 | 1.355 | 0 | NA |
| TRINITY_DN16616_c1_g4_i1 | -0.89782715 | 0.842130368 | 1 | 2.48 | 0 | 3.855 | 0 | NA |
| TRINITY_DN676_c0_g1_i4 | 0.897804028 | 0.592135164 | 0.999970678 | 1.381 | 0.158 | 0.544 | 0.181 | 2OG-FeII_Oxy |
| TRINITY_DN8981_c0_g1_i7 | -0.89775636 | 0.231656586 | 0.999970678 | 3.443 | 4.691 | 5.833 | 7.993 | NA |
| TRINITY_DN5632_c1_g1_i4 | -0.89769994 | 0.738606848 | 0.999970678 | 2.562 | 0 | 2.389 | 1.94 | NA |
| TRINITY_DN1794_c0_g1_i6 | 0.89765812 | 0.304293409 | 0.999970678 | 8.012 | 12.64 | 3.178 | 7.178 | NA |
| TRINITY_DN1336_c0_g1_i1 | -0.89744968 | 0.736908244 | 0.999970678 | 0 | 1.055 | 0.967 | 0.794 | NA |
| TRINITY_DN4770_c0_g1_i2 | -0.89728303 | 0.234961149 | 0.999970678 | 10.71 | 12.53 | 23.254 | 15.181 | NA |
| TRINITY_DN4624_c0_g1_i5 | 0.897208432 | 0.449404316 | 0.999970678 | 3.397 | 14.28 | 2.878 | 5.831 | MBD |
| TRINITY_DN4153_c0_g1_i15 | 0.897146308 | 0.479479572 | 0.999970678 | 0.372 | 1.202 | 0.289 | 0.493 | AMPK1_CBM |
| TRINITY_DN1831_c2_g1_i2 | -0.89710638 | 0.1806947 | 0.999970678 | 3.652 | 3.223 | 4.889 | 6.877 | FtsZ_C |
| TRINITY_DN923_c0_g1_i4 | 0.897100455 | 0.350300642 | 0.999970678 | 9.929 | 9.333 | 6.366 | 2.674 | NA |
| TRINITY_DN1634_c0_g3_i1 | 0.896983031 | 0.458315322 | 0.999970678 | 0.899 | 3.489 | 0.733 | 1.458 | NA |
| TRINITY_DN5973_c0_g1_i5 | 0.896961938 | 0.768333398 | 0.999970678 | 3.034 | 0 | 0.111 | 1.548 | NA |
| TRINITY_DN164747_c0_g1_i1 | -0.89692255 | 0.697089022 | 0.999970678 | 23.209 | 0.049 | 16.71 | 23.697 | NA |
| TRINITY_DN3551_c0_g1_i7 | 0.896785123 | 0.334852031 | 0.999970678 | 4.26 | 5.322 | 3.011 | 1.498 | Hyccin |
| TRINITY_DN1432_c0_g2_i4 | 0.896501107 | 0.417457353 | 0.999970678 | 0.627 | 1.114 | 0.533 | 0.292 | - |
| TRINITY_DN1277_c0_g1_i3 | -0.89647639 | 0.617569878 | 0.999970678 | 27.234 | 0.67 | 14.999 | 34.555 | NA |
| TRINITY_DN2500_c0_g1_i2 | 0.896447301 | 0.609442626 | 0.999970678 | 0.282 | 3.282 | 0.367 | 1.448 | NMO |
| TRINITY_DN2103_c0_g1_i2 | -0.89640324 | 0.225363504 | 0.999970678 | 2084.7 | 1194 | 2788.609 | 2694.471 | - |
| TRINITY_DN4185_c0_g1_i1 | 0.896400391 | 0.595854746 | 0.999970678 | 6.041 | 0.887 | 1.022 | 2.564 | NA |
| TRINITY_DN5051_c0_g1_i2 | -0.89637731 | 0.562635594 | 0.999970678 | 13.826 | 1.311 | 17.166 | 7.671 | NA |
| TRINITY_DN43290_c0_g1_i1 | 0.896224589 | 0.453570172 | 0.999970678 | 1.953 | 1.35 | 0.656 | 0.965 | NA |
| TRINITY_DN13221_c0_g1_i3 | 0.896201191 | 0.48379669 | 0.999970678 | 0.254 | 0.926 | 0.256 | 0.322 | NA |
| TRINITY_DN326_c0_g1_i2 | -0.89618948 | 0.436008556 | 0.999970678 | 2.216 | 5.263 | 2.789 | 10.396 | Bap31 |
| TRINITY_DN480_c0_g1_i2 | -0.89617272 | 0.737550086 | 0.999970678 | 2.407 | 0 | 1.567 | 2.674 | Bystin |
| TRINITY_DN7162_c0_g1_i2 | 0.89616657 | 0.476230494 | 0.999970678 | 1.535 | 1.242 | 1.033 | 0.241 | NA |
| TRINITY_DN3890_c0_g1_i3 | 0.896137549 | 0.733636425 | 0.999970678 | 1.363 | 2.661 | 0 | 2.151 | DUF1771 |
| TRINITY_DN1802_c0_g1_i1 | -0.8960338 | 0.400694164 | 0.999970678 | 0.5 | 0.67 | 0.644 | 1.408 | NA |
| TRINITY_DN698_c2_g1_i14 | 0.895839648 | 0.735582259 | 0.999970678 | 2.516 | 1.912 | 1.978 | 0 | DUF659 |
| TRINITY_DN5534_c0_g1_i5 | 0.895776496 | 0.737593151 | 0.999970678 | 0.445 | 0.345 | 0.356 | 0 | Malectin_like |
| TRINITY_DN319_c0_g1_i2 | -0.89577565 | 0.749133211 | 0.999970678 | 0.917 | 0 | 1.167 | 0.332 | MFS_1 |
| TRINITY_DN1680_c0_g2_i2 | -0.89577502 | 0.294265296 | 0.999970678 | 5.423 | 2.848 | 6.411 | 7.651 | - |
| TRINITY_DN234_c2_g1_i3 | 0.895707172 | 0.475397412 | 0.999970678 | 1.799 | 5.519 | 2.222 | 1.186 | NA |
| TRINITY_DN4974_c0_g1_i6 | -0.89569601 | 0.836120458 | 0.999970678 | 0 | 0.443 | 0 | 0.814 | Myb_DNA-bind_ |
| TRINITY_DN1435_c0_g1_i10 | -0.89567449 | 0.713166105 | 0.999970678 | 5.796 | 0.463 | 9.722 | 0.101 | GTP_EFTU |
| TRINITY_DN6965_c0_g1_i2 | -0.89561228 | 0.248468709 | 0.999970678 | 23.927 | 23.67 | 26.032 | 55.527 | NA |
| TRINITY_DN1421_c0_g1_i2 | 0.895532878 | 0.388106493 | 0.999970678 | 13.544 | 24.11 | 12.333 | 5.168 | DEAD |
| TRINITY_DN296_c0_g1_i1 | -0.89550807 | 0.368547398 | 0.999970678 | 0.663 | 0.542 | 1.289 | 0.684 | PPR |
| TRINITY_DN3025_c0_g1_i3 | -0.89547096 | 0.273592966 | 0.999970678 | 1.735 | 1.281 | 2.578 | 2.493 | NA |
| TRINITY_DN5804_c0_g1_i1 | 0.895408282 | 0.559522809 | 1 | 0.418 | 0.641 | 0.378 | 0.101 | NA |
| TRINITY_DN30164_c0_g3_i1 | -0.89538816 | 0.837790727 | 0.999970678 | 2.062 | 0 | 3.2 | 0 | NA |
| TRINITY_DN1022_c0_g1_i2 | 0.895253337 | 0.831099058 | 0.999970678 | 9.965 | 0 | 0 | 5.399 | NA |
| TRINITY_DN16781_c0_g1_i1 | 0.895202568 | 0.320148371 | 0.999970678 | 9.493 | 10.1 | 2.578 | 7.42 | bZIP_2 |
| TRINITY_DN8125_c0_g1_i4 | -0.89515993 | 0.236274018 | 0.999970678 | 1.671 | 1.932 | 2.411 | 3.75 | NA |
| TRINITY_DN1855_c0_g1_i1 | -0.89503348 | 0.377805255 | 0.999970678 | 24.736 | 14.23 | 45.031 | 18.318 | NA |
| TRINITY_DN16950_c0_g1_i5 | -0.89471079 | 0.832725148 | 0.999970678 | 2.425 | 0 | 3.8 | 0 | NA |
| TRINITY_DN2812_c0_g4_i1 | 0.894697664 | 0.3808856 | 0.999970678 | 1.417 | 3.469 | 0.678 | 1.8 | FKS1_dom1 |
| TRINITY_DN9558_c0_g1_i4 | -0.8946341 | 0.551322061 | 0.999970678 | 0.382 | 0.838 | 0.3 | 1.86 | PP2C |
| TRINITY_DN6364_c1_g1_i7 | -0.89462664 | 0.743865221 | 0.999970678 | 3.888 | 0 | 3.933 | 2.604 | Pkinase |
| TRINITY_DN24340_c1_g1_i4 | -0.89457942 | 0.430349876 | 0.999970678 | 45.556 | 36.36 | 22.088 | 125.902 | NA |
| TRINITY_DN4588_c1_g1_i3 | 0.894570084 | 0.368163783 | 0.999970678 | 105.7 | 37.82 | 41.009 | 28.201 | NA |
| TRINITY_DN423_c0_g1_i5 | -0.89446359 | 0.370016244 | 0.999970678 | 1.399 | 1.064 | 1.378 | 2.855 | NA |
| TRINITY_DN1347_c3_g1_i2 | 0.894442769 | 0.365107215 | 0.999970678 | 18.858 | 8.899 | 8.699 | 4.514 | NA |
| TRINITY_DN717_c0_g1_i6 | -0.89435352 | 0.427797818 | 0.999970678 | 93.119 | 20.14 | 85.395 | 109.434 | Band_7 |
| TRINITY_DN1794_c0_g1_i2 | -0.89433797 | 0.635794786 | 0.999970678 | 0.845 | 0.03 | 0.744 | 0.754 | NA |
| TRINITY_DN2751_c0_g2_i1 | 0.894324412 | 0.41979063 | 0.999970678 | 7.54 | 12.38 | 7.011 | 2.202 | NA |
| TRINITY_DN10399_c0_g3_i1 | -0.8943237 | 0.758450068 | 0.999970678 | 1.317 | 0 | 1.911 | 0.201 | COG6 |
| TRINITY_DN4969_c0_g1_i3 | 0.894233565 | 0.202697548 | 0.999970678 | 16.705 | 14.71 | 5.389 | 10.446 | NA |
| TRINITY_DN1180_c2_g1_i3 | -0.89416822 | 0.373078852 | 0.999970678 | 1.063 | 2.592 | 2.089 | 4.253 | NA |
| TRINITY_DN32070_c0_g1_i3 | -0.89416797 | 0.440466623 | 0.999970678 | 1.19 | 0.956 | 2.289 | 1.317 | NA |

| TRINITY_DN3894_c0_g1_i9 | -0.89406788 | 0.347608663 | 0.999970678 | 0.727 | 0.493 | 0.7 | 1.458 | - |
| --- | --- | --- | --- | --- | --- | --- | --- | --- |
| TRINITY_DN103_c0_g1_i5 | 0.894020054 | 0.13510404 | 0.999970678 | 62.652 | 61.93 | 31.265 | 29.357 | NA |
| TRINITY_DN3406_c0_g1_i1 | 0.894007231 | 0.484218344 | 0.999970678 | 2.244 | 11.9 | 2.733 | 4.223 | Response_reg |
| TRINITY_DN5203_c0_g1_i1 | -0.8939737 | 0.702574292 | 0.999970678 | 0.363 | 5.549 | 0.322 | 10.084 | NA |
| TRINITY_DN2536_c8_g2_i2 | -0.89391567 | 0.76165287 | 0.999970678 | 1.726 | 0 | 2.522 | 0.201 | NAD_binding_10 |
| TRINITY_DN9229_c0_g1_i2 | -0.89383632 | 0.832337542 | 0.999970678 | 9.729 | 0 | 15.021 | 0 | NA |
| TRINITY_DN2611_c0_g1_i2 | -0.89373476 | 0.752675405 | 0.999970678 | 41.786 | 0 | 33.965 | 37.4 | NA |
| TRINITY_DN1617_c0_g1_i14 | 0.893668901 | 0.278700245 | 0.999970678 | 1.98 | 2.158 | 0.989 | 1.056 | NA |
| TRINITY_DN1180_c0_g2_i1 | -0.89353649 | 0.246209163 | 0.999970678 | 6.014 | 11.57 | 14.077 | 15.493 | DSPc |
| TRINITY_DN584_c0_g1_i5 | -0.89342479 | 0.503029195 | 0.999970678 | 8.448 | 8.919 | 21.221 | 5.63 | NA |
| TRINITY_DN1509_c0_g1_i5 | -0.89342227 | 0.756299149 | 0.999970678 | 0 | 14.93 | 6.8 | 19.193 | NA |
| TRINITY_DN4516_c1_g1_i1 | -0.89338994 | 0.750568918 | 0.999970678 | 1.372 | 0 | 0.344 | 2.162 | - |
| TRINITY_DN4558_c0_g1_i3 | -0.89323596 | 0.765135768 | 0.999970678 | 4.342 | 0.325 | 0 | 8.807 | B56 |
| TRINITY_DN16887_c1_g1_i3 | -0.89308385 | 0.602897781 | 0.999970678 | 10.41 | 0.394 | 9.899 | 8.345 | NA |
| TRINITY_DN1679_c0_g1_i2 | 0.893057958 | 0.551877877 | 0.999970678 | 0.572 | 5.618 | 1.111 | 1.971 | SPC22 |
| TRINITY_DN1298_c0_g1_i1 | 0.892933363 | 0.757810661 | 0.999970678 | 4.905 | 0 | 0.422 | 2.192 | NB-ARC |
| TRINITY_DN229_c0_g1_i3 | 0.892917907 | 0.400123196 | 0.999970678 | 12.727 | 44.3 | 16.043 | 11.049 | AdoHcyase |
| TRINITY_DN29526_c0_g1_i1 | -0.89284909 | 0.614631705 | 0.999970678 | 347.71 | 7.963 | 302.448 | 305.079 | Aldedh |
| TRINITY_DN10084_c0_g1_i5 | -0.89277549 | 0.836674548 | 0.999970678 | 2.643 | 0 | 4.078 | 0 | Malate_synthase |
| TRINITY_DN11988_c0_g1_i3 | -0.8927356 | 0.470018931 | 0.999970678 | 1.69 | 1.636 | 1.211 | 4.605 | NA |
| TRINITY_DN4952_c0_g1_i7 | -0.89271408 | 0.842746734 | 1 | 0 | 0.266 | 0.4 | 0 | DUF659 |
| TRINITY_DN1899_c0_g1_i2 | 0.892663716 | 0.44690813 | 0.999970678 | 0.963 | 0.788 | 0.211 | 0.694 | NA |
| TRINITY_DN460_c0_g2_i1 | 0.892470047 | 0.258450283 | 0.999970678 | 9.293 | 5.558 | 3.878 | 3.368 | DOG1 |
| TRINITY_DN6967_c0_g2_i1 | 0.892302189 | 0.540667321 | 0.999970678 | 4.678 | 0.572 | 1.389 | 1.186 | NA |
| TRINITY_DN9829_c0_g1_i2 | 0.892208398 | 0.445569015 | 0.999970678 | 4.624 | 1.833 | 2.189 | 0.855 | NA |
| TRINITY_DN6024_c0_g1_i4 | -0.89220522 | 0.391756077 | 0.999970678 | 214.18 | 142.5 | 396.243 | 149.689 | NA |
| TRINITY_DN3942_c1_g1_i1 | 0.892201077 | 0.785469781 | 0.999970678 | 40.36 | 0 | 0.233 | 21.656 | NA |
| TRINITY_DN10578_c0_g1_i7 | 0.89207519 | 0.544309931 | 0.999970678 | 1.326 | 1.567 | 1.078 | 0.292 | NA |
| TRINITY_DN6811_c0_g1_i2 | -0.89206056 | 0.55276716 | 0.999970678 | 3.025 | 13.09 | 2.722 | 26.381 | NA |
| TRINITY_DN10032_c0_g2_i1 | 0.891934211 | 0.628744778 | 0.999970678 | 0.3 | 2.74 | 0.167 | 1.428 | NA |
| TRINITY_DN224_c0_g1_i4 | -0.89192742 | 0.754762551 | 0.999970678 | 0 | 2.247 | 3.089 | 0.392 | TRM |
| TRINITY_DN3819_c0_g1_i2 | 0.89188394 | 0.738068827 | 0.999970678 | 1.953 | 0.857 | 0 | 1.528 | Cellulose_synt |
| TRINITY_DN13094_c0_g1_i4 | 0.891847269 | 0.276359624 | 0.999970678 | 3.588 | 2.809 | 1.567 | 1.578 | NA |
| TRINITY_DN1150_c0_g1_i3 | 0.891773817 | 0.377385687 | 0.999970678 | 0.745 | 0.71 | 0.322 | 0.412 | NA |
| TRINITY_DN1334_c0_g1_i12 | -0.89168908 | 0.483461215 | 0.999970678 | 4.297 | 9.047 | 3.055 | 21.022 | NA |
| TRINITY_DN2649_c0_g1_i2 | 0.891598163 | 0.739395337 | 0.999970678 | 2.18 | 3.637 | 2.578 | 0 | NA |
| TRINITY_DN2661_c0_g1_i1 | 0.891597049 | 0.57164026 | 0.999970678 | 37.171 | 10.3 | 17.854 | 3.489 | NA |
| TRINITY_DN1357_c0_g1_i2 | 0.891573536 | 0.742318179 | 0.999970678 | 1.344 | 1.764 | 1.378 | 0 | Arm |
| TRINITY_DN5787_c1_g1_i4 | -0.8915262 | 0.832640279 | 0.999970678 | 3.216 | 0 | 4.989 | 0 | Spermine_synt_N |
| TRINITY_DN804_c1_g1_i3 | 0.891428766 | 0.418628542 | 0.999970678 | 1.508 | 1.104 | 0.444 | 0.845 | NA |
| TRINITY_DN9493_c1_g1_i1 | 0.891372771 | 0.746101992 | 0.999970678 | 1.135 | 3.784 | 0 | 2.594 | NA |
| TRINITY_DN22207_c0_g1_i1 | -0.89125773 | 0.415456917 | 0.999970678 | 0.436 | 0.355 | 0.622 | 0.714 | NA |
| TRINITY_DN13051_c0_g1_i1 | 0.891149473 | 0.531832699 | 0.999970678 | 2.662 | 4.129 | 2.611 | 0.483 | peroxidase |
| TRINITY_DN25279_c0_g1_i2 | -0.89114455 | 0.511429857 | 0.999970678 | 0.908 | 0.375 | 0.3 | 2.031 | Bromodomain |
| TRINITY_DN7932_c0_g3_i1 | 0.890991796 | 0.321578232 | 0.999970678 | 1.363 | 0.936 | 0.644 | 0.473 | Cation_ATPase |
| TRINITY_DN1605_c0_g1_i3 | 0.89098496 | 0.488368739 | 0.999970678 | 8.539 | 4.287 | 0.878 | 5.902 | NA |
| TRINITY_DN3672_c0_g3_i1 | -0.89094215 | 0.265678653 | 0.999970678 | 21.084 | 31.51 | 27.954 | 63.278 | NA |
| TRINITY_DN5572_c0_g1_i25 | -0.89093452 | 0.842314867 | 1 | 0.99 | 0 | 1.533 | 0 | NA |
| TRINITY_DN35791_c0_g1_i1 | -0.89093452 | 0.842314867 | 1 | 0.69 | 0 | 1.078 | 0 | ApbA |
| TRINITY_DN186_c6_g2_i3 | -0.8909256 | 0.32666664 | 0.999970678 | 191.27 | 454.9 | 530.59 | 454.085 | NA |
| TRINITY_DN7983_c0_g1_i12 | -0.8908457 | 0.840215631 | 1 | 0 | 1.005 | 0 | 1.81 | NA |
| TRINITY_DN4193_c0_g1_i2 | -0.89081019 | 0.726993704 | 0.999970678 | 15.633 | 0.848 | 25.476 | 0.06 | LTP_2 |
| TRINITY_DN9554_c0_g1_i4 | -0.89073859 | 0.579901581 | 0.999970678 | 0.118 | 1.232 | 1.189 | 1.056 | NA |
| TRINITY_DN147_c0_g1_i6 | -0.89073343 | 0.837962416 | 0.999970678 | 1.126 | 0 | 0 | 2.141 | NA |
| TRINITY_DN5125_c0_g1_i1 | -0.89069656 | 0.398070936 | 0.999970678 | 53.931 | 14.36 | 50.775 | 66.234 | AvrRpt-cleavage |
| TRINITY_DN964_c0_g1_i7 | 0.890676315 | 0.507365911 | 0.999970678 | 2.453 | 3.459 | 0.489 | 2.574 | NA |
| TRINITY_DN970_c1_g1_i5 | -0.89066318 | 0.758637108 | 0.999970678 | 0 | 0.877 | 1.189 | 0.161 | Myb_DNA-bindin |
| TRINITY_DN4524_c3_g1_i1 | 0.890610936 | 0.474304331 | 1 | 1.108 | 0.976 | 0.567 | 0.442 | NA |
| TRINITY_DN12760_c0_g1_i4 | 0.89059071 | 0.548706949 | 0.999970678 | 5.587 | 4.051 | 3.533 | 0.845 | NA |
| TRINITY_DN2433_c0_g1_i7 | -0.89058307 | 0.464447262 | 0.999970678 | 3.924 | 3.982 | 10.055 | 2.503 | NA |
| TRINITY_DN3835_c0_g1_i18 | -0.89055049 | 0.739229926 | 0.999970678 | 0 | 1.025 | 0.878 | 0.845 | NA |
| TRINITY_DN234_c0_g1_i9 | 0.890493634 | 0.48078648 | 0.999970678 | 1.508 | 5.223 | 2.178 | 0.985 | NA |
| TRINITY_DN39575_c0_g1_i3 | 0.890305645 | 0.38316835 | 0.999970678 | 212.62 | 72.41 | 44.697 | 100.949 | NA |
| TRINITY_DN3099_c0_g1_i6 | -0.89017408 | 0.74571087 | 0.999970678 | 8.112 | 0 | 5.755 | 8.294 | DAP3 |
| TRINITY_DN26339_c0_g3_i1 | 0.890153294 | 0.507322692 | 0.999970678 | 98.443 | 16.94 | 38.887 | 15.825 | NA |
| TRINITY_DN37_c0_g1_i1 | 0.890098223 | 0.547320856 | 0.999970678 | 0.182 | 0.986 | 0.322 | 0.231 | NA |
| TRINITY_DN14525_c0_g1_i1 | -0.89002191 | 0.589498054 | 0.999970678 | 0.581 | 4.159 | 5.6 | 1.8 | NA |
| TRINITY_DN1091_c1_g1_i1 | 0.889905152 | 0.534665699 | 0.999970678 | 2.316 | 0.631 | 1 | 0.392 | SNARE_assoc |
| TRINITY_DN4085_c0_g1_i6 | 0.889894646 | 0.558706843 | 0.999970678 | 84.163 | 8.022 | 23.487 | 19.745 | NA |
| TRINITY_DN6456_c1_g1_i3 | -0.8898722 | 0.475872628 | 0.999970678 | 0.663 | 1.252 | 0.8 | 2.503 | B_lectin |
| TRINITY_DN3844_c0_g1_i2 | -0.88983793 | 0.61560623 | 0.999970678 | 0.881 | 0.089 | 0.533 | 1.166 | NA |
| TRINITY_DN286_c0_g2_i1 | 0.889807215 | 0.440249244 | 0.999970678 | 1.935 | 2.927 | 0.689 | 1.769 | NA |
| TRINITY_DN3243_c0_g2_i1 | -0.88974446 | 0.539845603 | 1 | 1.008 | 2.424 | 1.467 | 4.414 | NA |
| TRINITY_DN520_c1_g2_i4 | -0.88963572 | 0.630939583 | 0.999970678 | 0.227 | 8.791 | 4.5 | 10.989 | LRR_1 |

| TRINITY_DN395_c1_g1_i4 | -0.88951349 | 0.739164645 | 0.999970678 | 1.79 | 0 | 1.522 | 1.548 | Glyco_transf_8 |
| --- | --- | --- | --- | --- | --- | --- | --- | --- |
| TRINITY_DN29210_c0_g1_i1 | 0.889498785 | 0.625326825 | 1 | 0.581 | 0.581 | 0.467 | 0.06 | NA |
| TRINITY_DN5771_c0_g1_i1 | -0.88937052 | 0.301720115 | 0.999970678 | 4.233 | 3.223 | 5.066 | 7.57 | NA |
| TRINITY_DN14401_c0_g1_i1 | 0.888961165 | 0.603274728 | 0.999970678 | 14.107 | 1.537 | 2.222 | 5.6 | NA |
| TRINITY_DN5882_c0_g2_i1 | 0.888950495 | 0.339362469 | 0.999970678 | 3.406 | 3.164 | 0.9 | 2.453 | PPR |
| TRINITY_DN6591_c0_g1_i10 | 0.888631703 | 0.51836403 | 0.999970678 | 6.513 | 1.419 | 1.533 | 2.473 | NA |
| TRINITY_DN4359_c0_g1_i2 | -0.88859408 | 0.756884192 | 0.999970678 | 0 | 0.828 | 1.089 | 0.181 | CDT1 |
| TRINITY_DN6303_c0_g1_i3 | 0.888314814 | 0.765618682 | 0.999970678 | 1.653 | 0 | 0.078 | 0.824 | NA |
| TRINITY_DN77101_c0_g1_i1 | 0.888197143 | 0.841044435 | 1 | 1.208 | 0 | 0.544 | 0 | NA |
| TRINITY_DN91067_c0_g1_i1 | 0.888197143 | 0.841044435 | 1 | 1.135 | 0 | 0.511 | 0 | Alk_phosphatase |
| TRINITY_DN7940_c0_g1_i8 | -0.88813507 | 0.788600456 | 0.999970678 | 8.266 | 0 | 12.655 | 0.101 | Ferritin_2 |
| TRINITY_DN9307_c0_g1_i3 | 0.888025091 | 0.208749656 | 0.999970678 | 3.933 | 4.563 | 2 | 2.172 | NA |
| TRINITY_DN2697_c0_g1_i1 | 0.88791097 | 0.750431922 | 0.999970678 | 3.979 | 0 | 0.878 | 1.126 | NA |
| TRINITY_DN11301_c0_g1_i1 | -0.8878513 | 0.199570037 | 0.999970678 | 17.241 | 28.12 | 37.187 | 38.656 | ADH_N_2 |
| TRINITY_DN325_c1_g1_i4 | -0.88778926 | 0.547231341 | 0.999970678 | 6.023 | 0.848 | 8.011 | 3.147 | Sugar_tr |
| TRINITY_DN9530_c0_g1_i4 | 0.887660107 | 0.504473454 | 0.999970678 | 0.554 | 0.276 | 0.278 | 0.121 | Kinesin |
| TRINITY_DN3160_c0_g1_i1 | -0.88742987 | 0.316254163 | 0.999970678 | 2.017 | 1.202 | 3.044 | 2.332 | Myb_DNA-bind_ |
| TRINITY_DN569_c0_g1_i2 | 0.887391054 | 0.777213436 | 0.999970678 | 22.901 | 0 | 0.478 | 12.024 | NA |
| TRINITY_DN4923_c0_g1_i1 | 0.887318583 | 0.555729318 | 0.999970678 | 4.288 | 0.828 | 1.689 | 0.724 | NA |
| TRINITY_DN3424_c0_g1_i3 | -0.88729628 | 0.528430404 | 0.999970678 | 0.409 | 1.202 | 2.044 | 0.493 | NA |
| TRINITY_DN40646_c0_g1_i1 | 0.887115064 | 0.446485817 | 0.999970678 | 0.636 | 0.532 | 0.389 | 0.171 | NA |
| TRINITY_DN14722_c0_g1_i8 | 0.88708931 | 0.341511258 | 0.999970678 | 0.581 | 0.788 | 0.356 | 0.312 | TAXi_C |
| TRINITY_DN6290_c0_g1_i4 | 0.887061598 | 0.227187994 | 0.999970678 | 1.126 | 1.045 | 0.5 | 0.583 | DIL |
| TRINITY_DN147_c1_g1_i1 | 0.887021846 | 0.297651796 | 0.999970678 | 12.254 | 19.45 | 7.6 | 7.671 | RRM_1 |
| TRINITY_DN136422_c0_g1_i1 | -0.8869369 | 0.846039067 | 1 | 0.382 | 0 | 0.589 | 0 | RHD3 |
| TRINITY_DN40377_c0_g1_i1 | -0.8869369 | 0.846039067 | 1 | 0.845 | 0 | 1.3 | 0 | NA |
| TRINITY_DN66473_c0_g1_i4 | -0.8869369 | 0.846039067 | 1 | 6.395 | 0 | 9.388 | 0 | NA |
| TRINITY_DN8502_c0_g1_i1 | -0.8869369 | 0.846039067 | 1 | 0.336 | 0 | 0.522 | 0 | MFS_1 |
| TRINITY_DN7177_c0_g1_i9 | 0.886923993 | 0.837110326 | 0.999970678 | 1.226 | 0 | 0 | 0.684 | Dynamin_M |
| TRINITY_DN9902_c0_g2_i2 | -0.88691107 | 0.389237156 | 0.999970678 | 30.468 | 27.89 | 70.118 | 23.284 | Ion_trans |
| TRINITY_DN1961_c0_g1_i4 | 0.886827054 | 0.350034302 | 0.999970678 | 6.204 | 2.464 | 2.133 | 2.151 | NA |
| TRINITY_DN29223_c0_g2_i3 | 0.886815405 | 0.476627022 | 0.999970678 | 0.999 | 1.557 | 0.278 | 1.025 | Proteasome |
| TRINITY_DN1747_c0_g1_i2 | -0.88679323 | 0.315910333 | 0.999970678 | 14.598 | 31.34 | 25.698 | 53.164 | NA |
| TRINITY_DN116671_c0_g1_i1 | 0.886767194 | 0.686521036 | 1 | 0.064 | 1.429 | 0.1 | 0.684 | NA |
| TRINITY_DN830_c0_g1_i2 | -0.8867516 | 0.445008661 | 0.999970678 | 7.958 | 2.286 | 5.489 | 12.396 | NA |
| TRINITY_DN11198_c0_g4_i1 | 0.886702646 | 0.561793473 | 0.999970678 | 2.262 | 0.443 | 0.922 | 0.382 | NA |
| TRINITY_DN439_c0_g1_i10 | 0.886682102 | 0.449985822 | 0.999970678 | 2.489 | 2.444 | 1.378 | 0.995 | NA |
| TRINITY_DN6017_c0_g1_i1 | 0.886667907 | 0.440733936 | 0.999970678 | 2.207 | 3.696 | 2.144 | 0.603 | DUF4283 |
| TRINITY_DN14278_c0_g2_i1 | 0.886660357 | 0.579906252 | 0.999970678 | 0.69 | 0.099 | 0.267 | 0.111 | ABC2_membrane |
| TRINITY_DN4824_c0_g1_i5 | -0.88663002 | 0.73905248 | 0.999970678 | 0 | 1.636 | 1.544 | 1.116 | Brix |
| TRINITY_DN2264_c0_g1_i3 | -0.88661698 | 0.413172882 | 0.999970678 | 4.124 | 1.37 | 3.389 | 6.143 | Patatin |
| TRINITY_DN10572_c0_g1_i16 | 0.886594022 | 0.833571828 | 0.999970678 | 4.887 | 0 | 2.211 | 0 | NA |
| TRINITY_DN716_c0_g3_i1 | -0.88650562 | 0.532618829 | 0.999970678 | 2.062 | 0.739 | 3.189 | 1.317 | Pirin |
| TRINITY_DN616_c2_g1_i2 | -0.88649769 | 0.75872672 | 0.999970678 | 0 | 26.32 | 27.376 | 14.819 | NA |
| TRINITY_DN2147_c0_g1_i2 | -0.88649184 | 0.742683006 | 0.999970678 | 9.366 | 0 | 4.5 | 11.873 | NA |
| TRINITY_DN1794_c0_g1_i7 | -0.88646162 | 0.758137691 | 0.999970678 | 0.273 | 1.33 | 2.433 | 0 | NA |
| TRINITY_DN860_c0_g1_i11 | -0.88640942 | 0.336561311 | 0.999970678 | 6.877 | 4.75 | 11.655 | 7.379 | DUF2052 |
| TRINITY_DN14461_c0_g1_i1 | -0.88631301 | 0.338046289 | 0.999970678 | 1.617 | 2.257 | 2.633 | 3.931 | NA |
| TRINITY_DN6712_c0_g1_i2 | 0.886278664 | 0.248406569 | 0.999970678 | 54.277 | 109 | 33.931 | 46.931 | NA |
| TRINITY_DN7838_c0_g1_i1 | -0.88624091 | 0.581189421 | 0.999970678 | 0.209 | 2.375 | 2.966 | 1.146 | PPR |
| TRINITY_DN3948_c0_g1_i1 | -0.88619002 | 0.275175279 | 0.999970678 | 5.114 | 4.011 | 9.011 | 5.992 | AAA |
| TRINITY_DN6135_c0_g2_i2 | -0.88600427 | 0.575863926 | 1 | 0.109 | 0.483 | 0.5 | 0.473 | NA |
| TRINITY_DN15081_c0_g4_i3 | 0.885808051 | 0.83520082 | 0.999970678 | 1.671 | 0 | 0.756 | 0 | NA |
| TRINITY_DN728_c0_g1_i12 | 0.88564396 | 0.673650945 | 0.999970678 | 0.999 | 2.976 | 1.733 | 0.07 | NA |
| TRINITY_DN5189_c0_g1_i2 | -0.88561984 | 0.74297453 | 0.999970678 | 0 | 1.981 | 2.211 | 0.945 | NA |
| TRINITY_DN9979_c0_g1_i4 | -0.88559376 | 0.47802874 | 0.999970678 | 0.345 | 0.286 | 0.244 | 0.885 | Pkinase |
| TRINITY_DN1246_c0_g3_i1 | -0.88533037 | 0.753357845 | 0.999970678 | 2.961 | 0 | 0.633 | 4.745 | NA |
| TRINITY_DN4540_c0_g1_i2 | -0.88524504 | 0.776922963 | 0.999970678 | 0 | 7.697 | 0.267 | 13.733 | Methyltransf_29 |
| TRINITY_DN12156_c0_g1_i1 | 0.885236245 | 0.841912067 | 1 | 0 | 0.453 | 0 | 0.241 | NA |
| TRINITY_DN1002_c0_g1_i2 | -0.88522297 | 0.643099243 | 0.999970678 | 0.527 | 3.853 | 0.278 | 7.641 | Amidase |
| TRINITY_DN7951_c0_g2_i3 | -0.88521212 | 0.580073808 | 0.999970678 | 0.291 | 0.67 | 0.189 | 1.528 | NA |
| TRINITY_DN1270_c1_g1_i5 | 0.88512264 | 0.751764091 | 1 | 1.154 | 0.355 | 0 | 0.814 | NA |
| TRINITY_DN1970_c0_g1_i14 | -0.8851042 | 0.449400183 | 0.999970678 | 0.863 | 0.719 | 1.4 | 1.227 | NA |
| TRINITY_DN7576_c0_g2_i1 | -0.88507774 | 0.842455594 | 1 | 2.571 | 0 | 3.966 | 0 | NA |
| TRINITY_DN10033_c0_g1_i1 | 0.884953861 | 0.539544307 | 0.999970678 | 145.75 | 1538 | 226.664 | 622.696 | PSI_PSAK |
| TRINITY_DN11870_c0_g1_i1 | 0.884898396 | 0.444487284 | 0.999970678 | 4.269 | 3.213 | 2.744 | 0.754 | NA |
| TRINITY_DN681_c0_g1_i1 | 0.884858929 | 0.833499341 | 0.999970678 | 15.57 | 0 | 0 | 8.536 | CRS1_YhbY |
| TRINITY_DN6000_c1_g1_i4 | -0.88483467 | 0.363387068 | 0.999970678 | 183.51 | 255 | 503.481 | 196.902 | CAAD |
| TRINITY_DN8823_c0_g1_i1 | -0.88469839 | 0.393074925 | 0.999970678 | 17.895 | 13.77 | 20.499 | 28.995 | Transferase |
| TRINITY_DN13584_c0_g2_i4 | 0.884648068 | 0.490714566 | 0.999970678 | 0.736 | 2.947 | 1.033 | 0.754 | NA |
| TRINITY_DN439_c0_g1_i5 | 0.884617989 | 0.254973392 | 0.999970678 | 4.406 | 4.859 | 1.744 | 2.895 | SAWADEE |
| TRINITY_DN7237_c0_g1_i2 | -0.88453329 | 0.633368335 | 0.999970678 | 1.626 | 0.266 | 0.189 | 3.318 | Malectin_like |
| TRINITY_DN1497_c0_g1_i2 | -0.88427006 | 0.374737315 | 0.999970678 | 5.75 | 11.64 | 7.644 | 22.651 | NA |
| TRINITY_DN3867_c0_g1_i2 | -0.88414589 | 0.381995883 | 0.999970678 | 3.043 | 3.834 | 7.522 | 3.579 | NA |

| TRINITY_DN4119_c0_g1_i11 | -0.88407891 | 0.753085784 | 0.999970678 | 3.261 | 1.399 | 7.122 | 0 | NA |
| --- | --- | --- | --- | --- | --- | --- | --- | --- |
| TRINITY_DN30236_c0_g1_i1 | -0.88404504 | 0.762880208 | 1 | 0 | 1.33 | 1.733 | 0.322 | NA |
| TRINITY_DN7766_c0_g1_i2 | 0.883884183 | 0.752116433 | 0.999970678 | 0 | 3.745 | 1 | 0.784 | NA |
| TRINITY_DN92130_c0_g1_i1 | 0.883798032 | 0.594131718 | 0.999970678 | 2.089 | 3.026 | 2.122 | 0.191 | Lipase_GDSL |
| TRINITY_DN3915_c0_g1_i7 | 0.883726114 | 0.841427497 | 0.999970678 | 2.098 | 0 | 0.955 | 0.02 | TRAPP |
| TRINITY_DN21707_c0_g1_i1 | 0.883726114 | 0.841427497 | 0.999970678 | 1.399 | 0 | 0.633 | 0 | DHQ_synthase |
| TRINITY_DN1368_c0_g1_i2 | -0.88371791 | 0.470208058 | 0.999970678 | 0.554 | 2.523 | 1.6 | 3.72 | NA |
| TRINITY_DN4784_c0_g1_i4 | 0.883614597 | 0.248480935 | 0.999970678 | 3.606 | 2.71 | 1.122 | 2.101 | FAD-oxidase_C |
| TRINITY_DN14387_c0_g1_i1 | -0.88352435 | 0.511525268 | 0.999970678 | 0.745 | 3.725 | 3.511 | 3.861 | NA |
| TRINITY_DN1895_c0_g1_i1 | -0.88343022 | 0.115130824 | 0.999970678 | 34.773 | 35.45 | 53.008 | 65.168 | NA |
| TRINITY_DN2416_c0_g1_i20 | 0.883246948 | 0.533765345 | 0.999970678 | 0.418 | 0.976 | 0.5 | 0.141 | NA |
| TRINITY_DN8689_c0_g1_i7 | 0.883222299 | 0.423961058 | 0.999970678 | 4.951 | 2.217 | 2.155 | 1.317 | NA |
| TRINITY_DN2671_c0_g1_i4 | -0.88319122 | 0.562957748 | 0.999970678 | 4.715 | 0.512 | 2.178 | 7.108 | NA |
| TRINITY_DN3470_c0_g1_i10 | -0.88313767 | 0.450986524 | 0.999970678 | 1.29 | 1.212 | 2.622 | 1.357 | Methyltransf_32 |
| TRINITY_DN6164_c0_g1_i8 | -0.88311485 | 0.404738377 | 0.999970678 | 24.3 | 50.41 | 26.687 | 105.493 | - |
| TRINITY_DN1496_c0_g1_i7 | -0.88307933 | 0.473710354 | 0.999970678 | 2.235 | 6.416 | 2.9 | 12.336 | NA |
| TRINITY_DN2575_c0_g1_i5 | 0.882982233 | 0.287919998 | 0.999970678 | 76.123 | 167.1 | 60.53 | 56.542 | Methyltransf_11 |
| TRINITY_DN1031_c1_g3_i1 | 0.882979509 | 0.512512719 | 0.999970678 | 2.943 | 13.09 | 1.478 | 6.826 | NA |
| TRINITY_DN15637_c0_g1_i6 | 0.882958429 | 0.560111531 | 1 | 0.254 | 0.207 | 0.167 | 0.05 | Laminin_G_3 |
| TRINITY_DN2103_c0_g1_i9 | -0.88278923 | 0.123180519 | 0.999970678 | 6309.6 | 7251 | 11087.21 | 11185.54 | NA |
| TRINITY_DN8228_c0_g1_i1 | -0.88278181 | 0.318091821 | 0.999970678 | 8.203 | 5.43 | 6.844 | 16.88 | NA |
| TRINITY_DN3539_c0_g1_i6 | 0.882753346 | 0.739909926 | 0.999970678 | 1.117 | 1.38 | 0 | 1.347 | Yippee-Mis18 |
| TRINITY_DN4_c0_g1_i9 | -0.8826488 | 0.477038645 | 0.999970678 | 4.778 | 9.54 | 4.3 | 20.761 | UDPGT |
| TRINITY_DN6865_c0_g1_i1 | 0.882611921 | 0.738216887 | 0.999970678 | 0.545 | 122 | 0.289 | 65.128 | NA |
| TRINITY_DN3305_c0_g1_i8 | -0.88260095 | 0.739335159 | 0.999970678 | 0 | 2.769 | 1.7 | 2.936 | NA |
| TRINITY_DN14219_c0_g1_i1 | 0.882538736 | 0.207188468 | 0.999970678 | 2.952 | 3.41 | 1.711 | 1.397 | Cohesin_HEAT |
| TRINITY_DN5119_c0_g2_i2 | -0.88245787 | 0.836717421 | 0.999970678 | 1.263 | 0 | 1.955 | 0 | - |
| TRINITY_DN6387_c0_g1_i15 | -0.88232206 | 0.608939888 | 0.999970678 | 0.227 | 2.375 | 2.378 | 1.86 | NA |
| TRINITY_DN11894_c0_g1_i3 | 0.882312566 | 0.752453192 | 0.999970678 | 0 | 4.06 | 0.633 | 1.408 | NA |
| TRINITY_DN2001_c0_g1_i2 | 0.882278271 | 0.355980402 | 0.999970678 | 43.112 | 27.39 | 8.699 | 27.849 | NA |
| TRINITY_DN498_c0_g1_i1 | 0.882277276 | 0.149894625 | 0.999970678 | 16.097 | 14.91 | 7.411 | 7.872 | - |
| TRINITY_DN640_c0_g2_i4 | 0.882132861 | 0.767135438 | 0.999970678 | 2.544 | 0 | 0.978 | 0.241 | AP2 |
| TRINITY_DN12775_c0_g1_i2 | 0.882110188 | 0.838045161 | 0.999970678 | 2.653 | 0 | 1.211 | 0 | HSP20 |
| TRINITY_DN11741_c0_g1_i1 | 0.882110188 | 0.838045161 | 0.999970678 | 3.316 | 0 | 1.511 | 0 | Catalase |
| TRINITY_DN5723_c0_g1_i3 | 0.881920541 | 0.788633448 | 0.999970678 | 12.999 | 0 | 0.122 | 6.927 | NA |
| TRINITY_DN2294_c0_g1_i3 | 0.881915949 | 0.641223574 | 0.999970678 | 1.363 | 10.22 | 0.2 | 5.952 | NA |
| TRINITY_DN1222_c0_g1_i3 | -0.88191557 | 0.56932297 | 0.999970678 | 2.08 | 7.342 | 1.811 | 14.829 | NA |
| TRINITY_DN2985_c0_g1_i5 | -0.88184337 | 0.59653146 | 0.999970678 | 0.154 | 1.212 | 0.356 | 2.061 | NA |
| TRINITY_DN1191_c0_g1_i2 | -0.88182477 | 0.779865815 | 0.999970678 | 0.145 | 5.893 | 9.033 | 0 | NA |
| TRINITY_DN16280_c0_g1_i2 | 0.881823571 | 0.757269598 | 0.999970678 | 0 | 2.878 | 0.333 | 1.166 | NA |
| TRINITY_DN403_c0_g2_i5 | 0.881653733 | 0.53518219 | 0.999970678 | 0.391 | 0.611 | 0.356 | 0.131 | NA |
| TRINITY_DN16778_c0_g1_i15 | 0.881552612 | 0.750086842 | 1 | 0.509 | 0.306 | 0.356 | 0 | DLD |
| TRINITY_DN118_c0_g1_i13 | -0.88149193 | 0.558566286 | 0.999970678 | 7.631 | 1.744 | 11.588 | 3.167 | NA |
| TRINITY_DN6814_c0_g1_i3 | -0.8814514 | 0.487477681 | 0.999970678 | 1.662 | 1.597 | 4.233 | 0.885 | NA |
| TRINITY_DN1504_c0_g1_i1 | -0.88140903 | 0.558373503 | 0.999970678 | 37.935 | 198.6 | 42.42 | 374.581 | - |
| TRINITY_DN289_c0_g1_i16 | 0.881402062 | 0.472156903 | 0.999970678 | 3.179 | 1.892 | 1.889 | 0.483 | DUF659 |
| TRINITY_DN482_c0_g1_i5 | 0.881359481 | 0.378401626 | 0.999970678 | 81.91 | 25.1 | 26.587 | 26.542 | EXS |
| TRINITY_DN808_c0_g4_i1 | 0.881245543 | 0.590556883 | 0.999970678 | 2.643 | 2.562 | 0.156 | 2.644 | ubiquitin |
| TRINITY_DN4204_c0_g1_i2 | 0.8812437 | 0.48648403 | 0.999970678 | 12.981 | 8.269 | 8.233 | 1.619 | NA |
| TRINITY_DN2348_c0_g1_i12 | -0.88121047 | 0.632326007 | 0.999970678 | 1.644 | 0.168 | 0.322 | 3.016 | NA |
| TRINITY_DN6724_c0_g1_i1 | 0.881087763 | 0.83958348 | 0.999970678 | 1.372 | 0 | 0.633 | 0 | Aft1_HRA |
| TRINITY_DN17850_c0_g1_i1 | -0.88108495 | 0.366641807 | 0.999970678 | 1.653 | 2.444 | 2.889 | 3.991 | NA |
| TRINITY_DN2717_c0_g1_i11 | -0.88097752 | 0.467375942 | 0.999970678 | 1.862 | 1.597 | 1.167 | 4.936 | NA |
| TRINITY_DN8508_c0_g1_i1 | -0.88067563 | 0.43811786 | 0.999970678 | 12.708 | 4.297 | 7.122 | 22.932 | p450 |
| TRINITY_DN10671_c1_g1_i5 | -0.8805889 | 0.840308726 | 0.999970678 | 0 | 0.414 | 0 | 0.744 | NA |
| TRINITY_DN1532_c0_g2_i1 | 0.880526969 | 0.57178465 | 0.999970678 | 31.267 | 1.971 | 6.722 | 10.134 | Aldo_ket_red |
| TRINITY_DN5510_c0_g1_i4 | 0.880517028 | 0.834690631 | 0.999970678 | 2.662 | 0 | 1.211 | 0 | HEAT |
| TRINITY_DN8972_c0_g1_i21 | -0.88042364 | 0.789758194 | 0.999970678 | 3.997 | 0.069 | 6.222 | 0 | NA |
| TRINITY_DN7231_c0_g1_i3 | -0.88034927 | 0.479894479 | 0.999970678 | 5.223 | 2.375 | 8.866 | 3.278 | NA |
| TRINITY_DN112_c0_g1_i1 | -0.88012352 | 0.464683128 | 0.999970678 | 2.88 | 5.075 | 9.944 | 2.564 | NA |
| TRINITY_DN11325_c0_g1_i1 | 0.880075253 | 0.45835615 | 0.999970678 | 1.762 | 3.893 | 0.955 | 1.88 | NA |
| TRINITY_DN4218_c0_g1_i19 | -0.88007167 | 0.370405632 | 0.999970678 | 0.936 | 1.222 | 2.222 | 1.287 | NA |
| TRINITY_DN2836_c1_g2_i3 | -0.88003517 | 0.840349696 | 0.999970678 | 0.845 | 0 | 1.3 | 0 | NA |
| TRINITY_DN5411_c1_g1_i4 | 0.879992008 | 0.371214168 | 0.999970678 | 0.99 | 0.69 | 0.433 | 0.402 | NA |
| TRINITY_DN23703_c0_g1_i4 | -0.87984566 | 0.525361447 | 0.999970678 | 0.972 | 0.719 | 2.111 | 0.553 | NA |
| TRINITY_DN4877_c0_g1_i3 | 0.879839296 | 0.482055728 | 0.999970678 | 11.773 | 3.4 | 2.611 | 5.027 | NA |
| TRINITY_DN14535_c0_g3_i1 | 0.879820663 | 0.441664124 | 0.999970678 | 2.053 | 2.957 | 1.544 | 0.834 | NA |
| TRINITY_DN5138_c0_g2_i2 | -0.87975229 | 0.690711507 | 0.999970678 | 1.372 | 0.217 | 2.4 | 0.04 | Glyco_transf_41 |
| TRINITY_DN1031_c0_g1_i12 | -0.87971075 | 0.167299628 | 0.999970678 | 14.098 | 16.09 | 20.465 | 30.392 | Fer2 |
| TRINITY_DN2287_c0_g2_i1 | 0.879628981 | 0.64272243 | 0.999970678 | 14.97 | 2.996 | 7.9 | 0.302 | NA |
| TRINITY_DN16903_c0_g1_i1 | -0.87957093 | 0.790470576 | 1 | 0.509 | 0 | 0.767 | 0.02 | NA |
| TRINITY_DN787_c0_g1_i3 | 0.879511815 | 0.279783088 | 0.999970678 | 1.09 | 0.946 | 0.4 | 0.633 | NA |
| TRINITY_DN776_c0_g1_i1 | -0.87947564 | 0.335181035 | 0.999970678 | 95.3 | 178.2 | 284.705 | 152.374 | Ribosomal_L18p |
| TRINITY_DN1752_c0_g1_i2 | -0.87944495 | 0.75607484 | 0.999970678 | 1.026 | 0 | 1.233 | 0.402 | - |

| TRINITY_DN3399_c0_g2_i1 | -0.87889751 | 0.277023162 | 0.999970678 | 32.92 | 23.07 | 54.919 | 36.073 | NA |
| --- | --- | --- | --- | --- | --- | --- | --- | --- |
| TRINITY_DN3216_c0_g1_i7 | 0.878862498 | 0.743761412 | 0.999970678 | 3.779 | 7.608 | 0 | 6.173 | Glyoxalase |
| TRINITY_DN1180_c0_g1_i4 | 0.878848148 | 0.759331142 | 0.999970678 | 12.972 | 0 | 4.266 | 1.991 | NA |
| TRINITY_DN3907_c1_g1_i1 | 0.878637452 | 0.446485611 | 0.999970678 | 0.572 | 1.36 | 0.544 | 0.392 | CTD_bind |
| TRINITY_DN15221_c0_g2_i1 | 0.878567644 | 0.292383736 | 0.999970678 | 2.135 | 1.567 | 1.067 | 0.734 | NA |
| TRINITY_DN4419_c0_g1_i1 | -0.87855585 | 0.424965552 | 0.999970678 | 14.289 | 30.36 | 15.032 | 63.449 | NA |
| TRINITY_DN9581_c0_g1_i1 | 0.878516399 | 0.712401319 | 0.999970678 | 7.194 | 0.03 | 2.866 | 0.513 | TspO_MBR |
| TRINITY_DN4962_c0_g1_i1 | -0.87848266 | 0.353876021 | 0.999970678 | 29.586 | 57.64 | 92.161 | 47.293 | NA |
| TRINITY_DN3270_c0_g2_i5 | 0.878306656 | 0.184673346 | 0.999970678 | 38.798 | 36.3 | 20.399 | 16.317 | NA |
| TRINITY_DN13513_c0_g1_i12 | 0.878236964 | 0.843197972 | 1 | 0 | 0.788 | 0.356 | 0 | NA |
| TRINITY_DN1549_c0_g1_i11 | -0.87788977 | 0.780824864 | 1 | 0 | 1.873 | 2.622 | 0.161 | NA |
| TRINITY_DN3912_c0_g2_i3 | -0.87781032 | 0.470729719 | 0.999970678 | 19.213 | 37.31 | 13.577 | 87.326 | Sugar_tr |
| TRINITY_DN9888_c0_g1_i2 | 0.877654992 | 0.552933508 | 0.999970678 | 0.136 | 0.729 | 0.133 | 0.312 | - |
| TRINITY_DN7428_c0_g1_i2 | -0.87760901 | 0.277032226 | 0.999970678 | 6.104 | 5.016 | 6.177 | 12.919 | NA |
| TRINITY_DN1360_c0_g1_i1 | 0.877600943 | 0.322513298 | 0.999970678 | 4.242 | 7.933 | 2.044 | 4.132 | TPT |
| TRINITY_DN826_c0_g1_i2 | -0.87757479 | 0.245826259 | 0.999970678 | 19.004 | 21.15 | 21.776 | 46.941 | NA |
| TRINITY_DN14264_c0_g1_i1 | 0.877568723 | 0.774801498 | 0.999970678 | 0 | 35.1 | 14.077 | 1.79 | NA |
| TRINITY_DN19626_c0_g1_i3 | 0.877566104 | 0.615202287 | 0.999970678 | 3.243 | 0.306 | 1.267 | 0.422 | NA |
| TRINITY_DN14632_c0_g3_i7 | -0.87752684 | 0.790262109 | 1 | 1.181 | 0 | 0.056 | 2.172 | NA |
| TRINITY_DN657_c0_g1_i1 | -0.87751468 | 0.759296626 | 0.999970678 | 3.534 | 0 | 4.889 | 0.674 | NA |
| TRINITY_DN594_c0_g1_i2 | 0.877473176 | 0.56556145 | 0.999970678 | 3.833 | 2.622 | 2.7 | 0.261 | NA |
| TRINITY_DN16912_c0_g1_i1 | -0.87736696 | 0.645922026 | 0.999970678 | 11.291 | 0.197 | 11.088 | 7.892 | Chloroa_b-bind |
| TRINITY_DN10033_c0_g1_i2 | -0.87736026 | 0.538822821 | 0.999970678 | 3.843 | 0.946 | 5.833 | 1.84 | PSI_PSAK |
| TRINITY_DN13725_c0_g1_i1 | -0.87732407 | 0.498806715 | 0.999970678 | 124.92 | 44.01 | 174.667 | 80.54 | NA |
| TRINITY_DN5614_c0_g1_i1 | -0.87717333 | 0.76846794 | 0.999970678 | 68.929 | 0 | 33.12 | 88.402 | NA |
| TRINITY_DN13126_c0_g1_i2 | 0.877169931 | 0.845124343 | 1 | 2.816 | 0 | 1.278 | 0 | NA |
| TRINITY_DN7642_c0_g2_i2 | 0.877169931 | 0.845124343 | 1 | 0.818 | 0 | 0.378 | 0 | NA |
| TRINITY_DN4232_c0_g1_i4 | -0.87713989 | 0.749353741 | 0.999970678 | 1.526 | 0 | 0.522 | 2.192 | NA |
| TRINITY_DN1284_c1_g2_i1 | -0.87713499 | 0.628553464 | 0.999970678 | 0.136 | 1.478 | 0.467 | 2.363 | NA |
| TRINITY_DN13282_c0_g1_i5 | -0.87704789 | 0.839053112 | 0.999970678 | 5.75 | 0 | 8.777 | 0 | Cyt-b5 |
| TRINITY_DN15404_c0_g1_i5 | 0.876964261 | 0.398368451 | 0.999970678 | 40.878 | 12.54 | 13.888 | 12.386 | NA |
| TRINITY_DN5819_c0_g1_i1 | -0.87689078 | 0.742933612 | 0.999970678 | 2.589 | 0 | 1.9 | 2.513 | NA |
| TRINITY_DN16229_c0_g1_i1 | -0.87681437 | 0.754860856 | 0.999970678 | 2.453 | 0.739 | 0 | 5.902 | NA |
| TRINITY_DN4594_c0_g1_i5 | -0.87666083 | 0.523567051 | 0.999970678 | 0.899 | 1.508 | 3.144 | 0.633 | NA |
| TRINITY_DN5310_c0_g1_i3 | -0.87663823 | 0.397417021 | 0.999970678 | 3.579 | 1.281 | 4.044 | 4.052 | XPG_I |
| TRINITY_DN4562_c0_g1_i1 | -0.87653588 | 0.339555072 | 0.999970678 | 64.46 | 93.43 | 172.223 | 79.535 | NA |
| TRINITY_DN1098_c0_g1_i1 | 0.876400566 | 0.838830095 | 0.999970678 | 52.06 | 0 | 0 | 28.593 | NA |
| TRINITY_DN478_c2_g1_i3 | 0.876398936 | 0.751761204 | 0.999970678 | 1.953 | 0.424 | 1.078 | 0 | NA |
| TRINITY_DN5433_c0_g2_i4 | -0.87632757 | 0.573856154 | 1 | 1.299 | 0.296 | 1.389 | 1.186 | NA |
| TRINITY_DN1411_c0_g1_i2 | -0.8761836 | 0.760539063 | 0.999970678 | 50.643 | 0 | 63.218 | 17.111 | NA |
| TRINITY_DN6888_c0_g1_i1 | 0.876124187 | 0.336596284 | 0.999970678 | 3.615 | 6.879 | 1.555 | 3.83 | NA |
| TRINITY_DN1848_c5_g1_i1 | -0.87607063 | 0.837181721 | 0.999970678 | 8.475 | 0 | 13.044 | 0 | Cellulase |
| TRINITY_DN7468_c0_g1_i6 | 0.876057285 | 0.585879067 | 0.999970678 | 0.327 | 1.557 | 0.611 | 0.292 | NA |
| TRINITY_DN997_c1_g1_i2 | 0.87604095 | 0.771761044 | 0.999970678 | 0.118 | 3.725 | 1.711 | 0 | NA |
| TRINITY_DN9449_c0_g1_i3 | 0.875774699 | 0.596863207 | 0.999970678 | 0.282 | 0.759 | 0.422 | 0.05 | DUF616 |
| TRINITY_DN19534_c0_g1_i3 | -0.87565187 | 0.835653885 | 0.999970678 | 3.198 | 0 | 4.9 | 0.01 | NA |
| TRINITY_DN924_c0_g1_i1 | -0.87564205 | 0.356983853 | 0.999970678 | 2.907 | 3.597 | 6.855 | 3.609 | Abhydrolase_1 |
| TRINITY_DN11026_c0_g1_i2 | -0.87553577 | 0.744232225 | 0.999970678 | 5.06 | 0 | 4.011 | 4.544 | - |
| TRINITY_DN2590_c0_g4_i1 | -0.87552753 | 0.513693137 | 0.999970678 | 0.863 | 1.675 | 0.9 | 3.549 | NA |
| TRINITY_DN6551_c5_g1_i4 | -0.87551548 | 0.367161618 | 0.999970678 | 401.55 | 129.9 | 378.555 | 526.844 | - |
| TRINITY_DN4324_c0_g1_i2 | -0.87551545 | 0.586478941 | 0.999970678 | 2.489 | 7.293 | 13.521 | 1.377 | NA |
| TRINITY_DN10177_c0_g1_i2 | -0.87549744 | 0.527647239 | 0.999970678 | 15.824 | 2.227 | 8.211 | 23.516 | NA |
| TRINITY_DN4284_c0_g1_i1 | 0.875492007 | 0.672584984 | 0.999970678 | 58.628 | 1.587 | 17.799 | 10.144 | NA |
| TRINITY_DN298_c0_g2_i2 | -0.87539414 | 0.539315962 | 0.999970678 | 1.69 | 2.474 | 0.644 | 6.877 | NA |
| TRINITY_DN5197_c0_g1_i1 | -0.8752472 | 0.763830997 | 0.999970678 | 26.434 | 0 | 9.666 | 37.561 | Aminotran_1_2 |
| TRINITY_DN21104_c0_g3_i3 | -0.87516569 | 0.642468788 | 1 | 1.008 | 0.108 | 1.067 | 0.724 | GST_C |
| TRINITY_DN3874_c0_g2_i6 | 0.875081203 | 0.538287979 | 0.999970678 | 2.035 | 0.69 | 0.955 | 0.362 | - |
| TRINITY_DN7431_c0_g1_i2 | 0.874971338 | 0.836457801 | 0.999970678 | 2.544 | 0 | 0 | 1.408 | F-box |
| TRINITY_DN1440_c1_g1_i7 | -0.87494758 | 0.373253232 | 0.999970678 | 24.663 | 9.53 | 21.032 | 36.666 | GST_C |
| TRINITY_DN17464_c2_g1_i1 | -0.87492969 | 0.553637849 | 0.999970678 | 170.33 | 60.37 | 84.239 | 285.334 | NA |
| TRINITY_DN14367_c0_g1_i1 | -0.87490337 | 0.414179622 | 0.999970678 | 9.929 | 10.65 | 8.911 | 25.758 | NA |
| TRINITY_DN24004_c0_g1_i2 | -0.87490315 | 0.791349753 | 1 | 0.527 | 0 | 0.8 | 0.02 | NA |
| TRINITY_DN1466_c0_g1_i6 | 0.874874957 | 0.584528946 | 0.999970678 | 2.398 | 2.858 | 0.167 | 2.664 | SQS_PSY |
| TRINITY_DN4847_c2_g2_i8 | -0.87465644 | 0.424616799 | 0.999970678 | 0.699 | 1.715 | 2.4 | 1.478 | NA |
| TRINITY_DN4289_c0_g2_i3 | -0.87463007 | 0.640023088 | 0.999970678 | 1.036 | 0.493 | 2.244 | 0.09 | NA |
| TRINITY_DN264_c0_g1_i4 | -0.87442778 | 0.264997691 | 0.999970678 | 8.139 | 6.238 | 9.899 | 14.035 | NA |
| TRINITY_DN545_c0_g1_i1 | 0.874384856 | 0.349250936 | 0.999970678 | 4.233 | 9.077 | 3.833 | 2.604 | NB-ARC |
| TRINITY_DN5544_c0_g1_i2 | -0.87432648 | 0.845808309 | 1 | 4.696 | 0 | 7.011 | 0 | NA |
| TRINITY_DN2057_c0_g2_i3 | -0.87432648 | 0.845808309 | 1 | 0.427 | 0 | 0.656 | 0 | NA |
| TRINITY_DN28315_c0_g1_i3 | -0.87432648 | 0.845808309 | 1 | 0.609 | 0 | 0.922 | 0 | NA |
| TRINITY_DN108938_c0_g1_i1 | -0.87432648 | 0.845808309 | 1 | 0.645 | 0 | 0.989 | 0 | NA |
| TRINITY_DN7509_c0_g4_i3 | -0.87413449 | 0.326732601 | 0.999970678 | 1.254 | 1.074 | 2.011 | 1.78 | NA |
| TRINITY_DN18_c0_g1_i4 | -0.8741144 | 0.284715387 | 0.999970678 | 154.45 | 155.6 | 145.547 | 391.008 | NA |
| TRINITY_DN2132_c0_g1_i2 | 0.874109151 | 0.593267331 | 0.999970678 | 46.964 | 13.31 | 23.71 | 3.408 | NA |

| TRINITY_DN11213_c0_g3_i1 | -0.87403421 | 0.782705877 | 1 | 0.327 | 0 | 0.489 | 0.03 | DUF4219 |
| --- | --- | --- | --- | --- | --- | --- | --- | --- |
| TRINITY_DN4242_c0_g2_i16 | -0.87394662 | 0.541181726 | 0.999970678 | 0.491 | 0.138 | 0.711 | 0.312 | NA |
| TRINITY_DN22237_c0_g1_i1 | 0.873939712 | 0.435998308 | 0.999970678 | 2.226 | 2.277 | 0.644 | 1.659 | RNase_H2_suC |
| TRINITY_DN2972_c0_g1_i4 | 0.873900204 | 0.846282756 | 1 | 1.09 | 0 | 0 | 0.603 | NA |
| TRINITY_DN247_c1_g3_i1 | 0.873797623 | 0.589630679 | 0.999970678 | 24.963 | 2.395 | 8.777 | 4.132 | NA |
| TRINITY_DN5156_c0_g2_i5 | 0.873779169 | 0.502753323 | 0.999970678 | 12.209 | 97.19 | 23.021 | 31.106 | MatE |
| TRINITY_DN3798_c0_g1_i6 | -0.87371578 | 0.467791278 | 0.999970678 | 9.32 | 6.16 | 18.043 | 6.203 | NA |
| TRINITY_DN17986_c0_g1_i1 | -0.8736947 | 0.840398334 | 0.999970678 | 13.335 | 0 | 20.488 | 0 | NA |
| TRINITY_DN5769_c0_g1_i6 | 0.87367269 | 0.569803974 | 1 | 1.572 | 1.055 | 0.989 | 0.261 | NA |
| TRINITY_DN4057_c0_g1_i9 | -0.87367138 | 0.338889775 | 0.999970678 | 0.273 | 0.503 | 0.6 | 0.694 | - |
| TRINITY_DN12158_c0_g3_i5 | 0.873634385 | 0.789699836 | 0.999970678 | 1.935 | 0 | 0.022 | 1.046 | DUF4371 |
| TRINITY_DN8095_c0_g1_i1 | 0.873540935 | 0.434533017 | 0.999970678 | 0.363 | 0.641 | 0.256 | 0.241 | EGF_2 |
| TRINITY_DN6387_c0_g1_i3 | 0.87352353 | 0.516425543 | 0.999970678 | 0.318 | 0.749 | 0.356 | 0.151 | G_glu_transpept |
| TRINITY_DN83_c0_g1_i4 | 0.873523355 | 0.55814498 | 0.999970678 | 1.617 | 11.74 | 2.578 | 4.072 | NA |
| TRINITY_DN2170_c0_g2_i4 | -0.87348118 | 0.748516659 | 0.999970678 | 0 | 1.666 | 1.933 | 0.653 | F-box |
| TRINITY_DN10939_c0_g1_i2 | 0.873383104 | 0.473715196 | 0.999970678 | 0.727 | 2.237 | 0.533 | 0.955 | NA |
| TRINITY_DN1191_c0_g1_i7 | 0.873363344 | 0.754172885 | 0.999970678 | 0 | 4.041 | 0.644 | 1.408 | NA |
| TRINITY_DN2441_c0_g1_i5 | 0.87329326 | 0.578628283 | 0.999970678 | 1.799 | 35.32 | 8.022 | 10.335 | NA |
| TRINITY_DN5272_c0_g2_i5 | 0.873280222 | 0.33457727 | 0.999970678 | 29.123 | 35.69 | 8.122 | 25.486 | DUF3007 |
| TRINITY_DN80282_c0_g1_i5 | -0.87327952 | 0.411956495 | 0.999970678 | 3.515 | 2.119 | 6.044 | 2.986 | NA |
| TRINITY_DN362_c0_g1_i4 | -0.87327268 | 0.667089909 | 0.999970678 | 0.309 | 49.21 | 37.942 | 43.593 | Bet_v_1 |
| TRINITY_DN4471_c0_g1_i4 | 0.873269971 | 0.839487579 | 0.999970678 | 13.208 | 0 | 0 | 7.359 | NA |
| TRINITY_DN424_c0_g1_i12 | -0.87321324 | 0.761093015 | 1 | 0.391 | 0 | 0.122 | 0.583 | NA |
| TRINITY_DN1344_c0_g1_i1 | 0.873200473 | 0.423259062 | 0.999970678 | 1.19 | 1.104 | 0.7 | 0.392 | NA |
| TRINITY_DN873_c0_g1_i2 | -0.87319498 | 0.837599759 | 0.999970678 | 0 | 2.947 | 4.433 | 0 | NA |
| TRINITY_DN8730_c0_g1_i2 | -0.87303983 | 0.748374615 | 0.999970678 | 0 | 1.951 | 0.667 | 2.704 | NA |
| TRINITY_DN706_c0_g2_i3 | 0.873019314 | 0.615331136 | 0.999970678 | 0.372 | 2.296 | 0.178 | 1.216 | NA |
| TRINITY_DN333_c0_g1_i2 | 0.87288846 | 0.493093694 | 0.999970678 | 0.581 | 1.419 | 0.211 | 0.845 | - |
| TRINITY_DN3040_c0_g1_i1 | -0.87278773 | 0.756109053 | 0.999970678 | 0 | 12.53 | 15.41 | 3.941 | NA |
| TRINITY_DN6168_c0_g1_i1 | 0.872722607 | 0.843914831 | 1 | 3.052 | 0 | 0 | 1.639 | Ribosomal_S17 |
| TRINITY_DN2119_c0_g1_i2 | -0.87236941 | 0.546340165 | 0.999970678 | 0.363 | 0.631 | 1.2 | 0.362 | NA |
| TRINITY_DN13468_c0_g1_i2 | -0.87234969 | 0.293699635 | 0.999970678 | 0.89 | 0.66 | 0.978 | 1.669 | bHLH-MYC_N |
| TRINITY_DN30602_c0_g1_i1 | -0.87230669 | 0.557787456 | 0.999970678 | 0.627 | 0.158 | 0.933 | 0.302 | NA |
| TRINITY_DN8279_c2_g1_i2 | -0.87229574 | 0.748666146 | 0.999970678 | 0 | 0.808 | 0.889 | 0.372 | NA |
| TRINITY_DN2882_c0_g1_i6 | 0.872173604 | 0.194035653 | 0.999970678 | 8.748 | 6.229 | 3.511 | 3.981 | NA |
| TRINITY_DN24811_c0_g1_i2 | 0.871979195 | 0.83924395 | 0.999970678 | 2.389 | 0 | 1.1 | 0 | Asp |
| TRINITY_DN706_c0_g1_i7 | -0.87197812 | 0.363511901 | 0.999970678 | 11.146 | 4.652 | 14.821 | 11.119 | NA |
| TRINITY_DN9561_c0_g1_i2 | 0.871934408 | 0.842180538 | 0.999970678 | 2.698 | 0 | 0 | 1.488 | NA |
| TRINITY_DN1613_c0_g1_i2 | -0.8717059 | 0.785055242 | 0.999970678 | 0.082 | 4.445 | 6.733 | 0 | NA |
| TRINITY_DN14975_c0_g1_i1 | -0.87163622 | 0.284271379 | 0.999970678 | 17.95 | 13.33 | 21.888 | 28.623 | NA |
| TRINITY_DN1308_c0_g1_i2 | -0.87161885 | 0.59959841 | 0.999970678 | 1.272 | 19.53 | 22.554 | 9.943 | NA |
| TRINITY_DN580_c0_g1_i8 | -0.87161641 | 0.667604341 | 0.999970678 | 5.886 | 0.177 | 7.9 | 1.538 | NA |
| TRINITY_DN3529_c0_g1_i1 | -0.87152524 | 0.159542547 | 0.999970678 | 50.743 | 42.76 | 77.218 | 76.589 | NA |
| TRINITY_DN3218_c0_g1_i5 | -0.8714265 | 0.752833502 | 0.999970678 | 10.428 | 0 | 6.1 | 11.974 | DUF1005 |
| TRINITY_DN2934_c0_g1_i4 | -0.87125717 | 0.339016178 | 0.999970678 | 1.108 | 0.966 | 1.078 | 2.503 | LRRNT_2 |
| TRINITY_DN6850_c0_g2_i3 | 0.870938306 | 0.740668516 | 0.999970678 | 1.771 | 3.193 | 0 | 2.694 | NA |
| TRINITY_DN794_c0_g1_i12 | -0.87084809 | 0.356386635 | 0.999970678 | 97.716 | 148.5 | 99.894 | 308.548 | Dimerisation |
| TRINITY_DN4333_c1_g1_i4 | -0.87082339 | 0.753160372 | 1 | 0.291 | 0 | 0.178 | 0.342 | NA |
| TRINITY_DN11661_c0_g2_i3 | -0.87070721 | 0.757105086 | 0.999970678 | 0.618 | 0.453 | 0 | 2.001 | Raffinose_syn |
| TRINITY_DN4_c0_g1_i8 | 0.870605097 | 0.692445231 | 1 | 3.098 | 44.67 | 1.944 | 21.123 | NA |
| TRINITY_DN867_c0_g1_i7 | 0.870592465 | 0.602396275 | 0.999970678 | 1.399 | 1.252 | 1.133 | 0.101 | ANF_receptor |
| TRINITY_DN86131_c0_g1_i1 | 0.870467986 | 0.475882913 | 0.999970678 | 0.79 | 0.926 | 0.267 | 0.613 | NA |
| TRINITY_DN3341_c0_g1_i9 | 0.870455981 | 0.348318505 | 0.999970678 | 3.915 | 2.05 | 1.044 | 2.021 | NA |
| TRINITY_DN13434_c0_g1_i5 | 0.870448831 | 0.757062215 | 1 | 10.592 | 4.317 | 6.633 | 0 | RVT_1 |
| TRINITY_DN632_c0_g1_i6 | -0.87040359 | 0.357937827 | 0.999970678 | 1.263 | 1.636 | 3.222 | 1.428 | Asp_protease_2 |
| TRINITY_DN37_c0_g1_i8 | -0.87026625 | 0.360998441 | 0.999970678 | 2.017 | 1.567 | 3.966 | 1.79 | NADHdh |
| TRINITY_DN68836_c0_g1_i1 | -0.87021487 | 0.553671108 | 1 | 0.227 | 0.503 | 0.267 | 1.005 | NA |
| TRINITY_DN4491_c0_g1_i3 | -0.87014155 | 0.761795566 | 0.999970678 | 0 | 44.37 | 40.975 | 30.181 | NA |
| TRINITY_DN16764_c0_g1_i5 | 0.870057755 | 0.433167788 | 0.999970678 | 1.163 | 3.055 | 1.355 | 0.664 | DNA_gyraseB |
| TRINITY_DN97043_c0_g1_i2 | -0.87005773 | 0.838023128 | 0.999970678 | 3.37 | 0 | 5.155 | 0 | NA |
| TRINITY_DN367_c0_g1_i3 | -0.87001774 | 0.311327177 | 0.999970678 | 8.321 | 12.91 | 16.632 | 17.514 | NA |
| TRINITY_DN5331_c0_g4_i3 | -0.86999644 | 0.748363793 | 0.999970678 | 0 | 13.25 | 11.388 | 9.109 | NA |
| TRINITY_DN64006_c0_g1_i1 | -0.86999412 | 0.598193244 | 0.999970678 | 16.496 | 160.6 | 206.743 | 48.539 | NA |
| TRINITY_DN1347_c1_g1_i1 | 0.869957606 | 0.479758759 | 0.999970678 | 0.309 | 0.522 | 0.267 | 0.131 | Gelsolin |
| TRINITY_DN4500_c0_g1_i8 | -0.86988666 | 0.753585803 | 0.999970678 | 1.308 | 0 | 1.578 | 0.533 | PSS |
| TRINITY_DN2334_c0_g1_i7 | 0.869782285 | 0.765190971 | 0.999970678 | 9.393 | 1.271 | 0 | 5.932 | NA |
| TRINITY_DN164497_c0_g1_i1 | 0.869757634 | 0.324332015 | 0.999970678 | 5.305 | 12.37 | 3.489 | 5.419 | MatE |
| TRINITY_DN182_c0_g1_i8 | -0.86967529 | 0.65301048 | 0.999970678 | 0.064 | 1.941 | 2.322 | 0.814 | NA |
| TRINITY_DN1477_c0_g2_i1 | -0.86963935 | 0.42849241 | 0.999970678 | 8.412 | 20.3 | 10.455 | 39.521 | NA |
| TRINITY_DN3289_c0_g1_i1 | 0.869573546 | 0.836140318 | 0.999970678 | 7.83 | 0 | 0 | 4.363 | WD40 |
| TRINITY_DN3263_c0_g1_i2 | -0.86956011 | 0.750925323 | 0.999970678 | 4.042 | 0 | 4.655 | 1.87 | NA |
| TRINITY_DN267_c0_g1_i9 | 0.869548985 | 0.641325723 | 1 | 0.581 | 1.646 | 0.1 | 1.076 | - |
| TRINITY_DN2655_c1_g1_i1 | -0.86953743 | 0.578164994 | 0.999970678 | 8.857 | 2.168 | 4.033 | 14.568 | Motile_Sperm |
| TRINITY_DN2289_c2_g1_i4 | -0.86953498 | 0.41843171 | 0.999970678 | 0.527 | 0.611 | 0.767 | 1.126 | Plant_zn_clust |

| TRINITY_DN1540_c1_g1_i6 | -0.86953283 | 0.264673857 | 0.999970678 | 1.453 | 1.961 | 2.444 | 3.267 | NA |
| --- | --- | --- | --- | --- | --- | --- | --- | --- |
| TRINITY_DN1145_c0_g1_i3 | -0.86951821 | 0.51297674 | 0.999970678 | 1.199 | 0.572 | 0.656 | 2.443 | NA |
| TRINITY_DN2858_c0_g1_i5 | -0.86950599 | 0.845716248 | 1 | 2.153 | 0 | 3.222 | 0 | F_bP_aldolase |
| TRINITY_DN1680_c0_g1_i4 | -0.86939717 | 0.530764665 | 0.999970678 | 0.672 | 0.572 | 1.467 | 0.493 | NA |
| TRINITY_DN779_c0_g1_i3 | -0.86930879 | 0.443826169 | 0.999970678 | 2.771 | 0.907 | 1.711 | 4.735 | NA |
| TRINITY_DN798_c0_g2_i6 | -0.86929148 | 0.381948759 | 0.999970678 | 2.071 | 4.947 | 3.689 | 8.304 | NA |
| TRINITY_DN1088_c0_g1_i1 | 0.869121315 | 0.454515027 | 0.999970678 | 50.434 | 58.4 | 8.122 | 49.424 | NA |
| TRINITY_DN30807_c0_g1_i1 | -0.86911775 | 0.753443318 | 0.999970678 | 20.639 | 0 | 18.021 | 16.317 | NA |
| TRINITY_DN3772_c0_g1_i6 | -0.86903714 | 0.576088572 | 0.999970678 | 3.615 | 0.739 | 1.267 | 6.434 | Cu_amine_oxid |
| TRINITY_DN5514_c0_g1_i1 | -0.86902408 | 0.747940456 | 0.999970678 | 3.279 | 0 | 2.966 | 2.493 | AMPK1_CBM |
| TRINITY_DN5514_c0_g1_i5 | -0.86902408 | 0.747940456 | 0.999970678 | 3.279 | 0 | 2.966 | 2.493 | AMPK1_CBM |
| TRINITY_DN28329_c0_g1_i1 | -0.86898932 | 0.847636086 | 1 | 0.663 | 0 | 0 | 1.227 | UDPGT |
| TRINITY_DN5165_c0_g1_i1 | 0.86897309 | 0.655464412 | 0.999970678 | 9.302 | 0.335 | 3.9 | 0.633 | WD40 |
| TRINITY_DN108265_c0_g1_i1 | 0.868825544 | 0.842701719 | 0.999970678 | 1.544 | 0 | 0.711 | 0 | NA |
| TRINITY_DN6322_c0_g1_i3 | 0.868801406 | 0.317843835 | 0.999970678 | 5.423 | 2.878 | 1.811 | 2.383 | B3 |
| TRINITY_DN50372_c0_g2_i1 | -0.86869412 | 0.553685303 | 0.999970678 | 1.917 | 5.095 | 2.411 | 9.37 | NA |
| TRINITY_DN165551_c0_g1_i1 | 0.868630385 | 0.43821989 | 0.999970678 | 5.932 | 1.951 | 2.122 | 1.78 | - |
| TRINITY_DN4023_c0_g1_i3 | 0.868584266 | 0.60108461 | 0.999970678 | 4.642 | 0.325 | 1.644 | 0.784 | NA |
| TRINITY_DN1218_c0_g1_i2 | 0.868558703 | 0.425848155 | 0.999970678 | 13.944 | 23.97 | 3.822 | 15.915 | NA |
| TRINITY_DN353_c1_g1_i9 | -0.86852308 | 0.485412482 | 0.999970678 | 30.985 | 6.061 | 36.32 | 23.797 | NA |
| TRINITY_DN586_c0_g1_i4 | -0.86842359 | 0.83597157 | 0.999970678 | 0 | 1.784 | 0 | 3.237 | Pkinase |
| TRINITY_DN70638_c0_g1_i1 | -0.86841297 | 0.842751799 | 0.999970678 | 0.745 | 0 | 1.133 | 0 | NA |
| TRINITY_DN7977_c1_g1_i3 | 0.868407783 | 0.501394662 | 0.999970678 | 2.889 | 1.104 | 1.3 | 0.613 | NA |
| TRINITY_DN1926_c0_g1_i1 | 0.868392773 | 0.362303209 | 0.999970678 | 45.565 | 15.79 | 14.599 | 16.227 | NA |
| TRINITY_DN19370_c0_g2_i1 | 0.868375254 | 0.29908617 | 0.999970678 | 5.378 | 10.01 | 3.322 | 4.353 | adh_short |
| TRINITY_DN2451_c1_g2_i7 | 0.868249292 | 0.483638379 | 0.999970678 | 0.309 | 0.197 | 0.189 | 0.05 | Ycf1 |
| TRINITY_DN1012_c0_g1_i8 | 0.868083719 | 0.414425693 | 0.999970678 | 8.666 | 7.648 | 4.689 | 3.036 | NA |
| TRINITY_DN72759_c0_g1_i3 | 0.868074206 | 0.47103717 | 0.999970678 | 7.93 | 35.32 | 13.721 | 6.907 | AA_permease_2 |
| TRINITY_DN2802_c0_g1_i2 | -0.86805493 | 0.19300204 | 0.999970678 | 9.611 | 7.835 | 14.366 | 14.427 | NA |
| TRINITY_DN8151_c0_g1_i1 | 0.868004822 | 0.468432289 | 1 | 0.309 | 0.315 | 0.156 | 0.161 | NA |
| TRINITY_DN46239_c0_g2_i3 | -0.86798251 | 0.295019171 | 0.999970678 | 23.473 | 21.32 | 46.342 | 25.657 | NA |
| TRINITY_DN604_c0_g1_i6 | -0.86797724 | 0.514938291 | 0.999970678 | 170.25 | 63.81 | 162.679 | 199.355 | NA |
| TRINITY_DN8477_c0_g1_i5 | 0.867939774 | 0.543107394 | 0.999970678 | 0.436 | 0.286 | 0.256 | 0.09 | PPR |
| TRINITY_DN12878_c0_g1_i1 | -0.8678454 | 0.596792764 | 1 | 0.545 | 0.099 | 0.422 | 0.684 | NA |
| TRINITY_DN5169_c0_g1_i3 | -0.86772376 | 0.473121351 | 0.999970678 | 3.425 | 4.395 | 7.8 | 4.695 | NA |
| TRINITY_DN194306_c0_g1_i1 | -0.86772189 | 0.385152251 | 0.999970678 | 0.472 | 0.66 | 0.8 | 1.106 | BRCT |
| TRINITY_DN393_c4_g1_i1 | -0.8677104 | 0.604039862 | 0.999970678 | 1.78 | 8.771 | 1.1 | 17.745 | NA |
| TRINITY_DN6644_c0_g1_i4 | 0.867628296 | 0.15680748 | 0.999970678 | 4.324 | 4.455 | 2.2 | 2.172 | zf-CW |
| TRINITY_DN13582_c0_g1_i1 | -0.86754879 | 0.837382053 | 0.999970678 | 2.989 | 0 | 4.566 | 0 | NA |
| TRINITY_DN7925_c0_g2_i5 | 0.867547813 | 0.624099612 | 0.999970678 | 0.254 | 0.798 | 0.056 | 0.523 | NA |
| TRINITY_DN9659_c0_g1_i1 | 0.86750947 | 0.193667369 | 0.999970678 | 9.983 | 12.34 | 6.055 | 4.936 | NA |
| TRINITY_DN12770_c0_g1_i1 | -0.86724933 | 0.384016887 | 0.999970678 | 11.428 | 4.652 | 13.599 | 12.648 | NA |
| TRINITY_DN48546_c0_g2_i1 | 0.867118007 | 0.607839406 | 1 | 0.563 | 0.572 | 0.456 | 0.08 | NA |
| TRINITY_DN3025_c0_g1_i2 | -0.86703273 | 0.494891065 | 0.999970678 | 6.159 | 1.892 | 6.133 | 6.997 | NA |
| TRINITY_DN1502_c0_g1_i10 | -0.86700613 | 0.516390868 | 0.999970678 | 3.361 | 2.681 | 7.144 | 2.292 | NA |
| TRINITY_DN1305_c0_g1_i8 | -0.86700135 | 0.756690595 | 1 | 1.744 | 0 | 1.044 | 1.87 | NA |
| TRINITY_DN4934_c0_g1_i5 | 0.866980964 | 0.263511995 | 0.999970678 | 1.681 | 1.725 | 0.889 | 0.794 | NA |
| TRINITY_DN1397_c0_g1_i15 | 0.866977027 | 0.571878711 | 0.999970678 | 0.863 | 1.212 | 0.856 | 0.101 | NA |
| TRINITY_DN15243_c0_g1_i4 | 0.866923676 | 0.574432641 | 0.999970678 | 0.563 | 4.307 | 1.111 | 1.297 | NA |
| TRINITY_DN5305_c0_g1_i2 | -0.8668423 | 0.466429019 | 0.999970678 | 0.618 | 0.453 | 1.044 | 0.653 | NA |
| TRINITY_DN89580_c0_g1_i1 | 0.866736423 | 0.84346853 | 0.999970678 | 1.926 | 0 | 0 | 1.066 | NA |
| TRINITY_DN7751_c0_g2_i3 | 0.866698143 | 0.757192467 | 1 | 2.044 | 0.966 | 1.344 | 0 | NA |
| TRINITY_DN3755_c0_g2_i2 | 0.866558705 | 0.759797586 | 0.999970678 | 0 | 3.361 | 0.922 | 0.694 | NA |
| TRINITY_DN19309_c2_g1_i1 | -0.86645816 | 0.421706636 | 0.999970678 | 0.209 | 0.414 | 0.456 | 0.583 | Mito_carr |
| TRINITY_DN729_c0_g2_i1 | -0.86641972 | 0.545296202 | 0.999970678 | 0.509 | 0.384 | 1.1 | 0.292 | BAG |
| TRINITY_DN6809_c0_g1_i5 | -0.86636666 | 0.763677187 | 0.999970678 | 3.134 | 0 | 3.111 | 2.061 | Chorein_N |
| TRINITY_DN581_c0_g1_i15 | 0.866335216 | 0.759661204 | 0.999970678 | 13.608 | 0 | 2.722 | 4.283 | PTS_2-RNA |
| TRINITY_DN1334_c0_g1_i3 | 0.866314984 | 0.755836725 | 0.999970678 | 1.653 | 7.283 | 4.011 | 0 | NA |
| TRINITY_DN17084_c0_g1_i1 | -0.86624122 | 0.292475599 | 0.999970678 | 1.644 | 2.109 | 2.333 | 3.991 | NA |
| TRINITY_DN5495_c0_g1_i2 | -0.86622575 | 0.395223306 | 0.999970678 | 0.545 | 0.798 | 0.8 | 1.448 | NA |
| TRINITY_DN13991_c0_g1_i5 | -0.86621943 | 0.755929673 | 0.999970678 | 1.244 | 0 | 0.322 | 1.92 | Golgin_A5 |
| TRINITY_DN4084_c0_g3_i2 | 0.866206182 | 0.536730697 | 0.999970678 | 1.181 | 0.936 | 0.744 | 0.271 | NA |
| TRINITY_DN800_c0_g1_i8 | 0.866164843 | 0.481505059 | 0.999970678 | 3.188 | 2.385 | 0.544 | 2.393 | NA |
| TRINITY_DN5263_c0_g2_i1 | 0.866097816 | 0.387665936 | 0.999970678 | 16.905 | 30.03 | 15.71 | 6.635 | DAP_epimerase |
| TRINITY_DN4454_c0_g1_i2 | 0.866001653 | 0.771302474 | 0.999970678 | 0.254 | 3.272 | 0 | 1.91 | NA |
| TRINITY_DN3435_c0_g1_i5 | -0.86593088 | 0.625442277 | 0.999970678 | 4.324 | 7.707 | 0.633 | 20.68 | NA |
| TRINITY_DN2379_c0_g1_i7 | 0.865922991 | 0.412142587 | 0.999970678 | 9.783 | 25.56 | 4.689 | 13.603 | S-methyl_trans |
| TRINITY_DN425_c0_g1_i2 | -0.86591419 | 0.232887035 | 0.999970678 | 4.151 | 6.367 | 8.422 | 8.908 | NA |
| TRINITY_DN3166_c0_g1_i2 | -0.86591157 | 0.461271774 | 0.999970678 | 1.771 | 7.263 | 9.022 | 5.419 | - |
| TRINITY_DN868_c0_g1_i6 | -0.86584608 | 0.688653176 | 0.999970678 | 9.229 | 0.108 | 12.155 | 2.483 | NA |
| TRINITY_DN3328_c0_g1_i5 | 0.865686869 | 0.778138801 | 0.999970678 | 0 | 1.271 | 0.033 | 0.653 | Cellulose_synt |
| TRINITY_DN6597_c0_g1_i8 | 0.865682414 | 0.74853675 | 0.999970678 | 0.745 | 0.325 | 0.489 | 0 | zf-RING_2 |
| TRINITY_DN60363_c1_g1_i7 | 0.865672142 | 0.655798249 | 1 | 1.744 | 0.168 | 0.711 | 0.181 | Abhydrolase_1 |
| TRINITY_DN4115_c0_g1_i1 | -0.8654644 | 0.375773684 | 0.999970678 | 2.226 | 1.813 | 4.155 | 2.343 | mTERF |

| TRINITY_DN158_c1_g1_i1 | 0.86544947 | 0.545660945 | 0.999970678 | 5.687 | 17.93 | 1.244 | 11.391 | - |
| --- | --- | --- | --- | --- | --- | --- | --- | --- |
| TRINITY_DN9140_c3_g1_i3 | -0.86541167 | 0.355224462 | 0.999970678 | 3217.8 | 2789 | 6715.871 | 2773.895 | NA |
| TRINITY_DN6198_c1_g1_i1 | 0.865407342 | 0.645712481 | 0.999970678 | 0.091 | 2.503 | 0.422 | 0.885 | NA |
| TRINITY_DN19083_c0_g1_i2 | -0.86538058 | 0.839102014 | 0.999970678 | 3.17 | 0 | 4.811 | 0 | - |
| TRINITY_DN13999_c0_g1_i1 | -0.8653804 | 0.838163954 | 0.999970678 | 2.68 | 0 | 4.1 | 0 | NA |
| TRINITY_DN1674_c0_g1_i3 | 0.865297314 | 0.757220413 | 0.999970678 | 10.928 | 0 | 1.911 | 3.76 | rRNA_processing |
| TRINITY_DN5899_c0_g1_i9 | 0.865173522 | 0.625986928 | 0.999970678 | 3.198 | 0.641 | 1.6 | 0.181 | NA |
| TRINITY_DN693_c0_g1_i6 | 0.865134879 | 0.200382962 | 0.999970678 | 12.381 | 12.09 | 5.033 | 7.259 | - |
| TRINITY_DN18227_c0_g3_i2 | -0.8650104 | 0.523267864 | 0.999970678 | 0.245 | 0.631 | 0.856 | 0.543 | TPT |
| TRINITY_DN3755_c0_g1_i3 | 0.864920766 | 0.374490835 | 0.999970678 | 1.099 | 1.133 | 0.567 | 0.553 | NA |
| TRINITY_DN7302_c0_g1_i1 | 0.864901392 | 0.575288838 | 0.999970678 | 0.627 | 3.666 | 0.4 | 1.85 | NA |
| TRINITY_DN1729_c0_g1_i7 | 0.86486684 | 0.558166925 | 0.999970678 | 3.77 | 1.34 | 1.989 | 0.432 | - |
| TRINITY_DN17379_c0_g1_i1 | -0.86482732 | 0.838255591 | 0.999970678 | 127.48 | 0 | 0 | 219.804 | S-AdoMet_synt_C |
| TRINITY_DN1017_c0_g1_i5 | 0.864776217 | 0.781559472 | 0.999970678 | 0.091 | 7.214 | 0 | 3.961 | NA |
| TRINITY_DN976_c2_g1_i3 | 0.864670096 | 0.773936933 | 0.999970678 | 0.827 | 0 | 0.322 | 0.07 | NA |
| TRINITY_DN964_c0_g1_i11 | 0.864515888 | 0.755199764 | 1 | 1.708 | 0.749 | 0 | 1.327 | NA |
| TRINITY_DN24930_c0_g1_i1 | -0.86447385 | 0.326527652 | 0.999970678 | 2.525 | 2.217 | 2.955 | 5.017 | NA |
| TRINITY_DN111246_c0_g1_i1 | -0.86434105 | 0.537745958 | 0.999970678 | 0.472 | 1.35 | 0.922 | 2.162 | - |
| TRINITY_DN11759_c0_g1_i4 | 0.864304088 | 0.754234775 | 1 | 0.345 | 0.572 | 0.422 | 0 | NA |
| TRINITY_DN4400_c0_g1_i1 | -0.86420289 | 0.394020525 | 0.999970678 | 7.185 | 14.71 | 23.954 | 10.687 | LRR_8 |
| TRINITY_DN43838_c0_g1_i1 | 0.864154801 | 0.613179626 | 0.999970678 | 0.781 | 1.488 | 0.955 | 0.09 | NA |
| TRINITY_DN13956_c0_g1_i6 | 0.863887593 | 0.845936577 | 1 | 7.276 | 0 | 3.378 | 0 | NA |
| TRINITY_DN8569_c0_g1_i5 | 0.863853869 | 0.760978615 | 1 | 0.372 | 0.099 | 0.222 | 0 | NA |
| TRINITY_DN62696_c0_g1_i5 | 0.863808869 | 0.784040257 | 0.999970678 | 0 | 1.833 | 0.756 | 0.06 | NA |
| TRINITY_DN687_c0_g1_i2 | -0.86375314 | 0.749612833 | 0.999970678 | 1.481 | 0 | 1.011 | 1.498 | NA |
| TRINITY_DN3515_c0_g2_i1 | 0.86373885 | 0.497881042 | 0.999970678 | 7.294 | 2.257 | 1.311 | 3.67 | RRM_1 |
| TRINITY_DN1917_c0_g1_i2 | 0.863654082 | 0.30071198 | 0.999970678 | 16.16 | 22.48 | 5.933 | 13.904 | NA |
| TRINITY_DN6633_c0_g1_i2 | -0.86360324 | 0.442764915 | 0.999970678 | 0.754 | 1.202 | 1.767 | 1.377 | NA |
| TRINITY_DN14189_c0_g1_i5 | -0.86346947 | 0.752299758 | 0.999970678 | 1.408 | 0 | 0.744 | 1.709 | DUF863 |
| TRINITY_DN6196_c0_g1_i18 | -0.86336238 | 0.752382082 | 0.999970678 | 1.335 | 0 | 0.633 | 1.729 | NA |
| TRINITY_DN13_c0_g1_i10 | -0.86290315 | 0.323804323 | 0.999970678 | 6.858 | 8.88 | 7.177 | 19.926 | NA |
| TRINITY_DN11311_c0_g1_i3 | -0.86284444 | 0.757751483 | 0.999970678 | 6.74 | 0 | 5.111 | 6.304 | NA |
| TRINITY_DN1202_c0_g2_i5 | 0.862827476 | 0.597858636 | 0.999970678 | 3.761 | 0.384 | 0.989 | 1.116 | Cellulose_synt |
| TRINITY_DN1711_c0_g2_i1 | -0.86266823 | 0.842851026 | 0.999970678 | 0.427 | 0 | 0.656 | 0 | NA |
| TRINITY_DN3186_c0_g1_i3 | -0.86262488 | 0.53406464 | 0.999970678 | 2.189 | 1.616 | 0.644 | 6.153 | NA |
| TRINITY_DN1078_c0_g3_i1 | 0.862595892 | 0.75524467 | 0.999970678 | 4.142 | 1.35 | 2.533 | 0 | Xan_ur_permease |
| TRINITY_DN633_c0_g1_i5 | -0.86256895 | 0.749893679 | 0.999970678 | 0 | 3.755 | 1.989 | 4.343 | NA |
| TRINITY_DN321_c0_g1_i6 | 0.862428846 | 0.760825027 | 0.999970678 | 4.469 | 0 | 0.9 | 1.418 | NA |
| TRINITY_DN1356_c0_g1_i5 | -0.86241172 | 0.451797243 | 0.999970678 | 33.874 | 130.2 | 72.329 | 207.046 | Cupin_1 |
| TRINITY_DN279_c0_g1_i1 | -0.86236181 | 0.747463141 | 0.999970678 | 8.248 | 0 | 5.089 | 8.857 | NA |
| TRINITY_DN6881_c0_g1_i1 | 0.862329741 | 0.542238772 | 0.999970678 | 0.581 | 3.055 | 0.7 | 1.136 | NA |
| TRINITY_DN1540_c1_g1_i13 | -0.86218126 | 0.307890748 | 0.999970678 | 4.778 | 6.702 | 6.2 | 13.18 | NA |
| TRINITY_DN19116_c0_g1_i7 | -0.86215317 | 0.768289862 | 1 | 0.527 | 0 | 0.7 | 0.131 | NA |
| TRINITY_DN1703_c0_g1_i4 | 0.862126308 | 0.581088848 | 0.999970678 | 1.971 | 3.41 | 2.255 | 0.241 | Glyco_transf_20 |
| TRINITY_DN6353_c0_g6_i1 | 0.8621177 | 0.760085192 | 0.999970678 | 0 | 12.19 | 4 | 1.769 | NA |
| TRINITY_DN18756_c0_g1_i1 | -0.86210193 | 0.435186812 | 0.999970678 | 1.508 | 1.764 | 1.389 | 4.243 | NA |
| TRINITY_DN13255_c0_g1_i2 | -0.86205825 | 0.759159941 | 0.999970678 | 0 | 0.355 | 0.411 | 0.121 | NA |
| TRINITY_DN5134_c0_g1_i10 | 0.862051074 | 0.745503142 | 0.999970678 | 1.263 | 3.124 | 0 | 2.393 | Mur_ligase_M |
| TRINITY_DN1058_c0_g3_i3 | -0.86204361 | 0.30890251 | 0.999970678 | 1.835 | 2.671 | 2.711 | 4.866 | PRA1 |
| TRINITY_DN9000_c0_g1_i5 | -0.86202552 | 0.487944929 | 0.999970678 | 0.836 | 1.084 | 2.3 | 0.714 | NA |
| TRINITY_DN13474_c0_g1_i12 | -0.86186609 | 0.663574609 | 0.999970678 | 0.681 | 0.503 | 0.067 | 2.071 | NA |
| TRINITY_DN3625_c0_g1_i3 | -0.86172384 | 0.753357546 | 0.999970678 | 0 | 1.39 | 0.522 | 1.87 | MT-A70 |
| TRINITY_DN5717_c0_g2_i1 | -0.86161587 | 0.692503218 | 0.999970678 | 1.054 | 0.512 | 0.078 | 2.785 | NA |
| TRINITY_DN387_c0_g1_i2 | -0.86157774 | 0.335958666 | 0.999970678 | 40.06 | 35.51 | 31.643 | 98.898 | NA |
| TRINITY_DN1171_c0_g1_i1 | -0.86157509 | 0.276349214 | 0.999970678 | 1.862 | 3.568 | 3.666 | 5.419 | Myb_Cef |
| TRINITY_DN26344_c0_g1_i4 | -0.86156239 | 0.695297896 | 1 | 3.761 | 0.641 | 6.533 | 0.322 | NA |
| TRINITY_DN907_c1_g2_i2 | -0.8615417 | 0.384917924 | 0.999970678 | 2.798 | 6.376 | 4.166 | 11.592 | 2OG-FeII_Oxy |
| TRINITY_DN30378_c0_g1_i13 | 0.861348095 | 0.846793304 | 1 | 0 | 1.932 | 0.889 | 0 | Sugar_tr |
| TRINITY_DN3673_c0_g2_i2 | 0.861279336 | 0.541570656 | 0.999970678 | 0.354 | 1.33 | 0.278 | 0.593 | EF-hand_5 |
| TRINITY_DN920_c0_g1_i5 | 0.861186379 | 0.52832148 | 0.999970678 | 4.524 | 3.055 | 3.044 | 0.503 | FBPase |
| TRINITY_DN32315_c0_g1_i2 | 0.861175938 | 0.420426051 | 0.999970678 | 1.708 | 2.316 | 0.556 | 1.518 | Myb_DNA-bind_ |
| TRINITY_DN814_c0_g1_i1 | -0.86114276 | 0.761433705 | 0.999970678 | 0.318 | 1.459 | 0 | 3.217 | - |
| TRINITY_DN5731_c0_g1_i2 | -0.86114177 | 0.400195003 | 0.999970678 | 9.602 | 8.978 | 6.311 | 26.2 | GRAS |
| TRINITY_DN4633_c0_g1_i1 | 0.861135401 | 0.498415393 | 0.999970678 | 14.543 | 2.385 | 3.2 | 5.57 | NA |
| TRINITY_DN5640_c0_g1_i1 | -0.86109848 | 0.658448099 | 0.999970678 | 0.781 | 37.03 | 46.508 | 11.019 | NA |
| TRINITY_DN7780_c0_g1_i1 | -0.86087255 | 0.514138009 | 0.999970678 | 4.106 | 2.02 | 1.589 | 9.199 | Asp_Glu_race |
| TRINITY_DN32960_c0_g1_i2 | -0.86071567 | 0.576066011 | 0.999970678 | 1.335 | 0.769 | 0.489 | 3.207 | NA |
| TRINITY_DN4352_c0_g1_i4 | -0.86068624 | 0.758581031 | 0.999970678 | 10.892 | 0 | 12.333 | 5.158 | NA |
| TRINITY_DN1293_c0_g1_i12 | 0.860581539 | 0.746098676 | 0.999970678 | 0.99 | 0.926 | 0.878 | 0 | NA |
| TRINITY_DN6863_c0_g1_i1 | -0.8604117 | 0.450526212 | 0.999970678 | 0.945 | 0.375 | 0.911 | 1.307 | NA |
| TRINITY_DN11037_c0_g2_i9 | -0.86041069 | 0.745470348 | 0.999970678 | 2.362 | 0.079 | 3.644 | 0.06 | NA |
| TRINITY_DN3943_c0_g1_i2 | 0.860396709 | 0.838536763 | 0.999970678 | 16.533 | 0 | 0 | 9.239 | NA |
| TRINITY_DN2895_c0_g1_i10 | 0.860343293 | 0.50709635 | 0.999970678 | 11.237 | 2.779 | 1.467 | 6.042 | PP2C |
| TRINITY_DN18242_c0_g1_i4 | -0.86022762 | 0.850168626 | 1 | 0.699 | 0 | 1.044 | 0 | NA |

| TRINITY_DN1080_c0_g1_i7 | 0.860007673 | 0.306618424 | 0.999970678 | 10.919 | 22.65 | 8.222 | 8.385 | LRRNT_2 |
| --- | --- | --- | --- | --- | --- | --- | --- | --- |
| TRINITY_DN220_c0_g2_i4 | -0.86000675 | 0.747911876 | 0.999970678 | 2.861 | 0 | 2.478 | 2.252 | NA |
| TRINITY_DN5822_c0_g1_i8 | -0.85998576 | 0.515793236 | 0.999970678 | 0.418 | 0.158 | 0.222 | 0.784 | NA |
| TRINITY_DN5115_c0_g1_i5 | 0.859867805 | 0.381054055 | 0.999970678 | 1.435 | 1.212 | 0.767 | 0.543 | NDK |
| TRINITY_DN4612_c0_g1_i7 | -0.85961567 | 0.74946444 | 0.999970678 | 0 | 1.646 | 1.7 | 0.895 | NA |
| TRINITY_DN2301_c1_g1_i5 | -0.85951224 | 0.400567816 | 0.999970678 | 3.57 | 1.212 | 3.566 | 4.444 | Anticodon_1 |
| TRINITY_DN53892_c0_g1_i1 | -0.85946824 | 0.207269617 | 0.999970678 | 8.457 | 7.165 | 10.677 | 15.392 | NA |
| TRINITY_DN3452_c0_g1_i4 | -0.85943394 | 0.781294765 | 1 | 0.327 | 0 | 0.467 | 0.05 | NA |
| TRINITY_DN15166_c0_g1_i1 | -0.85935337 | 0.404901084 | 0.999970678 | 0.5 | 0.926 | 0.644 | 1.8 | NA |
| TRINITY_DN11743_c2_g1_i1 | -0.85934289 | 0.774729062 | 1 | 0.845 | 0 | 0.133 | 1.408 | NA |
| TRINITY_DN5265_c2_g1_i3 | 0.859330611 | 0.761761226 | 0.999970678 | 0.372 | 3.361 | 0 | 2.031 | NA |
| TRINITY_DN6689_c0_g2_i5 | 0.859282736 | 0.619003151 | 0.999970678 | 1.008 | 1.675 | 1.1 | 0.151 | NA |
| TRINITY_DN7827_c0_g1_i2 | -0.85927785 | 0.838338938 | 0.999970678 | 0 | 2.543 | 3.755 | 0 | NA |
| TRINITY_DN7109_c0_g1_i1 | 0.859188552 | 0.752251473 | 0.999970678 | 0.236 | 0.345 | 0.267 | 0 | NA |
| TRINITY_DN12128_c0_g1_i9 | -0.85914875 | 0.761647045 | 1 | 0.6 | 0 | 0.567 | 0.382 | NA |
| TRINITY_DN81_c0_g1_i5 | -0.85913525 | 0.589173965 | 0.999970678 | 0.263 | 3.913 | 4.166 | 2.443 | Diphthamide_syn |
| TRINITY_DN1262_c1_g1_i1 | 0.859122555 | 0.765509863 | 0.999970678 | 0 | 19.64 | 6.444 | 2.865 | - |
| TRINITY_DN3423_c0_g1_i2 | 0.859103679 | 0.481321144 | 0.999970678 | 33.874 | 9.481 | 5.144 | 17.715 | RRM_1 |
| TRINITY_DN18690_c0_g2_i5 | 0.859100076 | 0.451369846 | 0.999970678 | 4.851 | 1.932 | 1.455 | 1.971 | NA |
| TRINITY_DN33226_c1_g1_i1 | -0.85909799 | 0.27565471 | 0.999970678 | 841.98 | 1510 | 2166.136 | 1619.952 | PsbY |
| TRINITY_DN25599_c0_g1_i1 | 0.859093908 | 0.794783504 | 1 | 0.045 | 1.725 | 0.789 | 0 | Lipase_GDSL |
| TRINITY_DN18530_c0_g1_i1 | -0.85908263 | 0.665451266 | 0.999970678 | 0.4 | 0.68 | 0.056 | 1.88 | p450 |
| TRINITY_DN556_c0_g1_i4 | -0.85905889 | 0.837902062 | 0.999970678 | 11.31 | 0 | 0 | 20.58 | NA |
| TRINITY_DN3595_c0_g2_i1 | -0.85901426 | 0.752032697 | 0.999970678 | 2.553 | 0 | 2.155 | 2.051 | NA |
| TRINITY_DN2951_c0_g1_i2 | 0.85894814 | 0.770741005 | 0.999970678 | 26.353 | 1.34 | 0 | 14.95 | NA |
| TRINITY_DN7873_c0_g1_i1 | -0.85891975 | 0.49852517 | 0.999970678 | 1.154 | 4.08 | 1.844 | 7.208 | NA |
| TRINITY_DN4021_c0_g1_i3 | -0.85888165 | 0.587235889 | 0.999970678 | 0.699 | 0.493 | 0.222 | 1.91 | NA |
| TRINITY_DN1091_c0_g1_i1 | 0.858849748 | 0.498509265 | 0.999970678 | 1.681 | 3.696 | 0.955 | 1.749 | NA |
| TRINITY_DN5750_c0_g1_i6 | 0.858769166 | 0.354857011 | 0.999970678 | 11.319 | 6.899 | 4.044 | 5.057 | NA |
| TRINITY_DN1498_c0_g1_i15 | -0.85871945 | 0.845407081 | 0.999970678 | 0.318 | 0 | 0.478 | 0 | - |
| TRINITY_DN2067_c0_g1_i2 | -0.85867221 | 0.761208636 | 0.999970678 | 17.514 | 0 | 17.654 | 10.858 | NA |
| TRINITY_DN3398_c0_g2_i1 | -0.85861821 | 0.46175873 | 0.999970678 | 4.315 | 1.084 | 3.166 | 6.032 | NIF |
| TRINITY_DN1863_c0_g1_i7 | -0.85861061 | 0.784907047 | 1 | 3.979 | 0 | 0.278 | 6.434 | Exostosin |
| TRINITY_DN13903_c0_g1_i22 | 0.858546708 | 0.451367521 | 0.999970678 | 0.518 | 0.798 | 0.278 | 0.402 | YL1 |
| TRINITY_DN59_c0_g3_i3 | 0.858512218 | 0.452810836 | 0.999970678 | 7.83 | 6.83 | 5.489 | 1.468 | NA |
| TRINITY_DN9209_c0_g1_i2 | 0.858503554 | 0.338241713 | 0.999970678 | 1.308 | 1.508 | 0.744 | 0.674 | NA |
| TRINITY_DN7734_c0_g1_i5 | -0.85836508 | 0.758949944 | 1 | 0 | 0.572 | 0.267 | 0.714 | WD40 |
| TRINITY_DN5492_c0_g3_i2 | 0.858314744 | 0.786506571 | 1 | 3.488 | 0.138 | 1.689 | 0 | NA |
| TRINITY_DN2021_c0_g1_i1 | 0.858275266 | 0.67381427 | 0.999970678 | 17.241 | 2.099 | 8.677 | 0.261 | NA |
| TRINITY_DN1960_c0_g1_i1 | 0.858174317 | 0.405729176 | 0.999970678 | 20.412 | 13.23 | 11.722 | 4.444 | NA |
| TRINITY_DN5957_c0_g1_i11 | -0.85813438 | 0.671376632 | 0.999970678 | 0.173 | 0.286 | 0.667 | 0.03 | NA |
| TRINITY_DN4254_c0_g1_i1 | 0.858087337 | 0.274062353 | 0.999970678 | 3.043 | 3.577 | 1.955 | 1.297 | AAA_11 |
| TRINITY_DN1658_c0_g1_i2 | 0.857985104 | 0.177365701 | 0.999970678 | 8.802 | 9.688 | 4.111 | 5.238 | NA |
| TRINITY_DN3154_c0_g1_i3 | -0.85797126 | 0.779341431 | 0.999970678 | 140.05 | 0 | 134.281 | 95.068 | NA |
| TRINITY_DN56446_c0_g5_i2 | -0.85782823 | 0.787046963 | 1 | 0.454 | 0.03 | 0.733 | 0 | Polysacc_deac_1 |
| TRINITY_DN793_c0_g1_i4 | -0.85754076 | 0.450320473 | 0.999970678 | 1.026 | 0.719 | 1.689 | 1.126 | adh_short |
| TRINITY_DN4093_c0_g1_i6 | 0.857421391 | 0.745902271 | 0.999970678 | 1.662 | 0.838 | 0 | 1.387 | - |
| TRINITY_DN7656_c0_g1_i3 | -0.85737322 | 0.571442054 | 0.999970678 | 1.072 | 0.197 | 0.489 | 1.709 | NT-C2 |
| TRINITY_DN645_c1_g1_i2 | -0.85734537 | 0.161639207 | 0.999970678 | 167.36 | 188.6 | 226.653 | 367.081 | NA |
| TRINITY_DN3671_c0_g1_i1 | -0.85732638 | 0.480034107 | 0.999970678 | 3.879 | 2.562 | 1.644 | 9.752 | Uso1_p115_C |
| TRINITY_DN1463_c0_g1_i4 | -0.85726111 | 0.646624058 | 0.999970678 | 139.25 | 21.42 | 232.575 | 9.641 | Cupin_1 |
| TRINITY_DN1260_c0_g1_i4 | -0.85718124 | 0.415513225 | 0.999970678 | 7.712 | 8.574 | 5.255 | 23.113 | NA |
| TRINITY_DN3440_c1_g1_i3 | 0.857047134 | 0.613603587 | 0.999970678 | 0.527 | 1.794 | 0.944 | 0.171 | DUF616 |
| TRINITY_DN681_c0_g1_i3 | 0.857030268 | 0.760038536 | 0.999970678 | 0 | 23.11 | 4.544 | 7.058 | CRS1_YhbY |
| TRINITY_DN25407_c0_g1_i5 | 0.856907521 | 0.427154435 | 0.999970678 | 2.235 | 4.179 | 0.822 | 2.523 | BT1 |
| TRINITY_DN1634_c0_g2_i13 | 0.856875945 | 0.599866507 | 0.999970678 | 3.07 | 0.306 | 1.267 | 0.362 | NA |
| TRINITY_DN254997_c0_g1_i1 | -0.85685596 | 0.839340842 | 0.999970678 | 2.071 | 0 | 3.155 | 0 | - |
| TRINITY_DN7163_c0_g1_i1 | 0.856855165 | 0.748828418 | 0.999970678 | 0.318 | 0.591 | 0.411 | 0 | RNA_pol_Rpb1_1 |
| TRINITY_DN1263_c0_g1_i3 | -0.85672395 | 0.284187308 | 0.999970678 | 12.872 | 11.88 | 23.932 | 15.613 | - |
| TRINITY_DN1318_c0_g1_i2 | -0.85669802 | 0.519677845 | 0.999970678 | 5.541 | 1.38 | 3.744 | 7.922 | NA |
| TRINITY_DN12395_c0_g1_i4 | 0.856697762 | 0.771781836 | 1 | 0.79 | 0.128 | 0.422 | 0 | Sugar_tr |
| TRINITY_DN5948_c1_g1_i2 | 0.856651801 | 0.756453546 | 0.999970678 | 1.499 | 9.441 | 0 | 5.912 | Auxin_inducible |
| TRINITY_DN29148_c2_g1_i1 | -0.85664881 | 0.778231491 | 1 | 2.098 | 0 | 2.8 | 0.442 | NA |
| TRINITY_DN3530_c0_g1_i3 | 0.856310397 | 0.760839792 | 0.999970678 | 1.036 | 0 | 0.233 | 0.292 | NA |
| TRINITY_DN3844_c0_g1_i6 | -0.85626365 | 0.57609169 | 0.999970678 | 2.489 | 0.779 | 3.833 | 1.247 | NA |
| TRINITY_DN364_c0_g1_i1 | 0.856207559 | 0.33921741 | 0.999970678 | 5.105 | 7.943 | 2.078 | 4.665 | Maf |
| TRINITY_DN14748_c0_g1_i2 | -0.85616332 | 0.650659135 | 1 | 0.064 | 0.355 | 0.533 | 0.101 | NA |
| TRINITY_DN1746_c0_g2_i1 | 0.85613835 | 0.638597662 | 0.999970678 | 3.733 | 0.118 | 0.6 | 1.438 | - |
| TRINITY_DN16377_c0_g1_i1 | -0.85603593 | 0.289741748 | 0.999970678 | 1024.1 | 2305 | 2501.204 | 2639.547 | NA |
| TRINITY_DN41134_c0_g1_i12 | -0.85598761 | 0.849674127 | 1 | 1.49 | 0 | 2.189 | 0 | Ribosomal_S8e |
| TRINITY_DN254958_c0_g1_i1 | -0.85598761 | 0.849674127 | 1 | 1.39 | 0 | 2.1 | 0 | NA |
| TRINITY_DN20104_c0_g3_i2 | -0.85598761 | 0.849674127 | 1 | 0.763 | 0 | 1.133 | 0 | - |
| TRINITY_DN251855_c0_g1_i1 | -0.85598761 | 0.849674127 | 1 | 0.79 | 0 | 1.2 | 0 | NA |
| TRINITY_DN6588_c0_g1_i5 | -0.85598112 | 0.839702656 | 0.999970678 | 3.942 | 0 | 5.989 | 0 | - |

| TRINITY_DN29319_c0_g1_i1 | -0.85598049 | 0.841541737 | 0.999970678 | 1.581 | 0 | 2.367 | 0 | NA |
| --- | --- | --- | --- | --- | --- | --- | --- | --- |
| TRINITY_DN698_c1_g1_i1 | -0.85597045 | 0.423671991 | 0.999970678 | 31.558 | 8.426 | 33.62 | 32.142 | NA |
| TRINITY_DN4527_c0_g1_i3 | -0.8558735 | 0.271551336 | 0.999970678 | 2.053 | 2.73 | 3.378 | 4.494 | PRAI |
| TRINITY_DN16185_c0_g1_i1 | -0.85561028 | 0.187632671 | 0.999970678 | 4.587 | 5.204 | 6.855 | 9.4 | NA |
| TRINITY_DN1643_c0_g2_i1 | 0.855602498 | 0.488106576 | 0.999970678 | 1.372 | 5.006 | 0.867 | 2.483 | NA |
| TRINITY_DN8389_c0_g1_i2 | 0.855499354 | 0.847344733 | 1 | 0 | 1.311 | 0 | 0.704 | NA |
| TRINITY_DN4238_c0_g2_i3 | 0.8554778 | 0.581493157 | 0.999970678 | 2.144 | 2.355 | 1.767 | 0.322 | NA |
| TRINITY_DN3215_c0_g1_i2 | -0.85543817 | 0.434112894 | 0.999970678 | 6.322 | 1.597 | 5.544 | 7.792 | ACP_syn_III_C |
| TRINITY_DN520_c1_g2_i5 | -0.85535619 | 0.314283997 | 0.999970678 | 19.857 | 9.55 | 23.721 | 24.441 | LRR_1 |
| TRINITY_DN266_c0_g1_i6 | 0.85535341 | 0.840769284 | 0.999970678 | 20.212 | 0 | 0 | 11.32 | NA |
| TRINITY_DN749_c0_g1_i1 | -0.85532691 | 0.391069393 | 0.999970678 | 3.815 | 10.39 | 10.955 | 12.064 | NA |
| TRINITY_DN9635_c0_g1_i1 | -0.85531954 | 0.483287415 | 0.999970678 | 0.372 | 0.65 | 0.9 | 0.684 | DUF702 |
| TRINITY_DN15114_c0_g1_i7 | -0.85519709 | 0.470239754 | 0.999970678 | 6.976 | 2.622 | 8.599 | 6.656 | NA |
| TRINITY_DN10005_c0_g1_i1 | 0.855000101 | 0.570166628 | 0.999970678 | 0.872 | 2.336 | 1.222 | 0.271 | DHHC |
| TRINITY_DN755_c0_g1_i3 | 0.854875844 | 0.268893214 | 0.999970678 | 3.843 | 2.582 | 1.467 | 1.81 | F-box-like |
| TRINITY_DN2156_c3_g1_i1 | -0.85476244 | 0.429497558 | 0.999970678 | 9.665 | 2.671 | 9.244 | 11.28 | NA |
| TRINITY_DN10289_c0_g1_i4 | -0.85475548 | 0.76625373 | 0.999970678 | 0 | 1.153 | 0.244 | 1.759 | NA |
| TRINITY_DN3258_c0_g1_i1 | -0.85464213 | 0.57257471 | 0.999970678 | 3.079 | 21.75 | 5.989 | 37.028 | PC-Esterase |
| TRINITY_DN214_c0_g2_i2 | 0.854622108 | 0.482338939 | 0.999970678 | 7.976 | 1.744 | 2.478 | 2.443 | NA |
| TRINITY_DN450_c0_g1_i6 | 0.854500558 | 0.337329806 | 0.999970678 | 1.408 | 1.468 | 0.733 | 0.704 | RCC1 |
| TRINITY_DN29_c1_g2_i5 | -0.85449053 | 0.841625984 | 0.999970678 | 1.653 | 0 | 2.511 | 0 | NA |
| TRINITY_DN5558_c0_g2_i6 | 0.854289971 | 0.466976218 | 0.999970678 | 0.518 | 0.473 | 0.311 | 0.181 | Pkinase |
| TRINITY_DN99452_c0_g1_i6 | -0.85413475 | 0.842988542 | 0.999970678 | 3.297 | 0 | 4.933 | 0 | NA |
| TRINITY_DN19713_c0_g1_i2 | -0.85404636 | 0.279136489 | 0.999970678 | 1.79 | 2.75 | 3.155 | 4.323 | EamA |
| TRINITY_DN492_c0_g1_i10 | -0.85398617 | 0.274801556 | 0.999970678 | 2.998 | 4.336 | 6.066 | 5.841 | NA |
| TRINITY_DN2829_c0_g1_i1 | -0.85395179 | 0.74774409 | 0.999970678 | 0 | 2.129 | 1.733 | 1.659 | Glyco_hydro_17 |
| TRINITY_DN6422_c0_g1_i5 | 0.853870245 | 0.426840959 | 0.999970678 | 0.79 | 2.139 | 0.811 | 0.633 | Glyco_hydro_2 |
| TRINITY_DN7853_c0_g1_i5 | 0.853779527 | 0.450016068 | 0.999970678 | 1.335 | 0.769 | 0.622 | 0.432 | NA |
| TRINITY_DN10011_c0_g1_i1 | -0.8536983 | 0.441305855 | 0.999970678 | 0.627 | 0.542 | 1.222 | 0.633 | NA |
| TRINITY_DN8846_c1_g2_i3 | -0.85362005 | 0.749817004 | 0.999970678 | 0 | 1.873 | 0.978 | 2.182 | NA |
| TRINITY_DN4343_c0_g1_i2 | -0.85351626 | 0.54172985 | 0.999970678 | 0.836 | 0.217 | 1.233 | 0.442 | NA |
| TRINITY_DN25645_c0_g1_i1 | 0.853486829 | 0.568998373 | 0.999970678 | 0.491 | 1.124 | 0.111 | 0.754 | LRRNT_2 |
| TRINITY_DN190_c1_g1_i1 | 0.853435097 | 0.467889729 | 0.999970678 | 29.159 | 102.8 | 44.775 | 18.268 | NA |
| TRINITY_DN1278_c2_g1_i2 | -0.85341342 | 0.839150304 | 0.999970678 | 2.189 | 0 | 0 | 4.032 | NA |
| TRINITY_DN3067_c0_g1_i5 | 0.853335352 | 0.539579575 | 0.999970678 | 0.509 | 1.656 | 0.633 | 0.442 | AAA |
| TRINITY_DN2802_c2_g1_i1 | -0.85327667 | 0.385993178 | 0.999970678 | 28.796 | 12.57 | 42.109 | 24.079 | Response_reg |
| TRINITY_DN61867_c0_g1_i1 | 0.853261385 | 0.609770056 | 0.999970678 | 18.613 | 269.8 | 27.554 | 110.601 | Chloroa_b-bind |
| TRINITY_DN20825_c0_g1_i2 | -0.85320328 | 0.758230196 | 0.999970678 | 0 | 1.922 | 0.644 | 2.604 | NA |
| TRINITY_DN4679_c0_g1_i5 | -0.85319924 | 0.489898335 | 0.999970678 | 78.894 | 12.91 | 72.373 | 79.314 | 40S_S4_C |
| TRINITY_DN6871_c0_g1_i4 | 0.853195734 | 0.407714754 | 0.999970678 | 4.342 | 5.075 | 2 | 2.614 | NA |
| TRINITY_DN832_c0_g1_i3 | -0.85301786 | 0.544599407 | 0.999970678 | 0.69 | 4.987 | 6.122 | 2.765 | NA |
| TRINITY_DN20215_c0_g1_i3 | -0.85294947 | 0.667430017 | 0.999970678 | 67.185 | 1.804 | 29.876 | 79.686 | NA |
| TRINITY_DN10139_c0_g1_i1 | 0.852892506 | 0.440555247 | 0.999970678 | 2.498 | 1.074 | 0.722 | 1.106 | Agenet |
| TRINITY_DN12436_c0_g1_i9 | 0.852890512 | 0.626621116 | 0.999970678 | 21.583 | 5.302 | 11.188 | 1.146 | LRRNT_2 |
| TRINITY_DN2967_c0_g1_i4 | 0.852664741 | 0.389863901 | 0.999970678 | 2.534 | 4.11 | 1 | 2.483 | NA |
| TRINITY_DN2591_c0_g1_i3 | 0.852649927 | 0.493509882 | 1 | 0.345 | 0.493 | 0.211 | 0.211 | DUF3453 |
| TRINITY_DN4474_c0_g1_i4 | 0.852334264 | 0.353706781 | 0.999970678 | 0.781 | 0.848 | 0.422 | 0.382 | Jas |
| TRINITY_DN3301_c0_g1_i1 | 0.852328944 | 0.841461 | 0.999970678 | 0 | 12.77 | 5.755 | 0 | NA |
| TRINITY_DN1125_c0_g1_i6 | 0.852291576 | 0.469338155 | 0.999970678 | 2.589 | 2.02 | 1.678 | 0.553 | FeThRed_A |
| TRINITY_DN7548_c0_g1_i3 | 0.852276432 | 0.461434986 | 1 | 4.469 | 3.922 | 1.833 | 2.373 | NA |
| TRINITY_DN2445_c0_g1_i4 | 0.85223563 | 0.566388652 | 0.999970678 | 10.501 | 1.459 | 4.5 | 1.267 | Pkinase |
| TRINITY_DN3558_c0_g1_i1 | -0.85190869 | 0.298089999 | 0.999970678 | 2.707 | 5.509 | 5.955 | 7.56 | NA |
| TRINITY_DN2772_c0_g1_i5 | -0.8518621 | 0.494788454 | 0.999970678 | 5.332 | 15.83 | 6.344 | 30.01 | NA |
| TRINITY_DN6076_c3_g1_i1 | -0.85180413 | 0.277680305 | 0.999970678 | 16.433 | 13.98 | 29.843 | 18.831 | NA |
| TRINITY_DN11410_c0_g1_i1 | -0.85179171 | 0.190493963 | 0.999970678 | 17.051 | 13.91 | 19.877 | 31.981 | NA |
| TRINITY_DN5386_c0_g1_i2 | 0.851710287 | 0.781119384 | 0.999970678 | 0 | 49.56 | 20.632 | 2.031 | - |
| TRINITY_DN3980_c0_g1_i2 | -0.85152827 | 0.487665664 | 0.999970678 | 12.036 | 11.59 | 29.876 | 6.384 | NA |
| TRINITY_DN8788_c0_g1_i1 | 0.851512881 | 0.386329723 | 0.999970678 | 4.115 | 4.947 | 1.344 | 3.348 | NA |
| TRINITY_DN3801_c0_g1_i2 | 0.851501431 | 0.474968611 | 0.999970678 | 0.972 | 0.798 | 0.6 | 0.241 | Myb_DNA-bindin |
| TRINITY_DN17227_c0_g1_i1 | 0.851455012 | 0.725020759 | 0.999970678 | 0.091 | 3.755 | 1.667 | 0.08 | PsbP |
| TRINITY_DN4562_c1_g2_i3 | -0.85144386 | 0.664234127 | 0.999970678 | 0.273 | 3.784 | 5.655 | 0.372 | NA |
| TRINITY_DN7050_c0_g3_i1 | -0.85127899 | 0.462855438 | 0.999970678 | 2.362 | 1.577 | 3.566 | 2.735 | NA |
| TRINITY_DN1665_c0_g3_i1 | -0.8512252 | 0.362636829 | 0.999970678 | 2.207 | 1.301 | 2.866 | 2.835 | NA |
| TRINITY_DN6719_c0_g1_i2 | -0.85116525 | 0.481249584 | 0.999970678 | 1.935 | 1.932 | 1.211 | 5.399 | NA |
| TRINITY_DN17550_c0_g1_i4 | -0.85102016 | 0.47884422 | 0.999970678 | 10.91 | 16.4 | 9.322 | 35.49 | NA |
| TRINITY_DN5682_c0_g1_i10 | 0.85100067 | 0.2871588 | 0.999970678 | 2.662 | 3.479 | 1.444 | 1.619 | NA |
| TRINITY_DN22_c0_g2_i5 | 0.850978487 | 0.555695315 | 1 | 0.436 | 0.424 | 0.089 | 0.372 | AA_kinase |
| TRINITY_DN20770_c0_g1_i2 | -0.8509542 | 0.71071292 | 0.999970678 | 0.545 | 0.118 | 0.989 | 0.02 | ABC_tran |
| TRINITY_DN367_c0_g1_i1 | -0.85082359 | 0.432506906 | 0.999970678 | 8.094 | 19.47 | 10.266 | 37.048 | Band_7 |
| TRINITY_DN11198_c0_g1_i2 | 0.850785021 | 0.608300044 | 0.999970678 | 9.157 | 0.542 | 3.3 | 1.458 | PGM_PMM_I |
| TRINITY_DN2162_c0_g1_i7 | 0.85076083 | 0.84818789 | 1 | 0 | 0.542 | 0.244 | 0 | NA |
| TRINITY_DN8375_c0_g1_i1 | -0.85071129 | 0.31844918 | 0.999970678 | 30.413 | 69.84 | 85.639 | 75.533 | LIM |
| TRINITY_DN234_c0_g1_i11 | 0.850702366 | 0.682055447 | 0.999970678 | 1.308 | 5.815 | 0.044 | 3.871 | NA |
| TRINITY_DN30547_c0_g1_i5 | 0.850603218 | 0.261719336 | 0.999970678 | 3.134 | 2.543 | 1.578 | 1.257 | OTU |

| TRINITY_DN1549_c0_g2_i1 | -0.85058111 | 0.84186188 | 0.999970678 | 7.14 | 0 | 10.655 | 0 | NA |
| --- | --- | --- | --- | --- | --- | --- | --- | --- |
| TRINITY_DN5973_c0_g4_i1 | -0.8505807 | 0.502369745 | 0.999970678 | 0.69 | 2.671 | 3.5 | 1.78 | NA |
| TRINITY_DN2555_c0_g1_i1 | 0.850561678 | 0.20129494 | 0.999970678 | 19.603 | 31.02 | 11.477 | 14.176 | Anticodon_1 |
| TRINITY_DN997_c2_g1_i1 | 0.850455579 | 0.67901031 | 0.999970678 | 4.024 | 7.549 | 5.089 | 0.171 | NA |
| TRINITY_DN321_c0_g1_i4 | 0.850438984 | 0.609295999 | 0.999970678 | 22.71 | 22.57 | 19.965 | 0.935 | NA |
| TRINITY_DN1426_c1_g1_i2 | 0.850423038 | 0.459623464 | 0.999970678 | 4.515 | 8.712 | 4.8 | 1.508 | NA |
| TRINITY_DN11427_c0_g1_i12 | -0.85041918 | 0.800634226 | 0.999970678 | 13.508 | 0.079 | 20.499 | 0 | NA |
| TRINITY_DN1854_c0_g1_i1 | 0.850378141 | 0.329846831 | 0.999970678 | 9.729 | 4.395 | 3.4 | 3.78 | Pyr_redox_2 |
| TRINITY_DN4349_c1_g1_i8 | -0.85036943 | 0.540667812 | 0.999970678 | 0.863 | 3.656 | 1.633 | 6.103 | NA |
| TRINITY_DN16451_c0_g1_i1 | 0.850241764 | 0.539680211 | 0.999970678 | 1.599 | 2.7 | 1.567 | 0.473 | NA |
| TRINITY_DN203_c0_g1_i5 | 0.850133158 | 0.850273504 | 0.999970678 | 31.712 | 0 | 0 | 17.966 | NA |
| TRINITY_DN247_c0_g1_i1 | 0.850086751 | 0.543435239 | 0.999970678 | 4.914 | 2.957 | 3.211 | 0.503 | NA |
| TRINITY_DN440_c0_g1_i4 | -0.8499388 | 0.331468912 | 0.999970678 | 12 | 7.746 | 10.077 | 23.335 | - |
| TRINITY_DN2507_c0_g2_i4 | -0.84987995 | 0.774646539 | 0.999970678 | 49.171 | 0 | 47.086 | 33.087 | NAF |
| TRINITY_DN2211_c0_g2_i3 | 0.849871447 | 0.757318792 | 0.999970678 | 0.917 | 3.459 | 0 | 2.413 | Plus-3 |
| TRINITY_DN6755_c0_g1_i1 | 0.849723626 | 0.36767343 | 0.999970678 | 6.94 | 3.528 | 2.755 | 2.483 | NA |
| TRINITY_DN5231_c0_g1_i5 | 0.849707684 | 0.386913088 | 0.999970678 | 1.562 | 0.907 | 0.522 | 0.754 | NA |
| TRINITY_DN24907_c0_g1_i1 | -0.84970592 | 0.620617664 | 1 | 0.118 | 0.71 | 0.433 | 0.905 | NA |
| TRINITY_DN11464_c0_g2_i1 | 0.849614417 | 0.579188644 | 1 | 0.354 | 0.532 | 0.344 | 0.101 | NA |
| TRINITY_DN679_c0_g1_i2 | 0.849569496 | 0.219744664 | 0.999970678 | 70.782 | 99.64 | 46.308 | 38.194 | NA |
| TRINITY_DN7164_c0_g1_i7 | 0.849526592 | 0.557689801 | 0.999970678 | 0.518 | 4.159 | 1.122 | 1.206 | NA |
| TRINITY_DN1459_c0_g1_i8 | -0.84947668 | 0.780768192 | 0.999970678 | 20.757 | 0 | 27.609 | 4.605 | NA |
| TRINITY_DN2593_c0_g1_i2 | -0.84935038 | 0.597030684 | 1 | 0.345 | 1.577 | 2.011 | 0.985 | Abhydrolase_1 |
| TRINITY_DN183_c1_g1_i1 | 0.849297693 | 0.516588063 | 0.999970678 | 23.282 | 178.8 | 51.052 | 47.504 | NA |
| TRINITY_DN1904_c0_g1_i10 | 0.849287889 | 0.374670994 | 0.999970678 | 12.772 | 7.756 | 6.722 | 3.308 | PMD |
| TRINITY_DN14813_c0_g1_i6 | -0.84926295 | 0.31189867 | 0.999970678 | 53.995 | 33.87 | 46.231 | 99.743 | NA |
| TRINITY_DN94575_c0_g2_i1 | 0.849240344 | 0.42717454 | 0.999970678 | 67.421 | 18.08 | 23.054 | 20.017 | Pkinase |
| TRINITY_DN34113_c0_g1_i3 | -0.84912165 | 0.763741135 | 0.999970678 | 0 | 4.632 | 6.022 | 0.935 | DLH |
| TRINITY_DN1952_c1_g1_i5 | 0.849099508 | 0.336984389 | 0.999970678 | 7.185 | 3.577 | 3.133 | 2.232 | LIM_bind |
| TRINITY_DN18991_c0_g1_i1 | -0.84897399 | 0.453888976 | 0.999970678 | 1.008 | 0.66 | 1.744 | 0.905 | NA |
| TRINITY_DN4644_c1_g1_i4 | 0.848949844 | 0.363893602 | 0.999970678 | 1.953 | 2.799 | 0.722 | 1.769 | GTP-bdg_M |
| TRINITY_DN133_c0_g2_i1 | -0.8489245 | 0.196030375 | 0.999970678 | 6.386 | 8.042 | 9.366 | 14.658 | zinc_ribbon_10 |
| TRINITY_DN9002_c0_g1_i1 | 0.848907408 | 0.399752408 | 0.999970678 | 4.606 | 9.924 | 2.333 | 5.137 | NA |
| TRINITY_DN2060_c0_g1_i6 | -0.84890145 | 0.675752064 | 0.999970678 | 0.164 | 1.439 | 2.089 | 0.261 | NA |
| TRINITY_DN3297_c0_g1_i7 | 0.848887594 | 0.747270959 | 0.999970678 | 3.043 | 3.371 | 0 | 3.569 | Ist1 |
| TRINITY_DN1875_c0_g1_i9 | -0.84886604 | 0.578996802 | 0.999970678 | 0.082 | 0.453 | 0.544 | 0.292 | PHD |
| TRINITY_DN3424_c0_g1_i1 | 0.848804449 | 0.346582132 | 0.999970678 | 7.73 | 3.982 | 3.466 | 2.363 | NA |
| TRINITY_DN16044_c0_g1_i1 | -0.84865668 | 0.682482082 | 0.999970678 | 0.027 | 0.719 | 0.767 | 0.392 | NA |
| TRINITY_DN14057_c0_g1_i3 | 0.84860847 | 0.287711799 | 0.999970678 | 2.861 | 2.208 | 1.022 | 1.588 | BSD |
| TRINITY_DN3763_c0_g1_i1 | -0.84858479 | 0.456102299 | 0.999970678 | 27.606 | 15.33 | 27.198 | 39.24 | NA |
| TRINITY_DN8270_c0_g1_i5 | -0.84858189 | 0.546618886 | 0.999970678 | 12.563 | 20.97 | 45.186 | 5.479 | Glyco_hydro_14 |
| TRINITY_DN10494_c0_g1_i1 | -0.84856467 | 0.464674144 | 0.999970678 | 0.481 | 0.867 | 1.422 | 0.684 | DEAD |
| TRINITY_DN2700_c0_g2_i2 | -0.84850173 | 0.766128134 | 1 | 0.881 | 0 | 0.944 | 0.462 | NA |
| TRINITY_DN5530_c0_g4_i2 | -0.84843098 | 0.451513052 | 0.999970678 | 1.635 | 2.858 | 5.155 | 1.83 | Cytochrom_C_as |
| TRINITY_DN15_c0_g1_i11 | 0.848394339 | 0.434689664 | 0.999970678 | 70.637 | 28.59 | 29.876 | 17.514 | NA |
| TRINITY_DN6499_c0_g1_i4 | -0.8480315 | 0.400006188 | 0.999970678 | 0.472 | 0.375 | 0.533 | 0.885 | NA |
| TRINITY_DN8342_c1_g1_i2 | -0.84798703 | 0.197435441 | 0.999970678 | 12.963 | 19.46 | 25.954 | 26.924 | GH3 |
| TRINITY_DN13445_c0_g1_i1 | 0.847936723 | 0.299616898 | 0.999970678 | 24.545 | 24.88 | 15.499 | 8.727 | NA |
| TRINITY_DN11871_c1_g2_i3 | 0.847916988 | 0.787404249 | 0.999970678 | 0.064 | 4.898 | 0 | 2.725 | NA |
| TRINITY_DN10224_c0_g1_i1 | -0.84783436 | 0.648113924 | 0.999970678 | 0.327 | 1.301 | 0.167 | 2.674 | VPS28 |
| TRINITY_DN10849_c0_g1_i5 | -0.84782682 | 0.753714104 | 0.999970678 | 1.354 | 0 | 0.7 | 1.659 | - |
| TRINITY_DN5717_c0_g4_i1 | 0.847803924 | 0.462017388 | 0.999970678 | 2.28 | 1.173 | 1 | 0.734 | NA |
| TRINITY_DN622_c0_g1_i3 | -0.8477571 | 0.563178746 | 0.999970678 | 2.525 | 0.463 | 1.167 | 3.991 | NA |
| TRINITY_DN2358_c2_g1_i3 | -0.84765168 | 0.426824087 | 0.999970678 | 55.603 | 53.23 | 32.065 | 157.501 | Oxysterol_BP |
| TRINITY_DN40564_c0_g1_i5 | 0.847542096 | 0.417483462 | 0.999970678 | 1.317 | 1.055 | 0.778 | 0.382 | NA |
| TRINITY_DN50319_c0_g1_i1 | 0.847512693 | 0.607770074 | 0.999970678 | 10.992 | 1.173 | 3.7 | 2.332 | NA |
| TRINITY_DN13755_c0_g1_i8 | 0.847477959 | 0.793873393 | 0.999970678 | 37.208 | 0 | 0.278 | 20.117 | NA |
| TRINITY_DN9091_c0_g1_i1 | -0.84742553 | 0.546922232 | 0.999970678 | 4.878 | 32.62 | 12.344 | 51.596 | PMEI |
| TRINITY_DN194_c0_g1_i1 | -0.84742434 | 0.63888897 | 0.999970678 | 1.19 | 0.68 | 2.589 | 0.211 | p450 |
| TRINITY_DN11784_c0_g1_i1 | -0.84734387 | 0.84207883 | 0.999970678 | 1.899 | 0 | 2.878 | 0 | GMC_oxred_C |
| TRINITY_DN7975_c0_g1_i1 | -0.84730836 | 0.302552172 | 0.999970678 | 5.441 | 11.82 | 12.799 | 15.412 | NA |
| TRINITY_DN8253_c0_g1_i1 | 0.847289565 | 0.485341799 | 0.999970678 | 2.425 | 7.313 | 3.466 | 1.196 | NA |
| TRINITY_DN3436_c0_g1_i4 | 0.847251831 | 0.557144703 | 0.999970678 | 0.927 | 0.463 | 0.556 | 0.101 | ACAS_N |
| TRINITY_DN1609_c0_g1_i9 | -0.84724359 | 0.576324721 | 0.999970678 | 0.627 | 3.173 | 4.4 | 1.418 | - |
| TRINITY_DN7700_c0_g2_i2 | 0.847219686 | 0.649900661 | 1 | 0.309 | 2.74 | 0.3 | 1.267 | NA |
| TRINITY_DN367_c0_g1_i12 | -0.84713904 | 0.41404049 | 0.999970678 | 82.737 | 118.9 | 66.052 | 272.083 | NA |
| TRINITY_DN2888_c0_g1_i1 | -0.84708152 | 0.234061271 | 0.999970678 | 22.029 | 35.35 | 36.353 | 58.784 | NA |
| TRINITY_DN23_c1_g1_i2 | 0.847002028 | 0.577306873 | 0.999970678 | 5.414 | 0.473 | 1.578 | 1.418 | NA |
| TRINITY_DN5637_c0_g1_i1 | -0.84691596 | 0.37405839 | 0.999970678 | 1.108 | 1.902 | 1.767 | 3.217 | Ribosomal_L21p |
| TRINITY_DN1119_c0_g1_i2 | 0.846891595 | 0.383920952 | 0.999970678 | 5.35 | 6.298 | 4.044 | 1.599 | Agenet |
| TRINITY_DN12124_c0_g1_i3 | -0.84686046 | 0.848604879 | 1 | 0.545 | 0 | 0.822 | 0 | NA |
| TRINITY_DN21660_c0_g1_i3 | -0.84672455 | 0.230640157 | 0.999970678 | 4.315 | 3.863 | 6.933 | 6.394 | WD40 |
| TRINITY_DN3366_c0_g2_i3 | -0.84663524 | 0.768045338 | 0.999970678 | 1.381 | 0.522 | 2.811 | 0 | NA |
| TRINITY_DN26675_c0_g1_i1 | -0.84654872 | 0.346209301 | 0.999970678 | 146.32 | 122.6 | 288.627 | 133.071 | Rubredoxin |

| TRINITY_DN4195_c0_g1_i9 | -0.84654084 | 0.760183036 | 0.999970678 | 0.209 | 0.266 | 0 | 0.865 | NA |
| --- | --- | --- | --- | --- | --- | --- | --- | --- |
| TRINITY_DN24196_c0_g1_i2 | 0.846491074 | 0.44606491 | 0.999970678 | 0.908 | 2.523 | 0.8 | 0.935 | TPT |
| TRINITY_DN21826_c0_g1_i1 | 0.846480976 | 0.84464827 | 0.999970678 | 4.079 | 0 | 1.9 | 0 | NA |
| TRINITY_DN8940_c0_g1_i2 | 0.846365774 | 0.32016689 | 0.999970678 | 0.827 | 1.419 | 0.611 | 0.513 | - |
| TRINITY_DN324_c0_g1_i5 | -0.84632037 | 0.364067785 | 0.999970678 | 22.51 | 14.25 | 38.309 | 19.635 | Ribosomal_L23 |
| TRINITY_DN8133_c0_g1_i2 | -0.84630089 | 0.759677184 | 0.999970678 | 6.159 | 0 | 2.167 | 8.636 | NA |
| TRINITY_DN974_c0_g1_i1 | -0.84616231 | 0.753786279 | 0.999970678 | 4.56 | 0 | 3.855 | 3.67 | Branch |
| TRINITY_DN9977_c0_g1_i2 | -0.84609429 | 0.543815504 | 0.999970678 | 26.007 | 7.244 | 9.233 | 46.609 | NA |
| TRINITY_DN19587_c0_g1_i5 | -0.84607704 | 0.844957151 | 0.999970678 | 1.372 | 0 | 2.055 | 0 | DUF4452 |
| TRINITY_DN5987_c0_g3_i2 | 0.846073206 | 0.407874855 | 0.999970678 | 9.72 | 3.646 | 3.944 | 2.714 | NA |
| TRINITY_DN8060_c0_g1_i1 | 0.845986997 | 0.517172737 | 0.999970678 | 1.244 | 1.055 | 0.844 | 0.271 | NA |
| TRINITY_DN4497_c1_g1_i1 | -0.84592976 | 0.435516485 | 0.999970678 | 1.862 | 1.804 | 2.522 | 3.458 | NA |
| TRINITY_DN2354_c0_g1_i5 | 0.845915661 | 0.427557539 | 0.999970678 | 7.876 | 3.913 | 1.433 | 4.886 | BURP |
| TRINITY_DN4527_c0_g1_i4 | 0.845853783 | 0.470286489 | 0.999970678 | 2.389 | 2.178 | 0.511 | 1.96 | PRAI |
| TRINITY_DN7438_c0_g1_i4 | -0.84577046 | 0.623592109 | 0.999970678 | 0.045 | 0.453 | 0.578 | 0.181 | Cytochrom_B561 |
| TRINITY_DN7660_c0_g1_i2 | 0.845754323 | 0.623076384 | 0.999970678 | 0.182 | 1.419 | 0.533 | 0.241 | NA |
| TRINITY_DN7951_c0_g2_i2 | -0.84572037 | 0.620609087 | 0.999970678 | 0.227 | 1.114 | 0.244 | 2.081 | NA |
| TRINITY_DN1933_c0_g1_i10 | 0.845626761 | 0.756745374 | 0.999970678 | 6.976 | 1.636 | 0 | 4.856 | p450 |
| TRINITY_DN377_c0_g1_i2 | 0.845547217 | 0.455345303 | 0.999970678 | 2.008 | 0.769 | 0.533 | 0.915 | NA |
| TRINITY_DN1568_c0_g1_i5 | 0.84545232 | 0.757854538 | 1 | 0.273 | 0.71 | 0 | 0.553 | Glyco_hydro_1 |
| TRINITY_DN5095_c0_g1_i1 | -0.84544202 | 0.753520558 | 0.999970678 | 1.653 | 0 | 0.933 | 1.91 | Utp12 |
| TRINITY_DN1215_c0_g1_i15 | 0.845390032 | 0.534029048 | 0.999970678 | 1.145 | 0.769 | 0.778 | 0.141 | TFIIIC_delta |
| TRINITY_DN1499_c0_g1_i17 | 0.845371215 | 0.386881483 | 0.999970678 | 13.344 | 4.977 | 4.966 | 4.283 | NA |
| TRINITY_DN1799_c0_g1_i10 | 0.845036966 | 0.706953099 | 0.999970678 | 0.027 | 1.971 | 0.222 | 0.834 | PPR |
| TRINITY_DN18958_c0_g1_i4 | -0.84493872 | 0.755384554 | 0.999970678 | 0.445 | 0 | 0.444 | 0.271 | Na_H_Exchanger |
| TRINITY_DN388_c0_g1_i4 | 0.844895745 | 0.664044537 | 1 | 0.318 | 4.139 | 0.378 | 1.94 | NA |
| TRINITY_DN2901_c0_g2_i1 | -0.84480324 | 0.625525353 | 1 | 0.136 | 1.114 | 1.211 | 0.784 | NA |
| TRINITY_DN63343_c0_g2_i1 | -0.84454064 | 0.481890756 | 0.999970678 | 1.045 | 2.01 | 3.455 | 1.297 | NA |
| TRINITY_DN1916_c0_g1_i1 | -0.84447725 | 0.446873095 | 0.999970678 | 17.55 | 29.16 | 13.51 | 67.43 | NA |
| TRINITY_DN1423_c0_g1_i2 | 0.844410023 | 0.324732982 | 0.999970678 | 55.185 | 25.32 | 15.455 | 26.532 | NA |
| TRINITY_DN2463_c0_g1_i1 | 0.844327049 | 0.322034583 | 0.999970678 | 8.021 | 18.83 | 6.644 | 6.867 | NA |
| TRINITY_DN12479_c0_g1_i4 | 0.844316825 | 0.557699033 | 0.999970678 | 14.116 | 6.091 | 7.744 | 1.89 | NB-ARC |
| TRINITY_DN3955_c0_g1_i2 | 0.844249638 | 0.520463347 | 0.999970678 | 1.508 | 0.424 | 0.289 | 0.754 | LRR_6 |
| TRINITY_DN266_c0_g1_i3 | 0.844179448 | 0.554349566 | 0.999970678 | 1.562 | 0.572 | 0.756 | 0.292 | NA |
| TRINITY_DN9045_c0_g1_i3 | -0.84413701 | 0.334488077 | 0.999970678 | 21.62 | 33.91 | 25.743 | 67.681 | ADK |
| TRINITY_DN528_c0_g1_i8 | -0.84389441 | 0.758870081 | 0.999970678 | 0 | 0.887 | 0.244 | 1.277 | LRRNT_2 |
| TRINITY_DN517_c0_g1_i3 | 0.843835611 | 0.314434232 | 0.999970678 | 9.656 | 4.977 | 3.911 | 3.469 | UDPGP |
| TRINITY_DN6869_c0_g1_i5 | 0.843724745 | 0.774607809 | 0.999970678 | 1.226 | 0 | 0.489 | 0.111 | NA |
| TRINITY_DN16095_c0_g1_i5 | 0.843658877 | 0.48793809 | 0.999970678 | 5.169 | 2.464 | 2.044 | 1.719 | NA |
| TRINITY_DN3806_c0_g1_i2 | -0.84350787 | 0.203663268 | 0.999970678 | 6.931 | 7.007 | 8.655 | 14.568 | Glyco_transf_34 |
| TRINITY_DN2881_c0_g1_i9 | 0.8434361 | 0.77532461 | 0.999970678 | 6.976 | 0 | 2.633 | 0.684 | NA |
| TRINITY_DN10220_c0_g1_i1 | -0.84340843 | 0.387918139 | 0.999970678 | 9.484 | 11.65 | 7.788 | 28.452 | NA |
| TRINITY_DN334_c0_g1_i3 | -0.84334225 | 0.408995101 | 0.999970678 | 32.729 | 61.69 | 34.398 | 127.27 | NA |
| TRINITY_DN6271_c0_g1_i1 | -0.84332337 | 0.434007828 | 0.999970678 | 4.587 | 4.06 | 3.055 | 11.692 | NA |
| TRINITY_DN5532_c0_g1_i6 | 0.843212732 | 0.85123005 | 1 | 3.288 | 0 | 1.578 | 0 | NA |
| TRINITY_DN8769_c0_g1_i2 | 0.843063316 | 0.771485331 | 0.999970678 | 0.936 | 0 | 0.322 | 0.151 | Retrotran_gag_2 |
| TRINITY_DN2328_c0_g1_i1 | -0.8430587 | 0.285378832 | 0.999970678 | 9.502 | 5.095 | 12.177 | 11.642 | NA |
| TRINITY_DN14637_c0_g1_i1 | -0.84290511 | 0.378590851 | 0.999970678 | 4.642 | 7.184 | 4.989 | 15.101 | Glyco_transf_10 |
| TRINITY_DN24032_c0_g1_i2 | 0.842898029 | 0.781685049 | 0.999970678 | 1.862 | 0.099 | 0.911 | 0 | FMO-like |
| TRINITY_DN17754_c0_g1_i2 | -0.84288423 | 0.311308961 | 0.999970678 | 5.532 | 4.11 | 6.289 | 9.551 | NA |
| TRINITY_DN784_c0_g1_i6 | 0.842786729 | 0.232357495 | 0.999970678 | 22.991 | 24.49 | 13.61 | 10.024 | CBM_20 |
| TRINITY_DN47198_c0_g1_i1 | -0.84276742 | 0.6350885 | 0.999970678 | 0.236 | 2.622 | 0.656 | 4.263 | Aa_trans |
| TRINITY_DN90042_c0_g1_i1 | 0.842766195 | 0.84367962 | 0.999970678 | 51.133 | 0 | 23.354 | 0 | NA |
| TRINITY_DN7776_c0_g1_i3 | -0.84276543 | 0.612633148 | 0.999970678 | 7.294 | 1.419 | 11.677 | 1.599 | NA |
| TRINITY_DN8899_c0_g1_i1 | 0.842758429 | 0.447682092 | 0.999970678 | 9.892 | 24.62 | 11.91 | 4.705 | Cellulase |
| TRINITY_DN2812_c0_g3_i3 | 0.84266296 | 0.461240456 | 0.999970678 | 4.106 | 8.071 | 4.278 | 1.588 | NA |
| TRINITY_DN14019_c0_g1_i1 | 0.842643581 | 0.696752324 | 0.999970678 | 5.414 | 684.7 | 67.351 | 255.816 | NA |
| TRINITY_DN6835_c0_g2_i1 | -0.84256762 | 0.32192471 | 0.999970678 | 20.857 | 50.14 | 52.019 | 63.137 | NA |
| TRINITY_DN13016_c0_g2_i1 | 0.842410905 | 0.321361657 | 0.999970678 | 2.534 | 1.577 | 0.833 | 1.307 | ANF_receptor |
| TRINITY_DN1299_c0_g1_i3 | 0.84240717 | 0.45986453 | 0.999970678 | 31.612 | 8.614 | 6.211 | 15.101 | NA |
| TRINITY_DN1087_c0_g3_i1 | 0.84237946 | 0.471936052 | 0.999970678 | 0.881 | 2.937 | 1 | 0.905 | NA |
| TRINITY_DN366_c0_g1_i3 | 0.842347996 | 0.287015669 | 0.999970678 | 119.25 | 59.09 | 42.853 | 48.338 | GST_C_2 |
| TRINITY_DN6722_c0_g1_i2 | 0.842179578 | 0.498539045 | 0.999970678 | 2.008 | 4.129 | 2.278 | 0.653 | NA |
| TRINITY_DN3902_c0_g2_i5 | 0.842148604 | 0.714377372 | 0.999970678 | 3.788 | 0.049 | 1.433 | 0.392 | NA |
| TRINITY_DN4235_c1_g1_i9 | 0.842147773 | 0.753500607 | 0.999970678 | 1.299 | 1.192 | 1.155 | 0 | HEAT |
| TRINITY_DN4647_c0_g1_i4 | 0.842094459 | 0.58674864 | 0.999970678 | 3.443 | 2.641 | 2.478 | 0.412 | NA |
| TRINITY_DN22525_c0_g4_i1 | -0.84203738 | 0.726998442 | 1 | 1.453 | 0.168 | 2.367 | 0.06 | HABP4_PAI-RBP1 |
| TRINITY_DN13343_c0_g1_i1 | 0.842006833 | 0.435398468 | 0.999970678 | 1.699 | 3.213 | 1.355 | 1.116 | NA |
| TRINITY_DN2136_c0_g1_i8 | 0.841720293 | 0.34843823 | 0.999970678 | 6.486 | 7.736 | 4.511 | 2.463 | NA |
| TRINITY_DN5263_c0_g1_i5 | -0.84170983 | 0.606269884 | 0.999970678 | 0.218 | 1.005 | 1.378 | 0.493 | DAP_epimerase |
| TRINITY_DN4540_c0_g1_i5 | -0.84169665 | 0.759022468 | 0.999970678 | 0 | 3.302 | 1.189 | 4.404 | Methyltransf_29 |
| TRINITY_DN254_c2_g1_i3 | 0.841502146 | 0.438791639 | 0.999970678 | 5.505 | 2.178 | 2.389 | 1.418 | NA |
| TRINITY_DN636_c2_g1_i6 | 0.84147011 | 0.487178676 | 0.999970678 | 1.463 | 4.356 | 0.989 | 2.031 | ANAPC10 |
| TRINITY_DN9022_c0_g1_i1 | 0.84112437 | 0.245017552 | 0.999970678 | 3.697 | 4.021 | 1.767 | 2.172 | NA |

| TRINITY_DN1915_c0_g1_i10 | -0.84110951 | 0.400851314 | 0.999970678 | 0.836 | 2.109 | 2.233 | 2.513 | - |
| --- | --- | --- | --- | --- | --- | --- | --- | --- |
| TRINITY_DN2693_c0_g1_i5 | 0.841094782 | 0.77246889 | 0.999970678 | 0 | 1.478 | 0.178 | 0.583 | NA |
| TRINITY_DN14141_c2_g3_i1 | -0.84104709 | 0.532142974 | 0.999970678 | 1.39 | 0.365 | 1.967 | 0.784 | NA |
| TRINITY_DN10377_c0_g1_i1 | -0.8410205 | 0.770589093 | 1 | 0 | 0.857 | 0.922 | 0.352 | NA |
| TRINITY_DN1523_c0_g1_i3 | -0.84098436 | 0.447138633 | 0.999970678 | 7.158 | 5.154 | 12.744 | 6.183 | NA |
| TRINITY_DN77152_c0_g2_i1 | -0.84092446 | 0.847804322 | 0.999970678 | 0.936 | 0 | 1.411 | 0 | NA |
| TRINITY_DN43986_c0_g2_i1 | 0.840910854 | 0.759767533 | 0.999970678 | 1.09 | 0.443 | 0.711 | 0 | NA |
| TRINITY_DN4176_c0_g1_i2 | 0.84084579 | 0.754908491 | 0.999970678 | 0.318 | 0.325 | 0.3 | 0 | KH_1 |
| TRINITY_DN29934_c0_g1_i1 | 0.840742832 | 0.847278249 | 0.999970678 | 1.39 | 0 | 0.644 | 0 | Peptidase_S8 |
| TRINITY_DN1940_c0_g1_i5 | -0.84071156 | 0.22370013 | 0.999970678 | 12.654 | 8.101 | 14.61 | 19.756 | Sugar_tr |
| TRINITY_DN5286_c0_g1_i3 | -0.8406705 | 0.410378668 | 0.999970678 | 51.624 | 116 | 66.64 | 216.687 | NA |
| TRINITY_DN8865_c0_g1_i1 | -0.84063599 | 0.405676676 | 0.999970678 | 12.654 | 21.56 | 12.633 | 45.664 | HR_lesion |
| TRINITY_DN6729_c0_g1_i1 | -0.8405901 | 0.473906426 | 0.999970678 | 3.307 | 9.087 | 4.489 | 16.548 | NA |
| TRINITY_DN2698_c0_g1_i4 | -0.84057138 | 0.75735527 | 0.999970678 | 2.144 | 0 | 0.867 | 2.845 | NA |
| TRINITY_DN7479_c0_g1_i5 | 0.840504298 | 0.58613206 | 0.999970678 | 1.372 | 4.711 | 2.455 | 0.392 | NA |
| TRINITY_DN3578_c0_g1_i1 | -0.84032496 | 0.800374292 | 1 | 1.535 | 0.089 | 2.367 | 0 | LTP_2 |
| TRINITY_DN11172_c0_g1_i12 | 0.84031963 | 0.7595149 | 1 | 0.463 | 0.325 | 0 | 0.432 | NA |
| TRINITY_DN220949_c0_g1_i1 | 0.840304927 | 0.451190381 | 0.999970678 | 0.436 | 0.67 | 0.256 | 0.302 | NA |
| TRINITY_DN15682_c0_g1_i6 | -0.8402866 | 0.842661564 | 0.999970678 | 2.171 | 0 | 3.244 | 0 | NA |
| TRINITY_DN13627_c0_g1_i2 | -0.84028533 | 0.549695701 | 0.999970678 | 9.62 | 7.382 | 3.866 | 24.35 | NA |
| TRINITY_DN16106_c0_g1_i3 | -0.84025952 | 0.390285033 | 0.999970678 | 9.829 | 20.95 | 13.577 | 38.335 | - |
| TRINITY_DN4420_c0_g1_i1 | 0.840166113 | 0.278965885 | 0.999970678 | 1.263 | 1.537 | 0.744 | 0.674 | BCAS3 |
| TRINITY_DN6273_c0_g1_i2 | 0.840134942 | 0.303454011 | 0.999970678 | 20.248 | 17.05 | 5.744 | 13.874 | NA |
| TRINITY_DN8461_c2_g1_i2 | -0.84000069 | 0.778927657 | 1 | 0.354 | 2.217 | 0 | 4.494 | NA |
| TRINITY_DN6564_c0_g2_i1 | -0.8399209 | 0.763671298 | 0.999970678 | 1.481 | 3.489 | 0 | 8.867 | NA |
| TRINITY_DN2365_c0_g1_i1 | 0.83986405 | 0.237987441 | 0.999970678 | 2.743 | 3.371 | 1.644 | 1.448 | NA |
| TRINITY_DN2066_c0_g1_i2 | -0.83982257 | 0.656235511 | 0.999970678 | 0.055 | 2.316 | 1.044 | 2.956 | NA |
| TRINITY_DN4903_c0_g1_i1 | -0.83981361 | 0.845361746 | 0.999970678 | 9.465 | 0 | 14.21 | 0 | NA |
| TRINITY_DN52424_c1_g2_i1 | 0.839760919 | 0.840893535 | 0.999970678 | 0 | 4.78 | 0 | 2.644 | AUX_IAA |
| TRINITY_DN3188_c0_g1_i3 | 0.839733947 | 0.760692663 | 1 | 0.209 | 0.473 | 0 | 0.382 | NA |
| TRINITY_DN3007_c0_g1_i7 | -0.83963452 | 0.760608102 | 0.999970678 | 1.989 | 0 | 1.155 | 2.232 | NA |
| TRINITY_DN8293_c0_g1_i8 | 0.839629847 | 0.395627858 | 0.999970678 | 1.699 | 2.247 | 0.756 | 1.287 | YIF1 |
| TRINITY_DN3990_c0_g1_i5 | -0.83952047 | 0.767537354 | 0.999970678 | 0 | 20.62 | 11.222 | 22.802 | NA |
| TRINITY_DN60573_c0_g1_i2 | -0.83951691 | 0.47831025 | 0.999970678 | 1.208 | 0.788 | 1.033 | 2.312 | NA |
| TRINITY_DN1035_c0_g1_i1 | 0.83948368 | 0.651026434 | 0.999970678 | 3.733 | 0.345 | 1.489 | 0.503 | NA |
| TRINITY_DN1035_c0_g3_i1 | 0.83948368 | 0.651026434 | 0.999970678 | 3.733 | 0.345 | 1.489 | 0.503 | NA |
| TRINITY_DN5776_c0_g2_i4 | -0.83948314 | 0.440793741 | 0.999970678 | 2.989 | 10.99 | 8.122 | 14.95 | NA |
| TRINITY_DN5387_c0_g1_i2 | -0.83943672 | 0.495232657 | 0.999970678 | 0.6 | 0.365 | 0.822 | 0.744 | WD40 |
| TRINITY_DN10337_c0_g3_i1 | -0.83941836 | 0.531057804 | 0.999970678 | 1.017 | 1.143 | 0.522 | 3.217 | NA |
| TRINITY_DN569_c0_g1_i5 | -0.8392925 | 0.843810106 | 0.999970678 | 0 | 5.854 | 8.522 | 0 | NA |
| TRINITY_DN1407_c1_g1_i2 | -0.83927942 | 0.296114889 | 0.999970678 | 47.909 | 44.28 | 43.697 | 112.35 | NA |
| TRINITY_DN4710_c0_g1_i2 | -0.83914441 | 0.63627482 | 0.999970678 | 1.199 | 1.567 | 0.278 | 4.544 | Topoisom_bac |
| TRINITY_DN2011_c0_g1_i7 | 0.838969597 | 0.620510096 | 0.999970678 | 0.89 | 0.128 | 0.4 | 0.101 | NA |
| TRINITY_DN10760_c0_g1_i11 | -0.83894935 | 0.759863465 | 0.999970678 | 1.181 | 0 | 1.078 | 0.814 | NA |
| TRINITY_DN4735_c0_g1_i1 | 0.838947971 | 0.438118779 | 0.999970678 | 2.353 | 1.488 | 0.467 | 1.578 | KH_1 |
| TRINITY_DN14816_c0_g1_i1 | -0.83874326 | 0.174503879 | 0.999970678 | 1626 | 1270 | 2235.72 | 2226.532 | NA |
| TRINITY_DN1760_c0_g1_i3 | -0.83869081 | 0.16101444 | 0.999970678 | 15.515 | 20.08 | 25.354 | 32.956 | NA |
| TRINITY_DN3479_c0_g1_i1 | 0.838608384 | 0.438791706 | 0.999970678 | 75.696 | 19.95 | 18.021 | 32.162 | NA |
| TRINITY_DN29552_c0_g1_i1 | -0.83851964 | 0.318297834 | 0.999970678 | 1.962 | 1.991 | 3.522 | 2.815 | Mito_carr |
| TRINITY_DN10578_c0_g1_i3 | 0.838461627 | 0.432206001 | 0.999970678 | 2.807 | 1.705 | 1.178 | 1.096 | NA |
| TRINITY_DN11082_c0_g3_i2 | -0.83842658 | 0.590230211 | 0.999970678 | 0.209 | 1.015 | 1.455 | 0.402 | NA |
| TRINITY_DN3186_c0_g1_i5 | 0.838414575 | 0.845134573 | 0.999970678 | 0 | 17.63 | 8.022 | 0 | NA |
| TRINITY_DN1552_c0_g1_i2 | -0.83837326 | 0.21282079 | 0.999970678 | 77.304 | 111.5 | 119.037 | 188.537 | Ribosomal_S27 |
| TRINITY_DN1197_c0_g3_i1 | 0.838274684 | 0.636097879 | 0.999970678 | 11.964 | 1.37 | 5.755 | 0.593 | NA |
| TRINITY_DN12933_c0_g1_i1 | -0.83823334 | 0.766357967 | 1 | 0.763 | 0 | 0.322 | 1.015 | NA |
| TRINITY_DN296_c0_g1_i8 | -0.83822517 | 0.755402228 | 0.999970678 | 4.887 | 0 | 3.422 | 4.705 | Glyco_transf_4 |
| TRINITY_DN4988_c0_g1_i3 | -0.83817453 | 0.499644484 | 0.999970678 | 6.622 | 8.476 | 3.255 | 22.983 | Hexapep |
| TRINITY_DN32890_c0_g1_i1 | -0.83808285 | 0.653705207 | 0.999970678 | 2.934 | 2.464 | 0.378 | 9.078 | LRRNT_2 |
| TRINITY_DN166119_c0_g1_i1 | -0.83800224 | 0.854770311 | 1 | 1.163 | 0 | 1.744 | 0 | adh_short |
| TRINITY_DN12192_c0_g1_i8 | -0.8380002 | 0.843474388 | 0.999970678 | 7.076 | 0 | 10.544 | 0 | SBDS |
| TRINITY_DN707_c0_g1_i12 | -0.83799932 | 0.847402064 | 0.999970678 | 0.164 | 0 | 0.233 | 0 | NA |
| TRINITY_DN5835_c0_g1_i7 | -0.83789966 | 0.76455688 | 1 | 0.945 | 0 | 0.589 | 1.056 | NA |
| TRINITY_DN1183_c0_g1_i5 | 0.837828178 | 0.765774875 | 1 | 0.245 | 0.601 | 0.389 | 0 | LRR_8 |
| TRINITY_DN620_c0_g1_i11 | -0.83772122 | 0.800938536 | 0.999970678 | 13.362 | 0 | 19.732 | 0.402 | DUF3458 |
| TRINITY_DN40498_c0_g1_i1 | -0.83772056 | 0.428643354 | 0.999970678 | 1.49 | 3.233 | 2.744 | 5.027 | NA |
| TRINITY_DN15263_c0_g1_i1 | -0.83752466 | 0.759143013 | 0.999970678 | 175.67 | 0 | 153.18 | 113.848 | NA |
| TRINITY_DN5480_c0_g1_i2 | 0.837508709 | 0.433655069 | 0.999970678 | 1.417 | 1.163 | 0.533 | 0.804 | NA |
| TRINITY_DN2586_c0_g1_i9 | -0.83750622 | 0.605787906 | 0.999970678 | 0.681 | 3.568 | 1.122 | 6.103 | Histone |
| TRINITY_DN2071_c0_g1_i2 | 0.837486784 | 0.842216504 | 0.999970678 | 0 | 7.076 | 3.222 | 0 | Abhydrolase_6 |
| TRINITY_DN27275_c0_g1_i2 | -0.83745249 | 0.645527305 | 0.999970678 | 1.045 | 0.473 | 0.189 | 2.534 | WRKY |
| TRINITY_DN7353_c0_g1_i2 | -0.83741958 | 0.763732371 | 0.999970678 | 0.318 | 0.424 | 1.1 | 0 | NA |
| TRINITY_DN9301_c0_g1_i3 | 0.837373461 | 0.509988006 | 0.999970678 | 0.936 | 3.213 | 1.367 | 0.653 | DRMBL |
| TRINITY_DN2881_c0_g1_i8 | 0.837355916 | 0.491881782 | 0.999970678 | 17.477 | 4.06 | 4.844 | 6.123 | NA |
| TRINITY_DN40449_c0_g1_i2 | -0.83730068 | 0.469045406 | 0.999970678 | 0.509 | 0.719 | 0.567 | 1.498 | NA |

| TRINITY_DN1882_c0_g1_i3 | 0.837087959 | 0.755845327 | 0.999970678 | 0.563 | 1.656 | 0 | 1.227 | NA |
| --- | --- | --- | --- | --- | --- | --- | --- | --- |
| TRINITY_DN9852_c0_g1_i21 | -0.83702666 | 0.852764679 | 1 | 0.254 | 0 | 0 | 0.462 | NA |
| TRINITY_DN277_c0_g1_i3 | -0.83688902 | 0.759052004 | 0.999970678 | 10.583 | 0 | 7.233 | 10.456 | NA |
| TRINITY_DN826_c0_g2_i2 | -0.83683967 | 0.359547915 | 0.999970678 | 7.758 | 8.495 | 7.233 | 20.148 | Peptidase_M16 |
| TRINITY_DN8100_c0_g1_i1 | -0.83675497 | 0.847224595 | 0.999970678 | 0.427 | 0 | 0.633 | 0 | CFEM |
| TRINITY_DN797_c0_g1_i5 | 0.836650602 | 0.446544257 | 0.999970678 | 2.534 | 1.971 | 1.622 | 0.573 | NA |
| TRINITY_DN750_c5_g1_i2 | 0.836649234 | 0.315244844 | 0.999970678 | 12.926 | 7.884 | 6.044 | 4.393 | NA |
| TRINITY_DN31779_c0_g1_i4 | -0.83655173 | 0.693767232 | 0.999970678 | 6.159 | 0.187 | 6.988 | 2.906 | NA |
| TRINITY_DN10484_c0_g1_i9 | -0.83649306 | 0.76646451 | 0.999970678 | 0 | 1.084 | 1.389 | 0.241 | NA |
| TRINITY_DN11972_c0_g1_i1 | 0.836439022 | 0.439572087 | 0.999970678 | 3.815 | 2.976 | 2.5 | 0.794 | VIR_N |
| TRINITY_DN12839_c0_g1_i1 | -0.8362523 | 0.780135015 | 0.999970678 | 934.72 | 0 | 278.017 | 1251.504 | NA |
| TRINITY_DN31962_c0_g1_i2 | -0.8360506 | 0.300677875 | 0.999970678 | 872.44 | 1513 | 2181.935 | 1425.906 | PSI_PsaH |
| TRINITY_DN14060_c0_g2_i1 | -0.83595417 | 0.337914451 | 0.999970678 | 6.341 | 4.445 | 5.9 | 12.024 | NA |
| TRINITY_DN3900_c0_g1_i3 | -0.83592532 | 0.363577303 | 0.999970678 | 0.754 | 0.719 | 1.133 | 1.267 | - |
| TRINITY_DN1880_c0_g1_i8 | -0.835821 | 0.790575839 | 0.999970678 | 0 | 1.439 | 2.011 | 0.07 | YEATS |
| TRINITY_DN3858_c0_g1_i5 | 0.835726561 | 0.279302528 | 0.999970678 | 41.895 | 60.38 | 29.965 | 20.691 | NA |
| TRINITY_DN2136_c0_g1_i2 | 0.835656695 | 0.606875232 | 0.999970678 | 17.278 | 3.459 | 1.444 | 9.822 | NA |
| TRINITY_DN3246_c0_g1_i3 | 0.835646428 | 0.31136509 | 0.999970678 | 5.923 | 4.464 | 3.044 | 2.141 | NA |
| TRINITY_DN10457_c0_g1_i1 | -0.83563593 | 0.843376418 | 0.999970678 | 5.895 | 0 | 8.822 | 0 | NA |
| TRINITY_DN56578_c0_g1_i1 | -0.83562961 | 0.853965848 | 1 | 0.5 | 0 | 0.744 | 0 | Mem_trans |
| TRINITY_DN3296_c0_g1_i7 | -0.83556278 | 0.483319865 | 0.999970678 | 45.283 | 11.28 | 56.419 | 32.112 | Ribosomal_L22e |
| TRINITY_DN7164_c0_g1_i4 | -0.83532083 | 0.765161576 | 0.999970678 | 0.836 | 0 | 0.311 | 1.126 | NA |
| TRINITY_DN782_c0_g2_i1 | -0.83526964 | 0.564802104 | 0.999970678 | 52.478 | 326.3 | 219.065 | 335.532 | NA |
| TRINITY_DN24340_c1_g2_i1 | -0.8351905 | 0.653632462 | 0.999970678 | 1.217 | 5.273 | 0.844 | 10.074 | NA |
| TRINITY_DN967_c3_g4_i1 | 0.835085927 | 0.642510768 | 1 | 7.222 | 4.425 | 0.511 | 5.58 | NA |
| TRINITY_DN15988_c0_g1_i3 | -0.83472978 | 0.487019552 | 0.999970678 | 0.799 | 0.828 | 1.667 | 0.965 | NA |
| TRINITY_DN1927_c0_g1_i3 | -0.83450404 | 0.578813628 | 0.999970678 | 28.024 | 51.4 | 8.144 | 129.813 | - |
| TRINITY_DN5587_c0_g1_i6 | -0.83440423 | 0.631584101 | 0.999970678 | 1.135 | 2.001 | 0.211 | 5.288 | mTERF |
| TRINITY_DN56450_c0_g1_i2 | -0.83431915 | 0.766182706 | 0.999970678 | 4.869 | 0 | 6.189 | 1.317 | NA |
| TRINITY_DN1215_c0_g1_i1 | 0.834259811 | 0.378523872 | 0.999970678 | 0.481 | 1.035 | 0.411 | 0.352 | TFIIIC_delta |
| TRINITY_DN45022_c0_g1_i1 | -0.83411836 | 0.299923848 | 0.999970678 | 511.71 | 931.4 | 1314.898 | 937.618 | - |
| TRINITY_DN3812_c0_g1_i6 | 0.834095597 | 0.851389177 | 1 | 3.007 | 0 | 0 | 1.649 | NA |
| TRINITY_DN5802_c0_g1_i1 | 0.834078521 | 0.593217566 | 0.999970678 | 0.863 | 0.237 | 0.411 | 0.141 | DUF641 |
| TRINITY_DN6478_c0_g1_i1 | -0.83404892 | 0.274380348 | 0.999970678 | 28.369 | 27.37 | 28.698 | 63.982 | NA |
| TRINITY_DN7653_c0_g1_i3 | 0.833978676 | 0.352700372 | 0.999970678 | 3.806 | 8.732 | 2.922 | 3.448 | NA |
| TRINITY_DN4380_c0_g2_i3 | 0.833977367 | 0.842574805 | 0.999970678 | 3.561 | 0 | 0 | 2.031 | Apt1 |
| TRINITY_DN34878_c0_g1_i5 | 0.833957577 | 0.591306772 | 0.999970678 | 0.554 | 2.277 | 0.278 | 1.237 | NA |
| TRINITY_DN274_c0_g1_i3 | -0.83366679 | 0.844631975 | 0.999970678 | 1.544 | 0 | 2.322 | 0 | RRM_1 |
| TRINITY_DN2160_c0_g1_i5 | -0.83352484 | 0.428416438 | 0.999970678 | 0.354 | 0.719 | 0.922 | 0.794 | NA |
| TRINITY_DN372_c0_g1_i1 | 0.833397522 | 0.785566816 | 0.999970678 | 44.893 | 0 | 19.532 | 1.387 | NA |
| TRINITY_DN854_c0_g1_i7 | -0.83322801 | 0.502761135 | 0.999970678 | 3.37 | 14.6 | 18.721 | 8.898 | CS |
| TRINITY_DN7686_c0_g1_i3 | -0.8330846 | 0.747751948 | 0.999970678 | 88.877 | 0.03 | 114.304 | 22.289 | CBFD_NFYB_HMF |
| TRINITY_DN4656_c0_g1_i2 | -0.83305617 | 0.783401126 | 0.999970678 | 7.058 | 0.375 | 0 | 13.452 | Yip1 |
| TRINITY_DN5242_c0_g1_i3 | -0.83301947 | 0.754019236 | 0.999970678 | 0 | 3.577 | 2.567 | 3.157 | LysM |
| TRINITY_DN5928_c0_g1_i4 | 0.832847094 | 0.715406683 | 0.999970678 | 0.754 | 0.079 | 0.378 | 0.02 | NA |
| TRINITY_DN7267_c0_g2_i2 | -0.83273365 | 0.760340064 | 0.999970678 | 1.063 | 0 | 0.422 | 1.408 | NA |
| TRINITY_DN3611_c0_g1_i1 | -0.83273282 | 0.551259541 | 0.999970678 | 0.69 | 1.124 | 0.444 | 2.664 | NA |
| TRINITY_DN3404_c0_g1_i8 | 0.832721065 | 0.411222568 | 0.999970678 | 9.475 | 5.223 | 4.822 | 2.443 | NA |
| TRINITY_DN17146_c0_g1_i3 | -0.83260173 | 0.482181894 | 0.999970678 | 361.74 | 129.3 | 572.21 | 187.753 | DnaJ |
| TRINITY_DN21985_c0_g1_i4 | 0.832581713 | 0.411594514 | 0.999970678 | 1.326 | 3.006 | 0.989 | 1.227 | NA |
| TRINITY_DN1913_c0_g1_i6 | -0.8325511 | 0.754820258 | 0.999970678 | 0 | 2.474 | 2.2 | 1.699 | NA |
| TRINITY_DN2496_c0_g1_i4 | 0.832375122 | 0.555043532 | 0.999970678 | 1.444 | 5.075 | 2.533 | 0.563 | NA |
| TRINITY_DN3282_c0_g1_i3 | 0.83228964 | 0.275226412 | 0.999970678 | 2.289 | 3.508 | 1.122 | 1.89 | Helicase_C |
| TRINITY_DN74_c0_g1_i4 | -0.83228679 | 0.409462418 | 0.999970678 | 16.951 | 28.95 | 18.554 | 56.602 | NA |
| TRINITY_DN34324_c0_g1_i3 | 0.832203355 | 0.375607329 | 0.999970678 | 18.322 | 6.84 | 6.389 | 6.535 | NA |
| TRINITY_DN4972_c0_g1_i6 | 0.832194891 | 0.641840222 | 0.999970678 | 4.869 | 1.36 | 2.755 | 0.181 | DUF3537 |
| TRINITY_DN723_c0_g1_i1 | 0.832104002 | 0.410344769 | 0.999970678 | 32.748 | 12.22 | 13.799 | 8.747 | NA |
| TRINITY_DN44466_c0_g1_i5 | 0.83206773 | 0.481473078 | 0.999970678 | 0.345 | 0.365 | 0.211 | 0.141 | NA |
| TRINITY_DN10602_c2_g1_i1 | 0.832043849 | 0.850689172 | 1 | 0 | 1.784 | 0.822 | 0 | DUF1308 |
| TRINITY_DN12914_c0_g1_i6 | -0.83201604 | 0.852513011 | 1 | 1.017 | 0 | 1.5 | 0 | Skp1 |
| TRINITY_DN8088_c0_g1_i10 | 0.831934312 | 0.774360857 | 0.999970678 | 0.754 | 0.128 | 0.411 | 0 | NA |
| TRINITY_DN8746_c0_g1_i12 | 0.831912974 | 0.849547675 | 0.999970678 | 0 | 0.719 | 0.322 | 0 | - |
| TRINITY_DN1835_c0_g1_i9 | -0.83164435 | 0.349961666 | 0.999970678 | 9.447 | 5.736 | 7.166 | 18.549 | NA |
| TRINITY_DN3426_c0_g1_i15 | -0.83161737 | 0.487113568 | 0.999970678 | 4.115 | 3.587 | 2.578 | 10.365 | NA |
| TRINITY_DN18568_c0_g1_i3 | 0.831600011 | 0.851363464 | 1 | 0.799 | 0 | 0.378 | 0 | NA |
| TRINITY_DN4087_c0_g1_i3 | 0.831595973 | 0.284440179 | 0.999970678 | 65.195 | 37.9 | 19.643 | 34.384 | Abhydrolase_1 |
| TRINITY_DN23741_c0_g1_i1 | -0.83159548 | 0.19894686 | 0.999970678 | 15.434 | 21.15 | 29.798 | 28.945 | - |
| TRINITY_DN144_c0_g1_i7 | 0.831543651 | 0.604534762 | 0.999970678 | 2.153 | 2.937 | 0.133 | 2.704 | EF-hand_5 |
| TRINITY_DN1876_c0_g1_i1 | 0.831527853 | 0.842848456 | 0.999970678 | 0 | 13.59 | 0 | 7.42 | NA |
| TRINITY_DN908_c0_g2_i1 | -0.83137813 | 0.514148102 | 0.999970678 | 26.262 | 4.001 | 21.188 | 28.643 | NA |
| TRINITY_DN1624_c0_g1_i14 | -0.83137688 | 0.60875263 | 0.999970678 | 22.019 | 2.01 | 30.009 | 6.977 | NA |
| TRINITY_DN47748_c0_g1_i1 | 0.831366031 | 0.755962272 | 0.999970678 | 6.213 | 7.983 | 0 | 7.58 | NA |
| TRINITY_DN7638_c0_g1_i1 | 0.831346499 | 0.765957605 | 0.999970678 | 0 | 6.396 | 0.967 | 2.393 | NA |
| TRINITY_DN4332_c0_g1_i1 | -0.83115181 | 0.540994019 | 0.999970678 | 0.254 | 0.414 | 0.256 | 0.875 | NA |

| TRINITY_DN6461_c0_g1_i1 | 0.831004875 | 0.249611491 | 0.999970678 | 3.188 | 3.548 | 1.233 | 2.302 | NA |
| --- | --- | --- | --- | --- | --- | --- | --- | --- |
| TRINITY_DN2940_c0_g1_i1 | 0.830992827 | 0.419871742 | 0.999970678 | 8.139 | 9.294 | 1.822 | 7.601 | Jacalin |
| TRINITY_DN2774_c0_g2_i1 | -0.83091503 | 0.755978917 | 0.999970678 | 0 | 2.878 | 1.8 | 2.785 | Pribosyltran |
| TRINITY_DN1281_c0_g1_i2 | 0.830815851 | 0.330566555 | 0.999970678 | 9.774 | 5.381 | 3.2 | 4.695 | HMG_box |
| TRINITY_DN911_c0_g1_i6 | 0.830791174 | 0.650452416 | 0.999970678 | 1.726 | 2.661 | 0.1 | 2.312 | NA |
| TRINITY_DN5475_c0_g1_i1 | 0.830781303 | 0.490790118 | 0.999970678 | 0.908 | 1.666 | 0.9 | 0.352 | NA |
| TRINITY_DN2190_c0_g1_i5 | 0.83076953 | 0.342686109 | 0.999970678 | 4.16 | 5.125 | 3.022 | 1.558 | NA |
| TRINITY_DN10132_c0_g1_i6 | -0.8306086 | 0.851945871 | 1 | 1.199 | 0 | 1.767 | 0 | NA |
| TRINITY_DN27722_c0_g1_i3 | -0.8306086 | 0.851945871 | 1 | 1.235 | 0 | 1.8 | 0 | - |
| TRINITY_DN5408_c0_g1_i5 | -0.83049767 | 0.509969472 | 0.999970678 | 1.036 | 3.646 | 2.355 | 5.389 | NA |
| TRINITY_DN4960_c2_g2_i3 | 0.830416826 | 0.248697337 | 0.999970678 | 6.849 | 8.278 | 2.789 | 5.147 | zf-C2H2 |
| TRINITY_DN3065_c0_g1_i1 | -0.83040127 | 0.517784564 | 0.999970678 | 0.581 | 2.346 | 1.811 | 2.946 | RRM_1 |
| TRINITY_DN982_c0_g1_i2 | -0.83027422 | 0.402404535 | 0.999970678 | 1.708 | 2.306 | 1.778 | 4.977 | NA |
| TRINITY_DN8326_c0_g1_i10 | 0.830270526 | 0.557451162 | 0.999970678 | 1.208 | 0.621 | 0.733 | 0.161 | ABC_tran |
| TRINITY_DN1720_c2_g3_i2 | 0.830247683 | 0.55535342 | 0.999970678 | 7.313 | 1.311 | 2.367 | 2.031 | WRKY |
| TRINITY_DN2626_c0_g1_i3 | 0.830087746 | 0.261074352 | 0.999970678 | 3.425 | 3.38 | 1.3 | 2.272 | - |
| TRINITY_DN152_c1_g1_i6 | 0.829987026 | 0.577585818 | 0.999970678 | 0.772 | 0.631 | 0.578 | 0.111 | NA |
| TRINITY_DN9944_c0_g1_i15 | 0.829957514 | 0.766397847 | 0.999970678 | 1.399 | 0 | 0.356 | 0.372 | NA |
| TRINITY_DN1657_c0_g1_i5 | -0.82982061 | 0.4560397 | 0.999970678 | 3.679 | 7.096 | 11.944 | 4.584 | NA |
| TRINITY_DN3171_c0_g1_i1 | 0.829779741 | 0.844340785 | 0.999970678 | 10.892 | 0 | 5.122 | 0 | Pro_isomerase |
| TRINITY_DN6789_c0_g1_i1 | 0.829706537 | 0.766063516 | 0.999970678 | 3.252 | 0 | 0.833 | 0.855 | NA |
| TRINITY_DN1946_c0_g1_i3 | -0.82970329 | 0.34425746 | 0.999970678 | 1.553 | 0.976 | 1.555 | 2.644 | NA |
| TRINITY_DN1437_c1_g1_i2 | -0.82969517 | 0.352744347 | 0.999970678 | 7.04 | 3.124 | 7.611 | 8.978 | NA |
| TRINITY_DN8848_c0_g1_i1 | -0.82958738 | 0.497920905 | 0.999970678 | 13.617 | 75.23 | 65.585 | 70.909 | NA |
| TRINITY_DN4889_c0_g1_i1 | -0.82955573 | 0.60826217 | 0.999970678 | 0.327 | 1.951 | 0.422 | 3.519 | Ion_trans |
| TRINITY_DN4908_c0_g2_i3 | 0.829491559 | 0.758728644 | 0.999970678 | 1.081 | 0.729 | 0.844 | 0 | B3 |
| TRINITY_DN1126_c1_g1_i2 | 0.829477975 | 0.52521429 | 0.999970678 | 3.915 | 6.061 | 4.044 | 0.714 | NA |
| TRINITY_DN2465_c0_g1_i4 | -0.82937754 | 0.695011863 | 0.999970678 | 11.518 | 1.892 | 19.099 | 0.593 | NA |
| TRINITY_DN152_c0_g2_i1 | -0.82924011 | 0.780548346 | 1 | 2.825 | 0.69 | 5.1 | 0 | NA |
| TRINITY_DN4459_c0_g1_i14 | 0.829198563 | 0.754014483 | 0.999970678 | 1.008 | 0.542 | 0 | 0.875 | NA |
| TRINITY_DN9816_c0_g1_i1 | -0.82915051 | 0.464702057 | 0.999970678 | 9.493 | 14.06 | 6.866 | 33.087 | NA |
| TRINITY_DN1372_c0_g1_i6 | -0.82913732 | 0.754686681 | 0.999970678 | 0 | 2.316 | 1.489 | 2.242 | NA |
| TRINITY_DN12368_c0_g1_i1 | -0.82907127 | 0.346791734 | 0.999970678 | 0.89 | 1.192 | 1.144 | 2.322 | DHFR_1 |
| TRINITY_DN14218_c0_g1_i3 | 0.829021311 | 0.794440716 | 1 | 0.481 | 0 | 0.211 | 0.03 | G-patch |
| TRINITY_DN11114_c0_g1_i1 | -0.8288484 | 0.846144072 | 0.999970678 | 7.276 | 0 | 10.777 | 0 | MAPEG |
| TRINITY_DN15764_c0_g1_i2 | 0.828842192 | 0.776568579 | 0.999970678 | 33.411 | 2.306 | 16.61 | 0 | NA |
| TRINITY_DN4256_c0_g1_i2 | -0.82881253 | 0.681662649 | 1 | 0.173 | 0.887 | 0.144 | 1.699 | - |
| TRINITY_DN3770_c0_g1_i1 | -0.82879672 | 0.394305771 | 0.999970678 | 4.778 | 11.58 | 12.666 | 13.231 | NA |
| TRINITY_DN267_c0_g1_i6 | 0.828704493 | 0.721404045 | 0.999970678 | 0.763 | 11.42 | 0.067 | 6.575 | - |
| TRINITY_DN4054_c0_g1_i4 | 0.828622024 | 0.574621311 | 0.999970678 | 0.554 | 0.71 | 0.489 | 0.131 | NA |
| TRINITY_DN10742_c0_g1_i2 | -0.82855605 | 0.533390996 | 0.999970678 | 0.654 | 0.237 | 0.478 | 0.985 | NA |
| TRINITY_DN10586_c0_g1_i2 | -0.82846949 | 0.550097684 | 0.999970678 | 6.195 | 2.306 | 10.199 | 2.825 | NA |
| TRINITY_DN10690_c0_g1_i1 | -0.82844952 | 0.403733491 | 0.999970678 | 20.375 | 32.02 | 18.988 | 69.803 | NA |
| TRINITY_DN4012_c0_g1_i3 | -0.82837008 | 0.768353188 | 0.999970678 | 12.445 | 0 | 6.044 | 15.151 | - |
| TRINITY_DN416_c2_g1_i1 | -0.82826594 | 0.447581912 | 0.999970678 | 2.353 | 4.287 | 2.489 | 8.717 | NA |
| TRINITY_DN15842_c0_g1_i1 | 0.828234013 | 0.622131751 | 0.999970678 | 0.927 | 10.61 | 2.322 | 3.428 | NA |
| TRINITY_DN2176_c0_g1_i36 | 0.828154559 | 0.781285762 | 0.999970678 | 0.836 | 0 | 0.056 | 0.402 | NA |
| TRINITY_DN4067_c0_g1_i9 | 0.828097567 | 0.389052557 | 0.999970678 | 4.36 | 8.131 | 3.211 | 3.117 | NA |
| TRINITY_DN9189_c0_g1_i4 | 0.828056321 | 0.3909298 | 0.999970678 | 0.754 | 0.591 | 0.256 | 0.452 | zf-U11-48K |
| TRINITY_DN11767_c0_g1_i1 | 0.827982333 | 0.651686129 | 1 | 0.763 | 0.237 | 0.422 | 0.06 | NA |
| TRINITY_DN21_c0_g1_i11 | -0.82797897 | 0.58746936 | 0.999970678 | 0.427 | 2.158 | 0.8 | 3.539 | NA |
| TRINITY_DN7234_c0_g1_i2 | 0.827939311 | 0.756844664 | 0.999970678 | 2.325 | 1.015 | 0 | 1.88 | NA |
| TRINITY_DN709_c0_g1_i3 | 0.827914558 | 0.620683393 | 0.999970678 | 0.945 | 1.941 | 1.244 | 0.131 | NA |
| TRINITY_DN10835_c0_g1_i5 | -0.82777173 | 0.844609653 | 0.999970678 | 4.033 | 0 | 6 | 0 | NA |
| TRINITY_DN23773_c0_g4_i2 | -0.82777133 | 0.75659437 | 0.999970678 | 0 | 4.09 | 2.178 | 4.474 | NA |
| TRINITY_DN2815_c0_g1_i1 | 0.827695597 | 0.400464266 | 0.999970678 | 0.563 | 0.986 | 0.289 | 0.513 | NA |
| TRINITY_DN17019_c0_g1_i5 | -0.82763477 | 0.787309123 | 0.999970678 | 0 | 40.34 | 26.854 | 38.274 | Cyclin |
| TRINITY_DN8624_c0_g1_i1 | -0.82752294 | 0.383620986 | 0.999970678 | 6.858 | 3.213 | 9.422 | 6.555 | NA |
| TRINITY_DN3831_c1_g1_i3 | 0.827514159 | 0.410331039 | 0.999970678 | 2.171 | 1.656 | 1.3 | 0.603 | NA |
| TRINITY_DN251075_c0_g1_i1 | 0.82741597 | 0.383264981 | 0.999970678 | 65.341 | 45.01 | 14.632 | 43.03 | NA |
| TRINITY_DN8903_c0_g3_i1 | 0.827348573 | 0.427123574 | 0.999970678 | 0.736 | 0.907 | 0.444 | 0.392 | NA |
| TRINITY_DN1750_c0_g1_i6 | 0.827319469 | 0.845909291 | 0.999970678 | 0 | 1.636 | 0 | 0.915 | SRP54 |
| TRINITY_DN1752_c0_g1_i4 | -0.8273085 | 0.555640324 | 0.999970678 | 1.099 | 0.917 | 2.489 | 0.583 | - |
| TRINITY_DN5484_c0_g1_i1 | 0.82729953 | 0.592145426 | 0.999970678 | 3.079 | 31.3 | 8.077 | 8.817 | 2OG-FeII_Oxy |
| TRINITY_DN1493_c0_g1_i2 | -0.8272966 | 0.50265524 | 0.999970678 | 24.181 | 4.179 | 19.499 | 27.175 | Rad60-SLD_2 |
| TRINITY_DN1564_c0_g1_i5 | -0.82729057 | 0.592112839 | 1 | 0.2 | 0.956 | 1.055 | 0.784 | CIAPIN1 |
| TRINITY_DN2405_c0_g1_i12 | 0.82725058 | 0.784457663 | 0.999970678 | 0 | 2.533 | 0.089 | 1.307 | Sugar_tr |
| TRINITY_DN3018_c0_g1_i1 | 0.827243695 | 0.844532577 | 0.999970678 | 0 | 8.269 | 3.778 | 0 | Glyco_hydro_17 |
| TRINITY_DN40152_c0_g1_i1 | -0.82720195 | 0.80329848 | 1 | 0.454 | 0 | 0.656 | 0.02 | Pkinase |
| TRINITY_DN4067_c0_g1_i13 | -0.82714027 | 0.576039294 | 0.999970678 | 13.045 | 3.134 | 3.355 | 24.762 | NA |
| TRINITY_DN685_c0_g1_i3 | -0.82709759 | 0.244478168 | 0.999970678 | 313.78 | 442 | 426.996 | 813.967 | - |
| TRINITY_DN750_c0_g1_i10 | 0.827050689 | 0.752188844 | 0.999970678 | 2.616 | 2.277 | 0 | 2.765 | NA |
| TRINITY_DN78552_c1_g1_i1 | 0.826914624 | 0.848095711 | 0.999970678 | 9.774 | 0 | 4.589 | 0 | Cyanate_lyase |
| TRINITY_DN17494_c0_g2_i2 | -0.82686588 | 0.765273707 | 0.999970678 | 2.162 | 0 | 2.6 | 0.744 | NA |

| TRINITY_DN1397_c1_g1_i1 | 0.826844685 | 0.844583796 | 0.999970678 | 4.606 | 0 | 0.011 | 2.654 | NA |
| --- | --- | --- | --- | --- | --- | --- | --- | --- |
| TRINITY_DN127_c0_g1_i1 | -0.82679614 | 0.768106313 | 0.999970678 | 80.802 | 0 | 31.131 | 102.628 | NA |
| TRINITY_DN16141_c0_g1_i1 | -0.8267877 | 0.772037154 | 1 | 4.442 | 0 | 1.522 | 5.992 | NA |
| TRINITY_DN6290_c0_g1_i5 | 0.826724588 | 0.760993106 | 0.999970678 | 0.254 | 0.207 | 0.222 | 0 | DIL |
| TRINITY_DN15144_c1_g1_i1 | -0.82669472 | 0.545800156 | 0.999970678 | 152.27 | 24.88 | 120.715 | 149.116 | NA |
| TRINITY_DN1391_c1_g1_i8 | 0.826685371 | 0.277268139 | 0.999970678 | 8.684 | 11.83 | 5.211 | 5.168 | CRAL_TRIO |
| TRINITY_DN5854_c0_g1_i10 | 0.826597855 | 0.479731714 | 0.999970678 | 13.39 | 5.962 | 2.278 | 8.113 | NA |
| TRINITY_DN269_c0_g1_i7 | 0.826550085 | 0.659216078 | 0.999970678 | 4.778 | 0.355 | 2.222 | 0.251 | ArgoL1 |
| TRINITY_DN3225_c0_g1_i4 | -0.82654409 | 0.497258776 | 0.999970678 | 0.872 | 3.035 | 2.478 | 3.82 | NA |
| TRINITY_DN31631_c0_g3_i3 | 0.826499226 | 0.372634645 | 0.999970678 | 10.574 | 24.63 | 5.955 | 12.537 | NA |
| TRINITY_DN491_c3_g1_i1 | -0.82642201 | 0.680451554 | 0.999970678 | 245.72 | 1.784 | 178.611 | 229.003 | NA |
| TRINITY_DN21318_c0_g1_i5 | 0.826390747 | 0.847438754 | 0.999970678 | 0 | 1.261 | 0.589 | 0 | PA |
| TRINITY_DN0_c0_g2_i5 | 0.826354417 | 0.79456194 | 0.999970678 | 6.631 | 0 | 3.066 | 0.08 | NA |
| TRINITY_DN15233_c0_g1_i1 | -0.82634668 | 0.39371511 | 0.999970678 | 13.526 | 18.21 | 19.266 | 29.839 | MMR_HSR1 |
| TRINITY_DN10978_c0_g1_i1 | -0.82605327 | 0.506690147 | 0.999970678 | 10.219 | 16.56 | 6.111 | 39.441 | PLAT |
| TRINITY_DN34612_c0_g1_i3 | 0.826042369 | 0.439826548 | 0.999970678 | 1.008 | 1.143 | 0.644 | 0.422 | NA |
| TRINITY_DN11516_c0_g1_i1 | 0.825941974 | 0.844340758 | 0.999970678 | 0 | 2.868 | 1.322 | 0 | Cation_ATPase |
| TRINITY_DN452_c1_g2_i2 | -0.82587525 | 0.784747284 | 0.999970678 | 0 | 3.173 | 4.411 | 0.201 | FA_desaturase |
| TRINITY_DN698_c2_g1_i2 | 0.825849872 | 0.518043042 | 0.999970678 | 3.852 | 2.257 | 2.4 | 0.563 | DUF659 |
| TRINITY_DN12659_c0_g1_i6 | -0.82557464 | 0.467471051 | 0.999970678 | 0.409 | 0.443 | 0.7 | 0.684 | NA |
| TRINITY_DN993_c0_g2_i7 | 0.825567927 | 0.848176707 | 0.999970678 | 2.98 | 0 | 1.422 | 0 | 2-Hacid_dh |
| TRINITY_DN10401_c1_g3_i1 | -0.82551602 | 0.621225875 | 0.999970678 | 4.487 | 0.926 | 6.289 | 2.121 | NA |
| TRINITY_DN2213_c0_g1_i4 | 0.825317936 | 0.757726065 | 0.999970678 | 2.271 | 1.37 | 1.711 | 0 | NA |
| TRINITY_DN2882_c0_g1_i2 | 0.825173853 | 0.779052037 | 0.999970678 | 44.457 | 41.07 | 0 | 48.489 | NA |
| TRINITY_DN16810_c0_g1_i3 | 0.82517315 | 0.76377733 | 1 | 0.509 | 0.375 | 0.411 | 0 | LON_substr_bdg |
| TRINITY_DN72_c1_g1_i1 | -0.82515645 | 0.734507816 | 0.999970678 | 0.009 | 1.685 | 2.333 | 0.131 | Hydrolase_4 |
| TRINITY_DN8666_c1_g1_i2 | 0.824993925 | 0.609133622 | 0.999970678 | 10.02 | 0.542 | 2.567 | 2.946 | DUF1475 |
| TRINITY_DN223_c1_g1_i9 | -0.82495292 | 0.54741938 | 0.999970678 | 1.335 | 3.568 | 0.989 | 7.47 | LRR_8 |
| TRINITY_DN1262_c0_g1_i3 | 0.824865605 | 0.768278655 | 0.999970678 | 2.616 | 0.67 | 1.555 | 0 | bZIP_1 |
| TRINITY_DN1880_c1_g1_i14 | 0.82475199 | 0.844561187 | 0.999970678 | 1.39 | 0 | 0 | 0.794 | NA |
| TRINITY_DN35464_c0_g1_i3 | -0.82473896 | 0.523721654 | 1 | 0.654 | 0.325 | 0.822 | 0.774 | NA |
| TRINITY_DN13470_c0_g1_i1 | -0.82468186 | 0.762931369 | 0.999970678 | 0.954 | 0 | 0.833 | 0.724 | NA |
| TRINITY_DN5252_c0_g1_i3 | -0.82468078 | 0.756763732 | 0.999970678 | 0 | 9.412 | 5.477 | 9.43 | NA |
| TRINITY_DN6346_c1_g2_i1 | -0.82467689 | 0.631365724 | 0.999970678 | 9.257 | 11.68 | 1.589 | 33.811 | NA |
| TRINITY_DN13547_c0_g1_i2 | -0.82467422 | 0.759382587 | 0.999970678 | 0 | 3.518 | 2.811 | 2.714 | NA |
| TRINITY_DN7337_c0_g1_i4 | 0.824525821 | 0.335152211 | 0.999970678 | 1.308 | 2.296 | 0.733 | 1.136 | - |
| TRINITY_DN93_c0_g1_i3 | -0.82448581 | 0.35349229 | 0.999970678 | 7.867 | 5.578 | 13.855 | 7.168 | DUF538 |
| TRINITY_DN2292_c0_g1_i2 | -0.8243982 | 0.787466282 | 0.999970678 | 109.33 | 0 | 83.073 | 96.254 | NA |
| TRINITY_DN3589_c0_g1_i11 | -0.82439654 | 0.433884111 | 0.999970678 | 5.314 | 2.178 | 4.278 | 8.043 | NA |
| TRINITY_DN12608_c0_g1_i1 | -0.82437568 | 0.845657376 | 0.999970678 | 4.506 | 0 | 6.7 | 0 | GMC_oxred_C |
| TRINITY_DN808_c0_g4_i3 | -0.82435968 | 0.61515412 | 0.999970678 | 0.436 | 1.498 | 0.344 | 2.956 | ubiquitin |
| TRINITY_DN10597_c0_g1_i1 | 0.824115486 | 0.795391162 | 1 | 1.226 | 0 | 0.544 | 0.05 | NA |
| TRINITY_DN9023_c0_g1_i3 | 0.8239149 | 0.469590223 | 0.999970678 | 0.609 | 0.286 | 0.156 | 0.332 | AAA_25 |
| TRINITY_DN9023_c0_g1_i1 | 0.8239149 | 0.469590223 | 0.999970678 | 0.609 | 0.286 | 0.156 | 0.332 | AAA_25 |
| TRINITY_DN3398_c1_g1_i2 | -0.82387598 | 0.762654784 | 0.999970678 | 0 | 3.538 | 3.355 | 2.021 | NA |
| TRINITY_DN490_c0_g1_i5 | 0.823873656 | 0.528386801 | 0.999970678 | 3.416 | 0.69 | 1.367 | 0.684 | Asp_protease_2 |
| TRINITY_DN996_c0_g1_i4 | -0.8238634 | 0.375307839 | 0.999970678 | 6.195 | 14.66 | 19.31 | 13.341 | MatE |
| TRINITY_DN14341_c1_g1_i12 | -0.8238465 | 0.614858347 | 1 | 1.78 | 0.375 | 2.3 | 1.106 | Sugar_tr |
| TRINITY_DN13183_c1_g1_i3 | -0.82384578 | 0.775857248 | 0.999970678 | 2.525 | 0 | 0.333 | 4.152 | NA |
| TRINITY_DN4024_c1_g1_i11 | -0.82384569 | 0.765887903 | 0.999970678 | 0.563 | 1.291 | 0 | 3.278 | Gal_Lectin |
| TRINITY_DN3053_c0_g1_i1 | 0.823766051 | 0.556318636 | 0.999970678 | 1.063 | 0.306 | 0.433 | 0.271 | CRAL_TRIO |
| TRINITY_DN9676_c0_g2_i2 | -0.82373574 | 0.761364981 | 0.999970678 | 0 | 2.02 | 0.733 | 2.644 | NA |
| TRINITY_DN951_c4_g1_i1 | -0.82371319 | 0.76475173 | 0.999970678 | 0 | 19.28 | 15.955 | 14.236 | NA |
| TRINITY_DN443_c0_g1_i8 | 0.823629995 | 0.630219163 | 0.999970678 | 1.998 | 1.981 | 1.778 | 0.09 | Amidohydro_1 |
| TRINITY_DN13_c1_g1_i3 | 0.823582853 | 0.48252668 | 0.999970678 | 2.952 | 1.577 | 1 | 1.307 | NA |
| TRINITY_DN2133_c1_g1_i5 | -0.82334673 | 0.650306931 | 0.999970678 | 3.279 | 0.099 | 1.689 | 4.062 | NA |
| TRINITY_DN1628_c0_g1_i6 | -0.82330767 | 0.497755321 | 0.999970678 | 1.026 | 4.336 | 4.255 | 4.253 | NA |
| TRINITY_DN1043_c0_g1_i2 | 0.823261139 | 0.844406709 | 0.999970678 | 0 | 10.25 | 0 | 5.65 | ADH_N |
| TRINITY_DN1127_c0_g2_i3 | -0.82325534 | 0.366243664 | 0.999970678 | 6.976 | 3.124 | 8.599 | 7.57 | NA |
| TRINITY_DN3048_c0_g1_i1 | -0.82325032 | 0.524274144 | 0.999970678 | 0.118 | 0.463 | 0.522 | 0.382 | NA |
| TRINITY_DN18024_c0_g1_i1 | -0.82323762 | 0.572221623 | 0.999970678 | 44.847 | 21.62 | 15.588 | 88.121 | NA |
| TRINITY_DN8737_c0_g2_i15 | -0.8231878 | 0.557076358 | 0.999970678 | 0.99 | 0.591 | 1.722 | 0.664 | NA |
| TRINITY_DN1346_c0_g1_i5 | 0.823153065 | 0.370154906 | 0.999970678 | 15.97 | 30.85 | 14.988 | 8.254 | NA |
| TRINITY_DN28377_c0_g1_i8 | -0.82310074 | 0.848602313 | 0.999970678 | 1.008 | 0 | 1.5 | 0 | NA |
| TRINITY_DN1782_c0_g1_i1 | 0.8230584 | 0.684293668 | 0.999970678 | 0.481 | 14.62 | 0.456 | 7.882 | DELLA |
| TRINITY_DN0_c1_g1_i5 | 0.82289824 | 0.84533569 | 0.999970678 | 0 | 5.351 | 2.455 | 0 | ACT |
| TRINITY_DN1930_c0_g1_i1 | 0.822778745 | 0.772940777 | 0.999970678 | 18.241 | 0 | 2.7 | 7.229 | NA |
| TRINITY_DN4175_c0_g1_i1 | 0.822750857 | 0.853290307 | 1 | 0 | 2.75 | 1.255 | 0 | NA |
| TRINITY_DN1697_c0_g1_i3 | -0.82274387 | 0.663390306 | 0.999970678 | 0.927 | 0.375 | 1.833 | 0.141 | Amidohydro_2 |
| TRINITY_DN7516_c0_g1_i2 | -0.82262039 | 0.493744832 | 0.999970678 | 1.308 | 0.611 | 1.611 | 1.458 | NA |
| TRINITY_DN222870_c0_g1_i1 | -0.82261676 | 0.616731532 | 0.999970678 | 1.444 | 3.922 | 0.678 | 8.536 | NA |
| TRINITY_DN4847_c2_g1_i4 | -0.82256896 | 0.853794681 | 1 | 0.699 | 0 | 0 | 1.227 | NA |
| TRINITY_DN2618_c0_g2_i1 | -0.8224942 | 0.441750721 | 0.999970678 | 1.326 | 0.7 | 1.744 | 1.438 | Dynein_light |
| TRINITY_DN36721_c0_g1_i1 | 0.822442377 | 0.718757221 | 0.999970678 | 9.792 | 1.124 | 5.011 | 0.08 | GTP_EFTU |

| TRINITY_DN4073_c0_g1_i1 | 0.822360051 | 0.382638779 | 0.999970678 | 1.281 | 2.434 | 0.833 | 1.086 | NA |
| --- | --- | --- | --- | --- | --- | --- | --- | --- |
| TRINITY_DN44924_c0_g1_i3 | 0.822231532 | 0.770680658 | 1 | 2.071 | 0.473 | 0 | 1.438 | NA |
| TRINITY_DN4930_c0_g1_i2 | -0.82213439 | 0.521991913 | 0.999970678 | 0.727 | 0.779 | 0.344 | 2.252 | NA |
| TRINITY_DN1657_c0_g1_i1 | -0.82212322 | 0.703425145 | 0.999970678 | 2.889 | 0.099 | 4.011 | 0.543 | NA |
| TRINITY_DN508_c0_g1_i4 | -0.82209829 | 0.207972634 | 0.999970678 | 23.146 | 22.64 | 38.731 | 33.69 | NA |
| TRINITY_DN3373_c0_g1_i7 | 0.822077975 | 0.845281649 | 0.999970678 | 2.689 | 0 | 0 | 1.558 | NA |
| TRINITY_DN152_c0_g2_i3 | 0.821920528 | 0.519140734 | 0.999970678 | 4.197 | 4.445 | 3.1 | 1.126 | NA |
| TRINITY_DN214_c0_g1_i3 | -0.82190341 | 0.420865019 | 0.999970678 | 9.729 | 5.618 | 5.822 | 20.218 | NA |
| TRINITY_DN8005_c0_g1_i4 | -0.82181018 | 0.165514329 | 0.999970678 | 5.632 | 5.194 | 8.122 | 9.36 | NA |
| TRINITY_DN6514_c0_g1_i4 | 0.821736934 | 0.605854661 | 0.999970678 | 2.226 | 0.473 | 0.356 | 1.076 | NA |
| TRINITY_DN8593_c0_g1_i4 | -0.82159319 | 0.764555602 | 0.999970678 | 0 | 15.73 | 16.188 | 7.631 | NA |
| TRINITY_DN172_c0_g1_i4 | -0.82158082 | 0.331736029 | 0.999970678 | 4.824 | 8.407 | 6.8 | 15.131 | NA |
| TRINITY_DN6604_c0_g1_i4 | -0.82157272 | 0.596954205 | 1 | 0.899 | 0.187 | 0.856 | 0.855 | GLTSCR1 |
| TRINITY_DN717_c0_g1_i2 | -0.82152946 | 0.293846401 | 0.999970678 | 1.426 | 2.336 | 2.966 | 3.036 | Band_7 |
| TRINITY_DN3205_c2_g1_i4 | -0.82125234 | 0.641159813 | 0.999970678 | 0.863 | 0.976 | 2.578 | 0.161 | EF_TS |
| TRINITY_DN8539_c0_g1_i3 | 0.821098259 | 0.468431672 | 0.999970678 | 0.972 | 0.71 | 0.589 | 0.251 | NA |
| TRINITY_DN3451_c0_g1_i1 | 0.821049162 | 0.475954009 | 0.999970678 | 1.608 | 6.16 | 1.511 | 2.513 | NA |
| TRINITY_DN1334_c0_g1_i4 | -0.82104075 | 0.769798394 | 0.999970678 | 11.954 | 0 | 13.877 | 4.635 | NA |
| TRINITY_DN17296_c0_g1_i1 | 0.821012472 | 0.756557167 | 0.999970678 | 1.517 | 3.183 | 0 | 2.654 | SPG48 |
| TRINITY_DN1173_c0_g1_i5 | 0.820958559 | 0.56384401 | 0.999970678 | 0.963 | 0.798 | 0.7 | 0.151 | NA |
| TRINITY_DN24615_c0_g2_i2 | -0.82094152 | 0.424804492 | 0.999970678 | 2.407 | 5.598 | 8.033 | 4.353 | NA |
| TRINITY_DN2717_c0_g1_i7 | -0.82091287 | 0.772453841 | 1 | 0 | 0.759 | 0.856 | 0.322 | NA |
| TRINITY_DN1183_c0_g1_i8 | 0.820808468 | 0.767961242 | 0.999970678 | 0 | 5.913 | 1.667 | 1.267 | LRR_8 |
| TRINITY_DN3559_c0_g1_i2 | -0.82077193 | 0.856312955 | 1 | 0 | 1.232 | 1.833 | 0 | NA |
| TRINITY_DN17867_c0_g1_i1 | -0.82077193 | 0.856312955 | 1 | 0 | 0.286 | 0.411 | 0 | NA |
| TRINITY_DN6981_c1_g2_i2 | -0.82077193 | 0.856312955 | 1 | 0 | 2.858 | 4.055 | 0 | NA |
| TRINITY_DN28519_c0_g3_i2 | 0.820707285 | 0.346332321 | 0.999970678 | 3.107 | 3.775 | 2.2 | 1.237 | CRM1_C |
| TRINITY_DN6368_c0_g1_i2 | -0.82065368 | 0.355887254 | 0.999970678 | 5.478 | 7.086 | 12.166 | 7.339 | Ribosomal_S9 |
| TRINITY_DN5592_c0_g1_i7 | 0.82060296 | 0.847307799 | 0.999970678 | 0 | 14.57 | 0 | 8.164 | Ribosomal_S5 |
| TRINITY_DN0_c0_g2_i4 | -0.82055341 | 0.690900998 | 0.999970678 | 1.263 | 0.483 | 2.544 | 0.04 | NA |
| TRINITY_DN40824_c0_g1_i1 | -0.82043948 | 0.407178152 | 0.999970678 | 2.117 | 1.685 | 3 | 3.036 | NA |
| TRINITY_DN15558_c0_g2_i5 | -0.82025601 | 0.698333997 | 0.999970678 | 13.408 | 1.449 | 20.643 | 0.945 | NA |
| TRINITY_DN2932_c0_g1_i1 | 0.820251993 | 0.558997787 | 0.999970678 | 1.408 | 1.39 | 1.133 | 0.211 | NA |
| TRINITY_DN548_c0_g1_i2 | 0.820246688 | 0.352196332 | 0.999970678 | 6.895 | 14.17 | 4.222 | 6.716 | HMA |
| TRINITY_DN207_c2_g1_i3 | 0.82015438 | 0.580967562 | 0.999970678 | 0.854 | 0.67 | 0.6 | 0.151 | PP-binding |
| TRINITY_DN5371_c0_g1_i6 | 0.820121517 | 0.51768374 | 0.999970678 | 2.634 | 0.641 | 1.067 | 0.573 | Cation_ATPase |
| TRINITY_DN2554_c0_g1_i1 | -0.81995383 | 0.325179925 | 0.999970678 | 2.18 | 1.281 | 2.489 | 3.137 | NTP_transf_2 |
| TRINITY_DN1474_c0_g1_i3 | 0.819838953 | 0.849593016 | 0.999970678 | 0 | 1.567 | 0.722 | 0 | NA |
| TRINITY_DN18907_c0_g2_i1 | -0.81979449 | 0.709150596 | 1 | 0.936 | 0.069 | 1.367 | 0.151 | NA |
| TRINITY_DN20844_c0_g1_i2 | 0.819689272 | 0.758358703 | 0.999970678 | 0.382 | 0.542 | 0 | 0.523 | Glyco_hydro_47 |
| TRINITY_DN921_c0_g5_i1 | 0.819619426 | 0.459924691 | 0.999970678 | 18.05 | 22.67 | 15.399 | 4.373 | NA |
| TRINITY_DN2099_c1_g1_i2 | -0.81951181 | 0.759356548 | 0.999970678 | 7.667 | 0 | 5.455 | 7.038 | NA |
| TRINITY_DN2096_c0_g1_i1 | 0.819460096 | 0.348568194 | 0.999970678 | 30.159 | 15.35 | 7.988 | 16.257 | Arm |
| TRINITY_DN836_c0_g2_i1 | 0.819389918 | 0.60419004 | 0.999970678 | 7.476 | 1.252 | 3.389 | 0.885 | NA |
| TRINITY_DN8436_c0_g1_i3 | -0.81936316 | 0.515419091 | 0.999970678 | 1.344 | 1.853 | 3.7 | 1.206 | NA |
| TRINITY_DN3769_c0_g1_i1 | -0.81928309 | 0.468670787 | 0.999970678 | 0.972 | 0.907 | 1.878 | 1.025 | NA |
| TRINITY_DN3117_c0_g1_i11 | -0.81926352 | 0.549734725 | 0.999970678 | 23.455 | 2.622 | 20.01 | 22.4 | DUF538 |
| TRINITY_DN5430_c0_g1_i1 | 0.81925001 | 0.462466772 | 0.999970678 | 35.064 | 15.1 | 6.277 | 20.399 | NA |
| TRINITY_DN5904_c0_g1_i4 | -0.81919437 | 0.533073432 | 0.999970678 | 17.305 | 5.805 | 25.932 | 9.179 | NA |
| TRINITY_DN1364_c0_g1_i2 | -0.8191366 | 0.268023055 | 0.999970678 | 10.873 | 12.74 | 21.621 | 15.432 | NA |
| TRINITY_DN1107_c0_g1_i5 | -0.8191127 | 0.790325325 | 0.999970678 | 0 | 8.012 | 0.322 | 13.261 | NA |
| TRINITY_DN1095_c0_g4_i1 | -0.81907801 | 0.796045011 | 1 | 4.887 | 0 | 6.866 | 0.322 | NA |
| TRINITY_DN906_c0_g3_i2 | 0.819010342 | 0.724491942 | 0.999970678 | 54.921 | 1.863 | 0.244 | 32.463 | NA |
| TRINITY_DN8449_c0_g1_i5 | 0.818928677 | 0.563172106 | 0.999970678 | 12.172 | 46.5 | 3.955 | 27.919 | Hpt |
| TRINITY_DN17002_c0_g1_i1 | -0.81889474 | 0.594211633 | 0.999970678 | 1.735 | 10.81 | 2.355 | 19.132 | - |
| TRINITY_DN76_c0_g1_i5 | -0.81883023 | 0.310939339 | 0.999970678 | 2.98 | 3.922 | 3.589 | 7.862 | NA |
| TRINITY_DN7972_c0_g1_i2 | 0.818792304 | 0.326509339 | 0.999970678 | 1.944 | 2.572 | 0.9 | 1.478 | NA |
| TRINITY_DN362_c0_g2_i1 | -0.81875178 | 0.489883382 | 0.999970678 | 1.544 | 5.085 | 3.466 | 7.369 | NA |
| TRINITY_DN785_c1_g2_i1 | -0.81852419 | 0.595697709 | 0.999970678 | 4.297 | 0.404 | 2.178 | 5.781 | NA |
| TRINITY_DN5169_c0_g1_i10 | -0.81841203 | 0.560205416 | 0.999970678 | 6.141 | 1.735 | 4.944 | 7.389 | NA |
| TRINITY_DN407_c0_g1_i2 | -0.818403 | 0.640219026 | 0.999970678 | 16.751 | 6.583 | 33.009 | 1.377 | NA |
| TRINITY_DN10046_c0_g1_i4 | -0.81835402 | 0.620711419 | 0.999970678 | 0.818 | 0.65 | 1.933 | 0.271 | NA |
| TRINITY_DN6902_c0_g1_i3 | 0.818352875 | 0.354449121 | 0.999970678 | 1.435 | 1.705 | 0.867 | 0.744 | NA |
| TRINITY_DN3240_c0_g1_i9 | 0.818346838 | 0.357785268 | 0.999970678 | 0.89 | 0.966 | 0.422 | 0.543 | NA |
| TRINITY_DN3269_c0_g1_i3 | -0.81812001 | 0.323754072 | 0.999970678 | 0.2 | 0.256 | 0.322 | 0.402 | NADH5_C |
| TRINITY_DN1735_c0_g1_i10 | -0.81810918 | 0.568265502 | 0.999970678 | 1.453 | 0.769 | 0.389 | 3.469 | NA |
| TRINITY_DN5030_c0_g1_i6 | 0.818045748 | 0.344641881 | 0.999970678 | 1.453 | 2.434 | 0.722 | 1.327 | NA |
| TRINITY_DN9376_c1_g1_i5 | -0.81804319 | 0.751909173 | 0.999970678 | 0.036 | 1.902 | 0.022 | 3.378 | NA |
| TRINITY_DN1225_c0_g1_i1 | -0.81803615 | 0.761241312 | 0.999970678 | 0 | 3.666 | 3.522 | 2.111 | NLE |
| TRINITY_DN4612_c0_g1_i6 | -0.81802085 | 0.647177036 | 0.999970678 | 1.526 | 0.128 | 0.511 | 2.363 | NA |
| TRINITY_DN14312_c0_g1_i9 | 0.818011755 | 0.5091431 | 0.999970678 | 4.542 | 7.845 | 3.655 | 2.483 | RbcS |
| TRINITY_DN61656_c0_g1_i1 | -0.81801033 | 0.783616211 | 0.999970678 | 1.526 | 0 | 0.156 | 2.534 | NA |
| TRINITY_DN9490_c0_g1_i1 | -0.81793983 | 0.48709609 | 0.999970678 | 1.235 | 0.463 | 1.711 | 0.975 | NA |
| TRINITY_DN1872_c0_g1_i3 | 0.817905777 | 0.458671358 | 0.999970678 | 6.722 | 1.981 | 2.6 | 1.84 | NA |

| TRINITY_DN441_c0_g1_i3 | 0.817736564 | 0.678456466 | 0.999970678 | 3.397 | 0.069 | 0.7 | 1.146 | NA |
| --- | --- | --- | --- | --- | --- | --- | --- | --- |
| TRINITY_DN4607_c0_g2_i4 | -0.81759224 | 0.462530035 | 0.999970678 | 9.611 | 14.26 | 26.354 | 9.521 | Pirin |
| TRINITY_DN7977_c0_g1_i1 | -0.8175467 | 0.765169864 | 0.999970678 | 0 | 1.892 | 2.155 | 0.674 | Glycos_transf_1 |
| TRINITY_DN2600_c0_g1_i1 | -0.8174633 | 0.476933754 | 0.999970678 | 47.073 | 11.06 | 53.83 | 37.943 | MinE |
| TRINITY_DN2811_c0_g2_i1 | -0.81746045 | 0.461512272 | 0.999970678 | 38.389 | 94.04 | 43.908 | 179.499 | AMP-binding |
| TRINITY_DN2944_c0_g1_i3 | -0.81743996 | 0.415081578 | 0.999970678 | 7.958 | 15.94 | 9.322 | 30.724 | Methyltransf_29 |
| TRINITY_DN1968_c0_g1_i7 | 0.817265413 | 0.420055132 | 0.999970678 | 2.189 | 1.744 | 0.989 | 1.036 | NA |
| TRINITY_DN582_c0_g2_i2 | -0.81712134 | 0.603451074 | 0.999970678 | 1.435 | 2.996 | 1.055 | 6.193 | NA |
| TRINITY_DN285_c2_g1_i3 | -0.81700292 | 0.661442924 | 1 | 0.908 | 0.059 | 0.711 | 0.855 | NA |
| TRINITY_DN2416_c0_g1_i3 | 0.816783719 | 0.765969656 | 0.999970678 | 0.409 | 0.197 | 0.289 | 0 | Pkinase |
| TRINITY_DN3051_c1_g3_i5 | -0.81677154 | 0.853290191 | 1 | 0 | 0.848 | 1.222 | 0 | NA |
| TRINITY_DN1562_c0_g1_i5 | -0.81675427 | 0.560764406 | 0.999970678 | 3.27 | 1.774 | 1.378 | 7.198 | NA |
| TRINITY_DN4920_c0_g1_i1 | -0.81673463 | 0.53961002 | 0.999970678 | 6.068 | 5.608 | 1.933 | 18.268 | NA |
| TRINITY_DN2840_c0_g1_i5 | 0.816730113 | 0.777037701 | 0.999970678 | 19.249 | 0 | 2.822 | 7.701 | PRP38 |
| TRINITY_DN21683_c0_g1_i1 | -0.81669324 | 0.845841698 | 0.999970678 | 4.487 | 0 | 0 | 8.023 | NA |
| TRINITY_DN616_c2_g1_i1 | 0.816628775 | 0.820346163 | 0.999970678 | 55.948 | 0 | 0.044 | 32.282 | NA |
| TRINITY_DN699_c0_g1_i1 | 0.816220196 | 0.762885492 | 0.999970678 | 11.128 | 10.95 | 0 | 12.527 | NA |
| TRINITY_DN49290_c0_g1_i1 | 0.816101664 | 0.610352106 | 0.999970678 | 0.808 | 7.924 | 1.278 | 3.328 | DUF1218 |
| TRINITY_DN4543_c2_g1_i2 | 0.816091651 | 0.629590626 | 0.999970678 | 2.771 | 5.972 | 0.2 | 4.715 | NA |
| TRINITY_DN51430_c0_g1_i3 | 0.816064023 | 0.481608729 | 0.999970678 | 0.99 | 3.242 | 0.7 | 1.538 | NA |
| TRINITY_DN72_c1_g1_i9 | -0.81605224 | 0.846744214 | 0.999970678 | 5.151 | 0 | 7.588 | 0 | Hydrolase_4 |
| TRINITY_DN4923_c0_g1_i2 | 0.816002712 | 0.185879321 | 0.999970678 | 62.634 | 49.88 | 27.576 | 30.483 | NA |
| TRINITY_DN725_c1_g1_i4 | 0.815901664 | 0.436064826 | 0.999970678 | 3.134 | 1.36 | 0.733 | 1.689 | EloA-BP1 |
| TRINITY_DN8717_c0_g1_i1 | -0.81586553 | 0.380856512 | 0.999970678 | 2.716 | 3.706 | 2.855 | 7.822 | NA |
| TRINITY_DN164_c0_g3_i2 | -0.81572788 | 0.183275631 | 0.999970678 | 10.274 | 13.09 | 16.51 | 21.093 | NAC |
| TRINITY_DN2052_c2_g1_i3 | 0.815720954 | 0.75555763 | 0.999970678 | 0.872 | 0.986 | 0 | 1.056 | NA |
| TRINITY_DN1440_c1_g1_i6 | -0.81543263 | 0.228779405 | 0.999970678 | 426.16 | 705 | 794.941 | 995.235 | GST_C |
| TRINITY_DN8982_c0_g1_i3 | 0.815423254 | 0.772069985 | 0.999970678 | 0 | 0.818 | 0.144 | 0.282 | FAT |
| TRINITY_DN17238_c0_g2_i1 | 0.81537269 | 0.553607504 | 0.999970678 | 1.235 | 0.503 | 0.622 | 0.231 | NA |
| TRINITY_DN11561_c0_g1_i1 | 0.8153043 | 0.497440494 | 0.999970678 | 603.25 | 112.9 | 205.81 | 161.472 | NA |
| TRINITY_DN2474_c0_g1_i2 | -0.81528427 | 0.472220964 | 0.999970678 | 0.908 | 3.4 | 4 | 2.694 | GCFC |
| TRINITY_DN3570_c0_g1_i3 | -0.81516451 | 0.851966115 | 0.999970678 | 0 | 1.823 | 2.611 | 0 | NA |
| TRINITY_DN4076_c0_g1_i2 | -0.81497762 | 0.767792674 | 0.999970678 | 8.866 | 0 | 9.299 | 4.564 | - |
| TRINITY_DN1268_c2_g1_i6 | 0.814936554 | 0.76738767 | 0.999970678 | 4.842 | 0.966 | 0 | 3.338 | NA |
| TRINITY_DN10320_c0_g1_i1 | -0.81482191 | 0.331166646 | 0.999970678 | 7.358 | 4.829 | 11.688 | 7.4 | DUF2921 |
| TRINITY_DN7423_c13_g1_i2 | -0.81476248 | 0.235058776 | 0.999970678 | 496.25 | 674.7 | 1023.727 | 817.979 | PsaD |
| TRINITY_DN7423_c13_g1_i1 | -0.81476248 | 0.235058776 | 0.999970678 | 496.25 | 674.7 | 1023.727 | 817.979 | PsaD |
| TRINITY_DN6318_c1_g1_i1 | -0.81475607 | 0.573027029 | 0.999970678 | 0.2 | 1.439 | 1.278 | 1.327 | NA |
| TRINITY_DN1824_c0_g1_i8 | 0.81471186 | 0.647651681 | 0.999970678 | 1.744 | 0.749 | 1.133 | 0.07 | NA |
| TRINITY_DN14727_c0_g1_i2 | 0.814697517 | 0.442144893 | 0.999970678 | 2.534 | 3.775 | 0.844 | 2.534 | zf-RING_2 |
| TRINITY_DN15166_c0_g1_i4 | -0.81452665 | 0.597495124 | 0.999970678 | 0.318 | 0.069 | 0.156 | 0.493 | NA |
| TRINITY_DN2368_c0_g1_i1 | -0.81450487 | 0.395988809 | 0.999970678 | 8.821 | 4.859 | 13.733 | 7.52 | DUF565 |
| TRINITY_DN4597_c0_g1_i8 | 0.81448484 | 0.848092973 | 0.999970678 | 0 | 0.877 | 0 | 0.503 | F-box |
| TRINITY_DN11036_c0_g1_i1 | -0.814424 | 0.726477213 | 1 | 1.853 | 0.118 | 2.567 | 0.342 | NA |
| TRINITY_DN2428_c0_g1_i2 | 0.81422747 | 0.62193702 | 0.999970678 | 0.409 | 0.631 | 0.433 | 0.07 | NA |
| TRINITY_DN1200_c0_g1_i5 | -0.81408187 | 0.418664755 | 0.999970678 | 139.41 | 250.5 | 421.075 | 167.665 | zf-B_box |
| TRINITY_DN1343_c0_g1_i4 | 0.814014381 | 0.773540788 | 0.999970678 | 0 | 1.439 | 0.289 | 0.452 | NA |
| TRINITY_DN728_c0_g1_i27 | 0.813902684 | 0.520819323 | 0.999970678 | 3.188 | 1.321 | 0.456 | 2.051 | Glyco_hydro_79n |
| TRINITY_DN947_c0_g1_i3 | 0.813825582 | 0.354572158 | 0.999970678 | 5.75 | 3.873 | 2.878 | 2.011 | NA |
| TRINITY_DN3264_c0_g1_i2 | -0.81381865 | 0.481281389 | 0.999970678 | 1.444 | 0.641 | 2.144 | 1.106 | NA |
| TRINITY_DN7903_c0_g1_i3 | -0.81349003 | 0.631768225 | 1 | 0.118 | 0.532 | 0.689 | 0.302 | NA |
| TRINITY_DN3263_c0_g1_i8 | -0.81342203 | 0.770260963 | 0.999970678 | 8.003 | 0 | 2.044 | 11.662 | NA |
| TRINITY_DN750_c0_g1_i3 | 0.813412847 | 0.333182974 | 0.999970678 | 9.983 | 8.091 | 5.7 | 3.418 | NA |
| TRINITY_DN2056_c0_g1_i4 | 0.813355565 | 0.789429292 | 0.999970678 | 0 | 4.07 | 1.8 | 0.121 | NA |
| TRINITY_DN6932_c0_g1_i3 | -0.81329476 | 0.550511523 | 0.999970678 | 2.144 | 0.779 | 1.711 | 3.066 | NA |
| TRINITY_DN5161_c0_g1_i4 | 0.813152701 | 0.851412108 | 0.999970678 | 3.652 | 0 | 1.755 | 0 | NA |
| TRINITY_DN28158_c0_g1_i1 | 0.813056232 | 0.575391327 | 1 | 1.662 | 1.961 | 0.389 | 1.558 | LRRNT_2 |
| TRINITY_DN190_c0_g1_i1 | -0.81303701 | 0.362293946 | 0.999970678 | 7.104 | 5.647 | 13.055 | 6.645 | CBS |
| TRINITY_DN10983_c1_g1_i8 | 0.813023474 | 0.650274808 | 0.999970678 | 0.427 | 2.789 | 0.267 | 1.508 | NA |
| TRINITY_DN15267_c0_g2_i1 | -0.81297943 | 0.294558664 | 0.999970678 | 11.518 | 8.495 | 18.754 | 12.637 | NA |
| TRINITY_DN17897_c0_g1_i1 | 0.812962857 | 0.769609483 | 1 | 0.363 | 0.237 | 0.289 | 0 | NA |
| TRINITY_DN8569_c0_g1_i8 | 0.812925646 | 0.764907951 | 0.999970678 | 0.563 | 0.256 | 0.389 | 0 | NA |
| TRINITY_DN5572_c0_g1_i15 | -0.81290426 | 0.849464391 | 0.999970678 | 12.99 | 0 | 18.788 | 0 | NA |
| TRINITY_DN32976_c0_g1_i1 | -0.81290398 | 0.849116366 | 0.999970678 | 1.626 | 0 | 2.389 | 0 | - |
| TRINITY_DN15191_c0_g1_i2 | -0.81290374 | 0.849283436 | 0.999970678 | 1.853 | 0 | 2.711 | 0 | But2 |
| TRINITY_DN4853_c0_g1_i17 | -0.81290127 | 0.852030285 | 0.999970678 | 6.94 | 0 | 10.088 | 0 | NA |
| TRINITY_DN10084_c0_g1_i2 | -0.81290044 | 0.853497369 | 0.999970678 | 1.917 | 0 | 2.789 | 0 | Malate_synthase |
| TRINITY_DN15902_c0_g1_i10 | -0.81290009 | 0.854790576 | 1 | 3.216 | 0 | 4.633 | 0 | Ribosomal_S17e |
| TRINITY_DN7940_c0_g1_i5 | -0.81289915 | 0.856430919 | 1 | 2.344 | 0 | 3.4 | 0 | Ferritin_2 |
| TRINITY_DN33264_c0_g1_i4 | -0.81289915 | 0.856430919 | 1 | 1.335 | 0 | 1.955 | 0 | NA |
| TRINITY_DN12911_c0_g1_i1 | -0.81289915 | 0.856430919 | 1 | 9.347 | 0 | 13.288 | 0 | NA |
| TRINITY_DN22587_c0_g1_i3 | -0.81289915 | 0.856430919 | 1 | 0.636 | 0 | 0.944 | 0 | vWA-TerF-like |
| TRINITY_DN14407_c0_g1_i3 | -0.81289838 | 0.858577895 | 1 | 0.454 | 0 | 0.678 | 0 | NA |
| TRINITY_DN231035_c0_g1_i1 | -0.81289838 | 0.858577895 | 1 | 1.09 | 0 | 1.6 | 0 | Lactamase_B |

| TRINITY_DN4119_c0_g1_i4 | -0.81262203 | 0.63284701 | 0.999970678 | 4.388 | 1.616 | 8.288 | 0.563 | NA |
| --- | --- | --- | --- | --- | --- | --- | --- | --- |
| TRINITY_DN4621_c0_g1_i2 | 0.8125301 | 0.515975848 | 1 | 1.681 | 1.202 | 0.811 | 0.684 | PP2 |
| TRINITY_DN17045_c0_g1_i2 | 0.812479426 | 0.553781823 | 0.999970678 | 36.808 | 8.82 | 17.477 | 4.936 | Lectin_legB |
| TRINITY_DN17012_c0_g1_i1 | -0.8124761 | 0.307851887 | 0.999970678 | 154.31 | 90.81 | 218.787 | 162.719 | NA |
| TRINITY_DN1_c0_g1_i1 | 0.812418971 | 0.52166751 | 0.999970678 | 24.345 | 34.55 | 24.032 | 4.363 | KH_1 |
| TRINITY_DN73651_c0_g1_i1 | 0.812396065 | 0.265032229 | 0.999970678 | 16.433 | 11.13 | 7.644 | 6.505 | NA |
| TRINITY_DN16106_c0_g1_i1 | -0.81239501 | 0.251422432 | 0.999970678 | 14.026 | 9.165 | 17.355 | 19.776 | - |
| TRINITY_DN775_c0_g1_i4 | -0.81226426 | 0.356558781 | 0.999970678 | 1.017 | 2.119 | 1.944 | 3.127 | NA |
| TRINITY_DN3701_c0_g1_i5 | -0.81226362 | 0.468730029 | 0.999970678 | 13.953 | 8.722 | 26.043 | 8.294 | GATA |
| TRINITY_DN14495_c1_g1_i3 | -0.81218905 | 0.248998029 | 0.999970678 | 2.789 | 2.444 | 3.744 | 4.725 | mTERF |
| TRINITY_DN581_c0_g1_i11 | -0.8121192 | 0.355534983 | 0.999970678 | 6.622 | 13.64 | 11.066 | 21.907 | Fcf1 |
| TRINITY_DN325_c0_g1_i2 | -0.81205142 | 0.406286004 | 0.999970678 | 267.45 | 91.97 | 207.999 | 384.785 | NA |
| TRINITY_DN3250_c0_g1_i12 | -0.81201589 | 0.395469765 | 0.999970678 | 1000.1 | 346.3 | 1113.266 | 1001.469 | NA |
| TRINITY_DN31763_c0_g2_i1 | -0.812002 | 0.482026228 | 0.999970678 | 0.718 | 0.365 | 0.8 | 0.925 | NA |
| TRINITY_DN1295_c0_g1_i7 | 0.811969431 | 0.768310759 | 0.999970678 | 12.209 | 10 | 0 | 12.718 | NA |
| TRINITY_DN535_c0_g1_i12 | -0.81192259 | 0.77030644 | 0.999970678 | 71.981 | 0.138 | 105.671 | 0.493 | NA |
| TRINITY_DN1264_c0_g1_i4 | -0.8118851 | 0.263135482 | 0.999970678 | 37.789 | 46.99 | 45.553 | 92.715 | Ribosomal_L32e |
| TRINITY_DN2126_c0_g2_i1 | 0.811732876 | 0.339718974 | 0.999970678 | 7.176 | 6.642 | 2.278 | 5.097 | Peptidase_C97 |
| TRINITY_DN2597_c0_g1_i12 | 0.811637061 | 0.778427713 | 0.999970678 | 2.044 | 0 | 0.789 | 0.221 | NA |
| TRINITY_DN1440_c1_g1_i4 | -0.81158094 | 0.312649689 | 0.999970678 | 190.95 | 212.1 | 185.267 | 472.614 | GST_C |
| TRINITY_DN1176_c1_g1_i4 | 0.811571281 | 0.628926313 | 0.999970678 | 4.705 | 0.65 | 0.589 | 2.373 | NA |
| TRINITY_DN2100_c0_g1_i16 | 0.811514806 | 0.856401149 | 1 | 0 | 0.946 | 0.433 | 0 | NA |
| TRINITY_DN3485_c0_g1_i2 | -0.81146534 | 0.541431025 | 0.999970678 | 0.836 | 1.468 | 2.755 | 0.684 | NA |
| TRINITY_DN2556_c0_g1_i1 | 0.811382299 | 0.422489132 | 0.999970678 | 45.647 | 80.4 | 44.631 | 17.594 | NA |
| TRINITY_DN1080_c0_g1_i4 | -0.81136596 | 0.666000846 | 0.999970678 | 13.108 | 1.192 | 1.144 | 24.099 | LRRNT_2 |
| TRINITY_DN5902_c0_g1_i4 | 0.811270391 | 0.318550784 | 0.999970678 | 1.59 | 1.744 | 0.756 | 0.985 | NA |
| TRINITY_DN1497_c0_g1_i3 | 0.811208919 | 0.575638875 | 0.999970678 | 40.66 | 4.356 | 7.377 | 16.89 | NA |
| TRINITY_DN11942_c0_g1_i2 | -0.8111656 | 0.588864719 | 0.999970678 | 1.081 | 2.405 | 1.089 | 4.735 | NA |
| TRINITY_DN6024_c0_g1_i8 | -0.81103531 | 0.212199217 | 0.999970678 | 53.232 | 64.05 | 77.106 | 104.609 | NA |
| TRINITY_DN3071_c1_g2_i2 | -0.81099166 | 0.545501061 | 0.999970678 | 4.615 | 3.607 | 2.9 | 10.345 | NA |
| TRINITY_DN638_c0_g1_i1 | 0.810889904 | 0.544058626 | 0.999970678 | 10.129 | 38.51 | 18.877 | 4.655 | NA |
| TRINITY_DN2138_c2_g2_i2 | -0.81073876 | 0.544101501 | 0.999970678 | 0.999 | 2.927 | 1.533 | 4.997 | Peroxidase_2 |
| TRINITY_DN14900_c0_g1_i1 | 0.8107247 | 0.315966223 | 0.999970678 | 2.852 | 3.538 | 1.811 | 1.458 | Pyridoxal_deC |
| TRINITY_DN4451_c1_g1_i4 | -0.81070274 | 0.804365427 | 0.999970678 | 1.008 | 0 | 0.033 | 1.759 | Aa_trans |
| TRINITY_DN4030_c0_g1_i2 | -0.81069992 | 0.366848903 | 0.999970678 | 4.606 | 2.917 | 4.289 | 8.033 | NA |
| TRINITY_DN8847_c0_g1_i1 | -0.81065306 | 0.447968712 | 0.999970678 | 0.454 | 0.503 | 0.8 | 0.704 | Glyco_hydro_14 |
| TRINITY_DN7316_c0_g1_i2 | 0.810581033 | 0.211084725 | 0.999970678 | 11.364 | 10.5 | 4.389 | 7.178 | DEK_C |
| TRINITY_DN1427_c0_g1_i5 | 0.810457153 | 0.568469514 | 0.999970678 | 0.663 | 0.177 | 0.122 | 0.332 | DUF4042 |
| TRINITY_DN221345_c0_g1_i1 | -0.810397 | 0.77743604 | 1 | 1.226 | 0 | 1.355 | 0.533 | NA |
| TRINITY_DN24257_c1_g1_i3 | 0.81035411 | 0.182981576 | 0.999970678 | 15.742 | 14.05 | 7.288 | 8.184 | Pkinase |
| TRINITY_DN2395_c0_g1_i1 | 0.810335836 | 0.352750767 | 0.999970678 | 3.897 | 2.365 | 1.178 | 2.172 | Methyltr_RsmB-F |
| TRINITY_DN12019_c0_g3_i2 | 0.810308414 | 0.805989244 | 0.999970678 | 1.908 | 0.02 | 0.922 | 0 | NA |
| TRINITY_DN9076_c0_g1_i1 | -0.81020909 | 0.668727409 | 1 | 2.562 | 0.256 | 3.289 | 0.965 | NA |
| TRINITY_DN4017_c0_g1_i1 | -0.8102011 | 0.402481239 | 0.999970678 | 15.543 | 32.87 | 49.353 | 24.883 | NA |
| TRINITY_DN539_c0_g1_i4 | -0.81020075 | 0.78633615 | 0.999970678 | 189.38 | 0 | 114.904 | 195.333 | - |
| TRINITY_DN11564_c0_g2_i1 | 0.810087054 | 0.435441806 | 0.999970678 | 5.341 | 2.306 | 1.222 | 2.926 | IBR |
| TRINITY_DN18261_c0_g1_i5 | 0.80993864 | 0.709249135 | 1 | 1.444 | 0.276 | 0.044 | 0.915 | NA |
| TRINITY_DN13390_c0_g1_i2 | -0.80974996 | 0.770823002 | 0.999970678 | 0.727 | 1.498 | 0 | 3.881 | NA |
| TRINITY_DN1823_c0_g1_i3 | -0.80965217 | 0.653212807 | 0.999970678 | 0.718 | 1.991 | 0.256 | 4.323 | F-box |
| TRINITY_DN1422_c0_g1_i4 | -0.80961093 | 0.38141067 | 0.999970678 | 1.045 | 0.848 | 1.644 | 1.387 | Kelch_3 |
| TRINITY_DN255_c1_g1_i3 | 0.809499584 | 0.590582388 | 0.999970678 | 3.098 | 0.838 | 0.378 | 1.78 | NA |
| TRINITY_DN18001_c0_g1_i4 | 0.809494998 | 0.852099325 | 0.999970678 | 1.526 | 0 | 0 | 0.885 | NA |
| TRINITY_DN1874_c0_g2_i3 | -0.80941977 | 0.458616388 | 0.999970678 | 4.596 | 1.429 | 3.4 | 6.505 | zf-CCCH |
| TRINITY_DN2950_c0_g1_i4 | -0.80936921 | 0.772461768 | 0.999970678 | 5.623 | 0 | 5.822 | 2.976 | AIG1 |
| TRINITY_DN16176_c1_g1_i1 | 0.80935721 | 0.697118553 | 0.999970678 | 1.008 | 26.62 | 1.933 | 12.316 | NA |
| TRINITY_DN2060_c0_g1_i11 | -0.8093003 | 0.502385967 | 0.999970678 | 5.668 | 8.761 | 5.689 | 17.383 | NA |
| TRINITY_DN46603_c0_g1_i1 | -0.80920793 | 0.848461611 | 0.999970678 | 6.086 | 0 | 0 | 10.637 | NA |
| TRINITY_DN3602_c0_g1_i2 | -0.80917809 | 0.674629804 | 0.999970678 | 0.5 | 0.424 | 1.278 | 0.06 | Sugar_tr |
| TRINITY_DN35858_c0_g1_i6 | 0.808956419 | 0.552750698 | 0.999970678 | 0.418 | 0.355 | 0.278 | 0.101 | NA |
| TRINITY_DN6597_c0_g1_i3 | 0.808952948 | 0.523129915 | 0.999970678 | 0.6 | 1.124 | 0.6 | 0.261 | zf-RING_2 |
| TRINITY_DN394_c0_g1_i8 | -0.80887665 | 0.388927672 | 0.999970678 | 62.616 | 25.38 | 79.673 | 58.372 | Polysacc_synt_4 |
| TRINITY_DN3501_c0_g1_i1 | -0.80887392 | 0.457363952 | 0.999970678 | 11.818 | 3.489 | 8.355 | 16.88 | NA |
| TRINITY_DN4032_c0_g1_i3 | 0.808832825 | 0.853789423 | 0.999970678 | 0 | 2.01 | 0.944 | 0 | NA |
| TRINITY_DN4180_c1_g1_i2 | 0.808825218 | 0.55970642 | 0.999970678 | 23.273 | 13.15 | 1.767 | 18.81 | NA |
| TRINITY_DN25677_c0_g1_i2 | 0.808721635 | 0.511391458 | 0.999970678 | 1.998 | 4.208 | 2.222 | 0.845 | NA |
| TRINITY_DN12450_c0_g1_i1 | -0.80871958 | 0.459785103 | 0.999970678 | 1.835 | 0.808 | 2.522 | 1.619 | NA |
| TRINITY_DN16597_c0_g1_i9 | -0.80856275 | 0.616108862 | 1 | 1.244 | 0.246 | 0.744 | 1.699 | NA |
| TRINITY_DN13879_c0_g1_i1 | 0.808474485 | 0.45997135 | 0.999970678 | 1.671 | 3.075 | 0.978 | 1.498 | NA |
| TRINITY_DN36635_c3_g1_i1 | 0.808437491 | 0.857507966 | 1 | 1.399 | 0 | 0.667 | 0 | Rotamase_3 |
| TRINITY_DN23718_c0_g1_i2 | -0.80843331 | 0.488189685 | 0.999970678 | 0.418 | 0.7 | 0.556 | 1.287 | NA |
| TRINITY_DN65193_c0_g1_i2 | 0.808303149 | 0.203716114 | 0.999970678 | 4.687 | 5.884 | 2.611 | 2.895 | NA |
| TRINITY_DN11331_c0_g1_i2 | 0.808274189 | 0.345461351 | 0.999970678 | 1.635 | 1.833 | 0.967 | 0.804 | Cation_efflux |
| TRINITY_DN810_c0_g1_i3 | -0.80819576 | 0.351463831 | 0.999970678 | 26.861 | 19.12 | 20.477 | 56.069 | NA |
| TRINITY_DN412_c0_g1_i5 | 0.808161722 | 0.679705039 | 0.999970678 | 1.39 | 1.35 | 1.278 | 0.02 | RRM_1 |

| TRINITY_DN1000_c0_g2_i10 | -0.80809009 | 0.416626169 | 0.999970678 | 0.481 | 0.394 | 0.633 | 0.754 | DS |
| --- | --- | --- | --- | --- | --- | --- | --- | --- |
| TRINITY_DN1352_c0_g1_i4 | -0.80807224 | 0.568694911 | 0.999970678 | 12.536 | 27.81 | 10.344 | 53.124 | NA |
| TRINITY_DN5184_c0_g1_i1 | -0.80800335 | 0.581702928 | 0.999970678 | 1.453 | 1.666 | 0.567 | 4.765 | p450 |
| TRINITY_DN6735_c0_g3_i2 | -0.80800063 | 0.467703667 | 0.999970678 | 0.799 | 0.621 | 1.133 | 1.116 | NA |
| TRINITY_DN6373_c0_g1_i4 | -0.80798692 | 0.579218683 | 0.999970678 | 1.862 | 9.737 | 2.455 | 17.212 | Metallophos |
| TRINITY_DN108_c0_g1_i4 | -0.80790056 | 0.514032565 | 0.999970678 | 171.14 | 100.5 | 337.313 | 69.763 | - |
| TRINITY_DN5310_c0_g1_i2 | 0.807865796 | 0.778292911 | 0.999970678 | 0.191 | 1.882 | 0 | 1.186 | XPG_I |
| TRINITY_DN1133_c0_g1_i1 | 0.807831242 | 0.276806195 | 0.999970678 | 9.702 | 5.913 | 3.644 | 4.554 | NA |
| TRINITY_DN3221_c0_g1_i4 | 0.807749248 | 0.256065621 | 0.999970678 | 6.94 | 10.07 | 3.422 | 5.56 | NA |
| TRINITY_DN33764_c0_g1_i1 | 0.807652369 | 0.36913673 | 0.999970678 | 2.489 | 3.952 | 2 | 1.267 | Gpi16 |
| TRINITY_DN16036_c0_g1_i1 | -0.80763945 | 0.802582202 | 0.999970678 | 112.99 | 0 | 15.144 | 183.208 | NA |
| TRINITY_DN420_c0_g1_i2 | -0.80749332 | 0.515816944 | 0.999970678 | 1.217 | 4.129 | 2.589 | 6.143 | NA |
| TRINITY_DN7545_c0_g1_i4 | -0.80736084 | 0.703345977 | 0.999970678 | 0.064 | 6.14 | 6.044 | 3.368 | NA |
| TRINITY_DN8230_c0_g1_i1 | -0.80724536 | 0.461214921 | 0.999970678 | 10.855 | 3.311 | 7.155 | 16.327 | LCM |
| TRINITY_DN1031_c2_g1_i3 | 0.807238249 | 0.85214789 | 0.999970678 | 0 | 2.355 | 1.111 | 0 | NA |
| TRINITY_DN11199_c0_g1_i1 | 0.807209916 | 0.851423986 | 0.999970678 | 4.569 | 0 | 2.189 | 0 | NA |
| TRINITY_DN4637_c0_g1_i2 | -0.80718498 | 0.682418066 | 1 | 0.69 | 0.118 | 1.067 | 0.161 | NA |
| TRINITY_DN4109_c0_g3_i1 | 0.807128677 | 0.561154264 | 0.999970678 | 7.44 | 5.233 | 4.955 | 1.206 | NA |
| TRINITY_DN2501_c0_g1_i1 | -0.80709819 | 0.553788815 | 0.999970678 | 1.054 | 3.065 | 1.1 | 5.821 | NA |
| TRINITY_DN5157_c0_g2_i2 | -0.80698958 | 0.764939084 | 0.999970678 | 1.235 | 0 | 0.767 | 1.267 | NA |
| TRINITY_DN340_c0_g4_i1 | -0.8068943 | 0.413445604 | 0.999970678 | 6.622 | 6.603 | 9.577 | 10.717 | NA |
| TRINITY_DN707_c0_g1_i8 | 0.806843581 | 0.85780605 | 1 | 0.154 | 0 | 0 | 0.09 | NA |
| TRINITY_DN13439_c0_g1_i5 | 0.806843581 | 0.85780605 | 1 | 2.507 | 0 | 0 | 1.448 | NA |
| TRINITY_DN5095_c0_g1_i3 | 0.806741597 | 0.853899622 | 0.999970678 | 0 | 0.552 | 0 | 0.312 | Utp12 |
| TRINITY_DN1345_c0_g1_i5 | -0.80673186 | 0.287483063 | 0.999970678 | 4.288 | 5.657 | 8.422 | 7.128 | LRRNT_2 |
| TRINITY_DN37_c0_g2_i2 | -0.80642019 | 0.342101186 | 0.999970678 | 31.094 | 76.14 | 77.484 | 92.615 | NA |
| TRINITY_DN7150_c0_g1_i7 | 0.806415769 | 0.576984802 | 0.999970678 | 2.08 | 0.7 | 0.955 | 0.462 | NA |
| TRINITY_DN3275_c0_g1_i6 | 0.806375319 | 0.765668969 | 0.999970678 | 0.754 | 0.759 | 0.7 | 0 | NA |
| TRINITY_DN107_c0_g1_i6 | -0.80636539 | 0.615013839 | 0.999970678 | 15.334 | 2.69 | 23.399 | 3.448 | NA |
| TRINITY_DN11079_c0_g1_i4 | -0.80628039 | 0.84850468 | 0.999970678 | 0 | 2.503 | 3.566 | 0 | NA |
| TRINITY_DN56983_c0_g1_i2 | -0.80625437 | 0.216177734 | 0.999970678 | 53.45 | 44.35 | 58.996 | 99.823 | NA |
| TRINITY_DN2408_c0_g1_i6 | 0.806185846 | 0.354545446 | 0.999970678 | 5.123 | 2.789 | 2.155 | 1.96 | NA |
| TRINITY_DN4934_c0_g1_i1 | 0.80616547 | 0.763907296 | 0.999970678 | 2.325 | 1.518 | 1.822 | 0 | NA |
| TRINITY_DN1254_c0_g1_i18 | -0.80615789 | 0.583701684 | 0.999970678 | 0.972 | 0.512 | 0.3 | 2.222 | NA |
| TRINITY_DN446_c0_g1_i3 | -0.80615347 | 0.32257755 | 0.999970678 | 15.652 | 11.78 | 26.121 | 16.388 | NA |
| TRINITY_DN525_c3_g1_i2 | 0.806146184 | 0.563307476 | 0.999970678 | 54.059 | 5.489 | 16.443 | 14.688 | zf-A20 |
| TRINITY_DN4052_c0_g1_i3 | -0.8061434 | 0.800520894 | 0.999970678 | 0.064 | 2.355 | 3.422 | 0 | NA |
| TRINITY_DN14705_c0_g1_i7 | -0.80613454 | 0.601325374 | 0.999970678 | 0.736 | 4.139 | 3.844 | 3.619 | NA |
| TRINITY_DN7806_c0_g3_i1 | -0.80611188 | 0.743533721 | 1 | 2.444 | 0.108 | 3.511 | 0.231 | NA |
| TRINITY_DN2287_c1_g2_i2 | -0.80604433 | 0.61952112 | 0.999970678 | 4.642 | 0.306 | 3.755 | 4.212 | NA |
| TRINITY_DN8386_c0_g2_i1 | -0.80590435 | 0.592987844 | 0.999970678 | 2.335 | 2.395 | 5.766 | 1.126 | NA |
| TRINITY_DN6710_c0_g1_i1 | 0.805901329 | 0.526032773 | 0.999970678 | 1.953 | 0.621 | 0.744 | 0.573 | Myb_CC_LHEQLE |
| TRINITY_DN246_c0_g1_i2 | -0.80583949 | 0.346012166 | 0.999970678 | 2.335 | 1.281 | 2.578 | 3.247 | NAM |
| TRINITY_DN5819_c1_g2_i3 | 0.805830269 | 0.778557911 | 0.999970678 | 25.499 | 0 | 6.566 | 6.837 | NA |
| TRINITY_DN8050_c0_g1_i20 | -0.80582551 | 0.769477368 | 0.999970678 | 0.309 | 0 | 0.222 | 0.282 | NA |
| TRINITY_DN4184_c0_g1_i3 | 0.80573927 | 0.1782437 | 0.999970678 | 14.48 | 14.16 | 7.255 | 7.631 | NA |
| TRINITY_DN11422_c0_g1_i3 | -0.80572477 | 0.853481541 | 0.999970678 | 0 | 0.65 | 0.922 | 0 | 5_nucleotid |
| TRINITY_DN3682_c0_g1_i8 | -0.80558978 | 0.744218686 | 0.999970678 | 2.689 | 0.039 | 3.878 | 0.181 | 2014/3/3 |
| TRINITY_DN8350_c0_g1_i5 | -0.80557073 | 0.855036401 | 1 | 0 | 0.759 | 1.1 | 0 | NA |
| TRINITY_DN238_c0_g1_i4 | 0.805506238 | 0.368871807 | 0.999970678 | 4.297 | 2.06 | 1.844 | 1.438 | DUF3752 |
| TRINITY_DN6471_c0_g2_i1 | -0.8054611 | 0.576804612 | 0.999970678 | 0.372 | 0.857 | 0.433 | 1.619 | Peroxidase_2 |
| TRINITY_DN203_c0_g1_i8 | -0.80545752 | 0.698740533 | 0.999970678 | 49.789 | 22.33 | 104.027 | 0.734 | NA |
| TRINITY_DN2140_c0_g2_i3 | 0.80523709 | 0.594939409 | 0.999970678 | 0.718 | 2.306 | 1.222 | 0.231 | CTD_bind |
| TRINITY_DN3048_c0_g1_i10 | 0.805056362 | 0.766380665 | 0.999970678 | 1.29 | 0.483 | 0.844 | 0 | NA |
| TRINITY_DN29295_c0_g1_i1 | -0.80498906 | 0.276926585 | 0.999970678 | 14.407 | 20.37 | 19.121 | 37.44 | BAG |
| TRINITY_DN23586_c0_g3_i2 | -0.80489649 | 0.61192249 | 0.999970678 | 1.335 | 3.686 | 1.411 | 6.766 | NA |
| TRINITY_DN8832_c0_g1_i6 | -0.80475208 | 0.670353187 | 0.999970678 | 7.276 | 0.838 | 11.377 | 0.593 | Glycolytic |
| TRINITY_DN18704_c0_g1_i2 | -0.8044584 | 0.770316588 | 0.999970678 | 1.208 | 0 | 0.633 | 1.347 | RNA_pol_Rpb2_1 |
| TRINITY_DN14089_c0_g1_i1 | 0.804200941 | 0.452442872 | 0.999970678 | 1.899 | 1.675 | 0.567 | 1.347 | AMP-binding |
| TRINITY_DN1962_c0_g2_i1 | -0.80418258 | 0.743943375 | 0.999970678 | 159.77 | 47.83 | 301.837 | 0.191 | Complex1_LYR |
| TRINITY_DN16967_c0_g1_i2 | -0.80416238 | 0.296357947 | 0.999970678 | 2.807 | 2.553 | 4.555 | 3.841 | - |
| TRINITY_DN2042_c0_g1_i10 | 0.804031283 | 0.686047977 | 0.999970678 | 0.863 | 0.059 | 0.378 | 0.09 | NA |
| TRINITY_DN4633_c0_g1_i3 | 0.804029879 | 0.580589661 | 0.999970678 | 1.135 | 0.266 | 0.467 | 0.241 | NA |
| TRINITY_DN372_c0_g1_i6 | -0.80402751 | 0.769755054 | 0.999970678 | 13.299 | 0 | 5.3 | 16.971 | NA |
| TRINITY_DN1031_c1_g2_i4 | 0.803950661 | 0.464041423 | 0.999970678 | 38.089 | 31.57 | 26.843 | 7.55 | Peptidase_M16 |
| TRINITY_DN24092_c0_g4_i2 | 0.803750684 | 0.856868522 | 1 | 3.152 | 0 | 1.511 | 0 | RRM_1 |
| TRINITY_DN6164_c0_g1_i9 | 0.803710798 | 0.437982566 | 0.999970678 | 0.472 | 0.237 | 0.156 | 0.221 | - |
| TRINITY_DN792_c0_g1_i1 | 0.803664378 | 0.232319189 | 0.999970678 | 2.916 | 2.434 | 1.311 | 1.498 | NA |
| TRINITY_DN4431_c0_g1_i4 | 0.803596936 | 0.423259204 | 0.999970678 | 0.672 | 0.936 | 0.333 | 0.513 | Sec1 |
| TRINITY_DN3341_c0_g1_i14 | 0.803351954 | 0.424946161 | 0.999970678 | 3.425 | 1.941 | 1.8 | 0.915 | NA |
| TRINITY_DN880_c1_g1_i4 | 0.803213506 | 0.346059954 | 0.999970678 | 12.236 | 7.382 | 4.722 | 5.469 | NA |
| TRINITY_DN479_c0_g1_i9 | 0.803204419 | 0.426188413 | 0.999970678 | 54.54 | 21.88 | 15.788 | 23.123 | DUF4228 |
| TRINITY_DN75342_c0_g1_i1 | 0.803201214 | 0.619291423 | 0.999970678 | 1.072 | 1.961 | 0.178 | 1.508 | - |
| TRINITY_DN39739_c0_g1_i1 | 0.802830487 | 0.782547671 | 0.999970678 | 0 | 1.922 | 0.2 | 0.834 | NA |

| TRINITY_DN5162_c0_g1_i1 | 0.80276181 | 0.622817219 | 0.999970678 | 4.715 | 3.666 | 3.8 | 0.221 | MafB19-deam |
| --- | --- | --- | --- | --- | --- | --- | --- | --- |
| TRINITY_DN794_c0_g1_i5 | -0.802705 | 0.281729606 | 0.999970678 | 124.49 | 128.4 | 126.259 | 287.375 | Dimerisation |
| TRINITY_DN4786_c0_g1_i2 | -0.80269175 | 0.537044064 | 0.999970678 | 0.291 | 0.581 | 0.911 | 0.392 | NA |
| TRINITY_DN1523_c0_g1_i4 | -0.80265743 | 0.798580672 | 1 | 2.044 | 0 | 0.2 | 3.388 | NA |
| TRINITY_DN509_c0_g1_i12 | -0.80263982 | 0.620221792 | 0.999970678 | 4.942 | 0.562 | 6.733 | 1.588 | NA |
| TRINITY_DN37435_c0_g1_i1 | 0.802591492 | 0.634388534 | 0.999970678 | 0.618 | 2.513 | 1.255 | 0.261 | NA |
| TRINITY_DN331_c0_g1_i5 | -0.80256919 | 0.851951503 | 0.999970678 | 0 | 8.259 | 11.733 | 0 | NA |
| TRINITY_DN1420_c0_g2_i1 | -0.80255963 | 0.40097516 | 0.999970678 | 7.185 | 11.36 | 16.232 | 11.873 | NA |
| TRINITY_DN13268_c0_g1_i4 | 0.802496664 | 0.665519649 | 0.999970678 | 21.865 | 6.879 | 0.311 | 16.277 | NA |
| TRINITY_DN5845_c0_g1_i1 | -0.80235257 | 0.338074746 | 0.999970678 | 2.044 | 3.42 | 3.733 | 4.977 | Cellulase |
| TRINITY_DN2771_c0_g1_i4 | 0.80232029 | 0.768619555 | 0.999970678 | 1.099 | 4.731 | 0 | 3.267 | NA |
| TRINITY_DN46136_c0_g1_i2 | 0.802203363 | 0.690232261 | 0.999970678 | 21.965 | 1357 | 82.617 | 599.874 | NA |
| TRINITY_DN9391_c0_g1_i2 | 0.802196999 | 0.54785363 | 0.999970678 | 1.526 | 2.957 | 0.322 | 2.182 | Med17 |
| TRINITY_DN12736_c0_g1_i4 | 0.802060748 | 0.445725642 | 0.999970678 | 0.845 | 0.719 | 0.322 | 0.503 | FPL |
| TRINITY_DN7717_c0_g2_i2 | 0.80192979 | 0.77355571 | 0.999970678 | 0 | 4.287 | 0.933 | 1.297 | NA |
| TRINITY_DN1063_c0_g1_i2 | -0.80192618 | 0.851750072 | 0.999970678 | 4.569 | 0 | 6.7 | 0 | NA |
| TRINITY_DN3808_c0_g2_i2 | 0.801709264 | 0.472063078 | 0.999970678 | 3.207 | 2.415 | 1.422 | 1.508 | NA |
| TRINITY_DN604_c1_g1_i2 | -0.80168301 | 0.493880056 | 0.999970678 | 2180.6 | 2538 | 1029.16 | 6378.286 | NA |
| TRINITY_DN3658_c0_g1_i6 | -0.80156583 | 0.706341745 | 1 | 3.079 | 0.108 | 1.2 | 3.951 | NA |
| TRINITY_DN5052_c0_g1_i1 | 0.801563685 | 0.611410019 | 0.999970678 | 8.957 | 11.95 | 0.567 | 11.27 | NA |
| TRINITY_DN30378_c0_g1_i17 | 0.801506969 | 0.848118832 | 0.999970678 | 0 | 9.116 | 0 | 5.127 | Sugar_tr |
| TRINITY_DN6034_c0_g5_i2 | 0.801431195 | 0.63394881 | 1 | 1.017 | 1.833 | 0.167 | 1.408 | NA |
| TRINITY_DN32204_c1_g2_i4 | 0.801424743 | 0.477072022 | 0.999970678 | 0.645 | 1.261 | 0.578 | 0.402 | DUF676 |
| TRINITY_DN1838_c0_g1_i2 | 0.801399106 | 0.849812907 | 0.999970678 | 5.868 | 0 | 0 | 3.438 | Myb_DNA-bindin |
| TRINITY_DN1834_c0_g1_i2 | 0.801317021 | 0.770674548 | 0.999970678 | 11.128 | 10.64 | 10.344 | 0 | NA |
| TRINITY_DN53993_c0_g1_i1 | 0.801286314 | 0.273823465 | 0.999970678 | 2.734 | 3.173 | 1.355 | 1.749 | Glyco_transf_8 |
| TRINITY_DN18966_c0_g1_i1 | 0.801247226 | 0.512803389 | 0.999970678 | 2.271 | 2.651 | 1.744 | 0.714 | NA |
| TRINITY_DN47302_c0_g1_i1 | 0.801172948 | 0.468444023 | 0.999970678 | 0.345 | 0.532 | 0.156 | 0.322 | NA |
| TRINITY_DN3603_c0_g1_i1 | -0.80116308 | 0.374624641 | 0.999970678 | 8.384 | 17.76 | 23.487 | 16.729 | NA |
| TRINITY_DN16835_c0_g2_i1 | -0.80111912 | 0.438610595 | 0.999970678 | 1.662 | 1.744 | 2.111 | 3.378 | NA |
| TRINITY_DN721_c1_g1_i5 | 0.80110122 | 0.505176595 | 0.999970678 | 8.53 | 23.96 | 3.211 | 14.638 | NA |
| TRINITY_DN63402_c0_g1_i1 | -0.80096683 | 0.792528659 | 1 | 0.718 | 0.099 | 1.189 | 0 | COesterase |
| TRINITY_DN98_c0_g1_i13 | 0.800919632 | 0.853899851 | 0.999970678 | 14.416 | 0 | 0 | 8.455 | Sigma70_r2 |
| TRINITY_DN1303_c0_g1_i7 | -0.80088325 | 0.426256927 | 0.999970678 | 7.104 | 12.3 | 17.821 | 11.431 | GUB_WAK_bind |
| TRINITY_DN2490_c0_g1_i1 | -0.80081716 | 0.530129735 | 0.999970678 | 0.545 | 1.271 | 0.633 | 2.403 | NA |
| TRINITY_DN808_c2_g1_i3 | -0.80070467 | 0.418344027 | 0.999970678 | 3.188 | 6.682 | 9.488 | 5.59 | Guanylate_kin |
| TRINITY_DN549_c0_g2_i1 | 0.80062987 | 0.259915194 | 0.999970678 | 28.442 | 34.28 | 11.177 | 22.52 | NA |
| TRINITY_DN3054_c0_g1_i1 | -0.8006094 | 0.575235408 | 0.999970678 | 1.699 | 2.75 | 1.167 | 6.173 | NA |
| TRINITY_DN11743_c0_g2_i2 | 0.800538717 | 0.419091984 | 0.999970678 | 1.099 | 1.478 | 0.833 | 0.483 | NA |
| TRINITY_DN9287_c0_g1_i2 | 0.800534564 | 0.395468223 | 0.999970678 | 1.962 | 3.104 | 1.5 | 1.086 | Lactamase_B |
| TRINITY_DN3100_c0_g1_i2 | 0.800511213 | 0.795848407 | 0.999970678 | 11.164 | 0.207 | 0 | 6.615 | NA |
| TRINITY_DN4087_c0_g1_i4 | -0.8004315 | 0.851118563 | 0.999970678 | 0 | 1.912 | 2.689 | 0 | Abhydrolase_1 |
| TRINITY_DN53574_c0_g1_i1 | -0.80040101 | 0.521736603 | 0.999970678 | 0.236 | 0.532 | 0.411 | 0.834 | Arm |
| TRINITY_DN35728_c0_g1_i1 | -0.80027811 | 0.310954239 | 0.999970678 | 1070.6 | 1158 | 2124.516 | 1189.875 | NA |
| TRINITY_DN994_c1_g1_i5 | 0.800273725 | 0.588735371 | 0.999970678 | 103.59 | 1376 | 252.862 | 501.679 | Chloroa_b-bind |
| TRINITY_DN6024_c0_g1_i2 | -0.80025636 | 0.475466759 | 0.999970678 | 44.729 | 94.61 | 43.62 | 178.091 | NA |
| TRINITY_DN896_c0_g1_i1 | 0.800173167 | 0.381068344 | 0.999970678 | 8.839 | 15.74 | 3.644 | 9.702 | NA |
| TRINITY_DN28418_c0_g1_i1 | -0.80011502 | 0.643032544 | 1 | 0.291 | 0.611 | 0.156 | 1.367 | NA |
| TRINITY_DN7154_c0_g1_i2 | -0.80010905 | 0.787521567 | 0.999970678 | 51.588 | 0 | 25.321 | 60.664 | TPR_8 |
| TRINITY_DN1945_c0_g1_i3 | 0.799982445 | 0.231004454 | 0.999970678 | 11.509 | 15.75 | 7.055 | 7.088 | EF-hand_1 |
| TRINITY_DN384_c0_g1_i2 | -0.79997564 | 0.297655851 | 0.999970678 | 29.105 | 46.44 | 41.031 | 81.083 | NA |
| TRINITY_DN80298_c0_g1_i1 | 0.799921692 | 0.441970172 | 0.999970678 | 2.344 | 3.696 | 1.244 | 1.92 | - |
| TRINITY_DN34945_c0_g3_i1 | -0.79984633 | 0.76233347 | 0.999970678 | 4.469 | 0.118 | 6.522 | 0.121 | NA |
| TRINITY_DN28115_c0_g2_i2 | -0.79983463 | 0.654848758 | 0.999970678 | 0.372 | 1.646 | 0.389 | 2.986 | NA |
| TRINITY_DN9270_c0_g1_i3 | -0.79965015 | 0.494646787 | 0.999970678 | 1.662 | 0.463 | 1.822 | 1.558 | NA |
| TRINITY_DN757_c1_g1_i3 | 0.799613154 | 0.646565228 | 0.999970678 | 7.076 | 2.622 | 4.466 | 0.221 | AUX_IAA |
| TRINITY_DN1374_c0_g1_i1 | 0.799591841 | 0.729265549 | 0.999970678 | 43.321 | 0.286 | 1.478 | 23.345 | NA |
| TRINITY_DN7094_c0_g1_i1 | 0.799558694 | 0.736732338 | 0.999970678 | 6.577 | 0.168 | 0.078 | 3.82 | - |
| TRINITY_DN1450_c0_g1_i4 | -0.79941327 | 0.285545546 | 0.999970678 | 697.12 | 1071 | 952.687 | 1911.097 | Chloroa_b-bind |
| TRINITY_DN1790_c0_g1_i1 | 0.799342454 | 0.276135512 | 0.999970678 | 3.852 | 3.134 | 1.422 | 2.302 | NA |
| TRINITY_DN12706_c0_g1_i1 | -0.79924932 | 0.398194893 | 0.999970678 | 10.401 | 17.63 | 11.344 | 34.916 | ANTH |
| TRINITY_DN2305_c0_g1_i3 | 0.799236884 | 0.849410757 | 0.999970678 | 0 | 6.633 | 3.1 | 0 | NA |
| TRINITY_DN2725_c0_g2_i7 | 0.79919684 | 0.676067604 | 0.999970678 | 6.595 | 1.991 | 4.011 | 0.151 | NA |
| TRINITY_DN680_c0_g3_i5 | 0.799162052 | 0.386162138 | 0.999970678 | 6.586 | 2.966 | 2.522 | 2.493 | Rrp15p |
| TRINITY_DN13722_c0_g2_i3 | -0.79909431 | 0.472334082 | 0.999970678 | 3.089 | 6.278 | 3.055 | 12.587 | NA |
| TRINITY_DN13357_c0_g1_i3 | -0.79902944 | 0.42310273 | 0.999970678 | 1.817 | 2.03 | 3.233 | 2.765 | NA |
| TRINITY_DN8887_c0_g1_i8 | 0.798936009 | 0.850939987 | 0.999970678 | 1.735 | 0 | 0 | 1.025 | - |
| TRINITY_DN863_c0_g2_i15 | 0.798930911 | 0.494650964 | 0.999970678 | 9.32 | 49.99 | 12.499 | 18.7 | NA |
| TRINITY_DN476_c1_g1_i1 | -0.79889874 | 0.4638046 | 0.999970678 | 1.49 | 2.779 | 1.822 | 5.168 | NA |
| TRINITY_DN11514_c0_g1_i3 | -0.79886073 | 0.852473513 | 0.999970678 | 10.537 | 0 | 15.388 | 0 | FMO-like |
| TRINITY_DN10062_c0_g2_i1 | -0.79881405 | 0.204998406 | 0.999970678 | 62.089 | 48.35 | 80.917 | 91.458 | NA |
| TRINITY_DN78_c0_g2_i2 | -0.7987199 | 0.362685444 | 0.999970678 | 78.322 | 41.41 | 60.463 | 134.046 | Calreticulin |
| TRINITY_DN1151_c0_g2_i7 | 0.798586112 | 0.772380759 | 0.999970678 | 1.036 | 6.238 | 0 | 4.142 | NA |
| TRINITY_DN3126_c1_g1_i5 | 0.798485126 | 0.796769585 | 0.999970678 | 15.497 | 0.296 | 0 | 9.219 | NA |

| TRINITY_DN7723_c0_g1_i2 | -0.79845351 | 0.775521904 | 1 | 0 | 0.463 | 0.256 | 0.483 | NA |
| --- | --- | --- | --- | --- | --- | --- | --- | --- |
| TRINITY_DN636_c0_g1_i1 | -0.79844513 | 0.361433577 | 0.999970678 | 29.832 | 13.38 | 36.82 | 31.066 | - |
| TRINITY_DN1283_c0_g2_i1 | 0.798434388 | 0.788958903 | 0.999970678 | 1.453 | 0 | 0.111 | 0.714 | NA |
| TRINITY_DN1543_c0_g1_i3 | 0.798369743 | 0.530951487 | 0.999970678 | 2.144 | 2.69 | 0.333 | 2.383 | Pept_tRNA_hydro |
| TRINITY_DN2957_c0_g1_i4 | -0.79824909 | 0.855903845 | 0.999970678 | 10.101 | 0 | 14.51 | 0 | EthD |
| TRINITY_DN2726_c0_g2_i1 | 0.798148714 | 0.855992753 | 0.999970678 | 16.142 | 0 | 7.644 | 0 | DUF1761 |
| TRINITY_DN9141_c3_g1_i6 | -0.79812205 | 0.533565636 | 0.999970678 | 1.063 | 2.681 | 0.978 | 5.328 | NA |
| TRINITY_DN761_c0_g1_i3 | -0.79808893 | 0.398938295 | 0.999970678 | 13.29 | 7.175 | 19.688 | 11.733 | NA |
| TRINITY_DN564_c0_g1_i2 | -0.79807929 | 0.455741678 | 0.999970678 | 51.851 | 16.13 | 64.818 | 40.426 | NA |
| TRINITY_DN3199_c0_g1_i1 | -0.79792283 | 0.510689461 | 0.999970678 | 0.173 | 0.424 | 0.344 | 0.603 | NA |
| TRINITY_DN1846_c0_g1_i2 | 0.797920595 | 0.363838605 | 0.999970678 | 4.833 | 5.578 | 2.966 | 2.363 | NA |
| TRINITY_DN1591_c0_g1_i1 | -0.79790781 | 0.340206512 | 0.999970678 | 3.97 | 5.056 | 4.378 | 10.375 | NA |
| TRINITY_DN4527_c0_g1_i1 | -0.79789927 | 0.453489128 | 0.999970678 | 3.216 | 1.468 | 2.211 | 5.57 | PRAI |
| TRINITY_DN1188_c0_g2_i3 | 0.797793296 | 0.479755953 | 0.999970678 | 0.663 | 1.478 | 0.522 | 0.593 | BTB |
| TRINITY_DN15940_c0_g1_i1 | -0.79777745 | 0.637433977 | 0.999970678 | 0.572 | 1.133 | 2.3 | 0.171 | NA |
| TRINITY_DN4997_c0_g1_i5 | -0.7977451 | 0.347552652 | 0.999970678 | 1.935 | 1.104 | 1.878 | 3.056 | BET |
| TRINITY_DN4541_c0_g1_i3 | -0.79771344 | 0.344583755 | 0.999970678 | 1.744 | 1.705 | 3.244 | 2.081 | TAF1_subA |
| TRINITY_DN9931_c1_g1_i1 | -0.79755624 | 0.217554908 | 0.999970678 | 542.75 | 372.9 | 650.961 | 807.312 | 2OG-FeII_Oxy |
| TRINITY_DN1999_c0_g1_i4 | 0.797481663 | 0.397732981 | 0.999970678 | 1.254 | 0.818 | 0.578 | 0.503 | NA |
| TRINITY_DN14350_c0_g2_i1 | 0.797477063 | 0.690526478 | 1 | 1.444 | 0.335 | 0.8 | 0.08 | Na_H_Exchanger |
| TRINITY_DN1800_c0_g2_i5 | -0.79744472 | 0.30389355 | 0.999970678 | 1.853 | 3.321 | 3.566 | 4.675 | Semialdhyde_dh |
| TRINITY_DN9540_c0_g1_i3 | 0.7974162 | 0.561751936 | 0.999970678 | 1.354 | 1.833 | 0.222 | 1.558 | NA |
| TRINITY_DN3592_c0_g1_i1 | -0.79738754 | 0.233520882 | 0.999970678 | 11.909 | 9.284 | 16.188 | 17.242 | NdhS |
| TRINITY_DN4848_c0_g1_i5 | 0.79737067 | 0.849575821 | 0.999970678 | 6.186 | 0 | 0 | 3.639 | PORR |
| TRINITY_DN28315_c0_g1_i2 | -0.79730875 | 0.850546501 | 0.999970678 | 1.599 | 0 | 2.344 | 0 | NA |
| TRINITY_DN169162_c0_g1_i1 | -0.79729806 | 0.856646557 | 0.999970678 | 2.135 | 0 | 3.1 | 0 | NA |
| TRINITY_DN236_c0_g2_i1 | 0.797240777 | 0.362383059 | 0.999970678 | 5.768 | 4.583 | 3.455 | 1.81 | HAT_KAT11 |
| TRINITY_DN3067_c1_g2_i3 | -0.7971617 | 0.386105895 | 0.999970678 | 5.805 | 7.372 | 5.955 | 15.493 | NA |
| TRINITY_DN14520_c1_g1_i2 | 0.797153037 | 0.799562034 | 1 | 0.609 | 0.02 | 0 | 0.372 | FAD_binding_8 |
| TRINITY_DN2268_c0_g1_i7 | -0.79693264 | 0.277414401 | 0.999970678 | 10.183 | 9.323 | 10.166 | 21.736 | Chloroa_b-bind |
| TRINITY_DN33395_c0_g1_i7 | -0.79690467 | 0.558981745 | 0.999970678 | 3.534 | 9.481 | 13.844 | 5.248 | NA |
| TRINITY_DN12788_c0_g2_i8 | -0.79689001 | 0.780562398 | 1 | 2.643 | 0 | 1.067 | 3.278 | NA |
| TRINITY_DN1977_c0_g1_i9 | -0.79678876 | 0.85031482 | 0.999970678 | 1.653 | 0 | 2.422 | 0 | Aldedh |
| TRINITY_DN2343_c0_g1_i8 | -0.79676055 | 0.61578856 | 0.999970678 | 1.49 | 1.794 | 0.389 | 5.228 | NA |
| TRINITY_DN1526_c0_g1_i6 | 0.796699218 | 0.570989852 | 0.999970678 | 1.199 | 7.214 | 1.722 | 2.654 | NA |
| TRINITY_DN546_c0_g1_i8 | 0.796538243 | 0.522228498 | 0.999970678 | 16.042 | 10.81 | 10.733 | 2.443 | ATP_bind_1 |
| TRINITY_DN97_c0_g1_i6 | 0.796437907 | 0.385346623 | 0.999970678 | 106.74 | 46.32 | 46.275 | 32.765 | NA |
| TRINITY_DN8019_c0_g1_i1 | 0.796379897 | 0.777286673 | 0.999970678 | 0 | 10.8 | 1.3 | 4.514 | NA |
| TRINITY_DN1952_c0_g2_i1 | 0.796276317 | 0.448105938 | 0.999970678 | 23.137 | 15.15 | 14.21 | 4.956 | NA |
| TRINITY_DN10260_c0_g1_i9 | -0.79621584 | 0.85748002 | 1 | 2.244 | 0 | 3.278 | 0 | NA |
| TRINITY_DN10979_c0_g3_i1 | -0.79621584 | 0.85748002 | 1 | 1.98 | 0 | 2.889 | 0 | NA |
| TRINITY_DN6346_c0_g1_i12 | -0.79613449 | 0.395516639 | 0.999970678 | 13.017 | 4.829 | 13.677 | 14.729 | S6PP |
| TRINITY_DN19125_c0_g1_i1 | 0.796096451 | 0.55204075 | 0.999970678 | 0.345 | 0.877 | 0.2 | 0.452 | HD |
| TRINITY_DN10351_c0_g1_i4 | -0.79601252 | 0.485306043 | 0.999970678 | 0.836 | 0.887 | 1.867 | 0.774 | NA |
| TRINITY_DN18308_c0_g1_i7 | 0.796003123 | 0.766843601 | 0.999970678 | 0.799 | 0.503 | 0 | 0.744 | Pro_isomerase |
| TRINITY_DN1841_c0_g2_i1 | -0.79580783 | 0.621857141 | 0.999970678 | 36.154 | 3.144 | 46.675 | 12.436 | Cyt-b5 |
| TRINITY_DN1841_c0_g1_i2 | -0.79580783 | 0.621857141 | 0.999970678 | 36.154 | 3.144 | 46.675 | 12.436 | NA |
| TRINITY_DN5_c1_g2_i3 | -0.79574423 | 0.219993262 | 0.999970678 | 10.583 | 14.61 | 16.521 | 23.656 | NA |
| TRINITY_DN6111_c0_g1_i7 | -0.79573541 | 0.620841871 | 0.999970678 | 3.307 | 1.468 | 0.622 | 7.48 | - |
| TRINITY_DN50126_c0_g1_i2 | 0.795640559 | 0.445643257 | 0.999970678 | 0.509 | 0.995 | 0.289 | 0.523 | AMP_N |
| TRINITY_DN38611_c0_g1_i1 | -0.7956225 | 0.852682545 | 0.999970678 | 0.781 | 0 | 1.144 | 0 | PHM7_cyt |
| TRINITY_DN238_c0_g1_i2 | 0.795493211 | 0.564049762 | 0.999970678 | 27.043 | 12.29 | 16.777 | 2.523 | NA |
| TRINITY_DN15229_c0_g1_i1 | 0.795284801 | 0.492890208 | 0.999970678 | 0.709 | 0.749 | 0.411 | 0.342 | NA |
| TRINITY_DN7861_c0_g1_i10 | 0.795112114 | 0.795550584 | 0.999970678 | 0.064 | 1.271 | 0.633 | 0 | Gln-synt_C |
| TRINITY_DN16781_c0_g1_i2 | 0.795015484 | 0.541137601 | 0.999970678 | 6.659 | 6.258 | 0.822 | 6.424 | bZIP_2 |
| TRINITY_DN29701_c0_g1_i1 | -0.79497081 | 0.858421357 | 1 | 0.454 | 0 | 0.656 | 0 | NA |
| TRINITY_DN3351_c0_g1_i1 | 0.79495284 | 0.785430993 | 0.999970678 | 1.626 | 0 | 0.544 | 0.261 | NA |
| TRINITY_DN3280_c0_g1_i1 | 0.794905744 | 0.780502211 | 0.999970678 | 4.051 | 0 | 1.511 | 0.553 | NA |
| TRINITY_DN24257_c1_g1_i1 | -0.79472692 | 0.457768011 | 0.999970678 | 0.936 | 1.133 | 0.844 | 2.574 | Pkinase |
| TRINITY_DN19259_c0_g1_i2 | -0.79471651 | 0.597484363 | 0.999970678 | 0.227 | 0.857 | 1.122 | 0.483 | NA |
| TRINITY_DN5602_c0_g1_i1 | 0.794620152 | 0.774361027 | 0.999970678 | 0 | 2.415 | 0.633 | 0.603 | NA |
| TRINITY_DN3708_c0_g1_i1 | 0.794608442 | 0.495046005 | 0.999970678 | 36.854 | 8.091 | 8.466 | 15.925 | ACT |
| TRINITY_DN8973_c0_g1_i2 | -0.79447382 | 0.506744218 | 0.999970678 | 7.667 | 3.449 | 11.355 | 5.389 | NA |
| TRINITY_DN973_c0_g1_i7 | 0.794375672 | 0.571169873 | 0.999970678 | 1.644 | 0.246 | 0.344 | 0.694 | Cyclin_N |
| TRINITY_DN8342_c0_g1_i3 | 0.794252605 | 0.774058237 | 1 | 0.672 | 0.71 | 0.667 | 0 | NA |
| TRINITY_DN1760_c0_g1_i9 | 0.794250066 | 0.78039231 | 0.999970678 | 0 | 3.715 | 0.611 | 1.337 | NA |
| TRINITY_DN5343_c1_g1_i1 | -0.79402881 | 0.230570483 | 0.999970678 | 17.777 | 23.79 | 25.576 | 40.768 | ADK |
| TRINITY_DN12826_c0_g1_i1 | 0.793984703 | 0.85165064 | 0.999970678 | 2.28 | 0 | 1.1 | 0 | LON_substr_bdg |
| TRINITY_DN956_c0_g2_i3 | -0.79382975 | 0.44397812 | 0.999970678 | 2.189 | 6.514 | 4.311 | 9.802 | NA |
| TRINITY_DN237_c3_g1_i5 | -0.79381574 | 0.779958705 | 1 | 0 | 0.867 | 0.911 | 0.352 | NA |
| TRINITY_DN2938_c0_g1_i1 | -0.79373176 | 0.332198625 | 0.999970678 | 10.91 | 6.298 | 15.466 | 11.31 | - |
| TRINITY_DN61997_c1_g4_i1 | 0.79369939 | 0.703464005 | 1 | 0.718 | 0.306 | 0.478 | 0.02 | NA |
| TRINITY_DN5833_c0_g1_i1 | -0.79369065 | 0.769130231 | 0.999970678 | 5.387 | 0 | 3.678 | 5.037 | NA |
| TRINITY_DN1702_c0_g1_i2 | 0.793643207 | 0.703371104 | 0.999970678 | 68.902 | 82.88 | 0.367 | 86.381 | NA |

| TRINITY_DN4894_c0_g1_i23 | -0.79344786 | 0.772429094 | 1 | 0 | 0.394 | 0.322 | 0.302 | ABC_membrane |
| --- | --- | --- | --- | --- | --- | --- | --- | --- |
| TRINITY_DN8428_c0_g1_i1 | 0.793415798 | 0.622253214 | 0.999970678 | 1.154 | 8.643 | 3.222 | 1.759 | NA |
| TRINITY_DN7288_c0_g1_i12 | 0.793399943 | 0.784740479 | 0.999970678 | 2.335 | 0 | 0.922 | 0.241 | NA |
| TRINITY_DN12453_c0_g2_i9 | 0.793292424 | 0.563928942 | 0.999970678 | 0.681 | 0.66 | 0.133 | 0.623 | RPAP3_C |
| TRINITY_DN3273_c0_g2_i1 | -0.79318544 | 0.279124087 | 0.999970678 | 3.089 | 2.809 | 4.366 | 4.936 | TCP |
| TRINITY_DN52424_c1_g1_i1 | 0.793165718 | 0.655189165 | 0.999970678 | 3.043 | 2.641 | 2.622 | 0.111 | NA |
| TRINITY_DN12351_c0_g1_i1 | -0.79314731 | 0.349172512 | 0.999970678 | 4.306 | 3.922 | 4.1 | 9.259 | NA |
| TRINITY_DN1548_c0_g1_i3 | -0.79312693 | 0.362557771 | 0.999970678 | 19.394 | 16.84 | 15.399 | 44.126 | NA |
| TRINITY_DN5265_c1_g2_i2 | 0.793092082 | 0.654874054 | 0.999970678 | 0.581 | 11.43 | 1.922 | 4.414 | NA |
| TRINITY_DN4541_c1_g1_i2 | 0.793047826 | 0.471974706 | 0.999970678 | 1.317 | 0.907 | 0.456 | 0.734 | TAF1_subA |
| TRINITY_DN27166_c0_g1_i1 | 0.792971209 | 0.855141046 | 0.999970678 | 11.046 | 0 | 5.289 | 0 | NA |
| TRINITY_DN12879_c0_g1_i2 | -0.79293503 | 0.557256135 | 0.999970678 | 1.017 | 2.553 | 1.022 | 4.926 | NA |
| TRINITY_DN4918_c0_g1_i5 | 0.79282207 | 0.387552339 | 0.999970678 | 17.623 | 7.539 | 7.488 | 5.6 | MFS_1 |
| TRINITY_DN47354_c0_g1_i1 | -0.79272028 | 0.854105592 | 0.999970678 | 2.562 | 0 | 3.711 | 0 | NAD_binding_10 |
| TRINITY_DN2558_c0_g1_i8 | -0.79272028 | 0.854105592 | 0.999970678 | 4.742 | 0 | 6.833 | 0 | Abhydrolase_1 |
| TRINITY_DN8388_c0_g1_i1 | 0.792651604 | 0.780931306 | 0.999970678 | 0 | 2.424 | 0.722 | 0.523 | NA |
| TRINITY_DN1183_c2_g1_i2 | -0.79259159 | 0.566783223 | 0.999970678 | 2.471 | 7.047 | 4.055 | 10.808 | NA |
| TRINITY_DN4067_c0_g1_i10 | -0.7925692 | 0.424637873 | 0.999970678 | 68.475 | 62.85 | 139.603 | 53.184 | NA |
| TRINITY_DN4184_c0_g1_i6 | 0.792415445 | 0.406059139 | 0.999970678 | 1.553 | 0.887 | 0.656 | 0.633 | - |
| TRINITY_DN4944_c0_g1_i7 | -0.79213552 | 0.77091242 | 0.999970678 | 0.808 | 0 | 0.656 | 0.623 | NA |
| TRINITY_DN18622_c0_g1_i1 | -0.79211962 | 0.512745158 | 0.999970678 | 1.026 | 1.114 | 2.422 | 0.774 | NA |
| TRINITY_DN5462_c0_g1_i2 | 0.791986257 | 0.580823903 | 0.999970678 | 0.436 | 1.399 | 0.2 | 0.824 | zf-C2H2 |
| TRINITY_DN2324_c0_g1_i1 | 0.79184079 | 0.798350259 | 0.999970678 | 0.127 | 4.524 | 2.167 | 0 | NA |
| TRINITY_DN91525_c0_g1_i1 | -0.79182825 | 0.860692877 | 1 | 3.779 | 0 | 5.422 | 0 | - |
| TRINITY_DN4843_c0_g1_i1 | -0.79182492 | 0.321758239 | 0.999970678 | 18.068 | 17.74 | 29.943 | 23.938 | NA |
| TRINITY_DN6020_c0_g1_i2 | 0.791815857 | 0.418574943 | 0.999970678 | 2.171 | 1.488 | 0.711 | 1.257 | NA |
| TRINITY_DN18866_c0_g1_i1 | -0.79161804 | 0.510098115 | 0.999970678 | 0.363 | 1.133 | 1.311 | 0.995 | Xan_ur_permease |
| TRINITY_DN6834_c0_g1_i2 | -0.79126766 | 0.46789045 | 0.999970678 | 0.845 | 0.414 | 0.856 | 1.156 | NA |
| TRINITY_DN5039_c0_g1_i4 | -0.79118547 | 0.76869897 | 0.999970678 | 3.007 | 0 | 2.511 | 2.252 | TFIIA |
| TRINITY_DN220_c0_g2_i14 | -0.79116666 | 0.775901964 | 0.999970678 | 2.725 | 0 | 3.244 | 0.875 | NA |
| TRINITY_DN2360_c0_g1_i7 | -0.7909641 | 0.624044974 | 0.999970678 | 1.344 | 0.483 | 2.344 | 0.342 | NA |
| TRINITY_DN2128_c0_g1_i4 | 0.790963485 | 0.43020029 | 0.999970678 | 13.308 | 4.415 | 4.5 | 4.886 | Zip |
| TRINITY_DN23240_c0_g2_i1 | -0.79095506 | 0.515389682 | 0.999970678 | 0.581 | 1.183 | 0.889 | 1.94 | NA |
| TRINITY_DN1046_c0_g1_i4 | -0.79086645 | 0.327994816 | 0.999970678 | 15.57 | 8.239 | 14.821 | 23.455 | NA |
| TRINITY_DN15_c0_g1_i19 | 0.790838625 | 0.66211389 | 0.999970678 | 272.88 | 12.55 | 36.576 | 109.143 | NA |
| TRINITY_DN4926_c0_g1_i2 | -0.79072921 | 0.309759615 | 0.999970678 | 57.856 | 30.96 | 56.574 | 85.657 | NA |
| TRINITY_DN9366_c0_g1_i1 | 0.79072605 | 0.559566971 | 0.999970678 | 2.416 | 2.572 | 0.389 | 2.413 | NA |
| TRINITY_DN16211_c0_g1_i1 | 0.790712189 | 0.58783511 | 0.999970678 | 1.481 | 4.563 | 2.311 | 0.674 | NA |
| TRINITY_DN9595_c0_g1_i2 | -0.79069251 | 0.772209681 | 0.999970678 | 3.025 | 0 | 1.133 | 3.941 | NA |
| TRINITY_DN10498_c0_g1_i5 | -0.79058904 | 0.301838682 | 0.999970678 | 1.59 | 1.459 | 1.933 | 2.926 | NA |
| TRINITY_DN11126_c0_g2_i1 | -0.79058423 | 0.174709545 | 0.999970678 | 16.151 | 14.56 | 22.932 | 25.496 | - |
| TRINITY_DN33680_c0_g1_i3 | 0.790515348 | 0.586378133 | 0.999970678 | 0.972 | 0.394 | 0.5 | 0.201 | NA |
| TRINITY_DN20249_c0_g1_i1 | -0.79044341 | 0.851503957 | 0.999970678 | 6.913 | 0 | 10.011 | 0 | NA |
| TRINITY_DN446_c0_g2_i2 | -0.79025898 | 0.429328092 | 0.999970678 | 2.616 | 8.495 | 8.299 | 9.068 | NA |
| TRINITY_DN1936_c0_g4_i1 | -0.79019163 | 0.798921682 | 1 | 2.453 | 0 | 3.222 | 0.362 | NA |
| TRINITY_DN1734_c0_g1_i2 | -0.7901824 | 0.794373768 | 0.999970678 | 46.973 | 0 | 17.666 | 61.368 | NA |
| TRINITY_DN415_c0_g1_i15 | 0.790071422 | 0.782251391 | 0.999970678 | 0 | 20.68 | 2.755 | 7.973 | NA |
| TRINITY_DN77753_c0_g1_i1 | -0.79003588 | 0.79887015 | 0.999970678 | 76.678 | 0 | 15.977 | 115.497 | - |
| TRINITY_DN41796_c0_g1_i5 | 0.789992714 | 0.299056276 | 0.999970678 | 2.825 | 3.095 | 1.655 | 1.418 | Asn_synthase |
| TRINITY_DN626_c0_g1_i6 | -0.78992651 | 0.471001818 | 0.999970678 | 2.961 | 7.973 | 4.244 | 13.683 | - |
| TRINITY_DN50289_c0_g1_i17 | 0.789916398 | 0.799088769 | 0.999970678 | 1.199 | 0 | 0.544 | 0.05 | ANAPC4 |
| TRINITY_DN14510_c0_g1_i7 | -0.78987979 | 0.602589183 | 0.999970678 | 0.136 | 0.65 | 0.889 | 0.261 | NA |
| TRINITY_DN7296_c1_g1_i8 | -0.78984553 | 0.585640443 | 0.999970678 | 0.79 | 0.907 | 0.433 | 2.383 | NA |
| TRINITY_DN9662_c0_g1_i1 | -0.7898305 | 0.514106308 | 0.999970678 | 2.071 | 5.785 | 2.678 | 10.235 | RRM_1 |
| TRINITY_DN5977_c0_g1_i1 | -0.78980379 | 0.862161864 | 1 | 2.571 | 0 | 3.644 | 0 | NA |
| TRINITY_DN138527_c0_g1_i1 | -0.78980379 | 0.862161864 | 1 | 0.963 | 0 | 1.389 | 0 | Methyltransf_31 |
| TRINITY_DN13841_c0_g1_i2 | -0.78980379 | 0.862161864 | 1 | 1.499 | 0 | 2.189 | 0 | NA |
| TRINITY_DN97589_c0_g1_i1 | -0.78980379 | 0.862161864 | 1 | 0.863 | 0 | 1.244 | 0 | NA |
| TRINITY_DN2346_c0_g1_i7 | 0.789796812 | 0.548577865 | 0.999970678 | 5.069 | 6.593 | 0.767 | 5.841 | NA |
| TRINITY_DN5465_c0_g1_i1 | -0.7897914 | 0.773221901 | 0.999970678 | 0.009 | 21.89 | 12.821 | 21.625 | NA |
| TRINITY_DN11427_c0_g1_i1 | 0.789782335 | 0.633061612 | 0.999970678 | 10.074 | 2.01 | 4.833 | 1.146 | - |
| TRINITY_DN4631_c0_g1_i6 | 0.789679588 | 0.599256346 | 0.999970678 | 7.431 | 4.307 | 4.744 | 1.005 | NA |
| TRINITY_DN5411_c0_g1_i1 | 0.789654148 | 0.376932538 | 0.999970678 | 1.344 | 3.371 | 1.267 | 1.186 | WRKY |
| TRINITY_DN428_c0_g1_i3 | 0.789617323 | 0.329110305 | 0.999970678 | 11.128 | 16.56 | 4.922 | 9.903 | GTP_EFTU |
| TRINITY_DN1633_c0_g1_i16 | 0.789560322 | 0.590140777 | 0.999970678 | 6.104 | 1.025 | 1.011 | 2.946 | F-box |
| TRINITY_DN5711_c0_g1_i2 | 0.789524473 | 0.547540494 | 0.999970678 | 2.453 | 0.986 | 1.255 | 0.483 | NA |
| TRINITY_DN13134_c0_g1_i3 | 0.789512337 | 0.851925322 | 0.999970678 | 6.54 | 0 | 3.2 | 0 | NA |
| TRINITY_DN7605_c0_g1_i1 | 0.789383726 | 0.602845616 | 0.999970678 | 1.299 | 13.41 | 2.933 | 4.796 | NA |
| TRINITY_DN92_c3_g1_i1 | -0.78937691 | 0.485653658 | 0.999970678 | 0.59 | 1.606 | 0.944 | 2.644 | NA |
| TRINITY_DN2714_c0_g1_i4 | 0.78937552 | 0.726427396 | 0.999970678 | 64.914 | 0.414 | 29.154 | 3.006 | PB1 |
| TRINITY_DN3353_c0_g1_i2 | 0.789347468 | 0.78137631 | 0.999970678 | 5.16 | 0.542 | 0 | 3.318 | NA |
| TRINITY_DN12389_c0_g1_i1 | -0.78930065 | 0.604906516 | 0.999970678 | 1.108 | 5.618 | 1.422 | 9.762 | DUF538 |
| TRINITY_DN2886_c0_g1_i2 | 0.789256351 | 0.85186266 | 0.999970678 | 4.833 | 0 | 0 | 2.865 | NA |
| TRINITY_DN8055_c3_g2_i1 | -0.78911288 | 0.804102163 | 0.999970678 | 2.135 | 0 | 0.044 | 3.7 | NA |

| TRINITY_DN4746_c0_g2_i4 | 0.789111429 | 0.588335514 | 0.999970678 | 1.753 | 2.07 | 0.211 | 1.94 | NA |
| --- | --- | --- | --- | --- | --- | --- | --- | --- |
| TRINITY_DN4746_c0_g1_i1 | 0.789111429 | 0.588335514 | 0.999970678 | 1.753 | 2.07 | 0.211 | 1.94 | SHMT |
| TRINITY_DN3287_c0_g1_i1 | -0.78904292 | 0.378528223 | 0.999970678 | 2.371 | 5.075 | 5.822 | 5.721 | PPR |
| TRINITY_DN3858_c0_g1_i4 | 0.788924936 | 0.610841093 | 0.999970678 | 1.081 | 16.16 | 4.889 | 3.921 | - |
| TRINITY_DN17197_c0_g1_i1 | 0.78888293 | 0.547298511 | 1 | 1.617 | 2.247 | 1.311 | 0.694 | NA |
| TRINITY_DN385_c0_g2_i1 | -0.78885091 | 0.862078457 | 1 | 0.472 | 0 | 0 | 0.824 | UDPGT |
| TRINITY_DN24122_c0_g1_i1 | -0.78876124 | 0.445540734 | 0.999970678 | 3.234 | 4.553 | 2.633 | 10.265 | NA |
| TRINITY_DN1976_c0_g1_i2 | 0.788727313 | 0.291145788 | 0.999970678 | 2.407 | 1.882 | 1.133 | 1.126 | NA |
| TRINITY_DN5910_c0_g1_i13 | 0.788698173 | 0.495508146 | 0.999970678 | 14.425 | 12.03 | 8.322 | 4.886 | NA |
| TRINITY_DN1136_c0_g1_i3 | -0.78869757 | 0.686285814 | 0.999970678 | 26.162 | 0.306 | 26.243 | 14.709 | MFS_MOT1 |
| TRINITY_DN581_c0_g1_i7 | -0.78863408 | 0.456989526 | 0.999970678 | 0.436 | 0.552 | 0.678 | 0.945 | NA |
| TRINITY_DN6865_c0_g1_i2 | -0.78859156 | 0.799014448 | 0.999970678 | 206.98 | 0 | 227.175 | 87.155 | NA |
| TRINITY_DN5236_c0_g1_i1 | 0.788527643 | 0.329713361 | 0.999970678 | 19.485 | 19.16 | 6.4 | 14.397 | NA |
| TRINITY_DN2833_c0_g1_i5 | -0.78847171 | 0.782573212 | 0.999970678 | 3.652 | 0 | 4.7 | 0.684 | NA |
| TRINITY_DN4908_c0_g1_i14 | 0.788447614 | 0.398805306 | 0.999970678 | 1.79 | 3.932 | 1.167 | 1.89 | B3 |
| TRINITY_DN3286_c0_g1_i2 | -0.78844463 | 0.200029459 | 0.999970678 | 197.55 | 209.1 | 243.585 | 408.612 | p450 |
| TRINITY_DN13793_c1_g5_i1 | 0.788361661 | 0.203841623 | 0.999970678 | 14.879 | 12.63 | 7.066 | 7.44 | NA |
| TRINITY_DN1969_c0_g1_i4 | 0.788290954 | 0.861171471 | 1 | 0 | 1.192 | 0.556 | 0 | tRNA-synt_1b |
| TRINITY_DN7499_c0_g1_i2 | 0.788249296 | 0.602131396 | 0.999970678 | 7.067 | 2.316 | 0.856 | 4.293 | NA |
| TRINITY_DN1424_c0_g2_i2 | 0.788237223 | 0.809652524 | 1 | 0 | 1.951 | 0.044 | 1.056 | NA |
| TRINITY_DN2984_c0_g1_i3 | -0.78821457 | 0.474437374 | 0.999970678 | 1.108 | 0.483 | 0.844 | 1.78 | GTP1_OBG |
| TRINITY_DN3039_c0_g1_i5 | -0.78800455 | 0.679311867 | 0.999970678 | 1.417 | 0.227 | 0.189 | 2.644 | HMA |
| TRINITY_DN5978_c0_g2_i1 | 0.787973641 | 0.480526708 | 0.999970678 | 1.744 | 0.798 | 0.744 | 0.583 | NA |
| TRINITY_DN85414_c0_g1_i1 | 0.787795031 | 0.632903372 | 1 | 2.207 | 2.365 | 0.3 | 2.282 | NA |
| TRINITY_DN2625_c0_g1_i2 | -0.78776647 | 0.353447699 | 0.999970678 | 9.447 | 5.755 | 7.511 | 17.333 | Adaptin_N |
| TRINITY_DN10395_c0_g1_i1 | 0.787621986 | 0.637601287 | 0.999970678 | 2.716 | 0.108 | 0.778 | 0.724 | Chromo |
| TRINITY_DN4564_c0_g1_i4 | 0.787375638 | 0.780074964 | 0.999970678 | 4.651 | 0 | 0.933 | 1.619 | Bromodomain |
| TRINITY_DN97950_c0_g1_i1 | -0.78735889 | 0.856738218 | 0.999970678 | 1.762 | 0 | 2.544 | 0 | NA |
| TRINITY_DN196395_c0_g1_i1 | -0.78735121 | 0.863534799 | 1 | 0.972 | 0 | 1.411 | 0 | - |
| TRINITY_DN11441_c0_g1_i1 | -0.78735121 | 0.863534799 | 1 | 0.781 | 0 | 1.133 | 0 | Spherulin4 |
| TRINITY_DN15494_c0_g1_i1 | -0.78733911 | 0.704848165 | 0.999970678 | 1.808 | 0.049 | 1.667 | 1.216 | NA |
| TRINITY_DN1088_c0_g1_i4 | -0.78721196 | 0.447366148 | 0.999970678 | 5.995 | 2.789 | 3.944 | 10.446 | NA |
| TRINITY_DN1572_c0_g1_i2 | -0.78720496 | 0.797094921 | 0.999970678 | 0 | 51.73 | 59.863 | 15.533 | 2-Hacid_dh |
| TRINITY_DN738_c0_g1_i4 | -0.7871305 | 0.276394527 | 0.999970678 | 31.467 | 39.94 | 60.419 | 47.514 | NA |
| TRINITY_DN3140_c0_g1_i1 | -0.78710007 | 0.587337527 | 0.999970678 | 2.262 | 0.838 | 3.844 | 0.744 | PGM_PMM_I |
| TRINITY_DN2925_c0_g1_i6 | 0.787069392 | 0.577923717 | 0.999970678 | 5.95 | 1.537 | 0.733 | 3.448 | CDP-OH_P_trans |
| TRINITY_DN1265_c0_g1_i7 | 0.787013559 | 0.851683208 | 0.999970678 | 0 | 12.55 | 5.911 | 0 | Sedlin_N |
| TRINITY_DN28616_c0_g1_i1 | -0.78701108 | 0.431187122 | 0.999970678 | 0.863 | 0.769 | 1.322 | 1.227 | NA |
| TRINITY_DN89264_c0_g1_i1 | -0.78697435 | 0.451653315 | 0.999970678 | 0.609 | 1.183 | 1.233 | 1.588 | NA |
| TRINITY_DN4782_c1_g1_i3 | -0.7869586 | 0.591066785 | 0.999970678 | 0.3 | 2.257 | 2.455 | 1.408 | NA |
| TRINITY_DN471_c0_g1_i5 | 0.786948921 | 0.851610902 | 0.999970678 | 0 | 2.848 | 1.344 | 0 | LRRNT_2 |
| TRINITY_DN18625_c0_g2_i2 | -0.78693815 | 0.509905753 | 0.999970678 | 3.316 | 1.774 | 4.155 | 3.619 | NA |
| TRINITY_DN41491_c1_g1_i1 | -0.78692982 | 0.700720105 | 1 | 0.055 | 0.749 | 0.967 | 0.201 | NA |
| TRINITY_DN242_c1_g1_i2 | 0.786905186 | 0.792551483 | 1 | 0.854 | 0.089 | 0.444 | 0 | B_lectin |
| TRINITY_DN2946_c1_g1_i6 | 0.78682641 | 0.582707284 | 0.999970678 | 1.962 | 1.922 | 1.533 | 0.422 | NA |
| TRINITY_DN2016_c0_g1_i3 | -0.7868232 | 0.481134505 | 0.999970678 | 0.318 | 0.375 | 0.478 | 0.603 | NA |
| TRINITY_DN855_c2_g1_i1 | -0.78676977 | 0.796611113 | 0.999970678 | 6.341 | 0 | 8.833 | 0.372 | NA |
| TRINITY_DN209_c1_g1_i2 | -0.78676213 | 0.480173363 | 0.999970678 | 1.88 | 0.798 | 1.3 | 3.076 | F-box |
| TRINITY_DN4797_c0_g1_i1 | -0.78670769 | 0.571539277 | 0.999970678 | 0.799 | 0.394 | 1.244 | 0.533 | NA |
| TRINITY_DN4952_c0_g1_i12 | 0.786638399 | 0.780776273 | 0.999970678 | 0.681 | 0.148 | 0.4 | 0 | DUF659 |
| TRINITY_DN8268_c0_g1_i12 | -0.78659509 | 0.782885561 | 1 | 0 | 1.222 | 0.422 | 1.538 | NA |
| TRINITY_DN26_c0_g1_i2 | 0.786559195 | 0.425929233 | 0.999970678 | 49.344 | 42.55 | 33.309 | 12.839 | zf-CDGSH |
| TRINITY_DN24297_c0_g1_i1 | 0.786533685 | 0.391706842 | 0.999970678 | 3.57 | 3.676 | 1.222 | 2.714 | NA |
| TRINITY_DN2324_c0_g1_i2 | -0.78651067 | 0.675388911 | 0.999970678 | 16.542 | 0.355 | 17.71 | 8.053 | NA |
| TRINITY_DN2798_c0_g1_i8 | 0.78650245 | 0.852941638 | 0.999970678 | 5.786 | 0 | 0 | 3.428 | - |
| TRINITY_DN490_c0_g1_i15 | -0.78649306 | 0.787038276 | 0.999970678 | 1.844 | 0 | 2.4 | 0.322 | NA |
| TRINITY_DN775_c0_g1_i5 | 0.786429451 | 0.545494717 | 0.999970678 | 4.07 | 0.749 | 1.5 | 1.025 | NA |
| TRINITY_DN6523_c0_g1_i6 | -0.78642599 | 0.854489653 | 0.999970678 | 1.635 | 0 | 2.378 | 0 | NA |
| TRINITY_DN15746_c0_g1_i2 | 0.786384336 | 0.320515971 | 0.999970678 | 2.171 | 2.651 | 1.344 | 1.176 | NA |
| TRINITY_DN899_c0_g1_i2 | -0.78595653 | 0.640056325 | 0.999970678 | 4.86 | 2.759 | 0.522 | 12.527 | NA |
| TRINITY_DN8046_c0_g1_i2 | 0.785914179 | 0.690478747 | 0.999970678 | 1.581 | 0.089 | 0.7 | 0.121 | NA |
| TRINITY_DN6795_c0_g1_i1 | -0.78584872 | 0.331065686 | 0.999970678 | 27.597 | 38.47 | 31.698 | 74.538 | NA |
| TRINITY_DN1056_c2_g1_i4 | 0.785825273 | 0.605213523 | 0.999970678 | 2.289 | 0.424 | 1.044 | 0.332 | NA |
| TRINITY_DN3323_c0_g2_i17 | -0.78576398 | 0.567153577 | 0.999970678 | 0.263 | 0.848 | 0.667 | 1.146 | NA |
| TRINITY_DN5546_c0_g1_i5 | 0.785696522 | 0.780415716 | 0.999970678 | 0.899 | 6.445 | 3.478 | 0 | Aldo_ket_red |
| TRINITY_DN1978_c0_g1_i7 | 0.785499901 | 0.377701386 | 0.999970678 | 1.953 | 1.192 | 0.822 | 0.824 | NA |
| TRINITY_DN11887_c0_g2_i1 | 0.7852643 | 0.370111152 | 0.999970678 | 3.443 | 2.139 | 1.144 | 1.87 | NA |
| TRINITY_DN28165_c0_g1_i1 | 0.785260106 | 0.554786584 | 0.999970678 | 1.281 | 0.286 | 0.344 | 0.503 | PIN_4 |
| TRINITY_DN650_c0_g1_i4 | -0.78521656 | 0.361478813 | 0.999970678 | 1.499 | 2.03 | 2.722 | 2.704 | Ssu72 |
| TRINITY_DN33321_c0_g1_i5 | -0.78513778 | 0.804449546 | 0.999970678 | 1.481 | 0 | 2.111 | 0.06 | Abhydrolase_1 |
| TRINITY_DN1909_c0_g1_i7 | -0.78510334 | 0.594600868 | 0.999970678 | 0.427 | 3.991 | 4.211 | 2.433 | DUF3475 |
| TRINITY_DN826_c0_g3_i7 | 0.785077737 | 0.852128018 | 0.999970678 | 8.33 | 0 | 0 | 4.836 | NA |
| TRINITY_DN1754_c0_g1_i2 | 0.785007534 | 0.429709442 | 0.999970678 | 2.916 | 1.222 | 1.189 | 0.975 | NA |
| TRINITY_DN1897_c0_g1_i2 | -0.78500051 | 0.315476906 | 0.999970678 | 18.813 | 33.42 | 34.631 | 45.724 | peroxidase |

| TRINITY_DN74442_c0_g1_i6 | 0.784966939 | 0.492075378 | 0.999970678 | 0.563 | 1.478 | 0.578 | 0.473 | NA |
| --- | --- | --- | --- | --- | --- | --- | --- | --- |
| TRINITY_DN12825_c0_g1_i3 | -0.78495288 | 0.585582564 | 0.999970678 | 0.709 | 2.779 | 0.889 | 4.876 | Aminotran_4 |
| TRINITY_DN3798_c0_g1_i14 | 0.784924195 | 0.48648 | 0.999970678 | 6.404 | 6.11 | 4.489 | 1.759 | NA |
| TRINITY_DN1642_c1_g1_i4 | 0.784893528 | 0.780308955 | 0.999970678 | 14.843 | 10.16 | 12.021 | 0 | NA |
| TRINITY_DN9322_c0_g3_i1 | -0.7848114 | 0.533907028 | 0.999970678 | 19.658 | 5.135 | 27.376 | 9.933 | - |
| TRINITY_DN305_c0_g3_i4 | 0.784773198 | 0.803478775 | 0.999970678 | 0 | 1.212 | 0.544 | 0.05 | NA |
| TRINITY_DN30815_c0_g1_i1 | -0.78474355 | 0.490698828 | 0.999970678 | 2.371 | 2.907 | 1.933 | 6.676 | - |
| TRINITY_DN180_c2_g1_i1 | 0.784595554 | 0.656060325 | 0.999970678 | 1.426 | 0.158 | 0.589 | 0.221 | RVT_2 |
| TRINITY_DN4503_c0_g1_i8 | 0.784533095 | 0.645582527 | 0.999970678 | 1.135 | 3.252 | 0.111 | 2.413 | PRP1_N |
| TRINITY_DN3154_c1_g1_i3 | -0.78450901 | 0.443005084 | 0.999970678 | 39.37 | 12.72 | 41.142 | 39.34 | Gamma-thionin |
| TRINITY_DN533_c0_g1_i8 | -0.78446418 | 0.775827766 | 0.999970678 | 0.99 | 2.04 | 0 | 5.208 | NA |
| TRINITY_DN77_c0_g1_i1 | -0.78442559 | 0.466117791 | 0.999970678 | 22.065 | 9.54 | 11.877 | 40.305 | NA |
| TRINITY_DN6372_c0_g1_i2 | -0.78438443 | 0.300812134 | 0.999970678 | 6.322 | 8.564 | 8.422 | 15.342 | - |
| TRINITY_DN583_c1_g1_i17 | 0.784296844 | 0.785327907 | 0.999970678 | 1.299 | 0 | 0.122 | 0.613 | Pkinase |
| TRINITY_DN12854_c0_g1_i1 | 0.784270564 | 0.564501232 | 0.999970678 | 0.881 | 0.631 | 0.567 | 0.191 | DUF4413 |
| TRINITY_DN1344_c1_g2_i3 | -0.78426698 | 0.773190588 | 0.999970678 | 1.935 | 0.01 | 0.856 | 2.363 | NA |
| TRINITY_DN41609_c0_g1_i6 | 0.784204324 | 0.555544443 | 0.999970678 | 1.19 | 0.443 | 0.256 | 0.633 | NA |
| TRINITY_DN1112_c0_g1_i3 | -0.78411947 | 0.777612666 | 0.999970678 | 0 | 11.98 | 12.11 | 4.956 | NA |
| TRINITY_DN1775_c0_g1_i1 | -0.78411219 | 0.519663918 | 0.999970678 | 1.244 | 3.065 | 4.677 | 1.719 | - |
| TRINITY_DN3333_c0_g1_i1 | 0.78408402 | 0.540139341 | 0.999970678 | 24.145 | 37.26 | 3.811 | 30.885 | UQ_con |
| TRINITY_DN758_c0_g1_i2 | 0.784012205 | 0.392807547 | 0.999970678 | 22.41 | 59.85 | 22.132 | 20.55 | Sugar_tr |
| TRINITY_DN544_c0_g2_i1 | 0.783961268 | 0.575377542 | 0.999970678 | 8.73 | 3.095 | 4.478 | 1.377 | NA |
| TRINITY_DN3526_c0_g1_i12 | -0.78394975 | 0.477892249 | 0.999970678 | 0.581 | 0.907 | 1.389 | 0.915 | MMR_HSR1 |
| TRINITY_DN27278_c0_g1_i7 | 0.783769726 | 0.606626878 | 0.999970678 | 2.344 | 2.148 | 1.967 | 0.231 | Pro_isomerase |
| TRINITY_DN2014_c0_g2_i2 | -0.78363867 | 0.357540962 | 0.999970678 | 1.163 | 0.818 | 1.6 | 1.518 | DEAD |
| TRINITY_DN51920_c0_g1_i3 | -0.78359651 | 0.797035263 | 0.999970678 | 0.345 | 8.288 | 0 | 14.588 | Dimerisation |
| TRINITY_DN414_c1_g1_i2 | -0.78353682 | 0.673416757 | 0.999970678 | 3.325 | 38.34 | 4.422 | 62.735 | NA |
| TRINITY_DN99_c0_g2_i6 | 0.783525472 | 0.23719838 | 0.999970678 | 79.73 | 77.13 | 45.064 | 36.274 | NA |
| TRINITY_DN17950_c0_g1_i1 | 0.783431101 | 0.29062934 | 0.999970678 | 2.752 | 2.158 | 1.433 | 1.136 | HSA |
| TRINITY_DN6007_c0_g1_i4 | -0.78332602 | 0.779085284 | 0.999970678 | 5.332 | 0 | 5.589 | 2.483 | NA |
| TRINITY_DN3589_c0_g1_i12 | -0.7833051 | 0.85572816 | 0.999970678 | 0.3 | 0 | 0 | 0.523 | NA |
| TRINITY_DN170_c1_g1_i3 | -0.78328479 | 0.46364084 | 0.999970678 | 27.261 | 10.04 | 37.309 | 19.363 | NA |
| TRINITY_DN3440_c0_g2_i1 | 0.783253642 | 0.548493219 | 0.999970678 | 1.49 | 4.632 | 0.622 | 2.775 | NA |
| TRINITY_DN21655_c0_g1_i1 | -0.78325076 | 0.644476241 | 0.999970678 | 0.291 | 2.553 | 0.567 | 4.152 | NA |
| TRINITY_DN79311_c0_g1_i2 | -0.78315125 | 0.525685377 | 0.999970678 | 7.076 | 10.14 | 4.389 | 23.757 | GUB_WAK_bind |
| TRINITY_DN2674_c0_g1_i3 | -0.78312309 | 0.852461799 | 0.999970678 | 1.899 | 0 | 0 | 3.318 | zf-FLZ |
| TRINITY_DN12863_c0_g1_i1 | 0.783089369 | 0.625058454 | 0.999970678 | 8.63 | 3.045 | 4.355 | 1.176 | Glyco_transf_5 |
| TRINITY_DN155_c0_g1_i14 | -0.78299505 | 0.793078764 | 0.999970678 | 0.127 | 2.079 | 0 | 3.79 | Ank |
| TRINITY_DN2051_c0_g1_i3 | 0.78298251 | 0.368201977 | 0.999970678 | 11.373 | 11.69 | 3.289 | 9.45 | RabGAP-TBC |
| TRINITY_DN4421_c0_g1_i2 | -0.78297367 | 0.782139376 | 0.999970678 | 0.191 | 0.424 | 0.878 | 0 | NA |
| TRINITY_DN8848_c0_g1_i6 | 0.782965994 | 0.717377565 | 0.999970678 | 0.927 | 58.89 | 25.154 | 3.278 | NA |
| TRINITY_DN9749_c0_g1_i4 | 0.7827929 | 0.462088036 | 0.999970678 | 7.122 | 4.642 | 4.322 | 1.639 | NA |
| TRINITY_DN3341_c0_g1_i3 | 0.782792571 | 0.854749276 | 0.999970678 | 12.536 | 0 | 0 | 7.43 | NA |
| TRINITY_DN39117_c0_g1_i4 | -0.78279073 | 0.357131545 | 0.999970678 | 2.707 | 2.05 | 4.144 | 3.187 | Myb_DNA-bindin |
| TRINITY_DN9353_c0_g1_i1 | 0.782656421 | 0.318445509 | 0.999970678 | 9.402 | 8.042 | 5.344 | 3.7 | NA |
| TRINITY_DN16159_c0_g1_i1 | 0.782580779 | 0.238392141 | 0.999970678 | 28.46 | 20.56 | 11.21 | 14.98 | THUMP |
| TRINITY_DN2769_c1_g1_i1 | 0.782539717 | 0.454723674 | 0.999970678 | 1.708 | 0.68 | 0.544 | 0.744 | Cation_efflux |
| TRINITY_DN189_c0_g1_i1 | 0.782505127 | 0.542236727 | 0.999970678 | 248.19 | 45.65 | 102.083 | 48.961 | NA |
| TRINITY_DN2922_c0_g1_i4 | -0.78249185 | 0.814270706 | 1 | 0.654 | 0 | 0.911 | 0.03 | NA |
| TRINITY_DN9558_c0_g1_i3 | 0.782464579 | 0.430838504 | 0.999970678 | 1.026 | 1.636 | 0.478 | 0.975 | PP2C |
| TRINITY_DN8683_c0_g1_i5 | 0.782401842 | 0.450103516 | 0.999970678 | 3.379 | 2.779 | 2.211 | 0.905 | NA |
| TRINITY_DN18768_c0_g1_i1 | -0.78229638 | 0.203219703 | 0.999970678 | 14.743 | 13.86 | 18.043 | 27.386 | NA |
| TRINITY_DN4459_c0_g1_i5 | -0.78215218 | 0.770908235 | 0.999970678 | 0 | 0.69 | 0.422 | 0.664 | NA |
| TRINITY_DN9842_c0_g1_i12 | 0.782135328 | 0.472610442 | 0.999970678 | 0.963 | 1.242 | 0.278 | 0.945 | ubiquitin |
| TRINITY_DN7549_c0_g1_i1 | 0.78211407 | 0.481284062 | 0.999970678 | 19.049 | 9.836 | 7.688 | 6.766 | NA |
| TRINITY_DN5236_c0_g1_i2 | -0.78209479 | 0.775934991 | 0.999970678 | 8.557 | 0 | 9.288 | 3.469 | NA |
| TRINITY_DN16674_c0_g1_i2 | 0.781987243 | 0.803640501 | 1 | 1.39 | 0.069 | 0 | 0.875 | NA |
| TRINITY_DN1571_c0_g1_i3 | 0.781912758 | 0.34656186 | 0.999970678 | 6.722 | 7.411 | 2.222 | 5.54 | ELK |
| TRINITY_DN226_c0_g1_i4 | -0.7818936 | 0.274602292 | 0.999970678 | 3.334 | 3.715 | 6.144 | 4.715 | ABC2_membrane |
| TRINITY_DN490_c0_g1_i13 | 0.78180807 | 0.50990364 | 0.999970678 | 4.097 | 2.759 | 2.744 | 0.694 | Asp_protease_2 |
| TRINITY_DN6056_c0_g1_i2 | -0.78180208 | 0.510249556 | 0.999970678 | 11.991 | 21.38 | 8.133 | 47.242 | Myb_DNA-bindin |
| TRINITY_DN5_c1_g2_i2 | -0.7816856 | 0.289235689 | 0.999970678 | 6.813 | 7.569 | 12.744 | 9.249 | NA |
| TRINITY_DN7915_c0_g2_i1 | -0.78167181 | 0.787512862 | 0.999970678 | 20.448 | 0 | 25.621 | 3.81 | p450 |
| TRINITY_DN11198_c0_g1_i5 | 0.78161373 | 0.801169519 | 0.999970678 | 0 | 66.66 | 27.298 | 5.178 | PGM_PMM_I |
| TRINITY_DN592_c0_g2_i1 | 0.781549568 | 0.773596124 | 0.999970678 | 15.107 | 32.51 | 0 | 27.175 | Ribosomal_L37 |
| TRINITY_DN17412_c0_g1_i2 | -0.78151663 | 0.789932429 | 0.999970678 | 0.917 | 0 | 1.155 | 0.171 | NA |
| TRINITY_DN7295_c0_g1_i1 | -0.7815019 | 0.573153769 | 1 | 0.2 | 0.404 | 0.611 | 0.312 | DALR_2 |
| TRINITY_DN232_c1_g1_i1 | -0.78138765 | 0.780101689 | 0.999970678 | 0.536 | 0.808 | 0 | 2.312 | Glyco_hydro_17 |
| TRINITY_DN106991_c0_g1_i1 | -0.7813294 | 0.224635522 | 0.999970678 | 9.029 | 7.805 | 12.266 | 14.055 | NA |
| TRINITY_DN3020_c0_g1_i7 | -0.78129873 | 0.773941889 | 0.999970678 | 2.825 | 0 | 1.167 | 3.529 | Autophagy_C |
| TRINITY_DN242_c1_g1_i4 | 0.781280672 | 0.487470256 | 0.999970678 | 2.998 | 0.936 | 1.133 | 0.925 | B_lectin |
| TRINITY_DN350_c0_g1_i2 | 0.781267113 | 0.272869986 | 0.999970678 | 10.637 | 11.64 | 6.622 | 4.967 | HEAT |
| TRINITY_DN1397_c0_g1_i5 | 0.781249171 | 0.853652748 | 0.999970678 | 1.59 | 0 | 0 | 0.945 | NA |
| TRINITY_DN1155_c0_g1_i2 | 0.781231301 | 0.612623167 | 0.999970678 | 1.226 | 0.601 | 0.811 | 0.101 | KH_1 |

| TRINITY_DN1412_c0_g2_i11 | -0.78122657 | 0.794190207 | 1 | 0.427 | 0 | 0.544 | 0.101 | Ras |
| --- | --- | --- | --- | --- | --- | --- | --- | --- |
| TRINITY_DN28447_c0_g1_i3 | 0.780930403 | 0.804541677 | 1 | 0.064 | 1.252 | 0.622 | 0 | Mito_carr |
| TRINITY_DN56119_c0_g1_i2 | -0.78092853 | 0.461337601 | 0.999970678 | 1.317 | 0.493 | 1.344 | 1.538 | PPR |
| TRINITY_DN1437_c2_g1_i11 | -0.78083168 | 0.297043356 | 0.999970678 | 4.56 | 6.288 | 7.3 | 9.722 | NA |
| TRINITY_DN10178_c0_g1_i1 | -0.78080026 | 0.271179318 | 0.999970678 | 1.662 | 2.02 | 2.889 | 2.845 | DEP |
| TRINITY_DN4_c0_g1_i11 | 0.780747284 | 0.573449678 | 0.999970678 | 5.232 | 0.907 | 0.978 | 2.443 | UDPGT |
| TRINITY_DN425_c0_g2_i2 | -0.78071894 | 0.270546556 | 0.999970678 | 37.78 | 36.46 | 39.175 | 80.037 | NA |
| TRINITY_DN656_c0_g1_i5 | 0.780628658 | 0.852853057 | 0.999970678 | 2.625 | 0 | 0 | 1.558 | NA |
| TRINITY_DN3152_c0_g1_i1 | -0.78061109 | 0.72711475 | 0.999970678 | 2.789 | 0.138 | 4.111 | 0.131 | NA |
| TRINITY_DN2910_c1_g1_i1 | -0.78053129 | 0.547355848 | 0.999970678 | 31.93 | 89.93 | 26.376 | 175.296 | NA |
| TRINITY_DN519_c1_g1_i7 | -0.78052814 | 0.77196684 | 0.999970678 | 4.569 | 0 | 3.766 | 3.438 | BPL_LplA_LipB |
| TRINITY_DN7113_c0_g1_i4 | 0.780493828 | 0.621416717 | 0.999970678 | 1.317 | 0.946 | 0.111 | 1.196 | NA |
| TRINITY_DN6571_c0_g1_i1 | -0.78049205 | 0.568697377 | 0.999970678 | 2.434 | 3.499 | 1.333 | 8.395 | Polyketide_cyc2 |
| TRINITY_DN1970_c0_g1_i1 | 0.780482931 | 0.778419014 | 0.999970678 | 0 | 3.853 | 0.756 | 1.317 | NA |
| TRINITY_DN11579_c0_g2_i4 | -0.78046711 | 0.860027126 | 0.999970678 | 1.017 | 0 | 1.5 | 0 | Enolase_C |
| TRINITY_DN9633_c0_g2_i1 | -0.78045916 | 0.505288898 | 0.999970678 | 0.372 | 0.522 | 0.533 | 0.824 | NA |
| TRINITY_DN3015_c0_g2_i1 | 0.780398979 | 0.587140291 | 0.999970678 | 2.734 | 4.642 | 0.533 | 3.589 | NA |
| TRINITY_DN8231_c0_g1_i1 | -0.78034597 | 0.861473876 | 0.999970678 | 46.41 | 0 | 66.785 | 0 | Ferritin_2 |
| TRINITY_DN5959_c0_g1_i1 | -0.78021684 | 0.341611072 | 0.999970678 | 89.922 | 123.3 | 204.165 | 117.035 | NA |
| TRINITY_DN3999_c0_g2_i3 | 0.780124406 | 0.303699247 | 0.999970678 | 6.195 | 4.74 | 2.689 | 3.137 | Pro_isomerase |
| TRINITY_DN20528_c0_g1_i2 | 0.780119691 | 0.781368088 | 0.999970678 | 23.791 | 7.332 | 0 | 18.278 | NA |
| TRINITY_DN2984_c0_g1_i6 | -0.77993629 | 0.549052973 | 0.999970678 | 4.188 | 1.104 | 2.067 | 6.645 | GTP1_OBG |
| TRINITY_DN1718_c0_g1_i2 | 0.779919931 | 0.654723798 | 0.999970678 | 0.854 | 5.943 | 2.922 | 0.392 | NA |
| TRINITY_DN1372_c0_g1_i2 | -0.77971058 | 0.852855104 | 0.999970678 | 2.507 | 0 | 0 | 4.404 | NA |
| TRINITY_DN74442_c0_g1_i5 | -0.77969338 | 0.487679366 | 0.999970678 | 4.151 | 1.37 | 2.5 | 6.585 | NA |
| TRINITY_DN1582_c0_g1_i6 | 0.77943798 | 0.605270945 | 0.999970678 | 0.736 | 0.177 | 0.311 | 0.161 | ORC3_N |
| TRINITY_DN1230_c0_g1_i1 | -0.77943323 | 0.397529002 | 0.999970678 | 1.917 | 1.478 | 2.555 | 2.674 | PsbP |
| TRINITY_DN842_c0_g1_i2 | 0.779421498 | 0.46571034 | 0.999970678 | 8.902 | 19.06 | 3.389 | 12.105 | Iso_dh |
| TRINITY_DN14896_c1_g1_i5 | 0.779387607 | 0.77986758 | 1 | 1.026 | 2.365 | 1.589 | 0 | NA |
| TRINITY_DN14552_c0_g1_i6 | 0.779379646 | 0.790544687 | 0.999970678 | 27.17 | 3.272 | 14.421 | 0 | Actin |
| TRINITY_DN8850_c1_g3_i1 | -0.77936179 | 0.675603405 | 0.999970678 | 3.724 | 0.375 | 4.989 | 1.036 | NA |
| TRINITY_DN956_c0_g1_i1 | -0.77928354 | 0.853454807 | 0.999970678 | 4.269 | 0 | 0 | 7.49 | NA |
| TRINITY_DN1928_c0_g1_i1 | -0.77910907 | 0.388195158 | 0.999970678 | 2.925 | 1.971 | 4.489 | 2.986 | NA |
| TRINITY_DN11827_c0_g1_i1 | 0.779020957 | 0.584366843 | 0.999970678 | 1.135 | 0.946 | 0.8 | 0.251 | NA |
| TRINITY_DN353_c1_g1_i4 | -0.7790037 | 0.490687376 | 0.999970678 | 1.88 | 1.597 | 2.067 | 3.448 | NA |
| TRINITY_DN2020_c0_g1_i4 | 0.77897827 | 0.305118333 | 0.999970678 | 14.68 | 9.284 | 4.755 | 8.314 | NA |
| TRINITY_DN9543_c0_g1_i5 | 0.778932455 | 0.751353998 | 0.999970678 | 17.196 | 0.315 | 8.177 | 0.332 | NA |
| TRINITY_DN1127_c0_g2_i6 | 0.778859473 | 0.483781029 | 0.999970678 | 0.799 | 1.902 | 0.7 | 0.724 | NA |
| TRINITY_DN7910_c0_g1_i6 | 0.77885643 | 0.478705392 | 0.999970678 | 10.274 | 6.504 | 6.366 | 2.111 | - |
| TRINITY_DN12768_c0_g1_i7 | 0.778843872 | 0.71303628 | 1 | 0.082 | 2.612 | 1.022 | 0.302 | NA |
| TRINITY_DN665_c1_g2_i2 | 0.778723593 | 0.481167046 | 0.999970678 | 139.3 | 69.56 | 74.895 | 27.577 | - |
| TRINITY_DN1350_c0_g1_i1 | -0.77869195 | 0.771665954 | 0.999970678 | 0 | 125.6 | 85.084 | 96.284 | NA |
| TRINITY_DN11526_c0_g1_i2 | 0.778361462 | 0.481336175 | 0.999970678 | 2.062 | 0.926 | 0.689 | 0.925 | zf-CCCH |
| TRINITY_DN175_c0_g1_i5 | -0.77827296 | 0.476139499 | 0.999970678 | 0.872 | 1.281 | 0.756 | 2.775 | NA |
| TRINITY_DN2484_c0_g3_i1 | -0.7782579 | 0.380683332 | 0.999970678 | 2.316 | 4.74 | 6.077 | 4.695 | NA |
| TRINITY_DN686_c7_g1_i3 | -0.77822214 | 0.562902332 | 0.999970678 | 41.314 | 336.1 | 179.256 | 423.743 | RPE65 |
| TRINITY_DN537_c0_g2_i2 | 0.778160137 | 0.800693187 | 0.999970678 | 0 | 6.682 | 3.044 | 0.151 | NA |
| TRINITY_DN15325_c0_g1_i1 | 0.778157239 | 0.383047966 | 0.999970678 | 1.88 | 1.626 | 0.644 | 1.277 | NA |
| TRINITY_DN6684_c0_g1_i4 | -0.77812134 | 0.861117679 | 1 | 3.425 | 0 | 4.889 | 0 | NA |
| TRINITY_DN213_c0_g1_i3 | -0.77808855 | 0.495002953 | 0.999970678 | 3.761 | 3.134 | 2.189 | 9.119 | Ribosomal_L22 |
| TRINITY_DN16741_c0_g2_i1 | -0.77807179 | 0.61290607 | 0.999970678 | 0.363 | 0.256 | 0.111 | 0.935 | LRR_8 |
| TRINITY_DN5032_c0_g1_i1 | -0.77802787 | 0.443038752 | 0.999970678 | 1.09 | 1.222 | 1.255 | 2.393 | NA |
| TRINITY_DN1506_c0_g1_i3 | -0.77798957 | 0.627996442 | 0.999970678 | 0.627 | 6.8 | 7.888 | 2.996 | NA |
| TRINITY_DN5589_c0_g1_i1 | 0.777949919 | 0.345172663 | 0.999970678 | 2.425 | 3.016 | 1.3 | 1.609 | NA |
| TRINITY_DN22119_c1_g1_i1 | -0.77790979 | 0.619640576 | 0.999970678 | 10.546 | 32.34 | 7.7 | 57.949 | NA |
| TRINITY_DN68_c0_g1_i2 | 0.777830553 | 0.787963598 | 0.999970678 | 47.727 | 76.47 | 0 | 71.974 | Ribonuc_L-PSP |
| TRINITY_DN11289_c0_g1_i1 | 0.777708157 | 0.40459679 | 0.999970678 | 1.826 | 4.888 | 1.433 | 2.172 | LRR_4 |
| TRINITY_DN2728_c0_g1_i4 | 0.77762616 | 0.692036849 | 1 | 0.127 | 0.976 | 0.067 | 0.533 | NA |
| TRINITY_DN1504_c0_g3_i3 | -0.77758944 | 0.809172749 | 0.999970678 | 6.386 | 0.099 | 0 | 11.3 | LRRNT_2 |
| TRINITY_DN5235_c0_g1_i2 | -0.777449 | 0.504083324 | 0.999970678 | 5.36 | 1.163 | 4.544 | 5.821 | NA |
| TRINITY_DN4302_c0_g1_i3 | 0.777377496 | 0.465338377 | 0.999970678 | 2.098 | 1.912 | 0.5 | 1.729 | NA |
| TRINITY_DN8516_c0_g1_i1 | 0.777290177 | 0.205564175 | 0.999970678 | 6.222 | 6.8 | 2.866 | 4.152 | NA |
| TRINITY_DN584_c0_g1_i7 | -0.7772136 | 0.558146558 | 0.999970678 | 5.033 | 1.035 | 5.744 | 3.509 | NA |
| TRINITY_DN1471_c6_g1_i1 | 0.777203372 | 0.241310421 | 0.999970678 | 5.968 | 8.712 | 3.9 | 3.851 | DUF3385 |
| TRINITY_DN3273_c0_g1_i4 | -0.77714601 | 0.524838459 | 0.999970678 | 0.309 | 0.65 | 0.856 | 0.583 | NA |
| TRINITY_DN21121_c0_g1_i1 | -0.77713224 | 0.546751804 | 0.999970678 | 2.171 | 1.035 | 0.8 | 4.574 | COBRA |
| TRINITY_DN5855_c0_g1_i1 | -0.777062 | 0.596684943 | 0.999970678 | 5.687 | 3.755 | 1.167 | 14.829 | UPF0114 |
| TRINITY_DN24455_c0_g1_i3 | -0.77703513 | 0.771865291 | 0.999970678 | 1.708 | 0 | 1.167 | 1.538 | Sec20 |
| TRINITY_DN20875_c0_g1_i2 | -0.77702652 | 0.854298055 | 0.999970678 | 1.481 | 0 | 2.133 | 0 | Carn_acyltransf |
| TRINITY_DN581_c0_g1_i14 | 0.776995761 | 0.78571373 | 0.999970678 | 10.447 | 0 | 1.989 | 3.589 | NA |
| TRINITY_DN10299_c0_g1_i1 | -0.77692935 | 0.778319728 | 0.999970678 | 1.435 | 0 | 0.578 | 1.79 | NA |
| TRINITY_DN18141_c0_g2_i1 | 0.776919982 | 0.775146199 | 0.999970678 | 0.936 | 3.361 | 0 | 2.483 | PQ-loop |
| TRINITY_DN922_c0_g1_i1 | -0.77684947 | 0.436451603 | 0.999970678 | 14.144 | 17.07 | 33.876 | 12.467 | DYW_deaminase |
| TRINITY_DN1516_c0_g1_i2 | 0.776812818 | 0.78389699 | 0.999970678 | 16.115 | 0 | 2.033 | 6.967 | BSD |

| TRINITY_DN16240_c0_g1_i2 | 0.776801407 | 0.515044979 | 0.999970678 | 1.354 | 1.37 | 0.467 | 1.005 | NA |
| --- | --- | --- | --- | --- | --- | --- | --- | --- |
| TRINITY_DN1746_c0_g1_i1 | -0.77673869 | 0.47676863 | 0.999970678 | 1.708 | 3.361 | 1.955 | 6.233 | Utp12 |
| TRINITY_DN24270_c0_g1_i1 | -0.77672473 | 0.552956439 | 0.999970678 | 2.035 | 6.012 | 2.144 | 11.069 | Pentapeptide |
| TRINITY_DN14264_c0_g1_i3 | -0.77672049 | 0.580254252 | 0.999970678 | 0.745 | 0.71 | 0.333 | 2.111 | NA |
| TRINITY_DN816_c0_g1_i1 | -0.77665119 | 0.419055025 | 0.999970678 | 1.317 | 3.489 | 3.578 | 3.871 | NA |
| TRINITY_DN5498_c0_g1_i1 | -0.77662906 | 0.584597681 | 0.999970678 | 12.245 | 3.656 | 3.778 | 22.39 | NA |
| TRINITY_DN6653_c0_g1_i1 | -0.7766139 | 0.791819134 | 0.999970678 | 0.009 | 52.17 | 43.02 | 36.022 | CorA |
| TRINITY_DN14188_c0_g1_i2 | 0.776484744 | 0.708207949 | 0.999970678 | 796.29 | 18.4 | 374.866 | 28.231 | NA |
| TRINITY_DN7720_c0_g1_i7 | 0.776384811 | 0.211752132 | 0.999970678 | 28.051 | 22.07 | 12.333 | 14.417 | NA |
| TRINITY_DN11035_c0_g1_i6 | 0.776381293 | 0.798396533 | 1 | 0.164 | 2.247 | 0 | 1.397 | NA |
| TRINITY_DN4187_c0_g1_i2 | -0.77632464 | 0.332769026 | 0.999970678 | 3.906 | 6.869 | 8.711 | 7.842 | zf-RING_2 |
| TRINITY_DN8720_c0_g1_i2 | -0.7762978 | 0.257306178 | 0.999970678 | 12.926 | 11.66 | 14.521 | 24.35 | NA |
| TRINITY_DN7883_c1_g3_i2 | -0.77626157 | 0.493654601 | 0.999970678 | 0.999 | 0.69 | 1.678 | 0.905 | NA |
| TRINITY_DN73651_c0_g1_i7 | -0.77626131 | 0.518563448 | 0.999970678 | 1.108 | 1.192 | 2.655 | 0.724 | NA |
| TRINITY_DN21418_c0_g1_i1 | -0.77622974 | 0.784923822 | 1 | 0.645 | 0 | 0.278 | 0.784 | NA |
| TRINITY_DN2752_c0_g1_i1 | 0.776224716 | 0.783601169 | 1 | 0.263 | 0.788 | 0.489 | 0 | NA |
| TRINITY_DN3550_c0_g1_i1 | -0.77620651 | 0.499592328 | 0.999970678 | 12.926 | 22.17 | 9.066 | 48.961 | NA |
| TRINITY_DN221088_c0_g1_i1 | -0.77618993 | 0.263090415 | 0.999970678 | 20.393 | 23.06 | 24.021 | 44.99 | NA |
| TRINITY_DN5156_c0_g2_i4 | 0.77615494 | 0.54419228 | 0.999970678 | 110.55 | 22.26 | 17.099 | 57.648 | MatE |
| TRINITY_DN22810_c0_g1_i1 | -0.7761192 | 0.858859046 | 0.999970678 | 1.935 | 0 | 0 | 3.328 | NA |
| TRINITY_DN4927_c0_g2_i1 | -0.77600864 | 0.170194424 | 0.999970678 | 217.9 | 264.2 | 344.735 | 406.059 | Plant_tran |
| TRINITY_DN12524_c2_g4_i4 | 0.775965007 | 0.400968057 | 0.999970678 | 5.023 | 4.1 | 2.522 | 2.262 | NA |
| TRINITY_DN8829_c0_g1_i4 | -0.77593491 | 0.857908986 | 0.999970678 | 0 | 1.301 | 0 | 2.192 | NA |
| TRINITY_DN1486_c0_g1_i21 | -0.77590395 | 0.686770568 | 0.999970678 | 4.397 | 0.128 | 1.922 | 5.459 | NA |
| TRINITY_DN4217_c0_g1_i1 | 0.77579799 | 0.382264918 | 0.999970678 | 3.579 | 5.095 | 2.855 | 1.609 | Cation_ATPase |
| TRINITY_DN2151_c0_g1_i2 | 0.775767525 | 0.853441667 | 0.999970678 | 14.116 | 0 | 0 | 8.254 | Myb_DNA-bindin |
| TRINITY_DN17433_c0_g2_i3 | -0.77574263 | 0.30563986 | 0.999970678 | 3.116 | 3.203 | 3.778 | 6.233 | NA |
| TRINITY_DN3405_c0_g1_i2 | -0.77537299 | 0.454715445 | 0.999970678 | 1.753 | 1.991 | 1.633 | 4.424 | DUF538 |
| TRINITY_DN3405_c0_g1_i3 | -0.77537299 | 0.454715445 | 0.999970678 | 1.753 | 1.991 | 1.633 | 4.424 | NA |
| TRINITY_DN6145_c0_g1_i3 | -0.77536551 | 0.523276045 | 0.999970678 | 0.345 | 0.197 | 0.289 | 0.593 | B_lectin |
| TRINITY_DN4530_c0_g1_i4 | 0.775322753 | 0.730117246 | 0.999970678 | 40.969 | 0.118 | 4.522 | 18.961 | WRKY |
| TRINITY_DN1978_c0_g1_i8 | 0.775276795 | 0.543339286 | 0.999970678 | 10.183 | 2.208 | 1.7 | 5.288 | NA |
| TRINITY_DN5022_c0_g3_i2 | -0.77516314 | 0.772361037 | 0.999970678 | 2.28 | 0 | 1.633 | 1.981 | NA |
| TRINITY_DN3436_c0_g1_i1 | -0.77509019 | 0.784281372 | 0.999970678 | 0 | 0.463 | 0.556 | 0.101 | ACAS_N |
| TRINITY_DN1902_c0_g1_i1 | -0.77506579 | 0.598088671 | 0.999970678 | 19.231 | 5.815 | 6.089 | 33.68 | NA |
| TRINITY_DN353_c1_g1_i11 | -0.77501029 | 0.237972376 | 0.999970678 | 15.125 | 12.52 | 20.31 | 22.4 | NA |
| TRINITY_DN1344_c0_g1_i5 | -0.774952 | 0.8131181 | 0.999970678 | 1.817 | 0 | 0.056 | 3.076 | NA |
| TRINITY_DN2536_c1_g1_i3 | 0.774928941 | 0.292915486 | 0.999970678 | 21.747 | 12.87 | 8.277 | 10.305 | Peptidase_S10 |
| TRINITY_DN1684_c0_g2_i2 | -0.77490026 | 0.451731793 | 0.999970678 | 3.952 | 2.622 | 2.889 | 7.691 | NA |
| TRINITY_DN15188_c0_g1_i1 | -0.77489304 | 0.457597931 | 0.999970678 | 7.803 | 4.869 | 11.677 | 7.249 | NA |
| TRINITY_DN996_c0_g1_i5 | 0.774891457 | 0.786308709 | 0.999970678 | 11.064 | 0 | 1.322 | 4.967 | MatE |
| TRINITY_DN26149_c0_g1_i2 | 0.77482456 | 0.607517815 | 0.999970678 | 0.345 | 2.878 | 0.878 | 0.794 | NA |
| TRINITY_DN1523_c0_g1_i2 | 0.774798854 | 0.466917778 | 0.999970678 | 2.907 | 5.815 | 1.878 | 2.664 | NA |
| TRINITY_DN7478_c0_g1_i2 | 0.774793186 | 0.469978732 | 0.999970678 | 1.562 | 2.897 | 1.555 | 0.724 | HAUS6_N |
| TRINITY_DN4657_c1_g1_i2 | -0.77478126 | 0.4417117 | 0.999970678 | 11.119 | 3.725 | 8.633 | 15.191 | PRP4 |
| TRINITY_DN20377_c0_g1_i4 | 0.774778294 | 0.548359428 | 0.999970678 | 3.534 | 1.971 | 2.267 | 0.493 | NA |
| TRINITY_DN18722_c0_g1_i1 | -0.77477723 | 0.576420939 | 0.999970678 | 4.669 | 7.717 | 15.421 | 2.393 | NA |
| TRINITY_DN1929_c0_g1_i1 | 0.774726521 | 0.419583664 | 0.999970678 | 28.996 | 26.65 | 20.199 | 8.123 | PRC |
| TRINITY_DN3504_c1_g1_i5 | 0.774623918 | 0.588198459 | 0.999970678 | 3.434 | 1.261 | 2 | 0.342 | DEAD |
| TRINITY_DN29797_c0_g1_i3 | -0.77460246 | 0.860255669 | 0.999970678 | 0.563 | 0 | 0 | 0.995 | RRM_1 |
| TRINITY_DN9829_c0_g1_i3 | 0.774584237 | 0.547466672 | 0.999970678 | 0.69 | 0.296 | 0.322 | 0.191 | NA |
| TRINITY_DN242_c0_g2_i1 | -0.77455696 | 0.439427175 | 0.999970678 | 2.325 | 2.897 | 2.544 | 5.761 | LRRNT_2 |
| TRINITY_DN2084_c0_g1_i5 | -0.77453324 | 0.526711767 | 0.999970678 | 6.695 | 10.9 | 3.811 | 25.456 | Methyltransf_29 |
| TRINITY_DN135_c0_g1_i2 | -0.77446578 | 0.207343822 | 0.999970678 | 12.963 | 14.01 | 18.521 | 23.485 | - |
| TRINITY_DN8535_c0_g1_i4 | -0.7744268 | 0.53309099 | 0.999970678 | 19.276 | 120 | 95.961 | 111.727 | NA |
| TRINITY_DN967_c1_g1_i2 | -0.77433562 | 0.801019435 | 0.999970678 | 0 | 9.215 | 12.51 | 0.362 | Myb_DNA-bindin |
| TRINITY_DN23_c2_g2_i1 | -0.77429712 | 0.707571592 | 1 | 14.271 | 0.394 | 9.255 | 12.979 | NA |
| TRINITY_DN1914_c0_g2_i2 | -0.77421715 | 0.856107051 | 0.999970678 | 203.18 | 0 | 281.05 | 0 | NA |
| TRINITY_DN29429_c0_g1_i2 | -0.77415264 | 0.471031197 | 0.999970678 | 0.545 | 1.035 | 0.955 | 1.528 | Rhomboid |
| TRINITY_DN3990_c0_g1_i1 | 0.773944983 | 0.255427503 | 0.999970678 | 27.561 | 30.28 | 10.922 | 20.64 | NA |
| TRINITY_DN12581_c0_g1_i1 | 0.773939922 | 0.570766849 | 0.999970678 | 1.426 | 1.941 | 1.222 | 0.493 | Inhibitor_I9 |
| TRINITY_DN6062_c0_g2_i1 | 0.773933777 | 0.676082161 | 0.999970678 | 1.026 | 0.207 | 0.544 | 0.08 | NA |
| TRINITY_DN5394_c0_g1_i2 | -0.77389292 | 0.566687107 | 0.999970678 | 0.636 | 1.33 | 2.011 | 0.925 | NA |
| TRINITY_DN2868_c0_g1_i1 | -0.77387574 | 0.617652663 | 0.999970678 | 13.935 | 0.926 | 9.011 | 14.839 | NA |
| TRINITY_DN465_c0_g1_i2 | -0.77383187 | 0.564347277 | 0.999970678 | 4.042 | 0.719 | 4.089 | 3.278 | Autophagy_act_C |
| TRINITY_DN2456_c1_g1_i3 | 0.773728556 | 0.291910556 | 0.999970678 | 3.706 | 5.095 | 2.011 | 2.704 | NA |
| TRINITY_DN5823_c0_g1_i2 | -0.77369904 | 0.814052604 | 0.999970678 | 77.005 | 0 | 1.644 | 131.07 | NA |
| TRINITY_DN533_c0_g1_i2 | -0.77369393 | 0.78732902 | 0.999970678 | 0 | 3.745 | 0.467 | 5.71 | NA |
| TRINITY_DN627_c0_g1_i10 | -0.77359461 | 0.775597821 | 0.999970678 | 1.971 | 0 | 1.955 | 1.056 | NA |
| TRINITY_DN2071_c0_g1_i1 | -0.77356277 | 0.775288848 | 0.999970678 | 7.158 | 0 | 4.566 | 6.897 | Abhydrolase_6 |
| TRINITY_DN4653_c0_g1_i1 | 0.773509728 | 0.858016285 | 0.999970678 | 0 | 11.47 | 0 | 6.645 | NA |
| TRINITY_DN3570_c0_g1_i11 | -0.7734487 | 0.864792988 | 1 | 0 | 0.729 | 1.022 | 0 | NA |
| TRINITY_DN15165_c0_g1_i1 | 0.77343308 | 0.493670267 | 0.999970678 | 0.618 | 0.926 | 0.467 | 0.332 | NA |
| TRINITY_DN17316_c0_g1_i4 | 0.773422179 | 0.558082148 | 0.999970678 | 0.491 | 1.33 | 0.322 | 0.664 | IFRD |

| TRINITY_DN4231_c0_g1_i8 | 0.773360825 | 0.621391738 | 0.999970678 | 0.854 | 3.489 | 0.278 | 2.192 | PDZ |
| --- | --- | --- | --- | --- | --- | --- | --- | --- |
| TRINITY_DN2745_c0_g1_i2 | 0.773284862 | 0.371545016 | 0.999970678 | 3.098 | 2.109 | 1.555 | 1.166 | NA |
| TRINITY_DN4452_c0_g1_i12 | 0.773185389 | 0.559932556 | 0.999970678 | 9.02 | 4.642 | 5.755 | 1.086 | NA |
| TRINITY_DN2404_c0_g1_i5 | 0.773010613 | 0.786001591 | 0.999970678 | 1.463 | 0 | 0.456 | 0.302 | - |
| TRINITY_DN10508_c0_g1_i1 | 0.772882515 | 0.393169465 | 0.999970678 | 6.168 | 15.23 | 6.277 | 4.896 | TamB |
| TRINITY_DN12013_c0_g1_i5 | 0.772835332 | 0.864507849 | 1 | 1.662 | 0 | 0.811 | 0 | NA |
| TRINITY_DN1541_c0_g1_i3 | -0.77281412 | 0.507365318 | 0.999970678 | 112.69 | 342.8 | 145.136 | 598.567 | Glyco_transf_5 |
| TRINITY_DN33213_c0_g1_i1 | 0.77277055 | 0.863424593 | 1 | 0.899 | 0 | 0.433 | 0 | NA |
| TRINITY_DN76347_c0_g1_i1 | 0.77277055 | 0.863424593 | 1 | 1.899 | 0 | 0.933 | 0 | NA |
| TRINITY_DN32882_c0_g4_i1 | 0.772742032 | 0.862937152 | 1 | 3.634 | 0 | 1.778 | 0 | NA |
| TRINITY_DN270_c2_g1_i1 | 0.772728125 | 0.652147919 | 0.999970678 | 9.075 | 1.705 | 0.522 | 5.71 | PB1 |
| TRINITY_DN9496_c0_g1_i15 | 0.772715651 | 0.862484254 | 1 | 1.354 | 0 | 0.678 | 0 | NA |
| TRINITY_DN7299_c0_g1_i1 | 0.772715651 | 0.862484254 | 1 | 0.999 | 0 | 0.489 | 0 | NA |
| TRINITY_DN13179_c0_g1_i1 | 0.772690995 | 0.862083005 | 1 | 3.143 | 0 | 1.533 | 0 | NA |
| TRINITY_DN40296_c0_g1_i1 | 0.772690995 | 0.862083005 | 1 | 1.435 | 0 | 0.7 | 0 | NA |
| TRINITY_DN13065_c0_g1_i1 | 0.772626781 | 0.860990158 | 0.999970678 | 1.181 | 0 | 0.578 | 0 | Calreticulin |
| TRINITY_DN28866_c0_g1_i13 | -0.77257774 | 0.815566599 | 1 | 2.544 | 0 | 3.611 | 0.09 | - |
| TRINITY_DN4093_c0_g1_i14 | -0.77257569 | 0.85480138 | 0.999970678 | 2.171 | 0 | 3.111 | 0 | - |
| TRINITY_DN11520_c1_g1_i3 | 0.772454439 | 0.254800365 | 0.999970678 | 2.316 | 2.937 | 1.389 | 1.397 | NA |
| TRINITY_DN5143_c0_g2_i2 | 0.772379982 | 0.617205901 | 0.999970678 | 2.598 | 0.818 | 1.278 | 0.452 | NA |
| TRINITY_DN10314_c0_g1_i3 | 0.77236433 | 0.638820191 | 0.999970678 | 0.799 | 0.089 | 0.211 | 0.282 | LRRNT_2 |
| TRINITY_DN2178_c0_g1_i1 | -0.77228022 | 0.485486047 | 0.999970678 | 10.91 | 14.7 | 6.933 | 35.319 | MFS_1 |
| TRINITY_DN11229_c0_g1_i8 | -0.77226943 | 0.648151382 | 1 | 0.6 | 0.118 | 0.744 | 0.312 | PPR |
| TRINITY_DN11372_c0_g1_i3 | 0.772259461 | 0.85539392 | 0.999970678 | 3.17 | 0 | 1.567 | 0 | NA |
| TRINITY_DN10363_c0_g1_i10 | -0.77224044 | 0.863741613 | 1 | 0.881 | 0 | 1.278 | 0 | NA |
| TRINITY_DN21025_c1_g1_i1 | 0.772205768 | 0.854973091 | 0.999970678 | 11.909 | 0 | 5.822 | 0 | 1-cysPrx_C |
| TRINITY_DN9516_c0_g1_i1 | -0.77217136 | 0.682768014 | 0.999970678 | 8.493 | 2.641 | 0.933 | 17.222 | NA |
| TRINITY_DN27336_c0_g4_i5 | -0.77214493 | 0.706184049 | 0.999970678 | 0.99 | 8.889 | 12.821 | 0.945 | NA |
| TRINITY_DN1188_c1_g1_i2 | -0.77213897 | 0.52965993 | 0.999970678 | 4.133 | 4.612 | 10.444 | 2.332 | SNARE |
| TRINITY_DN86203_c0_g1_i4 | -0.77213232 | 0.658941538 | 0.999970678 | 0.218 | 2.355 | 3.022 | 0.664 | - |
| TRINITY_DN5126_c1_g1_i12 | -0.77207696 | 0.581728208 | 0.999970678 | 0.509 | 2.148 | 2.889 | 1.005 | OPT |
| TRINITY_DN8615_c0_g1_i6 | 0.77200344 | 0.618678973 | 0.999970678 | 1.208 | 5.588 | 0.422 | 3.448 | NA |
| TRINITY_DN1684_c0_g1_i4 | -0.77172294 | 0.47098954 | 0.999970678 | 3.261 | 1.311 | 3.478 | 3.589 | NA |
| TRINITY_DN8337_c0_g2_i3 | 0.771695219 | 0.478622984 | 0.999970678 | 1.762 | 6.534 | 2.322 | 2.021 | NA |
| TRINITY_DN486_c0_g1_i12 | -0.77164463 | 0.42940708 | 0.999970678 | 6.413 | 7.904 | 5.622 | 17.373 | NA |
| TRINITY_DN3392_c0_g1_i4 | 0.771617623 | 0.525476774 | 0.999970678 | 6.586 | 5.45 | 1.122 | 5.68 | Use1 |
| TRINITY_DN9533_c0_g1_i3 | -0.77161402 | 0.16816578 | 0.999970678 | 237.67 | 253.2 | 367.3 | 389.52 | Ribosomal_L12 |
| TRINITY_DN50637_c0_g1_i7 | -0.7714092 | 0.589298081 | 0.999970678 | 2.698 | 10.38 | 15.943 | 2.845 | TPP_enzyme_C |
| TRINITY_DN7120_c0_g2_i3 | 0.771374377 | 0.556053081 | 0.999970678 | 1.036 | 0.68 | 0.678 | 0.191 | Glyco_transf_28 |
| TRINITY_DN21069_c0_g1_i2 | -0.77129156 | 0.739301358 | 0.999970678 | 4.024 | 0.197 | 5.789 | 0.322 | NA |
| TRINITY_DN2437_c0_g1_i1 | -0.77119494 | 0.503798802 | 0.999970678 | 2.171 | 3.627 | 1.744 | 7.761 | - |
| TRINITY_DN31637_c0_g1_i4 | -0.77117165 | 0.780262594 | 0.999970678 | 0.527 | 0 | 0.456 | 0.382 | Myb_DNA-bind_ |
| TRINITY_DN34082_c1_g1_i7 | 0.771096373 | 0.539560536 | 1 | 2.062 | 3.144 | 1.6 | 1.086 | NA |
| TRINITY_DN855_c0_g1_i5 | -0.77106799 | 0.860386774 | 0.999970678 | 0.736 | 0 | 1.044 | 0 | - |
| TRINITY_DN2039_c0_g1_i1 | 0.771042242 | 0.859017022 | 0.999970678 | 1.78 | 0 | 0 | 1.066 | NA |
| TRINITY_DN10583_c0_g1_i2 | -0.77103216 | 0.603898125 | 0.999970678 | 0.645 | 0.128 | 0.467 | 0.774 | NA |
| TRINITY_DN2217_c0_g1_i2 | -0.77091892 | 0.43218463 | 0.999970678 | 1.908 | 1.409 | 2.955 | 2.041 | PsbP |
| TRINITY_DN11987_c0_g1_i2 | 0.77089882 | 0.642733892 | 0.999970678 | 1.853 | 1.094 | 1.255 | 0.201 | NA |
| TRINITY_DN296_c0_g1_i11 | -0.77084937 | 0.468570252 | 0.999970678 | 0.409 | 0.207 | 0.5 | 0.473 | Nic96 |
| TRINITY_DN17514_c0_g1_i1 | -0.7708477 | 0.386609328 | 0.999970678 | 1.481 | 2.838 | 2.944 | 3.79 | NA |
| TRINITY_DN15202_c0_g1_i1 | -0.77081063 | 0.267573476 | 0.999970678 | 54.059 | 52.06 | 56.819 | 111.315 | Ctr |
| TRINITY_DN13631_c0_g2_i4 | 0.77078254 | 0.741361576 | 1 | 4.233 | 0.404 | 2.111 | 0.111 | Ribosomal_S26e |
| TRINITY_DN72474_c0_g1_i2 | -0.77077962 | 0.726518972 | 0.999970678 | 0.391 | 5.637 | 8.266 | 0.151 | NA |
| TRINITY_DN4912_c0_g1_i8 | 0.770713231 | 0.587707435 | 0.999970678 | 0.709 | 2.799 | 1.089 | 0.694 | NA |
| TRINITY_DN76448_c1_g1_i1 | -0.77068363 | 0.362604688 | 0.999970678 | 435.06 | 266.4 | 646.183 | 386.474 | NA |
| TRINITY_DN504_c0_g1_i16 | 0.77066901 | 0.498742328 | 0.999970678 | 1.989 | 0.907 | 1 | 0.493 | DUF3522 |
| TRINITY_DN6772_c0_g1_i1 | -0.77065501 | 0.587360055 | 0.999970678 | 0.363 | 0.877 | 0.467 | 1.538 | NA |
| TRINITY_DN5564_c0_g1_i1 | -0.77063416 | 0.363288704 | 0.999970678 | 24.745 | 15.58 | 36.109 | 24.601 | NA |
| TRINITY_DN11504_c0_g1_i2 | 0.77063007 | 0.399585016 | 0.999970678 | 3.134 | 7.717 | 2.278 | 3.579 | RabGAP-TBC |
| TRINITY_DN2024_c0_g1_i10 | 0.770598338 | 0.586179413 | 0.999970678 | 10.383 | 3.518 | 1.955 | 5.439 | NA |
| TRINITY_DN586_c0_g2_i1 | -0.77033574 | 0.61936868 | 1 | 78.767 | 21.41 | 46.286 | 101.06 | NA |
| TRINITY_DN5032_c0_g1_i4 | 0.770309754 | 0.587642858 | 0.999970678 | 21.329 | 14.13 | 15.444 | 2.051 | NA |
| TRINITY_DN194362_c0_g1_i1 | -0.77008263 | 0.857348606 | 0.999970678 | 1.59 | 0 | 2.278 | 0 | NA |
| TRINITY_DN13409_c0_g1_i2 | -0.76988321 | 0.864316339 | 1 | 0.254 | 0 | 0 | 0.442 | NA |
| TRINITY_DN12784_c0_g1_i5 | 0.769627976 | 0.65522231 | 0.999970678 | 8.602 | 8.505 | 7.4 | 0.875 | NA |
| TRINITY_DN7071_c0_g1_i1 | -0.76944707 | 0.676964449 | 0.999970678 | 1.508 | 1.577 | 4.189 | 0.251 | NA |
| TRINITY_DN31638_c0_g2_i4 | -0.7693729 | 0.27428771 | 0.999970678 | 26.162 | 32.01 | 31.943 | 59.548 | zf-FLZ |
| TRINITY_DN14029_c0_g1_i2 | 0.769339614 | 0.77524345 | 0.999970678 | 0.799 | 1.764 | 0 | 1.478 | - |
| TRINITY_DN9370_c0_g2_i1 | -0.76923051 | 0.7756888 | 0.999970678 | 0 | 2.385 | 1.011 | 2.775 | NA |
| TRINITY_DN15796_c2_g1_i4 | 0.769134213 | 0.519492807 | 0.999970678 | 0.972 | 0.867 | 0.556 | 0.402 | NA |
| TRINITY_DN1932_c0_g1_i3 | -0.76909187 | 0.428655602 | 0.999970678 | 14.398 | 35.86 | 24.054 | 55.466 | Ribosomal_S26e |
| TRINITY_DN22780_c0_g1_i1 | -0.76908532 | 0.532495742 | 0.999970678 | 0.872 | 0.532 | 0.5 | 1.8 | NA |
| TRINITY_DN1913_c0_g1_i8 | -0.7688837 | 0.446055305 | 0.999970678 | 1.617 | 0.917 | 2.433 | 1.387 | NA |
| TRINITY_DN544_c0_g2_i2 | -0.76883812 | 0.567955769 | 0.999970678 | 4.478 | 1.321 | 5.377 | 3.388 | NA |

| TRINITY_DN13234_c0_g1_i5 | 0.768785292 | 0.771570618 | 0.999970678 | 0.236 | 0.217 | 0 | 0.261 | NA |
| --- | --- | --- | --- | --- | --- | --- | --- | --- |
| TRINITY_DN7021_c0_g1_i1 | -0.76863939 | 0.565934055 | 0.999970678 | 0.391 | 1.478 | 1.733 | 0.995 | DUF3537 |
| TRINITY_DN1261_c0_g1_i6 | 0.768631155 | 0.557200651 | 0.999970678 | 4.769 | 0.798 | 1.611 | 1.357 | NA |
| TRINITY_DN3907_c1_g3_i2 | 0.768625183 | 0.64470329 | 1 | 0.609 | 0.936 | 0.1 | 0.784 | NA |
| TRINITY_DN1643_c0_g2_i2 | 0.768584581 | 0.531654299 | 0.999970678 | 1.299 | 1.981 | 1.267 | 0.382 | NA |
| TRINITY_DN2719_c0_g1_i1 | -0.76858383 | 0.339970599 | 0.999970678 | 0.99 | 1.478 | 1.989 | 1.79 | B_lectin |
| TRINITY_DN4442_c0_g2_i2 | 0.768573608 | 0.784492675 | 0.999970678 | 8.366 | 32.26 | 0 | 23.556 | NA |
| TRINITY_DN1644_c0_g1_i4 | 0.768551454 | 0.226810304 | 0.999970678 | 22.819 | 18.38 | 9.144 | 13.18 | PAP_fibrillin |
| TRINITY_DN222482_c0_g1_i1 | 0.768532039 | 0.515690017 | 0.999970678 | 1.208 | 0.601 | 0.511 | 0.422 | Glyco_transf_92 |
| TRINITY_DN2523_c1_g1_i6 | -0.76850042 | 0.857826451 | 0.999970678 | 3.888 | 0 | 5.533 | 0 | Aldo_ket_red |
| TRINITY_DN40571_c0_g1_i1 | -0.76848387 | 0.865400205 | 1 | 0.527 | 0 | 0.756 | 0 | NA |
| TRINITY_DN10998_c0_g1_i8 | -0.76848387 | 0.865400205 | 1 | 1.308 | 0 | 1.933 | 0 | NA |
| TRINITY_DN4781_c0_g1_i2 | 0.768424408 | 0.41065107 | 0.999970678 | 9.456 | 8.604 | 2.7 | 7.279 | CDI |
| TRINITY_DN14502_c0_g1_i1 | -0.76841875 | 0.571147374 | 0.999970678 | 5.623 | 27.93 | 9.711 | 44.478 | - |
| TRINITY_DN16160_c0_g1_i5 | 0.768378699 | 0.477645223 | 0.999970678 | 1.463 | 0.69 | 0.4 | 0.794 | Aminotran_5 |
| TRINITY_DN835_c3_g1_i5 | 0.768374545 | 0.544416768 | 0.999970678 | 1.308 | 2.434 | 1.511 | 0.362 | NA |
| TRINITY_DN7901_c0_g1_i1 | 0.768359013 | 0.514244488 | 0.999970678 | 18.159 | 18.46 | 12.31 | 5.791 | NA |
| TRINITY_DN412_c0_g1_i4 | -0.76828907 | 0.395714304 | 0.999970678 | 12.581 | 18.65 | 12.733 | 37.661 | RRM_1 |
| TRINITY_DN803_c0_g1_i6 | -0.76817692 | 0.435442225 | 0.999970678 | 2.416 | 1.666 | 3.722 | 2.443 | NA |
| TRINITY_DN5142_c0_g1_i1 | 0.768151801 | 0.497794005 | 0.999970678 | 2.089 | 2.119 | 1.522 | 0.623 | NA |
| TRINITY_DN1284_c0_g2_i5 | -0.7681446 | 0.580860534 | 0.999970678 | 1.499 | 7.076 | 6.377 | 6.284 | NA |
| TRINITY_DN228_c0_g1_i2 | -0.76809579 | 0.572705289 | 0.999970678 | 0.681 | 1.971 | 3.122 | 0.714 | UDPGT |
| TRINITY_DN10251_c0_g1_i4 | 0.768032697 | 0.783879281 | 0.999970678 | 0.309 | 1.725 | 0 | 1.186 | Dimerisation |
| TRINITY_DN1499_c0_g1_i9 | 0.767967702 | 0.787341228 | 1 | 1.317 | 0.434 | 0.856 | 0 | NA |
| TRINITY_DN19559_c0_g1_i4 | -0.76792193 | 0.855703607 | 0.999970678 | 5.968 | 0 | 8.511 | 0 | NA |
| TRINITY_DN11349_c0_g1_i1 | 0.767857031 | 0.275749698 | 0.999970678 | 2.643 | 2.72 | 1.411 | 1.448 | NA |
| TRINITY_DN872_c0_g1_i2 | 0.767784344 | 0.627150475 | 0.999970678 | 2.216 | 4.001 | 2.833 | 0.221 | NA |
| TRINITY_DN7913_c0_g1_i6 | 0.767774007 | 0.394588223 | 0.999970678 | 1.117 | 1.557 | 0.467 | 1.005 | CBM_20 |
| TRINITY_DN6017_c1_g2_i2 | 0.767526826 | 0.776646595 | 0.999970678 | 4.397 | 2.523 | 3.355 | 0 | NA |
| TRINITY_DN4376_c0_g1_i1 | -0.76743396 | 0.620808511 | 0.999970678 | 0.445 | 0.946 | 1.633 | 0.372 | NA |
| TRINITY_DN23912_c0_g1_i2 | 0.767339195 | 0.650103278 | 0.999970678 | 0.672 | 5.312 | 0.711 | 2.554 | NA |
| TRINITY_DN5520_c0_g1_i1 | -0.76733441 | 0.368989956 | 0.999970678 | 1.081 | 1.419 | 2.244 | 1.548 | ATP-synt_A |
| TRINITY_DN1008_c0_g1_i9 | -0.76732331 | 0.497821657 | 0.999970678 | 2.198 | 1.252 | 2.411 | 2.936 | NA |
| TRINITY_DN21737_c0_g1_i1 | -0.76718435 | 0.57984004 | 0.999970678 | 1.372 | 0.867 | 0.667 | 2.986 | NA |
| TRINITY_DN16228_c0_g1_i2 | -0.76718009 | 0.261815566 | 0.999970678 | 12.1 | 8.781 | 15.721 | 16.548 | NA |
| TRINITY_DN2212_c0_g1_i7 | 0.767141074 | 0.342698769 | 0.999970678 | 15.379 | 23.49 | 6.466 | 15.04 | NA |
| TRINITY_DN4432_c0_g1_i1 | 0.767125927 | 0.441751382 | 0.999970678 | 3.352 | 3.903 | 2.533 | 1.196 | PNRC |
| TRINITY_DN2527_c0_g1_i3 | 0.766979019 | 0.575094389 | 0.999970678 | 3.624 | 0.581 | 1.244 | 0.975 | BAAT_C |
| TRINITY_DN2820_c0_g1_i7 | -0.7669513 | 0.791071283 | 0.999970678 | 0 | 23.22 | 27.543 | 3.921 | NA |
| TRINITY_DN4792_c0_g1_i1 | -0.76693501 | 0.281165872 | 0.999970678 | 31.73 | 54.05 | 62.918 | 68.244 | NA |
| TRINITY_DN7188_c0_g1_i4 | 0.766932842 | 0.603551323 | 0.999970678 | 0.845 | 0.306 | 0.122 | 0.523 | NA |
| TRINITY_DN7841_c0_g2_i2 | 0.76693247 | 0.453530379 | 0.999970678 | 0.863 | 1.084 | 0.433 | 0.623 | DYW_deaminase |
| TRINITY_DN3227_c0_g1_i2 | 0.766923442 | 0.337223147 | 0.999970678 | 1.272 | 1.518 | 0.733 | 0.754 | Ank_2 |
| TRINITY_DN3671_c0_g1_i3 | 0.766909312 | 0.47607155 | 0.999970678 | 3.406 | 7.904 | 4.022 | 1.739 | Uso1_p115_C |
| TRINITY_DN713_c0_g1_i2 | 0.766907965 | 0.794765925 | 0.999970678 | 8.539 | 0 | 0.5 | 4.504 | NA |
| TRINITY_DN5597_c0_g1_i12 | -0.7668924 | 0.496765338 | 0.999970678 | 2.226 | 4.849 | 4.122 | 6.837 | NA |
| TRINITY_DN7881_c0_g1_i2 | 0.766881683 | 0.690282423 | 0.999970678 | 0.073 | 1.291 | 0.5 | 0.191 | NA |
| TRINITY_DN12988_c0_g1_i5 | -0.76677662 | 0.282901269 | 0.999970678 | 31.003 | 49.41 | 48.686 | 76.338 | PetM |
| TRINITY_DN9987_c0_g1_i7 | -0.7667722 | 0.519982116 | 0.999970678 | 48.981 | 177.4 | 97.361 | 229.023 | NA |
| TRINITY_DN80226_c0_g2_i3 | 0.766741203 | 0.71482845 | 0.999970678 | 2.653 | 1.843 | 0.011 | 2.624 | SRP40_C |
| TRINITY_DN8194_c0_g1_i2 | 0.76666077 | 0.650822075 | 0.999970678 | 1.472 | 0.345 | 0.722 | 0.201 | - |
| TRINITY_DN2730_c0_g1_i5 | 0.766591716 | 0.734336618 | 0.999970678 | 3.052 | 0.069 | 0.089 | 1.769 | DUF4413 |
| TRINITY_DN11044_c0_g1_i1 | -0.76639253 | 0.455973743 | 0.999970678 | 2.68 | 4.198 | 2.4 | 8.797 | NA |
| TRINITY_DN1567_c0_g1_i1 | -0.76626172 | 0.377181607 | 0.999970678 | 56.784 | 87.45 | 139.603 | 75.202 | Pterin_4a |
| TRINITY_DN2656_c0_g1_i6 | -0.76621831 | 0.482724709 | 0.999970678 | 2.017 | 2.158 | 4.455 | 1.689 | Amidohydro_1 |
| TRINITY_DN13_c0_g1_i1 | -0.76607514 | 0.649456534 | 0.999970678 | 5.132 | 0.838 | 0.8 | 9.33 | NA |
| TRINITY_DN12148_c1_g1_i1 | -0.76597307 | 0.631467973 | 0.999970678 | 0.727 | 0.65 | 0.189 | 2.131 | NA |
| TRINITY_DN3252_c0_g1_i5 | 0.765884318 | 0.862646622 | 1 | 1.726 | 0 | 0 | 1.056 | NA |
| TRINITY_DN31441_c0_g1_i1 | 0.765643829 | 0.681210902 | 1 | 1.481 | 0.404 | 0.833 | 0.111 | NA |
| TRINITY_DN16243_c0_g1_i2 | -0.76557815 | 0.862442884 | 0.999970678 | 0.763 | 0 | 1.111 | 0 | NA |
| TRINITY_DN4669_c0_g1_i4 | -0.76549284 | 0.675971108 | 0.999970678 | 0.745 | 0.108 | 0.978 | 0.292 | - |
| TRINITY_DN3646_c0_g1_i2 | -0.7654447 | 0.586112253 | 0.999970678 | 0.127 | 0.621 | 0.522 | 0.643 | Dimerisation |
| TRINITY_DN522_c0_g1_i4 | 0.765428433 | 0.644769102 | 0.999970678 | 52.569 | 10.18 | 28.665 | 2.604 | SPX |
| TRINITY_DN2481_c0_g1_i4 | 0.765385401 | 0.525576851 | 0.999970678 | 0.699 | 0.424 | 0.333 | 0.251 | NA |
| TRINITY_DN29541_c0_g2_i2 | -0.76535862 | 0.27698941 | 0.999970678 | 2.062 | 2.553 | 3.1 | 4.082 | NA |
| TRINITY_DN10505_c0_g1_i3 | 0.765279597 | 0.794427224 | 1 | 0.064 | 0.355 | 0.2 | 0 | RVP_2 |
| TRINITY_DN613_c0_g1_i7 | 0.765276708 | 0.415042729 | 0.999970678 | 3.997 | 7.579 | 2.289 | 3.981 | p450 |
| TRINITY_DN40532_c0_g1_i1 | -0.76523621 | 0.522468614 | 0.999970678 | 0.4 | 0.286 | 0.7 | 0.332 | NA |
| TRINITY_DN3265_c0_g1_i4 | 0.765205974 | 0.773230069 | 0.999970678 | 3.743 | 6.751 | 0 | 6.123 | NA |
| TRINITY_DN4941_c0_g1_i1 | -0.76520191 | 0.549780022 | 0.999970678 | 0.954 | 5.874 | 3.355 | 7.47 | CLTH |
| TRINITY_DN8201_c0_g1_i10 | -0.76519859 | 0.474101682 | 0.999970678 | 5.042 | 7.313 | 12.699 | 5.429 | NA |
| TRINITY_DN26799_c0_g2_i1 | 0.765158899 | 0.504477204 | 0.999970678 | 5.786 | 2.109 | 1.411 | 2.966 | - |
| TRINITY_DN10329_c1_g1_i1 | -0.76502071 | 0.695796491 | 0.999970678 | 0.236 | 1.281 | 0.178 | 2.312 | NA |
| TRINITY_DN650_c0_g1_i11 | 0.764910817 | 0.538438944 | 0.999970678 | 9.62 | 5.815 | 5.555 | 2.292 | NA |

| TRINITY_DN3691_c0_g1_i1 | -0.76482851 | 0.671936511 | 0.999970678 | 0.609 | 7.756 | 10.622 | 1.206 | Radical_SAM |
| --- | --- | --- | --- | --- | --- | --- | --- | --- |
| TRINITY_DN668_c1_g1_i2 | 0.764797759 | 0.8551326 | 0.999970678 | 0 | 19.15 | 0 | 10.969 | NA |
| TRINITY_DN3621_c0_g1_i2 | 0.764754446 | 0.557901864 | 0.999970678 | 26.734 | 21.37 | 6.211 | 18.65 | NA |
| TRINITY_DN3826_c0_g1_i8 | -0.76472453 | 0.543263744 | 0.999970678 | 0.818 | 2.306 | 3.078 | 1.599 | NA |
| TRINITY_DN67_c0_g1_i3 | -0.76466745 | 0.371615858 | 0.999970678 | 4.615 | 3.361 | 6.433 | 5.731 | Pet100 |
| TRINITY_DN70_c0_g1_i2 | -0.76458818 | 0.477657076 | 0.999970678 | 9.965 | 6.692 | 18.243 | 6.304 | UDPGT |
| TRINITY_DN1366_c0_g1_i3 | 0.7644543 | 0.440157415 | 0.999970678 | 3.743 | 11.42 | 3.611 | 4.484 | FH2 |
| TRINITY_DN3291_c0_g1_i2 | -0.76441976 | 0.785242813 | 0.999970678 | 15.697 | 0 | 7.966 | 17.443 | BES1_N |
| TRINITY_DN3475_c0_g1_i6 | 0.764230446 | 0.255404015 | 0.999970678 | 4.814 | 4.583 | 2.744 | 2.232 | Sec39 |
| TRINITY_DN564_c0_g1_i13 | -0.76418321 | 0.593757423 | 0.999970678 | 80.629 | 37.65 | 15.743 | 179.247 | NA |
| TRINITY_DN49_c0_g1_i3 | 0.7641044 | 0.788004076 | 0.999970678 | 7.44 | 15.04 | 0 | 13.241 | G-alpha |
| TRINITY_DN38295_c0_g1_i6 | -0.76403994 | 0.583134186 | 0.999970678 | 0.409 | 0.739 | 1.333 | 0.332 | NA |
| TRINITY_DN4788_c0_g1_i2 | -0.76396662 | 0.785120358 | 0.999970678 | 0 | 21.88 | 19.488 | 13.03 | NA |
| TRINITY_DN56791_c0_g1_i1 | -0.76396386 | 0.867359469 | 1 | 1.135 | 0 | 1.611 | 0 | ATP11 |
| TRINITY_DN24030_c0_g1_i2 | -0.76396386 | 0.867359469 | 1 | 0.472 | 0 | 0.656 | 0 | - |
| TRINITY_DN4820_c0_g1_i10 | -0.76396386 | 0.867359469 | 1 | 0.6 | 0 | 0.833 | 0 | DUF247 |
| TRINITY_DN971_c0_g1_i4 | 0.763779127 | 0.537977141 | 0.999970678 | 1.59 | 1.163 | 0.933 | 0.462 | NA |
| TRINITY_DN5562_c0_g1_i4 | 0.763619321 | 0.856079125 | 0.999970678 | 0 | 2.69 | 1.289 | 0 | DEAD |
| TRINITY_DN60919_c0_g2_i4 | 0.76360069 | 0.694081863 | 0.999970678 | 0.109 | 4.011 | 0.456 | 1.84 | NA |
| TRINITY_DN2512_c0_g1_i1 | -0.76355912 | 0.791226649 | 0.999970678 | 0 | 27.59 | 32.331 | 6.927 | Ribosomal_S9 |
| TRINITY_DN4584_c0_g1_i3 | 0.763539401 | 0.558463534 | 0.999970678 | 0.309 | 0.355 | 0.233 | 0.111 | NA |
| TRINITY_DN113_c0_g1_i10 | 0.763515718 | 0.856787937 | 0.999970678 | 2.389 | 0 | 1.178 | 0 | Pro_CA |
| TRINITY_DN5168_c0_g1_i3 | -0.76346869 | 0.560407971 | 0.999970678 | 0.282 | 1.183 | 1.489 | 0.684 | Bromo_TP |
| TRINITY_DN10208_c0_g1_i1 | 0.763381071 | 0.263953444 | 0.999970678 | 1.426 | 1.587 | 0.767 | 0.855 | NA |
| TRINITY_DN12882_c0_g1_i9 | -0.76332497 | 0.481018933 | 0.999970678 | 1.917 | 0.976 | 2.511 | 1.86 | NA |
| TRINITY_DN6442_c0_g1_i1 | -0.7632356 | 0.395993254 | 0.999970678 | 10.483 | 22.09 | 16.543 | 34.695 | NA |
| TRINITY_DN996_c0_g1_i2 | -0.76320393 | 0.450159177 | 0.999970678 | 0.845 | 0.512 | 0.9 | 1.227 | MatE |
| TRINITY_DN2573_c1_g1_i2 | 0.76320012 | 0.38719499 | 0.999970678 | 18.268 | 43.95 | 12.266 | 21.686 | NA |
| TRINITY_DN6288_c0_g1_i14 | -0.76319888 | 0.366848458 | 0.999970678 | 6.976 | 12.97 | 13.033 | 17.534 | NA |
| TRINITY_DN33730_c0_g1_i1 | 0.763176708 | 0.860086249 | 0.999970678 | 2.843 | 0 | 0 | 1.699 | NA |
| TRINITY_DN3623_c0_g1_i1 | 0.763173538 | 0.264304583 | 0.999970678 | 8.321 | 12.29 | 4.877 | 6.213 | NA |
| TRINITY_DN9412_c0_g1_i1 | -0.76298624 | 0.507578068 | 0.999970678 | 5.759 | 5.046 | 5.289 | 11.18 | NA |
| TRINITY_DN2325_c0_g1_i2 | 0.762949703 | 0.541537934 | 0.999970678 | 1.844 | 2.868 | 1.878 | 0.503 | NA |
| TRINITY_DN4743_c0_g1_i1 | 0.762949609 | 0.444459678 | 0.999970678 | 36.263 | 18.65 | 19.421 | 9.038 | DUF1298 |
| TRINITY_DN2590_c0_g1_i2 | 0.762673897 | 0.512484521 | 0.999970678 | 4.124 | 4.297 | 3.2 | 1.076 | DUF3223 |
| TRINITY_DN2990_c0_g1_i2 | 0.762601668 | 0.452933433 | 0.999970678 | 9.529 | 4.869 | 2.222 | 5.791 | NA |
| TRINITY_DN4990_c0_g1_i2 | -0.76253741 | 0.359339245 | 0.999970678 | 53.668 | 33.99 | 41.886 | 98.999 | NA |
| TRINITY_DN10176_c0_g1_i3 | 0.762521889 | 0.28643114 | 0.999970678 | 1.935 | 2.474 | 1.255 | 1.076 | NA |
| TRINITY_DN6595_c0_g1_i1 | -0.76250295 | 0.225989107 | 0.999970678 | 10.238 | 9.116 | 14.488 | 15.282 | RRM_1 |
| TRINITY_DN11990_c0_g1_i1 | -0.7624486 | 0.654289749 | 1 | 1.045 | 0.404 | 1.744 | 0.392 | NA |
| TRINITY_DN14998_c0_g1_i1 | 0.762311647 | 0.468661062 | 0.999970678 | 1.717 | 1.321 | 0.822 | 0.794 | NA |
| TRINITY_DN5345_c0_g1_i2 | -0.76230079 | 0.493157946 | 0.999970678 | 1.217 | 0.828 | 1.555 | 1.568 | IPP-2 |
| TRINITY_DN64028_c0_g1_i1 | -0.76225551 | 0.863665508 | 1 | 1.29 | 0 | 1.844 | 0 | NA |
| TRINITY_DN336_c0_g1_i22 | 0.762092783 | 0.688273525 | 0.999970678 | 1.826 | 1.035 | 1.344 | 0.07 | NA |
| TRINITY_DN703_c0_g1_i1 | 0.761729903 | 0.697531351 | 0.999970678 | 16.133 | 1.153 | 8.188 | 0.432 | NA |
| TRINITY_DN8800_c0_g2_i1 | 0.761691976 | 0.26351578 | 0.999970678 | 7.058 | 5.322 | 3.233 | 3.418 | HUN |
| TRINITY_DN1624_c0_g1_i11 | -0.76166453 | 0.3732559 | 0.999970678 | 3.17 | 6.692 | 7.633 | 7.389 | NA |
| TRINITY_DN8394_c0_g1_i1 | 0.761606282 | 0.395678069 | 0.999970678 | 0.59 | 0.621 | 0.389 | 0.261 | AAA |
| TRINITY_DN178_c0_g1_i8 | -0.76157403 | 0.556561386 | 0.999970678 | 69.519 | 14.06 | 88.972 | 35.54 | 2OG-FeII_Oxy |
| TRINITY_DN17143_c0_g1_i1 | 0.761559289 | 0.564710109 | 0.999970678 | 4.778 | 5.765 | 3.989 | 1.327 | NA |
| TRINITY_DN10485_c0_g1_i2 | 0.761526955 | 0.553225532 | 0.999970678 | 0.445 | 0.867 | 0.222 | 0.513 | NA |
| TRINITY_DN37895_c0_g1_i1 | -0.76137663 | 0.597195375 | 0.999970678 | 0.908 | 1.941 | 0.767 | 3.83 | AP2 |
| TRINITY_DN5877_c0_g1_i1 | -0.76121796 | 0.430423973 | 0.999970678 | 2.589 | 5.213 | 4.122 | 8.113 | NA |
| TRINITY_DN5381_c0_g1_i11 | -0.76121225 | 0.785059682 | 0.999970678 | 0.218 | 0.67 | 0 | 1.508 | ABC_membrane |
| TRINITY_DN10751_c0_g1_i1 | -0.76111702 | 0.524821671 | 0.999970678 | 1.862 | 2.622 | 1.222 | 6.103 | NA |
| TRINITY_DN7252_c0_g1_i7 | 0.761030522 | 0.601083181 | 0.999970678 | 0.554 | 0.522 | 0.456 | 0.08 | NA |
| TRINITY_DN782_c0_g1_i9 | 0.760836189 | 0.811053599 | 1 | 8.348 | 0 | 3.766 | 0.332 | NA |
| TRINITY_DN2390_c0_g1_i2 | -0.76078848 | 0.407561999 | 0.999970678 | 4.288 | 7.263 | 4.9 | 13.623 | NA |
| TRINITY_DN7300_c0_g1_i2 | 0.760776579 | 0.445788666 | 0.999970678 | 0.581 | 0.759 | 0.333 | 0.392 | NA |
| TRINITY_DN490_c0_g1_i11 | 0.760712201 | 0.66655477 | 0.999970678 | 3.815 | 3.548 | 3.5 | 0.111 | Asp_protease_2 |
| TRINITY_DN11104_c0_g1_i1 | 0.760599709 | 0.354070769 | 0.999970678 | 0.681 | 0.857 | 0.422 | 0.402 | U-box |
| TRINITY_DN197_c0_g1_i10 | -0.76059266 | 0.272818607 | 0.999970678 | 13.181 | 14.6 | 15.077 | 28.764 | NA |
| TRINITY_DN44013_c0_g1_i7 | 0.760560188 | 0.53103328 | 0.999970678 | 268.02 | 1624 | 428.585 | 500.312 | NA |
| TRINITY_DN5071_c0_g1_i1 | -0.76049111 | 0.599366731 | 0.999970678 | 0.627 | 0.424 | 1.2 | 0.362 | NA |
| TRINITY_DN804_c2_g1_i3 | -0.76034379 | 0.859915641 | 0.999970678 | 0 | 0.739 | 0 | 1.216 | NA |
| TRINITY_DN77929_c0_g2_i2 | -0.76028123 | 0.788001938 | 1 | 0.4 | 0 | 0.356 | 0.261 | DYW_deaminase |
| TRINITY_DN1325_c0_g1_i1 | 0.760269585 | 0.45283867 | 0.999970678 | 20.621 | 6.564 | 5.877 | 9.078 | CBS |
| TRINITY_DN12547_c1_g1_i2 | 0.760252846 | 0.63870666 | 0.999970678 | 2.271 | 3.045 | 2.444 | 0.181 | NA |
| TRINITY_DN4775_c0_g1_i5 | -0.76017366 | 0.4732496 | 0.999970678 | 1.78 | 4.08 | 4.777 | 3.991 | NA |
| TRINITY_DN536_c0_g1_i5 | 0.760102045 | 0.50028865 | 0.999970678 | 6.104 | 29.42 | 7.922 | 11.25 | NA |
| TRINITY_DN23313_c0_g1_i5 | -0.76008415 | 0.531304191 | 0.999970678 | 1.635 | 0.473 | 1.122 | 2.242 | PPR |
| TRINITY_DN2192_c0_g2_i1 | 0.760028394 | 0.657313363 | 0.999970678 | 6.84 | 0.907 | 0.556 | 3.921 | - |
| TRINITY_DN450_c0_g1_i3 | 0.759978466 | 0.857079681 | 0.999970678 | 2.135 | 0 | 0 | 1.287 | RCC1 |
| TRINITY_DN9033_c0_g1_i21 | -0.75989417 | 0.792302416 | 1 | 2.153 | 0 | 2.411 | 0.774 | NA |

| TRINITY_DN25110_c0_g1_i6 | -0.75984045 | 0.777896216 | 0.999970678 | 6.059 | 0 | 4.055 | 5.489 | NA |
| --- | --- | --- | --- | --- | --- | --- | --- | --- |
| TRINITY_DN11185_c2_g1_i1 | -0.75974998 | 0.817933807 | 0.999970678 | 1.481 | 0 | 2.078 | 0.03 | NA |
| TRINITY_DN37_c0_g1_i2 | -0.75966341 | 0.460432192 | 0.999970678 | 6.232 | 5.608 | 12.955 | 4.444 | NA |
| TRINITY_DN2445_c0_g1_i3 | 0.759452695 | 0.354297888 | 0.999970678 | 16.269 | 7.874 | 5.3 | 7.993 | Pkinase |
| TRINITY_DN8857_c0_g1_i1 | -0.75940104 | 0.8667787 | 1 | 0 | 0.197 | 0.278 | 0 | Myb_DNA-bind_ |
| TRINITY_DN1104_c0_g1_i4 | -0.75938558 | 0.450160848 | 0.999970678 | 0.681 | 1.124 | 1.611 | 1.096 | NA |
| TRINITY_DN34289_c0_g1_i1 | 0.75938 | 0.681518494 | 1 | 0.645 | 0.887 | 0.689 | 0.06 | NA |
| TRINITY_DN40097_c0_g1_i1 | 0.759162643 | 0.858827733 | 0.999970678 | 2.725 | 0 | 1.344 | 0 | Pyridoxal_deC |
| TRINITY_DN23465_c0_g1_i2 | -0.75912026 | 0.385084165 | 0.999970678 | 671.43 | 340.1 | 890.046 | 584.12 | NA |
| TRINITY_DN6013_c0_g1_i2 | 0.759081534 | 0.655980706 | 0.999970678 | 13.108 | 7.066 | 8.977 | 1.126 | ABC2_membrane |
| TRINITY_DN5719_c0_g1_i1 | -0.75901177 | 0.269559887 | 0.999970678 | 11.055 | 12.76 | 20.088 | 15.975 | SWIB |
| TRINITY_DN3770_c0_g1_i2 | 0.75896722 | 0.530288943 | 0.999970678 | 15.116 | 7.697 | 3.022 | 9.491 | NA |
| TRINITY_DN743_c0_g1_i10 | -0.75894823 | 0.597953705 | 0.999970678 | 2.989 | 10.13 | 2.167 | 19.454 | NA |
| TRINITY_DN8038_c0_g1_i1 | -0.7588658 | 0.449970426 | 0.999970678 | 1.29 | 2.089 | 3.333 | 1.659 | NA |
| TRINITY_DN651_c0_g1_i12 | -0.75886199 | 0.645078645 | 0.999970678 | 6.204 | 0.739 | 8.244 | 1.89 | NAD-GH |
| TRINITY_DN8789_c0_g1_i1 | -0.75876856 | 0.383445869 | 0.999970678 | 1.826 | 1.36 | 2.111 | 2.855 | - |
| TRINITY_DN8789_c0_g1_i2 | -0.75876856 | 0.383445869 | 0.999970678 | 1.826 | 1.36 | 2.111 | 2.855 | - |
| TRINITY_DN3124_c0_g1_i1 | 0.758734469 | 0.522489495 | 0.999970678 | 3.152 | 0.956 | 1.4 | 0.764 | DEAD |
| TRINITY_DN3249_c0_g1_i3 | -0.7586526 | 0.347842162 | 0.999970678 | 5.732 | 8.466 | 11.966 | 9.37 | NA |
| TRINITY_DN396_c0_g1_i4 | -0.75861855 | 0.751563683 | 0.999970678 | 3.97 | 0.473 | 6.055 | 0.161 | NA |
| TRINITY_DN182_c1_g1_i4 | 0.758571367 | 0.795835089 | 0.999970678 | 2.68 | 0 | 0.289 | 1.277 | NA |
| TRINITY_DN8658_c0_g1_i2 | 0.758539314 | 0.440285066 | 0.999970678 | 8.185 | 2.809 | 3.055 | 2.865 | SAM_decarbox |
| TRINITY_DN439_c0_g1_i6 | -0.7585374 | 0.784207652 | 0.999970678 | 16.633 | 0 | 5.033 | 22.048 | NA |
| TRINITY_DN1830_c0_g1_i2 | 0.758506617 | 0.79128464 | 0.999970678 | 0 | 16.81 | 5.144 | 3.549 | NA |
| TRINITY_DN1462_c0_g1_i9 | 0.758478781 | 0.499911931 | 0.999970678 | 2.834 | 1.744 | 0.656 | 1.9 | NA |
| TRINITY_DN1794_c0_g1_i9 | 0.75847 | 0.717854084 | 0.999970678 | 0.291 | 10.27 | 4.711 | 0.442 | NA |
| TRINITY_DN21397_c0_g1_i10 | -0.75844702 | 0.859049502 | 0.999970678 | 3.461 | 0 | 4.866 | 0 | NA |
| TRINITY_DN8443_c0_g1_i7 | 0.758323403 | 0.786024494 | 1 | 0.273 | 0.621 | 0 | 0.513 | NA |
| TRINITY_DN742_c0_g1_i1 | 0.758227839 | 0.28306078 | 0.999970678 | 12.245 | 11.58 | 7.077 | 5.54 | Pyr_redox_2 |
| TRINITY_DN9072_c0_g1_i5 | 0.758152037 | 0.430098707 | 0.999970678 | 4.478 | 4.524 | 1.2 | 3.881 | NA |
| TRINITY_DN8999_c0_g2_i1 | 0.75806216 | 0.685305036 | 0.999970678 | 0.808 | 0.237 | 0.478 | 0.05 | NA |
| TRINITY_DN3228_c0_g1_i19 | -0.75805257 | 0.519961048 | 0.999970678 | 0.799 | 2.208 | 3.078 | 1.317 | Serpin |
| TRINITY_DN2044_c1_g1_i3 | -0.75791895 | 0.33552128 | 0.999970678 | 28.26 | 20.55 | 35.42 | 36.736 | NA |
| TRINITY_DN3858_c0_g1_i7 | 0.757830776 | 0.333947935 | 0.999970678 | 122.24 | 101.3 | 36.564 | 88.633 | - |
| TRINITY_DN164964_c0_g1_i1 | -0.7577307 | 0.673601739 | 0.999970678 | 1.045 | 12.69 | 1.933 | 20.429 | DUF617 |
| TRINITY_DN8595_c0_g1_i1 | -0.75769959 | 0.233286133 | 0.999970678 | 8.321 | 9.5 | 12.833 | 14.497 | PCMT |
| TRINITY_DN5717_c0_g2_i2 | -0.75769875 | 0.669937075 | 0.999970678 | 20.693 | 24.42 | 1.5 | 73.694 | NA |
| TRINITY_DN415_c0_g1_i13 | -0.75767303 | 0.45367439 | 0.999970678 | 2.462 | 4.06 | 2.889 | 7.42 | NA |
| TRINITY_DN1389_c0_g1_i1 | 0.757597238 | 0.586598294 | 0.999970678 | 1.617 | 5.016 | 2.667 | 0.664 | Inhibitor_I29 |
| TRINITY_DN3372_c0_g1_i3 | 0.757585696 | 0.503588734 | 0.999970678 | 0.454 | 0.798 | 0.256 | 0.432 | PI3_PI4_kinase |
| TRINITY_DN4891_c0_g1_i4 | 0.757533847 | 0.795359943 | 0.999970678 | 10.892 | 0 | 4.522 | 1.066 | NA |
| TRINITY_DN1234_c0_g2_i2 | 0.757475869 | 0.281130026 | 0.999970678 | 4.315 | 4.326 | 2.433 | 2.182 | NA |
| TRINITY_DN6919_c0_g1_i4 | 0.75742664 | 0.425190384 | 0.999970678 | 15.924 | 6.199 | 6.255 | 5.6 | Pkinase |
| TRINITY_DN23_c0_g3_i2 | -0.75725766 | 0.40289136 | 0.999970678 | 2.48 | 4.346 | 3.178 | 7.661 | RVT_1 |
| TRINITY_DN5889_c0_g1_i2 | -0.75722913 | 0.50083989 | 0.999970678 | 0.899 | 0.552 | 1.455 | 0.704 | NA |
| TRINITY_DN573_c0_g1_i7 | -0.75722884 | 0.471951183 | 0.999970678 | 161.89 | 124.2 | 85.095 | 356.987 | NA |
| TRINITY_DN7019_c0_g1_i5 | 0.75719945 | 0.788609076 | 0.999970678 | 4.097 | 0.591 | 2.333 | 0 | PPR |
| TRINITY_DN17116_c0_g1_i1 | -0.75711748 | 0.458740165 | 0.999970678 | 2.008 | 3.164 | 5.366 | 2.242 | NA |
| TRINITY_DN5894_c0_g1_i3 | -0.75707322 | 0.560632529 | 0.999970678 | 1.063 | 1.37 | 2.889 | 0.593 | NA |
| TRINITY_DN3102_c1_g1_i1 | 0.757070951 | 0.419888629 | 0.999970678 | 9.602 | 4.74 | 2.567 | 5.419 | zf-RING_2 |
| TRINITY_DN4426_c0_g1_i5 | 0.757039537 | 0.568724274 | 0.999970678 | 0.818 | 4.011 | 0.967 | 1.629 | DUF247 |
| TRINITY_DN928_c0_g1_i7 | -0.75700432 | 0.812514029 | 1 | 2.534 | 0.237 | 3.866 | 0 | NA |
| TRINITY_DN2023_c0_g2_i1 | 0.756949028 | 0.80212148 | 0.999970678 | 16.587 | 1.281 | 8.855 | 0 | NA |
| TRINITY_DN8385_c0_g1_i1 | 0.756932578 | 0.42614669 | 0.999970678 | 3.098 | 6.386 | 1.889 | 3.298 | RNA_pol_Rpc4 |
| TRINITY_DN3819_c0_g1_i5 | -0.7568994 | 0.525036595 | 0.999970678 | 0.618 | 0.404 | 0.878 | 0.623 | Cellulose_synt |
| TRINITY_DN5845_c0_g1_i2 | 0.75679158 | 0.585480788 | 0.999970678 | 0.554 | 1.764 | 0.822 | 0.382 | Cellulase |
| TRINITY_DN274_c0_g1_i4 | -0.75677369 | 0.78086276 | 0.999970678 | 0 | 0.769 | 0.4 | 0.774 | RRM_1 |
| TRINITY_DN3322_c0_g1_i2 | -0.75677128 | 0.634717569 | 1 | 0.218 | 0.857 | 0.4 | 1.337 | NA |
| TRINITY_DN2104_c1_g1_i2 | -0.75668382 | 0.553159812 | 0.999970678 | 0.672 | 0.956 | 0.644 | 1.92 | NA |
| TRINITY_DN17518_c0_g1_i1 | 0.756651745 | 0.805696511 | 0.999970678 | 1.581 | 0 | 0.067 | 0.875 | NA |
| TRINITY_DN1795_c0_g1_i13 | 0.756255246 | 0.859187401 | 0.999970678 | 1.444 | 0 | 0 | 0.865 | NA |
| TRINITY_DN7691_c0_g2_i2 | -0.75613283 | 0.561467732 | 0.999970678 | 1.989 | 0.552 | 1.3 | 2.735 | NA |
| TRINITY_DN11129_c0_g1_i1 | 0.756041903 | 0.329605876 | 0.999970678 | 20.366 | 14.72 | 6.555 | 12.849 | NA |
| TRINITY_DN8606_c2_g1_i7 | 0.755872776 | 0.786203807 | 0.999970678 | 2.235 | 0.739 | 1.455 | 0 | NA |
| TRINITY_DN195607_c0_g1_i1 | -0.75585711 | 0.861652814 | 0.999970678 | 0.818 | 0 | 1.167 | 0 | ABC_tran |
| TRINITY_DN9676_c0_g2_i3 | -0.75572093 | 0.585098759 | 0.999970678 | 0.209 | 0.788 | 0.856 | 0.623 | NA |
| TRINITY_DN5756_c0_g1_i1 | -0.75567192 | 0.236334147 | 0.999970678 | 21.393 | 30.54 | 33.354 | 47.252 | Ribosomal_L5 |
| TRINITY_DN73_c0_g1_i2 | -0.75564526 | 0.514193803 | 0.999970678 | 5.223 | 1.557 | 5.089 | 5.328 | Thioredoxin |
| TRINITY_DN10657_c0_g1_i5 | 0.755599222 | 0.652076877 | 0.999970678 | 0.536 | 6.051 | 0.511 | 3.237 | Mem_trans |
| TRINITY_DN1152_c0_g1_i1 | 0.755567423 | 0.861128249 | 0.999970678 | 0 | 19.48 | 0 | 11.411 | CBS |
| TRINITY_DN5349_c0_g1_i4 | 0.755488022 | 0.520037185 | 0.999970678 | 0.672 | 1.922 | 0.889 | 0.462 | NA |
| TRINITY_DN1439_c0_g1_i4 | 0.75538226 | 0.658000477 | 0.999970678 | 1.408 | 12.98 | 6.133 | 0.975 | - |
| TRINITY_DN7328_c0_g1_i3 | -0.75526477 | 0.786814085 | 0.999970678 | 0 | 1.616 | 0.467 | 2.111 | Sec3-PIP2_bind |
| TRINITY_DN1242_c0_g2_i15 | -0.75516681 | 0.863545428 | 0.999970678 | 0.863 | 0 | 1.244 | 0 | NA |

| TRINITY_DN5927_c0_g1_i1 | 0.755008577 | 0.559868226 | 0.999970678 | 220.81 | 61.02 | 112.949 | 31.669 | NA |
| --- | --- | --- | --- | --- | --- | --- | --- | --- |
| TRINITY_DN26306_c0_g1_i2 | 0.754891122 | 0.594890566 | 0.999970678 | 0.291 | 0.798 | 0.378 | 0.181 | NA |
| TRINITY_DN86257_c0_g2_i5 | -0.75487285 | 0.781994417 | 0.999970678 | 2.752 | 0 | 2.633 | 1.528 | NA |
| TRINITY_DN1924_c0_g1_i6 | -0.75486634 | 0.591991998 | 0.999970678 | 8.366 | 1.695 | 2.633 | 13.985 | HSP70 |
| TRINITY_DN8164_c0_g1_i1 | -0.75484968 | 0.526761544 | 0.999970678 | 3.888 | 0.867 | 3.022 | 4.434 | NA |
| TRINITY_DN1845_c2_g1_i1 | -0.75477287 | 0.469398787 | 0.999970678 | 8.693 | 4.11 | 5.589 | 14.789 | NA |
| TRINITY_DN9236_c0_g1_i1 | 0.754763841 | 0.862087633 | 0.999970678 | 0 | 2.789 | 1.333 | 0 | RNase_P_Rpp14 |
| TRINITY_DN1207_c0_g1_i13 | -0.75475406 | 0.603377011 | 0.999970678 | 5.541 | 0.641 | 3.778 | 5.922 | NA |
| TRINITY_DN3328_c0_g1_i4 | 0.754736717 | 0.53770717 | 0.999970678 | 1.372 | 4.583 | 0.944 | 2.353 | Cellulose_synt |
| TRINITY_DN3254_c0_g1_i2 | -0.7545317 | 0.657772655 | 0.999970678 | 23.609 | 1.656 | 5.589 | 36.324 | - |
| TRINITY_DN25861_c0_g1_i1 | -0.75452195 | 0.742815629 | 1 | 9.911 | 0.394 | 1.655 | 14.487 | NA |
| TRINITY_DN186_c6_g1_i2 | 0.754343183 | 0.65453432 | 0.999970678 | 134.93 | 3126 | 378.766 | 1343.536 | NA |
| TRINITY_DN1568_c0_g1_i9 | 0.754288502 | 0.336645707 | 0.999970678 | 2.843 | 4.12 | 1.955 | 1.749 | Glyco_hydro_1 |
| TRINITY_DN2196_c0_g1_i4 | 0.754267573 | 0.265151709 | 0.999970678 | 16.66 | 18.98 | 10.299 | 8.646 | PPR |
| TRINITY_DN2935_c0_g1_i3 | -0.75421604 | 0.442347281 | 0.999970678 | 3.406 | 1.863 | 3.478 | 4.695 | NA |
| TRINITY_DN5651_c4_g1_i1 | 0.754184647 | 0.443970894 | 0.999970678 | 231.58 | 264.7 | 56.485 | 223.755 | NA |
| TRINITY_DN11591_c0_g1_i1 | 0.754170268 | 0.623756791 | 0.999970678 | 108.01 | 17.9 | 32.687 | 31.156 | NA |
| TRINITY_DN1212_c0_g1_i5 | 0.754161395 | 0.381882458 | 0.999970678 | 72.88 | 37.67 | 34.787 | 23.948 | NA |
| TRINITY_DN32407_c0_g1_i1 | -0.75414174 | 0.580737492 | 0.999970678 | 0.563 | 0.483 | 1.211 | 0.282 | NA |
| TRINITY_DN13040_c0_g3_i3 | -0.75399653 | 0.861049446 | 0.999970678 | 1.844 | 0 | 2.611 | 0 | - |
| TRINITY_DN2370_c0_g1_i19 | 0.753915843 | 0.596806169 | 0.999970678 | 1.472 | 1.281 | 1.055 | 0.352 | NA |
| TRINITY_DN296_c0_g1_i9 | -0.75390274 | 0.429883594 | 0.999970678 | 0.636 | 0.69 | 0.567 | 1.568 | PPR |
| TRINITY_DN11774_c0_g1_i3 | -0.75387911 | 0.37517237 | 0.999970678 | 2.407 | 4.287 | 3.889 | 6.535 | PrmA |
| TRINITY_DN5349_c0_g1_i5 | -0.75386413 | 0.500199871 | 0.999970678 | 1.463 | 4.662 | 5.955 | 3.056 | NA |
| TRINITY_DN4921_c0_g1_i4 | 0.753818336 | 0.819976657 | 0.999970678 | 2.434 | 0 | 1.189 | 0.04 | NA |
| TRINITY_DN7518_c0_g1_i1 | -0.75380075 | 0.623896771 | 0.999970678 | 1.708 | 0.345 | 1.733 | 1.387 | - |
| TRINITY_DN5304_c0_g2_i2 | 0.75376904 | 0.789254641 | 0.999970678 | 22.773 | 0 | 3.311 | 9.541 | NA |
| TRINITY_DN5740_c0_g2_i2 | -0.7536972 | 0.560565166 | 0.999970678 | 20.094 | 44.85 | 12.544 | 93.499 | NA |
| TRINITY_DN13664_c0_g1_i1 | 0.753599341 | 0.86685873 | 1 | 0 | 1.873 | 0 | 1.066 | NA |
| TRINITY_DN18217_c0_g2_i1 | 0.753557114 | 0.42732506 | 0.999970678 | 5.132 | 2.346 | 2.211 | 1.79 | NA |
| TRINITY_DN3607_c0_g1_i4 | -0.7535306 | 0.237678924 | 0.999970678 | 9.166 | 7.658 | 10.677 | 15.483 | Fer4 |
| TRINITY_DN5963_c0_g1_i5 | 0.753483732 | 0.482534528 | 0.999970678 | 3.089 | 1.183 | 1.111 | 1.196 | NA |
| TRINITY_DN16569_c0_g1_i4 | -0.75341035 | 0.812547498 | 1 | 0.672 | 0.049 | 1.044 | 0 | NA |
| TRINITY_DN34113_c0_g1_i1 | -0.75339504 | 0.424944036 | 0.999970678 | 45.529 | 20.13 | 60.519 | 38.053 | DLH |
| TRINITY_DN9849_c0_g1_i2 | 0.753357346 | 0.861895982 | 0.999970678 | 1.635 | 0 | 0.811 | 0 | NA |
| TRINITY_DN17012_c0_g2_i7 | 0.753354473 | 0.601995053 | 0.999970678 | 2.071 | 0.473 | 0.922 | 0.382 | DnaJ |
| TRINITY_DN2104_c1_g1_i1 | 0.753190793 | 0.655864473 | 1 | 0.2 | 0.7 | 0.367 | 0.09 | NA |
| TRINITY_DN8974_c0_g2_i2 | -0.75317523 | 0.784995707 | 0.999970678 | 5.677 | 0 | 1.989 | 7.309 | TP6A_N |
| TRINITY_DN1484_c0_g1_i1 | 0.753157302 | 0.342846115 | 0.999970678 | 4.024 | 3.765 | 1.422 | 2.906 | WRKY |
| TRINITY_DN6387_c0_g1_i16 | -0.75313346 | 0.792711869 | 0.999970678 | 0.282 | 1.055 | 1.833 | 0 | G_glu_transpept |
| TRINITY_DN8329_c0_g1_i4 | 0.753076177 | 0.457093549 | 0.999970678 | 1.099 | 0.552 | 0.311 | 0.613 | MCM3AP_GANP |
| TRINITY_DN2617_c0_g1_i1 | -0.75294997 | 0.489807677 | 0.999970678 | 6.531 | 1.961 | 5.833 | 7.329 | CarboxypepD_re |
| TRINITY_DN346_c0_g2_i1 | -0.75285638 | 0.405753597 | 0.999970678 | 30.577 | 33.93 | 25.443 | 75.926 | NA |
| TRINITY_DN28380_c0_g1_i3 | 0.752848994 | 0.862167092 | 0.999970678 | 1.726 | 0 | 0.856 | 0 | DUF4378 |
| TRINITY_DN4077_c0_g1_i1 | 0.752848994 | 0.862167092 | 0.999970678 | 1.317 | 0 | 0.656 | 0 | NA |
| TRINITY_DN2531_c0_g1_i2 | 0.752832094 | 0.80279926 | 0.999970678 | 0 | 72.98 | 23.932 | 13.713 | - |
| TRINITY_DN7714_c1_g1_i2 | -0.75281525 | 0.790129937 | 1 | 1.644 | 0 | 1.589 | 0.824 | NB-ARC |
| TRINITY_DN10200_c0_g1_i4 | -0.75277133 | 0.422620223 | 0.999970678 | 1.235 | 0.926 | 1.655 | 1.649 | NA |
| TRINITY_DN11045_c0_g1_i3 | -0.75276045 | 0.511163239 | 0.999970678 | 2.017 | 3.469 | 5.689 | 2.262 | DUF599 |
| TRINITY_DN33838_c0_g3_i2 | 0.752730783 | 0.678104957 | 0.999970678 | 0.654 | 2.109 | 0.1 | 1.508 | NA |
| TRINITY_DN812_c0_g1_i1 | -0.75267056 | 0.468674612 | 0.999970678 | 2.335 | 1.419 | 2.355 | 3.489 | NA |
| TRINITY_DN6603_c0_g1_i1 | -0.75265709 | 0.486553213 | 0.999970678 | 12.418 | 15.83 | 8.355 | 36.977 | NA |
| TRINITY_DN171_c0_g1_i9 | -0.75262033 | 0.403568822 | 0.999970678 | 2.68 | 4.859 | 3.622 | 8.294 | NA |
| TRINITY_DN30_c3_g1_i2 | -0.75259476 | 0.812692275 | 0.999970678 | 9.102 | 0 | 12.455 | 0.251 | NA |
| TRINITY_DN37539_c0_g1_i1 | -0.75256109 | 0.869521152 | 1 | 0 | 0.463 | 0.622 | 0 | Glyco_hydro_100 |
| TRINITY_DN4913_c0_g1_i2 | -0.75256109 | 0.869521152 | 1 | 0 | 0.552 | 0.778 | 0 | - |
| TRINITY_DN2116_c0_g1_i1 | -0.75250871 | 0.362631176 | 0.999970678 | 7.621 | 6.495 | 13.266 | 7.802 | Glyco_tran_28_C |
| TRINITY_DN5789_c0_g1_i3 | -0.75248008 | 0.337364213 | 0.999970678 | 4.533 | 2.868 | 4.478 | 7.098 | NA |
| TRINITY_DN2996_c0_g2_i1 | 0.752429467 | 0.545079889 | 0.999970678 | 0.908 | 1.823 | 0.889 | 0.533 | NA |
| TRINITY_DN164446_c0_g1_i1 | -0.75232797 | 0.660486988 | 0.999970678 | 149.14 | 11.55 | 28.187 | 233.849 | NA |
| TRINITY_DN967_c0_g1_i7 | -0.75219837 | 0.360148169 | 0.999970678 | 9.484 | 11.05 | 9.177 | 23.475 | AAA |
| TRINITY_DN284_c0_g1_i14 | 0.752031653 | 0.613838882 | 0.999970678 | 1.508 | 0.237 | 0.578 | 0.332 | NA |
| TRINITY_DN5117_c0_g1_i1 | 0.75197768 | 0.287584975 | 0.999970678 | 6.786 | 4.602 | 3.078 | 3.086 | DEAD |
| TRINITY_DN3098_c0_g1_i3 | -0.75194292 | 0.859838672 | 0.999970678 | 3.079 | 0 | 0 | 5.228 | NA |
| TRINITY_DN74442_c0_g1_i4 | 0.751904951 | 0.464710199 | 0.999970678 | 2.743 | 2.326 | 0.656 | 2.222 | NA |
| TRINITY_DN7528_c0_g1_i2 | -0.7518976 | 0.539368479 | 0.999970678 | 0.382 | 0.828 | 0.822 | 1.066 | NA |
| TRINITY_DN4537_c0_g2_i1 | 0.751894724 | 0.554357359 | 0.999970678 | 9.52 | 2.661 | 4.711 | 1.609 | NA |
| TRINITY_DN23225_c0_g1_i1 | -0.75186039 | 0.568930932 | 1 | 0.581 | 1.232 | 1.5 | 1.206 | NA |
| TRINITY_DN127_c1_g1_i6 | -0.7516821 | 0.858451854 | 0.999970678 | 0 | 208.7 | 0 | 312.951 | NA |
| TRINITY_DN27249_c0_g1_i1 | 0.751586541 | 0.483900541 | 0.999970678 | 0.699 | 0.483 | 0.222 | 0.432 | C2 |
| TRINITY_DN15342_c0_g2_i1 | 0.751570776 | 0.311687307 | 0.999970678 | 1.535 | 1.597 | 0.967 | 0.694 | Beach |
| TRINITY_DN575_c0_g1_i11 | -0.75150282 | 0.858834366 | 0.999970678 | 29.323 | 0 | 40.486 | 0 | GST_C |
| TRINITY_DN22782_c0_g3_i2 | -0.7514879 | 0.861756788 | 0.999970678 | 7.085 | 0.069 | 9.899 | 0 | NA |
| TRINITY_DN12802_c0_g1_i3 | -0.75147758 | 0.717544681 | 0.999970678 | 0.1 | 5.135 | 0.678 | 7.892 | ABC2_membrane |

| TRINITY_DN394_c0_g1_i5 | -0.75147654 | 0.424373386 | 0.999970678 | 8.748 | 3.38 | 7.966 | 10.918 | Polysacc_synt_4 |
| --- | --- | --- | --- | --- | --- | --- | --- | --- |
| TRINITY_DN3573_c0_g1_i2 | -0.75143746 | 0.781924646 | 0.999970678 | 4.669 | 0 | 3.966 | 3.167 | Gar1 |
| TRINITY_DN9549_c0_g1_i1 | -0.75138331 | 0.481475432 | 0.999970678 | 4.905 | 10.24 | 5.433 | 18.73 | - |
| TRINITY_DN1159_c1_g1_i2 | 0.751362803 | 0.472163207 | 0.999970678 | 3.17 | 2.139 | 1.867 | 0.905 | Pkinase |
| TRINITY_DN11739_c0_g1_i3 | -0.75128164 | 0.631250523 | 0.999970678 | 28.878 | 2.74 | 19.965 | 27.095 | NA |
| TRINITY_DN7673_c0_g1_i2 | -0.75125466 | 0.476489756 | 0.999970678 | 1.172 | 0.986 | 2.011 | 1.196 | mTERF |
| TRINITY_DN21488_c0_g1_i1 | 0.75117981 | 0.799413796 | 0.999970678 | 20.03 | 0 | 7.622 | 2.503 | NA |
| TRINITY_DN264_c0_g1_i10 | -0.7509446 | 0.521233207 | 0.999970678 | 2.071 | 4.376 | 2.433 | 7.802 | NA |
| TRINITY_DN8680_c0_g1_i1 | -0.75081967 | 0.452981867 | 0.999970678 | 17.278 | 27.4 | 29.954 | 34.645 | Histone |
| TRINITY_DN16778_c0_g1_i12 | 0.750701126 | 0.432294527 | 0.999970678 | 2.198 | 1.183 | 0.922 | 0.895 | DLD |
| TRINITY_DN3514_c0_g1_i1 | 0.750667656 | 0.540154627 | 0.999970678 | 125.76 | 21.32 | 41.364 | 38.375 | NA |
| TRINITY_DN1518_c0_g3_i2 | -0.75066023 | 0.38281773 | 0.999970678 | 15.397 | 9.461 | 23.043 | 14.125 | DYW_deaminase |
| TRINITY_DN3819_c0_g2_i8 | -0.75054195 | 0.793183538 | 0.999970678 | 0 | 1.025 | 0.2 | 1.458 | Cellulose_synt |
| TRINITY_DN1071_c0_g1_i6 | 0.750397009 | 0.484659106 | 0.999970678 | 3.061 | 11.2 | 3.078 | 4.685 | NA |
| TRINITY_DN2339_c0_g1_i1 | 0.750277918 | 0.457529353 | 0.999970678 | 1.535 | 4.543 | 1.455 | 1.83 | NA |
| TRINITY_DN2279_c0_g1_i1 | 0.750134999 | 0.472384265 | 0.999970678 | 38.707 | 24.48 | 7.177 | 29.045 | Cyclase |
| TRINITY_DN14600_c0_g1_i1 | -0.75010512 | 0.695462454 | 0.999970678 | 0.055 | 1.38 | 1.255 | 0.834 | NA |
| TRINITY_DN27892_c0_g1_i2 | 0.74999395 | 0.585684021 | 0.999970678 | 0.345 | 1.183 | 0.322 | 0.523 | NA |
| TRINITY_DN1209_c0_g1_i6 | 0.749949445 | 0.705780631 | 1 | 0.472 | 5.578 | 0.489 | 2.926 | NA |
| TRINITY_DN1459_c0_g1_i1 | -0.74994846 | 0.547787124 | 0.999970678 | 18.368 | 3.075 | 18.043 | 14.688 | NA |
| TRINITY_DN5455_c0_g1_i1 | 0.749857225 | 0.572135026 | 1 | 0.927 | 1.045 | 0.311 | 0.754 | NA |
| TRINITY_DN11030_c0_g1_i3 | -0.74971825 | 0.822196514 | 1 | 1.099 | 0.049 | 1.589 | 0 | - |
| TRINITY_DN6111_c0_g1_i2 | -0.74961011 | 0.808200188 | 0.999970678 | 0 | 3.42 | 4.5 | 0.161 | - |
| TRINITY_DN1718_c0_g1_i1 | -0.74957828 | 0.859441146 | 0.999970678 | 2.207 | 0 | 0 | 3.75 | NA |
| TRINITY_DN640_c0_g1_i10 | 0.749573251 | 0.789520636 | 0.999970678 | 0 | 5.43 | 1.044 | 1.92 | NA |
| TRINITY_DN17062_c0_g1_i5 | 0.749555839 | 0.800093344 | 1 | 14.634 | 2.533 | 8.366 | 0 | GTP_EFTU |
| TRINITY_DN93_c0_g2_i1 | -0.74952745 | 0.388816153 | 0.999970678 | 0.999 | 1.597 | 2.355 | 1.518 | NA |
| TRINITY_DN5045_c0_g1_i3 | -0.74946751 | 0.860794926 | 0.999970678 | 4.86 | 0.039 | 6.888 | 0 | NA |
| TRINITY_DN490_c2_g1_i8 | 0.749327641 | 0.534069999 | 0.999970678 | 1.562 | 4.425 | 2.133 | 0.975 | NA |
| TRINITY_DN335_c1_g1_i6 | 0.749221823 | 0.50215306 | 0.999970678 | 6.568 | 6.1 | 4.933 | 1.588 | LRRNT_2 |
| TRINITY_DN16223_c0_g1_i1 | 0.749218244 | 0.820505675 | 0.999970678 | 1.135 | 0.02 | 0.578 | 0 | EFG_C |
| TRINITY_DN545_c0_g2_i6 | -0.74919595 | 0.798757797 | 0.999970678 | 0 | 1.774 | 2.289 | 0.161 | NA |
| TRINITY_DN2380_c0_g1_i2 | 0.749134418 | 0.572577927 | 0.999970678 | 1.217 | 0.926 | 0.767 | 0.352 | NA |
| TRINITY_DN4686_c0_g3_i5 | 0.749116228 | 0.680694516 | 0.999970678 | 0.872 | 16.21 | 5.955 | 2.694 | NA |
| TRINITY_DN10863_c0_g1_i3 | 0.749107918 | 0.575921965 | 0.999970678 | 6.168 | 4.11 | 4.278 | 0.935 | Oxysterol_BP |
| TRINITY_DN7693_c0_g1_i5 | -0.74909041 | 0.752451741 | 0.999970678 | 1.908 | 0.059 | 2.733 | 0.08 | NA |
| TRINITY_DN5084_c0_g1_i3 | 0.749038792 | 0.434634674 | 0.999970678 | 14.316 | 9.323 | 5.533 | 7.048 | NA |
| TRINITY_DN7188_c0_g1_i2 | -0.74888574 | 0.535197442 | 0.999970678 | 0.273 | 0.897 | 0.867 | 0.925 | NA |
| TRINITY_DN8741_c0_g1_i1 | 0.748880216 | 0.443372668 | 0.999970678 | 3.316 | 1.754 | 0.9 | 1.95 | NA |
| TRINITY_DN18377_c0_g1_i3 | -0.74873964 | 0.868442866 | 1 | 4.778 | 0 | 6.633 | 0 | NA |
| TRINITY_DN38994_c0_g1_i1 | -0.74873964 | 0.868442866 | 1 | 0.727 | 0 | 1.022 | 0 | - |
| TRINITY_DN97659_c0_g1_i1 | -0.74850435 | 0.813362596 | 0.999970678 | 1.644 | 0 | 2.244 | 0.08 | NA |
| TRINITY_DN1544_c0_g1_i2 | -0.74847679 | 0.406517361 | 0.999970678 | 2.634 | 2.7 | 2.511 | 5.881 | Motile_Sperm |
| TRINITY_DN2094_c0_g1_i9 | 0.748183123 | 0.568803716 | 0.999970678 | 0.917 | 0.246 | 0.289 | 0.372 | NA |
| TRINITY_DN783_c0_g1_i4 | 0.748149144 | 0.481661889 | 0.999970678 | 1.508 | 0.877 | 0.733 | 0.543 | DUF4922 |
| TRINITY_DN2158_c0_g1_i10 | 0.748141166 | 0.795618617 | 0.999970678 | 9.611 | 0 | 3.889 | 1.086 | NA |
| TRINITY_DN702_c0_g1_i2 | -0.74809261 | 0.438693711 | 0.999970678 | 8.857 | 3.114 | 8.355 | 10.194 | PPR |
| TRINITY_DN2668_c0_g1_i2 | -0.74793241 | 0.786039306 | 0.999970678 | 9.901 | 0 | 4.566 | 11.331 | RRM_1 |
| TRINITY_DN2223_c0_g1_i1 | 0.747910403 | 0.316803302 | 0.999970678 | 12.309 | 8.426 | 5.744 | 5.409 | NA |
| TRINITY_DN8069_c0_g1_i3 | 0.747852783 | 0.786706366 | 0.999970678 | 0.772 | 0.177 | 0 | 0.573 | NA |
| TRINITY_DN2607_c0_g1_i1 | 0.747810861 | 0.714519416 | 1 | 8.412 | 6.761 | 7.022 | 0.342 | NA |
| TRINITY_DN6020_c0_g1_i1 | -0.74780151 | 0.591230269 | 0.999970678 | 2.416 | 0.857 | 3.689 | 1.046 | NA |
| TRINITY_DN2247_c0_g1_i1 | -0.74764381 | 0.691103137 | 0.999970678 | 0.772 | 0.246 | 0.1 | 1.599 | BSD |
| TRINITY_DN3277_c0_g1_i3 | 0.747624226 | 0.793293088 | 0.999970678 | 8.166 | 0 | 3.178 | 1.106 | NA |
| TRINITY_DN40619_c0_g1_i1 | -0.74761169 | 0.859553478 | 0.999970678 | 1.998 | 0 | 2.811 | 0 | NA |
| TRINITY_DN264_c0_g1_i3 | 0.747593052 | 0.63152243 | 0.999970678 | 0.908 | 1.597 | 0.111 | 1.367 | ThiC-associated |
| TRINITY_DN26802_c0_g1_i7 | 0.747556208 | 0.775044587 | 1 | 4.442 | 0.099 | 2.167 | 0.101 | NA |
| TRINITY_DN3086_c0_g1_i4 | -0.74751552 | 0.524481166 | 0.999970678 | 1.39 | 1.813 | 0.778 | 4.444 | BLM10_mid |
| TRINITY_DN6923_c0_g1_i2 | -0.74750058 | 0.470029619 | 0.999970678 | 1.254 | 1.36 | 0.933 | 3.247 | DUF2428 |
| TRINITY_DN37_c0_g3_i1 | -0.7474863 | 0.491247846 | 0.999970678 | 343.41 | 181.5 | 571.61 | 193.825 | NA |
| TRINITY_DN3654_c0_g1_i1 | -0.7474535 | 0.186340537 | 0.999970678 | 27.969 | 28.27 | 40.286 | 45.604 | zf-RING_2 |
| TRINITY_DN1139_c0_g1_i3 | 0.747381691 | 0.512759729 | 0.999970678 | 29.25 | 101.2 | 17.355 | 56.18 | Band_7 |
| TRINITY_DN7973_c0_g1_i2 | 0.747308157 | 0.489460423 | 0.999970678 | 2.117 | 7.786 | 2.955 | 2.292 | SBP |
| TRINITY_DN3981_c0_g1_i1 | -0.7470655 | 0.600182575 | 0.999970678 | 1.444 | 0.532 | 0.6 | 2.624 | LBP_BPI_CETP |
| TRINITY_DN4778_c0_g1_i5 | 0.746965784 | 0.32083297 | 0.999970678 | 19.349 | 13.93 | 7.255 | 10.848 | NA |
| TRINITY_DN2338_c0_g1_i4 | -0.74692944 | 0.570776676 | 0.999970678 | 86.997 | 26.59 | 118.593 | 40.024 | NA |
| TRINITY_DN18982_c1_g1_i6 | 0.746917718 | 0.782698448 | 0.999970678 | 0.563 | 1.547 | 1.044 | 0 | NA |
| TRINITY_DN5676_c0_g1_i7 | -0.74690716 | 0.650620463 | 0.999970678 | 0.1 | 0.601 | 0.789 | 0.241 | PPR_long |
| TRINITY_DN54485_c0_g1_i1 | 0.746780918 | 0.475481012 | 0.999970678 | 3.706 | 1.685 | 1.844 | 1.015 | NA |
| TRINITY_DN9926_c1_g1_i2 | -0.74673246 | 0.429587495 | 0.999970678 | 245.62 | 128.4 | 359.778 | 184.898 | NA |
| TRINITY_DN50637_c0_g1_i1 | -0.74664957 | 0.808472707 | 0.999970678 | 1.399 | 11.04 | 17.055 | 0 | TPP_enzyme_C |
| TRINITY_DN36_c1_g1_i4 | 0.746617836 | 0.462269429 | 0.999970678 | 2.198 | 1.143 | 0.622 | 1.257 | Asp_protease_2 |
| TRINITY_DN9672_c0_g1_i4 | -0.74658649 | 0.371814441 | 0.999970678 | 2.153 | 1.301 | 2.167 | 3.227 | NA |
| TRINITY_DN1191_c0_g1_i6 | 0.746582263 | 0.432727404 | 0.999970678 | 2.743 | 1.932 | 0.722 | 1.93 | NA |

| TRINITY_DN187_c0_g1_i7 | -0.74655546 | 0.374123743 | 0.999970678 | 27.433 | 60.5 | 50.364 | 85.557 | NA |
| --- | --- | --- | --- | --- | --- | --- | --- | --- |
| TRINITY_DN2358_c2_g1_i2 | -0.74640991 | 0.246878983 | 0.999970678 | 35.473 | 36.22 | 58.908 | 49.012 | Oxysterol_BP |
| TRINITY_DN24019_c0_g1_i4 | -0.74639826 | 0.367681789 | 0.999970678 | 1.017 | 1.429 | 1.478 | 2.312 | PORR |
| TRINITY_DN249812_c0_g1_i1 | -0.74638601 | 0.864478968 | 0.999970678 | 2.653 | 0 | 3.722 | 0 | NA |
| TRINITY_DN17152_c0_g1_i1 | -0.74638601 | 0.864478968 | 0.999970678 | 3.47 | 0 | 4.9 | 0 | Cation_efflux |
| TRINITY_DN5010_c0_g1_i3 | 0.74635842 | 0.471233005 | 0.999970678 | 2.743 | 3.568 | 2.378 | 0.895 | NA |
| TRINITY_DN18126_c0_g1_i6 | 0.746319611 | 0.802940484 | 1 | 0 | 0.887 | 0.344 | 0.09 | NA |
| TRINITY_DN3385_c0_g1_i4 | 0.746242891 | 0.859498221 | 0.999970678 | 5.886 | 0 | 0 | 3.579 | 3HCDH |
| TRINITY_DN2403_c0_g1_i2 | -0.7462361 | 0.5611597 | 0.999970678 | 8.621 | 18.83 | 6.222 | 37.551 | NA |
| TRINITY_DN2315_c0_g1_i1 | -0.74619056 | 0.434768951 | 0.999970678 | 129.05 | 193.2 | 329.924 | 138.902 | Rib_5-P_isom_A |
| TRINITY_DN52148_c0_g1_i1 | 0.746129225 | 0.626106286 | 1 | 5.895 | 5.42 | 4.511 | 1.166 | NA |
| TRINITY_DN4408_c0_g1_i4 | -0.74609984 | 0.699936297 | 1 | 1.753 | 0.256 | 0.478 | 2.735 | NA |
| TRINITY_DN8945_c0_g1_i1 | 0.746051702 | 0.317634989 | 0.999970678 | 9.329 | 6.78 | 3.722 | 5.097 | Bac_globin |
| TRINITY_DN7206_c4_g1_i1 | -0.74600944 | 0.36894091 | 0.999970678 | 1.453 | 1.941 | 1.833 | 3.469 | NA |
| TRINITY_DN9093_c0_g1_i8 | -0.74598335 | 0.696628482 | 0.999970678 | 0.527 | 0.03 | 0.611 | 0.211 | PPR |
| TRINITY_DN5383_c0_g1_i4 | -0.74597312 | 0.511219271 | 0.999970678 | 3.143 | 1.025 | 2.389 | 4.152 | PP2C |
| TRINITY_DN2599_c0_g1_i1 | 0.745870309 | 0.304673531 | 0.999970678 | 95.572 | 155.5 | 63.096 | 67.038 | UbiA |
| TRINITY_DN30326_c0_g1_i1 | 0.745864467 | 0.861309668 | 0.999970678 | 2.371 | 0 | 1.189 | 0 | - |
| TRINITY_DN3480_c0_g1_i5 | 0.745773237 | 0.565249624 | 1 | 1.417 | 1.587 | 0.944 | 0.583 | NA |
| TRINITY_DN6366_c0_g1_i1 | 0.745694257 | 0.511609718 | 0.999970678 | 2.471 | 5.588 | 2.944 | 1.216 | NA |
| TRINITY_DN2628_c0_g1_i5 | 0.745670035 | 0.866003658 | 1 | 8.466 | 0 | 0 | 4.946 | NA |
| TRINITY_DN16531_c0_g2_i1 | -0.74558637 | 0.669321775 | 1 | 1.635 | 0.197 | 1.322 | 1.478 | NA |
| TRINITY_DN7584_c2_g1_i3 | 0.745571319 | 0.704247087 | 1 | 0.182 | 0.838 | 0.044 | 0.563 | NA |
| TRINITY_DN8171_c0_g1_i27 | 0.745550755 | 0.864066791 | 0.999970678 | 0 | 0.857 | 0 | 0.513 | NA |
| TRINITY_DN2097_c0_g1_i2 | 0.745470628 | 0.306608379 | 0.999970678 | 4.833 | 6.347 | 2.378 | 3.79 | NA |
| TRINITY_DN281_c0_g1_i1 | 0.74538142 | 0.453266526 | 0.999970678 | 5.042 | 9.382 | 5.133 | 2.373 | NA |
| TRINITY_DN12870_c0_g2_i2 | -0.74537593 | 0.349125851 | 0.999970678 | 2.289 | 1.882 | 3.033 | 3.338 | zf-RING_2 |
| TRINITY_DN8279_c0_g1_i4 | -0.74532823 | 0.595183848 | 0.999970678 | 0.645 | 1.311 | 2.244 | 0.583 | NA |
| TRINITY_DN1563_c0_g1_i4 | 0.745326633 | 0.628676507 | 0.999970678 | 62.071 | 6.495 | 6.755 | 33.187 | HhH-GPD |
| TRINITY_DN90319_c0_g1_i1 | 0.745312319 | 0.467536811 | 0.999970678 | 0.654 | 0.887 | 0.511 | 0.302 | NA |
| TRINITY_DN4000_c0_g1_i1 | -0.745206 | 0.239395665 | 0.999970678 | 15.688 | 16.12 | 25.098 | 22.872 | - |
| TRINITY_DN743_c0_g1_i4 | -0.74520401 | 0.431819209 | 0.999970678 | 2.126 | 1.074 | 1.689 | 3.358 | NA |
| TRINITY_DN4022_c0_g1_i1 | -0.74517605 | 0.755998269 | 0.999970678 | 20.057 | 0.02 | 21.032 | 8.727 | DnaJ |
| TRINITY_DN1458_c0_g1_i2 | 0.745023779 | 0.332871164 | 0.999970678 | 46.782 | 46.11 | 15.866 | 35.801 | NA |
| TRINITY_DN29206_c0_g1_i1 | -0.74501654 | 0.552397239 | 0.999970678 | 0.4 | 0.907 | 0.678 | 1.357 | NA |
| TRINITY_DN1655_c0_g1_i7 | -0.74493745 | 0.54507681 | 0.999970678 | 5.714 | 5.213 | 4.666 | 11.863 | NA |
| TRINITY_DN8005_c0_g1_i1 | -0.74492387 | 0.364180606 | 0.999970678 | 19.558 | 27.49 | 43.72 | 25.788 | NA |
| TRINITY_DN4104_c0_g1_i6 | -0.74489176 | 0.654031679 | 1 | 1.817 | 10 | 5.244 | 12.326 | NA |
| TRINITY_DN15613_c0_g1_i1 | -0.74487181 | 0.346707165 | 0.999970678 | 4.351 | 2.917 | 5.133 | 5.972 | NA |
| TRINITY_DN1663_c0_g1_i2 | -0.74481421 | 0.603601079 | 0.999970678 | 0.309 | 0.907 | 0.5 | 1.428 | FolB |
| TRINITY_DN26799_c1_g1_i1 | 0.744762676 | 0.537998746 | 0.999970678 | 1.799 | 2.444 | 0.444 | 1.991 | WD40 |
| TRINITY_DN6096_c0_g1_i1 | -0.74465014 | 0.789714435 | 0.999970678 | 15.37 | 0 | 12.81 | 10.647 | NA |
| TRINITY_DN90053_c0_g1_i1 | 0.744626745 | 0.866441514 | 0.999970678 | 1.889 | 0 | 0.944 | 0 | NA |
| TRINITY_DN7093_c1_g1_i1 | 0.744535877 | 0.278671 | 0.999970678 | 2214.4 | 1783 | 1156.308 | 876.129 | NA |
| TRINITY_DN4543_c0_g1_i1 | 0.744521249 | 0.40350637 | 0.999970678 | 6.677 | 5.893 | 4.433 | 2.172 | DEAD |
| TRINITY_DN3664_c0_g1_i2 | -0.74450786 | 0.861129432 | 0.999970678 | 33.12 | 0 | 45.386 | 0 | NA |
| TRINITY_DN3493_c0_g1_i11 | -0.74448473 | 0.787519054 | 0.999970678 | 1.717 | 0 | 0.656 | 2.081 | NA |
| TRINITY_DN8803_c0_g1_i2 | -0.74443959 | 0.781088964 | 0.999970678 | 0 | 3.893 | 2.7 | 3.167 | Pkinase |
| TRINITY_DN54082_c0_g1_i1 | -0.74443322 | 0.631999237 | 0.999970678 | 22.128 | 4.711 | 4.378 | 39.491 | NA |
| TRINITY_DN4067_c0_g1_i2 | -0.74441367 | 0.533690908 | 0.999970678 | 1.381 | 4.1 | 3.178 | 5.178 | NA |
| TRINITY_DN12636_c0_g1_i4 | 0.744375542 | 0.538276469 | 0.999970678 | 0.382 | 1.183 | 0.456 | 0.382 | LRRNT_2 |
| TRINITY_DN1029_c0_g1_i1 | -0.74434637 | 0.795514134 | 0.999970678 | 49.916 | 0 | 27.721 | 50.962 | - |
| TRINITY_DN59_c0_g1_i3 | 0.744309153 | 0.540146959 | 0.999970678 | 4.133 | 6.928 | 0.933 | 5.469 | NA |
| TRINITY_DN1317_c0_g1_i1 | 0.744240956 | 0.319238998 | 0.999970678 | 318.57 | 181 | 137.503 | 132.85 | Chal_sti_synt_C |
| TRINITY_DN10002_c0_g1_i7 | 0.744097199 | 0.650447453 | 0.999970678 | 1.181 | 0.65 | 0.833 | 0.121 | Pkinase |
| TRINITY_DN5232_c0_g1_i1 | 0.743966031 | 0.790466568 | 0.999970678 | 0 | 1.192 | 0.311 | 0.322 | NB-ARC |
| TRINITY_DN14718_c0_g1_i7 | 0.743929304 | 0.784188947 | 0.999970678 | 0.427 | 1.587 | 0 | 1.186 | NA |
| TRINITY_DN2322_c1_g1_i1 | -0.74389106 | 0.524842757 | 0.999970678 | 0.336 | 0.227 | 0.544 | 0.292 | NA |
| TRINITY_DN17991_c1_g1_i2 | -0.74382182 | 0.420756374 | 0.999970678 | 40.614 | 52.83 | 94.305 | 41.793 | Acetyltransf_1 |
| TRINITY_DN2324_c0_g1_i8 | -0.7437016 | 0.790296546 | 0.999970678 | 23.173 | 0 | 11.088 | 25.818 | NA |
| TRINITY_DN26886_c0_g1_i1 | -0.74362606 | 0.609110857 | 0.999970678 | 0.845 | 0.217 | 0.7 | 0.945 | NA |
| TRINITY_DN2338_c0_g1_i7 | -0.74356162 | 0.444054908 | 0.999970678 | 106.16 | 42.56 | 131.992 | 86.733 | NA |
| TRINITY_DN1692_c0_g2_i1 | -0.74352377 | 0.428861058 | 0.999970678 | 27.025 | 45 | 71.451 | 33.569 | NA |
| TRINITY_DN4041_c0_g1_i2 | 0.743476705 | 0.507507424 | 0.999970678 | 2.662 | 1.971 | 1.778 | 0.623 | NA |
| TRINITY_DN843_c2_g1_i18 | -0.74337297 | 0.795318218 | 0.999970678 | 0.627 | 1.045 | 2.344 | 0 | NA |
| TRINITY_DN15348_c0_g2_i1 | -0.7433038 | 0.732983862 | 0.999970678 | 1.036 | 0.177 | 1.655 | 0.04 | NA |
| TRINITY_DN10902_c0_g1_i3 | -0.74327207 | 0.449225259 | 0.999970678 | 6.05 | 2.119 | 5.966 | 6.595 | DUF2828 |
| TRINITY_DN904_c0_g1_i14 | -0.74324642 | 0.247052296 | 0.999970678 | 110.82 | 116.8 | 177.156 | 153.269 | CENP-T_C |
| TRINITY_DN1481_c1_g1_i4 | -0.74324553 | 0.518452758 | 0.999970678 | 2.534 | 1.035 | 2.866 | 2.594 | NA |
| TRINITY_DN5090_c0_g1_i2 | -0.74320327 | 0.649883992 | 0.999970678 | 0.5 | 1.646 | 2.667 | 0.322 | ARID |
| TRINITY_DN1179_c0_g1_i4 | 0.743105699 | 0.529027096 | 0.999970678 | 47.018 | 54.76 | 42.397 | 9.461 | NA |
| TRINITY_DN6255_c0_g1_i4 | -0.7430632 | 0.363100067 | 0.999970678 | 1.889 | 2.247 | 3.233 | 3.036 | LysM |
| TRINITY_DN13722_c0_g1_i2 | -0.74302602 | 0.540852701 | 0.999970678 | 0.845 | 0.424 | 0.8 | 1.166 | NA |
| TRINITY_DN2134_c0_g1_i1 | -0.74302235 | 0.434818597 | 0.999970678 | 2.48 | 4.829 | 3.478 | 7.963 | NA |

| TRINITY_DN2436_c0_g1_i13 | -0.74288133 | 0.512979775 | 0.999970678 | 5.151 | 1.606 | 4.489 | 5.861 | NA |
| --- | --- | --- | --- | --- | --- | --- | --- | --- |
| TRINITY_DN11464_c0_g2_i5 | 0.742857774 | 0.58096943 | 0.999970678 | 0.382 | 1.202 | 0.311 | 0.573 | NA |
| TRINITY_DN6646_c0_g1_i2 | 0.742800204 | 0.513645755 | 0.999970678 | 1.163 | 3.982 | 1.522 | 1.196 | TPX2 |
| TRINITY_DN2547_c0_g1_i12 | 0.742765782 | 0.868860276 | 1 | 0.654 | 0 | 0 | 0.412 | NA |
| TRINITY_DN2547_c0_g1_i3 | 0.742765782 | 0.868860276 | 1 | 0.654 | 0 | 0 | 0.412 | NA |
| TRINITY_DN10560_c0_g1_i2 | -0.74276424 | 0.478123612 | 0.999970678 | 1.072 | 0.867 | 1.511 | 1.408 | NA |
| TRINITY_DN3793_c1_g3_i1 | -0.74273548 | 0.76235204 | 1 | 0.045 | 0.552 | 0.8 | 0.02 | NA |
| TRINITY_DN90_c0_g1_i2 | -0.74264224 | 0.445549513 | 0.999970678 | 206.31 | 680.7 | 625.218 | 691.584 | NA |
| TRINITY_DN6095_c0_g2_i2 | -0.74258137 | 0.490658486 | 0.999970678 | 1.435 | 1.892 | 1.078 | 4.233 | NA |
| TRINITY_DN7509_c0_g4_i2 | -0.74256613 | 0.488906896 | 0.999970678 | 0.863 | 1.232 | 1.1 | 2.162 | NA |
| TRINITY_DN2495_c0_g1_i2 | 0.742562844 | 0.255617595 | 0.999970678 | 6.976 | 7.707 | 4.266 | 3.629 | ABC_membrane |
| TRINITY_DN9733_c0_g1_i5 | -0.7424836 | 0.867592609 | 1 | 1.281 | 0 | 1.755 | 0 | NA |
| TRINITY_DN20018_c0_g1_i4 | -0.74247465 | 0.870607881 | 1 | 0.391 | 0 | 0.556 | 0 | - |
| TRINITY_DN32734_c0_g3_i2 | -0.74247465 | 0.870607881 | 1 | 0.491 | 0 | 0.689 | 0 | NA |
| TRINITY_DN6808_c0_g1_i9 | -0.74247465 | 0.870607881 | 1 | 0.309 | 0 | 0.433 | 0 | NA |
| TRINITY_DN29236_c0_g1_i2 | -0.74247465 | 0.870607881 | 1 | 1.054 | 0 | 1.511 | 0 | NA |
| TRINITY_DN1540_c1_g1_i7 | -0.74240437 | 0.588994437 | 0.999970678 | 1.372 | 0.414 | 1.889 | 0.714 | NA |
| TRINITY_DN527_c0_g1_i2 | -0.74236491 | 0.61952578 | 0.999970678 | 0.945 | 0.345 | 1.6 | 0.241 | A_deaminase |
| TRINITY_DN8222_c0_g1_i4 | -0.74233521 | 0.597666775 | 0.999970678 | 0.999 | 0.434 | 0.467 | 1.85 | NA |
| TRINITY_DN5788_c0_g1_i4 | 0.742307582 | 0.699947653 | 0.999970678 | 0.164 | 3.577 | 1.478 | 0.432 | MSP |
| TRINITY_DN885_c1_g1_i4 | 0.742251046 | 0.71352254 | 0.999970678 | 0.418 | 3.489 | 0.089 | 2.212 | NA |
| TRINITY_DN16107_c0_g2_i1 | 0.742246753 | 0.625204377 | 0.999970678 | 3.334 | 0.424 | 1.333 | 0.664 | Myb_DNA-bindin |
| TRINITY_DN1350_c0_g1_i5 | 0.742238996 | 0.584085505 | 0.999970678 | 137.75 | 738.5 | 87.528 | 392.838 | NA |
| TRINITY_DN10824_c0_g1_i3 | 0.742188208 | 0.266706925 | 0.999970678 | 7.04 | 7.638 | 3.466 | 4.544 | NA |
| TRINITY_DN470_c0_g1_i2 | 0.742148699 | 0.317682453 | 0.999970678 | 35.527 | 44.86 | 14.355 | 30.634 | Atg8 |
| TRINITY_DN6543_c0_g1_i6 | -0.74206572 | 0.787792815 | 0.999970678 | 0.681 | 0 | 0.589 | 0.442 | NA |
| TRINITY_DN3105_c0_g1_i2 | -0.74195213 | 0.421771768 | 0.999970678 | 6.023 | 6.061 | 12.033 | 5.65 | NA |
| TRINITY_DN225_c0_g1_i1 | 0.741945028 | 0.658687392 | 0.999970678 | 102.87 | 18.13 | 4.1 | 68.295 | NA |
| TRINITY_DN18836_c0_g1_i1 | -0.74176202 | 0.215740393 | 0.999970678 | 208.46 | 247.5 | 350.112 | 334.567 | NA |
| TRINITY_DN37473_c0_g1_i1 | -0.74150169 | 0.751473777 | 1 | 0.045 | 1.133 | 0.133 | 1.769 | NA |
| TRINITY_DN4695_c0_g1_i2 | -0.74144179 | 0.355157192 | 0.999970678 | 2.026 | 2.316 | 3.378 | 3.157 | NA |
| TRINITY_DN572_c0_g1_i7 | -0.74142047 | 0.440464268 | 0.999970678 | 1.699 | 2.809 | 2.933 | 3.911 | - |
| TRINITY_DN3439_c0_g1_i1 | 0.74141032 | 0.458521845 | 0.999970678 | 0.718 | 1.399 | 0.7 | 0.422 | NA |
| TRINITY_DN998_c0_g2_i3 | -0.74137508 | 0.553373101 | 0.999970678 | 2.453 | 14.55 | 13.832 | 11.341 | NA |
| TRINITY_DN186_c0_g1_i1 | -0.74136765 | 0.251224478 | 0.999970678 | 29.114 | 21.08 | 32.998 | 44.196 | Pkinase |
| TRINITY_DN2983_c0_g2_i1 | 0.741343382 | 0.571888498 | 0.999970678 | 5.777 | 4.632 | 4.5 | 0.794 | NA |
| TRINITY_DN536_c0_g1_i8 | 0.741185833 | 0.464898331 | 0.999970678 | 1.399 | 4.593 | 1.422 | 1.85 | NA |
| TRINITY_DN229_c0_g1_i1 | -0.74117687 | 0.565898451 | 0.999970678 | 39.452 | 155 | 51.286 | 260.411 | AdoHcyase |
| TRINITY_DN1215_c0_g1_i18 | 0.74107414 | 0.868613585 | 1 | 0 | 0.7 | 0.356 | 0 | WD40 |
| TRINITY_DN27_c1_g1_i1 | 0.741026402 | 0.661000833 | 0.999970678 | 7.785 | 0.936 | 3.511 | 1.005 | NA |
| TRINITY_DN483_c0_g2_i5 | -0.74102435 | 0.469405478 | 0.999970678 | 73.78 | 152 | 78.806 | 280.277 | Mito_carr |
| TRINITY_DN5682_c0_g1_i7 | 0.740998173 | 0.868235056 | 1 | 11.073 | 0 | 5.544 | 0 | NA |
| TRINITY_DN2212_c0_g2_i2 | -0.74097397 | 0.866412271 | 0.999970678 | 0 | 6.987 | 9.511 | 0 | RRM_1 |
| TRINITY_DN7315_c0_g1_i1 | 0.740856204 | 0.697856188 | 0.999970678 | 10.319 | 495.6 | 49.319 | 217.25 | Chloroa_b-bind |
| TRINITY_DN3566_c0_g2_i1 | -0.74080094 | 0.812367852 | 0.999970678 | 5.805 | 0.246 | 8.444 | 0 | NA |
| TRINITY_DN5466_c0_g2_i1 | 0.74077007 | 0.523502873 | 0.999970678 | 5.078 | 3.124 | 0.822 | 3.941 | NA |
| TRINITY_DN2063_c0_g1_i1 | -0.74067644 | 0.31310191 | 0.999970678 | 4.088 | 3.479 | 6.1 | 5.278 | NA |
| TRINITY_DN166_c1_g1_i1 | -0.74051751 | 0.512839054 | 0.999970678 | 250.51 | 146.4 | 444.706 | 125.581 | Ribonuclease_T2 |
| TRINITY_DN5226_c0_g1_i6 | 0.740500615 | 0.782665097 | 0.999970678 | 0.282 | 0.345 | 0 | 0.382 | - |
| TRINITY_DN21177_c2_g2_i1 | -0.7404475 | 0.490010208 | 0.999970678 | 0.427 | 1.035 | 1.022 | 1.186 | Arm |
| TRINITY_DN25274_c0_g1_i2 | 0.740399139 | 0.51415063 | 0.999970678 | 2.589 | 3.233 | 0.778 | 2.564 | NA |
| TRINITY_DN37203_c0_g1_i1 | -0.74038056 | 0.862110218 | 0.999970678 | 2.017 | 0 | 2.822 | 0 | - |
| TRINITY_DN341_c0_g1_i2 | -0.74037661 | 0.621893927 | 0.999970678 | 2.071 | 3.893 | 0.622 | 9.219 | VWA |
| TRINITY_DN262_c0_g1_i2 | 0.740366229 | 0.644306443 | 0.999970678 | 1.463 | 0.986 | 1.089 | 0.171 | NA |
| TRINITY_DN1649_c0_g1_i5 | -0.74032225 | 0.652030056 | 0.999970678 | 0.154 | 2.395 | 1.8 | 2.001 | NA |
| TRINITY_DN34933_c0_g1_i1 | -0.74028019 | 0.637792734 | 1 | 1.154 | 0.276 | 0.833 | 1.387 | NA |
| TRINITY_DN49_c0_g1_i1 | -0.74022598 | 0.561469399 | 0.999970678 | 8.176 | 2.148 | 11.188 | 3.881 | G-alpha |
| TRINITY_DN6353_c0_g5_i1 | -0.74019842 | 0.561077216 | 0.999970678 | 4.787 | 1.922 | 6.011 | 3.861 | NA |
| TRINITY_DN2112_c0_g1_i1 | -0.74013915 | 0.802058832 | 0.999970678 | 51.651 | 0 | 16.455 | 67.531 | ATP-synt_ab |
| TRINITY_DN12134_c0_g1_i2 | 0.740126718 | 0.63602837 | 0.999970678 | 0.945 | 3.873 | 0.322 | 2.453 | NA |
| TRINITY_DN7692_c0_g1_i1 | 0.740029832 | 0.659636202 | 0.999970678 | 0.391 | 1.37 | 0.1 | 0.925 | NA |
| TRINITY_DN51091_c1_g1_i1 | 0.739979453 | 0.863367764 | 0.999970678 | 1.917 | 0 | 0.967 | 0 | NA |
| TRINITY_DN4310_c0_g1_i2 | 0.739919929 | 0.592836225 | 0.999970678 | 0.454 | 1.666 | 0.667 | 0.462 | NA |
| TRINITY_DN4459_c0_g1_i3 | -0.73989988 | 0.788762227 | 1 | 0.427 | 0 | 0.278 | 0.402 | NA |
| TRINITY_DN6521_c0_g1_i1 | -0.7398439 | 0.621768941 | 0.999970678 | 1.408 | 0.296 | 1.522 | 0.975 | Myb_DNA-bindin |
| TRINITY_DN50585_c0_g1_i1 | 0.739793639 | 0.861100726 | 0.999970678 | 5.777 | 0 | 2.9 | 0 | NA |
| TRINITY_DN4821_c0_g1_i4 | -0.73978143 | 0.651323001 | 0.999970678 | 0.109 | 1.025 | 0.411 | 1.357 | MatE |
| TRINITY_DN4484_c0_g1_i3 | -0.73977949 | 0.496053475 | 0.999970678 | 11.555 | 43.6 | 48.253 | 32.876 | DUF3493 |
| TRINITY_DN5011_c0_g1_i1 | -0.73942701 | 0.452645337 | 0.999970678 | 5.096 | 2.503 | 6.866 | 4.424 | PPR |
| TRINITY_DN1560_c0_g1_i2 | 0.739397714 | 0.457339459 | 0.999970678 | 2.562 | 1.675 | 1.455 | 0.804 | GAD |
| TRINITY_DN1615_c0_g2_i1 | -0.73936988 | 0.250408311 | 0.999970678 | 90.749 | 66.82 | 101.161 | 138.52 | zf-FLZ |
| TRINITY_DN15295_c0_g1_i2 | 0.739286347 | 0.380552633 | 0.999970678 | 2.861 | 1.951 | 1.455 | 1.126 | HATPase_c_3 |
| TRINITY_DN45580_c0_g1_i2 | -0.73921038 | 0.550547535 | 1 | 0.645 | 1.104 | 1.011 | 1.689 | NA |
| TRINITY_DN7932_c0_g1_i1 | 0.739160876 | 0.53634442 | 0.999970678 | 20.53 | 5.312 | 3.955 | 10.798 | NA |

| TRINITY_DN19713_c1_g1_i1 | 0.739160829 | 0.339062824 | 0.999970678 | 1.853 | 1.36 | 0.744 | 1.036 | PHD |
| --- | --- | --- | --- | --- | --- | --- | --- | --- |
| TRINITY_DN894_c0_g1_i4 | 0.739103943 | 0.790725717 | 0.999970678 | 3.979 | 0 | 1.033 | 1.146 | p450 |
| TRINITY_DN6717_c0_g1_i1 | 0.739027907 | 0.630166721 | 0.999970678 | 13.417 | 1.311 | 5.533 | 2.252 | NA |
| TRINITY_DN26252_c0_g1_i2 | -0.73897076 | 0.527191197 | 0.999970678 | 1.354 | 1.675 | 1 | 3.871 | RNA_pol_Rpb2_1 |
| TRINITY_DN9645_c0_g1_i1 | 0.738955876 | 0.503036252 | 0.999970678 | 47.545 | 13.13 | 19.888 | 12.678 | Lipoxygenase |
| TRINITY_DN12805_c0_g1_i3 | -0.73888287 | 0.543243729 | 0.999970678 | 0.699 | 1.557 | 0.778 | 2.795 | NA |
| TRINITY_DN5196_c0_g1_i5 | -0.73888055 | 0.865291956 | 0.999970678 | 2.189 | 0 | 3.055 | 0 | Aldo_ket_red |
| TRINITY_DN14384_c0_g1_i4 | 0.738750631 | 0.86586649 | 0.999970678 | 2.171 | 0 | 0 | 1.307 | NA |
| TRINITY_DN881_c0_g1_i2 | 0.738711941 | 0.386928924 | 0.999970678 | 8.711 | 4.593 | 4 | 3.187 | LRR_5 |
| TRINITY_DN20906_c0_g1_i1 | -0.73870736 | 0.343428612 | 0.999970678 | 206.67 | 168.1 | 342.546 | 211.872 | NA |
| TRINITY_DN16472_c0_g1_i1 | 0.738689585 | 0.450101029 | 0.999970678 | 2.235 | 3.065 | 1.022 | 1.91 | Transcrip_act |
| TRINITY_DN975_c0_g1_i9 | -0.73856368 | 0.480546817 | 0.999970678 | 2.38 | 1.656 | 1.433 | 5.017 | RRM_1 |
| TRINITY_DN8241_c0_g1_i1 | -0.73848853 | 0.326946568 | 0.999970678 | 6.104 | 4.829 | 8.855 | 7.52 | DUF538 |
| TRINITY_DN5529_c0_g1_i1 | 0.738480844 | 0.498230594 | 0.999970678 | 0.954 | 0.532 | 0.378 | 0.432 | RabGAP-TBC |
| TRINITY_DN73312_c0_g1_i1 | -0.73846389 | 0.609092374 | 0.999970678 | 0.372 | 2.306 | 1.522 | 2.594 | PCO_ADO |
| TRINITY_DN2147_c0_g1_i6 | -0.73833953 | 0.793179739 | 0.999970678 | 0 | 2.631 | 0.811 | 3.338 | NA |
| TRINITY_DN680_c0_g3_i2 | 0.738289664 | 0.555948543 | 0.999970678 | 5.687 | 2.927 | 3.522 | 0.925 | Rrp15p |
| TRINITY_DN28081_c1_g1_i1 | 0.738152423 | 0.745094776 | 0.999970678 | 9.829 | 323.3 | 8.877 | 162.176 | NA |
| TRINITY_DN2141_c0_g1_i5 | -0.73813557 | 0.788395389 | 0.999970678 | 0.945 | 0 | 0.878 | 0.533 | NA |
| TRINITY_DN1017_c0_g1_i3 | 0.738135376 | 0.809071272 | 0.999970678 | 0.2 | 8.002 | 0 | 4.826 | NA |
| TRINITY_DN1218_c2_g1_i1 | 0.738068813 | 0.86264565 | 0.999970678 | 0 | 0.779 | 0 | 0.473 | ABC_membrane |
| TRINITY_DN2270_c0_g1_i4 | -0.73803981 | 0.525952677 | 0.999970678 | 0.509 | 1.409 | 0.978 | 2.021 | TPT |
| TRINITY_DN6710_c0_g2_i1 | -0.73803849 | 0.344710484 | 0.999970678 | 18.604 | 18.74 | 17.121 | 41.723 | NA |
| TRINITY_DN16029_c0_g1_i14 | -0.73792838 | 0.823313975 | 0.999970678 | 0.481 | 0 | 0.656 | 0.02 | NA |
| TRINITY_DN9695_c0_g3_i1 | -0.7378362 | 0.378723396 | 0.999970678 | 2.18 | 4.366 | 4.677 | 5.218 | NA |
| TRINITY_DN236_c0_g1_i1 | 0.737821261 | 0.682903302 | 0.999970678 | 1.535 | 1.626 | 1.533 | 0.04 | HAT_KAT11 |
| TRINITY_DN56188_c0_g1_i1 | -0.7377203 | 0.427926742 | 0.999970678 | 717.62 | 2046 | 2139.015 | 1833.593 | Chloroa_b-bind |
| TRINITY_DN22887_c0_g1_i3 | -0.73770836 | 0.804103898 | 0.999970678 | 1.463 | 0 | 0.167 | 2.262 | NA |
| TRINITY_DN265_c1_g2_i5 | -0.73767522 | 0.482096795 | 0.999970678 | 8.711 | 2.927 | 10.144 | 7.279 | NA |
| TRINITY_DN3058_c0_g1_i1 | 0.737604209 | 0.619181359 | 0.999970678 | 12.091 | 12.3 | 10.51 | 1.82 | NA |
| TRINITY_DN1168_c0_g1_i1 | -0.73757121 | 0.612672132 | 0.999970678 | 2.198 | 18.32 | 7.533 | 24.37 | Myb_DNA-bindin |
| TRINITY_DN286_c0_g1_i3 | -0.73748716 | 0.792428792 | 0.999970678 | 9.02 | 0 | 9.722 | 3.519 | NA |
| TRINITY_DN5798_c0_g1_i4 | -0.73738071 | 0.67768577 | 0.999970678 | 0.881 | 0.059 | 1 | 0.402 | APO_RNA-bind |
| TRINITY_DN58_c0_g2_i5 | -0.73735149 | 0.329967554 | 0.999970678 | 5.123 | 3.587 | 4.7 | 8.938 | UCH |
| TRINITY_DN5449_c1_g1_i2 | 0.737309563 | 0.513667429 | 0.999970678 | 1.208 | 1.015 | 0.644 | 0.533 | zf-Dof |
| TRINITY_DN3118_c0_g1_i8 | -0.73728615 | 0.796042458 | 1 | 0.418 | 0 | 0.456 | 0.161 | MatE |
| TRINITY_DN6320_c3_g1_i1 | 0.737214342 | 0.426891253 | 0.999970678 | 1.29 | 2.858 | 1.1 | 1.146 | TPR_1 |
| TRINITY_DN13100_c0_g1_i1 | 0.737211459 | 0.446974891 | 0.999970678 | 15.897 | 5.342 | 4.733 | 7.168 | - |
| TRINITY_DN1813_c2_g1_i1 | -0.73718772 | 0.558333549 | 0.999970678 | 21.12 | 61.68 | 19.688 | 113.426 | AUX_IAA |
| TRINITY_DN1941_c0_g1_i7 | 0.737159749 | 0.711403444 | 0.999970678 | 0.581 | 55.03 | 13.51 | 16.146 | NA |
| TRINITY_DN397_c0_g1_i3 | 0.737095359 | 0.587573247 | 0.999970678 | 5.614 | 8.742 | 6.344 | 0.915 | PUNUT |
| TRINITY_DN502_c0_g3_i3 | -0.73708437 | 0.35719177 | 0.999970678 | 23.246 | 43.92 | 36.253 | 67.661 | Tubulin |
| TRINITY_DN2810_c0_g3_i4 | -0.73707703 | 0.748256426 | 0.999970678 | 3.634 | 0.118 | 5.011 | 0.211 | - |
| TRINITY_DN1380_c0_g1_i8 | 0.737067397 | 0.795639291 | 0.999970678 | 5.596 | 0 | 1.644 | 1.387 | NA |
| TRINITY_DN19245_c0_g2_i3 | 0.736995007 | 0.787315975 | 1 | 0.2 | 0.227 | 0 | 0.251 | NB-ARC |
| TRINITY_DN9446_c0_g1_i3 | -0.73698592 | 0.607391442 | 0.999970678 | 1.19 | 1.074 | 2.722 | 0.452 | PK |
| TRINITY_DN190_c0_g1_i8 | 0.736982349 | 0.685008784 | 0.999970678 | 8.466 | 0.256 | 1.578 | 3.418 | CBS |
| TRINITY_DN549_c0_g1_i5 | 0.736966596 | 0.389702792 | 0.999970678 | 3.961 | 3.745 | 1.578 | 2.714 | NA |
| TRINITY_DN4914_c0_g1_i3 | 0.736955215 | 0.40166238 | 0.999970678 | 21.075 | 47.22 | 20.254 | 16.086 | NA |
| TRINITY_DN7428_c0_g2_i3 | -0.73686968 | 0.499298435 | 0.999970678 | 2.607 | 8.811 | 5.3 | 12.517 | NA |
| TRINITY_DN14384_c0_g1_i2 | -0.73683353 | 0.567722554 | 0.999970678 | 1.699 | 1.597 | 3.689 | 1.005 | NA |
| TRINITY_DN13285_c0_g1_i18 | 0.736802191 | 0.682010427 | 0.999970678 | 7.231 | 2.277 | 0.467 | 5.037 | NA |
| TRINITY_DN1024_c0_g1_i8 | 0.736740419 | 0.861537604 | 0.999970678 | 2.789 | 0 | 1.4 | 0 | HAT |
| TRINITY_DN6621_c0_g3_i2 | 0.736626617 | 0.385024043 | 0.999970678 | 1.363 | 1.951 | 0.989 | 0.794 | PPR |
| TRINITY_DN5_c1_g3_i1 | 0.736500926 | 0.358282545 | 0.999970678 | 9.429 | 6.12 | 4.444 | 3.971 | NA |
| TRINITY_DN3576_c0_g1_i7 | -0.73640379 | 0.790908981 | 1 | 1.508 | 0 | 1.067 | 1.297 | NA |
| TRINITY_DN6392_c0_g2_i2 | 0.736316074 | 0.48226964 | 0.999970678 | 6.731 | 6.376 | 5.133 | 1.679 | NA |
| TRINITY_DN8876_c0_g1_i3 | -0.73623981 | 0.567625438 | 0.999970678 | 0.654 | 0.591 | 0.478 | 1.488 | NA |
| TRINITY_DN5337_c0_g1_i9 | -0.73600851 | 0.420021413 | 0.999970678 | 1.372 | 0.867 | 1.911 | 1.458 | NA |
| TRINITY_DN24455_c0_g1_i1 | 0.735959712 | 0.795979713 | 0.999970678 | 1.326 | 0 | 0.389 | 0.352 | Sec20 |
| TRINITY_DN31821_c0_g1_i4 | 0.73590123 | 0.784439549 | 0.999970678 | 1.78 | 0.591 | 0 | 1.448 | - |
| TRINITY_DN3907_c0_g1_i2 | 0.735836323 | 0.805455212 | 0.999970678 | 6.177 | 26.07 | 15.855 | 0 | NA |
| TRINITY_DN5734_c0_g1_i1 | -0.73578309 | 0.650975462 | 0.999970678 | 1.39 | 0.926 | 2.9 | 0.372 | PMEI |
| TRINITY_DN2941_c1_g1_i11 | 0.735731966 | 0.807890639 | 1 | 0.845 | 0 | 0.067 | 0.432 | NA |
| TRINITY_DN21535_c0_g1_i1 | 0.73563357 | 0.655081575 | 0.999970678 | 2.407 | 1.764 | 1.811 | 0.292 | EF-hand_8 |
| TRINITY_DN9605_c1_g1_i1 | -0.73562257 | 0.509379326 | 0.999970678 | 0.282 | 0.601 | 0.6 | 0.744 | PTR2 |
| TRINITY_DN1006_c0_g2_i7 | -0.7353893 | 0.43071098 | 0.999970678 | 34.655 | 87.89 | 63.907 | 121.881 | Ribosomal_L40e |
| TRINITY_DN2181_c0_g3_i2 | -0.73536939 | 0.718375295 | 1 | 0.282 | 2.296 | 0.367 | 3.74 | NA |
| TRINITY_DN286_c0_g1_i2 | 0.735175382 | 0.861127876 | 0.999970678 | 7.222 | 0 | 0 | 4.404 | NA |
| TRINITY_DN5464_c0_g1_i3 | -0.73512606 | 0.507283711 | 0.999970678 | 4.315 | 6.13 | 2.789 | 14.045 | NA |
| TRINITY_DN795_c0_g1_i2 | 0.735110542 | 0.395125704 | 0.999970678 | 2.362 | 2.878 | 1.211 | 1.699 | NA |
| TRINITY_DN8367_c0_g1_i3 | 0.734978354 | 0.865132264 | 0.999970678 | 8.484 | 0 | 4.244 | 0 | NA |
| TRINITY_DN4953_c0_g1_i1 | -0.73496121 | 0.508493313 | 0.999970678 | 2.307 | 6.504 | 3.544 | 10.275 | RETICULATA-like |
| TRINITY_DN36096_c0_g1_i1 | -0.73485397 | 0.872560797 | 1 | 0.808 | 0 | 1.122 | 0 | Snf7 |

| TRINITY_DN4466_c0_g1_i5 | -0.73485397 | 0.872560797 | 1 | 0.645 | 0 | 0.911 | 0 | Peroxidase_2 |
| --- | --- | --- | --- | --- | --- | --- | --- | --- |
| TRINITY_DN54917_c0_g1_i1 | -0.73485397 | 0.872560797 | 1 | 0.645 | 0 | 0.9 | 0 | NA |
| TRINITY_DN10999_c0_g1_i8 | -0.73485397 | 0.872560797 | 1 | 0.273 | 0 | 0.378 | 0 | NA |
| TRINITY_DN10524_c0_g1_i13 | 0.734803693 | 0.521121175 | 0.999970678 | 1.671 | 0.719 | 0.5 | 0.834 | p450 |
| TRINITY_DN3498_c0_g1_i2 | -0.73473082 | 0.435913107 | 0.999970678 | 2.135 | 5.006 | 5.455 | 5.208 | DEAD |
| TRINITY_DN180_c1_g1_i6 | -0.73472277 | 0.352005601 | 0.999970678 | 2.38 | 1.754 | 3.411 | 2.765 | Inositol_P |
| TRINITY_DN46114_c0_g1_i1 | -0.73467009 | 0.823794254 | 1 | 0.018 | 0.946 | 1.244 | 0.04 | NA |
| TRINITY_DN1866_c0_g2_i1 | -0.73463709 | 0.613964865 | 0.999970678 | 2.071 | 0.414 | 1.867 | 1.89 | NA |
| TRINITY_DN964_c0_g1_i4 | 0.734616713 | 0.646664889 | 0.999970678 | 3.252 | 1.34 | 0.333 | 2.353 | NA |
| TRINITY_DN15719_c0_g1_i1 | 0.734602185 | 0.38652965 | 0.999970678 | 1.78 | 3.518 | 1.422 | 1.468 | Hira |
| TRINITY_DN7884_c0_g1_i6 | 0.734580693 | 0.696088202 | 0.999970678 | 2.307 | 0.256 | 1.2 | 0.111 | NA |
| TRINITY_DN28761_c0_g1_i1 | -0.73457137 | 0.573305066 | 1 | 0.545 | 1.173 | 1 | 1.548 | NA |
| TRINITY_DN596_c0_g1_i2 | 0.73452416 | 0.528516869 | 0.999970678 | 3.679 | 1.252 | 1.733 | 0.905 | XH |
| TRINITY_DN4526_c0_g1_i2 | 0.734347826 | 0.871453148 | 1 | 1.481 | 0 | 0.767 | 0 | NA |
| TRINITY_DN6023_c0_g2_i1 | -0.73430334 | 0.789150002 | 0.999970678 | 6.74 | 0 | 3.444 | 7.229 | NA |
| TRINITY_DN3091_c0_g1_i9 | 0.734254713 | 0.623932409 | 0.999970678 | 2.089 | 1.537 | 1.611 | 0.251 | NA |
| TRINITY_DN1331_c0_g2_i1 | -0.73422303 | 0.538055185 | 0.999970678 | 2.362 | 12.63 | 10.266 | 12.286 | NA |
| TRINITY_DN2602_c0_g1_i1 | 0.734160445 | 0.862141885 | 0.999970678 | 22.101 | 0 | 11.099 | 0 | NA |
| TRINITY_DN234_c2_g1_i1 | -0.73405801 | 0.303463581 | 0.999970678 | 9.066 | 14.21 | 15.599 | 19.615 | NA |
| TRINITY_DN13311_c0_g1_i2 | 0.734029335 | 0.353559196 | 0.999970678 | 4.478 | 4.366 | 2.789 | 1.96 | NA |
| TRINITY_DN866_c0_g1_i2 | -0.73402565 | 0.665753115 | 0.999970678 | 9.129 | 0.986 | 12.61 | 1.79 | NA |
| TRINITY_DN10306_c0_g1_i1 | 0.733998221 | 0.583632942 | 0.999970678 | 1.608 | 5.696 | 2.4 | 1.428 | NA |
| TRINITY_DN8330_c0_g1_i5 | -0.73399603 | 0.792784828 | 1 | 0.282 | 0 | 0.244 | 0.181 | NA |
| TRINITY_DN79_c3_g2_i2 | 0.73390399 | 0.700532448 | 0.999970678 | 0.472 | 33.82 | 10.41 | 7.721 | NA |
| TRINITY_DN1232_c0_g1_i4 | 0.733895358 | 0.555225051 | 0.999970678 | 4.787 | 2.03 | 0.867 | 3.036 | NA |
| TRINITY_DN8591_c0_g1_i2 | -0.73388161 | 0.471822793 | 0.999970678 | 0.981 | 0.512 | 0.922 | 1.397 | C2 |
| TRINITY_DN17586_c0_g1_i1 | 0.73376087 | 0.477365813 | 0.999970678 | 1.726 | 1.409 | 0.444 | 1.367 | RabGAP-TBC |
| TRINITY_DN2079_c0_g1_i10 | -0.73368475 | 0.68094833 | 1 | 0.382 | 2.72 | 2.922 | 1.347 | NA |
| TRINITY_DN13717_c1_g1_i3 | -0.73361043 | 0.599821563 | 1 | 1.199 | 3.006 | 2.044 | 4.373 | NA |
| TRINITY_DN5941_c0_g1_i1 | 0.733602729 | 0.248391021 | 0.999970678 | 8.275 | 6.958 | 4.255 | 4.062 | - |
| TRINITY_DN225_c0_g1_i3 | 0.733497838 | 0.819250092 | 0.999970678 | 33.32 | 0.365 | 16.866 | 0 | NA |
| TRINITY_DN6787_c0_g2_i2 | 0.733444413 | 0.261979678 | 0.999970678 | 3.388 | 4.642 | 2.011 | 2.403 | IKI3 |
| TRINITY_DN461_c2_g1_i4 | 0.733438593 | 0.479164851 | 0.999970678 | 31.022 | 10.26 | 12.91 | 9.38 | NA |
| TRINITY_DN9043_c0_g1_i8 | -0.73341406 | 0.593233212 | 1 | 0.636 | 0.286 | 0.411 | 1.005 | NA |
| TRINITY_DN3379_c0_g1_i2 | 0.733336223 | 0.551557164 | 0.999970678 | 3.752 | 8.722 | 4.955 | 1.468 | RRM_1 |
| TRINITY_DN234_c2_g1_i6 | -0.73332086 | 0.643714578 | 1 | 0.173 | 0.641 | 0.344 | 0.875 | NA |
| TRINITY_DN10021_c0_g1_i2 | 0.733175008 | 0.794078167 | 0.999970678 | 0 | 1.035 | 0.289 | 0.251 | NIF |
| TRINITY_DN225_c0_g1_i4 | -0.73307256 | 0.809707216 | 0.999970678 | 171.42 | 0 | 220.687 | 16.981 | NA |
| TRINITY_DN12932_c0_g1_i1 | -0.73297547 | 0.535236258 | 0.999970678 | 1.381 | 0.897 | 1.044 | 2.483 | NA |
| TRINITY_DN10212_c1_g1_i2 | 0.732931812 | 0.664771093 | 1 | 0.472 | 1.114 | 0.678 | 0.151 | GUB_WAK_bind |
| TRINITY_DN9507_c1_g1_i1 | 0.732816654 | 0.795203629 | 0.999970678 | 0 | 5.223 | 1.678 | 1.046 | NA |
| TRINITY_DN1849_c0_g1_i4 | 0.732778336 | 0.326472006 | 0.999970678 | 7.376 | 6.741 | 4.478 | 3.097 | - |
| TRINITY_DN1403_c0_g1_i5 | 0.732595279 | 0.365010689 | 0.999970678 | 14.534 | 26.83 | 12.577 | 9.611 | NA |
| TRINITY_DN371_c0_g1_i1 | 0.732478021 | 0.324173422 | 0.999970678 | 14.988 | 16.12 | 9.377 | 7.289 | Spermine_synt_N |
| TRINITY_DN13157_c0_g1_i1 | -0.73243122 | 0.350756497 | 0.999970678 | 8.03 | 12.42 | 16.677 | 13.502 | Ribosomal_L18A |
| TRINITY_DN3054_c0_g1_i2 | -0.73227272 | 0.645118556 | 0.999970678 | 0.563 | 2.602 | 1.022 | 3.911 | NA |
| TRINITY_DN6900_c0_g1_i2 | 0.73218652 | 0.346591382 | 0.999970678 | 3.752 | 2.493 | 1.7 | 1.719 | - |
| TRINITY_DN1352_c1_g1_i6 | 0.732183125 | 0.361812223 | 0.999970678 | 2.089 | 2.622 | 1.289 | 1.267 | NA |
| TRINITY_DN2542_c0_g1_i5 | 0.732077789 | 0.370280978 | 0.999970678 | 13.09 | 7.165 | 5.889 | 5.147 | NA |
| TRINITY_DN11547_c0_g1_i4 | 0.732077769 | 0.612746541 | 0.999970678 | 0.845 | 0.325 | 0.444 | 0.171 | PPR |
| TRINITY_DN15608_c2_g1_i2 | 0.732027579 | 0.80377099 | 0.999970678 | 0 | 58.75 | 23.099 | 6.776 | eIF-1a |
| TRINITY_DN7843_c0_g1_i11 | 0.732007332 | 0.800920755 | 0.999970678 | 1.59 | 0 | 0.611 | 0.241 | LRR_1 |
| TRINITY_DN2595_c0_g1_i4 | -0.73200319 | 0.594685582 | 0.999970678 | 1.036 | 2.346 | 0.733 | 4.725 | Dimerisation |
| TRINITY_DN828_c0_g1_i4 | 0.731965076 | 0.77712584 | 0.999970678 | 0.136 | 7.362 | 0.167 | 4.212 | NA |
| TRINITY_DN12725_c0_g1_i1 | -0.73187454 | 0.287210279 | 0.999970678 | 14.943 | 13.23 | 15.11 | 28.683 | NA |
| TRINITY_DN18032_c0_g1_i2 | -0.73184131 | 0.432336249 | 0.999970678 | 0.981 | 0.67 | 1.344 | 1.126 | NA |
| TRINITY_DN5168_c0_g1_i2 | 0.73179238 | 0.64663013 | 0.999970678 | 4.878 | 0.414 | 0.644 | 2.463 | Bromo_TP |
| TRINITY_DN509_c0_g1_i11 | -0.7317885 | 0.344038945 | 0.999970678 | 25.68 | 51.97 | 51.986 | 65.138 | NA |
| TRINITY_DN462_c0_g1_i7 | -0.73178149 | 0.604918167 | 0.999970678 | 3.679 | 0.601 | 3.855 | 2.523 | NA |
| TRINITY_DN8622_c0_g1_i4 | -0.73178108 | 0.788413696 | 0.999970678 | 1.154 | 0 | 1.155 | 0.553 | NB-ARC |
| TRINITY_DN4434_c0_g1_i2 | -0.73166513 | 0.306336607 | 0.999970678 | 11.964 | 12.67 | 15.144 | 21.756 | NA |
| TRINITY_DN2205_c0_g1_i4 | 0.731655839 | 0.656197103 | 0.999970678 | 12.963 | 6.071 | 9.088 | 0.533 | NA |
| TRINITY_DN3811_c0_g1_i2 | -0.73161758 | 0.495853933 | 0.999970678 | 1.708 | 4.09 | 3.2 | 5.57 | NA |
| TRINITY_DN38208_c0_g1_i2 | 0.731487133 | 0.669337046 | 0.999970678 | 2.071 | 0.276 | 0.233 | 1.126 | DUF647 |
| TRINITY_DN2812_c0_g3_i4 | -0.73142474 | 0.871247784 | 1 | 0.79 | 0 | 0 | 1.307 | NA |
| TRINITY_DN1530_c0_g1_i2 | 0.731366408 | 0.27563967 | 0.999970678 | 2.698 | 2.809 | 1.444 | 1.578 | NA |
| TRINITY_DN18308_c0_g1_i5 | -0.73124283 | 0.786365465 | 0.999970678 | 0 | 1.853 | 1.733 | 0.895 | Pro_isomerase |
| TRINITY_DN4333_c0_g2_i1 | -0.73120158 | 0.238449183 | 0.999970678 | 10.51 | 8.801 | 12.933 | 16.418 | NA |
| TRINITY_DN1263_c0_g1_i2 | -0.73116215 | 0.395743344 | 0.999970678 | 15.497 | 12.92 | 26.809 | 14.668 | - |
| TRINITY_DN11771_c0_g1_i1 | 0.731021585 | 0.489363178 | 0.999970678 | 1.217 | 3.242 | 1.378 | 1.005 | NT-C2 |
| TRINITY_DN257_c2_g1_i2 | 0.73097451 | 0.784032709 | 0.999970678 | 1.408 | 1.143 | 0 | 1.538 | NA |
| TRINITY_DN9296_c0_g1_i7 | 0.730948092 | 0.750593545 | 0.999970678 | 0.064 | 1.468 | 0.733 | 0.03 | Transket_pyr |
| TRINITY_DN7509_c0_g4_i1 | -0.73093317 | 0.577268783 | 0.999970678 | 0.681 | 0.276 | 0.644 | 0.794 | NA |
| TRINITY_DN1095_c0_g2_i5 | -0.73088354 | 0.70050211 | 0.999970678 | 1.254 | 0.187 | 1.678 | 0.352 | NA |

| TRINITY_DN1528_c1_g1_i7 | 0.730842034 | 0.497989053 | 0.999970678 | 3.897 | 2.385 | 2.344 | 0.955 | zf-C2H2 |
| --- | --- | --- | --- | --- | --- | --- | --- | --- |
| TRINITY_DN2603_c0_g1_i5 | -0.73077 | 0.865349404 | 0.999970678 | 8.13 | 0 | 0 | 13.11 | NA |
| TRINITY_DN80_c0_g1_i1 | -0.7306696 | 0.798956374 | 0.999970678 | 22.801 | 0 | 8.911 | 27.638 | RRM_1 |
| TRINITY_DN3632_c0_g1_i2 | -0.73063704 | 0.221619831 | 0.999970678 | 53.959 | 49.03 | 62.885 | 94.937 | EF1G |
| TRINITY_DN7653_c2_g2_i2 | 0.730458757 | 0.57321827 | 0.999970678 | 1.208 | 1.725 | 1.067 | 0.473 | NA |
| TRINITY_DN6523_c0_g1_i7 | -0.73040106 | 0.871280789 | 1 | 1.617 | 0 | 2.244 | 0 | NA |
| TRINITY_DN799_c0_g2_i11 | 0.730266206 | 0.61847834 | 0.999970678 | 45.202 | 216.4 | 16.243 | 137.243 | FA_desaturase |
| TRINITY_DN9748_c0_g1_i2 | -0.73021971 | 0.515381019 | 0.999970678 | 1.036 | 1.892 | 2.611 | 1.769 | Lin0512_fam |
| TRINITY_DN5110_c0_g1_i4 | 0.729990319 | 0.715231224 | 0.999970678 | 65.777 | 3.982 | 1.811 | 38.827 | NA |
| TRINITY_DN160_c0_g1_i10 | -0.72998941 | 0.862947368 | 0.999970678 | 14.843 | 0 | 20.51 | 0 | NA |
| TRINITY_DN2920_c0_g1_i2 | -0.72998646 | 0.862279489 | 0.999970678 | 0 | 3.371 | 4.555 | 0 | NA |
| TRINITY_DN46883_c0_g1_i2 | -0.72994531 | 0.800047663 | 0.999970678 | 0.082 | 0.266 | 0.467 | 0 | NA |
| TRINITY_DN772_c0_g4_i2 | 0.729940145 | 0.577040707 | 0.999970678 | 27.997 | 68.73 | 40.364 | 8.566 | - |
| TRINITY_DN340_c1_g1_i25 | -0.72992153 | 0.797862022 | 0.999970678 | 0.518 | 0 | 0.122 | 0.734 | NA |
| TRINITY_DN1422_c1_g1_i5 | -0.72983166 | 0.655832367 | 0.999970678 | 1.59 | 0.335 | 0.667 | 2.322 | NA |
| TRINITY_DN15645_c0_g1_i1 | -0.72983017 | 0.758894585 | 0.999970678 | 0.027 | 1.34 | 0.111 | 2.101 | NA |
| TRINITY_DN9130_c0_g1_i2 | 0.729804689 | 0.731705952 | 0.999970678 | 0.218 | 6.771 | 2.755 | 0.714 | NA |
| TRINITY_DN2331_c0_g1_i3 | 0.729756705 | 0.86432825 | 0.999970678 | 1.808 | 0 | 0 | 1.106 | NA |
| TRINITY_DN19869_c0_g1_i1 | -0.72971263 | 0.788528931 | 0.999970678 | 1.708 | 0 | 1.089 | 1.538 | NA |
| TRINITY_DN226_c0_g1_i6 | -0.72969638 | 0.441772579 | 0.999970678 | 7.24 | 7.086 | 5.011 | 17.815 | ABC2_membrane |
| TRINITY_DN203_c2_g1_i1 | -0.7296891 | 0.702457741 | 0.999970678 | 1.09 | 1.784 | 3.722 | 0.231 | NA |
| TRINITY_DN6535_c0_g1_i13 | 0.72966125 | 0.78645837 | 0.999970678 | 0.572 | 0.66 | 0 | 0.744 | NA |
| TRINITY_DN7157_c0_g1_i2 | 0.729586145 | 0.267001912 | 0.999970678 | 7.658 | 7.855 | 3.966 | 4.564 | EFP |
| TRINITY_DN21593_c0_g1_i1 | 0.729573605 | 0.702478788 | 0.999970678 | 1.272 | 7.677 | 0.422 | 4.675 | NA |
| TRINITY_DN1981_c0_g1_i1 | -0.72953649 | 0.417255953 | 0.999970678 | 44.584 | 26.15 | 66.763 | 36.907 | NA |
| TRINITY_DN16365_c0_g1_i1 | 0.729455604 | 0.343405909 | 0.999970678 | 2.171 | 1.685 | 0.933 | 1.206 | NA |
| TRINITY_DN581_c0_g1_i6 | -0.72933417 | 0.470567775 | 0.999970678 | 5.85 | 3.637 | 9.577 | 4.202 | Fcf1 |
| TRINITY_DN616_c5_g1_i1 | -0.72931874 | 0.792195277 | 1 | 4.987 | 0 | 2.833 | 4.655 | NA |
| TRINITY_DN9449_c0_g1_i1 | 0.729316095 | 0.332565264 | 0.999970678 | 2.28 | 1.813 | 1.2 | 1.036 | DUF616 |
| TRINITY_DN57070_c0_g1_i1 | 0.729280905 | 0.467928436 | 0.999970678 | 0.836 | 1.074 | 0.589 | 0.442 | FAR1 |
| TRINITY_DN361_c0_g2_i3 | -0.72923789 | 0.39573471 | 0.999970678 | 1.544 | 2.651 | 2.155 | 4.333 | F-box |
| TRINITY_DN28288_c0_g2_i1 | 0.729168989 | 0.818648998 | 1 | 5.123 | 0.256 | 2.689 | 0 | NA |
| TRINITY_DN2618_c0_g2_i2 | -0.72907775 | 0.528883626 | 0.999970678 | 3.443 | 1.34 | 4.666 | 2.363 | Dynein_light |
| TRINITY_DN9206_c0_g1_i1 | -0.72890533 | 0.648322477 | 0.999970678 | 0.827 | 0.246 | 1.211 | 0.322 | Plant_zn_clust |
| TRINITY_DN9000_c0_g1_i2 | -0.72888414 | 0.787715623 | 0.999970678 | 0 | 0.857 | 0.589 | 0.694 | NA |
| TRINITY_DN7756_c0_g1_i1 | -0.72885686 | 0.416897009 | 0.999970678 | 0.899 | 0.897 | 1.189 | 1.538 | NA |
| TRINITY_DN3916_c0_g1_i6 | 0.728816793 | 0.648651026 | 0.999970678 | 0.382 | 0.512 | 0.4 | 0.06 | BET |
| TRINITY_DN1365_c0_g1_i1 | -0.72880283 | 0.446477321 | 0.999970678 | 7.022 | 5.923 | 12.944 | 5.841 | SGL |
| TRINITY_DN3365_c0_g1_i2 | 0.728717541 | 0.343991718 | 0.999970678 | 8.403 | 13.68 | 4.522 | 7.822 | CorA |
| TRINITY_DN1843_c0_g1_i11 | -0.72866807 | 0.514399053 | 0.999970678 | 0.545 | 0.572 | 0.933 | 0.754 | zf-C3HC4_3 |
| TRINITY_DN7140_c0_g1_i2 | -0.72865396 | 0.872522366 | 1 | 0 | 0.552 | 0 | 0.895 | NA |
| TRINITY_DN11401_c0_g1_i7 | -0.7286491 | 0.869178338 | 1 | 0 | 2.907 | 0 | 4.655 | HsbA |
| TRINITY_DN3458_c0_g1_i15 | -0.72864473 | 0.865117058 | 0.999970678 | 0 | 1.163 | 0 | 1.9 | p450 |
| TRINITY_DN12334_c0_g1_i1 | 0.728508711 | 0.390977641 | 0.999970678 | 1.78 | 1.114 | 0.878 | 0.704 | INCENP_ARK-bin |
| TRINITY_DN30748_c0_g1_i6 | -0.72843176 | 0.687136162 | 0.999970678 | 214.83 | 55.5 | 348.79 | 15.01 | NA |
| TRINITY_DN1622_c0_g1_i16 | -0.7283585 | 0.725499254 | 1 | 2.471 | 0.099 | 2 | 1.86 | NA |
| TRINITY_DN8800_c0_g1_i5 | 0.72834101 | 0.473940099 | 0.999970678 | 1.771 | 1.468 | 1.144 | 0.573 | HUN |
| TRINITY_DN11807_c0_g1_i1 | 0.728188166 | 0.482336546 | 0.999970678 | 3.597 | 5.144 | 3.311 | 1.277 | NA |
| TRINITY_DN4875_c0_g1_i11 | 0.72818703 | 0.674844497 | 0.999970678 | 0.69 | 1.074 | 0.056 | 0.985 | HLH |
| TRINITY_DN1116_c2_g1_i3 | -0.72810951 | 0.789145133 | 0.999970678 | 2.816 | 0 | 2.489 | 1.689 | NA |
| TRINITY_DN4596_c0_g3_i4 | 0.728029861 | 0.491884855 | 0.999970678 | 1.081 | 1.902 | 1 | 0.583 | Choline_transpo |
| TRINITY_DN7510_c0_g1_i4 | 0.72781695 | 0.620267207 | 0.999970678 | 9.084 | 1.055 | 2.667 | 2.946 | NA |
| TRINITY_DN2296_c0_g1_i2 | 0.727803981 | 0.863619453 | 0.999970678 | 0 | 3.962 | 1.967 | 0 | - |
| TRINITY_DN1174_c0_g1_i6 | -0.72774566 | 0.795463699 | 0.999970678 | 1.699 | 0 | 1.989 | 0.473 | NA |
| TRINITY_DN97043_c0_g1_i3 | -0.72773992 | 0.863212906 | 0.999970678 | 8.057 | 0 | 11.177 | 0 | NA |
| TRINITY_DN1086_c0_g1_i2 | -0.72769956 | 0.616840335 | 0.999970678 | 0.463 | 1.537 | 1.978 | 0.834 | NA |
| TRINITY_DN4913_c0_g1_i1 | -0.7276128 | 0.292368072 | 0.999970678 | 12.654 | 13.66 | 14.077 | 26.512 | - |
| TRINITY_DN4885_c0_g1_i1 | 0.727597636 | 0.592127163 | 0.999970678 | 19.276 | 3.686 | 8.833 | 3.308 | Pkinase |
| TRINITY_DN17_c0_g1_i2 | 0.727418546 | 0.804239439 | 1 | 0 | 2.178 | 0.8 | 0.312 | NA |
| TRINITY_DN6636_c0_g1_i9 | 0.72726044 | 0.716402569 | 0.999970678 | 3.897 | 15.29 | 0.144 | 11.31 | NA |
| TRINITY_DN613_c0_g1_i4 | 0.727158722 | 0.634462612 | 0.999970678 | 5.832 | 24.27 | 1.655 | 15.875 | p450 |
| TRINITY_DN1812_c0_g3_i1 | 0.727114808 | 0.440877281 | 0.999970678 | 1.726 | 1.498 | 1.067 | 0.674 | NA |
| TRINITY_DN9409_c0_g1_i1 | 0.727098065 | 0.422709176 | 0.999970678 | 5.241 | 2.799 | 2.278 | 2.121 | NA |
| TRINITY_DN486_c0_g1_i4 | 0.727066837 | 0.419530558 | 0.999970678 | 10.946 | 12.12 | 3.722 | 9.229 | NA |
| TRINITY_DN25884_c0_g1_i5 | -0.72694209 | 0.367321705 | 0.999970678 | 2.371 | 2.74 | 2.689 | 5.178 | NA |
| TRINITY_DN748_c0_g1_i1 | -0.72687249 | 0.810549251 | 0.999970678 | 7.004 | 0.641 | 0 | 12.879 | NA |
| TRINITY_DN1970_c0_g1_i2 | 0.726851238 | 0.802102419 | 0.999970678 | 2.035 | 0 | 0.867 | 0.201 | NA |
| TRINITY_DN34_c2_g1_i1 | 0.726803592 | 0.79795568 | 0.999970678 | 0 | 5.953 | 2.333 | 0.714 | WD40 |
| TRINITY_DN218_c0_g1_i3 | 0.726715796 | 0.798526091 | 0.999970678 | 21.656 | 4.957 | 13.399 | 0 | KIX_2 |
| TRINITY_DN1039_c1_g1_i7 | -0.72661622 | 0.533759189 | 0.999970678 | 25.099 | 22.1 | 10.477 | 65.6 | B_lectin |
| TRINITY_DN790_c0_g1_i1 | 0.726601889 | 0.523563405 | 0.999970678 | 11.546 | 39.09 | 7.288 | 21.374 | NA |
| TRINITY_DN4812_c0_g1_i1 | 0.726578528 | 0.318507192 | 0.999970678 | 13.817 | 9.195 | 6.666 | 5.912 | NA |
| TRINITY_DN412_c0_g1_i3 | -0.7265629 | 0.592194835 | 0.999970678 | 0.309 | 1.37 | 0.722 | 1.91 | RRM_1 |
| TRINITY_DN4609_c0_g1_i4 | 0.726543778 | 0.869190981 | 1 | 0.6 | 0 | 0 | 0.372 | NA |

| TRINITY_DN830_c0_g1_i3 | -0.72652914 | 0.463670468 | 0.999970678 | 14.434 | 13.32 | 9.388 | 34.484 | NA |
| --- | --- | --- | --- | --- | --- | --- | --- | --- |
| TRINITY_DN3378_c4_g2_i1 | 0.726517689 | 0.42620015 | 0.999970678 | 869.58 | 385.6 | 403.276 | 276.839 | ABC_tran |
| TRINITY_DN2957_c0_g1_i8 | -0.72638002 | 0.865033005 | 0.999970678 | 36.318 | 0 | 48.442 | 0 | NA |
| TRINITY_DN1583_c0_g1_i2 | 0.726376196 | 0.797243016 | 0.999970678 | 30.259 | 26.7 | 0 | 34.444 | - |
| TRINITY_DN709_c0_g1_i5 | -0.72615356 | 0.564925807 | 0.999970678 | 29.55 | 4.731 | 26.532 | 25.094 | NA |
| TRINITY_DN786_c0_g1_i5 | 0.726145542 | 0.864751216 | 0.999970678 | 12.89 | 0 | 0 | 7.932 | DEAD |
| TRINITY_DN1285_c0_g1_i2 | 0.726100112 | 0.663488798 | 0.999970678 | 10.319 | 17.89 | 13.477 | 0.674 | CDC50 |
| TRINITY_DN12753_c0_g1_i1 | 0.726074494 | 0.811115369 | 1 | 2.344 | 0.217 | 1.3 | 0 | NA |
| TRINITY_DN7441_c0_g1_i3 | -0.72603988 | 0.704353922 | 0.999970678 | 1.254 | 0.069 | 1.422 | 0.462 | - |
| TRINITY_DN6318_c0_g3_i1 | 0.725982986 | 0.350782926 | 0.999970678 | 5.278 | 10.53 | 4.222 | 4.434 | Myb_DNA-bindin |
| TRINITY_DN2877_c0_g1_i4 | -0.72594506 | 0.796094421 | 0.999970678 | 2.144 | 0.739 | 0 | 4.796 | GST_C |
| TRINITY_DN969_c0_g1_i2 | 0.725847859 | 0.482950866 | 0.999970678 | 0.908 | 1.064 | 0.556 | 0.503 | Calmodulin_bind |
| TRINITY_DN9096_c0_g1_i2 | -0.72582792 | 0.761645391 | 0.999970678 | 0.027 | 0.848 | 0.067 | 1.337 | NA |
| TRINITY_DN9272_c0_g1_i1 | 0.72580829 | 0.448648324 | 0.999970678 | 0.627 | 1.252 | 0.522 | 0.503 | DEAD |
| TRINITY_DN25665_c0_g1_i3 | 0.725808073 | 0.794251572 | 1 | 0.799 | 0.365 | 0 | 0.714 | - |
| TRINITY_DN11395_c0_g1_i3 | -0.72576011 | 0.367899009 | 0.999970678 | 20.266 | 18.3 | 17.143 | 43.11 | NA |
| TRINITY_DN9829_c1_g1_i3 | 0.725675122 | 0.785955017 | 0.999970678 | 1.072 | 0.788 | 0.933 | 0 | MULE |
| TRINITY_DN7606_c0_g1_i2 | -0.72557385 | 0.541498986 | 0.999970678 | 1.29 | 5.608 | 4.4 | 5.942 | NA |
| TRINITY_DN1216_c0_g1_i3 | -0.72542451 | 0.383914798 | 0.999970678 | 9.293 | 8.052 | 7.9 | 19.062 | NA |
| TRINITY_DN14336_c0_g1_i2 | -0.72541412 | 0.64777395 | 0.999970678 | 0.736 | 5.765 | 4.911 | 4.494 | NA |
| TRINITY_DN11114_c0_g1_i6 | -0.72540968 | 0.868999004 | 0.999970678 | 13.235 | 0 | 18.299 | 0 | MAPEG |
| TRINITY_DN1922_c4_g1_i1 | 0.725392912 | 0.395017346 | 0.999970678 | 9.792 | 6.731 | 5.466 | 3.408 | NA |
| TRINITY_DN98_c0_g1_i6 | -0.72534274 | 0.348747624 | 0.999970678 | 3.57 | 6.84 | 7.222 | 8.405 | Sigma70_r2 |
| TRINITY_DN13873_c0_g3_i1 | -0.72529871 | 0.583032055 | 0.999970678 | 0.581 | 0.7 | 0.322 | 1.719 | adh_short |
| TRINITY_DN14197_c0_g1_i2 | 0.725237748 | 0.794160055 | 1 | 1.799 | 1.853 | 1.822 | 0 | NA |
| TRINITY_DN9505_c0_g1_i16 | -0.72501463 | 0.762540654 | 1 | 0.908 | 0.118 | 1.4 | 0.04 | NA |
| TRINITY_DN18145_c0_g1_i2 | 0.724673076 | 0.556463236 | 0.999970678 | 1.735 | 2.375 | 1.689 | 0.432 | NA |
| TRINITY_DN23538_c0_g1_i1 | 0.724614989 | 0.499545278 | 0.999970678 | 3.188 | 1.991 | 1.433 | 1.387 | Acetyltransf_1 |
| TRINITY_DN26172_c1_g2_i1 | -0.7245639 | 0.868801436 | 0.999970678 | 19.331 | 0 | 0 | 32.534 | NA |
| TRINITY_DN446_c0_g1_i4 | -0.72454313 | 0.370734859 | 0.999970678 | 33.974 | 64.47 | 52.663 | 96.968 | NA |
| TRINITY_DN1035_c0_g2_i5 | -0.7241474 | 0.701758533 | 1 | 3.697 | 0.286 | 3.322 | 2.493 | NA |
| TRINITY_DN2599_c0_g1_i4 | 0.724058643 | 0.311225703 | 0.999970678 | 173.82 | 143.6 | 93.483 | 72.135 | UbiA |
| TRINITY_DN265_c1_g2_i2 | -0.72403937 | 0.497726205 | 0.999970678 | 25.353 | 7.46 | 19.677 | 30.111 | NA |
| TRINITY_DN2863_c0_g1_i17 | -0.72388627 | 0.806600937 | 0.999970678 | 0 | 1.587 | 2 | 0.191 | Aa_trans |
| TRINITY_DN275_c0_g1_i3 | 0.723877206 | 0.80743865 | 0.999970678 | 1.453 | 0 | 0.667 | 0.09 | ABC_membrane |
| TRINITY_DN833_c0_g2_i2 | 0.723875314 | 0.581716243 | 0.999970678 | 3.525 | 2.03 | 0.467 | 2.815 | NA |
| TRINITY_DN28502_c0_g1_i2 | -0.72381042 | 0.602202439 | 0.999970678 | 6.976 | 40.99 | 17.088 | 54.752 | NA |
| TRINITY_DN5381_c0_g1_i4 | 0.723794201 | 0.512859776 | 0.999970678 | 0.854 | 0.907 | 0.656 | 0.271 | ABC_membrane |
| TRINITY_DN12257_c0_g1_i9 | -0.72379244 | 0.744788037 | 0.999970678 | 8.548 | 0.217 | 10.044 | 2.292 | NA |
| TRINITY_DN7484_c0_g1_i3 | 0.723772136 | 0.392743017 | 0.999970678 | 3.933 | 1.932 | 1.578 | 1.669 | Pkinase |
| TRINITY_DN8588_c0_g1_i4 | -0.72373858 | 0.596584864 | 0.999970678 | 1.054 | 0.414 | 1.255 | 0.845 | NA |
| TRINITY_DN72_c1_g1_i5 | -0.72372669 | 0.715302363 | 0.999970678 | 0.154 | 4.198 | 5.422 | 0.533 | Hydrolase_4 |
| TRINITY_DN153_c0_g2_i2 | -0.72369422 | 0.797396303 | 0.999970678 | 21.12 | 0 | 16.399 | 15.563 | Sina |
| TRINITY_DN1865_c0_g1_i6 | 0.723674525 | 0.359107126 | 0.999970678 | 10.501 | 5.578 | 3.655 | 5.399 | LRR_4 |
| TRINITY_DN3073_c0_g1_i1 | 0.723670507 | 0.571867614 | 0.999970678 | 2.198 | 1.124 | 0.544 | 1.367 | NA |
| TRINITY_DN6022_c0_g1_i9 | 0.723669713 | 0.401657243 | 0.999970678 | 0.781 | 1.409 | 0.578 | 0.623 | PNRC |
| TRINITY_DN4721_c0_g2_i4 | -0.72353982 | 0.201045633 | 0.999970678 | 24 | 23.42 | 32.009 | 39.581 | NA |
| TRINITY_DN11815_c0_g1_i2 | 0.723525347 | 0.863574433 | 0.999970678 | 12.3 | 0 | 0 | 7.56 | zf-CCCH |
| TRINITY_DN3287_c0_g3_i1 | 0.723484028 | 0.522033782 | 0.999970678 | 4.969 | 5.125 | 3.889 | 1.397 | tRNA-synt_1c |
| TRINITY_DN4235_c1_g1_i7 | 0.723296817 | 0.619504763 | 0.999970678 | 0.318 | 1.951 | 0.867 | 0.302 | HEAT |
| TRINITY_DN6245_c0_g1_i1 | -0.72321903 | 0.510738816 | 0.999970678 | 8.012 | 19.44 | 9.222 | 33.821 | NA |
| TRINITY_DN187_c0_g1_i10 | -0.72320285 | 0.485501204 | 0.999970678 | 7.04 | 20.82 | 13.599 | 28.904 | - |
| TRINITY_DN9672_c0_g2_i4 | 0.723182061 | 0.795984482 | 0.999970678 | 2.416 | 0.335 | 0 | 1.689 | NA |
| TRINITY_DN89327_c0_g1_i1 | 0.722977288 | 0.48724536 | 0.999970678 | 0.472 | 0.788 | 0.378 | 0.302 | - |
| TRINITY_DN24024_c1_g4_i1 | -0.72289578 | 0.641122375 | 0.999970678 | 0.127 | 0.266 | 0.433 | 0.121 | NA |
| TRINITY_DN13_c1_g2_i1 | 0.722711305 | 0.465500765 | 0.999970678 | 10.483 | 5.519 | 2.689 | 6.434 | NA |
| TRINITY_DN41490_c0_g3_i1 | -0.72265815 | 0.873643509 | 1 | 1.272 | 0 | 1.755 | 0 | EF-1_beta_acid |
| TRINITY_DN3651_c0_g2_i2 | -0.72265815 | 0.873643509 | 1 | 2.634 | 0 | 3.611 | 0 | Glyoxalase |
| TRINITY_DN9826_c0_g1_i1 | -0.72265815 | 0.873643509 | 1 | 1.145 | 0 | 1.578 | 0 | NA |
| TRINITY_DN1304_c0_g1_i2 | -0.72263635 | 0.794200453 | 0.999970678 | 0.418 | 0.552 | 1.333 | 0 | NA |
| TRINITY_DN9752_c0_g1_i6 | 0.722492809 | 0.868626732 | 0.999970678 | 241.99 | 0 | 0 | 132.789 | NA |
| TRINITY_DN7345_c1_g1_i1 | 0.722465422 | 0.770176776 | 0.999970678 | 0.055 | 51.8 | 3.355 | 26.723 | NA |
| TRINITY_DN1647_c0_g2_i1 | -0.72230518 | 0.827640785 | 0.999970678 | 3.207 | 0.049 | 4.489 | 0 | NA |
| TRINITY_DN1344_c0_g1_i13 | -0.72225077 | 0.42352918 | 0.999970678 | 25.726 | 47.73 | 31.676 | 81.897 | NA |
| TRINITY_DN1306_c0_g1_i5 | -0.72222028 | 0.681917632 | 0.999970678 | 2.925 | 37.61 | 5.433 | 59.076 | NA |
| TRINITY_DN6817_c0_g1_i9 | 0.722210993 | 0.593615023 | 0.999970678 | 6.976 | 4.307 | 4.911 | 0.955 | NA |
| TRINITY_DN4443_c0_g1_i2 | 0.722166446 | 0.468140735 | 0.999970678 | 0.636 | 0.512 | 0.344 | 0.292 | zf-C3HC4_3 |
| TRINITY_DN10395_c0_g1_i3 | 0.722083385 | 0.643063177 | 0.999970678 | 0.618 | 3.065 | 1.644 | 0.231 | Chromo |
| TRINITY_DN7425_c0_g1_i1 | -0.72207243 | 0.384313764 | 0.999970678 | 1.553 | 1.261 | 2.144 | 2.071 | BTB_2 |
| TRINITY_DN2640_c1_g1_i3 | 0.722059984 | 0.351383336 | 0.999970678 | 4.869 | 7.293 | 2.955 | 3.78 | NA |
| TRINITY_DN48422_c0_g1_i2 | 0.722010201 | 0.600084841 | 0.999970678 | 0.236 | 0.926 | 0.378 | 0.261 | NA |
| TRINITY_DN4647_c0_g1_i10 | 0.721975071 | 0.654070726 | 0.999970678 | 9.565 | 8.751 | 8.177 | 0.975 | NA |
| TRINITY_DN1148_c0_g1_i3 | 0.72191776 | 0.820648249 | 0.999970678 | 0 | 40.56 | 19.488 | 0.452 | Glyco_hydro_1 |
| TRINITY_DN1587_c0_g1_i1 | 0.721897907 | 0.473108013 | 0.999970678 | 1.244 | 0.69 | 0.667 | 0.382 | NA |

| TRINITY_DN2016_c0_g1_i1 | 0.721879714 | 0.41018445 | 0.999970678 | 2.671 | 4.524 | 1.4 | 2.664 | NA |
| --- | --- | --- | --- | --- | --- | --- | --- | --- |
| TRINITY_DN2269_c0_g2_i5 | -0.7218749 | 0.570032427 | 0.999970678 | 14.689 | 86.11 | 45.097 | 106.579 | NA |
| TRINITY_DN3011_c0_g1_i2 | 0.721739951 | 0.711558123 | 0.999970678 | 28.296 | 1.271 | 13.844 | 1.387 | NA |
| TRINITY_DN3874_c0_g2_i5 | 0.721677454 | 0.561344531 | 0.999970678 | 0.836 | 0.917 | 0.689 | 0.221 | Lactamase_B_2 |
| TRINITY_DN2305_c0_g1_i1 | -0.72165104 | 0.870958654 | 1 | 0 | 0.631 | 0.833 | 0 | NA |
| TRINITY_DN5714_c0_g1_i1 | -0.72156342 | 0.561603513 | 0.999970678 | 13.226 | 6.968 | 4.489 | 27.969 | NA |
| TRINITY_DN1329_c1_g1_i4 | -0.72143312 | 0.809580006 | 0.999970678 | 60.98 | 0 | 23.421 | 73.804 | NA |
| TRINITY_DN14236_c0_g1_i1 | -0.72142336 | 0.640859054 | 0.999970678 | 107.44 | 1484 | 556.311 | 1876.04 | Chloroa_b-bind |
| TRINITY_DN43753_c1_g1_i1 | 0.721344877 | 0.637398209 | 0.999970678 | 0.3 | 0.591 | 0.389 | 0.07 | NA |
| TRINITY_DN863_c3_g1_i1 | -0.7212652 | 0.697239346 | 0.999970678 | 0.59 | 1.34 | 2.478 | 0.131 | NA |
| TRINITY_DN10327_c0_g1_i1 | 0.721226585 | 0.488587922 | 0.999970678 | 4.079 | 4.336 | 3.233 | 1.206 | NA |
| TRINITY_DN2693_c0_g1_i3 | 0.721185754 | 0.808911385 | 0.999970678 | 1.254 | 0 | 0.533 | 0.121 | NA |
| TRINITY_DN4909_c2_g1_i2 | -0.7209931 | 0.390725362 | 0.999970678 | 594.13 | 1383 | 1505.476 | 1403.315 | PsaN |
| TRINITY_DN249_c0_g1_i3 | 0.720965924 | 0.663310525 | 0.999970678 | 0.454 | 3.439 | 0.422 | 1.81 | NA |
| TRINITY_DN7054_c0_g2_i2 | 0.72090342 | 0.58661403 | 0.999970678 | 1.517 | 0.857 | 0.244 | 1.146 | NA |
| TRINITY_DN2567_c0_g1_i1 | 0.720864987 | 0.458834572 | 0.999970678 | 0.609 | 0.552 | 0.222 | 0.452 | Hemerythrin |
| TRINITY_DN33333_c0_g1_i2 | 0.720683154 | 0.864908605 | 0.999970678 | 103.42 | 0 | 0 | 60.805 | Gp_dh_C |
| TRINITY_DN5093_c0_g1_i3 | 0.720665727 | 0.807482831 | 0.999970678 | 1.69 | 0.099 | 0 | 1.116 | NA |
| TRINITY_DN680_c0_g3_i1 | -0.72066472 | 0.560788949 | 0.999970678 | 3.161 | 0.897 | 3.966 | 1.93 | Rrp15p |
| TRINITY_DN668_c2_g1_i13 | 0.720661729 | 0.594570453 | 0.999970678 | 1.69 | 2.217 | 1.489 | 0.523 | DUF659 |
| TRINITY_DN6906_c0_g1_i8 | -0.72049555 | 0.389449842 | 0.999970678 | 5.36 | 8.919 | 6.733 | 15.372 | NA |
| TRINITY_DN12373_c2_g1_i3 | -0.72047979 | 0.798765771 | 0.999970678 | 0.745 | 0.848 | 2.2 | 0 | NA |
| TRINITY_DN62269_c0_g2_i1 | 0.720364754 | 0.492154562 | 0.999970678 | 16.442 | 13.6 | 11.877 | 3.931 | NA |
| TRINITY_DN9932_c0_g2_i1 | -0.72014706 | 0.655858835 | 0.999970678 | 0.663 | 3.706 | 0.867 | 6.042 | NA |
| TRINITY_DN2065_c0_g1_i3 | 0.720118587 | 0.865032113 | 0.999970678 | 5.823 | 0 | 0 | 3.609 | HAD_2 |
| TRINITY_DN3425_c0_g1_i7 | -0.72000279 | 0.797676499 | 0.999970678 | 3.752 | 2.326 | 8.077 | 0 | NA |
| TRINITY_DN5676_c1_g2_i1 | 0.719971715 | 0.333799333 | 0.999970678 | 72.608 | 49.86 | 37.898 | 28.784 | Cu-oxidase |
| TRINITY_DN58850_c0_g1_i1 | 0.719955492 | 0.867471903 | 0.999970678 | 4.66 | 0 | 2.367 | 0 | NA |
| TRINITY_DN21524_c0_g1_i2 | -0.71989545 | 0.547101267 | 0.999970678 | 0.908 | 0.68 | 1.533 | 0.734 | PC-Esterase |
| TRINITY_DN21397_c0_g1_i1 | 0.719861351 | 0.866090015 | 0.999970678 | 6.495 | 0 | 3.3 | 0 | NA |
| TRINITY_DN6768_c0_g1_i6 | 0.719829914 | 0.477010692 | 0.999970678 | 2.671 | 1.015 | 1.089 | 0.925 | AMP-binding |
| TRINITY_DN15172_c0_g1_i1 | 0.71975716 | 0.588021537 | 0.999970678 | 2.58 | 1.232 | 0.478 | 1.729 | SRF-TF |
| TRINITY_DN7796_c1_g1_i4 | -0.7197273 | 0.630033929 | 0.999970678 | 8.266 | 4.179 | 14.732 | 2.383 | NA |
| TRINITY_DN30697_c0_g1_i1 | 0.719632057 | 0.470863686 | 0.999970678 | 0.808 | 0.611 | 0.367 | 0.422 | DUF2439 |
| TRINITY_DN5712_c0_g1_i1 | -0.71962081 | 0.625946254 | 0.999970678 | 7.603 | 57.45 | 16.31 | 85.999 | NA |
| TRINITY_DN616_c1_g3_i2 | 0.719519577 | 0.677886077 | 0.999970678 | 1.69 | 0.542 | 0.1 | 1.247 | NA |
| TRINITY_DN1245_c0_g1_i4 | -0.71938146 | 0.311956032 | 0.999970678 | 8.466 | 12.11 | 11.966 | 19.203 | F-box |
| TRINITY_DN3943_c0_g1_i1 | -0.71920545 | 0.444974309 | 0.999970678 | 13.599 | 19.53 | 32.609 | 14.829 | NA |
| TRINITY_DN2499_c0_g1_i4 | -0.71918565 | 0.863965279 | 0.999970678 | 0 | 3.824 | 0 | 6.213 | NA |
| TRINITY_DN14327_c0_g1_i1 | 0.719124211 | 0.470518091 | 0.999970678 | 5.06 | 4.652 | 1.644 | 3.841 | - |
| TRINITY_DN484_c0_g1_i3 | 0.718911044 | 0.593947027 | 0.999970678 | 5.605 | 0.857 | 1.722 | 1.88 | NA |
| TRINITY_DN8610_c0_g2_i1 | 0.718882396 | 0.754955413 | 0.999970678 | 7.149 | 0.907 | 4.055 | 0.03 | NA |
| TRINITY_DN2264_c0_g1_i9 | 0.718820781 | 0.220465388 | 0.999970678 | 41.359 | 48.37 | 24.254 | 25.185 | Patatin |
| TRINITY_DN30378_c0_g1_i16 | 0.718659968 | 0.806925813 | 0.999970678 | 0 | 7.855 | 0.389 | 4.223 | Sugar_tr |
| TRINITY_DN15583_c0_g1_i1 | 0.7186201 | 0.562007236 | 0.999970678 | 0.59 | 1.419 | 0.389 | 0.734 | NA |
| TRINITY_DN4786_c0_g1_i6 | -0.71859928 | 0.545317871 | 0.999970678 | 2.752 | 1.468 | 4.389 | 1.629 | Abhydrolase_1 |
| TRINITY_DN1993_c2_g2_i1 | -0.71853529 | 0.489928846 | 0.999970678 | 2.698 | 6.357 | 8.322 | 4.755 | NA |
| TRINITY_DN19645_c1_g1_i1 | 0.718518844 | 0.568871767 | 0.999970678 | 0.6 | 1.754 | 0.744 | 0.523 | Mlo |
| TRINITY_DN1774_c0_g1_i2 | -0.71850215 | 0.699733738 | 0.999970678 | 57.538 | 0.926 | 32.831 | 57.96 | NA |
| TRINITY_DN7777_c0_g1_i2 | -0.71849794 | 0.593184433 | 0.999970678 | 16.56 | 11.27 | 33.842 | 4.997 | PABP |
| TRINITY_DN1430_c0_g1_i2 | -0.71843553 | 0.658307985 | 0.999970678 | 3.225 | 51.16 | 15.832 | 69.34 | 6PGD |
| TRINITY_DN26283_c1_g1_i1 | -0.71838233 | 0.720397683 | 1 | 1.154 | 0.079 | 1.344 | 0.432 | NA |
| TRINITY_DN7745_c0_g1_i1 | 0.718358278 | 0.284006892 | 0.999970678 | 7.213 | 7.51 | 4.444 | 3.599 | Syja_N |
| TRINITY_DN10657_c0_g1_i1 | -0.71823907 | 0.798188132 | 0.999970678 | 0.363 | 0.434 | 1.089 | 0 | Mem_trans |
| TRINITY_DN9854_c0_g1_i1 | 0.71821957 | 0.712000384 | 0.999970678 | 2.571 | 101 | 10.166 | 46.086 | NA |
| TRINITY_DN88657_c0_g1_i1 | 0.718166466 | 0.870938139 | 0.999970678 | 1.499 | 0 | 0.767 | 0 | Motile_Sperm |
| TRINITY_DN4826_c0_g1_i3 | 0.718141744 | 0.409709073 | 0.999970678 | 5.078 | 2.651 | 1.755 | 2.614 | NA |
| TRINITY_DN626_c0_g1_i5 | 0.718057121 | 0.509115305 | 0.999970678 | 1.026 | 2.129 | 0.489 | 1.317 | - |
| TRINITY_DN12633_c0_g1_i1 | -0.71803545 | 0.818398731 | 1 | 0.182 | 2.484 | 0 | 4.263 | NA |
| TRINITY_DN3772_c0_g1_i5 | 0.717900918 | 0.669864017 | 0.999970678 | 0.4 | 0.601 | 0.456 | 0.05 | Cu_amine_oxid |
| TRINITY_DN237_c0_g1_i2 | -0.71785132 | 0.613059014 | 0.999970678 | 2.035 | 1.518 | 4.3 | 0.633 | NA |
| TRINITY_DN16034_c0_g1_i10 | 0.717820854 | 0.586334232 | 0.999970678 | 1.19 | 1.892 | 1.311 | 0.292 | NA |
| TRINITY_DN1873_c3_g1_i8 | -0.71775123 | 0.868075388 | 0.999970678 | 1.39 | 0 | 0 | 2.322 | NA |
| TRINITY_DN5152_c0_g1_i3 | -0.71763539 | 0.568586182 | 0.999970678 | 5.123 | 1.143 | 3.211 | 6.485 | NA |
| TRINITY_DN61078_c0_g1_i1 | -0.71763318 | 0.24557359 | 0.999970678 | 739.3 | 1029 | 1110.088 | 1468.714 | NA |
| TRINITY_DN6355_c0_g3_i1 | -0.71760981 | 0.816552629 | 0.999970678 | 6.777 | 0 | 8.666 | 0.623 | NA |
| TRINITY_DN16343_c0_g1_i1 | -0.71754823 | 0.867128516 | 0.999970678 | 79.448 | 0 | 107.982 | 0 | NA |
| TRINITY_DN8047_c0_g1_i3 | -0.71748457 | 0.645367045 | 0.999970678 | 6.831 | 2.247 | 2.489 | 11.079 | NA |
| TRINITY_DN9731_c1_g1_i8 | 0.717422272 | 0.461486583 | 0.999970678 | 1.435 | 0.986 | 0.756 | 0.573 | COBRA |
| TRINITY_DN13608_c0_g1_i6 | -0.71737756 | 0.43163311 | 0.999970678 | 1.254 | 0.966 | 1.889 | 1.377 | NA |
| TRINITY_DN20225_c0_g1_i1 | 0.717299095 | 0.868251582 | 0.999970678 | 2.798 | 0 | 1.433 | 0 | NA |
| TRINITY_DN15558_c0_g2_i4 | 0.717299095 | 0.868251582 | 0.999970678 | 15.724 | 0 | 7.922 | 0 | NA |
| TRINITY_DN4_c3_g2_i1 | -0.71729009 | 0.554299892 | 0.999970678 | 8.775 | 1.981 | 5.166 | 11.592 | - |
| TRINITY_DN2336_c0_g1_i4 | 0.717284781 | 0.614644119 | 0.999970678 | 95.3 | 85.5 | 7.277 | 101.231 | NA |

| TRINITY_DN4598_c1_g1_i5 | 0.717259495 | 0.53127744 | 0.999970678 | 3.879 | 1.232 | 1.022 | 1.88 | Peptidase_C50 |
| --- | --- | --- | --- | --- | --- | --- | --- | --- |
| TRINITY_DN21144_c0_g1_i3 | 0.71725683 | 0.614469743 | 0.999970678 | 2.153 | 1.38 | 0.222 | 1.88 | PPR |
| TRINITY_DN604_c1_g2_i4 | 0.717210419 | 0.785299153 | 0.999970678 | 0.89 | 8337 | 1968.792 | 2246.488 | NA |
| TRINITY_DN19498_c0_g1_i1 | -0.71717219 | 0.63734398 | 1 | 2.589 | 0.769 | 3.144 | 1.749 | NA |
| TRINITY_DN9852_c0_g1_i5 | -0.71707388 | 0.805306684 | 1 | 0 | 0.286 | 0.311 | 0.08 | NA |
| TRINITY_DN9160_c0_g1_i4 | -0.71700623 | 0.486780285 | 0.999970678 | 1.553 | 0.897 | 2.211 | 1.357 | NA |
| TRINITY_DN1873_c5_g1_i3 | 0.716903464 | 0.39229851 | 0.999970678 | 4.279 | 4.277 | 1.433 | 3.479 | NA |
| TRINITY_DN16888_c1_g1_i4 | 0.716847816 | 0.379765721 | 0.999970678 | 2.035 | 1.922 | 0.822 | 1.428 | NA |
| TRINITY_DN15169_c0_g1_i1 | -0.71684758 | 0.433742008 | 0.999970678 | 1.926 | 1.104 | 2.278 | 2.252 | NA |
| TRINITY_DN23399_c0_g1_i10 | -0.7167422 | 0.6377169 | 0.999970678 | 0.1 | 0.631 | 0.589 | 0.473 | NA |
| TRINITY_DN1149_c0_g1_i10 | 0.716722428 | 0.81173494 | 0.999970678 | 105.2 | 0 | 36.931 | 20.188 | NA |
| TRINITY_DN6543_c0_g1_i9 | -0.71653129 | 0.797577064 | 0.999970678 | 1.662 | 0 | 1.744 | 0.664 | NA |
| TRINITY_DN3269_c0_g1_i7 | -0.71650764 | 0.410372401 | 0.999970678 | 2.207 | 2.996 | 4.955 | 2.564 | Proton_antipo_M |
| TRINITY_DN4877_c0_g1_i5 | 0.716497226 | 0.392170528 | 0.999970678 | 25.371 | 19.76 | 8.577 | 16.458 | NA |
| TRINITY_DN465_c0_g1_i3 | -0.71639688 | 0.471633691 | 0.999970678 | 11.927 | 4.395 | 13.255 | 10.878 | Autophagy_act_C |
| TRINITY_DN528_c0_g1_i6 | 0.7163334 | 0.560914425 | 0.999970678 | 26.552 | 5.943 | 11.877 | 5.65 | EGF_CA |
| TRINITY_DN4429_c0_g1_i5 | 0.716330375 | 0.660827771 | 0.999970678 | 0.808 | 14.95 | 2.155 | 6.897 | DUF5110 |
| TRINITY_DN5785_c0_g1_i2 | 0.716285007 | 0.86584821 | 0.999970678 | 13.68 | 0 | 0 | 8.455 | NA |
| TRINITY_DN6187_c0_g1_i3 | -0.71612664 | 0.423944712 | 0.999970678 | 0.808 | 1.163 | 1.222 | 1.739 | NA |
| TRINITY_DN67651_c0_g1_i2 | 0.716050083 | 0.871649299 | 1 | 1.444 | 0 | 0.744 | 0 | Complex1_49kDa |
| TRINITY_DN4080_c0_g1_i1 | -0.71603111 | 0.363796241 | 0.999970678 | 47.227 | 74.36 | 61.007 | 120.604 | NA |
| TRINITY_DN4236_c0_g1_i5 | -0.71601647 | 0.79797678 | 0.999970678 | 0.927 | 0 | 0.933 | 0.412 | NA |
| TRINITY_DN80553_c0_g1_i1 | -0.71596122 | 0.865417312 | 0.999970678 | 3.843 | 0 | 5.289 | 0 | NA |
| TRINITY_DN2404_c0_g1_i13 | 0.715905999 | 0.866658492 | 0.999970678 | 1.735 | 0 | 0 | 1.066 | - |
| TRINITY_DN7287_c0_g3_i1 | 0.715872164 | 0.273905846 | 0.999970678 | 16.242 | 16.83 | 6.988 | 11.692 | NA |
| TRINITY_DN1001_c0_g1_i3 | -0.71587019 | 0.651174528 | 0.999970678 | 3.997 | 0.246 | 2.966 | 3.519 | MAP70 |
| TRINITY_DN161_c0_g2_i1 | -0.71577963 | 0.342977674 | 0.999970678 | 69.61 | 70.25 | 65.296 | 149.81 | 2014/3/3 |
| TRINITY_DN395_c0_g2_i3 | -0.7156993 | 0.647139921 | 0.999970678 | 3.779 | 21.47 | 5.166 | 33.499 | NA |
| TRINITY_DN4333_c0_g1_i12 | -0.71564606 | 0.797615949 | 0.999970678 | 0.654 | 0.483 | 0 | 1.87 | NA |
| TRINITY_DN1853_c0_g1_i2 | -0.71552277 | 0.806660167 | 0.999970678 | 0.545 | 2.897 | 0 | 5.489 | NA |
| TRINITY_DN4591_c0_g1_i1 | 0.715436053 | 0.321279662 | 0.999970678 | 8.512 | 9.116 | 5.533 | 4.062 | NA |
| TRINITY_DN4355_c0_g1_i6 | -0.71532008 | 0.794483896 | 0.999970678 | 3.452 | 0 | 3.455 | 1.558 | NA |
| TRINITY_DN13828_c0_g2_i1 | 0.715292274 | 0.794672214 | 0.999970678 | 2.734 | 5.499 | 0 | 5.027 | NA |
| TRINITY_DN2451_c0_g1_i2 | -0.71523557 | 0.790069666 | 0.999970678 | 0 | 0.788 | 0.5 | 0.674 | NA |
| TRINITY_DN8622_c0_g1_i8 | 0.715150823 | 0.701858314 | 0.999970678 | 0.073 | 1.882 | 0.589 | 0.442 | NB-ARC |
| TRINITY_DN2771_c0_g1_i5 | -0.71514364 | 0.450115899 | 0.999970678 | 15.461 | 9.786 | 24.543 | 11.843 | NA |
| TRINITY_DN12850_c1_g1_i1 | -0.7151168 | 0.798518533 | 1 | 0 | 3.371 | 1.189 | 3.921 | NA |
| TRINITY_DN632_c0_g1_i1 | 0.715071492 | 0.503728669 | 0.999970678 | 5.659 | 6.573 | 1.389 | 5.791 | DAO |
| TRINITY_DN479_c0_g1_i4 | -0.71505013 | 0.455295538 | 0.999970678 | 27.234 | 14.33 | 37.809 | 21.756 | DUF4228 |
| TRINITY_DN50637_c0_g1_i6 | -0.71476211 | 0.597610603 | 0.999970678 | 20.693 | 6.11 | 5.544 | 37.872 | TPP_enzyme_C |
| TRINITY_DN6277_c0_g3_i2 | -0.7146705 | 0.552144573 | 0.999970678 | 0.763 | 1.261 | 2 | 0.895 | Cytochrom_C_as |
| TRINITY_DN34989_c0_g1_i1 | 0.714641502 | 0.867463185 | 0.999970678 | 4.769 | 0 | 2.433 | 0 | - |
| TRINITY_DN24305_c0_g1_i3 | -0.71453808 | 0.754390314 | 0.999970678 | 0.045 | 30.12 | 23.976 | 19.776 | NA |
| TRINITY_DN8985_c0_g1_i3 | 0.714441885 | 0.592544844 | 0.999970678 | 10.728 | 57.19 | 9.611 | 28.351 | NA |
| TRINITY_DN53848_c0_g1_i1 | 0.714357965 | 0.869113012 | 0.999970678 | 1.453 | 0 | 0.744 | 0 | DUF1996 |
| TRINITY_DN1186_c0_g1_i6 | 0.714347667 | 0.38183265 | 0.999970678 | 8.866 | 13.08 | 7.122 | 4.715 | NA |
| TRINITY_DN7428_c0_g2_i4 | -0.71430993 | 0.64520487 | 0.999970678 | 1.081 | 0.345 | 0.3 | 2.001 | NA |
| TRINITY_DN1795_c0_g1_i1 | 0.714282997 | 0.70679036 | 0.999970678 | 0.136 | 3.922 | 1.689 | 0.412 | NA |
| TRINITY_DN384_c0_g1_i1 | -0.71427229 | 0.814560985 | 0.999970678 | 25.944 | 0 | 1.778 | 40.888 | NA |
| TRINITY_DN539_c0_g1_i6 | -0.71426149 | 0.579204362 | 0.999970678 | 59.354 | 154.9 | 43.408 | 294.071 | NA |
| TRINITY_DN9008_c0_g1_i1 | -0.71419752 | 0.461571299 | 0.999970678 | 1.935 | 2.838 | 1.889 | 5.56 | Aa_trans |
| TRINITY_DN319_c0_g1_i9 | 0.714134534 | 0.453628428 | 0.999970678 | 1.889 | 1.557 | 1.111 | 0.754 | MFS_1 |
| TRINITY_DN3024_c0_g1_i2 | 0.714111001 | 0.384321603 | 0.999970678 | 2.325 | 2.434 | 1.044 | 1.649 | NA |
| TRINITY_DN2091_c0_g1_i5 | -0.71399638 | 0.432324389 | 0.999970678 | 5.142 | 4.829 | 3.955 | 11.582 | LRR_6 |
| TRINITY_DN3930_c0_g1_i7 | 0.713918602 | 0.282282896 | 0.999970678 | 81.946 | 59.16 | 39.475 | 38.335 | NA |
| TRINITY_DN4624_c0_g1_i4 | -0.71388473 | 0.311736824 | 0.999970678 | 8.112 | 5.45 | 8.666 | 11.823 | MBD |
| TRINITY_DN4295_c0_g3_i2 | -0.71380962 | 0.543850092 | 0.999970678 | 7.603 | 4.317 | 13.244 | 3.649 | NA |
| TRINITY_DN84880_c0_g1_i3 | 0.71375974 | 0.872412722 | 1 | 1.008 | 0 | 0.511 | 0 | NA |
| TRINITY_DN561_c0_g1_i5 | -0.71373082 | 0.868172244 | 0.999970678 | 2.516 | 0 | 3.466 | 0 | NA |
| TRINITY_DN4164_c0_g1_i3 | 0.713626787 | 0.708240498 | 0.999970678 | 0.463 | 0.207 | 0.322 | 0.02 | - |
| TRINITY_DN727_c0_g3_i1 | -0.71361765 | 0.253807111 | 0.999970678 | 21.965 | 18.4 | 29.665 | 30.242 | NA |
| TRINITY_DN3564_c0_g1_i1 | -0.71361337 | 0.235055468 | 0.999970678 | 10.138 | 12.09 | 15.144 | 18.097 | NA |
| TRINITY_DN3942_c0_g1_i5 | 0.713566709 | 0.535217515 | 0.999970678 | 0.527 | 0.7 | 0.3 | 0.402 | NA |
| TRINITY_DN4277_c0_g1_i2 | -0.71342851 | 0.543918316 | 0.999970678 | 0.581 | 0.887 | 1.455 | 0.643 | NA |
| TRINITY_DN13667_c0_g1_i1 | -0.71336517 | 0.490659445 | 0.999970678 | 2.135 | 1.38 | 3.366 | 1.699 | NA |
| TRINITY_DN4264_c0_g1_i2 | -0.71333819 | 0.870001533 | 0.999970678 | 1.808 | 0 | 2.522 | 0 | NA |
| TRINITY_DN224247_c0_g1_i1 | -0.71333386 | 0.870981393 | 0.999970678 | 0.781 | 0 | 1.078 | 0 | FMO-like |
| TRINITY_DN57012_c0_g1_i1 | -0.71332863 | 0.872262316 | 1 | 0.772 | 0 | 1.067 | 0 | NA |
| TRINITY_DN24118_c0_g1_i4 | -0.71332863 | 0.872262316 | 1 | 2.653 | 0 | 3.622 | 0 | NA |
| TRINITY_DN104920_c0_g1_i1 | -0.71331293 | 0.876243322 | 1 | 1.072 | 0 | 1.478 | 0 | - |
| TRINITY_DN201274_c0_g1_i1 | -0.71331293 | 0.876243322 | 1 | 0.627 | 0 | 0.867 | 0 | NA |
| TRINITY_DN4785_c0_g3_i7 | -0.71331293 | 0.876243322 | 1 | 0.681 | 0 | 0.922 | 0 | NA |
| TRINITY_DN4650_c0_g1_i2 | -0.71331293 | 0.876243322 | 1 | 0.4 | 0 | 0.544 | 0 | NA |
| TRINITY_DN35317_c0_g1_i2 | -0.71331293 | 0.876243322 | 1 | 0.927 | 0 | 1.322 | 0 | NA |

| TRINITY_DN50227_c0_g1_i6 | -0.71327581 | 0.805886329 | 1 | 0.472 | 0 | 0.456 | 0.211 | NA |
| --- | --- | --- | --- | --- | --- | --- | --- | --- |
| TRINITY_DN3450_c0_g1_i18 | 0.713239265 | 0.800407591 | 0.999970678 | 0 | 1.912 | 0.678 | 0.332 | NA |
| TRINITY_DN4109_c0_g1_i4 | -0.71317514 | 0.792090456 | 0.999970678 | 7.058 | 0 | 4.333 | 6.505 | NA |
| TRINITY_DN9550_c0_g1_i1 | -0.71297681 | 0.491664154 | 0.999970678 | 1.263 | 0.71 | 1.655 | 1.247 | Peptidase_M48 |
| TRINITY_DN18762_c0_g1_i2 | 0.712963987 | 0.581796119 | 0.999970678 | 3.824 | 19.59 | 6.122 | 6.555 | Glyco_hydro_14 |
| TRINITY_DN242_c1_g1_i13 | -0.71291639 | 0.551350641 | 0.999970678 | 12.899 | 4.918 | 5.744 | 22.229 | B_lectin |
| TRINITY_DN1490_c1_g1_i1 | 0.712909122 | 0.538515575 | 0.999970678 | 4.696 | 24.61 | 7.566 | 8.586 | PB1 |
| TRINITY_DN923_c0_g1_i5 | 0.712905311 | 0.390676386 | 0.999970678 | 17.823 | 39.83 | 13.899 | 18.107 | NA |
| TRINITY_DN8097_c0_g3_i1 | 0.712803947 | 0.786439999 | 0.999970678 | 29.05 | 0.217 | 14.832 | 0.121 | MIP |
| TRINITY_DN20094_c0_g1_i4 | 0.712764281 | 0.869578518 | 0.999970678 | 2.353 | 0 | 1.2 | 0 | NA |
| TRINITY_DN4730_c0_g1_i6 | -0.71274408 | 0.513349374 | 0.999970678 | 3.579 | 1.36 | 2.833 | 4.645 | - |
| TRINITY_DN3507_c0_g1_i2 | 0.712727144 | 0.387568252 | 0.999970678 | 5.014 | 10.18 | 3.433 | 5.107 | NA |
| TRINITY_DN1677_c0_g1_i8 | 0.712704894 | 0.346818564 | 0.999970678 | 1.762 | 1.232 | 0.856 | 0.794 | ANAPC4_WD40 |
| TRINITY_DN15348_c0_g1_i1 | -0.71269228 | 0.869733959 | 0.999970678 | 0 | 0.887 | 1.167 | 0 | NA |
| TRINITY_DN22614_c0_g1_i1 | 0.712492385 | 0.454498937 | 0.999970678 | 1.226 | 0.739 | 0.589 | 0.493 | NA |
| TRINITY_DN1459_c0_g1_i4 | 0.712404612 | 0.557465407 | 0.999970678 | 4.097 | 1.804 | 2.389 | 0.744 | NA |
| TRINITY_DN26252_c0_g1_i1 | 0.712347591 | 0.485335694 | 0.999970678 | 1.29 | 2.868 | 1.411 | 0.814 | RNA_pol_Rpb2_1 |
| TRINITY_DN5320_c0_g1_i3 | -0.71226626 | 0.444143905 | 0.999970678 | 0.827 | 1.202 | 1.178 | 1.89 | p450 |
| TRINITY_DN3145_c0_g2_i1 | -0.71223248 | 0.635214901 | 0.999970678 | 7.694 | 1.675 | 8.822 | 4.464 | NA |
| TRINITY_DN1070_c0_g1_i1 | 0.712163226 | 0.471719445 | 0.999970678 | 11.3 | 5.933 | 2.533 | 7.54 | Pkinase |
| TRINITY_DN1341_c0_g1_i2 | 0.712083682 | 0.423010971 | 0.999970678 | 48.454 | 131 | 46.042 | 53.315 | Hydrolase_6 |
| TRINITY_DN604_c1_g2_i6 | 0.712080945 | 0.814724399 | 0.999970678 | 0 | 325.8 | 50.408 | 132.779 | NA |
| TRINITY_DN16944_c0_g1_i2 | -0.7120251 | 0.572671026 | 0.999970678 | 22.365 | 64.08 | 57.852 | 57.849 | NA |
| TRINITY_DN6036_c0_g1_i2 | -0.71196987 | 0.867375389 | 0.999970678 | 0 | 1.35 | 0 | 2.182 | HLH |
| TRINITY_DN4300_c0_g1_i1 | 0.711901457 | 0.408909321 | 0.999970678 | 33.238 | 16.89 | 10.966 | 17.121 | - |
| TRINITY_DN686_c0_g1_i13 | 0.711758917 | 0.615909697 | 0.999970678 | 7.104 | 2.424 | 0.744 | 4.967 | NA |
| TRINITY_DN990_c1_g1_i2 | 0.711601456 | 0.38024333 | 0.999970678 | 60.154 | 133.3 | 45.853 | 61.82 | Methyltransf_24 |
| TRINITY_DN2264_c0_g1_i4 | -0.71159125 | 0.601738975 | 0.999970678 | 25.926 | 3.026 | 16.266 | 28.392 | Patatin |
| TRINITY_DN14075_c0_g1_i3 | -0.71143952 | 0.621999663 | 0.999970678 | 0.336 | 0.936 | 1.367 | 0.432 | NA |
| TRINITY_DN6403_c0_g1_i1 | 0.711430459 | 0.454800388 | 0.999970678 | 0.763 | 1.478 | 0.6 | 0.633 | NA |
| TRINITY_DN4343_c0_g1_i4 | 0.711276183 | 0.560043546 | 0.999970678 | 4.26 | 2.7 | 2.111 | 1.669 | NA |
| TRINITY_DN6145_c0_g1_i1 | -0.71115991 | 0.866013185 | 0.999970678 | 0 | 1.124 | 0 | 1.83 | B_lectin |
| TRINITY_DN6148_c0_g2_i2 | 0.711100449 | 0.575267768 | 0.999970678 | 1.644 | 9.294 | 1.944 | 4.263 | NA |
| TRINITY_DN2413_c0_g1_i1 | -0.71101557 | 0.866815255 | 0.999970678 | 3.07 | 0 | 4.2 | 0 | NA |
| TRINITY_DN12680_c0_g1_i1 | 0.710970559 | 0.799106426 | 0.999970678 | 0.118 | 0.601 | 0 | 0.432 | NA |
| TRINITY_DN15224_c0_g2_i2 | -0.71095389 | 0.268045481 | 0.999970678 | 6.74 | 6.347 | 9.799 | 9.581 | Bromo_TP |
| TRINITY_DN13225_c0_g1_i6 | 0.710907822 | 0.867501233 | 0.999970678 | 4.315 | 0 | 2.189 | 0 | Peroxidase_2 |
| TRINITY_DN3091_c0_g1_i6 | 0.710832727 | 0.578252316 | 0.999970678 | 1.09 | 3.775 | 1.444 | 1.206 | NA |
| TRINITY_DN3240_c0_g1_i18 | 0.710788437 | 0.497862778 | 0.999970678 | 4.805 | 1.804 | 2.067 | 1.568 | NA |
| TRINITY_DN6950_c0_g1_i4 | -0.7107731 | 0.434506789 | 0.999970678 | 16.923 | 10.43 | 25.421 | 14.105 | - |
| TRINITY_DN4890_c0_g1_i1 | -0.71064556 | 0.331198608 | 0.999970678 | 18.876 | 18 | 17.954 | 38.787 | NA |
| TRINITY_DN4019_c0_g1_i1 | 0.710590922 | 0.590603151 | 0.999970678 | 1.372 | 1.261 | 1.111 | 0.271 | Amino_oxidase |
| TRINITY_DN18722_c0_g1_i2 | -0.71055442 | 0.598560667 | 0.999970678 | 4.587 | 9.018 | 15.943 | 2.845 | NA |
| TRINITY_DN4546_c0_g2_i3 | -0.71045038 | 0.798121641 | 0.999970678 | 0.491 | 0 | 0.511 | 0.191 | ABC2_membrane |
| TRINITY_DN1219_c2_g1_i2 | 0.710360683 | 0.552808025 | 0.999970678 | 2.362 | 2.878 | 2.167 | 0.583 | NA |
| TRINITY_DN993_c0_g2_i2 | -0.71035696 | 0.773597667 | 1 | 0.808 | 0.089 | 1.178 | 0.02 | 2-Hacid_dh |
| TRINITY_DN12375_c0_g1_i4 | 0.710356456 | 0.654036919 | 1 | 0.727 | 0.434 | 0.1 | 0.593 | NA |
| TRINITY_DN4688_c0_g1_i4 | 0.710303297 | 0.655660406 | 0.999970678 | 1.917 | 0.631 | 1.089 | 0.231 | NA |
| TRINITY_DN14821_c0_g1_i2 | -0.71030133 | 0.280954445 | 0.999970678 | 38.116 | 40.46 | 42.242 | 77.454 | NA |
| TRINITY_DN4206_c0_g1_i1 | 0.71028721 | 0.326843948 | 0.999970678 | 5.014 | 4.395 | 2.944 | 2.212 | NA |
| TRINITY_DN772_c0_g3_i1 | 0.710222663 | 0.665444426 | 0.999970678 | 2.353 | 4.021 | 0.322 | 3.438 | PAE |
| TRINITY_DN4948_c1_g1_i1 | -0.71017962 | 0.544469678 | 0.999970678 | 2.625 | 5.292 | 2.433 | 9.802 | NA |
| TRINITY_DN7260_c0_g1_i11 | 0.710043646 | 0.527536342 | 0.999970678 | 2.453 | 3.045 | 0.756 | 2.433 | NA |
| TRINITY_DN18773_c0_g1_i5 | 0.710015602 | 0.806450266 | 0.999970678 | 0.772 | 0 | 0.089 | 0.372 | NA |
| TRINITY_DN73896_c0_g1_i1 | 0.710004761 | 0.668834121 | 0.999970678 | 18.477 | 57.57 | 3.466 | 38.013 | NA |
| TRINITY_DN2147_c0_g1_i4 | -0.70986575 | 0.790722254 | 0.999970678 | 0 | 15.67 | 10.144 | 12.617 | NA |
| TRINITY_DN4847_c0_g2_i4 | 0.709787246 | 0.519417242 | 0.999970678 | 12.227 | 28.91 | 9.299 | 12.557 | NA |
| TRINITY_DN10314_c0_g1_i1 | -0.70967728 | 0.451737664 | 0.999970678 | 0.618 | 1.459 | 1.189 | 1.96 | LRRNT_2 |
| TRINITY_DN10566_c0_g1_i1 | -0.70964562 | 0.339802164 | 0.999970678 | 8.384 | 11.27 | 10.133 | 19.826 | PP2 |
| TRINITY_DN1670_c0_g1_i1 | -0.70959853 | 0.710503797 | 0.999970678 | 4.56 | 0.079 | 2.244 | 5.007 | - |
| TRINITY_DN3532_c0_g1_i7 | 0.709549529 | 0.806379362 | 0.999970678 | 9.656 | 18.7 | 14.255 | 0 | - |
| TRINITY_DN15049_c0_g1_i1 | 0.709509198 | 0.504528283 | 0.999970678 | 1.09 | 1.38 | 0.544 | 0.824 | Aldo_ket_red |
| TRINITY_DN1137_c0_g1_i1 | 0.709497307 | 0.725150409 | 0.999970678 | 2.516 | 121.3 | 33.62 | 28.603 | NA |
| TRINITY_DN48776_c0_g1_i8 | -0.70937227 | 0.665302166 | 0.999970678 | 0.282 | 2.976 | 3.344 | 1.227 | NA |
| TRINITY_DN5136_c0_g1_i6 | 0.709370536 | 0.874345736 | 1 | 0 | 2.02 | 1.011 | 0 | NA |
| TRINITY_DN1440_c1_g1_i11 | -0.70925865 | 0.4550095 | 0.999970678 | 460.71 | 193.8 | 577.765 | 366.075 | GST_C |
| TRINITY_DN18546_c0_g1_i2 | 0.709152928 | 0.650272463 | 1 | 0.5 | 0.404 | 0.078 | 0.462 | NA |
| TRINITY_DN6001_c0_g1_i1 | 0.709147668 | 0.62658597 | 0.999970678 | 0.709 | 1.409 | 0.2 | 1.056 | NA |
| TRINITY_DN1993_c2_g1_i2 | -0.70907938 | 0.32384315 | 0.999970678 | 148.36 | 177.6 | 160.424 | 338.317 | TENA_THI-4 |
| TRINITY_DN1825_c0_g1_i3 | -0.70903042 | 0.801416066 | 0.999970678 | 15.17 | 0 | 8.911 | 14.407 | NA |
| TRINITY_DN8974_c0_g2_i1 | 0.708973767 | 0.802779039 | 0.999970678 | 0 | 11.59 | 4.333 | 1.739 | TP6A_N |
| TRINITY_DN10346_c0_g1_i2 | 0.708965933 | 0.518749594 | 0.999970678 | 39.561 | 16.72 | 7.255 | 25.758 | NA |
| TRINITY_DN1454_c0_g1_i2 | -0.70893197 | 0.628758021 | 1 | 0.127 | 0.384 | 0.233 | 0.543 | NA |
| TRINITY_DN31586_c1_g1_i1 | 0.708765767 | 0.800556511 | 0.999970678 | 8.148 | 0 | 2.533 | 1.96 | NA |

| TRINITY_DN688_c0_g1_i4 | 0.708673621 | 0.506055881 | 0.999970678 | 6.949 | 2.326 | 1.9 | 3.398 | Pkinase |
| --- | --- | --- | --- | --- | --- | --- | --- | --- |
| TRINITY_DN2996_c1_g3_i1 | 0.708667167 | 0.573171264 | 0.999970678 | 3.079 | 7.116 | 3.622 | 1.769 | NA |
| TRINITY_DN77286_c0_g1_i1 | 0.708567767 | 0.874107254 | 1 | 1.135 | 0 | 0.578 | 0 | NA |
| TRINITY_DN1676_c0_g1_i3 | 0.708539373 | 0.589275076 | 0.999970678 | 1.054 | 5.608 | 1.022 | 2.805 | NA |
| TRINITY_DN1144_c0_g1_i7 | 0.708493068 | 0.38013343 | 0.999970678 | 10.456 | 10.65 | 6.588 | 4.876 | - |
| TRINITY_DN4356_c0_g1_i10 | 0.708418554 | 0.872221524 | 1 | 1.844 | 0 | 0 | 1.156 | Pkinase |
| TRINITY_DN782_c1_g1_i2 | -0.70838075 | 0.402326947 | 0.999970678 | 238.12 | 257.6 | 197.81 | 561.971 | NA |
| TRINITY_DN19151_c0_g1_i3 | -0.70831341 | 0.678282193 | 1 | 2.716 | 0.995 | 4.2 | 0.925 | NA |
| TRINITY_DN13768_c0_g2_i1 | -0.70828652 | 0.797918407 | 0.999970678 | 1.326 | 0 | 1.355 | 0.553 | BRAP2 |
| TRINITY_DN1466_c0_g1_i7 | -0.70826519 | 0.290779812 | 0.999970678 | 5.777 | 4.543 | 6.955 | 8.485 | SQS_PSY |
| TRINITY_DN9000_c0_g1_i4 | 0.708216798 | 0.519860532 | 0.999970678 | 1.971 | 3.617 | 2.055 | 0.915 | NA |
| TRINITY_DN8096_c0_g1_i3 | 0.70812636 | 0.792881406 | 0.999970678 | 2.643 | 4.878 | 3.778 | 0 | NA |
| TRINITY_DN13268_c0_g1_i1 | 0.707981021 | 0.430467033 | 0.999970678 | 3.806 | 6.514 | 2.989 | 2.654 | NA |
| TRINITY_DN47116_c0_g1_i1 | -0.70790101 | 0.871358626 | 0.999970678 | 0.79 | 0 | 1.078 | 0 | Cu-oxidase |
| TRINITY_DN4621_c0_g1_i1 | 0.707898148 | 0.38874232 | 0.999970678 | 24.463 | 23.2 | 8.044 | 19.172 | PP2 |
| TRINITY_DN8182_c0_g1_i1 | 0.707869712 | 0.743051157 | 0.999970678 | 2.762 | 0.108 | 0.122 | 1.649 | NA |
| TRINITY_DN8727_c0_g1_i1 | 0.707861419 | 0.58407326 | 0.999970678 | 2.88 | 1.104 | 1.278 | 0.905 | NA |
| TRINITY_DN3446_c0_g1_i4 | -0.70784356 | 0.383755938 | 0.999970678 | 68.429 | 78.27 | 134.647 | 75.453 | Fer2 |
| TRINITY_DN4822_c0_g1_i3 | 0.707837709 | 0.525589101 | 0.999970678 | 1.199 | 0.453 | 0.6 | 0.302 | NA |
| TRINITY_DN4030_c0_g1_i7 | -0.70765498 | 0.529487669 | 0.999970678 | 4.342 | 1.399 | 3.711 | 4.866 | NA |
| TRINITY_DN12742_c0_g2_i1 | 0.707625175 | 0.570190076 | 0.999970678 | 2.098 | 2.079 | 1.567 | 0.664 | NA |
| TRINITY_DN679_c0_g1_i6 | 0.707604684 | 0.617156044 | 0.999970678 | 2.344 | 6.711 | 3.878 | 0.784 | NA |
| TRINITY_DN6424_c0_g2_i4 | -0.70749738 | 0.79606467 | 0.999970678 | 1.417 | 0 | 1.422 | 0.633 | NA |
| TRINITY_DN11058_c0_g3_i1 | 0.707472201 | 0.265319386 | 0.999970678 | 5.396 | 5.558 | 2.866 | 3.247 | FKBP_C |
| TRINITY_DN4335_c0_g1_i3 | -0.70745952 | 0.795071791 | 0.999970678 | 0.954 | 0 | 0.756 | 0.633 | NA |
| TRINITY_DN41349_c0_g1_i5 | -0.70734655 | 0.483414278 | 0.999970678 | 0.881 | 0.591 | 0.811 | 1.428 | - |
| TRINITY_DN4406_c0_g3_i1 | 0.707320922 | 0.679856072 | 1 | 1.354 | 0.68 | 0.144 | 1.116 | NA |
| TRINITY_DN2536_c0_g1_i3 | -0.70721405 | 0.35403853 | 0.999970678 | 6.786 | 9.865 | 13.955 | 10.245 | NA |
| TRINITY_DN2985_c0_g1_i10 | 0.707170398 | 0.547358232 | 0.999970678 | 11.718 | 57.49 | 18.966 | 18.65 | NA |
| TRINITY_DN197394_c0_g1_i1 | -0.70707065 | 0.872597776 | 0.999970678 | 1.235 | 0 | 1.689 | 0 | NA |
| TRINITY_DN6340_c0_g1_i1 | -0.70704253 | 0.868505653 | 0.999970678 | 2.353 | 0 | 0 | 3.881 | NA |
| TRINITY_DN2264_c0_g2_i2 | 0.706994637 | 0.582113229 | 0.999970678 | 7.095 | 20.53 | 2.544 | 13.703 | NA |
| TRINITY_DN2044_c1_g1_i2 | -0.70690582 | 0.821163074 | 1 | 0 | 51.17 | 3.655 | 68.777 | NA |
| TRINITY_DN2942_c0_g2_i1 | 0.706859642 | 0.866443133 | 0.999970678 | 9.011 | 0 | 0 | 5.61 | NA |
| TRINITY_DN13591_c0_g2_i2 | -0.70685134 | 0.690355707 | 0.999970678 | 0.727 | 0.581 | 1.667 | 0.131 | LRRNT_2 |
| TRINITY_DN5184_c0_g1_i5 | -0.70684442 | 0.525135721 | 0.999970678 | 7.349 | 18.8 | 8.511 | 32.172 | p450 |
| TRINITY_DN1943_c0_g1_i3 | 0.706836533 | 0.500116818 | 0.999970678 | 7.013 | 3.765 | 2.233 | 3.871 | NA |
| TRINITY_DN1649_c0_g1_i2 | 0.706470394 | 0.810228702 | 0.999970678 | 0 | 1.715 | 0.767 | 0.111 | NA |
| TRINITY_DN11153_c0_g2_i1 | -0.70633998 | 0.569270534 | 0.999970678 | 27.143 | 58.84 | 22.032 | 103.804 | NA |
| TRINITY_DN1258_c0_g1_i1 | 0.706317713 | 0.700917202 | 1 | 0.545 | 1.153 | 0.089 | 0.945 | - |
| TRINITY_DN5485_c0_g2_i1 | -0.70630157 | 0.352186134 | 0.999970678 | 11.646 | 9.323 | 10.533 | 21.495 | NA |
| TRINITY_DN1432_c0_g1_i6 | 0.706272211 | 0.684031485 | 0.999970678 | 1.971 | 2.336 | 2.1 | 0.09 | DUF1995 |
| TRINITY_DN1326_c1_g1_i4 | 0.706224434 | 0.583046511 | 0.999970678 | 4.17 | 18.43 | 2.722 | 10.436 | Glyco_hydro_17 |
| TRINITY_DN9773_c0_g1_i7 | 0.70619643 | 0.554699257 | 0.999970678 | 0.908 | 0.522 | 0.278 | 0.543 | NA |
| TRINITY_DN7252_c0_g1_i6 | -0.70609546 | 0.504570466 | 0.999970678 | 1.135 | 1.104 | 0.889 | 2.574 | NA |
| TRINITY_DN5083_c0_g1_i2 | 0.706077693 | 0.556868908 | 0.999970678 | 6.068 | 3.558 | 0.955 | 4.735 | SNARE |
| TRINITY_DN2040_c0_g1_i3 | 0.705991599 | 0.811436203 | 0.999970678 | 0 | 62.59 | 9.066 | 26.793 | Abhydrolase_1 |
| TRINITY_DN4559_c0_g1_i7 | -0.70597094 | 0.623747328 | 0.999970678 | 0.191 | 0.749 | 0.767 | 0.603 | NA |
| TRINITY_DN1589_c0_g1_i5 | 0.705847399 | 0.590745685 | 0.999970678 | 3.679 | 4.376 | 3.566 | 0.633 | Beta-Casp |
| TRINITY_DN8268_c0_g1_i4 | 0.705817468 | 0.602828032 | 0.999970678 | 3.643 | 4.198 | 3.178 | 0.925 | NA |
| TRINITY_DN1269_c0_g1_i6 | -0.70578477 | 0.656371109 | 0.999970678 | 34.083 | 3.942 | 44.253 | 9.199 | NA |
| TRINITY_DN4158_c0_g1_i1 | -0.70563857 | 0.404488722 | 0.999970678 | 15.425 | 33.24 | 38.831 | 31.9 | NA |
| TRINITY_DN2947_c0_g1_i8 | -0.70562617 | 0.808333708 | 1 | 6.332 | 0 | 1.944 | 7.601 | NA |
| TRINITY_DN46146_c0_g1_i1 | 0.705608619 | 0.875068992 | 1 | 1.435 | 0 | 0.733 | 0 | Pyr_redox_2 |
| TRINITY_DN9843_c0_g1_i1 | 0.705608619 | 0.875068992 | 1 | 4.733 | 0 | 2.411 | 0 | Redoxin |
| TRINITY_DN15627_c0_g1_i1 | 0.705597174 | 0.419015714 | 0.999970678 | 4.814 | 6.435 | 1.833 | 4.665 | NA |
| TRINITY_DN9659_c0_g4_i1 | -0.70558098 | 0.80859423 | 1 | 0 | 0.65 | 0.667 | 0.211 | NA |
| TRINITY_DN8230_c0_g1_i3 | 0.705289319 | 0.801297979 | 1 | 1.281 | 2.267 | 1.789 | 0 | LCM |
| TRINITY_DN15091_c0_g1_i1 | 0.705276816 | 0.491744878 | 0.999970678 | 2.28 | 0.887 | 0.789 | 1.005 | LRRNT_2 |
| TRINITY_DN393_c1_g1_i1 | -0.70527113 | 0.358429694 | 0.999970678 | 42.113 | 28.28 | 35.642 | 72.025 | NA |
| TRINITY_DN9251_c0_g2_i1 | 0.705258092 | 0.521738168 | 0.999970678 | 7.276 | 4.602 | 4.766 | 1.548 | NA |
| TRINITY_DN5355_c0_g1_i5 | -0.70523595 | 0.537088436 | 0.999970678 | 3.597 | 6.564 | 10.944 | 3.288 | OPT |
| TRINITY_DN9704_c0_g1_i4 | -0.70513618 | 0.7953284 | 0.999970678 | 0 | 1.222 | 1.111 | 0.593 | NA |
| TRINITY_DN9661_c0_g1_i1 | 0.705121985 | 0.80557154 | 0.999970678 | 0 | 7.953 | 0.778 | 3.82 | Auxin_inducible |
| TRINITY_DN10005_c0_g1_i3 | 0.705096325 | 0.678080802 | 0.999970678 | 3.252 | 6.849 | 0.222 | 5.912 | DHHC |
| TRINITY_DN4073_c0_g1_i2 | -0.70503127 | 0.402039259 | 0.999970678 | 1.535 | 2.888 | 2.822 | 3.79 | NA |
| TRINITY_DN1231_c0_g1_i7 | 0.70494704 | 0.634532542 | 0.999970678 | 9.874 | 1.232 | 4.1 | 1.92 | NA |
| TRINITY_DN6004_c0_g1_i19 | 0.704934277 | 0.874687841 | 1 | 0 | 2.03 | 0.989 | 0 | NA |
| TRINITY_DN1281_c0_g1_i3 | 0.704913542 | 0.802746621 | 0.999970678 | 6.786 | 1.005 | 4 | 0 | HMG_box |
| TRINITY_DN4921_c0_g1_i10 | 0.70489503 | 0.550636651 | 0.999970678 | 5.841 | 2.799 | 1.167 | 3.921 | NA |
| TRINITY_DN1175_c0_g1_i5 | 0.704858988 | 0.676249187 | 0.999970678 | 14.825 | 214.7 | 16.61 | 118 | NA |
| TRINITY_DN955_c0_g1_i7 | -0.7047625 | 0.470164353 | 0.999970678 | 4.715 | 9.313 | 13.088 | 6.907 | NA |
| TRINITY_DN2391_c0_g1_i1 | 0.70476032 | 0.268046089 | 0.999970678 | 18.722 | 16.85 | 10.355 | 9.36 | Paf1 |
| TRINITY_DN363_c0_g1_i1 | 0.704720929 | 0.424571277 | 0.999970678 | 1.072 | 1.685 | 0.5 | 1.076 | FH2 |

| TRINITY_DN7481_c0_g1_i12 | 0.704599631 | 0.516135477 | 0.999970678 | 4.16 | 7.165 | 2.189 | 4.112 | NA |
| --- | --- | --- | --- | --- | --- | --- | --- | --- |
| TRINITY_DN6636_c0_g2_i3 | 0.704542478 | 0.819785881 | 0.999970678 | 12.681 | 0 | 0.367 | 7.5 | NA |
| TRINITY_DN6905_c0_g1_i1 | 0.704528772 | 0.324044545 | 0.999970678 | 8.003 | 11.55 | 5.222 | 5.63 | NA |
| TRINITY_DN1404_c0_g1_i1 | -0.70445399 | 0.581805483 | 0.999970678 | 6.668 | 7.116 | 2.455 | 19.434 | Ion_trans_2 |
| TRINITY_DN2389_c0_g1_i3 | 0.70445285 | 0.870780437 | 0.999970678 | 0 | 20.35 | 0 | 12.336 | 3HCDH |
| TRINITY_DN34411_c0_g1_i4 | -0.70435138 | 0.870751806 | 0.999970678 | 1.535 | 0 | 2.089 | 0 | EHN |
| TRINITY_DN105400_c0_g1_i1 | -0.70432467 | 0.87641329 | 1 | 1.617 | 0 | 2.2 | 0 | NA |
| TRINITY_DN2056_c0_g1_i3 | 0.704301608 | 0.219413143 | 0.999970678 | 240.31 | 208.1 | 109.927 | 142.069 | NA |
| TRINITY_DN10702_c0_g3_i2 | 0.704230162 | 0.573249808 | 0.999970678 | 3.861 | 2.553 | 2.744 | 0.613 | Metallophos |
| TRINITY_DN3878_c0_g1_i10 | -0.70422782 | 0.621570826 | 0.999970678 | 0.291 | 0.562 | 0.911 | 0.271 | NA |
| TRINITY_DN2575_c0_g1_i11 | -0.7041992 | 0.712515026 | 0.999970678 | 0.509 | 1.518 | 2.567 | 0.151 | NA |
| TRINITY_DN14770_c0_g1_i1 | -0.7041535 | 0.230120503 | 0.999970678 | 8.166 | 9.087 | 11.755 | 13.894 | Glyco_hydro_77 |
| TRINITY_DN12113_c0_g2_i1 | 0.704114474 | 0.821627152 | 1 | 0.164 | 3.745 | 0 | 2.332 | NA |
| TRINITY_DN2562_c0_g1_i2 | 0.704055909 | 0.802407376 | 0.999970678 | 0 | 12.19 | 4.489 | 1.88 | NA |
| TRINITY_DN9217_c0_g2_i19 | -0.7039991 | 0.877248154 | 1 | 0.336 | 0 | 0 | 0.563 | DBD_Tnp_Mut |
| TRINITY_DN1709_c0_g2_i1 | -0.7039991 | 0.877248154 | 1 | 0.827 | 0 | 0 | 1.387 | NA |
| TRINITY_DN4722_c0_g2_i2 | 0.703936024 | 0.462496536 | 0.999970678 | 9.066 | 6.613 | 5.411 | 3.026 | NA |
| TRINITY_DN2836_c1_g1_i8 | -0.70391101 | 0.810012323 | 0.999970678 | 0 | 4.622 | 5.622 | 0.633 | DEAD |
| TRINITY_DN962_c0_g1_i2 | -0.70387082 | 0.805959172 | 0.999970678 | 1.199 | 0 | 0.278 | 1.639 | CCB2_CCB4 |
| TRINITY_DN1689_c0_g1_i3 | 0.703821315 | 0.799819881 | 0.999970678 | 4.015 | 0.69 | 0 | 2.926 | NA |
| TRINITY_DN250543_c0_g1_i1 | -0.70379955 | 0.33311107 | 0.999970678 | 43.712 | 47.05 | 77.673 | 53.305 | - |
| TRINITY_DN4880_c0_g2_i1 | 0.703755538 | 0.609928523 | 0.999970678 | 0.818 | 0.315 | 0.189 | 0.473 | AvrRpt-cleavage |
| TRINITY_DN2806_c0_g1_i1 | -0.70373589 | 0.712536068 | 0.999970678 | 0.164 | 0.907 | 0.111 | 1.588 | NA |
| TRINITY_DN5758_c0_g1_i1 | -0.70371077 | 0.364017455 | 0.999970678 | 89.922 | 46.68 | 87.628 | 117.115 | NA |
| TRINITY_DN3701_c0_g1_i2 | -0.70369917 | 0.502910598 | 0.999970678 | 1.408 | 1.055 | 2.267 | 1.257 | NA |
| TRINITY_DN3140_c0_g1_i7 | -0.70368098 | 0.570700685 | 0.999970678 | 20.775 | 6.347 | 28.987 | 9.501 | PGM_PMM_I |
| TRINITY_DN10277_c0_g1_i3 | -0.70364454 | 0.797996615 | 0.999970678 | 0 | 3.134 | 1.311 | 3.338 | NA |
| TRINITY_DN9641_c0_g1_i2 | 0.703600474 | 0.424316979 | 0.999970678 | 6.186 | 11.84 | 4.355 | 5.61 | NA |
| TRINITY_DN18692_c0_g1_i2 | -0.70357543 | 0.50265412 | 0.999970678 | 0.645 | 0.887 | 0.867 | 1.408 | NA |
| TRINITY_DN4180_c0_g1_i2 | 0.703528785 | 0.867761571 | 0.999970678 | 14.889 | 0 | 0 | 9.259 | NA |
| TRINITY_DN4661_c0_g1_i3 | -0.70349785 | 0.519703253 | 0.999970678 | 0.491 | 0.986 | 1.389 | 0.714 | BTB |
| TRINITY_DN167143_c0_g1_i1 | 0.70345905 | 0.613025209 | 0.999970678 | 47.055 | 10.07 | 5.489 | 28.462 | SOUL |
| TRINITY_DN48135_c0_g1_i1 | -0.7033586 | 0.369176376 | 0.999970678 | 78.131 | 74.38 | 67.351 | 167.786 | PHM7_cyt |
| TRINITY_DN6046_c0_g1_i1 | -0.7032954 | 0.237558921 | 0.999970678 | 80.129 | 94.71 | 105.56 | 155.169 | NA |
| TRINITY_DN1348_c0_g2_i1 | 0.703266961 | 0.356954211 | 0.999970678 | 5.114 | 3.43 | 2.122 | 2.684 | NA |
| TRINITY_DN13403_c0_g1_i1 | 0.703107458 | 0.607460383 | 0.999970678 | 5.423 | 3.233 | 3.889 | 0.633 | ABC1 |
| TRINITY_DN28085_c0_g1_i1 | -0.703026 | 0.262044848 | 0.999970678 | 39.224 | 51.28 | 54.663 | 80.198 | Glycos_transf_4 |
| TRINITY_DN12435_c1_g2_i1 | 0.703024354 | 0.714345886 | 0.999970678 | 56.166 | 3.134 | 7.977 | 24.611 | NA |
| TRINITY_DN15615_c0_g1_i2 | -0.70300861 | 0.672833371 | 0.999970678 | 0.954 | 3.41 | 5.155 | 0.794 | NA |
| TRINITY_DN9378_c0_g1_i1 | 0.702958523 | 0.689660359 | 0.999970678 | 7.494 | 5.647 | 6.144 | 0.553 | NA |
| TRINITY_DN27237_c0_g1_i1 | 0.702954284 | 0.558608609 | 0.999970678 | 0.663 | 0.463 | 0.4 | 0.201 | NA |
| TRINITY_DN10484_c0_g1_i17 | 0.702843111 | 0.686831541 | 0.999970678 | 0.345 | 2.208 | 1.111 | 0.231 | NA |
| TRINITY_DN87639_c0_g1_i4 | -0.70280892 | 0.338633532 | 0.999970678 | 130.23 | 111.6 | 207.776 | 141.315 | Ribosomal_L11 |
| TRINITY_DN27761_c0_g1_i2 | -0.70279643 | 0.553763287 | 1 | 6.477 | 4.583 | 7.833 | 7.651 | NA |
| TRINITY_DN100890_c0_g1_i1 | -0.70256901 | 0.804265562 | 1 | 0 | 0.532 | 0.156 | 0.643 | NA |
| TRINITY_DN446_c0_g2_i3 | -0.70255294 | 0.508462408 | 0.999970678 | 9.656 | 18.43 | 28.843 | 10.597 | NA |
| TRINITY_DN8098_c0_g1_i7 | -0.7025044 | 0.593726519 | 0.999970678 | 1.154 | 0.453 | 1.467 | 0.845 | NA |
| TRINITY_DN13955_c0_g2_i1 | -0.702467 | 0.644690408 | 0.999970678 | 5.45 | 0.887 | 3.066 | 6.505 | NA |
| TRINITY_DN375_c0_g2_i3 | -0.70240346 | 0.33025582 | 0.999970678 | 412.04 | 352.5 | 375.733 | 788.893 | CLP_protease |
| TRINITY_DN100054_c0_g1_i1 | 0.702360667 | 0.876090613 | 1 | 1.163 | 0 | 0.6 | 0 | NA |
| TRINITY_DN38877_c0_g1_i1 | 0.702360667 | 0.876090613 | 1 | 2.462 | 0 | 1.267 | 0 | Nitroreductase |
| TRINITY_DN57141_c0_g1_i2 | 0.702360667 | 0.876090613 | 1 | 2.498 | 0 | 1.255 | 0 | Rieske |
| TRINITY_DN108_c0_g1_i6 | -0.70233615 | 0.611678417 | 0.999970678 | 0.718 | 2.543 | 3.6 | 0.905 | - |
| TRINITY_DN24611_c0_g1_i1 | 0.702237079 | 0.722289187 | 0.999970678 | 0.245 | 1.528 | 0.8 | 0.08 | Glycolytic |
| TRINITY_DN3782_c0_g1_i5 | 0.702222636 | 0.31892294 | 0.999970678 | 14.289 | 19.24 | 6.933 | 12.185 | NA |
| TRINITY_DN41817_c0_g2_i2 | -0.70216753 | 0.617698613 | 1 | 0.218 | 0.729 | 0.622 | 0.764 | PORR |
| TRINITY_DN4688_c0_g1_i2 | -0.70210567 | 0.831931749 | 0.999970678 | 6.522 | 0 | 8.611 | 0.191 | NA |
| TRINITY_DN39_c4_g1_i1 | 0.702074066 | 0.794409663 | 0.999970678 | 2.371 | 5.144 | 3.8 | 0 | NA |
| TRINITY_DN101644_c0_g1_i1 | -0.70206006 | 0.808489324 | 0.999970678 | 0 | 38.73 | 26.687 | 29.779 | START |
| TRINITY_DN7033_c0_g1_i1 | 0.701974206 | 0.394359628 | 0.999970678 | 13.517 | 7.047 | 5.289 | 6.274 | - |
| TRINITY_DN40211_c1_g1_i1 | -0.70185423 | 0.50103413 | 0.999970678 | 32.702 | 124.2 | 101.816 | 125.52 | Chloroa_b-bind |
| TRINITY_DN1044_c0_g1_i2 | 0.701814463 | 0.601599422 | 0.999970678 | 6.368 | 1.971 | 3.478 | 0.945 | G-patch |
| TRINITY_DN1067_c0_g1_i7 | 0.701812435 | 0.874537464 | 0.999970678 | 0 | 26.1 | 13.066 | 0 | Peptidase_S9 |
| TRINITY_DN3609_c0_g1_i3 | -0.70174543 | 0.575595666 | 0.999970678 | 0.554 | 0.207 | 0.478 | 0.653 | NA |
| TRINITY_DN42295_c0_g2_i1 | -0.70172141 | 0.547038402 | 0.999970678 | 0.654 | 2.474 | 2.044 | 2.574 | AMP_N |
| TRINITY_DN4600_c1_g2_i1 | -0.70163401 | 0.452457389 | 0.999970678 | 2.398 | 3.706 | 2.633 | 6.676 | NA |
| TRINITY_DN93_c1_g1_i7 | 0.701574516 | 0.549587645 | 0.999970678 | 13.326 | 9.974 | 9.766 | 2.513 | NA |
| TRINITY_DN3058_c0_g1_i2 | -0.70154957 | 0.380264291 | 0.999970678 | 30.204 | 15.28 | 28.532 | 39.702 | NA |
| TRINITY_DN6270_c0_g1_i1 | 0.701520663 | 0.648257778 | 0.999970678 | 3.161 | 1.36 | 2.055 | 0.292 | NA |
| TRINITY_DN1308_c0_g2_i2 | -0.70151831 | 0.37660493 | 0.999970678 | 0.699 | 0.739 | 1.111 | 1.015 | NA |
| TRINITY_DN17313_c0_g1_i1 | -0.70146111 | 0.42075767 | 0.999970678 | 1.453 | 2.415 | 2.033 | 3.81 | NA |
| TRINITY_DN190_c0_g2_i4 | 0.701201945 | 0.794308858 | 0.999970678 | 1.099 | 0.808 | 0 | 1.186 | CLP_protease |
| TRINITY_DN954_c1_g1_i3 | 0.701082539 | 0.526009975 | 0.999970678 | 3.397 | 4.415 | 1 | 3.569 | NA |
| TRINITY_DN1039_c1_g1_i12 | 0.701071592 | 0.709706472 | 0.999970678 | 8.339 | 1.291 | 4.511 | 0.493 | B_lectin |

| TRINITY_DN2195_c4_g1_i2 | 0.701024741 | 0.510804419 | 0.999970678 | 1.553 | 5.529 | 1.533 | 2.483 | Kinesin |
| --- | --- | --- | --- | --- | --- | --- | --- | --- |
| TRINITY_DN4012_c0_g1_i2 | 0.700977664 | 0.592260352 | 0.999970678 | 2.071 | 12.25 | 4.911 | 2.785 | - |
| TRINITY_DN2014_c0_g1_i4 | -0.70093033 | 0.477511256 | 0.999970678 | 12.127 | 4.593 | 8.922 | 16.538 | - |
| TRINITY_DN1762_c0_g3_i2 | 0.700901977 | 0.592082865 | 0.999970678 | 7.785 | 16.53 | 1.844 | 12.607 | NA |
| TRINITY_DN569_c0_g1_i14 | 0.700782011 | 0.867693056 | 0.999970678 | 5.087 | 0 | 0 | 3.187 | NA |
| TRINITY_DN10644_c0_g2_i10 | -0.70076088 | 0.833780781 | 1 | 0.836 | 0.039 | 1.189 | 0 | Glyoxalase |
| TRINITY_DN5012_c1_g1_i5 | 0.700757076 | 0.797664476 | 0.999970678 | 0.218 | 0.562 | 0 | 0.483 | NA |
| TRINITY_DN82644_c0_g1_i2 | 0.700635373 | 0.616315762 | 0.999970678 | 11.319 | 1.725 | 4.844 | 2.252 | DNA_photolyase |
| TRINITY_DN2665_c0_g1_i2 | -0.70062163 | 0.372428341 | 0.999970678 | 119.26 | 124.3 | 219.864 | 129.019 | GST_C_2 |
| TRINITY_DN3446_c0_g1_i2 | 0.70062049 | 0.604673285 | 0.999970678 | 0.827 | 2.966 | 1.322 | 0.714 | NA |
| TRINITY_DN6899_c0_g1_i1 | 0.700599024 | 0.388953104 | 0.999970678 | 39.815 | 27.57 | 22.665 | 14.216 | NA |
| TRINITY_DN9309_c0_g1_i2 | -0.70048204 | 0.575237992 | 0.999970678 | 0.372 | 0.857 | 0.644 | 1.216 | Mito_carr |
| TRINITY_DN79_c0_g1_i23 | 0.700474757 | 0.869548461 | 0.999970678 | 2.135 | 0 | 0 | 1.337 | NA |
| TRINITY_DN12071_c0_g1_i1 | -0.70045832 | 0.470523073 | 0.999970678 | 3.761 | 4.415 | 3.344 | 9.139 | NA |
| TRINITY_DN754_c1_g1_i6 | -0.7003385 | 0.796773228 | 0.999970678 | 3.515 | 0 | 1.6 | 3.871 | LRR_6 |
| TRINITY_DN56820_c0_g1_i1 | -0.70033394 | 0.79439118 | 0.999970678 | 5.187 | 0 | 3.211 | 4.645 | NA |
| TRINITY_DN3402_c1_g1_i1 | -0.70029956 | 0.259881234 | 0.999970678 | 35.019 | 27.21 | 40.953 | 51.465 | Histone |
| TRINITY_DN11566_c0_g1_i1 | 0.70023429 | 0.388315373 | 0.999970678 | 7.73 | 4.326 | 3.189 | 3.599 | NA |
| TRINITY_DN1422_c0_g1_i3 | 0.700221628 | 0.237486186 | 0.999970678 | 12 | 11.97 | 5.633 | 7.993 | Kelch_3 |
| TRINITY_DN27611_c0_g1_i3 | -0.70019209 | 0.520835461 | 0.999970678 | 0.818 | 0.66 | 1.211 | 0.925 | Mak10 |
| TRINITY_DN5689_c0_g1_i4 | -0.7001244 | 0.871753597 | 0.999970678 | 0 | 0.335 | 0.444 | 0 | NA |
| TRINITY_DN1806_c0_g1_i4 | 0.70011984 | 0.598584421 | 0.999970678 | 2.562 | 7.411 | 0.944 | 4.926 | NA |
| TRINITY_DN6537_c0_g1_i1 | -0.70010277 | 0.474140522 | 0.999970678 | 13.426 | 22.87 | 12.777 | 43.402 | p450 |
| TRINITY_DN17333_c1_g1_i3 | -0.70001193 | 0.455078695 | 0.999970678 | 6.159 | 3.745 | 4.166 | 11.129 | NA |
| TRINITY_DN33191_c0_g1_i1 | -0.69996701 | 0.87441324 | 0.999970678 | 0.372 | 0 | 0.511 | 0 | CorA |
| TRINITY_DN2790_c0_g1_i1 | -0.69987604 | 0.47885152 | 0.999970678 | 44.993 | 86.31 | 127.792 | 56.793 | HNH |
| TRINITY_DN2906_c0_g1_i4 | -0.69985854 | 0.603826034 | 0.999970678 | 1.889 | 0.453 | 2.244 | 1.106 | - |
| TRINITY_DN397_c0_g2_i1 | -0.6996817 | 0.421314396 | 0.999970678 | 10.347 | 8.663 | 8.388 | 20.479 | NA |
| TRINITY_DN7260_c0_g1_i5 | -0.69954117 | 0.712169336 | 0.999970678 | 1.944 | 1.597 | 0.167 | 5.54 | NA |
| TRINITY_DN95_c0_g1_i4 | -0.69952749 | 0.646650556 | 0.999970678 | 2.044 | 0.325 | 2.578 | 0.804 | - |
| TRINITY_DN2351_c1_g1_i1 | 0.699158268 | 0.459885371 | 0.999970678 | 3.325 | 2.06 | 0.978 | 2.162 | - |
| TRINITY_DN6878_c0_g1_i1 | 0.699137427 | 0.653265173 | 0.999970678 | 0.763 | 0.769 | 0.089 | 0.824 | - |
| TRINITY_DN37539_c0_g1_i2 | -0.69911211 | 0.403444819 | 0.999970678 | 1.871 | 1.873 | 3.155 | 2.282 | Glyco_hydro_100 |
| TRINITY_DN2787_c0_g1_i3 | 0.69911019 | 0.60515915 | 0.999970678 | 0.572 | 0.818 | 0.567 | 0.181 | CIA30 |
| TRINITY_DN580_c0_g1_i5 | 0.69904386 | 0.725757769 | 0.999970678 | 0.082 | 1.744 | 0.7 | 0.251 | NA |
| TRINITY_DN7720_c2_g1_i3 | -0.69903796 | 0.716531716 | 0.999970678 | 2.671 | 1.37 | 0.167 | 6.334 | NA |
| TRINITY_DN37413_c0_g1_i1 | -0.69901188 | 0.724044889 | 0.999970678 | 0.391 | 1.587 | 0.122 | 3.016 | - |
| TRINITY_DN477_c0_g1_i7 | 0.698939187 | 0.516774058 | 0.999970678 | 2.298 | 3.637 | 2.233 | 0.945 | NA |
| TRINITY_DN1197_c1_g1_i1 | 0.698928923 | 0.603118599 | 0.999970678 | 19.44 | 2.405 | 5.966 | 6.434 | CLU_N |
| TRINITY_DN405_c0_g1_i2 | -0.69891666 | 0.285710157 | 0.999970678 | 65.377 | 71.33 | 107.738 | 88.955 | Ribosomal_L34e |
| TRINITY_DN17673_c0_g1_i1 | -0.69882289 | 0.320681136 | 0.999970678 | 308.2 | 186.4 | 344.279 | 385.801 | NA |
| TRINITY_DN55711_c0_g1_i1 | 0.69877776 | 0.877234009 | 1 | 2.498 | 0 | 1.289 | 0 | NA |
| TRINITY_DN77_c0_g2_i2 | 0.698766559 | 0.466086939 | 0.999970678 | 19.013 | 7.165 | 6.177 | 8.727 | NA |
| TRINITY_DN9709_c0_g1_i2 | -0.69871586 | 0.452973938 | 0.999970678 | 2.325 | 1.094 | 2.478 | 2.584 | F-box |
| TRINITY_DN7610_c0_g1_i1 | 0.698710181 | 0.549210964 | 0.999970678 | 8.439 | 4.317 | 3.944 | 2.986 | WRKY |
| TRINITY_DN6272_c0_g1_i2 | 0.698687301 | 0.44920156 | 0.999970678 | 1.88 | 2.119 | 0.611 | 1.719 | NA |
| TRINITY_DN975_c0_g1_i3 | -0.69864342 | 0.483960715 | 0.999970678 | 11.055 | 3.508 | 9.677 | 12.165 | RRM_1 |
| TRINITY_DN51053_c0_g1_i1 | -0.69858434 | 0.869571449 | 0.999970678 | 3.27 | 0 | 4.455 | 0 | NA |
| TRINITY_DN179422_c0_g1_i1 | -0.69856275 | 0.804182083 | 1 | 1.899 | 0 | 1 | 1.87 | NA |
| TRINITY_DN10773_c1_g1_i1 | -0.69855409 | 0.869818372 | 0.999970678 | 2.534 | 0 | 3.455 | 0 | FA_hydroxylase |
| TRINITY_DN1074_c0_g1_i1 | 0.698537477 | 0.731558304 | 0.999970678 | 45.874 | 19.93 | 0.256 | 40.607 | NA |
| TRINITY_DN4499_c1_g1_i2 | -0.69849491 | 0.398460253 | 0.999970678 | 0.818 | 0.542 | 0.8 | 1.247 | NA |
| TRINITY_DN2868_c0_g1_i2 | -0.69845527 | 0.868193229 | 0.999970678 | 0 | 4.819 | 6.355 | 0 | NA |
| TRINITY_DN7721_c0_g1_i4 | -0.69835188 | 0.803792005 | 0.999970678 | 0 | 5.361 | 6 | 1.327 | NA |
| TRINITY_DN15059_c0_g1_i4 | 0.698351614 | 0.589802601 | 0.999970678 | 5.932 | 1.774 | 3 | 1.166 | DUF971 |
| TRINITY_DN11883_c3_g1_i1 | -0.69835147 | 0.799288847 | 0.999970678 | 0.027 | 3.016 | 4 | 0.03 | - |
| TRINITY_DN12169_c0_g1_i1 | 0.698349665 | 0.355571751 | 0.999970678 | 8.194 | 12.2 | 5.155 | 6.253 | - |
| TRINITY_DN16886_c0_g1_i1 | -0.69827796 | 0.573435299 | 0.999970678 | 0.772 | 1.735 | 0.889 | 2.946 | zf-C3HC4_3 |
| TRINITY_DN7460_c0_g1_i1 | -0.69808029 | 0.645755043 | 0.999970678 | 0.954 | 8.771 | 2.722 | 12.336 | VWA_2 |
| TRINITY_DN5201_c0_g1_i1 | -0.69807785 | 0.71634623 | 0.999970678 | 1.099 | 0.365 | 1.878 | 0.121 | QRPTase_C |
| TRINITY_DN1662_c0_g1_i6 | -0.69793787 | 0.830445897 | 0.999970678 | 0.136 | 6.623 | 0 | 10.396 | NA |
| TRINITY_DN6843_c0_g1_i1 | -0.69791963 | 0.807567271 | 0.999970678 | 0 | 0.779 | 0.844 | 0.211 | - |
| TRINITY_DN2085_c0_g1_i1 | 0.697908726 | 0.324057782 | 0.999970678 | 5.532 | 4.602 | 2.555 | 3.167 | UDPGT |
| TRINITY_DN14920_c0_g1_i4 | 0.697879806 | 0.79840326 | 0.999970678 | 1.244 | 0.621 | 0.944 | 0.01 | NA |
| TRINITY_DN2807_c0_g1_i2 | -0.69783128 | 0.629505779 | 0.999970678 | 15.515 | 11.3 | 3.089 | 39.471 | p450 |
| TRINITY_DN2408_c0_g1_i2 | -0.69777416 | 0.868387163 | 0.999970678 | 0 | 2.602 | 3.433 | 0 | NA |
| TRINITY_DN4840_c0_g1_i3 | 0.697691266 | 0.474036664 | 0.999970678 | 11.21 | 20.05 | 10.822 | 5.942 | NA |
| TRINITY_DN1844_c0_g1_i1 | -0.69768753 | 0.814207147 | 0.999970678 | 3.779 | 0.355 | 5.611 | 0 | NA |
| TRINITY_DN1355_c0_g2_i2 | 0.697487097 | 0.764557641 | 0.999970678 | 10.628 | 0.177 | 5.122 | 0.462 | NA |
| TRINITY_DN3817_c0_g1_i1 | -0.69740966 | 0.563767884 | 0.999970678 | 8.848 | 2.227 | 8.877 | 7.259 | NA |
| TRINITY_DN2093_c0_g1_i3 | 0.697390845 | 0.869970757 | 0.999970678 | 1.19 | 0 | 0 | 0.754 | NA |
| TRINITY_DN1871_c0_g1_i2 | 0.697386097 | 0.722418506 | 0.999970678 | 4.887 | 0.187 | 2.267 | 0.432 | NA |
| TRINITY_DN23530_c0_g1_i1 | -0.69737446 | 0.876453213 | 1 | 0.527 | 0 | 0.722 | 0 | Cation_efflux |
| TRINITY_DN3975_c0_g1_i1 | -0.69734174 | 0.802550873 | 0.999970678 | 0 | 17.42 | 15.732 | 8.767 | NA |

| TRINITY_DN7368_c0_g1_i1 | 0.697166734 | 0.297678633 | 0.999970678 | 10.81 | 11.19 | 4.533 | 8.123 | Adaptin_N |
| --- | --- | --- | --- | --- | --- | --- | --- | --- |
| TRINITY_DN5111_c0_g1_i15 | -0.69714835 | 0.461200497 | 0.999970678 | 14.126 | 11.92 | 15.721 | 21.465 | NA |
| TRINITY_DN118_c0_g1_i17 | -0.69714746 | 0.569833324 | 0.999970678 | 3.997 | 2.129 | 6.6 | 2.001 | NA |
| TRINITY_DN2243_c0_g1_i2 | -0.69714729 | 0.391814345 | 0.999970678 | 14.598 | 11.92 | 12.155 | 28.08 | IF4E |
| TRINITY_DN36727_c0_g1_i1 | 0.696951637 | 0.639709066 | 0.999970678 | 25.471 | 4.425 | 10.866 | 5.037 | NA |
| TRINITY_DN6182_c0_g1_i4 | -0.6969337 | 0.802143022 | 0.999970678 | 5.886 | 0 | 6.2 | 2.192 | NA |
| TRINITY_DN609_c0_g1_i1 | -0.69673029 | 0.329696152 | 0.999970678 | 9.974 | 12.18 | 15.943 | 16.146 | NA |
| TRINITY_DN1230_c0_g1_i9 | 0.696656381 | 0.598754054 | 0.999970678 | 1.199 | 3.183 | 0.833 | 1.578 | PsbP |
| TRINITY_DN6826_c0_g1_i1 | 0.696625004 | 0.476872801 | 0.999970678 | 1.562 | 1.794 | 0.8 | 1.096 | NA |
| TRINITY_DN1875_c0_g1_i5 | 0.69651003 | 0.710808714 | 0.999970678 | 2.834 | 0.276 | 1.489 | 0.151 | PHD |
| TRINITY_DN5192_c0_g1_i4 | 0.696499088 | 0.81109936 | 0.999970678 | 0 | 5.154 | 0.3 | 2.765 | NA |
| TRINITY_DN9690_c0_g2_i1 | 0.696364292 | 0.491775952 | 0.999970678 | 9.874 | 7.135 | 6.544 | 2.594 | F-box-like |
| TRINITY_DN37200_c0_g1_i5 | 0.696302282 | 0.609934223 | 0.999970678 | 0.89 | 1.449 | 0.233 | 1.156 | Lipase_GDSL |
| TRINITY_DN4545_c1_g1_i3 | -0.69629565 | 0.451089286 | 0.999970678 | 1.435 | 2.04 | 1.622 | 3.67 | NA |
| TRINITY_DN91937_c0_g1_i1 | -0.69622843 | 0.645133429 | 0.999970678 | 0.609 | 2.168 | 0.833 | 3.418 | Thaumatin |
| TRINITY_DN2514_c0_g2_i4 | -0.69619152 | 0.661512485 | 0.999970678 | 8.339 | 0.71 | 5.444 | 8.113 | NA |
| TRINITY_DN3480_c0_g1_i7 | 0.696149558 | 0.64079914 | 0.999970678 | 1.771 | 5.785 | 1.122 | 3.086 | NA |
| TRINITY_DN717_c0_g1_i5 | -0.69610939 | 0.422979079 | 0.999970678 | 2.752 | 1.843 | 2.9 | 3.921 | UPF0160 |
| TRINITY_DN5335_c0_g1_i2 | -0.69600028 | 0.868437505 | 0.999970678 | 4.206 | 0 | 0 | 6.907 | Glyco_hydro_17 |
| TRINITY_DN3374_c0_g2_i1 | -0.69596331 | 0.258497178 | 0.999970678 | 8.021 | 6.544 | 9.155 | 12.637 | Nucleoporin2 |
| TRINITY_DN2709_c0_g2_i1 | -0.69586365 | 0.448556881 | 0.999970678 | 45.047 | 58.44 | 37.242 | 122.132 | IQ |
| TRINITY_DN960_c0_g1_i5 | 0.695834043 | 0.615485901 | 0.999970678 | 2.071 | 2.306 | 0.278 | 2.373 | Epimerase |
| TRINITY_DN1184_c0_g1_i1 | -0.69579252 | 0.598254776 | 0.999970678 | 0.654 | 1.192 | 2.089 | 0.473 | NA |
| TRINITY_DN1753_c0_g1_i7 | -0.69568724 | 0.361240001 | 0.999970678 | 26.498 | 14.43 | 29.954 | 30.543 | Ribosomal_60s |
| TRINITY_DN16888_c1_g1_i6 | -0.69559708 | 0.547730574 | 0.999970678 | 0.354 | 0.552 | 0.456 | 0.955 | NA |
| TRINITY_DN549_c0_g1_i1 | 0.695554787 | 0.354783842 | 0.999970678 | 6.313 | 10.74 | 5.033 | 4.434 | NA |
| TRINITY_DN164_c0_g1_i2 | -0.695552 | 0.802475627 | 0.999970678 | 2.662 | 0 | 2.911 | 0.834 | Glyco_hydro_18 |
| TRINITY_DN11581_c0_g1_i1 | 0.695542397 | 0.671755635 | 0.999970678 | 0.182 | 1.36 | 0.533 | 0.302 | FMO-like |
| TRINITY_DN4736_c0_g1_i1 | -0.69544419 | 0.621936362 | 0.999970678 | 7.276 | 50.02 | 16.943 | 70.899 | CBFD_NFYB_HMF |
| TRINITY_DN63988_c0_g1_i1 | -0.69531657 | 0.61592016 | 0.999970678 | 9.674 | 9.432 | 2.822 | 27.115 | EF-hand_1 |
| TRINITY_DN5415_c0_g1_i9 | -0.69531108 | 0.721928116 | 0.999970678 | 0.109 | 2.789 | 0.7 | 3.79 | NA |
| TRINITY_DN208_c0_g2_i3 | -0.69522723 | 0.367393577 | 0.999970678 | 16.56 | 12.73 | 17.866 | 24.511 | NA |
| TRINITY_DN2094_c0_g1_i5 | 0.695155426 | 0.658420645 | 0.999970678 | 0.627 | 0.266 | 0.378 | 0.101 | NA |
| TRINITY_DN2106_c2_g1_i3 | 0.695112094 | 0.434756445 | 0.999970678 | 1.98 | 2.681 | 1.567 | 0.985 | ABC_tran |
| TRINITY_DN20481_c0_g2_i1 | -0.69508415 | 0.342243154 | 0.999970678 | 109.91 | 138 | 210.854 | 144.884 | NA |
| TRINITY_DN10501_c0_g1_i6 | -0.69507484 | 0.719622634 | 1 | 0.481 | 0.089 | 0.656 | 0.131 | CLP1_N |
| TRINITY_DN11301_c0_g1_i4 | -0.69502307 | 0.693306436 | 0.999970678 | 0.091 | 0.897 | 0.822 | 0.573 | ADH_N_2 |
| TRINITY_DN14083_c0_g1_i2 | 0.694952718 | 0.473829987 | 0.999970678 | 2.434 | 1.143 | 1.011 | 0.995 | Reticulon |
| TRINITY_DN4545_c2_g1_i4 | 0.694866673 | 0.663721894 | 0.999970678 | 7.794 | 13.1 | 0.611 | 12.105 | NA |
| TRINITY_DN2601_c0_g1_i12 | -0.69482773 | 0.609896703 | 0.999970678 | 1.944 | 0.65 | 1.9 | 1.94 | NA |
| TRINITY_DN79264_c0_g1_i1 | 0.69480632 | 0.878514921 | 1 | 1.562 | 0 | 0.811 | 0 | NA |
| TRINITY_DN41974_c0_g1_i4 | 0.69480632 | 0.878514921 | 1 | 1.762 | 0 | 0.911 | 0 | NA |
| TRINITY_DN41713_c0_g1_i1 | 0.69480632 | 0.878514921 | 1 | 1.962 | 0 | 0.989 | 0 | NA |
| TRINITY_DN2728_c0_g1_i6 | -0.69477427 | 0.366009025 | 0.999970678 | 1.753 | 1.794 | 2.367 | 2.895 | NA |
| TRINITY_DN2855_c0_g1_i1 | 0.694773585 | 0.691858553 | 0.999970678 | 1.49 | 0.187 | 0.711 | 0.181 | EF-hand_5 |
| TRINITY_DN4707_c0_g2_i5 | -0.69476091 | 0.674321424 | 0.999970678 | 0.799 | 0.187 | 1.122 | 0.282 | NA |
| TRINITY_DN1699_c0_g2_i2 | -0.69473537 | 0.805325067 | 1 | 0.363 | 0 | 0.167 | 0.412 | Nucleotid_trans |
| TRINITY_DN1632_c0_g1_i1 | -0.69464389 | 0.63726493 | 0.999970678 | 0.118 | 0.552 | 0.667 | 0.261 | NA |
| TRINITY_DN3566_c0_g2_i5 | -0.69464058 | 0.8693625 | 0.999970678 | 30.84 | 0 | 41.153 | 0 | NA |
| TRINITY_DN3807_c0_g1_i3 | -0.69464023 | 0.473748802 | 0.999970678 | 4.86 | 2.415 | 4.066 | 6.867 | NA |
| TRINITY_DN4807_c0_g1_i10 | 0.694626366 | 0.627205949 | 0.999970678 | 8.557 | 3.794 | 4.7 | 1.9 | NA |
| TRINITY_DN1708_c0_g1_i2 | -0.69461547 | 0.685175851 | 0.999970678 | 33.374 | 2.148 | 8.411 | 47.373 | NA |
| TRINITY_DN691_c3_g1_i1 | -0.69459574 | 0.508362935 | 0.999970678 | 7.558 | 6.978 | 4.1 | 18.68 | PP2C |
| TRINITY_DN6663_c0_g2_i6 | 0.694487325 | 0.79797447 | 0.999970678 | 2.262 | 0.65 | 0 | 1.83 | NA |
| TRINITY_DN47_c0_g1_i2 | -0.69445406 | 0.516293861 | 0.999970678 | 15.297 | 6.504 | 7.588 | 26.512 | PTR2 |
| TRINITY_DN1257_c0_g1_i2 | -0.69441164 | 0.49119397 | 0.999970678 | 37.453 | 13.57 | 30.398 | 43.744 | NA |
| TRINITY_DN2362_c0_g1_i1 | 0.694393437 | 0.581770442 | 0.999970678 | 1.653 | 3.459 | 2.144 | 0.553 | NA |
| TRINITY_DN71227_c0_g1_i1 | -0.69437945 | 0.513535643 | 0.999970678 | 0.463 | 0.729 | 1.067 | 0.633 | NA |
| TRINITY_DN4486_c1_g1_i3 | 0.694373043 | 0.578292224 | 0.999970678 | 207.85 | 139.6 | 31.943 | 156.446 | NA |
| TRINITY_DN19274_c0_g1_i1 | 0.694282222 | 0.568473319 | 0.999970678 | 1.163 | 3.646 | 0.744 | 2.041 | HLH |
| TRINITY_DN3366_c0_g1_i4 | -0.69426782 | 0.683100568 | 0.999970678 | 2.58 | 0.256 | 2.8 | 1.247 | NA |
| TRINITY_DN5000_c2_g1_i1 | 0.694261378 | 0.612945728 | 0.999970678 | 2.489 | 13.52 | 2.055 | 7.269 | NA |
| TRINITY_DN5578_c0_g1_i8 | 0.694237158 | 0.799701983 | 0.999970678 | 0.818 | 2.671 | 1.767 | 0 | NA |
| TRINITY_DN1993_c0_g2_i3 | 0.694185941 | 0.537653114 | 0.999970678 | 4.969 | 11.82 | 2.055 | 7.822 | NA |
| TRINITY_DN11337_c0_g1_i1 | -0.69416091 | 0.32794774 | 0.999970678 | 27.37 | 24.63 | 26.098 | 52.681 | Epimerase |
| TRINITY_DN921_c0_g2_i2 | 0.694129022 | 0.356559131 | 0.999970678 | 15.606 | 12.63 | 7.688 | 8.023 | NA |
| TRINITY_DN10931_c0_g1_i5 | -0.69405241 | 0.537133694 | 0.999970678 | 0.3 | 0.355 | 0.489 | 0.473 | DYW_deaminase |
| TRINITY_DN1919_c0_g1_i3 | -0.69404787 | 0.5796144 | 0.999970678 | 42.159 | 8.899 | 49.73 | 23.274 | NA |
| TRINITY_DN10027_c0_g1_i1 | 0.693997497 | 0.729576656 | 0.999970678 | 13.499 | 1.38 | 6.755 | 0.955 | B_lectin |
| TRINITY_DN54056_c0_g1_i3 | -0.69396653 | 0.751804832 | 0.999970678 | 0.736 | 0.384 | 1.478 | 0.03 | Lipoxygenase |
| TRINITY_DN5117_c0_g1_i2 | 0.693964856 | 0.374060103 | 0.999970678 | 1.235 | 1.468 | 0.811 | 0.694 | - |
| TRINITY_DN840_c0_g1_i2 | -0.69392917 | 0.453728808 | 0.999970678 | 9.275 | 8.062 | 6.233 | 20.63 | Frigida |
| TRINITY_DN7363_c0_g1_i1 | -0.69389544 | 0.69615372 | 0.999970678 | 0.154 | 3.104 | 3.222 | 1.297 | NA |
| TRINITY_DN1031_c0_g1_i8 | -0.69384915 | 0.327812128 | 0.999970678 | 104.49 | 82.21 | 98.572 | 173.477 | Fer2 |

| TRINITY_DN3191_c0_g1_i14 | 0.693820556 | 0.871339373 | 0.999970678 | 2.871 | 0 | 0 | 1.82 | NA |
| --- | --- | --- | --- | --- | --- | --- | --- | --- |
| TRINITY_DN60363_c1_g1_i2 | -0.69369429 | 0.532059536 | 0.999970678 | 1.054 | 2.139 | 2.866 | 1.679 | Abhydrolase_1 |
| TRINITY_DN3022_c0_g1_i10 | -0.6936333 | 0.873624235 | 0.999970678 | 1.408 | 0 | 0 | 2.302 | NA |
| TRINITY_DN21674_c0_g1_i1 | -0.69362557 | 0.568922148 | 0.999970678 | 7.194 | 8.397 | 3.489 | 20.761 | NA |
| TRINITY_DN314_c0_g2_i8 | -0.69357516 | 0.351668984 | 0.999970678 | 4.478 | 6.978 | 7.355 | 9.541 | NA |
| TRINITY_DN14605_c0_g1_i3 | -0.69354019 | 0.879293695 | 1 | 0.845 | 0 | 1.155 | 0 | - |
| TRINITY_DN5867_c0_g1_i2 | 0.693519037 | 0.427517866 | 0.999970678 | 109.61 | 57.61 | 30.132 | 67.259 | NA |
| TRINITY_DN2942_c0_g1_i1 | -0.69348865 | 0.633455751 | 0.999970678 | 2.716 | 0.414 | 2.966 | 1.548 | NA |
| TRINITY_DN3875_c0_g1_i2 | -0.69348054 | 0.411731853 | 0.999970678 | 3.397 | 3.755 | 3.433 | 7.369 | - |
| TRINITY_DN5317_c0_g1_i1 | 0.693458647 | 0.577676503 | 0.999970678 | 137.85 | 22.9 | 33.576 | 59.488 | zf-C2H2_6 |
| TRINITY_DN1069_c0_g1_i7 | 0.693457611 | 0.611803139 | 0.999970678 | 0.89 | 0.355 | 0.222 | 0.503 | - |
| TRINITY_DN1997_c0_g1_i1 | -0.69345745 | 0.556517081 | 0.999970678 | 2.843 | 12.28 | 12.044 | 9.621 | RRM_1 |
| TRINITY_DN2915_c2_g1_i2 | 0.693343348 | 0.746163941 | 0.999970678 | 2.871 | 2.109 | 2.522 | 0.02 | NA |
| TRINITY_DN50563_c0_g1_i1 | -0.69334026 | 0.421747507 | 0.999970678 | 1.499 | 1.074 | 1.967 | 1.79 | Myb_DNA-bindin |
| TRINITY_DN2162_c0_g1_i11 | 0.693336453 | 0.498858779 | 0.999970678 | 1.671 | 1.892 | 1.344 | 0.573 | NA |
| TRINITY_DN5761_c0_g2_i1 | -0.69328959 | 0.771076301 | 0.999970678 | 15.561 | 0.01 | 11.233 | 12.044 | NA |
| TRINITY_DN7_c0_g1_i1 | -0.69327873 | 0.472153253 | 0.999970678 | 58.918 | 28.78 | 35.742 | 98.969 | CAF1 |
| TRINITY_DN1633_c0_g1_i8 | 0.693238153 | 0.820416806 | 0.999970678 | 0.218 | 5.351 | 0 | 3.358 | F-box |
| TRINITY_DN5812_c0_g1_i1 | 0.693221067 | 0.507003506 | 0.999970678 | 5.241 | 2.69 | 2.878 | 1.458 | WRKY |
| TRINITY_DN3026_c0_g1_i1 | -0.69316811 | 0.50242979 | 0.999970678 | 94.173 | 28.78 | 64.196 | 122.102 | Aconitase_C |
| TRINITY_DN2462_c0_g1_i2 | -0.69316456 | 0.556661174 | 0.999970678 | 3.207 | 1.064 | 4.1 | 2.021 | NA |
| TRINITY_DN2186_c0_g1_i1 | 0.693155904 | 0.875203416 | 0.999970678 | 4.46 | 0 | 2.333 | 0 | SnoaL_2 |
| TRINITY_DN12320_c0_g1_i8 | 0.693155904 | 0.875203416 | 0.999970678 | 1.453 | 0 | 0.756 | 0 | p450 |
| TRINITY_DN1416_c1_g1_i1 | 0.693117622 | 0.638983111 | 0.999970678 | 152.06 | 12.67 | 29.187 | 68.285 | GDPD |
| TRINITY_DN8342_c0_g2_i1 | 0.692925847 | 0.834525728 | 1 | 5.968 | 0 | 2.978 | 0.161 | NA |
| TRINITY_DN4416_c0_g1_i8 | 0.692898756 | 0.504059512 | 0.999970678 | 1.235 | 1.882 | 1.144 | 0.543 | NA |
| TRINITY_DN1353_c0_g1_i10 | 0.692870909 | 0.793162193 | 0.999970678 | 2.934 | 4.257 | 0 | 4.353 | RNase_PH |
| TRINITY_DN19645_c1_g2_i1 | -0.69283388 | 0.582031671 | 0.999970678 | 0.772 | 2.198 | 1.644 | 2.755 | NA |
| TRINITY_DN310_c0_g1_i4 | -0.69278465 | 0.747527772 | 0.999970678 | 2.235 | 9.54 | 15.266 | 0.221 | NA |
| TRINITY_DN758_c0_g1_i10 | 0.692738815 | 0.821958616 | 0.999970678 | 0.136 | 3.075 | 1.633 | 0 | Sugar_tr |
| TRINITY_DN3602_c0_g1_i1 | -0.69271122 | 0.8270662 | 0.999970678 | 0.836 | 0 | 1.078 | 0.06 | Sugar_tr |
| TRINITY_DN10342_c0_g1_i1 | -0.69269675 | 0.559351538 | 0.999970678 | 3.016 | 1.409 | 1.7 | 5.087 | Auxin_inducible |
| TRINITY_DN11714_c1_g1_i1 | -0.69264062 | 0.628441062 | 1 | 1.19 | 0.473 | 0.722 | 1.81 | NA |
| TRINITY_DN848_c0_g1_i3 | 0.692595666 | 0.486342974 | 0.999970678 | 9.429 | 25.64 | 11.755 | 7.359 | NA |
| TRINITY_DN31_c0_g1_i2 | 0.692507106 | 0.440890747 | 0.999970678 | 7.767 | 17.45 | 7.677 | 6.213 | AAA |
| TRINITY_DN8150_c0_g1_i10 | 0.692491452 | 0.580296124 | 0.999970678 | 1.762 | 1.163 | 1.089 | 0.493 | NA |
| TRINITY_DN3831_c1_g1_i4 | 0.692364464 | 0.389718682 | 0.999970678 | 3.07 | 2.316 | 1.733 | 1.237 | NA |
| TRINITY_DN4768_c0_g1_i2 | 0.692350645 | 0.655203611 | 0.999970678 | 4.678 | 2.158 | 0.322 | 3.871 | NA |
| TRINITY_DN28252_c0_g1_i1 | -0.69222428 | 0.623240386 | 0.999970678 | 1.608 | 1.715 | 0.756 | 4.414 | NA |
| TRINITY_DN1313_c0_g2_i3 | 0.692183906 | 0.632379428 | 0.999970678 | 2.698 | 21.03 | 3.022 | 10.808 | UDPGT |
| TRINITY_DN7069_c0_g1_i1 | -0.69204061 | 0.726735791 | 0.999970678 | 10.029 | 0.69 | 1.1 | 15.804 | NA |
| TRINITY_DN1492_c0_g3_i1 | 0.691939001 | 0.381660378 | 0.999970678 | 7.631 | 5.016 | 4.066 | 2.966 | NA |
| TRINITY_DN9333_c0_g1_i1 | 0.691894157 | 0.689774458 | 0.999970678 | 0.191 | 0.434 | 0.044 | 0.352 | NB-ARC |
| TRINITY_DN3270_c0_g1_i4 | -0.69185846 | 0.805250318 | 1 | 0.917 | 0 | 0.4 | 1.025 | NA |
| TRINITY_DN41435_c0_g1_i2 | -0.69183908 | 0.876466651 | 1 | 0.709 | 0 | 0.967 | 0 | CTP_transf_1 |
| TRINITY_DN219_c1_g2_i1 | -0.6916893 | 0.370420659 | 0.999970678 | 4.197 | 3.311 | 5.922 | 4.987 | PGK |
| TRINITY_DN9141_c3_g1_i5 | -0.69168324 | 0.266117173 | 0.999970678 | 5.95 | 7.736 | 9.166 | 11.019 | NA |
| TRINITY_DN1033_c0_g2_i4 | 0.691461469 | 0.726097779 | 1 | 0.363 | 1.735 | 0.1 | 1.206 | NA |
| TRINITY_DN8493_c0_g1_i2 | 0.691408287 | 0.870036362 | 0.999970678 | 1.69 | 0 | 0.878 | 0 | Acetyltransf_1 |
| TRINITY_DN1412_c0_g2_i16 | 0.691359764 | 0.79598159 | 0.999970678 | 4.042 | 1.646 | 0 | 3.539 | Ras |
| TRINITY_DN6671_c0_g2_i3 | -0.69134944 | 0.810100891 | 1 | 1.335 | 0 | 0.567 | 1.428 | LRRNT_2 |
| TRINITY_DN23213_c0_g1_i16 | 0.691253282 | 0.871196659 | 0.999970678 | 1.372 | 0 | 0 | 0.865 | Helicase_C |
| TRINITY_DN1373_c0_g1_i6 | 0.69125234 | 0.827292709 | 0.999970678 | 0 | 40.15 | 18.566 | 2.041 | NA |
| TRINITY_DN1803_c0_g1_i3 | 0.691221177 | 0.879572922 | 0.999970678 | 0 | 88.95 | 0 | 54.481 | Glyco_hydro_1 |
| TRINITY_DN9188_c0_g1_i7 | -0.69120453 | 0.761635257 | 0.999970678 | 2.752 | 0.118 | 3.689 | 0.261 | NA |
| TRINITY_DN20263_c0_g1_i2 | 0.691115567 | 0.811263725 | 0.999970678 | 0.854 | 0.099 | 0.489 | 0 | Cellulose_synt |
| TRINITY_DN5902_c0_g1_i3 | 0.691068825 | 0.535754769 | 0.999970678 | 3.797 | 3.459 | 0.778 | 3.569 | NA |
| TRINITY_DN19302_c0_g1_i1 | 0.69098766 | 0.458182071 | 0.999970678 | 3.125 | 3.242 | 1.867 | 1.669 | SPOUT_MTase |
| TRINITY_DN1505_c1_g1_i4 | -0.69097153 | 0.798944388 | 0.999970678 | 4.76 | 0 | 2.089 | 5.258 | NA |
| TRINITY_DN2161_c0_g1_i1 | -0.69096431 | 0.339678249 | 0.999970678 | 19.712 | 22.33 | 34.653 | 25.546 | NA |
| TRINITY_DN1027_c0_g1_i3 | -0.69095361 | 0.592097756 | 0.999970678 | 21.692 | 4.78 | 9.588 | 31.166 | NA |
| TRINITY_DN4067_c1_g1_i3 | -0.69092478 | 0.816283388 | 1 | 0 | 1.35 | 1.589 | 0.251 | NA |
| TRINITY_DN9499_c0_g1_i2 | -0.69087412 | 0.874683039 | 0.999970678 | 1.036 | 0 | 1.4 | 0 | NA |
| TRINITY_DN440_c1_g1_i1 | -0.69073991 | 0.433536532 | 0.999970678 | 55.721 | 29.81 | 38.864 | 92.011 | NA |
| TRINITY_DN207_c0_g2_i4 | -0.69073736 | 0.240612652 | 0.999970678 | 49.562 | 58.49 | 78.251 | 79.444 | NA |
| TRINITY_DN90832_c0_g1_i1 | 0.690685745 | 0.556210956 | 0.999970678 | 0.5 | 0.946 | 0.256 | 0.583 | NA |
| TRINITY_DN84923_c1_g1_i3 | -0.69063368 | 0.692677866 | 0.999970678 | 65.304 | 10.36 | 75.751 | 24.038 | NA |
| TRINITY_DN10742_c3_g1_i1 | -0.69050734 | 0.455798488 | 0.999970678 | 13.299 | 6.662 | 9.444 | 20.851 | VQ |
| TRINITY_DN10506_c0_g1_i2 | -0.69042065 | 0.564333258 | 0.999970678 | 0.89 | 0.493 | 0.578 | 1.548 | NA |
| TRINITY_DN8141_c0_g1_i1 | 0.690366788 | 0.365827088 | 0.999970678 | 2.453 | 4.297 | 1.778 | 2.011 | Xpo1 |
| TRINITY_DN4218_c0_g1_i5 | 0.690320332 | 0.813122315 | 0.999970678 | 1.944 | 0 | 0.8 | 0.241 | NA |
| TRINITY_DN1413_c0_g1_i1 | 0.69028567 | 0.630598948 | 0.999970678 | 24.954 | 2.7 | 9.544 | 5.801 | NA |
| TRINITY_DN256460_c0_g1_i1 | -0.69028229 | 0.870179728 | 0.999970678 | 5.523 | 0 | 7.466 | 0 | NA |
| TRINITY_DN39444_c0_g1_i3 | 0.690273661 | 0.470349566 | 0.999970678 | 2.289 | 1.38 | 0.789 | 1.337 | Iso_dh |

| TRINITY_DN370_c0_g1_i1 | -0.69026266 | 0.633178992 | 0.999970678 | 1.826 | 5.006 | 8.033 | 1.247 | NA |
| --- | --- | --- | --- | --- | --- | --- | --- | --- |
| TRINITY_DN143_c0_g1_i3 | 0.690230206 | 0.620872503 | 0.999970678 | 0.654 | 4.395 | 1.8 | 0.915 | NA |
| TRINITY_DN5915_c0_g1_i4 | -0.69021478 | 0.583684329 | 0.999970678 | 1.671 | 4.947 | 3.011 | 6.736 | NA |
| TRINITY_DN9162_c0_g1_i4 | 0.690205549 | 0.354615473 | 0.999970678 | 1.181 | 1.882 | 0.711 | 1.046 | RRM_1 |
| TRINITY_DN862_c0_g2_i1 | 0.69015472 | 0.804712331 | 0.999970678 | 0 | 5.627 | 1.244 | 1.87 | NA |
| TRINITY_DN11177_c0_g1_i2 | -0.69014178 | 0.674736563 | 0.999970678 | 14.135 | 86.07 | 10.466 | 147.256 | NA |
| TRINITY_DN282_c0_g1_i5 | 0.69005006 | 0.445737772 | 0.999970678 | 4.442 | 7.106 | 3.922 | 2.363 | - |
| TRINITY_DN991_c0_g1_i2 | -0.68993093 | 0.578457818 | 0.999970678 | 1.208 | 0.345 | 1.155 | 1.126 | GATA |
| TRINITY_DN11095_c0_g1_i1 | 0.689864738 | 0.417556329 | 0.999970678 | 2.553 | 2.089 | 1.222 | 1.397 | ubiquitin |
| TRINITY_DN4679_c0_g2_i2 | -0.68984643 | 0.35258484 | 0.999970678 | 7.194 | 6.012 | 7.533 | 12.054 | 40S_S4_C |
| TRINITY_DN3897_c0_g1_i5 | -0.68983571 | 0.591004548 | 0.999970678 | 2.58 | 0.808 | 1.178 | 4.082 | NA |
| TRINITY_DN7314_c0_g1_i3 | 0.689830179 | 0.46032968 | 0.999970678 | 2.707 | 1.232 | 0.944 | 1.327 | NA |
| TRINITY_DN15372_c0_g1_i4 | 0.689788536 | 0.550297612 | 0.999970678 | 0.409 | 0.946 | 0.278 | 0.493 | RRM_1 |
| TRINITY_DN33737_c0_g1_i2 | 0.689746921 | 0.663232609 | 0.999970678 | 2.952 | 33.77 | 6.455 | 13.965 | NA |
| TRINITY_DN2132_c0_g1_i3 | -0.68973301 | 0.367910965 | 0.999970678 | 5.977 | 4.326 | 8.677 | 6.203 | SHMT |
| TRINITY_DN29553_c0_g1_i1 | 0.68970452 | 0.371466495 | 0.999970678 | 5.36 | 6.081 | 3.878 | 2.413 | NA |
| TRINITY_DN1333_c1_g1_i9 | 0.689695227 | 0.597547462 | 0.999970678 | 0.863 | 5.44 | 1.467 | 2.091 | NA |
| TRINITY_DN6338_c0_g1_i1 | -0.68952422 | 0.537256069 | 0.999970678 | 10.946 | 3.853 | 8.666 | 13.231 | NA |
| TRINITY_DN2656_c0_g1_i3 | -0.68949535 | 0.466510572 | 0.999970678 | 1.908 | 2.316 | 1.8 | 4.645 | Amidohydro_1 |
| TRINITY_DN1304_c0_g1_i7 | -0.68942359 | 0.681446823 | 0.999970678 | 2.798 | 13.26 | 19.899 | 1.558 | NA |
| TRINITY_DN11298_c0_g1_i1 | 0.689392648 | 0.516199494 | 0.999970678 | 2.843 | 6.593 | 2.267 | 3.036 | NA |
| TRINITY_DN53690_c0_g1_i1 | -0.68924154 | 0.488770868 | 0.999970678 | 0.718 | 0.522 | 0.889 | 0.915 | PPR |
| TRINITY_DN1505_c1_g1_i2 | -0.68924018 | 0.479641577 | 0.999970678 | 2.525 | 3.804 | 5.244 | 3.78 | NA |
| TRINITY_DN3625_c0_g1_i5 | 0.689226849 | 0.49766132 | 0.999970678 | 3.933 | 3.676 | 3.011 | 1.086 | MT-A70 |
| TRINITY_DN9503_c0_g1_i1 | -0.68922024 | 0.824048279 | 0.999970678 | 3.288 | 0.108 | 4.611 | 0 | NA |
| TRINITY_DN114_c0_g1_i10 | -0.68915293 | 0.630015033 | 0.999970678 | 8.294 | 1.902 | 11.633 | 2.544 | NA |
| TRINITY_DN3739_c0_g1_i1 | 0.689018544 | 0.292016801 | 0.999970678 | 7.213 | 5.657 | 3.544 | 3.74 | Vta1 |
| TRINITY_DN283_c0_g2_i6 | -0.68899357 | 0.310749703 | 0.999970678 | 27.352 | 22.44 | 39.475 | 32.594 | Abhydrolase_1 |
| TRINITY_DN4369_c0_g1_i6 | 0.688952267 | 0.450933856 | 0.999970678 | 3.579 | 1.951 | 1 | 2.242 | NA |
| TRINITY_DN466_c1_g1_i2 | 0.688893636 | 0.512263452 | 0.999970678 | 1.326 | 1.675 | 0.444 | 1.327 | Glyco_transf_5 |
| TRINITY_DN405_c0_g1_i1 | -0.68871435 | 0.410310008 | 0.999970678 | 32.53 | 50.52 | 72.207 | 44.588 | Ribosomal_L34e |
| TRINITY_DN391_c0_g1_i2 | -0.68858542 | 0.281727094 | 0.999970678 | 25.562 | 36.31 | 41.82 | 48.409 | Ribosomal_L18 |
| TRINITY_DN9188_c0_g1_i9 | 0.688581347 | 0.762044312 | 0.999970678 | 4.369 | 0.532 | 2.522 | 0.09 | NA |
| TRINITY_DN2432_c0_g1_i1 | -0.68855561 | 0.802469156 | 0.999970678 | 0 | 6.938 | 6.177 | 3.549 | NA |
| TRINITY_DN1579_c0_g1_i1 | -0.68850868 | 0.391865007 | 0.999970678 | 5.441 | 8.466 | 11.722 | 8.174 | Hydrolase_4 |
| TRINITY_DN2014_c0_g5_i1 | 0.688484254 | 0.803788198 | 0.999970678 | 0 | 4.691 | 1.289 | 1.317 | NA |
| TRINITY_DN1322_c1_g1_i4 | 0.688472927 | 0.402210866 | 0.999970678 | 32.057 | 55.28 | 15.666 | 35.228 | NA |
| TRINITY_DN8593_c0_g1_i11 | 0.688450997 | 0.36606846 | 0.999970678 | 1.39 | 1.311 | 0.7 | 0.834 | NA |
| TRINITY_DN11536_c0_g1_i2 | 0.688427624 | 0.816320718 | 1 | 1.853 | 0.286 | 0 | 1.327 | NA |
| TRINITY_DN37140_c0_g1_i1 | 0.688358949 | 0.540646862 | 0.999970678 | 2.98 | 4.8 | 2.866 | 1.357 | NA |
| TRINITY_DN389_c0_g3_i3 | -0.68833253 | 0.392736221 | 0.999970678 | 6.795 | 11.8 | 9.333 | 18.609 | NA |
| TRINITY_DN1946_c0_g1_i7 | 0.6882505 | 0.606768648 | 0.999970678 | 4.587 | 1.537 | 0.578 | 3.137 | NA |
| TRINITY_DN78141_c0_g1_i1 | 0.688242011 | 0.661747993 | 0.999970678 | 0.263 | 0.976 | 0.478 | 0.191 | NA |
| TRINITY_DN1209_c0_g1_i5 | 0.688187657 | 0.707631806 | 0.999970678 | 0.699 | 5.184 | 0.356 | 3.066 | NA |
| TRINITY_DN4364_c0_g1_i1 | 0.688183609 | 0.879166655 | 1 | 0.263 | 0 | 0 | 0.171 | Med23 |
| TRINITY_DN7532_c0_g2_i1 | 0.688179209 | 0.464116766 | 0.999970678 | 9.384 | 7.214 | 6.266 | 2.765 | Chromo |
| TRINITY_DN2842_c0_g3_i3 | -0.68814249 | 0.587806767 | 0.999970678 | 9.565 | 1.764 | 10.488 | 5.811 | Sec1 |
| TRINITY_DN12575_c0_g1_i1 | 0.6881192 | 0.637469676 | 0.999970678 | 1.472 | 4.849 | 1.078 | 2.523 | Cu_bind_like |
| TRINITY_DN3357_c0_g1_i1 | 0.688093757 | 0.39639839 | 0.999970678 | 18.322 | 23.89 | 14.532 | 8.616 | LsmAD |
| TRINITY_DN39750_c0_g1_i3 | 0.688011663 | 0.471042436 | 0.999970678 | 2.335 | 1.055 | 0.856 | 1.076 | Dala_Dala_lig_C |
| TRINITY_DN1793_c0_g1_i2 | 0.687780394 | 0.802707947 | 1 | 0.263 | 0.621 | 0 | 0.543 | NA |
| TRINITY_DN7720_c0_g1_i9 | -0.68774795 | 0.596917387 | 0.999970678 | 11.864 | 1.725 | 8.799 | 11.491 | NA |
| TRINITY_DN397_c1_g1_i3 | 0.687612013 | 0.601897854 | 0.999970678 | 340.5 | 81.3 | 175.556 | 51.706 | PUNUT |
| TRINITY_DN1582_c0_g1_i4 | -0.68753835 | 0.876113036 | 0.999970678 | 0 | 0.404 | 0.533 | 0 | ORC3_N |
| TRINITY_DN2504_c0_g1_i2 | -0.68741849 | 0.574829766 | 0.999970678 | 1.962 | 6.367 | 4.777 | 7.048 | NA |
| TRINITY_DN23560_c0_g2_i1 | 0.68739297 | 0.361273872 | 0.999970678 | 4.524 | 6.012 | 3.311 | 2.534 | NA |
| TRINITY_DN54246_c0_g1_i2 | -0.68734728 | 0.872842216 | 0.999970678 | 1.49 | 0 | 2.011 | 0 | NA |
| TRINITY_DN1314_c0_g2_i1 | -0.68734214 | 0.873653024 | 0.999970678 | 0.572 | 0 | 0.767 | 0 | NA |
| TRINITY_DN4307_c0_g2_i2 | -0.68733289 | 0.8006664 | 0.999970678 | 5.223 | 0 | 2.966 | 4.946 | NA |
| TRINITY_DN14248_c0_g1_i7 | -0.68733075 | 0.806290105 | 1 | 1.09 | 0 | 0.933 | 0.633 | NA |
| TRINITY_DN18756_c0_g2_i2 | -0.687316 | 0.808940516 | 1 | 1.063 | 0 | 1.044 | 0.462 | NA |
| TRINITY_DN17160_c0_g2_i2 | -0.6873125 | 0.879028355 | 1 | 0.491 | 0.01 | 0.667 | 0 | NA |
| TRINITY_DN7437_c0_g1_i1 | 0.68727356 | 0.531731532 | 0.999970678 | 16.969 | 6.958 | 9.277 | 3.77 | Methyltransf_28 |
| TRINITY_DN1058_c0_g3_i1 | -0.68723831 | 0.625486674 | 0.999970678 | 0.872 | 1.114 | 0.411 | 2.694 | PRA1 |
| TRINITY_DN1978_c0_g1_i5 | -0.68696624 | 0.808956564 | 0.999970678 | 0 | 2.326 | 2.7 | 0.412 | NA |
| TRINITY_DN11195_c0_g1_i2 | -0.68690478 | 0.651439886 | 0.999970678 | 0.454 | 0.148 | 0.167 | 0.774 | NB-ARC |
| TRINITY_DN165740_c0_g1_i1 | 0.686896362 | 0.87695681 | 1 | 2.235 | 0 | 1.167 | 0 | DLH |
| TRINITY_DN4773_c0_g1_i3 | 0.68666449 | 0.871629181 | 0.999970678 | 36.899 | 0 | 0 | 23.013 | NA |
| TRINITY_DN3380_c0_g1_i3 | 0.686559214 | 0.602332166 | 0.999970678 | 0.518 | 1.409 | 0.356 | 0.754 | BTB |
| TRINITY_DN3932_c0_g1_i1 | -0.68654196 | 0.715385277 | 0.999970678 | 1.145 | 1.035 | 0.067 | 3.448 | Pkinase |
| TRINITY_DN2387_c0_g1_i3 | 0.686541404 | 0.613080749 | 0.999970678 | 2.634 | 1.547 | 0.556 | 1.93 | NA |
| TRINITY_DN13872_c0_g1_i1 | 0.686514498 | 0.342472505 | 0.999970678 | 4.506 | 4.297 | 2.589 | 2.343 | NA |
| TRINITY_DN1831_c1_g1_i2 | 0.686394813 | 0.66574892 | 0.999970678 | 0.917 | 3.371 | 0.3 | 2.302 | NA |
| TRINITY_DN96_c0_g3_i1 | -0.68635346 | 0.390120094 | 0.999970678 | 38.67 | 21.34 | 45.764 | 40.828 | - |

| TRINITY_DN3076_c0_g1_i3 | -0.68634714 | 0.472175011 | 0.999970678 | 2.026 | 4.721 | 5.189 | 4.544 | GrpE |
| --- | --- | --- | --- | --- | --- | --- | --- | --- |
| TRINITY_DN1720_c4_g1_i1 | 0.686335871 | 0.343713395 | 0.999970678 | 9.538 | 8.111 | 5.711 | 4.102 | NA |
| TRINITY_DN1880_c1_g1_i21 | -0.68626485 | 0.7028609 | 0.999970678 | 2.698 | 0.67 | 0.178 | 5.298 | NA |
| TRINITY_DN4665_c0_g1_i5 | 0.686188813 | 0.428462286 | 0.999970678 | 2.28 | 1.784 | 0.8 | 1.558 | NA |
| TRINITY_DN6644_c1_g1_i1 | 0.686187803 | 0.58539921 | 0.999970678 | 0.845 | 1.557 | 0.378 | 1.025 | NA |
| TRINITY_DN1872_c0_g1_i13 | 0.686128897 | 0.554746306 | 0.999970678 | 0.618 | 0.315 | 0.211 | 0.312 | NA |
| TRINITY_DN9048_c0_g2_i6 | 0.686063855 | 0.829749166 | 0.999970678 | 1.235 | 0.02 | 0.644 | 0 | NA |
| TRINITY_DN283_c0_g2_i9 | -0.68603436 | 0.368937101 | 0.999970678 | 20.63 | 37.85 | 35.365 | 49.997 | Abhydrolase_1 |
| TRINITY_DN1904_c0_g1_i1 | 0.686030237 | 0.598144923 | 0.999970678 | 9.756 | 4.967 | 1.533 | 7.259 | NA |
| TRINITY_DN3690_c0_g1_i2 | -0.68602154 | 0.608566895 | 0.999970678 | 2.035 | 0.591 | 1.244 | 2.755 | NA |
| TRINITY_DN3453_c0_g1_i1 | 0.685985082 | 0.871024171 | 0.999970678 | 26.062 | 0 | 13.521 | 0 | DUF1761 |
| TRINITY_DN24454_c0_g1_i1 | 0.685925433 | 0.807463727 | 1 | 0.672 | 0.315 | 0.511 | 0 | Cellulose_synt |
| TRINITY_DN1872_c0_g1_i2 | -0.68580846 | 0.80493859 | 0.999970678 | 3.007 | 0 | 1.111 | 3.589 | Cation_ATPase |
| TRINITY_DN1954_c0_g1_i1 | -0.68579768 | 0.435017138 | 0.999970678 | 40.206 | 101.3 | 84.095 | 122.082 | Ribosom_S12_S2 |
| TRINITY_DN1229_c0_g1_i2 | -0.68578562 | 0.562557484 | 0.999970678 | 2.598 | 5.204 | 2.289 | 9.581 | NA |
| TRINITY_DN976_c1_g1_i1 | 0.685728529 | 0.600248749 | 0.999970678 | 23.809 | 7.707 | 13.521 | 3.378 | ubiquitin |
| TRINITY_DN3780_c0_g1_i1 | -0.68569144 | 0.500581366 | 0.999970678 | 15.879 | 27.13 | 13.699 | 52.541 | NA |
| TRINITY_DN2696_c0_g1_i1 | -0.68558365 | 0.634926654 | 0.999970678 | 0.781 | 0.877 | 1.922 | 0.332 | zf-RING_2 |
| TRINITY_DN8435_c0_g1_i3 | 0.685540951 | 0.567174866 | 0.999970678 | 23.064 | 7.204 | 11.944 | 4.534 | NA |
| TRINITY_DN37368_c0_g1_i2 | -0.68548307 | 0.880950875 | 1 | 0 | 0.729 | 0.967 | 0 | NA |
| TRINITY_DN3341_c0_g1_i4 | 0.685453948 | 0.35671378 | 0.999970678 | 12.854 | 7.727 | 4.922 | 6.907 | NA |
| TRINITY_DN1831_c2_g1_i4 | -0.68545114 | 0.822649269 | 0.999970678 | 25.299 | 2.907 | 0 | 46.016 | FtsZ_C |
| TRINITY_DN23887_c0_g1_i6 | 0.685384791 | 0.547452127 | 0.999970678 | 6.677 | 9.51 | 2.711 | 6.535 | NA |
| TRINITY_DN3198_c0_g1_i14 | 0.685377093 | 0.426444381 | 0.999970678 | 11.464 | 24.45 | 8.155 | 12.225 | NA |
| TRINITY_DN10661_c0_g1_i3 | 0.685261317 | 0.806760467 | 0.999970678 | 2.08 | 0.384 | 1.278 | 0 | NA |
| TRINITY_DN12188_c0_g1_i2 | 0.685080648 | 0.675684958 | 1 | 0.727 | 0.532 | 0.544 | 0.121 | RRM_1 |
| TRINITY_DN2210_c0_g1_i5 | -0.68503965 | 0.37487907 | 0.999970678 | 99.678 | 78.4 | 155.057 | 99.471 | Cpn60_TCP1 |
| TRINITY_DN464_c0_g1_i2 | -0.68500047 | 0.327709614 | 0.999970678 | 7.058 | 7.017 | 9.466 | 11.019 | RRM_1 |
| TRINITY_DN4232_c0_g1_i7 | 0.68491101 | 0.657482446 | 0.999970678 | 0.327 | 1.045 | 0.133 | 0.684 | NA |
| TRINITY_DN34946_c0_g1_i1 | -0.68490996 | 0.540809088 | 0.999970678 | 0.89 | 0.562 | 1.022 | 1.106 | HhH-GPD |
| TRINITY_DN2094_c0_g1_i2 | 0.68484395 | 0.398421546 | 0.999970678 | 2.189 | 2.04 | 1.289 | 1.096 | NA |
| TRINITY_DN5588_c0_g1_i1 | -0.68481675 | 0.40156266 | 0.999970678 | 1.435 | 1.518 | 2.389 | 1.87 | NA |
| TRINITY_DN9148_c0_g1_i5 | 0.684811291 | 0.583290363 | 0.999970678 | 1.699 | 0.946 | 1.078 | 0.342 | Coilin_N |
| TRINITY_DN5038_c0_g2_i3 | 0.684754921 | 0.821439317 | 0.999970678 | 0 | 7.746 | 0.256 | 4.404 | SNARE |
| TRINITY_DN273_c0_g1_i1 | 0.684692136 | 0.591819106 | 0.999970678 | 0.781 | 1.261 | 0.2 | 1.036 | Aconitase |
| TRINITY_DN4524_c0_g1_i10 | 0.684675611 | 0.781786336 | 1 | 0.027 | 0.838 | 0.433 | 0.02 | HI0933_like |
| TRINITY_DN5324_c0_g2_i2 | -0.68458753 | 0.660202102 | 0.999970678 | 6.413 | 2.099 | 2.044 | 10.778 | LRRNT_2 |
| TRINITY_DN638_c0_g1_i10 | -0.6845493 | 0.578491781 | 0.999970678 | 1.045 | 2.493 | 3.678 | 1.216 | NA |
| TRINITY_DN69389_c0_g1_i7 | -0.68453429 | 0.516243753 | 0.999970678 | 1.453 | 0.877 | 0.933 | 2.624 | NA |
| TRINITY_DN257_c0_g1_i2 | -0.68448051 | 0.481277073 | 0.999970678 | 0.627 | 0.779 | 0.633 | 1.508 | Vps16_C |
| TRINITY_DN4384_c0_g1_i4 | -0.68436737 | 0.509840009 | 0.999970678 | 67.512 | 35.24 | 103.06 | 41.059 | Methyltransf_7 |
| TRINITY_DN8423_c0_g1_i2 | -0.68435803 | 0.410902007 | 0.999970678 | 22.01 | 12.04 | 19.91 | 30.03 | NA |
| TRINITY_DN18774_c0_g4_i1 | -0.68421976 | 0.799523956 | 0.999970678 | 1.653 | 0 | 1.178 | 1.277 | NA |
| TRINITY_DN22378_c0_g1_i1 | -0.68416908 | 0.708573743 | 0.999970678 | 0.055 | 1.439 | 1.189 | 0.935 | NA |
| TRINITY_DN3473_c0_g1_i5 | 0.684042844 | 0.60318339 | 0.999970678 | 1.29 | 0.601 | 0.678 | 0.342 | FGGY_C |
| TRINITY_DN200_c0_g1_i4 | -0.68400727 | 0.585189282 | 0.999970678 | 4.051 | 0.917 | 1.844 | 5.871 | NA |
| TRINITY_DN28536_c0_g4_i2 | -0.68400321 | 0.800960996 | 0.999970678 | 0 | 1.39 | 1.167 | 0.784 | NA |
| TRINITY_DN27374_c2_g3_i1 | -0.6839614 | 0.406035781 | 0.999970678 | 144.63 | 105.4 | 224.175 | 130.819 | NA |
| TRINITY_DN23_c0_g1_i2 | 0.683936253 | 0.733685972 | 0.999970678 | 25.181 | 1.439 | 13.288 | 0.704 | NA |
| TRINITY_DN4668_c0_g4_i3 | 0.683867664 | 0.814895186 | 0.999970678 | 0.818 | 0 | 0.3 | 0.141 | DUF659 |
| TRINITY_DN17835_c2_g1_i1 | -0.68382514 | 0.614569014 | 0.999970678 | 0.945 | 0.65 | 0.567 | 1.88 | NA |
| TRINITY_DN113_c0_g1_i8 | -0.68381635 | 0.469534211 | 0.999970678 | 16.569 | 7.204 | 12.033 | 23.656 | Pro_CA |
| TRINITY_DN13811_c0_g1_i2 | 0.683764646 | 0.622088262 | 0.999970678 | 0.59 | 0.443 | 0.111 | 0.513 | VEFS-Box |
| TRINITY_DN217_c0_g1_i3 | 0.683760335 | 0.593832857 | 0.999970678 | 16.551 | 14.75 | 14.088 | 2.453 | NA |
| TRINITY_DN2769_c0_g1_i5 | -0.68373732 | 0.564913032 | 0.999970678 | 2.48 | 2.237 | 4.7 | 1.91 | NA |
| TRINITY_DN5242_c0_g1_i5 | -0.68369341 | 0.812581538 | 0.999970678 | 5.396 | 0 | 0.756 | 7.922 | LysM |
| TRINITY_DN825_c0_g2_i1 | -0.68369299 | 0.60767363 | 0.999970678 | 2.544 | 2.73 | 5.911 | 1.287 | NA |
| TRINITY_DN443_c0_g1_i2 | 0.683610606 | 0.491904237 | 0.999970678 | 2.153 | 2.464 | 1.733 | 0.774 | Amidohydro_1 |
| TRINITY_DN13651_c0_g1_i1 | -0.68355759 | 0.51336044 | 0.999970678 | 7.222 | 4.188 | 11.155 | 4.856 | NA |
| TRINITY_DN79414_c0_g2_i2 | 0.683378304 | 0.877931759 | 1 | 0.745 | 0 | 0.389 | 0 | ABC_tran |
| TRINITY_DN4559_c0_g1_i9 | -0.68329948 | 0.596070422 | 0.999970678 | 0.872 | 0.394 | 1.122 | 0.664 | NA |
| TRINITY_DN2441_c0_g1_i7 | -0.68327784 | 0.819365958 | 0.999970678 | 33.938 | 0 | 17.443 | 34.404 | NA |
| TRINITY_DN4195_c0_g1_i10 | 0.683204615 | 0.50778078 | 0.999970678 | 1.653 | 2.198 | 1.389 | 0.704 | NA |
| TRINITY_DN5532_c0_g1_i4 | 0.682983205 | 0.872073702 | 0.999970678 | 11.155 | 0 | 5.8 | 0 | NA |
| TRINITY_DN2299_c0_g1_i1 | 0.682963527 | 0.514858466 | 0.999970678 | 22.083 | 60.87 | 12.944 | 35.299 | NA |
| TRINITY_DN24977_c0_g1_i2 | -0.68291479 | 0.690026687 | 0.999970678 | 1.154 | 0.118 | 1.344 | 0.432 | NA |
| TRINITY_DN6614_c0_g1_i1 | -0.68280537 | 0.395026086 | 0.999970678 | 9.683 | 18.13 | 15.81 | 25.094 | RsfS |
| TRINITY_DN9066_c0_g1_i1 | -0.68278617 | 0.579434951 | 0.999970678 | 49.144 | 10.13 | 24.821 | 65.902 | - |
| TRINITY_DN274_c0_g1_i17 | 0.682739108 | 0.804061545 | 0.999970678 | 0.291 | 0.68 | 0.5 | 0 | RRM_1 |
| TRINITY_DN6694_c0_g1_i2 | -0.68270952 | 0.534004181 | 0.999970678 | 1.599 | 0.7 | 2.067 | 1.206 | NA |
| TRINITY_DN4093_c0_g1_i8 | -0.68267548 | 0.80686002 | 0.999970678 | 0 | 1.764 | 1.944 | 0.432 | - |
| TRINITY_DN12796_c0_g1_i1 | -0.68267166 | 0.339045247 | 0.999970678 | 1.79 | 1.547 | 2.244 | 2.654 | RINT1_TIP1 |
| TRINITY_DN3825_c1_g2_i1 | -0.68247599 | 0.337631122 | 0.999970678 | 11.237 | 15.21 | 14.21 | 25.024 | NA |
| TRINITY_DN7893_c0_g1_i4 | -0.68245058 | 0.878795423 | 1 | 1.145 | 0 | 1.533 | 0.02 | FA_hydroxylase |

| TRINITY_DN22026_c0_g1_i2 | -0.68245058 | 0.878795423 | 1 | 1.335 | 0 | 1.822 | 0 | NA |
| --- | --- | --- | --- | --- | --- | --- | --- | --- |
| TRINITY_DN5025_c0_g1_i6 | -0.68239298 | 0.7090938 | 0.999970678 | 0.064 | 1.055 | 1.155 | 0.382 | Inhibitor_I9 |
| TRINITY_DN97_c0_g1_i2 | -0.68238695 | 0.572540206 | 0.999970678 | 8.094 | 15.78 | 23.676 | 8.616 | NA |
| TRINITY_DN7786_c0_g1_i5 | 0.682333635 | 0.658856263 | 1 | 0.554 | 0.552 | 0.489 | 0.121 | NA |
| TRINITY_DN12234_c0_g1_i5 | -0.6822168 | 0.837970193 | 1 | 3.534 | 0.158 | 4.889 | 0 | adh_short_C2 |
| TRINITY_DN2451_c1_g2_i5 | -0.68220809 | 0.491165356 | 0.999970678 | 0.463 | 0.394 | 0.833 | 0.362 | Ycf1 |
| TRINITY_DN1611_c0_g1_i2 | -0.68209272 | 0.8716641 | 0.999970678 | 0 | 6.435 | 8.411 | 0 | NA |
| TRINITY_DN11232_c0_g1_i1 | 0.682042883 | 0.414310573 | 0.999970678 | 9.084 | 9.895 | 3.122 | 8.063 | ALMT |
| TRINITY_DN575_c0_g1_i3 | -0.68197207 | 0.871733106 | 0.999970678 | 10.238 | 0 | 13.721 | 0 | GST_C |
| TRINITY_DN34292_c0_g2_i2 | 0.681942004 | 0.810762938 | 1 | 0.164 | 0.532 | 0.356 | 0 | DUF747 |
| TRINITY_DN17555_c0_g1_i2 | 0.681878655 | 0.813145769 | 1 | 0.827 | 0.177 | 0.533 | 0 | Cation_ATPase_N |
| TRINITY_DN18872_c0_g1_i1 | 0.681848506 | 0.583708526 | 1 | 1.29 | 1.084 | 0.733 | 0.583 | NA |
| TRINITY_DN8989_c0_g1_i2 | 0.681804549 | 0.578860876 | 0.999970678 | 1.98 | 4.445 | 0.711 | 3.097 | Pkinase |
| TRINITY_DN8847_c0_g1_i2 | 0.68175699 | 0.296966714 | 0.999970678 | 8.739 | 11.24 | 4.711 | 6.746 | Glyco_hydro_14 |
| TRINITY_DN18336_c1_g1_i1 | -0.68174409 | 0.490594137 | 0.999970678 | 140.61 | 60.98 | 82.506 | 224.921 | NA |
| TRINITY_DN18336_c1_g2_i1 | -0.68174409 | 0.490594137 | 0.999970678 | 140.61 | 60.98 | 82.506 | 224.921 | Cofilin_ADF |
| TRINITY_DN520_c0_g1_i7 | 0.681509393 | 0.804357069 | 0.999970678 | 0.382 | 0.246 | 0.322 | 0 | LRR_1 |
| TRINITY_DN20152_c0_g1_i9 | 0.681413375 | 0.879483451 | 1 | 1.626 | 0 | 0 | 1.015 | NA |
| TRINITY_DN13691_c0_g1_i1 | 0.68140675 | 0.481208555 | 0.999970678 | 13.998 | 5.085 | 5.2 | 5.7 | NA |
| TRINITY_DN8525_c0_g1_i1 | 0.681350304 | 0.502420559 | 0.999970678 | 3.025 | 3.026 | 2.278 | 1.015 | NA |
| TRINITY_DN239_c0_g1_i2 | 0.681295326 | 0.498337355 | 0.999970678 | 1.308 | 3.242 | 1.467 | 1.046 | NA |
| TRINITY_DN88874_c0_g1_i1 | -0.68128476 | 0.873322063 | 0.999970678 | 1.535 | 0 | 2.067 | 0 | DnaJ |
| TRINITY_DN509_c0_g1_i16 | -0.68125529 | 0.378640774 | 0.999970678 | 29.405 | 36.52 | 57.052 | 36.294 | NA |
| TRINITY_DN83_c0_g1_i5 | 0.681220057 | 0.500542828 | 0.999970678 | 2.553 | 5.085 | 2.089 | 2.242 | NA |
| TRINITY_DN9525_c0_g1_i1 | 0.681082218 | 0.501510831 | 0.999970678 | 0.927 | 2.464 | 0.711 | 1.257 | FPP |
| TRINITY_DN171_c0_g1_i3 | -0.68097694 | 0.409809573 | 0.999970678 | 5.614 | 8.949 | 7.133 | 14.648 | NA |
| TRINITY_DN4448_c0_g1_i3 | 0.680969125 | 0.508002929 | 0.999970678 | 2.189 | 2.454 | 1.455 | 1.126 | NA |
| TRINITY_DN15795_c0_g1_i1 | -0.68096522 | 0.638913979 | 0.999970678 | 6.086 | 10.12 | 1.955 | 23.395 | NA |
| TRINITY_DN29916_c0_g1_i11 | 0.680943844 | 0.608973368 | 0.999970678 | 0.173 | 0.621 | 0.256 | 0.191 | F-box-like |
| TRINITY_DN3915_c0_g1_i3 | -0.68091793 | 0.803190682 | 0.999970678 | 4.987 | 0 | 1.878 | 5.861 | TRAPP |
| TRINITY_DN27529_c0_g2_i2 | -0.68086404 | 0.651543384 | 0.999970678 | 0.536 | 2.06 | 0.967 | 2.936 | NA |
| TRINITY_DN157_c0_g1_i1 | -0.6808604 | 0.433064179 | 0.999970678 | 40.342 | 51.3 | 35.676 | 103.121 | HMA |
| TRINITY_DN4281_c0_g1_i5 | 0.680838545 | 0.541290864 | 0.999970678 | 1.526 | 4.573 | 1.989 | 1.377 | 5_3_exonuc |
| TRINITY_DN5604_c0_g1_i1 | 0.680805695 | 0.29772985 | 0.999970678 | 7.867 | 9.806 | 4.178 | 5.942 | Ran_BP1 |
| TRINITY_DN4370_c0_g1_i3 | -0.68073891 | 0.823407705 | 1 | 0.472 | 0 | 0.567 | 0.09 | p450 |
| TRINITY_DN6700_c0_g1_i1 | 0.680653292 | 0.312177179 | 0.999970678 | 3.334 | 2.71 | 1.633 | 1.81 | NA |
| TRINITY_DN836_c0_g1_i5 | -0.68055783 | 0.870990297 | 0.999970678 | 0 | 3.479 | 0 | 5.499 | RRM_1 |
| TRINITY_DN4394_c0_g1_i1 | 0.680526143 | 0.48043628 | 0.999970678 | 1.49 | 2.543 | 0.9 | 1.397 | zf-C3HC4_3 |
| TRINITY_DN519_c0_g2_i2 | -0.68050186 | 0.564749756 | 0.999970678 | 1.817 | 3.341 | 5.622 | 1.448 | NA |
| TRINITY_DN36107_c1_g1_i2 | 0.680467218 | 0.872775787 | 0.999970678 | 0 | 2.927 | 1.489 | 0 | NA |
| TRINITY_DN9162_c0_g1_i7 | -0.68043787 | 0.497843856 | 0.999970678 | 1.808 | 2.306 | 1.478 | 4.846 | RRM_1 |
| TRINITY_DN37001_c0_g1_i1 | -0.68041257 | 0.876413882 | 0.999970678 | 1.908 | 0 | 2.555 | 0 | - |
| TRINITY_DN35876_c0_g1_i2 | -0.68037487 | 0.881944219 | 1 | 3.488 | 0 | 4.544 | 0 | NA |
| TRINITY_DN15605_c0_g1_i1 | -0.68037487 | 0.881944219 | 1 | 2.743 | 0 | 3.644 | 0 | NA |
| TRINITY_DN15533_c3_g3_i1 | -0.68037487 | 0.881944219 | 1 | 0.718 | 0 | 0.955 | 0 | NA |
| TRINITY_DN11012_c0_g1_i13 | -0.68037487 | 0.881944219 | 1 | 0.627 | 0 | 0.833 | 0 | EF1G |
| TRINITY_DN5457_c0_g1_i1 | 0.680368596 | 0.509789761 | 0.999970678 | 1.889 | 1.409 | 0.6 | 1.337 | NA |
| TRINITY_DN8964_c0_g1_i3 | 0.680293516 | 0.880062246 | 1 | 0 | 1.912 | 0.989 | 0 | NA |
| TRINITY_DN8019_c0_g4_i1 | 0.68026533 | 0.754020882 | 0.999970678 | 0.3 | 5.982 | 0.178 | 3.619 | NA |
| TRINITY_DN23240_c0_g1_i5 | 0.680246624 | 0.592930582 | 0.999970678 | 18.122 | 3.164 | 6.988 | 4.967 | NA |
| TRINITY_DN4673_c0_g1_i1 | -0.68022138 | 0.413259788 | 0.999970678 | 2.262 | 3.893 | 3.289 | 5.851 | NA |
| TRINITY_DN8605_c0_g1_i9 | 0.680199373 | 0.343724281 | 0.999970678 | 5.35 | 6.485 | 3.678 | 2.946 | NA |
| TRINITY_DN193_c3_g1_i2 | -0.68010259 | 0.449884187 | 0.999970678 | 1.363 | 1.735 | 2.6 | 1.82 | Ank_2 |
| TRINITY_DN654_c1_g1_i1 | -0.680093 | 0.306270223 | 0.999970678 | 45.465 | 36.39 | 58.663 | 57.698 | NA |
| TRINITY_DN5772_c0_g1_i8 | -0.68006519 | 0.623417704 | 0.999970678 | 0.173 | 0.542 | 0.611 | 0.422 | NA |
| TRINITY_DN10510_c0_g1_i14 | 0.680020681 | 0.607197728 | 0.999970678 | 0.363 | 1.271 | 0.244 | 0.724 | CRT-like |
| TRINITY_DN11759_c0_g2_i1 | -0.6799478 | 0.481433982 | 0.999970678 | 0.491 | 0.552 | 0.955 | 0.513 | NA |
| TRINITY_DN11224_c0_g1_i1 | 0.679872087 | 0.438198192 | 0.999970678 | 3.497 | 5.578 | 2.789 | 2.242 | SRF-TF |
| TRINITY_DN6736_c1_g1_i3 | 0.679860163 | 0.405373219 | 0.999970678 | 3.425 | 5.736 | 1.978 | 3.328 | DUF3506 |
| TRINITY_DN3850_c0_g1_i5 | 0.679852401 | 0.8731158 | 0.999970678 | 1.59 | 0 | 0 | 1.005 | Pkinase |
| TRINITY_DN9124_c0_g1_i1 | -0.67978548 | 0.879840002 | 1 | 0 | 1.153 | 1.5 | 0 | NA |
| TRINITY_DN3214_c1_g1_i2 | -0.67978113 | 0.671946712 | 0.999970678 | 2.18 | 0.237 | 2.489 | 0.905 | NA |
| TRINITY_DN34082_c0_g1_i2 | 0.679663196 | 0.626968953 | 0.999970678 | 16.06 | 2.602 | 4.055 | 6.666 | NA |
| TRINITY_DN33981_c0_g1_i4 | 0.679657376 | 0.616617134 | 1 | 1.326 | 0.828 | 0.767 | 0.392 | Frigida |
| TRINITY_DN74314_c0_g1_i2 | 0.679536981 | 0.582344615 | 1 | 0.572 | 0.532 | 0.367 | 0.271 | DHHC |
| TRINITY_DN504_c0_g1_i10 | 0.679484728 | 0.803941257 | 0.999970678 | 0.418 | 0.296 | 0.367 | 0 | DUF3522 |
| TRINITY_DN13376_c0_g1_i1 | -0.6794465 | 0.337827707 | 0.999970678 | 24.072 | 15.27 | 24.41 | 33.62 | peroxidase |
| TRINITY_DN2384_c0_g1_i10 | 0.679435349 | 0.32191266 | 0.999970678 | 23.918 | 26.97 | 16.121 | 12.326 | RRM_1 |
| TRINITY_DN12547_c0_g2_i5 | -0.67932511 | 0.671699618 | 0.999970678 | 0.164 | 1.064 | 1.055 | 0.694 | - |
| TRINITY_DN2446_c0_g1_i1 | -0.67931229 | 0.808168451 | 0.999970678 | 6.322 | 7.293 | 0 | 21.786 | NA |
| TRINITY_DN2499_c0_g1_i7 | -0.67930984 | 0.872537657 | 0.999970678 | 2.589 | 0 | 3.466 | 0 | NA |
| TRINITY_DN3745_c0_g1_i1 | 0.679277111 | 0.354753997 | 0.999970678 | 2.026 | 2.247 | 1.244 | 1.156 | DWNN |
| TRINITY_DN1242_c0_g2_i3 | -0.67925843 | 0.81384778 | 0.999970678 | 0.3 | 1.212 | 2.022 | 0 | NA |
| TRINITY_DN5179_c0_g3_i1 | 0.679250711 | 0.72832026 | 0.999970678 | 6.313 | 0.168 | 1.567 | 2.182 | NA |

| TRINITY_DN8074_c0_g1_i1 | -0.67923793 | 0.483367695 | 0.999970678 | 13.317 | 10.07 | 7.955 | 27.969 | DDE_Tnp_4 |
| --- | --- | --- | --- | --- | --- | --- | --- | --- |
| TRINITY_DN16632_c0_g2_i1 | -0.67917732 | 0.803183626 | 0.999970678 | 2.653 | 0 | 1.055 | 3.016 | NA |
| TRINITY_DN7282_c1_g1_i1 | -0.67917649 | 0.582903606 | 0.999970678 | 1.444 | 1.419 | 2.978 | 1.015 | NA |
| TRINITY_DN1391_c3_g1_i1 | 0.679116053 | 0.458317782 | 0.999970678 | 4.787 | 8.515 | 2.811 | 4.816 | MBD |
| TRINITY_DN31847_c0_g1_i3 | -0.67907013 | 0.460919255 | 0.999970678 | 0.69 | 0.719 | 0.955 | 1.126 | Cupin_8 |
| TRINITY_DN9567_c2_g1_i2 | -0.67903454 | 0.48735901 | 0.999970678 | 37.135 | 106.8 | 69.162 | 145.165 | NA |
| TRINITY_DN9539_c0_g1_i3 | -0.67899736 | 0.809062783 | 0.999970678 | 1.272 | 2.769 | 0 | 6.404 | NA |
| TRINITY_DN666_c0_g1_i2 | -0.6789436 | 0.470289891 | 0.999970678 | 19.512 | 48.25 | 57.863 | 37.933 | NA |
| TRINITY_DN8965_c0_g1_i1 | 0.678837029 | 0.581968896 | 0.999970678 | 0.836 | 1.114 | 0.3 | 0.824 | NA |
| TRINITY_DN172_c0_g1_i3 | -0.67881064 | 0.347564744 | 0.999970678 | 6.186 | 6.15 | 7.077 | 11.089 | NA |
| TRINITY_DN53099_c0_g1_i1 | -0.67877303 | 0.672119916 | 0.999970678 | 0.1 | 0.641 | 0.278 | 0.824 | ELM2 |
| TRINITY_DN826_c2_g1_i2 | 0.678771453 | 0.619530163 | 0.999970678 | 8.176 | 8.121 | 7.366 | 1.247 | NA |
| TRINITY_DN1389_c0_g1_i2 | -0.67875651 | 0.416711735 | 0.999970678 | 0.709 | 1.015 | 1.267 | 1.247 | UPF0052 |
| TRINITY_DN1837_c0_g2_i3 | 0.678733072 | 0.381123033 | 0.999970678 | 24.754 | 37.9 | 20.243 | 14.518 | Longin |
| TRINITY_DN169157_c0_g1_i1 | -0.67871253 | 0.488560964 | 0.999970678 | 7.476 | 7.687 | 5.733 | 17.011 | - |
| TRINITY_DN13293_c0_g1_i1 | 0.678634765 | 0.620247757 | 0.999970678 | 12.018 | 2.897 | 1.933 | 6.957 | - |
| TRINITY_DN21069_c0_g1_i7 | -0.67862158 | 0.815781764 | 0.999970678 | 2.88 | 0.788 | 4.811 | 0 | NA |
| TRINITY_DN1914_c0_g1_i1 | -0.67860922 | 0.872684839 | 0.999970678 | 37.871 | 0 | 50.164 | 0 | NA |
| TRINITY_DN19507_c0_g1_i2 | -0.67857767 | 0.874159351 | 0.999970678 | 0.818 | 0 | 1.111 | 0 | DUF4219 |
| TRINITY_DN23029_c0_g2_i1 | 0.678539898 | 0.674630445 | 1 | 2.462 | 1.094 | 1.478 | 0.402 | NA |
| TRINITY_DN1464_c0_g1_i4 | 0.678478912 | 0.726347411 | 0.999970678 | 10.901 | 0.493 | 0.767 | 6.253 | NA |
| TRINITY_DN5250_c0_g2_i1 | 0.678402765 | 0.367811653 | 0.999970678 | 7.522 | 7.5 | 5.033 | 3.328 | NA |
| TRINITY_DN5095_c0_g1_i5 | 0.67839691 | 0.441652685 | 0.999970678 | 0.936 | 0.848 | 0.478 | 0.543 | Utp12 |
| TRINITY_DN99339_c0_g1_i2 | -0.6782074 | 0.819857311 | 0.999970678 | 0.845 | 0 | 0.1 | 1.257 | NA |
| TRINITY_DN131_c6_g1_i1 | -0.67814203 | 0.333905351 | 0.999970678 | 223.25 | 202.6 | 209.887 | 430.037 | Meth_synt_1 |
| TRINITY_DN5173_c0_g1_i1 | -0.67803032 | 0.801966792 | 0.999970678 | 6.359 | 0 | 3.333 | 6.213 | LANC_like |
| TRINITY_DN3269_c0_g1_i2 | -0.67802436 | 0.42534666 | 0.999970678 | 2.071 | 2.651 | 4.322 | 2.332 | NA |
| TRINITY_DN63343_c0_g2_i3 | -0.67790728 | 0.830229438 | 1 | 0.79 | 0 | 0.078 | 1.176 | NA |
| TRINITY_DN1457_c0_g1_i5 | -0.67787997 | 0.841294635 | 0.999970678 | 14.244 | 0 | 0.067 | 23.113 | NA |
| TRINITY_DN4616_c0_g1_i7 | 0.677879728 | 0.599385643 | 0.999970678 | 2.026 | 0.404 | 0.489 | 0.955 | CNH |
| TRINITY_DN3323_c0_g2_i1 | 0.677761843 | 0.582002615 | 0.999970678 | 0.99 | 2.651 | 1.222 | 0.784 | NA |
| TRINITY_DN10760_c0_g1_i3 | -0.67772761 | 0.878291808 | 1 | 0.6 | 0 | 0 | 0.985 | NA |
| TRINITY_DN23651_c1_g3_i1 | 0.677682373 | 0.595024578 | 0.999970678 | 0.436 | 1.271 | 0.367 | 0.623 | NA |
| TRINITY_DN5386_c0_g1_i1 | -0.67766011 | 0.488783724 | 0.999970678 | 78.885 | 41.9 | 44.653 | 139.787 | - |
| TRINITY_DN1654_c0_g1_i2 | 0.677586736 | 0.431193678 | 0.999970678 | 2.771 | 4.77 | 2.444 | 1.749 | NA |
| TRINITY_DN2035_c0_g1_i2 | -0.67753795 | 0.428362074 | 0.999970678 | 1.39 | 1.961 | 2.044 | 2.845 | HIT |
| TRINITY_DN3894_c1_g1_i2 | -0.67739452 | 0.483933618 | 0.999970678 | 0.872 | 1.133 | 0.922 | 2.091 | NA |
| TRINITY_DN24_c0_g1_i3 | 0.677307968 | 0.398919726 | 0.999970678 | 2.852 | 1.794 | 1.178 | 1.498 | AAA_22 |
| TRINITY_DN2509_c0_g1_i6 | -0.67730706 | 0.805766692 | 0.999970678 | 0.681 | 0 | 0.689 | 0.282 | RRM_1 |
| TRINITY_DN3765_c0_g1_i1 | 0.677290907 | 0.558257646 | 0.999970678 | 1.426 | 1.892 | 1.155 | 0.694 | BORCS7 |
| TRINITY_DN2762_c0_g2_i2 | 0.677090072 | 0.60706179 | 0.999970678 | 1.281 | 4.937 | 1.644 | 1.79 | Arf |
| TRINITY_DN4298_c0_g1_i8 | -0.67707599 | 0.462977083 | 0.999970678 | 2.107 | 2.109 | 1.955 | 4.333 | NA |
| TRINITY_DN18458_c0_g1_i1 | -0.67702272 | 0.874719193 | 0.999970678 | 11.091 | 0 | 14.866 | 0 | NA |
| TRINITY_DN14821_c0_g1_i1 | 0.677012513 | 0.468940974 | 0.999970678 | 2.089 | 2.286 | 0.811 | 1.749 | NA |
| TRINITY_DN5911_c0_g1_i3 | -0.67691558 | 0.506387167 | 0.999970678 | 55.975 | 23.86 | 74.929 | 37.631 | CcmH |
| TRINITY_DN19437_c0_g1_i1 | -0.6769145 | 0.439115057 | 0.999970678 | 2.662 | 2.198 | 2.9 | 4.273 | NA |
| TRINITY_DN89894_c0_g1_i1 | -0.67690218 | 0.874265391 | 0.999970678 | 1.935 | 0 | 2.589 | 0 | NA |
| TRINITY_DN190_c0_g2_i5 | -0.67689281 | 0.632285165 | 0.999970678 | 4.024 | 1.744 | 6.566 | 1.307 | CLP_protease |
| TRINITY_DN12517_c0_g1_i3 | 0.676866024 | 0.69411726 | 0.999970678 | 0.136 | 0.966 | 0.122 | 0.543 | NA |
| TRINITY_DN8137_c0_g1_i4 | -0.67683789 | 0.625824243 | 0.999970678 | 1.217 | 0.306 | 1.189 | 1.036 | Thg1 |
| TRINITY_DN24257_c1_g1_i2 | -0.67676235 | 0.705817725 | 1 | 0.2 | 0.68 | 0.133 | 1.247 | Pkinase |
| TRINITY_DN347_c0_g1_i1 | 0.676717391 | 0.544332781 | 0.999970678 | 1.09 | 0.976 | 0.733 | 0.392 | RRM_1 |
| TRINITY_DN21032_c0_g1_i1 | -0.67668039 | 0.873720441 | 0.999970678 | 1.572 | 0 | 2.1 | 0 | COesterase |
| TRINITY_DN8887_c0_g1_i3 | 0.676642131 | 0.801461619 | 0.999970678 | 1.154 | 2.277 | 1.767 | 0 | - |
| TRINITY_DN210_c0_g3_i1 | -0.67662629 | 0.521895716 | 0.999970678 | 3.198 | 1.429 | 4.422 | 2.101 | CCT |
| TRINITY_DN4660_c0_g1_i2 | 0.676595918 | 0.648665086 | 0.999970678 | 1.226 | 0.453 | 0.633 | 0.282 | NA |
| TRINITY_DN2384_c0_g1_i6 | 0.676536857 | 0.724421354 | 0.999970678 | 4.46 | 10.7 | 7.677 | 0.151 | RRM_1 |
| TRINITY_DN9449_c0_g3_i1 | -0.67650602 | 0.418806302 | 0.999970678 | 1.862 | 2.848 | 2.567 | 4.414 | NA |
| TRINITY_DN22177_c0_g1_i2 | 0.676474124 | 0.399588675 | 0.999970678 | 1.79 | 1.528 | 1 | 0.865 | NA |
| TRINITY_DN492_c0_g1_i9 | 0.676382481 | 0.593759731 | 0.999970678 | 4.051 | 3.104 | 3.189 | 0.633 | NA |
| TRINITY_DN159_c0_g1_i2 | -0.67634467 | 0.802661871 | 0.999970678 | 4.342 | 0 | 2.1 | 4.514 | NA |
| TRINITY_DN1001_c0_g2_i2 | 0.676327224 | 0.363326366 | 0.999970678 | 5.423 | 5.105 | 2.1 | 4.072 | MAP70 |
| TRINITY_DN98406_c0_g1_i4 | 0.676262283 | 0.802832814 | 0.999970678 | 0.799 | 0.394 | 0.622 | 0 | NA |
| TRINITY_DN782_c2_g2_i1 | -0.67622552 | 0.43656895 | 0.999970678 | 117.48 | 90.8 | 81.873 | 230.189 | ubiquitin |
| TRINITY_DN6321_c0_g1_i2 | -0.67611828 | 0.27327791 | 0.999970678 | 28.814 | 34.15 | 45.697 | 44.699 | Ribosomal_L7Ae |
| TRINITY_DN14419_c0_g1_i3 | 0.67609546 | 0.597067565 | 0.999970678 | 0.391 | 0.867 | 0.156 | 0.593 | AAA_11 |
| TRINITY_DN6997_c0_g1_i19 | -0.67609149 | 0.630789938 | 0.999970678 | 0.572 | 0.207 | 0.333 | 0.865 | NA |
| TRINITY_DN4425_c0_g2_i1 | -0.6760107 | 0.395585404 | 0.999970678 | 37.28 | 19.28 | 41.786 | 40.235 | NA |
| TRINITY_DN3276_c0_g1_i5 | 0.675924332 | 0.469359428 | 0.999970678 | 4.36 | 5.46 | 3.655 | 1.729 | GRAS |
| TRINITY_DN2331_c0_g1_i2 | -0.67585796 | 0.231192204 | 0.999970678 | 249.24 | 277.5 | 322.98 | 447.641 | NA |
| TRINITY_DN5992_c3_g1_i1 | -0.67584634 | 0.703282008 | 1 | 0.736 | 0.158 | 0.989 | 0.282 | NA |
| TRINITY_DN501_c1_g1_i1 | 0.675830078 | 0.724412212 | 0.999970678 | 0.981 | 2.385 | 1.611 | 0.131 | Methyltransf_11 |
| TRINITY_DN3198_c0_g1_i1 | -0.67579614 | 0.504267669 | 0.999970678 | 2.88 | 1.813 | 3.589 | 3.207 | NA |
| TRINITY_DN2002_c0_g2_i1 | -0.67576429 | 0.708374348 | 0.999970678 | 2.97 | 10.53 | 1.144 | 19.162 | NA |

| TRINITY_DN894_c0_g1_i9 | 0.675728641 | 0.835877479 | 0.999970678 | 15.27 | 0.089 | 0 | 9.782 | p450 |
| --- | --- | --- | --- | --- | --- | --- | --- | --- |
| TRINITY_DN32687_c0_g1_i2 | 0.675706407 | 0.404803424 | 0.999970678 | 4.596 | 7.017 | 3.933 | 2.523 | NA |
| TRINITY_DN9691_c0_g2_i3 | -0.67569014 | 0.802795079 | 0.999970678 | 1.417 | 0 | 0.733 | 1.418 | - |
| TRINITY_DN6134_c0_g1_i3 | 0.67565709 | 0.663937092 | 0.999970678 | 6.35 | 86.34 | 35.698 | 14.196 | FAD_binding_3 |
| TRINITY_DN8621_c0_g1_i4 | -0.67564912 | 0.810664402 | 0.999970678 | 2.244 | 9.363 | 0 | 17.775 | NA |
| TRINITY_DN1340_c0_g1_i1 | -0.67560904 | 0.490181464 | 0.999970678 | 3.579 | 3.213 | 5.9 | 3.599 | - |
| TRINITY_DN8194_c0_g1_i1 | -0.6755303 | 0.464334923 | 0.999970678 | 1.59 | 2.622 | 3.278 | 2.725 | - |
| TRINITY_DN5661_c0_g1_i1 | 0.675458894 | 0.756937684 | 0.999970678 | 0.309 | 4.671 | 2.478 | 0.07 | Gp_dh_C |
| TRINITY_DN92213_c0_g1_i1 | 0.675391643 | 0.880105968 | 1 | 1.444 | 0 | 0.756 | 0 | NA |
| TRINITY_DN39848_c0_g1_i1 | -0.67538938 | 0.873255069 | 0.999970678 | 4.669 | 0 | 6.244 | 0 | NA |
| TRINITY_DN854_c0_g1_i8 | 0.675337103 | 0.470907148 | 0.999970678 | 3.397 | 4.957 | 2.744 | 1.9 | CS |
| TRINITY_DN101241_c0_g2_i1 | -0.6753291 | 0.882085587 | 1 | 0.854 | 0 | 1.144 | 0 | NA |
| TRINITY_DN29393_c0_g1_i4 | -0.67527898 | 0.808349756 | 0.999970678 | 0.609 | 0 | 0.578 | 0.292 | Lipase_3 |
| TRINITY_DN3192_c2_g1_i3 | 0.675246455 | 0.678902132 | 0.999970678 | 0.609 | 10.53 | 2.222 | 4.182 | NA |
| TRINITY_DN3495_c1_g2_i4 | 0.675240791 | 0.557784618 | 0.999970678 | 3.588 | 4.366 | 2.911 | 1.428 | NA |
| TRINITY_DN3457_c0_g1_i2 | -0.6750976 | 0.567541395 | 0.999970678 | 0.59 | 2.188 | 1.544 | 2.523 | tRNA-synt_2 |
| TRINITY_DN4707_c0_g1_i2 | 0.675076494 | 0.549991205 | 0.999970678 | 0.363 | 0.365 | 0.256 | 0.151 | Ion_trans |
| TRINITY_DN473_c0_g1_i14 | 0.675020468 | 0.690867109 | 0.999970678 | 0.418 | 3.055 | 0.467 | 1.619 | NA |
| TRINITY_DN8050_c0_g1_i5 | 0.675015646 | 0.808313537 | 1 | 0.354 | 0.138 | 0 | 0.312 | NA |
| TRINITY_DN2517_c0_g1_i4 | 0.674890844 | 0.538669138 | 0.999970678 | 22.319 | 8.229 | 4.433 | 13.924 | NA |
| TRINITY_DN1752_c0_g1_i3 | -0.67487092 | 0.509653589 | 0.999970678 | 2.898 | 2.375 | 5.066 | 2.312 | - |
| TRINITY_DN636_c2_g1_i4 | 0.674806292 | 0.569470119 | 0.999970678 | 1.381 | 3.508 | 1.244 | 1.518 | ANAPC10 |
| TRINITY_DN17412_c0_g1_i1 | -0.67467111 | 0.702959542 | 0.999970678 | 8.185 | 0.443 | 9.266 | 2.755 | NA |
| TRINITY_DN3371_c0_g1_i3 | 0.674592902 | 0.592700766 | 0.999970678 | 5.959 | 37.18 | 13.955 | 9.863 | MFS_1 |
| TRINITY_DN948_c0_g2_i2 | -0.67453854 | 0.530779862 | 0.999970678 | 3.997 | 1.311 | 2.467 | 5.58 | TPR_MLP1_2 |
| TRINITY_DN7469_c0_g1_i3 | -0.67453009 | 0.838834349 | 0.999970678 | 2.68 | 0 | 3.566 | 0.03 | - |
| TRINITY_DN1047_c0_g1_i1 | -0.67440444 | 0.669793407 | 0.999970678 | 6.604 | 0.375 | 4.178 | 6.274 | NA |
| TRINITY_DN6216_c0_g1_i1 | -0.67428306 | 0.740709633 | 0.999970678 | 0.445 | 0.069 | 0.633 | 0.05 | NA |
| TRINITY_DN8767_c0_g1_i1 | -0.67426926 | 0.691878869 | 0.999970678 | 7.467 | 2.888 | 1.955 | 13.542 | NA |
| TRINITY_DN1335_c0_g1_i3 | 0.674260325 | 0.817968675 | 0.999970678 | 23.273 | 53.91 | 0 | 48.157 | ERp29 |
| TRINITY_DN5489_c1_g1_i2 | -0.67416368 | 0.592804636 | 0.999970678 | 65.032 | 467.7 | 343.257 | 422.155 | NA |
| TRINITY_DN4086_c0_g1_i6 | -0.67409636 | 0.812722328 | 1 | 2.398 | 0 | 2.067 | 1.357 | NA |
| TRINITY_DN2428_c0_g1_i5 | 0.674084723 | 0.66588367 | 0.999970678 | 0.509 | 0.256 | 0.067 | 0.402 | NA |
| TRINITY_DN3198_c0_g1_i9 | -0.67404745 | 0.754174071 | 1 | 0.182 | 1.37 | 0.178 | 2.071 | NA |
| TRINITY_DN297_c0_g1_i2 | -0.6740339 | 0.807185048 | 1 | 0 | 2.109 | 1.367 | 1.689 | NA |
| TRINITY_DN7625_c0_g1_i3 | 0.673969066 | 0.366630768 | 0.999970678 | 1.381 | 1.291 | 0.733 | 0.784 | FYRC |
| TRINITY_DN7631_c0_g1_i6 | 0.673965347 | 0.876469299 | 0.999970678 | 0 | 3.213 | 0 | 1.981 | NA |
| TRINITY_DN2080_c0_g1_i7 | -0.673924 | 0.704343808 | 0.999970678 | 15.688 | 1.606 | 3.6 | 22.319 | Lectin_legB |
| TRINITY_DN2723_c0_g1_i1 | -0.67387485 | 0.882536165 | 1 | 0 | 0.483 | 0.622 | 0 | NA |
| TRINITY_DN5482_c0_g1_i1 | -0.67387313 | 0.654268621 | 0.999970678 | 1.899 | 7.618 | 1.433 | 13.321 | MFS_1 |
| TRINITY_DN4403_c0_g1_i2 | -0.67380867 | 0.512363224 | 0.999970678 | 1.517 | 2.582 | 3.655 | 2.031 | DUF3593 |
| TRINITY_DN1278_c0_g1_i2 | 0.673773731 | 0.438319947 | 0.999970678 | 24.045 | 11.55 | 11.222 | 8.908 | NA |
| TRINITY_DN29958_c0_g1_i1 | 0.673750072 | 0.874895182 | 0.999970678 | 96.063 | 0 | 0 | 55.637 | NA |
| TRINITY_DN12064_c0_g1_i1 | -0.67362854 | 0.259707583 | 0.999970678 | 10.365 | 11.17 | 12.999 | 18.64 | NA |
| TRINITY_DN1932_c0_g1_i1 | -0.67362331 | 0.464677759 | 0.999970678 | 12.073 | 27.61 | 25.421 | 30.583 | Ribosomal_S26e |
| TRINITY_DN2805_c0_g1_i2 | -0.67361604 | 0.442613127 | 0.999970678 | 2.48 | 3.144 | 2.966 | 5.369 | NA |
| TRINITY_DN1988_c0_g1_i2 | 0.67361528 | 0.798168489 | 0.999970678 | 2.335 | 2.523 | 0 | 3.036 | NA |
| TRINITY_DN188_c0_g1_i9 | -0.67355164 | 0.670022403 | 0.999970678 | 12.372 | 1.597 | 6.755 | 13.412 | NA |
| TRINITY_DN5574_c1_g3_i1 | -0.67349683 | 0.775825529 | 0.999970678 | 0.173 | 7.608 | 0.222 | 11.883 | NA |
| TRINITY_DN282_c0_g1_i2 | -0.67347875 | 0.656706741 | 0.999970678 | 3.597 | 0.69 | 4.7 | 1.196 | - |
| TRINITY_DN493_c0_g1_i3 | 0.673474748 | 0.336520821 | 0.999970678 | 7.64 | 5.312 | 3.455 | 3.971 | NA |
| TRINITY_DN6666_c0_g1_i2 | -0.67334819 | 0.29350076 | 0.999970678 | 9.384 | 8.012 | 10.777 | 14.699 | NA |
| TRINITY_DN4053_c0_g1_i2 | -0.67323127 | 0.583958639 | 0.999970678 | 17.814 | 7.342 | 27.021 | 7.591 | Linker_histone |
| TRINITY_DN1614_c0_g1_i2 | 0.673228555 | 0.407020269 | 0.999970678 | 2.707 | 4.711 | 2.1 | 2.101 | 60KD_IMP |
| TRINITY_DN2573_c1_g1_i6 | 0.67319141 | 0.460385213 | 0.999970678 | 1.744 | 3.479 | 1.067 | 1.971 | NA |
| TRINITY_DN788_c0_g1_i7 | -0.672931 | 0.802952981 | 0.999970678 | 1.998 | 0 | 1.555 | 1.337 | NA |
| TRINITY_DN11405_c0_g1_i5 | -0.67292995 | 0.484924577 | 0.999970678 | 0.854 | 0.542 | 1 | 1.005 | NA |
| TRINITY_DN8699_c0_g1_i1 | -0.67287971 | 0.509769031 | 0.999970678 | 6.232 | 14.38 | 8.033 | 22.882 | DUF3223 |
| TRINITY_DN8_c1_g1_i1 | 0.672808788 | 0.623126059 | 0.999970678 | 2.235 | 8.19 | 1.089 | 5.137 | AUX_IAA |
| TRINITY_DN89591_c0_g1_i1 | 0.672769667 | 0.874629368 | 0.999970678 | 6.068 | 0 | 3.178 | 0 | NA |
| TRINITY_DN6368_c1_g1_i6 | 0.672723344 | 0.277366271 | 0.999970678 | 225.21 | 314.1 | 146.68 | 158.034 | NA |
| TRINITY_DN458_c0_g1_i11 | -0.67268319 | 0.750909293 | 1 | 0.109 | 1.271 | 0.222 | 1.88 | Epimerase |
| TRINITY_DN873_c1_g1_i11 | 0.672678373 | 0.598271743 | 0.999970678 | 0.363 | 0.168 | 0.167 | 0.121 | HNH |
| TRINITY_DN8331_c0_g1_i1 | 0.672624735 | 0.593831008 | 0.999970678 | 10.301 | 8.929 | 8.677 | 1.599 | IQ |
| TRINITY_DN14273_c0_g1_i1 | 0.672568585 | 0.614772381 | 0.999970678 | 1.635 | 6.79 | 1.767 | 3.046 | p450 |
| TRINITY_DN3306_c0_g1_i3 | -0.6725515 | 0.451124687 | 0.999970678 | 1.453 | 0.749 | 1.533 | 1.679 | UCH |
| TRINITY_DN255_c1_g1_i5 | -0.67236651 | 0.873160336 | 0.999970678 | 1.581 | 0 | 0 | 2.574 | NA |
| TRINITY_DN1737_c1_g2_i4 | 0.672366055 | 0.531678447 | 0.999970678 | 2.562 | 9.274 | 3.578 | 3.056 | NA |
| TRINITY_DN11117_c0_g1_i1 | 0.672293493 | 0.399676597 | 0.999970678 | 2.88 | 2.257 | 1.089 | 1.92 | DUF1336 |
| TRINITY_DN767_c1_g1_i2 | 0.672286761 | 0.811646175 | 0.999970678 | 8.53 | 0 | 1.233 | 3.861 | NA |
| TRINITY_DN563_c1_g1_i6 | -0.67228325 | 0.823273544 | 0.999970678 | 2.562 | 0 | 3.244 | 0.221 | NA |
| TRINITY_DN6636_c0_g2_i1 | 0.672275659 | 0.873427894 | 0.999970678 | 0 | 5.174 | 2.644 | 0 | NA |
| TRINITY_DN4541_c0_g1_i9 | 0.672243156 | 0.541008888 | 0.999970678 | 8.666 | 4.395 | 5.255 | 1.9 | AAA_11 |
| TRINITY_DN11397_c0_g1_i3 | -0.67215104 | 0.816351248 | 1 | 0 | 6.869 | 2.344 | 7.41 | 2OG-FeII_Oxy |

| TRINITY_DN2042_c0_g1_i11 | -0.67207296 | 0.746101696 | 0.999970678 | 2.098 | 0.059 | 1.544 | 1.578 | NA |
| --- | --- | --- | --- | --- | --- | --- | --- | --- |
| TRINITY_DN2542_c0_g1_i1 | 0.672020079 | 0.658493781 | 0.999970678 | 30.894 | 9.845 | 19.443 | 2.242 | NA |
| TRINITY_DN843_c1_g1_i10 | -0.67201203 | 0.47465848 | 0.999970678 | 4.042 | 1.764 | 4.066 | 4.363 | NAM |
| TRINITY_DN6014_c0_g1_i6 | 0.671937336 | 0.720108079 | 0.999970678 | 2.008 | 1.675 | 1.833 | 0.08 | NA |
| TRINITY_DN2263_c0_g1_i3 | -0.67174936 | 0.411611463 | 0.999970678 | 1.444 | 1.143 | 1.567 | 2.232 | NA |
| TRINITY_DN3645_c0_g1_i5 | 0.671696134 | 0.831358408 | 0.999970678 | 38.616 | 1.173 | 0 | 25.436 | NA |
| TRINITY_DN5299_c0_g1_i1 | 0.671637903 | 0.423507863 | 0.999970678 | 4.587 | 9.993 | 3.966 | 4.323 | Rxt3 |
| TRINITY_DN1118_c0_g1_i4 | 0.671571614 | 0.535157841 | 0.999970678 | 31.839 | 8.2 | 9.444 | 13.965 | NA |
| TRINITY_DN20425_c0_g2_i1 | -0.67156811 | 0.574023712 | 0.999970678 | 3.125 | 1.37 | 2.211 | 4.484 | NA |
| TRINITY_DN14394_c0_g1_i1 | 0.671534593 | 0.87719622 | 0.999970678 | 1.78 | 0 | 0.933 | 0 | NA |
| TRINITY_DN3129_c0_g1_i1 | 0.671497398 | 0.493937452 | 0.999970678 | 10.401 | 9.796 | 8.011 | 3.026 | NA |
| TRINITY_DN37864_c0_g1_i1 | -0.6714828 | 0.881471418 | 1 | 0.618 | 0 | 0.822 | 0 | MIP |
| TRINITY_DN9519_c0_g1_i1 | 0.671391791 | 0.590299935 | 0.999970678 | 0.845 | 0.936 | 0.311 | 0.744 | NA |
| TRINITY_DN7627_c0_g1_i4 | 0.671344036 | 0.721094202 | 0.999970678 | 3.47 | 0.572 | 0.189 | 2.322 | NA |
| TRINITY_DN10069_c0_g1_i2 | -0.67128994 | 0.873774622 | 0.999970678 | 4.088 | 0 | 5.455 | 0 | NA |
| TRINITY_DN14956_c0_g1_i3 | -0.67126641 | 0.875031969 | 0.999970678 | 0.736 | 0 | 0 | 1.206 | Choline_transpo |
| TRINITY_DN815_c0_g1_i1 | 0.671258482 | 0.593667001 | 0.999970678 | 26.353 | 7.559 | 4.044 | 16.498 | MMtag |
| TRINITY_DN1481_c0_g1_i4 | 0.67122641 | 0.601395431 | 0.999970678 | 2.28 | 3.479 | 2.533 | 0.533 | NA |
| TRINITY_DN166724_c0_g1_i1 | -0.67116142 | 0.462800419 | 0.999970678 | 4.687 | 2.848 | 3.766 | 7.43 | AP2 |
| TRINITY_DN9139_c0_g1_i3 | 0.671138439 | 0.531822105 | 0.999970678 | 3.715 | 2.405 | 2.044 | 1.357 | NA |
| TRINITY_DN3975_c0_g1_i6 | -0.67111324 | 0.490885646 | 0.999970678 | 1.244 | 1.981 | 1.333 | 3.499 | NA |
| TRINITY_DN5670_c0_g1_i4 | -0.67106676 | 0.342637736 | 0.999970678 | 3.252 | 3.568 | 3.866 | 6.143 | NA |
| TRINITY_DN2195_c0_g1_i4 | -0.67106243 | 0.418786028 | 0.999970678 | 2.589 | 4.218 | 5.866 | 3.75 | Carboxyl_trans |
| TRINITY_DN2971_c0_g1_i7 | 0.671041724 | 0.465027312 | 0.999970678 | 50.243 | 45.13 | 13.166 | 44.166 | NA |
| TRINITY_DN3885_c0_g1_i3 | -0.67101585 | 0.626908665 | 0.999970678 | 12.645 | 18.78 | 3.966 | 45.252 | NA |
| TRINITY_DN2985_c0_g1_i7 | -0.67099736 | 0.432393241 | 0.999970678 | 6.132 | 14.52 | 12.333 | 17.865 | NA |
| TRINITY_DN23610_c0_g2_i2 | -0.67099086 | 0.61099747 | 0.999970678 | 1.599 | 10.83 | 8.277 | 9.541 | NA |
| TRINITY_DN1370_c0_g2_i1 | -0.67094759 | 0.292480316 | 0.999970678 | 32.148 | 37 | 51.897 | 46.589 | KOW |
| TRINITY_DN1148_c0_g1_i8 | 0.67092465 | 0.616055852 | 0.999970678 | 12.427 | 75.35 | 11.155 | 41.21 | Glyco_hydro_1 |
| TRINITY_DN4050_c0_g1_i1 | 0.670919049 | 0.608107668 | 0.999970678 | 0.554 | 2.198 | 0.933 | 0.613 | NA |
| TRINITY_DN1685_c0_g1_i2 | -0.67090552 | 0.291125 | 0.999970678 | 86.879 | 73.36 | 90.417 | 143.718 | Ribosomal_L7Ae |
| TRINITY_DN10289_c0_g3_i4 | 0.670876151 | 0.602862416 | 0.999970678 | 3.143 | 0.897 | 1.089 | 1.237 | NA |
| TRINITY_DN73_c1_g1_i9 | -0.67078565 | 0.404790962 | 0.999970678 | 1.172 | 1.104 | 1.944 | 1.277 | NA |
| TRINITY_DN6003_c0_g1_i9 | -0.67077448 | 0.242038085 | 0.999970678 | 1128.8 | 1380 | 1557.617 | 2112.794 | LCAT |
| TRINITY_DN7809_c0_g1_i1 | -0.67060172 | 0.471470839 | 0.999970678 | 0.927 | 1.005 | 1.144 | 1.709 | NA |
| TRINITY_DN68301_c0_g1_i1 | 0.670574038 | 0.722020826 | 1 | 1.762 | 0.453 | 1.022 | 0.161 | NA |
| TRINITY_DN7557_c0_g1_i2 | 0.670502809 | 0.729829657 | 1 | 0.173 | 2.74 | 1.044 | 0.503 | Prefoldin |
| TRINITY_DN4517_c0_g1_i3 | -0.67049267 | 0.813624098 | 0.999970678 | 5.55 | 0 | 5.511 | 2.312 | NA |
| TRINITY_DN8981_c0_g1_i1 | -0.67048955 | 0.503801646 | 0.999970678 | 21.447 | 24.98 | 13.955 | 57.065 | NA |
| TRINITY_DN9733_c0_g1_i4 | -0.6704412 | 0.874727153 | 0.999970678 | 4.56 | 0 | 6.044 | 0 | NA |
| TRINITY_DN4357_c0_g2_i5 | 0.670363388 | 0.750331705 | 1 | 2.389 | 0.976 | 1.7 | 0.101 | NA |
| TRINITY_DN3263_c0_g1_i4 | -0.67031398 | 0.316786545 | 0.999970678 | 7.131 | 5.815 | 7.855 | 11.079 | NA |
| TRINITY_DN86_c0_g1_i3 | 0.670284279 | 0.684761808 | 0.999970678 | 12.182 | 0.641 | 2.689 | 4.916 | NA |
| TRINITY_DN3081_c0_g4_i2 | -0.67016694 | 0.537788545 | 0.999970678 | 4.796 | 3.775 | 9.011 | 2.785 | ATP-synt_ab |
| TRINITY_DN3607_c0_g1_i2 | -0.67008802 | 0.46502316 | 0.999970678 | 3.043 | 1.577 | 3.611 | 3.036 | Fer4 |
| TRINITY_DN890_c0_g1_i7 | 0.67007175 | 0.354153139 | 0.999970678 | 2.952 | 3.903 | 1.555 | 2.443 | NA |
| TRINITY_DN4946_c0_g1_i4 | 0.670024962 | 0.505961655 | 0.999970678 | 12.708 | 19.02 | 12.544 | 4.745 | Anticodon_1 |
| TRINITY_DN7109_c0_g1_i5 | 0.669986283 | 0.51642901 | 0.999970678 | 1.98 | 1.498 | 1.378 | 0.523 | Chorein_N |
| TRINITY_DN35194_c0_g1_i2 | 0.669908987 | 0.680154794 | 0.999970678 | 0.781 | 1.912 | 0.122 | 1.538 | Methyltr_RsmB-F |
| TRINITY_DN42_c0_g1_i2 | 0.669820785 | 0.573794801 | 0.999970678 | 1.69 | 5.47 | 1.367 | 2.785 | adh_short_C2 |
| TRINITY_DN5620_c0_g1_i1 | 0.669704141 | 0.315989999 | 0.999970678 | 19.712 | 14.6 | 9.255 | 10.396 | Tetraspannin |
| TRINITY_DN7395_c0_g1_i2 | 0.669641087 | 0.263804644 | 0.999970678 | 14.207 | 13.33 | 7.555 | 8.204 | NA |
| TRINITY_DN32460_c0_g1_i2 | 0.669474737 | 0.87925566 | 0.999970678 | 11.582 | 0 | 6.011 | 0 | NA |
| TRINITY_DN2949_c0_g1_i1 | 0.669392729 | 0.695327225 | 0.999970678 | 0.427 | 0.926 | 0.633 | 0.08 | Frigida |
| TRINITY_DN3107_c0_g1_i1 | -0.6693215 | 0.319443985 | 0.999970678 | 17.723 | 12.37 | 21.276 | 22.269 | NA |
| TRINITY_DN10900_c0_g1_i4 | -0.66930491 | 0.754273595 | 0.999970678 | 4.388 | 1.015 | 6.955 | 0.141 | NA |
| TRINITY_DN3284_c0_g2_i3 | -0.66921325 | 0.626850659 | 0.999970678 | 3.479 | 0.759 | 3.122 | 2.946 | NA |
| TRINITY_DN1080_c0_g1_i5 | -0.66921139 | 0.80416641 | 0.999970678 | 3.652 | 0 | 2.033 | 3.469 | NA |
| TRINITY_DN5589_c0_g1_i2 | -0.66916996 | 0.475346963 | 0.999970678 | 36.127 | 13.25 | 29.687 | 43.01 | NA |
| TRINITY_DN13078_c1_g1_i1 | -0.66915782 | 0.652028004 | 0.999970678 | 3.079 | 7.835 | 1.467 | 15.412 | NA |
| TRINITY_DN3857_c0_g1_i1 | -0.66910835 | 0.729353666 | 0.999970678 | 1.581 | 30.15 | 2.933 | 45.604 | NA |
| TRINITY_DN200_c0_g1_i3 | 0.669082247 | 0.433923613 | 0.999970678 | 1.544 | 1.38 | 0.767 | 0.925 | NA |
| TRINITY_DN1100_c0_g1_i1 | -0.66900572 | 0.46019718 | 0.999970678 | 2.689 | 3.715 | 4.211 | 4.856 | PUNUT |
| TRINITY_DN27934_c0_g1_i2 | -0.66897464 | 0.768832175 | 0.999970678 | 139.68 | 0.207 | 80.073 | 129.24 | NA |
| TRINITY_DN13045_c0_g1_i2 | 0.668939413 | 0.640063243 | 0.999970678 | 0.454 | 3.982 | 1.378 | 1.086 | Gal_mutarotas_2 |
| TRINITY_DN1791_c0_g2_i5 | -0.66892247 | 0.669746465 | 0.999970678 | 0.881 | 0.375 | 1.444 | 0.261 | HATPase_c_3 |
| TRINITY_DN801_c0_g1_i1 | 0.668873786 | 0.561069562 | 0.999970678 | 10.937 | 5.115 | 5.866 | 2.926 | NA |
| TRINITY_DN805_c0_g4_i1 | 0.668786668 | 0.874966382 | 0.999970678 | 0 | 6.652 | 3.4 | 0 | NA |
| TRINITY_DN7932_c0_g2_i1 | 0.668660603 | 0.421742183 | 0.999970678 | 8.348 | 6.958 | 5.433 | 3.086 | Cation_ATPase |
| TRINITY_DN44030_c0_g1_i1 | 0.668646352 | 0.251708243 | 0.999970678 | 13.208 | 15.4 | 7.866 | 8.515 | NA |
| TRINITY_DN8693_c0_g1_i1 | -0.66855624 | 0.451211805 | 0.999970678 | 3.761 | 1.754 | 4.1 | 3.871 | NA |
| TRINITY_DN7163_c0_g1_i2 | -0.66848227 | 0.464150636 | 0.999970678 | 1.363 | 1.37 | 1.055 | 3.076 | RNA_pol_Rpb1_1 |
| TRINITY_DN90_c0_g1_i5 | -0.66848208 | 0.616760879 | 0.999970678 | 3.252 | 4.257 | 8.233 | 1.9 | NA |
| TRINITY_DN10407_c0_g1_i4 | -0.66845345 | 0.881000364 | 1 | 0.254 | 0 | 0.344 | 0 | NA |

| TRINITY_DN7289_c0_g1_i2 | -0.66845345 | 0.881000364 | 1 | 0.382 | 0 | 0.5 | 0 | FPP |
| --- | --- | --- | --- | --- | --- | --- | --- | --- |
| TRINITY_DN1071_c0_g1_i2 | -0.66843619 | 0.809083305 | 0.999970678 | 0 | 3.233 | 0.811 | 4.102 | NA |
| TRINITY_DN3907_c0_g1_i5 | 0.668422054 | 0.379304232 | 0.999970678 | 3.797 | 6.564 | 3.055 | 2.815 | NA |
| TRINITY_DN33151_c1_g1_i1 | -0.66837721 | 0.841673641 | 1 | 0.391 | 0.02 | 0.544 | 0 | Cyt-b5 |
| TRINITY_DN2492_c0_g1_i2 | 0.668364562 | 0.425677939 | 0.999970678 | 2.008 | 3.262 | 1.211 | 1.85 | NA |
| TRINITY_DN50014_c0_g1_i2 | 0.668059655 | 0.618727042 | 0.999970678 | 1.317 | 0.986 | 1.044 | 0.191 | NA |
| TRINITY_DN3554_c0_g1_i3 | 0.667987607 | 0.493025208 | 0.999970678 | 4.051 | 8.604 | 4.389 | 2.624 | LBP_BPI_CETP |
| TRINITY_DN1126_c2_g1_i3 | 0.667872006 | 0.607825056 | 0.999970678 | 5.623 | 6.564 | 5.455 | 1.096 | NA |
| TRINITY_DN907_c0_g1_i6 | -0.66786464 | 0.689725679 | 0.999970678 | 3.161 | 2.533 | 6.922 | 0.593 | NA |
| TRINITY_DN1140_c0_g1_i4 | 0.66783665 | 0.80437637 | 0.999970678 | 7.358 | 2.957 | 0 | 6.515 | - |
| TRINITY_DN9288_c0_g1_i2 | -0.6676709 | 0.456004158 | 0.999970678 | 0.736 | 0.542 | 0.767 | 1.116 | NA |
| TRINITY_DN8171_c0_g2_i3 | 0.667664149 | 0.812180496 | 0.999970678 | 0.954 | 0 | 0.311 | 0.231 | NA |
| TRINITY_DN804_c1_g1_i6 | 0.6676526 | 0.499619808 | 0.999970678 | 0.645 | 1.084 | 0.478 | 0.503 | NA |
| TRINITY_DN7658_c0_g2_i3 | 0.667629854 | 0.877892733 | 0.999970678 | 15.27 | 0 | 0 | 9.812 | Aldose_epim |
| TRINITY_DN14697_c0_g1_i1 | -0.66753235 | 0.768580881 | 0.999970678 | 359.26 | 16.67 | 462.983 | 20.952 | NA |
| TRINITY_DN805_c0_g3_i2 | -0.66746196 | 0.29968614 | 0.999970678 | 20.412 | 30.35 | 32.065 | 41.743 | NA |
| TRINITY_DN6318_c1_g1_i3 | 0.667368233 | 0.389008887 | 0.999970678 | 3.052 | 3.095 | 1.822 | 1.689 | NA |
| TRINITY_DN12383_c1_g1_i1 | -0.66736459 | 0.470687748 | 0.999970678 | 432.64 | 373.2 | 781.253 | 334.305 | 1-cysPrx_C |
| TRINITY_DN11065_c0_g1_i1 | 0.667313977 | 0.708430525 | 0.999970678 | 2.925 | 4.415 | 0.167 | 4.363 | NA |
| TRINITY_DN1872_c1_g1_i1 | -0.66730656 | 0.365926978 | 0.999970678 | 1556.1 | 2001 | 2986.541 | 2023.678 | NA |
| TRINITY_DN11191_c0_g1_i2 | 0.667290721 | 0.539227703 | 0.999970678 | 4.288 | 11.99 | 2.378 | 7.339 | LRRNT_2 |
| TRINITY_DN1428_c0_g2_i4 | 0.667272777 | 0.818564583 | 1 | 0.59 | 0.148 | 0.378 | 0 | NA |
| TRINITY_DN45663_c0_g2_i1 | -0.66726822 | 0.882325658 | 1 | 0.845 | 0 | 0 | 1.387 | NA |
| TRINITY_DN987_c1_g1_i2 | -0.66725188 | 0.417345588 | 0.999970678 | 345.8 | 554.5 | 781.419 | 479.571 | PsbX |
| TRINITY_DN27770_c0_g1_i4 | 0.666943535 | 0.653096551 | 0.999970678 | 1.163 | 8.722 | 1.155 | 4.765 | NA |
| TRINITY_DN13605_c0_g1_i5 | 0.666891947 | 0.428024792 | 0.999970678 | 0.627 | 0.798 | 0.456 | 0.352 | zf-C3HC4_3 |
| TRINITY_DN2716_c0_g1_i2 | 0.66672693 | 0.527018242 | 0.999970678 | 1.544 | 4.455 | 1.6 | 1.8 | NA |
| TRINITY_DN9902_c0_g1_i1 | 0.666692311 | 0.807683891 | 0.999970678 | 5.977 | 3.597 | 5 | 0 | Hexapep |
| TRINITY_DN165760_c0_g1_i1 | -0.66658997 | 0.874829318 | 0.999970678 | 6.986 | 0 | 9.288 | 0 | NA |
| TRINITY_DN845_c0_g2_i3 | 0.666541105 | 0.585509358 | 0.999970678 | 4.279 | 14.95 | 2.9 | 8.345 | NA |
| TRINITY_DN4739_c0_g4_i1 | 0.666528272 | 0.513423465 | 0.999970678 | 0.845 | 2.306 | 0.811 | 1.005 | NA |
| TRINITY_DN3374_c0_g1_i3 | 0.666523506 | 0.746887013 | 0.999970678 | 8.975 | 0.69 | 4.889 | 0.231 | NA |
| TRINITY_DN8329_c0_g1_i1 | -0.66642734 | 0.712812031 | 0.999970678 | 0.045 | 0.355 | 0.411 | 0.131 | MCM3AP_GANP |
| TRINITY_DN1956_c0_g1_i2 | 0.666415282 | 0.880185184 | 1 | 0.409 | 0 | 0 | 0.261 | Filamin |
| TRINITY_DN39725_c0_g1_i1 | 0.66639859 | 0.838990485 | 1 | 0.027 | 0.907 | 0 | 0.583 | NA |
| TRINITY_DN2717_c0_g1_i1 | 0.666320174 | 0.807952842 | 0.999970678 | 0.727 | 0.7 | 0.744 | 0 | NA |
| TRINITY_DN8269_c0_g1_i1 | -0.66628562 | 0.399451174 | 0.999970678 | 1.944 | 3.351 | 3.744 | 3.851 | XG_FTase |
| TRINITY_DN7443_c0_g1_i2 | 0.666215469 | 0.880570628 | 1 | 0 | 0.207 | 0.111 | 0 | His_Phos_1 |
| TRINITY_DN31196_c0_g2_i1 | 0.666120303 | 0.645483332 | 1 | 0.808 | 0.375 | 0.167 | 0.553 | FPP |
| TRINITY_DN12939_c0_g1_i4 | -0.66604863 | 0.732705279 | 0.999970678 | 1.662 | 0.069 | 1.644 | 0.824 | NA |
| TRINITY_DN12812_c1_g1_i1 | 0.6660384 | 0.722059656 | 0.999970678 | 1.699 | 0.099 | 0.256 | 0.845 | - |
| TRINITY_DN10759_c0_g1_i6 | -0.66600674 | 0.880598319 | 1 | 1.29 | 0 | 1.722 | 0 | NA |
| TRINITY_DN5850_c0_g1_i1 | -0.66600674 | 0.880598319 | 1 | 1.235 | 0 | 1.667 | 0 | NA |
| TRINITY_DN9276_c0_g1_i6 | -0.66600674 | 0.880598319 | 1 | 4.315 | 0 | 5.644 | 0 | Inhibitor_I9 |
| TRINITY_DN6708_c0_g1_i4 | 0.66584968 | 0.377809796 | 0.999970678 | 3.416 | 4.474 | 2.533 | 1.92 | E1-E2_ATPase |
| TRINITY_DN2251_c0_g1_i16 | 0.665823348 | 0.882661418 | 1 | 0.382 | 0 | 0.2 | 0 | NA |
| TRINITY_DN32686_c0_g2_i4 | 0.665823348 | 0.882661418 | 1 | 4.351 | 0 | 2.289 | 0 | Ribosomal_L11 |
| TRINITY_DN12842_c0_g1_i12 | -0.66581715 | 0.819389894 | 1 | 0.218 | 0.099 | 0.411 | 0 | NA |
| TRINITY_DN7438_c0_g1_i5 | 0.665804541 | 0.48558137 | 0.999970678 | 1.063 | 1.577 | 0.911 | 0.553 | Cytochrom_B561 |
| TRINITY_DN2334_c0_g1_i2 | -0.66567768 | 0.65493161 | 0.999970678 | 0.645 | 0.108 | 0.711 | 0.332 | NA |
| TRINITY_DN4952_c0_g1_i14 | 0.665631359 | 0.607127631 | 0.999970678 | 1.953 | 0.414 | 0.744 | 0.613 | DUF659 |
| TRINITY_DN2893_c2_g1_i1 | -0.66551102 | 0.466209025 | 0.999970678 | 464.74 | 184.1 | 424.508 | 461.565 | NA |
| TRINITY_DN13115_c0_g1_i1 | 0.665487943 | 0.877308201 | 0.999970678 | 1.363 | 0 | 0 | 0.875 | UDPGT |
| TRINITY_DN1175_c0_g1_i9 | -0.6654855 | 0.680814754 | 0.999970678 | 2.425 | 36.19 | 11.355 | 46.488 | NA |
| TRINITY_DN5097_c0_g1_i1 | 0.665435519 | 0.688789972 | 0.999970678 | 0.718 | 0.276 | 0.456 | 0.101 | NA |
| TRINITY_DN598_c0_g2_i1 | -0.66542732 | 0.369910055 | 0.999970678 | 58.255 | 52.28 | 52.319 | 109.625 | NA |
| TRINITY_DN30542_c0_g1_i6 | -0.66540738 | 0.603043144 | 0.999970678 | 0.345 | 1.124 | 0.511 | 1.699 | RSB_motif |
| TRINITY_DN4825_c0_g1_i1 | -0.6654059 | 0.5776116 | 0.999970678 | 0.273 | 0.473 | 0.644 | 0.382 | NA |
| TRINITY_DN17018_c0_g1_i1 | -0.66538807 | 0.307252603 | 0.999970678 | 35.818 | 34.43 | 52.297 | 46.709 | NA |
| TRINITY_DN460_c0_g2_i2 | 0.66535011 | 0.439164002 | 0.999970678 | 18.64 | 8.614 | 8.122 | 7.48 | DOG1 |
| TRINITY_DN1671_c0_g1_i1 | -0.66532439 | 0.44272943 | 0.999970678 | 1.544 | 2.336 | 2.822 | 2.704 | zf-C3HC4_3 |
| TRINITY_DN2732_c0_g1_i2 | 0.665324031 | 0.366997911 | 0.999970678 | 44.375 | 72.76 | 36.209 | 29.859 | Sugar_tr |
| TRINITY_DN15329_c0_g1_i4 | -0.66531041 | 0.877736616 | 0.999970678 | 1.608 | 0 | 2.133 | 0 | Mito_carr |
| TRINITY_DN17261_c0_g1_i1 | 0.66523283 | 0.51888175 | 0.999970678 | 3.034 | 2.158 | 1.344 | 1.619 | NA |
| TRINITY_DN1040_c0_g1_i6 | 0.66518495 | 0.734059341 | 0.999970678 | 12.472 | 54.37 | 33.976 | 0.643 | Arginosuc_synth |
| TRINITY_DN4682_c0_g1_i1 | 0.665086766 | 0.476531482 | 0.999970678 | 57.52 | 35 | 14.088 | 41.27 | Ras |
| TRINITY_DN106866_c0_g1_i1 | -0.66497006 | 0.472250777 | 0.999970678 | 13.789 | 5.893 | 15.288 | 12.849 | NA |
| TRINITY_DN3452_c1_g2_i1 | -0.66492689 | 0.564640471 | 0.999970678 | 1.045 | 2.809 | 3.455 | 1.9 | NA |
| TRINITY_DN2961_c0_g3_i3 | 0.664919481 | 0.812162608 | 0.999970678 | 2.407 | 0.552 | 1.533 | 0 | FSH1 |
| TRINITY_DN11395_c0_g1_i2 | 0.664787455 | 0.33647415 | 0.999970678 | 28.197 | 32.62 | 19.577 | 14.709 | NA |
| TRINITY_DN10102_c0_g1_i4 | -0.66476033 | 0.784248287 | 0.999970678 | 4.987 | 0.138 | 6.355 | 0.503 | NA |
| TRINITY_DN6901_c0_g1_i1 | 0.664696252 | 0.677844343 | 0.999970678 | 1.099 | 16.15 | 2.278 | 8.013 | DUF296 |
| TRINITY_DN5485_c3_g1_i2 | 0.664578739 | 0.389387202 | 0.999970678 | 19.785 | 28.02 | 15.81 | 10.928 | NA |
| TRINITY_DN7908_c0_g1_i3 | -0.6645308 | 0.608051198 | 0.999970678 | 0.173 | 0.65 | 0.622 | 0.563 | Gal-bind_lectin |

| TRINITY_DN4347_c0_g1_i2 | -0.66443788 | 0.815976724 | 0.999970678 | 16.269 | 0 | 7.3 | 17.383 | - |
| --- | --- | --- | --- | --- | --- | --- | --- | --- |
| TRINITY_DN5275_c0_g2_i1 | 0.664433083 | 0.296715553 | 0.999970678 | 59.927 | 50.24 | 24.81 | 39.451 | - |
| TRINITY_DN173_c0_g1_i1 | 0.664367998 | 0.584615248 | 0.999970678 | 2.017 | 0.71 | 0.611 | 0.985 | 2OG-FeII_Oxy |
| TRINITY_DN12437_c0_g2_i1 | 0.664363225 | 0.651684825 | 1 | 12.327 | 10.95 | 9.222 | 2.986 | NA |
| TRINITY_DN4050_c0_g1_i4 | -0.66435102 | 0.654109613 | 0.999970678 | 2.534 | 20.71 | 6.266 | 28.784 | NA |
| TRINITY_DN3215_c0_g1_i3 | -0.6642066 | 0.464247315 | 0.999970678 | 2.852 | 6.672 | 6.577 | 7.068 | ACP_syn_III_C |
| TRINITY_DN9586_c0_g3_i1 | 0.664161241 | 0.819246173 | 0.999970678 | 3.316 | 0 | 1.389 | 0.442 | NA |
| TRINITY_DN3572_c0_g2_i2 | 0.664151312 | 0.714475838 | 1 | 0.1 | 0.68 | 0.089 | 0.382 | Phytochelatin |
| TRINITY_DN2500_c0_g1_i1 | 0.664146835 | 0.81434708 | 1 | 0.3 | 0.986 | 0 | 0.804 | NMO |
| TRINITY_DN4710_c0_g2_i1 | -0.66403338 | 0.357646707 | 0.999970678 | 9.157 | 13.1 | 15.599 | 16.086 | NA |
| TRINITY_DN1179_c0_g1_i1 | 0.664032724 | 0.428970957 | 0.999970678 | 77.095 | 86.95 | 59.719 | 31.267 | NA |
| TRINITY_DN13978_c0_g1_i2 | 0.663991319 | 0.34897002 | 0.999970678 | 40.751 | 32.23 | 23.265 | 18.016 | NA |
| TRINITY_DN14074_c0_g1_i1 | -0.66398843 | 0.880240328 | 0.999970678 | 1.081 | 0 | 1.444 | 0 | NA |
| TRINITY_DN63288_c0_g1_i1 | -0.66398843 | 0.880240328 | 0.999970678 | 0.736 | 0 | 0.978 | 0 | TRI12 |
| TRINITY_DN5508_c0_g1_i6 | -0.66393665 | 0.809168476 | 0.999970678 | 0 | 2.346 | 2.255 | 0.945 | NA |
| TRINITY_DN2396_c0_g1_i4 | 0.663867783 | 0.812145268 | 0.999970678 | 7.131 | 0 | 2.389 | 1.679 | NA |
| TRINITY_DN10519_c0_g1_i1 | 0.663861545 | 0.607365193 | 0.999970678 | 1.381 | 6.288 | 1.255 | 3.267 | NA |
| TRINITY_DN3540_c0_g1_i5 | -0.66379877 | 0.596886988 | 0.999970678 | 1.681 | 2.434 | 0.822 | 5.53 | NA |
| TRINITY_DN941_c0_g1_i2 | -0.66374247 | 0.327458378 | 0.999970678 | 13.054 | 18.8 | 23.343 | 22.068 | NA |
| TRINITY_DN12753_c0_g2_i5 | -0.6636863 | 0.834491344 | 0.999970678 | 0.917 | 0.059 | 1.278 | 0 | NA |
| TRINITY_DN2964_c0_g1_i1 | 0.663675146 | 0.813017727 | 1 | 0.282 | 0.227 | 0.278 | 0 | - |
| TRINITY_DN5679_c0_g1_i3 | 0.663672025 | 0.436580482 | 0.999970678 | 3.361 | 2.503 | 1.622 | 1.729 | NA |
| TRINITY_DN2273_c0_g1_i8 | -0.66366257 | 0.531588735 | 0.999970678 | 0.663 | 0.641 | 0.489 | 1.458 | CBS |
| TRINITY_DN5682_c1_g1_i1 | -0.66365667 | 0.667017909 | 0.999970678 | 1.481 | 0.207 | 1.6 | 0.754 | NA |
| TRINITY_DN6429_c1_g1_i1 | 0.663651019 | 0.381949283 | 0.999970678 | 3.961 | 4.12 | 2.689 | 1.86 | NA |
| TRINITY_DN5726_c0_g1_i2 | 0.663567225 | 0.644913036 | 0.999970678 | 2.498 | 1.754 | 0.344 | 2.212 | F-box-like |
| TRINITY_DN2304_c0_g1_i4 | -0.66354342 | 0.608243716 | 0.999970678 | 7.303 | 1.163 | 5.377 | 7.048 | NA |
| TRINITY_DN3404_c0_g1_i2 | 0.663447138 | 0.542456146 | 0.999970678 | 3.043 | 3.479 | 2.333 | 1.267 | NA |
| TRINITY_DN10112_c0_g1_i4 | -0.66337199 | 0.647254703 | 0.999970678 | 4.878 | 0.621 | 4.866 | 2.926 | NA |
| TRINITY_DN86203_c0_g1_i1 | 0.663337074 | 0.875329922 | 0.999970678 | 3.188 | 0 | 0 | 2.061 | - |
| TRINITY_DN12142_c0_g1_i10 | 0.663310091 | 0.627460762 | 0.999970678 | 0.354 | 1.143 | 0.544 | 0.282 | NA |
| TRINITY_DN1465_c0_g1_i4 | 0.663283535 | 0.832820295 | 0.999970678 | 0.154 | 8.2 | 4.278 | 0 | NA |
| TRINITY_DN1626_c0_g1_i7 | 0.663169255 | 0.513585037 | 0.999970678 | 6.631 | 2.937 | 2.2 | 3.358 | NA |
| TRINITY_DN681_c2_g1_i2 | 0.663164303 | 0.70358336 | 0.999970678 | 0.064 | 0.769 | 0.189 | 0.292 | NA |
| TRINITY_DN1873_c0_g1_i2 | -0.66312037 | 0.414159509 | 0.999970678 | 11.373 | 11.49 | 9.855 | 24.29 | Sld5 |
| TRINITY_DN2844_c0_g1_i14 | -0.66294232 | 0.387580304 | 0.999970678 | 2.943 | 2.641 | 4.411 | 3.509 | - |
| TRINITY_DN808_c0_g1_i2 | 0.662913752 | 0.668913542 | 0.999970678 | 11.091 | 2.079 | 6.122 | 1.005 | PDZ |
| TRINITY_DN31560_c0_g1_i2 | 0.662872889 | 0.589591025 | 0.999970678 | 0.645 | 1.557 | 0.333 | 0.985 | NA |
| TRINITY_DN1616_c0_g1_i4 | -0.66275656 | 0.486844638 | 0.999970678 | 3.27 | 1.606 | 2.367 | 4.896 | NA |
| TRINITY_DN10578_c1_g1_i11 | 0.662740094 | 0.722417561 | 1 | 2.98 | 2.109 | 2.433 | 0.241 | NA |
| TRINITY_DN2515_c0_g1_i3 | -0.66273195 | 0.751581578 | 0.999970678 | 0.154 | 1.124 | 0.122 | 1.84 | NA |
| TRINITY_DN14648_c0_g1_i1 | -0.66267952 | 0.456139736 | 0.999970678 | 0.781 | 1.34 | 1.2 | 1.91 | Glyco_trans_4_5 |
| TRINITY_DN2382_c1_g1_i6 | 0.662598375 | 0.874777396 | 0.999970678 | 1.899 | 0 | 0 | 1.216 | NA |
| TRINITY_DN704_c0_g1_i4 | -0.66257674 | 0.578937093 | 0.999970678 | 6.976 | 1.735 | 4.478 | 8.435 | NA |
| TRINITY_DN4238_c0_g2_i1 | 0.662531318 | 0.681951974 | 0.999970678 | 0.273 | 3.055 | 1.3 | 0.503 | FAR1 |
| TRINITY_DN545_c0_g1_i5 | 0.66240899 | 0.407997394 | 0.999970678 | 7.004 | 12.65 | 6.055 | 5.047 | NB-ARC |
| TRINITY_DN3938_c0_g1_i14 | -0.66231671 | 0.69804588 | 0.999970678 | 3.416 | 0.266 | 3.344 | 1.82 | NA |
| TRINITY_DN10046_c0_g1_i2 | -0.66230234 | 0.489806729 | 0.999970678 | 52.859 | 129.4 | 157.057 | 94.877 | NA |
| TRINITY_DN2795_c0_g1_i6 | 0.662293506 | 0.877968542 | 0.999970678 | 0 | 1.37 | 0.711 | 0 | NA |
| TRINITY_DN590_c0_g1_i6 | -0.66223267 | 0.464805337 | 0.999970678 | 252.34 | 626.5 | 435.118 | 850.311 | NA |
| TRINITY_DN1520_c0_g1_i1 | 0.662164137 | 0.486451656 | 0.999970678 | 5.832 | 13.31 | 3.5 | 7.772 | NA |
| TRINITY_DN6372_c0_g1_i3 | 0.661936209 | 0.390929933 | 0.999970678 | 3.379 | 3.39 | 1.933 | 1.93 | - |
| TRINITY_DN20031_c0_g1_i1 | -0.66171756 | 0.548134453 | 0.999970678 | 1.517 | 2.681 | 1.844 | 4.343 | - |
| TRINITY_DN1753_c0_g1_i6 | -0.66170489 | 0.278178456 | 0.999970678 | 48.626 | 67.16 | 73.596 | 93.841 | Ribosomal_60s |
| TRINITY_DN8626_c0_g1_i6 | 0.661637474 | 0.468278721 | 0.999970678 | 475.69 | 204.1 | 223.731 | 162.196 | MIP |
| TRINITY_DN12130_c0_g1_i2 | -0.66154575 | 0.508832639 | 0.999970678 | 0.509 | 0.808 | 1.144 | 0.694 | Adaptin_N |
| TRINITY_DN10102_c0_g1_i2 | -0.66149944 | 0.666100236 | 0.999970678 | 9.175 | 4.356 | 15.566 | 2.383 | NA |
| TRINITY_DN4797_c0_g1_i5 | -0.66148713 | 0.446191851 | 0.999970678 | 6.268 | 4.129 | 8.799 | 5.902 | NA |
| TRINITY_DN2082_c0_g1_i1 | -0.66141027 | 0.418419889 | 0.999970678 | 7.049 | 14.66 | 13.988 | 17.162 | NA |
| TRINITY_DN13713_c0_g1_i5 | 0.661375705 | 0.813650126 | 0.999970678 | 3.561 | 12.52 | 0 | 10.124 | NA |
| TRINITY_DN2253_c1_g1_i2 | 0.661333808 | 0.664456546 | 0.999970678 | 0.109 | 0.769 | 0.267 | 0.231 | CMAS |
| TRINITY_DN2358_c0_g1_i1 | 0.661328404 | 0.383890934 | 0.999970678 | 14.498 | 23.03 | 7.611 | 14.487 | NA |
| TRINITY_DN13208_c0_g1_i4 | 0.661272649 | 0.629084446 | 0.999970678 | 1.617 | 1.36 | 1.3 | 0.312 | - |
| TRINITY_DN39815_c0_g1_i1 | -0.66125626 | 0.580845554 | 0.999970678 | 0.818 | 1.754 | 2.078 | 1.518 | NA |
| TRINITY_DN5818_c0_g1_i17 | 0.661157172 | 0.660555103 | 0.999970678 | 0.091 | 0.867 | 0.2 | 0.362 | - |
| TRINITY_DN686_c7_g1_i1 | 0.661093737 | 0.710394932 | 0.999970678 | 62.943 | 589 | 20.51 | 383.528 | RPE65 |
| TRINITY_DN2147_c0_g1_i7 | -0.66107985 | 0.811679239 | 0.999970678 | 4.969 | 0 | 1.5 | 6.163 | NA |
| TRINITY_DN7726_c0_g1_i1 | 0.661059123 | 0.68526254 | 1 | 0.572 | 0.749 | 0.6 | 0.121 | NA |
| TRINITY_DN1439_c0_g1_i1 | -0.66099994 | 0.656537877 | 0.999970678 | 7.358 | 6.682 | 16.732 | 1.91 | - |
| TRINITY_DN6716_c0_g1_i2 | 0.66097969 | 0.803555384 | 0.999970678 | 3.37 | 6.475 | 0 | 6.163 | DUF1336 |
| TRINITY_DN102_c0_g1_i5 | -0.66097096 | 0.825883125 | 0.999970678 | 6.976 | 0 | 0.456 | 10.647 | NA |
| TRINITY_DN9987_c0_g1_i5 | 0.660882643 | 0.687215466 | 0.999970678 | 44.747 | 910.9 | 133.67 | 417.641 | NA |
| TRINITY_DN2776_c0_g1_i6 | -0.66087947 | 0.549124391 | 0.999970678 | 3.942 | 4.908 | 3.755 | 9.34 | NA |
| TRINITY_DN13361_c0_g1_i1 | -0.66085549 | 0.879670152 | 0.999970678 | 1.544 | 0 | 2.055 | 0 | NA |

| TRINITY_DN22223_c0_g1_i1 | -0.66081569 | 0.885292731 | 1 | 0.6 | 0 | 0.789 | 0 | NA |
| --- | --- | --- | --- | --- | --- | --- | --- | --- |
| TRINITY_DN15682_c0_g1_i4 | -0.66081569 | 0.885292731 | 1 | 1.553 | 0 | 2.122 | 0 | NA |
| TRINITY_DN5233_c0_g1_i4 | -0.66059054 | 0.681959968 | 1 | 0.554 | 2.553 | 1.244 | 3.207 | NA |
| TRINITY_DN104926_c0_g1_i1 | -0.66057975 | 0.649121992 | 0.999970678 | 1.399 | 4.937 | 1.889 | 7.48 | NA |
| TRINITY_DN19429_c0_g1_i4 | -0.66056232 | 0.632097376 | 0.999970678 | 0.736 | 0.414 | 0.356 | 1.397 | GMC_oxred_C |
| TRINITY_DN24965_c0_g1_i10 | -0.66053045 | 0.842187645 | 1 | 4.006 | 0.138 | 5.477 | 0 | NA |
| TRINITY_DN694_c0_g1_i6 | -0.66043496 | 0.661592189 | 0.999970678 | 3.906 | 0.591 | 4.511 | 1.739 | DapB_C |
| TRINITY_DN1270_c0_g1_i1 | 0.660395681 | 0.523506498 | 0.999970678 | 3.806 | 2.079 | 2.111 | 1.196 | Glyco_transf_17 |
| TRINITY_DN19425_c0_g2_i1 | 0.660291129 | 0.884130567 | 1 | 2.107 | 0 | 1.111 | 0 | NA |
| TRINITY_DN5537_c0_g1_i2 | 0.66025376 | 0.628367043 | 0.999970678 | 0.845 | 2.543 | 0.656 | 1.327 | NA |
| TRINITY_DN5121_c0_g1_i1 | 0.660250542 | 0.35865813 | 0.999970678 | 14.225 | 13.41 | 9.166 | 6.454 | DIL |
| TRINITY_DN8071_c0_g1_i2 | -0.66021469 | 0.710807635 | 1 | 0.245 | 0.562 | 0.9 | 0.141 | NA |
| TRINITY_DN473_c0_g1_i1 | -0.66016048 | 0.680738567 | 0.999970678 | 0.245 | 5.273 | 3.622 | 4.273 | AAA_11 |
| TRINITY_DN15456_c0_g1_i1 | 0.660144535 | 0.497091875 | 0.999970678 | 1.971 | 4.307 | 1.155 | 2.564 | Kinesin |
| TRINITY_DN1145_c0_g1_i4 | 0.66012139 | 0.42515654 | 0.999970678 | 35.827 | 53.97 | 15.532 | 37.812 | NA |
| TRINITY_DN2898_c0_g1_i2 | 0.660056598 | 0.651720552 | 0.999970678 | 0.663 | 2.267 | 1.1 | 0.533 | NA |
| TRINITY_DN1893_c0_g1_i7 | 0.660044034 | 0.882940527 | 1 | 0.672 | 0 | 0 | 0.432 | APG9 |
| TRINITY_DN715_c0_g1_i2 | 0.65998886 | 0.56228009 | 0.999970678 | 3.161 | 3.952 | 0.933 | 3.338 | NA |
| TRINITY_DN6557_c0_g1_i8 | 0.659879263 | 0.814187004 | 0.999970678 | 0 | 0.936 | 0.256 | 0.292 | NA |
| TRINITY_DN4963_c0_g1_i5 | 0.659877402 | 0.417520767 | 0.999970678 | 5.251 | 3.853 | 1.822 | 3.589 | NAF |
| TRINITY_DN4050_c0_g1_i2 | -0.65975354 | 0.68224722 | 0.999970678 | 0.236 | 2.079 | 0.733 | 2.704 | NA |
| TRINITY_DN1418_c0_g1_i1 | -0.65972757 | 0.52270764 | 0.999970678 | 150.68 | 42.42 | 116.404 | 165.604 | NA |
| TRINITY_DN91084_c0_g1_i1 | -0.65964049 | 0.876491929 | 0.999970678 | 1.299 | 0 | 1.722 | 0 | NA |
| TRINITY_DN1133_c0_g1_i7 | 0.65956502 | 0.876149475 | 0.999970678 | 2.58 | 0 | 0 | 1.659 | NA |
| TRINITY_DN96_c0_g1_i2 | -0.65956457 | 0.516962348 | 0.999970678 | 3.198 | 2.089 | 1.967 | 6.012 | - |
| TRINITY_DN53736_c0_g1_i4 | 0.659560867 | 0.603447382 | 0.999970678 | 4.478 | 7.421 | 1.011 | 6.294 | Prenyltrans |
| TRINITY_DN1452_c0_g3_i2 | 0.659375443 | 0.777383359 | 0.999970678 | 0.627 | 30.17 | 15.555 | 0.342 | NA |
| TRINITY_DN591_c0_g1_i3 | -0.65932482 | 0.511516249 | 0.999970678 | 21.447 | 37.44 | 19.043 | 69.853 | NA |
| TRINITY_DN21765_c0_g1_i1 | 0.659310504 | 0.876134146 | 0.999970678 | 5.36 | 0 | 2.844 | 0 | NA |
| TRINITY_DN2220_c0_g1_i3 | 0.659307121 | 0.880483535 | 0.999970678 | 0.672 | 0 | 0 | 0.432 | OKR_DC_1 |
| TRINITY_DN3063_c0_g1_i3 | -0.65928879 | 0.289744601 | 0.999970678 | 199.8 | 162.6 | 212.265 | 298.243 | Ribosomal_S21e |
| TRINITY_DN17189_c2_g1_i1 | 0.65922974 | 0.57315496 | 0.999970678 | 0.572 | 0.453 | 0.4 | 0.171 | PPR |
| TRINITY_DN449_c1_g1_i8 | -0.65901698 | 0.472042571 | 0.999970678 | 56.865 | 30.25 | 37.076 | 92.102 | Dimerisation |
| TRINITY_DN58890_c0_g1_i1 | -0.65900935 | 0.653545381 | 0.999970678 | 234.94 | 29.02 | 220.198 | 130.236 | NA |
| TRINITY_DN13903_c0_g1_i10 | 0.659008123 | 0.811777113 | 0.999970678 | 0.345 | 0.788 | 0.589 | 0 | YL1 |
| TRINITY_DN17444_c0_g1_i7 | -0.65881696 | 0.56882946 | 0.999970678 | 1.544 | 1.39 | 2.322 | 1.729 | NA |
| TRINITY_DN3266_c0_g1_i1 | -0.65880681 | 0.522279787 | 0.999970678 | 9.965 | 28.57 | 31.709 | 21.475 | V-ATPase_G |
| TRINITY_DN661_c0_g2_i1 | -0.65863616 | 0.392081733 | 0.999970678 | 3.388 | 2.286 | 4.055 | 4.102 | NA |
| TRINITY_DN2783_c1_g1_i5 | -0.65854902 | 0.466501866 | 0.999970678 | 992.28 | 2376 | 2726.379 | 1900.953 | PSI_PsaH |
| TRINITY_DN58838_c0_g1_i1 | -0.65853671 | 0.879232883 | 0.999970678 | 0.908 | 0 | 1.211 | 0 | Sugar_tr |
| TRINITY_DN15034_c0_g1_i5 | -0.65853671 | 0.879232883 | 0.999970678 | 1.199 | 0 | 1.555 | 0 | Glyco_hydro_16 |
| TRINITY_DN604_c1_g1_i4 | -0.65851133 | 0.577060732 | 0.999970678 | 351.33 | 417.5 | 153.257 | 1002.565 | NA |
| TRINITY_DN578_c0_g1_i6 | 0.6584102 | 0.56821078 | 0.999970678 | 1.308 | 0.71 | 0.7 | 0.442 | NA |
| TRINITY_DN4574_c0_g1_i3 | 0.658379295 | 0.814226854 | 0.999970678 | 2.607 | 0.848 | 1.8 | 0 | NA |
| TRINITY_DN26300_c0_g1_i3 | 0.658195805 | 0.566851896 | 0.999970678 | 0.845 | 2.464 | 0.678 | 1.257 | NA |
| TRINITY_DN4036_c0_g1_i1 | 0.658183088 | 0.307458436 | 0.999970678 | 14.098 | 16.61 | 6.666 | 11.441 | IQ |
| TRINITY_DN5762_c0_g1_i1 | 0.658153913 | 0.353962255 | 0.999970678 | 8.593 | 9.964 | 4 | 6.897 | Zip |
| TRINITY_DN2266_c0_g1_i2 | 0.658061012 | 0.70747987 | 0.999970678 | 7.44 | 3.262 | 0.2 | 6.595 | NA |
| TRINITY_DN269_c0_g1_i3 | -0.65805367 | 0.724235606 | 0.999970678 | 12.109 | 0.857 | 15.943 | 1.438 | ArgoL1 |
| TRINITY_DN11177_c0_g1_i1 | 0.658035963 | 0.644786569 | 0.999970678 | 8.848 | 60.36 | 7.377 | 34.434 | NA |
| TRINITY_DN1528_c1_g1_i12 | 0.658002919 | 0.809384149 | 0.999970678 | 5.423 | 1.971 | 0 | 4.735 | zf-C2H2 |
| TRINITY_DN5987_c0_g2_i5 | 0.657916941 | 0.806392811 | 0.999970678 | 0.572 | 1.143 | 0 | 1.086 | NA |
| TRINITY_DN6050_c0_g1_i1 | 0.657901101 | 0.822979578 | 0.999970678 | 0 | 3.272 | 1.5 | 0.221 | NA |
| TRINITY_DN9691_c0_g2_i1 | 0.657874319 | 0.754289389 | 0.999970678 | 0.036 | 3.282 | 1.289 | 0.523 | - |
| TRINITY_DN9976_c1_g1_i2 | 0.657846284 | 0.468867983 | 0.999970678 | 2.38 | 2.838 | 1.344 | 1.629 | NA |
| TRINITY_DN8160_c0_g1_i1 | -0.65777557 | 0.413370642 | 0.999970678 | 91.893 | 94.61 | 165.479 | 93.318 | Ribosomal_S6 |
| TRINITY_DN550_c0_g1_i8 | 0.657632395 | 0.621813157 | 0.999970678 | 1.281 | 6.573 | 1.267 | 3.398 | NA |
| TRINITY_DN707_c0_g1_i47 | -0.65758831 | 0.879054626 | 0.999970678 | 0.236 | 0 | 0.311 | 0 | SET |
| TRINITY_DN12439_c0_g1_i11 | -0.6575825 | 0.818855413 | 1 | 0.999 | 0 | 0.878 | 0.553 | NA |
| TRINITY_DN813_c3_g1_i7 | -0.65757023 | 0.816988191 | 0.999970678 | 1.508 | 0 | 1.655 | 0.412 | DUF659 |
| TRINITY_DN2096_c2_g1_i2 | 0.657532513 | 0.723247579 | 0.999970678 | 1.154 | 9.905 | 0.611 | 6.062 | zf-RING_11 |
| TRINITY_DN4369_c2_g1_i1 | -0.65744709 | 0.281634641 | 0.999970678 | 115.6 | 124.2 | 179.611 | 160.869 | NA |
| TRINITY_DN37028_c0_g1_i1 | -0.65736077 | 0.83707419 | 1 | 0.527 | 0.049 | 0.767 | 0 | - |
| TRINITY_DN18297_c0_g2_i2 | 0.657141547 | 0.882367872 | 1 | 1.472 | 0 | 0.8 | 0 | Ribosomal_S8e |
| TRINITY_DN1991_c0_g1_i3 | 0.657125058 | 0.586133885 | 0.999970678 | 1.399 | 4.405 | 0.989 | 2.483 | Serpin |
| TRINITY_DN3269_c0_g1_i5 | -0.65706368 | 0.581531123 | 0.999970678 | 0.6 | 0.335 | 0.989 | 0.292 | Proton_antipo_M |
| TRINITY_DN13449_c0_g1_i7 | -0.65704664 | 0.721758359 | 1 | 0.082 | 0.621 | 0.211 | 0.824 | FMO-like |
| TRINITY_DN3378_c4_g1_i1 | -0.65703814 | 0.764928661 | 0.999970678 | 5.877 | 13.61 | 0.178 | 29.095 | NA |
| TRINITY_DN3109_c0_g1_i1 | 0.65702514 | 0.542072088 | 0.999970678 | 68.438 | 86.54 | 16.332 | 76.69 | NA |
| TRINITY_DN903_c1_g1_i2 | -0.65702402 | 0.532299331 | 0.999970678 | 1.154 | 0.887 | 1.489 | 1.448 | NA |
| TRINITY_DN3955_c0_g1_i7 | 0.657016848 | 0.646661671 | 0.999970678 | 0.836 | 0.591 | 0.633 | 0.131 | LRR_6 |
| TRINITY_DN9367_c0_g1_i11 | -0.65687454 | 0.808738885 | 0.999970678 | 5.859 | 0 | 2.7 | 6.103 | NA |
| TRINITY_DN4848_c0_g1_i4 | -0.65679719 | 0.477184865 | 0.999970678 | 4.051 | 7.993 | 10.444 | 6.274 | PORR |
| TRINITY_DN2206_c0_g1_i1 | -0.65676327 | 0.704892044 | 0.999970678 | 2.562 | 50.66 | 28.043 | 43.754 | NA |

| TRINITY_DN10432_c0_g1_i1 | -0.65671587 | 0.883785482 | 1 | 1.054 | 0 | 1.389 | 0 | NA |
| --- | --- | --- | --- | --- | --- | --- | --- | --- |
| TRINITY_DN37748_c0_g4_i1 | -0.65671587 | 0.883785482 | 1 | 1.926 | 0 | 2.533 | 0 | Ribosomal_L6e |
| TRINITY_DN808_c2_g1_i4 | -0.65670658 | 0.524323982 | 0.999970678 | 2.189 | 1.183 | 2.755 | 2.011 | Guanylate_kin |
| TRINITY_DN67_c0_g1_i8 | 0.656662182 | 0.34244852 | 0.999970678 | 4.878 | 7.135 | 3.622 | 3.247 | DUF3535 |
| TRINITY_DN4461_c0_g1_i3 | -0.65662568 | 0.697071025 | 0.999970678 | 3.479 | 0.995 | 5.455 | 0.513 | NA |
| TRINITY_DN9621_c0_g2_i1 | -0.65661122 | 0.601634104 | 0.999970678 | 13.853 | 4.238 | 18.61 | 6.143 | Linker_histone |
| TRINITY_DN1318_c0_g1_i1 | -0.65658111 | 0.727739059 | 0.999970678 | 1.708 | 0.246 | 0.4 | 2.513 | NA |
| TRINITY_DN641_c0_g1_i3 | 0.656450056 | 0.401012593 | 0.999970678 | 1.79 | 2.129 | 1.044 | 1.206 | NA |
| TRINITY_DN4387_c0_g2_i1 | 0.656403552 | 0.44161918 | 0.999970678 | 2.689 | 2.838 | 1.033 | 2.262 | NA |
| TRINITY_DN3495_c2_g1_i1 | -0.65638762 | 0.511918764 | 0.999970678 | 4.406 | 6.229 | 3.366 | 12.708 | Castor_Poll_mid |
| TRINITY_DN8201_c0_g1_i9 | -0.65635209 | 0.362334103 | 0.999970678 | 11.019 | 9.244 | 11.255 | 18.056 | NA |
| TRINITY_DN1538_c1_g1_i2 | -0.65632373 | 0.402391604 | 0.999970678 | 94.473 | 185.3 | 152.846 | 251.704 | NA |
| TRINITY_DN26384_c0_g1_i1 | -0.65627883 | 0.563764447 | 0.999970678 | 0.454 | 0.581 | 0.622 | 0.875 | NA |
| TRINITY_DN8159_c0_g1_i1 | -0.6562706 | 0.620016521 | 0.999970678 | 2.616 | 10.02 | 4.211 | 14.397 | NA |
| TRINITY_DN2299_c0_g1_i4 | 0.656227758 | 0.778652593 | 0.999970678 | 125.15 | 0.266 | 8.911 | 69.36 | NA |
| TRINITY_DN5371_c0_g1_i4 | 0.656161492 | 0.806679395 | 0.999970678 | 0.263 | 0.315 | 0 | 0.362 | Cation_ATPase |
| TRINITY_DN2251_c0_g3_i1 | 0.656114924 | 0.760393947 | 0.999970678 | 15.579 | 1.252 | 8.622 | 0.312 | NA |
| TRINITY_DN17474_c0_g1_i1 | -0.65610343 | 0.550897064 | 0.999970678 | 0.709 | 2.316 | 2.167 | 2.131 | - |
| TRINITY_DN15814_c0_g1_i1 | -0.65609439 | 0.539997704 | 0.999970678 | 1.354 | 1.074 | 1.2 | 2.353 | NA |
| TRINITY_DN676_c0_g1_i6 | 0.656068401 | 0.486877967 | 0.999970678 | 38.289 | 16.47 | 18.821 | 12.266 | 2OG-FeII_Oxy |
| TRINITY_DN755_c0_g1_i1 | -0.65604914 | 0.80826784 | 0.999970678 | 0 | 1.035 | 0.744 | 0.704 | F-box-like |
| TRINITY_DN10638_c0_g1_i3 | 0.656023054 | 0.882980258 | 1 | 1.072 | 0 | 0 | 0.694 | - |
| TRINITY_DN4660_c0_g1_i4 | -0.65592276 | 0.532080035 | 0.999970678 | 7.158 | 2.907 | 8.799 | 5.268 | NA |
| TRINITY_DN1353_c2_g1_i1 | 0.655813698 | 0.365994624 | 0.999970678 | 6.431 | 7.648 | 4.689 | 3.278 | FAT |
| TRINITY_DN1084_c0_g1_i4 | -0.65573278 | 0.571412949 | 0.999970678 | 9.52 | 48.34 | 34.776 | 47.695 | NA |
| TRINITY_DN2436_c0_g2_i2 | -0.65571381 | 0.427275758 | 0.999970678 | 39.551 | 19.89 | 34.053 | 52.159 | NA |
| TRINITY_DN596_c0_g1_i4 | 0.655616146 | 0.631361631 | 0.999970678 | 1.172 | 2.769 | 0.3 | 2.121 | XH |
| TRINITY_DN9220_c0_g1_i7 | -0.6554717 | 0.578852759 | 1 | 1.308 | 1.064 | 1.622 | 1.719 | NA |
| TRINITY_DN2054_c0_g1_i16 | -0.65521343 | 0.680629623 | 0.999970678 | 33.438 | 4.593 | 43.897 | 7.44 | LRRNT_2 |
| TRINITY_DN8030_c0_g1_i6 | -0.65519538 | 0.810986312 | 0.999970678 | 0 | 4.169 | 2.322 | 3.68 | NA |
| TRINITY_DN9041_c0_g1_i9 | -0.65518635 | 0.401802894 | 0.999970678 | 9.611 | 10.15 | 9.111 | 19.947 | NA |
| TRINITY_DN314_c0_g1_i4 | -0.65512487 | 0.658333402 | 0.999970678 | 0.5 | 0.67 | 0.256 | 1.498 | NA |
| TRINITY_DN12595_c0_g1_i3 | -0.65510759 | 0.606786452 | 1 | 0.127 | 0.246 | 0.222 | 0.332 | PHD |
| TRINITY_DN3117_c0_g1_i4 | -0.65505472 | 0.475451742 | 0.999970678 | 22.701 | 12.22 | 14.766 | 37.37 | DUF538 |
| TRINITY_DN22352_c0_g1_i1 | 0.654995304 | 0.367640725 | 0.999970678 | 3.225 | 3.469 | 1.9 | 1.971 | Sgf11 |
| TRINITY_DN6312_c0_g1_i1 | -0.65480261 | 0.441744058 | 0.999970678 | 14.834 | 10.6 | 10.699 | 27.246 | Epimerase |
| TRINITY_DN721_c1_g1_i4 | 0.654685296 | 0.692018363 | 0.999970678 | 1.335 | 1.124 | 0.089 | 1.478 | NA |
| TRINITY_DN2979_c0_g1_i1 | -0.65463808 | 0.529799635 | 0.999970678 | 12.39 | 17.41 | 8.544 | 36.535 | Syntaxin-6_N |
| TRINITY_DN4470_c0_g1_i2 | 0.654623856 | 0.808687244 | 0.999970678 | 1.699 | 0.976 | 0 | 1.689 | Dev_Cell_Death |
| TRINITY_DN50244_c0_g1_i1 | -0.65461533 | 0.616623283 | 0.999970678 | 0.736 | 1.36 | 2.111 | 0.744 | F-box |
| TRINITY_DN3495_c1_g1_i1 | 0.654532914 | 0.436783394 | 0.999970678 | 12.518 | 6.613 | 6.066 | 4.886 | NA |
| TRINITY_DN1518_c2_g1_i1 | -0.65450635 | 0.529948534 | 0.999970678 | 5.278 | 11.31 | 5.6 | 19.283 | Pkinase_Tyr |
| TRINITY_DN8809_c0_g1_i2 | 0.654488259 | 0.481889149 | 0.999970678 | 11.655 | 4.928 | 4.511 | 5.147 | C2 |
| TRINITY_DN26849_c0_g1_i2 | 0.654462682 | 0.593459565 | 0.999970678 | 293.27 | 57.51 | 93.861 | 99.079 | Epimerase |
| TRINITY_DN3666_c0_g1_i4 | -0.65443751 | 0.398575477 | 0.999970678 | 2.053 | 2.503 | 2.544 | 4.052 | NA |
| TRINITY_DN13250_c0_g1_i1 | -0.65443026 | 0.443789549 | 0.999970678 | 11.464 | 7.993 | 8.366 | 20.6 | NA |
| TRINITY_DN4563_c0_g1_i1 | 0.654416458 | 0.409116583 | 0.999970678 | 19.076 | 29.98 | 15.91 | 11.703 | NA |
| TRINITY_DN26182_c0_g1_i3 | 0.654395748 | 0.563316748 | 0.999970678 | 19.004 | 20.97 | 13.755 | 7.641 | NA |
| TRINITY_DN285_c0_g1_i1 | -0.6542487 | 0.815424141 | 1 | 3.27 | 0 | 1.622 | 3.147 | NA |
| TRINITY_DN1457_c0_g1_i4 | 0.654241562 | 0.537053569 | 0.999970678 | 8.684 | 29.21 | 12.733 | 8.515 | NA |
| TRINITY_DN2887_c0_g1_i10 | 0.654232734 | 0.823225024 | 0.999970678 | 0.263 | 3.558 | 0 | 2.393 | 2OG-FeII_Oxy_2 |
| TRINITY_DN38802_c0_g1_i1 | -0.65423238 | 0.571754244 | 0.999970678 | 6.749 | 2.375 | 8.488 | 4.162 | - |
| TRINITY_DN2880_c0_g2_i1 | 0.654208518 | 0.814597865 | 0.999970678 | 0 | 1.843 | 0.6 | 0.442 | NA |
| TRINITY_DN95362_c0_g1_i1 | 0.654150603 | 0.885761465 | 1 | 1.544 | 0 | 0.822 | 0 | Sulfotransfer_1 |
| TRINITY_DN55528_c0_g1_i1 | -0.65414813 | 0.882504349 | 0.999970678 | 0.89 | 0 | 1.178 | 0 | - |
| TRINITY_DN5347_c0_g1_i2 | 0.654139319 | 0.61024557 | 0.999970678 | 57.91 | 51.68 | 7.122 | 60.594 | NA |
| TRINITY_DN9558_c0_g1_i5 | -0.65413614 | 0.469651432 | 0.999970678 | 0.827 | 1.419 | 1.422 | 1.82 | PP2C |
| TRINITY_DN6346_c0_g1_i6 | 0.654038211 | 0.72284719 | 1 | 0.709 | 0.148 | 0.389 | 0.09 | S6PP |
| TRINITY_DN24410_c0_g1_i1 | 0.654023461 | 0.658904581 | 1 | 0.581 | 0.729 | 0.556 | 0.171 | NA |
| TRINITY_DN4842_c0_g1_i1 | -0.65402124 | 0.392424268 | 0.999970678 | 43.249 | 23.46 | 39.731 | 57.547 | NA |
| TRINITY_DN152_c1_g1_i7 | -0.65397845 | 0.712167816 | 0.999970678 | 0.245 | 1.34 | 0.2 | 2.252 | NA |
| TRINITY_DN737_c0_g1_i3 | -0.65383861 | 0.252624514 | 0.999970678 | 48.799 | 51.91 | 65.007 | 78.318 | NA |
| TRINITY_DN525_c1_g2_i8 | -0.65383749 | 0.33776084 | 0.999970678 | 4.533 | 3.814 | 5 | 7.128 | FKBP_C |
| TRINITY_DN2647_c0_g1_i1 | -0.65376512 | 0.26834218 | 0.999970678 | 27.025 | 27.34 | 37.898 | 39.37 | CutA1 |
| TRINITY_DN8136_c0_g2_i2 | -0.65374258 | 0.676562236 | 0.999970678 | 1.272 | 5.351 | 0.878 | 9.229 | AA_permease_2 |
| TRINITY_DN2128_c2_g1_i1 | -0.65370786 | 0.420489472 | 0.999970678 | 6.55 | 10.73 | 11.01 | 13.462 | Zip |
| TRINITY_DN5394_c0_g1_i14 | 0.653675789 | 0.694833588 | 0.999970678 | 3.261 | 0.306 | 1.244 | 0.754 | NA |
| TRINITY_DN9204_c0_g1_i4 | -0.65358559 | 0.715290578 | 1 | 1.045 | 0.099 | 0.967 | 0.674 | NA |
| TRINITY_DN3068_c0_g1_i4 | -0.65357597 | 0.414408743 | 0.999970678 | 6.604 | 11.73 | 9.199 | 17.664 | Pkinase |
| TRINITY_DN165282_c0_g1_i1 | 0.653555923 | 0.877188081 | 0.999970678 | 13.462 | 0 | 7.144 | 0 | NA |
| TRINITY_DN6989_c0_g1_i1 | 0.653433838 | 0.501375835 | 0.999970678 | 3.87 | 8.249 | 4.289 | 2.513 | DUF547 |
| TRINITY_DN136892_c0_g1_i1 | 0.653419765 | 0.577510481 | 0.999970678 | 4.397 | 9.303 | 1.489 | 6.867 | NA |
| TRINITY_DN1759_c0_g1_i9 | -0.65333948 | 0.537882661 | 0.999970678 | 5.65 | 7.303 | 3.978 | 15.483 | Sedlin_N |
| TRINITY_DN3207_c0_g1_i2 | 0.653328238 | 0.876450565 | 0.999970678 | 4.079 | 0 | 0 | 2.644 | NA |

| TRINITY_DN18751_c0_g1_i4 | -0.65331392 | 0.724728711 | 0.999970678 | 0.127 | 1.429 | 0.467 | 1.86 | NA |
| --- | --- | --- | --- | --- | --- | --- | --- | --- |
| TRINITY_DN8625_c0_g1_i18 | 0.653260689 | 0.743118069 | 0.999970678 | 1.399 | 2.109 | 1.811 | 0.03 | NA |
| TRINITY_DN1126_c1_g1_i8 | -0.65322834 | 0.26816354 | 0.999970678 | 7.658 | 9.333 | 11.177 | 13.221 | NA |
| TRINITY_DN2981_c2_g2_i1 | 0.653165268 | 0.878407224 | 0.999970678 | 3.188 | 0 | 0 | 2.041 | NA |
| TRINITY_DN2666_c0_g1_i9 | 0.653132246 | 0.574344014 | 0.999970678 | 0.881 | 1.252 | 0.311 | 0.985 | NA |
| TRINITY_DN495_c0_g1_i4 | -0.65306817 | 0.816128551 | 1 | 4.469 | 0 | 2.622 | 3.901 | NA |
| TRINITY_DN57346_c0_g1_i1 | -0.65291997 | 0.656149508 | 1 | 0.164 | 0.414 | 0.222 | 0.633 | Gal-bind_lectin |
| TRINITY_DN2725_c0_g2_i2 | 0.652905406 | 0.815725866 | 0.999970678 | 0.336 | 1.863 | 1.144 | 0 | NA |
| TRINITY_DN2698_c0_g1_i1 | -0.65280503 | 0.488208415 | 0.999970678 | 187.62 | 188.1 | 122.359 | 441.397 | NA |
| TRINITY_DN3062_c0_g1_i5 | -0.65266698 | 0.536817381 | 0.999970678 | 6.004 | 11.61 | 5.977 | 20.158 | RraA-like |
| TRINITY_DN10867_c0_g1_i1 | -0.65262032 | 0.33847968 | 0.999970678 | 10.701 | 9.905 | 10.466 | 19.856 | AMP-binding |
| TRINITY_DN6391_c0_g1_i4 | 0.652565636 | 0.828781635 | 0.999970678 | 1.017 | 0 | 0.489 | 0.06 | NA |
| TRINITY_DN388_c0_g1_i2 | -0.65250631 | 0.556113158 | 0.999970678 | 8.348 | 17.4 | 9.288 | 27.859 | NA |
| TRINITY_DN8032_c0_g1_i1 | 0.65245552 | 0.812021183 | 0.999970678 | 0.273 | 0.384 | 0.344 | 0 | NA |
| TRINITY_DN2794_c0_g1_i1 | 0.652439979 | 0.807420335 | 0.999970678 | 5.741 | 2.681 | 0 | 5.389 | NA |
| TRINITY_DN20600_c0_g1_i3 | 0.652386352 | 0.566685684 | 0.999970678 | 3.307 | 12.02 | 2.633 | 6.505 | NA |
| TRINITY_DN13840_c0_g1_i1 | 0.652343369 | 0.883562744 | 1 | 1.426 | 0 | 0.767 | 0 | NA |
| TRINITY_DN57039_c0_g1_i2 | 0.65228472 | 0.654902544 | 0.999970678 | 1.653 | 1.646 | 0.311 | 1.739 | NA |
| TRINITY_DN1762_c1_g1_i7 | 0.652106878 | 0.470060648 | 0.999970678 | 2.189 | 3.272 | 1.922 | 1.136 | DNA_mis_repair |
| TRINITY_DN1372_c1_g1_i2 | -0.65196577 | 0.66606893 | 0.999970678 | 0.563 | 1.961 | 0.644 | 3.167 | AP2 |
| TRINITY_DN7640_c0_g1_i7 | 0.651871027 | 0.637323887 | 0.999970678 | 0.745 | 1.873 | 0.433 | 1.126 | NA |
| TRINITY_DN843_c0_g1_i10 | 0.651827375 | 0.529414609 | 0.999970678 | 8.103 | 25.91 | 9.333 | 10.024 | NA |
| TRINITY_DN740_c0_g1_i9 | -0.65174881 | 0.399409967 | 0.999970678 | 30.686 | 16.8 | 30.954 | 37.078 | NA |
| TRINITY_DN3715_c0_g1_i2 | -0.65172934 | 0.878092643 | 0.999970678 | 2.589 | 0 | 3.422 | 0 | Peroxidase_2 |
| TRINITY_DN813_c1_g1_i2 | 0.651696663 | 0.728116281 | 0.999970678 | 0.182 | 5.026 | 1.022 | 2.001 | NA |
| TRINITY_DN5537_c0_g1_i1 | -0.6516894 | 0.821020691 | 0.999970678 | 0.382 | 0.956 | 1.733 | 0 | NA |
| TRINITY_DN3556_c0_g1_i1 | -0.65166908 | 0.288718026 | 0.999970678 | 117.46 | 91.22 | 128.226 | 171.496 | - |
| TRINITY_DN3907_c0_g1_i1 | 0.651638128 | 0.4699869 | 0.999970678 | 19.785 | 21.03 | 5.855 | 19.012 | NA |
| TRINITY_DN28875_c0_g3_i1 | -0.65162611 | 0.616246048 | 0.999970678 | 0.409 | 0.345 | 0.789 | 0.231 | NA |
| TRINITY_DN11591_c0_g2_i2 | -0.65159627 | 0.593584234 | 0.999970678 | 10.274 | 5.233 | 12.466 | 8.254 | NA |
| TRINITY_DN3360_c0_g2_i2 | 0.651548142 | 0.666023646 | 0.999970678 | 9.148 | 1.005 | 3.611 | 2.162 | NA |
| TRINITY_DN16157_c0_g1_i1 | 0.651528852 | 0.430661076 | 0.999970678 | 5.396 | 3.558 | 3.033 | 2.061 | NA |
| TRINITY_DN1990_c0_g1_i2 | 0.651456035 | 0.379010092 | 0.999970678 | 5.641 | 5.568 | 2.422 | 4.243 | NA |
| TRINITY_DN975_c0_g1_i2 | 0.651419232 | 0.579461424 | 0.999970678 | 23.691 | 35.29 | 25.843 | 6.173 | RRM_1 |
| TRINITY_DN14795_c1_g1_i1 | -0.65135388 | 0.731679014 | 0.999970678 | 1.308 | 0.581 | 0.122 | 2.835 | NA |
| TRINITY_DN3984_c1_g1_i2 | 0.651347713 | 0.592196525 | 0.999970678 | 1.19 | 4.859 | 2.078 | 1.297 | NA |
| TRINITY_DN1176_c1_g1_i3 | -0.65129523 | 0.836717671 | 1 | 1.272 | 0 | 0.144 | 1.87 | NA |
| TRINITY_DN2369_c0_g1_i7 | -0.65127248 | 0.462862561 | 0.999970678 | 10.683 | 5.627 | 7.411 | 16.87 | LRR_1 |
| TRINITY_DN34437_c0_g1_i3 | 0.651272422 | 0.880818076 | 0.999970678 | 2.117 | 0 | 0 | 1.367 | NA |
| TRINITY_DN5524_c0_g1_i5 | 0.651097965 | 0.691125186 | 0.999970678 | 1.254 | 0.138 | 0.456 | 0.342 | EF_assoc_1 |
| TRINITY_DN42468_c0_g1_i1 | -0.65104065 | 0.551534964 | 0.999970678 | 425.17 | 1654 | 927.499 | 1967.739 | NA |
| TRINITY_DN653_c0_g1_i1 | -0.65099131 | 0.792187805 | 0.999970678 | 0.282 | 11.63 | 0.144 | 18.087 | NA |
| TRINITY_DN10421_c0_g1_i3 | -0.65098448 | 0.550848377 | 0.999970678 | 2.498 | 1.133 | 1.644 | 3.75 | DUF1677 |
| TRINITY_DN1039_c1_g1_i2 | -0.65095044 | 0.424476362 | 0.999970678 | 1.29 | 1.902 | 2.478 | 1.981 | B_lectin |
| TRINITY_DN570_c0_g1_i6 | 0.650902588 | 0.823099898 | 0.999970678 | 0 | 31.26 | 4.633 | 14.025 | mTERF |
| TRINITY_DN11411_c0_g1_i2 | 0.650901487 | 0.587370224 | 0.999970678 | 0.527 | 1.863 | 0.722 | 0.643 | NA |
| TRINITY_DN641_c0_g1_i5 | 0.650812199 | 0.698885421 | 0.999970678 | 3.252 | 1.045 | 2.144 | 0.161 | NA |
| TRINITY_DN119_c0_g1_i4 | 0.650704254 | 0.822706392 | 0.999970678 | 54.313 | 39.46 | 0 | 59.89 | Ferritin |
| TRINITY_DN9074_c0_g2_i1 | -0.6506211 | 0.880252034 | 0.999970678 | 2.653 | 0 | 0 | 4.192 | NA |
| TRINITY_DN76904_c0_g1_i1 | -0.65059157 | 0.880580956 | 0.999970678 | 4.542 | 0 | 5.944 | 0 | Sdh_cyt |
| TRINITY_DN1859_c0_g1_i2 | -0.65052391 | 0.391576298 | 0.999970678 | 37.689 | 46.57 | 37.92 | 86.261 | NA |
| TRINITY_DN923_c0_g1_i2 | 0.650517341 | 0.434503885 | 0.999970678 | 4.696 | 2.789 | 2.211 | 2.121 | NA |
| TRINITY_DN8660_c1_g1_i1 | -0.6504509 | 0.880352221 | 0.999970678 | 0 | 25.94 | 0 | 40.044 | NA |
| TRINITY_DN3815_c0_g1_i7 | -0.65034571 | 0.535160361 | 0.999970678 | 7.621 | 5.273 | 4.344 | 14.92 | NA |
| TRINITY_DN573_c0_g1_i4 | 0.650274241 | 0.497659897 | 0.999970678 | 79.911 | 35.25 | 22.232 | 44.588 | NA |
| TRINITY_DN112_c0_g1_i18 | -0.65024331 | 0.417467776 | 0.999970678 | 18.985 | 9.983 | 17.288 | 24.642 | NA |
| TRINITY_DN1131_c0_g2_i3 | 0.650102504 | 0.25778841 | 0.999970678 | 13.281 | 15.52 | 7.788 | 8.978 | Peptidase_S8 |
| TRINITY_DN34113_c0_g1_i5 | 0.650061058 | 0.817974521 | 0.999970678 | 0 | 17.92 | 6.577 | 3.247 | DLH |
| TRINITY_DN27908_c0_g1_i5 | -0.6499083 | 0.658972329 | 0.999970678 | 11.355 | 3.656 | 14.166 | 5.922 | LRRNT_2 |
| TRINITY_DN37441_c0_g1_i1 | -0.64980469 | 0.585984967 | 0.999970678 | 0.563 | 1.488 | 1.311 | 1.609 | rRNA_methylase |
| TRINITY_DN5481_c0_g1_i2 | 0.649759802 | 0.561426082 | 0.999970678 | 2.989 | 1.311 | 0.933 | 1.609 | NA |
| TRINITY_DN387_c0_g1_i7 | 0.649721065 | 0.30644372 | 0.999970678 | 107.86 | 86.21 | 56.674 | 54.541 | NA |
| TRINITY_DN4333_c0_g1_i2 | -0.64966209 | 0.813033967 | 0.999970678 | 0.654 | 1.281 | 0 | 3.036 | NA |
| TRINITY_DN405_c0_g1_i4 | 0.649661883 | 0.832721259 | 0.999970678 | 3.525 | 0 | 0.178 | 2.031 | Ribosomal_L34e |
| TRINITY_DN938_c0_g1_i15 | -0.64966108 | 0.595963568 | 0.999970678 | 4.469 | 1.114 | 3.466 | 4.605 | NA |
| TRINITY_DN1922_c3_g1_i4 | -0.64956259 | 0.641435671 | 0.999970678 | 0.672 | 0.187 | 0.656 | 0.583 | NA |
| TRINITY_DN11308_c0_g1_i2 | -0.64951518 | 0.80921333 | 0.999970678 | 3.824 | 0 | 2.378 | 3.157 | NA |
| TRINITY_DN3043_c0_g1_i5 | 0.649403985 | 0.404835986 | 0.999970678 | 17.196 | 15.74 | 6.189 | 13.472 | NA |
| TRINITY_DN17158_c0_g1_i4 | -0.64937134 | 0.879910075 | 0.999970678 | 1.317 | 0 | 1.733 | 0 | NA |
| TRINITY_DN1776_c1_g1_i13 | 0.649331626 | 0.654992434 | 0.999970678 | 2.008 | 3.321 | 0.4 | 2.875 | NA |
| TRINITY_DN3934_c0_g1_i1 | -0.64929521 | 0.815027223 | 0.999970678 | 22.483 | 0 | 13.233 | 19.585 | NA |
| TRINITY_DN3475_c0_g1_i4 | 0.649293071 | 0.877468439 | 0.999970678 | 0 | 1.025 | 0.533 | 0 | Sec39 |
| TRINITY_DN12560_c0_g1_i1 | 0.649283851 | 0.434974242 | 0.999970678 | 4.179 | 2.631 | 1.611 | 2.423 | Abhydrolase_6 |
| TRINITY_DN20691_c0_g2_i2 | -0.64925597 | 0.445524364 | 0.999970678 | 1.644 | 1.597 | 1.633 | 3.097 | Kelch_1 |

| TRINITY_DN3382_c0_g1_i1 | -0.64918273 | 0.279828191 | 0.999970678 | 14.889 | 16.79 | 18.654 | 27.095 | PP2C |
| --- | --- | --- | --- | --- | --- | --- | --- | --- |
| TRINITY_DN7035_c0_g1_i1 | 0.649134322 | 0.477889606 | 0.999970678 | 5.387 | 3.006 | 1.755 | 3.237 | NA |
| TRINITY_DN2270_c0_g1_i5 | 0.649066668 | 0.472206911 | 0.999970678 | 1.59 | 2.819 | 1.155 | 1.438 | TPT |
| TRINITY_DN29848_c0_g1_i1 | 0.649007665 | 0.699639986 | 0.999970678 | 6.831 | 1.419 | 3.678 | 0.824 | NA |
| TRINITY_DN912_c0_g1_i1 | -0.64896807 | 0.525435233 | 0.999970678 | 137.45 | 104.8 | 243.53 | 84.622 | NA |
| TRINITY_DN4524_c0_g2_i1 | 0.64887266 | 0.662099585 | 1 | 0.491 | 0.68 | 0.489 | 0.171 | HI0933_like |
| TRINITY_DN3965_c0_g1_i3 | 0.648827711 | 0.443903824 | 0.999970678 | 3.461 | 2.158 | 1.322 | 2.011 | NA |
| TRINITY_DN2121_c0_g1_i1 | 0.648731952 | 0.282863911 | 0.999970678 | 11.091 | 10.98 | 6.466 | 6.294 | NA |
| TRINITY_DN430_c0_g1_i2 | 0.648649726 | 0.640358279 | 0.999970678 | 10.21 | 8.682 | 1.044 | 10.788 | Proteasome |
| TRINITY_DN17487_c0_g1_i5 | -0.64855713 | 0.689979427 | 0.999970678 | 0.1 | 0.335 | 0.111 | 0.553 | KH_1 |
| TRINITY_DN20558_c0_g1_i5 | 0.648495247 | 0.605965321 | 0.999970678 | 1.063 | 0.591 | 0.722 | 0.191 | DUF3883 |
| TRINITY_DN1393_c0_g2_i1 | 0.64837864 | 0.552072897 | 0.999970678 | 4.887 | 5.302 | 3.833 | 1.82 | Melibiase_2 |
| TRINITY_DN9013_c0_g1_i2 | 0.648359949 | 0.737176718 | 0.999970678 | 0.763 | 4.583 | 2.633 | 0.191 | Chloroa_b-bind |
| TRINITY_DN7947_c0_g1_i5 | -0.64828728 | 0.635782354 | 0.999970678 | 8.33 | 1.784 | 7.455 | 6.495 | NA |
| TRINITY_DN19882_c0_g1_i2 | 0.648281302 | 0.583050879 | 0.999970678 | 0.99 | 0.788 | 0.633 | 0.362 | NA |
| TRINITY_DN18896_c0_g2_i2 | 0.648259543 | 0.817536228 | 0.999970678 | 0.154 | 0.828 | 0.511 | 0 | NA |
| TRINITY_DN8041_c0_g1_i8 | 0.647995045 | 0.574795993 | 0.999970678 | 2.298 | 2.592 | 1.533 | 1.206 | NA |
| TRINITY_DN94_c1_g1_i6 | -0.64793476 | 0.510820017 | 0.999970678 | 4.923 | 2.592 | 6.588 | 3.861 | NA |
| TRINITY_DN2057_c0_g1_i9 | 0.64793178 | 0.547774711 | 0.999970678 | 0.79 | 1.449 | 0.711 | 0.573 | NA |
| TRINITY_DN627_c0_g1_i6 | -0.64791994 | 0.580136067 | 0.999970678 | 3.179 | 1.764 | 2.989 | 4.062 | NA |
| TRINITY_DN1343_c0_g1_i15 | -0.64784644 | 0.882188211 | 0.999970678 | 0 | 0.788 | 1 | 0 | NA |
| TRINITY_DN55159_c0_g1_i1 | -0.64778295 | 0.630848966 | 0.999970678 | 0.336 | 0.256 | 0.589 | 0.191 | PWWP |
| TRINITY_DN1478_c0_g1_i15 | -0.64765703 | 0.816352749 | 0.999970678 | 3.706 | 0 | 1.1 | 4.595 | F-box |
| TRINITY_DN289_c4_g2_i1 | -0.64763937 | 0.598901809 | 0.999970678 | 2.625 | 10.71 | 4.933 | 14.688 | TBCA |
| TRINITY_DN46349_c0_g1_i1 | 0.647627593 | 0.826089016 | 0.999970678 | 0.164 | 1.636 | 0.933 | 0 | NA |
| TRINITY_DN1648_c0_g1_i2 | 0.647584085 | 0.707486699 | 0.999970678 | 0.736 | 11.75 | 1.267 | 6.344 | EamA |
| TRINITY_DN2245_c0_g1_i5 | 0.647284639 | 0.677693061 | 0.999970678 | 0.454 | 0.089 | 0.189 | 0.121 | Occludin_ELL |
| TRINITY_DN7259_c0_g1_i4 | -0.6472799 | 0.400731043 | 0.999970678 | 39.715 | 30.24 | 57.197 | 39.602 | NA |
| TRINITY_DN626_c0_g1_i1 | -0.6472224 | 0.503400426 | 0.999970678 | 4.179 | 8.16 | 4.755 | 13.512 | - |
| TRINITY_DN961_c5_g1_i1 | 0.647208214 | 0.82547828 | 0.999970678 | 34.128 | 0 | 9.499 | 10.667 | NA |
| TRINITY_DN835_c0_g2_i2 | -0.64715997 | 0.685208773 | 0.999970678 | 1.762 | 0.237 | 1.567 | 1.237 | NA |
| TRINITY_DN73_c1_g1_i3 | 0.647158261 | 0.821350874 | 0.999970678 | 0.627 | 0.079 | 0.378 | 0 | NA |
| TRINITY_DN88249_c0_g1_i1 | 0.647154489 | 0.667233958 | 0.999970678 | 5.814 | 9.136 | 7.233 | 0.744 | DUF4079 |
| TRINITY_DN4654_c0_g1_i7 | 0.647142832 | 0.678682824 | 0.999970678 | 1.217 | 0.237 | 0.2 | 0.694 | LRR_1 |
| TRINITY_DN99378_c0_g1_i1 | 0.64712696 | 0.884838968 | 1 | 1.39 | 0 | 0.744 | 0 | Peptidase_M16 |
| TRINITY_DN18196_c0_g1_i2 | 0.646937415 | 0.81211404 | 1 | 0.963 | 1.045 | 0 | 1.297 | NA |
| TRINITY_DN3867_c0_g1_i1 | -0.64690296 | 0.376367405 | 0.999970678 | 17.16 | 30.03 | 28.476 | 38.837 | NA |
| TRINITY_DN25996_c0_g1_i2 | 0.646881418 | 0.881025946 | 0.999970678 | 3.506 | 0 | 1.878 | 0 | Aldo_ket_red |
| TRINITY_DN63745_c0_g1_i1 | 0.646796353 | 0.388004258 | 0.999970678 | 11.564 | 14.51 | 8.844 | 5.972 | NA |
| TRINITY_DN3105_c0_g1_i1 | 0.646783826 | 0.352571868 | 0.999970678 | 47.554 | 29.96 | 20.621 | 24.762 | NA |
| TRINITY_DN386_c0_g2_i2 | 0.64675767 | 0.425464168 | 0.999970678 | 6.068 | 4.987 | 2.244 | 4.343 | NA |
| TRINITY_DN3934_c0_g1_i2 | -0.64675464 | 0.878695739 | 0.999970678 | 6.095 | 0 | 8.022 | 0 | NA |
| TRINITY_DN960_c0_g1_i1 | -0.64673761 | 0.839087963 | 0.999970678 | 97.68 | 0 | 8.522 | 144.964 | Epimerase |
| TRINITY_DN4029_c0_g2_i5 | -0.64671204 | 0.883949567 | 1 | 0.917 | 0 | 0 | 1.448 | NA |
| TRINITY_DN58_c0_g1_i3 | -0.64664012 | 0.61138557 | 0.999970678 | 1.154 | 0.67 | 0.611 | 2.172 | NA |
| TRINITY_DN7738_c0_g1_i1 | 0.646573643 | 0.68255873 | 0.999970678 | 6.477 | 9.638 | 8.066 | 0.513 | NA |
| TRINITY_DN1420_c0_g1_i4 | -0.64655518 | 0.551499675 | 0.999970678 | 5.895 | 9.974 | 15.388 | 5.972 | NA |
| TRINITY_DN1628_c1_g2_i1 | -0.64649973 | 0.820235994 | 0.999970678 | 1.417 | 0 | 1.467 | 0.452 | - |
| TRINITY_DN2963_c0_g1_i2 | -0.64649503 | 0.822504717 | 1 | 0.518 | 0 | 0.222 | 0.533 | NA |
| TRINITY_DN5408_c0_g1_i1 | -0.64640812 | 0.520632 | 0.999970678 | 4.406 | 2.168 | 4.811 | 4.383 | NA |
| TRINITY_DN36556_c0_g1_i1 | 0.646360083 | 0.383511548 | 0.999970678 | 11.764 | 17.43 | 9.244 | 7.44 | NA |
| TRINITY_DN1589_c0_g1_i1 | -0.64623151 | 0.436666973 | 0.999970678 | 3.307 | 2.789 | 2.667 | 6.354 | Beta-Casp |
| TRINITY_DN1943_c0_g1_i2 | 0.646227651 | 0.815831076 | 0.999970678 | 0 | 2.72 | 0.733 | 0.845 | NA |
| TRINITY_DN57_c0_g3_i1 | 0.646177192 | 0.702561943 | 0.999970678 | 1.635 | 0.355 | 0.955 | 0.131 | PTR2 |
| TRINITY_DN1933_c0_g1_i4 | -0.64598857 | 0.410448528 | 0.999970678 | 56.629 | 50.59 | 46.919 | 108.53 | p450 |
| TRINITY_DN590_c0_g1_i7 | -0.64598135 | 0.620845925 | 0.999970678 | 37.689 | 287.7 | 163.69 | 297.358 | Chloroa_b-bind |
| TRINITY_DN11530_c0_g1_i1 | -0.64571219 | 0.714904166 | 0.999970678 | 3.107 | 0.345 | 3.689 | 0.915 | NA |
| TRINITY_DN6845_c0_g1_i1 | -0.64569191 | 0.82959745 | 1 | 0 | 0.463 | 0.1 | 0.623 | DNA_pol_E_B |
| TRINITY_DN35114_c0_g1_i1 | -0.6456659 | 0.658818834 | 0.999970678 | 1.008 | 2.247 | 0.911 | 3.911 | NA |
| TRINITY_DN9268_c0_g1_i1 | -0.64554432 | 0.634383163 | 0.999970678 | 0.481 | 1.961 | 1.333 | 2.192 | NA |
| TRINITY_DN9990_c0_g1_i8 | -0.64543036 | 0.748730207 | 0.999970678 | 2.207 | 5.696 | 0.267 | 11.431 | NA |
| TRINITY_DN3269_c0_g1_i1 | -0.64541718 | 0.334222932 | 0.999970678 | 3.824 | 5.982 | 5.9 | 8.214 | NA |
| TRINITY_DN3402_c0_g1_i1 | -0.64541682 | 0.546235213 | 0.999970678 | 2.998 | 7.214 | 3.566 | 11.582 | NA |
| TRINITY_DN2253_c1_g1_i4 | -0.6453997 | 0.690168778 | 0.999970678 | 3.87 | 42.11 | 8.544 | 61.026 | CMAS |
| TRINITY_DN46009_c0_g1_i2 | 0.645344547 | 0.549033076 | 0.999970678 | 0.69 | 0.473 | 0.411 | 0.251 | NA |
| TRINITY_DN3930_c0_g1_i5 | -0.64528503 | 0.660892637 | 0.999970678 | 4.342 | 1.843 | 6.3 | 2.021 | NA |
| TRINITY_DN10641_c0_g1_i1 | 0.645253864 | 0.50337104 | 0.999970678 | 15.661 | 14.18 | 3.955 | 14.367 | TPR_1 |
| TRINITY_DN3229_c0_g1_i5 | 0.64524769 | 0.49006528 | 0.999970678 | 4.097 | 3.43 | 2.567 | 1.679 | NA |
| TRINITY_DN2901_c0_g2_i2 | 0.645159951 | 0.570653568 | 0.999970678 | 0.727 | 0.562 | 0.444 | 0.302 | NA |
| TRINITY_DN2738_c0_g1_i4 | -0.64504834 | 0.679549035 | 0.999970678 | 1.081 | 0.296 | 1.367 | 0.513 | NA |
| TRINITY_DN3462_c0_g1_i1 | 0.64493022 | 0.595528385 | 0.999970678 | 0.454 | 0.966 | 0.356 | 0.483 | SRP54 |
| TRINITY_DN14525_c0_g2_i1 | -0.64491299 | 0.51387771 | 0.999970678 | 59.009 | 108.7 | 57.041 | 188.899 | NA |
| TRINITY_DN4302_c0_g1_i5 | -0.64483414 | 0.370811522 | 0.999970678 | 53.65 | 57.96 | 92.017 | 63.208 | NA |
| TRINITY_DN12111_c0_g2_i1 | -0.64477429 | 0.351764049 | 0.999970678 | 6.577 | 4.829 | 8.211 | 7.973 | MAM33 |

| TRINITY_DN15990_c0_g1_i1 | -0.64476321 | 0.429047223 | 0.999970678 | 85.68 | 118.1 | 85.661 | 210.816 | NA |
| --- | --- | --- | --- | --- | --- | --- | --- | --- |
| TRINITY_DN299_c0_g2_i2 | -0.64475581 | 0.819026587 | 0.999970678 | 1.335 | 0 | 0.478 | 1.548 | Aldo_ket_red |
| TRINITY_DN2573_c0_g1_i5 | 0.644516894 | 0.471660244 | 0.999970678 | 20.148 | 50.42 | 21.088 | 19.233 | AP2 |
| TRINITY_DN19335_c0_g1_i1 | -0.64427366 | 0.830495818 | 1 | 0.672 | 0.128 | 1.033 | 0 | Sugar_tr |
| TRINITY_DN11935_c0_g1_i5 | -0.64427155 | 0.834038967 | 0.999970678 | 0 | 0.522 | 0.633 | 0.05 | DUF4101 |
| TRINITY_DN1226_c0_g2_i2 | -0.64420113 | 0.726210825 | 0.999970678 | 0.454 | 12.98 | 4.811 | 14.638 | NA |
| TRINITY_DN4658_c0_g1_i8 | 0.644178565 | 0.570331765 | 0.999970678 | 0.618 | 0.532 | 0.322 | 0.352 | NA |
| TRINITY_DN134_c0_g1_i4 | 0.644086394 | 0.626932669 | 0.999970678 | 0.736 | 0.325 | 0.389 | 0.201 | FAD_binding_8 |
| TRINITY_DN10498_c0_g1_i7 | 0.643980809 | 0.431363881 | 0.999970678 | 4.415 | 2.878 | 1.611 | 2.745 | NA |
| TRINITY_DN30_c3_g2_i1 | -0.64388748 | 0.320656972 | 0.999970678 | 62.87 | 76.32 | 106.016 | 89.156 | NA |
| TRINITY_DN11969_c0_g1_i1 | 0.643879189 | 0.423409009 | 0.999970678 | 3.924 | 5.263 | 3.033 | 2.182 | - |
| TRINITY_DN3648_c0_g1_i2 | 0.643842422 | 0.388404771 | 0.999970678 | 5.568 | 9.55 | 4.155 | 4.645 | NA |
| TRINITY_DN4320_c0_g1_i4 | 0.643809786 | 0.510509347 | 0.999970678 | 3.806 | 3.173 | 1.567 | 2.564 | NA |
| TRINITY_DN22680_c0_g1_i1 | 0.643710549 | 0.503384462 | 0.999970678 | 1.79 | 2.474 | 0.889 | 1.639 | Steroid_dh |
| TRINITY_DN981_c0_g1_i3 | -0.64370369 | 0.567548947 | 0.999970678 | 1.154 | 1.971 | 3.055 | 1.176 | NA |
| TRINITY_DN701_c0_g1_i5 | -0.64369415 | 0.553610434 | 0.999970678 | 1.072 | 1.133 | 2.233 | 0.764 | NB-ARC |
| TRINITY_DN84015_c0_g1_i1 | -0.64367516 | 0.881048612 | 0.999970678 | 1.808 | 0 | 0 | 2.845 | NA |
| TRINITY_DN10176_c0_g1_i1 | 0.643637977 | 0.645616919 | 0.999970678 | 0.936 | 2.74 | 0.411 | 1.86 | NA |
| TRINITY_DN6222_c0_g2_i1 | -0.64360091 | 0.52044542 | 0.999970678 | 0.391 | 0.572 | 0.489 | 0.895 | NA |
| TRINITY_DN7369_c0_g1_i3 | -0.64359937 | 0.450346937 | 0.999970678 | 1.19 | 0.907 | 1.1 | 1.92 | Peptidase_S24 |
| TRINITY_DN6292_c0_g1_i1 | -0.64356969 | 0.369482106 | 0.999970678 | 47.509 | 33.75 | 41.842 | 76.82 | WD40 |
| TRINITY_DN438_c0_g1_i4 | 0.643490881 | 0.653564367 | 0.999970678 | 2.434 | 10.13 | 4.478 | 2.463 | NA |
| TRINITY_DN866_c0_g1_i3 | -0.64348158 | 0.611171127 | 0.999970678 | 3.506 | 2.247 | 6.311 | 1.377 | NA |
| TRINITY_DN719_c1_g1_i1 | -0.64344984 | 0.724301473 | 0.999970678 | 0.927 | 0.394 | 0.111 | 1.95 | NA |
| TRINITY_DN5414_c0_g1_i5 | 0.643407744 | 0.609710862 | 0.999970678 | 0.709 | 0.838 | 0.256 | 0.674 | NA |
| TRINITY_DN10602_c1_g1_i3 | 0.643373985 | 0.491521043 | 0.999970678 | 15.197 | 10.7 | 4.311 | 11.24 | NA |
| TRINITY_DN29697_c0_g1_i1 | 0.643349245 | 0.48188709 | 0.999970678 | 0.854 | 0.857 | 0.356 | 0.664 | CDP-OH_P_trans |
| TRINITY_DN2114_c1_g3_i1 | -0.64329063 | 0.685907613 | 0.999970678 | 1.135 | 0.7 | 2.011 | 0.432 | NA |
| TRINITY_DN7497_c0_g1_i4 | 0.643247691 | 0.540178847 | 0.999970678 | 5.251 | 3.311 | 3.455 | 1.327 | Ist1 |
| TRINITY_DN15860_c0_g1_i7 | -0.64324754 | 0.73597702 | 0.999970678 | 7.276 | 11.68 | 0.489 | 28.11 | NA |
| TRINITY_DN6321_c0_g1_i4 | 0.643065848 | 0.81519882 | 0.999970678 | 0.363 | 1.212 | 0 | 1.005 | Ribosomal_L7Ae |
| TRINITY_DN3785_c0_g2_i1 | 0.643032626 | 0.579974135 | 0.999970678 | 7.403 | 1.705 | 2.722 | 2.604 | NA |
| TRINITY_DN3397_c0_g1_i3 | -0.64302551 | 0.593489597 | 0.999970678 | 0.709 | 2.079 | 2.033 | 1.8 | NA |
| TRINITY_DN12882_c1_g1_i1 | -0.64301946 | 0.788562019 | 1 | 1.099 | 0.039 | 1.378 | 0.131 | NA |
| TRINITY_DN12212_c2_g1_i1 | 0.64300549 | 0.655802021 | 0.999970678 | 0.454 | 0.631 | 0.111 | 0.553 | NA |
| TRINITY_DN7676_c0_g1_i4 | 0.642997311 | 0.628102408 | 0.999970678 | 0.345 | 0.67 | 0.133 | 0.493 | NA |
| TRINITY_DN336_c0_g1_i15 | 0.642942899 | 0.63854604 | 0.999970678 | 1.299 | 0.798 | 0.933 | 0.221 | DUF659 |
| TRINITY_DN20782_c0_g1_i1 | -0.64292759 | 0.883127656 | 0.999970678 | 1.553 | 0 | 2.033 | 0 | Mito_carr |
| TRINITY_DN11495_c0_g1_i1 | -0.64292759 | 0.883127656 | 0.999970678 | 1.962 | 0 | 2.544 | 0 | adh_short |
| TRINITY_DN50377_c1_g1_i1 | -0.64291784 | 0.884314994 | 0.999970678 | 0.699 | 0 | 0.9 | 0 | NA |
| TRINITY_DN78774_c0_g1_i1 | -0.64291784 | 0.884314994 | 0.999970678 | 1.653 | 0 | 2.155 | 0 | NA |
| TRINITY_DN4853_c0_g1_i6 | -0.64291225 | 0.885062632 | 1 | 5.523 | 0 | 7.255 | 0 | NA |
| TRINITY_DN25525_c0_g1_i5 | -0.64290554 | 0.88593865 | 1 | 10.828 | 0 | 13.999 | 0 | NA |
| TRINITY_DN109409_c0_g1_i1 | -0.64290554 | 0.88593865 | 1 | 0.69 | 0 | 0.911 | 0 | NA |
| TRINITY_DN16051_c1_g1_i7 | -0.64289759 | 0.886996138 | 1 | 2.943 | 0 | 3.7 | 0 | Thioredoxin |
| TRINITY_DN24102_c0_g1_i1 | -0.64289759 | 0.886996138 | 1 | 2.008 | 0 | 2.622 | 0 | Pyrid_ox_like |
| TRINITY_DN9826_c0_g1_i9 | -0.64288944 | 0.888290429 | 1 | 1.154 | 0 | 1.467 | 0 | NA |
| TRINITY_DN26470_c0_g1_i3 | -0.64288944 | 0.888290429 | 1 | 0.772 | 0 | 1.022 | 0 | - |
| TRINITY_DN18790_c0_g1_i1 | -0.64288944 | 0.888290429 | 1 | 0.854 | 0 | 1.1 | 0 | NA |
| TRINITY_DN38909_c0_g1_i1 | -0.64288944 | 0.888290429 | 1 | 0.436 | 0 | 0.578 | 0 | - |
| TRINITY_DN10573_c0_g1_i2 | 0.642889425 | 0.814255975 | 0.999970678 | 1.126 | 0.463 | 0 | 1.005 | NA |
| TRINITY_DN1606_c0_g1_i2 | 0.642854428 | 0.68851718 | 0.999970678 | 11.664 | 8.732 | 0.689 | 12.115 | Prefoldin_2 |
| TRINITY_DN1151_c0_g2_i8 | -0.64284512 | 0.569743663 | 0.999970678 | 1.163 | 0.976 | 0.833 | 2.312 | NA |
| TRINITY_DN713_c0_g1_i3 | 0.642842448 | 0.602384474 | 0.999970678 | 9.82 | 9.343 | 2.055 | 9.621 | NA |
| TRINITY_DN2218_c0_g1_i1 | -0.6428158 | 0.833530398 | 0.999970678 | 0 | 25.23 | 29.987 | 2.463 | VDE |
| TRINITY_DN73370_c0_g1_i1 | -0.64277908 | 0.360319821 | 0.999970678 | 225.77 | 385.2 | 383.466 | 439.93 | Chloroa_b-bind |
| TRINITY_DN4933_c0_g1_i2 | -0.64269853 | 0.319065108 | 0.999970678 | 64.714 | 55.32 | 90.139 | 78.298 | NA |
| TRINITY_DN11582_c0_g1_i6 | -0.64267095 | 0.692486662 | 0.999970678 | 2.298 | 0.315 | 2.755 | 0.794 | NA |
| TRINITY_DN2500_c0_g1_i3 | 0.642654472 | 0.737023694 | 0.999970678 | 0.391 | 10.45 | 0.756 | 5.932 | NMO |
| TRINITY_DN1350_c0_g1_i6 | 0.642573415 | 0.583400039 | 0.999970678 | 10.165 | 38.62 | 9.311 | 18.911 | NA |
| TRINITY_DN855_c0_g1_i14 | 0.64248574 | 0.647424196 | 0.999970678 | 0.627 | 3.715 | 0.722 | 1.86 | - |
| TRINITY_DN3713_c0_g1_i2 | 0.642446279 | 0.513355812 | 0.999970678 | 0.718 | 0.798 | 0.267 | 0.643 | NA |
| TRINITY_DN1053_c0_g1_i4 | 0.642420262 | 0.659448297 | 0.999970678 | 0.827 | 0.315 | 0.167 | 0.533 | Abhydrolase_1 |
| TRINITY_DN3295_c0_g1_i2 | 0.642384471 | 0.469641148 | 0.999970678 | 1.681 | 1.074 | 0.689 | 0.925 | KH_2 |
| TRINITY_DN72759_c0_g1_i4 | -0.64236505 | 0.879818617 | 0.999970678 | 6.304 | 0 | 0 | 10.044 | AA_permease_2 |
| TRINITY_DN5016_c0_g3_i1 | -0.6423608 | 0.338886173 | 0.999970678 | 5.405 | 6.327 | 7.644 | 8.998 | NA |
| TRINITY_DN2337_c0_g2_i2 | -0.64235901 | 0.423857845 | 0.999970678 | 15.815 | 13.23 | 12.433 | 30.443 | NA |
| TRINITY_DN15243_c0_g1_i2 | 0.642283542 | 0.423920109 | 0.999970678 | 7.531 | 4.149 | 2.722 | 4.263 | CoA_binding |
| TRINITY_DN7400_c0_g1_i1 | -0.64209382 | 0.443489843 | 0.999970678 | 26.207 | 15.59 | 33.942 | 24.028 | NA |
| TRINITY_DN586_c0_g1_i5 | 0.641982272 | 0.376519446 | 0.999970678 | 25.944 | 24.41 | 16.921 | 11.843 | Pkinase |
| TRINITY_DN2170_c0_g1_i2 | -0.64193865 | 0.87992612 | 0.999970678 | 0 | 1.025 | 1.311 | 0 | F-box |
| TRINITY_DN1473_c0_g1_i2 | -0.64192138 | 0.511207999 | 0.999970678 | 0.827 | 1.735 | 1.289 | 2.443 | ABC1 |
| TRINITY_DN566_c0_g1_i8 | -0.64189562 | 0.520733571 | 0.999970678 | 9.847 | 4.849 | 7.022 | 14.176 | Dirigent |
| TRINITY_DN8273_c0_g1_i8 | 0.641823446 | 0.658416115 | 0.999970678 | 13.871 | 45.83 | 6.666 | 26.723 | LRRNT_2 |

| TRINITY_DN1763_c0_g1_i2 | 0.641755215 | 0.290609018 | 0.999970678 | 10.755 | 12.36 | 6.522 | 6.937 | NAPRTase |
| --- | --- | --- | --- | --- | --- | --- | --- | --- |
| TRINITY_DN102112_c0_g1_i1 | 0.641723983 | 0.583109082 | 0.999970678 | 0.209 | 0.522 | 0.256 | 0.161 | MutS_I |
| TRINITY_DN12973_c0_g1_i1 | 0.641698679 | 0.424372262 | 0.999970678 | 21.72 | 11 | 8.322 | 11.029 | Biotin_carb_C |
| TRINITY_DN3363_c0_g2_i1 | -0.64165819 | 0.520030551 | 0.999970678 | 0.354 | 0.877 | 0.8 | 0.955 | NA |
| TRINITY_DN9718_c0_g1_i3 | -0.64164235 | 0.652597211 | 0.999970678 | 18.104 | 85.81 | 30.887 | 109.716 | NA |
| TRINITY_DN3807_c0_g1_i5 | 0.641633888 | 0.80896166 | 0.999970678 | 2.925 | 5.44 | 0 | 5.288 | NA |
| TRINITY_DN9139_c0_g1_i2 | -0.64148131 | 0.450520773 | 0.999970678 | 2.771 | 3.883 | 4.433 | 4.886 | - |
| TRINITY_DN3730_c1_g1_i1 | 0.641436571 | 0.395427958 | 0.999970678 | 5.632 | 7.204 | 4.278 | 3.056 | NA |
| TRINITY_DN654_c0_g2_i1 | -0.64133031 | 0.384917473 | 0.999970678 | 130.49 | 96.95 | 182.878 | 132.276 | NA |
| TRINITY_DN15729_c0_g1_i9 | -0.64105344 | 0.829946912 | 1 | 0 | 0.453 | 0.478 | 0.101 | O-FucT |
| TRINITY_DN2088_c0_g1_i1 | -0.64104603 | 0.824724354 | 0.999970678 | 21.865 | 0 | 4.233 | 29.387 | NA |
| TRINITY_DN15634_c0_g4_i1 | 0.640998371 | 0.531654528 | 0.999970678 | 22.692 | 33.7 | 6.933 | 27.648 | HLH |
| TRINITY_DN9353_c0_g1_i3 | -0.64092164 | 0.548423484 | 0.999970678 | 3.852 | 3.962 | 2.455 | 9.139 | NA |
| TRINITY_DN2915_c2_g1_i1 | -0.64088891 | 0.820033089 | 0.999970678 | 1.526 | 0 | 1.678 | 0.382 | NA |
| TRINITY_DN4405_c0_g1_i2 | 0.640819666 | 0.596934642 | 0.999970678 | 2.235 | 1.626 | 1.678 | 0.452 | NA |
| TRINITY_DN1174_c0_g1_i5 | 0.640815866 | 0.690507298 | 0.999970678 | 1.671 | 4.563 | 3.044 | 0.292 | NA |
| TRINITY_DN6703_c0_g1_i1 | -0.64077594 | 0.829107298 | 1 | 1.272 | 0 | 0.189 | 1.749 | NA |
| TRINITY_DN8609_c0_g1_i1 | 0.640666138 | 0.536951274 | 0.999970678 | 2.653 | 1.124 | 1.244 | 0.945 | NA |
| TRINITY_DN5676_c1_g1_i7 | 0.64049898 | 0.383040282 | 0.999970678 | 3.615 | 2.75 | 1.611 | 2.141 | NA |
| TRINITY_DN18386_c0_g1_i8 | -0.64042005 | 0.823246591 | 0.999970678 | 2.244 | 0 | 2.611 | 0.402 | NA |
| TRINITY_DN4925_c0_g1_i6 | 0.640326201 | 0.882506674 | 0.999970678 | 8.975 | 0 | 4.755 | 0 | NA |
| TRINITY_DN4031_c0_g1_i1 | 0.640231537 | 0.61401678 | 0.999970678 | 9.565 | 2.957 | 1.544 | 6.253 | NA |
| TRINITY_DN19926_c0_g1_i1 | 0.640207105 | 0.559915906 | 0.999970678 | 3.37 | 4.898 | 1.378 | 3.569 | NTF2 |
| TRINITY_DN84_c0_g1_i2 | -0.64017934 | 0.670781752 | 0.999970678 | 5.532 | 1.508 | 2.189 | 8.053 | NA |
| TRINITY_DN6733_c0_g1_i4 | 0.640157752 | 0.489634864 | 0.999970678 | 2.852 | 4.79 | 1.589 | 2.976 | Bax1-I |
| TRINITY_DN1086_c0_g1_i1 | 0.640143773 | 0.803533593 | 0.999970678 | 9.12 | 0.049 | 0.144 | 5.71 | NA |
| TRINITY_DN23710_c0_g1_i4 | -0.64006061 | 0.558618042 | 0.999970678 | 0.218 | 0.138 | 0.244 | 0.261 | Bromodomain |
| TRINITY_DN11115_c0_g1_i6 | 0.640028952 | 0.846670955 | 0.999970678 | 2.325 | 0.039 | 1.278 | 0 | NA |
| TRINITY_DN28_c0_g1_i3 | 0.640016704 | 0.648926479 | 0.999970678 | 8.83 | 2.651 | 0.889 | 6.384 | PP2C |
| TRINITY_DN1007_c0_g1_i8 | -0.63978547 | 0.429943628 | 0.999970678 | 10.256 | 9.363 | 8.333 | 20.53 | NA |
| TRINITY_DN10248_c0_g1_i1 | -0.63972825 | 0.814310535 | 0.999970678 | 0 | 1.064 | 0.7 | 0.754 | NA |
| TRINITY_DN3379_c1_g2_i1 | -0.63968983 | 0.87910805 | 0.999970678 | 9.702 | 0 | 0 | 15.262 | - |
| TRINITY_DN6018_c0_g1_i2 | 0.639689275 | 0.327542392 | 0.999970678 | 5.105 | 5.066 | 2.844 | 3.107 | WD40 |
| TRINITY_DN9285_c0_g1_i2 | 0.63946038 | 0.456095219 | 0.999970678 | 2.752 | 1.892 | 1.1 | 1.639 | B_lectin |
| TRINITY_DN8615_c0_g1_i1 | 0.639435344 | 0.737098285 | 0.999970678 | 3.379 | 0.453 | 1.889 | 0.161 | NA |
| TRINITY_DN13938_c0_g1_i1 | 0.639397305 | 0.493807384 | 0.999970678 | 0.699 | 0.522 | 0.322 | 0.392 | LRR_8 |
| TRINITY_DN2937_c0_g1_i8 | 0.639284642 | 0.660640305 | 0.999970678 | 27.552 | 18.51 | 2 | 27.145 | NA |
| TRINITY_DN466_c1_g1_i5 | 0.639199323 | 0.502229818 | 0.999970678 | 3.379 | 3.568 | 2.611 | 1.307 | Glyco_transf_5 |
| TRINITY_DN8885_c0_g1_i1 | 0.639177354 | 0.353793804 | 0.999970678 | 2.389 | 1.892 | 1.322 | 1.166 | Beach |
| TRINITY_DN1274_c0_g2_i1 | -0.63911139 | 0.820963583 | 1 | 0 | 0.384 | 0.322 | 0.181 | MatE |
| TRINITY_DN490_c1_g1_i2 | -0.63904624 | 0.507419615 | 0.999970678 | 434.28 | 683.8 | 367.711 | 1289.386 | - |
| TRINITY_DN2057_c0_g2_i6 | -0.63903686 | 0.723250271 | 0.999970678 | 1.217 | 1.823 | 3.766 | 0.151 | NA |
| TRINITY_DN1315_c0_g2_i3 | 0.639017558 | 0.61944093 | 0.999970678 | 6.45 | 2.001 | 3.466 | 1.287 | Prok-RING_4 |
| TRINITY_DN5261_c0_g1_i1 | -0.63901563 | 0.313748572 | 0.999970678 | 5.832 | 6.386 | 8.133 | 9.159 | Cation_efflux |
| TRINITY_DN5621_c0_g1_i3 | 0.639008582 | 0.442080592 | 0.999970678 | 4.669 | 9.638 | 4.389 | 3.851 | NA |
| TRINITY_DN3739_c0_g1_i2 | 0.638867307 | 0.698601255 | 0.999970678 | 0.772 | 0.365 | 0.544 | 0.06 | Vta1 |
| TRINITY_DN42085_c0_g1_i1 | 0.638821346 | 0.737905863 | 1 | 0.645 | 0.296 | 0.467 | 0.05 | NA |
| TRINITY_DN1526_c0_g1_i8 | -0.63870854 | 0.615435768 | 0.999970678 | 5.75 | 1.301 | 4.133 | 6.072 | NA |
| TRINITY_DN19357_c0_g5_i1 | 0.638618608 | 0.323333465 | 0.999970678 | 6.123 | 5.529 | 3.266 | 3.559 | NA |
| TRINITY_DN12988_c0_g1_i7 | -0.63857788 | 0.331158508 | 0.999970678 | 107.22 | 165.1 | 180.534 | 199.767 | PetM |
| TRINITY_DN11438_c0_g2_i1 | 0.638554348 | 0.456834687 | 0.999970678 | 69.883 | 43.22 | 40.553 | 23.988 | NA |
| TRINITY_DN2167_c0_g1_i2 | 0.638515686 | 0.571209581 | 0.999970678 | 1.126 | 2.237 | 0.478 | 1.568 | NA |
| TRINITY_DN12937_c0_g1_i1 | -0.63851028 | 0.888599343 | 1 | 0 | 1.35 | 0 | 2.081 | NA |
| TRINITY_DN10094_c0_g1_i7 | 0.638507209 | 0.55394627 | 0.999970678 | 2.008 | 6.918 | 2.889 | 2.202 | NA |
| TRINITY_DN2123_c1_g1_i4 | 0.638473294 | 0.693012948 | 0.999970678 | 14.625 | 22.13 | 0.878 | 22.52 | PCI |
| TRINITY_DN292_c1_g1_i1 | -0.63831984 | 0.819069277 | 0.999970678 | 1.844 | 0 | 0.489 | 2.322 | Peptidase_M14 |
| TRINITY_DN4900_c0_g1_i1 | -0.63826409 | 0.407395194 | 0.999970678 | 8.684 | 12.44 | 16.31 | 12.919 | NA |
| TRINITY_DN3180_c0_g1_i1 | 0.638229273 | 0.42759952 | 0.999970678 | 5.478 | 10.51 | 4.311 | 4.997 | NA |
| TRINITY_DN2546_c0_g1_i1 | -0.63819005 | 0.529631148 | 0.999970678 | 2.671 | 6.958 | 4.4 | 9.541 | NA |
| TRINITY_DN18641_c0_g1_i10 | -0.63814671 | 0.847019375 | 0.999970678 | 1.671 | 0 | 2.133 | 0.05 | NA |
| TRINITY_DN4373_c0_g1_i2 | -0.63800313 | 0.544047855 | 0.999970678 | 2.989 | 4.79 | 2.822 | 8.606 | NA |
| TRINITY_DN72_c0_g1_i9 | -0.63783551 | 0.442407133 | 0.999970678 | 16.642 | 13.15 | 26.076 | 14.94 | NA |
| TRINITY_DN1840_c0_g1_i1 | 0.637805323 | 0.617687716 | 0.999970678 | 6.05 | 5.972 | 5.566 | 0.995 | NA |
| TRINITY_DN5277_c3_g1_i3 | -0.63778983 | 0.742115763 | 0.999970678 | 0.536 | 0.315 | 1.055 | 0.05 | NA |
| TRINITY_DN17197_c0_g1_i7 | 0.637788921 | 0.75683248 | 0.999970678 | 1.363 | 0.079 | 0.133 | 0.754 | NA |
| TRINITY_DN713_c0_g1_i4 | -0.63777095 | 0.881734484 | 0.999970678 | 0.999 | 0 | 0 | 1.578 | NA |
| TRINITY_DN9494_c0_g1_i4 | 0.637756608 | 0.615206639 | 0.999970678 | 0.5 | 1.922 | 0.756 | 0.653 | NA |
| TRINITY_DN8422_c0_g1_i2 | 0.63767674 | 0.66833988 | 0.999970678 | 0.763 | 6.692 | 1.255 | 3.217 | ETF |
| TRINITY_DN193452_c0_g1_i1 | 0.637649087 | 0.885333622 | 0.999970678 | 0.872 | 0 | 0.467 | 0 | Fer4_20 |
| TRINITY_DN110974_c0_g1_i1 | -0.63755649 | 0.88137351 | 0.999970678 | 1.926 | 0 | 2.511 | 0 | NA |
| TRINITY_DN59_c0_g3_i4 | 0.637551245 | 0.531266815 | 0.999970678 | 4.378 | 3.508 | 3.155 | 1.277 | NA |
| TRINITY_DN19369_c0_g2_i10 | 0.637543704 | 0.886016991 | 1 | 0.745 | 0 | 0 | 0.493 | EF_assoc_1 |
| TRINITY_DN2858_c0_g1_i6 | -0.63752997 | 0.851502148 | 0.999970678 | 9.838 | 0 | 12.755 | 0.04 | F_bP_aldolase |
| TRINITY_DN7658_c0_g1_i1 | 0.637446102 | 0.556551509 | 0.999970678 | 8.848 | 34.28 | 8.611 | 17.121 | GDPD |

| TRINITY_DN6503_c0_g1_i1 | -0.63742784 | 0.429757957 | 0.999970678 | 2.026 | 2.375 | 3.389 | 2.755 | Branch |
| --- | --- | --- | --- | --- | --- | --- | --- | --- |
| TRINITY_DN2906_c0_g1_i9 | 0.637380941 | 0.529352015 | 0.999970678 | 2.807 | 2.7 | 0.822 | 2.554 | - |
| TRINITY_DN3837_c0_g1_i2 | 0.637376529 | 0.343161509 | 0.999970678 | 6.931 | 8.85 | 4.811 | 4.343 | RRM_1 |
| TRINITY_DN128_c0_g1_i1 | -0.63735686 | 0.557171476 | 0.999970678 | 12.99 | 7.027 | 6.355 | 23.355 | HSP20 |
| TRINITY_DN16228_c0_g1_i1 | -0.63722535 | 0.545298253 | 0.999970678 | 5.732 | 4.987 | 10.788 | 3.68 | NA |
| TRINITY_DN344_c0_g1_i7 | 0.637195274 | 0.882967753 | 0.999970678 | 5.378 | 0 | 0 | 3.448 | NA |
| TRINITY_DN693_c0_g2_i3 | -0.63715458 | 0.565243039 | 0.999970678 | 0.572 | 1.252 | 1.011 | 1.609 | NA |
| TRINITY_DN829_c0_g5_i2 | 0.637143648 | 0.704473816 | 1 | 1.272 | 4.918 | 0.689 | 3.066 | Arf |
| TRINITY_DN14_c0_g1_i2 | 0.63710561 | 0.34098251 | 0.999970678 | 8.021 | 5.716 | 3.589 | 4.544 | Gelsolin |
| TRINITY_DN476_c0_g4_i1 | 0.637075695 | 0.521985433 | 0.999970678 | 13.008 | 6.761 | 3.611 | 8.294 | NA |
| TRINITY_DN12595_c0_g1_i1 | 0.637073935 | 0.88555816 | 1 | 0 | 0.66 | 0.344 | 0 | PHD |
| TRINITY_DN78564_c0_g1_i5 | 0.637073935 | 0.88555816 | 1 | 0 | 3.607 | 1.911 | 0 | NA |
| TRINITY_DN843_c1_g1_i8 | -0.63702333 | 0.371378358 | 0.999970678 | 117.29 | 70.41 | 113.393 | 155.852 | NAM |
| TRINITY_DN1459_c0_g1_i7 | 0.636918844 | 0.645824027 | 0.999970678 | 7.331 | 1.577 | 3.833 | 1.156 | NA |
| TRINITY_DN8287_c0_g1_i2 | 0.636870629 | 0.287985693 | 0.999970678 | 16.397 | 17.11 | 9.988 | 9.521 | Ge1_WD40 |
| TRINITY_DN4543_c4_g1_i1 | -0.6368702 | 0.516494615 | 0.999970678 | 3.443 | 4.228 | 2.778 | 8.526 | NA |
| TRINITY_DN715_c0_g1_i6 | -0.6368296 | 0.630058008 | 0.999970678 | 2.553 | 5.874 | 1.733 | 10.959 | NA |
| TRINITY_DN8359_c0_g2_i4 | 0.636758351 | 0.615972071 | 0.999970678 | 1.354 | 1.084 | 1.044 | 0.342 | BRAP2 |
| TRINITY_DN3752_c0_g1_i1 | -0.63674779 | 0.816341281 | 0.999970678 | 6.395 | 0 | 3.078 | 6.394 | Brix |
| TRINITY_DN21137_c0_g1_i1 | -0.63671186 | 0.882108472 | 0.999970678 | 2.38 | 0 | 3.066 | 0 | NA |
| TRINITY_DN397_c0_g1_i13 | 0.636671721 | 0.564300782 | 0.999970678 | 4.724 | 2.602 | 3.033 | 1.076 | PUNUT |
| TRINITY_DN22296_c0_g1_i1 | -0.63662016 | 0.61466755 | 0.999970678 | 0.436 | 0.769 | 1.022 | 0.643 | NA |
| TRINITY_DN5566_c1_g1_i2 | -0.63660846 | 0.832819753 | 0.999970678 | 0 | 1.784 | 2.167 | 0.111 | NA |
| TRINITY_DN11182_c0_g1_i3 | 0.636571495 | 0.677559523 | 0.999970678 | 6.477 | 1.005 | 1.978 | 2.453 | Ank |
| TRINITY_DN577_c0_g1_i3 | -0.63656796 | 0.639458928 | 0.999970678 | 11.528 | 10.45 | 5.4 | 25.496 | NA |
| TRINITY_DN21826_c0_g2_i2 | 0.636544611 | 0.817378582 | 0.999970678 | 0.936 | 4.415 | 0 | 3.358 | LRRNT_2 |
| TRINITY_DN3049_c0_g1_i2 | -0.63649705 | 0.632786846 | 0.999970678 | 122.56 | 27.89 | 156.99 | 46.167 | - |
| TRINITY_DN254_c0_g1_i8 | -0.63649695 | 0.817188818 | 0.999970678 | 3.061 | 0 | 2.489 | 1.84 | PGI |
| TRINITY_DN491_c0_g1_i3 | -0.63649635 | 0.526727973 | 0.999970678 | 1.308 | 0.542 | 1.211 | 1.438 | NA |
| TRINITY_DN16560_c0_g1_i1 | -0.63636984 | 0.882412589 | 0.999970678 | 2.762 | 0 | 3.566 | 0 | NA |
| TRINITY_DN9349_c0_g1_i2 | 0.636324344 | 0.491735568 | 0.999970678 | 1.626 | 1.725 | 0.544 | 1.508 | NA |
| TRINITY_DN2101_c0_g1_i3 | 0.636290598 | 0.404235526 | 0.999970678 | 10.801 | 6.613 | 5.066 | 5.117 | NA |
| TRINITY_DN15979_c0_g1_i3 | 0.636076223 | 0.476280564 | 0.999970678 | 13.953 | 8.288 | 6.822 | 5.962 | ABC2_membrane |
| TRINITY_DN12966_c0_g1_i2 | -0.63605975 | 0.521836563 | 0.999970678 | 0.808 | 0.838 | 0.822 | 1.568 | NA |
| TRINITY_DN8337_c0_g2_i2 | -0.63605167 | 0.815836691 | 0.999970678 | 2.752 | 0 | 1.233 | 2.865 | NA |
| TRINITY_DN338_c2_g1_i2 | 0.635980772 | 0.812184683 | 0.999970678 | 1.989 | 4.711 | 0 | 4.243 | NA |
| TRINITY_DN5937_c0_g1_i3 | 0.635677384 | 0.603638896 | 0.999970678 | 1.59 | 3.459 | 0.989 | 2.001 | NA |
| TRINITY_DN6177_c0_g1_i4 | -0.63558783 | 0.517469499 | 0.999970678 | 2.407 | 4.326 | 5.877 | 3.328 | - |
| TRINITY_DN1343_c0_g1_i11 | 0.635516511 | 0.821914001 | 0.999970678 | 1.544 | 0 | 0.322 | 0.613 | NA |
| TRINITY_DN3678_c0_g1_i1 | -0.6353842 | 0.553638072 | 0.999970678 | 3.143 | 2.07 | 3.755 | 3.499 | NA |
| TRINITY_DN687_c0_g2_i2 | 0.635381285 | 0.825079315 | 0.999970678 | 4.36 | 0.473 | 2.622 | 0 | NA |
| TRINITY_DN9567_c5_g1_i1 | 0.635375361 | 0.684971789 | 0.999970678 | 0.236 | 2.099 | 0.778 | 0.543 | Glyco_tranf_2_3 |
| TRINITY_DN5362_c0_g1_i3 | 0.635330243 | 0.639363747 | 0.999970678 | 9.275 | 4.78 | 6.633 | 1.076 | PB1 |
| TRINITY_DN1528_c1_g1_i8 | 0.635302118 | 0.612066033 | 0.999970678 | 2.544 | 1.074 | 1.467 | 0.563 | zf-C2H2 |
| TRINITY_DN940_c0_g1_i3 | 0.63527268 | 0.391167679 | 0.999970678 | 6.686 | 5.529 | 2.889 | 4.373 | 40S_SA_C |
| TRINITY_DN3280_c0_g1_i3 | -0.63525659 | 0.526673642 | 0.999970678 | 1.935 | 1.025 | 1.344 | 2.986 | NA |
| TRINITY_DN1544_c0_g1_i10 | 0.635202327 | 0.594370575 | 0.999970678 | 11.373 | 2.69 | 3.978 | 4.303 | Motile_Sperm |
| TRINITY_DN3530_c0_g1_i7 | -0.6351904 | 0.689090537 | 0.999970678 | 0.391 | 0.049 | 0.244 | 0.402 | NA |
| TRINITY_DN401_c0_g1_i2 | -0.6351039 | 0.437665568 | 0.999970678 | 5.387 | 9.845 | 11.51 | 9.611 | - |
| TRINITY_DN32407_c1_g2_i3 | 0.635010082 | 0.757385395 | 1 | 0.454 | 0.414 | 0.433 | 0.02 | NA |
| TRINITY_DN32305_c0_g1_i5 | 0.635000222 | 0.884653122 | 0.999970678 | 3.461 | 0 | 1.867 | 0 | SUI1 |
| TRINITY_DN5933_c0_g1_i3 | -0.63498189 | 0.555474043 | 0.999970678 | 20.63 | 45.61 | 19.932 | 78.499 | DUF3675 |
| TRINITY_DN8464_c0_g1_i2 | -0.63497813 | 0.312475811 | 0.999970678 | 22.101 | 18.34 | 24.254 | 33.278 | NA |
| TRINITY_DN5104_c0_g2_i1 | -0.63492936 | 0.489052611 | 0.999970678 | 2.38 | 2.188 | 2.389 | 4.172 | KOW |
| TRINITY_DN1845_c0_g1_i12 | -0.63492433 | 0.812124493 | 0.999970678 | 0 | 0.759 | 0.5 | 0.553 | Cation_ATPase |
| TRINITY_DN1964_c0_g1_i4 | 0.634705152 | 0.654908352 | 0.999970678 | 3.87 | 0.788 | 1.778 | 0.905 | NA |
| TRINITY_DN42788_c1_g2_i4 | 0.634651665 | 0.628369054 | 0.999970678 | 0.263 | 0.384 | 0.244 | 0.111 | PPR |
| TRINITY_DN220_c0_g1_i19 | -0.63463899 | 0.650238701 | 0.999970678 | 2.425 | 1.34 | 3.855 | 1.126 | EGF_CA |
| TRINITY_DN2980_c0_g1_i2 | 0.634574845 | 0.546541201 | 0.999970678 | 312.5 | 1178 | 311.459 | 571.532 | NifU_N |
| TRINITY_DN1929_c0_g1_i3 | -0.63452999 | 0.321451294 | 0.999970678 | 6.059 | 8.229 | 9.522 | 10.647 | PRC |
| TRINITY_DN42306_c0_g1_i3 | -0.63452132 | 0.837868588 | 1 | 0.772 | 0.079 | 1.111 | 0 | NA |
| TRINITY_DN13956_c0_g1_i9 | -0.63451725 | 0.88406906 | 0.999970678 | 4.76 | 0 | 6.155 | 0 | NA |
| TRINITY_DN3841_c0_g1_i1 | -0.63436089 | 0.344121899 | 0.999970678 | 8.957 | 6.357 | 9.733 | 12.095 | B3 |
| TRINITY_DN9301_c0_g1_i7 | 0.634252219 | 0.822216314 | 0.999970678 | 0 | 2.237 | 0.3 | 1.056 | DRMBL |
| TRINITY_DN3621_c0_g1_i3 | 0.634248081 | 0.661734514 | 0.999970678 | 4.478 | 3.637 | 3.544 | 0.935 | NA |
| TRINITY_DN2445_c1_g2_i1 | 0.634188753 | 0.738389926 | 0.999970678 | 3.652 | 0.306 | 1.767 | 0.442 | NA |
| TRINITY_DN16528_c0_g1_i2 | 0.634078885 | 0.736282065 | 1 | 1.135 | 0.315 | 0.7 | 0.111 | Grp1_Fun34_Yaa |
| TRINITY_DN4477_c0_g1_i1 | -0.63406834 | 0.517803541 | 0.999970678 | 15.779 | 7.569 | 20.099 | 11.924 | NA |
| TRINITY_DN145_c0_g1_i3 | -0.63405694 | 0.813005081 | 0.999970678 | 0 | 2.02 | 0.989 | 1.89 | FoP_duplication |
| TRINITY_DN5783_c0_g1_i4 | 0.633939795 | 0.719716399 | 0.999970678 | 0.936 | 8.101 | 4.2 | 0.714 | Patatin |
| TRINITY_DN710_c0_g1_i3 | -0.63391198 | 0.654602097 | 0.999970678 | 70.255 | 631.2 | 246.618 | 767.479 | NA |
| TRINITY_DN6078_c0_g1_i4 | -0.63382658 | 0.423827916 | 0.999970678 | 7.14 | 12.33 | 9.488 | 18.72 | RNase_T |
| TRINITY_DN2989_c0_g1_i3 | -0.633796 | 0.880451044 | 0.999970678 | 2.752 | 0 | 0 | 4.353 | - |
| TRINITY_DN705_c1_g1_i1 | 0.633791003 | 0.559577786 | 0.999970678 | 7.994 | 5.47 | 5.711 | 1.81 | NA |

| TRINITY_DN305_c0_g3_i3 | 0.633695914 | 0.389798339 | 0.999970678 | 32.648 | 20.53 | 16.177 | 14.819 | NA |
| --- | --- | --- | --- | --- | --- | --- | --- | --- |
| TRINITY_DN1056_c0_g1_i1 | 0.633687582 | 0.517503658 | 0.999970678 | 9.511 | 12.36 | 8.644 | 3.619 | NA |
| TRINITY_DN1698_c0_g2_i2 | 0.63368581 | 0.754636864 | 0.999970678 | 26.062 | 5.322 | 16.443 | 0.432 | NA |
| TRINITY_DN1287_c0_g1_i3 | 0.633612252 | 0.472940653 | 0.999970678 | 20.584 | 46.46 | 21.91 | 16.468 | NA |
| TRINITY_DN2164_c0_g1_i7 | 0.633493227 | 0.885683513 | 1 | 15.688 | 0 | 0 | 9.742 | NA |
| TRINITY_DN164_c0_g1_i3 | -0.63348887 | 0.823966069 | 0.999970678 | 14.044 | 0 | 13.321 | 6.022 | Glyco_hydro_18 |
| TRINITY_DN6394_c0_g1_i7 | 0.633462412 | 0.883339931 | 0.999970678 | 0 | 4.622 | 0 | 2.916 | NA |
| TRINITY_DN44013_c0_g1_i2 | 0.633414333 | 0.742694684 | 0.999970678 | 10.992 | 649.1 | 93.339 | 267.931 | NA |
| TRINITY_DN17351_c0_g4_i1 | -0.63337313 | 0.444965266 | 0.999970678 | 24.082 | 29.18 | 46.053 | 26.341 | NA |
| TRINITY_DN7264_c0_g1_i5 | -0.63335135 | 0.562469163 | 0.999970678 | 18.032 | 77.65 | 51.952 | 84.089 | Annexin |
| TRINITY_DN98424_c0_g1_i1 | 0.633350367 | 0.408523184 | 0.999970678 | 1.081 | 1.518 | 0.711 | 0.814 | NA |
| TRINITY_DN28165_c0_g1_i2 | 0.633348413 | 0.418175097 | 0.999970678 | 15.797 | 10.74 | 8.933 | 6.384 | PIN_4 |
| TRINITY_DN909_c0_g1_i3 | -0.63332444 | 0.364230421 | 0.999970678 | 14.153 | 23.72 | 24.954 | 28.372 | NA |
| TRINITY_DN12080_c0_g1_i5 | 0.633259748 | 0.610777742 | 0.999970678 | 0.382 | 1.202 | 0.322 | 0.633 | NA |
| TRINITY_DN1624_c0_g1_i15 | -0.63320232 | 0.554030883 | 0.999970678 | 4.615 | 8.338 | 12.688 | 4.675 | NA |
| TRINITY_DN4847_c2_g3_i2 | 0.633065051 | 0.503003706 | 0.999970678 | 1.381 | 1.33 | 0.6 | 1.025 | NA |
| TRINITY_DN1186_c0_g1_i2 | 0.633062523 | 0.569811958 | 0.999970678 | 2.398 | 4.198 | 0.767 | 3.328 | NA |
| TRINITY_DN14270_c0_g2_i1 | 0.63304369 | 0.884145368 | 0.999970678 | 1.181 | 0 | 0.644 | 0 | NA |
| TRINITY_DN2179_c0_g1_i5 | -0.63290832 | 0.544291821 | 0.999970678 | 2.98 | 9.195 | 10.022 | 6.615 | NA |
| TRINITY_DN46566_c0_g1_i2 | 0.63286229 | 0.58640516 | 0.999970678 | 1.008 | 2.592 | 0.989 | 1.096 | Glyco_transf_9 |
| TRINITY_DN15882_c3_g1_i1 | -0.63267998 | 0.532586076 | 0.999970678 | 0.545 | 0.621 | 0.689 | 0.985 | DUF588 |
| TRINITY_DN4_c0_g1_i6 | 0.632601377 | 0.704160112 | 0.999970678 | 0.545 | 10.53 | 3.778 | 2.473 | - |
| TRINITY_DN3989_c0_g1_i3 | 0.632600769 | 0.844316043 | 0.999970678 | 31.985 | 0 | 0.367 | 20.499 | DUF760 |
| TRINITY_DN180_c0_g1_i1 | -0.63258502 | 0.633716416 | 0.999970678 | 9.738 | 3.331 | 13.021 | 4.434 | NA |
| TRINITY_DN351_c0_g1_i4 | 0.632424332 | 0.817361549 | 0.999970678 | 0.363 | 0.453 | 0.422 | 0 | NA |
| TRINITY_DN578_c0_g2_i2 | -0.63230174 | 0.48596716 | 0.999970678 | 2.398 | 1.478 | 2.2 | 3.368 | NA |
| TRINITY_DN2610_c0_g1_i1 | 0.632232518 | 0.331175559 | 0.999970678 | 79.512 | 100.7 | 39.586 | 68.415 | EGF_CA |
| TRINITY_DN9480_c0_g2_i2 | 0.632225699 | 0.528789727 | 0.999970678 | 0.572 | 0.71 | 0.333 | 0.422 | NA |
| TRINITY_DN14164_c0_g1_i2 | -0.63218486 | 0.733149938 | 0.999970678 | 68.311 | 8.614 | 77.64 | 20.53 | NA |
| TRINITY_DN9011_c1_g1_i1 | 0.632061397 | 0.791445015 | 1 | 0.064 | 0.936 | 0.5 | 0.03 | NA |
| TRINITY_DN250351_c0_g1_i1 | -0.63205417 | 0.406525251 | 0.999970678 | 8.602 | 14.61 | 12.455 | 20.741 | - |
| TRINITY_DN944_c0_g1_i7 | 0.631983666 | 0.647690378 | 0.999970678 | 2.662 | 0.542 | 0.511 | 1.458 | PPO1_DWL |
| TRINITY_DN5632_c0_g1_i4 | -0.63197251 | 0.761324797 | 0.999970678 | 0.936 | 3.124 | 0.1 | 6.072 | NA |
| TRINITY_DN2210_c0_g1_i4 | 0.631915554 | 0.813658984 | 0.999970678 | 6.468 | 3.164 | 0 | 6.183 | NA |
| TRINITY_DN1784_c1_g1_i1 | -0.63188886 | 0.341640087 | 0.999970678 | 94.782 | 142.4 | 167.323 | 162.287 | Redoxin |
| TRINITY_DN1848_c0_g1_i4 | 0.631864866 | 0.74243222 | 0.999970678 | 0.645 | 10.25 | 3.822 | 2.071 | NA |
| TRINITY_DN23547_c0_g1_i1 | 0.631801927 | 0.470854797 | 0.999970678 | 567.7 | 231.8 | 198.888 | 276.879 | NA |
| TRINITY_DN185_c1_g1_i9 | 0.631767914 | 0.36887955 | 0.999970678 | 6.059 | 5.962 | 3.922 | 3.036 | NA |
| TRINITY_DN2779_c0_g1_i2 | -0.63175199 | 0.390229964 | 0.999970678 | 9.711 | 11.07 | 9.888 | 20.188 | NA |
| TRINITY_DN29753_c0_g2_i1 | -0.63174471 | 0.824627966 | 0.999970678 | 0.227 | 0.897 | 0 | 1.709 | NA |
| TRINITY_DN18145_c0_g1_i4 | -0.63170066 | 0.624110559 | 0.999970678 | 1.254 | 0.936 | 0.478 | 2.845 | NA |
| TRINITY_DN8055_c0_g1_i5 | 0.63169371 | 0.615791658 | 0.999970678 | 0.463 | 1.606 | 0.489 | 0.724 | NA |
| TRINITY_DN21725_c0_g1_i1 | 0.631674175 | 0.540565014 | 0.999970678 | 10.274 | 3.824 | 3.789 | 4.524 | NA |
| TRINITY_DN1435_c0_g2_i3 | -0.63165735 | 0.382593049 | 0.999970678 | 279.76 | 482.7 | 417.697 | 674.824 | GTP_EFTU |
| TRINITY_DN10359_c0_g2_i3 | 0.631572152 | 0.824930067 | 0.999970678 | 0.118 | 0.729 | 0 | 0.553 | DUF4283 |
| TRINITY_DN30338_c0_g3_i1 | 0.631559346 | 0.884429546 | 0.999970678 | 0 | 31.52 | 16.543 | 0 | NA |
| TRINITY_DN27770_c0_g1_i5 | 0.631547495 | 0.662829512 | 0.999970678 | 0.609 | 4.09 | 0.733 | 2.141 | NA |
| TRINITY_DN8783_c0_g1_i2 | 0.631525671 | 0.544434035 | 0.999970678 | 2.171 | 6.948 | 1.822 | 3.66 | DUF3741 |
| TRINITY_DN6591_c1_g1_i1 | -0.63148779 | 0.467925525 | 0.999970678 | 30.44 | 17.48 | 40.664 | 25.305 | NdhO |
| TRINITY_DN3497_c0_g1_i1 | -0.63134316 | 0.740338074 | 0.999970678 | 0.082 | 4.504 | 3.955 | 2.222 | RabGAP-TBC |
| TRINITY_DN1429_c0_g1_i4 | 0.631326095 | 0.817978774 | 0.999970678 | 8.448 | 1.922 | 0 | 6.686 | NA |
| TRINITY_DN2269_c1_g1_i1 | 0.631279373 | 0.459411578 | 0.999970678 | 37.426 | 27 | 23.699 | 13.09 | ALIX_LYPXL_bnd |
| TRINITY_DN4494_c0_g1_i3 | -0.63126383 | 0.882843954 | 0.999970678 | 2.253 | 0 | 2.933 | 0 | Asp |
| TRINITY_DN2026_c0_g1_i4 | -0.6311235 | 0.831281135 | 0.999970678 | 0 | 34.55 | 8.077 | 43.01 | NA |
| TRINITY_DN640_c0_g2_i3 | 0.631072415 | 0.576104271 | 0.999970678 | 4.079 | 2.05 | 2.322 | 1.186 | AP2 |
| TRINITY_DN5030_c0_g1_i5 | 0.631062994 | 0.472221012 | 0.999970678 | 2.18 | 1.37 | 0.744 | 1.397 | NA |
| TRINITY_DN2629_c0_g1_i1 | -0.63104856 | 0.461085167 | 0.999970678 | 2.28 | 1.833 | 1.911 | 4.072 | bZIP_1 |
| TRINITY_DN15930_c0_g1_i1 | 0.631031025 | 0.735311099 | 1 | 0.109 | 0.522 | 0.044 | 0.352 | NA |
| TRINITY_DN6652_c0_g1_i4 | 0.630961808 | 0.494322748 | 0.999970678 | 1.208 | 1.547 | 0.944 | 0.633 | DEAD |
| TRINITY_DN2810_c0_g2_i2 | 0.630804644 | 0.83151759 | 0.999970678 | 5.251 | 0.601 | 3.155 | 0 | NA |
| TRINITY_DN15373_c2_g1_i4 | 0.630768221 | 0.75584473 | 0.999970678 | 2.398 | 0.079 | 0.756 | 0.714 | NA |
| TRINITY_DN241_c0_g1_i1 | 0.630628163 | 0.649304589 | 0.999970678 | 38.389 | 288.7 | 127.081 | 53.878 | NA |
| TRINITY_DN801_c3_g1_i6 | 0.630611011 | 0.821363568 | 0.999970678 | 2.453 | 0 | 0.767 | 0.694 | NA |
| TRINITY_DN705_c0_g1_i2 | 0.63059065 | 0.555160409 | 0.999970678 | 3.052 | 0.966 | 1.067 | 1.327 | NA |
| TRINITY_DN3203_c0_g1_i3 | 0.630580686 | 0.589971921 | 0.999970678 | 3.933 | 2.957 | 2.722 | 1.166 | NA |
| TRINITY_DN785_c1_g5_i1 | 0.630571279 | 0.585700068 | 0.999970678 | 3.897 | 6.13 | 2.489 | 3.378 | NA |
| TRINITY_DN7454_c0_g1_i5 | -0.6305582 | 0.847824481 | 0.999970678 | 4.315 | 0.128 | 0 | 6.796 | NA |
| TRINITY_DN1084_c0_g1_i8 | -0.63052215 | 0.848037373 | 0.999970678 | 0 | 13.12 | 16.355 | 0.191 | NA |
| TRINITY_DN13225_c1_g1_i4 | -0.63048931 | 0.848680215 | 0.999970678 | 1.036 | 0 | 1.322 | 0.01 | NA |
| TRINITY_DN15379_c0_g1_i8 | -0.63037475 | 0.311129687 | 0.999970678 | 4.933 | 5.736 | 7.355 | 7.631 | NA |
| TRINITY_DN5277_c3_g1_i1 | 0.630336791 | 0.584681095 | 0.999970678 | 0.927 | 1.892 | 0.378 | 1.357 | NA |
| TRINITY_DN11813_c0_g1_i5 | 0.630301623 | 0.64104787 | 0.999970678 | 9.039 | 2.75 | 1.089 | 6.344 | NA |
| TRINITY_DN18824_c0_g1_i1 | -0.63022909 | 0.734163454 | 1 | 0.064 | 0.463 | 0.511 | 0.181 | NA |
| TRINITY_DN4998_c0_g2_i4 | 0.630181849 | 0.88252677 | 0.999970678 | 17.859 | 0 | 0 | 11.713 | NA |

| TRINITY_DN10844_c0_g1_i1 | -0.63009984 | 0.50631224 | 0.999970678 | 3.924 | 3.489 | 2.644 | 8.294 | Nucleoside_tran |
| --- | --- | --- | --- | --- | --- | --- | --- | --- |
| TRINITY_DN3630_c0_g1_i1 | -0.63002111 | 0.342049758 | 0.999970678 | 43.185 | 61.03 | 57.896 | 89.85 | Ribosomal_L22e |
| TRINITY_DN14598_c0_g1_i2 | -0.62996346 | 0.887964039 | 1 | 0.927 | 0 | 1.2 | 0 | NA |
| TRINITY_DN6564_c0_g2_i6 | -0.62978242 | 0.772915147 | 0.999970678 | 0.645 | 1.518 | 0.022 | 3.288 | NA |
| TRINITY_DN3078_c0_g1_i1 | 0.629778543 | 0.27960265 | 0.999970678 | 45.538 | 55.97 | 26.132 | 33.921 | NA |
| TRINITY_DN1665_c0_g4_i1 | -0.62968484 | 0.404971384 | 0.999970678 | 118.57 | 208.2 | 168.823 | 296.835 | eIF-5a |
| TRINITY_DN3499_c0_g1_i6 | -0.62963004 | 0.884298488 | 0.999970678 | 0 | 1.153 | 1.467 | 0 | LRRNT_2 |
| TRINITY_DN1439_c0_g1_i6 | 0.629584569 | 0.669777907 | 1 | 0.945 | 0.976 | 0.833 | 0.282 | - |
| TRINITY_DN1036_c0_g1_i3 | -0.62958269 | 0.843862514 | 0.999970678 | 1.671 | 0 | 2.122 | 0.05 | NA |
| TRINITY_DN12292_c0_g1_i2 | 0.629542152 | 0.590783546 | 0.999970678 | 11.727 | 5.618 | 2.1 | 8.707 | PPI_Ypi1 |
| TRINITY_DN9926_c1_g2_i1 | -0.62953579 | 0.383293237 | 0.999970678 | 1649 | 1838 | 2839.973 | 1905.567 | NA |
| TRINITY_DN491_c3_g1_i3 | 0.629454985 | 0.525684964 | 0.999970678 | 62.198 | 205.7 | 81.806 | 72.608 | NA |
| TRINITY_DN29300_c0_g1_i1 | 0.629379866 | 0.7363433 | 1 | 2.053 | 3.095 | 2.511 | 0.241 | NA |
| TRINITY_DN9033_c0_g1_i7 | -0.62931512 | 0.821714135 | 0.999970678 | 8.811 | 0 | 9.122 | 2.584 | NA |
| TRINITY_DN2960_c0_g1_i1 | -0.62930771 | 0.584138555 | 0.999970678 | 10.047 | 12.44 | 5.244 | 28.17 | NA |
| TRINITY_DN2416_c0_g1_i11 | 0.629299719 | 0.730132641 | 1 | 0.318 | 0.532 | 0.044 | 0.503 | Pkinase |
| TRINITY_DN2417_c0_g1_i4 | 0.62928433 | 0.534361416 | 0.999970678 | 1.444 | 0.907 | 0.644 | 0.744 | NA |
| TRINITY_DN75_c2_g1_i1 | -0.62921234 | 0.455074007 | 0.999970678 | 5.269 | 6.593 | 10.377 | 5.791 | NA |
| TRINITY_DN5292_c0_g1_i8 | -0.62920733 | 0.82095902 | 0.999970678 | 0 | 2.158 | 2.044 | 0.794 | F-box |
| TRINITY_DN1716_c4_g1_i2 | -0.62917496 | 0.739965198 | 0.999970678 | 0.218 | 0.907 | 0.122 | 1.568 | Glyco_transf_8 |
| TRINITY_DN8814_c1_g1_i1 | -0.62916926 | 0.502052529 | 0.999970678 | 11.337 | 4.218 | 9.277 | 13.03 | Pkinase |
| TRINITY_DN1214_c0_g1_i1 | -0.62916868 | 0.826164393 | 0.999970678 | 13.935 | 0 | 9.777 | 10.114 | NA |
| TRINITY_DN4492_c0_g1_i3 | 0.629030918 | 0.659611914 | 0.999970678 | 3.879 | 4.691 | 1.022 | 4.112 | NA |
| TRINITY_DN627_c0_g2_i1 | 0.629019037 | 0.756429404 | 1 | 1.662 | 0.138 | 0.144 | 0.995 | NA |
| TRINITY_DN25959_c0_g1_i1 | 0.629013506 | 0.569879252 | 0.999970678 | 0.781 | 0.404 | 0.378 | 0.312 | FAR1 |
| TRINITY_DN55660_c0_g1_i1 | -0.62896501 | 0.594857789 | 1 | 0.327 | 0.325 | 0.456 | 0.462 | PPR |
| TRINITY_DN2214_c0_g1_i5 | -0.62892251 | 0.62042388 | 0.999970678 | 0.863 | 1.301 | 0.578 | 2.624 | NA |
| TRINITY_DN4212_c0_g1_i1 | -0.62888591 | 0.523204638 | 0.999970678 | 24.354 | 8.131 | 18.299 | 28.482 | NA |
| TRINITY_DN7316_c0_g1_i1 | 0.628831649 | 0.355415671 | 0.999970678 | 5.36 | 4.317 | 2.389 | 3.398 | DEK_C |
| TRINITY_DN12951_c0_g1_i1 | -0.62879898 | 0.886043638 | 0.999970678 | 0 | 1.153 | 1.444 | 0 | NA |
| TRINITY_DN517_c0_g1_i1 | -0.62867393 | 0.687293883 | 0.999970678 | 0.118 | 0.769 | 0.3 | 0.985 | UDPGP |
| TRINITY_DN13059_c0_g1_i1 | -0.6286314 | 0.419328569 | 0.999970678 | 13.099 | 13.78 | 21.21 | 15.422 | NA |
| TRINITY_DN1888_c0_g2_i1 | 0.628482184 | 0.74768399 | 0.999970678 | 0.418 | 11.06 | 1.3 | 5.54 | NA |
| TRINITY_DN9946_c0_g1_i14 | -0.62844163 | 0.329644392 | 0.999970678 | 25.662 | 33.5 | 42.609 | 39.581 | NA |
| TRINITY_DN7729_c0_g1_i5 | -0.62843574 | 0.884353799 | 0.999970678 | 0.781 | 0 | 1.011 | 0 | PHM7_cyt |
| TRINITY_DN5104_c0_g1_i1 | -0.62840529 | 0.819252747 | 0.999970678 | 0 | 3.164 | 3.055 | 1.166 | KOW |
| TRINITY_DN16064_c0_g1_i1 | -0.62839429 | 0.889216825 | 1 | 0.636 | 0 | 0.822 | 0 | NA |
| TRINITY_DN4690_c0_g1_i2 | 0.628328788 | 0.54753332 | 0.999970678 | 25.689 | 30.34 | 7.311 | 26.26 | NA |
| TRINITY_DN12701_c0_g1_i1 | 0.628283083 | 0.557227521 | 0.999970678 | 0.718 | 0.739 | 0.544 | 0.302 | NA |
| TRINITY_DN19195_c0_g2_i3 | -0.62827626 | 0.339247629 | 0.999970678 | 21.393 | 24.35 | 33.887 | 29.286 | NA |
| TRINITY_DN485_c0_g1_i9 | -0.62823361 | 0.752420178 | 0.999970678 | 0.936 | 69.78 | 15.344 | 89.267 | NA |
| TRINITY_DN2056_c0_g2_i1 | 0.628232175 | 0.632799774 | 1 | 0.709 | 0.542 | 0.2 | 0.573 | NA |
| TRINITY_DN116_c0_g1_i1 | -0.6281989 | 0.3965251 | 0.999970678 | 9.484 | 6.258 | 9.533 | 12.829 | TatD_DNase |
| TRINITY_DN9234_c0_g1_i1 | -0.6281289 | 0.63822621 | 1 | 0.781 | 0.463 | 0.522 | 1.247 | NA |
| TRINITY_DN2370_c0_g1_i9 | 0.628122285 | 0.641950349 | 0.999970678 | 1.826 | 4.78 | 2.811 | 0.865 | GST_C_2 |
| TRINITY_DN7007_c0_g1_i2 | 0.628094621 | 0.758355822 | 0.999970678 | 0.236 | 3.302 | 0.189 | 2.011 | NA |
| TRINITY_DN11568_c0_g1_i2 | -0.62803339 | 0.826601703 | 1 | 0 | 0.779 | 0.778 | 0.231 | NA |
| TRINITY_DN99387_c0_g1_i1 | 0.627884337 | 0.527509833 | 0.999970678 | 0.509 | 0.877 | 0.467 | 0.332 | - |
| TRINITY_DN1126_c1_g1_i7 | -0.62783882 | 0.487251239 | 0.999970678 | 0.681 | 0.887 | 0.778 | 1.518 | NA |
| TRINITY_DN4510_c0_g1_i1 | -0.62779009 | 0.355160632 | 0.999970678 | 4.887 | 5.618 | 7.277 | 7.44 | PRA1 |
| TRINITY_DN12686_c0_g1_i1 | 0.627735057 | 0.641776475 | 0.999970678 | 0.908 | 0.325 | 0.444 | 0.261 | NUDIX |
| TRINITY_DN3287_c0_g1_i7 | 0.62772374 | 0.722170664 | 0.999970678 | 4.778 | 2.385 | 3.711 | 0.181 | PPR |
| TRINITY_DN3898_c1_g1_i17 | 0.627718876 | 0.695033172 | 0.999970678 | 0.136 | 1.232 | 0.189 | 0.653 | MatE |
| TRINITY_DN11784_c0_g1_i2 | -0.62765415 | 0.800156003 | 0.999970678 | 6.186 | 0.158 | 8.177 | 0.07 | GMC_oxred_C |
| TRINITY_DN33822_c0_g5_i1 | -0.62759673 | 0.745141493 | 1 | 2.58 | 1.143 | 4.411 | 0.362 | Aldedh |
| TRINITY_DN1801_c0_g1_i2 | -0.62758896 | 0.412247911 | 0.999970678 | 4.024 | 5.509 | 4.933 | 8.707 | - |
| TRINITY_DN7164_c0_g1_i1 | -0.62757925 | 0.696008858 | 0.999970678 | 1.735 | 0.207 | 1.389 | 1.287 | NA |
| TRINITY_DN4256_c0_g1_i1 | -0.62751084 | 0.511045838 | 0.999970678 | 8.848 | 12.18 | 7.466 | 23.164 | - |
| TRINITY_DN4350_c0_g1_i1 | -0.62748732 | 0.587550862 | 0.999970678 | 1.908 | 2.424 | 1.433 | 4.906 | NA |
| TRINITY_DN6711_c0_g1_i4 | -0.62744682 | 0.519774144 | 0.999970678 | 4.142 | 2.72 | 2.689 | 7.339 | adh_short |
| TRINITY_DN1169_c1_g1_i10 | -0.62743027 | 0.58478811 | 0.999970678 | 15.697 | 6.573 | 21.876 | 8.073 | NA |
| TRINITY_DN9814_c0_g1_i1 | -0.62742315 | 0.601414693 | 0.999970678 | 0.291 | 0.512 | 0.633 | 0.483 | NA |
| TRINITY_DN5682_c1_g1_i2 | 0.62740663 | 0.4816824 | 0.999970678 | 4.251 | 8.052 | 2.322 | 5.137 | NA |
| TRINITY_DN5184_c0_g1_i7 | 0.627337103 | 0.589700666 | 0.999970678 | 1.263 | 2.503 | 1.155 | 0.985 | p450 |
| TRINITY_DN3027_c0_g1_i2 | 0.627242617 | 0.5231603 | 0.999970678 | 1.39 | 1.813 | 1.178 | 0.643 | ELYS |
| TRINITY_DN344_c1_g1_i4 | -0.62723348 | 0.561437919 | 0.999970678 | 24.027 | 89.7 | 63.374 | 90.272 | Pyrid_oxidase_2 |
| TRINITY_DN8336_c0_g1_i1 | 0.627216265 | 0.683922819 | 0.999970678 | 1.917 | 3.489 | 2.622 | 0.332 | WD40 |
| TRINITY_DN7020_c0_g1_i1 | 0.627196267 | 0.591696673 | 0.999970678 | 0.781 | 3.321 | 1.2 | 1.196 | RRM_1 |
| TRINITY_DN21706_c0_g1_i3 | 0.627185393 | 0.605202065 | 0.999970678 | 0.89 | 1.528 | 0.444 | 0.995 | 2OG-FeII_Oxy_2 |
| TRINITY_DN4948_c1_g1_i7 | 0.6271831 | 0.671388747 | 0.999970678 | 1.135 | 2.375 | 0.311 | 1.88 | NA |
| TRINITY_DN4383_c0_g1_i3 | 0.627017115 | 0.446067974 | 0.999970678 | 11.037 | 9.668 | 7.544 | 4.333 | CarD_CdnL_TRCF |
| TRINITY_DN14626_c0_g1_i1 | 0.626824382 | 0.70161943 | 0.999970678 | 2.471 | 0.867 | 0.278 | 1.759 | NA |
| TRINITY_DN616_c1_g6_i2 | -0.62679945 | 0.600946817 | 0.999970678 | 4.86 | 3.518 | 7.488 | 3.76 | NA |
| TRINITY_DN415_c0_g1_i17 | -0.62679658 | 0.362409091 | 0.999970678 | 13.971 | 14.61 | 14.777 | 26.009 | NA |

| TRINITY_DN3926_c0_g1_i1 | 0.626782017 | 0.685258601 | 0.999970678 | 3.361 | 0.71 | 1.878 | 0.382 | NA |
| --- | --- | --- | --- | --- | --- | --- | --- | --- |
| TRINITY_DN6769_c0_g1_i1 | -0.62677095 | 0.326491268 | 0.999970678 | 4.969 | 5.529 | 7.233 | 7.46 | NA |
| TRINITY_DN9892_c0_g2_i7 | 0.62672074 | 0.620798073 | 0.999970678 | 0.282 | 0.759 | 0.411 | 0.181 | NA |
| TRINITY_DN711_c0_g1_i7 | -0.62666163 | 0.643426469 | 0.999970678 | 108.48 | 16.39 | 117.082 | 53.234 | peroxidase |
| TRINITY_DN5815_c0_g1_i2 | -0.62651371 | 0.699209448 | 0.999970678 | 11.954 | 42.57 | 4.178 | 78.61 | zf-RING_11 |
| TRINITY_DN3127_c0_g2_i6 | -0.6265061 | 0.882821662 | 0.999970678 | 0 | 1.537 | 1.944 | 0 | 4HBT |
| TRINITY_DN1592_c0_g1_i3 | -0.62645899 | 0.88350085 | 0.999970678 | 34.165 | 0 | 43.02 | 0 | Pro_isomerase |
| TRINITY_DN17665_c0_g1_i1 | -0.62644032 | 0.88542394 | 0.999970678 | 1.626 | 0 | 2.1 | 0 | NA |
| TRINITY_DN926_c0_g1_i1 | 0.626430316 | 0.406531307 | 0.999970678 | 8.475 | 7.983 | 5.7 | 3.8 | WD40 |
| TRINITY_DN47168_c0_g1_i1 | -0.62639466 | 0.890757829 | 1 | 0.79 | 0 | 1.022 | 0 | NA |
| TRINITY_DN221786_c0_g1_i1 | -0.62639466 | 0.890757829 | 1 | 1.017 | 0 | 1.322 | 0 | DSBA |
| TRINITY_DN1564_c0_g1_i6 | 0.626323371 | 0.823223159 | 0.999970678 | 0.136 | 93.85 | 49.119 | 0.322 | CIAPIN1 |
| TRINITY_DN748_c1_g1_i1 | 0.626321766 | 0.822093081 | 0.999970678 | 0.391 | 1.804 | 1.155 | 0 | Thi4 |
| TRINITY_DN1258_c0_g1_i16 | 0.626004833 | 0.822464763 | 0.999970678 | 4.606 | 0 | 1.133 | 1.619 | - |
| TRINITY_DN1670_c0_g1_i5 | -0.62593102 | 0.824573469 | 0.999970678 | 9.229 | 0 | 2.167 | 11.863 | - |
| TRINITY_DN4681_c0_g1_i1 | 0.625912349 | 0.514046711 | 0.999970678 | 80.275 | 27.94 | 31.42 | 32.282 | NA |
| TRINITY_DN3439_c0_g3_i1 | 0.625874867 | 0.824583399 | 1 | 0.545 | 1.311 | 0.978 | 0 | NA |
| TRINITY_DN2946_c0_g2_i4 | 0.625850275 | 0.66638503 | 0.999970678 | 2.48 | 0.828 | 1.533 | 0.312 | Ank_2 |
| TRINITY_DN9748_c0_g1_i4 | -0.62583952 | 0.547719391 | 0.999970678 | 5.178 | 18.19 | 15.188 | 17.202 | Lin0512_fam |
| TRINITY_DN621_c0_g1_i5 | -0.62582665 | 0.666563331 | 0.999970678 | 1.535 | 0.345 | 1.733 | 0.834 | NA |
| TRINITY_DN3988_c0_g1_i15 | 0.625821821 | 0.707876319 | 0.999970678 | 6.659 | 0.552 | 3.278 | 0.784 | Phosphorylase |
| TRINITY_DN106792_c0_g1_i2 | -0.62581326 | 0.88224419 | 0.999970678 | 2.135 | 0 | 2.755 | 0 | NA |
| TRINITY_DN862_c0_g2_i3 | -0.62569251 | 0.823922592 | 1 | 1.544 | 0 | 0.844 | 1.438 | NA |
| TRINITY_DN452_c1_g2_i6 | -0.62560652 | 0.601963627 | 0.999970678 | 79.148 | 340 | 145.647 | 464.27 | FA_desaturase |
| TRINITY_DN13544_c0_g1_i2 | 0.625573883 | 0.737591488 | 0.999970678 | 0.491 | 0.581 | 0.544 | 0.04 | NA |
| TRINITY_DN1269_c0_g1_i5 | -0.62554395 | 0.693071512 | 0.999970678 | 47.99 | 18.01 | 78.495 | 6.686 | NA |
| TRINITY_DN525_c0_g1_i1 | -0.62551965 | 0.405261445 | 0.999970678 | 13.344 | 9.175 | 13.91 | 17.614 | NA |
| TRINITY_DN2826_c0_g2_i1 | -0.62529628 | 0.558183049 | 0.999970678 | 6.504 | 2.996 | 6.077 | 7.148 | NA |
| TRINITY_DN15932_c0_g1_i2 | -0.62522228 | 0.886073021 | 0.999970678 | 1.735 | 0 | 2.244 | 0 | NA |
| TRINITY_DN5046_c1_g2_i2 | -0.62521903 | 0.673650001 | 1 | 0.127 | 0.522 | 0.389 | 0.543 | NA |
| TRINITY_DN650_c0_g2_i1 | 0.625218389 | 0.364670444 | 0.999970678 | 14.952 | 10.17 | 7.355 | 7.47 | CwfJ_C_1 |
| TRINITY_DN2195_c1_g1_i4 | -0.62519841 | 0.590833747 | 0.999970678 | 20.984 | 10.32 | 32.087 | 9.631 | Cpn10 |
| TRINITY_DN57_c0_g1_i4 | 0.625140917 | 0.370609156 | 0.999970678 | 4.215 | 6.633 | 2.611 | 3.891 | NA |
| TRINITY_DN1178_c0_g1_i4 | 0.625085258 | 0.490121453 | 0.999970678 | 2.262 | 1.488 | 0.7 | 1.599 | Methyltr_RsmB-F |
| TRINITY_DN103_c0_g1_i11 | 0.624976121 | 0.497811433 | 0.999970678 | 44.239 | 17.83 | 19.899 | 16.448 | NA |
| TRINITY_DN543_c0_g1_i1 | -0.62493416 | 0.254468027 | 0.999970678 | 89.677 | 88.04 | 112.271 | 138.731 | PTR2 |
| TRINITY_DN3275_c0_g1_i10 | -0.62489822 | 0.694965655 | 0.999970678 | 1.108 | 3.568 | 5.422 | 0.593 | NA |
| TRINITY_DN10011_c0_g1_i6 | -0.62487677 | 0.630984409 | 0.999970678 | 2.198 | 0.848 | 2.944 | 1.136 | NA |
| TRINITY_DN37821_c0_g3_i1 | -0.62482036 | 0.882501699 | 0.999970678 | 7.249 | 0 | 9.344 | 0 | NA |
| TRINITY_DN13364_c0_g1_i1 | -0.62481834 | 0.531262726 | 0.999970678 | 21.538 | 9.254 | 26.832 | 15.241 | NA |
| TRINITY_DN250320_c0_g1_i1 | -0.62476106 | 0.503709676 | 0.999970678 | 37.498 | 33.19 | 23.321 | 81.133 | GRAS |
| TRINITY_DN17454_c0_g1_i1 | -0.62472579 | 0.550111271 | 0.999970678 | 295.73 | 259.8 | 146.558 | 653.48 | NA |
| TRINITY_DN6736_c1_g1_i2 | -0.62460943 | 0.681837806 | 0.999970678 | 12.481 | 0.838 | 8.677 | 10.375 | DUF3506 |
| TRINITY_DN21608_c0_g1_i4 | -0.62454456 | 0.529549528 | 0.999970678 | 1.908 | 0.798 | 1.878 | 1.93 | IF-2B |
| TRINITY_DN510_c0_g1_i8 | 0.624415799 | 0.882523354 | 0.999970678 | 9.22 | 0 | 4.977 | 0 | NA |
| TRINITY_DN26265_c0_g1_i4 | -0.62439648 | 0.596461363 | 0.999970678 | 2.08 | 4.021 | 2.555 | 6.153 | NA |
| TRINITY_DN22211_c0_g1_i3 | 0.624326386 | 0.888272003 | 1 | 0 | 0.995 | 0 | 0.633 | NA |
| TRINITY_DN961_c0_g1_i4 | -0.62428571 | 0.821031315 | 0.999970678 | 4.769 | 0 | 4.633 | 1.82 | ParA |
| TRINITY_DN92_c1_g1_i1 | -0.62426926 | 0.560536447 | 0.999970678 | 19.612 | 5.273 | 15.888 | 19.414 | Gly-zipper_Omp |
| TRINITY_DN1618_c0_g1_i1 | -0.62413253 | 0.821174675 | 0.999970678 | 5.786 | 0 | 5.177 | 2.805 | NA |
| TRINITY_DN22574_c0_g1_i1 | -0.62393535 | 0.53903618 | 0.999970678 | 0.481 | 0.769 | 0.933 | 0.794 | Glyco_hydro_17 |
| TRINITY_DN17117_c0_g1_i2 | -0.62393351 | 0.670246164 | 0.999970678 | 1.054 | 7.096 | 7.233 | 3.579 | NA |
| TRINITY_DN3685_c0_g1_i1 | 0.62386987 | 0.555789787 | 0.999970678 | 5.55 | 1.961 | 2.422 | 1.981 | NA |
| TRINITY_DN19299_c0_g1_i1 | 0.623815985 | 0.71561898 | 0.999970678 | 1.354 | 1.35 | 1.355 | 0.111 | MOR2-PAG1_C |
| TRINITY_DN19417_c0_g1_i15 | -0.62375777 | 0.891712447 | 1 | 0.418 | 0 | 0.544 | 0 | Aft1_HRA |
| TRINITY_DN22565_c0_g1_i2 | -0.62375777 | 0.891712447 | 1 | 0.818 | 0 | 1.033 | 0 | Catalase |
| TRINITY_DN17015_c0_g1_i4 | -0.62374316 | 0.605795776 | 0.999970678 | 0.336 | 0.187 | 0.378 | 0.342 | NA |
| TRINITY_DN3831_c1_g1_i7 | 0.623738592 | 0.643707986 | 0.999970678 | 5.469 | 2.395 | 3.622 | 0.784 | NA |
| TRINITY_DN41892_c0_g1_i8 | 0.623728082 | 0.820769089 | 0.999970678 | 1.308 | 0.473 | 0.978 | 0 | NA |
| TRINITY_DN4120_c0_g1_i1 | -0.62367778 | 0.674634254 | 0.999970678 | 1.063 | 14.09 | 9.188 | 11.934 | Aa_trans |
| TRINITY_DN17313_c0_g1_i2 | 0.623661842 | 0.514589453 | 0.999970678 | 1.19 | 2.03 | 1.055 | 0.804 | NA |
| TRINITY_DN63_c0_g2_i1 | -0.62361744 | 0.818338601 | 0.999970678 | 0 | 2.286 | 0.933 | 2.282 | zf-CCHC |
| TRINITY_DN3777_c0_g1_i3 | 0.623575646 | 0.538670704 | 0.999970678 | 1.426 | 1.202 | 0.833 | 0.694 | NA |
| TRINITY_DN11064_c0_g1_i10 | -0.62356753 | 0.830479355 | 0.999970678 | 1.49 | 0.384 | 2.4 | 0 | NA |
| TRINITY_DN31515_c0_g4_i1 | 0.623565163 | 0.678158399 | 1 | 0.59 | 0.798 | 0.133 | 0.734 | NA |
| TRINITY_DN9458_c0_g1_i1 | -0.62355289 | 0.654615408 | 0.999970678 | 0.709 | 5.923 | 3.211 | 6.223 | NA |
| TRINITY_DN4609_c0_g1_i6 | 0.623521376 | 0.816605983 | 0.999970678 | 1.49 | 1.833 | 1.789 | 0 | NA |
| TRINITY_DN13056_c0_g1_i1 | -0.62350682 | 0.733196842 | 0.999970678 | 0.3 | 8.988 | 2.9 | 10.556 | DUF538 |
| TRINITY_DN5773_c0_g1_i4 | -0.62350313 | 0.818499281 | 0.999970678 | 8.23 | 0 | 4.644 | 7.249 | PNP_UDP_1 |
| TRINITY_DN34727_c0_g1_i8 | 0.623431016 | 0.64470618 | 0.999970678 | 1.017 | 0.66 | 0.178 | 0.875 | PORR |
| TRINITY_DN5784_c0_g1_i1 | 0.62339029 | 0.491292303 | 0.999970678 | 16.469 | 7.116 | 7.4 | 6.465 | Pkinase |
| TRINITY_DN579_c0_g1_i7 | -0.62338549 | 0.474406515 | 0.999970678 | 53.504 | 106.6 | 72.396 | 152.706 | NA |
| TRINITY_DN16241_c1_g2_i1 | -0.62326136 | 0.496292105 | 0.999970678 | 61.435 | 61.83 | 115.104 | 50.268 | Psb28 |
| TRINITY_DN3759_c0_g1_i2 | -0.62322931 | 0.409738911 | 0.999970678 | 77.913 | 92.35 | 141.591 | 90.544 | NA |

| TRINITY_DN4692_c0_g1_i3 | 0.62307939 | 0.555736726 | 0.999970678 | 0.572 | 0.71 | 0.5 | 0.231 | NA |
| --- | --- | --- | --- | --- | --- | --- | --- | --- |
| TRINITY_DN13087_c0_g1_i3 | -0.62303454 | 0.817014185 | 0.999970678 | 0.927 | 0 | 0.611 | 0.704 | PIG-S |
| TRINITY_DN78564_c0_g1_i7 | -0.62303005 | 0.823865758 | 0.999970678 | 0 | 3.587 | 3.633 | 1.066 | NA |
| TRINITY_DN28264_c1_g2_i2 | -0.6230274 | 0.851112854 | 1 | 0.727 | 0.02 | 0.967 | 0 | Fasciclin |
| TRINITY_DN7491_c0_g1_i5 | 0.623009284 | 0.74763 | 0.999970678 | 8.285 | 1.419 | 0.522 | 5.429 | NA |
| TRINITY_DN3956_c0_g1_i8 | 0.622964617 | 0.834957558 | 1 | 0.109 | 0.897 | 0 | 0.664 | NA |
| TRINITY_DN8608_c0_g1_i2 | -0.62295792 | 0.469788495 | 0.999970678 | 0.981 | 1.646 | 1.678 | 2.001 | NA |
| TRINITY_DN4072_c0_g2_i1 | 0.622728618 | 0.693675965 | 0.999970678 | 5.051 | 0.739 | 1.078 | 2.463 | NA |
| TRINITY_DN2048_c0_g1_i5 | -0.6227127 | 0.272423252 | 0.999970678 | 45.865 | 46.61 | 58.085 | 71.18 | Ribosomal_S8 |
| TRINITY_DN10794_c0_g1_i5 | -0.6226624 | 0.705054161 | 0.999970678 | 0.173 | 1.143 | 0.467 | 1.408 | NA |
| TRINITY_DN682_c0_g2_i3 | -0.6226452 | 0.717468934 | 0.999970678 | 1.172 | 0.394 | 0.2 | 2.162 | DUF247 |
| TRINITY_DN8246_c0_g1_i2 | 0.622638378 | 0.712087808 | 0.999970678 | 0.409 | 1.537 | 0.955 | 0.101 | - |
| TRINITY_DN11910_c0_g1_i4 | -0.62262986 | 0.883552049 | 0.999970678 | 1.263 | 0 | 0 | 1.971 | CBM49 |
| TRINITY_DN2713_c0_g1_i1 | -0.62260457 | 0.644428373 | 0.999970678 | 0.709 | 0.798 | 0.356 | 1.87 | NA |
| TRINITY_DN831_c0_g1_i1 | -0.62250314 | 0.598754526 | 0.999970678 | 5.877 | 11.65 | 4.289 | 21.595 | NA |
| TRINITY_DN14894_c0_g1_i5 | 0.622473336 | 0.746726256 | 0.999970678 | 11.964 | 17 | 15.332 | 0.221 | NA |
| TRINITY_DN22177_c0_g1_i3 | -0.62246072 | 0.751836666 | 0.999970678 | 0.881 | 0.857 | 0.056 | 2.634 | NA |
| TRINITY_DN1464_c0_g1_i1 | 0.622329822 | 0.644044078 | 0.999970678 | 0.4 | 1.646 | 0.678 | 0.503 | NA |
| TRINITY_DN18748_c0_g1_i2 | 0.622256542 | 0.708218426 | 0.999970678 | 32.057 | 2.572 | 15.943 | 3.509 | Pkinase |
| TRINITY_DN386_c0_g1_i1 | -0.62222138 | 0.683756948 | 0.999970678 | 1.163 | 0.335 | 1.678 | 0.302 | NA |
| TRINITY_DN3715_c0_g1_i12 | -0.6221902 | 0.884092153 | 0.999970678 | 6.931 | 0 | 8.833 | 0 | Peroxidase_2 |
| TRINITY_DN20205_c0_g1_i7 | -0.62215553 | 0.887677167 | 0.999970678 | 1.317 | 0 | 1.689 | 0 | Fasciclin |
| TRINITY_DN14986_c1_g1_i1 | -0.62214617 | 0.823655843 | 1 | 0 | 0.946 | 0.456 | 0.875 | NA |
| TRINITY_DN6504_c0_g1_i1 | 0.622108571 | 0.576247291 | 0.999970678 | 0.5 | 0.71 | 0.3 | 0.432 | NA |
| TRINITY_DN6469_c0_g1_i8 | 0.622041736 | 0.825594174 | 0.999970678 | 3.034 | 0 | 0.856 | 0.965 | NA |
| TRINITY_DN9741_c0_g1_i1 | 0.622017828 | 0.459992715 | 0.999970678 | 3.779 | 3.706 | 1.378 | 3.207 | NA |
| TRINITY_DN381_c0_g1_i17 | 0.622009496 | 0.824605028 | 1 | 1.553 | 0.749 | 1.244 | 0 | NA |
| TRINITY_DN2167_c0_g1_i5 | 0.621968664 | 0.753164329 | 0.999970678 | 0.236 | 2.168 | 1.211 | 0.09 | NA |
| TRINITY_DN6576_c0_g6_i1 | 0.621932988 | 0.643240462 | 0.999970678 | 1.254 | 0.838 | 0.311 | 0.975 | NA |
| TRINITY_DN2705_c0_g1_i2 | 0.621895902 | 0.823905191 | 0.999970678 | 0 | 6.298 | 0.989 | 2.805 | NA |
| TRINITY_DN4699_c0_g1_i2 | 0.621890166 | 0.554852806 | 0.999970678 | 12.454 | 6.12 | 2.755 | 8.787 | Dehydrin |
| TRINITY_DN357_c0_g1_i6 | 0.621874296 | 0.555503693 | 0.999970678 | 17.16 | 32.66 | 19.832 | 8.093 | NA |
| TRINITY_DN1561_c0_g1_i1 | 0.621845759 | 0.645901379 | 0.999970678 | 0.999 | 5.608 | 2.033 | 1.78 | NA |
| TRINITY_DN14513_c1_g1_i8 | 0.621684807 | 0.827294375 | 0.999970678 | 1.081 | 0 | 0.2 | 0.462 | NA |
| TRINITY_DN3815_c0_g2_i2 | -0.62158736 | 0.577881495 | 0.999970678 | 7.767 | 14.05 | 9.744 | 19.756 | NA |
| TRINITY_DN74604_c0_g2_i1 | 0.621578414 | 0.36228429 | 0.999970678 | 346.37 | 345.6 | 141.18 | 278.487 | Glyco_hydro_16 |
| TRINITY_DN51116_c0_g1_i1 | 0.621570943 | 0.671287384 | 0.999970678 | 47.927 | 545.2 | 99.572 | 249.191 | NA |
| TRINITY_DN5726_c0_g1_i1 | -0.62151658 | 0.505651418 | 0.999970678 | 4.506 | 4.967 | 3.766 | 9.983 | F-box-like |
| TRINITY_DN88577_c0_g1_i1 | -0.62138749 | 0.34752186 | 0.999970678 | 29.15 | 21.73 | 32.065 | 38.968 | - |
| TRINITY_DN5739_c0_g1_i2 | -0.621385 | 0.891888161 | 1 | 0 | 0.7 | 0.889 | 0 | Pkinase |
| TRINITY_DN914_c0_g1_i6 | 0.621323646 | 0.351774627 | 0.999970678 | 7.403 | 8.071 | 3.766 | 5.499 | NA |
| TRINITY_DN11001_c0_g1_i1 | -0.62130186 | 0.504952142 | 0.999970678 | 1.708 | 2.799 | 2.111 | 4.363 | Mito_carr |
| TRINITY_DN5096_c0_g1_i1 | 0.621289075 | 0.658412953 | 0.999970678 | 2.353 | 2.976 | 0.322 | 3.066 | NA |
| TRINITY_DN7034_c0_g1_i1 | 0.621288933 | 0.534198269 | 0.999970678 | 18.976 | 7.234 | 8.966 | 6.294 | NA |
| TRINITY_DN40115_c0_g1_i7 | 0.621253654 | 0.88928607 | 1 | 1.263 | 0 | 0 | 0.834 | NA |
| TRINITY_DN11531_c0_g1_i1 | 0.621253654 | 0.88928607 | 1 | 0.954 | 0 | 0 | 0.633 | K_trans |
| TRINITY_DN32849_c0_g1_i1 | 0.621184012 | 0.775494008 | 0.999970678 | 8.33 | 2.01 | 5.444 | 0.131 | NA |
| TRINITY_DN1174_c0_g1_i7 | -0.62117081 | 0.615213405 | 0.999970678 | 106.62 | 23.99 | 122.081 | 55.084 | NA |
| TRINITY_DN3173_c1_g3_i1 | -0.6211034 | 0.828980664 | 0.999970678 | 3.161 | 0 | 3.555 | 0.623 | NA |
| TRINITY_DN9567_c0_g2_i1 | -0.62109987 | 0.369081354 | 0.999970678 | 4.024 | 4.731 | 6.444 | 5.68 | NA |
| TRINITY_DN21316_c1_g1_i2 | 0.621016079 | 0.65029058 | 0.999970678 | 0.382 | 0.552 | 0.1 | 0.493 | NA |
| TRINITY_DN1727_c0_g1_i8 | -0.62092223 | 0.679466185 | 0.999970678 | 1.026 | 0.414 | 0.233 | 1.95 | NA |
| TRINITY_DN1171_c0_g1_i4 | 0.620921963 | 0.446351553 | 0.999970678 | 3.425 | 2.513 | 2 | 1.448 | Myb_Cef |
| TRINITY_DN19082_c0_g2_i3 | -0.62086361 | 0.82931898 | 1 | 4.715 | 0 | 1.922 | 4.645 | NA |
| TRINITY_DN545_c0_g2_i3 | -0.6208601 | 0.758758356 | 0.999970678 | 3.234 | 12.79 | 0.222 | 24.3 | NA |
| TRINITY_DN12649_c0_g1_i1 | -0.62082476 | 0.88369501 | 0.999970678 | 6.922 | 0 | 8.922 | 0 | NA |
| TRINITY_DN108653_c0_g1_i1 | 0.620749218 | 0.891239081 | 1 | 1.145 | 0 | 0.622 | 0 | NA |
| TRINITY_DN2264_c0_g2_i3 | -0.6207355 | 0.648068025 | 0.999970678 | 82.391 | 9.156 | 61.33 | 68.064 | NA |
| TRINITY_DN3184_c0_g1_i2 | 0.620677279 | 0.517889915 | 0.999970678 | 23.382 | 8.692 | 7.633 | 11.743 | DUF1664 |
| TRINITY_DN4952_c0_g1_i17 | 0.620645515 | 0.623184884 | 0.999970678 | 0.3 | 0.818 | 0.189 | 0.483 | DUF659 |
| TRINITY_DN2823_c0_g1_i4 | -0.62063835 | 0.326546505 | 0.999970678 | 80.229 | 113.1 | 115.093 | 153.801 | NA |
| TRINITY_DN13991_c0_g1_i6 | 0.620626185 | 0.889310203 | 1 | 0.69 | 0 | 0.389 | 0 | Golgin_A5 |
| TRINITY_DN5871_c0_g1_i3 | -0.62054392 | 0.487008886 | 0.999970678 | 12.291 | 6.514 | 11.977 | 14.095 | NA |
| TRINITY_DN8608_c0_g1_i1 | 0.620535268 | 0.632432662 | 0.999970678 | 0.309 | 1.35 | 0.3 | 0.714 | - |
| TRINITY_DN6892_c0_g1_i2 | 0.620441885 | 0.496324792 | 0.999970678 | 3.806 | 2.217 | 1.933 | 1.629 | RRM_1 |
| TRINITY_DN10548_c0_g1_i2 | -0.62026074 | 0.528755888 | 0.999970678 | 1.126 | 3.075 | 2.211 | 3.76 | NA |
| TRINITY_DN2386_c0_g1_i3 | -0.62023354 | 0.884056478 | 0.999970678 | 1.699 | 0 | 2.178 | 0 | NA |
| TRINITY_DN13225_c1_g1_i3 | -0.62022533 | 0.884779329 | 0.999970678 | 1.544 | 0 | 2 | 0 | NA |
| TRINITY_DN8627_c0_g1_i1 | 0.620224081 | 0.632595349 | 1 | 0.527 | 0.512 | 0.211 | 0.422 | NA |
| TRINITY_DN4989_c0_g1_i1 | -0.6201942 | 0.822120274 | 0.999970678 | 0 | 17 | 16.799 | 5.238 | NA |
| TRINITY_DN20903_c0_g1_i8 | -0.62018787 | 0.888689243 | 0.999970678 | 16.687 | 0 | 20.965 | 0 | NA |
| TRINITY_DN1785_c0_g1_i5 | 0.620155297 | 0.817149965 | 0.999970678 | 0.836 | 1.36 | 1.167 | 0 | DUF659 |
| TRINITY_DN138252_c0_g1_i1 | -0.6201533 | 0.734371066 | 0.999970678 | 17.568 | 0.897 | 20.554 | 3.82 | NA |
| TRINITY_DN4359_c0_g1_i8 | 0.620122832 | 0.888890649 | 1 | 1.281 | 0 | 0 | 0.834 | CDT1 |

| TRINITY_DN16206_c0_g1_i8 | -0.62011939 | 0.845117227 | 0.999970678 | 1.508 | 0 | 1.878 | 0.06 | NA |
| --- | --- | --- | --- | --- | --- | --- | --- | --- |
| TRINITY_DN7767_c0_g1_i5 | -0.62010997 | 0.683496323 | 0.999970678 | 2.725 | 0.296 | 2.289 | 1.93 | NA |
| TRINITY_DN5441_c0_g1_i3 | 0.620108854 | 0.643075916 | 0.999970678 | 0.709 | 3.43 | 0.9 | 1.558 | NA |
| TRINITY_DN1192_c0_g1_i2 | -0.6200977 | 0.834134086 | 0.999970678 | 0 | 46.69 | 42.153 | 19.685 | NA |
| TRINITY_DN859_c0_g1_i3 | 0.620081177 | 0.625079091 | 0.999970678 | 6.395 | 25.93 | 13.244 | 4.776 | NA |
| TRINITY_DN15494_c0_g1_i5 | -0.62005321 | 0.355116981 | 0.999970678 | 4.705 | 4.395 | 5.033 | 7.902 | ATP_bind_1 |
| TRINITY_DN588_c0_g1_i3 | 0.62004007 | 0.525593755 | 0.999970678 | 6.931 | 19.7 | 5.589 | 10.456 | NA |
| TRINITY_DN40035_c0_g1_i1 | -0.62001516 | 0.740412514 | 1 | 1.117 | 0.227 | 1.478 | 0.292 | NAS |
| TRINITY_DN4339_c0_g1_i3 | -0.61991859 | 0.651645629 | 0.999970678 | 5.241 | 0.68 | 3.155 | 5.409 | NA |
| TRINITY_DN997_c1_g1_i3 | 0.619825911 | 0.82548189 | 0.999970678 | 6.213 | 0 | 1.744 | 2.011 | NA |
| TRINITY_DN3938_c0_g1_i11 | -0.61971513 | 0.820981959 | 0.999970678 | 0.282 | 0 | 0.144 | 0.251 | NA |
| TRINITY_DN2229_c0_g1_i3 | -0.6196673 | 0.583982778 | 0.999970678 | 1.481 | 0.739 | 1.5 | 1.588 | NA |
| TRINITY_DN1150_c0_g1_i15 | -0.61961988 | 0.563677241 | 0.999970678 | 3.143 | 1.281 | 2.4 | 3.901 | NA |
| TRINITY_DN27372_c0_g1_i3 | 0.619562222 | 0.597216918 | 0.999970678 | 2.117 | 3.095 | 2.022 | 0.925 | NA |
| TRINITY_DN165531_c1_g1_i1 | -0.61947628 | 0.554869235 | 0.999970678 | 107.37 | 119.9 | 59.208 | 275.009 | NA |
| TRINITY_DN326_c0_g1_i1 | -0.61942654 | 0.760315962 | 1 | 1.281 | 0.168 | 0.222 | 1.95 | NA |
| TRINITY_DN80_c0_g1_i3 | 0.619375608 | 0.888057023 | 0.999970678 | 0 | 45.43 | 24.054 | 0 | RRM_1 |
| TRINITY_DN5033_c0_g1_i1 | 0.619257532 | 0.355380198 | 0.999970678 | 30.776 | 23.89 | 17.232 | 14.91 | NA |
| TRINITY_DN30379_c0_g1_i1 | -0.61910362 | 0.756467612 | 1 | 0.436 | 0.03 | 0.456 | 0.171 | NA |
| TRINITY_DN10948_c0_g1_i2 | -0.61908636 | 0.825568061 | 0.999970678 | 7.185 | 0 | 1.778 | 8.797 | NA |
| TRINITY_DN7445_c0_g1_i1 | 0.619077936 | 0.397594988 | 0.999970678 | 2.989 | 2.227 | 1.311 | 1.83 | NA |
| TRINITY_DN5019_c0_g1_i1 | -0.61894956 | 0.408636281 | 0.999970678 | 2.462 | 3.272 | 3.866 | 4.132 | - |
| TRINITY_DN14094_c1_g1_i1 | -0.61892406 | 0.413611652 | 0.999970678 | 61.753 | 49.57 | 91.472 | 60.684 | NA |
| TRINITY_DN1471_c3_g1_i1 | -0.6188981 | 0.658113442 | 0.999970678 | 3.525 | 1.192 | 1.067 | 5.972 | NA |
| TRINITY_DN698_c1_g1_i10 | -0.61882186 | 0.681669008 | 0.999970678 | 1.835 | 0.749 | 0.6 | 3.257 | NA |
| TRINITY_DN8326_c0_g1_i6 | 0.618680289 | 0.641113875 | 0.999970678 | 2.071 | 1.212 | 1.433 | 0.412 | RVT_1 |
| TRINITY_DN1345_c1_g1_i1 | -0.61867929 | 0.722581503 | 0.999970678 | 8.602 | 2.848 | 13.988 | 0.734 | LRRNT_2 |
| TRINITY_DN2149_c0_g1_i1 | 0.618670086 | 0.447129154 | 0.999970678 | 10.229 | 22.03 | 7.844 | 11.461 | NA |
| TRINITY_DN9346_c0_g1_i1 | 0.618520798 | 0.831621464 | 0.999970678 | 1.635 | 12.79 | 0 | 8.757 | NA |
| TRINITY_DN694_c2_g2_i2 | -0.61848485 | 0.776337781 | 0.999970678 | 2.961 | 0.02 | 2.633 | 1.468 | Sugar_tr |
| TRINITY_DN8419_c0_g1_i1 | 0.618458412 | 0.37232383 | 0.999970678 | 68.039 | 80.55 | 49.608 | 36.857 | Alpha-amylase |
| TRINITY_DN1416_c1_g1_i3 | 0.618443218 | 0.833018226 | 0.999970678 | 2.362 | 0.207 | 1.411 | 0 | GDPD |
| TRINITY_DN966_c1_g1_i1 | 0.618436979 | 0.742744041 | 0.999970678 | 0.173 | 1.882 | 0.955 | 0.141 | NA |
| TRINITY_DN17653_c0_g1_i1 | -0.61837509 | 0.851210804 | 1 | 0 | 6.199 | 7.5 | 0.191 | NA |
| TRINITY_DN80292_c0_g1_i1 | 0.618348622 | 0.65646977 | 0.999970678 | 15.715 | 152.9 | 50.897 | 45.342 | Chloroa_b-bind |
| TRINITY_DN59_c0_g2_i2 | -0.61828208 | 0.509138248 | 0.999970678 | 1.135 | 1.321 | 1.878 | 1.498 | NA |
| TRINITY_DN1890_c0_g1_i4 | 0.618143535 | 0.827344698 | 0.999970678 | 0.627 | 3.242 | 2.067 | 0.03 | - |
| TRINITY_DN10389_c0_g1_i1 | -0.61808833 | 0.824480109 | 0.999970678 | 0.618 | 0 | 0.533 | 0.312 | NA |
| TRINITY_DN7509_c0_g5_i1 | -0.61806347 | 0.674891179 | 0.999970678 | 2.262 | 2.178 | 4.955 | 0.804 | NA |
| TRINITY_DN4365_c0_g2_i2 | -0.61798866 | 0.395296262 | 0.999970678 | 3.87 | 2.71 | 4.433 | 4.755 | ANAPC8 |
| TRINITY_DN1320_c0_g1_i17 | 0.617984108 | 0.888531155 | 0.999970678 | 0 | 28.59 | 15.155 | 0 | NA |
| TRINITY_DN14787_c0_g1_i2 | -0.61794412 | 0.59164619 | 0.999970678 | 2.752 | 2.799 | 2.233 | 5.66 | NA |
| TRINITY_DN8345_c0_g1_i1 | 0.617920962 | 0.398574071 | 0.999970678 | 6.15 | 8.574 | 3.133 | 5.811 | Peptidase_M28 |
| TRINITY_DN3290_c0_g2_i2 | 0.617849497 | 0.477850543 | 0.999970678 | 1.354 | 1.271 | 0.9 | 0.643 | HATPase_c |
| TRINITY_DN1247_c0_g1_i2 | 0.617840031 | 0.540992513 | 0.999970678 | 5.805 | 2.641 | 1.833 | 3.288 | NA |
| TRINITY_DN5306_c0_g1_i1 | -0.61783356 | 0.622985328 | 0.999970678 | 6.159 | 26.5 | 11.377 | 35.108 | NA |
| TRINITY_DN7737_c0_g1_i1 | -0.61780899 | 0.889873027 | 1 | 6.25 | 0 | 8.077 | 0 | Pro-kuma_activ |
| TRINITY_DN9967_c0_g1_i1 | -0.61776113 | 0.573185703 | 0.999970678 | 6.313 | 8.968 | 4.266 | 18.097 | NA |
| TRINITY_DN2193_c0_g1_i6 | 0.617704682 | 0.642299102 | 0.999970678 | 11.945 | 1.843 | 3.478 | 4.866 | adh_short |
| TRINITY_DN5515_c0_g1_i1 | -0.61763324 | 0.536247011 | 0.999970678 | 1.78 | 1.813 | 1.311 | 3.961 | NA |
| TRINITY_DN15859_c0_g2_i1 | -0.61763269 | 0.832158283 | 1 | 0 | 0.345 | 0.089 | 0.432 | Zw10 |
| TRINITY_DN10708_c0_g1_i7 | 0.617595053 | 0.738150321 | 1 | 0.391 | 1.951 | 1.089 | 0.211 | NA |
| TRINITY_DN5446_c0_g1_i1 | -0.61755857 | 0.282385843 | 0.999970678 | 45.865 | 43.63 | 53.763 | 71.824 | NA |
| TRINITY_DN4647_c0_g1_i14 | 0.617542466 | 0.560070647 | 0.999970678 | 9.938 | 6.189 | 6.622 | 2.564 | NA |
| TRINITY_DN8590_c0_g1_i9 | 0.617508825 | 0.550071067 | 0.999970678 | 14.843 | 6.376 | 7.8 | 4.414 | NA |
| TRINITY_DN24077_c0_g1_i1 | 0.617359143 | 0.84487065 | 1 | 1.072 | 0 | 0.056 | 0.643 | NA |
| TRINITY_DN16973_c0_g1_i1 | -0.61735019 | 0.57494034 | 0.999970678 | 210.28 | 275.1 | 119.459 | 535.138 | NA |
| TRINITY_DN353_c1_g2_i1 | -0.6172934 | 0.366947617 | 0.999970678 | 78.376 | 52.8 | 92.261 | 89.769 | NA |
| TRINITY_DN10435_c0_g1_i1 | 0.617282821 | 0.386517018 | 0.999970678 | 4.451 | 3.282 | 2.055 | 2.584 | NAGLU |
| TRINITY_DN23798_c0_g1_i2 | 0.617167046 | 0.62669713 | 0.999970678 | 1.163 | 1.626 | 0.311 | 1.438 | Granulin |
| TRINITY_DN2251_c1_g3_i3 | 0.617108429 | 0.337338442 | 0.999970678 | 8.957 | 6.928 | 4.011 | 5.56 | NA |
| TRINITY_DN1362_c0_g1_i4 | 0.617102555 | 0.456788037 | 0.999970678 | 3.079 | 3.873 | 1.3 | 2.966 | ARID |
| TRINITY_DN2217_c0_g1_i1 | -0.61708952 | 0.472961107 | 0.999970678 | 43.185 | 31.23 | 65.029 | 35.56 | PsbP |
| TRINITY_DN2798_c0_g1_i3 | 0.617078822 | 0.427570904 | 0.999970678 | 3.906 | 6.258 | 3.311 | 2.614 | - |
| TRINITY_DN6921_c0_g1_i1 | 0.617013666 | 0.689497297 | 1 | 0.227 | 0.207 | 0.189 | 0.06 | NA |
| TRINITY_DN13751_c0_g1_i8 | 0.616980733 | 0.81896745 | 0.999970678 | 0.463 | 0.562 | 0.556 | 0 | NA |
| TRINITY_DN70880_c0_g1_i2 | -0.61694961 | 0.633728944 | 0.999970678 | 0.799 | 1.606 | 2.222 | 1.015 | NA |
| TRINITY_DN9513_c0_g1_i1 | 0.616901766 | 0.432753439 | 0.999970678 | 18.359 | 10.15 | 7.977 | 8.998 | NA |
| TRINITY_DN30318_c0_g1_i3 | 0.616829441 | 0.887691392 | 0.999970678 | 3.897 | 0 | 0 | 2.513 | NA |
| TRINITY_DN7177_c0_g1_i7 | 0.616829441 | 0.887691392 | 0.999970678 | 0.945 | 0 | 0 | 0.623 | Dynamin_M |
| TRINITY_DN2724_c0_g1_i1 | 0.61678325 | 0.525563115 | 0.999970678 | 2.807 | 1.863 | 1.4 | 1.357 | EGF_CA |
| TRINITY_DN19976_c1_g2_i1 | -0.61667409 | 0.358733113 | 0.999970678 | 91.185 | 97.26 | 92.361 | 177.307 | Dimerisation |
| TRINITY_DN1131_c1_g1_i8 | 0.616607384 | 0.887466811 | 0.999970678 | 0 | 2.188 | 0 | 1.408 | NA |
| TRINITY_DN17152_c0_g1_i7 | 0.616551685 | 0.833554997 | 1 | 3.397 | 0.601 | 2.189 | 0 | NA |

| TRINITY_DN355_c0_g1_i2 | -0.61646445 | 0.636707145 | 0.999970678 | 1.372 | 0.532 | 1.1 | 1.578 | NA |
| --- | --- | --- | --- | --- | --- | --- | --- | --- |
| TRINITY_DN11049_c0_g3_i2 | 0.616437095 | 0.743284879 | 0.999970678 | 1.135 | 1.399 | 1.3 | 0.08 | NA |
| TRINITY_DN317_c0_g1_i1 | 0.616436963 | 0.826779628 | 0.999970678 | 6.15 | 4.849 | 5.944 | 0 | TIP120 |
| TRINITY_DN10706_c0_g1_i5 | -0.61643461 | 0.826191293 | 1 | 4.397 | 0 | 3.211 | 2.644 | NA |
| TRINITY_DN2181_c0_g1_i1 | -0.61637521 | 0.531120628 | 0.999970678 | 4.923 | 6.416 | 10.499 | 4.685 | DUF707 |
| TRINITY_DN697_c0_g1_i1 | -0.61634085 | 0.477883578 | 0.999970678 | 4.715 | 10.57 | 8 | 13.643 | NA |
| TRINITY_DN5917_c0_g1_i1 | -0.61631489 | 0.560272748 | 0.999970678 | 10.492 | 5.805 | 5.722 | 17.896 | NA |
| TRINITY_DN3128_c0_g1_i1 | -0.61627268 | 0.350298318 | 0.999970678 | 10.583 | 9.333 | 10.777 | 17.483 | RBFA |
| TRINITY_DN13671_c0_g3_i1 | 0.61625943 | 0.640794489 | 0.999970678 | 0.554 | 0.956 | 0.222 | 0.704 | XRN_N |
| TRINITY_DN19893_c0_g1_i3 | 0.616170171 | 0.817769901 | 0.999970678 | 1.453 | 3.528 | 0 | 3.217 | NA |
| TRINITY_DN341_c0_g1_i3 | 0.616161801 | 0.418136578 | 0.999970678 | 23.518 | 43.98 | 15.632 | 25.054 | NA |
| TRINITY_DN4707_c0_g1_i4 | 0.616142378 | 0.39081485 | 0.999970678 | 1.808 | 2.543 | 1.055 | 1.558 | Ion_trans |
| TRINITY_DN187_c0_g1_i3 | -0.61611313 | 0.41731656 | 0.999970678 | 9.92 | 18.56 | 17.988 | 21.646 | - |
| TRINITY_DN4681_c0_g1_i2 | 0.616071693 | 0.842777477 | 1 | 1.799 | 0 | 0.911 | 0.111 | NA |
| TRINITY_DN10064_c0_g1_i5 | 0.616008866 | 0.588618977 | 0.999970678 | 1.88 | 0.857 | 0.811 | 0.814 | NA |
| TRINITY_DN1447_c0_g1_i2 | -0.61597257 | 0.530512759 | 0.999970678 | 132.88 | 57.06 | 165.668 | 92.122 | NA |
| TRINITY_DN16003_c1_g1_i4 | -0.61594914 | 0.75343678 | 0.999970678 | 0.772 | 0.118 | 1.044 | 0.121 | NYN |
| TRINITY_DN8543_c0_g1_i1 | 0.615927844 | 0.579372883 | 0.999970678 | 0.745 | 2.316 | 0.7 | 1.136 | NA |
| TRINITY_DN3761_c0_g1_i1 | -0.61592486 | 0.517359384 | 0.999970678 | 32.448 | 39.88 | 22.965 | 83.204 | - |
| TRINITY_DN27931_c0_g1_i1 | 0.615903729 | 0.51845603 | 0.999970678 | 3106.6 | 1174 | 1207.438 | 1142.673 | NA |
| TRINITY_DN5015_c0_g1_i3 | 0.615866465 | 0.570777823 | 0.999970678 | 14.08 | 6.702 | 2.822 | 10.235 | NA |
| TRINITY_DN11815_c1_g1_i1 | -0.61574626 | 0.827257979 | 0.999970678 | 0.409 | 0.237 | 0.822 | 0 | NA |
| TRINITY_DN12204_c0_g2_i2 | -0.61573685 | 0.837124162 | 0.999970678 | 6.913 | 0 | 0.433 | 10.235 | NA |
| TRINITY_DN1640_c0_g3_i1 | -0.61570259 | 0.664996908 | 0.999970678 | 3.688 | 0.71 | 1.622 | 4.776 | NA |
| TRINITY_DN4713_c0_g1_i1 | 0.61564196 | 0.509241594 | 0.999970678 | 14.562 | 17.34 | 4.777 | 14.96 | NA |
| TRINITY_DN9011_c0_g1_i7 | -0.61555395 | 0.824252913 | 0.999970678 | 0.4 | 0.601 | 1.255 | 0 | NA |
| TRINITY_DN23760_c0_g1_i1 | -0.61553274 | 0.506706458 | 0.999970678 | 6.032 | 6.544 | 6.911 | 10.375 | NA |
| TRINITY_DN12032_c0_g1_i1 | 0.615528841 | 0.392786143 | 0.999970678 | 7.349 | 13.13 | 5.477 | 6.736 | NA |
| TRINITY_DN1394_c0_g1_i1 | 0.615515605 | 0.458477347 | 0.999970678 | 3.152 | 5.962 | 2.822 | 2.503 | Ribosomal_S4 |
| TRINITY_DN1235_c0_g1_i1 | -0.61529748 | 0.724585712 | 0.999970678 | 1.108 | 0.434 | 0.233 | 2.141 | NA |
| TRINITY_DN2253_c0_g1_i2 | -0.61517669 | 0.722240391 | 0.999970678 | 0.445 | 1.094 | 0.156 | 2.141 | TPX2 |
| TRINITY_DN2787_c0_g1_i7 | 0.615128408 | 0.45408942 | 0.999970678 | 5.432 | 3.4 | 1.911 | 3.479 | CIA30 |
| TRINITY_DN135_c0_g2_i1 | -0.61508479 | 0.65074453 | 0.999970678 | 1.699 | 0.601 | 2.367 | 0.694 | Oxidored_q4 |
| TRINITY_DN14800_c0_g1_i1 | 0.61498371 | 0.660410528 | 0.999970678 | 0.527 | 0.611 | 0.511 | 0.131 | NA |
| TRINITY_DN801_c3_g1_i10 | 0.614930682 | 0.641855049 | 0.999970678 | 3.379 | 1.449 | 2.144 | 0.563 | NA |
| TRINITY_DN2582_c0_g1_i5 | 0.614906233 | 0.632056343 | 0.999970678 | 1.708 | 7.687 | 3.8 | 1.488 | NA |
| TRINITY_DN11114_c0_g1_i7 | -0.61490418 | 0.888278058 | 0.999970678 | 8.466 | 0 | 10.755 | 0 | MAPEG |
| TRINITY_DN20175_c0_g1_i1 | -0.61487772 | 0.626178027 | 0.999970678 | 3.37 | 1.853 | 1.967 | 5.499 | NA |
| TRINITY_DN14790_c1_g1_i7 | -0.61486297 | 0.780958676 | 1 | 0.927 | 0.128 | 1.267 | 0.07 | NA |
| TRINITY_DN2307_c1_g1_i7 | -0.61482919 | 0.838376552 | 1 | 3.388 | 0 | 0.522 | 4.494 | NA |
| TRINITY_DN482_c0_g1_i4 | -0.61482887 | 0.580427851 | 0.999970678 | 2.889 | 3.035 | 1.489 | 7.289 | EXS |
| TRINITY_DN11870_c0_g1_i4 | -0.61478736 | 0.732980105 | 0.999970678 | 0.654 | 0.522 | 0.067 | 1.749 | NA |
| TRINITY_DN261_c0_g1_i5 | -0.61458423 | 0.490988272 | 0.999970678 | 15.434 | 6.435 | 12.488 | 18.629 | Metallophos |
| TRINITY_DN52_c0_g1_i1 | -0.61453316 | 0.370960267 | 0.999970678 | 25.353 | 18.96 | 31.354 | 29.618 | NA |
| TRINITY_DN2251_c0_g1_i23 | 0.614515443 | 0.596203841 | 0.999970678 | 0.808 | 0.414 | 0.389 | 0.332 | NA |
| TRINITY_DN14537_c1_g1_i2 | -0.61447359 | 0.584743603 | 0.999970678 | 0.645 | 0.512 | 0.989 | 0.563 | KH_1 |
| TRINITY_DN1966_c0_g1_i4 | 0.614244257 | 0.591471268 | 0.999970678 | 234.65 | 55.76 | 95.739 | 70.346 | NA |
| TRINITY_DN5419_c0_g2_i1 | -0.6142331 | 0.456399519 | 0.999970678 | 6.722 | 6.702 | 11.31 | 6.857 | mTERF |
| TRINITY_DN6451_c0_g1_i4 | 0.614144345 | 0.827240476 | 0.999970678 | 2.162 | 0 | 0.422 | 0.925 | NA |
| TRINITY_DN28257_c1_g1_i1 | 0.614101945 | 0.502957064 | 0.999970678 | 196.71 | 277.1 | 119.826 | 135.805 | NA |
| TRINITY_DN381_c0_g1_i5 | -0.61404523 | 0.606044451 | 0.999970678 | 7.467 | 2.759 | 9.677 | 4.021 | NB-ARC |
| TRINITY_DN39575_c0_g1_i4 | 0.613950401 | 0.399447084 | 0.999970678 | 138.33 | 80.78 | 54.552 | 77.856 | NA |
| TRINITY_DN4679_c0_g1_i2 | -0.6139333 | 0.274133019 | 0.999970678 | 146.06 | 143.3 | 194.077 | 207.217 | 40S_S4_C |
| TRINITY_DN1242_c0_g2_i11 | 0.613915422 | 0.425116719 | 0.999970678 | 8.394 | 6.633 | 4.811 | 4.001 | NA |
| TRINITY_DN15117_c0_g1_i1 | 0.613912597 | 0.886558512 | 0.999970678 | 2.607 | 0 | 0 | 1.749 | F-box |
| TRINITY_DN19653_c0_g1_i2 | -0.61387026 | 0.85458217 | 1 | 1.944 | 0.089 | 2.589 | 0 | SelR |
| TRINITY_DN8841_c0_g1_i1 | -0.61377292 | 0.598109806 | 0.999970678 | 3.634 | 6.002 | 2.478 | 11.652 | NA |
| TRINITY_DN17305_c0_g1_i1 | -0.6137636 | 0.610858468 | 0.999970678 | 11.237 | 6.534 | 3.989 | 22.41 | NA |
| TRINITY_DN12706_c0_g1_i3 | -0.61369641 | 0.383617523 | 0.999970678 | 4.96 | 4.445 | 5.166 | 8.174 | ANTH |
| TRINITY_DN43753_c1_g1_i2 | -0.61369489 | 0.622238245 | 0.999970678 | 0.309 | 0.65 | 0.556 | 0.804 | NA |
| TRINITY_DN14813_c0_g1_i1 | -0.61365784 | 0.638377395 | 0.999970678 | 37.408 | 5.075 | 31.365 | 27.949 | NA |
| TRINITY_DN24771_c0_g1_i1 | -0.61365231 | 0.462650545 | 0.999970678 | 1.726 | 1.754 | 2.633 | 2.121 | NA |
| TRINITY_DN255_c0_g2_i1 | 0.613641411 | 0.645522985 | 0.999970678 | 39.152 | 4.918 | 12.921 | 13.522 | DUF563 |
| TRINITY_DN35593_c0_g1_i3 | 0.613593887 | 0.673990027 | 1 | 3.179 | 1.429 | 0.722 | 2.162 | NA |
| TRINITY_DN30969_c0_g1_i1 | -0.61351985 | 0.402699115 | 0.999970678 | 2.498 | 1.932 | 2.511 | 3.77 | U3_assoc_6 |
| TRINITY_DN372_c0_g1_i4 | 0.613476313 | 0.826780564 | 0.999970678 | 12.545 | 0 | 4.478 | 2.875 | NA |
| TRINITY_DN4417_c0_g1_i1 | -0.6134203 | 0.425125871 | 0.999970678 | 127.78 | 246.4 | 202.61 | 322.141 | NA |
| TRINITY_DN2723_c0_g1_i3 | 0.613414129 | 0.427360908 | 0.999970678 | 10.537 | 9.924 | 4.011 | 8.505 | NA |
| TRINITY_DN1395_c0_g1_i1 | 0.613239338 | 0.827465085 | 1 | 0.536 | 0.177 | 0 | 0.483 | NA |
| TRINITY_DN297_c0_g1_i1 | 0.613204397 | 0.700667077 | 0.999970678 | 3.506 | 3.775 | 0.289 | 4.404 | NAM |
| TRINITY_DN13850_c0_g1_i2 | 0.613179077 | 0.605668794 | 1 | 0.427 | 0.572 | 0.267 | 0.312 | Kinesin |
| TRINITY_DN9945_c0_g1_i2 | -0.61314426 | 0.820061472 | 0.999970678 | 1.517 | 0 | 1.089 | 1.056 | NA |
| TRINITY_DN301_c0_g1_i7 | -0.61308621 | 0.388852831 | 0.999970678 | 9.565 | 7.175 | 8.833 | 14.99 | NA |
| TRINITY_DN2761_c0_g1_i1 | -0.61308491 | 0.364477861 | 0.999970678 | 11.809 | 17.79 | 17.021 | 24.561 | NA |

| TRINITY_DN73133_c0_g1_i4 | 0.613013782 | 0.701988906 | 0.999970678 | 4.696 | 19.41 | 1.055 | 14.306 | PMEI |
| --- | --- | --- | --- | --- | --- | --- | --- | --- |
| TRINITY_DN13991_c0_g1_i7 | 0.61299892 | 0.702298901 | 0.999970678 | 0.581 | 2.06 | 1.255 | 0.181 | Golgin_A5 |
| TRINITY_DN23937_c0_g2_i1 | 0.612968012 | 0.847432594 | 1 | 3.161 | 0.177 | 1.811 | 0 | Ribosomal_S17 |
| TRINITY_DN6932_c0_g1_i10 | 0.61287279 | 0.835031329 | 1 | 0.69 | 4.543 | 0 | 3.288 | NA |
| TRINITY_DN2145_c0_g1_i3 | -0.6128697 | 0.638642256 | 0.999970678 | 0.372 | 0.207 | 0.511 | 0.282 | NA |
| TRINITY_DN4386_c0_g1_i1 | 0.612761043 | 0.406243223 | 0.999970678 | 9.002 | 12.92 | 5.033 | 8.184 | NA |
| TRINITY_DN6706_c1_g1_i1 | -0.61270085 | 0.363636879 | 0.999970678 | 18.213 | 12.62 | 19.543 | 23.576 | NA |
| TRINITY_DN4346_c0_g2_i2 | -0.61269336 | 0.553090114 | 0.999970678 | 4.415 | 5.371 | 3.333 | 10.808 | EnY2 |
| TRINITY_DN18177_c0_g2_i3 | 0.612595937 | 0.587617837 | 0.999970678 | 0.618 | 0.818 | 0.356 | 0.503 | Ins134_P3_kin |
| TRINITY_DN9995_c0_g1_i4 | 0.612564282 | 0.438633458 | 0.999970678 | 1.49 | 1.774 | 1.044 | 0.865 | NA |
| TRINITY_DN103299_c0_g1_i1 | -0.61253895 | 0.889241091 | 0.999970678 | 0.963 | 0 | 1.233 | 0 | NA |
| TRINITY_DN7426_c0_g1_i5 | -0.61253411 | 0.538751203 | 0.999970678 | 1.481 | 1.646 | 2.033 | 2.232 | PPR |
| TRINITY_DN6984_c0_g1_i1 | 0.612486082 | 0.365283529 | 0.999970678 | 21.91 | 14.99 | 9.188 | 13.15 | CAF1A |
| TRINITY_DN680_c0_g3_i3 | -0.61233991 | 0.484677264 | 0.999970678 | 4.905 | 2.976 | 6.455 | 4.343 | Rrp15p |
| TRINITY_DN12106_c0_g1_i3 | 0.612316051 | 0.893202459 | 1 | 4.088 | 0 | 2.178 | 0 | Ribosomal_S27 |
| TRINITY_DN5775_c0_g1_i1 | 0.612316051 | 0.893202459 | 1 | 1.581 | 0 | 0.867 | 0 | NA |
| TRINITY_DN75037_c0_g1_i1 | 0.612316051 | 0.893202459 | 1 | 0.718 | 0 | 0.389 | 0 | NA |
| TRINITY_DN16604_c0_g1_i2 | 0.612316051 | 0.893202459 | 1 | 2.307 | 0 | 1.267 | 0 | NA |
| TRINITY_DN16995_c0_g1_i3 | 0.612316051 | 0.893202459 | 1 | 1.481 | 0 | 0.811 | 0 | NA |
| TRINITY_DN1215_c0_g1_i5 | -0.61226037 | 0.60043649 | 0.999970678 | 2.871 | 0.749 | 2.311 | 2.775 | WD40 |
| TRINITY_DN63155_c0_g2_i1 | -0.61217501 | 0.617041072 | 0.999970678 | 1.008 | 1.449 | 1.967 | 1.347 | - |
| TRINITY_DN92052_c0_g1_i1 | 0.611939581 | 0.549495667 | 0.999970678 | 1.481 | 1.232 | 0.778 | 0.834 | NA |
| TRINITY_DN4703_c0_g1_i8 | -0.6117443 | 0.592019858 | 0.999970678 | 2.689 | 4.563 | 2.033 | 8.556 | V-SNARE_C |
| TRINITY_DN1645_c0_g1_i6 | 0.611720774 | 0.778746673 | 0.999970678 | 40.015 | 13.52 | 28.987 | 0.141 | NA |
| TRINITY_DN2099_c0_g1_i7 | -0.61162093 | 0.853301567 | 0.999970678 | 5.668 | 0.049 | 0 | 8.847 | NA |
| TRINITY_DN12821_c0_g1_i1 | -0.61158178 | 0.519208316 | 0.999970678 | 43.203 | 47.37 | 85.117 | 34.836 | NA |
| TRINITY_DN2683_c0_g1_i3 | -0.61156128 | 0.704456576 | 0.999970678 | 12.972 | 1.114 | 3.411 | 17.725 | NAD_binding_10 |
| TRINITY_DN2351_c0_g1_i2 | -0.61147064 | 0.830436501 | 0.999970678 | 0.636 | 0 | 0.7 | 0.141 | NA |
| TRINITY_DN6692_c0_g2_i1 | -0.61138207 | 0.464654651 | 0.999970678 | 5.85 | 2.986 | 5.966 | 6.354 | NA |
| TRINITY_DN45093_c0_g1_i1 | -0.61136296 | 0.463867715 | 0.999970678 | 553.1 | 679.6 | 465.761 | 1205.76 | NA |
| TRINITY_DN1092_c0_g1_i4 | -0.61128214 | 0.82836245 | 0.999970678 | 15.261 | 0 | 7.7 | 14.367 | NA |
| TRINITY_DN5804_c0_g1_i5 | -0.61125189 | 0.65682544 | 0.999970678 | 0.345 | 1.547 | 0.867 | 1.79 | NA |
| TRINITY_DN105472_c0_g1_i1 | -0.61117005 | 0.893095478 | 1 | 0.709 | 0 | 0.9 | 0 | SH3_1 |
| TRINITY_DN40107_c0_g1_i1 | -0.61117005 | 0.893095478 | 1 | 0.845 | 0 | 1.067 | 0 | - |
| TRINITY_DN10250_c0_g1_i6 | -0.61117005 | 0.893095478 | 1 | 2.153 | 0 | 2.8 | 0 | NA |
| TRINITY_DN227616_c0_g1_i1 | -0.61117005 | 0.893095478 | 1 | 2.571 | 0 | 3.278 | 0 | NA |
| TRINITY_DN577_c2_g1_i1 | -0.61103552 | 0.512545397 | 0.999970678 | 9.765 | 14.25 | 9.477 | 24.42 | NA |
| TRINITY_DN481_c0_g3_i1 | 0.611018266 | 0.843597096 | 1 | 7.104 | 0.463 | 4.1 | 0 | NA |
| TRINITY_DN3543_c0_g1_i1 | -0.61084119 | 0.332215552 | 0.999970678 | 54.722 | 61.5 | 60.896 | 103.935 | Ribosomal_L7Ae |
| TRINITY_DN10286_c0_g1_i1 | -0.61079039 | 0.634364866 | 0.999970678 | 9.974 | 29.33 | 8.066 | 50.037 | Methyltransf_11 |
| TRINITY_DN4764_c0_g1_i2 | -0.61077864 | 0.45927584 | 0.999970678 | 22.746 | 38.18 | 26.654 | 60.403 | - |
| TRINITY_DN126_c0_g1_i2 | 0.610766828 | 0.49682002 | 0.999970678 | 11.927 | 6.475 | 6 | 4.765 | MRNIP |
| TRINITY_DN1724_c0_g1_i2 | 0.610741473 | 0.601341937 | 0.999970678 | 11.873 | 12.48 | 2.267 | 13.16 | NA |
| TRINITY_DN9010_c0_g1_i4 | -0.61071967 | 0.824922702 | 0.999970678 | 0.872 | 1.084 | 2.467 | 0 | NA |
| TRINITY_DN17867_c0_g1_i3 | -0.61063581 | 0.601022398 | 0.999970678 | 1.254 | 0.552 | 0.833 | 1.729 | NA |
| TRINITY_DN486_c0_g1_i7 | 0.610627518 | 0.884509375 | 0.999970678 | 24.717 | 0 | 0 | 16.096 | NA |
| TRINITY_DN6167_c0_g1_i2 | -0.61041661 | 0.595112252 | 0.999970678 | 2.062 | 8.416 | 5.455 | 9.219 | NA |
| TRINITY_DN1512_c0_g2_i1 | 0.610405189 | 0.426647236 | 0.999970678 | 26.707 | 17.03 | 12.077 | 13.723 | IGR |
| TRINITY_DN32305_c0_g1_i1 | -0.6103697 | 0.887166337 | 0.999970678 | 3.915 | 0 | 5.011 | 0 | SUI1 |
| TRINITY_DN2193_c0_g1_i2 | -0.6103493 | 0.593283442 | 0.999970678 | 258.08 | 93.2 | 106.005 | 405.325 | adh_short |
| TRINITY_DN4405_c0_g1_i8 | -0.61031981 | 0.537963739 | 0.999970678 | 1.036 | 1.518 | 2.289 | 1.136 | NA |
| TRINITY_DN2540_c0_g1_i2 | 0.610230344 | 0.707368626 | 0.999970678 | 0.799 | 0.976 | 0.867 | 0.121 | Pkinase |
| TRINITY_DN6039_c0_g3_i1 | -0.61022615 | 0.484928448 | 0.999970678 | 28.415 | 21.08 | 26.765 | 39.27 | NA |
| TRINITY_DN12548_c1_g1_i1 | 0.610195201 | 0.447471957 | 0.999970678 | 9.62 | 17.78 | 8.944 | 7.088 | NA |
| TRINITY_DN290_c0_g1_i2 | -0.6101816 | 0.71911482 | 0.999970678 | 5.078 | 0.986 | 0.878 | 8.154 | NA |
| TRINITY_DN16849_c0_g1_i6 | 0.6101767 | 0.558257317 | 0.999970678 | 3.543 | 2.631 | 2.533 | 0.985 | NA |
| TRINITY_DN758_c0_g1_i14 | -0.61015907 | 0.76187678 | 0.999970678 | 2.198 | 0.197 | 2.655 | 0.483 | NA |
| TRINITY_DN5194_c3_g1_i2 | 0.610076174 | 0.591242916 | 0.999970678 | 2.625 | 2.819 | 2.389 | 0.684 | NA |
| TRINITY_DN21619_c0_g1_i5 | 0.609986476 | 0.893195123 | 1 | 0 | 0.552 | 0.289 | 0 | Inhibitor_I9 |
| TRINITY_DN665_c1_g2_i1 | 0.609933945 | 0.419634338 | 0.999970678 | 112 | 70.75 | 46.964 | 58.151 | - |
| TRINITY_DN3218_c0_g1_i6 | 0.609917963 | 0.892700234 | 1 | 0 | 0.621 | 0.333 | 0 | DUF1005 |
| TRINITY_DN1004_c0_g1_i2 | 0.609879616 | 0.632073576 | 1 | 0.672 | 0.463 | 0.411 | 0.251 | Metallophos |
| TRINITY_DN6721_c0_g1_i1 | 0.609831428 | 0.680306052 | 0.999970678 | 0.981 | 0.828 | 0.867 | 0.131 | DRY_EERY |
| TRINITY_DN8328_c0_g1_i1 | -0.6097485 | 0.46229727 | 0.999970678 | 6.486 | 6.288 | 5.466 | 12.859 | NA |
| TRINITY_DN6454_c0_g1_i1 | 0.609726949 | 0.450992865 | 0.999970678 | 1.535 | 2.03 | 0.967 | 1.156 | Myb_DNA-bindin |
| TRINITY_DN527_c0_g3_i1 | -0.60969104 | 0.831067396 | 0.999970678 | 10.428 | 0 | 9.388 | 4.846 | A_deaminase |
| TRINITY_DN1307_c0_g1_i1 | -0.60966026 | 0.441422413 | 0.999970678 | 78.658 | 66.18 | 122.415 | 73.201 | NA |
| TRINITY_DN3667_c0_g1_i1 | 0.609656263 | 0.511143998 | 0.999970678 | 27.352 | 63.08 | 16.855 | 38.475 | NA |
| TRINITY_DN10441_c0_g1_i2 | 0.609617165 | 0.51295799 | 0.999970678 | 10.546 | 6.13 | 5.989 | 3.69 | Hydrolase_4 |
| TRINITY_DN5054_c0_g1_i2 | -0.60961518 | 0.548870836 | 0.999970678 | 0.863 | 0.956 | 1.478 | 0.995 | NA |
| TRINITY_DN4253_c0_g1_i1 | 0.609568701 | 0.425781051 | 0.999970678 | 14.889 | 10.88 | 5.589 | 10.084 | NA |
| TRINITY_DN8951_c0_g1_i2 | 0.609549414 | 0.738197838 | 0.999970678 | 0.463 | 4.346 | 0.267 | 2.805 | Lipase_GDSL |
| TRINITY_DN91_c0_g2_i1 | 0.609502557 | 0.320273832 | 0.999970678 | 21.029 | 16.7 | 10.71 | 11.883 | NA |
| TRINITY_DN1163_c0_g1_i11 | -0.60942605 | 0.733925356 | 0.999970678 | 5.632 | 0.355 | 5.589 | 2.393 | DUF3110 |

| TRINITY_DN7556_c0_g1_i1 | -0.60939072 | 0.738071023 | 0.999970678 | 10.21 | 0.237 | 8.899 | 5.429 | NA |
| --- | --- | --- | --- | --- | --- | --- | --- | --- |
| TRINITY_DN3789_c0_g1_i4 | 0.609384842 | 0.670740363 | 0.999970678 | 2.698 | 0.956 | 1.689 | 0.382 | NA |
| TRINITY_DN2211_c0_g2_i2 | -0.60938474 | 0.677416022 | 0.999970678 | 2.762 | 0.631 | 3.7 | 0.774 | Plus-3 |
| TRINITY_DN9496_c0_g1_i2 | -0.60934146 | 0.88623867 | 0.999970678 | 3.97 | 0 | 5.044 | 0 | NA |
| TRINITY_DN5113_c0_g2_i2 | 0.609275733 | 0.67092746 | 0.999970678 | 1.235 | 9.796 | 1.922 | 4.836 | HSP20 |
| TRINITY_DN10233_c1_g2_i3 | 0.609231822 | 0.88810783 | 0.999970678 | 0 | 2.454 | 1.333 | 0 | NA |
| TRINITY_DN29170_c0_g1_i1 | -0.6091713 | 0.445743105 | 0.999970678 | 3.025 | 2.986 | 3.222 | 5.228 | RNase_T |
| TRINITY_DN91611_c0_g1_i1 | 0.609072614 | 0.89054128 | 0.999970678 | 1.49 | 0 | 0.822 | 0 | NA |
| TRINITY_DN4539_c0_g1_i4 | 0.609049001 | 0.644929995 | 0.999970678 | 1.544 | 0.503 | 0.811 | 0.372 | NA |
| TRINITY_DN1337_c0_g1_i1 | -0.60903235 | 0.636004749 | 0.999970678 | 7.93 | 17.98 | 4.766 | 33.569 | p450 |
| TRINITY_DN367_c0_g1_i4 | -0.60901444 | 0.371801751 | 0.999970678 | 378.52 | 627.6 | 610.963 | 739.5 | - |
| TRINITY_DN3150_c0_g1_i2 | -0.60899538 | 0.629354056 | 0.999970678 | 1.998 | 3.696 | 1.367 | 7.017 | MIP |
| TRINITY_DN205_c0_g1_i11 | -0.60897492 | 0.888780674 | 0.999970678 | 1.281 | 0 | 1.667 | 0 | - |
| TRINITY_DN4462_c0_g1_i1 | 0.608752626 | 0.648215124 | 0.999970678 | 67.812 | 9.678 | 14.71 | 33.318 | NA |
| TRINITY_DN11260_c0_g1_i5 | 0.608752309 | 0.757930243 | 1 | 0.245 | 0.848 | 0.544 | 0.04 | NA |
| TRINITY_DN16693_c0_g2_i1 | 0.608749069 | 0.654358044 | 0.999970678 | 0.527 | 0.276 | 0.333 | 0.131 | NA |
| TRINITY_DN1915_c0_g1_i13 | 0.608682871 | 0.529416662 | 0.999970678 | 1.054 | 1.261 | 0.756 | 0.583 | - |
| TRINITY_DN728_c1_g1_i2 | 0.608644585 | 0.795247571 | 0.999970678 | 33.574 | 1.143 | 18.899 | 0.221 | Inhibitor_I29 |
| TRINITY_DN8630_c0_g1_i2 | 0.608635057 | 0.8850881 | 0.999970678 | 0 | 3.489 | 1.855 | 0 | - |
| TRINITY_DN74294_c0_g1_i1 | 0.608594805 | 0.831083759 | 1 | 0.118 | 0.591 | 0 | 0.462 | NA |
| TRINITY_DN7449_c0_g1_i9 | -0.60848866 | 0.524995261 | 0.999970678 | 1.935 | 1.882 | 1.733 | 3.71 | NA |
| TRINITY_DN50737_c0_g1_i1 | -0.60844612 | 0.612286621 | 0.999970678 | 1.29 | 1.015 | 0.711 | 2.644 | AA_permease_2 |
| TRINITY_DN5120_c0_g2_i5 | -0.60844201 | 0.842152368 | 1 | 1.226 | 0.227 | 1.811 | 0 | NA |
| TRINITY_DN262_c1_g1_i1 | 0.6084407 | 0.504634654 | 0.999970678 | 12.009 | 7.963 | 7.555 | 4.001 | NA |
| TRINITY_DN449_c0_g1_i7 | -0.60843226 | 0.645744313 | 0.999970678 | 4.724 | 1.271 | 5.166 | 2.956 | NA |
| TRINITY_DN2683_c0_g1_i1 | -0.60841709 | 0.642351684 | 0.999970678 | 1.254 | 2.326 | 3.822 | 0.814 | NAD_binding_10 |
| TRINITY_DN4630_c0_g1_i1 | -0.60840849 | 0.363586001 | 0.999970678 | 11.382 | 14.61 | 15.21 | 20.882 | NA |
| TRINITY_DN1956_c0_g1_i16 | -0.60839809 | 0.390823724 | 0.999970678 | 3.879 | 3.528 | 3.989 | 6.454 | NA |
| TRINITY_DN19687_c0_g1_i2 | -0.60837186 | 0.501625521 | 0.999970678 | 0.709 | 0.542 | 0.756 | 0.995 | DUF620 |
| TRINITY_DN8540_c0_g1_i1 | -0.6083157 | 0.585334915 | 0.999970678 | 4.569 | 7.116 | 3.111 | 13.985 | PAP2 |
| TRINITY_DN3560_c0_g1_i4 | -0.60825873 | 0.782368213 | 0.999970678 | 7.703 | 0.039 | 5.244 | 5.62 | - |
| TRINITY_DN72_c0_g1_i5 | 0.608255137 | 0.825120538 | 0.999970678 | 6.004 | 1.882 | 0 | 5.248 | NA |
| TRINITY_DN8108_c0_g1_i1 | -0.60818826 | 0.828088329 | 0.999970678 | 0.382 | 0.749 | 0 | 1.719 | p450 |
| TRINITY_DN15920_c0_g1_i3 | 0.608115025 | 0.55017044 | 0.999970678 | 1.889 | 1.183 | 0.889 | 0.965 | Motile_Sperm |
| TRINITY_DN3263_c1_g1_i4 | -0.6081134 | 0.548366735 | 0.999970678 | 1.017 | 0.562 | 0.9 | 1.337 | NA |
| TRINITY_DN18757_c0_g1_i3 | -0.60803958 | 0.823299959 | 0.999970678 | 0 | 9.195 | 7.655 | 4.484 | FKBP_C |
| TRINITY_DN3795_c0_g1_i2 | -0.60797771 | 0.69895796 | 0.999970678 | 0.345 | 0.739 | 0.233 | 1.367 | NA |
| TRINITY_DN8589_c0_g1_i1 | -0.60795812 | 0.417160943 | 0.999970678 | 55.258 | 59.86 | 94.072 | 60.976 | NA |
| TRINITY_DN8316_c1_g1_i1 | -0.60794186 | 0.886621263 | 0.999970678 | 1.799 | 0 | 2.3 | 0 | Sugar_tr |
| TRINITY_DN248_c1_g2_i1 | 0.607906845 | 0.34980027 | 0.999970678 | 6.268 | 5.026 | 3.189 | 3.579 | DENN |
| TRINITY_DN13805_c0_g1_i1 | 0.607903371 | 0.592589826 | 0.999970678 | 0.945 | 0.995 | 0.367 | 0.845 | NA |
| TRINITY_DN223_c0_g1_i2 | 0.607872893 | 0.521776745 | 0.999970678 | 13.39 | 14.68 | 3.9 | 13.733 | CUE |
| TRINITY_DN2371_c0_g1_i4 | 0.6078688 | 0.702911715 | 0.999970678 | 0.218 | 0.67 | 0.389 | 0.121 | NA |
| TRINITY_DN616_c1_g7_i1 | 0.607864916 | 0.885008465 | 0.999970678 | 5.378 | 0 | 0 | 3.589 | NA |
| TRINITY_DN1391_c0_g1_i4 | -0.60785501 | 0.360642443 | 0.999970678 | 446.12 | 293.3 | 475.316 | 557.508 | NA |
| TRINITY_DN449_c0_g1_i6 | 0.607761613 | 0.560498342 | 0.999970678 | 10.183 | 29.32 | 6.933 | 17.312 | NA |
| TRINITY_DN28273_c0_g1_i1 | -0.60766891 | 0.427846101 | 0.999970678 | 115.43 | 81.28 | 155.968 | 108.972 | RPS31 |
| TRINITY_DN43610_c1_g4_i1 | -0.60753993 | 0.820795832 | 0.999970678 | 0 | 15.58 | 8.299 | 13.14 | NA |
| TRINITY_DN3698_c0_g2_i3 | 0.607535939 | 0.572244729 | 0.999970678 | 1.49 | 4.809 | 1.489 | 2.312 | MatE |
| TRINITY_DN10244_c0_g1_i9 | -0.6075212 | 0.641359027 | 0.999970678 | 0.382 | 0.581 | 0.989 | 0.282 | DUF4283 |
| TRINITY_DN533_c0_g1_i35 | -0.6075189 | 0.844189809 | 0.999970678 | 0 | 6.189 | 7.488 | 0.221 | NA |
| TRINITY_DN7133_c0_g1_i1 | 0.607490673 | 0.640226008 | 0.999970678 | 2.961 | 0.769 | 1.467 | 0.724 | Methyltransf_29 |
| TRINITY_DN71929_c0_g1_i1 | 0.607393208 | 0.889034631 | 0.999970678 | 3.379 | 0 | 1.855 | 0 | adh_short |
| TRINITY_DN12095_c0_g1_i3 | -0.60738001 | 0.548630759 | 0.999970678 | 1.962 | 3.311 | 1.933 | 5.65 | NA |
| TRINITY_DN1045_c0_g1_i4 | -0.60732624 | 0.797879756 | 0.999970678 | 0.173 | 11.15 | 13.555 | 0.593 | NA |
| TRINITY_DN2176_c1_g1_i1 | 0.607189735 | 0.734772366 | 0.999970678 | 6.822 | 0.286 | 0.933 | 3.609 | NA |
| TRINITY_DN2159_c0_g1_i8 | -0.60701031 | 0.565022167 | 0.999970678 | 0.954 | 2.897 | 3.011 | 2.192 | NA |
| TRINITY_DN3242_c0_g1_i4 | 0.606970457 | 0.405048621 | 0.999970678 | 18.113 | 17.65 | 12.477 | 8.455 | JAB |
| TRINITY_DN10699_c0_g1_i1 | -0.60696649 | 0.643671302 | 0.999970678 | 1.217 | 0.857 | 0.767 | 2.212 | UDPGT |
| TRINITY_DN1423_c0_g1_i1 | -0.6069632 | 0.67584131 | 0.999970678 | 13.689 | 1.518 | 5.255 | 17.202 | NA |
| TRINITY_DN4149_c0_g1_i2 | -0.60695414 | 0.644622346 | 0.999970678 | 6.713 | 2.897 | 1.833 | 12.547 | Pkinase |
| TRINITY_DN1588_c0_g1_i1 | -0.60692738 | 0.72523488 | 0.999970678 | 2.698 | 3.775 | 0.278 | 9.491 | NA |
| TRINITY_DN8107_c0_g1_i2 | 0.606824171 | 0.888439656 | 0.999970678 | 0 | 1.912 | 0 | 1.227 | DUF573 |
| TRINITY_DN632_c0_g1_i3 | -0.60674368 | 0.614235609 | 0.999970678 | 1.344 | 0.434 | 1.133 | 1.317 | DAO |
| TRINITY_DN23610_c0_g1_i2 | -0.60667743 | 0.297291692 | 0.999970678 | 237.42 | 201.3 | 262.395 | 347.878 | NA |
| TRINITY_DN16767_c0_g1_i5 | 0.6066601 | 0.892505042 | 1 | 2.698 | 0 | 1.489 | 0 | NA |
| TRINITY_DN21_c0_g1_i15 | -0.60662902 | 0.606584252 | 0.999970678 | 2.035 | 7.106 | 3.266 | 9.833 | NA |
| TRINITY_DN19458_c0_g1_i6 | -0.60661845 | 0.83268391 | 0.999970678 | 3.824 | 0 | 0.844 | 4.806 | NA |
| TRINITY_DN7933_c0_g5_i1 | 0.60657314 | 0.831426949 | 0.999970678 | 5.387 | 0 | 2.178 | 0.965 | NA |
| TRINITY_DN1437_c1_g2_i1 | -0.60647175 | 0.501647066 | 0.999970678 | 5.914 | 10.18 | 6.411 | 16.689 | NA |
| TRINITY_DN432_c0_g1_i6 | -0.60646805 | 0.668943571 | 0.999970678 | 10.937 | 62.59 | 16.943 | 89.056 | NA |
| TRINITY_DN5107_c0_g1_i7 | 0.606465604 | 0.536964158 | 0.999970678 | 2.616 | 1.547 | 1.589 | 0.834 | NUFIP1 |
| TRINITY_DN245_c0_g1_i6 | 0.60645894 | 0.611684112 | 0.999970678 | 1.426 | 2.533 | 0.767 | 1.599 | Fe-S_biosyn |
| TRINITY_DN11768_c0_g3_i3 | 0.606418613 | 0.835424856 | 0.999970678 | 5.16 | 0.631 | 3.155 | 0 | NA |

| TRINITY_DN27080_c0_g1_i2 | -0.60639195 | 0.889682935 | 0.999970678 | 0.781 | 0 | 1 | 0 | NA |
| --- | --- | --- | --- | --- | --- | --- | --- | --- |
| TRINITY_DN1347_c1_g1_i6 | 0.606364717 | 0.609149675 | 0.999970678 | 0.763 | 2.346 | 1.211 | 0.583 | Gelsolin |
| TRINITY_DN21431_c0_g1_i1 | -0.60633659 | 0.894698686 | 1 | 4.469 | 0 | 5.611 | 0 | - |
| TRINITY_DN100326_c0_g1_i1 | -0.60633659 | 0.894698686 | 1 | 1.299 | 0 | 1.655 | 0 | NA |
| TRINITY_DN32516_c0_g1_i1 | -0.60633659 | 0.894698686 | 1 | 0.836 | 0 | 1.067 | 0 | MoCF_biosynth |
| TRINITY_DN77365_c0_g1_i1 | -0.60633659 | 0.894698686 | 1 | 1.572 | 0 | 2 | 0 | DUF1772 |
| TRINITY_DN10251_c0_g1_i3 | 0.606328238 | 0.820764136 | 0.999970678 | 0.981 | 1.064 | 0 | 1.347 | Dimerisation |
| TRINITY_DN9063_c0_g1_i2 | -0.60630232 | 0.48009322 | 0.999970678 | 21.92 | 23.21 | 21.321 | 39.642 | NA |
| TRINITY_DN437_c0_g1_i2 | -0.60623664 | 0.410756592 | 0.999970678 | 8.63 | 11.34 | 9.399 | 19.032 | B56 |
| TRINITY_DN420_c0_g1_i8 | 0.606207536 | 0.696523073 | 0.999970678 | 7.703 | 34.19 | 2.111 | 24.732 | NA |
| TRINITY_DN665_c1_g1_i17 | 0.606206559 | 0.639951516 | 0.999970678 | 77.304 | 34.78 | 8.722 | 63.258 | - |
| TRINITY_DN22710_c0_g1_i1 | 0.606119142 | 0.568506513 | 0.999970678 | 0.645 | 1.685 | 0.689 | 0.694 | Lzipper-MIP1 |
| TRINITY_DN434_c0_g1_i2 | -0.60606308 | 0.826425168 | 0.999970678 | 24.254 | 0 | 7.066 | 27.889 | NA |
| TRINITY_DN7067_c0_g1_i1 | -0.60603695 | 0.507355281 | 0.999970678 | 8.657 | 7.411 | 11.622 | 9.822 | NA |
| TRINITY_DN3202_c0_g1_i1 | 0.605898167 | 0.886780126 | 0.999970678 | 0 | 11.6 | 6.2 | 0 | NA |
| TRINITY_DN23639_c0_g1_i11 | -0.60588781 | 0.617764334 | 1 | 2.089 | 3.035 | 3.378 | 3.529 | NA |
| TRINITY_DN8131_c0_g1_i8 | 0.605820471 | 0.55124207 | 0.999970678 | 0.899 | 1.025 | 0.733 | 0.392 | NA |
| TRINITY_DN3598_c0_g1_i1 | 0.605692754 | 0.465029968 | 0.999970678 | 15.179 | 28.92 | 9.488 | 17.222 | Rhomboid |
| TRINITY_DN8095_c0_g1_i4 | 0.605651875 | 0.568272563 | 0.999970678 | 4.814 | 18.87 | 5.877 | 8.355 | EGF_2 |
| TRINITY_DN1024_c0_g1_i9 | 0.605629906 | 0.450680946 | 0.999970678 | 5.995 | 9.067 | 2.933 | 6.324 | HAT |
| TRINITY_DN4333_c2_g2_i1 | 0.605597513 | 0.490564253 | 0.999970678 | 5.905 | 6.751 | 4.866 | 2.433 | GYF |
| TRINITY_DN4479_c0_g1_i1 | 0.605564549 | 0.719761669 | 0.999970678 | 1.526 | 0.946 | 1.211 | 0.171 | NA |
| TRINITY_DN4833_c0_g1_i9 | -0.605501 | 0.695426219 | 0.999970678 | 3.207 | 0.306 | 1.6 | 3.479 | NA |
| TRINITY_DN192_c0_g1_i5 | -0.60546708 | 0.47657214 | 0.999970678 | 36.499 | 48.18 | 32.354 | 89.337 | Ribosomal_L3 |
| TRINITY_DN282_c2_g1_i1 | -0.60542744 | 0.470042285 | 0.999970678 | 133.25 | 60.28 | 126.381 | 139.676 | NA |
| TRINITY_DN1742_c0_g1_i2 | -0.60542678 | 0.659257782 | 0.999970678 | 1.989 | 0.296 | 1.689 | 1.508 | PPR |
| TRINITY_DN11038_c0_g1_i1 | -0.6054242 | 0.436255771 | 0.999970678 | 6.077 | 7.194 | 6 | 12.919 | Arm |
| TRINITY_DN49247_c0_g1_i1 | 0.605362776 | 0.734204157 | 0.999970678 | 56.738 | 1400 | 124.159 | 717.703 | Chloroa_b-bind |
| TRINITY_DN8013_c0_g1_i1 | -0.60532865 | 0.474127213 | 0.999970678 | 26.462 | 61.83 | 52.03 | 69.572 | NA |
| TRINITY_DN4698_c0_g1_i2 | -0.60523386 | 0.826642508 | 0.999970678 | 13.635 | 0 | 7.366 | 12.125 | NA |
| TRINITY_DN2522_c0_g1_i1 | 0.605211342 | 0.822451563 | 0.999970678 | 11.219 | 16.28 | 0 | 17.855 | NA |
| TRINITY_DN5904_c1_g1_i2 | 0.605094987 | 0.887977889 | 0.999970678 | 0 | 2.533 | 0 | 1.639 | NA |
| TRINITY_DN7913_c0_g1_i8 | 0.605012719 | 0.841434858 | 0.999970678 | 14.425 | 0 | 6.977 | 1.206 | CBM_20 |
| TRINITY_DN1371_c0_g1_i24 | -0.60501183 | 0.601558095 | 1 | 0.263 | 0.296 | 0.356 | 0.412 | Terpene_synth |
| TRINITY_DN4974_c0_g1_i5 | -0.6049664 | 0.427793066 | 0.999970678 | 3.743 | 4.563 | 3.922 | 7.882 | PolyA_pol |
| TRINITY_DN2133_c0_g1_i2 | -0.6048984 | 0.828294396 | 0.999970678 | 0.936 | 0.808 | 2.189 | 0 | NA |
| TRINITY_DN6615_c0_g1_i4 | 0.604875618 | 0.568644592 | 0.999970678 | 2.507 | 7.047 | 3.533 | 1.96 | HECT |
| TRINITY_DN3139_c0_g1_i2 | 0.604770136 | 0.886154305 | 0.999970678 | 15.243 | 0 | 0 | 10.134 | NA |
| TRINITY_DN5408_c0_g1_i2 | -0.60473641 | 0.477759899 | 0.999970678 | 2.226 | 1.705 | 2.811 | 2.624 | NA |
| TRINITY_DN7881_c0_g1_i6 | 0.604666028 | 0.885608991 | 0.999970678 | 13.39 | 0 | 0 | 8.857 | NA |
| TRINITY_DN4958_c0_g1_i3 | 0.604621169 | 0.438895184 | 0.999970678 | 4.969 | 6.544 | 3.944 | 2.785 | Trypsin_2 |
| TRINITY_DN38773_c1_g1_i1 | -0.60460916 | 0.655036946 | 0.999970678 | 0.59 | 1.695 | 0.6 | 2.725 | Sina |
| TRINITY_DN877_c0_g1_i5 | 0.604434976 | 0.426284416 | 0.999970678 | 11.982 | 7.628 | 6.311 | 5.318 | NA |
| TRINITY_DN7363_c0_g1_i5 | -0.60442035 | 0.461772384 | 0.999970678 | 20.212 | 26.5 | 19.099 | 47.644 | NA |
| TRINITY_DN9875_c0_g1_i5 | 0.604371632 | 0.619877857 | 0.999970678 | 0.936 | 0.749 | 0.667 | 0.302 | NA |
| TRINITY_DN382_c0_g1_i3 | 0.604329301 | 0.694811574 | 0.999970678 | 14.761 | 1.478 | 6.466 | 3.006 | NA |
| TRINITY_DN3547_c0_g1_i1 | 0.604310208 | 0.560741217 | 0.999970678 | 33.429 | 34.93 | 7.866 | 35.53 | FAF |
| TRINITY_DN2845_c0_g2_i9 | -0.60428364 | 0.894162217 | 1 | 0 | 0.345 | 0.411 | 0 | NA |
| TRINITY_DN94_c2_g1_i1 | 0.604204383 | 0.49649418 | 0.999970678 | 5.114 | 8.278 | 2.655 | 5.53 | Ribosomal_L18p |
| TRINITY_DN3796_c0_g1_i1 | 0.604199399 | 0.445957625 | 0.999970678 | 4.796 | 3.863 | 1.722 | 3.629 | NA |
| TRINITY_DN17117_c0_g1_i3 | 0.604110097 | 0.622834281 | 0.999970678 | 1.844 | 1.104 | 1 | 0.714 | NA |
| TRINITY_DN979_c0_g1_i2 | 0.604105893 | 0.730798347 | 0.999970678 | 12.336 | 192.1 | 12.277 | 117.789 | NA |
| TRINITY_DN383_c0_g1_i1 | 0.604062987 | 0.501110015 | 0.999970678 | 10.238 | 16.99 | 10.255 | 5.489 | NA |
| TRINITY_DN19787_c0_g1_i1 | -0.60396314 | 0.697635218 | 1 | 0.581 | 0.946 | 0.344 | 1.95 | - |
| TRINITY_DN21608_c0_g1_i5 | 0.60392066 | 0.642094772 | 0.999970678 | 2.416 | 0.936 | 0.4 | 1.709 | IF-2B |
| TRINITY_DN5400_c0_g1_i4 | 0.603862316 | 0.890264742 | 0.999970678 | 2.689 | 0 | 0 | 1.78 | NA |
| TRINITY_DN6731_c0_g1_i1 | -0.60381588 | 0.452377584 | 0.999970678 | 2.98 | 1.843 | 3.222 | 3.479 | - |
| TRINITY_DN407_c0_g1_i6 | -0.60377741 | 0.629915916 | 0.999970678 | 58.991 | 15.06 | 71.818 | 26.411 | NA |
| TRINITY_DN361_c0_g1_i3 | -0.60366064 | 0.539214327 | 0.999970678 | 1.463 | 0.798 | 1.522 | 1.599 | Ins134_P3_kin |
| TRINITY_DN1858_c0_g1_i1 | -0.60363421 | 0.517115639 | 0.999970678 | 13.035 | 32.31 | 21.432 | 42.226 | NA |
| TRINITY_DN569_c0_g1_i9 | 0.60360837 | 0.826906478 | 0.999970678 | 3.733 | 8.545 | 0 | 8.063 | NA |
| TRINITY_DN19119_c0_g2_i1 | -0.60358926 | 0.501853323 | 0.999970678 | 30.195 | 62.88 | 39.442 | 92.906 | Epimerase |
| TRINITY_DN8370_c0_g1_i1 | -0.60344554 | 0.545153491 | 0.999970678 | 1.081 | 0.749 | 0.955 | 1.619 | NA |
| TRINITY_DN13311_c0_g2_i1 | 0.603428567 | 0.631389542 | 0.999970678 | 1.499 | 7.253 | 1.733 | 3.599 | NA |
| TRINITY_DN205266_c0_g1_i1 | -0.60338296 | 0.890734129 | 0.999970678 | 2.462 | 0 | 3.133 | 0 | Pmp3 |
| TRINITY_DN205_c0_g1_i9 | -0.60337911 | 0.436319293 | 0.999970678 | 6.032 | 3.459 | 6.711 | 6.404 | - |
| TRINITY_DN53858_c0_g1_i1 | -0.60335764 | 0.85635796 | 1 | 0.445 | 0.02 | 0.589 | 0 | bZIP_1 |
| TRINITY_DN4098_c0_g1_i14 | -0.60327688 | 0.700113816 | 0.999970678 | 0.309 | 0.621 | 1 | 0.201 | NA |
| TRINITY_DN6801_c0_g1_i3 | 0.603272747 | 0.59217757 | 0.999970678 | 6.077 | 9.156 | 6.688 | 1.93 | - |
| TRINITY_DN3506_c0_g2_i1 | 0.603212713 | 0.391064453 | 0.999970678 | 35.927 | 50.2 | 28.387 | 22.349 | NA |
| TRINITY_DN16365_c0_g1_i3 | -0.60317409 | 0.891535352 | 1 | 0 | 0.483 | 0 | 0.734 | NA |
| TRINITY_DN743_c0_g1_i8 | -0.60317371 | 0.566544379 | 0.999970678 | 20.811 | 6.327 | 12.577 | 26.512 | NA |
| TRINITY_DN5938_c0_g1_i1 | -0.60315311 | 0.567465044 | 0.999970678 | 9.275 | 2.759 | 7.955 | 8.767 | Ribosomal_L7Ae |
| TRINITY_DN5938_c0_g1_i3 | -0.60315311 | 0.567465044 | 0.999970678 | 9.275 | 2.759 | 7.955 | 8.767 | Ribosomal_L7Ae |

| TRINITY_DN270_c0_g9_i1 | -0.60313186 | 0.6413559 | 0.999970678 | 2.453 | 0.769 | 2.4 | 1.95 | p450 |
| --- | --- | --- | --- | --- | --- | --- | --- | --- |
| TRINITY_DN2402_c0_g1_i2 | -0.6030978 | 0.604413534 | 0.999970678 | 0.872 | 2.947 | 3.2 | 1.9 | NA |
| TRINITY_DN1005_c0_g1_i10 | -0.60306671 | 0.836061753 | 0.999970678 | 0 | 3.351 | 0.356 | 4.595 | NA |
| TRINITY_DN717_c0_g1_i1 | 0.603033954 | 0.835071521 | 0.999970678 | 2.344 | 0 | 0.289 | 1.227 | UPF0160 |
| TRINITY_DN2949_c0_g1_i2 | 0.603016312 | 0.827854836 | 1 | 0.609 | 0.345 | 0.511 | 0 | Frigida |
| TRINITY_DN2981_c2_g2_i2 | -0.60299732 | 0.705118332 | 0.999970678 | 0.881 | 5.706 | 6.966 | 1.438 | NA |
| TRINITY_DN969_c0_g1_i1 | -0.60297111 | 0.513439033 | 0.999970678 | 23.954 | 15.5 | 14.277 | 43.02 | Calmodulin_bind |
| TRINITY_DN2441_c0_g1_i4 | 0.602931409 | 0.606962624 | 0.999970678 | 7.54 | 10.47 | 8.111 | 2.031 | NA |
| TRINITY_DN8607_c0_g1_i1 | -0.60293064 | 0.683694004 | 0.999970678 | 1.426 | 12.33 | 4.066 | 15.724 | TAXi_C |
| TRINITY_DN2506_c0_g1_i1 | 0.602883006 | 0.690705775 | 0.999970678 | 2.798 | 20.61 | 2.533 | 12.004 | NA |
| TRINITY_DN5791_c0_g1_i1 | 0.602678416 | 0.444311801 | 0.999970678 | 76.296 | 39.72 | 28.709 | 41.894 | SNAP |
| TRINITY_DN8216_c0_g1_i1 | 0.602661674 | 0.891975311 | 1 | 1.635 | 0 | 0.9 | 0 | NA |
| TRINITY_DN16741_c0_g1_i1 | 0.60266111 | 0.381730442 | 0.999970678 | 2.589 | 2.503 | 1.555 | 1.488 | LRR_8 |
| TRINITY_DN23_c0_g2_i4 | -0.60264307 | 0.541654306 | 0.999970678 | 52.987 | 26.58 | 72.385 | 34.183 | NA |
| TRINITY_DN6077_c0_g1_i1 | -0.60254735 | 0.443517679 | 0.999970678 | 2.353 | 4.11 | 3.544 | 5.509 | NA |
| TRINITY_DN185_c1_g1_i2 | 0.602546331 | 0.649029424 | 0.999970678 | 12.836 | 22.81 | 17.066 | 2.745 | - |
| TRINITY_DN90842_c0_g1_i1 | -0.6025326 | 0.618820132 | 0.999970678 | 0.572 | 0.355 | 0.456 | 0.855 | PPR |
| TRINITY_DN91063_c0_g1_i1 | 0.60245746 | 0.88892061 | 0.999970678 | 2.725 | 0 | 1.511 | 0 | Oxidored_FMN |
| TRINITY_DN9269_c0_g1_i1 | -0.60244982 | 0.296208309 | 0.999970678 | 116.37 | 127.3 | 138.769 | 200.37 | DIM1 |
| TRINITY_DN9073_c0_g1_i2 | -0.602426 | 0.58962926 | 0.999970678 | 13.317 | 3.262 | 8.433 | 15.282 | NA |
| TRINITY_DN2396_c0_g1_i14 | 0.602304394 | 0.887036743 | 0.999970678 | 4.869 | 0 | 2.678 | 0 | NA |
| TRINITY_DN551_c0_g1_i1 | 0.602256597 | 0.833440191 | 0.999970678 | 9.257 | 0 | 3.044 | 2.534 | Cellulose_synt |
| TRINITY_DN1436_c0_g1_i7 | -0.60224698 | 0.893269456 | 1 | 28.969 | 0 | 36.287 | 0 | NA |
| TRINITY_DN35531_c0_g1_i2 | -0.60224698 | 0.893269456 | 1 | 0.427 | 0 | 0.544 | 0 | NA |
| TRINITY_DN9035_c0_g1_i1 | -0.60216833 | 0.835835108 | 0.999970678 | 0 | 0.877 | 0.989 | 0.111 | DUF810 |
| TRINITY_DN11559_c0_g2_i3 | -0.60211824 | 0.825284476 | 0.999970678 | 2.062 | 0 | 0.789 | 2.212 | NA |
| TRINITY_DN994_c0_g1_i5 | 0.602060443 | 0.758895045 | 0.999970678 | 2.444 | 16.51 | 9.911 | 0.342 | NA |
| TRINITY_DN5120_c0_g1_i1 | 0.601940968 | 0.545932784 | 0.999970678 | 5.841 | 7.076 | 5.333 | 2.051 | NA |
| TRINITY_DN3050_c0_g1_i1 | -0.60178481 | 0.531432501 | 0.999970678 | 38.916 | 53.37 | 29.365 | 103.382 | DUF538 |
| TRINITY_DN12187_c0_g1_i4 | -0.60177113 | 0.610044167 | 0.999970678 | 2.162 | 0.798 | 1.411 | 2.785 | NA |
| TRINITY_DN4994_c0_g1_i5 | 0.601723761 | 0.478144387 | 0.999970678 | 3.961 | 4.869 | 1.7 | 3.76 | ADH_N |
| TRINITY_DN817_c0_g1_i3 | 0.601615549 | 0.501187879 | 0.999970678 | 1.017 | 1.202 | 0.711 | 0.613 | DUF4413 |
| TRINITY_DN436_c0_g1_i1 | -0.60161171 | 0.345608342 | 0.999970678 | 106.69 | 157.3 | 155.157 | 211.288 | WD40 |
| TRINITY_DN2808_c0_g1_i1 | -0.60157043 | 0.33159954 | 0.999970678 | 6.867 | 7.559 | 8.744 | 11.29 | NA |
| TRINITY_DN12395_c0_g1_i1 | -0.60153116 | 0.834060612 | 0.999970678 | 0.536 | 0.237 | 0.955 | 0 | Sugar_tr |
| TRINITY_DN6353_c0_g3_i3 | 0.601504629 | 0.828511722 | 0.999970678 | 0 | 6.426 | 2.3 | 1.367 | NA |
| TRINITY_DN7023_c0_g1_i1 | 0.601491187 | 0.4655118 | 0.999970678 | 13.889 | 12.09 | 9.677 | 5.469 | NA |
| TRINITY_DN2812_c0_g2_i3 | 0.601480098 | 0.462538286 | 0.999970678 | 4.242 | 8.16 | 3.611 | 3.75 | NA |
| TRINITY_DN28587_c0_g1_i2 | 0.601464 | 0.653272057 | 0.999970678 | 1.108 | 0.296 | 0.389 | 0.462 | HNH |
| TRINITY_DN196391_c0_g1_i1 | -0.601443 | 0.427830268 | 0.999970678 | 4.015 | 5.006 | 5.066 | 7.46 | NA |
| TRINITY_DN11564_c0_g3_i1 | -0.60142366 | 0.83061594 | 0.999970678 | 0 | 0.798 | 0.767 | 0.251 | AMP-binding |
| TRINITY_DN1095_c0_g2_i7 | -0.60140876 | 0.661983281 | 0.999970678 | 2.425 | 1.419 | 3.933 | 1.056 | NA |
| TRINITY_DN14229_c0_g1_i1 | -0.60137738 | 0.504747235 | 0.999970678 | 3.525 | 4.297 | 3.311 | 7.782 | TCTP |
| TRINITY_DN5789_c0_g1_i5 | 0.601334291 | 0.587401029 | 0.999970678 | 1.98 | 4.218 | 2.344 | 1.267 | NA |
| TRINITY_DN5097_c0_g1_i2 | -0.60131544 | 0.421060454 | 0.999970678 | 3.761 | 3.321 | 5.022 | 4.615 | NA |
| TRINITY_DN59735_c0_g1_i1 | -0.60126292 | 0.562303832 | 0.999970678 | 1.935 | 2.336 | 1.6 | 4.504 | NA |
| TRINITY_DN274_c0_g1_i7 | -0.60119805 | 0.838335569 | 0.999970678 | 0 | 1.498 | 0.144 | 2.071 | RRM_1 |
| TRINITY_DN2469_c0_g1_i2 | -0.60117458 | 0.369186988 | 0.999970678 | 186.53 | 195.7 | 185.455 | 354.252 | Glyco_hydro_14 |
| TRINITY_DN11551_c1_g2_i4 | -0.60110448 | 0.489506617 | 0.999970678 | 26.843 | 53.33 | 35.576 | 77.363 | NA |
| TRINITY_DN17546_c0_g1_i1 | -0.60106882 | 0.889484882 | 0.999970678 | 0 | 0.986 | 1.244 | 0 | - |
| TRINITY_DN70165_c0_g1_i2 | -0.60094458 | 0.888561769 | 0.999970678 | 1.499 | 0 | 1.911 | 0 | AA_permease |
| TRINITY_DN872_c0_g1_i4 | 0.600909481 | 0.689377678 | 0.999970678 | 32.866 | 29.82 | 32.065 | 2.584 | NA |
| TRINITY_DN4217_c0_g3_i1 | 0.600855012 | 0.720174543 | 0.999970678 | 0.836 | 10.31 | 1.144 | 5.871 | AA_kinase |
| TRINITY_DN1711_c0_g2_i3 | 0.600830659 | 0.359914044 | 0.999970678 | 4.306 | 3.794 | 2.433 | 2.413 | NA |
| TRINITY_DN31780_c0_g1_i2 | -0.6008127 | 0.840067624 | 1 | 0 | 1.064 | 1.178 | 0.181 | NA |
| TRINITY_DN2043_c0_g1_i1 | -0.60074765 | 0.831898165 | 1 | 0 | 0.394 | 0.322 | 0.191 | NA |
| TRINITY_DN2123_c0_g1_i3 | 0.600609371 | 0.565781692 | 0.999970678 | 1.172 | 3.4 | 1.389 | 1.337 | AICARFT_IMPCH |
| TRINITY_DN1288_c0_g1_i3 | -0.60059462 | 0.843059441 | 0.999970678 | 120.32 | 0 | 26.421 | 152.052 | Fe-S_biosyn |
| TRINITY_DN14312_c0_g1_i6 | 0.60055758 | 0.577886091 | 0.999970678 | 2.771 | 4.376 | 1.733 | 2.544 | RbcS |
| TRINITY_DN8416_c0_g1_i1 | -0.60055485 | 0.428209502 | 0.999970678 | 42.495 | 23.76 | 41.12 | 50.449 | WPP |
| TRINITY_DN12077_c0_g1_i4 | 0.600502841 | 0.826786155 | 0.999970678 | 1.699 | 3.026 | 2.567 | 0 | NA |
| TRINITY_DN6891_c2_g1_i1 | 0.600424036 | 0.748128968 | 1 | 1.553 | 0.237 | 0.833 | 0.181 | NA |
| TRINITY_DN168775_c0_g1_i1 | -0.60038973 | 0.584264355 | 0.999970678 | 0.718 | 0.729 | 1 | 0.985 | - |
| TRINITY_DN2295_c0_g1_i2 | -0.60037519 | 0.457096823 | 0.999970678 | 29.704 | 25.74 | 22.943 | 55.899 | NA |
| TRINITY_DN1552_c0_g1_i1 | -0.60037382 | 0.321744121 | 0.999970678 | 46.928 | 50.34 | 54.597 | 79.585 | Ribosomal_S27 |
| TRINITY_DN8002_c0_g1_i3 | 0.600358399 | 0.730381677 | 0.999970678 | 62.634 | 1978 | 294.704 | 954.719 | NA |
| TRINITY_DN1647_c0_g1_i4 | -0.6003117 | 0.835799444 | 0.999970678 | 1.145 | 0.325 | 1.867 | 0 | NA |
| TRINITY_DN303_c0_g1_i3 | -0.60027268 | 0.794454886 | 0.999970678 | 11.073 | 0.089 | 1.844 | 14.729 | PLAC8 |
| TRINITY_DN8352_c1_g1_i1 | -0.60026733 | 0.773493631 | 0.999970678 | 2.08 | 0.059 | 1.522 | 1.438 | LRRNT_2 |
| TRINITY_DN10589_c0_g1_i4 | -0.60021007 | 0.63613397 | 0.999970678 | 0.345 | 0.197 | 0.267 | 0.513 | NA |
| TRINITY_DN1188_c1_g1_i1 | 0.600162192 | 0.824007128 | 0.999970678 | 6.949 | 4.553 | 0 | 7.601 | SNARE |
| TRINITY_DN2347_c0_g1_i1 | 0.600120284 | 0.576688316 | 0.999970678 | 0.436 | 1.094 | 0.389 | 0.533 | NmrA |
| TRINITY_DN270_c0_g8_i1 | 0.599995026 | 0.490676199 | 0.999970678 | 48.79 | 36.27 | 31.631 | 17.463 | p450 |
| TRINITY_DN724_c0_g1_i6 | -0.5999755 | 0.836323708 | 0.999970678 | 2.616 | 0 | 0.333 | 3.619 | NA |

| TRINITY_DN1475_c1_g1_i2 | -0.59990111 | 0.887667795 | 0.999970678 | 1.117 | 0 | 0 | 1.749 | NA |
| --- | --- | --- | --- | --- | --- | --- | --- | --- |
| TRINITY_DN2726_c0_g4_i2 | -0.59983566 | 0.891957696 | 0.999970678 | 6.786 | 0 | 8.499 | 0 | NA |
| TRINITY_DN435_c0_g1_i2 | 0.599826285 | 0.455292924 | 0.999970678 | 3.161 | 5.568 | 2.333 | 2.926 | AA_permease_2 |
| TRINITY_DN6883_c0_g1_i1 | 0.599686371 | 0.891544684 | 0.999970678 | 4.17 | 0 | 2.3 | 0 | NA |
| TRINITY_DN1202_c0_g1_i6 | -0.59966921 | 0.514551787 | 0.999970678 | 54.031 | 82.44 | 123.104 | 57.437 | NA |
| TRINITY_DN50643_c0_g1_i2 | -0.59966445 | 0.845011082 | 1 | 1.717 | 0 | 0.178 | 2.423 | Ribosomal_S21 |
| TRINITY_DN7711_c0_g3_i2 | 0.599642774 | 0.675252958 | 1 | 3.388 | 1.252 | 0.856 | 2.001 | DUF4228 |
| TRINITY_DN3003_c0_g1_i3 | 0.599590394 | 0.531572707 | 0.999970678 | 5.587 | 9.727 | 5.944 | 2.885 | NA |
| TRINITY_DN845_c0_g1_i4 | 0.59958933 | 0.510303592 | 0.999970678 | 3.833 | 6.229 | 3.178 | 2.735 | NA |
| TRINITY_DN4334_c0_g1_i2 | 0.599506476 | 0.66825649 | 0.999970678 | 0.609 | 1.567 | 0.189 | 1.206 | NA |
| TRINITY_DN1934_c0_g1_i4 | -0.59941506 | 0.600612311 | 0.999970678 | 0.418 | 0.227 | 0.444 | 0.442 | Exo_endo_phos |
| TRINITY_DN1401_c0_g1_i3 | -0.59938324 | 0.426926704 | 0.999970678 | 18.159 | 26.84 | 21.043 | 42.587 | Ribosomal_S13 |
| TRINITY_DN4172_c0_g1_i2 | -0.59926671 | 0.40410472 | 0.999970678 | 6.313 | 5.568 | 5.911 | 10.848 | NA |
| TRINITY_DN3378_c0_g1_i7 | 0.599246236 | 0.44019614 | 0.999970678 | 140 | 237.1 | 77.54 | 154.023 | Chal_sti_synt_C |
| TRINITY_DN9942_c0_g1_i2 | 0.599186963 | 0.846899082 | 0.999970678 | 1.917 | 0 | 0.989 | 0.09 | NA |
| TRINITY_DN65171_c0_g1_i1 | -0.59916988 | 0.826214513 | 0.999970678 | 12.272 | 0 | 8.866 | 8.073 | NA |
| TRINITY_DN89952_c0_g1_i1 | 0.599071965 | 0.887579229 | 0.999970678 | 5.541 | 0 | 3.066 | 0 | NA |
| TRINITY_DN9230_c0_g1_i13 | 0.599057752 | 0.842030879 | 0.999970678 | 1.635 | 0 | 0.067 | 1.025 | PMD |
| TRINITY_DN1301_c0_g1_i3 | 0.599054718 | 0.560484689 | 0.999970678 | 32.266 | 19.28 | 7.611 | 24.239 | NA |
| TRINITY_DN3191_c0_g1_i22 | 0.599044606 | 0.674369721 | 0.999970678 | 0.554 | 0.739 | 0.6 | 0.131 | NA |
| TRINITY_DN9379_c0_g1_i1 | 0.599035456 | 0.612684267 | 0.999970678 | 9.138 | 10.59 | 8.622 | 2.513 | NA |
| TRINITY_DN8530_c0_g1_i1 | 0.598994016 | 0.671944071 | 0.999970678 | 0.818 | 1.912 | 0.267 | 1.468 | HD_4 |
| TRINITY_DN12163_c0_g1_i3 | -0.59893081 | 0.889981661 | 0.999970678 | 2.516 | 0 | 0 | 3.8 | NA |
| TRINITY_DN7471_c0_g1_i1 | -0.59892889 | 0.569666879 | 0.999970678 | 0.89 | 1.715 | 1.7 | 1.86 | mTERF |
| TRINITY_DN12529_c1_g1_i3 | 0.598833194 | 0.73563161 | 0.999970678 | 0.309 | 9.914 | 3.322 | 2.624 | Fasciclin |
| TRINITY_DN298_c0_g2_i7 | -0.59881977 | 0.324922482 | 0.999970678 | 10.737 | 9.747 | 13.81 | 14.357 | NA |
| TRINITY_DN8047_c0_g1_i5 | 0.598812263 | 0.613087621 | 0.999970678 | 25.726 | 10.87 | 10.788 | 10.104 | - |
| TRINITY_DN5765_c0_g1_i2 | -0.59872734 | 0.56539618 | 0.999970678 | 4.206 | 3.794 | 2.911 | 8.465 | B_lectin |
| TRINITY_DN6250_c0_g1_i2 | 0.598724667 | 0.823304876 | 0.999970678 | 1.526 | 3.952 | 0 | 3.599 | Glu_cyclase_2 |
| TRINITY_DN8797_c0_g1_i4 | 0.598719463 | 0.552828894 | 0.999970678 | 1.463 | 2.769 | 0.678 | 1.96 | Zein-binding |
| TRINITY_DN30278_c1_g1_i3 | 0.598662031 | 0.894299831 | 1 | 5.632 | 0 | 3.066 | 0 | - |
| TRINITY_DN1896_c0_g1_i1 | -0.5986618 | 0.314047892 | 0.999970678 | 29.005 | 34.16 | 36.164 | 51.696 | NA |
| TRINITY_DN194123_c0_g1_i1 | 0.59858987 | 0.399980272 | 0.999970678 | 17.559 | 15.11 | 10.055 | 9.28 | NA |
| TRINITY_DN1789_c0_g2_i2 | 0.598418236 | 0.3086241 | 0.999970678 | 150.05 | 123.6 | 72.884 | 92.645 | NA |
| TRINITY_DN3190_c0_g1_i4 | 0.598406019 | 0.449019234 | 0.999970678 | 5.278 | 3.755 | 2.922 | 2.453 | RVT_2 |
| TRINITY_DN1078_c1_g1_i2 | 0.598374127 | 0.338073283 | 0.999970678 | 8.748 | 11.95 | 5.911 | 6.535 | NA |
| TRINITY_DN7933_c1_g1_i2 | -0.59834101 | 0.59256989 | 0.999970678 | 17.359 | 55.46 | 24.621 | 79.786 | NA |
| TRINITY_DN5152_c0_g1_i2 | -0.5983113 | 0.677268962 | 0.999970678 | 0.69 | 0.217 | 0.889 | 0.322 | LRR_8 |
| TRINITY_DN3659_c0_g1_i1 | -0.59830119 | 0.463704185 | 0.999970678 | 6.695 | 13.14 | 9.988 | 17.865 | BTB |
| TRINITY_DN7693_c0_g2_i3 | 0.598275498 | 0.394144725 | 0.999970678 | 5.832 | 4.159 | 2.866 | 3.147 | NA |
| TRINITY_DN2326_c0_g2_i5 | -0.5982742 | 0.887395861 | 0.999970678 | 0 | 14.71 | 0 | 21.163 | NA |
| TRINITY_DN31185_c0_g1_i1 | -0.59827036 | 0.633328685 | 0.999970678 | 0.2 | 0.394 | 0.511 | 0.282 | NA |
| TRINITY_DN29726_c0_g1_i1 | -0.59824719 | 0.891038345 | 0.999970678 | 0.972 | 0 | 1.233 | 0 | NA |
| TRINITY_DN247_c0_g1_i13 | 0.598236226 | 0.7176923 | 0.999970678 | 6.504 | 7.037 | 6.366 | 0.975 | NA |
| TRINITY_DN4876_c0_g1_i2 | -0.59817579 | 0.726081994 | 0.999970678 | 2.825 | 1.143 | 4.611 | 0.462 | - |
| TRINITY_DN7299_c0_g2_i1 | -0.5981158 | 0.646030031 | 0.999970678 | 9.32 | 3.873 | 14.032 | 3.137 | NA |
| TRINITY_DN3507_c0_g1_i1 | -0.59810734 | 0.416331875 | 0.999970678 | 5.478 | 3.538 | 5.655 | 6.847 | NA |
| TRINITY_DN12095_c0_g1_i1 | 0.598066329 | 0.48048157 | 0.999970678 | 2.089 | 3.41 | 1.322 | 2.011 | NA |
| TRINITY_DN26844_c0_g1_i1 | 0.598062415 | 0.412132908 | 0.999970678 | 2.344 | 2.415 | 1.555 | 1.267 | NA |
| TRINITY_DN8181_c0_g1_i1 | 0.598022218 | 0.648842345 | 0.999970678 | 5.741 | 16.85 | 3.422 | 10.134 | NA |
| TRINITY_DN13420_c0_g1_i1 | -0.59801439 | 0.454249557 | 0.999970678 | 4.533 | 3.282 | 6.044 | 4.554 | NA |
| TRINITY_DN6829_c0_g2_i1 | 0.597982885 | 0.62258622 | 0.999970678 | 3.661 | 1.508 | 1.133 | 2.011 | NA |
| TRINITY_DN6535_c0_g1_i8 | 0.597930235 | 0.827193704 | 0.999970678 | 0.672 | 1.518 | 0 | 1.408 | NA |
| TRINITY_DN5896_c0_g1_i2 | -0.59784398 | 0.83760561 | 0.999970678 | 15.524 | 0 | 7.944 | 14.337 | SBF |
| TRINITY_DN5701_c0_g2_i3 | 0.597777254 | 0.579682692 | 0.999970678 | 0.917 | 0.69 | 0.3 | 0.694 | DYW_deaminase |
| TRINITY_DN322_c1_g1_i1 | -0.59775097 | 0.7870115 | 0.999970678 | 0.282 | 5.874 | 0.367 | 8.606 | NA |
| TRINITY_DN186_c0_g1_i4 | -0.59769071 | 0.438911686 | 0.999970678 | 680.29 | 1379 | 1167.185 | 1652.516 | Chloroa_b-bind |
| TRINITY_DN1108_c0_g1_i1 | 0.597678744 | 0.833097751 | 0.999970678 | 0 | 15.54 | 5.977 | 2.885 | La |
| TRINITY_DN2340_c0_g2_i12 | 0.597607939 | 0.829622469 | 0.999970678 | 2.734 | 0 | 0.711 | 0.985 | NA |
| TRINITY_DN2436_c0_g3_i1 | 0.597586086 | 0.604978796 | 0.999970678 | 8.057 | 32.7 | 9.199 | 15.161 | NA |
| TRINITY_DN744_c0_g1_i5 | -0.59758333 | 0.449589887 | 0.999970678 | 21.729 | 17.56 | 32.487 | 20.278 | DnaJ |
| TRINITY_DN2258_c0_g1_i3 | -0.5975442 | 0.773361322 | 0.999970678 | 21.838 | 673.2 | 819.995 | 40.496 | Chloroa_b-bind |
| TRINITY_DN2327_c0_g1_i1 | -0.59743345 | 0.414518369 | 0.999970678 | 10.465 | 17.71 | 17.155 | 21.545 | NA |
| TRINITY_DN3214_c0_g1_i7 | 0.597390969 | 0.88815015 | 0.999970678 | 0 | 2.316 | 1.244 | 0 | Rhodanese |
| TRINITY_DN15341_c0_g1_i2 | 0.597385506 | 0.891205425 | 0.999970678 | 5.541 | 0 | 3.044 | 0 | Peroxidase_2 |
| TRINITY_DN11705_c0_g1_i4 | 0.597372739 | 0.821989564 | 0.999970678 | 1.163 | 1.37 | 0 | 1.659 | - |
| TRINITY_DN3637_c0_g1_i7 | -0.59721186 | 0.887544442 | 0.999970678 | 0 | 5.539 | 0 | 8.274 | LRRNT_2 |
| TRINITY_DN10426_c0_g1_i7 | 0.597099194 | 0.667939269 | 0.999970678 | 33.32 | 26.13 | 5.866 | 28.955 | NA |
| TRINITY_DN19042_c0_g2_i1 | -0.59707196 | 0.895197013 | 1 | 1.926 | 0 | 2.4 | 0 | NA |
| TRINITY_DN10246_c0_g1_i6 | -0.59707196 | 0.895197013 | 1 | 0.936 | 0 | 1.2 | 0 | ADH_N |
| TRINITY_DN18419_c0_g2_i1 | 0.597070972 | 0.644582582 | 0.999970678 | 1.49 | 2.277 | 0.456 | 1.91 | RRM_1 |
| TRINITY_DN5986_c0_g1_i3 | 0.597017488 | 0.82988393 | 0.999970678 | 9.075 | 0 | 2.211 | 3.408 | NA |
| TRINITY_DN4163_c0_g1_i1 | 0.597006038 | 0.888121071 | 0.999970678 | 33.093 | 0 | 18.243 | 0 | NA |
| TRINITY_DN5965_c0_g1_i2 | 0.59700602 | 0.530903108 | 0.999970678 | 3.152 | 2.148 | 2 | 1.106 | NA |

| TRINITY_DN7554_c0_g1_i1 | 0.596940366 | 0.498391718 | 0.999970678 | 1.799 | 3.006 | 1.211 | 1.709 | Exostosin |
| --- | --- | --- | --- | --- | --- | --- | --- | --- |
| TRINITY_DN141_c0_g1_i4 | -0.59686931 | 0.375351682 | 0.999970678 | 3.634 | 4.849 | 5.533 | 6.133 | Pribosyl_synth |
| TRINITY_DN6817_c0_g1_i1 | -0.59683051 | 0.396080961 | 0.999970678 | 4.378 | 5.233 | 6.666 | 6.444 | NA |
| TRINITY_DN3682_c0_g1_i5 | 0.596792444 | 0.851033826 | 1 | 5.469 | 0.217 | 3.122 | 0 | 2014/3/3 |
| TRINITY_DN2365_c0_g1_i3 | 0.596591287 | 0.633773885 | 0.999970678 | 1.163 | 3.164 | 0.478 | 2.252 | NA |
| TRINITY_DN25290_c0_g1_i1 | -0.5965019 | 0.848211376 | 1 | 1.444 | 0.217 | 2.133 | 0 | MARVEL |
| TRINITY_DN16034_c0_g1_i15 | -0.59646878 | 0.716413447 | 0.999970678 | 0.372 | 0.897 | 0.133 | 1.739 | NA |
| TRINITY_DN16118_c0_g1_i5 | -0.59642349 | 0.724723273 | 0.999970678 | 1.526 | 0.128 | 1.378 | 0.925 | NA |
| TRINITY_DN4888_c0_g1_i6 | 0.596396312 | 0.76334649 | 0.999970678 | 0.118 | 6.179 | 0.8 | 3.137 | NA |
| TRINITY_DN6159_c0_g1_i8 | 0.596391509 | 0.369988949 | 0.999970678 | 4.342 | 3.42 | 2.2 | 2.493 | NA |
| TRINITY_DN3670_c0_g1_i5 | -0.59618431 | 0.671037323 | 0.999970678 | 1.026 | 0.325 | 0.433 | 1.548 | Myb_DNA-bindin |
| TRINITY_DN3658_c0_g1_i7 | -0.59614388 | 0.741057489 | 0.999970678 | 10.41 | 1.242 | 2.111 | 14.558 | NA |
| TRINITY_DN6476_c0_g1_i1 | -0.59607357 | 0.890023624 | 0.999970678 | 0.245 | 0 | 0 | 0.382 | DUF4283 |
| TRINITY_DN3175_c0_g1_i3 | 0.596051748 | 0.644970332 | 1 | 0.954 | 0.769 | 0.667 | 0.352 | NA |
| TRINITY_DN3809_c0_g1_i2 | -0.59599952 | 0.76245064 | 0.999970678 | 113.33 | 2.237 | 19.221 | 154.113 | Calreticulin |
| TRINITY_DN4848_c1_g1_i1 | -0.59599248 | 0.50600119 | 0.999970678 | 15.679 | 25.37 | 15.788 | 42.537 | NA |
| TRINITY_DN740_c0_g1_i8 | -0.59591309 | 0.484841982 | 0.999970678 | 24.227 | 52.99 | 39.52 | 67.149 | NA |
| TRINITY_DN2391_c0_g1_i2 | 0.595846281 | 0.639511153 | 0.999970678 | 9.465 | 2.464 | 5.011 | 1.93 | Paf1 |
| TRINITY_DN1799_c0_g1_i7 | -0.59584372 | 0.438013126 | 0.999970678 | 2.026 | 1.951 | 2.333 | 3.167 | PPR |
| TRINITY_DN2491_c0_g1_i1 | -0.59582211 | 0.491621323 | 0.999970678 | 3.016 | 4.08 | 5.644 | 3.921 | Ribosomal_L13 |
| TRINITY_DN8697_c0_g1_i2 | -0.595795 | 0.408463442 | 0.999970678 | 8.621 | 6.84 | 11.677 | 9.32 | NA |
| TRINITY_DN16849_c0_g1_i2 | 0.595788367 | 0.651603413 | 0.999970678 | 1.244 | 0.7 | 0.878 | 0.231 | NA |
| TRINITY_DN862_c0_g1_i8 | -0.59576311 | 0.89248412 | 1 | 0.709 | 0 | 0 | 1.096 | NA |
| TRINITY_DN10071_c0_g2_i2 | 0.595725406 | 0.893517003 | 1 | 1.572 | 0 | 0.867 | 0 | MFS_1 |
| TRINITY_DN24859_c0_g1_i2 | 0.595710461 | 0.332005577 | 0.999970678 | 10.91 | 11.36 | 6.522 | 6.837 | Per1 |
| TRINITY_DN391_c0_g1_i3 | -0.59559194 | 0.281012385 | 0.999970678 | 124.93 | 133.5 | 159.024 | 195.786 | Ribosomal_L18 |
| TRINITY_DN13442_c0_g1_i4 | -0.5955904 | 0.893397881 | 1 | 0.772 | 0 | 0.978 | 0 | Aconitase |
| TRINITY_DN11776_c0_g1_i2 | -0.59557009 | 0.544449379 | 0.999970678 | 0.4 | 0.631 | 0.767 | 0.633 | NA |
| TRINITY_DN5943_c0_g1_i1 | -0.5955411 | 0.826131224 | 0.999970678 | 7.122 | 0 | 5.144 | 4.725 | NA |
| TRINITY_DN879_c0_g1_i6 | -0.59553875 | 0.443303356 | 0.999970678 | 16.714 | 23.57 | 18.732 | 37.51 | NA |
| TRINITY_DN459_c0_g1_i6 | -0.59553802 | 0.721020468 | 0.999970678 | 8.403 | 12.46 | 1.078 | 30 | - |
| TRINITY_DN1262_c0_g2_i1 | -0.59549217 | 0.453680561 | 0.999970678 | 47.718 | 68.3 | 95.039 | 59.669 | NA |
| TRINITY_DN10292_c0_g2_i1 | -0.5954435 | 0.363023822 | 0.999970678 | 223.84 | 218 | 329.013 | 261.567 | NA |
| TRINITY_DN1970_c2_g1_i1 | -0.59543881 | 0.392361102 | 0.999970678 | 4.351 | 5.637 | 6.966 | 6.625 | p450 |
| TRINITY_DN5245_c0_g1_i2 | 0.595389667 | 0.887577144 | 0.999970678 | 5.387 | 0 | 0 | 3.629 | NA |
| TRINITY_DN2849_c0_g1_i1 | -0.59531764 | 0.60526429 | 0.999970678 | 6.986 | 2.592 | 5.755 | 7.319 | NA |
| TRINITY_DN9353_c0_g1_i4 | -0.59529987 | 0.378797949 | 0.999970678 | 3.833 | 4.09 | 4.955 | 5.962 | NA |
| TRINITY_DN833_c0_g1_i1 | -0.59525082 | 0.593045291 | 0.999970678 | 3.906 | 4.928 | 2.233 | 10.617 | Glyco_hydro_1 |
| TRINITY_DN2170_c0_g2_i1 | 0.595215154 | 0.826033065 | 0.999970678 | 1.744 | 2.868 | 2.522 | 0 | F-box |
| TRINITY_DN8403_c0_g1_i1 | 0.595089735 | 0.439310525 | 0.999970678 | 2.489 | 4.425 | 2.011 | 2.141 | Fucokinase |
| TRINITY_DN13398_c0_g4_i1 | -0.59504391 | 0.740578209 | 0.999970678 | 0.808 | 3.351 | 4.766 | 0.462 | HSP70 |
| TRINITY_DN3926_c0_g1_i3 | 0.59503785 | 0.62008242 | 0.999970678 | 113.5 | 60.73 | 79.884 | 19.353 | NA |
| TRINITY_DN27590_c0_g1_i1 | -0.59502292 | 0.444207874 | 0.999970678 | 59.282 | 31.13 | 60.296 | 63.921 | NA |
| TRINITY_DN8564_c0_g1_i1 | -0.59492728 | 0.548229342 | 0.999970678 | 17.041 | 9.116 | 22.299 | 12.306 | NA |
| TRINITY_DN37188_c0_g1_i1 | 0.594918443 | 0.653379065 | 1 | 3.098 | 3.676 | 2.567 | 1.247 | p450 |
| TRINITY_DN2130_c0_g1_i2 | 0.594876369 | 0.813872469 | 0.999970678 | 14.453 | 0.059 | 0.389 | 9.219 | p450 |
| TRINITY_DN7268_c0_g2_i1 | -0.59477422 | 0.810586412 | 1 | 2.507 | 0.089 | 3.1 | 0.191 | NA |
| TRINITY_DN31593_c0_g1_i1 | -0.59469229 | 0.888980424 | 0.999970678 | 1.889 | 0 | 2.4 | 0 | NA |
| TRINITY_DN7622_c0_g1_i3 | 0.594681983 | 0.547766052 | 0.999970678 | 0.799 | 1.015 | 0.367 | 0.744 | NA |
| TRINITY_DN41942_c0_g1_i1 | -0.5946551 | 0.892161076 | 0.999970678 | 1.853 | 0 | 2.344 | 0 | NA |
| TRINITY_DN31457_c0_g1_i1 | 0.594644135 | 0.391646713 | 0.999970678 | 3.624 | 2.996 | 1.633 | 2.423 | NA |
| TRINITY_DN913_c0_g2_i1 | 0.594641012 | 0.726305955 | 0.999970678 | 5.269 | 2.099 | 0.444 | 4.253 | NA |
| TRINITY_DN4112_c0_g1_i3 | 0.594573216 | 0.755774811 | 1 | 0.182 | 0.995 | 0.089 | 0.664 | NA |
| TRINITY_DN2384_c0_g2_i9 | 0.594516038 | 0.638028922 | 0.999970678 | 2.689 | 0.877 | 1.222 | 0.945 | NA |
| TRINITY_DN10580_c1_g2_i1 | 0.594511268 | 0.490318672 | 0.999970678 | 0.59 | 0.828 | 0.444 | 0.412 | - |
| TRINITY_DN5765_c1_g3_i1 | -0.59442678 | 0.707169153 | 0.999970678 | 1.408 | 0.197 | 0.711 | 1.609 | NA |
| TRINITY_DN2769_c0_g1_i4 | -0.59438599 | 0.625754181 | 0.999970678 | 2.008 | 3.696 | 5.533 | 1.82 | NA |
| TRINITY_DN22385_c0_g1_i1 | -0.59438545 | 0.857165003 | 0.999970678 | 0 | 8.062 | 9.799 | 0.09 | NA |
| TRINITY_DN4786_c0_g1_i5 | -0.59435954 | 0.624437866 | 0.999970678 | 1.444 | 1.882 | 0.744 | 4.122 | NA |
| TRINITY_DN10236_c0_g1_i7 | 0.594349374 | 0.484932131 | 0.999970678 | 18.704 | 41.55 | 13.577 | 23.294 | NA |
| TRINITY_DN1700_c0_g2_i3 | 0.594301285 | 0.43892934 | 0.999970678 | 1.599 | 1.478 | 0.811 | 1.056 | B_lectin |
| TRINITY_DN1827_c0_g1_i2 | -0.5942829 | 0.671498562 | 0.999970678 | 0.382 | 2.07 | 2.178 | 1.046 | AP2 |
| TRINITY_DN8171_c0_g1_i23 | 0.594232183 | 0.836942529 | 0.999970678 | 0.109 | 0.798 | 0.489 | 0 | NA |
| TRINITY_DN112_c0_g1_i11 | 0.594221518 | 0.760219885 | 0.999970678 | 46.328 | 0.67 | 19.743 | 7.711 | NA |
| TRINITY_DN5355_c0_g1_i2 | -0.59417966 | 0.548215271 | 0.999970678 | 1.036 | 2.779 | 2.722 | 2.423 | OPT |
| TRINITY_DN1436_c0_g1_i9 | -0.59404469 | 0.888685541 | 0.999970678 | 16.551 | 0 | 20.654 | 0 | NA |
| TRINITY_DN6582_c0_g1_i9 | 0.593985261 | 0.627551454 | 0.999970678 | 3.37 | 1.754 | 1.778 | 1.227 | NA |
| TRINITY_DN5987_c0_g2_i6 | 0.593963353 | 0.826205058 | 0.999970678 | 2.189 | 1.035 | 1.778 | 0 | ECH_2 |
| TRINITY_DN9129_c0_g1_i1 | -0.59387149 | 0.358720084 | 0.999970678 | 4.206 | 3.508 | 4.533 | 6.193 | NA |
| TRINITY_DN847_c0_g1_i4 | 0.593866407 | 0.607918922 | 0.999970678 | 28.042 | 131.7 | 31.365 | 66.757 | NA |
| TRINITY_DN1238_c0_g1_i4 | 0.593786359 | 0.41741386 | 0.999970678 | 5.087 | 6.179 | 2.467 | 4.454 | NA |
| TRINITY_DN502_c0_g2_i2 | -0.59376058 | 0.438789815 | 0.999970678 | 60.881 | 118.7 | 98.183 | 150.986 | Tubulin |
| TRINITY_DN11766_c0_g1_i9 | -0.59373178 | 0.843259122 | 1 | 1.581 | 0 | 1.767 | 0.322 | NA |
| TRINITY_DN1099_c0_g1_i12 | 0.593710084 | 0.829274815 | 0.999970678 | 0.409 | 1.439 | 1.011 | 0 | RNA_pol_Rpb1_1 |

| TRINITY_DN31407_c0_g1_i1 | 0.59364095 | 0.747631759 | 1 | 0.327 | 2.878 | 0.389 | 1.619 | DUF4228 |
| --- | --- | --- | --- | --- | --- | --- | --- | --- |
| TRINITY_DN6889_c0_g2_i1 | -0.59352524 | 0.842687722 | 1 | 0 | 1.114 | 1.133 | 0.292 | NA |
| TRINITY_DN11897_c0_g1_i1 | 0.593464935 | 0.681364477 | 0.999970678 | 2.553 | 3.568 | 0.478 | 3.418 | Bet_v_1 |
| TRINITY_DN94417_c0_g1_i3 | -0.59341324 | 0.639663828 | 0.999970678 | 1.417 | 1.498 | 2.633 | 1.227 | NA |
| TRINITY_DN13870_c0_g1_i1 | -0.59339014 | 0.493044909 | 0.999970678 | 4.914 | 4.445 | 3.778 | 9.551 | Mito_carr |
| TRINITY_DN4857_c0_g2_i1 | -0.59337868 | 0.759172629 | 1 | 0.173 | 1.843 | 0.678 | 2.141 | NA |
| TRINITY_DN82745_c0_g4_i1 | -0.593367 | 0.696240934 | 0.999970678 | 0.5 | 1.33 | 0.489 | 2.141 | NA |
| TRINITY_DN760_c0_g1_i1 | -0.59332768 | 0.70792235 | 0.999970678 | 2.08 | 9.619 | 2.378 | 14.105 | NA |
| TRINITY_DN3508_c0_g1_i4 | 0.593288165 | 0.303420046 | 0.999970678 | 6.813 | 7.529 | 3.989 | 4.715 | NA |
| TRINITY_DN4098_c0_g1_i1 | -0.59326873 | 0.842153036 | 0.999970678 | 0.69 | 0.118 | 1.011 | 0 | NA |
| TRINITY_DN10645_c0_g1_i1 | 0.593262755 | 0.536756556 | 0.999970678 | 18.677 | 7.451 | 7.822 | 7.882 | NA |
| TRINITY_DN9353_c0_g1_i2 | -0.59326094 | 0.653871856 | 0.999970678 | 3.061 | 0.926 | 1.122 | 4.705 | NA |
| TRINITY_DN1035_c0_g2_i4 | 0.593143173 | 0.838452213 | 0.999970678 | 0 | 7.037 | 3.344 | 0.543 | Pyrophosphatase |
| TRINITY_DN9066_c0_g1_i3 | 0.593111763 | 0.840939977 | 0.999970678 | 4.578 | 38.84 | 23.443 | 0 | - |
| TRINITY_DN460_c2_g1_i2 | 0.593093929 | 0.873627088 | 0.999970678 | 0 | 162.4 | 87.528 | 0.111 | TPR_17 |
| TRINITY_DN18871_c0_g2_i1 | -0.59299605 | 0.514369475 | 0.999970678 | 0.563 | 1.084 | 0.833 | 1.498 | ALMT |
| TRINITY_DN640_c0_g1_i7 | -0.59296682 | 0.617058817 | 0.999970678 | 1.108 | 1.36 | 0.967 | 2.534 | NA |
| TRINITY_DN12038_c0_g1_i1 | -0.59295013 | 0.479156804 | 0.999970678 | 3.715 | 5.312 | 4.011 | 8.767 | HLH |
| TRINITY_DN6753_c0_g1_i2 | 0.592878752 | 0.8901481 | 0.999970678 | 0 | 2.888 | 0 | 1.85 | NA |
| TRINITY_DN1689_c0_g1_i2 | -0.59284506 | 0.667003394 | 0.999970678 | 5.55 | 10.48 | 17.677 | 2.664 | NA |
| TRINITY_DN1507_c0_g1_i3 | 0.592628224 | 0.894035937 | 1 | 0 | 0.394 | 0 | 0.261 | BRCT_2 |
| TRINITY_DN12585_c0_g1_i5 | -0.59258981 | 0.549747434 | 0.999970678 | 3.116 | 4.809 | 3.166 | 7.993 | NA |
| TRINITY_DN13759_c0_g2_i1 | 0.592479308 | 0.644656343 | 0.999970678 | 0.327 | 0.542 | 0.133 | 0.412 | NA |
| TRINITY_DN3132_c0_g1_i3 | 0.5924559 | 0.889164616 | 0.999970678 | 5.614 | 0 | 0 | 3.8 | TPR_1 |
| TRINITY_DN612_c0_g1_i3 | 0.592452233 | 0.512093842 | 0.999970678 | 0.845 | 0.71 | 0.522 | 0.412 | - |
| TRINITY_DN8655_c0_g1_i8 | 0.592329798 | 0.626028595 | 0.999970678 | 0.572 | 1.409 | 0.589 | 0.603 | Gelsolin |
| TRINITY_DN17577_c0_g1_i2 | 0.59232279 | 0.563277268 | 0.999970678 | 2.316 | 1.636 | 1.511 | 0.804 | NTP_transferase |
| TRINITY_DN5231_c0_g1_i2 | -0.59231051 | 0.557955024 | 0.999970678 | 0.609 | 0.542 | 0.878 | 0.653 | NA |
| TRINITY_DN6660_c0_g1_i4 | -0.59217922 | 0.489249123 | 0.999970678 | 1.844 | 3.489 | 3.766 | 3.458 | NA |
| TRINITY_DN5685_c0_g1_i2 | -0.59216765 | 0.445210514 | 0.999970678 | 10.12 | 6.278 | 11.999 | 10.275 | Abhydrolase_1 |
| TRINITY_DN24071_c0_g1_i1 | -0.59214248 | 0.505387444 | 0.999970678 | 2.035 | 1.892 | 1.722 | 3.841 | NA |
| TRINITY_DN13140_c0_g1_i3 | -0.59207724 | 0.888846467 | 0.999970678 | 5.741 | 0 | 7.244 | 0 | NA |
| TRINITY_DN5411_c1_g1_i3 | 0.591994183 | 0.455814283 | 0.999970678 | 16.424 | 13.54 | 10.877 | 6.766 | NA |
| TRINITY_DN6997_c0_g1_i15 | 0.591898829 | 0.698084846 | 0.999970678 | 0.309 | 1.991 | 0.511 | 0.875 | NA |
| TRINITY_DN1463_c0_g1_i10 | -0.59187858 | 0.709757957 | 0.999970678 | 147.41 | 89.47 | 272.25 | 18.268 | Cupin_1 |
| TRINITY_DN4554_c0_g1_i5 | -0.5918294 | 0.839101142 | 1 | 1.653 | 0 | 1.722 | 0.473 | NA |
| TRINITY_DN16034_c0_g1_i4 | 0.59177352 | 0.729647282 | 0.999970678 | 0.209 | 4.001 | 0.822 | 1.739 | NA |
| TRINITY_DN1572_c0_g1_i5 | -0.59172392 | 0.897246195 | 1 | 1.463 | 0 | 0 | 2.212 | NA |
| TRINITY_DN11415_c1_g1_i1 | -0.59172392 | 0.897246195 | 1 | 1.671 | 0 | 0 | 2.523 | NA |
| TRINITY_DN1453_c0_g1_i3 | -0.59161129 | 0.83895356 | 0.999970678 | 0 | 7.874 | 8.466 | 1.448 | C2 |
| TRINITY_DN304_c0_g1_i12 | 0.591513793 | 0.742829344 | 0.999970678 | 0.145 | 1.488 | 0.644 | 0.292 | NA |
| TRINITY_DN6950_c0_g1_i5 | -0.59141881 | 0.856921664 | 1 | 0 | 1.133 | 0.056 | 1.649 | - |
| TRINITY_DN835_c0_g1_i1 | -0.59135427 | 0.523040535 | 0.999970678 | 1.544 | 0.749 | 1.655 | 1.488 | LRR_8 |
| TRINITY_DN469_c0_g1_i8 | 0.591278334 | 0.733291009 | 0.999970678 | 36.199 | 5.036 | 20.977 | 2.172 | p450 |
| TRINITY_DN890_c0_g1_i10 | 0.591273673 | 0.557262849 | 0.999970678 | 3.207 | 2.631 | 0.833 | 2.875 | NA |
| TRINITY_DN547_c0_g1_i2 | 0.591183888 | 0.442588105 | 0.999970678 | 35.609 | 33.27 | 25.043 | 15.553 | peroxidase |
| TRINITY_DN1049_c0_g1_i4 | -0.59109081 | 0.836124648 | 0.999970678 | 0 | 1.784 | 0.233 | 2.383 | NA |
| TRINITY_DN1283_c0_g2_i3 | 0.591047632 | 0.337740266 | 0.999970678 | 19.431 | 18.41 | 9.244 | 13.975 | NA |
| TRINITY_DN4458_c0_g1_i1 | 0.591020565 | 0.584691645 | 0.999970678 | 8.384 | 2.592 | 3.878 | 2.674 | NA |
| TRINITY_DN1505_c0_g2_i3 | 0.591002074 | 0.624017183 | 0.999970678 | 2.743 | 1.518 | 1.111 | 1.438 | NA |
| TRINITY_DN255_c1_g1_i13 | 0.590992648 | 0.853588907 | 0.999970678 | 64.332 | 0 | 5.877 | 36.515 | NA |
| TRINITY_DN2640_c0_g1_i7 | -0.59093533 | 0.732156283 | 0.999970678 | 4.179 | 0.453 | 0.978 | 5.841 | NA |
| TRINITY_DN5411_c0_g1_i5 | -0.59090358 | 0.61616236 | 0.999970678 | 0.972 | 2.06 | 0.767 | 3.629 | NA |
| TRINITY_DN169806_c0_g1_i1 | 0.590899543 | 0.602230581 | 0.999970678 | 0.609 | 1.38 | 0.422 | 0.804 | Pkinase_Tyr |
| TRINITY_DN2251_c1_g1_i1 | 0.590892674 | 0.837714766 | 0.999970678 | 1.826 | 0.168 | 0 | 1.347 | NA |
| TRINITY_DN15283_c0_g1_i1 | -0.59072631 | 0.585427275 | 0.999970678 | 21.674 | 41.73 | 26.832 | 55.748 | NA |
| TRINITY_DN20737_c0_g1_i2 | -0.59070728 | 0.724036033 | 0.999970678 | 2.253 | 0.483 | 3.089 | 0.432 | NA |
| TRINITY_DN94328_c0_g1_i4 | 0.590701655 | 0.802951082 | 0.999970678 | 2.852 | 0.039 | 1.433 | 0.231 | NA |
| TRINITY_DN2733_c0_g1_i10 | 0.590627098 | 0.492036265 | 0.999970678 | 2.144 | 1.271 | 1.178 | 0.865 | NA |
| TRINITY_DN9160_c0_g1_i1 | 0.590604003 | 0.689962205 | 0.999970678 | 0.481 | 1.035 | 0.678 | 0.191 | NA |
| TRINITY_DN2695_c0_g1_i1 | -0.59047057 | 0.471916807 | 0.999970678 | 299.19 | 494.8 | 336.446 | 775.602 | - |
| TRINITY_DN35968_c0_g1_i1 | -0.59045734 | 0.891495214 | 0.999970678 | 5.532 | 0 | 6.933 | 0 | NA |
| TRINITY_DN9237_c0_g1_i12 | -0.59039233 | 0.897434139 | 1 | 2.698 | 0 | 3.5 | 0 | NA |
| TRINITY_DN18072_c0_g1_i2 | -0.59039233 | 0.897434139 | 1 | 2.961 | 0 | 3.7 | 0 | Asp |
| TRINITY_DN7125_c0_g1_i1 | -0.59039233 | 0.897434139 | 1 | 1.145 | 0 | 1.467 | 0 | NA |
| TRINITY_DN18461_c0_g2_i2 | -0.59039233 | 0.897434139 | 1 | 6.54 | 0 | 7.955 | 0 | NAD-GH |
| TRINITY_DN25192_c0_g1_i2 | -0.59039233 | 0.897434139 | 1 | 0.6 | 0 | 0.756 | 0 | Fungal_trans |
| TRINITY_DN34436_c0_g1_i1 | 0.590236509 | 0.523506981 | 0.999970678 | 2.68 | 3.479 | 1.878 | 1.8 | NA |
| TRINITY_DN8427_c0_g1_i1 | -0.59022846 | 0.580027803 | 0.999970678 | 183.9 | 77.72 | 210.654 | 118.342 | NA |
| TRINITY_DN14894_c0_g1_i2 | 0.590210905 | 0.732902802 | 0.999970678 | 5.332 | 0.65 | 0.322 | 3.649 | NA |
| TRINITY_DN5327_c0_g1_i4 | -0.59019599 | 0.711999005 | 0.999970678 | 2.607 | 0.65 | 3.344 | 0.885 | Ribonuclease_3 |
| TRINITY_DN220635_c0_g1_i1 | -0.59015486 | 0.531525975 | 0.999970678 | 0.772 | 0.572 | 1.011 | 0.804 | NA |
| TRINITY_DN8008_c0_g1_i1 | 0.590073037 | 0.438284184 | 0.999970678 | 9.829 | 6.593 | 5.422 | 4.404 | NA |
| TRINITY_DN3175_c0_g1_i4 | 0.589987999 | 0.608930263 | 0.999970678 | 75.805 | 22.37 | 39.553 | 17.996 | NA |

| TRINITY_DN1041_c0_g1_i3 | -0.5899859 | 0.584784949 | 0.999970678 | 7.176 | 18.58 | 23.132 | 10.466 | NA |
| --- | --- | --- | --- | --- | --- | --- | --- | --- |
| TRINITY_DN140_c0_g2_i2 | 0.589898811 | 0.536183064 | 0.999970678 | 7.049 | 12.38 | 3.244 | 8.898 | ADH_N |
| TRINITY_DN4649_c0_g1_i2 | 0.589876884 | 0.414146007 | 0.999970678 | 4.951 | 7.569 | 4.033 | 3.438 | NA |
| TRINITY_DN6785_c1_g1_i9 | -0.58984781 | 0.666708402 | 0.999970678 | 0.754 | 0.246 | 0.522 | 0.885 | AUX_IAA |
| TRINITY_DN11911_c0_g1_i2 | -0.5898148 | 0.737043444 | 1 | 1.817 | 0.414 | 2.289 | 0.603 | NA |
| TRINITY_DN11036_c0_g4_i1 | 0.589539619 | 0.891651763 | 0.999970678 | 4.978 | 0 | 2.766 | 0 | NA |
| TRINITY_DN4799_c0_g1_i3 | -0.58949317 | 0.563901899 | 0.999970678 | 12.899 | 12.91 | 7.4 | 29.759 | NA |
| TRINITY_DN40382_c0_g1_i1 | 0.589428925 | 0.733058156 | 0.999970678 | 4.26 | 2.957 | 0.178 | 4.584 | NA |
| TRINITY_DN9884_c0_g1_i2 | 0.589334066 | 0.617847587 | 0.999970678 | 0.454 | 0.7 | 0.411 | 0.271 | B_lectin |
| TRINITY_DN114_c0_g1_i9 | 0.589220997 | 0.621849026 | 0.999970678 | 1.526 | 1.754 | 1.389 | 0.493 | NA |
| TRINITY_DN833_c0_g3_i1 | 0.589171508 | 0.718547883 | 0.999970678 | 0.273 | 1.941 | 0.322 | 1.086 | Glyco_hydro_1 |
| TRINITY_DN1715_c0_g1_i4 | -0.58913368 | 0.815520421 | 0.999970678 | 12.127 | 0.542 | 15.832 | 0.07 | NA |
| TRINITY_DN2024_c0_g1_i8 | 0.589125857 | 0.893575676 | 0.999970678 | 10.437 | 0 | 0 | 6.575 | NA |
| TRINITY_DN3207_c0_g2_i5 | 0.589125381 | 0.662203359 | 0.999970678 | 1.081 | 6.337 | 1.289 | 3.328 | - |
| TRINITY_DN15803_c0_g2_i2 | -0.58911823 | 0.831152603 | 0.999970678 | 1.39 | 0 | 0.989 | 0.925 | NA |
| TRINITY_DN1140_c0_g1_i1 | -0.58904618 | 0.376321597 | 0.999970678 | 23.918 | 19.26 | 22.621 | 37.651 | - |
| TRINITY_DN4356_c0_g1_i1 | 0.589026771 | 0.726793014 | 1 | 0.536 | 0.719 | 0.089 | 0.724 | BTB |
| TRINITY_DN5541_c1_g1_i8 | -0.58897181 | 0.708297752 | 0.999970678 | 8.811 | 1.725 | 10.466 | 3.237 | NA |
| TRINITY_DN2036_c0_g1_i1 | 0.588911735 | 0.645750707 | 0.999970678 | 20.185 | 27.29 | 6.211 | 21.646 | NA |
| TRINITY_DN471_c0_g1_i6 | -0.58888689 | 0.543045428 | 0.999970678 | 1.899 | 5.371 | 5.4 | 4.333 | LRRNT_2 |
| TRINITY_DN1921_c0_g1_i1 | 0.588783079 | 0.665925854 | 0.999970678 | 2.989 | 0.739 | 1.633 | 0.533 | NA |
| TRINITY_DN16_c0_g1_i1 | 0.588610344 | 0.673531932 | 0.999970678 | 70.382 | 7.086 | 24.71 | 22.369 | UDPGT |
| TRINITY_DN1865_c0_g1_i1 | 0.588590256 | 0.834414465 | 0.999970678 | 2.689 | 0.66 | 1.867 | 0 | NA |
| TRINITY_DN1662_c0_g1_i1 | -0.58853997 | 0.835806309 | 0.999970678 | 0 | 6.948 | 1.333 | 8.425 | NA |
| TRINITY_DN2164_c0_g1_i1 | 0.588527661 | 0.435229821 | 0.999970678 | 5.705 | 8.259 | 3.255 | 5.308 | NA |
| TRINITY_DN19745_c0_g1_i2 | -0.58850143 | 0.889967439 | 0.999970678 | 1.426 | 0 | 1.789 | 0 | NA |
| TRINITY_DN77018_c0_g1_i1 | -0.58850143 | 0.889967439 | 0.999970678 | 1.944 | 0 | 2.444 | 0 | NA |
| TRINITY_DN4719_c0_g3_i1 | -0.58846329 | 0.67690697 | 0.999970678 | 3.906 | 0.473 | 2.333 | 3.861 | NA |
| TRINITY_DN11891_c0_g1_i1 | -0.58845537 | 0.891691114 | 0.999970678 | 16.551 | 0 | 20.854 | 0 | BTB |
| TRINITY_DN8379_c0_g1_i3 | 0.588319721 | 0.599931656 | 0.999970678 | 2.471 | 1.991 | 0.633 | 2.212 | HSP20 |
| TRINITY_DN7509_c0_g1_i1 | -0.58828664 | 0.671518905 | 0.999970678 | 10.056 | 2.168 | 12.266 | 3.69 | NA |
| TRINITY_DN501_c0_g1_i1 | -0.58815816 | 0.313420414 | 0.999970678 | 117.98 | 139.7 | 146.847 | 207.066 | Ribosomal_L6 |
| TRINITY_DN29664_c1_g1_i1 | 0.588134333 | 0.627630698 | 0.999970678 | 2.444 | 1.163 | 0.589 | 1.699 | 2OG-FeII_Oxy_2 |
| TRINITY_DN3788_c0_g1_i5 | -0.58813135 | 0.634741667 | 0.999970678 | 0.836 | 2.277 | 0.944 | 3.499 | TAXi_C |
| TRINITY_DN6256_c0_g1_i1 | 0.588077396 | 0.614891317 | 0.999970678 | 12.154 | 8.673 | 2.089 | 11.32 | NA |
| TRINITY_DN6106_c0_g1_i1 | -0.58803506 | 0.440058838 | 0.999970678 | 32.748 | 51.77 | 40.32 | 76.971 | - |
| TRINITY_DN26439_c0_g1_i1 | -0.58799835 | 0.610645495 | 0.999970678 | 1.272 | 1.084 | 2.155 | 0.935 | F-box-like |
| TRINITY_DN15197_c0_g1_i1 | -0.58796801 | 0.667611218 | 1 | 0.263 | 0.601 | 0.444 | 0.704 | NA |
| TRINITY_DN1041_c0_g2_i2 | 0.587928777 | 0.636237693 | 0.999970678 | 3.924 | 2.02 | 2.355 | 1.146 | NA |
| TRINITY_DN7841_c0_g1_i1 | -0.58792524 | 0.684694153 | 0.999970678 | 0.472 | 0.414 | 0.222 | 1.056 | DYW_deaminase |
| TRINITY_DN15778_c0_g1_i1 | -0.5878107 | 0.833949099 | 0.999970678 | 13.372 | 0 | 5.066 | 14.286 | MOZ_SAS |
| TRINITY_DN4818_c0_g1_i3 | -0.58777771 | 0.478916866 | 0.999970678 | 2.316 | 3.075 | 4.222 | 2.996 | DUF3685 |
| TRINITY_DN1640_c0_g1_i10 | -0.58776776 | 0.63605113 | 0.999970678 | 0.363 | 0.956 | 1.078 | 0.653 | NA |
| TRINITY_DN14656_c1_g2_i1 | 0.587723205 | 0.829368819 | 0.999970678 | 0.645 | 2.277 | 0 | 1.91 | NA |
| TRINITY_DN3976_c0_g1_i1 | 0.587690542 | 0.767380788 | 1 | 0.1 | 0.995 | 0.489 | 0.121 | NA |
| TRINITY_DN9216_c0_g1_i1 | -0.58764804 | 0.466995022 | 0.999970678 | 6.268 | 9.964 | 7.544 | 15.161 | NA |
| TRINITY_DN5611_c0_g1_i1 | -0.58760571 | 0.832086527 | 0.999970678 | 6.15 | 0 | 4.133 | 4.393 | B_lectin |
| TRINITY_DN4901_c0_g1_i4 | -0.58749605 | 0.620509876 | 0.999970678 | 0.899 | 0.65 | 0.622 | 1.599 | NA |
| TRINITY_DN13499_c0_g3_i2 | -0.58745044 | 0.737092561 | 0.999970678 | 2.389 | 0.118 | 1.5 | 1.991 | F-box |
| TRINITY_DN1574_c0_g1_i2 | -0.58744861 | 0.472419609 | 0.999970678 | 5.478 | 4.8 | 8.288 | 5.419 | NA |
| TRINITY_DN4542_c0_g1_i3 | 0.587447791 | 0.730980391 | 0.999970678 | 0.527 | 3.154 | 0.289 | 2.071 | TRAP_alpha |
| TRINITY_DN15988_c0_g3_i2 | -0.58744356 | 0.508527971 | 0.999970678 | 7.213 | 3.203 | 6.866 | 7.43 | Acyltransferase |
| TRINITY_DN5468_c0_g1_i4 | 0.587353786 | 0.836257415 | 0.999970678 | 6.132 | 23.35 | 0 | 19.484 | Cyt-b5 |
| TRINITY_DN3222_c0_g1_i5 | 0.587271356 | 0.685113074 | 0.999970678 | 2.607 | 18.49 | 9.133 | 2.845 | Glycos_transf_1 |
| TRINITY_DN2503_c0_g1_i9 | -0.5871824 | 0.889482513 | 0.999970678 | 63.17 | 0 | 77.662 | 0 | NA |
| TRINITY_DN7203_c0_g1_i5 | 0.587175267 | 0.517366893 | 0.999970678 | 2.244 | 3.676 | 1.067 | 2.644 | Suf |
| TRINITY_DN7483_c0_g1_i4 | 0.587117069 | 0.652230111 | 0.999970678 | 2.934 | 5.529 | 1.467 | 3.609 | NA |
| TRINITY_DN14745_c0_g1_i1 | -0.58709536 | 0.891192089 | 0.999970678 | 6.704 | 0 | 8.455 | 0 | NA |
| TRINITY_DN4288_c0_g1_i4 | 0.587082138 | 0.838308864 | 0.999970678 | 0 | 2.129 | 0.211 | 1.156 | NA |
| TRINITY_DN343_c0_g1_i3 | -0.58695283 | 0.440641316 | 0.999970678 | 2.035 | 2.622 | 3.144 | 3.177 | D123 |
| TRINITY_DN17834_c0_g1_i1 | -0.58690649 | 0.505886939 | 0.999970678 | 27.679 | 49.83 | 32.22 | 75.101 | NA |
| TRINITY_DN3141_c0_g1_i6 | 0.586824097 | 0.891665288 | 0.999970678 | 34.265 | 0 | 19.121 | 0 | NA |
| TRINITY_DN724_c0_g1_i7 | 0.586816745 | 0.427741788 | 0.999970678 | 6.758 | 8.456 | 5.111 | 3.941 | NA |
| TRINITY_DN8649_c0_g1_i8 | -0.58669998 | 0.853496377 | 0.999970678 | 9.029 | 0 | 10.977 | 0.332 | Ribosomal_L1 |
| TRINITY_DN6785_c0_g1_i3 | -0.58652786 | 0.538067896 | 0.999970678 | 4.651 | 3.755 | 7.755 | 3.298 | NA |
| TRINITY_DN1663_c0_g1_i1 | 0.586480934 | 0.506823209 | 0.999970678 | 1.29 | 1.055 | 0.778 | 0.633 | NA |
| TRINITY_DN511_c0_g1_i2 | 0.586443173 | 0.576128892 | 0.999970678 | 1.19 | 1.853 | 0.711 | 1.166 | NA |
| TRINITY_DN14899_c0_g1_i1 | -0.58632589 | 0.893526298 | 0.999970678 | 24.445 | 0 | 29.732 | 0 | Redoxin |
| TRINITY_DN11969_c0_g1_i2 | 0.586268002 | 0.657109607 | 0.999970678 | 0.754 | 1.301 | 0.911 | 0.292 | - |
| TRINITY_DN70_c0_g1_i4 | -0.58622868 | 0.464091537 | 0.999970678 | 11.082 | 5.746 | 10.366 | 12.869 | UDPGT |
| TRINITY_DN8545_c0_g2_i1 | 0.586225708 | 0.591716419 | 0.999970678 | 2.943 | 8.87 | 3.5 | 3.549 | - |
| TRINITY_DN3117_c0_g1_i7 | 0.586193754 | 0.794276313 | 0.999970678 | 0.118 | 28.44 | 2.589 | 15.402 | NA |
| TRINITY_DN5854_c0_g1_i6 | 0.586179396 | 0.4991446 | 0.999970678 | 5.805 | 6.278 | 2.322 | 5.238 | NA |
| TRINITY_DN5413_c0_g1_i1 | 0.586162354 | 0.543338152 | 0.999970678 | 0.863 | 1.104 | 0.367 | 0.865 | GDI |

| TRINITY_DN3328_c0_g1_i3 | 0.586100547 | 0.449559111 | 0.999970678 | 32.312 | 66.99 | 26.687 | 33.73 | Cellulose_synt |
| --- | --- | --- | --- | --- | --- | --- | --- | --- |
| TRINITY_DN20506_c0_g1_i3 | 0.586049985 | 0.58931475 | 0.999970678 | 5.432 | 1.597 | 1.9 | 2.413 | NA |
| TRINITY_DN2352_c0_g1_i2 | -0.58604939 | 0.832298755 | 0.999970678 | 0 | 8.614 | 2.378 | 9.863 | Abhydrolase_1 |
| TRINITY_DN7833_c2_g1_i1 | 0.586035433 | 0.76922123 | 0.999970678 | 0.845 | 0.227 | 0.567 | 0.04 | NA |
| TRINITY_DN13482_c0_g1_i1 | -0.58596155 | 0.466686527 | 0.999970678 | 5.668 | 8.367 | 6.477 | 13.14 | NA |
| TRINITY_DN1527_c1_g2_i1 | 0.585884649 | 0.363222383 | 0.999970678 | 8.103 | 7.263 | 4.833 | 4.424 | AAA |
| TRINITY_DN10781_c0_g2_i1 | 0.585867627 | 0.704064981 | 0.999970678 | 0.336 | 2.582 | 0.867 | 0.885 | Methyltrans_RNA |
| TRINITY_DN71196_c0_g1_i1 | 0.585859549 | 0.890589846 | 0.999970678 | 2.58 | 0 | 1.433 | 0 | NA |
| TRINITY_DN2388_c0_g1_i1 | 0.585846758 | 0.521187433 | 0.999970678 | 2.18 | 1.725 | 0.978 | 1.428 | Chloroa_b-bind |
| TRINITY_DN5491_c0_g1_i1 | 0.585785333 | 0.362832933 | 0.999970678 | 7.167 | 9.707 | 5.166 | 5.007 | DDT |
| TRINITY_DN186_c1_g1_i3 | 0.58573845 | 0.69050093 | 0.999970678 | 0.354 | 3.026 | 0.8 | 1.247 | NA |
| TRINITY_DN147_c0_g1_i1 | -0.58573117 | 0.678733959 | 0.999970678 | 1.953 | 10.95 | 3.355 | 15.03 | NA |
| TRINITY_DN1209_c0_g1_i11 | 0.585670622 | 0.655953604 | 0.999970678 | 1.408 | 4.543 | 1.411 | 2.192 | NA |
| TRINITY_DN11311_c0_g1_i5 | 0.585437646 | 0.890442173 | 0.999970678 | 0 | 1.242 | 0.667 | 0 | NA |
| TRINITY_DN887_c0_g1_i1 | -0.58534016 | 0.892559451 | 0.999970678 | 12.236 | 0 | 15.399 | 0 | NA |
| TRINITY_DN994_c1_g1_i1 | -0.58526748 | 0.654371465 | 0.999970678 | 290.62 | 1869 | 1795.569 | 856.283 | Chloroa_b-bind |
| TRINITY_DN3803_c0_g1_i3 | -0.58525908 | 0.375266941 | 0.999970678 | 97.97 | 102.8 | 98.75 | 178.554 | zf-rbx1 |
| TRINITY_DN9987_c0_g1_i3 | 0.585239858 | 0.756564076 | 0.999970678 | 98.842 | 4401 | 404.787 | 2194.46 | NA |
| TRINITY_DN11759_c0_g3_i2 | -0.58523418 | 0.532945188 | 0.999970678 | 1.971 | 2.336 | 3.866 | 1.79 | NA |
| TRINITY_DN31638_c0_g2_i3 | -0.58523119 | 0.660322663 | 0.999970678 | 1.072 | 3.311 | 1.444 | 4.806 | zf-FLZ |
| TRINITY_DN8263_c0_g1_i1 | -0.58520305 | 0.347415498 | 0.999970678 | 24.845 | 20.19 | 29.676 | 31.669 | Ribosomal_S7e |
| TRINITY_DN2928_c0_g1_i2 | 0.585157287 | 0.788841835 | 0.999970678 | 14.97 | 0.246 | 3.966 | 5.137 | LRR_8 |
| TRINITY_DN1507_c1_g1_i1 | -0.58506784 | 0.434680696 | 0.999970678 | 4.106 | 3.893 | 3.9 | 7.259 | CbiQ |
| TRINITY_DN9025_c0_g2_i6 | -0.58504117 | 0.754587759 | 0.999970678 | 3.534 | 0.217 | 4.066 | 0.824 | Mito_carr |
| TRINITY_DN7209_c0_g1_i1 | 0.585038573 | 0.558744119 | 0.999970678 | 5.786 | 2.247 | 2.678 | 2.162 | NA |
| TRINITY_DN6997_c0_g1_i10 | 0.584900753 | 0.626259563 | 0.999970678 | 1.517 | 2.789 | 1.455 | 1.066 | NA |
| TRINITY_DN11738_c0_g1_i1 | 0.584784682 | 0.892653717 | 0.999970678 | 14.516 | 0 | 8 | 0 | NA |
| TRINITY_DN14060_c0_g3_i1 | -0.58471235 | 0.617330005 | 0.999970678 | 0.627 | 0.286 | 0.689 | 0.553 | NA |
| TRINITY_DN9521_c0_g1_i1 | 0.584702341 | 0.446685105 | 0.999970678 | 5.251 | 4.267 | 3.278 | 2.403 | NA |
| TRINITY_DN1628_c0_g1_i1 | 0.584699344 | 0.889981464 | 0.999970678 | 18.168 | 0 | 0 | 12.255 | NA |
| TRINITY_DN3931_c0_g1_i5 | -0.58463444 | 0.333349176 | 0.999970678 | 225.55 | 296.7 | 296.327 | 423.421 | NA |
| TRINITY_DN1663_c0_g1_i3 | -0.58459251 | 0.647404044 | 0.999970678 | 2.216 | 4.869 | 2.211 | 7.671 | FolB |
| TRINITY_DN525_c0_g1_i3 | -0.58456735 | 0.3430281 | 0.999970678 | 65.94 | 56.31 | 83.051 | 81.586 | NA |
| TRINITY_DN12975_c0_g1_i1 | -0.58451584 | 0.627051292 | 0.999970678 | 0.581 | 1.055 | 0.7 | 1.548 | NA |
| TRINITY_DN7053_c0_g1_i2 | -0.58450436 | 0.577580282 | 0.999970678 | 2.316 | 8.456 | 6.311 | 8.435 | PTH2 |
| TRINITY_DN85285_c0_g1_i1 | 0.584466625 | 0.658553669 | 0.999970678 | 292.38 | 487.9 | 48.43 | 459.575 | NA |
| TRINITY_DN7507_c0_g1_i1 | -0.58444581 | 0.416240945 | 0.999970678 | 8.866 | 14.63 | 13.555 | 18.67 | NA |
| TRINITY_DN4736_c1_g1_i1 | 0.584431542 | 0.539314056 | 0.999970678 | 6.431 | 13.12 | 7.388 | 4.052 | NA |
| TRINITY_DN237_c0_g1_i15 | 0.584421513 | 0.837096601 | 0.999970678 | 27.879 | 0 | 6.844 | 10.516 | NA |
| TRINITY_DN12023_c0_g1_i1 | 0.58433286 | 0.626663579 | 0.999970678 | 2.053 | 2.71 | 1.655 | 1.146 | NA |
| TRINITY_DN989_c0_g1_i1 | -0.58427654 | 0.841002316 | 0.999970678 | 0 | 35.36 | 32.098 | 13.351 | NA |
| TRINITY_DN4006_c0_g1_i4 | 0.584099325 | 0.649324837 | 0.999970678 | 2.071 | 1.39 | 0.356 | 1.87 | NA |
| TRINITY_DN247_c1_g2_i2 | 0.584059588 | 0.674709679 | 0.999970678 | 10.238 | 1.646 | 4.966 | 2.011 | Peptidase_S10 |
| TRINITY_DN13247_c0_g2_i2 | -0.58403564 | 0.6107338 | 0.999970678 | 0.209 | 0.227 | 0.333 | 0.241 | NA |
| TRINITY_DN1935_c0_g1_i13 | -0.58398154 | 0.735369485 | 0.999970678 | 10.728 | 1.488 | 13.366 | 2.151 | NA |
| TRINITY_DN16635_c0_g1_i6 | -0.58397942 | 0.897182282 | 1 | 0.554 | 0 | 0.689 | 0 | DinB_2 |
| TRINITY_DN165703_c0_g1_i1 | -0.58397942 | 0.897182282 | 1 | 0.618 | 0 | 0.778 | 0 | SH3_1 |
| TRINITY_DN5143_c0_g3_i3 | -0.58397942 | 0.897182282 | 1 | 0.409 | 0 | 0.5 | 0 | NA |
| TRINITY_DN4333_c0_g1_i5 | 0.583944675 | 0.851893227 | 0.999970678 | 9.475 | 0 | 5.111 | 0.241 | NA |
| TRINITY_DN4236_c0_g1_i7 | 0.583902849 | 0.889621065 | 0.999970678 | 4.351 | 0 | 0 | 2.956 | NA |
| TRINITY_DN16543_c0_g1_i2 | -0.58389241 | 0.653054693 | 0.999970678 | 0.354 | 0.158 | 0.433 | 0.231 | PPR |
| TRINITY_DN3223_c0_g1_i2 | -0.58385579 | 0.88974394 | 0.999970678 | 5.396 | 0 | 0 | 8.224 | WD40 |
| TRINITY_DN592_c0_g1_i1 | -0.58362402 | 0.482515171 | 0.999970678 | 12.172 | 24.59 | 19.743 | 30.453 | NA |
| TRINITY_DN361_c0_g3_i1 | -0.58355632 | 0.510948095 | 0.999970678 | 4.233 | 8.071 | 9.488 | 6.857 | NA |
| TRINITY_DN1669_c0_g1_i1 | 0.583453144 | 0.557293705 | 0.999970678 | 1.09 | 1.577 | 1.033 | 0.533 | ANF_receptor |
| TRINITY_DN5499_c0_g1_i5 | -0.58345163 | 0.843316496 | 1 | 1.381 | 0.335 | 2.144 | 0 | NA |
| TRINITY_DN6076_c1_g1_i1 | -0.5834354 | 0.644853961 | 0.999970678 | 9.238 | 2.503 | 11.399 | 3.971 | DNA_binding_2 |
| TRINITY_DN5457_c0_g1_i2 | 0.583398157 | 0.439974539 | 0.999970678 | 141.17 | 77.68 | 55.819 | 78.871 | NA |
| TRINITY_DN70585_c1_g1_i1 | -0.58337669 | 0.587804885 | 0.999970678 | 66.976 | 261.6 | 219.82 | 196.68 | NA |
| TRINITY_DN416_c0_g1_i8 | 0.583368864 | 0.669131213 | 0.999970678 | 233.72 | 84.65 | 148.18 | 28.05 | NA |
| TRINITY_DN5_c1_g2_i1 | 0.583357486 | 0.360732955 | 0.999970678 | 14.543 | 13.85 | 8.911 | 8.194 | NA |
| TRINITY_DN15905_c0_g1_i1 | 0.583163097 | 0.468930027 | 0.999970678 | 4.796 | 7.933 | 4.033 | 3.589 | NA |
| TRINITY_DN10184_c0_g1_i1 | -0.58315371 | 0.364588854 | 0.999970678 | 18.722 | 16.78 | 25.565 | 22.38 | NA |
| TRINITY_DN478_c0_g1_i1 | 0.583139359 | 0.623226362 | 0.999970678 | 12.836 | 5.046 | 7.622 | 2.775 | Seryl_tRNA_N |
| TRINITY_DN999_c0_g1_i3 | -0.58308397 | 0.859581891 | 1 | 0 | 1.597 | 0.111 | 2.212 | NA |
| TRINITY_DN15410_c0_g1_i1 | -0.58305667 | 0.848543833 | 1 | 1.135 | 0.177 | 1.611 | 0 | NA |
| TRINITY_DN5651_c1_g1_i6 | 0.583051397 | 0.3528975 | 0.999970678 | 14.825 | 15.99 | 7.466 | 11.542 | HAD_2 |
| TRINITY_DN4385_c0_g2_i1 | -0.58297305 | 0.638407955 | 0.999970678 | 0.681 | 1.035 | 1.555 | 0.664 | NA |
| TRINITY_DN1862_c0_g1_i8 | -0.58289354 | 0.638042377 | 0.999970678 | 0.6 | 1.754 | 2.2 | 0.845 | NA |
| TRINITY_DN1968_c0_g1_i2 | 0.582861488 | 0.461327205 | 0.999970678 | 29.559 | 59.47 | 23.821 | 29.87 | NA |
| TRINITY_DN7979_c0_g2_i1 | -0.58285216 | 0.331858345 | 0.999970678 | 59.373 | 67.74 | 87.406 | 83.868 | NA |
| TRINITY_DN104973_c0_g1_i1 | 0.582818461 | 0.838350305 | 0.999970678 | 0 | 9.116 | 3.4 | 1.9 | Ank_2 |
| TRINITY_DN5987_c0_g2_i3 | -0.58279058 | 0.684125656 | 0.999970678 | 0.291 | 2.06 | 1.811 | 1.307 | ECH_2 |
| TRINITY_DN12778_c0_g1_i1 | 0.582777286 | 0.3914985 | 0.999970678 | 4.015 | 4.543 | 2.555 | 2.644 | NA |

| TRINITY_DN553_c0_g1_i5 | -0.58274257 | 0.854865232 | 0.999970678 | 1.581 | 0 | 1.933 | 0.06 | dNK |
| --- | --- | --- | --- | --- | --- | --- | --- | --- |
| TRINITY_DN8440_c0_g1_i1 | 0.582728387 | 0.664701919 | 0.999970678 | 1.508 | 2.424 | 1.855 | 0.362 | NA |
| TRINITY_DN15268_c0_g2_i3 | -0.58252308 | 0.709112685 | 0.999970678 | 2.716 | 0.473 | 1.767 | 2.604 | NA |
| TRINITY_DN23122_c0_g2_i4 | 0.582490325 | 0.627237322 | 0.999970678 | 1.354 | 1.961 | 1.233 | 0.744 | NA |
| TRINITY_DN1992_c0_g1_i1 | -0.58244048 | 0.485154035 | 0.999970678 | 14.389 | 33.31 | 28.198 | 36.887 | Lipase_3 |
| TRINITY_DN11795_c0_g1_i1 | -0.5824342 | 0.506847452 | 0.999970678 | 33.656 | 15.48 | 36.176 | 30.01 | NA |
| TRINITY_DN3977_c0_g1_i2 | 0.582391814 | 0.549052866 | 0.999970678 | 0.799 | 0.857 | 0.378 | 0.643 | - |
| TRINITY_DN2114_c0_g3_i3 | 0.582377137 | 0.487818439 | 0.999970678 | 3.815 | 3.045 | 2.1 | 2.041 | NA |
| TRINITY_DN1584_c0_g1_i6 | -0.58232439 | 0.652553437 | 0.999970678 | 8.793 | 13.28 | 3.622 | 28.351 | NA |
| TRINITY_DN17157_c0_g1_i1 | -0.5822282 | 0.608270974 | 0.999970678 | 0.254 | 0.522 | 0.511 | 0.543 | DEP |
| TRINITY_DN304_c1_g1_i2 | -0.58220441 | 0.689802521 | 0.999970678 | 1.808 | 9.944 | 2.744 | 14.055 | HSF_DNA-bind |
| TRINITY_DN28331_c0_g1_i3 | 0.582084156 | 0.684919314 | 0.999970678 | 1.644 | 0.641 | 1.055 | 0.261 | NA |
| TRINITY_DN8000_c0_g1_i1 | -0.58207747 | 0.75565123 | 0.999970678 | 0.055 | 0.739 | 0.222 | 0.885 | NA |
| TRINITY_DN6881_c0_g2_i1 | 0.581936563 | 0.597676572 | 0.999970678 | 3.733 | 1.597 | 1.3 | 2.021 | NA |
| TRINITY_DN4741_c0_g1_i2 | -0.58193593 | 0.891471147 | 0.999970678 | 6.25 | 0 | 0 | 9.541 | LRR_4 |
| TRINITY_DN2978_c0_g1_i3 | -0.5819122 | 0.890756886 | 0.999970678 | 0 | 4.612 | 0 | 6.837 | NA |
| TRINITY_DN3720_c0_g2_i3 | 0.581895463 | 0.890425201 | 0.999970678 | 9.175 | 0 | 0 | 6.223 | C1_2 |
| TRINITY_DN14874_c0_g1_i1 | -0.58187261 | 0.646773643 | 1 | 0.709 | 0.434 | 0.6 | 0.975 | DUF716 |
| TRINITY_DN9220_c2_g1_i3 | -0.58185042 | 0.55079392 | 0.999970678 | 2.28 | 1.922 | 1.6 | 4.404 | UBA |
| TRINITY_DN35734_c0_g1_i1 | 0.581832575 | 0.848576217 | 0.999970678 | 51.324 | 3.045 | 29.465 | 0 | NA |
| TRINITY_DN30792_c0_g2_i2 | 0.581802278 | 0.826050112 | 0.999970678 | 0.581 | 0.453 | 0 | 0.694 | NA |
| TRINITY_DN8631_c0_g1_i2 | 0.581778758 | 0.734219646 | 0.999970678 | 0.709 | 5.026 | 0.4 | 3.288 | EMP24_GP25L |
| TRINITY_DN1275_c0_g1_i1 | 0.581740782 | 0.891634474 | 0.999970678 | 10.728 | 0 | 0 | 7.309 | Ribosomal_L24e |
| TRINITY_DN2400_c0_g2_i2 | 0.581740497 | 0.415099754 | 0.999970678 | 16.024 | 22.41 | 12.921 | 10.044 | PC-Esterase |
| TRINITY_DN2177_c0_g3_i1 | 0.581712795 | 0.830073744 | 0.999970678 | 0.927 | 1.163 | 0 | 1.387 | NA |
| TRINITY_DN1286_c0_g1_i1 | -0.58166627 | 0.692809483 | 0.999970678 | 2.216 | 8.436 | 1.611 | 13.864 | NA |
| TRINITY_DN70752_c0_g1_i1 | -0.58165544 | 0.844771335 | 1 | 1.79 | 0 | 0.3 | 2.332 | Lysine_decarbox |
| TRINITY_DN1158_c1_g1_i2 | 0.58162806 | 0.83517139 | 0.999970678 | 18.804 | 0 | 6.511 | 4.816 | MIF |
| TRINITY_DN12167_c0_g1_i1 | 0.581562383 | 0.843195219 | 1 | 0.554 | 0.138 | 0.378 | 0 | NA |
| TRINITY_DN4024_c1_g1_i9 | -0.58156072 | 0.842672619 | 0.999970678 | 0.236 | 2.493 | 0 | 4.042 | Gal_Lectin |
| TRINITY_DN2209_c0_g1_i6 | -0.58140429 | 0.660916381 | 0.999970678 | 0.863 | 0.217 | 0.911 | 0.513 | DnaJ |
| TRINITY_DN173_c0_g1_i2 | 0.581398459 | 0.45018424 | 0.999970678 | 22.156 | 12.37 | 9.877 | 11.22 | 2OG-FeII_Oxy |
| TRINITY_DN9318_c0_g3_i1 | 0.581338305 | 0.523157198 | 0.999970678 | 0.917 | 1.064 | 0.5 | 0.724 | NA |
| TRINITY_DN60363_c0_g1_i1 | -0.58133713 | 0.720636701 | 0.999970678 | 1.862 | 0.69 | 2.755 | 0.503 | NA |
| TRINITY_DN2257_c0_g1_i2 | -0.58133626 | 0.512384838 | 0.999970678 | 9.338 | 5.016 | 10.71 | 8.566 | NA |
| TRINITY_DN8077_c1_g1_i1 | 0.581329679 | 0.746791422 | 0.999970678 | 0.173 | 2.681 | 0.5 | 1.267 | NA |
| TRINITY_DN3426_c0_g1_i9 | -0.58132036 | 0.662284026 | 0.999970678 | 3.888 | 11.65 | 5.477 | 15.503 | NA |
| TRINITY_DN4339_c0_g1_i2 | 0.581309353 | 0.837276756 | 0.999970678 | 0 | 9.993 | 3.722 | 2.091 | NA |
| TRINITY_DN20207_c0_g1_i1 | 0.581177349 | 0.753657694 | 0.999970678 | 2.316 | 0.237 | 1.255 | 0.211 | Hydrolase_4 |
| TRINITY_DN24256_c0_g1_i1 | 0.581169628 | 0.54661743 | 0.999970678 | 1.635 | 1.084 | 0.711 | 0.955 | NA |
| TRINITY_DN3032_c0_g1_i10 | 0.58109501 | 0.748755352 | 0.999970678 | 1.008 | 0.207 | 0.622 | 0.08 | NA |
| TRINITY_DN1376_c0_g1_i2 | 0.581012166 | 0.890669497 | 0.999970678 | 8.375 | 0 | 0 | 5.69 | NA |
| TRINITY_DN3348_c0_g1_i3 | -0.58100059 | 0.503485449 | 0.999970678 | 1.917 | 1.666 | 2.978 | 1.78 | RRM_1 |
| TRINITY_DN1295_c0_g1_i4 | 0.580940674 | 0.890557455 | 0.999970678 | 0 | 7.175 | 3.922 | 0 | NA |
| TRINITY_DN1840_c0_g1_i4 | 0.580875953 | 0.688416413 | 0.999970678 | 8.094 | 0.867 | 3.366 | 2.001 | NA |
| TRINITY_DN9670_c0_g1_i1 | -0.58077674 | 0.84794981 | 1 | 1.399 | 0 | 0.289 | 1.769 | WRKY |
| TRINITY_DN7426_c0_g2_i1 | 0.580739301 | 0.618729627 | 0.999970678 | 1.308 | 1.153 | 0.822 | 0.653 | PPR |
| TRINITY_DN24052_c0_g1_i3 | -0.58059576 | 0.481733854 | 0.999970678 | 0.763 | 0.897 | 1.189 | 1.046 | NA |
| TRINITY_DN7302_c0_g2_i1 | -0.580583 | 0.48683398 | 0.999970678 | 4.46 | 3.499 | 6.344 | 4.253 | NA |
| TRINITY_DN2242_c0_g1_i1 | -0.58042095 | 0.646949615 | 0.999970678 | 0.263 | 0.67 | 0.7 | 0.553 | PP2C |
| TRINITY_DN29137_c0_g1_i3 | 0.580416968 | 0.831153958 | 0.999970678 | 2.344 | 1.508 | 0 | 2.483 | NA |
| TRINITY_DN6244_c0_g1_i2 | -0.58036072 | 0.61304322 | 0.999970678 | 1.544 | 2.02 | 0.944 | 4.182 | FAD_binding_4 |
| TRINITY_DN4435_c0_g1_i3 | 0.580313522 | 0.502166573 | 0.999970678 | 2.135 | 1.488 | 1.211 | 0.965 | WW |
| TRINITY_DN6392_c0_g1_i4 | 0.580286426 | 0.473065313 | 0.999970678 | 12.899 | 27.31 | 11.688 | 12.617 | NA |
| TRINITY_DN2011_c0_g3_i1 | 0.580219441 | 0.443498776 | 0.999970678 | 60.999 | 41.53 | 35.276 | 26.12 | IFRD |
| TRINITY_DN175_c0_g1_i24 | 0.58019178 | 0.746220278 | 0.999970678 | 4.533 | 2.986 | 3.878 | 0.322 | NA |
| TRINITY_DN21401_c0_g1_i12 | -0.58018398 | 0.893608726 | 0.999970678 | 0.936 | 0 | 1.178 | 0 | NA |
| TRINITY_DN1422_c3_g1_i2 | 0.580138208 | 0.555023845 | 0.999970678 | 1.39 | 2.286 | 1.344 | 0.824 | NA |
| TRINITY_DN582_c0_g1_i2 | 0.58012858 | 0.898915097 | 1 | 1.335 | 0 | 0 | 0.885 | NA |
| TRINITY_DN37357_c0_g1_i1 | 0.580093774 | 0.898482422 | 1 | 1.172 | 0 | 0.656 | 0 | Metallophos |
| TRINITY_DN24121_c0_g1_i1 | 0.580093774 | 0.898482422 | 1 | 3.025 | 0 | 1.689 | 0 | CsbD |
| TRINITY_DN1492_c0_g1_i2 | -0.58005461 | 0.55625939 | 0.999970678 | 3.57 | 5.243 | 8.066 | 3.408 | AP2 |
| TRINITY_DN9876_c0_g2_i1 | -0.58000216 | 0.644248756 | 0.999970678 | 1.526 | 0.828 | 2.055 | 1.056 | NA |
| TRINITY_DN30546_c0_g1_i1 | 0.579919084 | 0.895848483 | 0.999970678 | 2.053 | 0 | 1.155 | 0 | NA |
| TRINITY_DN34972_c0_g1_i1 | 0.579919084 | 0.895848483 | 0.999970678 | 10.383 | 0 | 5.744 | 0 | F1F0-ATPsyn_F |
| TRINITY_DN9141_c2_g1_i13 | -0.57987827 | 0.72936819 | 0.999970678 | 0.681 | 0.099 | 0.789 | 0.231 | NA |
| TRINITY_DN917_c1_g1_i7 | 0.579821939 | 0.654363594 | 0.999970678 | 21.038 | 24.36 | 22.121 | 3.579 | NA |
| TRINITY_DN13225_c1_g2_i1 | 0.579812918 | 0.894274617 | 0.999970678 | 2.207 | 0 | 1.222 | 0 | NA |
| TRINITY_DN28352_c0_g1_i6 | 0.579812089 | 0.795810063 | 0.999970678 | 0.109 | 2.897 | 1.5 | 0.221 | NA |
| TRINITY_DN10896_c0_g1_i1 | -0.57975907 | 0.538136325 | 0.999970678 | 2.198 | 4.159 | 2.766 | 6.123 | - |
| TRINITY_DN7909_c0_g1_i1 | 0.579751833 | 0.628978088 | 0.999970678 | 0.554 | 1.971 | 0.756 | 0.784 | FAD_binding_8 |
| TRINITY_DN1607_c0_g1_i15 | -0.57972542 | 0.543972324 | 0.999970678 | 0.4 | 0.562 | 0.533 | 0.804 | NA |
| TRINITY_DN2733_c0_g1_i4 | 0.579699891 | 0.588972398 | 0.999970678 | 1.472 | 1.409 | 1.244 | 0.432 | NA |
| TRINITY_DN5931_c1_g1_i1 | -0.5796821 | 0.719270229 | 0.999970678 | 0.445 | 5.933 | 5.177 | 3.127 | NA |

| TRINITY_DN7447_c0_g2_i15 | -0.5796141 | 0.894818545 | 0.999970678 | 1.39 | 0 | 0 | 2.081 | NA |
| --- | --- | --- | --- | --- | --- | --- | --- | --- |
| TRINITY_DN23310_c0_g1_i1 | 0.579557373 | 0.656544017 | 0.999970678 | 1.235 | 1.035 | 0.9 | 0.422 | NA |
| TRINITY_DN6514_c0_g2_i1 | -0.57952389 | 0.445047436 | 0.999970678 | 5.214 | 3.508 | 5.155 | 6.826 | NA |
| TRINITY_DN13671_c1_g1_i2 | 0.579514783 | 0.657754735 | 0.999970678 | 0.981 | 0.808 | 0.244 | 0.895 | DUF247 |
| TRINITY_DN1078_c1_g1_i1 | 0.57950843 | 0.891378039 | 0.999970678 | 4.233 | 0 | 0 | 2.895 | NA |
| TRINITY_DN1138_c0_g1_i3 | 0.579444215 | 0.802557622 | 0.999970678 | 0.309 | 16.07 | 8.711 | 0.251 | NA |
| TRINITY_DN9291_c0_g2_i1 | -0.57941765 | 0.440936967 | 0.999970678 | 2.126 | 1.675 | 2.655 | 2.493 | Spc97_Spc98 |
| TRINITY_DN211_c0_g1_i5 | -0.57940567 | 0.455608701 | 0.999970678 | 20.284 | 14.05 | 15.899 | 32.162 | NA |
| TRINITY_DN12827_c2_g1_i1 | -0.57938536 | 0.562408682 | 0.999970678 | 0.536 | 0.769 | 0.889 | 0.885 | DYW_deaminase |
| TRINITY_DN4408_c0_g1_i6 | -0.57930092 | 0.425589796 | 0.999970678 | 3.724 | 5.785 | 5.155 | 7.952 | NA |
| TRINITY_DN3020_c0_g1_i6 | -0.5792065 | 0.443709297 | 0.999970678 | 5.487 | 3.479 | 5.666 | 6.575 | Autophagy_C |
| TRINITY_DN37020_c0_g1_i3 | -0.5791835 | 0.619498362 | 0.999970678 | 1.235 | 1.133 | 0.911 | 2.413 | NA |
| TRINITY_DN1514_c0_g2_i1 | 0.579177186 | 0.738230022 | 0.999970678 | 0.409 | 11.24 | 2.4 | 4.755 | NA |
| TRINITY_DN2422_c0_g1_i2 | 0.5791588 | 0.640782906 | 0.999970678 | 5.514 | 22.8 | 4 | 13.944 | NA |
| TRINITY_DN1449_c0_g1_i2 | -0.5791218 | 0.581566257 | 0.999970678 | 29.759 | 8.239 | 24.121 | 27.979 | Abhydrolase_1 |
| TRINITY_DN5_c0_g1_i1 | 0.57912079 | 0.660979174 | 0.999970678 | 3.252 | 1.055 | 0.522 | 2.272 | EF-hand_1 |
| TRINITY_DN2381_c0_g1_i7 | 0.579117867 | 0.593951178 | 0.999970678 | 2.653 | 1.744 | 1.867 | 0.704 | PHD |
| TRINITY_DN2191_c0_g1_i2 | 0.579040735 | 0.724423366 | 0.999970678 | 0.409 | 6.031 | 2.522 | 1.216 | NA |
| TRINITY_DN962_c0_g1_i13 | -0.57903243 | 0.705790011 | 0.999970678 | 0.518 | 0.335 | 0.9 | 0.181 | CCB2_CCB4 |
| TRINITY_DN800_c0_g1_i7 | 0.578848225 | 0.684628539 | 0.999970678 | 14.443 | 21.07 | 1.8 | 21.344 | NA |
| TRINITY_DN108658_c0_g1_i1 | -0.57880996 | 0.892247277 | 0.999970678 | 2.607 | 0 | 3.255 | 0 | NA |
| TRINITY_DN3425_c0_g2_i1 | 0.578735441 | 0.840501235 | 1 | 7.612 | 2.7 | 5.644 | 0 | NA |
| TRINITY_DN10224_c0_g1_i3 | -0.57854308 | 0.430913638 | 0.999970678 | 33.084 | 48.26 | 38.487 | 74.337 | VPS28 |
| TRINITY_DN1041_c0_g2_i4 | 0.578523005 | 0.761260347 | 0.999970678 | 1.472 | 2.306 | 1.978 | 0.09 | NA |
| TRINITY_DN13086_c0_g1_i2 | -0.57851949 | 0.396427695 | 0.999970678 | 2.244 | 2.158 | 3 | 2.966 | ABC2_membrane |
| TRINITY_DN27007_c1_g2_i3 | -0.57849985 | 0.759456788 | 1 | 1.544 | 0.355 | 2.067 | 0.382 | F_bP_aldolase |
| TRINITY_DN4877_c0_g1_i4 | 0.57849942 | 0.611777985 | 0.999970678 | 4.715 | 6.8 | 2.233 | 4.735 | NA |
| TRINITY_DN1209_c0_g1_i7 | 0.578423573 | 0.623530599 | 0.999970678 | 34.855 | 104.8 | 17.766 | 69.461 | NA |
| TRINITY_DN746_c0_g1_i3 | 0.578295694 | 0.579589615 | 0.999970678 | 6.477 | 3.075 | 3.811 | 1.84 | NA |
| TRINITY_DN1269_c0_g1_i1 | 0.57824942 | 0.632747238 | 0.999970678 | 17.287 | 5.401 | 9.144 | 4.152 | NA |
| TRINITY_DN5980_c0_g1_i4 | -0.57824575 | 0.546894853 | 0.999970678 | 1.562 | 2.986 | 2.989 | 3.127 | Methyltransf_11 |
| TRINITY_DN3298_c0_g1_i3 | 0.578240956 | 0.837867435 | 0.999970678 | 0 | 6.071 | 2.555 | 0.875 | - |
| TRINITY_DN2106_c2_g2_i1 | -0.57822817 | 0.761706149 | 0.999970678 | 2.743 | 0.207 | 0.822 | 3.408 | NA |
| TRINITY_DN20263_c0_g1_i1 | -0.5781565 | 0.578940872 | 0.999970678 | 1.372 | 3.223 | 1.655 | 4.816 | Cellulose_synt |
| TRINITY_DN6463_c0_g1_i7 | 0.577955876 | 0.699673939 | 0.999970678 | 0.663 | 5.805 | 0.789 | 3.358 | NA |
| TRINITY_DN1367_c0_g1_i4 | 0.577952933 | 0.544000642 | 0.999970678 | 7.485 | 3.282 | 2.555 | 4.162 | HLH |
| TRINITY_DN10054_c0_g1_i1 | -0.57791709 | 0.771706145 | 0.999970678 | 10.556 | 0.197 | 8.233 | 6.103 | NA |
| TRINITY_DN2740_c0_g1_i10 | 0.577871562 | 0.766477571 | 0.999970678 | 0.881 | 0.197 | 0.567 | 0.05 | NA |
| TRINITY_DN158_c0_g1_i3 | 0.577799679 | 0.852699474 | 0.999970678 | 0 | 10.71 | 0.211 | 6.837 | Na_H_Exchanger |
| TRINITY_DN393_c0_g1_i2 | 0.577758811 | 0.544133386 | 0.999970678 | 5.632 | 2.661 | 1.989 | 3.177 | NA |
| TRINITY_DN3088_c0_g1_i2 | -0.57775385 | 0.734641264 | 0.999970678 | 0.881 | 3.065 | 0.756 | 4.796 | NA |
| TRINITY_DN3251_c0_g1_i2 | -0.57765138 | 0.565072838 | 0.999970678 | 1.163 | 0.65 | 1.267 | 1.176 | NA |
| TRINITY_DN8270_c0_g1_i4 | -0.57762808 | 0.838431493 | 0.999970678 | 0.481 | 0.404 | 1.078 | 0 | Glyco_hydro_14 |
| TRINITY_DN5607_c0_g1_i1 | -0.57761841 | 0.393105659 | 0.999970678 | 13.853 | 14.35 | 13.688 | 25.597 | p450 |
| TRINITY_DN38606_c0_g1_i3 | 0.577594877 | 0.515477805 | 0.999970678 | 2.126 | 1.961 | 1.422 | 1.025 | NA |
| TRINITY_DN1015_c0_g2_i1 | -0.57743714 | 0.718227778 | 0.999970678 | 1.835 | 0.375 | 0.689 | 2.493 | NA |
| TRINITY_DN11759_c1_g1_i2 | -0.57742438 | 0.656414776 | 0.999970678 | 0.418 | 0.266 | 0.644 | 0.251 | NA |
| TRINITY_DN5930_c0_g1_i3 | -0.57742165 | 0.678042294 | 0.999970678 | 1.426 | 1.153 | 2.622 | 0.734 | GFO_IDH_MocA |
| TRINITY_DN7019_c0_g1_i2 | -0.57733823 | 0.571096472 | 0.999970678 | 0.754 | 1.892 | 1.878 | 1.659 | PPR |
| TRINITY_DN166186_c0_g1_i1 | -0.57733555 | 0.727982486 | 0.999970678 | 1.135 | 0.118 | 0.656 | 1.116 | NA |
| TRINITY_DN25022_c0_g1_i1 | -0.57730343 | 0.755620097 | 1 | 1.308 | 0.158 | 1.322 | 0.583 | NA |
| TRINITY_DN47797_c0_g1_i4 | -0.57726733 | 0.588922719 | 0.999970678 | 1.135 | 0.512 | 1.022 | 1.216 | NA |
| TRINITY_DN14946_c0_g2_i1 | -0.57725238 | 0.838777188 | 0.999970678 | 2.089 | 0 | 2.055 | 0.674 | ABC_membrane |
| TRINITY_DN3021_c0_g1_i1 | 0.577236302 | 0.860398441 | 0.999970678 | 0.672 | 54 | 29.809 | 0 | NA |
| TRINITY_DN13547_c0_g1_i1 | 0.577093899 | 0.614444808 | 0.999970678 | 4.642 | 2.148 | 1.633 | 2.544 | NA |
| TRINITY_DN4879_c0_g1_i4 | -0.57708643 | 0.839781606 | 0.999970678 | 0 | 67.86 | 47.175 | 42.115 | NA |
| TRINITY_DN6956_c0_g1_i1 | 0.577084717 | 0.665590226 | 0.999970678 | 1.154 | 0.857 | 0.856 | 0.332 | NA |
| TRINITY_DN186_c6_g1_i4 | 0.577074855 | 0.748420389 | 0.999970678 | 57.847 | 1587 | 145.825 | 846.783 | NA |
| TRINITY_DN12232_c0_g1_i2 | 0.577042897 | 0.425100086 | 0.999970678 | 8.375 | 5.982 | 3.678 | 5.198 | NA |
| TRINITY_DN18548_c0_g1_i1 | -0.57703647 | 0.757850918 | 0.999970678 | 0.818 | 5.489 | 0.344 | 8.887 | ACP_syn_III_C |
| TRINITY_DN7199_c0_g1_i3 | 0.577012705 | 0.415919347 | 0.999970678 | 3.316 | 3.696 | 2.244 | 1.981 | NA |
| TRINITY_DN654_c0_g1_i4 | -0.57699902 | 0.580444485 | 0.999970678 | 17.923 | 43.28 | 22.399 | 61.056 | NA |
| TRINITY_DN6889_c0_g1_i5 | 0.576997054 | 0.427058004 | 0.999970678 | 7.022 | 4.583 | 3.233 | 3.911 | DEAD |
| TRINITY_DN237_c0_g1_i19 | 0.576954001 | 0.611470162 | 0.999970678 | 1.244 | 1.951 | 1.355 | 0.493 | NA |
| TRINITY_DN604_c1_g1_i1 | -0.57694771 | 0.643798551 | 0.999970678 | 214.5 | 191.8 | 68.807 | 476.354 | NA |
| TRINITY_DN1244_c0_g1_i3 | -0.57691932 | 0.475734493 | 0.999970678 | 3.697 | 2.553 | 4.344 | 4.092 | NA |
| TRINITY_DN12306_c0_g1_i4 | 0.576876406 | 0.630582482 | 0.999970678 | 24.363 | 6.83 | 10.522 | 7.973 | NA |
| TRINITY_DN17957_c1_g1_i1 | -0.57681905 | 0.622029413 | 0.999970678 | 2.089 | 1.912 | 3.2 | 2.051 | NA |
| TRINITY_DN5682_c0_g1_i5 | 0.576792784 | 0.727155758 | 0.999970678 | 31.049 | 18.5 | 25.487 | 1.89 | NA |
| TRINITY_DN1524_c0_g1_i9 | 0.576779614 | 0.592794786 | 0.999970678 | 11.164 | 8.929 | 7.033 | 4.625 | NA |
| TRINITY_DN28532_c0_g1_i1 | 0.576767889 | 0.58651663 | 0.999970678 | 0.536 | 0.818 | 0.467 | 0.342 | NA |
| TRINITY_DN29_c0_g1_i5 | -0.5767093 | 0.52461577 | 0.999970678 | 4.606 | 2.858 | 3.766 | 6.565 | Alliinase_C |
| TRINITY_DN19663_c0_g1_i1 | 0.576690496 | 0.534626316 | 0.999970678 | 8.884 | 10.15 | 5.644 | 5.509 | NA |
| TRINITY_DN1451_c0_g1_i2 | 0.576665766 | 0.68710451 | 0.999970678 | 4.315 | 3.499 | 3.911 | 0.523 | NA |

| TRINITY_DN5578_c0_g1_i13 | 0.57662078 | 0.892042115 | 0.999970678 | 1.453 | 0 | 0 | 0.995 | NA |
| --- | --- | --- | --- | --- | --- | --- | --- | --- |
| TRINITY_DN720_c0_g1_i1 | -0.57658857 | 0.566027085 | 0.999970678 | 2.044 | 0.808 | 1.911 | 2.001 | NA |
| TRINITY_DN165562_c0_g2_i1 | -0.57648415 | 0.891227641 | 0.999970678 | 0 | 12.5 | 0 | 18.318 | Ribosomal_S3Ae |
| TRINITY_DN7029_c0_g1_i3 | -0.57644042 | 0.856391831 | 0.999970678 | 7.885 | 0.118 | 0 | 12.135 | RRM_1 |
| TRINITY_DN313_c0_g1_i2 | 0.576393416 | 0.891145112 | 0.999970678 | 0 | 6.514 | 3.555 | 0 | RRM_1 |
| TRINITY_DN12778_c0_g1_i3 | -0.57622577 | 0.746448116 | 0.999970678 | 1.808 | 0.197 | 0.556 | 2.292 | NA |
| TRINITY_DN7188_c1_g1_i1 | -0.57618582 | 0.69957808 | 0.999970678 | 0.954 | 2.622 | 0.867 | 4.243 | NA |
| TRINITY_DN1410_c0_g2_i2 | -0.57615636 | 0.891618367 | 0.999970678 | 0 | 4.593 | 5.566 | 0 | NA |
| TRINITY_DN1782_c0_g1_i2 | -0.57612039 | 0.501685297 | 0.999970678 | 9.166 | 15.48 | 10.066 | 24.541 | DELLA |
| TRINITY_DN2590_c0_g1_i4 | 0.576112125 | 0.827485135 | 0.999970678 | 2.026 | 2.553 | 0 | 3.066 | DUF3223 |
| TRINITY_DN11150_c1_g1_i2 | -0.57608748 | 0.65114665 | 0.999970678 | 9.883 | 16.46 | 22.521 | 10.013 | NA |
| TRINITY_DN4989_c0_g2_i1 | -0.57607975 | 0.839886533 | 1 | 0 | 5.755 | 5.311 | 1.88 | NA |
| TRINITY_DN21319_c1_g1_i1 | 0.575786177 | 0.846041984 | 1 | 15.652 | 2.631 | 9.866 | 0 | NA |
| TRINITY_DN8046_c0_g1_i4 | -0.57575953 | 0.759088662 | 0.999970678 | 0.518 | 0.246 | 0.067 | 1.086 | NA |
| TRINITY_DN2856_c0_g2_i1 | -0.57575866 | 0.518656819 | 0.999970678 | 16.869 | 41.41 | 28.576 | 51.907 | Cyt-b5 |
| TRINITY_DN72728_c0_g3_i1 | -0.57574402 | 0.899767306 | 1 | 0.663 | 0 | 0.833 | 0 | - |
| TRINITY_DN9632_c1_g1_i1 | -0.57571006 | 0.430090628 | 0.999970678 | 10.165 | 16.16 | 18.777 | 16.408 | S4 |
| TRINITY_DN622_c0_g1_i4 | -0.57567933 | 0.533662938 | 0.999970678 | 1.444 | 0.877 | 1.322 | 1.88 | NA |
| TRINITY_DN98917_c0_g1_i1 | 0.575648228 | 0.893363142 | 0.999970678 | 1.081 | 0 | 0.611 | 0 | NA |
| TRINITY_DN5803_c0_g2_i1 | -0.57555474 | 0.723753624 | 0.999970678 | 0.372 | 1.449 | 0.467 | 2.071 | NA |
| TRINITY_DN3723_c0_g1_i4 | 0.575466636 | 0.828052641 | 0.999970678 | 5.45 | 8.387 | 0 | 9.139 | NA |
| TRINITY_DN23122_c0_g2_i5 | 0.575444673 | 0.666321967 | 0.999970678 | 2.662 | 1.518 | 1.922 | 0.493 | NA |
| TRINITY_DN18502_c0_g1_i2 | 0.575420549 | 0.465055936 | 0.999970678 | 4.015 | 3.371 | 2.322 | 2.151 | DUF220 |
| TRINITY_DN1179_c0_g2_i1 | 0.57540003 | 0.562596036 | 0.999970678 | 13.308 | 15.86 | 5.822 | 11.813 | NA |
| TRINITY_DN5546_c0_g1_i4 | 0.57539833 | 0.599972195 | 0.999970678 | 32.566 | 16.24 | 6.2 | 25.396 | Aldo_ket_red |
| TRINITY_DN1732_c0_g1_i5 | -0.57537963 | 0.715875399 | 0.999970678 | 2.18 | 0.779 | 3.366 | 0.352 | PWI |
| TRINITY_DN3837_c0_g1_i1 | -0.57533023 | 0.835018114 | 0.999970678 | 5.023 | 0 | 3.789 | 3.016 | RRM_1 |
| TRINITY_DN4258_c0_g1_i2 | -0.57526552 | 0.660805379 | 0.999970678 | 40.959 | 155.7 | 197.555 | 51.465 | NA |
| TRINITY_DN3041_c0_g1_i2 | 0.575249897 | 0.64299831 | 0.999970678 | 73.153 | 17.63 | 12.799 | 46.167 | La |
| TRINITY_DN14881_c0_g1_i1 | -0.57524677 | 0.447111303 | 0.999970678 | 2.525 | 3.282 | 3.144 | 4.826 | NA |
| TRINITY_DN102_c0_g1_i3 | -0.57517052 | 0.834447446 | 0.999970678 | 1.335 | 0 | 0.878 | 0.965 | NA |
| TRINITY_DN4617_c0_g1_i1 | -0.57516948 | 0.480628198 | 0.999970678 | 5.523 | 11.86 | 10.988 | 12.507 | DUF1232 |
| TRINITY_DN5158_c0_g1_i6 | 0.575095828 | 0.898511896 | 1 | 0 | 0.759 | 0.411 | 0 | NA |
| TRINITY_DN10230_c0_g2_i2 | -0.57506931 | 0.524924612 | 0.999970678 | 2.08 | 1.242 | 1.789 | 2.785 | NA |
| TRINITY_DN1743_c0_g1_i1 | 0.575062989 | 0.627895177 | 0.999970678 | 3.833 | 1.419 | 1.233 | 2.101 | NA |
| TRINITY_DN3730_c0_g1_i1 | 0.575061035 | 0.849739675 | 0.999970678 | 93.238 | 0 | 5.866 | 55.697 | NA |
| TRINITY_DN44013_c0_g1_i6 | 0.575002321 | 0.679101737 | 0.999970678 | 1246.3 | 12349 | 2554.501 | 5146.748 | NA |
| TRINITY_DN14481_c0_g1_i1 | 0.574997058 | 0.415795679 | 0.999970678 | 219.23 | 283.4 | 105.227 | 209.971 | NA |
| TRINITY_DN10960_c0_g1_i2 | 0.574951962 | 0.433483123 | 0.999970678 | 2.144 | 3.252 | 1.533 | 1.769 | NA |
| TRINITY_DN23895_c0_g1_i1 | 0.574947434 | 0.894239948 | 0.999970678 | 1.799 | 0 | 1.011 | 0 | NA |
| TRINITY_DN2911_c0_g1_i4 | 0.574924912 | 0.63015731 | 0.999970678 | 0.981 | 4.041 | 1.722 | 1.267 | NA |
| TRINITY_DN12527_c0_g1_i4 | -0.57484487 | 0.708493952 | 1 | 0.681 | 0.237 | 0.767 | 0.452 | NA |
| TRINITY_DN12719_c0_g1_i1 | -0.57483061 | 0.343909424 | 0.999970678 | 26.28 | 21.26 | 29.198 | 35.56 | NA |
| TRINITY_DN8892_c0_g1_i1 | -0.57480771 | 0.740860346 | 0.999970678 | 3.352 | 0.177 | 2.611 | 2.192 | NA |
| TRINITY_DN11398_c0_g1_i1 | 0.574721726 | 0.769456523 | 0.999970678 | 2.534 | 1.074 | 1.955 | 0.08 | PIR |
| TRINITY_DN31515_c0_g1_i1 | -0.57464203 | 0.834448599 | 0.999970678 | 1.453 | 0 | 1.255 | 0.684 | NA |
| TRINITY_DN2505_c0_g1_i1 | 0.574636742 | 0.737066682 | 0.999970678 | 0.336 | 6.317 | 2.011 | 1.96 | Hexapep |
| TRINITY_DN3108_c0_g1_i1 | -0.57458763 | 0.6786653 | 0.999970678 | 6.132 | 0.887 | 3.555 | 6.193 | Ribosomal_S8 |
| TRINITY_DN145_c0_g1_i1 | 0.574514373 | 0.845522077 | 1 | 0.136 | 0.749 | 0 | 0.593 | FoP_duplication |
| TRINITY_DN15889_c0_g1_i1 | 0.574514373 | 0.845522077 | 1 | 0.109 | 0.631 | 0 | 0.503 | NA |
| TRINITY_DN2891_c0_g1_i2 | 0.574440178 | 0.545987057 | 0.999970678 | 2.643 | 1.271 | 1.311 | 1.066 | NA |
| TRINITY_DN11353_c0_g1_i1 | -0.57442576 | 0.379445906 | 0.999970678 | 14.189 | 10.79 | 16.177 | 17.674 | NA |
| TRINITY_DN8886_c0_g1_i1 | 0.57437976 | 0.84088406 | 1 | 0.3 | 0.118 | 0.233 | 0 | RRM_1 |
| TRINITY_DN460_c0_g1_i2 | -0.57436315 | 0.703265978 | 0.999970678 | 0.963 | 0.877 | 0.2 | 2.513 | Lipase_GDSL |
| TRINITY_DN3443_c0_g1_i9 | -0.57425819 | 0.746633561 | 1 | 0.118 | 0.611 | 0.678 | 0.241 | NA |
| TRINITY_DN3911_c1_g1_i4 | -0.57421284 | 0.561865559 | 0.999970678 | 3.079 | 3.469 | 2.211 | 7.048 | NA |
| TRINITY_DN19787_c0_g1_i5 | -0.57419651 | 0.615230988 | 0.999970678 | 2.153 | 2.75 | 3.411 | 3.016 | NA |
| TRINITY_DN4324_c0_g1_i6 | -0.57413531 | 0.711727857 | 0.999970678 | 4.197 | 7.835 | 13.566 | 1.357 | NA |
| TRINITY_DN17569_c0_g1_i1 | 0.574075214 | 0.687372033 | 0.999970678 | 3.479 | 6.307 | 0.878 | 5.379 | NA |
| TRINITY_DN48659_c0_g1_i2 | 0.574018049 | 0.545724475 | 0.999970678 | 1.426 | 2.799 | 1.1 | 1.478 | - |
| TRINITY_DN518_c0_g1_i2 | -0.57401443 | 0.631836665 | 0.999970678 | 2.825 | 15.64 | 11.588 | 13.18 | Tubulin |
| TRINITY_DN47599_c0_g1_i1 | -0.57388788 | 0.666092847 | 0.999970678 | 1.708 | 2.444 | 0.822 | 5.168 | Y_phosphatase2 |
| TRINITY_DN1696_c0_g1_i3 | -0.57384359 | 0.551359936 | 0.999970678 | 0.609 | 1.163 | 1.022 | 1.387 | NA |
| TRINITY_DN3808_c0_g1_i3 | 0.573827035 | 0.596834084 | 0.999970678 | 0.472 | 0.838 | 0.256 | 0.563 | - |
| TRINITY_DN6694_c0_g2_i2 | 0.573822681 | 0.750439315 | 0.999970678 | 4.506 | 0.384 | 2.478 | 0.342 | NA |
| TRINITY_DN253_c0_g1_i2 | 0.573807377 | 0.371831137 | 0.999970678 | 5.096 | 6.692 | 3.589 | 3.589 | Fip1 |
| TRINITY_DN1370_c0_g1_i1 | -0.57377887 | 0.544096841 | 0.999970678 | 133.51 | 368.5 | 234.975 | 450.536 | KOW |
| TRINITY_DN89728_c0_g2_i1 | -0.57373567 | 0.61228219 | 0.999970678 | 1.453 | 1.557 | 1.089 | 3.157 | NA |
| TRINITY_DN2507_c0_g3_i2 | 0.573675089 | 0.583733694 | 0.999970678 | 1.372 | 1.34 | 1.111 | 0.483 | DBD_Tnp_Mut |
| TRINITY_DN864_c0_g1_i14 | 0.573628085 | 0.398109725 | 0.999970678 | 3.352 | 4.001 | 1.655 | 2.956 | PDCD2_C |
| TRINITY_DN885_c0_g1_i11 | -0.57358192 | 0.838452553 | 0.999970678 | 0 | 16.11 | 11.777 | 9.37 | CWC25 |
| TRINITY_DN3646_c0_g1_i4 | 0.573571548 | 0.691677843 | 0.999970678 | 0.218 | 1.242 | 0.356 | 0.543 | Dimerisation |
| TRINITY_DN9290_c0_g1_i1 | 0.573541118 | 0.600422587 | 0.999970678 | 19.621 | 48.66 | 10.477 | 31.8 | NA |
| TRINITY_DN3210_c0_g1_i2 | -0.5735398 | 0.510664953 | 0.999970678 | 2.771 | 6.022 | 6.033 | 5.721 | NA |

| TRINITY_DN6622_c0_g1_i1 | 0.57351117 | 0.473784021 | 0.999970678 | 4.142 | 6.672 | 3.811 | 2.664 | NA |
| --- | --- | --- | --- | --- | --- | --- | --- | --- |
| TRINITY_DN15211_c0_g1_i4 | 0.573510736 | 0.527044171 | 0.999970678 | 0.745 | 0.828 | 0.511 | 0.422 | NA |
| TRINITY_DN1422_c2_g2_i2 | -0.57350381 | 0.507250553 | 0.999970678 | 14.398 | 10.46 | 21.21 | 11.491 | NTF2 |
| TRINITY_DN1088_c0_g1_i6 | 0.573447465 | 0.706317518 | 0.999970678 | 2.371 | 0.404 | 0.589 | 1.146 | NA |
| TRINITY_DN2106_c1_g2_i1 | 0.573399625 | 0.899238794 | 1 | 0 | 2.69 | 1.467 | 0 | NA |
| TRINITY_DN225_c0_g1_i5 | 0.573370386 | 0.767181133 | 0.999970678 | 21.947 | 3.853 | 14.155 | 0.392 | NA |
| TRINITY_DN3898_c0_g1_i13 | 0.573364175 | 0.896676909 | 1 | 1.835 | 0 | 0 | 1.257 | NA |
| TRINITY_DN7337_c0_g1_i8 | 0.573299066 | 0.837225914 | 0.999970678 | 1.199 | 0 | 0.367 | 0.382 | - |
| TRINITY_DN1600_c0_g1_i4 | 0.573202712 | 0.532073624 | 0.999970678 | 5.905 | 4.198 | 4 | 1.991 | Pkinase_Tyr |
| TRINITY_DN4642_c0_g1_i3 | 0.572937035 | 0.605473097 | 0.999970678 | 0.672 | 1.537 | 0.856 | 0.442 | NA |
| TRINITY_DN3390_c0_g1_i5 | 0.572929124 | 0.679504441 | 0.999970678 | 0.818 | 1.715 | 0.256 | 1.408 | Cupin_1 |
| TRINITY_DN3884_c0_g1_i2 | 0.572728037 | 0.350560791 | 0.999970678 | 14.643 | 11.54 | 7.611 | 8.485 | Pkinase_Tyr |
| TRINITY_DN565_c1_g1_i2 | -0.57270729 | 0.712747524 | 0.999970678 | 0.345 | 1.055 | 1.422 | 0.322 | NA |
| TRINITY_DN2428_c0_g1_i6 | 0.57267977 | 0.616472622 | 0.999970678 | 3.752 | 2.75 | 0.756 | 3.458 | NA |
| TRINITY_DN1772_c0_g1_i4 | 0.572450325 | 0.58998722 | 0.999970678 | 1.326 | 2.139 | 1.422 | 0.613 | NA |
| TRINITY_DN2645_c0_g3_i2 | -0.57237651 | 0.642236164 | 0.999970678 | 0.908 | 3.538 | 1.6 | 4.645 | NA |
| TRINITY_DN2820_c1_g1_i1 | -0.57236275 | 0.33591217 | 0.999970678 | 22.728 | 20.81 | 24.921 | 34.565 | NA |
| TRINITY_DN7061_c0_g1_i12 | -0.57235884 | 0.892775281 | 0.999970678 | 5.632 | 0 | 6.955 | 0 | NA |
| TRINITY_DN26280_c0_g1_i1 | -0.57231121 | 0.566479353 | 0.999970678 | 3.316 | 8.16 | 7.022 | 8.294 | NA |
| TRINITY_DN75294_c0_g1_i1 | 0.572280455 | 0.897493606 | 1 | 1.208 | 0 | 0.678 | 0 | NA |
| TRINITY_DN2679_c0_g3_i1 | 0.572258061 | 0.617618557 | 0.999970678 | 9.62 | 31.4 | 16.177 | 7.691 | - |
| TRINITY_DN2679_c0_g2_i1 | 0.572258061 | 0.617618557 | 0.999970678 | 9.62 | 31.4 | 16.177 | 7.691 | NA |
| TRINITY_DN701_c0_g1_i2 | -0.57221345 | 0.833274341 | 0.999970678 | 2.153 | 0 | 1.3 | 1.669 | NB-ARC |
| TRINITY_DN6113_c0_g1_i1 | -0.57212892 | 0.49777892 | 0.999970678 | 7.74 | 8.712 | 6.533 | 16.488 | Chitin_bind_1 |
| TRINITY_DN6690_c0_g1_i1 | 0.572050573 | 0.894211581 | 0.999970678 | 17.214 | 0 | 9.711 | 0 | NA |
| TRINITY_DN772_c0_g1_i1 | -0.57204158 | 0.71942295 | 1 | 8.003 | 3.873 | 2.567 | 13.572 | NA |
| TRINITY_DN8743_c0_g1_i1 | -0.57193255 | 0.402117918 | 0.999970678 | 3.47 | 4.484 | 5.4 | 5.288 | NA |
| TRINITY_DN13340_c0_g1_i1 | -0.57193031 | 0.401666308 | 0.999970678 | 31.094 | 38 | 44.508 | 45.594 | NA |
| TRINITY_DN7345_c0_g1_i3 | -0.57188648 | 0.503845091 | 0.999970678 | 6.586 | 8.949 | 7.6 | 13.502 | NA |
| TRINITY_DN26985_c0_g1_i3 | -0.57185362 | 0.670088522 | 0.999970678 | 3.57 | 2.07 | 5.711 | 1.488 | NA |
| TRINITY_DN1031_c1_g1_i2 | -0.57182585 | 0.455090898 | 0.999970678 | 7.113 | 7.283 | 11.066 | 7.973 | NA |
| TRINITY_DN7203_c0_g1_i6 | -0.57179897 | 0.832996034 | 0.999970678 | 1.481 | 0 | 1.178 | 0.824 | Suf |
| TRINITY_DN10219_c0_g2_i9 | -0.57178807 | 0.899037995 | 1 | 3.207 | 0 | 3.922 | 0 | NA |
| TRINITY_DN14880_c0_g1_i6 | 0.571785641 | 0.768249275 | 0.999970678 | 1.499 | 2.562 | 2.211 | 0.04 | Methyltransf_4 |
| TRINITY_DN1786_c0_g1_i3 | 0.571767952 | 0.441920945 | 0.999970678 | 28.914 | 52.43 | 20.188 | 30.04 | NA |
| TRINITY_DN2436_c0_g2_i5 | 0.571738365 | 0.748680097 | 0.999970678 | 1.544 | 40.42 | 4.266 | 22.701 | NA |
| TRINITY_DN15602_c0_g1_i2 | 0.571728013 | 0.565756184 | 0.999970678 | 6.322 | 3.272 | 3.278 | 2.513 | DUF2870 |
| TRINITY_DN18203_c0_g2_i1 | 0.571724152 | 0.694501339 | 0.999970678 | 1.681 | 0.394 | 0.544 | 0.724 | NA |
| TRINITY_DN986_c1_g1_i3 | -0.57171998 | 0.500167085 | 0.999970678 | 0.981 | 1.212 | 1.278 | 1.739 | NA |
| TRINITY_DN38155_c0_g1_i1 | -0.57171118 | 0.422240026 | 0.999970678 | 2.698 | 3.154 | 3.622 | 4.303 | Presenilin |
| TRINITY_DN10870_c0_g1_i2 | -0.57169206 | 0.509586666 | 0.999970678 | 28.142 | 21.26 | 41.997 | 22.591 | NA |
| TRINITY_DN13228_c0_g1_i4 | 0.571649078 | 0.525015131 | 0.999970678 | 3.316 | 1.784 | 1.644 | 1.478 | AAA |
| TRINITY_DN443_c0_g1_i3 | 0.571605951 | 0.580204654 | 0.999970678 | 3.252 | 4.139 | 3.133 | 1.196 | Amidohydro_1 |
| TRINITY_DN1749_c0_g2_i2 | -0.57153939 | 0.42597113 | 0.999970678 | 3.797 | 3.134 | 4.344 | 5.087 | zf-met |
| TRINITY_DN1749_c0_g1_i2 | -0.57153939 | 0.42597113 | 0.999970678 | 3.797 | 3.134 | 4.344 | 5.087 | NA |
| TRINITY_DN21055_c0_g1_i2 | 0.571520636 | 0.900048253 | 1 | 0 | 0.286 | 0.156 | 0 | NA |
| TRINITY_DN18076_c1_g1_i3 | 0.571405186 | 0.739024782 | 0.999970678 | 0.2 | 1.124 | 0.644 | 0.121 | NA |
| TRINITY_DN10465_c0_g1_i3 | 0.571368437 | 0.525183877 | 0.999970678 | 6.413 | 6.317 | 2.4 | 5.6 | Vps55 |
| TRINITY_DN11005_c0_g1_i4 | -0.57132978 | 0.84264642 | 0.999970678 | 0 | 0.67 | 0.711 | 0.111 | NA |
| TRINITY_DN19696_c0_g1_i1 | 0.571256014 | 0.55268253 | 0.999970678 | 0.5 | 0.572 | 0.267 | 0.402 | NA |
| TRINITY_DN11746_c0_g1_i9 | 0.571245241 | 0.897533462 | 1 | 5.105 | 0 | 0 | 3.408 | NA |
| TRINITY_DN11062_c0_g1_i1 | -0.57120166 | 0.893766459 | 0.999970678 | 3.007 | 0 | 3.744 | 0 | NA |
| TRINITY_DN30607_c0_g1_i1 | -0.57108747 | 0.704883868 | 0.999970678 | 0.636 | 3.075 | 0.622 | 4.715 | - |
| TRINITY_DN8136_c0_g1_i3 | 0.57106448 | 0.619580429 | 0.999970678 | 2.171 | 9.175 | 2.633 | 4.424 | AA_permease_2 |
| TRINITY_DN100948_c0_g1_i1 | 0.571025289 | 0.898917736 | 1 | 1.154 | 0 | 0.656 | 0 | NA |
| TRINITY_DN12156_c0_g1_i4 | 0.571011623 | 0.755264227 | 0.999970678 | 0.118 | 0.926 | 0.089 | 0.573 | NA |
| TRINITY_DN10918_c0_g2_i4 | -0.57092754 | 0.848942128 | 0.999970678 | 1.008 | 0.079 | 0 | 1.649 | NA |
| TRINITY_DN19592_c0_g1_i10 | -0.57086759 | 0.660514888 | 0.999970678 | 8.702 | 2.759 | 2.933 | 13.532 | NA |
| TRINITY_DN2880_c0_g1_i4 | -0.57083714 | 0.737417561 | 0.999970678 | 1.081 | 0.108 | 0.722 | 0.935 | SURNod19 |
| TRINITY_DN1437_c2_g1_i10 | -0.57077914 | 0.538770476 | 0.999970678 | 5.841 | 5.568 | 4.355 | 11.612 | NA |
| TRINITY_DN590_c0_g1_i14 | -0.57070837 | 0.639471548 | 0.999970678 | 80.72 | 497.4 | 312.203 | 446.826 | NA |
| TRINITY_DN14001_c0_g1_i1 | -0.57066188 | 0.617324572 | 0.999970678 | 0.727 | 3.075 | 2.267 | 2.855 | DUF3657 |
| TRINITY_DN103_c0_g1_i10 | -0.57063331 | 0.656445427 | 0.999970678 | 1.808 | 0.414 | 1.8 | 1.156 | NA |
| TRINITY_DN955_c0_g1_i5 | -0.57060681 | 0.831765822 | 0.999970678 | 0 | 2.947 | 2.211 | 1.639 | NA |
| TRINITY_DN3318_c0_g1_i1 | 0.570583481 | 0.739020538 | 0.999970678 | 0.59 | 1.528 | 1.078 | 0.131 | NA |
| TRINITY_DN191_c0_g1_i6 | -0.57051632 | 0.850803495 | 0.999970678 | 62.97 | 0 | 59.785 | 22.641 | Inos-1-P_synth |
| TRINITY_DN694_c0_g1_i8 | -0.57039108 | 0.408116241 | 0.999970678 | 4.142 | 4.376 | 5.722 | 5.7 | DapB_C |
| TRINITY_DN9930_c0_g1_i2 | -0.57038889 | 0.833006796 | 0.999970678 | 1.535 | 0 | 0.844 | 1.307 | NA |
| TRINITY_DN12131_c0_g1_i1 | 0.570330986 | 0.520021289 | 0.999970678 | 7.967 | 14.95 | 4.677 | 9.652 | NA |
| TRINITY_DN7479_c0_g1_i1 | 0.570304568 | 0.757463393 | 0.999970678 | 0.227 | 8.456 | 1.5 | 3.941 | NA |
| TRINITY_DN799_c0_g2_i6 | 0.570283977 | 0.735932206 | 0.999970678 | 0.091 | 0.828 | 0.144 | 0.442 | FA_desaturase |
| TRINITY_DN112326_c0_g1_i1 | -0.57028246 | 0.894621324 | 0.999970678 | 3.089 | 0 | 3.833 | 0 | NA |
| TRINITY_DN19998_c0_g2_i3 | -0.57027864 | 0.424259467 | 0.999970678 | 40.969 | 50.25 | 42.709 | 81.948 | NA |
| TRINITY_DN557_c0_g1_i5 | -0.57018295 | 0.7287672 | 0.999970678 | 15.806 | 0.867 | 14.955 | 7.028 | Cpn60_TCP1 |

| TRINITY_DN119_c1_g1_i1 | -0.56997235 | 0.50726251 | 0.999970678 | 14.244 | 12.28 | 9.699 | 27.829 | NA |
| --- | --- | --- | --- | --- | --- | --- | --- | --- |
| TRINITY_DN3003_c0_g1_i4 | -0.56995762 | 0.662830327 | 0.999970678 | 0.781 | 0.986 | 1.833 | 0.422 | NA |
| TRINITY_DN11421_c0_g1_i8 | 0.569920099 | 0.849220849 | 0.999970678 | 0.436 | 8.052 | 0 | 5.459 | NA |
| TRINITY_DN4679_c0_g1_i4 | -0.5698668 | 0.400520837 | 0.999970678 | 106.03 | 118 | 109.738 | 184.938 | NA |
| TRINITY_DN28966_c0_g1_i3 | -0.56984362 | 0.687300935 | 1 | 2.107 | 5.824 | 4.344 | 5.972 | NA |
| TRINITY_DN1186_c0_g1_i4 | 0.569817612 | 0.565142723 | 0.999970678 | 2.353 | 4.642 | 2.589 | 1.568 | NA |
| TRINITY_DN212_c0_g1_i3 | -0.5698002 | 0.52937607 | 0.999970678 | 4.297 | 10.55 | 7.122 | 13.361 | Lycopene_cycl |
| TRINITY_DN1217_c0_g2_i1 | -0.5697578 | 0.393447841 | 0.999970678 | 8.657 | 7.313 | 9.944 | 11.592 | NA |
| TRINITY_DN3082_c0_g1_i1 | 0.569720666 | 0.688044333 | 0.999970678 | 0.981 | 1.301 | 1.133 | 0.161 | NA |
| TRINITY_DN5872_c1_g1_i2 | 0.569682656 | 0.834065342 | 0.999970678 | 0.445 | 0.276 | 0.4 | 0 | TMEM131_like |
| TRINITY_DN994_c0_g1_i7 | 0.569603174 | 0.741647043 | 0.999970678 | 2.135 | 11.51 | 6.866 | 0.744 | NA |
| TRINITY_DN180_c1_g1_i1 | 0.569466771 | 0.669751173 | 0.999970678 | 3.525 | 6.327 | 4.689 | 0.915 | Inositol_P |
| TRINITY_DN9845_c0_g1_i16 | 0.569449883 | 0.673356257 | 0.999970678 | 0.663 | 0.375 | 0.478 | 0.131 | NA |
| TRINITY_DN3110_c0_g1_i3 | -0.56936052 | 0.337697813 | 0.999970678 | 58.31 | 49.83 | 71.162 | 74.468 | Abhydrolase_2 |
| TRINITY_DN1150_c0_g1_i8 | -0.56933153 | 0.6622296 | 0.999970678 | 1.281 | 0.503 | 1.433 | 0.925 | NA |
| TRINITY_DN7326_c0_g1_i3 | 0.569232565 | 0.900828689 | 1 | 0.899 | 0 | 0.511 | 0 | DEAD |
| TRINITY_DN3167_c0_g1_i1 | 0.569232565 | 0.900828689 | 1 | 12.163 | 0 | 6.733 | 0 | NA |
| TRINITY_DN3440_c0_g1_i2 | -0.56916206 | 0.72442903 | 0.999970678 | 0.209 | 0.926 | 0.278 | 1.307 | NA |
| TRINITY_DN7016_c0_g1_i1 | -0.56913902 | 0.693754544 | 0.999970678 | 2.371 | 11.12 | 2.522 | 16.77 | - |
| TRINITY_DN22603_c0_g1_i4 | -0.56912903 | 0.667218029 | 0.999970678 | 1.054 | 1.232 | 0.578 | 2.614 | NA |
| TRINITY_DN12760_c0_g1_i5 | 0.569108502 | 0.692777188 | 1 | 1.145 | 1.025 | 0.933 | 0.292 | NA |
| TRINITY_DN9298_c0_g1_i1 | -0.56910662 | 0.502625852 | 0.999970678 | 9.093 | 18.6 | 17.654 | 19.092 | NA |
| TRINITY_DN90371_c0_g1_i1 | 0.56904856 | 0.623263813 | 0.999970678 | 3.261 | 2.947 | 1.089 | 2.835 | NA |
| TRINITY_DN5305_c0_g1_i6 | 0.569018935 | 0.643217515 | 0.999970678 | 0.981 | 2.474 | 1.111 | 0.965 | NA |
| TRINITY_DN15999_c0_g1_i4 | 0.568989937 | 0.560140718 | 0.999970678 | 3.897 | 3.784 | 2.533 | 2.061 | NA |
| TRINITY_DN3264_c0_g1_i1 | -0.56894577 | 0.500555345 | 0.999970678 | 18.077 | 11.36 | 23.887 | 14.98 | NA |
| TRINITY_DN2747_c0_g1_i2 | -0.56890209 | 0.633269499 | 0.999970678 | 4.942 | 13.8 | 5.477 | 20.761 | NA |
| TRINITY_DN56582_c0_g1_i1 | -0.56888223 | 0.898481157 | 1 | 0.709 | 0 | 0.889 | 0 | NA |
| TRINITY_DN4264_c0_g1_i5 | -0.56888223 | 0.898481157 | 1 | 0.936 | 0 | 1.155 | 0 | NA |
| TRINITY_DN167279_c0_g1_i1 | -0.56888223 | 0.898481157 | 1 | 0.808 | 0 | 1.011 | 0 | NA |
| TRINITY_DN4608_c1_g1_i1 | -0.56888159 | 0.695859394 | 0.999970678 | 0.827 | 0.335 | 1.1 | 0.432 | Cupin_8 |
| TRINITY_DN10456_c0_g1_i12 | 0.568822057 | 0.898488097 | 1 | 0.609 | 0 | 0 | 0.422 | NA |
| TRINITY_DN9137_c0_g1_i1 | -0.56879186 | 0.417210122 | 0.999970678 | 19.058 | 23.83 | 31.309 | 25.315 | Acetyltransf_1 |
| TRINITY_DN1838_c0_g3_i2 | 0.568784861 | 0.671103773 | 0.999970678 | 3.697 | 13.07 | 1.644 | 9.189 | NA |
| TRINITY_DN19530_c0_g1_i3 | -0.56877004 | 0.681116123 | 0.999970678 | 0.345 | 1.005 | 1.322 | 0.392 | ATP_bind_3 |
| TRINITY_DN4706_c0_g1_i9 | -0.56875102 | 0.676280243 | 0.999970678 | 0.327 | 0.443 | 0.156 | 0.955 | NA |
| TRINITY_DN167_c0_g1_i1 | -0.56871419 | 0.333907273 | 0.999970678 | 15.561 | 18.84 | 21.099 | 25.466 | EMP70 |
| TRINITY_DN9835_c0_g1_i1 | -0.56862771 | 0.730049149 | 0.999970678 | 0.273 | 1.528 | 1.533 | 0.774 | NA |
| TRINITY_DN2946_c0_g2_i2 | 0.56848843 | 0.52260423 | 0.999970678 | 40.605 | 23.7 | 24.51 | 13.965 | Ank_2 |
| TRINITY_DN1061_c0_g1_i5 | -0.5683994 | 0.516026244 | 0.999970678 | 30.885 | 19.75 | 19.643 | 51.756 | NA |
| TRINITY_DN2859_c0_g1_i2 | 0.568391766 | 0.558905213 | 0.999970678 | 2.271 | 3.331 | 1 | 2.554 | NA |
| TRINITY_DN2530_c0_g1_i5 | -0.56818671 | 0.683750953 | 0.999970678 | 0.372 | 0.217 | 0.167 | 0.684 | NA |
| TRINITY_DN319_c0_g1_i6 | -0.56818079 | 0.794250815 | 1 | 0.127 | 0.7 | 0.955 | 0.05 | MFS_1 |
| TRINITY_DN21605_c1_g1_i1 | -0.5681442 | 0.426709751 | 0.999970678 | 64.868 | 39.57 | 69.751 | 71.049 | NA |
| TRINITY_DN11724_c0_g1_i4 | 0.568134237 | 0.668113043 | 0.999970678 | 6.213 | 1.764 | 3.266 | 1.468 | Asp_protease_2 |
| TRINITY_DN3898_c1_g1_i1 | -0.56808907 | 0.836223493 | 0.999970678 | 0 | 1.547 | 1.433 | 0.523 | MatE |
| TRINITY_DN4710_c0_g1_i4 | 0.56796667 | 0.608552674 | 0.999970678 | 8.257 | 5.765 | 2 | 6.967 | zf-A20 |
| TRINITY_DN110861_c0_g1_i1 | -0.56791543 | 0.518673918 | 0.999970678 | 3.188 | 2.355 | 2.244 | 5.55 | NA |
| TRINITY_DN3188_c0_g1_i7 | 0.56785798 | 0.550422338 | 0.999970678 | 1.363 | 1.597 | 0.611 | 1.257 | NA |
| TRINITY_DN8953_c0_g1_i1 | -0.56776544 | 0.789358785 | 0.999970678 | 0.127 | 2.957 | 0.178 | 4.303 | NA |
| TRINITY_DN8247_c0_g1_i2 | 0.567761446 | 0.433932349 | 0.999970678 | 7.721 | 7.086 | 5.044 | 3.911 | NA |
| TRINITY_DN4572_c0_g1_i4 | 0.567741246 | 0.610610493 | 0.999970678 | 10.483 | 3.617 | 5.689 | 2.714 | ANF_receptor |
| TRINITY_DN444_c0_g1_i2 | -0.56771243 | 0.726282023 | 0.999970678 | 1.19 | 20.08 | 8.377 | 20.721 | NA |
| TRINITY_DN382_c1_g2_i1 | -0.56768949 | 0.449163362 | 0.999970678 | 4.07 | 6.209 | 7.077 | 6.635 | Glutaredoxin |
| TRINITY_DN3075_c0_g1_i1 | -0.56766264 | 0.623297864 | 0.999970678 | 4.569 | 1.399 | 3.889 | 4.162 | NA |
| TRINITY_DN23558_c0_g1_i2 | -0.5674523 | 0.844604919 | 1 | 1.917 | 0 | 0.644 | 2.031 | NA |
| TRINITY_DN57722_c0_g1_i9 | -0.56736853 | 0.895770646 | 0.999970678 | 0.808 | 0 | 1 | 0 | NA |
| TRINITY_DN2812_c0_g1_i1 | 0.567168652 | 0.392858868 | 0.999970678 | 2.062 | 2.434 | 1.1 | 1.709 | FKS1_dom1 |
| TRINITY_DN1323_c0_g1_i1 | -0.56715583 | 0.457505111 | 0.999970678 | 87.76 | 175.9 | 158.835 | 193.815 | Ribosomal_S19 |
| TRINITY_DN1004_c0_g1_i10 | -0.56710565 | 0.616477978 | 0.999970678 | 5.178 | 1.498 | 5.355 | 3.489 | Metallophos |
| TRINITY_DN1954_c0_g1_i5 | -0.5671056 | 0.519365639 | 0.999970678 | 2.97 | 4.395 | 4.777 | 5.077 | NA |
| TRINITY_DN2905_c0_g2_i1 | -0.56706723 | 0.457910778 | 0.999970678 | 5.532 | 9.993 | 10.233 | 10.546 | NA |
| TRINITY_DN142_c1_g2_i3 | -0.56692805 | 0.657416312 | 0.999970678 | 2.607 | 7.135 | 2.211 | 11.642 | F-box-like |
| TRINITY_DN3268_c0_g2_i1 | 0.566922066 | 0.651327519 | 0.999970678 | 0.309 | 0.148 | 0.156 | 0.131 | - |
| TRINITY_DN13160_c0_g1_i1 | -0.56682682 | 0.664284244 | 0.999970678 | 1.572 | 3.932 | 1.533 | 6.193 | zf-C2H2_6 |
| TRINITY_DN1874_c0_g1_i1 | 0.566749957 | 0.676290198 | 0.999970678 | 0.808 | 2.267 | 0.289 | 1.709 | - |
| TRINITY_DN9899_c0_g1_i4 | -0.5666902 | 0.856247072 | 0.999970678 | 0 | 2.168 | 0.089 | 3.036 | NA |
| TRINITY_DN466_c0_g1_i10 | 0.566676917 | 0.633671842 | 0.999970678 | 0.236 | 0.256 | 0.1 | 0.211 | NA |
| TRINITY_DN10373_c0_g2_i5 | -0.56665755 | 0.898007323 | 0.999970678 | 1.263 | 0 | 1.544 | 0 | NA |
| TRINITY_DN365_c0_g1_i3 | 0.566646781 | 0.760985802 | 0.999970678 | 60.181 | 1.498 | 26.61 | 9.792 | Thaumatin |
| TRINITY_DN2847_c0_g5_i2 | 0.566636837 | 0.401330386 | 0.999970678 | 5.596 | 8.269 | 3.633 | 4.967 | NA |
| TRINITY_DN637_c0_g1_i3 | -0.56652312 | 0.395466864 | 0.999970678 | 13.935 | 20.55 | 21.143 | 25.154 | PP-binding |
| TRINITY_DN109_c0_g1_i1 | -0.56647604 | 0.542944842 | 0.999970678 | 4.587 | 5.499 | 3.989 | 9.983 | NA |
| TRINITY_DN10589_c0_g1_i2 | -0.56639629 | 0.838122432 | 0.999970678 | 1.308 | 0.729 | 2.522 | 0 | NA |

| TRINITY_DN2258_c0_g1_i4 | -0.56638433 | 0.658239726 | 0.999970678 | 55.312 | 389.7 | 206.354 | 395.532 | Chloroa_b-bind |
| --- | --- | --- | --- | --- | --- | --- | --- | --- |
| TRINITY_DN3466_c0_g1_i1 | 0.566380287 | 0.439183837 | 0.999970678 | 33.356 | 41 | 26.343 | 18.308 | NA |
| TRINITY_DN758_c0_g1_i11 | 0.566267977 | 0.702623646 | 0.999970678 | 4.46 | 1.537 | 2.622 | 0.975 | Sugar_tr |
| TRINITY_DN2069_c0_g1_i2 | -0.56626492 | 0.787666057 | 0.999970678 | 3.052 | 0.059 | 1.511 | 2.875 | NA |
| TRINITY_DN1901_c0_g1_i7 | -0.56625415 | 0.563589655 | 0.999970678 | 3.397 | 2.375 | 2.111 | 6.012 | NA |
| TRINITY_DN1532_c0_g2_i2 | 0.56625059 | 0.624374383 | 0.999970678 | 28.015 | 54.1 | 37.009 | 10.345 | Aldo_ket_red |
| TRINITY_DN15961_c0_g1_i1 | -0.56624957 | 0.724684845 | 1 | 0.372 | 1.33 | 0.478 | 1.92 | NA |
| TRINITY_DN11197_c0_g1_i1 | -0.5662415 | 0.555794788 | 0.999970678 | 30.068 | 24.04 | 16.843 | 59.749 | NA |
| TRINITY_DN1065_c0_g1_i4 | 0.566215664 | 0.844660226 | 1 | 0.663 | 0.138 | 0.456 | 0 | CRS1_YhbY |
| TRINITY_DN913_c5_g1_i2 | 0.566171055 | 0.399590431 | 0.999970678 | 68.847 | 110.3 | 46.753 | 64.173 | Aldedh |
| TRINITY_DN665_c0_g1_i4 | 0.566140596 | 0.572218422 | 0.999970678 | 4.615 | 3.913 | 3.511 | 1.498 | LRR_8 |
| TRINITY_DN342_c0_g1_i4 | -0.56612123 | 0.53606656 | 0.999970678 | 1.708 | 1.784 | 2.8 | 1.79 | NA |
| TRINITY_DN9773_c0_g1_i9 | 0.566102265 | 0.648921999 | 0.999970678 | 0.445 | 1.133 | 0.456 | 0.503 | NA |
| TRINITY_DN1145_c0_g1_i1 | -0.56606177 | 0.661204634 | 0.999970678 | 21.974 | 12.39 | 5.833 | 43.754 | NA |
| TRINITY_DN18203_c0_g2_i2 | -0.5660463 | 0.788103528 | 0.999970678 | 2.325 | 0.365 | 3.266 | 0.111 | NA |
| TRINITY_DN1042_c0_g1_i8 | -0.56603938 | 0.753436781 | 0.999970678 | 3.506 | 0.197 | 0.844 | 4.554 | NA |
| TRINITY_DN52879_c0_g3_i5 | 0.566021266 | 0.899566903 | 1 | 1.299 | 0 | 0 | 0.885 | NA |
| TRINITY_DN224_c0_g1_i5 | 0.56593423 | 0.62108377 | 0.999970678 | 0.454 | 0.907 | 0.433 | 0.402 | TRM |
| TRINITY_DN6704_c0_g1_i1 | 0.565917474 | 0.777807715 | 0.999970678 | 1.671 | 88.4 | 5.311 | 53.677 | NA |
| TRINITY_DN221_c1_g1_i4 | 0.565812618 | 0.599958061 | 0.999970678 | 0.618 | 0.552 | 0.322 | 0.392 | NA |
| TRINITY_DN8377_c0_g1_i2 | 0.565773009 | 0.412226912 | 0.999970678 | 37.508 | 34.64 | 25.01 | 18.66 | ABC1 |
| TRINITY_DN8573_c0_g1_i1 | 0.565764462 | 0.843044276 | 0.999970678 | 4.26 | 0 | 0.533 | 2.272 | ATG101 |
| TRINITY_DN990_c1_g1_i1 | -0.56576386 | 0.84551368 | 0.999970678 | 52.56 | 0 | 31.865 | 40.295 | Methyltransf_24 |
| TRINITY_DN8182_c0_g1_i2 | -0.56575632 | 0.896006049 | 0.999970678 | 3.152 | 0 | 0 | 4.725 | NA |
| TRINITY_DN145_c0_g1_i7 | -0.56570352 | 0.840683627 | 0.999970678 | 5.432 | 0 | 1.122 | 6.786 | FoP_duplication |
| TRINITY_DN6244_c0_g1_i3 | -0.56566932 | 0.586326627 | 0.999970678 | 2.444 | 8.259 | 5.555 | 9.018 | FAD_binding_4 |
| TRINITY_DN8408_c0_g1_i1 | -0.56557414 | 0.893696254 | 0.999970678 | 6.25 | 0 | 7.755 | 0 | NA |
| TRINITY_DN4004_c0_g1_i9 | -0.56555121 | 0.894613747 | 0.999970678 | 2.998 | 0 | 0.022 | 4.454 | NA |
| TRINITY_DN12795_c0_g1_i11 | -0.56554906 | 0.894667203 | 0.999970678 | 0 | 0.286 | 0.344 | 0 | ABC2_membrane |
| TRINITY_DN20302_c0_g1_i8 | -0.56554906 | 0.894667203 | 0.999970678 | 0 | 0.532 | 0.644 | 0 | NA |
| TRINITY_DN14278_c0_g2_i2 | 0.565401453 | 0.332322586 | 0.999970678 | 10.338 | 9.993 | 6.144 | 6.364 | ABC2_membrane |
| TRINITY_DN1398_c0_g2_i1 | 0.565367105 | 0.445284114 | 0.999970678 | 8.448 | 11.37 | 6.844 | 5.097 | NA |
| TRINITY_DN8857_c0_g1_i3 | -0.56530712 | 0.649076922 | 0.999970678 | 0.781 | 0.345 | 0.333 | 1.277 | Myb_DNA-bind_ |
| TRINITY_DN6906_c0_g1_i5 | 0.56529295 | 0.507967714 | 0.999970678 | 4.042 | 7.904 | 4.144 | 3.046 | NA |
| TRINITY_DN657_c0_g1_i4 | 0.565247714 | 0.834577995 | 0.999970678 | 1.126 | 0.434 | 0 | 1.066 | DYW_deaminase |
| TRINITY_DN4848_c0_g1_i1 | -0.56523023 | 0.89307011 | 0.999970678 | 2.725 | 0 | 0 | 4.132 | PORR |
| TRINITY_DN1115_c0_g1_i1 | -0.56520788 | 0.613597003 | 0.999970678 | 14.534 | 47.7 | 21.999 | 63.891 | UQ_con |
| TRINITY_DN16196_c0_g1_i1 | 0.565199187 | 0.597582516 | 0.999970678 | 0.79 | 0.907 | 0.611 | 0.402 | Glyco_hydro_17 |
| TRINITY_DN10499_c0_g1_i2 | -0.56509744 | 0.595343159 | 0.999970678 | 6.05 | 1.744 | 4.366 | 6.384 | NA |
| TRINITY_DN2553_c0_g1_i4 | 0.565090507 | 0.40053781 | 0.999970678 | 2.671 | 2.089 | 1.355 | 1.599 | NA |
| TRINITY_DN4957_c0_g2_i1 | 0.565084064 | 0.747397898 | 0.999970678 | 3.815 | 8.367 | 0.611 | 7.098 | NA |
| TRINITY_DN3324_c0_g1_i1 | 0.565058419 | 0.641114816 | 0.999970678 | 85.589 | 15.86 | 22.454 | 42.165 | NA |
| TRINITY_DN7413_c0_g1_i4 | -0.5650572 | 0.583171443 | 0.999970678 | 3.425 | 5.135 | 2.633 | 9.461 | NA |
| TRINITY_DN6000_c1_g1_i1 | 0.564944879 | 0.73691563 | 0.999970678 | 7.458 | 225.4 | 68.307 | 71.432 | CAAD |
| TRINITY_DN47024_c0_g1_i1 | -0.5649407 | 0.894548876 | 0.999970678 | 3.479 | 0 | 4.3 | 0 | NA |
| TRINITY_DN10375_c0_g1_i6 | -0.56489802 | 0.897615999 | 0.999970678 | 3.434 | 0 | 4.278 | 0 | NA |
| TRINITY_DN166572_c0_g1_i1 | -0.56482779 | 0.90189601 | 1 | 0.69 | 0 | 0.856 | 0 | Phosphoesterase |
| TRINITY_DN13876_c0_g2_i1 | -0.56482779 | 0.90189601 | 1 | 0.627 | 0 | 0.778 | 0 | Glyco_hydro_16 |
| TRINITY_DN1848_c0_g1_i5 | -0.56482779 | 0.90189601 | 1 | 12.527 | 0 | 15.577 | 0 | NA |
| TRINITY_DN6875_c0_g1_i5 | -0.56482779 | 0.90189601 | 1 | 16.587 | 0 | 19.665 | 0 | NA |
| TRINITY_DN9001_c1_g1_i3 | -0.56482779 | 0.90189601 | 1 | 0.845 | 0 | 1.044 | 0 | NA |
| TRINITY_DN54235_c0_g1_i1 | -0.56482779 | 0.90189601 | 1 | 0.527 | 0 | 0.644 | 0 | NA |
| TRINITY_DN15868_c0_g1_i2 | -0.56482779 | 0.90189601 | 1 | 0.89 | 0 | 1.1 | 0 | ADH_N |
| TRINITY_DN1753_c0_g1_i3 | -0.56480834 | 0.381451178 | 0.999970678 | 244.11 | 168.9 | 260.162 | 299.761 | Lipoxygenase |
| TRINITY_DN1069_c0_g1_i2 | 0.564729942 | 0.396111739 | 0.999970678 | 11.927 | 15 | 6.522 | 10.305 | - |
| TRINITY_DN6992_c0_g1_i1 | -0.56472096 | 0.894034979 | 0.999970678 | 16.905 | 0 | 20.877 | 0 | NA |
| TRINITY_DN9832_c0_g1_i6 | -0.56467235 | 0.839396297 | 0.999970678 | 5.205 | 0 | 4.511 | 2.363 | NA |
| TRINITY_DN4747_c0_g1_i1 | 0.564660917 | 0.444650656 | 0.999970678 | 2.298 | 2.7 | 1.089 | 2.071 | - |
| TRINITY_DN1034_c0_g2_i1 | -0.56465777 | 0.418484815 | 0.999970678 | 12.681 | 19.85 | 18.232 | 25.788 | NA |
| TRINITY_DN8006_c0_g1_i1 | -0.56461089 | 0.57699501 | 0.999970678 | 4.006 | 1.685 | 2.989 | 4.816 | NA |
| TRINITY_DN77675_c0_g2_i1 | 0.564603373 | 0.89638749 | 0.999970678 | 2.98 | 0 | 1.689 | 0 | NA |
| TRINITY_DN5759_c0_g1_i10 | 0.564592729 | 0.518529107 | 0.999970678 | 1.117 | 1.114 | 0.756 | 0.603 | NA |
| TRINITY_DN2996_c0_g1_i2 | 0.564584764 | 0.638203645 | 0.999970678 | 4.269 | 24.09 | 8.588 | 8.596 | NA |
| TRINITY_DN26245_c0_g1_i5 | -0.56454394 | 0.846249877 | 0.999970678 | 0.6 | 0.177 | 0.978 | 0 | NA |
| TRINITY_DN89_c0_g1_i1 | 0.564492253 | 0.538257468 | 0.999970678 | 24.39 | 10.65 | 7.933 | 14.186 | NA |
| TRINITY_DN1046_c0_g1_i6 | 0.56441568 | 0.896880927 | 0.999970678 | 0 | 34.4 | 0 | 22.922 | NA |
| TRINITY_DN1953_c0_g1_i1 | 0.564307926 | 0.449541075 | 0.999970678 | 18.695 | 14.62 | 11.588 | 8.576 | 2OG-FeII_Oxy |
| TRINITY_DN1254_c0_g1_i5 | 0.564307518 | 0.459447775 | 0.999970678 | 4.115 | 4.731 | 3.133 | 2.202 | NA |
| TRINITY_DN11593_c0_g1_i4 | -0.56430613 | 0.616783541 | 0.999970678 | 704.94 | 263.4 | 900.257 | 341.695 | NA |
| TRINITY_DN13830_c0_g1_i2 | -0.56430342 | 0.893852007 | 0.999970678 | 0 | 1.626 | 1.967 | 0 | DnaJ |
| TRINITY_DN8206_c0_g3_i2 | -0.56410934 | 0.605933966 | 0.999970678 | 1.254 | 1.232 | 0.711 | 2.825 | K_trans |
| TRINITY_DN6317_c1_g1_i1 | 0.564093734 | 0.591811343 | 0.999970678 | 6.032 | 3.292 | 1.378 | 4.665 | CN_hydrolase |
| TRINITY_DN1758_c0_g1_i6 | 0.564017173 | 0.53660814 | 0.999970678 | 4.624 | 11.35 | 3.733 | 6.213 | O-FucT |
| TRINITY_DN3987_c0_g1_i5 | -0.56400655 | 0.715838281 | 0.999970678 | 2.907 | 0.365 | 3.055 | 1.176 | NA |

| TRINITY_DN1487_c0_g2_i1 | 0.564001418 | 0.831593414 | 0.999970678 | 3.779 | 3.183 | 0 | 4.675 | NA |
| --- | --- | --- | --- | --- | --- | --- | --- | --- |
| TRINITY_DN629_c0_g1_i1 | 0.563904013 | 0.625327692 | 0.999970678 | 2.625 | 5.874 | 1.289 | 4.162 | NA |
| TRINITY_DN4049_c0_g1_i2 | 0.563854607 | 0.731252976 | 0.999970678 | 0.799 | 13.87 | 5.522 | 3.076 | PAP2_C |
| TRINITY_DN5871_c0_g1_i1 | -0.56384903 | 0.442066284 | 0.999970678 | 14.38 | 15.63 | 22.165 | 17.111 | NA |
| TRINITY_DN3324_c2_g1_i2 | 0.563764819 | 0.815418085 | 1 | 2.171 | 0.138 | 1.267 | 0.05 | NA |
| TRINITY_DN14059_c0_g1_i2 | -0.56372685 | 0.75447288 | 1 | 1.853 | 0.227 | 0.844 | 1.971 | NA |
| TRINITY_DN8088_c0_g1_i1 | -0.56367107 | 0.849814622 | 0.999970678 | 0.645 | 0 | 0.722 | 0.08 | NA |
| TRINITY_DN19369_c0_g2_i9 | -0.56364992 | 0.842376113 | 1 | 0.509 | 0 | 0.389 | 0.271 | EF_assoc_1 |
| TRINITY_DN10156_c0_g1_i1 | -0.56353525 | 0.567376253 | 0.999970678 | 79.239 | 112.1 | 56.73 | 208.845 | NA |
| TRINITY_DN1949_c0_g1_i2 | 0.563510235 | 0.57694379 | 0.999970678 | 12.554 | 5.164 | 3.189 | 8.264 | Glyco_transf_8 |
| TRINITY_DN11344_c0_g1_i11 | -0.56347294 | 0.897297592 | 0.999970678 | 5.796 | 0 | 7.033 | 0 | Lactamase_B |
| TRINITY_DN2730_c1_g1_i13 | -0.56345096 | 0.778495128 | 1 | 0.836 | 0.079 | 0.856 | 0.322 | NA |
| TRINITY_DN5194_c0_g1_i1 | 0.563420957 | 0.852605092 | 0.999970678 | 85.107 | 0 | 23.532 | 30.061 | WRKY |
| TRINITY_DN1322_c0_g1_i5 | 0.563374878 | 0.625503466 | 0.999970678 | 22.91 | 22.46 | 4.322 | 25.436 | NA |
| TRINITY_DN65_c0_g1_i2 | 0.563365481 | 0.506055215 | 0.999970678 | 20.103 | 9.678 | 7.677 | 10.928 | LIM |
| TRINITY_DN242_c1_g1_i11 | 0.563358271 | 0.741095843 | 0.999970678 | 0.536 | 1.025 | 0.811 | 0.08 | B_lectin |
| TRINITY_DN5113_c0_g1_i1 | 0.563352003 | 0.682584651 | 0.999970678 | 1.154 | 7.796 | 2.911 | 2.453 | NA |
| TRINITY_DN2645_c2_g2_i2 | 0.563317408 | 0.602430925 | 0.999970678 | 1.063 | 3.035 | 1.155 | 1.357 | NA |
| TRINITY_DN2077_c0_g1_i7 | -0.56326883 | 0.678627126 | 0.999970678 | 1.045 | 2.01 | 0.5 | 3.921 | UDPGT |
| TRINITY_DN5107_c0_g1_i1 | 0.563260695 | 0.894211034 | 0.999970678 | 1.481 | 0 | 0.844 | 0 | NUFIP1 |
| TRINITY_DN4772_c0_g1_i1 | 0.563246363 | 0.639076536 | 0.999970678 | 1.653 | 0.719 | 0.911 | 0.513 | Aldedh |
| TRINITY_DN1814_c0_g1_i1 | 0.563195242 | 0.520063577 | 0.999970678 | 167.24 | 129.6 | 48.497 | 142.994 | NA |
| TRINITY_DN10359_c0_g2_i1 | -0.5631583 | 0.70971771 | 0.999970678 | 0.182 | 0.365 | 0.533 | 0.161 | DUF4283 |
| TRINITY_DN642_c0_g1_i1 | 0.563125876 | 0.455142382 | 0.999970678 | 5.614 | 4.218 | 3.033 | 3.006 | NA |
| TRINITY_DN7986_c0_g1_i8 | 0.563124851 | 0.837360606 | 0.999970678 | 0.345 | 1.114 | 0 | 0.985 | Glyco_hydro_47 |
| TRINITY_DN7468_c0_g1_i5 | 0.563102903 | 0.67221335 | 0.999970678 | 5.26 | 5.637 | 5.311 | 0.955 | NA |
| TRINITY_DN1131_c0_g1_i2 | -0.56299067 | 0.850336518 | 1 | 0 | 1.399 | 1.455 | 0.292 | NA |
| TRINITY_DN4529_c0_g1_i2 | 0.562971724 | 0.453379808 | 0.999970678 | 9.693 | 8.801 | 6.588 | 4.584 | ABC_membrane |
| TRINITY_DN2919_c0_g1_i2 | -0.56294056 | 0.763385369 | 0.999970678 | 61.598 | 10.47 | 2.667 | 104.377 | GHMP_kinases_C |
| TRINITY_DN22872_c4_g1_i1 | -0.56293457 | 0.760788951 | 0.999970678 | 1.79 | 5.844 | 0.367 | 10.657 | NA |
| TRINITY_DN9759_c0_g1_i1 | -0.562915 | 0.608636542 | 0.999970678 | 9.611 | 20.52 | 8.499 | 33.881 | FMN_red |
| TRINITY_DN3964_c0_g1_i1 | 0.562843801 | 0.426948233 | 0.999970678 | 6.486 | 5.805 | 2.922 | 4.786 | NA |
| TRINITY_DN747_c0_g1_i1 | -0.56273513 | 0.533135028 | 0.999970678 | 60.481 | 114.5 | 67.174 | 174.975 | Pro_isomerase |
| TRINITY_DN3195_c0_g1_i5 | 0.562721652 | 0.65331493 | 0.999970678 | 0.736 | 3.38 | 1.022 | 1.528 | Hydrolase_6 |
| TRINITY_DN7382_c0_g1_i2 | 0.562638586 | 0.839719462 | 0.999970678 | 0 | 1.991 | 0.611 | 0.573 | NA |
| TRINITY_DN28179_c1_g4_i1 | -0.56255065 | 0.838967969 | 0.999970678 | 1.199 | 0 | 0.856 | 0.764 | NA |
| TRINITY_DN2319_c0_g1_i2 | 0.562287268 | 0.631969434 | 0.999970678 | 4.006 | 8.604 | 1.478 | 6.696 | Carb_anhydrase |
| TRINITY_DN20020_c0_g1_i1 | 0.562274221 | 0.435390757 | 0.999970678 | 3.343 | 3.134 | 2.155 | 1.79 | NA |
| TRINITY_DN6141_c0_g1_i7 | -0.56216102 | 0.849402808 | 0.999970678 | 0 | 2.681 | 3.022 | 0.221 | NA |
| TRINITY_DN3282_c0_g1_i1 | 0.562130394 | 0.435630767 | 0.999970678 | 8.275 | 6.642 | 3.266 | 6.203 | Helicase_C |
| TRINITY_DN5485_c3_g2_i2 | 0.562108091 | 0.844406957 | 0.999970678 | 39.66 | 15.91 | 0 | 37.832 | Cullin |
| TRINITY_DN5194_c3_g1_i1 | -0.56206013 | 0.679457671 | 0.999970678 | 0.309 | 1.409 | 0.511 | 1.91 | NA |
| TRINITY_DN8986_c0_g1_i6 | -0.56204389 | 0.590464359 | 0.999970678 | 1.381 | 3.085 | 3.244 | 2.644 | Steroid_dh |
| TRINITY_DN665_c1_g1_i4 | 0.562023351 | 0.448291904 | 0.999970678 | 142.05 | 229.3 | 81.984 | 149.8 | - |
| TRINITY_DN12260_c1_g1_i1 | -0.56198451 | 0.736812807 | 0.999970678 | 5.568 | 1.37 | 6.944 | 1.689 | NA |
| TRINITY_DN187_c0_g1_i4 | -0.56197452 | 0.348367388 | 0.999970678 | 136.01 | 173.8 | 172.523 | 247.552 | - |
| TRINITY_DN1463_c0_g1_i11 | -0.56194542 | 0.754343206 | 0.999970678 | 27.924 | 11.25 | 45.586 | 2.192 | Cupin_1 |
| TRINITY_DN9079_c0_g1_i1 | 0.561900731 | 0.438336901 | 0.999970678 | 6.232 | 5.066 | 3.644 | 3.267 | NA |
| TRINITY_DN4482_c0_g1_i3 | 0.561828384 | 0.798484521 | 0.999970678 | 2.989 | 0.039 | 1.089 | 0.744 | NA |
| TRINITY_DN18533_c0_g1_i1 | 0.561816331 | 0.675405869 | 0.999970678 | 30.131 | 61.25 | 34.565 | 15.804 | NA |
| TRINITY_DN18658_c0_g1_i1 | -0.56173266 | 0.627688814 | 0.999970678 | 3.316 | 1.192 | 2.489 | 3.649 | NA |
| TRINITY_DN7251_c0_g1_i3 | 0.561730085 | 0.578491949 | 0.999970678 | 0.6 | 1.399 | 0.656 | 0.553 | NA |
| TRINITY_DN794_c1_g1_i1 | -0.56167122 | 0.842492646 | 1 | 222.07 | 0 | 183.9 | 92.997 | NA |
| TRINITY_DN56_c0_g1_i3 | 0.561642329 | 0.710033061 | 0.999970678 | 1.199 | 0.611 | 0.9 | 0.151 | NA |
| TRINITY_DN6908_c0_g1_i3 | 0.56158858 | 0.647485492 | 0.999970678 | 2.798 | 0.907 | 1.4 | 0.845 | NA |
| TRINITY_DN8011_c0_g1_i1 | -0.56153954 | 0.517067135 | 0.999970678 | 5.069 | 2.986 | 6.333 | 4.303 | Pkinase |
| TRINITY_DN50427_c0_g1_i1 | 0.561510397 | 0.601234249 | 0.999970678 | 1.381 | 0.66 | 0.567 | 0.704 | NA |
| TRINITY_DN11022_c0_g2_i1 | 0.561479536 | 0.568730127 | 0.999970678 | 2.689 | 1.508 | 1.555 | 0.975 | Myb_DNA-bind_ |
| TRINITY_DN2857_c2_g1_i4 | 0.561418662 | 0.850840808 | 0.999970678 | 0.109 | 1.311 | 0.789 | 0 | NA |
| TRINITY_DN8550_c0_g1_i4 | -0.56140286 | 0.546238368 | 0.999970678 | 2.289 | 1.705 | 3.222 | 1.991 | - |
| TRINITY_DN11247_c0_g1_i1 | -0.5613679 | 0.619081877 | 0.999970678 | 1.69 | 2.71 | 2.978 | 2.865 | NA |
| TRINITY_DN6589_c0_g1_i2 | -0.56128883 | 0.837330187 | 0.999970678 | 9.711 | 0 | 8.366 | 4.323 | - |
| TRINITY_DN4899_c0_g1_i8 | 0.561281321 | 0.837928586 | 0.999970678 | 2.262 | 0.532 | 0 | 1.92 | RRM_1 |
| TRINITY_DN136821_c0_g1_i1 | 0.561175733 | 0.901026428 | 1 | 1.908 | 0 | 1.078 | 0 | NAD_binding_11 |
| TRINITY_DN3182_c0_g1_i18 | 0.561175733 | 0.901026428 | 1 | 9.175 | 0 | 5.211 | 0 | NA |
| TRINITY_DN15028_c0_g1_i14 | 0.561175733 | 0.901026428 | 1 | 2.434 | 0 | 1.355 | 0 | NA |
| TRINITY_DN4026_c0_g1_i9 | -0.5610516 | 0.671858635 | 0.999970678 | 2.425 | 0.384 | 1.811 | 2.041 | DUF3741 |
| TRINITY_DN9922_c0_g1_i1 | -0.5610507 | 0.41384749 | 0.999970678 | 1972.6 | 1838 | 2883.903 | 2133.384 | NA |
| TRINITY_DN503_c0_g1_i8 | -0.56105001 | 0.465568851 | 0.999970678 | 12.936 | 24.63 | 22.388 | 27.899 | S10_plectin |
| TRINITY_DN25929_c0_g1_i1 | -0.56084361 | 0.694499343 | 0.999970678 | 2.207 | 0.512 | 2.055 | 1.538 | NA |
| TRINITY_DN9345_c0_g1_i2 | 0.560837786 | 0.639308291 | 0.999970678 | 0.899 | 1.311 | 0.733 | 0.603 | Cyt-b5 |
| TRINITY_DN3508_c0_g1_i1 | 0.560830493 | 0.516418688 | 0.999970678 | 0.781 | 0.562 | 0.422 | 0.402 | NA |
| TRINITY_DN73_c1_g1_i4 | 0.560812945 | 0.895845741 | 0.999970678 | 0.6 | 0 | 0.344 | 0 | NA |
| TRINITY_DN64_c0_g1_i1 | 0.560703116 | 0.616748664 | 0.999970678 | 1.135 | 1.468 | 0.444 | 1.247 | NA |

| TRINITY_DN3060_c0_g1_i3 | 0.560695632 | 0.842194835 | 0.999970678 | 0.182 | 0.69 | 0.489 | 0 | NA |
| --- | --- | --- | --- | --- | --- | --- | --- | --- |
| TRINITY_DN5170_c0_g2_i11 | 0.560688843 | 0.599266909 | 0.999970678 | 7.067 | 12.69 | 3.944 | 8.164 | NA |
| TRINITY_DN13896_c0_g1_i1 | -0.56067273 | 0.661436065 | 0.999970678 | 0.254 | 0.69 | 0.322 | 1.005 | NA |
| TRINITY_DN2641_c0_g1_i3 | 0.560638578 | 0.859824541 | 0.999970678 | 3.706 | 0.158 | 2.2 | 0 | - |
| TRINITY_DN6277_c0_g1_i3 | -0.56063202 | 0.612526761 | 0.999970678 | 0.645 | 0.917 | 1.455 | 0.543 | - |
| TRINITY_DN296_c1_g1_i1 | -0.5606157 | 0.609353512 | 0.999970678 | 26.452 | 6.721 | 22.854 | 21.666 | zf-RING_2 |
| TRINITY_DN6251_c0_g1_i1 | -0.56052074 | 0.419115556 | 0.999970678 | 63.942 | 43.92 | 56.797 | 90.162 | SNARE |
| TRINITY_DN698_c1_g1_i9 | -0.560499 | 0.839511066 | 0.999970678 | 0 | 1.468 | 1.411 | 0.442 | NA |
| TRINITY_DN23023_c0_g1_i1 | 0.56029194 | 0.690713475 | 0.999970678 | 25.044 | 3.706 | 12.399 | 4.735 | MFS_1 |
| TRINITY_DN7131_c0_g1_i2 | -0.56024319 | 0.520137653 | 0.999970678 | 1.499 | 2.612 | 2.478 | 3.046 | NA |
| TRINITY_DN4866_c0_g1_i2 | -0.5601299 | 0.770238542 | 0.999970678 | 1.944 | 2.878 | 5.7 | 0.231 | CRAL_TRIO |
| TRINITY_DN6624_c0_g1_i2 | -0.56004857 | 0.581179373 | 0.999970678 | 1.026 | 0.355 | 0.822 | 1.056 | EDR1 |
| TRINITY_DN3947_c0_g1_i8 | -0.56001335 | 0.902379942 | 1 | 0 | 0.591 | 0.7 | 0 | Ank_2 |
| TRINITY_DN1575_c0_g1_i1 | 0.55984096 | 0.855378604 | 0.999970678 | 0.645 | 13.94 | 0 | 9.812 | - |
| TRINITY_DN412_c0_g1_i2 | 0.559713494 | 0.845439449 | 0.999970678 | 0 | 0.719 | 0.111 | 0.352 | RRM_1 |
| TRINITY_DN2384_c0_g3_i1 | 0.559682716 | 0.648300706 | 0.999970678 | 3.597 | 7.805 | 5.122 | 1.468 | NA |
| TRINITY_DN7454_c1_g1_i1 | 0.559645322 | 0.897214141 | 0.999970678 | 9.747 | 0 | 0 | 6.424 | NA |
| TRINITY_DN333_c0_g1_i1 | 0.559615993 | 0.327369495 | 0.999970678 | 12.309 | 12.63 | 7.255 | 8.194 | - |
| TRINITY_DN443_c0_g1_i10 | -0.5595757 | 0.507175399 | 0.999970678 | 4.388 | 4.455 | 7.022 | 4.504 | Amidohydro_1 |
| TRINITY_DN272_c0_g1_i2 | -0.55956965 | 0.628410326 | 0.999970678 | 1.217 | 0.581 | 0.589 | 1.981 | - |
| TRINITY_DN4311_c0_g2_i4 | -0.55949393 | 0.680934534 | 0.999970678 | 0.972 | 1.912 | 2.966 | 0.653 | NA |
| TRINITY_DN10888_c0_g1_i2 | 0.559398958 | 0.639584263 | 0.999970678 | 0.436 | 1.35 | 0.422 | 0.704 | HATPase_c_3 |
| TRINITY_DN1220_c0_g1_i2 | -0.55937963 | 0.46476203 | 0.999970678 | 19.694 | 13.8 | 15.521 | 30.644 | eIF-6 |
| TRINITY_DN7142_c0_g1_i1 | -0.55931022 | 0.56840671 | 0.999970678 | 23.682 | 41.57 | 20.921 | 70.336 | - |
| TRINITY_DN58272_c0_g1_i10 | -0.55918407 | 0.750613381 | 1 | 0.309 | 0.148 | 0.511 | 0.07 | LRR_8 |
| TRINITY_DN20210_c0_g1_i3 | 0.559083003 | 0.896674789 | 0.999970678 | 0 | 1.685 | 0.933 | 0 | Metallophos |
| TRINITY_DN1296_c0_g1_i1 | -0.55906268 | 0.646435664 | 0.999970678 | 2.88 | 1.045 | 1.222 | 4.343 | - |
| TRINITY_DN99189_c0_g1_i2 | -0.55905016 | 0.900025696 | 1 | 1.217 | 0 | 1.511 | 0 | NA |
| TRINITY_DN1078_c0_g1_i6 | -0.55900295 | 0.788480373 | 0.999970678 | 7.095 | 0.039 | 4.089 | 5.741 | LRRNT_2 |
| TRINITY_DN18_c0_g1_i1 | 0.55899233 | 0.746343719 | 0.999970678 | 1.408 | 41.81 | 8.866 | 18.046 | NA |
| TRINITY_DN1294_c0_g1_i2 | 0.558983069 | 0.65977196 | 0.999970678 | 2.126 | 8.781 | 1.644 | 5.359 | Aminotran_1_2 |
| TRINITY_DN26220_c0_g1_i3 | -0.55898294 | 0.719250039 | 0.999970678 | 0.518 | 0.108 | 0.511 | 0.302 | NA |
| TRINITY_DN777_c0_g1_i6 | -0.55892162 | 0.839030666 | 0.999970678 | 14.961 | 0 | 4.955 | 16.016 | NA |
| TRINITY_DN7933_c0_g1_i1 | -0.55889421 | 0.83949564 | 0.999970678 | 4.506 | 5.44 | 0 | 14.668 | NA |
| TRINITY_DN5676_c3_g2_i1 | 0.55889405 | 0.523212838 | 0.999970678 | 6.777 | 3.903 | 2.8 | 3.861 | NA |
| TRINITY_DN1169_c0_g1_i1 | 0.558855002 | 0.677254866 | 0.999970678 | 11.391 | 3.922 | 6.622 | 2.363 | NA |
| TRINITY_DN223851_c0_g1_i1 | -0.55884287 | 0.894838456 | 0.999970678 | 5.042 | 0 | 6.222 | 0 | NA |
| TRINITY_DN11451_c0_g2_i1 | -0.55883414 | 0.613881643 | 0.999970678 | 2.189 | 6.475 | 5.689 | 5.67 | NA |
| TRINITY_DN9319_c0_g1_i3 | -0.5587948 | 0.725095155 | 0.999970678 | 0.363 | 1.804 | 0.6 | 2.423 | NA |
| TRINITY_DN6468_c0_g1_i1 | 0.55879147 | 0.411845108 | 0.999970678 | 9.366 | 10.63 | 6.788 | 5.389 | CRS1_YhbY |
| TRINITY_DN1064_c0_g1_i2 | 0.558730097 | 0.78475656 | 0.999970678 | 7.422 | 0.069 | 3.211 | 1.287 | LRRNT_2 |
| TRINITY_DN561_c0_g2_i3 | 0.558662807 | 0.561272999 | 0.999970678 | 3.952 | 5.46 | 3.844 | 1.739 | NA |
| TRINITY_DN6854_c0_g1_i2 | -0.55857521 | 0.743357323 | 0.999970678 | 1.572 | 6.307 | 0.922 | 10.184 | NA |
| TRINITY_DN1245_c0_g1_i7 | -0.55854889 | 0.641125782 | 0.999970678 | 1.608 | 5.43 | 2.155 | 7.741 | F-box |
| TRINITY_DN653_c1_g1_i2 | 0.558538589 | 0.576322098 | 0.999970678 | 4.978 | 10.58 | 3.144 | 6.615 | RBM39linker |
| TRINITY_DN807_c0_g1_i2 | -0.55849678 | 0.704463106 | 0.999970678 | 34.047 | 14.42 | 5.422 | 64.384 | NA |
| TRINITY_DN6133_c0_g1_i1 | 0.558474163 | 0.426252649 | 0.999970678 | 6.622 | 9.648 | 4.844 | 5.158 | NA |
| TRINITY_DN70182_c0_g1_i1 | 0.558418641 | 0.899523226 | 0.999970678 | 1.235 | 0 | 0.711 | 0 | Asp |
| TRINITY_DN15381_c0_g1_i2 | 0.558418641 | 0.899523226 | 0.999970678 | 0.981 | 0 | 0.556 | 0 | NA |
| TRINITY_DN13903_c1_g1_i1 | 0.558410431 | 0.757701901 | 0.999970678 | 7.967 | 4.159 | 6.322 | 0.523 | NA |
| TRINITY_DN11427_c0_g1_i10 | -0.55839946 | 0.792126388 | 1 | 3.652 | 0.818 | 5.255 | 0.352 | NA |
| TRINITY_DN1350_c0_g1_i9 | -0.5582906 | 0.680993064 | 0.999970678 | 11.809 | 2.227 | 9.933 | 8.355 | NA |
| TRINITY_DN26793_c0_g1_i7 | 0.558270813 | 0.749990197 | 0.999970678 | 0.5 | 0.049 | 0.222 | 0.121 | - |
| TRINITY_DN99013_c0_g1_i2 | 0.558139831 | 0.842310466 | 0.999970678 | 2.916 | 0 | 0.522 | 1.387 | NA |
| TRINITY_DN51920_c0_g1_i1 | 0.558120524 | 0.477713958 | 0.999970678 | 4.388 | 7.943 | 3.622 | 3.961 | Dimerisation |
| TRINITY_DN289_c0_g1_i14 | 0.558110931 | 0.494801264 | 0.999970678 | 3.743 | 2.523 | 2.2 | 1.609 | DUF659 |
| TRINITY_DN2922_c0_g3_i1 | -0.55810498 | 0.838404998 | 0.999970678 | 1.935 | 0 | 1.7 | 0.834 | NA |
| TRINITY_DN473_c0_g1_i10 | 0.558078739 | 0.782713868 | 0.999970678 | 19.748 | 4.642 | 12.655 | 0.855 | NA |
| TRINITY_DN1211_c0_g1_i5 | -0.55807651 | 0.73233226 | 0.999970678 | 0.136 | 0.601 | 0.167 | 0.875 | NA |
| TRINITY_DN14605_c0_g1_i1 | -0.55804831 | 0.896081044 | 0.999970678 | 2.834 | 0 | 3.455 | 0 | - |
| TRINITY_DN13344_c0_g2_i1 | -0.55804831 | 0.896081044 | 0.999970678 | 3.316 | 0 | 4.089 | 0 | NA |
| TRINITY_DN7850_c0_g1_i3 | 0.557863829 | 0.586160605 | 0.999970678 | 0.518 | 0.365 | 0.267 | 0.292 | PAS_9 |
| TRINITY_DN444_c0_g2_i1 | -0.55785574 | 0.612263935 | 0.999970678 | 9.611 | 10.82 | 5.055 | 23.777 | NA |
| TRINITY_DN2053_c0_g1_i1 | 0.55784059 | 0.719883684 | 0.999970678 | 13.226 | 1.951 | 7.211 | 1.659 | AP2 |
| TRINITY_DN1199_c0_g1_i2 | 0.557832662 | 0.534834426 | 0.999970678 | 7.185 | 8.062 | 5.922 | 3.167 | NA |
| TRINITY_DN19737_c0_g1_i1 | 0.557791742 | 0.818372593 | 0.999970678 | 0.027 | 9.018 | 4.566 | 0.523 | UDPGT |
| TRINITY_DN5254_c0_g1_i1 | -0.55778542 | 0.694927262 | 0.999970678 | 3.161 | 3.676 | 7.3 | 1.186 | zf-U1 |
| TRINITY_DN1815_c0_g1_i1 | 0.557775562 | 0.49330355 | 0.999970678 | 3.397 | 3.784 | 2.667 | 1.659 | SAC3_GANP |
| TRINITY_DN14064_c0_g1_i2 | 0.557763249 | 0.749666114 | 0.999970678 | 7.821 | 4.405 | 6.4 | 0.583 | NA |
| TRINITY_DN3868_c0_g1_i2 | -0.55776181 | 0.428967265 | 0.999970678 | 11.11 | 9.382 | 10.155 | 17.865 | NUDIX |
| TRINITY_DN10820_c0_g1_i1 | 0.557663187 | 0.649254613 | 1 | 0.309 | 0.345 | 0.2 | 0.221 | NA |
| TRINITY_DN10524_c0_g1_i1 | 0.557643785 | 0.770412935 | 0.999970678 | 2.325 | 0.276 | 1.344 | 0.151 | p450 |
| TRINITY_DN15705_c0_g3_i2 | -0.55758028 | 0.585869222 | 0.999970678 | 0.999 | 1.242 | 1.8 | 1.126 | DUF936 |
| TRINITY_DN295_c0_g3_i1 | 0.557566449 | 0.437842411 | 0.999970678 | 4.86 | 7.322 | 4.078 | 3.348 | HECT |

| TRINITY_DN3843_c0_g1_i8 | 0.55753473 | 0.634735414 | 0.999970678 | 1.49 | 0.591 | 0.411 | 0.925 | GatB_N |
| --- | --- | --- | --- | --- | --- | --- | --- | --- |
| TRINITY_DN6444_c0_g1_i6 | 0.557524755 | 0.607967626 | 0.999970678 | 0.545 | 1.311 | 0.489 | 0.674 | Castor_Poll_mid |
| TRINITY_DN3332_c0_g2_i1 | 0.557501607 | 0.368147021 | 0.999970678 | 8.448 | 8.209 | 4.766 | 5.57 | ABC1 |
| TRINITY_DN2388_c0_g1_i2 | 0.557410342 | 0.336499541 | 0.999970678 | 194.64 | 167.2 | 97.539 | 126.958 | Chloroa_b-bind |
| TRINITY_DN1210_c0_g1_i1 | -0.5574084 | 0.586921123 | 0.999970678 | 8.157 | 4.021 | 10.599 | 5.208 | NA |
| TRINITY_DN11991_c0_g1_i1 | -0.55735296 | 0.659540613 | 0.999970678 | 0.963 | 0.542 | 1.222 | 0.754 | NA |
| TRINITY_DN1065_c0_g1_i14 | -0.5572421 | 0.85758123 | 0.999970678 | 3.643 | 0.128 | 4.622 | 0 | CRS1_YhbY |
| TRINITY_DN13118_c0_g1_i1 | -0.55719295 | 0.528646128 | 0.999970678 | 4.115 | 2.227 | 4.589 | 3.841 | GRAS |
| TRINITY_DN7868_c0_g1_i1 | 0.557140376 | 0.439938391 | 0.999970678 | 9.175 | 5.785 | 4.444 | 4.856 | DUSP |
| TRINITY_DN15847_c2_g1_i3 | -0.55691805 | 0.759419039 | 0.999970678 | 1.808 | 0.158 | 2.1 | 0.402 | Aminotran_1_2 |
| TRINITY_DN7164_c0_g1_i6 | -0.55686813 | 0.853797528 | 1 | 0.572 | 0 | 0.1 | 0.724 | NA |
| TRINITY_DN1669_c0_g1_i5 | 0.556835754 | 0.698588123 | 0.999970678 | 0.209 | 1.587 | 0.511 | 0.593 | ANF_receptor |
| TRINITY_DN19245_c0_g2_i1 | 0.556792653 | 0.741064105 | 0.999970678 | 0.209 | 0.641 | 0.044 | 0.523 | NB-ARC |
| TRINITY_DN3380_c0_g1_i2 | -0.55678193 | 0.779647009 | 0.999970678 | 0.164 | 4.267 | 0.867 | 5.389 | BTB |
| TRINITY_DN517_c0_g1_i2 | 0.556735975 | 0.435490334 | 0.999970678 | 5.142 | 3.814 | 2.855 | 2.664 | UDPGP |
| TRINITY_DN964_c1_g2_i6 | 0.556725057 | 0.898524127 | 0.999970678 | 1.108 | 0 | 0.633 | 0 | NA |
| TRINITY_DN77465_c0_g1_i1 | 0.556725057 | 0.898524127 | 0.999970678 | 1.317 | 0 | 0.756 | 0 | NA |
| TRINITY_DN2983_c0_g1_i3 | 0.556656838 | 0.556091694 | 0.999970678 | 1.081 | 1.675 | 0.955 | 0.714 | ATG13 |
| TRINITY_DN13248_c0_g1_i2 | 0.55660195 | 0.509136649 | 0.999970678 | 2.616 | 2.72 | 1.411 | 1.92 | NA |
| TRINITY_DN8376_c0_g1_i1 | -0.55655328 | 0.392460528 | 0.999970678 | 19.294 | 15.23 | 21.121 | 25.004 | Nop16 |
| TRINITY_DN970_c1_g1_i7 | -0.55640395 | 0.4502055 | 0.999970678 | 3.007 | 4.563 | 4.344 | 5.831 | Myb_DNA-bindin |
| TRINITY_DN11602_c0_g1_i1 | -0.55637742 | 0.547641034 | 0.999970678 | 1.69 | 2.671 | 1.911 | 4.072 | NA |
| TRINITY_DN2390_c0_g2_i4 | -0.55635709 | 0.769512486 | 0.999970678 | 0.5 | 3.085 | 0.2 | 4.967 | NA |
| TRINITY_DN13183_c1_g1_i1 | -0.55627882 | 0.8375952 | 0.999970678 | 15.879 | 0 | 7.4 | 14.457 | NA |
| TRINITY_DN1074_c2_g1_i2 | 0.556229983 | 0.895038755 | 0.999970678 | 0 | 19.44 | 0 | 12.969 | GRAM |
| TRINITY_DN13475_c0_g2_i3 | -0.55621757 | 0.466045581 | 0.999970678 | 1.653 | 2.365 | 2.522 | 2.855 | PPR |
| TRINITY_DN3382_c0_g1_i2 | 0.556069008 | 0.83811637 | 0.999970678 | 8.412 | 4.415 | 0 | 8.777 | PP2C |
| TRINITY_DN81331_c0_g1_i2 | 0.556031151 | 0.844832639 | 1 | 0.618 | 0.197 | 0.467 | 0 | NA |
| TRINITY_DN507_c0_g1_i5 | 0.555989531 | 0.583928251 | 0.999970678 | 5.251 | 2.198 | 1.567 | 3.207 | NA |
| TRINITY_DN5844_c0_g1_i1 | 0.555858764 | 0.458488857 | 0.999970678 | 15.279 | 28.55 | 10.877 | 16.649 | FKS1_dom1 |
| TRINITY_DN5919_c0_g3_i4 | -0.55583223 | 0.495025481 | 0.999970678 | 1.771 | 2.553 | 2.744 | 2.996 | Tfb4 |
| TRINITY_DN13800_c0_g1_i2 | -0.55582948 | 0.842295096 | 1 | 0 | 1.449 | 0.744 | 1.186 | NA |
| TRINITY_DN12111_c0_g3_i3 | 0.555796945 | 0.579246012 | 0.999970678 | 5.886 | 2.464 | 2.978 | 2.121 | PORR |
| TRINITY_DN5866_c0_g1_i1 | -0.55578339 | 0.555585873 | 0.999970678 | 57.956 | 94.41 | 50.253 | 162.277 | EF-hand_1 |
| TRINITY_DN18674_c0_g1_i2 | 0.555554489 | 0.558488145 | 0.999970678 | 11.9 | 30.18 | 8.499 | 18.197 | NA |
| TRINITY_DN2926_c0_g1_i3 | -0.55552236 | 0.895896143 | 0.999970678 | 40.097 | 0 | 48.83 | 0 | Glutaredoxin |
| TRINITY_DN2237_c0_g1_i1 | -0.55545485 | 0.380338562 | 0.999970678 | 10.955 | 12.92 | 14.344 | 17.534 | NA |
| TRINITY_DN22912_c0_g1_i10 | 0.555397006 | 0.770732531 | 1 | 0.382 | 0.788 | 0.6 | 0.06 | GST_C |
| TRINITY_DN13545_c0_g2_i1 | 0.555393874 | 0.566484716 | 0.999970678 | 33.919 | 14.02 | 17.721 | 11.411 | NA |
| TRINITY_DN4344_c0_g2_i2 | -0.55534787 | 0.675971272 | 0.999970678 | 3.47 | 6.652 | 1.644 | 12.849 | Pkinase |
| TRINITY_DN13452_c0_g2_i1 | -0.55525081 | 0.675205792 | 1 | 1.163 | 0.562 | 1.144 | 1.156 | NA |
| TRINITY_DN5255_c0_g1_i7 | -0.55518905 | 0.700431777 | 0.999970678 | 15.316 | 4.721 | 21.399 | 3.72 | NA |
| TRINITY_DN5411_c1_g1_i5 | 0.555058832 | 0.503289939 | 0.999970678 | 1.544 | 1.35 | 0.689 | 1.156 | NA |
| TRINITY_DN4291_c0_g1_i1 | -0.5550551 | 0.689007579 | 0.999970678 | 2.153 | 19.04 | 15.521 | 11.934 | - |
| TRINITY_DN8658_c0_g1_i4 | -0.55503663 | 0.563623682 | 0.999970678 | 3.497 | 6.987 | 8.922 | 4.574 | SAM_decarbox |
| TRINITY_DN4274_c0_g1_i16 | -0.55503273 | 0.839649414 | 0.999970678 | 0 | 0.897 | 0.456 | 0.774 | NA |
| TRINITY_DN8762_c0_g2_i1 | 0.555032109 | 0.610778006 | 0.999970678 | 0.927 | 1.705 | 0.567 | 1.106 | NA |
| TRINITY_DN13140_c0_g1_i1 | 0.555020386 | 0.901163174 | 1 | 2.68 | 0 | 1.522 | 0 | NA |
| TRINITY_DN2076_c0_g2_i1 | 0.555018585 | 0.466376705 | 0.999970678 | 2.053 | 2.523 | 1.411 | 1.408 | DNA_pol_A_exo1 |
| TRINITY_DN137369_c0_g1_i1 | 0.554927032 | 0.484236102 | 0.999970678 | 5.014 | 5.864 | 2.833 | 3.931 | NA |
| TRINITY_DN645_c0_g1_i3 | -0.55487639 | 0.705061908 | 0.999970678 | 2.162 | 8.318 | 1.467 | 13.562 | NA |
| TRINITY_DN20359_c0_g4_i1 | -0.55483493 | 0.720348698 | 1 | 9.72 | 32.04 | 15.344 | 36.666 | NA |
| TRINITY_DN8487_c0_g1_i1 | -0.55478873 | 0.557841337 | 0.999970678 | 4.887 | 4.277 | 3.855 | 8.737 | NA |
| TRINITY_DN731_c0_g1_i6 | 0.55472505 | 0.896201632 | 0.999970678 | 2.598 | 0 | 0 | 1.81 | TraB |
| TRINITY_DN5428_c0_g1_i2 | -0.55468329 | 0.436177311 | 0.999970678 | 5.205 | 7.5 | 8.211 | 8.646 | NA |
| TRINITY_DN1207_c0_g1_i10 | 0.554628464 | 0.715969184 | 0.999970678 | 0.59 | 0.631 | 0.1 | 0.694 | Ank_2 |
| TRINITY_DN19651_c0_g1_i1 | -0.55462435 | 0.604118949 | 0.999970678 | 1.671 | 3.104 | 1.744 | 4.846 | NA |
| TRINITY_DN5905_c0_g1_i6 | 0.554615276 | 0.635979001 | 0.999970678 | 4.097 | 3.292 | 1.033 | 3.79 | NA |
| TRINITY_DN12160_c0_g1_i3 | 0.554569244 | 0.697297964 | 0.999970678 | 5.169 | 18.26 | 4.955 | 9.189 | NA |
| TRINITY_DN3579_c0_g1_i13 | 0.55452008 | 0.661832093 | 1 | 0.336 | 0.404 | 0.144 | 0.342 | NA |
| TRINITY_DN14626_c0_g1_i3 | 0.554483573 | 0.389969253 | 0.999970678 | 10.883 | 15.95 | 7.1 | 9.682 | NA |
| TRINITY_DN9449_c0_g1_i2 | 0.554432689 | 0.602165145 | 0.999970678 | 1.417 | 0.68 | 0.678 | 0.623 | DUF616 |
| TRINITY_DN6187_c0_g1_i1 | -0.55443164 | 0.443404239 | 0.999970678 | 10.628 | 17.35 | 14.444 | 23.516 | NA |
| TRINITY_DN964_c0_g1_i13 | -0.55442474 | 0.49659785 | 0.999970678 | 2.634 | 2.838 | 3.8 | 3.408 | NA |
| TRINITY_DN125_c0_g1_i1 | 0.554415173 | 0.605795905 | 0.999970678 | 8.412 | 3.459 | 2.011 | 5.71 | UDPGT |
| TRINITY_DN56058_c0_g1_i1 | -0.55439415 | 0.897118081 | 0.999970678 | 2.198 | 0 | 2.711 | 0 | NA |
| TRINITY_DN21218_c0_g1_i6 | 0.554353915 | 0.702098397 | 0.999970678 | 0.209 | 0.394 | 0.078 | 0.312 | RTT107_BRCT_5 |
| TRINITY_DN564_c0_g1_i12 | -0.55422362 | 0.450085067 | 0.999970678 | 5.814 | 5.992 | 7.3 | 8.435 | NA |
| TRINITY_DN1813_c5_g1_i1 | -0.55411827 | 0.77018418 | 0.999970678 | 5.023 | 0.641 | 5.966 | 1.086 | NA |
| TRINITY_DN11666_c0_g1_i3 | 0.554117426 | 0.731995551 | 0.999970678 | 64.696 | 15.26 | 36.042 | 9.038 | NA |
| TRINITY_DN4522_c0_g2_i5 | 0.554033582 | 0.641632994 | 0.999970678 | 0.227 | 0.276 | 0.189 | 0.111 | NA |
| TRINITY_DN8843_c0_g1_i2 | 0.554014759 | 0.849235483 | 0.999970678 | 0 | 4.553 | 0.422 | 2.493 | NA |
| TRINITY_DN11633_c0_g2_i2 | -0.55400377 | 0.647355875 | 0.999970678 | 0.999 | 2.7 | 1.355 | 3.73 | NA |
| TRINITY_DN21218_c0_g2_i2 | -0.55387647 | 0.848411035 | 1 | 0.981 | 0 | 0.933 | 0.332 | NA |

| TRINITY_DN3051_c1_g1_i2 | 0.553829213 | 0.538011918 | 0.999970678 | 8.966 | 3.982 | 3.578 | 4.564 | MFMR |
| --- | --- | --- | --- | --- | --- | --- | --- | --- |
| TRINITY_DN11648_c0_g1_i8 | -0.55381069 | 0.525276946 | 0.999970678 | 4.96 | 2.828 | 3.7 | 7.007 | NA |
| TRINITY_DN1691_c0_g1_i8 | -0.55380507 | 0.409986297 | 0.999970678 | 6.64 | 8.988 | 8.322 | 12.849 | Proteasome |
| TRINITY_DN835_c1_g1_i3 | 0.553775673 | 0.74958828 | 0.999970678 | 1.526 | 0.434 | 1.022 | 0.131 | NA |
| TRINITY_DN1868_c1_g1_i1 | -0.55376598 | 0.737615412 | 0.999970678 | 3.761 | 0.562 | 3.433 | 2.292 | NA |
| TRINITY_DN3308_c0_g1_i13 | 0.5537543 | 0.754374815 | 0.999970678 | 0.454 | 1.981 | 0.133 | 1.518 | NA |
| TRINITY_DN974_c0_g1_i2 | 0.553737694 | 0.504386485 | 0.999970678 | 4.896 | 4.386 | 3.389 | 2.242 | Branch |
| TRINITY_DN10592_c1_g1_i10 | 0.553686402 | 0.842856764 | 1 | 0.763 | 0.769 | 0.867 | 0 | NA |
| TRINITY_DN7124_c0_g1_i1 | 0.553664334 | 0.734018369 | 0.999970678 | 3.079 | 5.135 | 4.189 | 0.483 | Cu_bind_like |
| TRINITY_DN85362_c0_g1_i1 | -0.55361629 | 0.528564827 | 0.999970678 | 108.73 | 195 | 146.747 | 227.917 | NA |
| TRINITY_DN4452_c4_g2_i2 | 0.553520106 | 0.759467526 | 0.999970678 | 3.997 | 0.503 | 1.944 | 0.714 | NA |
| TRINITY_DN833_c0_g3_i4 | 0.553478482 | 0.671550724 | 0.999970678 | 1.426 | 7.608 | 1.367 | 4.464 | Glyco_hydro_1 |
| TRINITY_DN1522_c0_g1_i10 | -0.55345107 | 0.615582222 | 0.999970678 | 1.481 | 1.981 | 1.078 | 3.78 | NA |
| TRINITY_DN2048_c0_g1_i4 | 0.553432082 | 0.866661106 | 1 | 0 | 2.434 | 0.078 | 1.488 | Ribosomal_S8 |
| TRINITY_DN8688_c0_g1_i2 | 0.553362736 | 0.84929802 | 1 | 0.154 | 0.65 | 0 | 0.543 | NA |
| TRINITY_DN38_c0_g1_i4 | -0.55334241 | 0.851932891 | 0.999970678 | 1.181 | 0 | 1.355 | 0.131 | NA |
| TRINITY_DN727_c0_g3_i2 | -0.55332635 | 0.433002893 | 0.999970678 | 25.181 | 20.97 | 33.742 | 26.723 | NA |
| TRINITY_DN5107_c0_g1_i2 | 0.553286841 | 0.895924049 | 0.999970678 | 1.681 | 0 | 0.955 | 0 | NUFIP1 |
| TRINITY_DN6780_c0_g1_i3 | -0.55325913 | 0.856484474 | 1 | 0.154 | 1.232 | 0 | 1.991 | NA |
| TRINITY_DN4846_c0_g1_i2 | 0.553254707 | 0.427910788 | 0.999970678 | 3.652 | 5.48 | 2.855 | 2.775 | NA |
| TRINITY_DN4536_c1_g1_i7 | -0.5532543 | 0.837607353 | 0.999970678 | 1.299 | 0 | 0.867 | 0.885 | Exo_endo_phos |
| TRINITY_DN856_c0_g2_i9 | -0.55324527 | 0.797971071 | 1 | 21.42 | 0.552 | 19.765 | 8.264 | NA |
| TRINITY_DN2535_c0_g1_i14 | 0.553244526 | 0.65480457 | 0.999970678 | 1.771 | 1.892 | 1.478 | 0.724 | NA |
| TRINITY_DN19452_c0_g1_i2 | -0.55319446 | 0.83755422 | 0.999970678 | 7.485 | 0 | 4.433 | 5.68 | NA |
| TRINITY_DN8872_c0_g2_i1 | -0.55318237 | 0.507638957 | 0.999970678 | 2.516 | 1.646 | 2.522 | 3.066 | NA |
| TRINITY_DN32734_c0_g2_i12 | 0.553110685 | 0.89876343 | 0.999970678 | 1.59 | 0 | 0.922 | 0 | NA |
| TRINITY_DN967_c1_g1_i1 | -0.55302787 | 0.483710381 | 0.999970678 | 3.579 | 2.868 | 4.633 | 3.901 | Myb_DNA-bindin |
| TRINITY_DN1031_c4_g1_i1 | 0.553024005 | 0.712862615 | 0.999970678 | 1.363 | 11.89 | 1.744 | 6.826 | NA |
| TRINITY_DN11277_c0_g3_i1 | 0.552999143 | 0.901088946 | 1 | 2.789 | 0 | 0 | 1.87 | TMEM135_C_rich |
| TRINITY_DN6250_c0_g1_i4 | 0.552999143 | 0.901088946 | 1 | 1.444 | 0 | 0 | 1.005 | Glu_cyclase_2 |
| TRINITY_DN4189_c0_g1_i2 | 0.552999143 | 0.901088946 | 1 | 5.132 | 0 | 0 | 3.408 | NA |
| TRINITY_DN14011_c0_g1_i1 | 0.552948431 | 0.613782281 | 0.999970678 | 3.997 | 6.958 | 1.911 | 5.037 | NA |
| TRINITY_DN10575_c0_g1_i4 | 0.552868425 | 0.362830619 | 0.999970678 | 10.256 | 11.5 | 6.766 | 6.666 | SART-1 |
| TRINITY_DN3646_c0_g1_i6 | 0.552842795 | 0.388339614 | 0.999970678 | 24.554 | 20.02 | 13.555 | 13.995 | NA |
| TRINITY_DN16264_c0_g1_i1 | -0.55280945 | 0.521824909 | 0.999970678 | 30.658 | 26.78 | 49.153 | 24.923 | NdhN |
| TRINITY_DN16264_c0_g1_i4 | -0.55280945 | 0.521824909 | 0.999970678 | 30.658 | 26.78 | 49.153 | 24.923 | NdhN |
| TRINITY_DN1673_c0_g1_i3 | -0.55275445 | 0.67145672 | 0.999970678 | 9.32 | 17.37 | 4.233 | 33.821 | NA |
| TRINITY_DN651_c0_g1_i11 | -0.55267804 | 0.537549151 | 0.999970678 | 223.19 | 98.98 | 148.58 | 296.735 | NA |
| TRINITY_DN46136_c0_g1_i1 | 0.552635436 | 0.767919207 | 0.999970678 | 61.18 | 2362 | 220.953 | 1190.961 | NA |
| TRINITY_DN1531_c0_g1_i1 | -0.5525875 | 0.504088511 | 0.999970678 | 11.664 | 8.919 | 16.321 | 10.426 | NA |
| TRINITY_DN1314_c0_g2_i2 | 0.552579968 | 0.427383054 | 0.999970678 | 14.77 | 12.14 | 9.188 | 7.309 | NA |
| TRINITY_DN11107_c0_g2_i2 | 0.55257462 | 0.699343912 | 0.999970678 | 2.244 | 0.483 | 0.744 | 1.005 | NA |
| TRINITY_DN1360_c0_g1_i5 | -0.55255769 | 0.45948832 | 0.999970678 | 2.498 | 1.843 | 2.922 | 2.865 | TPT |
| TRINITY_DN51919_c0_g2_i1 | -0.55249203 | 0.86666507 | 1 | 0 | 3.213 | 0.133 | 4.323 | NA |
| TRINITY_DN805_c2_g1_i1 | -0.55245114 | 0.712305934 | 0.999970678 | 0.881 | 0.857 | 0.344 | 2.101 | NA |
| TRINITY_DN6807_c0_g1_i1 | 0.552403504 | 0.475042501 | 0.999970678 | 47.872 | 34.9 | 29.232 | 20.771 | NA |
| TRINITY_DN1692_c0_g1_i6 | 0.552316194 | 0.762595556 | 0.999970678 | 0.481 | 0.168 | 0.022 | 0.422 | NA |
| TRINITY_DN795_c0_g1_i4 | 0.552241791 | 0.640645798 | 0.999970678 | 14.752 | 3.715 | 3.811 | 8.073 | NA |
| TRINITY_DN1766_c0_g1_i1 | -0.55221006 | 0.446837486 | 0.999970678 | 22.919 | 21.98 | 20.143 | 41.301 | Aha1_N |
| TRINITY_DN3721_c0_g1_i4 | 0.552201722 | 0.672472025 | 0.999970678 | 0.563 | 1.124 | 0.722 | 0.271 | NA |
| TRINITY_DN8461_c0_g1_i2 | -0.55216339 | 0.497247383 | 0.999970678 | 254.48 | 188.1 | 176.3 | 434.048 | EF-hand_1 |
| TRINITY_DN12845_c0_g1_i3 | 0.552128848 | 0.746153424 | 0.999970678 | 0.418 | 0.148 | 0.289 | 0.05 | NA |
| TRINITY_DN28628_c0_g1_i1 | 0.552081599 | 0.646504638 | 0.999970678 | 3.016 | 1.597 | 1.244 | 1.649 | NA |
| TRINITY_DN3294_c0_g1_i4 | 0.552015893 | 0.37246161 | 0.999970678 | 8.384 | 6.968 | 4.389 | 5.198 | Syja_N |
| TRINITY_DN51116_c0_g1_i2 | -0.55199694 | 0.76893087 | 0.999970678 | 0.663 | 13.42 | 4.689 | 14.005 | NA |
| TRINITY_DN10616_c0_g1_i3 | -0.55181319 | 0.552492385 | 0.999970678 | 28.033 | 46.6 | 25.521 | 78.308 | vATP-synt_E |
| TRINITY_DN10616_c0_g1_i2 | -0.55181319 | 0.552492385 | 0.999970678 | 28.033 | 46.6 | 25.521 | 78.308 | vATP-synt_E |
| TRINITY_DN6305_c0_g1_i4 | 0.551771511 | 0.842959054 | 0.999970678 | 0 | 1.34 | 0.367 | 0.462 | NA |
| TRINITY_DN4486_c0_g1_i3 | -0.55173084 | 0.594043925 | 0.999970678 | 8.957 | 3.824 | 9.388 | 7.289 | NA |
| TRINITY_DN19_c0_g1_i6 | 0.551699435 | 0.548951564 | 0.999970678 | 3.697 | 5.342 | 3.5 | 1.91 | WHEP-TRS |
| TRINITY_DN519_c2_g1_i1 | 0.55168525 | 0.535165667 | 0.999970678 | 9.802 | 9.175 | 3.789 | 8.194 | NA |
| TRINITY_DN2195_c1_g1_i9 | -0.55160409 | 0.830289419 | 0.999970678 | 1.372 | 128.2 | 153.924 | 0.704 | Cpn10 |
| TRINITY_DN977_c0_g1_i2 | 0.551552852 | 0.469693931 | 0.999970678 | 16.269 | 9.905 | 7.944 | 8.284 | NA |
| TRINITY_DN2324_c0_g1_i3 | -0.55152765 | 0.745820537 | 0.999970678 | 1.49 | 0.177 | 1.511 | 0.633 | NA |
| TRINITY_DN7310_c0_g3_i1 | 0.551473107 | 0.691284235 | 0.999970678 | 1.508 | 0.384 | 0.622 | 0.543 | NA |
| TRINITY_DN999_c0_g2_i2 | -0.55146685 | 0.727556531 | 0.999970678 | 13.29 | 3.095 | 17.921 | 2.423 | NA |
| TRINITY_DN8924_c0_g1_i2 | -0.55135592 | 0.900391813 | 0.999970678 | 0.436 | 0 | 0 | 0.664 | LRR_8 |
| TRINITY_DN1039_c8_g1_i1 | -0.55129553 | 0.702675184 | 0.999970678 | 1.008 | 2.139 | 0.833 | 3.529 | NA |
| TRINITY_DN12018_c0_g1_i9 | 0.55125379 | 0.586102198 | 0.999970678 | 7.685 | 8.012 | 4.444 | 4.946 | NA |
| TRINITY_DN2556_c0_g1_i2 | -0.5512486 | 0.362460011 | 0.999970678 | 54.949 | 54.71 | 56.863 | 92.484 | NA |
| TRINITY_DN11231_c0_g1_i1 | -0.55118811 | 0.504274369 | 0.999970678 | 4.07 | 3.765 | 3.233 | 7.611 | NA |
| TRINITY_DN62954_c0_g1_i1 | 0.55118209 | 0.896494485 | 0.999970678 | 1.835 | 0 | 1.055 | 0 | HATPase_c |
| TRINITY_DN4301_c0_g1_i6 | -0.55108636 | 0.784277219 | 0.999970678 | 0.427 | 5.893 | 7.288 | 0.322 | WRKY |
| TRINITY_DN4147_c0_g1_i1 | -0.55096964 | 0.623642492 | 0.999970678 | 5.151 | 8.17 | 3.544 | 15.05 | NA |

| TRINITY_DN292_c0_g1_i1 | -0.55094826 | 0.512664406 | 0.999970678 | 29.377 | 40.19 | 26.387 | 69.622 | NA |
| --- | --- | --- | --- | --- | --- | --- | --- | --- |
| TRINITY_DN2399_c0_g1_i4 | -0.55094691 | 0.853407208 | 1 | 0 | 0.522 | 0.556 | 0.111 | Fer4_14 |
| TRINITY_DN23143_c0_g1_i1 | -0.55094638 | 0.539882479 | 0.999970678 | 3.034 | 2.217 | 4.333 | 2.493 | ATP-synt_DE |
| TRINITY_DN1922_c3_g1_i9 | -0.55071204 | 0.498054222 | 0.999970678 | 8.094 | 9.382 | 7 | 17.142 | NA |
| TRINITY_DN4324_c0_g1_i7 | -0.55068605 | 0.686019418 | 0.999970678 | 1.063 | 3.863 | 1.4 | 5.409 | NA |
| TRINITY_DN411_c0_g2_i1 | -0.55063404 | 0.712857192 | 0.999970678 | 3.043 | 1.685 | 0.822 | 5.871 | NA |
| TRINITY_DN12736_c0_g1_i8 | 0.550625412 | 0.653342256 | 0.999970678 | 1.235 | 1.38 | 1.189 | 0.352 | FPL |
| TRINITY_DN15411_c0_g1_i4 | 0.550566387 | 0.844808459 | 1 | 0.917 | 0.483 | 0.789 | 0 | Aldedh |
| TRINITY_DN4238_c0_g2_i2 | 0.550550202 | 0.58756161 | 0.999970678 | 1.244 | 0.68 | 0.7 | 0.493 | FAR1 |
| TRINITY_DN1334_c1_g1_i4 | 0.55052818 | 0.652784632 | 0.999970678 | 1.19 | 2.769 | 1.555 | 0.834 | NA |
| TRINITY_DN8767_c0_g1_i3 | -0.5504702 | 0.736741906 | 0.999970678 | 4.933 | 8.269 | 1.933 | 16.166 | NA |
| TRINITY_DN4999_c0_g1_i2 | -0.55046626 | 0.9009823 | 1 | 0.59 | 0 | 0 | 0.875 | NA |
| TRINITY_DN16912_c0_g1_i8 | -0.55045872 | 0.37547899 | 0.999970678 | 122.45 | 151.4 | 187.111 | 172.29 | Chloroa_b-bind |
| TRINITY_DN29288_c0_g1_i1 | 0.550389045 | 0.669215137 | 0.999970678 | 0.963 | 0.562 | 0.578 | 0.372 | NA |
| TRINITY_DN2846_c0_g1_i6 | -0.55035149 | 0.642238728 | 0.999970678 | 13.144 | 39.62 | 14.055 | 59.649 | NA |
| TRINITY_DN9331_c0_g1_i8 | -0.55033015 | 0.699540498 | 0.999970678 | 1.117 | 0.611 | 0.344 | 2.141 | NA |
| TRINITY_DN5024_c0_g1_i7 | 0.550319143 | 0.63449536 | 0.999970678 | 0.3 | 0.473 | 0.178 | 0.312 | NA |
| TRINITY_DN12105_c0_g1_i9 | 0.550309312 | 0.903352108 | 1 | 1.581 | 0 | 0.9 | 0 | - |
| TRINITY_DN20816_c1_g2_i1 | 0.550309312 | 0.903352108 | 1 | 1.535 | 0 | 0.878 | 0 | NA |
| TRINITY_DN8739_c0_g1_i4 | 0.550231481 | 0.737959321 | 0.999970678 | 0.6 | 0.345 | 0.478 | 0.07 | NA |
| TRINITY_DN512_c0_g1_i3 | 0.550220412 | 0.613515022 | 0.999970678 | 1.708 | 1.991 | 1.6 | 0.593 | CCT |
| TRINITY_DN7004_c1_g1_i1 | -0.5501913 | 0.611435992 | 0.999970678 | 1.98 | 3.42 | 1.822 | 5.65 | NA |
| TRINITY_DN4964_c0_g1_i2 | 0.550132764 | 0.591509565 | 0.999970678 | 17.032 | 23.23 | 5.444 | 20.53 | NA |
| TRINITY_DN7544_c0_g1_i3 | -0.5500611 | 0.590438429 | 0.999970678 | 0.827 | 1.498 | 1.311 | 1.78 | NA |
| TRINITY_DN5063_c0_g1_i8 | 0.550011302 | 0.772061525 | 0.999970678 | 10.02 | 26.92 | 1.178 | 22.098 | NA |
| TRINITY_DN576_c0_g1_i3 | -0.55000144 | 0.469624238 | 0.999970678 | 9.129 | 10.65 | 15.521 | 10.205 | NA |
| TRINITY_DN9108_c0_g2_i1 | -0.54984994 | 0.484276271 | 0.999970678 | 1.817 | 1.459 | 2.422 | 1.88 | AA_kinase |
| TRINITY_DN2043_c0_g1_i5 | 0.549828432 | 0.63534196 | 0.999970678 | 2.834 | 0.828 | 0.911 | 1.408 | NA |
| TRINITY_DN365_c0_g1_i5 | -0.54980399 | 0.627416237 | 0.999970678 | 10.165 | 8.308 | 4.444 | 21.525 | Thaumatin |
| TRINITY_DN15859_c0_g2_i3 | 0.549791784 | 0.519558198 | 0.999970678 | 2.398 | 1.429 | 1.167 | 1.206 | Zw10 |
| TRINITY_DN11775_c0_g1_i2 | -0.54977157 | 0.901488127 | 1 | 9.838 | 0 | 11.833 | 0 | NA |
| TRINITY_DN64310_c0_g1_i1 | -0.54975281 | 0.676693505 | 0.999970678 | 1.008 | 4.238 | 1.733 | 5.499 | NA |
| TRINITY_DN18267_c1_g1_i1 | 0.549746485 | 0.571754903 | 0.999970678 | 1.09 | 1.567 | 0.544 | 1.156 | BSD |
| TRINITY_DN20264_c0_g2_i1 | -0.5497431 | 0.90367544 | 1 | 7.703 | 0 | 9.266 | 0 | NA |
| TRINITY_DN40113_c0_g1_i1 | -0.5497431 | 0.90367544 | 1 | 0.572 | 0 | 0.711 | 0 | NA |
| TRINITY_DN11344_c0_g1_i2 | -0.5497431 | 0.90367544 | 1 | 3.307 | 0 | 4 | 0 | Lactamase_B |
| TRINITY_DN76875_c0_g1_i1 | -0.5497431 | 0.90367544 | 1 | 0.963 | 0 | 1.189 | 0 | ADH_N |
| TRINITY_DN6415_c0_g1_i2 | 0.549735368 | 0.380229433 | 0.999970678 | 3.861 | 3.715 | 2.244 | 2.473 | LRR_4 |
| TRINITY_DN7561_c0_g1_i6 | -0.5497195 | 0.90448842 | 1 | 1.308 | 0 | 1.633 | 0 | NA |
| TRINITY_DN41699_c0_g1_i5 | -0.5497195 | 0.90448842 | 1 | 7.948 | 0 | 9.544 | 0 | NA |
| TRINITY_DN53892_c0_g1_i2 | 0.549648883 | 0.502033001 | 0.999970678 | 34.237 | 31.78 | 25.243 | 14.457 | NA |
| TRINITY_DN488_c1_g1_i2 | 0.549414905 | 0.852636397 | 0.999970678 | 1.217 | 0 | 0.078 | 0.744 | UCH |
| TRINITY_DN7291_c0_g1_i2 | 0.549364984 | 0.41975424 | 0.999970678 | 3.815 | 3.272 | 2.2 | 2.192 | NA |
| TRINITY_DN4598_c0_g1_i7 | -0.54917503 | 0.896067035 | 0.999970678 | 8.357 | 0 | 0 | 12.356 | NA |
| TRINITY_DN528_c1_g1_i1 | -0.54913892 | 0.621525101 | 0.999970678 | 8.248 | 2.385 | 6.611 | 7.641 | EGF_CA |
| TRINITY_DN5986_c0_g1_i6 | 0.549092539 | 0.902340008 | 1 | 1.69 | 0 | 0 | 1.176 | NA |
| TRINITY_DN5992_c2_g2_i2 | -0.54909035 | 0.742478246 | 0.999970678 | 1.417 | 1.222 | 2.9 | 0.292 | NA |
| TRINITY_DN4708_c0_g1_i1 | 0.549047003 | 0.3991222 | 0.999970678 | 3.915 | 4.947 | 2.811 | 2.654 | NA |
| TRINITY_DN6915_c0_g1_i1 | 0.548997208 | 0.52432455 | 0.999970678 | 15.633 | 8.101 | 5.233 | 9.963 | CUE |
| TRINITY_DN10379_c0_g1_i2 | -0.54898287 | 0.601170192 | 0.999970678 | 1.299 | 0.887 | 1.633 | 1.237 | NA |
| TRINITY_DN20113_c1_g2_i1 | -0.54894243 | 0.573364147 | 0.999970678 | 1.417 | 4.001 | 3.566 | 3.569 | NA |
| TRINITY_DN7429_c0_g1_i3 | -0.54891498 | 0.604430806 | 0.999970678 | 1.399 | 3.144 | 3.322 | 2.574 | NA |
| TRINITY_DN3523_c1_g1_i1 | -0.54883363 | 0.717214988 | 0.999970678 | 7.812 | 0.631 | 3.489 | 8.335 | NA |
| TRINITY_DN2510_c0_g1_i6 | 0.548805999 | 0.579075903 | 0.999970678 | 5.232 | 4.987 | 1.589 | 5.067 | Mito_carr |
| TRINITY_DN11648_c0_g1_i11 | -0.54880318 | 0.602542043 | 0.999970678 | 19.167 | 6.278 | 10.91 | 24.35 | NA |
| TRINITY_DN20_c1_g1_i10 | 0.548755802 | 0.481862679 | 0.999970678 | 4.696 | 4.198 | 2.211 | 3.348 | NA |
| TRINITY_DN3989_c0_g1_i1 | -0.54859863 | 0.612550346 | 0.999970678 | 28.996 | 119.7 | 72.807 | 127.199 | DUF760 |
| TRINITY_DN2728_c0_g1_i5 | 0.548569576 | 0.83914005 | 0.999970678 | 0.827 | 2.405 | 0 | 2.192 | NA |
| TRINITY_DN1188_c0_g1_i2 | -0.54852485 | 0.567787623 | 0.999970678 | 24.527 | 24.75 | 14.721 | 54.27 | Pyridoxal_deC |
| TRINITY_DN13748_c0_g1_i4 | 0.54846159 | 0.518832117 | 0.999970678 | 38.916 | 23.27 | 23.176 | 14.678 | NA |
| TRINITY_DN1610_c0_g1_i11 | 0.548455502 | 0.869047124 | 0.999970678 | 0 | 1.192 | 0.656 | 0 | - |
| TRINITY_DN3627_c0_g1_i5 | -0.54836275 | 0.776386344 | 0.999970678 | 125.21 | 3.883 | 142.491 | 19.303 | Pro_CA |
| TRINITY_DN25646_c0_g1_i3 | -0.54834803 | 0.838954807 | 0.999970678 | 0 | 1.981 | 1.3 | 1.287 | NA |
| TRINITY_DN1329_c0_g3_i1 | 0.548331316 | 0.8971847 | 0.999970678 | 2.271 | 0 | 1.3 | 0 | Asparaginase_2 |
| TRINITY_DN2794_c0_g1_i2 | -0.5482769 | 0.558228491 | 0.999970678 | 5.087 | 10.54 | 12.344 | 7.761 | NA |
| TRINITY_DN2507_c0_g3_i1 | -0.54825625 | 0.668504579 | 0.999970678 | 0.845 | 1.508 | 0.478 | 2.875 | DBD_Tnp_Mut |
| TRINITY_DN1681_c0_g1_i5 | 0.548208848 | 0.582303677 | 0.999970678 | 21.765 | 10.95 | 5.233 | 16.237 | NAM |
| TRINITY_DN16764_c0_g1_i1 | 0.54813365 | 0.570179315 | 0.999970678 | 4.115 | 12.13 | 5.189 | 4.765 | DNA_gyraseB |
| TRINITY_DN344_c1_g1_i3 | -0.54808085 | 0.727780732 | 1 | 0.282 | 1.409 | 0.833 | 1.387 | NA |
| TRINITY_DN3124_c0_g3_i1 | -0.54808066 | 0.69889531 | 0.999970678 | 0.218 | 1.537 | 1.178 | 1.136 | - |
| TRINITY_DN7324_c0_g1_i17 | -0.5480754 | 0.846752155 | 0.999970678 | 2.78 | 0 | 0.478 | 3.549 | LRR_8 |
| TRINITY_DN645_c1_g1_i3 | -0.54806515 | 0.494577747 | 0.999970678 | 160.14 | 295.6 | 208.51 | 403.596 | NA |
| TRINITY_DN3308_c0_g2_i13 | -0.5480559 | 0.664231188 | 0.999970678 | 0.372 | 1.015 | 0.722 | 1.146 | NA |
| TRINITY_DN2624_c0_g1_i2 | -0.54797291 | 0.395439513 | 0.999970678 | 48.009 | 64.95 | 77.406 | 71.24 | NA |

| TRINITY_DN728_c0_g1_i14 | 0.547874669 | 0.840165535 | 0.999970678 | 0.772 | 0.375 | 0 | 0.804 | NA |
| --- | --- | --- | --- | --- | --- | --- | --- | --- |
| TRINITY_DN2110_c0_g1_i8 | -0.54771808 | 0.577929221 | 0.999970678 | 3.733 | 3.134 | 6.133 | 2.644 | NA |
| TRINITY_DN416_c0_g1_i1 | 0.547681582 | 0.699822339 | 0.999970678 | 58.028 | 35.42 | 45.853 | 6.927 | NA |
| TRINITY_DN13068_c0_g1_i6 | -0.5476617 | 0.619339837 | 0.999970678 | 1.135 | 0.69 | 1.233 | 1.176 | tRNA_int_end_N2 |
| TRINITY_DN3310_c0_g1_i1 | 0.547651924 | 0.572693629 | 0.999970678 | 13.308 | 8.702 | 9.199 | 4.001 | NA |
| TRINITY_DN1471_c1_g1_i1 | 0.54759579 | 0.611263476 | 0.999970678 | 1.889 | 4.908 | 2.067 | 2.121 | NA |
| TRINITY_DN1505_c0_g2_i4 | 0.547566564 | 0.84269066 | 0.999970678 | 0 | 2.868 | 0.955 | 0.774 | NA |
| TRINITY_DN513_c0_g1_i1 | 0.547559046 | 0.592987004 | 0.999970678 | 1.226 | 2.07 | 0.689 | 1.418 | CCT |
| TRINITY_DN2287_c1_g3_i2 | -0.54751187 | 0.568958002 | 0.999970678 | 9.066 | 3.873 | 9.333 | 7.681 | NA |
| TRINITY_DN4793_c0_g1_i7 | 0.547499471 | 0.634566056 | 0.999970678 | 3.77 | 1.725 | 1.111 | 2.413 | NA |
| TRINITY_DN11126_c0_g1_i2 | -0.54741419 | 0.723282252 | 0.999970678 | 10.138 | 1.114 | 5.989 | 9.129 | NA |
| TRINITY_DN13337_c0_g1_i2 | 0.54739332 | 0.581224291 | 0.999970678 | 1.681 | 5.273 | 2.1 | 2.192 | NA |
| TRINITY_DN1071_c0_g1_i5 | -0.54739212 | 0.492797476 | 0.999970678 | 74.97 | 43.03 | 55.841 | 104.91 | NA |
| TRINITY_DN8672_c0_g1_i11 | -0.54739091 | 0.640600189 | 0.999970678 | 0.718 | 1.084 | 1.555 | 0.804 | NA |
| TRINITY_DN7028_c0_g1_i2 | -0.54734932 | 0.852886911 | 0.999970678 | 1.299 | 0 | 1.433 | 0.191 | NUP50 |
| TRINITY_DN16921_c0_g1_i1 | 0.547337127 | 0.670915287 | 0.999970678 | 0.563 | 1.261 | 0.411 | 0.744 | NA |
| TRINITY_DN9001_c0_g2_i4 | -0.54716135 | 0.860068971 | 0.999970678 | 1.017 | 0.059 | 1.311 | 0 | NA |
| TRINITY_DN6929_c0_g1_i4 | -0.54715449 | 0.84617871 | 1 | 0 | 0.641 | 0.267 | 0.593 | NA |
| TRINITY_DN6063_c0_g1_i1 | 0.547149615 | 0.622097131 | 0.999970678 | 111.41 | 27.67 | 30.531 | 59.206 | NA |
| TRINITY_DN1326_c1_g1_i3 | 0.547130815 | 0.613461551 | 0.999970678 | 0.69 | 0.936 | 0.633 | 0.342 | Glyco_hydro_17 |
| TRINITY_DN4136_c0_g1_i7 | 0.547025705 | 0.399048645 | 0.999970678 | 3.525 | 3.292 | 1.944 | 2.322 | FHA |
| TRINITY_DN9744_c0_g1_i2 | -0.54699389 | 0.734845835 | 0.999970678 | 0.209 | 1.873 | 1.333 | 1.397 | Kelch_1 |
| TRINITY_DN10324_c0_g1_i3 | 0.546988036 | 0.618625557 | 0.999970678 | 9.12 | 16.21 | 3.244 | 13.321 | Ribosomal_L7Ae |
| TRINITY_DN17116_c0_g1_i2 | 0.546982904 | 0.632558028 | 0.999970678 | 1.608 | 1.587 | 1.422 | 0.473 | NA |
| TRINITY_DN108325_c0_g1_i1 | 0.546872253 | 0.900048452 | 0.999970678 | 21.484 | 0 | 12.077 | 0 | NA |
| TRINITY_DN19125_c0_g1_i2 | -0.54682743 | 0.569269214 | 0.999970678 | 3.543 | 6.899 | 3.855 | 10.546 | HD |
| TRINITY_DN5062_c0_g1_i3 | -0.54675821 | 0.851734795 | 0.999970678 | 20.33 | 0 | 17.71 | 8.767 | Calmodulin_bind |
| TRINITY_DN2380_c0_g1_i3 | 0.546738292 | 0.434326458 | 0.999970678 | 22.674 | 16.37 | 12.166 | 11.964 | NA |
| TRINITY_DN36_c1_g1_i2 | -0.54672282 | 0.676818964 | 0.999970678 | 2.244 | 0.512 | 1.044 | 2.825 | RVT_2 |
| TRINITY_DN17846_c0_g1_i1 | -0.54667694 | 0.569359485 | 0.999970678 | 3.234 | 7.313 | 4.311 | 10.134 | NA |
| TRINITY_DN12902_c0_g1_i1 | 0.546520228 | 0.657841324 | 0.999970678 | 4.851 | 4.721 | 4.578 | 1.025 | NA |
| TRINITY_DN8580_c0_g1_i3 | -0.54648279 | 0.392888459 | 0.999970678 | 2.68 | 2.996 | 3.333 | 4.273 | MMR_HSR1 |
| TRINITY_DN8580_c0_g1_i2 | -0.54648279 | 0.392888459 | 0.999970678 | 2.68 | 2.996 | 3.333 | 4.273 | MMR_HSR1 |
| TRINITY_DN2573_c0_g1_i8 | -0.54643488 | 0.641162294 | 0.999970678 | 83.218 | 141.5 | 45.764 | 268.755 | AP2 |
| TRINITY_DN9668_c1_g1_i1 | -0.54641937 | 0.51720881 | 0.999970678 | 4.306 | 5.627 | 5.089 | 8.164 | NA |
| TRINITY_DN8022_c0_g1_i2 | -0.54640521 | 0.643198473 | 1 | 0.282 | 0.197 | 0.3 | 0.342 | NA |
| TRINITY_DN231_c0_g1_i4 | -0.5462528 | 0.453267525 | 0.999970678 | 124.07 | 106 | 100.927 | 213.561 | Arf |
| TRINITY_DN5030_c0_g1_i4 | 0.546200202 | 0.636277861 | 0.999970678 | 1.426 | 3.666 | 0.878 | 2.413 | NA |
| TRINITY_DN26085_c0_g1_i4 | 0.546169809 | 0.844987108 | 1 | 0.391 | 0.581 | 0.011 | 0.684 | DEAD |
| TRINITY_DN97_c0_g1_i7 | 0.546138282 | 0.39167052 | 0.999970678 | 4.315 | 4.829 | 2.955 | 2.714 | NA |
| TRINITY_DN3273_c1_g1_i2 | 0.546130592 | 0.604873532 | 0.999970678 | 1.145 | 1.823 | 0.633 | 1.257 | AAA |
| TRINITY_DN7392_c0_g1_i1 | -0.54612931 | 0.83999153 | 0.999970678 | 1.671 | 0 | 1.044 | 1.206 | B3 |
| TRINITY_DN18448_c2_g1_i1 | -0.54611689 | 0.838444004 | 0.999970678 | 0 | 5.716 | 3.022 | 4.534 | NA |
| TRINITY_DN2641_c0_g1_i2 | -0.54603472 | 0.794955395 | 0.999970678 | 0.109 | 20.16 | 17.688 | 7.792 | - |
| TRINITY_DN6099_c0_g1_i2 | 0.546021965 | 0.490460027 | 0.999970678 | 44.611 | 22.46 | 18.143 | 24.31 | NA |
| TRINITY_DN2778_c0_g1_i9 | 0.545975049 | 0.671759747 | 0.999970678 | 1.017 | 4.169 | 1.789 | 1.377 | NA |
| TRINITY_DN3517_c1_g1_i10 | -0.54597133 | 0.772225098 | 0.999970678 | 1.408 | 0.049 | 0.656 | 1.337 | NA |
| TRINITY_DN19301_c0_g1_i1 | 0.545923032 | 0.897409145 | 0.999970678 | 3.452 | 0 | 1.989 | 0 | WSC |
| TRINITY_DN2407_c0_g1_i6 | -0.54587451 | 0.896736178 | 0.999970678 | 3.425 | 0 | 0 | 5.067 | EamA |
| TRINITY_DN88_c0_g3_i1 | -0.54584921 | 0.737484679 | 0.999970678 | 0.254 | 2.73 | 2.122 | 1.759 | NA |
| TRINITY_DN16092_c0_g1_i2 | 0.545715331 | 0.767877831 | 0.999970678 | 0.636 | 20.11 | 2.733 | 10.597 | NA |
| TRINITY_DN11643_c0_g1_i6 | 0.545710607 | 0.681258116 | 0.999970678 | 2.126 | 3.656 | 0.622 | 3.147 | NA |
| TRINITY_DN4270_c0_g1_i1 | -0.54570655 | 0.595753717 | 0.999970678 | 15.824 | 52.15 | 34.965 | 54.199 | - |
| TRINITY_DN4686_c0_g2_i3 | 0.545698243 | 0.848034624 | 0.999970678 | 13.144 | 0 | 2.311 | 6.354 | NA |
| TRINITY_DN209_c1_g1_i3 | 0.545650985 | 0.487083159 | 0.999970678 | 4.051 | 5.361 | 3.378 | 2.363 | F-box |
| TRINITY_DN715_c0_g1_i4 | -0.54563259 | 0.860480453 | 1 | 1.281 | 0.148 | 1.7 | 0 | NA |
| TRINITY_DN7162_c0_g1_i1 | 0.545600143 | 0.36415254 | 0.999970678 | 25.771 | 22.31 | 14.821 | 15.141 | NA |
| TRINITY_DN7405_c1_g2_i1 | 0.545582155 | 0.537544312 | 0.999970678 | 1.644 | 3.252 | 1.289 | 1.79 | WD40 |
| TRINITY_DN4412_c0_g1_i5 | 0.545570168 | 0.621048139 | 0.999970678 | 1.88 | 4.169 | 1.467 | 2.282 | NA |
| TRINITY_DN10113_c0_g1_i5 | 0.545566224 | 0.845339511 | 1 | 0.3 | 0.375 | 0.378 | 0 | NA |
| TRINITY_DN2661_c1_g1_i9 | 0.545490554 | 0.558989965 | 0.999970678 | 124.08 | 110.9 | 34.898 | 115.316 | - |
| TRINITY_DN2255_c0_g1_i1 | -0.54547245 | 0.507292263 | 0.999970678 | 10.628 | 7.579 | 14.377 | 9.249 | NA |
| TRINITY_DN8369_c0_g1_i6 | -0.54541479 | 0.857976891 | 0.999970678 | 0 | 1.912 | 0.078 | 2.654 | Exo70 |
| TRINITY_DN1158_c1_g1_i1 | -0.54528296 | 0.850428107 | 0.999970678 | 74.116 | 0 | 30.043 | 72.568 | MIF |
| TRINITY_DN2638_c0_g1_i1 | 0.545260524 | 0.663525886 | 0.999970678 | 1.017 | 0.779 | 0.311 | 0.845 | ENTH |
| TRINITY_DN460_c2_g1_i1 | -0.54513255 | 0.857465255 | 0.999970678 | 61.98 | 0 | 49.03 | 32.634 | TPR_17 |
| TRINITY_DN3888_c1_g2_i1 | 0.545038527 | 0.689053223 | 0.999970678 | 0.454 | 2.109 | 1.044 | 0.462 | TPR_16 |
| TRINITY_DN16206_c0_g2_i1 | 0.544776896 | 0.869125574 | 0.999970678 | 2.88 | 0 | 1.622 | 0.04 | NA |
| TRINITY_DN1343_c0_g1_i12 | 0.544707577 | 0.854869473 | 0.999970678 | 2.598 | 0 | 0.133 | 1.649 | NA |
| TRINITY_DN982_c0_g1_i4 | -0.54468743 | 0.447481393 | 0.999970678 | 18.268 | 23.83 | 19.332 | 37.983 | NA |
| TRINITY_DN3579_c0_g1_i8 | 0.544683268 | 0.729787632 | 0.999970678 | 0.699 | 1.498 | 0.189 | 1.247 | NA |
| TRINITY_DN7624_c0_g1_i1 | 0.54466798 | 0.374789021 | 0.999970678 | 3.661 | 3.627 | 2 | 2.594 | Nexin_C |
| TRINITY_DN3317_c0_g1_i2 | 0.544598967 | 0.480517089 | 0.999970678 | 2.743 | 3.558 | 1.622 | 2.353 | Peptidase_M24 |
| TRINITY_DN9558_c0_g4_i1 | 0.544571122 | 0.581598215 | 0.999970678 | 6.441 | 8.525 | 3.344 | 5.952 | NA |

| TRINITY_DN1939_c0_g2_i3 | -0.5445705 | 0.692241949 | 0.999970678 | 0.245 | 1.616 | 1.267 | 1.156 | NA |
| --- | --- | --- | --- | --- | --- | --- | --- | --- |
| TRINITY_DN3450_c0_g1_i13 | 0.544549511 | 0.903754481 | 1 | 0.5 | 0 | 0 | 0.342 | NA |
| TRINITY_DN211_c0_g1_i4 | -0.54448312 | 0.732290657 | 0.999970678 | 20.257 | 10.5 | 2.1 | 42.376 | NA |
| TRINITY_DN905_c0_g2_i6 | -0.54445354 | 0.709644235 | 0.999970678 | 3.861 | 5.893 | 9.977 | 1.95 | NA |
| TRINITY_DN2674_c0_g1_i1 | -0.54445348 | 0.346329068 | 0.999970678 | 19.412 | 18.74 | 24.199 | 26.421 | zf-FLZ |
| TRINITY_DN42_c1_g1_i1 | -0.54442004 | 0.464164261 | 0.999970678 | 15.888 | 13.89 | 13.333 | 27.235 | NA |
| TRINITY_DN1189_c0_g1_i5 | -0.54438988 | 0.856758908 | 0.999970678 | 73.589 | 0 | 54.441 | 43.181 | NA |
| TRINITY_DN2638_c0_g1_i3 | 0.544385965 | 0.505867041 | 0.999970678 | 44.193 | 22.44 | 16.288 | 26.17 | ENTH |
| TRINITY_DN1785_c0_g1_i7 | -0.54436401 | 0.756449184 | 0.999970678 | 0.863 | 0.108 | 1.011 | 0.231 | DUF659 |
| TRINITY_DN314_c0_g2_i9 | -0.54435755 | 0.775327316 | 0.999970678 | 2.725 | 6.918 | 0.256 | 13.623 | NA |
| TRINITY_DN7234_c0_g2_i2 | 0.544330397 | 0.740960494 | 0.999970678 | 1.944 | 0.453 | 0.211 | 1.418 | NA |
| TRINITY_DN1038_c0_g2_i1 | 0.544324464 | 0.699664722 | 0.999970678 | 1.017 | 1.774 | 0.244 | 1.599 | Mannosyl_trans3 |
| TRINITY_DN2454_c0_g1_i5 | -0.54430571 | 0.864012459 | 0.999970678 | 5.069 | 0.197 | 6.377 | 0 | SCP2 |
| TRINITY_DN2882_c0_g1_i1 | 0.544231905 | 0.624087829 | 0.999970678 | 0.881 | 2.237 | 0.744 | 1.216 | NA |
| TRINITY_DN6717_c2_g1_i10 | 0.544154025 | 0.848769419 | 1 | 0.581 | 0.168 | 0 | 0.533 | NA |
| TRINITY_DN9318_c0_g1_i1 | -0.5441411 | 0.56111645 | 0.999970678 | 0.59 | 0.779 | 1.033 | 0.754 | NA |
| TRINITY_DN1452_c0_g4_i1 | -0.54400027 | 0.619531109 | 0.999970678 | 2.707 | 1.124 | 2.1 | 3.076 | NA |
| TRINITY_DN9148_c0_g1_i1 | -0.54398099 | 0.507827874 | 0.999970678 | 0.836 | 0.995 | 1.067 | 1.357 | Coilin_N |
| TRINITY_DN1872_c1_g2_i2 | -0.5437058 | 0.846539408 | 0.999970678 | 164.52 | 0.581 | 0.289 | 244.818 | NA |
| TRINITY_DN2251_c0_g1_i11 | -0.54367359 | 0.848612207 | 0.999970678 | 0.763 | 0 | 0.789 | 0.171 | NA |
| TRINITY_DN3288_c0_g1_i7 | 0.543601384 | 0.856237412 | 0.999970678 | 3.17 | 0.306 | 2 | 0 | NA |
| TRINITY_DN387_c0_g1_i5 | -0.54354719 | 0.565513137 | 0.999970678 | 42.231 | 28.13 | 23.376 | 74.066 | NA |
| TRINITY_DN885_c0_g1_i6 | 0.543443369 | 0.757981827 | 0.999970678 | 15.633 | 0.867 | 8.011 | 1.79 | CWC25 |
| TRINITY_DN907_c1_g1_i4 | -0.5434166 | 0.853770308 | 0.999970678 | 1.099 | 0.168 | 0 | 1.84 | NA |
| TRINITY_DN1371_c0_g1_i4 | 0.54338904 | 0.571897173 | 0.999970678 | 1.735 | 1.114 | 0.967 | 0.794 | NA |
| TRINITY_DN249973_c0_g1_i1 | 0.543388648 | 0.660144215 | 0.999970678 | 5.151 | 12.09 | 2.244 | 8.877 | NA |
| TRINITY_DN28485_c0_g1_i1 | -0.54337083 | 0.900631318 | 0.999970678 | 1.19 | 0 | 1.444 | 0 | NA |
| TRINITY_DN222548_c0_g1_i1 | -0.54337083 | 0.900631318 | 0.999970678 | 1.681 | 0 | 2.055 | 0 | NA |
| TRINITY_DN6309_c0_g1_i1 | 0.543368493 | 0.647210129 | 0.999970678 | 1.072 | 4.898 | 1.455 | 2.312 | NA |
| TRINITY_DN276_c0_g1_i2 | 0.543335341 | 0.568145815 | 0.999970678 | 36.772 | 14.49 | 17.421 | 14.226 | NA |
| TRINITY_DN4069_c0_g1_i1 | 0.543321917 | 0.636502114 | 0.999970678 | 3.561 | 6.613 | 1.155 | 5.59 | ECH_2 |
| TRINITY_DN4069_c0_g1_i2 | 0.543321917 | 0.636502114 | 0.999970678 | 3.561 | 6.613 | 1.155 | 5.59 | ECH_2 |
| TRINITY_DN97540_c0_g1_i1 | -0.54328536 | 0.492652766 | 0.999970678 | 8.857 | 15.4 | 11.322 | 21.565 | 2-Hacid_dh |
| TRINITY_DN668_c0_g1_i19 | 0.543146411 | 0.792783704 | 0.999970678 | 2.207 | 0.877 | 1.744 | 0.03 | NA |
| TRINITY_DN13476_c0_g1_i1 | 0.543092133 | 0.900945748 | 0.999970678 | 20.966 | 0 | 12.066 | 0 | NA |
| TRINITY_DN2820_c0_g1_i9 | -0.54301835 | 0.67000671 | 0.999970678 | 1.29 | 3.646 | 3.122 | 3.408 | NA |
| TRINITY_DN6796_c0_g1_i3 | -0.54296032 | 0.455314844 | 0.999970678 | 1.235 | 1.409 | 1.911 | 1.558 | AIG1 |
| TRINITY_DN34574_c0_g1_i12 | -0.54273537 | 0.901238379 | 0.999970678 | 1.453 | 0.02 | 1.778 | 0 | NA |
| TRINITY_DN1653_c0_g1_i1 | 0.542734441 | 0.591350699 | 0.999970678 | 8.257 | 3.39 | 4.333 | 2.815 | NA |
| TRINITY_DN11564_c0_g1_i1 | 0.542695158 | 0.719164887 | 0.999970678 | 0.627 | 0.591 | 0.6 | 0.111 | NA |
| TRINITY_DN11664_c0_g1_i1 | 0.542683425 | 0.515447136 | 0.999970678 | 2.498 | 1.991 | 0.955 | 1.93 | NA |
| TRINITY_DN18748_c0_g1_i3 | -0.54266566 | 0.693665351 | 0.999970678 | 6.822 | 1.646 | 8.411 | 2.292 | Pkinase |
| TRINITY_DN502_c0_g2_i3 | -0.54250449 | 0.501610073 | 0.999970678 | 8.239 | 14.4 | 10.366 | 20.329 | Tubulin |
| TRINITY_DN2740_c1_g1_i8 | -0.54244037 | 0.586620693 | 0.999970678 | 3.688 | 1.468 | 2.489 | 4.554 | Lectin_legB |
| TRINITY_DN16973_c0_g3_i1 | -0.5424197 | 0.562560617 | 0.999970678 | 19.694 | 14.1 | 12.755 | 32.695 | NA |
| TRINITY_DN50289_c0_g1_i10 | -0.54235588 | 0.905692537 | 1 | 0 | 0.306 | 0 | 0.442 | ANAPC4 |
| TRINITY_DN5082_c0_g1_i1 | -0.54224889 | 0.569089155 | 0.999970678 | 19.612 | 19.64 | 11.888 | 42.869 | LEA_2 |
| TRINITY_DN385_c0_g1_i4 | 0.542234399 | 0.59655094 | 0.999970678 | 0.645 | 0.325 | 0.322 | 0.292 | RPAP1_C |
| TRINITY_DN27220_c0_g1_i1 | -0.54223211 | 0.614815393 | 0.999970678 | 48.118 | 18.93 | 36.253 | 49.032 | NA |
| TRINITY_DN8109_c0_g1_i1 | -0.5421991 | 0.707888501 | 0.999970678 | 2.135 | 1.094 | 3.3 | 0.724 | NA |
| TRINITY_DN3824_c0_g1_i2 | 0.542116387 | 0.656781079 | 0.999970678 | 0.736 | 2.641 | 0.533 | 1.669 | DHHC |
| TRINITY_DN3542_c0_g1_i1 | -0.54209713 | 0.667794909 | 0.999970678 | 16.624 | 61.17 | 18.877 | 89.377 | NA |
| TRINITY_DN15909_c0_g1_i5 | 0.542095621 | 0.495396815 | 0.999970678 | 1.481 | 2.099 | 1.167 | 1.056 | Helicase_C |
| TRINITY_DN11977_c0_g1_i1 | -0.54205623 | 0.720306762 | 0.999970678 | 0.154 | 0.976 | 0.411 | 1.156 | Inhibitor_I9 |
| TRINITY_DN15932_c0_g1_i1 | -0.54200837 | 0.898914783 | 0.999970678 | 2.961 | 0 | 3.611 | 0 | NA |
| TRINITY_DN8748_c0_g1_i1 | 0.541984987 | 0.634242457 | 0.999970678 | 0.772 | 1.528 | 0.922 | 0.462 | NA |
| TRINITY_DN6337_c0_g1_i3 | -0.54197678 | 0.6343701 | 0.999970678 | 2.625 | 0.946 | 1.633 | 3.267 | NA |
| TRINITY_DN22093_c0_g1_i2 | -0.54193207 | 0.543163534 | 0.999970678 | 56.584 | 57.46 | 37.887 | 120.122 | NA |
| TRINITY_DN604_c0_g1_i3 | -0.54192789 | 0.723067681 | 0.999970678 | 246.45 | 35.4 | 51.697 | 334.205 | NA |
| TRINITY_DN1624_c0_g1_i3 | -0.54191372 | 0.604088134 | 0.999970678 | 4.678 | 6.327 | 10.177 | 3.69 | NA |
| TRINITY_DN9296_c0_g1_i1 | -0.54181193 | 0.846875991 | 0.999970678 | 0.336 | 0.394 | 0.889 | 0 | Transket_pyr |
| TRINITY_DN876_c0_g1_i2 | -0.54174214 | 0.574030879 | 0.999970678 | 11.319 | 19.59 | 10.188 | 32.544 | Aminotran_5 |
| TRINITY_DN2205_c0_g1_i3 | 0.541736589 | 0.897592871 | 0.999970678 | 5.251 | 0 | 0 | 3.67 | NA |
| TRINITY_DN2515_c0_g1_i6 | 0.541704297 | 0.688846909 | 0.999970678 | 0.954 | 1.045 | 0.844 | 0.332 | NA |
| TRINITY_DN5750_c1_g1_i1 | -0.54169415 | 0.622534208 | 0.999970678 | 35.064 | 44.69 | 77.495 | 21.977 | NA |
| TRINITY_DN5750_c1_g2_i1 | -0.54169415 | 0.622534208 | 0.999970678 | 35.064 | 44.69 | 77.495 | 21.977 | DUF4079 |
| TRINITY_DN2224_c0_g1_i1 | 0.541653797 | 0.443302326 | 0.999970678 | 2.462 | 2.198 | 1.367 | 1.558 | Pkinase |
| TRINITY_DN12417_c0_g1_i8 | -0.54163722 | 0.853546529 | 1 | 0.236 | 0.089 | 0.4 | 0 | DEAD |
| TRINITY_DN11442_c0_g1_i5 | 0.541514033 | 0.561325773 | 0.999970678 | 0.636 | 1.005 | 0.489 | 0.523 | NA |
| TRINITY_DN4180_c0_g3_i1 | 0.541513787 | 0.847332051 | 0.999970678 | 22.71 | 5.874 | 0 | 19.756 | NA |
| TRINITY_DN255871_c0_g1_i1 | 0.54150124 | 0.753943096 | 0.999970678 | 6.004 | 0.434 | 2.9 | 0.965 | NA |
| TRINITY_DN17044_c0_g1_i1 | -0.54150085 | 0.792683345 | 0.999970678 | 511.09 | 6.544 | 499.536 | 123.49 | NA |
| TRINITY_DN9110_c0_g1_i2 | -0.54145751 | 0.443369766 | 0.999970678 | 6.886 | 4.78 | 7.6 | 7.862 | NA |
| TRINITY_DN45052_c0_g1_i1 | 0.541424968 | 0.785504883 | 1 | 1.435 | 10.92 | 0.722 | 6.887 | Glyco_transf_5 |

| TRINITY_DN2253_c0_g1_i1 | -0.54137679 | 0.787084955 | 1 | 0.273 | 0.749 | 0.067 | 1.347 | TPX2 |
| --- | --- | --- | --- | --- | --- | --- | --- | --- |
| TRINITY_DN13993_c0_g1_i1 | 0.54136817 | 0.898737793 | 0.999970678 | 7.049 | 0 | 4.022 | 0 | NA |
| TRINITY_DN164357_c0_g1_i1 | 0.541245737 | 0.903123668 | 1 | 2.28 | 0 | 1.311 | 0 | Porin_3 |
| TRINITY_DN74014_c0_g2_i2 | 0.541245737 | 0.903123668 | 1 | 1.735 | 0 | 1 | 0 | EF1G |
| TRINITY_DN6596_c2_g3_i3 | 0.541245737 | 0.903123668 | 1 | 2.244 | 0 | 1.289 | 0 | NA |
| TRINITY_DN14762_c0_g1_i1 | 0.541245737 | 0.903123668 | 1 | 0.345 | 0 | 0.2 | 0 | DIL |
| TRINITY_DN6727_c0_g1_i1 | -0.54122651 | 0.558763171 | 0.999970678 | 9.22 | 7.244 | 5.477 | 17.433 | NA |
| TRINITY_DN6556_c0_g1_i3 | 0.541223571 | 0.84232896 | 1 | 1.962 | 2.375 | 0 | 2.865 | Mur_ligase |
| TRINITY_DN6238_c0_g1_i4 | 0.541185301 | 0.534838289 | 0.999970678 | 4.424 | 3.735 | 1.755 | 3.438 | NA |
| TRINITY_DN1215_c0_g1_i9 | 0.541172686 | 0.673838018 | 0.999970678 | 0.4 | 0.986 | 0.156 | 0.764 | TFIIIC_delta |
| TRINITY_DN257_c2_g2_i5 | -0.54113169 | 0.905995177 | 1 | 0.699 | 0 | 0 | 1.015 | Sua5_yciO_yrdC |
| TRINITY_DN140357_c0_g1_i1 | -0.54101474 | 0.558663333 | 0.999970678 | 4.306 | 8.958 | 5.477 | 12.587 | PPR |
| TRINITY_DN10693_c0_g1_i16 | -0.54100085 | 0.902874929 | 1 | 1.163 | 0 | 1.422 | 0 | NA |
| TRINITY_DN7654_c0_g1_i6 | 0.5409989 | 0.692450511 | 1 | 0.781 | 0.67 | 0.211 | 0.724 | NA |
| TRINITY_DN13085_c0_g1_i1 | -0.54099622 | 0.675764786 | 0.999970678 | 2.107 | 3.351 | 0.989 | 6.736 | EamA |
| TRINITY_DN16050_c0_g1_i1 | -0.54088388 | 0.599526199 | 0.999970678 | 8.575 | 11.05 | 5.955 | 20.972 | - |
| TRINITY_DN2194_c0_g1_i4 | 0.540839481 | 0.856607823 | 0.999970678 | 40.106 | 16.17 | 0 | 39.149 | NA |
| TRINITY_DN1358_c0_g1_i2 | 0.540821348 | 0.772617325 | 0.999970678 | 2.035 | 2.622 | 2.578 | 0.08 | HECT |
| TRINITY_DN5948_c0_g3_i2 | -0.54081132 | 0.602786829 | 0.999970678 | 12.972 | 8.761 | 20.343 | 7.158 | Peptidase_S9 |
| TRINITY_DN35312_c0_g2_i2 | 0.540807263 | 0.742000051 | 0.999970678 | 1.344 | 0.187 | 0.656 | 0.282 | NA |
| TRINITY_DN452_c1_g2_i5 | 0.540781415 | 0.575995619 | 0.999970678 | 9.493 | 18.54 | 11.244 | 5.59 | NA |
| TRINITY_DN9941_c0_g1_i2 | -0.54071577 | 0.433518508 | 0.999970678 | 7.73 | 10.29 | 12.121 | 11.431 | Pro_isomerase |
| TRINITY_DN8836_c0_g1_i3 | -0.54065831 | 0.542241028 | 0.999970678 | 49.716 | 26.51 | 31.598 | 72.668 | NA |
| TRINITY_DN4587_c0_g1_i10 | -0.5406574 | 0.473290827 | 0.999970678 | 13.335 | 20.75 | 20.11 | 24.3 | NA |
| TRINITY_DN507_c0_g1_i3 | 0.54061902 | 0.390580899 | 0.999970678 | 19.848 | 26.54 | 11.766 | 17.705 | NA |
| TRINITY_DN943_c0_g1_i4 | 0.540562717 | 0.497254171 | 0.999970678 | 19.013 | 17.68 | 6.922 | 16.92 | NA |
| TRINITY_DN4697_c0_g1_i1 | 0.540521348 | 0.526021144 | 0.999970678 | 38.007 | 34.72 | 29.009 | 14.99 | NA |
| TRINITY_DN1966_c0_g1_i5 | 0.540472592 | 0.665651901 | 0.999970678 | 10.683 | 2.346 | 4.222 | 3.841 | NA |
| TRINITY_DN7614_c0_g1_i12 | -0.54044074 | 0.664900839 | 1 | 0.363 | 0.227 | 0.4 | 0.362 | NA |
| TRINITY_DN13476_c2_g2_i7 | 0.540411039 | 0.827353319 | 1 | 0.908 | 0.02 | 0.5 | 0.05 | NA |
| TRINITY_DN1912_c0_g1_i4 | 0.540333444 | 0.648150395 | 0.999970678 | 12.69 | 2.72 | 4.822 | 4.876 | NA |
| TRINITY_DN834_c0_g1_i25 | -0.54016751 | 0.846770783 | 0.999970678 | 0 | 9.126 | 1.855 | 10.667 | NA |
| TRINITY_DN19947_c0_g1_i1 | 0.540152303 | 0.477910392 | 0.999970678 | 3.852 | 4.238 | 2.978 | 1.981 | NA |
| TRINITY_DN1045_c0_g1_i1 | -0.5401414 | 0.749074142 | 0.999970678 | 1.917 | 13.06 | 15.866 | 2.162 | NA |
| TRINITY_DN1908_c0_g1_i2 | -0.54013056 | 0.642702999 | 0.999970678 | 2.226 | 0.552 | 1.567 | 2.202 | NA |
| TRINITY_DN6144_c0_g1_i3 | 0.540116053 | 0.485330283 | 0.999970678 | 2.398 | 2.484 | 1.2 | 1.9 | NA |
| TRINITY_DN22063_c0_g1_i1 | -0.54003788 | 0.687804827 | 0.999970678 | 12.454 | 4.819 | 12.321 | 9.089 | NA |
| TRINITY_DN536_c0_g1_i2 | 0.540023599 | 0.632371855 | 0.999970678 | 0.472 | 1.754 | 0.611 | 0.784 | NA |
| TRINITY_DN1694_c0_g1_i1 | 0.540006707 | 0.564306593 | 0.999970678 | 2.489 | 5.44 | 2.322 | 2.624 | Memo |
| TRINITY_DN2936_c0_g2_i2 | 0.539964527 | 0.898681262 | 0.999970678 | 8.412 | 0 | 0 | 5.902 | NA |
| TRINITY_DN1320_c0_g1_i5 | -0.53989871 | 0.870804895 | 1 | 0.154 | 3.577 | 4.444 | 0 | NA |
| TRINITY_DN2005_c0_g1_i3 | -0.53989111 | 0.869419188 | 1 | 0.055 | 1.153 | 0 | 1.709 | Aldo_ket_red |
| TRINITY_DN857_c0_g2_i4 | -0.53985716 | 0.693706918 | 0.999970678 | 0.59 | 1.912 | 1 | 2.413 | NA |
| TRINITY_DN13914_c0_g2_i3 | -0.53977403 | 0.903976783 | 1 | 2.353 | 0 | 2.833 | 0 | NA |
| TRINITY_DN8335_c0_g1_i5 | -0.53977403 | 0.903976783 | 1 | 0.554 | 0 | 0.678 | 0 | NA |
| TRINITY_DN393_c1_g3_i1 | 0.539745077 | 0.865427577 | 1 | 11.936 | 0 | 6.244 | 0.553 | NA |
| TRINITY_DN2466_c1_g1_i3 | -0.53974201 | 0.354223206 | 0.999970678 | 41.514 | 39.93 | 44.464 | 64.696 | UDPGT |
| TRINITY_DN11335_c0_g1_i6 | -0.53956697 | 0.669782551 | 0.999970678 | 0.218 | 0.325 | 0.444 | 0.241 | - |
| TRINITY_DN11335_c0_g1_i7 | 0.539523523 | 0.68877784 | 0.999970678 | 0.427 | 0.375 | 0.333 | 0.141 | - |
| TRINITY_DN282_c0_g1_i4 | 0.539391809 | 0.630887891 | 0.999970678 | 1.226 | 0.897 | 0.878 | 0.412 | - |
| TRINITY_DN665_c1_g1_i13 | 0.539374725 | 0.424837845 | 0.999970678 | 264.47 | 172.8 | 123.026 | 148.885 | - |
| TRINITY_DN5338_c0_g1_i1 | -0.539361 | 0.769434673 | 1 | 2.126 | 7.46 | 1.378 | 11.25 | NA |
| TRINITY_DN5617_c0_g1_i4 | -0.5392987 | 0.463717617 | 0.999970678 | 4.333 | 6.238 | 5.1 | 9.179 | Lectin_legB |
| TRINITY_DN343_c1_g1_i2 | -0.53929255 | 0.653469791 | 0.999970678 | 1.036 | 0.995 | 0.8 | 1.971 | NA |
| TRINITY_DN5246_c0_g1_i4 | 0.539282751 | 0.645147554 | 0.999970678 | 3.261 | 7.263 | 1.267 | 5.69 | COPIIcoated_ERV |
| TRINITY_DN950_c0_g1_i2 | -0.53922636 | 0.585933754 | 0.999970678 | 4.624 | 8.16 | 10.755 | 5.459 | Mito_carr |
| TRINITY_DN176_c1_g1_i13 | 0.539203088 | 0.905428859 | 1 | 2.516 | 0 | 0 | 1.749 | NA |
| TRINITY_DN8010_c0_g2_i1 | -0.53912204 | 0.633223046 | 0.999970678 | 1.572 | 6.564 | 5.566 | 4.997 | Glyco_hydro_17 |
| TRINITY_DN9974_c0_g1_i8 | -0.53908223 | 0.90057208 | 0.999970678 | 3.216 | 0 | 3.944 | 0 | NA |
| TRINITY_DN27402_c0_g1_i1 | -0.53908223 | 0.90057208 | 0.999970678 | 1.681 | 0 | 2.033 | 0 | NA |
| TRINITY_DN8992_c0_g1_i1 | -0.53904304 | 0.708991544 | 0.999970678 | 154.56 | 13.3 | 84.75 | 144.23 | NA |
| TRINITY_DN245_c0_g1_i1 | 0.539040936 | 0.726832364 | 0.999970678 | 1.045 | 6.741 | 1.367 | 3.559 | Fe-S_biosyn |
| TRINITY_DN489_c0_g1_i13 | -0.53900207 | 0.849960493 | 0.999970678 | 0 | 19.83 | 14.132 | 11.29 | NA |
| TRINITY_DN6979_c0_g1_i3 | -0.53896104 | 0.902104604 | 0.999970678 | 0.699 | 0 | 0 | 1.036 | NA |
| TRINITY_DN17835_c2_g4_i1 | -0.53893047 | 0.858338405 | 0.999970678 | 1.671 | 0 | 0.144 | 2.282 | NA |
| TRINITY_DN95804_c0_g1_i6 | -0.53881785 | 0.846344859 | 0.999970678 | 8.775 | 0 | 6.744 | 4.816 | NA |
| TRINITY_DN2775_c0_g1_i4 | 0.538695523 | 0.662043422 | 1 | 2.198 | 2.484 | 1.055 | 1.89 | GST_C |
| TRINITY_DN4450_c0_g1_i2 | -0.53865992 | 0.776122453 | 0.999970678 | 1.635 | 0.739 | 0.122 | 3.298 | NA |
| TRINITY_DN7014_c0_g1_i2 | 0.538658851 | 0.485514836 | 0.999970678 | 20.33 | 19.78 | 7.777 | 18.288 | NA |
| TRINITY_DN1848_c0_g1_i8 | -0.5385296 | 0.741071307 | 0.999970678 | 4.696 | 1.468 | 6.644 | 0.975 | NA |
| TRINITY_DN892_c0_g2_i3 | -0.53852826 | 0.773556139 | 0.999970678 | 7.531 | 0.296 | 1.411 | 9.833 | NA |
| TRINITY_DN5159_c0_g1_i2 | 0.538481904 | 0.584766153 | 0.999970678 | 1.463 | 2.03 | 0.567 | 1.729 | BT1 |
| TRINITY_DN5048_c0_g1_i1 | -0.53847789 | 0.68292979 | 0.999970678 | 93.992 | 28.93 | 124.426 | 29.357 | NA |
| TRINITY_DN3457_c0_g1_i1 | -0.53844803 | 0.650426973 | 0.999970678 | 6.322 | 19.39 | 23.743 | 8.345 | ABC2_membrane |

| TRINITY_DN4607_c0_g1_i1 | -0.53836964 | 0.71057906 | 0.999970678 | 2.126 | 0.838 | 2.644 | 1.096 | Pirin |
| --- | --- | --- | --- | --- | --- | --- | --- | --- |
| TRINITY_DN14048_c0_g1_i1 | -0.53832773 | 0.842713407 | 0.999970678 | 2.117 | 0 | 1.411 | 1.387 | EF-hand_7 |
| TRINITY_DN1334_c0_g1_i13 | 0.538277069 | 0.472541229 | 0.999970678 | 10.883 | 15.94 | 9.044 | 7.45 | NA |
| TRINITY_DN225351_c0_g1_i1 | 0.538259497 | 0.905920087 | 1 | 1.036 | 0 | 0.6 | 0 | NA |
| TRINITY_DN22391_c0_g1_i1 | 0.538259497 | 0.905920087 | 1 | 3.288 | 0 | 1.889 | 0 | Ribosomal_L13e |
| TRINITY_DN25722_c0_g2_i1 | 0.538259497 | 0.905920087 | 1 | 1.472 | 0 | 0.856 | 0 | ADH_N |
| TRINITY_DN3955_c0_g1_i6 | 0.538246728 | 0.62998258 | 0.999970678 | 8.748 | 5.815 | 6.655 | 2.031 | LRR_6 |
| TRINITY_DN25487_c0_g1_i11 | -0.53821597 | 0.901072496 | 0.999970678 | 2.934 | 0 | 3.566 | 0 | NA |
| TRINITY_DN46095_c0_g1_i1 | -0.53821597 | 0.901072496 | 0.999970678 | 1.78 | 0 | 2.167 | 0 | - |
| TRINITY_DN20563_c0_g1_i2 | -0.53815382 | 0.905380691 | 1 | 0.136 | 0 | 0.167 | 0 | DUF3444 |
| TRINITY_DN907_c2_g1_i1 | 0.538144779 | 0.514786042 | 0.999970678 | 4.796 | 4.336 | 3.555 | 2.011 | NA |
| TRINITY_DN1036_c1_g2_i2 | -0.53811437 | 0.846362299 | 0.999970678 | 2.544 | 0 | 0.689 | 2.936 | NA |
| TRINITY_DN1873_c0_g1_i1 | 0.538080137 | 0.735174214 | 0.999970678 | 17.378 | 12.81 | 14.299 | 2.775 | NA |
| TRINITY_DN27798_c1_g1_i3 | 0.538059324 | 0.903014873 | 0.999970678 | 2.816 | 0 | 1.655 | 0 | NA |
| TRINITY_DN29093_c0_g1_i1 | 0.538059324 | 0.903014873 | 0.999970678 | 4.506 | 0 | 2.589 | 0 | NA |
| TRINITY_DN199_c0_g1_i9 | -0.53791174 | 0.551045871 | 0.999970678 | 5.677 | 8.594 | 5.177 | 14.427 | NA |
| TRINITY_DN1316_c0_g1_i7 | -0.53787968 | 0.690941308 | 0.999970678 | 18.604 | 3.124 | 19.799 | 8.003 | LRRNT_2 |
| TRINITY_DN5171_c0_g1_i6 | -0.53787783 | 0.782973452 | 1 | 0.082 | 1.183 | 1.111 | 0.473 | NA |
| TRINITY_DN492_c0_g1_i7 | 0.537850346 | 0.843034327 | 0.999970678 | 0.491 | 1.705 | 0 | 1.518 | NA |
| TRINITY_DN1960_c0_g1_i2 | -0.53781499 | 0.657553964 | 0.999970678 | 3.406 | 10.31 | 3.455 | 15.644 | NA |
| TRINITY_DN5016_c0_g1_i2 | -0.53780808 | 0.648415955 | 0.999970678 | 3.198 | 5.282 | 2.155 | 9.611 | NA |
| TRINITY_DN8577_c0_g2_i1 | -0.53778456 | 0.459117725 | 0.999970678 | 98.116 | 114.1 | 162.646 | 110.862 | DUF2301 |
| TRINITY_DN297_c1_g1_i6 | 0.53778412 | 0.581018631 | 0.999970678 | 29.459 | 13.2 | 16.488 | 9.601 | NA |
| TRINITY_DN6867_c0_g1_i3 | 0.537776292 | 0.744315841 | 0.999970678 | 0.954 | 0.118 | 0.167 | 0.553 | TCP |
| TRINITY_DN1583_c1_g1_i1 | -0.53765302 | 0.627666386 | 0.999970678 | 2.598 | 2.503 | 4.844 | 1.548 | NA |
| TRINITY_DN334_c0_g1_i5 | 0.537648473 | 0.401997495 | 0.999970678 | 58.41 | 69.62 | 31.109 | 49.615 | NA |
| TRINITY_DN4297_c0_g1_i1 | 0.537624132 | 0.654123046 | 0.999970678 | 3.497 | 11 | 1.844 | 7.721 | AA_permease_2 |
| TRINITY_DN3590_c1_g1_i2 | -0.53758722 | 0.492971194 | 0.999970678 | 0.718 | 0.887 | 0.944 | 1.176 | NA |
| TRINITY_DN3253_c0_g1_i1 | 0.537563295 | 0.396399844 | 0.999970678 | 15.452 | 22.57 | 10.988 | 12.919 | ABC_membrane_ |
| TRINITY_DN189_c2_g1_i4 | 0.537553847 | 0.764755891 | 0.999970678 | 0.173 | 1.547 | 0.156 | 0.985 | NA |
| TRINITY_DN3087_c0_g1_i1 | -0.53754386 | 0.581720714 | 0.999970678 | 2.889 | 4.671 | 2.822 | 7.51 | NA |
| TRINITY_DN27050_c0_g2_i1 | -0.53750647 | 0.525525105 | 0.999970678 | 4.933 | 10.72 | 7.755 | 13.281 | Arm |
| TRINITY_DN5501_c0_g1_i2 | 0.537502621 | 0.421691805 | 0.999970678 | 13.462 | 9.609 | 6.455 | 8.164 | NA |
| TRINITY_DN14982_c0_g1_i11 | 0.537443227 | 0.784125024 | 1 | 0.736 | 4.08 | 2.378 | 0.342 | NA |
| TRINITY_DN24099_c0_g1_i1 | -0.53743586 | 0.651220886 | 0.999970678 | 0.445 | 0.296 | 0.322 | 0.704 | WD40 |
| TRINITY_DN299_c0_g1_i2 | -0.5374343 | 0.592279929 | 0.999970678 | 1.635 | 2.533 | 3.422 | 1.94 | Aldo_ket_red |
| TRINITY_DN21218_c0_g1_i5 | 0.537319501 | 0.487554313 | 0.999970678 | 1.88 | 3.203 | 1.533 | 1.659 | RTT107_BRCT_5 |
| TRINITY_DN17360_c0_g1_i2 | 0.537234864 | 0.689124783 | 0.999970678 | 0.618 | 3.075 | 1.211 | 1.056 | NA |
| TRINITY_DN5651_c3_g1_i2 | 0.537214557 | 0.522073102 | 0.999970678 | 1.635 | 1.449 | 0.789 | 1.186 | CCT |
| TRINITY_DN5096_c8_g2_i1 | -0.537166 | 0.843702191 | 0.999970678 | 9.747 | 0 | 6.877 | 5.61 | NA |
| TRINITY_DN5096_c8_g1_i1 | -0.537166 | 0.843702191 | 0.999970678 | 9.747 | 0 | 6.877 | 5.61 | NA |
| TRINITY_DN2566_c0_g1_i2 | 0.537130526 | 0.620706897 | 0.999970678 | 6.341 | 14.3 | 3.3 | 10.084 | NA |
| TRINITY_DN397_c0_g1_i4 | -0.53711405 | 0.637816388 | 0.999970678 | 0.754 | 0.266 | 0.756 | 0.583 | PUNUT |
| TRINITY_DN3898_c1_g1_i3 | -0.5371087 | 0.905176111 | 1 | 0 | 0.266 | 0.322 | 0 | NA |
| TRINITY_DN612_c0_g1_i7 | 0.537054686 | 0.528519888 | 0.999970678 | 0.799 | 0.976 | 0.411 | 0.724 | - |
| TRINITY_DN21656_c0_g1_i2 | -0.53704369 | 0.474857909 | 0.999970678 | 1.626 | 1.202 | 1.655 | 2.131 | NA |
| TRINITY_DN40366_c2_g1_i2 | -0.53699953 | 0.866685856 | 1 | 1.172 | 0.108 | 1.555 | 0 | NifU_N |
| TRINITY_DN11661_c0_g1_i9 | 0.536967435 | 0.589820467 | 0.999970678 | 32.784 | 18.93 | 21.199 | 9.933 | NA |
| TRINITY_DN3022_c0_g1_i2 | -0.53692216 | 0.69048409 | 0.999970678 | 0.327 | 1.557 | 0.822 | 1.749 | - |
| TRINITY_DN155_c2_g1_i3 | 0.536916309 | 0.624201123 | 0.999970678 | 1.699 | 3.834 | 0.955 | 2.644 | PsbP |
| TRINITY_DN7654_c0_g1_i5 | -0.5368948 | 0.591449774 | 0.999970678 | 1.853 | 1.055 | 1.411 | 2.503 | NA |
| TRINITY_DN3009_c0_g1_i3 | -0.53683726 | 0.452866765 | 0.999970678 | 6.35 | 6.465 | 5.889 | 11.511 | WD40 |
| TRINITY_DN342_c0_g1_i1 | -0.53681399 | 0.843204342 | 0.999970678 | 4.596 | 0 | 3.3 | 2.755 | NA |
| TRINITY_DN4144_c0_g1_i1 | 0.536793791 | 0.489913724 | 0.999970678 | 14.616 | 11.8 | 9.799 | 6.424 | NA |
| TRINITY_DN1150_c0_g1_i13 | -0.5367596 | 0.685032202 | 0.999970678 | 5.278 | 4.671 | 2.978 | 10.154 | NA |
| TRINITY_DN3235_c0_g1_i1 | -0.53671505 | 0.738323729 | 0.999970678 | 0.327 | 2.493 | 1.889 | 1.749 | NA |
| TRINITY_DN5922_c0_g2_i1 | 0.536698073 | 0.708887517 | 0.999970678 | 0.336 | 1.321 | 0.678 | 0.312 | NA |
| TRINITY_DN4860_c0_g1_i2 | -0.5366787 | 0.628601804 | 0.999970678 | 54.64 | 16.41 | 26.854 | 71.029 | NA |
| TRINITY_DN8732_c0_g1_i3 | 0.536606055 | 0.808902299 | 0.999970678 | 1.926 | 0.384 | 1.3 | 0.03 | NA |
| TRINITY_DN3590_c1_g1_i1 | -0.53657181 | 0.81843343 | 0.999970678 | 0.1 | 0.917 | 0.022 | 1.448 | NA |
| TRINITY_DN141039_c0_g1_i1 | 0.536533852 | 0.536420818 | 0.999970678 | 2.126 | 2.927 | 1.111 | 2.131 | Hexokinase_1 |
| TRINITY_DN813_c3_g1_i19 | 0.536519815 | 0.628498183 | 0.999970678 | 1.29 | 0.986 | 0.844 | 0.543 | DUF659 |
| TRINITY_DN3190_c1_g1_i2 | 0.536516963 | 0.60128013 | 0.999970678 | 4.042 | 6.347 | 1.689 | 5.087 | NA |
| TRINITY_DN28468_c2_g1_i1 | 0.536486518 | 0.798063531 | 1 | 0.954 | 0.512 | 0.8 | 0.04 | NA |
| TRINITY_DN12856_c0_g4_i1 | -0.53619497 | 0.419517222 | 0.999970678 | 19.93 | 19.58 | 18.866 | 34.595 | RRM_1 |
| TRINITY_DN2528_c0_g1_i3 | -0.53612185 | 0.843165829 | 0.999970678 | 1.054 | 0 | 0.756 | 0.643 | BAH |
| TRINITY_DN3048_c0_g1_i5 | 0.536115708 | 0.504110087 | 0.999970678 | 2.362 | 2.7 | 1 | 2.292 | NA |
| TRINITY_DN41_c3_g1_i1 | -0.53611319 | 0.365989216 | 0.999970678 | 43.757 | 49.19 | 61.107 | 60.463 | NA |
| TRINITY_DN335_c1_g1_i8 | -0.53607237 | 0.663985446 | 0.999970678 | 7.031 | 5.056 | 2.344 | 14.709 | LRRNT_2 |
| TRINITY_DN7844_c0_g1_i2 | -0.53606073 | 0.899803875 | 0.999970678 | 3.243 | 0 | 0 | 4.765 | NA |
| TRINITY_DN534_c0_g1_i4 | -0.53603235 | 0.846866183 | 0.999970678 | 0 | 1.587 | 1.233 | 0.784 | DPPIV_N |
| TRINITY_DN8096_c0_g1_i2 | -0.5359734 | 0.685171368 | 0.999970678 | 26.389 | 7.825 | 7.066 | 41.502 | NA |
| TRINITY_DN622_c0_g1_i1 | -0.53594932 | 0.449774443 | 0.999970678 | 42.913 | 29.28 | 36.087 | 61.388 | NA |
| TRINITY_DN7697_c0_g2_i4 | -0.53593189 | 0.77114385 | 0.999970678 | 436.98 | 12.46 | 103.438 | 506.887 | NA |

| TRINITY_DN7767_c0_g1_i9 | 0.535931886 | 0.905962906 | 1 | 0 | 0.848 | 0.478 | 0 | NA |
| --- | --- | --- | --- | --- | --- | --- | --- | --- |
| TRINITY_DN21683_c0_g1_i3 | 0.535926511 | 0.539399919 | 0.999970678 | 1.817 | 1.587 | 0.756 | 1.438 | NA |
| TRINITY_DN13113_c0_g1_i5 | -0.53591324 | 0.90685925 | 1 | 0.981 | 0 | 1.178 | 0 | NA |
| TRINITY_DN2934_c0_g1_i15 | -0.53591324 | 0.90685925 | 1 | 0.636 | 0 | 0.789 | 0 | - |
| TRINITY_DN2091_c0_g1_i6 | -0.53585287 | 0.844422483 | 0.999970678 | 1.199 | 0.966 | 0 | 3.157 | LRR_6 |
| TRINITY_DN4228_c0_g1_i1 | 0.535826526 | 0.527163836 | 0.999970678 | 6.095 | 4.1 | 3.6 | 2.684 | NA |
| TRINITY_DN9652_c1_g1_i4 | 0.535799963 | 0.657673468 | 0.999970678 | 1.644 | 0.572 | 0.411 | 1.046 | NA |
| TRINITY_DN4292_c0_g1_i17 | -0.53566411 | 0.903464752 | 1 | 0.736 | 0 | 0 | 1.076 | NA |
| TRINITY_DN16272_c0_g1_i1 | -0.53565936 | 0.586795657 | 0.999970678 | 3.979 | 5.401 | 3.222 | 9.682 | NA |
| TRINITY_DN224_c0_g1_i1 | 0.535565482 | 0.403613057 | 0.999970678 | 11.609 | 13.07 | 8.022 | 7.309 | TRM |
| TRINITY_DN18278_c0_g1_i1 | 0.535512017 | 0.775587942 | 0.999970678 | 0.191 | 0.66 | 0.444 | 0.04 | NA |
| TRINITY_DN6881_c0_g1_i9 | 0.535500671 | 0.676304112 | 0.999970678 | 0.618 | 0.404 | 0.444 | 0.181 | NA |
| TRINITY_DN6881_c0_g1_i19 | 0.535500671 | 0.676304112 | 0.999970678 | 0.618 | 0.404 | 0.444 | 0.181 | NA |
| TRINITY_DN14164_c0_g1_i3 | -0.53546586 | 0.769008494 | 1 | 8.185 | 1.951 | 9.911 | 2.483 | NA |
| TRINITY_DN42605_c0_g1_i1 | 0.535444321 | 0.902918013 | 0.999970678 | 0.69 | 0 | 0.4 | 0 | LisH |
| TRINITY_DN2114_c0_g3_i1 | 0.535152149 | 0.675046146 | 0.999970678 | 2.453 | 1.774 | 1.955 | 0.563 | NA |
| TRINITY_DN1091_c1_g1_i3 | -0.53513544 | 0.436402306 | 0.999970678 | 9.32 | 8.081 | 8.377 | 15.181 | ChaC |
| TRINITY_DN70640_c0_g1_i1 | -0.53507663 | 0.61447374 | 0.999970678 | 1.09 | 2.286 | 1.267 | 3.338 | NA |
| TRINITY_DN804_c0_g1_i6 | 0.534946298 | 0.564672063 | 0.999970678 | 8.375 | 6.16 | 5.955 | 2.865 | NA |
| TRINITY_DN484_c0_g1_i6 | -0.53491795 | 0.621881761 | 0.999970678 | 7.767 | 2.74 | 3.978 | 10.536 | NA |
| TRINITY_DN3820_c0_g1_i4 | 0.534917652 | 0.64252904 | 0.999970678 | 5.732 | 23.19 | 5.655 | 12.899 | zf-RING_2 |
| TRINITY_DN2506_c0_g1_i3 | 0.534895171 | 0.758184522 | 0.999970678 | 0.391 | 3.735 | 0.867 | 1.739 | NA |
| TRINITY_DN5922_c0_g1_i4 | -0.53488362 | 0.853454072 | 1 | 0.736 | 0 | 0.722 | 0.201 | FGGY_C |
| TRINITY_DN42381_c0_g1_i1 | 0.534876887 | 0.760831545 | 0.999970678 | 28.296 | 635.4 | 77.14 | 312.972 | NA |
| TRINITY_DN3386_c0_g1_i4 | -0.53487535 | 0.657136949 | 0.999970678 | 0.609 | 2.03 | 1.255 | 2.262 | NA |
| TRINITY_DN5731_c0_g1_i1 | 0.534875343 | 0.619589076 | 0.999970678 | 19.885 | 8.022 | 11.677 | 5.268 | GRAS |
| TRINITY_DN13545_c0_g1_i1 | 0.534868783 | 0.640846581 | 0.999970678 | 11.291 | 2.937 | 2.855 | 6.495 | - |
| TRINITY_DN8022_c0_g1_i1 | 0.534805146 | 0.641324834 | 0.999970678 | 2.707 | 12.72 | 4.911 | 4.615 | NA |
| TRINITY_DN3416_c0_g1_i6 | 0.534772834 | 0.708111851 | 0.999970678 | 0.872 | 0.276 | 0.222 | 0.543 | - |
| TRINITY_DN1184_c0_g1_i7 | -0.53474122 | 0.66937543 | 0.999970678 | 1.735 | 0.522 | 1.933 | 0.995 | NA |
| TRINITY_DN26962_c0_g1_i1 | 0.534686279 | 0.427261029 | 0.999970678 | 174.14 | 223.6 | 90.361 | 164.217 | NA |
| TRINITY_DN9166_c0_g1_i9 | 0.534659716 | 0.775314351 | 1 | 1.317 | 1.084 | 1.278 | 0.111 | NA |
| TRINITY_DN39_c4_g1_i7 | -0.53465066 | 0.866430637 | 0.999970678 | 28.996 | 0 | 28.354 | 8.465 | GATase_2 |
| TRINITY_DN9177_c0_g3_i2 | 0.534642943 | 0.491705294 | 0.999970678 | 0.699 | 0.907 | 0.578 | 0.422 | FYRC |
| TRINITY_DN16_c0_g1_i3 | 0.53463092 | 0.689440484 | 0.999970678 | 566.94 | 78.85 | 251.518 | 144.401 | UDPGT |
| TRINITY_DN7674_c0_g1_i1 | -0.53462244 | 0.384536191 | 0.999970678 | 19.194 | 17.19 | 19.41 | 29.296 | NA |
| TRINITY_DN7755_c0_g1_i3 | 0.53458923 | 0.631758907 | 0.999970678 | 0.936 | 0.848 | 0.733 | 0.342 | NA |
| TRINITY_DN3161_c0_g1_i1 | 0.534564485 | 0.847389887 | 0.999970678 | 0 | 1.271 | 0.278 | 0.533 | RRM_1 |
| TRINITY_DN102186_c0_g1_i1 | -0.53456137 | 0.900234743 | 0.999970678 | 4.033 | 0 | 4.877 | 0 | Esterase |
| TRINITY_DN916_c1_g1_i4 | 0.534551796 | 0.721357652 | 1 | 2.871 | 1.005 | 0.678 | 1.8 | NA |
| TRINITY_DN41168_c1_g1_i1 | 0.534488646 | 0.905462473 | 1 | 1.626 | 0 | 0.944 | 0 | Ribosomal_L14 |
| TRINITY_DN40136_c0_g1_i1 | 0.534488646 | 0.905462473 | 1 | 0.672 | 0 | 0.389 | 0 | Gal_mutarotas_2 |
| TRINITY_DN8299_c0_g2_i3 | 0.534445864 | 0.648143701 | 0.999970678 | 1.717 | 0.71 | 0.533 | 1.046 | NA |
| TRINITY_DN3140_c1_g1_i1 | 0.534372946 | 0.47689889 | 0.999970678 | 136.07 | 75.86 | 54.719 | 81.133 | NA |
| TRINITY_DN1760_c0_g1_i13 | 0.534334918 | 0.847283094 | 0.999970678 | 0.609 | 3.351 | 0 | 2.704 | NA |
| TRINITY_DN11141_c0_g1_i14 | 0.53426437 | 0.741205864 | 1 | 10.764 | 4.021 | 6.566 | 2.041 | NA |
| TRINITY_DN11177_c0_g1_i4 | 0.534245065 | 0.446916731 | 0.999970678 | 5.287 | 5.391 | 2.711 | 4.062 | NA |
| TRINITY_DN190_c0_g1_i5 | -0.5342047 | 0.447779442 | 0.999970678 | 5.977 | 6.978 | 6.144 | 11.331 | CBS |
| TRINITY_DN1333_c1_g1_i13 | -0.53416398 | 0.718483214 | 0.999970678 | 0.5 | 0.818 | 1.322 | 0.332 | NA |
| TRINITY_DN665_c0_g1_i5 | 0.534138185 | 0.690821297 | 0.999970678 | 4.923 | 3.439 | 4.178 | 0.724 | LRR_8 |
| TRINITY_DN29562_c0_g1_i2 | -0.53410516 | 0.630702572 | 0.999970678 | 5.341 | 5.736 | 6.022 | 7.973 | NA |
| TRINITY_DN10109_c0_g2_i1 | 0.534021691 | 0.480815654 | 0.999970678 | 4.142 | 6.82 | 3.378 | 3.448 | YTH |
| TRINITY_DN3307_c0_g2_i2 | 0.533909822 | 0.90041428 | 0.999970678 | 5.114 | 0 | 0 | 3.549 | NA |
| TRINITY_DN142525_c0_g1_i1 | 0.533844617 | 0.762555458 | 0.999970678 | 1.117 | 4.12 | 0.311 | 3.157 | - |
| TRINITY_DN4485_c0_g1_i8 | -0.53382383 | 0.795705489 | 0.999970678 | 0.972 | 0.089 | 1.167 | 0.131 | NA |
| TRINITY_DN3032_c0_g1_i6 | 0.533743126 | 0.717827675 | 0.999970678 | 0.59 | 0.71 | 0.633 | 0.121 | NA |
| TRINITY_DN3240_c0_g1_i7 | 0.533655877 | 0.619688493 | 0.999970678 | 1.862 | 1.656 | 0.5 | 1.83 | NA |
| TRINITY_DN5852_c0_g1_i7 | 0.533567751 | 0.593062097 | 0.999970678 | 4.733 | 5.233 | 2.055 | 4.263 | NA |
| TRINITY_DN2174_c0_g1_i5 | 0.533465709 | 0.515629413 | 0.999970678 | 2.135 | 2.661 | 1.1 | 1.971 | NA |
| TRINITY_DN3541_c1_g1_i7 | -0.53342217 | 0.484026584 | 0.999970678 | 59.918 | 31.78 | 56.43 | 65.299 | Glyco_hydro_32C |
| TRINITY_DN14203_c0_g1_i1 | 0.533406475 | 0.505423666 | 0.999970678 | 1.363 | 1.488 | 0.8 | 0.995 | NA |
| TRINITY_DN8463_c0_g2_i1 | 0.53332662 | 0.766601353 | 1 | 0.409 | 1.764 | 0.233 | 1.186 | NA |
| TRINITY_DN8530_c0_g1_i2 | 0.533304925 | 0.906165256 | 0.999970678 | 52.46 | 0 | 0 | 36.977 | HD_4 |
| TRINITY_DN2657_c0_g4_i2 | 0.53323891 | 0.48228543 | 0.999970678 | 3.606 | 4.277 | 2.778 | 2.101 | NA |
| TRINITY_DN1703_c2_g1_i1 | -0.53323654 | 0.677428862 | 0.999970678 | 3.634 | 18.38 | 6.933 | 23.174 | Acyltransferase |
| TRINITY_DN5958_c1_g1_i1 | -0.53320371 | 0.484934292 | 0.999970678 | 6.877 | 5.263 | 7.522 | 8.375 | NA |
| TRINITY_DN268_c0_g2_i1 | -0.53318535 | 0.337058342 | 0.999970678 | 63.724 | 65.88 | 76.706 | 94.143 | Ribosomal_S6e |
| TRINITY_DN41_c0_g1_i15 | 0.533170178 | 0.841129334 | 0.999970678 | 0.845 | 0.936 | 0 | 1.227 | NA |
| TRINITY_DN3102_c0_g1_i10 | -0.53315075 | 0.872818564 | 1 | 21.275 | 0.798 | 25.91 | 0 | NA |
| TRINITY_DN2029_c0_g1_i2 | 0.533121428 | 0.350957371 | 0.999970678 | 9.874 | 10.53 | 5.8 | 7.118 | PDZ_1 |
| TRINITY_DN5379_c0_g1_i3 | -0.53310702 | 0.637728113 | 0.999970678 | 0.79 | 2.257 | 1.989 | 1.981 | NA |
| TRINITY_DN32734_c0_g3_i6 | 0.533100457 | 0.797665023 | 0.999970678 | 2.925 | 0.089 | 0.222 | 1.84 | NA |
| TRINITY_DN38532_c0_g2_i1 | -0.53304753 | 0.503889315 | 0.999970678 | 308.99 | 469.6 | 318.636 | 725.545 | NA |
| TRINITY_DN4103_c0_g1_i1 | -0.53297518 | 0.474283766 | 0.999970678 | 15.034 | 19.6 | 26.187 | 18.358 | ATP-sulfurylase |

| TRINITY_DN5164_c0_g1_i1 | -0.5329747 | 0.577766169 | 0.999970678 | 7.258 | 6.13 | 4.411 | 14.025 | Hydrolase_4 |
| --- | --- | --- | --- | --- | --- | --- | --- | --- |
| TRINITY_DN3367_c0_g1_i2 | 0.532933258 | 0.56311133 | 0.999970678 | 3.252 | 5.174 | 2.411 | 2.805 | NA |
| TRINITY_DN7943_c0_g1_i3 | -0.53291246 | 0.630392898 | 0.999970678 | 5.905 | 1.912 | 6.488 | 3.569 | NA |
| TRINITY_DN209_c0_g1_i5 | -0.53285133 | 0.55090999 | 0.999970678 | 20.566 | 27.12 | 16.443 | 48.781 | NA |
| TRINITY_DN4652_c0_g1_i5 | 0.532815689 | 0.711203113 | 1 | 0.345 | 0.315 | 0.3 | 0.101 | Sigma70_r2 |
| TRINITY_DN7817_c0_g2_i11 | -0.53280166 | 0.77657084 | 0.999970678 | 0.899 | 0.7 | 1.767 | 0.131 | NA |
| TRINITY_DN13633_c0_g1_i4 | -0.53279763 | 0.902596128 | 0.999970678 | 0 | 1.429 | 0 | 2.051 | NA |
| TRINITY_DN2511_c0_g1_i1 | 0.532777452 | 0.632087557 | 0.999970678 | 10.928 | 4.583 | 6.6 | 2.805 | NA |
| TRINITY_DN7580_c0_g1_i1 | 0.532689575 | 0.437301462 | 0.999970678 | 17.314 | 18.03 | 12.21 | 9.662 | HlyIII |
| TRINITY_DN980_c0_g1_i14 | -0.53259024 | 0.688966891 | 0.999970678 | 0.654 | 4.445 | 2.478 | 4.323 | NA |
| TRINITY_DN13629_c0_g1_i3 | -0.532557 | 0.75107027 | 0.999970678 | 2.226 | 0.286 | 0.989 | 2.433 | GUB_WAK_bind |
| TRINITY_DN18690_c0_g1_i2 | 0.532540465 | 0.795348277 | 0.999970678 | 5.087 | 0.118 | 1.878 | 1.327 | DPBB_1 |
| TRINITY_DN1305_c1_g1_i1 | 0.532539145 | 0.629602639 | 0.999970678 | 8.484 | 2.572 | 3.2 | 3.81 | NA |
| TRINITY_DN1857_c0_g1_i3 | 0.532526126 | 0.61212113 | 0.999970678 | 17.986 | 7.49 | 10.455 | 5.087 | Homeobox |
| TRINITY_DN12236_c0_g1_i5 | -0.53238503 | 0.652545693 | 0.999970678 | 0.645 | 2.208 | 1.078 | 2.785 | TPR_16 |
| TRINITY_DN74349_c0_g1_i1 | -0.53237592 | 0.834971767 | 1 | 0.027 | 0.621 | 0.022 | 0.895 | NA |
| TRINITY_DN5555_c0_g3_i2 | -0.53232479 | 0.905758362 | 1 | 0 | 1.064 | 1.244 | 0 | NA |
| TRINITY_DN193497_c0_g1_i1 | -0.53230278 | 0.526121713 | 0.999970678 | 3.188 | 1.971 | 2.978 | 3.891 | - |
| TRINITY_DN5248_c0_g1_i1 | -0.53226957 | 0.489782949 | 0.999970678 | 30.567 | 29.81 | 47.797 | 29.568 | NA |
| TRINITY_DN2916_c0_g1_i2 | -0.53223256 | 0.858591459 | 0.999970678 | 9.901 | 0.641 | 0 | 15.332 | NA |
| TRINITY_DN812_c0_g1_i6 | -0.53202866 | 0.638431186 | 0.999970678 | 2.325 | 1.39 | 1.755 | 3.147 | NA |
| TRINITY_DN952_c0_g1_i1 | -0.53201578 | 0.441239728 | 0.999970678 | 5.214 | 7.924 | 7.377 | 10.044 | EF1_GNE |
| TRINITY_DN15440_c0_g1_i2 | 0.532001299 | 0.907074067 | 1 | 0 | 1.094 | 0.622 | 0 | NA |
| TRINITY_DN9475_c0_g1_i2 | 0.532001299 | 0.907074067 | 1 | 0 | 0.246 | 0.144 | 0 | NHL |
| TRINITY_DN9996_c0_g1_i1 | 0.531954117 | 0.651849067 | 1 | 0.727 | 0.739 | 0.478 | 0.462 | NA |
| TRINITY_DN2052_c2_g1_i6 | -0.53193711 | 0.617706865 | 0.999970678 | 4.951 | 5.075 | 2.7 | 11.22 | NA |
| TRINITY_DN4404_c0_g1_i2 | -0.53192832 | 0.671277387 | 0.999970678 | 103.42 | 27.57 | 122.581 | 42.236 | NA |
| TRINITY_DN2424_c0_g1_i2 | 0.531849762 | 0.611586914 | 0.999970678 | 4.724 | 6.376 | 4.478 | 2.172 | NA |
| TRINITY_DN3213_c0_g1_i3 | -0.53181196 | 0.508183483 | 0.999970678 | 16.297 | 10.37 | 16.721 | 17.775 | NA |
| TRINITY_DN1633_c0_g1_i15 | -0.53179109 | 0.575526033 | 0.999970678 | 2.625 | 1.449 | 2.044 | 3.398 | F-box |
| TRINITY_DN6737_c1_g1_i5 | -0.531781 | 0.68085462 | 0.999970678 | 0.091 | 0.266 | 0.244 | 0.221 | Exo_endo_phos |
| TRINITY_DN590_c4_g1_i1 | 0.531705623 | 0.75786324 | 0.999970678 | 38.543 | 1091 | 236.808 | 404.722 | NA |
| TRINITY_DN9312_c0_g1_i5 | -0.53168792 | 0.582096617 | 0.999970678 | 0.59 | 0.907 | 1.244 | 0.674 | AAA |
| TRINITY_DN45290_c0_g1_i2 | 0.531578344 | 0.905131955 | 1 | 1.39 | 0 | 0.8 | 0 | NA |
| TRINITY_DN3345_c0_g1_i1 | -0.53157102 | 0.694325468 | 0.999970678 | 0.972 | 0.434 | 0.333 | 1.619 | NA |
| TRINITY_DN12401_c0_g1_i4 | 0.531568182 | 0.5554519 | 0.999970678 | 1.135 | 1.242 | 0.767 | 0.714 | NA |
| TRINITY_DN7309_c0_g1_i5 | 0.531550209 | 0.661564786 | 0.999970678 | 0.963 | 0.522 | 0.522 | 0.422 | Myb_DNA-bind_ |
| TRINITY_DN1333_c1_g1_i28 | -0.53146191 | 0.636776122 | 0.999970678 | 0.4 | 0.788 | 0.622 | 0.945 | NA |
| TRINITY_DN19_c0_g1_i2 | -0.5314342 | 0.64716768 | 0.999970678 | 2.144 | 1.892 | 3.855 | 1.186 | WHEP-TRS |
| TRINITY_DN11712_c0_g1_i1 | -0.53135942 | 0.682048522 | 0.999970678 | 3.125 | 7.116 | 1.844 | 12.537 | NA |
| TRINITY_DN38174_c1_g1_i2 | 0.531321007 | 0.901545039 | 0.999970678 | 2.762 | 0 | 1.589 | 0 | Citrate_synt |
| TRINITY_DN5027_c0_g1_i4 | 0.531314793 | 0.588825449 | 0.999970678 | 3.697 | 6.229 | 1.711 | 4.786 | His_Phos_2 |
| TRINITY_DN1151_c1_g1_i1 | -0.53131402 | 0.386932991 | 0.999970678 | 13.98 | 15.31 | 19.599 | 18.609 | NA |
| TRINITY_DN505_c0_g1_i2 | -0.53120438 | 0.61839026 | 0.999970678 | 24.554 | 8.288 | 12.833 | 32.262 | NA |
| TRINITY_DN1087_c1_g1_i1 | 0.531161077 | 0.733421705 | 0.999970678 | 3.915 | 4.632 | 4.544 | 0.402 | NA |
| TRINITY_DN5542_c0_g3_i3 | 0.531131981 | 0.901532606 | 0.999970678 | 3.452 | 0 | 0 | 2.403 | NA |
| TRINITY_DN509_c0_g1_i10 | -0.53112282 | 0.703741062 | 0.999970678 | 1.244 | 4.563 | 1.422 | 6.625 | NA |
| TRINITY_DN581_c0_g1_i8 | -0.53109321 | 0.789875772 | 0.999970678 | 1.671 | 2.434 | 0.1 | 5.721 | NA |
| TRINITY_DN712_c0_g2_i5 | -0.53108047 | 0.568112445 | 0.999970678 | 3.252 | 4.001 | 2.444 | 7.51 | NA |
| TRINITY_DN254_c1_g1_i6 | -0.53107375 | 0.692359487 | 0.999970678 | 1.599 | 11.59 | 9.611 | 7.208 | NA |
| TRINITY_DN77_c0_g2_i3 | 0.531031407 | 0.714813282 | 0.999970678 | 1.145 | 0.483 | 0.689 | 0.322 | NA |
| TRINITY_DN5890_c0_g1_i1 | -0.53097082 | 0.544264512 | 0.999970678 | 1.572 | 2.602 | 1.722 | 3.941 | NA |
| TRINITY_DN4479_c0_g1_i8 | -0.53096241 | 0.831945867 | 0.999970678 | 0.045 | 1.813 | 2.111 | 0.09 | NA |
| TRINITY_DN20569_c0_g1_i1 | -0.53096239 | 0.419281643 | 0.999970678 | 8.757 | 12.63 | 12.055 | 16.277 | NA |
| TRINITY_DN22534_c0_g1_i4 | 0.5309601 | 0.778526704 | 1 | 17.368 | 5.026 | 10.944 | 1.689 | NA |
| TRINITY_DN8285_c0_g1_i1 | 0.530960078 | 0.721997482 | 0.999970678 | 2.289 | 29.69 | 10.788 | 8.787 | NA |
| TRINITY_DN24954_c0_g1_i1 | -0.53066185 | 0.657053595 | 0.999970678 | 1.063 | 4.238 | 3.344 | 3.539 | NA |
| TRINITY_DN1287_c0_g1_i2 | 0.530627862 | 0.720519966 | 0.999970678 | 3.37 | 0.857 | 2.011 | 0.523 | NA |
| TRINITY_DN10058_c0_g1_i7 | -0.53057374 | 0.504878937 | 0.999970678 | 508.7 | 403.8 | 360.567 | 886.344 | NA |
| TRINITY_DN24615_c0_g1_i2 | -0.53055041 | 0.718652553 | 0.999970678 | 1.562 | 3.962 | 0.811 | 6.967 | NA |
| TRINITY_DN1713_c0_g2_i1 | -0.5305357 | 0.411517583 | 0.999970678 | 3.588 | 4.662 | 4.489 | 6.505 | Clathrin |
| TRINITY_DN6117_c0_g1_i1 | 0.530529778 | 0.360556738 | 0.999970678 | 13.335 | 14.55 | 8.566 | 8.988 | NA |
| TRINITY_DN2064_c0_g1_i3 | 0.530528459 | 0.849738566 | 0.999970678 | 0 | 4.474 | 1.689 | 1.015 | Pkinase |
| TRINITY_DN4196_c0_g1_i3 | -0.53051385 | 0.84884006 | 0.999970678 | 0 | 8.83 | 6.933 | 4.192 | NA |
| TRINITY_DN12431_c0_g1_i4 | -0.53037444 | 0.521349354 | 0.999970678 | 2.017 | 2.346 | 2.989 | 2.644 | NA |
| TRINITY_DN18308_c0_g1_i3 | -0.53029755 | 0.523160799 | 0.999970678 | 13.29 | 15.63 | 23.587 | 13.19 | Pro_isomerase |
| TRINITY_DN6819_c0_g1_i2 | -0.53024614 | 0.654800169 | 0.999970678 | 0.6 | 2.07 | 1.267 | 2.312 | NA |
| TRINITY_DN287_c0_g1_i4 | -0.53019266 | 0.413046711 | 0.999970678 | 39.115 | 29.27 | 37.131 | 53.938 | NA |
| TRINITY_DN5163_c0_g1_i1 | 0.530146757 | 0.820846615 | 1 | 7.839 | 0.808 | 4.833 | 0.322 | NA |
| TRINITY_DN1635_c0_g1_i6 | -0.53014411 | 0.679452956 | 0.999970678 | 1.263 | 3.233 | 1.644 | 4.474 | - |
| TRINITY_DN6283_c0_g1_i1 | -0.53013202 | 0.732072347 | 0.999970678 | 1.063 | 5.371 | 0.989 | 8.013 | NA |
| TRINITY_DN18845_c1_g1_i1 | 0.530124367 | 0.762671765 | 1 | 0.545 | 0.365 | 0.467 | 0.07 | NA |
| TRINITY_DN255909_c0_g1_i1 | -0.53005455 | 0.900237822 | 0.999970678 | 5.014 | 0 | 6.077 | 0 | MFS_1 |
| TRINITY_DN1193_c0_g1_i4 | -0.53001044 | 0.850301917 | 0.999970678 | 11.809 | 0 | 10.166 | 5.047 | NA |

| TRINITY_DN5581_c0_g1_i1 | 0.529967285 | 0.516999185 | 0.999970678 | 20.73 | 21.56 | 16.577 | 9.27 | IF-2B |
| --- | --- | --- | --- | --- | --- | --- | --- | --- |
| TRINITY_DN8884_c0_g1_i1 | 0.529910103 | 0.900164122 | 0.999970678 | 32.893 | 0 | 0 | 22.721 | NA |
| TRINITY_DN1276_c0_g1_i9 | 0.529903581 | 0.856385498 | 0.999970678 | 2.153 | 0.187 | 0 | 1.629 | NA |
| TRINITY_DN8654_c0_g1_i1 | -0.52979888 | 0.866582765 | 0.999970678 | 1.208 | 0 | 0.044 | 1.719 | B3 |
| TRINITY_DN15427_c0_g1_i1 | 0.529789146 | 0.542875587 | 0.999970678 | 1.072 | 1.882 | 0.878 | 0.965 | BAH |
| TRINITY_DN1329_c0_g2_i2 | -0.52978388 | 0.683267604 | 0.999970678 | 1.535 | 10.24 | 8.277 | 6.796 | NA |
| TRINITY_DN91_c0_g1_i3 | 0.529772921 | 0.720997011 | 0.999970678 | 1.108 | 0.335 | 0.589 | 0.302 | NA |
| TRINITY_DN3846_c0_g1_i1 | -0.52977155 | 0.855544065 | 0.999970678 | 34.573 | 0 | 24.643 | 20.882 | TPR_12 |
| TRINITY_DN3188_c0_g1_i9 | 0.529768494 | 0.577696655 | 0.999970678 | 1.499 | 1.597 | 0.689 | 1.287 | NA |
| TRINITY_DN29223_c0_g2_i1 | -0.52974496 | 0.561661244 | 0.999970678 | 21.547 | 23.23 | 14.566 | 46.84 | Proteasome |
| TRINITY_DN2337_c1_g1_i4 | -0.52967841 | 0.686285127 | 0.999970678 | 116.51 | 279.4 | 62.807 | 490.208 | NA |
| TRINITY_DN686_c0_g1_i8 | -0.52965276 | 0.859955559 | 0.999970678 | 1.19 | 0 | 1.355 | 0.101 | NA |
| TRINITY_DN1090_c0_g1_i4 | -0.52964396 | 0.853519548 | 0.999970678 | 18.958 | 0 | 12.144 | 13.15 | NA |
| TRINITY_DN10324_c0_g1_i2 | -0.52961871 | 0.850847565 | 0.999970678 | 12.363 | 0 | 2.522 | 14.94 | Ribosomal_L7Ae |
| TRINITY_DN1498_c0_g1_i8 | 0.529582966 | 0.629959137 | 0.999970678 | 1.145 | 1.261 | 0.389 | 1.186 | - |
| TRINITY_DN6171_c0_g1_i14 | 0.52955622 | 0.704297796 | 0.999970678 | 4.097 | 14.38 | 1.733 | 10.587 | NA |
| TRINITY_DN3121_c0_g1_i2 | 0.529521754 | 0.581842109 | 0.999970678 | 15.225 | 6.396 | 6.255 | 7.43 | NA |
| TRINITY_DN9230_c0_g1_i1 | -0.52943338 | 0.744738097 | 0.999970678 | 3.588 | 1.064 | 4.589 | 1.106 | NA |
| TRINITY_DN6337_c0_g1_i1 | -0.52938801 | 0.901310509 | 0.999970678 | 0 | 1.143 | 0 | 1.629 | NA |
| TRINITY_DN2213_c0_g1_i5 | 0.529252124 | 0.678401278 | 0.999970678 | 13.462 | 6.071 | 9.433 | 2.192 | NA |
| TRINITY_DN74_c0_g1_i8 | -0.5292078 | 0.486863002 | 0.999970678 | 4.088 | 4.75 | 5.944 | 5.57 | NA |
| TRINITY_DN19262_c0_g1_i1 | 0.529139722 | 0.786961764 | 0.999970678 | 1.535 | 1.833 | 1.889 | 0.05 | NA |
| TRINITY_DN34650_c0_g1_i2 | 0.529049441 | 0.838587595 | 1 | 0.027 | 0.926 | 0.511 | 0.02 | NA |
| TRINITY_DN7051_c1_g1_i1 | 0.529041486 | 0.566184862 | 0.999970678 | 1.417 | 1.222 | 0.978 | 0.653 | PPR |
| TRINITY_DN1244_c0_g1_i1 | -0.52888428 | 0.900607596 | 0.999970678 | 2.852 | 0 | 0 | 4.192 | NA |
| TRINITY_DN4696_c0_g1_i4 | -0.52887514 | 0.652988934 | 0.999970678 | 0.445 | 1.064 | 0.711 | 1.307 | NA |
| TRINITY_DN8639_c0_g1_i2 | -0.52882359 | 0.529219478 | 0.999970678 | 1.135 | 1.478 | 1.367 | 2.121 | NA |
| TRINITY_DN21840_c0_g1_i2 | -0.52881905 | 0.634637872 | 0.999970678 | 9.284 | 12.85 | 5.255 | 25.335 | NA |
| TRINITY_DN161_c0_g1_i5 | -0.52872867 | 0.468503021 | 0.999970678 | 68.093 | 57.58 | 54.93 | 114.502 | 2014/3/3 |
| TRINITY_DN13571_c0_g1_i3 | -0.52871619 | 0.666794364 | 0.999970678 | 0.818 | 3.272 | 3.333 | 1.82 | NA |
| TRINITY_DN12811_c0_g1_i1 | -0.5287141 | 0.83361305 | 0.999970678 | 2.653 | 0.069 | 3.189 | 0.141 | NA |
| TRINITY_DN8869_c0_g1_i1 | 0.528711269 | 0.540924583 | 0.999970678 | 4.842 | 3.538 | 1.733 | 3.74 | AP2 |
| TRINITY_DN837_c0_g1_i5 | -0.52868867 | 0.496033679 | 0.999970678 | 20.457 | 11.44 | 19.754 | 22.138 | Ribosomal_L6e |
| TRINITY_DN7187_c0_g1_i1 | -0.52863644 | 0.425171077 | 0.999970678 | 40.378 | 56.93 | 51.186 | 76.79 | NA |
| TRINITY_DN202450_c0_g1_i1 | 0.528600006 | 0.714212854 | 0.999970678 | 2.716 | 6.761 | 0.833 | 5.449 | NA |
| TRINITY_DN11434_c0_g2_i1 | -0.52859527 | 0.485218111 | 0.999970678 | 22.882 | 20.62 | 33.642 | 22.088 | NA |
| TRINITY_DN19475_c0_g1_i2 | 0.528568851 | 0.603733125 | 0.999970678 | 2.616 | 2.789 | 2.167 | 1.116 | NA |
| TRINITY_DN15010_c0_g1_i1 | -0.52856015 | 0.629778108 | 0.999970678 | 0.781 | 2.139 | 2.144 | 1.588 | NA |
| TRINITY_DN685_c0_g1_i5 | 0.528542998 | 0.677050636 | 0.999970678 | 60.753 | 351.4 | 127.926 | 108.148 | NA |
| TRINITY_DN32709_c0_g1_i11 | 0.528539494 | 0.851588278 | 0.999970678 | 1.072 | 0.197 | 0.722 | 0 | NA |
| TRINITY_DN46566_c0_g1_i3 | -0.52853833 | 0.722684721 | 0.999970678 | 2.807 | 40.89 | 28.054 | 28.351 | Glyco_transf_9 |
| TRINITY_DN6869_c0_g1_i2 | 0.528449766 | 0.821455454 | 0.999970678 | 12.236 | 0.03 | 6.2 | 1.146 | NA |
| TRINITY_DN977_c0_g2_i1 | 0.528448927 | 0.671692278 | 0.999970678 | 17.959 | 4.021 | 8.622 | 4.916 | NA |
| TRINITY_DN9141_c1_g1_i9 | -0.5284095 | 0.848825455 | 0.999970678 | 0 | 1.084 | 1.022 | 0.302 | NA |
| TRINITY_DN5547_c0_g1_i5 | -0.52840907 | 0.641215325 | 0.999970678 | 0.563 | 1.005 | 1.089 | 0.935 | NA |
| TRINITY_DN917_c1_g1_i8 | 0.528396208 | 0.731290511 | 0.999970678 | 1.608 | 2.75 | 2.289 | 0.231 | NA |
| TRINITY_DN5696_c0_g1_i3 | -0.5283669 | 0.760861692 | 0.999970678 | 0.173 | 2.888 | 2.655 | 1.156 | p450 |
| TRINITY_DN12230_c0_g1_i1 | 0.528346188 | 0.717944805 | 0.999970678 | 0.59 | 3.587 | 0.578 | 2.202 | ALG11_N |
| TRINITY_DN476_c0_g1_i1 | -0.52828318 | 0.517462818 | 0.999970678 | 16.469 | 11.7 | 21.743 | 14.316 | - |
| TRINITY_DN10578_c0_g1_i13 | 0.528280506 | 0.738483065 | 0.999970678 | 4.351 | 2.917 | 3.666 | 0.593 | NA |
| TRINITY_DN6348_c0_g1_i1 | 0.528155717 | 0.421420501 | 0.999970678 | 6.05 | 7.618 | 4.033 | 4.595 | DRMBL |
| TRINITY_DN10806_c0_g1_i1 | -0.52808732 | 0.845308405 | 0.999970678 | 0 | 3.528 | 1.4 | 3.338 | UDPGT |
| TRINITY_DN6740_c0_g1_i1 | 0.528002194 | 0.578489332 | 0.999970678 | 6.322 | 10.64 | 3.644 | 7.158 | Erg28 |
| TRINITY_DN4803_c0_g1_i4 | -0.52795565 | 0.592798026 | 0.999970678 | 0.481 | 0.946 | 1.044 | 0.794 | F-box |
| TRINITY_DN4554_c0_g1_i7 | -0.52794736 | 0.900846266 | 0.999970678 | 3.079 | 0 | 0 | 4.484 | KH_1 |
| TRINITY_DN4484_c0_g1_i1 | 0.527945659 | 0.901701605 | 0.999970678 | 19.467 | 0 | 0 | 13.723 | DUF3493 |
| TRINITY_DN23081_c0_g2_i1 | 0.527824745 | 0.508282723 | 0.999970678 | 24.627 | 23.25 | 8.922 | 22.51 | Pkinase |
| TRINITY_DN1697_c0_g1_i2 | 0.527794907 | 0.514195675 | 0.999970678 | 10.674 | 5.854 | 5.522 | 4.846 | Amidohydro_2 |
| TRINITY_DN3625_c0_g1_i4 | 0.527792469 | 0.565858813 | 0.999970678 | 2.325 | 1.597 | 1.578 | 0.824 | MT-A70 |
| TRINITY_DN5083_c0_g1_i4 | 0.52772968 | 0.769021439 | 0.999970678 | 26.789 | 15.38 | 23.621 | 0.734 | SNARE |
| TRINITY_DN4387_c0_g3_i1 | -0.52772891 | 0.393321686 | 0.999970678 | 9.702 | 8.554 | 11.766 | 12.125 | NA |
| TRINITY_DN20442_c1_g1_i1 | 0.527667528 | 0.613772827 | 0.999970678 | 0.209 | 0.296 | 0.178 | 0.141 | Exo_endo_phos |
| TRINITY_DN3255_c0_g1_i3 | -0.52766232 | 0.56042759 | 0.999970678 | 34.047 | 14.68 | 22.965 | 42.879 | NA |
| TRINITY_DN14895_c0_g1_i1 | 0.527493051 | 0.497578424 | 0.999970678 | 5.705 | 8.15 | 5.111 | 3.438 | NA |
| TRINITY_DN225408_c0_g1_i1 | -0.52741307 | 0.904331266 | 0.999970678 | 2.162 | 0 | 2.611 | 0 | NA |
| TRINITY_DN1762_c0_g1_i2 | 0.527391744 | 0.698999315 | 0.999970678 | 1.562 | 3.676 | 2.289 | 0.845 | NA |
| TRINITY_DN2398_c0_g1_i7 | -0.5273876 | 0.722448855 | 0.999970678 | 6.241 | 10.17 | 2.911 | 19.193 | NA |
| TRINITY_DN100111_c0_g2_i1 | -0.52737323 | 0.907001421 | 1 | 0.454 | 0 | 0.544 | 0 | RGS |
| TRINITY_DN5115_c0_g1_i3 | -0.52734959 | 0.553882717 | 0.999970678 | 94.518 | 69.83 | 139.247 | 68.496 | NDK |
| TRINITY_DN11127_c0_g1_i1 | -0.52729212 | 0.459703909 | 0.999970678 | 70.891 | 85.79 | 69.329 | 140.51 | NAC |
| TRINITY_DN10290_c0_g1_i1 | -0.52712344 | 0.51888777 | 0.999970678 | 10.392 | 7.451 | 9.988 | 13.221 | Peptidase_M22 |
| TRINITY_DN5075_c0_g1_i2 | 0.527120964 | 0.900986974 | 0.999970678 | 0 | 18.06 | 0 | 12.336 | NA |
| TRINITY_DN8846_c0_g1_i1 | 0.5271188 | 0.85305893 | 0.999970678 | 7.331 | 0 | 1.322 | 3.589 | MMR_HSR1 |
| TRINITY_DN149_c0_g1_i1 | -0.52705408 | 0.452396191 | 0.999970678 | 54.894 | 49.61 | 77.084 | 56.964 | Ribosomal_S20p |

| TRINITY_DN221099_c0_g1_i1 | -0.52703976 | 0.740180421 | 1 | 2.544 | 1.281 | 3.711 | 0.995 | Glutaredoxin |
| --- | --- | --- | --- | --- | --- | --- | --- | --- |
| TRINITY_DN519_c0_g3_i1 | -0.52699023 | 0.578766981 | 0.999970678 | 3.77 | 3.873 | 6.677 | 2.966 | TPR_1 |
| TRINITY_DN45689_c0_g1_i6 | -0.52693183 | 0.848450859 | 0.999970678 | 0 | 1.547 | 0.422 | 1.689 | PARG_cat |
| TRINITY_DN10550_c0_g1_i2 | -0.52692308 | 0.553724584 | 0.999970678 | 1.417 | 1.537 | 2.278 | 1.508 | Glyco_transf_22 |
| TRINITY_DN8358_c0_g1_i1 | -0.52683553 | 0.439315245 | 0.999970678 | 15.979 | 14.86 | 16.599 | 23.767 | NA |
| TRINITY_DN34_c1_g1_i6 | 0.526775882 | 0.697690586 | 0.999970678 | 3.724 | 0.66 | 1.667 | 1.046 | F-box |
| TRINITY_DN16759_c0_g1_i7 | -0.52662974 | 0.603256621 | 0.999970678 | 0.4 | 0.611 | 0.567 | 0.774 | NA |
| TRINITY_DN5105_c0_g1_i1 | 0.526605115 | 0.591728202 | 0.999970678 | 1.499 | 2.395 | 1.255 | 1.166 | NA |
| TRINITY_DN512_c0_g3_i2 | 0.526536023 | 0.571429577 | 0.999970678 | 1.808 | 1.232 | 0.644 | 1.347 | EXS |
| TRINITY_DN796_c0_g1_i5 | -0.52647804 | 0.475322014 | 0.999970678 | 158.81 | 92.55 | 139.525 | 185.903 | NA |
| TRINITY_DN165372_c0_g1_i1 | -0.52645713 | 0.610354585 | 0.999970678 | 6.749 | 13.25 | 6.922 | 19.967 | NA |
| TRINITY_DN8353_c0_g1_i1 | -0.52640674 | 0.535112976 | 0.999970678 | 5.469 | 8.318 | 6.622 | 11.602 | NA |
| TRINITY_DN5092_c0_g1_i3 | 0.526405111 | 0.901241219 | 0.999970678 | 3.125 | 0 | 0 | 2.202 | NA |
| TRINITY_DN1400_c0_g3_i2 | -0.52635085 | 0.50700835 | 0.999970678 | 28.832 | 29.81 | 47.164 | 27.497 | NA |
| TRINITY_DN12962_c0_g1_i1 | 0.526296035 | 0.439949305 | 0.999970678 | 0.927 | 1.114 | 0.578 | 0.734 | NopRA1 |
| TRINITY_DN10343_c0_g1_i1 | -0.52623956 | 0.363809267 | 0.999970678 | 14.689 | 14.17 | 16.977 | 21.073 | NMT |
| TRINITY_DN7064_c0_g1_i1 | -0.52618755 | 0.660827522 | 0.999970678 | 2.335 | 0.67 | 2.289 | 1.588 | WD40 |
| TRINITY_DN2364_c0_g1_i1 | -0.52614347 | 0.56810851 | 0.999970678 | 9.947 | 5.686 | 12.421 | 7.54 | NA |
| TRINITY_DN106988_c0_g1_i1 | -0.52611061 | 0.376990972 | 0.999970678 | 68.802 | 77.84 | 96.75 | 93.429 | - |
| TRINITY_DN4804_c0_g1_i1 | 0.526053891 | 0.564812884 | 0.999970678 | 9.202 | 9.806 | 7.944 | 3.619 | NA |
| TRINITY_DN4654_c0_g1_i3 | 0.525963371 | 0.680065479 | 0.999970678 | 1.88 | 0.591 | 1 | 0.513 | LRR_1 |
| TRINITY_DN2220_c0_g1_i1 | -0.52592871 | 0.67345358 | 0.999970678 | 0.899 | 5.539 | 3.866 | 4.514 | OKR_DC_1 |
| TRINITY_DN1314_c0_g1_i2 | 0.525912565 | 0.538492407 | 0.999970678 | 10.565 | 5.558 | 5.722 | 4.343 | NA |
| TRINITY_DN8913_c2_g1_i1 | 0.525835694 | 0.712684771 | 0.999970678 | 0.972 | 0.798 | 0.867 | 0.181 | NA |
| TRINITY_DN1577_c0_g2_i1 | -0.52583139 | 0.524677974 | 0.999970678 | 21.92 | 33.45 | 21.799 | 53.224 | NA |
| TRINITY_DN4569_c0_g1_i14 | 0.52582697 | 0.784486762 | 0.999970678 | 2.752 | 1.409 | 2.289 | 0.111 | NA |
| TRINITY_DN28711_c0_g1_i12 | -0.52582104 | 0.90118147 | 0.999970678 | 3.67 | 0 | 4.411 | 0 | NA |
| TRINITY_DN18865_c0_g1_i1 | 0.525818307 | 0.778876862 | 0.999970678 | 0.699 | 0.296 | 0.544 | 0.03 | NA |
| TRINITY_DN10903_c0_g1_i1 | -0.52580488 | 0.692537769 | 0.999970678 | 4.288 | 1.439 | 3.755 | 3.609 | NA |
| TRINITY_DN8582_c0_g1_i2 | -0.52571522 | 0.846749436 | 0.999970678 | 0 | 1.941 | 1.633 | 0.794 | AARP2CN |
| TRINITY_DN10960_c0_g1_i1 | 0.52571138 | 0.849626376 | 0.999970678 | 0 | 1.656 | 0.322 | 0.744 | NA |
| TRINITY_DN190_c1_g2_i1 | 0.52561388 | 0.481792192 | 0.999970678 | 27.443 | 51.12 | 20.165 | 30.061 | NA |
| TRINITY_DN190_c0_g1_i9 | 0.525594774 | 0.499062066 | 0.999970678 | 6.949 | 4.248 | 2.866 | 4.343 | CBS |
| TRINITY_DN2483_c0_g1_i5 | 0.525592196 | 0.558901069 | 0.999970678 | 14.589 | 8.219 | 4.466 | 10.486 | NA |
| TRINITY_DN3223_c0_g1_i4 | 0.525571602 | 0.900887797 | 0.999970678 | 0 | 8.485 | 4.777 | 0 | WD40 |
| TRINITY_DN3683_c0_g2_i3 | 0.525550183 | 0.731705697 | 0.999970678 | 2.498 | 8.525 | 0.689 | 6.696 | NA |
| TRINITY_DN9496_c0_g1_i12 | -0.52554943 | 0.903605338 | 0.999970678 | 5.532 | 0 | 6.666 | 0 | NA |
| TRINITY_DN5634_c0_g1_i1 | 0.525543753 | 0.845026263 | 0.999970678 | 0.899 | 1.39 | 1.311 | 0 | NA |
| TRINITY_DN4171_c0_g1_i2 | 0.525537402 | 0.692449214 | 0.999970678 | 4.442 | 0.788 | 1.4 | 1.981 | zf-CCCH |
| TRINITY_DN3612_c0_g1_i3 | -0.52547681 | 0.689129765 | 0.999970678 | 0.718 | 3.962 | 2.089 | 4.172 | NA |
| TRINITY_DN16621_c0_g1_i2 | -0.52542784 | 0.366382987 | 0.999970678 | 29.405 | 31.99 | 32.976 | 48.63 | NA |
| TRINITY_DN1695_c0_g1_i2 | 0.525417045 | 0.85589963 | 1 | 0.654 | 0.197 | 0.5 | 0 | NA |
| TRINITY_DN2111_c0_g1_i3 | 0.525373489 | 0.426758765 | 0.999970678 | 3.416 | 3.814 | 2.378 | 2.162 | NA |
| TRINITY_DN4673_c0_g2_i2 | 0.525327056 | 0.702520104 | 0.999970678 | 1.335 | 2.641 | 0.344 | 2.322 | NA |
| TRINITY_DN3553_c1_g1_i1 | -0.5252838 | 0.409382868 | 0.999970678 | 6.304 | 5.933 | 6.566 | 9.702 | Aa_trans |
| TRINITY_DN13100_c1_g1_i1 | 0.525224039 | 0.504213784 | 0.999970678 | 5.251 | 4.297 | 3.544 | 2.373 | BRX |
| TRINITY_DN1898_c1_g1_i9 | 0.525209244 | 0.615990955 | 0.999970678 | 1.19 | 0.65 | 0.411 | 0.794 | NA |
| TRINITY_DN2911_c0_g1_i1 | -0.5251477 | 0.856152057 | 1 | 0.372 | 0.118 | 0 | 0.714 | NA |
| TRINITY_DN18723_c0_g1_i3 | -0.52505604 | 0.619555071 | 0.999970678 | 0.808 | 1.941 | 2.022 | 1.498 | NA |
| TRINITY_DN415_c0_g1_i2 | 0.525052635 | 0.389332305 | 0.999970678 | 138.31 | 109.9 | 76.406 | 80.259 | NA |
| TRINITY_DN555_c1_g1_i9 | -0.52503667 | 0.761773153 | 0.999970678 | 2.335 | 1.922 | 0.456 | 5.489 | NA |
| TRINITY_DN616_c0_g2_i2 | 0.525008928 | 0.511692822 | 0.999970678 | 1.535 | 1.143 | 0.678 | 1.046 | RRM_1 |
| TRINITY_DN3007_c0_g1_i5 | 0.524986008 | 0.852983418 | 0.999970678 | 1.617 | 0 | 0.589 | 0.452 | NA |
| TRINITY_DN164_c0_g2_i2 | 0.524936129 | 0.856673927 | 0.999970678 | 46.11 | 67.5 | 0 | 78.539 | NAC |
| TRINITY_DN3215_c0_g1_i4 | -0.52493232 | 0.468004334 | 0.999970678 | 3.579 | 4.573 | 3.755 | 7.219 | ACP_syn_III_C |
| TRINITY_DN7227_c0_g1_i4 | -0.52491468 | 0.720865648 | 0.999970678 | 1.045 | 0.384 | 0.3 | 1.709 | ArgoL1 |
| TRINITY_DN6178_c3_g1_i1 | 0.524902543 | 0.874016804 | 0.999970678 | 10.383 | 0.177 | 6.066 | 0 | NA |
| TRINITY_DN21033_c0_g1_i2 | -0.52482469 | 0.905444248 | 0.999970678 | 0.863 | 0 | 1.044 | 0 | PH |
| TRINITY_DN13505_c0_g1_i3 | -0.52482469 | 0.905444248 | 0.999970678 | 2.017 | 0.02 | 2.422 | 0 | NA |
| TRINITY_DN221169_c0_g1_i1 | 0.524823367 | 0.908687017 | 1 | 0.754 | 0 | 0.444 | 0 | RNB |
| TRINITY_DN57381_c0_g1_i1 | 0.524823367 | 0.908687017 | 1 | 0.936 | 0 | 0.544 | 0 | NA |
| TRINITY_DN2128_c0_g1_i6 | -0.52481605 | 0.458321521 | 0.999970678 | 27.279 | 28.87 | 24.932 | 50.52 | Zip |
| TRINITY_DN6865_c0_g1_i6 | 0.524783806 | 0.84669675 | 0.999970678 | 0.936 | 0.848 | 0 | 1.227 | NA |
| TRINITY_DN46566_c0_g1_i5 | -0.5247672 | 0.904182069 | 0.999970678 | 13.735 | 0 | 16.577 | 0 | Glyco_transf_9 |
| TRINITY_DN6198_c0_g1_i12 | -0.52468723 | 0.603682803 | 0.999970678 | 3.134 | 3.439 | 3.3 | 5.278 | NA |
| TRINITY_DN9570_c1_g1_i2 | 0.52464606 | 0.84704873 | 1 | 0.981 | 1.242 | 0 | 1.528 | NA |
| TRINITY_DN6323_c1_g1_i1 | 0.524635851 | 0.569398044 | 0.999970678 | 2.044 | 1.37 | 1.033 | 1.116 | NA |
| TRINITY_DN2805_c0_g1_i1 | 0.524600994 | 0.52003104 | 0.999970678 | 6.976 | 4.928 | 4.189 | 3.227 | NA |
| TRINITY_DN27003_c0_g1_i9 | 0.524500491 | 0.904171931 | 0.999970678 | 3.552 | 0 | 2.1 | 0 | - |
| TRINITY_DN2230_c2_g2_i3 | 0.524477108 | 0.799338323 | 0.999970678 | 0.145 | 2.484 | 1.344 | 0.171 | NA |
| TRINITY_DN2587_c0_g1_i1 | 0.524473807 | 0.565370997 | 0.999970678 | 5.069 | 4.455 | 3.889 | 1.94 | NA |
| TRINITY_DN1303_c0_g1_i6 | 0.524460213 | 0.697676616 | 0.999970678 | 7.058 | 5.755 | 5.589 | 1.95 | GUB_WAK_bind |
| TRINITY_DN1874_c0_g2_i2 | 0.524366363 | 0.848215279 | 0.999970678 | 2.562 | 0.641 | 0 | 2.272 | zf-CCCH |
| TRINITY_DN8567_c0_g1_i1 | -0.52432702 | 0.635624734 | 0.999970678 | 1.653 | 0.66 | 1.167 | 1.94 | NA |

| TRINITY_DN7933_c2_g2_i1 | 0.52427235 | 0.462299568 | 0.999970678 | 7.967 | 6.041 | 4.866 | 3.901 | Bromodomain |
| --- | --- | --- | --- | --- | --- | --- | --- | --- |
| TRINITY_DN6126_c1_g1_i7 | -0.5242298 | 0.86601212 | 1 | 1.235 | 0.138 | 1.655 | 0 | Ras |
| TRINITY_DN10212_c0_g1_i1 | 0.524214035 | 0.449843979 | 0.999970678 | 35.482 | 34.09 | 15.166 | 30.161 | Glyco_hydro_1 |
| TRINITY_DN16160_c0_g1_i4 | -0.52419945 | 0.763959858 | 0.999970678 | 0.463 | 0.079 | 0.544 | 0.161 | Aminotran_5 |
| TRINITY_DN119_c0_g1_i2 | -0.5241025 | 0.724498041 | 0.999970678 | 0.354 | 0.729 | 0.2 | 1.317 | Ferritin |
| TRINITY_DN799_c0_g2_i3 | 0.524099722 | 0.871989916 | 0.999970678 | 2.78 | 0.02 | 0 | 1.991 | Glyco_hydro_20 |
| TRINITY_DN3553_c0_g1_i1 | -0.52401822 | 0.57881024 | 0.999970678 | 6.041 | 4.336 | 8.933 | 4.182 | NA |
| TRINITY_DN1074_c1_g1_i4 | 0.524010042 | 0.711385063 | 0.999970678 | 11.4 | 2.198 | 6.422 | 1.79 | CH |
| TRINITY_DN6627_c0_g1_i3 | 0.523927363 | 0.860598209 | 0.999970678 | 0 | 15.58 | 0.822 | 9.31 | NA |
| TRINITY_DN7883_c2_g2_i8 | 0.523705105 | 0.746106935 | 0.999970678 | 0.273 | 3.075 | 0.633 | 1.528 | NA |
| TRINITY_DN583_c1_g1_i8 | 0.523665006 | 0.701550758 | 0.999970678 | 0.291 | 1.912 | 0.767 | 0.583 | Pkinase |
| TRINITY_DN12422_c0_g2_i1 | 0.52363951 | 0.629285394 | 0.999970678 | 5.578 | 20.77 | 6.611 | 10.064 | NA |
| TRINITY_DN13900_c1_g1_i2 | -0.52362699 | 0.854083826 | 0.999970678 | 0 | 20.75 | 17.788 | 7.842 | FAD_binding_6 |
| TRINITY_DN78191_c0_g1_i1 | -0.52361591 | 0.729773662 | 1 | 0.145 | 0.581 | 0.333 | 0.633 | NA |
| TRINITY_DN65419_c0_g2_i1 | 0.523605875 | 0.805568866 | 0.999970678 | 0.091 | 2.74 | 0.144 | 1.78 | NA |
| TRINITY_DN336_c0_g1_i14 | 0.523479313 | 0.779591473 | 0.999970678 | 1.69 | 0.769 | 1.344 | 0.07 | DUF659 |
| TRINITY_DN5664_c0_g1_i2 | 0.523464945 | 0.554746404 | 0.999970678 | 16.587 | 9.51 | 10.066 | 6.093 | NA |
| TRINITY_DN4089_c0_g1_i2 | -0.52342338 | 0.4078987 | 0.999970678 | 15.679 | 16.66 | 16.388 | 26.632 | Transferase |
| TRINITY_DN7658_c0_g1_i3 | 0.52334055 | 0.678693277 | 0.999970678 | 0.563 | 0.788 | 0.556 | 0.261 | GDPD |
| TRINITY_DN86206_c0_g1_i1 | -0.52333808 | 0.731796573 | 1 | 0.454 | 0.158 | 0.211 | 0.633 | NA |
| TRINITY_DN2812_c0_g2_i2 | 0.523324092 | 0.505756083 | 0.999970678 | 1.544 | 2.089 | 1.122 | 1.196 | DUF3444 |
| TRINITY_DN77103_c0_g1_i1 | 0.52330231 | 0.560819664 | 0.999970678 | 1.163 | 2.158 | 1.178 | 0.875 | NA |
| TRINITY_DN3180_c0_g1_i4 | -0.52330055 | 0.903655328 | 0.999970678 | 1.281 | 0 | 0 | 1.86 | NA |
| TRINITY_DN12422_c0_g2_i3 | 0.523287708 | 0.902920138 | 0.999970678 | 2.625 | 0 | 0 | 1.84 | NA |
| TRINITY_DN46_c0_g1_i4 | -0.52327871 | 0.664536452 | 0.999970678 | 6.849 | 1.37 | 5.255 | 5.6 | RRM_1 |
| TRINITY_DN22569_c0_g1_i5 | -0.52324727 | 0.909035785 | 1 | 1.081 | 0 | 1.3 | 0 | Grp1_Fun34_Yaa |
| TRINITY_DN3374_c0_g1_i2 | 0.523078644 | 0.672456474 | 0.999970678 | 6.059 | 1.163 | 2.389 | 2.192 | Nucleoporin2 |
| TRINITY_DN22924_c0_g1_i3 | -0.52305814 | 0.858921757 | 1 | 1.145 | 0 | 1.133 | 0.322 | NA |
| TRINITY_DN1418_c0_g1_i4 | -0.52305763 | 0.766009867 | 0.999970678 | 4.388 | 150.7 | 129.581 | 62.353 | NA |
| TRINITY_DN223372_c0_g1_i1 | -0.52300021 | 0.730370899 | 0.999970678 | 4.088 | 1.005 | 1.422 | 5.54 | NA |
| TRINITY_DN414_c0_g1_i1 | -0.52294157 | 0.550889005 | 0.999970678 | 4.46 | 3.065 | 3.266 | 6.867 | Oxidored_FMN |
| TRINITY_DN8273_c0_g1_i6 | -0.52291739 | 0.601070136 | 0.999970678 | 40.623 | 45.98 | 26.887 | 85.537 | LRRNT_2 |
| TRINITY_DN6174_c0_g1_i2 | 0.522916329 | 0.474787513 | 0.999970678 | 9.947 | 11.7 | 4.589 | 9.531 | NA |
| TRINITY_DN24012_c0_g2_i4 | -0.5228806 | 0.87516385 | 1 | 0.781 | 0.03 | 0.978 | 0 | Peptidase_M16 |
| TRINITY_DN2162_c0_g1_i9 | 0.522751771 | 0.623802349 | 0.999970678 | 0.781 | 0.414 | 0.389 | 0.362 | NA |
| TRINITY_DN7714_c0_g1_i2 | -0.52275172 | 0.76971407 | 1 | 4.469 | 1.833 | 6.522 | 0.995 | NA |
| TRINITY_DN4993_c0_g1_i4 | 0.522748403 | 0.724484239 | 0.999970678 | 3.416 | 6.869 | 1.033 | 5.59 | NA |
| TRINITY_DN6820_c0_g3_i5 | 0.522735136 | 0.849997689 | 1 | 0.382 | 0.798 | 0 | 0.824 | TerC |
| TRINITY_DN538_c0_g1_i4 | -0.52273381 | 0.491263233 | 0.999970678 | 13.008 | 7.746 | 13.399 | 13.703 | NA |
| TRINITY_DN5997_c0_g1_i4 | -0.52272485 | 0.696429558 | 0.999970678 | 1.826 | 5.263 | 1.344 | 8.505 | NA |
| TRINITY_DN3118_c0_g1_i11 | -0.52269827 | 0.848091659 | 0.999970678 | 0.836 | 0 | 0.422 | 0.734 | TPP_enzyme_C |
| TRINITY_DN37354_c0_g1_i1 | 0.522689535 | 0.907918456 | 1 | 1.344 | 0 | 0.778 | 0 | NA |
| TRINITY_DN195254_c0_g1_i1 | 0.522689535 | 0.907918456 | 1 | 1.254 | 0 | 0.733 | 0 | NA |
| TRINITY_DN9494_c0_g1_i3 | -0.52266859 | 0.718423108 | 1 | 0.154 | 0.503 | 0.467 | 0.392 | NA |
| TRINITY_DN2867_c0_g1_i1 | -0.5226571 | 0.652190452 | 0.999970678 | 0.636 | 1.587 | 0.811 | 2.172 | NA |
| TRINITY_DN282_c0_g3_i1 | -0.5226499 | 0.503901907 | 0.999970678 | 7.767 | 13.86 | 11.366 | 17.031 | NA |
| TRINITY_DN5850_c0_g1_i14 | -0.5225805 | 0.761764654 | 0.999970678 | 1.135 | 0.542 | 1.811 | 0.221 | NA |
| TRINITY_DN3730_c0_g2_i3 | 0.522557594 | 0.61188138 | 0.999970678 | 4.115 | 3.804 | 1.333 | 3.841 | NA |
| TRINITY_DN17744_c0_g1_i1 | 0.522547837 | 0.522043116 | 0.999970678 | 6.831 | 4.652 | 4.233 | 2.916 | NA |
| TRINITY_DN28079_c0_g1_i2 | -0.52250568 | 0.705627871 | 0.999970678 | 0.445 | 2.355 | 1.7 | 1.9 | NA |
| TRINITY_DN4847_c2_g2_i14 | -0.522489 | 0.61107022 | 0.999970678 | 2.952 | 1.725 | 3.811 | 2.131 | NA |
| TRINITY_DN1151_c0_g1_i5 | 0.522431405 | 0.683170901 | 0.999970678 | 4.015 | 1.39 | 1.622 | 1.749 | NA |
| TRINITY_DN6210_c0_g1_i1 | 0.522420327 | 0.584412812 | 0.999970678 | 4.315 | 9.5 | 4.844 | 3.69 | NUDIX |
| TRINITY_DN11348_c0_g1_i1 | -0.5224133 | 0.536388607 | 0.999970678 | 12.554 | 19.83 | 12.888 | 30.694 | AP2 |
| TRINITY_DN1699_c0_g2_i4 | -0.52239831 | 0.851289315 | 1 | 0.5 | 0 | 0.289 | 0.382 | Nucleotid_trans |
| TRINITY_DN1746_c0_g2_i3 | -0.52239155 | 0.865044284 | 0.999970678 | 0.781 | 0.049 | 0 | 1.206 | - |
| TRINITY_DN3452_c1_g1_i2 | 0.52236495 | 0.399494803 | 0.999970678 | 17.632 | 15.05 | 8.377 | 12.718 | NA |
| TRINITY_DN2426_c0_g1_i5 | 0.522356799 | 0.763057477 | 0.999970678 | 0.572 | 2.572 | 1.489 | 0.352 | NA |
| TRINITY_DN2154_c0_g1_i9 | 0.522331752 | 0.855043476 | 0.999970678 | 0.418 | 3.213 | 2.067 | 0 | NA |
| TRINITY_DN808_c2_g1_i6 | -0.52228046 | 0.678156139 | 0.999970678 | 5.005 | 4.149 | 9.066 | 2.111 | Guanylate_kin |
| TRINITY_DN3209_c0_g1_i6 | -0.52227459 | 0.904828667 | 0.999970678 | 0 | 1.045 | 0 | 1.468 | ADH_N |
| TRINITY_DN1350_c0_g1_i8 | -0.52224055 | 0.860688914 | 0.999970678 | 0 | 469.4 | 317.814 | 266.312 | NA |
| TRINITY_DN30003_c0_g1_i1 | -0.52221678 | 0.479966636 | 0.999970678 | 2.707 | 2.06 | 3.178 | 3.036 | O-FucT |
| TRINITY_DN809_c0_g1_i3 | 0.522124135 | 0.868586829 | 0.999970678 | 0 | 71.9 | 38.342 | 2.885 | DUF3493 |
| TRINITY_DN6750_c0_g1_i3 | -0.52207163 | 0.687452955 | 0.999970678 | 0.4 | 0.877 | 1.067 | 0.533 | NA |
| TRINITY_DN1194_c0_g1_i1 | -0.52195501 | 0.856349539 | 0.999970678 | 0 | 151.6 | 64.774 | 132.437 | NA |
| TRINITY_DN6049_c0_g1_i2 | -0.52195346 | 0.455880676 | 0.999970678 | 8.23 | 5.884 | 7.677 | 11.019 | ELMO_CED12 |
| TRINITY_DN1041_c0_g1_i2 | 0.521927512 | 0.655184126 | 0.999970678 | 10.565 | 40.74 | 19.754 | 11.22 | NA |
| TRINITY_DN1355_c0_g1_i1 | -0.52191912 | 0.818434852 | 0.999970678 | 3.924 | 9.363 | 15.532 | 0.09 | NA |
| TRINITY_DN158_c0_g1_i4 | 0.521893044 | 0.851214823 | 0.999970678 | 0.536 | 0.246 | 0.456 | 0 | Na_H_Exchanger |
| TRINITY_DN21318_c0_g1_i20 | 0.521880547 | 0.904860403 | 0.999970678 | 0 | 1.301 | 0.744 | 0 | PA |
| TRINITY_DN22390_c0_g1_i2 | 0.521736039 | 0.579146891 | 0.999970678 | 7.93 | 8.85 | 3.122 | 7.751 | NA |
| TRINITY_DN5187_c1_g1_i3 | 0.521709685 | 0.625702848 | 0.999970678 | 3.624 | 2.04 | 1.355 | 2.302 | - |
| TRINITY_DN6171_c0_g1_i10 | 0.521699371 | 0.705311367 | 0.999970678 | 1.045 | 2.809 | 0.5 | 2.031 | NA |

| TRINITY_DN7449_c0_g1_i5 | 0.521643899 | 0.903826001 | 0.999970678 | 2.135 | 0 | 0 | 1.518 | NA |
| --- | --- | --- | --- | --- | --- | --- | --- | --- |
| TRINITY_DN5464_c0_g1_i1 | 0.521624644 | 0.513319267 | 0.999970678 | 29.023 | 33.54 | 24.354 | 14.166 | NA |
| TRINITY_DN22115_c0_g1_i1 | 0.521618042 | 0.497312489 | 0.999970678 | 13.753 | 12.27 | 9.844 | 6.274 | DEAD |
| TRINITY_DN12409_c0_g1_i1 | -0.52160847 | 0.788376764 | 1 | 0.354 | 2.582 | 2.855 | 0.684 | NA |
| TRINITY_DN590_c1_g3_i1 | -0.52157888 | 0.756966915 | 0.999970678 | 249.56 | 3743 | 754.654 | 4041.224 | NA |
| TRINITY_DN4038_c0_g1_i3 | -0.52154943 | 0.780333308 | 0.999970678 | 0.227 | 2.05 | 0.444 | 2.644 | Sybindin |
| TRINITY_DN17320_c0_g1_i1 | -0.52148044 | 0.353818261 | 0.999970678 | 584.95 | 654.6 | 684.903 | 943.64 | ATP-synt_B |
| TRINITY_DN12975_c0_g1_i4 | -0.52139322 | 0.543496054 | 0.999970678 | 6.786 | 4.928 | 4.811 | 10.989 | NA |
| TRINITY_DN1268_c0_g1_i10 | -0.52134902 | 0.870830054 | 0.999970678 | 4.769 | 0 | 0.067 | 6.887 | NA |
| TRINITY_DN35534_c0_g1_i1 | -0.52133913 | 0.814439168 | 0.999970678 | 0.609 | 0.217 | 0.955 | 0.02 | Pkinase |
| TRINITY_DN2536_c0_g1_i5 | 0.521328321 | 0.507502076 | 0.999970678 | 11.373 | 7.539 | 6.777 | 5.047 | NA |
| TRINITY_DN5101_c0_g1_i1 | -0.52128457 | 0.523613034 | 0.999970678 | 3.316 | 4.652 | 5.655 | 4.544 | zf-HIT |
| TRINITY_DN20952_c0_g1_i1 | -0.52127146 | 0.902123196 | 0.999970678 | 5.777 | 0 | 6.933 | 0 | GST_C |
| TRINITY_DN251409_c0_g1_i1 | -0.52125939 | 0.506560584 | 0.999970678 | 8.112 | 10.69 | 8.111 | 17.071 | DUF1195 |
| TRINITY_DN3397_c0_g1_i1 | 0.521217251 | 0.656764157 | 0.999970678 | 3.397 | 10.28 | 3.766 | 4.906 | NA |
| TRINITY_DN563_c0_g1_i2 | -0.52111223 | 0.852154243 | 0.999970678 | 3.089 | 0 | 1.055 | 3.247 | PPR |
| TRINITY_DN1327_c0_g1_i1 | 0.521089353 | 0.862356566 | 0.999970678 | 2.253 | 0.187 | 1.411 | 0 | NA |
| TRINITY_DN60_c0_g1_i1 | -0.52099577 | 0.452812681 | 0.999970678 | 20.993 | 31.69 | 33.276 | 34.434 | Tryp_alpha_amyl |
| TRINITY_DN5152_c0_g1_i4 | 0.520977667 | 0.8485341 | 0.999970678 | 2.398 | 0.749 | 0 | 2.222 | LRR_8 |
| TRINITY_DN2033_c0_g2_i1 | -0.52094161 | 0.489373905 | 0.999970678 | 16.76 | 20.08 | 15.444 | 34.162 | Fer4_14 |
| TRINITY_DN6597_c0_g1_i7 | -0.52091274 | 0.722299407 | 0.999970678 | 4.406 | 1.311 | 1 | 7.028 | zf-RING_2 |
| TRINITY_DN4938_c0_g1_i1 | 0.520887094 | 0.43909876 | 0.999970678 | 11.119 | 14.84 | 8.188 | 8.133 | Bromodomain |
| TRINITY_DN6692_c0_g1_i6 | 0.520849788 | 0.663817795 | 0.999970678 | 3.17 | 8.899 | 1.6 | 6.434 | NA |
| TRINITY_DN4945_c0_g2_i1 | 0.520763658 | 0.478961277 | 0.999970678 | 2.008 | 2.148 | 1.4 | 1.206 | NA |
| TRINITY_DN1218_c1_g1_i1 | 0.520729144 | 0.655478117 | 0.999970678 | 176.05 | 36.71 | 74.673 | 59.126 | NA |
| TRINITY_DN6780_c0_g1_i14 | 0.520688133 | 0.864498823 | 0.999970678 | 0.164 | 2.996 | 0 | 2.151 | NA |
| TRINITY_DN8294_c0_g1_i13 | -0.52063453 | 0.905432476 | 0.999970678 | 1.026 | 0 | 1.233 | 0 | NA |
| TRINITY_DN4253_c0_g1_i5 | 0.520633593 | 0.517385179 | 0.999970678 | 3.788 | 3.528 | 2.122 | 2.534 | NA |
| TRINITY_DN85414_c0_g1_i2 | -0.52045408 | 0.661835371 | 0.999970678 | 2.925 | 1.35 | 2.844 | 2.684 | NA |
| TRINITY_DN109_c0_g2_i3 | -0.5204169 | 0.405018881 | 0.999970678 | 44.566 | 35.17 | 46.497 | 57.366 | NA |
| TRINITY_DN3445_c0_g1_i5 | -0.52035145 | 0.734593967 | 0.999970678 | 0.127 | 0.779 | 0.544 | 0.613 | NA |
| TRINITY_DN6081_c0_g1_i2 | -0.52030388 | 0.593773854 | 0.999970678 | 14.334 | 6.307 | 13.333 | 13.18 | NA |
| TRINITY_DN2072_c0_g1_i5 | 0.520290942 | 0.598413055 | 0.999970678 | 3.061 | 2.622 | 2.489 | 0.965 | FAT |
| TRINITY_DN3450_c0_g1_i22 | 0.520287874 | 0.717201083 | 0.999970678 | 0.981 | 0.69 | 0.144 | 1.005 | NA |
| TRINITY_DN7704_c0_g1_i5 | -0.52018585 | 0.819794479 | 1 | 0.354 | 7.529 | 8.033 | 1.267 | NB-ARC |
| TRINITY_DN1060_c0_g1_i1 | -0.52015073 | 0.902080094 | 0.999970678 | 0 | 7.401 | 8.577 | 0 | Complex1_LYR |
| TRINITY_DN6000_c1_g1_i3 | -0.52013382 | 0.866932724 | 0.999970678 | 239.44 | 0 | 84.828 | 245.079 | CAAD |
| TRINITY_DN7090_c0_g1_i1 | -0.52010845 | 0.902066011 | 0.999970678 | 8.148 | 0 | 9.777 | 0 | NA |
| TRINITY_DN2331_c0_g1_i1 | -0.52004172 | 0.850462087 | 0.999970678 | 0 | 2.661 | 2.444 | 0.794 | NA |
| TRINITY_DN589_c0_g1_i2 | -0.52002243 | 0.728490737 | 0.999970678 | 3.134 | 5.223 | 0.811 | 10.959 | NA |
| TRINITY_DN3809_c0_g1_i3 | 0.519981536 | 0.578145886 | 0.999970678 | 251.67 | 337 | 88.106 | 303.099 | Calreticulin |
| TRINITY_DN29_c1_g2_i1 | 0.519973293 | 0.77192055 | 0.999970678 | 0.59 | 4.208 | 2.478 | 0.302 | NA |
| TRINITY_DN4868_c0_g1_i2 | -0.51991116 | 0.524550339 | 0.999970678 | 30.813 | 19.44 | 37.964 | 26.15 | NA |
| TRINITY_DN7395_c0_g1_i1 | 0.519897527 | 0.674435862 | 0.999970678 | 0.972 | 3.972 | 1.833 | 1.196 | NA |
| TRINITY_DN2313_c0_g1_i1 | -0.5198945 | 0.790236571 | 0.999970678 | 1.562 | 32.31 | 2.333 | 45.081 | F-box |
| TRINITY_DN201_c0_g1_i2 | -0.5198927 | 0.58043982 | 0.999970678 | 4.251 | 7.52 | 9.577 | 5.228 | UDPGT |
| TRINITY_DN2687_c0_g2_i3 | -0.51980761 | 0.867430971 | 1 | 4.733 | 0.611 | 6.355 | 0 | Bac_rhodopsin |
| TRINITY_DN6344_c0_g2_i1 | 0.519783332 | 0.906777062 | 1 | 2.653 | 0 | 1.555 | 0 | - |
| TRINITY_DN3144_c0_g2_i1 | 0.519736172 | 0.578609187 | 0.999970678 | 2.643 | 3.804 | 2.578 | 1.357 | Castor_Poll_mid |
| TRINITY_DN284_c0_g2_i1 | -0.51969353 | 0.415958083 | 0.999970678 | 254.92 | 199.4 | 234.552 | 360.365 | NA |
| TRINITY_DN11421_c0_g1_i11 | -0.51966235 | 0.66659902 | 0.999970678 | 0.681 | 0.276 | 0.344 | 0.965 | HLH |
| TRINITY_DN5651_c0_g1_i5 | 0.519653155 | 0.684060994 | 0.999970678 | 0.836 | 0.779 | 0.211 | 0.875 | GDP_Man_Dehyd |
| TRINITY_DN4196_c0_g1_i1 | 0.51964427 | 0.415285148 | 0.999970678 | 5.796 | 5.755 | 3.066 | 4.353 | NA |
| TRINITY_DN2412_c0_g1_i1 | -0.51958856 | 0.532672167 | 0.999970678 | 7.767 | 5.066 | 9.188 | 7.289 | Methyltransf_16 |
| TRINITY_DN523_c0_g1_i2 | -0.51958396 | 0.853659805 | 1 | 0.845 | 0 | 0.622 | 0.452 | Mito_carr |
| TRINITY_DN76686_c0_g1_i1 | 0.519555176 | 0.669135986 | 0.999970678 | 0.3 | 0.453 | 0.289 | 0.171 | PPR |
| TRINITY_DN1731_c0_g2_i3 | 0.519502124 | 0.534705135 | 0.999970678 | 2.816 | 4.139 | 2.267 | 2.091 | Sigma70_r2 |
| TRINITY_DN750_c1_g1_i5 | -0.51947633 | 0.710002413 | 0.999970678 | 3.016 | 0.424 | 2.855 | 1.548 | zf-CCCH |
| TRINITY_DN11781_c0_g1_i1 | 0.519457756 | 0.489912912 | 0.999970678 | 3.27 | 3.223 | 2.244 | 1.82 | DEAD |
| TRINITY_DN5727_c0_g1_i1 | -0.5193919 | 0.5821317 | 0.999970678 | 2.053 | 3.666 | 3.189 | 4.263 | NA |
| TRINITY_DN1322_c0_g1_i1 | 0.519379668 | 0.611300463 | 0.999970678 | 85.235 | 25.54 | 36.453 | 33.64 | HMA |
| TRINITY_DN6428_c0_g1_i1 | 0.519313816 | 0.629256755 | 0.999970678 | 1.036 | 0.7 | 0.522 | 0.583 | DDT |
| TRINITY_DN1767_c0_g1_i13 | 0.519309201 | 0.595542055 | 0.999970678 | 3.752 | 6.288 | 1.867 | 4.695 | NA |
| TRINITY_DN395_c0_g1_i1 | -0.51913713 | 0.436080215 | 0.999970678 | 41.414 | 54.94 | 47.497 | 79.716 | NA |
| TRINITY_DN7461_c0_g1_i1 | 0.519057626 | 0.414503048 | 0.999970678 | 5.341 | 5.292 | 3.278 | 3.479 | Nsp1_C |
| TRINITY_DN1719_c1_g1_i2 | 0.519032181 | 0.532649936 | 0.999970678 | 15.552 | 26.51 | 15.61 | 10.375 | NA |
| TRINITY_DN4966_c0_g1_i8 | -0.5189757 | 0.694248621 | 0.999970678 | 6.431 | 18.91 | 10.244 | 21.394 | Aldedh |
| TRINITY_DN7804_c0_g1_i4 | -0.51889551 | 0.656890148 | 0.999970678 | 0.527 | 2.099 | 1.644 | 1.769 | - |
| TRINITY_DN15303_c0_g1_i4 | 0.518748774 | 0.906337567 | 0.999970678 | 3.47 | 0 | 2.022 | 0 | NA |
| TRINITY_DN5946_c0_g1_i2 | -0.51872919 | 0.487538363 | 0.999970678 | 4.733 | 5.903 | 5.411 | 8.626 | - |
| TRINITY_DN3783_c0_g1_i10 | -0.51872792 | 0.800879849 | 0.999970678 | 0.109 | 17.9 | 10.888 | 12.276 | NA |
| TRINITY_DN7459_c0_g1_i3 | -0.51869096 | 0.503192452 | 0.999970678 | 3.37 | 3.4 | 3.255 | 5.751 | NA |
| TRINITY_DN4219_c0_g1_i4 | 0.51867072 | 0.394841332 | 0.999970678 | 3.179 | 3.991 | 2.144 | 2.423 | NA |
| TRINITY_DN6617_c0_g1_i1 | -0.51858614 | 0.753228476 | 0.999970678 | 0.136 | 1.537 | 0.811 | 1.418 | NA |

| TRINITY_DN69_c0_g1_i4 | -0.51854221 | 0.460966198 | 0.999970678 | 12.445 | 8.377 | 11.81 | 15.593 | NA |
| --- | --- | --- | --- | --- | --- | --- | --- | --- |
| TRINITY_DN9209_c0_g2_i1 | 0.518510181 | 0.789020071 | 0.999970678 | 1.59 | 0.375 | 1.078 | 0.08 | Myb_DNA-bindin |
| TRINITY_DN3423_c0_g1_i1 | -0.5184901 | 0.902046413 | 0.999970678 | 0 | 6.662 | 7.788 | 0 | RRM_1 |
| TRINITY_DN4725_c0_g1_i1 | 0.518477641 | 0.750238775 | 0.999970678 | 3.007 | 18.67 | 1.144 | 13.633 | Mem_trans |
| TRINITY_DN64800_c0_g1_i2 | -0.5184635 | 0.809381769 | 1 | 10.21 | 0.71 | 11.333 | 1.81 | Lectin_legB |
| TRINITY_DN1158_c0_g5_i1 | -0.51841369 | 0.580740008 | 0.999970678 | 48.29 | 58.78 | 33.009 | 111.747 | Ribosomal_L28e |
| TRINITY_DN1646_c0_g1_i3 | -0.51840977 | 0.648105947 | 0.999970678 | 0.336 | 0.473 | 0.333 | 0.754 | NA |
| TRINITY_DN40720_c1_g1_i1 | -0.51833031 | 0.56105724 | 0.999970678 | 37.408 | 20.71 | 40.775 | 31.85 | NA |
| TRINITY_DN634_c0_g1_i3 | 0.518300916 | 0.901923457 | 0.999970678 | 9.52 | 0 | 0 | 6.726 | Caleosin |
| TRINITY_DN26339_c0_g3_i2 | 0.518297707 | 0.768195596 | 0.999970678 | 22.946 | 2.06 | 13.11 | 1.769 | NA |
| TRINITY_DN2791_c0_g1_i1 | 0.518254296 | 0.749333053 | 0.999970678 | 6.177 | 1.705 | 4.178 | 0.493 | NA |
| TRINITY_DN1980_c0_g1_i6 | -0.51823762 | 0.903357591 | 0.999970678 | 10.347 | 0 | 0 | 14.578 | NA |
| TRINITY_DN13513_c0_g1_i1 | 0.51822933 | 0.907392883 | 1 | 0.645 | 0 | 0 | 0.473 | NA |
| TRINITY_DN1115_c0_g1_i6 | 0.518229015 | 0.901986613 | 0.999970678 | 16.079 | 0 | 0 | 11.32 | UQ_con |
| TRINITY_DN823_c0_g1_i11 | 0.518221025 | 0.685739358 | 0.999970678 | 33.929 | 52.54 | 43.475 | 7.611 | FAD_binding_8 |
| TRINITY_DN5939_c0_g1_i1 | 0.518205243 | 0.645002505 | 0.999970678 | 73.762 | 28.24 | 14.555 | 53.827 | NA |
| TRINITY_DN2775_c0_g1_i2 | -0.51815208 | 0.544106663 | 0.999970678 | 3.525 | 4.257 | 3.366 | 7.048 | GST_C |
| TRINITY_DN6592_c0_g2_i4 | 0.518149388 | 0.543996329 | 0.999970678 | 5.078 | 3.164 | 3.066 | 2.071 | PH |
| TRINITY_DN1655_c0_g1_i6 | 0.518124678 | 0.438859234 | 0.999970678 | 23.219 | 24.36 | 13.61 | 16.126 | NA |
| TRINITY_DN1272_c0_g2_i5 | 0.518099068 | 0.49133557 | 0.999970678 | 9.584 | 10.33 | 7.255 | 5.137 | NA |
| TRINITY_DN5381_c0_g1_i6 | -0.51803168 | 0.675282747 | 0.999970678 | 0.6 | 0.857 | 0.644 | 1.307 | ABC_membrane |
| TRINITY_DN185_c0_g1_i2 | 0.517988123 | 0.787414669 | 1 | 2.262 | 0.434 | 0.144 | 1.709 | TPR_1 |
| TRINITY_DN1357_c0_g1_i3 | -0.51798739 | 0.767327205 | 0.999970678 | 0.273 | 1.094 | 0.078 | 1.84 | Arm |
| TRINITY_DN799_c0_g1_i4 | -0.51789959 | 0.683097661 | 0.999970678 | 39.987 | 22.34 | 63.885 | 12.416 | FA_desaturase |
| TRINITY_DN23937_c0_g1_i2 | 0.517895397 | 0.905965496 | 0.999970678 | 5.768 | 0 | 3.366 | 0 | Ribosomal_S17 |
| TRINITY_DN3825_c1_g2_i3 | -0.51787879 | 0.436026295 | 0.999970678 | 7.767 | 7.805 | 8.2 | 12.336 | NA |
| TRINITY_DN18226_c0_g1_i1 | -0.51786993 | 0.741289716 | 0.999970678 | 1.535 | 0.424 | 0.544 | 2.101 | NA |
| TRINITY_DN779_c0_g1_i8 | -0.51785161 | 0.704342109 | 0.999970678 | 1.117 | 1.242 | 0.367 | 2.946 | NA |
| TRINITY_DN2484_c0_g1_i3 | 0.517843374 | 0.505740482 | 0.999970678 | 7.676 | 5.322 | 3.244 | 5.168 | NA |
| TRINITY_DN849_c0_g1_i9 | -0.51784068 | 0.800587362 | 0.999970678 | 1.472 | 0.039 | 1.555 | 0.322 | NA |
| TRINITY_DN17302_c0_g1_i1 | -0.51779143 | 0.622336343 | 0.999970678 | 2.28 | 4.188 | 2.278 | 6.434 | NA |
| TRINITY_DN3589_c0_g1_i1 | 0.517788068 | 0.85921285 | 0.999970678 | 1.499 | 0 | 0.689 | 0.221 | NA |
| TRINITY_DN3518_c0_g1_i1 | -0.51772607 | 0.526797593 | 0.999970678 | 13.217 | 10.19 | 9.344 | 22.279 | NA |
| TRINITY_DN5876_c0_g1_i10 | -0.51772363 | 0.848538963 | 0.999970678 | 8.194 | 0 | 3.844 | 7.178 | NA |
| TRINITY_DN24902_c0_g1_i3 | 0.517719965 | 0.79453108 | 0.999970678 | 29.959 | 0.779 | 2.033 | 19.182 | NA |
| TRINITY_DN3855_c0_g1_i1 | -0.51765656 | 0.87588623 | 0.999970678 | 4.86 | 0.059 | 5.855 | 0 | NA |
| TRINITY_DN96_c0_g1_i5 | -0.51763522 | 0.613072133 | 0.999970678 | 3.443 | 10.63 | 6.455 | 12.185 | - |
| TRINITY_DN460_c1_g1_i2 | -0.51755006 | 0.495797963 | 0.999970678 | 16.714 | 24.26 | 30.143 | 21.887 | NA |
| TRINITY_DN235_c0_g2_i1 | -0.51753243 | 0.502354158 | 0.999970678 | 26.48 | 22.09 | 20.543 | 44.266 | NA |
| TRINITY_DN442_c0_g2_i3 | -0.51752932 | 0.598732025 | 0.999970678 | 25.417 | 12.49 | 13.766 | 37.611 | NA |
| TRINITY_DN3766_c0_g1_i2 | -0.51751206 | 0.518720998 | 0.999970678 | 47.909 | 50.1 | 36.831 | 96.335 | EFG_C |
| TRINITY_DN1931_c0_g1_i5 | 0.517480231 | 0.859510529 | 0.999970678 | 9.593 | 0.887 | 6.155 | 0 | NA |
| TRINITY_DN1183_c0_g1_i2 | 0.517479758 | 0.629910194 | 0.999970678 | 0.963 | 1.38 | 0.867 | 0.573 | LRR_8 |
| TRINITY_DN1179_c0_g3_i1 | 0.51747433 | 0.642980616 | 0.999970678 | 22.119 | 16.94 | 5.233 | 20.148 | NA |
| TRINITY_DN6346_c0_g1_i2 | -0.51743794 | 0.858493958 | 0.999970678 | 1.254 | 0 | 0.189 | 1.588 | S6PP |
| TRINITY_DN421_c0_g1_i1 | -0.51739751 | 0.551973467 | 0.999970678 | 33.22 | 69.84 | 44.064 | 93.55 | NA |
| TRINITY_DN3634_c0_g1_i1 | -0.5173819 | 0.904599712 | 0.999970678 | 5.36 | 0 | 6.433 | 0 | NA |
| TRINITY_DN7068_c1_g1_i2 | -0.51736955 | 0.608068431 | 0.999970678 | 1.926 | 2.819 | 2.789 | 3.388 | NA |
| TRINITY_DN15682_c0_g1_i7 | -0.51731922 | 0.908518321 | 1 | 0.972 | 0 | 1.133 | 0 | NA |
| TRINITY_DN26478_c0_g1_i3 | -0.51731922 | 0.908518321 | 1 | 0.999 | 0 | 1.189 | 0 | Catalase |
| TRINITY_DN790_c0_g2_i1 | -0.51729743 | 0.68770917 | 0.999970678 | 0.309 | 0.907 | 0.822 | 0.714 | UPF0203 |
| TRINITY_DN21_c0_g1_i2 | 0.517269242 | 0.857725571 | 1 | 0.454 | 1.537 | 0 | 1.367 | NA |
| TRINITY_DN6245_c0_g1_i2 | -0.51719225 | 0.579553667 | 0.999970678 | 3.852 | 3.489 | 5.889 | 3.358 | NA |
| TRINITY_DN19_c0_g1_i1 | 0.517033948 | 0.670735548 | 0.999970678 | 1.526 | 6.199 | 1.4 | 3.67 | WHEP-TRS |
| TRINITY_DN530_c0_g1_i1 | -0.51695952 | 0.467662588 | 0.999970678 | 38.498 | 53.91 | 42.453 | 80.631 | CS |
| TRINITY_DN9990_c0_g1_i2 | -0.51695941 | 0.707278692 | 0.999970678 | 83.981 | 143.4 | 27.943 | 271.731 | - |
| TRINITY_DN6235_c0_g1_i1 | -0.51694888 | 0.702340964 | 0.999970678 | 3.652 | 1.291 | 2.055 | 4.424 | NA |
| TRINITY_DN3420_c1_g1_i6 | 0.516878248 | 0.853519593 | 0.999970678 | 2.952 | 0 | 0.567 | 1.418 | NA |
| TRINITY_DN677_c0_g4_i1 | 0.516836474 | 0.688136785 | 0.999970678 | 13.689 | 19.86 | 2.5 | 20.389 | ADH_N_2 |
| TRINITY_DN3458_c0_g1_i14 | -0.51669753 | 0.648539952 | 0.999970678 | 2.662 | 0.828 | 1.667 | 3.046 | p450 |
| TRINITY_DN8622_c0_g2_i2 | 0.516684399 | 0.438234371 | 0.999970678 | 1.553 | 1.577 | 0.911 | 1.096 | Smg8_Smg9 |
| TRINITY_DN10175_c0_g1_i4 | 0.51665807 | 0.375905316 | 0.999970678 | 6.141 | 5.775 | 3.355 | 4.303 | NA |
| TRINITY_DN15050_c0_g1_i1 | 0.516642458 | 0.626546696 | 0.999970678 | 1.499 | 4.711 | 1.455 | 2.554 | ABC2_membrane |
| TRINITY_DN2881_c0_g1_i1 | 0.516533857 | 0.77578139 | 0.999970678 | 13.908 | 0.788 | 6.411 | 2.463 | NA |
| TRINITY_DN8850_c0_g1_i3 | -0.516443 | 0.399381916 | 0.999970678 | 30.94 | 39.06 | 37.831 | 53.878 | NA |
| TRINITY_DN12018_c0_g1_i13 | 0.516440959 | 0.754187598 | 0.999970678 | 7.004 | 3.745 | 5.422 | 0.804 | NA |
| TRINITY_DN504_c0_g1_i11 | 0.516232822 | 0.772785613 | 0.999970678 | 0.118 | 0.641 | 0.378 | 0.07 | DUF3522 |
| TRINITY_DN4239_c0_g1_i3 | -0.51617704 | 0.530957959 | 0.999970678 | 10.374 | 7.569 | 13.71 | 9.119 | NA |
| TRINITY_DN49428_c0_g1_i2 | 0.516125696 | 0.456157363 | 0.999970678 | 32.084 | 43.11 | 26.187 | 20.882 | Fer2_4 |
| TRINITY_DN9520_c0_g1_i1 | -0.51612082 | 0.855663665 | 0.999970678 | 1.526 | 0 | 1.378 | 0.553 | NA |
| TRINITY_DN9222_c0_g2_i2 | 0.516058033 | 0.696521444 | 0.999970678 | 0.927 | 3.479 | 1.089 | 1.769 | NA |
| TRINITY_DN11477_c0_g1_i2 | -0.51604442 | 0.458331699 | 0.999970678 | 32.203 | 47.3 | 54.819 | 46.911 | RRM_1 |
| TRINITY_DN13947_c0_g1_i2 | 0.516018185 | 0.853179015 | 0.999970678 | 0.409 | 1.981 | 0 | 1.649 | NA |
| TRINITY_DN2371_c1_g1_i15 | 0.516002729 | 0.804998183 | 1 | 3.806 | 2.079 | 3.322 | 0.131 | NA |

| TRINITY_DN730_c2_g1_i3 | -0.51591544 | 0.631418338 | 0.999970678 | 7.812 | 10.4 | 4.366 | 20.811 | zinc_ribbon_12 |
| --- | --- | --- | --- | --- | --- | --- | --- | --- |
| TRINITY_DN37_c2_g2_i1 | -0.5158678 | 0.465238488 | 0.999970678 | 13.308 | 11.24 | 17.81 | 13.643 | NA |
| TRINITY_DN2103_c0_g1_i5 | -0.51584474 | 0.620530182 | 0.999970678 | 1111.7 | 1666 | 2559.134 | 854.443 | NA |
| TRINITY_DN192_c0_g1_i6 | -0.51582076 | 0.640296386 | 0.999970678 | 1.29 | 4.612 | 2.811 | 5.007 | Ribosomal_L3 |
| TRINITY_DN2941_c1_g1_i7 | -0.51581686 | 0.85202185 | 0.999970678 | 0 | 0.591 | 0.511 | 0.231 | NA |
| TRINITY_DN1054_c1_g1_i6 | -0.51579876 | 0.590482646 | 0.999970678 | 5.55 | 2.119 | 5.022 | 4.987 | NA |
| TRINITY_DN3013_c0_g1_i5 | 0.515731076 | 0.869867939 | 1 | 4.742 | 0.217 | 0 | 3.418 | NA |
| TRINITY_DN23091_c0_g1_i1 | -0.51572623 | 0.5735611 | 0.999970678 | 1.917 | 1.922 | 2.733 | 2.162 | NA |
| TRINITY_DN9935_c0_g1_i1 | -0.51554094 | 0.571761669 | 0.999970678 | 2.28 | 2.789 | 2.389 | 4.263 | NA |
| TRINITY_DN7319_c0_g1_i1 | 0.51553174 | 0.481683663 | 0.999970678 | 2.798 | 2.355 | 1.789 | 1.458 | NA |
| TRINITY_DN18704_c0_g1_i4 | 0.515495855 | 0.785803784 | 0.999970678 | 3.27 | 6.544 | 5.555 | 0.101 | RNA_pol_Rpb2_1 |
| TRINITY_DN5625_c0_g3_i1 | -0.51548064 | 0.851242193 | 0.999970678 | 0 | 6.386 | 5.366 | 2.483 | SGTA_dimer |
| TRINITY_DN31813_c0_g1_i1 | 0.515451661 | 0.585596753 | 0.999970678 | 1.517 | 1.37 | 1.011 | 0.824 | NA |
| TRINITY_DN4247_c0_g1_i1 | 0.515337527 | 0.514746505 | 0.999970678 | 15.942 | 8.663 | 6.411 | 9.561 | NA |
| TRINITY_DN5865_c0_g1_i2 | -0.51518586 | 0.856083798 | 1 | 0.636 | 0.365 | 0 | 1.428 | Ras |
| TRINITY_DN3205_c0_g1_i6 | 0.515080935 | 0.563213666 | 0.999970678 | 1.199 | 1.468 | 0.644 | 1.076 | NA |
| TRINITY_DN13078_c0_g1_i1 | -0.51507502 | 0.358332335 | 0.999970678 | 66.594 | 66.64 | 76.718 | 96.646 | NA |
| TRINITY_DN13600_c0_g1_i2 | -0.51507096 | 0.714523647 | 0.999970678 | 5.759 | 1.173 | 6.389 | 2.262 | NA |
| TRINITY_DN37080_c3_g1_i1 | 0.515037577 | 0.871557679 | 1 | 6.068 | 0.315 | 3.7 | 0 | NA |
| TRINITY_DN11220_c0_g1_i4 | 0.515037577 | 0.871557679 | 1 | 1.126 | 0.049 | 0.7 | 0 | Bys1 |
| TRINITY_DN1683_c0_g1_i2 | 0.514970095 | 0.512502458 | 0.999970678 | 4.242 | 4.504 | 3.044 | 2.403 | NA |
| TRINITY_DN16836_c1_g1_i3 | 0.514793303 | 0.693557666 | 0.999970678 | 1.363 | 0.966 | 0.989 | 0.432 | NA |
| TRINITY_DN3298_c0_g1_i4 | 0.514788432 | 0.902579734 | 0.999970678 | 8.866 | 0 | 0 | 6.314 | - |
| TRINITY_DN28331_c0_g1_i2 | 0.5147815 | 0.706577916 | 0.999970678 | 2.698 | 0.976 | 1.411 | 0.875 | NA |
| TRINITY_DN1147_c2_g1_i1 | -0.51466268 | 0.755998163 | 0.999970678 | 0.164 | 2.355 | 1.389 | 1.87 | PLDc_2 |
| TRINITY_DN1172_c0_g1_i3 | 0.514623799 | 0.660413979 | 0.999970678 | 1.326 | 0.719 | 0.411 | 0.955 | NA |
| TRINITY_DN3420_c0_g1_i4 | -0.51461145 | 0.863947515 | 1 | 0 | 1.291 | 0.233 | 1.528 | Ribosomal_L12 |
| TRINITY_DN15473_c0_g2_i1 | -0.51454765 | 0.541611955 | 0.999970678 | 109.67 | 102.7 | 175.789 | 90.926 | FA_desaturase |
| TRINITY_DN14961_c0_g1_i3 | -0.51453883 | 0.85020211 | 0.999970678 | 3.288 | 0 | 2.255 | 2.031 | NA |
| TRINITY_DN4127_c0_g1_i3 | 0.514481148 | 0.673748677 | 0.999970678 | 5.096 | 17.98 | 3.133 | 12.245 | NA |
| TRINITY_DN5098_c0_g2_i2 | 0.514476056 | 0.554569717 | 0.999970678 | 4.524 | 3.558 | 3.189 | 1.81 | NA |
| TRINITY_DN687_c0_g1_i4 | 0.514471197 | 0.601037257 | 0.999970678 | 23.664 | 12.44 | 6.133 | 17.966 | NA |
| TRINITY_DN5570_c0_g1_i1 | -0.51438875 | 0.516411239 | 0.999970678 | 4.242 | 4.977 | 3.789 | 8.606 | NA |
| TRINITY_DN5269_c0_g1_i14 | 0.51431209 | 0.871528288 | 1 | 3.752 | 0.168 | 2.267 | 0 | NA |
| TRINITY_DN22814_c0_g1_i1 | 0.514306144 | 0.740597866 | 0.999970678 | 0.581 | 0.138 | 0.311 | 0.131 | NA |
| TRINITY_DN23769_c0_g1_i3 | 0.514286071 | 0.750941376 | 1 | 1.481 | 0.749 | 1.055 | 0.251 | NA |
| TRINITY_DN5127_c0_g1_i1 | -0.51427046 | 0.485475997 | 0.999970678 | 56.275 | 90.24 | 99.05 | 84.572 | NA |
| TRINITY_DN1543_c0_g1_i5 | 0.514242778 | 0.862099256 | 0.999970678 | 0 | 2.523 | 0.122 | 1.609 | Pept_tRNA_hydro |
| TRINITY_DN6514_c0_g1_i2 | 0.514178866 | 0.866232803 | 0.999970678 | 19.058 | 0.591 | 0 | 13.934 | NA |
| TRINITY_DN3205_c2_g1_i3 | -0.51415917 | 0.747515009 | 0.999970678 | 4.451 | 0.345 | 2.255 | 4.192 | EF_TS |
| TRINITY_DN4139_c0_g1_i1 | 0.514126846 | 0.378422009 | 0.999970678 | 12.3 | 11.05 | 6.466 | 8.596 | NA |
| TRINITY_DN1274_c0_g1_i1 | 0.514072635 | 0.730473758 | 0.999970678 | 0.672 | 7.598 | 1.855 | 3.489 | MatE |
| TRINITY_DN14695_c1_g1_i2 | 0.514053784 | 0.562394691 | 0.999970678 | 0.808 | 0.907 | 0.544 | 0.553 | NA |
| TRINITY_DN486_c0_g1_i2 | -0.51404562 | 0.648272387 | 0.999970678 | 7.294 | 8.19 | 8.311 | 11.059 | NA |
| TRINITY_DN2426_c0_g2_i1 | 0.513944198 | 0.630541521 | 0.999970678 | 1.699 | 5.213 | 2.489 | 1.8 | NA |
| TRINITY_DN7372_c0_g3_i1 | -0.51391372 | 0.515272252 | 0.999970678 | 18.985 | 22.03 | 16.31 | 38.626 | NA |
| TRINITY_DN1934_c0_g2_i1 | -0.51388465 | 0.598641639 | 0.999970678 | 399.29 | 1272 | 814.117 | 1376.924 | NA |
| TRINITY_DN161_c0_g1_i4 | -0.51384722 | 0.740970353 | 1 | 0.336 | 0.138 | 0.422 | 0.151 | DnaJ |
| TRINITY_DN3858_c0_g1_i2 | -0.51377067 | 0.869351476 | 0.999970678 | 101.25 | 0 | 73.318 | 58.402 | - |
| TRINITY_DN13120_c0_g2_i1 | 0.513737217 | 0.58330513 | 0.999970678 | 2.216 | 1.301 | 0.833 | 1.478 | NA |
| TRINITY_DN25172_c0_g2_i3 | 0.513690929 | 0.645746159 | 0.999970678 | 0.736 | 0.65 | 0.433 | 0.432 | NA |
| TRINITY_DN728_c0_g1_i26 | 0.513665454 | 0.862142008 | 0.999970678 | 1.235 | 0 | 0.611 | 0.141 | Glyco_hydro_79n |
| TRINITY_DN10008_c0_g1_i6 | -0.51364548 | 0.85385869 | 0.999970678 | 1.036 | 0.453 | 1.744 | 0 | NA |
| TRINITY_DN1513_c2_g1_i1 | 0.513638664 | 0.648218165 | 0.999970678 | 1.69 | 1.439 | 1.233 | 0.704 | NA |
| TRINITY_DN1772_c0_g1_i9 | -0.5136207 | 0.740671962 | 0.999970678 | 1.263 | 0.966 | 2.411 | 0.282 | NA |
| TRINITY_DN1056_c0_g3_i1 | 0.513610588 | 0.76488073 | 0.999970678 | 1880.2 | 255.4 | 1161.152 | 104.619 | Chloroa_b-bind |
| TRINITY_DN1634_c0_g2_i14 | -0.51351923 | 0.86873751 | 0.999970678 | 0.627 | 0.03 | 0.778 | 0 | NA |
| TRINITY_DN4625_c0_g1_i1 | -0.51346022 | 0.548303174 | 0.999970678 | 1.989 | 3.863 | 3.444 | 4.142 | NA |
| TRINITY_DN1375_c0_g1_i1 | 0.513412132 | 0.665351731 | 0.999970678 | 2.498 | 1.488 | 0.578 | 2.091 | NA |
| TRINITY_DN48_c0_g1_i3 | 0.513354297 | 0.588548012 | 0.999970678 | 7.576 | 3.006 | 2.9 | 3.971 | E1_dh |
| TRINITY_DN90828_c0_g1_i1 | -0.51328803 | 0.904728208 | 0.999970678 | 0.872 | 0 | 1.044 | 0 | NA |
| TRINITY_DN75576_c0_g1_i1 | -0.51323134 | 0.908115466 | 1 | 1.29 | 0 | 1.544 | 0 | NA |
| TRINITY_DN2246_c0_g1_i3 | -0.51321546 | 0.785255493 | 0.999970678 | 15.016 | 0.266 | 9.488 | 10.556 | Tom22 |
| TRINITY_DN9041_c0_g1_i3 | -0.51320527 | 0.738294845 | 0.999970678 | 8.43 | 1.725 | 10.21 | 2.192 | NA |
| TRINITY_DN6159_c0_g1_i7 | -0.51307046 | 0.84934544 | 0.999970678 | 0 | 1.045 | 0.422 | 0.975 | NA |
| TRINITY_DN7661_c0_g1_i1 | -0.51306795 | 0.526799533 | 0.999970678 | 3.77 | 2.799 | 4.178 | 4.303 | NA |
| TRINITY_DN3283_c0_g1_i4 | -0.51306062 | 0.849912207 | 0.999970678 | 1.363 | 0 | 0.833 | 0.955 | zf-SNAP50_C |
| TRINITY_DN7577_c0_g1_i2 | 0.513038954 | 0.522183787 | 0.999970678 | 9.111 | 12.2 | 4.2 | 9.873 | NA |
| TRINITY_DN714_c0_g1_i4 | -0.51292817 | 0.848192147 | 0.999970678 | 0 | 6.593 | 3.5 | 4.967 | NA |
| TRINITY_DN255_c0_g1_i3 | -0.51282188 | 0.447725147 | 0.999970678 | 4.56 | 3.932 | 4.611 | 6.575 | NA |
| TRINITY_DN30338_c0_g3_i2 | 0.512794199 | 0.671913664 | 0.999970678 | 102.02 | 25.57 | 19.899 | 66.234 | NA |
| TRINITY_DN1873_c5_g1_i2 | -0.51268593 | 0.737673401 | 0.999970678 | 0.372 | 0.266 | 0.622 | 0.141 | NA |
| TRINITY_DN14371_c0_g1_i3 | 0.512671766 | 0.767292292 | 1 | 0.572 | 1.163 | 0.111 | 1.056 | NA |
| TRINITY_DN930_c0_g1_i1 | -0.51260837 | 0.356092251 | 0.999970678 | 55.948 | 57.65 | 68.463 | 79.052 | ATP-synt_D |

| TRINITY_DN13254_c0_g1_i1 | 0.512582994 | 0.491339696 | 0.999970678 | 6.686 | 9.333 | 4.944 | 5.178 | NA |
| --- | --- | --- | --- | --- | --- | --- | --- | --- |
| TRINITY_DN945_c1_g1_i3 | 0.512512006 | 0.510048388 | 0.999970678 | 10.147 | 10.1 | 4.5 | 8.656 | Peptidase_S24 |
| TRINITY_DN2246_c0_g1_i6 | -0.5124807 | 0.903233839 | 0.999970678 | 0 | 7.845 | 9.099 | 0 | Tom22 |
| TRINITY_DN3132_c2_g3_i1 | 0.512471913 | 0.785746717 | 0.999970678 | 1.971 | 2.395 | 2.389 | 0.161 | TPR_1 |
| TRINITY_DN6607_c0_g1_i2 | -0.51244643 | 0.604686139 | 0.999970678 | 7.403 | 6.948 | 11.955 | 5.831 | Cellulose_synt |
| TRINITY_DN4238_c0_g2_i13 | -0.51242112 | 0.816980007 | 0.999970678 | 0.009 | 0.572 | 0.567 | 0.131 | FAR1 |
| TRINITY_DN1517_c0_g1_i14 | -0.51240195 | 0.856545462 | 0.999970678 | 0.654 | 0 | 0.156 | 0.764 | NA |
| TRINITY_DN1693_c0_g1_i1 | -0.51239834 | 0.632487042 | 0.999970678 | 2.008 | 0.867 | 2.067 | 1.639 | NA |
| TRINITY_DN14342_c1_g1_i1 | -0.51239633 | 0.90380451 | 0.999970678 | 3.724 | 0 | 4.466 | 0 | Glycogen_syn |
| TRINITY_DN7110_c0_g1_i1 | -0.51231582 | 0.437497507 | 0.999970678 | 43.058 | 30.36 | 46.953 | 48.238 | PCI |
| TRINITY_DN1702_c0_g1_i1 | -0.51231018 | 0.557817372 | 0.999970678 | 34.81 | 56.44 | 73.029 | 40.717 | NA |
| TRINITY_DN6240_c0_g1_i2 | 0.512271082 | 0.905051232 | 0.999970678 | 1.063 | 0 | 0 | 0.764 | TMEM135_C_rich |
| TRINITY_DN6626_c0_g1_i1 | 0.512150714 | 0.425166469 | 0.999970678 | 20.957 | 27.34 | 12.855 | 18.147 | NA |
| TRINITY_DN579_c0_g1_i4 | -0.51206059 | 0.549943497 | 0.999970678 | 15.116 | 17.1 | 23.887 | 15.875 | NA |
| TRINITY_DN122_c0_g1_i4 | -0.51189547 | 0.535015493 | 0.999970678 | 67.585 | 92.99 | 60.152 | 155.832 | AAA |
| TRINITY_DN9018_c0_g1_i3 | -0.51184617 | 0.76635317 | 0.999970678 | 1.026 | 0.798 | 1.933 | 0.241 | NA |
| TRINITY_DN8351_c0_g1_i3 | -0.51177441 | 0.908974785 | 1 | 0.899 | 0 | 0 | 1.297 | NA |
| TRINITY_DN10327_c0_g1_i2 | -0.51174951 | 0.853424605 | 0.999970678 | 0.509 | 1.518 | 0 | 2.875 | NA |
| TRINITY_DN287_c0_g2_i2 | 0.511723005 | 0.516719489 | 0.999970678 | 31.185 | 17.64 | 11.733 | 20.208 | BAG |
| TRINITY_DN9262_c0_g3_i3 | -0.51171863 | 0.832420582 | 0.999970678 | 1.608 | 0.039 | 0.122 | 2.202 | NA |
| TRINITY_DN5787_c0_g2_i3 | 0.511706687 | 0.87216571 | 0.999970678 | 12.3 | 0.266 | 7.322 | 0 | NA |
| TRINITY_DN1835_c0_g1_i2 | -0.51165746 | 0.555032514 | 0.999970678 | 21.22 | 10.76 | 23.554 | 17.343 | NA |
| TRINITY_DN3840_c0_g1_i4 | 0.511645039 | 0.767548252 | 0.999970678 | 0.545 | 4.435 | 0.344 | 3.046 | NA |
| TRINITY_DN10859_c0_g1_i3 | -0.51162374 | 0.679055516 | 0.999970678 | 0.354 | 0.838 | 0.633 | 0.895 | DNA_methylase |
| TRINITY_DN14741_c0_g1_i2 | 0.511601428 | 0.636214917 | 0.999970678 | 9.039 | 18.75 | 9.399 | 7.55 | NA |
| TRINITY_DN19_c0_g1_i7 | 0.511599901 | 0.69490026 | 0.999970678 | 0.509 | 1.33 | 0.744 | 0.362 | WHEP-TRS |
| TRINITY_DN9779_c0_g1_i11 | -0.511585 | 0.910761346 | 1 | 4.596 | 0 | 5.522 | 0 | NA |
| TRINITY_DN11024_c0_g1_i1 | -0.511585 | 0.910761346 | 1 | 1.217 | 0 | 1.467 | 0 | Ecl1 |
| TRINITY_DN11_c0_g1_i1 | 0.511563764 | 0.392003858 | 0.999970678 | 14.907 | 17.47 | 8.911 | 11.924 | NA |
| TRINITY_DN1084_c0_g1_i3 | -0.51134473 | 0.37783462 | 0.999970678 | 102.04 | 93.83 | 111.571 | 139.133 | NA |
| TRINITY_DN1957_c0_g1_i20 | 0.511323524 | 0.858777913 | 0.999970678 | 0.772 | 0 | 0.289 | 0.201 | NA |
| TRINITY_DN53595_c0_g1_i1 | -0.51129252 | 0.734187327 | 1 | 0.118 | 0.424 | 0.378 | 0.302 | BTB |
| TRINITY_DN2548_c0_g1_i1 | 0.511246797 | 0.768649687 | 0.999970678 | 7.685 | 1.311 | 4.289 | 1.106 | NA |
| TRINITY_DN11813_c0_g1_i1 | -0.51124636 | 0.635752179 | 0.999970678 | 4.824 | 8.742 | 3.666 | 14.869 | NA |
| TRINITY_DN1769_c0_g1_i2 | 0.511243263 | 0.670367017 | 0.999970678 | 1.354 | 4.809 | 1.778 | 2.141 | - |
| TRINITY_DN4123_c0_g1_i1 | 0.511157356 | 0.451876248 | 0.999970678 | 8.475 | 9.895 | 6.244 | 5.339 | NA |
| TRINITY_DN11448_c0_g1_i1 | 0.511115121 | 0.572530219 | 0.999970678 | 5.087 | 6.219 | 2.011 | 5.479 | TPT |
| TRINITY_DN7717_c0_g1_i6 | -0.51107192 | 0.759462365 | 0.999970678 | 0.236 | 0.67 | 0.133 | 1.106 | NA |
| TRINITY_DN548_c0_g3_i2 | 0.510971353 | 0.70088596 | 0.999970678 | 0.273 | 1.459 | 0.444 | 0.653 | HMA |
| TRINITY_DN460_c0_g1_i3 | -0.51089622 | 0.849245295 | 0.999970678 | 0 | 1.429 | 0.667 | 1.206 | Lipase_GDSL |
| TRINITY_DN8704_c0_g1_i6 | 0.510878314 | 0.697942714 | 0.999970678 | 1.19 | 2.277 | 1.689 | 0.382 | PPR |
| TRINITY_DN5000_c0_g2_i4 | -0.51087565 | 0.565756289 | 0.999970678 | 5.796 | 9.914 | 6 | 15.07 | NA |
| TRINITY_DN73_c1_g1_i2 | 0.510839665 | 0.870538264 | 0.999970678 | 0.763 | 0 | 0.433 | 0.02 | NA |
| TRINITY_DN11511_c0_g1_i2 | 0.510733324 | 0.426078237 | 0.999970678 | 6.704 | 8.673 | 4.189 | 5.741 | NA |
| TRINITY_DN61157_c0_g1_i1 | 0.510716707 | 0.661652651 | 0.999970678 | 1.817 | 0.907 | 0.922 | 0.794 | NA |
| TRINITY_DN13657_c0_g1_i1 | 0.510699312 | 0.786853421 | 1 | 1.889 | 1.271 | 1.678 | 0.171 | NA |
| TRINITY_DN1675_c0_g1_i8 | -0.51066745 | 0.629343345 | 0.999970678 | 5.587 | 1.873 | 4.444 | 5.298 | NA |
| TRINITY_DN11857_c0_g1_i1 | -0.51066594 | 0.50803776 | 0.999970678 | 3.434 | 4.248 | 3.922 | 6.143 | NA |
| TRINITY_DN835_c0_g2_i1 | 0.510503045 | 0.69822046 | 0.999970678 | 0.781 | 3.164 | 1.055 | 1.478 | NA |
| TRINITY_DN3161_c0_g1_i4 | 0.510482996 | 0.85897951 | 0.999970678 | 3.034 | 0 | 1.489 | 0.372 | RRM_1 |
| TRINITY_DN150_c0_g1_i1 | -0.51038373 | 0.489841688 | 0.999970678 | 18.231 | 11.31 | 17.754 | 20.56 | NA |
| TRINITY_DN1737_c1_g2_i3 | -0.51037301 | 0.621814436 | 0.999970678 | 3.361 | 12.16 | 9.066 | 10.989 | NA |
| TRINITY_DN1915_c0_g1_i16 | -0.51029478 | 0.911303343 | 1 | 0 | 4.701 | 5.489 | 0 | NA |
| TRINITY_DN4153_c0_g1_i5 | 0.51028175 | 0.629685992 | 0.999970678 | 26.879 | 61.21 | 12.755 | 46.207 | Pollen_Ole_e_I |
| TRINITY_DN4291_c0_g1_i3 | -0.51022306 | 0.871641332 | 0.999970678 | 0 | 3.065 | 3.422 | 0.121 | NA |
| TRINITY_DN47854_c0_g1_i2 | 0.510220675 | 0.526935967 | 0.999970678 | 1.653 | 2.336 | 1.344 | 1.176 | NA |
| TRINITY_DN17835_c3_g1_i3 | 0.510172046 | 0.850010636 | 0.999970678 | 0.627 | 0.808 | 0.833 | 0 | NA |
| TRINITY_DN1637_c0_g1_i11 | 0.510105734 | 0.857067191 | 0.999970678 | 9.465 | 1.537 | 0 | 7.822 | NA |
| TRINITY_DN3408_c0_g1_i3 | 0.510056308 | 0.852380596 | 0.999970678 | 0.645 | 2.73 | 0 | 2.353 | NA |
| TRINITY_DN1421_c0_g1_i1 | 0.510020772 | 0.636994707 | 0.999970678 | 3.007 | 8.012 | 2.822 | 4.172 | DEAD |
| TRINITY_DN2909_c0_g1_i3 | 0.509939391 | 0.515038909 | 0.999970678 | 12.836 | 9.993 | 8.544 | 5.731 | NA |
| TRINITY_DN45070_c0_g1_i1 | -0.50993924 | 0.904504805 | 0.999970678 | 1.008 | 0 | 1.2 | 0 | NA |
| TRINITY_DN462_c0_g1_i5 | -0.50988379 | 0.630043877 | 0.999970678 | 10.583 | 6.8 | 15.699 | 5.791 | NA |
| TRINITY_DN7676_c0_g1_i9 | -0.5098568 | 0.606037494 | 0.999970678 | 0.536 | 0.512 | 0.767 | 0.573 | NA |
| TRINITY_DN7278_c0_g1_i4 | 0.509843929 | 0.66802671 | 0.999970678 | 0.754 | 3.085 | 0.989 | 1.468 | EXS |
| TRINITY_DN5344_c0_g3_i1 | -0.50981272 | 0.43443124 | 0.999970678 | 9.293 | 9.432 | 9.377 | 15.322 | Pkinase |
| TRINITY_DN16649_c0_g1_i3 | 0.509797187 | 0.863421616 | 0.999970678 | 0 | 2.346 | 1.167 | 0.221 | NA |
| TRINITY_DN11_c0_g1_i2 | 0.509753569 | 0.851279818 | 0.999970678 | 1.69 | 3.804 | 3.178 | 0 | NA |
| TRINITY_DN1544_c0_g1_i6 | -0.50975067 | 0.576690554 | 0.999970678 | 7.449 | 7.46 | 5.077 | 15 | Motile_Sperm |
| TRINITY_DN4017_c0_g2_i6 | -0.50974329 | 0.545746099 | 0.999970678 | 1.117 | 0.818 | 1.455 | 0.995 | Utp13 |
| TRINITY_DN9984_c0_g1_i3 | -0.50972077 | 0.827874833 | 1 | 2.008 | 0.246 | 0.1 | 3.066 | NA |
| TRINITY_DN29_c0_g1_i4 | 0.509706254 | 0.65018149 | 0.999970678 | 0.845 | 2.602 | 0.978 | 1.216 | Alliinase_C |
| TRINITY_DN4801_c0_g1_i2 | 0.509694216 | 0.541029141 | 0.999970678 | 14.671 | 8.357 | 5.077 | 10.144 | NA |
| TRINITY_DN524_c0_g1_i1 | -0.50966572 | 0.507237922 | 0.999970678 | 22.492 | 44.16 | 39.831 | 45.875 | Ribosomal_L13 |

| TRINITY_DN46264_c0_g1_i1 | 0.509653495 | 0.910633783 | 1 | 2.289 | 0 | 1.344 | 0 | NA |
| --- | --- | --- | --- | --- | --- | --- | --- | --- |
| TRINITY_DN268_c1_g1_i1 | 0.509653495 | 0.910633783 | 1 | 2.771 | 0 | 1.633 | 0 | NA |
| TRINITY_DN4293_c0_g2_i2 | 0.509607788 | 0.868472542 | 0.999970678 | 3.561 | 0 | 0.189 | 2.252 | NA |
| TRINITY_DN37068_c0_g1_i1 | 0.509585086 | 0.909644791 | 1 | 4.469 | 0 | 2.611 | 0 | Sugar_tr |
| TRINITY_DN11092_c0_g1_i2 | 0.509585086 | 0.909644791 | 1 | 1.908 | 0 | 1.111 | 0 | NA |
| TRINITY_DN1329_c0_g3_i2 | 0.509577194 | 0.873506825 | 0.999970678 | 87.96 | 0.039 | 51.83 | 0.03 | Asparaginase_2 |
| TRINITY_DN10219_c2_g3_i1 | 0.509528772 | 0.908870036 | 1 | 3.207 | 0 | 1.878 | 0 | KOW |
| TRINITY_DN105227_c0_g1_i1 | 0.509528772 | 0.908870036 | 1 | 1.599 | 0 | 0.944 | 0 | NA |
| TRINITY_DN4547_c1_g1_i8 | -0.50944426 | 0.873739873 | 0.999970678 | 6.45 | 0.128 | 7.822 | 0 | Ribosomal_L16 |
| TRINITY_DN5032_c0_g1_i5 | 0.509428313 | 0.706857347 | 0.999970678 | 4.905 | 5.253 | 5.2 | 0.875 | NA |
| TRINITY_DN11630_c0_g1_i1 | 0.509427555 | 0.506923206 | 0.999970678 | 3.506 | 3.637 | 1.689 | 2.976 | NA |
| TRINITY_DN3400_c0_g1_i1 | -0.50939293 | 0.548299124 | 0.999970678 | 24.926 | 38.24 | 23.854 | 60.694 | NA |
| TRINITY_DN9931_c1_g1_i3 | -0.5093807 | 0.622171745 | 0.999970678 | 12.609 | 6.485 | 6.089 | 19.906 | 2OG-FeII_Oxy |
| TRINITY_DN877_c0_g1_i4 | 0.509365889 | 0.634540455 | 0.999970678 | 0.4 | 0.394 | 0.189 | 0.322 | NA |
| TRINITY_DN2848_c0_g1_i4 | -0.50930634 | 0.665911429 | 0.999970678 | 4.596 | 1.261 | 4.1 | 3.438 | NA |
| TRINITY_DN988_c0_g1_i2 | 0.509251375 | 0.682481697 | 0.999970678 | 8.021 | 32.98 | 17.832 | 6.957 | NA |
| TRINITY_DN16886_c0_g1_i5 | 0.509235169 | 0.632977194 | 0.999970678 | 0.509 | 0.562 | 0.4 | 0.282 | NA |
| TRINITY_DN3456_c0_g1_i1 | 0.509232685 | 0.59477741 | 0.999970678 | 3.942 | 3.952 | 2.144 | 2.885 | NA |
| TRINITY_DN19740_c0_g1_i2 | 0.509123038 | 0.694981164 | 0.999970678 | 1.481 | 0.611 | 0.789 | 0.513 | - |
| TRINITY_DN1506_c0_g1_i4 | -0.50910898 | 0.573106111 | 0.999970678 | 5.841 | 3.035 | 6.066 | 5.308 | NA |
| TRINITY_DN848_c2_g1_i1 | 0.509064954 | 0.417837531 | 0.999970678 | 13.526 | 18.72 | 9.522 | 11.129 | NA |
| TRINITY_DN3814_c0_g1_i7 | 0.509061049 | 0.632772594 | 0.999970678 | 2.553 | 6.081 | 3.489 | 1.81 | Cation_efflux |
| TRINITY_DN14510_c0_g1_i4 | 0.508932195 | 0.85870148 | 0.999970678 | 0.227 | 1.892 | 1.211 | 0 | NA |
| TRINITY_DN37380_c0_g1_i1 | -0.50888621 | 0.688896777 | 0.999970678 | 0.363 | 1.005 | 0.922 | 0.814 | DRMBL |
| TRINITY_DN14762_c0_g1_i2 | 0.508883144 | 0.444829539 | 0.999970678 | 6.25 | 6.367 | 4.378 | 3.599 | DIL |
| TRINITY_DN5433_c0_g1_i1 | 0.508864158 | 0.645261345 | 0.999970678 | 1.145 | 0.522 | 0.556 | 0.513 | zf-C2H2_jaz |
| TRINITY_DN1655_c0_g1_i3 | -0.50883582 | 0.601946743 | 0.999970678 | 5.786 | 3.242 | 5.833 | 5.69 | NA |
| TRINITY_DN3705_c0_g1_i2 | 0.508774843 | 0.677485878 | 0.999970678 | 6.241 | 10.49 | 2.033 | 9.068 | NA |
| TRINITY_DN2907_c0_g1_i1 | 0.508648418 | 0.744149301 | 0.999970678 | 0.709 | 11.11 | 2.755 | 4.886 | DUF3475 |
| TRINITY_DN37359_c0_g1_i2 | 0.508552601 | 0.592188103 | 0.999970678 | 0.936 | 0.719 | 0.533 | 0.523 | NA |
| TRINITY_DN2656_c0_g2_i7 | 0.508525231 | 0.682922868 | 0.999970678 | 0.863 | 2.828 | 0.533 | 1.94 | Telomere_reg-2 |
| TRINITY_DN17080_c0_g1_i1 | -0.50848761 | 0.766541237 | 0.999970678 | 1.453 | 3.844 | 5.733 | 0.523 | NA |
| TRINITY_DN31631_c0_g3_i2 | -0.50847749 | 0.783333079 | 0.999970678 | 0.127 | 1.39 | 0.478 | 1.538 | NA |
| TRINITY_DN985_c0_g1_i1 | 0.508447612 | 0.69428997 | 0.999970678 | 2.798 | 1.045 | 1.644 | 0.724 | zf-B_box |
| TRINITY_DN1316_c0_g1_i3 | -0.50843659 | 0.865978863 | 0.999970678 | 1.381 | 0 | 1.567 | 0.09 | LRRNT_2 |
| TRINITY_DN4970_c0_g1_i1 | -0.50832424 | 0.532291359 | 0.999970678 | 2.525 | 1.951 | 3.355 | 2.332 | NA |
| TRINITY_DN3346_c0_g1_i1 | 0.508290605 | 0.85601347 | 0.999970678 | 0 | 5.046 | 0.867 | 2.393 | PAC3 |
| TRINITY_DN1956_c0_g1_i3 | -0.50827957 | 0.62230303 | 0.999970678 | 0.672 | 1.133 | 1.522 | 0.714 | Filamin |
| TRINITY_DN12626_c0_g1_i1 | -0.50823983 | 0.743331462 | 0.999970678 | 1.354 | 0.512 | 1.767 | 0.513 | His_Phos_1 |
| TRINITY_DN2754_c0_g1_i2 | -0.50821481 | 0.476070754 | 0.999970678 | 23.391 | 31.49 | 25.154 | 47.283 | NA |
| TRINITY_DN4464_c0_g1_i1 | 0.508080986 | 0.553989228 | 0.999970678 | 6.132 | 9.569 | 2.978 | 7.44 | Lipoxygenase |
| TRINITY_DN2078_c0_g1_i1 | -0.5080533 | 0.495629799 | 0.999970678 | 5.023 | 5.115 | 4.6 | 8.847 | NA |
| TRINITY_DN5885_c0_g1_i1 | 0.508029231 | 0.639354607 | 0.999970678 | 71.818 | 17.69 | 26.454 | 31.528 | NA |
| TRINITY_DN3648_c0_g1_i1 | 0.508008658 | 0.598251041 | 0.999970678 | 26.489 | 79.44 | 36.242 | 30.171 | NA |
| TRINITY_DN137778_c0_g1_i1 | -0.50792068 | 0.909963038 | 1 | 1.726 | 0 | 2.055 | 0 | PBP |
| TRINITY_DN10030_c0_g1_i1 | -0.50787721 | 0.453707999 | 0.999970678 | 508.61 | 356.5 | 435.84 | 675.92 | ubiquitin |
| TRINITY_DN6078_c1_g1_i1 | -0.50787649 | 0.542523841 | 0.999970678 | 46.655 | 73.17 | 46.242 | 113.758 | Cullin_binding |
| TRINITY_DN4242_c0_g2_i17 | -0.50780366 | 0.733675855 | 0.999970678 | 0.818 | 0.246 | 1.022 | 0.302 | NA |
| TRINITY_DN4242_c0_g2_i7 | -0.50780366 | 0.733675855 | 0.999970678 | 0.818 | 0.246 | 1.022 | 0.302 | NA |
| TRINITY_DN4843_c2_g2_i2 | 0.507797965 | 0.59081161 | 0.999970678 | 11.555 | 7.529 | 3.4 | 9.29 | NA |
| TRINITY_DN848_c1_g4_i2 | -0.50779185 | 0.865315455 | 0.999970678 | 28.751 | 0 | 26.354 | 9.662 | Enolase_C |
| TRINITY_DN2620_c0_g1_i3 | -0.50775988 | 0.505045771 | 0.999970678 | 6.677 | 5.893 | 5.522 | 11.18 | NA |
| TRINITY_DN11301_c0_g1_i3 | -0.50771635 | 0.688406942 | 0.999970678 | 0.518 | 2.375 | 1.855 | 1.84 | ADH_N_2 |
| TRINITY_DN6972_c0_g1_i4 | 0.507629789 | 0.911397626 | 1 | 0 | 2.828 | 0 | 1.93 | NA |
| TRINITY_DN1074_c2_g1_i1 | -0.50755849 | 0.854280997 | 0.999970678 | 17.25 | 0 | 10.233 | 12.426 | GRAM |
| TRINITY_DN5680_c0_g1_i3 | 0.507495066 | 0.66130596 | 0.999970678 | 1.762 | 1.271 | 1.411 | 0.432 | Peptidase_S8 |
| TRINITY_DN29_c1_g2_i8 | 0.507403997 | 0.674280662 | 0.999970678 | 2.898 | 1.478 | 0.644 | 2.292 | NA |
| TRINITY_DN8168_c0_g1_i2 | -0.50737225 | 0.858155082 | 1 | 1.099 | 0 | 0.367 | 1.106 | NA |
| TRINITY_DN5947_c0_g1_i1 | -0.50730536 | 0.697959644 | 0.999970678 | 0.936 | 1.321 | 0.622 | 2.413 | NA |
| TRINITY_DN1746_c0_g1_i3 | -0.50728058 | 0.758432193 | 0.999970678 | 1.635 | 0.197 | 1.611 | 0.694 | Utp12 |
| TRINITY_DN5126_c1_g1_i1 | 0.507218604 | 0.58832088 | 0.999970678 | 156.27 | 154.8 | 135.092 | 55.617 | OPT |
| TRINITY_DN5634_c0_g1_i4 | 0.507141108 | 0.723188343 | 0.999970678 | 0.836 | 2.296 | 1.533 | 0.312 | NA |
| TRINITY_DN11586_c1_g1_i2 | -0.50704314 | 0.50301965 | 0.999970678 | 2.598 | 2.553 | 2.567 | 4.182 | Pkinase |
| TRINITY_DN9208_c0_g1_i1 | -0.50703472 | 0.580475784 | 0.999970678 | 6.168 | 6.317 | 4.1 | 12.808 | NA |
| TRINITY_DN214_c0_g1_i1 | -0.50700609 | 0.855950283 | 0.999970678 | 0.79 | 0.315 | 0 | 1.578 | NA |
| TRINITY_DN358_c0_g1_i1 | -0.50687439 | 0.792760504 | 0.999970678 | 0.091 | 5.805 | 3.455 | 4.052 | PPR |
| TRINITY_DN1260_c0_g1_i2 | -0.50686512 | 0.52640722 | 0.999970678 | 20.693 | 12.39 | 23.887 | 18.278 | NA |
| TRINITY_DN1039_c0_g3_i1 | -0.50685796 | 0.628703964 | 0.999970678 | 1.099 | 1.074 | 1.667 | 1.086 | B_lectin |
| TRINITY_DN393_c0_g2_i1 | -0.50685306 | 0.655416045 | 0.999970678 | 22.574 | 6.495 | 23.521 | 13.12 | NA |
| TRINITY_DN13723_c1_g1_i10 | -0.50667998 | 0.811834195 | 1 | 6.004 | 0.818 | 7.055 | 0.834 | NA |
| TRINITY_DN3552_c0_g1_i2 | -0.50667027 | 0.723526317 | 0.999970678 | 34.673 | 25.85 | 5.855 | 79.022 | NA |
| TRINITY_DN15157_c0_g1_i1 | 0.506670213 | 0.746843026 | 0.999970678 | 1.036 | 3.883 | 0.378 | 2.976 | NA |
| TRINITY_DN2740_c1_g1_i7 | 0.506448351 | 0.556562831 | 0.999970678 | 3.952 | 7.431 | 4.066 | 3.076 | Lectin_legB |
| TRINITY_DN1879_c0_g1_i1 | 0.506428217 | 0.384532513 | 0.999970678 | 65.631 | 56.16 | 34.231 | 44.498 | NA |

| TRINITY_DN933_c0_g2_i3 | -0.50641403 | 0.544720598 | 0.999970678 | 11.736 | 15.11 | 10.277 | 25.516 | LEA_2 |
| --- | --- | --- | --- | --- | --- | --- | --- | --- |
| TRINITY_DN4195_c0_g1_i4 | 0.506407299 | 0.908624123 | 0.999970678 | 0 | 0.503 | 0.289 | 0 | NA |
| TRINITY_DN4842_c0_g1_i3 | 0.506387581 | 0.563998192 | 0.999970678 | 68.747 | 178.3 | 76.618 | 79.947 | NA |
| TRINITY_DN7825_c0_g1_i1 | -0.50637737 | 0.507764075 | 0.999970678 | 21.102 | 14.44 | 25.654 | 19.514 | NA |
| TRINITY_DN21677_c0_g1_i2 | -0.50635518 | 0.573525346 | 0.999970678 | 14.28 | 6.189 | 14.266 | 12.014 | Sigma70_r2 |
| TRINITY_DN21793_c0_g1_i1 | -0.50631652 | 0.854641951 | 0.999970678 | 0 | 0.966 | 0.856 | 0.332 | NA |
| TRINITY_DN295_c0_g2_i1 | 0.506242821 | 0.447668288 | 0.999970678 | 8.039 | 12.65 | 6.144 | 7.138 | HECT |
| TRINITY_DN6043_c0_g1_i1 | -0.50622251 | 0.44916168 | 0.999970678 | 7.367 | 6.81 | 9.422 | 8.747 | MCU |
| TRINITY_DN7267_c0_g1_i1 | -0.50621048 | 0.745232259 | 0.999970678 | 1.508 | 0.355 | 0.711 | 1.769 | NA |
| TRINITY_DN10073_c0_g1_i3 | -0.50615235 | 0.83276459 | 0.999970678 | 2.816 | 0.158 | 3.366 | 0.171 | eIF-5a |
| TRINITY_DN8444_c0_g1_i2 | 0.506115134 | 0.730889546 | 0.999970678 | 0.354 | 2.208 | 0.911 | 0.704 | A_thal_3526 |
| TRINITY_DN3932_c0_g1_i11 | 0.506096489 | 0.877537787 | 0.999970678 | 0.836 | 0.01 | 0 | 0.603 | Pkinase |
| TRINITY_DN8201_c0_g1_i7 | -0.50609014 | 0.851389313 | 0.999970678 | 0 | 5.746 | 4.044 | 3.097 | NA |
| TRINITY_DN712_c0_g2_i1 | -0.50608218 | 0.605180912 | 0.999970678 | 2.934 | 1.941 | 1.633 | 4.987 | NA |
| TRINITY_DN4450_c0_g1_i1 | -0.50600855 | 0.730223462 | 0.999970678 | 72.408 | 9.313 | 20.699 | 91.83 | NA |
| TRINITY_DN33589_c0_g1_i1 | 0.50599617 | 0.855379118 | 1 | 0.291 | 0.65 | 0 | 0.664 | NA |
| TRINITY_DN9975_c0_g1_i1 | 0.50597271 | 0.567317393 | 0.999970678 | 7.476 | 4.494 | 4.611 | 2.895 | Noc2 |
| TRINITY_DN14761_c0_g1_i3 | 0.505930571 | 0.851919961 | 0.999970678 | 2.916 | 1.409 | 2.544 | 0 | NA |
| TRINITY_DN2881_c0_g1_i10 | 0.505892472 | 0.759020479 | 0.999970678 | 7.531 | 1.37 | 3.844 | 1.599 | NA |
| TRINITY_DN14899_c0_g1_i7 | -0.5058826 | 0.905474417 | 0.999970678 | 36.263 | 0 | 42.009 | 0 | Redoxin |
| TRINITY_DN4903_c0_g1_i6 | -0.50580665 | 0.905647327 | 0.999970678 | 6.995 | 0 | 8.355 | 0 | NA |
| TRINITY_DN2117_c0_g2_i13 | 0.505627186 | 0.78550228 | 0.999970678 | 0.699 | 1.34 | 0.067 | 1.357 | NA |
| TRINITY_DN10279_c0_g1_i1 | 0.505589747 | 0.710198726 | 1 | 0.627 | 1.084 | 0.311 | 0.824 | NA |
| TRINITY_DN13541_c1_g2_i1 | -0.50554204 | 0.912056849 | 1 | 0 | 0.857 | 0.978 | 0 | NA |
| TRINITY_DN17120_c0_g4_i1 | 0.505519461 | 0.549787577 | 0.999970678 | 1.399 | 1.843 | 0.822 | 1.297 | Myb_DNA-bind_ |
| TRINITY_DN64261_c0_g1_i9 | -0.50550041 | 0.634531402 | 0.999970678 | 126.49 | 45.14 | 136.114 | 74.377 | NA |
| TRINITY_DN4721_c0_g2_i3 | 0.505453404 | 0.67671283 | 0.999970678 | 0.972 | 1.281 | 0.456 | 1.005 | NA |
| TRINITY_DN11152_c0_g1_i1 | -0.50543594 | 0.489075221 | 0.999970678 | 7.385 | 12.51 | 10.299 | 15.734 | Ribophorin_I |
| TRINITY_DN11036_c0_g2_i8 | -0.50542018 | 0.906095393 | 0.999970678 | 3.752 | 0 | 4.455 | 0 | NA |
| TRINITY_DN77435_c0_g1_i2 | -0.50542018 | 0.906095393 | 0.999970678 | 1.572 | 0 | 1.867 | 0 | NA |
| TRINITY_DN2749_c0_g1_i1 | -0.50539428 | 0.856256405 | 0.999970678 | 0 | 15.86 | 12.677 | 6.806 | NA |
| TRINITY_DN9472_c0_g1_i1 | -0.50529937 | 0.375734707 | 0.999970678 | 25.644 | 27.23 | 29.609 | 39.169 | NA |
| TRINITY_DN18625_c0_g2_i1 | -0.5052538 | 0.587801531 | 0.999970678 | 20.139 | 23.65 | 37.831 | 16.307 | Glycolytic |
| TRINITY_DN5029_c0_g2_i8 | -0.50522327 | 0.71994823 | 1 | 2.126 | 0.729 | 2.033 | 1.558 | NA |
| TRINITY_DN632_c4_g1_i2 | -0.50517616 | 0.636374912 | 0.999970678 | 0.418 | 0.926 | 0.978 | 0.714 | - |
| TRINITY_DN5025_c0_g1_i1 | -0.5051495 | 0.738574159 | 0.999970678 | 0.327 | 3.942 | 2.811 | 2.594 | Inhibitor_I9 |
| TRINITY_DN582_c0_g2_i1 | -0.50508305 | 0.799028174 | 1 | 1.463 | 0.286 | 1.833 | 0.312 | NA |
| TRINITY_DN100_c0_g1_i1 | -0.5050582 | 0.666104807 | 0.999970678 | 1.09 | 0.926 | 0.711 | 1.981 | NA |
| TRINITY_DN1496_c0_g1_i2 | 0.505049425 | 0.48383417 | 0.999970678 | 78.967 | 53.85 | 45.864 | 38.194 | PB1 |
| TRINITY_DN9136_c0_g3_i2 | -0.50501403 | 0.868764394 | 1 | 3.388 | 0.503 | 4.644 | 0 | 2-Hacid_dh |
| TRINITY_DN3649_c0_g1_i4 | 0.504940129 | 0.459941605 | 0.999970678 | 4.151 | 5.42 | 2.333 | 3.921 | CHDCT2 |
| TRINITY_DN3316_c0_g1_i2 | 0.504923161 | 0.859066933 | 0.999970678 | 0 | 22.17 | 5.033 | 9.27 | NA |
| TRINITY_DN3037_c0_g1_i6 | -0.50491883 | 0.529216967 | 0.999970678 | 10.029 | 8.022 | 13.844 | 8.918 | Rho_N |
| TRINITY_DN2646_c0_g1_i2 | 0.504811111 | 0.681747923 | 0.999970678 | 0.818 | 0.601 | 0.278 | 0.664 | HMG_box |
| TRINITY_DN4516_c1_g1_i2 | -0.50477106 | 0.692102896 | 0.999970678 | 2.353 | 0.759 | 2.633 | 1.277 | NA |
| TRINITY_DN10639_c0_g1_i1 | -0.50468265 | 0.612841676 | 0.999970678 | 11.791 | 6.406 | 9.655 | 13.653 | NA |
| TRINITY_DN6896_c0_g1_i1 | 0.504671679 | 0.543723226 | 0.999970678 | 12.418 | 8.82 | 8.266 | 5.037 | NA |
| TRINITY_DN4587_c0_g2_i2 | -0.50464892 | 0.909121105 | 1 | 0.681 | 0 | 0 | 0.995 | NA |
| TRINITY_DN19530_c0_g1_i8 | 0.504540579 | 0.731146535 | 0.999970678 | 0.173 | 0.67 | 0.322 | 0.191 | ATP_bind_3 |
| TRINITY_DN9443_c0_g1_i1 | 0.504365485 | 0.659842472 | 0.999970678 | 5.459 | 21.07 | 4.877 | 12.637 | 2-oxoacid_dh |
| TRINITY_DN6564_c0_g2_i7 | 0.50436501 | 0.908004539 | 0.999970678 | 0 | 4.366 | 0 | 3.016 | NA |
| TRINITY_DN6143_c0_g1_i2 | -0.50428989 | 0.71077525 | 0.999970678 | 1.553 | 0.335 | 1.144 | 1.317 | mTERF |
| TRINITY_DN14425_c0_g1_i2 | 0.504286968 | 0.666157217 | 0.999970678 | 6.849 | 16.77 | 9.022 | 5.379 | NA |
| TRINITY_DN6132_c0_g1_i1 | -0.50418027 | 0.589778248 | 0.999970678 | 4.115 | 2.671 | 5.411 | 3.127 | EGF_CA |
| TRINITY_DN822_c0_g1_i1 | -0.50416346 | 0.857909695 | 0.999970678 | 10.41 | 0 | 4.578 | 9.491 | DUF3548 |
| TRINITY_DN1332_c0_g2_i2 | 0.504143583 | 0.856456973 | 0.999970678 | 2.008 | 0 | 0.767 | 0.513 | NA |
| TRINITY_DN9307_c0_g1_i6 | 0.504120846 | 0.909716393 | 1 | 1.026 | 0 | 0 | 0.744 | NA |
| TRINITY_DN5973_c0_g1_i4 | 0.504074772 | 0.862210301 | 1 | 0.872 | 0.217 | 0 | 0.764 | NA |
| TRINITY_DN16724_c0_g1_i1 | 0.504054773 | 0.832050275 | 1 | 0.082 | 1.005 | 0.611 | 0.03 | Inositol_P |
| TRINITY_DN937_c0_g2_i2 | -0.50404233 | 0.720696322 | 0.999970678 | 6.295 | 0.759 | 3.222 | 6.243 | Motile_Sperm |
| TRINITY_DN1610_c0_g1_i25 | 0.503986686 | 0.905975419 | 0.999970678 | 0 | 0.611 | 0.356 | 0 | NA |
| TRINITY_DN503_c0_g1_i3 | -0.5039408 | 0.609093268 | 0.999970678 | 10.855 | 30.97 | 19.032 | 35.429 | S10_plectin |
| TRINITY_DN6967_c0_g2_i2 | 0.503911322 | 0.576042658 | 0.999970678 | 34.11 | 13.46 | 14.532 | 16.237 | NA |
| TRINITY_DN41892_c0_g1_i4 | -0.50380283 | 0.642054117 | 0.999970678 | 1.435 | 2.789 | 1.289 | 4.424 | NA |
| TRINITY_DN2984_c0_g1_i7 | -0.50371166 | 0.865828591 | 0.999970678 | 0 | 0.581 | 0.6 | 0.08 | GTP1_OBG |
| TRINITY_DN5075_c0_g1_i1 | -0.50358808 | 0.513859531 | 0.999970678 | 86.879 | 47.39 | 88.55 | 83.808 | NA |
| TRINITY_DN1048_c0_g1_i1 | -0.50353975 | 0.789248865 | 0.999970678 | 0.327 | 2.819 | 3.378 | 0.332 | UDPGT |
| TRINITY_DN1485_c0_g1_i1 | -0.50350157 | 0.655780208 | 0.999970678 | 2.861 | 1.025 | 1.733 | 3.458 | NA |
| TRINITY_DN808_c2_g1_i5 | -0.50347685 | 0.908859057 | 0.999970678 | 1.326 | 0 | 1.567 | 0 | Guanylate_kin |
| TRINITY_DN15_c0_g1_i7 | -0.50345555 | 0.708444667 | 0.999970678 | 108.9 | 35.64 | 43.42 | 135.323 | NA |
| TRINITY_DN3537_c0_g1_i10 | 0.503444344 | 0.909469231 | 1 | 0 | 2.119 | 0 | 1.448 | NABP |
| TRINITY_DN26531_c0_g1_i10 | -0.50341698 | 0.626291695 | 0.999970678 | 2.035 | 1.419 | 1.5 | 3.086 | NA |
| TRINITY_DN4655_c0_g3_i2 | -0.50341338 | 0.53878343 | 0.999970678 | 2.952 | 2.444 | 3.244 | 3.77 | Med11 |
| TRINITY_DN16333_c0_g1_i1 | -0.50339555 | 0.584685616 | 0.999970678 | 26.062 | 24.23 | 25.454 | 36.264 | NAD-GH |

| TRINITY_DN140979_c0_g1_i1 | -0.5033878 | 0.912529153 | 1 | 0.699 | 0 | 0.833 | 0 | NA |
| --- | --- | --- | --- | --- | --- | --- | --- | --- |
| TRINITY_DN9982_c0_g1_i1 | -0.5033878 | 0.912529153 | 1 | 0.827 | 0 | 0.967 | 0 | NA |
| TRINITY_DN223400_c0_g1_i1 | -0.5033878 | 0.912529153 | 1 | 0.5 | 0 | 0.6 | 0 | Oxysterol_BP |
| TRINITY_DN8174_c0_g1_i1 | -0.50327846 | 0.532413079 | 0.999970678 | 91.621 | 45.1 | 88.328 | 87.809 | zf-C2H2_6 |
| TRINITY_DN16888_c1_g1_i14 | 0.503214834 | 0.874200567 | 1 | 0.79 | 0.039 | 0.5 | 0 | NA |
| TRINITY_DN3614_c0_g1_i3 | -0.50320108 | 0.861884617 | 0.999970678 | 36.89 | 0 | 21.354 | 27.266 | NA |
| TRINITY_DN1062_c0_g1_i1 | 0.503151205 | 0.748041828 | 0.999970678 | 37.726 | 4.11 | 4.933 | 23.465 | NA |
| TRINITY_DN1041_c0_g1_i5 | -0.50312029 | 0.761320605 | 0.999970678 | 34.101 | 1.35 | 23.187 | 22.953 | NA |
| TRINITY_DN77463_c0_g1_i1 | 0.503114868 | 0.675567782 | 0.999970678 | 0.327 | 0.67 | 0.411 | 0.191 | FKS1_dom1 |
| TRINITY_DN504_c0_g1_i2 | 0.503067498 | 0.598809303 | 0.999970678 | 0.908 | 1.567 | 0.922 | 0.633 | DUF3522 |
| TRINITY_DN47313_c0_g1_i1 | 0.502991174 | 0.708614818 | 0.999970678 | 1.254 | 3.39 | 1.722 | 1.156 | NA |
| TRINITY_DN1577_c0_g2_i2 | 0.502868105 | 0.421876815 | 0.999970678 | 57.538 | 44.55 | 32.131 | 33.338 | NA |
| TRINITY_DN653_c1_g2_i1 | 0.502818006 | 0.656691302 | 0.999970678 | 1.671 | 1.735 | 1.511 | 0.573 | NA |
| TRINITY_DN15824_c0_g1_i2 | -0.50276686 | 0.664979447 | 0.999970678 | 38.361 | 8.535 | 21.599 | 40.728 | NAR2 |
| TRINITY_DN14447_c0_g1_i2 | 0.502736415 | 0.55305853 | 0.999970678 | 2.216 | 1.291 | 1.189 | 1.056 | tRNA_lig_CPD |
| TRINITY_DN13373_c0_g1_i5 | -0.50269868 | 0.791902467 | 0.999970678 | 0.036 | 0.917 | 0.533 | 0.694 | DUF247 |
| TRINITY_DN13194_c0_g2_i2 | -0.50268445 | 0.545910637 | 0.999970678 | 3.497 | 3.962 | 5.555 | 3.871 | NA |
| TRINITY_DN20615_c0_g1_i1 | -0.50263552 | 0.618239511 | 0.999970678 | 3.434 | 5.549 | 2.944 | 9.109 | NA |
| TRINITY_DN939_c0_g1_i4 | -0.50255619 | 0.641212279 | 0.999970678 | 5.469 | 2.336 | 2.667 | 7.912 | NA |
| TRINITY_DN20962_c0_g1_i1 | 0.502546056 | 0.750982739 | 0.999970678 | 6.586 | 5.785 | 6.122 | 1.196 | NA |
| TRINITY_DN4879_c0_g3_i1 | -0.50252502 | 0.457623163 | 0.999970678 | 7.576 | 7.884 | 9.533 | 10.255 | NA |
| TRINITY_DN10094_c1_g1_i1 | 0.502376213 | 0.854379534 | 0.999970678 | 0.845 | 0.66 | 0.867 | 0 | NA |
| TRINITY_DN2829_c0_g1_i7 | 0.50236784 | 0.750406684 | 0.999970678 | 1.717 | 4.464 | 3.278 | 0.362 | Glyco_hydro_17 |
| TRINITY_DN1726_c0_g1_i3 | -0.50236595 | 0.86692195 | 0.999970678 | 0 | 2.562 | 2.833 | 0.151 | CTD_bind |
| TRINITY_DN52188_c1_g1_i2 | 0.502264707 | 0.818076542 | 1 | 20.203 | 2.306 | 11.855 | 1.156 | NA |
| TRINITY_DN15008_c0_g1_i1 | -0.50221944 | 0.688011391 | 0.999970678 | 9.12 | 6.909 | 2.844 | 19.223 | NA |
| TRINITY_DN39601_c0_g1_i4 | 0.502209938 | 0.801716727 | 0.999970678 | 0.345 | 4.179 | 0.144 | 2.976 | NA |
| TRINITY_DN4452_c0_g1_i10 | -0.50218385 | 0.694910711 | 0.999970678 | 2.698 | 0.631 | 2.733 | 1.448 | NA |
| TRINITY_DN1180_c0_g1_i2 | 0.502161414 | 0.757049644 | 0.999970678 | 7.885 | 0.542 | 2 | 3.609 | NA |
| TRINITY_DN6127_c0_g1_i1 | 0.502142335 | 0.491680988 | 0.999970678 | 16.66 | 10.85 | 8.733 | 8.908 | Epimerase |
| TRINITY_DN10057_c0_g2_i1 | -0.502098 | 0.65613213 | 0.999970678 | 0.709 | 1.222 | 1.344 | 1.096 | BSP |
| TRINITY_DN20060_c0_g1_i2 | 0.502085715 | 0.754822232 | 1 | 0.191 | 0.108 | 0.144 | 0.04 | NA |
| TRINITY_DN39618_c0_g1_i1 | -0.50207625 | 0.906026359 | 0.999970678 | 5.223 | 0 | 6.189 | 0 | NA |
| TRINITY_DN1338_c1_g1_i1 | -0.50206202 | 0.503750796 | 0.999970678 | 47.582 | 32.63 | 57.785 | 43.794 | Abhydrolase_1 |
| TRINITY_DN5991_c0_g1_i1 | -0.50203865 | 0.588523677 | 0.999970678 | 4.115 | 6.396 | 3.633 | 10.486 | muHD |
| TRINITY_DN2354_c0_g1_i4 | -0.50201416 | 0.493904553 | 0.999970678 | 425.66 | 438.7 | 355.356 | 795.509 | BURP |
| TRINITY_DN1165_c2_g1_i4 | 0.502007401 | 0.751977132 | 0.999970678 | 1.844 | 1.518 | 0.178 | 2.172 | NA |
| TRINITY_DN14286_c0_g1_i1 | 0.501930648 | 0.625743795 | 0.999970678 | 1.244 | 1.094 | 0.556 | 0.985 | NA |
[truncated: 1,799,645 more chars]
